# Supplementary material for: Investigation of Resistance Genes in Genus Vigna Reveals Highly Variable NLRome in Parallel Domesticated Member Species
Source: Genes (Basel). 2023 May 23;14(6):1129. doi: 10.3390/genes14061129 (PMC10297842; doi:10.3390/genes14061129)
Supplement: Supplementary file 1 [file genes-14-01129-s001.zip › Supplementary File S2.pdf]

1. NLR genes of *Vigna mungo*

>g403.t1

MSLLNNVADFNEAARLNNVKLIYNLFHLEKVNARWQKISSSSSHAINTYDVFVSFRGEDTRNNFTGFLFQALRTKGIHAF
KDDQHLNKGQSIAPELLQAIQASRLFIIVFSNNYASSIWCLRELAEILNCVQTSPTPVIPIFYDVDPSVVRKQSGCYEKAFA
EHENRFRENKAKMEEAKRWRKTLTEVANLSGWDIQNKLHNARALIILDNVDQVEQLRMFTGNRDTLLRECLGGGSKIII
ISRDEHILRTHGVDDVYQVQPLSWENAAQLFCRNAFKVNYILSDYEKLARDVLSHAQGHPLAIEVIGSSLFGGSVSQWK
SALARLKENKNKSIMDVLRISFDELDEGDKETFLDIYNGYEEVYVKEILSFRGFHPEFGMQVLIDKSLITNNYGRIYMHSLL
MDLGRSIVREKSPKEPRKWSRLWDYQDFHNVMSHNQEIDNLEAIVVKNEEWMFGRTTMRADGLSNIRQLKLLKLNV
NFSGSLSHLSNELGYLTWNKYPFKWLPPSFRSDKLVELKLRWSSIQ

>g967.t1

MDFLGPFGKVVEGVVDFVWKHGVRHMTYIVHYKKNVVELSDTVKDLRLEKEKIDHKCEEGTKNLHNIEGKVIEWVRKV
SEIETTVDVFENDDGHTRARSPNCFVFPYLWNRHRLGRQAKNLEVGVKKLIDVSPELDEISYRENVTSNDVTLSNSGFEQ
FSSTKSTVEKVMRELENSGVRMIGLYGEGGVGKSALIKEIARIAKDKKLFNVVVKVEVTANPNIQSIQEEIAYVFGLQLVG
EGENVRADCLRRRLKKEKGNTLLILDDLWHKLDLNKLGIPLDDNDDNDDLSNDTRDLDKSREKDKNNQKVLKREKIIGG
HKGCKILLTARQKRVLEVEMDVKSTFRVEPLDDKDALMFFQKLSEIHNMSDSRKEIVRKYCAGLPMAIITVAKALRGKSE
LVLESALGELQKQELVGIQTNMDISVKMSYDHLENEEIKSIFLLCAQMGHQPLIMDLVKCCYGLGILEGVFSLSEAREKIKI
TIQKLKDSGLLLDGNSDIHFNMHDIVRDAALSIAKKDKHVFTLRNGKLDQWPELEKCTSISICNCDIIVKLPIVNCSQLNFF
QIDTNNQSLTIPDKFFEGMKNLKVLILTGFHLKRFPPSIKGLLKLRMLCLERCTLEDNIAIGELEMLRIVSFSGSQLKSLPTEL
GCLDKLQLLDINDCSILEVNIPPNFLSSLTHLEELYIRKSLTKMLVEGEPNHVEESQAKNSGSEEDQKSDKAPLFGEQVEIP
NLESLNLCSLNMHKIWSDQFLSSFCFQNLIKLVVKECDELTYLCSLSVASSLKKLKSLIISECPYMEKIFETKENNADKVCVF
PKLEEIQLTKMKRLRDIWHTKVNDDSFSSLISVNIEECNKLDKIFPSNMEGWFESLDNLKVSRCKSVEVIFEIKDCEEIDVS
GEIDTNLQVILLEDLPKLKELWSKDPHGILNFKKLRTIDVSNCDELRNLFPASMAKDVSNLERMSVLDCDGMVEIVSSRD
ASEANTDPLEFPELINVRLDSLSKIKQFYAGRLPIKCPKLKELTVSGCWKLKTFCKEISKTTEEEEEEKEEEENFVFSAQKVLP
KLEYMEIDLKEAQKLLPDYPMHCLKELTLNSVKRSNRSFNCTKTTAFKLSILQTIEQLSYKDCEFEFSSLEILIVRECWKMEK
FSEKEAITPKLKNVFGVEGDEKSKWHWEDNLNDTIHKVFSDKVSFAYAESLRLDNDTGQLIIDQLWHRRHWVQQNSF
GYLKKLSAWHCHTLEHIIPSHLLSCFHNLEELQVDDCRAHVWEKDPEGIIGLQLLKKMSVSRCERLKSLFPASVAKELTRL
EVLEVTKCKELAEIFKKEGEGTTKELALGRLTTLMLEELPSLKYSIHCSNQVALSPPEISFGNAIKVIIDKDTHNQHQEDLES
HSKNQVGTEKCLSLGENGMKIILRGELERNLLDSLTALTLGFGSDVFEYEILKKVSNIEKLVVWDGSFKEMFCRESPDNVL
QQLKVLRLESLRELVSIGIENSWTHSFVGNIEIFEVINCWSLENLVACPVSFSNLTCLKVGSCHGLSYLLTSSTVKSLGKLKR
MEIKDCKSIEETVCKEDGEESDEDEIIFPQLTCLNLNMLRKLKRFYKGSLGFPSLEELSVKDSNKMFGMFGNRLQYPPAQL
QHICMSTGKNGMMMILRGEFERKLLESLKALTLIGSDVFRCKILEQFPNIEKLVVCDGSFKKMFCCESGNNVLQQLKVLR
LESLGELVSIGLENSWNDSLIRNIETFEVISCWSLKNLVASTVSFSNLSCLKVERCGSLSYLLTSSTAKRLDKLKRMEIKNCDS
IKEIVCQEDESVEDEIIFPQLTCLNLKGLKKLKMFYRGSLGFPSLEELSIKDCKKMITLCVGTIVTGKLSQVNLELDEYSEAIE
WEIDPNSTKMKKYLIETQRESLIDLKSRAGLHEIWRGSLSIPNFCFSELVTLIVDDCQFLSDAVIPFHLLPLLPKLNTLEVRN
CHSIKTIFDFNPSTQDTLVTLPLNNLVLLNLPNPENVWNKGLELEALVVEECERLVGIVADDNTDPNLQLTLPCPYLRSLKL
QGLQKFTYFYYCSHKSDIYTHLESPTEDQLPNEKCMSVGENGMKMILRGEFEKKLLDSLKVLTLCFDSDVFGCKILEQVP
NIEKLEVCDGSFKKMFCCESPNNVLQQLKVLRLESLGELVSIGLENSWTHSFVGNIKTFEVIKCLSLKNLIACTVSFSNLTSL
KVEHCNRLSYLLTSSTAKSLGQLKRMEIRYCSSIEDIVCKEDGKESDEDEIIFPQLTCLNLEGLEKLKRFYKGSLGFPSLEELSV
EDCDEMISLCVGTIVGGKMCEVKVGEFSEAIQLETELNSTMRKEYLRKIASSKWEKSLELRDRADLQEIWCLSLQIPDFCF
TYLETVIVNKCKISSDAVLPFTLLPLLPKLETFEVQNCDSVKTIFDVKCGQETLTFPLKKLVLRKLSNLETICNEVTEANPALP
KETDRKLTFPCVTSLALCDLPNFIYDDAAATFELIIPNLEHLTVGRNELKMIVDGEFQKNLLYKLKVIGVCFDIECDEFPEYG

FLQQLPNVKRLVVWNSSFKVIFCHQRPSNNELLLQLNELRGSLSFPSLEELSVRHCDEMVTLCPSTLKADKLSQVTIQYEE
VITGETDLYTTMQKEFGRKAQWELWIDLKSRGGLHEIWRGSLSIPNFCFSKLVTLIVDDCPFLSDAVLPFHLLPLLPKLETF
EVRNCDSVKTIFDVNPSTQDTSITFPLKKLVLSKLPNLENVWKEDPRGILSMHRHLEQVFVDTCKCLKSVFPASVAKELVK
LKDLVVEDCEGLMAIVAHESHENKEIIFERLQEVHVIKCSSMKSFSAVTKIDNATEWYYSEYERPRKETDLNSALHRNFEE
EAPDGASAIIPVLE

>g1205.t1

MQSVVNPMSFQVSHKIKYVACICLVFVTSSTSSIDTSQIKYDVFVSFRGEDVRRGFLSHLIEAFSQKQIAFFVDDSIQKGEE
LSEALFGAIEESYISLVIFSENYASSRWCLSELEKIMECRRKNGQTVVPIFYKVDPSDIRHQRRTYGDAFVKHERNYSLTTV
QTWRSALSESANLSGFHLSKFPDEAELVKRIVKFVWSTLNHVHQVKSKGLVGIGKRIAQVESLLQLETKDVRMIGIWGM
GGIGKTTIAQEVYNKLCFKYDSCCFLANIREESWRHGINSLKEKLFSTLLREEHLKIGRPDGFQKLADRRLHRMKVLIILDD
VGDAEQLENLARTNWFGSGSRIIVTTRDKQVLAEESASVYHVEALNFDESLRLFNLNAFKQKHIEAEYEELSKKAVEYAK
GIPFVLKVLGHRLHGKDREIWESELERQEVHNKKVKYIDFLLKDRDYSVAAGLERLKDKAFISISQENTVSMHDIIQETAW
QIAGEESIKNPRRNQIRLFVPDDIYEVLTYSKGNEAIRSIVVNLLRIKQLHLKPQVFTKMSKLHFLNIYTAGTRDIRFYEPWG
LYLHQGLESLPNELRYLGWMHYPLESLPSNFSAENLVELHLPYSRVKKLWHEVPDLVNLKVFMLYSSSNIKELPDFSKAP
NLEVIDLRSCVGLTSVHPSVFYLKKLEKLDLDECRSLTSLRSNVQMESLRYLSLFKCMELKDFSVTSKNMIMLNLENTDIKQ
LPSSFGSQSKLEDLNLAFSSIESLPASMKDLKRLQHLDLRHCRNLSSLPELPPSIETLDCRECVSLESVKFPSIAEQWKENKK
KVVFWNCLNLDERSLKAIETNARINMVKFAHRHLSTSGDAHAIYVYPGSQVPEWLTHKTTHDDDDGDDDYDDDDDD
DDSITFAPNPSHLGYIFCLILPAVQYSERVLKLTVSTGGEDEGDSVIVYLDRPHHTIKSDHVYLMYNKACSRFLASRAKQQ
PMLKIKVTVATLLISSKYIDVRLRGFGVSTISNFLQKQQLCDTRILKEHCEVCPDAL

>g1206.t1

MTYSNSFRVFDRIKRVIFVCVLLAAIDNNNSNNSSYPVTDFAYDNDSPQIKFDVFVSFRGTDIRQDFLSHLIEAFSQRHIN
AFVDNKVVRGDGMSEALIRAIEGSSISLIIFSQDYASSHWCLSELVKIVECRKKNGQIVLPVFYKVDPAHVRHQKGTYEHA
FAKHQIRYSLTTMQIWRTALTEAANLAGFHSSTFRDEAEFIKEIVKCVLSRLNQVQQGKSKGLVGVGKRIAHVESLLQSE
EPDVRIMGIWGMGGIGKTTIAQEVYDKLCFEYEGCCFLANIREESGRLGIISLKKKLFSTLLGGEDLKIDTPNGLPQYIERRL
RRMKVLIILDDVNDSDQLEVLAGTHDWFGSGSRIIITTRDKQVLAREFASIYEVEALNFDESLRLFNLNAFKQNHLESEYH
ELSKKVVNYAKGIPLVLKVLGHLLHGKDKETWESQLERLKKVQNKKVHDIIKLSYNDLDRDEKKIFLDIACFFDGLNLKVK
HMNFLLKDHDYSVVAGLERLKDKALISVSQENGVSMHNIIQETAWQIAREESIENPRSQIRLLDPEDIYHVLNYNKGDEA
IRSIVINLSRIKQLQLNPQVFARMSKLHFLDFYSKGSCSCLGDQGGLYLPQGLESLSNELRYLRWTHYPLESLPSKFSAENL
VELNLPNSRLKKLWQEAPDLVNLRVLILHSSTRLKELPNLSKATNLKVIDLRFCVRLTSVHSSIFSLRNIEKLYLGGCLSLRSL
RSNVHLDSLRYLSLYGCMSLKDFSVTSKNMVKLNLELTGIKQLPSSFGLQSNLQKLRLAYTYIDHLPTSIKHLTRLRHLDLR
YCRELLTLPELPASLETLDARGCISLETVTFPSTAGEQLKENKKRVAFWNCLKLDEPSLTAIELNAQINMMKFAHQHFSLF
GDAQSTYVYPGSKVPEWLVHKTTDDDFVIIDLSSVLSPHSSHIGFIFGFVVPEVPFGGSALEFKISTSGEGSHINVYMDRP
RHRITSDHVYLMYDQACSRYLNGLAKHEPRLKIKVALASRTLTSNVFD

>g1250.t1

MSSSMKKHDVFVSFRGEDTRTNFTSHLCKALEYKSIRAYIDRQLDRGESVWPALAKAIQDSRVSIVVFSENYACSKWCLE
ELVKILECRKKMGLAVIPIFYNIDPSDIRNQTGTYEKALTELLESNEEKGPKWKAALTEAANISGWDSRTHRDEAHVIENV
VNDVLQKLHLRCPTELKGLVASEENCRNVELLLKSCRVIGIWGMGGTGKSTIAKILFAKHFPQYDHVCFVTNAKEYSLDK
LFSTILKEEVSEKNVVGSSFHMRRLRSKKVFIVLDDVDMDSFEPLEYLCREYKAQHRDSKLVITTRDRQLLVERVDAIYEV
QKWKKTESLKLFCSEAFKKSYPDGGFDRLSESVVEYAGGVPLALKVLGSYLRSKSSSFWKSTIRKLSLYPNERIQEVLEMSY
TGLHDLEKKIFLDIVFFFREKQKDQVTRILDACGFEATSGIEVLADKALLTISYTNIIQMHDLLQQMGLEIVRLECTTDPGR
RSRLKDNDAHEVIEENKGTDAIQGIELDLSQVENLRLRSDTLTKMKTLRFLRFYNSSGQSSRNTYIDLPATLEPFSDKLRYIE

WIEYPFECLPSPFCAKFLVEIHMEHGKIKQLWQGIQELDNLEGINLSGCKQFEELPDLSKAPKLKWVNLYCCESLRYLHPS
VLSSATLVTLILHGCTKLESVKGEKRLKSLGYINVNGCLSLEEFAVSIVSIEIFDLSNKEIPMSGTPVRRKGKLKTNKILKELML
SNGRRLGKDLNNCNLPSMQHLYMLQLDRCNVTNLPEIIKNSGKLTFLSLENCDGFTHLPKLSSSINYLGVINCTSLVSVSD
LDNLANVMCGSSRFITFKNCLKLDEHSCKLIMKSVKLIMVCAAFDNLVRKSSDVHDYSYNSVELCLPGSKVPQEIKYRSTE
SSITIDLPKLSNLRGFIYSVVLSPSGEMKKHDTKIICKRHLRENTRESWVYSDIEGLKTDHVYIWYDPFHCDGILKYNEPSV
WFEFCVTNDKGEVDGSMCIKECGVGLISVSELPSVLEELDWHWDKKKDLVNRVELITGQRITLITSIEQSDERKNHFSAV
EEIINSTHKEVKTDSDTDCGQNTTKSTNAVKYEESGETAIKQDATLPETVESELDKENESKEKSSVKESIEYCASRATNATA
ERGPKEKSTKSTETVAYEHSQWLEESIKQVVGIHDTGNSVKYSSLDLENCLQQSDENPFKIFDLLSDELSPSLKQSSDATTL
LNEFRTLVFSTSLLKKIPDQSYQQQVTESLQKLHTYRGKITKEQEAGLDKFIELYNKAVDISQDKMLTEDNQAKLASEKRD
LYNKLEHSKLKVQRFDTTISTYKSQREILQKRQREIQEAITELEQENEALEKDSSTLEVLYSEQQTKKKETLESVNGEMSSST
KKHDVFVSFRGEDTRTNFTSHLYKALKDKSIGAYIDCQLDRGEAIWPTLAKAIQDSHVSIIVFSENYACSKWCLKELVKILE
CRKELGLVVIPVFYNIDPSDIRNHKGTYEKALAELLESNEEKGPKWKAALTEAANISGWDSRTHRDQAHVIENVVNDVL
QKLYLRWPTELNGLVGYEENCRNVELLLKSRKVIGIWGMGGIGKSTIAKILFAKHFPQYDHVCFVTSAKEYSLEKLSSTILK
EEVSEKNVVGSSFHMRRLRSKKVFIVLDDVDMDSFEPLEHLYREYEGQHSDSKLVITTRDRQLLVGRVDAIYEVQKWKK
TESLKLFCSESFKKSYPEGGYESLSESAVEYAGGVPLALKVLGSFLRSKQIREKEKDHVTMILDACGFEATSGIEVLADKALL
TISHTNIIQMHDLLQQMGWEIVRQECTADPGRRSRLKDKEAREVIEENKGTDAIQGIELDLSQVKNLRLRSDTLTKMKTL
RFLRFYNSSGQSSTNTYLDLPATLEPFSDKLRYIEWIGYPFECLPSPFCAKFLVEIHMQHGKVKQLWHGIQELDNLEGIDL
SGCKQFEELPDLSKAPKLKWVNLSCCESLRYLHPSVLSSTLVTLILNGCTKLESVKGEKHLKSLEKISVNGCLRLEEFAVSLDL
NKILYASSRGIQMSGTPVRPTGKLELTPTLSNLYMLKLDQCNVTTLPEIIKNSGNLRILSVENCLEFIHLPKLPSNIDYLGVIN
CTSLVSVSDLVNLANVMRGSTRFITFKNCSKLDEHSCKLIMKSVKLIMVCAAFDNLVRKSRDVHDYTYNTVELCLPGNKV
PQEIKYRSTESFFTIDLPKLSNLKGFIYSVVLSPSGEMKKHDTKIICKRHLRENTRESWVYSDIECLNTDHVYMWYDPFHC
HGILKYNEPSVCFEFCVINDKGEVDGSMCIKECGVDLISVSELPSVLEELDWHWDKKKDLVNRVESVTGQRITLTSIEQSD
EWKDHFSALEEIISSTHKEVKTDSGTDCGQNTTESTNAVVQVKYEEIGETPIKQDATLPETVESQLDKENESKEKSKSVED
NRGSKEYVYDVEESIEYCASSATNATAKRGPKEKSTKSTETVANEHSQRLEESIKQVVEIHDTDNSGIKYSSLDLENCLQQL
DENPFAILDLLYYELSPSLKQSNDATTLLNEFRTLVFSNSLLKKIPDQSYRQQVAESLQKLHTYRGKITKEQEAGLDTFRELY
NKAVDISQDKMVTEDNQAKLASEKRDLYNKLEHSKLKIQQFDTTISTCKSQRENLQKRQREIQKAIKTLQQENEALEKDS
SRLEVLYSEQQTKKKETLESVKXEKSFIVHLVSYDVVILGGSFLSLNPTLEIMESLEMSSTFGEKHDVFLSFRGADTRTNFTS
HLLNALTQKSISAFIDYEIIRGDYTWPALETAIEKSLLSIVVLSENYASSTWCLKELAHILECRRKRGMVVIPVFYEVDPSHV
RKLSGSFEKSFAKHERDSTSFDRISQRKDVSMWKAALKEVANISGWDSRSYRDEAQVIQNLVNDVLQKLHLRYPTELNG
LVRIKNTCAKVDLLLRKSRVIGIWGMGGIGKSTIAKALFAKYFPYFDHVCFMANANEFSLDKLFSELFREEVSASNVVGST
FDMRRLKRKRIFIVLDDMDCLDLLEYLCREYQNLDPNSKLIITTRDKQLLEGRVDQIYEVKKWETRASLKLFCLEAFKKRHP
KRGFESLSESAVEYAGGVPLALKVLGSYLRSKATSGIEILADKALLTISYRKIIHMHDLLQQMGLEIVRQESSGDPGRRSRLK
DKEAREVIEENKGTSAIQGIALDLSQIKGLILHADTFTKMKTLRFLKFYNKLGQSARDTYLDLPATLEPFSDKLRYIEWIGYP
FESLPSPFCAKVLVEIHMPHSKVKQLWQGIQELNYLEGINLRQCKQFEELPDLSKAPRLKWVNLSCCESLLYLHPSVLSSG
TLVTLILDKCTNLKSVKSEKHLKSLEKISVNGCLNLVEFAVSSDLIENLDLSNTGIQMLGTSIGSMHKLKSLNLEGLKLKPVLK
ELSCLTSLKVLKISDNGLVIDKQQIHVLFSGLRYLQILYLKDCSKLFELPDNINLLTQLQELRLDRSDLKRLPENIKNLQVLEIL
SLEDCKELLCLPTFPSLIKYLRVINCTSLVSVSNLKTLAIEMLGMTKRTTFKNSGKLDEDSLRIIMESLHLTMMSAAYHNVL
VRKTYGTYNSCNYTSVELCLPGGSVPEQIHYRSTQSSITIVLPPRSELLGFIYSVVLSPAGGKKTFGTKIFCKCHLPEEGIKAT
WLYGDIRGLKSDHVYGEVDGSIFIKECGVGLVNVLEMHSVLQELDFDSDKKKELVEGVESESKHRKLDWSESSSSDSLTT
PRSSSESDSEIVKPVSAENGKSTHKETKTDSGTSVVKYKNNKEVPKSDATLHETMESHSDNENQFREKSEVVEMIELENT
ESTCKETLSTAERGPKEKSRESTEIVADEHLQSTSLQASSQGGSEHLHDRLEESNKQVVETYDTEKFVTKYSYFDLESCLQ
QLDENPFAILDLLSNELSPPLKQSETCVQKVAQANDATTVLDEFRTLVFSTSMLEKLPDQSYRQQIEESLRKLHTYRREITK
EQEGVDKFIELYDKAANISQEKMLTENKQTKLASKKRNLYNKLQDSKLKVQQFDTAISTNKLEIENLQKRQREIQEAINKI
QQENEALEKERSALEVLYSEKQTKKNEILELVKHISTSVVYTTKQLEELEEKRLSLASAYEDLKEPYGRMKTKPPF

>g1964.t1

MVLVLALGDLHIPHRAPDLPAKFKSMLVPGKIQHIICTGNLCVKEIHDYLKTLCPDLHITRGEYDEETKYPETKTLTIGQFKL
GLCHGHQHLSVSIKGLWVGVVVIPAVIPWGDLDSLAMLQRQLDVDILVTGHTHQFTAYKHEGGVVINPGSATGAYSSI
TYDVNPSFVLMDIDGIDCHALGLSELREYTCTIIHCLRMDPRVWHKVAAISGVAALGLGTYGAHVFKPQNPAYKEVWH
TASLYHLVHTAALVAAPITKHPTVFGGLLTTGILAFSGTCYTVALLEDRKYSTLAPFGGFAFIAAWGSLYQPILYFIFVNHFP
FVLPMVHDLEISVSTDDVLNRLKATCECESFDMLRGESSSSSSDASSRGSYHVYLSFGMEGTHVDFANTLCVSLQRKGIS
TFRYDKLVERDVMLKKVQKAMEEYLVAIVLLSENYAASTWCLDELRKILDVGKPVIPVFYEVVPSDVRHQINSFAKAFEE
HERRSQEDQLKVQQWRKSLKEVADFSGWESKNRRREELIEDIIKSVWTKIRLRLPSYEEERVGIDSRVEKIRSLLKLELKDV
VHFTGIWGMGGIGKTTLARVVFKKICSQFDISCFLENVREISRKTHDMLTLQITLLSHMEVKEFTIQNLDEGKIVIGGILRN
NKVLLVLDDVDDARQLDSLGVNDQGKFGPGSRIIVTTRDMEKAFKRDKPTEQLLQLSKVAVQQAGGLPLALEMMGSS
FCGRNESQWKELLDMKEYSKKDIVMKKLTISYLGLPEIYQILFLDIACFFNGWVKERVEQILTICCRCPANGIDVLIGKSLVS
CDGSRLWMHDLLQEMGRKIVVEKCLIDASKRSRLWSPHDIDQALKRKKKNESIQGIVLKSSTEPYIANWDPEAFSKMYN
LRFLMINFPNIQFARGLKCLSSSLEVLQWTKCTLEALPLGVKLEKLVVLKMRYSKIKKIWSDSQVSSSVNILCIQFNTSIKSS
KANHFHTSVLIDQHFKRLKFIDLSHSEDLIESPIVSEVPCLEILLLEGCKNLVKVHQSVGLHKKLVVLNLKDCINLQILPTEFK
MDSLEELILSGCSKLKKLPKFANNVQCLSLLDLKKSENLICLPKSVCYVSGTKVRFENLKELSFNGGKELTSNSSICRKKLVSK
ELILPPLSSLTSLKFLNLSYCDLNDESMPDDLGSLSSLLGLDLSGNNFVRPTYCIRNLHALKSLTLIDCPRLESLPMLPPNVQ
CLCTANSTQTKPMNSDAYMLWKIFELHMNQVYSLYTPHSLPHLPPIHPNHFHKVCVYQMEGRPHFMFIIPGREIQKW
NEEYFLVDPSHHPYNMLGCDSAASIKVEAPQYCESSGWLGIAICLALERSQSHVSPHSKGNEETSIYFWACKAHDGEPD
LIFPIVPELSHSVHECNEEKCLLQLIFYVENHSKAWKPSIRKCGVHAKASMLSGESSSNSSRWTYHVFLSFRGEDTRLGFT
DHLYAALVRKGIITFRDDKNLEKGDKIDKELFKAIKESLAAIVILSENYASSSWCLDELNKILESNRALGRKVFPVFCGVSPSE
VQHQTTKSFEEAFQKHERRFDKDIEKVRQWRDSLKEVSQIAGWESKNYQHQTELIENIVESVWTKLRPEMPSFNDGLV
GIGSRVKKMDSLLRIESKDDARFIGIWGMGGIGKTTVARVVFRKIQHQFDISCFLDNVREISKESGGRLRLQGKLLSHLGI
KGLEIRDLDEGKNTIRELLFKKKVLLVIDDVDDTSQLESLADSLEWFGPGSRVIITTRDTHVLISHGIVENYKIDILNSDESLQ
LLSQKAFKRDKPDEHYLELTKAVAKYAGGLPLALELLGSFLCGRSESQWKEVVDMIKEVPPSHIAMKSLRISYNGLPLRYK
TLFLDIACFFKGRIKELVIQALEICERYPPVGIELLVEKSLATYDGFTIGMHDLLQESAREIVKEESYVDPAKRSRLWTLEETN
EANESIEGIVLNSPEKEEAIWDPEAFSRMYNLQLLIINYRVNLPTSLKCLCSSLKFLQWMNYPLESLPLGVLLDELVELKMH
SSRIKKIWNGNQDFAKLKFIDLSYSEDLIQTPIVSGAPSLERLLLIGCINLVEVHPSVGQHKRLVLLLLKDCKNLQIMPRKLE
MDSLEELILSGCSKIEKLPEFGENMKSLSLLNVENCINLLSLPNSICNLRSLRKLYVSGCSRISTLPDGMNENESLEEVHVSG
TDIREIPLCLEKLRELSFGGRKETTPKSQNLLQWISKFMGLQDMQESIVPPLSSLLALESLDLSYQGLTDESIPSDLGPLSLFK
RLDLSGNNFVNPPAQCIISLSMLHTLSFNDCPRLESLPLLPPNLQALYATNCPKLKPFHLVEDTLWKIFESHSHEDPIEGPE
LWFIIPGNEIPSWFNNQNSLAIDSSDETYEKLCCDSVTSITVDVPEDFQLSEWWGIAVCLVLEPLNMDVPSSSNARSTST
VNEEIGIYYWVCKAPDKDPDPNFPIAAKFGHMLYKFKDPYIHIIFLNADHVYIQHYLSGQQTQLEVILFVENFSESCKARIK
KCGCRVICKEKIEEWRKHSDDLIISRITETNHELEEEEPTSPNTSSPMEKTEGQTTLGK

>g2491.t1

MAELFLFSIAESLITKLASHAFQKASRVVGLYDDLRDLGNTLSFIKAVLLDAQQKQDHNHQLREWLTHLKTVFSEAEDLL
DEFEYQTLQNKVVKAHGNTKDKVSRFFSTSNPLLFRYKMTQQIKYINNRLDKVAAHRHKFSLQIIDVDTRVVHRRDMTH
SRVSDSDVIGRKNDKEKIIEHLMQQNPNDDDTSLSVIPIVGIGGLGKTTLAKFVFNDNRIQECFPLKMWVCVSDDFDIKQ
LIIKIINSANDEDVSPHQQNLNMLDLEELQKQLKRKLSGQKFLLVLDDVWNEDRVKWVELRNLIQVSGAGSKVVLTTRS
HSIASMMGTVPSHILEGLSEQDSLSLFVKWAFKEGEEEKHPHLVNIGREIVKKCRGVPLAVRTLGSLLFSKFEDSEWQYV
SDSEIWNLPQKKDDILPALKLSYDLLPSYLRQCFALFSLYPKDYLFPSYEIAAIWGSLGLIALPKTNRTLEDVANQYLHELLSR
SFLQDFENFGNKFRFRIHDLVHDLALFVATDECLHVSFKIQNIPDNVRHLSFAESSLFGNLVTKKSTVVRSVLFPNGAAAA
IDEALLNMFLSKFKYLRVLDLRGSTCKTLPRAIAKMKHLRYLDISENPNIKRLPDSICKLQSLQVLSLKTCKKLEILPKGFRKLI
SLRSLEFSTKQTILPVNEIAQLGSLEYLYIESCHNVESIFGDVKFPSLKTLCVGDCQGLKSLSLDGQNFPELETLLVKDCGNL

DLELCKGHHEEESPKLKLKLVLFFGLSQMVALPNWLQEAVKSLQRFIAESLVAKLASHAFQEAFRVVGLYDDLQDLTNTL
SLVKAVLLDAQQKQDHNHQLREWLTHLKTVFSEAEDVLDEFECQTLRNKVVKAHSSTKDKVAADRHKFNLQIIDVDTR
VVHRRDMTHSRVSNSDVIGRKNDKEKIIELLMQQNPNDDDTTPSVIPIVGIGGLGKTTLAKFAFNDNRIQECFPLKMW
VCVSDDFDIKQLIIKIINSANDEDVPPHQQNLNMLDLEQLQKQLKRKLSGQKFLLVLDDVWNEDRVKWVELRNLIQVSG
AGSKVVLTTRSHSIASMMGTVPSHILEGLSEQDSLSLLVKWAFKEGEEEKHPHLVNIGREIVKKCRGVPLAVRTVGSLLFS
RFEDSEWEYVSRNEIWNLPQKKDDILPALKLSYDLLPSYLRQCFALFSLYPKDYEFFSNDIASIWGSLGLITLPKTNRTLEDV
ANQYLHELLSRSFLQDFQNFGSSYSFRIHDLVHDLALFVAADECLHVSLNIQNIPYNVRHLYFAESSLLENLVTKKSAVVRS
VLFSNDAAAAIDEALLNTCLSKFKCLRVLVLSGSTFETLPCTIAKMKHLRYLVINNNLKIKRLPDSICKLQSLQVLSIKGCMEL
EVLPKGLRKLVSIRCLEFSTRQTVLPVNEIAQLGSLEFLYIDSCHNVESIFGGVKFPSLKILCIVSCQSLKSISLDGQNFPELETL
VLAACPNLELELWKGDHEKESPKLKLKLIGFNCLSQLVALPRGLQEAAKSLQCLFVSNCENIETLPDWLTTLTDLKTLSLID
CPKLASLPDNMHHLSALENLRIEDCVNLRKKYQPHVGEFWSKISHIKNIIIVEPEEPEN

>g2492.t1

MAESFLFSIAESLIAKLASHAFQEASRVVGLYDHLRDLTKTLSLVKAVLLDAQQKQDHNHQLREWLTQLKTVFSEAEDVL
DEFECQTLQNKVVKAHGSTKAKVSHFVSTSNPLVFRFKMAQQIKDINNRLNKVAANRHKFSLQIIDVDTRVVHRRDMT
HSRVSDSDVIGRKHDKENIIELLMQQNPNDDDTSLSVIPIVGIGGLGKTTLAKFVFNDNRVQECFPLKMWVCVSDDFDI
KQLIIKIINSANDEDVSPHQQNLNMLDLEELQKQLKRKLSGQKFLLVLDDVWNEERVKWVELRNLIQVGAAGSKVVVTT
RSPSIASMMGTVPSHILEGLSEEESLSLFVKWAFKEGEEEKHPHLVNIGREIVKKCRGVPLAVRTLGSLLFSKFEDSEWQY
VSDSEIWNLPQKKDDILPALKLTSVRTVLFPNGGVGANGETILNTCLSKFKCLRVLNLSGMAFETLPRAIAKMKHLSTKQT
VLPVNEMAKLVSLECLVIESCHNVESVFGGVKFPSLKTLYVRNCQSLKSMSLDGQNFPELETLVVANCGNLELELWKGD
HEEESPKLKLKLVLFIRLPQLVALPKWVEEAANSLGLPKFGISLR

>g2494.t1

MAESFLFSIAESLIAKLASHAFQEASRVVGLYDHLRDLTKTLSFIKAVLLDAQQKQDHNHQLHEWLTHLKTVFSEAQDVL
DEFECQTLRNKVVKVYDSTKDKVSHFLSTSNPIVFRFKMARKIKDINNRLNKVAADRHKFSLQIIDVDTRVVHRRDMTHS
RVSDSDVIGRKHDKEKIIELLMQQNPNDNDTSLSVIPIVGIGGSGKTTLAKFVFNDNRIRECFPLKMWVCVSVDFDIKQLI
IKIINSANGVDVPPHQQNLNMLDPEQLQNRLKSKLSGQKFLLVLDDVWNERRLKWVELRNLIQVSGAGSKVVVTTRNH
SIASMMGTVPSHNLEGLSEEESLSLFVKWAFKEGEEEKHPHLVNIGREIVKKCRGVPLAVRTLGSLLFSKFEDSEWKYVSD
SEIWNLPQEKDDILPALKLSYDLLPSYLRQCFALFSLYPKDHLFNSYKMTALWGAHGLITLPKTNRTLEDVANQYLHELLS
RSFVQDFDNFGTSYNFKIHDLVHDLALFVAKDECLHVRSNIQNIPENVGHLSFAESSLFDNLVIKKSAAVRTVLFPNGVG
ANGETILNTCLSKFKCLRVLNLSGLTLETLPRTIAKMKHLRYLDISNNLKIKRLPDSICKLQSLQVLLINGCMELEVLPKGLRK
LISLRYLFFSTKQTVLPVNEIAQLGSLEYLSIESCHNVESIFGGVKFPSLKTLYVRNCQSLKSMSLECQNFPELETLAVRTCDN
LDLKLWKGVHKKESPKLKLKLVFLDGLPQLVDLPEWVEEAANSLGGLIVSNCDNIKTLPYWLPTLTNLKSLHLLGCLKLIFE
FCYRIMMHTLLQTDLRFGVVYTDVVSAAVGCVDEVIKHESLVDDRFFLMCKGQERFRVNSVVRTKPYLVAQVTWLED
WPSPSTDLDLGRLATGEETDQNCDYETIYNWISEISRLKSYWEERRQAWYREMLETGSQNEDISRLVERGTQAHLVNS
QDYEENSQELMTFLQERLHSARISQKDGRDAIEEEDEDNADEHEEEHEHEDESLISNPYHEAGDYSNFSSSCSYRDNEG
GDDFDRVVSPSRQPYQSQSFYHSPIIIQWKWNSYDLRGHMEQLYHEMSELKKIIKGCMNMQMELQQTMKVEIKSGL
QKRKATTAFAEGQPYMESGRVWYAKDIGIPSRACRWMNNGFMAINTTGAGKAFIRSIYELYASWGVDFVKLDCVFGE
DLDLGEITYVSEILNGLNNSIVFSVSPGVSATPEMAEMVSGLVNMYRITKDDWDKWPAILSHFNVSRLP

>g2497.t1

MAELFLFSIAESLITKLASHAFHEASRGQKQDHNHQLREWLTHLKTVFSEAEDLLDEFEYQTLQNKVVKAHGNTKDKVS
RFFSTSSPLLFRYKMTQQIKYINNRLDKVAAHRHKFSLQIIDVDTRVVYRRDMTHSRVSDTDVIGRKHDKEKIVELLMQQ
NPNDDYTSLSVIPIVGIGGLGKTTLAKFVFNDKRIQECFPLKMWVCVSDDFDIKQLIIKIINSANDEDVTSHQQNLNMLDL

EQLQKQLKRKLSGQKFLLVLDDVWNEDRVKWVELRNLIQVNGAGSKVVMTTRSHSIASMMGTIPSHILEGLSEEESLSL
FVKWAFKEGEEEKHPHLVNIGREIVKKCKGVPLAVRTLGSLLFSKFEDSEWEYVSDSEIWNLPQKKDDILPALKLSYDLLP
SYLRQCFALFSLYPKDYLFNSYKMTALWGAHGLITLPKTNRTLEDVANQYLHELLSRSFLQDFENFGTSYSFRIHDLVHDL
ALFVATDECLHVRFNIQNIPENVGHLSFAENSLFDNLVIKKSATVRTVMCPNGAVGANGEAILNTCLSKFKCLRVLDLRG
SAFETLPRVIAKMKHLRYLDISNNLKIKRLPDSICNLQSLQVLLINGCIELEVLPKGLRKLISIRRLLFSTKQIVLPVNEIAKLGS
LKDLYIESCHNVESIFGGVKFPSLKTLCVSNCQSLKSMSLEGQNFPELETLVVAACDNLDMELWKGDQSPKLQLKLVYFA
GLPQLIGYQLSVI

>g2503.t1

MPITPDRFQTPKEWPEVLPSATLHTPREVSFFRIAYLNKPEILVLFMGTLAVATTGAVQPTMGLLLSNMINTFLEPAEELR
KDSKFWSLIFVALGWYGVMTVDYGCSGWCGGMVVDCGLPHGHSRGCVPIGLSTPNADQTIRGEHVFPSAPQSPPCV
FAASPIIVLAPVPAHLSSDLXKYVQSWNPFINEVDLETLFSLPISSLPFVFQTTMAESFLFSIAESLIAKLASHAFQEASRVVG
LYDDLRDLTNTLSSVKAVLLDAQQKQDHNHQLRGWLTHLKIVLSEAEDLLDEFECQTLRNKVVKADGSTKDKVSHFFST
SNPLVFRFKMAQQIKDINNRLDKVAADRDKFSLQIIDVDTRVVHRRDMTHSRVSDSDVIGRKDDKEKIIELLMQQNAN
DDDTSLPVIPIVGIGGLGKTTLAKFVFNDKRIQECFPSKMWVCVSDDFDIKQLIIKIINSANDVDVPPHQQNLNILDLEQL
QNQLKSKLSGKKFLLVLDDVWNEDRVKWVELRNLIQVSAAGSKVVVTTRSHSIASMMGTVPSHILEGLSKEESLSLFVK
WAFKEGEEEKHPHLVNIGREIMKKCRGVPLAVRTLGSLLFSKFEESEWEYVSDSEIWNLPQKKDDILPALKLSYDLLPSYL
RQCFALFSLYPKDYEFLSNEIAAIWGLLGLIALPKTNRTREDVANQYLHELLSRSFLQEVTNFATFYSFRIHDLVHDLALFVA
KDECLYVSSNIQNIPENVGHLSFAESSLFDNLEIKKSASVRTVLFPNGGVGANGEAILNTCLSKFKCLRVLDLSGSTFETLPR
SIAKLKHLRYLDISKNLKIKRLPDSICNLQSLQVLLISRCIELEVLPEGLRKLISIRDLAFSTKQIVLPVNEMAKLGSLEYLVIDSC
HNVESIFGGVKFPSLKTLSILNCQSLKSMSLDGQNFPELETLVVANCGNLELELWKGDHEEESPKLKLKLVYLVNLPQLVG
LPKWVEEAANSLRLPKFGISLR

>g2505.t1

MMMAESFLFSIADSLIAKLASQLYEEASRVMGLYHHLQEFTSTLSLVKAVLLDAEQKQEHNHELREWLRQLKRVFSDAQ
DVLDEFECQTLQNQVVKAYGTTKTKVSRFFSSSNPLVFRYKMAQQIKNISNRLNKIAADRHKFGLQTIDVDTRVVHRRE
MTHSHVRDSDVIGREHDKRKIIHVLTQKNPIDIDKNIFVMPIVGMGGLGKTTLAKFVFNDDRIDECFPLKMWVCVSEDF
DIKQMMVRIINSANDSASHSHATDFQQNLNILDMEQLQNQLRNKLVGKKFLLVLDDVWNEDRVKWVELRTLIQVGA
AAGSAVLVTTRSHSIASLMGTVSSHILQGLSLEDSVSLFVKWAFKEGEEENYPHLINIGIDIVKKCSGVPLAVRTIGSLLFSK
FEAKEWKYVRDSEIWNLPQKKEDILPTLKLSYDLMPSYLRQCFALFSLYPKDYKFVNLEVITLWGALGLLRSLKKNMTQE
DVANQYLYELQSRSFIQDFVSYGTAYTFQIHDLVHDLALFVAKDECLLVNSHIRSIPESIRHVSFVENDLDGNSISSKSVGV
RTILFPKDGVGAKSEAFFTLVSRYKYLHILNLSYSSVETLPDFIGKLKHLRSLCLYNNKKIRGLPDSICELQSLQELDLGGCME
LEALPKGLRKLISLRDFVITTKQAVLPENDIANLSSLQYLTIQYCDNVESLFSGIELHYLKALSVISCKRLESLSLDSKHFPAIET
LCIGNCDKLELSKGHEGQKFFLKLKTIHFLSLPQLRTLPHWLQGSIKTLLSLRLEHCSNLEVLPDWLPMLTCLKALDIKGCP
KLHSLPDGINYLSALERLEIVVCPELFRKFTLQLAEIIQLVAYNDADWEVV

>g2506.t1

MAESFLFSIAESLIAKLASQAFQKASRVVGLYDDLRDLTNTLSSVKAVLLDAQQKQDHNHQLRGWLTHLKIVLSEAEDLL
DEFDCQTLRNEVVKADGSTKAKVSHFFSTSNPLVFRFKMAQQIKDINNRLDKVATDRDKFSLQIIDVDTRVVHRRDMT
HSRVSDSDVIGRKDDKEKIIELLMQQNANDDDTSLPVIPIVGIGGLGKTTLAKFVFNDKRIQECFPSKMWVCVSDDFDIK
QLIIKIINSANDVDVPPHQQNLNMFDLEQLQNQLKSKLSGKKFLLVLDDVWNEERVKWVDLRNLIQVSGAGSKVVVTT
RSHSIASMMGTVPSHILEGLSEEESLSLFVKWAFKEGEEEKHPHLVNIGREIVKKCRGVPLAVRTLGSLLFSKFEDSEWEY
VSDSEIWNLPQKKDDILPALKLSYDLLPSYLRQCFALFSLYPKDHLFSSREMTALWGAHGLITLPKTNRTREDVANQYLHE
LHSRSFLQEVKNFATFYSFRIHDLVHDLALFVAKDECLYVSSNIQNIPENVGHLSFAESSLFDNLEIKKSASVRTVLFPNGG

VGANGEAILNTCLSKFKCLRVLDLSGSTFETLPRSIAKLKHLRYLDISKNLKIKRLPDSICNLQSLQVLFFNGCIELEVLPEGLR
KLISIRHLEFSTKQTDLPVNEIAKLVSLDCLVIESCHNVESVFGGVKFPSLKTLYVRNCQSLKSMSLDGQNFPELETLVVAN
CGNLELELWKGDHEEESPKLKLKLVYLVNLPQLVGLPKWVQEAANSLRLPKFGISLR

>g2509.t1

MAEYFVFDIAESLVRKLASFVCEEASRVYEVYDDVKGIKDTLSIVKGVLLDAEQKKEQRHGLREWIRQIQTVCLDAEDVL
DGLDCQNVRKQFLKASGSTRMKVRHFFSPSNSLVFRFQMARQVKNIRRRLDKIAADGNKFGLERIEVDNRLVQRREM
TYSHVDASGVIGRESDREHIIKLLMQPHPNGDGYGDQSVCVIPIVGIGGMGKTTLAKLVFNDNRMDDLFQLKMWVCIS
DDFDIRQILINIINSASVSASAPPTAFARQENIKNFDIEQLQTVLKLKLSGQKYLLVLDDIWNDNRAKWTELKDLIKVGGV
GSKILVTTRSNSIASMMGTVPSAEILILWESLGLLQSLRGNRKLENVARQYIDELHARSFLEDFEDFGYYYYFKVHDLVHD
LALFVAKEELLMLLLRREQLAFLEVLIIQSCGSLQFLPLHVLPKLEVLIVSRCEMLNLNYESAIHRLRMKFLHLEHCPKLHTLP
QWIEGSVDTLRTLLILNCHSLEMFPEWLTTMSSLKRLHIVNCHKLLCLPSEMHSLTALDDLTIEGCPELCRKCEPQNGEY
WSFINHIKRVSIGETRKGKLLVRMLQQMRLRLRDQ

>g2510.t1

MVECFVYDITESLLSKLASYVVYEEASRAYGVYNDLRGIKDTLSIVKGVLLDAEEKKEQNHGLREWLRQIRNVCFDAEDVLD
GLDCQNLRKQVLKASGNTRMKVDHFFSSSNSLVFRFRMAHQIKHVRCRLDKIAADGNKFGLERIDVDNRLVERREMTY
SHVDASGVIGRESDREKIIKILMQPHPNGDGYGDQSVCVIPIVGIGGLGKTTLAKLVFNDKRMDDLFQLKMWVCISDDF
DIRQILIKIINSASASAPTIALAHQENIKNFDIEQLQSRLRHRLSGQKYLLVLDDVWNDNRAKWTQLKDLIKVGAVGSKILV
TTRNNSIASMMGTVPSYVLEDYGFTGPEVVNLWVSLGLLRSEVGSQKPENAARLYIDELHSRSFLEDFEDFGKLYYFKVH
DLVHDLAQYVAKEEIFVVDSGTRNIPEQVRHLSVVENDSLRDSLFPKSSRLRTILFPTDGVGVDSVALLDAWIARYKFLRIL
DLSDSSFETFPNSIAKLEHLRALSLENNRRIKRLPQSICKLQNLQALTLRGCVELETLPKGFGMLISLRKLFITTKQSILPEDEF
ASLVNLNTLSFEFCDNLMFLFRGAQQFKSLEVLLVQSCRSLESFPFHILPKLEVLFVIRCEMLNLSLNCENPIERLRMKFLHI
EECPRQQTLPQWIEGAAETLQTLLISNLHSLEMLPEWLTAMTHLKMLHIVNCPQLLYLPSDMHRLGALEDLTIDGCSEL
CRKCEPQSGEYWSFIAHIKHLSIGETREGKLLFQMQKQIRLKLQDLKN

>g2511.t1

MAEYFVFEIAESRLGKLASNLYEQVSRAFDLYEDVQSFRDTLSIVKGVLLDAEEKKEKKHGLREWLRQIQNVCLDAEDVL
DGFECQNLRKQVLNASGTTRMKVNHFFSSSNSLVFRFRMARKIKDVTRRLDKIAADGNKFGLERIVIDHSPLRRREMTY
SHVDASGVIGRESDREQIIKLLMQPHPHGDGYGDRSVCVIPIVGIGGLGKTTLAKLVFNDNRMDHLFQLKTWVCISDDF
NIRQIIIKIINSATDPTISVVPQESMNNLDIEQLQSRLRHKLSCQKYLLVLDDVWNDDRAKWIELKDLIKVGAIGSKILVTTR
STSIASMMGTVPSYVLEGLSVENCLSLFLKWAFREGEEKEHPYLVDIGKEIVKKCRGVPLAVKTSGSSLFSVFDLQRWEIM
RDHELWNLKQHKDDILPSLKLSYDQMPSYLRHWAQAQLSSLEVLIIQSCGSLESLPLHILPKVEMLPEWLSTMTSLKMLH
IVNCPQLFHLPCDMHCLRALEDLIIDGCPELGRKCEPHSGRCILKHLGHNGSKIRLMRIGAATPRKSSGHPQGENSLEIDG
HFSFFNRIVSRVFLTLADDGGSAAMEIEHRKASSVAILGLGGATEKNWWFVMEDGGGARLLVVADSKVGGGEVGRDG
GRRQGAAARV

>g2512.t1

MAEYFVFEIAESLLGKLASNLYEQVSRAFDLYEDVQSFRDTLSIVKGVLLDAEEKKEMKHGLREWLRQIGNVCLDAEDVL
DGFECQNLRKQVLKASGTTRMKVNHFFSSSNSLVFRFRMARKVKNVTRRLNKITADGNKFGLERIDVDNRLVQKREMT
YSHVDASGVIGRESEREEIFKLLMQPHPNGDGYGDQSVCVIPIVGFGGLGKTTLAKLVLSDERMDGIFQLKMWVCISDD
FDIRHILIKIINSASDPTISVVPQESMNNLDIEQLQGRLRHKLSCQKYLLVLDDVWNDNRAKWIELKDLIKVGAVGSKILVT
TRSTSIASMMGTVPSYVLEGLSVENSLSLFLKWAFREGEEKEHPYLVDIGKEIVKKCRGVPLALKTSGSSLFSVFDSQRWEI
MRDHELWNLKQQKDDILPSLKLSYDQMPSYLRHWAQTQLSSLEVLIIQSCGSLESLPLHILPKLEVLIVTRCVMLNLSLNS

ERPIQKLKMKYLHIEHCPRQHTLPEWIQAASNTLRTLSILSCHCLETLPEWLSTMTHLKMLHIVNCSQLLHLPSNMHCLR
ALEDLIIDGCPELGRKCEPRSGEYWSFIAPIKCIAQNIVYIDIANNLWEDLKERFSMGDYFRISDMLQEIHSLKHRERSIRQF
YTDLKTLWEELESLRAVPDCICDIPCSCKEK

>g2513.t1

MASLVSSPFTLPSSKPDQLHSLAQKRLFLHSFLPKKAGYNGSSKSSLRVKCAAVGNGLFTQTSPEVRRIVPENDQNLPTV
KIVYVVLEAQYQSSLTAAVLALNSKRKHASFEVVGYLVEELRDASTYETFCKDLEDANVFIGSLIFVEELALKIKAAVEKERD
RLDAVLVFPSMPEVMRLNKLGSFSMSQLGQSKSPFFQLFKRKKPQSAGFADSMLKLVRTLPKVLKYLPSDKAQDARLYI
LSLQFWLGGSPDNLQNFLKMISGSYIPALKGTKIEYSEPVLYLDNGIWHPLAPCMYDDVKEYLNWYGTRRDANEKLKSP
NAPVIGLVLQRSHIVTGDDGHYVAVIMELEARGAKVIPIFAGGLDFSGPVEKFFIDPITKKPFVNSVVSLTGFALVGGPAR
QDHPRAVEALMKLDVPYIVALPLVFQTTEEWLNSTLGLHPIQVALQVALPELDGGMEPIVFAGRDPKTGKSHALHKRVE
QLCIRAIRWAELKRKSKEEKKLAITVFSFPPDKGNVGTAAYLNVFASIYSVMKELKQDGYNVDGLPETPEALIEDVIHDKE
AQFSSPNLNVAYKMSVREYQNLTPYSTALEENWGKPPGNLNADGENLLVYGKQYGNVFIGVQPTFGYEGDPMRLLFS
KSASPHHGFAAFYSFVEKIFKADAVLHFGTHGSLEFMPGKQVGMSDVCYPDSLIGNIPNVYYYAANNPSEATIAKRRSY
ANTISYLTPPAENAGLYKGLKQLSELISSYQSLKDTGRGAQIVSSIISTAKQCNLDKDVTLPDEGEEIPPKERDLVVGKVYSK
IMEIESRLLPCGLHVIGEPPSALEAVATLVNIAALDRPEDNISSLPSILAETVGRDIEDVYRGSNEGILKDVELLRQITEVSRG
AITAFVERTTNSKGQVVDVADKLTSILGFGINEPWIQYLSNTKFYRADREKLRILFMFLGECLKLVVADNEVGSLKQALEG
KYVEPGPGGDPIRNPKVLPTGKNIHALDPQAIPTTAAMQSAKIVVDRLIERQKVENGGKYPETIALVLWGTDNIKTYGES
LAQVLWMIGVLPVADTLGRVNRVEPVSLEELGRPRIDVVVNCSGVFRDLFINQMNLLDRAVKMVAELDEPAEQNYVR
KHALEQAQALGVEVREAATRIFSNASGSYSSNINLAVENSSWNDEKQLQDMYLSRKSFAFDCDAPGAGMTEKRKVFE
MALSTADATFQNLDSSEISLTDVSHYFDSDPTNLVQNLRKDGKKPSAYIADTTTANAQVRTLSETVRLDARTKLLNPKW
YEGMLSTGYEGVREIEKRLTNTVGWSATSGQVDNWVYEEANTTFIQDEQMLNKLMNTNPNSFRKLVQTFLEANGRG
YWETAEENIEKLRQLYSEVEDKIEGIDLLNSNPCEQIKNMAEYFVFEIAESMLGKLASNLYEQVSRAFDLYEDVQSFRDTL
SIVKGVLLDAEEKKEKKHGLREWLRQIQNVCLDAEDVLDGFECQNLRKQVLKASGTTRTKIAADGNKFGLERIVIDQSPL
RRREMTYSHVDASGMIGRESDREQIIKLLIQPHPNGDGYGDQSVCVIPIVGIGGLGKTTLAKLVFNDNRIDHLFQLKMW
VCISDDFNIRQIIIKIINSATDPTISVVPQESMNNLGIEQLQSRLRHKLSCQKYLLVLDDVWNDDRAKWIELKDLIKVGAIG
SKILVTTRSTSIASMMGTVPSYVLEGLSVENCLSLFLKWAFREGEEKEHPNLVDIGKEIVKKCRGVPLAVKTLGSSLFSVFD
SQRWEIMRDHELWNLKQQKDDILPSLKLSYDQMPSYLRHWAQTQLSSLEVLIIQSCGSLESLPLHILPKLEVLIVTRNTSV
KYGERSVRQFYTDLKTLWEELESLRAVPDCICDIPCSCKKE

>g2550.t1

MAESFLFSIAESLIAKLASRTFQEASRLVGLYDDLQDLTNTLSLVKDGLLDAQQKQEHNHELRRWLSQLKTVFSDAEDVL
DEFECQTLRKKVVKAHGSTKDKVSHFFSSSNPLVFRCKMAQQIKDINNKLDKVAANKHKFSLQMSDVDTRVVHRRDM
THSRVSDSDVIGRKHDKENIIELLMQQNPNDDDTSLSVIPIVGIGGLGKTTLAKFVFNDKKIQECFPLKMWVCVSDDFDI
KQLIIKIINSVNDSESDHAPFHQMNLNMLDLEQLQNQLNHKLSGQKFLLVLDDVWNEDRVKWYELKNLIQVSAGGSKI
LVTTRSHSIASMMGTIPSHVLEGLSEEDSISVLVKWAFKEGEEQKYPHLVNIGKEIVKKCGGVPLAVRTLGSLLFSKFEANE
WEYVSQNEIWNLPQKKNDILPALKLSYDLMPFYLRQCFALFSLYPKDYEHNNYEIIWFWRALGLIALPKTDITREDVANQ
YLHELLSRSFLQDFENFGTLYKFRIHDLVHDLALFVAMDECLHINSNIQNIPDNMRHLSFAESGLFSNLVTKKSAAVRSVR
FPNGVAAANCKAILKTCLEKFKCLRVLDLSYSTFEILPRKIGKLKHLRYLDISYNPNIKRLPNSICKLQNLQVLNLEGCVELEA
LPKGLRKLISLYCFEFSTKQTVLPLNEMANLRSIELLTVGSCHNVESIFGGMKFPTLKTLYVEDCQSLKSLLLEGKNFPQLET
LIVEECSNLDLELCKEDHEEQSPKLKLKFIEFSILSQLVTLPKWFQEAANSLQCLVVSDCHNFESFPEWLPVLTDMKTLDIR
NCPKLVSLPDNIHLFTALENLTIRGCAADLCKKYEPHVGEFWPKISHIKNILIDEWEDLDEE

>g2552.t1

MALSTQNCLSDAEDVLDEFECQTLRKKVVKAHGSTKDKVSHFFSSSNPLVFRCKMAQQIKDINNKLDKVAANKHKFSL
QMSDVDTRVVHRRDMTHSRVSDSDVIGRKHDKKQIIELLMQQNPDDDDTSLSVIPIVGIGGLGKTTLAKFVFNDKKIQE
CFPLKMWVCVSDDFDIKQLIIKIINSVNDLESDHAPFHQMNLNMLDLEQLQNQLNHKLSGQKFLLVLDDVWNEDRVK
WYELKNLIQVSAGGSKILVTTRSHSIASMMGTIPSHVLEGLSEEDSISVLVKWAFKEGEEQKYPHLVNIGREIVKKCGGIPL
AVRTLGSLLFSKFEANEWEYVSQNEIWNLPQKKEDILPALKLSYDLMPSYLRQCFALFSLYPKDYEYNSYEIMWFWRALG
LIALPKTDITREDVANQYLHELLSRSFLQDFENFGTLYKFRIHDLVHDLALFVAKDECLHMNSNIQNIPDNMRHLSFAESG
LFSNLVTKKSAAVRTVFFPKWGAAANCKAILKTCLEKFKCLRVLDLSYSTFETLPRKIGKLKHLRYLDISYNPNIKRLPDSICK
LQSLQALKLGGCVKLEALPKGLRKFISLYCFLFSTKQTVLPMNEIANLGWLEFLTVGSCHNVESIFGGMKFPTLKTLCVYD
CQSLKSLLLEGKNFPQLETLVVEDCSNLDLELWKGDHEEQSPKLKLKLVAFSHLSQLVTLPKWFQEAANSLKCLLVSYCH
NFESFPDWLLVLTDLKSLEIKNCPKLVSLPDNIHLFRALESLTIEGCAADLCRKYEPHVGEFWPKISHINHIFIDEAEDLDEE

>g2574.t1

MASVREAPSWKFHVFLSFRGEDTRNGFTDHLYAAFRGRGFAVFRDDEELERGEVISDALLKAIDESLCSVVVLSPHYASS
RWCLDELLRILESRANFDRNVLPIFYNVDPADVRHQRGTFDEAFAKHVERFGSDEVRMWRQALKDVAALSGWTSKDK
RETELIEEIVADVWEKLQTKLPSYDDELIGIDSRINSIYALLRMDSLEVRFMSIWGMGGIGKTTLASSDIQVENLAGKPEW
FGQGSRVIITTRDKHQLKSLHVCENYDVQVLNNYESLQLFCQKAFRGEKPEQDYLELSTSVVQYAGGVPLALKVLGSFLC
GRSVSVWEDALKMLRKDAQNDICKTLRISYDGLRDNEKAIFLDIACFFKGNTKDDVTRILENCDFNPLIGIEVLIEKSLVTY
DGLHLGMHDLLQEMGRNIVFQESPNDASKRSRLWTLKDIDQVLRNNNGTESIQAIVLNLPEPYEAFWNPDAFSKMSN
LRLLMILNKLQLPLGLKCLPSGLKVLVWKEYPLESLPVGPQLDELVELHMCQSKIKHLWGGTKFLENLKIINLRNCNNLHR
TPDFTGIPNLEKLDLEGCVNLVEIHASLGLLKKLSYLTFEVRKLPEFGESMKSLSVLALEETSIAELPVSVGHLTDASETAIRE
VPSSIVLLKNLRLLSLRGCKDLASNSWSSLLLPFENILRFNSHPTPKRLILPSFSSLSSLRKLDLSYCNLHDGSIPEDLGCLSSLV
TLDLSGNNFVCFPGSISELLKLERLLLKCCPRLESFPKLPPEVHYVNASDCRSMKPLSDPQQIWGHLASFAFDKFQDASNF
KTLLVSPGNEIPSSFFCQKHLNQVQDIEYLKENYIWADSTVSIPMDLAQLRHRNIALVGFQSLKSKLVIKKCGWHILSKED
AENWRTKLSECNTNSTNQCVENPRNCRLSPHFSSWRWISRLKVPQHCKTFLLAILCDRLPANERCSFCCLEGTVIHVLRD
CTRATAIWVQMVPPEVCDEFFSTSLHDWMHRFLLKLWFPDRDYYTDCLRFTITIWLLWKDRNSSIFKRNSPTDNDGLY
SMIQSLVKEYSILLHLKGEEGTSAANSLSQNSRLKLFVRLNVDGCCNGNPGNAGYGGLFRDVEGKWLGGFYGSLGLATN
VKAELYAICQGLIAAWDLGYRTVLVETDSLEAINLIKEANIEDSAYGGLLADIRSLMQRNWSLDLIHSLRQDNACADMLS
KLGTEQHEVYCFLAHPPQQLQLALAADALQVQLPCL

>g3139.t1

MTREIYGVLSEGNFDTISKPPTIKEIQQVLKDPNIYKIGLYGIDGMGKTTLVKELAREVENEGSFDVVAMAEVTDSPDVE
NIQGQIANALALKFDEETKEERVEKLNRRISKEKSILVILDDIRGKVDLAELGIPFGDDHKGCKLLLTSEHLNVLRRQMGTQ
KNFRVEVLSDEDSWKLFQKIAGQAIKLISTNKSPYELVEINSMVEDVPKYCNGFPLFVVVVAKALRTKNLSTWKDALKQL
RGLSEKGKFEEVVCPLELGYRYLESDELKTLFLFIVSLGPGRIHTGELFSCYWGLHGDSHELTEARNKYYQFIDDLRASSLLL
EVEIEYVRMHDSVRDTAKAISSRTHLTYEVQKFTQKEQWDIDQLKKCHYINLPSYNLDELPEKLDCPELKLMSLKSNLGHL
EIPDNFFAGMGEVKVLNLHRMSFAPSPPPSFRLLTNLRSLNLYECELDDITMVAELTSLEILSLERSKIQELPKEIGQLTQLR
MLNLTNCYQLKTIPRYLIYSLMCLEELYMGNCNIQWEAEGGKSQTNNASLGELRNLNRLTTLDLSIHDASVLPADMDVF
KQLRRYNIYIGNMWKWSSFWSGDAREISRTLKLVDSVNTEIFLNKGIQMLFTTVEDLSLAKINFADDVSYKLDREAFQHL
RHLYVQNSDAFCNLETLILCDLRNMYGPFAKQTLVLWNLHNMEDISYAPLATQCFENLQVFKVQDCRKLKKLLSYSVAK
NLAQLQQMEIFDCTAMEEIEMEIFNCTIMDEIIFDEIFEDESLHNVKDTGTEMKLEDGKEFPKIVLPKLHSLTLDTLPNLCS
FSLPLEIDKDDGSIPLPLFNQKVTCPNLDMLVIINMNRLNSIWYNQQAPNSELLQLQVLEISTSMIEMIVEDNYIQGPPD
NITFTKLEQLKLEYLPRLTKFCQESYNFKFPSLQTVDVIGCPNLKSSGHLNFTTIAQLEWRGKNGKKDDELNNLFNEKVA
MPNLENLRLSNIGSYGKIWGEKWRVPFFSENLKYLIEDEYHNHTESLFSSSTARELTKLKLLDIQSCPALVQIFVQQEKVTF
PNLETLLINDMSGLRSIWNNLQVVVAKELQQLQELEISRSINIENIVVKSHRSGAFGKHKNKKLYLENLPKLRSFCKKSDNF

KVPAFEKVTFPNLEKLIISGMSGLQSLWNKLQGPNSFHNLNKIQVTGCRALVHVFPVVVAKELQQLQVLEISRSINIENIV
VKSQSGGALGKHQNEKLYLENLPKLTSFCKESDNFNFPALEKVHVIGCPKMKTFCPGILITPSLSNVRIGRRKEDLRWHD
DLNTTLKIFHMELESNTEERRKSSDSGPIVELRMSWLGLLSFGCLGRAALFELRMSWSDCIAGLPSLGCLGRAALLAANE
GEIMPESRLPQCHHSCRREVVPMLEMQRILALSSEPSKKLRLNLKEETFSNFNPLGPRICTPCFGSSSKSNTDMGNALVR
KINAIIGTQLGSESERDFEAHEEQVKEYHHHENLKQYEPVG

>g4233.t1

MGETNQLVILTRFQDAMRMVMDIVERGRKNKRSKRILRSTLKNMTLVVQEIKQYNEHLNPPREEIINLVKEKDAGEELV
CNSCSRSLWWTKFLSWFSLYGEGLLHEKNDSLTADDKQVKDIKYTLYKLREIIELLDIENFEQKLKGSGTPIKCPYGVPENP
EFTVGFVPLLSKLKMEVLQEGVFTLLLSGLGGSGKTTLATILCKDKEVKGKFKNNILFVTFSQTPKLKNIVERLFEHCGYHV
PEFVSDEDAIKRLEILMRKIEGSPLLLVLDDVWPGSEALVEKFKFQMSDYKILVISRVAFPKFGTPFILKSLAHEDAMTLFR
HHALLEENSSKCSLSIPKEEIVQKVVRYCKGLPLAIKVIGRSLSHQSIELWQKMVEELSKGHCILDSNTELLTSLQKILDVLED
NPIIKECFMDLGLFPEDQRIPLPALIDMWAELYKLDDDGIEAMEIIKKLDSMNLANLLVARRNTGDSDNYCYNNHFVVL
HDLLRDLAIYQNNREPIDQRKRIITGINENQSQWWLGEKQQGTMSRLLSKYRGWCVKQTIQQVSARILSLSTDETCASY
WSDIQPSQAEVLILNLQTKKYTFPEFMEKMVKLKVLIVTNYGFHHSEVDNFQLLGSVSNLKRIRLERISVPHLSALKNLKKL
SLYMCSNISQVFENGTILVSDSFPSLLDLNIDYCKDMVSLPNGICDISSLKKLSITNCHKLCSLPQEIGQLLNLELLNLSTCTD
LEEIPDSIQNLSKLRLLNISNCISLSNLPEDIGNLCNLRNLNMTSCARCELPFSITNLENLKVVVCDEETAASWEAFEALLPN
LKVEVPQVDVNLNWLHSISS

>g4235.t1

MEETAEEIKRPFGIPEKPEFSVGLDVPLKKLKLNVLSEGASVIVLTGMGGSGKTTLATMLCWDEQVMGKFKENILFVTFS
KAPQLKIIVERLLEHFGYQVPELQSDGDAIHQLLLLRKINANPMLLVLDDVWPCSEAVVEKLKFQISDYKILVTSRIAFPRF
GTSLVLKPLVPEDAITLFRHHAFLKRASNIPDEDLVQKVVRHCKGLPLAIKVIGRSLSQQSIELWQKMVEELSEGHCILDSN
TELLSSLQKILDVLEDNPIIKECFMDLALFPEHQRIPVAALVDMWVELYGLDNYGIKAIAIVNRLDSMNLANVTVTRKNSS
DTDSYYYNNHFIVLHDILRDLAIYQSNKAQIELRKRLMIGINETKPEVCLGEKQHGRMIRTLSNNFRLCDEQKTQKIPVGT
LSISSDETRTSYWSHLRPAQAEVLILNIRTNQYSFPKYLKEMSKLKVLIVMNYGFHPCELFDFELLGTLSLLKRLRLERISLPS
FVTLKNLKKLSLYLCDTHEAFENSNILISDVFPNLEDLNIEYSKDMVGLPKGLCNITSLNVLSISNCHKLSALPQEIGNLENLK
LLRLSSCTDLQVIPNSVGRLSNLRHMDISNCINLPNLPEEFGNLCNLRILYMTSCERCELPSSIINLKNLKEVVCDEETAFSW
EAFKPMLPNLKIDVPQLDVNLNWLHAIHS

>g4236.t1

MKGPFGVPEKPEFCVGLDAPLRKVKMELLNRPKSIIVVTGFGGSGKTTLATLLCWDQQIKTPKFQNDEEGINQLGLLLRK
LKGSPTLMILDDVWPDSEDLVEKFKFHLSDYKLLVTSRVAFPRFGTPCVLKPLLHQDAMTFFFHYARLDSNCLNIPDEDTI
QKVVKSCMGLPLAIEMIGRSLSHRPNELWQKMVLELSHGNSILLDCNTELLTYLPKILDVLEDNTVIKECFMDLGLFPED
QRIPVTALIDMWAELYGLDNDGIEAMAIINKLESMNLANVLITRKNTSDTDNYYYNNHFIFLHDLLRELAIYESTLEPIEER
KRLIIHTNENESEGGLDEKQQGFVIRILSNCFKYCVKHKPQQITASTLSISIDEDVSSCWSHMHPTQAKVLILNLRVNQYSF
PESMKKMRKLKALIITNYNSHPSVLTNIELLGSLSNLKRIRLERIAVLSSFVTLKSLRKLSIYMSQAFQNGILLTSDTFPNLVD
LNIDYCKDMVVLPSGLCDIIPLKKLSVTNCHKLLALPQEIGKLVHLELLRLSSCTDLEALPESIGRLQNLRHLDISNCISLSNLP
EEVGKLCNLRNLRMINCERCELAYSVVNLVNLKTLSCDEETATSWEGFKAMLPNLLIEVSQVDVNLNWLPSVSS

>g4237.t1

MDQTTQGVVLPALFQERLRMVSEIVEKGKSSDTNKQILRSTLKDMNPVVQEIKQYNEHLNPPREEIKTLISEKDAKEELV
GKCLSKKFCLDKCLSFFLCRFGHKRDGSFAGGDKQALVAKDIEEKLYKVREILELLSKENFEQKLGGVGGPIRFPFGVPDN
PDFTVGLDVPLSKLKMEVLRDGVSVIMLTGLGGMGKTTLATKLCWDEQVKVPDFLSDEDAANQLGLLLRQIGRSSMLL

VLDDVWPGSEALVEKFKVQIPDYKILVISRVALLRSDMQCILKPLVHDDAETLFRHYTQLEESGSSIPDEVIQKVVRNCKG
LPLAIKVVGRSLCNQRSELWLQMMEELSQGRSILDSNVELITCLQKILDVLEDNLVIKGCFMDLGLFPEEQRIPVTALIDM
WAELYRLDDDGKEAMTIINKLDSMNLANVMVARKNATNTDSYYYNNHFIVLHDLLRELAIYQCSQEPMEQRKRLIIEIN
QNKHGEKTKFLSWCVKQKPQQVTAHTLSISTDENCPSDWPQMHLPQVEVLIFNLQTKQYSFPNCMEGMNKLKVLIVT
NYSYYPSEINNFELLGSSSNLRRIRLERISVPSFVAMKNLKKLSLYFCNTKQAFQNKDLLISYAFPNLEDLNIDYCKDMVGF
PKGLCDIISLKKLSITNCHKLSALPQDIGKLENLELLRLSSCTDLEGIPDSIGRLSNLRLLDISNCISLPNLPDDFGNLSNLQNLY
MTSCARCELPLSVTNLGNLKVVICDEETAASWENFKPMLPNLRIDVSQVDVNLNWLHTTFS

>g4239.t1

MAMVLDALVGHVLDELLSTVREMKDRAVKFRDTLEKLHSILEKVEPMARQIDGLNKRLDKPAKESQKLIDEMKKGKEL
VNECDKIDLWNCCYKASSQEKLQDLIDSISLYFQLDIQGNINVIVLENQMLLHQIHEKLVENVPRRIKGLCSPPEPPAFTV
GLDVHLRALKFKLLKNYHVGSVLTVTGTGGSGKSTLAKKFCSDEEVKGEFKDNIFFISLVEAPKLSTIVERLFEHNGYEKPQ
FQSDEGAVYQLENLLKQIGKNPILLVLDGVLPESASLVEKFVFQIPNYKILVTSRFTIKGFGLPHVLKSLNEADALNLFRHSA
SLDQTSSNIPDTIVQKIAKGCSGSPLALIVTGKSLSLEEPVVWHNRARTLSRGHSVLSYSSSGDGLLTCLQKSFDDLDAKLA
ESFTDLSLFPEAQKIPAAALVDIYAEQRDEDDDIAMENIHELVKRNVADLVVTRNTTSGTVDYNYHYVTQHGLLRDLAIH
QSRNLPTEKKHRLIIDLRGNNIPKWWTTQNEYHIAAHTLSISTDEEFTSEWCNLQPNEVKVLVMNLREKKRSLPPFMKK
MNKLKVLTITNYDVNRTELENLELLDYLSDLKRIRLEKVSIPFLSKTGVPLKNLNKFSFFMCNVNEAFKNSTIKVSDVLPNLK
EMNIDYCDMEELPDGLSDTVSLKKLSITNCHRLSKLPKEIGKLVKLESLRLTSCTKLEELPDTITSLHKLKFLDISDCVSLSML
AENIGELGSLERLNCRGCSRLSELPYSVTELESLRVIVCDEERAALWEPVISMFSDLKLEVVLTDFKLDPLL

>g4240.t1

MVGLTSLELCKLAERVESLDKRSRGLDHCLKIRSTSELEVWTTVSALAQQEGSLSERLKSGSTGSFIRSADWVAERVGWG
LGSPPLYLLSERALWLSDHLVAERAPGLVNDHDTLNNPISLVMAMFLDPLIGKAFDELLSIVKEKKDRAVKFRPTLEKLHSI
LEKVEPMARQINVLIQRLNKPDTESEKLIEELQKGKELVNECSKVDWWNCCYKASSQEKLQDLIDSISLYFQLDMQGNIY
VIGLESQMLLYEIHERLVENPRRIQGLCSPPEPPAFTVGLDDHLRELKFKLLKNHHVGPVLTVTGAEGSGKSTLAKKFCSD
EEVKGEFKDNIFFISLVEAPKLSTIVERLFEHNGYEKLQFQSDEDAVHRLENLLKQIGKNPILLVLDGVLPESASLVEKFVFQI
PNYKILVTSRCTIKGFGQPYVLKSLNEADALNLFRHSASLDQTSSTIPDTIVKKIAKGCSGSPLALIVTGKSLSLEEPVVWHN
RAKTLSRGHSVLSYSSSSDGLLTCLQKSFDDLDAMLAESFTDLSLFLEAQKIPAAALVDIYAEQRDEDDDIAMENILKLVKR
NVADLVVTRNTTSGTVDYNYHYVTQHGLLRDLAIHQSRNLPTEKKHRLIIDLRGNNIPKWWTTQNEYHIAAHTLSISTDE
EFTSEWCNLQPNEVKVLVMNLREKKRSLPPFMKKMNKLKVLTITNYDVNRTELENLELLDYLSDLKRIRLEKVSIPFLSKT
GVALKNLHKFSFFMCNVNEAFKNSTINVSDVLPNLKEMNIDYCDMEELPAGLSDTVSLKKLSIRNCHKLSKLPTGIGKLV
NLESLRLTSCTKLEELPDTITSLHKLNFLDISDCVSLSMLPENIGELGSLKWLNCRGCSRLSELPYSVTELESLRVVICDDERA
ALWEPIRSMFSDLKLEVVLTDFKLDSFL

>g5293.t1

MDIQEESSTTVGPLTTPSLRNMSSSSSAFFSANQSPFFSPRSSSCQLSESLQPDAPIDRIHLDVAAPSSSSGIPEPKSLVNV
GCTFSEVAASPAGCSAGDLQKLDRISSSVGISSCTVSGHFHPYDDSYSGQKDKRSKKGRNKRISLTPGSRSVSSYRLKSCD
VYIGLHGRKPPLIRFANWLRVELEIQGISCFVSDRAGYRNSGKLSIAEKAMDVASYGIVIITRKSFKNPYTIEELQFFSGKKN
LVPIYFDLSPADCLVRDIIEKRGELWEKHGGELWLLYGGLEQEWKDVVHGLSRVEERKLEAQDGNWRDCILMTVTLLA
MKLGRRSAAEHLTKWREKVKEEELPFTRNENFIGRKKELSQLEFMLFGDVTGDSRQDYIDLKARPKRRHLTICRSKSNVQ
EERHVGNGSREEKTPVLWKESEKEIEMQSIEFSHRRSRLKRGGKYTRRKKGMRILYGKGIACISGDSGIGKTELILEFAYRF
HQRYKMVLWIGGESRYIRQNYLNLRSFLEVDASVENSLEKTRIKGFEEQEEAAVSGIRKELMRNIPYLVIIDNLESEKDW
WDHKLVMDLLPRFGGETHVIISTCLPRVMNLEPMKLSYLSGVEAMSLMLGSGKEYPVAEVDALRTVEEKLGRLTLGLAI
VSGILSELPITPSRLLDTINRMPLKDMSWCNKKAHSFRQNTFLLQLLDVCFSIFDHADGARSLATRMVLVSGWFAPCAV

SVSLLALAAQKIPEKQKGTCFWRKLLQSLTCGFTSSHTKKSELEACSLLLRFNIARSSTKQGHIHFNEMIKLYARKREVTGS
AQAMVQAVMNQGSISESIEHLWAACFLLFAFGHNPAAVELEVSELLYLVKKVVLPLAIHTFITYSRCSAALELLHLCTNAL
EAADQALVTPVDKWFDKSLCWRSIQTNAQLNPCLWQELALCRATVLETRGKLMLRGAQFDIGDDLIRKAVFIRTSICGE
DHPDTISARETLSKLTRLIANVQIRASA

>g5411.t1

MAAEMVTGVLVSTFLERTIDTLASRLVHIFHQRKHKKQLSNLKMKLLAIDVVAFEAEQKQFTDPRVRDWLLRAKDVVID
AEDLLDEIDYEISKTQVEAESQSASKKVWNSLNSSFVNFFENEIESMMEQVIEDLEELATQSDFLGLKKGGGVGVGSGSG
SKLTHTSLPNESVIYGRDDDKEFVLNWLTSDTHKNLSILSIVGMGGMGKTSLAQHVFNDPRLEEANFDTKVWVSVPQE
FDVLKVSRAILDTITGSTDHSIQQEVIQKKLKEKLMGKKFLLVLDDVWNERPSKWEDVQKPLVFGGQGSRILVTTRSEKV
AVTMRSEKRLLQLLKKDYCWDLFAKHAFQIANPQPDSDFIEIGKKIVEKCNGLPLALKTMGSLLYNKSSLSEWQSIMKSEI
WDFSEDESGILPALRLSYFHLPSHLKTCFAFCALFPKGYWFDKEWLIQLWMAQNFLENPLQKKSPKEVGEEYCNDLLSW
SFFQQQSIFNGEKGFIIHDLLNDLAKYVCEDICIRLGVDEPKGIPKTTRHCSFSDSGFDGFGSSIDTQKLHTFIPTEWFWG
WDCKMSIDDLFSRFKLIRVLSLSHCHSLTEVPECVGNLKHLRSLDLSKTQIEKLPDSISLLYKLQILQLNYCENLKELPSCLYQ
LDNLRRLDLEGSGVQNVAAHLGKLKNLQVTMSSFHVEKSKENNFQQLGELDLHGSLTIDDLQNIENPSYALEVDLKNKP
HLVELRLVWNFSGSSSVDSEKAEDVIENLRPSKYLKKLSIRNYIGKQFPDWLLHNSLPNLVSLELEGCESCQRLPPLGLLPF
LNYLKISGFDETVSIDADFHGNNSSSFKSLKTLYFSNMRQWEKWDCQAVTGAFPCLQNFSIKNCPKLKGHLPKFVALKTL
RVVDCEQLEALISLRVEELSIYSPLESISDDCVSLRIFPLDFFPTLRTLELSGFPNLQMISQNHVHNHLHYMKIKECPKLESLP
ANMHMLLPSLRYLTIKECPRLEWFSEGGLPLNLKHIRLKNCFRLVGSLKRALGDSPSLESLTIEKVEAECFPDEGLLPLSLTR
LIIEDCRNLKKLNYKGLLELSSLEELKLWKCPNLECLPEEGLPKSISFLYISNCPLLEQRCQKEGGEDWEKISHIPQVFIRK

>g5649.t1

MDLEKFIEEGRKYVPLDRIATLPSNTLDMLSEKCINFDSRQSAYEQLLDAVKNNDVSMIGLYGMGGCGKTTLAMEVRKL
VEAEHLFEKVLFVAVSSTVEVRRIQEKVASSLQFEFPETEEMQRAQRLCSRLTQEKNIFIILDDVWEKLDLGRIGIPPSKHH
KGCKILITTRSESVCNSMDCQRKIYLPILTDEEAWTLFQNKALISEATSNTLKEMGRLICNECKGLPVAIAAVACSLKGKAE
TVWSVALNKFRRSNPINIERGLIDPYKCLQLSYDNLDDIEAKSLFLLCSVFPEDSEIPVEILTRCAIGLGVVGEVDSSEEARSE
VIAAKIKLVSCCLLLDADDEWVKMHDIVRDVAHIIAKNENKMIKCELEKDVRVEQNSNYELSDFSFLGGMKNLQSLSLCY
CLLPPFPELQTDFAITLKLLELRGCDIEVKNFEVMKRIPLLEELYIIDIQGKWYANSKDNIEFFKTFSIPETLQRCDELKHILTY
DEKSEDEFTRGLTQLKVLEIKECDKLEQIIGDIVPLTAQDRKEELDEIVEEGTLSSLASLKIKRCGKLGSIFTASIAKTLTSLEEL
FIEDCESLKDIVTHERVNRNQEESIVEDEHDCQSDISIFQSLKKLHISNCDLLKGIFPVSFVGELNDITNKEAAHLKDFSSRN
NTQIELPDLQVLELHHIRNTTIVGSYDVICPSLRTLSLDIGRYVGFFNVNCSTDASEATKTDSIAIKISNSDFVPPVQSVECLS
EQPHGLNLIMIHNIREIELNGFDKARYLFKLSIASSLMMLEILRIKECGGLEYIIDTDDIDEYGKENMKAIFPNLKELFVDDC
FKLKYMFGQYDVANKEYKEIRIQFSALEILYLRDLPKFVSICSNNTVTVTWPSLKEFQCYRCFYPFYGSVGCLTIPTNSREPII
TSTKDPKGIQNPLLTLQTLNIKNSDAERIFCLNEDEMIGQQVSLRLENMELYNLPQMTYIWVGPNNSLTFQHLTTLTISE
CEKLEVIFPKSVVRCLPELKKVTIRECMEFKQIMEEDGSEVEEIIGSDKKASNNYFAFPNLEKLEIIACDSLEVVFPKSVLRCL
PKLKLLKISKCKELRQIIEGDKNLSNILSPQPCFPKLEALYVDDCHKLKRLFSGSASNDLPNLHLLAINGANELEELVGCKQG
KIKVELPRLKLLIFMHLSNFGQEIELHNLKNCIVYKCPKLSLTSTTTLLELWEDFPYEGKYYI

>g5650.t1

MQRAQRLCLRLTQEKSVLIILDDVWEKLDFGRIGIPSSEHHKGCKILITTRSEAVCTLMDCQRKIYLPILTDEEAWTLFQNK
ALISEGSPYTLKNLGRLISNECKGLPVAIAAVACSLKGKPETVWSVSLNKLRHSKPLNIEKGLTDPYKCLRLSYDNLDDKEA
ESLFLLCSVFPEDSEIQVEVLTRCAIGLGVVGEVDSYEEARSEVIAAKFKLVSCCLLLDADDECVKMHDIVRDVAHIIAKNE
NKMIKCEVEKDVRVEQNSTKMKGFDGIFKRMGMLKVLILVNDGDFSFLGGMKNLQSLSLCYCLLPPFPELQTDFATLKL
LELRGCDIEVKNFEVMKRIPLLEELYIIDIKGEWWDANSEDNIEFFKTFSIPETLQRCDELKHILTYDEKSEDEFTRGLTQLK

VLEIEECDMLDQIIGDIVPLTEQDRKEELDEIVEEGTLSSLASLKIKRCGKLGSIFTASIAKTLTSLEDLFIEDCKSLKDIVTHERI
NKNQEESIVEDDYDCQSDISIFQSLKKLHISNCDLLEGIFPVSFVGELNDITNKDAADLENFFSRNNAQIKLPALQVLELGRI
RDRTIVGSYDVICKSLRTLSLDIGRYVGFFSINCSTDASEATKTDFIVIKISNSDFIPPVVECLSKQPRDLNLIMTNNIREIELK
GFDKSRYLFKLTIASSLMMLEILRIEKCEGLEYIIDTDDEYGKENMKAIFPNLKQLSVYDCFRLKYMFGQYHVTNKDYEEIHI
QFSALEILSLDDLPNFVSICSTNTVTVTWPSLKDFRCSKCLYPFYGSDSCLMIPTNSREPIMTSTKDPKGIQNHLHTLESLNI
SHCDVECILFLNEDEMSQQVSLRLEVIFPKSVVRCLPELKKVTIRECMELKQIMEEDGSNLLSIDGGSEVEEIIGWDKNLS
NILSPQPCFPKLEALHVDDCHKLKRLFSGSASNDLPNLLLLAINGANEVEELVGCKQGKIKEIEDEGNESIESSSTGVEDTSI
GDAVETHIESGGEDILALDSKVVEQDDKMTEGKPGIVASQGIQVQEGFNLLHKQQGRDVFPNNNIDISSDIRTRLGAYK
HFVDLDDAQISLLVEAITSYPHLWNASKKFSDRFQAWRLKILADMLSFLQKESVHSVIPQREKEFYKLCEEAIFVGFESSW
VEEMRQRVVARDPKLGEDIAKRQIDENAKRYSSGDMIEDSQAVEEGDGPKIRLEEGSDLVDKEGEIGVLSNDHILAPRN
EEPEQEFGAEVFTSEIPRIATPSNALVDTQLTSEPCQMKQKKPVGEIPKSIEQVDLEETIAKNTNMAASSILSESATSKLDP
SVTLLSKSHPYQGEINSSQIEARIAKESEGHPKIIQDFGADDIRSLFAPVVVGKEGEDKLVGKTLSELEKYLKMSLKDIVSSE
TNSLCLLSALNFLSNLPFKDVRVSDGLKHIINTMHRDFPSILCSFKQGFATIDKLAELEARANEVTIKKNFYDELQRKEVVLK
EQIIRLKEEIRVCEVALSSLEEEKSKCIAETVEYKTELENARKDKSEMLEVAHKWSVLCSQYELNRMAATNPS

>g5655.t1

MDCLVHQFCQKLRYLRSFNNFVKKLEQEEGDLILTRDDVQNSIEHVNGKTRVTSELVNKWLQDVMSEIGKKLANKAFD
LEKFIDEGKKHVSFGRIATLPSGTLDILLEKCMNFESRQSAYEQLLDAVKNNDVSMIGLYGMGGCDDVWEKLDFGRIGIP
SPEHHKGCKILITSRSEEVCTSMDCQRKIYLPILTDEEAWTLFQNKALISEATSDTLKDLGRLISNECKGLPVAIAAVACSLK
GKAETEWSVALNKLKHSEPINIERGLIDPYKCLQLSYDNLDTKEAKSLFLLCSVFPKDSGIPVETLTRCAIGLGVVGETLSYE
EARSEVIAAKIKLVSCCLLLIAGHERVKMHDIVRDVAHIIAQNENKMIKCEVEKDVTVEQNSVRYLWCAKISKDLDCSNLE
FLCLRTKMKEFDGILKRMGMLKVLIIDNDGDGKTPLPTISFKTLTNLRCLSILNCELSDFSFLRGMKNLQSLELYYCLLPSFP
QLQSDVAITLTNLKLLELNECDIKVKNYEVRKRIPLLEELYIIDIEGEWDANSEDNIEFFKTFSIPETLQSQDELTTGHPIQIIE
KDVEDKKLSNLVSPQPCFPKLEALHVDHCQKLKRFFSGSDSDDFPNLHLLAIKGMDLIMIPNIREIELKGFDNARYLFKLSI
ASLLMLEILRIEECGGLEHIIDTDYEYGKENMKAIFPNLRELSVRDCGQLKYIGEYPVANQDYKETHIHFSPLETLHLSRLPC
FVSIYATNTLTVTCPSNNYFAFLNLGKLEIIECDSLEVVFSKSVVRCLPKLNVLVIRKCNELRQIIEGDKNLANLVSPQPCFPK
LEALRVDHCHKLKRLFSGSASNDLPNLHLLAINGANELEELVGCKQGMTKVELPRLKLLIFRHLSKFRQEIELHNLKNYNV
YKCPKLSLTSTTTLEKLWLDFPYEDDVWEKLDFGRIGIPSTEHNKDCKILITSRSEAVCTLMDCQKKIYLPVLTDEEAWTLF
QTKAFVTEGTPDTLKDVGRLICNDCKGLPIAIAAVASSLKGKAETLSYDNLDTKEAKSLFLLCSVFPKDCEIKVEDLTRYAIG
LGVVGEVDSYEEARSEVIVAKIKLVTCCLLSDADNELTKMKEFDGIFKRMGTLKVLILVNDEDEETPLSTITFKTLTNLRYLFI
ENYELSDFSFLGDMKNLQSLELFGCSLPSFPELQTDVAITLTTLKLLELNDCDIKVKNFEGMKRIPLLEELYIIEGEWDANSE
DNIEFFKTFSFPETLQRCRKLEHILTYDEKSQDEFTSGHPVQIFQNLQDVKIKRCRELKHIFPANIVGGLTQLKVLRIEECDK
LEQIIGDIVPLTEQDRKEELDEIVEEGKHPHLYDTSFPTTTFVKQSPGTLSSLGRLKIKRCGKLGSIFTASIAKTLTSLEELFIED
CESLKDIVTHESIFEDDYDCQSDISIFQSLKKLHISECDLLEGIFPVSFVGELNDITNKEAADLKDISSRNNTQIELPALQVLEL
DLIRNRTIVGSYDVICPSLRTLSLDIGRYVGFFNVNCSTDASEATKRDFIAIKKREYHGDGVIRGYGEIGKGDGSNSEIAPLE
ATRTASTLIAKPWQLSPRRQHALGILVTMLAATLMAGDGGDLMLRFSGEEKS

>g5690.t1

MDLEKFIEKGRKYVPFDRIATLPSNTLDMLSEKCMNFESRQSAYEQLLDAVKNNDVSMIGLYGMGGCGKTTLAMEVR
KLVETEHLFEKVLFVPVSSTVEVRRIQEKIANDVWEKLDFGRIGIPSSEHHKGCKILITTRSVDVCISMDCQRKIYLPILTDD
EAWTLFQNKALIIEATSDTLKDLGRSISDECKGLPIAIAAVACSLKGKDETAWRVALNKLKHPEPINIERGLIDPYKCLQLSY
DNLDTKEAKSLFMLCSVFPEDSEIPVEALTRCAIGLGVVGETLSYEEARKEVITAKIKLASCCLLLIADHEGVKMHDIVRDV
AHIIAQNENKMIKCEVEKDVTVEQNSVRYLWCAKISKDLDCSNLEFLCLRTKMKEFDGIFKRMGMLKVLIIDNDGDGKT
PLPTISFKTLTNLRRLSILNCELSDFSFLSGLKNLQSLELYYCLLPSFPELQTDVAITLTTLKLLELNKCDIKVKNFEVMKRIPLL

EELYIIDIEGEWDANSGDNIEFFKTFSIPETLQRTLLLNHFDISNEVIKGLAKNAKNLFVANIDGGIKCLIDTRSHSSEVVTLF
SKLHSMRIDNMENLKAIWHCFLPANGSFENLEKLYLSWCPRLTSLFTHDAFITGHPIQIIEKDVEDKKLSNLVSPQPCFPK
LEALHVDHCQKLKRFFSGYDSDHFPNLHLLAIKGLDLIIIPNIREIELKGFDNARYLFKLSIASLLTLEILRIEECGGLEHIIDTDY
EYGKENMKAIFPNLRELSVRDCGQLKYIGEYPVANQDYRETHIHFSPLETLHLSRLPSFVSIYATNTLTVTCPSNNYFAFLN
LGKLKIIECDSLEVVFSKSVLRCLPKLKLLKIRKCKELREIIEGDKNLSNILSAQPCFPKLEALHVEDCHKLKRLFSESASNDLP
NLHLLVINGANEVEELVGCKQGMTKVELPRLKLLIFTHLENFSQEIELHNLKNCIVYKCPKLSLTSTTTLEKLRKYFPYKDFM
NTELGISTFLDILGRISYKYSQSTCLARDPKLEEDIAWRQIDENSKRCSSGDIVEEGDGPKIILEEGSDLVDKEGKIGVVSIDH
VLTPRNEEAEQEFVAEIFTSEIPRIATSLTNSQTVEKPTPSNTLVDPQKTSEPCLMKQKKPVGEIPKSIEQVALEETMTKNT
NMAASSILSESATSKVKLRAAKLRHALLKKVKVILKSFKTLGPMISGVYLHKLLWRKRVKIT

>g6135.t1

MVEQKVIGVVASSSYSPSVVSSKRYDVFLSFRGEDTRKKFTSHLYDALKQKKVETFIDNRLEKGEEISRTLIQVIEDSHISIVI
FSENYASSKWCLGELSKIMECKKEKGQIVIPVFYDIDPSHVRKQTGSYEKLFVTHKGEPMCNKWKAALTEAANLAAWDS
QTYRVESELLKDIVEDVLQKLAPIYPNCHKGLVGIEENYEKIESLLKVGSNEVKILGIWGMGGIGKTTLACALYDKLSHEFE
GHCFLENVREESDKHGVKALRNKLFSELLGNKNHCFDVAFSVTKFVLSRLGRKKVLIVLDDVATSEQLENLIEDFDFMGL
GSRVVVTSRNKQIFSQVDKIYEVKELSFHHSLQLFSLTVFREKQPKYGYEDLSRSATSYCKGVPLALKVLGASLRSRSKGA
WECELRKLQKFPNMKIHSVLKLSYDGLDHSQKDIFLDIACFFRGNQRDHVTNMLEAFDFSAISGIEVLLDRALITISGGNQ
LEMHDLIQEMGWEIVHQECVKDPGRRSRLWRHEEVHEVFKYKKGTDIVEGIILDLSKLIEDLYLSSDFLAKMTNVRFFKI
HSWSKFNIFNVYLPNGLNTLSHKMRYLHWDGFCLESLPANFCAEKLVELCMRCSKLKKLWDGAQNLVNLKTIDLWGS
RDLIEIPDLSLAENLENVSLCYCESLREVHVHSKSLRVLNLYGCSSLRKFSVTSEELTRLSLAFTSICSIPSSIWHKRKLKALYLT
GCRNLGKLTDEPRIHGSHKHSLTALASNAERFSMNIKSLSTLRMLWLDDCKKLVSLPKLPPSLGKLSASNCTSLDSYMTQ
RLVLQHMLQSRIPYLRTNDLRCYDEEYLFPGNHVIDECVFNTTETSITIPYLWKTELYGFIYCIILSKGSLLQSDVSCSVYQD
GIRVGWLQRLLEYESLTSNHVLCMYHDINEFDAITEEGHGHFFSNVTFIFENSKASIEEFGIFPIYGSESGLKLVGSR

>g7453.t1

MATSTSCDVGEIRHDVFISFAGIDVRSGLLSHLIRELRRKHIDAYADERLEKGGEMSSSLLRAIEGSKILLVVFSKHYASSH
WCLEELAKMVECMETNKQIVLPVFYNIDPSHVRHQYGDYGDALAEHEEEFKENMLKVQSWRSALKEACSFSGFHYPK
NYENESDLVEKIVEDISVKLSKFYPSESNGLVGIDQNVTQIQSLLRVESNEVLFVGIWGMGGIGKTTIARAIFDKCSLQYD
GCCFFNVREELERHGFSNLRERLIYELLEGEGLHINKARFFSSALRMLGRKKVMVVLDDVNTSEELRYLVTKPVCFGAGSR
VIITSRDQDVLTSGGLDRIHEVKEMKPLDSLKLFCLIAFNESQPKMGYEELTEEVLKIAQGNPLALKVLGAEFHSRSTINTW
QCALSKFKKYPNEKIQSVLRFSYDGLHEVEKKAFLDIAFFFQEDTKAYVTEQLDAWGLHGASGVEVLQRKALITVSNENII
QMHDLIRQMGCEIVRQECITHPGRRTRLRDKEEVYNVLRYKLGSDKVEGMQVDVLRIKDLSLKVGTFKKMPCLRFLKFY
LPDDNLFLPPNPDGNLWYGEYHFSLLLSAWCKDLMRVACEIQIKCVDYLYIDGCSHPSQLNKSSVTSTLGSHGMVTLST
ALISLNEPTGFLRDLECSDMLDQQFKILPDGLLCLRSTYYIKLSKKSTGQDSGKPKLHVLFDSLKFYERISGFKIEVGDFFKW
LREEARDNLPFHCWRRRGQSRLVVTPIEVSGPLPVNDVLVGCKFVPCPPVDVGVGGIKESFIMDEERIKIVVHHMGKFL
TDDNGVFKFDGEIVEWRCDADLLSYFGIVASVKELGYIDIKELWYGLGGQSVHPDMLELLTDDKEFIDWVGGEEGYGV
DVEVHTEGDGQKGGEVQVEGVDVQVEGGEVQVEDAGVDEVQDGEVQGEVQVEVQGEVHHVEEPEVHEVDDFEVE
DLGEDDDVEDSEGDELHENESEAESEKEDLHDISVECDIGYYKGNVREEHSSPVVESSESSDNENNMDAMRGLSDNE
WLSEELISDVEESEGEEDGGSSKSKTTFPTFSMPRSMDGYKWEVGTFFAEKKEFMDGIRTYALSNGRNMKFIKNDKKRII
VKCLGGKGKCNWYAYCAFRIDVNAWQLRKVVDTHSCSRDFNVKLMTSKWLSERMEKSVRENPTMKVMDIRDKVTR
KWNVGISRNMAFRARAMAKDKIEGSFHEQYRRLYDYGHELLKINPGSTVQMKVDNINGEVIFQRFYACLKACKDSFVS
YRPIVGLDGCFMKTKYGGELLTAVGRDGNEQMLPIAYAVVEVENKESVTWFFELLIEDLGGKDVCAGITFISDQQKGLLE
AFKDLLPGVEQRAAIATHPQQWEAEMRNIKAINIEAFKYLLSIPPRFWSRSRFTRRSQCDTLVNNMSEAFNSVLVDSRS
KPIISMLKDIRVYIMKRWAANKTKMTQYHASICPKVWNRFQKESSLGRYWLPRWSREKLFEVMHILEFGQQFVVNVD

TMDCTYYITHWFRKSTYEETYNNIIYPISGQHIWEVTPYSDILPPKKKTMPGRPKKKRRLEEWELKKNDSELRKGGQRKK
CGICHELGHNKKGCPQRPTAQSASEQTQGTQGPPTDVPLTQESTAIATESGHPQQLDPIVNDE

>g7723.t1

MAESFLFSIAESLIAKLASRAFQEASRLVGLYDNVQDLTNTLSLVKAVLLDAQQKQEHNHQLRQWLSQLKTVFSDAEDV
LDEFECHTLRKKVVKAHGSTKDKVSHFFSSSNPLVFRYKMAQQIKDISNRLDKVAADKHKFILQMSDVDTRVVHKRDM
TYSRVSDSDVIGRKHDKEKIIELLMQQNPNDDDTSLSVIPIVGIGGLGKTTLAKFVFNDKKIQECFPLKMWVCVSDDFDIT
QLIIKIINSANDSANAPSHQMNWNMLDPEQLQNELKNKLFGQKFLLVLDDVWNEDHVKWVELRNLIQVGTAGSKILV
TTRSHSIASMMSTIPSHILEGLSEEDSLSLLVKWAFKEGEKEKYPHLVNIGREIVKKCGGVPLAVRTLGSLLFSKFEASEWE
YVSKNEIWNLPQKKDDILPALKLSYDLMPSYLRQCFALFSLYPKDSEYSSDAIIRLWGTLGLMPLPKTNRTREDMANQYL
HELLSRSFLQVLENYGSVFTFRIHDLVHDLAIFVTRDECLYVSSNIENISDNVRHLSFAESILFSNLVTKKSAAVRTVMFPIG
VAGANCEDILKTCLAKFKCLRVLDLRCATFETLPRNIAKLKHLRYLDISQNPNIKRLPDSICKLQSLQLLIVTGCMELEELPK
GLKKLTSLNYFEFCTKQTVLPMNEIANMGSLETLNVESCHNVESIFGGVKFPTLKSLCVSECQSLKSLWLDGQYFPQLETL
IVDDCNDLDLELWNGHYEEESSKLKLKFLCFSDLSQLVALPEWVQEAANSLQSLFISKCHNIETLPDWLTTLTNLKTLAIR
DCSKLVSLPDDIHHLSALENLRIQGCGDLCVKYEPHIGEFWPKISHIQNISIQTK

>g7876.t1

MANHGDFTYDVFMSFKGENGTRYSFTDHLYRALLRHGINAFRDDQSLRSGDEIRPSLLQAIEDSRISLVVLCQNYASSS
WCLDELAKILHCYENKGKHVVAIFYLVQPSDVRYQKNSYATAMSKHESRYGKNSEKVKAWRSALSRVCDLTGIHYRNH
MYETEVIEKIVKDTSAKLPPMPLQIKHLVGLDSRFERVKSLIDVESNDEVCILGIYGVGGIGKTTFAVDLYNKIRHHFEAASF
LANVREKTNKSIKGLEDLQRTLLSEMGEETETIIGSTFKGSSEIKCRLGHKRVLLVVDDVDSVKQLEALAGGYDWFGPGSR
IIITTRDKDVLHKHDVEIKGYKMEELNYHESLELFCWYAFNRSRAAENFANISTSAVRYAKGIPLALRVIGSNLKGRGPEEC
ETELQKYRKVPDLEIQGVLEISYTSLSNLDQKIFLDIACFFKGERWDYVKRILDACDFNPDIKVFVSKCLITVDENGCLEMH
DLIQDMGKEIVRKESPSNPGYRSRLWCHKDVHERFEDLTFINLSHCQFITQIPDLSGAKSLKVFTLDKCYRLTRFDKSIGF
MPNLVYLSASECTLLTSFVPTMYLPSLEVLSFNFCRRLEHFPHVMQKMDKPLKIYMMSTAIKKIPESIGNLTGLEHIDMSV
CKELKNLPSSFFLLPKLVTLKVDECSQLGESFQRFQESRHSVANGSSNLVTLHFCETNPSYEDLCAILETFPKLEDLNVSHN
GFVALPNINGGSSHLKSLNVSFCRNLKEIPELPLSIQKVDARYCQSLTSEASSMLLSKVSEEIRRIQVVMPMLKREIPEWFD
CIGTKEIPHFWARRKFPVVALAFVFQESKKKLSDLEHAFQSAVESFTGFMNWHTVSLHLFIDGQEICGRDYHCFNVGED
HVLFCDLRVLFRDEEWQGLEASLGDDWKAIQVQYESDLILSHWGVNVYEQETNMEDIQFKLPTPSSTKNLIPSSLLVPK
GCPKQKMKHMLESFDPRDMFNKNLSLIESEEGPSRAGKVLLRTWRNAKAEITEEASVSVYGASLKQEHEESVDDVVQV
LEMIKENVPKHFSDSNPEEMQSFGGFVERLLRARVEVMKEDALDLGMPILLEYTDVGGSKYRRFWGVLQLKVGDPFYQ
AVLRKYIQLSLEFSTSDKAASSSGSWFENLRITIVLLKCLDPAMEEASDFGYEESLEEGYYDPELEELMMRIEQDGMGFNK
SYGKMKASIVRTDESVSPKYLFETLIFRRLIALGKLSMFGSATKFKITPYGNIRVEDDPFRIPKTIPVIGKILVCGWWLVMQI
LVSCKYLYHRMGKIMKIKKL

>g8295.t1

MGDPFSGGAVGALMGEALKGAISITKKGLAFKSTLDSNIETLNSLAPLVEEMKMCNMMLDRPSKEVEKLESLMRNSQE
LVRKCSKLGRWKMWSFPYFQSKLRSKEMALQTHLSMNMTAQNTVNLMKLVDDMRKVLEILLKEEFGRYPEYWLIDLC
GAPQEPECLGMDVPLRKLRIELLKDGVSVLVLTGLEGSGKSTLAKKICWDPQIKGDAMISKRPYDHKFGANIFFVTVSKT
PNLKTIVETLFEHCRCRVPKFQTDEDVVNRLEVLLRVFGKHPILLVLDDVCPGSEDLVEKFKIQIPDYKILVTSRVSFPRFGT
SYQLDKLDHVHAESLFRHFAQLKDKSSYIPEKTLVDEIVKGCKGSPLALKVIAGSLCNQPFEVWKNMKERLQRQSILESDS
TELLFRLQQSLDILETKFSVNEKECFMDLGLFPEDQRIPVAALIDMWVELYNLNEDGTNAMSIIHDLTTRNLINVIVTRKV
AKDTDMYYNNHFVLVHDLLRELAIHLSKEETFEQRERLIIELKGDNRPEWWVGLNPLGIIGSLFSYIMGMLYRQKQPKVA
ARILSISTDETFNSDWCDMQPDEAEVLVLNLLSSQYSLPEFTEKMHKLKVLIVTNYGFHPSELNMFERLGSLTNLKRIRLE

KVSIPPLCILKYLRKLSIHMCNTRQAFERYCISDAMPNLVEMSIDYCKDLVKLPDGLCNITPLKKLSITNCHKLSALPQDLAK
LENLEVLRLCSCSDLAEMPDSVKGLDKLSCLDISDCLNLTKLPDDIGELKLQKLYLKGCSKLRELPHSVVKFENLDHKIHVIC
DEEMASLWENSTIRNLKIEISTVEVNLNWLPGAHS

>g8744.t1

MCQRLPRVVGQERLAKRNLTLSSHPRVVNLTELGQEELCLKELNLKGPPWNISALAKYKLVFLGDQSVGKTSIITRFMYD
KFDNTYQATIGIDFLSKTMYLEDRTIRLQLWDTAGQERFRSLIPSYIRDSSVAVIVYDVASRQSFLNTAKWIEEVRTERGS
DVIIVLVGNKTDLVEKRQVSIEEGEGKARELNVMFIETSAKAGFNIKALFRKIAAALPGMETLSSAKQEDMVDVNLKSTT
GNAQSQPQSSGCAYTTKPLAEAPGMNDPVEVPRPPNATVSVFEFGSVVASNDKVTLAGYCPVLEDFECISRWSITPYTR
YSNTSQKKSKLAPLQERRMIDKCKIFAKAGDGGSGCSSLRKGRPDADDSAALMSVGPDKGGHGSSKNLIGSRGADKVA
RVPIGTVLHLVNGDISSVVKTQSLTDVDPWDIPGALVDDLPDPGYGSTSSDTRGEVKATHSFGCSSTQDEETNAKKSRH
DTSTDAFSQLSTSNGALEFCTKDIEENQEIIYNVAELTEEGQQIVIARGGEGGLGNVSSSKDSRKSMTMKAGACQQIPNL
QDPNSIFSSLHVGLPGFETVLILELKSIADVSFVGMPNAGKSTLLGVISRAKHAVGDYAFTTLRPNLGNLNYDDLSITVADI
PGLIKGAHQNRGLGHAFLRHIERTKVLAYVVDLAAALNGRKGIPHWEQLRELILELEYHQDGLSNRPSLIIANKIDEEGAD
EVYKGLKRRVQGVPIFPVCAILGEGITDLKVGLKMLVNAETSYKLCVDQILLATQRAIAGSKIVMMVFTKTDTKSTRRCIL
EVEKILECHQTFGQIVLSVFYEIDPLDVRFEKDGLGKALAEAALKSPSGKQLEYWWCRQPADIFGWDVRDFRHDAELVK
VIVSHVQTLLGYADMYIAQFPLGLESQVEKVIGCIENHSTDVCMIGIWGMGGSGKTTLAKAIYNRIYRPFIERGYVTEILN
GWRKHADIRITVLIELDLIKVGRNNKLEMHPLLQHMGREIIRQRCPQEPWKRSRLWFQDDVKVVLKNNTGTEATEGLS
LKLYSTGRDYFKTLAFKKLKRLRLLQLDHVQLIGDYGYLSKQLRWICWQGFPSKYIPNNFCMKNVIAMELKHSNLQLFW
KQPQELQVLERLKFLNLSHSKYLIETPDFSTLPSLERLILKDCPSLCKVHPSIGDLCNLLLLNLKNCTNLRSLPREIYKSKSLRTL
ILSGCFKIDILEEDIGQMKSLITLITENTAVRQDKCSVQLETEKALENNNWPDLASLRKGLATI

>g8762.t1

MALAVVGGALLSAFLDVLFDRLASPELVGLIRGKKPEKLLQKVENQLIVLRVVLADAENRQIKDSNVKKWLDVLRDIVYE
VDDLLDEVSTKAATQKEVSNSFSHIFNRKRIVSINKLEDIAERLDDILKQMESLNLKEIPVESNQPWKDQPTSLEDRYGMY
GRDKDKEAIMKLVLEDNTDGEEVSVIPIIGMGGVGKTTLARSVYNDGKLKQIFDLKTWVCVSDIFDTVKVTKTMIEEITK
MPCNLNDLNLLQLELMDKLKGKRFFIVLDDVWIEDCDSWNSLTKPFLSGIRGSKVLVTTRNESVAAVVPFNVVKVYHLN
QLSNEDCWLVFANHAFPLSEDSENRGTLEKIGKEIVKKCNGLPLAAQSLGGMLRRKHAIRDWINVLESDIWELPESQCKI
IPALRISYNHLPSHLKRCFVYCSLYPKDFKFEKDELILLWMAEDLLKAPRKEKTLEEVGEEYFDDLVSRSFFQCPIPDCFIMH
DLMHDLATFLGGQFYFRANELGKDTKIDRKTRHLSFARFSDPCSDIDVFETVKFSRSFLPINYKDCPFNNEKAPRIIVSML
KYLRVLKFSDFQGELVLPDSIGELTHLRYLNLNGTSIAMLPESLCNLYNLQTLKLVRCSNLTKLPRNIQNLVNLRHLQILSTSI
KEMPKRMGKLNQLHDLDCYVVGKHRENSIKELGELPNLHGSFYIKKLENVNASEEALEARIMDKKHITCLYLIWSVPND
DIIDFQIELDVLGKLQPHQDLKLLTIIGYRGTRFPEWVGNFSYQNITSLFLSRCNNCCMLPSLGQLPSIRNLNISNMNSVKT
IDASFYKKDDCLSVTPFPSLEYLSFYDMPCWEAWNAFDSEAFPVLKDLHITNCPILRGDLPKHLPALQTLRIENCELLVSSV
PVAPTLRELNIGKSNKVAFHEIPLLVKELMIEGRPVAEFMMEAITNIQPTCLQSLSLKNCSIAISFPGDRLPALLKSLYISGLN
KLKFPVQHKHELLESLTISNSCDSLKSLPLVNFPNLINLTIEDCENMESLLVLGSESLKSLNCFSIRRCPNFASFPGKGLCMP
NLTRLSVYDCDKLKSLPDQMETVFPKMEYLAISNCQQIESFPGGGMPPNLRTVFIVNCVKLLSNQAWVCMDMVTSLE
VWGPFDGIKSFPKESLLPPSLVSLHLRELSSLETLDCKGLLHLTSLQQLNIERCQKLENIAGEKLPLSLVKLKISKCALLEQLC
HKKNRQIWPKICHVRGIKIDGRWI

>g8871.t1

MSLSGSDDEMEMDFLRDRYHEKDGGNFEVFLSFRGEDTRASFTSHLYTALQNAGIFVFKDDESLPRGKQISPSLQLAIEE
SRISIVVFSKNYAESLWCMKELEKIMECHRTTGHVVLPVFYDVDPSEVRHQRGDFGKAFQRLLTKFLKEEDEKVLDWKK
RWMKTLGLKEYTNRRTIGKRHFLRGRMKIADSIEFLIGRWMAGLFEGAEIPFRELEIDDGMDFLLKQWREVFCEPVSILK

VALLDPCGETEIANAIKLHVEHCREVLFEAAGISGDLVLNSCGEMEITNAIELHMKHWRDAFSEEAGTVLDSNYYGSGLR
TVHSEIWHLRHHWREKVREAYDRSGDDRLYSRREIDIERHLEHCTDALIEAINISTAAVFQSSFQKLKAYNRIESLVEYWRI
ALCKAAEISRLVVQHYRGITDDEINYIEKHARDALREAAGISGVVILNSRNESEAVKNIVKYVTSLLDKTELFVANNPVGVE
SRVQEMVQLLEQKQSNDVLLLGVWGMGGIGKTTIAKAIYNKIGRNFEGRSFLADIREVWGQEAGHVCLQEQLLFDIHK
ENNTKIHNIESGKVILRERLRHKRILLILDDVNKLQQLNALCGNREWFGSGSRIIITTRDIRLLRGKRVDQVFSMTGMNVD
ESIELFSWHAFKQASPKEDFIELSRNVVAYAGGLPLALEVLGSYLFDMEVTEWKSVLEKLRKIPNDEVQEKLKISYDGLSD
DTEKGIFLDIACFFIGKDRNDVIHILNGCGLFAENGLRVLVERSLVTVDDKNQLGMHDLLRDMGREIIRSKSPMELEERSR
LWFHEDVLDVLSKETGTKFIEGLTLKLPRTNTKSLRTKAFMNMKKLRLLQLSGVELVGDFEYLSKDLRWLCWHGFPFAFI
PTSFYQGSLVSIELENSKITMVWKATQVGPL

>g8877.t1

MVQLLEEKQSNDVLLLGVWGMGGIGKTTIAKAIYNKIGRNFEGRSFLANIREGWGQEAGHVALQEQLLLDIYKENSTKI
HNIESGKVILRERLRRKRILLILDDVNKLQQLNAFCGSREWFGSGSRIIITTRDIHLLRGKRVDRVFAMTGMNEDESVELFS
WHAFKQASPKEDFIELSRNVVAYAGGLPLALQVLGSYLFDMEVTEWKSVLEKLRKIPNDEVQEKLKISYDGLSDDTEKGI
FLDIACFFIGKDRNDVIHILNGCGLFAENGIRVLVERSLVTVDDRNQLGMHDLLRDMGREIVRSKSPMELEERSRLWFHE
DVLDVLSKKTGTKFIEGLTLKLPRSNTKTLCTTTFMNMKKLRLLSRKSGFY

>g8881.t1

MSLSGSDDEMEMDFLRDRIERVDKFEDHWKEKLLEIVQISKFLDLDPRGRMKIAGSIEFFVRQSMAGLFKGAEIPFRKEK
IDDAMDFLVKQWREVFCEPFSRSEVALLDPCLKFFNAVSHCSSLFRHYMPLSATVQRCRLPPRAATLSIFVRHEQACTVY
APPSARQIAITTPSNHLLQRYSKSTKDIQILTSHEVPSMETLSTRLLRVLVSQTQEAHEVVEPSIMVVTRGRGGCHIREISN
VIKLRMKYCREKLFEAAGISGDLVLNSCGEMEITNAIELHMERWSNAFSEQAGTDLDSDDFSSGLSMVRSEIQYLRDHW
WEKVREAYGRSGEYQLYSRAIDIKLHLGNCAKVLLEDIISTGAVSISSVQKLRAYNRIESLRKYWSIAFCKAAEISRSVVQHR
GITDNEINYIEKNARDALREASGIAGVVILNSRNESEAVKNIVKNVTSLLDKTELFVAHNPVGVESRVHEMVQLLQQKQS
NDVLLLGVWGMGGIGKTTIAKAIYNKIGRNFEGRSFLADIREVWGQEAGHVCLQEQLIFDIHKENNTRIHNIESGKVILR
ERLRRSRIIITTRDIHLLRGKRVDQVFAMTGMNVDESIELFSWHAFKQASPKEDFIELSRNVVAYAGGLPLALEVLGSYLF
DMEVTQWKSVLEKLRKIPNDEVQEKLKISYDGLSDDTEKGIFLDIACFFIGKDVIHILNGCGLFPENGIRVLVERSLVTVDD
KNQLGMHDLLRDMGREIIRSKSPMELEERSRLWFHEDVLDVLSKETGTKFIEGLALKLHRTNTKSLCTKAFMNMKKLRL
LHLSGVEFVGDFEYLSKDLRCYVGMDFLLPSYQQAFIKEV

>g8884.t1

MHSRNFEVFLSFRGKDTRASFTSHLYTALQNAGIFVFKDDESLPRGKQISPSLQLGIEESQISIVVFSKNYAESFWCLKELE
KIMECHRTIGNVVLPVFYDVDPSEVRHQRGDFGKAFQRLLSKFSKEEEEKVLDWKQRWWKTLHKLDNRWKKTLLEAV
QISKILDLNPRGKMKIADSIEILVWQSIAGLFNGAEIPFRKEKVDDAMDFLVKQWREVFCEPVSISEVALLDPCLLGTTRST
TNVDPPPRPTIQLGEGGGRTNNTTWSTKSTREPGLQGPSGLQGPSNLQGPSSLQVSSESLSTQFKSQMLSKFEMSDLG
ELNYFLGIQFSKTQYGTVMHQFRCLQELLIKFNMRKSNPTCTPTEVEYIVASDVACHAAWLGYVMKELKVEFMYKTKLL
VDNRSTIDLARHRASHGKINDGKLVIQHCKSEVQFAHIFTKALKRESGETEIANAIKLHMEHCREALFEAAGISGDAVLNS
CEEMEITNAIEFHMKHWRDAFSKDAGTVLHSDYPCSGFGAVNSEIMLLKNHWEEKVLKAYGNELYSRRTSDIERHLAH
CASTLIEATDISTAFQPSFQKLQAYNRIDSLVECWRMALCEAAEIWRLVVQHYRGITDNEINYIEKRARDALREAAGISGV
VILNSRNESEAVKNIVKSVTSLLDKTELFVANNPVGVESRVQEMVQLLEEKQSNDVLLLGIWGMGGIGKTTIAKAIYNKI
GLNFEGRSFLADIREAWGQEAGHVCLQQQLLSDIHKGNNTKIHNSESGKFILRERLRRKRILVILDDVNELQQLNALCGN
REWFGSGSRIIITTRDIHLLRGKRVDQVFAMTGMNVDESIELFSWHAFKQASPKEDFIDLSRNVVAYAGGLPLALEVLGS
YLFGMEVTEWKSVLEKLRKIPDEKVQEKLKISYDGLSDDTEKGIFLDIACFFIGKDRNDVIHILNGCGHFAENGIRVLIDDK
NQLGMHDLLRDMGREIIRSKSPMELEERSRLWFHEDVLDVLSKETGTKFIEGLTLKLPRTNTKSLFPKAFMNMKKLRLLQ

LSDVELVGDFEYLSKDLRWLCWHGFPLAFIPTSFYQGSLVSIELESSKITMVWKATQKTEESSDESHGSINDNKGGWRE
NAEGERDGERSEITPNRRRRVELPTCEGLDTLNWINREEKFFEIQKEGLKEALLIRFESRHRVRQTVTMEVYVREFEALAG
QTKEFSDNELLREGAHGDGDGDSALAAIFVGSEEVVKEVEEDEDLKKIVADITKDPDKHSSFTMENGRLHYKGRLVILAK
SDWVPKLLAEYHTTPTGGHSGVYRTYRRIAQSLFLIGMKVVTEFVAQCLVCQQHKYLAASPQGLLQPLPIPQAIWEDIS
MDFIVRLPKSKGCDAILVVVDRLSKYGHFIPLKHPYTARIVAEAFVREVVKLHRVPKSIVSDRDPWFLSSFWRELFKLKGT
QLQMSTSYHPEIDDQIEVLNRVLEGYLSYQGAARSTPFEVVYGRTPLALARFVPGEMMVEVVAQELQTRDEAVQQLRV
IQKVGEVAFRLLLPESATIHPVFHVSQLKLAIGTKKVEKELPADLQMSGPSCWPTRVIDRCVQQQGGDQPGEGG

>g8892.t1

MSLSDSDDEMEMDFLRDRYHEKDGGNFEVFLSFRGEDTRASFISHLYTALQNAGIFVFKDDESLPRGKQISPSLRLAIEES
RISIVVFSKNYAESWWCLKELEKIMECHRTIGNVVLPVFYDVDPSEVRHQRGDFGKAFQRLLSKFSKEEVKNQNWWW
KPPREIGGITALERVYQLEDHWKEALLEVVQISKILDLNPRGKMKIAESIEILVGRWMAGIFKGAGIPFRKEKVNDAMDF
VWKQWREVFCEAVNISEVALLDPCGETDIANAINLHVEHCREALFEAAGMSGDLVLNSCGEMEITNAIELHMEHWRD
AFSEEADTIFDSNYIGSGLRTVHSEIRLLKLHWQEKVLKAYGNELYCRRTSDIERHLEHCASALIEATDISTAFQSSFQKLQA
YNRIESLVEYWRAALCETAEILRLVVQHYRGITDNEINYIEKHARDALREAAGISGVVILNSRSESEAVKNIVKNVTSLLDKT
ELFVAHNPVGVESRVQEMVQLLQQKQSNDVLLLGVWGMGGIGKTTIARAIYNKIGRNFEGRSFLADIREVWGQEAGH
VCLQEQLLFDIHKENNTKIHNIESGKVILRERLRHKKILLILDDVNKLQQLNALCGNREWFGSGSRIIITTRDIHLLRGQRVD
QVFAMTGMNVDESIELFSWHAFKQASPKEEFIELSRNVVAYAGGLPLALQVLGSYLFDMEVTQWKSVLEKLRKIPNDE
VQEKLKISYDGLSDDTEKGIFLDIACFFIGKDRNDVIHILNGCGLFAENGIRVLVERSLVTVDDRNQLGMHDLLRDMGREI
VRSKSPMELEERSRLWFHEDVLDVLSKETGTKFIEGLTLKLPRTNTKSLRTKAFMNMKKLRLLQLSGVELVGDFEYLSKDL
RWLCWHGFPFAFIPTSFYQGSLVSIELENSKITMVWKATQIDKLEEDLEQMESLTTLVADRTAIRRVPFSIVRSKINSLSSR
VQTLVDMASLVSLDIQNSSSNQLSYISEELPKLQSLWIECGSDLQLSRDTTSILDALNARNSEESESSGTTSQVKNNITTSD
GGDCLVPGDCYPDWLTFSSEGSSVTFEIPQVNGRSLKTMMCHIHYSSSDSITSDGLKNLLVINHTKSTIQLYKRNALASFD
DEEWQRVLSNIEPGNKVQIVVVFWSRITVNKTSIYLIYEAIDEKVEHYHAPNMNVPRSIFPAVESMEDMRGASVKSLTK
RLLIKFLSGKYFCKGKVEKKINQG

>g10551.t1

MLDFVGPVLDIIIRLWDCCAYVRDYEENLSCLRDMTSDLLGLWVDVSVKVQLAEDQHLRRLNEVNDWLVKAEAMQRE
VQAIQQRVSHASRCLINFPTSFRMGRIVSKKIGEIRELIDKGHFDVVAQEMPYAVVDEIPLEVTIGKTTLLKKFNNEFLPTK
FYDVVIWVVVSKEADVGSVQQSIGNKLNVPVGKWGGKSIHDRAIVLYNFLKRKKFVLLLDDLWERIDLLKLGIPLPDTRN
GSKVIFTTRSMDVCRNMEANTCIKVDCLAPNEAFALFREKVGEESLNSHPEIFHLAKIVAKECEGLPLALITVGRSMARKT
LPEWKRAARTLKIYPSRFSGMVDYVYCLLEFSYDSLPSASHKSCFLYCSIFPEDYDIRKDELIQLWIGEGLLAEFGDDVYEA
RIQGEEIIASLKFACLLEDSERENRIKMHDVIRDMALWLACDHGSKTRFLVRDGACSGSVETYNQAKWKEVEKLSMWR
QSIQKLSGKQDCSNLLTMLVRNTKITNFPDEIFLTANHLRVLDLSGNKRVRELPSSIGELVQLQHLDLSGSGIRKLPRELQN
LKKLRCLLLNYICNRLVFPRNLISSLVSLQVFSKLPWEDQFILPALGEPEEAVLLKELECLEYLQDISIALFCVSSMQVLLNSSK
LQRCIRHLRVLSPFTSTPHIILFSLLTKMQHLEVLSMSVSSPSSLDHVRKKGSPSSQCSMTECIPMSSKLTEHDYIVGLRELS
LEGCGMQNLNWLTRAQSLQLLRIYNCSSLEEVIDCPKLIKLPFDSNSARNSLKHINGQKSWWRKLQWEDEATRDHFAS
RYRSKERLLNNLRILSDDTKALYLTNYIEANDEDVEIYVEHVPSETQVINFIVVSVVDGNMGKEAEARHVEAMEEKVNVV
KEKVNMAEEEANVAEEEVNMTEEEANVAEEEVNIV

>g10591.t1

MASQNPKWQVRISLDSSTIVQMNGLLRDVHVMVCRWARHDVNFRLKLEWYIGKRVRNMPQIIAAFDLFDEGIEGVG
GGHEEPMGGAAEEVLNEEGVGGGHEEPVGGAAKEMLNEEGVGGGHEEPVGGVDEEVGGRVEEDRVVTFNEGVLV
AEEPRNEVVNVVEIDDDGVHDEEAETPVPLAIVPLPAIAGDPKSTVNADQTVIAASVRDRPQRKLVSHLHSVLLQAQVK

TLVNEENVQEGMKLEERMGAIAVSKIAIIVFSKTYTESTCCLLELEKIIECLETFGQVVLPVFYEIEPLDVRDQKDDFGKALE
ETAKKSYLGEQVEHALSRWRRALTTAASMNSWDVRDFRDDAELVEVIVRRVQALLDYKDLFVTQFPVGLELHVEKVIEC
IQNHNTKVCIIGIWGMVGTGKTTIAKAIYKRIYRLFIGKSFVENIREFWSRLFRTYVDLQEHLLDDVLKNKFEVGSDEMGR
NLIETKLSRKKLLIVLDDVTEFGQLENLCGNCEWFGRGTVIIITTRDFKMLNRLKVNYVYKMDAMNKNDSLELFSWYAFR
EAKPRKELNEISRNIVANCGGLPLALKVLGGWTMQIPYAQVQEWKCVSLRLPNAFAQVREKLKISFDGLGDMEKDIFLD
VCCFFIGKERGYVTEILNACGLEADIGITVLIERDFIKVERNNKLGMHPLLRDMGREITRRDWPQEPGKRSRLWFHEDVK
DILKTKSIDILEEDIVQLESLITLVTENTVAKQLPCSIVSSKSIGYISLRGFEASSHNIFPSIIRSWMSPIVNLQSYISPYCMDME
NNNGRDLTPLLSVLANIQTVLVQCDTEVQLSKQVKKILVECSVNFKDSRISKRHLRFSLNCVGSYNDFFNTLNNSVSEGLT
SSKCCDVSLPGDNYPYWLVHMGEGHSVSFTVPRNSDMKKMILRVVYLSTPGIMATECLRSVLIVNYTKFTLQIHNHGSV
ISFNDEDWHVIMANLGSGDKVEIFVTVDHGLVRLSDMW

>g10593.t1

MELASSTFKPQMCDVLINFTGEDIRRKFVSHLDSALSAVGLTTFLHEETAVKGMHIQQPILNMCRATIVVFTKTYSQSA
WCLHELQQIIKWHETYSRHVLPVYYEIQPSDVRLQKGDFGKSFKATAQQTFSGEQLEHGMLKWSHALTKAANFFGWD
ESNYRSDAELVDTIVKSVLILPVLSATKFPVRLQSHVKDMIQVIKNKSTEVCIIGICGEEGSGKTTLAKAVYHQVHSTFTYKS
FIEDIAEVSQTRGLVGLQEQLLSDVLKTKIKLHSVQMGISMIRERLSGKRMLIVLDGTNEYDPIGLWNSHVWFGKGTVMI
ITTREEGLLRIPEVDSVLLIELLNTNESLELLSWHAFREAKPREEYNDLAKRVVACCGGLPLTLEVIGSRTYVTQILNGCGVD
ADSGIRILIERSLIQVKRNNKLGIQPLLQKMGKEIVVEILERRLRNNTQLRFRLDENYVVSDNTLFSSQQTKVIQILPSKLFLT
RTDLLQPCRAKVSDTSRIFELVEHSNSDEYLSKKLRWISLQGFPSKYLPNDFYLHDSIAIDLKHSSLRFVWKQPQVLPWLK
VLNLSHSKYLRKTPDFSGLSSLEQLILKDCPRLGQVHQSIGCLCYLRLLNLKDYWPNRKRYSANGILNNSNCGKYSCETSA
FFNYMEDNSWDDFVPLLRTLENLRSVLVQCDSEFQLSKQVKTILVEHGVNITETGTPKPHFRYSLIGVGRCKEFFNAVSD
SISEVLASSESCDVCLPGDNDPYWLAHIGEGLSASFNLPQDRVMNGMALCVVHLSTSKTIEPTITTVLIVNYEKCTLQIHN
YGTLVSFTDEDWHCIMSNLGSGDKVEIFVTLDYGFVIKNTAVYLIYGVP

>g10614.t1

MLKSGLQRFWDRFGSRSSGSTSSSSYDSSDTIQNQHYRYDVFISFRGPDTRNSFVDHLCSHLLRKGIFVFKDDHNLQKGE
SISPQLLQAIQLSRLSIIVFSKNYASSTWCLDEMTAIASCKQQLSQIVFPIFYDVDPSHVRHQNGVYKNDFLSHRWKFGKD
RDKVLGWKRAMTDFANSVGWDMRDKPEFEQIQSIVLEVIKKLGRKFSRSVNDLIGIQPRVQALEDKLRLSSNSDDVQV
LGIWGMNGIGKTTHAAVLYDKISHRFHASCFIEDVNKLYRDGGHTAVQKQIIHQTLRENIDMSNPIEISGIVENRLHSIKV
LIVLDNVDELEQLENLAIKPKLLLKGSRMVITTTDEHILKAYEGDVLIHKLPLLNDADARELFCRKAFKYEDQSSNCSALIHE
VLKYAQCLPLAIRVLGSFLCTRDADEWRDVLNRLENSPDDKIMNVLQISVDGLQPEEKQIFLHIACFFKGEKVDYVKRILK
CCGLHPDIGISRLTEKSLITISDEEIHMHELLQELGKKMVRDQSPEEPGSWSRIWLHKDFLHALTAETVTSYISKLRNREGE
SHSIK

>g10616.t1

MMMTMIDKLGFRRFWDWLGSRSSSTSVISSESATSSSDHSSDTIQNQDHRYDVFISFRGSDTRNSFVDHLYSHLLRKGI
FVFKDDHKLRKGESISSQLLQAIRRSRISIIVFSQDYPSSSWCLEEMATIADCKQQSNQTVFPVFYDVDPSHVRHQNGVY
ENAFLSLRQKFKGKPDKVYRWERAMNWFGTLAGWDVRNRPEFGEIENIVQAVIQTLGHKFSGFVDDLIGIQPRVQALE
DKLRLSSKSDDVLVLGIWGMGGIGKTTHAAVLYDKMSHRFDASCFIEDELAINSKLHFRGSRMIIISRDEHILKVYGAHVI
HKVSLMSDKDARELFYTKAFKNEEQNSGCVELIPEVLKYAQCLPLAVRVIGSFLCRRNSSEWRDTLDRFENNPDDNKIM
EGTNDVKAIVLNKKGAISESYGLVELNMPNSSIKCLWEGRKHFPCLKRMDLSNSKYLTETPDFTGIPNLERVHLSGCTGLS
FVHPSIGLLQQLAFLSLRNCTNLISIKFGNGLNLNSLRVIHFSGCAKLENTPDFTWTTNLEYLDFDGCTSLSSVHESIGVLTK
LTFLSLRDCTSLAFVFALPFTKMYFINGKVVRNGSRDLVINWMDPLNYQELTWLVRLAKVRTLFSFPFSIFG

>g10618.t1

MVKTKAGLKRFWDRFGGRSSGYTSSSSSYDSSDTIQNQDYRYDVFISFRGPDTRNSFVDHLCSHLLRKGIFVFKDDHSLQ
KGKSISPQLVQAIQLSRLSIIVFSKNYASSTWCLDEMAAIASCKQRSNHIVFPIFYDVDPSHARHQNGVYENDFVSHRRKF
RKDRDKVRGWKRAMTDLANSAGWDVRDKPEFEQIQNIVQAVIKKLGHKFSWFVDDLIGIQQRVQTLEDKLKLSSNSD
DVQVLGIWGMNGIGKTTHAAVLFDKISHRKAFKSEEQSSSCEALIPEVLKYAQCLPLAIRVLGSFLCTRDADEWRDVLNR
LENSPDDKIMNVLQISVDGLQHSLRNHSSLLAMKKFICMNCYKNWERKWFGTNLHKNQDSGVEYGFIKTSCKS

>g10619.t1

MCVLMRLSLVMLKMPKMLEMLDMLEMLEMRGTMKLGGGMRIEKREERREKKLDKLGFRRFWGRLGSRSSSTSVISS
ESASSSSDHSSDTIQNQDHRYDVFISFRGSDTRNSFVDHLYSHLLRKGIFVFKDDHKLRKGESISSQLLQAIRGSRISIIVFS
QDYPSSSWCLDEMATIADCKQQSNQTVFPVFYDVNPSYVRHQNGAYENAFVLHRSKFKEELNKVLRWEKAMTYLANL
AGWDIGIKPEFGEIENIVQAVIQTLGHKFSGFVNDLIGIQPRVQALEDKLRLSSKTDDVQVLGIWGMGGIGKTTHAAVLY
DKMSHRFDASCFIEDVSKLYRDGGHTAVQKQIIHQTLGEKCLDTYSPIEISGIVRNRLHKIKVLVVLDNVNELEQLQELAIN
SKLLSRGSRMIIISRDEHILKVYGAHVIHKVSLMNDKDARELFYTKAFKSEEQNSSCVELIPERLRYLLWHDYPFASLPSSFA
APGLVELNMPDSSINCLWEGRKHFPCLKRMDLSNSKYLTETPDFTGVPCLERLDLSGCTDLSFVHPSIGLLQHLAFLSLRN
CSNLISIKFGNGFNVSSLRVLHFSGCTKLENTPDFTWTTNLEYLDFDGCTSLSSVHESIGVLAKLKFLSLRDCTSLVGFCNLL
EVPDAIGDLLLQRLPKLSAISSSSTGRYFKTVSGSRDHRSGLYFFDCPKIDWMDPLDYQEFRWLVRLAKNPCYFRCGFDIV
VPWDLDLPYWLNQRFKGDSVIRIVEFNENDDWMGFVFCVVFERNNGPVVGRSSSHPFYLSFESEDTEEYFDMPMNLE
RDKVIGSKHLWIIYISREHCHFAKTGAHISFKAHPSLEINAWGMSSIFREDEENNFECKGDVNFDFVEKSSTNSGPKFQLP
YNWPLIKDKNSLLEASSTISFNNLEILGEIVFVVCRRPCFVHQRWVGFGMCDGDMNMMKVAKYLGKVHLYVVHKVNE
PEVADNENEILYLCQGPAKSGPVDCDGDLERDDGVQVNIEGGVEVDEDGVVGVEQDAAVGFEQVGAVEVEEVGGVD
VEQDGDLEESDEEYKVEDSGRGLSDDEWESDELLTPENSGSEKDDSADRPSRGPFPTFGKKKSMVDYKWGLGTIFTDK
DEFKEAIRSYAIHARRALKGCNETRNFKTHVNGGFGVERCFETAERSGRTTTNIAPINGGSSVNGPLPVIDQLWSREHFH
VKDGKLMADPETGEFCSSPPKLLCIEKGQAEPVELVRGIDKLKPQIIRVIEGFEALLKRQVVNVKGLLKADPVREEPQPHI
DCTGHLQNCEMMRQDIQAILTILKDCKIDNGGKRRDGSESKRVELPMFEGGEPWNWIGRVEKFFEEGLKRALGIRFGG
GTRGTVYEKLSAIRQTGAVEDYVRDFEVLVGQTTQILEQQILGYFMAGLREEVRDHARPHDPPDLMTAMRVAREVEN
GPFSPGHRCPERGLRMLIMVDEEEEEGGETETVNLAGMELSDFGASHNFIDRRLVEELKMAVAETQPYMVSLGDGQR
RRISGCCEQVELELGDAKLVKRFFPFELGGVEMILGVEWLEKLGKFLVDWRQMTMEYRQGGREVTVKGDPTLERKVV
GSRAWLNIERPTAGFMVWELGSMEAQNKAPREMEHHTQLKEGTDSVKVPPYRYPHAMKAEIERQVTEMLRTKSNR
DAQTRGGIAESVGSVKKDATQGEGVERTTGDGRERNIHKLPYSEFVKIERRVDASGVGGLSALVIGA

>g11602.t1

MPSKAIMECSSSSSSQVTTIYDVFVSFRGEDTRNNFTDFLFQALRRKGFHAFKDDADLKKGESIAPELQQAIEGSRIFIVVF
SKNYASSTWCLRELAHICYFVEAPGRHILPIFYDVDPSDVRKQSGYFEKPFVELEERFREDNERMEEVQRWREALTQVA
NLSGWDIRNKPQYAGIEEVVQKVTDILGHKFSSLPNDNLVGMKFRVEELAKLLYSESSNDVRVFGITGMDGIGKTTLVR
ALFEGISHQYDFTCYIDDVNQIYGDSSTLGVQKQLLSHSLNEKCEEICNVSQGTYLIWKRLQGKKALIVLDNVDEVEQLKIF
TGNRDTMLRDCLGRGSIIMIISRDEQILRIHGVDEVHQVQPLENTEAVQLFCRHAFKANVIMNGFEKMTNEVLWHAQ
GHPFAIEILGSSLFGLIKNGYQEPMEDENQTVAQIVALRKTQVKEKSTLYLLYKVVDESSFEKIAKAASSKEAWEILDDRVK
QVQL

>g11621.t1

MEEAERWREALTEVANLSGWDIRNKLHNVRALVVLDNVDEVEQLKMFTGNRDTLLRECLGGGSIIIIVSRDEHILRTHG
VDDIYQVPPLNDENAMQLAIPWQLKCIVKEESHKEPLKRSRLCDYQDFHKAMSNNQETKMLEVVAVNLQCSSKPLRV

NGLSKIRHLKFLRFETDLIVQDVNFSGSLSHLSKELGYLTWHKYPFECLPQSFQPHKLVELKLRCSNIRRLWSDTKVLPNLK
RLYLSYSKKLVEMPDVAEALNLEVIDLKGCIQLRKLSPSIGFLRKLTILNLENCKNLVSLPNSILGLNSLEYLSVSGCSNLFNN
ELLDQARNTEHLVKLCLVKGLIHSQSTSPSMKKMLWWTLDLLYSRAQRESVSCLLPSSPLFA

>g11633.t1

MACNKSQRSSSSHTKNFDVFVSFRGADTRNGFTNHLFAALQRKGVVAFRDDQTIQKGDFLESELLLAIEGSRVFIVVFSK
DYASSTWCMKELTKIVDWIEVTGRSLLPIFYDVTPSEVRKQSGEFSKAFSEHEERFKDDLEMVKGWRAALKTSCDRCG
WDVQNKQQYEEIENVVEEVINILSRNQIWNFGDDLVDMHSRLKKLEELLDLSANDIVHLVGICGMGGIGKTTLATALFN
KISPQFNACCYLDDLSKIYCNFGAASAQKQLLCQALNQGNIEIHNASHGTMLIRTRLCHLKALVVVDNVDQVEQLKKLG
LQSEYLGAGSRIIIISRNCRILQNYGVNKVYEVQVLDKTQSLQLLRKKAFRSNDIGKEHEGLTLDILKYVNGLPLAIEVLGSFL
LDRDVCEWRSALTRMEENPSKDIMDVLRISYDGLENIEKEIFLDIACFFSDNNSYSWEPAVMKLLDYRQCYPDIGMKVLI
EKSLISCQNATIKMHDLLKELGKSIVREKAPKEPRKWSRLWNYKDLQKVMKINKEAENVEAIFIEQHQKEFLQGRIRVDA
LSKMDHLEMLILRNVNYYGTLNFVSNELRYLVWDHFPWLSLPSTFFADQLVELILHHSNIKQLWEGKKCLPNLRNLDLR
HSKNLIEVPDLSEVPRLTDLNLEGCIQLVHIHPSLGILRDLRSLSLKSCKNLIEVSDLSEVYLDPQMEIVIPGSEIPKWFSKQK
AGTSISMDPSAVIDDPNWIDTMHDLQAVKFETMVKSLPGLRFMVKNCGYRLVFKEDLQQFNSNKFFNRNSSSRKRKLL
TSE

>g11836.t1

MPVLETLGGALFGAVLQVLFHKLDSHQVLGFFHGRNLDEKLLKKLKRKLMDVNALIDDAEQKQFSNSLVKEWLDEVRD
VLYDAEDMLDQIDYEFSKTKLGAEFQTSSSKVHNFESKIIALLDDQESLLNQKIVKDFKIYSGVKSGLGNKVFEKKVESTSL
VAEEVIYGRDEDKDMIFSWLTSDTNDTKLSILSIVGMGGMGKTTLAQHIYNDPKTDEAKFDEKAWVCVSDAFDALRVS
KAIIGAFTNSRDDSEDLEMVHGKLKKRLSGRKFLLVLDDVWNEDRNKWKALQTPLTFGAKGSKILVTTHSQKVASIMQS
TYMHPLKQLDEDHSWQTFTKHAFQDENSTLNSEIKEIGIKIVEKCQGLPLAVETIGCLLQSKSSVSEWEGVLTSEIWDLPI
EDSKIVPTLLLSYYHLPSHLKRCFAYCALFPKDHRFDKKSLIFLWMAQNFLHCSQQSMSPEEVGENYFNDLVSRLGVDGE
KRVSRKTRHLSYISGPIQYYTSLCDAKGLRSFINFQRCCEMSIEELIVNYKFLRVLSWHWCIKVPDTIGDLIHLRSLDLSGTDI
ERLPDSTCSLYNLQELKLNKCLNLKELLLTLHELTNLRRLEPMGTTLTKVSLRLGKLKNLHVWIDKFEVGKNNGLDQIVRID
DEFYGNSSFAFESLKTLSFTDMKEWEEWQYMKGAFPRLQSLSLRNCPKLKGNLPEQLSLLKKLDIIDCGQLGAPFPRADT
LEYLIIYSCPGINIPINHWYHSLVELDIRFACDSLTTFPLDLFPKLRKLCLLECRNLQMISQEHPHNHLERLSIEKCSEFESFPN
EGLFAHQLKTFFIAELEKLKLMPKCMSALLPSLNPLYIRNCPVVELSDGCLPSNVKEMYLRNCSKLVASLKGAWGTNPSL
NILYIGNMDVECFPGEGLLPLSISNLEIADCSNLKKLDYRGLCHLSSLQKLILRNCSILQCLPDEGLPESISQLNIIGSMSLEQ
RCKKQEGEDWEKIAHIKSIIVDYKQVNI

>g11837.t1

MEIVEKCQGLPLALETVGCLLQSKSCVSDWEGVLRSNIWDLPIEDSKIIPALLLSYYHLPSQLKRCFAYCALFPKDHEFDKE
NLILLWMAQDFLQCSQQGKSPEEVGEQYFNDLLSRLGVDRVKTVPKKARHFSAVSDPVEFSTVECHEYRSLCDATRLRT
FVSISMDCGMSIQELISNFKFLRLLSLNGCDNIKEVPDSVGNLIHLRSLDLSGTDIERLPDSTCSLCNLQVLKLNDCDNLQE
LPSTLHELTNLRRLELMETTLRKVPVLLGKLKNLRVWMDSLLVGETSESNIQHRGELDLHGQLSITNLENVVNPCDALVA
DLKNRTHLVGLRLEWSLMRNNEDSMKEREILENLQPSRYLEQLSIDGYCGTEFPRWLSDNYLSNVVSLILNNCKYCLWL
PSLGLLTVLKHLTIGRLDQIVRINADFYGNSSSAFASLETLTFSNMKEWKEWQSMPGAFRNLQSLRVTKCPKLKGHLPKQ
LSHLKKLTIGKCKQFVASIPRIVEIQDVQMEPSSFDMTGPLVSDIPLESLSIYSSPGMNISINHCYHFLLQLSILGNESLKNLP
LDLFPKLWKLALGNCRNLQMISQRHPHSHLKSLAIEKCCQFESFPNEGLFAPQLETFRIMGLEKLKTFPKLMPSRLSSLNH
LTIHGCHAVELSDGCLPSNLKDMCLLNCSKFVVSLKGALGANCSLRSLSISGVDVESFPDEGFLPLSLTELLIEDCPNL

>g11839.t1

MPVLETLGGALFGAVLQVLFDKLDSHQVLSYFHRRNLDDKLLKKLKRKLMDVNAVIDDAEEKQFSNPLVKEWLNEVRD
ALYDAEDLLEQIDYVSKSELKAEIQTSATKVCSLESKMIEVLDDLESLLDQKVVQAFKISSIVRSGLGNKVLDRKNESSSLVA
EDVIYGRDEDKEMILNWLTSDNSNNNPLLILSIVGMGGMGKTTLAQHVYNDPKIKEAKFELKAWVCVSDEFDVLTVSK
AIIGAFTKSKDDSRDLEMVHGKLKEKLAGKKFLLVLDDVWNEDRNQWKTLQTLVFKAAATYSASAVDCATVACFFDAQ
DKIPDPSEKAYPDVLFMSSTNPAQSLSV

>g11840.t1

MPVIETLGGALFGAVLQLLFDRFDSHHVFDYFRRRKLNEKLLYKLKVKLLSINNVIDDAEQKQFSNSYVKAWLDEVKDAV
FDAEDLLDEIDYEFCKWKLRAESHTRSKKVWKFFKPSSKSFFDKKIESRMEQVLEQIEFLSSQKGDLGLEYVSCVGVGPES
VSKVTQKLPSTSLVVESVIYGREDEKEMILNWLGSDTDNHSQPAVLSIVAMGGMGKTTLAQHVYNDPRIEEAKFDLKV
WVCVSYEYDAFKVAREILQAIHNSIDDSRNLEIVQVRLKEKLTGKKFLLVLDDVWNEDRDRWKPLQTPLKYGAKGSKILV
TTRSSKVATTVQSHKVHELKQLEEDYSWQVFAKHAFQDNNRHLNVEVEKIGKRIVEKCKGLPLALETIGCLLHTKSSVSE
WESVLISKIWDLPREDSKIIPALLLSYYHLPSHLKRCFAYCALFPKDQEFHKESLILLWMAENFLQCSQQGRSPEVVGELYF
DELLSRFGVDEEKKTLVKTRHLSLVTNGDQYSDGFRSLYDAKGLRTIMPTSRRINIDYYWDCKMSMDELFSKFKFLRVLSL
SCCRGLKEVPGLVGDLKHLRSLDLSGTLIEKLPDSTCSLYNLQILKLNSCFNLKVLPSNLHKLTNLRRLELMKTNVIKMPVNI
GKLNNLQVLMSSFRVSESSEFTIQQLGDLSLRGGLTIEDLQNILNPLDAMAADLKKNCNFCTRLPPLGLFPYLKDLTISGM
GEVVSINADFFGSTSSSFSSLETLKFSFMYGWENWECQAVTVTDAFPRLQHLSIRHCPKLKGNLPEKLLQLRKLLICECKE
LVASAPVAQEIFELDLQDCGKLQFDYHPTSLRRLTVTGDSSMQLSLDFLSKSKTNIPVSSYDFLETLDINDGCDSLMSISLD
CFPKLLRLCLKYCHNLHTISQGRIHNHLKDLQIIRCPQFQSFPDEGLSAPMLESFSMKRLPKLKSLPQQMHILLPSLTSLLIH
DCPRVKILSDEGLPSNLENMNISNCSRLVASLKGPLGANTSLQTLSVQEVDVESFPSEGFLPISLTCLIIRDCPHLKTLNYKG
LCNPSSLKKLTLFDCPNIRCLPEEGLPKSISTLRILGNCPKLKESSQKPQGQD

>g12400.t1

MADSIVVFLIDKLTRLLVEEAKLLTGVRDQVTSLQSELRFMNLFLRNSQGKRKEHDMVAELVSQIRDVAHEAEDVIDTYV
ACIIKQSRRNVIGKVGFRGVDHALMLHQVAVKVDGIKARIKEIFDNKERYGIEDGRRGSEEEAERIRRQRREVEEEEVVG
FALDSKVVIEKLMVSDSRLKVVSVVGMGGLGKTTLARKVYNSNRVKNVFPCRAWGYVSNDYRPREFFLSLLKCLLSTSKY
SSLFKKREETSVSDEELKMKVRECLNRSKYLVVVDDVWQKQVWNEVKGAFPDDQNGSRILMTTRSAEVASHAGPVPP
YALPFLTKEESWELLSKKVFRGEDCPSDLESLGKLIADSCDGLPLALVVMAGILANKKSPRDWSRIKDHVNWHLGRDNTL
KDILKLSYDSLPARLKPCFLYFGMYPEDYRIPVKQLIQLWISEGLLTQETSGGQDIPEPEYIAEEYLDELVDRSLIQVVSRTN
DGGVKTCRIHDLLRDLCISESREDKFFEVCGEIDIQNLNSCPRKLSLQGTLFHFSSSIVSDYTISATRSLLCFGQEVYKVKAN
HWRWLLKSFRLARVLDLGRMNVNSIPTDLEKLIHLRYLRIHSHNLETVPPSICRLWNLETLDLRGSPIKSFSGELWQLKQL
RHLLLFGPVGLPEMPSESKTMPNLQTLSTVALDPRTASLLDSRRFPGMTKLGIHYERRDKCNARIQLQSLHRLSHLRKLK
VIGTTEIPQNANVFPSNITKISLTKFGFFNSTVMHTLGKLPNLQVLKLSSQTNDTRFDLHCATGGFLQLQVFEMIAIKVKM
WRVDRGSMPRVRRLVVRSCKSLTQLPKEVWSLNTLREVQVLWPCTELAKGLQNLVMNNACKLVVYPLSANDELDFL

>g12436.t1

MADSVVGFLVQRLSQLLESEIKLLSGVEDKIKSLRNELKFIDIFIKSSEGKYKDAVVKEVEPLLHIYQLPFLPEDQSWELFCKV
FRGEECPSELEPLGKSIVEATCGGLPLAIVILGGLFAKKEKSQREWSRMKKMSWNSTADKNEVMDILRLSYDNLPPTLKP
CFLYFGIYAEDHKINAREVIRLWGAEGFVQPQENAELEEVGDFYLDELVDRSLVQVTERRSDGGVKICQVHDVLRDFCIS
ESKFCKFMDVYRESNIDTFKCFI

>g12572.t1

MGCEVLPHDFVLEGDRIIKMVEAVVSFAVDRLGDLLIEEARLLSGVSNKVKSMQNELKMMQCFLRDAESRQDESDTIK
NYISEVRKLAYDAEDVIEIYAIKVAFGISIGTKNPLSRAKNIHKVGSELITINSRISDLTRSLQTYGLTATKDNEEASEVKRQLR

WSYSHIVDEFIVGLDKDINKVAEWLINENQDCRFVYICGMGGLGKTTLAKSIYHYNAIRRNFDGFAWAYISQQCKKRDV
WEGILLKLISPTKEERDEITKMKDDELARKLFKVQQEKKCLIILDDIWSNEAWDILSPAFPSQNTKSKIVFTSRNKDISLHV
NPEGLLHEPSCLNAEDSWALFKKKAFPRQDNPESTTSDDFKRLGREMVAKCAGLPLAIIVLGGLLATKESVSEWEKIHRH
LSSYLIGAEVRDRRRLDEVLDLSYQDLPCQLKPCFLYLSQFPEDSEIPKTKLLQLWVAEGVVSSQYESDRDETMEDVAERY
LGNLISRCMVQIGQMGSTGRIKTYRLHDLMRDLCLSKARKENFLYIINGSQQNSTVIATGSSNVSDARQINEVRRLAVYL
DQHVDQLIPQDKQVNERLRSLVFFHDKKCRMENWDLVRGVFVKFKLLRVLDLEGIKGLKGQSLPKEVGNLLWLKFLSLK
RTRIQVLPSSLGNLENLQFLNLQTVNKVSWDSTVEIPNVICKLKRLRHLYLPNWCGNIVNNLQLDNLTNLQTLVNFPASK
CDVKDLLKLRRLRKLVLNDPRHFQKFSESFSPPNKRFDCLQSLSLRTDMLSFPENVVDVEKLVLGCPFLRKLQVEGRMER
LPDASLFPQHISKLTLWGCRLVEDPMVTLEKLPNLKFLNGWDMFVGKKMTCSPNGFLQLKFLVLRGLPKLDEWTIENQ
AMRNLYRLSISDCNNLKTVPDGLKYITSLRELEIRWMPKSFKTRLGTAGEDYPKVQHVPSIIFLN

>g12671.t1

MHYCQLIGIAFVAEDCFHTVLNQIRDVAHLAEDVIDTFVAKVAIYKRRTILGRMLRGFGQARLLHHVAEKIDKLKTTLNEI
RDNKDKYDAFREATNQSAAEEEEEKERLQSLHKLRRDVEEEHVVGFVHDSKDVVKRLLEGNSNRKVISIVGMGDWERP
LLPERSTIATRGNKKDKKNVEDVNNLSEDELKKLVRNCLEGKMYLLVVDDLWKSQDWDEVQDAFPDNNNGSRILITSR
LKEVALHAGDDVPHYLQFLNEEESWELFCRKVFKGENCPSDLETLGKQMVQSCSGLPLSIVVLAGILGKKEKSHREWSK
VVGHINWYLTRDETQVKDIVLKLSYDNLPRALKPCFLYLGLFPEDFEIPVMSLLQKWVAEGFIQDTGNRNSDDVAEDYLY
ELIDRSLVQVARVGLNGGLEKCQVHDLVRDLCISESKEEKVFEVCTDNNILISTKPRRLSIQSDMGHYISSSNNDHSCIRSL
FFFGPEYDVGGREWKWLLDDFKLVRVLEFGPNSCQKIPSNLGNFIHLRYLRINSTRARFVPDSILDLWNLQTIDLVVSAF
NIPISFPVQIWKLKHLRHLSAQGSIILRGRCSKSYQKMWNLQTISHLVLNKQATSLIKKGTFPNIKKMGLYANGYEDQLLN
LLQNL

>g12736.t1

MEESVVTFVLDHLAQLVAREANLLYGVEDRVQSLQNELQMIKELLNTTKRKKGMEHTVLNQIRDVAHLAEDVIDTFVA
KVAIYKRRTILGRMLRGFGQLRLLHHVADKIDTIKATLNEIRDNKDKYDAFKETNNQSAAEEAEEEKRAQSLHKLRRNVE
EEDVVGFIHDSKDVINLLLEGGSNRKAVSIVGMGGLGKTTLARKVYNSSQVMSHFDCRAWVYVSNECRVKDLLIGLFKH
LMPNFEQQRRGNKRGKKNVGDINDLSEEELKKLVRSCLEWKRYLVVVDDLWKKQDWDEVLDAFPDNNRNSRILITSR
LKEVALHAGHDVPHYLQFLNEEESWELFRRKVFRGEDYPSDLEPLGKQMVRSCRGLPLSIIVLAGLLANKEKSHREWSKV
VGHVNWYLTQDETQVKDIVLKLSYDNLPKRLKPCFLYLGLFPEDFEIPVTPLLQKWVAEGFIQNTGNRDPDDVAEDYLY
ELIDRSLVQVVRVETNAGVETCQIHDLLRDLCILESKEDKVFEVCTDHNILISAKPRRLSIHGMMDHYISLSNNEHSCVRSL
FFFGSHYYIRGRDWKWIFKNLKLVRVLEFGLNGSNKMPSNLGNFIHLKYLRINIEYIMFVPDSILKLWNLQTIDLSPPRGN
VPISFPAKIWKLRHLRHLNTPRPIKLRGSCSGSYEKMWNAQTVSSLVLNSQARSLIERGTFPNVKRLGLRVTSECEAELPK
LLQSLQQLSYLNKLVIVLRDRDDEGVKDLTDESVKRNNGFRPQEVLQSLGQFNCLTILTIENAFDLLTCELTFPPTVTELTLS
EIDCISDEGINGLGNHTKLKKLRLLGDVSLSTGESFDLNCVGGGFSQLEVIEMENLKLEKWKLDNGAMSRLQSVMIHNC
ERLDDLPNELWSLSGLRKVQVMKPSKEMARMLRNLEIKNEVQLVTEDYQPRFQSRAAAESIAGAPWRSPSSLPSRTKT
REAASHLTSDLQKLAAREANLLYGVEDRVQSLHYELQMIKDLLNTTTRKKGMEHTVLNQIRDVAQLAEDVIDTFVAKVSI
YKRRTILGRMLRGFGQARLLHHVSHKIDNIKTTLNEIRDNKDKYDAFKETNNQSAEEEEQKRTQSVHKLRRNVEEEDVV
GFVQDSKDVIKRLLEGGSNRKVISIVGMGGLGKTTLARKVYNNSQVKQYFKSRAWVYVSNECRVKELLLGLLKHLMPKF
EQQRRGNKKGMKSAGDISSLNEEELKKLVRNCLEWKRYLVVVDDLWKKQDWDEVLDAFPDNNRGSRILLTSRLKEVAL
HAGHDVPHYLQFLNKEESWELFQRKVFRGEDYPSDLEPLGQQIVQSCQGLPLSIIVLAGLLANKEKSYREWSKVVGHVN
WYLTRDEIQVKDIVLKLSYDNLPRRLKPCFLYLGLFPEDLEIPVMPLLQKWVAEGFIQDTRNRDPDDVAEDYLYELIDRSL
VQVARVEMNGGLETCRVHDLLRDLCISKSKEDKVFEVCTDNNILIPTKPRRLSIHSKMTHYISSSNNDHSCIRSLLFFGPDY
CVDRREWKWLLESFKLVRVLELEPNRCGKIPSKLGKFIHLRYLRIDSEEVKFVPASILDLWNLQTIDLGPWSHDIPISFPMQ
IWKLKHLRHLNSRRPIKLRGSSSGSNEKMWNLQTISPLELVI

>g12737.t1

MTESVVTFVLDHLAKLATRETNLLFGVKDKVESLRSELEMIKELLNTTKRKEGTEQIALDQIRDVAYSAEDVIDTFVAQVV
LYKRRTIMGKMLHGFGQVKLLFHVAHEIDKLKTRLNEILDNKEKYHVSKTANESAVEEEEEKKRLRWLHKLRRNVEEKDV
VGFVQDSKDVVKRLLGDSSNHKVISIVGMGGLGKTTLARKVYNNSQVKNHFDCLAWVYVSNECRVRELLLGLLKQLMP
NFEQQCRDINSLNEEELSNMVRNCLEEKRYLVVVDDLWKRQDWDEVKDAFPDNNRGSGILITSRSKEVATHAGHDVP
HYLKFLNEEESWELFRKKVFSDEDCPSDLKSLGKQMVKRCRGLPLSIIVLAGLLANKEKSHREWSKVVDHVNWYLIRDET
QVNDIVLKLSYDNLPKRLKPCFLYLGLFPEDFEIPVASLLQKWVAEGFIQDTGDRDPDDVAEDYLYELIDRSLVQVARVKL
NGGLEKCQVHDLLRDLCISESKKDKVFEVCTKNNNVDSTKPRRLSIHSAMRDYISSSKNDHSCIRSLFFFGLHYDAWKWL
LDNFKLLRVLEFGLNSRHEIPSNLGNFIHLRYLRIKCVYFIPDSILNLWNLQTIDLVTSRDMLRVSFPVKMWKLKYLRHLNT
SEPIEFRGTCSQSVEKMWNLQTISPLILNKQAISLIKNETFPNIKRMGMYVNCEGEGELSNILQNLQQLEHLSSLVILPRTL
LGCKPQELVQRLGQFSRLTILVIDNAKNLLTSELIFPPNIIELTLSNIHSISNEGMNGLGNHSKLKILRLLGNIMVSMDPTDL
NCVGFPQLEVLEMKLLRLRKWALGNGTLQRLQHVIIDRCRPLDNLPTELCCLNGLRKLHLNNYPSEVTGQMDDILRILET
NNRVKVFRGHFPSWDEENNWIS

>g12738.t1

MEEIVVSFVLDHLAQLVAREVNLLYGVEDGVESLRRELEMIKELLNTTRRERGMEHIVLNQIRDMAYLAEDVVDTFVAK
VSIYKRRTILGKTLHGFGQAWLLHRLSDKIDYIKNTLKEIRDNKDTYDAFRETTNESAAEEERERLEWLRTLRRDVEVKDV
VGFVHDSEVVVNRLMGDSSKLKVVSIVGMGGLGKTTLARKVFNNIQVKNHFDCLAWVYVSNECRFRELLLGLLHQFM
PNFEQECRDINSRNEDELKNMVKNCLEGKRYLVVVDDLWNIEDWDKVKGAFPDNNKGSRILITSRSKEVATHAGLDVP
HYLPFLNEEESWELFHKKVFIDGDCPSDLEPLGKQMVQRCCGLPLSIIVLAGLLANKEKSHTVWSKVVDHVNSYINQDKT
QVNEIVLKLSYDNLPRRLKPCFLYLGLFPEDSEILVEPLLQKWVAEGFIQDTGDRDPDDVAEDYLYELIDRSLVQVAGVKL
NGSLEKCQIHDLLRDLCISQSKKDKVFEVCTNNNIVDSTKPRKLSIQSATSDYISSSKRDHSCVRSLLLFGTHDDEWKWLV
DEFKLVRVLQFGSIYLQAIPSILGNFIHLRYLRIENVLVYFVPDSILNLWNLQTIDLGIFKYRQAVSFPAQMWKLKYLRHW
NSSGPIELRGRCLQSVEKMWNLRTMSPLTLNKQAISLIRNGTFPNIKRMGLSGSHDCKDELPNLLQSLQQLEHLNSLVIF
PQGFFDINPQELVQRLEQYSHLTTLRIDWVKDLLTSELIFPPNIIELTLSRIEHISDEGMNGLGNHSKLKILRLLGNLDRFND
PIDLNCVGFPQLEMLKMKGLKLRNWKLSNDAMRKLQRVIIKFCGPYINLPTELCSLNGLKQVHIESNIVQVTEQVHDILRI
LETDNRVQVFRSFYPALDEENDWESHY

>g12739.t1

MADNVVTFVLDHLAQLASREANLLYGVEDRVQSLHYELQMIKDLLNTTKRKKGMEHTVLNQIRDVAHLAEDVIDTFVT
KVSIYKRRTILGMMLCGFGQVRLLHHVGHKIDKLKTTLNEIRDNKDKYDVFKETTNQSAAEKEEEKERLQSLHKLRRDVE
EEHVVGFVQESKDVAKRLLGGGSNRKVVSIVGMGGLGKTTLARKVYNSSQVMNHFDCHAWVYVSNECRVTKLLLDLL
KHLMPNFEQQCRDNNKDVNNLGKEELKKHVWNCLERKRYLVVVDDLWKRRDWDEVQDAFPDNNS

>g12741.t1

MLILLRSDNRGENCPSDLETLGKEMVQSCRGFPLSIIVLAGLLANKEKSYKEWKKVADDVHWYLTRDETQVKDIVLKLSY
DNLPKRLKPCFLYLGLFPEDFEIPVTPLLQKWIAEGFIQDTGNRDPDDVAEDYLYELIDRSLVQVARVESNGDLKMCQVH
DLLRDLCISKSKEDKVFEVCTDNNILIPTKARRLSIHSDMGHYISSSNNDHSCIRSLFFFGPYYQVHRREWKWLLDKFKLVR
VLEFGSNNYEKIPSNLGNFIHLRYLRIDSRLVSFVPDSILNLWNLQTIDLGISRHNNPISFSIQRRNLKDTRPNRRDIPISFPI
QMWKLKHLRHLNTKGPIELRGNSSQSDEKMWNLQTISLLMLNKQATSLIKKGTFPNIKRMGLEVDGYEELTLSGIKCIT
DEGMNGLGNHSKLKILRLLGNIFESGDSIYLNCVEGSFPQLEVLQMKYLRLQKWELGNGAMRRLQNVLIQELRTGKKM
MVKARDEDGGAWRWSQRHHGNDILGFVCRRYVVIAVVA

>g12742.t1

MAESVVSFVLDHLAQLASREVNLLYGVEDRVESLQYELQMIKELLNITKRKKGMEHAVLNQIRDVAHLAEDVIDKFVAK
VSIYKRRTIMGRMLRGFGQARLLHHVAHKIDKLKTTLNDIRDNKDKYDAFKETTNQSAAEEEEEEKERLQSLHKLRRDVE
QEHVVGFVQDSKDVAKKLLRGGSNRKVVSIVGMGGLGKTTLARKVYNSSQVKNHFNCHAWVYVSNDCRVKELLLGLL
KHLMPNFEQQRTGNTKGKKSAEEDVDNLSEEELKKMVWNYLDRKRYLVVIDDLWKRRDWDEVQGAFPDNNKGSRIL
ITSRLKEVALHAGHDVPHYLQFLSTEESWELFCRKVFKGENCPSGLETLGKQMVQSCRGLPLSIIVLGGLLANKEKSDRE
WSKVVGHVNWYLTRDETQVKDIVLKLSYDNLPRRLKPCFLYLGLFPEDFEIHVTPLLQKWVAEGFIQDTGNRDSDDVAE
DYLYELIDRSLVQVTRVRSNGSLKTCQVHDVLRDLCISESKEEKVFEVCTDNNIIIPTKSRRLSIHSDIGHYISSSNKDHSCIRS
LFFLGPSYSLLGKEWKWLLNDFKLVRVLEFGPNSCQKIPSTLGNFIHLRYLRIDSMNATFVPDSILNLWNLQTIDLGILKGH
SPISFPIQMWKLKHLRNLNTAGPIKLRGRCSESDEKMWNLQTISPLILNKQATSLIKKGTFPNIKTMSFSVKAKDYDGELP
NLLQNLQQFKHLNKLVILPRFWCGCKPRELVQSLGQLSCLTILMIDNVLDLLTSELIFPPNIT

>g12743.t1

MAESVVSFVLDHLAQLASREANLLYGVDDRVQSLQFELQMIKDLLDTTTRKKGMEHTVLNQIRDVAHLAEDVIDTFVA
KVSIYKRRTILGRMLRGFGQARLLHHVANKIEKLRTTLNDIRDNKDKYDAFKETTNQSAAEEEEEVERTQSQLKKLRRNV
EEEDVVGFDQDSKDIIKRLLEGGSNRKTVSIIGMGGLGKTTLARKVYNNGQVKNHFNCHTGSKKGKKSVGDINILSEEEL
KKQVRNCLERKRYLVVIDDLWKRRDWDEVQDAFPNNNRGSKILITSRLKEVALHAGDDVPHYLQFLNEDESWELFCKK
VFRGENCPSDLEILGKQMVQSCRGLPLSIIVLARVLPYKEKSNKEWKKVADDVNWYLTRDETQVKDIVLKLSYDNLPRRL
KPCFLYLGLFPEDFEIPVTPLLQKWVAEGFVQDTGNRDPDDVAEDYLYELIDRSLVQVARVKSYGGLETCQIHDLLRYLCI
SESKEDKMFEVCTGNNILIPTKPRRLSIQSDMGHYISSSNNDHSCIRSLLFFGPCYHIHEREWKWLLDDFKLVRVLEFGPN
RCQKIPSNLGNLNHLRYLRIDSRSATFVPDSILNLWNLQTIDLELTLSGIKYITDEGMNSMGNHSKLKFLRLLGIMSESGDS
IDLNCVDGGFPQLEVLEMKYMSIRKWELGNDAMRRLQKLFIDCEQLDNLPTELCSLKELRKVQIRSNGSHLNNFENKK
WS

>g12744.t1

MADSVVSFVSDHLGQLVACEASLLYGVKNKVQSLQNELEMIKELLNTTNIKEGTEHITLDQIRDVAYSAEDVIDTFVAQV
AIYNRRTIREKMLHGFGQVKLLFDVAHEIDKLKTRINEILNNKDKYHVSKTVDECKAEKEEEKKRLEWLQRLRRDVEVKD
VVGFVRDSEDVVKRLLGDSSNRKVVSIVGMGGLGKTTLARKIFNEVKSHFDCFAWVYVSNECRVRELLLGLLKELMPDF
EKQCGGINNQNEEELKNMVEKCLEGKKYLVVVDDLWEKEHWDKVEGAFPDNNRSSKILITSRSKEVATHAGLDVPHYL
QFLNEEESWELFRKKVFINGDCPSDLEELGQQIVKSCRGLPLSIVVLAGLLANKEKSHTVWFKVVDHVNSYINQDKTQV
NEIVLKLSYDNLPRRLKPCFLYLGLFPEDFEIPVAPLLQKWVAEGFIQDTGDRDPDDVAEDYLYELIDRSLVQVTRVKLNG
SLEKCQVHDLLRDLCILESKKHKVIEVCTNNNIVDTTKPRRLSIHSAMHDYISTSKRDHSCIRSLFFFGTHYDEWKWLVDD
FKLVRVLEFGPISHQAFPYNIGNFIHLRYLRIKDVDNHFVPDSILYLWNLQTIDLGPIELRGTCSKPVEKMWNLQTMSPLV
LNKQAIFLIRNGTFPNIKRMGMYGNRDGEELTLSKLYWIRNEGMNGLGNHSKLKILRLLGVFECLIDLNCVGFPQLEVLE
MQDVSLRKWEIDNGAMRKLQHVIIDRCQPLEFKSLSEKFHFGMKRQIGYLKYC

>g12745.t1

MADNVVTFVLDHLAQLAAREANLLYGVEDRVQSLRYELEMIKELLNTTKREKGMEHTVLNQIRDVAYLAEDVIDTFVAK
VVIYKRRTILGRMLHGFGQARLLHHVADKIDTLKTTLNEIRDNKDKYDAFKETTNQSAAEEEEKERLQSLHKLRRDVEEE
HVVGFVQDSKDVVKRLQGGGSNRKVISIVGMGGLGKTTLARKVYNSSQVKNHFDCRAWVYVSNESRVRELLLGLLKHL
MPNFEQQRRGNKKGKKSAEDINILSEEELKKQVWNCLERKRYLVVVDDLWKRRDWDEVQDVFPDNDTGSRILITSRLK
EVALHAGDDVPHYLQFLSEEESWELFRGKVFKGEKCPSSLETLGKQMVQSCRGLPLSIIVLAGLLANKEKSHREWSKVVG
HVNWYLTRDETQVKDIVLKLSYDNLPRRLKPCFLYLGLFSEDFEIPVTPLLQKWVAEGFIQDTGNRDPDDVAEDYLYELID
RSLVQVARVEMNGSLATCQVHDLLRDLCISESKEDKIFEVCTDNNILIPTKPRKLSIHSDMGHYISSSNNDHSCIRSLFFFG
PHYRVRGREWKWLLDNFKLVRVLEFGLDSFKKIPSNFGNFIHLRPISFPIQMWKLKHLRHLNTSGPIKLRGRCSESDEKM

WNLQTISPLILNKQATSLIKKGAFPNIKRMGFFFETDDYEGRRSCSGGCVDRPTAKLSLVLQPEVTSQRCYCDVRDFLDLI
Y

>g12758.t1

MAESVVSFVLDHLAQLAAREANMLYGVEDRVQSLQYELQMIKELLNTTERKKGMEHTVLNQIRDVAQIAEDVIDTFVA
KVVIYKRRTIMGKMLGAFGQAKLLCHVAHKIDKLKTTLHDIRDNKDKYEAFKITINQSTAEEEEEKERLQLLHKLRRDVEK
ENVVGFVQDAKDVVKRLHGSGSNRKVVSIVGMGGLGKTTLARKVYNSSQVMIHFDCRAWVYVSNKCRVRELLLGLLK
QLMPNFEQLRRGNKKSKKSAEDVNSLSEEELKEQVWNCLERKRYLVVVDDLWKRRDWDEVQDAFPNNNRDSRILITT
RLKEVALHAGDDVPHHLQFLSQEESWELFCRKVFRGENCPSGLETLGKQMVQSCHGLPLSILVLGGMLAKKEKSHREW
SKVVDHVNWYLTRDETLVKDIVLMLSYDNLPKRLKPCFLYVGLFPEDFEIPVTPLLQKWVAEGFIQDTGNRDPDDVAED
YLYELIDRSLVQVARMDFNTGLETCQVHDLLRDLCISESKEDKVFEVCTDNNIQISTKPRRLSIHSDMGHYISSSNNDHSG
IHSLFFFGPRYHVHEREWKWLLDDFKLIRVLEFGPNSYQTIPSNLGNFIHLRYLRINIVNATFVPDSILNLWNLQTIDLTPM
MFKISISFPVQMWKLKHLRHLNTSRPIELRGRCLKSNEKMWNLQTISLLMFNEQAASLIKEGTFPNIKRMGLYVEGHDY
EASELMLPPNITELTLTRIRCITDEGMNALGNHSKLKILRLMGRVGSVSYETFTNSKMGIRQWCNAKASKCDY

>g12760.t1

MAENVVTFVLDHLAQLASREANLLCGVEDRVQSLQYELQMIKELLNTTKRKKGMEHTVLNQIRDVAHLAEDVIDTFVA
KVSVYKRRTIIGRMLRGFGQVRLLHHVAHKIDKLKTTLNEIRDNKDKYDAFKETTDQSAVEEEEKERLQLLHELRRDVEEE
NVVGFVHDSKDVVKRLLGGRSNREVVSVIGMGGLGKTTLARKVYKSSQVMSHFDCHAWVYVSNECRVHDLLIDLLKH
LMPNFEQQRRGKKNAGDIKDVSKEELKKHVCNYLERKRYLVVVDDLWKRRDWDEVKDAFPDNNKGSRILITSRLKEVA
LHAGDDVPHHLQFLSEEESWKLFCRKAFRGKNCPSDLEPLGKQMVQSCRGLPLSIIVLGGLLANKEKSDREWSKVVDH
VNWYLTRDETQVKDIVLKLSYDNLPRRLQPCFLYLGLFPEDFEIPVTSLLRKWVAEGFIQDTGNRDPDDVAEDYLYELIDR
SLVQVATVKLNGSLETCQVHDLLRDLCISESKEDKVFEVCTDNDILIPIKPRRLSIPSDMGHYISSSNKDHSCIRSLFFFGPYY
YLDEKEWKWLLDDFKLVRVLEFGSNIGGNYLPSNLGNFVLLRYLKIDSIFGFVPDSILNLWNLQTIELGCWDRVSWDGI
MPVSFPIQMWKLKHLRHLNSQGPVKLRGSCSPSSYEKMWNLQTIFPLLLNKQATSLIEKGTFPNIKRLRFVDVDGFEELT
LSGIKCITDEGMNSLGNHSKLKVLRLRGKVMELEGSYDLNCADGGFPQLEIQIYTLVHAVMEEIVVSFVLDHLAQLVSRE
VNLLYGVEDRVESLQRELEMIKELLNTTRREKGMEHIVLNQIRDLAYLAEDVIDTFVAKVSIYKRKTIMGKMLRCFGQVRL
LHRLSDKIDYIKTTLKEIRDNKDTYDAFRETTNESAAEEERERLEWLQRLRRDVEVKDVVGFVHDSEVVVNRLLGDSLNR
KVVSIVGMGGLGKTTLARKVFNKSEVKNHFDCLAWVCVSKECRVREFLNGLLHQFMPNFEQQCGGINNRNEDELKN
MVENFLEGKRYLVVVDDLWNIEDWDKVKGAFPDNNRGSRILITSRSKEVATHAGLDVPHYLPFLNEEESWELFRKKVFI
NGDCPSDLEPLGKQMVQRCCGLPLSIIVLAGLLANKEKSHTVWSKVVDHVNSYINQDTQVNEIVLKLSYDNLPKRLKPC
FLYLGLFPEDFEIPVASLLQKWVAEGFIQDTGDKDPDDVAEDYLYVLIDRSLVQVTRVKLNGSLEKCQVHDLLRDLCISQS
KKDKVFEVCTNNNIVDSTKPRKLAIQSDMRDYISSSKRDHSCVRSLFFFGTNHDEWKWPLDDFKLVRVLKFGSITSQEIP
SNLGKFIHLRYLKINFIFGSTFVPDSILNLWNLQTIDLGLIELRGRCLQSVEKMWNLQTMSPLILNKQAISLIRNGTFPNIKR
MGLSCSDECEDELPNLLQSLQQLEHLNSLVIFPQGLPDFKAQELVQRVYFDKD

>g12761.t1

MADSVVSFVLDHLAQLAAREANLLRGVEDRVQSLHYELQMIKELLNTTKRKKGMEHTVLNQIRDVAHLAEDVIDTFVA
KVSIYKRRTILGRMLRGFGQARLLHHVAHKIDKLKTTLNEIRDNKDKYDAFREATNQSTVEEEEEKERLQSLHKLRRDVEE
EHVIGFVQDSKDVVKRLREGGSNRKVISIVGMGGLGKTTLARKVYNSSKVKNHFDCRAWFYVSNECRVKELLLGLHKHL
MPNFEQQHRGNKKGKKSVEDVNSLSEEELKKQVRNCLEGKRYLVVLDDLWKRRDWDEVQDAFPDNNRESRILITSRL
KEVALHAGDDVPHYLQFLSEEESWELFCEKVFRSEKCPSDLEILGKQMVQSCRGLPLSIIVLGGLLVKKEKSDREWSKVV
GHVNWYLTRDETQVKDIVLKLSYDNLPRRLKPCFLYLGLFPEDFEIPVTPLLQKWVAEGFIQDTGNRDPDDVAEDYLYDL

IDRSLVQVARVEMNGVLKTCQVHDLLRDLCISESKEDKVFEVCTDNNIPIPTKPRRLSIHSYMSHYISSNNNDHSCIRSLFY
FGPHYDVHGREWKWLLDDFKLVRVLEFGASSCRKIPFDLGNFIHLRHKSF

>g12762.t1

MAAKSISGDVVHAGSAAAPSFLLPRGRGKYRRPSVMRPTITSKASRPRIDGWWDNVMRKLVLLVAGGKERDSTLMCD
EEIGKEIGWTIMYDYRNCKSLVLFFHHQPHIQIYTLVQVVMADSVVTFVLDHLAQLAAREADLLCGVEDRVQSLQYELQ
MIKELLNTTKRKKGMEYTEENHSGEDAPWLWPSKVAPPRSRQNRQAQNHSQEILDNRDNYDAFKETTNQSVAEEEEE
KERLQSLHKLRRDVEEEHVVGFVQDSKDIVQRLQGGGSNRNVVSIVGMGGLGKTTLARKVYNSSQVMNQFDCRAWV
YVSNECRVRELLLGLLKLVMPNFEQQSKGNKKGQKSVEDVNNLSEEELKKQVWNCLERKRYLVVVDDLWKRRDWDEV
QDAFPDNNIGSRILITSRLKEVALHAGDDVPHYLQSLSAEESWELFCRKVFKGENCPSGLETLGKQMVQSCHGLPLSIIVL
GGLLAKKEKSDREWSKVVGHVNWYLTRDETQVKDIVLKLSYDNLPRRLKPCFLYLGLFPEDFEIPVTPLLQKWVAEGFIQ
DTGNRDPDDVAEDYLYELLDRSLVQVARVKSNGSLAKCRIHDLLRDLCISESKEDRVFEVCTDNNILIPTKPRRLSIHSDM
GHYISSSDNDHSCIRSLFFFGQQYDLRGREWKWLLDSFKLVRVLDLGRNRCRKIPSNLGNFIHLRYFRIRSMRTTFVPDSI
LNLWNLQTIELKPIKLRGICSKSNEKMWNLQTISDLLLNTQSTSLIKKGTFPNIKRLSLYVKADSYEELMLTGIKCITDEGM
NVSHNWKCWK

>g12864.t1

MEEFFLSIATKIAEYAVHPILHHAQYLCCFNNFASNLPKAKEQLELTRDGVKERIREAINRVEKVEPTVEKWLKDVQKVLE
EVQMLEERILSLNKSYFRRQCQYSFAKEIEKKTTEMIQLHRNSKFKPFSRITELPGMQYYSSKDFFMFNSTEASYKKLLEAL
NNKSAFIIALVGLGGSGKTTLAKEVGKKAEKMKLFEKVVFATVSQPLNIRSIQDQIADQLSIELKEASEIGRAQRLSERLRK
GTTLLILDDIWEILNFEALGIPLDESSKACCVLITTRNKEVCTSMQCQSTIELHLLSDEEAWTLFRRYANITDDSSEALKGVA
RKIVNECKGLPIAIVTVGSTLKYKIIANFESALSRLQNSKPPHIPEGSTSPYVCLELSYKNLTNPLAQSLLLLCSMFPEDCEIDL
EDLFRFGREFDIIGTMFGTMENARREMDAAIDMLKNCFLLMHAKEKQRVKMHDLVRDVALWIASKSGKAIFTRTEVD
PRALADDETLKDRKAIAVWGLKNYDVLNYTINRPILETLLLSFYVTGGVKVSDGCLQSLENLKTLAILNSVWWKHDALPL
QESLKSLKNLRTLCLRGFDLGDISFVEGLQALEILDLRGSYFDDLPIGIVELKKLRLLDLYECRIMKDKNVEFYENVGKCLQL
EELYLYLRYCRKPSPRDVSLSRLQRYVIETGRSDYETVRSDSDTTQLMKKYGQPRSLIMNWFDVAPQSFISLPIKDLFIRAE
FLCLKDLREDYKNIIPSMDPQGMNQLIALTLYNGSVECLVDSTEKDKTEVIAFSSLVYLSLVGIYSLREVFCDPSSRCSLKNL
QELKIQNCGELYSISFPRNSKLCNLKVLSISECPMLTCLFTSSVVQTLELLEDLRIYCCTSLRHIIEEENDVLSNTQSHSSLKLP
KLRNIDIIECDNLEYVFSVVLFEGLSNLECVNIRSNPKLKYVFGSEKEHNVAGRME

>g14171.t1

MVVAMEVARDGCHGSLQARLHGDMVTLDDLCDNDGVRKRLASVFTTIKATLADAEEQQFSNRSIKDWLQKLKEAAYI
LDDILDECAYEALRLEYQGVKFCPSNKVKCSGLSIFHPKRTVFRYKIAKKMKRICERLEEIADERTKFHLAEMVPERRSGVIE
WRQTSSFISEPHVYGREEDRDKLIDFLVHDASHDENLSVYPIIGLGGLGKTTLSQLIFNHERVRKHFEPRVWICVSEDFGL
RRITKAIIEAVSGHACEDLDLDPLQRKLQDLLQNKRYLLVLDDVWDDDPESWERLKSVLACGALGASVLVTTRLTKVAAI
MGTVPPHELSNLSDNDCWRLFKHRAFVVDEVEREELVVVGKEIVKKCGGVPLAAKVLGGLLRFKREVREWLKVKESNI
WGLTHNIMPALRLSYLNLPIHLKQCFAYCAIFPKDERIEKQYLVELWMTNGFISSDGKLDAEDVGDGIWNELYWRSFFQ
DIEKDEFGEVESFKMHDLVYDLAQFVAEEVCCITNDDDIHALFERKRIHHFSDYGWEFHPAQLHQVKSLRTYLGKNVQL
SPDVVKCYSLRLLQFKPRKELLSAIGDLKHLSLVRLKALQQLSLKDCKLLSRLAPHIGKLNSLRNLSMYLVGEGRGFLLAELG
PLKLKGCIDIKHLERVKSINDAKESNMSSKQLNKLTLRWKGFREGELARNDEEVLEALKPCTQTLQSLRLEGYQGLNFPQ
WMSSPCFKSLTYLKLWFCRNYIKLPVLRKLPSLKRLVIGGAKYVKYVQEESNDDDDDHVAFMALEYLSLESLPSLIRLSSED
GENQFPCLSTLKVNDCPNFSLHGLHSLKTLEIMRLPKLKVWAGLQCLTSLEVLGITGCDEVEGLQYMTALKKLSLRNLPN
MESLPDCFGELSLLRELSIVGCYKLMCLPTSLSLSTVEVLSILDCNIELSKRCEKENGEDWPIIAHIPHLYV

>g14491.t1

MAESAVSFASKHVLPKFLEAVKMLRDLPKEVAEVVDELESFQDFIHDADKVAEAEEDKNRRDRIRKRLMKLREAAFRME
DVIDDYVICDEKQPEEDPRCAALLCETVEIIKTQIHRLQIAYQIQDVKSLVRAERDGFKNHFPIGPRSDGSRRSENFTWQK
LRIDPLFIKEDEVVGFEGPIHTLKKWLTEGRKERTVISIVGMAGLGKTTLSKQVFDRVHTDFECHALITVSRSYTVEELLRD
MTNKLCKERMEDPPRDVATMNQMSLIEEVRNRLCNKRYVVLFDDVWNETFWDDIELALIDNKNGSRILITTRDEKVVE
FCKKALFFEVHKLQPLSKAKSLELLCKKAFGYGFDGCCPKDYEAVGLDIVKKCECLPLAIVAIGSLLYRKCKSPSEWHLFSQ
NLSSELQSNSELHSVTKILSLSYDDLPHNLKSCLLYFGMYPEDYEVKCGRLIKQWIAEGFVKHESGRNLEEVGQQHLMELI
SRSLVLVASFTTDDKAKTCRVHDSIHEMIREKIKNTGFCEYIDEHNHLESSGIIRRLTIARNSNGLSGSIEESQYVRSILIFTNE
VSSKDFTRRLLAKYMRLRVLDFGYAPLYDVPENLGSLIHLKYLSFRGTFISGFPKSISKLQNLETLDVRALGEIEVPKEITKLR
KLRCLLGNPISSIAVKDSLGSITSLEKMHVLIIDPDGVVIRELGKLKQLRDLRLTMLRGDQADTLCSSVNEMPLLERLHISLK
YGTEALDLHIKLSLSKLRKLHLYGSLKEFPNWIPRLQNLVKLSLVESRLTNIRLTDLGSMPNLLLLSFDSNSYDGETLHFENG
GFQKLKELQLNGLQQLSSILIDSGALESLEKLQIMSIPQLKAVPSGIQHLKKLQVLDILYMPTEFQQRIDPNGGKEHWMIK
HVPDVHFVTKNRALLLAERAAEIFSSRL

>g14495.t1

MMRDLSKEVAEITDEHGSLLDFIDDADKLADAEEDVKRRDRMKERLMRLREATFRTEDVIDEYCIRVKEKQPQHGPRW
AASLSKTVHSIKTFILRLQIACKIQDEKSVVRDEKVGFESQYFSEKILNSSRENKNVTFNQLRMDPLFMKEEEVVGLAGPTE
RLTDWLTNGREERTVISVVGIAGVGKTTLAKHVFDRVHRYFECHALITVSQSYTVEELLRNMVRKLCKERKEDPHCKIST
MDRGSLIEEVRDRLHQKRYVVLFDDVWNEKFWDDIQSALIDDNNKSRIIITTRHEKVAVFCKKSSFVEVHKLEEPLKDES
FRLFCRKAFKYGSAGGCPEELKDLSLEIVRKCKGLPLAIVAIGGVLSQKDENAKEWRLFSQNLSSELKRNPDLNIITKIIGLSY
DNLPSHLRLCLLYFGMYPKDYEIESERLVRQWVAEGFVTHEEGKTLEEVAHEYLLGLVRRSLVQVSSFSMEGKVKKCRVH
DLIHDMIRTKMKDKCFGEYVGGGHDQSETSELVRRLTIQANNDLNRRIKRSHIRSIIVIPGKKEELLSVHLVRKIPTDYMLL
KVLDFEDCGLLCVPKKLGNLIYLRYLSFRGTQIKVLPKSIGKLVNLETLDIRQTQVCEVPKEIRKLRKLRHLLTPDSVSSSIQW
KDIGGMTMLEKIPQVRMEDDGVAIREVGKLKQLRNLRVLCFDGEHITLLFYSINEMEHLQALRIEKSNDGIVVDLNTTSTI
SELRKLFLDLKLEKLPNWIPQLHNLERLTLRHSDLTNDPLESLKDMPSLLVLSLSHAYEGQSLHFQPGGFKKLRMLNLEHL
WNLNSILIAETALQSLEHLKLKGLDELRTVPDGIQHLKKLKFLLVPSMPTQFVKEIDRIGKNQHWNINYGYM

>g14496.t1

MAEQVVLAAGECILKLLWEAVKRLKNLPDDVGKMRDELDELKINLEDKEADGENVATMRNKKKQALKATFLTEDVIDE
YLILVEEKQPQDDRGWAAFPSKAAYHIQTLIPRFQISYKIESNLSVVRRTNESFRNNRPQSTQNNNVTFNQLRMEGLFM
EEQEVVGLAGPTEILKDWLKNGQEERTVIFVVGVPGVGKTTLAKHVFNKVCSDFEHHAFFTVSQSNTVEELWGSMYER
YMKSKPPSDNCILIEEVRGYLRGKRYVLLFDDVWEENFWDDIKKLEEPLTEEECLRLLHKNAGYSSDCPEELKDISLEIVRK
CRALPLAIAVAGGVLDFEDCGLLCVPKKLGNLIYLRYLSFRGTQIKVLPKSIGKLVNLETLDIRQTQVCEVPKEIRKLRKLRHL
LTPDSVSSSIQWKDIGGMTMLEKIPQVRMEDDGVAIREVGKLKQLRNLRVLCFDGEHITLLFYSINEMEHLQALRIEKSN
DGIVVDLNTTSTISELRKLFLDLKLERLPNWIPQLHNLERLTLRHSDLTNDPLESLKDMPSLLVLSLSHAYEGQTLHFQSGG
FKKLRKLNLEHLWNLNSILIDETALQCLEHLNLKGLDELRKVPDGIKHLKKLKFLLVPSMPTQFVQEIDRIWRINYGYMSF
AVLISTPELRFLL

>g14561.t1

MAESAVTILALLAENLLKLLKDEGRILRGVHKDVEQIENLVNKIIPFMKNAEEKVLIEESVKIWMNGLRKVVFRMEDVVH
LYLFKVAKRDGMMKKIITVKHRHRISSEIKDIRKTLDDLFSISKGLQLQPFHGDTLPNIIPRPHFVEESQLVGIEHNMQKFR
DWLAKAKSPLLVVVGPPGIGKTSIVKNVYNKQTKLNQQKKKKDFHFCVWITMTQADSWYPIMEIKEEILKADPRGSTSL
RSATRENLIEKLREYFTDKRCLIVLDDVKELKIWNVIQFAIPQHRVIITTQRDDFPNNIGSDTTVEKIMLEPLSLENALKLFH

QKVKHVQFPELSQFSKEFMEKCNGVPLAIVAISSLLSTMKSANEWQRGYSISCKRLIRLWVAEDFVEGDTQNKLMEEFG
GEYLAELICRGLVHASQVDFDGIPRSCHVYNLMHEIIASICKDQMFCHVLEDVRTPVNSNMDFIHRRLSIIKKNDSATME
RDQKWGKVRSCFVFDDAKKWLLNNHFFSSFEFLIRLDLSDACLSVDVLPEQVGNLLNLKYLSLRNTNIISLPKSIGNLVNL
QTLDLKQTKLHEVKIDKLVKLRHLLAYYVSDQSSELYCLEGLRLSEGVQNLESLQNLSYLDVSGGRIIKGLQKLTKLRKLGIIK
LEAKHGEALCNSIEHMINLCSLSIGALGKQGMLKLQSLKPPLSLKRLYLYGRLGELPTWISTLPNLIRLYLKWSDLKQDPLH
YLKDLPQLLHLELYDAYKGERLHFGNGWLKLKVLYLGLLPNLKTIEIGKGKVPCLEVLKIGRCHQMIRLPKDILNLKHLEKLY
LYDMHEQGISRGYLKGVAVIGTR

>g15197.t1

MMAQAVVSFIVQSLGDLLIQEAVFLYGVEDQVLQLQTELRMMRSYLQDADRRQDENESLRSWISEIREAAYDSDDVIE
SYALREASRRNLPGVWNLIRRYASIINRFIEIHMVGSRVDNVKARISSLTRSLKTYGIKPEKEEPSNSMHARQILRRSYSHVI
EEDIIGVDDDVKKLESYLVDPSKRVVAVCGMGGLGKTTLAKKVYHSVDVRNSFKSWAWAYISQHCQARDVWIGILFRLI
SPSQEQRQEIDNMRDEELAKMLYQVQMEKSCLVVLDDIWNAETWNKLKPAFPHGTSVSAVGSKILLTSRNIDVAFQM
DPSCYLHTPKCLNEVDSWELFQKKSFLKIDDPDYREKEKLGREMVGRCGGLPLAIIVLGGLLASKPTFYEWDTVCQNINS
YLRRANGQEQRLEEVLALSYYELPYQLKPCFLHLAHFPENLEIPTKKLIRIWVAEGIISLAHSEGEGEEALEDVAQRYLTELV
ERCMIQVVEKSSSGRIRSCQMHNLMRELCVDRAYLENFLLEINSRNVDEYRGTSRARSVGKVRRIALFLDQDVDRFFLS
QLKSHHHLRSLLCFHEKTARLSEWGLMKSFFEKCRLLRVLNLEGMQGLGGKLPKEIGYLIHLRFLSLRNTKIDELPTSIGNL
KCLMTLDLLTGNSTVQIPNVIGNMQKMRHLYLPESCGNGIERWKLDNLKNLQTLINFPAEKCHVRDLMKLTNLRKLVID
DPNFGGIFRYPDVQFRHLESLFFVSYEDISVVQVALGCPNLYKLHIEGPIKNFPEPHQLSSKLLKLKLTGSGLLVDPMPTLE
KLPNLRLLELQLDSFVGKQLHCSSKGFAQLKSLVIHDLFNLEEWRLDKGAMPCLRELKIENCTKLEKVPDGLRFLTTLQHL
EIRSMFAAFRTKLEKGGEDHYKIQHVSAVADDGTIVVCCVLASGLTLARWHSKGAAVVHCKFGLRAVFFYVVGDNAWS
SDDVRNGEVHWLLVSRKNLSFSQWWRSGKPTPVLGIYM

>g15372.t1

MEVIAQIVLQNLNSFAQEEFGIIWNLKDDVQQMKRSVSAIKAVLLDAEGKANNLQISNWLEELKDVLYDADDLLNGISS
EAMKRKVIGARKILRKIRVFFSQENHIVYSFKLGHQMKAIQKRLEAIAKNKITLQLTDRPMETPIAYRKQRQTYSFVREDD
VIGRKEEKKLLESYLLDTKVSVIDNVSVLAIVGFGGLGKTTLAQLVFNDNAVQCSFEQKMWVCVSDEFDISKIAEKMIGN
DKNSEIEQLQQDLRNKVRGKKFLLVLDDVWNEDRELWLKFKSLVVEGGKGSAIIVTTRSRTVAKIMATHPPLFLKGLDLE
RSWKLFSRVAFDEGKEPNDMELLAMGRDIVKKCAGVPLAIRTIGSLLYSRNMGRSDWLYFSEVEFSKIDQHKDKIFAILK
LSYDHLPSFLKKCFAYCSLFPKDFVFHKKTLIQLWVAEGFIQPSRDNRCEEDVGHEYFMNLLSMSLFQDVTLDDCGGIFT
CKMHDLIHDLAQLVVGKEYAVAEGKKEHNESRTRYLSSCTSLHFPEKTSSSSNKLRTFILLGQPVYGSQNLGPPPSLQFPF
LLSIKCLRVLTLCGLHLITIPNSIRELKQLRYLDLSMNRFLESLPPDVTSLHNLQTLKLSGCGKLKELPSDINKSLRHLELNDCG
KLTCMPCGLGQLTNLQTLTHFILDSKSKNVDISELSGLNNLRGKLVIKCLDSLRENAALVESANILLEKQHLQDLELRWVL
GETEYWTDPIEIRRKEEKNTWVKDENQMKDEKILKGLQPHHSIKRLVIDGYCGNSVPDWIANLSSLLSLEISNCYCLKSIP
DGIRNLVSLQRLCIYNCSMLEGRCARGHGEEWSKIAHIPLVIVSAFNPTDLRYIN

>g15376.t1

MEVIAQIVLQNLNSFAQEEFGIIWNLKDDVQQMKRTVSAIKAVLLDAEGKTNNLQNSNWLEELKDVLYDADDLLNDIS
SEAMKRKVIGARKILRKIQVFFSQENQIVYSFKLGHQMKAIQKRLDAIAKNKITLQLTDRPMETPIAYRRQRQTYSFVRED
DVIGRKEEKKLLESYLLDTKVSLIDNVSVLAIVGFGGLGKTTLAQLVFNDNAVQCSFEQKMWVCVSDEFDISKIAEKMIG
NDKNSEIEQLQQDLRNKVRGKKFLLVLDDVWNEDRELWLKLKSLVVEGGKGSAIIVTTRSRTVAKIMATHPPLFLKGLDL
ERSWKLFSRVAFDEGKEPNDMELLAKGRDIVKKCAGVPLAIRTIGSLLYSRNLGRSDWQYFSEVEFSKIDQHKDNIFSILK
LSYDHLPSFLKKCFAYCSLFPKDFVFHKKTLIQLWVAEGFIQPSQDNRCEEDVGHEYFMNLLSMSLFQNVTLDDCGDILT
CKMHDLIHDLAQLVVGKEYAFVEGKKEHIENRTRYLSSCTSLHFSEKTSSSSNKLRTFILLGQPLYGRQNLGPPPSHQFPFL

LSIKCLRVLTLCGLHLITIPNSIRELKQLRYLDLSMNKFLVSLPPDVTSLHNLQTLKLSRCGKLKELPSDINKGLRHLELNDCG
ELRCMPCGLGQLTNLQTLTHFILDSKSKNGDISELSGLNNLRGKLVIKWLDSLRENAAVVESANILLEKQHLQDLELRWV
LGETEYWTDPIEISRTQEKNTWVKDENQMKDEKILKGLQPHHSIKRLVIDGYRGNSLPDWIANLSSLLSLEISNCYCLKSIP
DGIRNLVSLQRLCICNCSMLEGRCARGHGEEWSKIAHIPLVIVSAFNPTDLRRQQPLVGVNHHFLAMAP

>g15413.t1

MAVECISGAFLAAAFQVTLDKLASRDIQDYFHGSKLKDKMLKKLDIVLNSINQVLEDAEERQYKSSNVMNWLDQLKEAI
YEAELLLDEVANEASRQKLEAEFQPATSKVRGFFKAFVNPFDMEIASRVEELLENIQFLASQKDMLGLRKGIFSGNEVGV
SWKQSKQLPTTSLVDESSICGREEDKEEIVKILLSHNFTCNQVPIISIVGMGGMGKTTLSQLVYNDQRVLEQFDLKAWVY
VSQDFDVVAITRAILKALGSKGAEEKDLNLLQLELKQRLMGKRYFLVLDDVWNEDYSSWGVLQIPFIYGPSGSRILITTRN
EKAALVMNSSHLYHLKPLEKEDCWKLFSDVAFHDKDATKYPYLVSVGSKIVDKCGGLPLALKALGNILRVKFSQHEWVKI
LESDMWHLSDNDANINPALRLSYHNLPSYLKRCFAHCSIFPKGYEFDRDQLIQLWMAEGLLNCCQINKSEEELGAEFFN
DLVARSFFQQSRRRASCFTMHDLLNDLAKSVSGEYCSQISGSLEKIITKRARHISWSSKINIDDKFLEHISKCNRLRCFVAFK
WEFGRGGLINTDKQRVLLSTLKYLRVLSFHDCLLTELVDDIGNLKLLRYLDLSYTKIKRLPDSICRLHNLQTLILLWCYHLDE
LPIDLHKLVNLRHLDLRMSGINKMPNHIGRLKDLRTLTSFFIRNHDVKELGNLSNLQGTLSIFWLENVTDPADAMEANLK
GKKHLDGLVLNWGNKFGRCNENEDSIIERQVLEALQPNGNLKKLSVLRYDGTSFPRWFGGSHLPNLVSVALTESKFCFV
LPPFGQLPSLKELSISCFYGIEVIGPEFCGNDSSNIPFRSLEILKFEEMSAWKEWCSFEGHSEEGQGLSCLKELSLRRCPWLR
RALPQHLPSLQKLEICECQHLEDSVPKAVSIHEVKFRLCEKLFLKDLPSSLKKATIHGTCIIEACIHQILVNNPFLEELKIHNFH
GPNKKWSSLDLHRQDSLVTLSITSWYSSSLPFALHLFSNLHSLLFHDCPYLESFPEGGLPSSLRKLEIEDCPKLVASREKWG
LFKLHSLTDLRVSDDFENVESFPEDGLLPPTLNVLYLIGCSKLTNTNYMGLLHLKSLNSFYILCCPLLQSLPEAALSNSLSVLY
ILDCPLLKQRYQRDGEHWRKIQHIPSVIIS

>g15664.t1

MAEVAVSTVATKLAELLVEQAAVAVSQLAGVRGQVENLKNELGWMQSFLRDADAKQEGSDRVRLWVSEIRDVAFEA
EELIETYVYNTTMQRQLDKVFRPFHLYKVRTRIDKILSKIKSISDRRETYGVVMTGDVGNNSNERLRQWRQPSPSSEEEYL
IELEDDMELFLTQLLALEPNPYVVSIVGMGGLGKTTLAKKLYNHNKITNHFDCKAWVYVSKEYRRIDVLQRILRDVDGAP
RHEMGRIPEEEFINKLRTVLSEKRYLVVLDDIWGMEVWDGLKSAFPRGKMGSKILLTTRNWDVALHADACSNPHQLRP
LTADESLRLLSNKAFPGTNGIPSELKDLATEIVVKCGGLPLAVVVVGGLLSRKLKSSGEWKRVLQNISWYLLEEQEKIARIL
ALSYNDLPSHLKSCFLYLGLFPEVVNIQTKKLIRLWVAEGFLPQEGEETAEGVAQKYLNELIGRCMIQVGTVSSLGRVKTIR
IHHLLRDLSLSKGKEEYFLKIFQGDVAGPSTKARRQSMHSCDERYDFLKHNAHHSRSLLFFNREYNADIARKPWLPLNFQ
QEKKLNFIYRKFKLLRVLELDGVRVVSLPSTIGDLIQLRYLGLRKTNLEEELPLSIGNLLNLQTLDLSYCCFLKKIPNVIWKLVN
LRHLLLYTPFDSPDSGHLRLDTLTNLQTLPHIEAGNWIVDGGLANMANLRQLGICELSGQLVNSVLSTAQGLRNLYSLSL
SLQSEEDEFPIFMQLSQCTHLQKLSLNGKIKKLPDPHEFPPNLLKLTLHNSHLQKESIAKLERLPNLKMLVLGKRAYNWPE
LTFNSEGFSQLHILRLVLLKELEDWKVEQSSIPRLEYMVIDRCEKLKAIPEGLKAITSLKKLKIIGMPVEFEHKLRTKDISEFA
NTPINRWRNVGDNDNKRPTPSSMEWKHLQLLTLNRSYSFSQSLGMASGN

>g16004.t1

MDNASSLYKLPRMYDVLINFTGEDINRKFVSHLNSVLSTVGLTTFLHHPNAVKSTHIQQPILSHCPVVIVVFTQTYSKSA
WCLNQLQQIIKWQETYCRHVLPVYYEIQPSDVRLQKGGFGKALKTTAQQTFSGQELEDGMSRWRHALTKAANFFGW
DERNHRSDAEVVDKIVKSVVNLPVLSATKFPVRLQSYVEDLIQTIENNSTKVCIIGIGGERGSGKTTLAKAIYNQIHRTFKD
KSFIEGVSTVRGIRQHLRLVEKLLLDVLKQKVEMPSIDVGRTMIRERLSGKRMLIVLDDVSIFSLFNICDCRKWFVDGTVIIL
TSTNKEPLIGSDSVFWVRRMNEEESLELLGNEQCS

>g16005.t1

MDIASSSYKLPRKYDVLINFTGEDIHRKFVSHLNSAFSTVGLTTFLHHPNAVKTTHIQEPILSHCRVVIVIFTQTYSQSAWC
LNQLLQIIKWHQTYCRHVLPVYYEIQPSHVRLQKGYFGKALRATARQTFSGQELKHGMSRWSHVLTKAANFFGWDES
NHRNDAELVEKIVKSVLNLPVLSATKFPVRLQLYVEDLIRTIKTKSTEVCIIGIGGPKGYGKTTLAKAIYNQIHWNFKEKSFIE
NISHVRGIRGLLLLQEQLLLDVLKQKVEIPSVDVGRTMIRERLLGKRVLIVLDDVDNSNNLFDLRDCRKWLVEGTGTEDT
QWMPVKLPSVLINFISVQPTVNSQYLLKKLRWISLHEFSSERLPKNFYEHDAIAIDLKRSLLRFVWKTPQVLRSVKVLNLS
HSKYLTTTPDFTGLPSLEHLIFKYCSRLRKLHRSIGSLNSLILLNLKDCTSLYNLPTEIYDLKSLRTFILSGCSKIDIKEKDIAKLES
LITLIAENTAVKHVPFSIVLASSESGDVCLPAVNDPYCLAHMESGDKVEIFVNFGNGLVVKNTTVYLIYGESKNMRKAFES
KKHGLIRFVKKVVM

>g16007.t1

MDNASSSYKLQRRYDVLINFTGEDIPKKIVSHLNSALSTVGLTTFLHHPNAVKSTHIQEPILSRCRVAIVVFTQTYSQSAW
CLHQLQQIIKWQETYCRHVLPVYYEIQPSDVRLQMGDFGKALKATARQTFSGQELEHGMSRWRHALTKATNFFGWD
ESNHRSDAELVDKIVKSVVNLPILSATKFPVGLQSRVEDLIRTIKKKSNEVYMIGIVGQRGSGKTTLAKAIYNQIHWIFNKK
SFIENISQVIEISGQLRLQEQLLLDILNQKVEIPSIDVGRSIIRERLSGKRVLIVLDDLPGNDELWNCSHNWFSEGTVIIITALP
GVMCNYPVDSVFSIKLMNKEESLELLSWHAFREAKPKEEYEDLARRVVSCCEGLPLALEVVGSCLFEKTKEEWNSLLFKS
PIAVVDIYTGDIYTGEEIIKGTEAMPWLPVKLLTSIESVRPTEISGYLLNKIRWLSSHGLSSECLPNNFYEDKATAIDLKRSLL
RFLWKTPQVLRSLKVLNLGHSNHLTTTPDFTGLPSLEQLVLKYCSRLSKVHRSIGSLNSLTLLNSKYCTSLNNLPTEIYDLKS
LRTFILSGCSKIHIMDKDIAKLESLITLIAENTAVKRVPFSIVSSKESGISKQHLRCSLIGVGAYHQFFNAVSDNIYQVLASSES
GDVCLPAVNDPYCLSHMGEGHSVSFVVPEDRDMKGMTLCVVYLSNPKIIEPEFTTVIIVNYTKCTFQIHHHGTVISFKDE
DWHDIMSNLECGDNVEIFVNFGNGLVVKNTAVYLICGESKDMEKASEPKKHGLIRFVKKVVM

>g16011.t1

MDNTSSSYKLPRKYDVLINFTGEDIRRKFVSHLNSAFFTVGLTTFLHHPNTVKSTHTQEPILSHCRVAIVVFTQTYSQSAW
CLHQLQQIIKCHETYGQHILPVYYEIQPSDVRLQKGYFGKALKENAQQTFSGEELEDGMSRWSHALTKAANFFGWDES
NHRSDAELVDKIVKSVVNLPVLSATKFPVGLQSHGIMRTYPVDFVFRLEGMNAEESLELLSWHAFREAKPKDEYEDLAR
RVVSYCGGLPLALEVVGNSLFRKIQERNMGCDCGLIMQDGIEKMDWFPVKPLPLVTAFRNSPLYVNSEYLLMKLRWLS
LHGFSSECLPDNNFDLHDSIAIDLKRSLLRLFWKTPQVLRSLKVLNLSHSKYLTITPDFTGLPSLEQLILKDCPGLREVHRSIG
CLCYLLLLNLKDCTNLSNLPTGIYELKSLRTLILSGCSKIDLMENDIVQMESLITLIAENKAVKQVVPFSIHFRSSLISVGAYHE
FFNAVSDNIYEVLARSESCDVSLPAVNDPFCLTHMGEGHSVSFIVPKDRDMKAMTLCVVYLSNPKIIEPEFTTIVIVNYTK
CSIQIHNHGTVISFQDKDWHSIMSNLESGDNVEIFVSFGNGLVVKNTTMYLICGESKNMEKASELKKLSLIRFIKKVVSHP
F

>g16012.t1

MDNASSSNKLPRRKYDVLINFTGEDIHRKFVSHLNSAFSTVGLTTFLHHPNAVKSTHIQQPILSHCHVGIVVFTQTYSKSA
WCLDQLQQIIKWHETYCRHVLPVYYEIQPSDVRLQKGDFGKALKETAQQTFSGEELEDGMSRWRHALTKAANFFGW
DERNHRSDAELVDKIVKSVINLPVLSATKFPVGLQSHVEDLIRTINSKSTEVCYIEIRGEEGSGKTTLAKAIYNQIHWTFKKK
SFIENISQVSGIRGRLRLQEQLLLDVLKQKVEIPSIDLGRSMIRKRLSGKRVLIVLDDVSYFSLFDLWNCGKWFGEGTVIIVT
STFEKVVKDHANSFFRVEPLNAEESLELLSWHAFREAKPKEEYEDLARRVVSYCGGLPLALEVIGSTLFEKTKEEWNSLLFK
FRIDGLRPDPEIIKVSIEGSLNEMEKDIFLDICCFFVGESRAYVRKILNGCGVDADIAIRVLIQLKDIGKNRRLWFDKDAKYG
TEPLQWLPVEPPSVRIALGEPIVNSEYLLKKLRWIRLHGFSSECLPNNSYEDNSTTIDLKRSLLRFVWKTPQIHIMDKDIVQ
MESLITLIAGNKAVKEVPFSIHLRCSLIGVGAYHQFFNVVSDNIYQVLSRSESCDVCLPAVNDHYCLAHMGEGHSVSFVV
PEDGILQQHCRCSFIGVGAYHQFFNVVSHTYMRKELYYHSVCQDSSEVISHPRIYSQPLSGSNCCFHPNLFSIWCLHQLH
QIIKWHETYCRHVLPVYYEIQPSDVRLQNGDFGKALKATAQQAFSGQELEHGMSRWRHALTKAANFFGWDESNHRS
DAEPVDKIVKSVINLSVLSATKFPVGLQSQVEDLIRTIKNKSNEVYMIGKGGEEGSGKTTLAKAIYNQIHWKFNKKSFIENI

SQVSEIRGQLRLQEKLLLDVVKQKVEIPNIDVGRSIIRERLSEKRVLIVLDDLPANYELLALWNYSHNWFGEGTVIIITITEFG
VMSKYPVDSVFRIKLMNEEESLELLSWHAFREAKPKEEYEDLARRVVSYCGGLPLALEVIGRKDLWKNRGLWFDYDVTY
VPETLQCLPVEPPSVRIAFGEPIVNSEYLLKKLRWIRLHGFSSECLPNNSYENDSTTVDLKRSLLRFMWKTPQIHIMDKDIA
KLKSLITLIADNTAVKEVPFSIIHNHGTVISFKDEDWHDIMSNLESGDNVEIFVNFGNGLVVKNTTVYLICGESMNMRKA
FEPKKHGLIRFVKKLVM

>g16027.t1

MDDASSSYKLPQKYDVLINFTGEDIHRKFVSHLNSALSTVALTTSLHHPNAVKSTHIQQPILSHCQVVIIVFTQTYSQSAW
CLHQLQQIIKWHETYCRHVLPVYYEIQPSDVRLQKGDFGKALKATAQQTFSGQELEHGMSRWNHSLTKAANFFGWDE
SNHRSDAELVDKIVKSVVNLPVLSATKFPVGLQSHVEDVIRTIKNKSTEVCMIKIVGEHGSGKTTLAKAIYNQIHGTFKEKS
FIENISHVSGIRGLLRLQEQLLLDVLKQKVEIPSVDVGRTMIRERLLGKRVLIVLDDVVSFDLFDVLDCLKLLVEGTVIIVTST
YGIGLGDQANSVFRVKVMNEEESIQLLSWHAFREPKPKEEYKDLARKVVSYCGGLPLALEVVGSTLFERTKEEWNSLLFK
FAMLDMHIVGEIIKSRSYVRKILNGCGVDADIAIRVLIQRKELRKNRRLWFDKDAKYGTEAMHWLPVNAFVGAKPTVN
SQYLLKKLRCISWHGLSSEGLPNNFYEHDAIAIDLKRSLLRFLWKTPQVLRSLKVLNLSHSKHLTTTPDFTGLPSLEHLILKC
CSRLSKVHQSIGSLNSLIFINLKDCTSLNNLPIEIYDLKSLRTFILSGCSKIDIMDKDIAKLESLITLIAENTAVKEVPFSIDNTIDI
APLLSTLANIRSVLVACDTEFQLSKQVTNILDEYFPNITESGISKQHFRCSLIGVGAYHQFFNAVSDNISELLSSSESGDVCL
PAVNDPYCLAYMGEGHSVSFVVPEDCDMKGMILCVIYLSTPNIIEAEFTTVVIVNYTKCRFQIHNHGTAISFKDEDWHGI
MSNLESGDNVEIFVNFGNGLVRCI

>g16029.t1

MDIASSSYKLPRKYDVLINFTGEDINRKFVSHLNSVLSTVGLTSFLHHHNAVKSTHIQEPILSHCRVAIVVFTQTYSQSAW
CLHQLQQIINWHETYFRHVLPIYYEIQPSDVRLQKGDFGKALEETAQQTFSGQELEHGMSRWSHALTKAANFFGWDES
NHRSDAELVEKIVKSVVNLPVLSVTKFPVGLQSHVENLIRTIKNKSTEVCMIRIHGDEGSGKTTLAKAIYNQIHWTFKEKSF
IENISRVSGIRGLLRLQEQLLLDVLKQKVEIPSVDVGRTMIREKLLGKRVLIVLDDVFYSNLFDLTDCTKWLVEGTVIIVTSTY
GIGLGDQANSVFRVGRMNEEESLELLSWHAFREAKPKEEYKDLARKVVSYCEGLPLALEVVGSTLFEKTKEEWNNVLFAI
DGLRPDPEIIKVSIEGSLNEMEKDIFLDICCFFVGKSRSYVRRILNGCGVDADIGIRVLMERNLIKINKNNKFGMHPLLQEI
GLRIIRENYGKDLGNRRLWFDKDTKYGIEALQWLPINAFVNVQQTVNSEYLLKKLRWISWHGLSSERLPNNFYEHDAIAI
DLKRSLLPFLWKTPQVMTSLKVLNLSHSKHITTTPDFTGLPKSGISKQHLRCSLIGVGAYHQFFNAVSDNISELLGSSASG
DVCLPAVNDPYCMAHMGEGHSVSFTVPQDHNVEIFVNFGNGLVVKNTTVYLIWGEAENMKKASEPKKHSLIRFIKKV
CKGVAGKVKMNKKDDKLVRGIMGVDREGLGVDREGW

>g16030.t1

MEEHFANLSKQLEDMSLAQKAHFEQIFKWQQNHEDYVADQFHEFDVCLGNIENRLNLQRPERPPRRLFLHCSGSFIKG
KCRLLASGSLVEGSTLLTCDEGIFVSHLNYALSTVGLTTFLHHHNAVKSSHIQQPILSHCCVAIVVFTQTYSQSAWCLHQLL
QIIKWHQTYCRHVLPVYYEIQQSDVRLQKGDFGKALKSTAQQTFSSQELKHGMSRWSHALTKAANFFGWDESNHRSD
AELVEKIVKSVVNLPVLSATKFPVGLQSHVEDLIRTIKNKSTEVCTIGIGGQRGSGKTTLAKAIYNQIHWTFKKKSFIENISQ
VSGIRGHLRLQEQLLSDVLKEKVEIPSIDHYGTVVIIGSEKELAYVRKILNGCGVDADIGIRVLIQRKDIGKNRRLLFDKDTKY
GTEAMQWLPVKQPSVFAYNEIVHPTVNSEYLLKKLKWISSHGLSSKRLPNNFYEHDEIAIDLKRSLLRFLWKTPQVLRAL
KVLNLSHSKYLTTTPDFTGLPCLEHLIFKYCSRLREVHQSIGSLNSLILLNLKDCTSLNSLPTEIYDLKSLRTFILSGCSKIHIMD
EDIAKLESLITLMAENTALKHIPFSIVLASSESGEVCLPAVNDPYCLAHMGDGHSVSFIVPQDRDMKGMTLCVVYLSNPE
AIEPQFTNVLIANNTKCTFHIHNHGTLISFNDEDWHSIMSNLESGDNVGIFVNFGNGLVVKNTLVYLICGESKNMEKASE
PKKHSLIRFIKKVVM

>g16033.t1

MSSSIPTMEFTFSSSKLQRRYDVLINFTGDDIRKKFVSHLHSALSAVGFNTFLPEENAVKEMHIQEPILDLCHVAIVVFTKN
YAQSAWCLHQLQEIIRWHQTYSRHVLPIYYEVEPSDVRLQKGDFGKAFKETAHQTFSAQQLEDGMSRWSQALNKAAN
FFGWDERNHRSDAELVDKIVKSLLNLPVLSVTKFPVGLHSRAEDVIRIIKSKSKEVCIIGICGKEGSDLWHCRKSFDEGTVII
LTTRYESLMKDKVNCFYRVELMNAEESLELISWHAFREAKPKEEYKDLARRVVSHCEGLPLVLEVIGSTLFERTEEEWHSD
KAYATKILNGCAVDTDIGIRLLMERNLIKVNKNNKLGMHHLLIRMGITIVLDNSKKEHGKNTRLWFDKYERYGTKANKW
FLPERNGEVSVSEYLFEYLLLDDYERYGTKSNKWFLPELNEIEMSANSEYLSQELRWIIEHGFPSQYLRSEFCVHDAIAIDLK
HSLLRLVWKKPQVLRWLKVLNLSHSKYLKETPDFSGLPSLEQLILKDCPGLREIDIMENDIVQMESLITLIAENTAVKEVPF
SIVLANSESSDVSIVAYNDPHWLAHTGEGHSVSFTIPQDRDMKGIALCILYLSNPYKITANECLKSVLIVNYTKCTFQIHNH
GTAISFKDEDWHGIMSNLEYGDKVEIFVSIDPGFVVKNTTVYLIYGESKNMEKESLPKKHSLVRFIKKIVR

>g16034.t1

MDNTSSSYKLPRKYDVLINFTGEDIHRKFVSHLNSALSTVGLTTFLHHPNAVKSSHIQEPILSHCRVAIVVFTQTYSQSAW
CLDQLQQIINWHETYCPHVLPVYYEIEPSDVRLQKGDFGKALKATAQQTFSGQELVHGMSRWRHALTKAANFFGWD
GSNHRSDAELVNKIVESVVNLPVLSATKFPVGLQSHVEDVIRIIKNKSTEICMIEIIGEHGSGKNTLAKAIYNQIHWMFKEK
SFIENISRVSGIRGLLRLQEQLLLDVLKQKVEIPSVELGRTMIRERLLGKRVLIVLDDVFYSENLFDLRDCLKLLVEGTVIIVTSI
YGIRDQADSVFRINLMNEEESLELLSWHAFREQKPKEEYEDLARLVVSYCEGLPLTLEVVGSSLFERTKEEWNSVLFKYAII
DMQMVNKHIVGEIIKSRAYVRKILNGCGIDADIGISVLIQRKDLGKNRRLWFDKDTKYGTEALQWLPVNAFQSLQPTVN
YEYLLKKLRWISWHGLSSERLPNNFYEHDAIAIDLKRSLLPFLWKTPQVLRSLKVLNLSHSKHLTTTPDFTGLPSLEQLIFKY
CSRLSKIDIKDKDIAKLESLITLIAENTAVKEVPFSIVLASSESGDVCLPDVDDPYCLAHMGEGHSVSFVVPEECDMKGMIF
KFQAYAL

>g16035.t1

MDNASSSSYKLPRKYDVLINFTGEDIHRKFVSHLNSVLSTVGLTTFLHYHNAVKSSHIQEPILSHCRVAIVVFTQTYSQSA
WCLDQLQQIINWHETYCRHVLPVYYEIEPSDVRLQKGDFGKALKATAQQTFSGQELKHGMSRWRHALTKAANFFGW
DESNHRSDAELVDKIVKSVVYLPVLSATKFPVGLQSHVEDLIRTIKNKSTDVCMIWICGEKGSGKSTLAKAIYNQIHWTFK
EKSFIESVSRVHGISQHLRVVEKLLLDVLKQKVEIPSTNVGRKMIQERLSGKRMLIVLDDVSNFSLFDIWDCRNWFVEGT
VIILTSTSKEPPRGGNSVFWVERMHAEESLELLSWHAFREAKPKEEYENLARTVASYCGGLPLALEVVGSSLFEKTKEEW
NTVLFKFAKDGRAYVRKILNGCGVDADIAIRVLMERNLIKINKNYKFGMHTLLKEIGVQIIRENSGKDLGKNRRLWFHKN
AKYGTEAMQWSPVNAFDSVHPTVNSEYRLKKLRWIDWNGLSSERLPNNFYEHDAIAIDLKRSLLRFLWKTPQIDIKDKD
IAKLESLITLIAENTVGKEVPFSIVLASTKSCDVCLLAVNDSYCLAHMGEGHSVSFVVPEDDSRYTTMAQQFPLKMKIGM
A

>g16036.t1

MFQIPDANLYCLGWSENRGSSPIADRLSGEKSFYRCTTGVFCPDCSQNRGSIPISEKLAGENSSLQVGSRVFCLGFLTTE
MVSIVSDLAKSTMEKLINATIEQSRYICCFTCITDDYEKERENLIGKKETWEEYARVATRRGDNIRKDVTHWQKQAKELIE
EDTKMKVKCFFGWCPNCIWQYSRGKELESKTEKIKSLMECNFENVGITRDVPDIEYHSSQNYISFKSRKLKFEELLNAFKD
DNSYMVGLQGMGGTGKTTLAKEVGPLGLSLKGYTESERPRKLMDRLTNADKILLILDDVWGDISFEEIGIPFKDNHNNC
RILVTTRDIRICNKMECEKIIQLDILPEEEGWILFQKHAALSDKSSKSILDIGRKISKECKGLPIAIVVIARSLKGQTRKKEWDL
ALKSLQKSMRIPGDDENWSEVYTCLKYSYDNMKNKTAKNLFLLCSLFREDEEIPEELLVRLAIGAGLVEQIDVDYNYDAY
RNEVIVAKYKLIDSCLLLNEEFESVKMHDLVREMALSIANKEIVAVNTSNKNAMTMVEKGKNIKYLLCEGKSKDLFSLKFD
GSKLDILIIYLNEMEDYYVEVPDPFFENVSRFRVLYLSNNTYKESTMSLPQSIQLLTNLRSLLLESFTLGDISILGKLHGLETLE
LVKCWLVELPREIAKLGKLQLLKLNDCMFKRNNSFEVIESCSSLEELYLIHEGISKTSHKVQLPNYQRFCILDFSCFFMTKEF
FARNSLNMFMADEVFSKETFKNLVQKAEILCLNGLEGVWKSLIPDIIFPDDDEEVLVIVKASMLEEVFEGNSDEQVEIPNL
KTAVFAELPSLRQKIEFLTVKHSWVKNGPKFSLSSSLPSFEDILASSKGYFKEDRDISERLIVILCGLYLFGEIGSRMPIGYEVA

TQGEVTSISSKWRHAKIRDSPILKQIDEDNQRQNDVGSNELEEVENQSQETIEDEHNLRIPSIISPIETPMYKTDSPENEE
QMTIPSSNLNMELATTKNVNDGEFERTSNSVVSQNTNTTGSELTYHHVKNISLEMADNLDEDSKTEVASGSELTASKGI
KRTCEEGTISPHAETIVLSTDSESEHGEARVSVPFSVVNIELEDVGVGDIQEASPEKLDEITEVL

>g16040.t1

MVSIVSDLAKSNLEKLINATIEQSRYICCFTCITDDFEKEREDLIAKRKTWEEYARLATRRGDNIREDVTHWQKQAKELIEE
DTKMKVKCFFGWCPDCKWQYSRGKELESKTKEIRRLAERNFENVGITRDVLDIEYHSSQNYISLESRKLKFEELFNALKDD
NNYMIGLQGMGGIGKTTLAKEVGKELKKSKCFNQVVDTTISNTPDIKKIQDDIAGPLGLSLKDYTESERPRKLKDRLTKG
DRILLILDDVWEKISFEEIGIPDNHKNCKILVTTRHIRTCDVMGCKKIIEVDILPEEEGWILFQKYAGLSDNSSKSILYKGSKIS
KECKGLPIAIEIIASSLKGARPLEEWDVALKSLEKSMHVHNADESLRKVYTCLKYSYDNMKDETAKKLFLLCSLFREDEEISE
ELLVRLAKGATLIEKIDDDYSYDECRKKVIVAKYKLIDSCLLLSCKSGRVKMHDLVREMALWIANEEIVAVNTTKKNEMT
MVNEPNANDVIAQEMSVKLENLSLNDTSKILFTLQNVTELRALNIYRCNELKRIIGEDAKNQRKPFFPRLKALIIRECNKLK
CIFPISTSEMVPMLEALLIMEASLLEEVFEGNSDEKVKIQNLKTAGFVELPSLHQKIEFFTVKHCLVRNCPKLCISSFPQDLE
HFSYSIIEEYFKDWHLDKIIRRVLDDHKRQEIRNENRWREDSAIGTEVEAASFKGSELTSSQNNANQSTLANIDEDNKQH
EDVVKESTERVEEEQQIVGKASSMPISPTDFPLHKTHSPVKEEEEMAIPSSNLNTEPPITKDVNDGDFQGTSNSVVTNSQ
NTYSTDQRIKKILTEIAGDLDEDSKTTEIRSQNEMTKAKEDFSIDAKAEVLSGTDFTSSPYEKTIALSTDSESEHGEAQISVP
FPVVNIELETTEDVGVGDIQEASPDKLAEITQDSSSGYTVRRELEELVSKKHLGSENLALLSNFLVKHPSLRLADDAMSERY
KGFAYTCLAQLLKFLQSHSVFDMLGSSHSEFLELLKELRNCGFNKDWLDDVEKLKNVI

>g16319.t1

MAETGTAVASTLIERVVDAIIGRARCLFYFKKSVVDLRKSKRELEKTLQHMRERVKEATTNAEKIVQPVEEWLKDVERVL
EDVQGPEERVEESECCLNMTFKKPVYEEILQAIQDERSNVIGVVGVEDVKLRDIQSQIADHLSFSLMEETELGRALRLSHR
LKTEKILIILDSVWEKLDLEAIGIPLNENEKKDEDEGWDLFKQCAQIDDDSPEELREVAKRVFDKCKGLLVAIVAVARTLKG
KTFTNWELALFRLETSESIDVQEGLTSTYECNELEEIICLDWKDAGQQLRYLYAPSQPVFPKLRKILIKGCIKLRKSSSQAWF
QASHC

>g16357.t1

MSFRTNPKKAIPFVLKRLRMTRTTKTLDKSCDDKLEKLTSQLDQIKDLFMAVKWNEDELLDTLALLDRHLRTIGNIEKEKF
ETDMETISQRIKDSTEKLLPKGWPSQRGTQASVSPKVTTYQDHKTGKLHSPPSSSQHKDELHEENLSQNIEVSFEKLEGY
LKPCLLSLLVFPEDAVINKRHTIYWWIGEGFLRNSGEKTAEEVGEGVVDELLNCQMIVAHGNGLNPVVKKFKVNPRIRG
ELLTLDSEKTLQHLGLSFQEFWNQRAWLKQRKVVLGDDKDNLKPNHWKSIFNVGASYLSFEPEWLAKVKSLEVLQLGR
WQDSPSHHIEVASEEFLKELRGDKQLKYLSLRGISRIPELPPSIAQLENLEILDLKACHSLEALPENIASMKSLTHLDVSECYL
LDSMPRGIEKLTQLQVLKGFVIGSSMKTPCRISDLANLKKLKRFSVHIGSEAVIQEMKFESLKDLTAVKCLKISWGVSGEK
YSDIQVTFPSSLEKLDLEGFPGTAIPEWLKPSRVPGSMRKLYINGGKLKSLDHGEICHKWHVEILRLRYLKQLQIEERKLHK
LFPSLRYVERTKVLNRSFQEWRLEE

>g16360.t1

MSIRTNRMKAVPSLQRRLTSVKSREERNISEKLDRLMFDLTAIKDLFSTVKSNEEELLDTLKLVDDLLRNFKTEKFNEISSRI
LETEKADPQKGSDEPSSSQKAEEKSGKLDETIDEKLENLEGKFKNLKANFSTLKKNEELRTKLTELENFLRNFNSKLADDAS
MEEMNQAFTGAGTDKPSSSQLTKDNKSIVEVKSSSGIAKPSQDSDQSETESSELPRQKSRDEILHQDLKSTGIEKPPSSQ
ETQDEKSIAEKDRTIDEKFESFMSKLDKIKGMSSKENEELRKKLGVMEDLLRKFNSNQSETKNLEPPTQIKRDESVDKGFK
ASGVDKPASNQEPKDENFQEKYVPIDEKLVSLKVELDNIKGKFSPAKENEEMSKTLRTLEDLLRSIDIPAEDAKPTETKGV
DKPSSSQEVQDKESVKKEVSDDEKVESLKSERDNKKADLVTLNKNEENLLKNTNKKVSDENVKRKDSKEEVVDKVVKAI
NKKIKKSTKKLGVEANESSEKGKSSKTVDYPILSLSRDDKTLELVLKRFQASYDALNSHSKVCCLSLSIFPANVVIMKRHIIY

WWIGEGFVKRSTEKTSEEEGENVFDELLNSNLIVAHGSGKCPVVNKFKINPWIRHMLLSSVLGENKQPFGIYSQITTSSH
GNHADYGFLVLDPKKVKIGGDFASKYDHWRSVFNLGARYLTIEPQWMAKMKKLVVLQLGRWHESPEHHIEVAGTEF
MTATKAQKHLKYLSLRGISRISALPPSIAQLVSLEILDLKACHNLETLPNEITSLKKLTHLDVSQCYLLESMPKGIGKLTELQV
LKGFVVGSSDKTPTISDLANFKKLKRLSIHIGSDAVIHAKEFDNLRNSEVKCLKISWGVSSNTKYKNIDILLPQGLEKLNLEG
FPGEKTPTWLKLNLLPSDLKRLYIIGGKLSSIEEDKIKCKVEILRLKYLKNLQIDIHHLRKLFPSLKYAEVYHMDRAHHETERA
SYELNSLGRKTILSRHSSELGFTEDFLFSVITAKPVHLSCTPVRSPPRRTGKDRRLRQDQRTSSRRSAYFPLFRCRFTQSRVA
GQILEANTSYNVLLGRPCLNSFGAIVSTPHLAMKYPTSRGTICTIRADQKVARECYAVGLKMYPKEARRRVSGSEVAMA
DLDPRTNMDGPTKCTFGVAADKFLGFMLTSRGIEANPDKCEAILKMKSPSTLKEVQRLVGRLKALSRFIPKLAERLRPILK
KMKKNSNNAWDDDCESTFCDIKDILVSPPIMNRSTSGEELQVYLGISDAAISAVLLQEQLTPKLIYFVSRTLNEPETHYQQ
VEKVALALLNAARRLRPYFQSHQVVVRTDHPVSKILRKPDLAGPEIPLTNEYEWNLYVDGASGRTISGAGVVLEGPNGFL
MEHSLVFKFKAFNNQAEYEALLAGLQLAKDMGARRLTCRTDSQLVVGQMNGDFPEQNSRADMLSKLSAGNEKGQLT
TIIRQVLLQPLVECHAITISEIADWRTDIRGLMKKQDDGETLRQAETRKIARFILIGDDLYRRGFSTPLLKCLARDEIQYVM
DELHNDICGFHTGGRALKARILRAGYYWPTMENNAMAFTHWCVQCQAHANNKHLHLRLCTPSSHPGRSLSGELPEVL
WAYRCTPHGTTRETPFNLTYGTDAMLPVELGEPSPCRNMEDLRQNDQELRVELDAIDERRDRAVLRAEACRRMVERK
YNTKVRPRSFQEGDLVWRKTGEARRIAAHRKLAAKWEGPFKIIESLGNGAYRLTKLDGRHITNTWNASHLKLYFS

>g16366.t1

MASDTAEVLKENFLEFFKDSESFNLSPTISSILKEIKEEFSEYKGFWSPPPVESLCDLYQLDHAINECRANKKTFSMFSRLDP
VERDNETALNHIKKNLHDMRNSKININEYSTFPLFSSCRYPTGNDAPDLSVFDGEMEKILKWLESNNGFKAIGVHGMCG
TGKTTLAKMVLSDPRVRDKYKKPIWVCLYDLQSKEEMDIRIVKEMLALLDDDPDLLAEEPEDKWLVKNLHDKLLDQKKY
LIVLDAVWHCNDWFNNLFCVDQDGGDTSKELFSQALPKDTGGAVIVTSRQKEVTTKLVREENLIHLKPWGDKKLKNFV
KQCLKKQDKITQENIDCVAYHCHGIPFVAATISGWIAEQITKTSYSN

>g16371.t1

MKAAVVVHQLGNLISNRSILEKLKTDLETLQDQRKKVQENLIWEDEEFQTQSEWVKRVDKILGQGDKLLNSYEGKSTCT
NILLRYKIGKQSRKMQPEILALILEGDSHVSAKTRKHYERPASRDETIGDIMEALKNPDIQAVGVWGLGGLGTTSLAENIR
KKAKEQKLFDAMVFITVTDKPNQEQVQNAIADELGVQFTNGESLVKRRNKLRQRIKKEQSTLIIVDDTWGELNPEEFDL
EEFGVPPGNEHEGCKVLVTSGNLNFIQYLKGASKLNKVFQLEELQKEEARMLFEKMVGSFDEDQSFIVEEIVRSCEGSISL
IYALAKALENKGIDALMQLKENISPAKLLSYCLEENEEHKALLYLLTIRERRFINSYSIYIDMWTGVFKNLETADSARKKRESL
ISDLKAYGLVVENGKDWVKVDDYIYQTAYRMAQHDRRASVISREWPPEELLTDLHFCNLHPVGDLKLRATLQCPNLKH
LLISRENSTIDVPNSFFEETKLLKVLDFVSFHCPNLPHSFVVLKDLEALSMYKCVLGDITEVCELTNLRMLGLLGSSIQQLPA
QIVKLQKLLFLDLRDTNLKVIPPNVLSKLASLEELYLRNSFCNWEIEMSTSENKNASMKELTDLEHLAYIEDMYVPDPRA
WPVDLFFGNLRSYTIFIGEGWDRAHYGDHELKTLKLKLNRRFQSENGIKKMLKEVQVLYLDTMNGVQNVVNDMECD
GFPQLQSLFIQHNAEVKCIATGSGIDPLDTFPNLESLSLTILSNLEYICHAGSLTDKSFFKLRVIKIEKCNAMRCLFSVSMIEG
IPHIATLEVSQCISIKAIVLFEGAENRPIEFPELCSLTLQGLPALISFCSSEGSSSATLFHDKVSCPKLETMVISEVSELTTIWNE
EYDAENSFGKLKNVIIKDCEKLRTVFPVNLSKNLDNLKTLEVRNCRLMTSIFTVMRQDSTKPGLQLSIPMIEITLTGLPKLE
YVCVTTGFEALKKKFEEEWYAGLPGLSVRVASLLLRFSCRFHQCVASSYRAPSFQGCRFSIDCTTASEALALELCWCPRCR
ISAHALGD

>g16628.t1

MDIQEESPVFGSLTAMTTRNMSSSSSVFFSANQSPFFSPRSPSSCQLSHSARLDTQNNTVHLGLAPSSTTLEIPEPNSTV
NVRCNVSDVSASPAGCNSGGLMKLDRKSSPVGISSSSISSYSNCHDDGYSGQRERRIKKDRNHRTSSTPGSTSFSSYRLR
SCDVFIGLHGSKPPLLRFAKWLCGELEIQGISCFVSDRARTRSSRKLGIAERAMDAASFGIVIITKKSFKNQYTIEELNFFCR
RKNLIPIYFDLSPADCLFRDIIEKRGELWEKHGGELWLSYEGLEQEWKDAVHGLSRVDECKLEAQDGNWRDCILRAVTLI

AMRLGRRSIAERVTKWREKVEKEEFPFIRNENFIGRKKELSQLEFILFGDVTGDAEQDYIELKARPRRKSVRIGWGKSNMI
DERWNDRRKEKEPVVWKESEKDIEMQGVEFSHRHNHPRLKRGKYAKRKNGMKILYGKGIACVSGDSGIGKTELILEFA
YRFHQRYKMVLWIGGESRYIRQNYLNIRSFLEVDVGVENSLDKTKIRSFEEQEVAAISRVRKELMKNIPYLVIIDNLESEKD
WWDHKLVMDLLPRFGVETHVIVSTRLPRIMNLEPLKLSYLSGVEAMSLMVGSSKDYSVAEVDALRSIEEKVGRLTLGLAI
ISAILSELPITPSRLLDTINRMPLKEMPWSDKEALSFTKNTFLLQLFDVCFSIFDHADGPRSLATRMVLVSGWFAPGAIPIS
LLALAAEKVPERCQGKCFWRKMLQLLSCGFPSSYAKKPELEASSLLLRFNIARNSTKQDYIHINEVFKLYARKRENTGAAQ
AMIQAIISNGSISQNLDHLWAACFLLFGFGHDPVIVELKVSELLYLVKRVVLPLAIHTFITYSRCTAALELLRLCTNALEAAD
QAFVTPVDKWFDKSLCWRSIQTNAQLNPCLWQELALTRATVLETRAKLMLRGAQFDVGDDLIRKAVFIRTSICGEDHP
DTVSARETLSKLTRLNANVQIHTST

>g17049.t1

MIGLYGMGGCGKTTLAMEVKKIAEAEHLFHKVVFVPVSSTVEVPRIQEKIASSLQYPFPENQEMERAQRLCMRLTQEKI
LMILDDVWEKLDFGRIGIPSSEYHKGCKILITTRLKEVCISMDCQKNIYLPFLTDEESWTLFQNKALISKDTPENINHLAISIS
KECKGLPVAIVAVASSLKGKEEVIWRAALNKLKSSKQINIGKGLRDPYKCLKLSYDNLDDEEAKSLFLLCSAFPEDYEISVEC
LIRCAIGLGVAGEVGTYEEARTEMTAVKMNLVSSCLMMEAGVERVKMHDLVRDVAHWIAKNENKMIKCEVEKGVAV
VNNVFQIINKSPLSSPSILGIERYFISERYEETTKSFLSWLFIAFLP

>g17050.t1

MDCQKNIYLPILTDEEARTLFQNKALISKDTPENIKHLAISISKECKGLPVAIVAVASSLKGKQEVIWRAALNKLKSSKPINIR
KGLRDPYKCLKLSYDNLDDEEAKSLFLLSSAFPEDYEISVECLIRCAIGLGVAGEVDTYEEARTEVTATKIKLVAVVNNVFQII
NKSPLSDPSKLGFERHFIFTTLTNLKLLEFKSCDIESNIFEEIKRIPFLEELYIYKVYDYGNDRKEESVKFSNSFSVPQKLQSITG
GLTQLKELHIINCKVLEQIIEDVVPRAHHEETNEIVEEDVQSTSGSFSLPGLAVLSIKYCQMLESLFTISIAKTLTSLEELDIVQ
CHGLKHLVTLARVKRNKKENMVEDEHEFKSDLSMFSSLKRVHIWGCDSLQDIFTTPFVGDVVNIQNYFPTQQTLRMSN
AYNGGWMMGQQHRFIHNCPKLSLTSTATPPELERNLRLEGLYSKYIESQMDEIKKLDEVSTSNSSTELSSSQINEKSDKR
FTEKDYGPKELAHATSINSEVDEKSNREKFSQELVEEKSPTESYLTDQQNPLGETESTIKMSQEWDPTPKNTSPFQMNR
EDPSTSEIKPCSSQVNDNNQSMLESRVEMVGHHNKIETKTQAPEIEEFQKIERKGEMTSEPQAIEQSFPVISSPNMAQH
IDEIEANNIGKTTTSDKLAIPTSVSESIEEIGRERSRGGAATEGATIKTLSIGGDNISFVSSVTIHKSSAPNILPQDSQVIALFW
SPFLPIFMSDYSSNILQLLQILVQDWKSISISLI

>g17057.t1

MKFDSRKYASNQLMEALKDDGIAMIGLYGMGGSGKTTLAMEVKKIAEAEHLFDSVIFVPVSSTVEVPRIQEKIASSLHYT
FPENQEMERAHRLSMRLTEEKKILMILDDVWEKLDFGRIGIPLSEYHKGRKILITTRSEEVCISMDCQKKIYLPILTDEEAW
TLFQEKALISKDTPENIKHLAISISNECKGLPVAIVAVASSLKGKEKVIWRNALNKLRSSKPINIDRGFRDPYKCLKLSYDNLD
SEEAKSLFLLCSVFPEDYEISVECLIRCAIGLGVAGEVDTYEEARTEVTAAKIKLVAVVNNVFQIINKSPLSDPSKMEFERRLI
FTTLTNLKLLEFKSCDIESNMFEEIKRIPFLEELYIYEDHWNDRKEESVKFSNSFSVLQKLQSITGGLTQLKELDIRNCNMLE
QIIEDVVPPAHHEETNEIVEEDVQSTSGSFSLSSLAVLSIESCRKLESLFTISVAKTLTSLEELKIIHCHGLENFRIPDWQISSW
MDEIKKLDEVSRSNNSTELPSSQINEKLDKNVTEKDYGSKEVAPATSINAEVDGKSNRETYSKDWDPTPKNTSPLQMNR
EDPSTSQIKPFSSQVNDNNQSMLESRVEMRTDKIVANNLGKTTTSDKLAIPTSASEEIGRERSGGGATTEGAPIKTLSIGG
DNINLVSGVTIYKSSSANILIQDSEFVKQDNEMNEDKTEIAPYKNIKIQEGVNLLNKTEGVGIVSNNDIVVTPDTRTRLEKY
KHFVDLNDSQISLLVEAIEAYPHLWNACEKFTDRFRAWMLKTLADMLLFLRSESVGSINPHREKEFLKLCDEAVQLGFER
SWVDEMRQRVLGRDPKLDHAKVFHSSKTAKMVRGEGILMLKSVTDSSRGY

>g17205.t1

MPPESHVTISSYRLCWDVFLSFRGTHTGHTFTMRLYHALHGRGVRVFRNDDGLERRAEIQKKLLEAVEDSAAAVVVISP
DYASSHWCLEELAKICEVGRLILPVFYWVNPSHVRKQEGPFEEWFVWHAQRFPKERVEQWRNAMKKVGGLAGFVLD
EKSDKSDELIQILVQNLMKQLRNTPLSVAPFTVGVDDRVEVLKNLLDLKSNDVRVLGLYGMGGVGKTTLAKSLFNNLVV
HNFERRSFIPNVRSQVSKHHGLVSLQNKIRGDLCGRKEDLINDISDGISAIQKIVQENRVLLILDDVDDVEQLNFLMGKRE
WFYKGSRVVITTRDKEILHGSYVDVDFEVKELEFSEAMELFCFHAIRRKEPAEGFLDVSKQIVEKTGGLPLALEVFGSFLFD
KRTEREWKDALEKLKQIRPPCLQEVLKISFDALDEQEQCIFLDIACLFVQMEMKRDDVVDILNGCDFRGEIAVAVLSARC
LIKIIGDGKVWMHDQVRDMGRQIVRSESLTDPGLRSRLWDRDEILTVLKNMKGTRNVQGIVLDCVKRRMSIPRDRSA
DEITRENFRRKPSCKSAFEYIKERLEGQFICLPPKIKWLQWKQCPLSYMPSSYNPLELAVMDLSESLIETLWKGRSNKVAV
HLMVLNLSRCHRLTGTPDLSGYLSLKKLNLEECSHLTRIHESLGNLNSLVHLNLRLCYNLIELPGDVSGLKHLEDLVLSDCW
KLKALPKDLSCMVSLRRLLLDSTSITELPVSIFHLTKLEKLSANGCHLLKRLPTCTGKLCSLQELSLNHTALQELPDSVGSLEK
LEMLSLMGCKSLSVIPNSTGKLISLTRLYLDGSGIKELPASIGALSYLRKLSVGDCTSLYKFPVSMEALVSIVELKLDGTKVSN
FPEEIFVGMKMLEKLEMGKVQHLKFVPVSFGCLSALTILDMHDANITELPESIGMLENLIRLRLDKCKQLQRLPDSIGNLK
SLRWLMMKETALTRLPESFGMLRSLVELDMKRLPYLNGAGNNVSTGTIIPEIREQPSSEAILTSFCNLSLLEKLNAHGWGI
YGKIPDEFEKLSSLETLSLGHNNICSLPASMTGLSYLKKLLLSDCRELMFVPPLPSSLEELNLENCVAVQYIHDISNLERLEEF
NLTNCEKVVDVPGLEHLKSLRRLYMSGCIGCSLAVKRRFSKNIAENQREGLELVDVQGKIFNLTSEVFSTTIRLLRVPRRNE
DHIFLRRFGARTPLVFQLKDRYTLHLQRRNPPRIEGLELNNCRIHLVFYGDDDYEGDEGSLEESQYSVSQKLAKFFNFDAD
DPDV

>g17510.t1

MSSSRSQPERDILSSRDEGKPHKPSRKSPPESTKDFHGFLKGKKGLDSFVSSMERMLATAESQTPAAEELRDYLERQSKA
TEQFGVPIEDSASHVDHIMLIAEGELTYLRTVLKNFYHSKISDPDQMNLLVDITSVVNYAKRVMDSMLIKTYKKKIQKEIE
YSVGKPHVVRTLERTIKRMREIRERIKNNMQTELVVTVETPCLGREGPKPVVAIALEMLDYILNQNLITREDVIETVLRFRE
GYLGDAQSIILSSFNSLEDRSQRSERVWLQEVKDICDYTVSVAEKFIKRRERSRNVWGVLYPFEQHVVEMNLLKHMEHI
NSQFGDALYRRWTFGDGGKIVMGDIKGSRSKPLTFTCFPQFVYSWRSLDHNLITTRRYLTVMEAFLDDIDGTVEGLNQ
RQKVWVEQLRVVARKGHSLVEEYREETGKEIYGIANIQGRRRESLSWGPSIQGTGREIVDEVKDEDDNSGAEENPLIAQ
PDSFLHRHITELEKEFQSIKGEKELMNALFDDVQKIGYEKLDERSKIWVDQMKEIAPEIDLVIEECKDELKHVTMLNIILES
KAGLHIMDKIKRIRRKIKDVYRSRKAYSLVQRQSRAESLCRKIKPILITKESRMVGFDEDVEILIAQLLSGEKQRCICSIVGTK
GTGKTELARFIYRSEVVLSSFDYVIWVTPSSTAERLKYKIAKKAAKTMGYDQDNWPRRPVLGILSFRRYLIVVDGIQTPHV
LDTLREAIPDRLTASRFLLTTRKAIIAQRAGTSSSFVHPLRLLDDKNSWILFTRNLSVEMNSEPMLQEIGKKLVAKCGGLPL
EILKMSKQLLSEDVTEEESAKELEQNPWSETVDTVNTNLPSYLRRCLFYFELFPADFEIPVRRLVALWVAEGLVTLGEDQE
GPPELVAETYLTELVDLDMVQIAKRKPNGKIKTCRFPNGLRQIFKSTESRIPEVGTSKDLEAVPKNSRIRRVADHLEEKDI
WHTHIHGNSNISARDCKSLRTYYQNVFSFLSFDTRHGSKPGQDIGNFLNLCIWNNCLLQLRVLDLEGVYKPKLPKDIAKL
SGLSSIWKMKLRHLFLSESNRTRFPPKPIGIDMGSSLHDLQTLWGVFVDEETPVKGGLDKLVNITKLGITCQSKSSQQEA
MESQLNAVADWIVKLQYLQTLRLKSRDKQGRPWKIHLKNLQHHTNLTDLYLLGCLSIPLNQLFLPPTLVVLTLSYSKLED
DPMQILKDVPNLRSLSLLAESYLGRTIVCTSGSFSHLRVLRVWKLEQLEEWNRADSTSLSETTGDPIMSTHEILKCTTFHP
TVKSTKMTPSNMRSSLSCQEGSHTGASENSGREENSHAPPDSFSYRNMTEVERELRSIRGEKQLMEALFRDVKDIGWE
KLDERSKIWVEQLQEVALHNILEWKTRFNIVDKITKIGHKIQDVSRSIKAYMVYFNVNLGRMKDSETIKDYADKLLDIANK
IRLLGTKIPDSRIIQEILVTIPKRYETTLTSLENSKDLSTITLAELLATLQAPEQRRLMREEDKWRATNKLQDVGGPKRIPSGK
KVKLVSVKVGESQIKPGKLEEEEHPKTTVKQKLKMRSNKAILKRVWVSVSSRYSYTVEQLREEIAKMAAKVIMGDQTET
WTTKDVLETLESKKHLIIFYGIETLHFLDTLRDTIAACVKSLEAGALRLLVYKPEGTPLSETTGNPIVSGFDKASF

>g17743.t1

MDLEKFIEKGRKYVPFDRIATLPSNTLDMLSEKCMNFESRQYAYEQLLDAVKNNDVSMIGLYGMGGCGKTTLAMEVR
KLVEAEHLFEKVLFIAVSSTVEVRRIQEKIANDVWEKLDFGRIGIPSSEHHKGCKILITTRSEAVCTLMDCQRKIYLPILTDEE

AWTLFQNKALISEGTPHTLKNLGRLISNECKGLPVAIAAVACSLKGKAETVWSVALNKLRRSKPINIERGLIDPYKCLQLSY
DNLDTKEAKSLFLLCSVFPEDSEIQVEILTRCAIGLGVVGEVDSYEEARSEVIAAKIKLVSCCLLLDADNERVKMHDIVRDV
AHIIAQNENKKIKCEVEIDVTVEQNSVRYLWCSKFPNDLDCSNLEFLCLHTIMKGFEGIFKRMGKLKVLLLCNDEDEKTPL
STTSLKTLTNLRDLFISNFELSDFSFLSGTKNLQTLSLYDCSLPSFPELQTDVAITLKLLELNECYIKVKNFEVMKRIPLLEELYII
EIEGEWDANSEDNIEFFKTFSVPQTLQRYGIVLGSDNFYHYNDGDIYIDGRTLLLNHFDISNEVIKGLAKKAKDLFVRNID
GGAKNMIPDIFEIEGGGLNELNTLEIRDSAGIKILIDTRSHSSEVVTLFSKLHTLEMKRMENLKAIWHCFLPTNGPFENLEN
LYLTNIVGGLTQLKVLEIRECDKLDQIIGDIVPLTKQHRKEELDEIVEEGTLPSLTSLKIIYCGKLGSIFTASKAKTLTSLEELFIH
WCKSLKDIVTHEKVNKNQEESIVEDDHISECDSLEVVFPKSYIWADPKNSLTLQHLTTLTIWKCGNMEVIFPKSVVRCLPE
LKKVTIRECMELKQIIEEDGSNLLSIDGGSEVEEIIGCDKEASKNYFAFPNLEKLEIIECVKLEVVFPKSVLRCLPKLKLLKIIQC
KELSQIIEGDINLSNILSPQPCFPKLEALHVDDCHKLKRFFSGSASNDLPNLHLLAINGTYELEELVGCKQGMTKVELPRLKL
LIFMHLPNFRQEIELHNLKNCIVYKCPKLSLTSTTSLEKLLEDFPHKDFINTELGPSELKGIVRSVSEYSTINGSSEFTSSQEIE
VVGNENIKEGSLVEGSKTKSSSTTGVEDIGIGSGVEDIGIYEDATSGSSELTSSQEIEDVGNESIKSSSTGVEDIGIGDAVAT
HIESGGEDILALDSKVVEQDDKMNEGKPRIVAGQRIQVEEGLNLLHKQEGIDVVPNNNIDISSDIRTRLGAYKHFVDMD
DAQISLLVEAITTYPHLWNASKKFSERFQAWRLKILADMLLFLQKESVDSIIPQREKEFHKLCEEAIEVGFESSWVEEMRQ
RVVARDPKLEEDIAKRQIHENSKRCSSGDMVQDSQAVEQGDGPMISLEEGSSLVDKEGEIHVVSNDHILAPRNEEPEQE
FVSEIFTSEIPRIATSLTNSQTVEKPTSSNALVDTQISEPCLMNQKKPLGEIPKSIDQVAVEETIAKNTNMAASSILSESATSK
LDTSVTLQRKSHPHSEIRSSQNEVRITKESEGHPELIQDFGSNDMIPIALGKEGEDNIVVKTLFELENYLKMSLKDIVSSET
NTLRLFSTLNFLSNLPFKDVTLTDQLKHIIETMHQHFPTILCSFKQRFATTHKLAELEARQNEVAVKISEAENFNDEARLKE
VVLKEEIIRLKEEIKVCEATLSSLNEGKNKCIVETIKYKKELENVRKNKSQMVEDQRKVEQELFEMAYKWSVLCSEYELNRI
AARNPS

>g17928.t1

MASAVFVSFNDEDTHDITAFVLDPLRSRGIQVFVKGESTTFDLFQVIERSRLFIVVLSKNYASSICCLRELVAIINTVKSSPRS
ILTIFYGVQESEVLFQDGCYGQAISKHEERFREHKQRMEEVQRWREALTRVACFPGWHMENAKGVEGRRFVQHAVDI
LSREFSTPQNETIFNRYGKFEDIVQYKDGDEVSYKELRGLIRLRDFISLVLTYPVKLEEDGVLRSWISAFPEWKAQLFSTYRF
HGICKQWSLLTEKALGCMKGFMPVEMMAEIESGERSLFNRHNDVIPEGLLPLTVDLAVDQLIQTLFSGDYSARYIRLLTR
NSSEKESVVNKIHTALKDKYNMFGIDKDFLTDVWINALTCETDAEVHVQEEINKVMVSISMTEDDDMLTTVDTDKKKR
SNRLLVIVVDADSNRRLDLQKVQFPTGIVVLITTESSIQAVKDDDFGIASTMDLNIWTQDHMLPWKLFNTYVGSFISCST
VGSSMTIQKIAVEIVKKSRGHLLAIVLVAKHLRYVKDNKYWDLVLDKLSNPNPFYDYQDCDLIGISRVMVNAFVNIIWED
IDDELKLCLQLSLPVHNIKNGVRDDILVSYWANTLRYTQELGECKRQLQYYLEELLDCFVLLKFESRDVYLPIETYDIIKSLHI
SKPSIIWLGALGLTDIGQWHSLIQIEVVNNNICELPESPDCPKLEVLLLQGNADLLDIPDSFFDHMPLLQHLDLSYTSIRDL
PPSLTKLMQLKKLYLKGCDLIMEISPQIFQLKNLEELDLDGTLITHLPKDIRELINLQRLALCFDAYHHVLSRGNKGKQISKT
MIIPSGVISNLTQLNYLSLDVDPEDEQWSGNVNSVLVEILGLEKLKTVSIYVPKADLLELIPAEKSLNFRLVVGHHMRRLIS
RVPPELESKFKHFDYSMKFVNGVNVPNGVKMNLGRFEALYLDRHMTIKSLSDFNLNNVRRILRSFGSAASGGAHPCCA
LSLSHSKEDMRFYVRDLTTRMKIGDLRMKRQALRNLLEVVLEDEKYVKVVVDVGDVVHLLVGFLGSNEVEIQEESAKVV
SVVAGFDSYKGVLVGAGVIAPLVKVLDYGSDSRKVAAARFLTVEKMTNNVVHGDGNQVRTSDGGFVMVVDGVNGV
AREMVMVARGGTHDDEEGDASGCCGGQTMVLVTMPQHGSLLVSGGMILGGVVVHDV

>g17931.t1

MSGLELVGPLVGEMGSIGVGEMHTTIGSKIAFSKNLDDNYNILVKDTEMLHAIKKDKEMKAQRNSHKDTTNAYKLWT
NRVSEATAEVQKLKLKYEEKTLPWWRIQKRSHLSEEMEKKSNCVRQLMNDECLKDFLVDKPPEPVLKELNVPQISGYPT
LQGALDNTLVLLKNNKIKIVGVCGTKGVGKTTIMRNLNNNEEIAKLFEIVIFVKVTSNDRKLQEKIAHRLMLDKGINKEDS
DDVARRIHRELEKKRYLLILDEVEDAINLELLGIPRDNNNGSKVVIVTRFPLVYKLNRVQRVIKVAELSPDEAWKMFRDTV
HAFNPKIDSPDIQPTAKLVCKRCSRLPLLIYNIANSFKLKESASSWWAGLEDLKPWPELQNQGLEELYSCLKFCYDELNDK

RKQKCFLYTSLYPADSKVYSDYLVECWAAQGLLGDINDTRSYQSARNCGIDILEHLANVSLLEKGEAMIYVNMNHCMR
QLALHISSKDPECSFYLQDGEESENLSNSRAWQQARWVSMRQVHELRRSQDCSPILTLLLRKNPELTALPECFFENMSN
LLLLDLYSSMITQLPSSLSKLTVLRGLFLNRCELLESLSSEIGLLQFLEVLDIRDTKVIFIPLQIGFLTKLRCLRIPFIASEDNEAQ
NVHVISKLHRLEELTIQVISYEQWCNDANNVLAHVASLENVTHLRCCFPSSIILGEFLSRSRSWHNKQNSFRFTVGCQNS
RRPQILESFEYKITNYLRYCNGGQRDDPAIIEVLPKTDAFELVCHKDIRKLSNFAGIACLERIRGLLIKRCNQVLTIVAGETSS
NVMNGIQIETAVILPNLEQLYLENLLNLKCAFRGPLHSGTFSRLQTLSLKNCPRLSQIFSNGAIQHFSELQKLKLEDCSKIEE
LIGEDIERERDVLPKLEILLLVNLPNFKSICATHTLAWSSLELLRIHNCHRFKTLPLDSVNAVNLKSIKGQQEWWANLDWT
NNEEVLQRLQPIFVASNEYFS

>g18317.t1

MAAELVGGALLSAFLQVAFDRLASPQFLDFFRGRKLDEKLLDHLNIMLHSINALAHDAEQKQFTDPHVKAWLFSVKEA
VFDAEDLFGEIDYELTRSQVEAESEPQTFTYKVSNFFNSTFNSFNKKIESEMKALLEKLEYLAKQKGALGLKESTYSCDSSG
GKVSQKLPSSSLVVESVIYGRDADKEMIFNWLTSETDNHNHLSILSIVGMGGLGKTTLAQHVYNDPKMETEFDIRAWV
CVSDHFDILTVTKTILEAITKFKDDSGDLEMVHGRLKEKVSGKKFLLVLDDVWSERREEWEAVQTPLSYGAPGSRILVTTR
VGKVAANMRSKVHLLKQLEEDECWKVFEEQALKGDDVELNDEKKEIGRRIVEKCNGLPLALKTIGNLLRTKSSLSDWKS
VLKSDIWDLPKEVEILPALLLSYQHLPSNLKRCFAYCALFPKNYEFDKDELILLWMAEGFLHYSPQNNNLEEIGEQYFDDLL
TRSFFLRSNIKMHFSMHDLLNDLAKYVYAEFCFRLNFDKGDCVPKSTRHFSFALTDLMFCDVKCFDCFGSLRDAKRLRSF
FPTIVHDYGKTLSSRFEIREFSKLKFLRVLSLNGYYHIKEVPDCIGDLKHLHSLDLSRTMIQKLPDSVGLLYNLLILKLNYCSNL
KELPSCLHKLTKLRCLEFEYTEVTKMPIHFGKLKNLHVLNVFCVGRNSEFSIKQLGGINLHEKLSIKELQNIENPLDALEANL
KNKHFVELKLIWNQNHIPDDPRKEKKVLENLQPSDQLERFSIDNYGGIQFPSWVFDNSLSNLVSLELIDCKYCLYLPPLGL
LSSLKTLKITRLDGIVSIGAEFYGSNSSSFKSLERLEFCDMKEWEEWECKTTSFPRLRYLFIVLCPKLKGLSEQLLHLKELHIYL
CPNLIISEHIEDTSALELLRTRSCPLVNIPMTHYDFIEDMIIFSSCDSLSIFQLNLFPVLRVLCLSGCQNLQRISLEHAHNYLKK
MTICECPQFESFPSEGLSAAFPSLTELEIIGCPKVEKFPDGGLPSHVKRMSLSSIKLIASLRETLDVNTCLQSLSIVSLDVECFP
DEVLLPPSLTSLTIHRCRNLKRLNYKLLHNLSSLSFYDCPNLQCLPEEGLPKSISSLRIWDCPLLEQRCQKPEGEDWRKIAHI
EKTEIFLIR

>g18318.t1

MAAELVGGALLSAFLQVSFDKLASPQFLDFFRGRKLDDKLLGNLNIMLHSINALAHDAELKQFTDPHVKAWLFSVKEAV
FDAEDLLAEIDYELTRSQVEAQSEPQTFTYKVSNFFNSTFKTFNKKIESEMKELLEKLEYLAKQKGALGLREGTYSGDRSGS
KVSQKLPSSSLLVESVIYGRDADKQMICNWLTSETDNNNHPSILSIVGMGGLGKTTLAQHVYNDTDIEEAKFDIRAWVC
VSDHFDILTVTKTILEAITKSKDDSGDLEMVHGRLKEKVSGKKFLLVLDDVWSERRQEWEAVRTPLSYGALGSRILVTTRV
EKVASNMRSKVHHLKQLEEDECWKVFEEQALKDDDLQLNDEKREIGRRIVEKCKGLPLALKTIGSLIRTKSSLSDWKSVLE
SDIWELPKEVEIIPALLLSYQNLPSHLKRCFAYCALFPKNYEFEKENLILMWMAEGFLHYSPQNNNLEEIGEQYFDDLLTR
SFFLRSTIKMHFSMHDLVNDLAKYVCADFCFRLKFDKGNCIPKTTRHFLFAFDDLRYFDGSGSLIDAKRLRSFVPITNDFV
HGAFPCQIKILIRELFSKWKFLRILSLNGNYDFQEVPDSVGDLKHLYSLDLSRTMIRKLPDSVGLLYNLLRLKLNGCSFLKEL
PSNLHKLTNLRCLEFEDTGVTKMPMHFGELKNLHVLNMFCVGRNSDFSTKQLGGINLHGRLSINELQNIVNPLDALEAN
LKNKQLLKLKLIWNWNHIPDDPRKDKKVLENLQPSNQLEHLSIRSYCGTKFPSWVFDNSLSNLVSLELEDCKYCLCLPPLG
LLSSLRTLKIRGFDGIVSIGAEFYGSSSSSFKSLEILKFYNMKEWEEWKCKTTSFPRLQHLVIVRCSKLKGLSQQLLHLKELIIE
SCPNLIIREPNEDTSTIELLRTRSCPRVNIPMTHYDFIEEMTIDDACDSLTIFQLNFFPMLRLLVLIECQNLQRISQEHPHNH
LKKMSIRGCPQFESFPSEGLSAAFPSLTELEITWCRKVEKFPDAGLPSNVKHMTLSSLKLITSLREILDVNTCLQSLCIEYLD
VESFPDEVLLPPSLTSLRIYHCSNLKRLDYKLLHNLSSLTLFDCRNLQCLPEEGLPKSISSLHIMCCPLLEQRCQKPEGKDWR
KIAHIQNLRV

>g19277.t1

MDMVILSEAISYTLQCATTFLSPPATRLSSNEIQQFEDNLKRILHFVQKAMHSNIQDPSVLSLWLTNVKDVVNDLNDFM
EDHRHNKETATATISLIKAGQNMAHRHKFKHQIKDATEELKRLSNEAENLVISEEGRQNERKLIRKSEEFEYVEVVVREN
VKKDIIDQLMKMFVNSNVVSVPVVTIVGVAGIGKTKLARLVYGDEQVKGLFASRIWVNLETFNVESIATRVIETTSKGKR
FLLVLDDLRVENGEGCLQKLQDRLAEAGVGGAVVVTTRSNFVAKKIAKIGTVKLEPHVLQELNEEESWSLFQKIHGPGS
GKINDVGRRVVREYCGGVPMKIIVIARLLEDLDSPVPEIELKKKFLREIRFTYYDELSPQQKLCFAYCSLFPQEHEIDAGRLIR
LWMAEGFLWRNLYSDPQEFGLACFNDFVPFVFQEMVSDEFGVVKRYKMNRLMHELARTVAWDENIIVDSAEVEVHE
RVVRSSFHFALDVQCGIPKALFENAKKLRSILLLGKTNKSRLPHEVKMTISTCEKILETFKCLRVLDFHDLGIKMVPSSIGDL
KYLRLLDLSHNNIKKLPSSITKLFHLQTLKLSQCHVLEELPKDLENLSSLIHLYLEGCLDLTHMPRGIGKLSSLQTLSLFVVGK
NYQVGGLRELTDLDGLRGHLEILHLEQLNFSAPLEAKDKYLREKKHLRCLTLRWDHEEKEEEDEKKRNVIAEKDKESLECL
DPNPNLAVLSVFGYYGKTFSNWLSSIKCLVKFSLNDCYNCQYLPALDHLQHLRFLELRRLDSLEYVSKNSDQISSDTEASSS
SSNTPFFPSLKELTISDCPKLRSWWETAKWEPNRPFFTRISKLDVQCCPELHCMPLYPYLDEELVVVDSSVKSMRDTVHA
SISEDFLPFSKLKTMLIARITQTPPERWLKNFISLKTLQIRDCSKLLYLPQGFKSLSSLESLTIERCAELDLDRSKTEWEGLKRL
RFLIIKEIPKLKSLPWGVEDVTSLEELELHECPALLNLPETIANLTSLTKLVISKCGELDSLPKGLGKIESLQTLAITDCPLLTPRC
QPETGDDWPQIGHIGNIILKQSSQDLRDLWSHGRIGKGRYF

>g19300.t1

MQFSLSSSTNGITCCWPAPPLAAGGTVNDGKGSYKRCDNGNKCTELMAESAVGFLLQRLAPVFENKVKLFTGVQAEVI
CLKGQLELIRSFLRVADALEESDDELKVWVKQVRDVVHEAEDLLDELELVQVHNHTNGFSIYLRIRNMKARYRIAHDLKS
INSRLKAISSSRKRFLSKLDSSSSVASNSINTGNAWHDQRGDALLLDNTDLVGIDRPKKQVIGWLINGCPGRKVISVTGM
GGIGKTTLVKKVYDDPDVKKNFKACAWVTVSQSCKIEELLKDLAKKLFSEIRRPIPEGMESMCSDKLKMMIKDLLQRKSY
LVVFDDVWHMYEWEAVKYALPNNNCSSRIMITTRRSDLAFTSTIESNGKVYNLQPLKQDEAWDLFCRNTFQAISGVLA
TKDKRRIDDWGMICHNLGVEIQGNGKLDNFKTVLNLSFNDLPYHLKYCFLYLSIFPQDYLIQRMRLIRLWIAEGFIEAKEG
KTKEDVAHDYLKELLNRNLIQVAGTTTDGRVKTLRVHDLLREIIILKSKDQNFASIVNEQSAAWPEKIRRLSVHGTLPYRQ
QHRSVSQLRSFLMFGVGEYIPLGKLFPSGFKLLSVLDYQDAPLKKFPLAVIDLYHLRYLSLRNTKVKTVPGHIIGKLHNLETL
DLKKTSVRELPVDILKLQKLRHLLVYQLKFKGYAQFDSKDGLKAPSEIGKLKSLQKLCFVEANQDCGMIIRQLGELSQLRRL
GILKLREEDGMAFCLSIERLTNLHALAVASEGESKVIDLTFLCSPPPFLQRLYLSGRLQELPSWIQSLHSLARLFLKWSCLKY
DPLVYLQDLPNLAHLELLKVYDGDTLHFRSGKFKKLKVLGLDKFDGLKEVTVGKDAMNCLEKLSIGRCELLKKVPSGIENL
TKLKVLEFFDMPDELMKTICPHGPGKDYCKVLHIPNVYSTYWRDGGWDVYALDTFSRDCSPRSGTLIRSHEPRIQWKV

>g19302.t1

MADSSVSFLLDKLNALLQEEVNLQRGVREDVQHIKYELERHKAILRVADAMEDRDPELKAWVKGVRDVAHDMEDAID
EFNLRLVDQHGQGNGSSLHRFIFGLKTMRARRRIALDMQSIKSKVNVISQGRPELPGIGSRSSQRLSSRLDSQGDALLLE
EADLVGIDKPKRQLCDLLFNEEPGRAVIPIYGMGGLGKTTLAKQVYDDPKVKKRFRIHAWINVSQSFKLQELLKDLVQQL
HNVIGKPAPEAVGQMKSEELKELIKNLLQSSRYLIVLDDVWHVKVWDSVKLALPSSNRGSRVMITTRKKDIALYSCAELG
KDFDHEFLPEEEAWYLFCKKTFQGNSCPPHLEEVCRKILKMCGGLPLAIVAIGGALATRERANIEEWQMVCRSFGSEIEG
NDKLEDMKKVLSLSFNELPYYLKSCLLYLTIFPEFHAIEHMRLIRLWIAEGFVNGEDGRTREEVADSYLKELLDRSLLQVVS
KTSDGRMKTCRMHDLLREIVNLKAKDQNFATIAKDQDIIWPDKVRRLSIINTLDNVRQNRASFQLRSLLMFDLSDPLEH
FSIRGLCSTGYKLIRVLDLQDAPLEVFPAEIVNLYLLKYLSLKNTKVKSIPGTIKKLQQLETLDLKQSLVTVLPVEIVELQQLRH
LLVYRYEIESYAYFHSRHGFKVAAPIGLMRSLQKLCFIEADQALMVELGKLTQLRRLGIRKMRQQDGAALSLSIEKMINLR
SLSITAIEKHEIIDIHNIFRPPPYLHQLYLSGRLDIFPRWISSMKNLVRVFLKWSRLTEDPLVHLQDLPNLRHLEFLQVYVGE
TLNFKAKGFPSLKVLGLDDLNELKSMTVEEGAMPGLKRLIIQRCGSLKQLPFGIEHLTKLKSIEFFDMPEELITRLHPKGGQ
DYLRAKNVPAVYSSYWRDGGWDVYSLETLEERETDSSRSTAMRSLENCPLWKV

>g19338.t1

MAEALLGIVIQNLQSFGQDQLATFWGVDQHTQKLSSNLTAIRAVLRDAERKQITSHAVKDWLQKLTDAAYVLDDILDE
CSIHSTKVNFDDRHTPCLSRLHPKDVLLRFNIGKRMKDITQRFHDIHEERSMFNLVPGVTEVQTIHDNWRQTSSDITEPI
LYGRDQDREKILKFLLEDVSDSEDLSIYPIVGMGGLGKTTLAKQVFNDNRVRKHFDLTIWVCVSDDFNTMTILQSIIECITR
ESSNLISLEAMRKKLEEVLYGMRYLLVLDDVWNEDQEKWKQLKGKLQCARAAKGATVLVTTRLEEVASIMQTHPAYHL
KELSGDESWSLFKYHAFGPNREEMEELVSIGKEIVRKCIGSPLAIKTLGSLLRDESNVKQWQNVKESEIWDMREESGSTT
GEENSIMRALKLSYFNLELSLRRCFSFCAIFPKDFVIEKEDLIHLWMANGFIESERNIEVEDVGNKVWNKLYRRSFFQEAK
CDEFGIVTSFKMHDLFHDLARSIMGEECAAFVEGRLTPLSTRVHYSSLFNSDVSVDMTALKKVESLRTFRHYGSIGPVPS
NHCLRALQTSSSLLSPLKDLTLLRYLSLRFSRSSEESINNSICQLPKLQILKLCYCLELHGLPKDLTRLQDLRHIVIDGMDPTQ
EMPPNIGKLRHLRTLSIFVVGSKPGCGLAELHSLNLGGRLRIIGLENVPNEWDAKQANLISKKELNRLYLSWDGRANPKG
SNVNVERVLEALEPPSTLKSFEMNGYEGRKLSSWMRNRAVLRDLVDVRLSNCDNCEELSPLGKLPHLKRLKVSGMKNV
KWIDGETYDGVEEKAFPSLEELSLYNLPKLERLLRDEGVEMVPRLSKLIIYGVLNFKVPHLPCVEKLHAERIGEAASFMEGV
GKNIACLKTLSIKFINGVVVLADEFRRLSSLQELYIEEWYDVEYFPEHVLEGLTSLRTLDIYDCKKLKSLSEGVGHLACLESLSI
SKCPELVALPSNMSQLSALRKVSIKYCCTLPYGLQFVPSLRTLDIGRCFSTSLPDWLGGMTTLEELSIWDCEELRSVPSSIQ
RLTNLSYLRIHYCPHLKKRCKREIGEDWQYINHIPRLELYFWKKPTFCDELKSVLFTSRRNVSLCL

>g19339.t1

MAEALLGIVIQNLQSFGQDQLATFWGVDQHTQKLSSNLTAIRAVLRDAERKQITSHAVKDWLQKLRDAAYVLDDILDE
CSIHFTKMHSDDGHTSCLSRLHPNDILFRFNIGKRMKDITQRFHEIYEERSMFNLVPGVTEVQTIHDNWRQTSSDITEPV
VYGRDQDREQIVKFLLEDASNSEELSIFPIVGMGGHGKTTLAKQVFNDNRVCKHFDLTIWVCVSDDFNTMPILQSIIECI
TGQNPNLNSLEALRKKVEEVLHGSRYLLVLDDVWNEDPEKWKQLKGKLQCARAAKGATILVTTRLEEVASTMQTNPAY
HLKELSGDDSWSLFKHHAFGPNREETEELVSIGKEIVTKCVGSPLAIKTLGSLLRDESDVKQWQNVKESEIWDIREESSSA
TGEENSIMRALKLSYFNLELSLRRCFSFCAIFPKDFEIDKEELIHLWMANGFIKCEGNVEVEDVGNKVWKKLYSRSFFQEA
KYDEFGMITTFKIHDLFHDLAQSIMGEECVVIVKERLTPLPTRVHYSSLLNFGVSGSMTAFKQRMTTALKKVESLQTFLDF
GGIGPVPSSHCLRALRTSSSLLSPLRDLTHLRYLSLRFSMSWEESLNNSICQMPKLQILKLFRCHELHGLPKDLTRLMDLRH
IVIDGMDTIQEMPPNIGKLRHLRTLSIFVVGSKPGCGLAELHSLNLGGTLRIRGLENVPNEWDAKEANLIGKKELNILRLS
WDGNGNPKGSNVSVERVLEALEPPSTLKSFEMNGYQGRQFSSWMRNSGVLRDLVEVKLLGCENCEELPPLGKLPHLK
RLKVSGMKNVKWIDGETYDGVEEKAFPSLEELSVYNLPKLERLLRDEGVEMVPHLSKLTIDGVLNFKVPRLPCVETLYAR
GIEAVTSFMEVVGNMACLKTLDIYCIKGVVVLPDEFRRLGALQELYIEEWYDVEYFPEHVLEGLTSLRTLDIYECKKLKSLS
EGVGHLACLESLRISKCPELVALPSNMSQLSALRNVLIQYCCAAILDGLQRVPSLRVLDIECCTCTSLPDWLGDMNTLEEL
SIWDCKELRSLPSSIQRLTNLSYLTIFECPHLKKRCKRETGKDWQYINHIPKLFVYGIEGCPDLKKRCKRETGEDWQYINHI
PRCGLAELHSLNLGGSLRIRGLENVPNEGDAKQANLMSKKELNRLHLSWGSSANSEGSNVSVERVLEALEPPSALKSFG
MRGYQGRQLSNWMKSVLVMRDLVEVELLDCDNCEELPPLGKLAHLKRLKVSGMKNVKWIDGETYDGVEEKAFPSLE
KLTVRNLPNLERMLREEGVEMLPRLSQLTIYGVFNFKVPRLPCVEKLRAERIGEAASFMEVVGNMACVKTLSIEEWHDV
EHFLEHVMEGLTSLRTLSIQYCEKLKSLSEGVGHLACLESLRISKCPKLVALTSNMSQLTALREVSIMYCCTLPYGLQRVPSL
RLLCIYECTSTSLPDWLGDMISLQQLRIVCCKELRSLPNMSSSEEAMQ

>g19341.t1

MAEVLLGIVIQNLQSFGQDQLATCWGVHQHTQMLSSNLSAIRAVLRDAESKQITSYAVKDWLQKLTDAAYVLDDILDE
CSIHFTKMRSDDGHTSCVPRLHPNDILFRFNIAKKIKDITQRFHDIHEEKSRFNLEHGVTEVQRVDDDWRQTSSDITEPV
VYGRDHDREQIVKFLLEDASNSEDLSIFLIVGMGGLGKTTLAKQVFNDHRVCKHFDLTIWVCVSDDFNTMTILQSIIECIT
GQNPNLNSLEAMRKKVEEVLHGSRYLLVLDDVWNEDQEKWKQLKGKLQCARAAKGATILVTTRLEEVAFTMQTHPAY
RLKELPGDESWSLFKYHAFGPNREETEELVEIGKEIVRNCIGLPLAIKTLGSLLRDQSEEAKCDEYGMVKSFKMHDLFHDL
AQSIMGEECAAFVEGRLTPLSSRVHYSTLLSSNVSFGGLKHAFKKVESLRTFLDLRASVVLVVGTLPKISKLRHLRTLSIFVV
GSKPGCGLAELHSLNLGGTLRIRGLENVPNEWDAKEANLIGKKELNILHLSWDGSGNPKGSNVSVERVLEALEPPSTLKS

FEMNGYQGRKLSSWMRNRAVLRDLVDVRLSNCDNCEELSPLGKLPHLKRLNVSGMKNVKCIDGETYEGVEEKAFPSLE
ELRVDNLPNLERLLKDEGVEMVPRLSKLRIEGVLNFKVPRLPCVETLHATGIEGVTSFMEGVGESMACLKTLSIKFIKGVV
VLPDEFRRLGALQELYITEWNDVEYFPEHVVEGLTSLRTLSIQYCEKLKSLSEGVRHLVCLESLTIGNCPELVALPSNMSQL
TALRKVSINYCCTLPDGLQRVPFLRTLDISCCKCSSLPDWLGDMTSLQKLSIAYCKELTSLPSSIQHLTNLSHLRIDGCPDLK
KRCKRETGEDWQYIKHIPQLELYFRRETTFLS

>g19342.t1

MEGRMVVVEGHLEAVEVAIAETKADTGALRQETAALRQDVQAILKALGERNHNHHGKRQGESESSVNDNGGGPKGE
GGRGGDGNQGGLSQWGKRVELPVFEGGEPWVWIGRAEKFFEVQKVAEEEKLELAFISMDGYTESWFRFWREKTKNL
SWDGLKRALGIRFGGGTRGTVYKRLSTIRQSGPVEEYIRDFEVLVGQTTQIPEEQIMGYFLSGLREEVGDHVRPHDPPDL
MTAMRVARDVEKLCTKTGGGWLSKNPNSWGKASGSVTRVEPNRESTVRVGTAESVGSVNKDATQGRNKGVATSSG
GAVNVLMQGRNPIAYFSKALSEGKLSKSIYEKEMMALVLAIQHWRPYLVGQHFVVHTDQKSLRHLLEQRITTQNQQD
WIAKLLGYDFEIVYKTGAANKAADALSRQFEGEDEGEKELSVIARPFWQDFGEIMQEVEVDEFLTYRHIAQSLYWVGM
KKDVTEFVAKCLVCQQHKYLTSSPQGLLQPLPVPNAIWEELSMDFIVRLPKSQGYDAVLVVVDRLSKYAHFLPLKHPYSA
KTVAEIFIREIVRLHGIPQSIVSDRDPLFLSIFWKELFKGQGTQLKMSTAYHPETDGQTEVLNRVLEGYLRCFCSEQPKGW
MTVLSWAEYWYNTSYQGAIRCTPFEAVYGRAPPSLHRFIPGESLVEADLMVRQANKKRKAANVEVGDWVYLKIRPHR
QTSMQTRLHPKLAARYFGPFLVIQKVGENACKLQLPETARIHPVFHVSQVKKAIGEKRVERDLPHDLQAEGPSFWPVNI
LGRRQLQEGGESVPQMLVEWQEGGQEGATWENEITIREQYPDFNLGDKVGVQGAGIDRDNRRCNLGRLFLTLIIFLLT
MADFFLTMAYCSICGFFSTSSSPSSSCGSNSQSLITTKQLSSNLKAIRAILRDAERKQITSYAVKDWLQKLTDAAYVFDDIL
DECSIHSTKMHSVDAHTSCLSRLQPKDILFRFHIEKRMKDITQRFHNIHEERLTFELRVGVTEKQTVNDDDDDWHQTSS
VITEPIFCGRDKDREKIVKFLLEDASNSEDFSIYPIVGMGGLGKTILAKQVFNDHEISKKFKLRIWICVSENFNVKRILQSIIEC
TIGQNPNLSDLEARLKKVEEALQNKRYLLVLDDVWNENTEKWKELKGMLECARGTKGATILVTTRLEEVVSIMGTHSAY
RLKALSEDDSWSLFKHHAFGPNREEREEFVTIGKEIMRKRVGSPLAIKTLTSRLHDESESIMGEECVVIETSSRTLLSARVH
YSSFLESGISFDLTDFKPGLISTFKKVESMRTFLDFGCTGMVPLNHYLRALCLRSSPLFSPFKDLVHLRHLSLHFSLQRSLDN
LIGQMPKLQILKLTSCPFLELLPKTLTQLQDLRHVMIDKCASIVELPPNISKLRYLRTLGIFVSGSKPGCGLRELHRLNLGGTL
KIKGLENVPNERNAKQANFIGKKDLNILHLSLGGKANSEGNYVSVERVLEALEPPSTLKSFGMNGY

>g19343.t1

MAEALLGIVIQNLQSFGQDQLATFWGVHQHTQMLSSNLTAIRAVLRDAERKQITSHAVKDWLQKLTDAAYVLDDILDE
CSIHSTKLNFDHRHTSCLSRLHPKDILFRFNIGKRIKDIIQRFHDIHEERSMFNLVPGVTEVQTIHDNWRQTSSDITEPIIYG
RDQDREQILKFLLEDVSDSEDLSIYPIVGMGGLGKTTLAKQVFNDNGICKHFDLTIWVCVSDDFNTMTILQSIIECITGQN
PNLNSLEAMRKKVEEVLHGKRYLLVLDDVWNEDQEKWKQLKGKLQCARAAKGATILVTTRLEEVASTMQTHPAYHLK
ELSGDDSWSFFKHHAFGPNREEKEELVSIGKEIVAKCVGSPLAIKTLGSLLRDESDVKQWQNVKESEIWDIREESSSATGL
GNVSNEWDAKEANLKSKKELNRLYLSWGGGANSEGNNVSAERVLEALEGPSTLKSFGMEGY

>g19346.t1

MAEALLGSVIENLGSFVQDQLGTYLGVEQQIQKLSNNLTAIRAVLRDAERKQITSEVLKNWLQKLTDAAYVLDDILDECSI
HFTKMPSDDGHTSSLSRLHPKDILFRFNIGKRMKDISQRFHDIHEEKSRFNLEHGVTEVQAVDDDWRQTSSDITEPVVY
GRDQDREQIVKFLLEDASNSEELSIFPIVGMGGLGKTTLVKQVFNDHRVCKHFDLTIWVCVSDDFKTMTILQSIIECITGQ
NPNLNSLEAMRKKVEEVLHGKMYLLVLDDVWNEDPEKWKQLKGKLQCARAAKGATILVTTRLEEVASIMQTHPAYHL
KELPGDESWSLFKYHAFGPNREEMEELVAIGKEIVRNCIGLPLAIKTLGSLLRDQSEEAKCDEFGILKSFKMHDLFHDLAQ
SIMGEECAAFVEERLTPLSSRVHYLSSNVSYRGLKHAFKKVESLRTFLDLRPYFRVSDSDPVPSNHSLRALCTKSSLLSPLKD
LTHLRYLIYLRGLPKNLTQLQDLRHIVMDGCDSVEETLPKISKLRHLRTLSIFVVGSKPGCGLTELHSLNLGGTLRIRGLENV
PNEWDAKEANLIGKKELNILRLSWDGNGNPKGSNVSVERVLEALEPPSTLKSFEMNGYQGRQFSSWMRNSGVLSDLV

EVKLLGCENCEELPPLGKLPHLKRLEVSGMKNVKWIDGETYEGVEEKAFPSLEELRVENLPNMERFLRDEGVEMVPHLS
KLRIEGVLNFKVPRLPCVEELDARKIEAGTSFMEGVGENMACLKTLSIKFIKGVVVLPDEFRRLGALQELYIGEWYDLEYFP
EHVMEGLTSLRTLSIEFCEKLKSLSEGVGHLACLERLTISKCPELVALPSNMSQLSALREVLIKYCCTLPDGLQRVPFLRTLDI
SCCKCSSLPDWLGDMTSLQKLGIWFCKELRSLPSSIQRLTNLSHLWIAQCPHLKKRFKRETGEDWQYINHIPKLELLSLXL
TERVESLNERLRVWITVGVFAWGVGSLSEVGSSGPLFVHSHNELSH

>g19347.t1

MHSHDGHTSCLSRLHPNDILFRFNIGKRMKDITQRFHDIHEEKSTFNLEHGITEVQTVDDFWRQTSSDITEPVVYGRNQ
DREKIVKFLLEDASNSEELSIFPIVGIGGLGKTTLAKQVFNDHRVCKHFDLTIWVCVSDDFNTKTILQSIIECITGQNPNLNS
LEAMRKKVEEVLHGMRYLLVLDDVWNEYQEKWKQLKGKLQCARAAKGATILVTTRLEEVASTMQTHPTYHLKKLSGD
ESWSLFKYHAFGPNREETEELVAIGKEIVRNCIGLPLAIKTLGSLLRDQSEEAKCDEYGMVKSFKMHDLFHDLAQSIMGE
ECAAFVEGRLTPLSSRVHYSTLLSSDVSSNVSFRQFMHGFKKVESLRTFLDLRTYFTLGDFDPVPSNHSIRALRTKSSWLSP
LKDLTHLRYLSLNFDYKANLNNLICQMRKLQTFKLQRSTYLRGLPKTLTQLQDLRHIVMDDCMSVVETLPKISKLRHLRTL
SIFVVGSKPGCGLAELHSLNLGGRLRIRGLENVPNEWDAKQANLIGKKDLNILHLSWDGSINPKGKAVIELDEKQCSFET
WWMLHSPTVTTCIDGETYDGVEEEAFPSLEELRVKSLPNLERLLSDEGVEMVPRLSQLRSEGVLNFKVPRLPCVEILYAR
GIEAGTSFIEGVGENMVCLKTLSIEIIKGSVVLPDEFRRLGALQELYIAECYDLEYFPEHVLEGLTSLRTLSINYCKKLKSLSEG
VRHLACLESLMIRDCPALGTLPSNMSQLTALRNISIEGYSTLPYGLQRVPSLRTLDISYCECTSLPDWLGDMNTLEELSIW
YCKELRSLSSSIQRLTNLSYLRIEGCPHLMKRCKRETGEDWQYINHIPKLKLEE

>g19350.t1

MADPLLGIVFQDLQSVVQSQLATYWGIDQQAQKLSSNLTAIRAVLTDAERKQIASLAVKDWLQKLTDAAYVLDDILDEC
SIHSREMLSDDGHTSCLARAHPKDILFRFHIGKRMKNITQRFHDINEERRMFELRDGVTEKPTVDDGEDDQTTSVITEPI
FCGRDEDREKVVKVLLEANNNEDLTIYPIVGMGGLGKTALAKHVFHDQRVCKHFDLTIWVYVSVDFNVKAILQSIIEYAT
GQNPNLHTLETMRKKVEEVLQSKRYLLVLDDVWNEDQEKWKHLEGILRFARGAKGATVLVTTRLEEVASNMETHPAY
HLTELSEYDSWSLFKSYAFGPNREEKKELVAIGEDIVKKCVGSPLAIKTLGSLLRDQTAVTQWENIKESEIWDIRSESSSMT
GKENSIMRALKLSYFNLELSLRRCFSFCAIFPKGFEIVKEELIHLWMANGFVKSEGNVEVEDVANNVWRKLYRRSFFQEA
KSDKFGMITSCKIHDLFYDLAKSIMGEECVMVEEGRLSQLSTRVHHLHFLTYNMSVDVAAFKKVESLRTFLFGNIRQLPS
NRFLRALCIRSSMLPPLNDLAHLRYLSVHDSSVRNLNDSICQLSKLQILKLESCFDFYEITKKLTQLQDLRHILINKCGSVPD
MPPNIGKLRHLRTLSLFDAGSTPGYGLTELSGLRLGGRLHIRGLGNVSNEWDAKEANLKSKKELNRLYLSWGGGANSEG
NNVSAERVLEALEAPSTLKGFGMEGYQGRKLPSWMGSVVVLKDLVEIILFDCNNCEELPPLGKLPHLKRVHVRGMKNV
KWIDGESYEGVEEKAFPSLEKLSVENLPKLERMLRDEGVEMVPHLSQLKIDGVFNFKVPHLPCVEELHATRIVAATSFIEG
VGDNMVSLKTLSVRLIKGLKVLPDQLRRLGALKDLRIGFWYDLEHFPEHVSEGLTSLRSLSISNCPKLVALPSNMSKLTSLR
TVSIYDCSTLPYGLQFVPSLRTLYIVSCMFTSLPDWLGDMTTVEELWIWSCKELRSLPSSIERLTNLSYLTIYYCPHLRKRC
MRETGEDWQYIKHIPEIKLDSYSLPEEKTLTLSGESIRRLSCSWNCFKTARQPAKGVVSSNLFDLVLEE

>g19351.t1

MAEALVESLIENLGSLVQDQLAIYWGVDQQTQKLSSNLRAIRVVLRDAERKQITSYAVKDWLQKLTDAAYVLDDILDEC
SIHSTKMHSVDAHTSRLSRLHPKDILFRFRIGKKMKDITQIFHNIHEERLTFELRVGVTEKQAVNDDDDDWRQTSPVITE
PIFCGRDKDREKIVKFLLEDASNSEDLSIYPIVGMGGLGKTTLAKQVFNDHQISKHFDLKIWICVSDDFNVKRILQSIIECTI
GQNPNLGDLEARRRKVVEALQNKRYLLVLDDVWNENTERWKELKAMLECANGAKGTTILVTTRLEEVASIVGTHSAYR
LKVLSEDDSWKVRFGRYGTEVVLRQEAKCDEFGILKSFKMHDLFHDLAQSIMGEECAAFVEERLTPLSSRVHYLSSNVSY
RGFMHGFKKVESLRTFLDLGPSFRVSDSDPVPSNHSLRALCTKSSLLSPLKDLTHLRYLSLNCDYRANLNNNSICQMRKL
QILKLQSCRYLRGLPKNLTQLQDLRHIVMNDCREVEKSLPKISKLRHLRTLSIFVVGSKPGCGLAELHSLNLGGRLRIRGLE
NVPNEWDAKQANLIGKKDLNILELSWDGSANPKGSNVSVERVLEALEPPSTLKSFEMNGYEGRKLSSWMRNRAVLRD

LVDVRLSNCDNCEELSPLGKLPHLRRLKVSGMKNVKWIDGDMYDGVEEKAFPSLEKLRVENLPNMERLLRDEGVEMLP
RLSQLTIYGVLNFKVPRLPCVETLDARRIEAGTSFMEGVVENMACLNTLIIEMIKGVVVLPDEFRRLGALQQLYIREWYD
VEYFPEHVVEGLTSLRTLSIFNCEKLKSLSEGVRHLARLESLRISNCPELVALPSNMSQLTALREVSINYCCTLPDGLQRVPS
LRLLNIYKCTCTSLPDWLGDITSLEGLTIEYCEELRSLPSSIQRLTNLSYLHISGCPHLKKRCKRETGEDWQYIKQIPKLVVEE
NDVL

>g19352.t1

MLENAKGAKGATILVTTRLQEVASIMGTRPAYSLTALSEDDSRSLFKHHAFGLNREEREQLVAIGKEIVRKCVGSPLAIKT
LGSCLRDVSEVKQWHNIKESEIWNIWEASSDENSIMRALKLSYFNLELSFRRCFSFCAIFPKDFEIDKEELIHLWMANGFI
KREGNVEVEDVGNKGWKRLYDRSFFQEAKANRIGMIKTLKMHDLFHDLAQSIMGEECVIYGERKLTRLPNRDLRHLVI
NKCPSIAEMPRNIGKLRHLRTLKTFVVGSKLEYGLAELHGLSLVGKLHIRGLENVSNEWEAKEANLKSKKELNRLYLSCGG
SANSQGSNVSVERVLEDLEPPSTLKSFEMQGYQGRKLSSWMRSVVVLKDLVEVILLDCNNCEELPPLGKLPHLKKIEVRG
MKNVKWIDGESYEGVEEKAFPSLEKLSVKNLPNMERLLRDEGVEMVPRLSQLRIDGVLNFEVPHLPCVEELDARRIKAA
TCFMEGVVENMTCLKSLLIAFMKDLKVLPDKLSSLSALQKLEILGCEELRSLPSSFQRLTNLSKLSIFSIVLG

>g19353.t1

MAEALLGTVNQNLQSFGQDQLATFWGVHQQTQKLSSNLTAIRAVLRDAERKQITSYAVKDWLQKLTDAAYVLDDILD
ECSIHSTKLNFDDAHTSCLTRLHPKDILFRFNIGKRMKDITQRFQDIHEERSMFNLVPGVTEVQTIDDNWRQTSSDITEP
VVYGRDQDRDQILKFLLEEVSDSEDLSIYPIVGMGGLGKTTLAKQVFNDNRVCKHFDLTIWVCVSDDFNTMTILRSIIECI
TGQKSKLKSLEAIWKKVEEVLHGMKYLLVLDDVWNEDQEKWKQLKGKLQCARAAKGATILVTTRLEEVASTMQTHPA
YHLKELLGDDSWSLFKHHAFGPNREETEELVSIGKEIVTKCIGSPLAIKTLGSLLRDESDVKLWQNVKESEIWHIREESSFA
TDLEIDKEELIHLWMANGFIESERNIDVEDVGNKVWNKLYRRSFFQEAKCDEFGMIKSFKMHDLFHDLAQSIMGEECV
VVVKGRLTTLSTKVHYSSLFNSGESDDMTAQRVHYSSLFNSGESDDMTAQRFMTALKKVESLRTFLDYEGIGPMPSNP
CLRALQTSSSLLSPLKDLTLLRYLSLSLTFNRSSEENLNNSICQLPKLQTLKLCNCHQFHGLPKNLTRLEDLRHIVINGRDSIQ
EMPSNIGKLRQLRTLNIFVVGLKPGCGLAELHSLKLGGTLRIRGLENIPNEWDAKEANLMTKKELNRLHLSWGGSANSE
GSNVSAEKVLEALQPPSTLKSFEMRGYRGRQLSHWMRSVVVLRDLVEVNLLDCDNCDELPPLGKLPLLKRLEVSGMKN
VKWIDGETYDGVEEKAFPSLEKLTMRNLPNLERMLREEGVEMLPRLSELRIFCVSNLKFPRLSSVEKLVAVSIDEVASFME
GVVGNLPSLKTLEIQSVKGAVVLPNQLRGLDALQELCIGDWQDVEYFTEHVLEGLTSLRTLSINYCKKLKSLSEGVQHLAC
LESLTISGCPELMNLSSNMSQLTTLWTVSIKVCSRLPYGLQHVPSLRTLDITSCKCTSLPDWVGDMTSLQKLSIRFCRELRS
LPNSIQHLTNLSHLSIHGCPRLKKRCRRETGEDWQYIKHIPKIELFHCETDLLW

>g19354.t1

MAEALLGIVIQNLQSFGRHQLGTYWGVEQQTQKLSHNLTAIRAVLRDAERKQITSEVVKNWLQKLTDAAYVLDDILDE
CSIHFTKMHSDDGQTSCLSRLHPNDILFRFNIGKRIKDITQRFHDIHEEKSKFNLEPGVTEVVQTVDDDWRQTSSDITEPV
VYGRDHDREQIVKFLLEDANSEDLSIYPIVGMGGLGKTTLAKQVFNDHRVCKHFDLTIWVCVSDDFNTKTILQSIIECITR
QNPNLNSLEAMRKKLEEVLHGMRYLLVLDDVWNEDQEKWRELKGKLQCARAAKGSTILVTTRLEEVASTMQTHPAYH
LKKLSGDDRWSLFKYHAFGPNREDIEELVAIGIEIVRKCIGLPLAIKTLGSLLRDESEDLRHIVISDCDLIQEMPANIGKLRHL
RTLSIFVVGSKPGCGLAELHSLNLGGTLRIKGLENVRNEWDAKQANLIDKKELNILHLSWDGRANPKGSNVSVERVLEAL
EPPSTLKSFAMNGYQGRQISSWMRNRAVLRDLVDVRLSNCDNCEELPPLGISIVGELGCGEFTKLERLLRDEGIEILPCLS
RLRINGVFNLKIPRLPSIEELDAKWISEAASFMEVVGFMACLKTLRIEIIKGGKVPSDQLSMLDALEYLHIGLWYDLEYFPE
NVLESLTSLQTLSIANCKNLKSLSEGVGHLACLQNLIISECPELVALPSNMSQLTVLRKVSIDSCSTLPYGLQFVPSLRTLDIE
SCMSTSLPDWLGDMTTLEELSIGFCKKLRSLPSSIQRLTNLSYLSIHCCLI

>g21401.t1

MAIGSRSSSFTYDVFLSFRGEDTRHGFTGHLYKALHDRVITFIDDEELQRGEEITPALVKAIEESRIAITVDPSDVRHQKGS
YGEALAKHEQRFNHSMEKLENWKKALHQVANLSGFHLKHGDGYEHEFIGRIVELVSSKINHAPLPVADYPVGLESQALA
VRKLLDVGSDDVQMIGIYGIEKSNKHGLQHLQSILLWEMLGEKEAKFASVGQGASVIHHRFQRKKVILILDDVDKYEQL
QAIIGRPCLFGPGSRVIITTRDKQLLSSYGVIRTYEVNLLDKNNGLDLLSWKAFKTKKIDASYKEVLNDVVIYAYGLPLALEV
IGSNLFGKSIEEWKSAIKQYKRIPNNQILEILKFSRYSQETSIALHSLIQYMGKEIVRQESPKDPGKRSRLWLPEDIIQVLEDD
KIPDISVLQNLEELSFQCCVNLITVHHSVGFLDKLKTLRACSCEKLRSFPPIRLTSLEKLDLSCCYSLKGFPEVLAKMENIRKL
NLAKCPITELPLSFQNLTGLPSLEMLFSTNAIVKVPNSIIMMPKLTDIYARGLKGLRWLKQEEGEEEMGPVVVPSKVEWL
TASNCNLYDDFFKIDFTRFAHVKNLWLPKNNFIILPECLKKCQFLSYLDVSNCKHLREIRDIPPNLKRFFAINCKSLAYSRKR
KLLKQELHEAGNTVFCLPGFNIPKWFNHQSQGTSISFWFRNKFPDKVQCVLVAPMQDNCFRPRVFINGKEYTSYSHFN
LKGNHHKYLFDLREASFRKSPYEVPFDSEWNHAKVTFPEGKYTSINHAKIGIHIVKHENNMEDVRFTDPCSKRKSDVDIN
TSDSESLPVAKYGVGKIPLPENSSPNFSLSIHTLSFSASDGSTPVKVKPYRYLHSQKDEIERLVHGVAMETSKLEAINNWP
QPMSLKQLRGFLGVTGYYRRFIRNYASMAGPLTDLLKKNSFHWSELASKAFLQLKQAMTFAPVLAIPNFKEPFILETDAS
SSEPTSTWLIKVAEETKKDNTLLELLKQHAHGVKIDHNYEVKDGLVFRKGKLMILPDSLLRQQILQEFHDTKIGGHAGKTT
VARICTQFYWPKMQEDIKAYIKNCSICQQAKSDQALPVGLLQLLPVPTQIWEDIAMDFITHLPSSHGYATIMVVHSIAM
SSFKYVFGREDPSVNPYQIHSKDPVSLQEILIKRDKLLQQLKDNLFKAQNYMKFQTDKKRRDVQLAVGDLALMKLQPYR
QHSMALRKNQKLGMRFFGPFKVVQRIGSVAYKLELLESARIHSVFHISLLKKFYGNP

>g21673.t1

MATPSSRRFTYDVFLSFRGEDTRYGFTGNLYKALCDRGLHTFIDDDKLQSGDEITPALQKAIEESRIAIAVLSHNYASSSFC
LDELATILRCKKSKGLLVIPVFYKVDPSYVRHQKGSYEEALAKHQKKFKAQKDKLQKWKMALRQVADFSGYHFKDGQEY
QYEFTGRIVDRVSREINRSPLHVADYPVGLLSQVLHVKKLLDVGSDEVVHMIGIHGMGGLGKTTLSLAVYNLIADDFDSS
CFLQNVREESNKHGLKHLQTLLLSEILGEKDINLTSVQRGISVIQQRLRRKKVLLILDAVDNRKQLQAFAGRSDWFGPGSR
VIVTTRDEQLLKCHEIERTYEVKELNNNDSLQLLIWNAFKGEKVDPSYEDVLKRVVTYASGLPLALEVIGSNLVGKSVEEW
ESAIQHYKRIPNGEILEVLKVSFDALGEEEKNVFLDMACCLKGSSLTEVEQILGVLYDNNMKHHIGVLVKKSLIKIGRGRFD
VIEMHDLIEDMGRQIDLQNSPKEVGKRRRLWLGKDIIHVLKDNKKFENLTVLNFDECKFLTQTPDMSDLRNLVEVSFRG
CESLVEVHDSIGFLSKLKILNAEDCIKPMSFPPLNLPTLERLELSFCSNLEKFPEILGKMGNIRGWQWVKSEDAEDNVGS
MVPSKIDWFSALSCNLDDDFFSAGFMRLAQVGSLFLRYNNFKYLPECIKEFHNLSILDQLHEARKTEFWFPGASFPEWV
DLQSSGPSCSFWFRNKFPAKVLSLRIAPVGDKDVFDFIRPMVFIDGKVQESRPFYFKEIERMFEFDQTYLFNLQRLSIYSN
LSELPLEKEWKHVKVTYEGVIETSIIKATGIHVFKDENSMMEDIRFDNPYTNNKLDKDLNVSQSQNHLLRTIALGFDGVR
SFTDIQSLEVPDLVLVLNLVLVFDLNCVQPFKGFQLKCQCYQIYSPQRCKDLGCRLQQHIMMEACLGLELAGSNSKTEHT
HPNNCLALDAALPYISLLEYHVYSKFDSIPNTTLTIITPYFVLMATPSSRRFTYDVFLSFRGEDTRYGFTGNLYKALCDRGLH
TFIDDDKLQSGDEITPTLLKAIEESRIAIVVLSHNYASSSFCLDELATILHCKKSKGLLVIPVFFKVDPSYVRHQKGTYDEALA
KHQKRFKAQKEKLQKWKIALRQVADFSGYHFQDGHEYQYEFIGRIVERVSREINRTPLHVADYPVGLLSQVLHVKKLLD
VGSDDVVHMIGIRGMGEESNKHGLKHLQTVLLSEILGENDINLASIQRGISVIQQRLRRKKVLLILDDVDNRKQLQALAG
RSDWFGPGSRVIVTTRDEQVLKCHEIERTYEVKELNKNDSLQLLIWNAFKGEKVDPSYEDVLKRVVTYASGLPLALEVIGS
NLVGKSVEEWESAIQHYKRIPNGEILEILKVSFDALGEEEKNVFLDMACCMKGSSLTEVEQILGVLYDNNMKHHIGVLVK
KSLIKVGRGRFDVIEMHDLIEDMGRQIDLQNSPKEVGKRRRLWLGKDIIHVLKDNKGTSETEIICLDLSISEKEETIEWNA
NAFRRMKNLKILIIRNGKFSNGPNYFPESLRVLEWHGYPSNCLPSNFDPNKLVTCKLPHSHFASFGFLDSSKASLKSKFEN
LTVLNFDWCKFLIQTPDMSVLGNLEEVSFKGCESLVEVHDSVGFMSKLKILNAEGCIKLMSFPPLNLPTLERLELSFCSNLE
KFPEILGKMGNIRVLQLEELPIKELPLSFQNLNGLEDLSLSCKIVHLPSSIVTMPNLFDFCVTNCKGWQWVKSEDAEDNV
SSVVPSKVDWFSALSCNLDDDFFSAGFMRLAQVRSLFLRDNNFKHLPECIEEFHNLCTLDVSHCRHLQEIRGFPPKLELF
KAINCISLTSSSLSMLLNKQLYEARKTEFWFPGASFPEWVDLQSSGPSCSFWFRNKFPAKVLSLLIAPVGDKDEFGFIRPM
VFIDGKVQESRPFYFQEIERMFEFDQTYLFDLQRLPIYSNLFELPLEKEWKHVKITYEGVIETSIVKATGIHVFKDENSIIEDI
RFDDPYTNNKLDKDLNASQSQNHLLRTIGIGFDRNSAVFCRIL

>g21674.t1

MATPSFRRFTYDVFLSFRGKDTRYGFTGNLYGALCDRGLHTFIDDDKLQSGNEITPALLKAIEESRIAIVVLSHNYASSSFC
LDELATILRCKKSSYEEALAKHQKKFKAQKDKLQKWKIALRQVADFSGYHFKDGNEYQYEFIGKIVERVSREINRAPLHVA
DYPVGLLSQTLLLSEILGEKDINLASVQRGISVIQQRLRRKKVLFILDDVDNRKQLQAFAGRSDWFGPGSRVIVTTRDEQL
LKSHEIERTYEMEELNENDSLQLLKWNAFKREKVDSSYEDVLKRVVTYASGLPLALEVIGSNLVGKSVEEWESAIQHYKRI
PNGEILEILKESFDALGEEEKNVFLDMACCLKGSSLTEVEQILGVLYDNNMKHHIGVLVKKSLLKVERGFDVIKMHDLIED
MGRQIDQQKSPKEVGKRRRLWLGKDIIHVLKDNKGTSETEIICLELSISEKEETIEWNANAFRRMKNLKILIIRNGKFSKGP
NYLPKSLRKFENLTVSNFDRCKLLTQTPDMSDLGNLEEVSFKGKLMSFPPLNLPILKRLELSFCPNLEKFPEILGKMGNIKV
LQLDGLPIKELPLSFQNLIGLEDLSLSCEIVHLPSSITTMPNLFNFRATNCKGWQWVKSEDAAEDNVGSMVPSKVGWFS
ALSCNLDDDFFSAGFMRLTQEIRGFPPNLRHFNAINCISLTSVSLSMLLNKQPHEARKTEFRFPGASFQSGLISRAVDLLV
LSGFVISFLPKCFLFL

>g21733.t1

MSGSSSSFSVSTSPTKHDVFLSFRGEDTRDNFISHFHAALRRKRIEAYIDDRLQTGEEISSALQTAIEESKIYVLVFSKNYASS
SWCLNELTKILDCKNRYGRDVIPVFYKVDPSTVRKQKERYKAAFEKHEQLFKDDMDRVQGWKNALTEAAGLSGWDSN
VIRNNVHGLGSRGVEICQRNSISSPNFGLITLWQNKRSMGKGHEESVVQEKLDDCGFSSKIGMDILKDRCLISISHGIIVM
HDLIQEMGQEIVRQECPHYPGKRSRLFKTDEILEVLKKNKSLPKLKRLDLSYSKKLSRIQDLSLSPNIEEIILNDCPKLIKVHSS
ILLTKLTCLSLEGCYDLKSVTVPSNILSRSPGLILLSSCEKLKMFSTSQTQICYSPPSSSLIAPIFPHRNGAHISSVEDLLRSYFSE
IFSISFDRYSEADGGKERPILLTRRGPLSVLIFKKICIIDLTNCSSFTIFPFDLSEMKFLNQLCLRRCSKFENFPEIEDTMEDLAIL
ILDNTAIKALPSSLWRLVGLQELSLSGCLNLEIIPSCIGRLTRLCKLDLTYCQSLQTFPSTIFKLKLRKLNLCDCLRLRTFPEITEE
AQTFSHINLTKTAMKDLRFSFVNLVNLRSLQLDDCPNLESLPDSIVNLNLLSLLDCSGCAKLTEIPRHIGRLTSLVELSLGES
GIVNLPESIAHLSSLKSLDLSNCKKLECIPQIPPFLKQLVALDCPSIRRVMSNQNLSDSKEGVFKFHFTNAQQLDLGARANI
EGDARLRMTEDAYRSVFFCLPGCAVPQWFPFHGKGRSVTINEDLSFCSNDRLIGFALCVVFGLLNTNDINGRYGSFSYTL
KLESDDDGTQIISNNDVLINYFNWNGKDRFINQDHTFMWKLNLESLRTRGVSLRLCDARSFTFEISPLDSRDIRSTGREF
QSVVTIRECGLCPLYSSESNFAESSRETKE

>g21734.t1

MSGSSSSFVMPTSVIRYDVFLNFRGEDTHENFISHLYGALQRKHIETYIDYGLQRCEEISPAIQTAIEESKIYVVVFSENYAS
STWCLNELTKILDCKKRYGRNVIPVFYKVDPATVRKQEERYKEAFEEHEQQFKEDLSKVQRWKDALTEAAGLSGWDSNI
ISYDGLREEEKNIFLDIACFYKGHNEIVVAETLSDCGFSSKIGMDALKDKCLISILDGRIVMHDLIQEMGREIVSKECLQHP
GKRSRLFKANEIHEIVRDNKGSDAIQCILQDIDEMKEVKVHGEAFKMMNNLRMLMLYSYSSCWHRNLFLESSLVSLPDT
LKILYWTGFHQKSLTSNFCPQNLVRLEMPGCHLEQLWQGDQSLPKLKRLNLSYSKKLTTIPDLSMSPNIEEIILNDCPELIK
VHSSILLTKLTSLRLDNCYDLNSVIVPSNILSRSPGFILLSCGCSKLKNFPEIENTMEYLAVLLLDKTAIQALPSSLWRLVGLQ
ELSLSRCWNLEIIPSSIGSLTRLCKLDLTFCQSLQTFPSTIFKLKLRKLDLRGCLRLRTFPEITEPAQTFSQINLTDTAVKELPSS
LGNLANLRSLQLNRCTDLESLPNSIVNLKHLCKLDCSGCAKLTEIPTHIGRLSSLMELSLSGSGIVNLPESMGHLSGLKTLNL
SDCKKLECIPHIPPFLKQLVALDCTSIRGAMSNSPVRNLSNSKDSFFRFHLTNAQQLDSGARANIEEDARLRMSNDAYES
VCFCFPGSAVPHWFPFRSEGPSVTINEDLSFCSDDRLIGFALCVAFGVLDTNDIKGRRSSFVYSLKFECDDDDGTQIIPNN
DVLNTYFRWNLSDRLIDKDHTFLWKFNLESLRRRGMNLRRLSDARSFTFEISTSDYGYLWPDYITVLTIKECGLCPLYRSG
SNVGESSMETEEDRKRKAESYL

>g21735.t1

MAESSSSFAVSTSPTRYDVFLNFRGEDTRENFISHLYAALQRKHIETYIDYRLQRGEEISPALQTAIEESKIYVLVFSENYASS
TWCLNELTKILNCKKRYGRDVIPIFYKVDPSTVRKHEERYKDAFEEHELRFKENMDKVQEWKDALTEAAGFAGWDSNVI

RCLISILDGRIVMHDLIQEMGQEIVRKECPQHPGKRSRLFKADEIHEILRNNKGSDAIQCILQDIDEMEEVKVHAKAFKK
MKNLRMLMFYSYSSSWHLDVFLESSLSLPKLKRLDLSYSDKLTRIQDLSLSPNIEEIILNNCPKLIKVHSSILLTKLTSLRLDEC
HNLNSAIVPSNILSRSPGLILLSSCEKLKMFSTSVPRFPHVKLERQRGAISRSLLMKPEYEPGTVSSFPRLIRQSNGRRAFPR
SLIRDLSNCSSLTIFPFDLSQIKFLKKLRLSGCSKLEYFPEIEDTIENLAVLILDDTAIQALPSSLWRLVGLQELSLSRCKNLEIIP
SSIGSLTRLCKLDLTYCESLQTFPSTIFKLKLKKLDLCGCLRLRTFPEIMEPAQSFAHINLTKTLIKELPSSFGNLVNLRSLQLN
NCSDLESLPNSIVNLKCLSKLDCSGCAKLTEIPTHIGRLSSLMEMSLRKSGIVNLPESMAHLSSLKSLDLSDCKKLECIPPIPP
FLKQLVALDCPSIRRVMPNSLFRNLSDSEEGVFKFHFTNAQQLNSGARASIEEDARLRMCDDAYESVCFCFPGSAVPH
WFPFRSEGPSVSINEDLSFCSDDRLIGFALCVVFELLDTNDIEGRYGSFSYSLKFECDDDGTQIIPNNDVLNNYFQWTHKD
RLLDQSHTFVWKFNLEPLKTSGMSLRLCDATSFTFEISPYDYNFRWPNYDSVVNELKSVVKIKECGMCPLYSSGSNVAQ
CSRETKEDKKRKAES

>g21736.t1

MAESSSSLAVPTSPTKYDVFLSFRGEDTRYNFISHLYDALHRNHIEAYMDERLQRGEEISPALQHAIEESKIYVLVFSENYA
SSTWCLNELIKILNCKKIYERDVIPVFYKVDPSTVRKQEERYKAAFDEHEQRFKDEMDKVQRWKDALTEASGLSGWDSN
VIRCDSFGQGSRIIITSRDRQVLKNVGAEDIYEVKELNGVDSLKLFRSDAIQCILFDFELIKEVIVHGQAFLKMDNLRMLILE
GYYGPGFFESNMFLSSSLVSLPDTLKILYWNGFSERSLPPKFCPENLVRLEMPSCHLEQLWEEDQIKFLKKLCLDGCSKLQI
FPEIENRMENLTVLILDGTAIQALPSSLWRLVGLEQLSLRGCKNLEIIPSSIGSLTRLCKLDLSFCESLQTFPGTIFKLKLRKLD
LCGCFNLRTFPEITEPAQTFAHINLTGTAIKDLPSSLGNLAKLRSLKLNRCTDLESLPNSIVNLKHLCKLDCSGCAKLTEIPTH
IGSLSSLMELSLSESGIVNLPESIAHLSSLKSLDLSGCKNLECIPQIPPFLKQLVALDCTSIRRVMSNGNLSDSKEGVYKFHFT
NAQQLDSDARANIEEDARLRMTHDACRSVFFCLPGTGVPHWFPFRGKGRSVTTNEDLSFCSDDRLIGFALCMVFGVLD
TNGIKDRFVYFGYSLKFECDDDGTQIIPNNDKLNNYFRWGYSYRVLDKDHTFMWKFNLESVRRSGMNPRLSDARSFTF
EISPKAYNFLLPDYHSVVTIKECGMCPLYSSGSNVAESSS

>g22149.t1

MSIRTNKMKAVGVLMKQLTTSRRKFHERGRDESFDGELEKLWLVLNNIKDVFVEVKKNEENLLDTLAEVYNHLHRLDS
RKLHQDMHSICERIKYSARNLLPTLVFDESYKEEDDRGGKISHSSQELVQSHQKQSWTAKDFNLQDDRLKVCLLRLLLIFP
ENAIIRKRIAINLWIAEGLIENTEKTAEELGEDVIHNLLKLKVIVRYGSTKDPRVNKFQIPPDIRSQLKLYLDGDLKSIRPSCEL
KNLEVLQLGRWQDSPLYHVEVGSEEFLKELRTLKQLKYLSLRGISRIFELPSSIGELESLAMPKGIEKLTNLQVLKGFLITTSE
KTPCRIWDLAENLTDLRRLSIRIGSEAVIRDGEFKSLENFPALKHLKISWSVSDPRRKNGKYE

>g22150.t1

MSIRTNKMKAVAVLLKELMTARSKFDERGKDESFDRKLEKLRLDLSKIKDVFVRVKKNEEELLDILAEVYGHLHKLDREKL
NEDMDGICERIRDSAHNLLPKDAFNELSKMEDHSSEELVQPHQKKSWTAEDFNLLGLPSRFCMFSLLIFPENAVIRKRN
SIHLWIEEGLITNTEKKTAEEKGEDVIDDLLKFKVIVRYGSTKDPIVNKFQILPHVRSQLKLYLDGNSEYIRPSRLLLDRKKVI
VGGVDTKNVTLRNIYNIGASYLNFGPQWVTDQWKNLEMLQLGRWQDSPLHHIEVGSQEFLRELRTLKLLKYLSLRGISR
IFELPSSIVELESLQIIDLKACHNLETLPDDISSMKSLTHLIMFECCLLEVMPKGIEKLTNLQVLKGSEAVIKDWEFSSLRNFP
TLKHLKISWSVSDPRMFYTSRSDAM

>g22154.t1

MKAVGVLMKELTTSRRKFHERGRDESFDGKLEKLWLVLNNIKDVFVVVKKNEEKLLDTLAEVYNHLHRLDRKKLDEDM
KHICKRIRDSADNLLSKDASDELSKEEDHKGGKIYHSLEELVQPHLNEIWTEKDFNQLGYPLKPCLFCLLIFPENAVIRKRIAI
NMWIAEGLVRNTEKKTAEEKGEDVIDDLLKFKVLQLGRWQDSSLHHIEVGSQEFLRELRTLKQLKYLSLRGISNIFELPSSI
TGLERLLILDLKACHNLERLPDDISSMKSLTHLIMSDCCLLEGMPKGIEKLTNLQVLKGFLISTSPCRISDLAKNLRKLRRLSI

RIGSEAVIKDWEFSSLGTLSALKHLKISWSVSDPRYSKIGVILPISLTKLHLECFPGKSFEECFALPNLMPCELNITGGKLESM
IPQDLLWKQEDLLRNVEILRLKYLKQLNVDINNLKEWFPALKYVEIK

>g22237.t1

MEAVVSTTTENALQIAGRVVKRQLSYFFNYNDKYEEVKRYVEMLDNTRKRIQHQVNNAEMNAEEIEDDVQHCLKQLD
EKIEKYEQFIHDEYHSKTRCSIGFFPSNLSLRYRLGRNATKMVEEMKVEELWNKTFEEVSYRVLPSINVSLTNISYESFASRT
KTIDMFMQALEDSTVNMIGLYGVGGVGKTTLVKEVAKKAQEKKLFTVVVMANITRNPNIIAIQGQIAEILGMRLEEESEI
VRADRIRKRLKKEKENTLIILDDLWDRLDLNRLGIPNSDEDDGSQQDVNDISNSGYHKMEKEGLSSDFNNMTEEKLSGN
NKRCKILLTSRRKQVLCNQMDVKERSTFSIGVLDENEAKTLLKKVAGIQIQNFAYDDKAIEIARMCDGLPIALVSIGRTLKN
KSSFVWEDVYQQMKKQSYIEGKEPIEFSIKLSYDHLENELLKCIFLQCARMGNDALVMDLVKFCIGLGLLQGVHTIREAR
NKVNMLIEELKESSLLLESYSSNRFNMHDIVRDVALSISSKEKQVFFMKNSILDEWPHKNQLESYTAIFVHSCYIVDDLPG
SIYCPRLEVLQIESKDQLLKIPDDFFKDMIELRVLILTGLNLPCLPSSLIFLTKLRMLSLEKCTLGQNLSIIGELKKLRILTLSGSNI
ECFPCEFGQLDKLQLLDLSSCSKLRLIPSNVISRMNILEEFYMRDSLIRWETEENIQSQNGSLCELSHLNQLRNLDIHIQNV
VCVPQNLFFDELDSYKIIIGEFNMLTEGEFKIPDKYEVVKLLVLNLKEDEGRNINKDIIIEIEQDGTSSCHSLFNEKGNIDCVF
PKLKKMEIMCMEKLNTIWQSHISLHSFSKTMKEIVASDKGSDENVITFKFPHLKTVSLRSLFELVSFYGGTHTLEWPSLKD
LSILRCGKLEGITTNISNSQAKPIVLATQKVIYNLEYMAMSLGEVEWLQKYIINVHKMHNLQSVVLHGLKNVEVLFWFLH
RLPNLKRLIMRFCQMRRIWAPLTDISREKIGVVMQLKELELRDMWSLEEIGFEHDMLLQRVFFDHSKDMKLVDYPEMK
EVRYGKPVFSDNFFGSLKKLEFDAVSKREIVLPSHVLPYLKNLEELNVHSCKLARLKLFKSGFHDSPLQHPMFSIEEVVPKL
KELTLSEKNIILLDDGHSPQDLLHKLNYLQISFEDYDDKKDTFPFDFLHKGKSFGNIKKLEFDGKSKGDTVIPSNVLSHLKSL
EELNVHNSDEVQTHEKQRYDNLVEIAGKEDAIENGTTEVLMFEFPCLSLLTLYKLTNLNCFYPEKHHLECPKLEIMHVAYC
PKLKLFTSKIHDSHKEAIAEAPISCLQQPLFIVEKVVPKLQGLTLNEKNMMLMSDAHVPEDYLSKLNILRLCFEDDKNEKG
TLPFDFLHKVPNLEDFQVQRCFGIKEIFSSQKLQVHDGIPATLNALTLFELNQLESIGFEHPWVEKSACDTEHLKFGDHSH
LQEIWLGVAPIPTNNSFNNLKSLTVVECESFPNVIPFHLLPFLCNLKEIEVSNCQSVKAIFDVNGEGADMKPISLPLKKLVL
NQLPNLEHIWNLNPDEILSLQDLQQVSISNCQTLKSLFPTSVANHLVKLHVRACATLVEIFAVADAAINGETKQFNFHCL
TSLTLWELPELKYLYPGKHTLEWPMLTHLDIYHCDQLKLFKTEHHSGEFADTKDQLGISIHQQAAFSVEKVMPSIEHQEIT
WKDTMIGQGQFGANVAHLLQNLKLLKLMCYHEDDKSNIFSSGLLEEIPNIENLEVVCSSFTEIFCSQGPTTDCSKVLSKLK
RLHLKNLSQLNAIGLEHSWVEPLLKTLETLEVFSCPTMKILVPSTLSFSNLTSLNVGECHGLIYVFTSSTAKRLGQLKHISIRD
CQAIQEIVSKEEDHESEDEDEDITFDQLSLLSLESLPNIIGIYSGTFKLKFPCLDQVTLKECPQMKYSCVPDLHEFKPQEQT

>g22244.t1

MVEEIIEDELWKTSFDNVSYQEFSSIDATFSSNGYESFASRTKTMEMIMKALQDSTVGTIGVYGPGGVGKTTLVTEIANI
AREKKLFKIIIIANITENPDFKKIQEQIAGMLGMKLEEESEIARVDRIRKRLKNEKENTLIILDDLWGGLDFNKLGIPCNDDA
SQQEVNDMSDFGLNNDISDFGVLDEKESENLLKKVAGVKNSEFDRNATEIAKWSAGFPIALVSIGRTLKNKSLSTWEDV
CQQIKRQNFTSEWGFTDFSIKLSYDHLRNEELKCIFLHCARMGNDALIVDLVKFCVGLNLLPGVHTITDARKRVKEVIKEL
EESSLLVKSYSIDRFNMHDIVQYVALSISSKEKHVLYMKNVILDEWPHENNFERLNSYKIVIGEFNLFNLLKVGEFKVPDKY
EEVKFLALNLEEGHENTNIVTNTKHGVTNSCLPLFNEKVSTPKLEWLELSSINIHKIWSDQCGVVQLKELELDNMWSLEEI
GFEHDVLLQRVSFEYSKDINLVDYPEGKVRHGKFAFPDNFFGCLKKLKFDEACKRDTLIPSHVLPYLKNLEELNVEKCESA
QLIFDTDESEIQTYGMVFRLKKLSLKHLSNLKCVWKENTEGIVRFSNLKSVDVDGCGSLVTLFPLSLAKNLGKLKILDIQEC
EKMVEIVGREDEMEHGTTIMFEFPSLSYLYLENMPLLSCFYRGKHDLECPLLDRLYVECCPELKVFSSSFDDDSKKEVLEA
PTNLLQQPLFSIKKVSRKLEELTLNEENIKLTSDACFPQDLLCKLKGLILSFEDDKNGKDSLPFDFFHKLPNLERLTVHKCFV
RVYRFRAPVGTNILQKA

>g22253.t1

MLEEITGDELRKTFFDNVSYQEFPSIDATFSNNGYESFSSRTKTMEMIMKALQDSTVGMIGVYGPGGVGKTTLVKEIAN
KAREKNLFEIVIIANITRNPDFKKIQQQIAGMLGMKLEEESEIARVDRIRKRLKNEKENTLIILDDLWGGLDFNKLGIPCND
DASQQEVNDISDFGSNNDISDFGYNKKEMKELSKVDQDKIKKEKLSNDYRGGKILLTSRNKQVLCNEMDVQQRSIFSVG
VLEGKESEALLKKVAGVKNSEFDRNATEIAKWSAGSPHNYICEEKSEGDDKRARRIEFAGSNIENLPNEFGQLDKLQFLDI
SNCLKLRQIASNIIPRMGILEEFYIRDNLIIWEAEENLKSENASLSELNSYKIVIGEFNLFNLLKVGEFKVPDKYEEVSTPKLE
WLELSSINIRDKDKWYWEGDLNATLQTHFTNQVSFEYSKDINLVDYPERKYRRGKFGFSDNFFGCLKKLEFDSLSCKRDT
LIPSHVLPYLKNLEELDVEKCKSAQLIFDIDESEIQTYGMVFRLKKLSLKHLSNLKCVWKENLEGIVSFSNLKRVDVDGCGSL
VTLFPLSLAKSLGKLETLDIQTCEKMVEIVEKEDEMERGMTLTLEFPRLTMLSLCNMPLLSCFYPGKHHLECPLLNKLDVA
FCPKLKIFRSSFDDDSKREVLEAPISLLRQPLFSIQKVSCKLEKLTLNEKNIKLMSDAHLPQDLLLWKCFGLKEIFPSQKLQV
HEKVLAGLKQLDLFALPELECIGLEHTWVQPYTEKLELLFLYECPLVERIVSCAVSFINLEYLFVIHCQRMEYLFTFATLKSLV
KLKILCIENCESIKEIVRNEEEDGCVEIIFGRLRSIKLEYLPRLISFYSGNATLQCPCLQIVTVAECPNMITFSEGVIKLAMFLGI
QTSEDSDFTFHVDLNATVESPVVAGPMVAGSVVAGPVVVVMEVDVPVGHHWVLDFELEYFGGLKSLVQLRLPKAEG
MAGGRQESLETFQAANHIVVLAI

>g22267.t1

MEMIIKALQDSTVGMIGVYGSGGVGKTTLVKEIANKAREKKLFKIVIIANITGNPDFKNIQEQIAGMLGMKLEEESEIARV
DRIRKRLKNEKENTLIILDDLWGGLDFNKLGIPCNDDASQQEVNDMSDFGSNNDISDFGYNKTEMKELSKVDLDKMKK
EKLSNDYRGGKILLTSRNKQVLCNEMDVHESSIFSVGVLDEKESETLLKKVAGVKNSEFDRNATEIAKWSAGFPIALVSIG
RTLKNKSLSTWEDVCQQIKRQNFTSEWGFTDFSIKLSYDHLKNEELKCIFLHCARMGNDALIMDLVKFCVGLNLLPGVH
TISDARKRVKEMIRELEESSLLVRSYSIDRFNMHDIVRDVALSISSKEKLVLYMKNAILDEWPHENDFKRLNSYKIVIGEFNL
FNFLKVEEFKVPDKYEEVKFLALNLKEVCDCDSLKEIVSKESQTHTINDDKIEFPHAHSLQDQVFQQRNKDIVVDVEHRVT
NSCLPLFNEKVSTPKLEWLELSSINIYKIWSDQCSHCFQNLLTLNVTDCSNLKYLLSFSMAESLVNLQSLFVSECEMMEDIF
RPEDAEIAKSSELDSYRLRIG

>g22268.t1

MVEEMEAEELWNKKFDEVSYRVLPSINAALTNTSYESFASREKTINVCMQALEDSTINMIGLYGVGGVGKTTLVKEVAK
KAQEKKLFNVVVMANITRNPNIIKIQGQIAEMLGMRLEEESEIVRADRIRKRLNKEKENTLIILDDLWDGLDLNRLGIPISD
ENDGSQQDVNDISDSSYDKMEKEELSSDFNNMTEENLSEDQKRCKILLTSRRKQVLCNQMDVKERSTFSVGVLNEIEG
KTLLKRVAGINIQNLMYDEKAIEIARMCDGLPIALVSIGRTLKNKSSVVWEDVYQQMKKKSFMEGQEPIEFSIKLSYDHLK
NEQLKCIFLHCARMGNDALVMDLVKFCIGLGLIQGVHTIGEARNKVNMLIEELKESSLLLESYSSNRFNMHDIVRDVALSI
SSKEKQMFFMKNGIIDEWPHKDQLERYTAIFLHYCYINDDLPVSIYCPRLEVLHIDNKDQFLKIPDDFFKDMIELRVLILTG
FNLPCLPSSIICLSKLRMLSLEKCTLGQNLSIIGELKKLRILTLSGSNIESLPFEFGQLDKLQLLDLSNCSKLSAIPSNVISRMNIL
EELYMRDTLILWEAKEMIQNENASLSELRHLNQLRNLDLHIQNVSHVPQNLFFDKLDSYKIIIGEFNMLTEGEFKIPDKYE
VVKLLVLNLKEVCDCDSLKDIVAIERQPHTDSDDNIEFPQLRTLTLKSLPAFTCLYTNDKMPCSAQSLEEKGRNRNRDIIVE
VEQDDSNSCLSLFNEKGNIDYVFPKLKKMEITCMEKLNSIWQPHIVAGSPNLNHLFPLSITNDLENLEFLDVRNCRAMKE
IVGSDKAHTLEWPSLKRFLILRCGKLEGINTDISNSQVKPIGLAAEKVIYNLEFMAMSFREVEWLQNYIINVHRMQNLQT
VVLHGLKNVEVLFWLLHRLPNLKRLTLGFCHLKRIWTPASLISREKIGVVMQLQALELKNIWPLEEIGFEHECDLKFPVME
NLVVSECPKMTKFSKVQSAPNLQKVQVVATEKDKWYWEASKREIVLPSYVLPYLKNLEELNVESCKSARVIFDIDDCEIK
DTVFRLKKLTLKDLSNMKWIWNKNPEGIASFPNLEEVLVNSCGTLVTLFPSTLARNLSKLKTLTIHNCCKLVEIVEKEEEM
EDEITEMFEFPCLSKLFLWNLPMLICFYPRQHHLKCPMLERLHVAYCLKLKLFTSQPHHSLPHPMFLIEEVVPKLKEVILNE
KNITLLKDGHAPDLLHKLNYLDLASEDYENKKDSLPFDFLQKGFFEYSKQMILLDYLEMRGFGPVRLVFPPKIFGSLKKLEF
DGASKGDTVIPSNVLSHLKSLEELNVHSSDEVQVIFGMDHNSRAKSKETVFHLKKLTLKDLSNLKCILNKNLQGSVSFPNL
QELFIDGCGSLVTLFARKLQTLEMQKCDKLVEIVGNEDATTETFAFEFPCLSSLTLYNLTDLSCFYPGKHHLECPQLEILHV
AYCPKLNLFKSKIHDSHKETVAVAPINWLQQPLFMVEKVVPKLRGLTLNEKNMMLLSDEHVPEVNLTKLNLLRLCFEDD

KNEKDSLPFEFMNKVLNLEHLRVQRCFGVKEIFPSQKFQVHDGIPASLKGLTLFELNELESIGFEHPWQKTYLEDLPRSTS
KGIGYEKSTVANKATLQAYQSVLCSSTLQTSTEPKMPNSTQTSSPHKEHSTMTISQGHV

>g22270.t1

MVKEIIGDELWKTSFDNVSYQEFPSIDATFSNNGYESFSSRTKTMEMIMKALQDSTVGLIGVYGPGGVGVLDEKESETLL
KKVAGVKNSEFDRNATEIAKWSAGFPIALVSIGRTLKNKSLSTWEDVCVHTITDARKRVKEMIKELEESSLLVKSYSIDRFN
MHDIVRDVALSISTKEKHVLYMKNAILDEWPHENDFERLNSYKIFIGEFNLFNLLKEGEFKVPDKYDEEIVSKESQTHTISD
DKIEFPHAHSLQEQVFKRRNKDIAVEVEQRVTNSCLPLFNEKVSTPKLEWLELSSINIHEIWSDQCGVMQLKELQLLNIW
SLEEIGFEHENLEELNVEKCESAQLIFDIDETEIQTSLVTLFPSSLAKNLGKLKTLYIEDCEKMVEIVGREDEMEDGTTIMFEF
PYLSCLRLQNMELLSCFYPGKHDLECPLLDQLYVTCCPKVKLFRSSFDDDSKKEVLEGPTNLLQQPLFSIEKVSPKLEGLTL
NEEKIKLMSDERSPQDLLCKLKGLILDDDNNGKDSLPFDFFHKLPNLEQLTIRKCFGLKEIFPSQKLQVHDNVLAGLKGLC
LLELKELKCIGLEHTWVQPYTEKLKQLLLYVCPLVERIVYYAVSFINLKDIYVMGCERMEYLFTFATLKRLVKLETLYIGYCGS
MKEIARNEEEDGCDEIIFGRLKWIKLEYLWRLISFYSGNATFQCPCLQTAIVAECPI

>g22271.t1

MVEEIIGDELWKTSFDNVSYQAFPFIDDTFSNNGYESFGSRTKTMEMIMKALQDSTVGMIGVYGPGGVGKTTLVKEIA
NKAREKMLFKIVIIANITGNPDLKKIQEQIAGMLGMKLEGESEIARVDRIRKRLKNEKENTLIILDDLWGGVDFNKLGIPC
NDDANQQEVNDMYDFGSSNNISDFGYNKTEIKELSKVDLDEMKKEKLSNDYRGGKILLTSRNKQVLCTEMDVQERSIF
SVGVLDEKESETLLKKVAGVKNSEFDRNATEIAKWSAGFPIALVSIGRTLKNKSLSTWEDVCQQIKRQNFTSEWGFTDFS
VKLSYDHLKNEELKCIFLQCARMGNDALIMDLVKFCVGLNLLPGVHTITDARKRIKEMIKELEESSLLVKSYSIDRFNMHDI
VRDVALSISSKEKHVLYMKNEILDEWPHENDFERYSAIFLHFCDITDELPERSNIENLPLEFGQLEKLQFLDISNCLKLRQIT
SNIIPRMAILEEFYMRDNLIIWEAEENMKSENASLSELRHLNQLQNLDIQIHSSSHFPQNLFFDRLNSYKIVIGEFNLFNLL
KVGEFKVPDKYEEVRFLALNLKEGEKDKWYWEGDLNATLQTHFTNQVSFEYSKDINLVDYPERKVRHGKFDFPDNFFA
CLKKLEFDEACERDTLIPSHVLPYLKNLEELNVEKCKSAQIIFDIDESEIQTYGMVFRLKKLILNDLLNLKCVWKENLEGIVSF
SNLQEVEVYGCGSLVTLFPSSLAKNLGKLKRLIIGECEKMVEIIGREDKMEHGTTIMFEFPCLSYLLLEKMPLLSCFYPEKH
HLECPLLACLYVACCPKLKLFRSSSDDDSKREVLPTNLLQQPFFSIEKVSPKAVGLTLNEENIKLMSDASLPQDLLCKLKYLIL
SFEDDNNRKDSLPFDFFHKLPNLEWLRVQKCFGLKEIFPSQKLQVHDRGLEGLKELLLIDLKELELVGLEHPWVKPYSEKL
QVLELDNCPQLQKIVHCAVSFINLKELRVALCERMEYLFTFATVKSLVKLETLIIQSCESIKEIIKHENEDGYAEMVFGRLKSI
KLKSLPRLVRFYSGKATLQCSYLKVVMVVKCPSMITFSEGVMKMPMFSGIQTSKDSDLTFHDDLNTTIKKLFHEEVEKSA
CDVEHLKFGDHPNLEEIWLDVVPISRNDCFNNLKSLAVVECESLSNVIPFDILRFLSNLKEIEVSNCQSVKAIFDVNGEGA
DMRPISLPLKKLVLNQLPNLEHIWNLNPDEILSLQDLQQVSISNCQTLKSLFPTSVANHLVKLDVRACATLVEIFVEAEIAF
EGETKQLIFHCLNSLTLRELSELKYLYSGKHTLEWPMLTHLDIYHCDQLKLFKTEHHSDEFADTKDQFGISIHQQAAFSVE
KEHQAITCKESMIGEGEFGTKAAHLLQNLEVLKLMCYHEDDESNIFSSGLLEEIPTIENLEVVCSSFTEIFSSQRPRDDCSKV
LSKLKRLHLKSLQKLISIGLEQSWVEPLVKTVETLEVFCCPSLKSVAPSTVCFSNLTSLNVGECDGMVYLLTSSTAKSLSQLR
RMYVRDCQAIEEIVWNDEEDDESNDEEIAFEKLSDMSLSSLPSIVGICSEALKLKFPSLDHVTLTECPNMKYSYIPDLQQF
KPRHQI

>g22273.t1

MVKEIIGDELWKTSFDNVSYQEFPSIDATFSNNGYESFGSRTKTMEMIMKALQDSTVGLIGVYGPGGVGKTTLVKEIAR
KALEKKLFKIVIIANITGNPDLKKIQEQIAGMLGMKLEGESEIARVDRIRKRLKNGKENTLIILDDLWGGLDFNKLGIPCND
DASQQEVIDMSDFGSNISDFGYNKTEMKELSKVDLDKMEKEKLSNDYRRGKILLISRNKQVLCNEMDVQQSSIFSVGVL
DEKESETLLKKVVGVKNSEFDKNATEIAKWSAGFPIALVSIGRTLKNKSLSTWEDVCQQIKRQNFTSEWGFTDFSIKLSYD
HLKNEELKCIFLHCKNGK

>g22275.t1

MVKEIIGDELWKTSFDNVSYQEFPSIDATFLNTGYESFGSRTKTTAMIMKALQDSTVGLIGVYGPGGVGKTTLVKEIARK
ALEKKLFKIVIIANITGNPDLKKIQEQIAGMLGMKLEGESEIARVDRIRKRLKNEKENTLIILDDLWGGLDFNKLGIPCNDD
ASQQEVIDMSDFGSNISDFGYNKTEMKELSKVDLDKMEKEKLSNDYRGGKILLTSRNKQVLCNEMDVQQRSIFSVGVL
DEKESEALLKKVAGVKNSEFDRNATEIAKWSAGFPIALVSIGRTLKNKSLSTWEDVCQQIKRQNFTSEWGFTDFSIKLSYD
HLKNEELKCIFLHCARMGNDALIMDLVKFCVGLNLLPGVHTIAHARKRVKEMIKELEESSLLVKSYSIDRFNMHDIVRDV
ALSISSKEKHVLYMKNAILDEWPDENDFERLNSYKIVIGEFKLFNLLKVGEFKVPEKYEEEKFLALNLIEDQVFQQRNKDIV
VDAEHRVTNSCLSLFNEKVSTPKLEWLELSSINIHKIWSDQYLHELRSFYSGIHTLEWPPLKKLDIVDCSMLEGLTSEITNSK
EQPIVLATKKVHKVRSDRNQMFFF

>g22277.t1

MVDEIIGDELWKTSFDNVSYQECPSIDATFSNNGYESFASRTKTLEMIMEALQDSTVGMIGVYGPGGVGKTTLVKEIAR
KAQEKKLFKIVIIASITGNPDFKKIQEQIAGMLGMKLEEESEIARLDRIRKRLKNEKENTLIILDDLWSGLDFNKLGIPCNDD
ASQREVNDMSDFGSNNDISDFGYNKTEMKELSKVDLDMMKKEKLSNDYRGGKILLISRNKQVLCNEMDVPQRSIFSV
GVLDEKESETLLKKVASVKNSEFDRNATEIAKWSAGFPIALVSIGRTLKNKSLSTWEDVCQQIKRQNFTSEWGFTDFSIKL
SYDHLKNEELKCIFLHCARMGNDALIMDLVKFCVGLNLLPGVHTITYARKRVKEMIKELEESSLLVKSYSIDCFNMHDIVR
DVALSISSKEKNVLYMKNVILDEWPHENDFERLNSYKIVIGEFNLFNLLKVGEFKVPDKYQEIPGSAHSLQDKVFQQRNR
DTVVDVEHRVTNSCLPLFNEKVSTPKLEWLELSSINIHKIWSDQSGEKDKWYWEGDLNATLQKHFSYQVSFEYSKHARL
VDYPERKDVRLGKSAFPENYFGCLKKLEFGEACTRNILIPSHVLPYLKILEELNVENCKPTQVIFDIDESELKMKGMIFRLKK
LNLNKLSNLKCVWKESSGGIVSFPNLHRVDVDGCGSLVTLFPLSLARNIKKLETLQITECEKLVEIVGKEVMEHGTTIIFNF
PCLSWLFVHNVPLLSCFYPGKHYLKCPLLQSLFVASCPKLKLFTSEFDDNQKATIIEAPVIPIRPLFSVEILASPKLKKLSLNEE
NVMLLRDAHLPKDLFCNLNVLRLYFEDDNNQKDSLPFDFFHRLPSLTCLIVQKFFGLREIFPYQKLQVHDKVLAGLRELLL
LKLKELESIGLEHTWVQPYSEKLELLRIHSCPRLQKIVSCAVSFINLKELQVTFCDQMEYLFTFATVKSLVKLECLIVKYCESIK
EIVKSEDGDDCDEILVFGRLRSIELDSLPRLLRFYSGNATLRCLCLKTVMVAKCPNMITFSLGTINVPLFSGINVSSKDSDLT
FDGDLNTTIETFFHEQEFFNLSKHMILDEYLEMTGVQHIKPAISDNFFGSFKELEFDAVCKRAIVIPFHVLPYLKNLEKLNV
HSSDAVKVIFDIDEIEDKTKGIVSSLRKLTLNNLSNLKHVWKENSTGIISFHNLQEVVVNGCGSLITLFSSSLARNLRKLKTLE
ITECGKLVEIVEKEDGMERGTKIMFELPCLSWLYLENMPLLSCFYPGKHDLDCPLLETLLVCYCPKLKLFTSDFDKNQKGDI
ETQISPLQQPMFSVEKDDNNEKDSLPFDFFHKLPNLFFLTIEKCFGLREIFPSQKLQVHDRGLAELKQLALMDLKDLELLG
LEHPWVQPYSEKLEVLKLKKCPQLQKLVYCAVSFINLKELQVKLCERMEYLFTFATVKSLVKLETLIINSCESIKEIIKHENED
GCAEMVFGRLKSIKLKSLPRLVRFYSGNATLQCSYLKIVMVVKCPSMKTFSKGVIKVPMILGIQTSKDSDLTFHDDLNTTI
KKLFHEEVEKSTCDVEHLKFSDHPNLEDIWLGVVSIPRNDCFNNLKSVAAVECESLSNAIPFYSLRFLSKLKEIEVSNCQSV
KEIFDVNSAAADMKPISLPLKKLILNQLPNQENIWNLNPDEILSLQDLQQVSLSNCQTLKSLFPTSVANHLVKLDVRACAT
LVETFLDAETSFEGETKQFNSIV

>g22653.t1

MATRILEGLVKTSEIYSLILDILGGFNGFNDNVQMLEMKLEELCCLEHDINRELEIEELEHGKKRKREVENWLRNVKRKKT
EVHGMVQELRDCGMFRHLKLIVQVRKLTGQVTDLVARGRFPGGIVGSAQESRGCALLTTELAGAMFQKNVRKIWDW
LVNDGVLMIGVYGMGGVGKTSMLMHIHNMLLTAVTNFESVFWVTISKSFSIHKLQCDVAKIVGIDISKESDERKRAARL
SRALVRRKRCVLFLDDVWNHFPLERVGIPVRADGLKLVLTSRSLDVCRRMNCKNSVKVEPLSMEEAWTLFVDNLGQQT
TLSPEVKQVARSVAKQCAGLPLAIITMARSMRGVEEICEWRHTLEELKNTEAKHEEMDMELLRVLRFSYDHLNDKILQQ
CFLCCALYPEDFEIDRDVLIETFVDEGLVNGMKSLEAMFDEGNTIVNKLENICLLGKVENFVGGKKCMGLQDASSHIAIS
MMKRGCQHVELMNNVEGYYVGSQLVKMHDLVRAMAINVIKENNNFLVKAGLQLTKIPDEVEWSEDLEKVSLMCNW
IHEIPTEISPRCPKLRTLILKHNESLTRISDSFFVHMSALEVLDLSFTDIEVLPKSVSDLSTLTALLLTSCKRLKHMPSLAKLQAL
LRLDLSFTAITEMPQGLEMLVNLKWLNLYAKELVSSGKEVAKLTSLQFLILHWWSRKIKVKVEYASCLRKLETFAANLYN

MHHFNSYVKTMHIYGPRSYLLQLDTEESLGNSPWCCFAEVCFRKDVIISNCKIRTGESPLMLPLDIQRLKVERCNDIRSLC
DVMSLKNANSLKRCEIADCDGPEYMFSLSCSSSCCTSLHSLESLELYSLKNLHGLCKDGEVEAQTFPPGRAFTCLKYFFIYR
CPLIKKLLTPRLLAYLPNLEEITVHNCKSMEEIISVDGIDYESFGGKKSHVTNRDTIVVTHSKLVSLSLKHLPELKSISSAQMV
CESLKNFRIFKCPKLARFPKTATPVQILYDSF

>g22793.t1

MVEEIIGDELWKTFFDNVSYQEFPSIDATFSNNGYESFASRTKTMKMIMKALQDSTVGMIGVYGPGGVGKTTLVKEIA
NKAREKNLFEIVIIANITGNPDIKKIQEQIAGMLGMKLEEESEIARVDRIRKRLKNEKENTLIILDDLWGGLDFNKLGIPCN
DDASQQDVNEISDFGYNKIEIKELSKVDLDKIKKEKLSNDYRRGKILLTSRNKQVLCNEMDVQQRSIFSVGVLDEKESETLL
KKVAGVKNSEFDRNATEIAKWSAGFPIALVSIGRTLKNKSLSTWEDVCQQIKRQNFTSEWGFTDFSIKLSYDHLKNEELK
CIFLHCARMGNDALIMDLVKFCVGLDLLPEVYTITDARKRVKEIIQELEESSLLVKSYSNDRFNMHDIVRDVALSISSKEKQ
VLYKKNAILYEWPHENDFERLNSYKIVIGEFNLSNLLKVGEFKVPDKYEEVSTPKLEWLELSSINIHKIWSDQFAKDLGKLK
TLDIKECEKMIEIVGREDEREHGTTIMFEFPCLSYLNLENMPLLSCFYPGKHHLECPLLDKLYVACCPKLKVFRSSFDDDSK
KEVLEAPSNLLQQPLFSIQKISPKPVGLTLNEENIKLMSDARLPQDLLCKLKFLILSFEDDNNGKDSLPFDFFHKLPNLESLT
VQKCSGLKEIFPSQKIQVHDNVLAGLKGLFFLELSELESIGLEHTWVQPYTEKLELLYLLNCPQVENIVSCAVSFINLKDLFV
TFCEKMEYLFTSATLKSLVKLETLLIGYCGSIKEIARNEEEDGCDEIIFGRLRSINLECLPRLVSFYSGNATLQCPCLQNVMVT
ECPNMITFSEGVIKLAMFSGIQTFEDSDFTFHVDLNTTVESLFHEKVPDCTTTFYRHLGFISKRSVPNLNLGELFEQEFFNH
SKHMILDEYLEITGAQHIKPDIADNFFGSFKELEFNAACKRAIVIPFHLLPYLKNLEKLNVHSSDAVNVIFDFDESEDKTKGI
VSSLKELTLKNLSNLKCVWKENIEGIVSFPNLEKVTVTGCRSLVTLLSSSLAKSLEKLKTLNMERCEKLEEIVGEEDEREHG
MTLTHKEEVIEAPISPLQQPLFLVEKVIFPSQKLQVHNKVLAGLKQLSLLRLRELESIGLEHEWVQPYSRKLEVLKLDTCPQ
SVMVAKCPNMITFSEGVINMPILSGIKTSKDSVVVFHDNLNTTIETLLHEQEFMEYSKRMILEDYLGMSGVHHRKPVVS
DNFFGSFKKLEFDGACNRTILIPSHVLPYLKNLEELNVKNSDAMQIIFDIDESEVKTKGVVFGLKKLTLNKLSNLKHVWKE
NSTGIISFHNLQEVVVNGCGGLIRLFSSSLARNLWKLEELRIKECGKLVEIVEKEDGTENGTKIMFEFPCLTSLYLKNMPLLS
CFYPGKHDLDCPLLELLLVYFCPKLKLFTSDFDENQKGAIEAQISPLQQPMFSVEKEIFPSQKLQVHDGGLAGLKELFLVDL
KELELVGLEHSWVQPYSEKLQVLSLKSMKTFSEGVMKVPKFSGIQTSKDSDLIFHEDLNTTIKKLFHEEVEKSACDLEHLKF
GDHPNLEEIWLGVVPIPRNDSFNNLQSLAVVECESLSNVIPFYLLHFLSNLKEIEVSNCQSVKAIFDVKGEGADMKPISLSL
KKLILNQLPNLEHIWNLNPDEILSLQDLQQVSISNCQTLKSLFPTAVANHLVKLDVRACATLVEIFVEAETAFEGETKQFNF
HCLTSITLWELPELKYLYPGKHTLEWPMLAHLDIYHCDQLKLFKIEHHSDEFSDTKDQLGISIHQQPAFSVKKVMPSIEHQ
AITWKDTRIGQGQFGANVAHLLQNLKLLKLMCYHEDDKSNIFSSGLLEEIPNIENLEVVCSSFTEIFCSQGPTHDCSKVLS
KLKRLHLKNLPQLNAIGLENSWVEPLLKTLETLEVFSCPTMKILVPSTLSFSNLTSLSVDECHGLLYLFTSSTAKRLRQLKHIS
IRDCQAIQEIVSKEEDHESEDEDITFDQLSLLSLQSLPNIVVIYSGTFKLKFPCLDQVTIKECPQMKYSYVPDLHEFKPQERT

>g22881.t1

MYSNVFNIIRRILRYVKEPYRIMSGMSDGMTVFDPTVPIGPSVRASDATIHHTSSSSHAYDVFVSFRGEDTRNNFTSFLF
AALRRQRILAFKDDECIKKGDFIAPELQQAIQGSQVFVVVLSKNYASSTWCLRELLEILNCCETSARPLIPIFYDVEPTTVRN
QKGCYEKAFAEHENRFREDKVKMEEVQTWKEALKKVADISGSEIGNKDELVGIEDRVQDLGNILRFDLFNDVRVVGISG
MGGIGKTTLARALYERICHQYNHRCFIDDSKLNNAKALIVFDNVDEVQQLRMFSGNRDNLLRECLGGGSRIIIVSRDEQI
LKIHGVDDIYQVRPLIWKDAIQLFCRNAFKVNYILSDYEELAYEILSHVEGHPLAIETIGSSLFGRNSIQVLIDKSLITRDFDG
RIHMHSLLVDLGRCIVREVSPKEPLKWSRLWTGKDLYDAMSENEETRNLEAIVLESRVEIRGLKIFGCPELVDVEQCTAKS
FSWAIQILEAAYRRQFPESIIPGSQIPSLFNNEFVNGDIDGECLTFDPPPVTHDNNLIGLLGCVIFRLYNEQIPMDFGRYH
MWLHYEELINMDGSGIYPWVNHILELLGYDTYSIKVDVKKFQYRWKIHFEVKNVGSIIMTLFTMASAQSLNYVNDDCH
NSTTKEQALTLTFRTNLNTTLSRLSSDAATSKGFNNTTTGNGSTLADDAVYGLYDCRGDVTGPFCQFCVCTAASDILQQ
CPNRSSAVIWYNYCILRYSNHNFFGNLTTTPSWEIVENSKNTSDPQEIQQAESYMQSLKREATVESNKLYAMGGFNLSD
GEERYGLVQCSRDLTSDECSQCLEAMIEKIPQCCAAKRAWQVLAPSCLIKYDDFMFYQITDQTSSPLPNSDGLMSVNTP

RSFHGHVQREEALNADLPTIPLIWIQQSTNYFSDLSKLGEGGFGPVYKGNLEDGTEVAVKRLSKASNQGLEEFKNEVIFIA KLQHRNLVRLLGCCIEENEKLLVYEYMPNSSLDFHLFSMF

>g22883.t1

MLSGARSQWDSSHAIIHRTSSSSNAYDVFVSFRGEDTRNNFTSFLFGALRRQGILAFKDDQYIKKAYNAAQSKRCYEKAF AEHKNRFREDKVKMEEVQKWKEALRKVADISGSEIGNKDELVGIEDRVQDLRTILRFDLRNDVRVVGISGMGGIGKTTL ARSKLNNAYRDDNVDEVQQLKIFSGNRDNLLRECLGFRGFHPEDSIQVLIDKSLITISTFGFIHMHSLLVDLGRCIVREVSP KEPIKRSRLWTPKDLHDAMSENEATRNLEAIVLENHVEIRDIRGPKS

>g22884.t1

MYSSVFNILRPILRCVFNILRRIFRCVEEPDRIMSGMSEGMTVSHPTAPIGPSVASDAIIHHTPSSSHAYDVFVSFRGEDTR NNFTSFLFGALRRQGILAFKDDQCIKKGDFIAPELLQAIQGSQVFVVVLSKNYASSTWCLRELVEILNCCETSARPLIPIFYD VEPTTVRNQKGCYEKAFAEHENRFREDKVKMEEVQRWKEALRKVADISGSEIGNKAQNEQIEKIVREITNNLRPKILNLP RDELVGIEARVQDLGNILHFDLHNDVRVVGISGMGGIGKTTLARALYERICHQYNYRCFIDDSKLNNAKALIVFDNVDEV RQLRIFSGNRDNLLRECLENVKEILNFRGFHPEDSIQVLIDKSLITRDFDGRIHMHSLLVDLGRCIVREVSPKEPIKWSRLW TRKDLRNAMSENEATRNLEAIVLENHVVIRDETIRADGLSKIKHLKLLFVKNLNVNFSGSLSHLSDELGYLVWYEYPFECLP QSFQPHNLLELVLYGSSIQRLWEGKKYCKGLKYLPDLPSRTVLPSEPFTSRFPSSILKSHLTSLKFTGLIIFGCPELVEIEQLTT KSFSWTIQIIEATYQLPFSETYMPDSIMPGSQIPSLFNNEFVNGDIENLEEYLGEEYLTIDRPPVPHDNNFIGVLYCVIFCLY NIEQIPMNFGRDHMWLHYEELNMDRSGIYPCLTRIIELLGHTTYSSTVYVKKFQYRWVNEQDLINLKMMHCANFTVGK RKISAIEENG

>g22912.t1

MAITLSEGVSSSSGFDRGWTYDVFLSFRGEDTRRSFTGFLYHGLCQRGINVFIDDEKLRRGEDLSPTLLGAIQESRIAIIVFS QNYAFSTWCLDELAKIIDCYKTRGQLVWPVFFHVDPSVVRHQRGTFQTAMAQHEVRFKGNVEKLQKWKKALFEASNF SGWNLENGYEFQIIQDIVEEASRKLSHTSLHIAEYPVGIETRISEVMPLLQIEPGEDIRVIGIYGLGGIGKTTIARALYNMIAD QFEATSFLSDIRESSNQRQGLVQLQESLLFDTVGDKNIKLGSIYKGIPIIKKRLCCKKVLLIIDDVDRLEQLQALAGGRDWF GFGSVIIITTRDKQLLSAHQVDKTYEVKKLNYGEAFELFTWSAFKRKAPDAGYLEVSNRVVLYAEGLPLALKIMGSNLFGK TVEEWKSALGKYERIPNKEVQNVLRITYDNLEENEKEIFLDVACFFKGETAEYVEKTLQACGFYPTIGISVLIDRSLVSIDEY NRLRMHDLIQDMGREIVREVSPLEPGKRSRLWYHEDVLEVLTENKGTYRIQGMMVDLPDDYMVHLKDDSFKKMKNL KILIVRNGNFFGSPQHLPNSLRLLDWMKYPSSLPSSFQPKKLVVLNLSGSRFTMQEPFKYLDSLTSMDLSSCELLTKLPEIA GVPNLTQLTLDYCTNLEEVHESVGFLEKLVEFRAYGCTKLKVFPNAIRLTSLRSLILNWCSSLQNFPAILGKMDNLISISIEG TGIKELPPSIGNLVGLQELSMTSCLNLKELPHNFDMLQSLTNLDMEGCPHLRNFLTKLANIGESTHTFGNILSLNETFEG WELQAMVPGTMVPEWFDQITKGEYMTFWVREKFPAIIICFVLEVESEMKKIFNCEIRFYINGEEVYELQIPRGFSDMVT DHVWLYDLRTRSSINWRSLDLYLMDGWNQVEISCEKISGASNVTVSWSGVHVVKQEANMKDILLTDPDPDLDSVIAS GSNTLVYDHPVKAQPQSQVTSFILQTPQNNNSSTIVLPTTVQTSLTVNDADMEAFYAVLDDEISVVSLNNDSTMVSKLT NQRPSEETEKALKTLQAHVTKEFSALLGPNEYSTMNDTLEYLTNLPAEDGISVEIRSLIIQVSRQFTRWSRDYTSENKKIES TTAKLLKADELEKCLEANKTNFKQVMCMENELSNDLAYLEQRKRELEEQINAVKANISASEAAKNMATQIKREIFGEAKI LKAQRDELREQVPHLRDEQELAKKIQSNIRDEWSKLGEKFNYGLRHGKID

>g22919.t1

MAEASISFALGEVFQILKEEKNLLSGINTEFLDIRDELESIQAFLKDADRKAADEANTNDGIRTWVKQVRQVSVRIEDVID EYLRVIHQVPCHGFGASICKITNLIRKLLSRHQIAVEIQDIKLSLSLIKERSERYKFQVSQEKSTSSSTGRIEGSGWNDHRMG SLFIEETEIVGFELPRDELLSLLLEGKKERTLISVVGMGGLGKTTLAKHVFDSENVKVHFDCRACITVSQSYTVRGIFTDMIK QFCTETKDPLPEMLEEMDEKTLISELRQYLEHKRYLIFFDDVWHEDFCNQVELAMPSNNRSSRIIITTRMIHVVEFFKKSF

PLHVHNLQPLPSEKAWELFCKKAFKFELDGQCPAELKGMSNEIVGKCKGLPLAIVAIGGLLSTKSKTVFEWQKVSQNLNL
ELHRNAHLTRLTKILSLSYDDLPYYLKPCILYFGIYPEDSSINHKRLTRQWIAEGFVKSDGRTLEQIADEYLSELIYRSLVQVS
GVGFEGKVKSCRVHDLLHELIVRKMKDLCFCHFVHEGDDESATSASTRRLSIDTSINNVLKSTNFTHIRALHAFGKGGAV
ELFTGLLASKSRVLKVLDLESTSLNHVPRNLGNIFHLKYLNLKSTKIRSIPKSVGRLQNLETLDIRETLVHELPSEINKLKKLRH
LLAFHRNYEAAYSLLGFTTGVLMKKGIKNLTSLQNLCYVEVEHGGIDLIRELIFLKQLRKLGLRRVRREHGKAICASVAEMR
HLESLNITAIGEDEIIDLNSISSIPQLQRLHLKARLEKMPNWISKLEFLVKMRLALSNLKDDPLRSLENLPNLLKLTIWDNAY
GGEILHFQSGGFRKLKELNLARLNTVSAILIDKGALVSLEYVKITKITHLKKVPSGIKALYNLKVIDFCDMPTELVESIDPQN
GHDYWIINHVPLVFIRRWMGPKLNDFEVRIVHLSTKESLTN

>g22920.t1

MADSAVSFVVEQLYQLLREEGNLLKGLGNDFADIKHELESIKAFLKDADRRAGDEGGEGDTHEGIKTWVKQLREISFCIE
DVIDEYIMDVAYRANHHPPCIASLQKIAHKIKTLKSRHRIASNIQDIKSAVQGIKERSERYKFQSTFEDGSLNSSKGAKDFK
WDDPRMASHFIEETEVVGFELPKDELIGCLIKGTDQLSLISVVGMGGLGKSTLAKHVFNNQQVKRHFYCRSFITVSQSYT
VREILTEMVQKFCKDANEPIPKGLHNMDDETLVTELRQYLQSKRYLVLFDDVWKENFCDEIEHALPNNRKGSRIIITTRK
MHVAEYFKKSVVVHVHRLQHLSPDKAWELFCKKAFRFEPSEQCPTELEDMSKEIVQKCGGLPLAIVCMGGLLATKEKSI
LEWRKVCQNLRMELERNTHLNSLKWILSLSYDDLPHNLKSCMLYFGVYPEDYSISRKRLTRQWMAEGFIKNEERRPVED
VAEEYLTQLTSRSLVQVSRVGFDGKVKSCQVHDLLRDIIIRKMNELSFCHLMREDDELDTVEKTRRFSIASCSKNVLRETS
NSGIRAIYVFKKSELPEDFVGSLSAKFKLLKVLDFESTMLNSVPNNLGNLFHLRYLNLSHTKVKILPKSVGKLLNLETLDLRQ
TQVQVLPREIKNLTKLRLLPVYYRKYEGQYSMLNFTTGVKMQKGIGCLKSLQKLYFLEADHGGLELMQELKMLKQLRKL
GIRRVKTEYANALSSAIGEMKHLESLNVSAKDQDEIIDLNFLSTPTSLLVLNLKARVTKFPDWIPKLKYLVKLRLGLSNLEGD
PLDSLKDLPSLLRLNMWDNAYIGESLHFKRGGFRRLKELDLTRLSRLNSISIDEGALLGLEHFRFKDNPQMKVVPHGLKHL
KNLQFLGFADMPAELVESIDPEKDGEDYSVIKHIPLVLIRQNVGPKFHDYELRAIPTLVTV

>g22921.t1

MLSGARSQWDSSHAIIRHTSSSSNAYDVFVSFRGEDTRNNFTSFLFGALRRQGILAFKDDQCIKIGDFIAPELLQAIHGSQ
VFVVVLSKNYASSTWCLRELVEILNCCETSARPVIPIFYDVDPTTVRNQKGCYEKAFAEHENRFREDKVKMEEVHTWKE
ALRKVADISGSEIGNKDELVGIEDRVQDLGNILRFDLLNDVRVVGISGMGGIGKTTLARALYERICHQYNHCCFIDDSKLN
NAKALIVFDNVDEIQQLRIFSGNRDNLLRECLGKGSRIIIVSRDEQILKIHGVDDIYQVRPLIWKAAIQLFCRNAFKDSIQVL
IDKSLITRDFDERIHMHSLLVDMGRCIVREVSPKEPIKWSRLWTRKDLRDAMSENEATRNLEAIVVRYHLDEVGVKIPDD
TIKADGLSKIKHLKLLDVRNVKFSGSLSHLSNEIGYLIWYRYPFECLPQSFQPHKLVELCLYGRSIQRLWEGKKAAYQLSFP
RTYIPESIMPGSQIPSLFNNEFVNVDIQYPDNNFITIYPPPVPHDNNFIGVLCCVIFRLHNKHIPMDFRRDHMWLHYKDV
SMDGRDIHPYPFRLRSINALLDDRDFAALDVKKFQYRWVNEQDLINFKITHCANLTARKISVIEENG

>g23530.t1

MIVVTTRSKEVASTMLSKVYFLEQLQEDDSSELFVKHAFPNSECRDIDKKISKKCKGLPLALKTIGSLLDNKSSVSEWETVF
QNDIWELPKDRFDIVPALALSYIHLPPHLKNLKVMMNPFIVGHSKEFGIHRLGELNLDGSISIEELENIENSLDALEEDLKN
KTCLVKLKLRRDSRRNIDSKKEDDVIENMQPSKNLKELSIFSYGEKQFPNWLLENSLWNMMSLVLEECESCQSLPPLGLL
PFLKDLMG

>g23533.t1

MAVQLIIDAALSKFFEKTFDNLFSRFGDIFRGDKSKKKQISNLKGKLLAIDVVADDAEQKQFTNPRVRDWLLAAKDAVYD
AEDLLEEIDHDAKEVWNPLTSSFTSFFENKFETRMEQLIEDLEDLASQSHVLGLKKADDVGVSGWVSKLRSTYLPNESVIY
GRDDDKKFVFEWLTSNTHNNLSILSIVGMPGVGKTTLAQHLFNDPRMDKNKFDVKVWVCVSDEFDVFKVSRAILEDV
TGSPDDSRDTEMVHKRLKEKFTRKKFLLVLDDVWNENQSKWEEVQKPLVFGAKGSKILVTTRNKEVATIMQSEEHYLE

QLPYKHSWRLFAKHAFRNDDTEPNPEYKDIDVNIVKKCKGLPLALKTMGSLLYNKSSLPEWQTVFQSEIWEFSQERCDI
VPALALSYIHLPSHLKVCFAYCALFPKDYEFRKEDLIQLWMSENFLHSHQHSKTPEEACQQYFNDLKSRYLEELPSNLNSL
TTLCRLEFIETKVIKVPNLKELKNLKVMMSSFKVGCSSELGIQRLGELNNLHESLSIEELEKIEKPRDASEANLKNNTHLVKL
ELKWNSKRGENSIDSKKEEDVIEKLQPSKNLKELSIFSYGGRQFPDWLLEDSLWEMVSLALKECKSCQRLPPLGLWKYLK
DLKIAGLDGIVSIDADFYGNNSSSFNFLENLEFSDMKQLKKWKCKAVRGAFPSLKRLSISNCPKLEGDLPEQLVPLNDLKIS
QCDQALTVTALKVRWPTMKRLKIDGDNLEASMVKIVWHFTSLEVLDINSHSKANSDESVSPWTFPLHFFPTLNALNLR
GFLNLQMISQDDRHNHLKYLTIEKCPKFESLPESMHTLLPSLMRLSIYDCQKLESFPHGGLPSNLNFVKLQNCCSTLIGSLK
GALGANTLKSLWIGLVDAECFPVENLLPLSLTSLHIYRFENLKNLNYEGLRQLSSLKKLSLRDCPNLERLPEEGLPESISQFSI
SGDCPLLKHRCQREVGEDWEKIAHIQNLDVEVIFR

>g23535.t1

MAAEKIAGALVSTFVERTIDNLASQFVDIFRGNKSNKKQLSNLKVKLLAVDAVADDAEQKQFTDPHVRDWLLAAKDV
MLDTEDLLEEIDYALSKSLVEAESQSSTKKVRNSLKSSFVSFFKNEIESRMEELIENLEYLATQSHALGLKKADDVGDRSGS
GSKLRSTYLPNESVIYGRDDDKEFVFNWLTSDIPNNLSILSIVGMGGVGKTTLAQHVFNDPRVNEAKFDVKAWEDDSW
ELLAKHAFRDGTEPNPKCREIGKKIVKKCTGLPLALKTIGSLLYNKSSLSEWETVLLNKIWELPNGDIVPALALSYIHLPSHLK
TCFAYCALFRKDYEFKKEELTQLWMTENFQQHSKTPDETCQQYFNDLLSRSFFQRSSDVEELFVMHDLLNDLAKYVAG
DIYFRFTKVRKVPQGLEKLKNLVVMMNVFYVDYSMESGIQRLGKLNNLSEHLEICGLQCIKNHEDALEVNLKNKTHFVR
LTLAWERTENSIDSRKAKDVIENLKPSKNLKELSILNYGGSKFPNWLLQDSLPNLVSLVLHNCESCQHLPPLGLLSFLKKLYI
SGFDEIVSIDADFHGNNSSSFQSLKKLEFSDMKQWEKWECQAVTGAFPNLQILSIKKCPKLKGQLPKLLVPLKTLKITRCQ
QLEAFPPRTLELDLRHCGKLQLDWATMEWPRMDGHHMRALFSESDGSHTLDDLEIVEVISDDSIPLTTFPLDSFPTVKR
LVLSWLRNLEMISQDQAHHDHLLDLAIRRCPKFESLPGNMHMLSLTTLWIEDCPRFKSIPFGGLPSNLENLTIKGCPKFES
LPDSVGNLKLLRSLDLSHTAIRKLPEKICSLSRLQILELNYCIYLEELPINLHLLTNLCRLEFMFTKVKKFPPNLEKLKNLIIIKEW
GIQGKFNNFYEDLSIEMHDIENPHYVFETDLKNKTDIESLTLGWERTGNSIDSKKAEDVLENLQPSKTSKELSIFNHGENK
FPNWLLRTSIWNMVSLELDKCKSCQSLPPLGLLPFLKKLVISGFDQIVSIDADFHGKNSSSFKSLETLYFSDMRQWEKWE
CKVVTGAYPCLQHLSISFCPKLKGQLPEQLVPLETLHITYCEQLEDYAPMALDLEIRDCGKLRLDCPTMKRLKMGGHNTE
ASLPKIVRSDTLEHLDISSSLESISDDCVSVRTISLDCFPTLRTLNLSGFGNLQKILQDHAHNHLQDLTIKKCPKFESVSGIMH
MLSLKSLWIEDCPRLVSFPDGGLPSNLNDMRLNNCSRLVCSLKGAFGDLSCLKSLWIEGINAECFPGEGLLPLSLTSLTICD
CPNLEKLDYKGLYQLSSLRRLTLVSCPNLQCLPEEGLPRSISYLCIGDCPLLEQRCQTEGGEDWEKISHIQNLNIL

>g23538.t1

MALEFVGGALLSAFLQVAFQKLASPQILDFFRENKLDQKLLTKLETKLNSINSLADDAERKQFTDPHVRNWLLQVKDVVL
DAEDLLDDIQMLSESQVDAESESQGCSCKVFIFFKSSPISSFIKEIEYRMEQTLDGLEFLSSQKGDLGLEIVSRVAYGLSNKL
PQKSQTTSLVVGTDIYGRDHDKEVILDWLTSDINRNQPSILSIVGMGGVEEFDVFKVSRAIIEGVTKSTDDNRDMEMVH
RRLKEKLMGKKFLLVLDDVWNENQPKWEEVKKPLVFAAQGSRILVTTRSKEVASIMREIGMKIVKKCKGLPLALKTMGS
LLYNKSSVSEWETLFQSEIWEFSKEHCDIMPALALSYIHLPSHLKICFSYCALFPKDYEFKKEHLIQLWMTENLLHCRQHSK
TPEEVCQQYFNDLLSRSFFQQSDEKLEAFVMHDLLSDLAKYVGGDIYFRWDVDQAEKIQKVTRHFSVELGDNKSFDGF
GTLCNTKRLRTFMPTSRRVNDLGDRWHCYLSIRELLTKFKFLRILSLSPCFYLRELPDSINNLEHLQLPSNLHLLTTLCRLEFI
FTGVRKLPSGKLNNLYHDLGIWGLQDIENPEDALEADLKNKTHLVNLKLGWKRTRSSIDSKKIEDVLENLQPSKNLKVLSI
FNYGGNKFPKWLVDDSLPNLVFLELGNCESCQHLPPLGLFPLLKKLKIIDSPRLVGSLKGAFGDSSSLESLEISELDAECFPY
ENLLPLSLAKLTIYGFPNLEKLDYKGISQLSSLQSLALIHCPKLQCLPEQGLPQSISNLRIFCCPLVEQRCKEGGEDWEKIAHI
QCLDMDY

>g23541.t1

MAAELITGTLVSTFVQMTIDNLASRFVDIFRGNKSNKKQLSNLKMKLQAIDVVADDAEQKQFTDLRVRDWLLTAKGIM
LDTEDLLEEIDHALSKSQVEAESQSSAKKVWNSLKSSFVSFFKKEIESRMEKLIENLEYLVTQSHDLGLKTADDVGVGSVF
GSKLRSTYLPNECVIYGRDDDKEFVINWLTSDIPNNLPILSIVGMGGVGKTTLAQHVFNDPRVDEAKYDVKAWVCVSVE
FDVLKVSRKILEDVTGSPDQSTNTYMVHRRLKENLSGKKFLLVSDDVWNENLSNWEEVQKPLLFGIQGSRIVVTTRSKE
VASTMRSKEHFLKQLQEEHSSKLFAKHAFHDDTQPNSECREIDMKIIKKCKGLPLALKTIGSLLYNKSSVSEWETVFQNDI
WELPKERCNIVPALALSYIHLPSHLKACFAYCAMFPKDYEFKKERLIQLWMTENFLQQSKTPEETCQQYFNDLLSRSFFQ
RSGDVEELFVMHDLLNDLAKYVAGDIYFSLLEELPSNLHLIITLCRLEFIFTKVRKVPPGLEKLENLKVIMNTFGVDHSMES
GIQRLGKLNNLYEDLRIWGLQDIENPEDALEANLKNKTHLMSLTLGWERTGNSIDSKKAENVLQNLQPSKNLEVLSIFNY
SGNKFPNWWLLDDSFPKLVTLVLCNCESCEHLPPLGLLPFLKNLYISGFDEIVSIDANFHGNNSSSFQSLKNLEFSDMKQ
WEKWECQTVTGAFPTLQILSIKDCPKLKGQLPKLPVPLGTLKITGCQQLEAFLPRTRALDLRDSGKLQLDWATMKWPR
MGGHHMKALFSEGDWSHTLDDLEIEESISDYSIPLTTFPLDSFPTVKTLVLSGFGNLQIISQDDVHNHLQYLLIKECPELES
LPANMHMLLPSLWMLEIIDCPRLKLLSKGCLPSNLERMKFINCSRFVGSLKGALGDISSLERLEIFKLDAACFPDESLLPPSL
AELIFRDCPNLERLDHKGLSQLSSLQSLVLEDCPKLQCLPEQGLPPSISNLKMNNCPLLKQRCQRGGQDWEKIAHIQYLN
MY

>g23545.t1

MMQRESSSRIHMSETGCLRSKMLSLMQNILDAEDLLDDIQKLSESKVDAESESQTSTGCTCKVLNFFKSSPIGSFNKEIES
RMEQTLDALEFLSSQKGDLGLKTVSRVGYGLGNELPQKSQTTSLLVGTDIYGRDDDKEVILDWLTSDINRNQPSILSIVG
MGGVGKTALAQHVFNDPRVDEPKFNIKVWVCVSEEFDVFKVSRAVLEAVTKSTDDSRDMEMKPLVFAAQGSRILVTT
RSKEVASTMRSEVHYLKQLHEDHCWKLFAKHAFRDDDDTQPNSECREIGMKIVKKCKGLPLALKTMGSLLYNKSSVSE
WETLFQSEIWEFSKEHCDIIPALALSYIHLPSHLKACFAYCALFPKDYEFKKEHLIQLWMTENLLHCHKHSKTPEEVCQQY
FNDLLSRSFFQQSGEKVGLFVMHDLLNDLAKYVGGDIYFRWEVDQAEKIQKVTRHFSIELGDNKYFDGFETLCNTERLR
TFMPTSRRVDDFGYRWHCYLSIHELFSKFKFLRILSLSYCADIIVVPDSVVNLGQLRLLDLSGTAIRKLSQKICSLSYLQILKL
NYCTHLHELPSNLHLLTNLCRLEFIYTKVKEVPPHLGRLKNLKVVMNSFNVGHNRESGIQQLGELNLDGSLSIGELQNIEN
SLDALEANLKNKKCLMKLKLRWDSRWNGNSIDSVKEGEVIENLQPPKNIKKLSIFSYGGKQFPNWFMENSLWNMVSL
VLDECESCQHLPPLGVLPFLRVLKIIKLDGILSIDSVFHGNNSCSFKSLEKLIFSNMRQWEKWECQAVTGAFPRLQSLSIKN
CPNLKGQLPNQHVPLKTLQITDCKQLEASAPRSLHFELQGCGKVLFDWATMKRFTLGGHNMEASLLEMVRHIIPDNSI
EHLEIHSYNLSDFPINDDSVSLWTFPLHFFPTLRMVVLRGFGNLQMISQDHTNNHLQDLTIKECSKFESLPGNMHMLLP
SLTRLCIYDCPRLESFPDGGLPSKLNFMKLKNCSRLIGSLKGAFGDSPSLKSLWIEKLDAESFPDEDLLPLSLTSLTISDCPNL
EKLDHRGLYQLSSLQSLSLCYCPKLQCLPEEGLPKSISGLEIDDCPLLEQSCRKSGGKDWEKIAHIKKLYIGE

>g23546.t1

MALEFVGGSLLSAFLQVAFEKLASPKILNFFRAKKLDQKLLTKLETKLHSINSLADDAERKQFTDPHVRSWLFKVKDAVLD
AEDLLDDIQMLSKTQVDAESESQTSTGCTCKVLNFFKSSPIFSFNKEIEFRMKQNIDDLKFLSSQKDALGLKTASCVGYG
MSNELSQKSQTTSLILGTDIYGRDDDKELIFDWLTSDINNRNQPSILSIVGMGGVGKTALAQHVFNDPRVDEPKFNIKV
WVCVSEEFDVFKVSRAILESVTKSTDDSRDMEMVHTRLKEKLIGKKFLLVLDDVWNENQPKWEEVQKPLVFAAQGKRI
LVTTRNKEVASIMRSEVHSLKQLHEDHCWKLFAEHAFRDDDDTQPNPECREIGRKIVKKCKVLPLALKTMESLLYNKSSV
SEWETVFQSEIWEFSKEQCDIIPVLALSYIHLPSHLKACFAYCALFPKDYEFEKEHLIQLWMTENFLQHRNIPEETCEQYFN
DLLSRSFFQRSGGNLVKQIRYKK

>g23547.t1

MALEFVGGALLSVFLEVAFQKLASPQILDFFGARNLDQKLLNKLETKLHSIHSLADDAEGKQFTDPHVRNWLLKVKDAV
LDAEDLLDDIQMLSKRKVDDDSESQTFTGCTCKVLNFFKSSPFSSLNKEIESRMEQILDDLEFLSSQKGDLGLKTASGVGS
GLSNDLPHKSQTTSLVVGTDIYGRDHDKELIFDWLISDTNIQNQPSILSIVGMGGVGKTTLAQHLFNDLRVDEAKFDVKA

WVCVSDKFDVFKVSRAILEAVTESTDHSRDLEMIHRRLKENLTEKKFLLVLDDVWNKNQSKWEDVQKSLVYGAKGSRIL
VTTRSKEIASTMRSKDHSLKQLQEDDCWKLFAKHAFRDDDTQPNPECKEIGMKIVKKCKGLPLALKTMGSLLYKKSSISE
WKSVFQSEIWKFSQDRCDIVPALALSYIHLSSHLKVCFAYCALFPKDYEFRKEDLFQLWMTENFLYCSHHSMTPEDVCQ
QYFNDLLSRFLPNTQDPQCQMISHDHAHHHLQDLTISECPKFESLPVNMHILLPSLSWLYIKHCPRLELFPDGGLPSNLE
DKENMQMCINYFSLSLVPSVMTRIYPSHAQSMPCALMDEIIQYFLTDIGKMLSFTQRFNPIYPRLTMALEFVGGALLSAF
LQVAFEKLASPQILDFFCAKKLDQKLLTKLETKLHSIHSLADDAERKQFTDPHVRNWLLKVKDVVLDAEDLLDDIQKLSKS
QMDAESESQTSTGCTCKVLNFFKSSSIISSFNKEIESRMEQNLDDLEFLSSQKGALGLKTANSVGYGLSNELPQKSQTTSL
VVGTDIYGRDHDKEVIFDWLTSDINRNQPSILSIVGMGGVGKTTLAQHVFNDPRVDEPKFNVKVWVCVSQEFDVFKLS
RAILEAVTKSTDDSRDLEMVHRRLKEKLMGKKFFLVLDDVWNENQPKWEEVKKPLVFVAQESRILVTTRSKEVASTMR
SEVHSLKQLHEDHCWKLFAKHAFRGDDDTQPNPECRDIGMKIVKKCKGLPLALKTMGSLLYNKSSVSEWKIVFQSEIW
EFSKEQCDIIPALALSYIHLPSHLKVCFVYCALFPKDYEFKKEHLIHLWMTENLLHCRQHSKTPEEVCQQYFNDLLSRSFFQ
QSGEKVGLFVMHDLLNDLAKYVGGDMYFRWEVNQAEKMQKVTRHLSLELGDNKYFDGFGTLCKTERLRTFLPTSDRK
LVYLYWYCNMSIHELFIKFKFLRILSLSHCSNIKELPDSVGNLEHLRSLLEELPSNLYLLTTLCRLEFIFTKVRKVPPGLEKLKNL
KVMMTTFEVDHSMESGIEVLGKLNVLYKDLSIEGLQHIENPEDALEADLKNKTHVVRLTLRWERTGNSMDSKKVEDVIE
NLQPSKNLKELSISNYGGNKFPKWLLDDSLPNLVSLELTNCEFCPSLPPLGLLPFLKTLYISGFEKVVSIDADFHGNNSSSFQ
SLQRLEFSNMRQWEKWECQSVTSAFQNLRILSLKDCPKLKGQLPELLVPLETLEITGCQQLEVLPPRTLELDLRHCGKLQL
DWAPMEWPRMGGHHMKALFSKGDGSRTLDKLEIEDSIDDSIPLTTFPLDSFPTVKRLVLSRFGNLRMISQDQAHHDLG
DLIISECPKLESFPANIHMLLPSLWRLRIMDCPRLTLLSSGGLPSNLEEMKLINCSRLVGSLKGAFGDSSSLESLEISELDAEC
FPDESLLPPSLAKLTLRDCLNLEKLDYKGLSKLSSLQSLALLHCPKLQCLPEQGFPQSISNLKIINCPLLKQRCQRGGQDWE
KIAHIEWEKCSAGVILCGVPPFWVETKQGVALAILRGEIDFKRERRLPLAKSYTPNQKSVPTFSQTPRTSGPPTGSSLTFD
QSVFPGSEHIDFAGILRVGNYVRRLKGILKMLPFRSLPSQSSAPYLNKEEVKETVSFCCPP

>g23556.t1

MAAEMVTGVIVPTFLGRTIDTLASRLVHIFSQRKHKKQLNNLKMKLLAIDVVAFDAEQKQFTDQRVRDWLLKAKDAVF
DAEDLLEEIDYELSKSQVEAESQSDGKKVWNSLNSSFLSFENEIESMMEKVEDLFLDLEYLANESNILGLEKGGGVGVGS
GSGSKLTYTSLPNESFIYGRDDDKKFVLNWLTSDTHNNLSILSIVGMGGLGKTSLAQHVFDDQRLEGKFDMKAWVSVS
QEFDVLKVSKAILGTITGSTDHSIQEVIQKRLKEELMRKKFLLILDDVWNEDQFKWEDVQKPLVFGGQGSRILVTTRSKK
VAAAMRSEKHFLQVLKGDDCWDLFAKHAFQNANPQPDPDFIEIGKKIVEKCDGLPLALKTMGSLLHNKSSLWEWKSI
MKSEIWHLSENESGIFPALKLSYFHLPSHLKKCFAFCALFPKGYWFDKEYLIQLWTTQNFLENPLHKKSLKEVGEEYFSDLL
SRSFFQPSTNGVEKHFIMHDLLNDLAKYVSEDICIRLGVDEPKEKGLAWDCKMTIDDLFSRFKLIRVLSLNHCRSLTENIE
NPSDTIEVDLKNKPHLVGLWLKWNFIGSSSVDSEKVEDLEGCKSCQRLPPLGLLPFLKDLSISGFDEIAMGKVRMPSCDR
CFSTSTTFMDTKLSEAERTPAKCGKLQLERSTMKKLTRDGHDMAASLVATVGHMLFDTSLEILKIGSDLESKSDDCVSLRI
FPLDFFPTLRSLELGGFPDLQMISQDHVHNHLQDLTIKDCPRLELFPEGGLPSNLNSIELINCFRLVGSLKRVFGDSSSLESL
GISTPDAECFPDEGLLPLSLSSLMIYDCPNLNKLNYKGLLELSSLESLDLWYCPNLQCLPEEGLPKSISSLHIHNCLLLEQRYH
KGGEDRKKIAHIRNISIW

>g23557.t1

MAAEMVTGVIVPTFLGRTIDTLASRLVHIFSQRKHKKQLNNLKMKLLAIDVVAFDAEQKQFTDQRVRDWLLRAKDAVF
DAEDLLDEIDYELSKSQVEAESQSDGKKVWNSLNSSFLSFENEIESMMEKVEDLFLDLEYLANESNILGLEKGGGVGVGS
GSGSKLTYTSLPNESFIYGRDDDKKFVLNWLTSDTHNNLSILSIVGMGGLGKTSLAQHVFDDQRLEGKFDMKAWVSVS
QEFDVLEVSKAILGTITGSTDHSIQEVIQKRLKEELMRKKFLLILDNVWNESPSKWEDVQKPLVFGGQGSRILVTTRSQKV
AAAMRSEKHFLQVLKEDDCWDLFAKHAFQNGNPQPDPDFIEIGKKIVEKCDGLPLALKTMGSLLHNKSSLGEWESIMK
SEIWHFSENESDIFPALKLSYFHLPSHLKKCFAFCALFPKGYRFDKEYLIQQWIAQYFLENPLQKKSPKEVGEEYFSDLLSRF
FFQPSTNEEEKHFIMHDLLNDLAKYVSEDICIRLGVDEPKEEGWVWNCKMSIDDLFSRFKLIRVLSLNHCRSLTEVPKSIG

NLKHLRSLDLSWTQIEKLPDSISLLHKLQILQLNYCHRLKELPSCLHQLDNLRCLELERTGVKNVVAYLGKLKNPQVSISSFH
VEKNKEMNIRQLGKLNLHGSLTIDDLQNIENPSDTIEVDLKNKPHLVRLELKWNFIGSSSVDSEKVEDVIENLRPSKYLKN
LSISNYIGKQFPNWLLHNSLPNLVSLVLEGCKSCQRLPPLGLLPFLKYLNISGFDEIVSIDADFHGNNSSSFKSLQTLSFYDM
RQWEKWECQAVTGAFPRLQRLRIQNCPKLKEHLPKFVALKDLYVFNCEQLEALIVSVIELRLEECGKLQLERSTMKKLTR
DGHDMAASLVATVGHMLFDTSLESLNIGSALESKSDDCVSLRIFPLDFFPTLRILELGGFPDLQMISQDHVHNHLQNLKI
KDCPRLELFPEGGLPSNLKYIELINCFRLVGSLKRVFGDSSSLESLSISTPDAECFPDEGLLPLSLSSLRLYDCPNLNKLNYKGL
LELSSLGSLDLMNCLNLQCLPEEGLPKSISSLDIHNCTLLEQRYHKGGEDRKKIAHIRSIFIW

>g23558.t1

MAAEMVTGVLVPAFLGRTIDTLASRLVHIFSQRKHKKQLNNLKMKLLAIDVVAFDAEQKQFTDQRVRDWLLRAKDAV
FDAEDLLDEIDYELSKSQVEAESQSDGKKVWNSLNSSFLSFENEIESMMEKVEDLFLDLEDLANESNILGLEKGGGVGVG
SGSGSKLTYTSLPNESFIYGRDDDKKFVLNWLTSDTHNNLSILSIVGMGGLGKTSLAQHVFDDPRLEGKFDMKAWVSVP
QEFDVLEVSKAILGTITGSTDHSIQEVIQKRLKEELMRKKFLLILDNVWNESPSKWEDVQKPLVFGGQGSRILVTTRSQKV
AAAMRSEKHFLQVLKGDDCWDLFAKHAFQNANPQPDPDFIEIGKKIVEKCDGLPLALKTMGSLLHNKSSLWEWKSIM
KSEIWHLSENESGIFPALKLSYFHLPSHLKKCFSFCALFPKGYWFDKEYLIQQWMAQYFLENPLQKKSPKEVGEEYFSDLL
SRSFFQPSTNQEEKYFIMHDLLNDLAKYVSEDICIRLGVDEPKGIGVENVVAHLGKLKNVQVSMSSFHVEKNKEMNIQQ
LGKLNLHGSLTIDDLQNIENPSDTIEVDLKNKPHLVGLELKWNFIGSSSVDSEKVEDVIENLRPSKYLKRLSISNYIGKQFPN
WLLDNSLPNLVSLVLEGCKSCQRLPPLGLLPFLNYLKISGFDEIVSIDADFHGNHSSSFKSLQDLYFFDMRQWEKWECQA
VTGAFPRLQHLSITNCPKLKGHLPALKYLYVFNCEQLEALIVSVIELRLEECGKLQLERSTMKKLTRDGHDMAASLVATVG
HMLFDTSLEILKIGSALESKSDDCVSLRIFPLDFFPKLRTLELDGFPDLQMISQDHVHNHLQDLKIKDCPRLELFPEGGLPS
NLKYIKLINCFRLVGSLKRVFGDSSSLESLKIEKVEAECFPDEGLLPLSLSSLKISDCPNLNKLNYKALLELSSLRRLNLINCPNL
QCLPEEGLPKSILSLDIRKCPLLKQRCDKGGEDRKKIAHIPYIFIWW

>g23559.t1

MAAELVGGALLSAFLQVAFDRLASPQVLHFFRGRKLDQKLLKRLKRKLRSIDALADDADIQQFINKRVKAWLADVIDALF
EAEDLLDEIYEFSMREVEDESQRTFNKVWNLNLQISSVSFFIDENEIETRMEQVLDDLDELVNEGSRIGLKEASGVGVGV
DSGSNSKVSQKSSSTSLLSERDDIYGRDDDKDIIFNWLAPDNDNGDKLLILSIMGMGGLGKTTLAQHKALDSGAHGSRIL
ATTRSKKVALSMRSKEHHLKELEEDYCWQLFSNHAFQNVTQANTDCKEIGKQIVEKCRGLPLALKTMGSLLHNKSSISE
WENVLKSEIWELEDNDLIPALLLSYNHLPSHLKRCFAYCALFPKDYEFDKNCLIQLWIAENFVQFHNHKSPEDIGEQYFD
DLLSRLEVDKTKDWSWHCKMSIDDLFSKFKFLHVLSMSNCSNLTKVPDSVGDLKHLRSLDLSNTHIERLPESTCSLYNLQI
LKLNNCPLLKELPSNLHKLTNLRRLEFMDTELLKVPEHLEKLKNLKVFMSSFDVGSKEFSIQQLGQLHHHGRLSIGELQNIE
NPSDAKAVDLKNKTHLRELMLKWNRDQVPVDALKERDVNVMENLQPSKYLEKLSISHYDGTKFPTWFGDYSLMNVVS
LSLFQCKQCKCLPSLGHLPFLKDLTISGLDGVVGIDADFYGSGSCSFPSLETLKFSRMKGWEKWDCEAMRGAFPRLHHL
AIDYCPKLKERLPVQILHFETVYIRHCKQLLGYDGMVKMNGKEVTIFRVHNMEAWFVEWIGKMISHDSVEDLKVYSCP
NMSVLMSQQYNFLVSLTVHDSCDSLTTFPIDFFPTLRSLYLLSCCNLQTILQVHAHNHLQDLAIIDCPQFESFPERVHILLP
CLEFLSIRDCPRFESFSDGCLPSNIKRMYLTNSSKLVASLKGSFGNNPSLETLFIIGKLDAESFPDEGFLPRSLTSLGIYDCPHL
KKLDYKGLCHLPSLKELLLVNCPRLQCLPDEGLPKSISYLTISGNCPLLKQRCKYPGGQDWGKIAHVQNLSIL

>g24058.t1

MAAISCSYAFSHDVFLSFRGSDTRHGFVGNLYKALDDKGIHTFIDDEKLQRGEEITPALMKAIEESRIAITVLSHNYASSSF
CLDELVHIIACAKEKGLLVLPVFYDLNPSDVRHQKGSYAEALTRHEERFKDKKESFSHNMERLEKWKMALHHVANLSGY
HFKQGYEYEYEYEFIRRIVELVSSKINRTPLHVADYPVGLEAQMVEVMKLLDVGTDDGVHMVGIHGIGGIGKTTLALAIYNL
VFDHFDGLCFLENVRENSDKHGLQHLQSILLAELVKEKRINIASVQEGISMIQHRLQGKKVLLIADDVDKHEQLQAIVGR
SDWFGSGSRIIITTRDEQLLASHEVKRMYEVKELNKKDALQLLTWKAFRTYEADPSYEEVLKRVVAYASGLPLALEVIGSN

LFGKSIEEWKSAIKQYERIPNNQILKILKVSFDALEEEEKNVFLDIACCFKGYELEEVQDILHAHYGDCMKYHIGVLVDKSLL
KFSMHGMMVTMHDLVEDMGKEIVRKESPKDPGKRSRLWLHEDIIQVLEDNTGTSEIEIIRLDFAQLDKEEMVEWNRK
AFKKMRNLKTLIIKSDFHSKKLAMCKLPQSCFTSHELKFMGMRFLNLDKSKSLTQIPDVSGLPNLEKLSFQHCQNLTAIH
NSIGFLCKLKILSAFGCTKLVSFPPIKLTALEKLNLSRCYSLENFPEILGKMENIRELQLEYTAIKEFPYSFQNLTRLQELQLSN
CGVVQLPSSIAVMPELTDLIGWKWKGWQWLKHEENEEKEGSSIVSSNVEYLWASECNLCDDFFSIGFMRFAHVKDLD
LSKNNFTMLPECIKEFQFLRKLNVNDCKLLQEIRGIPPSLKHFLATNCKSLTPSSTSMFLNQELHEAGKTQFYLPGERVPE
WFDHQSNGPSISFWFRNRFPEKVLCLVIGPVNDSGMFRPMVVINGNKCFLGSGYFMMGMDHTYIFDLLTIEFEDNLY
GVPLDNEWNHAEVKFVGLEEISILKESGIHVFKQEGGMEDIWFSDPYCKRKLEDDLNSFESQNQQLLKKHRFVDMEAL

>g24089.t1

MVHGRLKGKVFGKKFLLVLDDVWNERREEWEAMRTPLSYGASGSRILVTTRVEKVSSNMRSKVHHLKQLEKDECWKF
FNKQALKDDDLELNDEKKEIGRRIVEKCKGLPLALKAIGSLLHTKSLILDWQSVLESNIWDLPKEVEIIPALLLSYQHLPSHL
KRCFLHHSQLNKNVQEIGEQYFDDLLTRVKFDKGNCIPKTTRHFAFAFNDVRYFDGFGSLTDAKRLCPFVPIRHIWNHTP
SSWTFKISIHDLFSKIKFVRVLSLSCCSRFREVPDSVGDLKHLHSLDLTGSLWLANCKYCLCLPPFGLLSSLKTLTIMGFDGIV
SIGAEFYGTGSFSFKSLEILVFSNMKEWECKTTNFPHLKHLSIDRCRKLKGLSDQLLHSEQLTIDSCDELIVSENSMNTSSIE
NLHIYSCPLVSIPTTHNDFLEVIKIGGGCDSLTIFPLDFFPKLRLLQLRRCQNLRRISQEHTHNHLNELTIIHCPQFESFPNEG
LFAPCLEIIAIEGAENLKLLPKHMKIQLQSLKNLRIIDCPQVEMLSDEGLPRNVKYVSLSSLKLMASLRESFDENTWLERLCI
EKMDVECFPNEGLLPRSLISLQILNCPNLKKLNYKGLCHLSSLTLTHCHNLQCLPVEGLPKSISSLTIRGCPLLQLRCQNPEG
EDWVKIAHIQELIIGE

>g24612.t1

MALRIASSLSSSRPTWIYDVFLSFRGEDTRFHFTDNLYHSLCQKGIRTFIDREGLRKGEEITPALFHAIQNSRISIVVFSKNY
ASSTYCLNELVKILECAKKEGRSIYPIFYGVDPSEIRHQTGIYAEALSKHEAKFYNDADNEKAQKWRKALREAANLSGWHF
QHGSQQEYEFISTIVEEISENINYIPLHVADNAIGLEYAVQGVKSLLGDGSDVNMIGIYGIGGIGKTTIARAVYNAIFWHY
QGSCFLSDIREKAINKHGIVQLQEILLSETFKESIKVGDVNRAIPILKGRLQHKKVLLVLDDVDKLEQLKALAGGYDWFGSG
SIIIITTRDKHLLDAHGIVNLYERAVSYACGLPLALEVIGSHLFGKNIDECSSALDKYESIPHEKIHEILKVSYDGLEENEKGIFL
DIACFFNTWELGREIVRQESTIEPGRRSRLWFNEDIVHVLEENTGSDKIEFIKLEGYNNVQVQWDGKAFKEMKNLRILIIE
DSRFSTGLKHLPNSLRVLDWSCYPSPCLPHDFNPKRFEILLLPESFLQMLKPQKACLSLKFFGLTMSSTLKDNNLTHSVFM
LQMLESLSVISLQDCKFLTDLPSLRDAPFLTTLRLDNCCNLVNIDESIGFLDKLRFLSASYCTKLKTLAPCIMLTSLETLDLGR
CFSLESFPEVLGKMEKIRTIYLDCTGIDKLPFSIGNFVWLELLSLTECERLHQLPGSISIMPKVKVLVGYGHGAYQIFQEQLS
SEVSPRAMLIGDSDLYLDVYYSCIGPNNSIQLCSPDPLFHSDFNLLFPKLGREEDWLCFRRESSMHFSFRNKFPKIALCCSI
LLPLLKIIMVLNLKFRVFINDTLQFSALCNFILREWDTILWCDLEGKVEGVFSDEEWNKAEIVFELDFPMRRNSRNGNTRR
SIGIGSLGWSLMGVYEEGNNTEDIEFKDPMIGKVVHTA

>g24804.t1

MLAGDSKSSSRLRQSSCSCPPRMVKISAQPSSSSSFSSTARLKRHDVFISFRGEDTRNSFTSHLYAAFQHKNIQAFIDNRL
QKGDVISPSIFEAIKHSNVSLVVLSKHYASSTWCLRELAKILQLRKRGGHIVIPVFYKIDPSYVRKQTGTYGMAFQKYKEDV
KQNMAMLQKWKAALTEVADIVGWESKNFRTENELIQEIVKDVMQKLNHMYPTEVKTLVGIDQNLAPIESLLRLRSKEV
RIIGIWGMGGLGKTTIARALFDKLSSQFEASCFLANVMEEHEKKGLDYLRNKLLSEILEDVNPHISTSKVRSTFVMKRLRQ
KKVLIVLDDVDDPKKLEDLVAQHGCLGSGSRVIVTTRDKHVLSKGVDAIYEVKGLSLDHAVRLFSLKAFGKTYPERGFEM
LSKQAVDHANGNPLALKVLGSVLHSRNEQQWDNAMRKFKKEMGWEIVHQESIKDPGRRSRLWDLKDVYDVLKNNR
GTEAVEGIILDVSQIRVLLLSCETFSRMINMRFLKFYMGKGRKCNLHLPSGLQSLPNKLMYLQWDGYPSKSLPSTFCPDN
LVVLSMMESHVEKLWDGTKCLPSLKEMNLHASKKLTNLPDLSQAPNLETIDVSSCTSLLHVPLSIQYVKKLLLFNLESCKS
LKSLPRNIHLSSLGMFILRRCSSLDEFSLTSENMTRLDLRETKIADFPESVWQHLNKLVYLNLESCNKLKSLTSKSHLKSLQR

LNLRDCSILEEFSVTSESMEYLNLRGTSIRELPTSVWRNNKLYTLVLHSCKKLVNFPERPKLEDLPLVSSSERPNMDELWTL
SSLADLSLKGSTVENLPESIKDLPSLKKLTLTECKKLRSLPSLPPSLEDLSLDESNIVCLPVSIKDLSHLRKLALINHKKLLTPLLS
ESKVDPQLVSMKGLSQLQMFPQVKWKMFHSLPELPPFLEEFSLSESNIKFIPESIKNLSHMRKLAFTKCTRLQYLPELPPN
LEDLFVSGCDIESLPTSIRDVVHLRKITLIECKKLKALPELPQCLQSLCAADCTSLKIVRSTKNILIEDRYTFYWNCINLDQKSR
NNIIADAPFEEAFTSLKERTPLGPLISICLPGTEIPDWFSHQSTNSSLDMEIPLEWFVDSMFLGFALCLVIGGFQQNSYEGY
DPDINCYHFVKPASYSGPSVPFLGHCTTVMQVPRGFNSDHIFICYYPSFNASILQDFKDLSLYYDANDLRLRVIFKFKGPS
QRLDIVKKCGVRPLLIANTERLHIESELQPE

>g25049.t1

MAAACVGGALLSAFLQVAFDRLASPEVLHFFRRRKLNEALLSKLNIKMLSINSLADDAEQKQFRDTRVKAWLFAVKDAV
LEAEDLLDEIDYELTKCEMEAQSESQSLSKKVSSFFHSTFSSFNRKIDSGLKQVLEKLEYLASQKGDLGLKEATYSGLRPGSV
VQQKLPSTSLVAENVIYGRDDDKETIFNWLTYETDNRNQLSILSIVGMAGVGKTTLAQHVYNDPRTEEAGFSIKAWVCV
SDDFDVLLVTSTILEAITKSKDDSRNLEMAHGRLKEKLSGKKFFLVLDDVWNERREKWEAVQTPLNFGASGSKILVTTRS
EKVASTMRSSKVHRLMHLQEDHCWDVFAKHALQDDHPQLNAELKDIGIKIVKKCKGLPLALKAIGSLLHTKSSFSEWEC
VLVSKIWDTPIEENEIMPALLLSYHHLPSHLKRCFAYFALFPKDYKFDKQSIILLWIAENCLQCPQHNKSPEEIGELYFDDLL
SRLRTFIPLPTFVIVSSLNDPWQCKIPVHELFSKFKFIHTLSLLCCSGLLEVPDSIGDLKHLRSLDLSRTDIRKLPDSSCLLYNL
QILKLNFCLLLKELPSNLYKLNNLRCLEFIATSVRKVPMHMGKMKNLQVLSSFCVGKSSEFGIQQLVGLNLHGGLPIGDM
QNIVNPSDALQVDLKIKSTL

>g25735.t1

MAAEMVTGILLSTFLERTIDTLASRLVHILFQRKHKKQLNNLKMKFLAIDVVALDAERKQFKDPRVRDWLLRAKDVVFD
AEDLLDEIDYELSKSQAEVESQTTNKVWNSLNSSFASFFEIEIESRMEQVIEDLEDLANESYILGLEKGGGVGVRSVSGSKL
TYTSLPNESVIYGRDDDKEFVFNWLISDTPNNLSILSIVGMGGMGKTSLAQHVFNDPRLEGKFDTKVWVSVPQEFDVLK
VSRSILDTITASTDHSIQQEVIQKRLKEELMGKKFFLVLDDVWNERPSKWEDLQKPLVFGGQGSRILITTRSEKVAITIRSE
KHFLQVLKEDYCWDLLAKHAFENANRQPHPDFIEIGKKIVQKCNGLPLALKTMGSLLHNKSYLWEWESIMKSEIWHFSE
NESDILPALRLSYFHLPSHLKKCFAFCALFPRGYRFDKECLIQLWMAQNFLENPLEKKSPKEVGEEYFNDLLSWSFFQQSS
NEEEKRFIMHDLLNDLAKYVCEDICIRLGVDEPKDIPKTTRHCSFSSSKLCFDGFGSSIDSQKLHTFTATDQGWHWNCK
MSIDDLFSRFKLIRVLSLCYCRSLTEVPKSVGNLKHLRSLDLSWTQIENLPESISSLFKLQTLKLNNCRKLKELPSCLHQLDNL
RCLELVGIGVKNVVTHLGKLKNPQVSISSFHVEKSKKINIQQLGKLNLYGSLTIDDLQNIENPSYALEVDLKNKPHLVELRLT
WNFIGSSSVDSEKAEDVIENLRPSKYLKKLSVSNYIGKQFPNWLLHNSLPNLLSLVLEECKSCQRLPPLGLLPFLKYLQISGF
DEIVSIDADFHGNNFCSFKSLQKLYFSNMMQWEKWDCQVVTGAFPRLEFFSIGNCPKLKGHLPKFVALKSLYVFHCEQL
EALMVSALELRLQDCGKLQLERSTMKKLTMDEDGMAASLVAMVERSRSQSSPASDNQRLSKT

>g26273.t1

MLISISRKSLLCRASADQLITYIHELLPTIEEIKYSGVELPALRQSQLHRLSELLRSGVELSHKVLASSRWNVYRNLHLAKKM
DKLEKTVSKFLLGPMQAHIMADVHHTRFEMAERFDRVDNSVRRLEQYFGNIKIGVGGGGWVEEAVRSVDENVVDGS
SAVGLEFGKIKVREMIVGREDLWVVGISGIGGSGKTTLAREVCKDDQVRRYFRERILFLTVSQSPNVEQLRTKIWGYIMG
NERLDSNYVVPQWVPQFECKSEAARTLIVLDDLWTFSVMEQLVCRIPGCKYLVVSRTKFQTVLNYEVELLSEEDALSLFC
HHAFGQKSIPLGANENLVKQVVTECGRLPLALKVIGASLRDQPEMFWLSVKNRLSQGQSIGESHEINLIERMAISINYLPE
KVKECFLDLCSFPEDKKIPLDVLINMWVEIHDIPETEAYAIVVELSNKNLLTLMKEPRAGGMYSSCFEISVTQHDILRNLAI
NLSNRDSINERRRLVMPKRENAIPKEWLRYKHKPFEAQIVSIHTGEMKDVDWCNMEFPKTEVLIINFTSTEYFLPLFINR
MPNLRALIIINYSATYACLHNISVFKNLPNLRSLWLEKVSTPELSGTVLENLGKLFIVLCKINNRLVEKEVDLAKVFPNLFDL
TLDHCDDLTQLPSSICGMNSLQNLSLTNCHNLNQLPLELGKLKSLEILRLYACPDLKTLPNSICEMMRLKYIDISQCVNLTC
FPEEIGRLVSLEKIDMRECSMIRNVPKSALSLQSLLLVICDEELSGIWKEVEKAKPNVLIQVSEQHFDLDWLKE

>g26274.t1

MALTDLFTGEIASDLWKMLITISRKALRCKSSAEQLITYVREILPTIEEIKYSGVELPAPRQSQLDRLSEILRSGVELSHQALS
SSRWNVYRNFQLAKKMEKLEKHVTRFLQVPMQAHILADVHHARFEMAERFDRVEASNRRMERFLEEMKIGVNGGG
WVEEAVKSMQEDETWVEGCNGNNGFGVGLDFGKKKVMEMVFSNNDADWIVGICGIGGSGKTTLARELCRDDQVR
CYFKDRILFLTVSQSPNVEQLRARIWGHIMGNQGLNGNYVVPQWMPQFECKGEAQVLVVLDDVWSLSVLEQLVWKI
PGCKFLVVSRFRFPTFFSATYHVELLGEEDALSLFCHHAFGQKYIPLGANVSLVKQVVAECGKLPLALKVIGASLRDQNE
MFWLSVKSNLSQGHSIGESYEINLIDRMAISTNYLPEKIKECFLDLCSFPEDRKIPLEILINMWVEIHDIREAEAYAIAVELS
NKNLLTLVKEARAGGMYSSSFDISVTQHDTLRDLALILSKRGSIHEHRRLVMAQREENGLLPREWSRYQDRPFEAQIVSI
NTGEMTEMDWFELDFPKAEVLIINFTSSDYFLPPFISKMPNLRALIIVNYSTSYARLHNVSVLRNLTNLRSLWLEKVSTPPL
SGTVLKSLSKLFVVLCQINNGLDGKQFPNLSELTLDHCNDLTYLPSSICGIKSLRNMSLTDCHNLSELPDEFGNLKSLEILRL
YACPDLETLPPSMCDMKKLKYIDISQCTNLTCFPKEIGRLVSLEKIDMRECPMIRYLPKSAVSLHSLQLVICDEEVYGTWR
DVAEMAKSNVHIQVPEQHFDLDWLQE

>g26392.t1

MATPSFRRFTYDVFLNFRGEDTRYGFTGYLYKALCDRGVHTFMDDDKLQSGEEITPALQKAIQESRIAIVVLSHNYASSSF
CLDELATILDYKSKGLLVIPVFYKVDPSYVRHQKGSYEEALAKHQMRFKGQKEKLHKWKMALHEVADFSGYHFKDGDE
YQYEFIGRIVERISRVINHAPLHVADYPVGLLSQVLKVKKLLDVGSDDVVHMIGIHGMGGLGKTTLSLAVYNLIADDFDSS
CFLQNVREESNKHGLKHLQNLLLSEILGEKDINLASVQRGISVIQQRLRRKKVLLILDDVDNRKQLQAFAGRSDWFGPGS
RVIITTRDEQLLKSHEIERTYVVKELNKNDSLQLLKWNAFKREKVDPRYEDVLKGVVTYASGLPLAIEVIGSNLVGKSVEE
WESAIEHYKRIPNCEILEILKVSFDALGKEEKNVFLDIACCFKGSSLKEVESILGVLYDNNMKHHIGVLVEKSLIKVGRGWF
DVIEMHDLVEDMGRQIDLQKSPKEPGKRKRLWLGKDIIHVLKVNKKFENLTVLNFDWCMFLTQTPDMSDLGNLEEVS
FKGCESLVAVHDSVGFMNKLKILNVEGCIKLMSFPPLNLPTLERLELSFCFNLENFPEILGKMGNIRVLQLQDLPIKELPLSF
QNLIGLEELSLSCENVHFSSSIATMPNLFRFSVTNCRGWQWVKSEDAEDNLGSMVPSKVEWFAAWSCNLDDDFFSAG
LMLLAQVRCLFLRDNNFKHLPECIKELHNLWALDVSHCKHLEEIRGFPPKLEHFKAINCISLTSSSLSMLLNKELHEARKTE
LWFPGASFPEWFDLKSSGPSCSFWFRNKFPARVLSLLIAPVGDDEDGFSLIRPVVFINGKVQPRPFYFKKIKRMIQFDHS
YLFDLQTIPIYSNLFKLPLEKEWKHVKVTYEGVIETSVVKATGIHIFKEGNNIMEDIRFDDPYTNSKLDKYLNSSPSQNHLLR
TLGHFRYRDHYTRLSRSPRQTINFDTTTSIMIPILKVFLTKGIEVDPSDVRHQKGSYGEALTKHQKRFKYKKEKLQNWKM
TLRQVADLSGYHFKDGIEYEYKFIGSIVERVCSEIGRGCCLHVADYPVGLDSRVLEVRMLLEVGCYDGVHMIGIHGMGG
VGKSTLARAVYNGLIAENFDSLCFLENVREKSNKHGLEHLQSILISQVLGKEDINLTSKQQGISMIKCRLERKKILLILDDVD
NLGQLRALAGGCDWFGPGSRIIITTRDKQLLATHQVTRTYEVRELNEKDALRLLTLKAFKKEKADLDYELILNPVVTYASG
LPLALEVIGSNLFGKSVKEWESATRQYKRIPKKEILEILKVSYEALEEEEKVVFLDIACCFKGYALREVEDILGALYDDCMKH
HIGVLVEKSLIKVSLLSTVEMHDLIEDMGRKIDQKESATELGNRRRLCMDNEELQSGEEITPALLKAIEESRIAIVVLSHNYA
SSSFCLDELVTILHCQSKGMLVIPVFYKVDPSNVRHQKGSYEEALAKLQNRFQGQEEKLHKWKMALRQVADLSGYHFG
DGYEYQYKFIGSIVERVSKEIKRAPLHVADYPVGLPSQVLKVEKLLDLGSHDVVHMIGIRGMGWQEGASMIQQRLGRK
KVLLILDDVDNRKQLQAFAGRADWFGPGSRVIVTTRDEQLLKSHEIERTYEVEKLNDNDSLQLLIWNAFKREKVDPSYED
VLKRVVTYASGLPLALEVIGSNLVGKSVEEWESAIEHYKRIPKGEILKILEVSYYALEEEQKSVFLDIACFFKGSSLEEVEHILG
VLYDNNMKHHVGVLVDKSLIKVWWVDEGFGVIEMHDLIEDMGRHIDRQKSPKKPGKRRRLWLGKDILHVLKHNKVR
N

>g26393.t1

MKEKLGASTKSRKERPTIEDRSSVNANRSLANLRTRTLRDIYNTTNESLLENFVKKLQSPTMYVDSSNMEKVEDFKVRDL
VRSKPSLGTRPSCDWNSVGRESLAIVHSVQDFRYLELACCSRKRKWITHYTDVERVSSFKVGQYVSFLLMAVTSPSQAFS
YDVFLNFRGSDTRQGFTGYLYKALLDSGIHIFIDDEGIQSGKIITPELKEAIQKSRIAITVLSTNYASSSFCLDELAIILDCSKRT
GLLVLPVFYKVPPCHVRHQQGSYKEALARPEQRLHHNMENWKMALKEVAGLSGFHFEDGKEYEHKLIKKIVERVFSFIS

NVEERLHVADYPVGLESQVLEIRKLLDVGDDDGANMIGIHGMGGVGKSALARAVYNLITDQFEDKHKQLHGLVGSPD
WFGPGSIVIITTRDKQLLASHEVKRTHEVKELNKSDALELLKFKAFRMEKVDPSYKEVLNQVVTYASGIPLALEVIGSNLFG
KSVQEWESAIKQYKRIPNNKILEILKVSFDSLGEEEKSVFLDIACCFKGYKLSEIEETLRALYDNCMKYHIGVLVEKSLIKISH
GERVTFHDLIEDMGKRIDRQQSPREPGKRRRLWLQEDIIQVLKDKSVSE

>g26394.t1

MAAPSTYDVFLSFRGGDTRHGFVGNLYKALCERGINTFMYKALCERGINTFMDDEMILKGKVPIATLLNAIEGSRIAIVV
LSHNYASSYFCLEELATILHCQTKGLLVIPVFYKVHPSDVRHQKGSYGEALTMHQKKFEDMEKVQKWKMALCQVANLS
GYHFEDGVGNEYKFIKRIVDDVYDKITRDFFTHVSDYPIGVQSQVLEVRKLLDFGFDDGVHMIGIHGMGGVGKSTLARA
VYNDLIGEKDINFTSTQQGISTMKYRLKMKKVLLVLDDVDKPDQLQAVAGGRDWFGPGSRIIITTRDKQLLANHRVTM
MYEVKGLNDNDALQLLKWKAFRNEKADPDYELALNRLVTYASGLPLALEVVGGSLVGKSIEGWKTAIKQYEMIRKREIQ
NILKVSFDALEEEQKSVFLDIACFLKEHALREVEDILHAYYGRSMKHHIDMLVERSLLKFDYFDNRIKMHDLLRDMSRIVR
KELQKEPGNKSRLWLHKDVIHVSNNNKYETLTVMNFDECRILTQLPDVSDLPNLIELSFKGKLISFPSLNFNSLERLELSNC
SNFKNFQRFTTEEWECAINENDPVGLESRSDAFCYDVFLSFRDLDTLYGFTGYLYKALHDSGIHTFIDDENLQRGEEITPT
TVKAIEESKIAITVLSINYASSTWCLDELVTILDCLKRKRLLVLPVFYHVDPTQVQLQQGRFGEALTKHEERLKNNMEKLVK
WKIALYQVAKLFSFHIYHGAGYEYEFIGKIVEWVSKKINRAHYPIGLESQVQEVMKLLDVGCDGGAHMIGIHGTGGVGK
STLAQEVYNNLISERFDASCFIENVREKSNKHGLQYLQSTILENLLGEKDIKLTSVQQGILMIQRRLQKKKVLLILDDVDRQ
QQLQAVVGRADWFGRGSRVIITTRDEQLLASHDVQITYEVKKLNKKDALVLLKWKAFKKHYFDPRYEKLLNRAVTFSSGI
PLALEVIGSNLCGKSIEEWKYVIHQLEKCPNNPVETVLKASFDSLEEKERSVLLDLACCYKGYELAEVEDILQAHYGQNMK
CYIDSLVDKSLVKLSHGTKPCYDRVTLHDMIEDMGKDIVRQESLTGPGERTRLWLLEDVRQLLENNRGTSKTEIICLDFSIF
DQEEKVEWDGKAFQNMQNLKTLIIRHAKGTCHMQVTLQ

>g26613.t1

MGCVGFSYDVFISFRGEDTRDNFVGHLRKELGRRGILTFHDDRDMGIGESLSPALNNAMEESRIFIVVFSENYASSTWCL
DELVRIMELSKMREKKQVVFPVFYHVHPSDILQDRNSFGKHMRAHENTFGKQSQRMQAWRSALSQAVHLPRKHITT
GCENNFIEEIVGEVYKNIAPKPLYIGQKPLGLEPHIEEVMSLLDMKPDDKTVRMLGIYGLGGIGKTELAKALYDKIVQHFD
AASFLAGVREKSNTINGMEDLQKTLLSEMFEESETKLGSTNKGIYEIKRKLCRKKVLLVLDDIDDKEELEKLAGGCDWFGP
GSRIIITTREKDVLIAHHVGNIYEMKELDEQHSLELFCWNAFRQGCPKTGFQDVSVRAVDYAKGLPLALKVIGSDLATLHE
ESLDSWEDALEEYEKTPPNKKIQDVLKISYDRLDDDAKQVFLDIACFFKGERMEYVSKILDESGSASKIKVLVNKSLITVDK
GCLKMHDLIQDMGRQIVRQEAPNPGERSRIWDYEDVIEILNEDCGSDKIQGIMLDPPQQEKVKWSGTEFEKMKCLRILI
VRNTSFSSEPQHLPNNLRLLDWDNYPSKSFPPKFHPKKIVVFNLPRSCLTLEEPFKKFPCLTNMDLSYNQRIIEIPDVSELQ
NLRELRLDHCGNLIAVHETVGFLKRLSHLKIMKEMTKPLKIHMINTAIQELPESISKLTGLVSLDISNNRELKYLPRSLFMLP
NVDSFIVKACSKLGESIRSLVQHPSKPNVHKKLRLLNFENGNLSDEDLLAIICYFPKLEELIVSENNFVSIPSCIKECGDLTSLD
LNGCKKLKKIPELTSLRILDVHHCLDLEEISELPSTVQKVDARFCFKLTKETSDMLWNQVKKGVGGIEMVMPFLTEIPEWF
NFVGVGRIPRFWVRGKFPKMVVAMIFHFEKESQRDKFDGRGLVDLRLLINGRYAPRKGYRNFRIEAEHMLLCDVGVLC
SEKEWVGNAVMEHEWNLVEVSYDATSSLMISGWGAFVFEEGTNMEDLLFASPNNLIVNGDSTSVHKGSLEDDEDYV
PLPEGIAWEFLFEGIKDGIVEAWNKFPSMDVAEISVAALKKNCKIEWTVEGMEGIPSAENRTYMTGLYGVLLEAKLRFP
DLDVGAALTTVANRKGIKGTFKSPLQQNLRIPRLDWSTVRLPPSHDPLMQIFMMMMKQQSTSESELKTKTFWKLKES
HLILRNRLSHKTAAQNSASSWKNQYDELIQKFNMQYDAFVGKRVDNLYGVAKYERDSGVLKERAEEIERELDAVVGRL
QKSEEFGDEVRQPVIPPLSRTGGCRCSVKPASRGALLSRNQETHTFAKDPWLNSQLGVGRPASCNLQTSLASHSLVQSP
CCPHSGHKLTLPSSANSHDCQKIYKD

>g26614.t1

MGKRGEEEECVGFSYDVFISFRGEDTGNNFVGHLRKELGRKGMVSLNDESDMRTEEGLSAAVCEAIEESNIFIVVFSEN
YASSTWCLDELVKIIQRTHINSKQVVYPVFYHVDPSDIRKMRNSFGKHMRAHENEIGKESQRMQAWRSALSEAVNLPG
MHVTTGYENNFIEKIVKKVRKSIAPKPLSTGENLVGLEPHIEEVMSLLDMNNDKTVRMLGIYGLGGIGKTELAKALYDKV
VQNFDAASFLAGVREKSNTINGMEELQKTLLSEMLEESETMLGSTNKGIYEIKRKLRRKKVLLVLDDVDDKEELENLAGG
CDWFGPGSRIIITTREKDVLIAHHVGNIYEMKELDEQHSLELFCWNAFGQGCPKTGFQDVSVRAVDYAKGLPLALKVIG
SDLATLDEESLDAWEDALEEYEKTPSKKEIEDVLKISYDRLDDDAKQVFLDIACFFKGERMKYVKKILKEFCSTSNMKVLV
NKSLITIENDRLKMHDLIQDMGRQIIRQEAPDNPGQRSRIWDYEDVIEILNEDSGSDKIQGIKLDPPKQAEVKWSGTAF
GKMKWLRILIVRNTSFSSELQHLPNHLRLLHWDNYPSKSFPQKFHPRKIVVFNLPRSSLTFQEPFKKFPCLSNMDFSYNQ
RIIEIPDVSELQNLRELRLDHCRNLIAVHQSVGFLKRLSHLSVSECTKLQNFLSRMFLPSLEVFDLNLCESVGHFPEIMQEM
TKPLKIYVINTGIQELPESISKLIGLVFIDISNNRQLKYLPSSLFMLPNVDSFKIEACSKLGQSFRSLVQHPSKVNVRPKLRSLN
FQKGNLSDEDLLAILCYFPKLEELIVSENNFVYIPSCIKECGDLTSLDLNGCKKLKKIPELTSLRILDVHHCFYLEEISELPSTVQ
KVDARFCFKLTKETSDMLWCQVKKRVGGIEMVMPFITEIPEWFNFVGVGRIPSLWIRGKFPNIVLAMIFHFQNESERNK
FVRRRLVDLRLLINGRNAPGKGYRDFEIEAEHILICDLRVLFSEKEWFGLDALVENEWNPVQVEYEASSSLMISGWGAFV
YDEGSNMKDVAFTCPNPMTSDKIPQTIIPEKDPMEKYKKRIRELRLDEIFKKTLTEWQENKERGGDRSHDDCIRMALGQ
IKKISEDAEDALNSKGSALEDPNSYLRWLLDTLENDDGKPKEIIKGDLALVVQKHSATGKKKDNVGEASCSGHQGRKEEE
EGYDPVVEAPSIPFYTSVVREQRVNDSMEADLPEDIVMELLCEGMRDGLVEAQNEFPSLDIAETSNAVLEKGYKVRWA
PEVEEKISVESRIYMSGIYSGLKEAKLRFLDLDMWATINTVAKRKGIEGIFVPPSQANLEFPYLDWSTVKVPPSEDPLMQI
FMKMKQQSNFEGEVMSKLFWKLKGEHEVLRNKFAELDDGNENEYDECVEKREEKHDGVGKYEEISGVLRGRGEEIERL
YEAGVEGLKRSEEFEDVMGAIYLNGLRAGLLEAHALLLNLLTPHRN

>g26708.t1

MLVIPVFYKVDPSNVRHQKGSYEEALAKHQKRFKGQEEKLNKWKMALRQVADLSGYHFKDGYEYQYKFIGGIVERVSK
MIINRAPLHVPDYLVGLPSQELEVKKLLDLGSNDVVHMIGIHGMGGTKSNKDDGLQYLQSILLSKMLMGENIILTSWQE
GASIIQQRLGRKKVLLILDDVDNREQLQAFAGRADWFGPGSRVIITTRDEQLLKSHEIERTYEMEGLNENDSLQLLIWNA
FKREKVDPSYEDVLKRVVTYASGLPLALKVIGSNLYSKSVEEWESAIEHYKGIPDGEILEILKVSYVDLGEEEKSVFLDIACFF
KGSSLEEVEHILGVLYDNNVKQHIRVLVEKSLIKVWWDGRFGVIEMHDLIEDMGKHIDRQKSPKEPGKRRRLWLGKDIL
HVLKHNKGTTETETICLDLSIYEKEDTLEWNANAKFENLTVLKFDAYKFLTQTPDMSDLLKSEDAEDKIGSMVPSKLHLF
WALSCNLDDDFFSAGNTKSPPTLQPHFCQALDDF

>g26710.t1

MTTPSSGTFTYDVFLSFRGKDTRYGFTGDLYEALRHKGVHTFMDDEELQSGEEITPTLLKAIEESRIAIVVLSQHYASSSFC
LDELATILDCKSKGMLVIPVFYKVDPSYVRHQKGSYEEALAKHQKRFQGQKEKLHKWKMALRQVADLSGYHFKDGND
YKYKFIESIVERVSRVINRVPLHVPDYLVGLPSQELKVKKLLDLGSNDVVHMIGIHGMEKNIILTSWQEGASMIQQRLGR
KKVLLILDNVENREQLEALAGRADWFGPGSRVIITTRDEHLLKYHQIERTYKMEGLNDNDSLQLLKWNAFKREKVDPSY
EDVLKRVVTYASGLPMALKVIGSNLYNKSVEEWESAIEHYGRIPNNEILEILKVSYVALGEEEKNVFLDIACFFNENSLEEV
EGILRVLYGNNMKHHIGVLVDKSLIDFWTDGEDYIEMHNLIEDMGTHIDQQESPKEPGKRRRLWSEEDILHVLKHDKEL
YEARKVTTFTFPGESFPEWFDLKSSGPSCGGHYFLEQTKFEFDQTYLSDLNMYDNLFEQPLEKEWNHVKVTYSTPTGKV
KGTGIHAIKESMIAITVLSQNYASSSFCLDELAAILDCKNKGLLVIPVFLLISMQLIAIVVLSQNYASSSFCLDELATIIHCQSK
GLLVIPVFYKVDPSNVRHQKGSYEEALAKHRRRFKAQKEKFQKWKMALRQVADLSGCHFKDGDGYEYKFIGSVVDRVY
HKINPCPLHVADYPVGLEPQVRELRKLLNVESGDGFHMIGISMIQQRLQRKKVLLILDDVDKCEQLQALAGSPDWFGP
GSRVIITTRDTQLLASHQVKRTYEVKTLNREHALQLLIWKAFKTGHVDQSYVEVLNHAVTYASGLPLALEAIGSNLFGKRV
EQWKSAINQYKRIPNDNILGILKVSFEALNREEKSVFLDIACCFKGYALREVEDILEVLYDDCMKYHICVLVDKSLIKTSCGR
VEIHDLIEEMGRQIDQQESPEGSGKRRRISLPKDIIQVLKENTGTSMIEILCLDISIFNKGETLYWNGNAFGNMRKLKTLIIR
NCKFSRGPNCFPGSLRVLEWHGYPSNCLPSKFDPNKLVICKLPNSRLRSLGLLAHQRQV

>g26711.t1

MATSSSRRFTYDVFLSFRGEDTRDGFTGDLYNALCDNGVNTFMDDEELQSGEEITPALLKAIEESRIAIVVLSHNYASSSF
CLDELATILHCQSKGMLVIPVFYKVDPSNVRHQKGSYEEALAKHQKRFKGQEEKLNKWKMALSQVADLSGYHYGDGN
DYKYKFIGSIVEQVSRKISRVPLHVPDYFVGLLSQELKVKKLLDLESNAVVHMIGIHGMGGLGKTTLALTVYNSIADDFDS
SCFLQNVREESNKQGLKHLQSILLSKMLREKNIILTSWQEGASMIQQRLRRKKVLLILDDVDNREQLEAFAGRADWFGP
GSRVIITTRDEQLLKSHKIERTYEMEGLNENDSLLTFILLTVRSFITALSAKDKVEFVLGSAPQPSKTDASFPTWFRCNSMV
VSWLTHSVSPSIRESIIWMDLAIDIWTDLKHRFAQGDLARISALQMEATTLSQGELSVTKFFTKLRVIWDELDCFRPDPV
YTCQTKCSCTVSTVLSQHKNEDRAMQLLRGLNDHYTNIQSHILLLDPIPPISKIFSLVIQQERQLMNDHITASVTTTPFPSS
TPNSVTCNYCNRVGHQENTCFKKHGFPNQEHKTVKTTNNNSRKICTYCHRNGHTIDICFKKHGYPPGHKFSTKSGQVH
NVISSTNADNQLKSLDTERSSLETIQLTPQQYQILAELFKQSTTNNSNVHINQVGTVSTNMSPAKHAVFLINQMPTHVL
QNETPHERLHGSPCDLSMLRVFGCLCYANTITSHRKKFDDRAIPARLVAKGYNQIEGLDYLDTFAPVAKITTVRLLLAIAA
SKCWSLKQLDVNNAFLHGDLHEEVARNSKGIHLSQRKYTLDLLAETGLLDSSPVPTPMVPKQSTTNTVQSLDDTDAASY
RRLIGKLIYLTTTRPDITFVVNNLSQFMSAPTTAHQQATSRILRYLKGTPGAGIFLPSTSPIQLKAFCDSDWAACPNTTSHF
TSFGILGSSKASLNSKFENLTVLKFDAYKFLTQTPDMSDLLKSEDAEDKVGSMVPSKLHLFWALSCNLDDDFFSAARGTR
RTEPILGSRLMSEKGDGANDGERNEPNGDVVGGLNGVEGGGPNGKSSNGSDGVGRPEEQSNWRKRRSYDLPTSVW
KAVRGFGSGFGRLGQLLGEPISERFVAIRIDEGIRANDSHKDGKGCGGNPRRYEDGQWEYEQNTILGQTERNRNTNRK
QPFQPNPYGNCNRELNGAHGEWGDWRKRRNAQERPNRQQQGKKRHQCPERSLRMTILAEDEEDEDEEEGATELDH
IQMELSAFSAGGLTQPRTMKLHGQIGGKQVLILIDSGASHNFISNELVRALGLAVEDTPPYSVSLGDGQKKKTQGRCEG
VTIEEVEAWMIVWELGWTKKKIRGLPPDRGMVHQIPLKEGTDPVNVRPYCYPHMMKGEIEKQVSEMLKTGVIRPSTS
PYSSPVILVKKKDGSWRFCVDYRALNRATVPDKFPIPLIEELLDELKGAKYFSKVDMKSGYHQIRMGERDIEKTAFRTHQ
GHYEFMVMPFGLTNAPATFQSAMNNLMQSYLRKFVLIFFDDILVYSRSWEEHLGHVALVLRRLKENHWVANRKKCEF
GKVQIGYLGHQISEKGVEMDPDKVRAVMDWEKPKTVKALRGFLGLTGQFTWTKQAEEAMASLKRVVTTAPVLVLPD
FNQPFHIECDASGRGIGAVLTQGRRPIAFFSKALSERSLSKSVYEKELMALVLAIQHWRHYLLGQKFVVHTDQRSLRYLLE
QRITTQNQQNWIAKLLGYDFEIVYKSGITNRVADALSRKEEEDTEETRELGMVARPFWQDFGEILEEVEKDEALKKVIED
LRKDPNSHPSFTMENDRLHYKGRLVISAHSTWVPKLIAEFHTTQTGGHSGVYRTYRKVAQSLYWIGIKKAVTDFVASCL
VCQQHKYLSSSPQGLLQPLPIPNAIWEEISMDFIVKLPKSHGYDAVLVVVDRLSKYGHFIPLKHPYSARTIAEVFVKEVVRL
HGGTQLKMSTTYHPESDGQTEVVNRVLEGYLRCFCLEQPKGWSVVLPWAEYWYNTSYQEAARCTPFDLYFAWVIWE
SFRGPGVAGDSLSMGRVEPWRYIWVAPCNLKFKRKAGGERGVERRSWEITSVENFNAGQVDDRQVGREESFTGNIYE
ALSDNEVHTFIDDDKPQSREEITPALQKAIEEYKIKYKIRSITLIFKQNTLFKQNTLSSR

>g26713.t1

MATPSSRRFTYDVFLNFRGEDTRCGFTGHLYEALRHKGVHTFMDDEELQSGEKITPALLKAIKESRIAIVVLSHNYASSSF
CLDELATILDCQSKGMLVIPVFYKVDPSNVRKQEGSYEEALAKHQKRFKGQKEKLNNWKMALRQVADLSGYHFKDGD
EYEYKFIGIIVDRVSRVINRVPLDVAAYPVGLPSRVLVVKILLDVGSDDVVHMMGIHGMGGLGKTTLALAVYNSIADNFD
ESCFLQNVREESNKHGLKHLQSILLSKMLGEKNIILTSWQDGASMIQQRLGRKKVLLILDDVDNREQLQAFAGRADWF
GPGSRVIITTRDEQLLKSHKIERTYEMKGLNENDSLQLLIWNAFKRENVDRSYEDVLKGVVTYASGLPLALEVIGSNLFGK
SVEEWESAIELYKRIPNKDILKILKVSYDALGEEEKNVFLDMACFFKGSSLEEVEHILGAFYDNNMKYHIGVLVEKSLIKVR
DKRFGVIEMHDLIEDMGRDIDRQESPKEPENRRRLWLGKDIIHVLKHNKKFENLTVLKFDWCIFLTQTPDMSVLRNLEE
VSFNWCQSLVAVHDSIGFMNKLKILHAERCIKLTSFPPLNLPTLEKLKLSYCSNLEKFPEISGKMENIKELGLAGLPIKELPLS
FQNLIGLERLSLSCEILHLRSSILRMPNLIEFSVTNCKEWQWIKSDAEDNVGSMVPSKLKQFSAESCNLDDNFFSAGFMR
LAQVRTLILKDNNFKHLPECVKEFHNLNSLDVTHCKHLEEIRGFPPKLEYFTAPNCISLSSTSLDMLLNKELYEARKADFYF
PGASFPKWFDLKSSGPSFFLGCTLMGNLNQAQATI

>g26714.t1

MTSPTFTYDVFLSFRGEDTRYGFTGNLYKALRDKGIHTFIDDDKLERGEEITPALMKAIQESMIAITVLSQNYASSSFCLDE
LETILDYKNKGLLVIPVFYKVDPSDVRHQKGSYGEALTKHQKRFKDKKEKLQNWKMTLRQVADLSGYHFKDGIEYEYKFI
GSIVERVYSEICRGCCLHVADYPVGLDSRVLEVRKLLEVGCYDGVHMIGIHGMGGVGKSTLARAVYNGLITENFESLCFL
ENVREKSNKHGLEHLQSILISQVLGKEDINLTSKQQGISMIKCRLKRKKILLILDDVDNLGQLRALAGGRDWFGPGSRIIIT
TRDKQLLATHQVTRTYEVRELNENDALRLLTLKAFKKEKADLDYELILNRVVTYASGLPLALEVIGSNLFGKSVKEWESAT
RQYKRIPKKEILEILKVSYEALEEEEKVVFLDIACCFKGYALREVEDILGALYDDCMKHHIGVLVEKSLIKVSLLSTVEMHDLI
EDMGRKIDQKESAKEPGNRRRLWLPKDIIQALKYKMGTGKIEIICLDFSISENEETIECDENTFMEMENLKILINRNVKFSK
VPNYFPKSLKVLEWHRYPSNCLPSNFHPNNLVICKLPDSCFASFGFHGSSKKFENLTSLNFDDCKLLTQITDVSDLQNLEK
LSFEWCESLAVVHNSVGFLQLAQVSYLWLQESDITFLPECIKEFHNLHVLDKLHEGGGTVFIFPGGSVPEWLDKQSKGPS
ISFWFRNKFPAKVLCLLIAPVLGPFDSAIPMVYIDGKIRKSLFHMNKEGKILELDYTHLFDIKELNFEDDLMGVSEKEWKQ
VEVTYEGLFDTSVIKATGIHVVEEESRSMEDIRYDDPYSNKEDNHLNTSQSQNHSLNSIAPSGSGVERHFLPLSGGVLESR
ALSARPLALSSDDTDVSKCEGEAQMEHEGAAKKLGKGRGRVLVESVRGQMVGVMRRGMGGSMKMVMGDEDVKV
VRVRGAEGGEPNECVSAIATYLVLMATPSSRRFTYDVFLSFRGEDTRYGFTGNLYKALCDRGIHTFMDDDKLQTGEEITP
ALQKAIEESRIAIVVLSHNYASSSFCLDELATILDCKSKGLLVIPVFYKVDPSYVRHQKGSYEEALAKHQKRFKGQKEKLHK
WKMALHQVADFSGYHFKDGDEYQYEFIGRIVERISRVINHAPLHVADYPVGLLSQVLKVKKLLDVGSDDVVHMIGIHG
MGGLGKTTLSLAVYNLIADDFDSSCFLQNVREESNKHGLKHLQNLLLSEILGEKDINLASVQRGISVIQQRLRRKKVLLILD
DVDNRKQLQAFAGRSDWFGPGSRVIITTRDKQLLKSHEIERTYEMKELNENDSLQLLIWNAFKRKKVDPSYENVLKGVV
TYASGLPLALEVIGSNLVGKSVEEWESTIELYKRIPNDEILEILKVSYDALGKEEKSVFLDIACFFKGSSLEEVEHILGVLYDNN
MKHHIGVLVEKSLIKVRDGRFGVIEMHDLIEDMGRDIDQQKSPEEPGKRRRLWLGKDIIHVLKHNKGWQWVKSEDAE
DNVGSMVPSKLDWFSAESCNLDDDFFSTGFMWLAHVHSLYLDHNNFKHLPECIKEFHNLNELNELYEARKTEFRFPGA
SFPEWFDIKSSGPSCSFWFRNKFPARVLSLLITHTDTDMYSIITPSVYINGKYRTGSRKRNGII

>g26746.t1

MSASFSYDVFLSFRGSDTRYGFTGNLYKALCDKGIHVFIDDEELQRGDEIGEALIEAIKQSRMAIVVFSKNYASSSFCLDEL
VKIIDCVKEKGRLILPIFYDVRPSHVRGLSGSYAEALAMHQERFKSSNQSLNDNMGRLQKWKMALNQAANVSGKHYKL
GNEYEHEFIGKIVKEVSDKINRRPLHVADYPVGMECRVQKVKSLLQFGSDTGVHIVGIHGIGGMGKTTLARAVYNSIAD
QFEGLCFLDGVRENAVKQGLVHLQEMLLSEIVGEKDIRIGSVSKGISIIKHRLHRKKVLLILDDVDKLEQVRATVGERNWF
GSGSRVIITTRDKHLLQGVDGKYEVEDLNEEEALELLSWNAFKDHKVDPSYKDISNQAVAYASGLPLALEVIGSLLFGKGT
REWESALDQFKKIPNRRIQEILKVSYNALEENQQRIFLDIACCLKGYELEEVEDILGVHYGVCMKYDIGVLVDKSLIKIKNG
CVTLHELIEVMGKEIDRQESPKELGKHRRLWFHKDIIQVLAENTGTSEIEIICLDFPLFEEDEEVFVEWDGEAFKKMENLK
TLIIRNSHFSKGPAYLPNSLRVLEWWTYPLQDLPTDFHPSKLAICKLPRSCFTSLELATISKACKFMNLTVLNFDGTESIHGS
VGFLDKLKILSAFGCSKLTSFPPIKLISLERLDISSCSSLESFPEILGKMENITQLELKYTPLKEFPFSFRNLARLQDLVLVDCGN
VQLPSSIVMLPELAEIFALGCKGWLLPKQDENDEEKVSLVSSNVKCLCLSGCNLSDEYFPMVLAWFGNVKELELSSNSFT
FLPECIKQCRSLKLLNLDNCEHLREIRGTPPNLEYFSAGNCKSLSFCCSAMLLDQELHMAGNTMFCLPGSRIPEWLEQQS
IGPSLSFWFREKFPVMDLCFVIGPMGKDSILFRPIMTINGNTMEIQSLTDKRFCFDFPALDYHILIIGTKYMKFGDNLDKP
LSKNEWNHVVVSIALDFEPTPKEIIVKQTALHVIKPESSMDDIQFTDPCNQPSFIEKQRLVDTVDYQTSLASLEPHVRQGR
NSLSLIPPQACKNNPNWDSFSTGTSSIASVQAAEKIGYFGFTTTTTHGFIGFTATKGQRIAFIALLSIIGVDGLMAEEMHH
HFPRWFYIDLTDWLTLATIGYKSLQEQHLPSTIKENFEGCNGINEAKVNEVTRCKNNDGNDNERNPLVEQLLLTKFPRQ
KRIPEELIPMEFEEAHNNFLAIRLLYRLLEDNSIALQNSNPKHLVEWARDLLKSLLDVAVESVFETHLKWQESNSLVAANIS
QSSVKYPTCSKMTSEKDEQPQLRLISKKQLPELSKFSISDDGGTKCKTLGCIGEESSTMQQNAVKATGVSDDIDSLPYTESI
HSKQQQNDVNPLYGDGEDAKKSLQMFDAKDDEQNEENNVILLNRIHEAQRNSDAAGQVEHLNQKGEFSDDLVNAIK
RIESRILAFQICSNVMDSTKNSAGHLTTLEAANSDGPAMQRNSGVSGSQMSSGKSLLKGHRLLSQKTFKSSCKDENSISE
SAFEESLFPGKESLNQSQMANQNFCLQNSAESAKNIDVPKQSGTQMVLRGECLSNHERIQNSERNIAIDSVKPIDGLVS
GDAYFETRASKGIPGFRVPLNQDNNSKKHSMLLSKTKRENSERKNPVAWSKTDQNPRERISESSYTQKSTRSKTIITRRE

KPPPHQMVMKPTLLDQKSSDIKVNSHKHRDRRRTLNTSHLEPRNTRVLQQHFEREESSSSSSSKSDSDSDSSRWNSQQ
GSANSCSIDSEDYSLSDGTQSPSSSGRMVDAAYEGSSEETINSYLEMDDDPSPRLGSFKSYRHHHERYHKETTGRLRRLR
NKLGLIFHHHHHHHHHHADHGNTHSREGHGPTRWNNLQNVFHHKDKHGVLTKNVEKTKGGHAAKVLSGNQVGQF
NRLAEGVLRHIRHSKKPKPPMFDVVKQSRNKPHGHNQKKLPATVSEPPLTLVKTFSRLCLTAQPKTFACKTWTFCLQNP
SHSLMEKMTHENAIGIEIDYEYDDCNNSKRPKMTSKVWEEMQRIQTTEGSKVLCRHCGKLLQDNCGTSHLKRHLVICP
KRPKRLDAITRDSMASGCFRGPSSARESGLNTVLMVRPLKIEPESQVPCFFPTSNNRVPTIASTENAPNSIQELHAKSSSP
LMLSTIESPKNQEELTLDDVEMKAFYASLDAETSVMSPSQDTTVVTELSNSTPSEETKKALKTLQDLLSKDFTDLLHTGQS
GTIKSSIEYLAKLSADDGISAEMRLLILEVSREFTRWSCDYNDANRKIESANANILKADKLEENLEANKKEFKEVSSLENELS
NQLSCLEKRKKELEEQINALKANISVFQSAKVTSTKRKREAFEEAKILKAQRDELKEQVAMFLHLLLLLALNTQPLLAAEPE
PVVDKQGNPLVPGVGYYVWPLWADEGGLTLGQTRNKTCPLDVIRDPSFIGSPVAFYAEGLGHIPTLTDLTIDFPVFTVC
NQPTVWRLSKQGAGFWFVSTRGNPEDITSKFKIERLEGDHAYEIYSFKFCPSVPGTLCAPVGTFEDVDGTKVMAVGDNI
EPYYVSLDQLKRIHALCVTLGFLHTQNLQQSLSCKLLQCYKNVGKTEQAQRVFDQINDPDIVSWTCLLNLYLHSGLPSKSL
YAFSRCLHVGLRPDSFLIVAALSSCGQCKDLVRGRVVHGMLLRNGFEENPVVGNSLIDMYCRNGVMGMAALVFDNM
GSKDVFSWTSLLNGYMLCNNVSCAREVFDAMPERNLVSWTAMITGCVRAGAPVQALEMFKQMEGDDGGLHCTDL
MVAVLSACADIGALDFGQCMHGCVHKRRLELDVAVSNALMDMYSKSGRLGLAVRIFDEILKKDVFSWTTMISGYAYH
GEGHLALEAFCRMLESSVNPNEVTLLSVLTACSHAGLVVEGKVLFNRMIQCRYMKPKIEHYGCLVDLLGRAGLLEEAKE
VIGMMPFSPDAAMWRSLLTACLVHENLRMAQIAGKKLIELEPNDDGVYMLLWNMYCVADMWKEASEIRKFMRERG
VRKNPGRSMVDVNGVVQEFFAKDSSLHVEFLFYLSDDL

>g26748.t1

MAQMELSSSLRNYTHSYDVFLSFRGVDTRTGFTGHLYSALCQRGILTFMDDDALSKGEEITPSLLNAIRKYPSEVRHQTG
SYAEAFEKYEQERFKNDQEKVQNWRGEYEHVIIKRILEKIFCKINRPLLQIADYPVGLEARMRYVLSLMNAELDTKVKML
GIHGIGGIGKSTLARAIYNLIAHQFEHSCFLANCGLDWFGIGSMVIITTRDKHLLDIHGVEKQYEVDIFNVTEAMKLFRW
NALRKKVVDPSCTEIIKREIYYTAGLPLALETIGSNLLGKTLDEWKSALDAYEKLSSRGIQEILQVCCDGLEENEKDIFLDIAC
FFKGCTVKKVTDMLNACGFHAEYGISVLRNKSLVNILMSGEHDGEIVTMHDLIQCMGKEIVRQRSTLPEKRSRFSNPYP
PNTTFPTDHGTRNQLDIQDSFDPVVSMHTRMLDKLAYLSTRQWRLPFMYPEWKEPLDNVLSPNLSESDLSDVIIGQIEE
ISSEMEEIPLHNPYPTETVHDSHCSTCHENEVFRKLVDCRIKSSSSNLHLGTSSTVFPTKIEPSERNQIEQTQEDHEEMDTF
YASLGESHVSHSRHKTATDVLSKMDREALKIVDDFISDDALVLLHPERFIIVKNSLAYLSTLSADDGMSIAMEALIAEALGK
FTHWRRDYTEASMKIETTASELQRADELEAG

>g26751.t1

MEKEEEEEAVAPMVKLGSYGGEVRLMVGGEESAAEETMLLWGILQPTLSKPNAFVSQSSLQLSLDTCGHSLSILQSPSS
LVSFSLTLPALVKMIIGKKALILILGTPGVTGAVMWDSGVVLGKFLEHSVDSGMLVLQGKKIVELGSGCGLVGCIAALLGG
EVVVTDLPDRLRLLRKNIETNMKHVSLRGSITATELTWGEDPDSELIDPTPDFVLGSDVVYSEGAVVDLLETLMQLSGPN
TTIFLAGELRNDVILEYFLEAAMNNFTIGRVDQTLWHPDYCSNPAVAWGAGEPLVMEEVEVSPPQPMEIRIKVVSTSLC
RSDLSAWESHAIFPRIFGHEASGIVESVGLGVTEFKVGDHVLTVFIGECMTCRHCTSGKSNICQVLGLERRGLMHSDQKT
RFSVKGKPVYHYCAVSSFSEYTVIHSGCAVKVSPHAPLEKICLLSCGVAAGLGAAWNVADVSKGSTVVIFGLGTVGLSVA
QAAKLRGASRIIGVDNNPQKCENAKHFGITEVVDPNSCEEPIAQVIKRITDGGADFSFECVGDTDMITTALQSCCDGWG
LTVTLGVPKAKPEMSAHYGLLLMGRTLKGSLFGGWKPKSDLPSLVEKYVNKEIQIDDYITHNLPFDDINKAFNLMKEGKF
LMAIRPRSSSFTYDVFLNFSGEDTRHGFTGHLYKALHDRGIYTFIDDDELQRGEEITPALVKAIGESRIAITVDPSDVRHQK
GSYGEALAKHEQRFNHSMEKLENWKKALHQVANLDGYEHEFIGRIVELVSSKINHGPLPVADYPVGLESQALTVRKLLD
VGSDDVHMIGIYGIGGIGKSTLALAHGLQNLQSILLREMLGEKEANFASIEQGASVIHHRFQRKKVILILDDVDKYEQLQA
IVGRPCLFGPGSRVIITTRDKQLLSSYGVIRTYEVPSSIIMMPKLAGIYARGLKGLQWLKQEEGEEEMGPVVVPSMVERLT
ASDCNLYDDFFSIDFARFSHVKNLWLPKNNFTILPECLKQCQFLSYLDVSYCKHLREIRGIPPNLKRFSAINCESLASSSKR

MLLKQELHEAGNTVFCLPGFSIPKWFNHQNRGISISFWFRNKFPDKVQCVLVAPIQHDYFCPRVFINGKQYTHYSRCRL
TGKHHKYLFDLREASFRKSPYEVPFDSEWNHAKVTFVHGKHTFINRAKIGIHVVKQENSMEDVRFTDPYCKRKLDVDIN
SFDSESIPVLDLELS

>g26771.t1

MWILPVFYDVEPSQVRHQTGTYQEAFAKHAERFKDDIQKLQQWRLALRHAANLSGLHFKTGEKYESEIIQELMGGEFD
KKVTMLGIHGMGGIGKSTLARAMYNLMADQFEGSHFLANVREKSDKDGLIHIQETMLSELVGERNIKLGDVHRGIPIL
QHRLCDKKVLLVLDDIRKIEQLQATAGGLDWFGPEQAIYYANGLPLALETIGSNLFGKTLEEWESALETYERIPNRDVQK
VLGVSYDSLNAYEKEMFLDIACFFRGCSLSLVTNILEARGFLPKFSLRVLEEKSLIKIRECQNKTVEMHDMIKCMGKEIVRQ
QSTLPHKRNRLWFYEDIDCVLEKNMENDRIEAMMLIPEYQEEMQCNPKFDKMKSLRMLIIDKKNSKVLRDLILRGCKNI
RRIPDISGFPNLTRLLVEECTNLFEIHDSVGSLLNLKVFRAVGCTKLIIGPSRIKWISMEHLSLRDCSNLVMFPEVAKSQYILT
TLAGETLTMDNKLVLLGEASDTQQQEQSYALFNPTTSLESAIGEQPLNYEYNISDSKESASTVDSNIQGVLVDDQPILAPS
SAETQNAEVKREIAPLISEVSSEFTAQVSIKIESTASQSPKVDEVDADLETQNQFRNTILLENQIHEKLTRIRKMKEELGEKL
SAIKADISAIEANNSSIKANISDIKTIFLQIDQRKNN

>g26774.t1

MNGSEGYESEMVKKITEKVSAKLNRPPLHIADYPVGLKVRMQQIQKLTGDTKVTMLGIHGMGGIGKSTLSRAMYNLM
AHQFEASHFLANVREKSDKDGLVHIQETMLSELVGERNIKLGDVHRGIPILQHRLCGKKVLLVLDDISKKEQLQATAGGL
DWFGPGSIIIITTRDKHLLDVHGVQKQYMVGPVNCMEALELFKWNAFKNKEVDPCYKEVIKRAMYYANGLPLALETIGS
NLFGKTLNEWESALETYERIPNRDIQEVLRVSYDSLDAYEKEIFLDIACFFRGCSLRYVTDMLEARGFPPKFGLRVLEEKSLI
KIRECQHETVVMHDMIRCMGKEIVRQQSTLPHKRNRLWFYEDIVCVLEKNMENDKIEAMMLIPEHQEEMQCNPKFG
KMKSLRMLLIEKKVGFLRSPAALPNSLRVLEWRGYPATSLPRNFHLKNLVILNLSHSYFGWDKPLQAFGSLLNLKEFCAE
GCTKLTIGPSRIKLISLEHLCLRGCSSLVMFPEVLAPMHKLKHVDLGGTGIRNLPQSMQNLEDITILPASIEKCHSLKFLHVG
NCKNLQEIRGLPLSIKGFSAANSPVEANSLTLKLRQAIDSATMKVCVLQSRKIPELFDHSSRGNSLNFWFRKELPTLAVCAI
IGVWDNVKPPFAADFSYIIILNLQNDFQHPLNSDIQKVLLTNEWIPGKILWRINPESDSSKLAEIRRTGVYVNRTFSRMED
VRFEDPYDLNKASTMDNKLMLVGEASHTQQQEQSSALFNPTTKLESVIGEQPLNYVYNSDSKESASTVYSNKQDGVVV
GDQPILAPSSAETQIAEAKREIASLISEVSSEFTAQVSMKIESTASQSPKVDEVEAGLKTQNQFRNTMVLENQIHQRLTRI
KKMKEELGEKLSAIKADISAIEANNSSIKANISDIKVIFLQIDQRNLLPAFIYE

>g26777.t1

MAERQAPSSTSSVSNYSYDVFLSFRGPDTRFGFTGNLYSALSRRGIFTFIDDEGLRMGEEITSSLRKAIQESRISIIVFSKTYA
FSTFCLDELLHIIECHKKQNMCILPVFYDVDPSQVRHQTGTYQEAFAKHAERFKDDIQKLQQWKLALRHAANLSGLHFK
TGEEYESEIVKKITEEISTKLNRPPLHIADYPVGLKVRMQQIQELMGGEFGKKVTMLGIHGMAGIGKSTLSRAMYNLMA
HQFEASYFLANVREKSEKDGLVHIQESMLSELVGETNIKLGDVHRGIPILQHRLCGKKVLLVLDDINKKEQLQATAGGLD
WFGPGSIIIITTRNKHLLDVHGVEKQYMVGEINFKESLELFKWNAFKNKEVDPCYEEVTERAMYYANGLPLALETIGSNL
FDKTLDEWESAIETYERIPNRDVQEVLRVSYDSLDAYEKEIFLDIACFFRGYSLEYVTDMLEARGFRPKFGLRVLEEKSLIKIR
ECPHETVEMHDMIRCMGIEIVRQPSTLPHKRNRLWFYEDIVCALEKNMNSKVLRHLILKGCKNIRRIPDISGFPNLTELC
VGECTNLFEIHDSVGSLLNLKEFCAEGCTKLTICPSRIKLISLEHLCLRGCSSLVTFPEVLAPMNKLTHVDLGGTGIRNLPLS
MQNLKDITIRPASFEECHCLKFLLVTNCKKLQEIRGLPLSIKEFSAADSPVEANSLTLKLRQAIDSAVMRTCVLPGRKIPELF
DHSSKGNSQRFWFRKELPTLAVCAIIGIWDNVKPPFIVCHIIILNLQNDFQHPLNSDIQKALLTNEWIPGEILLRIHPQSDL
RKSGEIRRTGVYVNRTLSRMEDVRFEDPYDLNKASTMENKLVLVGEASDKKQQEQSSALFNPTTSLESVIGEQPLNYEY
NNSDSKDSASTVDSNNQGVLVDDQPILAPSSAETQIAEAKREMAPLISEVSSEFTAQVSIKMESTASQSPKVDEVEAGLE
TQNQFRNTMVLENQIHQRLTRIKKMKEELGEKLSAMKADISAIEANNSSIKANISDIKAIFLQTDQRYNN

>g27308.t1

MVDSSTPPPSQSKDQSQPSTQPKCRRATRLKDLTISRSADKRLPIQFDMSTGKVFGNNRARFTSFVALLGRSKASILIDD
WVHVPEQVKNQIWQTIMLTYDVPNAILLRTKWISYAGQRWRCFKSDLTSRYIYGKLSHKNPCDIYQFLDEETWQAFRD
KRLHPTFQEKRKAAQDMVKKNVHPHRLSRGGYHRLEEIMICEASSSAPDDQSDLISPPPRHDKWKRARTKPSGEYTSEE
TRLIAERIDDLVEKSSQGSFTQQGREDILATAIGRPEHPGYVRGVGGGVGIKQFFGGTTRKVTLAQLSESDKNALRNEFK
KELFPQLRDELLSEIKSEIASLGLAVQGPPKETPPIVASTKGSCPLHEESGDGVDVPVDCKIYNLGSTIHHNTIKDDMLRVV
VVDIKDCSARVSVPNEEVQTVGQAPGNFIIWPSRLTKPILDVSKKTNIVTPPQPQLSPLQQLGAAVVTMGNKTIEIDMP
PELTCKTATTTLFVCHRDICEIVIGNDLLSTTVLQVWNLYLHHLCIERKNDTIYGFLDPVIIQSVGNKSEEVQKYLTEMFEKA
SKEVYLAPYLHKSLEAHSRLRGIPSVSRKKMQIVTPNCRRQLGSYECGYYVMKHMHTIICTNITDSWSKIFNDSSPMEAPI
IEDIRTKTHKAIEDSHVSIVIISENYAFSKWCLEELSKILECWKNQGQIVIPVFYNIDPSHGSKQTGSYEQAFVKHQEDFRRR
CEQRLWSSNELQDDEPGTSIRALKEVAILSTLNANGDHQRVRRTKWKRIKETCNVAGLSALNYQRSKKWTPWAESRR
QRNFPSTERDEMKEDEAGIYLYGERRELLLDPLLAEEDQTSKLEDEGLRNLVKPHDSRDLLTAMAQARDVERAEIIPRGS
DGMARKGGPMWGRYSSSSGTIARTKKYRGSSEGFINADGGQMMKKDGVSSNLRKWRKVWKNKTKNPYWTELREA
LIRRFGERDRGFILEKLAQTAEVREAVAEKVVSIAAHEVANASISRGDSGGGCDVYVTIVKKGEDTGKKAKSGGNRVTIM
VGNKVEDAVVLSGEKIWLSHTYKTPEDQWQGYFFARLQFNIQRKQLAQHDDDEEIGWSGHQVLSFLASKMNKSGQG
EGLATGAAIKVQKRIESELLMDIVADILRKLTARCPTQLRGLVGIEENYEQIESLLRIGSREVRTLGIWGMGGIGKITLATAL
YARFSPEFEVGCFLSNVRENSSRQGGLEALRSKLFTELLENENHCFDAPLLIPQVVMRRLGHKRVFIALDDVATSEQLERLI
IDYGLLGPGSTVIVTTRDKQFFRPNDDIYEVKELSIHHSHELFSLTAFEEKIPKHGYEDLSRRAISYCKGMPLALKVLGGTEV
VEAMTLDMCKLNRDLNLSSNSFTKMVSMRFLKIHSSYCSSRFNVHLPSGLESLSDNLRYFRWDGFFHESLSSNFHAEHL
VELDMRCSKLKKLWEGVQSLVNLENIYLEASRDLVEIPDLSKAEKLRRIISLIVKACILDLDGCSSLKQLSVTSHKMEFLDLS
YTAISPDHFERTQHNANLSFVQSLPNIIGHLNLVSLPELPSSLEMLTAYNCTSLDTVFTQLLKDERVRIKECGVFSVYASES
GLKLFGSCSIEFFELESITQSFNESQSRAIGVGVRCSTGENGLESFVEVSNNEFSFETLNGEFHGTCKLANFVGRVRVVHKI
VCGVQKNSARENTFSPCHTPTVTVHRNRSPQVATPRLAVGCQLCLSCCGSHLNETFSSPNATLSPSNTTITDRDKKTLLS
VKQPLYLFGPAWVTGLGNDFKETAMERMMAIWGHIVLWIVIPARDKLIWEGMFGLAIPVRTKSIRE

>g27658.t1

MSPERDVISATPGAFRLRWDVFLSFRGTDTRGTITKGLYKSLQARGVRVFLDDEGLERGEAVANGLMEGIDDSAAFIVII
SENYASSHWCLEELTKICDTGRLLLPVFYRVDPSQVRHVSGSFGSGFESHEKRFEKNTVSRWKEALKKVGGVAGWVFN
HSEEDDLIQRLVRRVLKELSNTPMGVPEFAVGLDERVERVMEVLQVQSNGVKVLGLYGMGGVGKTTLAKALFNALVN
RFEHRCFISDVRQVSSKHDGLVSLQSKIIKDLFSGAGSASIGDVNDGISAIKGRLSENRVLLVLDDVDEVKQLDALIGKRE
WFYDGSCVIITTRDTKVLTQNHITVSYEVRELYASEARVLFSYHALRRSAPPENLLSLSEEIISLTGRMPLALEVFGSFLFGKR
REEEWEDAVKKLRLIRPHHLQDVLKISYDALDEEEKCIFLDIACLFVQMDMKRDGVIDVLRGCGFRGEIAITVLVQKCLM
KITPDNTVWMHDQIRDMGRQIVMDESFVDPGSRSRLWDRAQIMTVLKGHKGTRCVQGIVLDFEEERFYKGKVGSVF
PKKFQWRPSLRNISCYIKQCLNKNHPEPQAEENTEFVLHTKSFEPMVNLRQLQINNLKLQGKFLPSELKWLQWQGCPL
ERMPLKSWPGELAVLDLQNSKKMETLWGWNGYNKVPQKLMVLNLSNCIQLTAIPDLSGCQCLEKIDLENCINLTKIHE
SIGSLSTLRSLNLTRCSSLINLPIDVSGLKQLESLFLSGCSKLKSLPENIGILKSLKALHANDTAIAELPLSIFRLTKLEQLVLERC
QYLRRLPNSLGHLCSLQELSLYHSGLEELPESVGSLNNLETLNLMGCESLTVIPDSVGNLMSLTELLVDRTAIKELPTTVGS
LSYLRELSVGNCKLLTQLPNSVKTLASVVELQLDGTAITNLPDEIGEMKLLRILKLMNCKNLEYLPESIGHLASLTTLNTVNG
NIKELPESTGRLENLVNLRLNKCRMLRKLPASIGDLKSLYHIFMEETSVSSLPESFGMLSSLRTLKMAKRPELDTNESSFLA
EPKENHSPFVLTSSFCNLTLLTELDARAWKISGKIPDEFEKLSLLETLKLDRNDFHSLPSSLKGLSILKVLSLSNCTQLNSLPSL
PSNLIKLNVQNCSSLETIHDMSNLESLQELNLTNCVKVGDIPGLESLKSLRRLYLSGCIACSSQIRKRLSKVALRNLQNLSM
PGSKLPEWFSGQTVSFSKRKNLELKSVLVGVIISINHSIDIPNMKRDDMPGLIDVEANVLKGGRTLFRTVLNICGMPRTD
EEHMHLCRFHDYHQLVAFLKDADTFSVSKRNPPFDTGLELRKCGVYLIFEGDDDYDGGEESLDKGLQSVSEKLANFFSTP
EGGAQGNSADVGGVDTVQTSKKDLEIEEEDDGKGVENIQVEKKALNIEEENGGKGVSPESKTGVDDKLERGNIKKKW

HLVFLSMKVAVGISWMPNEKRKSHPKVRQIMITVDRAKTVIFQLFQAIFQCLEEDIGWDEIEDIESNDENKGDVVGSAS
RIHLRKRFNIVSVGLVPLHNFSTSGHFACFLCRWVEVGWVPAKKALTLKSVPLSSSMGSVGGEGGGSSSGSRFSSSNLGS
VSRGERSDRSGGSLGSSEPSGNRIINGVSLFLLERGIEVDAMRAEPAGRWPALGGYGWASHDVGSYESEYKSRDDLLE
WANQSFLARDEEDARLIRLSVSYPNERVFHGKGTSVKDLFFPYYESFKNFKTRDPTRISHVSTDEMYLAEVEAVKTINNLP
CRLHVRQLVDCLCHEHFTRVAFSMMLVSAHLRSPVMKERFMADRFQTSAAQSQTKPAVETIPVVKPPVVMEVPPVV
NLESSGAVVAASAQVVPSSSKNKRKSNEGEMSSSKKSRQESTPQPLPGGLVDPTIGVSDRVDFRMSSSQRAAVDFRMS
SPLVALC

>g28937.t1

MALAVVGGALLSAFFDVLFDRLASPEVLNFIRGKKPDKLLQKVKTQLIVVRVVLADAEKRQIADSNVKDWLDLLRDVVY
EVDDLLDEISTKAAIQKEVSNSFSHLFKSKKIVSITKLEDIVERLDDILKQKESLDLKEIPVESYHPWKVQPTSLEDGYAIYGR
DKDKEAIMKMVLEDSTNGETVSVIPIVGMGGVGKTTLARSVFNDGKLKQQIFDLKAWVCVSDIFDIVKVTRTMIEEITR
KPCKLSDLNALQLELMDKLKCKRLLIVLDDVWIEDCDNWSSLTKPFLSGNRGSKVLITTRNINVVAAVPFHTVEVYHLNK
LSNEDCWSVFANHAFPLSESSETRGTLEKIGKEIVKKCNGLPLAARSLGGMLRRKQTIRDWNNVLESDIWELPEIKFART
FLLIDYKDSPFNNEKAPLIIVSRLKYLRVLSLCKFQSQLALPDSVAELIHLRYLNLSQTSIETLPESLCNLCNLQTLKLSYCSELT
KLPIAMQNLVNLRYLEILCTRIEEMPKRMGKLNQLRNLNYYVVGKHIENSIKELGGLPNLRGSFSIEALENVTKGEEALEA
RIMDKNHISDLSLEWSKANDNNIDFQVELDVLSNLEPHQDLKSLSIKGYKGNIFPEWVGNFSYRYMTSFYKKDDCSSVT
PFPSLKSLSISNMPCWEVWNSFDSEAFPVLKDLYISHCPNLKGNLPNHLPALQTLIIRNCELLDSSVPGPLTLRTLELRKCN
KVAFHEFPLLVESIEVEGDPMVESMMEAISNIQPTCLQSLKLENCSSAISFRGGRLPASLKILDITGINKLKLPLQYKHELLE
SLSIYNSCDSLTSLPLAILPNLTHLDIGYCENMESLLVSGSDSLSLSYFKISHCPNFVSFPGEGLCMPNLTSFSVYYCDKLKW
LPDQMGTLVPKMKYLDISNCQQIESFPKGGMPPNLTVRIRNCEKLLRGLGWKSMDMVTSLIVWGPCDDIKSFPKERLL
PPSLVSLYLFNLSSLEMLDCKGLLHLTSLQELNIERCQKLEKIAGEKLPLSLIKLTIYECPLLQKRCNRKDRQIWPKICHVRGIK
IDSRWI

>g28941.t1

MRVHHSIIKMPISGRGQLCHAGTSLRTTLGKTNAQGQNTMSRILSTMEKGCEMFVKKCMSCQSHENDLRTPPEMLH
HLVSPWPFAQCGMDIAGPLPTRRSQCKFLLVFIERKLEDFLQGLGIKHVTSSGEHLQAEATNKAIVSELKKRLGEAKGLW
VEELPEVPWAYRCTHCSIGKTPFNLTYGTNAMLPVEVDEPFLRRNIQNMNLNEEQLHHGISCGGGALLSAFFDVLFDRL
ASPEVLNFIRGKKPDKLLQKVKTQLIVVRVVLADAEKRQITDSNVKEWLDLLRDVVYEVDDLLDEISTKAAIQKEEIPVESY
QPWKAQPTSLEDGYAMYGREKDKEAIMKMVLEDSTNGETVFVIPIVGMGGVGKTTLTRSVFNDGKLKQQIFDLKAW
GKRLLIVLDDVWIEDCENWSSLTKPFLSGNKASKVVITTRNENVATAVPFHTVEVYNLNKLSNEDCWLVFANHAFPPSE
ASETKETLEKIGKEIVKKCNGLSLAARSLGGMLRRKQTIRDWNNVLQSDIWELPENELIQLWMAEDLVKATKKGKTLKE
VGQEYFDDLVSRCFIQSSSGSGGDYFVMHDLIHDLATFLGGDFYLRIDELEKETIIDRKTRHLSLTRFSDPASDIEAFDTVKF
PRTFLLIDYKDSPFNNEKAPRIIVSRLKYMRVLSLCNFESQLALPDSVGFYKKDDCSSVTPFPSLVSLSISNMPCWEVWNSI
DSEAFPVLKDLCIEECPNLKGDLPNHLPALQTLSIRNCELLDCSVPGPLTLRTLEIRKCNTVAFHKFPHLVEPISFRGGRLPA
SLKTLDICGSEMSKGLNSFAIGHCPNFVSFLGEGLCMPNLTRFSVYNCDKLKSLPDQMGTLVPKMEYLDISNCQQIESFP
GGGVNPISELLLCTHSNALIVQFLGHVISAGGISVDPAKVQAVLQWERPKTIDSLTRKDHLFAWTDRCEESFQELKQRLT
SAPVLVIPDTGKPFEVFCDASHQGLGCVLMQEKKVVAYASRQLKIHEKNYPTHDLELAAVVFALKIWRHYLYGAQFQVF
SDHKSLKYLFDQKELNMRQQRWMKFLKDYDFDLLYHPGKANVVADALSRKTIHISTLMVREMELVESFSDLKLEVEIKP
DCIRCCKLVISSDVFERIMEEQLSDPELMNSRALIGTEQGKWFNTGTDGMLRFKVDVARFVSSCLTCQKVKIEHQHPGG
MLQQLDVPEWKWDSIAMDFVTHLPRTARSKSNIFSKMTTIREMSANFHKLDKFEGVGFRRWQKKMHFLLSALNVAY
VLSSPQPIESENETLEEQRRRNKWENDDYVCRGHILNDMSDPLFDIYQYVNSAKELWDQLESKYISEDASSKKFLRDCTI
WKRKMAKNGGKQGQGESSQGQPKKGASRHVCKDRCLFKTFKEDDNGEVLYMGNDSMAKVLEEDSLTYQEAIRSQD
ATFLKEAINDEMESILGNNTWILVDLPPKHQVNLTKTFLSSYFSMKDMGEADVILEDHSSTSSWIFLLGGGAISWSSKKQ

TCMTDSTMASEFIALASAGKEAKWLRNLMFEIPLLPKPISPIAIHCDNAATLAKAYSQVYNGKSRHIGVRHSYVQGLIKD
GVITVDFVRTKLNLADGFTKALATDSISRMTIGIGMEPTINHNQLQMSSAYHPQTDGQSERTIQTLEDLLRTCVLDHRG
AWDEILPLVEFTYNNSFQARIGMAPFEALYGRRCRTPLCWYQEGEAVLTGPEIVQQTTEKVKLIKERMKASQSRQKSYA
DKRRRPLEFEAGNHVFIRVTTTTGVGRALRAKKLSPRYIGPYQITRRIGPVAYEIAMPPQLANLHPVFHVSQLRKYVFDPS
HMLEVDDVQVKEDLTLHVQPIWIEECRNKQKWGKTIRLVKVIWDDRIGDFTWELEEEMKERSLKNPGATGFDSTEQL
GVSSGEHFFPFAFIREP

>g29397.t1

MQSSPSSFPYRFRYDVFLSFRGKDTRQGFTGNLYKSLCDRGIHTFIDDHHLPRGDQISSELENAIQDSRIFIIVLSQNYASS
SFCLNELHYILRFIKHKGRLIFPVFYGVDPSHVRHHTGTFREALAHHQNKLNSYSNWDKLETWKMALHQVANLSGYHFK
HGEGYEYEFIERIVELVCSKINRAALHVADYPVGIESQVLELKLLLDVGCDDVVHMVGFHGLGGVGKTTLAAAVYNSIAD
HFEALCFLENVRETSSKHGLLHLQSNLLSETVGEIKLTSVKKGISVIQHRLQQKKVLLILDDVDKEEQLQALAGRPHWFGL
GSRVIITTRDKQLLKCHRVRRTYEVKELNEENALELLTWKAFKFEQFDPSYKDVLNLAVTYASGLPLALEVIGSNLFGRNIE
QWKYALDQYKKIPKMDIQDTLKVSYDALEEDEQSVFLDIACFFKNNDLAEVEDILRAHHGHSVKHHIDVLVEKSFIKISLD
GKVTVHNLIEDMGREIVRRESPKEPGKRSRLWFPQDIVQVLEDNKGTGQIEIICVDFPSFEELEIEWDGDAFKKMKNLRT
LIIRNGHFSKGPKHLPNSLRVMEWWRYPSQNFPQDFHPKKLTIFKLPYYAEGCSRLKSFPPIKLTSLEQLKLRYCHSLENFP
EVLGNMEYVRELDLKETPVKKFPSSFRNLTRLQKLHLCLSVRVMKSGRDGLPLSSICMMPELVDIAANEWEGGLFREAD
EGAEKVSSILSTNVQYLQLRCCNLTDDFFPTLLPWFANMKNLDLSGNNFTVIPECIKEFHFLTRLNLNFCERLEEIRGIPPN
LKYFFAIDCQSLTSSCRSMLLNQELHEAGSTFFYLPGAKIAEWFEFQTLELPISFWFRGKLPAMAICLAMERVCEYSSSEG
GKYRPLVIHSTFRLMSPIVIINGNEHLLETWEMMDDCTCVFDLRETNLKNDLDEQLVENEWNHAEVTCRCVSLGQTLIK
HGIHVFKKESSVAEIRFTDPSRKRNLDMVELNSLRSQHQQLLNKQR

>g29402.t1

MQSSPSSFPYRFRYDVFLSFRGKDTRQGFTGNLYKSLCDRGIHTFIDDHHLPRGDQISSELENAIQDSRIFIIVLSQNYASS
SFCLNELHYILRFIKHKGRLIFPVFYGVDPSHVRHHTGTFREALAHHQNKLNSYSNWDKLETWKMALHQVANLSGYHFK
HGEGYEYEFIERIVELVCSKINRAALHVADYPVGLESQVIEVKLLLDVGCDDVVHMVGFHGLGGVGKTTLAAAVYNSIAD
HFEALCFLENVRETSSKHGLLHLQSNLLSETVGEIKLTSVKKGISVIQHRLQQKKVLLILDDVDKEEQLQALAGRPHWFGL
GSRVIITTRDKQLLKCHRVRRTYEVKELNEENALELLTWKAFKFEQFDPSYKDVLNLAVTYASGLPLALEVIGSNLFGRTIE
QWKYALDQYKKIPKMDIQDTLKVSYDALEEDEQSVFLDIACFFKNNDLAEVEDILRAHHGHSVKHYIDVLVEKSFIKISLD
GKVTLHNLIEDMGREIVRRESPKEPGKRSRLWFPQDIVQVLEDNKGTGQIEIICVDFPSFEELEIEWDGDAFKKMKNLRT
LIIRNGHFSKGPKHLPNSLRVMEWWRYPSQNFPQDFHPKKLTIFKLPYYAEGCSRLKSFPPIKLTSLEQLKLRYCHSLENFP
EVLGNMEYVRELDLKETPVKKFPSSFRNLTRLQKLHLCLSVRVMKSGRDGLPLSSICMMPELVDIAANEWEGGLFREAD
EGAEKVSSILSTNVQYLQLRCCNLTDDFFPTLLPWFANMKNLDLSGNNFTVIPECIKEFHFLTRLNLNFCERLEEIRGIPPN
LKYFFAIDCQSLTSSCRSMLLNQELHEAGSTFFYLPGAKIAEWFEFQTLELPISFWFRGKLPAMAICFAMERVCEYSSSEG
GKYRPLVIHSTFRLMSPIVIINGNEHLLETWEMMDDCTCVFDLRETNLKNDLDEQLVENEWNHAEVTCRCVSLGQTLIK
HGIHIFKKESSVAEIRFTDPSRKRNLDMMNSIAQDLNTNGC

>g30137.t1

MAESLLFSFAELLIGKLATAAVQEASLAFGVHSELQQMKATMALIKGVLLDVENSQSNALREWLEQVKRVFYDAEDIVD
DFECEALRKHVVNTYGSFSRKVRRFFSKNNPVVYRLRMAHHIQDINTRLARLAADRNMFGLQIIDHDTRVVHVREMTH
SHVNPSDVTGREHDKNKIVNLLVQDGDCQSLSVISIVGMGGLGKTTLAKLVFNDTKIDECFQLKMWVCVSNDFELRNV
LIKILNSAPNSTPKNFKNFETEKLQNLLRKKLEGKKFLLMLDDVWNEDPARWHELKEIIDVGVKGSKVLVTTRNHAVAVI
MRTKSSNLYLLGCLSEKDSLSLFVKFAFDDGEEKKHPELLEIGKEIMEKCDGLPLAVKTVGSSLFSRYDKKEWESIRDSEIW

NLKQNEEGILPALKLSYDQLPSYLKPCFASFSLYPKDVAIHCSHISSMWEALGFLPPPEENESMMDVANHLLHELWSRSF
LSDYLDLGSDSSFRLHDLVHDLATDIAKGLRSVSLPEGMNNEALLIRLVSRCKNLSIV

>g30268.t1

MEFASSLSSSSSSFLTSEPHFIHDVFINFGGEEIGRRFVSHLYSVLLQAQVKTFISQENLYEGMKLEEHMRAIGHTKITIIVFS
KSYAESIWCLLELEKIIECHETFGQIVLPVFYEIDPLDVLHQKDDFGKALEETAHKTYSGELLKYAFSNNWSLALTTAAGMS
GWDVRNFRHDAELVDVIVNRVKTLLDYRDLFITKYSVGLESRVEEVIKCIENESSKVCMIGIWGMGGSGYVALQENLVS
DVLKSKLEVKSVEIGRTMIDNRFSRKKLLIVLDDVNESAKLENLCGSREXDYVTEILNGCGLHADIGIAVLIERRLINVERNK
LQMHPLLQHMGREIIRQECPEKPGKRSRLWFHDDVEDVLKENSGTEAILSLKLHSGIRDCFIKAHAFKEMQRLRLLQLV
DVLERLKFLNLSHSKYLIGTPDFSTLPSLEQLILKDCPSLRKVHQSIADLSSIRVINLKDCTSLSYLPREVYKLTSLKTLILSGCSK
LRPLEKI

>g30269.t1

MKLEEQIRAIRGTKITIIVFSKSYAESIWCLLELEKIIECHETFGQIVLPVFYEIDPLDVRHQKDDFGKALEETARKTYPAELLT
TAAGMSGWDVRNFRHDAELVDVIVSRVKTLLDYKDLFITKYPVGLESRVEEVIKCIENESSKVCMIGIWGMGGSGKTTI
AKAIYNRIYRQFIDKSFIENIRYEGYVAMQENLLSDVLISKLKVKSFEMGRTMIENGFSRKKLLIVLDDVNGSGQLENLYGS
LKSFGHGTVIIITTSNVSLLNRLEVNHVYRTHLLNLNESLELFSWHAFRKAKPRKEWSLLARNVVVYCGGLPLALQFLGSFL
CDRRIEVWESVLLKLERIPPDELLSVLKVSFEDLSDTEKDIFLDVCCFFIGKEKDYVTEILNGCGLHADIGIRVLIERGLIKVER
NNKLQMNLLLQEMAREIIRQECPEKPGKRSRLWFPDDVEDVLKENTGTETILSLKLDSSIIDYFEAHAFKEMKRLRLLQLD
NIHSKEIPFGKCNCNGFKEKSSSTRLETTTLEKTPGFKAVKIP

>g30271.t1

MGGWKTANGRRVSGGGSAAIMVTSGHEMVRGGWWVVLQNAFRVLARPNRNTILGGWKTVDVRRVSGGGSGGH
RWAAATRRQWVCEMDKRRRFVKWVRDGIPSMEFASSSLSSSSSSSFLTSEPHFIHDVFINFGGEEIGKRFVSHLHSVLL
QAQVKTFISQENLQEGMKVEDQIRAIEGSKITIIVFSKSYTESACCLLELEKIIECHETFDQIVLPVFYEIDPPDDFGKALEES
AHKSYSEKLLKYALHKWSRALTTAGMSGWDVRNFRHDAELVERIVSRVERLLDYKDLFITKYPVGLESRVEEVIKCIENKS
SKVCMIGIWGMRGSGKTTISKAIYNRISREFIDKSFIENIRYEGYVALQENHLSDFLKSNLGVKSVEMGRTMIRNGFFRKK
LLIVLDDVSRFDQLENLYGNRKWFGQGTVIIITTRNVSLWNRLKVNHVYQTHLLNENESLELFSWHAFREAIPRKELNEL
ARKVVVYCGGLPLALQFLGSFLCDRKIEVWESVLLKLERIPPDELLSVLKVSFEDLRYTEKDIFLDVCCFFIGKDRDYVREILN
GCGLHADIGITVLIERGLIKVGRNNKLQMHPLLQEMAREIIRQECPEKPGKRSRLWLKDDVKDVLRGNTGTEAILSLKLD
SSIGDCFEALAFKEMKRLRLLQLDDVKLSGDCGHISKHIVIFNSSGNNHSGNNPRFYSG

>g30421.t1

MAAEMFTGALVSTFVESTIDTLASRFAHIFRARKHHKKQLSHLKTKLLAIDVVAFDAEQKQFTDPRVRDWLLRAKDAVF
DAEDLLDEIDYELSKSQEEADSQSATKKVWNSLKSSFVSFFKNEIESMMEQVIEDLEDLANKSNILGLEKGAGVGVGSGS
GSKLAHTSLPNESVIFGRDDDKEFVLNWLTLDTHKNLSILSIVGMGGMGKTSLAQHVFNDPRLEEANFHTKVWVSVPQ
EFDVLKVSRTILDTITGSTDHSIQLEVIQKRLKEEFMGKKFLLVLDDVWNERPSKWEAVQKPLVFGGQGSRILVTTRSEKV
AVTMRSEKRLLQVLKRDYCWELFAKHAFQNANPQPDSDFIEIGKKIVEKCNGLPLALKTMGSLLYNKSSLSEWESIMKSE
IWDFSENESDILPALRLSYFHLPSHLKKCFAFCALFPKGYWFDKEWLIQLWMAQNFLENPLQKKSPKEVGEEYCNDLLS
WSFFQQSRDEEEFIMHDLLNDLAKYICEDICIRLGVDEPKGIPKTTRHCSFSDSGFDGFGSSIDTQKLHTFTPIHQDFGWD
CKMSIDDLFSRFKLIRVLSLYNCRGLRELNYCLKLEELPSSLHQLDKLQILQLNYCENLKELPSCLYQLDNLRRLDLEGSGVQ
NVAAHLGKLKNLQVTMSSFHVEKSKENNFQQLGELDLHGSLTIDDLQNIENPSYALEVDLNNKPHLVGLQLEWNFIGSS
SVDSEKAEDVIENLRPSKYLKKLSIRNYIGKHFPDWLLHNSLPNLVSLELEGCESCQRLPPLGLLPFLNYLKISGFDETVSIDA
DFHGNNSSSFKSLDTLYFSDMRQWEKWDCQAVTGSFPCLEVFSIENCPKLKGHLPKFVALKTLRVVDCEQLEALISLRVE

ELSVCSDLESISDDYVSLRIFPLDFFPTTLRTLELSGFPNLQMISQNHVQNHLEYLTIKECPKLESLPANMHMLLPSLRYLTI
KECPRLESFSDGGLPLNLKFITLKNCFRLVGSLKRALGDSPSLTTLSIGKVEAECFPDEGLLPLSLTQLIINDCPNLKKLNYKG
LLELSSLEQLRLWECPNLQCLPEEGLPKSISLFEISNCPLLKQRCHKEGGEDWEKISHIPHVFL

>g30422.t1

MAAEMFTGALVSTFVESTIDTLASRFAHIFRARKHHKKQLSHLKTKLLAIDVVAFDAEQKQFTDPRVRDWLLRAKDAVF
DAEDLLDEIDYELSKSQEEADSQSATKKVWNSLKSSFVSFFKNEIESMMEQVIEDLEDLANKSNILGLEKGAGVGVGSGS
GSKLAHTSLPNESVIFGRDDDKEFVLNWLTLDTHKNLSILSIVGMGGMGKTSLAQHVFNDPRLEEANFHTKVWVSVPQ
EFDVLKVSRTILDTITGSTDHSIQLEVIQKRLKEEFMGKKFLLVLDDVWNERPSKWEAVQKPLVFGGQGSRILVTTRSEKV
AVTMRSEKRLLQVLKRDYCWELFAKHAFQNANPQPDSDFIEIGKKIVEKCNGLPLALKTMGSLLYNKSSLSEWESIMKSE
IWDFSENESDILPALRLSYFHLPSHLKKCFAFCALFPKGYWFDKEWLIQLWMAQNFLENPLQKKSPKEVGEEYCNDLLS
WSFFQQSRDEEEFIMHDLLNDLAKYICEDICIRLGVDEPKGIPKTTRHCSFSDSGFDGFGSSIDTQKLHTFTPIHQDFGWD
CKMSIDDLFSRFKLIRVLSLYNCRGLRELNYCLKLEELPSSLHQLDKLQILQLNYCENLKELPSCLYQLDNLRRLDLEGSGVQ
NVAAHLGKLKNLQVTMSSFHVEKSKENNFQQLGELDLHGSLTIDDLQNIENPSYALEVDLNNKPHLVGLQLEWNFIGSS
SVDSEKAEDVIENLRPSKYLKKLSIRNYIGKHFPDWLLHNSLPNLVSLELEGCESCQRLPPLGLLPFLNYLKISGFDETVSIDA
DFHGNNSSSFKSLDTLYFSDMRQWEKWDCQAVTGSFPCLEVFSIENCPKLKGHLPKFVALKTLRVVDCEQLEALISLRVE
ELSVCSDLESISDDYVSLRIFPLDFFPTTLRTLELSGFPNLQMISQNHVQNHLEYLTIKECPKLESLPANMHMLLPSLRYLTI
KECPRLESFSDGGLPLNLKFITLKNCFRLVGSLKRALGDSPSLTTLSIGKVEAECFPDEGLLPLSLTQLIINDCPNLKKLNYKG
LLELSSLEQLRLWECPNLQCLPEEGLPKSISLFEISNCPLLKQRCHKEGGEDWEKISHIPHVFL

>g30829.t1

MTPSSAYEHASSSQNVYRPTMDFQNEQQSIQRQDEVLVDIGFMIIEEAMFFHAFRNALTDDELGLEILHIALPTTLALAA
DPIASLIDTAFIGHIGPVELAAVGVSIAIFNQISKITIIPLVSVTTSLVAEEDAAEQNHQSEKAILLEESNVNVKVDIEDKTGNT
FSINADKKAAVGRGKAYIPSASSGIVIGGVLGVLQTLFLIFTAKPMLNYMGVDSNSPMFKPAQQYLTLRSFGAPAVIISM
AIQGVFRGIRDTKTPLYATVMGDATNILLDPLLMFVVRLGVSGAAIAHIFSQYLIAIMLLWSLMKQVVLLPPTMKDFQFG
KILKNGFLLLVKVGAVTFCVTLSASLAARKGSTTMAAFQICLQIWMATSLLADGLAVAGQAIIASEFAKKDYKKVIESASR
VLQLGLILGLVLSVLLMSLLPFGSKLFTNDRSVLQLISIGIPYVAATQPINALAFVFDGINYGASDFTYSAYSMIMVALVSICS
LYVLFSSFGFTGIWIALSIYMALRIFAGFWRIGTASGPWSFLTENSNLFQILHLEFHVSTGSTQIYWESEGKFELRYLAALEL
QQLIGKLASGAVHEASLALGVHADLQQMKTNQASVLRQHEKQEILKLLLQHDHDGHDKSLSVVSILGFGGLGKTTLAKL
AFNDTTIDECFPLKMWVCVSGDFELRNVLIKILNSALNPTNENFKNIETEQLQNRLRNTLQRQKFLLVLDDVWNEYQAR
WDDLKEILDVGVEGCKILVTTRSHLTATMMCTKSSNSYHLERLSEDDSFFLFVKTAFKEGEEERYPQLLEIGKEIVIKCGGI
PLAVKTLASSLFSVVGKSKWEAMRGDKIWNLPQKEKDILPALEISYNQLPSHLKPCFVCFSLFCEGSEFFSFYVAKLWEAL
GFFPPPKENETMHDVSIQFLRELWSRSFLTGFIELSHGYSFKLHDLVHDLAMYAAKGEFQTIYPRSSKISPNARHLAFSDN
ILLDQAVIPTGMRTIIFPDEATNEAFLNKLVSRCKYLRFLELSNSMYESLPPSIGKLKHLRYLSLFGNKNLKGLPRSVCNLQN
LETLNLNQCTELQELPKGISKLISLRQLHTTTRQLHFPDQEISTLTSLETLTFNSCDNLESLLKGIQLSSLKNLSLHDCGKLKSV
SSHVITNLENLVIENCCELELSMSFGNQIPDLMLKSLAFKSLQQLVTLPRWLQGSVNKLHSLVIANCNNLKELPEWLSSMI
CLKLLVIEYCSSLQSLPDNLKNLENLLINSCPELCKRYQPGVGQDYDKISHIQKVFVGELEE

>g31482.t1

MDTIASVASSVAAPLLRNITYVLMYSTYLTELETEIKRLQSEEKEVRHTVEAAKRGGEEIEDTVRDWFERVRAAVEQGQA
FLEEEERERVGCMDVYSKYTNSQRARTLVEIVREVRKETFDRVSYRCALRCNVGPAAREYVAIQSRTVMLNDVVKMLKD
GGVDIVGVYGVAGVGKTAMVKELAWQVEKDGLFDVVVVVTVTNSLDVGRIRNEIADGLGLKFDELTELGRASRLRQRI
RQEQRILVVLDDLWGKLDLTKIGVPFGEDYKGCRCQLLVTSRNRNVLSSNFGSGKFCRLEVLSEDESWELFEKRAGDAV
KDPSIQSVAKKVAKSCGGLPLLIVTVVEELKNKDLYAWKDALEQITSFELEGCLYSPLRSAIELSYDHLESQELKTFFLLLGSI

GNGCSTRDLLVFGWCLGLHKQVDSLADGRNRLHKLIDNLRAACLLLDEGKRDSVVALEVVRHVANSIATRVKPFFTVQR
NKEFKWPRMDFLGSCHHIFLDWCYIRDLPEVLECPKLKILQINSQGNCLKIPDDFFVHMKELKVLSLGGLDCTPSLPPSLS
LLTDLQALYLCECKLEDIATVGEITNLEILNLEKSELKELPAKIGGLSNLRLLDLTDCPTLGGIPGNVISRLTRLEELYMGNCD
VQVEAKERKSQNNDSSLSELKHLNQVKILNVQIEDTSVFPRDMLSFGRLESYKILIGDGWKWSGVESENYKTSRLLKLNS
GADPTILKDYGIKMLINKAEELYLAELKGVREVLYELNDEGFSQLKHLCILNCAEMESIIGSTEWAYSDHAFPNLESLILHNL
INMERICSDPLPAQAFRKLQVIKVKDCDRMEFVFSHSMVKHLSELVEIEISECKSMTNILSGQRQEDADAGQTNKITLINL
RSLTLQCLPSLVSFSPDSSTEASESGNGFSSQLFSNKVEFPNLETLKLYSINIQMIWINHHHSYFENLTSLTVDGCERLTYIFS
YPVAVKLVKLQHLLLSSCKFVEKIFVLDENLGHVHHFRKSIHTELVPIFPNLETFVISQMDNLKAIWPALLPQNSFCKLKKM
EIESCNNLLNVFPCHVLDKLQSLESLNVWKCMALEVVYEIDGINTEQEGLDIPLRTLSLGNLPKLKHLWNKDPEGNIKFQ
NLFMVQASKCQSLKYVFPLSLAKDLLHLQFLELSDCGVEEIIASDKEGVGVAFGFPLLNQFFAVECPRMETFSQGILRASIL
RKIHLTRKGDEWYWQGDLNTTIRKLFNRVLVEHVRKLCFDIKKAMENTLKSDSRASGGWLVDAGLRVLWLGLKTVVW
GFFAKIR

>g31493.t1

MPVLETLGGALFGAVLQVLFHKLDSHQVLDYFRGRMLDEKLLKNLKRKLVSMNAVVDHAELKQFRNEYVKTWLDDVR
DVLLDTEDLLDEIELEYQSSASKVRTFESKLKEVLDDLEFLLSQKDDLGLKNAGGVGYELGNKVLERKNESSSLVAEHIICGR
DEDKEIILNWLTSSHDNLNQLSILAIVGMGGMGKTTLAQHLYNDPKMKEAGFDDKAWVCVSDEFDVLKVSKAIIGALT
KSKDDSEDIEMVHGKLKEKLTGRKFLLVLDDVWNEDRNLWKTLQTPLRYGAKGSKILLTTRSNKVASIMQSSYVHQLKQ
LQKHYSWQVFAKNALQDDTSMLNCELEEIGMKIVEKCKGLPLALETLGCLLHTKSSVSEWEGVLTSEIWDLSIEDSKIIPA
LLLSYYHLPSHLKRCFAYCALFPKDHKFDKETLILLWMAENFLQCSQQSKCPEEVGELYFNDLLSRSFFQQSIKDNETCFL
MHDLLNDLAKYVSGEICFRLGVDRAERVPKTTRHFSTVINPIQYPKSLCDAKGLRTFISFWGNCEISIQELISNFKFLRVLSL
PCFKVKDVSDTIADLKHLRSLDLSNTGITKLPDSTCSLCNLQVLKLNKCFNLQELPSNLHQLTSLRRLELVGTTLRKAPVHL
GKLKNLRVWMGRFEVGKSSQSNIQLGEIELHGQLSIRSLENIVNPYDALAANLKNKKHLVGLNLEWSLKRNNDDSIIERE
VLENLQPFRKLKHLLIDGYGGTQFPRWLSDNSLLNVESLTLNNCRHCQLLPSLGLLTFLKHLTIHGLDCIVRIDADFYGNSS
FAFASLETLSFNDMKEWEEWQCMTGAFPSLHCLSVMNCPKLKGNLPVQLSRIKKLTIGQCKQLVASIPKAIEIEGVKME
PSSFDMIEPLVFDTPLESLSIYSCPGMNIPTNHWYPLLVELDISQCCDSLTNFPLDIFPKLCDLCLDECHNLQMISQEHCH
NNLKTLSIEKCSQFESFPNEGLFAQKLERFHIEGLEKLKSMPKSMSVLLPSLNYLSIRNCPGVELSEGCLPTNLKEMRLKNC
SKLVASLKGVWGTNTSLKSLYIGEVDVEFFPYEGLLPLSLTNLAIYDCPNLKKLSCNALSRLSSLEKLDLLSCPGLQCLSEEGL
PKSISELRIKNCPLLKQRCKKQEGEDWEKIAHIKYIDFD

>g31494.t1

MPVLETLGGALFGAVLQVLFDKLDSHQVLDFFRGRNLNDKLLKKLKRKLMDVNSVIDDAEQKQFSNSLVKEWLDEVRD
VLYDAEDLLEQIHYEFFKNESEAELHTSASKVRNFESKMIEVLDDLESLLSQKVVQDFKISTGVRSGFGNKVSEKKVESSSL
MAKDAVIYGRDKDKEMIFSWLTSDTNDNKLSILSIVGMGGMGKTTLAQHVYNDSKTEEAKFDEKAWVYVSDAFDALR
VSKAIIGAFTNSRDDSGNLEMVHGKLKKKLSGRKFLLVLDDVWNEDRNQWKALETPLTFGAKESKILVTTRSHKVASIM
QSTCIHQLKQLDEDRSWQIFAKHAFQDENSKLNSELKEIGMKIVEKCQGLPLALETIGCLLQSKSSVSEWEGALTSEIWD
LPIEDSKIVPTLLLSYYHLPSHLKRCFAYCALFPKDHRFDKESLIFLWMAQNFLDCSEQSKSPEEVGENYFNDLVSRSFFKQI
IWYNKTYFVMHDLLNDLAKYVSGEICYRLGVDGEKRVSRKTRHLSYVSGPIQYYTSLCDAKGLRTFITFRWREMSIEELIS
NCKFLRVLSWRWCIKVPDSIGDLIHLRSLDLSGTDIERLPDSTCSLYNLQELKLNNCVNLKELPLTLHELTNLRRLELKGTTL
TKAPLRLGKLKNLHVWIDKFEVGKSREFSIQQLGELDLHGELSIKNLESITNPYETILKNKAHIVRLSLQWNLKRNNADSM
KQREVLENLQPSRHLKQLVIYCYGGTKFPRWLSDNSLTNVVSLTLKNCKYCLFLPSLGLLTFLKHLTIDGLDQIVRIDAEFY
GNSSSAFACLETLRFTDMKEWEEWQCMTGAFPSLQSLFLTNCPKLKELPDNLCHLKKLTVKNCGQFGAPIPRDVENHC
MNMRPSSFDMSRPPQISDIINHSYNSLVELYINGCDSLTTFPLDLFPKLRKLSLLECCNLQMISQGHPHNHLERLSIEKCSE
FESFPNEGLLASQLRTFFIEGLEKLKSMPKCMSALLPSLNDLYIRNCPVVELSEGCLPSNVKTMRLRYCSKLVASLKGAWG

TNPSLKVLNIGNVDLECFPGEGLLPFSLPELFIRDCPNLKKLDYRGLCHLSSLKKLTLDNCPILQCLPEEGLPKSISKLIIEDCPL
LKQRCKKQEGEDWEKIAHIKSIIVDNKEVNI

>g31510.t1

MCILSRLVDIFRQRKYKKQLNNLKMKLLAVDVVAFDAEQKQFTDPRVRDWLLRTKDVVIDAEYLLDEIDYELSKTQVEAE
SQGAARKVSNSLNSSFVCFFDNEIESMMEQVIEDLEDLAAKIDFLGLKKGCGVGVGLGSGSKLTYTSLPNESVIYGRDDD
KNFVFNWLTSNTHNNLSILCIVGMGGLGKTSLAQHVFNDSRIEGNFDIKPWISVPQEFDVFNVSRAIIEGVTTLTDDSRD
LEMVQKRLKEILMGKKFLLVLDDNWNENQSKWEQVHKALHFGIQGSRILVTTRSEKVALTMRSEKHLLQVFKEDYCW
DLFAKHALQSANSHSDPDFIEIGKKIVKKCNGLPLALKTMGSLLHNKSSLCDWESIMKSEIWDFSENESDILPALRLSYLHL
PPHLKKCFAFCGLFPKGHRFDRDLLIQLWMAENFLESPSQKKSPQEIGEEYFNDLLYWSFFKRSDNNEIEESFIMHDLLN
DLAKYICKDNCIRLGVDKPKGISKITRHCSFSNHGLCFDGFGSLIDAQKLHTFTETTWSGSCKISINDLFSKFKCIRVLSLARC
GELTEVPESVGNMKHLRSLNLSYSNIEKLPDSLSLLYKLQILNYCLKLEELPSYLHQLDNLRRLEFVQTRVKNVPAHLGKLK
DLQVLMSSFCVKKSKEFGIQQLGELDLQGSLIIDELQNIENPSDALEANLKNKLHLTWVALTWKLIVGSPVDSTKVENVIE
NLRPSKCLKKLSVRNYVGKQFPNWLLNNSLPNLVYLGLHNCKNCRRFPPLGLLPFLKELMISECDAIVNIDADFHGNNSC
SFKSLEKLELSCMVQCEKWECKAVTGAFPRLQHLSIDKCPKLKGHLPEKHFPLITLKITCCQQLEASTPRTIALEVHHCGKL
QFEWATMKSLRMSDTNTEASLLEHITSDISLQHMDIYSSLTSMSVNSVSLRTFSLDFFPKLRTLSITEFPNLQTISQDHLH
NHLQNVTITKCPKFESFPTNMHTLLPSLTKLHIADCPRLESFPKGCLPSNLKSMSLYNCSRLVSSLKGALANALLEILLIGKV
DAECFPEEGLLPLSLTELMISGCPNLKEIDHKGLYQLSSLQTLRLLNCPNLQRLPAEGLPKSISHLQLWDCPNLKEIDHKGL
YQVSSFQTLKFLNCPNLLSLPAEDLP

>g31511.t1

MRIRDIVAQLKALEVTMSDSFLGMQNLRRPVRSEQYIYSGSKMSSHVEAIGTLGHISIERIKRLVNEGVLSTLDFTDFETY
VDCIKVEIQYGKQIKIVRSDRGGEYYGRYTENGHVRIYNPQEKKLDPRTISEYFIGYAEKSKGYRFYCPSHNTRIVESRNAR
FLENDLVSGSDQFPNIGNERDHYEAQPSSSSDRLVVIHTPQAQMGVRQTIIEASQHAENDLTDQVANKEQVEQPTEQL
LEQQVPQENDEATLRRSTRVKRPSIPSDYIVYLQEVDYNIGAANDPETFALNLTLHMLLEFWEDIRVIQVLTTGKLQRRV
VDSISRPLRLYCDNSAAVFMAKNYKSGCRTDMVSGALVSVFLERTVDILASRFADILHARKYKKQLSDLKMKLLAIDVVA
FDAEQKQFTDPRVRDWLLRTKDVVFDAEDLLDEIDYELYKNQKEGESQSATKKVWNPLNSFFEIEIESRMEQVIEDLEDL
ATKIDFLGLKKGSGVVVESGFGSKLTHTSLPNESVIYGRDDDKDFVFNWLTSYTQSNLSILSIVGMGGLGKTSLAQHVFN
DPRIEGHFDINAWISVPQEFDVFNVSRAIIEGVTTLTDDSRDLEMVQKRLKEKLMGKKFLLVLDDNWNENQSKWEQV
QKVLGFGIQGSRILVTTRSEKVADTMRSEKHLLQVLKEDYCWDLFAKHAFRSANSQPDPDFIQVGKKIVEKCNRLPLALK
TMGSLLHNKSSLCEWESIMKSEIWDFSENESHILPALRLSYLQLPPHLKKCFAFCGLFPKGYRFNKDLLIQLWMAENFLES
PLQKKSPKEIGEEYFNDLLSWSFFQRSGNNEMKKSFIMHDLLNDLAKYICEDICIRLGVDQPKGNLKHLRSLNLSHTNIEK
LPDSLSLLYKLQILKLNCCQKLKELPSYLHQLDNLCRLEFVHTRVKNVPPYLGKRKVLQVLMSSFCVKKSKEFGIQQLGELD
LHGSLTIDELQNIENPSDALEADLRNKSHLMGLELTWKLIVGSPVDSTKVENVIENLRPSKGLKKLSVINYVGKQFPNWLF
NNSLPNLVYLVLLNCKNCKRFPPLGLLPFLMKLVISRCDEIVNIDADFHGNNSCSFKSLETLEFSFMMQWEKWECKAVT
GAFPRLQHLSIHDCPKLKGHLPEKLVPLKTLKITSCQQLEASTTRTIALEVHHCGKLQLEWATMKRLKMGCHSTEASLLE
NITSDISLQHMDIYSSLTSMSVNSVSLRTFSLDFFPKLRTLSITEFPNLQTISQDHLHNHLQNVTITKCPKFESFPTNMHTLL
PSLTKLHIADCPRLESFPKGGLPSNLKSMSLNNCSRLVGSLKGAFGANALLEMLSIEEVDAECFPEEGLLPLSVTSLTIDHCL
NLKEIYYKGLYQLSSLQTLRLLNCPNLQRLPDEGLPKSISSLEIANCPLLKQRCKEEEGEDREKVNV

>g31515.t1

MAAEMVTGVLVSTFLESTIDTLASRLIDTFRRRNHKQKLSNLKMKLLAIDVVAFDAEQKQFTDPRVRHWLLRAKDVVLD
AEDLLDEINYELSKTQLEAKSQSATTKVWNSSQSSFGFFFESEIESMMEQVIDDLEDLATRSDFLGLKKGSGAGVGSGLG
SKLTHTSLPNESVIYGRDDDKDFVFNWLISDTLNKLSILSIVGMGGLGKTSLAQHVFNDPRIEGKFDIKAWVSVPQEFDV

LNVSKAILNTITGSTDDSVQQELIQRRLKEKLMGTKFLLVLDDVWNERPSKWEDVQKPLVFGGQGSRILVTTRSEKVVA
AMRSEKHLLQVLKEDYCWDLFAKHAFENPNPQPDPDFIEMGKKIVQKCNGLPLALKTMGCLLYNKSSLWEWESIMES
EIWDFSENESDILPALKLSYFHLPSHLKKCFAFCALFPRGYRFDKECLIQLWMAQNFLENPLQKKSPKEVGEDYFNDLLS
WSFFQQSSNEEEKHFIMHDLLNDLAKFVCEDICIKLGVDEPKGIPKTTRHCSFSSSKLSFDGLGSSIDTKKLHTFTPRDWD
WNCKMSIDDLFSKFKFIRVLSLSHYRKLTEVPNSVGNLKHLRSLDLSYTNIEKLPDSISLLYKLQILKLNYCHRLKELPSCLHQ
LDNLRCLELVDIGVKNVVAYLGKLKNAEVWMSSFHVEKSKEINIQQLGELNLHGSLAIYDLQNIENPSYTIEVNLKNKPHL
VGLRLKWNFIDSSSVDSEKVEDVIENLQPSKYLKKLSISNYIGKQFPNWLLHNSLPNLVSLVLERCESCQRLPPLGLLPFLK
DLNIARLDGIVSIDADFHGNNSCSFKSLQTLYFSDMRQWEKWDCEAVTGAFPCLQYFSIENCPKLKEHLPKFVALKTLRV
IHCEQLEALIVSALEECGKLQLEWSTMKKLTMDEHGMAASLVAMAASLEKLSICSSMESISDDCVSLRIFPLDFFPTLRTL
ELIGFPNLRMISQDHIHNHLQYLTMKDCPKFESLPANMHILLPSLTILWIEECPGLESMSDGGLPSNLRYITLYNCFRLVGL
LKEALRDSSSLETLRISTPDAECFPDDGLLPLSLTSLTIYGCPNLERLNCKSLLELSSLERLTLSKCEKLQCLPEEGLPKSITHLYI
NECPLLEERCHKGGEDREKIAHIRNVFIWE

>g31994.t1

MDTVLKIIDPAIEFVRDQCIKQVTYIFCYAKNFEELKKKVKRLGEVKERLDRQHDEAKRKGDIVEARVEEWFGEVGEFESS
VENYRKNAGHKKTRGLYYLFPYYRHKLGRQAKKMEIEALRLTDESPNVDEVSHAEKVTSFDLTSSNSGYIEFNSRKSIME
DIMKKLKDPNMKIIGLRGSQGMGKSTLIKKIANKAKDEGLFDRVAEIDVTINLNPLKIQEEIAHVLGLPLTGDSENVRADY
LRRWLKIENVSILIILDNLHERLDLNRLGIPVDDDYDLRKKNELSISKQDTIGDTKVFPNEIGVESNFCIKELDENDALKLFDK
EILQIPMDCDKVKFLRLHTLTLQSLPSITCFNAKVKTSCRQHSTEAKTTNRDHTEISAEEDDHSDNAPPLFGELVEVPNLQ
NLNLSSLNICKIWSDQHLSSFYFQNLISLKRLKSLVISGCHIMENIFEIEGISANKVCVFPKLEEIHLSNMKRLTNIWQTKVS
VDSFCSLISSMKEIFEVTDFEEIDEYGGIDTNLQVILLEDLPKLKELWSKDPHGILNFKKLRTVDVSNCEELRNLFPASMAK
DVSKLERMSILHCERMVEIVSSKDASEANNDPLEFPELTYVRLYELQNIKQFYKGRHPIKCPKLKELSIGKCMKLKTFSQET
SDEKFVFSAQVVFSKLEYLEIEFEEAQNLLSKYQMHNLKELIILRAVKSPDLFYPFLYKMPNLEKLKLTFSYYYESLESLRSTNI
GQHDRLGVVLQLKQLFIRSSNIKDLGFDGDQVLQRLELLSLKDCYGLNTLAPSSVSLSYLTCLKLKNCLRLRNLMASSTAKS
MVQLKTMKVINCYNIEEIVSNEETEEGKVMKIVFRKLISIELVGLMNMASFCSYKDCEFEFPSLEILIIRGCPKMEKFSEKR
AIAPKLKDVFGVEGDQKAKWQWKGDLNATIQNVFNHKVSFAYTEYLWLDNTDDFIIKQVWDDTHWGQQNKFGYLK
RLSVWGCDTLKHIIPSHLLSCFHNLEDLSVWDCSKAEVIFNMDAENRVMTKPSGIFRLKTLYLERLPKLKHVWEKDPEGII
GLQLLKEMRVEYCKRLKSLFPASVAKDLTRLEVLKVKDCEELVEIFGKDEKDEERTTQEFVFRDLNSLTLEKLPALKYSIHCS
KQQERISNLRLREIQELCLGSRLIPISCFGLLDSLIVDGCKFSSDVLLPFTLLSVLAKLETLEVRNCVSVKVIFDVKCTTQDMIT
FPLKKLTLSQLPDLKNVWNEDPNGILNMRHLQQVHVKNCKGLESVFPTSVAKDLVELESLVVKDCERLMTIVAEDNTNI
EFTFPCPCVRSLELRGLPQFKYFYYCSLKSHTFTPLVDTERCLILGKYGMEKISGEEFQRILLNNLKVFTLSFGWDVFQYEIL
EQVSNIEKLVVCYGSFKEMFCGESLNNVNYSGLLLQLKELKLESLGELVSIGLEKSWTESFVKNLETFEVINCSKLENVVTS
KVFLSNLTYLKLESCSSLQYLFTSSTAKTLDHLKTMEIENCDSIKEIVLKEEGKKSDEDEIIFPKLTRLNLDYLRKLQSFYRGRLS
FPSLEELSVTDCNQIETLCSGTITASKLSQVKLGRYSEAIPIEDDLNQTMRKEFRKKTLGESWIDLKSNKRLHKIWKGSMHI
PRFNPHGILSISHLEEVSVDSCKCLKTVFPASVAKDFGTPIDLTVQDCEGLMTIVADDNTDLRGTNVEVTSPCYCVRSLKL
RGLPKLKYFYYYTHLELETEKCMTVGENGMKMILRGELERKVLDSLKTLTLCFDSGVFPCKLLEVVPNIDKVVVCDGSVKK
MFCCESGNNVLQLLKVLRLESLGELVSIGLENSWTDSFVRNIETFEVIRCKSLENLVGCTVSFSNLRYLKVEHCNSLSYLLTS
SAAKRLGQLKRMEIKWCDSIEEIVYKETDEDEIIFPQLTSLNLDSLWMLKRLYRGSLGFPSLEELSVTYCDEMITLCEGSIEA
GNLSQVILDKYSDAIQLETELNSTMRKKYLTKIASWNWKERLEFRDRADLQEIWCLSLQITFRYLKTLIMNKCKISSDDAV
LPFTLLSLLPKLETLEVQNCDSVKTIFDVKCTTQTSTTFPLKNLVLRKLPNLETVCNEVTEANQALSEGTNPNLTFSCLTSFT
VLDLPNFKHNTIYCIHDDAAPTFELIIPNLEHLTVGKNELKMIVDGGFQRNLLHNLKVLTLCFDIECDEFPEYGFLQELPNV
MKLVVCDSSFKVIFSDQRPNNSEIVLQVKELRLESLQELVSIGLENSWTEPFVGNLETFEVISCSNLKNLVPCRVSFSNLICL
KVENCDNLSYLFTSSTAKSLAKLQRMEIEKCESIEEIVSKEEEESDEDEIIFPKLNCLNLAYMKNLVRFYKGSLSFPLLEELSIA
KCDDMVRLCGGTLEASKLCEVELERSNTTLETGLNSTMKKEFLKKGDSDEGIGYMFKIVMVGDSRVGKSQLLNRFMKN

EFMLFKSGCIDEMLERDSDFPFDDFTGEIVFGMCWHWKNGVAWELLHVVMRLGGAVSVVMRLGGAVVISKLYELDL
ESRRGLQEIWNGSLHIPDLCFSMLAKLTVNDCQFLSDTVLPFHLLPLLPQLGILEVRNCNHVTVIFDVNPSTQYTLITLPLK
KLVLSNLPNVENVWNEDPRGILSMQHLEQVFVDKCKCLKTVFLESVAKDIVKLEELVVEDCEALMTIVANESKEREELVE
DEIIFSQLIYLEVKSCNSLPYLFRSSTAKCLGKLKSMKIIECKSVEEIISKEKEESDENVEIKFKQLQDLSLEKLDELKCFYDGNF
TLSFPCLKEVHVIECSFMKTFSAFNKIDNPTKWYYSEYARPRKEIDLNSALCRTSEEEAPDASSTSNT

>g31997.t1

MATTLSAKNRAQFIDGAVAKPTRGNHSHNAWKRYNNVVEKQLMNNNFLHGLECKSVIATISTVVCSFCGQNGHINT
MCFKKHGFPGKTSSSKKSCSNCGKTSHTIDQYQALIDLINPTVDTNAASTAQIGSMISSSQDIDSTTHHKIDSADLYAGLY
VLYGPILNVSPSIHSTSSQKPHTATNNNIWHNRLAHHQVAFRILPYIKNTPGQVIFLKASSNVTLKAYNGSDLAGCPDPR
KTTTAYIVYLGDSPISWTSKKQSIVSLQVTYIFRYEKHFEELNKEVKHLGEVKKGLHRLRDEAKSKGDIVEDRVEEWFGEV
GEFESRVENYIKDEGHKKTRGLYHVFPYYRHKLGRKAKKMENEASLLRDASPKVDEVSYAKKVTSFDLTSSNLGYIEFDSR
KSIVEDVMTKLKNPNMKIIGLHGSQGMGKSTLIKEIANKAKDEGLFDRVAEIDVTINPNPLKIQEDIAHVLGLPLSGESEN
VRADYLRRWLKIENVSILIILDDLHERLDLNRLGIPVDDDYDLRKKNELSISSSEQGSNANLNSTKELDDNDSLMLFENEA
GRDTNKGQNSFLSELTNLHQLKVVDLSIPCVSSFPNQLIFDKLTYYKIEIGDFEMISVGEFWMPNKYEELKVLALQLKNDT
DIRSKEGIKLLLKTVHSLLLGEAEETSEISDCNSYMNEFLASLEMIEVYVFPKLEEIHLSKMNGLIDIWQTKVSADSFSNLISV
TIEECNQLNKIFPSQMEGWFESLINLRVSKCKSVIEIFEINDSHEINASGIDTNLQVILLEGLAKLKQLWSKDPDGILNFKKL
RTIDVRNCDELTNLFPTSVAKDVSKLERMSVFRCKKMVEIVASKDASEDMKDPLKFPELTYVRLYGLSNMKQFYEGRHPI
KCPKLKELSIDKCVKLQTFLQEKSETRNEENFIFLAEEATLKSSYPVFP

>g32061.t1

MSDSNVPEIKYDVFVSFRGKEIRDGFLSHLTEAFDMKKINGFVDDKLEKGEEIWPSLVTAIERSAISLIIFSPDYASSRWCL
DEVVKIFECKEKYERIVIPVFYKVAPTDVRHQSGSYENAFANHRLNYTTKVQMWKDALKKSADLSGIESSKFRNDAEVVK
EIVNLVDKRLNKPQVIRKELVGIHEKIATLESWISDEPKTTQLIGIWGMGGIGQEVDIDTPNYLPENIVRRIGCMKVLVVL
DDVNDSDHIEKLVGTPENFGKGSTVIVTTRDEQVLRINRVSKTYKVKEFSSDEALELFNLNAFSGSYKEKEYDEISKSVVHY
AKGNPLVVIVLAKLLRGINKEEWESVLAKLKTMPPRKVYDVRDLKCLLKDNESDNSVAFELRRLEDKALITISEDDNTVSM
HDSLQEMAWEIVRQESVEDPGSRSRLWDPNDINQALENEKVSEAIRSIHLTSDTAITSTKKQMLRAQFLATELRFVCWD
EFPMKCLPESFNAEKLVILELRISRMRKLWDGVKNLANLKRLILHWAIKLKELPDLSGAIRLEELYVRGCESLRSLHSSIFSLP
KLEILDLNNCMSITILSSKSEKRVSSLWGNSELRCLNMSSCEKLETIAELPPFLKTLDVSFSTSLKTLPKLPLSLQILNVSFCQS
LESLPELPPLLSTLNVSSCYSLQIPSNLPLSLQIVNLCDCRLFQTLVQHIRSVKTKDTKNSRLVHQTLSKLPLPSEVSCSCNSIK
TGAVSLESFTSLIEDCLSSLKFMTEEE

>g32072.t1

MLNFCGSYSFIRVLFSFVSWLFGGLKGAEEKPTTNNNDPQIKHDVFVSFRGKDVHRGFLSHLTEAFQRRNIDAFVADNN
LEKGEEIWPSLVEAIEGSSISLVIFSQNYSSSRWCLEELVTILECREKYGTSVLPVFYYVEPTYVRHQSEVQRWRHALNLAS
RLAGINSSKFSDDAELVKEIVNEVLKRLVKQSINTKGLVGIDEKIAAIESLIREEPEQTRLIGIWGMGGVGKTTLAEEVFNKL
QSEYEGSYFVASETDRSNKHELISLKEKMFSKLLGYDVKIDTPNSLPKGIVRRIGCMKVLIVLDGVNESEQLEKLLGTLDNF
GSGSRIIVTSRDEQVLRANKAHKIYQLKEFNFDKALELFHLNAFKKNDHVQEYKELSKRVVDYAQGIPLVVKVLAGLLHG
KNKEEWESMLGKLKKMPPKEVYEVMKLSYDSLDRKGKQIFLDLACFFLRTHVAVNVDHLKSLLKDDDSDNSVAFELGRL
KDKALITISDDNTVSIHDSLQEMAWEIVRQESVEHPGNRSRLWELNDICEALKNDKVKEAIRSIRIHLPTIKEQKLVPQILEI
NMSKLKFLEVFVCDRYDELTLLKGPQFLATELRFLSWYQYPLKSLPENFSAEKLVILKLRNGKMEKLWDGVKNMVNLKQ
VDLSRSQNLKKLPDLSKATNLEVLLLMSCSSLTSVHPSIFSLPKLVKLDLLCCTSLTTLGSSSSSCNLSFLNLGRCTNLRKFLLI
SENIKKLRLGNTMVKELPSSISNFNQLLHLDISFCSKLRTIPKLPLSLETLKARECQSLKTILFPSTAVEQLKENRKEIRFWKC
MNLDNQTLVGIGLNVQINVMNYANQHISTASHNHVEHCDDDNCDSYSGVYVYPGSRVPKWLEYKTRKDSIIDLSSAP

PSPLIGFIFCFVLGEYHNADVDRFEVVITIDGEDEGNSISVPIYIDYGYEKTESDHVCVMYEERCSTFLNNRAKNQTRGEDI
RDGFLGHLTEAFDVKKINAFVDDKLEKGEELWPSLLQAIQGSSISLIIFSPDYASSPWCLKELVTILQCKEKYGQTVIPVFYH
VKPTDVRHQSSDGYRKAFTEHERKHKNEVQLWRDVFEKSAYLSGIESSKFRNDADLLKAIVDLVLRRLTKSLVNSKGLVG
IDKRIADVESLIHRESEKARLIGLWGMGGIGKTTLAEEVYNKLRSKYEGDVKIDTPNSLPEYIVRRISQMKVLIVLDDVNDS
DHIGDLLGALDNFGSGSCIIVTTRDEQVLKANKTDEIYHLREFTSDEALELFNLNAFNQRVHQREYDELSKRIVHYAKGLP
LILKVLAHRLHGKNEEVWESELDKLKNVPPTKVYDITTDYLKYLLKDGERDNSVVVSLERLKDKALITFSQDNVVCMHDSI
KEMAWEIVRQESPEDPGNRSRLWDPDDIYEAFKNDKVNEAIRSIRINSVLLRWQECVPHIAKMSRLRFVEIYGADFSRC
RQLSAEGQGLQFWGTEIRFLSWVCYPLKSLPDKFSGEKLVILKLELGRMEKLWDGVKNLVNLKQLDLTDSHSLKELPDLS
KAKNIEVLCLGSCSGLTRLPSSFANHTQLLYLDISHCPKILTIPELPLSLETLYAGSCKSLKTVLFHSTAVEQTKENRKQVLFD
GCMNLDEPSLEAIGLNVRINVMKFANQHLSAPKQDDFQNYNDYDKNFYSYQAFYGYPGSSIPEWLEYKSKKYSVIIDLSS
APPSPVYGFILCFVLLGEVDKIRFNIVISDCEGKGVMERFRMKLFRNTWRSIESQKVVVMYDQRCSNFLNNIAKRLTRFKI
MVRRATKDDGSFYSKRIMVGLSGFGVSIIRTSTYSSYMQQMELCDSMYHLR

>g32075.t1

MKVLIVLDDVNDSDHIEDLLGILDNFRSGSSIIVTTRDEQVLKANKADEIYHLREFTSNEALELFNLNAFNQSVHQRQYNK
LSKRIVQYAKGLPLILKVLAHRLYGKNEEIWESELDKLKKMPPTKVYDVIKLSYDDLDRKEKQIFLDLACFFCISFQKITIGYL
KYLLKDGERDNSVVVSLERLKDKALITFSQDNVVYIHDSIKEMAWEIVRQESPEDPGNRSRLWAMDDIYEAFKNDKVSE
ATRSIQINLVLLPWPEFVSHIAKMSRLRFVEINGADFSHCRQLSAEGQGLQFWGTEIRFLSWVCYPLKSLPDKFSGEKLVI
LKLKVGRMEKLWDGVKNLVNLKQLDLTDSYSLKELPDLSKATNIEVLRLVCCVRLTRLPSSFASNTQLLHLNVTGCEEIRTI
PELPLSLQTLHARNCVSLKTVLFNSTAVEQTKENRKQVLFAGCKNLDEPSLEAIGLNARINVMKFANQHLSAPKQDDFQ
NYNDYEKNYDSYQAFYEYPGSSVPEWLEYKSKKNSVIIDLSSAPPSPVYGFILCFVPFEDVYDIEFNIVISDCEGKGITDRVR
MKLNHGFAFVRLPKVVVMYDQRCSDFLNNIATSLTRFEIMAWQRRGIGIPCSLSSSIFQLRGFGVSIIRTWTYSSFMQQI
KEAR

>g32331.t1

MAMELVAGPLMGAIFNVLLDRIASTEVVNFFKNKNCEKLLKRLKIILLSVNVVLNDAEEKQMKNGAVKEWLEELKDVAF
AAEDLLDEIYTDAKIKAKQVNTLHSGHMSFYGKGVEEKIEDVHERLEFIMRQKEVLDLKVGKEVKMAHKTPTSSVMEAC
DVFGRDNDKEFLVDLVLTHDEKIGVIPIVGMGGIGKTTLAQLIYNDQRVQKEFDLKAWIYVSEEFDICKITKTLLEAVTSCS
CDVEDLNFLQRDLKMHVMKKKFLFVLDDVWNENYDNWDKFRSPFKHAGEHGSKIIVTTRSGCVASIMQTVSPYNLRE
LSNEDSWNLFSKHAFDYGDSRLQLHQSLDKVGREIVRKCKGLPLAVKTLAGLLRCKSDRQEWCKVLDSEMWDLQDSES
NILPALRLSYHYLPSHLKRCFAYCAIFPKDYEFEKENLVLLWMAEGFLQQSKRHRRIEEVGNEYFCELVSRSFFQQSRRGK
SCFLMHHLVNDLAQFVSGTFSIRMECSNSNEIKERTRHLSHIIADSSSYVNLKDVSKANCLRTFLQIRPVGTSIDLFNNMP
NDLLTKLRCLRVLSLVGTHIYSLPNSVGELKHLRYLEVADTEIVRLPESICNLFSLQTLKLVGCHNLIELPASIHKLVNLRHLDI
RGTSLRWMPLQINELNSLQNLSDFFVGKGCGSSLGELGELSCLHGELFIHFLEHIVSDKDCEKAKLKEKHGLEKLSLDWC
GNGETENSQKEKTILNSLQPHTNLKKLDIYDYPGTEFPEWLGDQSFYNLVSLMLNGCKYCYKLPPLGQVPMLKELQISRF
EGLVSVGSEFLGNRTSYVSDYFPALEILRIESMPLWEKWYPNAENAGSKAFFHLREFHIGNCPKLRGDLPDNLPSLTLLVI
RDCKRLLCSLPNSPSLRVLNIQNCQSLEFKVHSPLGHQSLTSLFLHGSCDSLVFLPLDLFPNIKSLDIWGCKNLEALTISESD
AARPNLKSLHSLRVRHCPNFTSFPKGGFAASKLTLLTINYCQKLNSLPEQMHDLMPSLKEVQLRGCPKIESSSMRPLRIRI
CSKHVEGKQNLSDPLFARLKGLATDQSPSSS

>g32796.t1

MESEREDNNNGTDSTHAAISSTLKIKIVSCSKSGESLDNSNLPSPNINNGIESSPYGSPLVSPPSSAFVSALQSPYISPRAIIP
DPPNGSPLENQPPLLTITTSTTNPSTPEDVPSSSYTPPSDQYEFSDDTADTRLKYVTCVPEPAPPRISFSFPVPRISFAKGPI
SPATNAKLRSCDVYIGFHGQNPNLVRFCRWLKSELELQGIDCMLADRAKYSDSQSHEIADGVICSVAFGVVVVTSSSFLN

HFSMEEVRFFAQKKNLIPLLFDTGPAEIMALLNCKSIDKECKEAIDGLMKCNEFNIEANDGNWRSCIAKAAGILRARLGR
KNAEQKDNVQGHENLPFPRNTYFVGREKEIMEIEGLFFGRGNCMEQVQDHCRVFTKGEASGSGQSEGLADEESEPVI
ARCGRYISLEMGRSKEPTLEAWVEPIMGNNSLKRLKNKKSKSGSYKSVCSSVICINGVSGIGKSELALEFAHRYHQRYKM
VLWVGGEARYLRQNLLNLSLNLGLDVGADSEMERGRIRSFEDQEFEAFKRVKRELFGETPYLLIIDNLETEVEWWEGKD
LYDLIPRNTGGTHVIVTTRLSKVMSYDTIQLPPLPLSDAMILMIGRKRKEYSADEIDLLEKINEKLGRLSIGLWMIGSLLSEL
SIGLSCLYEAINQVPLDEDSNSCYMSIAEGQWCKSNPFLMKTLLFCLETLEKTKAKGNLLAIRMLLVSGWFSPSPISASLLA
NAAKSIPRVESRLKKWTKSLSLTTSCLSPRTWKNEEESAMILVKMGLARRANQHDGCWLHFHPITQAFAKRKGGLQYA
KAAIQGVRKMGSQVNSDHLWASAFLVFGFKSEPPLVQLKAIDMVLYIKRTALPLAIQAFTIFSRCNSSLELLRVCTNALEE
VEKSFVSQIQDWSSHGSVCWKRRLQRGQKVDEYVWQDVTLLKATLLETRAKLLARGGHLDSAKELCRTCISIRTVMLG
HNHAQTLAAQDTLARLVRMRSKI

>g33694.t1

MENVKLVSPLLSPLLSLAWPYLKPFLYKLLPYEIRDGHLAKDINKLQKLRKRVQHKVRDEENRIERPIVGDVIKWLDEVDD
VISDYEDFLEDEDRPYALYSDGYLPKPSIRYEMFESRNQIMGDIMKALADSSIGMIGVYGWSGVGKTSLIKEVVKKVKGN
MFDVVIMVNITSRSDIRIIQGQIAKKLGMKLEGESESERAAHLRERLKDPKEKTLIVLDNMEVKLDFNMLGIPTENNDAS
QTNHKMKEISARRNYALKNEEFHGSILRKVEDPFTRYKGCKILMISKSEQLLLRQMGGKAVQTFCVQALTEKEAETMFK
AMSEIDNENSLFKTLAAQISKKCKGLPMTIVATANALKNKSLLVWEDAYRNLERQNLTAVQEFSTKLSYNLLENDELKHT
LLVCAWMSHDASLTDLVRNCIGLGLFQGIYTVREGKDRIHALVAELKELSLLSDSFSSDHFTMQDIIRDAALSIESQQMH
AFVLTKGKLEEWPDQDKLERYTVISLQHCDVTDIMKKFPEKIDCFRLRVFRLDNKDPHLEIPDNFFIGMKELRVLILIGVHL
SCLPSSIECLKKLRMLCLEQCKLGENLSIIGHLETLRVLSFSGSVIDKLPAKLSQLAKLQIFDISNCFKLREIPLDVLSKLDDLEE
LYVGNSPIQWIDGGQGNSILSELRKLDHLTTLDIQIPEITHMPKNMFFDKLHSYNIVIRDSDAYSLLDINMLEMCETSRFL
ALQLENGFDIHHRKDIKILFERVENLLLGQLNDVEDIFYELNYEGFPYLKCLSIVSNSKIKSIINSRNQKHLEKVFPRLESMFL
YEVHNMEHICYSQLTSDSFCKLKIIKLNMCGQLKYVFFSSTIKHLSALEAIEVSECNTLKEIVTSEAENREQIIFPELRSLTLQS
LSELIGFYTYGVSLGEQVSNELFDKKLVPPISHGEDKSGVVPKLKSLKVRVLDFKGMSKSEHHSTFLDAISSPILQQILLQNG
KEERCHGKGDLNVATSNMCQALDPTNVVASNPYKPLENSRLKILKLANCELGSHAIPTDVFPSLKNLEELEGFPEQLLEKI
PNGEYLQISQYSGFKKLSPSLPEQGDSSHPEQDLKLLELIDVWELKSIGSGDVPWLDTICKKLPRLYVANCPHFMPLMHS
SSETINHIVFGYSLMLQEIWKSETLPEGYFHNLTSMVVEGCEFLSDAILPSHLLHLLSNLKRLQVRRCNSLKAVFIHTKITN
MQPQGSLAHLEELHVENCVELGTIVAKVEAEIDEANKQIAIFSTITLLRLSNLPNLRCIYPEMHMLKWDMLKELHVENCQ
KLKFFATEYQNPTDLNQDDQDRFSTDQQAIGT

>g33702.t1

MAALIACFQFLTSSPRAKAWLKKQLIQLDLYETRVGEVKDVVEKLKNKRDAIQHTVEEEERRHGRRIHDEVKEWMESV
DKLIRAYEDFHNDEICHKCAVFDFFDSGYLPKPGIRYRRSRKANDITKQANWLLQNAKFDILSYWSGPPSMATFFSNLGY
ESYSSRNETVKKITAEFQKPDVRMIGLHGLSGVGKTSLVKEVVKKALKDKMFEVVTMASVTKNPDVRKIQGQIADMLG
VVLDEESDIARAARIHQILNNENNSTLIILDDLWEQVNFNLLGIPCEIGKEDGVTNVQGKSLDVDSLKNVSDGKSLDFDIL
KNVVGDGKSPILGSSTSFRKGMLQGADGSKNVNKGKSLGTADSVNVKKGGFLGGSLKNVNEGKSPLDASERVKAENS
VPQYKGCKILMISEIKQVLLSQMEGKEECIFPVEILKEEEAEKLFKKKAGISGKNSEYDKLAAQIASKCKGLPMTIVTTARAL
KNKSLSVWDETNRKFESQKLTGAPEFSTKLSYELLEDEDLKYTFLLCARMGHDALIMDLVKYCIGIGFLRGINTARQTRDR
VYTLIAKLKESGLLSDSYSSDHFTMPDTVRRAALSIAHKGNQLFTMTKGKLDEWPDKLESYKIVIGDLSSFLETGFQMPER
YETLKFLAVQLENGSDIHSLMGIKMLFEGVENLFLELNTVHEKHNSVREARNIVHDLFYRLNLKGFPYLRHLWIVNNSTIQ
SLIHPKDRQHPEKAFPKLESLHLYNLKMDEICSCKLSEPSFGKLKVIKINLCGELNSVFSISMVGLLKVLETIEVSECSSLKEII
NVNLENAELVMLPELRYLKLQSLSEFIGFDVIPHIEEGERKLFHEKVGVSKLERLELSSIKIDSLVNLQSLYVSECGKMTSIFL
SEQDTEKDIMGSFFPELKNMKLHNMNSLSKIWNPKLPSDYFKKLETLIIEECHRLENALDGIFGSLCNLRVRNCRSMQAI
FNIREQVGDVANNLQDVHLETLPKLELVWKMNNKDLVGIPKFNNLKRIWTQGCESLECIFPFYVAKNLDNLESLVVCDC

DGLSKIVAEKEVTNTDTAKFNFPKLSTIKFSHLPRLTSFYPTTYDLSCPLNELSIEFCDNLEPFNNGTEHAQTNPVHAFFPEE
VINNLKSMQIEFWHAKSPNSYMGKGNHRRDNLEELSLSRLMNTEILYSFLYRNPNLKSLSLNTCFFEKIVPLKEDTEIENL
GVVPNLKSLMLIDLLNLKELSFEPDVILERLEFLILKNCRLMITIAPSSVSFTRLTNLEVVNCDRLQSLMSAYTAKSLAQLNT
MKVVKCESLVEIVRKDGEKSDRIVFPQLKALELVSLKNLKSFCVSDCDFEFPSLEKLVKFFEGMEEISLSEHQELQETWQQ
GAGLQKKNSWFYSLKILKLEKCVIQPFAIPSNILPYLRSLKELQVQGCNNVEVIFEMNAEEGTGSTFHLQKLTLQELPKLKD
VWERNGKGTESFQNLKLVNVRNCENLQTVFPLTLAKSLKKLDELQIIDCEGLLEIVRKEEDTAAVFVFPCLTTLTLADLPEL
IYFYPESFTLECSALHKMIVWNCPALELFGSTNTHSIFFDLKHICNLEVLVLGWEHTLGLRTMLGEPMDNLEYLNDIQLVF
VVDENGRHDLPIQILKKMPNLTKMSIRHCSSLEIFQTQIPEIVEKRVLTHLKTLKLYDVSKLQSIGSENSPWLNVICDSERL
QQLYVLDCPDLKTLVHSTPSVSFRYVKEIYIDNCQNMKYLFTLSAVNKLENLEYILVKNCESMEAIVLKDKDDISEEIKLQQ
LKCIDLYILSNLECFYSDNDTLQLPSLMQVDIWMCPKMKFFSRGEIQLNSSFRGIQALNFSSDELVFYHNLNSSVEKVFLQ
EEFFNAVDTDCYDLNIKCYPDSEIGVENNLFANLETMKLKNCTQSYAIPSFILALLKNLKELEVRDSDQVEAIFDINDDTEIK
ETEFHLKILTLNGLSKLKRVWEKDTHKIIIFRNLQEVVVSDCAKLQTLFPTSLAKCLKDLKRLKIDNCENFQDFVEQEETTFV
TEKFVFPCLEDLNLNDLPHVTCPKMFTLEFPSVKFLSVRDCDGLGLFQSVYDPMGEGTSSRTLPLISDPNVISNLEKLTLD
WKQILALSLWFKSQQSTKGLTNLNSISLCFFRAKENEMPMLPVEILKAPILIEMDISNCQSLENFLAQNPKIGEEEMLGQL
TILKICNVSITQFFELEYSSCLNMFERLHKLSVSHCPHLTTLGVHSTSTMSFSCLKEVHIYKCPNLKYLFTSSAAKMLMNLQ
EISVTECELLTEIVVKEGDATSEAIEFERLHTIYLQSLTSLICFYSGRDTLQLPSLKTVTIWSCPNMEIFSQGIESLMGITLSIDL
EPDNLPPPQDLNTRIKGISQRKEFIEAVDKVCFSDYLDLQEDPHCNFRLQNQWLGDLVSLKLQNCTLPYAIPSAILALLKSL
KELEVRDSATVDVLFYINDAETTQISSQLKMLTIEGLSKMTRVWEKKKNGVVIFPNLQQVESLTLALLDKLESFYTGSSTL
NFPSLRSVWVDKCFTTKIFRRHDKVPPKFKVDIDGIRCKGDKKALIMQQVEEEAS

>g34592.t1

MSLAVPDKLLQKVENQLIVLRVVLADAENRQITDSNVKKWLDVLRDIVYEVDDLLDEVSTKAACRKEDSLNLKEIPVESN
QPRKDQPTSLEDRYGMYGRDKDKETIMKLVSEDNTVGQEVSVIPIIGMGGVGKTTLARSVYNDRKLKQIFDLMTWVC
VSDIFDTVKVTKTMIEEITKMPCNLNDLNLLQLELMDKLKGKRFLIVLDDVWIEDCDSWNSLTKPFLSGIRETKVLVTNR
NESVAAVIPFDVVKVYHLNQLSNEDCWLVFANHAFPLSENSENRGTLEKIGKEIVKKCDGLPLAAQSLGGMLRRKQAIR
DWNNVLESDIWELPESQCKIIPALRISYNHLPSHLKRCFVYCSLYPKDYEFEKDELILLWMAEDLLKTSRKEKSLEEVGEEY
FDDLVSRSFFQYSTSGTRGIGFVMHDLMHDLATFLGGEFYFRVDELGKETKIDRKTRHLSFARFSDPVSDIDVFETAKFPR
TFLQIKYEKSPFNNEKAPLIIVSMLKNLRVLKFSDYQSELVLPDSIGELIHLRYLNLSHSSIAILPESFYVEGGPMVESMMEAI
SNIQPTCLQSLRLQNCSSAISFRGGRLPLSLKTLAITGSELSKRMNSFTIGHCPNFVSFPGEELCMPNLTSFSVYNCNKLKW
LPDQMETLIPKMEYLGISNCQQIESFPGGGMPPNLRTVEIKNCEKLLRGLGSKSMDMVTSLSVWGPCDGIKSFPKESLL
PPSLVYLDLRDLSSLETLDCKGLLHLTSLQQLKIARCQKLENIAGEKLPLSLIKLTIYGCPLLEQQCHKKDRQIWSKICHVREH

>g34594.t1

MALAVVGGALLSAFLDVLFDRLASPELVSLIRGKKPDKLLQKVENQLIVLRVVLADAENRQITDFNVKKWLDVLRDIVYE
VDDLLDEVSTKATTRKEVSNSFSHIFNMKRIVSINKLEDIAERLDDILKQKESLNLKEIPVESNQPRKNQPTSLEDRYGMYG
RDKDKDTIMKLVSEDNTVGQEVSVIPIIGMGGVGKTTLARSVYNDGNLKQIFDLMTWVCVSDIFDTVKVTKTMIEEITK
MPCNLNDLNLLQLELMDKLKGKRFLIVLDDVWIEDCDSWNSLTKPFLSGIRGSKILVTTRNESVAAVVPFDVVKLYHLN
QLSNEDCWLVFANHAFPLSEDSENRGTLEKIGKEIVKKCNGLPLAAQSLGGMLRRKHAIRDWNNVLESDIWELPESQC
KIIPALRISYSHLPPHLKRCFVYCSLYPKDYEFQKNELILLWMAEDLLKAPRKEKSLEEVGEEYFDDLVSRSFFQCSSRTRGL
YFVMHDLMHDLATFLGGEFYFRANELGKETKIDTKTRHLSFARFSDPVSHIDVFETVKFPRTFLQIKNEDSPFNNQKAPRI
IVSMLKYLRVLKFSDYQSEFVLPDSIGELIHLRYLNLSHSSIAMLPESLCNLYNLQTLKLISCFNLTKLPRDMQNLVNLRHLQ
IFWTPIKEMPKRMGKLNQLRYLDRYVVGKHKENSIKELGGLPNLHGWFCIEKLENVTKGEEALEARIMDKKHITSLWLE
WSERNNDIIDFQIELDALGKLQPHQDLKSLKISCYRGTRFPEWLPSLRNLYISNMNLLKTIDAGFYKKDDCSSVSPFPSLES
LDISNMPCWEVWNTFDSEAFPVLKNLFIQDCPKLKGDLPNHLPALQKLWIRNCELLVSSIHGPPTLLELNIQNINKIAFHE

FPLLVESIDVEGGPVVESMMEAISNIQPTCLQSLRLQNCSSAISFRGGRLPPSLKTLAITGSELSKRMHSFTIGHCPNFVSF
PGEELCMPNLTSFSVYNCNKLKWLPDQMETLIPKMEYLGISNCQQIESFPGGGMPPNLRTVEIKNCEKLLRGLGSKSMD
MVTSLSVWGPCDGIKSFTKESLLPPSLVSLKLDKQFRLLHFTQNSFSLPSPPSRPICKVLSLGQPASAAPAIPLYRACHCAS
TSLPPLFSRRASSTVAAAGPTWSSLPFSPS

>g34606.t1

MALAVVGGALLSAFIDVLFDRLASPELVNFIRGKKPDKLLQKMKSQLLVVKVVLADAEKRQITDTNVKEWLDLLNDVVY
RADDLLDEVYTKAATQKEVSNSFSHLFKRNKVVKFSKLEDIVERLDDILKQKESLDLKEIAVENSQPWNAPTTSLEDGYG
MYGRDEDKEAIMKLVLEDSSGDEKVSVIPIVGMGGVGKTTLTRSIYNDGKLNEIFKWKAWICVSDRFDILKVTKTMLEEI
TKKPCELSDLNLIQHDLFEKLKGTKFLIVLDDVWIEDCDSWSTLRKPFLSGISGSKVIVTTRNEKVAAVVPFHNVKVYHLN
KLSNEDCWLVFASHALPLSEDSENRETLEKVGKEIVKKCNGLPLAAQSLGGMLRRKHKIEDWNDVLESDIWELPESELIH
LRYLDLSFTGIETLPESLCNLYNLQTLKLYGCFRLTKLPSAMQNLENLRHLEIRDSSIKEMPKRMGKLNQLQNLDFYIVGKH
KENSIKELGGFPNLHGRFSIEKLENVTNGEEALEARIMDKKYINSLELQWSLXRPMVESMMEAIIDIQPTCLKDLSLSDCS
SAISFPGSESLKSLDTFVIGDCPNFVSFPGEGFSAPNLTYFSVKDCAKLKSLPHQMGTLLPKMEHLSISNCQQIECFPEGG
MPPNLTTVKIRNCEKLLISLGRKPIDMVTSLYVYGPCDGINAFPMEGFLPPSLTSLYLSDFSSLDTLDCEGLLHLTSLQTLNI
ENCKKLENIEGERLPVSLVQLIIYECPLLQKRCHVKVRQIWPKICHVRGIKVDGRWI

>g35045.t1

MADALLGIVFQDLQSVVQSQLATYWGIDQQAQKLSSNLTIIRAVLRDAERRKIASHAVKDWLQKLIDAAYVLDDILDEC
SIHSREVLSEDEHTSCLARAHPKDILFRFHIGKRMKNITQRFHDINEERRMFQLRDGDTEKPIVDDGEDDQTTSVITEPIF
CGRDEDREKVVKVLLEANNNEDLTIYPIVGMGGLGKTALAKHVFHDHRVCKHFDLTIWVYVSVDFNVKEILQSIIEYATG
QNPNLHTLETMRKKVEEVLQSKRYLLVLDDVWNEDQEKWKHLEGMLRFARGAKGATVLVTTRLEEVASTMETHPAY
HLKELSEHDSWSLFKSYASGPNKEEKEELVAIGEDIVKKCVGSPLAIKTLGSLLRDQTTVTQWENIKCTRDHRPVAGIRDH
RTDSALEDQECKQARGPSGHRHPTVVPSGHLEYGSTRGSQIADRPIGRNCVGLELGPIPEHFGAHRGAECLDPMVTTR
NTTVEDPIEAIRALRQQMEDMRWQHEQELSAVREECAARITREREAREKDRTWSLEDTELEGSWARTEKVTSTVNTTP
AGGAPTVKEEETVEGLPFTRAIMDVHISEHFVPPQLSIYDGTTDPDDHIQAFSTRMAFRTGNRAIWCRAFSLSLEGEALE
WFNSLPSGSIQSFEGLKEMFGRQFTGSRAEDPTVFELSNLRQGKYETLKAFMDRYQKMVRRVKGLNVELALQYVMPA
LRPGPFKDSVCRKRPKTMEELRERAADEMRPREAPRGAKFQQYTALNAPPERILQEALSVNLIPPLKKRPTPAGADGNK
HCLYHQNMGHTTEECVTLRDKIEELIHAGHLKQYVKTPPAEPPRRREPSPAPRRDQGRINTISGGFAGGGPSTSARKRH
VRALRSVNSVRATRKSMLPITFTDDDFHAPDLEQDDPMVITAEIARYEVGNVLVDQGSSANILYWKTFLQMDLSEELIV
PFHEQIVGFAGERVDTKGYVDLRTRLGTGRDGDEKKVRYLLVDANTSYNVLLGRPCLNSFGAIVSTPHLTLKYPNERRKII
TVRADQKTARECYAAGLRMYPRVPRAKVPRSEVAMADLDPRLGTEDRMEPHGMVQPAKLGPREDQVTTMAGGLDP
VTEDDLRRILWRNINLFAWIAADMPGIHPSIMTHRLALFREARPVAQKKRRMGAEKERAVEEEMKKLKEAGFIREVTYT
TWLANMVMVKKSSGKWRMCTDYTDLNKACPKDSHPLPNIDSLVDGASGHRLLSFLDAYSGYNQIPMHEADREKTTFI
TNRGNYCYDVMPFGLKNAGATYQRLMDKIFADQIGRCMEVYVDDMVVRSTTLEAHLRDLEEVFQQRDRSQPRQVH
DHPRHATSCQPEGDPAAGWTTDGPCPIHSEVGRPDPTDLKKDEKGDEGGVWDADCERAFAAVKDLLTNPVVMNRP
LPNTKLQIYLGVSHEVISAALVQDAREPRLIYFVNRVLQPAETRYQLVEKVALALLHASRRLRPYFQSHQVVVKTDHPVSK
ILRKPDIAGRMVSWAVELSEFGLRFEPRGSIKGQHLADFALELPQAQSPEGWSLYVDGAAGRASAGAGVVLEGPGGFLI
EQSLVFKFKASNNQAEYEMVGEFQVKDDQLLRYFHKASTLAKNFQPFIIKHIPREENAWADMLSKLSAGKEKGQLTTVI
RQVLTEPSVECMALGGVSLKPADARRLARYVTIGGDLYRRGFSVPLLKCVSPDQAAYVLNELHNGICGLHTGARTLRAR
ALRAGYYWPTMESDAKAFTAKCEQCQAHTNIPHGPPAELRTIVSPWPFAKWGVDIVGPFPPGRAQKKFILVAVDYFTK
WVEAEPLATISARQVQSFFWKIICRFGLPRTVVTDNGRQFIDKKLQTFFQGLGIKHITSSVEHPQTNGQAEAANKAIVAE
LKRRLGERKGAWVDELPEVLWAYRCSPYGTTGESPFNLTYGTDAMLPVELGEPSLRRQIQDLQLNEEELRVELDSVEER

RDRPVLHAEACWRLVERRYNTKVRPRSFQEGDLVWRKTGDARKEQAHGKFAAKWDDPFRVLENLQNGAYCLSAPG
GRPLRNTWNASHLKFYFS

>g35048.t1

MAEALLGFVLQNLESFVQYQRATYWGVNQQTDKLSSNLTAIRAVLRDAERKQITSELLKIWLQKLTDAAYVLDDILDECS
IHFTKMHSHDGHTSCLSRLHPNDILFRFNIAKRMKDITQRFHDIHEERRTFELRDGVTEKPTVDDGEDDQTTSVITERRFY
GRDDDQEKIVKVLLEANNNEDLTVYPIVGMGGLGKTTLAKHVFHDDRVCKHFDLTIWVYVSVDFNVKAILQSIIEYATG
QNPNLHTLEAMGKKVEEVLQSKRYLLVLDDVWNENQEKWKDLEGMLRFARGAKGATVLVTTRLEEVASIMETHPAY
HLTELSEYDSWSLFKSYAFGPNREEKKELAAIGEDIVKKCVGSPLAIKTLGSLLHDQTAVTQWENIKESEIWDIRSESSSMT
GKENSIMRALKLSYFNLELSLRRCFSFCAIFPKGFEIVKEELIHLWMANGFIKSEGNVEVEDVANNVWRKLYRRSFFQEAK
SDKFGTITSCKIHDLFYDLAKSIMGEECAAFVDRSLRIRGLENVPNEGDAKQANLMSKKELNRLHLSWGSSANSEGSNVS
VERVLEALEPPSALKSFGMRGYQGRQLSNWMKSVLVMRDLVEVELLDCDNCEELPPLGKLPHLKILYISEMKNVKWID
GETYDGVEEKAFPSLEKLTVRNLPNLERMLREEGVEMLPRLSKLRIDGVLSFKVPRLPCVETLHATGIEDVTSFMEGVGE
NMACLKTLSIYCIKGVVVLPDEFRRLGALQVLYIGEWYDLEYFPEHVMEGLTSLRTLSIEFCEKLKSLSEGVGHLACLERLTI
SKCPELVLPSNMSQLTALRKVSINYFCTLPDGLQRVPSLRVLDICGCTSLPDWLGDMTTLEELSIRYCDELRSLPSSIQRLT
NLSHLTIHYCPHLKKRCKRKTGEDWQYINHIPNLELLS

>g35049.t1

MAEALLGIVIQNLQSFGQVQLATFWGVHQHTQMLSSNLTAIRAVLKDAERKQITSHAVKDWLQKLTDAAYVLDDILDE
CSIHSTKLNFDHRHTSCLSRLHPKDILFRFNIGKRIKDITQRFHDIHEERSMFNLVPGVTEVQTIDDNWRQTNSDITEPVV
YGRDQDREQIVKFLLEDDSNSEELSIFPIVGMGGLGKTTLAKQVFNDNRVCKHFDLTIWVCVSDDFNTMTILQSIIECITG
QNPNLNSLEAMRKKVEEVLHGMRYLLVLDDVWNEDQEKWKQLKGKLQCARAAKGATILVTTRLEEVASTMQTHPSY
HLKELSGDDSWSLFKHHAFGPNREEKEELVSIGKEIVTKCVGSPLAIKILGSLLRDESDVKQWQNVKESEIWDIREESSYA
TGEENSIMRALKLSYFNLELSLRRCFSFCAIFPKDFEIDKEELIHLWMANGFIKCEGNIEVEDVGNKVWKKLYSRSFFQEAK
YDEFGMIRNFKIHDLFHDLAQSIMGEECVVIVKGRLTPLPTRVHYSSLFNSGVSVDMTAFKRFTTALKKVESLQTFLDFG
GIGPAPSNHSLRALKTSSSLLSPLKDLTLLRYLSLSFDRSLEESLNNSICQLSKLQILKLCYCGGLHWLPKDLTRLQDLRHIVIG
GLDSIQEMPPDIGKLRHLRTLNTFVVGLKPGCGLAELHSLKLGGTLRIRGLENIPNEWDSKQANLMSKKELNRLHLSWG
SSANSEGSNVSVERVLEALEPPSALKSFGMRGYQGRQLSNWMKSVLVMRDLVEVELLDCDNCEELPPLGKLAHLKRLR
VSGMKNVKWIDGETYDGVEEKAFPSLEKLTVRNLPNLERMLREEGVEMLPQLSELIIFYASNLKFPRLSSVEKLHAVGIYE
VASFMEGVVGNLPSLKTLNIQIVKGAVVLPDQLSGLDALQQLHIRGWHDVEYFTEHVLEGLTSLRTLDIHYCKKLKSLSE
GVRHLARLESLTILGCPELIALPSNMSQLTALRNVSILDQSTLPYGLQRVPSLRMLNIHYCTFTSLPDWLGDMTSLQKLIIM
ECYELRSLPSSIRHLTNLSHLRISECPHLKKRCKRETGEDWQYIKHIPKINLSW

>g35050.t1

MAEALLGSVIENLGSFVQDQLGTYLGVEQQIQKLSNNLTAIRAVLRDAERKQITSEVLKNWLQKLTDAAYVLDDILDECSI
HFTKMPSDDGHTSSLSRLHPKDILFRFNIGKRMKDISQRFHDIHEEKSRFNLEHGVTEVQAVDDDWRQTSSDITEPVVY
GRDQDREQIVKFLLEDASNSEELSIFPIVGMGGLGKTTLVKQVFNDHRVCKHFDLTIWVCVSDDFNTMTILQSIIECITGQ
NPNLNSLEAMRKKVEEVLHGKMYLLVLDDVWNEDPEKWKQLKGKLQCARAAKGATILVTTRLEEVASIMQTHPAYHL
KELPGDESWSLFKYHAFGPNREEMEELVAIGKEIVRNCIGLPLAIKTLGSLLRDQSESIMGEECAAFVEGRLTPLSSRVHYL
SSNVSFRGLKHAFKKVESLRTFLDLRYLRGLPKNLTQLQDLRHIMMDDCDSVVETLPKISKLRHLRTLSIFVVGSKPGCGL
AELHSLNLGGTLRIRGLENVPNEGDAKEANLIGKKELNILRLSWDGNGNPKGSNVSVERVLEALEPPSTLKSFEMNGYQ
GRQFSSWMRNSGVLRDLVEVKLLGCENCEELPPLGKLPHLKRLKVSGMKNVKWIDGETYDGVEEKAFPSLEKLTVRNL
PNLERMLREEGVEMLPRLSKLRIDGVLSFKVPRLPCVETLHATGIEDVTSFMEGVGENMACLKTLSIYCIKGVVVLPDEFR
RLGALQVLYIGEWYDLEYFPEHVMEGLTSLRTLSIEFCEKLKSLSEGVGHLACLERLTISKCPELVLPSNMSQLTALRKVSIN

YFCTLPDGLQRVPSLRVLDICGCTSLPDWLGDMTTLEELSIRYCDELRSLPSSIQRLTNLSHLTIHYCPHLKKRCKRKTGED
WQYINHIPNLELLS

>g35332.t1

MIVKGYTQLEGLDYLETFALVSKLTTLRLLLAIAAYNQWILKQLDVNNVFFMVTYMKKSICNFLLASPLTIQIKCANFKDLY
MSSNRLDDNGMKNFPLFFDIIIIHALMLIIPFFLKHDNCHTTAILIYVDDILAGKNATKIQHITTSLDSLFHIKNLGDLTYFLG
LEVARNSSGIHLSQRKYTLDLLTEAGMLHCAPMPIPMAHSSRLTSQGNLLNDEDASSYRRLIGRLIYLTNTRLDITFSINNL
SQFIPLLPTYINKLLTAFLDISREVLVMVSLSKATTPISSKPIVILTGSLVLNPENLSLVIPSTLAISSSWKSKKQQTVSRSSSKA
EYRALATVTCELQWLTYVLRDLRILTFQLAIVYCDNCYTIQIVSNQVFHERTKHIAIDCHIVRNKLINGLLKLLPISTTEQAA
DLFTKPLARVVFKVGKKNSYPWIYAGKIRFRYLIPVGKTYPWVSRLCSSERVLRRLPHQVPWWNESSATPSSPCSTPVVQ
VELGTEEDLFRKGGKADDEVEDVILGVCEGWSWVAHLTDAGLKHLPIPSQSSQQVPFFTSESKGCYIGTIMTQLPSSSCS
SSSTCEGTHDVFLSFRGDDTRSGFTGNLYKSLCDRGIHTFIDDEGLRKGEEIRPALFKAIEQSRIAIVVFSENYADSTYCLEEL
IVILECILRKGRLVWPVFYGVTPSYVRFQKGSYGKALAKHGERFKNDQEKLQKWKLALHVAAGLSGSHFKLKQGYEHELI
RTIVEEVSKKINRSPLHVANYPIGLESRVQDVKLLLDVGSNRGVSMVGIFGIGGIGKTAIACAVYNAIADQFDVQCFLGDI
RQKSMKYDLVQLQETVLSEMVGEKSIKLGSINRGMAVMKSKLQRKKVLLILDDVDKLEQLKALAGDPSWFGDGSKIIVT
TRNRHFLRVHGVERTYEAKGLDDKEALELFSWHAFKRNEVGPGYLNISKRAVFRCNGLPLALEIIGSNLNGITMSEWEA
ALDTIERIPDEDIQEKLKVSYDGLKGNEKEIFLDMACFFRGYHLKDVINLLLQSRGFSPEYVIRMLVDKSLIKIDQYGFVQM
HNLVEDMGREIVRQESPSEPGKRSRLWLYEDIVDVLENDKGTDTIEVIMLHLPKNREVLWNGSELKKMTNLKMLTIEN
ADFSRGPEYLPSSLRVLKWRGYPTQSLPPEYDPRRLVMLDLSMSRNILGKQLNLMKFESLSEMVLRGCRFIKQAPDMSG
AKNLRKLCLDNCKNLVEVHNSIGLLDKLTWFTAIGCTSLRTLPHSFKLTSLEYLSLRKCSSLQRLPNISEEMKHMKNLDLCG
TAIEQLPYSFRKLTGLKYLVLDKCKRLNQIPINILMLPKLERLTAVKCGRYVNLILGKSEEQVRLASSESLIDFRINYNDLMPT
SFPNVEFLVLTGCSFKVLPECISQCRFLKNLVLDNCKELQEIRVVPPKIKYLSAINCTLLSHESQNMLLNQRLHEGGGTDFS
LPGTRLPEWFDHCARGPSLSFWFRNKFPRMTLAVVGVLDKQGSFPMSRFHLLINGIQKLHCHFTVQSKLITYHIFLSDV
QLKSYNGGLESVYGEDGWNHVEVSYVGPRVFPHSCRTKKGTIKWMGVHVQKQKTSMQDIRFINPWFPKRAHSEVSK
ADLQESFQSLPKRIRVSHRKEICEAPQTKQHEANSSYHGVSQRLWLAICSIAAPLNVKVLMWNICQDDLPTFEYLFRRKL
VLSPLCPICGTEPESVEHVFLFCPWTRPLWFGSDFQWCINVKEVQSFQLWLWHKLMEIQRVYPENANQISAQVGSIC
WSIWKGRNEFVLEGKPVNPLILR

>g36087.t1

MAESSSSFTASASPTSYEVFLNLSGEDTRENFISHLYAALERKHIETYIDYRLQRSEEISPALQTAIEESKIYVLVFSENYASST
WCLNELTKILDCKKRYGRDVIPVFYKVHPTTVRKQEERYKEAFEEHKLRFKEDMGKVQGWKDALTEAAGLSGWDSNLI
RGHNEIVVVETLNDCGFSSKIGMDVFKDKCLISILDGRIVMHHLIQEMGQEIVRKECPQHPGKRSRLFNADEIHEILRTNK
LCSIVTQLKESHRLISPGLILLSQCSKLEMFSTCQPRFPHVKLERQRGTFSRSLREKPEYQPGTVSSFLRIERRALSRSFVRDG
LGEPDYFDELYLSNCSSLTIFPFDISEIKFLKKLRLSGCSKLENFPEIENTMEDLSVLLLDCTAIHTLPSSLCRLVGLQELSLRSC
SNLEIIPSSIGRLTRLCKLDVTYCESLQTFPSTIFKLKLRKLDLCGCFKLRTFPEIMEPTQTFVHIDLTKTLIKELPSSFGNLVKLR
SLQLNSCSALQSLPNSIVNLKHLCKLDCSRCYKLTEIPRHIGRLTSLVELSLSESGIVNLPESIAHLSSLKSLDLSDCKKLECIPHI
PPFLKQLVALDCTSIRGVMSNSNSLVPNLSNSKEGFFRFHFTNAQQLDSGARANIEEDARFRMRDYVYGSVCFCFPGSA
VPHWFPFRNEGPSVSINEDLSLCSDDRLIGFALCVVLGVLDTNDIKSRYGSFGYSLKFECDDDGTQIIPNNDVLNNYFTW
GYSPRLLDKDHIFMWKFNLESLRRRGMNLRLCDARSFTFEISPYDYNSQWPDYDSVVNELKSVVTIKECGMCPLYSSGS
NVAESSR

>g36470.t1

MAESLIVAVAESLITKLASRAVEQASLTLGVYQELQQMKKTMALVKAFLLDAEQKKQQNNALSEWLRQIRQVFAHAED
IVDNFECEVLRKHVVSSHGSFCRKVCRLLSTSNPVVYRYRMGREIKGIKKQLEKVATDGHMFGLQSSDKDTKVLHAREM

THSHVNVSNVVGREHDKQKIIELLLQDDHHDRSLSVISIVGFGGLGKTTLAKVVFNDTAIEECFTLKMWVCVSNDFELRN
VLIKILNSAPNPTNEKFKNLDTDQLQIRLRSSLQREKFLLVLDDVWNEDRVKWDELKEIIEMVGNKGSKILVTTRSHSINA
MMRTKSSNSYILKGLSEEDSMSLFVKSAFDDGEGKNHPELMEIGRQIVRKCGGIPLAVRTLGSSLFSRVDRKEWENIRD
NEIWNLKQNENDILPALELSYDQLPSHLKRCFACFSLTSKDFDVSSSYVALLWEALGFLSPPKQNETTHDVANEYLRELW
SRSFLTDFLDMGSTCRFKLHDLVRDLAVYVAKGEFQILYPHSTSISEHAQHLSFIENDTLGQDLVPIGARTIIFPMAATND
AFLNTLVLRCKYLRVLDLSYSEYESLPSCIGKLKHLRYLNLSGNKKLKELPDSVCKLQNLQTLDLRGCIKLQKLPKGIRKLVSL
RRLLVTTRQPDFPDKEISKLASIETVKLYSCDNLESLFQAIQPRSLKFLHLSGCGGLKSLSFHVITNLESLVIFKCSKMELSMG
LSNLNSIPDSRLKLLVLQSLPQLVTLPEWLQGSVNTLHSLLLVDCNNLEELPEWLSTLTSLKLLIIEHCPRLISLPDSTHHLRN
LEHLEINDCPELCKRCQPGVGLDWQKISHIKQVIIDFSLEVVGLKKLPDGLQKDSNIVVLSAPPSSPMRKKGAATMPQM
KLKWRIDEPPGWREGNVLIVLISPFHCRWLFPVWLWSSFFPIVDPIFFVWLFAYVAGESEEASLVVVSDLLYLLWLSAPF
GSKWLVIRLSGLGNYCWGSIVYRYLVWLCDKLIPPKGEIQKSPRILHWMNVHLGDNIVKQALESGVVVDEFDVGGTKD
FKDDEPTMESKADKSPSAKKLLKRSLKDSVMQTLVCQQKLVAGLKMKVVELDKEVAFEKARRRGAEEGGPSVAREDR
WFSPGGISPEGISSRPMSTDAPAFQTFVRKGSRPRFKSKALRTPFTGSSRIKDV

>g36471.t1

MAESLLFSFAESLTGKLATAAVQEASLALGVHSELRQMKASMALIKGVLLDVERKNQQSSALSEWLRQVKHVCCDAEDI
VDDFECEALRKQVVNSYGSCSRKVRRFFSKVILLFIVLEWRITFKISTPGREHDKNEIIKLLVQDGDCKTLSVISIAGMGGLG
KTTLAKLVFNETNIDECFQLKMWVCVSNDFELRNVLIKILNSAPNLTGENLNNFETEQLQNRLRNTLKGKKFLLAQVFPH
IGLRSVSLPEGMNNEAFLIRFVSRCKYLRILELWSSSRVCLTLLES

>g36474.t1

MAESLLFRIAESVLGKLATAAVHEASLALGVRSELQQMKENMEIIRGVLLDAEKKTTQSSGLSEWLRQVKRLFSDAEDIV
DDFECEALRKHVVNTYGSCSRKVRRFLSKSNPVVYRLRMAHHIQDINTRLAKLAADRNMFGLQIIDHDTRVVHVREMT
HSHVNPSNVTGREHDKNEIVKLLVQDGDFQSLSVISIVGMGGLGKTTLAKLVFNDTNIDECFQLKMWVCVSNDFELRN
VLIKILNSAPNLTRENFNNFETEQLQNLLRNRLEGKKFLLVLDDAWNEDPARWHELKEIIDVDVEGSKVLVTTRSHAAATI
MHTKSSNLYLLGCLSKEDSLSLFLKFAFDDGEENKHPELLEIGKEIVEKCGGLPLAVKTVGSSLFSKFDKKEWESIRDNEIW
SLKQNEEGILPALKLSYDQLPSYLKPCFASFSLCPQNAIIICSQISTMWEALGFLPPPKESESMVDVANHLVYELWSRSFLA
DYFDFGGDSSFTLHDLVYDLVVHIAKGNEQ

>g36487.t1

MAESLLFSSAESLLGKLASAALQEASLAFGVHRHLQHMKETMELIRGVLLDAEKKNPQSSALSEWLTQIKRVFSDAEDIV
DDFECEALRKDVVNTYGSCSRKVHRFFSSSNPVVYRLRMAHHIQNINIRLAKLATQRSMFDLQVIHQDTRVVHVREMT
HSHVNPSNVIGREHDKQEIIKLLLQDDHGQSLSVISIVGMGGLGKTTLSKLVFNDTTIHACFPLRMWVCASNDFELRNVL
IKILNSAPNPTGENFNNFETEQLQIRLRNTLHGQKFLLALDDVWNEDPVRWDELKEIIDMGVEGSKILVTTRSQKVTTIM
HTKSSNSYLLQCLSKEDSLSLFVKYAFEDGDEIKHPQLLKIGEGIVKKCGGLPLAVKTVGSSLFSRVDEKEWESVRDNEIW
NLKQNEKDILPALKLSYDQLPSYLKPCFASFSLYPEDTYIFSSQVCLFWGVLGFLPTPKASESMIDVATQLLHELWSRSFLS
DYEDFGGDCRFKLHDLVVDLAEYVAKALLPSSLRTISFPSGANNEDFLNTLVSSSCTMNDENNHPSSFLYLHPSESPATSL
VSPLLDSNNYHAWNKSVTIALSAKNKAQFIDGTTTKPPREDPSHNAWKRRNNMVVSWLVYSVSPYIRHNILWMDDA
QTIWNDLKSRFFQGDLLRISELHKEASSLKQGSQTISELYTKLCIIWDELDSFILDPICTCNTKCSCKMLSIISQRKHEDRALQ
FLRD

>g37319.t1

MFNSTEVSYKKLQETLKNKSASIIGLVGLGGLGKTTLAKEVGKKAEDMKIFEKVVWATVSQPLNIRTIQDQIVDQLCFKLI
EESEIGRAQRLSERLRKGTTLIILDDVREKLNFEALGIPLDESSKACCVLITTRSKEVCTSIQCQSIIELNLLSDEEAWTLFKHY

ANINDDSSEALKVVARKIVNESLWIASKSGQAIFTRTEVDLRVLADDEVMKDMKAIALWGLKNDHLLNYKINCPMLEILL
LSFNASGGVKVSDGCLQSLENLKTLAILKSAHWSRGALPLQESLKSIKNLRTLCLRGHDLGDISFVEGLQALEILDLRGSIFD
DLPIGIVELKKLRLLDLYECSIRKNKIGQPYEVLGKCLQLEELYLRLRHGREPSPHDVFLSRLQKVCYRN

>g37328.t1

MATPSFRRFTYDVFLSFRGEDTRYGFTGYLYKALCDRGLHTFMDDDKLQSGEEITPTLQKAIEESRIAIVVLSHNYASSSFC
LDELETILHCQSKGMLVIPVFYKVDPSYVRYQQGSYGEALAKHQKRFKAQKKLHKWEMALRQVADFSGYHFKYGDEYQ
HEFIGRIVERVSREINRAPLHVADYPVGLLSQLLKIKELLDVRSDDVVHMIGIHGMEESNKHGLKHLQTVLLSEILGEKDM
NLASVQRGISVIQQRLRRKKVLLILDDVDNRKQLQAFAGRSDWFGPGSRIIITTRDRQLLKSHEIERTYEVEELNENDSLQ
LLIWNAFKRENVDPRYEDVLKRVVTYASGLPLALEVIGSNLVGKSVKEWESALEHYKRIPNGEILGILKVSFDALGEEEKN
VFLDIACCLKGNSLKEVEHILGVLYDNNMKHHIGVLVEKSLIKGTCVTEFICLDLSISEKEETLVWNANAFRRMQNLKILIIR
NGFMRLAHVSSLFLWDNNFKHLPECLKEFHNLYELDVSHCKRLEEIRGFPPKLECFNATNCISLTSSSLSMLLNKELYKAG
QTMFRFPGASFPEWFDLRSSEPSCGCLYFKKEERMLECDQTYLLHLQRHGASQQTFQKMVVIEQEESEHRNASRFNAA
GAANDASDGFEIASEADLASDDDDGDGGVDRQQEHNHPEEHHHQQQIEQQPEHDVPQSPTESSENALITEESPENSL
INEE

>g38090.t1

MQFTRNNFTGFLFEALRRKGIDAFKDDEDLKKGESIAPELLHAIQGSRLFIVVFSKNYASSTWCLRELAEIRNCVETLARPV
IPVFYDVDPSVVRKQSECYEKAFAEYEKRFREDKAKMEEAERWREALTEVANLSGWDIRNKLHNVRALVVLDNVDEVE
QLRMFTGNRDTLLLEYLSGGSIIIVISRHEHILRTYGVDDIYQVQPLNKDNAMQLFCRNAFKVTYILSGYEKLARDILSHAQ
GHPLAIEDLGRCIVKEESPKEPLKRSRLCNYQDFCKAMSKNQTTEILEVIAVNAEASKTTRVDGLSKIRHLKLLKFWNHCK
RLKYLPDLPSRTHLPLEVYRVDVHIIEDDEHKEGLIIFNCPKIVERERCTSMSVSWMLQIVQAKRLTSTLFRLSVESVIPGSE
LNTNVVQQSTCKGLEALSFSPFEVFFGCSKRSFHPFEISSVLKLFVAMACSPDIDSSNKVWFYDRFIPSNAFVHKYPRILH
WMTVSVGDKFIKDAMETGLMVDDYGATRKEMGESSVKASLHQSGQDTKKGVRLNKMEKDKVLADLDQVLEEQDG
VIDELEEELYALKAVLVEMNKKDESGYVTPTKENDFNYDFPCEGGIYSNGSVKKGGQC

>g38523.t1

MDFLGPFGKVVEGVVDFVWKHGVRHINYIVHYKQNVVELSDTIKDLRFEKEKIDHKCEEDTKNLNNIEGKVIEWVRKVS
ETETTVEVFENDDGHTRARSPNCFVFPYLWNRHRLGRQAKNLEVGVKKLINESPELDEISYKENVTSNDATLSNFGFEEF
GSTKSTMEKVMRELENSSVRMIGLYGEGGVGKSALIKEIARIAIDKKLFNVVVKVEITDNPNLQSIQEEIAYVFGLQLEGE
GENVRADCLRRRLKKEKGNTLLILDDLWHKLDLNKVGIPLDDNDDNDDLKPLDDKDALMFFQKLSGIHNMSESRKEIVR
KYCAGLPMAIITVAKALRGKSELVLESALGELKKQELVGVQTNMDISVKMSYDHLENEEIKSIFLLCAQMGHQPLIMDLV
KCCFGLGILEGVFSLWEARDKIKITIQKLKDSGLLLDGNSDIHFNMHDIVRDAALSIAKKNKNVFTLRNGKLDEWPELEKC
TSISIFNCDIIDKLPIVNCSRLNFFQIDTNDQSLTIPEKFFKGMKNLKVLILTGFHLKIFPPSIKGLLKLRMLCLERCTLEDNIAI
RELKKLRILSFSGSQLKSLPTELGCLDKLQLLDINDCSIMEVNIPPNVLSSLTHLEELYVRKSLTKMLVEGESSHGQNSVLCE
LKNLHQLKVVDLSIPCFSILPNHLFFAKLKDYKIVVGDVEMFSIIGFKMLDKHETFRVLALQLNHDTNIHSQEDIKLLFKTV
QSLLLGKVDDVVKVVNELNMDGFPDLKHLSIIDSNAIKYVNSMELSNCINVFPNLESICLYNLKNLEMISYGPLTAASFAK
LKSIKVNMCDLLVSLYSVYMVEFANSEEPCEIIECNSYLDKFCASLETIEVSECESLREILQIPMNCDKVKFLKLQTLTFQSLP
SFTRFYTELEESCWPHPRKPHAKNNGTEEDQKNDKAPLLFGEQVCVFPKLEEIQLSKMRRLRDIWHTKVNIDSFSSLISV
NIEECNKLDIIFPSNMEGWFESLINLKISKCKSVKEIFEVNDSEEIDVSGGIETNLQVILLEDLPKLKELWSKDPHGVLNFKN
LRTIDVSDCDELRNLFPASMAKDLSKLERMSIWYCKKMVEIISSKDASEANNDPLEFPELTYVFPNLEYMEIDFEEAQKLL
PKYQMHRLKELSLVSVKNVDFLNQFPYRMPNLEKLKLVFPYSLEVLKPRTNLAQQERLVIALELKELVVMNARIKDLGFPI
LGRLEVLSLIECGKLRNLDPPSLSFTYLTCLKLNNCSGLRNLMTSSTAKSMVQLKTLKVIDCSKVEQIVSNELSEEGTEIKIVF
SKLITIELVGLKNMKSFCTYKDYEFEFPSLEILIVRECLKMEKFSEREAITPKLKNVFGVEGDEKSKWHWEGNLNATIHQIFT

DKLPNLEHVWDEDPEGIIGLQLLKEMRVEECESLKSLFPASVAKELTRLEVLQVRKCKELAEIFKKEGEGTTPELVLGRLNT
LMLKELPSLKYSIHCSNQRVTYSLKEMEFTTTKL

>g38655.t1

MVVFALNNGFRAERCFETPGRSTPTMADTTQVNGDGPVNDPLSMNDCGQWRFVDEMTFSLVLVVCKTLTATAMA
GVAEATTGDTGAPLLALKLSSTLLFSAATRMSLIMNKIQPSKLAEEQRNATRLFKELHTQIQTLITIGNPTKKDVKSSIEKVL
ALDRDYPLPLLGAMLDKFPQKYEPVVWCPSSQSQEKSTTQERKGLIMDGVKKDSEGWREKDYSISPHVQFTFSRRPQA
IASPMPLTQDVFTNSLVKEWLDEVRDVLYDAEDLLEQIHYEFSKTKLEAEFQITSSKVHSFESDIIALLDYLESLLNRKIVTDF
KIYSGIRFGLGNKVSEKKVESTSLVAEEVIYGREEDKETIFTWLRSDTSDNKLSILSIVGMGGMGKTTLAQHVYNDPKTDE
AKFDEKAWVCVSDVFDALRVSKAIIGAFTNSRDDSEDLEMVHGKLKKRLSERKFLLVLDDVWNEDRNQWKALQTPLTF
GAKGSKIIVTTRSHKVASIMQSTYIHQLKQLDEDHSWQTFAKHAFQDQDSKLKSELKEIGMKIVKKCQGLPLALETIGCLL
RSKSSVTDWEGVLTSEIWDLPIEDSKIVPTLLLSYYHLPAHLKRCFAYCALFPKDHKLNKDSLIFLWMAQNFLHCSQQSKS
PEEVGEDYFNDLVSRLGVDDEKRVSGKIRHMSYVSGPIMSLCDAKGLRTFITFWKNDEMSIELISDFKFLRVLSLRWCIKV
PDNIGDLIHLRSLNLSGGSIEKLPDSTCSLYNLQELKLNLLAMDWKHRDLVPNSKHSKM

>g38657.t1

MPVLETLGGALFGVVLQMLFDKLDSHQVLGFFHDAEQKQFTNSLVKEWLDEVRDVLYDAEDLLDQIECEFSKTKLEAEF
HTSSSKSTSLVAEEVIYGRDEDKEMIFNWLRSNTNDNKLSILSIVGMDGMGKTTLAQHVYNDPKTEEAKFDEKAWVCV
SETFDALRVSKAIIGDFTNSRDDSGDLEMIHGKLNKRLSGRKFLLVQDDVWNEDRNQWKALQTPLMFGAKGSKVIVTT
RSHKVASIMQSTYIHQLKQLDEDHSWQTFAKHSFHDEKLKPELKEIGMKIVEKWQGLPLALDTIGCLLQLKSSVSEWEG
VLTSEIWDLPIEDSKIVPSLLLSYYHLPSHLKRCFAYCALFPKDHRFDKESLILLWMAQNFVHCSQQSKPPEEVGENYFND
LVSRLGVDGEKIVSRKTRHIYVPDTIGDLIHLRSLDLSGTDIERLTDSICSLYNLQELKLNNCVKLKELPFTLHALTNLRLMHA
TRQRAVCVVHSSIFGVWDMGVEYLVEKLEINEQRNRKDHREKCQYLKDEENHPSDMGYGSM

>g38659.t1

MTKLEAEFQTSSSKVHSFESNIIALLDDLESLLNQNIVKDFKIYSGVRSGLGNKVSEKKIESTSLVAEEVIYGRDEDKEMIFT
WLRSDANDNKLSILSIVGMGGMGKTTLAQHVYNDPKIEEAKFDEKAWVCVSDAFDVLRVSKAIIGAFTNSTDDSGDLE
TIHGKLTKRLSGRKFLLVLDDVWNEDRNQWKALQTPLTFGAKGSKILVTTRIHKVASIMQSTYILQLKQLDEDHSWQTF
AKHAFQDQDSKLKSELKEIGMKIVKKCQGLPLALETIGCLLQLKSSVSEWEGVLTSEIWDLSIEDSKIVPTLLLSYYYLPSHL
KRCEICYRLGVDGEKRVSRKTRHLSYVRDPIQDYTSLYDAKGLRTFITFKGLPTFTFLRHYAMSELISNFKFLRVLSWRWCI
KVPDTIGDLIHLRSLDLSGSSIESLPDSICLLYNLQVLKLNNCFGLKELPLTLHELTNLRRLELKGTTLTKAPLRLEKLKNLHV
WMGIFKVGKSSEFSIQQLGELNLHGELSIRNLENIANPYEMNLKNKTHIVSLSLQWNLKRNNEDSLKEREVLENLQPSRH
LKHLSIEGYGGTQFPRYFSCKSLSNVVSLKLYHCKNCLWLPSLGLLTFLEELRIHGLDCIGRVDADFYGNSSCAFASLKTLSF
EDMKEWEEWQCMTGVFPSLQSLSLTNCPKLKELPDILCHLKKLLIENCGQIGAPITRAGEIECVNMRPSSFDMTRPPHIP
ISHWYHSLVELVIRYGCGSLTTFPLDLFPKLGELSLIQCCNLRMISQEHPHNHLKYLMIRHCPEFESFPNEGLFAPQLVSFTI
FGLGKLKSMPKCMYALLPSLNRLYIRNCPALESSEECLASNIKEMHLLNCSKLVASLKGVWGTNPSLKDLYISFVDLECFP
GEGLLPLSLVKLEIYNCPNLKKLDYRGLCHLSSLQTLSFRYCPILQCLPEEGLPKSISELKIVGCRLLKQRCKKEEGSPHYLLKR
LHRDT

>g38957.t1

MALAVVGGALLSAFIDVLFDRLASPEVVNFIRGKKPDKLLQKVKTQLIVVRVVLADAENRQITDPNVKEWLDLIRDVVYE
VDDLLDEVSTKAATQKEVSNAFSRLFKMKKMVNISKLEDIVERLDDILKQKESLDLKEIPVESYRPWKAQPTSLDDGYAIY
GRDKDKEAIMKLLLEENTNGEKVSMIPIVGMGGVGKTTLARSVFNDDKLKQQTFDLKAWVCVSDIFDIVKVTRSMIEEI
TRKACKLSDLNALQLELMDKLKGKRLLIVLDDVWIEDCDNWRSLTKPFLSGIRGSKVLITTRNENVAVAVPFHNVEAYHL

SKLSNEDCWLVFANHAFPLSEASETRGTLEKIGKEIVRKCNGLPLAAQSLGGMLRRKQTIKDWNNVLQSDIWELPGEDE
LIQLWMAEDIVKPPKNGKTLEEVGHEYFDDLVSRSFFQRTNGNTFVMHDLAAFLGGEFYFRADELGKKTKINRKTRHLS
FTRFSDPVSDIEVFDTVKFPRTFLLIKFKESPFNNETAPRIIVSRLKYLRALSLCNFQSQLALPDSVGELIHLRYLNLSSTSVET
LPESLSNLCNLQTLKLSFCLKLTKLPSAMQNLVNLRHLEIHATSIKEMPKRMGKLNQLQNLEFYIVGKHIENSIKELGGLSN
LRGSFSIKALENVTKGEEALEARIMDKSYINHLSLEWSIANDSSIDFQYQLDVLSRLEPHQDLESLSINGYKGTRFPEWVGS
FSYRYMTFIGLSNCKNCCILPSLGQLPSLKHLSISDMNSVKTIDAGFYRKVDGSSVIPFASLESLHISSMPCWEVWNAIDSE
AFPVLQNLCIVDCQNLRGDLPKHLPALKTLTIINCEFLVSSVPMAPTLRTLDIRNSNKVEFHEFPLLVESINVEGGPVVESI
MKAFTIIQPTCLHFLFLKNCSSDISFPGSELSKSLNYVEISHCPNFVSFLGEGLCMPNLTRFCVYNCDKLKSLPDQMGTFLP
KIEFLNISNCHQIESFPGGGMPPNLRTVEIRNCEKLLRGLGWKSMDMVTSLRVYGPSDGIKSFPKESKDRAYVTEILNAC
ELCADVGIPVLIERSLIKVDKNNKLGMHPLLQEMGREIIRENSRKDPGKHSRLWFEKEVVEVLTKNTGTKAIEGLVLKMH
LTSRDCFKADSFLKMERLRLLQLHHVQLAGNYGYLSKQLRWISWHGFRSNYLPNSFCMDDVIAIDLKYSHLRFVWKQS
QDCTSLSNLPKEIYKLKSVKTFIPSGCSKFDKLEEDIAQMESLTTLIGDNTAVKQVPVSVVRSKSIGYISLCGFEGLARNVFP
SIIQSWMSPAMNPLLSIRPFFGTSCSLVSMNTQDNTLVELAPMLRSLPHLRSLLLRYETESQLSKDVETFLVEHVVNVAEL
RISRHHLRSSLIGVGSYKAFFDILNDTISKGLVTNEACEVLLPIDNYPYWLAHIGEGQSVFFTVPEDCGMKGMTLCIVYLSK
TEIKPTECLTSSVLIANYTKRTLQIHRQETVTSFNDEDWQEIISHLAGGDKVEIFVTFGHHLVVKKTAVYLMYGESNNIEIE
PTNCESNDVEIELLHCESNNIEIKAICEPNDIESKPLHCEANDIKITAMNSYRKRKCPDFEDKEPPRNNRESNLGQTKLTEK
GDRVILLHQNNTHDKSKGISFELKRKGEIGKGQNMSLIHGLLEFKCRLSNKISHKDQLQLQKQKLLGEDALGFFKEEIALN
QCLKALTIRACYYWPTMEEGAKRFVQSCLSCQEHANNAHVPPFVLHNIVSPWPFVQWGMNIVGPFLVGRAHKKFLLV
AIDYFTKWMEADALATITAQQFIGKRVAKFYKSLGIKHVSNSMKDLHTNGQAETINKTIVAKLKRRLGEKKGAWVDEVP
KILWAYKCTRHRTTGETPFNLTYATDVMLPIKVGEPSCVDSWKTSG

>g38959.t1

MECVVSATAAADSSSSSSSKSKTHWRYDVFLNFRGGDTRSTFVSHLHSALSNAGIHTFIDVGLHKGQNLGPELLRAIEGS
QIAIVVFSRNYAESSWCLEELAKIAECHRTHGQVVVPIFYDVDPSVVRRQTGDFGKAMEAVAVRTFPSKTLLDSLVRWS
DALNQVANFTGWDMRQCRDEAEQVKKIVQGVLAALDNTSLSITQFPVGLESRVKEVIMFIENQSSKACKIGIWGMGG
SGKTTTAKSIYNQIRRKYDYRSFIENIRQVCEQDSKGYIHFQEQLLLDVLKSEVKVHSTEAGTTLIEERFRGKSTLVVLDDVT
KLEQVHALCGNRKWISSGSVFIVTSRDIRLLHLLEVDYIYKVEEMNESESLELFSWHAFREVTPTHDFIELSRNVVSYCGGL
PLALEVLGSYLHKRTKQEWKSVLSKLRIIPNDQVQKKLRISFDGLEDQMERDIFLDICCFFIGKERGYVTEILNGCGLHADI
GITVLIERSLVKVEENNKLGMHDLVRDMGREIIRHSSPKDPGKRSRLWFHEDVLDVLTNNTGTEAIEGLALKSHGTGRD
CFEANAFKEMKRLKLLKLHSVQLIGSFGHLSKHLRWICWQGFPLKYIPVNFHLGSVVAINLKHSHLKLVWKESQLLEWLK
ILNLSHSKNLTITPDFSKLPNLEKIILKDCPSLCKLHQSIGDLCKLLLLNLKDCTNLSNLPRRTYKLKSVKTLILSGCLKMDKLD
EDIVQMESLTTLVAENIAVKQIPFSIINPLSGIYPCWSMSSSLTSIDVQSNNLGDLASTLTSLSKLRSVLLHCDTEFQLSKELR
TILDDLYDEATMKSSILSTRAYQRDSLPMSFVMFFFRVTTIHFG

>g38961.t1

MELASSTFKPQMCDVLINFTGEDIRRKFVSHLDSALSAVGLTTFLHEETAVKGMHIQQPILNMCRATIVVFTKTYSQSA
WCLHELQQIIKWHETYSRHVLPVYYEIQPSDVRLQKGDFGKSFKATAQQTFSGEQLEHGMLKWSHALTKAANFFGWD
ESNYRSDAELVDTIVKSVLILPVLSATKFPVRLQSHVKDMIQVIKNKSTEVCIIGICGEEGSGKTTLAKAVYHQVHSTFTYKS
FIEDIAEVSQTRGLVGLQEQLLSDVLKTKIKLHSVQMGISMIRERLSGKRMLIVLDGTNEYDPIGLWNSHVWFGKGTVMI
ITTREEGLLRIPEVDSVLLIELLNTNESLELLSWHAFREAKPREEYNDLAKRVVACCGGLPLTLEVIGSRTYVTQILNGCGVD
ADSGIRILIERSLIQVKRNNKLGIQPLLQKMGKEIVVEILERRLRNNTQLRFRLDENYVVSDNTLFSSQQTKVIQILPSKLFLT
RTDLLQPCRAKVSDTSRIFELVEHSNSDEYLSKKLRWISLQGFPSKYLPNDFYLHDSIAIDLKHSSLRFVWKQPQVLPWLK
VLNLSHSKYLRKTPDFSGLSSLEQLILKDCPRLGQVHQSIGCLCYLRLLNLKDYWPNRKRYSANGILNNSNCGKYSCETSA
FFNYMEDNSWDDFVPLLRTLENLRSVLVQCDSEFQLSKQVKTILVEHGVNITETGTPKPHFRYSLIGVGRCKEFFNAVSD

SISEVLASSESCDVCLPGDNDPYWLAHIGEGLSASFNLPQDRVMNGMALCVVHLSTSKTIEPTITTVLIVNYEKCTLQIHN
YGTLVSFTDEDWHCIMSNLGSGDKVEIFVTLDYGFVIKNTAVYLIYGVP

>g38963.t1

MASQNPKWQVRISLDSSTIVQMNGLLRDVHVMVCRWARHDVNFRLKLEWYIGKRVRNMPQIIAAFDLFDEGIEGVG
GGHEEPMGGAAEEVLNEEGVGGGHEEPVGGAAKEMLNEEGVGGGHEEPVGGVDEEVGGRVEEDRVVTFNEGVLV
AEEPRNEVVNVVEIDDDGVHDEEAETPVPLAIVPLPAIAGDPKSTVNADQTVIAASVRDRPQRKLVSHLHSVLLQAQVK
TLVNEENVQEGMKLEERMGAIAVSKIAIIVFSKTYTESTCCLLELEKIIECLETFGQVVLPVFYEIEPLDVRDQKDDFGKALE
ETAKKSYLGEQVEHALSRWRRALTTAASMNSWDVRDFRDDAELVEVIVRRVQALLDYKDLFVTQFPVGLELHVEKVIEC
IQNHNTKVCIIGIWGMVGTGKTTIAKAIYKRIYRLFIGKSFVENIREFWSRLFRTYVDLQEHLLDDVLKNKFEVGSDEMGR
NLIETKLSRKKLLIVLDDVTEFGQLENLCGNCEWFGRGTVIIITTRDFKMLNRLKVNYVYKMDAMNKNDSLELFSWYAFR
EAKPRKELNEISRNIVANCGGLPLALKVLGGWTMQIPYAQVQEWKCVSLRLPNAFAQVREKLKISFDGLGDMEKDIFLD
VCCFFIGKERGYVTEILNACGLEADIGITVLIERDFIKVERNNKLGMHPLLRDMGREITRRDWPQEPGKRSRLWFHEDVK
DILKTKSIDILEEDIVQLESLITLVTENTVAKQLPCSIVSSKSIGYISLRGFEASSHNIFPSIIRSWMSPIVNLQSYISPYCMDME
NNNGRDLTPLLSVLANIQTVLVQCDTEVQLSKQVKKILVECSVNFKDSRISKRHLRFSLNCVGSYNDFFNTLNNSVSEGLT
SSKCCDVSLPGDNYPYWLVHMGEGHSVSFTVPRNSDMKKMILRVVYLSTPGIMATECLRSVLIVNYTKFTLQIHNHGSV
ISFNDEDWHVIMANLGSGDKVEIFVTVDHGLVRLSDMW

>g39186.t1

MEFASSLSSSSSSSFLKSESQFIYDVFINFGGEDTGRKFVSHLHSALLLAQVKTFISKENLQEGIKLEEYTREIASSKIAIIVFSK
TYTESPSWRLELEKIIQCRQTFGQIVLPVIYDIVRLDERHQKVVRVLEKATPGSYSGDQMEHPMSSWSRALTKAAGITG
WDFRDFRHDAELVEEIVKRIQTLLDYTEMSITRFPVGLESHVEKVIGLIEEQSTEVCMIGIWGMGGLVDRRHVHLQENLL
YDVLKSKFEVESDRAGRTMIETKLSRKKLLIVLDGVNEFGQLENLCGNREWFGQGTVIIITTRDVRMLNRLKVNHVYKM
DVMNENDSLELLSWHAFRESKPRKDLNELARNIVAYCGGLPLALQFLERGLIKVEGANKLQMHPLLRDMGREIIRQRCP
EEPGKRSRLWFQNDIKDVLKKNTGTKAIQGLSLKLHSTGRDCFEVRAFKEMKRLRLLQLDNVQLTGDYGYFSKQLRWIS
WQGLPSKYIPNNFHMENVIAIDLKHSYLQLVWQQPQVLEQLKFLNLSHSKYLRETPDFSRLPSLERLILKDCPSLCKIDILE
EDIVQMESLITLVTENTVVKQVPFSIEVKTLVEYLVNFTESGISNHHLRFSLIGVGSYNKLHSTLSDSIFEGLASSESCEVSLP
GDNHPYWLAYMGEGHSVSFTVPQDCDMKGMIMCIVYLSNHGIVPTECLLQVMAHLDDSDDAREDVATCKDFAR

>g39187.t1

MRLSIPSMELASSVSKLPRMYDVLINFTGEDIRRKFVSHLDSVLSSVGLTTFLQHDNAVQPKHIQEPILNLCRVAIVVFTET
YSQSACCLHQLQQIIEWHETYGRHVLPVYYEIQPSDVRLQKGDFGKAFRETAHQTFSAQELEHGMSRWSHAITKAANF
FGWDDCNYRSDAELVDTIVKSVLNLPVLIGICGMGGSGKTTLAKAIYSQIYVTFTEKSFIEDISEVSRRRGPVHLQRQLLSD
VLKTNIEIHNVEMGKSLILERLYRKRVLIVLDDVNEHCPLDLWESRGWFGEGSVIIITTRDEDLLRKHEVRSVFRIDLMNE
NQSLELLSWHAFREAKPKAEYIDLAKRVVATCGGLPLALEVIGSKAYVREILNGCGVDADRGIRVLIESNLIKVKKKNRLGV
HPLLRKIGIEVILEISRKEPRNKNRLWLDKDMHHALLENTENKIIHRCPMERDLFERYPPLEISGPLTLVKITRDSELPPKKLK
WTSRQGFPTEYLPKELYLHDAIVIDLKYSLLRFLWKEPQVLVSLKVLNLSHSMYLTETPDFSRLPSLEQLILKDCPRLRQVH
QSIGCLCNLTLLNVKDCTSLDNLPEEIYRLKSLKTLILSGCSKISLIEKDVGQMESLITLIAENTAVKQVPFSICWMSQTLNPL
SYIHSFCMDKEHNSGDDIIPLFNTLANLRSVLVQCDTEFPLSKQVRTTLVEYVVNISESGIPKHHFRLYLIGFGRHTEFFNT
VSDSVSQVFTSSELSDISLPGDNDPYWLAHMGEGHSVSFTLPQDRVMKGMALKGAGAKEKFPRISGNETSVGKSVFLF
QHDEHYDLKDLLPMSLMMFSSRVATVHFGLLTQVAKK

>g39188.t1

MAFTTSPSFSKSKHQRIHDVFINFRGEDTRRKFVSHLHYALSNAGVNTFFDEENLVKGMQLQELMRAVEGSQIAIVVFS
QSYTESTWCLDELEQIIKCNQTQGQSVLPVFYEIDPSDVRHQKGDFGKSLEEAARRTYSGEQLERALSRWSRALNKAAGI
SGWDVRNFRNEAELVRQIVDRVQKLLDYEVLSITEYPVGLESRAQDVIGLIETRSTQVCMIGIWGMGGSGKTTVAKAVY
NQIHRRFMDKSFIENIRETCENHGRGYIPLQEQLLSNVLKTKVEIHSVGMGTTMIENRLDGKTALIVLDDVNEYNQLKAV
CGNRKWIGQGSVIIITTRDVGLLTRLDVDYVYGMDKMDEDESLQLFSFHCFGDAKPKEDFSELSRNVVAYCGGLPLALE
VLGSYLFDKTSKKVWLGVLSILEKIPNDEVQRKLRISFDSLSNDMEKDIFLDVCCFFIGKDRGYVTEILNGCELCADVGIPVL
IERSLIKVEKNNKLGMHPLLQEMGREIIRENSRKDPGKHSRLWSQKEVVEVLTKNTGTEAIEGLVLKMHLTSRDCFKTDS
FQKMERLRLLQLHHVQLDGNYGYLSKQLKWISWHGFPSNCLPNSFCMNDVIAIDLKYSHLRFVWKQSQIDKLEEDIAQ
MESLTTLIADNTAVKRVPVSIVISKSIGYISLCGFEGLARNVFPSIIQSWMSPTMNPLLFICPYPGASCSLVSMNTQNNTLG
ELAPMLRSLPNLRSVLLRYETESQLSKLVETFLVERDVNVSELGISRHQLRSSLIGVGSYKAYFDILNDKISKGLVTNEACEV
SLPIDNKPYWLAHTGEGQSVFFTVPEDCGMKGMTLCIVYLSNPEIKPTECLTSVLIANYTKRTLQIHRQETVTSFNDEDW
QEIISHLAGGDKVEIFVTFGHDLVVKKTAVYLMYGESNDIEIEPTNCESNDVEIELPHCESNNIEIKAICIPNDIESKPMHCE
ANDIKIAAMNSESNVLEMKSDPKPNGNGCIRFFKKFAMCDW

>g39189.t1

MAFESSSPSAKSEWIYDVFINFRGVDTRKKFVSHLHSSLSKAGVKTFLDEENLLKGMELQELFRAIKVSQIAIIVFSKRYAES
SWCLDELQKIFQCRQTCGLRVVPVFYYVEPSEVRHQTGDFGDALRTAAESRYEGEYLEFVFSSWRRTLTDAANLSGWN
ATDQRTEAELVRDIVNHVIANLDYNAFSITKFPVGLDHPVQEVIRFIERTKSCCKIGIWGMGGSGKTTIAKVIYNKLHRLFE
NKSFIENIREVCQTDRRRGLIRLQEKLLSDILKVKVEIQNVGIGQGMIENRFIGKRALIVLDDVNEFDQVEALCGNTEWM
GERSVIIITTRDLHVLKRFEVNYVYEMKEMEANESLELFSRHAFRESKPREDFNELAKDAVSYCGGLPLALEVLERGLIKVE
KNNKLRMHHLLQDMGREIIRELSRKEPGKRSRLWFQEDVRDVLTNSTGTDAVEGLALKLNLNNRECFKADAFEEMRSL
RLLQLHHVELMGDYGHLSKQLRWIYWQGFPSKHIPDNFYLEDAIAINFKHSNLRQVWKEPKVLLMLKFLNLSHSKYLTE
TPDFSGLPYLEKLILKYCPSLRCVHKSIGDLCNIVLINLKNCTSLSSLPREIYKLKSLKTLILSGCTKIDKLEEDIAEMKSLTTLIAE
NAVVKKVPFSIVSSKSIGYLFPCEFEGLSHDVLPSIIWCWLSPTMNPLSSTLPLCGISASLVSMNMQNIDLGDLAPILTNLLN
LRSVWVQCDTEFQITKQVRKILNDVRWVHFTELGIASCTSEISDNSLRSYLIGIGSYKEEDFNTLNKSISKELATSGSCHVFL
PGGNYPFWLAHTGEGHSVSFTVPQDWDMKGMALCVLYLATPETAATECLISVVMVNYTKYTIQLYKRDTVISFNDAD
WQGIISHLEAGDEVEIFLSFRNELVIKNTTVYLKNYEFIDMVGKAVSKAPVMKVYSRKRNIKDQESSRKRQW

>g39190.t1

MASASSSSSFSKFRIEGDVFIHCLGYEIRKNFVSHLSSALLQAGVKPFLLAVEMQQEQFVASIEGFQIGIVVLNKSYFESFQ
CIDELVRIIECHETHGLTVMPVFYEMDRSDFGNMLKATAREVMKVEHIIWSGEYQKTWFQRWNVALTKAGTLPTWEE
SEHRSDAELVEEIVKSVLAKLDCPLAITKFPVGLEPHVKNVIGFFENQPTKVCMIGIWGMGGSGKTTVAKAIYNQIPYAF
DDKSFIQGIREVCETDDRGLVHLQEKLPSDTLKTNVKIESGEIKKITLESRLSGKKLFIVVDDVNEIDQLKDLLGNDKRFGQ
GSVIIITTRNLELLYQLNVDYVYEMDKMDVNDSVELFNRHAFREAKPREDFIKLARSAVSYCGGLPLALEVLGSYLSKRDY
VTEILKGCGLHADIGIKVLIERGLIKIEKNNELGMHPLLRDMGREIVRQTSTIQPGKRRRLWLHKDVLDVLTKNTGTEAIE
GLSLNCQLTSSGFLKACAFEKMKRLRFLQLDHVEMNGDYGYLSKQLRWIYWRGFPLKYIPNNFYLKEAVVIDFQHSNLR
LMWKQTQVLRWLKILNLSHSMFLIETPDFSKLRSLEKLIVKNCPRLRKGSENSASCDVFLPGDKVPYWLAHMGEGHSVT
FTIPEDRRIKGMTLCAIYRRRTVQSFSDVDWQGIITNLGPGDKVEVTACGCGNHYSPKYKDVNKVYEHRWLLIDFRSMA
SSSLSFFLKLVSGEVFIHCLEDDIHENFVSHLRSALLQAGVEPSLVAVGVLSEKFMPSITRFQIGIVVFTKAYIESSRCVEDLA
RIIGCHETDGLMIIPVFYDTDRSSLDRVWQENFRKARRAQRRTEAETKIRGEIYNKSMRQKAKMMQNFSLSRALYRVTN
LPTWDESKPRDDAELVEEIVKSVLAKLDRALPVTKFPVELETHVKNVIGLFENQPHKVCMIGIWGMGGSGKTSLAKAIY
NKIPFTFGDKSFIQDIRKVCQTDGRRGLLQLQEQLLSDVLKYVKIDSSEMGKTTIENRLYGKKLFIVLDDVNEIDQLKHLCG
NGIWFRAGSVIIITTRHLDLLYQQKVDYVYEMDELDENDSVKLFSWHAFREAKPREDFNKLARSAVAYCGGLPLALEVL
GSYLRRDYVTEILNGCGLYADIGIKVLIERGLVKIEKNNKLGMHHLLRDMGREIVRQTSTMQPGKRSRLWLPKDVLDVLI

KNTGTEAIVGLSLNSKLTNSDSFKTDAFKEMKKLRFLQLDHVRLTGDYGYLSKQLRCISWQGFSLEHIPNNFYLEGVIVM
DFQHSNLRLLWNEPTVLPWLKILNLSHSKYLTETPDFSKLPSLEKLILKHCMSLGKIDIFKEHILHMKSLKTLISENTAVKQV
PISVVSSKSIGYIHVDEEKGLSLTVLHSIILSWMSHSFNPRYRIRPFRGISSSLVSMNVEHNDLVDLAPILRSILNLRTVLVQC
VTEYPILQQVTAILEEVQQVRRVTWTTSTQLSNHPFSSYSIQFGGYQEEVFNTLRKSIYDEELAARQTGKVFVPSDNYPH
WLAYKNEGHSVNFTVPDNFDMDGMILA

>g39194.t1

MSYTSSSSKSKLRWIYDAFISFRGEDTRKNFVSHLYSALANAGVNTFLDDEKLAKGQQLKTELFHAIEGSQISIVVFSENYI
SSRWCLDELVKIMECHASRGQVVLPVFYGLFPSFLRRLHDVNFEVILEKNSDLHRVKQWKKALGDAADFAGWEVSKYR
SDDFVVKEIVSEVLERLDRTYMSITDFPVGLECRVEHCIGLLRKETRGAYILGIWGMGGIGKTTIAKAIYNEIRYEFKHKSFL
ANIREVWQRDQGQIDLQERLLSDILKTEKVKVYSSDWGKAMIKETLCTKRVLVVLDDVNTLEQLSALCGNGNGIVQGS
VIIITTRDVRLLNVLEVDHVYEVEEMNEIESLELFSWHAFKEANPSKAFLELSKQVVTYCGALPLALEVLGSYLYKRREKEW
QSVLSKLKEIPNDKIQEKLKISYDGLTDDTEKDIFLDICCFFIGKDRGYVTEILNGCGLHAEIGITVLIERSLIKVEKNNKLGIHD
LLRDMGREIVRRSSPLEPQKRSRLWVHDDVLDILTEHTGTGVIEGLALKMQRTSGVCFGTETFGKMKRLRLLKLDNVQV
AGDYGHLPKQLRWVDWKAFSLTHIPENFYQENIVAIDLKYSYLKLVWKVPQFLERLKFLNLSHSKYLSKTPDFSKLPNLEK
LILKGCPSLNEVHHSIGDLSNLLLLNLKDCTCLGNLPMVIYKLKSLQTLILSGCSNIDKLEEDIGQMESLTTLIADNTSLKQVP
FAIVRSKQIGYISLCGYEGLARDVFPSLIWTLMSHTRGTLSCFQPFGIMPTSIVSMNIQDNNLVNLLSKVGEFSKLRSISVQ
CDSDFQLTQELRSILHKLCNVNSSEPESAHQSQIPDNSMASYLIGMGSYQQVFDMLSNSISKVLRTNSCTDFVLPGDNY
PYWLAYTGEGYSVPFQVPEDSDCRMKGMLLCVVYSSTPGNMATQNLTNIFIFNYTKCTFQIYKQATTMFFSDEDWQG
VISNLGPGDNVEIFVGVGDGITAKKTAVYLIYGQSITMRLESLGLSAQESPELSVILLPKMSARPTNDVEMKAKKPKKNIIS
KIRSIVRVCCSCL

>g39195.t1

MEFEYFSSSRSDHIHDVFINFRGEDTRTNFVSHLYAALSNAGVNTFLDETDFPKGMDLRVGLLETIERSLIVVFSRNYTESS
WCLDELERIIDCHKIHGRIVLPIFYHVVPSQVRHQTGDFGNVLKASAQQRSWDEYMMSRWNTALTAATNFSGWDVN
NTRNQAQLVNEIVEDVLKKLDNAFMSITEFPVGLESRVQEVTRFIKREFSTVCIIGIWGMGGLGKTTTAKATFNRIHRRFT
DKCFIEDIREVCETDRRGHVHLQEQLLSGILKAKVNITSVGMGRDMLKNKLSVRKALIVLDDVNEFGQLKNLCGNRKWF
GHGSVIIITTRDVRLLKRLKVDYVYKIEEMNENESLELFSWHAFREEKPIEDFNEVARTVVGYCGGLPLALEVLGSYLSERT
KKEWKNRDYVTEILNGCGLHADIGISVLIERSLIKVEKSNKLGMHDLLRDMGREIIRESSPKRPGKRSRLWFHEDVVEVM
TKNTGTEAIEGLTLKLPLTSTGCFNTSVFKKMQRLRLLQLDHVKLNEDYRFLPKQLRWIYWKRFPLKYIPNNFYLGRVIAID
LRHSNLRLVWKEPQVFPWVKVLNLSHSKHLIKTPDFSKLPSLEKLILKDCTSLCEIDKLEEDIGQMESLRTLLAKNTAVKQV
PFSVVRSKSIGYISLCGFEGLSRNVFPSIIWSWMSPRMSPLSRIHSFSSTSASLVSKDMQNNDLSDLAPVLNNLSNLRSVL
VQCNTEFVLSKQLRTVLDGVYDVNFPGLEVTSYTSQISKHSLKSYLIGIGSYEEVFNTICKSVSEVVKIKSYLETFDFWPITLI
PSNESTTVASSQLGHSTDSEILLAQANFANTLHDISGRTSALESHSSSNPPHHPSTRPIKFDLPTFDDSETLGWIFKVTQFF
EFHQTPIGQRIQVASFYLVGPVLA

>g39199.t1

MASSIPSTEFASSSSKLPWMYDVLINFTGDDIRNKFVSHLDSALSSVGFTTFLHYHNAVKPMHIQQPILDLCRVAIVVFTK
TYSQSAWCLHELQQIIEWHKTYCRHVLPVYYEISPSDVRLQKGDFGKAFEATARQTFSRQQLKHGMSRWGQALTKAA
NFFGWDESNYRSDAELVDKIVKTVLNLSVLSATKFPVALQPKVEDLIQTIENKSTEVCTIGIYGVGGSGKTTLAKAIYHQIH
GTFTEKCFIEADVEDKGLRSCVNLQEQLLSDVLKIKVKIPTEEMGRSMIRERLNGKRVLIVLDDMNDYSLLHLLGGSSCFG
EGTVIIITTTVGGMLNINPVDYAFRIKLMNTNESLELLSWHAFREAKPKEEYDYLARRVISCCGGLPLTLEVIGAYLFEMNT
NKWETVLFILEKISDDNVTKILKISFDGLHNEMEKDLFLDLCTFFVGKSKVYVTKVLNGCGVDVDWGIRVFMERNLIKLTR
NNKFGIHPLVQGMGRKIVAQRGDARYALGETDVFSSERTKILRECPDEKLFLTRKRCIEPFLLRHRPRLLKHAGNPGDLR

NSSEFLPYDFYLQDAISIDLRHSLLRLDWKNLQCAPEFQLSQQVKIILVEYGVNITESRISKHCLRSSLVGVGRYNEFLSTVL
ESSESGDVSLPSDNDPYWLAHIGPGDKVEIFVTFGHGLMVKNTTVYLICCESQDLEREPALNKNSLITFFKKIVM

>g39200.t1

MEFGSSSTKLPRYYDVLINFNGEDIRRKFVSHLDFALSSVRLTTFLHHQDAVESMHVQQPILNLCRVVIVVFTKTYSESA
WCLHQLQQIIQWHQTYCRHVLPVYYEIQPSDVRLQKGDFGKALKATAHQTFSAQHLEHGMSRWSHALTKAANFFG
WDESNHRSDAELVDKIVKSVLNLSVFSATKFPVGLQDQVQYYIRTIKEKSRDVCTIGIYGMEGSGKTTLAKAIYHQIHDVL
KTKVKIDTVEMGRNMIRDRLFGKRVLIVLDDMDDYVPLLDLWKIRSXGYATKILNGSRIEADSKTSGLHGFRVDADSGIR
VLMERNLINVKRNNKFEMHPLLQEMGIAISREISGEKRWKNGQLLFDVDAEYALKVNAGRKDYEVLPVLLSTRKEPSRL
SKDGVNSENLSPKLRWISFRGFSIEYLPNYINAHDTIAIDLKHSLLQFFLEEPQVLRSLKVLNLSHSMYLTHTPDFSRLPNLE
ELILKDCPRLREVHQSIGHLCYLILLNLKDCKRLSNLPQEIYRLKSLRTLILSGCSNIDPMEKDIMQTKSLITLVAENTTVKKV
PFSIIWRIIVGMILRHCLATWQIFEVFWCNVTPSFNYLIKYSLIGVGAYHEFFNAVSNNISEVLASSESSDVSLPGDNPPYW
LAHMGEGDSVSFTVPPDNAMKGMFLYTCMAKQSPLMI

>g39405.t1

MTLSTMNVAEHNLLQENLVERLYGGISLMYTLKWESRCLKLFSRSNNKLSQKLEVSAFLSTTDDPMLRAMMICVLSRG
NNQPHSDNDRVMTRRGFDKSVASEAAMTRLREAFFLSSSLGPTKFRRIGSEVLSRDAVRHSSGNLRRGKQIWQPACTR
WGGKVEEETLKKGLGLAHLEEEVLFMAVRSHSQEFTYDVFLNFRGSDTRYGFAGNLYKALDDRGIHTFIDDEKLQGGDE
LAPALVKAIQESRIAITVLSDTYASSSFCLDELVYILDRAEEKELLVLPVFYNVDPSFVRHQKGSYGEALARHEERLKADNKE
RLNRHMEKLEKWKMALHQVANLSGFHYKYGEGYEYKFIGKIVEWVSSEINRAPLHVADYPVGLDSQVLKVMKLLDVGS
SDGVLLIGIYGMGGVGKTSLASAVYNLIASHFDGSCFLQNVREKSRKHGLEHLQSIILSKILGDKRIVFASEQQGISMIQHR
LQKKRLLLILDDVDKHEQLQTLVGRPDCFGPGSKVIITTRDKQLLASHHVQKTYKVKKLNKNHALQLLTWKVFKSEHVYP
EYEEVLNRAVTYACGLPLALEVIGANLCGKSIQECKSVIDQYKRIPNNRIQETLKVSFDALQEEEKRVFLDIACCFKGYKLTE
VEDILHAHHGACIKYHIGVLAEKSLIKIGQYDRVTLHDLVEDMGKEIVRQESAEEPGKRSRLWLPKDIIQVLEDDTLQSSIV
MMPELTLIEAWGWKGWQWIKWEEDEEKDGSMLDVSCCKHLREIRGIPPKLKHFNATNCLSLTSSSTRMFLNEDLHEN
RKTMLVLPGSRKPEWFDHISYGPSSSFWIRNKFPGKVLCHFVAPKDRDISDYVKPMLLINEKVYVCFFDRLKFLKLGAEH
TFLFDLRNLIFTNNLYEVPLENEWNHVKVIYFDSTAASMPTCFQAGIHVFKQESSDEEIMFTDPYAKKRKLEMIQPVSPP
SIFDFDLNIDPAFNQWDLNSW

>g39529.t1

MSSTAIVQYTISSSHAINRYDVFVSFRGEDTRNNFTGFLFQALRRKGIHAFKDDQDLRKGESIAPELLHAIQSSRLFIVVFS
NNYASSTWCLRELAEIRNCVQTSPRPVIPVFYDVDPSVVRKQSECYEKAFAEHEKRFREDKAKMEEAERWRKALTDVA
NLSGWDIRNKLHNVRALVVLDNVDEVEQLRMFTGKRDTLLRECLGEGSIIIVVSRDEHILRTHGVDDIYQVQPLNYENA
MQLFCRNAFKVNHILSDYEKLAWDVLSYAQGHPLAIEDLGMYIVKEESPKEPLKRSRLCDYQDFCKAMSNNQTTEILEAI
IVFNFHKSSKTVRVDGLSKIRHLKFLRLEDVNCSGSLSHLSSELGYLTWNNYPFECLPQSFQPHKLVELKLRGSSIQRLWSD
TKVLPNLKRLDLSYSQNLVEIPDVAEALNLEWIVLERCIQLRKLSPSIGLLRKLTTLNLNDCKNLMKQGTQSI

>g39535.t1

MSSNAIVQYTASSSHTINRYDVFVSFRGEDTRNNFTGFLFQALRTKGIHAFKDDEDLKRGESIAPELLHAIQSSRLFIVVFS
KNYASSTWCLRELAEIRNCVQTSPRPVIPVFYDVDPSVVRKQSECYEKAFAEHEKRFREDKAKMEEAERWREALTQVAN
LSGWDLRNKLHNVRTLVVLDNVDKVEQLRISTGNRDTLLRECLGGGSIIIIVSRDEHILRAHGVDDIYQVQPLNNENAM
QLLCRNAFKVNHILSDYEKLAWDVLSYADGHPLAIELDEEDKEIFLDIASALCYYDEKYVKEVLKFRGFHPEYGLQVLLDKS
LILKAASGYIHMHGLLMDLGRLIVKEESPKEPLKRSRLWDYQDFAKAMSNNQVDGLSKIRNLKFLRLENMKCSGSLSHLS
SELGYLTWNNYPFKCLPQSFQPHKLVELKLRGSSIQRLWSGTKVLPSLKCLDLSYSEKLVEMPDVAEALNLEWIVLERCIQ

LRKLSPSIGLLSKLTILNLKDCENLISLPNSILGLNSLEYLSVYGCSNLFNNELLDEGRNTEHLKKLCLVEGPIQPHSTSPLIKKA
RRESVSCLLPSSPTLPCLRELDLRFCNLVKIPDAIGKLRCLEKLNLKGNNFVTLPNLKDLFRLYYLNLQHCKRLKYLPDLPSRT
HLPLNIYSYPMRYLSTFNYQVPNEDECMAGLRMFNCPEIVERERCTSMTVSWMIQIVQSWYNSEDLPFSFITTLGSSIP
GSEIPMWFNNQLVSMDNSIIIDVSPFVHDNNWVGVVCCAILSEDLYRTRILEDLRRDLSVEHSDHILTTSRVINISEDIFV
GFNCTLGGGNVTHQSP

>g39575.t1

MEKVMRELENSSVRMIGLYGEGGVGKSALIKEIARIARDKKLFNVVVKVEITDNPNLQSIQEEIAYVFGLQLEGEGENVR
ADCLRRRLKKEKGNTLLILDDLWHKLDLNKVGIPLDDNDDNDDLKPLDDKDALMFFQKLSGIHNMSESRKEIVRKYCAG
LPMAIITVAKALRGKSELVLESALGELKKQELVGVQTNMDISVKMSYDHLENEEIKSIFLLCAQMGHQPLIMDLVKCCFG
LGILEGVFSLWEARDKIKITIQKLKDSGLLLDGNSDIHFNMHDIVRDAALSIAKKNKNVFTLRNGKLDEWPELEKCTSISIF
NCDIIDKLPIVNCSRLNFFQIDTNDQSLTIPEKFFKGMKNLKVLILTGFHLKIFPPSIKGLLKLRMLCLERCTLEDNIAIRELKK
LRILSFSGSQLKSLPTELGCLDKLQLLDINDCSIMEVNIPPNVLSSLTHLEELYVRKSLTKMLVEGESSHGQNSVLCELKNLH
QLKVVDLSIPCFSILPNHLFFAKLKDYKIVVGDVEMFSIIGFKMLDKHETFRVLALQLNHDTNIHSQEDIKLLFKTVQSLLLG
KVDDVVKVVNELNMDGFPDLKHLSIIDSNAIKYVNSMELSNCINVFPNLESICLYNLKNLEMISYGPLTAASFAKLKSIKVN
MCDLLVSLYSVYMVEFANSEEPCEIIECNSYLDKFCASLETIEVSECESLREILQIPMNCDKVKFLKLQTLTFQSLPSFTRFYT
ELEESCWPHPRKPHAKNNGTEEDQKNDKAPLLFGEQVCVFPKLEEIQLSKMRRLRDIWHTKVNIDSFSSLISVNIEECNK
LDIIFPSNMEGWFESLINLKISKCKSVKEIFEVNDSEEIDVSGGIETNLQVILLEDLPKLKELWSKDPHGVLNFKNLRTIDVS
NCHELRNLFPASMAKDLSKLERMSIWYCKKMVEIISSKDASEANNDPLEFPELTYVFPNLEYMEIDFEEAQKLLPKYQM
HRLKELSLVSVKNVDFLNKFPYRMPNLEKLKLAFAYSSNVLKPRTNLAQQERLVIALELKELVVMNARIKDLGFPILGRLEE
FNDILHRQKHVSTQNYEGNQLSSSRGNSKE

>g39587.t1

MEGSGKTTIAKAIYNRIYREFIGKSFMENIRYESQENLHERMKLKEHMRAIRHTKITIIVFSKSYAESIWCLLELEKIIECHEIF
GQIVVPVFYEIDQNQVHDFVKALTESAHKIYSEEQVERVLFKWGDALTTAAGMTGWDVRSFRHDAALVEVIVRRVKKL
LDYKDLFITQYPVALLESRVEDVIKYIENQSTKVCIIGLWGMEGSGKTTLAKAIYNRIYREFIGKSFIENIPDEKYVALQENLL
SDVLKSKLKVGDVGIGRIMIQNKFCRKKLLIVLDDVNEFGQLENLCWSPEWLGQGTVIIITTRDLYLLKQLENSYVYENESL
EAKPRKELKELARDIVDYFGGLPLALQFLGDTEKDILLDVCCFFKGQEINYVTDILNGCGLRADIGITVLIKRGLIKVERNNK
LQMHPLLQHMGREIIRQVCPEEPGKRSRLWLQDDVKDVLIQNTVHPSIADLFNIRVINLKDCTSLRYLPRER

>g39589.t1

MEFASSSLSSSSSSFLTSEPHFIHDVFINFGGEDIGRRFVSHLHYALLQAQVKTFISQENLNDEGMKLEEHMRAIGHTKISII
VFSKSYTESVCCFLELEKIIECHETFGQIVLPVFYEIDPSDVRHQKNDFVKALEEITHNSYLGEQLQHALSKWSLPLSTAAGI
SVWDVRIFRDDAALVERIVSHVKTLLDYKDLSITEFPVGLESRVEDVIKLIENQSTKVCVIGIWGMVGSGKTTIAKATYNRI
YREFIGKSFIENIHYEGYLSLEEKLLSDVLKSKLEHRSVGMGITMIDNRFSRKKLLIVLDDVNESAKLENLCGSHEWFGQGT
VIIITTRDFHLLNQLRVNYVYEMDLLNENESLELFSWHAFGEAISKKEWSELARNVVVYCRGLPLALQFLGKEKDYVTEIL
NGCGLDADFGVTVLIERGLIKVERNNKLQIHPLLQDMGREIIRQVCPEEPGKRSRLWLQDDVKDVLIQNTGTEAIQGLSL
ELHSTSRDCFKTHAFKQMKKLRLLRLDHVQVVGDYGHISKELRWICWKGFPSKYIPNNFYMGNVIAIDLRHSHLQLVW
KQPLWKQPQVLERLKFLNLSHSKYLKETPDFSRLPSLEQLILKDCPSLRKLHQSIADLSNIVLINLKDCTSLSYLPRELTSISLH
HSFTISAAYHPNFQQSHIAKNLQICLASTLHCSIGVHQPYCSIQSLTSWIYNLHHTLMYQVVFRARFKLEP

>g39591.t1

MEFASSSLSSSSSSFLTSEPHFIYDVFINFGGEDIGRKFVSHLHSVLLQAQVKTFISQQNLHEGMKLEEHMRAIGHTKITIIV
FSKSYAESACCLLELEKIIESHETFGHIVLPVFYGIDPSDVRHQKDDFGKALEEIAHETYSGEQLEDALCSWSHALTTTAGIT

GWDDRDFRHDAELVERIVSRIKTLLDYKDLFITQFPVGLESRMEEVIKCIEKQSNEVCMIGIWGMGGSGKTTIAKATYNR
ICREFIGKCFFENISYAWDPENKNEWYVALQENLLSDVLKSKLEVKSVGMGRTMIQNGFSRKKLLIVFDDVNEFGQLENL
CGSREWFGQGTVIIITTRDISLLNRLEVNYIYEMHLLNENESLELLSWHAFGEAKPRKGLNELARDIVAYSGGLPLALEFLG
SYLWNRRTKEEWEKRDYVTEILNGCELHADIGITVLIERGLIKIERNNELQMHPLLRDMGREIIQECSKGKGKRSRLWLQ
DDVKDVLKKNTGTDAIQGLYLKLPSSSRDCLEARAFKEMKRLRLLQLDNVLERLKFLNLSHSKYLKETPDFSGLPRLEKLILK
DCPNLCEVHQSIGRLRNLLVINLKDCISLSYLPKEVYELRSLKILILSGCLKFPPIDIAKVKSLATIIAENTKPSAL

>g39593.t1

MKLEEHMRAIGRTKITIIIFSKRYTESTCCLLELEKVIECHQSFGQIVLPVFYEIEALDVRHHKNDFGKAMEETAHKSYSGEL
VEHALSRWSRALFTAAGITGLDARDFWHDAQFVKVIVGRVRTLLDYKDLLITQYPVGLESRVEDVIKCIENQSTKVCVIGI
WGMGGSGKTTIAKAIYNRIYREFIAKSFMENITEIWVPKNKNYVGLQRHLLFDVLKSWFDVDSIGMGRTMIEKELSQKT
LLIVLDDLNEFSQFENLCGNCEWLGQGSVIIITTRDVHLLNGLKVNYVYKIDGLNEKDSLELLCWHGLGEGKPRKDLDDL
ARKIAAYCRGLPLALKLLGSFLYDKTMDEWESVWSKLNRTPMYHIESILEAMEKDIILEVCRFYIGKERGYVTDILNAWGL
HANIGLTVLIERGLIKVDSDNKLEMDPLLQYIGKEISRERSTRGIGSIMDSK

>g39594.t1

MEFGSSSLSSSSSCFLKSEPHFIHDVFINFGGEDFGRRFVSHLHSVLLQNQVKTFISQKNLYEGMKQEEHLRAIGHTKISIIV
FSKSYTESACSLLELEKIIECHETFGQIVVPVFYEIDPLDVRHQKDDFGKALEDTTHRSYSGEQLQHAWSRWSSALNRVAG
MTGWDVRDFRHDAELVEQIVSRVKPIDLSITEYPVGLESRVEEVIKCIENQSTKVCMIGIWGMGGSGKTTIAKAIYNRIY
REFIATSFIENIREVRDPEYDLQEYLLSDVLKAKWKVNSIAMGRTMIETQLSRKRLLIVLDDVNEFGQLENLCGSRKWFG
QGTVIIITTRDFHLLNQLRVNYVYEMDLLNENESLELFSSHAFGDAKPKENFSEVSRNVVAYCGGLPLALEVLGSYLFDKT
SKRVWEGVLSILSKIPNDEVQRKLRISFDSLSDDMEKDIFLDGTQAIEGLVLKMHLTSRDCFKTDSFQKMERLRLLQLHH
VQLAGNYGYLSKQLRWISWHGFPSNCLPNSFCMDDVIAIDLKHSHLRFVWKQSQDLKWLKVLNLSHSSCSKFDKLEED
IAQMESLTTQIADNTAVKQVPVSINNTLGDLHQFLEAFQIFEVFCCEMRQSLNYLNT

>g39657.t1

MAAELVGGALLSAFLQVAFDRLASPQFVDFFRRRKLDDKLLGNLNIMLHSINALAHDAEQKQFTDPHVKAWLFSVKEA
VFDAEDLLSEIDYELTRSQVEAQSEPQTFTYKVSNFFNSTFNSFNKKIESEMKALLEKLEYLTKQKGALGLKEGTYSCDSSG
GKVSQKLPSSSLLVESIIYGRDADKEKIFNWLTSETDNHNHPSILSIVGMGGLGKTTLAQHVYNDPKIEEAKFDIKAWVCI
SDHFDVLTVTKTILEAITKSKDDSGDLEMVHGRLKEKISGRKFLLVLDDVWNERQEEWEAVRTPLSYGAPGSRILVTTRIE
KVASNMRSEVHHLKQLEEDECWKVFEKQALKDDDLRLNGEKKEIGKRIVEKCKGLPLALKTIGSLLRTKSSSSYWRSVLES
DIWELPKEVEIIPALLLSYQHLPSHLKRNNEVINIKQLEGLNLHGKLSINELQNIVNPLDALEANLKNHHLVELELRWSLNHI
PDDPRKEQKVLENLQPSKQLKSLKINNYGGTQFPSWVFDNSLSNLVSLWLADCKYCLCLPPFGLLSSLRMLHIIGFDGIVS
IGAEFYGSSPSSFKSLKVLEFYNMKEWEEWECKTTSFPLLQHLSIHKCPKLKSLPKQLLHLKNLDINKCDKLVISVNSMFISS
LQLWSIILCPLVEIPMIHYDCIEAMEINNDCVSFTIFQLDCFPKLRLLQLSGCQNLRRISQGHTHNHLKVLRIYGCRQFESF
PSEGLSAPWLQIISIRKAENLKLLPKRMQILLPSLTELEIIDCPQIEMFPGGGLPSNIKGMSLSSLKLIASLKDTLDVNTCLKSL
TIEKYGCSLLRKDMKQIVITLDSSDIVYLEKQGDKQALAKASLESIKKQIGEGISQINSAKESSNANKGSSSGFGLIIDGKSLD
YSLNKNLERSFFELAINCASVICCRSSPKQKARVTRLVKLGTGKTTLSIGDGACFRRLILELVSVVLKGCRIWWSDPILQPLD

>g39658.t1

MAAELVGGALLSAFLRDAFDRLASPQFLDFFRGRKLDEKLLGNLNIMLHSINALAHDAEQKQFTDPHVKAWLFSVKEAV
FDSEDLLAEIDYELTRSQVEAQSQPQTFTYKVSNFFNSTFNSFNKKIESEMKEVLEKLEYLAKQKGALGLKEGTYSGDRSG
GKVSQKLPSSSLMVQSVIYGRDADKEMIFNWLTSENDNHNHLSILSIVGMGGLGKTTLAQHVYNDPKMETKFDIRAW
VREEWEAIRTPLSYGAPGSRILVTTRVEKVASNMRSKVHRLKQLDEDECWKVFEEHALKGDDLELNDEKKEIGRRIVEKC

KGLPLALKTIGSLLRTKSSLSDWKIVLKSDIWDLPKEVEIIPALLLSYQHLPSHLKRIQKLPDSVGLLYNLLILKLDCCFYLKELP
SSLRKLTKLRCLEFEDTKVTKMPMHFEQVKNLYVLNMFCVGRNSEFSIKQLGGINLHERLSINELQNIENPLDALEANLK
NKHFVELKLIWNRNHIPDDPRKEKKVLENLEPSNQLEHLSIRSYCGTQFPSWVFDNSLSNLVSLELKDCKYCLYLPPLGLLS
SLKTLEIRGLDGIVSIGAEFYGSNSSSFKSLERLEFENMKEWEEWECKTTSFPRLRDLYMIQCTKLKGLSEQLVHLGWLYIE
SCPNLIIKEHSKNTSALKYMTIIRGCDSLTIFQLNFFPMLRYLVLKGCQNLQRISQEHAYNHIEMMSICECPQFESFPGEGL
SAAFPSLTELHIVNCPKVEKFPDGGLPSKVKDMSLSSLKLIASLRQILDVNTCLQSLDISYLDVECFPDEVLLPPSLTSLRIMD
CPNLQKLDCKVLYNLSSLSLGGCPNLQCLPEEDLPKSISSLHIWDCPLLQERCQKPEGKDWRKIAHIENLTELVGGALLSA
FLQAAFDKLASPQFVDFFRRRNFDEKLLGNLNIILHSINSLADDAEQKQFRDPHLKAWLFAVKEAVFDAEDILDEINYELT
KCEVEAESQSQSITYKVSNFFNATFSSFNKKTDSELKQVLEKLEYLSKQKGALGLKECAYSSVGSCSNILQKLPSTSLVVESV
IYGRDADKEIIFNWLTSETKNQNHPSILSIVGMGGLDHFDVLTVTKTILEAIDNKKDDSGNLEMVHKKLKEKLTGRKFLLV
LDDVWNEKREEWEAVRTPLSYGAPGSRILVTTRAERVASNMRSEVHRLKQLEEDECWKVFIKHALKDDDLKLNDEQKE
IGRRIVEKCKGLPLALKTIGSLLHTKSSISDWKSVLASDIWDLPKEDSEIIPALFLSYHYLPSHLKRCFAYCAIFPKDYEFVKKE
LIFLWMAENILHCPQQIRQPVEVGEEYFNDLLSRLKFDKGECIPKATRHFSFAFDDVKCFDGFGSLTEAKRLRSFIPLTKFG
KGYYLDYSWKFKISIHDLCSKMKFLRVLSFNCCSDLREVPDSVGDLKHLRSLDLSSTEIRKLPDSTCLLYNLLILKLNYCLNLE
EFPSNLHKLTNLCCLEFKRTKVTKMPMDFGELKNLQVLNTFIVDKNSEFSTKQLGGLDIHGRLSIKELQNIKNSSDALVM
DLKNKTHLVTLKFKWNKNHILDDPRKEKKVFENLQPSKQLETLGINNYGGTEFPNWVFDNSLSNLVFLQLKDCKYCLCM
PPLGLLSSLKTLKIIGLDGIVSIGDEFYGRESSSFKSLERLEFYNMKEWEEWECKTDSFPRLQTLCMNECPKMKGLSEQLLH
LKKLIIRSCEILIINEHYMDPSSLKILRIYSCPLTNVPITLYNFLEEMEINGGCDFLTTFPLDLFPKLCSLKLTRCRNLQRISQEHT
HNHLKYLIVEKCPQFESFPSIGLSAPWLQTIEIRGAENLKLLPRHMQKLLPSITNLHIIDCPQVEMFPDGGLPSNVKYVSLS
SFKLIASLRESLNANTCLERLCITNVDMEFFPGEVLLPHSLTSLQISCCPNLKKIDYNGLPKSISALNIWDCPLLQQSCQNSE
DKD

>g39660.t1

MAAELVGGALLSAFLQVAFDRLTSPQFLDFFRRRKLDDKLLGNMNIMLHSINALAHDAEQKQFTDPHVKAWLFSVKEA
VFHAEDLFGEIDYELTRSQVEAESEPQTFTYKVSNFFNSTFNSFNKKIESEMKALLEKLEYLAKQKGALGLKEGTYSCDSSG
GKVSQKLPSSSLLVESIIYGRDADKEKIFNWLTSETDNHNHPSVLSIVGMGGLGKTTLAQHVYNDPKIEEAKFDIKAWVCI
SDHFDVLTVTKTILEAITKSKDDSGDLEMVHGRLKEKISGRKFLLVLDDVWNERQEEWEAVRTPLSYGAPGSRILVTTRIE
KVASNMRSEVHHLKQLEEDECWKVFEKQALKDDDLRLNGEKKEIGKRIVEKCKGLPLALKTIGSLLRTKSSNSYWKSVLE
SDIWELPKEVEIIPALLLSYQHLPSHLKSEVHDSVGDLKHLRSIDLSHTDIQKLPNSIGLDGLVSIGAEFYGSNSSSFKSLEILK
FYNMKEWEEWECKTTSFPHLRHLVIVRCPKLKSLPEQHLPLKNLDIISCNKLAISINNMFRSSLQLLSITSCSIVHIPMTNY
DFLEEMELNSDCYSLTIFQLDFFPKLRLLRLSRCQNLRRVSQGHTHIHLKVLRINECRLFESFPSEGLSAPLLQIISIREAENLK
LLPKRMQILLLSLTELEIIDCPKVEKFPEGGLPSNVKKASLSSLKLIASLRDTLDANTCLKSLTIEKLEVESFPGEVLLPRSLTSLS
ILFCPNLKKLDYKGLRNYYRISDFLKQVTCNDGYTSLPMSSSLQVIALGIFCHVVSAKFCLKKTDRMERDLILLGATAVEDR
LQKGVPECIEKLARAKIKLWVLTGDKMETAVNIGYACSLLRKDMKQIVITLDSSDILYLEKQGDKQALAKASLESIKKHIGE
GISQINSAKESSNANKGSSSGFGLIIDRKSLDYSLNKNLERSFFELAINCASVICCRSSPKQKARVTRLVKLGTGKTILSIGDG
ANDVGMLQEADIGVGISGAEGMQV

>g39661.t1

MAAELVGGALLSAFLQVAFDKLASPQFVDFFRRRKFDEKLLANLNIMLHSINSLADDAEQKQFRDPHVKAWLFAVKEA
VFDAEDLLDEINYELTRCEVEAESQSQSITYKVSNFFNSTFSSFNKKTDSGLKDVLEKLEYLSRQKGALGLKECSYSSVGSCS
NISQKLPSTSLVVESVIYGRDADKEIIFNWLTSKTENHNQPSILSIVGMGGLGKTTLAQHVYNDPKIDNIKFDIKVWVCVS
DHFDVLTVTKTILEAIDNKKDDSGNLEMVHKKLKEKLSGRKFFLVLDDVWNEKREEWEAVRTPLTYGAPGSRIIVTTRAE
RVASNMRSEVHRLKQLEEDECWKVFIKHALKDDDLKLNDEQKEIGRRIVEKCKGLPLALKTIGSLLHTKSSISDWKSVLAS
DIWDLPKEDSEIIPALFLSYHYLPSHLKRCFAYCAIFPKDYEFVKKELIFLWMAENILHCPQQIRQPVEVGEEYFNDLLSRSF

FQQSRSKRHFVIHDLLNDLAKYVCADFCFRLKFDKGKCIPKATRHFSFAFDDVKCFDGFGSLTEAKRLRSFIPLTKFGKGYY
LDNSWNFKISIHDLCSKMKFLRVLSFNCCSDLREVPDSVGDLKHLRSLDLSSTEIRKLPDSTCLLYNLLILKLNYCLNLKEFPS
NLHKLTNLCCLEFKRTKVTKMPMDFGELKNLQVLNTFIVDKNSEFSTKQLGGLDLHGRLSIKELQNIKNSSDALVMDLKN
KTHLVTVKFKWNKNHILDDPRKEKKVFENLQPSKQLETLGINNYGGTEFPNWVFDNSLSNLVFLQLKDCKYCLCLPPLGL
LSSLKTLKIIGLDGIVSIGDEFYGSESSSFKSLERLEFYNMKEWEEWECKTDSFPLLQTLSMNECPKMKGLSEQLLHLKKLII
RSCEILIINEHYMDPSSLKILRIYSCPLTNVPITLYNFLEEMEINGGCDFLTTFPLDLFPKLCSLKLTRCRNLQRISQEHTHNHL
KYLIVEKCPQFESFPSIGLSAPWLQTIEIRGAENLKLLPRHMQILLPSLTNLHIIDCPQVEMFPNGGLPSNVKYVSLSSFKLIA
SLRETLDANTCLEWLYITNGDMEFFPGEVLLPHSLTSLQISCCPNLKKIDYKALPQSISTLNIWDCPLLQQCCQNSEDKD

>g39662.t1

MAAELVGGALLSAFLQVAFDRLASPQFVDFFRGRKLDEKLLGNLNIMLHSMNALAHDAEQKQFTDPHVKAWLFSVKE
AVFDAEDLLAEIDYELTRCQVEAESEPQTFTHKVSNFFNSSFNSFNKKIESEMKELLEKLEYLSKQKGALGLKEGTYSGDRS
GDKMSQKLPSSSLMVESVIYGRDADKEMIFNWLTSETNNQNLSILSIVGMGGLGKTTLAQHVYNDQKIETKFDIRAWV
CVSDHFDILAVTKTILEAITKSKDDSGNLEMVHGRLKEKVSGKKFLLILDDVWSERREEWEAVQTPLSYGAPGSRILVTTRI
EKVASNMRSKVHRLKQLEEDECWKVFEDQALKGDDIELNDEKKEIGRRIVEKCKGLPLALKTIGSLLCTKSSLSDWKSVLK
SDIWDLPKEVEIIPALLLSYQHLPSNLKRCFAYCALFPKNYEFDKDELILLWMAEGFLHYSPQNNNLEEIGEQYFDDLLMR
SFFLRSNIKMHFSMHDLLNDLAKYVSAEFCFRLNFDKGDCVPKTTRHFSYALSDLNSCDVKYFDGLGSLRDAKRLRSFLPI
VSNYGDGLFSRFEILIREFFKLKFLRVLSLNGYHHFKEVPDSVGDLKHLHSLDLSYTWIQKLPDSVGLLYNLLILKLNGCFYL
KELPSSLHNLTKLRCLEFEDTEVTKMPMHFKQVKNLHVLNMFCVSRNSEFSIKQLGGINLHKRLSINELQNIENPLDALEA
NLKKKQLVRLKLIWNLNHIPDDPMKEKKVLENLQPSNQLEHLSIRSYCGTQFPSWVFDNSLSNLVSLELEDCKYCLYLPPL
GLLSSLKSLEIKGLDGIVSIGAEFYGSNSSSFKSLEILRFFYMKEWEEWECKTTSFPCLRRLFVVQCPKLKGLSEQLLHLEQLY
IESCPNLIISEHIEDTLTLELLTIIRGCYSLTIFQLNFFPMLRFLHLQGCQNLQRISQHPHNHLETMSICACPQFESFPGEGLS
VAFPSLTELRIINCPKVEKFPDVGLPSNVKHLSLSSLKLITSLREILNVNTCLEMLYIDSLDVECFPDEVLLPPSLTSLRISNCR
NLKKLDYKLLYNLSSLTLSECRNLQFLPEEGLPKSISFLDIWDCPLLEQRCQKPEGKDWRKIAHIENLGVGS

>g39663.t1

MAAELVGGALLSAFLQVAFDRLASPQFVDFFRRRKLDDKLLGNLNIMLHSINALAHDAEQKQFTDPHVKAWLFSVKEA
VFHAEDLLVEIDYELTRSQVEAQSEPQTFTYKVSNFFNSTFNSFNKKIESEMKEILEKLEYLAKQKGALGLKEGNYSGDRS
GGKVSQKLPSSSLVVESVIYGRDADKEMIFNWLTSENENHNHLSILSIVGMGGLVTKTILEAITKSKDDSEDLEMVHGRL
KEKLSGKKFLLILDDVWSERREEWEAVRTPLSYGAPGSRILVTTRVEKVAHNMRSRVHRLKQLEEDEGWTVFEEQALKG
DDVELNVEKMEIGRRIVEKCKGLPLALKTIGSLLRTKSSILDWKSVLESDMWDLQKEVEIMPALLLSYQHLPSHLKICFAYY
FCFRLKFDKGNCIPKTIRHFSFAFRDVGYFDGFGSLTDAKRLRSFVQITNNTVYSAFPYQIEILIRELFSKFKFLRVLSLTGIYG
LKELEDCKYCLCLPPLELLSSLKTLCIIGLDGIVSIGAEFYGSNSSSFKSLERLEFYDMNEWEEWECKTTSFPRLLYLFIIQCRK
LKGLSEQLLHLEKLHIDSCPNLIISEHNEDISGLERLRTRSCPLVNIPMTHYDFIEDMKIIRGCDSLTIFHLNFFPMLRFLDLV
GCKNLQRISQQHHHNHLEVMSIRECLQFESFPGEGLSAAFPSLIELRIIDCPKVEKFPDGGLPSKVKYMCLSSLKLIASLRE
NLDVNTCLQSLTIENLEVESFPDEVLLPPSLTSLSINHCPNLKKLDYKVLVNLSSLTLIDCGNLQFLPEEGLPKSISSLYTFGCP
LLEQRCQGKK

>g39664.t1

MAAELVGGALLSAFLQVAFDRLSSPQFVDFFRGRKLDDKLLGNLNIMLHSINALAHDAEQKQFTDPHVKAWLFSVKEA
VFDAEDLLGEIDYELTRSQVEAESEPQTFTYKVSNFFNSTFNSFNKKIESEMRELLEKLEYLAKQKGALGLKEGTYSGDRSG
SKVSQKLPSSSLVVQSVVFGRDVDKEMIFNWLSETDNHNHLSILSIVGMGGLGKTTLAQHVYNDPKMEEAKFDIRAWV
CVSDHFDILTVTKTILEAITKSKDDSGDLEMVHGRLKEKISGKKFLLVLDDVWSERREEWEAVRTPLSYGAPGSRILVTTR
VEKVASNMRSKVHRLKQLEKDEGWKVFEEQALKDDDLELNDDKKEIGRRIVEKCKGLPLALKTIGSLLHTKSSISDWKSV

LESDIWDLPKEVEIMPALLLSYQHLPSHLKRCFAYCALFPKDYEFDKKELSLLWMAEGFLHYSPQNNNLEEIGEQYFDDLL
TRSFFLRSNIKMHFSMHDLLNDLANYVYAEFCFRFKFDKGNCIPKTTRHFSFASCDARYLYGFGSLTDAKRLRSIVPINYFY
SAWKFNISIHDLFSKLKFLRVLSLNRYCDLKEIPDSVGDLKHLHSLDLSYTWIQKLPDSVGLLYNLLILKLNGCFYLKELPSSL
HNLTKLRCLEFEDTEVTKMPMHFGELKNLQVLSTFCVNKNTEVINTKQLGGINLHGRLSINELQNIVNPLDALEANLKNK
QLVELNLIWNLNHIPDDPRKEKKVLENLQPSNQLEHLSIRSYCGTQFPSWIFDNSLSNLVSLELKDCKYCLCLPPLGLLSSL
KTLKIRGFDGIVSIGAEFYGSNSSSFKSLEILKFYNMREWEEWECKTTSFPLLKYLSIKTCPKLKSLPEQLLHLKELNIKSCDN
LIISEHSVDTSSLELSTTSLCPPLNIFMTYYDFIEDLRIGGGCDSLKIFELDFFPMLSLLVLSGCQNLQRISQEHTHNNLKVISII
ECPQFESFPGEGLSVAFPSLTALEIIDCPKVKKFPDVGLPSNVEHVSLSSLKLITSLRETLDVNTCLQRLSIVSLDVECFPDEV
LLPPSLTSLTIDRCRNLKRLNYKLLHNLSSLSLYHCPNLQCLPEEGLPKSISYLEIWDCPLLEQRCQEPEGKDWRKIAHIEKLR
FF

>g39665.t1

MAAELVGGALLSAFLQVAFDRLASPQFVDFFRGRKLDDKLLRNLNIMLHSVNALADDAEQKQFTDPHVKSWLFSVKEA
VFDAEDLLDEIDYEITRCQVEAESEPQTIIYKVSNFFNATFRSFNRKVDSGLKEVLEKLEYLTGQKGALGLKEGTYSGDRSG
STISQKLPSTSLVAESVIYGRDTDKEMIINWLTSETDICDQPSILSIVGMGGLGKTTLVQHVYNDPKMDDAKFDSKAWVC
VSDHFNALTVAKTILEAITDEKDESGNLEMVHKKLKEKLKGKKFLLILDDIWNQRRDEWEAVQTPLSYAAPGSKILVTTR
DEKVASNMQSKVHRLKQLREDECWKVFEKHASKDYNIELNDELKEIGSRIVDKCKGLPLALKTIGCLLRTKSSISDWKSVL
VSDIWDLPNEDNEIIPALFLSYHHLPSHLKRCFAYCALFPKDYEFVKEELILLWMAESFLQCSQIRHPEEVGEQYFNDLLSR
SFFQQSTTEKRFVMHDLLNDLAKYVCGDICFRLKFDKGKYIPKTTRHFSFEFDHVKCCDGFGSLTDAKRLRSFLPITEIERT
YLGYYPWQFKISVYDLFSKFKFLRILSFYNCLGLTKLPDSIGDLKHLRSLDFSHTAIQKLPDSTCLLYNLLVLRLNHCLRLEELP
SNLHKLTKLRCLEFKDTKVTKMPIHFGELKNLQVLNMFFVDKNNEFSTKQLGRLRLHGRLSINEVQNITNPLDALEANLK
NQHLVELELKWNSKHILNDPKKEKKILENLQPPKQLEGLGISNYGSTHFPSWLFNNSLTNLVFLRLEDCKYCIFLPPLGLLS
SLKTLEIVGLDGIVSIGDEFYGSNASSFMSLERLEFYDMKELREWKCKSTSFPRLQHLSMDHCPELKVLSEHLLHLKKLVIG
YCDKLIISRNNMDTSSLELLKICSCPLTNIPMTHYDFLEEMEIDGGCDFLTTFSLDFFPNLRSLQLTRCRNLQRFSHEHTHN
HLKYFIIEKCPLVESFFSEGLSAPLLQRIEIRGAENLRLLPKRMEILLPSLIELLIIDCPKVETFPEGGLPSNVKHASLSSLKLIASL
RESLDANTCLESLSIGKLDVESFPDEVLLPHSLTSLQIFDCPNLEKMEYKGLCDLSSLTLLHCPGLQCLPEEGLPKAISSLTIW
DCPLLKQRCQNPEGEDWGKIGHIEKLIIR

>g39666.t1

MPGGSSKRRIHFSKLYSFSCLKSPFRDGHSQIGQKGYSRVVYCNDPDNPEAVQLSYGGNYVSTTKYTAFNFIPKSLFEQF
RRVANIYFLIVACVSFSPLAPFTALSIVAPLLVVIGATMAKEAVEDWRRRKQDVEANNRKVQVYGRNYTFTETRWKKLR
VGDIIKVYKDEYFPADLLLLSSSYGDGVCYVETMNLDGETNLKLKHALEVTVHLHDEKSLQKFRAMVKCEDPNENLYSFI
GTLQHDGKEYPLSLQQILLRDSKLKNTDFIYGIVIFTGHDTKVMQNSTDPPSKRSKIERKMDKIIYILFSTLVLISFIGSVFFGI
ETKKDISGGRYTRWYLRPDNSTVFYDPRRATLAAVLHFLTAIMLYGYLIPISLYVSIEIVKVLQCIFINQDQEMYHEESDRP
AHARTSNLNEELGQVDTILSDKTGTLTCNSMEFVKCSIGGIAYGRGMTEVEKALARRGKGGESDDVDSGSSDFLGQNN
ESVDSLHPVKGFNFSDERLVNGQWVNEPYPDFIQKFFRVLAICHTAIPDKDKESGEISYEAESPDEAAFVIAARELGFEFF
ARTQTSISLHELNYESGKKVDSRVYQLLHVLEFSSSRKRMSVIVRNEENQILLLCKGADSVMFERLSQHGRQFEVETRDHI
KRYAEAGLRTLVVTYRELDEEEYKLWDKEFSKVKTSVTEDQDALVDAAADRMERDLILLGATAVEDRLQKGVPECIEKLA
RAKIKLWVLTGDKMETAVNIGYACSLLRKDMKQIVITLDSSDIIYLEKQGDKQALAKASLESIKKQIGEGISQINSAKESSN
ANKGSSSGFGLIIDGKSLDYSLNKNLERSFFELAINCASVICCRSSPKQKARVTRLVKLGTGKTTLSIGDGANDVGMLQEA
DIGVGISGAEGMQAVMASDFAIAQFRFLERLLLVHGHWCYRRISMMAYASFSGQAAYNDWYMSFYNVFFTSLPVIAL
GVFDQDVSAKLCLKYPFLYLEGVEDTLFSWPRILGWMLNGVLSSLVIFFLTTNSVLNQAFRRDGKVVDFEILGVTIIAFWY
VFVLVYGYLSPAISTTSYMVFVEACAPSGPRKTNTPQREIEAKGVMKXLVGGALLSAFLQVAFDKLASPQFLEFFRGRKL
DDKLLGNLNIMLHSINALAHDAEQKQFTDPHVKAWLFSVKEALFDAEDLLVEIDYELTRSQVEAESEPHTFTYKVSNFFN

STFNSFNKKIESEMKEVLEKLEYLAKQKGALGLKEGTYSGDRSGIKVSQKLPSSSLAVESVIYGRDADKEMIFNWLTSDTD
NHNHLSILSIVGMGGSGKTTLAQHVYNDQRMETKFDIRAWVCVSDHFDVFTVTKTILEAITKSKDDSGDLEMVHGRLK
GKVSGKKFLLVLDDVWNEKLEEWEAVRTPLSYGAPGSRILVTTRIEKVASNMRSEVHHLKQLEENECWKVFNKQALKD
DDLELNDEKKEIGRRIVEKCKGLPLALKTIGSLLRTKSSILDWKSVLESHIWDLSKEVELMPALLLSYQHLPSHLKRCFAYW
YYGLKEVSDSVGNLKHLHSLDLSSTRIQKLPDSVGVTKMPMHFAELKNLHVLNMFCVGRNSEFSTKELGGINLHGRLSIN
ELQNIVNPSDALEANLKNKHLVELNLIWNSNHIPDDPRKEKKVLENLQPSNQLERFSIRSYCGTQFPSWVFDNSLSNLVS
LELKDCKYILCLPPLGLLSSLKTLEITALNGIVSIGVEFYGNNTSSFKSLERLLFFDMKEWEEWECKTTSFPRLEELVIHDCPK
LKGLSEQLLHLKALSIETCDNLIIREHNVDTSALQWLKTHSCPLVNIPMTHYDFIQDMTIVGGCDSLTIFQLDFFPMLSLLV
LSGCQNLQKISQEHAHNHLKELSIYECPQFESFTNEGLSAPLVQIIDIRQAENLKLLPKRMQILLPSLTELEIINCPKVEKFPD
GGLPSNVKHLSLSSLKLIAFLRENLDVNTCLESLSIDSLDVESFPDDVLLPPSLTSLKIIRCQNLKKLDYKLLYHLSSLELGDCP
NLQCLPEEGLPKSISSLQIWSCPLLEQRCQKPEGKD

>g39702.t1

MRIHVHAGPTFVFHPSSFSSIQLNSLYFNHNHNLSFDSSTSSLSDPMPPESHVTISSYRLCWDVFLSFRGTHTGHTFTMR
LYHALHGRGVRVFRNDDGLERRAEIQKKLLEAVEDSAAAVVVISPDYASSHWCLEELAKICEVGRLILPVFYWVNPSHVR
KQEGPFEEWFVWHAQRFPKERVEQWRNAMKKVGGLAGFVLDEKSDKSDELIQILVQNLMKQLRNTPLSVAPFTVGV
DDRVEVLKNLLDLKSNDVRVLGLYGMGGVGKTTLAKSLFNNLVVHNFERRSFIPNVRSQVSKHHGLVSLQNKIRGDLCG
RKEDLINDISDGISAIQKIVQENRVLLILDDVDDVEQLNFLMGKREWFYKGSRVVITTRDKEILHGSYVDVDFEVKELEFSE
AMELFCFHAIRRKEPAEGFLDVSKQIVEKTGGLPLALEVFGSFLFDKRTEREWKDALEKLKQIRPPCLQEVLKISFDALDEQ
EQCIFLDIACLFVQMEMKRDDVVDILNGCDFRGEIAVAVLSARCLIKIIGDGKVWMHDQVRDMGRQIVRSESLTDPGL
RSRLWDRDEILTVLKNMKGTRNVQGIVLDCVKRRMSIPRDRSADEITRENFRRKPSCKSAFEYIKERLEGQFICLPPKIKW
LQWKQCPLSYMPSSYNPLELAVMDLSESLIETLWKGRSNKVAVHLMVLNLSRCHRLTGTPDLSGYLSLKKLNLEECSHLT
RIHESLGNLNSLVHLNLRLCYNLIELPGDVSGLKHLEDLVLSDCWKLKALPKDLSCMVSLRRLLLDSTSITELPVSIFHLTKLE
KLSANGCHLLKRLPTCTGKLCSLQELSLNHTALQELPDSVGSLEKLEMLSLMGCKSLSVIPNSTGKLISLTRLYLDGSGIKEL
PASIGALSYLRKLSVGDCTSLYKFPVSMEALVSIVELKLDGTKVSNFPEEIFVGMKMLEKLEMGKVQHLKFVPVSFGCLSA
LTILDMHDANITELPESIGMLENLIRLRLDKCKQLQRLPDSIGNLKSLRWLMMKETALTRLPESFGMLRSLVELDMKRLP
YLNGAGNNVSTGTIIPEIREQPSSEAILTSFCNLSLLEKLNAHGWGIYGKIPDEFEKLSSLETLSLGHNNICSLPASMTGLSYL
KKLLLSDCRELMFVPPLPSSLEELNLENCVAVQYIHDISNLERLEEFNLTNCEKVVDVPGLEHLKSLRRLYMSGCIGCSLAV
KRRFSKNIAENQREGLELVDVQGKIFNLTSEVFSTTIRLLRVPRRNEDHIFLRRFGARTPLVFQLKDRYTLHLQRRNPPRIE
GLELNNCRIHLVFYGDDDYEGDEGSLEESQYSVSQKLAKFFNFDADDPDV

>g39784.t1

MAAEMVTGVLVSTFLERTIDTLASRLVDIFHQRKYKKQLSNLKMKLLAIDVVAFEAEQKQFTDPRVRDWLLRAKDVVID
AEDLLDEIDYELSKTQVEAESLSDAKKVWNSLNSSFVSFFENEIESMMEQAIEDLEDLATQSDFLGLKKGGGVGVGSGSG
SKLTHTSLPNESVIYGRDDDKEFVLNWLTSDTHKNLSILSIVGMGGMGKTSLAQHVFNDPRLEEANFDTKVWVSVPQE
FDVLKVSRAILDTITGSTDHSIQQEVIQKKLKEKLMGKKFLLVLDDVWNERPSKWEDVQKPLVFGGQGSRILVTTRSEKV
AVTMRSEKRLLQLLKKDYCWDLFAKHAFQIANPQPDSDFIEIGKKIVEKCNGLPLALKTMGSLLYNKSSLSEWQSIMKSEI
WDFSEDESGILPALRLSYFHLPSHLKTCFAFCALFPKGYWFDKEWLIQLWMAQNFLENPLQKKSPKEVGEEYCNDLLSW
SFFQQQSIFNGEKGFIIHDLLNDLAKYVCEDICIRLGVDEPKGIPKTTRHCSFSDSGFDGFGSSIDTQKLHTFTSRDEEEFIIH
DLLNDLAKYVCEDICIRLGVDEPKGIPKTTRHCSFSDSGFDGFGSSIDTQKLHTFIPTEWFWGWDCKMSIDDLFSRFKLIR
VLSLSHCHSLTEVPECVGNLKHLRSLDLSKTQIEKLPDSISLLYKLQILQLNYCENLKELPSCLYQLDNLRRLDLEGSGVQNV
AAHLGKLKNLQVTMSSFHVEKSKENNFQQLGELDLHGSLTIDDLQNIENPSYALEVDLKNKPHLVELRLVWNFSGSSSV
DSEKAEDVIENLRPSKYLKKLSIRNYIGKQFPDWLLHNSLPNLVSLELEGCESCQRLPPLGLLPFLNYLKISGFDETVSIDADF
HGNNSSSFKSLKTLYFSNMRQWEKWDCQAVTGAFPCLQNFSIKNCPKLKGHLPKFVALKTLRVVDCEQLEALISLRVEE

LSIYSPLESISDDCVSLRIFPLDFFPTLRTLELSGFPNLQMISQNHVHNHLHYMKIKECPKLESLPANMHMLLPSLRYLTIKE
CPRLEWFSEGGLPLNLKHIRLKNCFRLVGSLKRALGDSPSLESLTIEKVEAECFPDEGLLPLSLTRLIIEDCRNLKKLNYKGLL
ELSSLEELKLWKCPNLECLPEEGLPKSISFLYISNCPLLEQRCQKEGGEDWEKISHIPQVFIRK

>g39789.t1

MSSSSSSQPQWIYDVFINFRGGDTRRDFVSHLYCALSNAGVNTFFDDENLLKGTPLEELTRAIEASQIAIVVFSETYTEST
WCLTELQKIIDCNESYGQIVVPIFHGVEPSILRNPKGRFREALEAAAKKKFSEEHREYGLSRWKNVLKKAANFSGWDVKN
HRNTAKLVKDIVDDILTKLDYALLSITEFPVGLESRVQEVVGIIENQSAKVCTIGIWGMGGSGKTTMAKAIYNQIHRRFND
KSFIENIREVSETYGRGHVHLQEKLLSDVLKTKVKIHSVGMGTTVIENRLSKKKVFIIFDDVNDFGQLKDLCGNRKWFGKE
SVIIITTRDLHLLDLLKVDYVYKMEEMDKNESLELFSWHAFREAKPREDFDELARNVVAYCAGLPLALEVLGSYLIERTKKD
WESVLLKLEKIPNDQVQEKLRISFDGLCDDMEKDIFLDVCCFFIGKDKADVIEILNGCGLHADIGITVLIERSLLKVEKDNKL
GMHQLLRDMGREIICESSRKDPGKRSRLWLHEDVLDVLTKNTGTETIEGLALKLHFIGKDCFKAYAFEEMKKLRLLQLDH
VQLTGNYGYLSKQLSNLQLFWKEPQILRWLKILNLSHSKYLTVTPDFSKLPNLEKLILKDCPRLCKVHESIGGLRNLLLINLE
DCKSLGNLPRGVYKLKSVKTLILSGCLKIDKLEEDIVQMESLQTLIAENTAMKQVPFSIVKTKSIVYISLCGFEGFSRNVFPSII
RSWMSPTMNPLSYVHPFCSTSSYLVSMDMQSYNLGDLEPMLSSLSNLRSVLVQCDTESQVSMQIRALLGNSVNFTQV
EIASQISKHYLRSYLIGIGNYQEVFNILSASISEGLASSESCDAFQPGDNDPFWLAHTGEGNSVYFTVPEDRRMKGMTLC
VVYLSAPEITATEYLISVLMVNYTRYTIQVFKRETVFSFNDVDWQGIISHSGPGDKVEIFVNFGHGLEVKKTVIYLMCDESI
DKEVKPSPEPKKEQKKNPKSVWDTYTYSWGEEGYLCYIDILEIFINNIPQSSLPVVLASWFLKAFSCSALKQNDVVTGVEK
MAYSLKLKLFTYVGWNVMIVHSLLLMAVCSVQVMDFVFQLAD

>g39792.t1

MSTRLHPKLAPHYYGPFLVLKQVGPVAFRLSLPDATRIHLVFHVSQLKTTIGSHLVEKELLAEVHDPKSSCFPSKVLGYRV
QQFHVISVLQVLIQWNTCRLKGPTWEDEDTIQDKFPKFHHLADKVARNAEGDNDPYWLAHIGEEHSVSFTVPQDRV
MKGIVLYVVYLSIPGIVTTECFTSVLIVNYTKCTLLMHNHGTVISSNDEDWHGIMSNLGSGDKVEIFVTFGHGLVIKNTAV
YLIYCELEDLERESALNKNSLITFIKKIFFTSSIPSMEFASSSSKLPRYYDVLINFNGEDILTKFVSHLDSVLSSVGLTTFLHHQN
AVESTHVQQPILDLCRVVIVVFTKTYSESAWCLHQLQQIIQWHQTYCRHVLPVYYEIQPSDVRFQKGDFGKAFKETAHQ
TFSAQQLQHGMSRWSHALTKAANLFGWDESNHRSDAELVHEIVKSVRNSSVFSATKFPVGLQYPVEELIQTIKDKSMD
VCTIGICGMRGSGKSTLAKAIYHQIHGTFMHKSFIEGIAQFSEPRGGINFQEQLLSDVIKTKDRDYATKILSSSVVEADSET
GVLHDFRVDADSGIRVLMERNLIKVKRNNKFEMHPLLQEMGIAISREISGEKRWKNGQLLFDVDAEYALKVNAGRKDY
EVLLSTRREPSRLPKDGVNSENLYPKLRWISFRGFSIEYLPNYFNAHDTIAIDLKHSLLRFFLEEPQVFKSLKVLDLSHSMYL
THTPDFSRLPNLEELILKDCPRLQKIDPIENDIVQMKSLITLVAENTTVKKVPFSIVLASSECSDVFLPGDNRPYWLAYMGE
GDSVSFTVPPDNDIKGMMEEAKEWRPKRKTVPSYSKDQKGEDTRRNFVSHLYSALKDTGVNAFLDDEKLDKGEELKSE
LLHAIEGSQITIIVFSQNYIHSTWCLDELLKIMECHAFRGQVIMPVFYDIGPSFLRDTEDISFEVISDQQSRIKLWKKALTQA
ANLAGWDLRNYKLSRSASATNTAAERILRNENDLMKEIVCKVLKRLDRTYLSITNFPVGLECHHMEKDIFLDICCFFIGKD
RGYVTEILNGCGLHADIGIPVLVERSLIKVEKNNKLGIHDLLRDMGREIVRQSSPLPQKRSRLWVHDHVLDILTEHTGTEV
IEGLALKLHRPGRVHFSAETFENMKRLRILQFDHVQLAGDYGHISKHLTWVYWRGFSLKYIPENFYQGNVVAIDMKHS
NLKLVWKEPLQQAGKAKVHQS

>g39793.t1

MASSNPSIEFASSSSKLPRMYDVLINFTGDDIRIKFVSHLDSALSNVGFTTFLHHHNAVKPMHIQQQPILDLCRVAIVVFT
KTYSQSAWCLHELQRIIKWHETYCRHVLPVYYEISPSDVRFQKGDFGKAFEATARQTFTSQQLEHGISKWSHALTKAAN
FFGWDESNSRSDAELVDKIVKTVLNLSVLSATKFPVGLQPKVEELIRIIKNKSTEVCTIGICGVVGSGKTTLAKAIYHQIHGT
FTEKSFIEVTLEGKGIRSCVSLQEQLLSDVLKTKVKIPGDEMGRSMIRERLNGKRVLIVLDDVNQYGPLDLWESSSCFGED
KAYVTKILKGCGVDIDCGIRVLIERNLIKLTRNNKFGMHPLLQEMGREIVARGGHARYALGETDVFTFKRTNIIRRCPEKLF

LTRKRCIEPYPLRIRHKSRLLKLAGNPGYLRNSSEFLLYDCYLHDAISIDLRHSVLRLDWKKLQCDPEFPLSKQVKTILVEYG
MDITESRISKHCLRSSLVGVGRYNEFLNTDKEVKPEVTG

>g39794.t1

MEFASSSSKLPRYYDVLINFNGEDIRRKFVSHLDSVLSSVGLTTFLHHQNAVESTHLQQPILDLCRVVIVVFTKTYSESAW
CLHQLQQIIQWHQTYCRHVLPVYYEIQPSDVRLQKGDFGKAFKATAHQTFSAKQLQHGMSRWSHALTKAANFFGWN
GSNSRSDAELVDQIVKSVLNSSVLSATKFPVGLQYKVEKLIQTIKDKSMDVCIIGICGMEGSGKTTLAKVIYHQIHGTFLHK
SFIEDIAQVSESRGRVHLQEQLLSDVLNTKVKIDSLEMGRNMIRDRLFGKRVLIVLDDMDNYIPLLDLSKIRSWLSEGTVII
MTTSYKELLNKYQIHPYFVVNPMKENESLELLSWHAFREAKPNEEYDYLARRVISYCGGLPLVLEVIGSTLFERTEEEWH
NVLFELEKIPIIDVLPKLQISLNRLRNQMEMDLFLDVCCFFVGKDRVYATKILNGSRVEADSDTSVLHGFRVDAENGIRVL
MDRNLIKVKSNNKFGIHPVLQEIGLAIFRKISRKKGWKNGQLLFDVDTEYALEVNDESKDYEVLPVLLSTRREPSRLPKDA
VNSENLPLKLRWISFHGFSIQDLPNVFNAHDAIAIELKHSLLQFFWEEPQVLKSLKVLNLSHSLYLTRTPDFSRLPNLEELIL
KDCPRLREVHQSIGHLSYLILLNLKDCKCLSNLPQEHLRSSLIGVGAYHEFFHAVSDNISEVLASSVSSDISLPGDNQPYWL
GYMESLYLWN

>g39795.t1

MTSPIPSMEFASSTTELPRKYDVLINFNGEDIGKKFVSHLDSVLSAVGLTTFLHHDNGVKSMNEPILNLYRVAIVVFTKTY
SESAWCLHQLQQIIQWHQTYSRHVLPVYYEIQPFDVRFQKGDFGKAFKRAAHQTFSRQELEHGMSKWSNALTKAANF
FGWDESNYRSDAELVDKIVKSVLNLPVLFATKFPVGLQSNVEDLTQTIKDKSSEVCIIGITGAGGSGKTTLAKAIYNKIHET
FTEKSFIEDIGQVLRTRGHHRLQEQLLLDVLKAKVKITSVDMGTRMIRERLTGKRVLIVLDDVTENFTLLDLWGCREWFG
GGTVIIITTRDVDLPRILKVDSVFGIRLMNENESLELLSWHAFREAKPKEEYNYLAKGVVTHCGGLPLTLEGRAYVTKMLN
GWGVDADSGIRVLIECSLIKVKRNSKLGMHPLLQEMGREIINEICEEEFETEWLLRFDDAQYVLTDNTGRRAIERVPVKL
RSVRREPSRILKHTENSDYISKKLRWISLDWFSSEYLPDKFYLHDAMTIHLKHSSLRFLLKESQELSWLKVLNLSHSRYLTGT
PDFSGLPSLEQLILKGCTGLREVHPSIGCLCNLTLLNLKDCTSLSNLPREIYKLISLETLILSGCSKIDLLEKDVVRMESLITLIAE
NTIVKHVPFSIGSSKSIGHISLQGFERLSCNIFPSIIRSWMSPVMNPISYIHSLCMDIDNSWENIGPLLSSLENLRSVLVQCD
TEYQLSKQVNSILVEYFANFAESGISKQQFRSSFIGLGTYHDEFFNAVCDNISKVLL

>g39820.t1

MVEEIIGDELWKTSFENVSYQECPSIDATFSNNGYESFSSRTKTLEMIMEALQDSTVGMIGVYGPGGVGKTTLVKEIARK
AQEKKLFKIVIIANITGNPDFKKIQEQIAGMLGMKLEEESEIARLDRIRKRLKHEKEKALIILDDLWGRLDFNKLGIPCNDDA
SQREANDIFDFGSNNDISDFGYNKTEMKVLSKVDLDKMKKEKLSNDYRGGKILLISRNKQVLCNEMDVQQRSIFSVGVL
DEKESETLLKKVASVKNSEFDRNATEIAKWSAGFPIALVSIGRTLKNKSLSTWEDVCQQIKRQNFTSEWGFTDFSIKLSYD
HLKNEELKCIFLHCTRMGNDALIMDLVKFCVGLNLLPGVHTITNARKRVKEMIKELEESSLLVKSYSIDRFNMHDIVRDVA
LSISSKEKNVLYLKNVILDEWPHENDFERLNSYKIVIGEFNLFNLLKVGEFKVPDKYEEMKFLALNLKEVCDCDSLKEIVSKE
SQTYTIGDGRIEFAQVTNSCLPLFNEKVSTPKLEWLELSSINIHKIWSDQXSHTLEWPSLKKLDIADCSMLEGFTLEIINSQ
DQPITLATKKVSFEYSKHARLVDYPERKDVRLGKSAFPDNFFGCLKKLEFGEACTRNILIPSHVLPYLKILEELNVENCKPTQ
VIFDIDESELKMKGMIFRLKKLNLNKLSNLKCVWKENSGGIVSFPNLHRVDVDGCGNLVTLFPLSLARNIKKLETLQITECE
KLVEIVGKEVMEHGTTLFTSEFDDNQKATIIEAPVIPIRPLFSVEILASPKLKKLSLNEENVMLLRDAHLPKDLFCNLNVLRL
YFEDDNNQKDSLPFDFFHRLPSLTCLIVQKFFGLREIFPYQKLQVHDEVKILLLLKLKELESIGLEHTWVQPYSEKLELLRIHS
CPRLQKIVSCAVSFINLKELQVTFCDQMEYLFTFATVKSLVKLECLIVKYCESIKEIAKSEDGDDCDEILVFGRLRSIELDSLPR
LLRFYSGNATLRCLCLKTVMVANCPNMITFSLGTINVPLFSGINVSSKDSDLTFDGDLNTTIETLFHEQEFFNLSKHMILDE
YLEMTGVQHIKPAISDNFFGSFKELEFDAVCKRAIVIPFHVLPYLKNLEKLNVHSSDAVKVIFDIDEIEDKTKGIVSSLRKLTL
NNLSNLKHVWKENSTGIISFHNLQEVVVNGCGSLITLFSSSLARNLRKLKTLEITECGKLVEIVEKEDGMERGTKIIGRGGD
HDGTGRRRISLATRQHARREAANLVTVATARQGAWMVRSPRSLHHGSRLAETIRMVWSSVDSGTLAAATAAVTTAT

MEYYCMLASVFAFKCCMKFWVVNVLFMYVLASTALLRSSVLFRSASTKKRGRTSPLNGGYLALNVIPVKPYSVIFAYALL
NSGVNLKQLDVESCEEMKNLFTFSTAKSLVQLEILTVLNCESMKEIVKNEDEDAYKEIILGRLKELKLNFLSKLVSFYSGNA
MLQLPCLSTVTIVKCPKMKKFSEGGLNVPMFSGIKTSLMDSDFHFHNDFNSTVQWLHQHVSDEHSKHLTLADDSKLEK
ILHSKAAFHDNNFSSLRSLVVNNVTKDHLISSQVLLCLKSLEELECVWNKNPRGIVSFPNLEEVFVSDCGELEALFPSSLAR
NLVKLEELDIENCEKLPRLSCFYPGKHHLGCPLLETLDVSYCPKLKLFTSEFHDSHKESVIEIQVSSTITISRLQHPLFLIEKVVP
KLKELTVNEESIILLSHANLPQDLFRKLNLLLLCQEDEDEDNRKDTLPFDFLLKTVKGWSIYSHFPLPKAWCNLWTYVEGD
SVMESTDRGSTDGDN

>g39822.t1

MDPSIIVSTATESVFKFGENLVTRHLGYFCNYNGKFEEVKHRIEMLDDTRKRVQNDVMVAEMNAEEIEEDVKHWLKH
VDEKIKEYENFLCDKRHEKTRGSIGFFPNNLQLRYRLGRKATKIVEEIIADELLNKKFSKVSYHIGPSMDAALSNTGYESFTS
RKKIMGMIMQALEDSTISMIGVYGVGGVGKTTLVKEIAKQAKERKLFNKVVMANITRNPDIKKVQGQIAEMLGMRLEE
ESEIVRADRIRKRLKKEKENTLIILDDLWNGLDLNTLGIQRNEDDGVSQKVAKDVAEFGVLDQKEGEALLKKMAEISVTN
SAFDDKVTEISKMCAGLPIALISIGKTLKNKSHYVWEDVCRQIERQNFTGGQEPIEFSARLSYDHLKTEELKHIFLQCARM
GSDFSIMDLVKFCIGLDMLQGVFTIRETKSRVNVLMEELTESSLLMKSYSNDCFNMHDIVRDVALSISSKEKNVFFMKN
GKLNEWPHKDKLERQLDKLQFLDLSNISQLRAIPSNMILGMNSLEEFYMRDNLILRETNEEIQSKNASLSELRHLKQLRSL
DIHIPSVSRFPQNLFFDKLDSYKIIIGEINMLSVGEFKIPDKYEVVKFLALNLKDEDQVQNRELKEITTVAGQDTNACFSLFN
GKMEEFMVLISFPFDWVAMPKLEFLELSSINIPQIWNEKSLHCFQSLITLNSLFVSGCELMEDIFCAEDAMVVAKGLEKLE
TLDVCNCWEMEEVVACNYQSNVNLITFRLPNLESITLKNCLFEGVWASTSLAAHEKVGVVVQLKELIIDNLSYLTYLEVT
NCLGLRNLMTSSTAMTLVQLTIMKVSLCQGIEKIVAEEEKTQVIEFRHLKAIELVSLPSLTCFCSSEKCDLKFPSLENLVVSD
CLLMETFSEVQSAPNLRKIHVLVGEKDRWYWEGDLNSTLQKLSTDKVSFKHSKHLTITEDSELEEIWHSKAAFQDNYFHS
LKTLVVMDITKDHVIPSHVLPCLKNLEELEVESCGAVEVIFDVNDIDTKKKGTVARLKKLTLTMLPNLSRVWKRNPQRIVS
FPNLQEVSVFDCGQLALLFPSSLAKNLLKLQTLEIQWCDNLVEIVEKEDALELGTAEMFKFPCLFWLLLYNLPLLTCFYPGK
HRLECHMLDVLDVSYCPMLKLFTSKFHDSYKEAVTESQASVPITTTWSQQPLFSVEEVVPKLKELTVNEESIILLSHAHLP
QDLLCKLNYLQLCFEDENNKKDTFPFHFLQKVPSLEHLLVCECFGLMEIFPSQTLQYHEIILVRLRKLTLNNLPELDTIGLEH
SWIKPYTKKLEFLKLEKCPRLEKLVSDVVSFSNLKQLDVESCEEMKNLFTFSTAKSLVQLEILTVLNCESMKEIVKNEDEDA
YKEIILGRLKELKLNFLSKLVSFYSGNAMLQLPCLSTVTIVKCPKMKKFSEGGLNVPMFSGIKTSLMDSDFHFHNDFNSTV
QWLHQHVSDEHSKHLTLADDSKLEKILHSKAAFPDNNFSSLRSLVVNNVTKDHLIPSQVLLCLKSLEELEVKSCKEMGKIF
DVNDLDTEKKGIVSRLKKLNLDTLPNLKCVWNKNPRGIVSFPNLEEVFVSDCGELEALFPSSLARNLVKLEELDIENCCHD
LVAFILENIICHKESVIEIQVSSTITISRLQHPIFLIEKVVPKLKELTVNEESIILLSHANLPQDLFRKLNLLLLCQEDEDEDNRKD
TLPFDFLLKVEEDSVMESTDRGSSDGDN

>g40040.t1

MDNASSSYKLPRKYDVLINFTGEDIHRKFVSHLNSAFSTVGLTTFLHHHNAVESTHIQQPILSNCRVAIVVFTQTYSQSA
WCLHQLQQIIKCHETYGQHILPVYYEIQPSDVRLQKGYFGKALKETAQQTFPGEELEDGMSRWSHALTKAANFFGWDE
SNHRSDAELVDKIVKSVVNLPVLSATKFPVGLQSRVEDLIRTIKSKSTEVCIVEICEDQGYGKTTLAKAIYNQIHWTFKKKSF
IENISQVSGIRGYLRLLEQLVLDILKQKVEIPTIDVGRRMIRETLYGKKVFIVLDDVPNNYELLALWTYNHYWFSKGTVIIITT
SDEDLRMNQPIDSIFRIERMNAEESLELFSWHAFREPKPKDEYEDLARRVISYCGGLPLALEIVGSSLFEKTKEEWNSLLFK
YPIIGWHRVREIIKISIEGSLNEMEKDIFLDICCFFVGKSRAYVTKILNGCGVDADIGLRVLIQRNLIKINKNNKFGMHPLLQ
EIGIQIISEKSGKDLGKNRRLWFDKDAKYGTEALQWLPVKLPSVNNGLQNVQPTVNFEYLLKKLRWISLHAFSSECLPNN
FYQHDPIAIALKRSLLRFLWKTSQVLRSVKVLNLSYSKYLTTTPDFTGLPXSGISKQHFRCSLTGVGTYHQFFNAVSDNIYQ
VLGSSESGDVCLPAVHDPYCLSHMGEGHSVSFVVPEDGDMKGMILNFVAALQVFLDWCWSIPSILQCYEGYYGLRGR
SDDFGKALKETAQQTFSGEELEDGMSRWSHALTKAANFFGWDERNHRYITHSINHLLRLKFHFVMTASITAIELEGVML
N

>g40041.t1

MDIASSSYILPWKYDVLINFTGEDIHRKFVSHLNSAFSTVGLTTFLHHPNAVKSTHIQQPILSHCHVVIVVFTQTYSKSAW
CLDQLQQIIKWHETYCRHVLPVYYEIQPSDVRLQKGDFGKALKATAQQTFSGEELEHGMSRWNHALTKAANFFGWDE
SNHRSDAELVDKIVKSVVNLPILSATKFPVRLQSYVEDLIRTIKSKSTEVCIIGIGGEEGSGKTTIAKAIYNQIHWTFKDKSFIE
NISQVSGIRGHLRLVEKLLLDVLKQKVEIPSIDVGRTIIREKLSGKRMLIVLDDVSNFILFDIWDFRKWSVEGTVIILTSTYIEP
LRGGDSVFWVKRMNAEESLELLSWHAFREVKPKEEYKELATSVVRYCGGLPLALEVVGSTLFEKTKEEWNRLLIKYAIIDT
HLDSEIIKSRAYVTKILNECGVDADIAIRVLIQRNLIKINKNYKFGMHPLLQEIGIKIIRENSGKDLGKNRRLWFDKDTKYGT
EALQWLPVKLLTAIESLQPTAISSDLLNKIRWISSHGLSSECLPNNIYEDDATAIDLKRSLLRFLWKTPQVLRSLKVLNLSHS
KHLTTTPDFTGLPSLEHLIFKYCSRLRKLHQSIGSLNSLILLNLKDCTSLNNLPTEIYDLKSLRTFILSGCSKIDIMDKDIAKLESL
ITLIAENTAVKHVPFSIVSSKESGISKQHLRCSLIGVGAYHQFFNVVSDNIYQVLASSESGDVCLPAVNDPYCLAHMGEGH
SVSFVVPEDRDMKGMTLCVVYLSNPKIIEPEFTTVIIVNYTKCTFQIHHHGTVISFKDEDWHDLMSNLESGDNVEIFVNF
GNGLVVKNTTVYLICAESKNMGKASEPKKHSLIRFVKKVVI

>g40228.t1

MAAEMVTGALVSTFVESTIDTLASRFAHIFRARKHHKKQLSHLKMKLLAIDVMAFDAEQKQFTDPRVRDWLLRAKDAV
FDAEDLLDEIDYELSKSQEEAESQSATKKVWNSLKSSFVSFFKNEIQSMMEQVIEDLEDLANESNILGLEKGAGVGVGSG
SGSKLAHTSLPNESVIFGRDDDKEFVLNWLTSDTHKNLSILSIVGMGGMGKTSLAQHVFNDPRLEQANFHTKVWVSVP
QEFDVLKVSRTILDTITRSTDHSIQLEVIQKRLKEELMGKKFLLVLDDVWNERPSKWEDVQKPLVFGSQGSRILVTTRSEK
VAVTMRSEKHLLQVLKVDYCWDLFVKHAFQNVNPQPDSDFIEIGKKIVEKCNGLPLALKTMGSLLYNKSSLSEWKGIMK
SEIWDFSENESDILPALRLSYFHLPSHLKKCFAFCALFPKGYWFDKEWLIQLWMTENFLENPLQKKSPKEVGEEYCNDLL
SWSFFQQSIHEEIKSFIMHDLLNDLAKYVCEDICIRLGVDEPKGIPKTTRHCSFSYSKSYFDGFGSSIDTQKLHIFTPTDRIG
GWDCKMSIDDLFSRFKLIRVLSLCNCNSLTEVPKSVGNLKHLRSLDLSFTEIEKLPDSISLLYKLQILQLNYCRKLKELPSCLH
QLDKLQILQLNYCKNLKELPSCLYQLDNLRRLDLKGTEVQNVVAHLGKLKNLQVTMSSFHVEKSKEINFQQLGELDLHG
RLTIDDLQNIENPSYALEVDLKNKPHLVELQIEWNFIGRSSVDSEKAEDVIENLRPSKYLKKLSIRNYIGKQFPDWLLHNSL
PNLVSLELEGCESCQRLPPLGLFLFLKHLSIAGLDGIVSIDADFHGNNSSSFKSLQTLYFSDMRQWEKWDCQAVTVIHCE
QLEALIVSAVELRLHDCGKLSLEQSLVATVGHMLFDTSLEKLSICSESISDDCVSLRIFPLDFFPTLRTLELSGFPNLQMISQN
HVHNHLWHLEIKECPKFESLPANMHMLLPSLRSLEIKECPRLEWFSEGGLPLNLKDITLNNCFRLVGSLKRALGDNPSLRS
LRIERVKAKCFPDEALPQRRRRRLGKDFSHSKPINTVKDEKCCSKDSEGHEVLCVRAYGSSFFTLGINIRNGRFLLQSSQNI
VVSSALIECEEALNQGSMTAVEVFISLRSKSILHLFASIGRVLGLEVYLPVFSPLDCLQAYYLGFRIPFKV

>g40231.t1

MAANMVTGVLVSTFLERTIDTLVSRLVDIFHQRKHKKQLNNLKMKLLAIDVVALDAEQKQFTDPRVRDWLLRSKDVVI
DAEDLLDEIDYELSKTQVEAESLSDAKKVWNSLNSSFLSFFENEIESMMEQVIEDLEDLANESNILGLEKGAGVRVGSGS
GSKLTHTSLPNDNVIYGRDDDKEFVLNWLTSDTHNSLSILSIVGMGGMGKTSLAQHVFNDPRLEQANFDTKVWVSVP
QEFDVLKVSRAILNTITGSTDHSIQHEVIQKRLKEELIGKKFLLVLDDIWNERPSKWEDVQKPLVFGGQGSRILVTTRSEKV
AVTMRSEKHLLQVLKEDYCWDLFAKHAFQNVNPQPDSDFIEIGKKIVEKCNGLPLALKTMGSLLHNKSSLSEWKSIMKS
EIWDFSENESDILPALRLSYFHLPSHLKKCFAFCALFPKGYWFDKEWLIRLWMTENFLENPLQKKSPKEVGEEYCNDLLS
WSFFQQSRFKGEKGFIMHDFLNDLAKYVCEDICIRLGVDEPKGIPKTTRHCSFPESGFDGFGSSIDTQKLHTFTPTKLMP
RGRFWSWDCKMSIDDLFSRFKLIRVLSLCNCGILTEVPKSVGNLKHLRSLDLSSTPIEKLPDSISLLYKLQILQLNYCQKLKEL
PSCLHQLDNLRRLELLGTEVKNVGAHLGKLKNLQVFNSFHVGKSKEINFQQLGELDLDGSLTIDDMQDIENPSYALELDL
KNNPHLVKLRLEWNFIGSSSVDSEKAEDVIENLRPSKYLKKLSIRNYIGKQFPDWLLHNSLPNLVSLKLEGCESCQRLPPLG
LFLFLKDLGIARLDGIVSIDADFHGNNSSSFKSLQRFYFSDMRQWEKWDCQAVTGAFPCLEEFSIENCPKLKGHLPKFIAL
KTLRVIHCEQLEALIRLRVEELSVCSPLESISDYCLSLRIFPLDFFPTLRTLELDGFPNLQMISQNHVQNHLEYLTIKECPKLES
LPANMHMLLPSLTNLYIKDCPRLESFSDGGLPLNLKEITLKNCFRLVGSLKRALGDSPSLTTLSIGKVEAECFPDEGLLPLSL

TQLIINDCPNLKKLNYKGLLELSSLEQLCLWKCPNLQCLPEEGLPKSISHLEIEKCPLLEQSYHKGGEDWEKVSHIPKIFIWE
NDFV

>g40233.t1

MSLGGIPSGEMPPGEKEVSSWATLGPPSYEMRQPHQDRRDDTPTPRTKNDGESEEMRHQRRGIVVSKYGEAKTISKQ
KFHSSSLFPIVIMAAEMVTGVLVSTFLERTIDTLASRLVDIFHQRKYKKQLSNLKMKLLAIDVVAFEAEQKQFTDPRVRD
WLLRAKDVVIDAEDLLDEIDYELSKTQVEAESLSDAKKVWNSLNSSFVSFFENEIESMMEQAIEDLEDLATQSDFLGLKK
GGGVGVGSGSGSKLTHTSLPNESVIYGRDDDKEFVLNWLTSDTHNNLSILSIVGMGGMGKTSLAQHVFNDPRLEEANF
DTKVWVSVPQEFDVLKVSRAILDTITGSTDHSIQQEVIQKKLKEKLMGKKFLLVLDDVWNERPSKWEDVQKPLVFGSQ
GSRILVTTRSEKVAVTMRSEKHLLQVLKVDYCWDLFVKHAFQNVNPQPDSDFIEIGKKIVEKCNGLPLALKTMGSLLYNK
SSLSEWQSIMKSEIWDFSEDESGILPALRLSYFHLPSHLKKCFAFCALFPKGYCFDKEWLIQLWMAQNFLENPLHKKSPK
EVGEEYYNDLLSWSFFQQSINEEEYIIHDLLNDLAKYVCEDICIRLGVDEPKGILKTTRHCSFSNSVFDGFGSSIDTQKLHTLI
PTERFWGWDCKMSIDDLFSRFKLIRVLSLYHCCSLKEVPKSVGNLKHLRSLDLSFTEIEKLPDSISLLYKLQILQLNYCVNLK
ELPSCLHQLDKLQILQLNYCKNLKELPSCLYQLDNLRRLDLEGSGVQNVAAHLGKLKNLQVTMSSFHVEKSKENNFQQL
GELDLHGSLTIDDLQNIENPSYALEVDLKNKPHLVGLRLEWNFIDNSSVDSEKAEDLKGCESCQRLPPLGLLPFLNYLKISG
FDETVSIDADFHGNNSSSFKSLKTLYFSNMRQWEKWDCQAVTALISLRVEELSIYSPLESISDDCVSLRIFPLDFFPTLRTLE
LSGFPNLQMISQNHVHNHLHYMKIKECPKLESLPANMHMLLPSLRYLTIKECPRLEWFSEGGLPLNLKHIRLKNCFRLVG
SLKRALGDSPSLESLTIEKVEAECFPDEGLLPLSLTRLIIEDCRNLKKLNYKGLLELSSLEELKLWKCPNLECLPEEGLPKSISFL
YISNCPLLEQRCQKEGGEDWEKISHIPQVFIRK

>g40363.t1

MTSPTFTYDVFLSFRGEDTRYGFTGNLYKALRDKGIHTFIDDDKLERGEEITPALMKAIQESMIAITVLSQNYASSSFCLDE
LETILDYKNKGLLVIPVFYKVDPSDVRHQKGSYGEALTKHQKRFKDKKEKLQNWKMTLRQVADLSGYHFKDGIEYEYKFI
GSIVERVYSEICRGCCLHVADYPVGLDSRVLEVRKLLEVGCYDGVHMIGIHGMGGVGKSTLARAVYNGLITENFESLCFL
ENVREKSNKHGLEHLQSILISQVLGKEDINLTSKQQGISMIKCRLKRKKILLILDDVDNLGQLRALAGGRDWFGPGSRIIIT
TRDKQLLATHQVTRTYEVRELNENDALRLLTLKAFKKEKADLDYELILNRVVTYASGLPLALEVIGSNLFGKSVKEWESAT
RQYKRIPKKEILEILKVSYEALEEEEKVVFLDIACCFKGYVLREVEDILGALYDDCMKHHIGVLVEKSLIKVSLLSTVEMHDLI
EDMGRKIDQKESATELGNRRRLWLPKDIIQVLKYKMGTGKIEIICLDFSISESEETIECDENTFMEMENLKILINRNVKFSK
VPNYFPESLKVLEWHRYPSNCLLSNFHPNKLVICKLPDSCFASFGFHGSSKKFENLTSLNFDDCKLLTQIPDVSDLQNLEKL
SFEWCESLTVVHNSGWEWEKSEEGDGQVGSIVSSKLDDFIALSCNLNDDFLSAGFSQLAQVSYLWLQESDITFLPECIKD
FHNLHVLDKLHEGGGTVFIFPGGSVPEWLDKQSKGPSISFWFRNKFPAKVLCLLIAPVLGPFDLAIPMVYIDGKIRKSLFQ
MNKEGKILELDYTHLFDIKELNFEDDLMGVSEKEWKKVEVTYEGKFTLQKAIKESKIVILVLSNNYASSSFCLDELATILHC
QSKGMLVIPVFYKVDPSNVRHQKGSYEEALAKHQKRFKGQEEKLNKWKMALSQVADLSGYHYGDGNDYKYKFIGSIV
EQVSRKISRVPLHVPDYFVGLLSQELKVKKLLDLESNAVVHMIGIHGMGGLGKTTLALTVYNSIADDFDSSCFLQNVREE
SNKQGLKHLQSILLSKMLREKNIILTSWQEGASMIQQRLRRKKVLLILDDVDNREQLEAFAGRADWFGPGSRVIITTRDE
QLLKSHEIERTYEVEKLNDNDSLQLLIWNAFKREKVDPSYEDVLKRVVTYASGLPLALKVIGSNLYSKSVEEWESAVEHYK
RIPDGEILEILKVWWDGRFGVIEMHDLIEDMDRHIDRQKSPKEPGKRRRLWLGKDILHVLKHNKKFENLTVLKFDAYKFL
TQTPDMSDLLKSEDAEDKTGNTKSPPTLQPPFCQALDDF

>g40416.t1

MSSNAIVQYTASSSHTINRYDVFVSFRGEDTRNNFTGFLFQALRTKGIDAFKDDEDLKKGESIAPELLHAIQSSRLFIVVFS
NNYASSTWCLRELVEIRNCVQTSPRPVIPIFYDVDPSVVRKQSECYENAFAEHEKRFRADKAKMEEAERWREALTQVAN
LSGWDIRNKLHNVRALVVLDNVDEVEQLRMFTGNRGTLLRECLGGGSIIIVVSRDEHILRTHEVDDIYQVQPLNYENAM
KLFCRNAFKVNHILTDYEKLARDVLYYAQGHPLAIEDLGRYIVKEESPKEPLKRSRLCNYQDFNKAMSNNQTTEILEVVAV

NSRETVRVDGLSKMRHLKFLRLENVKCSGSLSHLSNELGYLTWNKYPFECLPQSFQPHKLVRLKLRCSNIQRLWSGTKVL
PNLKHLDLSNSQKLVEMPDVAGALNLERIILEECIQLRKLSPSIGLLRKLTILNLRNCKNLHCKRLEYLPDLPSRTHLPLKVYS
YQRQYGEFIFEMPLGDDIYVTGLRMYNCPEIVEKERCSSMTVSWMLQIYQSWYKSKCLTSLSFLTSLGSMIPGSEIPKWF
NNQL

2. NLR genes of Vigna radiata

>XP_014489569.1

MAAEKIAGALVSTFVERTIDNLASRFVDIFRGNKSNKKQLSNLKVKLLAVDAVADDAEQKQFTDPHVRDWLLAAKDVM
LDTEDLLEEIDYALSKSQVEAESHSSTKKVRNSLKSSFVSFFKNEIESTMEELIKNLEYLATQSHVLGLKKADDVGVRSGSGS
KLRSTYLPNESVIYGRDDDKEFVFNWLTSDIPNNLSILSIVGMGGLGKTTLAQHVFNDPRVNEAKFDVKAWVCLSYEFD
VFKVSRKILEDVTRSPDHSSDTDMVHRRLREKLTGKKFLVVLDNVWNVTLSNWEEVQKPLLFGAQGSRIIVTTRSLQVA
SDMLSEIRFLMLLPEDDSWELFAKYAFRDGIQLNPECREIGKKIVKKCTGLPLALKTIGSLLYNKSSVPEWETVLLNKIWEL
PNCDIVPALALSYIHLPSHLKTCFAYFALFRNNYEFKKEELTQLWMTENFQHHSKTPDETCQQYFNDLLSRSFFQRLGDA
EELFVMHDLLNDLAKYVAGDLYFRCEDSQTNNIQKVTRHILFELPNFGRFREFGTLCKTESLRTFLPTPDRKLDYFHWFCT
MSIHELFSEFKFLRILSLSHCSNLLELPDSVDNLKLLRSLDLSHTAIRKLPEKICSLFHLQILELNYCTYLEELPINLHLLTNLCRL
EFRFTKIRKVPPGLEKLKNLIVMMNVFYIDHNMESGIQLLGKLNNLSEYLEIWGLECIKNPEDALEVNLKNKTHLVRLTLA
LERTGNSIDSKNAEVIENLQPSKNLKELSILNYGGSKFPKWLLQDSLPNLVSLVLRNCESCQHLPPLGLLPFLKKLYISGFDV
IVSIDADFHGNNSSSFQSLKKLEFSDMKQWEKWVCQAVTGAFPNLQILSIKKCPKLKGHLPELLVPLKTLKITRCQQLEAF
PPRTLELDLRHCGKLQLDWATMEWPRMDGHHMTALFSESDGSHTLDDLEIVEVISDDSIPLMTFPLDSFPTVKRLVLS
WLRNLEMISQDQAHHDHLLDLTITRCPKFESLPGNMHMMSLTSLWIEDCPRFKSIPYGGLPSNLENLTIKGCPKFESLPD
SVDNLKLLRSLDLSDTAIRKLPEKICSLSRLQILELNYCTYLEELPINLYLLTNLCRLEFRFTKVRKVPPGLEKLKNLIVIMSNFD
LDLESDIQRLGKLNNLYEDLSIGELQSPSDAFEADLKNKTHIESVTLGWERTENSIDSKTAEDVLENLQPSKTLKELSIFNH
GENKFPNWLLQTSIWNIVSLELDKCKSCQSLPPLGLLPFLKKLVISGFDQIVSIDADFHGKNSSSFKSLETLYFSDMRQWE
KWECKVVTGAYPRLQHLSISFCPKLKGQLPEQLVPLETLHITYCEQLEAFAPRALDLELCDCGKLQLDSAMKRLVMGGH
NTEASLLEMVGSNTLEHLDISSSLESMSDDCVSVRTFSLDFFPILRTLNLRGFGNLQKVLQDHAHNHLQDLTIKKCPKFES
LSGIMHMLSLKSLWIEDCPRLVSFPEGGLPSNLNDMRLNNCSRLVCSLKGAFGDRSSLESLWIEGMDAECFPGEGLLPL
SLTSLTICDCPNLEKLDYKGLYQLSSLRRLTLVSCPNLQCLPEEGLPRSISYLCIGDCPLLEQRCQTEGGEDWEKIAHIQNLN
IL

>XP_014489745.1

MEFASSSSSSSSLYFNSEPRFIYDVFINFGGEDIGRRFVSHLHSALLQAQLKTLINEENLPEGLELDDQIGAIGGAKITIIVFSK
SYTESTCSLRHLEKIIECHETFGQIVLPVFYEIDPLDVRHQKDDFGKALEETAHRCYSGEQLEHALSRWSSALNRVAGITG
WDVRDFRHDAELVKVIVDSVRRLLDHIDWYINQFPLGLDSIWEDEIESTENQSTEVIGIFGIGGLGKTTLAMKIYRIEFIGG
KLVDWNYDAKVSGWLKKPGKRSRLCLQDDVKDVLEENIETEAIEGLSPKLHSSSRDCLEAHTFKKLKRLRLLLSADYGQIS
KQLREFPYKYMPNNFHLENAIAL

>XP_014489746.1

MEFASSSSSSSSLYFNSEPRFIYDVFINFGGEDIGRRFVSHLHSALLQAQLKTLINEENLPEGLELDDQIGAIGGAKITIIVFSK
SYTESTCSLRHLEKIIECHETFGQIVLPVFYEIDPLDVRHQKDDFGKALEETAHRCYSGEQLEHALSRWSSALNRVAGITG
WDVRDFSVRRLLDHIDWYINQFPLGLDSIWEDEIESTENQSTEVIGIFGIGGLGKTTLAMKIYRIEFIGGKLVDWNYDAKV
SGWLKKPGKRSRLCLQDDVKDVLEENIETEAIEGLSPKLHSSSRDCLEAHTFKKLKRLRLLLSADYGQISKQLREFPYKYMP
NNFHLENAIAL

>XP_014490253.1

MDPIVSTATQSALNITVSVVKRQVGYFFNYKDKYKELESYIEKLEHNKERLQHQVDSALRSAEEIENDVQRCLTLMDDKI
KEYKSYINDDCHAKTICSIGFLPNNFRLRHQLGRKATKMVEEIIEDELWKTSFDNVSYQEFPSIDATFSNNGYESFASRTKT
MEMIMKALQDSTVGMIGVYGPGGVGKTTLVKEIANKAREKKLFKIVIIANITGNPNFKKIQEQIAGMLGMKLEEESEIAR
VDRIRKRLKNEKENTLIILDDLWGGLDFNKLGIPCNDDASQQEVIESDFGSNNDISDFGFNKTEIKELSKIDLDKMKNEKL
SNDYRGGKILLTSRNKQVLCNEMDVQESSIFSVGVLDEKESETLLKKVAGVKNSEFDRNATEIAKWSAGFPIALVSIGRTL
KNKSLSTWEDVCQQIKRQNFTSEWGFTDFSIKLSYDHLKNEELKCIFLHCARMGNDALIIDLVKFCVGLNLLPGVHTFTD
ARRRVKEMIKELEESSLLVKGYSIDRFNMHDIVRDVALSISSKEKHVLYMKNAILDEWPHENDFERYTAIFLHSCDIKDELP
KRLHCPRIEVLHIDNDTESFKIPDYLFKFMIRLKVLVLTGVNLSRLPSSIKCLKKLRMLCLERCTLGKNLSIIGELNNLRILSLSG
SNIKNLPPQFGKLDKLQFLDISNCLKLRQIASNIIPRMGILEEFYMRDNLIIWEAEENMKSENASLSELRHLNQLQNLDIHI
HSSSHFPKNLFFDRLNSYKIVIGEFNLFNLLKVGELKVPDKYEEVKFLALNLKKGIVQKWIKMLFKSVECLLLGELNDVEDIF
YELNVEGFPNLKHLSIVNNFGIKYIINPRERFHSLXAFPKLESIWLYKLDNLEIICDNQLVETSFRNLKVIKIQTCIKLINLFPFS
MVRLLTMLETIEVCDCDSLKEIVSKESQTHTISDDKINFPHLRLLTLKYLPTFISLYNVDKIPSSAHSLQDQVFQQRNKDIVV
EVEHRVTNSCLPLFNEKVSTPKLEWLELSSINIHKIWSDQCNHCFKNLLTLNVTDCGNMKYLLSFSMAGSLVNLQSISVSE
CEMMEDIFRPEDAEYIDVFPKLKKMEIICMGKLCTIWKSDIGLHSFCNLNSLMIRECRKLVTIFPTYMGQRSQSLQSLTVT
DCELVENIFDFANIPHPSDIIEANLGNIFLENLPNLVNVWKGDTGEILKCNNLQSIRVDESPKLKYLFPVSIANDLEKLEVLE
VRNCWAMTEIIGLDKHSSETAITFKFPHLNILSLKDLHSLRSFYSGIHTLEWPPLKKLEIVDCSMLEGLTSEITNSKEQPIVLA
AKKAIYNLEYMSVSLKEAEWLQKYIVNIHRMHRLEEVTLYGLKNNKILFWFLHRLPNLKILTLELCLMKRIWTPQSLRSGE
KIGCAMQLKELDLRSMWFLEEIGFEHDVLLQRVERLIISGCKKLRNLASSSVSFSYLTSIEVVNCMMKNLMTLSTAKSLVQ
LTTMKVSSCPLIVEIAAENEEENVQEVEFKQLKSLELVSLQNLTCFSNVEKCDLKFPLLEKLVVSECPQMTKLCEVQSAPKL
QKVHVEAGDKDKWYWEGDLNATLQTHFTNQVSFEYSKDINLVEYPEGKVRHDKFAFPDNFFGCLKKLKFDEACKRDTL
IPSHVLPYLKNLEELNVEKCESAQLIFDIDESEIQTYGMVFRLKTLTLKHLSNLKCVWKENIEGVVSFSNLKRVHVDGCGSL
VTLFPLSLAKHLGKLNTLYINNCVKMVEIVRREDDMEHRPTIMFEFPCLSYLILENMRLLSCFYPGKHHLECPQLDSLHVA
WCPKLKLFRSSLLRKPLFSIQEVSPKLEGLTLNKQNIKLMSDSRLPEDLLYKLKYLILIFEDDYNGKYSLPFDFFHKLPNLRWL
QVQKCFGLKEIFPSQKLQVHDKVLAGLKQLNLFELKELECIGLEHTWVQPYTEKLEKLVLYKCPLVERIVSCEVSFINLKYLY
VMYCERMEYLFTFATLKSLVKLETLSIGYCGSIIEIARNEEEDGCDEIIFGRLRWIKLEYLPRLISFYSGNATLQCQCLQAMIV
TECPNMITFSGGVIKLAMFSGIQTSEDSDFTFNVDLNTTIESLFHEKDFSNYSKLMILHDYLGMMRVQHTKPTASDNFF
GSFRRLEFDITCNRSFVIPSHIFPYLKNLEELKVHSSDEVEVIFDTDEIEVETKGIIFGLKKLILYDLSNLKCVWRENLEEIVSFS
NLQEVDVDGCGSLVTLFPLSVAKNLGKLESLDIKKCEKMVEIVGREDEMEHGTTIMFEFPCLSYLYLEKMPLLSCFYPGKH
DLECPMLDKLYVECCPKLKVFRSSFDDDSKNEEVLEAPTNLLQQPLFSIEK

>XP_014490509.1

MDNASSSYKLPRKYDVIINFTGEDIHTKFISHLNSALSTVGLTTFLHHPNALLQIIKWHQTYCRHVLPVYYEIQPSDVRLQK
GDFGKALKATAEQTFSGEELKHGMSRWRHALTKVANLFGWDESNHRSDAELVDKIVKSVVNLPALSATKYQHGSGKT
TLAKAIYNQIHWTFKDKSFIENISRVSEIRGLLRLQEQLLLDVLKQKVEIPSVDVGRTMIRERLLGKRVLIVLDDFSDFYSEN
LFDLRDCLKLLFEGTVIIVTSTYGTRLIDQADSVFRVERMNEEESLELLSWHAFRKPKLKEEYKDLARSVVIYCGGLPLALEV
VGSTLFEKTKEEWKSLLFKYPIIGWHRVSEIIKVSIEGSLNEMEKDIFLDICCFFVGKSIAYVRKILNGCEVDVVIGIRVLIQRN
LIKIDNNNKFEMHPLLQEIRLQIIGDKFAGDFEKNRLPWKYETEEALQLLPSKLLTAIGSVQPTVNSQYLVEKLRWISFHGF
SSECLPNNFDYDPIAIELKRSLLRFLWKTPQVLRSLKILNLSHSKHLTTTPDFTGLPSLVHLIFKYCSRLRKVHQSIGSLNSLILL
NLKDCTSLSNLPREMYELKSLRTLILSGCSKIDLMENDMVQMESLITLIAENKAVKEVRFSIVSSKSIGYISLPGFEGLSHNL
FPSIIRSWMSPIMNPISYIHSLCMDTEDNTIDIAPLLSTLSNIRIRKALYGLK

>XP_014490533.1

MCMQDPPSSFTLLHSPAYNSLYFNHNHNLSFDSSTSSLSDPMPPESDVTISSYRLCWDVFLSFRGTHTGHTFTMRLYHA
LHGRGVRVFRNDDGLERRGEIQKKLLEAVEDSAAAVVVISPDYASSHWCLEELAKICEVGRLILPVFYWVNPSHVRKQE
GPFEEWFVWHVQRFPKERVEQWRNAMKKVGGLAGFVLDEKSDKSDELIQILVQNLMKQLRNTPLSVAPFTVGVDDR
VEVLKNLLDLKSNDVRVLGLYGMGGVGKTTLAKSLFNNLVVHNFERRSFIPNVRSQVSKHHGLVSLQNKIRGDLCGRKE
DLINDISDGISAIQKIVQENRVLLILDDVDDVEQLNFLMGKREWFYKGSRVVITTRDKEILYGSYVDVDFEVKELEFSEAM
ELFCFHAIRRKEPAEGFLDVSKQIVEKTGGLPLALEVFGSFLFDKRTEREWKDALEKLKQIRPSCLQEVLKISFDALDEQEQ
CIFLDIACLFVQMEMKRDDVVDILNGCDFRGEIAVAVLTARCLIKIIGDGKVWMHDQVRDMGRQIVRSESLTDPGLRSR
LWDRDEILTVLKNMKGTRNVQGIVLDCVKRRMSIPRDRSADEITRENFRRKPSCKSAFEYIKERYKKYVEDRKEKSKEVIL
QPKHFQPMVSLRMLQINYSRLEGQFICLPPKIKWLQWKQCPLRYMPSSYNPLELAVMDLSESLIETLWKGRSNKVAVH
LMVLNLSRCHRLTATPDLSGYLSLKKLNLEECSHLTRIHESLGNLNSLVHLNLRLCYNLIELPGDVSGLKHLEDLVLSDCWK
LKALPKDLSCMVSLRQLLLDSTSITELPVSIFHLTKLEKLSANGCHLLKKLPTCTGKLCSLQELSLNHTALEELPDSVGSLEKL
EMLSLMGCKSLSVIPNSTGKLISLTRLYLDGSGIKELPASIGALSYLRKLSVGDCTSLHKFPVSMEALVSIVELKLDGTKVSN
FPEEIFVGMKMLEKLEMGKVQHLKFVPVSFGCLSALTILDMHDANITELPESIGMLENLIRLRLDKCKQLQRLPDSIGNLK
SLRWLMMKETAVTRLPDSFGMLRSLVELDMKRLPYLNGAGNNVSTGTIIPEIREQPSSEAILTSFCNLSLLEKLNAHGW
GIYGKIPDEFEKLSSLETLCLGHNNICNLPASMTGLSYLKKLLLSDCRELMFLPPLPSSLEELNLENCVAVQYIHDISNLERLE
EFNLTNCEKMVDVPGLEHLKSLRRLYMSGCIGCSLAVKRRFSKVLLKKLEILIIPGSRVPDWFTAEPVVFSKRSNRELKGVI
FFGVISFKNIAENQREGLELVDVQGKIFNLTSEVFSTTFRLLRVPRRNEDHIFLRRFGARTPLVFQLKDRYTLHLQRRNPPR
IEGLELNNCRIHLVFYGDDDYEGDEGSLEEIQYSVSQKLAKFFNFAADYPDV

>XP_014490773.2

MSLSGSDDEMEMDFLGDRYRKMDGGNFEVFLSFRGEDTRASFTSHLYAALQNAGIFVFKDDESLPRGKQISPSLRLAIE
ESPISIVVFSKNYAESLWCLKELEKIMECHRTTGHVVLPVFYDVDPSEVRHQRGDFGKAFQRLLSKSSREEEEKVLDWKQ
RWRKILRELDGFSVVEILNSRIGRGDNLEEHWKEALFEVVQISRERMEIADGIGFLVSQLMAGLFEGAKIPFREVKIDDRI
DFLVKQWRELLCEAVRISEVALLDPCGETEVANAIKLHMKHCREALFEASGISGDAVLNSCGEMEITNAIELHMKHWRD
AFSENAGTISGSDYSRFIGGRNWYDVSSSGFRTVHYEIWHLQHYWREKVREAYDRSGDDQLYSRREIDIERHLEHCASA
VNKAIDSSTASRSSFQKLQAYNRIESLVKYWRKALCEAAEISRWVVQHYRGITDNEINYIEKNARDALREAAGISGVVILN
SRNESEAVTNIVKNVTSLLDKTELFVAHNPVGVESRVQEMVQLLQQKQSNDVLLLGVWGMGGIGKTTIAKAIYNKIGR
DFEGRSFLADIRQVWGQEAGHVCLQEQLLFDIHKESNTKIRNIESGKVLLRERLSRKRILLILDDVNKLQQLNALCGNRE
WFGSGSRIIITTRDIHLLRGKRVDQVFAVTGMNVDESIELFSWHAFKQASPKQDFIELSRNVVAYAGGLPLALEVLGSYLF
DMEVTEWKSVLEKLRKIPNDEVQEKLKISYDGLSDDTEKGIFLDIACFFIGKDRNDVIHILNGCGLFAENGIRVLVERSLVT
VDDKNQLGMHDLLRDMGREIIRSKSPMELEERSRLWFHEDVLDVLSKETGTKFIEGLTLKLPRTNTKSLCTKAFMNMKK
LRLLQLSCVELVGDFEYLSKDLRWLCWHGFPFAFIPTSFYQGSLVSIELENSKITMVWKATQLMEKLKILNLSHSHYLTKT
PDFLNLPNLEKLVLMDCPRLSEVSYTIGHLTKVLLINFQDCISLCNLPRSIYKLKSLKTLILSGCLKIDKLEEDIEQMESLTTLV
ADKTAIRRVPFSIVRSKSIGYISLCGYEGFSRNVFPSIIWSWMSPVNSLSSRVQTLVDMSSLVSLDVQNSSSNQLSYISDEL
PKLQSLWIECGSDLQLSRDTASILDALNATNSEESESYGTTSQMKNVFTLIECNSRSKLFEKTLLIQMGISWEITHILKERIL
QHIYLYFYRM

>XP_014490775.2

MSLSGSDDEMEMDFLGDRYRKMDGGNFEVFLSFRGEDTRASFTSHLYAALQNAGIFVFKDDESLPRGKQISPSLRLAIE
ESPISIVVFSKNYAESLWCLKELEKIMECHRTTGHVVLPVFYDVDPSEVRHQRGDFGKAFQRLLSKSSREEEEKVLDWKQ
RWRKILRELDGFSVVEILNSRIGRGDNLEEHWKEALFEVVQISSGETEVANAIKLHMKHCREALFEASGISGDAVLNSCG
EMEITNAIELHMKHWRDAFSENAGTISGSDYSRFIGGRNWYDVSSSGFRTVHYEIWHLQHYWREKVREAYDRSGDDQ
LYSRREIDIERHLEHCASAVNKAIDSSTASRSSFQKLQAYNRIESLVKYWRKALCEAAEISRWVVQHYRGITDNEINYIEKN
ARDALREAAGISGVVILNSRNESEAVTNIVKNVTSLLDKTELFVAHNPVGVESRVQEMVQLLQQKQSNDVLLLGVWG

MGGIGKTTIAKAIYNKIGRDFEGRSFLADIRQVWGQEAGHVCLQEQLLFDIHKESNTKIRNIESGKVLLRERLSRKRILLILD
DVNKLQQLNALCGNREWFGSGSRIIITTRDIHLLRGKRVDQVFAVTGMNVDESIELFSWHAFKQASPKQDFIELSRNVV
AYAGGLPLALEVLGSYLFDMEVTEWKSVLEKLRKIPNDEVQEKLKISYDGLSDDTEKGIFLDIACFFIGKDRNDVIHILNGC
GLFAENGIRVLVERSLVTVDDKNQLGMHDLLRDMGREIIRSKSPMELEERSRLWFHEDVLDVLSKETGTKFIEGLTLKLP
RTNTKSLCTKAFMNMKKLRLLQLSCVELVGDFEYLSKDLRWLCWHGFPFAFIPTSFYQGSLVSIELENSKITMVWKATQL
MEKLKILNLSHSHYLTKTPDFLNLPNLEKLVLMDCPRLSEVSYTIGHLTKVLLINFQDCISLCNLPRSIYKLKSLKTLILSGCLKI
DKLEEDIEQMESLTTLVADKTAIRRVPFSIVRSKSIGYISLCGYEGFSRNVFPSIIWSWMSPVNSLSSRVQTLVDMSSLVSL
DVQNSSSNQLSYISDELPKLQSLWIECGSDLQLSRDTASILDALNATNSEESESYGTTSQMKNVFTLIECNSRSKLFEKTLLI
QMGISWEITHILKERILQHIYLYFYRM

>XP_014490883.1

MHNKKQAKKPSEIVDKWLEDAINDVHNVNQLLEEAKTHKYCCFGHCPNWIGRYHVGKKLANKTMDLEKFIKEGRKYV
PFDRIATLSSNTLDMLSEKCMTFESRQSASEQLLDALKNNDVSKIGLYGMGGYGKTTLAMEVRKIVEAEHLFEKVLFVPV
SSTVEVRRIQEKIASSLQFEFPETEEMQRAQRLCSRLIEEKNIFIILDDVWEKLDFGRIGIPSSDHHKGCKILITSRSEEVCILM
DCQRKIYLPILTDEEAWTLFQNKALITEATFDTLKDMGRLISNECKGLSVAISAVACSLKGKAETEWRVALNKLRHSKPM
NIQRGFINPYKCLQLSYDNLDTKEAKSLFLLFNKIEIGLGAVGEAHSYDEARSEVIAAKIKLIRCCLLLDADDEYVKMHDVV
RDVAHIIAQNENKIIKCEEEKDVIVEQNSVRYLWCVKFPNDLDCSNLEFLCLRTEMKEFDGIFERMGMLKVLSLVNYGDE
KTPLSTSSFKTLTNLRDLLISGCELSDFSFLSGMKNLQSLSLDNCLLPSFPEIQIGVAITLKLLELNECAIKARNFEVMKRIPHL
EELYIIEWYASEDNIEFFKTFSVPETLQRYGIVLGSRNFYHYNDRDIYIDGRTLLLNHFDISNEVIKGLAKKAKNLFVANIQG
GATNIIPDIFQIEGGDLNELKKLEIHDCEELECLIDTSSRSSEVVTLFSKLHTLIIENMKSLKAIWNCFIPANGPFEKLEKLDIS
DCPRLTSLFIYVVARNLVKLKILKISGCNELKHILADDEKTEKGHDEFSSGHPVQIFQNLQEVEVYSCPELKHIFSAKIVGGL
GQLQVLKIERCEMLNQIIGDRKEVDEIVEEGTLSSLASLEIKRCGKLGSIFTASIAKTLTSLEELFIEDCKSLKDIVTHESIVEDE
HDSQSDISIFQSLKKLHISECDLLEDVDFKDFSSRNNTQIELPALQVLELYRIRNRTIVDSYDVICPSLRKLSLDIGRYVGFFNI
NCSTDASEATKRDSISIKISNSDFVPPTERL

>XP_014490936.2

MSSSSWSTNESGDEGQGYADGVAASSSSSGSSSTSPSGSPDRRGVVYEDVEFNVDNSRVWTSLIPPPAVQRYDWAPH
EVRNYLPYYNTTKSIRHLLEWVDLLADLRDADQYSLVVCRSNERACHDFFGGMEQHQSFARIMAIKNRLGIAGEGTSSP
AKQTGFSSNTPVHLVNPALKIVDTPTPAADTKPINPEAVKVKAKRKPSQEKTPSPPKKRRIHAPLLTGPLDPNVHVAERL
QFNLNVEEKKPFKVMSLSESLNMAYELIARASVCMNYFAGTTKPLLVAELETTRKDLEEVKKENTTLSSCLKEVTKAVED
DRVKAANSLKEAQDEITHLKQSTDNLRLDLQKASSKNKKLIKERDAAIANRDKMATENVTLGDELCEERQCGFDQGVA
QCHYFFNTPLEHEGFDIMKVYVDGQLVKLSVPEADTDSAAVIKTIPLVHPIVTGEVVDISSTEKNPKASNPSFFLRSSELLQ
LLFLLCRTIVTCKLAHQRSSQQSHSQYNVKESCIWWNKLMDCFVDFASSVSRDLVCGALNQLRYLYSFNNFVKKLEQGE
DDLIVTRDDVQKSVEDAKRKTRETSGVVVKWLKDAMSDIGKVNQLLEEARTKKMCCFGYCPNWIWRYRIGKKLAYKFF
DLEKFIDQGKKYVSFNRIPALPSGTLHILSEKSMNFESRQSAYEQLLDAVKSNDVSMIGLYGMGGCGKTTLAMEVMKLV
EADHLFEKVLFVPVSSTVDVRKIQEKIASSLQFKFRETEEMQRAQRLCLRLILEKNIFIILDDVWEKLDFGRIGIPSSEHHKG
CKILITSRSEAVCTSMDCRRKFYLPILTDEEAWTLFQNKALITEATSDTLKEMGRLISNECKGLPVAIAAVASSLKGKVETV
WSVALNKLRHSKPINIERGLTDPYQCLQLSYDNLDDKDAKSLFLLCSVYPQGSEIPVEHLTRYAIGLGVVGEVDSYEEGRS
EVIAAKIKLVSCCLLLDADDECVKMHDLVHDVAHIIAKNENKMIKCEMEKNVRVGQNSVRYLWCVKFPNDLDCSNLEFL
YLETKMKEFDGIFKRMGTLKVLILVNDEDGKTPLSTLSFKTLTNLSCLFIYSFELSDFSFLSGMKNLQSLSLYYCLLPSFPELQ
TDVTALKLLELNECDIKVKNFEGMKRIPLLEELYIIEGEWDANSEDNIEFFKTFSFPETLQRYGIVLGSDNFYNFNDGDIYIH
GRTLLLNHFDISNEVIKGLAKKAKDLFVRNIHGGAKNMIPDIFEIEGGGLNELNKLEIRYSKELECLIDTSSHSSEVVTLFSNL
EKLDLSNCRRLKFLFTYVVAQNLVQLKILKISRCRKLEHILTYDEKSQDEFTSGHPVQIFQNLEDVKIKRCRELKHIFPANIV
GGLTQLKVLKIERCEKLEQIIGDIVPSTEQDRKEELDEIVEEGKLPHLYNTSFPTTTFVKQSPGTLSSLGRLKIKCCGKLGSIFT

ASIAKTLTSLEELFIEDCESLKDIVTHESIFEDDYDCQSDISIFQSLKKLHISECDLLEGIFPVSLVGELNDITNKEAADLKDISSR
NNTQIELPALQVLELDLIRNRTIVGSYDVICPSLRTLSLDIGRYVGFFNINFSTDASEATKRDFIAIKISNSDFDPPIGSVECLS
KQPHGLNLIMTQNIREIELKGFDSAKYLFKLSNVSSLMMLEILIIKECDGLEYIIDTDDEYGKENMKAIFPNLKQLSVINCSQ
LKYIIGQHHVANKDYKEIYIQFSALEILYLWNLPNFVSICSTNTISVTWPSLKVFESYSCLYPLYGSVSCLTIPTNSREPIITSTK
DPKGIQNHLLTLQTLNIAYSDAEHIFCLNEDEMIGQQVSLRLENLGLQFLSQMTYIWVAPNNSLTLQHLTTLTIWECEKL
EVIFPKSVVRFLPQLKLLKIMKCKELRQIIEMDKKFSTVVSPQPCFPKLEALHVDDCHKLKRLFSGSSSNDLPNLLLLAINGA
NELEELVGCKQEKIKIELPRLKLLIFMHLPNLSQEIELHNLKNCIVYKCPKLSLTSTTTLQKLREDFPYEEDFINAEVGSWEFE
AIVRSIDDKFISSQDIEDLGNKSIKSSSTGVEDTGIEDAVATHIDSKVVEQDDKVTEGKPGIVASQGIQVQERLNLLHKQEG
IDFVPNNNIDISSASSADIRTRLGAYKHFVDLDDTQISLLVEAITTYPHLWNASKKFSERFQAWRLKILADMLLFLQMESV
HSVIPQKEKEFYKLCEEAILVGFESSWVEEMRQRVVARDPKLGEDIARRQIDENSKSNLSLLNRYSSGDMIEYSQAVEEG
DGPKISLEEGSDLVDKEGEIGVVSNDHIVALRNEEAEQEFVAEVFTSEIPRIGTSLTNSQTVEKPTPSCPLVDTQQTGEQC
QMKQKKPVGEIPKSIEQVALEETIAKNTNMAASSILSESATSKLDPTVTLQRKSHLHSEIRSSQIEARITKESESHPELIQDF
GGNDMIPIALGKEGEDNIVVKTLVELENYLKMSLKDIVGSETNTLRLFSTLNFLSNLPFKDVTLSDRLKHIIKTMHQHFPTI
LCSFKQRFVTTDKLEELEARQNEVAIKISEAKNFNDEAQLKEVVLKEQINRLKEEIKVCETALSSLDEGKDKCIAETIRYKME
LENVRKSKSQMVEDQRKVEQELFEVAYKWSVLCSEYELDRMVARNPS

>XP_014491132.1

MAAELVGGALLSAFLQEIGRRIVEKCKGLPLALKTIGSLLRTKSSSSYWKSVLESDIWDLPKEVEIIPALLLSYQNLPSHLKRC
FAYCALFPKDYEFDKKELILLWMAEGFLHHSKQIKNVQEIGEQYFDDLLTRSFFLQSSFIRKFVMHDLMNDLAKYVCADF
CFRLKFDKGNCIPKTTRHFSFASADLRFFDDFGSLTDAKRLRSFVQITNYAIYPGFPGQFEIIRELFTKLKFLRVLSLNGYSNL
KEVPDSLGDLKHLHSLDLSHTNIEKLPDSIGLLYNLLILRLNYCSCLKELPSSLHKLSKLRCLEFEHTEVTKMPMHFGELKNL
HVLDMFCVSRNSEFSTKQLGGLNLHGSLSINEVQNIVNPLDALEAKLKNKHLVELKLIWNSNHIPDDPRKEKEVLENLQP
SNKLEHFSIKGYCGTQFPSWVFDNSLSNLVSLQLKDCKYCLCLPPLGLLSSLETIEIRGFDGIVSIGAEFYGSNSSSFKSLEILK
FYNMKEWEEWECKTTSFPRLQRLFVVQCPKLKGLSEQLLHLKELYIESCDNLIISEHSVDASALECLKTASSPLVNIPMTHY
DFIEKMTIDDGCDSLTIFQLNFFPMLRLLHLEGCQNLQRISQEHAHYNLKKMTMWKCPQFESFPSGGLSAPWLQIISIRE
VRNLKLLPKLMQILLPSLTELRIINCPKVEKFADGGLSSKIKDMSLSSLKLIASLRETLDVNTCLKKLSIEYLDVESFPDEVLLP
PSLTSLTIIRCRNLKKLDYKLLYHLSSLELGDCPNLQCLPEEEGLPIAISSLTIWDFPLLKQRCQNPEGEDWGKIGHIEKLIIR

>XP_014491799.1

MVRNDAELVKSIFKTVVTRLVKPSIQSKGLVGIEEKITDVEQLIRKDPKDTSFIGIWGMAGIGKTTLAEQVFHRLRSEYEG
HAVKIDTPTSLPQEILRRMSCMKVLIVLDDVNDLDHTEKLLGTLDNFGSGTRIIVTTRDKQVLKANKVDKIYQLKEFSSKD
ALELFNMIAFDQSDHQMEFNELSQRVVDYAHGIPLLVKVLARLLCGRNKEVWESQLHKLKKMSLTEVYDVMKLSYNGL
DRKEKQIFLDLACFFLRSHVRVNSGDLKYLLKDDESDDTIVVGLERLKDKALITFDDNSISMHDALQEMAWEIVHQESSK
PGSTNWLLDPNGDVYQTLKNDKGLGGIRSLRIHLTTTGKKKLIHGLFAEMSRLQFLEISVENNDDLFDQVYALAKELQFL
ETELRFLCWLNYPLNSLPENFSTDKLVILKLQYGRMEKLWDGFKNLVNLKELDLMHSKKLKKLPDLSQATNLEVLVLLGC
SMLTSVDSSIFSLPKLESINLSGCKSLTLLTSNSHFCNFSYLNLDFCKNLREFSLISQNMKELRLGFTKVKVLPSSFECHSELK
SLHLTRSDIEMLPSSFNNLTQLQHLDINNCNRLRTIVELPPSLKTLEVSKCKSLQNLPNLPSSLKTLNAIECKSLKTVSFPSTA
DEQLTEFKKRFLFWNCRNLDESSAEAIGLNAQMNLMELANQPLHTPSQEHHELYNDYEYNYHSYQGIYVYPGSSVPA
WFKHTETNGDIIIDLSSASPFELFGFIFCFVLNKFHDTDIIGRLEFNITISDVDDVGEGKMGSVKIYIDCYSDWSVAPYHVC
VMFDQRCSATLNNIARKQKRFKMNVSVGARIEFYDNYHELAQEVLKGVGVSPISISAYNIQQIEL

>XP_014491800.1

MVRNDAELVKSIFKTVVTRLVKPSIQSKGLVGIEEKITDVEQLIRKDPKDTSFIGIWGMAGIGKTTLAEQVFHRLRSEYEG
HAVKIDTPTSLPQEILRRMSCMKVLIVLDDVNDLDHTEKLLGTLDNFGSGTRIIVTTRDKQVLKANKVDKIYQLKEFSSKD

ALELFNMIAFDQSDHQMEFNELSQRVVDYAHGIPLLVKVLARLLCGRNKEVWESQLHKLKKMSLTEVYDVMKLSYNGL
DRKEKQIFLDLACFFLRSHVRVNSGDLKYLLKDDESDDTIVVGLERLKDKALITFDDNSISMHDALQEMAWEIVHQESSK
PGSTNWLLDPNGDVYQTLKNDKGLGGIRSLRIHLTTTGKKKLIHGLFAEMSRLQFLEISVENNDDLFDQVYALAKELQFL
ETELRFLCWLNYPLNSLPENFSTDKLVILKLQYGRMEKLWDGFKNLVNLKELDLMHSKKLKKLPDLSQATNLEVLVLLGC
SMLTSVDSSIFSLPKLESINLSGCKSLTLLTSNSHFCNFSYLNLDFCKNLREFSLISQNMKELRLGFTKVKVLPSSFECHSELK
SLHLTRSDIEMLPSSFNNLTQLQHLDINNCNRLRTIVELPPSLKTLEVSKCKSLQNLPNLPSSLKTLNAIECKSLKTVSFPSTA
DEQLTEFKKRFLFWNCRNLDESSAEAIGLNAQMNLMELANQPLHTPSQEHHELYNDYEYNYHSYQGIYVYPGSSVPA
WFKHTETNGDIIIDLSSASPFELFGFIFCFVLNKFHDTDIIGRLEFNITISDVDDVGEGKMGSVKIYIDCYSDWSVAPYHVC
VMFDQRCSATLNNIARKQKRFKMNVSVGARIEFYDNYHELAQEVLKGVGVSPISISAYNIQQIEL

>XP_014491807.1

MDTIASVASSVAAPLLRNITYVLMYSTYLTELETEIKRLQSEEKEVRHTVEAAKRGGEEIEDTVRDWFDRVRAAVEQGQA
FLEEEERERVGCMDVYSKYTNSQRARTLVEIVREVRKETFDRVSYRRALRCNVGPAAREYVAIQSRTVMLNDVVKMLKD
GGVDIVGVYGVAGVGKTAMVKELAWQVEKDGLFDVVVMATVTNSLDVGRIRNEIADGLGLKFDELTELGRASRLRQR
IRQEQRILVVLDDLWGKLDLTKIGVPFGEDYKGCRCQLLVTSRNRNMLSSNFGSGKFCRLDVLSEDESWELFEKRAGDA
VKDPSIQSVAEKVAKSCGGLPLLIVTVVEELKNKDLYAWKDALEQITSFELEGCLYSPLRSAIELSYDHLESQELKTFFLLLGS
IGNGCSTRDLLVFGWCLGLHKQVDSLADGRNRLYKLIDNLRAACLLLDEGKRDSVVTLEVVRHVANSIATRVKPFFTVQR
NKEFKWPRMDFLGSCHHIFLDWCYIRELPEVLECPKLKILQINSQGNYLKIPDDFFVHMKELKVLSLGGLDCTPSLPPSLSL
LTDLQALYLCECKLEDIATVGEITSLEILNLEKSELKELPAKIGGLSNLRLLDLTDCPTLGGIPGNVISRLTRLEELYMGNCDV
QVEAKERKSQNNDSSLSELKHLNQVTILNVQIEDTSVFPRDMLSFGRLESYKILIGDGWKWSGVESENYKTSRLLKLNSG
ADPTILKDYGIKMLMNKAEELYLAELKGVREVLYELNDEGFSQLKHLCILNCAEMESIIGSTEWAYCDHAFPNLESLILHN
LINMERICSDPLPAQAFRNLQVIKVKDCDRMEFVFSHSMVKHLSELVEIEISECKSMTNILSGQRQEDADAGQTNKISLI
NLRSLTLQCLPSLVSFSPDSSTQASENGNGFSSQLFSNKVEFPNLETLKLYSINIQMIWINHHHSYFENLTSLTVDGCERLT
YIFSYPVAVKLVKLQHLLLSSCKFVENIFVPDENLGHVHHFRKSIHKELVPIFPNLETFVISQMDNLKAIWPDLLPQNSFCK
LKKMEIESCSNLLNVFPCHVLDKLQSLESLNVWKCMALEVVYEIDGINTEHEGSSQEGLDIPLRTLSLGNLPKLKHLWNK
DPQGNIKFQNLFMVQASKCQSLKYVFPLSLAKDLLHLQFLEINDCGVEEIIASDKGGVGAAFGFVFPKLVSIKFFNLPDLR
CFCDGNHNLRFPLLNQFFAVECPRMETFSRGILRASILRKIHLTREGDQWYWQGDLNTTIRKLFNRGTQVLVV

>XP_014491808.1

MPVLETLGGALFGAVLQVLFDKLDSHQVLDFFRGRNLDDKLLKKLKRKLMDVNSVIDDAEQKQFSNSLVKEWLDEVRD
VLYDAEDLLEQIHYEFFKSESKAKLQASASKVRNFESKMIEVLDDLESLLSQKVVQDFKLSTGVRSGFGNKVSEKKVESSSL
MAKDSVIYGRDKDKEMIFSWLRSDTNDNKLSIFSILGMGGMGKTTLAQHVYNDSKTEEAKFDEKAWVCVSDAFDALR
VSKAIIGAFTNSRDDSENLEMVHGKLKKKLFGRKFLLVLDDVWNEDRKQWKALQTPLTFGAKGSKILVTTRSHKVASIM
QSTYIHQLKQLDEDRSWQIFAKHAFQDENSKLNSNLKEIGVKIVEKCQGLPPALETIGCLLQLKSSVSEWEGVLRSEIWDL
PIEDSKIVPTLLLSYYHLPSHLKRCFAYCALFPKDHKFDKENLIFLWMAQNFLDCSQQSKSPEEVGENYFNDLVSRSFFKQI
IWYNKTYFVMHDLLNDLAKYVSGEICYRLGVDGEKKVSRKTRHLSYLSGPTQYYKSLCDAKGLRTFITFRWREMSIEELIS
NCKFLRVLSCRWCIKVPDSIGDLIHLRSLDLSGTDIGRLPDSTCSLYNLQELKLNNCVNLKELPLTLHELSNLRRLELKGTILT
KAPLRLGKLKNLHVWIDKFEVGKSREFSIQQLGELDLHGELSINNLESITNPYETNLKNKAHIVRLSLQWNLKRNNADSM
KQREVLENLQPSRHLKQLVIYCYGGTKFPRWLADNSLTNVVSLTLKNCKYCLFLPSFGLLTFLKHLTIDGLDQIVRIDAEFY
GNSSSAFASLETLRFTNMKEWEEWQCMTGAFPSLQSLFLTNCPKLKELPENLCHLTKLTVKNCSQFGAPIPRAVEIQCV
NMWPSSSDIINHSYNSLVELYINGCDSLTTFPLDLFPKLRKLSLLECCNLQMISQGHPHNHLERLSIEKCSKFESFPNEGLF
ASQLETFFIEGLEKLKSMPKCMSALLPSLNDLYIRNCPVVELSEGCLPSNVKTMRLRYCSKLVASLKGAWGTNPSLKVLNI
GNVDLECFPGEGLLPFSLPELFIRDCPNLKKLDYRGLCHLSSLKKLTLDNCPILQCLPKEGLPKSISTLIIEDCPLLKQRCKKQE
GEDWKKIAHIKSIVVDNKEVNI

>XP_014491948.1

MALAVVGGALLSAFIDVLFDRLASPEVVNFIRGKKPDKLLQKVKTQLLVVRVVLADAENRQITDPNVKEWLDLIRDVVYE
VDDLLDEVSTKASAQKEVSNAFSRLFKTKKMVSISKLEDIVERLDDILKQKESLDLKEIPVESYRPWKAQPTSLEDGYAIYG
RDKDKEAIMKLVLEDNTNGENVSMIPIVGMGGVGKTTLARSVFNDDKLKQQTFDLKAWVCVSDIFDIVKVTRSMIEEIT
RKACKLSDLNALQLELMDKLKGKRLLIVLDDVWIEDRDNWRSLTKPFLSGIRGSKVLITTRNENVAAAVPFHNVEVYHLS
KLSNEDCWLVFANHAFPHSEASETRGTLENIGKEIVKKCNGLPLAAQSLGGMLRRKRTIRDWNNVLQSDIWELPGGQC
EIIPALRISYNYLPPHLKQCFVYCSLYPKDYEFLKDELIQLWMAEDIVKPPKNGKTLEEVGHEYFDDLVSRSFFQCTNKHW
GGFFVMHDLMHDLAAFLGREFYFRADELGKKTKINRKTRHLSFTRFSDPVSDIEVFDTVKFPRTFLLINFKESPFNNETAP
RIIVSRLKYLRVLSLCNFQSQLALPDSVGELIHLRYLNLSSTNIETLPESLCNLCNLETLKLSFCSELTKLPSAMQNLVNLRHLE
IHGTPIKEMPKRMGKLNQLQNLEFYIVGKHIENSIKELGGLSNLRGSFSIKALENVTKGEEALEARIMDKNYIIHLSLEWSI
ANDSSIDFQNELDVLSRLEPHQDLELLSINGYKGSRFPEWMGSFSYHYMTFIGLYNCKNCCILPSLGQLPSLKDLIISDMN
SVKTIDAGFYGKVDGSSVIPFASLESLHILRMPCLEVWNAFDSEAFPVLKDLYITDCHNLKGDLPKHHPALETLRIRNCQY
LVSSVPMAPALRTLEILNSNKVEFHEFPLLVESINVEGGPVVESIMEAFTIRQPTFLDSLFLQNCSSAISFPGDRLPASLKSL
CISGLTKMKFPMQHKHELLDSLSINNSCDSLTSLPLAIFPNLISLQIQNCENMESLLVSGSELSKSLNSFEIRNCPNFVSFPG
EGLCMPNLTRFSVYKCDKLKSLPDQMGTFLPKIEYLKISNCHQIDSFPGGGMPPNLRTVCIKNCVKLLSNPAWVCMDM
VTSVDVWGPCDGIKSFPKESLLPPSLVYLDLYDLSSLETLDCKGLLHLTSLQELYIRRCQKLENIAGEKLPFSLIKLIIDECPLLK
QRCHKKDRQIWPKICHVRGIKIDGRWI

>XP_014492420.1

MALAVVGGSLLSAFIEVVFDKLASPELVNFIRWKKPDKLLQKMRSQLLVVKVVLDDAEKRQITDSNVKEWLDLLNDVVY
EVDDLLDEVSTKVATQKKVTNSLSHLFNRKKIVSISKLEDIVGRLDDILKQKQNLDLKDIPVENNQPWKPQPTSLEDRYDI
YGRYEDKETIMKLVLEDSSDGEEVSVIPIVGMGGIGKTTLARSVYNDGKLKQIFDLKAWVCVSDIFDIVKVTKTMIEEIIQK
PCKLNDLNTIQLDLLDKLRGKRFLIVLDDVWMEDCDSWSSLTKPFLSGIRGSKVLMTTRNENVAAVVPFHSVKVYHLNK
LSNEDCWLVFANHAFPLSRSSGNRGTLEKIGKEIVKKCNGLPLAAQSLGGMLRRKHAIRDWNNVLESDIWELPESQCKII
PALRISYNYLPPHLKRCFVYCSLYPKDYKIQKHELILLWMAEDLVKAPKKGKTLEQVGKEYFDELVSRSFFQSSSHPTSGNC
FLMHDLMHDLATFLGGEFYFRADELRKGTKINRKTRHLSFTRFSDPVSDIEIFETVKFSRTFLPIDYKDFPFNNEKAPRVIG
SMLKYLRVLSFRDFQSVLALPDSIGELIHLRYLNLSYTGIATLPESLCNLYNLQTLKLYCCSELTKLPGAMQNLVNLRHLEIL
NTSIKEMPKGMGKLNQMQNLDFYIAGKHIENSINQLRGLPNLHGSFCIQKLENVTQAEEALEARLMDKKHINDLSLEW
SVCNNNSTNFQIELDVLSNLQPHQDLKSLSISGYKGTRFPEWMENCSYYYTTILSLHNCNNCSKLPSLGQLLSLRRLHISN
MISVKTIDAGFYKKNDCSSVTPFPSLESLYIYNMPCWETWVAFDSEAFLVLKDLYIQNCPKLKGDLPDHLPALQTLAIRNC
ELLVSSVPGTPTLRTLEISESNKLAFHSFPLLVERIEIEGSPVVESMMEAITNIQPTCLHYLSIKDCSSDISFPGDHLPISLKTLII
SGLNKLKFPMQHKHELLESLSVNSSCDSLTSLPLVSFPNLIRLQIINCENMESLSVLGSDSFKSLSSFEIGRCPNFVSFSGEGL
SAPNLTRFIVYDCDKLKSLPDQMCTLLPKMEYLSISNCQQLESFPEGGMPPNLRIVEINNCEKLLSGQPWVSKDIFTYLKV
WGPCDGINSFPKEGLLPPSLTSLQLFGFSSLETLECKGLLHLTSLREFHIQSCKKLANIVGERLPISLIKLSINRCPLLQKRCHI
KDRQIWPKISHVRGINIDGRWIQ

>XP_014492421.1

MRSQLLVVKVVLDDAEKRQITDSNVKEWLDLLNDVVYEVDDLLDEVSTKVATQKKVTNSLSHLFNRKKIVSISKLEDIVG
RLDDILKQKQNLDLKDIPVENNQPWKPQPTSLEDRYDIYGRYEDKETIMKLVLEDSSDGEEVSVIPIVGMGGIGKTTLAR
SVYNDGKLKQIFDLKAWVCVSDIFDIVKVTKTMIEEIIQKPCKLNDLNTIQLDLLDKLRGKRFLIVLDDVWMEDCDSWSSL
TKPFLSGIRGSKVLMTTRNENVAAVVPFHSVKVYHLNKLSNEDCWLVFANHAFPLSRSSGNRGTLEKIGKEIVKKCNGLP
LAAQSLGGMLRRKHAIRDWNNVLESDIWELPESQCKIIPALRISYNYLPPHLKRCFVYCSLYPKDYKIQKHELILLWMAED
LVKAPKKGKTLEQVGKEYFDELVSRSFFQSSSHPTSGNCFLMHDLMHDLATFLGGEFYFRADELRKGTKINRKTRHLSFT
RFSDPVSDIEIFETVKFSRTFLPIDYKDFPFNNEKAPRVIGSMLKYLRVLSFRDFQSVLALPDSIGELIHLRYLNLSYTGIATLP

ESLCNLYNLQTLKLYCCSELTKLPGAMQNLVNLRHLEILNTSIKEMPKGMGKLNQMQNLDFYIAGKHIENSINQLRGLP
NLHGSFCIQKLENVTQAEEALEARLMDKKHINDLSLEWSVCNNNSTNFQIELDVLSNLQPHQDLKSLSISGYKGTRFPE
WMENCSYYYTTILSLHNCNNCSKLPSLGQLLSLRRLHISNMISVKTIDAGFYKKNDCSSVTPFPSLESLYIYNMPCWETW
VAFDSEAFLVLKDLYIQNCPKLKGDLPDHLPALQTLAIRNCELLVSSVPGTPTLRTLEISESNKLAFHSFPLLVERIEIEGSPV
VESMMEAITNIQPTCLHYLSIKDCSSDISFPGDHLPISLKTLIISGLNKLKFPMQHKHELLESLSVNSSCDSLTSLPLVSFPNLI
RLQIINCENMESLSVLGSDSFKSLSSFEIGRCPNFVSFSGEGLSAPNLTRFIVYDCDKLKSLPDQMCTLLPKMEYLSISNCQ
QLESFPEGGMPPNLRIVEINNCEKLLSGQPWVSKDIFTYLKVWGPCDGINSFPKEGLLPPSLTSLQLFGFSSLETLECKGLL
HLTSLREFHIQSCKKLANIVGERLPISLIKLSINRCPLLQKRCHIKDRQIWPKISHVRGINIDGRWIQ

>XP_014492422.1

MRSQLLVVKVVLDDAEKRQITDSNVKEWLDLLNDVVYEVDDLLDEVSTKVATQKKVTNSLSHLFNRKKIVSISKLEDIVG
RLDDILKQKQNLDLKDIPVENNQPWKPQPTSLEDRYDIYGRYEDKETIMKLVLEDSSDGEEVSVIPIVGMGGIGKTTLAR
SVYNDGKLKQIFDLKAWVCVSDIFDIVKVTKTMIEEIIQKPCKLNDLNTIQLDLLDKLRGKRFLIVLDDVWMEDCDSWSSL
TKPFLSGIRGSKVLMTTRNENVAAVVPFHSVKVYHLNKLSNEDCWLVFANHAFPLSRSSGNRGTLEKIGKEIVKKCNGLP
LAAQSLGGMLRRKHAIRDWNNVLESDIWELPESQCKIIPALRISYNYLPPHLKRCFVYCSLYPKDYKIQKHELILLWMAED
LVKAPKKGKTLEQVGKEYFDELVSRSFFQSSSHPTSGNCFLMHDLMHDLATFLGGEFYFRADELRKGTKINRKTRHLSFT
RFSDPVSDIEIFETVKFSRTFLPIDYKDFPFNNEKAPRVIGSMLKYLRVLSFRDFQSVLALPDSIGELIHLRYLNLSYTGIATLP
ESLCNLYNLQTLKLYCCSELTKLPGAMQNLVNLRHLEILNTSIKEMPKGMGKLNQMQNLDFYIAGKHIENSINQLRGLP
NLHGSFCIQKLENVTQAEEALEARLMDKKHINDLSLEWSVCNNNSTNFQIELDVLSNLQPHQDLKSLSISGYKGTRFPE
WMENCSYYYTTILSLHNCNNCSKLPSLGQLLSLRRLHISNMISVKTIDAGFYKKNDCSSVTPFPSLESLYIYNMPCWETW
VAFDSEAFLVLKDLYIQNCPKLKGDLPDHLPALQTLAIRNCELLVSSVPGTPTLRTLEISESNKLAFHSFPLLVERIEIEGSPV
VESMMEAITNIQPTCLHYLSIKDCSSDISFPGDHLPISLKTLIISGLNKLKFPMQHKHELLESLSVNSSCDSLTSLPLVSFPNLI
RLQIINCENMESLSVLGSDSFKSLSSFEIGRCPNFVSFSGEGLSAPNLTRFIVYDCDKLKSLPDQMCTLLPKMEYLSISNCQ
QLESFPEGGMPPNLRIVEINNCEKLLSGQPWVSKDIFTYLKVWGPCDGINSFPKEGLLPPSLTSLQLFGFSSLETLECKGLL
HLTSLREFHIQSCKKLANIVGERLPISLIKLSINRCPLLQKRCHIKDRQIWPKISHVRGINIDGRWIQ

>XP_014492432.1

MALAVVGGALLSAFIDVLFDRLASPEFVNFIRGKKPDKLLQKMKSQLLVVKVVLADAEKRQITDSNVKEWLDLLNEVVY
EVDDLLDEVYTKAATQKEVSNSFSHLFKRNKIVNISKLEDIVERLDDILKQKESLDLKEIAVENSQPWNAQTTSLEDRYGM
YGRDKDKEAIMKLVLDDSSDGEEVSVIPIVGMGGVGKTTLTRSVYNDGKLKEIFKLKAWICVSDIFDIVKVTKTMLEEITQ
KPCKLSDLNLIQLDLLEKLKGKKFLIVLDDVWIXDCDXWSSLTKPFLSGISGSKVIVTTRNERVAAVVPFHYVKVYHLNKLS
NEDCWLVFASHALPLSEDSENREILEKIGKEIVKKCNGLPLAAQSLGGMLRRKHEIKDWNNVLESDIWELPENQCKIIPAL
RISYNYLPPQLKRCFVYCSLYPKDYEFQKHDLILLWMAEDLVKASKKGKTLEEVGQEYFDDLVSRSFFQRSSTRNWGNYF
VMHDLMHDLATFLGGEFYFRADEHGNETKIDRKTRHLSFTRFSDSVSDAEVLDRVKFSRTFLPTYDNYSPFKNRNTPCII
VSMLKYLRVLSFSNLQCQLVLPDSIGELIHLRYLDLSFTGIETLPESLCNLYNLQTLNLSGCFSLTKLPSSMQNLENLRHLEIG
HSSIKEMPKRMGKLNQLQNLDLYIVGKHKENSIKELGGLPNLHGYVSIQKLENVTNGEEALEAGILNKKYINTLDLQWSL
CNDSSINFQIELDVLDKLQPHQDLKCLRVNGYNGTRFPKWVDNFSYRSMKSLILKNCNNCCMLPSLGQLPALKHILISD
MNSVKTIDAGFYKKEDCSSMAPFPSLEFLYIFNMPCWEMWSSVDSKAFPVLKDLEIKNCPKLKGDLPNHLPTLQTIKIKS
CQLLVSSFPRAPVLRTLKIWESNKVELHAFPQSVESIKISGRPMVESMIEAITDIQPTCVKELSLSDCSSAISFPGDRLPSSLK
TLNINGLNKLSFPVLHKHKLLESLSIKNSCDSLKSFPPAIFPNLTSLKIENCENLESLLVSGPESLKSLNSFVIGDCPNFVSFPV
EGFSAPNLTRFSVYDCAKLKSLPHQMGTLLPKMEYLNISNCQQIECFPEGDMPPNLRTLEIRNCEKLLISLGWISIDMVTS
LEVYGPCDSINAFPKEGLLTPSLTTLYLIDFSSLDTLDCKGLLHLTSLQTLFIKNCKKLKNIAGERLPVSLVELIITECPLLQKRC
HVKDREIWPKICHVRGIKVDGRWI

>XP_014492437.1

MALAVVGGALLSAFLDVLFDRLASPELVSLIRGKKPEKLLRKVENQLIVLRVVLADAENRQITDFNVKKWLDVLRDIVYEV
DDLLDEVSTKAATRKEVSNSFSHIFNMKRIVSINKLEDIAERLDDLLKQKESLNLKEIPVESNQPWKNQPTSLEDRYGMY
GRDKDKEAIMKLVLEDNTDGEEVSVIPIIGMGGVGKTTLARSVYNDGKLKQIFDLMTWVCVSDIFDNVKVTKAMIEEIT
KMPCNLNDLNLLQLELMDKLKGKRFLIVLDDVWIEDCDSWNSLTKPFLSGIRGSKXLVTTRNESVAAVVPFHVVKLYHL
NQLSNEDCWLVFANHAFPLSEDSENRGTLEKIGKEIVXKCNGLPLAAQSLGGMLRRKHAIRDWNNVLQSDIWELPESQ
CKIIPALRISYSHLPPHLKRCFVYCSLYPKDYEFQKNELILLWMAEDLLKAPRKEKSLEEVGEEYFDDLVSRSFFQCSSRTRG
LYFVMHDLMHDLATFLGGEFYFRANELGKETKIDRKTRHLSFARFSDPVSDIDVFETVKFPRTFLQIKNEDSPFNNEKAPR
IIVSMLKYLRVLKFSDYQSECVLPDSIGELIHLRYLNLSHSSIAMLPESLCNLSNLQTLKLISCFNLTKLPRDMQNLVNLRHL
QIFWTPIKEMPKRMGKLNQLRYLDRYVVGKHKENNIKELGGLPNLHGWFCIEKLENVTKVEEALEARIMDKKHITSLWL
EWSERNNDIIDFQIKLDALGKLQPHQDLKSLKISCYRGTRFPEWVGHFYYQNITNIRLHHCNNCCMLPSLGQLPSLRNLY
ISNMNSLKTIDAGFYKKDDCSSVTPFPSLESLDISNMPCWEVWNTFDSEAFPVLKNLFIQDCPKLKGDLPNHLPALQKL
WIRNCELLVSSIHGPPTLLELNIQNINKIAFHEFPLFVEKIIVEGGPMVESMMEVISNIQPTCLQYLMLENCSSAISFRGGR
LPPSLKTLVIRDINKLKFPLKHKHELLESLSINNSCDSLTSLPLSIFPNLTRLYITNCENVESVSVSGSEMSKGLNSFEIGHCPN
FVSVPGEGLCMPNLTSFIIYNCDKLKWLPDQMGTLVPKMEYLEISNCQQIESFPGGGMPPNLRTVEIRNCEKLLRGLGW
KSMDMVTSLSVWGPCDGIKSFPKESLLPPSLVSLKLGDFSSLETLDCKGLLHLTSLQQLYIERCQKRENIAGEKPPLSLIKL
MISECPLLKQRCHKKDRQIWPKICHVRGIKIDGRWI

>XP_014492438.1

MALAVVGGALLSAFLDVLFDRLASPELVSLIRGKKPEKLLRKVENQLIVLRVVLADAENRQITDFNVKKWLDVLRDIVYEV
DDLLDEVSTKAATRKEVSNSFSHIFNMKRIVSINKLEDIAERLDDLLKQKESLNLKEIPVESNQPWKNQPTSLEDRYGMY
GRDKDKEAIMKLVLEDNTDGEEVSVIPIIGMGGVGKTTLARSVYNDGKLKQIFDLMTWVCVSDIFDNVKVTKAMIEEIT
KMPCNLNDLNLLQLELMDKLKGKRFLIVLDDVWIEDCDSWNSLTKPFLSGIRGSKXLVTTRNESVAAVVPFHVVKLYHL
NQLSNEDCWLVFANHAFPLSEDSENRGTLEKIGKEIVXKCNGLPLAAQSLGGMLRRKHAIRDWNNVLQSDIWELPESQ
CKIIPALRISYSHLPPHLKRCFVYCSLYPKDYEFQKNELILLWMAEDLLKAPRKEKSLEEVGEEYFDDLVSRSFFQCSSRTRG
LYFVMHDLMHDLATFLGGEFYFRANELGKETKIDRKTRHLSFARFSDPVSDIDVFETVKFPRTFLQIKNEDSPFNNEKAPR
IIVSMLKYLRVLKFSDYQSECVLPDSIGELIHLRYLNLSHSSIAMLPESLCNLSNLQTLKLISCFNLTKLPRDMQNLVNLRHL
QIFWTPIKEMPKRMGKLNQLRYLDRYVVGKHKENNIKELGGLPNLHGWFCIEKLENVTKVEEALEARIMDKKHITSLWL
EWSERNNDIIDFQIKLDALGKLQPHQDLKSLKISCYRGTRFPEWVGHFYYQNITNIRLHHCNNCCMLPSLGQLPSLRNLY
ISNMNSLKTIDAGFYKKDDCSSVTPFPSLESLDISNMPCWEVWNTFDSEAFPVLKNLFIQDCPKLKGDLPNHLPALQKL
WIRNCELLVSSIHGPPTLLELNIQNINKIAFHEFPLFVEKIIVEGGPMVESMMEVISNIQPTCLQYLMLENCSSAISFRGGR
LPPSLKTLVIRDINKLKFPLKHKHELLESLSINNSCDSLTSLPLSIFPNLTRLYITNCENVESVSVSGSEMSKGLNSFEIGHCPN
FVSVPGEGLCMPNLTSFIIYNCDKLKWLPDQMGTLVPKMEYLEISNCQQIESFPGGGMPPNLRTVEIRNCEKLLRGLGW
KSMDMVTSLSVWGPCDGIKSFPKESLLPPSLVSLKLGDFSSLETLDCKGLLHLTSLQQLYIERCQKRENIAGEKPPLSLIKL
MISECPLLKQRCHKKDRQIWPKICHVRGIKIDGRWI

>XP_014492651.1

MALELVGGALLSVFLEVAFQKLASPQILDFFRARKLDQKLLNKLETKLHSIHSLADDAEGKQFTDPHVRNWLLKVKDVVL
DAEDLLDDIQMLSKRKVDDDSESQTFTGCTCKVLDFFKSSPFSSLNKEIESRMKQILDDLEFLSSQKGDLGLKTASGVGSG
LSNDLPHKSQTTPLVVGTDIYGRDHDKELIFDWLISDNNIQNQPSILSIVGIGGVGKTTLAQHVFNDPRVDEAKFDVKA
WVCVSEEFDVFEVSRVILEAVSESTDHSKDLEMIHRRLKENLIEKKFFLVLDDVWIENQSKWEEVQKSLVFGAKGSRILVT
TRTKEVASTMGSKDHSLKQLEEYDSWKLFAKHAFRDDDTQPNAEFKEIGMKIVKKCKGLPLALKIMGSLLYKKSSISEWK
SVFQSEIWEFSQNRCDIVPALALSYIHFSSHLKVCFAYCALFSKDYEFRKEDLFQLWMTENFLHCSHHSRTPEDVCQQYF
NDLLSRSFFQQLDEKEEVFVMHDLVHDLAKYVGGDMYFMWEVGKSDKIQKDTRHFSVKLRYNQYFDGFGKLCNSERL

RTFMPTGFLLINMSIHELFSKFKLLRMLSLCFVLDLQELPDSIGNLEHLRSLDLSNTAIKKLPEKICSLTHLQILKLNFCRDLE
DLPSNLYLLTNLYRLEFIETKVRKVPPHLEKRKNLEVVMNFSVGHGREFFTHLWSQ

>XP_014492652.1

MALEFVGGALLSAFLQVAFEKLASPQILDFFCAKKLDQKLLTKLETKLNSINSLADDAERKQFTDPHVRNWLLKVKDAVL
DAEDLLDDIQKLSKSQLDAESESQTSTGCTCKVLNFFKSSPISSFNKEIESRMEENLDDLEFLSSQKGALGLKTTNSVGYGL
SNELPQKSQTTSLVVGTDIYGRDHDKEVIFEWLTSDINRNQPSILSIVGMGGVGKTTLAQHVFNDPRVDEPKFNVKVW
VCVSQEFDVFKLSRAILEAVTKSTDDSRDMEMVHRRLKEKLMGKKFFLILDDLWNENQPKWEEVKKPLVFAAQGSRIL
VTTRSKEVASTMRSEVYSLKQLHEDHCWKLFAKHAFRGDDDTQPNPECRDIGMKIVKKCKGLPLALKTMGSMLYNKSS
LSEWKIVFQSEIWEFSKEQCDIIPALALSYIHLPSHLKACFVYCALFPKDYEFKKEHLIHLWMTENLLHCRQHSKTREEVCQ
QYFNDLLSRSFFQQSGEKVGLFVMHDLLNDLAKYVGGDIYFRWEVDQAEKMQKVTRHLSLELGDNKYFDGFGTLFKTE
RLRTFLPTSDRKLVYLYWYCNMSIHELFTKFKFLRILSLSHCSNLKELPDSVGNLKHLRSLDLSWTEIKKLSEKICSLPHLQIM
KLNCCSLLEELPSNLHLLTTLCRLEFIFTKVRKVPPGLEKLKNLKVMMTTFEVDHGMESGIQVLGKLNILYKDLSIGGLQYV
ENPEDALEADLKNKTHLVRLTLGWERIGNSMDSKKAEVIENLQPSKNLKELSISDYGGNKFPKWLFDDSLPNLVSLELAN
CEFCPSLPPLGLLPFLKTLYISGFDKVVSIDADFHGNNSSSFQSLQRLEFSYMRQWEKWECQFVTSAFQNLRILSLKDCPK
LKGQLPELLVPLETLEITGCQQLEVLPPRTLELDLRHSGKLQLDWAPMEWPRMGGHHMKALFSKGDGSRTLHKLEIEDS
IDDSIPLTTFPLDSFPTVKRLVLSRFGNLHMISQDQAHQDLGDLIISECPKLESFPANIHMLLPSLWRLRIMDCPRLKLLSS
GGLPSNLEEMKLINCSRLVGSLKGAFGDSSSLESLEIAELDAECFPDESLLPPSLAKLTLRDCLNLEKLDYKGLSQLSSLQSLA
LLHCPKLQCLPEQGFPQSISNLKIINCPLLKQRCQRGGQDWEKIAHIECLNMW

>XP_014492654.1

MAAETITSALVSTFLVRTIDTLASRFVHIFRARTRHKKQLSNLKMKLLAIDVVAFDAEQKQFKDPRVRDWLLRAKDAVFD
AEDLLDEIDYELSKSQEEAESQSATEKVWNSLKSSFVSFFENEIESRMDQVIEDLENLATESNILGLEKGGGVGVGSGSGT
KLTYTSLPNESVIYGRDNDKEFVFNWLISDTLNKLSILSIVGMGGMGKTSLAQHVFNDPRLEEAKFDTKIWVSVPEEFDV
LNVSRVILGTITGSTDYSVEHELIQRRLKEELSGKKFLLILDDVWNENPFKWKDVQKPLVFGSQGSRILVTTRSEKVADAM
RSEKHLLQVLKEDYCWDLFAKQAFQGANPQPDPDFIEIGKKIVEKCKGLPLALTTMGSLLYNKSSLYEWKSIMKSEIWDF
SENESGILPALRLSYLHLPSHMKKCFAFCALFPKRYRFDKESLIQLWMAENFLESPLQKKSSKEVGEQYFNDLLSWSFFQQ
VSNEEENYFIMHDLLNDLAKYVSQDICIRLRVDKPQGIPKTTRHCSFSYSELGFNGFGSSIDTQKLHTFTPIERGWVWDC
KMSIDDLFSKFKFIRVISLSHYRNLTEVPKSIGNLKHLRSLDLSWTEIEKLPESISLLFKLQILKLNQCYRLKELPSCLHQLDNL
RCLELEHAGVKNVVAYLGKLKNPQVSISSFHVEKSKEINIRQLGELNLHGCLTIDDLQKIENPSDAIEVDLKNKPHLVGLRL
KWNFIDNSSVDSEKAEDIIENLQPSKYLKELSISNYIGKQFPNWLLDNLLPNLVNLVLEGCESCPRLPPLGLLPFLKDLRME
GLDAIVNIDADFHGNNSFSFKSLQTLSFSDMRQWEKWECQAVTGAFPCLQLFSIKNCPKLKGHLPKFVALESLHVFNCK
QLEALIVSAVELHLEECGTLQLERSTMKKLTTDGHGMAASLVAMAASLEILSICSALESISDDCVSLRIFPLDFFPTLRSLKLS
GFPNLQMISQNHVHNHLRYLEIKECPRLELFPEGGLPSNLKYIQLNNCFRLVGSLKRAFGDSSSLESLRIEKVEAECFPDEG
LLPFSLTELRISNSPNLNKLNYKGLLELSSLESLYLWNCPNLQCLPEEGLPKSISLLDIRACPLLEQRYHEGGEDRKNIAHIQN
IYLYGSDDVNFV

>XP_014492660.1

MAAEMVTGVLVPTFLGRTIDTLASRLVHIFSQRKHKKQLSNLKMKLLAIDVVAFDAEQKQFTDQRVKDWLLRAKDVVF
DVEDLLDEIDYELSKSQAEAESQSATNKVWNSLNSSFVSFFEIEIESKMEQVIEDLEDLATESNILGLEKGGGVGVGSGSG
SKLTYTSLPNDSFIYGRDDDKKFVLNWLTSDTHNNLSILSIVGMGGLGKTSLAQHVFRDPRLEGKFDMKAWVSVPQEF
DVLKVSKAILGTITGSTDHSIQQEMIQKRLKEELMRKKFLLILDDVWNENPSKWEDVQKPLVFGGQGSRILVTTRSQKVA
AAMRSEKHFLQGLKGDYCWDLFAKHAFQNANPRPDPDFIEIGKKIVEKCDGLPLALKTMGSLLHNKSFLWEWESIMKS
EIWHFSENESGIFPALKLSYFHLPSHLKKCFAFCALFPKGYRFDKEYLIQLWMTQNFLENPLQKKSLKEVGEEYFSDLLSW

SFFQPSTNKEENRFIMHDLLNDLAKYVSEDFCIRLGVDEPKGIPKTTRHFSFSFSSSKSCFYGFGSSIDTQKLHTFTPIGDG
WVWDCKMSIDDLFSRFKLIRVLSLNHCSLTEMPKSIGNLKHLRSFDLSYTEIEKLPDSISLLYKLQILKLNYCRKLKEIPSCLH
ELDNLRCLELEGIGVKNVVAHLGKLKNPQVSISSFHVEKSKEINIRQLGELNLHGSLTIDDMQNIENSSDTIEVDLKNKPHL
VELELKWNFIDSSSVDSEKVEDVIENLRPSKYLKKLSISNYIGKQFPNWLLDNSLQNLVSLVLEGCESCQRLPPLGLLPFLNY
LRISGFDEIVSIDADFHENNSCSFKSLKTLYFSDMRQWEKWDCQAVTGAFPRLQHFSIRNCPKLKGYLPKFVTLKYLYVS
NCEQLEALIVSAVELRLEECGKLQLERSTMKKLTRDGHDMAASLVATVGHMLFDTSLEILDIGSALESKSDDCVSLKIFPL
DFFPTLRTLELGGFPNLQMISQDHVHNHLKYLTIKDCPRLELFPDGGFPLNLNSITLNNCFRLVGSLKRVFGDSSSLEKLSIE
NVEAECFPDEGLLPLSLSSLIISDCRNLKKLNYKGLLELSSLKTLGLWNCPNLQCLPEESLPKSVSLLYIHDCPLLEQRYHKGG
EDRKKIAHIRDIFIW

>XP_014492694.1

MQLEEHMRAIACSKIAIIVSSKTYTKSTCCLRELEKIIECRETFGQIVLPVFYEIDPFDVRDPKDDFKKALEESAHKSYSGEQL
EDVLSRWSRALTIAAGINGWNVTDFRHDAQLVEVAVSRVQTLLGYGDLSHAEFPIGLESHVKKVVGSIENHSAKVCMV
GIWGMGGSGKTTIAKAIYNRIYRQFIGKSFIENIRQENRHVDLQNKVIYQALKFKFKVKSDWMGRSIIENKLSRKKLLIVL
DDVNEFWQLANVCGNREWFGQGTVIIITTRDVRLLSKLKVDFVYKMNLMNENDSLELLCWHAFKNAKPREDLNELGR
EIVICCGGLPQALVAVGSYIFSKRFAPWKRTILARKIFADYHVLENLEKSFRGLRDDMEKDIFLDVCCFFIGKDRGCVTEILN
GCGQHADVGITVLIERGLVKVERNNKLEMHPLLRDMGREIIGQRWPMEPEKRSRLGFHDDVKHVLRNKTGTQATQGL
SLKLHSTSRDCFKAHAFKEMKRLRLLQVDHVLKWLKFLNLSNSKYLRKTPDFSGLPSLEQLILKYCPSLLKVHPSIGDLCNLL
LINLKDCTRLSSLPREVYKLKSVKTFILGCFKIDILEEDIMQMKSLITLISENTAVKQVPCSIVNSKHMGYISLRGFEGLSHNIL
SSIIRSWMSPIMNSRSYIRPLCMDMENDNWRNLAPLHSCLANIRSISVQCDTEFQLSKQLNTIFVEYGVNFTESTNHSLR
FSLIGVGSRNQFLNILSDSVSKVPSLSLTFVCIQHELILIQYLNKLE

>XP_014492699.1

MALAAVGGALLSAFFDVLFDRLASPEVLNFIRGKKPDKLLQKVKTQLIVVRVVLADAEKRQITDSNVKEWLDLLRDVVYE
VDDLLDEISTKAAIQKEVSNSFSHLFKSKKIVSITKLEDIVERLDDILKQKENLDLKEIPVESYQPWKAQPTSLEDGYAMYGR
DKDKEAIMKMVLEDSTNGETVSVIPIVGMGGVGKTTLARSVFNDGKLKQQIFDLKAWVCVSDIFDIVKVTRTMIEEITR
KPCKLNDLNALQLELIDELKGKRLLIVLDDVWIEDCENWSSLTKPFLSGNKGSKVVITTRNENVATAVPFHTVEVYHLNKL
SNEDCWLVFANHAFPQSEASETKETLEKIGKEIVKKCNGLPLAARSLGGMLRRKQTIRDWNNVLQSDIWELPESQCKIIP
ALRISYNHLPPHLKRCFVYCSLYPKDYEFKKDDLIQLWMAEDLVKATKKGKTLEEVGQEYFDDLVSRCFFQSSSGSVDDY
FVMHDLIHDLATFLGGDFYFRTDELEKETNIDRKTRHLSLTRFSDPASDIEAFDTVKFPRTFLLIDYKDSPFNNEKAPRIIVS
RLKYLRVLSLYKFQSQLALPDSVGELIHLRYLNLSYTSIETLPESLCNLCNLQTLKLSSCSELTKLPSAMQNLVNLRHLEIFGT
PITEMPKRMGKLNKLRNLDCYVVGKHTENSIKELGGLPNLHGMFYIEKLENVTKGEEALEARIMDKNHISHLSLEWSIAN
DNSIDFQVELDILSNLEPHQDLESLSIKGYKGNIFPEWVGNFSYRYMTRINLLKCNNCCMLPSLGQLPSLSHLHISKMYSV
KTIDAGFYKKDDCSSVTPFPSLKYLSISNMPCWEVWNAFDSEAFPVLKVLCIEECPNLKGDLPNHLPALQTLRIRNCELLD
CSVPGPRTLRTLEIQKCKTVAFHEFPHLVRRIDVEGGPMVESMMEAISNFQPTCLYFLRLRNCSSAISFRGGRLPASLKIL
DITGINKLKFPLQDKHELLESLSIYNSCDSLTSLPLAIFPNLTSLRITNCENMESLLVSGSEISKRLESFEIRHCPNFVSFPGEGF
CMPNLTRFRVYNCEKLKSLPDQMGTLFPKMEYLYISNCQQIESFPGGGMPPNLRTVNIIDCVKLLSNQAWVCMDMVT
SLNVWGPCDGIKSFPKESLLPPSLVSLDLFNLCSLEMLDCKGLLHLTSLQELNIRSCQKLENIAGEKLPLSLIKLTIDECPLLK
QRCHKKDRQIWPKICHVRGIKIDGRWI

>XP_014492701.1

MALAVVGGALLSAFFDVLFDRLVSPEVLNFVRGKKPDKLLQKVKTQLIVVRVVLADAEKRQITDSNVKEWLDLLRDVVY
EVDDLLDEISTKAATQKEVSNSFSHFFKSKKIVSITKLEDIVERLDDILKQKESLDLKEIPVESYQPWKAQPTSLEDGYSMYG
RDKDKEAIMKMVLEDSTNGETVSVIPIVGMGGVGKTTLARSVFNDGKLKQQIFDVKAWVCVSDIFDIVKVTRTMIEEIT

RKPCKLNDLNALQLELMDELKGKRLFIVLDDVWIEDCDNWSSLTKPFLSGNKGSKVVITTRNENVAAAVPFHTVEVYHL
NKLSNEDCWLVFANHAFPPSETSETRGTLEKIGKQIVKRCNGLPLAAQSLGGMLRRKQTIRDWNNVLQSDIWELPENQ
CKIIPALRISYNYLPPHLKRCFVYCSLYPKDYEFEKDELIQMWIAEDLVKAAKKGKTLEEVGQEYFDDLVSRCFFQSPRWSE
GGYFVMHDLIHDLATFLGGDFYFRTDELEKETNIDRKTRHLSLTRFSDPVSDIEAFDTVKFARTFLLIDYKDSPFNNEKAPL
IIVSRLKYLRVLSLYKFQSQLALPDSVGELIHLRYLNLSYTSIETLPESLCNLCNLQTLKLSSCSELTKLPSAMQNLVNLRHLEIL
GTSIKEMPKRMGKLNKLRNLDCYVVGKHTENSIKELGGLPNLHGMFSIEKLENVTKGEEALEARIMDKNHISHLSLEWSI
DNDNSIDFQVQLDVLSRLEPHQDLKMLSIHGYKGTRFPEWVGNFSYRFMTHIYLRNCNNCCMLPSLGQLPSLSHLGISN
MNVKTIDAGFYKKDDCSSVTPFPSLKHLSIYHMPCWEVWNAFDSEAFPVLKFLCIEECPNLKGDLPNHLPALQTLRIRNC
ELLDCSVPGPRTLRTLEIQKCKTVAFHEFPHLVRRIDVEGGPMVESMMEAISNFQPTCLYFLRLRNCSSAISFRGGRLPAS
LKILDITGINKLKFPLQDKHELLESLSIYNSCDSLTSLPLAIFPNLTSLRITNCENMESLLVSGSEISKRLESFEIRHCPNFVSFPG
EGFCMPNLTRFRVYNCEKLKSLPDQMGTLFPKMEYLYISNCQQIESFPGGGMPPNLRTVNIIDCVKLLSNQAWVCMD
MVTSLNVWGPCDGIKSFPKESLLPPSLVSLDLFNLCSLEMLDCKGLLHLTSLQELNIRSCQKLENIAGEKLPLSLIKLTIDECP
LLKQRCHKKDRQIWPKICHVRGIKIDGRWI

>XP_014492807.1

MSNNSSPKIKYDVFVSFRGEDVRDGFLGHLTEAFDVKKINAFVDDKLEKGEELWPSLVQAIEGSSISLIIFSPHYASSPWCL
KELVTILQCKDKYGHTVIPVFYHVKPTDVRHQSSDAYRKAFAEHARTHENEVQLWRDVFKKSADLSGIESSKFRNDADLL
KEIVDLVLRRLTKSLVNSKGLVGIDKRIADVESLIHRESEKARLIGLWGMGGIGKTTLAEEVYNKLRSKYEGCYFLANVREQ
LSRHGKDCLRNVIFSALLLRDVKIDTPNSLPEYIVRRICQMKVLIVLDDVNDSDHIGDLLGARDKFGSGSSIIVTTRDEQVL
KANEADEIYHLREFTSDEALELFNLNAFNQSVHQRQYDKLSKRIVQYAKGLPLILKVLAHRLHGKNEEVWESELDKLKKK
VLPTKVYDVIKLSYDDLDRKEKQIFLDLACFLCISSVKITTGYLKYLLKEDERDNSVVVSLERLKDKALITFSKDNVVCMHDSI
KEMAWEIVRQESPEDPGNRSRLWDPEDIYKAFKNDKVEA

>XP_014492988.1

MAAEMVTGALVSNFVDRTIDTLASRFAHIFRARKHHKKQLSNLKMKLLAIDVVAFDAEQKQFIDPRVKDWLLRAKDAV
FDAEDLLDEIDYELSKSQKEAESQSATKKVWNSLKSSFVSFFENEIESMMEQVIEDLEDLANESNILGLKKGGGVGVGSV
SGSKLTHTSLPNDSVIYGRDDDKAFVFNWLTSDTHSNLSILSIVGMGGLGKTSLAQHVFNDPRLEEANFDTKVWVSVPQ
EFDVLKVSRAILDTIIGSTYHSMQQEVIQKRLKEELMGKKFLLVLDDVWNERPSKWEDVQKPLVFGGQGSRILVTTRSEK
VADIMRSEKRLLQVLEDDYCWDLFAKHAFQNANPQPDSDFIEIGKKIVEKCNGLPLALKTMGSLLYNKSSFSEWKSIMK
SEIWDFSENESDILPALKLSYFHLPSHLKKCFAFCALFPKGDWFDKEWLIQLWMAQNFLENPLQKKSPEEVGEEYCNDLL
SWSFFQPSSNEKYFMMHDLLNDLAKYVCENICIRLGVDEPKGIPKTTRHCSFPYSKLGFDGFGSSIDTQKLHTFTPTTRIW
YWDCKMSIDDLFSRFKFIRVLSLSHCDSLTKVPKSVGNLKHLRSLDLSRTQIEKLPDSISLLYKLQILLLNYCKNLKELPSCLY
QLDNLRRLDLEGSGVQNVVAHLGKVKNLQVAMSSFHVEKIKENNFQQLGELDLHGSLTIDDLQNIENPFYALEVDLKNK
PHLEELLLEWNFIGSSSVDSEKAEDVIENLRPSKYLKKLSIKNYIGKQFPDWLLHNSLPNLVWLKLEGCESCQRLPPLGFFL
FLKYLTIARLDEIVSIGADFHGNNSSSFKSLQELYFYDMRQWEKWDCQAVTGAFPCLQYFSIRNCPKLKGHLPKFVALKT
LRVIHCQQLEELSVCSAPESISDDCVSLRIFQLDFFPTLRDLTLRGFPNLQMISQTYDQNHLEYLKIEECPKLESLPANMHM
LLPSLRELHIKECPRLESFSDGGLPLNLKFIRLDYCFRLVGSLKRVLEDHPSLLKTLMIGKVEAECFPDEGLLPLCLSELRIYDC
RNLKKLNYKGLLELSSLETLCLWKCPNLECLPEEGLPKSISRLGIEKCPLLEQRYHKEGGEDWEKVSHVPQLFIYENDFV

>XP_014493032.2

MSSNAIVQYTVSSSHTINRYDVFVSFRGEDTCNNFTGFLFQALRRKAIDVFKDDEDLKKGESIAPELLHAIQSSRLFIVVFS
KNYASSTWCLRELAEIRNCVQTSPRPIIPVFYDVDPSVVRKQSECYEKAFAEHEKRFREDKAKMEEAERWREALTEVANL
SGWDIRNKPQYVEIEKIVQTVTNILDPKICSLPKDELVRIEHTLEELVGIERRLESINNVRVIGISGMGGIGKTILARALYERIY
HQYDFHCFIDDVSKIYRYSSSLGVQKQLVSQSLNEKNQEISNSFEGTCLTWSRLHNVRALVVLDNVDEVEQLRMFTGNR

DTLLRQCLGGGSIIIVVSRDEHILRTYGVDYIYQVQPLNHKNAMQLFCKNAFKVNHISSDYEKLAWDVLSHAQGHPLAIE
VIGSSLFGRNVSQWKSALARLKENKTKNIMDVLRVSFDQLDEDHKEIFLDIACVLCDCDEKYTREVLNFRGFHVDYSLQV
LLEKSLISKMGTRIYMHNLLKDLGRYIVKEESPKEPLKRSRLWDYQDFCKAMLNNQTTQILEVIAVNSGWISGWKSETVK
IDGLSKIEKLKFLRLENVKCSGSLSHLSNELGYLTWNNYPFECLPQNFEPHKLVKLKLRNSRIQRLWSGTKELPNLKHLDLS
YSEKLVEMPDVAKALNLEEILLEDCIELRKLSPSISFLRKLTILSLRNCINLVSVPNNILGFNSLEYLSVYGCSKLFNNELLEEAS
NTEHLKKLCSVEGPLQSHSTSPLIKKARRESVSCLLPSFPTLPYLRELDLSWCKLVKIPDVIGKLSCLEKLNLKGNHFVTLPNL
TDLYRLYYLNLEHCSRLKYLPDLPSQTNLPPKVYWHEEQYMEDIFVCLRANDIYVAGLRMFYCPEIVEWERCTDMAISW
MLQILQVCMFLPYTFLLLISFDVEKQLLTYNITQSWYKSKCLTSLPFLKSLGSTIDGSEILKWFNNQLVSMDNSIIIDASPFV
HDNNWVGVVCCAILNKHAYKSRIYAYSGSDLSVYDFDHMCLFYYSRQQFLHEQHIFRGKLNTRDLSLTRDLVRIFDVLLC
RKRKDGNQVFPKFKVKKYGYRWVTVQDLHDPWLKFGSSIPSEDTKLLL

>XP_014493153.1

MAMVLDALVGHVLDELLSTVRQMKDRAVKFRDTLENLHSILEKVEPMARQIDGLNKRLDKPAKESQKLIDEMKKGKEL
VNECGKVDWWNCCYKASSQEKLQDLIDSISLYFQLDMQGNINVIVLENQMLLHQIHEKLVENVPRRIAGLCSPPEPPAF
TVGLDVHLRALKFKLLKNYHVGSVLTVTGTGGSGKSTLAKKFCSDEEVKGEFKDNIFFISLAEAPKLSTIVERLFEHNGYEK
PQFQSDEGAVYLLENLLKQIGKNPILLVLDGVLPESASLVEKFVFQIPNYKILVTSRFTIKGFGQPYVLKSLNEADALNLFRH
SASLDQTSSNIPDTIVHKIAKGCSGSPLALIVTGKSLSLEEPVVWHNRARTLSRGHSVLSYSFIGDGLLTCLQKSFDDLDAKL
AESFTDLSLFPEAQKIPAAALVDIYAEQRDXDDDIAMENIHELVKRNVADLVVTRNTTSGTVDYNYHYVTQHGLLRDLAI
HQSRNLPTEKKHRLIIDLRGNNIPKWWTTQNEYHIAAHTLSISTDEXFTSEWCNLQPNEVKVLVMNLREKKRSLPPFMK
KMNKLKVLTITNYDVNRAELENLELLDYLSDLKRIRLEKVSIPFLSKTGVPLKNLXKFSFXMCNVNEAFKNSTIKVSDXLPNL
KEMNIDXCDMEELPAGLSDTVSXKKXXXTNCHKLSKLPKEIGKLVNLESLRLTSCTKLEELPDTI

>XP_014493779.1

MADSVVSFVLDHLSQLAAREANLLYGVEDRVQSLQYELQMIKDLLNTTTRKKGMEHTVLNQIRDVAHLAEDVIDTFVA
KVAIYKRRTILGRMLRGFGQARLLHHVAEKIDKLKTTLNEIRDNKDKYDAFREATNQSAAEEEEKKERLQSLHKLRRDVEE
EHVVGFAQDSKDVVKRLQGGGSNRKVVSIVGMGGLGKTTLARKVYNSSQVKHHFDCRVWVYVSNEWRVRELLLGLL
KHLMPNFEQQRRGNKRGKKNVEDVNSPSEEELKKQILNCLERKKYLVVVDDLWKRRDWDEVRDAFPDNNNGSRILIT
SRLKEVALHAGDDVPHYLQFMSEEESWELFCRKVFRGEDYPSDLESLGKQMVQSCRGLPLSIIVLAGLLAKKGKSHREW
SRVVGHVNWYLTRDETQVKDIVLKLSYDNLPRELKPCFLYLGLFPEDFEIPVTSLLQKWVAEDFIQDTGNRDPDDVAEDY
LYELIDRSLVQVSRMESNGGLATCHIHDLLRDLCISESKEEKVFEICTDNNILIPTKPRRLSIHSDMGHYISSNNNDHSCIRS
LFFFGPHYRVHGKEWKWLLDDFKLVRVLEFGLNSCHKIPSNLGNFIHLRYLRIHRFHTFVPDSILNLWNLQTIELGFYPRDI
PVSFPVQIWKLKHLRHLNTQGPIELRGRCSESYQKMWNLQTISLLVLNKQATYLIKKGTFPNIKRMEFYMSGYEDELPHL
LPNLQLLKPLNKLVIWQLERSLLEQMIHILQILETNNGVEVVFVTQPPPQDFEIDFIF

>XP_014493977.1

MPSKAIMECSSSSSSLVTRTYDVFVSFRGEDTRNNFTDFLFQALRRKGFHAFKDDADLKKGESIAPELQQAIEGSRIFIVV
FSKNYASSTWCLRELAHICYFVAAPGRHILPIFYDVDPSDVRKQSGYFEKPFVELEERFREDNERMEEVQRWREALTQVA
NLSGWDIRNKPQYAGIEEVVQKVTDILGHKFSSLPNDNLVGMKFRVEELAKLLYSESSNDVRVFGITGMDGIGKTTLVR
ALYEGISHQYDFTCYIEDVNQIYGDSSTLGVQKQLLSHSLNEKCEEICNVSQGTYLIWKRLRGKKALIVLDNVDEVEQLKIF
TGNRDTMLRDCLGRGSIIIIISRDEQILRIHEVDEVHQVQPLKDTEAVQLFCRHAFKANVIMNGFEKMTNEVLWHAQG
HPFAIEILGSSLFGLSVSQWRSALAMLRENKSKKIMDLLRVSYEALDYTNKQIFLDISCFLHGYAVEHVVEILNFRGFYPEY
GLQVLINKSLLIVEDGRIKMHRLLMDLGQSIVREISPKEPRNWSRVWSYKNLQNILSNNMPAENLECVVCEFALFKTTLR
ADGFSNMRHLKLLKLNYVEFSGSLDYLPSELGYLCWHGYPFKSLPPRFLPYKLVSLSLANSKIKQLWEDTKPLHNLKRLDL
SSSEDLIKVPDLGEAINLEWLSLKNCTKIREIHPSIGLLRKLSYVTLEGCKSLIKLPHFEETQNLEILDLERCIKLEKIHPSIGLIRK

LKFLNLKNCESLTMLPHFREDLNLEILNLEGCRKLGQINPSIHHLKKLTVLNLKGCKRLVSLPNSILCLNSLSYLNLSACSNLS
YIQLLEKPRDEGHSKRPCIGEASLRSSIMKRWFKSPLHLFNSRKHKDSFSHLFPSPNFSCIRELDLSFCALLQIPDVIGNLHS
LERLNLSGNSFCKLPNLKELSKLYRLVLRHCRQLKYLPELPSQTYLPSVIYERPWPTTESPVWVNAPEKPLLHFINSGLEVL
NCPELVEIERERCTRMTVSWMIQILQAQRRIDPLSIPMNPKFSSVIPGSEIPRLFNHQEYSTLTRIDESPFGHDYVAAVFC
VLFEARFKRGIVSSICPPKAKGKFNDSKISMVFDSDLAIADYVSDYLWILFLDTDEFIQYQYAPLYDEIVDLVGNDHYDIDV
KKCGYRLIYDLDLELSNLTMMHGGNLPALKNDLLAIEDNK

>XP_014494614.1

MAESVVSFVLDHLSQLAAREANLLYGVEDRVQSLQYELQMIKDLLNTTKRKKGMEHTVLNQIRDVAHLAEDVIDTFVTK
VAIYKRRTILGRMLHGFGQAMLLHHVAHKIDKFKTTLNEIRENKDKYDAFIEATNQSAVEEEEEKERLQSLHKLRRDVEEE
HVVGFVQDSKDVVMRLQEGGSNRRVVSIVGMGGLGKTTLARKVYNSSQVKNQFNCRAWVYVSNECRARELLLGLLK
HLMPNFQQQRNDNKRGKKNAEDVNNPSEEELKKQVFDCLESKKYLVVVDDLWKMRDWDKVQDAFPNNNNGSRILI
TSRLKEVALHAGDDVPHYLKFLSEEESWELFCRKVFKGENRPSDLETLGKQMVQSCRGLPLSVIVLAGLLAKKEKSHREW
SKVEGHVNWYLTRDETRVRDILKLSYDNLPRRLKPCFLYLGLFPEDFEIPVTSLLQIWVAEGFIQDTGNRDPDDVAEDYLY
ELIDRSLVQVVKVKLNGGLATCQVHDLVRDLCISESKKDNMFEVCTDNNILIPTKPRRLSIQSDMGHYISSSNNDHSCIRS
LFFFDPWHYIHRREWKWLLDDFKLVRVLEIESHWCYKFPSKLGNFINLRYLRIESVSSFVPDSILNLCNLQTINMGIWKDE
NPISFPIQMWKLKHSRHLNSVCSLKLRGRCSDEKMWNLQTISPLRLNTEATSLIKKGTFPNIKRIGLYVNGCEDELSKLLP
NLQQFKHLNKLMIHKALKLLTSEVIFPSSITELSLSMIRCITDEGMNSLRNHSKIKILRLSGDDKWVGSTIDLNCVDGSFPQ
LEVLEMESLLVRKWELGHGAMQRLQDVFIHRCPVLDNLPTELCSLKGLRKVQITGSSIEEMAHILRILETDNGVQVITKN
PPPRDDSDFILNIY

>XP_014494843.1

MAESFLFSIAESLLTKLASLAFQEAFRVVGLYEDLRDLTNTLSLVKAVLLDAQQKQDHNHQLREWLTHLKTVFSEAEDVL
DEFECQTLRNKVVKAHSSTKDKVSHFFSTSNPLLFRCKMAQQIKNINNRLNKVAADRHKFNLQIIDVDTRVVHRRDMT
HSRVSNSDVIGRKNDKEKIIELLMQQNPNDDDTTLSVIPIVGIGGLGKTTLAKFVFNDNRIQECFPLKMWVCVSDDFDIK
QLIIKIINSANDEDVPPHQQNLNMLDLEQLQKQLKRKLSGQKFLLVLDDVWNEDRVQWVELRNLIQVSAAGSKVVVTT
RSHSIASMMGTLPSHILEGLSEQDSLSLLVKWAFKEGEEEKHPHLVKIGKEIVKKCKGVPLAVRTVGSLLFSKFEDSEWEY
VSRNEIWNLPQKKDDILPALKLSYDLLPSYLRQCFALFSLYPKDYEFFSNDIASIWGSLGLITLPKTNWTLEDVANQYLHEL
LSRSFLQDFQNFGSSYSFRIHDLVHDLALFVAADECLHVSLNIQNIPDNVRHLYFAESSLLENLVTKKSAVVRSVLFSDDAA
AATDEALLNTCLSKFKLLRVLVLSGSTFETLPCTIAKMKHLRYLVINNNLKIKRLSDSICKLQSLQVLSIKGCMELEVLPKGLR
KLVSIRCLEFTTKQTVLPVNEIAQLGSLEYMYIDSCQNVESIFGGVKFPSLKILCIVSCQSLKSISLDGQNFPELETLVLAACP
NLDLELWKGDPEKESPKMKLKLIGFNCLSQLVALPRGLQEAAKSLQCLFVSNCYNIETLPDWLTTLTDLKTVSLINCPKLV
SLPDNMHHLSALENLRIEDCVNLHKKYQPHVGEFWSKISHIKNIIIVEPEEPEN

>XP_014494886.1

MAESFLFSIAESLIAKLASHALQEASRVVGVYDDLRNLGKTLSLVKAVLLDAQQKQDHNHQLREWLTQLKIVFSEAEDVL
DEFECQTLQNKVVKAHGSTKDKVSHFFSTSNSLLFRYKMAQQIKYINNRLDKVAADRHKFSLQIIDVDTRVVHQRDMT
HSRVSDSDVIGRKNDKEKIIKLLMQQNPNDDDTSLSVTPIVGIGGLGKTTLAKFVFNDNRIEECFPLKMWVCVSDDFDIK
QLIIKIINSANDADVPPHQQNLNMLDLEQLQNQLKSKLSGKKFLLVLDDVWNEDRVKWVELRNLIQVGAAGSKVVVTT
RSHSIASMMGTVPSHILEGLSEEESLSLFVKWAFKEGEEEKHPHLVNIGREIVKKCKGVPLAVRTVGSLLFSKFEXSEWXY
VSXXEIXNLPQKKDDILPALKLSYDLLPSYLRQCFALFSLYPKDYXFXSXEIXXIWGSLGLIALPKTNRTLEDVANQYLHELLS
RSFLQDFENFGTSYSFRIHDLVHDLSLFVATDECLHVSFNIQNIPENIRHLSFAESSLFDNLVIKKSAAVRTVLFPKGAVGA
DGETILNTCLSKFKCLRVLNLDGLTFETLPRTIAKMKHLRYLNISNNRKIKRLPDSICKLQSLQVLIISGCIELEVLPKGLGKLI
NIRHLLFSTKQVVLPVNEMAKLGSLEYLSVTSCHNVESIFGGIKFPSLKTLCIMNCQSLKSMSLDGQNFPELETLVVVACD

NLDMELWKGDHEEESPKLKLKLVFFSNLSQLVALPKWVQEAANSLGCLIVSNCHNIETLPDWLPALTNLKSLHIIGCLNL
VSISDNIHLLTALEILKISGCPNLYKKYEPNVGEFWPKISHIKDIVIYKPKEPKKN

>XP_014494937.1

MMMAESFLFSIADSLIAKLASQLYEEASRVMGLYHXLQEFTNTLSLVKAVLLDAEQKQEHNHELREWLRQLKRVFSDAQ
DVLDEFECQTLQNQVVKAYGTTKTKVSRFFSSSNPLVFRYKMAQQIKDISNRLDKVAADRHKFGLQTIDVDTRVVHQRE
MTHSHVKDSDVIGREHDKRKIIHVLTQKNPIDIDKNISVIPIVGIGGLGKTTLAKFVFNDDRINECFPLKMWVCVCEDFDI
KQMMVRIINSANDSASHSHAPDFQQNLNILDMEQLQNQLRXKLVGKKFLLVLDDVWNEDRVKWVELRTLIQVGAAA
GSAVLVTTRSHSIASLMGTVSSHILQGISLENSVSLFVKWAFKEGEEENYPHLINIGIDIVKKCSGVPLAVRTIGSLLFSKFEA
KEWKYVRDSEIWNLPQKKEDILPTLKLSYDLMPSYLKQCFALFSLYPKDYKFVNLEVITLWGALGLLRSLKKNMTQEDVA
NQXLYELQSRSFIQDFVSYGTAYTFKIHDLVHDLALFVAKDECLLVNSHIRNIPESIRHLSFVENDLDGNSISSKSVGVRTILF
PKDGVGAKSEXFFTLVSRYKYLHILNLSYSSVETLPDFIGKLKHLRSLCLYNNKKIRRLPDSICELQSLQELDLGGCMELEALP
KGLXKLISLRDFVITTKQAVLPENDIANLSSLQYLTIQYCDNVESLFSGIELPYLKALSVISCKRLQSLSIDSKHFPAIETLCIGN
CDKLELSKGHEGQKFFLKLKTIHFLSLPQLRTLPHWLQGSIKTLLSLRLEHCSNLEVLPDWLPMLTCLKALDIKGCPKLHSLP
DGINYLSALERLEIVVCPELFRKFTLQVREHWDQMSHIKRISILESK

>XP_014494938.1

MAEYFVFEIAESLLGKLASNLYEEVSRAFDLYEDVQSFRDTLSIVKGVLLDAEEKKEKKHGLREWLRQIQNVCLDAEDVLD
GFECQNLRKQVLKASGTTRMKVNHFFSSSNSLVFRFRMARKIKNVTRRLDKIAADGNKFGLERIVIDHSPLQRREMTYS
HVDASGVIGRESDREQIIKLLMQPHPNGDGYGDQSVCVIPIVGIGGLGKTTLAKLVFSDERMDGLFQLKMWVCXSDDF
DIRHILIKIINSASDPTISVVPQESMNNXDIEQLQGRLRHKLSXQKYLLVLDDVWNDNRAKWIELKDLIKVGAVGSKILVTT
RSTXIASMMGTVPSYVLXGLSVENCXSLFLKWAFREGEEKEHPYLVDXGKEIVKKCRGVPLAVKTSGSSLFSVFDSQRWE
IMRDHELWNLKQQKDDILPSLKLSYDQMPSYLRHCFAFFSLYPKDFGFTSAEXANFWATLGLLRSPFGSQKIENIGKAYI
NELYSRSFLEDFEDFGTIYYFKLHDLVHDLSLYVAKEEFLMVNSNSRNIPEQVRHMSVVENDSLSHSLFPKSRCVRTIIFPV
NGVGVGSESLLETWIERYKYLRHLDLSNSSFEKLPNSIAQLEHLRALSLDNNSKIKKLPNSICRLQNLQMLSLRRCLGLETL
PKRLGMLISLRKMYITTKQSILSEDEFASLINLHTLIFEYCDNLKFLFRGAQVQLSSLEVLIIQSCGSLESLPLHILPKLEVLIVTR
CVMLNLSLNSERPIQKLKMKYLHIEHCPRQHTLPEWIQAASDSLRTLLILNFHCLEMLPEWLSTMTSLKMLHIVNCPQLF
HLPCDMHCLSALEDLIIDGCPELGRKCEPHSGEYWSFIAHIKCVSIGETRKRKLLFQMLSRLGLNCT

>XP_014494978.1

MGIHDLVHDLALFVAKDECLYVSSNIQNIPENVRHLSFAESSLFDNLVIKKSAAVRTILFPNGAVGANGEAMLNTCLSKFK
CLRVLDLNGSTFETLPRAIAKMKHLRYLDISKNFKIKGLPDSICNLQSLQVLFCSECIELEVLPKGLRKLISIRHLEFSTKQTVL
PVNEITKLVSLECLAIESCHNVESVFGGVKFPSLKTLHVRNCGSLKSMSLDGQNFPKLETLVVENCDNLDLELCKGDGEEE
SPKLKLKLVFLVGLPQLVALPKWVQEAANSLGCLVVFNCENIETLPDWLPTLTNLKSLYLLDCPNLVSLSDNIHHLTALETL
KIGDCANLYKKYEPNVGEFWPKISHIKDFVIHEP

>XP_014494980.1

MAEYFVFEIAESLLGKLTSNLYEEVSRAFDLYEDVQTYINELYSRSFLEDFEDFGTVYYFKLHDLVHDLSLYVAKEEFLMVN
SNSRNIPEQVRHISVVENDSLSHSFFPKSRGLRTIIFPVNGVGVGSESLLETWIKRYKYLRHLNLSNSSFATLPNSISKLEHLR
ALILDKNFKIKRLPNSICRLQNLQKLSLRRCSGLETLPKRLGMLISLRKLFITTKQSVLSEDEFASLNNLHTLIFEYCDNLKYLF
RGAQAQLSSLEVLIIQSCGSLESLPLHILPKLEVLIVTRCVMLNLSLNSERPIPRSKMKYLHIEHCPRQHTLPEWIQAASNTL
RTLSILNCHCLEMLPEWLSTMTHLKMLHIVNCSQLFHLPSNMHCLRALEDLIIDGCPELGRKCEPHSGEYWSVIAHIKCV
SIGETRKKKLLFQLHSRMGLNCT

>XP_014494986.1

MAASFTYDVFLSFRGADTRYGFVGNLYKALCERGIHTFTDQMLLKRKESIATFLSAIEESRIAIVVLSHNYASSYFCLDELA
NILHCQTKGLLVIPVFYKVHPSDVRHQRGSYGEALTMHQKRFKDMEKVQKWKMALHQVANLSGYHFEDGVGYEYKFI
KRIVDDVYHKITRDSLKHVSDYPIGVQSQVLEVRKLLDFGFEDGVHMIGIHGMGGVGKSTLARAVYNHLIGENFDGLCF
LENVREKTNKHGLKHLQSILLSQILGEKDINFTSMQQGISTIKYRLKMKKVLLVLDDVNKPDQLQAVAGGRDWFGPGSR
IIITTRDKQLLANHRVTMMYEVKGWNDNDALQLLKWKAFRNEKADPDFELALNRLVNYASGLPLALEVIGGSLVGKSIE
GWKTVIKQYESIRKREILNILKVSFDALEEEEKIPFLDIACFLKEHALREVEDILHAYYGRSMKQHIGMLVERSLLKFDYFDN
RLTMHDLVRDMPRFVWKELWKELGKNSRLWLHKDMINVSNNNVSEKL

>XP_014495007.1

MLLAFRCFEEEWESAINKNDPVGLESRSDAFCYDVFLSFIGIDTLYGFTGHLYKALNDSGIHTFIDDENHQTGEEITPTTVK
AIEESRIAITVLSINYASSTWCLDELATILDCLNRERLLVLPVFYYVDPTQVQLQKGRFGEALTKHEERLKHNMEKLLKWK
MALHQVANLFFFHIDHGAGYEYEFIGKIVEWVSKKINHAHYPIGLESQVQEVMKLLDVGCDGGAHMIGIHGTGGVGKS
TLAQEVYNNLISERFDASCFIENVREKSNKHGLQYLQSTILENLLGEKDIKLTSVEQGILMIQRRLQKKKVLLILDDVDRQE
QLQAVVGRADWFGRGSRVIITTRDEQLLASHDVQITYEVKKLNNKDALVLLKWKAFKNHDFDPRYEELLNLAVTFSDGI
PLALEVIGSNLCGKSVEEWKSVIHQLEKCPSNPVETVLKASFDSLEEKERSVFLDLACCYKGYELAEVEDILQAHYGQNMK
CYIDSLVEKSLVKLSHGTKPCYDRVTLHDMIEDMGKDIVRQESLXGPGERTRLWLLEDVRQLLENNRGTSKIEIICLDFSIF
DQEEKVEWDGKAFQNMQNLKTLIIRHGSFSKGPEYLPDNLRVIEWWRYPSNCLPSDFQPKELAICKLPCSSISTIEMKNL
LKKSVNLRVLKFSNTKV

>XP_014495024.1

MLELYEARKATNFKFPGESFPEWFDLKSSGPSCSFWFRNKFPARVLSLLIIHMNKYDNLSIFHPEVRINGKWQTGGGHY
LLEQTKFEFDLTYLSDLEVYGNLLEQPLEKEWNHVKVTYSTPTGNSWIKATGIHVFKEENSIMEDIRFDDPYIMEEEEGRR
GKKRKQHHGRHSRFDDPYSNKKLGKDLNPSQSQNHLLRTIVRRKKVLLILDDVDNRKQLQAFAGRSDWFGPGSRXIITT
RXEQLLKSHEIXRTXXVXELNENDSLQLLVWNAFKREKVDPRYEDVLKRVVTYACGLPLAIEVIGSNLVGKSVEEWESAIE
HYKRIPNGEILEILKVSFDGLGEEEKNVFLDIACCFKGSSLKEVESILSVLYDNNMKHHIGVLVEKSLIKVWGEIEMHDLIED
MGRHIDRQKSPEEPGKRRRLWLGKDIIDVLKHNKGTSKTEIICLNLSISEKEEMIEWNANAFRSMKNLKILIIRNGKFSKG
PNYFPESLRVLEWHGYPSNCLPSNFDSSKLVTCMLPHSHFTSFGFLGSSKKFENLTVLNFDGCKFLTQTPDMSVLGNLEE
VSFNWCENLVAVHDSIGFMNKLKILHAQHCIKLMSFPPLNLPTLKSLELSFCSNLEKFPEILGKMEKIRVLELEGLPIKELPP
SFQNLIGLEELYLLCKNVHLSSIIATMPNLYYFDSTNSKGWQWVKSEDAEDNVGSMVPSKVDLFSALSCILDDNFFSTGF
KGRCTYDVFLSFRGKDTRYGFIDNLYQALCNMGIHPFTDKELSKGEEIVPALQKAIAESRIAIVVFSHDYASSSFCLDELATI
LHHKSKGLLVIPVFYNVYPSYVRRQKGSYEEAFANHQERFKGQEEKLHEWKMALRQVADLSGYYFTDGDEYHQYEFIQ
RIVERVSRVISGSALDVANNPVGLLSQVVKVKKLLDVGSDDVVHMIGIL

>XP_014495318.1

MAQKQETSSASFMSKYSYDVFLSFRGPDTRFGFTGNLYSXLSQXGIFTFXDDEALRKGEEIXPSLRKAIQESRISIIXFSKXY
AFSTFCLXELLHIIQCHKNQNMWILPVFYDLEPSQLRNQTGSYQEAFEKHAERFKDDIQKLQXWRLALXXAAXXXGLHF
KTGEXYESEIVKKITEEXSTXLNRPPLHIADYPVGLKXRMXQIQELMGGEFDKKVTMLGIHGMGGIGKSTLSRAMYNLM
AHQFEASHFLANVREKSXKDGLVHIQETMLSELVGETNIKLGDVHRGIPILQHRLCGKKVLLVLDDISRKEQLRATAGGL
DWFGPGSIIIITTRDKHLLDVHGVQKQYMVGEINFKEALELFKWNAFKNKEIDPSYKEVTKRAMYYANGLPLALETIGSN
LFGKTLNEWESALETYERIPNRDIQEVLRVSYDSLDAYEKEIFLXIACFFRGCSLRYLTNLLEARGFPPEFGLRVLEEKSLIKIR
KCSHETVVMHDMIRCMGKEIVRQQSTLPHKRNRLWFYEDIVCVLEKNMENDKIQAMMLDMPEHQEEMQCIPKFQK
IKSLQMLLIEKNVGFLRSPAALPNNLRVLEWRGYPATSLPSNFHFKNLVILNLSHSYFGWDKPLQNSKVLRHLILRGCMNI

RRIPDISGFPNLTRFFVGECTNLFEIHDSVGSLLNLKEFCAEGCTKLTIGPSRIKLISLEHLCLRGCSSLLMFPEVLAPMHKLK
HVNLVGTGIRNLPLSMQNLEGIQVLSLGKGKMLEINESSNFFQNLPMFFPNLTKLYLRNLDITILPASIEECHXLKFLHVTN
CKKLQEIKGLPSSINEFSAANSPVEANSLTLKLRQAIHSAAMRIFVVPGLKIPELFDHSSRGSSVSFWFRKELPTLAVCAIIG
VWDNVKPPFIARYNFFVRVKKKISKCVSCFRCGNINWTTEDSHVIIFNRQYDYQHPLNSDIQKALLTNEWIPGKILLRINP
ENDSSKLGEIRRTGVYVNRTLSRMEDVRFENPFDLYKATTRENKLVVLGEASDTQEQEQSSALFNPTTTLESVIGEQPLN
YEYNNSDSTESTSTVDSNNQDGVLVDDQPILAPSSAETQNSEAKREIAPLISELSSESNAHVSMEIESTASQSPKVDEVEA
GLETQNQFRNTMMVLENKIHEKLTRIRKMKEELGEKLSAIKADISAIEANNSSIKANISDIKAIFLQTDQRKNN

>XP_014495419.1

MAESFLFSIAESLIEKLSSHAFQEACRVVDLYDDLQDLTKTLSSVKAVLLDAQQKQDHNHQLREWLTQLKTVFSDAEDVL
DEFECQTLRNKVVKAHGSTKDKVSHFFSTSNPLLFRFKMAQQIKDINNKLDKVAANRHKFSLQIIDVDTRVVHRRDMTH
SRVSDSDVIGRKHDKEKIIELLMQQNPDDDDTSLSVIPIVGIGGLGKTTLAKFVFNDNRIQECFPLKMWVCVSDDFDIKQ
LIIKIINSANDEDVSPHQQNLNMLDLEQLQKQLKRKLSGQKFLLVLDDVWNEDRVKWVELRNLIQVGAVGSKVVVTTR
NHSIASMMGTVPSHILEGLSEEESLSLFVKWAFKEGEEEKHPHLVNIGREIVKKCKGVPLAVRTVGSLLFSKFEXSEWXYV
SXXEIXNLPQKKDDILPALKLSYDLLPSYLRQCFALFSLYPKDYEFGSNEIAAIWGSLGLIALPKTNRTLKDVANQYLHELLSR
SFLQDFENFGTSYSFRIHDLVHDLALFVAKDECLHVRFNIQNIPENVRHLSFAESSLFDNLVIKKSAAVRTVLFPNGAGGA
NGETILNTCLSKFKCLRVLDLRGSAFETLPRGIAKMKHLRYLDISHNLKIKRLPDSICNLQSLLVLIIFRCMELEVLPKGLRKLI
SIRHLMFSTKQTVLPVNEIAKLVSLEYLVIESCHNVESIFGGVKFPSLKTLYVWNCRSLKSLSLYGQCFPELETLVVGDCGNL
DLELWKGDHEEESPKLKLKMIEFSNFSQLVGLPKWIQETANSLGCLAVSNCENIETLPDWLPTLTNLKTLHLIGCPNLVSL
SDNIHHLTALETLKIEDCANLYKKYEPNVGEFWPKISHIKDFVIYN

>XP_014495438.1

MAECFVFDITESLLSKLASYVYEEASRAYGVYDDLRGIKDTLSIVKGVLLDAEEKKEQNHGVREWLRQIRNVCFDAEDVL
DGLDCQNLRKQALKASGNTRMKVDHFFSSFNSLVFRFRMAHQIKHVRRRLDKIAADGNKFGLERIDVDNRLVQRREM
TYSHVDASGVIGRESDREKIIKLLMQHHPNGDGYGDQSICVIPIVGIGGLGKTTLAKLVFNDKRMDDLFQLKMWVCISD
DFDIRQILIKIINSASASAPTIALAHQENIKNFDIEQLQSRLRHRLSGQKYLLVLDDVWNDNRPKWTQLKDLIKVGAVGSKI
LVTTRSNSIASMMGTVPSYALEGLSLENCYALFNKWAFKEGEEKKYSNLVEIGKEIVKKCIGVPLAVRTLGSSLFLNFNLER
WEFVRDHGIWNLKQEKDDILPALKLSYDQMPSYLRHCFAFFSLYPKDYGFTGAKVVNLWVSLGLLRSEVGSQKLENAA
RLYIDELHSRSFLEDFEDFGNIYYFKVHDLVHDLAQYVAKEEILVVDSGTRNIPEQVRHLSVVENSLRDALFPKSSRVRTILF
PTDGVGVDSVALLDAWIARYKFLRILDLSDSSFETFPNSIAKLEHLRALSLENNRRIKRLPQSICKLQNLQVLTLRGCVELET
LPKGFGMLISLRQLSITTKQSILSEDEFASLINLNTLSFEFCDNLMFLFRGTQQLKSLEVLIVQSCRSLESFPFHILPKLEVLFVI
RCEMLNLSLNCENPIERLRMKFLHIEECPRQQTLPQWIEGAAETLQTLLISNLHSLEMLPEWLTSMTHLKMLHIVNCPQL
LYLPSDMHRLGALEDLTIDGCSELCQKCEPQSGEYWSFIAHIKHLSIGETREGKLLFQMQKQIRLKLQDLQN

>XP_014496055.1

MAESFLFSIAESLIAKIASHAFQEASRVVGLYDDLRDLTKTLSSVKAVLLDAQQKQDHNHQLREWLTQLKIVFSEAEDVLD
EFECQTLRNKVVKVHGSTKDKVSHFFSTSNPLLFRFKMAQQIKDINNRLDKVAENRHKFSLQIIDVDTRVVHRRDMTHS
RVSDSDVIGRKHDKEKIIELLMQQNLNDDDTSLSVIPIVGIGGLGKTILAKFVFNDNRIQECFPLRIWVCVSDDFDIKQLIIK
IINSANDVDVPPHQQNLNMLDLEQLQNQLKSKLSGKKFLLVLDDVWNEDRVKWVELRNLIQVGAAGSKVVVTTRSHSI
ASMMGTVPSHILESLSEEESLSLFVKWAFKEGEEEKHPHLVNIGREIVKKCKGVPLAVRTVGSLLFSKFEDSEWEYVSDSEI
WNLPQKKDDILPALKLSYDLMPSYLRQCFALFSLYPKDYLFDSNEIVAIWGSLGLIALPKTNRTLEDVANQYLHELLSRSFL
QDFQNFGTSYNFRIHDLVHDLALFVAKDECXYVSSNIQXIXENVRHLSFAESSLFDNLVXKKSAAVRTVRFPNGAGGAN
GETILNTCLSKFKCLRVLXLXGSXFETLPXXIAKMKHLRYLDISHNLKIKRLPDSICKLQSLQVLXXSXCXEXEVLPKGLRKLISI
RYLKFSTKQTILPVNEIXXLXSLEXLXXXSCHNVESXFGGVKFPSLKTLXVXNCXSLKSXSLDGQNFPXLEXLXVENCXNLXL

XLXKGDXEEESPKLKLKLVLFISLPQLVGLPKWVQEAANSLGCLVVSNCDNIETLPDWLPTLTNLKSLYLVGCPNLASLSD
NIHHLTALETLKIEDCANLYKKYEPNVGEFWPKISHIKDIVIHEP

>XP_014496168.1

MAELFLFSIAESLITKLASHAFQEASRVVGLYDDLRDLGKTLSFIKAVLLDAQQKQDHNHQLREWLTHLKTVFSEAEDLLD
EFECQTLQNKVVKAHGNTKDKVSRFFSNSNPLLFRYKMTQQIKYINNRLDKVAAHRHKFSLQIIDVDTRVVYRRDMTHS
RVSDLDVIGRKNDKEKIIELLMQQNPNDDDTSLFVIPIVGIGGLGKTTLAKFVFNDNRIQECFPLKMWVCVSDDFDIKQLI
IKIINSANDEDVSPHQQNLNMLDLEQLQKQLKRKLFGQKFLLVLDDVWNEDRVKWVELRNLIQVSAAGSKVVVTTRSH
SIASMMGTVPSHILEGLSEHDSLSLFVKWAFKEGEEEKHPHLVNIGREIVKKCRGVPLAVRTLGSLLFSKFEDSEWEYVRD
SEIWNLPQKKDDILPALKLSYDLLPSYLKQCFALFSLYPKDYLFPSFEIAAIWGSLGLIALPKTNRTLEDVANQYLHELLSRSF
LQDFENFGNKFRFRIHDLVHDLALFVATDECLHVSFKIQNIPDNVRHLSFTESSLFGNLVTKKSTVVRSVLFPNGAAAAID
EALLNMFLSKFKYLRVLDLRGSTCKTLPRAITKMKHLRYLDISENLNIKRLPDSICKLQSLQVLSLKTCKKLEILPKGLRKLISL
RSLEFSTKQTILPVNEIAQLGSLEYLYIESCHNVESIFGGVKFPSLKTLCVGDCQRLKSLSLDGQNFPELETLLVKDCGNLDL
ELCKGHHEEESPKLKLKLVLFFGLSQMVALPNWLQEAVKSLQRLYVLNCDNIETLPDWLTTLTDLKALGLINCPNLVSLA
DNIHHHTALESLRIEGCANLHKKYEPNVGEFWPKISHIKKIVIVEPEELKKN

>XP_014496280.1

MAESFLFSIAESLIAKLASHALQEASRVVGVYDDLRDLTKTLSSVKAVLLDADQKQDHNHQLREWLTQLKIVFSEAEDVL
DEFECQTLQNKVVKAHGSTKDKVSHFFSTSNSLLFRYKMAQQIKYINNRLDKVAADRYKFSLQIIDVDTRVVHQRDMTH
SRVSDSDVIGRKNDKEKIIKLLMQQTPNDDDTSLSVIPIVGIGGLGKTTLAKFVFNDNRIQECFPLKMWVCVSDDFGIKQ
LVIKIINSANDADVPPHQQNLNMLDLEQLQNQLKSKLSGKKFLLVLDDVWNEDRVKWVELRNLIQVGAAGSKVVVTTR
SHSIAFMMGTVPSHILEXLXEEESLSLFVKWAFKEGEEEKHPHLVNIGREIVKKCKGVPLAVRTIGSLLFSKFEASEWEYVS
RNEIWNLPQKKDDILPALKLSYDLMPSYLRQCFALFSLYPKDFVLSSYKMTALWGAHGLITLQKTNRALEDVANQYLHEL
LSRSFLQDFQNFGTSYNFRIHDLVHDLALFVAKDECLYVSSNIQNIPENVRHLSFAESSLFDNLVIKKSAAVRTVQFPNGA
VGVNGEAILNTCLSKFKCLRVLDLRGSAFETLPRAIAKMKHLRYLDISNNRKIKRLPDSICKLQSLQVLLINGCMELEVLPK
GIRKLISIHYLMFSTKQTVLPVNEIAKLVSLEYLVIESCHNVESIFGGVKFPSLKSLRILDCGSLKSISLDGQNFPELETLVVAD
CSNLDLELWKGDREEESPKLKLKLVFLIGLPQLVTLPKWVQEAANSLGCLVVFNCENIETLPDWLPTLTNLKSLHLLDCPN
LVSISDNIHHLTALETLKIGGCANLYKKYEPNVGEFWPKISHIKDIVIEEPEEPE

>XP_014496281.1

MAESFLFSIAESLIAKLASHALQEASRVVGVYDDLRDLTKTLSSVKAVLLDADQKQDHNHQLREWLTQLKIVFSEAEDVL
DEFECQTLQNKVVKAHGSTKDKRDMTHSRVSDSDVIGRKNDKEKIIKLLMQQTPNDDDTSLSVIPIVGIGGLGKTTLAKF
VFNDNRIQECFPLKMWVCVSDDFGIKQLVIKIINSANDADVPPHQQNLNMLDLEQLQNQLKSKLSGKKFLLVLDDVW
NEDRVKWVELRNLIQVGAAGSKVVVTTRSHSIAFMMGTVPSHILEXLXEEESLSLFVKWAFKEGEEEKHPHLVNIGREIV
KKCKGVPLAVRTIGSLLFSKFEASEWEYVSRNEIWNLPQKKDDILPALKLSYDLMPSYLRQCFALFSLYPKDFVLSSYKMT
ALWGAHGLITLQKTNRALEDVANQYLHELLSRSFLQDFQNFGTSYNFRIHDLVHDLALFVAKDECLYVSSNIQNIPENVR
HLSFAESSLFDNLVIKKSAAVRTVQFPNGAVGVNGEAILNTCLSKFKCLRVLDLRGSAFETLPRAIAKMKHLRYLDISNNR
KIKRLPDSICKLQSLQVLLINGCMELEVLPKGIRKLISIHYLMFSTKQTVLPVNEIAKLVSLEYLVIESCHNVESIFGGVKFPSL
KSLRILDCGSLKSISLDGQNFPELETLVVADCSNLDLELWKGDREEESPKLKLKLVFLIGLPQLVTLPKWVQEAANSLGCLV
VFNCENIETLPDWLPTLTNLKSLHLLDCPNLVSISDNIHHLTALETLKIGGCANLYKKYEPNVGEFWPKISHIKDIVIEEPEEP
E

>XP_014496804.1

MSIRTNKMKAIGVLMKQLTTSRRKFQERGKEESFDGKLEKLRLVLNNIKDVFVEVKKNEEKLLDTLAEVYDHLHRLDRRK
LHQDMHSICERIRDSARNLLPTLVFDESYKEEDNRGDKISPSSQELVHLHQKKSWTAEDFNLQDDRLKACLLRLLIFPENA
VIRKRNAINLWFEEGLIENTEEKTADELGEDVVDDLLKFKVIVRYGSTKDPIVNKFQVLPHVRSQLKLYLDGDSEYIRPSRL
LLDRKKVTVGGVDTKNVTLRNIYNIGASYLNFEPQWVTDSIKNLEXLQLGRWQDSPLHHIEVVSQEFLRELRTLKELKYLS
LRGISNIFELPSSITELESLVILDLKACHNLERLPDDISSMKSLTHLIMSECCLLEVMPKGIEKLTNLQVLKGFLISTSEKTPCRI
WDLAENLTELKRLSIRIGSEAVIRDEEFKSLKNFKKLKHLKISWSGSYPRYAKIPIILPLYLKKLHLECFPGKSLKEVFTSVQVS
FIIPPAISELNITGGKVESIHESILGRNMVEILRLKYLKQLNINIDDLKTFFPLLKYVEIKQISNHSYIVREFE

>XP_014496805.1

MSIRTNKMKAVAVLLKELMTARSKFHERGKDESFDRKLEKLRLDLSKIKDVFVRVKKNEEELLDILAEVYGHLHKLDREKL
NEDMDGICERIRDSAHNLLPKDAFNMLSKMEDHSSEELVQTHQKKSWTAEDFNLLGLPSRFCMFSLLIFPENAVIRKRN
AIHLWIEEGLITNTEKKTAEEKGEDVIHDLLKFKVIVRYGSTKDPIVDKFQILPHVRSQLKLYLDGDSEYIRPSRLLLDRKKVT
VGGVDTKNVTLRNIYNIGASYLNFGPQWVSDQWKNLEVLQLGRWQDSPLHHIEVGSEEFLRELRTMKLLKYLSLRGISR
IFELPSSIVELENLEILDLKACHNLETLPDDISSMKSLTHLIMSECSLLEVMPKGIEKLTNLQVLKGFLITEKTPCRMSDLVNL
RKLRRLSINIGSEAMIKDGEFSSLRNFLTLKHLKISWSVSDPRYSKIGVILPISLTKLHLECFPGKSFEECFTLPDLIPCELNITG
GKLETMSPQALLWNVEILRLKYLKQLNVDINNLKTFFPWLTYIEIKQISNYSYIEHVYL

>XP_014496807.1

MKAVGVLMKELTTSRRKFHERGRDESFDGKLEKLRLVLNNIKDVFVEVKKNEEKLLDTLAEVXDHLHRLDRKKLHQDME
GICKRIRDSAHNLLPTLVFDESYKEEDHRDGKISHLSEELVLPHLIELWTEKYFNLQRYPSKSCLFCLLIFPENAVIRKRIAIN
MWIAEDMVRNTMEKTAEELGEDVIDDLLKFKVIVRYGSIKDPIVNKFQILPHVRSHLKLSLERELGYMIQSRLLLDRKKVT
IGGVDTKTTTLRILFNIGTSYLNFGSQWVTHSFKNIEMLQLGRWQDSPLHHIEVGSEEFLKELRTLKQLKYLSLRGISRIFEL
PSSIGELESLVILDLKACHNLERLPDDISSMKSLTHLIMSDCCLLEVMPKGIEKLTNLQVLKGFLITTSEKTPCRISDLAENLIK
LKRLSIRIGSEAVIKDTEFESLINFTALKHLKISWSVSDPRYAKIPIILPLHLRKLHLECFPGKSLKEVFIVYFTGPRELNITGGKL
ESLSLDLTGNWKVEILRLKYLKQLNVDIDYLKSLIPRLKYVEIKQISNHSYIEREYE

>XP_014496808.1

MSIRTNKMKAISVLMKQLXTSRRKFHERGRDESFDGQLEKLRLVLNNIKDVFVEVKKNEEKLLDTLAEVYDYLHRLDRRK
LHQDMHSICERIRDSARNLLPTLVFDESYKEEDXRGXKISXSSQELVXXHQKXSWTAEDFXLQDVRLKACLLRLLIFPENAI
IRKRNAINLWIAEGLIENTEKKTEELDEDVGLIENTEKKTAEELGEDVVDNLLESNIIVRYGSTKDPRVNKFQIPTDIRSQLK
LYLDGDLKSIRPSWLLXDRKXVTIXTKNVSLRNIFNIGASYLNFKPQWVTELKNLEVLQLGRWQDSPLHHIEVGSEEFLKE
LRTLKQLKYLSLRGISRIFELPSSIAELESLVILDLKACHNLERLPDDISSMKSLTHLIMSDCCLLEGMPKGIEKLTNLQVLKGF
LITTSEKTPCTISDLAENLIELKRLSIRIGSENVIMDEEFKSLKNFKKLKHLKISWSGSYPRYAKIPIILPLHLRKLHLECFPGKSL
KEVLTSSQVYFLKPPAISELNITGGKVESMTPSFLQRLPVYILRLKYLKQLNVGIDYLKVCFPELKYVEIKQISNHSYIVRKYE
DYIDLNRLLNEEW

>XP_014496818.1

MKNRPGFQEILDHNDGAYYNDIEFFFLVEPLLYVCSVANTPQGCCWQHIFILGYHSIKTKQEQPEFLVPLVVVSQAITFSV
LCHFCFLEFDLSMLDFVGPVLEIIIRLWDCCACVRDYEENLSCLRDMTSDLLGLWVDVSVKVQLAEEQQLRRLNEVNDW
LVKAEAMQREVQAIQQRVAHAQETRIRCLINFPTSFRMGRIVSKKIGEIRELIDKGHFDVVAQEMPYAVVDEIPLEVTIGL
ESTFDELGERFDDNNVGIIGLFGMGGVGKTTLLKKFNNEFLPTKFYDVVIWVVVSKEADVGSVQQSIGNKLNVPVGKW
GGKSIDDRAIVLYNFLKRKKFVLLLDDLWERIDLLKLGIPLPDTENGSKVIFTTRSMDVCRNMEANTCIKVECLAPNEAFAL
FREKVGEETLNSHPEIFHLAKIVAKECEGLPLALITVGRSMARKTLPEWKRAARTLKIYPSRFSGMVDYVYCLLEFSYDSLP
SASHKSCFLYCSIFPEDYDIRKDELIQLWIGEGLLAEFGDDVYEARIQGEEIIASLKFACLLEDSERENRIKMHDVIRDMAL

WLACDHGSNTRFLVRDGASSGSVETYNQAKWKEVEKLSMWRQSIQKLSGKQDCSNLLTMLVRNTEITNFPDEIFLTAN
HLRVLDLSGNKRVRELPSSIGELVQLQHLDLSGTGIRKLPRELQNLKKLRCLLLNYICNRLVFPRNLISSLVSLQVFSKLPWE
DQFILPALGEPEEAVLLKELECLEYLQDISIALFCVSSMQVLLNSSKLQRCIRHLRVLSPFTSTPHTILFSLLTKMQHLEVLSM
SVSSPSSLDHVRKKGSPSSQCSMTECIPMSSKLTEHDYIVGLRELSLEGCGMLNLNWLTRAQSLQILRIYNCPSLEEVIGEE
FGHSENVFSSLEIVDLDSLPKLRSICSQVLQFPCLKEICVADCPKLIKLPFDSNSARNSLKHINGQKSWWRKLQWEDEATR
DHFASRYVPLRKILKIR

>XP_014496898.2

MDICNEAESGNTAITLQRNLDEFISNLKREEDDLKQQLQYLKSRGKKHKRVVDEWFDKLQNMKQRAMDMKNSLNES
GWPNFNVQGEIRDLIKEMKNHKKCKPMVLSNEFVGKKFERNVKKMWKFLQDDRVFIIGIYGKGGVGKTFLATYIQSEI
KRSKVFENVLWVTLSHDFNIFKLQEYIAKQIKVNLCEYDDENSRAIILESELGKGKNTIIILDNIWKYIDLAKVGIPLGVGVK
GIKVVMTSRLRHVCQEMQCLSDNMIEVGVFYDDEYEEAWELFLLGLQHFGTPPKLSPQVRDIARCVVSKCDGLPLAIRV
MARTMRGKTSIHEWRYALNKFDKWEMGVTMREEVLTVLRRSYDNLAGMKKRFLHSALLPKFFVKEDLIMMMVDM
GLLNGDRSLKEIFDEGNDIVNELINHSLLLEHDSILRMQGLVKKMAWDILKENNANMMLKCNQYMRNIYEILEWSTDL
ETVSLANNSIEEIPYGTSPYCPRLSTLLLFDNSIGHIPESFFTYMNALKTFGLSEKHNLTCLPRSLSNVRSLTSLMLHECSKLN
DIPPLGELRSLLRLQISGCSIQALPEGLKNLINLKWLDLSNNVNLELVLGSFLSSLTNIQYLNLWICSGGIKVEDVKRMTML
ECFAGTFVDKDNLNRYVREILDNGNGPQTYLIHYLDWKQNGRKMPLWGREYRLSTFMCRTMSFRDCKELSHVLPRDL
MKLLLDCNNHWVCVCDALSSNDSSPLEEICIRDWTKLTSLFCSSCSLCTRIRNFQSLKLDYLESLTTIFKELPSRDRKIELLTE
VPDKGKKRKHLKNFRRLLQGIKTQFLQGIASNVYVLYD

>XP_014496900.1

MADSVVGFLVQRLSQLLESEIKLLSGVEEKIKSLRNELKFMDIFIKSSEGKYKDAVVKEVVTQIRDVAHKAEDVVDTYILNI
AKHKRRNKLCRLFHLKEKLVVPHEVDAEIEKIRSRIDEIYKNQERYGIKEGEFQSEEAVAIEWRRKRRMDVEEEDVVGLVN
ESDTVIQQLQKDDVRLNVASIVGMGGLGKTTLARKIYNKDVVKNIFPCRAWGNVSNDYRPKEVFQSLLSCLNLSGFENL
SEENLKKEVAKGLKGKKYLIVLDDIWETRVWDDIKGAFPDDNTGSRVLITSREMHVARHAGTTSPYELPFLTEDKSWELF
FKKVFRGEKCPSELEPLGRSIVKETCGGLPLAIVILGGLFAKKEKSQREWSRMKKMSWNSTADKNEVMDILRLSYDNLPP
TLKPCFLYFGIYPEDHKISAREVIQLWGAEGFVQPQENAEPEEVGDFYLDELVDRSLVQVTERRTDGGVKICQVHDVLRD
FCISESKFCKFMDVYRESNIDTLSDTNPRRLSLLCQPQSSISVMTFDKSMSSTRSVFVFTEASKSVDDLVKRFMLARVIHG
FQPSLFPDNLKRMIHLRYLKILFVDSLPACVGNLWNLETLDVSYKKRVSSKIWKLKRLRRLCLRGRGKLPVLPKGTRMENL
QSLWLYGWSSEIKSLVDNGIFPRLVKLALSPMTLLRIDEDEEVDFLSGVQCLNHLGSLKIDEIGNLPSDNNVFPSKITKITFK
QILSWSFMKTLGQLPNLQILKLEYESGILRNLDIGKGEFCQLQVFHIKFLSIKSWRLEESAMPRLRHLRIINCYYLFQLPQQL
WSLRTLQLVHIVRPSQELTSNLQIVEFKNNCKLVLEDMW

>XP_014496944.1

MEVVVSTATENAMQIAVRVVKRQFSYFFNYNDKFEEVKCYIERLDNTRKRIQHQVNNAEMNAEEIEDDVQHCLKQLD
EKIEKYEQFINDEYHSKTRCSIGFFPNNLSLRYRLGRNATKMVEEMEAEELWNKKFDEVSYRVLPSINAALTNTSYESFAS
REKTINVCMQALEDSTINMIGLYGVGGVGKTTLVKEVAKKAQEKKLFNVVVMANITRNPNIIKIQGQIAEMLGMRLEEE
SEIVRADRIRKRLNKEKENTLIILDDLWDGLDLNRLGIPISDENDGSQQDVNDISDSSYDKMEKEELSSDFNNMTEENLSE
DQKRCKILLTSRRKQVLCNQMDVKERSTFSVGVLNETEGKTLLKRVAGIQIQNLVYDEKAIEIARMCDGLPIALVSIGRTL
KNKSSFVWEDVYQQMKKKSFMEGQEPIEFSIKLSYDHLKNEQLKCIFLHCARMGNDALVMDLVKFCIGLGLIQGVHTIG
EARNKVNMLIEELKESSLLLESYSSNRFNMHDIVRDVALSISSKEKQMFFMKNGIIDEWPHKDQLERYTAIFLHYCYINDD
LPVSIYCPRLEVLHIDNKDQFLKIPDDFFKDMIELRVLILTSFNMPCLPSSIICLSKLRMLSLEKCTLGQNLSIIGELKKLRILTLS
GSNIESFPFEFGQLDKLQLLDLSNCSKLSVIPSNVISRINILEELYMRDTLILWEAKEMIQNKNASLSELRNLNQLRNLDLHI
QNVSHVPQNLFFDKLDSYKIIIGEFNMLTEGEFKIPDKYEVVKLLVLNLKEGIDIHSETWVKMLFKNVEYLLLGELIDVHDV

FYELNVEGFQKLKHLSIVNNIGLQYIINSVERFHPLLAFPKLESLYLYKLYNLEKLCNNQLLEASFCRLKTIKIKSCDKLESLFPF
FMVGLLTMLENIEVCDCDSLKDIVAIERQPHTDSDDNIQFPQLRTLTLKSLPAFTCLYTNDKMPCSAQSLEEKGRNRNRD
VIVEVEQDDTNSCLSLFNEKISIPKLESLELSSVNIQKIWSDQSQHCFQNLLTLNVTDCDNLKYLLSFSMAVCLVNLQSLSV
SECEMMEDIFRPKDDEGTIDYVFPKLKKMEITCMEKLSSIWQPHIGLHSFHSLDSLIIRECHKLVTIFPSFMGQKFQSLQSL
TITNCKLVENIFDFEMIPQTSDINETQLHKIVLQNLSNLVSIWKDDTCEILKYNNLQSITVAGSPNLNHLFPLSITNDLENLE
FLDVRNCRAMKEIVGSDKGKNENVITFKFPRLNYVSLQSLFELVSFYGRAHTLEWPSLKRLLILRCGKLEGINTDISNSQM
KPIGLAAEKVIYNLEYMAMSFREVEWLQNYIINVHRMQNLQTVVLHGLKNVEVLFWLLHRLPNLKRLTLGFCHLKRIWA
PASLISREKIGVVMQLQALELKNIWPLEEIGFEHEVLLQRVERLTIQRCTKLKILASSSVSFGYLTYLEVENCMLRSLMTCST
AKTLVQLKTMKVSSCPKLVEIISENEGEEIQEIEFKLLRSLELVSLQNLTSFMNVDKCDLKFPVMENLVVSECPKMTKFSKV
QSAPNLQKVQVVATEKDKWYWEGDLNATLKKHFTHQVSFEYSKHMKLKDYPEMKDVCQGKLVFQDNFFGSLKKLEF
DAASKREIVLPSYVLPYLKNLEELNVESCKSARVIFDIDDCEIKDTVFRLKKLTLKDLSNMKWIWNKNPEGIASFPNLEEVL
VNSCGTLVTLFPSTLARNLSKLKTLTIHNCCKLVEIVEKEEEMEDEITEMFEFPCLSKLFLWNLPMLICFYPRKHHLKCPILE
RLHVAYCRKLKLFTSQPHHSLPHPMFLIEEVVPKLKEVILNEKNITLLKDGHSPDLLHKLNYLDLASEDYENKKDSLPFDFL
QKVPNLEYLVVRQCFGLKEIFPSKKLDGDHDGILLAGLNKLSLNKLLELESIGLDHPWVKPYTEKLQGLAVIKCPRLERLVN
CVTSFINLKQLIVKNCKRMKYLFTFSTAKSLGKLETLRIENCESMKEIIEKEDENGYDEIIFGRLTKLWLYSLPKLVSFYSGND
TLQFSSLQIMRLFKCPNMKTFSQGNTNAPMFYGIKSSSDSDLTFHSDLNMTVESLFHEQGFFEYSKQMILLDYLEMRGF
GPVKHVFPSKFFGSLKKLEFDGASKGDTVIPSNVLPYLKSLEELNVHSSDEVQVIFGMDHNSRAKSKETVFHMKKLTLKD
LSNLKCILNKNLQGSVSFPNLQELFIDGCGSLVTLFARKLQTLEMQKCDKLVEIVGNEDATTETFVFEFPCLSSLTLYNLTDL
SCFYPGKHHLECPQLEILHVAYCPKLNLFKSKIHDSHRQTVAEAPINWLQQQPLFMVEKVAPKLRGLTLNEKNMMLLSD
EHVPEVNLTNLNLLRLCFEDDKNEKDSLPFEFMNKVLNLEHLRVQRCFGVKEIFPSQKLQVHDGIPASLKGLTLFELNELE
SIGFEHPWVSPYSEKLQTLRVVNCPMLQKLGCHAMSFLNLKELYVKDCDRMEYLFTFSTAKCLVKLETLIIKNCESIKEIAN
IEDEDGCDKIIFGKLTTLRLYSLPRLQSFISGNVTLQFSYLRNATVIDCPNMKTFAERVLNVPRILSIETSLEDSDLFLHNDLP
ASNTGKSILGRLASFYKQRNRL

>XP_014496947.1

MDPIVSTATESALNITASLVKRQVGYFFNYKDKFKELESYIEKLEHNKERLQHQVDSALRSAEEIEKDVQRCLTLMDDKIK
EYKSYINDECHAKTICSIGFFPNNFRLRYQLGRKATKMVEEIIGNELWKTSFDNVSYQEFPSIDATFSNNGYESFGSRTKT
MEMIMKALQDSTVGMIGVYGPGGVGKTTLVKEIANKAREKNLFETVIIANITGNPDFKIIQEQIAGMLGMKLEEESEIAR
VDRIRKRLKNEKENTLIILDDLWGGVDFNKLGIPCNDDASQQEVNDMSDFGSNNDISDFGYYKTEMKEFSKVDLDKMR
KEKLSKDYRGGKILLTSRNKQVLCNEMDVQESSIFSVGVLDEKESETLLKKVAGVKNSEFDRNATQIAKWSAGFPIALVSI
GRTLKNKSLSTWEDVCQQIKRQNFTSEWGFTDFSVKLSYDHLKNEELKCIFLQCARMGNDALIMDLVKFCVGLNLLPGV
HTITDARKRVKEMIKELEESSLLVKSYSIDRFNMHDIVRDVALSISSKEKHVLYMKNAILDEWPHENDFERYSAIFIHFCDI
NDELPESIHCPRLEVLHIDNQNESFQIPDQLFKSMVRLRVLVLTGITLSCLPSSIKCLKKLRMLCLERCTLGENLSIIAELKNIR
ILTFSGSNIKNLPLDFGKLDKLQFLDISNCLKLRQVTSNIIPRMGILEEFYIRDNLIIWEAEENMTSENASLSELRHLNKLQSL
DIHIHCSSHFPQNLFFDRLNNYKIVIGEFNLFNLLKVGEFKVPDKYEEVKFLAVNLKEGIDIHSEKWIKMLFKSVEHLLLGEL
NDVEDIFYELNVEGFPNLKHLSIVHNFGIKYIINPTERFHSFVAFPKLESIWLYKLYNLEIICDNQLIKTSFHNLKVIKIQTCIKL
VNLFLVSMVRLLTMLETIEVCDCDSLKEIVSNESQTHTIRNDKIEFPRLRLLILKYLPTFICLYNVDKIRGSAHPMQDQVIQ
QRNKDIVVDDDHRVTNSCLPLFNEKVSTPKLEWLELSSINIHKIWSDQCNHFFQNLLTLNVTDCGNLKYLLSFSMAESLA
NLQSIFVSECEMMEDIFRPEDAEYIDVFPKLKKMEIICMEKLSTIWKSDIGLHSFSNLNSLVIRECHKIVTIFPNYMGQRLQ
SLQSLTVRDCELVENIFDFANIRHTYDIIEANLVNIFLEKLPNLVNVWKGDTDEILKCNNLQRIEVYKSPKLKYLFPFCIAND
LQKLEVLEVWYCWAMTEIIGLDIHSSETPITFKFPHLNTLSLIDLHELRSFYSGIHTLEWPPLKILEIVDCSMLEGLTSEITNS
KEQPIVLATKKAIYNVENMSVSLKEAEWLQKYIVNVYRMHKLEELTLYRLKNNKILFWFLHRLPNLKSLRLGLCHMKRIW
TPESLSSRGKIGGVIQLKELGLYNMLSLEDIGFDHDVLLQRVERLIISGCKKLTNLASSRVSFSNLTSLKVVNCMMRNLMT
LSTAKTLVQLTTLKVCSCPMIVEIVAENEEEKVQQVEFKQLKSLKLVSLQNLTSFSSVEKCNLKFPLLEKLVVSECPQMTKL

SKVQSAPNLQKVHVEEGDKDKWYWEGDLNATLQTHFTDQVSFEYSKDINLVDYPERKVRHGKFSFPDNFFGCLKKLEF
DEACKRDTLIPSHVLPYLKNLEELNVEKCESAQLIFDVDESEIQMHGMVFRLKKLTLKHLSNLKCVWKENIEEIVSFYNLER
VHVDGCGSLVTLFPLSLAKHLGKLNTLYIEECEKMVEIVGREDKMEHGTTIMFEFPCLSYLYLEKMPLLSCFYPGKHHLEC
PLLYMLCVECCPKLKVFRSNFDDDGKKEVLKAPTNLQQPLFSIEKVFPKLEGLVLNEEYIKLMTDARLPQDLLCKLRILTLFF
EDVNNGKESLPFDFFHKLPNLEGLGVRKCFGLKEIFPSQKLQVHDKVLAGLKRLYLSELSELECIGLEHPWVQPYSKKLKLL
NLYSCPRVENIVLCAVSFINLKELCVNLCEKMEYLFTFATLKSLVKLENLSIKKCESIKEVVKKDDEDGCDEIVFGRLRSITLKY
LPRLIIFYSGNATLQCSCLQNVMVVECPNMKTFSEGVTKLAIFSGIQTSDDSDFTFYVDLNTTVQRLFHEKDFFNYSKFMI
LHDYLGMMRVQHTKPTISDHFFGSFTKLEFDITWNRSFAIPSHILPYLKNLKELNVHSCDVVQVIFDTDEIEVETKGIIFGL
KKLTLKHLSNLKCIWKENLQGIVSFSNLKQVNVDGCGSLVTVFPLSLAKHLGKLKILDIQECEKMVEIVGREDEMEHGTTI
MFEFPCLSYLNLEKMPLLSCFYPKKHDLECPLLDHLYVECCPKLKVFRSSFDDDSKKEVLEAPTNFLQQPLFSIEKVSPKLE
GLTLNEESIKLMSDARLSQDLLCKLESLILSFEDDNNEKDSLPFDFLHKLPNLEWLTVQKCFGLKEIFPSQKLQVHDKVLAG
LKRLFLLELSELECIGLEHPWVQPFINLTKLSVKLCEKVEYLFTFSTLKSLVKLETVKIQKCDSIKEIVKKDDEDDCDEIVFIRLR
SIELNSLPKLISFYSGNATLQCSYLKNVMVAECPNMITFSQGFIKVPTFLKIQTSKDSDSTFDGDLNTTIQKLFHNQVIHES

>XP_014498041.1

MGCCEVLPHDFVLEGDRIIKMVEAVVSFAVDRLGDLLIEEARLLSGVSNKVKSMQNELKMMQCFLRDAESRQDESDTI
KNYISEVRKLAYDAEDVIEIYAIKVAFGISIGTKNPLSRAKNIHKVGSELITINSRISDLTRSLQTYGLTATKDNEEASEVKRQL
RWSYSHIVDEFIVGLDQDINKVAEWLINENQDCRFVYICGMGGLGKTTLAKSIYHYNAIRRNFDGFAWAYISQQCKKRD
VWEGILLKLISPTKEERDEITKMKDDELARKLFKVQQEKKCLIILDDIWSNEAWDILSPAFPSQNTRSKIVFTSRNKDISLHV
NPEGLLHEPSCLNAEDSWALFKKKAFPRQDNPESTTSDDFKRLGREMVAKCAGLPLAIIVLGGLLATKESVSEWEKIHRH
LSSYLIGAEVRDRRRLDEVLDLSYQDLPCQLKPCFLYLSQFPEDSEIPKTKLLQLWVAEGVVSSQYESDRDETMEDVAERY
LGNLISRCMVQIGQMGSTGRIKTYRLHDLMRDLCLSKARKENFLYIINGSQQNSAIIATHSSNISEATQIDEVRRLAVYLD
QHVDQLIPQDKQVNERLRSLVFFHDKKCRMENWDLVRGVFVKFKLLRVLDLEGIKGLKGQSLPKEVGNLLWLKFLSLKR
TRIQVLPSSLGNLENLQFLNLQTVNKVSWDSTVEIPNVICKLKRLRHLYLPNWCGNIVNNLQLDNLTNLQTLVNFPASKC
DVKDLLKLKRLRKLVLNDPRHFQKFSESFSPPNKRLDCLQSLSLRTDMLSFPENVVDVEKLVLGCPFLRKLQVEGRMDRL
PDASLFPRHISKLTLWGCRLVEDPMVTLEKLPNLKFLNGWDMFVGKKMTCSPNGFLQLKVLVLRGLPKLDEWTIENQA
MRNLYRLSISDCNNLKRVPDGLRYITSLRELEIRWMPKSFKTRLGTSGEDYPKVQHVPSIIFLN

>XP_014498042.1

MVEAVVSFAVDRLGDLLIEEARLLSGVSNKVKSMQNELKMMQCFLRDAESRQDESDTIKNYISEVRKLAYDAEDVIEIYA
IKVAFGISIGTKNPLSRAKNIHKVGSELITINSRISDLTRSLQTYGLTATKDNEEASEVKRQLRWSYSHIVDEFIVGLDQDINK
VAEWLINENQDCRFVYICGMGGLGKTTLAKSIYHYNAIRRNFDGFAWAYISQQCKKRDVWEGILLKLISPTKEERDEITK
MKDDELARKLFKVQQEKKCLIILDDIWSNEAWDILSPAFPSQNTRSKIVFTSRNKDISLHVNPEGLLHEPSCLNAEDSWA
LFKKKAFPRQDNPESTTSDDFKRLGREMVAKCAGLPLAIIVLGGLLATKESVSEWEKIHRHLSSYLIGAEVRDRRRLDEVL
DLSYQDLPCQLKPCFLYLSQFPEDSEIPKTKLLQLWVAEGVVSSQYESDRDETMEDVAERYLGNLISRCMVQIGQMGST
GRIKTYRLHDLMRDLCLSKARKENFLYIINGSQQNSAIIATHSSNISEATQIDEVRRLAVYLDQHVDQLIPQDKQVNERLR
SLVFFHDKKCRMENWDLVRGVFVKFKLLRVLDLEGIKGLKGQSLPKEVGNLLWLKFLSLKRTRIQVLPSSLGNLENLQFL
NLQTVNKVSWDSTVEIPNVICKLKRLRHLYLPNWCGNIVNNLQLDNLTNLQTLVNFPASKCDVKDLLKLKRLRKLVLND
PRHFQKFSESFSPPNKRLDCLQSLSLRTDMLSFPENVVDVEKLVLGCPFLRKLQVEGRMDRLPDASLFPRHISKLTLWGC
RLVEDPMVTLEKLPNLKFLNGWDMFVGKKMTCSPNGFLQLKVLVLRGLPKLDEWTIENQAMRNLYRLSISDCNNLKR
VPDGLRYITSLRELEIRWMPKSFKTRLGTSGEDYPKVQHVPSIIFLN

>XP_014498043.1

MVEAVVSFAVDRLGDLLIEEARLLSGVSNKVKSMQNELKMMQCFLRDAESRQDESDTIKNYISEVRKLAYDAEDVIEIYA
IKVAFGISIGTKNPLSRAKNIHKVGSELITINSRISDLTRSLQTYGLTATKDNEEASEVKRQLRWSYSHIVDEFIVGLDQDINK
VAEWLINENQDCRFVYICGMGGLGKTTLAKSIYHYNAIRRNFDGFAWAYISQQCKKRDVWEGILLKLISPTKEERDEITK
MKDDELARKLFKVQQEKKCLIILDDIWSNEAWDILSPAFPSQNTRSKIVFTSRNKDISLHVNPEGLLHEPSCLNAEDSWA
LFKKKAFPRQDNPESTTSDDFKRLGREMVAKCAGLPLAIIVLGGLLATKESVSEWEKIHRHLSSYLIGAEVRDRRRLDEVL
DLSYQDLPCQLKPCFLYLSQFPEDSEIPKTKLLQLWVAEGVVSSQYESDRDETMEDVAERYLGNLISRCMVQIGQMGST
GRIKTYRLHDLMRDLCLSKARKENFLYIINGSQQNSAIIATHSSNISEATQIDEVRRLAVYLDQHVDQLIPQDKQVNERLR
SLVFFHDKKCRMENWDLVRGVFVKFKLLRVLDLEGIKGLKGQSLPKEVGNLLWLKFLSLKRTRIQVLPSSLGNLENLQFL
NLQTVNKVSWDSTVEIPNVICKLKRLRHLYLPNWCGNIVNNLQLDNLTNLQTLVNFPASKCDVKDLLKLKRLRKLVLND
PRHFQKFSESFSPPNKRLDCLQSLSLRTDMLSFPENVVDVEKLVLGCPFLRKLQVEGRMDRLPDASLFPRHISKLTLWGC
RLVEDPMVTLEKLPNLKFLNGWDMFVGKKMTCSPNGFLQLKVLVLRGLPKLDEWTIENQAMRNLYRLSISDCNNLKR
VPDGLRYITSLRELEIRWMPKSFKTRLGTSGEDYPKVQHVPSIIFLN

>XP_014498044.1

MADSIVVFLIDKLTRLLVEEAKLLTGVRDQVTSLQSELRFMNLFLRNSQGKRKEHDMVAELVSQIRDVAHEAEDVIDTYV
ACIIKQSRRNVIGKVGFRGVDHALMLHQVAVKVDGIKARIKEIFDNKERYGIEDGRRGSEEEAERIRKQRREVEEEEVVG
FALDSKVVIEKLMVSDSRLKVVSVVGMGGLGKTTLARKVYNSNRVKNVFPCRAWGYVSNDYRPREFFLSLLKCLLSTSKY
SSLFKKREETSVSDEELKMKVRECLNRSKYLVVVDDVWQKQVWNEVKGAFPDDQNGSRILMTTRSAEVASHAGPVPP
YALPFLTKEESWELLSKKVFRGGEDCPSDLESLGKLIADSCDGLPLALVVMAGILANKMSPRDWSRIKDHVNWHLGRD
NTLKDILKLSYDSLPARLKPCFLYFGMYPEDYRIPVKQLIQLWISEGLLTQETSGGQDIPEPEYIAEEYLDELVDRSLIQVVSR
TNDGGVKTCRIHDLLRDLCISESREDKFFEVCGEIDIQNLNSCPRKLSLQGTLFHFSSSMVSDYTISATRSLLCFGQEVYKV
KANHWRWLLKSFRLARVLDLGRMNVNSIPTDLEKLIHLRYLRIHSHNLETIPPSICRLWNLETLDLRGSPIKSFSGELWQL
KQLRHLLLFGPVGLPEMPSESKTMPNLQTLSTVALDPRTASLLDSRRFPGMTKLGIHYERRDKCNARIQLQSLHRLSHLR
KLKVIGTTEIPQNANVFPSNINKISLTKFGFFNSTVMHTLGKLPNLQVLKLSSQTNDTRFDLHCATGGFLQLQVFEMIAIK
VKMWRVDRGSMPRVRRLVVRSCKSLTQLPKEVWSLNTLREVQVLWPCTELAKGLQNLVMNNGCKLVVYPLSANDEL
DFL

>XP_014498240.1

MGRPKDEYYWNQVDREEDGGLKCKHCGLKFKGGVSRIKAHIDLIEGKGIRICPTSPKCITSSDHSHQDINAITLSQGVES
NLEMDGGASTSLAASFEGNEEQCDTGVLTTLQSKLDELRSDLTREEKDIQGQLQLLESHGKRCKRKVDLWLNEIQNMK
QRAIDMKNSLNQFRCSDFYVPQGEMYSAEESQKKIQHLTEEIQKHKKLKPLVLSNEYFGRKFEKNVETLWKLMRDDRV
FIIGIYGMGGVGKTFLAKYMQSEIKRTKTFENVLWFTVPYSFSIFSLQEDIAKIIKVRFHTYDETERAMILTKELEKRGNIILIL
DDVWRYVDLEMVGISLRINGIKLIITSRLRDIFQQMDCLSINIIEVSPFYYYHHYNSCDIYYRNDYDDDSDEDDDKYDEALK
LFLLKLGSYGTPLTLSPEVRDIVRYVVKECDGLPLGISVMAQTMKGKTDIHWWRRVQNKFDKLKMGVEMQDKVFTVL
RRSYFNLKEKDWQKCFLYIALLPNFMRRNCLIKKLVDTGQLEGNGSLEEIFDEANVLVDKLVNDSLLEANKEKEIKHLSSY
KNDLVLSMHGLVRKMALNILKQSVNNMMIKCNGNMTKIPYTEKWATDLEVVSLAHNNIQVIPEGTSPNCPRLSTLLLF
ENSIYHIPECFFAHMEALKTLDLSKNKSLTCLPHSLSNLTSLTSLMLHECSELNYIPPLGELHSLLRLQISSCSIKAPPQGLENL
INLKWLDLSMNKNLKLVPGSFLPSLTKIHYLDLLGCAGGIEVEDVKGMTMLECFAGTFVVQDNYKDNFSRYVREILDSTN
GPQTYFINLDNKRNMSTLIEYPFSRFKDRTMSFSDCKELIHFLPRDLLKLELKYNDHWICLCYGLSSYDNSLLEEIRIYDWK
KLKSLFCSSCYLCTDIKNLQSLNLNCLESLTVICKKLPENDMFSSLKTLCVHKCHQMKILLTSKLVRQLQNLESITVSHCNSIE
QIFGVAYDKDKDEEDENEDYEDEEDENEDYEDEDDEGDSNIIILRKLTWLSITYLPQLKTVYKGILICTSGFKSSIENCPQLC
KPRIEYEAS

>XP_014498241.1

MGRPKDEYYWNQVDREEDGGLKCKHCGLKFKGGVSRIKAHIDLIEGKGIRICPTSPKCITSSDHSHQDINAITLSQGVES
NLEMDGGASTSLAASFEGNEEQCDTGVLTTLQSKLDELRSDLTREEKDIQGQLQLLESHGKRCKRKVDLWLNEIQNMK
QRAIDMKNSLNQFRCSDFYVPQGEMYSAEESQKKIQHLTEEIQKHKKLKPLVLSNEYFGRKFEKNVETLWKLMRDDRV
FIIGIYGMGGVGKTFLAKYMQSEIKRTKTFENVLWFTVPYSFSIFSLQEDIAKIIKVRFHTYDETERAMILTKELEKRGNIILIL
DDVWRYVDLEMVGISLRINGIKLIITSRLRDIFQQMDCLSINIIEVSPFYYYHHYNSCDIYYRNDYDDDSDEDDDKYDEALK
LFLLKLGSYGTPLTLSPEVRDIVRYVVKECDGLPLGISVMAQTMKGKTDIHWWRRVQNKFDKLKMGVEMQDKVFTVL
RRSYFNLKEKDWQKCFLYIALLPNFMRRNCLIKKLVDTGQLEGNGSLEEIFDEANVLVDKLVNDSLLEANKEKEIKHLSSY
KNDLVLSMHGLVRKMALNILKQSVNNMMIKCNGNMTKIPYTEKWATDLEVVSLAHNNIQVIPEGTSPNCPRLSTLLLF
ENSIYHIPECFFAHMEALKTLDLSKNKSLTCLPHSLSNLTSLTSLMLHECSELNYIPPLGELHSLLRLQISSCSIKAPPQGLENL
INLKWLDLSMNKNLKLVPGSFLPSLTKIHYLDLLGCAGGIEVEDVKGMTMLECFAGTFVVQDNYKDNFSRYVREILDSTN
GPQTYFINLDNKRNMSTLIEYPFSRFKDRTMSFSDCKELIHFLPRDLLKLELKYNDHWICLCYGLSSYDNSLLEEIRIYDWK
KLKSLFCSSCYLCTDIKNLQSLNLNCLESLTVICKKLPENDMFSSLKTLCVHKCHQMKILLTSKLVRQLQNLESITVSHCNSIE
QIFGVAYDKDKDEEDENEDYEDEEDENEDYEDEDDEGDSNIIILRKLTWLSITYLPQLKTVYKGILICTSGFKSSIENCPQLC
KPRIEYEAS

>XP_014498528.2

MFVPNFIWQRMEVIAQIVLQNLNSFAQEEFGIIWNLKDDVQQMKRTVSAIKAVLLDAEGKANNLQISNWLEELKDVLY
DADDLLNDISSEAMKRKVIGARKILRKIQVFFSQENQIVYSFKLGHQMKAIQKRLDAIAKNKITLQLTDRPMETPIAYRRQ
RQTYSFVREDDVIGRKEEKKLLESYLLDTKVSVIDNVSVLAIVGFGGLGKTTLAQLVFNDNAVQCSFEQKMWVCVSDEF
DISKIAEKMIGNDKNSEIEQLQQDLRNKVRGKKFLLVLDDVWNEDRELWLKFKSLVVEGGKGSAIIVTTRSRTVAKIVAT
HPPLFLKGLDLERSWKLFSRVAFDEGKEPNDMELLAMGRDIVKKCAGVPLAIRTIGSLLYSRNLGRSDWQYFSEVEFSKI
DQHKDNIFSILKLSYDHLPSFLKKCFAYCSLFPKDFEFDKKTLIQLWVAEGFIQPSRDNRCEEDVGHEYFMNLLSMSLFQN
VTLDDCGDILTCKMHDLIHDLAQLVVGKEYAFVEGKKEHIENRTRYLSSCTSLHFSEKTSSSSNKLRTFILLGQPVYGSQNL
GPPPSLQFPFLLSIKCLRVLTLCGLHLITIPNSIRELKQLRYLDLSMNRFLVSLPPDVTSLHNLQTLKLSGCGKLKELPSDINKS
LRHLELNDCGKLCMPCGLGQLTNLQTLTHFILDSESKNVDISELSGLNNLRGKLVIKCLDSLRKNAAVVESANILLEKQHL
QDLELRWGLGETKYWVGPIEIRRKEDENTWVENETTLYINDKYQMKDEKILQGLQPHHSIKRLVIDGYHGNSLPDWIG
NLSSLLSLKISNCYGLKSIPDGIRNLVSLQRLCIYRCSMLERRCARGHGGEWSKIAHIPEVLLPAFNPIAFNATDLRQQPLV
CVNHHFLAMAPWFMLLVEKMTMWKHPIDRQKLQIVACIINQILMVNGSASSMNSLR

>XP_014498529.2

MEVIAQIVLQNLNSFAQEEFGIIWNLKDDVQQMKRTVSAIKAVLLDAEGKANNLQISNWLEELKDVLYDADDLLNDISS
EAMKRKVIGARKILRKIQVFFSQENQIVYSFKLGHQMKAIQKRLDAIAKNKITLQLTDRPMETPIAYRRQRQTYSFVRED
DVIGRKEEKKLLESYLLDTKVSVIDNVSVLAIVGFGGLGKTTLAQLVFNDNAVQCSFEQKMWVCVSDEFDISKIAEKMIG
NDKNSEIEQLQQDLRNKVRGKKFLLVLDDVWNEDRELWLKFKSLVVEGGKGSAIIVTTRSRTVAKIVATHPPLFLKGLDL
ERSWKLFSRVAFDEGKEPNDMELLAMGRDIVKKCAGVPLAIRTIGSLLYSRNLGRSDWQYFSEVEFSKIDQHKDNIFSILK
LSYDHLPSFLKKCFAYCSLFPKDFEFDKKTLIQLWVAEGFIQPSRDNRCEEDVGHEYFMNLLSMSLFQNVTLDDCGDILT
CKMHDLIHDLAQLVVGKEYAFVEGKKEHIENRTRYLSSCTSLHFSEKTSSSSNKLRTFILLGQPVYGSQNLGPPPSLQFPFL
LSIKCLRVLTLCGLHLITIPNSIRELKQLRYLDLSMNRFLVSLPPDVTSLHNLQTLKLSGCGKLKELPSDINKSLRHLELNDCG
KLCMPCGLGQLTNLQTLTHFILDSESKNVDISELSGLNNLRGKLVIKCLDSLRKNAAVVESANILLEKQHLQDLELRWGLG
ETKYWVGPIEIRRKEDENTWVENETTLYINDKYQMKDEKILQGLQPHHSIKRLVIDGYHGNSLPDWIGNLSSLLSLKISNC
YGLKSIPDGIRNLVSLQRLCIYRCSMLERRCARGHGGEWSKIAHIPEVLLPAFNPIAFNATDLRQQPLVCVNHHFLAMAP
WFMLLVEKMTMWKHPIDRQKLQIVACIINQILMVNGSASSMNSLR

>XP_014498731.1

MMAQAVVSFIVQSLGDLLIREAVFLYGVEDQVLQLQTELRMMRSYLQDADRRQDENESLRSWISEIREAAYDSDDVIE
SYALREASRRNLPGVWNLIKRYASIINRFIEIHMVGSRVDNVKARISSLTRSLKTYGIKPEKEEPSNSMHGRQILRRSYSHVI
EEDIIGVDDDVKKLESYLVDPSKRVVAICGMGGLGKTTLAKKVYHSVDVRNSFKSWAWAYISQHCQARDVWIGILFRLI
SPSQEQRQEIDNMRDEELAKMLYQVQMEKSCLVVLDDIWNAETWNKLKPAFPHGTSVSAVGSKILLTSRNIDVAFQM
DPSCYLHTPKCLNEVDSWELFQKKSFLKIDDPDYREKEKLGREMVGRCGGLPLAIIVLGGLLASKPTFYEWNTVCQNINS
YLRRANGQEQCLGEVLALSYYELPYLLKPCFLHLAHFPENLEIPTKKLIRIWVAEGIISLAHSEGEGEEALEDVAQRYLTELV
ERCMIQVVEKSSSGRIRSCQMHNLMRELCVDRAYLENFLLEINSRNVDESRGTSRARSVGKVRRIALFLDQDVDRFFLSQ
LKSHHHLRSLLCFHEKTARISEWGLMKSFFEKCRLLRVLNLEGMQGLGGKLPKEIGYLIHLRFLSLRNTKIDELPTSIGNLKC
LMTLDLLTGNSTVQIPNVIGNMQKMRHLYLPESCGNGIERWQLDNLKNLQTLINFPAEKCHVTDLMKLTNLRKLVIDD
PNFGGIFRYPDVQFRHLESLFFVSYEDISVVDVALGCPNLYKLHIEGPIKNFPEPHQLSSKLLKLKLTGSGLLADPMPTLEKL
PNLRLLELQLDSFVGKQLHCSSKGFAQLKSLVIHDLFNLEEWRLDKGAMPCLRELKIENCTKLEKVPDGLRFLTTLQHLEIR
SMFAAFRTKLEKGGEDHYKIQHVSAVVFCYCDY

>XP_014498827.1

MAVECISGAFLAAAFQVTLDKLASRDIQDYFHGSKLKDKMLKKLDIVLNSINQVLEDAEERQYKSSNVMNWLDQLKEAI
YEAELLLDEVANEASRQKLEAEFQPATSKVRGFFKAFVNPFDMEIASRVEELLENIQFLASQKDMLGLRKGIFSGNEVGV
SWKQSKQLPTTSLVDESSICGREEDKEEIIKILLSDNVTCNQVPIISIVGMGGMGKTTLSQLVYNDQRVLEQFDLKAWVY
VSQDFDVVAVTRAILKALGSKGAEEKDLNLLQLELKQRLMGKRYFLVLDDVWNEDYSSWGVLQIPFIYGPSGSRILITTR
NEKVALVMNSSHLYHLKPLDKEECWKLFSDVAFHDKDATRYPYLVSVGSKIVDKCGGLPLALKALGNILRLKFSQHEWV
KILESDMWHLSDNDANINPALRLSYHNLPSYLKRCFAHCSIFPKGYEFDRDQLIQLWMAEGLLNCFQINKSEEELGTEFF
NDLVARSFFQQSRRRASCFTMHDLLNDLAKSVSGEFCSQISGSLEKIITKRARHISWSSKINIDDKFLEHISKCNRLRCFVA
FKWEFGRGGLINTDKQRVLLSTLKYLRVLSFHDCLLTELVDDIGNLKLLRYLDLSYTKIKRLPDSICRLHNLQTLLLLWCYHL
DELPIDLHKLVNLRHLDLRMSGINKMPNHIGRLKHLRTLTSFFIRKHDVKELGNLSNLQGTLSIFQLENVTDPKDAMEAN
LKGKKHLDGLVLNWGNKFGRCNENEDSIMERQVLEALQPNGNLKKLSVLRYDGTSFPRWFGGSHLPNLVSIALTESKFC
FVLPPFGQLPSLKELSISCFYGIEIIGPEFCGNDSSNIPFRSLEILKFEEMSAWKEWCSFEGHIEEGQGLSCLKELSVRRCPW
LRRALPQHLPSLQKLEICECQNLEDSVPKAVSIHEVKFRLCEKLFLKDLPSSLKKATIHGTCIIEACLHQILVNNPFLEELKIRN
FHGPNKKWSSLDLHRQDSLVTLSITSWYSSSLPFALHLFSKLHSVLFHDCPYLESFPEGGLPSSLRKLEIEDCPKLVASREK
WGLFKLHSLRVKS

>XP_014498828.1

MAVECISGAFLAAAFQVTLDKLASRDIQDYFHGSKLKDKMLKKLDIVLNSINQVLEDAEERQYKSSNVMNWLDQLKEAI
YEAELLLDEVANEASRQKLEAEFQPATSKVRGFFKAFVNPFDMEIASRVEELLENIQFLASQKDMLGLRKGIFSGNEVGV
SWKQSKQLPTTSLVDESSICGREEDKEEIIKILLSDNVTCNQVPIISIVGMGGMGKTTLSQLVYNDQRVLEQFDLKAWVY
VSQDFDVVAVTRAILKALGSKGAEEKDLNLLQLELKQRLMGKRYFLVLDDVWNEDYSSWGVLQIPFIYGPSGSRILITTR
NEKVALVMNSSHLYHLKPLDKEECWKLFSDVAFHDKDATRYPYLVSVGSKIVDKCGGLPLALKALGNILRLKFSQHEWV
KILESDMWHLSDNDANINPALRLSYHNLPSYLKRCFAHCSIFPKGYEFDRDQLIQLWMAEGLLNCFQINKSEEELGTEFF
NDLVARSFFQQSRRRASCFTMHDLLNDLAKSVSGEFCSQISGSLEKIITKRARHISWSSKINIDDKFLEHISKCNRLRCFVA
FKWEFGRGGLINTDKQRVLLSTLKYLRVLSFHDCLLTELVDDIGNLKLLRYLDLSYTKIKRLPDSICRLHNLQTLLLLWCYHL
DELPIDLHKLVNLRHLDLRMSGINKMPNHIGRLKHLRTLTSFFIRKHDVKELGNLSNLQGTLSIFQLENVTDPKDAMEAN
LKGKKHLDGLVLNWGNKFGRCNENEDSIMERQVLEALQPNGNLKKLSVLRYDGTSFPRWFGGSHLPNLVSIALTESKFC
FVLPPFGQLPSLKELSISCFYGIEIIGPEFCGNDSSNIPFRSLEILKFEEMSAWKEWCSFEGHIEEGQGLSCLKELSVRRCPW
LRRALPQHLPSLQKLEICECQNLEDSVPKAVSIHEVKFRLCEKLFLKDLPSSLKKATIHGTCIIEACLHQILVNNPFLEELKIRN
FHGPNKKWSSLDLHRQDSLVTLSITSWYSSSLPFALHLFSKLHSVLFHDCPYLESFPEGGLPSSLRKLEIEDCPKLVASREK
WGLFKLHSLRVKS

>XP_014498829.1

MAVECISGAFLAAAFQVTLDKLASRDIQDYFHGSKLKDKMLKKLDIVLNSINQVLEDAEERQYKSSNVMNWLDQLKEAI
YEAELLLDEVANEASRQKLEAEFQPATSKVRGFFKAFVNPFDMEIASRVEELLENIQFLASQKDMLGLRKGIFSGNEVGV
SWKQSKQLPTTSLVDESSICGREEDKEEIIKILLSDNVTCNQVPIISIVGMGGMGKTTLSQLVYNDQRVLEQFDLKAWVY
VSQDFDVVAVTRAILKALGSKGAEEKDLNLLQLELKQRLMGKRYFLVLDDVWNEDYSSWGVLQIPFIYGPSGSRILITTR
NEKVALVMNSSHLYHLKPLDKEECWKLFSDVAFHDKDATRYPYLVSVGSKIVDKCGGLPLALKALGNILRLKFSQHEWV
KILESDMWHLSDNDANINPALRLSYHNLPSYLKRCFAHCSIFPKGYEFDRDQLIQLWMAEGLLNCFQINKSEEELGTEFF
NDLVARSFFQQSRRRASCFTMHDLLNDLAKSVSGEFCSQISGSLEKIITKRARHISWSSKINIDDKFLEHISKCNRLRCFVA
FKWEFGRGGLINTDKQRVLLSTLKYLRVLSFHDCLLTELVDDIGNLKLLRYLDLSYTKIKRLPDSICRLHNLQTLLLLWCYHL
DELPIDLHKLVNLRHLDLRMSGINKMPNHIGRLKHLRTLTSFFIRKHDVKELGNLSNLQGTLSIFQLENVTDPKDAMEAN
LKGKKHLDGLVLNWGNKFGRCNENEDSIMERQVLEALQPNGNLKKLSVLRYDGTSFPRWFGGSHLPNLVSIALTESKFC
FVLPPFGQLPSLKELSISCFYGIEIIGPEFCGNDSSNIPFRSLEILKFEEMSAWKEWCSFEGHIEEGQGLSCLKELSVRRCPW
LRRALPQHLPSLQKLEICECQNLEDSVPKAVSIHEVKFRLCEKLFLKDLPSSLKKATIHGTCIIEACLHQILVNNPFLEELKIRN
FHGPNKKWSSLDLHRQDSLVTLSITSWYSSSLPFALHLFSKLHSVLFHDCPYLESFPEGGLPSSLRKLEIEDCPKLVASREK
WGLFKLHSLRVKS

>XP_014498999.1

MKDKAVSDNSVPRIKHDVFVSFRSEDIRRGFLTHLTNTFQEKKIKAFVDGQTLKGEAIWPSIVGAIEASSISVIIFSPGYVSS
EWCLEELAKIFECRVKYGHTIIPVFYHVEQADILYRSGRYRRSPFAKDDSKHESKLQSWRSALEKSAQLSGIESSKIRDDDK
LVKEIVNHVLTRLRMVNSKGQVGIDKKIEEVVSWIKENPKQTRLIGIWGKGGVGKTTLVEEVYNKLQSQYEGCYFLAHV
TEELSRHGILSLKENCFSKLLGYDVKIVSPNSLPKDIVRRIESMKVLIVLDDVNDSEHLEKWLEILGNFGSGSRIIVTTRHLEV
LEANKADKKYQLGELSSDEALTLFNLNAYNQSDYRREYNRLSECVVLYAKGIPLVIKVLAGHLRGKKKEEWQRELDKLEK
MPQRKVYDVMKLSYDGLEHKQKQMFLDLACFFLRTHERVNVGYLKSLLKDDESDNSIDFELGRMKDKALITFSEDNNF
VSMHDNLQQMAWEIVRQESIEDPGKRIRFWNPSETYEALKNVKGIDSIRSIVLHWPAIKKERLRPHIFAKMSRLQFLEID
DLYHYNSVKPFDIHGANTCWPKWKKARIADILAEGLHFLATELRFLCWYHCPLESLPENFSAEKLVILRLPHSRMEKLWL
GVKNLVNLKELDLSKSKKLKGLPDLSKAINLEVLILRSCSMLTSVHPSIFSLPKLEKLDLERCESLTILASCSNLHSLSHLHLDF
CENLMKFSLISDNMKELRLRCSKIKALPPSFGHQRKLELLHLEESGIERLPSLKNLTQLLHLDLSFCDKLQTIAELPPFLENLN
VQYCTLLQTIPKLPSSLKTLNTEACTSLETLPELPPSIEILNVESCSSLGTIPELPPCLQTLNVQCCHLLRTIPKLPTCLKTLHTK
NCTSLQNLPQLPPFLETLNATYCKSLKRVLFLTAKDEKLKENMKMLLFWNCSNLDKHSLVAIRLNMKINMMKFVANHIS
APDHSHVEDYSDYEYNYHSYQAVYAYPGSNFPEWLEYKTTEDYIIIGLPSSMSSSLAGFVFCFVLGEFQHTDIIRRVEFKIT
VSDGGGKDEGERESVRMYMDYWDDTIESDNICVMYDQRCSHFLLSRPRRLTSFKIQITMEARISFDESFYVPLQKVLKG
FGVSPISFDKLQSSNYKFR

>XP_014499526.1

MADSAVSFVVEQLYQLLREEGNLLKGLGNDFSDIKHELESIKAFLKDADRRAGDEGGEGDTHEGIKTWVKQLREISFCIE
DVIDEYIMDVAYRANHHPPCIASLQKIAHQIKTLKSRHRIASNIQDIKSAVQGIKERSERYKFQSTFEDGSLNSSKGAKDFK
WGDPRMASHFIEETEVVGFELPRDELIGCLIKGTDQLSLISVVGMGGLGKSTLAKHVFNNQQVKRHFYCRSFITVSQSYT
VREILTEMVQKFCKDANEPIPKGLHNMDDETLVTELRQYLQSKRYLVLFDDVWKENFSDEIEHALPNNRKGSRIIITTRN
MDVAEYFKKSVVVHVHRLQHLSPDKAWELFCKKAFRFEPSEQCPTELEDMSKEIVQKCGGLPLAIVCMGGLLATKEKSI
LEWRKVCQNLRMELERNTHLNSLKWILSLSYDDLPHNLKSCMLYFGVYPEDYSISRKRLTRQWMAEGFIKNEERRPME
DVAEEYLTQLISRSLVQVSRVGFDGKVKSCQVHDLLRDIIIRKMNELSFCHLMREDDELDTVEKTRRFSIASCSKNVLRETS
NSGIRAIYVFKKSELPEDFVGSLSAKFKLLKVLDFESTMLDSVPNNLGNLFHLRYLNLSHTKVKILPKSVGKLLNLETLDLRQ
TQVQVLPREIKNLTKLRLLPVYYRKYEGQYSMLNFTTGVKMQKGIGCLKSLQKLYFLEADHGGLELMQELKMLKQLRKL
GIRRVKTEYANALSSAIGEMKHLESLNVSAKDQDEIIDLNFLSTPTSLLVLNLKARVTKFPDWIPKLKYLVKLRLGLSNLEGD

PLDSLKDLPSLLRLNMWDNAYIGESLHFKRGGFRRLKELDLTRLSRLNSISIDEGALLGLKHFRFKDNPQMKVVPHGLKH
LKNLQFLGFADMPAELVESIDPEKDGQDYSVIKHIPLVLIRQNVGPKFHDYELRAIPTLVTVSTTEMAEAAISFALGEVFQI
LKEEKNLLSGINKEFLDIRDELESIQAFLKDADRKAADEANTNDGIRTWVKQVREVSVRIEDVIDEYLRVIHQVPRHGFGA
SICKITNLIRTLLSRHQIAVEIQDIKLSLSLIKERSERYKFQISQEKPSSSSTGRIEGSGRNDHGMGSLFIEETEIVGFELPRDEL
LSLLLEGKKERTLISVVGMGGLGKTTLAKLVFDSENVKVHFDCRACITVSQSYTVRGIFTDMIKQFCRETKDPLPEMLEEM
DEKTLISELRQYLEHKRYLIFFDDVWHEDFCDQVELAMPSNNRSSRIIITTRMIHVVEFFKKSFPLHVHNLQPLPSEKAWE
LFCKKAFKFELDGQCPAELKGMSNEIVGKCKGLPLAIVAIGGLLSTKSKTVFEWQKVSQNLNLELHRNAHLTRLKKILSLSY
DDLPYYLKPCILYFGIYPEDSSINHKRLTRQWIAEGFVKSDGRTLEQVADEYLSELIYRSLVQVSWVGFEGKVKSCRVHDLL
HELIVRKMKDLCFCHFVHEGDDESATSASSRRLSIDTSINNVLKSTNFTHIRALHAFGKGGAVELFTGLLASKSRVLKVLDL
ESTSLNHVPRNIGNIFHLKYLNLKRTKIRSIPKSVGRLQNLETLDIRETLVHELPSEINKLKKLRHLLAFHRNYEAAYSLLGFTT
GVLMKKGIKNLTSLQNLCYVEVEHGGIDLIQELIFLKQLRKLGLRSVRREHGKAICASVAEMTHLESLNITAIGEDEIIDLNSI
SSIPQLQRLHLKARLEKMPNWISKLEFLVKMRLALSNLKDDPLRSLENLPNLLKLTIWDNAYGGEILHFQSGGFRKLKELN
LARLNTVSAILIDKGALLSLEYVKITKITRMKKVPSGIKALYNLKVIDFCDMPTELVESIDPQNGQDYWIINHVPLVFIRRW
MGPKLNDFEVRIVHSSTKESLTN

>XP_014499546.1

MESEREDNNNGTDSTHAAISSTLKIKIVSCSKSGESLDNSNLPSPNINNGIESSPYGSPLVSPPSSAFVSALQSPYISPRAIIP
DPPNGSPLENQPPLLTITTSTTNPSTPEDVPSSSYTPPSDQYEFSDDTADTRLKYVTCVPEPAPPRISFSFPVPRISFAKGPI
SPATNAKLRSCDVYIGFHGQNPNLVRFCRWLKSELELQGIDCMLADRAKYSDSQSHEIADGVICSVAFGVVVVTSSSFLN
HFSMEEVRFFAQKKNLIPVLFDTGPAEIMALLNCKSIDKECKEAIDGLMKCNEFNIEANDGNWRSCIAKAAGILRARLGR
KNAEQKDNVQGHENLPFPRNTYFVGREKEIMEIEGLFFGRGNCMEQVQDHCRVFTKGEASGSGQSEGLADEESEPVI
ARCGRYISLEMGRSKEPTLEAWVEPIMGNNSLKRLKNKKSKSGSYKSVCSSVICINGVSGIGKSELALEFAHRYHQRYKM
VLWVGGEARYLRQNLLNLSLNLGLDVGADSEMERGRIRSFEDQEFEAFKRVKRELFGETPYLLIIDNLETEVEWWEGKD
LYDLIPRNTGGTHVIVTTRLSKVMSYDTIQLPPLPLSDAMILMIGRKRKEYSADEIDLLEKINEKLGRLSIGLWLIGSLLSELSI
GPSCLYEAINQVPLDEDSNSCYMSIAEGQWCKSNPFLMKTLLFCLETLEKTKAKGNLLAIRMLLVSGWFSPSPISSSLLAN
AAKSIPTVESRLKKWTKSLSLTTSCLSPRTWKNEEESAMLLVKMGLARRANQHDGCWLHFHPITQAFAKRKGGLQYAK
AAIQGVRKMGSQVNSDHLWASAFLVFGFKSEPPLVQLKAIDMVLYIKRTALPLAIQAFTIFSRCNSSLELLRVCTNALEEV
EKSFVSQIQDWSSHGSVCWKRRLQRGQKVDEYVWQDVTLLKATLLETRAKLLARGGHLDSAKELCRTCISIRTVMLGH
NHAQTLAAQDTLARLVRMRSKI

>XP_014499943.1

MAESSSSLAVPTSPTKYDVFLSFRGEDTRYNFISHLYDALHRNRIKAYRDDRLQRGEEISPALQTAIEESKIYVLVFSENYAS
STWCLNELTKILNCKKIYERDVIPVFYKVDPSTVRKQEERYKAAFEEYEQRFKDDMDKVQGWKDALTEAAGLSGWDSN
VIWSENILVEEIVKDIVKKLDLYSSSYDQGIIGIEKHIERIRFLMHFESSDIRIIGICDMGGIGKTTISEQIYHTLGMQFDSRSL
VLDTKRKIERDGIDIVRKKYMSEYSERLKRMRILIILDDVTDSVQLKQLLGRHDSFGQGSRIIITSRDRQVLKNVGADDIYD
VKELNDLDSLKLFSLHAFKQNSSQEITYKDLTEKVLRYAKGIPLVLQTLGSLLYGRTREAWESQLQKLEKCQHLEIFNVLKLS
YDGLDEEEKNIFLDIACFYRGDEESEVVERLDECGFSSKIGMDVLKDRCLISIFDGRIVMHDLLQEMGQEIVRKECPEYPG
KRSRLFKDEEIHEVLKKNKGSDAIQCILLGTWEIKKDVIVDGQAFQKMDNLRMLMLSDYLSYEPQSESRVFLASSLVTLPD
TLKILYWKGFPQRSLPPNFCPENLVRLEMPHCHLEQLWEEDQSLPKLKRLDLSWSSKLTRIQDLSMSPNIEEIILNYCRKLI
KVHSSILLTKLSYLSLDECHNLKSVIIPSNILTKSPGLILLSRCGELEMFSTTQTQIYYSPPSSSLIGPHVKLRRQRRNSFNELQS
FASLPSRFYSNEIFSITFDRYWGTDEEKEVTNDEMPIQLTGGVSLNLSLKKLCLIDLSHCSSLTTFPFVLSEMKFLKKLFLSGC
SKLENFPEIEDTMENLAILSLNNTSIKALPSSLGRLVGLQELNLYGCRNLEIIPSSIGRLTRLCKLHLTNCHSLQTFPNTIFKLK
LRKLDLYRCLRLRTFPKITEQAQTFSYIDLSETAIKELPSSFDNLVSLRSLQLNKCRNLESLPNSIVNLKHLCKLDCSGCHKLTE
IPRHIGRLTSLMELLLNDSGIVNLPESIAHLSSLKSLDLSDCKRLECIPQIPSCLKQLVALDCPSIRQVMSNRNLSGSKEGVFK

FHFTNAQQLDSGARANIEEDARLRMTDYAYRSVFFCYPGTAIPHWFPFRGKGCSVTINEDLSFCSDDRLIGFALCVAFGL
FDSNDINGRRGSFRYRLKFECDDYGTQIIPNNDLFQNYFKWNGKNRFVNQDHTFMWKFNLESLRRSCMNLRLCDACR
FTFEIIRYYGEMVREFRSVVTIKECGLCPLYRSGSNVGESSMETKEDSKRKAESYL

>XP_014500013.1

MAITLSEGASSSSGFDRGWTYDVFLSFRGEDTRRSFTGFLYHGLCQRGINVFIDDDKLRKGEDLSPTLLGAIQESRIAIIVF
SQNYAFSTWCLDELAKIIDCYKTRGQLVWPVFFHVDPSVVRHQRGTFQTAMAQHEVRFKGNVEKLQKWKKALFEASN
FSGWNLENGYEFQIIQDIVEEASRKLSHTILHIAEYPVGIEIRISEVMPLLQIEPGEDIRVIGIYGLGGIGKTTIARALYNMIAD
QFEATSFLSDIRESSNQRQGLVQLQESLLFDTVGDKNIKLGSIYKGIPIIKKRLCCKKVLLIIDDVDRLEQLQALAGGRDWF
GFGSVIIITTRDKHLLSAHQVDKTYEVKKLNYGEAFELFTWSAFKRKAPDAGYLEVSNRVVLYAEGLPLALKIMGSNLFGK
TVEEWKSALGKYEKIPNKEVQNVLRVTYDNLEENEKEIFLDVACFFKGETVEYVENTLQGCGFYPTIGISVLIDRSLVSIDEY
NRLRMHDLIQDMGREIVREVSPLEPGKRSRLWYHEDVFEVLTENKGTYRIQGMMVDLPDDYMVHLKDDSFKKMKNL
KILIVRNGNFFGSPQHLPNSLRLLDWMKYPSSLPSSFQPKKLVVLNLSGSRFTMQEPFKYLDSLTSMDLSSCELLTKLPEIA
GVPNLTQLTLDYCTNLEEVHESVGFLEKLVEFRAYGCTKLKVFPNAIRLTSLRSLILNWCSSLQNFPAILGKMDNLISISIEG
TGIKELPPSIGNLVSLQELSMTSCLNLKELPHNFDMLQSLTNLDMEGCPHLRNFLTKLANMGESTHTFGNILSLNVENCG
LIDEDLPIIFNNFPNLASVVLSGNHFKALPSCIQQCPCLELLHLDNCKNIQEISAFPPNMQYINAQNCISLSEESSNLLFNKE
TFEGWELQAMVPGTMVPEWFDHITKGEYMTFWVRERFPAIIICFVLEVESEMKKIFNCEIRFYINGEEVYELQIPRGFSD
MVTDHVWLYDLRTHSSINWRSLDLYLMDGWNQVEISCEKISGASNLTVSWCGVHVVKQEANMKDILLTDPDPDLDS
VIASGSNTLVSDHPVKAQPQSQVTSFTLQTPQNNNSSTIVLPTTVQTSLTVNDADMEAFYAVLDDEISVVSLNNDSTMV
SKLTNQRPSEETKKALKTLQAHVTKEFSALLGPNEYSTVNDTLEYLTNLPAEDEISVEIRSLIIQVSRQFTRWSRDYTSENKK
IESTTAKLLKADELEKCLEANKTNFKQVMCMENELCNDLAHLEQRKRELEEQINAVKANISASEAAKNMASEIKRELFGK
AKILKAERDELREQVPHLRDEQELAKKIQSNIRDEWSKLGEKFNYGLRHGKID

>XP_014500600.2

MLSGARSQWHSSHAIIHQTSSSSHAYDVFVSFRGEDTRNNFTSFLFGALCRQGILAFKDDQCIRKGDFIAPELLQAIQGS
QVFVVVLSKNYASSTWCLRELVEIFNCCETSARPVIPIFYDVEPTTVRYQKGCYEKAFAEHENRFREDKVKMEEVQTWKE
ALRKVADISGSEIGNKPQNEQIEKIVQEIINNLRPKILNLPRDELVGIEDRLQDLGNILHFDLLNDVRVVGISGMGGIGKTT
LARALYERICHQYNYRCFIDDVSKIFLDSRSLGLXKQLISQTLNEKNVEICNVIEGTCLVQSKLNNAKALIVFDNVDEVQQL
RIFSGNRDNLLRECLGKGSRIIIVSRDEQILKIHGVDDIYQVRPLNRKDAIQLFCRNAFKXNXILSDYEKLAREILSHVEGHPL
AIETIGSSLLGRSLSHWKSVLEGLKENKSKNIMGILRISYIQLEEKYKQTFLDIACFFNSYYEKDVKEILNFRGFHPEDSIQVLI
DKSLITKDFKGYIRMHSLLVDLGKCIVREVSPKVPRKWSRLWTYKDFHDAMSENKTTRNLEAIVLRYHLDEDEDDVEIPD
KTIKADGLSKIKHLKLLYVENVKANVNFSGSLNHLLNELGYLTWYNYPFECLPQSFQPHKLVELKLRWSSIQRLWEGTKSL
PNLKRLDLLHCQSLVEMPDVTEAPNLQSIHLEGCGQLQKLNPSIESLRKLVLLNLRYCKKLVILSNTILGLKSLEYLDVSYCSI
IGSNWLNTENLIRCLSPSSPLSCLCELNLRFCNLVQIPDYIGKLGCLESLNLEENNFVRLPNLKDLLRLYYLNLQDCKRLKYLP
DLPSRTVTRSSSMFLKVAGFIIFGCPELVEIKQCITKSFSWAIQIFEATYQYQWIARVTIMPGSQIPSLFNNEFVNVDQEYL
DEKNLIVDPPPVPHDNNFIGVLCCVIFRLHNRHIPMDFRRDHMCLHYGKVSIDELKNEQIPMDFRRDHMCLHYEKVSM
DERGIHPYHFRLRMIDLLLSYKTSRNFVTVKKFQYRWVNEEDLINFKMTHCANLTGRKGKISVIEENG

>XP_014500603.1

MMYSPVFNILRRILRFVKEPDRIMSGMSDCMTVSDPTAPIGPSVPASDAIIHRTSSSSNAYDVFVSFRGEDTRNNFTSFL
FGALHRQGILAFKDDQCIKKGDFIAPELLQAIRDSQVFVVVLSKNYASSTWCLRELVEILNCSETSARPLIPIFYDVEPTTVR
NQKGCYEKAFAEHENRFREDKVKMEEVQRWKEALRKVADISGSEIGNKSQNEQIEKIVQEIINNLRPKILNFPRDELVGI
EDRVQDLGNILRFDLRNDVRVVGISGMGGIGKTTLARALYERICHQYNYRCFIDDVSKIFVDSRSLGLQKQLISQTLNEKN
VEICNVIEGTCLVQSKLNNAKTLIVFDNVDQVQQLRIFSGNRDNLLRECLGKGSRIIIVSRDEQILKIHGVDDIYQVRPLIRE

DAIQLFCRNAFKDNYILSDYEKLADEILSHVEGHPLAIETIGSSLLGRSLSQWKSVLEGLKENKSKNIMDILRISYIQLEEKYK
QTFLDIACFFNFYHEEHLKEILNFRGFHPEDSIQVLIDKSLITRHGRSIGMHSLLVDLGRCIVREVSPKEPIKWSRLWTCKDL
HDAMSENEVTRNLEAIVLEDDVTDYETIKADGLSKIKHLKLLYVGYVKFSGSLSHLSNELGYLVWRRYPFECLPQSFQPHK
LVDLILYGSSIQRLWEGTKPLPNLKRLDLSFCESLVEMPDVAEALNLEWIILLGCIQLQKLTPSIGSLRKLVGLILRDCKKLVIL
SNTILGLNSLKYLDVSGCSIIDSKCLQDETRNTEYLISCLSPSSPLSCLCELDLSFCNLAQIPDYIGKLSSLEKLNLKGNNFVTLP
NLKDLWRLYYLNLQDCKQLKYLPDLPSGSVLSSKLSSSRFLLSSIFESRYFLNKEEGIYFWNVGLNIFGCPELVEIEECIRKSF
SWTIQIIQATYQLPFPITHMPDSIMPGSQIPNLFNNEFVNVDIQYLDKENLDEKYLTVDLPPVPHDNNFIGVLCCVIFRLD
HEEIPMDFRTDHMWLHYQDVSMNARGINRFRFSLSRITTLLDYHSFFVTVDVKKFQYRWVNEQDLINFKMTXCANLT
ARKRKISVIEENG

>XP_014501487.1

MAVASFSSSFTYDVFLSFRGEDTRYSFTGNLYRALRDRGIHTFIDDEKLPKGDEITSALEKAIEGSRIFIIVFSRNYASSSFCL
NELAYILPYANRNGLLVLPLFYDVVPSHVRHHMGSYGEALDTHENRFKATSQGFELNMEKLNKWKMALRGTANLSGY
HFKHGEEYEYEFIKRIVDLVSNKINRAPLHVADYPVGLETRVLEVKLLLDIGSDDGVHMVGIHGLGGVGKTTLALAVYNSI
ADHFEGLCFLENVRENSNKHGLQHLQRILLSQMVGENNVNITSVRQGISMMQHRLRQKKILLILDDVDKHEQLQAIVG
RPDWFGPGSRVIITTRDKHLLSCHLIEKLYKVKKLKKNNAHRLLSWKAFRTEEVDTSYLNVMDRVLAYASGHPLALEVIGS
KLFRKSVKEWESAIKQYEKIPNNQILEVLKISFDALEEVEKSVFLDISCCFKGYALSEVEDILRAHYGDCMKYHIGVLVEKSLI
KYGWSCVVTMHDLIEDMGKEIVRQKSPNRPGKRSRLWSPEEIIKVLEDNLGSGEIEIICLNSSLPDKEEIVEWNRKAFKK
MKNLKTLIIKNGNFSEGPEYLPNSLRVLEWLKYPSHGLPPDFRSKKLSLCKLPSSCFGSLELAEFSKKFMNMTLLNFDECE
GLTQIPDLSGMPNLERFSFKNCKSLITIHDSIGFLGKLNSLNAVGCSKLRSFPPLKLTSLENLELSYCYSLESFPEILGKMGQI
TELVLEDCHIKELPISFQNLTELRTLQLRSCPTLRLPSSIVMMPKLANIIAWESKGWLFPKQVEREEKIGSMVSSNVDCLVL
SGCKLSDDFFPIILEWFANVKDLNLSRNNFTVLPECIANCHLLCKLTLDACHSLREIRGIPPNIRQLLARNCKSFTSSCRRTLL
NQKLHEAGNTMFSFSGARFPEWFDHHSRGASCSFWVGKKFPSIALCIAIGPTHLEHLEIVGPIMIINCIECSFDWEENPY
LYMLPHHTYIFDLQNIVFPDYLDRFVSENEWNHVEITYSVKQRFKGKDKHEVTPISIENGIYVFKQKSSMEDIQFTDPHKK
RRLDVDPEL

>XP_014501521.1

MLSGARSQWDSSHAIIHRTSSSSNAYDVFVSFRGEDTRNNFTSFLFGALRRQGILAFKDDQCIKKGDFIAPELLQAIQGS
QVFVVVLSKNYASSTWCLRELVEIFNCCQTSARPVIPIFYDVEPTTVRNQKGCYEKAFAEHENRFREDKVKMEEVQRWK
EALRKVADISGSEIGNKPQNEQIEKIVQEIIYNLRPKILNLPRDELVGIEDRVQNLGNILRFDLLNDVRVVGISGMGGIGKT
TLARALYERICHQYNYRCFIDDVSKIFLDSRSLGLQKQLISQTLNEKNVEICNVIEGTCLVQSKLNNAKALIVFDNVDEVQQ
LRIFSGNRDNLLRECLGKGSRIIIVSRDEQILKIHGVDDIYQVRPLIWKDAIQLFCRNAFKVNYILSDYEKLAHEILSHVEGHP
LAIETIGSSLFGRSLSQWKSVLKGLEENNKSKNIMDILRISYIQLEEKYKQTFLDIACFFNYFHEERLKEILNFRGFHPEDSIQ
VLIDKSLITRDFDERIHMHSLLVDMGRCIVREVSPKEPIKWSRLWTRKDLRDAMSENEATRNLEAIVLRYHLDEVGVKIP
DETIKADGLSKIKHLKLLDVRNVKFSGSLSHLSNEIGYLVWYKYPFECLPQSFQPHKLVELNLYGSSIQRLWEGTKPLSNLK
HLDLSLCKSLVEMPDVTEALNLESINLEGCGQLQNLNPSIGSLRKLVVLKLRDCKKLVILSNTILGLNSLKYLDVYGCSKIGS
NCLLDETGNTEHMISCLSPSSPLSCLCELDLSFCNVVQIPDYIGKLSCLEKLNLKGNNFVKLPNLKDLLRLYFLDLRNCKRLK
YLPDLPSQTVLPSKPLMLSRFPSSMFHDLPYYMDFRLRGLIIFGCPELVEIEECTTKSILWTIQIIEAAYQLSFPRTYIPESIMP
GSQIPSLFNNEFVNVDIQYPDNNFITIYPPPVPHDNNFIGVLCCVIFRLHNKHIPMDFRTDHMCLHYKDVSMDGRDIHP
YPFRLRSINALLDDRDFAAVDVKKFQYRWVNEQDLINFKITHCANLTARKISVIEENG

>XP_014501522.1

MLSGARSQWDSSHAIIHRTSSSSNAYDVFVSFRGEDTRNNFTSFLFGALRRQGILAFKDDQCIKKGDFIAPELLQAIQGS
QVFVVVLSKNYASSTWCLRELVEIFNCCQTSARPVIPIFYDVEPTTVRNQKGCYEKAFAEHENRFREDKVKMEEVQRWK

EALRKVADISGSEIGNKPQNEQIEKIVQEIIYNLRPKILNLPRDELVGIEDRVQNLGNILRFDLLNDVRVVGISGMGGIGKT
TLARALYERICHQYNYRCFIDDVSKIFLDSRSLGLQKQLISQTLNEKNVEICNVIEGTCLVQSKLNNAKALIVFDNVDEVQQ
LRIFSGNRDNLLRECLGKGSRIIIVSRDEQILKIHGVDDIYQVRPLIWKDAIQLFCRNAFKVNYILSDYEKLAHEILSHVEGHP
LAIETIGSSLFGRSLSQWKSVLKGLEENNKSKNIMDILRISYIQLEEKYKQTFLDIACFFNYFHEERLKEILNFRGFHPEDSIQ
VLIDKSLITRDFDERIHMHSLLVDMGRCIVREVSPKEPIKWSRLWTRKDLRDAMSENEATRNLEAIVLRYHLDEVGVKIP
DETIKADGLSKIKHLKLLDVRNVKFSGSLSHLSNEIGYLVWYKYPFECLPQSFQPHKLVELNLYGSSIQRLWEGTKPLSNLK
HLDLSLCKSLVEMPDVTEALNLESINLEGCGQLQNLNPSIGSLRKLVVLKLRDCKKLVILSNTILGLNSLKYLDVYGCSKIGS
NCLLDETGNTEHMISCLSPSSPLSCLCELDLSFCNVVQIPDYIGKLSCLEKLNLKGNNFVKLPNLKDLLRLYFLDLRNCKRLK
YLPDLPSQTVLPSKPLMLSRFPSSMFHDLPYYMDFRLRGLIIFGCPELVEIEECTTKSILWTIQIIEGFCCSGCEEISVSLGK

>XP_014501523.1

MLSGARSQWDSSHAIIHRTSSSSNAYDVFVSFRGEDTRNNFTSFLFGALRRQGILAFKDDQCIKKGDFIAPELLQAIQGS
QVFVVVLSKNYASSTWCLRELVEIFNCCQTSARPVIPIFYDVEPTTVRNQKGCYEKAFAEHENRFREDKVKMEEVQRWK
EALRKVADISGSEIGNKPQNEQIEKIVQEIIYNLRPKILNLPRDELVGIEDRVQNLGNILRFDLLNDVRVVGISGMGGIGKT
TLARALYERICHQYNYRCFIDDVSKIFLDSRSLGLQKQLISQTLNEKNVEICNVIEGTCLVQSKLNNAKALIVFDNVDEVQQ
LRIFSGNRDNLLRECLGKGSRIIIVSRDEQILKIHGVDDIYQVRPLIWKDAIQLFCRNAFKVNYILSDYEKLAHEILSHVEGHP
LAIETIGSSLFGRSLSQWKSVLKGLEENNKSKNIMDILRISYIQLEEKYKQTFLDIACFFNYFHEERLKEILNFRGFHPEDSIQ
VLIDKSLITRDFDERIHMHSLLVDMGRCIVREVSPKEPIKWSRLWTRKDLRDAMSENEATRNLEAIVLRYHLDEVGVKIP
DETIKADGLSKIKHLKLLDVRNVKFSGSLSHLSNEIGYLVWYKYPFECLPQSFQPHKLVELNLYGSSIQRLWEGTKPLSNLK
HLDLSLCKSLVEMPDVTEALNLESINLEGCGQLQNLNPSIGSLRKLVVLKLRDCKKLVILSNTILGLNSLKYLDVYGCSKIGS
NCLLDETGNTEHMISCLSPSSPLSCLCELDLSFCNVVQIPDYIGKLSCLEKLNLKGNNFVKLPNLKDLLRLYFLDLRNCKRLK
YLPDLPSQTVLPSKPLMLSRFPSSMFHDLPYYMDFRLRGLIIFGCPELVEIEECTTKSILWTIQIIEIEVD

>XP_014502210.1

MEVIAQIVLQNLNSFAQEEFGIIWNLKDDVQQMKRTVSAIKAVLLDAEGKANNLQISNWLEELKDVLYDADDLLNDISS
EAMKRKVIGARKILRKIQVFFSQENQIVYSFKLGHQMKAIQKRLDAIAKNKITLQLTDRPMETPIAYRRQRQTYSFVRED
DVIGRKEEKKLLESYLLDTKVSVIDNVSVLAIVGFGGLGKTTLAQLVFNDNAVQCSFEQKMWVCVSDEFDISKIAEKMIG
NDKNSEIEQLQQDLRNKVRGKKFLLVLDDVWNEDRELWLKFKSLVVEGGKGSAIIVTARSRTVAKIVATHPPLFLKGLDL
ERSWKLFSRVAFDGGKEPNDMELLAMGRDIVKKCAGVPLAIRTIGSLLYSRNLGRSDWQYFSEVEFSKIDQHKDNIFSIL
KLSYDHLPSFLKKCFAYCSLFPKDFEFDKKTLIQLWVAEGFIQPSRDNRCEEDVGHEYFMNLLSMSLFQNVTLDDCGDILT
CKMHDLIHDLAQLVVGKEYAFVEGKKEHIENRTRYLSSCTSLHFSEKTSSSSNKLRTFILLGQPEPLYGRQNLGPPPSLHFP
FLLSIKCLRVLTLCGLHLITIPDSIRKLKQLRYLDLSFNRFLVSLPPDVTSLHNLQTLKLSRCGKFKELPSDINKSLRHLELNDC
GELRCMPCGLGQLTNLQTLTHFILDSESKNVDISELSGLNNLRGKLVIKCLDSLRENAAVVESANILLEKQHLQDLELRWG
FRETKYRKDPVKNRRKEEENRWVDDENKIKDEKILKGLQPHHSIKRLVIDGYCGNSLPDWIGNLSSLLSLEISNCYCLKSIP
DGIRNLVSLQRLCIYNCSMLEGRCARGHGEEWSKIAHIPLAIVSAFNPTDLRYIN

>XP_014502868.1

MAAEMVTGVLVSTFLERTIDTLAPRLVHIFPQRKHKKQLSNLKMKLLAIDVVALDAEQKQFTDPRVRNWLLKAKDVVF
DAEDILDEIDYELSKSQADAESQSATNKVRNSLNSSFFSFFEIEIESRMEQVIEDLEDLANESYILGLXKGGGVGVGSGSGS
KLTYTSLTNENVIYGRDDDKEFVFNWLTSNTHNNLSILSIVGMGGMGKTSLAQHVFNDPRLEDKFDTKVWVSVPQEFD
VLKLSRAILDTITGSTDHSIQHEVIQKRLKEELMGKKFLLVLDDVWNERPSKWEDVQKPLIFGGQGSRLLVTTRSEKVAVT
MRSEKHLLKVLKEDYCWDLFAKHAFENANPQLDPDFVETGKKIVEKCNGLPLALKTMGVLLHNKSSLWEWESIMKSEI
WHLSENESDILPALKLSYFHLPSHLKKCFAFCALFPRGYRFDKEYLIQLWMAQNFLENPLQKKSPKEVGDEYFNDLLSWS
FFQQSSNEEKKRFIMHDLLNDLAKYVCEDICIRLGVDEPKGIAKTTRHCSFSTSKLCFDGFGSSIDTQKLHTFTPRDWGW

DWNCKMSIVDLFSKFKFIRVLSLSHYRNLKEVPKSVGNLKYLRSLDLSYTDIEKLPDSISLLYKLQILQLNYCQRLKELPLYLH
QLDNLRCLEFIKTAVKNVPAHLGKLKNLQVSMSSFYIEKGKEFSIKHLRELSLHGSLTLDDLQNIENPSYALEVDLKNKSHL
VDLRLKWNFIGSSSVYSAKVEDVIENLQPSKYLKKLLIKNYIGKQFPNWLLHNSLPNLVSLVLEKCESCQRLPPLGLLPFLKK
LEISGFDEIVSIDADFHGNDSCSFKSLQTLYFSNMRQWEKWDCQAVTGAFPHLQLFSIENCPKLKGYLPKFVALKTLSVIH
CEQLEALIVSAVELRLEDCEKLDGHGMAASLVAIVGHMLFDTSLENLSICSALESISDDYVSLRIFPLDFFPTLRSLKLGSFP
NLQMISQDHVHNHLQYLEIKECPKFESLPANMHMLLPSLTRLWIEDCPRLASFPEGSLPSNLKYITLYNCFRLVGLLKGAL
GDSSSLERLGISTPDAECFPDEGLLPLSLSFLIISDCRNLKKLNYKGLLELSSLKRLNLWKCPNLQSLPEEGLPKSISFLEIRECP
LLEQRCNKRGKDRGKIAHIRKVFI

>XP_014502892.1

MATLSSRRFTYHVFLSFRGEDTRYGFTGYLYKALWDRGIHTFMDDDKLQSGEEITPALQKAIQESRIAIVVLSHNYASSSF
CLDELATILDCQSEGLLVIPVFYKVDPSNVRHQNDSYKEALAKHQERFKGQEEKLNKWKMALRQVVDLCGYHFEDGND
YKYKFIGSIVERVSRVINRVPLKVXDYLVGLPSQELKVKKLLDLGSNAVVHMIGIHGMGGLGKTTLSLVVYNSIADNFDCS
CFLHIVRKKSNKDRLEDLQSILLSKMVXEENIMLTSWQEGASMIQQRLGRKKVLLILDNVDNRXQLEAFAGRADWFGP
GSRVIITTRDEQLLKSHQIERTYKMEELNDNDSLQLLKWNAFKREKVDPSYEDVLKRVVTYASGLPLALKVIGSNLYNKSV
EEWESAIEHYERIPNDEILQILKVSYVALGEEEKSVFLDIACFFNESSLEEVEGILRVLYGNNMKHHIGVLLDKSLIDFWSDD
EDCIVMHDLIEDMGTHIDQQESPKEPGKRRRLWSEEDILHVLKNDKGTSKTEIIILDSEQEKTLEWNPNAFRRMKNLKILI
IRNGKFSKGPNYFPESLRLLEWHGYPSNSLPSNFDPSNLVTCMLPNSHFTSFEFLGSSKKFENLSVLNFDWCKFLTHIPD
MSDFVNLEEVSFKGCESLVAVHDSIGFMSKLKILNAECCIKLMSFPPLNLPTLERLQLSDCSSLEKFPEILGKMENIKGFEL
AGLPIKELPLSFQNLIGLEELSLSCKIVHLRSSIFTMPNLIYFIVTDCMEWKWVKSDDAEDNLGSTVPSKLEVFSAPSCNLD
DNFFSAGFMQLAQVRSLILQNNNFKNLPECIKEFHNLKHLDVTHCKHLEEIRGFPPKLEYFSAPNCISLSSTSLDMLLNKEL
YEVRKATNFKFPGESFPEWFDLKSSGPSCSFWFRNKFPARVLSLLIIHMNKYENLSIFDPEVCINGKWQTGGGHYLLEET
KFEFDQTYLCDLKMYGNFFKQPFEKEWNHVKVTYSTPTGKSWIKATGIHVFKEENNIMEDIRFDDPYIMEEEEKEEEKEE
GEEEEEEKEEKAEEENSIMEDTMEEEGRRGKRRKLHHGRHSWFDDPYTNKKRKKGKEKRNKKKKKNNMQKKKKIASW
KTLHLMLYHGRHHGRREKKKEENNIMKDTMEEEGRRGKRRKQQHGSHSV

>XP_014502918.2

MATPSSRRFKYDVFLSFRGEDTRYGFTGDLYEALRHKGVHTFMDDEELQSGEEITPALLKAIQESRIAIVVLSHNYASSSF
CLDELATILHCQSEGLLVIPVFYKVDPSKVRHQKGSYEEALKRFEGQEEKLNKWKMALRQVADLCGYHFEDGNDYKYKFI
GSIVERVSRVINRVPLKVXDYLVGLPSQELKVKKLLDLGSNAVVHMIGIHGMGGLGKTTLSLVVYNSIADNFDCSCFLHIV
RKKSNKDRLEDLQSILLSKMVXEENIMLTSWQEGASMIQQRLGRKKVLLILDNVDNRXQLEAFAGRADWFGPGSRVIIT
TRDEQLLKSHQIERTYKMEELNDNDSLQLLKWNAFKREKVDPSYEDVLKRVVTYASGLPLALKVIGSNLYNKSVEEWESA
IEHYERIPNDEILEILKVSYVALGEEEKSVFLDITCFFKGSSLEEVEGILRVLYGNNMKHHIGVLVDKSLIDFWTDDEDYIVM
HDLIEDMGTHIDQQESPKEPGKRRRLWSEEDILHVFKHDKGTSKTEIIILDFKQEKTLEWNRNAFRRMKNLKILIIRNGKF
SKGPNYFPESLRLLEWHGYPSNSLPSNFDPSNLVTCKLPYSHFTSFEFLGSSKKFENLSVLNFDWCRFLTHIPDMSDFVNL
EEVSFEGCESLVAVHDSIGFMSKLKILNAEGCIKLMSFPPLNLPTLERLKLSDCSSLEKFPEILGKMENIKKLRLAGLPIKELPL
SFQNLIGLEELVLSCEIVHLPNFSTLSVTEDNVGSMVPSKLKRFSAQSCNLDDNFFSAGFMWLAQVCELSLQKNNFKHLP
ECIKEFHNLKHLDVTHCKHLEEIRGFPPKLESFSAPNCISLSSTSLDMLLNKELYEARKATNFKFPGEIFPEWFDLKSSGPSC
SFWFRNKFPARVLSLVIIHMNKYDNLSIFHPEVRINGKWQTGGGHYLKEQTKFEFDLTYLCDVKMYGNLLEQPLEKEW
NHVKVTYRTLSRESWIKATGIHIFKEENNIMEDIRFDDPYIMEDIMEEEEEEKEEEKEEEEEEENNRFKNNRFVHVQNFHR
PFSFYFRAFIFYFIYQPQHQPRV

>XP_014502928.2

MCLPLIFFSLSCFSLQIVPFSVFLLHAPLHVADYPVGLPSQVEKVKKLLDVGSDDVVYMIGIHGMGGLGKTTLSLAVYNSI
ADDFDSSCFLQNVREESNQHGLKHPQSILLSKMLRENIILTSWQEGASMIQERLGRKKVLLILDDVDNRKQLQAFAGRA
DWFGPGSRVIITTRDEQLLKSHEIKRTYEVEKLNDNDSLQLLIWNAFKREKVDPSYEDVLKRVVTYASGLPLALEVIGSNL
VGKSVEEWESAIEHYKRIPNGEILEILKVSYDALGEEEKNVFLDIACFFKGSSLEEVEHILGVLYDNNMKHHIGVLVEKSLIK
VQDERFGVIQMHDLIEDMGRDIDRQESPKEPENRMRLWLGKDILHVLKHNKGISKTEIIRLDLSKSEREETLEWNPNAF
RRMKSLKILIIRNGKFSKGPNYLPKSLRVLEWHGYPSNCFPSNFDPRNLVTCKLPHSRFTSFGFLGSSKKFENLTVLKFDW
CIYLTHAPDMSDLGNLEHVSFQWCENLVAVHDSIGFLSKLKILNARNCKKLMSFPPLNLPTLESLQLSYCSSLEKFPEILGK
MGNIMELNLYLPIKELPLSFQNLIGLKKLYLSYEIDHLRSSIVTMPNLIDFRVTNCKRWEWVKSEDAEDNLGSMVPSELDT
FAAKSCNLDDDFFAVGFMRLAHVRWLCLSFNNFKHLPECIKEFHNLNQLDVSHCKHLEEIRGFPPKLEYFNATNCISLTP
TSLSMLLDKELYEARKPHFRFPGESIPKWFDLKSSGPSCSFWFRNRFPARVLSLLIIHMDIDKFSFVTPEVYINGKCHGSSG
TYLLKEERMFEFDQTYLSDLGRLHNLFEQPLEKEWNHVKVTYDESFLNMSRNKVTIKATGIHVFKEENMEDIRFDDPYIR
EDIIEEEDEKEKEEEKEEEEELNFR

>XP_014502932.1

MEKHGEEEECSRYDVFISFRGEDIRHTFLRQLREGLGRRGILSFHDDRDMRIGTDLSIALHEAIEESNVFIVVFSENYASST
WCLDELVKIIHRSHTNIKQVVLPVFYHVDPSDIQKMRNSFRKHMEAHEKEFGKESQRMQAWRSALSQAANLPRMHIT
TTTGFEKDYIEEIVGKVQMNLCRKPLYTGQNPLGLEPHIEKVMSLLDIDDNTVKMLSIYGLGGIGKTELAKALYNKIVRHF
DAASFLAGVREKSNTINGMEDLQKTLLSEMFEESETKLGSTNKGIYEIKPKKKVLLVLDDVDDKEKLEKLAGGCDWFGPG
SRIIITTRERDLLIVGNVYEMKELDEQHSLELFCWNAFGQGCPKTGFEDVSMRAVNYAKGLPLALKVLGSDLATLHGESL
DAWEDALEEYKKTPNKKIQDNVLQVCYDRLDYNAKQVFLDIACFFKGEKVEYVNEILEEFYSESKMKELVNKSLITIENGC
LKMHDLIQDMGRQIVRQEAPNPGERSRIWDYEDVLQILTEDSGSDKIQGIMLDPPQQEKVKWSGTEFQKMKCLRILIV
RNTSFSSEPEHLPNHLRLLDWDNYPSNSFPTKFHPKKIVVFNLPRSSLTLEELSFKKFPCLTNMDFSYNQRIIEIPDVSELQ
NLRELRLDHCRNLIAVHESVGFLKRLSHLSVSECTKLQIFMSRMFLPSLEVFDLNLCESIDHFPEIMQEMTKSLKIKMKNT
GIQELPESISNLTGLVSIDISSNKELKYLPRSVFMLPNLEELIVSENNFVSIPSCIKQCGDWTSLDLNGSKKLKKISEPTSLRIL
DVHHCLDLEEISELPSTVQKVDARFCFKLTKETSNMLWCQVKKGVGGIEMVMPFLTEIPEWFNFVGVERIPGFWVRGK
FPNIVLAMIFHFENPNQREEYGRRHLVDLRLLINGRYAPRKGYQNFRIEAEHILVCDLRVLFSEKEWFGLDALLEDEWNL
VEVAYDATSSLMISGWGAFVYEEEINMEHLLFTYPYPVIVNEASTSGHMESEEEEKGYFAYLEGIAWETLYEGVRDGLFE
ARNRFPSMDIDEIFTATLEKGPRIEWTAEGMELIPSAEHRTYFNGIYSGLLEAKLRFPDLDVWATLNTVANRRGIKGTFES
PLQQNLWIPRLDWSTVTLPPSHDPLMQIFMMMMKQQSTSESELKTKTFWKLKESHQILRNRLSHKTAPQNSASSSKN
QYDELIQKFNMQYDAFVGKRLDNLYGVAKYXXDSXVLKERAEELERVLDAVVGRLQNSEEFGDVMTAMFLNGVRDGI
LEARAILVALRTEDKKK

>XP_014502938.1

MAAELVGGALLSAFLQVAFDKLTSPQFVDFFRGRKLDEKLLGNLNIMLHSINALAYDAEQKQFTDPHVKAWLFSVKEAV
FDAEDLLAEIDYELTRSQVEAESEPQTFTYKVSNFFNSTFSSFNKKTESEMKEILEKLEYLAKQKGALGLKEGTYSGDRSGS
KVSQKLPSSSLVVESVIYGRDADKEIIFNWLTSETDNHNHLSILSIVGMGGLGKTTLAQHVYNDSKMEAKFDIRAWVCVS
DHFDVLTVTKTILEAITKSKDDSRELEMVHGRLKGEVSGKKFLLVLDDVWNERQEEWEAVRTPLSYGAPGSRILVTTRAE
RVASNMRSEVHRLKQLEEDECWKVFIKHALKGDDLKLNDEQKEIGRRIVEKCKGLPLALKTIGSLLHTKSSISDWKSVLES
DIWELPKKVKIIPALLLSYQHLPSHLKKCFAYCALFPKDYEFVKKELILLWMAEGFLHHSHENKNVEEIGEQYFDDLLTRSF
FLQSTVIKKFVMHDLLNDLAKYVCGDFCFRFKFDKGNSIPKTTRHFSFAYADFSFASADVRFFDGFGSLKRLRSFVQITNN
VRGPGIPYLFESIHELFSKLKFLRVLCLSGYNDMKEVPNSVGDLKHLHSLDLSYTCIQNLPDSVGLLYNLLILRLNGCFYFKE
LPSTLHKLTKLRCLEFEGTQVTKMPMHFGELKNLQVLSTFCVNRNNEVTSIKELGGLNLHGRLSINEVQNIVNPLDALEA
NLKNKHLMELNLIWHRNQIPDDPRKEKKVLENLQPSNQLEHLSIRSYCGTQFPSWIFDNSLSNLVSLELENCKYCLCLPPL
GLLSSLKTLKITGLDGIVSIGAEFYGSNSSSFKSLERLEFYCMKEWEEWECKTTSFPLLRQVGIVQCPKLKSLPEQLLHLKDL

SIQSCDILIISEHSVVTSTLEVLQTYSCPLVNIHMTHYDFIEVLIINGGCDSLTIFQLNFFPMLRLLYLHGCQNLQRISQEHVH
NHLFEMSIYECPLFESFPNEGLSAPLVQIIKIGRAGNLKLLPKNMQILLPSLTELEIIDCPKVEKFPDGGLPPNVKDMSLSSL
KLTASLRENLDVNTCLESLYIKYLDVESFADEVLLPPSVTSLTIYGCRNLKKLDYKLLYNLSSLTLFDCGNLQFLPEEGLPKSIS
SLCILDCPLLKQRCQKPEGKDWRKIAHIENLRV

>XP_014502954.1

MAAELVGGALLSAFLQVAFDRLASPQFVDFFRRRKLDEKLLRNLNIMLHSVNALADDAEQKQFTDPHVKSWLFSVKEA
VFDAEDLLDEIDYEITRCQAEAESEPQTIIYKVSNFFNATFRSFNKKVDSGLKEVLEKLEYLTGQKGALGLKEGTYSGDRSG
STISQKLPSTSLVAESVIYGRDTDKEMIINWLTSETDICDQPSILSIVGMGGLGKTTLVQHVYNDPKMDDAKFDSKAWVC
VSDHFNALTVAKTILEAITDEKDESGNLEMVHKKLKEKLKGKKFLLILDDIWNQRRDEWEAVQTPLSYAAPGSKILVTTR
DEKVASNMQSKVHRLKQLREDECWKVFEKHASKDYNIELNDELKEIGSRIVDKCKGLPLALKTIGCLLRTKSSISDWKSVL
VSDIWDLPNEDNEIIPALLLSYHHLPSHLKRCFAYCALFPKDYEFVKEELILLWMAESFLQCPQIRHPEEVGEQYFNDLLSR
SFFQQSTTEKRFVMHDLLNDLAKYVCGDICFRLKFDKGKYIPKTTRHFSFEFDHVKCFDGFGSLTDAKRLRSFLPITEIERT
YLGYYPWQFKISVHDLFSKFKFLRILSFYNCLGLAKLPDSIGDLKHLRLLDFSHTGIQKLPDSTCLLYNLLILRLNHCLRLEELP
SNLHKLTKLRCLEFKDTKVTKMPLHFGELKNLQVLNMFFIDKNNEFSTKQLGRLRLHGRLSINEVQNITNPLDALEANLK
NQHLVELELKWNSKHILNDPKKEKKILENLQPPKQLEGLGISNYGSTHFPSWLFNNSLTNLVFLRLEDCKYCIFLPPLGLLS
SLKTLEIVGLDGIVSIGDEFYGSNASSFMSLERLEFYDMKELREWKCKTTSFPRLQHLSMDHCPELKVLSEHLLHLKKLVIG
YCDKLIISRNNMDTSSLEFLKICSCPLTNIPMTHYDFLEEMDIDGGVDFLTTFSLDFFPNLRSLQLTRCRNLQRFSHEHTHN
HLKYFIIEKCPLVESFFSEGLSAPLLQRIEIRGAENLQLLPKRMEILLPSLIELLIIDCPKVETFPEGGLPSNVKHVSLSSLKLIASL
RESLDANTCLESLSIGKLDVESFPDEVLLPHSLTSLQIFDCPNLKKMEYKGLSDLSSLTLLHCPGLQCLPDEGLPKAISSLTIW
DCPLLKQRCQNPEGEDWGKIGHIEKLIIR

>XP_014502961.1

MAAELVGGALLSAFLQVAFDRLASPQFVDFFRRRKLDEKLLGNLNIMLHSINALAHDAEQKQFTDPHVKAWLFSVKEA
VFDAEDLLAEIDYELTRXQVEAXSEXXTFTXKVSNFFNSTFSSLNKKIELEMKELLEKLEYLAKQKGSLGLKESTYSGDRSGS
KLSQKLPSSSLVVESVIYGRDVDKEMIINWLTSETDNHNHLSILSIVGMGGLGKTTLAQHVYNDPKIEEAKFYIRAWVCVS
DHFDVLTVTKSILEAITKSKDDSGDLEMVHGRLKEKISGRKFLLVLDDVWNERLEKWEAVRTPLSYGAPGSRILVTTRVEK
VASNMRSKVHHLKQLEEDECWKVFNKQALKDDDLELNDVKKEIGRRIVEKCKGLPLALKTIGSLLRTKSSILDWQSVLEN
NIWDLPKEVEIIPALLLSYQHLPSHLKRCFAYCALFPKDYEFDKKKLILLWMGEGFLHHSQQNKNVQEIGEQYFDDLLTRS
FFLQSSSRMRFLMHDLLNDLAKYVCADFCFRLKFDKGSCIPKTTRHFSFSFKYARYFDGFGSLTNAKRLRSFVHITNIRGG
GPPCLFERIHELFSKLKYLRVLSLNGYIELDEVPDSVGDLKHLHSLDLSLTSIQKLPDSVGLLYNLLILRLNGCFHFKELPSSLH
KLTKLRCLEFERTQVTKMPMHFGELKNLQVLNKFYVNRNNEIKQLGGLNLHGRLSISDLQNIVNPLDALEANLKNKHLV
ELKLIWNHNHIPDDPRKEKKALENLQPSKQLKSLKINNYGGTQFPSWVFDNLLSNLVSLELKDCKYCLCLPSLGLLSSLKTL
KITGLDGIVSIGAEFYGSNSSSFKCLERLIFFDMKEWEEWECKTTSFPRVEQLVIHDCPKLKGLSEQLLHLKALSIETCDNLII
REHNVDTSALESLNTHSCPLVNIPMTHYDFIQDMIIVGGCHSLTIFQLNFFPMLRFLSLEGCQNLQRISQEHAHNHLKK
MSICKCPQFESFAPFVQIIDIRQEENLELLPKRMQILLPSLIELHIIGCPKVEKFPDGGLPSNLKLVSLSSLKLIASLRDTLDVN
TCLQSLYIKSLDVESFSDEVLLPPSLTSLTINCCQNLKKLDYKLLYNVSSLTLGDCPNLQCLPEEGLPKSISSLDIWRCPLLKHS
CQKTEGKDWEKISHIQNLSVQ

>XP_014502968.1

MAAELVGGALLSAFLQVAFDRLASPQFVDFFRRRKLDEKLLGNLNIMLHSINALAHDAEQKQFTDPHVKAWLFSVKEA
VFDAEDLLAEIDYELTRXQVEAXSEXXTFTXKVSNFFNSTFSSLNKKIELEMKELLEKLEYLAKQKGSLGLKESTYSGDRSGS
KLSQKLPSSSLVVESVIYGRDVDKEMIINWLTSETDNHNHLSILSIVGMGGLGKTTLAQHVYNDPKIEEAKFYIRAWVCVS
DHFDVLTVTKSILEAITKSKDDSGDLEMVHGRLKEKISGRKFLLVLDDVWNERLEKWEAVRTPLSYGAPGSRILVTTRVEK

VASNMRSKVHHLKQLEEDECWKVFNKQALKDDDLELNDVKKEIGRRIVEKCKGLPLALKTIGSLLRTKSSILDWQSVLEN
NIWDLPKEVEIIPALLLSYQHLPSHLKRCFAYCALFPKDYEFDKKKLILLWMGEGFLHHSQQNKNVQEIGEQYFDDLLTRS
FFLQSSSRMRFLMHDLLNDLAKYVCADFCFRLKFDKGSCIPKTTRHFSFSFKYARYFDGFGSLTNAKRLRSFVHITNIRGG
GPPCLFERIHELFSKLKYLRVLSLNGYIELDEVPDSVGDLKHLHSLDLSLTSIQKLPDSVGLLYNLLILRLNGCFHFKELPSSLH
KLTKLRCLEFERTQVTKMPMHFGELKNLQVLNKFYVNRNNEIKQLGGLNLHGRLSISDLQNIVNPLDALEANLKNKHLV
ELKLIWNHNHIPDDPRKEKKALENLQPSKQLKSLKINNYGGTQFPSWVFDNLLSNLVSLELKDCKYCLCLPSLGLLSSLKTL
KITGLDGIVSIGAEFYGSNSSSFKCLERLIFFDMKEWEEWECKTTSFPRVEQLVIHDCPKLKGLSEQLLHLKALSIETCDNLII
REHNVDTSALESLNTHSCPLVNIPMTHYDFIQDMIIVGGCHSLTIFQLNFFPMLRFLSLEGCQNLQRISQEHAHNHLKK
MSICKCPQFESFAPFVQIIDIRQEENLELLPKRMQILLPSLIELHIIGCPKVEKFPDGGLPSNLKLVSLSSLKLIASLRDTLDVN
TCLQSLYIKSLDVESFSDEVLLPPSLTSLTINCCQNLKKLDYKLLYNVSSLTLGDCPNLQCLPEEGLPKSISSLDIWRCPLLKHS
CQKTEGKDWEKISHIQNLSVQ

>XP_014502978.1

MAAELVGGALLSAFLQVAFDRLASPQFVDFFRGRKLDEKLLGNLNIMLHSINALAHDAEQKQFTDPHVKAWLFSVKEA
VFDAEDLLAEIDYELTRCQVEAESEPKTFTHKVSNFFNSSFNSFNKKIESEMKELLEKLEYLXKQKGALGLKEGTYSGDRSG
DKMSQKLPSSSLMVESVIYGRDADKEMIFNWLTSETDNQNLSILSIVGMGGLGKTTLAQHVYNDQKIETKFDIRAWVC
VSDHFDILTVTKTILEAITKSKDDSGNLEMVHGRLKEKVSGKKFLLVLDDIWSERREEWEAVQTPLSYGAPGSRILVTTRV
EKVASNMRSKVHRLKQLEEDECWKVFEEQALKGDDIELNDDKKEIGRRIVEKCKGLPLALKTIGSLLCTKSSLSDWKNVL
KSDIWDLPKEVEIIPALLLSYQHLPSNLKRCFAYCALFPKNYEFDKDELILLWMAEGFLHYSPQNNNIEEIGEQYFDDLLTR
SFFLRSNIKMHFSMHDLLNDLAKYVSAEFCFRLNFDKGDCVPKTTRHFSYALSDLNSCDVKYFDGLGSLRDAKRLRSFLPI
VSNYGDGLFSRFEILIREFFKLKFLRVLSLNGYHHFKEVPDSVGDLKHLHSLDLSYTWIQRLPDSVGLLYNLLILKLNGCFYL
KELPSSLRNLTKLRCLEFEDTEVTKMPMHFEQVKNLHVLNMFCVSRNSEFSIKQLGGINLHKRLSINELQNIDNPLDALEA
NLKKKQLVRLKLIWNLNHIPDDPMKEKKVLENLQPSNQLEHLSIRSYCGTQFPSWVFDNSLSNLVSLELEDCKYCLYLPPL
GLLSSLKSLEIKGLDGIVSIGAEFYGSNSSSFKSLEILRFFYMKEWEEWECKTTSFPRLRRLFVVQCPKLKGLSEQLLHLEQLY
IESCPNLIISEHIKDTLALELLTIIRGCNSLTIFQLNFFPMLRFLHLQGCQNLQRISQEHPHNHLETMSICACPQFESFPGEGL
SEAFPSLTELRIINCPKVEKFPDVGLPSNVKHLSLSSLKLITSLREILNVNTCLEMLYIDSLDVECFPDEVLLPPSLTSLRISNCR
NLKKLDYKLLYNLSSLTLSECRNLQFLPEEGLPKSISFLDIWDCPLLEQRCQKPEGKDWRKIAHIENLGVGS

>XP_014502986.1

MAAELVGGALLSAFLQVAFDRLASPQFVDFFRGRKLDEKLLGNLNIMLHSINALAHDAEQKQFTDPHVKAWLFSVKEA
VFDAEDLLAEIDYELTRCQVEAESEPKTFTHKVSNFFNSSFNSFNKKIESEMKELLEKLEYLXKQKGALGLKEGTYSGDRSG
DKMSQKLPSSSLMVESVIYGRDADKEMIFNWLTSETDNQNLSILSIVGMGGLGKTTLAQHVYNDQKIETKFDIRAWVC
VSDHFDILTVTKTILEAITKSKDDSGNLEMVHGRLKEKVSGKKFLLVLDDIWSERREEWEAVQTPLSYGAPGSRILVTTRV
EKVASNMRSKVHRLKQLEEDECWKVFEEQALKGDDIELNDDKKEIGRRIVEKCKGLPLALKTIGSLLCTKSSLSDWKNVL
KSDIWDLPKEVEIIPALLLSYQHLPSNLKRCFAYCALFPKNYEFDKDELILLWMAEGFLHYSPQNNNIEEIGEQYFDDLLTR
SFFLRSNIKMHFSMHDLLNDLAKYVSAEFCFRLNFDKGDCVPKTTRHFSYALSDLNSCDVKYFDGLGSLRDAKRLRSFLPI
VSNYGDGLFSRFEILIREFFKLKFLRVLSLNGYHHFKEVPDSVGDLKHLHSLDLSYTWIQRLPDSVGLLYNLLILKLNGCFYL
KELPSSLRNLTKLRCLEFEDTEVTKMPMHFEQVKNLHVLNMFCVSRNSEFSIKQLGGINLHKRLSINELQNIDNPLDALEA
NLKKKQLVRLKLIWNLNHIPDDPMKEKKVLENLQPSNQLEHLSIRSYCGTQFPSWVFDNSLSNLVSLELEDCKYCLYLPPL
GLLSSLKSLEIKGLDGIVSIGAEFYGSNSSSFKSLEILRFFYMKEWEEWECKTTSFPRLRRLFVVQCPKLKGLSEQLLHLEQLY
IESCPNLIISEHIKDTLALELLTIIRGCNSLTIFQLNFFPMLRFLHLQGCQNLQRISQEHPHNHLETMSICACPQFESFPGEGL
SEAFPSLTELRIINCPKVEKFPDVGLPSNVKHLSLSSLKLITSLREILNVNTCLEMLYIDSLDVECFPDEVLLPPSLTSLRISNCR
NLKKLDYKLLYNLSSLTLSECRNLQFLPEEGLPKSISFLDIWDCPLLEQRCQKPEGKDWRKIAHIENLGVGS

>XP_014502992.1

MAAELVGGALXSAFLQVAFDRLASPQFVDFFRRRKLDXKLLGNLNIMLHSIXALAXDAEQKQFTDXHVKAWLFSVKEA
VFDAEDLLVEIDYELTRSQVEAESKPQTFTYKVSNFFNSTFNSFNKKIESEMKEVLEKLEYLAKQKGALGLKEGNYSGDRS
GGKVSQKLPSSSLVVESVIYGRDADKEMIFNWLTSENENHNHLSILSIVGMGGLGKTTLAQHVYNDPKLETKFDIRAWV
CVSDHFDILTVTKTILEAITKSKDDSVDLEMVHGRLKEKVSRKKFLLILDDVWSERREEWEAVRTPLSYGALGSRILVTTRV
EKVAHNMRSRVHRLKQLEEDEGWTVFEEQALKGDDVELNDEKMEIGRRIVDKCKGLPLALKTIGSLLRTKSSILDWKSV
LESDIWDLQKEVEIMPALLLSYQHLPSHLKSCFAYCALFPKDYEFDKMELILLWMAEGFLHHSQPNKNVEEIGEQYFDDL
LTRSFFLQSSSELRFVMHDLLNDLAKYVCADFCFRLKFDKGNCIPKTTRHFSFAFRDVGYFDGFGSLTNAKRLHSFVQITK
NTVYSAFPCQIEILIRELFSKFKFLRVLSLTGIYGLKEVPDSVGDLKHLHSLDLSYTNIQKLPDSICLLYNLLILRLNGCFYFKEL
PLSLHKLTKLRCLEFERTQVTKMPMHFGELKNLHVLNMFCLNGNNEVINIKQLGGLNLHGKLSINDLQNIVNPLDALEA
NLKNKPLVELNLIWYRNQIPDDPRKEKKVLENLQPSNQLEHLSIRSYCGTQFPCWLFDNSLSNLVSLQLEDCKYCLCLPPL
GLLSSLKTLCIIGLDGIVSIGAEFYGSNSSSFKSLERLEFYNMNEWEEWECKTTSFPRLRGLFIHECPKLKVLAKQLLHLEKLH
IESCPNLIISEHNEDISGLELLRTRSCPLVNIPMTHYDFIEDMKIIRGCDFLTIFHLNFFPMLRFLDLVGCKNLKRISQQHHH
NHLEVMSIRECPQFESFLGEGLFAAFPSVIELHIINCPKVEKFPYGGLPSKVKYMCLSSLKLIASLRETLDVNTCLESLTIENL
DVESFPDEVLLPPSLTSLSINHCPNLKKLDYRLLYNLSSLTLFDCGNLQFLPEEGLLKSISSFEIWYCPLLEQCC

>XP_014503040.1

MEYILGILCHYAGKIAEYTLGSVCVCYINDFTLNVLDVKKSFEWIRGIMKEQIREITNGTEKFEPDFLKHVENLLKAAENVS
ERQQLLEERISNTNKSYFRRQRLYFLAKEIERETHKIQELLLDVKKIESLSRITELSNDLVAFKSPEEAYTEILAALKDRSVSIIG
LVGFGGSGKTTLAKEVGKKAEEIKLFEKVVWATVSQLLNIRSIQDQIADQLNVKLKEEEASEIGRADRISEGLRKRTTLVIL
DDVWEKVNFEALGIPLDESSKGCCVLITTRSKEVCTSMKCQNIIEVNPLHYRKVRALFKFHANISNDSPEALKVLAERIVTK
CNGSSTAIATLGSTLKDKTIEEFESAWLRLQNSEQLSSLKGLTSYQLCLKVSYDNLTNQLAKSLLLLCSIFPKNHEIDLEDLFR
FGRGLGVIWRFGRMEKERRVMHAAINILKNSYMLAYVKEKEKVKMPDWIRDVALWIAAESGQAILTCTAVDPRVLIDD
EITKDKNVIALWDMKNGELLNYEWNCPSLEILLLHSPEVGFTISNALIERLKMLKLLAFLKFEYGWKLPFETETPSWYTSPL
SQSIESLKNLNTLSLRGYKLGDISVLESLEALEILDLRGSSFKQLPNGIVALQKLKLLDLYGCLIEKNNAYEVIGRCLRLEELYLY
LLPSKEEFPHDSLSRDCRVYLHLKYIEGGYKNVIPSMRSQGMNQIVVLILEHCLDIEFLFDGTFTNSNVDMLHTKTVFSNL
GTVRLHQMHGLREVFHDPSSQCSLERLQELSIDSCNQLYNTSFPRNSNLCSLKVLKLEFAQC

>XP_014503041.1

MEDLFLSIATKIAEYTVHPILHHAQYLCCFNNFASNLPNAKEQLEFTRDGVKERIREARNRVEKVEPTVEKWLKDVEKVLE
EVQMLEERILSVNKSYFRRQCQYSLAKQIEKKTTKMIQLHRNSKFEPFSRITELPGMQYYSSKDFFMFNSXXASYXKLLEA
LNNKSAFIIGLVGLGGSGKTTLAKEVGKKAKEMKLFEKVVWATVSQSLNIRSIQDQIADQLSIDLKEASEIGRAQRLSERLR
KGTTLLVLDDIWEILNFEXXGIPLXEXSKACCILITTRSKEVCTSMQCQSIIELNLLSDEEAWTLFRHYANINDDSSEALKGV
ARKIVNXCKGLPIAIVTVGSTLKDKTIANFELALSRLENSKPLDIPKGLTSPYVCLELSYNNLTNPLAQSLLLSCSMFPEDYEI
DLEDLFRFGMRFDIIGTFGTMENARREMDAAIDMLKNCFLLMHAKEKQRVKMHDLVRDVALWIASKSGKAIFTGTEV
DPRALADDETLKDRKAIAVWGLKSYDVLNYKINRPILETLLISFNVRDSVKVSTGCIQSLENLKTLAILNSAWWKINALPLQ
ESLKSLKNLRTLCLRGFGLGDISFVEGLQALEILDLRGSYFDDFPIWILELKKLRLLDLYECHIMKYKNSQFYEVVGKCLQLEE
LYLCLSYGIKPSPYDVSFSRLQRYVIETGHNRRSFDDTTELMKKYGQPRSLIMNWWFEVVPESFISLPIKDLFIRAEFLCLRF
LRNYKNIIPSMDPQGMNQLIALRLYDCSNIECLVDSTISFGLLQTEVIAFSALVYLSLVNLHDLREVFCDPSSRCSLKNLQEL
EIKNCGKLYSISFPRNSKLCNLKVISISECPMLTSLFTSSVGQTLELLEDLKVYYCRSLRHIIEEENDVLSSTQSHSSFTLPKLRN
IAIFGCDNLEYVFSVFLVEGLLSLKSVNIVGNQKLKYVFGSEKEHNVAGYSNFQQTNTRRNLFNLNTLELRFLPNLIAIWPE
YFRAHLPSLNYLYCDDCPKLSIHKAMNASDIQQQQTTPTVIY

>XP_014503042.2

MLEDLFLSIATKIAEYAVHPILHHSQYLCCFNNFASNLPNAKEQLELTRDGVKERIREAINMVEKVEPTVEKWLKDVENVL
EEVQMLEERILCVNKSYFRRQCRYSLAKQIERKTTEMIQLHRNSKFEPFSRITELPGMQYYSSKDFFMFNSTVASYNKLLE
ALNNKSAFIIGLVGLGGSGKTTLAKEVGKKAEDMKLFGKVVWATVSQPLNIRSIQDQIVDQLGFKLTEDSDIGRARRLSE
RLKKGTTLIILDDIWEILNFEALGIPLNESSKACCVLITTRSKEVCTSMQCQSIIELNLLSDQEVWTLFRHYANITDDSSEVLK
GVARKIVHECKGLPIVIVTVGSMLKDKTIANFELACSRLENYEPLDIPKGLISLYVCVELSYSNLTSQLAQSLLLSCSMFPED
CEIDLEDLFRFGRGFGTIGTFGTMEDARGWINAAIDMLKNCFLLMDAKEKQRVKMHDVVRDAALWIASKSGKAIFTRT
EVDPRALEDDETLKDMKAIAVWGLKSYDVLNYKINCPILETLLLSFNVSDGVKVSDGCLQSLENLKTLAILNSVGWKHDE
LPLQESLKSLKNLRTLCLRSCNLGDISFVESLQALEILDLRGSYFDYLPVGIVELKKLRILDLYKCLVKKNENVEAYEVVGKCL
QLEELYLHLMNYENAFPRDVSFSRLQRRMKLPSHSTNWMFYCLIPNSNLEIFG

>XP_014503047.1

MEDLFLSIATKIAEYTVHPILHHAQYLCCFNNFASNLPNAKEQLEFTRDGVKERIRAIYRIEKVEPTVEKWLKDVEKVLEEV
QMLEERILSVNKSYFKRQCQYSLAKQIERKTSEMIQLHSNSKFEPFSRITELPGMKYYSSKDFFMFNSTEVSYKKLLEALKN
KSAFIIGLVGLGGSGKTTLAKEVGKKAEDMKIFEKVVWATVSQPLNIRTIQAQIADQLGFKLNEESEIGRAQRLSERLRKG
TTLVILDDVWEKLNFEALGIPLDESNKDCCVLITTRSKEVCTSIQCQSTIELNLLSDEEAWTLFRHYANITDDSSEALKGVA
RKIVNECKGLPIAIVTVGSTLKDKTIANFELALSKLEISEPLDIPQGFRSPYVCLELSYNNLTNPLAQSLLLLCSMFPEDCEIDL
EDLFRFGRGFGTIGTMFGTMEKARREMDAAIDMLKNCFLLMHAKEKYKVKMHDLVRDVALWIASKSGQAIFTRTKVD
PIVLADDDEVMKDMKAIAVWGSKNDHLLNYKINCPILEILLLSFNESGGVKVSDECLQSLEKLKTLAILESALWNRGALPL
QESLKSLKNLRTLCLRGYGLGDISFVERLQALEILDLSFSSFDDLPGGIVKLKKLKLLDLYQCWIQKKENVNPYEVVGKCLQL
EELYLHLINYEKAFPPDVSFSRLQRYVIETRPDRSSDGDTTDLMKKYGHSRSLIMNGFDVAPQSFISLPIKDLFIRAEFLCLR
DLREDYKNIIPSMDPQGMNQLIALRLYYGSVECLVDSTEKDKTEVIAFSELVYLSLVRIYSLREVFCDPSSRCSLKNLQELEI
KNCGNLYSISFPRNSKLCNLKVISISECPMLTSLFTSSVGQTLELLEDLIISKCRSLRHIIEEENDVLSSTQSHSSFTLPKLRNISI
FGCDNLEYVFSVVLFEGLLSLKSVNIGRNRALKYVFGSEKEHNVAGYPSFQQTNTITNLFNLNTLTFSDLPNLIAIWPEYFR
AHLPSLNYLCCDDCPKLSIHKAMNASDIQQQQTTPTENEILWLITNTLNQFGDDPLSHPQLKVVLKFRELELIDIRIKGIFQ
FQMGEEGGTTELVPLNLDIEYLCLFNLPELNFIWKGPTEECSQLHKAFAFSEEEMGKDGKQVLLPNLKDIRLINLPNLEEI
HHGFKLKEDVEQTLEECPKYSPSSYLHQEENEVTLEDALERNENIDAAP

>XP_014503234.1

MDIASSSYKLPRKYDVLINFTGEDIHRKFVSHLNSAFSTVGLTTFLHHPNAVNTTHIQQPILSHCRVAIVIFTQTYSQSAWC
LNQLLQIIKWHQTYCRHVLPVYYEIQPSDVRLQKGDFGKALKETAQQTFSGQELDHGMSRWSHALTKAANFFGWDES
SYRSDAELVDKIVKSVVNLPVLSATKFPVGLQSHVEDLIRTIKNKSTEVCIIEILGEQGSGKTTLAKAIYNQIHWTFKEKSFIK
NTSHVTGIRGQLRLQEQLLLDVLKQKVEIPSVDVGRTMIRERLLGKMVLIVLDDVNYFTLFDVCDCRKLLVEGTVIIVTSTY
GIRLGDQANSVFWLEGMNTEESLELLSWHAFREPKPKEEYEDLARRVVSYCEGLPLALEVVGSSLFEKTKEEWNNLLFAI
YGMRCVPKIIKISIEGSLNEMEKDIFLNICYFYVGKSRGYVRKILNGCGVDADIGIRVLIQRNLIKINKNNKFGMHHLLQNI
GMKIIHENSGKDLGKNRRLWFDKDAKYVRTFFTCGLKQGTEAMQWLPVNAFVLRSLKVLNLSHSEYLTTTPDFTGLPSL
EQLIFKYCSRLSKVHQSIGSLNSLILLNLKYCTSLNNLPTEIYELKSLRTFILSGCSKIDIMDKDIAKLESLITLIAENTAVKEVPF
SIVSSKSIGYISLSGFEGLSHNLFPSIIRSWMSPIMNPISYIHSLCMDTEDNTIDIAPLLSTLSNIRIRKALYGLK

>XP_014504582.1

MDILYGFLSGVLKDVACGATNQLQYSFCFNSFVKELEKEEDNLIEMIRSVEDRVIHARRQTLKTTEVIDKWLENANIDSEY
VNRLLRETNAKKSCFFEFCPNWIWRYRLGKKLAIKKADLQKIIQDGRQYIQLQRIASIPSNTFDILTEKSMNFDSRKFAFD
QLMEAVKDDGVAMIGLYGMGGCGKTTLAMEVKKIAEVEHLFDRVIFVPVSSTVEVPRIQEKIASSLHYTFPENQEMERS
QRLCMRLTQEKKILMILDDVWEKLDFGRIGISSSEYHKGCKILITTRSEDVCILMDCQKKIYLPILTDEEAWTLFQNKALMS
KDTPENMQHLAISISKECKGLPVAIVAVASSLKGKKEAIWHSALNKLRSSKPINISRGLQDPYMCLKLSYDNLDTDEAKSL

FLLCSVFPEDYEISVECLIRCAIGLGVAGEVDIYEEARTEVTAAKIKLVSSCLMLEAGDERVKMHDLVRDVAHWIAKNENK
IIKCELEKDVSLEEGSIRYLWCVKFPDGMDCSNLEFLSIQTKLEVSDGIFERMGKLRVLIITNKNGYRLQLSTTSFKSLTNLR
CLVLQYFALRDISFVRDLKKLQSLSFHRCSCSSFLDLQTDVAVTTLTNLKLLEFISCDIESNIFEEIKRIPFLEELYILENGQRYN
NEENVKFFNSFSVPHTLQRFGIILGYDIWQLEFCSYERTLRINYFDISNEVIKGLAGKARELYVGNIEGGAKNMMPDIFEIE
EGMNELKELRIRDCEEIVCLVDTSNHLSKMGNIFSKLRLMRIMSMNNLKTLWHGCLPANGCFEKLENMYIEDCHQLTC
LFTNIIVPTNDKTKKSEDQFRNGHSMQSKIFQNLQEVRIYHCRELKHVFSTSISGDLSQLKMLEIKYCHMLEQIIEEVLPPP
AHHEETNEIVEEDVQSSSGSFFLSSLALLKISSCSMLESLFTISVAKTLTSLEKLKISNCHGLKHIVPPARVKRNKNMVEDEH
EFENDLSMFSNLKSIFIRECVSLQDIFATPVNECFEKLEKVYIDNCPQLTYLFTYVAQGLTELKILHIYNCDILKHIITYDDKTK
KSEDQFTSRHSVQSRIFQNLEEVMLDECGELKHVFSANIIGGLPQLKKLEIENCNMLQQIIVEEDECQHFESNQVKVSPES
ISIPSVSTVNNISGSFSLSSLALLSIKYCLMLDSLFTTSVAKTLTSLEELDISNCDGLKHIVSPERVKRNKNMVEDEHEFESNLS
MFSSLKWVKISRCDSLQDIFIMPFVGGTVNIQNHFSTQQTLRMSNANNGDWMMGQQVSLKLEFLKLSYLHEMTHIW
VATNNSFTLQHLNSLIIEECEKLEVIFPQSMLRSLPELNYLQVSECKELRQIIEEDLEDKSLFAQPCFPKLDSLVIERCHKLKCF
TSVIASNALSNLRILIIKEATELQEFIACEYDETVKTKVELPQLKLIIFMDLSNFQQESIFSNVKHRIIRNCPKLSLTSTITPQEL
QQNYPLEGLGMSETIRLEISSLMYEIKKLDEVSTNNNSTELPSSQINEKLDKNVTEKDYGSKEVAPATTASYFTDQQSPLG
QTQSTIKMSQQDWDPTPKNTSPLQMNREDQSTSQIKPFSSQVNDNNQSMLESRVEMVGQHNKIETKTQAPEIEEFQ
KIDRKNEMASDPQAMDQNFPVISSPNMTQRTDEIETDNLGKTTTSDKPAIPTFVSENIEEIGRERRRGGPAIEGVTIKTLP
IGGDNISLVSGVTIHNSSGANILSQDSQIVKQDNELNEDKTEIAPHTNIKIQERVNLLDKTEGVRIVSNNDVVVTSASTDT
RTRLEKYKQFVDLNDSQISLLVEAIEAYPHLWNACEKFTDRFRAWMLKTLVDMLLFLRSESVGSINPHREKEFLKLCDEA
VQLGFERSWVDQMRQRVLGRDPKLDHAKVRINELLKRHDHLTWELDNIKKELRSLNDFLNAQAKCFDFL

>XP_014504583.1

MDILYGFLSGVLKDVACGATNQLQYSFCFNSFVKELEKEEDNLIEMIRSVEDRVIHARRQTLKTTEVIDKWLENANIDSEY
VNRLLRETNAKKSCFFEFCPNWIWRYRLGKKLAIKKADLQKIIQDGRQYIQLQRIASIPSNTFDILTEKSMNFDSRKFAFD
QLMEAVKDDGVAMIGLYGMGGCGKTTLAMEVKKIAEVEHLFDRVIFVPVSSTVEVPRIQEKIASSLHYTFPENQEMERS
QRLCMRLTQEKKILMILDDVWEKLDFGRIGISSSEYHKGCKILITTRSEDVCILMDCQKKIYLPILTDEEAWTLFQNKALMS
KDTPENMQHLAISISKECKGLPVAIVAVASSLKGKKEAIWHSALNKLRSSKPINISRGLQDPYMCLKLSYDNLDTDEAKSL
FLLCSVFPEDYEISVECLIRCAIGLGVAGEVDIYEEARTEVTAAKIKLVSSCLMLEAGDERVKMHDLVRDVAHWIAKNENK
IIKCELEKDVSLEEGSIRYLWCVKFPDGMDCSNLEFLSIQTKLEVSDGIFERMGKLRVLIITNKNGYRLQLSTTSFKSLTNLR
CLVLQYFALRDISFVRDLKKLQSLSFHRCSCSSFLDLQTDVAVTTLTNLKLLEFISCDIESNIFEEIKRIPFLEELYILENGQRYN
NEENVKFFNSFSVPHTLQRFGIILGYDIWQLEFCSYERTLRINYFDISNEVIKGLAGKARELYVGNIEGGAKNMMPDIFEIE
EGMNELKELRIRDCEEIVCLVDTSNHLSKMGNIFSKLRLMRIMSMNNLKTLWHGCLPANGCFEKLENMYIEDCHQLTC
LFTNIIVPTNDKTKKSEDQFRNGHSMQSKIFQNLQEVRIYHCRELKHVFSTSISGDLSQLKMLEIKYCHMLEQIIEEVLPPP
AHHEETNEIVEEDVQSSSGSFFLSSLALLKISSCSMLESLFTISVAKTLTSLEKLKISNCHGLKHIVPPARVKRNKNMVEDEH
EFENDLSMFSNLKSIFIRECVSLQDIFATPVNECFEKLEKVYIDNCPQLTYLFTYVAQGLTELKILHIYNCDILKHIITYDDKTK
KSEDQFTSRHSVQSRIFQNLEEVMLDECGELKHVFSANIIGGLPQLKKLEIENCNMLQQIIVEEDECQHFESNQVKVSPES
ISIPSVSTVNNISGSFSLSSLALLSIKYCLMLDSLFTTSVAKTLTSLEELDISNCDGLKHIVSPERVKRNKNMVEDEHEFESNLS
MFSSLKWVKISRCDSLQDIFIMPFVGGTVNIQNHFSTQQTLRMSNANNGDWMMGQQVSLKLEFLKLSYLHEMTHIW
VATNNSFTLQHLNSLIIEECEKLEVIFPQSMLRSLPELNYLQVSECKELRQIIEEDLEDKSLFAQPCFPKLDSLVIERCHKLKCF
TSVIASNALSNLRILIIKEATELQEFIACEYDETVKTKVELPQLKLIIFMDLSNFQQESIFSNVKHRIIRNCPKLSLTSTITPQEL
QQNYPLEGLGMSETIRLEISSLMYEIKKLDEVSTNNNSTELPSSQINEKLDKNVTEKDYGSKEVAPATTASYFTDQQSPLG
QTQSTIKMSQDWDPTPKNTSPLQMNREDQSTSQIKPFSSQVNDNNQSMLESRVEMVGQHNKIETKTQAPEIEEFQKI
DRKNEMASDPQAMDQNFPVISSPNMTQRTDEIETDNLGKTTTSDKPAIPTFVSENIEEIGRERRRGGPAIEGVTIKTLPI
GGDNISLVSGVTIHNSSGANILSQDSQIVKQDNELNEDKTEIAPHTNIKIQERVNLLDKTEGVRIVSNNDVVVTSASTDTR

TRLEKYKQFVDLNDSQISLLVEAIEAYPHLWNACEKFTDRFRAWMLKTLVDMLLFLRSESVGSINPHREKEFLKLCDEAV
QLGFERSWVDQMRQRVLGRDPKLDHAKVRINELLKRHDHLTWELDNIKKELRSLNDFLNAQAKCFDFL

>XP_014504584.1

MDILYGFLSGVLKDVACGATNQLQYSFCFNSFVKELEKEEDNLIEMIRSVEDRVIHARRQTLKTTEVIDKWLENANIDSEY
VNRLLRETNAKKSCFFEFCPNWIWRYRLGKKLAIKKADLQKIIQDGRQYIQLQRIASIPSNTFDILTEKSMNFDSRKFAFD
QLMEAVKDDGVAMIGLYGMGGCGKTTLAMEVKKIAEVEHLFDRVIFVPVSSTVEVPRIQEKIASSLHYTFPENQEMERS
QRLCMRLTQEKKILMILDDVWEKLDFGRIGISSSEYHKGCKILITTRSEDVCILMDCQKKIYLPILTDEEAWTLFQNKALMS
KDTPENMQHLAISISKECKGLPVAIVAVASSLKGKKEAIWHSALNKLRSSKPINISRGLQDPYMCLKLSYDNLDTDEAKSL
FLLCSVFPEDYEISVECLIRCAIGLGVAGEVDIYEEARTEVTAAKIKLVSSCLMLEAGDERVKMHDLVRDVAHWIAKNENK
IIKCELEKDVSLEEGSIRYLWCVKFPDGMDCSNLEFLSIQTKLEVSDGIFERMGKLRVLIITNKNGYRLQLSTTSFKSLTNLR
CLVLQYFALRDISFVRDLKKLQSLSFHRCSCSSFLDLQTDVAVTTLTNLKLLEFISCDIESNIFEEIKRIPFLEELYILENGQRYN
NEENVKFFNSFSVPHTLQRFGIILGYDIWQLEFCSYERTLRINYFDISNEVIKGLAGKARELYVGNIEGGAKNMMPDIFEIE
EGMNELKELRIRDCEEIVCLVDTSNHLSKMGNIFSKLRLMRIMSMNNLKTLWHGCLPANGCFEKLENMYIEDCHQLTC
LFTNIIVPTNDKTKKSEDQFRNGHSMQSKIFQNLQEVRIYHCRELKHVFSTSISGDLSQLKMLEIKYCHMLEQIIEEVLPPP
AHHEETNEIVEEDVQSSSGSFFLSSLALLKISSCSMLESLFTISVAKTLTSLEKLKISNCHGLKHIVPPARVKRNKNMVEDEH
EFENDLSMFSNLKSIFIRECVSLQDIFATPVNECFEKLEKVYIDNCPQLTYLFTYVAQGLTELKILHIYNCDILKHIITYDDKTK
KSEDQFTSRHSVQSRIFQNLEEVMLDECGELKHVFSANIIGGLPQLKKLEIENCNMLQQIIVEEDECQHFESNQVKVSPES
ISIPSVSTVNNISGSFSLSSLALLSIKYCLMLDSLFTTSVAKTLTSLEELDISNCDGLKHIVSPERVKRNKNMVEDEHEFESNLS
MFSSLKWVKISRCDSLQDIFIMPFVGGTVNIQNHFSTQQTLRMSNANNGDWMMGQQVSLKLEFLKLSYLHEMTHIW
VATNNSFTLQHLNSLIIEECEKLEVIFPQSMLRSLPELNYLQVSECKELRQIIEEDLEDKSLFAQPCFPKLDSLVIERCHKLKCF
TSVIASNALSNLRILIIKEATELQEFIACEYDETVKTKVELPQLKLIIFMDLSNFQQESIFSNVKHRIIRNCPKLSLTSTITPQEL
QQNYPLEGLGMSETIRLEISSLMYEIKKLDEVSTNNNSTELPSSQEVAPATTASYFTDQQSPLGQTQSTIKMSQQDWDP
TPKNTSPLQMNREDQSTSQIKPFSSQVNDNNQSMLESRVEMVGQHNKIETKTQAPEIEEFQKIDRKNEMASDPQAM
DQNFPVISSPNMTQRTDEIETDNLGKTTTSDKPAIPTFVSENIEEIGRERRRGGPAIEGVTIKTLPIGGDNISLVSGVTIHNS
SGANILSQDSQIVKQDNELNEDKTEIAPHTNIKIQERVNLLDKTEGVRIVSNNDVVVTSASTDTRTRLEKYKQFVDLNDS
QISLLVEAIEAYPHLWNACEKFTDRFRAWMLKTLVDMLLFLRSESVGSINPHREKEFLKLCDEAVQLGFERSWVDQMR
QRVLGRDPKLDHAKVRINELLKRHDHLTWELDNIKKELRSLNDFLNAQAKCFDFL

>XP_014504586.1

MDILYGFLSGVLKDVACGATNQLQYSFCFNSFVKELEKEEDNLIEMIRSVEDRVIHARRQTLKTTEVIDKWLENANIDSEY
VNRLLRETNAKKSCFFEFCPNWIWRYRLGKKLAIKKADLQKIIQDGRQYIQLQRIASIPSNTFDILTEKSMNFDSRKFAFD
QLMEAVKDDGVAMIGLYGMGGCGKTTLAMEVKKIAEVEHLFDRVIFVPVSSTVEVPRIQEKIASSLHYTFPENQEMERS
QRLCMRLTQEKKILMILDDVWEKLDFGRIGISSSEYHKGCKILITTRSEDVCILMDCQKKIYLPILTDEEAWTLFQNKALMS
KDTPENMQHLAISISKECKGLPVAIVAVASSLKGKKEAIWHSALNKLRSSKPINISRGLQDPYMCLKLSYDNLDTDEAKSL
FLLCSVFPEDYEISVECLIRCAIGLGVAGEVDIYEEARTEVTAAKIKLVSSCLMLEAGDERVKMHDLVRDVAHWIAKNENK
IIKCELEKDVSLEEGSIRYLWCVKFPDGMDCSNLEFLSIQTKLEVSDGIFERMGKLRVLIITNKNGYRLQLSTTSFKSLTNLR
CLVLQYFALRDISFVRDLKKLQSLSFHRCSCSSFLDLQTDVAVTTLTNLKLLEFISCDIESNIFEEIKRIPFLEELYILENGQRYN
NEENVKFFNSFSVPHTLQRFGIILGYDIWQLEFCSYERTLRINYFDISNEVIKGLAGKARELYVGNIEGGAKNMMPDIFEIE
EGMNELKELRIRDCEEIVCLVDTSNHLSKMGNIFSKLRLMRIMSMNNLKTLWHGCLPANGCFEKLENMYIEDCHQLTC
LFTNIIVPTNDKTKKSEDQFRNGHSMQSKIFQNLQEVRIYHCRELKHVFSTSISGDLSQLKMLEIKYCHMLEQIIEEVLPPP
AHHEETNEIVEEDVQSSSGSFFLSSLALLKISSCSMLESLFTISVAKTLTSLEKLKISNCHGLKHIVPPARVKRNKNMVEDEH
EFENDLSMFSNLKSIFIRECVSLQDIFATPVNECFEKLEKVYIDNCPQLTYLFTYVAQGLTELKILHIYNCDILKHIITYDDKTK
KSEDQFTSRHSVQSRIFQNLEEVMLDECGELKHVFSANIIGGLPQLKKLEIENCNMLQQIIVEEDECQHFESNQVKVSPES

ISIPSVSTVNNISGSFSLSSLALLSIKYCLMLDSLFTTSVAKTLTSLEELDISNCDGLKHIVSPERVKRNKNMVEDEHEFESNLS
MFSSLKWVKISRCDSLQDIFIMPFVGGTVNIQNHFSTQQTLRMSNANNGDWMMGQQVSLKLEFLKLSYLHEMTHIW
VATNNSFTLQHLNSLIIEECEKLEVIFPQSMLRSLPELNYLQVSECKELRQIIEEDLEDKSLFAQPCFPKLDSLVIERCHKLKCF
TSVIASNALSNLRILIIKEATELQEFIACEYDETVKTKVELPQLKLIIFMDLSNFQQESIFSNVKHRIIRNCPKLSLTSTITPQEL
QQNYPLEGLGMSETIRLEISSLMYEIKKLDEVSTNNNSTELPSSQIRMSLRKIMVQRKWLLQRLLHTSQTNKVHLDKLKV
PSKCLSRIGILRQKTLLPSK

>XP_014504587.1

MDILYGFLSGVLKDVACGATNQLQYSFCFNSFVKELEKEEDNLIEMIRSVEDRVIHARRQTLKTTEVIDKWLENANIDSEY
VNRLLRETNAKKSCFFEFCPNWIWRYRLGKKLAIKKADLQKIIQDGRQYIQLQRIASIPSNTFDILTEKSMNFDSRKFAFD
QLMEAVKDDGVAMIGLYGMGGCGKTTLAMEVKKIAEVEHLFDRVIFVPVSSTVEVPRIQEKIASSLHYTFPENQEMERS
QRLCMRLTQEKKILMILDDVWEKLDFGRIGISSSEYHKGCKILITTRSEDVCILMDCQKKIYLPILTDEEAWTLFQNKALMS
KDTPENMQHLAISISKECKGLPVAIVAVASSLKGKKEAIWHSALNKLRSSKPINISRGLQDPYMCLKLSYDNLDTDEAKSL
FLLCSVFPEDYEISVECLIRCAIGLGVAGEVDIYEEARTEVTAAKIKLVSSCLMLEAGDERVKMHDLVRDVAHWIAKNENK
IIKCELEKDVSLEEGSIRYLWCVKFPDGMDCSNLEFLSIQTKLEVSDGIFERMGKLRVLIITNKNGYRLQLSTTSFKSLTNLR
CLVLQYFALRDISFVRDLKKLQSLSFHRCSCSSFLDLQTDVAVTTLTNLKLLEFISCDIESNIFEEIKRIPFLEELYILENGQRYN
NEENVKFFNSFSVPHTLQRFGIILGYDIWQLEFCSYERTLRINYFDISNEVIKGLAGKARELYVGNIEGGAKNMMPDIFEIE
EGMNELKELRIRDCEEIVCLVDTSNHLSKMGNIFSKLRLMRIMSMNNLKTLWHGCLPANGCFEKLENMYIEDCHQLTC
LFTNIIVPTNDKTKKSEDQFRNGHSMQSKIFQNLQEVRIYHCRELKHVFSTSISGDLSQLKMLEIKYCHMLEQIIEEVLPPP
AHHEETNEIVEEDVQSSSGSFFLSSLALLKISSCSMLESLFTISVAKTLTSLEKLKISNCHGLKHIVPPARVKRNKNMVEDEH
EFENDLSMFSNLKSIFIRECVSLQDIFATPVNECFEKLEKVYIDNCPQLTYLFTYVAQGLTELKILHIYNCDILKHIITYDDKTK
KSEDQFTSRHSVQSRIFQNLEEVMLDECGELKHVFSANIIGGLPQLKKLEIENCNMLQQIIVEEDECQHFESNQVKVSPES
ISIPSVSTVNNISGSFSLSSLALLSIKYCLMLDSLFTTSVAKTLTSLEELDISNCDGLKHIVSPERVKRNKNMVEDEHEFESNLS
MFSSLKWVKISRCDSLQDIFIMPFVGGTVNIQNHFSTQQTLRMSNANNGDWMMGQQVSLKLEFLKLSYLHEMTHIW
VATNNSFTLQHLNSLIIEECEKLEVIFPQSMLRSLPELNYLQVSECKELRQIIEEDLEDKSLFAQPCFPKLDSLVIERCHKLKCF
TSVIASNALSNLRILIIKEATELQEFIACEYDETVKTKVELPQLKLIIFMDLSNFQQESIFSNVKHRIIRNCPKLSLTSTITPQEL
QQNYPLEGLGMSETIRLEISSLMYEIKKLDEVSTNNNSTELPSSQIRMSLRKIMVQRKWLLQRLLHTSQTNKVHLDKLKV
PSKCLRIGILRQKTLLPSK

>XP_014504640.1

MEKHGEEEEFDGFSYDVFISFRGEDAGKNFVGHLRRELGREGINTFNDDRDMRTEEGLSPAVCEAIEESNIFIVVFSENY
ASSTWCLDELVKIIQRTHINSKQVVYPVFYHVDPSDTRKIKDSFEKHMMAHENEIGKESQRIQAWRSALFEAASLPGMH
VPTGNDENDIIEKIVEKVRRSIAPKPLYTGENVVGLKPRVEEVMSLLDMKPNDKTVRMLGIYGLGGIGKTKLAKALYDKIV
HNFDAASFLADVRENSKRINGMEDLQKTLLSEMFEELETELGSAYKGIYEIKRKLHRKKLLLVLDDVDDKEELEKLAGGCD
WFGPGSRIIVTTREKDVLNAHHVGNIYEMKELDEQHSLELFCWNAFGQGCPKTGFQDVSVRAAGYAKGVPLAIQVIAS
DLANLHEESLDAWEDALEEYEKTPSKKEIQDVLKISYDRLDVDAKQVFLDIACFFKGERMEYVKKILKEFCSTSNMKVLVN
KSLITIENDRLKMHDLIQDMGRQIVRQEAPDNPGQRSRILDYEDVIEILTKDSGSDKIQGIKLDPPKQAEVKWSGTEFGK
MKWLRILIVRNTSFSSELQHLPNHLRLLHWDNYPSKSFPQKFHPKKIVVFNLPRSSLTFQDPFKKFPCLTNMDFSYNQRII
EIPDVSELQNLRELRLDHCRNLIAVHQSVGFLKKLSHLSVSECTKLQNFLSRMFLPSLEVFDLNLCERVGHFPEIMQEMTK
PLKIYMINTGIQELPESISKLIGLVFIDISNNRQLKYLPSSLFMLPNVDSFKIEACSKLGQSFRSLVQHPSKVNVRPKLRSLNF
QKGNLSDEDLLAILCYFPKLEELIVSENNFVYIPSCIKECGDLTSLDLNGCKKLKKIPELTSLRILDVHHCSDLEEISELPSTVEK
VDARFCFKLTKETSDMLWCQVKKGVGGIEMVMPLITEIPEWFNFVEVGTIPGFWVRGKFPNIVLAMIFHFPNQSQSDE
FVRRRHVDLRLVINGRNAFGKGYHNFIIEAGHVLICDLRVLFSEKEWFGLDALLEKEWNVVQVEYEATSSLMISSWGVFL
YEEGSNMEDLLFNCPNHMDSEKMPPAIVPEKDPMEKYRKMIRELRLDETFKNTLTEWQEDKERGGAGVHDDCIRTVL

GESKKISKDAEDALKSNGSALEDPNSYLRWLLDTVENDDGKPKVIFKDDLALIRLEEPVTGKKKDNVGEASSSGHQESKE
EEEGYDIPFYTNFVVRKQTVDDSLPEDIVMELLCEGMRDGLVEAQNGFPSLDIAETSNAVLEKGDKVRWAPEVEEQISV
ESRIYMTGIYSGLKEAKLRFPDLDIWATINTVAKRKGIEGIFVSASQEKLGFPHLDWSTVKVPPSEDPLMQIFMKMKQQS
NFEGEVMNKLFWKLKEEHEVLRNKFAELDDGNENEYDECVKKREEKLDGVGKYEEISGVLRERGEEIERLYDAGVEGFK
RSEEFEDVMGAIYLNGLRAGLLEAQALLLNLLTPHRN

>XP_014504729.1

MAVRSQSQEFTYDVFLNFRGSDTRYGFAGNLYKALDDRGIHTFIDDEKLQGGDELAPALVKAIQESRIAITVLSHTYASSS
FCLDELVYILDRAEEKKLLVLPVFYNVDPSFVRHQKGSYGEALARHEERLKADNIERLNRHMEKLEKWKMALHQVANLS
GFHYKYGEGYEYKFIGRIVERVSSEINRAPLHVADYPVGLDSQVLKVMNLLDVGSNDGVHLIGIYGMGGVGKTSLSSAV
YNLIACHFDGSCFLQNVREKSRKHGLEHLQSIILSEILGDKRIMFASEQQGISRIQHRLQNKRLLLILDDVDKHEQLQTLVG
RPDCFGPGSKVVITTRDKQLLASHHVQKTYKVKKLNKNHALQLLTWKVFKSEHVYPAYVEVLNRAVTYACGLPLALEVIG
ANLCGKSIQQCISVIDQYKRIPNNRIQETLKVSFDALQEEEKRVFLDIACCFKGYKLTEVEEILHAHHGACMKYHIGVLAEK
SLIKIGQYDRVTLHDLVEDMGKEIVRQESAEEPGKRSRLWLPKDIIQVLEDNTKLRNLRVLNFECCECLTQIPEAVSDLQN
LEELSFQNCVNLVRVHNSVGLLEKLRILEASGCIKLRNFPPLKLTSLEKLELSHCSSLESFPEIIGKMENIRELRLLGTFIKELPL
SFKNLTRLKKLSLLFCGIVQLQSSIVMMPELTFIEAWGWKGWQWIKWEEDEEKDGSMISSKVELLWAPKCNLRDDFFQ
IGFTRFAHVKDLDLSNNNFTHLPECIKECQFLKKLDVSCCNHLREIRGIPPKLKHFNATNCLSLTSSSISMFLNQDLHANRK
TMLVLPGSWKPEWFDHISYGPSCSFWIRNKFPGKVLCHFVAPEDRDIRDYVKPMLCINDKVYLCFFDRLKFLKLGAEHT
FLFDLRNLIFTNNLYEVPLQNEWNPVKVIFFNLKAASMPTQVPIQIGIHVFKRESSNEDIMFTNPYTKKRKLEKILTGFPNT
SNYILDFDLSGYWFSQYMQAFHQDQILNNGKIKLLTG

>XP_014504854.1

MALTDLFTGEIASDLWKMLITISRKALRCKSSAEQLITYVREILPTIEEIKYSGVELPAPRQSQLDRLSEILRSGVELSHQALS
SSRWNVYRNFQLAKKMEKLEKHVTRFLQVPMQAHILADVHHARFEMAERFDRVEASNRRMERFLEEMKIGVNGGG
WVEEAVKSMQEDETWVEGCNGNNGFGVGLDFGKKKVMEMVFSNNDADWIVGICGIGGSGKTTLARELCRDDQVR
CYFKDRILFLTVSQSPNVEQLRARIWGHIMGNQGLNGNYVVPQWMPQFECKGEAQVLVVLDDVWSLSVLEQLVWKI
PGCKFLVVSRFRFPTFFSATYHVELLGEEDALSLFCHHAFGQKSIPLGANVSLVKQVVAECGKLPLALKVIGASLRDQNEM
FWLSVKSNLSQGHSIGESYEINLIDRMAISTNYLPEKIKECFLDLCSFPEDRKIPLEILINMWVEIHDIREAEAYAIAVELSNK
NLLTLVKEARAGGMYSSCFEISVTQHDTLRDLALILSKRGSIHEHRRLVMAQREENGLLPREWSRYQDQPFEAQIVSINT
GEMTEMDWFELDFPKAEVLIINFTSSDYFLPPFISKMPNLRALIIVNYSTSYARLHNVSVLRNLTNLRSLWLEKVSTPQLS
GTVLKSLSKLFVVLCQINNSLDGKQFPNLSELTLDHCNDLTYLPSSICGIKSLRNMSLTDCHNLSELPDEFGNLKSLEILRLY
ACPDLETLPPSMCDMKKLKYIDISQCTNLTCFPKEIGRLVSLEKIDMRECPMIRYLPKSAVSLHSLQLVICDEEVYGTWRD
VAEMAKSNVHIQVPEQHFDLDWLQE

>XP_014504979.1

MAESSSSLAVSTSPTKYDVFLSFRGEDTRYNFISHFYDALHRNHIEAYMDQRLQRGEEISPALQTAIEESKIYVLVFSENYA
SSTWCLNELIKILNCKKIYERDVIPVFYKVDPSTVRKQEERYKAAFDEHEQRFKDDMDKVQRWKDALTEASGLSGWDSN
VIRSENTLVEEIVKDIVKKLDLYSISYDQGIIGIDKHIERIRFLMHFESTDIRIIGICGMGGIGKTTISEQIYHTFAKQFDSRSLV
LDTQQKIERDGIDTVREKYMSEYSERLKRKKILIILDDVTDSVQPKQLLGRCDSFGQGSRIIITSRDKQVLKNVGADDIYEV
EKLNELNSLKLFTLHAFKQNSSQETTYMDLVEEVLRYAKGIPLALQILGSLLYGRTREAWESELQKLKKCQHLKIFSILKLSY
DGLDDEEKNIFLDITCFYRGHHESEVVERLDDCDFSSTIGMDVLKDRCLISISNGRIEMHDLIQEMGKEIVRQECPQYHEK
RSRLFKVNEIHEVLKKNKGLDAIQCILLHLREIKEVIIHGQAFEKMDNLRMLIVNDYEDLHKSKVVLASSLVSLPDTLKILFW
KDFPQRSLPPNFCPENLVRLEMPNCHLEQLWEEDQSLPKLKRLNLSYSQKLTRIQDLSLSPNIEEIILNDCPKLIKVHSSILL
TKLTCLSLDRCHNLNSVTVPCNILSRSPGYINLSWCPKLVMFSIGQTQICYSPPSSSSICSMFPHAKLASQLHHSIKANLAR

RKNLRRSCYSPIFSITFDRSWEADEEKQLTNNNKYAYDEMRLTGGLPLNFLKNLCFINLSNCFSLTIFPFDLSEMKFLKKLYL
CGCLKLQNFPEGTMENLAVGLQELSLRSCWNLESIPSSIGRLTRLCTLDLSYCESLQTFPSTIFKLKLRKLDLCGCLRLRTFPE
ITEQTQTFAHINLTETAIKDLPSSFGNLVNLRSLQLNKCMDLESLPNSIVNLKHLCKLDCSGCAKLTEIPTHIGSLSSLMELSL
SESGIVNLPESIAHLLSLKSLDLSGCKNLECIPQIPPSLNQLVALDSLSIRQVMSNRNLSESKEGVFKFYFTNAQQLDSGAR
ANIEEDARLRMTDDTYKCVVFCFPGSAVPHWFPFRNKGPSVSINEDLSFCSDDRLIGFTLCVVFGLLDTNDIKSRYGSFG
YNLKFECDDDGTQIIPNNDVLNNYFTWGYSPGLLDKDHIFMWKFNLESIRRSGMNLRLSDARSFTFEISPYDYNFQWP
DYDSVVNELKSVVTIKECGMCPLYSSRSNVAESSRETKEDRKRKAESDLVG

>XP_014505252.1

MALNDFFAGEIATELLKMLISISRKSLLCRASADQLITYIHELLPTIEEIKYSGVELPALRQSQLHRLSELLRSGVELSHKVLAS
SRWNVYRNLHLAKKMDKLEKTVSKFLLGPMQAHVMADVHHTRFEMAERFDRVDNSVRRLEQYFGNIKIGVGGGGW
VEEAVRSVDENVVDGSSAVGLEFGKIKVREMIVGREDLWVVGISGIGGSGKTTLAREVCKDDQVRRYFRERILFLTVSQS
PNVEQLRTKIWGYIMGNERLDSNYVVPQWVPQFECKSEAARTLIVLDDLWTFSVMEQLVCRIPGCKYLVVSRTKFQTV
LNYEVELLSEEDALSLFCHHAFGQKSIPLGANENLVKQVVTECGRLPLALKVIGASLRDQPEMFWLSVKNRLSQGQSIGE
SHEINLIERMAISINYLPEKVKECFLDLCSFPEDKKIPLDVLINMWVEIHDIPETEAYAIVVELSNKNLLTLMKEPRAGGMYS
SCFEISVTQHDILRNLAINLSNRDSINERRRLVMPKRENAIPKEWLRYKHKPFEAQIVSIHTGEMKDVDWCNMEFPKTEV
LIINFTSTEYFLPLFINRMPNLRALIIINYSATYACLHNISVFKNLPNLRSLWLEKVSTPELSGTVLENLGKLFIVLCKINNRLVE
KEVDLAKVFPNLFDLTLDHCDDLTQLPTSICGMNSLQNLSLTNCHNLTQLPLELGKLKSLEILRLYACPDLKTLPNSICEM
MRLKYIDISQCVNLTCFPEEIGRLVSLEKIDMRECSMIRNVPKSALSLQSLRLVICDEELSGIWKEVEKAKPNVLIQVSEQH
FDLDWLKE

>XP_014505407.2

MNALTLLENSPFLKTNGARTKRCNDSDNHTDNDNEGEQGKEKDQRELDGEHKVIKQKPNSYIYDVLINFTGEDIHRLFV
SHLNSVLSTVGLTTFLHHHNAVKSTHIQEPILSHCRVAIVVFTQTYSQSAWCLHQLQQIINWHETYFQHVLPIYYEIQPSD
VRLQKGDFGKALEETAQQTFSGQELEHGMSRWSHALTKAANFFGWDESNYRSDAELVEKIVKSVVNLPVLSATKFPVG
LQSHVENLIRTIKNKSTEVCMIWIHGDEGSGXTTLAKAIYNQIHWTFKDKSFIENISRVSGIRGLLRLQEQLLLDVLKQKVE
IPSVDVGRTMIRERLLGKRVLIVLDDLFYSNLFDLRDCTKWLVEGTVIIVTSTYGIGLRDQANSVFRVERMNEEESLELVS
WHAFREAKPKEEYKDLARKVVSYCEGLPLALEVVGSSLFEKTKEEWNNVLFAIDGLRPDPEIIKVSIEGSLNEMEKDIFLDI
CCFFVGKNRAYVRKILNGCGVDADIAIRVLIQHNLIKINKNNKFGMHPLLQEIGLRIIRENYGKDLGNRRLWFDKDTKYG
TEALQWLPVNAFVNVQQTVNSEYLLKKLRWISWHGLSSERLPNNFYEHDAIAIDLKRSLLRFLWKTPQVMTSLKVLNLS
HSKHLTTTPDFTGLPSLEHLILKYCSRLSKVHQSIGSLNSLILLNVKYCTSLYNLPTEIYELKSLRTFILSGCSKIDIMDKDIAKLE
SLITLIAENTAVKEAPFSIVSSKRIGYISPTWI

>XP_014505866.1

MDIQEESSTTVGPLTTPSLRNMSSSSSAFFSANQSPFFSPRSSSCQLSESLQPDAPIDRIHLDEAAPSSSSGIPEPKSLVNV
GCTFSEVAASPAGCSAGDLQKLDRISSSVGISSCTVSGHFHPYDDSYSGQKDKRSKKGRNKRISSTPGSRSVSSYRLKSCD
VYIGLHGRKPPLIRFANWLRVELEIQGISCFVSDRAGYRNSCKLSIAEKAMDVASYGIVIITRKSFKNPYTIEELQFFSGKKN
LVPIYFDLSPADCLVRDIIEKRGELWEKHGGELWLLYGGLEQEWKDVVHGLSRVEERKLEAQDGNWRDCILMAVTLLA
MKLGRRSAAEHLTKWREKVKEEELPFTRNENFIGRKKELSQLEFMLFGDVTGDSRQDYIDLKARPKRRHLTICRSKSNVQ
EERHVGNGSREEKTPVLWKESEKEIEMQSIEFSHRRSRLKRGGKYTRRKKGMRILYGKGIACISGDSGIGKTELILEFAYRF
HQRYKMVLWIGGESRYIRQNYLNLRSFLEVDASVENSLEKTRIKGFEEQEEAAVSGIRKELMRNIPYLVIIDNLESEKDW
WDHKLVMDLLPRFGGETHVIISTCLPRVMNLEPLKLSYLSGVEAMSLMLGSGKEYPVAEVDALRTIEEKLGRLTLGLAIVS
GILSELPITPSRLLDTINRMPLKDMSWCNKKAHSFRQNTFLLQLLDVCFSIFDHADGARSLATRMVLVSGWFAPCAVSV
SLLALAAQKIPEKQKGTCFWRKLLQSLTCGFTSSHTKKSELEACSLLLRFNIARSSTKQGHIHFNEMIKLYARKREVTGSAQ

AMVQAVMNQGSISKSIEHLWAACFLLFAFGHNPAAVELEVSELLYLVKKVVLPLAIHTFITYSRCSAALELLHLCTNALEA
ADQALVTPVDKWFDKSLCWRSIQTNAQLNPCLWQELALCRATVLETRGKLMLRGAQFDIGDDLIRKAVFIRTSICGED
HPDTISARETLSKLTRLIANVQIRASA

>XP_014506380.1

MQSSPSSFPYRFRYDVFLSFRGKDTRQGFTGNLYKSLCDRGIHTFIDDHHLPRGDQISSELENAIQDSRIFIIVLSQNYASS
TFCLNELHYILRFIKHKGRLIFPVFYGVDPSHVRHHTGTFREALAHHQNKLNSYSNWEKLETWKMALHQVANLSGYHFK
HGEGYEYEFIERIVELVCSKINRAALHVADYPVGLESQVLEVKLLLDVGCDDVVHMVGFHGLGGVGKTTLAAAVYNSIA
DHFEALCFLENVRETSSKHGLLHLQSNLLSETVGEIKLTSVKKGISVIQHRLQKKKVLLILDDVDKEEQLQALAGRPHWFG
LGSRIIITTRDKQLLKCHRVKRTYEVKELNEENALELLTWKSFKFEKFDPSYKDVLNLAVTYASGLPLALEVIGSNLFGKNIE
QWKYALDQYKKIP

>XP_014506475.2

MEERRKGVGFGRMKGPFGVPEKPEFCVGLDAPLSKVKMELLNRPKSIIVVTGFGGSGKTTLATLLCWDQQIKSKFRENI
FFVTFSKTPKLKIMVERVFEHLGYEVPKFQNDEEGINQLGLLLRKLKGSPTLMILDDVWPDSEDLVEKFKFHLSDYKLLVT
SRVAFPRFGTPCVLKPLLHQDAMTFFFHYARLDSNTLNIPDQDTIQKVVKSCMGLPLAIKVIGRSLSHRPNELWQKMVL
ELSDGNSILLDCNTELLTYLPKILDVLEDNTVIKECFMDLGLFSEDQRIPVTALIDMWAELYGLDNDGIEAMAIINKLESM
NLANVLITRKNSSDTDNYYYNNHFIVLHDLLRELAIYESTLEPIEERKRLIIHTNENESEGGLDEKQQGFVIRILSNCFKYCVK
HKPQQITARTLSISIDENISSCWSHMHPTQAKVLILNLRVNQYSFPESMKKMRKLKALIITNYNSHPSELTNIELLGSLSNL
KRIRLERIAVLSSFVTLKSLRKLSIYMSQAFQNGILLTSDAFPNLVDLNIDYCKDMVVLPSGLCYITPLKKLSVTNCHKLLALP
QEIGKLVHLELLRLSSCTDLEALPESIGRLQNLRHLDISNCISLSNLPEDVGKLFNLRNLRMINCERCELAYSVVNLVNLKTV
SCDEETATSWEGFKAMLPNLLIEVSQVDVNLNWLPSVSS

>XP_014506476.1

MEETAQEIKRPFGIPEKPEFTVGLDVPLKKLKLNVLSEGSSVIVLTGLGGSGKTTLATVLCWDEQVLGKFKENILFVTFSKA
PQLKIIVERLLEHFGYQVPELQSDGDAIDQLVLLLRKINANPMLLVLDDVWPCSEAVVEKLKFQISDYKILVTSRIAFPRFG
TSLVLKPLVPEDAITLFRHHAFLKRASSNIPEEDLVQKVVRHCKGLPLAIKVIGRSLSQQSIELWQKMVEELSEGHCILDSN
SELLSSLQKILDVLEDNPIIKECFMDLALFPEHQRIPVAALVDMWVELYGLDNYGIKAIAIVNRLDSMNLANVTVTRKNSN
DTDSYYYNNHFIVLHDILRDLAIYQSNKAQIELRKRLMIGINETKPEVCLGEKQHGRMIRTLSNNFRLCDKQKTQKIPVGT
LSISSDETCTSYWSHLRPAQAEVLILNIPTNQYSIPKYLKEMSKLKVLIVMNYGFHPCELFDFELLGTLSLLKRLRLERISIPSF
VTLKNLKKLSLYLCDTREAFENSNILISDAFPNLEDLNIEYSKEMVGLPKGLCNITSLNVLSISNCHKLSALPQEIGNLENLKL
LRLSSCTDLQVIPNSVGRLSNLRHMDISNCINLPNLPEEFGNLCNLRTLYMTSCERCELPSSIINLKNLKEVVCDEETAFSW
EAFKPMLPNLKIDVPQLDINLNWLHAIHS

>XP_014506669.1

MASSAFVSFHDEDIHFITDFVLDPLRSRGFHVFVKGGSTTFDLFQAIERSRFHIVVFSKNYPSSICCLRELMAIINAVESSP
WSTNFLPIYHGVQQSEVLFQTGCYGQVFSKYEERFREHKQRMEEMQRWREALTRVAGFPGLNMENVTRHGGFDFV
QYAARILGREFLTSQNETISSRYGKFDDIVQYKDGDEVSYKELRGLIRLRDFISLVLTYPVKLEEDGVLRSWISAFPEWKNQ
LFSSDGFPVICKPCRLLTEKALGCMKGLMPVEIMAEIETGERSLFNRHNDVIPEGLLPLIVDLAVNQLIQTLFSGDYCVRYI
RLLTRNSSEKESVVNKILTALEDKHDMFGIDKDFLMAVWINALTCETDAEVQVQEEINMIMGSISTTEGDDLLTTVDTD
KKKRINRLLVIVVDADSNKRLDLQKVQFPTGMVVLITTESSTQAVKDDDFGIACTMDLNIWTQDHMLPWKLFYTYVGS
CINCSSMTIQKIAVEIVKKSHGHLLAIVLVAKHLRYVKDDKYWDFVLDKLSNPNPFYDYQDCDRIGFSRVMVNAFVNIIW
EGIDDELKLCLQLSLPVHNIKNGVRDDILVCYWANILRYTQELGEYKRQLQYYLEELLDCFVLLKFESGDIYLPIETYEIIKSLH
ISKPSIIRHGALGLTEIGQWHSLIQIELVNSNICELPESPDCPKLKVLLLQGNADLLDIPDSFFDQMPLLQHLDLSYTSIRDLP

PSLTKLMQLKKLYLKSCDLIMEISPQIFQLKNLEELDLDGTLITHLPRDIGKLINLQRLALCFDAYHHVLSRGKKGKQISNTM
AIPLGVISNLTQLNYLSLDVDPEDEQWSENVNSVLVEILGLEKLKTVSIYVPKADLLELIPAEKSLDFRLVVGHHMRRLISRV
TPEIETKFKDFDYSMKFVNGVNVPNGVKMNLGRFKALYLDRHMSIKSLSDFNVKDVRRLKVDNYCFALQRILPLPT

>XP_014508886.1

MGETNQLVILTRFQDAMRMVMDIVERGRKNKRSKRILRSTLKNMTLVVQEIKQYNEHLNPPREEIITLVKEKDAGEELV
CNSCSRSLWWTKFLSWFSLYGEGLLHEKNDSLTADDKQVKDIKYTLYKLREIIELLDIENFEQKLKGSGTPIKFPYGVPENP
EFTVGFVPLLSKLKMEVLQEGVFTLLLSGLGGSGKTTLATMLCKDKEVKGKFKNNILFVTFSQTPKLKNIVERLFEHCGYH
VPEFVSDEDAIKRLEILMRKIEGSPLLLVLDDVWPGSEALVEKFKFQMSDYKILVISRVAFPKFGTPFILKSLAHEDAMTLF
RHHALLEENSSKCSLSIPDEEIIQKVVRYCKGLPLAIKVIGRSLSHQSIELWQKMVEELSKGHCILDSNTELLTSLQKILDVLE
DNPIIKECFMDLGLFPEDQRIPLPALIDMWAELYKLDDDGIEAMEIIKKLDSMNLANLLVARRNTGDSDNYCYNNHFVV
LHDLLRDLAIYQNNREPIDQRKRIITGINENQSQWWLGEKQQGTMSRLLLKYRGWCVKQTIQQVSARILSLSTDETCAS
YWSDIQPSQAEVLILNLQTKKYTFPEFMEKMIKLKVLIVTNYGFHHSEVDNFQLLGSVSNLKRIRLERISAPHLSALKNLKK
LTLYMCSNISQVFENDTILVSDSFPSLLDLNIDYCKDMVSLPNGICDISSLKKLSITNCHKLCSLPQEIGQLLNLELLNLSTCT
DLEEIPDSIQNLSKLRRLNISNCISLSNLPEDIGNLCNLRNLNMTSCARCELPYSITNLENLKVVVCDEETAASWEAFEALLP
NLKVEVPQVDVNLNWLHSISS

>XP_014508887.1

MGETNQLVILTRFQDAMRMVMDIVERGRKNKRSKRILRSTLKNMTLVVQEIKQYNEHLNPPREEIITLVKEKDAGEELV
CNSCSRSLWWTKFLSWFSLYGEGLLHEKNDSLTADDKQVKDIKYTLYKLREIIELLDIENFEQKLKGSGTPIKFPYGVPENP
EFTVGFVPLLSKLKMEVLQEGVFTLLLSGLGGSGKTTLATMLCKDKEVKGKFKNNILFVTFSQTPKLKNIVERLFEHCGYH
VPEFVSDEDAIKRLEILMRKIEGSPLLLVLDDVWPGSEALVEKFKFQMSDYKILVISRVAFPKFGTPFILKSLAHEDAMTLF
RHHALLEENSSKCSLSIPDEEIIQKVVRYCKGLPLAIKVIGRSLSHQSIELWQKMVEELSKGHCILDSNTELLTSLQKILDVLE
DNPIIKECFMDLGLFPEDQRIPLPALIDMWAELYKLDDDGIEAMEIIKKLDSMNLANLLVARRNTGDSDNYCYNNHFVV
LHDLLRDLAIYQNNREPIDQRKRIITGINENQSQWWLGEKQQGTMSRLLLKYRGWCVKQTIQQVSARILSLSTDETCAS
YWSDIQPSQAEVLILNLQTKKYTFPEFMEKMIKLKVLIVTNYGFHHSEVDNFQLLGSVSNLKRIRLERISAPHLSALKNLKK
LTLYMCSNISQVFENDTILVSDSFPSLLDLNIDYCKDMVSLPNGICDISSLKKLSITNCHKLCSLPQEIGQLLNLELLNLSTCT
DLEEIPDSIQNLSKLRRLNISNCISLSNLPEDIGNLCNLRNLNMTSCARCELPYSITNLENLKVVVCDEETAASWEAFEALLP
NLKVEVPQVDVNLNWLHSISS

>XP_014508888.1

MGETNQLVILTRFQDAMRMVMDIVERGRKNKRSKRILRSTLKNMTLVVQEIKQYNEHLNPPREEIITLVKEKDAGEELV
CNSCSRSLWWTKFLSWFSLYGEGLLHEKNDSLTADDKQVKDIKYTLYKLREIIELLDIENFEQKLKGSGTPIKFPYGVPENP
EFTVGFVPLLSKLKMEVLQEGVFTLLLSGLGGSGKTTLATMLCKDKEVKGKFKNNILFVTFSQTPKLKNIVERLFEHCGYH
VPEFVSDEDAIKRLEILMRKIEGSPLLLVLDDVWPGSEALVEKFKFQMSDYKILVISRVAFPKFGTPFILKSLAHEDAMTLF
RHHALLEENSSKCSLSIPDEEIIQKVVRYCKGLPLAIKVIGRSLSHQSIELWQKMVEELSKGHCILDSNTELLTSLQKILDVLE
DNPIIKECFMDLGLFPEDQRIPLPALIDMWAELYKLDDDGIEAMEIIKKLDSMNLANLLVARRNTGDSDNYCYNNHFVV
LHDLLRDLAIYQNNREPIDQRKRIITGINENQSQWWLGEKQQGTMSRLLLKYRGWCVKQTIQQVSARILSLSTDETCAS
YWSDIQPSQAEVLILNLQTKKYTFPEFMEKMIKLKVLIVTNYGFHHSEVDNFQLLGSVSNLKRIRLERISAPHLSALKNLKK
LTLYMCSNISQVFENDTILVSDSFPSLLDLNIDYCKDMVSLPNGICDISSLKKLSITNCHKLCSLPQEIGQLLNLELLNLSTCT
DLEEIPDSIQNLSKLRRLNISNCISLSNLPEDIGNLCNLRNLNMTSCARCELPYSITNLENLKVVVCDEETAASWEAFEALLP
NLKVEVPQVDVNLNWLHSISS

>XP_014508889.1

MGETNQLVILTRFQDAMRMVMDIVERGRKNKRSKRILRSTLKNMTLVVQEIKQYNEHLNPPREEIITLVKEKDAGEELV
CNSCSRSLWWTKFLSWFSLYGEGLLHEKNDSLTADDKQVKDIKYTLYKLREIIELLDIENFEQKLKGSGTPIKFPYGVPENP
EFTVGFVPLLSKLKMEVLQEGVFTLLLSGLGGSGKTTLATMLCKDKEVKGKFKNNILFVTFSQTPKLKNIVERLFEHCGYH
VPEFVSDEDAIKRLEILMRKIEGSPLLLVLDDVWPGSEALVEKFKFQMSDYKILVISRVAFPKFGTPFILKSLAHEDAMTLF
RHHALLEENSSKCSLSIPDEEIIQKVVRYCKGLPLAIKVIGRSLSHQSIELWQKMVEELSKGHCILDSNTELLTSLQKILDVLE
DNPIIKECFMDLGLFPEDQRIPLPALIDMWAELYKLDDDGIEAMEIIKKLDSMNLANLLVARRNTGDSDNYCYNNHFVV
LHDLLRDLAIYQNNREPIDQRKRIITGINENQSQWWLGEKQQGTMSRLLLKYRGWCVKQTIQQVSARILSLSTDETCAS
YWSDIQPSQAEVLILNLQTKKYTFPEFMEKMIKLKVLIVTNYGFHHSEVDNFQLLGSVSNLKRIRLERISAPHLSALKNLKK
LTLYMCSNISQVFENDTILVSDSFPSLLDLNIDYCKDMVSLPNGICDISSLKKLSITNCHKLCSLPQEIGQLLNLELLNLSTCT
DLEEIPDSIQNLSKLRRLNISNCISLSNLPEDIGNLCNLRNLNMTSCARCELPYSITNLENLKVVVCDEETAASWEAFEALLP
NLKVEVPQVDVNLNWLHSISS

>XP_014508890.1

MGETNQLVILTRFQDAMRMVMDIVERGRKNKRSKRILRSTLKNMTLVVQEIKQYNEHLNPPREEIITLVKEKDAGEELV
CNSCSRSLWWTKFLSWFSLYGEGLLHEKNDSLTADDKQVKDIKYTLYKLREIIELLDIENFEQKLKGSGTPIKFPYGVPENP
EFTVGFVPLLSKLKMEVLQEGVFTLLLSGLGGSGKTTLATMLCKDKEVKGKFKNNILFVTFSQTPKLKNIVERLFEHCGYH
VPEFVSDEDAIKRLEILMRKIEGSPLLLVLDDVWPGSEALVEKFKFQMSDYKILVISRVAFPKFGTPFILKSLAHEDAMTLF
RHHALLEENSSKCSLSIPDEEIIQKVVRYCKGLPLAIKVIGRSLSHQSIELWQKMVEELSKGHCILDSNTELLTSLQKILDVLE
DNPIIKECFMDLGLFPEDQRIPLPALIDMWAELYKLDDDGIEAMEIIKKLDSMNLANLLVARRNTGDSDNYCYNNHFVV
LHDLLRDLAIYQNNREPIDQRKRIITGINENQSQWWLGEKQQGTMSRLLLKYRGWCVKQTIQQVSARILSLSTDETCAS
YWSDIQPSQAEVLILNLQTKKYTFPEFMEKMIKLKVLIVTNYGFHHSEVDNFQLLGSVSNLKRIRLERISAPHLSALKNLKK
LTLYMCSNISQVFENDTILVSDSFPSLLDLNIDYCKDMVSLPNGICDISSLKKLSITNCHKLCSLPQEIGQLLNLELLNLSTCT
DLEEIPDSIQNLSKLRRLNISNCISLSNLPEDIGNLCNLRNLNMTSCARCELPYSITNLENLKVVVCDEETAASWEAFEALLP
NLKVEVPQVDVNLNWLHSISS

>XP_014508891.1

MGETNQLVILTRFQDAMRMVMDIVERGRKNKRSKRILRSTLKNMTLVVQEIKQYNEHLNPPREEIITLVKEKDAGEELV
CNSCSRSLWWTKFLSWFSLYGEGLLHEKNDSLTADDKQVKDIKYTLYKLREIIELLDIENFEQKLKGSGTPIKFPYGVPENP
EFTVGFVPLLSKLKMEVLQEGVFTLLLSGLGGSGKTTLATMLCKDKEVKGKFKNNILFVTFSQTPKLKNIVERLFEHCGYH
VPEFVSDEDAIKRLEILMRKIEGSPLLLVLDDVWPGSEALVEKFKFQMSDYKILVISRVAFPKFGTPFILKSLAHEDAMTLF
RHHALLEENSSKCSLSIPDEEIIQKVVRYCKGLPLAIKVIGRSLSHQSIELWQKMVEELSKGHCILDSNTELLTSLQKILDVLE
DNPIIKECFMDLGLFPEDQRIPLPALIDMWAELYKLDDDGIEAMEIIKKLDSMNLANLLVARRNTGDSDNYCYNNHFVV
LHDLLRDLAIYQNNREPIDQRKRIITGINENQSQWWLGEKQQGTMSRLLLKYRGWCVKQTIQQVSARILSLSTDETCAS
YWSDIQPSQAEVLILNLQTKKYTFPEFMEKMIKLKVLIVTNYGFHHSEVDNFQLLGSVSNLKRIRLERISAPHLSALKNLKK
LTLYMCSNISQVFENDTILVSDSFPSLLDLNIDYCKDMVSLPNGICDISSLKKLSITNCHKLCSLPQEIGQLLNLELLNLSTCT
DLEEIPDSIQNLSKLRRLNISNCISLSNLPEDIGNLCNLRNLNMTSCARCELPYSITNLENLKVVVCDEETAASWEAFEALLP
NLKVEVPQVDVNLNWLHSISS

>XP_014509422.1

MSPERDVISATPGAFRLRWDVFLSFRGTDTRGTITKGLYKSLQGRGVRVFLDDEGLERGEAVANGLMEGIDDSAAFIVII
SENYASSHWCLEELTKICDTGRLLLPVFYRVDPSQVRHVSGPFGSGFESHEKRFEKNTVSKWKEALKKVGGVAGWVFN
HSEEDDLIQRLVRRVLKELSNTPMSVPEFAVGLDERVEKVMEVLQVQSNGVKVLGLYGMGGVGKTTLAKALFNALVNR
FQHRCFISDVRQVSSKHDGLVSLQSKIIKDLFPGEGSPSIGNVNVGISAIKGRVSENRVLLVLDDVDEVKQLDALIGNREW
FYDGSCVIITTRDTKVLTQNHVTVSYEVRELLYGSEARELFSYHALRRSAPPENLQSLSEEIISLTGGMPLALEVFGSFLFGK

RREEEWEDAVKKLRLIRPHHLQDVLKISYDALDEEEKCIFLDIACLFVQMEMKRDGVIDVLRGCGFRGEIAITVLVQKCL
MKITPDNTVWMHDQIRDMGRQIVMDESFVDPGSRSRLWDRAQIMTVLKGHKGTRCVQGIVLDFEEERIYKGKVGSV
FPKKFQWRPSLRNISCYIKQCLNKNHPEPQAEENTEFVLHTKSFEPMVNLRQLQINNLKLQGKFLPSELKWLQWQGCP
LERMPLKSWPGELAVLDLQNSKKMKTLWGWNGYNKVPQKLMVLNLSNCIQLTAIPDLSGCQCLEKIDLENCINLTKIH
ESIGSLSTLRSLNLTRCSSLINLPIDVSGLKQLESLFLSGCSKLKSLPENIGILKSLKALHANDTAIAELPLSIFRLTKLEQLVLER
CQYLRRLPNSLGHLCSLQELSLYQSGLEELPESVGSLNNLETLNLRGCESLTVIPDSVGNLMSLTELLVDRTAIKELPTTVGS
LSYLRELSVGNCKLLTQLPNSIKTLASVVELQLDGTAITNLPDEIGEMKLLRILNLMNCKNLEYLPESIGHLASLTTLNTVNG
NIKELPESTGRLENLVDLRLNKCRMLRKLPASIGDLKSLYHIFMEETSVSSLPESFGMLSSLRTLKMAKRPELYTNEGSFLA
EPEENHSPFVLTSSFCNLTLLTELDARARKISGKIPDEFEKLSLLETLKLDRNDFHSLPSSLKGLSILKVLSLSNCTQLNSLPSLP
SNLIKLNAQNCSLLETIHDMSNLESLQELNLTNCVKVGDIPGLESLKSLRRMYLSGCIACSSQIRKRLSKVALRNLQNLSMP
GSKLPEWFSGQTVSFSKRKNLELKSILVGVIISINHSIDIPNMKRDDMPGLIDVEANVLKGGRTLFKTVLNICGVPRTDDE
HMHLCRFHDYHQLVVFLKDADTFSVSKRNPPFDTGLELRKCGVYLIFEGDDDYDGGEESLDKGLQSVSEKLANFFSTPE
GGDQVSVNGIGHAGT

>XP_014509437.1

MLGGDSKRSSRLRQSSCSCPPRMVKISAQPSSSSSSSSSFSSTAQLKRHDVFISFRGEDTRNSFTSHLYAAFQHKNIQAFI
DNRLQKGDVISPSIFEAIEHSNVSLVVLSKYYASSTWCLRELAKILQLRKRGGHIVIPVFYKIDPSHVRKQTGTYGMAFQKY
KEDVKQNMAMLQKWKAALTEVADIVGWESKNFRTENELIQEIVKDVMQKLNHMYPTEVKTLVGIDQNLAPIESLLRL
RSKEVRIIGIWGMGGLGKTTIARALFDKLSSQFEASCFLANVMEEHEKQGLDYLRNKLLSEILEDVNPHISTSKVRSTFVM
KRLRQKKVLIVLDDVDDPKKLEDLVAQHGCLGSGSRVIVTTRDKHVLSKGVDAIYEVKGLSLDHAVRLFSLKAFGKTYPER
GFEMLSKQAVDHANGNPLALKVLGSVLHSRNEQQWDNAMRKFKKVPNTEIQNVLRWSYDGLDYEQKNMFLDIACFF
RGENKENVIRLLDICGFYAYIGIKILLEKGLITFSDDGDVCMHELIQEMGWEIVHQESIKDPGRRSRLWDLNEVYDVLKN
NRGTEAVEGIILDVSQIRVLLLSCETFSRMINMRFLKFYMGKGRKCNLHLPSGLQSLPNKLMYLQWDGYPSKSLPSTFCP
DNLVVLSMMESHVEKLWDGTKCLPSLKEMNLHASKKLTNLPDLSQAPNLETIDVSNCTSLLHVPLSIQYVKKLLLFNLES
CKSLKSLPRNIHLSSLGMFILRRCSSLDEFSLTSENMTRLDLRETKIADFPESVWQHLNKLVYLNLESCNKLKSLTSKIHLKSL
QRLNLRDCSILEEFSVTSESMEYLNLRGTSIRELPTSVWRNNKLYTLVLHSCKKLVNFPERPKLEDLPLLSSSERPNMDEL
WTLSSLADLSLKGSTVENLPASIKDLPSLKKLTLTECKKLRSLPSLPPSLEDLSLDESNIVCLPVSIKDLSHLRKLALINHKKLLT
PHDLPPSLKAPLLSESKVDPHLVSMKGLSQLQMFPQVKWKMFHSLPELPPFLEEFSLSESNIKFIPESIKILSHMRKLAFTK
CTRLQYLPELPPNLEDLFVSGCDIESLPTSIRDLVHLRKITLIECKKLKALPELPQCLQSLCAADCTSLKIVRSTKNILIEDRYTF
YWNCINLDQKSRNNIIADAPFEAAFTSLKERTPLGPLISICLPGTEIPDWFSHQSTNSSLDMEIPLEWFVDSMFLGFALCL
VIGSFQQNSYEGYDPDINCYHFVKSASYSGPSVPFLGHCTTVMQVPRGFNSDHIFICYYPSFNASILQDFKDLSLYYDAN
DIRLRVIFKFKGPSQRLDIVKKCGVRPLLIANTERLRIESELQPE

>XP_014509495.1

MDQTTQRVVLPTLFQERLRMVSEIVEKGKSSDSNKQILRSTLKDMNPVVQEIKQYNEHLNPPREEIKTLISEKDAKEEPK
KFCLDKCLFWFLCRFGHKRDGSFAGGDKEALVAKDIEEKLYKVREILELLSKENFEQKVGGVGGPIRFPFGVPDNPDFTV
GLDVPLSKLKMEVLRDGVSVIMLTGLGGMGKTTLATKLCWDEQVKGKFGGNILFVTVSKTAKLKIIVERLFQHCGYEVP
DFLSDEDAANQLGLLLRQIGRSSMLLVLDDVWPGSEALVEKFKVQIPDYKILVISRVALLRSDMQCILKPLGHDDAETLFR
HYTHLEESGASIPDEVIQKVVRNCKGLPLAIKVVGRSLCNQRSELWLQMMEELSQGRSILDSNVELITCLQKILDVLEDNL
AIKGCFMDLGLFPEEQRIPVTALIDMWAELYRLDDDGKEAMAIINKLDSMNLANVMVARKNATNTDSYYYNNHFIVLH
DLLRELAIYQCSQEPMEQRKRLIIEINQNKHGEKTKFLSWCVKQKPQQVTAHTLSISTDENFPSDWPQMQLAQVEVLIF
NLQTKQYSFPDCMEGMNKLKVLIVTNYSYYPSEINNFELLGSSSNLRRIRLERISVPSFVAMKNLKKLSLYFCNMKQAFQ
NKDLLISYAFPNLEDLNIDYCKDMVGFPKGLCDIISLKKLSITNCHKLSALPQDIGKLENLELLRLSSCTDLEGIPDSIGRLSNL

RLLDISNCIGLPNLPDDFGNLSNLQNLYMTSCARCELPLSVTNLGNLKVVICDEETAASWENFKPMLPNLRIDVSQVDV
NLNWLHTTFS

>XP_014509923.1

MAESSSSLAMPTSPTKYDVFLSFRGEDTRYNFISHLYDALHRNHIEAYMDQRLQRGEEISPALQTAIEESKIYVLVFSENY
ASSTWCLNELIKILHCKKIYERDVIPVFYKVDPSTVRKQEERYKAAFDEHEQRFKDDMDKVQRWKDALTEASGLSGWDS
NVIRSENTLVEEIVKDILKKLDLYSISYDPGIIGIEKHIESIRLLMRFESSDIRIIGICGMGGIGKTTISEQIYHTFAKQFDSRSLV
LDTQQKIERDGIDTVRKKYMSELLNEVPSPSLYYSERLKQVRILIILDDVTDSVQLKQLLGRCNSFGQGSRIIITSRDIQVLK
NVGADDIYEVEKLNELNSLKLFTLHAFKQNSSQETTYMDLVEEVLRYAKGIPLALQILGSLLYGRTREAWESELQKLKKCQ
HLKIFSVLKLSYDGLDEEEKNIFLDIACFYRGDEESVVQERLDDCGFSSKIGMDILKDRCLISISHGIIEMHDLIQEMGQEIV
RQECPHYPGKRSRLFKGEESLEVLKKNKGSDAIQGILLNLREIKEVIVHGQTFEKMDNLRMFMLFDYLSYGSRVASSIVTL
PDTLKIFYWHGFPQRSLPPNFCPENLVRLEMPYCDLEQLWEEDQSLPKLKRLDLSYSDKLSRIQDLSLSPNIEEIILNDCPK
LIKVHSSILLTKLTSLRLDDCHNLNSAIVPSNILSRSPPGCINLSWCGKLEMFSISQTQICYAPPSSSLIGPIFPHVKPRPQRR
NFLGPPRILFPENLFRYNVTEISSITFDRYWESDEEKEVTNKICFIDLSNCSSLTIFPFDLSQIKFLNKLCLRDCSKLQNFPEIE
NTMENLAVLILDGTAIQALPSSLWRLVGLEQLSLRGCKNLEIIPSSIGSLTRLCKLDLTFCESLQTFPSTIFKLKLRKLDLCGCF
NLRTFPEITEPAQTLAHINLTHTAVKELPSSFDNLAKLRSLQLNRCTDLESLPNSIVNLKLLCKLDCSECAKLKEIPAHIGSMS
SLMELSLSESGIVNLPESIAHLSSLKSLDLSGCKNLECIPQIPPFLKQLVALDCTSIRRVMSNQNLSDSKEGVFKFHFTNAQ
QLDSNARANIEEDARLRMTHDACRSVFFCFPGTAVPHWFPFRGKGSSVTTNEDLSFCSDDRLIGFALCIVFGVLDTNAIK
DRFLYFGYSLKFECDDDGTQIIPNNDELINYFRWGYSYRVLDKDHTFMWKFNLESVRRSGMNLRRLSDARSFTFEISPKA
CNFLLPDYRSIMTIKECGMCPLYSSGSNVA

>XP_014509938.1

MANHGDFTYDVFMSFKGENGTRYSFTDHLYRALLRHGINAFRDDQSLRSGDEIRPSLLQAIEASRISLVVLCQNYASSS
WCLDELAKILHCYENKGKHIVAIFYLVEPSDVRYQKNSYATAMSKHESRYGKDSEKVKAWRSALSRVCDLTGIHCRNHM
YETEVIEKIVKDTLAKLPPMPLQIKHLVGLDSRLERVKSLIDVESNDEVCILGIYGVGGIGKTTFAVDLYNKIRHHFEAASFL
ANVREKTNKSIKGLEDLQRTLLSEMGEETETIIGSTFKGSSEIKCRLGHKRVLLVVDDVDSVKQLEALAGGYDWFGPGSRI
IITTRDKDVLHKHDVEIKGYKMEELNYHESLELFCWYAFNRSRAAENFANISTSAVRYAKGIPLALRVIGSNLKGRGPEEC
ETELQKYRKVPDLEIQGVLEISYTSLSNLDQKIFLDIACFFKGERWDYVKRILDACDFNPDIKVFVSKCLITVDENGCLEMH
DLIQDMGREIVRKESPSNPGYRSRLWCHKDVHEVLKENSGSDTVEGIMLYPPKIEKIDYWTVTAFKKMKNLRILIVRNAI
FSSGPSYLPNSLRLIDWKGYPSKSFPPDFYPHRIVDFKLPHSSLIFRKPFQRFEDLTFINLSHCQLITQIPDLSGAKSLKVFTL
DNCYRLTRFDKSIGFMPNLVYLSASECTLLTSFVPTMYLPSLEVLSFNFCRRLEHFPHVMQKMNKPLKIYMMSTAIKKIPE
SIGNLTGLEHIEMSVCKELKNLPSSFFLLPKLVTLKVDECSQLGESFQRFRESRHSVANGSSNLVTLHFCETNPSYEDLCAIL
EIFPKLEDFNVSHNGFVALPNIIGGSSHLKSLNVSFCRNLKEIPELPLSIQKVDARYCQSLTSEASSMLWSKVSEEIPRIQVV
MPMLKREIPEWFDCIGTKEIPHFWARRKFPVVALAFVFQESRKKLSDLEHAFQSAVESFTGFMNWHTVSLHLFIDGQEI
CGRDYHCFNVGEDHVLFCDLRVLFRDEEWQGLEASLGDDWKAIQVQYESDLILSHWGVNVYEQETNMDDIQFKFPTP
SSTRNLIPSSLLVPKGCPKQKMKHMLESFDPRDMFTKNLSLIESEEGPSRAAKVLLRTWRNAKAEITEEASVSVYGASLK
QEHEECVDDVVQVLEMIKENVLKHFSDSNPEEMQSLGRFVERLLRARVEVMKEDALELGMPILLEYTDVGGSKYRRFW
GVLQLEVGDPFYKAVLRKYIQLSLEFSTSNKAASSSGSWFENLRISIVLLKCLDTAMEEASGFGYEESLEEGYYDPELQELM
MRIEQDGMGFNKSYGKMKASIVRTDESVSPKYLFETLIFRRLIALGKLSMFGSATKFKITPYGNIRVEDDPFRIPKTCFWSL
ILVLYLLFFIIIRLFCLVGYVGLLICRIPVIGKILVCGWWLVMQILMSCKYLYHGMGKIMKIKKKDL

>XP_014509939.1

MANHGDFTYDVFMSFKGENGTRYSFTDHLYRALLRHGINAFRDDQSLRSGDEIRPSLLQAIEASRISLVVLCQNYASSS
WCLDELAKILHCYENKGKHIVAIFYLVEPSDVRYQKNSYATAMSKHESRYGKDSEKVKAWRSALSRVCDLTGIHCRNHM

YETEVIEKIVKDTLAKLPPMPLQIKHLVGLDSRLERVKSLIDVESNDEVCILGIYGVGGIGKTTFAVDLYNKIRHHFEAASFL
ANVREKTNKSIKGLEDLQRTLLSEMGEETETIIGSTFKGSSEIKCRLGHKRVLLVVDDVDSVKQLEALAGGYDWFGPGSRI
IITTRDKDVLHKHDVEIKGYKMEELNYHESLELFCWYAFNRSRAAENFANISTSAVRYAKGIPLALRVIGSNLKGRGPEEC
ETELQKYRKVPDLEIQGVLEISYTSLSNLDQKIFLDIACFFKGERWDYVKRILDACDFNPDIKVFVSKCLITVDENGCLEMH
DLIQDMGREIVRKESPSNPGYRSRLWCHKDVHEGSDTVEGIMLYPPKIEKIDYWTVTAFKKMKNLRILIVRNAIFSSGPSY
LPNSLRLIDWKGYPSKSFPPDFYPHRIVDFKLPHSSLIFRKPFQRFEDLTFINLSHCQLITQIPDLSGAKSLKVFTLDNCYRLT
RFDKSIGFMPNLVYLSASECTLLTSFVPTMYLPSLEVLSFNFCRRLEHFPHVMQKMNKPLKIYMMSTAIKKIPESIGNLTG
LEHIEMSVCKELKNLPSSFFLLPKLVTLKVDECSQLGESFQRFRESRHSVANGSSNLVTLHFCETNPSYEDLCAILEIFPKLE
DFNVSHNGFVALPNIIGGSSHLKSLNVSFCRNLKEIPELPLSIQKVDARYCQSLTSEASSMLWSKVSEEIPRIQVVMPMLK
REIPEWFDCIGTKEIPHFWARRKFPVVALAFVFQESRKKLSDLEHAFQSAVESFTGFMNWHTVSLHLFIDGQEICGRDY
HCFNVGEDHVLFCDLRVLFRDEEWQGLEASLGDDWKAIQVQYESDLILSHWGVNVYEQETNMDDIQFKFPTPSSTRN
LIPSSLLVPKGCPKQKMKHMLESFDPRDMFTKNLSLIESEEGPSRAAKVLLRTWRNAKAEITEEASVSVYGASLKQEHEE
CVDDVVQVLEMIKENVLKHFSDSNPEEMQSLGRFVERLLRARVEVMKEDALELGMPILLEYTDVGGSKYRRFWGVLQL
EVGDPFYKAVLRKYIQLSLEFSTSNKAASSSGSWFENLRISIVLLKCLDTAMEEASGFGYEESLEEGYYDPELQELMMRIE
QDGMGFNKSYGKMKASIVRTDESVSPKYLFETLIFRRLIALGKLSMFGSATKFKITPYGNIRVEDDPFRIPKTCFWSLILVL
YLLFFIIIRLFCLVGYVGLLICRIPVIGKILVCGWWLVMQILMSCKYLYHGMGKIMKIKKKDL

>XP_014510098.1

MTQLPSSSSSSSSTYEGTHDVFLSFRGDDTRSGFTGNLYKSLCDRGIHTFIDDEGLRKGEEIRPALFKAIEQSRIAIVVFSEN
YADSTYCLEELIVILECILRKGRLVWPVFYGVTPSYVRFQKGSYGKALAKHGERFKNDQEKLQKWKLALQVAAGLSGSHF
KLKQGYEHELIRTIVEEVSKKINRSPLHVANYPIGLESRVQDVKLLLDVGSNRGVSMVGIFGIGGIGKTAIACAVYNAIADQ
FDVQCFLGDIRQKSMKYDLVQLQETVLSEMVGEKSIKLGSINRGMAVMKSKLQRKKVLLILDDVDKLEQLKALAGDPS
WFGDGSKIIVTTRNRRFLRVHGVERTYEAKGLDDKEALELFSWHAFKRNEVGPGYLDISKRAVFRCNGLPLALEIIGSNLN
GITMSEWEAALDTIERIPDEDIQEKLKVSYDGLKGNEKEVFLDMACFFRGYHLKDVINLLLQSRGFSPEYVIRMLVDKSLI
KIDQYGFVQMHNLVEDMGREIVRQESPSEPGKRSRLWLYEDIVDVLENDKGTDTIEVIMLHLPKNREVLWNGSELKKM
TNLKMLTIENADFSRGPEYLPSSLRVLKWRGYPTQSLPPEYDPRRLVMLDLSMSRNILGKQLNLMKFESLSEMVLRGCR
FIKQAPDMSGAKNLRKLCLDNCKNLVQVHNSIGLLDKLTWFTAIGCTSLRTLPHSFKLTSLEYLSLRKCSSLQRLPNISEEM
KHMKNLDLCGTAIEQLPYSFRKLTGLKYLVLDKCKRLNQIPINILMLPKLERLTAVKCGRYVNLILGKSEEQVRLASSDSLID
FRLNYNGLTPTSFPNVEFLVLTGCSFKVLPECISQCRFLKNLVLDNCKELQEIRVVPPKIKYLSAINCTLLSHESQNMLLNQR
LHEGGGTDFSLPGTRLPEWFDHCTRGPSLSFWFRNKFPRMTLAVVGVLDKQGSFPMSRFHLLINGIQKLHCHFTVQSK
LITYHIFLSDVLLKSYNGGLESVYGEDGWNHVEVSYVGPRVFPHSCRTKKGTIKWMGVHVHKQKTNMQDIRFINPWFP
KRAHSEVSKADLQESFQSLPKRIRVSHRKEICEAPQTKQHEANSSYHGVSQRLWLAICSIAAPLNVKVLMWNICQDDLP
TFEYLFRRKLVLSPLCPICGTEPETVEHVFLFCPWTRPLWFGSDFQWCIDVKEVQSFQLWLWHKLMEIQRVYPENANRI
SAQVGSICWSIWKGRNEFVLEGKPVNPLILR

>XP_014510240.1

MSGLELVGPLVGEMGSIGVGEMHTTIASKIAFSKNLDDNYNILVKDTEMLHAIKKDKEMKAQRNSHKDTTNAYKLWT
NRVSEATEEAQKLKLKYEEKTLPWWRIQKRSHLSEEMEKKSNCVRQLMNDECLKEFLVDKPPEPVLKELNVPQISGYPTL
QGALDNTLVLLKNNKIKIIGMCGTKGVGKTTIMRNLNNNEEIAKLFEIVIFVKVTSNDRKLQEKIAHRLMLDKGVNNEDS
DDVARRIHRELEKKRYLLILDEVEDAIDLELLGIPSDNNNGSKVVIVTRFPLVYKLNRVQRVIKVAELSPDEAWKMFRDTV
HAFNPKIDSPDIQPTAKLVCKRCSRLPLLIYNIANSFKLKESASSWWAGLEDLKPWPELQSQGLEELYSCLKFCYDELNDK
RKQKCFLYTSLYPADSKVYSDYLVECWAAQGLLGDINDTRSYQSARNCGIDILEHLANVSLLEKGEAMIYVNMNHCMR
QLALHISSKDPECSFYLQDGEESENLSNSRAWQQARWVSMRQVHELRRSQDCSTILTLLLRKNPELTALPECFFENMSS
LLLLDLYSSMITQLPSSLSKLTVLRGLFLNRCELLESLSSEIGLLQFLEVLDIRDTKVIFIPLQIGFLTKLRCLRIPFIASEDNEAQ

NVHVISKLHRLEELTIQVISYEQWCNDAKNVLAHVASLENVNHLRCCFPSSIILGEFLSRSKSWHRKQNSFRFTVGCQNS
RRPQILESFEYKITNYLRYCNGGQKDDPAIIEVLPKTDAFELVCHKDIKKLSNFAGIACLERIRGLLIERCNQVLTIVSGETSR
NVMNGIQIETAVILPNLEQLYLENLLNLKCAFRGPLHSGTFSRLQTLSLKNCPRLSQIFSNGAIQHFSELQKLKLEDCSKIEE
LVGEDIERERDVLPKLEILLLVNLPNFKSICATHTLAWSSLELLRIHNCHKFKTLPLDSVNAVNLKSIKGQQEWWANLDWT
NNEEVLQRLQPIFVASNEYFS

>XP_014510241.1

MLHAIKKDKEMKAQRNSHKDTTNAYKLWTNRVSEATEEAQKLKLKYEEKTLPWWRIQKRSHLSEEMEKKSNCVRQL
MNDECLKEFLVDKPPEPVLKELNVPQISGYPTLQGALDNTLVLLKNNKIKIIGMCGTKGVGKTTIMRNLNNNEEIAKLFEI
VIFVKVTSNDRKLQEKIAHRLMLDKGVNNEDSDDVARRIHRELEKKRYLLILDEVEDAIDLELLGIPSDNNNGSKVVIVTRF
PLVYKLNRVQRVIKVAELSPDEAWKMFRDTVHAFNPKIDSPDIQPTAKLVCKRCSRLPLLIYNIANSFKLKESASSWWAG
LEDLKPWPELQSQGLEELYSCLKFCYDELNDKRKQKCFLYTSLYPADSKVYSDYLVECWAAQGLLGDINDTRSYQSARNC
GIDILEHLANVSLLEKGEAMIYVNMNHCMRQLALHISSKDPECSFYLQDGEESENLSNSRAWQQARWVSMRQVHELR
RSQDCSTILTLLLRKNPELTALPECFFENMSSLLLLDLYSSMITQLPSSLSKLTVLRGLFLNRCELLESLSSEIGLLQFLEVLDIR
DTKVIFIPLQIGFLTKLRCLRIPFIASEDNEAQNVHVISKLHRLEELTIQVISYEQWCNDAKNVLAHVASLENVNHLRCCFPS
SIILGEFLSRSKSWHRKQNSFRFTVGCQNSRRPQILESFEYKITNYLRYCNGGQKDDPAIIEVLPKTDAFELVCHKDIKKLSN
FAGIACLERIRGLLIERCNQVLTIVSGETSRNVMNGIQIETAVILPNLEQLYLENLLNLKCAFRGPLHSGTFSRLQTLSLKNC
PRLSQIFSNGAIQHFSELQKLKLEDCSKIEELVGEDIERERDVLPKLEILLLVNLPNFKSICATHTLAWSSLELLRIHNCHKFKT
LPLDSVNAVNLKSIKGQQEWWANLDWTNNEEVLQRLQPIFVASNEYFS

>XP_014510449.1

MASAVFVSFNDEDTRDTTVFVLDPLRSRGIQVFVKGESTTFDLFQVIERSRLFIVVLSKNYASSICCLRELVAIINAVESSPR
FLLPIFYGVHQSNVLSQNGCYVQAFSKHEERFREHKERMEEVQRWKEALTRVAAFPGWHMENAKGVEGRRFVQHAV
DILSREFSTPQNETIFNRYGKFEDIVQYKDGDEVSYKELRGLIRLRDFISLVLTYPVKLEEDGVLRSWISAFPEWKAQLFSTY
GFPGICKPWSLLTEKALGCMKGFMPVEMMAEIESGQRSLFNRNNDVIPEGLLPLTVDLAVDQLIQTLFSGDYRARYIRLL
TRNSSEKESVVNKIHTALKDKHNMFGIDKDFLTDVWINALTCETDAEVYIQEEINKVMVSISMTEGDDMLVDTDKKKRS
NRLLVIVVDADSNRKLDLQKVQFPSGIVVLIATESSTQAVKDDDFGIACTMDLNIWTQDHMLPWKLFYTYVRSCISCSTL
GSSTTIQEIAVEIVKKSRGHLLAIVLVAKHLRYVKDDKYWEFVLDKLSNPSPFYDYQDCDRIGISRVMVNAFVNIIWEDID
DELKLCLQLSLPVDNIKNGVRDDILVSYWANTLRYTQELGEYKRQLPYYLEELLDCFVLLKFESRVVYLPIETYDIIKSLHISK
PSIIWHGALGLTDIGQWHSLIQIELVNKNICELPESPDCPKLEVLLLQGNADLLDIPDLFFDHMPLLQHLDLSYTSIGDLPP
SLTKLMQLKKLYLKGCDLIMEISPQIFQLKNLEELDLDGTLITHLSKDIRELINLQRLVLCFEAYHHVRSRGKKGNQISNTMI
IPPGVISNLTQLNYLSLDVDPEDEQWSENVNSVLLEILGLEKLKTVSIYVPKADLLELIPAEKSLNFRLVVGRHMRRLISRVT
PELETKFKDFDYSMKFVNGVNVPNGVKMNLGRFEALYLDRHMTIKSLSDFNLNNVRRLKVCILAECNEMETIVDGDNS
PQLFCLELLSVFYMKNLRSICQRCDPFSYLKYMALHTCPMLTTIFTLHTFFILPFLEEISVEDCPKVTTLISHNSPKLELTFFLP
KLRVISLLYLPNLVNIFNGLRVEHVLEEMICYCCPKLQSLSRSELSSSYLKFIKGERMWWEALEWRVSEWGFGGRPKFFD
QFFKPIKVEADMMNPPAAHQDTQVNEYHGTMYQGVSSSTELMSKLHLETPLLLPSTPFPLSDSKEGEAQKRKAVIEPIV
VPPLLRRKPNQGGYKRRLLPQAVKEIRTDQTLDDGFSWRKYGQKDVLGAKYPREYYRCPHKSTQGCPALKGVQRLDGD
PTTFEVAYRGTHSCTQKQDLIAESGDAEVLEDFGGKANSASSSNKSSITVLEEISAEPEELQMSLP

>XP_014510914.2

MISRSIQTKPRYPCISFPQISLKNIMAESLLFTFAESLIGKLASGAVHEASLALGVHADLQQMKMSMSLIKAFLLDAEQKK
PRSNSLSEWLIQIKQVFSHAEDLVDDFECEALRKHVVNTRGGFTRQVRRFFSTSNPIIYRIRMAHEIKDIKERLQKVAADG
NTFGLQIIDQDTRVVHVTDTTYSHVNPSNVIGRHHEKQEILKLLLQHDHDGYDKSLSVVSILGFGGLGKTTLAKLVFNDTT
IDECFPLKMWVCVSGDFELRNVLIKILNSALNPTNENFKNIETEQLQNRLRNTLQRQKFLLVLDDVWNEYQARWDDLK

EILDVGVEGCKILVTTRSHLTATMMCTKSSNSYHLERLSEDDSFFLFVKTAFKEGEEERYPQLLEIGKEIVIKCGGIPLAVKTL
ASSLFSVVGKSKWEAMRDDKIWNLPQKEKDILPALEISYNQLPSHLKPCFVCFSLFCEGSEFFSFYVAKLWEALGFLPPPK
ENETMHDIAIQFLRELWSRSFLTDFIELGHGYSFKLHDLVHDLAMYTAKGEFQTIYPRSSKISPNARHLAFSDNNLLDQA
VIPTGLRTIIFPDEATNEAFLNTLVSRCKYLRFLELSNSIYESLPPSIGKLKHLRYLSLFGSKNLKGLPRSVCNLQNLETLNLNQ
CTELQELPKGISKLISLRQLHITTRQLHFPDQEISTLTSLETLTFNSCDNLESLLKGIQLSSLKNLSLHDCGKLKSLSSHVITNLE
NLVIDNCCELELSMGFGNQIPDLRLKSLAFKSLQQLVTLPQWLQGSMNKLHSLVIADCNNLKELPEWLSSTICLKLLVIEY
CSSLQSLPDNLKNLENLLINSCPELCKRYQPGVGQDYHKISHIQKVFVGELEE

>XP_014511272.1

MAMELVAGPLMGAIFNVLLDRIASTEVVNFFKNKNCEKLLKRLKIILLSVNVVLNDAEEKQMKNGAVKEWLEELKDVAF
AAEDLLDEIYTDAKIKAKQVNMLHSGHMSFYCKGVEEKIEDVHERLEFIMRQKEVLDLKVGKEIKMAHKTPTSSVMEAS
DVFGRDNDKEFLVDLVLTHDEKIGVIPIVGMGGIGKTTLAQLIYNDQRVQKEFDLKAWIYVSEEFDICKITKTLLEAVTSCS
CDVEDLNFLQRDLKMHVMKKKFLFVLDDVWNENYDNWDKFRSPFKHAGEHGSKIIVTTRSWCVASIMQTVSPYNLRE
LSNEDSWNLFSKHAFDYGDSSLQLHQSLDKVGREIVRKCKGLPLAVKTLAGLLRCKSDRQEWCKVLDSEMWDLQYSES
NILPALRLSYHYLPSHLKRCFAYCAIFPKDYEFEKENLVLLWMAEGFLQQSKRHRRIEEVGNEYFSELVSRSFFQQSRRGKS
CFLMHHLVNDLAQFVSGTFSIRMECSNSNEIKERTRHLSHIIADSSSYVNLKDVSKANCLRTFLQIRPVGTSIDLYNNMPN
DLLTKLRCLRVLSLVGTHIYSLPNSVGELKHLRYLEVADTEIVRLPESICSLFNLQTLKLVGCHNLIELPASIHKLVNLRHLDIR
GTSLRWMPLQINELNSLQNLSDFFVGKGCGSSLGELGELSCLHGGLFIHCLEHIVSIKDCEKAKLKEKHGLEKLSLDWCG
NGETENSQKEKTILNSLQPHTNLKKLDIYDYPGTEFPEWLGDQSFYNLVSLMLNGCKYCYKLPPLGQLPMLKELQISKFE
GLVSVGSEFLGNRTSYVSDYFPALEILRIESMPLWEKWYPNAENAGSKALFHLREFHIGNCPKLSGDLPDNLPSLALLVIR
DCKRLLCSLPNSPSLRVLNIQNCESLEFQVHSPRCHQSLTSLFLHGSCDSLVFLPLDLFPNIKSLDIWGCKNLEALTVSESDT
NRPNLKSLHSLRIRHCPNFTSFPKGGFAASKLTLLTINYCQKLNSLPEQMHDLMPSLKEVQLRGCPKIESSSMRPLRIRICS
KHMEGKQNLSDPLFARLKGLATDQSPSSS

>XP_014511283.1

MAESLIVAVAESLITKLASRVVEQASLALGVHQELQQMKKTMALVKAFLLDAEQKKQQNNALSEWLRQIRQVFAHAE
DIVDNFECEVLRKHVVSSHGSFCRKVCRLFSTSNPVVYRYRMGREIKGIKKQLEKVATDGHMFGLQSSDKDTKVLHARE
MTHSHVNVSNVVGREHDKQKIIELLLQDDHHDRSLSVISIVGFGGLGKTTLAKVVFNDTAIEECFTLKMWVCVSNDFEL
RNVLMKILNSAPNPTNEKFKNLDTDQLQIRLRSCLQREKFLLVLDDVWNEDRVKWDELKEIIEMVGNKGSKILVTTRSHS
INAMMRTKSSNSYILKGLSEEDSMSLFVKSAFDDGEGKNHPELMEIGRQIVRKCGGIPLAVRTLGSSLFSRVDRKEWENI
RDNEIWNLKQNENDILPALELSYDQLPSHLKRCFACFSLTSKDFDVSSSYVALLWEALGFLSAPKQNETTHDVANEYLRE
LWSRSFLTDFLDMGSTCRFKLHDLVRDLAVYVAKGEFQILYPHSTSISEHAQHLSFIENDMLGQDLVPIGARTIIFPMEAT
NDAFLNTLVSRCKYLRVLDLSYSEYESLPSCIGKLKHLRYLNLSGNKKLKGLPNSVCKLQNLQTLDLRGCIKLQKLPKGIRKLI
SLRRLLVTTRQPDFPDKEISKLASMETVELYSCDNLESLFQAIQPRSLKFLHLSGCGGLKSLSFHVITNLESLVIFKCSKMELS
MGLSNLNSIPDSRLKLLVLQSLPQLVTLPQWLQGSVNTLHSLLLVDCNNLEELPEWLSTLNCLKLLIIEHCPKLISLPDTTHR
LRNLEHLEINDCPELCKRCQPGVGLDWHKISHIKQVIIGEPEE

>XP_014511381.1

MAESLLFSFAESLIGKLATAAVQEASLAFGVHSQLQQMKATMDLIKGVLLDAELKNLQSSALSRWLRQVKRVFSDAEDI
VDDFECEALRKYVVVNTYGSCSRKVRRFFSSSNPVVYRLRMTHHIQDINTRLAKLAVDRNMFGLQIIDHDTRVVHVREMT
HSHVNPSNVKGREHDKNEIVKLLVQDGDYQSLSVISIAGMGGLGKTTLAKLVFNDKNIDECFQLKMWVCVSNDFELRN
VLIKILNSAPNLTQENFNNFETEQLQIRLRSTLEGKKFLLVLDDVWNEDPTRWHELKEIIDVDVEGSKVLVTTRSHAATAI
MRTKPSNLYLLQCLSEEDSLSLFVKYAFDDGEEKKHPELLEIGKEIVEKCGGLPLVVKTVGSSLFSRFDKKEWESIRDMRFG
I

>XP_014511382.1

MVDVANHLVYELWSRSFLSDYLDLGGDSTFTLHDLVYDLAVHIAKGEFERIDLRNKKNSENAQHLVFEENNLLAQAFPH
TGLRSVYLPLGMNNEAFLIRLVSRCKYLRILELCSSEFESLPYFIGKLKHLRHLNLQFNKKLRRLPDSVCELQNLQTLNIFGC
MELQKLPKGMRNLVSLRNLFITTKQVDFPEKDIAVLTSLEVLNISDSHNLESLFNGIQLSTLKKLVLNRCESLNLVAFHAIR
NINVLSICKCDKMELSMGIGSEIPDSRLKLVIFQDLPQLVTLPQWIQGSANFLQSLFIIGCINLKELPGWLPTLVYLKLLSIC
NCPNLLSLPDNIHQLTNLENIEMSGCPELWKRFKPGVGQDWYKISHINIVYNVQEEEDEEEDEEKDDEE

>XP_014511384.2

MIHQPTILVTNVPIAFFSPMAESLLFSYGESILGRLANAAIEEASLALGVHSELQQMKETMTLIRSVLLDAQQKTPHSSALI
EWLRQVNCVFCDAEDIIDDFERESLQKNVVNTSGSFSSKVRRFFSSSNPVVYRLRMTHRIQDINTRLAKFAADRNMFAL
QIIDHDTCVVHVREMTHSYVNPSNVIGREHDKNEIVNLLVEDGDRESLSVIPIVGIEGLGKTTLAKLIFNDSNINAFFLLKV
WVCVSDDFELKNVLVKILNSIPNPTRENFNDFEIEQLQNHLRNTLKGKKFLIVLDDVWNEEYEKWDELKEIIDVGVQGSK
ILVTTRSHAVAAMMCTKSSNSYLLECLSEENSLSLFVKYAFEDGEEKNHPELLEIGKEIVEKCARLPLALKTVGSSLFSRVDK
KEWESIRDNEIWCSK

>XP_014511386.1

MAESLLFSFAESLLRKLATVAIQEAALALGVRSELHQMKETMSLIRGVLLYAQQKTPQSMALSEWLRRVKRVFSDAEDIV
DDFECEALRKHVVNTYGSLSMKVRRFFSSSNSVVYRLRMAHRIQDINTRLAKLAADRNMFGLQIIDHDTRVVHVRDMT
HSYVKPSNVTGREHDKNEILNLLMQDGDRPSLSVISIVGMGGLGKTTLAKLVFNDTNIDACFPLKMWVCVSNDFELRN
VLVKILNSIPNPTRENFNNLEIEHLQNHLRNTLKGKKFLLVLDDVWNEDPARWHELKEIIDSDVEGSKVVVTTRSHAVAT
MMQTKSSNPYLLECLSEEDSLSLFVKYAFVDGEEKNHPELLEIGKEIVEKCGRLPLALKTVGSSLFSKVVKEDWESIRDNEI
WNLEQNEKGILPPLKLSYDQLPSYLKPCFASLFLYEYDINVLCYDLSMLLDSLGFLPPPKEGESMSDVANQVLRELRTRSFL
SDDLDFGVDSCFEMHDLVSDLAVYIGKGEFERVNRRNPKISENAQHLVFEENDFCGEDLVPTGVRSVVFRNGGNDINF
LNTLVSRCKYLRLLDLRYSEYENLPQCIGKLKHLRFLCLAKNEKLKELPDSVCKLQNLQTLILSGCTNLQKLPKGMKNLVSLR
HLAITTAQTDFPEELIANLTSLENLSFTECDNLESLFEGVQLSTVKSLILSECGSLKSVSLHAFRNLEVLMIADCIKLELSMSLS
YQIPDSKLKYLFLDDLPQLVKLPQSFQGSADSLQHLAISDCVNLDELPEWLPTLSCLKVLDICSCPNLLSLPDNIHHLTNLE
NLEIVDCPELCRRFKPKVGQDWHKISHIKQVHIEDSDNEEELSE

>XP_014511391.2

MIQLQKPLPIAFLFLPLLKLMAESLLFSSAESLLGKLASAALQEASLAFGVNRDLELMKETMTFIRGVLLDAEKKNSQSSAL
KEWLIQVKCVFSDAEDIVDDFECEALRKHTVNTYGSCSRKVCRFFSSSNPVVYRLRMAHHIQDINIRLTKLASQRSMFSL
QIIDQDTRVVHVREMTHSHVNPSNVIGREHDKQEIIKLLVQGDHGQSLSIIPIVGMGGLGKTTLAKLVFNDPIIHACFPLR
MWVCVTNEFELRNVLIKILNSVPNPTRENFNNFEIEQLQILLRNTLEGYKSLLVLDDVWNEDPARWDELREIIDIGVEGSK
ILVTTRSQKVAAIMHTKSSKSYLLGCLSEKDSLSLFMKYAFEDGDEMKHPQLLKIGKEIVKKCGGLPLAVKTVGSSLFLMV
DEREWESVRDNEIWNLKQNVKGILPALKLSYDQLPSYLKPCFASLSLYQKDIYFYSSQVCMLWGGLGFLPPPKASESMA
NVATQLLHELWSRSFLSEYEDFGGDCRFKLHDLVFDLAIYIAKGEFDIIHPNPNLYKNAHHLLFMNDNLLDQALLPSSLRI
FIFTGGPSNENFLNTLVSRCKFLRILILDWSEYVSLPRFIGKLKHLRFLSLWNNENLMEVPDSVCKLQNLQTLNLEGCIKLQ
KLPKGLANLVSLRYLGITTIEPTFPEKEVASLTSLEDLRFCRCDNLESLFKGIQLHTLKGLSLQDCKSLKMVSFHAIKNLEVLV
IAQCNKLELSMGLSNEILDSRLKLLILRGLPSLATLPRWLQGSANSLQSLVIEECMNLEELPDWLPTLNCLQQLVVRYCPN
LLSLPDNIHHVTNLKVINVTGSSELWKRYRPRVGQDWHKISHVNLVCYDESENEKEFS

>XP_014511410.1

MAETLLFSFAESLIGKLATAAVQEASLALGVHSELQQMKATMALIKGVLLDAEKKNLRSSALSEWLTQIKRVFSDAEDIV
DDFECEALRKHVVNTYGSCSRKVRRFFSKSNPAVYRLRMAHHIQDINTRLAKLATDRNMFGLQIIDHDTRVVHVREMT
HSHVNPSNVTGRKHDKNEIVKLLVQDSERQSLSVISIAGMGGLGKTTLAKLVFNDTNIDECFPLKMWVCVSNDFELRNV
LIKILNSVPNPTQENSNNFGMEQLQNLLRNRLEGKKFLLVLDDVWNEDPARWHELKEIIDVGVEGSKVLVTTRSHGVAV
IMRTKPSHLYLLGCLSEEDSLSLFVKYAFDDGEEKKHPELLKIGKEIVKKCGGLPLAVKTVGSSLFSRFDKKEWESIRDNEIW
NLTQIENDILPALKLSYDQLPSYLKPCFASFSLYPEDTVIRCSEISTMWEALGFLPPPKESESMVDVANHLLYELWSRSFLL
DYFDFGGDSAFMLHDLVYDLAVHIAKGEFERIDLRHKKFSENVKHLAFMENNLLAQVFPHTGLRSVYLPRRMNNEAFLI
TLLSRCKYLRILELRSSELESLPYFIGKLKHLRHLNLQFSKKLKRLPDSVCELQNLQTLNLIGCVELQKLPKGMRNLISLRNLRI
TTKQVDFPDKDIAVLTSLEELKIKDSHNLESLFNGIQLSTLKKLALYGCKSLKSVSFHAIRNINVLAICKCDKLELSMGVGSQI
PNSRLKLVSLEDLPQLVTLPQWIQGSGNYLRSLFIVGCINLKELPDWLPTLVYLKLLSITNCPNLLSLPDNMHQLTNLESLEI
TSCPELWKRFKPGVGQDWYKISHIKDVYNLQEKKMKRKTKKKNKTIQS

>XP_014511477.1

MSIRTNKMKAVAVLMKQLTTSRRKFHERGRDESFDGELEKLRLVLNNIKDVFVEVKKNEENLLDTLAEVYDYLHRLDHK
KLHQGMKDICNRIKCSARNLLPKLDFDESKKEEIFHSSKELLQPRETSWSLRDFASSEGYLYSLLVFPKNAVIRKRNAINLW
IGEGLLKNTGNKTTEELGNDVIRTLLKFNLIVSYGNGKCPLVNKFQIVPSIRNQMEGGFSIINVDHPGNKLAAGFSERNVD
HRGQYFIPNLDFEGTTERLTLERKKVTLGGGDWYFSDRPLCTVFNISASYLNFRPQWVTELKNLEVLQLGRWQDSPLHH
IEVGSEELLKELRYLQKLKYLSLRGISRIFELPSSIVELESLLILDLKACHNLERLPNDISLMTNLTHLIMSDCCLLESMPKGIEK
LTNLQVLKGFLITTSEKTPCKISDLVNNLRKLKRLSIRIGSEAVIKDGEFQSLASFSALEHLKISWSVFDPRYANISIHLPTDLR
KLHLECFPGKSLPPSLERSMTRPREINITGGKLESIDPNIILLYKVEILRLKYLEHLKVDIDNLKAFIPKLKYVEIRKIQNHSYIER
AYEYDSD

>XP_014511784.1

MALAVVGGALLSAFLDLLFDRLASPELVGLIRGKKPDKLLQKPPHLKRCFVYCSLYPKDYEFHKNEVILLWMAEDLLKAQ
RKEKTLEEVGEEYFDDLVSRSFFQCSSRQPWSNFFIMHDLMHDLATFLGGEFYFRANELGKETKFDRKTRHLSFARFSDP
VSDINAFETAKFPRTFLQINNEDCPFNNEKAPRIIVSMLKYLRVLKFGLYQSELVLPDSIGELIHLRYLNLNGTSIEMLPESLC
NLYNLQTLKLGSCSNLTKLPRNFQNLVNLRHLQIVGTYIKEMPKRMGKLNQLHNLDCYVVGKHKENSIKELGGLPNLHG
SFSIKKLENVTKGEEAIEARIMDKKHIRHLSLIWSEGNDDIIDFQIELDVLAKLQPHQDLKLLGISGYKGTRFPEWVGNFSY
QNITSLYLNSCNNCCVLPSLGQLPSLSDLDISYMNSVKTIDAGFYKKDDCSSVTPFPSLKYLRIYDMPCWEMWNAFDSEA
FPVLKDLDIRNCPNLRGDLPKHLPALQTLYIGNCELLVSSVPMAPTLQELNILNSNKVTFHEFPLLVKDLRIEGRPVVELM
MEAISKIQPTCLQSLSLEDCSTAISFPGDRLPASLKSLHISGLKKLKFPVQHKHELLKSLRIINSCDSLKSLPLINFPNLINLTIE
DCEYMESLLVLGSESLKSLNYFAIRRCPNFASFPGEGLCMPNLTHLSVYDCDKLKSLPDQMETVFPRMEYLEISNCQQIES
FPGGGMPPNLRTVSIKNCVKLLSNQAWECMDMVTSLEVWGPCDGIKSFPEESLLPPSLVSLHLSDFSSLETLDCKSLLHL
TSLQQLNIQRCPKLENIAGEKLPLSIIKLMIYKCPLLELRCHKKDRQIWPKISHVRGIKIDHGWI

>XP_014512172.1

MAVTSPSQATSYDVFLNFRGSDTRQGFTGYLYKALFDSGIHIFIDDEGIQSGQIIPESLKEAIKKSRIAITVLSPDYASSSFCL
DELVTILECSKNSGLLVLPVFYKVPPGHVRHQRGSYGEAFAIHEQGLHPNMEKWKKALKDVANISGFPLDYGNEYEHELI
KKIVERVIRFLNNDEKSLHVPDFLVGVGSQVEEIRNLLAVRDNGVNVNMIGIHGMGGVGKSTLACLVYNLITAGFEGSC
FLQNVREEANKHGLKHLHSIILSQLVGMKEINVASEQQGISIIKNRLKRMKVLLILDDVEKHEQLQGIVGSADWFGPGSIV
IITTRDKQLLASHGVKRTHEVKELNKRDALKLLKFKAFKMEEVDPSYKEVLNQVVTYASGIPLALEVIGSNLFGKSVQEWE
SAIRQYKRIPNNKILEILKVSFDSLGEEEKSVFLDIACCFKGYKLSEIEETLRALYDNCMKYHIGVLVEKSLIKISHDERVTFHD
LIEDMGKRIDRQQSPREPGKRRRLWLQEDIIQVLKDKSGTSQIKIICLHFSISDVQVVEWDGITDMKSLKILIVRNGIFSQI
WNYLPEGLKVLEWRA

>XP_014512377.1

MAEAVIEVVLGNLNSLVQKELGLFLGFDQDLERLASVFTTIKATLADAEEQQFSSRSIKDWLQKLKEAAYILDDILDECAY
EALRLEYQGVKLCPSNKVKCSGLSTFHPKRTVFRYKIAKKMKRICERLEEIADERTKFHLAEMVPERRSGVIEWRQTSSFIS
EPHVYGREEDRDKLIDFLVHDASHDENLSVYPIIGLGGLGKTTLSQLIFNHERVRKHFEPRVWICVSEDFGLRRMTKAIIE
AVSGHACEDLDLDPLQRKLQDLLQNKRYLLVLDDVWDDDPESWERLKSVLVCGALGASVLVTTRLTKVAAIMGTVPPH
ELSNLSDNDCWRLFKHRAFVADEVELEELVVVGKEIVKKCGGVPLAAKVLGGLLRFKREVREWLKVKESNIWGLTHNIM
PALRLSYLNLPIHLKQCFAYCAIFPKDERIEKQYLVELWMTNGFISSDGKLDAEDVGDGIWNELYWRSFFQDIEKDEFGE
VESFKMHDLVYDLAQFVAEEVCCITNDDDIHALFERKRIHHLSDYGWEFHPAQLHQVKSLRTYLGKNVQLSPDVVKCYS
LRLLQFKPRKELLSAIGDLKHLRYLNLSHGNFQTLPESLCRLLNLQILKLDYCYTFKKLPDSLVRLKALQQLSLKDCKLLSRLA
PYIGKLTSLRNLSMYLVGEGRGFLLAELGPLKLKGCIDIKHLERVKSMNDAKESNMSSKQLNKLTLRWKGFREGELARND
EEVLEALKPCNQTLQSLRLEGYQGLNFPQWMSSPCFKSLTYLKLWFCRNCIKLPVLRKLPSLKRLVIGGAKYVKYVQEESN
DDDDHVAFMALEYLSLESLPSLIRLSSEDGENQFPCLFTLKVNDCPNFSLHGLHSLKTLEIMRLPKLKVWPGLQCLTSLEV
LGITGCDEVEGLQYMTALKKLSLRNLPNMESLPDCFGELSLLRELSIVGCYKLMRLSTSLSLSSVEVLSILDCNLELSKRCEK
ENGEDWPIIAHIPHLYV

>XP_014512378.1

MAEAVIEVVLGNLNSLVQKELGLFLGFDQDLERLASVFTTIKATLADAEEQQFSSRSIKDWLQKLKEAAYILDDILDECAY
EALRLEYQGVKLCPSNKVKCSGLSTFHPKRTVFRYKIAKKMKRICERLEEIADERTKFHLAEMVPERRSGVIEWRQTSSFIS
EPHVYGREEDRDKLIDFLVHDASHDENLSVYPIIGLGGLGKTTLSQLIFNHERVRKHFEPRVWICVSEDFGLRRMTKAIIE
AVSGHACEDLDLDPLQRKLQDLLQNKRYLLVLDDVWDDDPESWERLKSVLVCGALGASVLVTTRLTKVAAIMGTVPPH
ELSNLSDNDCWRLFKHRAFVADEVELEELVVVGKEIVKKCGGVPLAAKVLGGLLRFKREVREWLKVKESNIWGLTHNIM
PALRLSYLNLPIHLKQCFAYCAIFPKDERIEKQYLVELWMTNGFISSDGKLDAEDVGDGIWNELYWRSFFQDIEKDEFGE
VESFKMHDLVYDLAQFVAEEVCCITNDDDIHALFERKRIHHLSDYGWEFHPAQLHQVKSLRTYLGKNVQLSPDVVKCYS
LRLLQFKPRKELLSAIGDLKHLRLAIQVGARVTFSNGVLKKTLMKKTVQLRGKGG

>XP_014513527.1

MTAEMLTGVLVSTFLERTIDTLASRLVHILRQRKHKKQLNNLKMKFLAIDVVALDAEQKQFKDLRVRDWLLRAKDVVFD
AEDLLDXIDYELSKSQPEVESQSATNKVWNSLNSSFVSFFEIEIESRMEQVIEDLEDLANESYILGLEKGGGVGIGSVSGSK
LTYTSLPNESVIYGRDDDKEFLFNWLTSDTPSNLSILSIVGMGGMGKTSLAQHVFNDPRLEGKFDTKVWVSVPQEFDVL
KVSRAIIDTITASTDHSIQQEVIQKKLKEELMGKKFLLVLDDVWNERPSKWEDVQKPLVFGGQGSRILVTTRSEKVAVTM
RSEKHLLQILKEDHCWDLFVKHAFKNVDPQPDPDFIEIGKNIVQKCNGLPLALKTMGSLLHNKSFLWEWESIMKSEIWH
FSENESDILPALRLSYFHLPSHLKKCFAFCALFPRGYRFDKECLIQLWMAQNFLENPLQKKSPKEVGEEYFNDLLSYSFFQ
QSSNEEEKRFIMHDLLNDLAKYICEDICIRLGVDEPKGIPKTIRHCSFSSSKLCFDGFGSSIDPQKLHTFTPTDRGWHWNC
KMSVDDLFSRFKLIRVLSLCHCRSLVEVPKSVGNLKHLRSLDLSWTQIKNLPESISSLFKLQTLKLNNCRKLKELPSCLHQLD
NLRCLELVGIGVKNVVTHLGKLKNPQVSISSFHVEKSKKINIQQLGKLNLHGSLTIDDLQNIENPSYALEVDLKNKLHLVEL
RLTWNFIGSSSVDSEKVEDVIENLQPSKYLKKLSVGNYIGKQFPNWLLHNSLPNLVSLVLEECESCQRLPPLGLLPFLKYLQ
ISGFDEIMSIDADFHGNNSCSFKSLRKLYFSNMRQWEKWDCQAVTGAFPRLEFFSIGNCPKLKGHLPKFAALKSLYVFH
CEQLEALMVSALELHLQDCGKLQLERSTMKKLTMDESMLFSMAASLVATVESMLFDTSLENLSFCSLLESISDDCVSLRIF
PLDFFPTLRTLELSGFPNLHMISQDHVHNHLQHLTIKDCPRLESYGDTVLPWKA

>XP_014515021.1

MEFFSSSSSSSSSSFLESEPHFIYDVFINFGGKDIGRKLVSHLHSVLLQAQVKTLVNEENLQEGMKLEEHMGAIAVSKIAIIV
FSKTYTESTCCLLELEKIIECLETFGQVVLPVFYEIERLDVRDQKDDFGKALEETANKSYLGEQVEHALSRWRRALTTAAG

MTSWDVRDFRDDAELVEVIVGHVQTLLDYKDLFITQFPVGLESHVEKVIGCIQNHDTKVCIIGIWGMVGTGKTTIAKAIY
NQIYRLFIGKSFVENIREFWSRLFRTYVDLQEHLLDDVLKYKFELENDEMGRTLIETKFSRKKLLIVLDDVSEFGQLENLCG
NREWFGRGTLIIITTRDVKMLNRLKVNYVYKMDAMNKNDSLELLSWHAFGEAKPRKEFNVIARNIVANCGGLPLALKV
LSGWTIQIPSVQVQEWKYVSLLPNAFAKITEKLKISFDGLGDMEKDIFLDVCCFFIGKERGYVTEILNACGLQADIGITVLIE
RDFIKVERNNKLGMHPLLRDMGREITREDWPQEPGKRSRLWFHEDVKDILKTKSGTKATQGLSLKLYSTSRDCFEAQAF
KNMKSLRLLQLDHVQLTGDYGYLSKQLRWISWKGFPSKYIPNNFCMENVVAMDLKHSHLQLLWKQPLRKQPQVLKQ
LKFLNLSHSKYLRETPDFSILPSLEQLILKDCPSLCNVHPSIGDLCNLFLINLKDCTSLSCLPREVYKLKSLRTFILSGCFKIDILEE
DIVQLESLTTLVTENTVAESTAVKQLPCSIVSSKSIGYISLRGFEASSHNIFPSIIRSWMSPIVNPQSYISPYCMDMENNNG
RDLAPLLSVLEKIRTVLVQCDTEFQLSKQVKKILVEHSVNFTASRISKRHLRFSLNCVGSYNDFFNTLNNSISEGLASSESCD
VSLPGDNYPYWLVHMGEGHSVSFNVPQNSGMKRMILCVVYLATPGIMATECLRNVLIVSYTKCTLQIHNHGSVISIND
EDWHGIMANLGSGEKVEIFMTFDHGLVVYMICGESNFEIKPYQAKGKYLKEIH

>XP_014515045.1

MTSAIPSMEFASSTTVLPQKYDVLINFNGEDIGRKFVSHLDSVLSAIGLTTFLHHDNGEKSMNEPLLNLYRVAIVVFTKTY
SQSPWCLHQLQQIIQWHQTYSRHVLPVYYEIQPFDVRFQKGDFGKAFKRAAHQTFSRQELEHGMSKWSHALTKAAN
FFGWDESNYRSDAELVDKIVKSVLNLPVLSATKFPVGLQSNVEDLIRTIKDKSSEVCIIGITGAGGSGKTTLAKAIYNKIHET
FTEKSFIEDIGQVRRTRGHHRLQEQFLLDVLKTKVEIPSVDMGTRMIREGLTGKRVLIVLDDVTENFTLLDLWGCREWFG
GGTVIIITTRDVDLPRILKVDSVFGIRLMNENESLELLSWHAFREAKPKEEYNYLAKGVVTHCGGLPLALEVIGNCLFKRTK
EEWNSVLLKLEKIPLHNVQQKLKISFDGLRNQIEKNLFLDICCSFVGEGRAYVKKMLNGCGVDADSGIRVLIECSLIKVKR
NSKLGMHPLLQEMGREIINEIYEEEFETEWLLRFDDVQYVLTDNTGRRAIERVPVKLRSVKREPSRILKHTENSDYISKKLR
WISLDWFSSVPDKFYLHDAMTIHLKHSSLRFLLKEPQELSWLKVLNLSHSKYLTGTPDFSGLPSLEQLILKGCTGLREVHPS
IGCLCNLTLLNLKDCTSLSNFPREIYKLISLETLILSGCSKIDLLERDVVRMESLITLIAENTIVKHVPFSIGSSKSIGHISLQGFER
LSCNIFPSIIRSWMSPVMNPISYIHSLCMDIDNSWESIGPLLSSLVNLRSVLVQCDTEYQLSKQVKSILVEYFANSAESGISK
QQFRSSFIGLGTYHEFFNAVSDNISEVLLNSDSCDVSLPVDNLPNWLAYMGEGNSVSFSVPWDRDMKGMALSVVHLS
TGEIVATECLRSVLIVNYTKCTLQIHKYGTIISFNDIDWQGIMSNLGPEDRVEIFVTFGHGLVVKNTILYLICGESNYLKKEP
ESEKNYLLRFIMKIVMCDFW

>XP_014515047.1

MFCPYITKFSHLMYAFKRVILEKHSKELHTKLSHDKNWSMACPSGATHSPKLQISLDGMRAITGITGAGGSGKTTLAKAI
YNKIHETFTEKSFIEDIGQVRRTRGHHRLQEQFLLDVLKTKVEIPSVDMGTRMIREGLTGKRVLIVLDDVTENFTLLDLWG
CREWFGGGTVIIITTRDVDLPRILKVDSVFGIRLMNENESLELLSWHAFREAKPKEEYNYLAKGVVTHCGGLPLALEVIGN
CLFKRTKEEWNSVLLKLEKIPLHNVQQKLKISFDGLRNQIEKNLFLDICCSFVGEGRAYVKKMLNGCGVDADSGIRVLIEC
SLIKVKRNSKLGMHPLLQEMGREIINEIYEEEFETEWLLRFDDVQYVLTDNTGRRAIERVPVKLRSVKREPSRILKHTENSD
YISKKLRWISLDWFSSVPDKFYLHDAMTIHLKHSSLRFLLKEPQELSWLKVLNLSHSKYLTGTPDFSGLPSLEQLILKGCTGL
REVHPSIGCLCNLTLLNLKDCTSLSNFPREIYKLISLETLILSGCSKIDLLERDVVRMESLITLIAENTIVKHVPFSIGSSKSIGHIS
LQGFERLSCNIFPSIIRSWMSPVMNPISYIHSLCMDIDNSWESIGPLLSSLVNLRSVLVQCDTEYQLSKQVKSILVEYFANS
AESGISKQQFRSSFIGLGTYHEFFNAVSDNISEVLLNSDSCDVSLPVDNLPNWLAYMGEGNSVSFSVPWDRDMKGMAL
SVVHLSTGEIVATECLRSVLIVNYTKCTLQIHKYGTIISFNDIDWQGIMSNLGPEDRVEIFVTFGHGLVVKNTILYLICGESN
YLKKEPESEKNYLLRFIMKIVMCDFW

>XP_014515186.1

MTYSNSFRVFDRIKRVIFVCVLLAAIDNNNSNNSSYPVTDFAYDNDSPQIKFDVFVSFRGTDIRQDFLSHLIEAFSQRHIN
AFVDNKVVRGDGMSEALIRAIEGSSISLIIFSQDYASSHWCLSELVKIVECRKKNGQIVLPVFYKVDPAHVRHQKGTYEHA
FAKHQIRYSLTTMQIWRTALTEAANLAGFHSSTFRDEAEFIKEIVKCVLSRLNQVQQGKAKGLVGVGKRIAHVESLLQSE

EPDVRIMGIWGMGGIGKTTIAQEVYDKLCFEYEGCCFLANIREESGRLGMISLKKKLFSTLLGGEDLKIDTPNGLPQYIER
RLRRMKVLIILDDVNDSDQLEVLAGTHDWFGSGSRIIITTRDKQVLAREFASIYEVEALNFDESLRLFNLNAFKQNHLESE
YHELSKKVVNYAKGIPLVLKVLGHLLHGKDKETWESQLERLKKVQNKKVHDIIKLSYNDLDRDEKKIFLDIACFFDGLNLK
VKHMNFLLKDHDYSVVAGLERLKDKALISVSQENGVSMHNIIQETAWQIAREESIDNPRSQIRLLDPEDIYHVLNYNKG
DEAIRSIVINLSRIKQLQLNPQVFARMSKLHFLDFYSKGSCSCLRDQGGLYLPQGLESLSNELRYLRWTHYPLESLPSKFSA
ENLVELNLPNSRLKKLWQEAPEDLVNLRVLILHSSTRLKELPNFSKATNLKVIDLRFCVRLTSVHSSIFSLRNLEKLYLGGCLS
LRSLRSNVHLDSLRYLSLYGCMSLKDFSVTSKNMVKLNLELTGIKRLPSSFGLQSNLQKLRLAYTYIDHLPTSIKHLTRLRHL
DLRYCRQLRTLPELPASLETLDARGCISLETVTFPSTAGEQLKENKKRVAFWNCLKLDEPSLTAIELNAQINMMKFAHQH
FSLFGDAQSTYVYPGSKVPEWLVHKTTHDDFVIIDLSSVLSPHSSHIGFIFGFVVPEVPFGGSALEFKISISGEGSHINVYM
DRPRHRITSDHVYLMYDQACSRYLNGRAKHEPRLKIKVALASRTLTSKYVPLKLRAFGISAINTTDFLSFVQKVKFGDNVP
NVPILSRFFCTFCIVVFVGTFNICIRRLV

>XP_014515208.1

MASSNPSIEFGSSTSKLPQKYDVLINFNGEDIRRKFVSHLDYALSTVGLTTFLHEENAVKGMHIQQPILNLCRVAIVVFTKT
YSQSAWCLHELQQIIKWQETYSRHVLPVYYEIQPSDVRLQKGDFGKSFKATAQETFPWQQLEHGMSRWSHALTKAAN
FFGWDERNYRSDAELVDIIIKGVVNLPVLSATKFPVGLQSRVKDVIQIIKNKSTEVCVIGIWGEGGSGKTTLAKAIYHQLH
GTFTEKSFIEDISQFSQTRGHVHLQEQLLSDVLNTKIEIRSIEMGKCMIREKLSGKKLLIVLDDTKYDPLLDLCDSHVWLAK
GSVIIITAREESLLRIPEIGSVISINLLSTNESLELLSWHAFREAKPEEEYNDLAKSVAVCCGGLPLALEVIGSRLFEKTKEEWK
SVLLELKEIHNHDVHRKLKISFDGLSNEMEKDLFLDVCCFFVGKGRAFVTKILNDCGVDADSGIRILIERGLIQVKKNNKLG
MQPMLQKMGRKIIRQISGKELGKNPQLWFGQDAEYELLENTLFSSQQTKVIRKLPLKMFLIATRELFEHPSVVRDKSRLL
KLTRDFGKLRWISLQGFSSEYLPEDFYLQDAMVIDLKHSLLRFVWKEPQVLMWLTVLNLSHSKYLRETPDFSGLPRLEQLI
LKDCSRLRKVHHSIGCLNNLVLLNLKDCTSLSNLPREVYKLKSLTTLILSGCLKIDPLEKYIVEMESLIILIAENAAVKQVPFSIV
SSKSIGYIFLRGFEGLSCNLFPSIIRSWMSPIMNPLSYIHSFCMDIEDNGWNDRLPLLSTLANLRSVLVQCDTEFQLCKQVE
TFLIEYGVNVTKSGISQKNLKYSLIGVGRCKDFFDAVSDSISQVFASNESRDVSLPGDNDPYWLGHMGKGHSVSFTVPR
DRDLKGMALCVIYLSTPEIVATECLRSVLIVNYTKCTLQIHNHGTVISFNDIDWQGIIPNLGSGDKVEICVTFAHELVVENI
VVYLICSELNDSQKEPAPKKNSLIRFIKKVVM

>XP_014515214.1

MAFESSSPSSESEWIHDVFINFRGVDTRRTFVSHLHSSLSKAGVKTFLDEEHLLKGMKLQELFRAIQVSQIAIVVFSKGYP
DSSWCLDELQKIFQCSQTCGLRVFPVFYYVEPSEIRNQTGDFGDALRAAAERDYAGEHLEFTLSSWRDILTDAANLCGW
DAKDRRRTEAELVRDIVDYVIDQLDYNVLSITQFPVGLDHPVQVVIRFIKRIKKSCKIGIWGMGGSGKTTMAKAIYNKLH
RSFENKSFIENIREVCQTDRRRLVRLQEKLLSDILKVKMEIRSVGIGQVMIDNRFNGKRAFIVLDDVNKFDQLEALSGNTE
WMGERSVIIITTRDLHLLKRFEVDYVYEMKEMQANESLTLFSRHAFREEKPREDFNELAKEAVNYCGGLPLALEVLGSYL
SNRTMTEWRSVLSKLQISPNPEVQEKLRISYDLCDEMEQEIFLDVCCVFNGKDRSCVTEVLNGCELCADIGITVLLERSLIK
IVKNNKLKMHQLLQEMGRDIIRGGSRREPGKYSRLWFQKDVRDVLTNSTGTDAVEGLALKFDLNDKEHFKADAFKEM
RSLRLLQLHYVELMGDYCHLSKQLRWIYWEGFLSEHIPDNFYLENAIAINFKHSHLRQVWKEPKDLSMLKFLNLSYSNYL
TETPDFSKLKNLEKLILKYCRSLRCVHKSIGDLRNIVFINLKDCTSLTSLPREIYKLKSLKTLILSGCTKIDKLEEDISEMKSLTTLI
AENAVVKVPFSIVSSKSIGYLFPCGFEGFSHDVLPSIIWCWSSPTMNPLSRILPLCGISASLVSMNMQNIDLGDLAPILTNL
LNLRSVCMQCDTEFQITKQVRKFLNELASCTSEISDNSLRSYLIGIGSYQEEDFNTLNKSISKELATSGSCHVFLPGGNYPF
WLAHTGEGHSVSFTVPQDWDMKGMALCVLYLATPETAATECLISVVMVNYTKYTIQLYKRDTVISFNDADWQGIISHL
EAGDEVEIFLSFRNELVIKNTAVYLINYEFIDMEVKAVSKPPVMKVYSRKRNLTDQESSRKRQW

>XP_014515215.2

MDIASSSYKLSRKYDVLINFTGEDIHRKFVSHLNSVLSTVGLTTFLHHHNAVKSSHIQEPILSRCRVAIVVFTQTYSQSAWC
LNQLQQIINWHETYCRHVLPVYYEIQPSDVRLQKGDFGKALKETAQQTFSRQELEHGMSRWRHALTKAANFFGWDES
NHRSDAELVDKIVKSIINLPVLSATKFPVGLQSQVEDLIRTIKNKSNEVYMIGIGGEEGSGKTTLAKAIYNQIHWKFNKKSF
IENISQVSDIRGQLRLQEKLLLDVLKQKVEIPNIDVGRSIIRERLSGKRVLIVLDDLPANNELLALWNYSHNWFGEGTVIIITI
TEFGVMSKCPVDSVFRIKLMNEEESLELLSWHAFREPKPKEEYEDLARRVVSYCGGLPLALEVIGSTLFEKIKEEWNNLLF
KFPIAMVDIHTVRETIKVSIEGSLNEIEKDIFLDVCCFFVGESRAYATKILNGCGVDADIGIRVLIQRNLIKINKNNKFGMHP
LVQEIGIKIIYENSEKDPGKNRRLWFDKDAKYVPETLQWLPVEPPSVRIAFGEPILNSEYLLKKLRWIRLHGFSSECLPNNS
YEDDSTAIDLKRSLLRFLWKTPQVLRSIKVLNLSHSKYLTTTPDFTGLPSLEYLILKYCSRLSKVHQSIGSLNSLILLNLKYCTSL
NNLPTEIYDLKSLRTFILSGCSKIDIKDKDIAKLESLITLIAENTAVKLVPFSIVSSKSIGYISLSGFEGLSHNLFPSIIRSWMSPI
MNPISYIHFFCMDMEDNTIDIAPLLSTLANIRSVLVACDTDFQLSKQVKNILDEYFPSITESGISKQHLRCSLIGVGAYHQL
FNAVNDNIYEVLANSESGEVCLPAVNDPYCLAHMGDGHSVSFIVPEDRYMKGMTLCVVYLSNPEAIEPQFTTVIVINYT
KCTFQIHNHGTVISFKDEDWHDIISNLESGDNVEIFVNFGNGLVVKNTTVYLICGESKNLRKAFEPKKHGLIRFVKKVVM

>XP_014515217.1

MSRWSHALTKAANFFGWDESNYRSDAELVHRIVKSVLNSSVFSATKFPVGLQYPVEELIQTIKNKSMDVCTIGICGMRG
SGKTTLAKAIYHKIHGTFMHKSFIEGIAQFSEPRGGIHFQEQLLSDVIKTKVKIDTLEMGRNMIRDRLLGKRLLIVLDDMD
DYLSLLYLRESRSWLSKGTVIIMTTTDDDLFRKYQVQSVFRINPMNENRSLELLSWHAFREAKPKEEYDYLARRLISLCGG
LPLVLEVIGSILFERTEEEWYSVLFKLEKIPYYNVQQKLKISLDRLRNQLERNLFLDLCCFFVGKDRAYATKILSSSVVEADSE
TGVLHDFRVDFDSGIRVLLKRNLIKVKRNNKFGMHPLLQEMGRAIFRETSRGKRWKNGQLLFDVDAEYALKVNAGRKD
YQVLLSTRRKPSRLPKDAVNSENLSPRLRWISFHGFSMQYIPNVFNAHDTIAIDLKHSLPQFFWQELQVLRSLKVLNLSH
SIYLTEIADFSRLPNLEELILKDCPRLREIHQSIGHLCFLILLNLKDCKRLSNLPQEIYNLKSLRTLILSGCSNIDPMEKDIIQTKS
LITLVAENTTVKKVPFSIVSSKAIGYISLRGFERLSCDPFPSIIIRSWMLPIMNSISYINSNCVDMEDNSRYDIAPLLGNLANL
RSVLVQCDTEFQLSNQVKNILVDYFSNITESEISKQHFRSSLIGVGAYHEFFNAVSNNISEVLASSESSDVSLPSDNPPYWL
AHMGEGDSVSFTVPPDNAMKGLFLCVFYVSTPEIVATEGLRSVLIVNYTKCTLQIHMHGKIISFNDIDWEAIRSNLGSGD
KVEIFVTFSQGVVVKNTHVYLICGESHYREKVFTQKKMISIDS

>XP_014515315.2

MGWLAPLVKVGVEAISFVWRQVTYIVRYKQSVHELKDSIKDLENEKDKIDHQCDEADKNLKNIEGKVTEWNRKVSEINI
TVEEFENDDGHEKAQSLNCYVFPYLWNRHRLGRKAKKMEVGVKKLINESPGLDEISYKENVTSNDATLSNSGFEEFGST
KSTMEKVMRELENSSVRMIGLYGEGGVGKSALIKEIARIARDKKLFNVVVKVEITDNPNLQSIQEEIAYVFGLQLEGEGEN
VRADCLRRRLKKETGNTLLILDDLWHKLDLNKLGIALDDNDDNDDLSNDTRDLDDKFREKDKNNKDLNQKVLKREKIIG
GHKRCKILLTARQKRVLEVEMDVKSTFRVEPLDDKDALMFFQKLSGIHNMSDSRKEIVRKYCAGLPMAIITVAKALRGKS
ELVWEVALEKLEKQELVGVQTYMDISVKMSYDHLENEEIKSIFLLCAQMGHQPLIMDLVKCCYGLGILEGVFSLWEARD
KIKITIQKLKDSGLLLDGNSDTHFNMHDIVRDAALSIAKKEKNVFTLRNRKLDEWPELEKCTSISICNCDIIDELPIVNCSRL
NFFQIDTNNQSLTIPEKFFEGMKNLKVLILTGFHLKRFPPSIKGLLKLRMLCLELCTLEDNIAIGELKKLRILSFSGSQLKSLPT
ELGCLNKLQLLDINDCSILELNIPPNILSSLKHLEELYIRKSLTKMLVEGESSYGQNSVLCELKNLHQLKVVDLSIPCFSILPNH
LFFAKLKHYKIVVGDVEMFSIIGFKMFDKHETFRVLALQLNHDTNIHSQEDINLLFKTAQSLLLGKVEVVKVVNELNIDGF
PDLKHLSIIDSSAIKYVNSMKLSNCINVFPNLESLCLYNLKNLEMISYGPLTVASFTKLKSIKVNMCDRLVTLYSVYMVEFA
DSEEPCENIECNFYSDKFCASLETIEISECESLKEILQIPMNCDKVKFPKLQTLTFQSLPSFTCFDTEVEESRWPHPTNPQAK
NSGSEEDPKSDKAPPLFGEQVEIPNLESLNLSSLNIHKIWSDQLTSSFYFQNLIKLVVKECDKLTYLCSLSVASNLKKLKSLIIS
DCPIMEKIFETKENNAEKVCVFPKLEEIQLSKMRRLRDIWHTKVNIDSFSSLISVNIEECNKLDMIFPSNMEGWFESLINLK
ISKCKSVKEIFEVNDSEEIDVSGGIETNLQVILLEDLPKLKELWSKDPHGIVNFKKLRTIDVSNCDELRNLFPASMTKDISKLE
RLSIWKCKRMVEIVSSKDASEANNDPLEFPELTYVRLHSLPNIKHLCKGRHPIKCPKLNELTVNHCLKLKTFSKGINKTSKE

EESFVFSTQKVLSKLEYMEIDFKEAQNLLPEYPMHYLKELSLISVESVDFLNQFPYRMPNLEKLKFTSSYESKELEPASEERL
RITLELKELVLRKLRIKDLTGVSIVRKLELLSLVFCEELNNLGPSSVSFT

>XP_014515321.1

MEIVSKIFDPAIEFVRDHGIRQVTYIFCYTKNFKELQKKVKRLREIKERLVRQHDEAKRKGDIVEGTVEEWFVEVGEFESSV
ENYSKNAGHKKTRGLYYLFPYYRHKLGRQAKKMEIEALRLXDESPKIDEVSHAEKVTSFDLTSSNSGYIEFNSRKSIMEDI
MKKLKDPNMKIIGLHGSQGMGKSTLIKKIANKAKYEGLFDRVAEIDVTINLNPLNIQEDIAYVLGLQLTGESENVRADYLR
RWLKIENVSILIILDNLHERLDLNRLGIPIDDDYDLRKKNELSIRSSKKDKDDNTQGTDEKVLKKANFLADYKGCKVLLSSRY
KKVFPNEVDVESNFCLKELDGNDSLKLFEKVTGGGNKMSMPKEEIQNYCAGLPMRIVTFAMAFKNWSESESEPTLEKF
KKQGLVKWKESSEIPNKIKYDLPENKEHKFIFLLCAQMGHLPLVNDLVKYCFGLGILEGVSSLSAARKKINEVMQELKNLS
LVSYENPNIHFSMPHMVRDDAISNALMDDNVFAIRDGKLDYWPNLEKCIFISICNSDITYRFPQVINCPQLKFLQIETNN
PSLKIPERFFSNMRNLLVLILRGFHLSCLPYSIEDLLNLRMLCLERCTLXCNLSVLRKFKDLSILSFSGSQLKNLPVELQYSDXL
XLLDIXDCFELEIIPPNLISNLTXLEELYXGKSLMKMXVEREESKGQNAFLSELXNLHELKVVDLSIPCVSILPNHLFFDRLKYY
KIEIGDFEMFSIGEFKMPNKYEELKVFALQLKDDTDIHSHKGIKLLFKTAQSLLLGKVGVQNVVNELNIDGFQDLKHLSIIN
NNDIKYINSTCLSNSVNIFPNLESLCLYNMMNLKMICGSSITLESFAKLKTIKVKMCYQLENLFSFYAIKISTSTRSSEINECN
SYKKKVLASLEMIEVCECESLKEILQIPMDCDKVKFLRLHTLTLQSLPSLTSFNTKVKRSCWPHSIDAQTTNRDHTEISTEQ
DGHSDNAPPLFGELVEVPNLQNLNLSSLNIPKIWSDQHLSSFYFQNLIRLVVKDCDKLTHLCSLSMASSLKRLKSLVXSGC
RIMENIFEIEGISANKVCVFPKLEEIHLSNMKRLTNIWQTEVSVDSFCSLISVSIEECNELDKIFPSHMEGWFESLINLKVSN
CKSVKEIFEVTDYEEIDEYGGIDTNLQVILLEDLPKLKELWSKDPHGILNFKNLRTIDVSNCEELRNLFPASMAKDVSKLER
MSILHCERMVEIVSSKDASEANNDPLEFPELSYVRLYELQNIRQFYKGRHPIKCPKLKELSIGKCMKLKTFSQETSDEKFVF
SAEVVFPKLEYMEIDFLEAQNLLSKYEMHNLKELIILRAIECPDLFYPFLYKMPNLEKLKLTFGYYSSYYRLQSRNIGQHDRL
GVVLQLKQLIIRSSDINDIGFDRDQVLQRLELLSLKECNELKTLAPSSVSLSYLTCLKVKNCEGLRNLMASSTAKSMVRLKT
MKVINCDKVKEIVSHEENEEDKVMKIVFSKLISIELVGLNKLVSFCSYKDCEFEFPSLEILIVRECPKMEKFSEKRAIAPKLKD
VFGVEGDEKAKWQWRGDLNATIQKVFNDKVSFAYTEELWWLDYNIIKQLWNNTHWGQQNNFGYLKRLSLSGCDTL
TRIIPYHLLSCFHNLEELYVFSCSNAEVIFNMNGENRVMTKSSGIFRLKILSLSFLPALEQVWDKDPEGVIGLQLLREMRVE
DCKSLKSLFPASVAKHLTRLDVLIVTDCEELVEIFEKDEKDEKGEGATHESVFPRLTTLTLKQLPLLKYSIQCSKQQESISNLR
FKEIQELCLGSRPIPNSCFGLLDSLIVDGCKFSSDVLLPFTLLSFLAKLESLEVRKCDSVKVIFDVKCTTQDMITFPLKKLTLSQ
LPDLKNVWNEDPHGILSMHHLQELHVKECKGLKSMFPASGAKEVLLPFNLLPFLPNLVTLEVRNCDGVKAIFDVKCTTQ
GRDMTSQPFSLKNLTLSKLPKLKTIWNNPHGILNMRHLRQVHVEECKCLKSVFPASVAEDLLELESLVVLHCERLMTIVV
EDNTNGSQEFTVPCPCVRSVKLRGLPKFKYFYYYSLTSHTYKCLESNTENQVDTEKSLILAKYEMEKISGEEFHRKLLNNLK
VLTLSFGWDVLQYKILEQVPNIEKLVVCYGSFKEMFCLESPNNVNYSGLLLQLKTLRLESLEELVSIGLEKSWTESFVKNLE
TFEVISCSKLKNVVESKVFLSNLTYLKLESCNSLQYLFTSSTAKTLGHLKAMEIDSCYSIEEIVFKEDGEKSDEEEIIFPKLIRLN
LYYLRKLQLFYRGRLCFPSLEELLVPGCTEMETFCIGTITACKLSQVKLGRYSQAIPIEDDLNQTMRKEFLKETLGETRIDLKK
KSKTTQDMEQLNADPSFQ

>XP_014515322.1

MEIVSKIFDPAIEFVRDHGIRQVTYIFCYTKNFKELQKKVKRLREIKERLVRQHDEAKRKGDIVEGTVEEWFVEVGEFESSV
ENYSKNAGHKKTRGLYYLFPYYRHKLGRQAKKMEIEALRLXDESPKIDEVSHAEKVTSFDLTSSNSGYIEFNSRKSIMEDI
MKKLKDPNMKIIGLHGSQGMGKSTLIKKIANKAKYEGLFDRVAEIDVTINLNPLNIQEDIAYVLGLQLTGESENVRADYLR
RWLKIENVSILIILDNLHERLDLNRLGIPIDDDYDLRKKNELSIRSSKKDKDDNTQGTDEKVLKKANFLADYKGCKVLLSSRY
KKVFPNEVDVESNFCLKELDGNDSLKLFEKVTGGGNKMSMPKEEIQNYCAGLPMRIVTFAMAFKNWSESESEPTLEKF
KKQGLVKWKESSEIPNKIKYDLPENKEHKFIFLLCAQMGHLPLVNDLVKYCFGLGILEGVSSLSAARKKINEVMQELKNLS
LVSYENPNIHFSMPHMVRDDAISNALMDDNVFAIRDGKLDYWPNLEKCIFISICNSDITYRFPQVINCPQLKFLQIETNN
PSLKIPERFFSNMRNLLVLILRGFHLSCLPYSIEDLLNLRMLCLERCTLXCNLSVLRKFKDLSILSFSGSQLKNLPVELQYSDXL

XLLDIXDCFELEIIPPNLISNLTXLEELYXGKSLMKMXVEREESKGQNAFLSELXNLHELKVVDLSIPCVSILPNHLFFDRLKYY
KIEIGDFEMFSIGEFKMPNKYEELKVFALQLKDDTDIHSHKGIKLLFKTAQSLLLGKVGVQNVVNELNIDGFQDLKHLSIIN
NNDIKYINSTCLSNSVNIFPNLESLCLYNMMNLKMICGSSITLESFAKLKTIKVKMCYQLENLFSFYAIKISTSTRSSEINECN
SYKKKVLASLEMIEVCECESLKEILQIPMDCDKVKFLRLHTLTLQSLPSLTSFNTKVKRSCWPHSIDAQTTNRDHTEISTEQ
DGHSDNAPPLFGELVEVPNLQNLNLSSLNIPKIWSDQHLSSFYFQNLIRLVVKDCDKLTHLCSLSMASSLKRLKSLVXSGC
RIMENIFEIEGISANKVCVFPKLEEIHLSNMKRLTNIWQTEVSVDSFCSLISVSIEECNELDKIFPSHMEGWFESLINLKVSN
CKSVKEIFEVTDYEEIDEYGGIDTNLQVILLEDLPKLKELWSKDPHGILNFKNLRTIDVSNCEELRNLFPASMAKDVSKLER
MSILHCERMVEIVSSKDASEANNDPLEFPELSYVRLYELQNIRQFYKGRHPIKCPKLKELSIGKCMKLKTFSQETSDEKFVF
SAEVVFPKLEYMEIDFLEAQNLLSKYEMHNLKELIILRAIECPDLFYPFLYKMPNLEKLKLTFGYYSSYYRLQSRNIGQHDRL
GVVLQLKQLIIRSSDINDIGFDRDQVLQRLELLSLKECNELKTLAPSSVSLSYLTCLKVKNCEGLRNLMASSTAKSMVRLKT
MKVINCDKVKEIVSHEENEEDKVMKIVFSKLISIELVGLNKLVSFCSYKDCEFEFPSLEILIVRECPKMEKFSEKRAIAPKLKD
VFGVEGDEKAKWQWRGDLNATIQKVFNDKVSFAYTEELWWLDYNIIKQLWNNTHWGQQNNFGYLKRLSLSGCDTL
TRIIPYHLLSCFHNLEELYVFSCSNAEVIFNMNGENRVMTKSSGIFRLKILSLSFLPALEQVWDKDPEGVIGLQLLREMRVE
DCKSLKSLFPASVAKHLTRLDVLIVTDCEELVEIFEKDEKDEKGEGATHESVFPRLTTLTLKQLPLLKYSIQCSKQQESISNLR
FKEIQELCLGSRPIPNSCFGLLDSLIVDGCKFSSDVLLPFTLLSFLAKLESLEVRKCDSVKVIFDVKCTTQDMITFPLKKLTLSQ
LPDLKNVWNEDPHGILSMHHLQELHVKECKGLKSMFPASGAKEVLLPFNLLPFLPNLVTLEVRNCDGVKAIFDVKCTTQ
GRDMTSQPFSLKNLTLSKLPKLKTIWNNPHGILNMRHLRQVHVEECKCLKSVFPASVAEDLLELESLVVLHCERLMTIVV
EDNTNGSQEFTVPCPCVRSVKLRGLPKFKYFYYYSLTSHTYKCLESNTENQVDTEKSLILAKYEMEKISGEEFHRKLLNNLK
VLTLSFGWDVLQYKILEQVPNIEKLVVCYGSFKEMFCLESPNNVNYSGLLLQLKTLRLESLEELVSIGLEKSWTESFVKNLE
TFEVISCSKLKNVVESKVFLSNLTYLKLESCNSLQYLFTSSTAKTLGHLKAMEIDSCYSIEEIVFKEDGEKSDEEEIIFPKLIRLN
LYYLRKLQLFYRGRLCFPSLEELLVPGCTEMETFCIGTITACKLSQVKLGRYSQAIPIEDDLNQTMRKEFLKETLGETRIDLKK
KSKTTQDMEQLNADPSFQ

>XP_014515398.1

MDFLGPFGKVIEGLIDFVWKHGVRQVTYIVNYNNNVFELKDSVKDLELEKERINHQRDEAEKNLNNIEGKVIEWDRKLS
EIETAVEVFENDDGHTRARSPNCFLFPFLRNRHRLGRQAHKMKEDVKRLIGESPELDEVFYRQNVTCNDATLSNCGFVE
FSSIKSIIEKVMIQLQDSTVRMIGLYGRGGVGKSTLVKEIARKAKEKKMFDVVVKVEITADPNPHKIQEEIAYVLGLRLEGE
GENVRADCLRRRLKKEKGNILLILDDLWHKLDLNKLGIPVEDYDDDEDFNRQKPDNKDSNNDPSSKVSKKENIPGGHKG
CKILLTSRDKNVLCVEMDVKSTFCIRELDDKDALILFQKLAGIDNEMSSSKQEIVKKYCEGLPMAIVVVARALRNKSESVW
EATIKRHKKHELMGEGTSMDISVKMSYDHLENEEIKYIFLLCAQMGRRALIMDLVKYSFGLGIFEGVSSLWEAREKIKTSI
QKLKDSGLLLDESSNNHFNMHDMVRDTALSIAHKDHNAFNLRNGKLDDWPELEKCTSIFMCNSDIIDGLEVINCPHLKL
FQIDTNDPYLKIPKSFFKRMKNLRVLIMTGFCVSNLPSSIQYLSKLRMLCLQRCTLDCNLSIIGKLKKLRILSFSGSILKNLPIE
LQCLDKLRLLDISDCSELKIIPPNVISSLTCLEELYIRESLIKMLVERETNKGQDLFLSELKNLHQLKVVELSIPRVSNFPNHLFF
DKLRDYNIVIGDFDFFSLGEFKMLNKHETSRVLAVQLKDNTNIHSQENIKLLFKTVQTLLLGKTNGVREVVNDLNIDGFQ
DLKHLSIINNNDFKYVNSTKLCNYVNVFPNLESLCLYNLGKLDMICYGPVTVVSFAKLRTIKVEMCHRLKNLYSLDMAKFP
IGAQTCDISECNSYMDIFLSSIEIIEVSECKSLKEILQIPKHYRKVEFLKLHTLTLRLLPLLSSFYTKVDKFFWPHLTKAQTTIM
GHKDLTSEEDKQSDEEPSLFGELVEIPNLETLNLSSLKIHKIWSDQNSSSFLFQNLIKLVVKDCDKLTHLCSLSMAKSLKKLK
SLVISECPVMEKIFETERNNADKVCIFPKLEEIHVTKMNKLTDIWQTKVSVDSFSNLISVKIEECNKLDKIFPCHMEGWFES
LDNLKVYWCESVEVIFEINDSQEIDEFGGIDTKLQIILLEGLPKLKQLWSTDQNGILSFKKLRTIDVDSCDELKNLFPVSVAK
DVPKLEHMSVLDCDKMEEIVGSQDASDANPDLLVFPELIYVRLHRLPNMKYFYKKKYPIKCPKLKELSVTKCVKLKTFVKD
TINTTNKVGNFVFSIEEVFPKLEHMEIEFKEAQELLPKYQMQRLKELSLISVESVDLLNQFPYRTPNLEKLKLESYDFKELAP
KANNGRQKGLGIVLQLKELVFLDSNIKDLGFERGQVLQRLEVLRLEDCSKLSNLAPPSVSLNYLTHLELKYCFGLKHLMAS
STAKSMVQLKTMKVSYCDKIEQIISMEESEEGKVMKIVFSKLISIELVGLNNLASFCSHKECEFEFSSLETLIVRECPKMEKF
SEKRSIAPKLRNIFGVEGDEKTKWQWEGDLNGTIHKIFDDKVSLAYTENLWLDIDNDQYIIEQLWHDRHWVQQNSFGY

LKTLFVWRCDTVVHVIPSHLHSCFPNLEGLDVRYCSNAEVIFNMNDENRVMTKPSGIFRLKILNLYNLPKLEHVWDKDL
EGIIDLDALEEVRVENCRSLRTFFPAWVAKDLNRLEVFQITKCDELEEIFRSGEEEEGSLPDSVFHQLTTLTLQQLPRLKYSI
HRSKQQESTSYLSERDIKGLCFGSQVIPNSNFCLLESLKVDGCQFLSDILLPFNLLPSLANLEILEVQNCMFIKTIFDVKCTTE
SKDVTSTGPTLFSLKNVTLSNLPNLKNVWNEDPDGILRMHHLEEVYVENCKCLRSVFPESIAKDVVELKNLEVEDCEGLT
TIVAENNNADPRGTNQDLPCPLVRSLKLKSLPMFKYFYHCSLQCDNFANLESHSENQVGTEKLKWLSLGNNGVEMILH
GEFQRNFLENLKVHTLCLFSDAFGCEILEEVPNIEKLVVRGGSLKEMFCCQSPNNEDYSGLLLQLKELRLESLQELVSIGLE
NSWTEPFVRNLETFEVISCSSLKNLVTCRESFSNLICLKFENCDSLSYLFTSSTAKSLAKLQRMKIKKCKSIEEIVSKEEEESDK
DEIIFPKLNCLKLKYLKNLRRFYKGSLSFPLLEELSIRDCDDMVSLCQGTLEASKLSQVIPLEIDLSSAIWKEFMSKIGRRSSVE
FKDRADLNEIWRLSLQFQDFCFSNLETLVVDGCQLLSYVIPFHVLPLLPELQTLEVRNCDSVKTIFDVKSSQHTFTFPLKKL
VLLKLPNLETIWNEDPAEIVTEPNPTHPEQTNPKLITPNLEHLTVGENELKIIVDGKFQRNLLHKLKVLGLCFDMECDEFLE
YGFLQQLPNVMKLMVCDSSFKVIFCYHIPNNSEILIQLKELRLESLQELVSIGLENSWTEPFVRNLETFEVISCSILKNLVTC
RVSFSNLICLKVENCDSLTYLFTSLTAKSLAKLQRMDIKKCESIEEIVSKEGEEADEGKIIFPKLNCLKLKYLKNLQRFYKGSLS
FPLLEALSIRDCDDMVRLCGGTLEASKLSEVKLEESNTKLKTVLNSTMRKEFLKKISELDKLDLKSRPLQKIWNGLLDISDLC
FSGLAILTVNDCPFLSDAVLPFHLLPLLPRLKTLEVGNCDYVKTIFDVKCTTKDTSFTFPLKKLVLSKLPNLENVWNEDPHKI
LCMQHLEEIHVKECKDLKTVFPPSAAKSLVELEDLVVEDCEGLMAIVADESKEGNELDKNGIIFPRLSYLKVESCNTLPYLF
TSSTAKGLAELKTMKIKQCKSIEEVVSKEGEVSDEDEEIIFKQLEDLYLEKLDELGCFYSGNLTLSFPSLEEVHVIKCSSMKTF
SAVNKINHPTKWYCSEYERPQKETDLNSAVLKTSAKEAPDASGAIISVLQ

>XP_014515469.1

MPVLETLGGAHQILDFFRGRNLDENLLKKLKRKLMDVTSVINDAEQKQFTNPLVKEWLDEVRDVLYDAEDLLEQIEYEF
SKTKLEAEFQTSSSKVHSFESKIIALLDDLESLFNQNIVKDFKIYSGVRFGLGNKVSEKKVESMSLVAEEVIYGRNEEKEIIFT
WLRSNTNDNKLQILSTVGMGGMGKTTLAQHVYNDPKTEEAKFDEKAWVCVSDAFDVLRVSKTIIGAFTNSRDDSGDL
EXVHGKLKKRLSGRKFLLVLDDVWNEDRNQWKALQTPLTSGAKGSKILVTTRSHKIIRVYVMLKGYRHL

>XP_014515492.1

MEFASSSFLTSEPHFIHDVFINFGGEDIGRRFVSHLHSVLLQNQVKTFISQENLHEGMKLEEHMRAIGHTKITIIVFSKSYA
ESIWCLLELEKIIECDETFGQIVLPLFYEIDPSDVRHQKDDFGKALEEAAHKSYSGEQLEHALSKWSRALTTAARITGWDFR
NFRHDAELVERIVSHVKTLLDYKDLSITQYPVGLESQVEDVIKCIENESSKVCMIGIWGMGGSGRTTLAKAIYNRIYREFIG
KSFIENIGEVWDSKIERYVALQENLLSDVLKSKLEVKSVGIRRTMIQNGISRKKLLIVLDDVNEIGQLKSLCGSREWFGQGT
VIILTTRDKHLLNQHLVDYVYQMDRLNENESLELFSWHAFRDAIPKKEWSELARNVIVYCRGLPLALQFLGSYLCDKTIEV
WESVLRELQRNPPNELLNLLNMSFDGLRDTEKDIFLDVCCFFKGKEKDYVTEILNGCGLHADIGITLLIDRGLIKVERNNKL
ELHPLLRDMGRQIIHQECSKEPGKRSRLWLQDDVKDVLNENTGTEAIQGLSLKLHSRRRDGFEAHAFKKMKRLRLLQLD
HVQLSGDFGNISEELRWICWRGFPYEHIPSNFHLQNAIAIDLKHSHLQLLWKQPPGFRAVKIP

>XP_014515501.1

MTIYFNVSCIHEKLDLQQYEGHNCYHRLRLLVVEKLREDPVEEKLTIEFRKAKIYRVTNSALSTVPLTTFLLYPNAVKSTHIQ
EPILSHCRVTIVVFTQTYSQSAWCLHQLQQIIDWHETYCRHVLPVYYEIQPSDVRFQKGDFGKALKATAQQTFSGQELE
HGMSRWSHALTKAANLFGWDESNHRSDAELVDKIVKSVVNLPVLSATKFPVGLQSHVEDLIRTIKNKSTEVCIIEISGEE
GSGKTTLAKAIYNQIHWKFKDKSFIENISHVSGTRGLLRLQEQLLLDVLKQEVEIPSVDVGKTMIRERLLGKRVLIVLDDVN
YSNNLFDLRDCLKLLVEGTVIIVTSTYGIHISDQANSDFWVERMNEEESLELLSWHAFREPKPKEEYEDLARRVVSYCGGL
PLALEVVGSTLFEKTKKEWNSSLFAIDGLLPDPEIIKLSIEGSLNEMEKDIFLDICCFFIGKSRAYVTKILNGCGVDADNGIRV
LIERXLIKINKNNKLGMHPLLQEIGIRIIQKNSGKDLGKNRRLWFDKDTKYGTEAKQWLPVKQPSVFTPFESVQPTVNSEY
LLKKLRWISSHGLSSERLPNNFYQHDVIAIDLKRSLLRFLWKTPQVMTSLKVLNLSHSKHLTTTPDFTGLPSLEHLILKYCSR
LSKVHQSIGSLNSLILLNLKDCTSLNNLPTEIYELKSLRTFILSGCSKIHIMDKDIAKLGSLITLIAENTVVKEVPFSIVSSKSIGYI

SLRGFEGLSHNLFPSIIRSWMSPIMNPISYIHSFCMDTEDNTIDIAPLLSTLANIRSVLVVCDTEFQLSKQVKNFLVEYFSNI
TESGISKQHFRCSLIGVGAYHQLFNAVSDNISEVLSSSESCDVCLPAVNDPYCMAHMGEGHSVSFIVPEDGDMKGMILC
VVYLSTPKIIKPEFTTVVIVNYTKCTFQIHNHGIAISFKDEDWHGIMSNLESGDNVEIFVNFGNGLMVKNTIVYLIWGEAE
NMEKASEPKKHSLIRFIKKVVM

>XP_014515505.1

MSYTSSSSKSKLRWIYDVFISFRGEDTRKNFVSHLYSALANAGVNTFLDDEKLAKGQQLKTELFHAIEGSQISIVVLSENYI
YSTWCLDELVKIMECHAFRGQVVLPVFYGVFPRFLRNLNDVSFEVGDLHRVKQWKKALGEVAGFAGWDVSNYRNEN
FVVKEIVSEVLERLDRTYMSITDFPVGLECRVEHCIGLLRKETRGAYILGIWGMGGIGKTTIAKAIYNEIRYEFKHKSFLANX
REVWQRDQGQIDLQERLLSDILKTEKVKVYSSDWGKAMIKETLCTKRVLVVLDDVNTLEQLSALCGNGNGIVQGSVIIIT
TRDVRLLNVLEVDHVYEVEEMNEIESLELFSWHAFEEANPPEDFLELSKQVVTYCGALPLALEVLGSYLYKRKEKEWQSV
LSKLKEIPNDKIQEKLKISYDGLSDHTEKDIFLDICCGTGVIEGLALKMQRTSGVCFGTETFGNMKRLRLLKLDNVQVAGD
YGHLPKQLRWVDWKAFSLTHIPENFYQENIVAIDLKYSYLKLVWKVPQFLERLKFLNLSHSKYLSKTPDFSKLPNLEKLILK
DCPSLYEVHHSIGDLXNLLLLNXKXCTCLGNLPMVIYKXKSLQTLILSGCSNIDKLEEDIGQMESLTTLIADNTSLKQVPFAI
VRSKQIGYISLCGYEGLARDVFPSLIWTLMSHTRGTLSCFQPFGIMPTSIVSMNIQDNNLVNLLSKVGEFSKLRSISVQCD
SDFQLTQELRTILHKLCNINSSEPESAYQSQIPENSMASYLIGMGSYQQVFDMLSNSISKVLRTNSGTDFVLPGDNYPYW
LAYTGEGYSVPFQVPEDSDCRMKGMLLCVVYSSTPDNMATQNLTNVFIFNYTKCTIQIYKQATTMFFSDEDWQGVISN
LGPGDNVEIFVGVGDGITAKKTAVYLIYGQSITMRMESLGLSAQESPELSVILSPKMSAQPTTDVEMKAKKPKKNIFAKIR
NIVRVCSCLE

>XP_014515506.1

MSYTSSSSKSKLRWIYDAFISFRGEDTRKNFVSHLYSALANAGVNTFLDDEKLAKGQQLKTELFHAIEGSQISIVVFSENYI
SSRWCLDELVKIMECHASRGQVVLPVFYGVFPWFLRDLNDVTFEVILEKASDLHRVKQWKKALGEAAGFAGWDVSNY
RNENFVVKEIVSEVLERLDRTYMSITDFPVGLECRVEHCIGLLRKETRGAYILGIWGMGGIGKTTIAKAIYNEIRYEFKHKSF
LANIREVWQRDQGQIDLQERLLSDILKTEKVKVYSSDWGKAMIKETLCTKRVLVVLDDVNTLEQLSALCGNGNGIVQGS
VIIITTRDVRLLNVLEVDHVYEVEEMNEIESLELFSWHTFKEANPPEGFLELSKQVVTYCGALPLALEVLGSYLYKRREKEW
QSVLSKLKEIPNDKIQEKLKISYDGLTDHTEKDIFLDICCFFIGKDRGYVTEILNGCGLHAEIGITVLIERSLIKVEKNNKLGIH
DLLRDMGREIVRQSSPLEPQKRSRLWVHDDVLDILTEHTGTGVIEGLALKMQXTSGVCFGTETFGKMKRLRLLKLDNVQ
VAGDYGHLPKQLRWVDWKAFSLTHIPENFYQENIVAIDLKYSYLKLVWKVPQFLERLKFLNLSHSKYLSKTPDFSKLPNLE
KLILKDCPSLYEVHHSIGDLSNLLLLNMKDCTCLGNLPMVIYKLKSLQTLILSGCSNIDKLEEDIGQMESLTTLIADNTSLKQ
VPFAIVRSKQIGYISLCGYEGLARDVFPSLIWTLMSHTRGTLSCFQPFGIMPTSIVSMNIQDNNLVNLLSKVGEFSKLRSIS
VQCDSDFQLTQELRTILHKLCNINSSEPESAYQSQIPENSMASYLIGMGSYQQVFDMLSNSISKVLRTNSSTDFVLPGDN
YPYWLAYTGEGYSVPFQVPEDSNCRMKGMLLCVVYSSTLGNMATQNLTNVFIYNYTKCTIQIYKQATTMFFSDEDWQ
GVISNLGPGDNVEIFVGVGDGITAKKTAVYLIYGQSITMRMESLGMGAQESPELSVILSPKMNARSTTDVEMKAKKPKK
KIFSKIGSIVRGCMIVEMNSDAPVIATPGDTAKVDGRTRFGGLGNYGSDVDDEDNEIVCETNFVNAQIKTMSLPSNDSV
SDQLHDDKGHQDMNCLERALEGKKQQIIIQVGKAEGPDRNIHVRQRNYTESSWCLDELEAIIDCHKFHGHIVLPILYHV
VPSQVRHQTGDFGNVLKAFAQKRSWDEYMMSRWNTAFTGATNFSGWDTPFKSLTTDSPTMQNNVDVAFNWPRG
FPSGIVR

>XP_014515516.2

MDVSSSLLFFTKFRVLADVFIHCFGDDVRRNFVSHLSSALLQAGVKPYLLVDMEMLSEKFMSSITRFQIGIVVLTKAYIESS
QCVEDLVRIIECLETHCLIVMPVFYDIDPSDVLDITNYSSHVRDQKEREFQRPIDLSKDNSRDGRTKWTPIKAERFNSPVIV
HTYAASYALNRVANLSTWDESKHRNDAELVEEIVKSVLAKLDRVLPVTKFPIELETHVKNVIGLFENQPNKVCMIGIWGI
GGSGKTTLAKAIYNKIPFTFGDKSFIQDIREVCQTDGRRGLVHLQEQLLSDVLKNVKTESGDMEKTTIENSLSRRKLFIVLD

DVHEIDQLKHLCGNSKWFGAGSVIIITSRHLDLLYHHKVDYVYEMNELDENDSVELFSWHAFREAKPRENFNKLARTAV
AYCDGLPLALEVLGSYLSKRSENEWRSVLSKXEIIPNTQVQNILRISFDGLCSEMEKDIFLDVXCFFIGKERXYVTEILNGCG
LXADXGXXVLIERGLXKIEKNXKLGMHHLLRDMGREIVRQTSXMQPGKRXRLWLPKDVLDVLTKNTGTEAIVGLSLNSE
LTNSDSFKADADADAFKKMKMLRFLQLDHVXLTGDYGCLSKQLRCISWKGFSLEYIPNNFYLEGAIVIDFQHSNLILVWK
EPTVLPLLKILNLSHSKYLTETPDFSKLPSLEKLILKHCVNLDKVHQSIGDSHNLLLINLKGCTNLSNLPSETYKLKSLKTLILSG
CLKIDIFKEDILHMKSLKTLFSENTAVKQVPISVVSSKGMGYIHVDEEKGLSVTVLHTVILSWISRNFNPLYRIRPFRGISSTL
VSMNVEHNDLVDLAPILRSFLKLSTVLVQCVTEYSILQQVTVFLEEVQQVGPVTWTRSTQLSNHPFSPYLIQFGGYQEEV
FNTLRRRLYDEGLAVRQTRKVFQSSHNYPHWFAYKSEGHSVNFTVPDNFHMDGMILCVEHLSRLGDPTQYLICVLMV
NYTKCIIQFFNRQTITSWKDVDWQGAISPHLRSGDNLKIFIIFEKGFEIQKTAVYLVCNGSTDRKSM

>XP_014515520.2

MKGMILCIVYLSKPEIKPTECLTGVLDANYTKRTLQIHRQETVISFNDEDWQEIISHLAGGDMVEIFVAFGHDLVVKKTIV
YLIYTEMDEAEQVKKIVQGVLAALDNTSLSITQFPVGLESRVKEVIMFIINQSSKACKIGIWGMGGSGKTTTAKSIYNQIR
RTYDYRSFIENIRQVCEQDSKGYIHFQEQLLLDVLKSEVKVHSTAAGTTLIEERLRGKSTLVVLDDVTKLEQVHALCGNRK
WIGSGSVFIVTSRDKRLLHLLEVDYIYKVEEMNESESLELFSWHAFREVTPTHDFIELSRNVVSYCGGLPLALEVLGSYLHK
RTKQEWRSVLSKLRIIPNDQVQKKLRISFDGLEDQMERDIFLDICCFFIGKERGYVTEILNACGLHADIGITVLIERSLVKVE
ENNKLGMHDLVRDMGREIIRHSSPKDPGKRSRLWFHEDVLDVLTNNTGTEAIEGLAFKSHGTGRDSFEANAFKEMKRL
KLLKLHSVQLTGSFGHLSKQLRWICWQGFPLKYIPVNFHLGSVVAINLKHSHLKLVWKESQLLEWLKILNLSHSKNLTITP
DFSKLPNLEKLILKDCPRLYKLHQSIGDLCKLLLLNLKDCTSLSNLPRRTYKLKSVKTLILSGCLKMDKLDEDIVQMESLTTLV
AENIAVKQIPFSIVRSKSIGYISLCGHKGLSRNVFPSIIWSWMSPKINPLSGIYPCWSMSSSLTSIDVQSNNLGDLASTLTSL
SKLRSVLLHCDTEFQLSKELRRILDDVYDVSSIKLEIRSNESQITNQSLRPYLIGIGRYHEVFNTLNKSISEGFTTNDCDVFLP
GDNYPLWLTHTGEGHSVLFRVPEDRNCLMTGMFLCVVYSSPLERVAKECLLSVLIVNYTKCTIQIYKRDTVISFNEEDWK
DIISQLGFGDHVEIVVTFGHELIVMKMAIYLLYTESTDMPINPSLEPKENALLIFLKRIMEFASSSSSSSSSFLKSEPHFIYDVF
INFGGEDIGRRFLSHLHYALLQAQVKIFISEENGQEGTKMEEHLRAIAASKIAIIVFSKTYAESTCCLLELEEIIECLKTFGQIV
LPVFYEIDPLDVRDQTNDFGKALGQTANKSYSGEELEHALSRWSRALAIASGITGWDLRNFRHDAEFVEVIVNRVQTLL
DYKDLVITQFPVGLEFHVEKVIECIENHSTKVCMIGIWEMVGSGKTTIAKAIYNRIYHLFIGKSFVENLRKVWEPVDRWHL
RLQEEFLYDVLKSKFRLQTTWMGRIMIEKELSRKKLLIVLDWFGKGTAIIITTRDVHLLNRLKLNYVYRMSDMKENESLGC
HGFSEAKPRKDLNKLVRNIVVYCGGLPLALIFGRLVLSVQRSSLTLFHYCFLFQHFITSSIPSMELASSTFKPQMCDVLINFT
GEDIRRKFISHLDSALSAVRLTTFLHEETAVNGMHIQQPILNMCRATIVVFTKTYSQSAWCLHELQQIIKWHETYSRHVL
PVYYEIQPSDVRLQKGDFGKSFKATAQQTFSGEQLEHGMLKWSHALTKAANFFGWDESNYRSDAELVDIIVKSVLILPV
LSATKFPVRLQSHVKDMIQVIKNKSTEVCIIGICGEEGSGKTTLAKAVYHQVHSTFTYKSFIEDIAQVSQTRGLVGLQEQLL
SDILKTKIKLHSIQMGISMIRERLSGKRMLIVLDGTNEYDPIGLWNSHVWFGKGTVMIVTTREEGLLRIPEVDSVLLIELLN
ANESLELLSWHAFREAKPREEYNDLAKRVVACCGGLPLTLEVIGSSLFERPKEEWKSILLELEKIPKHDVHQKLKISFQGLR
NEMEKNLFLDVCYFFVGKGRTYVTQILNGCGVDADSGIRNLIEGSLIQVKRNNKLGMQPLLQKMGKEIVLEILERTLQN
NPQLRFRLDENYVVSDNTLFSSQQTKVIQILPSKLFLTRTDLLEPCRAKVSDTSRIFELVEHSNSDEYLSKKLRWISLQGFPS
KYLPNDFYLHDSIAIDLKHSSLRFVWKQPQVLPWLKVLNLSHSKYLRKTPEFLISVKNKYFYTYKLP

>XP_014515525.1

MEFASSSSSSSSSFLKSEPHFLYDVFISFGEEEIGRKFVSHLHSALLQAQVKTFIDVENLSKEMKLEEHMRAIERTKITIIIFSK
RYTESACCLLELEKVIECHQSFGQIVLPVFYEIEAFDVRHHKNDFGKAMEETAHKSYSGELVEHALSRWSRALFTAAGIIG
WDVTDFWHDAQLVKKIVGRVQTLLDYKDLFITQYPVALLESRVEDVIKYIENQPSKVCIIGIWGMRGSGKKTIAKAIYNRI
YREFIGKSFIENITEFGVPDNKSYVGLQRHLHFDVLKSWFDVDSIGIGRTMIEKELSQKKLLIVLDDVNDFVQFEIVCGNCE
WLGQGSVIVITTRDVHVLNGIKVNYVYKMDGMNENDSLELLSWHAFGEGKPRKDLDDLARNIAAYCRGLPLALELLGS

FLYDKTMNERESVWSKLNRTPMYDIESILEICFELEAMEKDIFLEVCRFYIGKERGYVTDILNALGLHANIGITVLIERGLIKV
DSDNKLEMDPLLQHIGKEISRERSSRGIGSIMDSE

>XP_014515542.1

MLAKKEKSHREWCKVVGHVNWYLTRDETQVKDIVLTLNYDNLPSRLKPCFLYLGLFPKDFEIPVTPLLQKWVAEGFIQD
TGNRDPDDVAKDYFYELIDRSLVQVTKVKFIGDLRKCQVHDLLRDLCISETKEEKVFDVCTDNNILIPTKPRSLSIHGDMG
HYISSSNNDHSCMRSLFFFGPEYYVQRRGWKWLLDDFKLVRVLEFGHNRCLEIPSNLGNFIHLRYLRINSTLATFVPNSIL
GLWNLQTIDLGILEYDSPISFPIQMWKLKHLRHLNTSRPIKLRGRYSESNEKMWNLQTISPLILNTEATSLIKKGTFPNIKR
MGLYLETDGYEGELPNLFQNLQQLKHLNTLMIYNVLNLLTFEVIFPPSITELWLSRIKCITDEGMNGLKNHSKIKILRLLGV
EWSENSIDLNCVDGSFPQLEVLEMKHLSLRKFKLGNGAMQRLQNVIIQDCQYLYHLLVEFQSLNGLRKIEITETPLEQLD
YFLQILERYHGVQVVRGYYPKMSVIGF

>XP_014515580.1

MVSIVSDLAKSNLEKLINATIEQSRYICCFTCITDDFEKERENLIAKKETWEEYAIVATRKGDNIRKDVTHWQKQAKELIGE
DTKMKVTCFLGWCPNCKWQYSRGKELESKTKEIRRLVERNFENVGISRDVPDIEYHSSQNYISFESRKLKFEELFNALKD
DNNYMIGLQGMGGTGKTTLAKEVGKELKKSKCFNQVIDTTISNTPDMKKIQDDIAGPLGLSLKDFTESERPKKLKDRLT
NGENILLILDDVWGDINFEEIGIPFKDDHNNCRILVTTRNMKICQQMECDKTIELHILPEKEGWILFQKYAGLSDNSSKSIL
DKGRKISKECKGLPIAIAVIARSLKGPRPLEEWDVALKSLQKSMHIHDDEDESWRKVYTCLKYSYDNMKDETAKKLFLLCS
LFREDEEISEELLVRLAKGANLIEKIDEDYSYDECRKKVIVAKYKLTDSCLLLNCESGGVKMHDLVREMALLIANKEIVAVNT
SKKNEMTMVEKGKNIKYLLCEGKINDLFSSKFNGSKLDILIVYMNDDGIVEVPNSFFENIPGLQVLILSNNQNKKPILSLPQ
SIKLLTNIKSLYLKHFSLGNSSILGNLQGLETLELVACKMYELPREIGKLVNLKLLKLNYCRFEMSNVFKVIKSCSSLEELHLVT
TYDNKIFKDCHLELPNYQRFCIGEHDDWFRAKYPEWFLRNSLDVDNAMEFFSEETLKNLVQKAEILRLRGPIGVWKNLI
PDIIFPEDESTSNLMEISLEDNSELTCLIDNNDSLVQESALSRLVTLKLEGMKNLKEICNGPLPSELLKYLETISLDNCNHLQ
GALFKSKISLCNLKDLSICYCPMLTCLFELSTTQSLLSLEKLSIVSCEQLKSIIRDENKWKDSREEIIDTHNNNKSTISIFPNLTT
LLVQRCHLLHFVLPVTQKIPKLEDITIVNCDRLKYLFGRYQHEHEEEDLHQELKDVIFTSPKHVYLHILPNFVDIFKKCDESFI
SSKKSTSKDETKGKIESKSIKCKVLHWTHKYKNKWRTTKIPFDSKDQLRDSSLSMVNEPNANDVIAQDMRVRLENMSL
NDTSKILFTLQNVTELHIERCEKVEVLFCASMLECLPYLGALHIYRCNELKQIIGEDAKNQRKPFFPRLKAVIIRECNKLKCIF
PISTSEMVPMLEALLIMEASMLEEVFEGNSDEKVEIPNLKTAGFVELPSLRQKIEFLTVKHCLVRNCPKLYFSSFPQDLQHF
SYSITEYFKDMNLEYSIPEALRDLKSEEIRNEEDSAIGTEVEAASFRGSELTSSQNNANQSSLAKIDEDNKHEDIVKESKERV
EEEQQIVVGKASSMPISPTDFRLHTTHSPENEEEMAIPSSNLNTEPPITKDVNDGDFQGTSISVVTNSQNTNSTEATDQS
IKKILTEITDDLDEDSKTTDIRSQNEMTKAKEDFSIDAKAEVLSGSDFTSSQETKKTCEDGTTSPYEKTIALSTDSESEHGEA
QISVPFPVVNIELETTEDVGVGEIQEASPEKLDEITQDSSSGYTVRRELEELVSKKHLGSENLALLSNFLVKHPSLRLADDA
MSERYKGFAYTCLAELLKFLQSHSVYDMLGSSHSEFVELLKDLRNCGFNKDWLDDVERLKTVL

>XP_014515674.2

MXKIIAELEKPSVRMIGLHGLSGMGKTTLVKEVVKVALEAKKIDVVTMASVTRNPDIRKIQGQIADSLGIILDEESXVARA
ARIXKRLKXEKEKTLIILDDLWEKVDFNMLGIPYEIHNHTGLKXVKEGKSVHVDSLMNQKEGSPKEPHGASXELKTEKTRS
QYKGCKVLLISESKTVLLTQMGRKENCVFCLEALKEKETEMLFKKMAGMGDYNSKFQKLGAQIANKCNGLPMTIVTTA
RALKKQSPFVWEDILRKLEWQKLTGAPELSTKLSYDLLEDDELKYTFLLCARMGHDALIMDLVKYCIGLGFLQGIYTVRET
RDRVCTLVEKLKESGLLSDSYSRDQFTMQNIIRNAALSIANKEKHLFTVTKGKIDEWPEKLDEYAAISLHHCNFIEGFPRRI
KYPGLRVFHFNNNDPHLKVPTNFFKGMKGLKVLILTGIHLSLLESSVSSLIELRMLCLEQCTLEELSIIGMLKKLRILSFSRSDI
EKLPVELQKLKKLQIFDLSNCSKLKEIPSGVISNLVCLEELYMRNTLIHWEKEELTRQSKIGLLSELKHLSQLTTLDIQIPEVSH
LPKTLFFDKLYSYKIVIGDLSALLETDFKIPEMYETSRFLAIRLKDGFDIHSLKGVKMLFEGVQSLLLEELNTVHDLFYRLNLK
GFPYLKHLFIVNNFTIQSLIYPKDRQQSQHPEKAFPKLESLHLYNLKKIKNICSCKLSEPSFGKLKVIKINLCGQLKSVFSISM

VNLLTALETIEVSECYSLTEIVSVEESQSNTQESKLVFPKLLYLKLQSLSLCGKFYSITEEKLFNEKVDVSKLERMELSSIPITEI
WNYHHSNRSLFKNLTHLDVNGCWKLEHLISFSMAKCLGSLQSLFVSECGNMKGIFPDSMQMEGDIFPKLKNIKLSSMK
SLRKIWPSDLPSKSFSKLDTLIIEKCDKLVSVFPHYYEGIFHSLCNLTVTKCTQMQTIFDTYDKMRYVGVVSLQDLHLEILPN
LKHVWKLEKGKGGILELKNLQKIMVQNCYCLENIFPFSVAENLGNLEYLVVSYCVELREIVAKSAAVNTAKLLFKFPKLRSI
KFSVLPRLTSFYPGAFQLCCPTLNDLSIEYCDLLEPFQKETVDVEGKCVLFSEEVLNKLKSMQIESWHAKSQNSCMNEGN
HRRDSLEELCLSRLSDTEILYSFLHSNPNLESLSLSDCLFEEIVPLEKNTKIETLGVVPKLKNLKLINCKLKDLGFEEDIILRRVEL
LLLKRCPCMTTVVSSSTSLSSLVNLEVDNCDGLENLMSPSTAESLGQLNTMKVTKCKNLMEIVGKDAGNASKVVFKKLK
TLELVSLKKLQSFCNSESCEFEFPSLERLVVSACYNMKKFSDKVTRRSTPILQNVYAILEQEKKRYCWEGDLNATIHKMFL
DKKFFEGMNMMSFSDDGKLKQEMMSLSDHLELNRAWLGGEVDPQSIHSWFYSLKVLKLEKCEIQQCAIPSNILPYLKN
LNELMVQDCNNVEVVFEMNVTQGTGTTFQLQKLTLNRLSKLKNVWKGNGEETRSFQNLREVAVLECHMMQTLFPA
ALVKNLKKLHTLMIGRSWGLEIVGKEDNAALATQNLVFPCLTTLVLVDLPELNYLYPEPFTVECPVLKFLRVLDCPKLDLFP
RFLDLKTVVANLEQLVLEGKHSLVLESSLQDLEYCFEDLNSISLRFDVDENEKSNLPIQILQRVPNLHEMSISSCHCLDAFRT
EISEMNKKVMLTDLKRLDLNDVWELKSIGSGDVPWLDMICKKLHRLYVANCPRFMAVMHSPSAQFFSCLKVLSVFNC
QRLEYLFTSSAAEKLTQLERIHLYKCRSIKEIVAKEQGETTSGGFKLQQLRCVSLSYLSSLECFYSGNRTLQLPSLIEVRIIECPK
MEIFCKGSMGPNLCKEIRTSYTSNNGLVIVHDDLNSTVKKVFRQQNHIVFGDSRMLQDIWKSVTLPEGYFHNLTSMVV
EGCEFLSDAILPSHLLHLHQPSTSTTFDPNNVVSKDAYDSLLARFENLENLVRTLVPQQGHTAPSSSQQPPFQTTIVQDE
DSDDSDDRDDPNPDADVACESEASSNELKKELDLLGKKFGCSSSMTNNIISDGSELRYDNPVIGTIPKIVEASVDVLVRSP
VAVKRKG

>XP_014515708.1

MAAISCSYAFSHDVFLSFRGSDTRHGFVGNLYKALDDKGIHTFIDDEKLQREEEITPALMKAIEESRIAITVLSHNYASSSFC
LDELVHIIACAKKKGLLVLPVFYDLNPSDVRHQKGSYAEALTRHEERFKDKKESFSHNMERLEKWKMALLHVANLSGYH
FKQGYEYEYEFIRRIVELVSSKINRTPLHVADYPVGLEAQMVEVMKLLDVGIDDGVHMVGIHGIGGIGKTTLALAIYNLVF
DHFDGLCFLENVRENSDKHGLQHLQSILLAELVKEKRINIASVQEGISMIQHRLQRKKVFLIVDDVDKHEQLQAIVGRSD
WFGSGSRIIITTRDEQLLASHEIKRMYEVKELNKKDALQLLTWKAFRTDEADPSYEEVLKRVVAYASGLPLALEVIGSNLF
GKSIEEWKSAIKQYERIPNNQILKILKVSFDALEEEEKNVFLDIACCFKGYELEEVQDILHAHYGDCMKYHIGVLVDKSLLKF
SMHGMMVTMHDLVEDMGKEIVRKESPKDPGKRSRLWLHEDIIQVLEDNTGTSEIEIIHLDFALLDKEEMVEWNRKAF
KKMRNLKTLIIKSGNFSAGPKYLPNSLRVLEWWRYPSHDLPSDFHSKKLAMCKLPQSCFTSHELVSLLKKFMGMRFLNL
DKSKSLTQIPDVSGLPNLEKLSFQHCQNLTAIHNSIGFLCKLKILSAFGCTKLVSFPPIKLTALEKLNLSRCYSLENFPEILGK
MENIRELQLEYTAIKEFPNSFQNLTRLQELQLSNCGVVQLPSSIAVMPELTDLIGWKWKGWQWLKQEEDKEKEGSSIVS
SNVEYLWASECNLCDDFFSIGFMRFAHVKDLDLSKNNFTMLPECIKEFQFLRKLNVNDCKLLQEIRGIPPSLKHFLATNCK
SLTPSSTRMFLNQELHEAGKTQFYLPGERVPEWFDHQSNGPSISFWFRNRFPEKVLCLVIGPVNDSGMFRPMVVINGN
KNFLGSGYFMMGMDHTYIFYLLTVEFEDNLYGVPLDNEWNHAEVKYVGLEEISILKESGIHVFKQGGMEDIWFSDPYG
KRKLEDDLNSFESQNQQLLKKHRFVDMEAL

>XP_014515821.1

MESTSSSFLTSDPNFIYDVFINFGGEDVGRRFVSHLHSVLLQNQVKTLISQENLHEGMELEEQMRGIGGTKITIIVFSKSYA
ESACCLLELEKIIECHETFGQIVLPVFYEIDLFDILHQMFDFGQALEEAAHKSYSGEQVEHALCKWSRALTTAAGMTGWD
VRKFRHDAELVDVIVNRIKTLLDYKDLFITKYPVGLESRVEEVIKCIENESSKVCMIGIWGMGGSGKTTIAKAIYNRIYRQFI
GKSFIENIRYERYVTLQENLLSDVLKSKLEVNSVGMGRTMIQNGFSRKKLLIVLDDVSGFDQLENLYGSREWFGQGTVIII
TTRNVSLWNRLEVNYVYQTHLLNENESLELFSWHTFRVAKPKKEWSLLARNVVVYCRGLPLALQCLSSCLCDRTIEVWE
SVLLKLQRIPPNELLSVLKISFEDLRDTEKDIFLDVCCFFIGKDRDYVTEILNGCGLHADIGITLLIERGLIKVEKNNKLQMYSL
LQDMGREIIRQECPEKPGKRSRLWFQNDVEDVLKENTGTEAIKGLSLKHSRIRGCFEAHAFKEMKRLRLLQLDNVQLRG

DCGHISKQLRWICWRGFPYRYIPKDFHLENLIAIDFKHSLLYLWEQRVVLERLKFLNLSHSKYLKETPDFSRLPSLEQLILKD
CPRLLKVHSSIGDLSNIVLINLKDCRSLSYLPREMHKLRSLKTLIVSGCCSKLRPLEKI

>XP_014515822.1

MFDFGQALEEAAHKSYSGEQVEHALCKWSRALTTAAGMTGWDVRKFRHDAELVDVIVNRIKTLLDYKDLFITKYPVGL
ESRVEEVIKCIENESSKVCMIGIWGMGGSGKTTIAKAIYNRIYRQFIGKSFIENIRYERYVTLQENLLSDVLKSKLEVNSVG
MGRTMIQNGFSRKKLLIVLDDVSGFDQLENLYGSREWFGQGTVIIITTRNVSLWNRLEVNYVYQTHLLNENESLELFSW
HTFRVAKPKKEWSLLARNVVVYCRGLPLALQCLSSCLCDRTIEVWESVLLKLQRIPPNELLSVLKISFEDLRDTEKDIFLDVC
CFFIGKDRDYVTEILNGCGLHADIGITLLIERGLIKVEKNNKLQMYSLLQDMGREIIRQECPEKPGKRSRLWFQNDVEDVL
KENTGTEAIKGLSLKHSRIRGCFEAHAFKEMKRLRLLQLDNVQLRGDCGHISKQLRWICWRGFPYRYIPKDFHLENLIAID
FKHSLLYLWEQRVVLERLKFLNLSHSKYLKETPDFSRLPSLEQLILKDCPRLLKVHSSIGDLSNIVLINLKDCRSLSYLPREMH
KLRSLKTLIVSGCCSKLRPLEKI

>XP_014515875.1

MASASSSSSFSKFRIEGDVFIHCLGYDIRRNFVSHLSSALLQAGVKPCLLAVEMQQEQFVASIEGFQIGIVVLNKSYFESFQ
CVDELVRIIECHETHGLTVMPVFYEMDRSDFGNMLKATAREVMKVEHIIWSGEYQKTWFQRWNVALTKAGTLPTWE
ESEHRSDAELVEKIVKSVLAKLDCPLAITKFPIGLEPHVKNVIGFFENQPTKVCMIGIWGMGGSGKTTVAKAIYNQIPYAF
DDKSFIQGIREVCETDDRGLVHLQEKLPSDALETNVKIESGEMKKISFEDRLSGKKLFIVLDDVNEIDQLKDLLGNDKRFG
QGSVIIITTRNLELLYQLNVDYVYEMDTMDENDSVELFNWHAFGEAKPREDFIKLARSAVSYCGGLPLALEVLGSYLSKRS
VNEWRSVLSKLELIPNTQVQNILRTSFDGLCLMEKDIFLDVCCFFIGKDRDYVTEILKGCGLHADIGIKVLIERGLIKIEKNN
ELGMHPLLRDMGREIVRQTSTIQPGKRRRLWLHKDVLDVLTKNTGTEAIEGLSLNCQFTSSDFDFVKACAFEKMKRLRF
LQLDHVQVNGDYGYLSKQLRWIYWQGFPLEYIPINFYLKKAIVIDFQRSNLTLMWREPQVLRWLKILNLSYSMFLIETPD
FSKLRSLEKLIVKHCPRLRNVHQSIGDLRKLVLINLKGCTKLRNLPTETYKLKSLKTLILSGCLKIHIYEKDILCMGSLTTLISEN
TAVKQVPFSIVSSKSIGHILLGGNNGLSFTVFQSIISSWISPKINFLSGIRPFRGISSSLVSMSMEDNDLGDLAPILSSDLNIL
NVLVQCDVEFQRYQQVMAILDEIRGLNLTEFEIRPSTPETSKHPLRPYLIEFGSYQEEVFDILSKSIFEGSENSASCDVFLPG
NKIPYWLEHMGEGHSVTFTIPEGRRIKGMTLCAVNLYNPRHIATTEYLISILMVNYTKCTIQIYRRRTVQSFSDVDWQGII
TNLGPGDKVEIFVIFGDKFLVKKTAVYLMCDGLIDKEMNPSLDPKNID

>XP_014515932.1

MEFASSSLSSSSSSFLTSEPHFIHDVFINFGGEDIGRRFVSHLHSVLLQNQVKTFISQQNLHEGMELEEQMRGIGGTKITII
VFSKSYAESACCLLELEKIIECPQTFGQIVLPVFYEIDPLDVRHQNDDFRKALEETAGRXYALYKWNRALNTAASMTGWD
VRNFRHDAELVDVIVNRVNTLLDYKDLFITEYPVGLESRVEDVIKCIKNQTTKVCMIEICGREGSGKTTLAKAIYNRIYREFI
GKSFIQNIRYEGYVALQENLLSDVLISKPGVKSVGMGRTMVENGFSREKLLIVLDDVNDFDQLRNLCGSREWFGQGTVII
VTSRDFHLLNQFRDNYVYKMDILNENESLKLFSWHAFRDAIPEKEWYELARNVVVYCGGLPLALEFLGSYLCDRTIEVW
ESVLLKLQRIPPNELLSVLKISFEDLRDTEKDIFLDVCCFFIGKEREYVTEILNGCGLHADIGITLLIERGLIKVESNNKLQMHP
LLQEMGREIIRQECPEKPGKRSRLWFPDDVEDVLNKNTGTKAILSLKLHSSIGDWFEAHAFKEMKRLRLLQLDNVKLSG
NYGHISKQLRWICWRGFPYRYIPNNFNLENVIAMDLKHSHLQLIWKQPLWKKPQVLERLKFLNLSHSKYLKETPDFRGL
PSLEQLILKDCPSLLKVHQSIADLSNIVVINLKDCTSLSYLPIEIYKLRSLKTLILSGCSKLDSLENPLKL

>XP_014515979.1

MEFASSSFLTSEPHFIHDVFINFGGEDIGRRFVSHLHYALLQAQVKTFISQENLHDEGMKLEEHMRAIGHTKISIIVFSKSY
TESVCCFLELEKIIECHETFGQIVLPVFYEIDPSVVRHQKNDFVKALEEITHNSYLGKQLQHALSKSSLPLTTAAGIAVWDV
RIFRHDAALVERIVSHVKTLLDYKDLSITEFLVGLESRVEDVIKLIENQSTNVCVIGIWGMEGSGKTTIAKATYNRIYREFIG
KSFIENTHYEEYLDLEAKLISDVLKSELEGQSGGMGTTMIQNRLYRKKLFIVLDDVNHIGQLENLCVSRERFGQGTVIIITT

RDFQLLNQLRVNYVYEMDLLNGNESLELFSWHAFGEAIPKKEWSELARSVVVYCRGLPLALQFLGSYLFDSTIQVWKSV
LSILQRIPPDNLLNVLKICFDDSHNTEKDILLDVCCFFKGKEKDYVTEILNGCGLYADFGVTALIERGLIKVERNNKLEIHPLL
QDMGREIIRQVCPEEPGKRSRLWLQDDVKDVLKENTGTEAIQGLSLXLHSTSRDCFKTHAFKEMKKLRLLRLDNVHLVG
DYGHISKELXWICWKGFPSKYIPXNFYMGNVIAIDLRHSHLQRVWKQPLWKQSQVLERLKFLNLSHSKYLKETPDFSRLP
SLEQLILKDCPSLLKLHPSIGDLSNILLINLKDCTSLSYLPREISKLRSLKTLILSGCSKLGPIDIAQVKSLVTIIA

>XP_014516055.2

MGSSIPSQEFASSSSKLPWMYDVLINFTGDKIRNKFVSHLDSALSSVGFTTFLHHHNAVKPMHIQQQQPILDLCRVAIVV
FTKTYSQSAWCLYELQQIIEWHEFYCRHVLPVYYEISPSDVRLQKGDFGKAFEATACQTFSRQQLEHGISRWSHALTKAA
NFFGWDESNYRSDAELVDKIVKTVLNLSVLSATKFPVGLQPKVQDLXRTIKXKSTEVCTIGICGMEGSGKTTLAKAIYHQI
HGTFTEKSFIEVTLEGKGIRSCVSLQEQLLSDVLKTKVKIPGDEMGRCMIRERLNGKRVLIVLDDVNQYGPLDLWETSSCF
GEGTVIIITTRDEYLLRRHQADSVFRINLMNENESLELLSWHAFREAKPKEEYDYLARRVIFHCRGLPLTLEVIGAYLFETSR
KKWEVVWYKLGKIPYDNVQLILKISIDGLRNEMEKALFLDVCRFFVGKSKAYVTKILDGCKVDVDNGIRVLIERNLIKLKR
NNKFGMHHLLPGMAMQIVAQEHPSYAREETDVFWRFPDKLFSTGKDCFKPYPLEIRHKSRVLKLAGNHGYLRNSSEFV
AYDFNLYDDAVSIDLRHSLLGHDWKKLQVLAWLKVLNLSHSKYLTKTPDFSRLPSLEQLILKDCPRLRQLHQSIGCLCNLT
LLNLKDCICLSNLPRKIYMLKSLKTLILSGCSKIYLMEKDIVQMESLISLISENTAIKQVPFSIVSSRNIGYISLHRFEGLSRNLF
HFIIRSRMSLTINPLSYIHSFMDTEDNSWDVIAPFFSSLVNLRSVLVHCDPEFQLSQQVKTILVEYGVNITESRISKHCFRSS
LVGVGRYNEFLSTVLESSESGDVSLPSDNDPYWLAHIGEGHSVSFTVPQDRVMKGIVLYVVYLSTPGIVTTECFTSVLIVN
YTKSTLLMHNHGTVISFNDEDCHDIMSNLGSGDKVEIFVTFGHRLMVKNTAVYLICCESQDLEREPALNKNSLITFFKKIV
M

>XP_014516167.2

MRLSIPSMELASSVSKLPRMYDVLINFTGEDIRRKFVSHLDYVLSSVGLTTFLQHDNAVQPKHIQEPILNLCRVAIVVFTET
YSRSACCLHQLQQIIEWHETYGRHVLPVYYEIQPSDVRLQKGDFGKAFRETAHQTFSAQELEHGMSRWSHAITKAANF
FGWDDCNYRSDAELVDTIVRSVLNLPVLSATQLLSLAEEVIQIIKNKSTGLCRIGICGMGGSGKTTLAKAIYSQIHGTFVEK
SFVKDISEVSRRRGPVHLQRQLLSDVLKTKVEIHNVEMGRSMILERLYRKRVLIVLDDVNEHCPLDLWESRGWFGEGSVI
IITTRDEDLLRKHEVRSVFRVDLMNENKSLELLS

>XP_014516243.1

MDPIVSATTESALNITTSVVKRQVGYFFNYKDKFKELKSYIEKLEHNKERLQHQVDSALRSGGEIEKDVQRCLTLMDDKIK
EYKSYINDDCHAKTICSIGFFPNNFRLRYQLGRKATKMVEEIIGDELWKTFFDNVSYQEFPSIDAAFSNNGYESFASRTKT
MKMIMKALQDSTVGMIGVYGPGGVGKTTLVKEIANKAREKNLFEIVIIANITGNPDXKKIQEQIAGMLGMKLEXESEIA
RVDRIRKRLKNEKENTLIILDDLWGGLDFNKLGIPCNDDASQQEVNDISDFGYNKIEIKELSKVDLDKMKKEKLSNDYRRG
KILLTSRNKQVLCNEMDVQQRSIFSVGVLDEKESETLLKKVAGVKNSEFDRNATEIAKWSAGFPIALVSIGRTLKNKSLST
WEDVCQQIKRQNFTSEWGFTDFSIKLSYDHLKNEELKCIFLHCARMGNDALIMDLVKFCVGLDLLPEVYTITDARKRVKE
IIQELEESSLLVKSYSNDRFNMHDIVRDVALSISSKEKQVLYKKNAILYEWPHENDFERYSAIFVHFCDINDKLPESIHCPRL
EVLHIDNKNESFEIPDEFFKSMVRLRVLVLTGIHFSCLPSSIKCLKKLRMLCLERCTLGENLSIIGELKNLRILSFSGSNIENLPL
EFGQLDKLQFLDISNCLKLRQITSNIIPRMGILEEFYIRDNLIIWEAEENMKSENASLSELRHLNQLQNLDIHIHCSSYFPQN
LFFDRLNSYKIVIGEFNLSNLLKVGEFKVPDKYEEVKFLALNLKEGIDIHSEKWIKMLFKSVECLLLGELNDVQDIFYELNVE
GFPNLKHLSIVNNFDINYIINPKERFHSLVAFPKLESIWLYKLDNLEIICNNQLVETSFRNLKVIKIQTCLKLVNLFLFSMVRLL
TLLETIEVCDCDSLKEIVSKESQTNTISDDKIQFPQLRLLTLKYLPTFIYLYNVVDKIPGSSHSLQDQVFQQRNKDIVVDVEH
MVTNSCLPLFNEKVSTPKLEWLELSSINIHKIWSDQCNHCFQNLLTLNVTDCSNLKYLLSFSMAESLVNLQSIFVSECAIM
EDIFRPEDAEYIDVFPKLKKMEIICMDKLCTIWKSDIGLHSFSCLNTLMIRECHKLVTIFPNYMGQRLQSLQSLTVTDCKLV
ENIFDFANIPHTYDIIETNLGKIILEDLPNLVNVWKGYTGEILKYNNLQSIRVYESPKLKYLFPVSIANDLEKQEVLEVRNCG

AMTEIIALDKHSSETFITFKFPHLNTLSLIDLHDLRSFYSGIHTLEWPPLKKLDIINCSMLEGLTSEITNSKEQPIVLATKKAIY
NLEYMSVSLKEAEWLQKYIVNVHRMHKLEELTLYGLKNNEILFWFLHRLPNLKRLTLELCHLKRIWALKSLISREKIGGVV
QLKELKLESMWSLEEIGFEHEVLLQRVERISIHHCTKLKNLVSSSVTFSYLTYLEVMNCKSMRSLMTCSAAKTLVQLTTLK
VCSCPMIVEIVADNKVEKVREIEFKQLKSLELVSLQNLTSFSKVEKCDLKFPLLEKLVVSECPQMTKLSEVQSAPNLQKVH
VEAGEKDKWYWEGDLNATLQTHFTNQVSFEYSKYINLVDYPEKKVPHDKFAFPDNFFGYLKKLEFDEACKRDTLIPSHV
LPYLKNIEELNVEKCKSAQLIFDIDESEIQTYGMVFRLKNLTIKNLSNLKCVWKENLEGIVSFSNLQKVDVDGCGSLLTLFPL
SVAKDLGKLKTVDIKECEKMIEIVGREDEREHGTTIMFEFPCLSYLNLDNMPLLSCFYPGKHHLECPLLEKLYVACCPKLKL
FRSSFDDDSKKEVLEAPTNLLQQPLFSIQKVSPKPVGLTLNEENIKLMSDARLPQDLLCKLKFLILSFEDDNNGTDSLPFDF
FHKLPNLESLTVQKCSGLKEIFPSQKLQVHDSILVGLKGLFLFELSELESIGLEHTWVQPYTEKLELLYLLLTPQVENIVSCAV
SFINLKDLFVMFCEKMEYLFTSTTLKSLVKLETLLIGYCGSIKEIARNEEEDGCDEIIFGRLRSIKLEYLPRLISFYSGNATLQCP
CLQNVMITECPNMITFSEGVIKLAMFSGIQTFEDSDFTFHDDLNTTVESLFHEKEFFNHSKHMILDEYLEITGAQHIKPDI
ADNFFGSFKELEFNAACKRAIVIPFHLLPYLKNLEKLNVHSSDAVNVIFDFDESEDKTKGIVSSLKELTLKNLSNLKCVWKE
NLEGIVSFPNLEKVTVTGCRSLVTLLSSSLAKSLEKLKTLHMARCEKLEEIVGEEDEREHGMTLTFEFPCLTILFLFDMPLLSC
FYPGKHYLECPILDTLYVSYCPKLKLFTSDTDDSHKEEVIEAPISPLQQPLFLVEKVSPKLKKLALNEKNIMLLRDGCLLHDLL
CKLSHLWFLFEDYKIENDTLPFDFFHKLPGLEYLLLQNCFGLKVIFPSQKLQVHNKVLAGLKQLSLLRLRELESIGLENEWV
QSYSRKLEVLKLDTCPQVENIVSCSVSFINLTKLSVKLCEKMEYLFTFATLKSLVKLQTLSIKNCESIKEIVKKEDEDACDEIVF
EQLRSIKLNSLPNLLSFYSGNATLKCLCLQSVMVAKCPNMITFSEGVINMPILSGIKTSKDSVVVFHDNLNTTIETLLHEQE
FMEYSKRMILEDYLGMSGVHHRKPVVSDNFFGSFKKLEFDGACNRTILIPSHVLPYLKNLEELNVKNSDAMQIIFDIDESK
VKTKGVVFGLKKLTLNKLSNLKHVWKENSTGIISFHNLQEVVVNGCGGLIRLFSSSLARNLWKLEELRIKECGKLVEIVEKE
DGTENGTKIMFEFPCLTSLYLKNMPLLSCFYPGKHDLDCPLLEILLVCFCPKLKLFTSDFDENQKGAIEAQISPLQQPMFSV
EKFSPKIKVLALNEENIMLFREVQFLQDILCNIVGLVLCFEDDNIEKDSLPFDFFHTLPNVFILAIKKCFGLKEIFPSQKLQVH
DGGLAGLKELFLVDLKELELVGLEHPWVQPYSEKLQVLSLKRCPQLQKLVYCAVSFINLKKLRVKLCERMEYLFTFATVKSL
VKLETLIINSCESIKEIIKHENEDGCAEMVFGRLKSIKLKSLPRLVRFYSGKATLQCSYLKIVMVVKCPSMKTFSEGVMKVPK
FSGIQTSKDSDLIFHEDLNTTIKKLFHEEVEKSACDLEHLKFGDHPNLEVIWLGVVPIPRNDSFNNLQSLAVVECESLSNVI
PFYLLRFLSNLKEIEVSNCQSVKAIFDVKGEGADMKPISLSLKKLILNQLPNLEHIWNLNPDEILSLEDLQQVSISNCQTLKS
LFPTSVANHLIKLHVRACATLVEIFVEAETAFEGETKQFNFHCLTSITLWELPELKYLYPGKHTLEWPMLAHLDIYHCDKLK
LFKIEHHSDEFSDTKDQLGISIHQQPAFSVKKVFPKLVQLSLKKEDAMAILQGQLQVMPSIEHQAITWKDTRIGQGQFG
ANVAYLLQNLKLLKLMCYHEDDKSNIFSSGLLEEIPNIENLEVVCSSFTEIFCSQGPTHDCSKVLSKLKRLHLKNLPQLNAIG
LENSWVEPLLKTLETLEVFSCPAMKILVPSTVSFSNLTSLSVDECHGLLYLFTSSTAKRLGQLKHISIRDCQAIQEIVCKEED
HESEDEDITFDQLSLLSLQSLPNIVVIYSRTFKLKFPCLDQVIIKECPQMKYSYVPDLHEFKPQEQA

>XP_014516245.1

MDPIVSATTESALNITTSVVKRQVGYFFNYKDKFKELKSYIEKLEHNKERLQHQVDSALRSGGEIEKDVQRCLTLMDDKIK
EYKSYINDDCHAKTICSIGFFPNNFRLRYQLGRKATKMVEEIIGDELWKTFFDNVSYQEFPSIDAAFSNNGYESFASRTKT
MKMIMKALQDSTVGMIGVYGPGGVGKTTLVKEIANKAREKNLFEIVIIANITGNPDXKKIQEQIAGMLGMKLEXESEIA
RVDRIRKRLKNEKENTLIILDDLWGGLDFNKLGIPCNDDASQQEVNDISDFGYNKIEIKELSKVDLDKMKKEKLSNDYRRG
KILLTSRNKQVLCNEMDVQQRSIFSVGVLDEKESETLLKKVAGVKNSEFDRNATEIAKWSAGFPIALVSIGRTLKNKSLST
WEDVCQQIKRQNFTSEWGFTDFSIKLSYDHLKNEELKCIFLHCARMGNDALIMDLVKFCVGLDLLPEVYTITDARKRVKE
IIQELEESSLLVKSYSNDRFNMHDIVRDVALSISSKEKQVLYKKNAILYEWPHENDFERYSAIFVHFCDINDKLPESIHCPRL
EVLHIDNKNESFEIPDEFFKSMVRLRVLVLTGIHFSCLPSSIKCLKKLRMLCLERCTLGENLSIIGELKNLRILSFSGSNIENLPL
EFGQLDKLQFLDISNCLKLRQITSNIIPRMGILEEFYIRDNLIIWEAEENMKSENASLSELRHLNQLQNLDIHIHCSSYFPQN
LFFDRLNSYKIVIGEFNLSNLLKVGEFKVPDKYEEVKFLALNLKEGIDIHSEKWIKMLFKSVECLLLGELNDVQDIFYELNVE
GFPNLKHLSIVNNFDINYIINPKERFHSLVAFPKLESIWLYKLDNLEIICNNQLVETSFRNLKVIKIQTCLKLVNLFLFSMVRLL
TLLETIEVCDCDSLKEIVSKESQTNTISDDKIQFPQLRLLTLKYLPTFIYLYNVVDKIPGSSHSLQDQVFQQRNKDIVVDVEH

MVTNSCLPLFNEKVSTPKLEWLELSSINIHKIWSDQCNHCFQNLLTLNVTDCSNLKYLLSFSMAESLVNLQSIFVSECAIM
EDIFRPEDAEYIDVFPKLKKMEIICMDKLCTIWKSDIGLHSFSCLNTLMIRECHKLVTIFPNYMGQRLQSLQSLTVTDCKLV
ENIFDFANIPHTYDIIETNLGKIILEDLPNLVNVWKGYTGEILKYNNLQSIRVYESPKLKYLFPVSIANDLEKQEVLEVRNCG
AMTEIIALDKHSSETFITFKFPHLNTLSLIDLHDLRSFYSGIHTLEWPPLKKLDIINCSMLEGLTSEITNSKEQPIVLATKKAIY
NLEYMSVSLKEAEWLQKYIVNVHRMHKLEELTLYGLKNNEILFWFLHRLPNLKRLTLELCHLKRIWALKSLISREKIGGVV
QLKELKLESMWSLEEIGFEHEVLLQRVERISIHHCTKLKNLVSSSVTFSYLTYLEVMNCKSMRSLMTCSAAKTLVQLTTLK
VCSCPMIVEIVADNKVEKVREIEFKQLKSLELVSLQNLTSFSKVEKCDLKFPLLEKLVVSECPQMTKLSEVQSAPNLQKVH
VEAGEKDKWYWEGDLNATLQTHFTNQVSFEYSKYINLVDYPEKKVPHDKFAFPDNFFGYLKKLEFDEACKRDTLIPSHV
LPYLKNIEELNVEKCKSAQLIFDIDESEIQTYGMVFRLKNLTIKNLSNLKCVWKENLEGIVSFSNLQKVDVDGCGSLLTLFPL
SVAKDLGKLKTVDIKECEKMIEIVGREDEREHGTTIMFEFPCLSYLNLDNMPLLSCFYPGKHHLECPLLEKLYVACCPKLKL
FRSSFDDDSKKEVLEAPTNLLQQPLFSIQKVSPKPVGLTLNEENIKLMSDARLPQDLLCKLKFLILSFEDDNNGTDSLPFDF
FHKLPNLESLTVQKCSGLKEIFPSQKLQVHDSILVGLKGLFLFELSELESIGLEHTWVQPYTEKLELLYLLLTPQVENIVSCAV
SFINLKDLFVMFCEKMEYLFTSTTLKSLVKLETLLIGYCGSIKEIARNEEEDGCDEIIFGRLRSIKLEYLPRLISFYSGNATLQCP
CLQNVMITECPNMITFSEGVIKLAMFSGIQTFEDSDFTFHDDLNTTVESLFHEKEFFNHSKHMILDEYLEITGAQHIKPDI
ADNFFGSFKELEFNAACKRAIVIPFHLLPYLKNLEKLNVHSSDAVNVIFDFDESEDKTKGIVSSLKELTLKNLSNLKCVWKE
NLEGIVSFPNLEKVTVTGCRSLVTLLSSSLAKSLEKLKTLHMARCEKLEEIVGEEDEREHGMTLTFEFPCLTILFLFDMPLLSC
FYPGKHYLECPILDTLYVSYCPKLKLFTSDTDDSHKEEVIEAPISPLQQPLFLVEKVSPKLKKLALNEKNIMLLRDGCLLHDLL
CKLSHLWFLFEDYKIENDTLPFDFFHKLPGLEYLLLQNCFGLKVIFPSQKLQVHNKVLAGLKQLSLLRLRELESIGLENEWV
QSYSRKLEVLKLDTCPQVENIVSCSVSFINLTKLSVKLCEKMEYLFTFATLKSLVKLQTLSIKNCESIKEIVKKEDEDACDEIVF
EQLRSIKLNSLPNLLSFYSGNATLKCLCLQSVMVAKCPNMITFSEGVINMPILSGIKTSKDSVVVFHDNLNTTIETLLHEQE
FMEYSKRMILEDYLGMSGVHHRKPVVSDNFFGSFKKLEFDGACNRTILIPSHVLPYLKNLEELNVKNSDAMQIIFDIDESK
VKTKGVVFGLKKLTLNKLSNLKHVWKENSTGIISFHNLQEVVVNGCGGLIRLFSSSLARNLWKLEELRIKECGKLVEIVEKE
DGTENGTKIMFEFPCLTSLYLKNMPLLSCFYPGKHDLDCPLLEILLVCFCPKLKLFTSDFDENQKGAIEAQISPLQQPMFSV
EKFSPKIKVLALNEENIMLFREVQFLQDILCNIVGLVLCFEDDNIEKDSLPFDFFHTLPNVFILAIKKCFGLKEIFPSQKLQVH
DGGLAGLKELFLVDLKELELVGLEHPWVQPYSEKLQVLSLKRCPQLQKLVYCAVSFINLKKLRVKLCERMEYLFTFATVKSL
VKLETLIINSCESIKEIIKHENEDGCAEMVFGRLKSIKLKSLPRLVRFYSGKATLQCSYLKIVMVVKCPSMKTFSEGVMKVPK
FSGIQTSKDSDLIFHEDLNTTIKKLFHEEVEKSACDLEHLKFGDHPNLEVIWLGVVPIPRNDSFNNLQSLAVVECESLSNVI
PFYLLRFLSNLKEIEVSNCQSVKAIFDVKGEGADMKPISLSLKKLILNQLPNLEHIWNLNPDEILSLEDLQQVSISNCQTLKS
LFPTSVANHLIKLHVRACATLVEIFVEAETAFEGETKQFNFHCLTSITLWELPELKYLYPGKHTLEWPMLAHLDIYHCDKLK
LFKIEHHSDEFSDTKDQLGISIHQQPAFSVKKVFPKLVQLSLKKEDAMAILQGQLQVMPSIEHQAITWKDTRIGQGQFG
ANVAYLLQNLKLLKLMCYHEDDKSNIFSSGLLEEIPNIENLEVVCSSFTEIFCSQGPTHDCSKVLSKLKRLHLKNLPQLNAIG
LENSWVEPLLKTLETLEVFSCPAMKILVPSTVSFSNLTSLSVDECHGLLYLFTSSTAKRLGQLKHISIRDCQAIQEIVCKEED
HESEDEDITFDQLSLLSLQSLPNIVVIYSRTFKLKFPCLDQVIIKECPQMKYSYVPDLHEFKPQEQA

>XP_014516317.1

MESLEMSSTFGEKHDVFLSFRGADTRTNFTSHLLNALTQKSISAFIDYELIRGDYTWPALETAIEKSLLSIVVLSENYASST
WCLKELAHILECRRKRGMVVIPVFYEVDPSHVRKLSGSFEKSFAKHERDSTSFDRISQRKDVSMWKAALKEVANISGWD
SRSYRDEAQVIQNLVNDVLQKLHLRYPTELNGLVRIKNTCAKVDLLLRKSRVIGIWGMGGIGKSTIAKALFAKYFPYFDHV
CFMANANEFSLDKLFSELFREEVSASNVVGSSFDMRRLKRKRIFIVLDDMDCLDLLEYLCREYQSLDPNSKLIITTRDKQLL
EGRVDQIYEVKKWETRASLKLFCLEAFKKRHPKRGYESLSESAVEYAGGVPLALKVLGSYLRSKGINFWESTIRKLSMYPN
ERIQKVLEVTYTGLHDLEKNIFLDIVFFFKEKQKDHVITILDACGFEATSGIEILADKALLTISYRKIIHMHDLLQQMGLEIVR
QESSGDPGRRSRLKDKEAREVIEENKGTSAIQGIALDLSQIKGLILHADTFTKMKTLRFLKFYNKLGQSARDTYLDLPATLE
PFSDKLRYIEWIGYPFESLPSPFCAKVLVEIHMPHSKVKQLWQGIQELNYLEGINLRQCKQFEELPDLSKAPRLKWVNLSC
CESLCYLHPSVLSSGTLVTLILDKCTNLKSVKSEKDLKSLEKISVNGCLNLVEFAVSSDLIENLDLSNTGIQMLGTSIGSMHKL

KSLNLEGLKLKPVLKELSCLTSLKVLKISDNGLVIDKQQIHVLFSGLRYLQILYLKDCSKLFELPDNINLLTQLQELRLDRSDLK
RLPENIKNLQMLEILSLEDCKELLCLPTFPSLIKYLRVINCTSLVSVSNLKTLAIEMLGMTKRITFKNSGKLDEDSLRIIMESLH
LTMMSAAYHNVLVRKTYGTYNSCNYTSVELCLPGGSVPEQIHYRSTQSSITIVLPPHSELLGFIYSVVLSPAGGKKTFGTKI
FCKCHLPEEGIKATWLYGDIRGLKSDHVYVWYDPLHCDSILKYYKQSKVCFEFCVANDKGEVDGSICIKECGVGLVNVLE
MHSVLQELDFDSDKKKELVEGVESESKHRKLDWSESSSSDSLTTPRSSSESDSEIVKPVSAENGKSTHKETKTDSGTSVVK
YKNNKEVPKSDATLHETMESHSDNQNQFREKSVTQSEIVEMIELENTESTCKETLSTAERGPKEKLRESTKIVADEHLQST
SLQASSQGGSEHLHDRLEESKKQVVETYDTEKFVTKYSYFDLESCLQQLDENPFAILDLLSNELSPLKQSETCVQRVAQAN
DATTVLNEFRTLVFSTSLLEKLPDQSYRQQIEESLRKLHTYRREITEEQEGVDKFIELYEKAANISQEKMLTEDKQTKLASKK
RDLYNKLQDSKLKVQQFDTAISTDKSEIENLQKRQREIQEAINKLQQENEALEKERSALEVLYSGKQTKKNETLELVKHIST
SVVYTTKQLEELEEKRLSLASAYEDLKEPYGTMKTKPPF

>XP_014516318.1

MSSSTKKHDVFVSFRGEDTRTNFTSHLCKALEYKSIGAYIDRQLDRGETVWPALAKAIQDSRVSIVVFSENYACSKWCLE
ELVKILECRKKMGLAVIPIFYNIDPSDIRNQKGTYEKALTELLESNEEKGPKWKAALTEAANISGWDSRTHRDEAHVIENV
VNDVLQKLHLRCPTELKGLVASEENCRNIXLLLKSCRVIGIWGMGGIGKSTIAKILFAKHFPQYDHVCFVTNAKEYSLDKL
FSTILKEEVSEKNVVGSSFHMRRLRSKKVFIVLDDVDKDSFEPLEYLCXXYXXQXSDSKLVITTRDRQLLVXRVDAIYEVQK
WKKTESLKLFCSEAFKKNCPERGYESLSESAVEYSGGVPLAVKVLGSYLRSKGXSFWXXTIRKLXXYPNEXIQXVLEMSYT
GLHDLEKXXFLDIVFFFREKQXXXXTRILDACGFEATSGIEVLADKALLTISYTNIIQMHDLLHQMGLEIVRLECTTDPGRRS
RLKDSDAHEVIEENKGTDAIQGIELDLSQVENLRLRSDTLTKMKTLRFLRFYNSSGQSSRNTYIDLPATLEPFFDKLRYIEWI
GYPFECLPSPFCAKFLVEIHMEHGKIKQLWQGIQELDNLEGINLSGCKHLEEFPDLSKAPRLKWVNLYCCESLRYLHPSVL
SSATLVTLILHGCTKLESVKGEKRLKSLGYINVNGCLSLEEFAVSIVSIEIFDLSNKEIPMSGTPVRRKGKLKTNKILKELMLSN
GRRLGKDLNNCNLPSMQHLYMLQLDRCNVTNLPEIIKXSGKLTFLSLENCDGFTHLPKLSSGINYLRVINCTSLVSVSDLD
NLANVMYGSSRFITFKNCXKLDEHSCKLIMKSVKLIMVCAAFDNLVRKSSDVHDYSYNSVELCLPGSKVPQEIKYRSTESS
ITIDLPKLSNLRGFIYSVVLSPSGEMKKHDTKIICKRHLRENTRESWVYSDIQGLNTDHVYXWYDPFHCDGILKYNEPSVC
FEFCVTNDKGEVDDSKCIKECGVGLISVSELPSVLEELDWHWDKKKDLVNRVKLVTGQRITLITSIEQSDERKNHFSAVEE
IINSTHKEVKTDSGTDCGQNTTKSTNAVKYKESGETAIKQDATLPETVESELDKENESKEKSMMVELESVEDNRGSKEYF
SGVKENIEYCASRATNATAERGPKEKSTKSTETVAYEHSQRLEESIKQVVEIHDTGNSVIKYSSLDLENCLQQSDKNPFAIL
DLLSNELSPSLKQSSDATTLLNEFRTLVFSTSLLKKIPDQSYQQQVTESLQKLHTYRGKITKEQEAGLDTFIELYNKAVDISQ
DKMLTEDNQAKLASEKRDLYNKLEHSKPKVQQFDTTISTYKSQREILQKRQREIQEAIKELEQENEALEKDSSTLEVLYSE
QQTKKKETLESVKCISISVVQTTKQLEELEKKRLSLASAYEGLEEPYQRMKTKPPF

>XP_014516350.2

MQSVVNPMFFQVSHKIKYVAFICLVFVTSSVTSNDTSQIKYDVFVSFRGEDVRRGFLSHLIEAFSQKQIAFFVDDSILKGE
ELSEALFGAIEESYISLVIFSENYASSRWCLSELEKIMECRRKNGQTVVPIFYKVDPSDIRHQRRTYGDAFIKHERNYSLATV
QTWRSALSESANLSGFDLSKFPDEAELVKRIVKFVWSTLNHVHQVNSKGLVGIGKRIAQVESLLQLETKDVRMIGIWGM
GGIGKTTIAQEVYNKLCFKYDSCCFLANIREESWRHGINSLKEKLFSTLLREEHLKIGRPDGFQKLVERRFHRMKVLIILDD
VGDAEQLENLARTNWFGCGSRIIVTTRDKQVLAEESASVYHVEALNFDESLRLFNLNAFKQKHTEAEYEELSKKAVEYAK
GIPFVLKVLGHRLHGKDREIWESELERQEVHNKRVHDIIKSSYYDLEEDEKRIFLDIACFFYGQQLQVKYIDFLLKDRDYSV
PAGLERLKDKAFISISQENTVSMHDIIQETAWQIAGQESIENPRRSQIRLFVPDDIYEVLTYNKGNEAIRSIVVNLLRIKQLH
LKPQVFTKMSKLHFLNIYTAGTRDIRFYEPWGLYLHQGLQSLPNELRYLGWMHYPLESLPSNFSAENLVELHLPYSRVKK
LWHEVPDLVNLKVLMLYSSSNIKELPDFSRAPNLEVIDLRSCVGLTSVHPSVFSLKKLEKLDLDECRSLTSLRSNVQMESLR
YLSLFKCMELKDFSVTSKNMIMLNLENTDIKQLPSSFGSQSKLEDLNLAFSSIESLPADMKDLKGLQHLDLRHCRNLSSLP
ELPPSIETLDCRECVSLGSVTFPSIAEQWKENKKKVVFWNCLNLDERSLKAIETNARINMVKFAHRHLSTSGDAHAIYVYP
GSQVPEWLTHKTTHDDDDDDDGDDDEDSITFAPNPSHLGYIFCLILPAVQYSERVLKLTVSTEGEDEGDSMIVYLDRPH

HTIKFDHVYLMYNEACSRFLTSRAKQQPMLKIKVTVATLTLSSKYTEVQLRGFGVSTISNFLQKRQLYDTPIPMEHSEGFPDALWNR

>XP_014516380.1

MEFASSSLSSSSSSFLKSEPHFIHDVFINFGGEDIGRRFVSHLHSVLLQNQVKTFISQENLHEGMKKEEHMRAIRRTKITIIVFSKSYTESACSILELEKIIECHETFGQIVLPVFYEIDPLDVRHQKEDFGKALEETAHRSYLGEQLQHARSRWSSALNRVAGMTGWDVRNFRHDAELVKQIVSRVQMLLDYKELFITQYTVGLESHVEDVIGCIENNSTKLCMIGIWGMGGSGKTTLAKVIYNRIYREFIGKSFIENISEVYDPENERYVDLQENLLSDVLKSKYEVESVRMGRTMIENVFSRKKLLIVLDDVTAFGQLENLCGSREWFGQGTVIIITTRNYELLDRLKVNYVHLIGLMNEKDSLELFRCHAFREGKPRKYFNELARNVADYSRGHPLALNVLGSFLCDRTMKEWKSVLSKLKRTARSMDNHVLDVLKISFEGLRDTEKDIFLDVCCFFIGKERSYVTDILNGCGLDADIGITILIDCGLIKIGKNNTLEMRPLFRDMGREIIQERCSEEPGKMSRLWFQDDVKDVLKKNTGTEAIQGLSLKLHSTSRDFFKANAFKEMKRLRLLQLYHVQLVGDYGCLSKELRWICWKGFSSKYIPNNFHMKNVISIDLRHSHLQLVWKQPLWKKPQVLERLKFLNLSHSRYLIETPDFSVLPSLEQLILKDCPSLLKVHQSIGDLSNILLINLKNCTSLSYLPREIYKLTSLKTLILSGCSKLDPIDITQVKSLVTIIA

>XP_014516381.1

MEFASSSLSSSSSSFLKSEPHFIHDVFINFGGEDIGRRFVSHLHSVLLQNQVKTFISQENLHEGMKKEEHMRAIRRTKITIIVFSKSYTESACSILELEKIIECHETFGQIVLPVFYEIDPLDVRHQKEDFGKALEETAHRSYLGEQLQHARSRWSSALNRVAGMTGWDVRNFRHDAELVKQIVSRVQMLLDYKELFITQYTVGLESHVEDVIGCIENNSTKLCMIGIWGMGGSGKTTLAKVIYNRIYREFIGKSFIENISEVYDPENERYVDLQENLLSDVLKSKYEVESVRMGRTMIENVFSRKKLLIVLDDVTAFGQLENLCGSREWFGQGTVIIITTRNYELLDRLKVNYVHLIGLMNEKDSLELFRCHAFREGKPRKYFNELARNVADYSRGHPLALNVLGSFLCDRTMKEWKSVLSKLKRTARSMDNHVLDVLKISFEGLRDTEKDIFLDVCCFFIGKERSYVTDILNGCGLDADIGITILIDCGLIKIGKNNTLEMRPLFRDMGREIIQERCSEEPGKMSRLWFQDDVKDVLKKNTGTEAIQGLSLKLHSTSRDFFKANAFKEMKRLRLLQLYHVQLVGDYGCLSKELRWICWKGFSSKYIPNNFHMKNVISIDLRHSHLQLVWKQPLWKKPQVLERLKFLNLSHSRYLIETPDFSVLPSLEQLILKDCPSLLKGLVTNED

>XP_014516394.1

MDIASSSYKLPWKYDVLINFTGEDIHRKFVSHLNSAFSTIGLTTFLHHPNAVKSTHIQQPILSNCRVAIVVFTQTYSQSAWCLHQLQQIIKWHETYCRHVLPVYYEIQPSDVRLQKGDFGKALKATAQQTFSGEELVHGMSRWSHALTKAANFFGWDESNHRSDAXLVEKIVKSVVNLPILSATKFPVRLQSYVEDLIQTIKNKQSEVCIIGIGGEEGSGKTTIAKAIYNQIHWTFKEKSFIENISQVSGIRGHLRLLEQLLLDVLKQKVEIPSIDVGRTIIREKLSGKRMLIVLDDVSNFSLFDIWDFRKWSVEGTVIILTSTYIEPLRGGDSVFWVKRMNAEDSLELLSWHAFKEPKPKEEYKDLARSVVRYCAGLPLALEVVGSALFEKTKEEWNNLLFAIYGMRCVPKIIKISIEGSLNEMEKDIFLDICFFFVGKSRAYVRKILNGCGVDADIGIRVLIQRNLIKINKNNKFGMHHLLQNIGMKIIHENSGKDLGKNRRLWFDKDTKYGTEALQWLPVKLLTAIESLQPTAISYDLLNKIRWISSHGLSSEYLPNNIYEDDATAIDLKRSLLRFLWKTPQVLRALKVLNLSHYEHLTTTPDFTGLPSLEHLIFKYCSRLREVHRSIGSLNSLILLNLKDCTSLNYLPIEIYDLKSLRTFILSGCSKIDIMDKDIAKLESLITLIAENTAVKHVPFSIVISKSIGYISLRGFEGLSHNLFPSIIRSWMSPIMNPISYIHSFCMDTEDNTIDIAPLLSTLANIRSVLVACDTEFQLSKQVKNILVEYFANITESGISKQHFRYSLIGVGAYHQFFNAVSDNIYQVLSSSESGDVCLPAVNDPYCMAHMGEGHSVSFVVPEDGDLKGMIFCVVYLSTPKIIEPQFTTVVIVNYTKCTFHIHNHGTTISFKDGDWHGIMSNLECGDNVEIFVNFGNGLVVKNTTVYLIRGESENMEKASETKKHSLIRFIKKVVM

>XP_014516395.1

MECPGGAMHSPKLQISLDGMRAITVRLQSYVEDLIQTIKNKQSEVCIIGIGGEEGSGKTTIAKAIYNQIHWTFKEKSFIENISQVSGIRGHLRLLEQLLLDVLKQKVEIPSIDVGRTIIREKLSGKRMLIVLDDVSNFSLFDIWDFRKWSVEGTVIILTSTYIEPLRGGDSVFWVKRMNAEDSLELLSWHAFKEPKPKEEYKDLARSVVRYCAGLPLALEVVGSALFEKTKEEWNNLLFAIYGM

RCVPKIIKISIEGSLNEMEKDIFLDICFFFVGKSRAYVRKILNGCGVDADIGIRVLIQRNLIKINKNNKFGMHHLLQNIGMKII
HENSGKDLGKNRRLWFDKDTKYGTEALQWLPVKLLTAIESLQPTAISYDLLNKIRWISSHGLSSEYLPNNIYEDDATAIDL
KRSLLRFLWKTPQVLRALKVLNLSHYEHLTTTPDFTGLPSLEHLIFKYCSRLREVHRSIGSLNSLILLNLKDCTSLNYLPIEIYD
LKSLRTFILSGCSKIDIMDKDIAKLESLITLIAENTAVKHVPFSIVISKSIGYISLRGFEGLSHNLFPSIIRSWMSPIMNPISYIHS
FCMDTEDNTIDIAPLLSTLANIRSVLVACDTEFQLSKQVKNILVEYFANITESGISKQHFRYSLIGVGAYHQFFNAVSDNIY
QVLSSSESGDVCLPAVNDPYCMAHMGEGHSVSFVVPEDGDLKGMIFCVVYLSTPKIIEPQFTTVVIVNYTKCTFHIHNH
GTTISFKDGDWHGIMSNLECGDNVEIFVNFGNGLVVKNTTVYLIRGESENMEKASETKKHSLIRFIKKVVM

>XP_014516446.1

MKQEEHMRAIGHTKITIIVFSKSYTESACSLLELEKIIECHETFGQIVLPVFYEIDPLDVCHQKDDFGKALEDTAHRSYAGE
QLQHARSRWSSALKRVAGMTGWDHRDFRHDAELVEQIVSRVKPIDLFITEYPVGLESRVEEVIKCIENQSTKVCMIGIW
GMGGSGKTTIAKAICNRIYRKFLANSFIQNIKEAWDPEYEENVRLQEYLLSDVLKSKFKIQSVRIGRTMIQNELSRRKLLIVL
DDVNEFGQLEILCGCREWFGQGTVIIITTRDVRLLNQLKVNYVHKMDFLDENKSLELFSWHAFRDAKPRKEFKELARDT
VDYCGGLPLALEVLGSYLCYKTIEVWKSVLLKLQRIPPDDLVRALKISFEDLRDTEKDIFLDVCCFFIGKDRDYVTEILNDCG
LHADIGITVLIERGLIKVESNNKLEMHILLQEMGREIIRQVYPEEPGKRSRLWLQDDVKDVLKENTGTEAIQGLSLELHSTS
RDCFKTHAFKEMKKLRLLRLDHVQVVGDYGHISKELRWICWKGFPSKYIPNNFYMGNVIAIDLRHSHLQLVWKQPQVL
ERLKFLNLSHSKYLKETPDFSRLPSLEQLILKDCPSLRKLHQSIGDLSNILMINLKDCTSLRCLPREIYKLRSLKTLILSGCSKLSS
IDITQVKSMVNIIG

>XP_014516449.1

MTGWDHRDFRHDAELVEQIVSRVKPIDLFITEYPVGLESRVEEVIKCIENQSTKVCMIGIWGMGGSGKTTIAKAICNRIY
RKFLANSFIQNIKEAWDPEYEENVRLQEYLLSDVLKSKFKIQSVRIGRTMIQNELSRRKLLIVLDDVNEFGQLEILCGCREW
FGQGTVIIITTRDVRLLNQLKVNYVHKMDFLDENKSLELFSWHAFRDAKPRKEFKELARDTVDYCGGLPLALEVLGSYLC
YKTIEVWKSVLLKLQRIPPDDLVRALKISFEDLRDTEKDIFLDVCCFFIGKDRDYVTEILNDCGLHADIGITVLIERGLIKVESN
NKLEMHILLQEMGREIIRQVYPEEPGKRSRLWLQDDVKDVLKENTGTEAIQGLSLELHSTSRDCFKTHAFKEMKKLRLLR
LDHVQVVGDYGHISKELRWICWKGFPSKYIPNNFYMGNVIAIDLRHSHLQLVWKQPQVLERLKFLNLSHSKYLKETPDF
SRLPSLEQLILKDCPSLRKLHQSIGDLSNILMINLKDCTSLRCLPREIYKLRSLKTLILSGCSKLSSIDITQVKSMVNIIG

>XP_014516450.1

MKQEEHMRAIGHTKITIIVFSKSYTESACSLLELEKIIECHETFGQIVLPVFYEIDPLDVCHQKDDFGKALEDTAHRSYAGE
QLQHARSRWSSALKRVAGMTGWDHRDFRHDAELVEQIVSRVKPIDLFITEYPVGLESRVEEVIKCIENQSTKVCMIGIW
GMGGSGKTTIAKAICNRIYRKFLANSFIQNIKEAWDPEYEENVRLQEYLLSDVLKSKFKIQSVRIGRTMIQNELSRRKLLIVL
DDVNEFGQLEILCGCREWFGQGTVIIITTRDVRLLNQLKVNYVHKMDFLDENKSLELFSWHAFRDAKPRKEFKELARDT
VDYCGGLPLALEVLGSYLCYKTIEVWKSVLLKLQRIPPDDLVRALKISFEDLRDTEKDIFLDVCCFFIGKDRDYVTEILNDCG
LHADIGITVLIERGLIKVESNNKLEMHILLQEMGREIIRQVYPEEPGKRSRLWLQDDVKDVLKENTGTEAIQGLSLELHSTS
RDCFKTHAFKEMKKLRLLRLDHVQVVGDYGHISKELRWICWKGFPSKYIPNNFYMGNVIAIDLRHSHLQLVWKQPQLK
K

>XP_014516484.1

MAFESSSPSAKSEWIYDVFINFRGVDTRKKFVSHLHSSLSKAGVKTFLDEENLLKGMELQELLRAIQVSQIAIVVFSKRYAE
SSWCLDELQKIFQCRQTCGLRVVPVFYYVEPSEVRHQTGDFGDALRAAAESSYAGEHLEFALSSWRRTLTDAANLSGW
NATDRRTEAELVRDIVNHVIANLDYNAFSITKFPVGLDHPVQEVIRFIERTKGCCKIGIWGMGGSGKTTIAKVIYNKLHRL
FENKSFIENIREVCQTDRRRGLVRLQEKLLSDILKVKVEIQNAGIGQGMIDNRFIGKKALIVLDDVNEFDQLEALCGNTEW
MGERSVIIITTRDLHLLKRFEVNYVYEMKEMEVDESLELFSRHAFREAKPREDFNEVAKDAVSYCGGLPLALEVLGSYLSN

RTMTEWRSVLSKLKISPNTQVQEKLRISFDNLCDQMEKEIFLDVCCVFIGKDRGCVTEVLNGCGLYADIGITVLLERGLIK
VEKNNKLRMHHLLQDMGREIIRELSRKEPGKRSRLWFQEDVRDVLTNSTGTDAVEGLALKLNLNNRECFKADAFEEMR
SLRLLQLHHVELMGDYGHLSKQLRWICWQGFPSKHIPENFYLEDAIAINFKHSNLRQVWKEPKVLLMLKFLNLSHSKYLT
ETPDFSGLPYLEKLILKYCPSLRCVHKSIGDLCNIVLINLKNCTSLSSLPREIYKLKSLKTLILSGCTKIDKLEEDIAEMKSLTTLIA
ENAVVKVPFSIVSSKSIGYLFPCGFEGLSHDVLPSIIWCWLSPTMNPLSSTLPLCGISASLVSMNMQNIDLGDLAPILTNLL
NLRSVWVQCDTEFQITKQVRKILNDVGWVHFTELGIASCTSEISDNSLRSYLIGIGSYQEEDFNTLNKSISKELATSGSCHV
FLPGGNYPFWLAHTGEGHSVSFTVPQDWDMKGMALCVLYLATPETAATECLISVVMVNYTKYTIQLYKRDTVISFNDA
DWQGIISHLEAGDEVEIFLSFRNELVIKNTAVYLINYEFIDMEVKAVSKPPVMKVYSRKRNLTDQESSRKRQW

>XP_014516506.1

MSSSSSSQPQWIYDVFINFRGGDTRRDFVSHLYCALSNAGVNTFFDDENLLKGTPLEELTRAIEASQIAIVVFSETYTEST
WCLTELQKIIDCNESYGQIVVPIFHGVEPSILRNPKGRFREALEAAAKKKFSEEHREYGLSRWKNVLKKAANFSGWDVKN
HRNTAKLVKDIVEDILTKLDYALLSITEFPVGLESRVQEVVGIIENQSAKVCTIGIWGMGGSGKTTMAKAIYNQIHRRFND
KSFIENIREVSETYGRGHVHLQEKLLSDVLKTKVKIHSVGMGTTVIENRLSKKKVFIVLDDVNDFGQLKDLCGNRKWFGK
ESVIIITTRDLHLLDLLKVDYVYKMEEMDKNESLELFSWHAFREAKPREDFDELARNVVAYCAGLPLALEVLGSYLIERTKK
DWESVLLKLEKIPNDQVQEKLRISFDGLCDDMEKDIFLDVCCFFIGKDKADVIEILNGCGLHADIGITVLIERSLLKVEKDN
KLGMHQLLRDMGREIICESSRKDPGKRSRLWFHEDVLDVLTKNTGTETIEGLALKLHFIGKDCFKAYAFEEMKKLRLLQL
DHVQLTGNYGYLSKQLRWVCWKGFPSKYIPKKFFLEGAVVIDLKHSNLQLFWKEPQILRWLKILNLSHSKYLTLTPDFSKL
PNLEKLILKDCPRLCKVHESIGGLRNLLLINLEDCKSLGNLPRGVYKLKSVKTLILSGCLKIDKLEEDIVQMESLQTLIAENTA
MKQVPFSIVKTKSIVYISLCGFEGFSRNVFPSIIRSWMSPTMNPLSYVHPFCSTSSYLVSMDMQSYNLGDLEPMLSSLSN
LRSVLVQCDTESQVSMQIRALLGNSVNFTQVEIASQISKHYLRSYLIGIGNYQEVFNILSASISEGLASSESCDAFQPGDND
PFWLARTGEGNSVYFTVPEDRRMKGMTLCVVYLSAPEITATEYLISVLMVNYTRYTIQVFKRETVFSFNDVDWQGIISHS
GPGDKVEIFVNFGHGLEVKKTVIYLMCDESIDKEVKPSPEPKKEPKKNVFERFIKKILT

>XP_014516507.1

MSSSSSSQPQWIYDVFINFRGGDTRRDFVSHLYCALSNAGVNTFFDDENLLKGTPLEELTRAIEASQIAIVVFSETYTEST
WCLTELQKIIDCNESYGQIVVPIFHGVEPSILRNPKGRFREALEAAAKKKFSEEHREYGLSRWKNVLKKAANFSGWDVKN
HRNTAKLVKDIVEDILTKLDYALLSITEFPVGLESRVQEVVGIIENQSAKVCTIGIWGMGGSGKTTMAKAIYNQIHRRFND
KSFIENIREVSETYGRGHVHLQEKLLSDVLKTKVKIHSVGMGTTVIENRLSKKKVFIVLDDVNDFGQLKDLCGNRKWFGK
ESVIIITTRDLHLLDLLKVDYVYKMEEMDKNESLELFSWHAFREAKPREDFDELARNVVAYCAGLPLALEVLGSYLIERTKK
DWESVLLKLEKIPNDQVQEKLRISFDGLCDDMEKDIFLDVCCFFIGKDKADVIEILNGCGLHADIGITVLIERSLLKVEKDN
KLGMHQLLRDMGREIICESSRKDPGKRSRLWFHEDVLDVLTKNTGTETIEGLALKLHFIGKDCFKAYAFEEMKKLRLLQL
DHVQLTGNYGYLSKQLRWVCWKGFPSKYIPKKFFLEGAVVIDLKHSNLQLFWKEPQILRWLKILNLSHSKYLTLTPDFSKL
PNLEKLILKDCPRLCKVHESIGGLRNLLLINLEDCKSLGNLPRGVYKLKSVKTLILSGCLKIDKLEEDIVQMESLQTLIAENTA
MKQVPFSIVKTKSIVYISLCGFEGFSRNVFPSIIRSWMSPTMNPLSYVHPFCSTSSYLVSMDMQSYNLGDLEPMLSSLSN
LRSVLVQCDTESQVSMQIRALLGNSVNFTQVEIASQISKHYLRSYLIGIGNYQEVFNILSASISEGLASSESCDAFQPGDND
PFWLARTGEGNSVYFTVPEDRRMKGMTLCVVYLSAPEITATEYLISVLMVNYTRYTIQVFKRETVFSFNDVDWQGIISHS
GPGDKVEIFVNFGHGLEVKKTVIYLMCDESIDKEVKPSPEPKKEPKKNVFERFIKKILT

>XP_014516508.1

MSSSSSSQPQWIYDVFINFRGGDTRRDFVSHLYCALSNAGVNTFFDDENLLKGTPLEELTRAIEASQIAIVVFSETYTEST
WCLTELQKIIDCNESYGQIVVPIFHGVEPSILRNPKGRFREALEAAAKKKFSEEHREYGLSRWKNVLKKAANFSGWDVKN
HRNTAKLVKDIVEDILTKLDYALLSITEFPVGLESRVQEVVGIIENQSAKVCTIGIWGMGGSGKTTMAKAIYNQIHRRFND
KSFIENIREVSETYGRGHVHLQEKLLSDVLKTKVKIHSVGMGTTVIENRLSKKKVFIVLDDVNDFGQLKDLCGNRKWFGK

ESVIIITTRDLHLLDLLKVDYVYKMEEMDKNESLELFSWHAFREAKPREDFDELARNVVAYCAGLPLALEVLGSYLIERTKK
DWESVLLKLEKIPNDQVQEKLRISFDGLCDDMEKDIFLDVCCFFIGKDKADVIEILNGCGLHADIGITVLIERSLLKVEKDN
KLGMHQLLRDMGREIICESSRKDPGKRSRLWFHEDVLDVLTKNTGTETIEGLALKLHFIGKDCFKAYAFEEMKKLRLLQL
DHVQLTGNYGYLSKQLRWVCWKGFPSKYIPKKFFLEGAVVIDLKHSNLQLFWKEPQILRWLKILNLSHSKYLTLTPDFSKL
PNLEKLILKDCPRLCKVHESIGGLRNLLLINLEDCKSLGNLPRGVYKLKSVKTLILSGCLKIDKLEEDIVQMESLQTLIAENTA
MKQVPFSIVKTKSIVYISLCGFEGFSRNVFPSIIRSWMSPTMNPLSYVHPFCSTSSYLVSMDMQSYNLGDLEPMLSSLSN
LRSVLVQCDTESQVSMQIRALLGNSVNFTQVEIASQISKHYLRSYLIGIGNYQEVFNILSASISEGLASSESCDAFQPGDND
PFWLARTGEGNSVYFTVPEDRRMKGMTLCVVYLSAPEITATEYLISVLMVNYTRYTIQVFKRETVFSFNDVDWQGIISHS
GPGDKVEIFVNFGHGLEVKKTVIYLMCDESIDKEVKPSPEPKKEPKKNVFERFIKKILT

>XP_014516510.1

MSSSSSSQPQWIYDVFINFRGGDTRRDFVSHLYCALSNAGVNTFFDDENLLKGTPLEELTRAIEASQIAIVVFSETYTEST
WCLTELQKIIDCNESYGQIVVPIFHGVEPSILRNPKGRFREALEAAAKKKFSEEHREYGLSRWKNVLKKAANFSGWDVKN
HRNTAKLVKDIVEDILTKLDYALLSITEFPVGLESRVQEVVGIIENQSAKVCTIGIWGMGGSGKTTMAKAIYNQIHRRFND
KSFIENIREVSETYGRGHVHLQEKLLSDVLKTKVKIHSVGMGTTVIENRLSKKKVFIVLDDVNDFGQLKDLCGNRKWFGK
ESVIIITTRDLHLLDLLKVDYVYKMEEMDKNESLELFSWHAFREAKPREDFDELARNVVAYCAGLPLALEVLGSYLIERTKK
DWESVLLKLEKIPNDQVQEKLRISFDGLCDDMEKDIFLDVCCFFIGKDKADVIEILNGCGLHADIGITVLIERSLLKVEKDN
KLGMHQLLRDMGREIICESSRKDPGKRSRLWFHEDVLDVLTKNTGTETIEGLALKLHFIGKDCFKAYAFEEMKKLRLLQL
DHVQLTGNYGYLSKQLRWVCWKGFPSKYIPKKFFLEGAVVIDLKHSNLQLFWKEPQILRWLKILNLSHSKYLTLTPDFSKL
PNLEKLILKDCPRLCKVHESIGGLRNLLLINLEDCKSLGNLPRGVYKLKSVKTLILSGCLKIDKLEEDIVQMESLQTLIAENTA
MKQVPFSIVKTKSIVYISLCGFEGFSRNVFPSIIRSWMSPTMNPLSYVHPFCSTSSYLVSMDMQSYNLGDLEPMLSSLSN
LRSVLVQCDTESQVSMQIRALLGNSVNFTQVEIASQISKHYLRSYLIGIGNYQEVFNILSASISEGLASSESCDAFQPGDND
PFWLARTGEGNSVYFTVPEDRRMKGMTLCVVYLSAPEITATEYLISVLMVNYTRYTIQVFKRETVFSFNDVDWQGIISHS
GPGDKVEIFVNFGHGLEVKKTVIYLMCDESIDKEVKPSPEPKKEPKKNVFERFIKKILT

>XP_014516511.1

MSSSSSSQPQWIYDVFINFRGGDTRRDFVSHLYCALSNAGVNTFFDDENLLKGTPLEELTRAIEASQIAIVVFSETYTEST
WCLTELQKIIDCNESYGQIVVPIFHGVEPSILRNPKGRFREALEAAAKKKFSEEHREYGLSRWKNVLKKAANFSGWDVKN
HRNTAKLVKDIVEDILTKLDYALLSITEFPVGLESRVQEVVGIIENQSAKVCTIGIWGMGGSGKTTMAKAIYNQIHRRFND
KSFIENIREVSETYGRGHVHLQEKLLSDVLKTKVKIHSVGMGTTVIENRLSKKKVFIVLDDVNDFGQLKDLCGNRKWFGK
ESVIIITTRDLHLLDLLKVDYVYKMEEMDKNESLELFSWHAFREAKPREDFDELARNVVAYCAGLPLALEVLGSYLIERTKK
DWESVLLKLEKIPNDQVQEKLRISFDGLCDDMEKDIFLDVCCFFIGKDKADVIEILNGCGLHADIGITVLIERSLLKVEKDN
KLGMHQLLRDMGREIICESSRKDPGKRSRLWFHEDVLDVLTKNTGTETIEGLALKLHFIGKDCFKAYAFEEMKKLRLLQL
DHVQLTGNYGYLSKQLRWVCWKGFPSKYIPKKFFLEGAVVIDLKHSNLQLFWKEPQILRWLKILNLSHSKYLTLTPDFSKL
PNLEKLILKDCPRLCKVHESIGGLRNLLLINLEDCKSLGNLPRGVYKLKSVKTLILSGCLKIDKLEEDIVQMESLQTLIAENTA
MKQVPFSIVKTKSIVYISLCGFEGFSRNVFPSIIRSWMSPTMNPLSYVHPFCSTSSYLVSMDMQSYNLGDLEPMLSSLSN
LRSVLVQCDTESQVSMQIRALLGNSVNFTQVEIASQISKHYLRSYLIGIGNYQEVFNILSASISEGLASSESCDAFQPGDND
PFWLARTGEGNSVYFTVPEDRRMKGMTLCVVYLSAPEITATEYLISVLMVNYTRYTIQVFKRETVFSFNDVDWQGIISHS
GPGDKVEIFVNFGHGLEVKKTVIYLMCDESIDKEVKPSPEPKKEPKKNVFERFIKKILT

>XP_014516512.1

MSSSSSSQPQWIYDVFINFRGGDTRRDFVSHLYCALSNAGVNTFFDDENLLKGTPLEELTRAIEASQIAIVVFSETYTEST
WCLTELQKIIDCNESYGQIVVPIFHGVEPSILRNPKGRFREALEAAAKKKFSEEHREYGLSRWKNVLKKAANFSGWDVKN
HRNTAKLVKDIVEDILTKLDYALLSITEFPVGLESRVQEVVGIIENQSAKVCTIGIWGMGGSGKTTMAKAIYNQIHRRFND

KSFIENIREVSETYGRGHVHLQEKLLSDVLKTKVKIHSVGMGTTVIENRLSKKKVFIVLDDVNDFGQLKDLCGNRKWFGK
ESVIIITTRDLHLLDLLKVDYVYKMEEMDKNESLELFSWHAFREAKPREDFDELARNVVAYCAGLPLALEVLGSYLIERTKK
DWESVLLKLEKIPNDQVQEKLRISFDGLCDDMEKDIFLDVCCFFIGKDKADVIEILNGCGLHADIGITVLIERSLLKVEKDN
KLGMHQLLRDMGREIICESSRKDPGKRSRLWFHEDVLDVLTKNTGTETIEGLALKLHFIGKDCFKAYAFEEMKKLRLLQL
DHVQLTGNYGYLSKQLRWVCWKGFPSKYIPKKFFLEGAVVIDLKHSNLQLFWKEPQILRWLKILNLSHSKYLTLTPDFSKL
PNLEKLILKDCPRLCKVV

>XP_014516528.1

MIGIWGMGGSGKTTIVKAIYNRIYRQFIGKSFIENIRHEGYIALQENLLSDVLKSKFKVKSVGMGRTMIQNRFSRKKLLIVL
DDVNEFAKLENLCGSREWFGQGTVIIITTRDFQLLKQLRVNYVYKMHLLNENESLELFSWHAFRDAIPKKEWSELARNV
VVYCGGLPLALEFLGSYLCDKTIEVWKSVLLKLQRIPPDELLSVLKISFEDLHDAEKNIFLDVCCFFIGKEREYVTEILNGCGL
NADIGITVLIERGLIKVERNNKLQMHPLLQEMGREIIRQECPEKPGKRSRLWFQDDVEDVLKENTGTEAILSLKSDSSIGD
CLESRAFKEMKRLRLLQLDHVQLSGDLGHISKQLRWICWRGFRYRYIPKNFHLENVIAIDLKRSLLHLVWQGRVVLERLK
FLNLSHSKYLKETPDFSGLPSLEQLILKDCARLRKVHPSIGVLSNIRVINLEDCTSLRYLPREIYKLRSLKTLILSGCSNLRSREKI

>XP_014516597.2

MDFLGPFGKVVEGVVDFVWKHGVRHMTYIVHYKKNVVELSDTVKDLRFEKEKIDHKCEEGTKNLHNXEGKVIEWVRK
VSEIETTVDVXENDDGHTRARSPNCFVFPYLWNRHRLGRQAKNLEVGVKKLIDESPELDEISYRENVTSNDVTLSNSGFE
QFSSTKSTVEKVMRELENSGVRMIGLYGEGGVGKSALIKEIARIAXDKKXFNVVVKVEVTANPNIQSIQEEIAYVFGLQLV
GEGENVRADCLRRRLKKEKGNTLLILDDLWHKLDLNKLGIPLDDNDDNDDLSNDMRDLDDKFLKKEKNNKDFNQKVLK
REKIIGGHKGCKILLTARQKRVLEVEMDVKSTFRVEPLDDKDALMFFQKLSEIHNMSDSRKEIVRKYCAGLPMAIITVAKA
LRGKSELVWEAALGELKKQELVGIQTNMDXSVKMSYEHLENEEIKSIFLLCAQMGHQPLIMDLVKCCYGLGILEGVFSLS
EAREKIKIXIQKLKDSGLLLDGNSDIHFNMHDIVRDAALSIAKKDKHVFTLRNGKLDQWPELEKCTSISICNCDIIVKLPIVN
CSQLNFFQIDTNHQSLTIPDKFFEGMKNLKVLILTGFHLKRFPPSIKGLLKLRMLCLERCTLEDNIAIGELEMLRILSFSGSQL
KSLPTELGCLDKLQLLDINDCSILEVNIPPNFLSSLTHLEELYIRKSLTKMLVEGEPNHGKNSILCELKNLHQLKVVDLSIPSFS
VLPNHLFYDKLQDYKIVVGEVEKFSVIGFKMPDKYETSRVLALHDVXKIDSHKDINLLFKTAQSLLLGKVEVVKVVNELSID
GFPDLKHLSIIDSNAIKYVNSMQLSNCINVFPNLESLCLYNLKNLKMISIGPLKVASFSKLKSIKVNMCEGLVSFYSVSMVE
FSNSEEPCEIIECNSYLDNFCASLETIEVSECKSLKEILKIPKKCHKVKFLKLQTLTLQSLPSFKCFYTKVEESQAKNSGSEEDX
KSDKAPLFGEQVEIPNLESLNLCSLNMHKIWSDQFLSSFCFQNLIKLVVKECDELTYLCSLSVASSLKKLKSLIISECGIMEKI
FETKENNADKVCVFPKLEEIQLTKMKRLRDIWHTKVNDDSFSSLISVNIEECNKLDKIFPSNMEGWFESLDNLKVSSCESV
EVIFEIKDCEEIDVSGEIDTNLQVILLEDLPKLKELWSKDPHGILNFKKLRTIDVSSCYKLRNLFPASMTKDVSKLERMSVFN
CRGMVGIVSSRDASEANTDPLEFPELINVRLLSLSNIKQFYAGRLPIKCPKLKELTVYRCSKLKTFSKEISKTTEEEEEKEEEKE
EEENFVFSAQKVLPKLEYMEIDLKEAQNLLPEYPMHCLKELSLISVESVDFLNQFPYRMPNLEKLKLTSSYESKELEPASEE
RLRITLELKEIVLSSLKINDLTGVSIVRKLQLLSLAHCSELNNLGPSSLSFTYLTYLELKYCRELKYLMTPSTAKSMVQLKTMK
VINCRKVEEIVSNELSEEGTVMKIAFGKLITIELVGLKNLESFCRCKDCEFEFSLLEILIVRECWKMEKFSEREAITPKLKNVF
GVEGDEKSKWYWEGNLNSTIRKIFTDKVSFACTENLFLGYDDTGQLIIDQLWHRRHWVQQNSFGYLKKLSVWECDTLE
HIIPSHLLSCFHNLEELSVGLCSKAEVIFNMNDENRVMTKPSGIFRLKTLSLFKLPNLKHVWEKDPEGIIGLQLLKEMRVKE
CKRLKSLFPASVAKELTRLEVLXVTNCKELAEIFKKEGEGRTQELALGRLTTLMLXELPSLKYSIHCSNQESISNLSERNIQEL
CLGSRCIIPNSNFGLLESLILDGCKFLSDVLLPCNLLPFLKKLETLEVRNCDTVKIIFEVKCITQDRGVVSMGPTLHFSLNKLTL
FNLPELENVCNEDNCCPFVRSLEVQQVPNLKYFYYSSMYCEIFTDQESHSKNQVGTEKCLSLGENGMKIILRRELERNLL
DSLTALTLGFGSDVFEYEILKKVPNIEKLVVWDGSFKEMFCRESPDNVLQQLKVLLLESLGELVSIGLENSWTHSFVRNIET
FEVISCWSLKNLVACTVSFSNLRCLKVKHCNSLSYLLTSSTAKCLGKLKRMEITWCYSIEEIVCKEDGEESDDDEIIFQQLTS
LKLHWLGNLKRFYKGSLGFPSLEELSVQDCDEMISLCVGSIVGGKMCEVKVGKFSQAIQLETELNSTMRKEYLRKIASSEE
SLEFRDMADLQELWCLSQQIPHFRFTYMETLIVNKCKISSDAVLPFTLLPLLPKLETFEVRNCDSVKIIFDVKCAQEILTFPL

KRLVLRKLSNLETICNEVTEANPALPKETNPKLTFPSVTSLALCDLPNFIYNDAAPTFELIIPNLEDLTVGRNELKMIVDGEF
QRNLLDKLKVLGVCFDSECDEFPEYGFLQQLPNVKRLVVWNSSFKVIFCHQRPSNNELLLQLNELRLESLQGLVSIGLENS
WIEPFLENLQNFEVITCSSLKNLVTCRVCFSNLTCLKVENCDGXSYLFTSSTAKSLGQLQRMXIKECKSIEEIVSKEREESDE
DEIIFSQLSCLKLDELLNLRWFYRGSLSFPSLAEISVIDCSEMVTLCPSTLKADKLSQVTIENEEVITVETDLYSTMQKKFGRK
TQRKSRIDLKSRGGLHEIWRGSSLPIPNFCFSELDTLIVDDCQFLSDAVLPFHLLPLLPELETLEVRNCDSVKTIFDVNPSTQ
DTSITFPLKKLVLSKLPNLENVWKEDPRGILSMQSHLEQVFVDTCKCLKSVFPASVAKELVKLEDLVVEDCEGLMAIVADE
SHENQEIIFERLQVLDLKRLKQLTCFCTGKFTLSFPSLEEVHIIKCSSMKSFSAVTKIDNPTKWYYSEYERPRKETDLNSALH
RNFEEEAPDGASAIIPVLQ

>XP_014516653.1

MDFTSSKSKHQRIHDVFINFRGEDTRRKFVSHLHYALSNAGVNTFFDEENLVKGMQLQELMRAVEGSQIAIVVFSQTYT
ESTWCLDELEQIIKCNQTQGQSVLPVFYEIDPSDVRHQKGDFGKSLEEAARRTYSGEELERALSRWSRALNKAAGISGW
DVRNFRNEAELVRQIVDRVQKLLDYEVLSITEYPVGLESRAQDVIGLIETRSTQVCMIGIWGMGGSGKTTVAKAVYNQI
HRRFMDKSFIENIRETCENHGRGYVPLQEQLLSNVLKTKVEIHSVGMGTTMIENRLDGKTALIVLDDVNEYNQLKAVCG
NRKWIGQGSVIIITTRDVGLLTRLDVDYVYGMDKMDEDESLQLFSFHCFGDAKPKEDFRELSKNVVAYCGGLPLALEVL
GSYLFDKTSKRVWEGVLSILEKIPNDEVQRKLRISFDSLSNDMEKDIXLDVCCFFIGKDRGYVTEILNGCELCADVGIPVLIE
RSLIKVEKNNKLGMHPLLQEMGREIIRENSRKDPGKHSRLWSQKEVVEVLTKNSGTEAIEGLVLKMHLTSRDCFKTDSF
QKMERLRLLQLHHVQLAGNYGYLSKQLRWISWHGFPSNCLPNSFCMDDVIAIDLKHSHLRFVWKQSQDLKWLKVLNL
SHSRYLTETPDFSRLPSLEQLILKDCPSLLAIHNSIGDLCNILLINLKDCTSLSNLPKEIYKLKSVKTFILSGXSKIDKLEEDIAQM
ESLTTLIADNTAVKQVPVSIVTSKSIGYISLCGFEGLARNVFPSIIQSWMSPTMNPLLFIRPFSATSCSLVSMNTQNNTLGE
LAPMLRSLPNLRSVLLRYETESQLSKHVKTFLVEHVVNVAELGISRHHLRSSLIGVGSYKAYFDILNDRISKELVTDEACDVS
LPIDNYPYWSAHTGEGQSVFFTVPEDCGMKGMTLCIVYLSNPEIKPTECLTSVLIANYTKRTLQIHKQETVISFNDEDWQ
GIISHLAGGDKVEIFVTFGHDLVVKKTAVYLMYGESNDIEIETTNCELNDVEIEPPHCESNNVEIKAVCIPNDIESKPMHYE
ANDIKIAAMNSESNVLEMKSDPKPNGNGCIRFFKKLAMCDWNASG

>XP_014516682.1

MEFASSSSLSSSSSSFLTSEPHFIHDVFINFGGEDIGRRFVSHLHSVLLQSQVKTFISQENLHDEGMKLEEHMRAIGHTKIT
IIVFSKSYTESACSLLELEKIIECHETFGQIVLPVFHEIDPLDVRHQKDDFGKALEDTAHRSYSGEQLQHARSRWSSALNRV
AGMTGWDVRHFGHDAALVDVIVNRVKTLLDYRDLFITSYPVGLESRVEDVIKCIENQSTKVCMIGIWGMGGSGKTTLA
KAIYNRIYRKFIGKSFIENIRKVWDLKYEKYVLLQEYLLSDVLESKLKVKSVGMGRTMIQNEFSRKKLLIVLDDVNEFGQLE
NLCGRREWFGQGTVIIITTRDFHLLNQLKVNYVYEMDLLNENESLELFSSLAFGDAKAKEDFSELSRNVVAYCGGLPLALE
VLGAYLFDQTSKRVWEGVLSILEKIPNKEVQRKLRISFDSLSNDMEKDILLDVCCFFIGKDRGYVTEILNGCELCADVGIPV
LIERSLIKVEKNNKLGMHPLLQEMGREIIRENSRKDPGKHSRLWSQKEVVEVLTKNTGTEAIEGLVLKMDLTSRDCFKTD
SFKKMERLRLLQLHHVQLDGNYAYLSKQLKWISWHGFPSNSLPNSFCMNDVIAIDLKYSHLRFVWKQSQDLKWLKFLN
LSHSMYLRETPDFSRLPSLEQLILKDCPSLLGIHKSIGDLCNILLINLKDCTSLSNLPKEIYKLKSVKTFILSGCSKIDKLEEDIAQ
MESLTTLIADNTAVKQVPVSIVTSKSIGYISLCGFEGLARNVFPSIILSWMSPTMNPLLFMRPFSGTSCSLVSMNTQDNTL
SELAPMLRSLPNLRSVLLRYETESQLSKDVETFLVERAVNVAELGISRHHLRSSLIGVGSYKAFFEILNDRISKGLVTNEASE
VSLPIDNKPYWLAHIGEGQSVFFTVPEDCGMKGMTLCIVYLSNPEIKPTECLTSVLIANYTKRSLQIHRQETVISFNDEDW
QEIISHLAGGDKVEIFVTFGHDLVVKKTAVYLMYGESKDIEIELTNCESNGVEIEPPHCESIVSKMRQYVYQMILKVC

>XP_014516683.1

MEFASSSSLSSSSSSFLTSEPHFIHDVFINFGGEDIGRRFVSHLHSVLLQSQVKTFISQENLHDEGMKLEEHMRAIGHTKIT
IIVFSKSYTESACSLLELEKIIECHETFGQIVLPVFHEIDPLDVRHQKDDFGKALEDTAHRSYSGEQLQHARSRWSSALNRV
AGMTGWDVRHFGHDAALVDVIVNRVKTLLDYRDLFITSYPVGLESRVEDVIKCIENQSTKVCMIGIWGMGGSEYLLSD

VLESKLKVKSVGMGRTMIQNEFSRKKLLIVLDDVNEFGQLENLCGRREWFGQGTVIIITTRDFHLLNQLKVNYVYEMDLL
NENESLELFSSLAFGDAKAKEDFSELSRNVVAYCGGLPLALEVLGAYLFDQTSKRVWEGVLSILEKIPNKEVQRKLRISFDS
LSNDMEKDILLDVCCFFIGKDRGYVTEILNGCELCADVGIPVLIERSLIKVEKNNKLGMHPLLQEMGREIIRENSRKDPGK
HSRLWSQKEVVEVLTKNTGTEAIEGLVLKMDLTSRDCFKTDSFKKMERLRLLQLHHVQLDGNYAYLSKQLKWISWHGF
PSNSLPNSFCMNDVIAIDLKYSHLRFVWKQSQDLKWLKFLNLSHSMYLRETPDFSRLPSLEQLILKDCPSLLGIHKSIGDLC
NILLINLKDCTSLSNLPKEIYKLKSVKTFILSGCSKIDKLEEDIAQMESLTTLIADNTAVKQVPVSIVTSKSIGYISLCGFEGLAR
NVFPSIILSWMSPTMNPLLFMRPFSGTSCSLVSMNTQDNTLSELAPMLRSLPNLRSVLLRYETESQLSKDVETFLVERAV
NVAELGISRHHLRSSLIGVGSYKAFFEILNDRISKGLVTNEASEVSLPIDNKPYWLAHIGEGQSVFFTVPEDCGMKGMTLC
IVYLSNPEIKPTECLTSVLIANYTKRSLQIHRQETVISFNDEDWQEIISHLAGGDKVEIFVTFGHDLVVKKTAVYLMYGESK
DIEIELTNCESNGVEIEPPHCESIVSKMRQYVYQMILKVC

>XP_014516829.1

MDNGSSSYKLPRKYDVLINFTGEDINRKFVSHLNSAISTVGLTTFLHHHNAIESTHIQQPILSHCRVAIVVFNQTYSQSAW
CLNQLLQIIDWHETYGRHVLPVYYEIQPSDVRLQKGDFGKAFKATAQKTFSGQELEHGMSRWSHALTKTANFFGWDE
SNHRSDAELVEKIVKSVLNLPVLSATKFPVRLQLYVEDLIRTIKTKSTETCIIGIGGSGGYGKTTLAKAIYNQIHGTFKKKSFIE
NISHVRGIRGLLGLQEQLLLDVLKQKVEIPSVDVGRTMIRERLLGKRVLIVLDDVSYFSLFDLWDCRKWLVEGTVIIVTHEI
PLTGQVNFVFWLEPMNEEESLELLSWHAFREPKPKEEYEDLARSVVSYCEGLPLALEVVGSCLFEKTKEEWNTLLIKYAIV
GRHRVSEIIKVSIEGSINEMEKDIFLDICCFFVGKSRAYATKILNGCGVDADMGIRVLIQRNLIKINNNNKFGIHPLLQEIGV
QIIRENLETDHEKNRLWFDKDVKHGTKAMQWLPVNAYGSAQPTVNSDYLLKKLRWISWHGLSSERLPNNFYEHNAIAI
DLKRSLLRFLWKTPQVLRSLKVLNLSHSKHLTTTPDFTGLPSLEHLSFKYCSRLRKLHRSIGSLNSLILLNLKYCTSLNNLPKEI
YELKSLRTFILSGCSKIHIMDKDIAKLESLITLIAENTAVKEVPFSIVSSKSIGYISLPGFEGLSHNLFPSIIRSWISPIMNPISYIH
SFCMDTEDNTIDIAPLLSTLANIRSVLVACDTDFQLSKQVKNILDEYFSSITKSGISKQHLRCSLIGVGAYHQFFNAVRDNIY
EVLANSESGDVCLPAVNDPYCLAHMGDGHSVSFIVPQDRYMKGMTLCVVYLSNPEAIEPQFTTVVVINYTKCRFQIHN
YGTVISFKDEDWHDIISNLESGDKVEIFVNFGNGLVVKNTTVYLICGESKNMRKAFEPKKHGLIRFVKKVVM

>XP_014517203.1

MSFRTNPKKAIPFVLKRLRMTRTTKTLDKSCDDKLEKLTSQLDQIKDLFMAVKWNEDELLDTLALLDRHLRTIGNIEKEKF
ETDMETISQRIKDSTEKLLPKGCPSQGGTQASVSPKVTRYQDHKTGKLHSPPSSSQHKDELHEENLNQNIEVSFEKLEGH
LKPCLLSLLVFPEDAVINKRHTIYWWIGEGFVRNSGEKTAEEVGEGVVDELLNCQMIVAHGNGLNPVVKKFKVNPRIRG
ELLTLDSEKTLQHLGLSFKEFWNQRAWLKQRKVVLGDDKDNLKPNHWKSIFNVGASYLSFEPEWLAKVKSLEVLQLGR
WQDSPSHHIEVASEEFLKELRGDKQLKYLSLRGISRIPELPPSIAQLENLEILDLKACHSLEALPENIASMKSLTHLDVSECYL
LDSMPRGIEKLTQLQVLKGFVIGSSLKTPCRISDLANLKKLQRFSVHIGSEAVIQEMEFESLKDLTAVKCLKISWGVSGEKY
SDIQVIFPSSLEKLDLEGFPGTAIPEWLKPSRVPGSMRKLYINGGKLKSLDHGEICHKWHVEILRLRYLKQLQIEERKLHKLF
PSLRYVERTKVLNRSFQEWRLEE

>XP_014517204.1

MSIRTNRMKAVPTLQRRLTLVKSGEEPNISDKLDRLMSDLTAIKDLFSTVKSNEEELLDTLKLVDDLLRNFKTEKKFDEISS
RILEPEKADPQKGNDEPSSSQKAEEKSGKKDETIDEKLKNLEGTFKNLKANFSTLKKNEELRTKLTELENFLQNFNSKLAED
ASMKEMNQDFTGAGTDKPSSGQLTKDNKSIVEEVKSSSEIAEPSQDSDQSETKSSELPRQKSTDKSLDQDLKATGIEMP
PSSQETQDEKSIAEIDRTIEEKFESFMSKLDKIKGMSSKENEELRKKLEEMEDLLRKFNSNQSETKNLELPTQIERDESVDK
GFKATGVDKPASNQEPKDENFQEKYVPIDEKLESLKVELDNMKAKFSPAKENEEMSKTLRTLEDLLQSIDIPAEDANPPE
TKGSELTSQISRGESENQVLKGVDKPSSSQDVQDKESVEKDVSDDEKVESLNVKRKDSKEEVVEKVVNAINKKIKKSTKKL
GDSEIKIQPNESLEKGKTSKTYPKLSLSRDDKTLELVLKRFQASYDALNSHSKVCCLSLSIFPENVVIMKRQIIYWWIGEGFV
KRSTEKTSEEEGENVFNELLNSNLIVPHGSGKCPVVNKFKINPWIRHMLVSSVLGENKQPFGFYSQITTSSHRNHADYGC

LVLDRQNVEIGGHFDWKSVHSRSVFNLGARYLTIEPRWMAKMKKLVVLQLGRWQESPEHHIEVAGTEFMTATKAQK
HLKYLSLRGISRISALPPSIAQLVSLEILDLKACHNLETLPNEITSLKKLTHLDVSQCYLLESMPKGIGKLTELQVLKGFVVGSS
KETLTISDLANFKKLKRLSIHIGSEAVIQKEFESLRNSEVKCLKISWGASSYTKYKDIDIVLPIGLEKLNLEGFPSEKTPTWLKL
NLLPSDLKRLYIIGGKLSSIEEEKSIKRNCKVEILRLKYLKNLQIDIDHIIKLFPSLKYAEVKKFKEPSHEWSI

>XP_014517221.1

MAAELVGGALLSAFLQVSFDKLASPQFLDFFRGRKLDEKLLGNLNIMLHSINALAHDAEQKQFTDPHVKAWLFSVKEAV
FDAEDLLAEIDYELTRSQVEAESEAQTFTYKVSNFFNSTFKTFNKKIESEMKELLEKLEYLAKQKGALGLREGTYSGDRSGS
KVSQKLPSSSLVVESVIYGRDADKQMICNWLTSETDNNNHPSILSIVGMGGLGKTILAQHVYNDTDIEEAKFDIRAWVC
VSDHFDILTVTKTILEAITKSKDDSGDLEMVHGRLKEKVSGKKFLLVLDDVWSERRQEWEAVRTPLSYGAPGSRILVTTRV
EKVASNMRSKVHHLKQLEEDECWKVFEEQALKDDDLQLNDEKKEIGRRIVEKCKGLPLALKTIGSLLRTKSSXXXWXXVL
EXDIWXLPKEVEIIPALLLSYQNLPSHLKRCFAYCALFPKNYEFEKENLILMWMAEGFLHYSPQNNNLEEIGEQYFDDLLT
RSFFLRSTIKMHFSMHDLVNDLAKYVCADFCFRLKFDKGNCIPKTTRHFLFAFDDLRYFDGSGSLTDAKRLRSFVPITNDF
VHGAFPCQIKILIRELFSKWKFLRVLSLNGNYDFQEVPDSVGDLKHLHSLDLSRTMIRKLPDSVGLLYNLLILKLNDCSFLKE
LPSNLHKLTNLRCLEFEDTGVTKMPMHIGELKNLHVLNMFCVDRNSEFSTKQLGGINLHGRLAINELQNIVNPLDALEA
NLKNKQLVKLKLIWNWNHIPDDPRKDKKVLENLQPSNQLEHLSIRSYCGTQFPSWVFDNSLSNLVSLELEDCKYCLCLPP
LGLLSSLRTLKIRGFDGIVSIGAEFYGSSSSSFKSLEILKFYNMKEWEEWKCKTTSFPRLQHLVIVRCSKLKGLSQQLLHLKEL
IIESCRNLIIREHNEDTSTIELLRTRSCPRVNIPMTHYDFIEEMTIDDACDSLTIFQLNFFPMLRLLHLEGCQNLQRISQEHP
HNHLKKMSIRRCPQFESFPNEGLSAAFPSLTELEITWCRKVEKFPDGGLPSNVKHMTLSSLKLIASLRETLDVNTCLESLSI
QYLNVESFPDEVLLPPSLTSLRICHCSNLKTLDYKLLHNLSSLTLFDCRNLQCLPEEGLPKSISSLHIVYCPLLKQRCQKPEGK
DWRKIAHIQNLRV

>XP_014517261.2

TKLEETSMPFARRYLPPKPFEALKMMRDFSKEVAEITDEHGSLLDFLDDADKLADAEEDVKRRDRMKERLMRLREAAFR
MEDVIDEYCIRVKEKQPQHGPRWAASLSKTVHSIKTFILRLQIACKIQDEKSVVRDEKVGFESQYFSEKILNSSRENKNVTF
NQLRMDPLFMKEEEVVGLAGPTERLTDWLTNGREERTVISVVGIAGVGKTTLAKHVFDKVHDDFECHALITVSQSYTVE
ELLRKMVRKLCKERKEDPPCKISTMDRGSLIEEVRDRLHQKRYVVLFDDVWNEKFWDDIQSALIDDNNKSRIIMTTRHE
KVAIFCKKSSFVEVHKLEEPLKDESFRLFCRKAFKYGSAGGCPEELKDISLEIVRKCKGLPLAMVAIGGFLSQKDESAKEWR
LFSQNLSSELKRNPDLNIITKIIGLSYDNLPSPLRLCLLYFGMYPKDYEIESERLVRQWVAEGFVTHEDGKTLEEVAHEYLLG
LVRRSLVQVSSFSMEGKVKKCCVHDLIHDMIRTKVKDTCFGEYIGGGHDQSESSELVRRLTIQANNDLNRRIKRSHIRSII
VIPGKKEELLSVHLVRKIPTDYMLLKVLDFEGCGLLCVPKKLENLIYLRYLSFRGTQIKVLPKSIGKLVNLETLDIQQTQVCKV
PKEITKLRKLRHLLTPDSVSSSIEWKDIGGMTMLQKIPQVRMEDDGVAIREVGKLKQLRNLRVLCFSGEHITLLFSSINEM
EHLQALRIEKSGDRSVVDLNTTSPMFPRSRLRKLFLDIKLEKLPNWIPQLHDLERLILRHSDLTNDPLESLKDMPSLLVLSLS
HAYEGQTLHFKSGGFKKLRKLNLEHLWNLNSILIDETALQSLEHLNLKGLDELRTVPDGIQHLKKLKFLLVPSMPTQFVKEI
DRIGKNQHWDINYGYM

>XP_014517962.1

MEDVALAAGECILKLLWETVKRLKDLPDDVREIRDELDKLKNFIDLEDKEADGENDGTKRNKKKQDLMQLRKATFLTED
VIDEYLIRIEEKQPQDDRGWAAFHIQTLIPRFKISFKIESNLSVVRRTNERFRNNRPQSTQNNNNVTFHQCRMDPLFMEE
QEVVGLAGPTEILKDWLKNGQRERTVIFVVGVPGVGKTTLAKHVFNKVCGDFEHHARFTVSQSNTVEELWRVMYKHY
MKSEPPSDNSILIEEVRGYLRGKRYVLLFDDVWEDEFWKSIEKALIDNKNKSRIIITTRKENVANSCRNSPFFKEYKLEEPLT
EEECLRLLHKNARYSSDCPEELKDISLEIVRKCRALPLAIAVAGGVLKEKDGSAHEWGLFSEELKRNPDLNIITKIVGLSYDN
LPSHLRLCLLYFGMYPKDYEIKYGRLVRQWVAEGFVTDEGRKTLEEVAHGYLLYLVERSLVQVTSYPRKEKVKKCRVTDSI
HDMILTKVKDTCFGEYIGGGHDQSESSDLVRRLTIETNNDLNRRFKRSHIRSIISIPGKQEESWSVDLERNILKDDMPLKV

LDFEGCGLFCVPKKLGNLIYLRYLSFRGTQITVLPKSIGKLVNLKTLDIRQTQVLVLPKEITKLRKLRHLLTPSSAFSSIEWKDI
GGMTMLQKIPQVRMEDDGEAIKEVGKLKQLRNLRVLCFGGEHITLLFSSINEMEHLQALRIEKSGDRSVVDLNTTLPRF
PWSKLRKLFLDMKLERLPNWIPQLKNLERLTLRHSDLTNDPLESLKDMPCLLVLSLSHAYEGQTLHFQPGGFKKLRMLNL
QHLWNLNSILIAETALQALEDFELTCLDDLRTVPDGIQHLMKLKSVRVPYMPTQFVEEIVRIGKDKPWRINYGYM

>XP_014518072.1

MASDTAEVLKENFLEFFKDSESFNLSPTISSILKEIEAEFSEYKGFWSPPPVESLHYLYQLDHAIAECRANKKTFSMFSRLDP
VERANETVLKNIKEYLQDTRNFKNNINEYSTFPLFSSCRYPTGNDAPDLSVFDGEMEKIVKWLESNNGFKAIGIHGMCG
TGKTTLAKMVLNDPRVRDKYKKPIWVCLYDLQSKEEMDIRIVKEMLALLDDDPDLLAEEPEDKWLVKNLHDKLLDQKN
YLIVLDAVWHCNDWFNNLFCVDQDGGDTSKELFSQALPKETGGAVIVTSRQKEVTTKLVREENLIHLKPWDDEKLKNF
VKQCLKKQDKITQENIDCVAYHCHGIPFVAATISGWIAEQITKTSDSN

>XP_014518510.1

MAETAVSFASKHVLPKFLEAVKMLRDLPKEVAEVADELESFQDFIHDADKVAEAEEDKNRRDRIRKRLMKLREAAFRM
EDVIDDYVICDEKQPEEDPRCATLLCETVEIIKTQILRLQIAYQIQDVKSLVRAERDSFKNHFPIGPRSDGSRRNENFTWQK
LRIDPLFIKEDEVVGFEGPIHTLKKWLTEGRKERTVISIVGMAGLGKTTLSKQVFDRVHTDFECHALITVSRSYTVEELLRD
MTNKLCKERMEDPPRDVATMNQMSLIEEVRNRLCNKRYVVLFDDVWNETFWDDIELALIDNKNGSRILITTRDEKVV
NFCKKALFFEVHKLQPLSKAKSLELLCKKAFGYGFDGCCPKDYEEAGLDIIRKCECLPLAIVAIGSLLYRKCKSSSEWHLFSQ
NLSLELQSNSELHSVTKILSLSYDDLPHNLRSCLLYFGMYPEDYEVKCGRIIKQWIAEGFVKHESGRNLEEVAQQHLMELI
SRSLVLVASFTTDDKAKTCRVHDSIHEMIRGKIKNTGFCEYIDEHNHLESSGIIRRLTIARNSNGLSGSIEESQNIRSILIFTNE
VSSEDFTRRLLAKYMRLRVLDFGYAPLHDVPEDLGSLIHLKYLSFRGTFISGLPKSISKLQNLETLDVRAFGEIEVPKEITKLR
KLRYLLGNPISSIAVKDSLGSITSLEKMHVLIIDPDGVVIRELGKLKQLRDLRLTMLRGDQADTLCSSINEMPLLERLHISLKY
GTETIDLHIRLSLSKLRKLRLYGSLKELPNWIPRLQNLVKLSLVESRLTNIRLADLGSMPNLLLLSFDSNSYDGETLHFENGG
FQKLKELHLNGLQQLSSIFIDSGALQSLEKLQIMSILQLKAVPCGIQHLKKLQVLDILYMPTEFQQRIDPNGGKEHWMIKH
VPDVHFVTKNRALLLAERAAEIFSSRL

>XP_014519090.1

MVDQKVIGVVAASSYSPSVVSSKRYDVFLSFRGEDTRKKFTSHLYDALKQKKVETFIDNRLEKGEEISTTLIQVIEDSHISIVI
FSENYASSKWCLGELSKIMECKKEKGQIVIPVFYDIDPSHVRKQTGSYEKLFVTHKGEPMCNKWKAALTEAANLAAWDS
QTYRVESELLKDIVEDVLQKLAPIYPNCHKGLVGIEENYEKIESLLKVGSNEVKILGIWGMGGIGKTTLARALYDKLSHEFE
GHCFLENVREESDKHGVKALRNKLFSELLGNKNHCFDVAFSVTKFVLSRLGRKKVFIVLDDVATSEQLENLIEDFDFMGL
GSRVVVTSRNKQIFSQDKIYEVKELSFHHSLQLFSLTVFREKQPKYGYEDLSRSATYYCKGVPLALKVLGATLRSRSKGAW
ECELRKLQKFPNMKIHSVLKLSYDGLDHTQKDIFLDIACFFRGNQRDHVTNMLEAFDFSAISGIEALLDRALITVSGCNQL
EMHDLIQEMGWEIVHQECVKDPGRRSRLWKHEEVHEVFKYNKGTDIVEGIILDLSKLIEDLYLSSDFLAKMTNVRFFKIH
SWSKFNIFNVYLPNGLNTLSHKMRYLHWDGFCLESLPANFCAEKLVELCMRCSKLKKLWDGVQNLVNLKTIDLWGSRD
LIEIPDLSLAEKLENVSLCYCESLREVHVHSKSLRVLNLYGCSSLRKFSVTSEELTRLSLAFTSICSIPTSIWHKRKLKALYLTGC
RNLGKLTEEPRIHGSHKHSLTALASNAERFSMNIKSLSTLRMLWLDDCKKLVSLPKLPPSLGKLSASNCTSLDSYMTQRLV
LQHMLQSRIPYLRTNDLRCYDEEYLFPGNHVIEECVFNTTETSMTIPYLWKTELYGFIYCIILSKGSLLQSDVSCSVYQDGIR
VGWLQRLLEYESLTSNHVLCMYHDINEFDAINEEGHGHFFSNVTFIFENSKASIEEFGVFPIYGSESGLKLVGSR

>XP_014519091.1

MVDQKVIGVVAASSYSPSVVSSKRYDVFLSFRGEDTRKKFTSHLYDALKQKKVETFIDNRLEKGEEISTTLIQVIEDSHISIVI
FSENYASSKWCLGELSKIMECKKEKGQIVIPVFYDIDPSHVRKQTGSYEKLFVTHKGEPMCNKWKAALTEAANLAAWDS
QTYRVESELLKDIVEDVLQKLAPIYPNCHKGLVGIEENYEKIESLLKVGSNEVKILGIWGMGGIGKTTLARALYDKLSHEFE

GHCFLENVREESDKHGVKALRNKLFSELLGNKNHCFDVAFSVTKFVLSRLGRKKVFIVLDDVATSEQLENLIEDFDFMGL
GSRVVVTSRNKQIFSQDKIYEVKELSFHHSLQLFSLTVFREKQPKYGYEDLSRSATYYCKGVPLALKVLGATLRSRSKGAW
ECELRKLQKFPNMKIHSVLKLSYDGLDHTQKDIFLDIACFFRGNQRDHVTNMLEAFDFSAISGIEALLDRALITVSGCNQL
EMHDLIQEMGWEIVHQECVKDPGRRSRLWKHEEVHEVFKYNKGTDIVEGIILDLSKLIEDLYLSSDFLAKMTNVRFFKIH
SWSKFNIFNVYLPNGLNTLSHKMRYLHWDGFCLESLPANFCAEKLVELCMRCSKLKKLWDGVQNLVNLKTIDLWGSRD
LIEIPDLSLAEKLENVSLCYCESLRE

>XP_014519514.1

MDIQEESPVFGSLTAVTTRNMSSSSSVFFSANQSPFFSPRSPSSCQLSHSARLDTQNNTVHLGLAPSSTTLEIPEPNSAVN
VRCNVSDVSASPAGCNSGGLMKLDRKSSPVGISSSSISSYSNCHDDGYSGQRERRIKKDRNHRTSSTPGSTSFSSYRLRSC
DVFIGLHGSKPPLLRFAKWLCGELEIQGISCFVSDRARSRSSRKLGIAERAMDAASFGIVIITKKSFKNQYTIEELNFFCRRK
NLIPIYFDLSPADCLVRDIIEKRGELWEKHGGELWLSYEGLEQEWKDAVHGLSRVDECKLEAQDGSWRDCILRAVTLIA
MRLGRRSVAERVTKWREKVEKEEFPFIRNENFIGRKKELSQLEFILFGDVTGDAEQDYIELKARPRRKSVRIGWGKSNMI
DERWNDRRKEKEPVVWKESEKDIEMQGVEFSHRHNHPRLKRGKYAKRKNGMKILYGKGIACVSGDSGIGKTELILEFA
YRFHQRYKMVLWIGGESRYIRQNYLNIRSFLEVDVGVENSLDKTKIRSFEEQEVAAISRVRKELMKNIPYLVIIDNLESEKD
WWDHKLVMDLLPRFGVETHVIVSTRLPRIMNLEPLKLSYLSGVEAMSLMVGSSKDYSVAEVDALRSIEEKVGRLTLGLAI
ISAILSELPITPSRLLDTINRMPLKEMPWSDKEALSFTKNAFLLQLFDVCFSIFDHADGPRSLATRMVLVSGWFAPGAIPIS
LLALAAEKIPERCQGKCFWRKMLQLLSCGFPSSYAKKPELEASSLLLRFNIARNSTKQGYIHINEVFKLYARKRENTGAAQ
AMIQAIISNGSISQNLDHLWAACFLLFGFGHDPVIVELKVSELLYLVKRVVLPLAIHTFITYSRCTAALELLRLCTNALEAAD
QAFVTPVDKWFDKSLCWRSIQTNAQLNPCLWQELALTRATVLETRAKLMLRGAQFDVGDDLIRKAVFIRTSICGEDHP
DTVSARETLSKLTRLNANVQIHT

>XP_014519671.1

MADALLGIVFQDLQSVVQSQLATYWGIDQQAQKLSSNLTAIRAVLRDAERKQIASHAVKDWLQKLTDAAYVLDDILDE
CSIHSREVLSDDGHTSCLARAHPKDILFRFHIGKRMKNINQRFHDINEERRMFELRDGVTEKPTVDDGEDDQTTSVITEP
IFCGRDEDREKVVKVLLEANNDKDLTVYPIVGMGGLGKTTLAKHVFHDHRVCEHFDLTIWVYVSVDFNVKAILQSIIEYA
TGQNPNLHSLETMRKKVEEVLQSKRYLLVLDDVWNEDQEKWKHLEGMLRFARGAKGATXLVTTRLEEVASIMQTHPA
YHLXELSEYDSWSLFKSYAFGPNREEKEELMAIGEDIVKKCVGSPLAIKTLGSLLRDQTAVTQWENIKESEIWDIRSESSS
MTGKENSIMRALKLSYFNLELSLRRCFSFCAIFPKGFEIVKEELIHLWMANGFIKSEGNVEVEEVANNVWRKLYRRSFFQ
EAKSDKFGMITSCKIHDLFYDLAKSIMGEECVMVEEGRLTQVSTRVHHLHFLSYNMSVDVAAFKKVESLRTFLFGNTRQ
LPSNRFLRALCIRSSMLPPLNDLAHLRYLSVHDSSVRNLNDSICQLSKLQILKLESCFDFYEIPKKLTQLQDLRHILINKCASV
PDMPPNIGKLRHLRTLSLFDAGSTPGYGLTELSGLRLGGKLHIRGLGNVSDEWDAKEANLKSKKELNRLYLSWGGGANS
EGNNVSAERVLEALEAPSTLKGFGMEGYQGRKLPSWMRSVVVLKDLVEIILFDCNNCEELPPLGKLPHLKRVHVRGMK
NVKWIDGESYEGVEEKAFPSLEKLSVENLPKLERMLRDEGVEMIPHLSQLTIDGVFNFKVPHLPCVEELHATRIVAATSFI
EGVGDNMVCLKTLRVSLIKGLKVLPDQLRRLGALEYLRIGFWYDLEHFPEHVLEGLTSLRGLSISNCENLKSLPEGVRHLT
CLRRLSIDNCPKLVALPSNMSKLTSLRTVYMDDCSTLPYGLQFVPSLRTLDIERYMSTSLPDWMGEMTSLQELTILDCGE
LRSLPSSIQRLTNLSYLTIHGCPHLRKRCVGGEDWQYINHIPQIELDSYSLPEEKTLTISGESIRRLSCSWNCFKPARQPAKG
VVSSNSFDRVLEE

>XP_014519672.1

MAEALVESVIENLGSLFQDQLAIYWGVDQQTEKLSSNLRAIRAVLRDAERKQITSYAVKDWLQKLTDAAYVLDDILDECS
IHSTKMHSVDGHISRLSRLHPKDVLFRFHIGKRMKDITQRFHNIHEERLTFELRVGVTEKQAVNDDDDDWRQTSSVIT
EPIFCGRDKDREKIVKFLLEDASNSEDLSIYPIVGMGGLGKTTLAKQVFNDHEISKHFDLRIWICVSEDFNVKRILQSIIECTI
GQNPNLGDLEARRRKVEEALQNKRYLLVLDDVWNENTEKWKELKGMLECANGAKGTTILVTTRLEEVASIVGTHSAYR

LKVLSEDDSWSLFKHHAFGPNREEREELVTIGKQIMRKCVGSPLAIKTLASCLRDETEVRQWENVKKSEIWNIQEESSSS
MTGEENSIMRALKLSYSNLKSSMKRCFSFCAIFPKDFEIDKEELIHLWMANGFIKCEGNVEVEDVGNKVWKKLYRRSFF
QEAKYDEVGMITTFKIHDLFHDLAQSIMGEECVVIVKGRLTQLPTRVHYSSLLNSGVSGYMTAFKQRLTTALKKVKSLQS
FLDFGGIGPVPSNHSLRALQTSSSLLSPLKDLTLLRYLSLRFSQSSEENLNNSICQLPKLQILKLFHCPELHGLPKDLTRLQDL
RHIVIDGMDTIQEMPPNIGKLRHLRTLSIFVVGSKPGCGLAELHSLNLRGTLSIRGLENVLNELDAKEANLIGKKELNILELS
WDGSGNPKGSNVSVERVLEALEPPSTLKSFQMDGYQGRQFSSWMRSSLVLRDLVNVILSDCENCEELPPLGKLPHLKR
LIVSGMKNVKWIDGETYDGVEEKAFPSLEELIVDNLPNLERLLRDEGVEMLPRLSQLTIEDVLNFKVPRLPCVETLDARGI
EAGTSFMEGVGENMACLETLIIKIIKGVVVLPDEFRRLGALQELYIGEWYDVEYFPEHVVEGLTSLRTLSIKNCEKLKSLSE
GVGHLACLERLWIEKCPELVALPSNMSQLSALRNVSIQLCCTLPDGLQRVPSLRVLFIEKCTCTSLPDWLGDMTTLERLFI
WHCEELRSLPSSIQRLTNLSYLSIEGCPHLKKRCKRETGEDWQYINHIPQLFVDGNDVL

>XP_014519673.1

MAEALLGTLNQNLQSFGQDQLATFWGVDQQIQKLSSNLTAIRAVLRDAERKQITSYAVKDWLQKLTDAAYVLDDILDE
CSIHSTKLNFDDAHTSCLTRFHPKDILFRFNIGKRMKDITQRFQDIHEERSMFNLEPGVTEVQTINDNWRQTSSDITEPV
VYGRDQDREQILKFLFEEVSDSEGLSIYPIVGMGGLGKTTLAKQVFNDNRVCKHFDLTIWVCVSDDFNTMTILRSIIECIT
GQKSKLKSLEAIRKKVEEVLHGMKYLLVLDDVWNEDQEKWKQLKGKLQCARAAKGATILVTTRLEEVANTMQTHPAY
HLKELSGDDSWSLFKHHAFGPNREETEELVSIGKEIVTKCIGSPLAIKTLGSLLRDESDVKQWQNVKESEIWHIREESSFAT
GEENSIMRALKLSYFNLELSLRRCFSFCAIFPKDLEIDKEELIHLWMANGFIESERNIEVEDVGNKVWNKLYRRSFFQEAKC
DEFGMIKSFKVHDLFHDLAQSIMGEECVVVVKGRLTTLSTKVHYSSLFNSGESDDMTAQRVHYSSLFNSGESDDMTAQ
RFMTALKKVESLRTFLDYEGIGPMPSNPCLRALQTSSSLLSPLKDLTLLRYLSLSLTFNRSSEENLNNSICQLPKLQTLKLCN
CHQFHGLPKNLTRLEDLRHIVINGRDSIQEMPSNIGKLRQLRTLNIFVVGLKPGCGLAELHSLKLGGTLRIRGLENIPNEW
DAKQANLMSKKELNRLHLSWGASANSEGSNVSAEKVLEALQPPSTLKSFEMRGYRGRQLSHWMRSVVVLRDLVEVNL
LDCDNCDELPPLGKLAHLKRLEVSGMKNVKWIDGETYNGVEEKAFPSLEKLTMRNLPNLERMLREEGVEMLPRLSELRT
FCVSNIKFPRLSSVEKLIAVSIDEVASFMEGVVGNLPSLKTLEIQSVKGAVVLPNQLRVLDALQELRIGDWHDVEYFTEHV
LEGLTSLRTLSINYCKKLKSLSEGVRHLACLESLTISGCPELMDLSSNMSQLTTLWTVSIKVCSRLPYGLQHVPSLRTLDITSC
KCTSLPDWVGDMTSLQKLSIRFCRELRSLPNSIQHLTNLSHLSIHGCPHLKKRCKRETGEDWQYIKHIPKIELFHCETDLL
W

>XP_014519682.1

MAEALLGTVIQNLQSFGKDQLATFWGVDQQTQKLSSNLTAIRAVLRDAERKQITSHAVKDWLQKLTDAAYVLDDILDE
CSIHSTKLNFDDRHTSCLSRLHPKDILFRFNIGKRMKDITQRFHDIHEERSMFNLVPGVTEVQTIHDNWRQTSSDITEPIV
YGRDQDREQILKFLLEDVSDSEELSIYPIVGMGGLGKTTLAKQVFNDNRVCKHFDLTIWVCVSDDFNTMTILQSIIECITG
QNPNLNSLEAMRKKVEEVLHGSRYLLVLDDVWNEDQEKWKQLKGKLQCARAAKGATILVTTRLEEVASTMQTHPAYY
LKELSGDDSWSLFKHHAFGPNREEKEELVSIGKEIVIKCVGSPLAIKTLGSLLRDESDVKQWQNVKESEIWDLQEESSSGT
GEENSIMRALKLSYFNLELSLRRCFSFCAIFPKDFEIDKEELIHLWMANGFIKCEGNIEVEDVGNKVWKKLYSRSFFQEAKY
DEFGMITTFKMHDLFHDLAQSIMGEECVVIVKGRLTPLPTRVHYSSLLHSGVSDDMTAFKQRLTTALKKVESLQTFLDFG
GIGPVPSNHCLRALQTNSSLLSPLKDLTHLRYLSLRFSSSSEESLNNSICQLTKLQILKLCNCPELHGLPKDLTRLQDLRHIVI
DGMDTIQGMPPNIGKLRHLRTLSIFVVGSKPGCGLAELHSLNLGGRLRIRGLENVPSEWDAKQANLMSKKELNRLHLS
WGSSANSEGSNVSVERVLEALEPPSALKSFGMKGYQGRQLPNWMKSYVVLRDLVEVGLLDCENCEELPPLGKLPHLKR
LKVSGMKNVKWIDGETYDGVEEKAFPSLKELSLYSLPNLERLLKDEGVEMLPRLSRLTIDGVFNFKVPRLPCVEKLSAKRI
GEVGSFMEGVVGNLPSLKTLDIYCVKGAVVLPDQLSGLDALQQLHIDVWHDVEYFTEHVLEGLTSLRTLTIQDCKKLKSL
SEGVRHLACLESLRISKCPELVALPSNMSQLSALREVSINSCCTLPDGLQRVPSLRVLYIYECTCTSLPDWLGDMTTLEKLTI
LYCEELRSLPSSIQRLTNLSYLRIQYCPHLKKRCKRETGEDWQYINHIPNIQVLFGRKPTFCGEFKSVLFTSPRNVSLFENFV

>XP_014520718.1

MAAEMVTGVLVSTFLERTIDTLASRLLNIFHQRKHKKQLSNLKMKLLAIDVVAFEAEQKQFTDPRVRDWLLRAKDVVID
AEDLLDELDYELSKTKVEAESQSASKKVWNSLDSSFFEIEFDPMMEQVIEDLEELAIQSDFLGLKKVGGVGVGSVSDSKLI
YTSLPNESVIYGRDDDKEFVLNWLTSDTHNNLSILSIVGMGGMGKTSLAQHVFNDPRLEEASFDTKVWVSVPQEFDVL
KVSRAILDTITGSTDHSIQQEVIQKRLKEKLMGKKFLLVLDDVWNERSSKWEDVQKPLIFGGQGSRILVTTRSEKVVVAM
RSEKRLLQVLKKDYCWDLFAKHAFQNGNPQPDSDFIEIGKKIVEKCNGLPLALKTMGSLLHNKSSVSEWRSIMKSEIWD
FSENESDILPALRLSYFHLPSHLKKCFAFCALFPKGYRFDKEWLIQLWMAENFLENPLQKKSPEEVGEEYCNDLLSWSFFQ
QQSGYEGKKGFIMHDLLNDLAKYVCEDICIRLGVDEPKHIPKTTRHCSFSDSGFDGFGSSIDSQKLHIFTPTERIWDWVC
KMSIDDLFSRFKLIRVLSLYNCRSLTEVPESVGNLKHLRSLDLSWTRIEKLPDSIILLYKLQILLLNYCEKLKELPSCLYQLDNLR
RLDLEGSGVQNVVAHLGKLRNLQVAMSSFHVKKSKEINFQQLGELDLHGSLTIDDLQNIESPSYALEVDLKNKPHLVKLR
LEWNFIGSSSVDSEKAEDVIENLHPSKYLKKLSIRNYIGKQFPDWLLHNSLPNLVSLELEGCESCQGLPPLGLLLFLKHLSIA
RLDGILSIDADFHGNNSSSFKSLQTLYFSDMRQWEKWDCQAVTGAFPCLQEFSIKNCPKLKGHLPKFGALKTLRAIHCQ
QLEALIRLRVEELSVCSALESISDDCVSLRIFPLDFFPTLRTLELSGFPNLQMISQNHVHNHLWHLYIKECPKLESLPANMH
MLLPSLRELQIKECPRLESFSEGGLPLNLKEITLNNCFRLVGSLKRALGDSPSLISLSIKKVEAECFPDEGLLPLSLTQLIINDSP
NLKKLNYKGLLELSSLRSLKLSKCPNLECLPEEGLPKSISFFQIFNCPLLEQRCHKEGGEDWEKVSHIPELLIW

>XP_014520932.1

MASNKRQRSSSSHTKNFDVFVSFRGADTRNGFTNHLFAALERNGVVAFRDDQTIQKGNFLESELLLAIEGSRVFIVVFSK
DYASSTWCMKELTKIVDWVEVTGRSLLPIFYDVTPSEVRKQSGEFAKAFAEHEERFKDDLEMVKGWRAALKTSCDRCG
WDVQNKQQYEQIENVVEEVINILGRNQIWNFGDDLVDMHSRVKKLEELLDLSANDIVHLVGICGMGGIGKTTLATALF
NKISPQFDACCYLDDLSKIYCNFGAASAQKQLLCRALNRGNIEIHNASHGTMLIRTRLCHLKALVVVDNVDQVEQLKKLG
LQSEYLGAGSRIIIISRNRRILQNYGVNKVYEVQVLDXTXSLQLLXKKAFRSNDIGKEHEXLTLDILKYVNGLPLAIEVLGSFL
LDRDVCEWRSALTRMEENPSKDIMDVLRISYDGLENIEKEIFLDIACFFSNNNLYSWEPTVKKLLDYRQFYSDIGMKVLIE
KSLIRCQDETIIMHDLLKELGKSIVREKAPKEPRKWSRLWNYKDLQKVTKINKEAEKVEAILIEQHEKEFLQGRIRVDALSK
MDHLEMLILQNVNCYGTLNFISNELRYLFWNHFPWLSLPSTFFPDQLVELILRDSNIKQLWEGKKCLPNLRNLDLSHSKN
LIEVPDLSEVPRLTDLSLEGCIQLVHIHPSIGILRDLRLLNLKNCKTLVLNLNILFGISSLEILNISGCSQLLNSKMLMDPSDTK
HLEKVDKNTNIIQLPTSSVYKLLMLPFHFFYPPKPQDSIGSLLSSFSFSVPCLFNLDISFCNLLQIPDEIGNLRSLEILNLGGNK
FVTLPSTIKQLSYLHYLNLTHCKELKYLPELPTIQEKTIDRYIRGLYTFDCPKLSDLEHCYSIVFSWMKKNLEVYLVPRMEIVI
PGSEIPKWFSKQNASASISMDPSAVIDDPNWIGVSICVLFVTHESPMNLGEGDYLIDSPLFYGVNNVNFETKWYSGVPII
FKKDLVTVGLDYLLIVFYSRQEFFHLLNGHSDTMHDLQALKFETWGRSLLDLRFMVKKCGYRWVFKEDLQQLNSDKFFS
RNSASRKRKLLTSE

>XP_014521060.1

MECLLGFVTSFSRDLVCGALNQLRYPCCFKNFVKRLEEEESNLIITKDSVQKFVTHDKKQARKPSEIVDKWLEDAINDVH
NVNQLLEEARTKKHCCFGHCPNWIWRYHVGKKLANKTMDLEKFIEKGRKYVPFDRIATLPSNTLDMLSEKCMNFESRQ
YAYEQLLDAVKNSDVSMIGLYGMGGCGKTTLAMEVRKLVEAEHIFEKVLFIAVSSTVEVRRIQEKIASSLQFEFPETEEM
QRAQRLCSRLTQEKNIFIILDDVWEKLDFGRIGIPSFEHHKGCKIFITTRSEAVCTLMDCQRKIYLPILTDEEAWTLFQNKA
LISEGTPHTLKNLGRLISNECKGLPVAIAAVACSLKGKTETIWSVALNKLRRSKPINIERGLIDPYKCLQLSYDNLDTKEAKSL
FLLCSVFPEDSEIQVEILTRCAIRLGVVGEVDSYEEARSEVIAAKIKLVSCCLLLDGDNERVKMHDIVRDVAHIIAQNENKKI
KCEVEIDVTVEQNSVKYLWCSKFPNDLDCSNLEFLCLHTIMKGFEGIFKRMGKLKVLLLGNDEDEKTPLSTTSLKTLTNLR
DLFIFNFELSDFSFLSGMKNLQTLSLYDCSLPSFPEFQTDVAITLKLLELNECYIKVKNFEVMKRIPLLEELYIIDITGEWYANS
EDNIEFFKTFSIPKTLQRYGIVLGSDNFYHYNDRDIYIHSRTLLLNHFDISKNEVIKGLAKKAKDLFVRNIHGGAKNMIPDIF
EIEGGGLNDLNTLEIRDSAGIKCLIDTRSHSSEVVTLFSKLHTLEMKRMENLKAIWHCFLPANGPFENLENLYLSNCPRLTF
LFTYVVARNLVQLKILKISGCDELKHILTYDEKSQDEFTTGHPIQIFQNLLDVKIKRCRELKHIFPANIVGGLTQLKVLEIREC
DMLDQIIGDIVPLTEQDRKQELDEIVEEGTLPSLTSLKITYCGKLDSIFTASKAKTLTSLEELFIEDCKSLKDIVTHERVNKNQ

EESIVEDEHISECDSLEVVFPNWTGPKNSLTLQHLTILTIWKCGNMEVIFPKSVVRCLPELKKVTIRECMELKQIIEEDESNL
LSIDGGFEVEEIIGCDKEASKNYFAFPNLEKLEIIECEKLEVVFPKSILRCLPKLKLLKISQCKELSQIIEEDKNLSNILSPQPCFP
KLEALHVDDCHKLKRLFSGSASNDLPNLHLLAINGTYELEELVGCKQGKTKVELPRLKLLIFMHLPNFRQEIELRDLKNCIIY
KCPKLSLTSTTTLEKLLEDFPHKDFIHTELGPSELKGIVRSIYEYSTFNGSSEFTSSQEIEVVGNENIEEGPLVEGSKTKSSSTG
VEDIGIGSGVEDIGIYEDSTSGSSELTSSQEIEDVGNESIKSSSTGVEDIGIGGATHIESGGEDILALDSKVVEQDDKMNEGK
PGIVASQGIQVQEGLNLLHKQDGTDVVPNNNIDISSDICTRLGAYKHFADLDDAQISLLVEAITTYPHLWNASKKFSERF
QAWRLKILADMLLFLQKESVDSIIPQREKEFHKLCEEAIEIGFESSWVEEMRQRVVARDPKLEEDIAKRQMDENSKSLLN
RCSSGDVVEEGDGPKIRLEEGSDLVDKEGEVGVVSNDHILAPRNEEPEHEFVAEVSTSEIPRIATSLTNSQTVEKQTPSHL
YIPVRETSSNALVDTKQTSEPCLMKQKKPLGEIPKSIDQVVEEETIAKNTDMAASSILSDSTTSKLDTTVTLQRKSHPHSEI
RSSQNVVRITKESEGHLELIQDFGGNDMIAISLGKEGDDNIVVKTLVELENYLKMSLKDIVSSETNALRLFSTLNFLSNLPF
KDVTLSDRLKHIIKTMHQHFPTILCSFKQRFATTDKLAELEARQNEVAIKIFEAENFNDEARLKEVVLKEEIIRLKEEIKVCEA
ALSSLDEGKNKCIVETIRYKKELENVRKNKSQMVKDQRKVEQELFEVAYKWSVLCSEYELNRIAARNPS

>XP_014521061.1

MECLLGFVTSFSRDLVCGALNQLRYPCCFKNFVKRLEEEESNLIITKDSVQKFVTHDKKQARKPSEIVDKWLEDAINDVH
NVNQLLEEARTKKHCCFGHCPNWIWRYHVGKKLANKTMDLEKFIEKGRKYVPFDRIATLPSNTLDMLSEKCMNFESRQ
YAYEQLLDAVKNSDVSMIGLYGMGGCGKTTLAMEVRKLVEAEHIFEKVLFIAVSSTVEVRRIQEKIASSLQFEFPETEEM
QRAQRLCSRLTQEKNIFIILDDVWEKLDFGRIGIPSFEHHKGCKIFITTRSEAVCTLMDCQRKIYLPILTDEEAWTLFQNKA
LISEGTPHTLKNLGRLISNECKGLPVAIAAVACSLKGKTETIWSVALNKLRRSKPINIERGLIDPYKCLQLSYDNLDTKEAKSL
FLLCSVFPEDSEIQVEILTRCAIRLGVVGEVDSYEEARSEVIAAKIKLVSCCLLLDGDNERVKMHDIVRDVAHIIAQNENKKI
KCEVEIDVTVEQNSVKYLWCSKFPNDLDCSNLEFLCLHTIMKGFEGIFKRMGKLKVLLLGNDEDEKTPLSTTSLKTLTNLR
DLFIFNFELSDFSFLSGMKNLQTLSLYDCSLPSFPEFQTDVAITLKLLELNECYIKVKNFEVMKRIPLLEELYIIDITGEWYANS
EDNIEFFKTFSIPKTLQRYGIVLGSDNFYHYNDRDIYIHSRTLLLNHFDISKNEVIKGLAKKAKDLFVRNIHGGAKNMIPDIF
EIEGGGLNDLNTLEIRDSAGIKCLIDTRSHSSEVVTLFSKLHTLEMKRMENLKAIWHCFLPANGPFENLENLYLSNCPRLTF
LFTYVVARNLVQLKILKISGCDELKHILTYDEKSQDEFTTGHPIQIFQNLLDVKIKRCRELKHIFPANIVGGLTQLKVLEIREC
DMLDQIIGDIVPLTEQDRKQELDEIVEEGTLPSLTSLKITYCGKLDSIFTASKAKTLTSLEELFIEDCKSLKDIVTHERVNKNQ
EESIVEDEHISECDSLEVVFPNWTGPKNSLTLQHLTILTIWKCGNMEVIFPKSVVRCLPELKKVTIRECMELKQIIEEDESNL
LSIDGGFEVEEIIGCDKEASKNYFAFPNLEKLEIIECEKLEVVFPKSILRCLPKLKLLKISQCKELSQIIEEDKNLSNILSPQPCFP
KLEALHVDDCHKLKRLFSGSASNDLPNLHLLAINGTYELEELVGCKQGKTKVELPRLKLLIFMHLPNFRQEIELRDLKNCIIY
KCPKLSLTSTTTLEKLLEDFPHKDFIHTELGPSELKGIVRSIYEYSTFNGSSEFTSSQEIEVVGNENIEEGPLVEGSKTKSSSTG
VEDIGIGSGVEDIGIYEDSTSGSSELTSSQEIEDVGNESIKSSSTGVEDIGIGGATHIESGGEDILALDSKVVEQDDKMNEGK
PGIVASQGIQVQEGLNLLHKQDGTDVVPNNNIDISSDICTRLGAYKHFADLDDAQISLLVEAITTYPHLWNASKKFSERF
QAWRLKILADMLLFLQKESVDSIIPQREKEFHKLCEEAIEIGFESSWVEEMRQRVVARDPKLEEDIAKRQMDENSKRCSS
GDVVEEGDGPKIRLEEGSDLVDKEGEVGVVSNDHILAPRNEEPEHEFVAEVSTSEIPRIATSLTNSQTVEKQTPSHLYIPV
RETSSNALVDTKQTSEPCLMKQKKPLGEIPKSIDQVVEEETIAKNTDMAASSILSDSTTSKLDTTVTLQRKSHPHSEIRSSQ
NVVRITKESEGHLELIQDFGGNDMIAISLGKEGDDNIVVKTLVELENYLKMSLKDIVSSETNALRLFSTLNFLSNLPFKDVT
LSDRLKHIIKTMHQHFPTILCSFKQRFATTDKLAELEARQNEVAIKIFEAENFNDEARLKEVVLKEEIIRLKEEIKVCEAALSS
LDEGKNKCIVETIRYKKELENVRKNKSQMVKDQRKVEQELFEVAYKWSVLCSEYELNRIAARNPS

>XP_014521076.1

MGDLFSGGAVGALMGEALKGAISIIKKGLAFKSTLDSNIETLNSLAPLVEEMKMCNMMLDRPSKEIEKLESLMRNSQEL
VRKCSKLGRWKMWSFPYFQSKLRSKEVALQTHLSMNMTAQNTVNLMKLVDDMRKVLEILLKEEFGRYPEYRLIDLCG
APQEPECLGMDVPLRKLRIELLKDGVSVLVLTGLGGSGKSTLAKKICWDPQIKDKFGANIFFVTVSKTPNLKTIVETLFEHC
RCRVPKFQTDEDVVNRLEVLLRVFGKHPILLVLDDVWPGSEDLVEKFKIQIPDYKILVTSRVSFPRFGTSYQLDKLDHVHA

ESLFRHFAQLKDKSSYIPERNIVDEIVKGCKGSPLALKVIAGSLCNQPFEVWKNMKERLQKQSILESDSTDLLFRLQQSLDI
LETKFSVNEKECFMDLGLFPEDQRIPVAALIDMWVELYNLNEDGTNAMSIIHDLTTRNLINVIVTRKVAKDTDMYYNNH
FVLVHDLLRELAIHLSKGETFEQRERVMIELKGDNRPEWWVGLNPLGIIGRLFSYIMGMLYRQKQPKVAARILSISTDETF
NSDWCDMQPDEAEVLVLNLLSSQYSLPEFTEKMHKLKVLIVTNYGFHPSELNMFERLGSLNNLKRIRLEKVSIPPLCILKN
LRKLSIHMCNTRQTFERYSISDAMPNLVEMSIDYCKDLVKLPDGLCNITPLKKLSITNCHRLSALPRDLAKLENLEVLRLCS
CSDLAEMPDSVKGLNKLSCLDISDCLNLTKLPDDIGELKLQKLYLKGCSKLRELPHSVVKFENLDHKIHVICDEEMASLWE
SSTIRNLKIEISTVEVNLNWLPGAHS

>XP_014521204.1

MADSVVSFVLDHLAQLAAREANLLYGVEDRVQSLQYELQMIKELLNTTKRKKGMEHTVLNQIRDVAHLAEDVIDTFVA
KVAIYKRRTIMGRMLRGFGQARLLHQVADKIDKLKTTLNEIRDNKDKYDAFREATNQSSREEEEEEERSQSLHKLRREVE
EEHVVGFFHDSKDVIQRLQEGGSNRKVVSIVGMGGLGKTTLARKVYNSSQVMNHFNCRAWVYVSNECRVKELLLGLF
KHLMPNFEQQRRGNKKGNKSVEDVNSLSEEELKKHVLKCLERKRYLVVVDDLWKSRDWDEVQDAFPDNNNGSRILIT
SRLKEVVLHAGDDVPHYLQFLSEEESWELFCGKVFRGENCPSDLEILGKQMVQSCRGLPLSIIVLAGMLAKKEKSDREWS
KVVGHVNWYLTRDETQVKDIVLKLSYDNLPRRLKPCFLYLGLFPEDFEIPVMPLLQKWVAEGFIQDTGNRDPNDVAEDY
LYELIDRSLIQVARMKSNGGLARCQIHDLLRDLCISESKEDKVFEICTDNNILIPIKPRRLSIQSGMGHYISSSNNDHSCIRSL
FFFGSEYNVGRREWKWLLDDFKLVRVLYFGSNRCQNIPSNLGNFVHLRYLKINSRDVNFIPDSILNLWNLQTIELGTYMF
DGQISFPGQIWKLKHLRHLNTSRPIKLRGRCSESDEKMWNLQTISLLILNTQTTFLIKKGTFLNVKRMRLYSDMSRYEPNL
LQNLQQLKHLNKLIIDNVWNLLTSEVIFPPSITELSLSRIKCITDEGMNGLRNHSKIKILKLLGFEIYMRSIDRRYTSSIDLNCV
DGDFPQLEVLEMKYLTLGKWKLGNGAMQRLQNVIIHDCQQLDDLLPDFCSLNGLRKVQISKSPLEQIAHILQILETNNG
VQVVRDNHPKVDESDWILNIY

>XP_014521305.1

MADSSVSFLLEKLNALLQEEVNLQRGVREDVQYIKYELERHKAILRVADAMEDRDPELKAWVKGVRDVAHDMEDAID
EFNLRLVDQHGQGNGSSLHRFIFGLKTMRARRRIALDMQSIKSKVNVISQGRPELPGIGSRSSQRLSSRLDSQGDALLLE
EADLVGIDKPKRQLCDLLFNEEPGRAVIPIYGMGGLGKTTLAKQVYDDPKVKKRFRIHAWINVSQSFKLQELLKDLVQQL
HNVIGKPAPEAVGQMKSEELKELIKNLLQSSRYLIVLDDVWHVKVWDSVKLALPSSNRGSRVMITTRKKDIALYSCAELG
KDFVHEFLPEEEAWYLFCKKTFQGNSCPPHLEEVCRKILKMCGGLPLAIVAIGGALATRERANIEEWQMVCRSFGSEIEG
NDKLEDMKKVLSLSFNELPYYLKSCLLYLTIFPEFHAIEHMRLIRLWIAEGFVNGEDGRTREEVADSYLKELLDRSLLQVVS
KTSDGRMKTCRMHDLLREIVNLKAKDQNFATIAKDQDIIWPDKVRRLSIINTLDNVRQNRASFQLRSLLMFDLSDPLEH
FSIRGLCFTGYKLIRVLDLQDAPLEVFPAEIVNLYLLKYLSLKNTKVKSIPGTIKKLQQLETLDLKQSLVTVLPVEIVELQQLRH
LLVYRYEIESYAYFHSRHGFKVAAPIGLMRSLQKLCFIEADQALMVELGKLTQLRRLGIRKMRQQDGAALSLSIEKMINLR
SLSITAIEKHEIIDIHNIFRPPPYLHQLYLSGRLDIFPRWISSMKNLVRVFLKWSRLTEDPLVHLQDLPNLRHLEFLQVYVGE
TLNFKAKGFPSLKVLGLDDLNELKSMTVEEGAMPGLKRLIIQRCGSLKQLPFGIEHLTKLKSIEFFDMPEELITRLHPKGGQ
EDYLRAKNVPAVYSSYWRDGGWDVYSLETLEERETDFSRSTAMRSLENCPLWKA

>XP_014521310.1

MAEVAVSTVATKLAELLVEQAAVAVSQLAGVRGQVENLKNELGWMQSFLRDADAKQEGSDRVRLWVSEIRDVAFEA
EELIETYVYNTTMQRQLDKVFRPFHLYKVRTRIDKILSKIKSISDRRETYGVVMIGDDGNNSNERLRQWRQPSPSSEEEYLI
ELEDDMELFLTQLLALEPNPYVVSIVGMGGLGKTTLAKKLYNHNKITNHFDCKAWVYVSKEYRRIDVLQRILRDVDGAP
RHEMGRIPEEEFINKLRTVLSEKRYLVVLDDIWGMEVWDGLKSAFPRGKMGSKILLTTRNWDVALHADACSNPHQLRP
LTADESLRLLSNKAFPGTNGIPSELKDLATEIVVKCGGLPLAVVVVGGLLSRKLKSSGEWKRVLQNISWYLLEEQEKIARIL
ALSYNDLPSHLKSCFLYLGLFPEVVNIQTKKLIRLWVAEGFLPQEGEETAEGVAQKYLNELIGRCMIQVGTVSSLGRVKTIR
IHHLLRDLSLSKGKEECFLKIFQGDVAGPSTKSRRQSMHSCDERYDFLKHNAHHSRSLLFFNREYNADIARKPWLPLNFQ

QEKKLNFIYRKFKLLRVLELDGVRVVSLPSTIGDLIQLRYLGLRKTNLEEELPLSIGNLLNLQTLDLSYCCFLKKIPNVIWKLVN
LRHLLLYTPFDSPDSGHLRLDTLTNLQTLPHIEAGNWIVDGGLANMANLRQLGICELSGQLVNSVLSAAQGLRNLYSLSL
SLQSEEDEFPIFMQLSQCTHLQKLSLNGKIKKLPDPHEFPPNLLKLTLHNSHLQKESIAKLERLPNLKMLVLGKRAYNWPE
LTFNSEGFSQLHILRLVLLKELEDWKVEQSSIPRLEYMVIDRCEKLKAIPEGLKAITSLKKLKIIGMPVEFEHKLRTKDISEFA
NTPVIESTTDILAID

>XP_014521330.1

MAESAVGFLLQRLAPVFENKVKLFTGVQAEVICLKGQLELIRAFLRVADALEESDDELKVWVKQVRDVVHEAEDLLDEL
ELVQLHNHTNGFSIYLRIRNMKARYRIAHDLKSINSRLKAISSSRKLDSSSVASTSINTGTAWHDQRGDALLLDNTDLVGI
DRPKKQVIGWLINGCPGRKVISVTGMGGIGKTTLVKKVYDDPDVKKHFKACAWVTVSQSCKIEELLKDLAKKLFSEIRRP
IPEGMESMCSDKLKMMIKDLLQRKRYLVVFDDVWHMYEWEAVKYALPNNNCSSRIMITTRRSDLAFTSTIESNGKVYN
LQPLKQDEAWDLFCRNTFQGDSCPSYLIDICKYILRKCEGLPLAIVAISGVLATKDKRRIDEWGMICHSLGAEIQGNGKLD
NFKTVLNLSFNDLPYHLKYCFLYLSIFPQDYLIQRMRLIRLWIAEGFVEAKEGKTKEDVAHDYLKELLNRNLIQVAGTTTDG
RVKTLRVHDLLREIIILKSKDQNFASIVNEQSAAWPEKIRRLSVHGTLPYRQQHRSVSQLRSFLMFGVGEYVPLGKLFPSG
FKLLSVLDYQDAPLKKFPLAVIDLYHLRYLSLRNTKVKTVPGHIIGKLHNLETLDLKKTCVRELPVDILKLQKLRHLLVYQLKF
KGYAEFHSKDGLKAPSEIGKLKSLQKLCFVEANQDCGMIIRQLGELSQLRRLGILKLREEDGMAFCLSIERLTNLHALSVAS
EGESKVIDLTFLCSPPPFLQRLYLSGRLQELPSWIQSLNSLARLFLKWSCLKYDPLVHLQDLPNLAHLELLKVYDGDALHFR
SGKFKKLKVLGLDKFDGLKEVTVGKDAMNCLEKLSIGRCELLKKVPSGIENLTKLKVLEFFDMPDELMKTICPHGPGKDYC
KVLHIPNVYSTYWKDGGWDVYALDTFSTDCSPRSGTLIRSHEPRIQWKV

>XP_014521331.1

MAESAVGFLLQRLAPVFENKVKLFTGVQAEVICLKGQLELIRAFLRVADALEESDDELKVWVKQVRDVVHEAEDLLDEL
ELVQLHNHTNGFSIYLRIRNMKARYRIAHDLKSINSRLKAISSSRKLDSSSVASTSINTGTAWHDQRGDALLLDNTDLVGI
DRPKKQVIGWLINGCPGRKVISVTGMGGIGKTTLVKKVYDDPDVKKHFKACAWVTVSQSCKIEELLKDLAKKLFSEIRRP
IPEGMESMCSDKLKMMIKDLLQRKRYLVVFDDVWHMYEWEAVKYALPNNNCSSRIMITTRRSDLAFTSTIESNGKVYN
LQPLKQDEAWDLFCRNTFQGDSCPSYLIDICKYILRKCEGLPLAIVAISGVLATKDKRRIDEWGMICHSLGAEIQGNGKLD
NFKTVLNLSFNDLPYHLKYCFLYLSIFPQDYLIQRMRLIRLWIAEGFVEAKEGKTKEDVAHDYLKELLNRNLIQVAGTTTDG
RVKTLRVHDLLREIIILKSKDQNFASIVNEQSAAWPEKIRRLSVHGTLPYRQQHRSVSQLRSFLMFGVGEYVPLGKLFPSG
FKLLSVLDYQDAPLKKFPLAVIDLYHLRYLSLRNTKVKTVPGHIIGKLHNLETLDLKKTCVRELPVDILKLQKLRHLLVYQLKF
KGYAEFHSKDGLKAPSEIGKLKSLQKLCFVEANQDCGMIIRQLGELSQLRRLGILKLREEDGMAFCLSIERLTNLHALSVAS
EGESKVIDLTFLCSPPPFLQRLYLSGRLQELPSWIQSLNSLARLFLKWSCLKYDPLVHLQDLPNLAHLELLKVYDGDALHFR
SGKFKKLKVLGLDKFDGLKEVTVGKDAMNCLEKLSIGRCELLKKVPSGIENLTKLKVLEFFDMPDELMKTICPHGPGKDYC
KVLHIPNVYSTYWKDGGWDVYALDTFSTDCSPRSGTLIRSHEPRIQWKV

>XP_014521504.1

MADSVITFVLDHLAQLAAREADLLCGVEDRVQSLQYELQMIKELLGTTKRKKGMEYTVLKQIRDVAHLAEDVIDTFVAK
VSIYRRRTILGRMLRGFGQARLLRHVADKIDKLKTTLNEIRDNKDKYDAFKETTNQSVAEEEEEKERLQSLHKLRRDVEEE
HVVGFVHDSKDIVQRLQGGGSNRKVVSIVGMGGLGKTTLARKVYNSNQVMDQFDCRAWVYVSNECRVRELLLGLLK
LLMPNFEQQRRGNKKGKESAEDVNNLSEEELKKQVWNCLERKRYLVVVDDLWKRQDWDEVQDAFPDNNRGSRILIT
SRLKEVALHAGDDVPHYLQFLSAEESWELFCRKVFKGENCPSGLETLGKQMVQSCCGLPLSIIVLGGLLAKKEKSDREWC
KVVGHVNWYLTRDETQVKDIVLKLSYDNLPRRLKPCFLYLGLFPEDFEIPVTPLLQKWVAEGFIQDTGNRDPDDVAEDYL
YELLDRSLVQVARVNSNGSLATCQVHDLLRDLCISESKEDRVFEVCTDNNILIPTKPRRLSIHSDMGHYISSSDNDHSCIRS
LIFFGQQCDIRGREWKWLLDDFKLVRVLEFGRNRCRKIPFNLGNFIHLRYLRIDSMNATFVPDSILNLWNLQTIELRISYYI
GYIEISFPGEIWKLKHLRHLNISEPIKLRGRSSKSNEKMWNLQTISYLLLNRQSTSLIKKGTFPNIKRLSLYVKADGYEGELP

NLLQNLQQLKHLNKLGIWSKLWLGYKPQELVQSLGQFSCLTFLVIGNVLDLLTSELIFPPNITELMLTGIRCITDKGMNGL
GNHFKLKILRLGGSLLEDSVDLNCADGGFPQLEVLEMRYMRLRKWKLGNGAMRRLQNVFIHHCQPLDNHPIELCSLNG
LKKVHIRDSFSEQMAHIFRNLETNNGVQIVIGNYLYLDDENDWNIFH

>XP_014521516.1

MAESVVSFVLDHLGELAAREANLLYGVEDRVQSLHYELQMIKELLNTTKRKKGMEHIVLNQIRDVAHLAEDVIDTFVAK
VSIYKRRTILGRMLHGFGQARLLHHVAHKIDNIRTTLNELRDNRDKYDAFKETNNQSATEEEEKERFQSLHKLRRDVEEE
HVVGFVQDSKDVIKQLLEGGSNRKVVSIVGMGGLGKTTLARKVYNNNQVKQCFKCRAWVYVSNECRVKELLLGLLKHL
MPKFEQQRRGNKKGMKSAGDISSLNEEELKKLVRNCLEWKRYLVVVDDLWKKQDWDEVQDAFPNNDRGNRILLTSR
LKEVALHAGHDVPHYLQFLNKEESWELFRRKVFRGENYPSDLEPLGQHIVQSCQGLPLSIIVLAGLLANREKSYREWSKV
VGHVNWYLTRDETQVKDIVLKLSYDNLPKRLKPCFLYLGLFPEDLEIPVMPLLQKWVAEGFIQDTGNRDPDDIAEDYLYE
LIDRSLVQVARVEMNGGLETCRVHDLLRDLCISKSKEDKVFEVCTDNNILIPTKPRRLSIHSKMAHYISSSNNDHSCIRSLL
FFGPDYCVDRREWKWLLEGFKLVRVLELEPKRCGKIPSKLGKFIHLRYLRIDSEEVKFVPASILDLWNLQTIDLGPWSHDIP
ISFPLQIWKLKHLRHLNSRRPIKLRGSSSGSNEKMWNLQTISPLELNKQTTFLVKNETFPNLKRMGLKVDSGCKGELPNLL
ESLQQLSHLNKLEILLPDSYDASLNVEGSVHESVERNNGCKPQELLQNLGQFNYLSVLKIENALNLLISAVTFPPNITELKLS
GISCISDEGISGLGNHSKLKILRLLGDVTWSGDSSVFYCVEGSFPELEIFRMGVLAVTKWKLSNGAMQKLESVMIHNCE
MLDDLPNELWSLSGLRKVQITKPSEQMVRMLRNLKINNGIQLLIDAEEPFSFENKF

>XP_014521705.2

MMGNDSAMCETKCRYDVFLSFRGEDTRHTFTCHLYHALRRKGIITFMDDGELKLGDQIGPTLLRAIEESSISIVVLSENYA
DSSWCLDELVKILECKESNNQLVWPIFYKVNPSDVRHQKGSYGEAMTNHEKRFGKDSKKVLQWRSTLTAIADMTGEH
LKSEQGRDESKFIEELVSMIFMKVSPKYLSSDEHIVGREYRVEELKSLLNLESPNIKLLGIHGTGGIGKTTLAKALYDSIYKQF
QGSCFLFNVRENSNQINGIEFLQQRLLSEILEDSKIQLASKAEGTSRIKQSLRSKRILIVLDDVDDIEQFQNLAGKRFWYGK
GSRIIVTTRDKKLLDVGGVENRYEVKGLNNQESLELFCQSAFGNSCPETNYEDLSNRAIGCCKGLPLALKVLGSHMIGKD
FGGWKTALDRYEKGPHQDVQKVLRISYDSLPFNEKNIFLDIACFFKGQRSEYVKRVLDACDFNSGDGIDTLVNKSLLTVD
SQSECLEMHDLIQDMGKEIVKEEAWTEIGERSRLWFHEDVLRVLADDMGSGKIQGLMLDPPQRGRINCTGTVFEKMK
NLRILIVRNTSFSHEPSCLPNNLRLLDWKNYPSKSFPSEFYPRNIGAFNLSRSVLALKEPFERFQHLTYMNISYCNMVTKFP
DVSGAKNLRELRLDGCEKLVTIHESVGLLANLVFLSASECILLKKFFSTMYLPSLEYLSLNLCTKLAFFPEISGTMNSKLKIN
MLDTAITELPESIEKLTGLTYLDMTDCKELQHVPGSLFMLPNFVTLKIGGCRRLRESITRFKGSDSVRPKLETLHFDNAYLS
EKDVRTIIYHFPNLKDLNISGNPVVYLPACIKESTNLTSLHLRYCDVLQEIPALPSSVQKVDAKYCHSLSSKSLNILWNQVRK
ETKRLEVVMPKREIPEWFDHVNKGGFPEFTARRKFPALALALVFGEVDAIADREIMEGRWRTVGMHLFIQGKRRRYHN
FSVAENHVLLCDLRVLFSLEKWEDVGFGVDEDWKTLRVSCETNLSLCSWGVYVYKTETNMDDINFNSQHPSSSLVWSD
DELISDMDKIVSKTRSLNLPEMHREYRMWSTGSRKRAYFKGESSQGLEKASRRVMALRRVEGKSGMNIEDEKDKDLDA
MLVVLGTAELDKHFHYPNSHTAESFTSKYCCIL

>XP_014521820.2

MCYQVFLSLLFSSTQHCLLFFLVPNQHYSSTLVESMDMVIVSEAISYTLQCATTFLSPPATRLSSDDIQQFEDNLKRILHIV
QKAMHSNIQDPSVLSLWLKNVKDVVNDLNDFMEDHRHNKETAATTLSLIKAGQNMAHRHKFKHQIKDATEELKRLSN
EAENLVISEEARQNERKLTRKSEEFEYVEVVVRENLKKDIVDQLMKIFVNSNVVSVPVVTIVGVAGIGKTKLARLVYGNEE
VKALFASRIWINLETFNVESIATRVMETTNKGKRFLLVLDDLRVENGEGCLQKLQDRLAEAGVGGAVVVTTRSNFVAKKI
AKTGTVKLKPHVLQELNEEESWSLFQKIRGPGSGKINEDVGRRVVREYCGGIPMKIIAIARLLEDLDSPVPEIELKKKFLREI
RFTYYDELSPQQKLCFAYCSLFPQEHEIDAGRLIRLWMAEGFLWRNLYSDPQEFGLACFNDFVPFVFQETGSDEFGVVK
RYKMNRLMHELARTVAWDENIIVDSGEVEVHERVVRSSFHFALDVQCGIPKALFENAKKLRSILLLGKTNKSRLPHEVK
MTISTCEKILETFKCLRVLDFHDLGIKMVPSSIGELKYLRLLDLSHNNIKKLPSSITKLFHLQTLKLSQCHVLEELPKDLENLSS

LIHLYLEGCLDLTHMPRGIGKLSFLQTLSLFVVGKNYQVGGLRELTDLDGLRGHLEILHLEQLNFSAPLEAKDKYLREKKHL
RCLTLRWDHEEKEEEDEKKRNVIAEKDKESLECLDPNPNLAVLSVVGYYGKTFSNWLSSIKCLVKFSLNDCYNCQYLPAL
DHLQHLRVLELRRLDSLKYVSKNSDQVSADTEASSSSSTPFFPSLKELTISDCPKLQSWWETAKWEPNRPFFTRISKLDV
QCCPELHCMPLYPYLDEELVVVDSSVKSMRDTVHASISADFLPFSKLKTMLIARITQTPPERWLKNFISLKTLQIRDCSKLL
YLPQGFKSLSSLESLTIERCAELDLDRSKTEWEGLKRLRFLIIKEIPKLKSLPWGVEDVTSLEELELHECPALLNLPETIANLTSL
TKIVICKCGELDSLPKGLGKIESLHTLAITDCPLLTPRCQPETGDDWPQIGHIRNIILKQSSQDLRDLWSHGRIGKGRYF

>XP_014522058.1

MIMTDKLGFRRLWGRLGRRSSSSNVIPSESASASSSSNHSSDTIQNQDHKYDVFISFRGSDTRNSFVDHLYSHLLRKGIFV
FKDDHKLRKGESISSQLLQAIRGSRISIIVFSQDYPSSSWCLDEMATIADCKQQSNQTVFPVFYDVDPSHVRRQSGAYEN
AFVLHRSKFKEPNKVLRWEKAMTDLANLAGWDIRNKSEFGEIENIVQAVIQTLGHKFSGFVNDLIGIQPRVQALEDKLKL
SSKSDDVQVLGIWGMGGIGKTTHAAVLYDKMSHRFDASCFIEDVSKLYRDGGHTAVQKQIIHQTLGEKCLDTYSPIEISG
IVRNRLHKIKVLLVLDNVDELEQLQELAINSKLLFGGSRMIIISRDEHILKVYGAHVIHKVSLMNDKDARELFYTKAFKSEEQ
NSSCVELIPEVLKYSQCLPLAVRVIGSFLCGRNSSEWRDTLDRFENHPDNNKIMDVLQISFDGLHYMEKEIFLHIACFFKE
EREDYVKRILECCGLHPHIGIPRMIEKSLITIKDQQIHMHDMLQELGKKIVRNQCPEEPESWSRIWLYEDFFHVLTTQTGT
NDVKAIVLNKNEAISEFSIDGLSKMKKLRLLILYHKSFSGSLNFLSQSLRYLLWHDYPFASLPSSFAALGLVELNMPNSSINC
LWEGRKHFPCLKRMDLSNSKYLIETPDFTGVPNLERLDLSGCTDLLFVHPSIGLLQQLAFLSLRNCSNLISIKFGNGFNLSS
LRVLHFSGCTKLENTPDFTWTTNLEYLDFNGCTSLSSIHESIGVLTKLTFLSLRDCTSLVSIPSNNNIMKSLQTLDFSGCFQL
TDYSLWRSFISLSMISLILLDIGFCNLLKVPDAIGDLFCLERLNLQGNNFVFIPSSIGKLCNLAYLNVSHCHRLQRLPYLSTRS
SSSTGRYFKTVSGSRDHRSGLYFFDCPEINWIDPPYYQELTWLLRLAKNPCYFRCGFDIVVPWGLELPYWLNHRFKGDS
VIRIVEFNEDDDWMGFVFCVIFEQKNGPMVGRSSSHPFYLSFESEETEEYFDMQLNLERDKMDGSKHLWIIYISRKHCH
FVKTGAHISFKAHPSLEINAWGINSILREDEEINLECKGDVNFDFVEKSSTKSGPKFQLPYNWLVTDEEEVENINAKAKEN
NLSYAGL

>XP_014522062.1

MTDLANSAGWDIRNKPEFGEIENIVQAVIQTLGHKFSGFVNDLIGIQPRVQVLEDKLKLSSKSDDVQVLGIWGMGGIG
KTTHAAVLYDKMSHRFDASCFIEDVSKLYRDGGHTAVQKQIIHQTLGEKCLDTYSPIEISGIVRNRLHKIKVLLVLDNVDEL
EQLQELAINSKLLFGGSRMIIISRDEHILKVYGAHVIHKVSLMNDKDARELFYTKAFKSEEQNSSCVELIPEVLKYSQCLPLA
VRVIGSFLCGRNSSEWRDTLDRFENHPDNNKIMDVLQISFDGLHYMEKEIFLHIACFFKEEREDYVKRILECCRLHPHIGIP
RMIEKSLITIKDQQIHMHDMLQELGKKIVRNQCPEEPESWSRIWLYEDFFHVLTTQTGTNHVKAIVLNKKEAISECSIDGL
SKMKNLRLLILYHKSFSGSLNFLSQKLRYLLWHDYPFASLPSSFAASDLVELNMPNSSINCIWEGSKYFRCLKRMDLSNSK
YLTETPDFSEVPNLERLDLSGCTGLSFVHPSIGLLQRLAFLSLRNCSNLISIKFGNGFNLSSLRVLHFSGCTKLENTPDFTWT
RNLEYLDFDECTSLSSVHESIGVLAKLKFLSLRDCTSLVSIPSNYKMMKSLQTLDFSGCFQLTDLSLGKSFIFLSMISLDIGFC
NLLEVPDAIGGLLRLERLNLQGNNIVSIPHSITQLSCLAYLNVSHCHRLQRLPKLSAISSSSTGRYFKTVSGSRDHRSGIYVF
DCPNYVVLKSFKDLNGEEFKIREHEWLKRLAMKPCSFRCGFDIVVPWVLDHLPWWLEKGFKGDSVIRIVEFNENDYW
MGFVFCVIFEQKNGPVVGRSSSHPFYLSFESEETEEYFDMPMDLERDKVDGSRHLWIIYISREHCHFAKTGAHISFKAHP
SLQINAWGMSMFCKDVVRRIRYPDHHMNIDFVEKSSTKSGPKFQLPYNWLVTHEDEVENINAKEKENNLSYAGLNLI

>XP_014522065.1

MLKSGLKWFWDRFGSRSSGSTSSSSSSYDSSSDTIHNQDYRYDVFISFRGPDTRNSFVDHLCSHLLRKGIFVFKDDHSLQ
KGESISPQLLQAIQLSRLSIIVFSKNYASSTWCLDEMTAIAFCKQQSSQIVFPIFYDVDPSHVRHQNGVYENDFVSHRCKF
QKDRDKVRGWERAMTDLANSAGWDVRDKPEFEQIQSIVQAVIKKLRHKFSWFVNDLIGIQPRVQELEDKLRLRSNSD
DVQVLGIWGMNGIGKTTHAAVLFDKISHRFDASCFIEDVSKLYRDGGHTAVQKQIIHQTLRENIDMSNPIEISGIVENRL
HSIKVLVVLDNVDELEQLENLAIKPKLLLKGSRMVITTTDEHILKAYEGDVLIHKLPLLNDADARELFCRKAFKCEDQSSNC

AALIPEVLKYAQCLPLAIKVLGSFLCTRDADEWSDVLNRLEDSPDDKIMNVLEISVEGLQREEKQIFLHIACFFKGERVDYV
KRILECCGLYPHIGISRLIEKSLITISDEEIHMHELLQELGKKMVRDQSPEEPGSWSRIWLHKNFLHALRAETGTEKVKAIVL
NKKEEMSECIVDGLSRMKGLTLLILYHTRVSGRLEFLSDRLQYLLWHDYPFASLPPYFTASDIVELNMPNSHITHFWEGR
KSCPNLKRIDLSNSKYLSETPDFSRIIKLERLDLSGCTSLSYVHSSIGLLKKLAFLNLRNCCNLVSIDFGYVGNMSSLRVLHLS
GCSKLESTPDFTRATHLEYLDMDACTSLSTIHESIGVLSSLTFLSLRKCEELVSIPYDINFLVSLQTLDLYGSFNRMDPIPGQS
YMSHLEYLDRTNPILRQAFSSHLVSLIFLDISYCNLKEVPDTIGELRCLERLNLEGNKFVSIPDSFSQLHCLAYLNLSHCPNLK
SLPDLPIEGDKSGGKYFKTVSGSRDHRSGFYLLNSSNVSHDRLEFVGLEGFFHLIKEPCNFRCGFDLVLPWDMGFRPIFGE
TFLRNSIIRILRCGMNDNWIGFGFFVIFKRLGGYLPYHSSFSSSSLYLSFESEYTEECSPLYLSFESEYTEECFDMRLNSRRDQ
TYGSLHIWIIYISREHCHFVKTGAHITFKAQPNVVIEEWGMRPILKQDISDSKGQRYIKFSKLNHDHVDFEYVEKSNTGSG
PKIQLPYNWYVTEEEEVENIDAKVKENNLSNAGL

>XP_014522066.1

MLKSGLKWFWDRFGSRSSGSTSSSSSSYDSSSDTIHNQDYRYDVFISFRGPDTRNSFVDHLCSHLLRKGIFVFKDDHSLQ
KGESISPQLLQAIQLSRLSIIVFSKNYASSTWCLDEMTAIAFCKQQSSQIVFPIFYDVDPSHVRHQNGVYENDFVSHRCKF
QKDRDKVRGWERAMTDLANSAGWDVRDKPEFEQIQSIVQAVIKKLRHKFSWFVNDLIGIQPRVQELEDKLRLRSNSD
DVQVLGIWGMNGIGKTTHAAVLFDKISHRFDASCFIEDVSKLYRDGGHTAVQKQIIHQTLRENIDMSNPIEISGIVENRL
HSIKVLVVLDNVDELEQLENLAIKPKLLLKGSRMVITTTDEHILKAYEGDVLIHKLPLLNDADARELFCRKAFKCEDQSSNC
AALIPEVLKYAQCLPLAIKVLGSFLCTRDADEWSDVLNRLEDSPDDKIMNVLEISVEGLQREEKQIFLHIACFFKGERVDYV
KRILECCGLYPHIGISRLIEKSLITISDEEIHMHELLQELGKKMVRDQSPEEPGSWSRIWLHKNFLHALRAETGTEKVKAIVL
NKKEEMSECIVDGLSRMKGLTLLILYHTRVSGRLEFLSDRLQYLLWHDYPFASLPPYFTASDIVELNMPNSHITHFWEGR
KSCPNLKRIDLSNSKYLSETPDFSRIIKLERLDLSGCTSLSYVHSSIGLLKKLAFLNLRNCCNLVSIDFGYVGNMSSLRVLHLS
GCSKLESTPDFTRATHLEYLDMDACTSLSTIHESIGVLSSLTFLSLRKCEELVSIPYDINFLVSLQTLDLYGSFNRMDPIPGQS
YMSHLEYLDRTNPILRQAFSSHLVSLIFLDISYCNLKEVPDTIGELRCLERLNLEGNKFVSIPDSFSQLHCLAYLNLSHCPNLK
SLPDLPIEGDKSGGKYFKTVSGSRDHRSGFYLLNSSNVSHDRLEFVGLEGFFHLIKEPCNFRCGFDLVLPWDMGFRPIFGE
TFLRNSIIRILRCGMNDNWIGFGFFVIFKRLGGYLPYHSSFSSSSLYLSFESEYTEECSPLYLSFESEYTEECFDMRLNSRRDQ
TYGSLHIWIIYISREHCHFVKTGAHITFKAQPNVVIEEWGMRPILKQDISDSKGQRYIKFSKLNHDHVDFEYVEKSNTGSG
PKIQLPYNWYVTEEEEVENIDAKVKENNLSNAGL

>XP_014522068.1

MLKSGLKWFWDRFGSRSSGSTSSSSSSYDSSSDTIHNQDYRYDVFISFRGPDTRNSFVDHLCSHLLRKGIFVFKDDHSLQ
KGESISPQLLQAIQLSRLSIIVFSKNYASSTWCLDEMTAIAFCKQQSSQIVFPIFYDVDPSHVRHQNGVYENDFVSHRCKF
QKDRDKVRGWERAMTDLANSAGWDVRDKPEFEQIQSIVQAVIKKLRHKFSWFVNDLIGIQPRVQELEDKLRLRSNSD
DVQVLGIWGMNGIGKTTHAAVLFDKISHRFDASCFIEDVSKLYRDGGHTAVQKQIIHQTLRENIDMSNPIEISGIVENRL
HSIKVLVVLDNVDELEQLENLAIKPKLLLKGSRMVITTTDEHILKAYEGDVLIHKLPLLNDADARELFCRKAFKCEDQSSNC
AALIPEVLKYAQCLPLAIKVLGSFLCTRDADEWSDVLNRLEDSPDDKIMNVLEISVEGLQREEKQIFLHIACFFKGERVDYV
KRILECCGLYPHIGISRLIEKSLITISDEEIHMHELLQELGKKMVRDQSPEEPGSWSRIWLHKNFLHALRAETGTEKVKAIVL
NKKEEMSECIVDGLSRMKGLTLLILYHTRVSGRLEFLSDRLQYLLWHDYPFASLPPYFTASDIVELNMPNSHITHFWEGR
KSCPNLKRIDLSNSKYLSETPDFSRIIKLERLDLSGCTSLSYVHSSIGLLKKLAFLNLRNCCNLVSIDFGYVGNMSSLRVLHLS
GCSKLESTPDFTRATHLEYLDMDACTSLSTIHESIGVLSSLTFLSLRKCEELVSIPYDINFLVSLQTLDLYGSFNRMDPIPGQS
YMSHLEYLDRTNPILRQAFSSHLVSLIFLDISYCNLKEVPDTIGELRCLERLNLEGNKFVSIPDSFSQLHCLAYLNLSHCPNLK
SLPDLPIEGDKSGGKYFKTVSGSRDHRSGFYLLNSSNVSHDRLEFVGLEGFFHLIKEPCNFRCGFDLVLPWDMGFRPIFGE
TFLRNSIIRILRCVNTQKSALHFIFLLKVNTQKSALICDLIRGEIKPMDLYIFG

>XP_014522415.1

MEDFLLSIAAKIAEYAVDPILHHAQYLCCFNNFGLYLPNVKEQLELTRDRVNEQITEAIIKVEKVEPSVEKWLKDVEKVLEE
VQMLEQRILSVNKNYFKRQCQYSLAKEIERKTTEMIQLHHNSKFEPFSRITELPGMQYYSSNDFFMFNSTEASYKKLLEAL
KNKCSFIIGLIGLGGSGKTTLAKEVGKKAEQMKLFEKVVLATVSQPLNIRSIQDQIVDQLGFKLMEESDIGRAQRLSERLRK
GTTLVILDDVWEILNFEALGIPLNESSKACCVLITTRSKELCTSLQCQSIIELNLLNDEEAWTLFRHYANITDDSSETLKCVAR
KIVNECKGLPIAIVTVGSTLKHKTIANFKLALSRLENSKPLDIPKGLTSPYVCLELSYNNLTNPLAQSLLLLCSVFPEDCEIDLE
DLFRFGRGFGTIGTFGMMEDARREMDSAIDMLKNSLWIASKSGQAIFTRTKVDPRVLEDDEVMKDMKTIAVWDFKH
DHLNHKLNCPSLEILVLSVNVSGGLKVSDGCLQSLEKLKTLAFLNPDVRYIGALSLQESLMSLKNLQTLCLRRQDLGDVSF
VEQLQALEILDFCGSYFDDLPVGIIKLKKLRLLDLYKCVIKKNKNVEFYEVVGKCLQLEELYLCLINYEKAFPHDVSFSKLQRY
VIQSGHYQYSYETTVILEKYGQIRSLLINRFDVVAQRFASLAIKDLFIRAEFLCLTNLMGDYKNIIPSMDPQGMNQLIALQL
QHCKEIECLIDGTINNRTSFGLLQTEVVFSTLVYLSLVDLPKLRELFCDPSSRCCLQNLEELEINMCNELYSISFPRNSKLCNL
KVVSISGCPMLTSLFTSSVVQTLELLEDLKIYKCRSLRHIIEEENGVLSITQSHSSLKLRKLRFIGIYECENLEYMFSVFLVEGLL
SLKRVAIFNNPKLKYVFGSEKEHNVAGYPCFQQTNTKRNLLNLNTLELIELPNLTAIWPEYCRPHLLSLNELFCLGCPKLSN
SSIYKVINASDFQQQTTPTENEILWSFTNTSNQLEDLLLHPQLKTFLKFRVLSLTDVRIKGIFQFQIGEGTTELVSLNLDIESL
YLCDIPELSFIWKGPTTFLSLQNLFDIRVDGCPKLKTIFSTTVVTSLPMLTYLHISDCDELEEIFDLGDPHQLKSLYSSQQVYF
PNISDIEVNKCNKLKCLFYNLSACHFTSLIRLEIEECTQLHKAFGFEHEPDGGGLEKMGKDGKQVLFQNLKFISLINLPNFE
DIHHGFKLKEDVDQTIKECPMYSPSLYLHP

>XP_014522481.2

MDSLIAGLHKFLTSPLLKAWLNEQLIHLKSYEARVRELKGVVENLKNVRDEIQHKVDEEELRRGREIPVEEKEWIERVEELI
SEYVDFIKDPDHKLALFDLWESGYLPKPGIRYRQSRKAYDITRKANGLLQRANFDTFSYWSGPPSMAAFFSNVGYESFPS
REETMKKIIAELEKPSVRMIGLHGLSGMGKTTLVKEVVKVALEAKKIDVVTMASVTRNPDIRKIQGQIADSLGIILDEESXV
ARAARIXKRLKXEKEKTLIILDDLWEKVDFNMLGIPYEIHNHTGLKXVKEGKSVHVDSLMNQKEGSPKEPHGASDELKTE
KTRSQYKGCKVLLISESKTVLLTQMGRKENCVFCLEALKEKETEMLFKKMAGMGDYNSKFQKLGAQIANKCNGLPMTI
VTTARALKKQSPFVWEDILRKLEWQKLTGAPELSTKLSYDLLEDDELKYTFLLCARMGHDALIMDLVKYCIGLGFLQGIYT
VRETRDRVCTLVEKLKESGLLSDSYSRDQFTMQNIIRNAALSIANKEKHLFTVTKGKIDEWPEKLDEYAAISLHHCNFIEGF
PRRIKYPGLRVFHFNNNDPHLKVPTNFFKGMKGLKVLILTGIHLSLLESSVSSLIELRMLCLEQCTLEELSIIGMLKKLRILSFS
RSDIEKLPVELQKLKKLQIFDLSNCSKLKEIPSGVISNLVCLEELYMRNTLIHWEKEELTRQSKIGLLSELKHLSQLTTLDIQIP
EVSHLPKTLFFDKLYSYKIVIGDLSALLETDFKIPEMYETSRFLAIRLKDGFDIHSLKGVKMLFEGVQSLLLEELNTVHDLFYR
LNLKGFPYLKHLFIVNNFTIQSLIYPKDRQQSQHPEKAFPKLESLHLYNLKKIKNICSCKLSEPSFGKLKVIKINLCGQLKSVFS
ISMVNLLTALETIEVSECYSLTEIVSVEESQSNTQESKLVFPKLLYLKLQSLSLCGKFYSITEEKLFNEKVDVSKLERMELSSIPI
TEIWNYHHSNRSLFKNLTHLDVNGCWKLEHLISFSMAKCLGSLQSLFVSECGNMKGIFPDSMQMEGDIFPKLKNIKLSS
MKSLRKIWPSDLPSKSFSKLDTLIIEKCDKLVSVFPHYYEGIFHSLCNLTVTKCTQMQTIFDTYDKMRYVGVVSLQDLHLEI
LPNLKHVWKLEKGKGGILELKNLQKIMVQNCYCLENIFPFSVAENLGNLEYLVVSYCVELREIVAKSAAVNTAKLLFKFPK
LRSIKFSVLPRLTSFYPGAFQLCCPTLNDLSIEYCDLLEPFQKETVDVEGKCVLFSEEVLNKLKSMQIESWHAKSQNSCMN
EGNHRRDSLEELCLSRLSDTEILYSFLHSNPNLESLSLSDCLFEEIVPLEKNTKIETLGVVPKLKNLKLINCKLKDLGFEEDIILR
RVELLLLKRCPCMTTVVSSSTSLSSLVNLEVDNCDGLENLMSPSTAESLGQLNTMKVTKCKNLMEIVGKDAGNASKVVF
KKLKTLELVSLKKLQSFCNSESCEFEFPSLERLVVSACYNMKKFSDKVTRRSTPILQNVYAILEQEKKRYCWEGDLNATIHK
MFLDKKFFEGMNMMSFSDDGKLKQEMMSLSDHLELNRAWLGGEVDPQSIHSWFYSLKVLKLEKCEIQQCAIPSNILP
YLKNLNELMVQDCNNVEVVFEMNVTQGTGTTFQLQKLTLNRLSKLKNVWKGNGEETRSFQNLREVAVLECHMMQT
LFPAALVKNLKKLHTLMIGRSWGLEIVGKEDNAALATQNLVFPCLTTLVLVDLPELNYLYPEPFTVECPVLKFLRVLDCPKL
DLFPRFLDLKTVVANLEQLVLEGKHSLVLESSLQDLEYCFEDLNSISLRFDVDENEKSNLPIQILQRVPNLHEMSISSCHCLD
AFRTEISEMNKKVMLTDLKRLDLNDVWELKSIGSGDVPWLDMICKKLHRLYVANCPRFMAVMHSPSAQFFSCLKVLSV
FNCQRLEYLFTSSAAEKLTQLERIHLYKCRSIKEIVAKEQGETTSGGFKLQQLRCVSLSYLSSLECFYSGNRTLQLPSLIEVRII
ECPKMEIFCKGSMGPNLCKEIRTSYTSNNGLVIVHDDLNSTVKKVFRQQNHIVFGDSRMLQDIWKSVTLPEGYFHNLTS

MVVEGCEFLSDAILPSHLLHLLSNLKRLQVRRCNSLKAVFIHTKITNMGPQGSLTHLEELHVENCVELVAIVAKFEAEIDEA
NKEITIFSTITLLRLSHLPKLRCIYPEMHMLKWDMLKELHVEHCQKLKFFATEYQNSKYLNQDHQDRVSTAQQEVVSLEK
VTPCLKVMSLGKEEAVMIEQGKLQIDLPKLSSLKLQCFDDEQGDAFPFIFGSKMSLSLPTIKKLVLLHCAFKEIFPTQALG
MDCTKILSQLKTLELVSLSQLKSIGLEHSWVMPLLQNLKTLLVRDCHYLSNLTPSTVSFSNLIKLNVKHCSKLKYLFTFSTTK
TLVVLKEIYITNCKSLKTIVVEDRDGGHLIEDNEVEEEGEGDGNEDDDEEEEAQHESEDKEIDDDDDDDDDEDKEKMDE
AKTNAEGGGDEGENEGGNDAKHGMEKEKEGDGAGEDDKNEAKANVKVEGDGDGNENENAGDGVNDGNECEDEI
TFKKLERLTLRSLPKLRSFYSGISTLNFLSLEEVSVSKCCTAKLFCRDDKIPKKLRVKIDDCKRDMNFVKMQQMRQRHHDS
C

>XP_014522482.1

MEYVKLLGPLLGPSWPYLKPFLYKLVPYEIRDAHLAKDINKLQKVKSMVQDKVRDEENRIEIPIIGDIIKWLDEVDEVISDY
EDFLDDEDRPYALYSDGYLPKPSIRYRLSKIVNDIANRVRVLLHTSNDADFSRWLGLPSYDVDFDNIDSSVGMIGVYGGS
GVGKTSLMKEIVKKVKGNMFDVVIMVNITSRVDIKRIQEQIAKKLGMKLEGESESKRAAHLQERLNDPREKTLIVVDNV
EVKLDFNMLGIPTENNDDSKVNHRMKDISARRNYALKIEEFDDSILRKVEDPLAKYKGCKILMISKSEQLLLRQMDGKAI
QTFCVKALTEKEAEKMFKAMSGIDNENSLFKALAAQISKKCKGVPMRIVATAKALKNKSLLVWEDAYRNLERQNLTSV
QEFSTKLSYNLLENDELKHTLLVCARMSHDASLTDLVRNCIGLGLLQGIYTVREAKDRVHALVAELKELSLLSDSLSSDRFT
MQDIIRDAVLSIESQHMHAFALTKGKLEEWPDKDKLERYTAISLQNCDVTDMMKKFPESIDCFRLRVFHLDNKDPHLEI
PDNFFIGMKELRVLILIGVHLSYLPSSIEYLKKLRMLCLEQCKLGENLSIIGHLETLRVLSFSGSEIDKLPAELSQLAKLQIFDIS
NCFKLRQIPVDVLSKLDDLEELYVGNSPIQWIDGRQGNSILSELRKLDHLTTLDIQIPEITHMPKHLFFDKLHSYNIVIRDSD
DSSVWDIKMLEMCETSRFLALQLENGFDIHHRKDIKILFERVENLLLGQLNDVEDXFYELNYEGFPYLKXLSIVSNSKXKXX
INSENQKHLEKVFPRLESLFLHEVNNMEHICNSQLTSDSFCKLKIIKLNMCGQLKCVFFSSTIKHLSALEVIEVSECNTLKEIV
TSEAENREQIIFPELRSLTLQSLYELIGFYTYGVSMGEQVSNELFDKKVTNCMSMEEIFDLKDCQKQDVEDMIRLQNVHA
EVLPKLQHVWNKDPKGILNLKNLKKIRIQECLNLKYIFPVSTAMSLEELEYLEVWNCGELKEIVSRGETNNVSSISFEFPKLT
TVRFSKLPSLEGFYGGAHELHCSALINLCVERCHKLKLFSKENTNSEIKPVFLPEKLIPSISHGEDMTGVVPKLKSLKALDPT
NVVASNPYKPLENSRLKILKLANCELGSHAIPTDVFPSLKNLEELEVSNTNVEAIFDIMNDEMKGYILSLKKMTLDKLSKLT
QVWKDKNPQASFKFQNLREVIVNNCKILETLFPIELAKRVKKLEKLKIGYCKKFSRIVEQENAITEATTEFSFPRLTSLNLCM
LPQLSCFYPKRFTMECPHLKHLEVLFCGDFETFQIHQEAQSSTSVNRQPLFYEEKANFILESVKLDWKNTMILLNGKFPFE
MFHQLVQFEIHFADANEVQGFPEQLLEKIPNGEYLQISKYSGFEKLSTSHPEQEKIVAKEQGETTSGEFKLQQLCSVSLSD
LSSLECFYSGNRTLQLPSLIEVRMIECPKMEIFSQGSVGPNLCREIRTSYTSNNGLVIVHDDLNSTLKKVFLQQNHIVFGYS
LMLQEIWKSETLPEGYFHNLTSMCGDVSNSLKAVFIHTKITNMQPQGSLAHLDELHVENCVELGTIVAKVEAEIDEANK
QIAIFSTITLLRLSNLPNLRLLPALITLPLKELVLLYLPDLENVWNKDPPHRILCMQHLKKVKVKECESLRSVFPASVAKDLKL
EDLVIEECERLVAIVAEDNTDPNQ

>XP_014522592.1

MIKLQLLKVFIYSLQLEPLLIYTFEYSFLCDCKLHSIAFFWCLSKSLLVFFHHQPRTIIQPQIQIYTLLHAVMADSVVSFVLDHL
AQLAAREANLLYGVEDRVQSLQYELQMIKDLLNTTKRKKGMEHTVLDQIRDVAHLAEDVIDTFVAKVSVYKRRTILGRM
LRGFGQVRLLHHVADKIDKLKTTLNEIRDNKNKYDAFKETTDQSAVEEEEKERLQLLHELRRDVEEENVVGFVDDSKDV
VQRLLRGGSNREVVSVVGMGGLGKTTLARKVYNSSQVVNHFDCHAWVYVSNECRVHDLLIDLLKHLMPIFEQQLRGN
KSAVDIKDLSKEELKKHVWNYLERKRYLVVVDDLWKRRDWDEVKDAFPDNNRGSRILITSRLKEVALHAGDDVPHHLQ
FLSEEESWELFCRKAFRGKNCPSDLEPLGKQMVQSCRGLPLSIIVLGGLLANKEKSDREWSKVVGHVNWYLTRDETQVK
DIVLKLSYDNLPRRLKPCFLYLGLFPEDFKIPVTPLLQKWVAEGFIQDTGNRDPDDVAEDYLYELIDRSLVQVARMDFNG
GLKTCQVHDLLRDLCISESKEDKMFEVCTNNNILLRTKPRRLSIHIDTGHYISSSNNDHSCIRSLFFFGPGYHVRGREWKW
LLDDFKLVRVLEFGPYDRNKIPSNLGNFIHLRYLRIKDVFVYSIPDSILNLWNLQTIDLGSWEGLMPVSFPIQMWKLKHLR
HLNTQGPIKLRGRCSPSSYEKMWNLQTISLVLNMQATSLIKKGTFPNIKWLRLKEEYGFVGELPNLSQNLELNHLNKLLIL

SPFRRGHKLQEQLQSLGQLSCLTILIITDVLDLLTSTLILPPNITELTLSGIKCITDEGMNSLANHSKLKVLRLRGKVTAFEDSV
DLNCADGGFPQLEVFKMRDIKLRKWRLGNCAMQKLQNVFILDCRPLDHLPTELCSLNELREVHITEYPSKQMTHILEILK
TNNGVRVIIGIFSTPDVEWSHVSGETYEIYYSK

>XP_014522600.1

MFFSMRSIASYTLYCFTDALVNLSYSSSITNHKPFQPQIQIYTLVHAVMEEIVVSFVLDHLAQLVAREVNLLYGVEDGVES
LRRELEMIKELLNTTRRERGMEHIVLNQIRDMAYLAEDVVDTFVAKVSIYKRRTILGKTLHGFGQAWLLHRLSDKIDYIKN
TLKEIRDNKDTYDAFRETTNESAAEEERERLEWLRTLRRDVEVKDVVGFVHDSEVVVNRLLGDSSKLKVISIVGMGGLGK
TTLARKVFNNIQVKNHFDCLAWVYVSNECRFRELLLGLLHQFMPNFEQQCRDINSRNEDELKNMAKNCLEGKRYLVVV
DDLWNIEDWDKVKGAFPDNNRGSRILITSRSKEVATHASLDVPYYLPFLNEEESWELFRKKVFIDGDCPSDLEPLGKQM
VQRCCGLPLSIIVLAGLLANKEKSHTIWSKVVDHVNSYINQNKTQVNEIVLKLSYDNLPRRLKPCFLYLGLFPEDSEILVEPL
LQKWVAEGFIQDTGDRDPDDVAEDYLYELIDRSLVQVARVKLNGSLEKCQIHDLLRDLCISQSKKDKVFEVCTNNNIVDS
TKPRKLSIQSATSDYISSSKRDHSCVRSLLLFGTHDDEWKWLVDEFKLVRVLQFGSIYLQAIPSILGNFIHLRYLRIENVIVYF
VPDSILNLWNLQTIDLGIFKYRQAVSFPAQMWKLKYLRHWNSSGPIELRGRCLQSVEKMWNLRTMSPLTLNKQAISLIR
NGTFPNIKRMGLSGSHDCEDELPNLLQSLQQLEHLNSLVIFPQGFFDINPQELVQRLEQYSHLTTLRIDWVKDLLTSELIF
PPNIIELTLSRIEHISDEGMNGLGNHSKLKILRLLGNLDRFNDPIDLNCVGFPQLEVLKMKELKLRNWKLGNGAMRKLQH
VIIKFCGPYINLPTELCSLNGLKQVHIESNIVQVTEQVHDILRILETDNRVQVFRSFYPALDEENDWESHY

>XP_014522609.1

MEESVVTFVLDHLAQLVAREANLLYGVEDRVQSLQNELQMIKELLNTTKRKKGMEHTVLNQIRDVAHLAEDVIDTFVA
KVAIYKRRTILGKMLRGFGQLRLLHHVADKIDTIKATLNEIRDNKDKYDAFKETNNQSAAEEAEEEKRAQSLHKLRRNVE
EEDVVGFIHDSKDVINLLLEGGSNRKAVSIVGMGGLGKTTLARKVYNSSQVMNHFDCRAWVYVSNECRVKDLLIGLFK
HLMPNFEQQQRRGNKRGKKSAGEINDLSEEELKKLVRNCLEWKRYLVVVDDLWKKQDWDEVLDAFPDNNKGSRILIT
SRLKEVALHAGHDVPHYLQFLNEEESWELFRRKVFRGEDYPSDLEPLGKQMVRSCRGLPLSIIVLAGLLANKEKSHREWS
KVVGHVNWYLTQDETQVKDIVLKLSYDNLPKRLKPCFLYLGLFPEDFEIPVTPLLQKWVAEGFIQNTGNRDPDDVAEDY
LYELIDRSLVQVVRVETNAGVETCQIHDLLRDLCILESKEDKVFEVFTDHSILISAKPRRLSIHGKMDHYISLSNNDHSCVRS
VFFFGSHYYIRGRDWKWIFENLKLVRVLEFGLNGSNKIPSNLGNFIHLKYLRIHIEYVMFVPDSILKLWNLQTIDLSPPRAN
VPISFPAQIWKLRHLRHLNTPRPIKLRGSCSGSYEKMWNAQTISSLLLNSQAMSLIKRGTFPNVKRLGLRVTSECEAELPK
LLQSLHQSSYLNKLVIVLRDRDDEGVKDLTDESVKRNNGFKPQEVLQSLGQFNCLTILTIKNAFDLLTCELTFPPTVTELTLS
EIDCISDEGINGLGNHTKLKKLRLLGDVSLSTGESFDLNCVGGGFSQLEVIEMENLNLEKWKLDNGAMSRLQSVMIHNC
ERLDDLPNELWSLSGLRKVQVMKPSKEMARMLRNLEIKNGVQLVTEDYQPRFQCRLNSIDSMDFNTFEYISDI

>XP_014522630.1

MEESVVTFVLDHLAELVAREVNLLYGVEDRVQSLQYELQMIKELLNTTKRKKGMEHVVLNQIRDVAHLAEDVIDTFVAK
VSIYKRRTILGRMLRAFGQARLLHHVAHKIDKLKTTLNEIHNNKDKYDAFKETTNQSAEEEKEENERLQSQLKKLRRNVEE
EDVVGFIHDSKDVINLLLEGGSNRKAVSIVGMGGLGKTTLARKVYNSSQVMSHFDCRAWVYVSNECRVKDLLIGLFKHL
IPNFEQQRRGNKRGKKNVGDINDLSEEELKKLVRSCLKWKSYLVVVDDLWKKQDWDEVLDAFPDNNRNSRILITSRLKE
VALHAGHDVPHYLQFLNEEESWELFRRKVFRGEDYPSDLELLGKQMVRSCRGLPLSIIVLAGLLANKEKSHREWSKVVG
HVNWYLIQDETQVKDIVLKLSYDNLPRKLKPCFLYLGLFPEDFEIPVIPLLQNWVAEGFIQDTGNRDPDDVAEDYLYELID
RSLVQVTRVNFNGGLKTCHVHDLLRDLCIAESKEDKMFEVCTNYNILIPTKPRRLSIHGDMGHYISSSNNDHSCIRSLFFF
GPRYDVRRREWKWLLDNFKLVRVLEFGASKCREIPSNLGNFIHLRYLRINSKHVTFVPDSILNLWNLQAIDLGIWKQDDP
ISFPIQMWKLKHLRHLNTQGPIKLRGRCSPSSYEKMWNLQTIPPLILNAQATSLIEKGIFPNIKSMGLDVKGHGYEELMLS
RINCITDEGMNSLGNHSKLKFLRLVGNPVFEDSIDLNCVGGSFPQLEVLQMECLNLQKWELGNGAMRRLHNVIIHACQ
LLDNLPTELCSLNRLRKVHITDSPSKQMTRILEILETNNGVEVVIGDSSPWYDDVYFKLHTTFLA

>XP_014522641.1

MEESVVSFVLDHLAQLVASEVNLLYGVEDRVQSLQRELEMIKELLNTTRREKGMEHIVLNHIRDMAYLAEDVIDTFVAK
VLIYKRRTILGRMLHGFGQARLLHRLSDKIDYIKTILKEIRDNKDTYDAFRETTNESAAEEVRERLEWLQRLRRDVEVKDVV
GFVHDSEVVVKRLLGDSSNRKVVSIVGMGGLGKTTLARKVFNNSQVKHHFYCHAWVYVSNECRVRELLLGLLKQLMP
NFEQQCEGIKSPNEEELKNMVENCLEGKRYLVVVDDLWKIEDWDKVKGAFPDNNRGSRILITSRSREVATHAGLDIPHY
LPFLNEGESWELFRKKVFIDGDCPSDLEPLGKQMVERCCGLPLSIVVLAGLLANKEKSHTLWSKVVDHVNSYINQDKTQ
VNDIVLKLSYDNLPRRLKPCFLYLGLFPEDFEIPVASLLQKWVAEGFIQDTGDKDPDDIAEDYLYELIDRSLVQVTRVKLNG
SLEKCQVHDLLRDLCISQSKKDKVFEVCTNNNIVDSTKPRKLAIQSDMRDYISSSKRDHSCVRSVFFFGTNYDEWKWPLD
NFKLVRVLEFGPTTRLEIPSNLGNFIHLRYLRIDSLFPKFVPDSILNLWNLQTIDLGIFKNGLQVSFPAQMWKLKYLRHWN
SSGPIELRGRCLQSVEKMWNLQTMSPLTLNKQAISLIRNGTFPNIKRMGLKGSHDCKDELPNLLENLTQLKHLNTLVIFP
EGLLDFKPQELVQRLGQFNRLTILVIDCVNLLTSELIFPPNIIELTLSVIKRISDEGMNGVGNHSKLKILTLLGYIMEFKDPFN
LNCVGFPKLEVLQLKYLPLRNWKLSNGAMQKLQHVIIYCCEPLDNLPIELCSLNGLKKMHIQDYRTKVIEQMVTSYEYW
KQIIELKSLENMLHLGLMH

>XP_014523767.2

MDFVSDRYQKIDGGNFEVFLSFKGEDTRASFTSHLYTALQNAGIFVFKDDESLPQGKQISRSLRQAIEESRISIVVFSKNYA
ESWWCLKELEIIMECHKTTGNVVLPVFYDIYPSEVRHQKGDFGEAFQRLLSKFSKEEEQKVLDWKHCWWNTLREICNIS
AVEILSPSRIERVDKLDNQWEKTLLEVVQISKILDLNPRGKMKIADSIEILVRQSKEGLFKGAEISFREEKVYDKMDFLMKQ
WREVFCEPFRNSEVALDPCFLVLHVKHFRETLFEAADISGDLVLNSCGEMEITYAIELHMKHWRNAFSEEAGTILDSIYIR
YLRDKSVVGMVHSEIRNLRDHWREKVCEAYDRSGDNQLYSRRKNDIELHLENCTKALLEATDISKRAVFVSSYKKLKAYN
RIESLVKYWKIALCEAVEISRLVVEHYRGITDNEVNYIEKHARDALREAAGLPGNVILNFRNESEAVKNIVKDVTRLLDKTE
LFVPNNPVGVESRVQEMVQLLEQKQSNDVLLLGIWGMGGIGKTTIAKAIYNKIGRNFEGRSFLADIREVWGQEAGHVS
LQQQLLSDVYKENRIKIHNIESGKVILRERLRRKRILLILDDVNKLQQLNALCGNREWFGAGSRIIITTRDIHLLRGKRVDQV
FTMTGMNVDESIELFSWHAFKQASPKEDFIELSRNVVAYAGGLPLALEVLGSYLFDMEVTEWKSVLEKLRKIPNDEVQE
KLKISYDGLSDDTEKGIFLDIACFFIGKDRNDVIHILNGCGLFAENGIRVLVERSLVTVDDKNQLGMHDLLRDMGREIIRSK
SPMELEERSRLWFHEDVLDVLSKETGTKFIEGLTLKLPRSNTKSLSTKAFMNMKKLRLLQLSGVELVGDFEYLSKDLRWLC
WHGFPFASIPTSFYQGSLVSIELENSKITMVWKATQLMEKLKILNLSHSHYLTKTPDFLNLPNLEKLILVDCPRLSEVSYTIG
HLTKVLLINFQDCISLCNLPRSIYKLKSLKTLILSGCLKIDKLEEDLEEMESLTTLFADKTAIRRVPFSIVRSKSIGYISLCGYEGF
SRDVFPSIIWSWMSPVNSLSSRVQTLVDMSSLVFLDVQNSSSNQLSYISEELPKLQSLWIECGSDLQLSRYTKIILDALNAT
NSEESESYGTTSQMQNVFTLIECSSRSKLFEKTLLIQMGGSWEITHILKQRILQNMTTSDGGDCLLPGDSYPDWLSFSSE
GSSVTFEIPEVNGRSLKTMMCHIHYSSPDSITSDGLKNLLVINHTKSTIQLYKRNALAAFDDEEWQRVLSNIDTGNQVQI
VVVFWSRITVKKTSIYLIYEAIDEKVEHYHASNMNVPVSISPEVESMEDLRGAFVKSLTKFLSCCFGT

>XP_014523874.1

PALRISYNHLPPHLKRCFVYCSLYPKDYEFEKDELILLWMAEDLLKAPRKEKTLEEVGEEYFDDLVSRSFFQRSNDCFIMH
DLMHDLATFLGGQFYFRANELGKKTKIDRKTRHLSFARFSDLCSDIDVFETVKFSRTFLMINYRDYPFNKEKAPRIIVSML
KYLRVLKFSDYKGELVLPDSIGELIHLRYLNLNGTSIAMLPESLCNLYNLQTLKLGSCFNMTKLPRNIQNLVNLRHLQILNT
PIEEMPKRMGKLNQLQDLDYYVVGKHKENSIKELGGLPNLHGWFFIQKLENVTKGEEALEARIMDKKHIAHLYLIWSEG
NYDIIDFQIELDVLAKLQPHQDLKSLEIRGYRGKRFPEWVGNFSYQNITVLHLKHCNNCCMLPSLGQLPSLSNLVISNMN
SVKTIDAGFYNKDDCSYVTPFPFLKYLSFYDMPCWEVWNAFDSEAFPVLENLYIEKCPKLRGGLPDHLPALKTLSIDNCEL
LVSSVPMAPTLRTLNIANINKVAFHEFPLLVKDLRIEGRPVVEFMMEAITNIQPTSLQ

>XP_014524058.1

MATRILEGLVKTSEIYSLILDILDGFNGFNDNVQMLEMKLEELCCLEHDINRELEIEELEHGKKRKREVENWLRNVKRKKT
EVHGMVQELRDCGMFRHLKLNVQVRKLTGQVTDLVARGRFPGGIVGSAQESRGCALLTTELAGAMLQKNVRKIWD
WLMNDGVLMIGVYGMGGVGKTSVLMHIHNMLLTAVTNFESVFWVTISKSFSIHKLQCDVAKIVGIDISKESDERKRAA
RLSRALVRRKRCVLFLDDVWNHFPLERVGIPVRADGLKLVLTSRSLDVCRRMNCKNSVKVEPLSMEEAWTLFVDNLGQ
QTNLSPEVKQVARSVAKQCAGLPLAIITMARSMRGVEEICEWRHTLEELRNTEAKHQEMEMEVLRVLRFSYDHLNDKI
VQQCFLCCALYPEDFEIDRDVLIESFVDEGLVKGMKSLEAMFDEGNTIVNKLENICLLGKVENFVGGKKCMGLQDASSHI
AISMMKRGCQHVEPMNNVEGYYVGSQLVKMHDLVRAVAINVIKENNNFLVKAGLQLTKIPDEVEWSEDLEKVSLMC
NWIHEIPTEISPRCPKLRTLILKHNESLTRISDSFFVHMSALEVLDLSFTDIEVLPKSVSDLSTLTALLLTSCKRLKHMPSLAKL
QALLRLDLSFTAITEMPQGLEMLVNLKWLNLYAKDLVSSGKEVSKLTSLQFLILHWWSRKIKVKVEYTSCLRKLETFAANL
YNMQHFNSYVKTMHIYGPRSYLLQLDTEESLGNSPWCCFAEVCFRKDVIISNCKIRTGESPLMLPLDIQRLKVERCHDIRS
LCDVMSLKNATSLKRCEIADCDGPEYMFSLSCSSSCCTSLHSLESLELYSLKNLHGLCKEDEVAAQTFPPGRAFTCLKYFFIY
HCPLIKKLLKPRLLAYLPNLEEITVHNCKSMEEIISVDGIDYESFGGKKSYVTNRDTIVVTHSKLVSLSLKHLPELKSISSAQM
VCESLKNFRIFKCPKLARFPETATPVQILYDSF

>XP_014524125.1

MSFNAIVQYTISSSHAINRYDVFVSFCGEDTRNNFTGFLFQALRRKGIDAFKDDEDLKKGESIAPELLHAIQSSRLFIVVFSK
NYASSTWCLRELAAIRNCVQTSPRPVIPVFYDVDPSVVRKQSECYENAFAEHEKRFREDKAKMEEAERWREALTQVAN
LSGWDIRNKSQYAQIEEIVQNITNILGPKICSLPKDELVGIERGLEILANLVCFESFNNVRVVGISGMGGIGKTTLARALYEK
IYHQYDFHCFIDDVSKIYRDSSSLGVQKQLISQFLNEKNLEISNSFEGTCLMWSRLHNVKALVVLDNIDEVEQLRIFTGNR
DTLLRECLGGGSIIIVVSRDEHILRTHGVDDIYQVQPLNYEYAMQLFCRNAFKVNHILSDYEKLAWDILSYAQGHPLAIKVI
GSSLFRRNVSQWESALARLKEKKSKHIMDVLRISFDQLDXEDKQIFLDIACALYYYDEKYVTEVLKFRGFHPEYGLQVLHD
XSLISKMGRCIYMHNLLKDLGXXIVKEESPKEPLKRSRLCNYQDFLKAMSKNQTTEILEVVAVNSYGSSKTVRVDGLSKIK
HLKFLRLENMNCSGSLSHLSSELGYLIWNNYPFECLPKSFQPHKLVKLKLKGSSIQRLWSGTKVLPNLKRLDLSYSKELVE
MPDVAEALNLEGIDLEGCIQLRKLSPSIGLLSKLTTLNLKNCENLVSLPNSILSLNSLEYLSVYGCSNLFNNELLDEASNTEHL
KKLCSLKGPIESHSTSPLIKKARRESVSCLLPSSPTLSCLRELDLRFCNLVKIPDAIGKLSCLEKLNLKGNNFVTLPNLKDLFRL
YYLNLEDCKRLKYLPVLPSRTHLPLNLYSYLLQYFEDFIYQVPNEDEFMAGLTMFNRPKIVERERCTSMTVSWMLQIIQS
WYNSEYLSFSSSLRSIIPGSEIPMWFNNQLLSMDNSIIDVSPFVHDNNWVGVVCCAILGENLYRTRILEDLRRDLFVEHS
DHMCLFYYSRQQFCHEEGIFWGKLNTHDLSLTFKLHDVIILCANMKDRDEGFYEFEVKKYGYRWVKKQDLQ

>XP_014524498.1

MACNKSQRSSSSHTKNFDVFVSFRGADTRNGFTNHLFAALQRKGVVAFRDDQTIQKGDFLESELLLAIEGSRVFIVVFSK
DYASSTWCMKELTKIVDWVEVTGRSLLPIFYDVTPSEVRKQSGEFAKAFAEHEERFKDDLEMVKGWRAALKTSCDRCG
WDVQNKQQYEEIENVVEEVINILGRNQIWNFGDDLVDMHSRVKKLEELLDLSANDIVHLVGICGMGGIGKSTLATALF
NKISPQFDACCYLDDLSKIYCNFGAASAQKQLLCQALNQGNIEIHNASHGTMLLRTRLCHLKALVVVDNVDQVDQLKKL
GLQSEYLGAGSRIIIISRNRRILQNYGVNKVYEVQVLDXTXSLQLLXKKAFRSNDIGKEHEXLTLDILKYVNGLPLAIEVLGSF
LLDRDVCEWRSALTRMEENPSKDIMDVLRISYDGLENIEKEIFLDIACFFSKNIFYPLEPTVKKLLDYRQFYPDIGLKVLIEKS
LIRCQDQIIKMHDLLKELGKSIVREKAPKEPRKWSRLWNYKDLQKVMKINKEAENVEAIFIEQYELQGRIRVDALSKMDH
LELLILQNVNCYGTLNFISNELRYLFWNHFPWLSLPSTFFPDQLVELILPHSNIKQLWEGKKCLPNLRNLDLRHSKNLIEVP
DLSEVPRLTDLNLKGCIQLVHIHPSIGILRDLRCLNLKNCKNLVLNFGISSLNSKMLMDPSDTKHLEKVDKNTNIIQLPTSS
VYKLLMLPFHFFYPPKPQDSIGSLLSSFSFSVPCLFNLDISFCNLLQIPDEIGNLRSLEILNLGGNKFVTLPSTIKQLSYLHYLN
LTHCKELKYLPELPTIQEKTIGRYSNQLSTFDCPKLSDLEHCYSIVFSWMTKNLEVFLDLQMEIVVPGSEIPKWFSKQKAST
SISMDPSAVIDDPNWIGVSICVLFVTHEGPMNLGERGYPTDTLFYGVNNVNFERKLYSEFLIILKKDLVTVGLDYLLIVFYS
RQEFIHLLNGHSDTMHDLQALKFETLVEIYPGLHESHPGLHFMVKKCGYRWVFKEDVQQFNSDKFFSRNSSSRKRKLLT
SE

>XP_014524544.1

SKGLVGIDKRIADVESLIHRESEKARLIGLWGMGGIGKTTLAEEVYNKLRXKYEGCYFLANVREQLSRXGKXXLRNXIFSAL
LLRDVKIDTPNSLPEYIVRRIXXMKVLIVLDDVNDSDHIXDLLGXXDXFGSGSSIIVTTRDEQVLKAXXADEIYHLREFTSDE
ALELFNLNAFNQSVHQRQYDKLSKRIVQYAKGLPLILKVLAHRLNGKNEEIWESELDKLKKVPPTKVYDVIKLSYDDLDRK
EKQIFLDLACFLCISFAKITIGYLKYLLKDDERDNSVVVSLERLKDKALITFSKDNVVCMHDSIKEMAWEIVRQESPEDPGN
RSRLWDPEDIYKAFKNDKVSEAIRSIQINLLTLKGQKLEPHIAKMSRLRFVEIYGETYSFIRYLRERYFYNSYNGERYSYNRY
NGERCSYNRCRHPSAEGQGFQFSATETRFLLWECYPLKSLPDNFSGEXLVILKLVLGRMEKLWDGVKVNMSLIILC

>XP_022631314.1

MECLLGFVTSFSRDLVCGALNQLRYPCCFKNFVKRLEEEESNLIITKDSVQKFVTHDKKQARKPSEIVDKWLEDAINDVH
NVNQLLEEARTKKHCCFGHCPNWIWRYHVGKKLANKTMDLEKFIEKGRKYVPFDRIATLPSNTLDMLSEKCMNFESRQ
YAYEQLLDAVKNSDVSMIGLYGMGGCGKTTLAMEVRKLVEAEHIFEKVLFIAVSSTVEVRRIQEKIASSLQFEFPETEEM
QRAQRLCSRLTQEKNIFIILDDVWEKLDFGRIGIPSFEHHKGCKIFITTRSEAVCTLMDCQRKIYLPILTDEEAWTLFQNKA
LISEGTPHTLKNLGRLISNECKGLPVAIAAVACSLKGKTETIWSVALNKLRRSKPINIERGLIDPYKCLQLSYDNLDTKEAKSL
FLLCSVFPEDSEIQVEILTRCAIRLGVVGEVDSYEEARSEVIAAKIKLVSCCLLLDGDNERVKMHDIVRDVAHIIAQNENKKI
KCEVEIDVTVEQNSVKYLWCSKFPNDLDCSNLEFLCLHTIMKGFEGIFKRMGKLKVLLLGNDEDEKTPLSTTSLKTLTNLR
DLFIFNFELSDFSFLSGMKNLQTLSLYDCSLPSFPEFQTDVAITLKLLELNECYIKVKNFEVMKRIPLLEELYIIDITGEWYANS
EDNIEFFKTFSIPKTLQRYGIVLGSDNFYHYNDRDIYIHSRTLLLNHFDISKNEVIKGLAKKAKDLFVRNIHGGAKNMIPDIF
EIEGGGLNDLNTLEIRDSAGIKCLIDTRSHSSEVVTLFSKLHTLEMKRMENLKAIWHCFLPANGPFENLENLYLSNCPRLTF
LFTYVVARNLVQLKILKISGCDELKHILTYDEKSQDEFTTGHPIQIFQNLLDVKIKRCRELKHIFPANIVGGLTQLKVLEIREC
DMLDQIIGDIVPLTEQDRKQELDEIVEEGTLPSLTSLKITYCGKLDSIFTASKAKTLTSLEELFIEDCKSLKDIVTHERVNKNQ
EESIVEDEHISECDSLEVVFPNWTGPKNSLTLQHLTILTIWKCGNMEVIFPKSVVRCLPELKKVTIRECMELKQIIEEDESNL
LSIDGGFEVEEIIGCDKEASKNYFAFPNLEKLEIIECEKLEVVFPKSILRCLPKLKLLKISQCKELSQIIEEDKNLSNILSPQPCFP
KLEALHVDDCHKLKRLFSGSASNDLPNLHLLAINGTYELEELVGCKQGKTKVELPRLKLLIFMHLPNFRQEIELRDLKNCIIY
KCPKLSLTSTTTLEKLLEDFPHKDFIHTELGPSELKGIVRSIYEYSTFNGSSEFTSSQEIEVVGNENIEEGPLVEGSKTKSSSTG
VEDIGIGSGVEDIGIYEDSTSGSSELTSSQEIEDVGNESIKSSSTGVEDIGIGGATHIESGGEDILALDSKVVEQDDKMNEGK
PGIVASQGIQVQEGLNLLHKQDGTDVVPNNNIDISSDICTRLGAYKHFADLDDAQISLLVEAITTYPHLWNASKKFSERF
QAWRLKILADMLLFLQKESVDSIIPQREKEFHKLCEEAIEIGFESSWVEEMRQRVVARDPKLEEDIAKRQMDENSKSNLS
LLNRCSSGDVVEEGDGPKIRLEEGSDLVDKEGEVGVVSNDHILAPRNEEPEHEFVAEVSTSEIPRIATSLTNSQTVEKQTP
SHLYIPVRETSSNALVDTKQTSEPCLMKQKKPLGEIPKSIDQVVEEETIAKNTDMAASSILSDSTTSKLDTTVTLQRKSHPH
SEIRSSQNVVRITKESEGHLELIQDFGGNDMIAISLGKEGDDNIVVKTLVELENYLKMSLKDIVSSETNALRLFSTLNFLSNL
PFKDVTLSDRLKHIIKTMHQHFPTILCSFKQRFATTDKLAELEARQNEVAIKIFEAENFNDEARLKEVVLKEEIIRLKEEIKVC
EAALSSLDEGKNKCIVETIRYKKELENVRKNKSQMVKDQRKVEQELFEVAYKWSVLCSEYELNRIAARNPS

>XP_022631315.1

MECLLGFVTSFSRDLVCGALNQLRYPCCFKNFVKRLEEEESNLIITKDSVQKFVTHDKKQARKPSEIVDKWLEDAINDVH
NVNQLLEEARTKKHCCFGHCPNWIWRYHVGKKLANKTMDLEKFIEKGRKYVPFDRIATLPSNTLDMLSEKCMNFESRQ
YAYEQLLDAVKNSDVSMIGLYGMGGCGKTTLAMEVRKLVEAEHIFEKVLFIAVSSTVEVRRIQEKIASSLQFEFPETEEM
QRAQRLCSRLTQEKNIFIILDDVWEKLDFGRIGIPSFEHHKGCKIFITTRSEAVCTLMDCQRKIYLPILTDEEAWTLFQNKA
LISEGTPHTLKNLGRLISNECKGLPVAIAAVACSLKGKTETIWSVALNKLRRSKPINIERGLIDPYKCLQLSYDNLDTKEAKSL
FLLCSVFPEDSEIQVEILTRCAIRLGVVGEVDSYEEARSEVIAAKIKLVSCCLLLDGDNERVKMHDIVRDVAHIIAQNENKKI
KCEVEIDVTVEQNSVKYLWCSKFPNDLDCSNLEFLCLHTIMKGFEGIFKRMGKLKVLLLGNDEDEKTPLSTTSLKTLTNLR
DLFIFNFELSDFSFLSGMKNLQTLSLYDCSLPSFPEFQTDVAITLKLLELNECYIKVKNFEVMKRIPLLEELYIIDITGEWYANS
EDNIEFFKTFSIPKTLQRYGIVLGSDNFYHYNDRDIYIHSRTLLLNHFDISKNEVIKGLAKKAKDLFVRNIHGGAKNMIPDIF

EIEGGGLNDLNTLEIRDSAGIKCLIDTRSHSSEVVTLFSKLHTLEMKRMENLKAIWHCFLPANGPFENLENLYLSNCPRLTF
LFTYVVARNLVQLKILKISGCDELKHILTYDEKSQDEFTTGHPIQIFQNLLDVKIKRCRELKHIFPANIVGGLTQLKVLEIREC
DMLDQIIGDIVPLTEQDRKQELDEIVEEGTLPSLTSLKITYCGKLDSIFTASKAKTLTSLEELFIEDCKSLKDIVTHERVNKNQ
EESIVEDEHISECDSLEVVFPNWTGPKNSLTLQHLTILTIWKCGNMEVIFPKSVVRCLPELKKVTIRECMELKQIIEEDESNL
LSIDGGFEVEEIIGCDKEASKNYFAFPNLEKLEIIECEKLEVVFPKSILRCLPKLKLLKISQCKELSQIIEEDKNLSNILSPQPCFP
KLEALHVDDCHKLKRLFSGSASNDLPNLHLLAINGTYELEELVGCKQGKTKVELPRLKLLIFMHLPNFRQEIELRDLKNCIIY
KCPKLSLTSTTTLEKLLEDFPHKDFIHTELGPSELKGIVRSIYEYSTFNGSSEFTSSQEIEVVGNENIEEGPLVEGSKTKSSSTG
VEDIGIGSGVEDIGIYEDSTSGSSELTSSQEIEDVGNESIKSSSTGVEDIGIGGATHIESGGEDILALDSKVVEQDDKMNEGK
PGIVASQGIQVQEGLNLLHKQDGTDVVPNNNIDISSDICTRLGAYKHFADLDDAQISLLVEAITTYPHLWNASKKFSERF
QAWRLKILADMLLFLQKESVDSIIPQREKEFHKLCEEAIEIGFESSWVEEMRQRVVARDPKLEEDIAKRQMDENSKSNLS
LLNRCSSGDVVEEGDGPKIRLEEGSDLVDKEGEVGVVSNDHILAPRNEEPEHEFVAEVSTSEIPRIATSLTNSQTVEKQTP
SHLYIPALVDTKQTSEPCLMKQKKPLGEIPKSIDQVVEEETIAKNTDMAASSILSDSTTSKLDTTVTLQRKSHPHSEIRSSQ
NVVRITKESEGHLELIQDFGGNDMIAISLGKEGDDNIVVKTLVELENYLKMSLKDIVSSETNALRLFSTLNFLSNLPFKDVT
LSDRLKHIIKTMHQHFPTILCSFKQRFATTDKLAELEARQNEVAIKIFEAENFNDEARLKEVVLKEEIIRLKEEIKVCEAALSS
LDEGKNKCIVETIRYKKELENVRKNKSQMVKDQRKVEQELFEVAYKWSVLCSEYELNRIAARNPS

>XP_022631418.1

MCFWCFIFTATHKLSSNLTAIRAVLRDAERKQMTNHAVKDWLQKLTDAAYVLDDILDECSIHFAKMHSDDGHTSCLSR
LHPNDILFRFNICKRMKDITQRFHDIHEEKSTFNLEPGLTEVVQRVDDDWRQTSSDITEPVVYGRNKDREQIVKFLLEDA
NNREELSIFPILGMGGLGKTTLAKQVFNDHRVCKHFDLTIWVCVSDDFNTMTILQSILECITGQNPNLNSLEAMRKKVEE
VLHGKRYLLVLDDVWNEDQEKWEQLKGKLQCARAAKGSTILVTTRLEEVASIMQTHPAYHLKELPGDESWSLFKYHAF
GPNREETEELVAIGKEIVRNCIGLPLAIKTLGSLLRDQSEVRQWINVKESSSMTDEDNSIMRALKLSYSNLELSLRRCFSFC
AIFPKDFEIDKEELIYLWIANGFIESERNTEVEDVGNKVWNKLYRRSFFQEAKCDEFGMVKSFKMHDLFHDLAQSIMGE
ECAAVVEGRLTPLSSRVHYLSSNVSYRGFMHAFKKVESLRTFLDLRTSIYNFCPVPSNHSLRALCTKSSLLSPLKDLTHLRYL
SLTCHSKASLNNFICQMPKLQILKLKSSAIRGLPKNLTQLQDLRHIVIDDYDSVVETLPKISKLRHLRTLSIFVVGSKPGCGLA
ELHSLNLGGRLRIIGLENVPNEWDAKEANLIGKKELNILHLSWDGNANPKGSNVSVERVLEALEPPSTLKSFQMYGYQG
RQISSWMRNKAVLRDLVDVRLSNFRNCEELPPLGKLPHLKRINVRGMKNVKWIDGETYDGVEEKAFPSLEEVRMDNLP
NLERLLKDEGVEMLPRLSQLTIKDVLNFKVPHLPCVEKLEARGIEAGASFIEGVGENMACLETLIIKIIKGVVVLPDEFRRLG
ALQELYIAEWYDVEYFPEHVLEGLTSLRILTIENCKKLKSLSEGVRHFACLESLRISKCPELVALPSNMSQLSALRVFSIYYCC
TLPDGLQRVPSLRLLVIFGCTCTSLPDWLGDMTTLEQLTIAYCNELRSLPSSIQRLTNLSYLFIQGCPDLKKRCKRETGEDW
QYINHIPKIDLW

>XP_022631464.1

MDIQEESPVFGSLTAVTTRNMSSSSSVFFSANQSPFFSPRSPSSCQLSHSARLDTQNNTVHLGLAPSSTTLEIPEPNSAVN
VRCNVSDVSASPAGCNSGGLMKLDRKSSPVGISSSSISSYSNCHDDGYSGQRERRIKKDRNHRTSSTPGSTSFSSYRLRSC
DVFIGLHGSKPPLLRFAKWLCGELEIQGISCFVSDRARSRSSRKLGIAERAMDAASFGIVIITKKSFKNQYTIEELNFFCRRK
NLIPIYFDLSPADCLVRDIIEKRGELWEKHGGELWLSYEGLEQEWKDAVHGLSRVDECKLEAQDGSWRDCILRAVTLIA
MRLGRRSVAERVTKWREKVEKEEFPFIRNENFIGRKKELSQLEFILFGDVTGDAEQDYIELKARPRRKSVRIGWGKSNMI
DERWNDRRKEKEPVVWKESEKDIEMQGVEFSHRHNHPRLKRGKYAKRKNGMKILYGKGIACVSGDSGIGKTELILEFA
YRFHQRYKMVLWIGGESRYIRQNYLNIRSFLEVDVGVENSLDKTKIRSFEEQEVAAISRVRKELMKNIPYLVIIDNLESEKD
WWDHKLVMDLLPRFGVETHVIVSTRLPRIMNLEPLKLSYLSGVEAMSLMVGSSKDYSVAEVDALRSIEEKVGRLTLGLAI
ISAILSELPITPSRLLDTINRMPLKEMPWSDKEALSFTKNAFLLQLFDVCFSIFDHADGPRSLATRMVLVSGWFAPGAIPIS
LLALAAEKIPERCQGKCFWRKMLQLLSCGFPSSYAKKPELEASSLLLRFNIARNSTKQGYIHINEVFKLYARKRENTGAAQ
AMIQAIISNGSISQNLDHLWAACFLLFGFGHDPVIVELKVSELLYLVKRVVLPLAIHTFITYSRCTAALELLRLCTNALEAAD

QAFVTPVDKWFDKSLCWRSIQTNAQLNPCLWQELALTRATVLETRAKLMLRGAQFDVGDDLIRKAVFIRTSICGEDHP
DTVSARETLSKLTRLNANVQIHT

>XP_022631465.1

MDIQEESPVFGSLTAVTTRNMSSSSSVFFSANQSPFFSPRSPSSCQLSHSARLDTQNNTVHLGLAPSSTTLEIPEPNSAVN
VRCNVSDVSASPAGCNSGGLMKLDRKSSPVGISSSSISSYSNCHDDGYSGQRERRIKKDRNHRTSSTPGSTSFSSYRLRSC
DVFIGLHGSKPPLLRFAKWLCGELEIQGISCFVSDRARSRSSRKLGIAERAMDAASFGIVIITKKSFKNQYTIEELNFFCRRK
NLIPIYFDLSPADCLVRDIIEKRGELWEKHGGELWLSYEGLEQEWKDAVHGLSRVDECKLEAQDGSWRDCILRAVTLIA
MRLGRRSVAERVTKWREKVEKEEFPFIRNENFIGRKKELSQLEFILFGDVTGDAEQDYIELKARPRRKSVRIGWGKSNMI
DERWNDRRKEKEPVVWKESEKDIEMQGVEFSHRHNHPRLKRGKYAKRKNGMKILYGKGIACVSGDSGIGKTELILEFA
YRFHQRYKMVLWIGGESRYIRQNYLNIRSFLEVDVGVENSLDKTKIRSFEEQEVAAISRVRKELMKNIPYLVIIDNLESEKD
WWDHKLVMDLLPRFGVETHVIVSTRLPRIMNLEPLKLSYLSGVEAMSLMVGSSKDYSVAEVDALRSIEEKVGRLTLGLAI
ISAILSELPITPSRLLDTINRMPLKEMPWSDKEALSFTKNAFLLQLFDVCFSIFDHADGPRSLATRMVLVSGWFAPGAIPIS
LLALAAEKIPERCQGKCFWRKMLQLLSCGFPSSYAKKPELEASSLLLRFNIARNSTKQGYIHINEVFKLYARKRENTGAAQ
AMIQAIISNGSISQNLDHLWAACFLLFGFGHDPVIVELKVSELLYLVKRVVLPLAIHTFITYSRCTAALELLRLCTNALEAAD
QAFVTPVDKWFDKSLCWRSIQTNAQLNPCLWQELALTRATVLETRAKLMLRGAQFDVGDDLIRKAVFIRTSICGEDHP
DTVSARETLSKLTRLNANVQIHT

>XP_022631466.1

MDIQEESPVFGSLTAVTTRNMSSSSSVFFSANQSPFFSPRSPSSCQLSHSARLDTQNNTVHLGLAPSSTTLEIPEPNSAVN
VRCNVSDVSASPAGCNSGGLMKLDRKSSPVGISSSSISSYSNCHDDGYSGQRERRIKKDRNHRTSSTPGSTSFSSYRLRSC
DVFIGLHGSKPPLLRFAKWLCGELEIQGISCFVSDRARSRSSRKLGIAERAMDAASFGIVIITKKSFKNQYTIEELNFFCRRK
NLIPIYFDLSPADCLVRDIIEKRGELWEKHGGELWLSYEGLEQEWKDAVHGLSRVDECKLEAQDGSWRDCILRAVTLIA
MRLGRRSVAERVTKWREKVEKEEFPFIRNENFIGRKKELSQLEFILFGDVTGDAEQDYIELKARPRRKSVRIGWGKSNMI
DERWNDRRKEKEPVVWKESEKDIEMQGVEFSHRHNHPRLKRGKYAKRKNGMKILYGKGIACVSGDSGIGKTELILEFA
YRFHQRYKMVLWIGGESRYIRQNYLNIRSFLEVDVGVENSLDKTKIRSFEEQEVAAISRVRKELMKNIPYLVIIDNLESEKD
WWDHKLVMDLLPRFGVETHVIVSTRLPRIMNLEPLKLSYLSGVEAMSLMVGSSKDYSVAEVDALRSIEEKVGRLTLGLAI
ISAILSELPITPSRLLDTINRMPLKEMPWSDKEALSFTKNAFLLQLFDVCFSIFDHADGPRSLATRMVLVSGWFAPGAIPIS
LLALAAEKIPERCQGKCFWRKMLQLLSCGFPSSYAKKPELEASSLLLRFNIARNSTKQGYIHINEVFKLYARKRENTGAAQ
AMIQAIISNGSISQNLDHLWAACFLLFGFGHDPVIVELKVSELLYLVKRVVLPLAIHTFITYSRCTAALELLRLCTNALEAAD
QAFVTPVDKWFDKSLCWRSIQTNAQLNPCLWQELALTRATVLETRAKLMLRGAQFDVGDDLIRKAVFIRTSICGEDHP
DTVSARETLSKLTRLNANVQIHT

>XP_022631467.1

MDIQEESPVFGSLTAVTTRNMSSSSSVFFSANQSPFFSPRSPSSCQLSHSARLDTQNNTVHLGLAPSSTTLEIPEPNSAVN
VRCNVSDVSASPAGCNSGGLMKLDRKSSPVGISSSSISSYSNCHDDGYSGQRERRIKKDRNHRTSSTPGSTSFSSYRLRSC
DVFIGLHGSKPPLLRFAKWLCGELEIQGISCFVSDRARSRSSRKLGIAERAMDAASFGIVIITKKSFKNQYTIEELNFFCRRK
NLIPIYFDLSPADCLVRDIIEKRGELWEKHGGELWLSYEGLEQEWKDAVHGLSRVDECKLEAQDGSWRDCILRAVTLIA
MRLGRRSVAERVTKWREKVEKEEFPFIRNENFIGRKKELSQLEFILFGDVTGDAEQDYIELKARPRRKSVRIGWGKSNMI
DERWNDRRKEKEPVVWKESEKDIEMQGVEFSHRHNHPRLKRGKYAKRKNGMKILYGKGIACVSGDSGIGKTELILEFA
YRFHQRYKMVLWIGGESRYIRQNYLNIRSFLEVDVGVENSLDKTKIRSFEEQEVAAISRVRKELMKNIPYLVIIDNLESEKD
WWDHKLVMDLLPRFGVETHVIVSTRLPRIMNLEPLKLSYLSGVEAMSLMVGSSKDYSVAEVDALRSIEEKVGRLTLGLAI
ISAILSELPITPSRLLDTINRMPLKEMPWSDKEALSFTKNAFLLQLFDVCFSIFDHADGPRSLATRMVLVSGWFAPGAIPIS
LLALAAEKIPERCQGKCFWRKMLQLLSCGFPSSYAKKPELEASSLLLRFNIARNSTKQGYIHINEVFKLYARKRENTGAAQ

AMIQAIISNGSISQNLDHLWAACFLLFGFGHDPVIVELKVSELLYLVKRVVLPLAIHTFITYSRCTAALELLRLCTNALEAAD
QAFVTPVDKWFDKSLCWRSIQTNAQLNPCLWQELALTRATVLETRAKLMLRGAQFDVGDDLIRKAVFIRTSICGEDHP
DTVSARETLSKLTRLNANVQIHT

>XP_022631468.1

MDIQEESPVFGSLTAVTTRNMSSSSSVFFSANQSPFFSPRSPSSCQLSHSARLDTQNNTVHLGLAPSSTTLEIPEPNSAVN
VRCNVSDVSASPAGCNSGGLMKLDRKSSPVGISSSSISSYSNCHDDGYSGQRERRIKKDRNHRTSSTPGSTSFSSYRLRSC
DVFIGLHGSKPPLLRFAKWLCGELEIQGISCFVSDRARSRSSRKLGIAERAMDAASFGIVIITKKSFKNQYTIEELNFFCRRK
NLIPIYFDLSPADCLVRDIIEKRGELWEKHGGELWLSYEGLEQEWKDAVHGLSRVDECKLEAQDGSWRDCILRAVTLIA
MRLGRRSVAERVTKWREKVEKEEFPFIRNENFIGRKKELSQLEFILFGDVTGDAEQDYIELKARPRRKSVRIGWGKSNMI
DERWNDRRKEKEPVVWKESEKDIEMQGVEFSHRHNHPRLKRGKYAKRKNGMKILYGKGIACVSGDSGIGKTELILEFA
YRFHQRYKMVLWIGGESRYIRQNYLNIRSFLEVDVGVENSLDKTKIRSFEEQEVAAISRVRKELMKNIPYLVIIDNLESEKD
WWDHKLVMDLLPRFGVETHVIVSTRLPRIMNLEPLKLSYLSGVEAMSLMVGSSKDYSVAEVDALRSIEEKVGRLTLGLAI
ISAILSELPITPSRLLDTINRMPLKEMPWSDKEALSFTKNAFLLQLFDVCFSIFDHADGPRSLATRMVLVSGWFAPGAIPIS
LLALAAEKIPERCQGKCFWRKMLQLLSCGFPSSYAKKPELEASSLLLRFNIARNSTKQGYIHINEVFKLYARKRENTGAAQ
AMIQAIISNGSISQNLDHLWAACFLLFGFGHDPVIVELKVSELLYLVKRVVLPLAIHTFITYSRCTAALELLRLCTNALEAAD
QAFVTPVDKWFDKSLCWRSIQTNAQLNPCLWQELALTRATVLETRAKLMLRGAQFDVGDDLIRKAVFIRTSICGEDHP
DTVSARETLSKLTRLNANVQIHT

>XP_022631613.1

MAEALLGSVIENLGSFVQDQLGTYLGVEQQIQKLSNNLTAIRAVLRDAERKQITSEVLKNWLQKLTDAAYVLDDILDECSI
HFTKMPSDDGHTSCLSRLHPKDILFRFNIAKRMKDITQRFHDIHEEKSRFNLEHGVTEVQAVDDDWRQTSSDITEPVVY
GRDHDREQIVKFLLEDASNSEELSIFPIVGMGGLGKTTLAKQVFNDHRVCKHFHKTIWVCVSDDFNNMTILQSIIECITG
QNPNLNSLEAMRKKVEEVLHGMRYLLVLDDVWNEDPEKWEQLKGKLQCARAAKGSTILVTTRLEEVASIIQTHPAYHL
KKLSGDESWSLFKYHAFGPNRKEMEELVAIGKEIVRNCIGLPLAIKTLGSLLRDQSEDIFVDCPKT

>XP_022631659.1

MAEALLGFVLQNLGSFVQYQLTSCWGVDQQTHKLSSNLTAIRAVLRDAERKQMTNHAVKDWLQKLTDAAYVLDDIL
DECSIHFAKMHSDDGHTSCLSRLHPNDILFRFNICKRMKDITQRFHDIHEEKSTFNLEPGLTEVVQRVDDDWRQTSSDIT
EPVVYGRDHDRDQIVKFLLEDANNREELSIFPIVGMGGLGKTTLAKQVFNDHRVCKHFDLTIWVCVSDDFNTMTILQSI
LECITRQNPNLNSLEAMRKKVEEVLHGKRYLLVLDDVWNEDREKWEQLKGKLQCERAAKGATILVTTRLEEVAFIMQT
HPAYHLKELSGDESWWLFKYHAFGPNREEMGELVSIGKEIVRKCVGSPLAIKTLGSLLRDESEVKQWQNVKESEIWDIR
EESSSATGEENSIMRALKLSYFNLELSLRRCFSFCAIFPKDFEIDKEELIHLWMANGFIESKRNIEVEDVGNKVWNKLYRRS
FFQEAKRDEFGIITTFKMHDLLHDLAQSIMGEECAAFVEGRLTPFSSRVHYSTLLSSDVSSNVSFRQFMHAFKKVQSLRT
FLDLGPSFRVSDSDLVPSNHSLRALCTQFSWLSPLKNLTHLRYLSLIHFQGSLNNSICQMRKLQILKLQRCRYLCGLPKNLT
QLQDLRHIVMNDCREVEKSLPKISKLRHLRTLSIFVVGSKPGCGLAELHSLNLGGTLRIRGLENVLNELDAKEANLIGKKEL
NILELSWDGNANPKGSNVSVERVLEALEPPSTLKSFEINGYQGRQLSSWMRNSGVLRDLVDVRLSDCENCEELPPLGKL
AHLKRLKVSGMKNVKWIDGETYDGVEEKAFPSLDELSVYNLPNLERLLSDEGVEMLPRLSQLRINGVLNFKVPRLPCVET
LDARGIEDVTSFMEGVGENMACLKTLRIINIKGVVVLPDEFSRLGALQELYIEEWYDVEYFPEHVLEGLTSLRILTIENCKKL
KSLSEGVRHLACLHGLRISKCPELVALPSNMSQLSALREVSIESCSTLPDGLQRVPSLRVLHIEECTCTSLPDWLGDITTLET
LGLFYCEELRSLPSSIQRLTNLSHLIIEYSPYLKKRCNKETGEDWQYISHIPKLELFFTRRLTFCGF

>XP_022631956.1

MVKTKARLKRFWDRFGGRSSGSSSSSSSYDSSDTIQNQNYRYDVFISFRGPDTRNSFVDHLCSHLLRKGIFLFKDDHSLQ
KGESISPQLLQAIQLSRLSIIVFSKNYASSTWCLDEMAAIASCKQRSNHIVFPIFYDVDPSHARHQNGVYENDFVSHRRKF
RKDRDKVRGWERAMTDLANSAGWDMRDKPEFEQIQNIVQAVIKKLGHKFSWFVDDLIGIQQRVQTLEDKLKLSSNSD
DVKVLGIWGMDGIGKTTHAGVLYDKISHRFDACCFIENVSKLYSDGGHTAVQKEIIYQTLGQKGLDMCSPVQISGIVRT
RIHNIRVLIVLDNVDELEQLENLAIKPKLLLKGSRMVITTTDMHILKVYEGGVLIHKVSLLNDKVARELFCRKAFKSEERSSS
CEALIPEVLKYAQCLPLAIRVLGSFLCTRDAVEWRDVLNRLQSSPDKKIMNVLQLSVDGLNHEEKQIFLHIACFFKGERVD
FVKRILDCCELYPHIGISRLIEKSLVTISNEEIHMHELLQELGKKMVWDQSPQEPRFWSRIWLHKDFLQVLTAEAGTEKVK
AIVLNKEEEMSECSIGGLSRMKELTLLILYHTKVSGSLEFLSDRLRYLLWHDYPFDSLPPYFTVSNLVELNMPNSHIISLWH
GSKSCPNLKRIDLSNSKDLCETPDFSRIIKLERLDLSGCTSLSYVHSSIGLLKKLAFLNLRNCCNLVFIDFGCVGNMSSLRVLH
LSGCSKLESTPDFTRATHLEYLDMDACTSLSTIHESIGVLSSLTFLSLRVCIKLVSIPNDINSLVSLQTLDLCSCLNLKDMLQR
QASSSHLKSLIFLDLSFCNIEEVPDAIGDLRCLERLNLQGNKFVSIPDSFRKLHCLAYLNLSHCHKLKSLPDLPTEGDKSGGK
YFKTVSGSRDHRSGFYIFDSSNVSHDRLEFVGLERFFHLIKEPCNFRCGFDLVLLRDRRLRPVFGETFLGNSIIRILQCVKND
NWIGFSFFVIFRQNTVGGFPPHSSFSSSHPLYLSFESEYTEEYFDMRLNLKADQSEESTHIWIIYISREHCHFVKTGALITFK
AQPYIKIDAWGMRPILKQDIHDSKGKKYIQFFKLNYDHVDFEYVEKSNTGSGPKIQLPYNWYVTAEEQVENIDAKAKEN
NLSNAGL

>XP_022631957.1

MVKTKARLKRFWDRFGGRSSGSSSSSSSYDSSDTIQNQNYRYDVFISFRGPDTRNSFVDHLCSHLLRKGIFLFKDDHSLQ
KGESISPQLLQAIQLSRLSIIVFSKNYASSTWCLDEMAAIASCKQRSNHIVFPIFYDVDPSHARHQNGVYENDFVSHRRKF
RKDRDKVRGWERAMTDLANSAGWDMRDKPEFEQIQNIVQAVIKKLGHKFSWFVDDLIGIQQRVQTLEDKLKLSSNSD
DVKVLGIWGMDGIGKTTHAGVLYDKISHRFDACCFIENVSKLYSDGGHTAVQKEIIYQTLGQKGLDMCSPVQISGIVRT
RIHNIRVLIVLDNVDELEQLENLAIKPKLLLKGSRMVITTTDMHILKVYEGGVLIHKVSLLNDKVARELFCRKAFKSEERSSS
CEALIPEVLKYAQCLPLAIRVLGSFLCTRDAVEWRDVLNRLQSSPDKKIMNVLQLSVDGLNHEEKQIFLHIACFFKGERVD
FVKRILDCCELYPHIGISRLIEKSLVTISNEEIHMHELLQELGKKMVWDQSPQEPRFWSRIWLHKDFLQVLTAEAGTEKVK
AIVLNKEEEMSECSIGGLSRMKELTLLILYHTKVSGSLEFLSDRLRYLLWHDYPFDSLPPYFTVSNLVELNMPNSHIISLWH
GSKSCPNLKRIDLSNSKDLCETPDFSRIIKLERLDLSGCTSLSYVHSSIGLLKKLAFLNLRNCCNLVFIDFGCVGNMSSLRVLH
LSGCSKLESTPDFTRATHLEYLDMDACTSLSTIHESIGVLSSLTFLSLRVCIKLVSIPNDINSLVSLQTLDLCSCLNLKDMLQR
QASSSHLKSLIFLDLSFCNIEEVPDAIGDLRCLERLNLQGNKFVSIPDSFRKLHCLAYLNLSHCHKLKSLPDLPTEGDKSGGK
YFKTVSGSRDHRSGFYIFDSSNVSHDRLEFVGLERFFHLIKEPCNFRCGFDLVLLRDRRLRPVFGETFLGNSIIRILQCVKND
NWIGFSFFVIFRQNTVGGFPPHSSFSSSHPLYLSFESEYTEEYFDMRLNLKADQSEESTHIWIIYISREHCHFVKTGALITFK
AQPYIKIDAWGMRPILKQDIHDSKGKKYIQFFKLNYDHVDFEYVEKSNTGSGPKIQLPYNWSLSCL

>XP_022632074.1

MDCLVGFASSVSRDLVCGALNQLRYLRSFNKFVKKLEQEEGDLILTRDGVQKSVEQVKRKTRETSELVNKWLEDAISDIG
KVNQLLEEARTKKMCCFGYCPNWIWRYRIGKKIANKVFDLEKFIDEGKKYVSFDSIVTLPSGTLHILSEKCMNFESRQSVY
EQLLDAVKNNDVSMIGLYGMGGCGKTTLAMEVMKLVEVEHLFDKVLFVPVSSTVDVRKIQEKIASSLQVEFPETEEMQ
RAQRLCLRLIQEKNIFIILDDVWEKLDFGRIGIPSSDHHKGCKILITTRSEEVCTSMDCQRKIYLPILTDEEAWTLFENKAFIT
KGSPDTWKDLGRLISNECKGLPVAIAAVACSLKGKAETVWRVAFNKLRHSKPINIERGLTDPYKCLQLSYDNLDTKEAKS
LFLLCSVFPEDFEIQVELLTRCAIGLGVVGEVDSYEEARSEVIAAKIKLVSCCLLLDADDECVKMHDLVRDVAHIIAKTENK
MIKCEVERHVTVEQNSVRYLWCSKFPNVLDCSNLEFLCLEANMKGFDGIFKRMGMLKVLILANDEDGKTQMSTISFKT
LTNLRYLFIENYELSDFSFLGGMKNLQSLKLFDCLLPSFPELQTDVAITLKLLELNKCDIKVKNFEGMKRIPLLQELYIIDIEGE
WYANSEDNIEFFNTFSIPETLQRYGIVLGSRYFYHHSDGDIYIHGRTLLLNHFDISNEVIKGLAKKAKDLFVGNIHGGAKNI
TPDIFQIEGGGLNELKKLEIIDSEELECLIDTSSHSSEVVTLFSKLHTLKMVRMENLKAIWHCFLPANGPFENLEKLDLSDCP
RLTSLFTYVVALNLVKLKILKLSRCHELKHILTDDEKTEKSHNEFTGGHPVQIFQNLQDVKIDSCPELKYIFSANIVGGLGQL

KVLKIERCDMLDQIIGDRKEELDEIVEEGTLSSLSSLTITCCGKLGSIFTSSIAKTLTSLEELFIDDCKSLKDIVSHERDNKNQEE
SIVEDEHDCQCDISIFQSLERLDIRNCDLLKKLRIFPVSFVGGMKKLNDITNKEAADLKDFSSRNNTQNEEILNHFLILQTLV
IWRSGLECIFFLNGYGMIDQQVSLRLEKLDLYNLPQMTYIWVGPNNSLTLQHLITLEITDCGKLEVIFPNSVIRCLPELAWL
TISKCMELKQIIEEDESNIDGGSEVEEIIECNKEPSNNYFDFPNLERLVIVECAKLEVVFSKSVLRCLPKLNVLVIKKCNELRQI
IEGDKNLSNILSPQPCFPKLEALQVDHCQKLKRVFSGYASNDLPNLHLLAINGANELEELVGCKQEKIKVELPRLKLLIFMH
MENFSQEIDLHNLKNCFVYKCPKLSLTSTTTFWKVLEHFPSQGKLVYEESNSEGGFPYQYALEEAIVQGSFQTLESLNRIF
SICNTQEIEDLRNESVKSSSTGVEDIGIEDAVATHTDSGGEDILALDSKVVEQDDKMNESKPGIMASQGIQVEEGLNLLH
KQEGIDVVPNNNIDISSASADIRTRLGAYKHFVDLDDEQISLLVEAITTYPHLWNASKKFSERFQAWRLKILEDMLSFLQE
SGDSIPEREKEFHKLCEEAIEIGFDSSWIEEMRQRVVARDLKPGEDIARRQIDENSKSLLHRCSSGDMVPDSQSVERGDG
SKISLEEGSKLVDKESEISIVSNDRIVAPRNEELEMEFVAKVSTSEIPSIAATLTNSQPVERPTPSCLNMPLCETPANALVDK
QRIIEPCLMNQQKPFGNISEITSSQTEVGTAKEIAHPKIIQGFGSNDVISLFASEKESEDSLVGKTLTELEKFLKMSLKDVVS
SETDTLRSTLNLLSNIPFKDVTLSDGLKHIIETMRQHFPTILRSFKQGFATIDKLGKLEACQNEVATTLVSKISEADNFYNEA
QMKEEVLKEQIKVCEAALSSFEREKNKCIAETIGYKKELDNVMKDRSQILEDKRKVEQELFEVAYKWSVLCSQYELNRMV
ATNIP

>XP_022632083.1

MPHDAHPMGVLVNAMSALTVFHTDANPALRDVYFYQLKEFSSNDALELFNLIAFDQSDHRMEFNELSQRVVDYAHGI
PLLVKVLARLLCGRNKEVWESQLNKLKKMSVKNG

>XP_022632194.1

MAENAVTLLALLAENLLTLLKEEGKTLSGVHKDVKQIENLVKQIKPFMKNAEEKVLIEESVKNWMNGLREVMFRMEDV
VDLYLFKVAKRDGVRYGMTKKIKSVKHRHRISSEIKDIRLTLDDLLISISTRLQVLPSHGDALPITIPRPHFVKESQLVGIEHN
MQKFRDWLAKANSPLLVVVGPPGIGKTSIVKNVYNKQRKLNQPKRKKDFDFCVWITMTQADSWYPIMQIKEEILMAD
PSGSTSLRSATRENLTEKLGEYFIDKRCLIVLDDVKELKIWDVIQFAIPQHRVIITTQRDKFPNNIGSDTSVEKIKLEPLSSED
ALKLFHQKVKHVQFPELSQLSKEFMEKCNGVPLAILAISSLLSTMKSAIEWRRVRDDLGSLLRSHHHLEMVRHVLLQSYR
ELPYRLKQCFLYFGLFPQGYSISCKRLIRLWVAEDFVEGDTQNKSMEEFGGEYMAELICRGLVHASRVDFDGIPRSCHVY
NLMHEIIASICKDQMFCHVLEDVRTPVNSNMDFFHRRLSIIKKNDSATMERDQNWGKVRSCFVFDDAKKWQVNNHF
FSSFEFLIRLDLSHACLSVDVLPEQVGNLLNLKYLSLRNTNIMSLPESIGNLVNLQTLDLKQTKLHEVKIDKLVKLRHLLAYY
VSYQSSEFYCLEGLRLSEGVQNLESLQNLSYLDVSGGRIVKGLQKLTKLRKLGIIKLEAKHGEALCISIEHMINLCSLSIGALG
KQGMLKLQSLKPPLSLKRLYLYGRLGELPTWISTLPNLIRLYLKWSDLKQDPLHYLKDLPQLLHLELFCT

>XP_022632284.1

MKEVLEKLEYLAKQKGALGLRESIYSGDRSGSKVSQKLPSSSLIVESVIYGRDTDKEMIFNWLTSETDNHNHLSILSIVGM
AGLGKTTLAQHVYNDPKMETKFDIRAWVCVSDHFDILTVTKKILEAITKSKDDSEDLEMVHGRLKEKLSGKKFFLVLDDV
WSESREEWEAVRTPLSYGAPGSRIFVTTRDEKVASNMRSKVHRLKQLEEEECWKVFEEQALKDNDFELNDEKKEIGRRI
VEKCKGLPLALKTIGSLLRTKSSILDWKSVLESDLWELPKEIEIMPALLLSYQHLPSHLKRCFAYCALFPKDYEFDKKELILLW
MAEGFLHHSQQNKNVQEIGEQYFNDLLTRSFFLPSTLIMKFLMHDLLNDLAKYVCADFCFRLKFDKGNSIPKTTRHFSFA
SNDVRYFNRFGSLTDAKRLRSFVPIRHMNSGWKFNISIHDLFSELKFLRVLSLNGYYDLKEVPDSIGDLKHLHSLDLSGTM
IQKLPDSVGLLYNLLILKLNNCSYMKELPWNLHKLTKLRCLEFEDTKVTKMPMHFGELKNLHVLSTFCVNRDPEIINIKQL
GGLNLRGKLSINELQNIVNPLDALEANLKNKHIVQLNLRWNSNHMPDDPRKEKKVLENLKPSNQLEHVSIWSYCGTQF
PSWVFDNSLSNLVSLQLRDCKYCLCLPPLGLLSSLKVLYIVGLDGIVSIGAEFYGSNSSSFMSLETLVFYNMKEWEEWECK
TTSFPRLRHLAIVRCPKLKGLSEQLLHLKKLSIESCDNLIISDHSINTSTLELLSIRSCPLVNIPMTHYGFIQEMKIDDGCDSLTI
FQLNFFPMLRLLFLKGCQNLQRISQEHPHNHLKEMSIYACAQFESFPIEGLSAPWLQTIKIQGAENLKLLPKRMKILLPSLI

ELEIIDCRNVEKFPEGGLPSKIKDISLSSLKLIASLRETLDVNTCIRSLSIEYLDVESFADEVLLPPSLTSLSINHCRNLKRLDYKV
LYNLSSLSLGDCPNLQCLPEEGLPKSISSLHIWRCPLLEQRCQNPEGKDWRKIAHIENLRVK

>XP_022632558.1

MDFVSDRYQKIDGGNFEVFLSFKGEDTRASFTSHLYTALQNAGIFVFKDDESLPQGKQISRSLRQAIEESRISIVVFSKNYA
ESWWCLKELEIIMECHKTTGNVVLPVFYDIYPSEVRHQKGDFGEAFQRLLSKFSKEEEQKVLDWKHCWWNTLREICNIS
AVEILSPRIERVDKLDNQWEKTLLEVVQISKILDLNPRGKMKIADSIEILVRQSKEGLFKGAEISFREEKVYDKMDFLMKQ
WREVFCEPFRNSEVALDPCFLVLHVKHFRETLFEAADISGDLVLNSCGEMEITYAIELHMKHWRNAFSEEAGTILDSIYIR
YLRDKSVVGMVHSEIRNLRDHWREKVCEAYDRSGDNQLYSRRKNDIELHLENCTKALLEATDISKRAVFVSSYKKLKAYN
RIESLVKYWKIALCEAVEISRLVVEHYRGITDNEVNYIEKHARDALREAAGLPGNVILNFRNESEAVKNIVKDVTRLLDKTE
LFVPNNPVGVESRVQEMVQLLEQKQSNDVLLLGIWGMGGIGKTTIAKAIYNKIGRNFEGRSFLADIREVWGQEAGHVS
LQQQLLSDVYKENRIKIHNIESGKVILRERLRRKRILLILDDVNKLQQLNALCGNREWFGAGSRIIITTRDIHLLRGKRVDQV
FTMTGMNVDESIELFSWHAFKQASPKEDFIELSRNVVAYAGGLPLALEVLGSYLFDMEVTEWKSVLEKLRKIPNDEVQE
KLKISYDGLSDDTEKGIFLDIACFFIGKDRNDVIHILNGCGLFAENGIRVLVERSLVTVDDKNQLGMHDLLRDMGREIIRSK
SPMELEERSRLWFHEDVLDVLSKETGTKFIEGLTLKLPRSNTKSLSTKAFMNMKKLRLLQLSGVELVGDFEYLSKDLRWLC
WHGFPFASIPTSFYQGSLVSIELENSKITMVWKATQLMEKLKILNLSHSHYLTKTPDFLNLPNLEKLILVDCPRLSEVSYTIG
HLTKVLLINFQDCISLCNLPRSIYKLKSLKTLILSGCLKIDKLEEDLEEMESLTTLFADKTAIRRVPFSIVRSKSIGYISLCGYEGF
SRDVFPSIIWSWMSPVNSLSSRVQTLVDMSSLVFLDVQNSSSNQLSYISEELPKLQSLWIECGSDLQLSRYTKIILDALNAT
NSEESESYGTTSQMQNVFTLIECSSRSKLFEKTLLIQMGGSWEITHILKQRILQNMTTSDGGDCLLPGDSYPDWLSFSSE
GSSVTFEIPEVNGRSLKTMMCHIHYSSPDSITSDGLKNLLVINHTKSTIQLYKRNALAAFDDEEWQRVLSNIDTGNQVQI
VVVFWSRITVKKTSIYLIYEAIDEKVEHYHASNMNVPVSISPEVESMEDLRGAFVKSLTKFLSCCFGT

>XP_022632559.1

MDFVSDRYQKIDGGNFEVFLSFKGEDTRASFTSHLYTALQNAGIFVFKDDESLPQGKQISRSLRQAIEESRISIVVFSKNYA
ESWWCLKELEIIMECHKTTGNVVLPVFYDIYPSEVRHQKGDFGEAFQRLLSKFSKEEEQKVLDWKHCWWNTLREICNIS
AVEILSPSRIERVDKLDNQWEKTLLEVVQISKILDLNPRGKMKIADSIEILVRQSKEGLFKGAEISFREEKVYDKMDFLMKQ
WREVFCEPFRNSEVALDPCFLVLHVKHFRETLFEAADISGDLVLNSCGEMEITYAIELHMKHWRNAFSEEAGTILDSIYIR
YLRDKSVVGMVHSEIRNLRDHWREKVCEAYDRSGDNQLYSSYKKLKAYNRIESLVKYWKIALCEAVEISRLVVEHYRGIT
DNEVNYIEKHARDALREAAGLPGNVILNFRNESEAVKNIVKDVTRLLDKTELFVPNNPVGVESRVQEMVQLLEQKQSN
DVLLLGIWGMGGIGKTTIAKAIYNKIGRNFEGRSFLADIREVWGQEAGHVSLQQQLLSDVYKENRIKIHNIESGKVILRER
LRRKRILLILDDVNKLQQLNALCGNREWFGAGSRIIITTRDIHLLRGKRVDQVFTMTGMNVDESIELFSWHAFKQASPKE
DFIELSRNVVAYAGGLPLALEVLGSYLFDMEVTEWKSVLEKLRKIPNDEVQEKLKISYDGLSDDTEKGIFLDIACFFIGKDR
NDVIHILNGCGLFAENGIRVLVERSLVTVDDKNQLGMHDLLRDMGREIIRSKSPMELEERSRLWFHEDVLDVLSKETGT
KFIEGLTLKLPRSNTKSLSTKAFMNMKKLRLLQLSGVELVGDFEYLSKDLRWLCWHGFPFASIPTSFYQGSLVSIELENSKI
TMVWKATQLMEKLKILNLSHSHYLTKTPDFLNLPNLEKLILVDCPRLSEVSYTIGHLTKVLLINFQDCISLCNLPRSIYKLKSL
KTLILSGCLKIDKLEEDLEEMESLTTLFADKTAIRRVPFSIVRSKSIGYISLCGYEGFSRDVFPSIIWSWMSPVNSLSSRVQTL
VDMSSLVFLDVQNSSSNQLSYISEELPKLQSLWIECGSDLQLSRYTKIILDALNATNSEESESYGTTSQMQNVFTLIECSSR
SKLFEKTLLIQMGGSWEITHILKQRILQNMTTSDGGDCLLPGDSYPDWLSFSSEGSSVTFEIPEVNGRSLKTMMCHIHYS
SPDSITSDGLKNLLVINHTKSTIQLYKRNALAAFDDEEWQRVLSNIDTGNQVQIVVVFWSRITVKKTSIYLIYEAIDEKVEH
YHASNMNVPVSISPEVESMEDLRGAFVKSLTKFLSCCFGT

>XP_022632561.1

MDFVSDRYQKIDGGNFEVFLSFKGEDTRASFTSHLYTALQNAGIFVFKDDESLPQGKQISRSLRQAIEESRISIVVFSKNYA
ESWWCLKELEIIMECHKTTGNVVLPVFYDIYPSEVRHQKGDFGEAFQRLLSKFSKEEEQKVLDWKHCWWNTLREICNIS

AVEILSPSRIERVDKLDNQWEKTLLEVVQISKILDLNPRGKMKIADSIEILVRQSKEGLFKGAEISFREEKVYDKMDFLMKQ
WREVFCEPFRNSEVALDPCFLVLHVKHFRETLFEAADISGDLVLNSCGEMEITYAIELHMKHWRNAFSEEAGTILDSIYIR
YLRDKSVVGMVHSEIRNLRDHWREKVCEAYDRSGDNQLYSRRKNDIELHLENCTKALLEATDISKRAVFVSSYKKLKAYN
RIESLVKYWKIALCEAVEISRLVVEHYRGITDNEVNYIEKHARDALREAAGLPGNVILNFRNESEAVKNIVKDVTRLLDKTE
LFVPNNPVGVESRVQEMVQLLEQKQSNDVLLLGIWGMGGIGKTTIAKAIYNKIGRNFEGRSFLADIREVWGQEAGHVS
LQQQLLSDVYKENRIKIHNIESGKVILRERLRRKRILLILDDVNKLQQLNALCGNREWFGAGSRIIITTRDIHLLRGKRVDQV
FTMTGMNVDESIELFSWHAFKQASPKEDFIELSRNVVAYAGGLPLALEVLGSYLFDMEVTEWKSVLEKLRKIPNDEVQE
KLKISYDGLSDDTEKGIFLDIACFFIGKDRNDVIHILNGCGLFAENGIRVLVERSLVTVDDKNQLGMHDLLRDMGREIIRSK
SPMELEERSRLWFHEDVLDVLSKETGTKFIEGLTLKLPRSNTKSLSTKAFMNMKKLRLLQLSGVELVGDFEYLSKDLRWLC
WHGFPFASIPTSFYQGSLVSIELENSKITMVWKATQLMEKLKILNLSHSHYLTKTPDFLNLPNLEKLILVDCPRLSEVSYTIG
HLTKVLLINFQDCISLCNLPRSIYKLKSLKTLILSGCLKIDKLEEDLEEMESLTTLFADKTAIRRVPFSIVRSKSIGYISLCGYEGF
SRDVFPSIIWSWMSPVNSLSSRVQTLVDMSSLVFLDVQNSSSNQLSYISEELPKLQSLWIECGSDLQLSRYTKIILDALNAT
NSEESESYGTTSQMQNVFTLIECSSRSKLFEKTLLIQMGGSWEITHILKQRILQNMTTSDGGDCLLPGDSYPDWLSFSSE
GSSVTFEIPEVNGRSLKTMMCHIHYSSPDSITSDGLKNLLVINHTKSTIQLYKRNALAAFDDEEWQRVLSNIDTGNQFVV
VRWCGGVVRCGCSCCSW

>XP_022632562.1

MDFVSDRYQKIDGGNFEVFLSFKGEDTRASFTSHLYTALQNAGIFVFKDDESLPQGKQISRSLRQAIEESRISIVVFSKNYA
ESWWCLKELEIIMECHKTTGNVVLPVFYDIYPSEVRHQKGDFGEAFQRLLSKFSKEEEQKVLDWKHCWWNTLREICNIS
AVEILSPSRIERVDKLDNQWEKTLLEVVQISKILDLNPRGKMKIADSIEILVRQSKEGLFKGAEISFREEKVYDKMDFLMKQ
WREVFCEPFRNSEVALDPCFLVLHVKHFRETLFEAADISGDLVLNSCGEMEITYAIELHMKHWRNAFSEEAGTILDSIYIR
YLRDKSVVGMVHSEIRNLRDHWREKVCEAYDRSGDNQLYSRRKNDIELHLENCTKALLEATDISKRAVFVSSYKKLKAYN
RIESLVKYWKIALCEAVEISRLVVEHYRGITDNEVNYIEKHARDALREAAGLPGNVILNFRNESEAVKNIVKDVTRLLDKTE
LFVPNNPVGVESRVQEMVQLLEQKQSNDVLLLGIWGMGGIGKTTIAKAIYNKIGRNFEGRSFLADIREVWGQEAGHVS
LQQQLLSDVYKENRIKIHNIESGKVILRERLRRKRILLILDDVNKLQQLNALCGNREWFGAGSRIIITTRDIHLLRGKRVDQV
FTMTGMNVDESIELFSWHAFKQASPKEDFIELSRNVVAYAGGLPLALEVLGSYLFDMEVTEWKSVLEKLRKIPNDEVQE
KLKISYDGLSDDTEKGIFLDIACFFIGKDRNDVIHILNGCGLFAENGIRVLVERSLVTVDDKNQLGMHDLLRDMGREIIRSK
SPMELEERSRLWFHEDVLDVLSKETGTKFIEGLTLKLPRSNTKSLSTKAFMNMKKLRLLQLSGVELVGDFEYLSKDLRWLC
WHGFPFASIPTSFYQGSLVSIELENSKITMVWKATQLMEKLKILNLSHSHYLTKTPDFLNLPNLEKLILVDCPRLSEVSYTIG
HLTKVLLINFQDCISLCNLPRSIYKLKSLKTLILSGCLKIDKLEEDLEEMESLTTLFADKTAIRRVPFSIVRSKSIGYISLCGYEGF
SRDVFPSIIWSWMSPVNSLSSRVQTLVDMSSLVFLDVQNSSSNQLSYISEELPKLQSLWIECGSDLQLSRYTKIILDALNAT
NSEESESYGTTSQMQNVFTLIECSSRSKLFEKTLLIQMGGSWEITHILKQRILQNMTTSDGGDCLLPGDSYPDWLSFSSE
GSSVTFEIPEVNGRSLKTMMCHIHYSSPDSITSDGLKNLLVINHTKSTIQLYKRNALAAFDDEEWQRVLSNIDTGNQFVV
VRWCGGVVRCGCSCCSW

>XP_022632563.1

MDFVSDRYQKIDGGNFEVFLSFKGEDTRASFTSHLYTALQNAGIFVFKDDESLPQGKQISRSLRQAIEESRISIVVFSKNYA
ESWWCLKELEIIMECHKTTGNVVLPVFYDIYPSEVRHQKGDFGEAFQRLLSKFSKEEEQKVLDWKHCWWNTLREICNIS
AVEILSPSRIERVDKLDNQWEKTLLEVVQISKILDLNPRGKMKIADSIEILVRQSKEGLFKGAEISFREEKVYDKMDFLMKQ
WREVFCEPFRNSEVALDPCFLVLHVKHFRETLFEAADISGDLVLNSCGEMEITYAIELHMKHWRNAFSEEAGTILDSIYIR
YLRDKSVVGMVHSEIRNLRDHWREKVCEAYDRSGDNQLYSRRKNDIELHLENCTKALLEATDISKRAVFVSSYKKLKAYN
RIESLVKYWKIALCEAVEISRLVVEHYRGITDNEVNYIEKHARDALREAAGLPGNVILNFRNESEAVKNIVKDVTRLLDKTE
LFVPNNPVGVESRVQEMVQLLEQKQSNDVLLLGIWGMGGIGKTTIAKAIYNKIGRNFEGRSFLADIREVWGQEAGHVS
LQQQLLSDVYKENRIKIHNIESGKVILRERLRRKRILLILDDVNKLQQLNALCGNREWFGAGSRIIITTRDIHLLRGKRVDQV

FTMTGMNVDESIELFSWHAFKQASPKEDFIELSRNVVAYAGGLPLALEVLGSYLFDMEVTEWKSVLEKLRKIPNDEVQE
KLKISYDGLSDDTEKGIFLDIACFFIGKDRNDVIHILNGCGLFAENGIRVLVERSLVTVDDKNQLGMHDLLRDMGREIIRSK
SPMELEERSRLWFHEDVLDVLSKETGTKFIEGLTLKLPRSNTKSLSTKAFMNMKKLRLLQLSGVELVGDFEYLSKDLRWLC
WHGFPFASIPTSFYQGSLVSIELENSKITMVWKATQLMEKLKILNLSHSHYLTKTPDFLNLPNLEKLILVDCPRLSEVSYTIG
HLTKVLLINFQDCISLCNLPRSIYKLKSLKTLILSGCLKIDKLEEDLEEMESLTTLFADKTAIRRVPFSIVRSKSIGYISLCGYEGF
SRDVFPSIIWSWMSPVNSLSSRVQTLVDMSSLVFLDVQNSSSNQLSYISEELPKLQSLWIECGSDLQLSRYTKIILDALNAT
NSEESESYGTTSQMQNVFTLIECSSRSKLFEKTLLIQMGGSWEITHILKQRILQNMTTSDGGDCLLPGDSYPDWLSFSSE
GSSVTFEIPEVNGRSLKTMMCHIHYSSPDSITSDGLKNLLVINHTKSTIQLYKRNALAAFDDEEWQRVLSNIDTGNQFVV
VRWCGGVVRCGCSCCSW

>XP_022632835.1

MADSVITFVLDHLAQLAAREADLLCGVEDRVQSLQYELQMIKELLGTTKRKKGMEYTVLKQIRDVAHLAEDVIDTFVAK
VSIYRRRTILGRMLRGFGQARLLRHVADKIDKLKTTLNEIRDNKDKYDAFKETTNQSVAEEEEEKERLQSLHKLRRDVEEE
HVVGFVHDSKDIVQRLQGGGSNRKVVSIVGMGGLGKTTLARKVYNSNQVMDQFDCRAWVYVSNECRVRELLLGLLK
LLMPNFEQQRRGNKKGKESAEDVNNLSEEELKKQVWNCLERKRYLVVVDDLWKRQDWDEVQDAFPDNNRGSRILIT
SRLKEVALHAGDDVPHYLQFLSAEESWELFCRKVFKGENCPSGLETLGKQMVQSCCGLPLSIIVLGGLLAKKEKSDREWC
KVVGHVNWYLTRDETQVKDIVLKLSYDNLPRRLKPCFLYLGLFPEDFEIPVTPLLQKWVAEGFIQDTGNRDPDDVAEDYL
YELLDRSLVQVARVNSNGSLATCQVHDLLRDLCISESKEDRVFEVCTDNNILIPTKPRRLSIHSDMGHYISSSDNDHSCIRS
LIFFGQQCDIRGREWKWLLDDFKLVRVLEFGRNRCRKIPFNLGNFIHLRYLRIDSMNATFVPDSILNLWNLQTIELRISYYI
GYIEISFPGEIWKLKHLRHLNISEPIKLRGRSSKSNEKMWNLQTISYLLLNRQSTSLIKKGTFPNIKRLSLYVKADGYEGELP
NLLQNLQQLKHLNKLGIWSKLWLGYKPQELVQSLGQFSCLTFLVIGNVLDLLTSELIFPPNITELMLTGIRCITDKGMNGL
GNHFKLKILRLGGSLLEDSVDLNCADGGFPQLEVLEMRYMRLRKWKLGNGAMRRLQNVFIHHCQPLDNHPIELCSLNG
LKKVHIRDSFSEQMAHIFRNLETNNGVQIVIGNYLYLDDENDWNIFH

>XP_022632837.1

MAVQLIIDAALSKFFEKTFDNLFSRFGDIFRGDKSKKKQISNLKGKLLAIDVVADDAEQKQFTNPRVRDWLLAAKDAVYD
AEDLLEEIDHDAKEVWNPLTSFISFFENKFKTRMEQLIEDLEDLASQSRVLGLKKADDVGVSGWVSKLRSTYLPNESVIYG
RDDDKKFVFEWLTSNTHNNLSILSIVGMPGVGKTTLAQHLFNDPRMDKNKFDVKVWVCVSDEFDVFKVSRAILEDVT
GSRDDSRDTEMVHKRLKEKFTKKKFLLVLDDVWNENQSKWEEVQKPLVFGAQGSKILVTTRNKEVATIMQSEEHYLEQ
LEYKHSWRLFAKHAFRNDDTEPNPEYKDIDVKIVKKCKGLPLALKTMGSLLYNKSSVSEWETVFQSEIWKFSQERCDIVP
ALALSYIHLPSHLKVCFAYCALFPKDYEFRKEDLIHLWITENFLHSHQHSKTPEEACQQYFNDLKSRSLFQQSGEKKEVFV
MHDLLNDLAKYVAGGIYFRCKIDQTKKIQEVTRHFSFELDCNRDFEGLGTLYKTQRLRTFMPTETKVIKVPNLKELKNLKV
MMSSFKVGCSSELGIQRLGELNNLHESLSIDELEKIEKPRDASDANLKNNTHLMKLELKWNSKREENSIDSKKEEDVIDKL
QPSKNLKELSIFSYGGTQFPDWLLEDSLWEMVSLALKECKSCQSLPPLGLWKYLKDLKIAGLDGIVSIDADFYGNNSSSFN
SLENLEISDMKQLEKWECKAVRGAFPSLKRLSISNCPKLEGDLPEQLVPLNDLKISQCDQALTVTALKVRWPTMKRLKID
GDNLEASMVKIVWHFTSLEVLDINSHSKANSDESVSPWTFPLHFFPTLSTLNLRGFLNLQMISQVDRHNHLKHLTIEKCP
KFESLPESMHTMLPSLMRLSIYDCQKLESFPHGGLPPNLNFVKLQNCCSTLIGSLKGALGANTLKSLWIGLVDAECFPVE
GLLPLSLTSLHIYRFENLKNLDYEGLRQLSSLEKLSLRDCPNLERLPEKGLPESILQFSISGKCSLLKHRCQEGGEDWEKIAHI
QNLDVGMTSGCQH

>XP_022632839.1

MIKELLGTTKRKKGMEYTVLKQIRDVAHLAEDVIDTFVAKVSIYRRRTILGRMLRGFGQARLLRHVADKIDKLKTTLNEIR
DNKDKYDAFKETTNQSVAEEEEEKERLQSLHKLRRDVEEEHVVGFVHDSKDIVQRLQGGGSNRKVVSIVGMGGLGKTT
LARKVYNSNQVMDQFDCRAWVYVSNECRVRELLLGLLKLLMPNFEQQRRGNKKGKESAEDVNNLSEEELKKQVWNC

LERKRYLVVVDDLWKRQDWDEVQDAFPDNNRGSRILITSRLKEVALHAGDDVPHYLQFLSAEESWELFCRKVFKGENC
PSGLETLGKQMVQSCCGLPLSIIVLGGLLAKKEKSDREWCKVVGHVNWYLTRDETQVKDIVLKLSYDNLPRRLKPCFLYL
GLFPEDFEIPVTPLLQKWVAEGFIQDTGNRDPDDVAEDYLYELLDRSLVQVARVNSNGSLATCQVHDLLRDLCISESKED
RVFEVCTDNNILIPTKPRRLSIHSDMGHYISSSDNDHSCIRSLIFFGQQCDIRGREWKWLLDDFKLVRVLEFGRNRCRKIP
FNLGNFIHLRYLRIDSMNATFVPDSILNLWNLQTIELRISYYIGYIEISFPGEIWKLKHLRHLNISEPIKLRGRSSKSNEKMW
NLQTISYLLLNRQSTSLIKKGTFPNIKRLSLYVKADGYEGELPNLLQNLQQLKHLNKLGIWSKLWLGYKPQELVQSLGQFS
CLTFLVIGNVLDLLTSELIFPPNITELMLTGIRCITDKGMNGLGNHFKLKILRLGGSLLEDSVDLNCADGGFPQLEVLEMRY
MRLRKWKLGNGAMRRLQNVFIHHCQPLDNHPIELCSLNGLKKVHIRDSFSEQMAHIFRNLETNNGVQIVIGNYLYLDD
ENDWNIFH

>XP_022632880.1

MEESVVTFVLDHLAELVAREVNLLYGVEDRVQSLQYELQMIKELLNTTKRKKGMEHVVLNQIRDVAHLAEDVIDTFVAK
VSIYKRRTILGRMLRAFGQARLLHHVAHKIDKLKTTLNEIHNNKDKYDAFKETTNQSAAEEKEENERLQSQLKKLRRNVE
EEDVVGFDQDSKDIIKRLLEGGSNRKAVSIIGMGGLGKTTLARKVYNNNQVKQRFMCRAWVYVSNECRVKELLVNLLK
HLMPNFKQQSKGKKSDGDINSLSEEELKELVRSCLERKRYLVIVDDLWKRQDWDEVQDAFPDNNTGSRILITSRLKEVA
LHAGHDVPHYLQFLNDEESWELFRRKVFRGEDYPFDLEPLGKQMVQSCRGLPLSIIVLAGLLANKEKSHREWCKVVGH
VNWYLTQDETQVKDIVLKLSYDNLPRRLQPCFLYLGLFPEDFEIPVTPLLQKWVAEGFIQHTGNRDPYDVAEDYLYELID
RSLIQIARLKTNAGVKTCRVHDLLRDLCISESKEDKVFEVCTDNNILVPTKPRRLSIHDYRVHRRERKWLLKDFKLVRVLEL
APMSCKKIPSNIGNFMHLKYLRIDSQYITFVPDSILHLWSLQTIDLGPWSQNTPTSFPVQVWKLKHLRHLSTRGPIKLRGS
CLGSSEKMYNLQTISPLVLNKQATYLIEKGTFPNIKSIGLSVVFGKGQLSKLLQSVQLLRYLNEFVIVLPYRYDTIVTLHEGVE
RKNGCKPQELFESLGQFNCLTVLEINNVIDLLTCAITLPSNVTELKLKGISCISDEGMKCLGNHAKLKILRLFGFMLFSTQDS
YDLTCVGGSFPQLEVFQMESLKIEKWNLGNGAMPRLQSLVISFCGRLDDLPNELWSLSDLKKVHVTKPSEQMIRILQNL
EINNAVQLVIEDLPSWMDESDWKFFEYLMQG

>XP_022632889.1

MEESIVSFALDHLAQLVADEVNLLYGVKDRVQSLQRELEMIKELLNNTRREEGMEHIVLNQIRDMAYLAEDVIDTFVVE
VDIYKRKTILQKMLCCFGQVRLLHRLSAKIDYIKTTLKEIRDNKDIYDAFRETTNESAAEEKRERLEWLQRLRRDVEVKDVV
GFVRDSEVVVNQLLGDSSNRKVVSIVGMGGLGKTTLARKVFNKSQVKNHFHCLTWVNVSNDCRVKELLLGLLKQLMP
NFEQQCGGINNWNEDELKNKVENCLEGKRYLVVLDDLWNIRDWDKVKSAFPDNNRGSRILITSRSKEVAMHAGLDVP
HYLPFLKEEESWELFRKKVFIDGDCPSDLEPLGKLMVQRCCGLPLSIIVLAGLLANKEKSHILWSEVVDHVNSYINRDKTQ
VNDIVLKLSYDNLPRRLKPCFLYLGLFPEDFEIPVAPLLQKWVAEGFIQDTGDRDPDNVAEDYLYELIDRSLVQVTRVKSN
GSLEKCEVHDLLRDLCISQSKKEKVFEVCTNNNIVGIAKPPTPDKPRRLSIHSAMHDYISASKRDHSCVRSLFFFGTHYDQ
WKWLVDDFKLVRVLEFGPISRQENLSNLGNFIHLRYLRIKDVYVYFVPDSILNLLNLQTIDLGISRGGGRVSFPIQIWKLKY
LRHWNSSGPIELRGNCSQSVKKMWNLQTMSPLVCNKQAISLIKNRTFPNIKRMGLDGSRECGDELHNLLHNLQQLEH
LNSLVIIPQGFLDFKPQELVQGLGEFSHLTILEIHCVKDLLTSEHIFPPNVTELTLSGIHCISDEGMNGLGNHSKLKILRLLGN
LLMFKNPTDLNCVGFPQLEVLKMKFLRLQKWELDNGAMRRLQHVIIDRCGPLGNLPTELCYLNGLKKVHINSFPSKEVI
GQMADILRTLETNNGVQVLAGHIYIPPWDEENDWVHDY

>XP_022633156.1

MKEVLEKLEYLAKQKGALGLRESIYSGDRSGSKVSQKLPSSSLIVESVIYGRDTDKEMIFNWLTSETDNHNHLSILSIVGM
AGLGKTTLAQHVYNDPKMETKFDIRAWVCVSDHFDILTVTKKILEAITKSKDDSEDLEMVHGRLKEKLSGKKFFLVLDDV
WSESREEWEAVRTPLSYGAPGSRIFVTTRDEKVASNMRSKVHRLKQLEEEECWKVFEEQALKDNDFELNDEKKEIGRRI
VEKCKGLPLALKTIGSLLRTKSSILDWKSVLESDLWELPKEIEIMPALLLSYQHLPSHLKRCFAYCALFPKDYEFDKKELILLW
MAEGFLHHSQQNKNVQEIGEQYFNDLLTRSFFLPSTLIMKFLMHDLLNDLAKYVCADFCFRLKFDKGNSIPKTTRHFSFA

SNDVRYFNRFGSLTDAKRLRSFVPIRHMNSGWKFNISIHDLFSELKFLRVLSLNGYYDLKEVPDSIGDLKHLHSLDLSGTM
IQKLPDSVGLLYNLLILKLNNCSYMKELPWNLHKLTKLRCLEFEDTKVTKMPMHFGELKNLHVLSTFCVNRDPEIINIKQL
GGLNLRGKLSINELQNIVNPLDALEANLKNKHIVQLNLRWNSNHMPDDPRKEKKVLENLKPSNQLEHVSIWSYCGTQF
PSWVFDNSLSNLVSLQLRDCKYCLCLPPLGLLSSLKVLYIVGLDGIVSIGAEFYGSNSSSFMSLETLVFYNMKEWEEWECK
TTSFPRLRHLAIVRCPKLKGLSEQLLHLKKLSIESCDNLIISDHSINTSTLELLSIRSCPLVNIPMTHYGFIQEMKIDDGCDSLTI
FQLNFFPMLRLLFLKGCQNLQRISQEHPHNHLKEMSIYACAQFESFPIEGLSAPWLQTIKIQGAENLKLLPKRMKILLPSLI
ELEIIDCRNVEKFPEGGLPSKIKDISLSSLKLIASLRETLDVNTCIRSLSIEYLDVESFADEVLLPPSLTSLSINHCRNLKRLDYKV
LYNLSSLSLGDCPNLQCLPEEGLPKSISSLHIWRCPLLEQRCQNPEGKDWRKIAHIENLRVK

>XP_022633165.1

MCMQDPPSSFTLLHSPAYNSLYFNHNHNLSFDSSTSSLSDPMPPESDVTISSYRLCWDVFLSFRGTHTGHTFTMRLYHA
LHGRGVRVFRNDDGLERRGEIQKKLLEAVEDSAAAVVVISPDYASSHWCLEELAKICEVGRLILPVFYWVNPSHVRKQE
GPFEEWFVWHVQRFPKERVEQWRNAMKKVGGLAGFVLDEKSDKSDELIQILVQNLMKQLRNTPLSVAPFTVGVDDR
VEVLKNLLDLKSNDVRVLGLYGMGGVGKTTLAKSLFNNLVVHNFERRSFIPNVRSQVSKHHGLVSLQNKIRGDLCGRKE
DLINDISDGISAIQKIVQENRVLLILDDVDDVEQLNFLMGKREWFYKGSRVVITTRDKEILYGSYVDVDFEVKELEFSEAM
ELFCFHAIRRKEPAEGFLDVSKQIVEKTGGLPLALEVFGSFLFDKRTEREWKDALEKLKQIRPSCLQEVLKISFDALDEQEQ
CIFLDIACLFVQMEMKRDDVVDILNGCDFRGEIAVAVLTARCLIKIIGDGKVWMHDQVRDMGRQIVRSESLTDPGLRSR
LWDRDEILTVLKNMKGTRNVQGIVLDCVKRRMSIPRDRSADEITRENFRRKPSCKSAFEYIKERYKKYVEDRKEKSKEVIL
QPKHFQPMVSLRMLQINYSRLEGQFICLPPKIKWLQWKQCPLRYMPSSYNPLELAVMDLSESLIETLWKGRSNKVAVH
LMVLNLSRCHRLTATPDLSGYLSLKKLNLEECSHLTRIHESLGNLNSLVHLNLRLCYNLIELPGDVSGLKHLEDLVLSDCWK
LKALPKDLSCMVSLRQLLLDSTSITELPVSIFHLTKLEKLSANGCHLLKKLPTCTGKLCSLQELSLNHTALEELPDSVGSLEKL
EMLSLMGCKSLSVIPNSTGKLISLTRLYLDGSGIKELPASIGALSYLRKLSVGDCTSLHKFPVSMEALVSIVELKLDGTKVSN
FPEEIFVGMKMLEKLEMGKVQHLKFVPVSFGCLSALTILDMHDANITELPESIGMLENLIRLRLDKCKQLQRLPDSIGNLK
SLRWLMMKETAVTRLPDSFGMLRSLVELDMKRLPYLNGAGNNVSTGTIIPEIREQPSSEAILTSFCNLSLLEKLNAHGW
GIYGKIPDEFEKLSSLETLCLGHNNICNLPASMTGLSYLKKLLLSDCRELMFLPPLPSSLEELNLENCVAVQYIHDISNLERLE
EFNLTNCEKMVDVPGLEHLKSLRRLYMSGCIGCSLAVKRRFSKVCPRFYLRN

>XP_022633246.1

MSLSGSDDEMEMDFLGDRYRKMDGGNFEVFLSFRGEDTRASFTSHLYAALQNAGIFVFKDDESLPRGKQISPSLRLAIE
ESPISIVVFSKNYAESLWCLKELEKIMECHRTTGHVVLPVFYDVDPSEVRHQRGDFGKAFQRLLSKSSREEEEKVLDWKQ
RWRKILRELDGFSVVEILNSRIGRGDNLEEHWKEALFEVVQISRERMEIADGIGFLVSQLMAGLFEGAKIPFSGETEVAN
AIKLHMKHCREALFEASGISGDAVLNSCGEMEITNAIELHMKHWRDAFSENAGTISGSDYSRFIGGRNWYDVSSSGFRT
VHYEIWHLQHYWREKVREAYDRSGDDQLYSRREIDIERHLEHCASAVNKAIDSSTASRSSFQKLQAYNRIESLVKYWRK
ALCEAAEISRWVVQHYRGITDNEINYIEKNARDALREAAGISGVVILNSRNESEAVTNIVKNVTSLLDKTELFVAHNPVGV
ESRVQEMVQLLQQKQSNDVLLLGVWGMGGIGKTTIAKAIYNKIGRDFEGRSFLADIRQVWGQEAGHVCLQEQLLFDI
HKESNTKIRNIESGKVLLRERLSRKRILLILDDVNKLQQLNALCGNREWFGSGSRIIITTRDIHLLRGKRVDQVFAVTGMNV
DESIELFSWHAFKQASPKQDFIELSRNVVAYAGGLPLALEVLGSYLFDMEVTEWKSVLEKLRKIPNDEVQEKLKISYDGLS
DDTEKGIFLDIACFFIGKDRNDVIHILNGCGLFAENGIRVLVERSLVTVDDKNQLGMHDLLRDMGREIIRSKSPMELEERS
RLWFHEDVLDVLSKETGTKFIEGLTLKLPRTNTKSLCTKAFMNMKKLRLLQLSCVELVGDFEYLSKDLRWLCWHGFPFAF
IPTSFYQGSLVSIELENSKITMVWKATQLMEKLKILNLSHSHYLTKTPDFLNLPNLEKLVLMDCPRLSEVSYTIGHLTKVLLI
NFQDCISLCNLPRSIYKLKSLKTLILSGCLKIDKLEEDIEQMESLTTLVADKTAIRRVPFSIVRSKSIGYISLCGYEGFSRNVFPSI
IWSWMSPVNSLSSRVQTLVDMSSLVSLDVQNSSSNQLSYISDELPKLQSLWIECGSDLQLSRDTASILDALNATNSEESE
SYGTTSQMKNVFTLIECNSRSKLFEKTLLIQMGISWEITHILKERILQHIYLYFYRM

>XP_022633266.1

MDCLVGFASSVSRDIVCGALKELRYLRSFNNFVEKLKQEEDDLNVRIDDVHKSVEDAKRKTRETSGDVDKWLQDAISNI
GEVNKLLEEARTKKNWCFGHCPNWIGRYHVGKKLANKTMDLEKFIEEGRKHVSFDRIATLPSNTLDMLSEKCINFESRQ
SAYEQLLDAVKNNDVSMIGLYGMGGCGKTTLAMEIRKFVEAEHLFEKVLFVPVSSSVEVLRIQEKIRSSLQHEIPETEEM
QRAQRLCSILTKEKSALLILDDVWEKLDFVRIGIPSSEHRKGCKILITTRSEEVCTLMDCQRKIYLPHLTDEEAWTLFQNKAL
ITEGTPHTLKDLGRSISDECKGLPVAIAAVACSLKGKTETVWRVALNKLKHSNRINIGRGLTDPYECLQLSYVNLDDKEAK
SLFLLCSVFPEDFEIPIEVLTRCAIGLGVVGEAHSYEEARSEVIAAKIKLVSCCLLLDADDGCVKMHDIVRDVAHIIAKNENK
TIKCEEEKDVTVEQNSIRYLWCVKFPRDLDCSNLEFLFLRTKMKEFDGIFKTMGMLKVLILVNDGDGKTPLPTISFKTLTN
LRCLYILNCELSDFSFLSGMKNLQSLELYYCLLPSFPELQIDVAIPLTTLKLLVLNECDIKVKNFEVMKRIPLLEELYIIDIQGQ
WYANSEDTIQFFKTFSIPETLQRYGIVLGSCSFDGGDFKKLSCHHRTLLLNYFDVSNEVMKGLAKNAKDLFVGNIHGGAK
NMIPDIFQIEGGGLNELNTLEIHDSVEIKCLIDTRSHSSEVVTLLSNLHTLKISCMENLKAIWNCFLPINGPFKNLEKLDLSV
CPQMTSLFTYVVARSLVKLKILKISRCDELKHILTYDEKSQDEFTSGHPLQIFQNLEEVEVYSCPELKHMFPANIVGGLTHL
KVLKIEGCDMLDQIIGDIVSLIEHDRKEELDEIVEEGKHPHLYNTSIPTTIVVKQSPGTLSNLASLKITSCGKLGSIFTASIAKTL
TSLEELYIGGCKSLKDIVTHERVNKNQEESIVENEHDCQSDISIFQSLKKIHISDCDLLEGIFPVSFVGELNDITNKEGFDFKN
FSSRNNTQIELPTLEVLELDHIRNRTIVGSYDVICPSLRTLSLDIGRYVGFFNINCSTDASEPTKTDFIAIKISNSDFDPPVESV
ECLSKEPHGLNLIMIHNI

>XP_022633299.1

MECLFGFASSVSRDLVCGALNQLRYPCCFKNFVKRLQEEESNLIITRDSVQKFVTHGKKQARKPNEIVDKWLEDAINDV
HNVNQLLEEARTKKHCCFGHCPNWIWRYHVGKNLANKTMDLEKFIEKGRKYVPFDRIATLPSNTLDMLLEKCMNFESR
QTAYEQLLDAVKSNDVSMIGLYGMGGCGKTTLAMEVRKLVESEHLFEKVLFVPVSSTVEIRRIQEKIASSLQFEFPETEE
MQRAQRLCSRLTQEKSVLLILDDVWEKLDFGHIGIPSSEHHKGFKILITSRSEAVCTLMDCQRNIYLPILTDEEAWTLFQN
KALISEATPHTLKNLGRLISDECKGLPVAISAVACSLKGKVETEWRVALNKLRHSKPINIERGLIDPYKCLQLSYDNLDTKEA
KSLFRLCSVFPEDSEIQVEVLTRCAIGLGVVGEVDSYEEARSEVIAAKIKLVSCCLLLDTDYEGVKMHDIVRDVAHIIAKNE
NKMIKCEVEKDVRVEQNSVRYLWCAKFPNDLDCSNLEFLCLRTKMKGFDEIFKRMGRLKVLIFINDEDGKTPLSTISFKTL
TNLRYLFIKNYELSDFSFFGGMKNLQSLKLHGCSLPAFPEAITLKLLELKKCDIKVKNFEVMKRIPHLEELYIIDIEGEWWDA
NSEDNIEFFKTFSIPETLQRYGIVLGSYNFDHYNNDRDIYIHGRTLLLNHFDISNEVMKGLAKNAKDLFVGNIHGGAKNMI
PDIFQIEGGGLNELNMLVIRDSEELECLIDTHSHSSEVVTLFSKLHTLKILNMENLKAIWHCFLPANGPFENLEKLYLSDCP
WLTFLFTYVVAQNLVQLKILKISRCDELKHILTYDEKSEDEFSSGHPVQIFQNLQEVEVYSCRELKHIFLANIVGGLTQLKVL
LIEECDMLDQIIADIVPLRDQDRKEELDEIIEKGTLSSLGSLKIKRCGKLGSIFTASIAKTLTSLEELFIEHCKSLKDIVTHERINK
NQEESIVEDDYDCQSDISIFQSLKKLHISNCDLLEGIFPVSFVGELNDITNKEAAHLKDFSSPNNTQIELPALQVLELHYIRN
RTIVGSYDVICPSLRTLSLDIGRYVVFFNINCSTDASEATKRDFIAIKISNSDFVPPVESVECLSELPHGLNLIMTHNIREIELK
GFDKASYLFKLSIASSLMMLEILRVKECDGLEYIIDTDDDEYGKDNMKAIFPNLKELSLYECFQLKYMFGKYHVANKDYKEI
HIQFSALEILYFRNLPKFVSICSTNNLILTWPSLKDFECNECFYPFYSFVSCLTIPSNSREPIITSTKDPKGIQNHLPTLQTLNIK
HSRAERIFCLNEHEMIGQQVSLRLEKLDLYNLPEMTYIWVGPNNSLTLQHLTTLEIRDCGKLEVIFPKSVVRCLPELKNVTI
IRCMELKQIIEEDESNPLSIDGDSEEEIIGCHKKASKNYFAFPNLEELEIFECAKLEVVFPKSVLRCLLKLKILKISKCKEVRQIIE
KDVEDKNLSNILSPRPCFPKLEALQVDDCHKLKRLFCGSASNDLPNLHLLVINGANELEELVGCKQGKIKVELPRLKLLIFIH
LENFSQEIDLHNLKNCIVYECPKLSLTSTTTLEKLCEDFPSEDFINTELGSWEFERIVRISTITDSSEFTSSQEIEDLGNESIKSS
STGVEDIGTGDAVATHIDSKVVEQDDKLNEGKAGTVESQVEEGLNLFHKQERIDVFPNNNIDISSASADIRTRLGAYKHF
VDLDDAQISLLVEAITTYPHLWNAPKKFSERFQAWRFKILADMLLFLQKESVHSVIPQRQKEFDRLCEEAIEIGFESSWVE
EMRQRVVARDSKLGEDIAKRQMDENSKRCSKGDMVPDSQAAERGDGPKISLEEGSDLVDKEAEIGVVSNHHIVAMR
NEEAKKEFVAEVFTSEIPRIATSLTNSQTVEKPTPSNELVDTQQTSEQCQMKQKKPLGEIPKSIEQVALEETIAKNTNMAA
SSILSESATSKLDPTVTLLSKSYPYLSEIKSSQIEARIAKESEGHPKIIQDFGANDITSLFAPVIVGKEGEDKLVGKTLAELEKY
MKMSLKDIVSSETNSLCLLSALNFLSNLPFKDVKVSDGLKHIIDTMHRHFPSILCSFKQGFATTHKLAELEARANEVTIKKN

LYDELQRKEVVLKEQIIRLKEEIRVCEVALSSLEEEKNKCIAETVEYKTKLENARKEESQMLEVAHKWSILCSQYELNRMATINPS

>XP_022633300.1

MECLFGFASSVSRDLVCGALNQLRYPCCFKNFVKRLQEEESNLIITRDSVQKFVTHGKKQARKPNEIVDKWLEDAINDVHNVNQLLEEARTKKHCCFGHCPNWIWRYHVGKNLANKTMDLEKFIEKGRKYVPFDRIATLPSNTLDMLLEKCMNFESRQTAYEQLLDAVKSNDVSMIGLYGMGGCGKTTLAMEVRKLVESEHLFEKVLFVPVSSTVEIRRIQEKIASSLQFEFPETEEMQRAQRLCSRLTQEKSVLLILDDVWEKLDFGHIGIPSSEHHKGFKILITSRSEAVCTLMDCQRNIYLPILTDEEAWTLFQNKALISEATPHTLKNLGRLISDECKGLPVAISAVACSLKGKVETEWRVALNKLRHSKPINIERGLIDPYKCLQLSYDNLDTKEAKSLFRLCSVFPEDSEIQVEVLTRCAIGLGVVGEVDSYEEARSEVIAAKIKLVSCCLLLDTDYEGVKMHDIVRDVAHIIAKNENKMIKCEVEKDVRVEQNSVRYLWCAKFPNDLDCSNLEFLCLRTKMKGFDEIFKRMGRLKVLIFINDEDGKTPLSTISFKTLTNLRYLFIKNYELSDFSFFGGMKNLQSLKLHGCSLPAFPEAITLKLLELKKCDIKVKNFEVMKRIPHLEELYIIDIEGEWWDANSEDNIEFFKTFSIPETLQRYGIVLGSYNFDHYNNDRDIYIHGRTLLLNHFDISNEVMKGLAKNAKDLFVGNIHGGAKNMIPDIFQIEGGGLNELNMLVIRDSEELECLIDTHSHSSEVVTLFSKLHTLKILNMENLKAIWHCFLPANGPFENLEKLYLSDCPWLTFLFTYVVAQNLVQLKILKISRCDELKHILTYDEKSEDEFSSGHPVQIFQNLQEVEVYSCRELKHIFLANIVGGLTQLKVLLIEECDMLDQIIADIVPLRDQDRKEELDEIIEKGTLSSLGSLKIKRCGKLGSIFTASIAKTLTSLEELFIEHCKSLKDIVTHERINKNQEESIVEDDYDCQSDISIFQSLKKLHISNCDLLEGIFPVSFVGELNDITNKEAAHLKDFSSPNNTQIELPALQVLELHYIRNRTIVGSYDVICPSLRTLSLDIGRYVVFFNINCSTDASEATKRDFIAIKISNSDFVPPVESVECLSELPHGLNLIMTHNIREIELKGFDKASYLFKLSIASSLMMLEILRVKECDGLEYIIDTDDDEYGKDNMKAIFPNLKELSLYECFQLKYMFGKYHVANKDYKEIHIQFSALEILYFRNLPKFVSICSTNNLILTWPSLKDFECNECFYPFYSFVSCLTIPSNSREPIITSTKDPKGIQNHLPTLQTLNIKHSRAERIFCLNEHEMIGQQVSLRLEKLDLYNLPEMTYIWVGPNNSLTLQHLTTLEIRDCGKLEVIFPKSVVRCLPELKNVTIIRCMELKQIIEEDESNPLSIDGDSEEEIIGCHKKASKNYFAFPNLEELEIFECAKLEVVFPKSVLRCLLKLKILKISKCKEVRQIIEKDVEDKNLSNILSPRPCFPKLEALQVDDCHKLKRLFCGSASNDLPNLHLLVINGANELEELVGCKQGKIKVELPRLKLLIFIHLENFSQEIDLHNLKNCIVYECPKLSLTSTTTLEKLCEDFPSEDFINTELGSWEFERIVRISTITDSSEFTSSQEIEDLGNESIKSSSTGVEDIGTGDAVATHIDSKVVEQDDKLNEGKAGTVESQVEEGLNLFHKQERIDVFPNNNIDISSASADIRTRLGAYKHFVDLDDAQISLLVEAITTYPHLWNAPKKFSERFQAWRFKILADMLLFLQKESVHSVIPQRQKEFDRLCEEAIEIGFESSWVEEMRQRVVARDSKLGEDIAKRQMDENSKRCSKGDMVPDSQAAERGDGPKISLEEGSDLVDKEAEIGVVSNHHIVAMRNEEAKKEFVAEVFTSEIPRIATSLTNSQTVEKPTPSNELVDTQQTSEQCQMKQKKPLGEIPKSIEQVALEETIAKNTNMAASSILSESATSKLDPTVTLLSKSYPYLSEIKSSQIEARIAKESEGHPKIIQDFGANDITSLFAPVIVGKEGEDKLVGKTLAELEKYMKMSLKDIVSSETNSLCLLSALNFLSNLPFKDVKVSDGLKHIIDTMHRHFPSILCSFKQGFATTHKLAELEARANEVTIKKNLYDELQRKEVVLKEQIIRLKEEIRVCEVALSSLEEEKNKCIAETVEYKTKLENARKEESQMLEVAHKWSILCSQYELNRMATINPS

>XP_022633301.1

MECLFGFASSVSRDLVCGALNQLRYPCCFKNFVKRLQEEESNLIITRDSVQKFVTHGKKQARKPNEIVDKWLEDAINDVHNVNQLLEEARTKKHCCFGHCPNWIWRYHVGKNLANKTMDLEKFIEKGRKYVPFDRIATLPSNTLDMLLEKCMNFESRQTAYEQLLDAVKSNDVSMIGLYGMGGCGKTTLAMEVRKLVESEHLFEKVLFVPVSSTVEIRRIQEKIASSLQFEFPETEEMQRAQRLCSRLTQEKSVLLILDDVWEKLDFGHIGIPSSEHHKGFKILITSRSEAVCTLMDCQRNIYLPILTDEEAWTLFQNKALISEATPHTLKNLGRLISDECKGLPVAISAVACSLKGKVETEWRVALNKLRHSKPINIERGLIDPYKCLQLSYDNLDTKEAKSLFRLCSVFPEDSEIQVEVLTRCAIGLGVVGEVDSYEEARSEVIAAKIKLVSCCLLLDTDYEGVKMHDIVRDVAHIIAKNENKMIKCEVEKDVRVEQNSVRYLWCAKFPNDLDCSNLEFLCLRTKMKGFDEIFKRMGRLKVLIFINDEDGKTPLSTISFKTLTNLRYLFIKNYELSDFSFFGGMKNLQSLKLHGCSLPAFPEAITLKLLELKKCDIKVKNFEVMKRIPHLEELYIIDIEGEWWDANSEDNIEFFKTFSIPETLQRYGIVLGSYNFDHYNNDRDIYIHGRTLLLNHFDISNEVMKGLAKNAKDLFVGNIHGGAKNMIPDIFQIEGGGLNELNMLVIRDSEELECLIDTHSHSSEVVTLFSKLHTLKILNMENLKAIWHCFLPANGPFENLEKLYLSDCP

WLTFLFTYVVAQNLVQLKILKISRCDELKHILTYDEKSEDEFSSGHPVQIFQNLQEVEVYSCRELKHIFLANIVGGLTQLKVL
LIEECDMLDQIIADIVPLRDQDRKEELDEIIEKGTLSSLGSLKIKRCGKLGSIFTASIAKTLTSLEELFIEHCKSLKDIVTHERINK
NQEESIVEDDYDCQSDISIFQSLKKLHISNCDLLEGIFPVSFVGELNDITNKEAAHLKDFSSPNNTQIELPALQVLELHYIRN
RTIVGSYDVICPSLRTLSLDIGRYVVFFNINCSTDASEATKRDFIAIKISNSDFVPPVESVECLSELPHGLNLIMTHNIREIELK
GFDKASYLFKLSIASSLMMLEILRVKECDGLEYIIDTDDDEYGKDNMKAIFPNLKELSLYECFQLKYMFGKYHVANKDYKEI
HIQFSALEILYFRNLPKFVSICSTNNLILTWPSLKDFECNECFYPFYSFVSCLTIPSNSREPIITSTKDPKGIQNHLPTLQTLNIK
HSRAERIFCLNEHEMIGQQVSLRLEKLDLYNLPEMTYIWVGPNNSLTLQHLTTLEIRDCGKLEVIFPKSVVRCLPELKNVTI
IRCMELKQIIEEDESNPLSIDGDSEEEIIGCHKKASKNYFAFPNLEELEIFECAKLEVVFPKSVLRCLLKLKILKISKCKEVRQIIE
KDVEDKNLSNILSPRPCFPKLEALQVDDCHKLKRLFCGSASNDLPNLHLLVINGANELEELVGCKQGKIKVELPRLKLLIFIH
LENFSQEIDLHNLKNCIVYECPKLSLTSTTTLEKLCEDFPSEDFINTELGSWEFERIVRISTITDSSEFTSSQEIEDLGNESIKSS
STGVEDIGTGDAVATHIDSKVVEQDDKLNEGKAGTVESQVEEGLNLFHKQERIDVFPNNNIDISSDIRTRLGAYKHFVDL
DDAQISLLVEAITTYPHLWNAPKKFSERFQAWRFKILADMLLFLQKESVHSVIPQRQKEFDRLCEEAIEIGFESSWVEEM
RQRVVARDSKLGEDIAKRQMDENSKRCSKGDMVPDSQAAERGDGPKISLEEGSDLVDKEAEIGVVSNHHIVAMRNEE
AKKEFVAEVFTSEIPRIATSLTNSQTVEKPTPSNELVDTQQTSEQCQMKQKKPLGEIPKSIEQVALEETIAKNTNMAASSIL
SESATSKLDPTVTLLSKSYPYLSEIKSSQIEARIAKESEGHPKIIQDFGANDITSLFAPVIVGKEGEDKLVGKTLAELEKYMKM
SLKDIVSSETNSLCLLSALNFLSNLPFKDVKVSDGLKHIIDTMHRHFPSILCSFKQGFATTHKLAELEARANEVTIKKNLYDE
LQRKEVVLKEQIIRLKEEIRVCEVALSSLEEEKNKCIAETVEYKTKLENARKEESQMLEVAHKWSILCSQYELNRMATINPS

>XP_022633302.1

MTRNKRENPVKLWTSGLKMLSMMYTMSISCWKRLKHTNIVALGTSQIGFGDTMLEKKLANKTMDLVKFIENGRKYVP
FDRIATLPSNTLDMLSEKCMNFESRQSASEQLLDAVKNNDVSMIGLYGMGGCGKTTLAMEVRKLVEAEQKVLFVPVSS
TVEVRRIQEKIASSLPFEFPETEEMQRAQRLCSRLTEEKNIFIILDDVWEKLDFGRIGIPSSQHHKGCKILITTRLEDVCISMD
CQRRIYLPILTDEEAWTLFQNKAFISEDTPDTLKNLGRLISNQCKGLPVAIAAVACSLKGKAETVWSVALNKLRRSKPINIE
RGLIDPYKCLQLSYDNLDTKEAKSFFLLCSVFPEDFEIPIELLTRYAIGLGVVGEVDSYEEARSEVIAAKIKLVSCCLLLDADHE
CIKMHDIVRDVAHIIAKNENKMIMCEVEKDVRVEQNSARYLWCAKFPNNLDCSNLEFLYLRTKMKGFDEIFKRMGTLK
VLILVNDEGGKTRLSTISFKTLTNLRSLLIENYELSDFSFFGGMKNLQSLSLYYCSLPSFPESQTDVAITLKLLELNECDIKVKN
FEVMKRIPLLEELYIIDIKGEWDANSEDNIEFFKTFSIPETLQRYGIVLASYNFEDFDDGDIYIHGRTLLLNHFDISNEVIKGL
AKKAKDLFVRNIDGGAKNMIPDIFEIEGGGLNELNKLEIRDSAEIKCLIDTRSHSSEVVTLFSKLHTLRIGSMENLKAIWNC
FLPANGPFENLEKLYLSDCPRLTFLFTYVGARNLVQLKILKISGCDELKHILTYDEKSQDEFTTGHPVQIFQNLQEVEVDSC
PELKHIFPANIVGGLGQLKELKIERCDMLDQIIGDIVPLTDQDRKEELYETIEEGTLSSLVSLKINCCGKLGSIIKASIAKTLISL
EELFIQDCKSLKDIITHERIIENQEESIVEDEHDSQSDISIFQSLKKIHISQCDLFEGIFPVSFVGELNDITNKEAADLKDFSSRN
NTQIELPALQVLELDHIRNRTIVGSYDVICPSLRTLSLDIGRYVGFFNINCSTDASEATETDSVAIKISNSDFDPPVESIECLS
QQPHGLNLIMIHNIREIALRGFDKARYLFKLSIASSLMMLEILRIEVWKGLEYIIDIDDDEYSKENMKAIFPNLKELSVSWCY
QLKYMFGQYDEANKDYKEIHIQFSALEILSLKFLPKFVSICSTNTVTVTWPSLKVFKCGRCLYPFYGSVSCLTIPTNSREPIIT
STKDPKGIQNHLPTLQTLNIKHSRAERIFCLNEHEMIGQQVSLRLEKLDLYNLPEMTYIWVGPNNSLTLQHLTTLEIRDCG
KLEVIFPKSVVRCLPELKNVTIIRCMELKQIIEEDESNPLSIDGDSEEEIIGCHKKASKNYFAFPNLEELEIFECAKLEVVFPKSV
LRCLLKLKILKISKCKEVRQIIEKDVEDKNLSNILSPRPCFPKLEALQVDDCHKLKRLFCGSASNDLPNLHLLVINGANELEEL
VGCKQGKIKVELPRLKLLIFIHLENFSQEIDLHNLKNCIVYECPKLSLTSTTTLEKLCEDFPSEDFINTELGSWEFERIVRISTIT
DSSEFTSSQEIEDLGNESIKSSSTGVEDIGTGDAVATHIDSKVVEQDDKLNEGKAGTVESQVEEGLNLFHKQERIDVFPN
NNIDISSASADIRTRLGAYKHFVDLDDAQISLLVEAITTYPHLWNAPKKFSERFQAWRFKILADMLLFLQKESVHSVIPQR
QKEFDRLCEEAIEIGFESSWVEEMRQRVVARDSKLGEDIAKRQMDENSKRCSKGDMVPDSQAAERGDGPKISLEEGSD
LVDKEAEIGVVSNHHIVAMRNEEAKKEFVAEVFTSEIPRIATSLTNSQTVEKPTPSNELVDTQQTSEQCQMKQKKPLGEI
PKSIEQVALEETIAKNTNMAASSILSESATSKLDPTVTLLSKSYPYLSEIKSSQIEARIAKESEGHPKIIQDFGANDITSLFAPVI
VGKEGEDKLVGKTLAELEKYMKMSLKDIVSSETNSLCLLSALNFLSNLPFKDVKVSDGLKHIIDTMHRHFPSILCSFKQGF

ATTHKLAELEARANEVTIKKNLYDELQRKEVVLKEQIIRLKEEIRVCEVALSSLEEEKNKCIAETVEYKTKLENARKEESQML
EVAHKWSILCSQYELNRMATINPS

>XP_022633327.1

MECLLGFASSVSRDLVCGALNQLRYLCCFKNFVKRLEEEESSLIITRDSVQKFVKHVKKQARNPDAIVDKWLEDAINDVH
NVNQLLEEARTPKHCCFGHCPNWIWRYHVGKKLANKTMDLKKFIDEGRKYVPFDRIATLPSNTLDMLLEKCMNFESRQ
SAYEQLLDAVKNNDVSMIGLYGMGGCGKTTLAMEVRKFVEAEHLFEEVLFVPVSSTAEVRRVQEKIASSLQFEFPEPEE
MQRAQRLCSRLTQEKNIFIILDDVWEKLDLGHIGIPSSEHHKGCKILITSRSEAVCTLMDCQRKIYLPILTDEEAWTLFQNK
ALISEATSDTLKEMGRLISNECKGLPVTIAAVACSLKGKTEMVWSVTLNKLRHSKPINIERGLTDPYKCLRLSYDNLDAKEA
KSLFLLCSVFPEDCEILVELLTRCAIGLGVVGEVHSYEEARSEVIAAKIKLVSCCLLLDEDHERVKMHDLVRDVAHLIAKNEN
KMIKCEVEKDVMVEQNSVRYLYCVKFPNDLDCSNLEFLRLRTKMKEFDGIFKRMGMLKVLILDNGGDGKTPLSTICFKT
LTNLRDLIIFNYELSDFSFLSGMKNLQSLSLFGCLLPSFPELQTDVAITLKLLELTGCDIKVKNFEVIKRIPFLEELYIIDIQGEW
DANSEDNIKEWWNAETNIEFFKTFSVPQTLQSYGIVLGSYDFEHFNYGDIYTHRRTLLLNHFDVSNEVMKGLAKKAKDL
FVRNIHGGAKSMIPDIFQIEGGYLNELNKLEVRFSQELECLIDTRSHSSEVVTLFSKLHTLIIDNMENLRVIWHCFLPANGP
FENLENLSLSYCPGLTSLFTYGVARSLVKLKILKISRCDGLKHILTDEEKAEKSQDEFTSGRHPVQIFQNLQEVEVFRCREVK
HIFPANIVGGLTQLKVLEIEECEKLEEIIGDIVPLTEQDRKELDEIVEEGTLSSLASLRIKGCGKLGSIFTTSIAKTLTSLEELFIES
CKSLKDIVTHESIVEDEHDCESDISIFQSLKKLHISYCALLVGIFPVSFVGELNEIDTDDEYGKENMRAIFPNLKELSVYNCSQ
LKYMIDQYDVANKDYKEIRIQFSALEILSLQYVPKFVSICSINTLTVTWPSLKDFHCYSCLTIITSTKDPKGIQNHLPTLQTLN
IKHSAVERIFCLNEHEMIGQPVSLRLENMELYNVPQLTYIWVGPNNSLTLQHLTTLYISYCGKLEVIFPKSIVRCLPQLKWL
TITECMELKQIMEEDESILLSFGCDKEASKNYFAFPNLEKLEIIECAKLEVVFPKSVLRCLPKLKLLKIRKCKELRQIIEGDKNL
SNLVSPQPCFPKLEALLVDHCPKLKRCVSESASHDLPNLHLLAINGAYELEELVGCKEGKIKVELPRLKLLIFMHLSNFSQEI
ELHNLKNCIVYKCPKLSLTSTTTLQKLWLHFPYKDFINTEVRSWEFEDIVRSIDDYSTINGSSEFASSQVRINQSMNMNIN
KIKKSR

>XP_022633328.1

ESCICWNKLMECLYGFASSVSRDLVCRALNQLRYPCCFKNFVKKLEEEESHLIITKDSVQKFVTHNKKQARKPSEIVDKWL
EDAINDVHNVNQLLEEARTQKHCCFGHCPNWIWRYHVGRKLANKTMHLEKFIEEGRKYVPLDRIATLPSNTLDMLSEK
CINFDSRKSAYEQLLDAVKNNDVSMIGLYGMGGCGKTTLAMEVRKLVEAEHLFEKVLFVPVSSSVDVRRIQEKIASSLQF
EFPETEEMQRAQRLCSRLTQEETIFIILDDVWEKLDFGRIGIPSSEHHKGCKILITTRSEAVCTLMDCQRKIDLPILTDEEAW
TLFQNKASISEDTPDTLKNLGRLISSECKGLPVAIAAVACSLKGTAETVWSDALNKLRRSKPIYIERGLINPYKCLQLSYDNL
DAKEAKSLFLLCSVFPEDFEIPVEVLTRCAMGLGVVGEAHSYEEARSEVIAAKIKLVRCCLLLDEDDGCVKMHDIVRDVA
HIIAENENKMIKCEVEKHVTVEQNSVRYLWCEKFPNDLDCSNLEFLCLWTKMEEFDGIFKRMGMLKVLILYNDGAGKT
PLSTSSFKTLTNLRDLIISNCELSDFSFLNGTKNLLSLSLFGCSLPSFLELQTDVTFTLKLLELNECDIKVKNFEVMKRIPLLEEL
YIIEGEWDANSEDNIEFFKTFSFPETLQRYGIVLGSDNFYNFNDGDIYIHGRTLLLNHFDISNEVIKGLAKKAKDLFVGNIH
GGAKTMIPDVFEIEGGGLNELNMLEIRDSAEIKCLIDTRSNSSEVVTLFSNLHMLRIKSMENLEAIWYCFLPANGPFENLE
KLYLSDCPRLTSLFTYVVARNLVKLKTLQISICDELKHIIADDEKAQKSEDEFTSGHPVQIFQNLQEVQVYSCPKLKHIFSVNI
VRGLGQLKMLNIQRCDMLDQIIGDIVPLTEQDRREELDKIVEEGTLSNLASLKITWCMNLGSIFTASIAKTLTSLEELYIYG
CTSLKDIVTHERVNKNQEESIVEDDNEMIGQQVSLRLKKFELEFLPQMTYIWVGPKNSLTLQHLTTLTITECEKLEVIFSKS
VVRFLPELKLLKIRKCKELREIIEGDDLVSPQPCFPKLEALHVGHCHKLKRCFSESTSDDLPNLHFLAINGANELEELVGCKQ
GMTKVELPRLKLLIFMHLPNFRQEIELHNLKNRIVYKCPKLSFTSTTTLEKLRYDFPHKDFINTELDRLEFEDIVRLTHIDSTIS
DSSKFTSSQEIEDVGNESIKSSSTSVEDTGIGDVVAFHIDSKVVEQDDKMNEGKPGIVASQGIQVQEGLNLLHKQQEIDF
VHPNNNIDISSDIRTRLGAYRRFVDLDDAQISLLVEAITSYPHLWNASKKFSDRFQAWRLKILADMLSFLQKESVHSVIPQ
REREFYKLCEEAMFVGFESSWVEEMRQRVVARDPKLGEDIAWRQINENSKRSSSGDVVPDSQAFEQGDGLKISLEEGS
DLVDKEGEIGVVSNDHILALRNEEAEEEFVAEVSTSEIPRIATSLTNSQPVERPTPSCLNMPLRETPANALVDKQRISEPCL

MNQQKPFGEIRTKQGSVEETIAKNTNMAASSILSEPDSSQLEQKITFQSKSHPHNEITSTQLEAGSGKEIDHPKIIQGFGT
NDVISLFASEKESEDSPVGKTLTELEKFLTMSLKDVVSSESDTLRLWSTLNLLSNLPFKNVTLSDGLKRIIDTMLQHFPTILR
SFKQGFAATDKLGKLEACQNEVATTLVSKISEAENFYNET

>XP_022633348.1

MLLAFRCFEEEWESAINKNDPVGLESRSDAFCYDVFLSFIGIDTLYGFTGHLYKALNDSGIHTFIDDENHQTGEEITPTTVK
AIEESRIAITVLSINYASSTWCLDELATILDCLNRERLLVLPVFYYVDPTQVQLQKGRFGEALTKHEERLKHNMEKLLKWK
MALHQVANLFFFHIDHGAGYEYEFIGKIVEWVSKKINHAHYPIGLESQVQEVMKLLDVGCDGGAHMIGIHGTGGVGKS
TLAQEVYNNLISERFDASCFIENVREKSNKHGLQYLQSTILENLLGEKDIKLTSVEQGILMIQRRLQKKKVLLILDDVDRQE
QLQAVVGRADWFGRGSRVIITTRDEQLLASHDVQITYEVKKLNNKDALVLLKWKAFKNHDFDPRYEELLNLAVTFSDGI
PLALEVIGSNLCGKSVEEWKSVIHQLEKCPSNPVETVLKASFDSLEEKERSVFLDLACCYKGYELAEVEDILQAHYGQNMK
CYIDSLVEKSLVKLSHGTKPCYDRVTLHDMIEDMGKDIVRQESLXGPGERTRLWLLEDVRQLLENNRGTSKIEIICLDFSIF
DQEEKVEWDGKAFQNMQNLKTLIIRHGSFSKGPEYLPDNLRVIEWWRYPSNCLPSDFQPKELAICKLPCSSISTIEMKNL
LKKSVNLRVLKFSNTKV

>XP_022633627.1

MKLETSCMMQKICWSKYTMSSSKVSRKLSCKPVLARDKDKEMIFSWLRSDTNDNKLSIFSILGMGGMGKTTLAQHVY
NDSKTEEAKFDEKAWVCVSDAFDALRVSKAIIGAFTNSRDDSENLEMVHGKLKKKLFGRKFLLVLDDVWNEDRKQWK
ALQTPLTFGAKGSKILVTTRSHKVASIMQSTYIHQLKQLDEDRSWQIFAKHAFQDENSKLNSNLKEIGVKIVEKCQGLPP
ALETIGCLLQLKSSVSEWEGVLRSEIWDLPIEDSKIVPTLLLSYYHLPSHLKRCFAYCALFPKDHKFDKENLIFLWMAQNFL
DCSQQSKSPEEVGENYFNDLVSRSFFKQIIWYNKTYFVMHDLLNDLAKYVSGEICYRLGVDGEKKVSRKTRHLSYLSGPT
QYYKSLCDAKGLRTFITFRWREMSIEELISNCKFLRVLSCRWCIKVPDSIGDLIHLRSLDLSGTDIGRLPDSTCSLYNLQELKL
NNCVNLKELPLTLHELSNLRRLELKGTILTKAPLRLGKLKNLHVWIDKFEVGKSREFSIQQLGELDLHGELSINNLESITNPY
ETNLKNKAHIVRLSLQWNLKRNNADSMKQREVLENLQPSRHLKQLVIYCYGGTKFPRWLADNSLTNVVSLTLKNCKYC
LFLPSFGLLTFLKHLTIDGLDQIVRIDAEFYGNSSSAFASLETLRFTNMKEWEEWQCMTGAFPSLQSLFLTNCPKLKELPE
NLCHLTKLTVKNCSQFGAPIPRAVEIQCVNMWPSSSDIINHSYNSLVELYINGCDSLTTFPLDLFPKLRKLSLLECCNLQMI
SQGHPHNHLERLSIEKCSKFESFPNEGLFASQLETFFIEGLEKLKSMPKCMSALLPSLNDLYIRNCPVVELSEGCLPSNVKT
MRLRYCSKLVASLKGAWGTNPSLKVLNIGNVDLECFPGEGLLPFSLPELFIRDCPNLKKLDYRGLCHLSSLKKLTLDNCPIL
QCLPKEGLPKSISTLIIEDCPLLKQRCKKQEGEDWKKIAHIKSIVVDNKEVNI

>XP_022633636.1

MDTIASVASSVAAPLLRNITYVLMYSTYLTELETEIKRLQSEEKEVRHTVEAAKRGGEEIEDTVRDWFDRVRAAVEQGQA
FLEEEERERVGCMDVYSKYTNSQRARTLVEIVREVRKETFDRVSYRRALRCNVGPAAREYVAIQSRTVMLNDVVKMLKD
GGVDIVGVYGVAGVGKTAMVKELAWQVEKDGLFDVVVMATVTNSLDVGRIRNEIADGLGLKFDELTELGRASRLRQR
IRQEQRILVVLDDLWGKLDLTKIGVPFGEDYKGCRCQLLVTSRNRNMLSSNFGSGKFCRLDVLSEDESWELFEKRAGDA
VKDPSIQSVAEKVAKSCGGLPLLIVTVVEELKNKDLYAWKDALEQITSFELEGCLYSPLRSAIELSYDHLESQELKTFFLLLGS
IGNGCSTRDLLVFGWCLGLHKQVDSLADGRNRLYKLIDNLRAACLLLDEGKRDSVVTLEVVRHVANSIATRVKPFFTVQR
NKEFKWPRMDFLGSCHHIFLDWCYIRELPEVLECPKLKILQINSQGNYLKIPDDFFVHMKELKVLSLGGLDCTPSLPPSLSL
LTDLQALYLCECKLEDIATVGEITSLEILNLEKSELKELPAKIGGLSNLRLLDLTDCPTLGGIPGNVISRLTRLEELYMGNCDV
QVEAKERKSQNNDSSLSELKHLNQVTILNVQIEDTSVFPRDMLSFGRLESYKILIGDGWKWSGVESENYKTSRLLKLNSG
ADPTILKDYGIKMLMNKAEELYLAELKGVREVLYELNDEGFSQLKHLCILNCAEMESIIGSTEWAYCDHAFPNLESLILHN
LINMERICSDPLPAQAFRNLQVIKVKDCDRMEFVFSHSMVKHLSELVEIEISECKSMTNILSGQRQEDADAGQTNKISLI
NLRSLTLQCLPSLVSFSPDSSTQASENGNGFSSQLFSNKVEFPNLETLKLYSINIQMIWINHHHSYFENLTSLTVDGCERLT
YIFSYPVAVKLVKLQHLLLSSCKFVENIFVPDENLGHVHHFRKSIHKELVPIFPNLETFVISQMDNLKAIWPDLLPQNSFCK

LKKMEIESCSNLLNVFPCHVLDKLQSLESLNVWKCMALEVVYEIDGINTEHEGSSQEGLDIPLRTLSLGNLPKLKHLWNK
DPQGNIKFQNLFMVQASKCQSLKYVFPLSLAKDLLHLQFLEINDCGVEEIIASDKGGVGAAFGFVFPKLVSIKFFNLPDLR
CFCDGNHNLRFPLLNQFFAVECPRMETFSRGILRASILRKIHLTREGDQWYWQGDLNTTIRKLFNRGTQVLVV

>XP_022633637.1

MDTIASVASSVAAPLLRNITYVLMYSTYLTELETEIKRLQSEEKEVRHTVEAAKRGGEEIEDTVRDWFDRVRAAVEQGQA
FLEEEERERVGCMDVYSKYTNSQRARTLVEIVREVRKETFDRVSYRRALRCNVGPAAREYVAIQSRTVMLNDVVKMLKD
GGVDIVGVYGVAGVGKTAMVKELAWQVEKDGLFDVVVMATVTNSLDVGRIRNEIADGLGLKFDELTELGRASRLRQR
IRQEQRILVVLDDLWGKLDLTKIGVPFGEDYKGCRCQLLVTSRNRNMLSSNFGSGKFCRLDVLSEDESWELFEKRAGDA
VKDPSIQSVAEKVAKSCGGLPLLIVTVVEELKNKDLYAWKDALEQITSFELEGCLYSPLRSAIELSYDHLESQELKTFFLLLGS
IGNGCSTRDLLVFGWCLGLHKQVDSLADGRNRLYKLIDNLRAACLLLDEGKRDSVVTLEVVRHVANSIATRVKPFFTVQR
NKEFKWPRMDFLGSCHHIFLDWCYIRELPEVLECPKLKILQINSQGNYLKIPDDFFVHMKELKVLSLGGLDCTPSLPPSLSL
LTDLQALYLCECKLEDIATVGEITSLEILNLEKSELKELPAKIGGLSNLRLLDLTDCPTLGGIPGNVISRLTRLEELYMGNCDV
QVEAKERKSQNNDSSLSELKHLNQVTILNVQIEDTSVFPRDMLSFGRLESYKILIGDGWKWSGVESENYKTSRLLKLNSG
ADPTILKDYGIKMLMNKAEELYLAELKGVREVLYELNDEGFSQLKHLCILNCAEMESIIGSTEWAYCDHAFPNLESLILHN
LINMERICSDPLPAQAFRNLQVIKVKDCDRMEFVFSHSMVKHLSELVEIEISECKSMTNILSGQRQEDADAGQTNKISLI
NLRSLTLQCLPSLVSFSPDSSTQASENGNGFSSQLFSNKVEFPNLETLKLYSINIQMIWINHHHSYFENLTSLTVDGCERLT
YIFSYPVAVKLVKLQHLLLSSCKFVENIFVPDENLGHVHHFRKSIHKELVPIFPNLETFVISQMDNLKAIWPDLLPQNSFCK
LKKMEIESCSNLLNVFPCHVLDKLQSLESLNVWKCMALEVVYEIDGINTEHEGSSQEGLDIPLRTLSLGNLPKLKHLWNK
DPQGNIKFQNLFMVQASKCQSLKYVFPLSLAKDLLHLQFLEINDCGVEEIIASDKGGVGAAFGFVFPKLVSIKFFNLPDLR
CFCDGNHNLRFPLLNQFFAVECPRMETFSRGILRASILRKIHLTREGDQWYWQGDLNTTIRKLFNRGTQVLVV

>XP_022633684.1

MSPNPIVQYTASSSHTIYRYDVFVSFRGEDTRYNFTGFLLEALCRKGIDAFKDDENLKKGEFIAPELLHAIQSSRLFIVVFSK
NYASSTWCLRELAEIRNCIQTSPRRVIPVFYDVDPSVVRKQSECYENAFAEHEKRFREDKAKMKEAERWREALKEVADIS
GWDIRNKPQYVEIEEIVQNVRNILGPKICSLPKDELVGIERGLEKLANLVCFESVNNVRVVGISGMPGIGKSILTRALYERIY
HQYEFHCFIDDVSKIYRDSSLLGLQKQLISQSLNEKNLAISNSIEGTCLMWSRLHNVRALVVLDNVDEFVQLKMFTGNRN
TLLRECLAGGSIIIVVSRDEHILKRYGVDYIYQVQPLNRENAMQLFCRNAFKVNHILSAYEMLAWDILSLANGHPLAIEVI
GSSLFNRDVSQWESALARLKEKNSNDIMDVFRVSFDQLDEDHKEIFLDIACVLCDYDEKYVKEVLNFRGFHADYSLQVLL
EKSLISKMGTCIYMHSLLKDLGRYIVKEESPKEPLKRSRLWDYKDFCKAMLNNQTTEILEVIAVNSGGWIPGWKSETVRI
NGLSKIEKLKFLRLENVKCSGSLSHLSNELGYLTWNKYPFDCLPQSFEPEKLVKLKLRESSIQRLWSGTKGLPNLKHLDLSYS
NKLVEMPDVAEAPNLEGILLEDCIQLRKLSPSIGLLSKLTILNLKGCKNLVGLPNSILGLNSLEYLSVYGCSKLFNNVLLDEAS
NTEHLKNLCSLKGPIQSHSTFPLRKEARRESVSCLLPSSPTLPCLRELDLRWCNLVKIPDVIGKLCCLEKLNLEGNNFITLPN
LKDLYRLYYLNLQHCTLLKYLPDLPSQTNLPPKVYSHEEQYREAIFVYSRKNNIYVAGLKMVDGPEIEMLDWDRCTSMAI
SWMLQILQSWYNSDCLTSLPFLKSLGSSTHGYEIPKWFNNEFVSMDDSIIIDASPFVHDNNWVGVVCCAILSQTRYLGK
YKLGNMCLFYYSRQQFCHEQRIFRDKLNTRDLSLTRDLVRIFDVQFPKFKVKKYGYRWVSVQDLHDPWLKFGSSIPSED
TKLLL

>XP_022633771.1

MKEAERWREALKEVADISGWDIRNKPQYVEIEEIVQNVRNILGPKICSLPKDELVGIERGLEKLANLVCFESVNNVRVVGI
SGMPGIGKSILTRALYERIYHQYEFHCFIDDVSKIYRDSSLLGLQKQLISQSLNEKNLAISNSIEGTCLMWSRLHNVRALVV
LDNVDEFVQLKMFTGNRNTLLRECLAGGSIIIVVSRDEHILKRYGVDYIYQVQPLNRENAMQLFCRNAFKVNHILSAYE
MLAWDILSLANGHPLAIEVIGSSLFNRDVSQWESALARLKEKNSNDIMDVFRVSFDQLDEDHKEIFLDIACVLCDYDEKY
VKEVLNFRGFHADYSLQVLLEKSLISKMGTCIYMHSLLKDLGRYIVKEESPKEPLKRSRLWDYKDFCKAMLNNQTTEILEV

IAVNSGGWIPGWKSETVRINGLSKIEKLKFLRLENVKCSGSLSHLSNELGYLTWNKYPFDCLPQSFEPEKLVKLKLRESSIQ
RLWSGTKGLPNLKHLDLSYSNKLVEMPDVAEAPNLEGILLEDCIQLRKLSPSIGLLSKLTILNLKGCKNLVGLPNSILGLNSL
EYLSVYGCSKLFNNVLLDEASNTEHLKNLCSLKGPIQSHSTFPLRKEARRESVSCLLPSSPTLPCLRELDLRWCNLVKIPDVI
GKLCCLEKLNLEGNNFITLPNLKDLYRLYYLNLQHCTLLKYLPDLPSQTNLPPKVYSHEEQYREAIFVYSRKNNIYVAGLKM
VDGPEIEMLDWDRCTSMAISWMLQILQSWYNSDCLTSLPFLKSLGSSTHGYEIPKWFNNEFVSMDDSIIIDASPFVHDN
NWVGVVCCAILSQTRYLGKYKLGNMCLFYYSRQQFCHEQRIFRDKLNTRDLSLTRDLVRIFDVQFPKFKVKKYGYRWVS
VQDLHDPWLKFGSSIPSEDTKLLL

>XP_022633810.1

MIWFPDPFFQYSTSGTWGINFVMHDLMHDLATFLGGEFYFRVDELGKETKIDRKTRHLSFARFSDPVSDIDVFETAKFP
RTFLQNTNKDSPFNNEKALRITVLMFKYLRVLKFSDYQSEFVLPDSIGELIHLRYLNLSGTSIAILPESLCNLYHLQTLKLGFC
FNLTKLPRDMQNLVNLRHLEILIKEMPKRMGKLNQLRKLSYYVVGKHKENSIKELGGLPNLHGWFCIEKLENVTKGEEAL
EARIMDKKHIADLFLEWSLHNDDIIDLQIELDVLDKLQPHQDLKSLEISGYRGTRFPEWVGNFSYQNITNLHLQNCNNCC
MLPSLGQLPSLNNLIISNMNSVKTIDADFYKKDDCSSVTPFPSLEYLSILNMPCWEVWNGFDSEAFPVLKHLGIEQCPKL
MGDLPNHLPALQKLTIINCELLVSSIHGPPTLRTLEIGNSNKIAFHEFPLLVESIDVEGGPMVESMMESLIFNLLACNL

>XP_022633859.1

MAATSVLVSTFLERTIDTLASRLVHIFHQRKHKKQLSNLKMKLLVIDVVALDAEQKQFKDPRVRDWLLRAKDVVFDAED
LLDEIDYELSKSQAEAQSQSATNKVWNSLNSSFVSFFEIEIESKMEQVIEDLEDLAKESNILGLEKSGGVGVGSGSGSKLTY
TSLPNESFIYGRDDDKKFVFNWLTSDTHNNLSILSIVGMGGLGKTSLAQHVFXDXRLEGKFDMKAWVSVPQEFDVLKV
SKAILGTITGSTDHSIQQEVIQKRLKEELMRKKFLLILDDVWNENPFKWEDVQKPLVFGGQGSRILVTTRSEKVAAAMRS
EKHFLQVLKGDDCWDLFAKHAFQNANTQANTDCKEIGKQIVEKCRGLPLALKTMGSLLHNKSSISEWENVLKSEIWGL
EDNDLIPALALSYNHLPSHLKRCFAYCALFPKDYEFDKNGLIQLWIAENFVQFHNHRSPEDIGEQYFDDLLSRSFFQQSN
RYKTCFVMHDLLNDLAKYVCEDICFRLGVDKTKGIPKTTRHFTFATNHVEYFDRFGSIHDVERLHTFMPTDWSWHCKM
SIDDLFSKFKFLRVLSVSNCSNLTKVPDSIGNLKHLRSLDLSNTHIERLPESTCSLYNLQILKLNNCPLLKELPSNLHKLTNLRR
LEFMDTELLKVPEHLEKLKNLQVFMSSFDVGSKEFSIQQLGQLDHHGRLSIGELQNIENPSDAKAADLKNKTHLRELMLK
WNRDQVPVDALKERDVNVMQNLQPSKYLEKLSISHYDGTKFPTWFGDYSLLNVVSLSLFECKQCKCLPSLGHLPFLKDL
TISGLDGVVGIDADFYGSGSCSFPSLETLKFSRMKGWEKWDCEAMRGAFPRLHHLAIDYCPKLKERLPVQILQFETVYIR
HCKQLLGYDGMVKMNGKEVTIFRVHNLEAWFVGWIGKMISHDSVEDLKVYSCPNMSILMSQQYNFLVSLTVHDSCD
SLTTFPIDFFPTLRSLYLLSCCNLQTILHAHNHLQDLAIIDCPQFESFPERVHILLPCLEFLSIRDCPRFESFADGCLPSNIKRM
YLTNSSKLVASLKGSFGDNPSLETLFIIGKLDAESFPDEGFLPRSLTSLGIYDCQHLKKLDYKGLCHLPSLKELLLVNCPNLQC
LPDEGLPKSISYLTISGNCPLLKQRCKYPGGQDWGKIAHVQNLSIL

>XP_022633873.1

MALAAVGGALLSAFFDVLFDRLASPEVLNFIRGKKPDKLLQKVKTQLIVVRVVLADAEKRQITDSNVKEWLDLLRDVVYE
VDDLLDEISTKAAIQKEVSNSFSHLFKSKKIVSITKLEDIVERLDDILKQKENLDLKEIPVESYQPWKAQPTSLEDGYAMYGR
DKDKEAIMKMVLEDSTNGETVSVIPIVGMGGVGKTTLARSVFNDGKLKQQIFDLKAWVCVSDIFDIVKVTRTMIEEITR
KPCKLNDLNALQLELIDELKGKRLLIVLDDVWIEDCENWSSLTKPFLSGNKGSKVVITTRNENVATAVPFHTVEVYHLNKL
SNEDCWLVFANHAFPQSEASETKETLEKIGKEIVKKCNGLPLAARSLGGMLRRKQTIRDWNNVLQSDIWELPESQCKIIP
ALRISYNHLPPHLKRCFVYCSLYPKDYEFKKDDLIQLWMAEDLVKATKKGKTLEEVGQEYFDDLVSRCFFQSSSGSVDDY
FVMHDLIHDLATFLGGDFYFRTDELEKETNIDRKTRHLSLTRFSDPASDIEAFDTVKFPRTFLLIDYKDSPFNNEKAPRIIVS
RLKYLRVLSLYKFQSQLALPDSVGELIHLRYLNLSYTSIETLPESLCNLCNLQTLKLSSCSELTKLPSAMQNLVNLRHLEIFGT
PITEMPKRMGKLNKLRNLDCYVVGKHTENSIKELGGLPNLHGMFYIEKLENVTKGEEALEARIMDKNHISHLSLEWSIAN
DNSIDFQVELDILSNLEPHQDLESLSIKGYKGNIFPEWVGNFSYRYMTRINLLKCNNCCMLPSLGQLPSLSHLHISKMYSV

KTIDAGFYKKDDCSSVTPFPSLKYLSISNMPCWEVWNAFDSEAFPVLKVLCIEECPNLKGDLPNHLPALQTLRIRNCELLD
CSVPGPRTLRTLEIQKCKTVAFHEFPHLVRRIDVEGGPMVESMMEAISNFQPTCLYFLRLRNCSSAISFRGGRLPASLKIL
DITGINKLKFPLQDKHELLESLSIYNSCDSLTSLPLAIFPNLTSLRITNCENMESLLVSGSEISKRLESFEIRHCPNFVSFPGEGF
CMPNLTRFRVYNCEKLKSLPDQMGTLFPKMEYLYISNCQQIESFPGGGMPPNLRTVNIIDCVKLLSNQAWVCMDMVT
SLNVWGPCDGIKSFPKESLLPPSLVSLDLFNLCSLEMLDCKGLLHLTSLQELNIRSCQKLENIAGEKLPLSLIKLTIDECPLLK
QRCHKKDRQIWPKICHVRGIKIDGRWI

>XP_022633874.1

MELASSSSSSSSSFLKSKPHFIYDVFINFWGEEMQRKFVSHLHSVLLQAQVKTLINMENLEEGMQLEEHMRAIASSKIAII
VFSKTYTESTCCLRELEKIIECRETFGQIVLPVFYEIDPFDVRHQKDDFRKALEESAQKSYSGEQLEHVLSRWSRALTTAAGI
NGWDVTDFRHDAELVEVAVSRVQTLLGYEDLSHTEFPVGLESHVEKMVGSIENHSAKVCMVGIWGMGGSGKTTIAK
AIYNQIYRQFIGKSFIENVGQVHRHIGLEEELIYQVLKPKFKWKGDWMGRTIVKNELSNKKLLIVLDDVNKFWQLQNVC
GNHELFGQGTVIIITTRDVRLLNKLKLDFVYKMDLMNENDSLELLCWHAFRDAKPRKNFNELARKIVTCCGGLPQALVA
LGSDLYLKTFAPWEPTILPRQIFADYHVLENLEKSFDGLCNYMQRDIFLDVCCFFIGKDRGYVTKILNDCGLRADIGITVLI
QRGLIKVERNNKLQMHPLLQDMGREIICRSWPMEPEKRSRLWFHEDVKHVLRNKTGTAATEGLSLKLHSTRDCFKADT
FKEMKRLRLLELDHVQLVGDYGYLSEELRWICWKGFPSQYIPDNFRMKNVIAIDLKHSHLQLVWKQAQVLKWLKFLNL
SHSKYLRETPDFAGLPSLEQLILKDCPSLCKVHPSIGDLCNILLINLKDCTSLSSLPKEVYKLKSLQTFILSGCLKIDILEEDIVQ
MKSLITLVSENTAVKQVPCTIVNSKHTGYISLRRFEGLSHNILSSIIRSWMSPIMNSRSYIRPLCIDMENDNLRDLAPLLSCL
ANIRSILVQCDTQSQLSKQLNTIFVEDGVNFTESTNHSLRFSLIGVGSCNEFLNTVRDSVSKGLESSECCDISLPCENNPYW
LGHTGEGHSVSFSVPKDCDIKGMTLCVVYSSTPEIVATECLRSVLIVNYTKCTYQIHKHGTVMSFNDEDWHGIISNLESE
DKVEIFVSFGHGLVVKNTTVYFINREPVKTLSQEYERFFCLDWLACIMQPFFLK

>XP_022633897.1

MLNFCGSFIRVLFSFVSWLFGGLKGAEEKPKTDNNAPQIKHDVFVSFSGKDVHRGFLSHLTEAFRRRNIDAFVADKNLE
KGEEIWPSLVEAIEGSSISLVIFSQNYASSDWCLEELVTILECREKYGTSVLPVFYYVEPTYVRHQSEVQRWRHALNLASRL
AGINSSKFSGDAELVKEIVNEVLKRIVKQSINTKGLVGIDEKIAAIESLIREEPKQTRLIGIWGMGGVGKTTLAEEVFNKLQS
EYEGSYFVASETDRSNKHELISLKENMFSKLLGYDVKIDTPNSLPKGIVRRIGCMKVLIVLDGVNESEQLEKLLGTLDNFGS
GSRIIVTSRDEQVLRANKADKIYQLKEFNFDKALELFYLNAFKKNDHVQEYKELPKRVVDYAQGIPLVVKVLAGLLHGKNK
EEWESMLGKLKKMPPKEVYEVMKLSYDSLDRKGKQIFLDLACFFLRTHVAVNVDYLKSLLKDDDSDNSVAFELGRLKDK
ALITISDDNTVSIHDSLQEMAWEIVRQESVEQPGNRSRLWELNDICEALKNNKVKEAIRSIRIHLPTIKEQKLVPQILDINM
SKLKFLEVFVCDRYDELTLLKGPQFLATELRFLSWYQYPLKSLPENFSAEKLVILKLRNGRMEKLWDGVKNMVNLKQVDL
SRSQNLKKLPDLSKATNLEVLLLMSCSSLTSVHPSIFSLPKLVKLDLLCCTSLTTLGSSSSLCNLSFLNLGRCTNLRKFLLISENI
KKLRLGNTMVKELPSSISYFNQLLHLDISFCSKLRTIPKLPLSLETLNARECQSLKTILFPSQAVEQLKENRKWISFWKCMNL
DNQTLVAIGLNVQINVMNYANQHISIPSHNHVEHCDDDNCDSYSGVYVYPGSRVPKWLEYKTRKDSIILDLSSAPPSPLI
GFIFCFVLGKYHNTDVDRFEVMITIDGEDEGNSISVPIYIDYGYEKTESDHVCVMYEQRCSTFLNNRAKNQTRFKIQVTVE
EALSHKFLKGFGVSLISSSTYKNFIQEMQLRDSQYQFQKKPMSNNNSPQIKYDVFVSFRGEDIRDGFLSHLTEAFDVKKIN
AFVDDKLEKGEELWPSLVQAIQGSSISLIIFSPDYASSPWCLKELVTILQCKEKYGQTIIPVFYHVKPTDVRRQSSDGYRKAF
ADHERKHKHEVQLWKDVFEKSAYLSGIESSKFRNDADLLKAIVDLVLRRLTKSHKQIFLDLACFLCISSVKITTGYLKYLLKE
DERDNSVVVALERLKDKALITFSKDNVVCMHDSIKEMAWEIVRQESPEDPGNRSRLWDPDDIYEAFKNDKVSEATRSI
QINLLTLKGQKLKPHIAKMSRLRFVEIHGEEDNNAYYGRFGAEGQGLKFSATEIRFLFWKCYALKSLPDKFSFEKVVILKLE
LGRTEKLWDGVKNLVSLKELDLTGSRSLKKLPDLSKAKNIEVLCLGSCSGLTRLPSSLGNHTQLLYLDISHCPKILTIPELPLS
LETLYAINCESLKTVSFHSTAVEQTKENRKQVSFDNCMNLDEPSLEAIGLNARFNFMKFSNQHQSAAKQDYFQNYNDY
DKNYDSYQAFYVYPGNSVPEWLEYKSKKYSVIIDLSSAPPSPVYGFILCFVLLGELDKIRVNIVISDCEGKGVMDRFRMKL

NRKFRNSFVHESSVESQKVVVMYDQRCSNFLNNIAKRLTRFKIMVRRATKDDGSFYSKRIMIGLSGFGVSIIRTSTYSSFMQQMELCDSMYHIR

>XP_022633937.1

MECLLGFASSVSRDLVCGALNQLRYPCGFKNFVKRLEEEESNLIITKDSVQKFVTHNKKQARKPSEILDKWLEDAINDVHNVNQLLEEAKTQKQCCFGHCPNWIWRYHVGKKLANKTMHLEKVIEEGRKYVPFDRIATLPSNTLDMLLEKCMNFESRQSAYDQLLDAVKNNDVSLIGLYGMGGCGKTTLAMEVRKLVEAEHLFEKVLFIPVSSTVEVRRIQEKIASSLQFEFPEIEEMQRAQRLCSRLTLEKNILIILDDVWEKLNFGRIGIPSSQHHKGCNILIITRSEAVCTSMDCQRKIYLPILTDEEAWTLFQNKALITEATSDALKDLGRLISNECKGLPVAIAAVGCSLKGKAETVWSVALNKLRRSNPINIERGLIDPYKCLQLSYDNLDTKEAKSLFLLCSVFPEDSEIPVEHLTRCAIGLGVVGEVDSYEEARSEVIVAKIKLVSCCLLLDADDECVKMHDIVRDVAHIIAKNENKMIKCEVEKDVRVEQNSVRYLWCVKFPNDLDCSNLEFLYLETKMKEFDGIFKRMGMLKVLILVNDEDGETRLSTISFKTLTNLRFLFIENYELSDFSFLGGMKNLQSLSLNHCLLPSFPELQPDVTTLKLLEVIECDINVKNFEVMKRIPLLEELYIIDIEGEWDANSEDNIEFFKTFSIPETLQRYGIVLGSNNFMDFNDRDMYIHGRTLLLNHFDISNEVIKGLAKKAKDLFVANIDGGVKNMIPDIFEIEGGGLNELNKLEIIDSEELECLIDTSSRSSEVVTLFSKLHTLIIGNMKNLRVIWHCFLPANGPFENLENLYLSYCSRLTSLFTYVVARSLVKLKILKITRCDELKHILTNEEKVEKSQDEFTSGHPVQIFQNLQEVDVDSCPELKHIFSANIVGGLGQLKVLNIEECAMLDQIIGDIVSLAKQDRKEPDEIVEEGTLSSLASLKIKHCGNLGSIFTASIAKTLTSLEELFIEDCKSLKDIVTQERVNKNLEESIVEDEHDCESDISIFQSLKKLHISKCDLLEGIFPVSFIGELNDITNKETADLKDFSSRNNTQIELPALQVLELHHIPVGSYDVICPSLRTLSLYIGRYVGFFNINCSTDASAATKSDFIAIKILNSDFEPPVESVECLSKQPLGLNLITTHNIREIELKGFDKARYLFKLSIASSFMLEILRIKECHGLEYIIDTDDEYGKENIKAIFPNLKQLSVNKCFQLKYMIGQHHVANKDYKEIHVQFSALEILSLWNLPKFVSICSTNTLSVTWPSLKDFECYNCFYPFYGFDSCLTIPTNSREPIITSTKDTKGIQNHLLTLQTLNIVDSDAEGIFCLNEHEMIGQQVSLRLENLELRFLPEMTYIWVGPNSSLTLQHLTTLEIWECEKLEVIFPKSVVRCLPELKLLKIRKCKELREIIEGDKNLSNILSPQPCFPKLEALHVDHCHKLKRLFSGSTSNDLPNLHLLAINGANELEELVGCIQGRIKVELPRLKLLIFMNLENFSQEIELHNLKNCIVYKCPKLSLTSTTTLQKLCEYFPHKDFTNTELHRGTFDIIVGFIYAEYSQEIEDVGNESIKSSSTGVANIGIGNAVATHIDFKVVEQDDKMTEGKPGIVASQGIQVEEGLNLLHKQEGKDVVPNSNIDISSAYSADIRTRLGAYKHFVDLDDAQISLLVETITTYPHLWNACERFSERFQAWRLKILADMFLFLQKESAHSVIPQREKEFDRLCEEAIEIGFESSWVEEMRQLVVARDPKLGEDIARRQIDENSKRCSSGDMIPDSQAVEEGDGPKISLEEGSDLVDKEGEIGVVSNHHILAPRNDEPEQEFVAEVFTSEIPRIATSLTNSQTVEKPTPSNALLDTQQTNESCLMEQKKPLGEIPKNIEQVALEETIAKNTNIAPSSILSEYATSKLDPRITLERKSHPHGEINSSQIEPRIAKESESNLKIIQNFGANDITSLFAPVVVGKKGEDNLVGKTLAELEKYLKMSLKDIVSSETNSLCLLSTLNFLSNLPFKDVKVSDGLKHIIDTMHQQFPSILSSFKQGFVTTDKLAELEVRANEVIIKKNFYDELQLKEVVLKEQIIRLKDEIRVCEVALSSLEEEKNKCIAEIVEYKTELENARKDESQMLEVAHKWSIQCSQYELNRMAATNPS

>XP_022634008.1

MSSNAIVQYTVSSSHTINRYDVFVSFRGEDTCNNFTGFLFQALRRKAIDVFKDDEDLKKGESIAPELLHAIQSSRLFIVVFSKNYASSTWCLRELAEIRNCVQTSPRPIIPVFYDVDPSVVRKQSECYEKAFAEHEKRFREDKAKMEEAERWREALTEVANLSGWDIRNKPQYVEIEKIVQTVTNILDPKICSLPKDELVRIEHTLEELVGIERRLESINNVRVIGISGMGGIGKTILARALYERIYHQYDFHCFIDDVSKIYRYSSSLGVQKQLVSQSLNEKNQEISNSFEGTCLTWSRLHNVRALVVLDNVDEVEQLRMFTGNRDTLLRQCLGGGSIIIVVSRDEHILRTYGVDYIYQVQPLNHKNAMQLFCKNAFKVNHISSDYEKLAWDVLSHAQGHPLAIEVIGSSLFGRNVSQWKSALARLKENKTKNIMDVLRVSFDQLDEDHKEIFLDIACVLCDCDEKYTREVLNFRGFHVDYSLQVLLEKSLISKMGTRIYMHNLLKDLGRYIVKEESPKEPLKRSRLWDYQDFCKAMLNNQTTQILEVIAVNSGWISGWKSETVKIDGLSKIEKLKFLRLENVKCSGSLSHLSNELGYLTWNNYPFECLPQNFEPHKLVKLKLRNSRIQRLWSGTKELPNLKHLDLSYSEKLVEMPDVAKALNLEEILLEDCIELRKLSPSISFLRKLTILSLRNCINLVSVPNNILGFNSLEYLSVYGCSKLFNNELLEEASNTEHLKKLCSVEGPLQSHSTSPLIKKARRESVSCLLPSFPTLPYLRELDLSWCKLVKIPDVIGKLSCLEKLNLKGNHFVTLPNLTDLYRLYYLNLEHCSRLKYLPDLPSQTNLPPKVYWHEEQYMEDIFVCLRANDIYVAGLRMFYCPEIVEWERCTDMAISWMLQILQVCMFLPYTFLLLISFDVEKQLLTYNITQSWYKSKCLTSLPFLKSLGSTIDGSEILKWFNNQLVSMDNSIIIDASPFV

HDNNWVGVVCCAILNKHAYKSRIYAYSGSDLSVYDFDHMCLFYYSRQQFLHEQHIFRGKLNTRDLSLTRDLVRIFDVLLC
RKRKDGNQVFPKFKVKKYGYRWVTVQDLHDPWLKFGSSIPSEDTKLLL

>XP_022634009.1

MSSNAIVQYTVSSSHTINRYDVFVSFRGEDTCNNFTGFLFQALRRKAIDVFKDDEDLKKGESIAPELLHAIQSSRLFIVVFS
KNYASSTWCLRELAEIRNCVQTSPRPIIPVFYDVDPSVVRKQSECYEKAFAEHEKRFREDKAKMEEAERWREALTEVANL
SGWDIRNKPQYVEIEKIVQTVTNILDPKICSLPKDELVRIEHTLEELVGIERRLESINNVRVIGISGMGGIGKTILARALYERIY
HQYDFHCFIDDVSKIYRYSSSLGVQKQLVSQSLNEKNQEISNSFEGTCLTWSRLHNVRALVVLDNVDEVEQLRMFTGNR
DTLLRQCLGGGSIIIVVSRDEHILRTYGVDYIYQVQPLNHKNAMQLFCKNAFKVNHISSDYEKLAWDVLSHAQGHPLAIE
VIGSSLFGRNVSQWKSALARLKENKTKNIMDVLRVSFDQLDEDHKEIFLDIACVLCDCDEKYTREVLNFRGFHVDYSLQV
LLEKSLISKMGTRIYMHNLLKDLGRYIVKEESPKEPLKRSRLWDYQDFCKAMLNNQTTQILEVIAVNSGWISGWKSETVK
IDGLSKIEKLKFLRLENVKCSGSLSHLSNELGYLTWNNYPFECLPQNFEPHKLVKLKLRNSRIQRLWSGTKELPNLKHLDLS
YSEKLVEMPDVAKALNLEEILLEDCIELRKLSPSISFLRKLTILSLRNCINLVSVPNNILGFNSLEYLSVYGCSKLFNNELLEEAS
NTEHLKKLCSVEGPLQSHSTSPLIKKARRESVSCLLPSFPTLPYLRELDLSWCKLVKIPDVIGKLSCLEKLNLKGNHFVTLPNL
TDLYRLYYLNLEHCSRLKYLPDLPSQTNLPPKVYWHEEQYMEDIFVCLRANDIYVAGLRMFYCPEIVEWERCTDMAISW
MLQILQVCMFLPYTFLLLISFDVEKQLLTYNITQSWYKSKCLTSLPFLKSLGSTIDGSEILKWFNNQLVSMDNSIIIDASPFV
HDNNWVGVVCCAILNKHAYKSRIYAYSGSDLSVYDFDHMCLFYYSRQQFLHEQHIFRGKLNTRDLSLTRDLVRIFDVLLC
RKRKDGNQVFPKFKVKKYGYRWVTVQDLHDPWLKFGSSIPSEDTKLLL

>XP_022634010.1

MSSNAIVQYTVSSSHTINRYDVFVSFRGEDTCNNFTGFLFQALRRKAIDVFKDDEDLKKGESIAPELLHAIQSSRLFIVVFS
KNYASSTWCLRELAEIRNCVQTSPRPIIPVFYDVDPSVVRKQSECYEKAFAEHEKRFREDKAKMEEAERWREALTEVANL
SGWDIRNKPQYVEIEKIVQTVTNILDPKICSLPKDELVRIEHTLEELVGIERRLESINNVRVIGISGMGGIGKTILARALYERIY
HQYDFHCFIDDVSKIYRYSSSLGVQKQLVSQSLNEKNQEISNSFEGTCLTWSRLHNVRALVVLDNVDEVEQLRMFTGNR
DTLLRQCLGGGSIIIVVSRDEHILRTYGVDYIYQVQPLNHKNAMQLFCKNAFKVNHISSDYEKLAWDVLSHAQGHPLAIE
VIGSSLFGRNVSQWKSALARLKENKTKNIMDVLRVSFDQLDEDHKEIFLDIACVLCDCDEKYTREVLNFRGFHVDYSLQV
LLEKSLISKMGTRIYMHNLLKDLGRYIVKEESPKEPLKRSRLWDYQDFCKAMLNNQTTQILEVIAVNSGWISGWKSETVK
IDGLSKIEKLKFLRLENVKCSGSLSHLSNELGYLTWNNYPFECLPQNFEPHKLVKLKLRNSRIQRLWSGTKELPNLKHLDLS
YSEKLVEMPDVAKALNLEEILLEDCIELRKLSPSISFLRKLTILSLRNCINLVSVPNNILGFNSLEYLSVYGCSKLFNNELLEEAS
NTEHLKKLCSVEGPLQSHSTSPLIKKARRESVSCLLPSFPTLPYLRELDLSWCKLVKIPDVIGKLSCLEKLNLKGNHFVTLPNL
TDLYRLYYLNLEHCSRLKYLPDLPSQTNLPPKVYWHEEQYMEDIFVCLRANDIYVAGLRMFYCPEIVEWERCTDMAISW
MLQILQVCMFLPYTFLLLISFDVEKQLLTYNITQSWYKSKCLTSLPFLKSLGSTIDGSEILKWFNNQLVSMDNSIIIDASPFV
HDNNWVGVVCCAILNKHAYKSRIYAYSGSDLSVYDFDHMCLFYYSRQQFLHEQHIFRGKLNTRDLSLTRDLVRIFDVLLC
RKRKDGNQVFPKFKVKKYGYRWVTVQDLHDPWLKFGSSIPSEDTKLLL

>XP_022634011.1

MSSNAIVQYTVSSSHTINRYDVFVSFRGEDTCNNFTGFLFQALRRKAIDVFKDDEDLKKGESIAPELLHAIQSSRLFIVVFS
KNYASSTWCLRELAEIRNCVQTSPRPIIPVFYDVDPSVVRKQSECYEKAFAEHEKRFREDKAKMEEAERWREALTEVANL
SGWDIRNKPQYVEIEKIVQTVTNILDPKICSLPKDELVRIEHTLEELVGIERRLESINNVRVIGISGMGGIGKTILARALYERIY
HQYDFHCFIDDVSKIYRYSSSLGVQKQLVSQSLNEKNQEISNSFEGTCLTWSRLHNVRALVVLDNVDEVEQLRMFTGNR
DTLLRQCLGGGSIIIVVSRDEHILRTYGVDYIYQVQPLNHKNAMQLFCKNAFKVNHISSDYEKLAWDVLSHAQGHPLAIE
VIGSSLFGRNVSQWKSALARLKENKTKNIMDVLRVSFDQLDEDHKEIFLDIACVLCDCDEKYTREVLNFRGFHVDYSLQV
LLEKSLISKMGTRIYMHNLLKDLGRYIVKEESPKEPLKRSRLWDYQDFCKAMLNNQTTQILEVIAVNSGWISGWKSETVK
IDGLSKIEKLKFLRLENVKCSGSLSHLSNELGYLTWNNYPFECLPQNFEPHKLVKLKLRNSRIQRLWSGTKELPNLKHLDLS

YSEKLVEMPDVAKALNLEEILLEDCIELRKLSPSISFLRKLTILSLRNCINLVSVPNNILGFNSLEYLSVYGCSKLFNNELLEEAS
NTEHLKKLCSVEGPLQSHSTSPLIKKARRESVSCLLPSFPTLPYLRELDLSWCKLVKIPDVIGKLSCLEKLNLKGNHFVTLPNL
TDLYRLYYLNLEHCSRLKYLPDLPSQTNLPPKVYWHEEQYMEDIFVCLRANDIYVAGLRMFYCPEIVEWERCTDMAISW
MLQILQVCMFLPYTFLLLISFDVEKQLLTYNITQSWYKSKCLTSLPFLKSLGSTIDGSEILKWFNNQLVSMDNSIIIDASPFV
HDNNWVGVVCCAILNKHAYKSRIYAYSGSDLSVYDFDHMCLFYYSRQQFLHEQHIFRGKLNTRDLSLTRDLVRIFDVLLC
RKRKDGNQVFPKFKVKKYGYRWVTVQDLHDPWLKFGSSIPSEDTKLLL

>XP_022634012.1

MSSNAIVQYTVSSSHTINRYDVFVSFRGEDTCNNFTGFLFQALRRKAIDVFKDDEDLKKGESIAPELLHAIQSSRLFIVVFS
KNYASSTWCLRELAEIRNCVQTSPRPIIPVFYDVDPSVVRKQSECYEKAFAEHEKRFREDKAKMEEAERWREALTEVANL
SGWDIRNKPQYVEIEKIVQTVTNILDPKICSLPKDELVRIEHTLEELVGIERRLESINNVRVIGISGMGGIGKTILARALYERIY
HQYDFHCFIDDVSKIYRYSSSLGVQKQLVSQSLNEKNQEISNSFEGTCLTWSRLHNVRALVVLDNVDEVEQLRMFTGNR
DTLLRQCLGGGSIIIVVSRDEHILRTYGVDYIYQVQPLNHKNAMQLFCKNAFKVNHISSDYEKLAWDVLSHAQGHPLAIE
VIGSSLFGRNVSQWKSALARLKENKTKNIMDVLRVSFDQLDEDHKEIFLDIACVLCDCDEKYTREVLNFRGFHVDYSLQV
LLEKSLISKMGTRIYMHNLLKDLGRYIVKEESPKEPLKRSRLWDYQDFCKAMLNNQTTQILEVIAVNSGWISGWKSETVK
IDGLSKIEKLKFLRLENVKCSGSLSHLSNELGYLTWNNYPFECLPQNFEPHKLVKLKLRNSRIQRLWSGTKELPNLKHLDLS
YSEKLVEMPDVAKALNLEEILLEDCIELRKLSPSISFLRKLTILSLRNCINLVSVPNNILGFNSLEYLSVYGCSKLFNNELLEEAS
NTEHLKKLCSVEGPLQSHSTSPLIKKARRESVSCLLPSFPTLPYLRELDLSWCKLVKIPDVIGKLSCLEKLNLKGNHFVTLPNL
TDLYRLYYLNLEHCSRLKYLPDLPSQTNLPPKVYWHEEQYMEDIFVCLRANDIYVAGLRMFYCPEIVEWERCTDMAISW
MLQILQSWYKSKCLTSLPFLKSLGSTIDGSEILKWFNNQLVSMDNSIIIDASPFVHDNNWVGVVCCAILNKHAYKSRIYAY
SGSDLSVYDFDHMCLFYYSRQQFLHEQHIFRGKLNTRDLSLTRDLVRIFDVLLCRKRKDGNQVFPKFKVKKYGYRWVTV
QDLHDPWLKFGSSIPSEDTKLLL

>XP_022634013.1

MPFKALDFSLLSSQRTMLPPPGACVNWQRYVTAFKLPQDLLYLSFMTLILRWCANKVNVMRKHLQNTKRDSEKTKRR
WRKLRDGEKLSQKWPIFLVGIFEIRNRDTLLRQCLGGGSIIIVVSRDEHILRTYGVDYIYQVQPLNHKNAMQLFCKNAFK
VNHISSDYEKLAWDVLSHAQGHPLAIEVIGSSLFGRNVSQWKSALARLKENKTKNIMDVLRVSFDQLDEDHKEIFLDIAC
VLCDCDEKYTREVLNFRGFHVDYSLQVLLEKSLISKMGTRIYMHNLLKDLGRYIVKEESPKEPLKRSRLWDYQDFCKAML
NNQTTQILEVIAVNSGWISGWKSETVKIDGLSKIEKLKFLRLENVKCSGSLSHLSNELGYLTWNNYPFECLPQNFEPHKLV
KLKLRNSRIQRLWSGTKELPNLKHLDLSYSEKLVEMPDVAKALNLEEILLEDCIELRKLSPSISFLRKLTILSLRNCINLVSVPN
NILGFNSLEYLSVYGCSKLFNNELLEEASNTEHLKKLCSVEGPLQSHSTSPLIKKARRESVSCLLPSFPTLPYLRELDLSWCKL
VKIPDVIGKLSCLEKLNLKGNHFVTLPNLTDLYRLYYLNLEHCSRLKYLPDLPSQTNLPPKVYWHEEQYMEDIFVCLRANDI
YVAGLRMFYCPEIVEWERCTDMAISWMLQILQVCMFLPYTFLLLISFDVEKQLLTYNITQSWYKSKCLTSLPFLKSLGSTI
DGSEILKWFNNQLVSMDNSIIIDASPFVHDNNWVGVVCCAILNKHAYKSRIYAYSGSDLSVYDFDHMCLFYYSRQQFLH
EQHIFRGKLNTRDLSLTRDLVRIFDVLLCRKRKDGNQVFPKFKVKKYGYRWVTVQDLHDPWLKFGSSIPSEDTKLLL

>XP_022634344.1

MSFNAIVQYTISSSHAINRYDVFVSFCGEDTRNNFTGFLFQALRRKGIDAFKDDEDLKKGESIAPELLHAIQSSRLFIVVFSK
NYASSTWCLRELAAIRNCVQTSPRPVIPVFYDVDPSVVRKQSECYENAFAEHEKRFREDKAKMEEAERWREALTQVAN
LSGWDIRNKSQYAQIEEIVQNITNILGPKICSLPKDELVGIERGLEILANLVCFESFNNVRVVGISGMGGIGKTTLARALYEK
IYHQYDFHCFIDDVSKIYRDSSSLGVQKQLISQFLNEKNLEISNSFEGTCLMWSRLHNVKALVVLDNIDEVEQLRIFTGNR
DTLLRECLGGGSIIIVVSRDEHILRTHGVDDIYQVQPLNYEYAMQLFCRNAFKVNHILSDYEKLAWDILSYAQGHPLAIKVI
GSSLFRRNVSQWESALARLKEKKSKHIMDVLRISFDQLDXEDKQIFLDIACALYYYDEKYVTEVLKFRGFHPEYGLQVLHD
XSLISKMGRCIYMHNLLKDLGXXIVKEESPKEPLKRSRLCNYQDFLKAMSKNQTTEILEVVAVNSYGSSKTVRVDGLSKIK

HLKFLRLENMNCSGSLSHLSSELGYLIWNNYPFECLPKSFQPHKLVKLKLKGSSIQRLWSGTKVLPNLKRLDLSYSKELVE
MPDVAEALNLEGIDLEGCIQLRKLSPSIGLLSKLTTLNLKNCENLVSLPNSILSLNSLEYLSVYGCSNLFNNELLDEASNTEHL
KKLCSLKGPIESHSTSPLIKKARRESVSCLLPSSPTLSCLRELDLRFCNLVKIPDAIGKLSCLEKLNLKGNNFVTLPNLKDLFRL
YYLNLEDCKRLKYLPVLPSRTHLPLNLYSYLLQYFEDFIYQVPNEDEFMAGLTMFNRPKIVERERCTSMTVSWMLQIIQV
CMFLPYTYHLLISFGVEKQLLMHNITQSWYNSEYLSFSSSLRSIIPGSEIPMWFNNQLLSMDNSIIIDVSPFVHDNNWVG
VVCCAILGENLYRTRILEDLRRDLFVEHSDHMCLFYYSRQQFCHEEGIFWGKLNTHDLSLTFKLHDVIILCANMKDRDEG
FYEFEVKKYGYRWVKKQDLQ

>XP_022634345.1

MSFNAIVQYTISSSHAINRYDVFVSFCGEDTRNNFTGFLFQALRRKGIDAFKDDEDLKKGESIAPELLHAIQSSRLFIVVFSK
NYASSTWCLRELAAIRNCVQTSPRPVIPVFYDVDPSVVRKQSECYENAFAEHEKRFREDKAKMEEAERWREALTQVAN
LSGWDIRNKSQYAQIEEIVQNITNILGPKICSLPKDELVGIERGLEILANLVCFESFNNVRVVGISGMGGIGKTTLARALYEK
IYHQYDFHCFIDDVSKIYRDSSSLGVQKQLISQFLNEKNLEISNSFEGTCLMWSRLHNVKALVVLDNIDEVEQLRIFTGNR
DTLLRECLGGGSIIIVVSRDEHILRTHGVDDIYQVQPLNYEYAMQLFCRNAFKVNHILSDYEKLAWDILSYAQGHPLAIKVI
GSSLFRRNVSQWESALARLKEKKSKHIMDVLRISFDQLDXEDKQIFLDIACALYYYDEKYVTEVLKFRGFHPEYGLQVLHD
XSLISKMGRCIYMHNLLKDLGXXIVKEESPKEPLKRSRLCNYQDFLKAMSKNQVLPNLKRLDLSYSKELVEMPDVAEALN
LEGIDLEGCIQLRKLSPSIGLLSKLTTLNLKNCENLVSLPNSILSLNSLEYLSVYGCSNLFNNELLDEASNTEHLKKLCSLKGPIE
SHSTSPLIKKARRESVSCLLPSSPTLSCLRELDLRFCNLVKIPDAIGKLSCLEKLNLKGNNFVTLPNLKDLFRLYYLNLEDCKRL
KYLPVLPSRTHLPLNLYSYLLQYFEDFIYQVPNEDEFMAGLTMFNRPKIVERERCTSMTVSWMLQIIQVCMFLPYTYHLLI
SFGVEKQLLMHNITQSWYNSEYLSFSSSLRSIIPGSEIPMWFNNQLLSMDNSIIIDVSPFVHDNNWVGVVCCAILGENLY
RTRILEDLRRDLFVEHSDHMCLFYYSRQQFCHEEGIFWGKLNTHDLSLTFKLHDVIILCANMKDRDEGFYEFEVKKYGYR
WVKKQDLQ

>XP_022634346.1

MEEAERWREALKEVADLSGWDIRNKSQYAQIEEIVQNITNILGPKICSLPKDELVGIERGLEILANLVCFESFNNVRVVGIS
GMGGIGKTTLARALYEKIYHQYDFHCFIDDVSKIYRDSSSLGVQKQLISQFLNEKNLEISNSFEGTCLMWSRLHNVKALV
VLDNIDEVEQLRIFTGNRDTLLRECLGGGSIIIVVSRDEHILRTHGVDDIYQVQPLNYEYAMQLFCRNAFKVNHILSDYEKL
AWDILSYAQGHPLAIKVIGSSLFRRNVSQWESALARLKEKKSKHIMDVLRISFDQLDXEDKQIFLDIACALYYYDEKYVTE
VLKFRGFHPEYGLQVLHDKSLISKMGRCIYMHNLLKDLGRYIVKEESPKEPLKRSRLCNYQDFLKAMSKNQTTEILEVVAV
NSYESSKTVRIDGLSKIKHLKFLKLVGVNCSGSLSHLSSELGYLTWNKYPFECLPQSFQPHKLVKLKLRESSIQRLWSDTKVL
PNLKLLDLSYSKELVEMPDVAEALNVEEIVLEGCIQLRKLSPSIGLLSKLTILNLKDCENLVSLPNSILGLNSLEYLSVYGCSNL
FNNELLDEASNTEHLKKLCSVEGPIHSHSIFPSMKKARRESVSCLLPSSPILPCLRELDLRFCNLVKIPDAIGKLSCLEKLNLK
GNNFVTLPNLKDLFRLYYLNLQCCKRLKYLPDLPSRTHLPLNLYSYPMQYIEDYFRIEDRKDYPGLMMFNCPEIVERERCT
SMTISWMLQILQVCMFLPYTYLLLIYFWC

>XP_022634347.1

MSSNAIVQYTASSSHAINRYDVFVSFRGEDTRNNFTGFLFQALRRKGIHAFKDDEDLKKGESIAPELLHAIQSSRLFIIVFS
KNYASSTWCLRELAEIRNCVQTSPRPVIPVFYDVDPSVVRKQSECYEKAFAEHEKRFRENKAKMEEAERWREALKEVAD
LSGWDIRNKSQYAQIEEIVQNITNILGPKICSLPKDELVGIERGLEILANLVCFESFNNVRVVGISGMGGIGKTTLARALYEK
IYHQYDFHCFIDDVSKIYRDSSSLGVQKQLISQFLNEKNLEISNSFEGTCLMWSRLHNVKALVVLDNIDEVEQLRIFTGNR
DTLLRECLGGGSIIIVVSRDEHILRTHGVDDIYQVQPLNYEYAMQLFCRNAFKVNHILSDYEKLAWDILSYAQGHPLAIKVI
GSSLFRRNVSQWESALARLKEKKSKHIMDVLRISFDQLDXEDKQIFLDIACALYYYDEKYVTEVLKFRGFHPEYGLQVLHD
KSLISKMGRCIYMHNLLKDLGRYIVKEESPKEPLKRSRLCNYQDFLKAMSKNQVKLIDSIIILRCYFFRSSKTVRIDGLSKIKH
LKFLKLVGVNCSGSLSHLSSELGYLTWNKYPFECLPQSFQPHKLVKLKLRESSIQRLWSDTKVLPNLKLLDLSYSKELVEMP

DVAEALNVEEIVLEGCIQLRKLSPSIGLLSKLTILNLKDCENLVSLPNSILGLNSLEYLSVYGCSNLFNNELLDEASNTEHLKKL
CSVEGPIHSHSIFPSMKKARRESVSCLLPSSPILPCLRELDLRFCNLVKIPDAIGKLSCLEKLNLKGNNFVTLPNLKDLFRLYYL
NLQCCKRLKYLPDLPSRTHLPLNLYSYPMQYIEDYFRIEDRKDYPGLMMFNCPEIVERERCTSMTISWMLQILQSWYKSE
CLSFSSSLRSIIPGSEIPMWFNNQLLSMDNSIIIDVSPFVHDNNLIGVVCCAILSEDFYRTRISVEHSDHVCLFYYSRQQFCD
KEGIFRGKLNTHDLSLKFKLHDVVILCAKMKDRDEGCYEFEVKKYGYRWVKKQDLH

>XP_022634385.1

MASNKRQRSSSSHTKNFDVFVSFRGADTRNGFTNHLFAALERNGVVAFRDDQTIQKGNFLESELLLAIEGSRVFIVVFSK
DYASSTWCMKELTKIVDWVEVTGRSLLPIFYDVTPSEVRKQSGEFAKAFAEHEERFKDDLEMVKGWRAALKTSCDRCG
WDVQNKQQYEQIENVVEEVINILGRNQIWNFGDDLVDMHSRVKKLEELLDLSANDIVHLVGICGMGGIGKTTLATALF
NKISPQFDACCYLDDLSKIYCNFGAASAQKQLLCRALNRGNIEIHNASHGTMLIRTRLCHLKALVVVDNVDQVEQLKKLG
LQSEYLGAGSRIIIISRNRRILQNYGVNKVYEVQVLDXTXSLQLLXKKAFRSNDIGKEHEXLTLDILKYVNGLPLAIEVLGSFL
LDRDVCEWRSALTRMEENPSKDIMDVLRISYDGLENIEKEIFLDIACFFSNNNLYSWEPTVKKLLDYRQFYSDIGMKVLIE
KSLIRCQDETIIMHDLLKELGKSIVREKAPKEPRKWSRLWNYKDLQKVTKINKEAEKVEAILIEQHEKEFLQGRIRVDALSK
MDHLEMLILQNVNCYGTLNFISNELRYLFWNHFPWLSLPSTFFPDQLVELILRDSNIKQLWEGKKVYLVPRMEIVIPGSEI
PKWFSKQNASASISMDPSAVIDDPNWIGVSICVLFVTHESPMNLGEGDYLIDSPLFYGVNNVNFETKWYSGVPIIFKKDL
VTVGLDYLLIVFYSRQEFFHLLNGHSDTMHDLQALKFETWGRSLLDLRFMVKKCGYRWVFKEDLQQLNSDKFFSRNSA
SRKRKLLTSE

>XP_022634386.1

MACNKSQRSSSSHTKNFDVFVSFRGADTRNGFTNHLFAALQRKGVVAFRDDQTIQKGDFLESELLLAIEGSRVFIVVFSK
DYASSTWCMKELTKIVDWVEVTGRSLLPIFYDVTPSEVRKQSGEFAKAFAEHEERFKDDLEMVKGWRAALKTSCDRCG
WDVQNKQQYEEIENVVEEVINILGRNQIWNFGDDLVDMHSRVKKLEELLDLSANDIVHLVGICGMGGIGKSTLATALF
NKISPQFDACCYLDDLSKIYCNFGAASAQKQLLCQALNQGNIEIHNASHGTMLLRTRLCHLKALVVVDNVDQVDQLKKL
GLQSEYLGAGSRIIIISRNRRILQNYGVNKVYEVQVLDXTXSLQLLXKKAFRSNDIGKEHEXLTLDILKYVNGLPLAIEVLGSF
LLDRDVCEWRSALTRMEENPSKDIMDVLRISYDGLENIEKEIFLDIACFFSKNIFYPLEPTVKKLLDYRQFYPDIGLKVLIEKS
LIRCQDQIIKMHDLLKELGKSIVREKAPKEPRKWSRLWNYKDLQKVMKINKEAENVEAIFIEQYELQGRIRVDALSKMDH
LELLILQNVNCYGTLNFISNELRYLFWNHFPWLSLPSTFFPDQLVELILPHSNIKQLWEGKKCLPNLRNLDLRHSKNLIEVP
DLSEVPRLTDLNLKGCIQLVHIHPSIGILRDLRCLNLKNCKNLVLNFGISSLNSKMLMDPSDTKHLEKVDKNTNIIQLPTSS
VYKLLMLPFHFFYPPKPQDSIGSLLSSFSFSVPCLFNLDISFCNLLQIPDEIGNLRSLEILNLGGNKFVTLPSTIKQLSYLHYLN
LTHCKELKYLPELPTIQEKTIGRYSNQLSTFDCPKLSDLEHCYSIVFSWMTKNLEMEIVVPGSEIPKWFSKQKASTSISMDP
SAVIDDPNWIGVSICVLFVTHEGPMNLGERGYPTDTLFYGVNNVNFERKLYSEFLIILKKDLVTVGLDYLLIVFYSRQEFIHL
LNGHSDTMHDLQALKFETLVEIYPGLHESHPGLHFMVKKCGYRWVFKEDVQQFNSDKFFSRNSSSRKRKLLTSE

>XP_022634387.1

MSSNAIVQYRTSSSHAIQTYDVFVSFRGEDTRNNFTGFLFEALRRKGIDAFKDDEDLKKGESIAPELLHAIQGSRLFIVVFS
KNYASSTWCLRELAEIRNCVETSPRRVIPVFYDVDPSVVRKQSECYEKAFAEYEKRFRDDQAKMEEAQRWRETLTKMA
NLSGWDIQNKPQYVQIEEIVQNVTNILGPKICSLQKDELVGIEYGLENLANLVCYESVNNVQVVGISGMGGIGKTTLVRA
LYERIYHQYDFHCFIDDVSKIYRDSSSLGVQKQLISQSLNEKYLEISNSFEGTCLMWSRLHNVRALVVLDNVDEVEQLKMF
TGNRDTLLHECLGGGSIIIIVSRDEHILRTHGVDDIYRVPPLNNENAMQLLCRKAFKLNYILSDYERLAFDVVSHAQGHPL
AIEVIGSSLFGRNVSQWKSAVARLKGIKNKNIMDVLRISFDQLDEEDKEIFLDIACALCKHDEKYVKEVLNFRGFHPEYSL
QVLHDKSLIIKEDGHIYMHRLLNDLGRCIVKEESHKEPLKRSRLCDYQDFHKAMSNNQTTEILEVVAVDLQWSSKPMRV
NGLSKIRHLKLLRLENTLLCSDVNFSGSLSHLSNELGYLTWYKYPFECLPQSFQPHKLVELKLRYSSIRRLWSDTKVLPNLKR
LYLSNSKKLVEMPDVAEALNLEEIDLEGCIQLRKLSPSIGFLRKLTILNLENCKNLVSLPNSILGLKSLEYLNVSGCSNLFNNE

LLDQARNTEHLEKLCLVKGLIHSHSTSPSIKKMLWWTLDLLYSRAQRELPSSPTLPCLRELDLRFCNLVTIPDAIGKLRCLEK
LNLKGNNFVILPNLRNLFRLYYLNLQHCKHLKYLPDLPSRIHLPLKVYEISREYFASVYIITLEEDEEKAGLLIFNCPEIVKRKR
CTSMSVLWMLQIVQVCIFLLYPFPSFNLFWCWKTITNI

>XP_022634653.1

MNSLRAKRKPSLRVLLKSVIYGRDDDKAFVFNWLTSDTHSNLSILSIVGMGGLGKTSLAQHVFNDPRLEEANFDTKVWV
SVPQEFDVLKVSRAILDTIIGSTYHSMQQEVIQKRLKEELMGKKFLLVLDDVWNERPSKWEDVQKPLVFGGQGSRILVT
TRSEKVADIMRSEKRLLQVLEDDYCWDLFAKHAFQNANPQPDSDFIEIGKKIVEKCNGLPLALKTMGSLLYNKSSFSEWK
SIMKSEIWDFSENESDILPALKLSYFHLPSHLKKCFAFCALFPKGDWFDKEWLIQLWMAQNFLENPLQKKSPEEVGEEYC
NDLLSWSFFQPSSNEKYFMMHDLLNDLAKYVCENICIRLGVDEPKGIPKTTRHCSFPYSKLGFDGFGSSIDTQKLHTFTPT
TRIWYWDCKMSIDDLFSRFKFIRVLSLSHCDSLTKVPKSVGNLKHLRSLDLSRTQIEKLPDSISLLYKLQILLLNYCKNLKELP
SCLYQLDNLRRLDLEGSGVQNVVAHLGKVKNLQVAMSSFHVEKIKENNFQQLGELDLHGSLTIDDLQNIENPFYALEVD
LKNKPHLEELLLEWNFIGSSSVDSEKAEDVIENLRPSKYLKKLSIKNYIGKQFPDWLLHNSLPNLVWLKLEGCESCQRLPPL
GFFLFLKYLTIARLDEIVSIGADFHGNNSSSFKSLQELYFYDMRQWEKWDCQAVTGAFPCLQYFSIRNCPKLKGHLPKFV
ALKTLRVIHCQQLEELSVCSAPESISDDCVSLRIFQLDFFPTLRDLTLRGFPNLQMISQTYDQNHLEYLKIEECPKLESLPAN
MHMLLPSLRELHIKECPRLESFSDGGLPLNLKFIRLDYCFRLVGSLKRVLEDHPSLLKTLMIGKVEAECFPDEGLLPLCLSEL
RIYDCRNLKKLNYKGLLELSSLETLCLWKCPNLECLPEEGLPKSISRLGIEKCPLLEQRYHKEGGEDWEKVSHVPQLFIYEND
FV

>XP_022634858.1

MTYSHVDASGVIGRESDREKIIKLLMQHHPNGDGYGDQSICVIPIVGIGGLGKTTLAKLVFNDKRMDDLFQLKMWVCIS
DDFDIRQILIKIINSASASAPTIALAHQENIKNFDIEQLQSRLRHRLSGQKYLLVLDDVWNDNRPKWTQLKDLIKVGAVGS
KILVTTRSNSIASMMGTVPSYALEGLSLENCYALFNKWAFKEGEEKKYSNLVEIGKEIVKKCIGVPLAVRTLGSSLFLNFNL
ERWEFVRDHGIWNLKQEKDDILPALKLSYDQMPSYLRHCFAFFSLYPKDYGFTGAKVVNLWVSLGLLRSEVGSQKLENA
ARLYIDELHSRSFLEDFEDFGNIYYFKVHDLVHDLAQYVAKEEILVVDSGTRNIPEQVRHLSVVENSLRDALFPKSSRVRTIL
FPTDGVGVDSVALLDAWIARYKFLRILDLSDSSFETFPNSIAKLEHLRALSLENNRRIKRLPQSICKLQNLQVLTLRGCVELE
TLPKGFGMLISLRQLSITTKQSILSEDEFASLINLNTLSFEFCDNLMFLFRGTQQLKSLEVLIVQSCRSLESFPFHILPKLEVLF
VIRCEMLNLSLNCENPIERLRMKFLHIEECPRQQTLPQWIEGAAETLQTLLISNLHSLEMLPEWLTSMTHLKMLHIVNCP
QLLYLPSDMHRLGALEDLTIDGCSELCQKCEPQSGEYWSFIAHIKHLSIGETREGKLLFQMQKQIRLKLQDLQN

>XP_022634859.1

MTYSHVDASGVIGRESDREQIIKLLMQPHPNGDGYGDQSVCVIPIVGIGGLGKTTLAKLVFSDERMDGLFQLKMWVCX
SDDFDIRHILIKIINSASDPTISVVPQESMNNXDIEQLQGRLRHKLSXQKYLLVLDDVWNDNRAKWIELKDLIKVGAVGSK
ILVTTRSTXIASMMGTVPSYVLXGLSVENCXSLFLKWAFREGEEKEHPYLVDXGKEIVKKCRGVPLAVKTSGSSLFSVFDS
QRWEIMRDHELWNLKQQKDDILPSLKLSYDQMPSYLRHCFAFFSLYPKDFGFTSAEXANFWATLGLLRSPFGSQKIENI
GKAYINELYSRSFLEDFEDFGTIYYFKLHDLVHDLSLYVAKEEFLMVNSNSRNIPEQVRHMSVVENDSLSHSLFPKSRCVR
TIIFPVNGVGVGSESLLETWIERYKYLRHLDLSNSSFEKLPNSIAQLEHLRALSLDNNSKIKKLPNSICRLQNLQMLSLRRCL
GLETLPKRLGMLISLRKMYITTKQSILSEDEFASLINLHTLIFEYCDNLKFLFRGAQVQLSSLEVLIIQSCGSLESLPLHILPKLE
VLIVTRCVMLNLSLNSERPIQKLKMKYLHIEHCPRQHTLPEWIQAASDSLRTLLILNFHCLEMLPEWLSTMTSLKMLHIV
NCPQLFHLPCDMHCLSALEDLIIDGCPELGRKCEPHSGEYWSFIAHIKCVSIGETRKRKLLFQMLSRLGLNCT

>XP_022635009.1

MATPSFRRFTYDVFLSFRGEDTRYGFTGNLYKTLCDRGVHTFMDDDKLQTGEEITPALQKAIEESRIAIVVLSHNYASSSF
CLDELATILDCKSKWLLVIPVFYKVDPSYVRHQKGSYEEALAKHQKRFKGQEEKLHKWKMALRQVADLSGYHFGHGDE

YQYKFIGSIVDRVSRVINRAPLHVADYPVGLLSHVLKVKKLLDVGSDDVVHMIGICGMGGLGKTTISLAVYNLIADDFDSS
CFLQNVREESNKHGLKHLQTVLLSEILGEKDISLASVQRGISVIQQRLRRKKVLLILDDVDNRKQLQAFAGRSDWFGPGS
RVIITTRNEQLLKSHEIQRTYEVEELNENDSLQLLVWNAFKRXK

>XP_022635041.1

MVIITRDKHLLDIHGVEKQYEVDIFNVTEAMKLFRWNALRNKVVDPSCTEIIKREIYYTGGLPLALETIGSNLLGKILDEWK
SALDACEKLPSRGIQEILQVSYDGLEENEKDIFLDIACFFKGCTVKKVTDMLNACGFHTEYGISVLRNKSLVNILMSGEHD
GEIVTMHDLIQCMGKEIVRQRSTLTEKCSRLWFYQDISCVLEKNMAIDSSTRKFYVLPGERVPKWFDHSTKGNSLCFWF
WRDFPSITVCTILGVLDNIEGPFFVKFNFFVTINDIEIPFLFDFNYILDTDHMFMFNNFTYPMKLDCGGLVSENEWSYGE
VLSVIPPNSGSSESIKSSGVYVNRTCTTTMENVRFSNPYSPNTTFPTDHGTRNQLDIQQDSFDPVVSMHTRMLDKLAYL
SNRQWRRPFMYPEWKEPFDNVLSPNLSESDLSMSS

>XP_022635184.1

MQCLIALATGTLTKLGESLVAPIGNQFGYLVHYKKNIKDLKRELKTLEGRKQGVQGVVDEDRRNGRQIVSIVQDWLFKV
ERIIDEIEKFNDFQVENNKCLDIWSPNLVSRYSLSKKAKILMMSVTKLNEEKFDIISYSLPTPKLGSTFSNVIKSFPSRKSTITE
ILETLKDDDFKILGICGMGGVGKTTLVKEIIKILEICKLFDEVVMVVVSQNLDYVKIQGQIADALGLRFEKETIQGRACQLH
ERLKGVNNILIVLDDVWIDFDFESIGIPSYEHHKNCKILFTSRNEDVCYKMGSQKNFTISILSPNESWDLFHDMVGRNLST
KLDILHIAKEVSNECGGLPIAIVTMAKALANKEKHVWEDALDQLKRSSITSLLEMKASVYSSIKLSYNFLDTAEEKNVLFLC
CLFAEDFEIPIEVLLRHGMGLQLFNNNINELWKVRNRVHTIVDKLKRRFMLLDGNVEECVKMHDVIRDVIISVVSTEECD
FMVQCDGYQMEQSKEETCYHSTVISLISEEAKEHCKVLNCPKLKLLQIASKKKGLVPDNFFQCVNNLMVLSLQNVHIHS
MSLVVQALGNIHTLLLEDCQVRDISIIGKQLKRLEILSFSNSNIKELPEEIGQLSSLRLLDLTECNDLTQISSNVFASLSRLEEL
YIRVRSLSYKETNHILFELQSLSHHLKVIEIVILTNEELPIDLFFNNIERFWIYLGDSSTIFHGIVRKGYLHPNILKLNNAYYKYIK
RSVTIRQLLEKVEILNLVAIKNMKSVILELNERGFPFLKHLSIEFCNNLEYVVDAVDGLIFPQLLSISLSNLDNLKEIFNVSSY
MSQTSVFAKVKSISSSQCFGTLTKLKIEYCNKLKTVFTLFPRTISLATLQCLHVVESYGIECIISSNSKIDEKSMIEFSNLVELKL
QDLPNLIGYTNVIHDRCSSTIQIHESSDQLIEDGQLLSSKYVDQIVPIATLFESNCMQLFPKLEKLFLHACSSLDMVFDLQK
SQFHGESTAFLFPQLKEIEISWLSKLRHIWGNVPSYIQGFQNVKSIKVKKCDSLGFLLTPNIARALTQLQKMVIHSCQSME
KIVGKEENLNGDDEEKNVETLVFGQLESLKLIDLPNLMSICSDSYEVMWPSLRFLCIDGCPQLVTSSMFTQTVARQENFN
GSSCCSTTCDVANGTFKEDSPKFLQCCLGCTPHVFSNLKFKASSTEKVPSVSNIYSKSETVSSIPILEQMQVKGWDSLQVL
FLLKQNQLSNDSNTNCLVKLILAQTPKSSVEITAFNNLTVLTIDSCHKLRYLFSYSIAKLLVKLQEIKVSNCKVIKQLVQREGE
DSLTLFLPQSSSVKVFENSSSTDHATSSQEVCALEWSSLKRISISHCGVLEVVIGEIGEKIDTIIASFAQLQSLTLSHLPNAVSF
CLSHCASESPSFENIHGTGYQNHEATNNEEMRIMRVDPLINGFIFPNLTYLAISSCNKVGNLFSPSTSTSFVRLVELDISSC
REIEEIVSAEETQGNVIKIVFHSLQRLKLENLPKLKAFCQGSYGFDFPSLHQVLIKNCQMMETFSHDPLYTPKLETVTMEIG
SITKNMWMGDLNDTVPLCKGLLAFQTSETLGWIKQDTCIQRYFTHEKHLTVESFQRLLKLVPSNVIHIFQNLKELTIKNC
GSLVEVFESHGVDAKQMHAMVHYKLEALNLYFLPKLINLWKNYGGVLGFQKLRILNVQHCGNLCNLFPPSIARSLVQLR
HLRVHSCHMMEEITTKDEELEGANNAKIVFPLLNKLELRYVPNLKCFSSGNFNIDLPSCEEMIIEKCPKMTTFCYGSVTAA
KLPHIYKGSYEYVDIMGDLNMTIYHANESLKVAQQTSETITFIEHGQELQPYLRSDTELVVQGNEKLLHCIPSNMLHRFQ
HLKQLKVYDCGSLVEIFESEEVDESEDEGGTTTPYNFDVQELHLYDLPKLMHIWKYHSRILSFMNLRKLKIQHCHSLKNVL
SPEMARSLSQLQELSVHECELMEEIISRDEKLSEEPNKVKIIFQALQWLTLYRLPSLRCFCSSTYHFELPSCHDITITECPKM
EACHGNRGTSELPSASFKTNGLWREI

>XP_022635602.1

MDPVVSTATESALIITANVVKRQVGYFFNYKEKFKKLESYIEKLEHNKVRLQHQVDSALRSGEEIENDVQHCLKLMDEKIK
EYKSYINDDCHAKTICSIGFFPNNFRLRYQLGRKATKMVEEITGGELWKTSFDNVSYQEFPSVDATFSNNGYESFASRTQ
TMEMIMKALQDSTVGLIGVYGPGGVGKTTLVKEIANKVREKNLFEIVIIANITGNPDFKKIQEQIAGMLGMQLEEESEIA

RVDRIRKRLKNEKENTLIILDDLWGRLDFNKLGIPCNDDASQQEVNDMSDFGSNNDISDFGYNKKEMKELSKVDLTKM
KKEKLSNDYRGGKILLTSRYKQVLCNEMDVQESSIFSVGVLDEKESETLLKKVAGVKNSEFDRNATEIAKWSAGFPIALISI
GRTLKNKSLSTWEDVCQQIKRQNFTSEWGFTDFSIKLSYDHLKNEELKCIFLHCARMGNDALIMDLVKFCVGLNLLPRV
HTLTDARKRVKEMIQELEESSLLVKSYSIDRFNMHDIVRDVALSILSKEKDVLYMKNEILDEWPHENDFERYTAIFLHSCDI
KDELPKRLHCPRIEVLHIENDTESFKIPDYFFKFMIRLKVLVLTGVNLSRLPSSIKCLKKLRMLCLERCTLGKNLSIIGELNNLR
ILSLSGSNIQNLPPEFGQLDKLQFLDISNCLKLHITSNIIPRMGILEEFYIRDNLIIWEAEENMKSENASLSELRHLNQLQNL
DIRIHSSSHFPQNLFFDRLNSYKIVIGEFNLFNLIKVGEFKVPDKYEEVKFLALNLKEGIDIHSEKWIKMLFKSVECLLLGDLN
DVEDIFYELNVEGFPNLKHLSIVSNFGIKYIINPRERFHSLVAFPKLESIWLYKLDNLEIICDNQLVETSFRNLKVIKIQTCIKLI
NLFPFCIVRLLTKLETIEVCDCDSLKEIVSKESQTHTISNDKIEFPQLRILTLKYLPTFIYLYNADKIPGSAHSLQDQVFQQRN
QDIVVDVEHRVTNSCLPLFNEKVSTPKLEWLELSSINICKIWSDQYNHCFQNLLTLNVTDCGNLKYLLSFSMARSLVNLQ
SILVSECEMMEDIFRPEDAEYIDVFPKLKKMEIICMEKLSTIWKSDIGLHSFSNLNSLIIRECHKLVTIFPNFMGQRLQSLQS
FAIKDCELVENIFDFTHILHTFDIVEANLGNIILEDLPNLVNVWKGDTGEILKCNNLQSIKVDESPKLKYLFPVSIANDLQKL
EVLEVRNCWAMTEIIGLDKHSSESAITFKFPHLNTLSLIDLHDLRSFYSGIHTLEWPPLKKLEIVDCSMLQGLTSEITNSSEQ
PIVLATKKAIYNLEYMSVSLKEAKWLQKYIVNVHRMHKLEELTLYGLNNNKIFFWFLHRIPNLKSLTLGHCHMKRIWTPK
SLSSCEKIGGVMQLKELELRSMWSLEEIGFEHDVLLQRVERLIIRGCKKLRNLASSLVSFSCLTSLEVVNCMMRNLMTLST
AKTLNQLTTMKVSSCPLILEIVAENEEENIQEVEFKQLKSLELVSLQNLTSFSSVEKCDLKFPLLEKLVVSECPKMTKLSEVQ
SAPNLQKVHVEAGEKDKWYWEGDLNGTLQTHFTNQVSFEYSKDIKLVDYPERKVRRGKFAFPENFFGCLKKLEFDEGC
KRDTLIPSHVLPYLKNLEELNVEKCESAQLIFDIDESEIQTYGMVFHLEKLTLKNLSNLKCVWKKNLEGIVSFYNLETVHVD
SCGSLVTLFPLSLGKNLGKLKTLKIKECEKMEEIVGREDEIEHGTTIMFEFPCLSCLNLEKMPLLSCFYPGKHHLECLLLDRLY
VAWCPKLKVFRSSFDDDSKKEVLKAPSNLLQQPLFSIEKVSLKLEGVTLNEENIKVMSDARLPQDLLCKLKILILSFEDDNN
GKDNLPFDFFHKVPNLENLIVEKCFGLKEIFPSQKLQVHDKVMAGLKRLLLSELSELECIGLEHSWVQPYSKKLELLSLYSC
PLVENIVSCAVSFINLKDLYVIDCERMEYLFTFTTLKSLVKLETLSIGYCGSIKEIARNEEEDGCDEIIFGRLRSIKLEYLPKLISF
YSGNATLQCPYLQIVMVAECPNMITFSEGVIKLAMFSGIQTSEDSDFTFDVDLNTTVESLFHEKEFFNHSKHMIVDEYLE
MTEVQHIKPAISDNFFGSFKELEFDAACKRTVVIPFHVLPYLKNLEELNVHSSDAVKVIFDIDENEDKTKGIVSSLKKLTLNK
LSNLKHVWKENSTGIISFHNLQEVVVNDCGSLITLFSSSLARNLGKLKKLEITDCGKLVEIAEKEDGMEHGTKIMFELPCLS
SLFLVNMPLLSCFYPDKHDLDCPSLEALFVGYCPKLKLFISDIDENQKGAIEAQISPLQQPMFSVEKEIFPSQKLQVHDREL
VGLKELFLLDLRELELVGLEHPWVKPYSEKLQVLKLHNCPQLQKLVHCAVSFINLKELRVEDCERMEYLFTFATVKSLVKLE
TLIINSCESMKEIIKDENEDGCAEMVFGQLKSIKLESLPRLVRFYSGKATLQCSYLKMVMVVECPSMITFSEGVIKVPMLS
GIQTSKDSKLIFHEDLNTTIEKLFHQEEFMEYSKRMILEDYLGMSGVYHTKPIVSDNFFGSFKKLEFDAACNRTILIPSHVLP
YLKNLQELNVKKSDAMQIIFDINECEVKTKGVVFGLKKITLKNLSNLKHIWKENSSGIVSFQNLQKVNVNGCGSLVTLFPL
SLAKNLGKLETLDIKKCEKMVEIVGREDEMEHGTTIMFAFPCLSYLYLEMMPLLSCFYPGKHHLECPLLDKLYLECCPKMK
LFRSSFDDDSKKEVLETPINLLQQPLFSIEKVSPKPEGLTLNEENIKLMSDARLPEDLLCKLKYLILSFEDDNNGKDSLPFDFF
HKLPNLEWLTVRKCFGLKEIFPSQKFQVHDKVLAGLKGLCLLELKELKCIGLEHTWVQPYSKKLELLRLHNCPGVESIVSCA
VSFINLKELYVKLCEKMEYLFTFATLKSLVKLETLSIKKCESIKEVVKKDDEDGCDEIVFGKLRSIKLNSLPKLVSFYSGNATLQ
CSYLKNVMVAECPNMITFSQGLIKVSMFLEIQTSKDSDSIFDGDLNTTIQKLFHNQLAVIAALHSQRWRNNDDTSIMNK
SLF

>XP_022635603.1

MEVVVSTTTESVLQIATRVMKQQLSYFFNYNDKLDEVKCYIEMLDNTRKRIQHQVNNAEMNAEEIEDDVQHCLKLLDE
KIKTYENFIHDEHHSKTRCSIGFFPNNLQLRYRLGRKATKMVEEMKAGELWNKRFDEVSYRVLPSIDVALTNTIYENFASR
TKTMDMVMQALEDCTVNMIGIYGVGGVGKTTLVKEVAKIAQEKKLFNVVVMANIARIPNIIKIQGQIAEMLGMRLEEE
SEIVRADRIRKRLTKTKENTLIILDDLWDGLDLNRLGIPCSDEDDGSQHDVNDIPNSSYDKMEKEELSSDLIEEKLSDNQKR
CKILLTSRRKQVLCNKMDVQEKSTFSVGVLPENEAKTLLKKVAGIHDQNLVYDEKALEIARMCDGLPIALVSIGKTLKNKS
SFVWVDVYQQMKKQSFVDGKEPIEFSIKLSYDHLENEQLKCIFLHCARMGNDALVMDVVKFCIGLGLLQGVHTIREAR

NKVTMLIEELKESSLVLESYSSYCFNMHDIVRDVALLISSKEKHIFFMKNGILDEWPHKDQLERYTAIVLQYCYINDDLPESI
YCPRLEVLHIDNKDHFLKIPDDFFRGMIELRVLILNGLSFPCLPSSIMCLTKLRMLTLKKCTIGQNLSIIGKLKKLRILNLSGSN
IECFPFEFGQLVKLQLLDLSNCSKLKVIPSNVISKMNSLEEFYMQDSLIQWETEENIQSQNGSLCELRHLNQLRNLDIHIQ
NVVHVPRNFFLDELDSYKIIIGESKMLTEGDFKIPNKYEMVKLLALNLKEGIAIHTETWIKMLLKDVEYLWLGELIDVHDVF
YELNVEGFQKLKHLSIVNNIGLQYIINSVERFHPLLAFPKLESLYLYKLYNLKKLCNNQLLEASFYRLKTIKIKSCDKLESLFPFS
MVKHLTMLETIEVCDCDSLMDMVSVGTQTHTNNGDNIEFPQLRLLTLKSLHVFTCLYTNDKIPCAQPFEDKGQSISKDII
TGVYQDGTNSCLSLFNEKVSIPKLEWLELSSTNIQKIWSDQSQHCFQNLLTLNVTDCGNLKYLVSFSMAGHLVNLQTLSV
SECEMMEDIFRPEEVEGTIDYMFPKLKKMEITCMEKLNTIWQPQFGLHSFCGLDSLIIRECHKLDTIFPSFMGQRFQSLQ
SLTITNCKSVENIFVFANIPQTCDRNETNLHKIIIQGLPNLVSIWKHGTGEILKHNNLQRITVSGCPNLKYVFPLSITNDLEN
LEFLEVRNCRTMKEIVAWDRDSNKNDITFKFAKLENVSLQSLFELVSFYGGTHTLEWPSLKKLSILRCGKLEGITTKISNSQ
AKPIVLATEKVIYNLEHMAMSFREVEWLQNYIVNVHRMLNLQSIALHGLKNAEILFWFLHRIPNLKRLTVGLCHLRRIW
GPATHISQEKIGVVMQLKELVLKSMWSLEEIGFEHEVLLQRVQRLIILRCTKLKNLASSTVSFNRLTYLEVANCMMKNLM
TYSTAKTLVQLTTMKVSSCPMIAVIVAKNEGENVQEIEFKQLRSLELVSLPNLTSFLSVDKCVLKFSLLENLVVSECPQMTK
FSEVLSTPNLQKVHAVTGEKDKWYWEGDLNATLQKHFPHQVFFKYSKHMKLKDYPEMKEVRYGKPVFSDNFFGSLKK
LEFDVASKRDIVIPSHVLPYLKNLEELKVERCKPAEVIFDLYESETKTIVFQLKKLTLKDLSNLKCVWNKNPKGIINFPNLEEV
FIYECETLATLFPLTLAKNLGNLKTLTIHKCFKLIEIVEKKEETERGTIETFEFPRLLKLFLWNLPQLNCFYSGQHHLKCPMLE
RLHVAYCHKVKLFKSGFQHSPLQHPMFSIEEVVPKLKELMLSEKNIILLNDRHSPQDLLHKINYLDISFEDHDSMKNTLPF
DFLHKVPNLENLVVRGYCGLKELFPSQKLDGHDGMLPELNKLSLQMVFELESIGLDHPWVKPYTEKLKVLAVGKCHRLE
RLVSCATSFIDLKQLVVKDCKRMKNLFTFPTAKSLMNLETLVIENCASIKEIIEKEDEDVNDEILLGRLSNLSLHSLPRLVSFY
SGNATLNFSSLQQVKLFNCPSMKTFCETNINAPMLYGIKSSTDDFNLDLFHDLNTTIESFFYEKDFFEYSKHTILLDYFEMR
GIGSVKQASQGKSFGNIKKLEFDGKSTGDTVIPSNVLSHLKSLEELNVHNSDEVQVIFGMNDSHTKTKETVFHLKILTLKD
LSNLKCIVSKNPQESVSFPNLQKLFVDSCGSLVTLIARNLGKLNTHEMQRYDKLVEIVGKEDAIENRTTEVLMFEFPCLSLL
TLYNLTNLSCFYPEKHHLECPKLEIMHVAYCPKLKLFTSKIHDSHKEAITEAPISCLQQPLFIVEKVVPKLKGLTLNEKNMML
FSDARMPQDYLSKLSLLRLCFEDDKNEKGTLPFDFLHRVPNLEHFRVQRCFGTKEIFSSKKLKVHDGIPATLNALTLFELNE
LESIGFEHPWVKPFSEKLQTLRVISCPWLEKLGRGALSFINLKELYVMDCGRIEYLFTFSTAKSLVLLETLIVRNCESIKEIAM
KEDEDDCDEIIFERLTTLTLNSLPRLQSFLAGNAIMQFSCLKNAYVINCPNMKTFSDGVLTAPRFLGIKTSYQDSDLFFHD
DLNTSFQRLFQTQVEKSACDTEHLKFGDHSHLQEIWLGVAPISTNNSFNNLKSLTVVECESFPNVIPFHLLPFLCNLKEIEV
SNCQSVKAIFDVNGEGADMKPISLPLKKLVLNQLPNLEHIWNLNPDEILSLQDLQQVSISNCQTLKSLFPTSVANHLVKL
HVRACATLVEIFAEADEAINGETKQFNFHCLTSLTLWELPELKYLYPGKHTLEWPMLTQLDIYHCDQLKLFKTEHHSDEF
AHTKDQLGISIHQQAAFSVEKVFPKLVQLSLKKEDAVAISQAQLQVMPSIEHQEITWKDTMIGQGQFGANVAHLLQNL
KLLKLMCYHEDDNSNIFSSGLLEEIPNIENLEVVCSSFTEIFCSQGPATDCSKVLSKLKRLHLKNLSQLNAIGLEHSWVEPLL
KTLETLEVFSCPTMKILVPSTLSFSNLTSLNVGECHGLIYVFTSSTAKRLGQLKNISIRDCQAIQEIVSKEEDHESEDEDEEITF
DQLSLLSLESLPNIIGIYSGTFKLKFPCLDQVTLKECPQMKYSYVPDLHEFKPQEQT

>XP_022635604.1

MEVVVSTTTESVLQIATRVMKQQLSYFFNYNDKLDEVKCYIEMLDNTRKRIQHQVNNAEMNAEEIEDDVQHCLKLLDE
KIKTYENFIHDEHHSKTRCSIGFFPNNLQLRYRLGRKATKMVEEMKAGELWNKRFDEVSYRVLPSIDVALTNTIYENFASR
TKTMDMVMQALEDCTVNMIGIYGVGGVGKTTLVKEVAKIAQEKKLFNVVVMANIARIPNIIKIQGQIAEMLGMRLEEE
SEIVRADRIRKRLTKTKENTLIILDDLWDGLDLNRLGIPCSDEDDGSQHDVNDIPNSSYDKMEKEELSSDLIEEKLSDNQKR
CKILLTSRRKQVLCNKMDVQEKSTFSVGVLPENEAKTLLKKVAGIHDQNLVYDEKALEIARMCDGLPIALVSIGKTLKNKS
SFVWVDVYQQMKKQSFVDGKEPIEFSIKLSYDHLENEQLKCIFLHCARMGNDALVMDVVKFCIGLGLLQGVHTIREAR
NKVTMLIEELKESSLVLESYSSYCFNMHDIVRDVALLISSKEKHIFFMKNGILDEWPHKDQLERYTAIVLQYCYINDDLPESI
YCPRLEVLHIDNKDHFLKIPDDFFRGMIELRVLILNGLSFPCLPSSIMCLTKLRMLTLKKCTIGQNLSIIGKLKKLRILNLSGSN
IECFPFEFGQLVKLQLLDLSNCSKLKVIPSNVISKMNSLEEFYMQDSLIQWETEENIQSQNGSLCELRHLNQLRNLDIHIQ

NVVHVPRNFFLDELDSYKIIIGESKMLTEGDFKIPNKYEMVKLLALNLKEGIAIHTETWIKMLLKDVEYLWLGELIDVHDVF
YELNVEGFQKLKHLSIVNNIGLQYIINSVERFHPLLAFPKLESLYLYKLYNLKKLCNNQLLEASFYRLKTIKIKSCDKLESLFPFS
MVKHLTMLETIEVCDCDSLMDMVSVGTQTHTNNGDNIEFPQLRLLTLKSLHVFTCLYTNDKIPCAQPFEDKGQSISKDII
TGVYQDGTNSCLSLFNEKVIYNLEHMAMSFREVEWLQNYIVNVHRMLNLQSIALHGLKNAEILFWFLHRIPNLKRLTVG
LCHLRRIWGPATHISQEKIGVVMQLKELVLKSMWSLEEIGFEHEVLLQRVQRLIILRCTKLKNLASSTVSFNRLTYLEVANC
MMKNLMTYSTAKTLVQLTTMKVSSCPMIAVIVAKNEGENVQEIEFKQLRSLELVSLPNLTSFLSVDKCVLKFSLLENLVVS
ECPQMTKFSEVLSTPNLQKVHAVTGEKDKWYWEGDLNATLQKHFPHQVFFKYSKHMKLKDYPEMKEVRYGKPVFSD
NFFGSLKKLEFDVASKRDIVIPSHVLPYLKNLEELKVERCKPAEVIFDLYESETKTIVFQLKKLTLKDLSNLKCVWNKNPKGII
NFPNLEEVFIYECETLATLFPLTLAKNLGNLKTLTIHKCFKLIEIVEKKEETERGTIETFEFPRLLKLFLWNLPQLNCFYSGQHH
LKCPMLERLHVAYCHKVKLFKSGFQHSPLQHPMFSIEEVVPKLKELMLSEKNIILLNDRHSPQDLLHKINYLDISFEDHDS
MKNTLPFDFLHKVPNLENLVVRGYCGLKELFPSQKLDGHDGMLPELNKLSLQMVFELESIGLDHPWVKPYTEKLKVLAV
GKCHRLERLVSCATSFIDLKQLVVKDCKRMKNLFTFPTAKSLMNLETLVIENCASIKEIIEKEDEDVNDEILLGRLSNLSLHS
LPRLVSFYSGNATLNFSSLQQVKLFNCPSMKTFCETNINAPMLYGIKSSTDDFNLDLFHDLNTTIESFFYEKDFFEYSKHTIL
LDYFEMRGIGSVKQASQGKSFGNIKKLEFDGKSTGDTVIPSNVLSHLKSLEELNVHNSDEVQVIFGMNDSHTKTKETVFH
LKILTLKDLSNLKCIVSKNPQESVSFPNLQKLFVDSCGSLVTLIARNLGKLNTHEMQRYDKLVEIVGKEDAIENRTTEVLMF
EFPCLSLLTLYNLTNLSCFYPEKHHLECPKLEIMHVAYCPKLKLFTSKIHDSHKEAITEAPISCLQQPLFIVEKVVPKLKGLTLN
EKNMMLFSDARMPQDYLSKLSLLRLCFEDDKNEKGTLPFDFLHRVPNLEHFRVQRCFGTKEIFSSKKLKVHDGIPATLNA
LTLFELNELESIGFEHPWVKPFSEKLQTLRVISCPWLEKLGRGALSFINLKELYVMDCGRIEYLFTFSTAKSLVLLETLIVRNC
ESIKEIAMKEDEDDCDEIIFERLTTLTLNSLPRLQSFLAGNAIMQFSCLKNAYVINCPNMKTFSDGVLTAPRFLGIKTSYQD
SDLFFHDDLNTSFQRLFQTQVEKSACDTEHLKFGDHSHLQEIWLGVAPISTNNSFNNLKSLTVVECESFPNVIPFHLLPFL
CNLKEIEVSNCQSVKAIFDVNGEGADMKPISLPLKKLVLNQLPNLEHIWNLNPDEILSLQDLQQVSISNCQTLKSLFPTSV
ANHLVKLHVRACATLVEIFAEADEAINGETKQFNFHCLTSLTLWELPELKYLYPGKHTLEWPMLTQLDIYHCDQLKLFKTE
HHSDEFAHTKDQLGISIHQQAAFSVEKVFPKLVQLSLKKEDAVAISQAQLQVMPSIEHQEITWKDTMIGQGQFGANVA
HLLQNLKLLKLMCYHEDDNSNIFSSGLLEEIPNIENLEVVCSSFTEIFCSQGPATDCSKVLSKLKRLHLKNLSQLNAIGLEHS
WVEPLLKTLETLEVFSCPTMKILVPSTLSFSNLTSLNVGECHGLIYVFTSSTAKRLGQLKNISIRDCQAIQEIVSKEEDHESED
EDEEITFDQLSLLSLESLPNIIGIYSGTFKLKFPCLDQVTLKECPQMKYSYVPDLHEFKPQEQT

>XP_022635605.1

MEAVVSTTTENALQIAGRVVKRQLSYFFYYNDKYEEVKRYVEMLDNTRKRIQHQVNNAEMNAEEIEDDVQHCLKQLD
EKIEKYEQFIHDEYHSKTRCSIGFFPSNLSLRYRLGRNATKMVEEMKVEELWNKTFEEVSYRVLPSINVSLTNISYESFASRT
KTIDMFMQALEDSTVNMIGLYGVGGVGKTTLVKEVAKKAQEKKLFTVVVMANITRNPNIIAIQGQIAEILGMRLEEESEI
VRADRIRKRLKKEKENTLIILDDLWDRLDLNRLGIPNSDEDDGSQQDVNDISNSGYHKMEKEGLSSDFNNMTEERLSGN
NKRCKILLTSRRKQVLCNQMDVKERSTFSIGVLDENEAKTLLKKVAGIHIQNFAYDDKAIEIARMCDGLPIALVSIGRTLKN
KSSFVWEDVYQQMKKQSYIEGKEPIEFSIKLSYDHLENELLKCIFLQCARMGNDALVMDLVKFCIGLGLLQGVHTIREAR
NKVNMLIEELKESSLLLESYSSNRFNMHDIVRDVALSISSKEKQVFFMKNSILDEWPHKNRLESYTAIFVHSCYIVDDLPGS
IYCPRLEVLQIESKDQFLKIPDDFFKDMIELRVLILTGLNLPCLPSSLIFLTKLRMLSLEKCILGQNLSIIGELKKLRILTLSGSNIE
CFPCEFGQLDKLQLLDLSSCSKLRLIPSNVISRMNILEEFYMRDSLIRWETEENIQSQNGSLCELSHLNQLRNLDIHIQNVV
CVPQNLFFDELDSYKIIIGEFNMLTEGEFKIPDKYEVVKLLVLNLKEGIDIHSEIWIKMLLKSVEYLWLGELINVHDVFYELN
VEGFLKLKHLSIVNNFGIQYIINSMEQLHPLLAFPNLESLYLYKLDNLEKICNNQLLEASFSRLIIIKIKSCGKLENIFPFSMVG
HLNKLETIEVCDCDSLENIISVESQTHINNGDNIEFPELRLLTLKSLHAFTSLYTNDKMPCAQSFEDEGRNINKDIITEIEQD
GTSSCHSLFNEKVLIPKLEWLELSSINIQKIWSDQSQHCFKNLLTLNVRDCGNLKYLLSFSMAKHLENLQSLFVGECEMM
EDIFCPEDVEGNIDCVFPKLKKMEIMCMEKLNTIWQSHIGLHSFSNLGYLIIRECHKLETIFPSFMRQRFQNLQSLTITNCK
LVENIFDFANISQTCDKSETNLHNIVLQGLPNLVSIWKDDTGEILKHNNLQSIKIIGSPNLKYVFPLSVTYDLENLESLEVFN
CRTMKEIVASDKGSNENVITFKFPHLKTVSLRSLFELVSFYGGTHTLEWPSLKDLSILRCGKLEGITTNISNSQAKPIVLATQ

KVIYNLEYMAMSLGEVEWLQKYIINVHKMHNLQSVVLHGLKNVEVLFWFLHRLPNLKRLIMRFCQMRRIWAPLTDISR
EKIGVVMQLKELELRDMWSLEEIGFEHDMLLQRVQHLIIERCTKLKTLASSSVSFRRLTYLEVVNCMMKNLMTYSTAKTL
DQLTTMKVSSCPMIVAIVEKNEEENVQEIEFKQLRSLELFALPNLTSFFTADKSVLKFSLLENLVVSECPQMTKFSEVLSAP
HLQKVHVVAGEKDKWYWEGDLNATLQKYFPYQVFFDHSKDMKLVDYPEMKEVRYGKPVFSDNFFGSLKKLEFDAVSK
REIIVPSHVLPYLKNLEELNVEKCKSARVIFDLDESETRTKGIVFRLKKLTLKNLSNLKCVWNKHSQGMVNFSNLQEVFVY
DCGTLVTLFPLTLAKNLGNLKTLTIQVCFKLIAIVEEKEETVHGTTEKFEFPCLSKLFFWKMPQLICFYSGQHHLKCLMLESL
HVSYCRKLKLFKSGFHDSPLHHPMFSIEEVVPKLKELTLNEKNIILLDDGHSPQDLLHKLNYLQISFEDYDDKKDTFPFDFL
HKVPNLESLTVRRCFGLKELFPSQKLDGHDGIPTKLNTLRLVNLSELESIGLHHPWIKPYIEKLEVLAVLWCSRLNRLVCGA
TSFINLKLLFVRNCRKIKCLFTSSTAKSLLNLETLIIQNCESIQEITEKEDVDGEIVFGRLTILSMSSLPRLVSFYSGNATLHFSSL
QQVTLSECPNMTTFSQANINAPMLYGIKSSINDSNLTFFYDLNTTIQSLFYHKDFFEYSKHTILLDYFETRGIGSVKHAFQE
KSFGNIKKLEFDGKSKGDTVIPSNVLSHLKSLEELNVHNSDEVQVIFGMNDSHTETKETVFHLKKLILKDLSNLKCILNKNP
QESVSFPNLYELFVDGCGSLVTLFARNLGKLKTHEKQKYDNLVEIAGKEDAIENGATEVLMFEFPCLSLLTLYKLTNLNCFY
PEKHHLECPKLEIMHVAYCPKLKLFTSKIHDSHKEAITEAPISCLQQPLFIVEKVVPKLQGLTLNEKNMMLMSDAHVPED
YLSKLNILRLCFEDDKNEKGTLPFDFLHKVPNLEDFQVQRCFGIKEIFSSQKLQVHDGIPATLNALTLFELNELESIGFEHP
WVKPFSEKLQTLRVGSCPRLEKLGRGAMSFINLKELYVKDCGRIEYLFTFSTAKSLVQLETLIVKNCELIKGIAIKEDEDDCD
EIIFERLTKLTLNCLPRLQSFLSGNATMQFSCLKNAYVINCPNLKTFSEGVLTAPRFLGIKTSSNSDLFFHDDLNTSFQRLFQ
TQVEKSACDIEHLKFGDHSHIQEIWLGVAPIPTNNSFNNLKSLTVVECESFPNVIPFYLLPFLCNLKEIEVSNCQSVKAIFDV
NGAVADMKPISLPLKKLVLNQLPNLEYIWNLNPDEILSLQDLQQVSISNCQTLKSLFPTSVANHLVKLHVRACATLVEIFA
EADEAINGETKQFNFHCLTSLTLWELPELKYLYPGKHTLEWPMLTQLDIYHCDQLKLFKTEHHSDEFAHTKDQLGISIHQ
QAAFSVEKVFPKLVQLSLKKEDAVAISQAQLQVMPSIEHQEITWKDTMIGQGQFGANVAHLLQNLKLLKLMCYHEDDK
SNIFSSGLLEEIPNIENLEVVCSSFTEIFCSQGPATDCSKLLSKLKRLHLKNLSQLNAIGLEHSWVEPLLKTLETLEVFSCPTM
KILVPSTLSFSNLTSLNVGECHGLIYVFTSSTAKRLGQLKHISIRDCQAIQEIVSKEEDHESEDEDITFDQLSLLSLESLPNIIGI
YSGTFKLKFPCLDQVTLKECPQMKYSYVPDLHEFKPQEQT

>XP_022635606.1

MDPIVSTATESVLKITATVVKRQVGYFFNYKDKFKELESYIEKLEHNKDRLQHQVDNALRSAEEIENDVQRCLTLMDDKI
KEYKSYVNDENHAKTIFSIGFFPNNFRLRYQLGRKATKMVEEIIGDELWKTSFDNVSYQEFPFIDDTFSNNGYESFGSRTK
TMEMIMKALQDSTVGMIGVYGPGGVGKTTLVKEIANKAREKMLFKIVIIANITGNPDLKKIQEQIAGMLGMKLEGESEI
ARVDRIRKRLKNEKENTLIILDDLWGGLDFNKLGIPCNDDASQQEVNDMYDFGSSNNISDFGYNKTEIKELSKVDLDEM
KKEKLSNDYRGGKILLTSRNKQVLCNEMDVQESSIFSVGVLDEKESETLLKKVAGVKNSEFDRNATEIAKWSAGFPIALVS
IGRTLKNKSLSTWEDVCQQIKRQNFTSEWGFTDFSVKLSYDHLKNEELKCIFLQCARMGNDALIMDLVKFCVGLNLLPG
VHTITDARKRIKEMIKELEESSLLVKSYSIDRFNMHDIVRDVALSISSKEKHVLYMKNEILDEWPHENDFERYSAIFLHFCDI
TDELPESIHCPRLEVLHIDNINESFEIPDECFKSMVRLRVLVLTGINLSCLPSSIKCLKKLRMLCLEGCTLGENLSIIGELKNLRI
LTFSGSNIENLPLEFGQLEKLQFLDISNCLKLRQITSNIIPRMAILEEFYMRDNLIIWEAEENMKSENASLSELRHLNQLQNL
DIQIHSSSHFPQNLFFDRLNSYKIVIGEFNLFNLLKVGEFKVPDKYEEVRFLALNLKEGIDIHSEKWVKMLFKSVECLLLGEL
NDVEDIFYELNVEGFPNLKHLSTVNNFGIKYIINPRERFHSLVAFPKLESIWLYKLDNLEIICDNQLVETSFRNLKVIKIQTCIK
LINLFPFCIVRLLTKLETIEVCDCDSLKEIVSKESQTHTIRDDKIEFPQLRRLTLKYLPTFIYLYDVDKIPGTAHSLQNQVFQQR
NKDIVVDVEHRVTNSCLPFFNEKVSTPKLEWLELYSINIHKIWSDQCSHCFQNLLTLNVTDCSNLKYLLSFSMAESLVNLQ
SILVSECEMMEDIFCPENAEYIDVFPKLKKMEIICMEKLSTIWKSDIGLHSFHNLNSLIIRECRKLVTIFPNCMGQILQSLQS
LTVKDCKLVENIFDFANIPHTCDIIEANLGNIFLENLPNLVNVWKGDIDEILKCNNLQNVRVDESPKLKYLFPVSIANNLVK
LEVLEVRDCWAMTEIIGLDKHSSETAITFKFPHLNTLSLIGMHELRSFYSGIHTLEWPPLKKLEIVDCSMLEGLTSEITSSSE
QPIVLATKKAIYNLEYMSVSLKGAEWLQKYIVNVHRMHKLEELTLYGLKNNEILFWFLHRLPNLKSLTLGLCHMKRIWTP
ESLSSREKIGGVMQLKELELRSMWFLEEIGFEHDVLLQRVECLTIHGCKKLRNLASSSVSFSYLTSLEVVNCMMRNLMTL
STAKALNQLTTVKVCSCPLVVEIVAENEEEKVQVVEFKQLKSLELVSLQNLTSFSSVEKCDLKFPLLEKLVVSECPQMKKLC

EVQSAPKLQKVHVEAGEKDKWYWEGDLNATLQTHFTNQVSFEYSKDINLVDYPERKVRQGKFAFPDNFFGCLKKLEFD
EACERDTLIPSHVLPYLKNLEELNVEKCKSAQIIFDIDESEIQTYGMVFRLKKLILSDLLNLKCVWKENLEGIVSFSNLQEVEV
YCCGSLVTLFPSSLAKNLGKLKRLIIGECEKMVEIIGREDKMEHGTTIMFEFPCLSYLLLEKMPLLSCFYPEKHHLECPLLACL
YVACCPKLKLFRSSSDDDSKREVLPTNLLQQPFFSIEKVSPKPVGLTLNEENIKLMSDASLPEDLLCKLKYLILSFEDDNNRK
DSLPFDFFXKLPNLEWLRVQKCFGLKEIFPSQKLQVHDRGLEGLKELLLVDLKELELVGLEHPWVKPYSEKLEVLVLDNCP
QLQKIVHCAVSFINLKELQVVLCERIEYLFTFATVKSLVKLETLIIQSCESIKEIIKDENEDGYAEMVFGRLKSIKLKSLPRLVRF
YSGKATLQCSYLKVVMVVKCPSMITFSEGVMKMPMFSGIQTSKDSDLTFHDDLNTTIKKLFHEEVEKSACDVEHLKFGD
HPNLEEIWLDVVPISRNDCFNNLKSLAVVECESLSNVIPSYILRFLSNLKEIEVSNCQSVKAIFDVNGAAADMKPISLPLKKL
ILNQLPNLEHIWNLSPDEILSLQDLEQVSISNCQTLKSLFPTSVANHLVKLDVRACATLVEIFVEAETAFEGETKQFNFNCL
TSITLWELPELKYLYLGKHTLEWPMLTHLDIYHCDQLKLFKTEHHSDEFADTKDQFGISIHQHAAFSVEKVFPKLVQLSLK
KEDAMAITQAQFQEHQAIICKDSMTGEGEFGTKAAHLLQNLEVLKLMCYHEDDESNIFSSGLLEEIPTIENLEVVCSSFTE
ISSSQTPRADCSKVLSKLKRLHLKSLQKLISIGLEQSWVEPLVKTVETLEVFCCPSLKSVVPSTVCFSNLTSLNVGECDGMV
YLLTSSTAKSLSQLRRMYVRDCKAIEEIVWNDEEDDESNDEEIAFEKLSDMCLASLPSIVGICSEALKLKFPSLDQVTLTECP
HMKYSYVPHLHQFKPL

>XP_022635607.1

MDPVVSAVTESFLKKLGQVVKKQWGYFFNYKSKFEKLKSYVEELEGNRESLQHNVDKALENAEEIENEVQRCLKLMDE
KIKKYKSYKDGRTLEEIMKICSNGFLPNNFHLKYQLGRKVTKMVEEIIEDGLWKKKFDKVSYQEYPSDDYAFSNSGYVSFA
SRTRTLEEIMEKLKDSTVDMIGVYGRSGMGKTFLVKEIAKKAKEKLFKRVIIANITGNPDFEKIQGQIAGMLDITLEDKNEI
ARANRIRKKLMKEKKNTLIILDDLWDRLDLNRLGISCDDEEEDDASRRDANDIDNNFGYNKTENNEALKVDLNKMKKEK
PSNSYKGCKILLTSRKKEVLCDQMGVQESSTFLVEGLNEKEAETLVKKVADVKISEFDRNAIEIAKWSGGLPMALVSIGKT
LKNKSLPAWENICQKIKRQSFTEELGFTDFSIKLSYDELKSEQLKCMFLQCARMGNDALIMDLVKFCIGLNLLQGVQTITD
ARKMVQKMIEELEESSLLVRSYSIDRFNMHDIVRGYALSICKEKQVLFMKNDILDEWPHEDDFERYTAIFLHSCDIKDELP
KRLHCPRVEVLHIDNITESFKIPDYLFKFMIRLKVLVLTGVNLSRLPSSIKCLKKLRMLCLERCTLRKNLSIIGELKSLRILSLSG
SNIESLPLEFGQLDKLQLFDISNCPKLRQIPSSIIPRMNILEEFYTRESLILWEAEETESKNASLSELRHLNHLQNLELHIQSYA
HFPQNLFLDKLNSYKIVIGEFNLLNLLTVGEFKVPDKYEEVKFLALELKEGIDIHYEKWIKMLFKSVECLLLGELNDVRDIFYE
LNVEGFPNLKHLSIVNNFGIKYIINPRERFHSLIAFPKLESVWLYKLDNLEIICDNQLVETSFRNLKVIKIQTCIKLVNLFPFC
MVRLLTELETIEVCDCDSLKEIVSKERKTETMSDEKIEFPKLRQLTLKSLPTFICLYGVDEMLGSVPLLQGQENTNIVNNAE
HGVNNSCLPLFNEKVSIPKLESLKLSSINIQKIWSDQYDHCFENLLTLNVTDCSNLKYLLSFSMAESLMNLQSIFASECEM
MEDIFRPEDAEYIDVFPKLKKMEIICMEKLSTIWKSDIGLHSFNNLNSLMISECHKLVTIFPNYMGHTLQSLQSLTVTDCKL
VENIFDFANIPHTCDIIEANLGIIFLEKLPNLVNVWKGDTGEILKCNNLQSIRVDESPKLKYLFPVSIANDLQKLEVLEIWDC
WEMIEIIALDKHSSETAITFKFPHLNTLSLIDLHELRSFYSGIHTLEWPPLKKLEIVDCSMLQGLTSEITNSSEQPIVLATKKAI
YNLEYMSVSLKEAEWLQKYIVNVHRMHKLEELFLYRLKNNEVLFWFLHRLPNLKNLTLGLCHMKRICTPESLNSPEKIGG
VMQLKELVLHNMWSLEEIGFEHDVLLQRVECLTIHGCTKLRNLASSSVSFSYLTSLEVVNCMMRNLMTLSTARTLNQLT
TMKVSSCPFIVEIVAENEEEKVQEVEFKQLKSLELVSLQNLKSFSSVEKCDLKFPLLKKLVVSECPQMKKLSEVQTAPNLQK
VHVEAGEKDKWYWEGDLNATLQTHFTNKVSFEYSKDINLVDYPERKIRHGKFAFPDNFFGCLKKLVFDEAYKRDTLIPS
HVLPYLKNLEELNVEKCESTQLIFDVDESEIQMHGMVFRLKKLTLKHLSNLKCVWKENRKGIVSFPNLNTVVVTDCEGLV
TLFPSSLARNFKKLKTLFIWCNKKLEEIVGKEEGTEHEKTIIFEFPCLSELAIVDMPLLSCFYPGKHQLECPLLETLYVAYAPKL
KLFTSNSQKGDMEAPIRSLQQALFLVEKVPPKLTDLALNEENIMLFREENLPQNLLCKLSRLFLCFEDDKNEKNSLPFDLF
HKLPNLEWLTVRKCFGLKEIFPSQKLQVHDKVLAGLKQLNLFQLKELECIGLENMWIQPYSIKLELLQLHGCLLVERIVYCA
VSFINLKYLYVMHCERMEYLFTFATLKSLVKLETLSIGYCGSIKEIARTEEEDGSDEIIFGRLTSIKLEYLPRLISFYSGNATLQC
PSLQTVMVADCPNMITFSEGVIKLAMFPGIQTSEDSDFTFHVDMNTTVESLFHEKEFMEYSKRMILEEYLGMSGVHHT
KPIVLDNFFGSFKKLEFDAACNRTILIPSHVLPYLKNLEELNVKNSDAIQIIFDIDESEVKTKGVVFGLKKLTLNKLSNLKHV
WKENSTGIISFHNLPEVVVNGCGSLITLFPLSLAKNLGKLKKLELEECEKMVEIVGREDEIEHGTPIMLEFPCLSRLGLEKM

PLLSCFYPGKHHLECPLLDELYVACCPKLKLFTSNFDDDSKKEVSEAPTNLLQQPLFSIEKVCPNLMGLTFNEENMKLMS
DARLPQDLLCKLNLLYFCFEDDNNEKGILPFDFFHRVPNLEELYIDKCFGIKEIFPSQKVEVHDKVLVRFKNLVLMELKELE
WVGLEHPWVQPYTEKLELLKLCSCPLVEKLVSCAVSFINLKELYVKLCEKMEYLFTFATLKSLVKLETVKIQKCESIKEIVKK
DDEDDCDEIVFIRLRSIELNSLPKLISFYSGNATLQCSYLKNVMVAECPNMITFSQGIIKVPMFLDIRTSKHSDLTFHSDLYT
KIQKLFPNQVIHES

>XP_022635608.1

MEVVVSTATENAMQIAVRVVKRQFSYFFNYNDKFEEVKCYIERLDNTRKRIQHQVNNAEMNAEEIEDDVQHCLKQLD
EKIEKYEQFINDEYHSKTRCSIGFFPNNLSLRYRLGRNATKMVEEMEAEELWNKKFDEVSYRVLPSINAALTNTSYESFAS
REKTINVCMQALEDSTINMIGLYGVGGVGKTTLVKEVAKKAQEKKLFNVVVMANITRNPNIIKIQGQIAEMLGMRLEEE
SEIVRADRIRKRLNKEKENTLIILDDLWDGLDLNRLGIPISDENDGSQQDVNDISDSSYDKMEKEELSSDFNNMTEENLSE
DQKRCKILLTSRRKQVLCNQMDVKERSTFSVGVLNETEGKTLLKRVAGIQIQNLVYDEKAIEIARMCDGLPIALVSIGRTL
KNKSSFVWEDVYQQMKKKSFMEGQEPIEFSIKLSYDHLKNEQLKCIFLHCARMGNDALVMDLVKFCIGLGLIQGVHTIG
EARNKVNMLIEELKESSLLLESYSSNRFNMHDIVRDVALSISSKEKQMFFMKNGIIDEWPHKDQLERYTAIFLHYCYINDD
LPVSIYCPRLEVLHIDNKDQFLKIPDDFFKDMIELRVLILTSFNMPCLPSSIICLSKLRMLSLEKCTLGQNLSIIGELKKLRILTLS
GSNIESFPFEFGQLDKLQLLDLSNCSKLSVIPSNVISRINILEELYMRDTLILWEAKEMIQNKNASLSELRNLNQLRNLDLHI
QNVSHVPQNLFFDKLDSYKIIIGEFNMLTEGEFKIPDKYEVVKLLVLNLKEGIDIHSETWVKMLFKNVEYLLLGELIDVHDV
FYELNVEGFQKLKHLSIVNNIGLQYIINSVERFHPLLAFPKLESLYLYKLYNLEKLCNNQLLEASFCRLKTIKIKSCDKLESLFPF
FMVGLLTMLENIEVCDCDSLKDIVAIERQPHTDSDDNIQFPQLRTLTLKSLPAFTCLYTNDKMPCSAQSLEEKGRNRNRD
VIVEVEQDDTNSCLSLFNEKISIPKLESLELSSVNIQKIWSDQSQHCFQNLLTLNVTDCDNLKYLLSFSMAVCLVNLQSLSV
SECEMMEDIFRPKDDEGTIDYVFPKLKKMEITCMEKLSSIWQPHIGLHSFHSLDSLIIRECHKLVTIFPSFMGQKFQSLQSL
TITNCKLVENIFDFEMIPQTSDINETQLHKIVLQNLSNLVSIWKDDTCEILKYNNLQSITVAGSPNLNHLFPLSITNDLENLE
FLDVRNCRAMKEIVGSDKGKNENVITFKFPRLNYVSLQSLFELVSFYGRAHTLEWPSLKRLLILRCGKLEGINTDISNSQM
KPIGLAAEKVIYNLEYMAMSFREVEWLQNYIINVHRMQNLQTVVLHGLKNVEVLFWLLHRLPNLKRLTLGFCHLKRIWA
PASLISREKIGVVMQLQALELKNIWPLEEIGFEHEVLLQRVERLTIQRCTKLKILASSSVSFGYLTYLEVENCMLRSLMTCST
AKTLVQLKTMKVSSCPKLVEIISENEGEEIQEIEFKLLRSLELVSLQNLTSFMNVDKCDLKFPVMENLVVSECPKMTKFSKV
QSAPNLQKVQVVATEKDKWYWEGDLNATLKKHFTHQVVPKLKEVILNEKNITLLKDGHSPDLLHKLNYLDLASEDYENK
KDSLPFDFLQKVPNLEYLVVRQCFGLKEIFPSKKLDGDHDGILLAGLNKLSLNKLLELESIGLDHPWVKPYTEKLQGLAVIK
CPRLERLVNCVTSFINLKQLIVKNCKRMKYLFTFSTAKSLGKLETLRIENCESMKEIIEKEDENGYDEIIFGRLTKLWLYSLPK
LVSFYSGNDTLQFSSLQIMRLFKCPNMKTFSQGNTNAPMFYGIKSSSDSDLTFHSDLNMTVESLFHEQGFFEYSKQMIL
LDYLEMRGFGPVKHVFPSKFFGSLKKLEFDGASKGDTVIPSNVLPYLKSLEELNVHSSDEVQVIFGMDHNSRAKSKETVF
HMKKLTLKDLSNLKCILNKNLQGSVSFPNLQELFIDGCGSLVTLFARKLQTLEMQKCDKLVEIVGNEDATTETFVFEFPCL
SSLTLYNLTDLSCFYPGKHHLECPQLEILHVAYCPKLNLFKSKIHDSHRQTVAEAPINWLQQQPLFMVEKVAPKLRGLTLN
EKNMMLLSDEHVPEVNLTNLNLLRLCFEDDKNEKDSLPFEFMNKVLNLEHLRVQRCFGVKEIFPSQKLQVHDGIPASLK
GLTLFELNELESIGFEHPWVSPYSEKLQTLRVVNCPMLQKLGCHAMSFLNLKELYVKDCDRMEYLFTFSTAKCLVKLETLII
KNCESIKEIANIEDEDGCDKIIFGKLTTLRLYSLPRLQSFISGNVTLQFSYLRNATVIDCPNMKTFAERVLNVPRILSIETSLED
SDLFLHNDLPASNTGKSILGRLASFYKQRNRL

>XP_022635609.1

MDPIVSTATESALNITASLVKRQVGYFFNYKDKFKELESYIEKLEHNKERLQHQVDSALRSAEEIEKDVQRCLTLMDDKIK
EYKSYINDECHAKTICSIGFFPNNFRLRYQLGRKATKMVEEIIGNELWKTSFDNVSYQEFPSIDATFSNNGYESFGSRTKT
MEMIMKALQDSTVGMIGVYGPGGVGKTTLVKEIANKAREKNLFETVIIANITGNPDFKIIQEQIAGMLGMKLEEESEIAR
VDRIRKRLKNEKENTLIILDDLWGGVDFNKLGIPCNDDASQQEVNDMSDFGSNNDISDFGYYKTEMKEFSKVDLDKMR
KEKLSKDYRGGKILLTSRNKQVLCNEMDVQESSIFSVGVLDEKESETLLKKVAGVKNSEFDRNATQIAKWSAGFPIALVSI

GRTLKNKSLSTWEDVCQQIKRQNFTSEWGFTDFSVKLSYDHLKNEELKCIFLQCARMGNDALIMDLVKFCVGLNLLPGV
HTITDARKRVKEMIKELEESSLLVKSYSIDRFNMHDIVRDVALSISSKEKHVLYMKNAILDEWPHENDFERYSAIFIHFCDI
NDELPESIHCPRLEVLHIDNQNESFQIPDQLFKSMVRLRVLVLTGITLSCLPSSIKCLKKLRMLCLERCTLGENLSIIAELKNIR
ILTFSGSNIKNLPLDFGKLDKLQFLDISNCLKLRQVTSNIIPRMGILEEFYIRDNLIIWEAEENMTSENASLSELRHLNKLQSL
DIHIHCSSHFPQNLFFDRLNNYKIVIGEFNLFNLLKVGEFKVPDKYEEVKFLAVNLKEGIDIHSEKWIKMLFKSVEHLLLGEL
NDVEDIFYELNVEGFPNLKHLSIVHNFGIKYIINPTERFHSFVAFPKLESIWLYKLYNLEIICDNQLIKTSFHNLKVIKIQTCIKL
VNLFLVSMVRLLTMLETIEVCDCDSLKEIVSNESQTHTIRNDKIEFPRLRLLILKYLPTFICLYNVDKIRGSAHPMQDQVIQ
QRNKDIVVDDDHRVTNSCLPLFNEKVSTPKLEWLELSSINIHKIWSDQCNHFFQNLLTLNVTDCGNLKYLLSFSMAESLA
NLQSIFVSECEMMEDIFRPEDAEYIDVFPKLKKMEIICMEKLSTIWKSDIGLHSFSNLNSLVIRECHKIVTIFPNYMGQRLQ
SLQSLTVRDCELVENIFDFANIRHTYDIIEANLVNIFLEKLPNLVNVWKGDTDEILKCNNLQRIEVYKSPKLKYLFPFCIAND
LQKLEVLEVWYCWAMTEIIGLDIHSSETPITFKFPHLNTLSLIDLHELRSFYSGIHTLEWPPLKILEIVDCSMLEGLTSEITNS
KEQPIVLATKKAIYNVENMSVSLKEAEWLQKYIVNVYRMHKLEELTLYRLKNNKILFWFLHRLPNLKSLRLGLCHMKRIW
TPESLSSRGKIGGVIQLKELGLYNMLSLEDIGFDHDVLLQRVERLIISGCKKLTNLASSRVSFSNLTSLKVVNCMMRNLMT
LSTAKTLVQLTTLKVCSCPMIVEIVAENEEEKVQQVEFKQLKSLKLVSLQNLTSFSSVEKCNLKFPLLEKLVVSECPQMTKL
SKVQSAPNLQKVHVEEGDKDKWYWEGDLNATLQTHFTDQVSFEYSKDINLVDYPERKVRHGKFSFPDNFFGCLKKLEF
DEACKRDTLIPSHVLPYLKNLEELNVEKCESAQLIFDVDESEIQMHGMVFRLKKLTLKHLSNLKCVWKENIEEIVSFYNLER
VHVDGCGSLVTLFPLSLAKHLGKLNTLYIEECEKMVEIVGREDKMEHGTTIMFEFPCLSYLYLEKMPLLSCFYPGKHHLEC
PLLYMLCVECCPKLKVFRSNFDDDGKKEVLKAPTNLQQPLFSIEKVFPKLEGLVLNEEYIKLMTDARLPQDLLCKLRILTLFF
EDVNNGKESLPFDFFHKLPNLEGLGVRKCFGLKEIFPSQKLQVHDKVLAGLKRLYLSELSELECIGLEHPWVQPYSKKLKLL
NLYSCPRVENIVLCAVSFINLKELCVNLCEKMEYLFTFATLKSLVKLENLSIKKCESIKEVVKKDDEDGCDEIVFGRLRSITLKY
LPRLIIFYSGNATLQCSCLQNVMVVECPNMKTFSEGVTKLAIFSGIQTSDDSDFTFYVDLNTTVQRLFHEKDFFNYSKFMI
LHDYLGMMRVQHTKPTISDHFFGSFTKLEFDITWNRSFAIPSHILPYLKNLKELNVHSCDVVQVIFDTDEIEVETKGIIFGL
KKLTLKHLSNLKCIWKENLQGIVSFSNLKQVNVDGCGSLVTVFPLSLAKHLGKLKILDIQECEKMVEIVGREDEMEHGTTI
MFEFPCLSYLNLEKMPLLSCFYPKKHDLECPLLDHLYVECCPKLKVFRSSFDDDSKKEVLEAPTNFLQQPLFSIEKVSPKLE
GLTLNEESIKLMSDARLSQDLLCKLESLILSFEDDNNEKDSLPFDFLHKLPNLEWLTVQKCFGLKEIFPSQKLQVHDKVLAG
LKRLFLLELSELECIGLEHPWVQPFINLTKLSVKLCEKVEYLFTFSTLKSLVKLETVKIQKCDSIKEIVKKDDEDDCDEIVFIRLR
SIELNSLPKLISFYSGNATLQCSYLKNVMVAECPNMITFSQGFIKVPTFLKIQTSKDSDSTFDGDLNTTIQKLFHNQVIHES

>XP_022635610.1

MDPIVSTATESALNITASLVKRQVGYFFNYKDKFKELESYIEKLEHNKERLQHQVDSALRSAEEIEKDVQRCLTLMDDKIK
EYKSYINDECHAKTICSIGFFPNNFRLRYQLGRKATKMVEEIIGNELWKTSFDNVSYQEFPSIDATFSNNGYESFGSRTKT
MEMIMKALQDSTVGMIGVYGPGGVGKTTLVKEIANKAREKNLFETVIIANITGNPDFKIIQEQIAGMLGMKLEEESEIAR
VDRIRKRLKNEKENTLIILDDLWGGVDFNKLGIPCNDDASQQEVNDMSDFGSNNDISDFGYYKTEMKEFSKVDLDKMR
KEKLSKDYRGGKILLTSRNKQVLCNEMDVQESSIFSVGVLDEKESETLLKKVAGVKNSEFDRNATQIAKWSAGFPIALVSI
GRTLKNKSLSTWEDVCQQIKRQNFTSEWGFTDFSVKLSYDHLKNEELKCIFLQCARMGNDALIMDLVKFCVGLNLLPGV
HTITDARKRVKEMIKELEESSLLVKSYSIDRFNMHDIVRDVALSISSKEKHVLYMKNAILDEWPHENDFERYSAIFIHFCDI
NDELPESIHCPRLEVLHIDNQNESFQIPDQLFKSMVRLRVLVLTGITLSCLPSSIKCLKKLRMLCLERCTLGENLSIIAELKNIR
ILTFSGSNIKNLPLDFGKLDKLQFLDISNCLKLRQVTSNIIPRMGILEEFYIRDNLIIWEAEENMTSENASLSELRHLNKLQSL
DIHIHCSSHFPQNLFFDRLNNYKIVIGEFNLFNLLKVGEFKVPDKYEEVKFLAVNLKEGIDIHSEKWIKMLFKSVEHLLLGEL
NDVEDIFYELNVEGFPNLKHLSIVHNFGIKYIINPTERFHSFVAFPKLESIWLYKLYNLEIICDNQLIKTSFHNLKVIKIQTCIKL
VNLFLVSMVRLLTMLETIEVCDCDSLKEIVSNESQTHTIRNDKIEFPRLRLLILKYLPTFICLYNVDKIRGSAHPMQDQVIQ
QRNKDIVVDDDHRVTNSCLPLFNEKVSTPKLEWLELSSINIHKIWSDQCNHFFQNLLTLNVTDCGNLKYLLSFSMAESLA
NLQSIFVSECEMMEDIFRPEDAEYIDVFPKLKKMEIICMEKLSTIWKSDIGLHSFSNLNSLVIRECHKIVTIFPNYMGQRLQ
SLQSLTVRDCELVENIFDFANIRHTYDIIEANLVNIFLEKLPNLVNVWKGDTDEILKCNNLQRIEVYKSPKLKYLFPFCIAND

LQKLEVLEVWYCWAMTEIIGLDIHSSETPITFKFPHLNTLSLIDLHELRSFYSGIHTLEWPPLKILEIVDCSMLEGLTSEITNS
KEQPIVLATKKAIYNVENMSVSLKEAEWLQKYIVNVYRMHKLEELTLYRLKNNKILFWFLHRLPNLKSLRLGLCHMKRIW
TPESLSSRGKIGGVIQLKELGLYNMLSLEDIGFDHDVLLQRVERLIISGCKKLTNLASSRVSFSNLTSLKVVNCMMRNLMT
LSTAKTLVQLTTLKVCSCPMIVEIVAENEEEKVQQVEFKQLKSLKLVSLQNLTSFSSVEKCNLKFPLLEKLVVSECPQMTKL
SKVQSAPNLQKVHVEEGDKDKWYWEGDLNATLQTHFTDQVSFEYSKDINLVDYPERKVRHGKFSFPDNFFGCLKKLEF
DEACKRDTLIPSHVLPYLKNLEELNVEKCESAQLIFDVDESEIQMHGMVFRLKKLTLKHLSNLKCVWKENIEEIVSFYNLER
VHVDGCGSLVTLFPLSLAKHLGKLNTLYIEECEKMVEIVGREDKMEHGTTIMFEFPCLSYLYLEKMPLLSCFYPGKHHLEC
PLLYMLCVECCPKLKVFRSNFDDDGKKEVLKAPTNLQQPLFSIEKVFPKLEGLVLNEEYIKLMTDARLPQDLLCKLRILTLFF
EDVNNGKESLPFDFFHKLPNLEGLGVRKCFGLKEIFPSQKLQVHDKVLAGLKRLYLSELSELECIGLEHPWVQPYSKKLKLL
NLYSCPRVENIVLCAVSFINLKELCVNLCEKMEYLFTFATLKSLVKLENLSIKKCESIKEVVKKDDEDGCDEIVFGRLRSITLKY
LPRLIIFYSGNATLQCSCLQNVMVVECPNMKTFSEGVTKLAIFSGIQTSDDSDFTFYVDLNTTVQRLFHEKDFFNYSKFMI
LHDYLGMMRVQHTKPTISDHFFGSFTKLEFDITWNRSFAIPSHILPYLKNLKELNVHSCDVVQVIFDTDEIEVETKGIIFGL
KKLTLKHLSNLKCIWKENLQGIVSFSNLKQVNVDGCGSLVTVFPLSLAKHLGKLKILDIQECEKMVEIVGREDEMEHGTTI
MFEFPCLSYLNLEKMPLLSCFYPKKHDLECPLLDHLYVECCPKLKVFRSSFDDDSKKEVLEAPTNFLQQPLFSIEKVSPKLE
GLTLNEESIKLMSDARLSQDLLCKLESLILSFEDDNNEKDSLPFDFLHKLPNLEWLTVQKCFGLKEIFPSQKLQVHDKVLAG
LKRLFLLELSELECIGLEHPWVQPFINLTKLSVKLCEKVEYLFTFSTLKSLVKLETVKIQKCDSIKEIVKKDDEDDCDEIVFIRLR
SIELNSLPKLISFYSGNATLQCSYLKNVMVAECPNMITFSQGFIKVPTFLKIQTSKDSDSTFDGDLNTTIQKLFHNQVIHES

>XP_022635611.1

MDPIVSTATESALNITASLVKRQVGYFFNYKDKFKELESYIEKLEHNKERLQHQVDSALRSAEEIEKDVQRCLTLMDDKIK
EYKSYINDECHAKTICSIGFFPNNFRLRYQLGRKATKMVEEIIGNELWKTSFDNVSYQEFPSIDATFSNNGYESFGSRTKT
MEMIMKALQDSTVGMIGVYGPGGVGKTTLVKEIANKAREKNLFETVIIANITGNPDFKIIQEQIAGMLGMKLEEESEIAR
VDRIRKRLKNEKENTLIILDDLWGGVDFNKLGIPCNDDASQQEVNDMSDFGSNNDISDFGYYKTEMKEFSKVDLDKMR
KEKLSKDYRGGKILLTSRNKQVLCNEMDVQESSIFSVGVLDEKESETLLKKVAGVKNSEFDRNATQIAKWSAGFPIALVSI
GRTLKNKSLSTWEDVCQQIKRQNFTSEWGFTDFSVKLSYDHLKNEELKCIFLQCARMGNDALIMDLVKFCVGLNLLPGV
HTITDARKRVKEMIKELEESSLLVKSYSIDRFNMHDIVRDVALSISSKEKHVLYMKNAILDEWPHENDFERYSAIFIHFCDI
NDELPESIHCPRLEVLHIDNQNESFQIPDQLFKSMVRLRVLVLTGITLSCLPSSIKCLKKLRMLCLERCTLGENLSIIAELKNIR
ILTFSGSNIKNLPLDFGKLDKLQFLDISNCLKLRQVTSNIIPRMGILEEFYIRDNLIIWEAEENMTSENASLSELRHLNKLQSL
DIHIHCSSHFPQNLFFDRLNNYKIVIGEFNLFNLLKVGEFKVPDKYEEVKFLAVNLKEGIDIHSEKWIKMLFKSVEHLLLGEL
NDVEDIFYELNVEGFPNLKHLSIVHNFGIKYIINPTERFHSFVAFPKLESIWLYKLYNLEIICDNQLIKTSFHNLKVIKIQTCIKL
VNLFLVSMVRLLTMLETIEVCDCDSLKEIVSNESQTHTIRNDKIEFPRLRLLILKYLPTFICLYNVDKIRGSAHPMQDQVIQ
QRNKDIVVDDDHRVTNSCLPLFNEKVSTPKLEWLELSSINIHKIWSDQCNHFFQNLLTLNVTDCGNLKYLLSFSMAESLA
NLQSIFVSECEMMEDIFRPEDAEYIDVFPKLKKMEIICMEKLSTIWKSDIGLHSFSNLNSLVIRECHKIVTIFPNYMGQRLQ
SLQSLTVRDCELVENIFDFANIRHTYDIIEANLVNIFLEKLPNLVNVWKGDTDEILKCNNLQRIEVYKSPKLKYLFPFCIAND
LQKLEVLEVWYCWAMTEIIGLDIHSSETPITFKFPHLNTLSLIDLHELRSFYSGIHTLEWPPLKILEIVDCSMLEGLTSEITNS
KEQPIVLATKKAIYNVENMSVSLKEAEWLQKYIVNVYRMHKLEELTLYRLKNNKILFWFLHRLPNLKSLRLGLCHMKRIW
TPESLSSRGKIGGVIQLKELGLYNMLSLEDIGFDHDVLLQRVERLIISGCKKLTNLASSRVSFSNLTSLKVVNCMMRNLMT
LSTAKTLVQLTTLKVCSCPMIVEIVAENEEEKVQQVEFKQLKSLKLVSLQNLTSFSSVEKCNLKFPLLEKLVVSECPQMTKL
SKVQSAPNLQKVHVEEGDKDKWYWEGDLNATLQTHFTDQVSFEYSKDINLVDYPERKVRHGKFSFPDNFFGCLKKLEF
DEACKRDTLIPSHVLPYLKNLEELNVEKCESAQLIFDVDESEIQMHGMVFRLKKLTLKHLSNLKCVWKENIEEIVSFYNLER
VHVDGCGSLVTLFPLSLAKHLGKLNTLYIEECEKMVEIVGREDKMEHGTTIMFEFPCLSYLYLEKMPLLSCFYPGKHHLEC
PLLYMLCVECCPKLKVFRSNFDDDGKKEVLKAPTNLQQPLFSIEKVFPKLEGLVLNEEYIKLMTDARLPQDLLCKLRILTLFF
EDVNNGKESLPFDFFHKLPNLEGLGVRKCFGLKEIFPSQKLQVHDKVLAGLKRLYLSELSELECIGLEHPWVQPYSKKLKLL

NLYSCPRVENIVLCAVSFINLKELCVNLCEKMEYLFTFATLKSLVKLENLSIKKCESIKEVVKKDDEDGCDEIVFGRLRSITLKY
LPRLIIFYSGNATLQCSCLQNVMVVECPNMKTFSEGVTKLAIFSGIQTSDDSDFTFYVDLNTTVQRLFHEKC

>XP_022635613.1

MEAVVSTTTESALQITGRVVKRQLSYFFNYNDKFEEVKRYIEMLNNTRKRIQHQVNNAEMNPEEIEDDVQHCIKQLDEK
IEKYEQFIQDEYHSKKRCSIGFFPRNMSLRYRLGRNATKMVEEIKVEELWNKRFDEVSYRVLPSINVSLTDISYESFASRTK
TIDMFMQALEDSTVNMIGLYGVGGVGKTTLVKEVAKKAQEKKSFPVAVMANITRNPNIITIQGQIAEMLGMRLEEEIV
RVDRIWKRLKKEKENILIILDDLWDRLDLNRLGIPNSDEDDGNQN

>XP_022635764.1

MGCCEVLPHDFVLEGDRIIKMVEAVVSFAVDRLGDLLIEEARLLSGVSNKVKSMQNELKMMQCFLRDAESRQDESDTI
KNYISEVRKLAYDAEDVIEIYAIKVAFGISIGTKNPLSRAKNIHKVGSELITINSRISDLTRSLQTYGLTATKDNEEASEVKRQL
RWSYSHIVDEFIVGLDQDINKVAEWLINENQDCRFVYICGMGGLGKTTLAKSIYHYNAIRRNFDGFAWAYISQQCKKRD
VWEGILLKLISPTKEERDEITKMKDDELARKLFKVQQEKKCLIILDDIWSNEAWDILSPAFPSQNTRSKIVFTSRNKDISLHV
NPEGLLHEPSCLNAEDSWALFKKKAFPRQDNPESTTSDDFKRLGREMVAKCAGLPLAIIVLGGLLATKESVSEWEKIHRH
LSSYLIGAEVRDRRRLDEVLDLSYQDLPCQLKPCFLYLSQFPEDSEIPKTKLLQLWVAEGVVSSQYESDRDETMEDVAERY
LGNLISRCMVQIGQMGSTGRIKTYRLHDLMRDLCLSKARKENFLYIINGSQQNSAIIATHSSNISEATQIDEVRRLAVYLD
QHVDQLIPQDKQVNERLRSLVFFHDKKCRMENWDLVRGVFVKFKLLRVLDLEGIKGLKGQSLPKEVGNLLWLKFLSLKR
TRIQVLPSSLGNLENLQFLNLQTVNKVSWDSTVEIPNVICKLKRLRHLYLPNWCGNIVNNLQLDNLTNLQTLVNFPASKC
DVKDLLKLKRLRKLVLNDPRHFQKFSESFSPPNKRLDCLQSLSLRTDMLSFPENVVDVEKLVLGCPFLRKLQVEGRMDRL
PDASLFPRHISKLTLWGCRLVEDPMVTLEKLPNLKFLNGWDMFVGKKMTCSPNGFLQLKVLVLRGLPKLDEWTIENQA
MRNLYRLSISDCNNLKRVPDGLRYITSLRELEIRWMPKSFKTRLGTSGEDYPKVQHVPSIIFLN

>XP_022635783.1

MGRPKDEYYWNQVDREEDGGLKCKHCGLKFKGGVSRIKAHIDLIEGKGIRICPTSPKCITSSDHSHQDINAITLSQGVES
NLEMDGGASTSLAASFEGNEEQCDTGVLTTLQSKLDELRSDLTREEKDIQGQLQLLESHGKRCKRKVDLWLNEIQNMK
QRAIDMKNSLNQFRCSDFYVPQGEMYSAEESQKKIQHLTEEIQKHKKLKPLVLSNEYFGRKFEKNVETLWKLMRDDRV
FIIGIYGMGGVGKTFLAKYMQSEIKRTKTFENVLWFTVPYSFSIFSLQEDIAKIIKVRFHTYDETERAMILTKELEKRGNIILIL
DDVWRYVDLEMVGISLRINGIKLIITSRLRDIFQQMDCLSINIIEVSPFYYYHHYNSCDIYYRNDYDDDSDEDDDKYDEALK
LFLLKLGSYGTPLTLSPEVRDIVRYVVKECDGLPLGISVMAQTMKGKTDIHWWRRVQNKFDKLKMGVEMQDKVFTVL
RRSYFNLKEKDWQKCFLYIALLPNFMRRNCLIKKLVDTGQLEGNGSLEEIFDEANVLVDKLVNDSLLEANKEKEIKHLSSY
KNDLVLSMHGLVRKMALNILKQSVNNMMIKCNGNMTKIPYTEKWATDLEVVSLAHNNIQVIPEGTSPNCPRLSTLLLF
ENSIYHIPECFFAHMEALKTLDLSKNKSLTCLPHSLSNLTSLTSLMLHECSELNYIPPLGELHSLLRLQISSCSIKAPPQGLENL
INLKWLDLSMNKNLKLVPGSFLPSLTKIHYLDLLGCAGGIEVEDVKGMTMLECFAGTFVVQDNYKDNFSRYVREILDSTN
GPQTYFINLDNKRNMSTLIEYPFSRFKDRTMSFSDCKELIHFLPRDLLKLELKYNDHWICLCYGLSSYDNSLLEEIRIYDWK
KLKSLFCSSCYLCTDIKNLQSLNLNCLESLTVICKKLPENDMFSSLKTLCVHKCHQMKILLTSKLVRQLQNLESITVSHCNSIE
QIFGVAYDKDKDEEDENEDYEDDEGDSNIIILRKLTWLSITYLPQLKTVYKGILICTSGFKSSIENCPQLCKPRIEYEAS

>XP_022635784.1

MPSLYHKIFFFFVYISCIEGVESNLEMDGGASTSLAASFEGNEEQCDTGVLTTLQSKLDELRSDLTREEKDIQGQLQLLESH
GKRCKRKVDLWLNEIQNMKQRAIDMKNSLNQFRCSDFYVPQGEMYSAEESQKKIQHLTEEIQKHKKLKPLVLSNEYFG
RKFEKNVETLWKLMRDDRVFIIGIYGMGGVGKTFLAKYMQSEIKRTKTFENVLWFTVPYSFSIFSLQEDIAKIIKVRFHTY
DETERAMILTKELEKRGNIILILDDVWRYVDLEMVGISLRINGIKLIITSRLRDIFQQMDCLSINIIEVSPFYYYHHYNSCDIYY
RNDYDDDSDEDDDKYDEALKLFLLKLGSYGTPLTLSPEVRDIVRYVVKECDGLPLGISVMAQTMKGKTDIHWWRRVQN

KFDKLKMGVEMQDKVFTVLRRSYFNLKEKDWQKCFLYIALLPNFMRRNCLIKKLVDTGQLEGNGSLEEIFDEANVLVDK
LVNDSLLEANKEKEIKHLSSYKNDLVLSMHGLVRKMALNILKQSVNNMMIKCNGNMTKIPYTEKWATDLEVVSLAHNN
IQVIPEGTSPNCPRLSTLLLFENSIYHIPECFFAHMEALKTLDLSKNKSLTCLPHSLSNLTSLTSLMLHECSELNYIPPLGELHS
LLRLQISSCSIKAPPQGLENLINLKWLDLSMNKNLKLVPGSFLPSLTKIHYLDLLGCAGGIEVEDVKGMTMLECFAGTFVV
QDNYKDNFSRYVREILDSTNGPQTYFINLDNKRNMSTLIEYPFSRFKDRTMSFSDCKELIHFLPRDLLKLELKYNDHWICL
CYGLSSYDNSLLEEIRIYDWKKLKSLFCSSCYLCTDIKNLQSLNLNCLESLTVICKKLPENDMFSSLKTLCVHKCHQMKILLTS
KLVRQLQNLESITVSHCNSIEQIFGVAYDKDKDEEDENEDYEDEEDENEDYEDEDDEGDSNIIILRKLTWLSITYLPQLKTV
YKGILICTSGFKSSIENCPQLCKPRIEYEAS

>XP_022635793.1

MADSVVGFLVQRLSQLLESEIKLLSGVEEKIKSLRNELKFMDIFIKSSEGKYKDAVVKEVVTQIRDVAHKAEDVVDTYILNI
AKHKRRNKLCRLFHLKEKLVVPHEVDAEIEKIRSRIDEIYKNQERYGIKEGEFQSEEAVAIEWRRKRRMDVEEEDVVGLVN
ESDTVIQQLQKDDVRLNVASIVGMGGLGKTTLARKIYNKDVVKNIFPCRAWGNVSNDYRPKEVFQSLLSCLNLSGFENL
SEENLKKEVAKGLKGKKYLIVLDDIWETRVWDDIKGAFPDDNTGSRVLITSREMHVARHAGTTSPYELPFLTEDKSWELF
FKKVFRGEKCPSELEPLGRSIVKETCGGLPLAIVILGGLFAKKEKSQREWSRMKKMSWNSTADKNEVMDILRLSYDNLPP
TLKPCFLYFGIYPEDHKISAREVIQLWGAEGFVQPQENAEPEEVGDFYLDELVDRSLVQVTERRTDGGVKICQVHDVLRD
FCISESKFCKFMDVYRESNIDTLSDTNPRRLSLLCQPQSSISVMTFDKSMSSTRSVFVFTEASKSVDDLVKRFMLARVIHG
FQPSLFPDNLKRMIHLRYLKILFVDSLPACVGNLWNLETLDVSYKKRVSSKIWKLKRLRRLCLRGRGKLPVLPKGTRMENL
QSLWLYGWSSEIKSLVDNGIFPRLVKLALSPMTLLRIDEDEEVDFLSGVQCLNHLGSLKIDEIGNLPSDNNVFPSKITKITFK
QILSWSFMKTLGQLPNLQILKLEYESGILRNLDIGKGEFCQLQVFHIKFLSIKSWRLEESAMPRLRHLRIINCYYLFQLPQQL
WSLRTLQLVHIVRPSQELTSNLQIVEFKNNCKLVLEDMW

>XP_022635881.1

MSIRTNKMKAVGVLMKQLTTSRRKFHERGRDESFDGQLEKLRLVLNNIKDVFVEVKKNEEKLLDTLAEVYDHLHRLDRK
KLHQEMEGICKRIRDSAHNLLPTLVFDNRGGKISHSSQELVQLHQKESWTPEYYYVAPFLDIPSRFCLWSLLIFPANAIIRK
RNAINLWIEEGLIENTEKKTADELGEDVVNNLLKFKMIVRYGRGKDPLVNKFEIPSQVHSQLTLYGDRAIGYIRPSRLLLDK
KNVTIGGVDTKSVTLHNIYNIGASYLNFGPQWVTNQLKNLEVLQLGRWQYSPLHHIEVGSQEFLRELRTLKQLKYLSLHG
ISRIFELPSSIGELESLVILDLKACHNLERLPDDISSMKSLTHLIMSDCCLLEGMPKGIEKLTNLQVLKGLLISTPEKTPCRISDL
AKNLTNLKRLSIRIGSEAVIRDGEFKSLENFRKLKHLKISWGVSDPRYAEIPINLPLHLRKLHFECFPGKRLIKCLPRSDKWPC
ELNISGGKLESLSVHMNRNWTVEILRLKYLKQFNVDINNLKVWFPALKYVEIKQISNHSYIEHQWNLPYESKYRMKREEI

>XP_022635884.1

MDPIVSTATESALNITASVVKRQVGYFFNYKDKIRELESYIEKLEHNKERLQHQVDNALRNAEEIEKDVHHCLTLMDDKIK
EYKSYINDECHAKTICSFGFFPNNFRLRYQLGRKATKMVKEIIGDELWKTSFDNVSYQEFPSIDATFSNNGYESFGSRTKT
MEMIMKALQDSTVGLIGVYGPGGVGKTTLVKEIAKKAREKKLFKIVIIANITGNPDFKNIQEQIAGMLGMKLEGESEIAR
VDRIRKRLKNEKESTLIILDDLWARLDFNKLGIPCNDDASQQEVNDMSDFGSNNDPSDFGYNKTEIKELSKVDLDKMKK
EKLSNDYRRGKILLISRNKQVLCNEMDVQQSSIFSVGVLDEKESETLLKKVVGVKNSEFDKNATEIAKWSAGFPIALVSIG
RTLKNKSLSTWEDVCQQIKRQNFTSEWGFTDFSIKLSYDHLKNEELKSIFLHCARMGNDALIMDLVKFCVGLNLLPGVH
TITNARKRVKEIIQELEESSLLVKSYSIDRFNMHDIVRDVALSILSKEKHVLFMKNAVLDEWPHENDFERLNSYKIVIGEFNL
LNLLTVGEFRVPDKYEEVKFLAINLKEGIDIHSEKWIKMLFKTVERLNS

>XP_022635982.1

MDSKIGGSMTKYVRSQLGYITSYKSDLEKLKTEVETLKVGKSRVQETVDKAKGNGEKILNNVQIWLKKVDATIAEANNLI
FNDVQENYPIHIPNIQFRLRHSKKLQKMTREIYGVLSEGNFDKISKPPTIKEIQQVLKDPKIYKIGLYGIDGVGKTTLVKELA

REVEKEGSFDVVAMAEVTDSPDVENIQCQIANALALKFDEESKEERVEKLRRRISKEKSILVILDDIWGKVDLAELGIDKG
CKLLLTSERLNVLRCQMGTEKNFKVEVLSDEDSWKLFQKIAGQAIKLISTNKSPYELVEINSMVEDVPKYCNGFPLFVVVV
AKALRTKNLATWKDALKQLRGLSEKGKFEEVVCPLELGYRYLESDELKTLFLFIVSLGPGRIHTGELFSCYWGLHGDSHELT
EARNKYYQFIDDLRASSLLLEVEIEYVRMHDSVRDTAKAISSRTHLTYEVQKFTQKDQWDIDQLKKCHYINLPSYNLDELP
EKLDCPELKLMSLKSDLGHLKIPDHFFAGMGEVKVLNLHRMSFAPSPPPSFRLLSNLRSLNLYECVLDDITMVAELTSLEIL
SLERSKIQELPKEIGQLTQLRMLNLTNCYQLKTISRYLIYSLMCLEELYMGNCNIQWEAEGGKSQTNNASLGELRNLNRLT
TLDLSIHDTSVLPADMDVFKQLRRYNIYIGNMWKWSSFWSGDAREISRTLKLVDSLNTEIFLNQGIQMLFTTVEDLSLAK
INFADDVFYKLNREAFQHLRHLYVQNSDAFCNLETLILCDLRNMYGPFAKQTLVLWNLHNMEDISYAPLATQCFENLQ
VFKVQGCRKLKKLLSYSVAKNLAQLQQMEIFDCTAMEEIVCEEKLEDENLHNIKDNAIHCFEKLRVIKVHGCHKLKKLLSY
SLAKNLFELQEMEIFNCTIMDEIIFEEKFQDESLHNVKDTGTVIFEKLQVIKVHGCHKLKKLLSYSLAKNLFQLQEMEIFNC
TIMDEIIFEEKFEDESLHNVKDTGRVFFEKLQVIKVHGCHKLKTLLPYSLAKNLSQLQEIEIFDCTNMEEIISKKKIEDENLHN
VKDFDTHFLEKLQVLKVQGCYNLKILLPYSLAKNLSQLREIEIFDCTNMEEIISEENFEDENLNHMEDIGTHFFEKLQVINV
KSCYKLKKLLPYALAKNLSQLQEMKLEDGKEFPKIVLPKLHSLTLDTLPNLCSFSLPLEIDKDDGSIPLPLFNQKVTCPNLDM
LVIINLNRLNSIWYNQQAPSSVRNLKTIKITRCNALHHVFPTAVAKELLQLQVLEISTSMIEMIVEDNYIQGPPDNITFTKLE
QLKLEYLPRLTKFCQESYNFKFPSLQTVEVIGCPNLKFSGHLNFTSIAQLEWRAKNGRKDDELNNLFNEKVAMPNLENLR
LSNIGSYGKIWDEKWRVPFFSENLKYLIEDEYHNHTESLFSSSTARELTKLKLLDIQSCPALVQIFVQQEEVTFQNLETLLIN
DMSGLRSIWNNLQGPSSFHKLNKIQIIGCHALHHVFPVVVAKELQQLQELQISRSINIENIVVKSHRGGAFGKHKNKSHP
FFKKIEENTDDDDDDDDEVFMKFKENKGDDANEIVFMKLKELYLENLPKLRSFSKKSDNFKFPAFEKVTFPNLEKLIISGM
SGLQSLWNKLQGPNSFHNLNKIQVTGCRALVHVFPVVVAKELQQLQVLEISRSINIENIVVKSQSGGALGKHQNESHPF
FKKFEENTDDDDVVFMKVKENKGDDVIDIVFIKLKELKVKTSIFQPSKKYM

>XP_022636911.1

MYSCVFNILRRIFRCVEEPDRITSGMSEGMTMSHPTAPIGPSIASDAIIHHNPSSSNNAYDVFVSFRGEDTRNNFTSFLFG
ALRRQGILAFKDDQCIKKGDFIAPELLQAIQGSQVFVVVLSKNYASSTWCLRELVEILNCCQTSARPLIPIFYDVEPTTVRN
QKGCYEKAFAEHEKRSREGKVEMEEVQRWKEALRKVADISGSEIGNKPQNEKIEKIVQEITNNLRPKILNLPRDELVGIET
RVQDLGNILRFDLRNDVRVVGISGMGGIGKTTLVRALYERICHQYNYRCFIDDVSKIFLDSHLLGLQKQLISQTLNEKNVEI
CNVIEGTCLVQSKLNNAKALIVFDNVDEVQQLRIFSGNRDNLLRECLGGGSRIIIVSRDEQILKIHGVDDIYQVQPLNRED
AIQLFCRNAFKVNYILSDYEKLADEIPSHVEGHPLAIETIGSSLFGRSLSQWESVLEGLKENKSKNIMDILRISYIQLEEKYKQ
TFLDIACFFNSYEEEHLKEILNFRGFHPEDSIQVLIDKSLITRSFGCFIHMHSLLVDLGRCIVREVSPKEPIKWSRLWTRKDL
HDAMSENEATRNLEAIVLKRGNDVGILDKTIKADGLSKIKHLKLLDVGDVNVEFSGSLSHLSNELGYLIWYRYPFECLPQS
FQPHKLVQLDLRWSSIQRLWEGTKDCKRLKYLPDLPSRTVLPSEPFTLRFLSAILNTNLPPIQRTGLIIFGCPELVEIEECIRK
SFSWTIQIIEVWMLFPSSFCFLIILLCRRTTTNQ

>XP_022636914.1

MYSYVFNILRCILRRFVRRVEEPDRIMSGMSEGMTVSHPTAPIGGPSVASDAIIHHTPSSSHAYDVFVSFRGEDTRNNFT
SFLFGALCRQGIVAFKDDECIKKGDFITPKLLQAIKGSQVFVVVLSKNYASSTWCLRELVEIFNCCETSARPVIPIFYDVEPT
TVRHQKGCYEKAFAEHENRFRKDKVKMEEVQRWKEALRKVADISGSEIANKLQNEQIEKIVQEIKNHLRPKILNLPRDEL
VGIEDRAQNLRNILRFDLPNDVRVVGISGMSGIGKTTLIRALYERICHQYSYSCFIDNVNKIFVDSGSMGLQKQLISQTLN
EKNVEIRNLTEGTCLVQSKLNNANALIVFDNVDQVEHLNIFSGNRVNLLRECLGGGSRIIIVSRDEQILKIHGVDDIYQVQ
PLNWEDAIQLFCRNAFKDNYILNGYEKLSYEILSHVDGHPLAIETIGSSLFGRSLSQWKSVLEALEENKSKNIMDILRISYIQ
LEERYQQTFLDIACFFNYQDEEEVKEILNFRGFHPEDSIEVLIDKSLVIRDFEGFIYMHNLLEDLGRCIVKEVSPKEPIKWSRL
WTCKDLHDAMSENKVTQNLEAIFLTKDDVDDDDDDVVDDDDDEIRDDETIKADGLSKIKHLKLFYVRNVNVKFSGSLS
HLSKELGYLVWYRYPFECLPQTFQPHKLVELNLFGSSIQRLWEGTKRLPHLKRLDLSYCTSLIEMPNVSKALNLEWIILEGC
VQLQELDPSIWSLRKLVDLNLRDCKKLVILSNTILGLHSLKYLDFSGCSIIDSKDETRNTEDLISSLPPSSPLSCLCELDLNFCN

LVQIHDYIGKLRFLERLNLKGNNFVTLPNLKDLSRLYVLNLQDCKRLKYLPDLPSRTVRNSKPFMLSRLRSIKFYSPHYWNE
ENTGFTFPGLIIFGCPKLVEIEQCIRKSFSWTIQILKATYPWPFPQSIIPGSQIPSLFNNEFVNGDIEYLDKEYLTVDPPPVPH
YNNFIGVLCCVIFRLYNKQIPIDFGRDHMWLHYEKVYMDERGIHPCRLRVRSIGERLGYENSVTVDVKKFQYFWVNEQ
DLINLKMMHCATRKRKISVIEENG

>XP_022637169.1

MAAELVGGALLSAFLQVAFDRLASPQFVDFFRRRKLDEKLLGNLNIMLHSINALAHDAEQKQFTDPHVKAWLFSVKEA
VFDAEDLLAEIDYELTRXQVEAXSEXXTFTXKVSNFFNSTFSSLNKKIELEMKELLEKLEYLAKQKGSLGLKESTYSGDRSGS
KLSQKLPSSSLVVESVIYGRDVDKEMIINWLTSETDNHNHLSILSIVGMGGLGKTTLAQHVYNDPKIEEAKFYIRAWVCVS
DHFDVLTVTKSILEAITKSKDDSGDLEMVHGRLKEKISGRKFLLVLDDVWNERLEKWEAVRTPLSYGAPGSRILVTTRVEK
VASNMRSKVHHLKQLEEDECWKVFNKQALKDDDLELNDVKKEIGRRIVEKCKGLPLALKTIGSLLRTKSSILDWQSVLEN
NIWDLPKEVEIIPALLLSYQHLPSHLKRCFAYCALFPKDYEFDKKKLILLWMGEGFLHHSQQNKNVQEIGEQYFDDLLTRS
FFLQSSSRMRFLMHDLLNDLAKYVCADFCFRLKFDKGSCIPKTTRHFSFSFKYARYFDGFGSLTNAKRLRSFVHITNIRGG
GPPCLFERIHELFSKLKYLRVLSLNGYIELDEVPDSVGDLKHLHSLDLSLTSIQKLPDSVGLLYNLLILRLNGCFHFKELPSSLH
KLTKLRCLEFERTQVTKMPMHFGELKNLQVLNKFYVNRNNEIKQLGGLNLHGRLSISDLQNIVNPLDALEANLKNKHLV
ELKLIWNHNHIPDDPRKEKKALENLQPSKQLKSLKINNYGGTQFPSWVFDNLLSNLVSLELKDCKYCLCLPSLGLLSSLKTL
KITGLDGIVSIGAEFYGSNSSSFKCLERLIFFDMKEWEEWECKTTSFPRVEQLVIHDCPKLKGLSEQLLHLKALSIETCDNLII
REHNVDTSALESLNTHSCPLVNIPMTHYDFIQDMIIVGGCHSLTIFQLNFFPMLRFLSLEGCQNLQRISQEHAHNHLKK
MSICKCPQFESFAPFVQIIDIRQEENLELLPKRMQILLPSLIELHIIGCPKVEKFPDGGLPSNLKLVSLSSLKLIASLRDTLDVN
TCLQSLYIKSLDVESFSDEVLLPPSLTSLTINCCQNLKKLDYKLLYNVSSLTLGDCPNLQCLPEEGLPKSISSLDIWRCPLLKHS
CQKTEGKDWEKISHIQNLSVQ

>XP_022637171.1

MAAELVGGALLSAFLQVAFDRLASPQFVDFFRRRKLDEKLLGNLNIMLHSINALAHDAEQKQFTDPHVKAWLFSVKEA
VFDAEDLLAEIDYELTRXQVEAXSEXXTFTXKVSNFFNSTFSSLNKKIELEMKELLEKLEYLAKQKGSLGLKESTYSGDRSGS
KLSQKLPSSSLVVESVIYGRDVDKEMIINWLTSETDNHNHLSILSIVGMGGLGKTTLAQHVYNDPKIEEAKFYIRAWVCVS
DHFDVLTVTKSILEAITKSKDDSGDLEMVHGRLKEKISGRKFLLVLDDVWNERLEKWEAVRTPLSYGAPGSRILVTTRVEK
VASNMRSKVHHLKQLEEDECWKVFNKQALKDDDLELNDVKKEIGRRIVEKCKGLPLALKTIGSLLRTKSSILDWQSVLEN
NIWDLPKEVEIIPALLLSYQHLPSHLKRCFAYCALFPKDYEFDKKKLILLWMGEGFLHHSQQNKNVQEIGEQYFDDLLTRS
FFLQSSSRMRFLMHDLLNDLAKYVCADFCFRLKFDKGSCIPKTTRHFSFSFKYARYFDGFGSLTNAKRLRSFVHITNIRGG
GPPCLFERIHELFSKLKYLRVLSLNGYIELDEVPDSVGDLKHLHSLDLSLTSIQKLPDSVGLLYNLLILRLNGCFHFKELPSSLH
KLTKLRCLEFERTQVTKMPMHFGELKNLQVLNKFYVNRNNEIKQLGGLNLHGRLSISDLQNIVNPLDALEANLKNKHLV
ELKLIWNHNHIPDDPRKEKKALENLQPSKQLKSLKINNYGGTQFPSWVFDNLLSNLVSLELKDCKYCLCLPSLGLLSSLKTL
KITGLDGIVSIGAEFYGSNSSSFKCLERLIFFDMKEWEEWECKTTSFPRVEQLVIHDCPKLKGLSEQLLHLKALSIETCDNLII
REHNVDTSALESLNTHSCPLVNIPMTHYDFIQDMIIVGGCHSLTIFQLNFFPMLRFLSLEGCQNLQRISQEHAHNHLKK
MSICKCPQFESFAPFVQIIDIRQEENLELLPKRMQILLPSLIELHIIGCPKVEKFPDGGLPSNLKLVSLSSLKLIASLRDTLDVN
TCLQSLYIKSLDVESFSDEVLLPPSLTSLTINCCQNLKKLDYKLLYNVSSLTLGDCPNLQCLPEEGLPKSISSLDIWRCPLLKHS
CQKTEGKDWEKISHIQNLSVQ

>XP_022637174.1

MAAELVGGALLSAFLQVAFDRLASPQFVDFFRRRKLDEKLLGNLNIMLHSINALAHDAEQKQFTDPHVKAWLFSVKEA
VFDAEDLLAEIDYELTRXQVEAXSEXXTFTXKVSNFFNSTFSSLNKKIELEMKELLEKLEYLAKQKGSLGLKESTYSGDRSGS
KLSQKLPSSSLVVESVIYGRDVDKEMIINWLTSETDNHNHLSILSIVGMGGLGKTTLAQHVYNDPKIEEAKFYIRAWVCVS
DHFDVLTVTKSILEAITKSKDDSGDLEMVHGRLKEKISGRKFLLVLDDVWNERLEKWEAVRTPLSYGAPGSRILVTTRVEK

VASNMRSKVHHLKQLEEDECWKVFNKQALKDDDLELNDVKKEIGRRIVEKCKGLPLALKTIGSLLRTKSSILDWQSVLEN
NIWDLPKEVEIIPALLLSYQHLPSHLKRCFAYCALFPKDYEFDKKKLILLWMGEGFLHHSQQNKNVQEIGEQYFDDLLTRS
FFLQSSSRMRFLMHDLLNDLAKYVCADFCFRLKFDKGSCIPKTTRHFSFSFKYARYFDGFGSLTNAKRLRSFVHITNIRGG
GPPCLFERIHELFSKLKYLRVLSLNGYIELDEVPDSVGDLKHLHSLDLSLTSIQKLPDSVGLLYNLLILRLNGCFHFKELPSSLH
KLTKLRCLEFERTQVTKMPMHFGELKNLQVLNKFYVNRNNEIKQLGGLNLHGRLSISDLQNIVNPLDALEANLKNKHLV
ELKLIWNHNHIPDDPRKEKKALENLQPSKQLKSLKINNYGGTQFPSWVFDNLLSNLVSLELKDCKYCLCLPSLGLLSSLKTL
KITGLDGIVSIGAEFYGSNSSSFKCLERLIFFDMKEWEEWECKTTSFPRVEQLVIHDCPKLKGLSEQLLHLKALSIETCDNLII
REHNVDTSALESLNTHSCPLVNIPMTHYDFIQDMIIVGGCHSLTIFQLNFFPMLRFLSLEGCQNLQRISQEHAHNHLKK
MSICKCPQFESFAPFVQIIDIRQEENLELLPKRMQILLPSLIELHIIGCPKVEKFPDGGLPSNLKLVSLSSLKLIASLRDTLDVN
TCLQSLYIKSLDVESFSDEVLLPPSLTSLTINCCQNLKKLDYKLLYNVSSLTLGDCPNLQCLPEEGLPKSISSLDIWRCPLLKHS
CQKTEGKDWEKISHIQNLSVQ

>XP_022637178.1

MAAELVGGALLSAFLQVAFDRLASPQFVDFFRRRKLDEKLLGNLNIMLHSINALAHDAEQKQFTDPHVKAWLFSVKEA
VFDAEDLLAEIDYELTRXQVEAXSEXXTFTXKVSNFFNSTFSSLNKKIELEMKELLEKLEYLAKQKGSLGLKESTYSGDRSGS
KLSQKLPSSSLVVESVIYGRDVDKEMIINWLTSETDNHNHLSILSIVGMGGLGKTTLAQHVYNDPKIEEAKFYIRAWVCVS
DHFDVLTVTKSILEAITKSKDDSGDLEMVHGRLKEKISGRKFLLVLDDVWNERLEKWEAVRTPLSYGAPGSRILVTTRVEK
VASNMRSKVHHLKQLEEDECWKVFNKQALKDDDLELNDVKKEIGRRIVEKCKGLPLALKTIGSLLRTKSSILDWQSVLEN
NIWDLPKEVEIIPALLLSYQHLPSHLKRCFAYCALFPKDYEFDKKKLILLWMGEGFLHHSQQNKNVQEIGEQYFDDLLTRS
FFLQSSSRMRFLMHDLLNDLAKYVCADFCFRLKFDKGSCIPKTTRHFSFSFKYARYFDGFGSLTNAKRLRSFVHITNIRGG
GPPCLFERIHELFSKLKYLRVLSLNGYIELDEVPDSVGDLKHLHSLDLSLTSIQKLPDSVGLLYNLLILRLNGCFHFKELPSSLH
KLTKLRCLEFERTQVTKMPMHFGELKNLQVLNKFYVNRNNEIKQLGGLNLHGRLSISDLQNIVNPLDALEANLKNKHLV
ELKLIWNHNHIPDDPRKEKKALENLQPSKQLKSLKINNYGGTQFPSWVFDNLLSNLVSLELKDCKYCLCLPSLGLLSSLKTL
KITGLDGIVSIGAEFYGSNSSSFKCLERLIFFDMKEWEEWECKTTSFPRVEQLVIHDCPKLKGLSEQLLHLKALSIETCDNLII
REHNVDTSALESLNTHSCPLVNIPMTHYDFIQDMIIVGGCHSLTIFQLNFFPMLRFLSLEGCQNLQRISQEHAHNHLKK
MSICKCPQFESFAPFVQIIDIRQEENLELLPKRMQILLPSLIELHIIGCPKVEKFPDGGLPSNLKLVSLSSLKLIASLRDTLDVN
TCLQSLYIKSLDVESFSDEVLLPPSLTSLTINCCQNLKKLDYKLLYNVSSLTLGDCPNLQCLPEEGLPKSISSLDIWRCPLLKHS
CQKTEGKDWEKISHIQNLSVQ

>XP_022637180.1

MAAELVGGALLSAFLQVAFDRLASPQFVDFFRRRKLDEKLLGNLNIMLHSINALAHDAEQKQFTDPHVKAWLFSVKEA
VFDAEDLLAEIDYELTRXQVEAXSEXXTFTXKVSNFFNSTFSSLNKKIELEMKELLEKLEYLAKQKGSLGLKESTYSGDRSGS
KLSQKLPSSSLVVESVIYGRDVDKEMIINWLTSETDNHNHLSILSIVGMGGLGKTTLAQHVYNDPKIEEAKFYIRAWVCVS
DHFDVLTVTKSILEAITKSKDDSGDLEMVHGRLKEKISGRKFLLVLDDVWNERLEKWEAVRTPLSYGAPGSRILVTTRVEK
VASNMRSKVHHLKQLEEDECWKVFNKQALKDDDLELNDVKKEIGRRIVEKCKGLPLALKTIGSLLRTKSSILDWQSVLEN
NIWDLPKEVEIIPALLLSYQHLPSHLKRCFAYCALFPKDYEFDKKKLILLWMGEGFLHHSQQNKNVQEIGEQYFDDLLTRS
FFLQSSSRMRFLMHDLLNDLAKYVCADFCFRLKFDKGSCIPKTTRHFSFSFKYARYFDGFGSLTNAKRLRSFVHITNIRGG
GPPCLFERIHELFSKLKYLRVLSLNGYIELDEVPDSVGDLKHLHSLDLSLTSIQKLPDSVGLLYNLLILRLNGCFHFKELPSSLH
KLTKLRCLEFERTQVTKMPMHFGELKNLQVLNKFYVNRNNEIKQLGGLNLHGRLSISDLQNIVNPLDALEANLKNKHLV
ELKLIWNHNHIPDDPRKEKKALENLQPSKQLKSLKINNYGGTQFPSWVFDNLLSNLVSLELKDCKYCLCLPSLGLLSSLKTL
KITGLDGIVSIGAEFYGSNSSSFKCLERLIFFDMKEWEEWECKTTSFPRVEQLVIHDCPKLKGLSEQLLHLKALSIETCDNLII
REHNVDTSALESLNTHSCPLVNIPMTHYDFIQDMIIVGGCHSLTIFQLNFFPMLRFLSLEGCQNLQRISQEHAHNHLKK
MSICKCPQFESFAPFVQIIDIRQEENLELLPKRMQILLPSLIELHIIGCPKVEKFPDGGLPSNLKLVSLSSLKLIASLRDTLDVN

TCLQSLYIKSLDVESFSDEVLLPPSLTSLTINCCQNLKKLDYKLLYNVSSLTLGDCPNLQCLPEEGLPKSISSLDIWRCPLLKHS
CQKTEGKDWEKISHIQNLSVQ

>XP_022637182.1

MAAELVGGALLSAFLQVAFDRLASPQFVDFFRRRKLDEKLLGNLNIMLHSINALAHDAELKQFTDPHVKAWLFSVKEAV
FDAEDLLAEIDYELTRSQVEAQSEPQTFTYKVSNFFNSTFNSFNKKIESEMKEVLEKLEYLANQKGALGLKEGTYSADRSG
GKVSQKLPSSSLLVESVIYGRDADKEKICNWLTSETDTHNHPSILSIVGMGGLGKTSLAQHVYNDPKIEEAKFDIKXWVCI
SDHFDVLTVTKTILEAITKSKDDSGDLEMVHGRLKEKISGMKFLLVLDDVWNERQEEWEVVRTPLSYGAPGSRILVTTRI
EKVASNMRSEVHHLKQLEEDECWKVFEKQALKDDDLRLNGEKKEIGKRIVEKCKGLPLALKTIGSLLRTKSSSSYWKSVLE
SDIWELPKEVEIIPALLLSYQHLPSHLKRCFAYCALFPKDYEFDKKELILLWMAEGFLPHSKMIKNVQEIGEQCFDDLLRRS
FSLESSFEMRFVMHDLLNDLAKYVCADFCFRLKFDKGSCIPNTIRHFSFSLDDVEDFDGLGSLTDAERLRSFYSITNNCSY
GINPCQFTILVHESFSKFKFLRVLSLNGYSELSEVHDSVGDLKHLHSIDLSYTLIQNLPNSIGFLYNLLILKLNFCSFLEELPSNL
HKLTKLHCLEFEHTKVTKMPMHFGELKNLQVLSTFFVNRNNEVISIKQLGRLNIHGRLSINELQNIVNPLNAIEANLKNH
HLEELELKWSLNHIPDDPRKEQKVLENLQPSKQLKSLKINNYGGTQFPSWVFDNSLSNLVSLRLADCKYCLCLPPFGLLSS
LRMLHIIGFDGIVSIGAEFYGSSPSSFKSLKVLEFYNMKEWEEWECKTTSFPLLQHLSIQTCPKLKSLPEQLLHLKNLDIHSC
DKLIISVNSMFISSLQLWSIILCPLVEIPMIHYDCIEAMEINNDCVSFTIFQLDCFPKLRLLQLSGCQNLRRISQGHTHNHLK
VLKIYGCHQFESFPSEGLSAPWLQIISIRKAENLKLLPKRMQILLPSLTELEIIDCPQIEMFPGGGLPSNIKGMSLSSLKLIASL
RDTLDVNTCLKSLTIEKLDVESFPGEVLLPRSLTSLRFLFCLNLKKIDYKGLCNVSSFTYCGCPNLHL

>XP_022637185.1

MAAELVGGALLSAFLQVAFDRLASPQFVDFFRRRKLDEKLLGNLNIMLHSINALAHDAELKQFTDPHVKAWLFSVKEAV
FDAEDLLAEIDYELTRSQVEAQSEPQTFTYKVSNFFNSTFNSFNKKIESEMKEVLEKLEYLANQKGALGLKEGTYSADRSG
GKVSQKLPSSSLLVESVIYGRDADKEKICNWLTSETDTHNHPSILSIVGMGGLGKTSLAQHVYNDPKIEEAKFDIKXWVCI
SDHFDVLTVTKTILEAITKSKDDSGDLEMVHGRLKEKISGMKFLLVLDDVWNERQEEWEVVRTPLSYGAPGSRILVTTRI
EKVASNMRSEVHHLKQLEEDECWKVFEKQALKDDDLRLNGEKKEIGKRIVEKCKGLPLALKTIGSLLRTKSSSSYWKSVLE
SDIWELPKEVEIIPALLLSYQHLPSHLKRCFAYCALFPKDYEFDKKELILLWMAEGFLPHSKMIKNVQEIGEQCFDDLLRRS
FSLESSFEMRFVMHDLLNDLAKYVCADFCFRLKFDKGSCIPNTIRHFSFSLDDVEDFDGLGSLTDAERLRSFYSITNNCSY
GINPCQFTILVHESFSKFKFLRVLSLNGYSELSEVHDSVGDLKHLHSIDLSYTLIQNLPNSIGFLYNLLILKLNFCSFLEELPSNL
HKLTKLHCLEFEHTKVTKMPMHFGELKNLQVLSTFFVNRNNEVISIKQLGRLNIHGRLSINELQNIVNPLNAIEANLKNH
HLEELELKWSLNHIPDDPRKEQKVLENLQPSKQLKSLKINNYGGTQFPSWVFDNSLSNLVSLRLADCKYCLCLPPFGLLSS
LRMLHIIGFDGIVSIGAEFYGSSPSSFKSLKVLEFYNMKEWEEWECKTTSFPLLQHLSIQTCPKLKSLPEQLLHLKNLDIHSC
DKLIISVNSMFISSLQLWSIILCPLVEIPMIHYDCIEAMEINNDCVSFTIFQLDCFPKLRLLQLSGCQNLRRISQGHTHNHLK
VLKIYGCHQFESFPSEGLSAPWLQIISIRKAENLKLLPKRMQILLPSLTELEIIDCPQIEMFPGGGLPSNIKGMSLSSLKLIASL
RDTLDVNTCLKSLTIEKLDVESFPGEVLLPRSLTSLRFLFCLNLKKIDYKGLCNVSSFTYCGCPNLHL

>XP_022637195.1

MAAELVGGALLSAFLQAAFDKMASPQFVDFFRRRKFDEKLLANLNIMLHSINSLADDAEQKQFRDPHVKAWLFAVKE
AVFDAEDLLDEINYELTRCQMEAESQSQSITYKVSNFFNSTFSSFNKKTDSGLKEVLEKLEYLSRQKGALGLKECTYSSVGS
RGNISQKLPSTSLVVESVIYGRDADKEIIFNWLTSETKNHHNHPSILSIVGMGGLGKTTLVQHVYNDPKIDTAKFDIKVW
VCVSDHFDVLTVTKTILEAIDNKKDDSGNLEMVHKKLKEKLSGRKFLLILDDVWNEKREEWEAVRTPLSYGAPGSRVIVT
TRAERVASNMRSEVHRLKQLQEDECWKVFIKHALKDDDLKLNDDQKEIGRRIVEKCKGLPLALKTIGSLLHTKSSISDWK
SVLASDIWDLPKEDSEIIPALYLSYHYLPSHLKRCFSYCAIFPKDYEFVKKELIFLWMAENILQCPQQIRHPVEVGEEYFNDL
LSRSFFQQSRSKRHFIIHDLLNDLAKYVCADFCFRLKFDKGKCIPKATRHFSFAFDDVKYFDGFGSLTEAKRLRSFIPLTKFG
KGYYLDYSWKFKISIHDLCSKMKFLRVLSFNCCSDLREVPDSVGDLKHLRSLDLSSTEIQKLPDSTCLLFNLQILKLNYCLNI

VELPSNLYKLTNLCCLEFKRTRVTKMPMHFGELKNLQVLNTFIVDKNSEFSTKQLGRLDLHGRLSIKELQNIKNSSDALVM
DLKNKRHLVTLKFKWNKNHILDDPRKEKKVFENLQPSKQLETLGINNYGGTEFPNWVFDNSLSNLVFLQLKDCKYCLCL
PPLGLLSSLKTLKIIGLDGIVSIGNEFYGSESSSFKSLERLEFYNMKEWEEWDCKTDSFPRLQTLSMNECPKMKGLSEQLLH
LKKLIIRSCEILVINEHYMDPSTLKILRIYSCPLTNVPITLYNFLEEMEINGGCDFLTTFPLDLFPKLCSLKLVRCRNLQRISQEH
THNHLKYLIIEKCPQFESFPSIGLSAPWLQTIEIQGAENLKLLPRHMQILLPSLTDLHIIDCPQVEMFPDGGLPSNVKYVSLS
SFKLIASLRESLNANTCLERLCITNGDMEFFPGEVLLPHSLTSLQISCCPNLKKIDYKGLPKSISTLNIWDCPLLQQCCQNSE
DKD

>XP_022637201.1

MAITLSEGASSSSGFDRGWTYDVFLSFRGEDTRRSFTGFLYHGLCQRGINVFIDDDKLRKGEDLSPTLLGAIQESRIAIIVF
SQNYAFSTWCLDELAKIIDCYKTRGQLVWPVFFHVDPSVVRHQRGTFQTAMAQHEVRFKGNVEKLQKWKKALFEASN
FSGWNLENGYEFQIIQDIVEEASRKLSHTILHIAEYPVGIEIRISEVMPLLQIEPGEDIRVIGIYGLGGIGKTTIARALYNMIAD
QFEATSFLSDIRESSNQRQGLVQLQESLLFDTVGDKNIKLGSIYKGIPIIKKRLCCKKVLLIIDDVDRLEQLQALAGGRDWF
GFGSVIIITTRDKHLLSAHQVDKTYEVKKLNYGEAFELFTWSAFKRKAPDAGYLEVSNRVVLYAEGLPLALKIMGSNLFGK
TVEEWKSALGKYEKIPNKEVQNVLRVTYDNLEENEKEIFLDVACFFKGETVEYVENTLQGCGFYPTIGISVLIDRSLVSIDEY
NRLRMHDLIQDMGREIVREVSPLEPGKRSRLWYHEDVFEVLTENKGTYRIQGMMVDLPDDYMVHLKDDSFKKMKNL
KILIVRNGNFFGSPQHLPNSLRLLDWMKYPSSLPSSFQPKKLVVLNLSGSRFTMQEPFKYLDSLTSMDLSSCELLTKLPEIA
GVPNLTQLTLDYCTNLEEVHESVGFLEKLVEFRAYGCTKLKVFPNAIRLTSLRSLILNWCSSLQNFPAILGKMDNLISISIEG
TGIKELPPSIGNLVSLQELSMTSCLNLKELPHNFDMLQSLTNLDMEGCPHLRNFLTKLANMGESTHTFGNILSLNVENCG
LIDEDLPIIFNNFPNLASVVLSGNHFKALPSCIQQCPCLELLHLDNCKNIQEISAFPPNMQYINAQNCISLSEESSNLLFNKV
CIFFKSSMLSLVTTHFHLNFLVQETFEGWELQAMVPGTMVPEWFDHITKGEYMTFWVRERFPAIIICFVLEVESEMKKIF
NCEIRFYINGEEVYELQIPRGFSDMVTDHVWLYDLRTHSSINWRSLDLYLMDGWNQVEISCEKISGASNLTVSWCGVH
VVKQEANMKDILLTDPDPDLDSVIASGSNTLVSDHPVKAQPQSQVTSFTLQTPQNNNSSTIVLPTTVQTSLTVNDADM
EAFYAVLDDEISVVSLNNDSTMVSKLTNQRPSEETKKALKTLQAHVTKEFSALLGPNEYSTVNDTLEYLTNLPAEDEISVEI
RSLIIQVSRQFTRWSRDYTSENKKIESTTAKLLKADELEKCLEANKTNFKQVMCMENELCNDLAHLEQRKRELEEQINAV
KANISASEAAKNMASEIKRELFGKAKILKAERDELREQVPHLRDEQELAKKIQSNIRDEWSKLGEKFNYGLRHGKID

>XP_022637226.1

MAAELVGGALLSAFLQVAFDRLASPQFVDFFRRRKLDDKLLGNLNIMLHSINALAHDAEQKQFTDPHVKAWLFSVXEA
QSEPQTFTYKVSNFFNSTFNSFNKKIESEMKEVLEKLEYLAKQKGALGLKEDTYSGDRSGIKVSQKLPSSSLVVESIIYGRD
ADKEIIFNWLTSETHNQNLSILSIVGMGGLGKTTLAQHVYNDQRMETKFDIRAWVCVSDHFDVFTVTKTILEAITKSKDD
SGDLEMVHGRLKGKVSGKKFLLVLDDVWNEKLEEWEAVRTPLSYGAPGSRILVTTRIEKVASNMRSEVHHLKQLEENE
CWKVFNKQALKDDDLELNDEKKEIGRRIVEKCKGLPLALKTIGSLLRTKSSILDWKSVLESHIWDLSKEVEIMPALLLSYQH
LPSHLKRCFAYCALFPKDHEFLKEELILFWMGEGFLYHSQQNKNLLEIGEQYFDDLLTRSFFFPSNFELHFVMHDLLNDLA
KYVCADFCFRLKFDKGNCIPKITRHFSFAYDDIRYFDSFGSLTDAKRLRSFVQITNDCLFPGLPGPFEILISEVFSKLKFLHVLS
LKGYYGLKEVPDSVGNLKHLHLLDLSSTRIQKLPDSVGLLYNLLILRLNGCSNLKELPSSLHKLTKLRCLEFERTGVTKMPM
HFAELKNLHVLNMFCVGRNSEFSTKQLGGINLHGRLSINELQNIVNPSDALEANLKNKHVVELNLIWNSNHIPNDPRKE
KKVLENLQPSNQLERFSIRSYCGTQFPSWVFDNSLSNLVSLELKDCKYCLCLPPLGLLSSLKTLEITGLDGIVSIGAEFYGSNS
SSFKCLERLLFFKMKEWEEWECKTTSFPRLEQLVIHDCPKLEGLSEQLLHLKDLSIETCDSLIIREHNVDTSTLECLKTYSCPL
VNIPMTHYDFLQDMTIVGGCDSLTIFQLDLFPMLGSLHLGRCQNLQRISQVHAHNNLKEMSIYECPRFESFPNEGLSAP
LVQIIDIRQVENLMLLPKRMQILLSSLTELKIIDCPKVEKFPDEGLPSKVKDMSLSSLKLIASLRENLDVNTCLESLSIESLDVE
SFPDNVLLPPSLTSLRIIHCQNLKKLDYKLLYHLSSLELGDCPNLQCLPEEGLPKSISYLDIWRCPLLEQRCQKPEGKDWIKI
AHIENLSVG

>XP_022637356.1

MDILNGFLSDVLKEVVCGAVNQLQYSFCFNSFVKELEKEKENLIETKISVKDRVIHAQRQTLKTAEVIDKWLENANTSSES
VNRLLRETNTKKSCFFEFCPNWIWRYRLGKKLAIKKADLQKIIQEGRQYIQLERIASIPSNTFDILTEKSINFDSRKNAFDQL
VKALKDDGIAMIGLYGMGGCGKTTLAMEVKKIAEAEHLFDKVVFVPVSSTVEVPRIQEKLASSLQYQFPENQEMERAQ
RLCMRLTQENKILMILDDVWEKLDFGSIGIPSSEYRKGCKILITTRLEEVCNSMDCQKNIYLPILTDEESWTLFQNKALISK
DTPENIKHLAKSISNECKGLPVAIVAVASSLKGKKEVIWHSALNKLRSSKPINISRGLQDPYMCLKLSFDNLDTEEAKSLFLL
CSVFPEDYEISVECLIRCAIGLGVTGEVHSYEEARTEVIAAKIKLVSSCLMLEVNDECVKMHDLVRDVAHWIAKNENKMI
KCEVEKDVSLEEGPIRYLWCVKFPDSMDCSNLEFLSIQTNLEVSDGIFERMGKLRVLIITNKSIPTQLLTMSFKSLINVRCLV
LQYWKLRDISFVRDMKKLQSLSFHNCSWPSFLDLQTDVAVTTLTNLKLLEFISCDIESNIFEEIKRIPFLEEFYIYKDNNYWN
DPKEESVKFSNSFSVSQMLQRYGIVLGYSSFRNSYRFQEFCSCERTLVMNYFDISNEVIKGLAKKAKELSVGNIEGGAKN
MMPDIFKIEEGMNELKELKMHNCEEIECLIDNSSNLSKMGNIFSKLRYMEIFNINHLKTLWHGCPRVNGCFEKMEELYI
RDCPQLISLFTYVIAPTNDKTKKSEDQFTHGHLMPSKIFQNLQKMELFNCGELRHVFSASITGSLTQLKILTIEECNMLEQI
FEDALPPTHHEETNEIVEEDVQSSSGTFSILHIS

>XP_022637412.1

MGCVGFSYDVFISFRGEDTRDNFVGHLRKELGRRGILTFHDDRDMGIGESLSPALNNAMEESRIFIVVFSENYASSTWCL
DELVRIMELSKMREKKQVVFPVFYHVHPSDILQDRNSFGKHMRAHENTFGKQSQRMQAWRSALSQAVHLPRKHITT
GCENNFIEEIVGEVYKNIAPKPLYIGQKPLGLEPHIEEVMSLLDMKPDDKTVRMLGIYGLGGIGKTELAKALYDKVVQHF
DAASFLAGVREKSNTINGMEDLQKTLLSEMLEESDTKLGSTNKGIYEIKRKLRRKKVLLVLDDVDDKEELEKLAGGCDWF
GPGSRIIITTREKDVLIAHHVGHIYEMKELDEQHSLELFCWNAFGQGCPKTGFEDVSMRAVNYAKGLPLALKVIGSDLAT
LHEESLDAWEDALEEYEKTPPNKKIQDVLKISYDRLDDDAKQVFLDIACFFKGERMKYVKKILKEFCSTSNMKVLVNKSLI
TXENDRLKMHDLIQDMGRQIVRHEATNPGERSRIWDYEDVIEILNEDCGSDKIQGIMLDPPQQEKVKWSGTEFQKMK
CLRILIVRNTSFSSELQHLPNHLRLLDWDNYPSKTFPPKFHPKKIVVFNLPRSCLTLEEPFKKFPCLTNMDFSYNQRIIEIPD
VSELQNLTELRLDHCGNLIAVHESVGFLERLSHLSVSECKKLQIFMSRMFLPSLQIFNLNFCESLGHFPEIMKEMTKPLKIH
MINTAIQELPESISKLTGLVSIDISNNRELKYLPRSLFMLPNVDSFIIKACSKLGESIRSLLQHPSKANVHQKLRLLNFKNGNL
SDKDLLAIICYFPKLEELIVSENNFVSIPSCIKECGDLTSLDLNGCKKLKKIPELTSLRILDVHHCLYLEEISELPPTVQKIDARFC
FNLTKETSDMLWCQVKKGVGGIEMVMPFLTEIPEWFNFVGVGRIPSFWVRGKFPKMVVAMIFHFQNESQRDKFDGR
GLVDLRLLINGRYAPRKGYRNFRIEAEHILLCDVGVLCSEKEWVGNAVMEHEWNLVQVSYDATSSLMISGWGAFVFEE
GTNMEDLLFASPNNLIVNGDSTSVHKGSLEDDEDYVPLPEGIAWEFLFEGIKDGIVEAWNKFPSMDVAEISVAALKKNC
KIEWTVEGMEGIPSAENRTYMTGLYGVLLEAKLRFPDLDVGAALTTVANRKGIKGTFKSPLQQNLRIPRLDWSTVTLPPS
HDPLMQIFMMMMKQQSTSESELKTKTFWKLKESHQILRNRLSHKTAPQNSASSSKNQYDELIQKFNMQYDAFVGKRL
DNLYGVAKYERDSGVLKERAEEIERELDAVVGRLQNSEEFGDVMTAMFLNGVRDGILEARAILLALRTEAKKK

>XP_022637413.1

MGKRGEEEECVGFSYDVFISFLGEDTGNNFVGHLRKELGRKGMVTLNDESDMRTEEGLSPAVCEAIEESRIFIVVFSENY
ASSTWCLDELVKIIERTHTNSKQVVYPVFYHVDPSDIRKMRNSFGKHMTAHENEIGKESQRMQAWRSALSEAVNLPG
MHVTTGYENNFIEKIVKKVRKSIAPKPLSTGENLVGLEPHIEEVMSLLDMNNDKTVRMLGIYGLGGIGKTELAKALYDKIV
QNFDAASFLAGVREKSNTINGMEELQKTLLSEMLEESETMLGSTNKGIYEIKRKLCRKKVLLVLDDVDDKEELENLAGGC
DWFGVGSRIIITTREKDVLIAHHVGHIYEMKELDEQHSLELFCWNAFGQGERMEYVKKILKEFCSTSNMKVLVNKSLITIE
NDRLKMHDLIQDMGRQIVRQEAPDNPGQRSRILDYEDVIEILTKDSGSDKIQGIKLDPPKQAEVKWSGTEFGKMKWLR
ILIVRNTSFSSELQHLPNHLRLLHWDNYPSKSFPQKFHPKKIVVFNLPRSSLTFQDPFKKFPCLTNMDFSYNQRIIEIPDVSE
LQNLRELRLDHCRNLIAVHQSVGFLKKLSHLSVSECTKLQNFLSRMFLPSLEVFDLNLCERVGHFPEIMQEMTKPLKIYMI
NTGIQELPESISKLIGLVFIDISNNRQLKYLPSSLFMLPNVDSFKIEACSKLGQSFRSLVQHPSKVNVRPKLRSLNFQKGNLS
DEDLLAILCYFPKLEELIVSENNFVYIPSCIKECGDLTSLDLNGCKKLKKIPELTSLRILDVHHCFYLEEISELPSTVEKVDARFC
FKLTKETSDMLWCQVKKRVAGIEMVMPFITEIPEWFNFVGVGRIPSFWIRGKFPNIVLAMIFHFQNESERNKFVRRRLV

DLRLLINGRNAPGKGYRDFEIEAEHILICDLRVLFSEKEWFGLDALVENEWNLVQVEYEASSSLMISGWGAFVYDEGSN
MKDVAFTCPNPMYSDKMPQTIIPEKDPMEKYKKRIRELRLDEIFKKTLTEWQENKERGGDRSHDNCIRMALGQIKKISE
DAEDALNSKGSALEDPNSYLRWLLDTLENDDGKPKEIIKGDLALVVQKHSATGKKKEDNVGEASCSRHHGRKEEEEGYD
PVVEAPSIPFYTSVVREQRVNDSVEADLPEDIVMELFCEGMKDGLVEAQNGFPSLDIAETSNAVLEKGDKVRWAPDVE
EQISVESRIYMTGIYSGLKEAKLRFPDLDMWTTINTVAKRKGIEGIFVSASQEKLGFPHLDWSTVKVPPSEDPLMQILMK
MKQQSNFEGEVMSKLFWKLKEEHEALRNKFAELDDGNENEYDECVEKREEKHDGVGKYEEISGVLRGRGEEIERLYDA
GVEGFKRSEEFEDVMGAIYLNGLRAGLLEAQALLLNLLTPHRN

>XP_022637427.1

MLRGESSSSSSDASSRGSYHVYLSFGMEGTHVDFANTLCLSLQRKGISTFRYDKLVERDVMLKKVQKAMEECLVAIVLLS
ENYAASTWCLDELRKILDVGKPVIPVFYEVVPSDVRHQRNSFAKAFEEHERRSEEDQLKVQQWRKSLQEVADFSGWES
KDRRREELIEDIIKSVWTKIRLRLASYEEERVGIDSRVEKIRSLLKLELKDVVHFTGIWGMGGIGKTTLARVVFKKICSQFDIS
CFLENVREISGKTHDMLTLQITLLSHMEVKEFTIQNLDEGKIVIGGILRNNKVLLVLDDVDDARQLDSLGVNDQGKFGPG
SRIIVTTRDMEVLKPLGNFEICKIDLLNSDESLQLFCQKAFKRDKPSEQLLQLSKVAVQQAGGLPLALEMMGSSFCGRNE
SQWKTLLDMKEYSKKDIVMEKLIISYHGLPESYQILFLDIACFFNGWVKEHVEEILTICCRCPANGIDVLIGKSLVSCDGSRL
WMHDLLQEMGRKIVVEKCLVDASKRSRLWSPHDIDQALKRKKKNESIQGIVLKSSTEPYIANWDPEAFSKMYNLKFLII
NFHNIQFPRGLKCLSSSLEVLQWTKCTLEALPLGVELEKLVVLKMRYSKIKKIWSDSQVSISVNILRIQFSTSTKSSKANHFH
TSVLIGQHFKRLKFIDLSHSEDLIESPIVSEVPCLEILLLEGCKNLVKVHQSVGLHKKLVVLNLKDCINLQILPTEFKMDSLEEL
ILSGCSKLKKLPEFDLLTGLEIFMPSNHFGLNFEHKLNYFCPRVHAKASMLSGESSSNSSRWTYHVFLSFRGEDTRLGFTD
HLYAALVRKGIITFRDDKNLEKGDKIDKELFKAIEESLGAIVILSENYASSSWCLDELNKILESNRALGRKVFPVFCGVSPSEV
QHQTTKSFEEAFQKHERRFEKDTEKVRQWRDSLKEVSQIAGWESKNYQHQTELIENIVESVWTKLRPEMPSFNDGLVG
IGSRVKKMDSLLRIESKDDASFIGIWGMGGIGKTTLARAVFRKIQHQFDISCFLDNIREISKESGGRLRLQGKLLSHLGIKG
LEIRDLDEGKNTIRELLFKKKVLLVLDDVDDTSQLESLAERLEWFGPGSRVIITTRDTHVLISHGIVENYKIDVLNSDESLQLL
SQKAFKRDKPDEHYLELSKAVAKYAGGLPLALELLGSFLCGRSESQWKEVVDMIKEVPPSHIAMKSLRISYNGLPLRYKTL
FLDIACFFKGRIKELVIQALEICERYPPVGIELLVEKSLATYDGFTIGMHDLLQESAREIVKEESYVDPAKRSRLWTLEETNEV
LKHNKANESIEGIVLNSPEKEEAIWDPEAFSRMYNLQLLIINYRLNLPTSLKSLCSSLKFLQWMNYPLESLPLGVLLDELVEL
KMHSSRIKKIWNGNQDFAKLKFIDLSYSEDLIETPIVSGAPSLERLLLIGCINLVEVHPSVGQHKRLVLLLLKDCKNLQIMPR
KLELDSLEELILSGCSKIEKLPEFGENMKSLSLLNVENCINLLSLPDSICNLRSLRKLYVSGCSRISTLPDGMNENESLEEVHV
SGTDIREIPLCLEKLRELSFGGRKETTPKSQNLLQWISKFMGQLDMQESMVPPLSSLLALESLDLSYRGLTDESIPSDLGPF
SLLKRLDLSGNNFVNPPAQCIISLSMLHTLSFNDCPRLESLPLLPPNLQALYATNCPNLEPFHLVGDTLWKIFESHSHEDPI
EGPELWFIIPGNEIPSWFNNQNSLAIDSSDETYEKLCCDSVTSITVDVPEDFQLSEWWGIAVCLVLEPLNMDVPSSCNAR
STSTVNEEIGIYYWVCKAPDKDPDPNFPIAPKFGHMLYKFKEPYIHIIFLNADHVYIQHYLSGEQTQLEVILFVENFSESCK
ARIKKCGCRVICKEKIEEWRKHSDDLNISRITETNQDEERHELEEEEPTSPNTSSPVEKTEGQTTLGK

>XP_022637489.1

MDILYGFLSGVLKDVACGATNQLQYSFCFNSFVKELEKEEDNLIEMIRSVEDRVIHARRQTLKTTEVIDKWLENANIDSEY
VNRLLRETNAKKSCFFEFCPNWIWRYRLGKKLAIKKADLQKIIQDGRQYIQLQRIASIPSNTFDILTEKSMNFDSRKFAFD
QLMEAVKDDGVAMIGLYGMGGCGKTTLAMEVKKIAEVEHLFDRVIFVPVSSTVEVPRIQEKIASSLHYTFPENQEMERS
QRLCMRLTQEKKILMILDDVWEKLDFGRIGISSSEYHKGCKILITTRSEDVCILMDCQKKIYLPILTDEEAWTLFQNKALMS
KDTPENMQHLAISISKECKGLPVAIVAVASSLKGKKEAIWHSALNKLRSSKPINISRGLQDPYMCLKLSYDNLDTDEAKSL
FLLCSVFPEDYEISVECLIRCAIGLGVAGEVDIYEEARTEVTAAKIKLVSSCLMLEAGDERVKMHDLVRDVAHWIAKNENK
IIKCELEKDVSLEEGSIRYLWCVKFPDGMDCSNLEFLSIQTKLEVSDGIFERMGKLRVLIITNKNGYRLQLSTTSFKSLTNLR
CLVLQYFALRDISFVRDLKKLQSLSFHRCSCSSFLDLQTDVAVTTLTNLKLLEFISCDIESNIFEEIKRIPFLEELYILENGQRYN
NEENVKFFNSFSVPHTLQRFGIILGYDIWQLEFCSYERTLRINYFDISNEVIKGLAGKARELYVGNIEGGAKNMMPDIFEIE

EGMNELKELRIRDCEEIVCLVDTSNHLSKMGNIFSKLRLMRIMSMNNLKTLWHGCLPANGCFEKLENMYIEDCHQLTC
LFTNIIVPTNDKTKKSEDQFRNGHSMQSKIFQNLQEVRIYHCRELKHVFSTSISGDLSQLKMLEIKYCHMLEQIIEEVLPPP
AHHEETNEIVEEDVQSSSGSFFLSSLALLKISSCSMLESLFTISVAKTLTSLEKLKISNCHGLKHIVPPARVKRNKNMVEDEH
EFENDLSMFSNLKSIFIRECVSLQDIFATPVNECFEKLEKVYIDNCPQLTYLFTYVAQGLTELKILHIYNCDILKHIITYDDKTK
KSEDQFTSRHSVQSRIFQNLEEVMLDECGELKHVFSANIIGGLPQLKKLEIENCNMLQQIIVEEDECQHFESNQVKVSPES
ISIPSVSTVNNISGSFSLSSLALLSIKYCLMLDSLFTTSVAKTLTSLEELDISNCDGLKHIVSPERVKRNKNMVEDEHEFESNLS
MFSSLKWVKISRCDSLQDIFIMPFVGGTVNIQNHFSTQQTLRMSNANNGDWMMGQQVSLKLEFLKLSYLHEMTHIW
VATNNSFTLQHLNSLIIEECEKLEVIFPQSMLRSLPELNYLQVSECKELRQIIEEDLEDKSLFAQPCFPKLDSLVIERCHKLKCF
TSVIASNALSNLRILIIKEATELQEFIACEYDETVKTKVELPQLKLIIFMDLSNFQQESIFSNVKHRIIRNCPKLSLTSTITPQEL
QQNYPLEGLGMSETIRLEISSLMYEIKKLDEVSTNNNSTELPSSQINEKLDKNVTEKDYGSKEVAPATTASYFTDQQSPLG
QTQSTIKMSQQDWDPTPKNTSPLQMNREDQSTSQIKPFSSQVNDNNQSMLESRVEMVGQHNKIETKTQAPEIEEFQ
KIDRKNEMASDPQAMDQNFPVISSPNMTQRTDEIETDNLGKTTTSDKPAIPTFVSENIEEIGRERRRGGPAIEGVTIKTLP
IGGDNISLVSGVTIHNSSGANILSQDSQIVKQDNELNEDKTEIAPHTNIKIQERVNLLDKTEGVRIVSNNDVVVTSASTDT
RTRLEKYKQFVDLNDSQISLLVEAIEAYPHLWNACEKFTDRFRAWMLKTLVDMLLFLRSESVGSINPHREKEFLKLCDEA
VQLGFERSWVDQMRQRVLGRDPKLDHAKVRINELLKRHDHLTWELDNIKKELRSLNDFLNAQAKCFDFL

>XP_022637490.1

MDILYGFLSGVLKDVACGATNQLQYSFCFNSFVKELEKEEDNLIEMIRSVEDRVIHARRQTLKTTEVIDKWLENANIDSEY
VNRLLRETNAKKSCFFEFCPNWIWRYRLGKKLAIKKADLQKIIQDGRQYIQLQRIASIPSNTFDILTEKSMNFDSRKFAFD
QLMEAVKDDGVAMIGLYGMGGCGKTTLAMEVKKIAEVEHLFDRVIFVPVSSTVEVPRIQEKIASSLHYTFPENQEMERS
QRLCMRLTQEKKILMILDDVWEKLDFGRIGISSSEYHKGCKILITTRSEDVCILMDCQKKIYLPILTDEEAWTLFQNKALMS
KDTPENMQHLAISISKECKGLPVAIVAVASSLKGKKEAIWHSALNKLRSSKPINISRGLQDPYMCLKLSYDNLDTDEAKSL
FLLCSVFPEDYEISVECLIRCAIGLGVAGEVDIYEEARTEVTAAKIKLVSSCLMLEAGDERVKMHDLVRDVAHWIAKNENK
IIKCELEKDVSLEEGSIRYLWCVKFPDGMDCSNLEFLSIQTKLEVSDGIFERMGKLRVLIITNKNGYRLQLSTTSFKSLTNLR
CLVLQYFALRDISFVRDLKKLQSLSFHRCSCSSFLDLQTDVAVTTLTNLKLLEFISCDIESNIFEEIKRIPFLEELYILENGQRYN
NEENVKFFNSFSVPHTLQRFGIILGYDIWQLEFCSYERTLRINYFDISNEVIKGLAGKARELYVGNIEGGAKNMMPDIFEIE
EGMNELKELRIRDCEEIVCLVDTSNHLSKMGNIFSKLRLMRIMSMNNLKTLWHGCLPANGCFEKLENMYIEDCHQLTC
LFTNIIVPTNDKTKKSEDQFRNGHSMQSKIFQNLQEVRIYHCRELKHVFSTSISGDLSQLKMLEIKYCHMLEQIIEEVLPPP
AHHEETNEIVEEDVQSSSGSFFLSSLALLKISSCSMLESLFTISVAKTLTSLEKLKISNCHGLKHIVPPARVKRNKNMVEDEH
EFENDLSMFSNLKSIFIRECVSLQDIFATPVNECFEKLEKVYIDNCPQLTYLFTYVAQGLTELKILHIYNCDILKHIITYDDKTK
KSEDQFTSRHSVQSRIFQNLEEVMLDECGELKHVFSANIIGGLPQLKKLEIENCNMLQQIIVEEDECQHFESNQVKVSPES
ISIPSVSTVNNISGSFSLSSLALLSIKYCLMLDSLFTTSVAKTLTSLEELDISNCDGLKHIVSPERVKRNKNMVEDEHEFESNLS
MFSSLKWVKISRCDSLQDIFIMPFVGGTVNIQNHFSTQQTLRMSNANNGDWMMGQQVSLKLEFLKLSYLHEMTHIW
VATNNSFTLQHLNSLIIEECEKLEVIFPQSMLRSLPELNYLQVSECKELRQIIEEDLEDKSLFAQPCFPKLDSLVIERCHKLKCF
TSVIASNALSNLRILIIKEATELQEFIACEYDETVKTKVELPQLKLIIFMDLSNFQQESIFSNVKHRIIRNCPKLSLTSTITPQEL
QQNYPLEGLGMSETIRLEISSLMYEIKKLDEVSTNNNSTELPSSQINEKLDKNVTEKDYGSKEVAPATTASYFTDQQSPLG
QTQSTIKMSQQDWDPTPKNTSPLQMNREDQSTSQIKPFSSQVNDNNQSMLESRVEMVGQHNKIETKTQAPEIEEFQ
KIDRKNEMASDPQAMDQNFPVISSPNMTQRTDEIETDNLGKTTTSDKPAIPTFVSENIEEIGRERRRGGPAIEGVTIKTLP
IGGDNISLVSGVTIHNSSGANILSQDSQIVKQDNELNEDKTEIAPHTNIKIQERVNLLDKTEGVRIVSNNDVVVTSASTDT
RTRLEKYKQFVDLNDSQISLLVEAIEAYPHLWNACEKFTDRFRAWMLKTLVDMLLFLRSESVGSINPHREKEFLKLCDEA
VQLGFERSWVDQMRQRVLGRDPKLDHAKVRINELLKRHDHLTWELDNIKKELRSLNDFLNAQAKCFDFL

>XP_022637491.1

MDILYGFLSGVLKDVACGATNQLQYSFCFNSFVKELEKEEDNLIEMIRSVEDRVIHARRQTLKTTEVIDKWLENANIDSEY
VNRLLRETNAKKSCFFEFCPNWIWRYRLGKKLAIKKADLQKIIQDGRQYIQLQRIASIPSNTFDILTEKSMNFDSRKFAFD
QLMEAVKDDGVAMIGLYGMGGCGKTTLAMEVKKIAEVEHLFDRVIFVPVSSTVEVPRIQEKIASSLHYTFPENQEMERS
QRLCMRLTQEKKILMILDDVWEKLDFGRIGISSSEYHKGCKILITTRSEDVCILMDCQKKIYLPILTDEEAWTLFQNKALMS
KDTPENMQHLAISISKECKGLPVAIVAVASSLKGKKEAIWHSALNKLRSSKPINISRGLQDPYMCLKLSYDNLDTDEAKSL
FLLCSVFPEDYEISVECLIRCAIGLGVAGEVDIYEEARTEVTAAKIKLVSSCLMLEAGDERVKMHDLVRDVAHWIAKNENK
IIKCELEKDVSLEEGSIRYLWCVKFPDGMDCSNLEFLSIQTKLEVSDGIFERMGKLRVLIITNKNGYRLQLSTTSFKSLTNLR
CLVLQYFALRDISFVRDLKKLQSLSFHRCSCSSFLDLQTDVAVTTLTNLKLLEFISCDIESNIFEEIKRIPFLEELYILENGQRYN
NEENVKFFNSFSVPHTLQRFGIILGYDIWQLEFCSYERTLRINYFDISNEVIKGLAGKARELYVGNIEGGAKNMMPDIFEIE
EGMNELKELRIRDCEEIVCLVDTSNHLSKMGNIFSKLRLMRIMSMNNLKTLWHGCLPANGCFEKLENMYIEDCHQLTC
LFTNIIVPTNDKTKKSEDQFRNGHSMQSKIFQNLQEVRIYHCRELKHVFSTSISGDLSQLKMLEIKYCHMLEQIIEEVLPPP
AHHEETNEIVEEDVQSSSGSFFLSSLALLKISSCSMLESLFTISVAKTLTSLEKLKISNCHGLKHIVPPARVKRNKNMVEDEH
EFENDLSMFSNLKSIFIRECVSLQDIFATPVNECFEKLEKVYIDNCPQLTYLFTYVAQGLTELKILHIYNCDILKHIITYDDKTK
KSEDQFTSRHSVQSRIFQNLEEVMLDECGELKHVFSANIIGGLPQLKKLEIENCNMLQQIIVEEDECQHFESNQVKVSPES
ISIPSVSTVNNISGSFSLSSLALLSIKYCLMLDSLFTTSVAKTLTSLEELDISNCDGLKHIVSPERVKRNKNMVEDEHEFESNLS
MFSSLKWVKISRCDSLQDIFIMPFVGGTVNIQNHFSTQQTLRMSNANNGDWMMGQQVSLKLEFLKLSYLHEMTHIW
VATNNSFTLQHLNSLIIEECEKLEVIFPQSMLRSLPELNYLQVSECKELRQIIEEDLEDKSLFAQPCFPKLDSLVIERCHKLKCF
TSVIASNALSNLRILIIKEATELQEFIACEYDETVKTKVELPQLKLIIFMDLSNFQQESIFSNVKHRIIRNCPKLSLTSTITPQEL
QQNYPLEGLGMSETIRLEISSLMYEIKKLDEVSTNNNSTELPSSQINEKLDKNVTEKDYGSKEVAPATTASYFTDQQSPLG
QTQSTIKMSQQDWDPTPKNTSPLQMNREDQSTSQIKPFSSQVNDNNQSMLESRVEMVGQHNKIETKTQAPEIEEFQ
KIDRKNEMASDPQAMDQNFPVISSPNMTQRTDEIETDNLGKTTTSDKPAIPTFVSENIEEIGRERRRGGPAIEGVTIKTLP
IGGDNISLVSGVTIHNSSGANILSQDSQIVKQDNELNEDKTEIAPHTNIKIQERVNLLDKTEGVRIVSNNDVVVTSASTDT
RTRLEKYKQFVDLNDSQISLLVEAIEAYPHLWNACEKFTDRFRAWMLKTLVDMLLFLRSESVGSINPHREKEFLKLCDEA
VQLGFERSWVDQMRQRVLGRDPKLDHAKVRINELLKRHDHLTWELDNIKKELRSLNDFLNAQAKCFDFL

>XP_022637492.1

MDILYGFLSGVLKDVACGATNQLQYSFCFNSFVKELEKEEDNLIEMIRSVEDRVIHARRQTLKTTEVIDKWLENANIDSEY
VNRLLRETNAKKSCFFEFCPNWIWRYRLGKKLAIKKADLQKIIQDGRQYIQLQRIASIPSNTFDILTEKSMNFDSRKFAFD
QLMEAVKDDGVAMIGLYGMGGCGKTTLAMEVKKIAEVEHLFDRVIFVPVSSTVEVPRIQEKIASSLHYTFPENQEMERS
QRLCMRLTQEKKILMILDDVWEKLDFGRIGISSSEYHKGCKILITTRSEDVCILMDCQKKIYLPILTDEEAWTLFQNKALMS
KDTPENMQHLAISISKECKGLPVAIVAVASSLKGKKEAIWHSALNKLRSSKPINISRGLQDPYMCLKLSYDNLDTDEAKSL
FLLCSVFPEDYEISVECLIRCAIGLGVAGEVDIYEEARTEVTAAKIKLVSSCLMLEAGDERVKMHDLVRDVAHWIAKNENK
IIKCELEKDVSLEEGSIRYLWCVKFPDGMDCSNLEFLSIQTKLEVSDGIFERMGKLRVLIITNKNGYRLQLSTTSFKSLTNLR
CLVLQYFALRDISFVRDLKKLQSLSFHRCSCSSFLDLQTDVAVTTLTNLKLLEFISCDIESNIFEEIKRIPFLEELYILENGQRYN
NEENVKFFNSFSVPHTLQRFGIILGYDIWQLEFCSYERTLRINYFDISNEVIKGLAGKARELYVGNIEGGAKNMMPDIFEIE
EGMNELKELRIRDCEEIVCLVDTSNHLSKMGNIFSKLRLMRIMSMNNLKTLWHGCLPANGCFEKLENMYIEDCHQLTC
LFTNIIVPTNDKTKKSEDQFRNGHSMQSKIFQNLQEVRIYHCRELKHVFSTSISGDLSQLKMLEIKYCHMLEQIIEEVLPPP
AHHEETNEIVEEDVQSSSGSFFLSSLALLKISSCSMLESLFTISVAKTLTSLEKLKISNCHGLKHIVPPARVKRNKNMVEDEH
EFENDLSMFSNLKSIFIRECVSLQDIFATPVNECFEKLEKVYIDNCPQLTYLFTYVAQGLTELKILHIYNCDILKHIITYDDKTK
KSEDQFTSRHSVQSRIFQNLEEVMLDECGELKHVFSANIIGGLPQLKKLEIENCNMLQQIIVEEDECQHFESNQVKVSPES
ISIPSVSTVNNISGSFSLSSLALLSIKYCLMLDSLFTTSVAKTLTSLEELDISNCDGLKHIVSPERVKRNKNMVEDEHEFESNLS
MFSSLKWVKISRCDSLQDIFIMPFVGGTVNIQNHFSTQQTLRMSNANNGDWMMGQQVSLKLEFLKLSYLHEMTHIW
VATNNSFTLQHLNSLIIEECEKLEVIFPQSMLRSLPELNYLQVSECKELRQIIEEDLEDKSLFAQPCFPKLDSLVIERCHKLKCF
TSVIASNALSNLRILIIKEATELQEFIACEYDETVKTKVELPQLKLIIFMDLSNFQQESIFSNVKHRIIRNCPKLSLTSTITPQEL

QQNYPLEGLGMSETIRLEISSLMYEIKKLDEVSTNNNSTELPSSQINEKLDKNVTEKDYGSKEVAPATTASYFTDQQSPLG
QTQSTIKMSQQDWDPTPKNTSPLQMNREDQSTSQIKPFSSQVNDNNQSMLESRVEMVGQHNKIETKTQAPEIEEFQ
KIDRKNEMASDPQAMDQNFPVISSPNMTQRTDEIETDNLGKTTTSDKPAIPTFVSENIEEIGRERRRGGPAIEGVTIKTLP
IGGDNISLVSGVTIHNSSGANILSQDSQIVKQDNELNEDKTEIAPHTNIKIQERVNLLDKTEGVRIVSNNDVVVTSASTDT
RTRLEKYKQFVDLNDSQISLLVEAIEAYPHLWNACEKFTDRFRAWMLKTLVDMLLFLRSESVGSINPHREKEFLKLCDEA
VQLGFERSWVDQMRQRVLGRDPKLDHAKVRINELLKRHDHLTWELDNIKKELRSLNDFLNAQAKCFDFL

>XP_022637493.1

MDILYGFLSGVLKDVACGATNQLQYSFCFNSFVKELEKEEDNLIEMIRSVEDRVIHARRQTLKTTEVIDKWLENANIDSEY
VNRLLRETNAKKSCFFEFCPNWIWRYRLGKKLAIKKADLQKIIQDGRQYIQLQRIASIPSNTFDILTEKSMNFDSRKFAFD
QLMEAVKDDGVAMIGLYGMGGCGKTTLAMEVKKIAEVEHLFDRVIFVPVSSTVEVPRIQEKIASSLHYTFPENQEMERS
QRLCMRLTQEKKILMILDDVWEKLDFGRIGISSSEYHKGCKILITTRSEDVCILMDCQKKIYLPILTDEEAWTLFQNKALMS
KDTPENMQHLAISISKECKGLPVAIVAVASSLKGKKEAIWHSALNKLRSSKPINISRGLQDPYMCLKLSYDNLDTDEAKSL
FLLCSVFPEDYEISVECLIRCAIGLGVAGEVDIYEEARTEVTAAKIKLVSSCLMLEAGDERVKMHDLVRDVAHWIAKNENK
IIKCELEKDVSLEEGSIRYLWCVKFPDGMDCSNLEFLSIQTKLEVSDGIFERMGKLRVLIITNKNGYRLQLSTTSFKSLTNLR
CLVLQYFALRDISFVRDLKKLQSLSFHRCSCSSFLDLQTDVAVTTLTNLKLLEFISCDIESNIFEEIKRIPFLEELYILENGQRYN
NEENVKFFNSFSVPHTLQRFGIILGYDIWQLEFCSYERTLRINYFDISNEVIKGLAGKARELYVGNIEGGAKNMMPDIFEIE
EGMNELKELRIRDCEEIVCLVDTSNHLSKMGNIFSKLRLMRIMSMNNLKTLWHGCLPANGCFEKLENMYIEDCHQLTC
LFTNIIVPTNDKTKKSEDQFRNGHSMQSKIFQNLQEVRIYHCRELKHVFSTSISGDLSQLKMLEIKYCHMLEQIIEEVLPPP
AHHEETNEIVEEDVQSSSGSFFLSSLALLKISSCSMLESLFTISVAKTLTSLEKLKISNCHGLKHIVPPARVKRNKNMVEDEH
EFENDLSMFSNLKSIFIRECVSLQDIFATPVNECFEKLEKVYIDNCPQLTYLFTYVAQGLTELKILHIYNCDILKHIITYDDKTK
KSEDQFTSRHSVQSRIFQNLEEVMLDECGELKHVFSANIIGGLPQLKKLEIENCNMLQQIIVEEDECQHFESNQVKVSPES
ISIPSVSTVNNISGSFSLSSLALLSIKYCLMLDSLFTTSVAKTLTSLEELDISNCDGLKHIVSPERVKRNKNMVEDEHEFESNLS
MFSSLKWVKISRCDSLQDIFIMPFVGGTVNIQNHFSTQQTLRMSNANNGDWMMGQQVSLKLEFLKLSYLHEMTHIW
VATNNSFTLQHLNSLIIEECEKLEVIFPQSMLRSLPELNYLQVSECKELRQIIEEDLEDKSLFAQPCFPKLDSLVIERCHKLKCF
TSVIASNALSNLRILIIKEATELQEFIACEYDETVKTKVELPQLKLIIFMDLSNFQQESIFSNVKHRIIRNCPKLSLTSTITPQEL
QQNYPLEGLGMSETIRLEISSLMYEIKKLDEVSTNNNSTELPSSQINEKLDKNVTEKDYGSKEVAPATTASYFTDQQSPLG
QTQSTIKMSQQDWDPTPKNTSPLQMNREDQSTSQIKPFSSQVNDNNQSMLESRVEMVGQHNKIETKTQAPEIEEFQ
KIDRKNEMASDPQAMDQNFPVISSPNMTQRTDEIETDNLGKTTTSDKPAIPTFVSENIEEIGRERRRGGPAIEGVTIKTLP
IGGDNISLVSGVTIHNSSGANILSQDSQIVKQDNELNEDKTEIAPHTNIKIQERVNLLDKTEGVRIVSNNDVVVTSDTRTR
LEKYKQFVDLNDSQISLLVEAIEAYPHLWNACEKFTDRFRAWMLKTLVDMLLFLRSESVGSINPHREKEFLKLCDEAVQL
GFERSWVDQMRQRVLGRDPKLDHAKVRINELLKRHDHLTWELDNIKKELRSLNDFLNAQAKCFDFL

>XP_022637835.1

MAESSSSFDVSTSPTSYDVFLNFRGEDTRENFISHLYAALERKHIEVYIDYRLKRGDEISPALQTAIEESKIYVLVFSENYASS
TWCLNELIKILDCKKRYGRDVIPVFYKVDPSTVRKQEKRYKEAFKEHELRFKEDMDKVQGWKDALTEAAGLSGWDSNVI
RLEYVLVEEIVEDILKKLNCYYVTYDQGIIGTEKHIERIQSLLHLESSDIRIVGICGMGGIGKTTISEQLYHTLAKQFDSHSLILD
VQEKLQREGIDNIRRKYQSELLKEASSTRLSYYNERIKRSKVLLILDDVTDSTLLNKLIGERDSFGQGSRIIMTSRDRQVLKN
AGANDIYELKELNFYDSLKLFNLHAFKQNSSEEITYMDLSIKVLRYAKGIPLTLQILGSLLYGRQRAEWESQLQKLEKGQDV
ETFNVLKLSYDGLDEEQKNIFLEIACFYRGHNEIVVVEXLXXCGXSSXIGMXXLKDXXLISILDGRIVMHDLIQEMGQEIVR
KECPQHPGKRSRLFKADEIHEILRNNKGSDTIQCILQDIDEMEGVKVHAKAFKKMNNLRMLMFYSYSCSWYRDVFLESS
LVSLPDTLKILYWFGFHQKSLSRNFCSQNLVRLEMPYCHLEKLWKDQSLPKLKRLNLSYSQKLTRIPDLSMSPNIEEIILND
CSKLIKVHSSILLTKLTSLRLDKCYDLNSVIVPSNILYRSPGLILLSSCEKLKMFSTSVPRFPHVKLERQRGTISRSLLMKPEYKP
GTVSSFPRLIRRSNRRRAFPRSSVRNGLGEGTCYWPCNDTHYPLSKISFLREIFSIIFVKYEEEKEVINDNIYVYDESPVQLIG

GVPLNFQSLKKLCLIDLSHCSSLTIFPFDLSEIKFLKKLCLSGCSKLENFPEIENTMEDLAVLLLDGTAIQALPSSLWRLVGLQ
ELSLHCCRNLEIIPSSIGSLTRLCKLDLTFCESLQSFPSTIFKLKLRKLDFCGCLRLRTFPEIMEPAQSFAHINLTKTLIKELPSSF
GNLVNLRSLQLNNCSDLESLPNPIVNLKLLCKLDCSGCAKLTEIPTHIGRLSSLMEMSLRKSGIVNLPESMAHLSSLKSLDL
SDCEKLECIPHIPPFLEQLVALDCLSIRRVMTNSLVPNLSDSKEGVFKFHFTNAQQLNSGARASIEEDARLRMTDDAYESV
CFCFPGSAVPHWFPFCSEGPSLTINQDLSFCSDDRLIGFALCVVFGVLDTNDIKGRRGSFVYNLKFESDDNGTQIIPNND
VLKNYFTWDCNHRVLDKDHTFIWKFNLESLRRSGMNLRLSDACSFTFEISPYDYNFQWPDYDSVLNELRSVVTIKECGM
CPLYSSRSNVAESSS

>XP_022637881.1

MNSPKARQMLSLRVLQTRDDDKEFVFNWLTSNTHNNLSILSIVGMGGMGKTSLAQHVFNDPRLEDKFDTKVWVSVP
QEFDVLKLSRAILDTITGSTDHSIQHEVIQKRLKEELMGKKFLLVLDDVWNERPSKWEDVQKPLIFGGQGSRLLVTTRSEK
VAVTMRSEKHLLKVLKEDYCWDLFAKHAFENANPQLDPDFVETGKKIVEKCNGLPLALKTMGVLLHNKSSLWEWESI
MKSEIWHLSENESDILPALKLSYFHLPSHLKKCFAFCALFPRGYRFDKEYLIQLWMAQNFLENPLQKKSPKEVGDEYFND
LLSWSFFQQSSNEEKKRFIMHDLLNDLAKYVCEDICIRLGVDEPKGIAKTTRHCSFSTSKLCFDGFGSSIDTQKLHTFTPRD
WGWDWNCKMSIVDLFSKFKFIRVLSLSHYRNLKEVPKSVGNLKYLRSLDLSYTDIEKLPDSISLLYKLQILQLNYCQRLKEL
PLYLHQLDNLRCLEFIKTAVKNVPAHLGKLKNLQVSMSSFYIEKGKEFSIKHLRELSLHGSLTLDDLQNIENPSYALEVDLK
NKSHLVDLRLKWNFIGSSSVYSAKVEDVIENLQPSKYLKKLLIKNYIGKQFPNWLLHNSLPNLVSLVLEKCESCQRLPPLGL
LPFLKKLEISGFDEIVSIDADFHGNDSCSFKSLQTLYFSNMRQWEKWDCQAVTGAFPHLQLFSIENCPKLKGYLPKFVALK
TLSVIHCEQLEALIVSAVELRLEDCEKLDGHGMAASLVAIVGHMLFDTSLENLSICSALESISDDYVSLRIFPLDFFPTLRSLK
LGSFPNLQMISQDHVHNHLQYLEIKECPKFESLPANMHMLLPSLTRLWIEDCPRLASFPEGSLPSNLKYITLYNCFRLVGL
LKGALGDSSSLERLGISTPDAECFPDEGLLPLSLSFLIISDCRNLKKLNYKGLLELSSLKRLNLWKCPNLQSLPEEGLPKSISFL
EIRECPLLEQRCNKRGKDRGKIAHIRKVFI

>XP_022637882.1

MTPEMFTGAFVSTFVERTIDSLASRFAKILRARKCDKNQLSDLKMKLLAIDVVADDAEQKQFTDPRVRDWLLRAKDVV
VDAEDILDEIDYELSKSKVEAESQSAATKVWLNSVKSSFVGFFENEIGSRMEQVIENLKDLETESRYLSLKRSSGLGVGSGS
KLTYTSLPNESVIYGRDDDKESIFNWLTSELTSDTDINLSILSIVGMGGLGKTSLAQHVFNDPRLEGKFNIKAWVSVPQEF
DVLNVSKAILESVSTSSVDSTNLEGVHKKLKEKLTGKKFFLVLDDNWNENKSKWEQVQKALEFGAPGSRLLVTTRSEKVV
VTMRSEKHLLQVLRKDYCWDLFAKHAFQSPNSQLDPDFMEIGKKIVKKCNGLPLALKTMGNLLYNKPSLREWESIMKS
EMWDFSENESDILPALKLSYLHLPSHLKKCFAFCALFPKGCVFDKECLIQLWMAQNFLESSSTIKSLKEVGEQYFNDLLSW
SFFQQLNKEEENAFIMHDLLMDLAKYLCQDICIRSGVDEPQGIHKITRHFSFATDLSLIFYGFGNLTDSRKLHTFVPTVRT
GWRRYWWCKSIDVLFSKLKYVRFLSLNGFLGLTEVPKSIGNLKHLRLLDLSYTKIEKLSDSIGLLYKLQILKLNHCERLKELPS
CFLQLEKLCFLELFNTEVKNVHILGEMKSLQVSMNLQVLMNSFCVDMHKELSIQQLGQINLHGSLTISGLQNIENPFHAT
EAYLKNKPHLVKLVFNWNWNHNSSLDSTKDKDVIENLQPSKHLKELSIRSYMGEQFPNWLLDNSLPYLVSLELNECKYC
ERLPPLGLLPSLKNLRIEKLHGIVNIDADFYGNNSSSFKSLEKLYFYHMLEWENWECKDLTDAFPRLQKLSIEYCPKLKGQL
PELLVSLKTLVIINCEKLEVLAPRALDLYLEENEKVWFDWATVKSFNLAGYGMEAPFLDMVLDNISNNSIQRLEINISSCG
EINGSSCGSPPNIGDSVSLWTFPLHFFPTLKKLTLWHLNNLQMISQNHAQNHLECLDIGWCPKIESLPKNMDMLKSLHI
RDCPKLEPFTEGDLPSNLEEITLSNCSRLLGSLKGALGDNPSLKTLHIEHLDAECFPDEGFSINSRLLKV

>XP_022637899.1

MEDFFLSIATKIAEYAVHPILHHAQYLCCFNKFASNLPNAKEQLELTRDEVKERIREAINRVEKVEPTVEKWLKDVEKVLEE
VQMLEERILSLNKYYFRRQCQYSLAKQIERKTSEMIQLHRNNKFEPFSKITELPGMQYYSSKDFFMFNSTEASYKKLLEAL
NNKSAFIIGLVGLGGSGKTTLAKEVGKKAEDMKLFEKVVWATVSQPLNIRSIQDQIVDQLGFKLTEDSDIGRARRLSERLK
KGTTLIIMDDIWEILNFETLGIPLNESSKACCILITTRSKEVCTSMQCQSIIELNLLSDEEAWTLFRHYTNITDDSSEALKGVA

RKIVNECKGLPIAIVTVGSTLKDKTIANFELALSRLENSKPLDIPKGLTSPYVCLELSYNNLTNPLAQSLLLSCSMFPEDYEIDL
EDLFRFGMRFDIIGTFGTMENARREMDAAIDMLKNCFLLMHAKEKQRVKMHDLVRDVALWIASKSGKAIFTGTEVDP
RALADDESLKDKKAIAVWGLKSYDVLNYKINRPILETLLLSFNVSGGVKVSDGCLQSLENLKTLAILKSRWKSDALSLQESL
KSLKNLRTLCLRGFDLGDISFVEILQALEILDLRDSYFDDLPIGIVELKKLRLLDLYECWMTKNENVEVYKVVGKCLQLEELY
FRLSYNYKKDFPYDVSFSRLQRYVIEIGDNRRTLDDITKLMKKYGQPRSLIMNGFDVAPQSFISLPIKDLFIRAEFLCLRYQR
NYKNIIPSMDPQGMNQLIALRLYHASGIECLVDNTEKDKTEVIAFSGLVYLSLVYNYSLREVFRDPSSRCSLKNLQELEIKN
CGSLYSISFPRNSKLCNLKVISISDCYMLISLFTSSVVQTLELLEDLRISKCRSLRHIIEEENDVLSSTQSHSSLTLPKLRFLFIEYC
DNLEYVFSVFLVQGLLSLESVDIMRNRALKYVFGNEKEHNVAGYPSFQQTNTITNLFNLNTLTFSDLPNLIAIWPEYFRAH
LPSLNDLSCRYCPKLSIHKAMNASDIQQQTTPTENGILWLITNTLNRLEDDPLSHPQLKAVLKIIRLHLKDVRMKGIFQFQ
MGEEGGTTELLPLNLDINYLILENLPELNFIWKGPTSFLSLQNLHLIYVDGCPKLKTIFSTTVVTSLPMLKYLNIRNCDELEQI
FDLGDAYQLNSPYSSQQVCFPTLSSITVQKCNKLKCLFYNLSACHFTSLRRLKIQECSQLHKAFAFSEEEEMGKDGEQVLL
QKLKNIRLINLPNLEEIHQGFKLKEDVEQTLEECPKYSPSLYLHQDM

>XP_022638557.1

MATSTSCDVSEIRHDVFISFTGIDVRRGLLSHLKRELHRKHIDAYADERLDKGGEISSSLLRAIEASKILLVVFSKHYASSHW
CLEELAKMIECMETNKQIVLPVFFDIDPSEVRHQYGDYGDALAEHEEEFKENMQIWRSALKTAGCLSGFHYPTNYENES
DLVEKIVEDISVKLSKFYPSESNGLVGIDQNVTQIQSLLRVESNEVLFVGIWGMGGIGKTTIARAIFDKCSLQYDGCCFFN
VREESERHGISNLRERLIYELLEGEGLHINKARFFRSAPRMLGRKKVMVVLDDVNTSEELGYLVTKPVCFGAGSRVIITSRD
QDVLISGGLDQIHEVKEMKPLDSLKLFCLIAFNESQPKMGYEKLTEEVLKIAQGNPLALKVLGAEFHSRSTIDTWKCALRK
FKKYPNEKILNAIRFSYDGLHEVEKKAFLDIAFFFQEDTKAYVIEQLDAWGLHGASGVEVLQRKALITVSNDNIIQMHDLI
RQMGCEVVRQECITHPGRRTRLRDKEDVYSVLRYELGSDKMEGMQVDVFRIKDLPLKVGTFKKMPCLRFLKFYLPDDN
IFLPPNPDGTLWYEKRHFPLLLSAWCKDLMRVACEIQIKCVDYVYFDGYSHPSLTSTLRNRGREIRSSTSMSLNKLTGSLG
DVECSDILDQQFKILPDGLLCLRSTYYLKLSKKSTGEDSAKPKLHVLFDGLRFYERISVSKLKNSDIEGNRTLFLYFAGLIFLFY
PFLLKRPWFHFLFSFRFAFFCTLFLYLLWKTIL

>XP_022638866.1

MTQLPSSSSSSSSTYEGTHDVFLSFRGDDTRSGFTGNLYKSLCDRGIHTFIDDEGLRKGEEIRPALFKAIEQSRIAIVVFSEN
YADSTYCLEELIVILECILRKGRLVWPVFYGVTPSYVRFQKGSYGKALAKHGERFKNDQEKLQKWKLALQVAAGLSGSHF
KLKQGYEHELIRTIVEEVSKKINRSPLHVANYPIGLESRVQDVKLLLDVGSNRGVSMVGIFGIGGIGKTAIACAVYNAIADQ
FDVQCFLGDIRQKSMKYDLVQLQETVLSEMVGEKSIKLGSINRGMAVMKSKLQRKKVLLILDDVDKLEQLKALAGDPS
WFGDGSKIIVTTRNRRFLRVHGVERTYEAKGLDDKEALELFSWHAFKRNEVGPGYLDISKRAVFRCNGLPLALEIIGSNLN
GITMSEWEAALDTIERIPDEDIQEKLKVSYDGLKGNEKEVFLDMACFFRGYHLKDVINLLLQSRGFSPEYVIRMLVDKSLI
KIDQYGFVQMHNLVEDMGREIVRQESPSEPGKRSRLWLYEDIVDVLENDKGTDTIEVIMLHLPKNREVLWNGSELKKM
TNLKMLTIENADFSRGPEYLPSSLRVLKWRGYPTQSLPPEYDPRRLVMLDLSMSRNILGKQLNLMVCTIDIFLEIFCLFYIFI
FINSMWKFPKLIAFSLAYQKFESLSEMVLRGCRFIKQAPDMSGAKNLRKLCLDNCKNLVQVHNSIGLLDKLTWFTAIGCT
SLRTLPHSFKLTSLEYLSLRKCSSLQRLPNISEEMKHMKNLDLCGTAIEQLPYSFRKLTGLKYLVLDKCKRLNQIPINILMLPK
LERLTAVKCGRYVNLILGKSEEQVRLASSDSLIDFRLNYNGLTPTSFPNVEFLVLTGCSFKVLPECISQCRFLKNLVLDNCKE
LQEIRVVPPKIKYLSAINCTLLSHESQNMLLNQRLHEGGGTDFSLPGTRLPEWFDHCTRGPSLSFWFRNKFPRMTLAVV
GVLDKQGSFPMSRFHLLINGIQKLHCHFTVQSKLITYHIFLSDVLLKSYNGGLESVYGEDGWNHVEVSYVGPRVFPHSCR
TKKGTIKWMGVHVHKQKTNMQDIRFINPWFPKRAHSEVSKADLQESFQSLPKRIRVSHRKEICEAPQTKQHEANSSYH
GVSQRLWLAICSIAAPLNVKVLMWNICQDDLPTFEYLFRRKLVLSPLCPICGTEPETVEHVFLFCPWTRPLWFGSDFQW
CIDVKEVQSFQLWLWHKLMEIQRVYPENANRISAQVGSICWSIWKGRNEFVLEGKPVNPLILR

>XP_022639044.1

MEETAQEIKRPFGIPEKPEFTVGLDVPLKKLKLNVLSEGSSVIVLTGLGGSGKTTLATVLCWDEQVLGKFKENILFVTFSKA
PQLKIIVERLLEHFGYQVPELQSDGDAIDQLVLLLRKINANPMLLVLDDVWPCSEAVVEKLKFQISDYKILVTSRIAFPRFG
TSLVLKPLVPEDAITLFRHHAFLKRASSNIPEEDLVQKVVRHCKGLPLAIKVIGRSLSQQSIELWQKMVEELSEGHCILDSN
SELLSSLQKILDVLEDNPIIKECFMDLALFPEHQRIPVAALVDMWVELYGLDNYGIKAIAIVNRLDSMNLANVTVTRKNSN
DTDSYYYNNHFIVLHDILRDLAIYQSNKAQIELRKRLMIGINETKPEVCLGEKQHGRMIRTLSNNFRLCDKQKTQKIPVGT
LSISSDETCTSYWSHLRPAQAEVLILNIPTNQYSIPKYLKEMSKLKVLIVMNYGFHPCELFDFELLGTLSLLKRLRLERISIPSF
VTLKNLKKLSLYLCDTREAFENSNILISDAFPNLEDLNIEYSKEMVGLPKGLCNITSLNVLSISNCHKLSALPQEIGNLENLKL
LRLSSCTDLQVIPNSVGRLSNLRHMDISNCINLPNLPEEFGNLCNLRTLYMTSCERCELPSSIINLKNLKEVVCDEETAFSW
EAFKPMLPNLKIDVPQLDINLNWLHAIHS

>XP_022639796.1

MEKLVKWKIALYQVAKLFSFHIYHGDGYEYELIGKIVEWVSKKINRAHYPIGLESQVQEVMKLLDVGCDGGPHMIGIHG
TGGVGKSTLAQEVYNNLISERFDASCFIENVREKSNKHGLQYLQSTILENLLGEKDIKLTSVEQGILMIQRRLQKKKVLLILD
DVDRQEQLQAVVGRADWFGRGSRVIITTRDEQLLASHDVQITYEVKKLNNKDALVLLKWKAFKKHYFDPRYEXLLNXA
VTFSXGIPLALEVIGSNLCGKSVEEWKSVIHQLEKCPNNPVETVLKASFDSLEEKERSVFLDLACCYKGYELAEVEDILQAH
YGQNMKCYIDSLVDKSLVKLSHGTKPCYDRVTLHDLIEDMGKDIVRQESLTGPGERTRLWLLEDVRQLLENSRGNSKIEII
CLDFSIFDQEERVEWDGKAFQNMQNLKTLIIKHGSFSKGPEYLPDNLRVLEWWRYPSNCLPSDFHPKELAICKLPCSSIST
IELTNILKASLMSALFVN

>XP_022639811.1

MATPSSRRFKYDVFLSFRGEDTRYGFTGDLYEALRHKGVHTFMDDEELQSGEEITPALQKAIEESKIAIVVLSHNYASSSF
CLDELATILDCNSKGLLVIPVFYKVDPSYVRHQKGSYEEALAKHQKRFKGQEEKLHKWKMALRQVADLCGYHFGDGYEY
QYKFIGSIVERVSRVINRVPLHVAVYPVGLPSQVLKVKKLLDLGSHDVVHMIGIRGMXGLGKTTLSLAVYNSIADDFDESC
FLQNVREESNEEGLKHLQSILLSKMLGEKNIILTSWQEGASMIQERLRRKKVLLILDDVDNRKQLQAFAGRADWFGPGS
RVIITTRDEQVLKSHEIERTYEMEELNENDSLQLLIWNAFKREKVDPSYEDVLKRVVTYASGLPLALEVIGSNLVGKSVEE
WESAIEHYKRIPNGQILEILKVSFDGLGEEEKNVFLDMACFFKGSSLEEVEHILGILYDNNMKHHIGVLVEKSLIKVRYGRF
RVIEMHDLIEDMGRDIDRQESPKERENHRRLWLGKDILHVLKHNKGTSKTEIIRLDLSISEREETLEWNPNAFRRMKNLK
ILIIRNGXFSKGXNYLPKSLRVLEWHGYPSNCFPSNFDPNNLVTCKLPNSHFTSFGFLGSAKKFENLTVLKFDGCKLLTQTP
DMSVLRNLEEVSFNRCESLVAVHDSIGFMSKLKILHAKHCIKLTSFPPLNLPTLEKLKLSYCSNLEKFPEILGKMGNIKELEL
RELPIKELPLSFQNLIGLERLSLSCENVHLRSSILTMPNLIEFSVTNCKEWQWIKSDAEDNVGSMVPSKLKRFSAQSCNLD
DNFFSAGFMQLAQVRSLILLNNNFKHLPECIKEFHNLNELNVTHCKHLEEIRGFPPKLVYFSAPNCISLTSTSLDMLLNKEL
YEARKATNFTFPGESFPEWFDLKSSGPPCSFWFRNKFPARVLSLLILHMNKYDYFIFYPKVYINGKWQTGSSRYFLEHTKF
EFGLTYLCDLNLMYGNLFEQPLEKEWNHVEVTYSTPIGKSWVKAIGIHIFKEENNIMEDIRFDDPYIMEDIMEEEEKEEEK
EEGEEKEEKEEKAKEEEKKEKAEEEENSIMEDTMEEEGRRGKRRKLHHGRHSRFDDPFTNKKRKKGKEKRNKKKKKNN
M

>XP_022639818.1

MATPSSRRFTYDVFLSFRGEDTRYDFTGYLYKALCDGGVHTFMDDGKLQSGEEITPALQKAIQESKIAIVILSHNYASSSF
CLDELATILDCKSKGLLVIPVFYKVDPSYVRHQKGSYEEALTKHQKRFKGQEEKLHKWKTALRQVADLSGYHFGDGYEYQ
YKFIGSIVERVSRVINRVPLHVAVYPVGLPSQVLKVKKLLDLGSHDVVHMIGIRGMGGLGKTTLSLAVYNSIADDFDESCF
LQNVREESNKEGLKHLQSILLSKMLREKNIILTSWQEGASXIQERLGRKKVLLILDDVDNRKQLQAFAGRADWFGPGSRII
ITTRDEQLLKSHEIERTYEMEELNENDSLQLLIWNAFKREKVDPSYEDVLKRVVTYASGLPLALEVIGSNLVGKSVEEWES
AIEHYKRIPNGQILEILKVSFDXLGEEEKNVFLDXACFFKGSSLEEVEHILGILYDNNMKHHIGVLVEKSLIKVRXGRFXVIE
MHDLIEDMGRDIDRQESPKEXENHRRLWLGKDILHVLKHNKGTSKTEIIRLDLSISEREETLEWNPNAFRRMKNLKILIIR

NGXFSKGXNYLPKSLRVLEWHGYPSNCFPSNFDPNNLVTCKLPNSHFTSFGFLGSAKKFENLTVLNFDWCMFLTQTPD
MSDLGNLEEVSFNWCVSLDAVHDSIGFMSKLKILHANHCINLTSFPPLNLPTLESLELSFCYSLEKFPEILGKMGNIRVLQL
LDLPIKELPLSFQNLIGLEQLSLSCEIVHLRSSIFTMPNLIKFSVTNCKEWQWIKSEDAEDNVGSVVPSKLDWFSAWSCNL
DDDFFSAGFMRLARVRDLYLQDNNFKHLPECIKEFHNLNSLDVTHCKHLEEIRGFPPKLKYFSAPQCISLTSTSLDMLLNK
ELYEARKTAIFTFPGESFPEWFDLKSSGPSCSFWFRNKFPARVLSLLIIHMNKYENLSTIFEVHINGELQTGGGHYLLEVAK
FEFGQTYLSDLKMYVNFFEQPFGKEWNHVEVTYYTPSRKSWIKATGIHVFKEEMEDIRFDDPYIVEDIMEEEEKEEEKEE
GEEKEEKAKEEEKKEKAEEEENSIMGHHGRRRKKGKKKKTASWKTFLV

>XP_022639823.1

MATPSSRRFTYDVFLSFRGEDTRYDFTGYLYKALCDGGVHTFMDDGKLQSGEEITPALQKAIQESKIAIVILSHNYASSSF
CLDELATILDCKSKGLLVIPVFYKVDPSYVRHQKGSYEEALTKHQKRFKGQEEKLHKWKTALRQVADLSGYHFGDGYEYQ
YKFIGSIVERVSRVINRVPLHVAVYPVGLPSQVLKVKKLLDLGSHDVVHMIGIRGMGGLGKTTLSLAVYNSIADDFDESCF
LQNVREESNKEGLKHLQSILLSKMLREKNIILTSWQEGASXIQERLGRKKVLLILDDVDNRKQLQAFAGRADWFGPGSRII
ITTRDEQLLKSHEIERTYEMEELNENDSLQLLIWNAFKREKVDPSYEDVLKRVVTYASGLPLALEVIGSNLVGKSVEEWES
AIEHYKRIPNGQILEILKVSFDXLGEEEKNVFLDXACFFKGSSLEEVEHILGILYDNNMKHHIGVLVEKSLIKVRXGRFXVIE
MHDLIEDMGRDIDRQESPKEXENHRRLWLGKDILHVLKHNKGTSKTEIIRLDLSISEREETLEWNPNAFRRMKNLKILIIR
NGXFSKGXNYLPKSLRVLEWHGYPSNCFPSNFDPNNLVTCKLPNSHFTSFGFLGSAKKFENLTVLNFDWCMFLTQTPD
MSDLGNLEEVSFNWCVSLDAVHDSIGFMSKLKILHANHCINLTSFPPLNLPTLESLELSFCYSLEKFPEILGKMGNIREWQ
WIKSEDAEDNVGSVVPSKLDWFSAWSCNLDDDFFSAGFMRLARVRDLYLQDNNFKHLPECIKEFHNLNSLDVTHCKH
LEEIRGFPPKLKYFSAPQCISLTSTSLDMLLNKELYEARKTAIFTFPGESFPEWFDLKSSGPSCSFWFRNKFPARVLSLLIIHM
NKYENLSTIFEVHINGELQTGGGHYLLEVAKFEFGQTYLSDLKMYVNFFEQPFGKEWNHVEVTYYTPSRKSWIKATGIHV
FKEEMEDIRFDDPYIVEDIMEEEEKEEEKEEGEEKEEKAKEEEKKEKAEEEENSIMGHHGRRRKKGKKKKTASWKTFLV

>XP_022639827.1

MATPSSRRFTYDVFLSFRGEDTRYDFTGYLYKALCDGGVHTFMDDGKLQSGEEITPALQKAIQESKIAIVILSHNYASSSF
CLDELATILDCKSKGLLVIPVFYKVDPSYVRHQKGSYEEALTKHQKRFKGQEEKLHKWKTALRQVADLSGYHFGDGYEYQ
YKFIGSIVERVSRVINRVPLHVAVYPVGLPSQVLKVKKLLDLGSHDVVHMIGIRGMGGLGKTTLSLAVYNSIADDFDESCF
LQNVREESNKEGLKHLQSILLSKMLREKNIILTSWQEGASXIQERLGRKKVLLILDDVDNRKQLQAFAGRADWFGPGSRII
ITTRDEQLLKSHEIERTYEMEELNENDSLQLLIWNAFKREKVDPSYEDVLKRVVTYASGLPLALEVIGSNLVGKSVEEWES
AIEHYKRIPNGQILEILKVSFDXLGEEEKNVFLDXACFFKGSSLEEVEHILGILYDNNMKHHIGVLVEKSLIKVRXGRFXVIE
MHDLIEDMGRDIDRQESPKEXENHRRLWLGKDILHVLKHNKGTSKTEIIRLDLSISEREETLEWNPNAFRRMKNLKILIIR
NGXFSKGXNYLPKSLRVLEWHGYPSNCFPSNFDPNNLVTCKLPNSHFTSFGFLGSAKKFENLTVLNFDWCMFLTQTPD
MSDLGNLEEVSFNCVTNCKEWQWIKSEDAEDNVGSVVPSKLDWFSAWSCNLDDDFFSAGFMRLARVRDLYLQDNN
FKHLPECIKEFHNLNSLDVTHCKHLEEIRGFPPKLKYFSAPQCISLTSTSLDMLLNKELYEARKTAIFTFPGESFPEWFDLKSS
GPSCSFWFRNKFPARVLSLLIIHMNKYENLSTIFEVHINGELQTGGGHYLLEVAKFEFGQTYLSDLKMYVNFFEQPFGKE
WNHVEVTYYTPSRKSWIKATGIHVFKEEMEDIRFDDPYIVEDIMEEEEKEEEKEEGEEKEEKAKEEEKKEKAEEEENSIMG
HHGRRRKKGKKKKTASWKTFLV

>XP_022639939.1

MAESSSSLAMPTSPTKYDVFLSFRGEDTRYNFISHLYDALHRNHIEAYMDQRLQRGEEISPALQTAIEESKIYVLVFSENY
ASSTWCLNELIKILHCKKIYERDVIPVFYKVDPSTVRKQEERYKAAFDEHEQRFKDDMDKVQRWKDALTEASGLSGWDS
NVIRSENTLVEEIVKDILKKLDLYSISYDPGIIGIEKHIESIRLLMRFESSDIRIIGICGMGGIGKTTISEQIYHTFAKQFDSRSLV
LDTQQKIERDGIDTVRKKYMSELLNEVPSPSLYYSERLKQVRILIILDDVTDSVQLKQLLGRCNSFGQGSRIIITSRDIQVLK
NVGADDIYEVEKLNELNSLKLFTLHAFKQNSSQETTYMDLVEEVLRYAKGIPLALQILGSLLYGRTREAWESELQKLKKCQ

HLKIFSVLKLSYDGLDEEEKNIFLDIACFYRGDEESVVQERLDDCGFSSKIGMDILKDRCLISISHGIIEMHDLIQEMGQEIV
RQECPHYPGKRSRLFKGEESLEVLKKNKGSDAIQGILLNLREIKEVIVHGQTFEKMDNLRMFMLFDYLSYGSRVASSIVTL
PDTLKIFYWHGFPQRSLPPNFCPENLVRLEMPYCDLEQLWEEDQVFQSLPKLKRLDLSYSDKLSRIQDLSLSPNIEEIILND
CPKLIKVHSSILLTKLTSLRLDDCHNLNSAIVPSNILSRSPPGCINLSWCGKLEMFSISQTQICYAPPSSSLIGPIFPHVKPRPQ
RRNFLGPPRILFPENLFRYNVTEISSITFDRYWESDEEKEVTNKICFIDLSNCSSLTIFPFDLSQIKFLNKLCLRDCSKLQNFPEI
ENTMENLAVLILDGTAIQALPSSLWRLVGLEQLSLRGCKNLEIIPSSIGSLTRLCKLDLTFCESLQTFPSTIFKLKLRKLDLCGC
FNLRTFPEITEPAQTLAHINLTHTAVKELPSSFDNLAKLRSLQLNRCTDLESLPNSIVNLKLLCKLDCSECAKLKEIPAHIGSM
SSLMELSLSESGIVNLPESIAHLSSLKSLDLSGCKNLECIPQIPPFLKQLVALDCTSIRRVMSNQNLSDSKEGVFKFHFTNAQ
QLDSNARANIEEDARLRMTHDACRSVFFCFPGTAVPHWFPFRGKGSSVTTNEDLSFCSDDRLIGFALCIVFGVLDTNAIK
DRFLYFGYSLKFECDDDGTQIIPNNDELINYFRWGYSYRVLDKDHTFMWKFNLESVRRSGMNLRRLSDARSFTFEISPKA
CNFLLPDYRSIMTIKECGMCPLYSSGSNVA

>XP_022639940.1

MAESSSSLAMPTSPTKYDVFLSFRGEDTRYNFISHLYDALHRNHIEAYMDQRLQRGEEISPALQTAIEESKIYVLVFSENY
ASSTWCLNELIKILHCKKIYERDVIPVFYKVDPSTVRKQEERYKAAFDEHEQRFKDDMDKVQRWKDALTEASGLSGWDS
NVIRSENTLVEEIVKDILKKLDLYSISYDPGIIGIEKHIESIRLLMRFESSDIRIIGICGMGGIGKTTISEQIYHTFAKQFDSRSLV
LDTQQKIERDGIDTVRKKYMSELLNEVPSPSLYYSERLKQVRILIILDDVTDSVQLKQLLGRCNSFGQGSRIIITSRDIQVLK
NVGADDIYEVEKLNELNSLKLFTLHAFKQNSSQETTYMDLVEEVLRYAKGIPLALQILGSLLYGRTREAWESELQKLKKCQ
HLKIFSVLKLSYDGLDEEEKNIFLDIACFYRGDEESVVQERLDDCGFSSKIGMDILKDRCLISISHGIIEMHDLIQEMGQEIV
RQECPHYPGKRSRLFKGEESLEVLKKNKGSDAIQGILLNLREIKEVIVHGQTFEKMDNLRMFMLFDYLSYGSRVASSIVTL
PDTLKIFYWHGFPQRSLPPNFCPENLVRLEMPYCDLEQLWEEDQVFQSLPKLKRLDLSYSDKLSRIQDLSLSPNIEEIILND
CPKLIKVHSSILLTKLTSLRLDDCHNLNSAIVPSNILSRSPPGCINLSWCGKLEMFSISQTQICYAPPSSSLIGPIFPHVKPRPQ
RRNFLGPPRILFPENLFRYNVTEISSITFDRYWESDEEKEVTNKICFIDLSNCSSLTIFPFDLSQIKFLNKLCLRDCSKLQNFPEI
ENTMENLAVLILDGTAIQALPSSLWRLVGLEQLSLRGCKNLEIIPSSIGSLTRLCKLDLTFCESLQTFPSTIFKLKLRKLDLCGC
FNLRTFPEITEPAQTLAHINLTHTAVKELPSSFDNLAKLRSLQLNRCTDLESLPNSIVNLKLLCKLDCSECAKLKEIPAHIGSM
SSLMELSLSESGIVNLPESIAHLSSLKSLDLSGCKNLECIPQIPPFLKQLVALDCTSIRRVMSNQNLSDSKEGVFKFHFTNAQ
QLDSNARANIEEDARLRMTHDACRSVFFCFPGTAVPHWFPFRDGVIVTESLIKITHLCGNLTWRA

>XP_022639941.1

MSGSSSSFVMPTSVTRYDVFLNFRREDTCENFISHLYGALQRKHIEAYIDYELQRCEEISPAIQTAIEESKIYVVVFSENYAS
SKWCLNELTKILDCKNRYGRNVIPVFYKVDPATVRKQEERYKEAFEEHEQQFKEDISKVQRWKDALTEAAGLSGVEEIIE
DILKKLNPYSISYNQGIIGIEKHIEEIRFLLRLESENTRIIGICGIGGIGKTTISKQLYHKFAMHFDSHSLILDVQEKLQREGIHDI
IIKYQSELLKEAPSHLSHYNERIKRTKALLILDDVTDSTILSKVMRERSFGKGSRIIMTSRDRQVLKNAGADDIYELKELIFHD
SLKLFNLHAFKQNSSEEITYMDLSVKMLRYAKGVPLALQILGSLLYGRQREAWESQLQNLEKCQDHDIFNILKLSYDGLRE
EEKNIFLDIACFYKGHNEIVVGETLSDCGFSSKIGMDVLKDKCLISILDGRIVMHDLIQAMGREIVSKECLQHPGKRSRLFK
ANEIHEILRNNKGVPLNFQSLKKLCIIDLSNCSSLTIFPFDLSEIKFLNKLRLSGCSKLKNFPEIENTMEYLAVLLLDATAIQAL
PSSLWHLVGLQELSLSRCWNLEIIPSCIGRLTRLCKLDLAYCLSLQTFPSTIFKLKLRKLDLSECVKLRTFPEITEPAQTFTQIN
LTDTAVKELPSSLGNLAKLRSLKLNRCTDLESLPNSIVNLKHLCKLDCSGCAKLTEIPTHIGSLSSLMELSLSNSGIVNLPESM
AHLSGLKSLDLSDCKKLECIPHIPPFLNQLVALDCTSIRGAMSNSPVRNLSNSKDSFFRFHLTNSQQLDSGGRANIEEDAR
LRMSNDAYESVCFCFPGTAVPHWFPFRSEGPSLTINEDLSFCSDDRLIGFALCVVFEVLDTNDIKGRCSSFVYSLKFECDD
DGTQIIPNNDVLNTYFTWHLSDRVLDKDHTFMWKFNLESLRRRGMNLRRLSDARSFTFEISICTSNYDNLWPDYITVLTI
KECGVCPLYRSGSSVGESSKETKEDRKRKA

>XP_022639957.1

MSASFSYDVFLSFRGSDTRYGFTGNLYKALCDKGIHVFIDDEELQRGDEIREALIEAIKQSRMAIVVFSMNYASSSFCLDEL
VKIIDCVKEKGRLILPIFYDVRPSHVRGLSGSYAEALAMHQERFKSSNQSLNDNMGRLQKWKMALNQAANVSGKHYKL
GNEYEHEFIGKIVKEVSNKINRRPLHVADYPVGMECRVQKVKSLLQFGSDTGVHIVGIYGIGGMGKTTLARAVYNSIAD
QFEGLCFLDGVRENAVKQGLVHLQEMLLSEIVGEKDIRIGSVSKGISIIKHRLHRKKVLLILDDVDKLEQVRATAGERNWF
GSGSRVIITTRDKHLLQGVDGKYEVEDLNEEEALELLSWNAFKDHRVDPSYKDISNQAVAYASGLPLALEVIGSLLFGKGT
REWESALDQFKKIPNRRIQEILKVSYNALEENQQRIFLDIACCLKGYELEEVEDILGVHYGVCMKYDIGVLVDKSLIKIENG
CVTLHELIEAMGKEIDRQESPKELGKHRRLWFHKDIIQVLAENTGTSDIEIICLDFPLFEEDEEVFVEWDGEAFKKMENLK
TLIIRNSHFSKGPAYLPNSLRVLEWWTYPLQDLPTDFHPSKLAICKLPRSCFTSLELATISKACVMGSFPRSFEDYSVGSYLF
SLFLLQKFMNLTVLNFDGTECLTKIPDISSLQNLEKLTFECCENLVAIHGSVGFLDKLKILSAFGCSKLTSFPPIKLISLERLDIS
SCSSLESFPEILGKMENITQLELKYTPLKEFPFSFRNLARLQDLVLVDCGNVQLPSSIVMLPELAEIFALGCKGWLLPKQDE
NDEEKVSLVSSNVKCLCLSGCNLSDEYFPMVLAWFVNVKELELSSNSFTFLPECIKQCRSLKLLNLDNCEHLREIRGTPPNL
EYFSAGNCKSLSFCCSAMLLDQELHVAGNTMFCLPGSRIPEWLEQQSIGPSLSFWFRENFPVMDLCFVIGPMGKDSILF
RPIMTINGNTMEIQSLTDKRFCFDFPALDYHILIIGTKYMKFGDNLDKPLSKNEWNHVVVSIALDFEPTPKEIIVKQTALH
VIKPESSMDDIQFTDPCNQPSFKEKQRLVDTVDCHRQFMQHQTTLVSLEPHVRQGRNSLSLIPPHACKNNLNWDSFST
GTSSIASVQEYEIASKKLRLDMGILQFVQQRKRLAILGLQQQRRMASLDLLQRRGRELLSLLCSPSLELMVSWQRRCITTF
QGLQEQHLPSTIKQNFEGCNGINEAKVNEVTRCKNNDGNDNERNPLVEQLLLIKFPRPKRIPEELITMEFEEAHNNFLAI
RLLYRLLEDNSIALQNSNPKHLVEWARDLLKSLLDVAVESVFETHLKVHICMVHHFPAGPFFFGWQESNSLVAANISQSS
VKYPTCSKMTSEKDEQPQFRLISKKKLPELSKFSISDDGGTKCKTLGCIGEESSTMQQNAVKATGLSDDIDSLPYTESIHSK
QQQNDVNRLYGDGEDAKESLQMFDAKDDEQNEENNVILLNRIHEAQRNSDAAGQVEHLNQRGEFSDDLVNAIKRIES
RILAFQICSNVMDSTKNSAGHLTTLEAANSDGPAMQRNSGVSGSQISSGKSLLKGHRLMSQKTFKSSCKDENSIAESAF
EEPLFPGKESLNQSQMANQNFCLQNSAESAKNIDVPKKSGTQMVSRGECLSNHERIQNSERNIAIDSVKPIDGLVSGDA
YFETRASKGIPGFRVPLNQDNNSKKHSMLLSKTNRENSARKNPVAWSKTDQNPREMISESSYTQKSARSKTIITRREKPP
PHQMVMKPTLLDQKSSDIKVNSHKHRGRRRTFNTSHLEPRNTRVLQQHFEREESSSSSSKSDSDSSCWSSQQGRANS
SSIDSEDYSLSDGTQSPSSSGRMVDAAYEGSSEETINSYLEMDDDPSPRLGSFKSYRHHDERYHKETTGRLRRLRNKLGLI
FHHHHHHHHHHDHGNTHSREGHGPTTWNHLKNVFHHKDKHGVLTKNVEKTKGGHAAKVLSGNQVGQFNRLAEGL
LRHIRHSKKPKPPKFDVVKQSRNKPHGHNQKKLRWLQMLRKQRGVKMKNQCRTKMGFMSQNSLKNY

>XP_022639959.1

MSASFSYDVFLSFRGSDTRYGFTGNLYKALCDKGIHVFIDDEELQRGDEIREALIEAIKQSRMAIVVFSMNYASSSFCLDEL
VKIIDCVKEKGRLILPIFYDVRPSHVRGLSGSYAEALAMHQERFKSSNQSLNDNMGRLQKWKMALNQAANVSGKHYKL
GNEYEHEFIGKIVKEVSNKINRRPLHVADYPVGMECRVQKVKSLLQFGSDTGVHIVGIYGIGGMGKTTLARAVYNSIAD
QFEGLCFLDGVRENAVKQGLVHLQEMLLSEIVGEKDIRIGSVSKGISIIKHRLHRKKVLLILDDVDKLEQVRATAGERNWF
GSGSRVIITTRDKHLLQGVDGKYEVEDLNEEEALELLSWNAFKDHRVDPSYKDISNQAVAYASGLPLALEVIGSLLFGKGT
REWESALDQFKKIPNRRIQEILKVSYNALEENQQRIFLDIACCLKGYELEEVEDILGVHYGVCMKYDIGVLVDKSLIKIENG
CVTLHELIEAMGKEIDRQESPKELGKHRRLWFHKDIIQVLAENTGTSDIEIICLDFPLFEEDEEVFVEWDGEAFKKMENLK
TLIIRNSHFSKGPAYLPNSLRVLEWWTYPLQDLPTDFHPSKLAICKLPRSCFTSLELATISKACVMGSFPRSFEDYSVGSYLF
SLFLLQKFMNLTVLNFDGTECLTKIPDISSLQNLEKLTFECCENLVAIHGSVGFLDKLKILSAFGCSKLTSFPPIKLISLERLDIS
SCSSLESFPEILGKMENITQLELKYTPLKEFPFSFRNLARLQDLVLVDCGNVQLPSSIVMLPELAEIFALGCKGWLLPKQDE
NDEEKVSLVSSNVKCLCLSGCNLSDEYFPMVLAWFVNVKELELSSNSFTFLPECIKQCRSLKLLNLDNCEHLREIRGTPPNL
EYFSAGNCKSLSFCCSAMLLDQELHVAGNTMFCLPGSRIPEWLEQQSIGPSLSFWFRENFPVMDLCFVIGPMGKDSILF
RPIMTINGNTMEIQSLTDKRFCFDFPALDYHILIIGTKYMKFGDNLDKPLSKNEWNHVVVSIALDFEPTPKEIIVKQTALH
VIKPESSMDDIQFTDPCNQPSFKEKQRLVDTVDCHRQFMQHQTTLVSLEPHVRQGRNSLSLIPPHACKNNLNWDSFST
GTSSIASVQEYEIASKKLRLDMGILQFVQQRKRLAILGLQQQRRMASLDLLQRRGRELLSLLCSPSLELMVSWQRRCITTF
QGLQEQHLPSTIKQNFEGCNGINEAKVNEVTRCKNNDGNDNERNPLVEQLLLIKFPRPKRIPEELITMEFEEAHNNFLAI

RLLYRLLEDNSIALQNSNPKHLVEWARDLLKSLLDVAVESVFETHLKWQESNSLVAANISQSSVKYPTCSKMTSEKDEQP
QFRLISKKKLPELSKFSISDDGGTKCKTLGCIGEESSTMQQNAVKATGLSDDIDSLPYTESIHSKQQQNDVNRLYGDGED
AKESLQMFDAKDDEQNEENNVILLNRIHEAQRNSDAAGQVEHLNQRGEFSDDLVNAIKRIESRILAFQICSNVMDSTK
NSAGHLTTLEAANSDGPAMQRNSGVSGSQISSGKSLLKGHRLMSQKTFKSSCKDENSIAESAFEEPLFPGKESLNQSQM
ANQNFCLQNSAESAKNIDVPKKSGTQMVSRGECLSNHERIQNSERNIAIDSVKPIDGLVSGDAYFETRASKGIPGFRVPL
NQDNNSKKHSMLLSKTNRENSARKNPVAWSKTDQNPREMISESSYTQKSARSKTIITRREKPPPHQMVMKPTLLDQK
SSDIKVNSHKHRGRRRTFNTSHLEPRNTRVLQQHFEREESSSSSSSKSDSDSSCWSSQQGRANSSSIDSEDYSLSDGTQS
PSSSGRMVDAAYEGSSEETINSYLEMDDDPSPRLGSFKSYRHHDERYHKETTGRLRRLRNKLGLIFHHHHHHHHHHDH
GNTHSREGHGPTTWNHLKNVFHHKDKHGVLTKNVEKTKGGHAAKVLSGNQVGQFNRLAEGLLRHIRHSKKPKPPKF
DVVKQSRNKPHGHNQKKLRWLQMLRKQRGVKMKNQCRTKMGFMSQNSLKNY

>XP_022639960.1

MSASFSYDVFLSFRGSDTRYGFTGNLYKALCDKGIHVFIDDEELQRGDEIREALIEAIKQSRMAIVVFSMNYASSSFCLDEL
VKIIDCVKEKGRLILPIFYDVRPSHVRGLSGSYAEALAMHQERFKSSNQSLNDNMGRLQKWKMALNQAANVSGKHYKL
GNEYEHEFIGKIVKEVSNKINRRPLHVADYPVGMECRVQKVKSLLQFGSDTGVHIVGIYGIGGMGKTTLARAVYNSIAD
QFEGLCFLDGVRENAVKQGLVHLQEMLLSEIVGEKDIRIGSVSKGISIIKHRLHRKKVLLILDDVDKLEQVRATAGERNWF
GSGSRVIITTRDKHLLQGVDGKYEVEDLNEEEALELLSWNAFKDHRVDPSYKDISNQAVAYASGLPLALEVIGSLLFGKGT
REWESALDQFKKIPNRRIQEILKVSYNALEENQQRIFLDIACCLKGYELEEVEDILGVHYGVCMKYDIGVLVDKSLIKIENG
CVTLHELIEAMGKEIDRQESPKELGKHRRLWFHKDIIQVLAENTGTSDIEIICLDFPLFEEDEEVFVEWDGEAFKKMENLK
TLIIRNSHFSKGPAYLPNSLRVLEWWTYPLQDLPTDFHPSKLAICKLPRSCFTSLELATISKKFMNLTVLNFDGTECLTKIPDI
SSLQNLEKLTFECCENLVAIHGSVGFLDKLKILSAFGCSKLTSFPPIKLISLERLDISSCSSLESFPEILGKMENITQLELKYTPLK
EFPFSFRNLARLQDLVLVDCGNVQLPSSIVMLPELAEIFALGCKGWLLPKQDENDEEKVSLVSSNVKCLCLSGCNLSDEYF
PMVLAWFVNVKELELSSNSFTFLPECIKQCRSLKLLNLDNCEHLREIRGTPPNLEYFSAGNCKSLSFCCSAMLLDQELHVA
GNTMFCLPGSRIPEWLEQQSIGPSLSFWFRENFPVMDLCFVIGPMGKDSILFRPIMTINGNTMEIQSLTDKRFCFDFPAL
DYHILIIGTKYMKFGDNLDKPLSKNEWNHVVVSIALDFEPTPKEIIVKQTALHVIKPESSMDDIQFTDPCNQPSFKEKQRL
VDTVDCHRQFMQHQTTLVSLEPHVRQGRNSLSLIPPHACKNNLNWDSFSTGTSSIASVQEYEIASKKLRLDMGILQFVQ
QRKRLAILGLQQQRRMASLDLLQRRGRELLSLLCSPSLELMVSWQRRCITTFQGLQEQHLPSTIKQNFEGCNGINEAKV
NEVTRCKNNDGNDNERNPLVEQLLLIKFPRPKRIPEELITMEFEEAHNNFLAIRLLYRLLEDNSIALQNSNPKHLVEWARD
LLKSLLDVAVESVFETHLKVHICMVHHFPAGPFFFGWQESNSLVAANISQSSVKYPTCSKMTSEKDEQPQFRLISKKKLP
ELSKFSISDDGGTKCKTLGCIGEESSTMQQNAVKATGLSDDIDSLPYTESIHSKQQQNDVNRLYGDGEDAKESLQMFDA
KDDEQNEENNVILLNRIHEAQRNSDAAGQVEHLNQRGEFSDDLVNAIKRIESRILAFQICSNVMDSTKNSAGHLTTLEA
ANSDGPAMQRNSGVSGSQISSGKSLLKGHRLMSQKTFKSSCKDENSIAESAFEEPLFPGKESLNQSQMANQNFCLQNS
AESAKNIDVPKKSGTQMVSRGECLSNHERIQNSERNIAIDSVKPIDGLVSGDAYFETRASKGIPGFRVPLNQDNNSKKHS
MLLSKTNRENSARKNPVAWSKTDQNPREMISESSYTQKSARSKTIITRREKPPPHQMVMKPTLLDQKSSDIKVNSHKH
RGRRRTFNTSHLEPRNTRVLQQHFEREESSSSSSSKSDSDSSCWSSQQGRANSSSIDSEDYSLSDGTQSPSSSGRMVDA
AYEGSSEETINSYLEMDDDPSPRLGSFKSYRHHDERYHKETTGRLRRLRNKLGLIFHHHHHHHHHHDHGNTHSREGHG
PTTWNHLKNVFHHKDKHGVLTKNVEKTKGGHAAKVLSGNQVGQFNRLAEGLLRHIRHSKKPKPPKFDVVKQSRNKPH
GHNQKKLRWLQMLRKQRGVKMKNQCRTKMGFMSQNSLKNY

>XP_022639962.1

MSASFSYDVFLSFRGSDTRYGFTGNLYKALCDKGIHVFIDDEELQRGDEIREALIEAIKQSRMAIVVFSMNYASSSFCLDEL
VKIIDCVKEKGRLILPIFYDVRPSHVRGLSGSYAEALAMHQERFKSSNQSLNDNMGRLQKWKMALNQAANVSGKHYKL
GNEYEHEFIGKIVKEVSNKINRRPLHVADYPVGMECRVQKVKSLLQFGSDTGVHIVGIYGIGGMGKTTLARAVYNSIAD
QFEGLCFLDGVRENAVKQGLVHLQEMLLSEIVGEKDIRIGSVSKGISIIKHRLHRKKVLLILDDVDKLEQVRATAGERNWF

GSGSRVIITTRDKHLLQGVDGKYEVEDLNEEEALELLSWNAFKDHRVDPSYKDISNQAVAYASGLPLALEVIGSLLFGKGT
REWESALDQFKKIPNRRIQEILKVSYNALEENQQRIFLDIACCLKGYELEEVEDILGVHYGVCMKYDIGVLVDKSLIKIENG
CVTLHELIEAMGKEIDRQESPKELGKHRRLWFHKDIIQVLAENTGTSDIEIICLDFPLFEEDEEVFVEWDGEAFKKMENLK
TLIIRNSHFSKGPAYLPNSLRVLEWWTYPLQDLPTDFHPSKLAICKLPRSCFTSLELATISKACVMGSFPRSFEDYSVGSYLF
SLFLLQKFMNLTVLNFDGTECLTKIPDISSLQNLEKLTFECCENLVAIHGSVGFLDKLKILSAFGCSKLTSFPPIKLISLERLDIS
SCSSLESFPEILGKMENITQLELKYTPLKEFPFSFRNLARLQDLVLVDCGNVQLPSSIVMLPELAEIFALGCKGWLLPKQDE
NDEEKVSLVSSNVKCLCLSGCNLSDEYFPMVLAWFVNVKELELSSNSFTFLPECIKQCRSLKLLNLDNCEHLREIRGTPPNL
EYFSAGNCKSLSFCCSAMLLDQELHVAGNTMFCLPGSRIPEWLEQQSIGPSLSFWFRENFPVMDLCFVIGPMGKDSILF
RPIMTINGNTMEIQSLTDKRFCFDFPALDYHILIIGTKYMKFGDNLDKPLSKNEWNHVVVSIALDFEPTPKEIIVKQTALH
VIKPESSMDDIQFTDPCNQPSFKEKQRLVDTVDCHRQFMQHQTTLVSLEPHVRQGRNSLSLIPPHACKNNLNWDSFST
GTSSIASVQEYEIASKKLRLDMGILQFVQQRKRLAILGLQQQRRMASLDLLQRRGRELLSLLCSPSLELMVSWQRRCITTF
QGLQEQHLPSTIKQNFEGCNGINEAKVNEVTRCKNNDGNDNERNPLVEQLLLIKLVEWARDLLKSLLDVAVESVFETHL
KVHICMVHHFPAGPFFFGWQESNSLVAANISQSSVKYPTCSKMTSEKDEQPQFRLISKKKLPELSKFSISDDGGTKCKTL
GCIGEESSTMQQNAVKATGLSDDIDSLPYTESIHSKQQQNDVNRLYGDGEDAKESLQMFDAKDDEQNEENNVILLNRI
HEAQRNSDAAGQVEHLNQRGEFSDDLVNAIKRIESRILAFQICSNVMDSTKNSAGHLTTLEAANSDGPAMQRNSGVS
GSQISSGKSLLKGHRLMSQKTFKSSCKDENSIAESAFEEPLFPGKESLNQSQMANQNFCLQNSAESAKNIDVPKKSGTQ
MVSRGECLSNHERIQNSERNIAIDSVKPIDGLVSGDAYFETRASKGIPGFRVPLNQDNNSKKHSMLLSKTNRENSARKN
PVAWSKTDQNPREMISESSYTQKSARSKTIITRREKPPPHQMVMKPTLLDQKSSDIKVNSHKHRGRRRTFNTSHLEPRN
TRVLQQHFEREESSSSSSSKSDSDSSCWSSQQGRANSSSIDSEDYSLSDGTQSPSSSGRMVDAAYEGSSEETINSYLEMD
DDPSPRLGSFKSYRHHDERYHKETTGRLRRLRNKLGLIFHHHHHHHHHHHDHGNTHSREGHGPTTWNHLKNVFHHKD
KHGVLTKNVEKTKGGHAAKVLSGNQVGQFNRLAEGLLRHIRHSKKPKPPKFDVVKQSRNKPHGHNQKKLRWLQMLR
KQRGVKMKNQCRTKMGFMSQNSLKNY

>XP_022639964.1

MSASFSYDVFLSFRGSDTRYGFTGNLYKALCDKGIHVFIDDEELQRGDEIREALIEAIKQSRMAIVVFSMNYASSSFCLDEL
VKIIDCVKEKGRLILPIFYDVRPSHVRGLSGSYAEALAMHQERFKSSNQSLNDNMGRLQKWKMALNQAANVSGKHYKL
GNEYEHEFIGKIVKEVSNKINRRPLHVADYPVGMECRVQKVKSLLQFGSDTGVHIVGIYGIGGMGKTTLARAVYNSIAD
QFEGLCFLDGVRENAVKQGLVHLQEMLLSEIVGEKDIRIGSVSKGISIIKHRLHRKKVLLILDDVDKLEQVRATAGERNWF
GSGSRVIITTRDKHLLQGVDGKYEVEDLNEEEALELLSWNAFKDHRVDPSYKDISNQAVAYASGLPLALEVIGSLLFGKGT
REWESALDQFKKIPNRRIQEILKVSYNALEENQQRIFLDIACCLKGYELEEVEDILGVHYGVCMKYDIGVLVDKSLIKIENG
CVTLHELIEAMGKEIDRQESPKELGKHRRLWFHKDIIQVLAENTGTSDIEIICLDFPLFEEDEEVFVEWDGEAFKKMENLK
TLIIRNSHFSKGPAYLPNSLRVLEWWTYPLQDLPTDFHPSKLAICKLPRSCFTSLELATISKACVMGSFPRSFEDYSVGSYLF
SLFLLQKFMNLTVLNFDGTECLTKIPDISSLQNLEKLTFECCENLVAIHGSVGFLDKLKILSAFGCSKLTSFPPIKLISLERLDIS
SCSSLESFPEILGKMENITQLELKYTPLKEFPFSFRNLARLQDLVLVDCGNVQLPSSIVMLPELAEIFALGCKGWLLPKQDE
NDEEKVSLVSSNVKCLCLSGCNLSDEYFPMVLAWFVNVKELELSSNSFTFLPECIKQCRSLKLLNLDNCEHLREIRGTPPNL
EYFSAGNCKSLSFCCSAMLLDQELHVAGNTMFCLPGSRIPEWLEQQSIGPSLSFWFRENFPVMDLCFVIGPMGKDSILF
RPIMTINGNTMEIQSLTDKRFCFDFPALDYHILIIGTKYMKFGDNLDKPLSKNEWNHVVVSIALDFEPTPKEIIVKQTALH
VIKPESSMDDIQFTDPCNQPSFKEKQRLVDTVDCHRQFMQHQTTLVSLEPHVRQGRNSLSLIPPHACKNNLNWDSFST
GTSSIASVQEYEIASKKLRLDMGILQFVQQRKRLAILGLQQQRRMASLDLLQRRGRELLSLLCSPSLELMVSWQRRCITTF
QGLQEQHLPSTIKQNFEGCNGINEAKVNEVTRCKNNDGNDNERNPLVEQLLLIKLVEWARDLLKSLLDVAVESVFETHL
KWQESNSLVAANISQSSVKYPTCSKMTSEKDEQPQFRLISKKKLPELSKFSISDDGGTKCKTLGCIGEESSTMQQNAVKA
TGLSDDIDSLPYTESIHSKQQQNDVNRLYGDGEDAKESLQMFDAKDDEQNEENNVILLNRIHEAQRNSDAAGQVEHL
NQRGEFSDDLVNAIKRIESRILAFQICSNVMDSTKNSAGHLTTLEAANSDGPAMQRNSGVSGSQISSGKSLLKGHRLMS
QKTFKSSCKDENSIAESAFEEPLFPGKESLNQSQMANQNFCLQNSAESAKNIDVPKKSGTQMVSRGECLSNHERIQNSE

RNIAIDSVKPIDGLVSGDAYFETRASKGIPGFRVPLNQDNNSKKHSMLLSKTNRENSARKNPVAWSKTDQNPREMISES
SYTQKSARSKTIITRREKPPPHQMVMKPTLLDQKSSDIKVNSHKHRGRRRTFNTSHLEPRNTRVLQQHFEREESSSSSSS
KSDSDSSCWSSQQGRANSSSIDSEDYSLSDGTQSPSSSGRMVDAAYEGSSEETINSYLEMDDDPSPRLGSFKSYRHHDE
RYHKETTGRLRRLRNKLGLIFHHHHHHHHHHDHGNTHSREGHGPTTWNHLKNVFHHKDKHGVLTKNVEKTKGGHAA
KVLSGNQVGQFNRLAEGLLRHIRHSKKPKPPKFDVVKQSRNKPHGHNQKKLRWLQMLRKQRGVKMKNQCRTKMGF
MSQNSLKNY

>XP_022639967.1

MSASFSYDVFLSFRGSDTRYGFTGNLYKALCDKGIHVFIDDEELQRGDEIREALIEAIKQSRMAIVVFSMNYASSSFCLDEL
VKIIDCVKEKGRLILPIFYDVRPSHVRGLSGSYAEALAMHQERFKSSNQSLNDNMGRLQKWKMALNQAANVSGKHYKL
GNEYEHEFIGKIVKEVSNKINRRPLHVADYPVGMECRVQKVKSLLQFGSDTGVHIVGIYGIGGMGKTTLARAVYNSIAD
QFEGLCFLDGVRENAVKQGLVHLQEMLLSEIVGEKDIRIGSVSKGISIIKHRLHRKKVLLILDDVDKLEQVRATAGERNWF
GSGSRVIITTRDKHLLQGVDGKYEVEDLNEEEALELLSWNAFKDHRVDPSYKDISNQAVAYASGLPLALEVIGSLLFGKGT
REWESALDQFKKIPNRRIQEILKVSYNALEENQQRIFLDIACCLKGYELEEVEDILGVHYGVCMKYDIGVLVDKSLIKIENG
CVTLHELIEAMGKEIDRQESPKELGKHRRLWFHKDIIQVLAENTGTSDIEIICLDFPLFEEDEEVFVEWDGEAFKKMENLK
TLIIRNSHFSKGPAYLPNSLRVLEWWTYPLQDLPTDFHPSKLAICKLPRSCFTSLELATISKACVMGSFPRSFEDYSVGSYLF
SLFLLQKFMNLTVLNFDGTECLTKIPDISSLQNLEKLTFECCENLVAIHGSVGFLDKLKILSAFGCSKLTSFPPIKLISLERLDIS
SCSSLESFPEILGKMENITQLELKYTPLKEFPFSFRNLARLQDLVLVDCGNVQLPSSIVMLPELAEIFALGCKGWLLPKQDE
NDEEKELHVAGNTMFCLPGSRIPEWLEQQSIGPSLSFWFRENFPVMDLCFVIGPMGKDSILFRPIMTINGNTMEIQSLT
DKRFCFDFPALDYHILIIGTKYMKFGDNLDKPLSKNEWNHVVVSIALDFEPTPKEIIVKQTALHVIKPESSMDDIQFTDPC
NQPSFKEKQRLVDTVDCHRQFMQHQTTLVSLEPHVRQGRNSLSLIPPHACKNNLNWDSFSTGTSSIASVQEYEIASKKL
RLDMGILQFVQQRKRLAILGLQQQRRMASLDLLQRRGRELLSLLCSPSLELMVSWQRRCITTFQGLQEQHLPSTIKQNF
EGCNGINEAKVNEVTRCKNNDGNDNERNPLVEQLLLIKFPRPKRIPEELITMEFEEAHNNFLAIRLLYRLLEDNSIALQNS
NPKHLVEWARDLLKSLLDVAVESVFETHLKVHICMVHHFPAGPFFFGWQESNSLVAANISQSSVKYPTCSKMTSEKDE
QPQFRLISKKKLPELSKFSISDDGGTKCKTLGCIGEESSTMQQNAVKATGLSDDIDSLPYTESIHSKQQQNDVNRLYGDG
EDAKESLQMFDAKDDEQNEENNVILLNRIHEAQRNSDAAGQVEHLNQRGEFSDDLVNAIKRIESRILAFQICSNVMDST
KNSAGHLTTLEAANSDGPAMQRNSGVSGSQISSGKSLLKGHRLMSQKTFKSSCKDENSIAESAFEEPLFPGKESLNQSQ
MANQNFCLQNSAESAKNIDVPKKSGTQMVSRGECLSNHERIQNSERNIAIDSVKPIDGLVSGDAYFETRASKGIPGFRV
PLNQDNNSKKHSMLLSKTNRENSARKNPVAWSKTDQNPREMISESSYTQKSARSKTIITRREKPPPHQMVMKPTLLDQ
KSSDIKVNSHKHRGRRRTFNTSHLEPRNTRVLQQHFEREESSSSSSSKSDSDSSCWSSQQGRANSSSIDSEDYSLSDGTQ
SPSSSGRMVDAAYEGSSEETINSYLEMDDDPSPRLGSFKSYRHHDERYHKETTGRLRRLRNKLGLIFHHHHHHHHHHD
HGNTHSREGHGPTTWNHLKNVFHHKDKHGVLTKNVEKTKGGHAAKVLSGNQVGQFNRLAEGLLRHIRHSKKPKPPK
FDVVKQSRNKPHGHNQKKLRWLQMLRKQRGVKMKNQCRTKMGFMSQNSLKNY

>XP_022639969.1

MSQRDWSEGSYYGLTQVLHIICIEKIFTPSSSNFKHLAHGNEYEHEFIGKIVKEVSNKINRRPLHVADYPVGMECRVQKV
KSLLQFGSDTGVHIVGIYGIGGMGKTTLARAVYNSIADQFEGLCFLDGVRENAVKQGLVHLQEMLLSEIVGEKDIRIGSV
SKGISIIKHRLHRKKVLLILDDVDKLEQVRATAGERNWFGSGSRVIITTRDKHLLQGVDGKYEVEDLNEEEALELLSWNAF
KDHRVDPSYKDISNQAVAYASGLPLALEVIGSLLFGKGTREWESALDQFKKIPNRRIQEILKVSYNALEENQQRIFLDIACC
LKGYELEEVEDILGVHYGVCMKYDIGVLVDKSLIKIENGCVTLHELIEAMGKEIDRQESPKELGKHRRLWFHKDIIQVLAE
NTGTSDIEIICLDFPLFEEDEEVFVEWDGEAFKKMENLKTLIIRNSHFSKGPAYLPNSLRVLEWWTYPLQDLPTDFHPSKL
AICKLPRSCFTSLELATISKACVMGSFPRSFEDYSVGSYLFSLFLLQKFMNLTVLNFDGTECLTKIPDISSLQNLEKLTFECCE
NLVAIHGSVGFLDKLKILSAFGCSKLTSFPPIKLISLERLDISSCSSLESFPEILGKMENITQLELKYTPLKEFPFSFRNLARLQD
LVLVDCGNVQLPSSIVMLPELAEIFALGCKGWLLPKQDENDEEKVSLVSSNVKCLCLSGCNLSDEYFPMVLAWFVNVKE

LELSSNSFTFLPECIKQCRSLKLLNLDNCEHLREIRGTPPNLEYFSAGNCKSLSFCCSAMLLDQELHVAGNTMFCLPGSRIP
EWLEQQSIGPSLSFWFRENFPVMDLCFVIGPMGKDSILFRPIMTINGNTMEIQSLTDKRFCFDFPALDYHILIIGTKYMKF
GDNLDKPLSKNEWNHVVVSIALDFEPTPKEIIVKQTALHVIKPESSMDDIQFTDPCNQPSFKEKQRLVDTVDCHRQFM
QHQTTLVSLEPHVRQGRNSLSLIPPHACKNNLNWDSFSTGTSSIASVQEYEIASKKLRLDMGILQFVQQRKRLAILGLQQ
QRRMASLDLLQRRGRELLSLLCSPSLELMVSWQRRCITTFQGLQEQHLPSTIKQNFEGCNGINEAKVNEVTRCKNNDG
NDNERNPLVEQLLLIKFPRPKRIPEELITMEFEEAHNNFLAIRLLYRLLEDNSIALQNSNPKHLVEWARDLLKSLLDVAVES
VFETHLKVHICMVHHFPAGPFFFGWQESNSLVAANISQSSVKYPTCSKMTSEKDEQPQFRLISKKKLPELSKFSISDDGG
TKCKTLGCIGEESSTMQQNAVKATGLSDDIDSLPYTESIHSKQQQNDVNRLYGDGEDAKESLQMFDAKDDEQNEENN
VILLNRIHEAQRNSDAAGQVEHLNQRGEFSDDLVNAIKRIESRILAFQICSNVMDSTKNSAGHLTTLEAANSDGPAMQR
NSGVSGSQISSGKSLLKGHRLMSQKTFKSSCKDENSIAESAFEEPLFPGKESLNQSQMANQNFCLQNSAESAKNIDVPK
KSGTQMVSRGECLSNHERIQNSERNIAIDSVKPIDGLVSGDAYFETRASKGIPGFRVPLNQDNNSKKHSMLLSKTNREN
SARKNPVAWSKTDQNPREMISESSYTQKSARSKTIITRREKPPPHQMVMKPTLLDQKSSDIKVNSHKHRGRRRTFNTSH
LEPRNTRVLQQHFEREESSSSSSSKSDSDSSCWSSQQGRANSSSIDSEDYSLSDGTQSPSSSGRMVDAAYEGSSEETINS
YLEMDDDPSPRLGSFKSYRHHDERYHKETTGRLRRLRNKLGLIFHHHHHHHHHHDHGNTHSREGHGPTTWNHLKNV
FHHKDKHGVLTKNVEKTKGGHAAKVLSGNQVGQFNRLAEGLLRHIRHSKKPKPPKFDVVKQSRNKPHGHNQKKLRWL
QMLRKQRGVKMKNQCRTKMGFMSQNSLKNY

>XP_022640019.1

MAIRSRSSSFTYDVFLSFRGEDTRHGFTGHLYKALHDRGIYTFIDDEQLQRGEEITPSLVKAIEESRIAITVLSINYASSSFCL
NELEYILECFNKKYMXVLPVFFKIDPSDVRHQKGSYGEALAKHEQRFNHSMXKLENWKKALHQVANLSGFHLKHGDGY
EHEFIGRIVELVSSKINHAPLPVADYPVGLESQALAVRKLLDVGSDDVHMIGIYGIGGIGKSTLALAVYNLIVHHFDCSCFL
QNVREKSSKHGLQHLQSILFREMLGEKEANFASVEQGASVIHHRFQRKKVILILDDVDKYEQLQAIVGRPCLFGPGSRVII
TTRDKXLLSSYGVIRTYEVNLLDKNNALDLLSWKAFKTKXIDASYNEVLNDVVIXAYGLPLALEVIGSNLFGKSIEEWKSAIK
QYKRIPNNQILEILKVSFDSLEEEEKSVFLDICWLNRYPLSKVEDLLLAHYGDCMKYHIGVLVDKSLIKLSRYTLETRIAMHS
LIQDMGKEIVRQESPKDPGKRSRLWLPEDIIQVLEDDKGTSEIXIICLDXPKXDKEAIVEFNTKAFKTMKNLKTLILRNVNF
SEGPEHLPNSLRVLEWLGYPSHCLPSDFHPKKLVLCKLSRSSISSLEFSKDFMNLRVLNFDWCKCLTQIPDMSLLQNLEEL
SFKYCVNLITVHDSVGFLDKLKTLNAFSCEKXSCFPPMKLTSLEKLELSLCYSLKGFPEVLVKMENIRELNLEGCPITELPISF
QNLTGLPSLEMFFSTSSIVKVPSSIIMMPKLARIFARGLKGLQWLKQEEGEEQMGPVVVPSKVEWLTASNCNLYDDFFSI
DFARFSHVKNLWLPKNNFTILPECLKQCQFLSYLDVSYCKHLREIRGIPPNLKLFSAINCESLASSSKRMLVKQELHEAGNT
MFCLPGFSIPKWFNHQNRGTSISFWFRNKFPDKVQCVLVAPMLHNSFHPTVFINGKEYTLYGHFCWTGNHHKYLFDL
REASFRIRPYEVPFDSEWNHAKLTFPPGTYTSFDRAKMGIHIVKQENSMEDVRFTDPCSKRKSDVDINSSDSESSSC

>XP_022640021.1

MAIRSRSSSFTYDVFLSFRGEDTRHGFTGHLYKALHDRGIYTFIDDEELQRGEEITPALVKAIEESRIAIIVLSINYASSSFCLN
ELEYILECFNKKYMLVLPVFFKIDPSDVRHQKGSYGEALAKHEQRFNHSMEKLENWKKSLHQVANLSGFHLKHGDGYE
HEFIGRIVELVSSKINHSPLPVADYPVGLESQXLAVRKLLDVGSDDVHMIGIYGTGGIGKSTLALAVYNLIVHHFXCSCFLQ
NVREKSNKHGLQHLQSILLREMLGEKEANFXSVEQGASVIHHRFQRKKVILILDDIDKYEQLQAIVGRPCLFGPGSRVIITT
RDKQLLSSYGVIRTYEVNLLDKNNALDLLSWKAFKTKEFDASYNEVLNDVVIYAYGLPLALEVIGSNLFGKSIEEWKSAIKQ
YKRIPNNQILEILKVSFDSXEEEEKSVFLDICWLNRYPLSKVEDLLLAHYGDCMKYHIGVLVDKSLIKLSRYTLETRIALHSLIQ
DMGKEIVRQESPKDPGKRSRLWLPEDIIQVLEDDKDFMNLRVLNFDFCKCLTQIPDMSVLQNLEELSFKYCVNLITVHDS
VGLLDKLKTLSAFSCEKLRSFPPIKLTSLEKLDLSGCDSLKGFPEVLVKMENIRELNLEGCPITELPISFQNLTGLPSLEMFFST
SAIVKVPSSIIMMPKLADIDARGLKGLQWLKHEEGEEQMGAVVVPSKIEFLTASNCNLYDDFFSIDFTRFAHVKNLWLPK
NNFTILPECLKQCQFLYDLDVSDCKHLREIRGIPPNLKRFFAINCKSLASSSKRMLLKQELHEAGNTVFCLPGFSIPKWFNH

QNRGTSISFWFRNKFPDKVQCVLVAPMQDKFFRPRVFINGKQYTSYGHFGLTGNHHKYLFDLREASFKTKSPYRVPFDS
EWKHAKLSFPPGTYTSFDRAKMGIHIAKQENSMEDVRFTDPCSKRKTDVDISSSDSESIPVAKEHRFLDFDL

>XP_022640078.1

MDIQEESSTTVGPLTTPSLRNMSSSSSAFFSANQSPFFSPRSSSCQLSESLQPDAPIDRIHLDEAAPSSSSGIPEPKSLVNV
GCTFSEVAASPAGCSAGDLQKLDRISSSVGISSCTVSGHFHPYDDSYSGQKDKRSKKGRNKRISSTPGSRSVSSYRLKSCD
VYIGLHGRKPPLIRFANWLRVELEIQGISCFVSDRAGYRNSCKLSIAEKAMDVASYGIVIITRKSFKNPYTIEELQFFSGKKN
LVPIYFDLSPADCLVRDIIEKRGELWEKHGGELWLLYGGLEQEWKDVVHGLSRVEERKLEAQDGNWRDCILMAVTLLA
MKLGRRSAAEHLTKWREKVKEEELPFTRNENFIGRKKELSQLEFMLFGDVTGDSRQDYIDLKARPKRRHLTICRSKSNVQ
EERHVGNGSREEKTPVLWKESEKEIEMQSIEFSHRRSRLKRGGKYTRRKKGMRILYGKGIACISGDSGIGKTELILEFAYRF
HQRYKMVLWIGGESRYIRQNYLNLRSFLEVDASVENSLEKTRIKGFEEQEEAAVSGIRKELMRNIPYLVIIDNLESEKDW
WDHKLVMDLLPRFGGETHVIISTCLPRVMNLEPLKLSYLSGVEAMSLMLGSGKEYPVAEVDALRTIEEKLGRLTLGLAIVS
GILSELPITPSRLLDTINRMPLKDMSWCNKKAHSFRQNTFLLQLLDVCFSIFDHADGARSLATRMVLVSGWFAPCAVSV
SLLALAAQKIPEKQKGTCFWRKLLQSLTCGFTSSHTKKSELEACSLLLRFNIARSSTKQGHIHFNEMIKLYARKREVTGSAQ
AMVQAVMNQGSISKSIEHLWAACFLLFAFGHNPAAVELEVSELLYLVKKVVLPLAIHTFITYSRCSAALELLHLCTNALEA
ADQALVTPVDKWFDKSLCWRSIQTNAQLNPCLWQELALCRATVLETRGKLMLRGAQFDIGDDLIRKAVFIRTSICGED
HPDTISARETLSKLTRLIANVQIRASA

>XP_022640079.1

MDIQEESSTTVGPLTTPSLRNMSSSSSAFFSANQSPFFSPRSSSCQLSESLQPDAPIDRIHLDEAAPSSSSGIPEPKSLVNV
GCTFSEVAASPAGCSAGDLQKLDRISSSVGISSCTVSGHFHPYDDSYSGQKDKRSKKGRNKRISSTPGSRSVSSYRLKSCD
VYIGLHGRKPPLIRFANWLRVELEIQGISCFVSDRAGYRNSCKLSIAEKAMDVASYGIVIITRKSFKNPYTIEELQFFSGKKN
LVPIYFDLSPADCLVRDIIEKRGELWEKHGGELWLLYGGLEQEWKDVVHGLSRVEERKLEAQDGNWRDCILMAVTLLA
MKLGRRSAAEHLTKWREKVKEEELPFTRNENFIGRKKELSQLEFMLFGDVTGDSRQDYIDLKARPKRRHLTICRSKSNVQ
EERHVGNGSREEKTPVLWKESEKEIEMQSIEFSHRRSRLKRGGKYTRRKKGMRILYGKGIACISGDSGIGKTELILEFAYRF
HQRYKMVLWIGGESRYIRQNYLNLRSFLEVDASVENSLEKTRIKGFEEQEEAAVSGIRKELMRNIPYLVIIDNLESEKDW
WDHKLVMDLLPRFGGETHVIISTCLPRVMNLEPLKLSYLSGVEAMSLMLGSGKEYPVAEVDALRTIEEKLGRLTLGLAIVS
GILSELPITPSRLLDTINRMPLKDMSWCNKKAHSFRQNTFLLQLLDVCFSIFDHADGARSLATRMVLVSGWFAPCAVSV
SLLALAAQKIPEKQKGTCFWRKLLQSLTCGFTSSHTKKSELEACSLLLRFNIARSSTKQGHIHFNEMIKLYARKREVTGSAQ
AMVQAVMNQGSISKSIEHLWAACFLLFAFGHNPAAVELEVSELLYLVKKVVLPLAIHTFITYSRCSAALELLHLCTNALEA
ADQALVTPVDKWFDKSLCWRSIQTNAQLNPCLWQELALCRATVLETRGKLMLRGAQFDIGDDLIRKAVFIRTSICGED
HPDTISARETLSKLTRLIANVQIRASA

>XP_022640085.1

MKQKPKHFASFVILDQKQATSSASFMSKYSYDVFLSFRGPDTRFGFTGNLYSALSQKRIFTFIDDEALKKGEEITPSLRKSI
QESRISIIVFSKNYAFSTFCLDKLLHIIQCHKKQNMWILPVFYDIEPSQVRHQTGTYQEAFAKHAERFKDDIQKLQQWRLA
LRHAADLAAFPLKTGEEYESEIVKKITKEISIKLNRPPLHIADYPVGLKVRMQQIQELMGGELDKKVTMLGIHGMGGIGKS
TISRAMYNLMAHQFEASHFLANVREKSDKDGLVHIQETMLSELVGEXNIKLGDVHRGIPILQHRLXGKKVLLVLDDISKK
EQLQATAGGLDWFGPGSIIIITTRDKHLVDVHGVQKQYMVGEINFMEALELFKWNAFKNKEVDPCYREVTERAMYYA
NGLPLALETIGSNLFGKTLDEWESALETYERIPNRDIQEVLRVSYDSLDAYEKEIFLDITCFFRGCSLIYLTNLLEARGFPPEF
GLRVLEEKSLIKIRECPHKTVAMHDMIRCMGKEIVRQQSTLPHKWNRLWFYEDIVCVLEKNMENDKIEGMMLIPEHQE
EMQCNPKFGKMKSLRMLLIEKKVGFLRSLVALPNSSKILMISIKQVLWKINLCWLVTHLIHRNRNNPLLYSTSHPH

>XP_022640193.1

MPTSMTSYDVFLNFRGEDTRDNFISHLYAALQRKHVETYIDYRLQRGEEISPALQTAIEESKIYVLVFSENYASSTWCLNEL
TKILDCKKRYGRNVIPVFYKVDPATVRKQEERYKEAFQEHEERFKEDMEKVQGWKDALTEAAGLSGWDSNVIRPEHALI
EEIIEDILKKLNRYSVSYDEGIIGIEKHIEEIRFLLRLESTDIRIIGICGMGGIGKTTISKQLYHTLAMHFDSHSLVLDVQEKLERE
GIQNIKKKYQSELLKEAPSNHLSYYNERLKRTKALLILDDVSDSTLLSKLMRVGSFGQGSRIIMTSRDRQVLKNAGTDDIYE
LKELNFYDSLKLFNLHAFRQNSSKEITYMDLSIKVLRYAKGVPLALQILGSLLYDRQREAWESQLQNLDKCQDHHLFNVL
KLSYDGLREEEKNIFLDIACFYRGHNEIVVAETLSNCCFSSKIGMDVLKDKCLISILDGRIVMHDLIQEMGREIVRKECLQH
PGKRSRLFKANEIHEILKKNKGSDVIQCILQDINEMEEVKVHAKAFKKMSNLRMLMLNSYPRPWYNVFLKSSLVSLPDTL
KILNWTGFHQKSLPPNFCPQNLVRLEMSGCHLEQLWEEDQVFHVIYVPLCLFHIIRFDIAFIRIPQLYIYSLPKLKRLNLSYS
KKLTRIQDLSLSPNIEEIILNDCPKLIKVHSSILLTKLSFLRLDRCHNLKSVTIPGNILSRSPGFILLFSCFKLEMFSTSVPRFPHM
KLERQHGTFSRSPHKKPEYQPATFSSFPRFKQHFHGRRAFRRSSVRDGLGELDTFSGSSVDRNYVSNSFSRIPFLSEIFSIIF
MRYEEEEKEVTNNNIYVHDERPIQLTGGVPLNFHSLKNLCFIDLSNCSSLTVFPFDLSQIKFLKKLRLSGCSKLENFPEIENT
MEDLAVLLLDATAIQALPSSLWRLVGLQELSLSRCRNLEIIPSCIGRLTRLCKLDLTYCQSLQTFPSTIFKLKLRKLDLCGCLKL
RTFPEIMEPAQTFAHINLRATTIKELPSSFGNLVMLRSLKLYNCSDLESLPNSIVNLKRLCKLDCSGCAKLTEIPTHIGRLSSL
MEMSLKKSGIVNLPESMAHLSGLKSLDLSDCKKLECIPHVPPFLKQLVALDCTSIRRVMPNSLVRNLSESKEDVFKFHFTN
AQQLDSGARANIEEDARLRMTDDAYESVCFCFPGSAVPHWFPFRNEGASVSINEDLSFCSDDRLIGFALCVVFGVLDTN
DIKGKRGSFGYSLKFECDDDGTQIIPNNDVLKNYFTWVYSHRVLDKDHIFMWKFNLESLRRSGMNLRLSDARSFTFEISP
YNYNFRWPDYDSVVTIKECEMCPLYNSRSNVAESSS

>XP_022640251.1

MTAEMLTGVLVSTFLERTIDTLASRLVHILRQRKHKKQLNNLKMKFLAIDVVALDAEQKQFKDLRVRDWLLRAKDVVFD
AEDLLDXIDYELSKSQPEVESQSATNKVWNSLNSSFVSFFEIEIESRMEQVIEDLEDLANESYILGLEKGGGVGIGSVSGSK
LTYTSLPNESVIYGRDDDKEFLFNWLTSDTPSNLSILSIVGMGGMGKTSLAQHVFNDPRLEGKFDTKVWVSVPQEFDVL
KVSRAIIDTITASTDHSIQQEVIQKKLKEELMGKKFLLVLDDVWNERPSKWEDVQKPLVFGGQGSRILVTTRSEKVAVTM
RSEKHLLQILKEDHCWDLFVKHAFKNVDPQPDPDFIEIGKNIVQKCNGLPLALKTMGSLLHNKSFLWEWESIMKSEIWH
FSENESDILPALRLSYFHLPSHLKKCFAFCALFPRGYRFDKECLIQLWMAQNFLENPLQKKSPKEVGEEYFNDLLSYSFFQ
QSSNEEEKRFIMHDLLNDLAKYICEDICIRLGVDEPKGIPKTIRHCSFSSSKLCFDGFGSSIDPQKLHTFTPTDRGWHWNC
KMSVDDLFSRFKLIRVLSLCHCRSLVEVPKSVGNLKHLRSLDLSWTQIKNLPESISSLFKLQTLKLNNCRKLKELPSCLHQLD
NLRCLELVGIGVKNVVTHLGKLKNPQVSISSFHVEKSKKINIQQLGKLNLHGSLTIDDLQNIENPSYALEVDLKNKLHLVEL
RLTWNFIGSSSVDSEKVEDVIENLQPSKYLKKLSVGNYIGKQFPNWLLHNSLPNLVSLVLEECESCQRLPPLGLLPFLKYLQ
ISGFDEIMSIDADFHGNNSCSFKSLRKLYFSNMRQWEKWDCQAVTGAFPRLEFFSIGNCPKLKGHLPKFAALKSLYVFH
CEQLEALMVSALELHLQDCGKLQLERSTMKKLTMDESMLFSMAASLVATVESMLFDTSLENLSFCSLLESISDDCVSLRIF
PLDFFPTLRTLELSGFPNLHMISQDHVHNHLQHLTIKDCPRLESYGG

>XP_022640252.1

MTAEMLTGVLVSTFLERTIDTLASRLVHILRQRKHKKQLNNLKMKFLAIDVVALDAEQKQFKDLRVRDWLLRAKDVVFD
AEDLLDXIDYELSKSQPEVESQSATNKVWNSLNSSFVSFFEIEIESRMEQVIEDLEDLANESYILGLEKGGGVGIGSVSGSK
LTYTSLPNESVIYGRDDDKEFLFNWLTSDTPSNLSILSIVGMGGMGKTSLAQHVFNDPRLEGKFDTKVWVSVPQEFDVL
KVSRAIIDTITASTDHSIQQEVIQKKLKEELMGKKFLLVLDDVWNERPSKWEDVQKPLVFGGQGSRILVTTRSEKVAVTM
RSEKHLLQILKEDHCWDLFVKHAFKNVDPQPDPDFIEIGKNIVQKCNGLPLALKTMGSLLHNKSFLWEWESIMKSEIWH
FSENESDILPALRLSYFHLPSHLKKCFAFCALFPRGYRFDKECLIQLWMAQNFLENPLQKKSPKEVGEEYFNDLLSYSFFQ
QSSNEEEKRFIMHDLLNDLAKYICEDICIRLGVDEPKGIPKTIRHCSFSSSKLCFDGFGSSIDPQKLHTFTPTDRGWHWNC
KMSVDDLFSRFKLIRVLSLCHCRSLVEVPKSVGNLKHLRSLDLSWTQIKNLPESISSLFKLQTLKLNNCRKLKELPSCLHQLD
NLRCLELVGIGVKNVVTHLGKLKNPQVSISSFHVEKSKKINIQQLGKLNLHGSLTIDDLQNIENPSYALEVDLKNKLHLVEL
RLTWNFIGSSSVDSEKVEDVIENLQPSKYLKKLSVGNYIGKQFPNWLLHNSLPNLVSLVLEECESCQRLPPLGLLPFLKYLQ

ISGFDEIMSIDADFHGNNSCSFKSLRKLYFSNMRQWEKWDCQAVTGAFPRLEFFSIGNCPKLKGHLPKFAALKSLYVFH
CEQLEALMVSALELHLQDCGKLQLERSTMKKLTMDESMLFSMAASLVATVERSRSQSSPASHNQRLSKT

>XP_022640257.1

MAVRSQSQEFTYDVFLNFRGSDTRYGFAGNLYKALDDRGIHTFIDDEKLQGGDELAPALVKAIQESRIAITVLSHTYASSS
FCLDELVYILDRAEEKKLLVLPVFYNVDPSFVRHQKGSYGEALARHEERLKADNIERLNRHMEKLEKWKMALHQVANLS
GFHYKYGEGYEYKFIGRIVERVSSEINRAPLHVADYPVGLDSQVLKVMNLLDVGSNDGVHLIGIYGMGGVGKTSLSSAV
YNLIACHFDGSCFLQNVREKSRKHGLEHLQSIILSEILGDKRIMFASEQQGISRIQHRLQNKRLLLILDDVDKHEQLQTLVG
RPDCFGPGSKVVITTRDKQLLASHHVQKTYKVKKLNKNHALQLLTWKVFKSEHVYPAYVEVLNRAVTYACGLPLALEVIG
ANLCGKSIQQCISVIDQYKRIPNNRIQETLKVSFDALQEEEKRVFLDIACCFKGYKLTEVEEILHAHHGACMKYHIGVLAEK
SLIKIGQYDRVTLHDLVEDMGKEIVRQESAEEPGKRSRLWLPKDIIQVLEDNTGTSEIKIICLDFPLFENKMVEWNGMAF
QKMQNLKTLIIRNGIFSDDPKCFPNSLRVLEWWRYPSHCLPSNFQPKQLAICKLPHSLFMSFEMDGLSKKLRNLRVLNFE
CCECLTQIPEAVSDLQNLEELSFQNCVNLVRVHNSVGLLEKLRILEASGCIKLRNFPPLKLTSLEKLELSHCSSLESFPEIIGK
MENIRELRLLGTFIKELPLSFKNLTRLKKLSLLFCGIVQLQSSIVMMPELTFIEAWGWKGWQWIKWEEDEEKDGSMISSK
VELLWAPKCNLRDDFFQIGFTRFAHVKDLDLSNNNFTHLPECIKECQFLKKLDVSCCNHLREIRGIPPKLKHFNATNCLSL
TSSSISMFLNQDLHANRKTMLVLPGSWKPEWFDHISYGPSCSFWIRNKFPGKVLCHFVAPEDRDIRDYVKPMLCINDK
VYLCFFDRLKFLKLGAEHTFLFDLRNLIFTNNLYEVPLQNEWNPVKVIFFNLKAASMPTQVPIQIGIHVFKRESSNEDIMFT
NPYTKKRKLEKILTGFPNTSNYILDFDLSGYWFSQYMQAFHQDQILNNGKIKLLTG

>XP_022640259.1

MAVRSQSQEFTYDVFLNFRGSDTRYGFAGNLYKALDDRGIHTFIDDEKLQGGDELAPALVKAIQESRIAITVLSHTYASSS
FCLDELVYILDRAEEKKLLVLPVFYNVDPSFVRHQKGSYGEALARHEERLKADNIERLNRHMEKLEKWKMALHQVANLS
GFHYKYGEGYEYKFIGRIVERVSSEINRAPLHVADYPVGLDSQVLKVMNLLDVGSNDGVHLIGIYGMGGVGKTSLSSAV
YNLIACHFDGSCFLQNVREKSRKHGLEHLQSIILSEILGDKRIMFASEQQGISRIQHRLQNKRLLLILDDVDKHEQLQTLVG
RPDCFGPGSKVVITTRDKQLLASHHVQKTYKEEEKRVFLDIACCFKGYKLTEVEEILHAHHGACMKYHIGVLAEKSLIKIG
QYDRVTLHDLVEDMGKEIVRQESAEEPGKRSRLWLPKDIIQVLEDNTGTSEIKIICLDFPLFENKMVEWNGMAFQKMQ
NLKTLIIRNGIFSDDPKCFPNSLRVLEWWRYPSHCLPSNFQPKQLAICKLPHSLFMSFEMDGLSKKLRNLRVLNFECCECL
TQIPEAVSDLQNLEELSFQNCVNLVRVHNSVGLLEKLRILEASGCIKLRNFPPLKLTSLEKLELSHCSSLESFPEIIGKMENIR
ELRLLGTFIKELPLSFKNLTRLKKLSLLFCGIVQLQSSIVMMPELTFIEAWGWKGWQWIKWEEDEEKDGSMISSKVELLW
APKCNLRDDFFQIGFTRFAHVKDLDLSNNNFTHLPECIKECQFLKKLDVSCCNHLREIRGIPPKLKHFNATNCLSLTSSSIS
MFLNQDLHANRKTMLVLPGSWKPEWFDHISYGPSCSFWIRNKFPGKVLCHFVAPEDRDIRDYVKPMLCINDKVYLCFF
DRLKFLKLGAEHTFLFDLRNLIFTNNLYEVPLQNEWNPVKVIFFNLKAASMPTQVPIQIGIHVFKRESSNEDIMFTNPYTK
KRKLEKILTGFPNTSNYILDFDLSGYWFSQYMQAFHQDQILNNGKIKLLTG

>XP_022640285.1

MIQLQTPLSIPFFFLPHLKVMAELLLFSSAESLLGKLASAALQEVSLAFGVHRHLQHMKETMELIRGVLLDAEKKNPHSSA
LSEWLTQIKRVFSDAEDIVDDFECEALRKDVVNTYGSCSRKVHRFFSSSNPVVYRLRMAHHIQNINTRLAKLAAQRSMF
GLQVIHQDTRIVHVREMTHSHVNPSNVIGREHDKQEIINLLLQDDHGQSLSVIPIVGMGGLGKTTLAKLVFNDTTIHACF
PLRMWVCVSNDFELRNVLIKILNSAPNPIGENFNNFETEQLQIRLRNTLHGQKFLLALDDVWNEDPARWDELKEIIDM
GVEGSKILVTTRSQKVAAIMHTKSSNLYLLQCLSKEDSLSLFVKYAFEDGDVMKHPQLLKIGEEIVKKCGGLPLAVKTVGS
SLFSRVDEKEWESVRDNEIWNLKQNEKDILPALKLSYDQLPSYLKPCFASFSLYPEDTYIFSSQVCLFWGVLGFLPTPKASE
SIIDVATQLLHELWSRSFLSDYEDLGGDYRFKLHDLVVDLAEYVAKGEFEIIQNHNPNLNKNAHHLVLMNNNLLDKALLP
SSLRTISFPTGANNEDFLNTLVSRTPGFNLQTSKFAKFEP

>XP_022640781.1

MATPSFPRFTYDVFLSFRGEDTRYGFTGNLYKALCDRGVHTFIDDDKLQSGDEITPALLKAIEESRIAIVLLSHNYASSSFCL
DELATIFHCKKSKGLLVIPVFYKVDPSYVRHQKGTYEEALVKHQKKFKAQKDKLQKWKMALRQVADFSGYHFKDGHEY
QYEFIGKIVERVSREINRAPLHVADYPVGLLTQVLHVKKLLDVGSDDVVHMIGIHGMGGLGKTTLSLAVYNLIADDFDSS
CFLQNVREESNKHGLKHLQTLLLXEILGEKDIDLTSVQRGISVIQQRLRRKKVLLILDDVDXRKQLQAFAGRSDWFGPGSR
VIVTTRDEQVLKCHDIERTYEVKELNKNDSLQLLMWNAFKREKVDPSYEXVLKRAVTYASGLPLALEVIGSNLVGKSVQE
WESAIQHYKRIPNSEIVEILKVSFDALGDEEKNIFLDMACCLKGSSLKEVEQILGVLYDNNMKHHIGVLVKKSLIKVGRGR
FNAIEMHDLIEDMGRQIDLQSSPKEVGKRRRLWLGKDIIHVLKDNKGTSETEIICLDLSISEKEETIEWNANAFRRMKNLK
ILIIRNGKFSKGPNYFPESLRVLEWHGYPSNCLPSNFDPNKLVTCKLPXSHFTSFGFLGSSKKFENLTVLNFDKCKFLTQTP
DMSDLGNLEEVSFKGCESLVEVHHSIGFMSKLKILNAGGCRKLMSFPPLNLPTLERLELSFCPNLEKFPEILGKMGNIKLL
QLNGLPIKEFPLSFENLIGLEELSLSCEVVHLPSSITTMPKLFNFRATNCKGWQWVKAEDGEDNVGSMVPSKVDWFSAL
SCNLDDDFFSAGFMRLAQVCVLFLRDNNFTHLPECIKEFHNLFFLDVSHCEHLQEIRGFPPKLEYFKAINCISLTSFSLSML
LNKQLHEARNTEFWFPGANFPEWVDLQSSGPSCSFWFRNKFPAKVLSLLIAPVGDKHQLDFIIPMVFIDGKVQESEDFY
FKEIEIERMLEFDQTYIFDLQNLPIYSKLFELPIEKEWKHVKVTYEGVIETSIVKATGIHVFKDENSIMEDIRFDDPYTNNKLD
KDLNTSQSQNHLLRTIGFFMCKFFIGLFLFLFSAFIFYFIYRPQHQPK

>XP_022640784.1

MATPSFPRFTYDVFLSFRGEDTRYGFTGNLYKALCDRGVHTFIDDDKLQSGDEITPALLKAIEESRIAIVLLSHNYASSSFCL
DELATIFHCKKSKGLLVIPVFYKVDPSYVRHQKGTYEEALVKHQKKFKAQKDKLQKWKMALRQVADFSGYHFKDGHEY
QYEFIGKIVERVSREINRAPLHVADYPVGLLTQVLHVKKLLDVGSDDVVHMIGIHGMGGLGKTTLSLAVYNLIADDFDSS
CFLQNVREESNKHGLKHLQTLLLXEILGEKDIDLTSVQRGISVIQQRLRRKKVLLILDDVDXRKQLQAFAGRSDWFGPGSR
VIVTTRDEQVLKCHDIERTYEVKELNKNDSLQLLMWNAFKREKVDPSYEXVLKRAVTYASGLPLALEVIGSNLVGKSVQE
WESAIQHYKRIPNSEIVEILKVSFDALGDEEKNIFLDMACCLKGSSLKEVEQILGVLYDNNMKHHIGVLVKKSLIKVGRGR
FNAIEMHDLIEDMGRQIDLQSSPKEVGKRRRLWLGKDIIHVLKDNKGTSETEIICLDLSISEKEETIEWNANAFRRMKNLK
ILIIRNGKFSKGPNYFPESLRVLEWHGYPSNCLPSNFDPNKLVTCKLPXSHFTSFGFLGSSKKFENLTVLNFDKCKFLTQTP
DMSDLGNLEEVSFKGCESLVEVHHSIGFMSKLKILNAGGCRKLMSFPPLNLPTLERLELSFCPNLEKFPEILGKMGNIKLL
QLNGLPIKEFPLSFENLIGLEELSLSCEVVHLPSSITTMPKLFNFRATNCKGWQWVKAEDGEDNVGSMVPSKVDWFSAL
SCNLDDDFFSAGFMRLAQVCVLFLRDNNFTHLPECIKEFHNLFFLDVSHCEHLQEIRGFPPKLEYFKQLHEARNTEFWFP
GANFPEWVDLQSSGPSCSFWFRNKFPAKVLSLLIAPVGDKHQLDFIIPMVFIDGKVQESEDFYFKEIEIERMLEFDQTYIF
DLQNLPIYSKLFELPIEKEWKHVKVTYEGVIETSIVKATGIHVFKDENSIMEDIRFDDPYTNNKLDKDLNTSQSQNHLLRTI
GFFMCKFFIGLFLFLFSAFIFYFIYRPQHQPK

>XP_022640788.1

MAEAVIEVVLGNLNSLVQKELGLFLGFDQDLERLASVFTTIKATLADAEEQQFSSRSIKDWLQKLKEAAYILDDILDECAY
EALRLEYQGVKLCPSNKVKCSGLSTFHPKRTVFRYKIAKKMKRICERLEEIADERTKFHLAEMVPERRSGVIEWRQTSSFIS
EPHVYGREEDRDKLIDFLVHDASHDENLSVYPIIGLGGLGKTTLSQLIFNHERVRKHFEPRVWICVSEDFGLRRMTKAIIE
AVSGHACEDLDLDPLQRKLQDLLQNKRYLLVLDDVWDDDPESWERLKSVLVCGALGASVLVTTRLTKVAAIMGTVPPH
ELSNLSDNDCWRLFKHRAFVADEVELEELVVVGKEIVKKCGGVPLAAKVLGGLLRFKREVREWLKVKESNIWGLTHNIM
PALRLSYLNLPIHLKQCFAYCAIFPKDERIEKQYLVELWMTNGFISSDGKLDAEDVGDGIWNELYWRSFFQDIEKDEFGE
VESFKMHDLVYDLAQFVAEEVCCITNDDDIHALFERKRIHHLSDYGWEFHPAQLHQVKSLRTYLGKNVQLSPDVVKCYS
LRLLQFKPRKELLSAIGDLKHLRFFGETL

>XP_022640879.1

MAESSSSLAASASPTRYEVFLNFRGEDTRENFISHLYAALERKHIETYIDYRLQRGEEISPALQTAIEESKIYVLVFSENYAFS
TWCLNELTKILDCKKRYGRDVIPVFYKVHPTIVRKQEERYKEAFEEHKLRFKEDMGKVQGWKDALTEAAGLSGWDSNLI
RSEHILVEEIIDDILKKLNRYSVSYNQGIIGIEKHIEEIGSLLDLESPDIRTIGICGMGGMGKSTISEQLYHTLAVKFDSHSLVL
DVQEKLQREGIHNIITKYRSELLKETSSPHLSYYNERLKRTKVLLILDDVTDSTLLNKLIGGCDRFGLGSRIIITSRDRQVLKN
AGTDDIYELKELNFYDSLKLFNLHAFKQNSSKEITYMDLSIKVLRYAKGVPLALQSLGSLLYGRQKEEWESQLQKLEKGQD
VETFNVLKLSYDGLDEEQKNIFLEIACFYRGHNEIVVVETLKDCGFSSEIGMDVLKDKCLISILDGRIVMHDLIQEMGQEIV
RKECPQHPGKRSRLFKADEIHEILRTNKGVPLNFQSLKRLCFIDLSNCSSLTIFPFDLSEIKFLKKLRLSGCSKLENFPEIENTM
EDLAVLLLDCTAIHTLPSSLYRLVGLQELSLRSCSNLEIIPSSIGRLTRLCKLDITYCASLQTFPSTIFKLKLRKLDLCGCFKLRTF
PEIMEPTQTFVHIDLTKTLIKELPSSFGNLVKLRSLQLNSCSALESLPNSIVNLKHLCKLDCSGCYKLTEIPRHIGRLTSLVELS
LSESGIVSLPESIAHLSSLKSLDLSDCKKLECIPHIPPFLKQLVALDCTSIRGVMLNSNSLVPNLSNSKEGFFRFHFTNAQQLD
SGARANIEEDARLRMSDDAYGSVCFCFPGSAVPHWFPFRNEGPSVSINEDLSFCSDDKLIGFALCVVLGVLDTNDIKSRY
SSFGYSLKFECDDDGTHIIPNNDVLNNYFTWGYSPRLLDKDHIFMWKFNLESLRRRGMNLRLCDARSFTFEISPYHYNS
QWPDYYSVVNELKSVVKIKECGMCPLYSSGSNVAESSR

>XP_022640992.1

MRSFVLSQAKYPPNTYLYLNQSHSFNRSSNTVNSNEKPTTAACVGGALLSAFLQVAFDRLASPKVLHFFRRRKLDEALLS
KLNIKMLSINSLADDAEQKQFRDTRIKAWLFAVKDAVLDAEDLLDEIDYELTKCEVEAQSESQSLSKKVSSFFHSTFTSFN
RKINSGLKQVLEKLEYLSSQKGDLGLKEATYSGLRPGSVAQQKLPSTSLVAENVIYGRDDDKETIFNWLTYETDNRNQLSI
LSIVGMGGVGKTTLAQHVYNDPRMEEADFSIKAWVCVSDDFDVMVVTTTILEAITKSIDDSRNLEMAHGRLKEKLLGK
KFFLVLDDVWNERREKWEAVQTPLNFGASGSKILVTTRSEKVASTMRSSKVHRLMHLQEDHCWDVFAKHALQDDHP
QLNAELKDIGIKIVKKCKGLPLALKAIGSLLRTKSSFSEWECVLVSKIWDTPIEENEIMPALLLSYHHLPSHLKRCFAYFALFP
KDYKFDKQSIILLWIAENFLQCPQRNKSPEEIGELYFDDLLSRSFFQQSSGLESCFVMHDLLNDLAKYVCGDIYFNLEVDK
ALCIPKMARHFSFAIKDVKYFDSLHEAKRLRTFIPLPTFVIVSYFNDPWQCKIPVHELFSKFKFIHTLSLFCCSGLLEVPDSIG
DFKHLRSLDLSRTDIRKLPDSSCLLYNLQILKLNFCLLLKELPSNLYKLNNLRCLEFIATSVRKVPMHMGKMKNLQVLSSFY
VGKSSEFGIQQLVGLNLHGGLSIGNMQNIVNPSDALQVDLKNKKHLIKLELEWNSNHIPDNPRNEKQVLEYLQPPKHLK
NFSISHYGGTQFPSWLFDNTLSNLVSLSLIGCKYCLHLPPLGLLPFLKQLVIIELDGIVAVGAEFHGSSSSSFTCLETLYFYNM
KEWEEWDCETDFPRLQHLSIVHCPKLKGLPKQLLHVKQIIICECERLTITGHNMESSITNNSLEVLHIYSCLYMNIPLRVRY
NLLVTLEIDGAFDSPMTFPLDFFPKLCSLKLGCCNLQMISQDHTHNHLKDLCISNSPQFESFPREGLSAPRLVKFSIKELM
NLKLLPKRMDILLPSLTDLQILDCPQVELFSDGGLPSNLNTMDLSACSKFMASMKMALGANNSLEVMSVRKLDVESFP
DEGFLPFSLTSLEIRNCSDLKNLDYNGICHLSSLQKLFLFNCPSLQCLPEEGLPNSILELKIVGCPLLEQCQG

>XP_022641228.1

MAAELVGGALLSAFLQVAFDRLASPQFLDFFRGRKLDEKLLGNLNIMLHSINALAHDAEQKQXTDPHVKAWLFSVKEA
DECWKVFEKQALKDDDLRLNGEKKEIGKRIVEKCKGLPLALKTIGSLLRTKSSSSYWKSVLESDIWELPKEVEIIPALLLSYQ
HLPSHLKRCFAYCALFPKDYEFDKKELVLLWMAEGFLHHSKIIKNVQEIGEQYFDDLLTRSFFLQSSLEIRQTKNAREIGEK
HLDGLLTRSFSLELGFEMRFVMHDLLNDLAKYVCADFCFRLKFDKGNCIPNTTRHFSFSLDYVGYFDGLRSLTDAERLRSF
HSITNGTRYHFDLRQFNILVHELFSKFKFLRVLSLNGYSELSEVHDSVGDLKHLRSIDLSHTDIQKLPNSIGLLYNLLILKLNY
CSFLEELPSNLHMLTKLHCLEFEHTKVTKMPMNFGELKNLQALSTFCVNRNNEVISIKQLGGLNLHGTLSIMEVQNIVNP
LNALEANLKNKHLVVLELKWNSNHVSDDPAKENKVLENLEPSKHLEHLSIHNYGGTQFPSWVFDNSLPNLVSLWLADC
KYCLCLPPLGQLSSLKTLQITGLDGLVSIGAEFYGSNSSSFKSLEILKFYNMKEWEEWECKTTSFPHLRHLVIVRCPKLKSLP
EQHLPFKNLDINSCNKLAISINNMFRSSLQLLSIISCSIVHIPMTNYDFLEEMEINSDCYSFTIFELDFFPKLRLLRLSRCQNLR
RVSQGHTHIHLKVLRINECRLFESFPSEGLSAPLLQIISIREAENLKLLPKRMQILLPSLTELEIIDCPKVEKFPEGGLPSNVKK
VSLSSLKLIASLRDTLDANTCLKSLTIEKLEVESFPGEVLLPRSLTSLSILFCPNLKKLDYKGLRNVSSFEIYGCPNLHLR

>XP_022641303.1

MIQLQTPLPIAFLFLPLLKLMAESLLFSSAESLLGKLASRAFQEASLAFGVHRDLQQMKETMELIRGVLLDAEKKNPQSSA
LSEWLIQIKRVFSDAEDIVDDFECETLRKHVVNTYGSCSRKVSRFFSSSNPVVYRLRMAHHIQDINTRLTKLASQRSMFDL
QVIHKDTRVVHVREMTHSHVNPSNVTGREHDKQEIIKLLLQDDHGQSLSVISIVGMGGLGKTTLAKLVFNDPIIHACFPL
RMWVCVSNEFELRNVLIKILNSTPNPTRENFNNFETEQLQIHLRNTLQGQKSLLVLDNVWNEDPTRWDELREIIDVGVE
GSKILVTTRSHKVATIMHIKSSNLYLLGCLSEEDSLSLFVKYAFKDGDEMKHPQLLKIGEEIVKKCGGLPLAVKTVGSSLFSK
VDEKEWKSVRDNEIWNLEQNEKDILPALKLSYDQLPSYLKPCFASFSLYTKDTSVFGTQVCTLWGALGFLPTPKASESM
MDVATQLLHELWSRSFLSEYEDYGGECRFKLHDLVIDLAVYIAKGEFEIIDNHNPKLYKNAHHLRFVENNLLDQALLPSSL
RTIIFPRGANNEDFLNTLVSRCKFLRVLNLNFSEYASLPRCIGKLKHLRSLSLMENENLTELPDSICKLQNLETLLLVWCMKL
QKLPKGLANLVSLRYLGITTIEPACPEKDIASLTSLEKLGFYRCDNFESLFKEILLPTLKEFSLSDCKSLKTVSFHAIKNLEVLMI
SNCNKLELSMGLNNQILDLRLKLLILSDLPRLVTLPRWLQGSANSLQSLYIQHCINIYELPDWLPTLNCLQGLSFLYCPNLLS
LPDNMHRLTNLKEINVTGSYELWKRYKPDVGQDWHKISHVNLVCNDYQSESEEELTDKS

>XP_022641554.1

MPVIETLGGALFGAVLQLLFDRPDSHHVFDYFRRRKLNEKLLYKLKVKLLSINNVIDDAEQKQFSNSYVKAWLDEVKDAV
FDAEDLLDEIDYEFCKCKLKAESHTRSKKVWKFFKPSSKSFLDKKIESRMEQVLEQIEFLSSQKGDLGLEYVSCVGVGPESV
SKVTQKLPSTSLVVESVVYGREDEKEMILNWLSSDTDNHSQPAVLSIVAMGGMGKTTLAQHVYNDPRIEEAKFDLKAW
VCVSDEYDAFKVAREILQAINNSIDDSRNLEMVQVRLKEKLTGKKFLFVLDDVWNEDRDRWKPLQTPLKYGAKGSKILV
TTRSSKVATTVQSHKVHELKQLEEDHSWQVFAKHAFQDNNYHLNVEVEKIGKKIVEKCKGLPLALETIGCLLHTKSSVSE
WESVLISKIWDLPREDSKIIPALLLSYYHLPSHLKRCFAYCALFPKDQEFHKESLILLWMAENFLQCSQQGRSPEVVGEQY
FDELLSRSFFQKLIQDNKTYFVMHDLLNDLAKYVCGDICFRFGVDEEKKTLVKTRHLSLVTNEDQYSDGFRSLYDAKGLRT
IMPTSRRINIDYYWDCKMSMNELFSKFKFLRVLSLSCCRGLKEVPGLVGDLKHLRSLDLSGTLIEKLPDSICSLYNLQILKLN
SCFNLKVLPSNLHKLTNLRCLELMKTNVIKMPVNIGKLNNLQVLMSSFRVGESSEFTIQQLGDLSLRGGLTIEDLQNILNP
LDAVAADLKSKRNLVELTLKWNHSWNLADLIKEREVLENLQPSKHLEKLSIWNYGGIQFPSWLANTSLSFMVSLSLENC
NFCTRLPPLGLFPYLKDLTISGMGEVVSINADFFGSTSSSFSSLETLKFSFMYGWENWECQAVTVTDAFPRLQHLSIRHCP
KLKGDLPEKLLQLRKLLICECKELVASAPVAQEIFELDLQDCGKLQFDYHPTSLKRLTVTGDSSMQLSLDFLSKSKTNIPVSC
YDFLETLDINDGCDSLMSISLDCFPKLLRLCLKYCHNLHTISQGRIHNHLKDLQIIACPQFQSFPDEGLSAPMLESFAMKRL
PKLKSLPQHMHILLPSLTSLLIHDCPQMKILSDEGLPSNLENMNISNCSRLVASLKGPLGANTSLQTLSVQEVDVESFPSE
GFLPISLTCLIIRDCPHLKTLNYKGLCNPSSLKKLTLFDCPNIRCLPEEGLPKSISTFRILGNCPLLKESSQKPQGQD

>XP_022641627.1

MNPSFFLWHDAELVDIIVSRVKTLLDYKDLFITEYPVGLESRVEDVIKCIENESSKVCMIGIWGIGGSGKTTIAKAIYNRIYR
QFIGKSFIEFNRYQGHVALQQNLLSNVLKSKLKVASVEIGRTMVQNGFSRKKFLLVLDDVNSSGQFENLYGSYRWFGQG
SVIIITSRNVSLLNRLEVNYVYQTHPLNENESLELFSWHAFKEAKPRKEWSLLARNVVVYCGGLPLTLQFLGSYLYDRTIEV
WESVLLKLQRIPPDELLSVLKICFEDLRDTEKDIFLDVCCFFVGKERDYVTEILNDCGLHADIGITVLIQRGLIKVERNNKLQ
VHPLLQHMGREIIRQECPEKPGKRSRLWFQDDVKDVLIHNTGTEAIKGLSLKLHSTSRDCFKAHSFKKMKRLRLLQLDH
GQLSGDYGHISEQLRCICWRGFPYKHIPSNFHLQNVIAMDLKHSHLQLLWKQPQVLERLKFLNLSHSKYLIQTPDFSGLP
SLEQLILKDCPSLLKVHQSIADLSNIVLINLKDCTSLSYLPREIYKLTSLKTLILSGCSKLRPLEKI

>XP_022641628.1

MDNAFSSYKLSGKYDVLINFTGEDIRRKFVSHLNSAFSTVGLTTFLHHPNAVKSTHIQQPIHSHCRVAIVVFTQTYSQSA
WCLHQLHHIINWHETYCRHVLPVYYEIQPSDVRLQKGDFGKAFKATAQQTFSEQELEHGMSRWSHALTKAANFFGW
DESNHRSDAELVDKIVKSVVNLLVLSATKFPVGLQSRVEDLIRTIKNKSTEVCIVEICGDQGYGKTTLAKAIYNQIHWTFKK
KSFIENISQVTGIRGYLRLLEQLVLDILKQKVEIPTIDVGRRMIRETLYGKSKGTVIIITTSDEDLRMNQPIDSIFRLERMNAE
DSLELFSWHAFREPKPKDEYEDLARRVISYCRGVTLALEIVGSSLFEKTKEEWNSLLFKYLIIGWHRVPDIIKVSIEESGISKQ

HFRCSLIGVGTYHQFFNAVSDNIYQVLSSSESYDVCLPAVDDPYCMAHMGEGHSVSFVVPEDGDMKGMILCVIYLSTP
KIIEPQFTTVVIVNYTKCTFHIHNHGTAISFKDEDWHDIMSNLECGDNVEIFVNFGNGLVVKNTTVYLIWGEAENMEKV
SEPKKQSLIRFIKKVVM

>XP_022641629.1

FVSILILGYFCCRNENDLMKEIVCKVLKRLDRTYLSITNFPVGLECRLQHAIDFIRNKKIGTCILGIWGMGGIGKTTIAKSIYN
EIRHEFKYRSFLANIREVWGGDRGPIDLQEQLLSDILKTTKTNVHSIDWGKGKIKEMLCTKKVLVVLDDVNIFEQLIALCG
NRNEISRGSVIIITTRNVRLLEEIGVDYVHGVEKMNKIESLELFSWHAFRRANPTRDFFEVSKEVITYCGGLPLALEVLGSYL
YKRKKEEWQSVLSKLKEIPNDKIQEKLKISYDGLSDHMEKDIFLDICCFFIGKDRGYVTEILNGCGLHADIGIPVLVERSLIKV
EKNNKLGIHDLLRDMGREIVRQSSPLPQKRSRLCVHDHVLDILTEHTGTEAIEGLALKLHRPGRVHFSAKTFENMKRLRIL
QFDHVQLAGDYGHISKHLTWVYWRGFSLKYIPDNFYQGNVVAIDMKHSNLKLVWKEPLQQRLERLKFINLSHSKLLSKT
PDFSKLPNLERVILKDCPNLSELHHSIGNLPNLLILNLKDCICLRNLPVIKSKSLRVLILSGCSKIDKLEEEIVQMESLTTLRAEN
TSLTQVPFSIIRLKKIGFISLCGYEGLASVLFPSIIWSWMSPTTGRVSSMQSLGSISTSLVSVHLQDNNLGNLLSKLSEFSKLR
SICVQCNSDLQLTQELRRIVDDFCKVHSAMETTYAPQILENSMVSRLIGFGSHYRVMDMLSNDMTEVDSISLISRLSFSL
CV

>XP_022641630.1

MEGSGKTTLAKAIYHQIHGTFIHKSFIEDIAQAREPRGRIHLQKQLLSDVLETKAKINTVEMGRNMIRDRLFGKRVLIVLD
DMDDYLPLLDISKSRSWLSAGTVIIMTTKDEDLFSKYQVDSVVRINLMNENESLELLSWHAFREAKPQKKYDYLARSVVT
HCAGLPLVLEVIGSTLFERTEEEWYSVLYELHKIPMHRVQRKLEISLDRLRNQMERDLFLDLCCFFVGKDRAYATKILNGP
RVEADSGIRVLMKRNLIKVKRNNKFGMHPLLQEMGIANFCEISREKRGKNRQLLFDVDAEYSLQVYAIRTFFERKDYEVL
PVLLSTRREPSRLPKDGVNSENLSPKLRWISFRGFSIEYLPNYFNAHDTTAIEIKHSLLQFFWEEPQVLRSLKVLNLSHSMY
LTETPDFSRLPNLEELILKDCPRLQEVHQSIGHLCYLILLNLKDCKRLSNLPQEIYRLKSLKTLILSGCSKIDPMEKDIVQMKSL
ITLVAENTTVKKVPFSIVSSKAIGYISLQGFERLSRNSFLSIIRSLISPTMNPISYIHSLCMDIDNNWEDIGPLLSSLENLRSVL
VQCDTEFQLSEQVKNILVYYFSKFTQSEISKQNSRSSLIGVGAYLEFFNAVRDNISEVLASSESSDVNRPYWLAYMGEGDS
VSFTVPLDNDIKGMVLCVVYVSTPEIEATECLRSVLIVNYTKCTLQIHMHGKLISFNDIYWQTIRSNLGSGDKVKIFVNFSQ
GLVVKNTYVVYLICGDSHYLEKEPTPNKN

>XP_022641632.1

MASVSSSLSFFSKLVSEVFIHCLEDDIHENFVSHLRSALLQAGVEPSLVAVGKLSKKFMPSVTRFQIGIIVFTKAYIQSCRCV
QDLARIIECHETHGLMVIPVFYDIDPSYDQKADVDPSHVPDQKRDIDPSHVDDQKRDVDPFHISSVSTNPFRELEEAETK
TGRNDAELVEEIVKSVLAKLDRALPVTKFPVELEIHVKNVIGLFENQPNKVCMIGIWGMGGSGKTTLAKAIYNKIPFTFG
DKSFIQDIRKVCQTDGIRGLVQLQEQLLSDVLKYVKIESGDMEKTTIENRLSRKKLFIVLDDVNEIDQLKHLCGNGKWFGA
GSVIIITTRHLDLLYQHKVDYVYEMDELDENDSVELFSWHAFRKAKPREDFNELARSAVDYCGGLPLALEVLGSYLSKRSE
NEWRSVLSKLEIIPNTQIQNILRVSFDGLCSEMEKDIFLDVCCFFIGKERDYVTEILNGCGLRADIGIKVLIERGLVKIEKNNK
LGMHHLLRDMGREIVRQTSTMQPGKRSRLWFLKDVRHVLTKNTGTEAILGLSLNTDWRL

>XP_022641633.1

MEFASSSSKLPRKYDVLISFTGEDIRRKFVSHLDSVLSSVGLTTFLQHDNAVQPKHIQEPILNLCRVAIVVFTETYSQSACCL
HQLQQIIEWHETYGRHVLPVYYEIQPSDVRLQKGDFGKAFIETAHQTFSAQELEHGMSRWSHAITKAANFFGWDDCN
YRSDAELVDTIVKSVLNLPVLSVTKFPVELQSLAEEVIQIIKNKSTGLCRIGICGMGGSGKTTLAKAIYSQIHGTFVEKSFIEDI
SEVSQTRGHVNLQRQFLSDVLKTKVEIHNVEMGRSMILERLYRKRVLIVLDDVNEHCPLDIWESRGWFGEGSVIIITTRD
EDLLRKHEVRSVFRIDLMNENQSLELLSWHAFREAKPKTEYIDLAKRVVARCGGLPLALEVIGSSLFERTKEEWKTVVSKL
DNIPQHEVLQKLKISFDSLHNQMEKDLFLDVCCFFVGKGKAYVRKILNGCGVDADSGIRVLIERNLIKVKKKNRLGVHPLL

RKMGREVILEISRKEPRNKNQLWLDKDMHHALLENTLFSSQENKIIHRWPIERDLFVRYPPFEISVRLTLVNLTRDSEFHP
KKLKWISRQGFPTEYLPSELYLHDAIVIDLKYNLLRFFWKEPQVLVSLKVLNLSHSMYLTETPHFSRLPSLEQLILKDCPRLR
QVHESIGCLCNLTLLNVKDCTSLNNLPKEIYRLKSLKTLILSGCSKISLIEKDIGQMESLITLITENTAVKQVPFSIVSSKCIGRIS
LYYFDGLLYNLFPSIIRCWMSQTMNPLSYIHSFCMDKEHNSGDDIIPLFNTLANLRSVLVQCDTDFHYLSK

>XP_022641635.1

MEFASFSPSSSSSSSFLKSEPQFIYDVFINFGGEDIGRKFVSHLHSALLLAQVKTFISKENLEGIKLEAYRRAIASSKIAIIVFSK
TYTESTYWRLELEKIIECRQTFGQIVLPLIYDIVRLDERHQMVVRVLEKATPGSYSGEQLERPMSRWRRALTKAAGITGW
DFRDFRHDAELVEEIVRHIQTLLDYTDMSITQFPVGLESHVEKLIGMIEEQSTEVCMIGIWGMGGSGKTTIAKAIYNQIY
HSFIGKSFIENIGQFRNRVDRRHVHLQENLLYDVLKSKFEVESDRVGRTMIETQLSRKKLLIVLDGVNEFVQLENLCGNRE
WFGQGTVIIITTRDVTVLNQLKVNHVYTMDVMNENDSLELLSWHAFREAKPRKELNELARNIVGYCGGLPLALEFLGSY
LCDRTKEEWESVSSKLKINPFNQIQEKLEMTSFDGLDDMVKDIFLDICCFFIGKERSYVTKILNGCGLYADIGITVLIERGLIK
VEGANKLQMHPLLRDMGREIIRKRCPEEPGKRSRLWFQNDIKDVLKKNTGTKAIQGLSLKLHSTGRDCFEAHAFKEMK
RLRFLQLDHVQLTGDYGYLSKQLRWISWQGFPSKYIPNKFHMENVIAIDIKHSYLQLVWQQPQVLEQLKFLNLSHSKYL
RETPDFSRLPSLERLILKDCPSLCKVHPSIGHLYNLLLINLKNCTSLSSLPREVYKLKSLRTLILSGCFKIYIFEQDIVQMESLITL
VTENTVVKQVPCSIVSSKSIAYICLHRFEGLPENIFPSIIQSWMPPTMNHHYYNSPVCMGMENYNSRDLAPLHRCLANL
RSVLVQCDTKFQLHKEVKTILVEYLVNFTESRISNHHLRFSLIGVGSYNNLQNTLSDSISEGLASSESCEVSLPGDNHPYWL
AYMGEGHSVSFTVPQDCDMKGMIICIVYLSNHGIVPTECLTSVLIVNYTKCTLHIHKHSAVISFNDEDWHVIISNLGSGD
KVEIFMTFDHRLVVKNTAVYLICDESNGLEMGPCPGKCSE

>XP_022641643.1

MVSIVSDLAKSTLEKLINATIEESRYICCFTCITDDFEKERENLIAKKETWEEYARVVTRRGDNIRKDVTHWQKQAKELIEE
DTKMKVKCFFGWCPNCIWQYSRGKELESKTEKIKKLMECNFENVGITRDVPDIEYHSSQNYISFKSRKLKFEELFNALKD
DNNYMVGLQGMGGTGKTTLAKEVGKELKKSKCFNQVIDTTISNTPDIKKIQDDIAGPLGLSLKGYTESERPRKLMDRLT
NGDKILVILDDVWGDISFEEIGIPFKDNHKNCRILVTTRDIRVCNKMECEKIIQLDILPEEEGWILFQKHAALSDKSSKSILDI
GRKISKECKGLPIAIVVIARSLKGQTRKKEWDLALKSLQKSMHIPGDDENWSEVYTCLKYSYDNMKNKTAKNLFLLCSLF
REDEEIPEELLVRLAIGAGLVEQIDVDYNYDVYRNEVIVAKYKLIDSCLLLNEEFESVKMHDLVREMALSIANKEIVAVKTS
NKNAMTMVEKGKNIKYLLCEGKSKDLSSLKFDGSKLDILIIYLNEMEDYYVKVPDPFFENVSRIRVLYLSNNTYKESTMSLP
RSIQLLTNLRSLLLESFTLGDISILGKLQGLETLELVKCWLVELPREIAKLGKLQLLKLNDCMFKRNNSFEVIESCSSLEELYLI
HEGISKTSHKVHLPNYQRFCILDFSCFFMTKEFFARNSLNMFMADEVFSKETFKNLVQKAEILCLNGLEGVWKSLIPDIIF
PDDDEVDPFPLNF

>XP_022641644.1

MNALTLLENSPFLKTNGARTKRCNDSDNHTDNDNEGEQGKEKDQRELDGEHKVIKQKPNSYIYDVLINFTGEDIHRLFV
SHLNSVLSTVGLTTFLHHHNAVKSTHIQEPILSHCRVAIVVFTQTYSQSAWCLHQLQQIINWHETYFQHVLPIYYEIQPSD
VRLQKGDFGKALEETAQQTFSGQELEHGMSRWSHALTKAANFFGWDESNYRSDAELVEKIVKSVVNLPVLSATKFPVG
LQSHVENLIRTIKNKSTEVCMIWIHGDEGSGKTTLAKAIYNQIHWTFKDKSFIENISRVSGIRGLLRLQEQLLLDVLKQKVE
IPSVDVGRTMIRERLLGKRVLIVLDDLFYSNLFDLRDCTKWLVEGTVIIVTSTYGIGLRDQANSVFRVERMNEEESLELVS
WHAFREAKPKEEYKDLARKVVSYCEGLPLALEVVGSSLFEKTKEEWNNVLFAIDGLRPDPEIIKVSIEGSLNEMEKDIFLDI
CCFFVGKNRAYVRKILNGCGVDADIAIRVLIQHNLIKINKNNKFGMHPLLQEIGLRIIRENYGKDLGNRRLWFDKDTKYG
TEALQWLPVNAFVNVQQTVNSEYLLKKLRWISWHGLSSERLPNNFYEHDAIAIDLKRSLLRFLWKTPQVMTSLKVLNLS
HSKHLTTTPDFTGLPSLEHLILKYCSRLSKVHQSIGSLNSLILLNVKYCTSLYNLPTEIYELKSLRTFILSGCSKIDIMDKDIAKLE
SLITLIAENTAVKEAPFSIVSSKRIGYISPTWI

>XP_022641645.1

MDNAFSSYKLPRKYDVLINFTGEDINRKFVSHLNYAFSTVGLTTFLHHPNAVKSTHIQEPVLSHCRVVIVVFTQTYSQSA
WCLDQLQQIIKWHETYCRHVLPVYYEIQPSDVRLQKGNFGKALKATAQQTFSGQELKHGMSRWSQAITKAANLFGW
DESNHRSDAELVEKIVKSVVNLPALSVTKFPVGLQSHLEDLFRTIKNKSTEFCMIEICGEEGSGKTTLAKAIYNQIHWTFKE
KSFIENISQVTGTRGYIRLLEQLLLDVLKQKVEIPSVDVGNKMIRERLYGKRLLIVLDNVLHSYELLEVWTYNHYRFSKGTVI
IITTSDENLLMGQPTYSMFRIERMNAEESLELFSWHAFREPKPKEEYEDLARRVVSYCGGLPLVLEIIGSSLFERTEEEWHR
VLFAIDGRHRVSEIIKVSIEGSLNEMEKYIFVDVCCLFVGKSRAYVRKILKGCGVDADIAIRVLIQRNLIKIDNNKKFRVHPLL
QNIGGQIIHEKVVGDFEKNRPPSKYETEALQWLPAKLLAAIESVQPTLNSQYLFKKLRWISSYGLSSECLPNNFYWDDAT
VIDLKRSLLQFLWKTPQVLRSLKVLNLSHSTDLTTTPDFTGLPSLEHLILKYCSRLRKLHQSIGSLNSLILLNLKFCTSLNNLPT
EIYELKSLRTFILSGCSKIHIMDKDIAKLESLITLIADTPVKHVPFSIMTGSYTQIIWPIRKFTVRTIHNLAGRSLPSSRATLREG
HSRRPAILDVKHLAGWSLPSSSDPGR

>XP_022641649.1

MVSIVSDLAKSTLEKLINATIEESRYICCFTCITDDFEKEKENLKAKKETWEEYARVATRRGDNIRKDVTHWQKQAKELIEE
DTKMKVKCFFGWCPNCIWQYSRGKELESKTEKIKKLMECNFENVGITRDVPDIEYHSSQNYVSFKSRKLKFEELFNALKD
DNNYMIGLQGMGGIGKTTLAKEVGKELKKSKCFNQVIDTTISNSPYIKRIQDEIAGPLGLSFENCTESERARKLMGRLTN
ADKILLILDDVWGNISFGEIGIPFKDNHNNCKILVTTRYIRACNEIGCEKIIQLDILPEEEGWILFQKYAGLSDNSSKSILYKG
RKISKECKGLPIAIEIIASSLKGTLPLEEWDLALKSLQKSMHVHYDDESLRKIYTCLKYSYDNMKDQTTKKLFLLCSMFREDE
EISEELLVRLAKGATLIEKIDDDDSYDECGKKVIVAKYKLIDSCLLLNCKSGRVKMHDLVREMALWIVNEEIVAVNTSKKNE
MTMVEKGKDIKYLLCEGKISDLFSSKFDGSKLDILIVNMNDHGPVEVPNSFFENIPGLQVLILSNNRYKRAILSLPQSIQLLT
NIKSLYLKQFSLGDISILGNLQTLETVEFVKCGMDELPREIGKLVKLKLLKLNYCRFERSNVFEVIKSYSSLEELHLVTTYDNKI
FKDCHLELPNYQRFCIGEHFRDIYPEWFLRNSLDVDNAMEIFSEETLKNLVQKAEILRLRGPIGVWKNLIPDIIFPEDESTN
NLMEIALEDNSELTCLIDNNDSLVQSALSRLVTLELEGMKNLKEICNGPLPSEFLKNLETFHLNDCIHLQGALFKSKISLCNL
KTLSIRSCPMLTSLFELSTTQSLLSLETLHIMSCEQLKSIIRDENKWKDSGEEIVDTHNNNKSTISIFPNLTTLYVYECRLLHFV
LPVTQKIPKLESITIRNCDGLKYLFGRYQHEHEEEDLHQELKDVIFTSLKYVYLDNLPNFVDIFQKCDESFCSLKKSTSKDET
KGKIESKSRKYKVLHWTHKYKSKWRTTKIPYDSKDQLQDSSLSMVNEAHANDVIAQYMRVELENMSLNDTSKILFTLQ
NVTELHINRCT

>XP_022641665.1

MAALIACFQFLTSSPRAKAWLKKQLIQLDLYETRVGEVKDVVEKLKNKRDAIRHTVEEEERRHGRRIHDEVKEWMESVD
KLIRAYEDFHNDEICHKCAVFDFFDSGYLPKPGIRYRRSRKANEITKQANGLLQNAKFDILSYWSGPPSMATFFSNLGYES
YSSRKETVKKITAEFQKPDVRMIGLHGLSGVGKTSLVKEVVKKALKDKMFEVVTMASVTKNPDVRKIQGQIADMLGVV
LEEESDIARAARIHQILNNENNSTLIILDDLWEQVNFNLLGIPCEIGKEDGVTNVQGKSLDVDSLKNVSDGKSLDVDILKN
VVGDGKSPILGSSTSFRKGMLQGADGSKNVNKGKSLGTADSVNVKKGGFLGGSLKNVNEGKSPLDASERVKAENSVP
QYKGCKILMISEIKQVLLSQMEGKEECIFPVEILKEEEAEKLFKKKAGISGKNSEYDKLAAQIASKCKGLPMTIVTTARALKN
KSLSVWDETNRKFESQKLTGAPEFSTKLSYELLEDEDLKYTFLLCARMGHDALIMDLVKYCIGIGFLRGINTARQTRDRVY
TLVAKLKESGLLSDSYSSDHFTMPDTVRRAALSIAHKGNQLFTMTKGKLDEWPDKLERYAAISLHHCDFIEDFSGRIKYPR
LRVLQIVNNIPRPKIPKNFFKGMKELRVLILTGIHLPLIDSSFSSLHKLRMLCLEQCCMLDEELSILGGLKKLRVLSFSGSDIKS
LPGELNELKMLQIFDISNCSKLKKIPRGVISSLVSLEELYMRNTSIQWEDEEQTCQSKTALLSDLKHLNQLTTLDIQIPNVSY
LPKNLFFDKLDSYKIVIGDLSSFLETGFQMPERYETLKFLAVQLENGSDIHSLMGIKMLFEGVENLFLELNTVHEKHNSVR
EARNIVHDLFYRLNLKGFPYLRHLWIVNNSTIQSLIHPKDRQHPEKAFPKLESLHLYNLKMDEICSCKLSEPSFGKLKVIKIN
LCGVLNSVFSISMVGLLKVLETIEVSECSSLKKIINVGPPINLENTELLMLPELRYLKLQSLSEFIGFDAIPPIDQGERKLFHEK
ERVCKELNFKKRRRLEFQN

>XP_022641675.1

MECPGGAMHSPKLQISLDGMRAITGIGGEEGSGKTTIAKAIYNQIHWTFKEKSFIENISQVSGIRGHLRLLEQLLLDVLKQ
KVEIPSIDVGRTIIREKLSGKRMLIVLDDVSNFSLFDIWDFRKWSVEGTVIILTSTYIEPLRGGDSVFWVKRMNAEDSLELLS
WHAFKEPKPKEEYKDLARSVVRYCAGLPLALEVVGSALFEKTKEEWNNLLFAIYGMRCVPKIIKISIEGSLNEMEKDIFLDI
CFFFVGKSRAYVRKILNGCGVDADIGIRVLIQRNLIKINKNNKFGMHHLLQNIGMKIIHENSGKDLGKNRRLWFDKDTKY
GTEALQWLPVKLLTAIESLQPTAISYDLLNKIRWISSHGLSSEYLPNNIYEDDATAIDLKRSLLRFLWKTPQVLRALKVLNLS
HYEHLTTTPDFTGLPSLEHLIFKYCSRLREVHRSIGSLNSLILLNLKDCTSLNYLPIEIYDLKSLRTFILSGCSKIDIMDKDIAKLE
SLITLIAENTAVKHVPFSIVISKSIGYISLRGFEGLSHNLFPSIIRSWMSPIMNPISYIHSFCMDTEDNTIDIAPLLSTLANIRSV
LVACDTEFQLSKQVKNILVEYFANITESGISKQHFRYSLIGVGAYHQFFNAVSDNIYQVLSSSESGDVCLPAVNDPYCMA
HMGEGHSVSFVVPEDGDLKGMIFCVVYLSTPKIIEPQFTTVVIVNYTKCTFHIHNHGTTISFKDGDWHGIMSNLECGDN
VEIFVNFGNGLVVKNTTVYLIRGESENMEKASETKKHSLIRFIKKVVM

>XP_022641721.1

MDAALKIIDPVIVFLWDHGIRQVTYIFRYEKHFEELNKEVKHLGEVKKGLHRLRDEAKSKGDIVEDRVEEWFGEVGEFES
RVENYIKDAGHKKTRGLYHVFPYYRHKLGRKAKKMENEASLLRDASPKVDEVSYAKKVTSFDLTSSNLGYIEFDSRKSIVE
DVMTKLKNPNMKIIGLHGPQGMGKSMLIKKIAIKTKEEGLFDKVAEIDVTVNPNPLKIQEDIAYVIGLPLVGESENVRAD
CLRRWLKIENVSILIILDDLHERLDLNRLGIPVDDDYDLRKKNELSISSSEQGSNANLNSTKVTVGGTRGTDEKVLKRENFL
SSYKGCRVLLSSRHKEVFPDEVDLESIFSLKELDDNDSLMLFENEAGRGNKMSMPKEEIKNYCTGTPMTIIRCAKRFKSWI
ESESEPTLDKFKFEWEKSMKITNETKHHFPKDEELKFIYLLCAQMGPLPLIMDLVKYCFGLGIFEGVSLLSEARHKINKSIQE
LQDLGLVSYKSPNTHFKMSHIIRGDALSNAHKDHNIFALRDGKLDDWPELEKCTSISICNSDIIDGLPQFINCPHLKFLQID
TSDPSLEIPESFFKRMENLKVLILTGFRLSSLPYSIECLSNLRMFSLERCTLDCNLSLVRKLKKLRILSFSGSQLKTLPTELRYLD
KLRLLDISDCFELKIIPPNLLSSLTCLEELYISKSLIKMLVETDTNKGQNSFLSELTNLHQLKVVDLSIPCVSSFPNQLIFDKLTY
YKIEIGYFEMISVGEFWMPNKYEELKVLALQLKNDTDIRSKEGIKLLLKTVHSLLLGEAGVQNVVNDLNMDGFLNLKHLSI
INNKDIEYVNSTKLPNCGNVFPNLEYICLYNMMNLKMICYGEVTVASFAKLKTIKVEMCYRLENLFSFYTIKTSTCAEETSE
ISDCNSYMNEFLASLEMIEVCKCESLKEIFQIPVNYDKVEFLMLRTLTLQSLPSFTCFSTKVESSRGSNLTKTQTTNRGQTK
NSTEEDDHSDKAPPLFGELVEVPNLENLNLSSLNIHKIWSNQYSSSVCFQNLIKLVVKDCVKLTHLCSLPMASHLKKLKSL
VISGCPIMEKIFETEGISAENVYVFPKLEEIHLSKMNGLIDIWQTKVSADSFSNLISVTIEECNQLNKIFPSQMEGWFESLIN
LKVSKCKSVIEIFEINDSHEINASGIDTNLQVILLEGLPKLKQLWSKDPDGILNFKKLQSIDVRYCDELTNLFPTSVAKDVSKL
ERMSVFQCEKMVEIVASKDASEEIKDPLKFPELIYVRLYGLSNMKQFYKGRHPIKCPKLKELSIDKCLKLQTFPQEKSETRN
EENFIFLAEEVFPNLEYMEIDLKEAQKLLQKYQMQCLKELIILGSVKNTDLLYPFMYRMPNLEKLKLTFSRFAEESLTSSNIE
PQELGTVLKLKQLFVCFSDIKDIGFERYQVLQRLELLRLKGCDKLNTLAPSSVMLTYLTCLKLKYCCGLKNIMASSTAKSMV
QLKTMKVINCPEVQEIVSMEESEEGKVMKIVFSKLISIELVGLKKLASFCSHKECEFEFSSLEMFIVRECPKMEKFSEKRSIA
PKLKNIFGVKGNEKSKWQWEGHLNATIQKIFNDKVSFAYTEYLWLDNTDDFIIKQVWDDTHWRQQNNFGYLKRLSV
WDCHTLKHIIPSHLLSCFHNLEELDIYNCGGAEVIFNMNDENRVMTKPSGIFRLKTLQLYDLPQLKHVWDKDPKGIIGLQ
LLKEMSVSSCNCLKSLFPASVAKDLTRLEVLRVRNCKELVEIFWKDEKDEEEEGEGTTHESLFPRLTTFTLEELPRLKYSND
CSKQQESTSNLSERDIQELCIGSRRIPNSNFSLLESLIVDGCKFVSDVVLPFSLLPFLTNLETLVVRNCDFVKAIFDVKSTTQS
RDVTSMGQTLPFSLKKLTVSKLPNLKNVWNEDPQVIISMHHLQEVIVEECEGLTSVFPASENEYLLKLESLVVKDCKGLM
SIFAEDIIDPRTKLELMFSCPFVRSLELKDLPMFKYFYYCSLPCDNFAHLESHTENQVGTEKCMSVGENGMKMILRGEFE
RKLLDSLKTLTLCFGSDVFRYKLLEVVPNIEKLVVCDGSFKKMFCCESGNNVLKQLKVLRLESLEELVSIGLENSWTDSFVR
NLETFEVIRCKSLKNLVGCTVSFSNLTCLKVEDCDSLSYLLTSSAAKRLGKLKRMEIKDCDSIEEIVYKETDEDEIIFPQLSCLD
LDSLRMLKRFYKGSLGFPSLEELSVTYCNEMITLCEGSIEAANLSQVKLDPFSDAIQLETELNSTMRKKYLTEIAPRRESIEF
RDREDLQXIWCLSLQIPDFCFTXLVTLIVNKCKISSDAVLPFTLFPLLPKLETLEVQNCDSVKTIFDVNCTKQGTLTLPLKKLV
LRKLPNLETVCSEVKETNTTHPEGTNPELTFPCVTSFXLLDLPNFNAVPTFELIIPNLEHLTVGKNELKMIVNGEFQRNLLH
KLKVLTLCFDIECDEYPEYGFLQQLPNVMKLVVCDSSFKVIFCDQRPNNSEILLQLKELRLESLQELVSIGLENSWTEPFVR

NLETFEVISCSNLKNLVPCRVSFSNLICLKVENCDSLSYLFTSSTAKSLVKLQRMEIKRCESIEEIVSKEGEESDEDEITFLELN
CLKLKYLKNLRRFYKGSLSFPLLEELSIRWFDEMVSLCGGTLEASMLSEVKLDWSTTTLETGLNSTMKKEFLKKISELDELD
LESGPRLQEIWNGSLHIPDLCFSELAKLTVNDCQFLSDAVLPFHLLPLLPKLEILEVRNCNYVTVIFDVNPSTRYPLITLPLKK
LVLSNLPNVENVWNEDPRGILSMQHLQQVFVDKCKCLKTVFPASVARDLVKLEDLVLEDCEGLMAIVADESDEKEEIIFE
RLQVLDLKRLKELTCFYDGNFTLSFPSLKEVHVIECSSMKTFSAFNKIDNPTRWYYSEHARPRKETDLNSALHKTCEEEAP
DASTTSNPSDTEDSGYRTNTSDTEDSEYGTNTSDTEDSGPVVHQILTTAIAIVNLF

>XP_022641780.1

MAFESSSPSAKSEWIYDVFINFRGVDTRKKFVSHLHSSLSKAGVKTFLDEENLLKGMELQELLRAIQVSQIAIVVFSKRYAE
SSWCLDELQKIFQCRQTCGLRVVPVFYYVEPSEVRHQTGDFGDALRAAAESSYAGEHLEFALSSWRRTLTDAANLSGW
NATDRRTEAELVRDIVNHVIANLDYNAFSITKFPVGLDHPVQEVIRFIERTKGCCKIGIWGMGGSGKTTIAKVIYNKLHRL
FENKSFIENIREVCQTDRRRGLVRLQEKLLSDILKVKVEIQNAGIGQGMIDNRFIGKKALIVLDDVNEFDQLEALCGNTEW
MGERSVIIITTRDLHLLKRFEVNYVYEMKEMEVDESLELFSRHAFREAKPREDFNEVAKDAVSYCGGLPLALEVLGSYLSN
RTMTEWRSVLSKLKISPNTQVQEKLRISFDNLCDQMEKEIFLDVCCVFIGKDRGCVTEVLNGCGLYADIGITVLLERGLIK
VEKNNKLRMHHLLQDMGREIIRELSRKEPGKRSRLWFQEDGTDAVEGLALKLNLNNRECFKADAFEEMRSLRLLQLHH
VELMGDYGHLSKQLRWICWQGFPSKHIPENFYLEDAIAINFKHSNLRQVWKEPKVLLMLKFLNLSHSKYLTETPDFSGLP
YLEKLILKYCPSLRCVHKSIGDLCNIVLINLKNCTSLSSLPREIYKLKSLKTLILSGCTKIDKLEEDIAEMKSLTTLIAENAVVKVP
FSIVSSKSIGYLFPCGFEGLSHDVLPSIIWCWLSPTMNPLSSTLPLCGISASLVSMNMQNIDLGDLAPILTNLLNLRSVWV
QCDTEFQITKQVRKILNDVGWVHFTELGIASCTSEISDNSLRSYLIGIGSYQEEDFNTLNKSISKELATSGSCHVFLPGGNY
PFWLAHTGEGHSVSFTVPQDWDMKGMALCVLYLATPETAATECLISVVMVNYTKYTIQLYKRDTVISFNDADWQGIIS
HLEAGDEVEIFLSFRNELVIKNTAVYLINYEFIDMEVKAVSKPPVMKVYSRKRNLTDQESSRKRQW

>XP_022641887.1

MDFLGPFGKVIEGLIDFVWKHGVRQVTYIVNYNNNVFELKDSVKDLELEKERINHQRDEAEKNLNNIEGKVIEWDRKLS
EIETAVEVFENDDGHTRARSPNCFLFPFLRNRHRLGRQAHKMKEDVKRLIGESPELDEVFYRQNVTCNDATLSNCGFVE
FSSIKSIIEKVMIQLQDSTVRMIGLYGRGGVGKSTLVKEIARKAKEKKMFDVVVKVEITADPNPHKIQEEIAYVLGLRLEGE
GENVRADCLRRRLKKEKGNILLILDDLWHKLDLNKLGIPVEDYDDDEDFNRQKPDNKDSNNDPSSKVSKKENIPGGHKG
CKILLTSRDKNVLCVEMDVKSTFCIRELDDKDALILFQKLAGIDNEMSSSKQEIVKKYCEGLPMAIVVVARALRNKSESVW
EATIKRHKKHELMGEGTSMDISVKMSYDHLENEEIKYIFLLCAQMGRRALIMDLVKYSFGLGIFEGVSSLWEAREKIKTSI
QKLKDSGLLLDESSNNHFNMHDMVRDTALSIAHKDHNAFNLRNGKLDDWPELEKCTSIFMCNSDIIDGLEVINCPHLKL
FQIDTNDPYLKIPKSFFKRMKNLRVLIMTGFCVSNLPSSIQYLSKLRMLCLQRCTLDCNLSIIGKLKKLRILSFSGSILKNLPIE
LQCLDKLRLLDISDCSELKIIPPNVISSLTCLEELYIRESLIKMLVERETNKGQDLFLSELKNLHQLKVVELSIPRVSNFPNHLFF
DKLRDYNIVIGDFDFFSLGEFKMLNKHETSRVLAVQLKDNTNIHSQENIKLLFKTVQTLLLGKTNGVREVVNDLNIDGFQ
DLKHLSIINNNDFKYVNSTKLCNYVNVFPNLESLCLYNLGKLDMICYGPVTVVSFAKLRTIKVEMCHRLKNLYSLDMAKFP
IGAQTCDISECNSYMDIFLSSIEIIEVSECKSLKEILQIPKHYRKVEFLKLHTLTLRLLPLLSSFYTKVDKFFWPHLTKAQTTIM
GHKDLTSEEDKQSDEEPSLFGELVEIPNLETLNLSSLKIHKIWSDQNSSSFLFQNLIKLVVKDCDKLTHLCSLSMAKSLKKLK
SLVISECPVMEKIFETERNNADKVCIFPKLEEIHVTKMNKLTDIWQTKVSVDSFSNLISVKIEECNKLDKIFPCHMEGWFES
LDNLKVYWCESVEVIFEINDSQEIDEFGGIDTKLQIILLEGLPKLKQLWSTDQNGILSFKKLRTIDVDSCDELKNLFPVSVAK
DVPKLEHMSVLDCDKMEEIVGSQDASDANPDLLVFPELIYVRLHRLPNMKYFYKKKYPIKCPKLKELSVTKCVKLKTFVKD
TINTTNKVGNFVFSIEEVFPKLEHMEIEFKEAQELLPKYQMQRLKELSLISVESVDLLNQFPYRTPNLEKLKLESYDFKELAP
KANNGRQKGLGIVLQLKELVFLDSNIKDLGFERGQVLQRLEVLRLEDCSKLSNLAPPSVSLNYLTHLELKYCFGLKHLMAS
STAKSMVQLKTMKVSYCDKIEQIISMEESEEGKVMKIVFSKLISIELVGLNNLASFCSHKECEFEFSSLETLIVRECPKMEKF
SEKRSIAPKLRNIFGVEGDEKTKWQWEGDLNGTIHKIFDDKVSLAYTENLWLDIDNDQYIIEQLWHDRHWVQQNSFGY
LKTLFVWRCDTVVHVIPSHLHSCFPNLEGLDVRYCSNAEVIFNMNDENRVMTKPSGIFRLKILNLYNLPKLEHVWDKDL

EGIIDLDALEEVRVENCRSLRTFFPAWVAKDLNRLEVFQITKCDELEEIFRSGEEEEGSLPDSVFHQLTTLTLQQLPRLKYSI
HRSKQQESTSYLSERDIKGLCFGSQVIPNSNFCLLESLKVDGCQFLSDILLPFNLLPSLANLEILEVQNCMFIKTIFDVKCTTE
SKDVTSTGPTLFSLKNVTLSNLPNLKNVWNEDPDGILRMHHLEEVYVENCKCLRSVFPESIAKDVVELKNLEVEDCEGLT
TIVAENNNADPRGTNQDLPCPLVRSLKLKSLPMFKYFYHCSLQCDNFANLESHSENQVGTEKLKWLSLGNNGVEMILH
GEFQRNFLENLKVHTLCLFSDAFGCEILEEVPNIEKLVVRGGSLKEMFCCQSPNNEDYSGLLLQLKELRLESLQELVSIGLE
NSWTEPFVRNLETFEVISCSSLKNLVTCRESFSNLICLKFENCDSLSYLFTSSTAKSLAKLQRMKIKKCKSIEEIVSKEEEESDK
DEIIFPKLNCLKLKYLKNLRRFYKGSLSFPLLEELSIRDCDDMVSLCQGTLEASKLSQVIPLEIDLSSAIWKEFMSKIGRRSSVE
FKDRADLNEIWRLSLQFQDFCFSNLETLVVDGCQLLSYVIPFHVLPLLPELQTLEVRNCDSVKTIFDVKSSQHTFTFPLKKL
VLLKLPNLETIWNEDPAEIVTEPNPTHPEQTNPKLITPNLEHLTVGENELKIIVDGKFQRNLLHKLKVLGLCFDMECDEFLE
YGFLQQLPNVMKLMVCDSSFKVIFCYHIPNNSEILIQLKELRLESLQELVSIGLENSWTEPFVRNLETFEVISCSILKNLVTC
RVSFSNLICLKVENCDSLTYLFTSLTAKSLAKLQRMDIKKCESIEEIVSKEGEEADEGKIIFPKLNCLKLKYLKNLQRFYKGSLS
FPLLEALSIRDCDDMVRLCGGTLEASKLSEVKLEESNTKLKTVLNSTMRKEFLKKISELDKLDLKSRPLQKIWNGLLDISDLC
FSGLAILTVNDCPFLSDAVLPFHLLPLLPRLKTLEVGNCDYVKTIFDVKCTTKDTSFTFPLKKLVLSKLPNLENVWNEDPHKI
LCMQHLEEIHVKECKDLKTVFPPSAAKSLVELEDLVVEDCEGLMAIVADESKEGNELDKNGIIFPRLSYLKVESCNTLPYLF
TSSTAKGLAELKTMKIKQCKSIEEVVSKEGEVSDEDEEIIFKQLEDLYLEKLDELGCFYSGNLTLSFPSLEEVHVIKCSSMKTF
SAVNKINHPTKWYCSEYERPQKETDLNSAVLKTSAKEAPDASGAIISVLQ

>XP_022641888.1

MDFLGPFGKVIEGLIDFVWKHGVRQVTYIVNYNNNVFELKDSVKDLELEKERINHQRDEAEKNLNNIEGKVIEWDRKLS
EIETAVEVFENDDGHTRARSPNCFLFPFLRNRHRLGRQAHKMKEDVKRLIGESPELDEVFYRQNVTCNDATLSNCGFVE
FSSIKSIIEKVMIQLQDSTVRMIGLYGRGGVGKSTLVKEIARKAKEKKMFDVVVKVEITADPNPHKIQEEIAYVLGLRLEGE
GENVRADCLRRRLKKEKGNILLILDDLWHKLDLNKLGIPVEDYDDDEDFNRQKPDNKDSNNDPSSKVSKKENIPGGHKG
CKILLTSRDKNVLCVEMDVKSTFCIRELDDKDALILFQKLAGIDNEMSSSKQEIVKKYCEGLPMAIVVVARALRNKSESVW
EATIKRHKKHELMGEGTSMDISVKMSYDHLENEEIKYIFLLCAQMGRRALIMDLVKYSFGLGIFEGVSSLWEAREKIKTSI
QKLKDSGLLLDESSNNHFNMHDMVRDTALSIAHKDHNAFNLRNGKLDDWPELEKCTSIFMCNSDIIDGLEVINCPHLKL
FQIDTNDPYLKIPKSFFKRMKNLRVLIMTGFCVSNLPSSIQYLSKLRMLCLQRCTLDCNLSIIGKLKKLRILSFSGSILKNLPIE
LQCLDKLRLLDISDCSELKIIPPNVISSLTCLEELYIRESLIKMLVERETNKGQDLFLSELKNLHQLKVVELSIPRVSNFPNHLFF
DKLRDYNIVIGDFDFFSLGEFKMLNKHETSRVLAVQLKDNTNIHSQENIKLLFKTVQTLLLGKTNGVREVVNDLNIDGFQ
DLKHLSIINNNDFKYVNSTKLCNYVNVFPNLESLCLYNLGKLDMICYGPVTVVSFAKLRTIKVEMCHRLKNLYSLDMAKFP
IGAQTCDISECNSYMDIFLSSIEIIEVSECKSLKEILQIPKHYRKVEFLKLHTLTLRLLPLLSSFYTKVDKFFWPHLTKAQTTIM
GHKDLTSEEDKQSDEEPSLFGELVEIPNLETLNLSSLKIHKIWSDQNSSSFLFQNLIKLVVKDCDKLTHLCSLSMAKSLKKLK
SLVISECPVMEKIFETERNNADKVCIFPKLEEIHVTKMNKLTDIWQTKVSVDSFSNLISVKIEECNKLDKIFPCHMEGWFES
LDNLKVYWCESVEVIFEINDSQEIDEFGGIDTKLQIILLEGLPKLKQLWSTDQNGILSFKKLRTIDVDSCDELKNLFPVSVAK
DVPKLEHMSVLDCDKMEEIVGSQDASDANPDLLVFPELIYVRLHRLPNMKYFYKKKYPIKCPKLKELSVTKCVKLKTFVKD
TINTTNKVGNFVFSIEEVFPKLEHMEIEFKEAQELLPKYQMQRLKELSLISVESVDLLNQFPYRTPNLEKLKLESYDFKELAP
KANNGRQKGLGIVLQLKELVFLDSNIKDLGFERGQVLQRLEVLRLEDCSKLSNLAPPSVSLNYLTHLELKYCFGLKHLMAS
STAKSMVQLKTMKVSYCDKIEQIISMEESEEGKVMKIVFSKLISIELVGLNNLASFCSHKECEFEFSSLETLIVRECPKMEKF
SEKRSIAPKLRNIFGVEGDEKTKWQWEGDLNGTIHKIFDDKVSLAYTENLWLDIDNDQYIIEQLWHDRHWVQQNSFGY
LKTLFVWRCDTVVHVIPSHLHSCFPNLEGLDVRYCSNAEVIFNMNDENRVMTKPSGIFRLKILNLYNLPKLEHVWDKDL
EGIIDLDALEEVRVENCRSLRTFFPAWVAKDLNRLEVFQITKCDELEEIFRSGEEEEGSLPDSVFHQLTTLTLQQLPRLKYSI
HRSKQQESTSYLSERDIKGLCFGSQVIPNSNFCLLESLKVDGCQFLSDILLPFNLLPSLANLEILEVQNCMFIKTIFDVKCTTE
SKDVTSTGPTLFSLKNVTLSNLPNLKNVWNEDPDGILRMHHLEEVYVENCKCLRSVFPESIAKDVVELKNLEVEDCEGLT
TIVAENNNADPRGTNQDLPCPLVRSLKLKSLPMFKYFYHCSLQCDNFANLESHSENQVGTEKLKWLSLGNNGVEMILH
GEFQRNFLENLKVHTLCLFSDAFGCEILEEVPNIEKLVVRGGSLKEMFCCQSPNNEDYSGLLLQLKELRLESLQELVSIGLE

NSWTEPFVRNLETFEVISCSSLKNLVTCRESFSNLICLKFENCDSLSYLFTSSTAKSLAKLQRMKIKKCKSIEEIVSKEEEESDK
DEIIFPKLNCLKLKYLKNLRRFYKGSLSFPLLEELSIRDCDDMVSLCQGTLEASKLSQVIPLEIDLSSAIWKEFMSKIGRRSSVE
FKDRADLNEIWRLSLQFQDFCFSNLETLVVDGCQLLSYVIPFHVLPLLPELQTLEVRNCDSVKTIFDVKSSQHTFTFPLKKL
VLLKLPNLETIWNEDPAEIVTEPNPTHPEQTNPKLITPNLEHLTVGENELKIIVDGKFQRNLLHKLKVLGLCFDMECDEFLE
YGFLQQLPNVMKLMVCDSSFKVIFCYHIPNNSEILIQLKELRLESLQELVSIGLENSWTEPFVRNLETFEVISCSILKNLVTC
RVSFSNLICLKVENCDSLTYLFTSLTAKSLAKLQRMDIKKCESIEEIVSKEGEEADEGKIIFPKLNCLKLKYLKNLQRFYKGSLS
FPLLEALSIRDCDDMVRLCGGTLEASKLSEVKLEESNTKLKTVLNSTMRKEFLKKISELDKLDLKSRPLQKIWNGLLDISDLC
FSGLAILTVNDCPFLSDAVLPFHLLPLLPRLKTLEVGNCDYVKTIFDVKCTTKDTSFTFPLKKLVLSKLPNLENVWNEDPHKI
LCMQHLEEIHVKECKDLKTVFPPSAAKSLVELEDLVVEDCEGLMAIVADESKEGNELDKNGIIFPRLSYLKVESCNTLPYLF
TSSTAKGLAELKTMKIKQCKSIEEVVSKEGEVSDEDEEIIFKQLEDLYLEKLDELGCFYSGNLTLSFPSLEEVHVIKCSSMKTF
SAVNKINHPTKWYCSEYERPQKETDLNSAVLKTSAKEAPDASGAIISVLQ

>XP_022641889.1

MDFTSSKSKHQRIHDVFINFRGEDTRRKFVSHLHYALSNAGVNTFFDEENLVKGMQLQELMRAVEGSQIAIVVFSQTYT
ESTWCLDELEQIIKCNQTQGQSVLPVFYEIDPSDVRHQKGDFGKSLEEAARRTYSGEELERALSRWSRALNKAAGISGW
DVRNFRNEAELVRQIVDRVQKLLDYEVLSITEYPVGLESRAQDVIGLIETRSTQVCMIGIWGMGGSGKTTVAKAVYNQI
HRRFMDKSFIENIRETCENHGRGYVPLQEQLLSNVLKTKVEIHSVGMGTTMIENRLDGKTALIVLDDVNEYNQLKAVCG
NRKWIGQGSVIIITTRDVGLLTRLDVDYVYGMDKMDEDESLQLFSFHCFGDAKPKEDFRELSKNVVAYCGGLPLALEVL
GSYLFDKTSKRVWEGVLSILEKIPNDEVQRKLRISFDSLSNDMEKDIXLDVCCFFIGKDRGYVTEILNGCELCADVGIPVLIE
RSLIKVEKNNKLGMHPLLQEMGREIIRENSRKDPGKHSRLWSQKEVVEVLTKNSGTEAIEGLVLKMHLTSRDCFKTDSF
QKMERLRLLQLHHVQLAGNYGYLSKQLRWISWHGFPSNCLPNSFCMDDVIAIDLKHSHLRFVWKQSQDLKWLKVLNL
SHSRYLTETPDFSRLPSLEQLILKDCPSLLAIHNSIGDLCNILLINLKDCTSLSNLPKEIYKLKSVKTFILSGXSKIDKLEEDIAQM
ESLTTLIADNTAVKQVPVSIVTSKSIGYISLCGFEGLARNVFPSIIQSWMSPTMNPLLFIRPFSATSCSLVSMNTQNNTLGE
LAPMLRSLPNLRSVLLRYETESQLSKHVKTFLVEHVVNVAELGISRHHLRSSLIGVGSYKAYFDILNDRISKELVTDEACDVS
LPIDNYPYWSAHTDTQARNSDFL

>XP_022641968.1

MEFASSSLSSSSSSFLTSEPHFIHDVFINFGGEDIGRRFVSHLHSVLLQNQVKTFISQQNLHEGMELEEQMRGIGGTKITII
VFSKSYAESACCLLELEKIIECPQTFGQIVLPVFYEIDPLDVRHQNDDFRKALEETAGRXYALYKWNRALNTAASMTGWD
VRNFRHDAELVDVIVNRVNTLLDYKDLFITEYPVGLESRVEDVIKCIKNQTTKVCMIEICGREGSGKTTLAKAIYNRIYREFI
GKSFIQNIRYEGYVALQENLLSDVLISKPGVKSVGMGRTMVENGFSREKLLIVLDDVNDFDQLRNLCGSREWFGQGTVII
VTSRDFHLLNQFRDNYVYKMDILNENESLKLFSWHAFRDAIPEKEWYELARNVVVYCGGLPLALEFLGSYLCDRTIEVW
ESVLLKLQRIPPNELLSVLKISFEDLRDTEKDIFLDVCCFFIGKEREYVTEILNGCGLHADIGITLLIERGLIKVESNNKLQMHP
LLQEMGREIIRQECPEKPGKRSRLWFPDDVEDVLNKNTGTKAILSLKLHSSIGDWFEAHAFKEMKRLRLLQLDNVKLSG
NYGHISKQLRWICWRGFPYRYIPNNFNLENVIAMDLKHSHLQLIWKQPLWKKPQYVCRF

>XP_022641989.1

MTYSNSFRVFDRIKRVIFVCVLLAAIDNNNSNNSSYPVTDFAYDNDSPQIKFDVFVSFRGTDIRQDFLSHLIEAFSQRHIN
AFVDNKVVRGDGMSEALIRAIEGSSISLIIFSQDYASSHWCLSELVKIVECRKKNGQIVLPVFYKVDPAHVRHQKGTYEHA
FAKHQIRYSLTTMQIWRTALTEAANLAGFHSSTFRDEAEFIKEIVKCVLSRLNQVQQGKAKGLVGVGKRIAHVESLLQSE
EPDVRIMGIWGMGGIGKTTIAQEVYDKLCFEYEGCCFLANIREESGRLGMISLKKKLFSTLLGGEDLKIDTPNGLPQYIER
RLRRMKVLIILDDVNDSDQLEVLAGTHDWFGSGSRIIITTRDKQVLAREFASIYEVEALNFDESLRLFNLNAFKQNHLESE
YHELSKKVVNYAKGIPLVLKVLGHLLHGKDKETWESQLERLKKVQNKKVHDIIKLSYNDLDRDEKKIFLDIACFFDGLNLK
VKHMNFLLKDHDYSVVAGLERLKDKALISVSQENGVSMHNIIQETAWQIAREESIDNPRSQIRLLDPEDIYHVLNYNKG

DEAIRSIVINLSRIKQLQLNPQVFARMSKLHFLDFYSKGSCSCLRDQGGLYLPQGLESLSNELRYLRWTHYPLESLPSKFSA
ENLVELNLPNSRLKKLWQEAPEDLVNLRVLILHSSTRLKELPNFSKATNLKVIDLRFCVRLTSVHSSIFSLRNLEKLYLGGCLS
LRSLRSNVHLDSLRYLSLYGCMSLKDFSVTSKNMVKLNLELTGIKRLPSSFGLQSNLQKLRLAYTYIDHLPTSIKHLTRLRHL
DLRYCRQLRTLPELPASLETLDARGCISLETVTFPSTAGEQLKENKKRVAFWNCLKLDEPSLTAIELNAQINMMKFAHQH
FSLFGDAQSTYVYPGSKVPEWLVHKTTHDDFVIIDLSSVLSPHSSHIGFIFGFVVPEVPFGGSALEFKISISGEGSHINVYM
DRPRHRITSDHVYLMYDQACSRYLNGRAKHEPRLKIKVALASRTLTSKYVPLKLRAFGISAINTTDFLSFVQKVKFGDNVP
NVPILSRFFCTFCIVVFVGTFNICIRRLV

>XP_022641990.1

MTYSNSFRVFDRIKRVIFVCVLLAAIDNNNSNNSSYPVTDFAYDNDSPQIKFDVFVSFRGTDIRQDFLSHLIEAFSQRHIN
AFVDNKVVRGDGMSEALIRAIEGSSISLIIFSQDYASSHWCLSELVKIVECRKKNGQIVLPVFYKVDPAHVRHQKGTYEHA
FAKHQIRYSLTTMQIWRTALTEAANLAGFHSSTFRDEAEFIKEIVKCVLSRLNQVQQGKAKGLVGVGKRIAHVESLLQSE
EPDVRIMGIWGMGGIGKTTIAQEVYDKLCFEYEGCCFLANIREESGRLGMISLKKKLFSTLLGGEDLKIDTPNGLPQYIER
RLRRMKVLIILDDVNDSDQLEVLAGTHDWFGSGSRIIITTRDKQVLAREFASIYEVEALNFDESLRLFNLNAFKQNHLESE
YHELSKKVVNYAKGIPLVLKVLGHLLHGKDKETWESQLERLKKVQNKKVHDIIKLSYNDLDRDEKKIFLDIACFFDGLNLK
VKHMNFLLKDHDYSVVAGLERLKDKALISVSQENGVSMHNIIQETAWQIAREESIDNPRSQIRLLDPEDIYHVLNYNKG
DEAIRSIVINLSRIKQLQLNPQVFARMSKLHFLDFYSKGSCSCLRDQGGLYLPQGLESLSNELRYLRWTHYPLESLPSKFSA
ENLVELNLPNSRLKKLWQEAPEDLVNLRVLILHSSTRLKELPNFSKATNLKVIDLRFCVRLTSVHSSIFSLRNLEKLYLGGCLS
LRSLRSNVHLDSLRYLSLYGCMSLKDFSVTSKNMVKLNLELTGIKRLPSSFGLQSNLQKLRLAYTYIDHLPTSIKHLTRLRHL
DLRYCRQLRTLPELPASLETLDARGCISLETVTFPSTAGEQLKENKKRVAFWNCLKLDEPSLTAIELNAQINMMKFAHQH
FSLFGDAQSTYVYPGSKVPEWLVHKTTHDDFVIIDLSSVLSPHSSHIGFIFGFVVPEVPFGGSALEFKISISGEGSHINVYM
DRPRHRITSDHVYLMYDQACSRYLNGRAKHEPRLKIKVALASRTLTSKYVPLKLRAFGISAINTTDFLSFVQKVKFGDNVP
NVPILSRFFCTFCIVVFVGTFNICIRRLV

>XP_022641991.1

MTYSNSFRVFDRIKRVIFVCVLLAAIDNNNSNNSSYPVTDFAYDNDSPQIKFDVFVSFRGTDIRQDFLSHLIEAFSQRHIN
AFVDNKVVRGDGMSEALIRAIEGSSISLIIFSQDYASSHWCLSELVKIVECRKKNGQIVLPVFYKVDPAHVRHQKGTYEHA
FAKHQIRYSLTTMQIWRTALTEAANLAGFHSSTFRDEAEFIKEIVKCVLSRLNQVQQGKAKGLVGVGKRIAHVESLLQSE
EPDVRIMGIWGMGGIGKTTIAQEVYDKLCFEYEGCCFLANIREESGRLGMISLKKKLFSTLLGGEDLKIDTPNGLPQYIER
RLRRMKVLIILDDVNDSDQLEVLAGTHDWFGSGSRIIITTRDKQVLAREFASIYEVEALNFDESLRLFNLNAFKQNHLESE
YHELSKKVVNYAKGIPLVLKVLGHLLHGKDKETWESQLERLKKVQNKKVHDIIKLSYNDLDRDEKKIFLDIACFFDGLNLK
VKHMNFLLKDHDYSVVAGLERLKDKALISVSQENGVSMHNIIQETAWQIAREESIDNPRSQIRLLDPEDIYHVLNYNKG
DEAIRSIVINLSRIKQLQLNPQVFARMSKLHFLDFYSKGSCSCLRDQGGLYLPQGLESLSNELRYLRWTHYPLESLPSKFSA
ENLVELNLPNSRLKKLWQEAPEDLVNLRVLILHSSTRLKELPNFSKATNLKVIDLRFCVRLTSVHSSIFSLRNLEKLYLGGCLS
LRSLRSNVHLDSLRYLSLYGCMSLKDFSVTSKNMVKLNLELTGIKRLPSSFGLQSNLQKLRLAYTYIDHLPTSIKHLTRLRHL
DLRYCRQLRTLPELPASLETLDARGCISLETVTFPSTAGEQLKENKKRVAFWNCLKLDEPSLTAIELNAQINMMKFAHQH
FSLFGDAQSTYVYPGSKVPEWLVHKTTHDDFVIIDLSSVLSPHSSHIGFIFGFVVPEVPFGGSALEFKISISGEGSHINVYM
DRPRHRITSDHVYLMYDQACSRYLNGRAKHEPRLKIKVALASRTLTSKYVPLKLRAFGISAINTTDFLSFVQKVKFGDNVP
NVPILSRFFCTFCIVVFVGTFNICIRRLV

>XP_022641992.1

MTYSNSFRVFDRIKRVIFVCVLLAAIDNNNSNNSSYPVTDFAYDNDSPQIKFDVFVSFRGTDIRQDFLSHLIEAFSQRHIN
AFVDNKVVRGDGMSEALIRAIEGSSISLIIFSQDYASSHWCLSELVKIVECRKKNGQIVLPVFYKVDPAHVRHQKGTYEHA
FAKHQIRYSLTTMQIWRTALTEAANLAGFHSSTFRDEAEFIKEIVKCVLSRLNQVQQGKAKGLVGVGKRIAHVESLLQSE

EPDVRIMGIWGMGGIGKTTIAQEVYDKLCFEYEGCCFLANIREESGRLGMISLKKKLFSTLLGGEDLKIDTPNGLPQYIER
RLRRMKVLIILDDVNDSDQLEVLAGTHDWFGSGSRIIITTRDKQVLAREFASIYEVEALNFDESLRLFNLNAFKQNHLESE
YHELSKKVVNYAKGIPLVLKVLGHLLHGKDKETWESQLERLKKVQNKKVHDIIKLSYNDLDRDEKKIFLDIACFFDGLNLK
VKHMNFLLKDHDYSVVAGLERLKDKALISVSQENGVSMHNIIQETAWQIAREESIDNPRSQIRLLDPEDIYHVLNYNKG
DEAIRSIVINLSRIKQLQLNPQVFARMSKLHFLDFYSKGSCSCLRDQGGLYLPQGLESLSNELRYLRWTHYPLESLPSKFSA
ENLVELNLPNSRLKKLWQEAPEDLVNLRVLILHSSTRLKELPNFSKATNLKVIDLRFCVRLTSVHSSIFSLRNLEKLYLGGCLS
LRSLRSNVHLDSLRYLSLYGCMSLKDFSVTSKNMVKLNLELTGIKRLPSSFGLQSNLQKLRLAYTYIDHLPTSIKHLTRLRHL
DLRYCRQLRTLPELPASLETLDARGCISLETVTFPSTAGEQLKENKKRVAFWNCLKLDEPSLTAIELNAQINMMKFAHQH
FSLFGDAQSTYVYPGSKVPEWLVHKTTHDDFVIIDLSSVLSPHSSHIGFIFGFVVPEVPFGGSALEFKISISGEGSHINVYM
DRPRHRITSDHVYLMYDQACSRYLNGRAKHEPRLKIKVALASRTLTSKYVPLKLRAFGISAINTTDFLSFVQKVKFGDNVP
NVPILSRFFCTFCIVVFVGTFNICIRRLV

>XP_022641993.1

MTYSNSFRVFDRIKRVIFVCVLLAAIDNNNSNNSSYPVTDFAYDNDSPQIKFDVFVSFRGTDIRQDFLSHLIEAFSQRHIN
AFVDNKVVRGDGMSEALIRAIEGSSISLIIFSQDYASSHWCLSELVKIVECRKKNGQIVLPVFYKVDPAHVRHQKGTYEHA
FAKHQIRYSLTTMQIWRTALTEAANLAGFHSSTFRDEAEFIKEIVKCVLSRLNQVQQGKAKGLVGVGKRIAHVESLLQSE
EPDVRIMGIWGMGGIGKTTIAQEVYDKLCFEYEGCCFLANIREESGRLGMISLKKKLFSTLLGGEDLKIDTPNGLPQYIER
RLRRMKVLIILDDVNDSDQLEVLAGTHDWFGSGSRIIITTRDKQVLAREFASIYEVEALNFDESLRLFNLNAFKQNHLESE
YHELSKKVVNYAKGIPLVLKVLGHLLHGKDKETWESQLERLKKVQNKKVHDIIKLSYNDLDRDEKKIFLDIACFFDGLNLK
VKHMNFLLKDHDYSVVAGLERLKDKALISVSQENGVSMHNIIQETAWQIAREESIDNPRSQIRLLDPEDIYHVLNYNKG
DEAIRSIVINLSRIKQLQLNPQVFARMSKLHFLDFYSKGSCSCLRDQGGLYLPQGLESLSNELRYLRWTHYPLESLPSKFSA
ENLVELNLPNSRLKKLWQEAPEDLVNLRVLILHSSTRLKELPNFSKATNLKVIDLRFCVRLTSVHSSIFSLRNLEKLYLGGCLS
LRSLRSNVHLDSLRYLSLYGCMSLKDFSVTSKNMVKLNLELTGIKRLPSSFGLQSNLQKLRLAYTYIDHLPTSIKHLTRLRHL
DLRYCRQLRTLPELPASLETLDARGCISLETVTFPSTAGEQLKENKKRVAFWNCLKLDEPSLTAIELNAQINMMKFAHQH
FSLFGDAQSTYVYPGSKVPEWLVHKTTHDDFVIIDLSSVLSPHSSHIGFIFGFVVPEVPFGGSALEFKISISGEGSHINVYM
DRPRHRITSDHVYLMYDQACSRYLNGRAKHEPRLKIKVALASRTLTSKYVPLKLRAFGISAINTTDFLSFVQKVKFGDNVP
NVPILSRFFCTFCIVVFVGTFNICIRRLV

>XP_022641994.1

MTYSNSFRVFDRIKRVIFVCVLLAAIDNNNSNNSSYPVTDFAYDNDSPQIKFDVFVSFRGTDIRQDFLSHLIEAFSQRHIN
AFVDNKVVRGDGMSEALIRAIEGSSISLIIFSQDYASSHWCLSELVKIVECRKKNGQIVLPVFYKVDPAHVRHQKGTYEHA
FAKHQIRYSLTTMQIWRTALTEAANLAGFHSSTFRDEAEFIKEIVKCVLSRLNQVQQGKAKGLVGVGKRIAHVESLLQSE
EPDVRIMGIWGMGGIGKTTIAQEVYDKLCFEYEGCCFLANIREESGRLGMISLKKKLFSTLLGGEDLKIDTPNGLPQYIER
RLRRMKVLIILDDVNDSDQLEVLAGTHDWFGSGSRIIITTRDKQVLAREFASIYEVEALNFDESLRLFNLNAFKQNHLESE
YHELSKKVVNYAKGIPLVLKVLGHLLHGKDKETWESQLERLKKVQNKKVHDIIKLSYNDLDRDEKKIFLDIACFFDGLNLK
VKHMNFLLKDHDYSVVAGLERLKDKALISVSQENGVSMHNIIQETAWQIAREESIDNPRSQIRLLDPEDIYHVLNYNKG
DEAIRSIVINLSRIKQLQLNPQVFARMSKLHFLDFYSKGSCSCLRDQGGLYLPQGLESLSNELRYLRWTHYPLESLPSKFSA
ENLVELNLPNSRLKKLWQEAPEDLVNLRVLILHSSTRLKELPNFSKATNLKVIDLRFCVRLTSVHSSIFSLRNLEKLYLGGCLS
LRSLRSNVHLDSLRYLSLYGCMSLKDFSVTSKNMVKLNLELTGIKRLPSSFGLQSNLQKLRLAYTYIDHLPTSIKHLTRLRHL
DLRYCRQLRTLPELPASLETLDARGCISLETVTFPSTAGEQLKENKKRVAFWNCLKLDEPSLTAIELNAQINMMKFAHQH
FSLFGDAQSTYVYPGSKVPEWLVHKTTHDDFVIIDLSSVLSPHSSHIGFIFGFVVPEVPFGGSALEFKISISGEGSHINVYM
DRPRHRITSDHVYLMYDQACSRYLNGRAKHEPRLKIKVALASRTLTSKYVPLKLRAFGISAINTTDFLSFVQKVKFGDNVP
NVPILSRFFCTFCIVVFVGTFNICIRRLV

>XP_022641995.1

MTYSNSFRVFDRIKRVIFVCVLLAAIDNNNSNNSSYPVTDFAYDNDSPQIKFDVFVSFRGTDIRQDFLSHLIEAFSQRHIN
AFVDNKVVRGDGMSEALIRAIEGSSISLIIFSQDYASSHWCLSELVKIVECRKKNGQIVLPVFYKVDPAHVRHQKGTYEHA
FAKHQIRYSLTTMQIWRTALTEAANLAGFHSSTFRDEAEFIKEIVKCVLSRLNQVQQGKAKGLVGVGKRIAHVESLLQSE
EPDVRIMGIWGMGGIGKTTIAQEVYDKLCFEYEGCCFLANIREESGRLGMISLKKKLFSTLLGGEDLKIDTPNGLPQYIER
RLRRMKVLIILDDVNDSDQLEVLAGTHDWFGSGSRIIITTRDKQVLAREFASIYEVEALNFDESLRLFNLNAFKQNHLESE
YHELSKKVVNYAKGIPLVLKVLGHLLHGKDKETWESQLERLKKVQNKKVHDIIKLSYNDLDRDEKKIFLDIACFFDGLNLK
VKHMNFLLKDHDYSVVAGLERLKDKALISVSQENGVSMHNIIQETAWQIAREESIDNPRSQIRLLDPEDIYHVLNYNKG
DEAIRSIVINLSRIKQLQLNPQVFARMSKLHFLDFYSKGSCSCLRDQGGLYLPQGLESLSNELRYLRWTHYPLESLPSKFSA
ENLVELNLPNSRLKKLWQEAPEDLVNLRVLILHSSTRLKELPNFSKATNLKVIDLRFCVRLTSVHSSIFSLRNLEKLYLGGCLS
LRSLRSNVHLDSLRYLSLYGCMSLKDFSVTSKNMVKLNLELTGIKRLPSSFGLQSNLQKLRLAYTYIDHLPTSIKHLTRLRHL
DLRYCRQLRTLPELPASLETLDARGCISLETVTFPSTAGEQLKENKKRVAFWNCLKLDEPSLTAIELNAQINMMKFAHQH
FSLFGDAQSTYVYPGSKVPEWLVHKTTHDDFVIIDLSSVLSPHSSHIGFIFGFVVPEVPFGGSALEFKISISGEGSHINVYM
DRPRHRITSDHVYLMYDQACSRYLNGRAKHEPRLKIKVALASRTLTSKYVPLKLRAFGISAINTTDFLSFVQKVKFGDNVP
NVPILSRFFCTFCIVVFVGTFNICIRRLV

>XP_022641996.1

MTYSNSFRVFDRIKRVIFVCVLLAAIDNNNSNNSSYPVTDFAYDNDSPQIKFDVFVSFRGTDIRQDFLSHLIEAFSQRHIN
AFVDNKVVRGDGMSEALIRAIEGSSISLIIFSQDYASSHWCLSELVKIVECRKKNGQIVLPVFYKVDPAHVRHQKGTYEHA
FAKHQIRYSLTTMQIWRTALTEAANLAGFHSSTFRDEAEFIKEIVKCVLSRLNQVQQGKAKGLVGVGKRIAHVESLLQSE
EPDVRIMGIWGMGGIGKTTIAQEVYDKLCFEYEGCCFLANIREESGRLGMISLKKKLFSTLLGGEDLKIDTPNGLPQYIER
RLRRMKVLIILDDVNDSDQLEVLAGTHDWFGSGSRIIITTRDKQVLAREFASIYEVEALNFDESLRLFNLNAFKQNHLESE
YHELSKKVVNYAKGIPLVLKVLGHLLHGKDKETWESQLERLKKVQNKKVHDIIKLSYNDLDRDEKKIFLDIACFFDGLNLK
VKHMNFLLKDHDYSVVAGLERLKDKALISVSQENGVSMHNIIQETAWQIAREESIDNPRSQIRLLDPEDIYHVLNYNKG
DEAIRSIVINLSRIKQLQLNPQVFARMSKLHFLDFYSKGSCSCLRDQGGLYLPQGLESLSNELRYLRWTHYPLESLPSKFSA
ENLVELNLPNSRLKKLWQEAPEDLVNLRVLILHSSTRLKELPNFSKATNLKVIDLRFCVRLTSVHSSIFSLRNLEKLYLGGCLS
LRSLRSNVHLDSLRYLSLYGCMSLKDFSVTSKNMVKLNLELTGIKRLPSSFGLQSNLQKLRLAYTYIDHLPTSIKHLTRLRHL
DLRYCRQLRTLPELPASLETLDARGCISLETVTFPSTAGEQLKENKKRVAFWNCLKLDEPSLTAIELNAQINMMKFAHQH
FSLFGDAQSTYVYPGSKVPEWLVHKTTHDDFVIIDLSSVLSPHSSHIGFIFGFVVPEVPFGGSALEFKISISGEGSHINVYM
DRPRHRITSDHVYLMYDQACSRYLNGRAKHEPRLKIKVALASRTLTSKYVPLKLRAFGISAINTTDFLSFVQKVKFGDNVP
NVPILSRFFCTFCIVVFVGTFNICIRRLV

>XP_022642034.1

MDFLGPFGKVVEGVVDFVWKHGVRHMTYIVHYKKNVVELSDTVKDLRFEKEKIDHKCEEGTKNLHNVEGKVIEWVRK
VSEIETTVDVFENDDGHTRARSPNCFVFPYLWNRHRENVTSNDVTLSNSGFEQFSSTKSTVEKVMRELENSGVRMIGLY
GEGGVGKSALIKEIARIARDKKLFNVVVKVEVTANPNIQSIQEEIAYVADCLRRRLKKEKGNTLLILDDLWHKLDLNKLGIP
LDDNDDNDDLSNDMRDLDDKFLKKEKNNKDFNQKVLKREKIIGGHKGCKILLTARQKRVLEVEMDVKSTFRVEPLDDK
DALMFFQKLSEIHNMSDSRKEIVRKYCAGLPMAIITVAKALRGKSELVWEAALGELKKQELVGIQTNMDISVKMSYEHL
ENEEIKSIFLLCAQMGHQPLIMDLVKCCYGLGILEGVFSLSEAREKIKITIQKLKDSGLLLDGNSDIHFNMHDIVRDAALSIA
KKDKHVFTLRNGKLDQWPELEKCTSISICNCDIIVKLPIVNCSQLNFFQIDTNNQFLTIPDKFFEGMKNLKVLILTGFHLKEF
PPSIKGLLKLRMLCLERCTIEDNIAIGELEMLRILSFSGSQLKSLPTVLGCWDKLTLLDINDCSILEVNIPPDILSWLTHLEELYI
RKSIGQNSILCELKNLHQLKVVDLSIPSFSVLPNHLFYDKLQDYKIVVGEVEKFSVIGFKMPDKYETSRVLALHDVXKIDSH
KDINLLFKTAQSLLLGKVEVVKVVNELSIDGFPDLKHLSIIDSNAIKYVNSMQLSNCINVFPNLESLCLYNLKNLKMISIGPL
KVASFSKLKSIKVNMCEGLVSFYSVSMVEFSNSEEPCEIIECNSYLDNFCASLETIEVSECKSLKEILKIPKKCHKVKFLKLQTL
TLQSLPSFKCFYTKVEESQAKNSGSEEDQKSDKAPLFGEQVEIPNLESLNLCSLNMHKIWSDQFLSSFCFQNLIKLVVKEC

DELTYLCSLSVASSLKKLKSLIISECPYMEKIFETKENNADKVCVFPKLEEIQLTKMKRLRDIWHTKVNDDSFSSLISVNIEEC
NKLDKIFPSNMEGWFESLDNLKVSRCKSVEVIFEIKDCEEIDVSGEIDTNLQVILLEYLPKLKELWSKDPHGILNFKKLRTID
VSSCHKLRNLFPASMTKDVSKLERMSVLNCTEMVEIVSEANTDPLEFPELINVRLLLLPNIKQFYAGRLPIKCPKLKELTVY
NCLKLKTFSKEISKTTEEEEEEEEEEEEEEENFVFSAQKVHNVPLFLEFTKRKCVVNFFFFYIKRSE

>XP_022642037.1

MDIVWKIFDPAIDFVRDHGINQVTYIFCYTKNFEELKKKVKRLGEVKERLDRQHDEAKRKGHIVEATVEEWFGEVGEFES
SVENYSKNAGHKKTRGLYYLFPYYRHKLGRQAKKMEIEALRLTDESPKIDEVSHAEKVTSFDLTSSNSGYIEFNSRKSIMED
IMKKLKDPNMKIIGLHGSQGMGKSTLIKKIANKAKYEGLFDRVAEIDVTINPNPLKIQEDIAYVLGLPLAGESENVRADYL
RRWLRIENVSILIILDNLHERLDLNRLGIPIDDDYDLRKKNELSIRSSKQGSNPNLNRTQDTVGGTRGTDKKVLKKTNFLAD
YKGCKVLISSCYKKVFPNEIDVESNFCLKELDGNDALKLFEKVTGGGDKMSMPKEEIQNYCTGLPMRIVTFAVAFKNWS
ESESEPTLEKFKKQGLVEWKESSEIPNKIKYDLPKNKELKFIFLLCAQMGHLPLVNDLVKYCFGLGILEGVSSLSAAREKINE
VIQELKNLSLVSYENPNIHFSMPYMVRDYALSNALIDNNVFVFRDGKLDYWPNLEKCISISICNSDIIDGFPQVINCPQLN
FLQIETKDPSLEIPERFFSSMKNLLVLILRGFHLSCLPYSIEDLLNLRMLCLERCTLDCNLSVLRKFKKLRILSFSGSQLKNLPV
ELRFLNKLRLLDISDCFELKTIPPNLISNLACLEELYIRKSLITEESKGQNAFLSELMNLHQLKVVDLSIPCVSIMPNPLFFDML
KDYKIEIGDFEMISVGEYMMTNKYEELKVLALQSNDDIDIHSHKGIKLLLKTAKSLLLGNVRVQNVVNELNIDGFQDLKHL
SIINNNDVKYLNSTNLSNCVNIFPNLESLWLYNMMNLKMICDGPITLESFAKLKTIKVEMCYQLENLFSYAIEISTSTRSSEI
IECNSYMKKVLASLEMIEVCECVSLKEILQIPMDCKKVKFLRLHTLTLQSLPSITCFNAKVKTSCRQHSTEAQTTNRDHTEI
SAEEDDHSDNAPPLFGELVEVPNLENLNLSSLNICKIWSDQHLSSFYFQNLIRLVVKDCDKLTHLCSLSMASSLKRLKSLVL
SGCRIMENIFEIEGISANKVCVFPKLEEIHLSNMKRLTNIWQTKVSVDSFCSLISVSIEKCNQLDKIFPSHMEGWFESLINLK
VSNCKSVKEIFEVTYYEEIDEYGGIDTNLQVILLEDLPKLKELWSKDPHGILNFKKLRTIDISNCEELRNLFPASMAKDVSKL
ERMSILHCERMVEIVSSKDASEANNDPLEFPELSYVRLYELENMKQFYKGRHPIKCPKLKELSIGKCTKLKTFSQETSDEKF
VFSAEVVFPKLEYLEIDFEEAQNLLSKYQMQNLKELIILSAVNSPDLFYPFLYKMPNLEKLKLTFNPFYKSIESLRSTNIGQH
DRLGVVLKLKQLFIRSSNIKDLGFDRDQVLQRLELLSLKYCYGLNTLAPSSVSLSYLTCLKVKNCMGLRNLMASSTAKSMV
QLKTMKVINCYDIEEIVSNEETEEGKVMKIVFSKLISIELVGLKNMASFCSYKECEFEFPSLEILIIRGCPKMEKFSEKRAIAAK
LKDVFDVEGDQKAKWQWRGDLNATIQKVFNDKQVWDDTHWRQQNKFGYLKRLSVWGCHTLKHIIPSHLLSCFHNL
EELKVWKCKNAEVIFKMNDENRVMTKPSGIFRLKTLSLSFLAKLKHVWEKDPEGIIGLQLLKEMHVQYCKSVKSLFPASV
AKDLTRLEVLEVTECEELAEIFKKDEKDEERTTQEFVFRDLTSLTLEELPALKYSIHCSKQQVILKTLPLRGHSKVI

>XP_022642038.1

MDIVWKIFDPAIDFVRDHGINQVTYIFCYKKNFEELKKKVKRLGEVKERLDRQHDEAKRKGHIVEARVEEWFGEVGEFE
SSVENYSKNAGHKKTGGRYYFFPYYRHKLGRQAKKMEIEALRLIDESPKIDEVSHAEKVTSFDLTSSNSGYIEFSSRKSIME
DIMTKLKDPNMKIIGLHGSLGMGKSTLVKEIANKAKYERLFDRVAEINVTINPNPLKIQEEIAHVLGLPLTGESENVRADY
LRRWLKIENVSILIILDNLHERLDLNRLGIPIDGDYDLRKKNELSIRSSKKDTDDNTQGTDEKVLEKANFLSKYKGCKVLLSS
RDKKVFPDEVDVESNFCLKELDGNDSLKLFEVHGGGNKMSMPTEEIQKYCAGLPMSIVRFAKLFKNWSESESEPTLEKF
KKQGLVEWKESSEIPNKIKYDLPKNKELKFIFLLCAQMGHLPLVNDLVKYCFGLGILEGVSSLSAAREKINEVMQELKKLSL
VSYENPNIHFSMSHMVRDYALSNALMDHNVFSFRDGKLDYWPNLEKCISISICNSDITCRLKYYKIGIGDFEKFSIGEFKM
PNKYEELKVLALQLKDDTDIHFHKGIKLLFKTAQSLLLGKIGVQNVVNELNIDGFQDLKHLSIINNNDIKYVNSTDLSNTVN
IFPNLESLCLYNMMNLKMICDSSITLESFAKLKTIKVEMCYQLENLFSIYAIKISTSTRTSEIFECNSYMKKFLASLETIEVCEC
ESLKEILQIPMDCDKVEFLKLGTLTLHSLSSLTCFNTKVKRSCWSHSTEAQTRNKGHTEISTDDDHSDNVPLLFGESVEVP
NLENLNLSSLNIHKIWSDQHLSSFYFQNLIKLVVKDCNKLTHLCSLPMASSLKKLKSLIISGCFIMESIFEIEGTSANKVFVFP
KLEEIHLSNMKRLRDIWHTRVNIDSFSSLISVNIEECNQLDKIFPSHMEGWFESLINLKVSNCKSVKEIFEISDYEEIDEYGGI
DTNLQIILLKDLPKLKELWSKDPHGILNFKKLRTIDINNCEELRNLFPASMAKDVSKLERMSILHCKRMVEIVSSKDASEAN
NDPLEFPELSYVRLYELENMKQFYKGRHPIKCPKLKELSIGKCMKLKTFSQETSDEKFVFSAQVVFPKLEYMEIDFKEAQN

LLSKYQMHNLKELIILNVQPSNIFYRFLYRMPNLEKLKLTGYYSESLEPLWSTNGQQDRLGVVLQLKQLFIHSSNIKDLGFD
GDQVLQRLELLSLKYCYELNTLAPSSVSMSYLTCLKLKNCWGLRNLMASSTAKSMVQLKTMKEHDKFLQLQEL

>XP_022642085.1

MESLEMSSTFGEKHDVFLSFRGADTRTNFTSHLLNALTQKSISAFIDYELIRGDYTWPALETAIEKSLLSIVVLSENYASST
WCLKELAHILECRRKRGMVVIPVFYEVDPSHVRKLSGSFEKSFAKHERDSTSFDRISQRKDVSMWKAALKEVANISGWD
SRSYRDEAQVIQNLVNDVLQKLHLRYPTELNGLVRIKNTCAKVDLLLRKSRVIGIWGMGGIGKSTIAKALFAKYFPYFDHV
CFMANANEFSLDKLFSELFREEVSASNVVGSSFDMRRLKRKRIFIVLDDMDCLDLLEYLCREYQSLDPNSKLIITTRDKQLL
EGRVDQIYEVKKWETRASLKLFCLEAFKKRHPKRGYESLSESAVEYAGGVPLALKVLGSYLRSKGINFWESTIRKLSMYPN
ERIQKVLEVTYTGLHDLEKNIFLDIVFFFKEKQKDHVITILDACGFEATSGIEILADKALLTISYRKIIHMHDLLQQMGLEIVR
QESSGDPGRRSRLKDKEAREVIEENKGTSAIQGIALDLSQIKGLILHADTFTKMKTLRFLKFYNKLGQSARDTYLDLPATLE
PFSDKLRYIEWIGYPFESLPSPFCAKVLVEIHMPHSKVKQLWQGIQELNYLEGINLRQCKQFEELPDLSKAPRLKWVNLSC
CESLCYLHPSVLSSGTLVTLILDKCTNLKSVKSEKDLKSLEKISVNGCLNLVEFAVSSDLIENLDLSNTGIQMLGTSIGSMHKL
KSLNLEGLKLKPVLKELSCLTSLKVLKISDNGLVIDKQQIHVLFSGLRYLQILYLKDCSKLFELPDNINLLTQLQELRLDRSDLK
RLPENIKNLQMLEILSLEDCKELLCLPTFPSLIKYLRVINCTSLVSVSNLKTLAIEMLGMTKRITFKNSGKLDEDSLRIIMESLH
LTMMSAAYHNVLVRKTYGTYNSCNYTSVELCLPGGSVPEQIHYRSTQSSITIVLPPHSELLGFIYSVVLSPAGGKKTFGTKI
FCKCHLPEEGIKATWLYGDIRGLKSDHVYVWYDPLHCDSILKYYKQSKVCFEFCVANDKGEVDGSICIKECGVGLVNVLE
MHSVLQELDFDSDKKKELVEGVESESKHRKLDWSESSSSDSLTTPRSSSVTQSEIVEMIELENTESTCKETLSTAERGPKEK
LRESTKIVADEHLQSTSLQASSQGGSEHLHDRLEESKKQVVETYDTEKFVTKYSYFDLESCLQQLDENPFAILDLLSNELSP
LKQSETCVQRVAQANDATTVLNEFRTLVFSTSLLEKLPDQSYRQQIEESLRKLHTYRREITEEQEGVDKFIELYEKAANISQ
EKMLTEDKQTKLASKKRDLYNKLQDSKLKVQQFDTAISTDKSEIENLQKRQREIQEAINKLQQENEALEKERSALEVLYSG
KQTKKNETLELVKHISTSVVYTTKQLEELEEKRLSLASAYEDLKEPYGTMKTKPPF

>XP_022642096.1

MASSNPSIEFGSSTSKLPQKYDVLINFNGEDIRRKFVSHLDYALSTVGLTTFLHEENAVKGMHIQQPILNLCRVAIVVFTKT
YSQSAWCLHELQQIIKWQETYSRHVLPVYYEIQPSDVRLQKGDFGKSFKATAQETFPWQQLEHGMSRWSHALTKAAN
FFGWDERNYRSDAELVDIIIKGVVNLPVLSATKFPVGLQSRVKDVIQIIKNKSTEVCVIGIWGEGGSGKTTLAKAIYHQLH
GTFTEKSFIEDISQFSQTRGHVHLQEQLLSDVLNTKIEIRSIEMGKCMIREKLSGKKLLIVLDDTKYDPLLDLCDSHVWLAK
GSVIIITAREESLLRIPEIGSVISINLLSTNESLELLSWHAFREAKPEEEYNDLAKSVAVCCGGLPLALEVIGSRLFEKTKEEWK
SVLLELKEIHNHDVHRKLKISFDGLSNEMEKDLFLDVCCFFVGKGRAFVTKILNDCGVDADSGIRILIERGLIQVKKNNKLG
MQPMLQKMGRKIIRQISGKELGKNPQLWFGQDAEYELLENTLFSSQQTKVIRKLPLKMFLIATRELFEHPSVVRDKSRLL
KLTRDFGKLRWISLQGFSSEYLPEDFYLQDAMVIDLKHSLLRFVWKEPQVLMWLTVLNLSHSKYLRETPDFSGLPRLEQLI
LKDCSRLRKVHHSIGCLNNLVLLNLKDCTSLSNLPREDNGWNDRLPLLSTLANLRSVLVQCDTEFQLCKQVETFLIEYGVN
VTKSGISQKNLKYSLIGVGRCKDFFDAVSDSISQVFASNESRDVSLPGDNDPYWLGHMGKGHSVSFTVPRDRDLKGMA
LCVIYLSTPEIVATECLRSVLIVNYTKCTLQIHNHGTVISFNDIDWQGIIPNLGSGDKVEICVTFAHELVVENIVVYLICSELN
DSQKEPAPKKNSLIRFIKKVVM

>XP_022642102.1

MGQPTYSMFRIERMNAEESLELFSWHAFREPKPKEEYEDLARRVVSYCGGLPLVLEIIGSSLFERTEEEWHRVLFAIDGR
HRVSEIIKVSIEGSLNEMEKYIFVDVCCLFVGKSRAYVRKILKGCGVDADIAIRVLIQRNLIKIDNNKKFRVHPLLQNIGGQII
HEKVVGDFEKNRPPSKYVRTFFTCGLKLLLKAFSFSCAVQLFFFYLLFSSQETEALQWLPAKLLAAIESVQPTLNSQYLFKKL
RWISSYGLSSECLPNNFYWDDATVIDLKRSLLQFLWKTPQVLRSLKVLNLSHSTDLTTTPDFTGLPSLEHLILKYCSRLRKL
HQSIGSLNSLILLNLKFCTSLNNLPTEIYELKSLRTFILSGCSKIHIMDKDIAKLESLITLIADTPVKHVPFSIVSSKGIGYMSLLG
FEGLSYNLFPSIIRSWMSPTMNSISYIHSFCMDIEDNTIDIAPLLCTLAHIRSVLVACETEFQLSKQVTNILVEYFANITELGIS

KQHLRCSLIGVGAYHQFLNAISDNIYELLGSSESGDVCLPTVNDPYCMAHMGEGHSVSFTVPEDRDMKGMALCVVYLSNPKIIEPEFTTVIIVNYTKCTFQIHHHGTVISFRDEDWHDIMSNLESGDNVEIFVNFGNGLVVKNTTVYLIWGEAENMEKASEPKKHSLIRFVKKVVM

>XP_022642103.1

MGQPTYSMFRIERMNAEESLELFSWHAFREPKPKEEYEDLARRVVSYCGGLPLVLEIIGSSLFERTEEEWHRVLFAIDGRHRVSEIIKVSIEGSLNEMEKYIFVDVCCLFVGKSRAYVRKILKGCGVDADIAIRVLIQRNLIKIDNNKKFRVHPLLQNIGGQIIHEKVVGDFEKNRPPSKYETEALQWLPAKLLAAIESVQPTLNSQYLFKKLRWISSYGLSSECLPNNFYWDDATVIDLKRSLLQFLWKTPQVLRSLKVLNLSHSTDLTTTPDFTGLPSLEHLILKYCSRLRKLHQSIGSLNSLILLNLKFCTSLNNLPTEIYELKSLRTFILSGCSKIHIMDKDIAKLESLITLIADTPVKHVPFSIVSSKGIGYMSLLGFEGLSYNLFPSIIRSWMSPTMNSISYIHSFCMDIEDNTIDIAPLLCTLAHIRSVLVACETEFQLSKQVTNILVEYFANITELGISKQHLRCSLIGVGAYHQFLNAISDNIYELLGSSESGDVCLPTVNDPYCMAHMGEGHSVSFTVPEDRDMKGMALCVVYLSNPKIIEPEFTTVIIVNYTKCTFQIHHHGTVISFRDEDWHDIMSNLESGDNVEIFVNFGNGLVVKNTTVYLIWGEAENMEKASEPKKHSLIRFVKKVVM

>XP_022642132.1

MDVSSSLSIFSKFIVAGEVFIHFLGDDIHRNFVSHLSSSLLQAGVKPSLIAVEMLSEKFMPSITRFQIGIVVFTKAYIESSRCVEDLLRIIECHENHGLIVMPVFYDIDPSDLLDLKMDSSSYVLYPMKGELKKNSSPEQRSAKRKVKTTVKWTQMKGERLNYPIILYTSAGSRALNRVANLPTWDESKHRNDAELVEEIVKSVLSKLECALPVTKFPVELETQAKNVIGLFENQPKDFCTIGIWGMGGSGKTTLAKAIYNKIPFTFGDKSFIQDIRKVCQTDGRRGLVQLQEQLLSDVLKYVKIEKGEMEKTTIENSLSGRKLFIVLDDVNEIDQLKNLCGNGKWFRAGSVIIITTRQLDLLYQQKVDYVYEMDELDENDSVELFSWHAFRKATPRTELNNLARSAVAYCGGLPLALEVLGSYLSKRSENEWRSVLSKLEIIPNTQIQNILRISFDGLCSEMEKDIFLDVCCFFIGKERDYVTEILNGCGLRADIGIKVLIERGLVKIEKNNKLGMHHLLRDMGREIVRQTSTMQPGKRSRLWFLKDVRHVLTKNTGTEAILGLSLNTDWRL

>XP_022642186.1

MEFASSSLSSSSSFLTSQPHFIHDVFINFGGEEIGRRFVSHLHSVLLQAQVKTTISQENLQEGMKLEEQIRAIGGTKITIIVFSKSYAESTCCLLELEKIIECHQTFGQILVPVFYEINPWVVRHQMHDFGKALEEAAHKSYSGEQVEHALSKWRRALTAAAGMTGWEFRNYRHDAELVDKIVNRVKTLLDYKDLFITEYPVGLESRVEDVIKCIENQSTKVCMIGIWGMRGSGKTTIAKAIYNRIYREFIGKSFIENSGYEGYVAMQENLLSDVLKSKLEVKNVQMGRTKIQNGFSRKKLLIVLDDVNHIGQLENLCGSREWFGQGTVIIITSKDIHLLNQFRSNYVYKMHLLNENESLELFSWHAFRDAIPEKEWSVGKCIVEKLARNVVVYCGGLPLALEFLGSYLGDRTIEVWESVLLKLKRNPPNEPLSVLKVSFEDLRDTEKDIFLDVCCFFIGKDRDYVTEILNGCGLHADIGITVLIERGLIKVERNNKLQMHPLLQEMAREIIRQECLEKPGKRSRLWFHDDVEDVLKENSGTESILSLKLDFGIADCFEAHAFKKMKRLRLLQLDHVQLSGDFGNISDQLRWICWRGFPYKDFPRNFHMQNVIAMDLKHSLLLHLGWQRGVVLERLKFLNLSHSKYLIGTPNFSRLPSLEQLILKDCPSLLKVHQSIADLSNIVLINLKDCTSLSYLPREIYKLRSLKTLILSGCSKLGPIDIKQVKSLVNIIA

>XP_022642187.1

MEFASSSLSSSSSFLTSQPHFIHDVFINFGGEEIGRRFVSHLHSVLLQAQVKTTISQENLQEGMKLEEQIRAIGGTKITIIVFSKSYAESTCCLLELEKIIECHQTFGQILVPVFYEINPWVVRHQMHDFGKALEEAAHKSYSGEQVEHALSKWRRALTAAAGMTGWEFRNYRHDAELVDKIVNRVKTLLDYKDLFITEYPVGLESRVEDVIKCIENQSTKVCMIGIWGMRGSGKTTIAKAIYNRIYREFIGKSFIENSGYEGYVAMQENLLSDVLKSKLEVKNVQMGRTKIQNGFSRKKLLIVLDDVNHIGQLENLCGSREWFGQGTVIIITSKDIHLLNQFRSNYVYKMHLLNENESLELFSWHAFRDAIPEKEWSVGKCIVEKLARNVVVYCGGLPLALEFLGSYLGDRTIEVWESVLLKLKRNPPNEPLSVLKVSFEDLRDTEKDIFLDVCCFFIGKDRDYVTEILNGCGLHADIGITVLIERGLIKVERNNKLQMHPLLQEMAREIIRQECLEKPGKRSRLWFHDDVEDVLKENSIALKLMLSRK

>XP_022642189.1

MDPIVSATTESALNITTSVVKRQVGYFFNYKDKFKELKSYIEKLEHNKERLQHQVDSALRSGGEIEKDVQRCLTLMDDKIK
EYKSYINDDCHAKTICSIGFFPNNFRLRYQLGRKATKMVEEIIGDELWKTFFDNVSYQEFPSIDAAFSNNGYESFASRTKT
MKMIMKALQDSTVGMIGVYGPGGVGKTTLVKEIANKAREKNLFEIVIIANITGNPDXKKIQEQIAGMLGMKLEXESEIA
RVDRIRKRLKNEKENTLIILDDLWGGLDFNKLGIPCNDDASQQEVNDISDFGYNKIEIKELSKVDLDKMKKEKLSNDYRRG
KILLTSRNKQVLCNEMDVQQRSIFSVGVLDEKESETLLKKVAGVKNSEFDRNATEIAKWSAGFPIALVSIGRTLKNKSLST
WEDVCQQIKRQNFTSEWGFTDFSIKLSYDHLKNEELKCIFLHCARMGNDALIMDLVKFCVGLDLLPEVYTITDARKRVKE
IIQELEESSLLVKSYSNDRFNMHDIVRDVALSISSKEKQVLYKKNAILYEWPHENDFERYSAIFVHFCDINDKLPESIHCPRL
EVLHIDNKNESFEIPDEFFKSMVRLRVLVLTGIHFSCLPSSIKCLKKLRMLCLERCTLGENLSIIGELKNLRILSFSGSNIENLPL
EFGQLDKLQFLDISNCLKLRQITSNIIPRMGILEEFYIRDNLIIWEAEENMKSENASLSELRHLNQLQNLDIHIHCSSYFPQN
LFFDRLNSYKIVIGEFNLSNLLKVGEFKVPDKYEEVKFLALNLKEGIDIHSEKWIKMLFKSVECLLLGELNDVQDIFYELNVE
GFPNLKHLSIVNNFDINYIINPKERFHSLVAFPKLESIWLYKLDNLEIICNNQLVETSFRNLKVIKIQTCLKLVNLFLFSMVRLL
TLLETIEVCDCDSLKEIVSKESQTNTISDDKIQFPQLRLLTLKYLPTFIYLYNVVDKIPGSSHSLQDQVFQQRNKDIVVDVEH
MVTNSCLPLFNEKVSTPKLEWLELSSINIHKIWSDQCNHCFQNLLTLNVTDCSNLKYLLSFSMAESLVNLQSIFVSECAIM
EDIFRPEDAEYIDVFPKLKKMEIICMDKLCTIWKSDIGLHSFSCLNTLMIRECHKLVTIFPNYMGQRLQSLQSLTVTDCKLV
ENIFDFANIPHTYDIIETNLGKIILEDLPNLVNVWKGYTGEILKYNNLQSIRVYESPKLKYLFPVSIANDLEKQEVLEVRNCG
AMTEIIALDKHSSETFITFKFPHLNTLSLIDLHDLRSFYSGIHTLEWPPLKKLDIINCSMLEGLTSEITNSKEQPIVLATKKAIY
NLEYMSVSLKEAEWLQKYIVNVHRMHKLEELTLYGLKNNEILFWFLHRLPNLKRLTLELCHLKRIWALKSLISREKIGGVV
QLKELKLESMWSLEEIGFEHEVLLQRVERISIHHCTKLKNLVSSSVTFSYLTYLEVMNCKSMRSLMTCSAAKTLVQLTTLK
VCSCPMIVEIVADNKVEKVREIEFKQLKSLELVSLQNLTSFSKVEKCDLKFPLLEKLVVSECPQMTKLSEVQSAPNLQKVH
VEAGEKDKWYWEGDLNATLQTHFTNQVSFEYSKYINLVDYPEKKVPHDKFAFPDNFFGYLKKLEFDEACKRDTLIPSHV
LPYLKNIEELNVEKCKSAQLIFDIDESEIQTYGMVFRLKNLTIKNLSNLKCVWKENLEGIVSFSNLQKVDVDGCGSLLTLFPL
SVAKDLGKLKTVDIKECEKMIEIVGREDEREHGTTIMFEFPCLSYLNLDNMPLLSCFYPGKHHLECPLLEKLYVACCPKLKL
FRSSFDDDSKKEVLEAPTNLLQQPLFSIQKVSPKPVGLTLNEENIKLMSDARLPQDLLCKLKFLILSFEDDNNGTDSLPFDF
FHKLPNLESLTVQKCSGLKEIFPSQKLQVHDSILVGLKGLFLFELSELESIGLEHTWVQPYTEKLELLYLLLTPQVENIVSCAV
SFINLKDLFVMFCEKMEYLFTSTTLKSLVKLETLLIGYCGSIKEIARNEEEDGCDEIIFGRLRSIKLEYLPRLISFYSGNATLQCP
CLQNVMITECPNMITFSEGVIKLAMFSGIQTFEDSDFTFHDDLNTTVESLFHEKEFFNHSKHMILDEYLEITGAQHIKPDI
ADNFFGSFKELEFNAACKRAIVIPFHLLPYLKNLEKLNVHSSDAVNVIFDFDESEDKTKGIVSSLKELTLKNLSNLKCVWKE
NLEGIVSFPNLEKVTVTGCRSLVTLLSSSLAKSLEKLKTLHMARCEKLEEIVGEEDEREHGMTLTFEFPCLTILFLFDMPLLSC
FYPGKHYLECPILDTLYVSYCPKLKLFTSDTDDSHKEEVIEAPISPLQQPLFLVEKVSPKLKKLALNEKNIMLLRDGCLLHDLL
CKLSHLWFLFEDYKIENDTLPFDFFHKLPGLEYLLLQNCFGLKVIFPSQKLQVHNKVLAGLKQLSLLRLRELESIGLENEWV
QSYSRKLEVLKLDTCPQVENIVSCSVSFINLTKLSVKLCEKMEYLFTFATLKSLVKLQTLSIKNCESIKEIVKKEDEDACDEIVF
EQLRSIKLNSLPNLLSFYSGNATLKCLCLQSVMVAKCPNMITFSEGVINMPILSGIKTSKDSVVVFHDNLNTTIETLLHEQE
FMEYSKRMILEDYLGMSGVHHRKPVVSDNFFGSFKKLEFDGACNRTILIPSHVLPYLKNLEELNVKNSDAMQIIFDIDESK
VKTKGVVFGLKKLTLNKLSNLKHVWKENSTGIISFHNLQEVVVNGCGGLIRLFSSSLARNLWKLEELRIKECGKLVEIVEKE
DGTENGTKIMFEFPCLTSLYLKNMPLLSCFYPGKHDLDCPLLEILLVCFCPKLKLFTSDFDENQKGAIEAQISPLQQPMFSV
EKFSPKIKVLALNEENIMLFREVQFLQDILCNIVGLVLCFEDDNIEKDSLPFDFFHTLPNVFILAIKKCFGLKEIFPSQKLQVH
DGGLAGLKELFLVDLKELELVGLEHPWVQPYSEKLQVLSLKRCPQLQKLVYCAVSFINLKKLRVKLCERMEYLFTFATVKSL
VKLETLIINSCESIKEIIKHENEDGCAEMVFGRLKSIKLKSLPRLVRFYSGKATLQCSYLKIVMVVKCPSMKTFSEGVMKVPK
FSGIQTSKDSDLIFHEDLNTTIKKLFHEEVEKSACDLEHLKFGDHPNLEVIWLGVVPIPRNDSFNNLQSLAVVECESLSNVI
PFYLLRFLSNLKEIEVSNCQSVKAIFDVKGEGADMKPISLSLKKLILNQLPNLEHIWNLNPDEILSLEDLQQVSISNCQTLKS
LFPTSVANHLIKLHVRACATLVEIFVEAETAFEGETKQFNFHCLTSITLWELPELKYLYPGKHTLEWPMLAHLDIYHCDKLK
LFKIEHHSDEFSDTKDQLGISIHQQPAFSVKKVFPKLVQLSLKKEDAMAILQGQLQVMPSIEHQAITWKDTRIGQGQFG
ANVAYLLQNLKLLKLMCYHEDDKSNIFSSGLLEEIPNIENLEVVCSSFTEIFCSQGPTHDCSKVLSKLKRLHLKNLPQLNAIG

LENSWVEPLLKTLETLEVFSCPAMKILVPSTVSFSNLTSLSVDECHGLLYLFTSSTAKRLGQLKHISIRDCQAIQEIVCKEED
HESEDEDITFDQLSLLSLQSLPNIVVIYSRTFKLKFPCLDQVIIKECPQMKYSYVPDLHEFKPQEQA

>XP_022642190.1

MDPIVSATTESALNITTSVVKRQVGYFFNYKDKFKELKSYIEKLEHNKERLQHQVDSALRSGGEIEKDVQRCLTLMDDKIK
EYKSYINDDCHAKTICSIGFFPNNFRLRYQLGRKATKMVEEIIGDELWKTFFDNVSYQEFPSIDAAFSNNGYESFASRTKT
MKMIMKALQDSTVGMIGVYGPGGVGKTTLVKEIANKAREKNLFEIVIIANITGNPDXKKIQEQIAGMLGMKLEXESEIA
RVDRIRKRLKNEKENTLIILDDLWGGLDFNKLGIPCNDDASQQEVNDISDFGYNKIEIKELSKVDLDKMKKEKLSNDYRRG
KILLTSRNKQVLCNEMDVQQRSIFSVGVLDEKESETLLKKVAGVKNSEFDRNATEIAKWSAGFPIALVSIGRTLKNKSLST
WEDVCQQIKRQNFTSEWGFTDFSIKLSYDHLKNEELKCIFLHCARMGNDALIMDLVKFCVGLDLLPEVYTITDARKRVKE
IIQELEESSLLVKSYSNDRFNMHDIVRDVALSISSKEKQVLYKKNAILYEWPHENDFERYSAIFVHFCDINDKLPESIHCPRL
EVLHIDNKNESFEIPDEFFKSMVRLRVLVLTGIHFSCLPSSIKCLKKLRMLCLERCTLGENLSIIGELKNLRILSFSGSNIENLPL
EFGQLDKLQFLDISNCLKLRQITSNIIPRMGILEEFYIRDNLIIWEAEENMKSENASLSELRHLNQLQNLDIHIHCSSYFPQN
LFFDRLNSYKIVIGEFNLSNLLKVGEFKVPDKYEEVKFLALNLKEGIDIHSEKWIKMLFKSVECLLLGELNDVQDIFYELNVE
GFPNLKHLSIVNNFDINYIINPKERFHSLVAFPKLESIWLYKLDNLEIICNNQLVETSFRNLKVIKIQTCLKLVNLFLFSMVRLL
TLLETIEVCDCDSLKEIVSKESQTNTISDDKIQFPQLRLLTLKYLPTFIYLYNVVDKIPGSSHSLQDQVFQQRNKDIVVDVEH
MVTNSCLPLFNEKVSTPKLEWLELSSINIHKIWSDQCNHCFQNLLTLNVTDCSNLKYLLSFSMAESLVNLQSIFVSECAIM
EDIFRPEDAEYIDVFPKLKKMEIICMDKLCTIWKSDIGLHSFSCLNTLMIRECHKLVTIFPNYMGQRLQSLQSLTVTDCKLV
ENIFDFANIPHTYDIIETNLGKIILEDLPNLVNVWKGYTGEILKYNNLQSIRVYESPKLKYLFPVSIANDLEKQEVLEVRNCG
AMTEIIALDKHSSETFITFKFPHLNTLSLIDLHDLRSFYSGIHTLEWPPLKKLDIINCSMLEGLTSEITNSKEQPIVLATKKAIY
NLEYMSVSLKEAEWLQKYIVNVHRMHKLEELTLYGLKNNEILFWFLHRLPNLKRLTLELCHLKRIWALKSLISREKIGGVV
QLKELKLESMWSLEEIGFEHEVLLQRVERISIHHCTKLKNLVSSSVTFSYLTYLEVMNCKSMRSLMTCSAAKTLVQLTTLK
VCSCPMIVEIVADNKVEKVREIEFKQLKSLELVSLQNLTSFSKVEKCDLKFPLLEKLVVSECPQMTKLSEVQSAPNLQKVH
VEAGEKDKWYWEGDLNATLQTHFTNQVSFEYSKYINLVDYPEKKVPHDKFAFPDNFFGYLKKLEFDEACKRDTLIPSHV
LPYLKNIEELNVEKCKSAQLIFDIDESEIQTYGMVFRLKNLTIKNLSNLKCVWKENLEGIVSFSNLQKVDVDGCGSLLTLFPL
SVAKDLGKLKTVDIKECEKMIEIVGREDEREHGTTIMFEFPCLSYLNLDNMPLLSCFYPGKHHLECPLLEKLYVACCPKLKL
FRSSFDDDSKKEVLEAPTNLLQQPLFSIQKVSPKPVGLTLNEENIKLMSDARLPQDLLCKLKFLILSFEDDNNGTDSLPFDF
FHKLPNLESLTVQKCSGLKEIFPSQKLQVHDSILVGLKGLFLFELSELESIGLEHTWVQPYTEKLELLYLLLTPQVENIVSCAV
SFINLKDLFVMFCEKMEYLFTSTTLKSLVKLETLLIGYCGSIKEIARNEEEDGCDEIIFGRLRSIKLEYLPRLISFYSGNATLQCP
CLQNVMITECPNMITFSEGVIKLAMFSGIQTFEDSDFTFHDDLNTTVESLFHEKEFFNHSKHMILDEYLEITGAQHIKPDI
ADNFFGSFKELEFNAACKRAIVIPFHLLPYLKNLEKLNVHSSDAVNVIFDFDESEDKTKGIVSSLKELTLKNLSNLKCVWKE
NLEGIVSFPNLEKVTVTGCRSLVTLLSSSLAKSLEKLKTLHMARCEKLEEIVGEEDEREHGMTLTFEFPCLTILFLFDMPLLSC
FYPGKHYLECPILDTLYVSYCPKLKLFTSDTDDSHKEEVIEAPISPLQQPLFLVEKVSPKLKKLALNEKNIMLLRDGCLLHDLL
CKLSHLWFLFEDYKIENDTLPFDFFHKLPGLEYLLLQNCFGLKVIFPSQKLQVHNKVLAGLKQLSLLRLRELESIGLENEWV
QSYSRKLEVLKLDTCPQVENIVSCSVSFINLTKLSVKLCEKMEYLFTFATLKSLVKLQTLSIKNCESIKEIVKKEDEDACDEIVF
EQLRSIKLNSLPNLLSFYSGNATLKCLCLQSVMVAKCPNMITFSEGVINMPILSGIKTSKDSVVVFHDNLNTTIETLLHEQE
FMEYSKRMILEDYLGMSGVHHRKPVVSDNFFGSFKKLEFDGACNRTILIPSHVLPYLKNLEELNVKNSDAMQIIFDIDESK
VKTKGVVFGLKKLTLNKLSNLKHVWKENSTGIISFHNLQEVVVNGCGGLIRLFSSSLARNLWKLEELRIKECGKLVEIVEKE
DGTENGTKIMFEFPCLTSLYLKNMPLLSCFYPGKHDLDCPLLEILLVCFCPKLKLFTSDFDENQKGAIEAQISPLQQPMFSV
EKFSPKIKVLALNEENIMLFREVQFLQDILCNIVGLVLCFEDDNIEKDSLPFDFFHTLPNVFILAIKKCFGLKEIFPSQKLQVH
DGGLAGLKELFLVDLKELELVGLEHPWVQPYSEKLQVLSLKRCPQLQKLVYCAVSFINLKKLRVKLCERMEYLFTFATVKSL
VKLETLIINSCESIKEIIKHENEDGCAEMVFGRLKSIKLKSLPRLVRFYSGKATLQCSYLKIVMVVKCPSMKTFSEGVMKVPK
FSGIQTSKDSDLIFHEDLNTTIKKLFHEEVEKSACDLEHLKFGDHPNLEVIWLGVVPIPRNDSFNNLQSLAVVECESLSNVI
PFYLLRFLSNLKEIEVSNCQSVKAIFDVKGEGADMKPISLSLKKLILNQLPNLEHIWNLNPDEILSLEDLQQVSISNCQTLKS

LFPTSVANHLIKLHVRACATLVEIFVEAETAFEGETKQFNFHCLTSITLWELPELKYLYPGKHTLEWPMLAHLDIYHCDKLK
LFKIEHHSDEFSDTKDQLGISIHQQPAFSVKKVFPKLVQLSLKKEDAMAILQGQLQVMPSIEHQAITWKDTRIGQGQFG
ANVAYLLQNLKLLKLMCYHEDDKSNIFSSGLLEEIPNIENLEVVCSSFTEIFCSQGPTHDCSKVLSKLKRLHLKNLPQLNAIG
LENSWVEPLLKTLETLEVFSCPAMKILVPSTVSFSNLTSLSVDECHGLLYLFTSSTAKRLGQLKHISIRDCQAIQEIVCKEED
HESEDEDITFDQLSLLSLQSLPNIVVIYSRTFKLKFPCLDQVIIKECPQMKYSYVPDLHEFKPQEQA

>XP_022642191.1

MDPIVSATTESALNITTSVVKRQVGYFFNYKDKFKELKSYIEKLEHNKERLQHQVDSALRSGGEIEKDVQRCLTLMDDKIK
EYKSYINDDCHAKTICSIGFFPNNFRLRYQLGRKATKMVEEIIGDELWKTFFDNVSYQEFPSIDAAFSNNGYESFASRTKT
MKMIMKALQDSTVGMIGVYGPGGVGKTTLVKEIANKAREKNLFEIVIIANITGNPDXKKIQEQIAGMLGMKLEXESEIA
RVDRIRKRLKNEKENTLIILDDLWGGLDFNKLGIPCNDDASQQEVNDISDFGYNKIEIKELSKVDLDKMKKEKLSNDYRRG
KILLTSRNKQVLCNEMDVQQRSIFSVGVLDEKESETLLKKVAGVKNSEFDRNATEIAKWSAGFPIALVSIGRTLKNKSLST
WEDVCQQIKRQNFTSEWGFTDFSIKLSYDHLKNEELKCIFLHCARMGNDALIMDLVKFCVGLDLLPEVYTITDARKRVKE
IIQELEESSLLVKSYSNDRFNMHDIVRDVALSISSKEKQVLYKKNAILYEWPHENDFERYSAIFVHFCDINDKLPESIHCPRL
EVLHIDNKNESFEIPDEFFKSMVRLRVLVLTGIHFSCLPSSIKCLKKLRMLCLERCTLGENLSIIGELKNLRILSFSGSNIENLPL
EFGQLDKLQFLDISNCLKLRQITSNIIPRMGILEEFYIRDNLIIWEAEENMKSENASLSELRHLNQLQNLDIHIHCSSYFPQN
LFFDRLNSYKIVIGEFNLSNLLKVGEFKVPDKYEEVKFLALNLKEGIDIHSEKWIKMLFKSVECLLLGELNDVQDIFYELNVE
GFPNLKHLSIVNNFDINYIINPKERFHSLVAFPKLESIWLYKLDNLEIICNNQLVETSFRNLKVIKIQTCLKLVNLFLFSMVRLL
TLLETIEVCDCDSLKEIVSKESQTNTISDDKIQFPQLRLLTLKYLPTFIYLYNVVDKIPGSSHSLQDQVFQQRNKDIVVDVEH
MVTNSCLPLFNEKVSTPKLEWLELSSINIHKIWSDQCNHCFQNLLTLNVTDCSNLKYLLSFSMAESLVNLQSIFVSECAIM
EDIFRPEDAEYIDVFPKLKKMEIICMDKLCTIWKSDIGLHSFSCLNTLMIRECHKLVTIFPNYMGQRLQSLQSLTVTDCKLV
ENIFDFANIPHTYDIIETNLGKIILEDLPNLVNVWKGYTGEILKYNNLQSIRVYESPKLKYLFPVSIANDLEKQEVLEVRNCG
AMTEIIALDKHSSETFITFKFPHLNTLSLIDLHDLRSFYSGIHTLEWPPLKKLDIINCSMLEGLTSEITNSKEQPIVLATKKAIY
NLEYMSVSLKEAEWLQKYIVNVHRMHKLEELTLYGLKNNEILFWFLHRLPNLKRLTLELCHLKRIWALKSLISREKIGGVV
QLKELKLESMWSLEEIGFEHEVLLQRVERISIHHCTKLKNLVSSSVTFSYLTYLEVMNCKSMRSLMTCSAAKTLVQLTTLK
VCSCPMIVEIVADNKVEKVREIEFKQLKSLELVSLQNLTSFSKVEKCDLKFPLLEKLVVSECPQMTKLSEVQSAPNLQKVH
VEAGEKDKWYWEGDLNATLQTHFTNQVSFEYSKYINLVDYPEKKVPHDKFAFPDNFFGYLKKLEFDEACKRDTLIPSHV
LPYLKNIEELNVEKCKSAQLIFDIDESEIQTYGMVFRLKNLTIKNLSNLKCVWKENLEGIVSFSNLQKVDVDGCGSLLTLFPL
SVAKDLGKLKTVDIKECEKMIEIVGREDEREHGTTIMFEFPCLSYLNLDNMPLLSCFYPGKHHLECPLLEKLYVACCPKLKL
FRSSFDDDSKKEVLEAPTNLLQQPLFSIQKVSPKPVGLTLNEENIKLMSDARLPQDLLCKLKFLILSFEDDNNGTDSLPFDF
FHKLPNLESLTVQKCSGLKEIFPSQKLQVHDSILVGLKGLFLFELSELESIGLEHTWVQPYTEKLELLYLLLTPQVENIVSCAV
SFINLKDLFVMFCEKMEYLFTSTTLKSLVKLETLLIGYCGSIKEIARNEEEDGCDEIIFGRLRSIKLEYLPRLISFYSGNATLQCP
CLQNVMITECPNMITFSEGVIKLAMFSGIQTFEDSDFTFHDDLNTTVESLFHEKEFFNHSKHMILDEYLEITGAQHIKPDI
ADNFFGSFKELEFNAACKRAIVIPFHLLPYLKNLEKLNVHSSDAVNVIFDFDESEDKTKGIVSSLKELTLKNLSNLKCVWKE
NLEGIVSFPNLEKVTVTGCRSLVTLLSSSLAKSLEKLKTLHMARCEKLEEIVGEEDEREHGMTLTFEFPCLTILFLFDMPLLSC
FYPGKHYLECPILDTLYVSYCPKLKLFTSDTDDSHKEEVIEAPISPLQQPLFLVEKVSPKLKKLALNEKNIMLLRDGCLLHDLL
CKLSHLWFLFEDYKIENDTLPFDFFHKLPGLEYLLLQNCFGLKVIFPSQKLQVHNKVLAGLKQLSLLRLRELESIGLENEWV
QSYSRKLEVLKLDTCPQVENIVSCSVSFINLTKLSVKLCEKMEYLFTFATLKSLVKLQTLSIKNCESIKEIVKKEDEDACDEIVF
EQLRSIKLNSLPNLLSFYSGNATLKCLCLQSVMVAKCPNMITFSEGVINMPILSGIKTSKDSVVVFHDNLNTTIETLLHEQE
FMEYSKRMILEDYLGMSGVHHRKPVVSDNFFGSFKKLEFDGACNRTILIPSHVLPYLKNLEELNVKNSDAMQIIFDIDESK
VKTKGVVFGLKKLTLNKLSNLKHVWKENSTGIISFHNLQEVVVNGCGGLIRLFSSSLARNLWKLEELRIKECGKLVEIVEKE
DGTENGTKIMFEFPCLTSLYLKNMPLLSCFYPGKHDLDCPLLEILLVCFCPKLKLFTSDFDENQKGAIEAQISPLQQPMFSV
EKFSPKIKVLALNEENIMLFREVQFLQDILCNIVGLVLCFEDDNIEKDSLPFDFFHTLPNVFILAIKKCFGLKEIFPSQKLQVH
DGGLAGLKELFLVDLKELELVGLEHPWVQPYSEKLQVLSLKRCPQLQKLVYCAVSFINLKKLRVKLCERMEYLFTFATVKSL

VKLETLIINSCESIKEIIKHENEDGCAEMVFGRLKSIKLKSLPRLVRFYSGKATLQCSYLKIVMVVKCPSMKTFSEGVMKVPK
FSGIQTSKDSDLIFHEDLNTTIKKLFHEEVEKSACDLEHLKFGDHPNLEVIWLGVVPIPRNDSFNNLQSLAVVECESLSNVI
PFYLLRFLSNLKEIEVSNCQSVKAIFDVKGEGADMKPISLSLKKLILNQLPNLEHIWNLNPDEILSLEDLQQVSISNCQTLKS
LFPTSVANHLIKLHVRACATLVEIFVEAETAFEGETKQFNFHCLTSITLWELPELKYLYPGKHTLEWPMLAHLDIYHCDKLK
LFKIEHHSDEFSDTKDQLGISIHQQPAFSVKKVFPKLVQLSLKKEDAMAILQGQLQVMPSIEHQAITWKDTRIGQGQFG
ANVAYLLQNLKLLKLMCYHEDDKSNIFSSGLLEEIPNIENLEVVCSSFTEIFCSQGPTHDCSKVLSKLKRLHLKNLPQLNAIG
LENSWVEPLLKTLETLEVFSCPAMKILVPSTVSFSNLTSLSVDECHGLLYLFTSSTAKRLGQLKHISIRDCQAIQEIVCKEED
HESEDEDITFDQLSLLSLQSLPNIVVIYSRTFKLKFPCLDQVIIKECPQMKYSYVPDLHEFKPQEQA

>XP_022642192.1

MDPIVSATTESALNITTSVVKRQVGYFFNYKDKFKELKSYIEKLEHNKERLQHQVDSALRSGGEIEKDVQRCLTLMDDKIK
EYKSYINDDCHAKTICSIGFFPNNFRLRYQLGRKATKMVEEIIGDELWKTFFDNVSYQEFPSIDAAFSNNGYESFASRTKT
MKMIMKALQDSTVGMIGVYGPGGVGKTTLVKEIANKAREKNLFEIVIIANITGNPDXKKIQEQIAGMLGMKLEXESEIA
RVDRIRKRLKNEKENTLIILDDLWGGLDFNKLGIPCNDDASQQEVNDISDFGYNKIEIKELSKVDLDKMKKEKLSNDYRRG
KILLTSRNKQVLCNEMDVQQRSIFSVGVLDEKESETLLKKVAGVKNSEFDRNATEIAKWSAGFPIALVSIGRTLKNKSLST
WEDVCQQIKRQNFTSEWGFTDFSIKLSYDHLKNEELKCIFLHCARMGNDALIMDLVKFCVGLDLLPEVYTITDARKRVKE
IIQELEESSLLVKSYSNDRFNMHDIVRDVALSISSKEKQVLYKKNAILYEWPHENDFERYSAIFVHFCDINDKLPESIHCPRL
EVLHIDNKNESFEIPDEFFKSMVRLRVLVLTGIHFSCLPSSIKCLKKLRMLCLERCTLGENLSIIGELKNLRILSFSGSNIENLPL
EFGQLDKLQFLDISNCLKLRQITSNIIPRMGILEEFYIRDNLIIWEAEENMKSENASLSELRHLNQLQNLDIHIHCSSYFPQN
LFFDRLNSYKIVIGEFNLSNLLKVGEFKVPDKYEEVKFLALNLKEGIDIHSEKWIKMLFKSVECLLLGELNDVQDIFYELNVE
GFPNLKHLSIVNNFDINYIINPKERFHSLVAFPKLESIWLYKLDNLEIICNNQLVETSFRNLKVIKIQTCLKLVNLFLFSMVRLL
TLLETIEVCDCDSLKEIVSKESQTNTISDDKIQFPQLRLLTLKYLPTFIYLYNVVDKIPGSSHSLQDQVFQQRNKDIVVDVEH
MVTNSCLPLFNEKVSTPKLEWLELSSINIHKIWSDQCNHCFQNLLTLNVTDCSNLKYLLSFSMAESLVNLQSIFVSECAIM
EDIFRPEDAEYIDVFPKLKKMEIICMDKLCTIWKSDIGLHSFSCLNTLMIRECHKLVTIFPNYMGQRLQSLQSLTVTDCKLV
ENIFDFANIPHTYDIIETNLGKIILEDLPNLVNVWKGYTGEILKYNNLQSIRVYESPKLKYLFPVSIANDLEKQEVLEVRNCG
AMTEIIALDKHSSETFITFKFPHLNTLSLIDLHDLRSFYSGIHTLEWPPLKKLDIINCSMLEGLTSEITNSKEQPIVLATKKAIY
NLEYMSVSLKEAEWLQKYIVNVHRMHKLEELTLYGLKNNEILFWFLHRLPNLKRLTLELCHLKRIWALKSLISREKIGGVV
QLKELKLESMWSLEEIGFEHEVLLQRVERISIHHCTKLKNLVSSSVTFSYLTYLEVMNCKSMRSLMTCSAAKTLVQLTTLK
VCSCPMIVEIVADNKVEKVREIEFKQLKSLELVSLQNLTSFSKVEKCDLKFPLLEKLVVSECPQMTKLSEVQSAPNLQKVH
VEAGEKDKWYWEGDLNATLQTHFTNQVSFEYSKYINLVDYPEKKVPHDKFAFPDNFFGYLKKLEFDEACKRDTLIPSHV
LPYLKNIEELNVEKCKSAQLIFDIDESEIQTYGMVFRLKNLTIKNLSNLKCVWKENLEGIVSFSNLQKVDVDGCGSLLTLFPL
SVAKDLGKLKTVDIKECEKMIEIVGREDEREHGTTIMFEFPCLSYLNLDNMPLLSCFYPGKHHLECPLLEKLYVACCPKLKL
FRSSFDDDSKKEVLEAPTNLLQQPLFSIQKVSPKPVGLTLNEENIKLMSDARLPQDLLCKLKFLILSFEDDNNGTDSLPFDF
FHKLPNLESLTVQKCSGLKEIFPSQKLQVHDSILVGLKGLFLFELSELESIGLEHTWVQPYTEKLELLYLLLTPQVENIVSCAV
SFINLKDLFVMFCEKMEYLFTSTTLKSLVKLETLLIGYCGSIKEIARNEEEDGCDEIIFGRLRSIKLEYLPRLISFYSGNATLQCP
CLQNVMITECPNMITFSEGVIKLAMFSGIQTFEDSDFTFHDDLNTTVESLFHEKEFFNHSKHMILDEYLEITGAQHIKPDI
ADNFFGSFKELEFNAACKRAIVIPFHLLPYLKNLEKLNVHSSDAVNVIFDFDESEDKTKGIVSSLKELTLKNLSNLKCVWKE
NLEGIVSFPNLEKVTVTGCRSLVTLLSSSLAKSLEKLKTLHMARCEKLEEIVGEEDEREHGMTLTFEFPCLTILFLFDMPLLSC
FYPGKHYLECPILDTLYVSYCPKLKLFTSDTDDSHKEEVIEAPISPLQQPLFLVEKVSPKLKKLALNEKNIMLLRDGCLLHDLL
CKLSHLWFLFEDYKIENDTLPFDFFHKLPGLEYLLLQNCFGLKVIFPSQKLQVHNKVLAGLKQLSLLRLRELESIGLENEWV
QSYSRKLEVLKLDTCPQVENIVSCSVSFINLTKLSVKLCEKMEYLFTFATLKSLVKLQTLSIKNCESIKEIVKKEDEDACDEIVF
EQLRSIKLNSLPNLLSFYSGNATLKCLCLQSVMVAKCPNMITFSEGVINMPILSGIKTSKDSVVVFHDNLNTTIETLLHEQE
FMEYSKRMILEDYLGMSGVHHRKPVVSDNFFGSFKKLEFDGACNRTILIPSHVLPYLKNLEELNVKNSDAMQIIFDIDESK
VKTKGVVFGLKKLTLNKLSNLKHVWKENSTGIISFHNLQEVVVNGCGGLIRLFSSSLARNLWKLEELRIKECGKLVEIVEKE

DGTENGTKIMFEFPCLTSLYLKNMPLLSCFYPGKHDLDCPLLEILLVCFCPKLKLFTSDFDENQKGAIEAQISPLQQPMFSV
EKFSPKIKVLALNEENIMLFREVQFLQDILCNIVGLVLCFEDDNIEKDSLPFDFFHTLPNVFILAIKKCFGLKEIFPSQKLQVH
DGGLAGLKELFLVDLKELELVGLEHPWVQPYSEKLQVLSLKRCPQLQKLVYCAVSFINLKKLRVKLCERMEYLFTFATVKSL
VKLETLIINSCESIKEIIKHENEDGCAEMVFGRLKSIKLKSLPRLVRFYSGKATLQCSYLKIVMVVKCPSMKTFSEGVMKVPK
FSGIQTSKDSDLIFHEDLNTTIKKLFHEEVEKSACDLEHLKFGDHPNLEVIWLGVVPIPRNDSFNNLQSLAVVECESLSNVI
PFYLLRFLSNLKEIEVSNCQSVKAIFDVKGEGADMKPISLSLKKLILNQLPNLEHIWNLNPDEILSLEDLQQVSISNCQTLKS
LFPTSVANHLIKLHVRACATLVEIFVEAETAFEGETKQFNFHCLTSITLWELPELKYLYPGKHTLEWPMLAHLDIYHCDKLK
LFKIEHHSDEFSDTKDQLGISIHQQPAFSVKKVFPKLVQLSLKKEDAMAILQGQLQVMPSIEHQAITWKDTRIGQGQFG
ANVAYLLQNLKLLKLMCYHEDDKSNIFSSGLLEEIPNIENLEVVCSSFTEIFCSQGPTHDCSKVLSKLKRLHLKNLPQLNAIG
LENSWVEPLLKTLETLEVFSCPAMKILVPSTVSFSNLTSLSVDECHGLLYLFTSSTAKRLGQLKHISIRDCQAIQEIVCKEED
HESEDEDITFDQLSLLSLQSLPNIVVIYSRTFKLKFPCLDQVIIKECPQMKYSYVPDLHEFKPQEQA

>XP_022642193.1

MDPIVSATTESALNITTSVVKRQVGYFFNYKDKFKELKSYIEKLEHNKERLQHQVDSALRSGGEIEKDVQRCLTLMDDKIK
EYKSYINDDCHAKTICSIGFFPNNFRLRYQLGRKATKMVEEIIGDELWKTFFDNVSYQEFPSIDAAFSNNGYESFASRTKT
MKMIMKALQDSTVGMIGVYGPGGVGKTTLVKEIANKAREKNLFEIVIIANITGNPDXKKIQEQIAGMLGMKLEXESEIA
RVDRIRKRLKNEKENTLIILDDLWGGLDFNKLGIPCNDDASQQEVNDISDFGYNKIEIKELSKVDLDKMKKEKLSNDYRRG
KILLTSRNKQVLCNEMDVQQRSIFSVGVLDEKESETLLKKVAGVKNSEFDRNATEIAKWSAGFPIALVSIGRTLKNKSLST
WEDVCQQIKRQNFTSEWGFTDFSIKLSYDHLKNEELKCIFLHCARMGNDALIMDLVKFCVGLDLLPEVYTITDARKRVKE
IIQELEESSLLVKSYSNDRFNMHDIVRDVALSISSKEKQVLYKKNAILYEWPHENDFERYSAIFVHFCDINDKLPESIHCPRL
EVLHIDNKNESFEIPDEFFKSMVRLRVLVLTGIHFSCLPSSIKCLKKLRMLCLERCTLGENLSIIGELKNLRILSFSGSNIENLPL
EFGQLDKLQFLDISNCLKLRQITSNIIPRMGILEEFYIRDNLIIWEAEENMKSENASLSELRHLNQLQNLDIHIHCSSYFPQN
LFFDRLNSYKIVIGEFNLSNLLKVGEFKVPDKYEEVKFLALNLKEGIDIHSEKWIKMLFKSVECLLLGELNDVQDIFYELNVE
GFPNLKHLSIVNNFDINYIINPKERFHSLVAFPKLESIWLYKLDNLEIICNNQLVETSFRNLKVIKIQTCLKLVNLFLFSMVRLL
TLLETIEVCDCDSLKEIVSKESQTNTISDDKIQFPQLRLLTLKYLPTFIYLYNVVDKIPGSSHSLQDQVFQQRNKDIVVDVEH
MVTNSCLPLFNEKVSTPKLEWLELSSINIHKIWSDQCNHCFQNLLTLNVTDCSNLKYLLSFSMAESLVNLQSIFVSECAIM
EDIFRPEDAEYIDVFPKLKKMEIICMDKLCTIWKSDIGLHSFSCLNTLMIRECHKLVTIFPNYMGQRLQSLQSLTVTDCKLV
ENIFDFANIPHTYDIIETNLGKIILEDLPNLVNVWKGYTGEILKYNNLQSIRVYESPKLKYLFPVSIANDLEKQEVLEVRNCG
AMTEIIALDKHSSETFITFKFPHLNTLSLIDLHDLRSFYSGIHTLEWPPLKKLDIINCSMLEGLTSEITNSKEQPIVLATKKAIY
NLEYMSVSLKEAEWLQKYIVNVHRMHKLEELTLYGLKNNEILFWFLHRLPNLKRLTLELCHLKRIWALKSLISREKIGGVV
QLKELKLESMWSLEEIGFEHEVLLQRVERISIHHCTKLKNLVSSSVTFSYLTYLEVMNCKSMRSLMTCSAAKTLVQLTTLK
VCSCPMIVEIVADNKVEKVREIEFKQLKSLELVSLQNLTSFSKVEKCDLKFPLLEKLVVSECPQMTKLSEVQSAPNLQKVH
VEAGEKDKWYWEGDLNATLQTHFTNQVSFEYSKYINLVDYPEKKVPHDKFAFPDNFFGYLKKLEFDEACKRDTLIPSHV
LPYLKNIEELNVEKCKSAQLIFDIDESEIQTYGMVFRLKNLTIKNLSNLKCVWKENLEGIVSFSNLQKVDVDGCGSLLTLFPL
SVAKDLGKLKTVDIKECEKMIEIVGREDEREHGTTIMFEFPCLSYLNLDNMPLLSCFYPGKHHLECPLLEKLYVACCPKLKL
FRSSFDDDSKKEVLEAPTNLLQQPLFSIQKVSPKPVGLTLNEENIKLMSDARLPQDLLCKLKFLILSFEDDNNGTDSLPFDF
FHKLPNLESLTVQKCSGLKEIFPSQKLQVHDSILVGLKGLFLFELSELESIGLEHTWVQPYTEKLELLYLLLTPQVENIVSCAV
SFINLKDLFVMFCEKMEYLFTSTTLKSLVKLETLLIGYCGSIKEIARNEEEDGCDEIIFGRLRSIKLEYLPRLISFYSGNATLQCP
CLQNVMITECPNMITFSEGVIKLAMFSGIQTFEDSDFTFHDDLNTTVESLFHEKEFFNHSKHMILDEYLEITGAQHIKPDI
ADNFFGSFKELEFNAACKRAIVIPFHLLPYLKNLEKLNVHSSDAVNVIFDFDESEDKTKGIVSSLKELTLKNLSNLKCVWKE
NLEGIVSFPNLEKVTVTGCRSLVTLLSSSLAKSLEKLKTLHMARCEKLEEIVGEEDEREHGMTLTFEFPCLTILFLFDMPLLSC
FYPGKHYLECPILDTLYVSYCPKLKLFTSDTDDSHKEEVIEAPISPLQQPLFLVEKVSPKLKKLALNEKNIMLLRDGCLLHDLL
CKLSHLWFLFEDYKIENDTLPFDFFHKLPGLEYLLLQNCFGLKVIFPSQKLQVHNKVLAGLKQLSLLRLRELESIGLENEWV
QSYSRKLEVLKLDTCPQVENIVSCSVSFINLTKLSVKLCEKMEYLFTFATLKSLVKLQTLSIKNCESIKEIVKKEDEDACDEIVF

EQLRSIKLNSLPNLLSFYSGNATLKCLCLQSVMVAKCPNMITFSEGVINMPILSGIKTSKDSVVVFHDNLNTTIETLLHEQE
FMEYSKRMILEDYLGMSGVHHRKPVVSDNFFGSFKKLEFDGACNRTILIPSHVLPYLKNLEELNVKNSDAMQIIFDIDESK
VKTKGVVFGLKKLTLNKLSNLKHVWKENSTGIISFHNLQEVVVNGCGGLIRLFSSSLARNLWKLEELRIKECGKLVEIVEKE
DGTENGTKIMFEFPCLTSLYLKNMPLLSCFYPGKHDLDCPLLEILLVCFCPKLKLFTSDFDENQKGAIEAQISPLQQPMFSV
EKFSPKIKVLALNEENIMLFREVQFLQDILCNIVGLVLCFEDDNIEKDSLPFDFFHTLPNVFILAIKKCFGLKEIFPSQKLQVH
DGGLAGLKELFLVDLKELELVGLEHPWVQPYSEKLQVLSLKRCPQLQKLVYCAVSFINLKKLRVKLCERMEYLFTFATVKSL
VKLETLIINSCESIKEIIKHENEDGCAEMVFGRLKSIKLKSLPRLVRFYSGKATLQCSYLKIVMVVKCPSMKTFSEGVMKVPK
FSGIQTSKDSDLIFHEDLNTTIKKLFHEEVEKSACDLEHLKFGDHPNLEVIWLGVVPIPRNDSFNNLQSLAVVECESLSNVI
PFYLLRFLSNLKEIEVSNCQSVKAIFDVKGEGADMKPISLSLKKLILNQLPNLEHIWNLNPDEILSLEDLQQVSISNCQTLKS
LFPTSVANHLIKLHVRACATLVEIFVEAETAFEGETKQFNFHCLTSITLWELPELKYLYPGKHTLEWPMLAHLDIYHCDKLK
LFKIEHHSDEFSDTKDQLGISIHQQPAFSVKKVFPKLVQLSLKKEDAMAILQGQLQVMPSIEHQAITWKDTRIGQGQFG
ANVAYLLQNLKLLKLMCYHEDDKSNIFSSGLLEEIPNIENLEVVCSSFTEIFCSQGPTHDCSKVLSKLKRLHLKNLPQLNAIG
LENSWVEPLLKTLETLEVFSCPAMKILVPSTVSFSNLTSLSVDECHGLLYLFTSSTAKRLGQLKHISIRDCQAIQEIVCKEED
HESEDEDITFDQLSLLSLQSLPNIVVIYSRTFKLKFPCLDQVIIKECPQMKYSYVPDLHEFKPQEQA

>XP_022642194.1

MDPIVSATTESALNITTSVVKRQVGYFFNYKDKFKELKSYIEKLEHNKERLQHQVDSALRSGGEIEKDVQRCLTLMDDKIK
EYKSYINDDCHAKTICSIGFFPNNFRLRYQLGRKATKMVEEIIGDELWKTFFDNVSYQEFPSIDAAFSNNGYESFASRTKT
MKMIMKALQDSTVGMIGVYGPGGVGKTTLVKEIANKAREKNLFEIVIIANITGNPDXKKIQEQIAGMLGMKLEXESEIA
RVDRIRKRLKNEKENTLIILDDLWGGLDFNKLGIPCNDDASQQEVNDISDFGYNKIEIKELSKVDLDKMKKEKLSNDYRRG
KILLTSRNKQVLCNEMDVQQRSIFSVGVLDEKESETLLKKVAGVKNSEFDRNATEIAKWSAGFPIALVSIGRTLKNKSLST
WEDVCQQIKRQNFTSEWGFTDFSIKLSYDHLKNEELKCIFLHCARMGNDALIMDLVKFCVGLDLLPEVYTITDARKRVKE
IIQELEESSLLVKSYSNDRFNMHDIVRDVALSISSKEKQVLYKKNAILYEWPHENDFERYSAIFVHFCDINDKLPESIHCPRL
EVLHIDNKNESFEIPDEFFKSMVRLRVLVLTGIHFSCLPSSIKCLKKLRMLCLERCTLGENLSIIGELKNLRILSFSGSNIENLPL
EFGQLDKLQFLDISNCLKLRQITSNIIPRMGILEEFYIRDNLIIWEAEENMKSENASLSELRHLNQLQNLDIHIHCSSYFPQN
LFFDRLNSYKIVIGEFNLSNLLKVGEFKVPDKYEEVKFLALNLKEGIDIHSEKWIKMLFKSVECLLLGELNDVQDIFYELNVE
GFPNLKHLSIVNNFDINYIINPKERFHSLVAFPKLESIWLYKLDNLEIICNNQLVETSFRNLKVIKIQTCLKLVNLFLFSMVRLL
TLLETIEVCDCDSLKEIVSKESQTNTISDDKIQFPQLRLLTLKYLPTFIYLYNVVDKIPGSSHSLQDQVFQQRNKDIVVDVEH
MVTNSCLPLFNEKVSTPKLEWLELSSINIHKIWSDQCNHCFQNLLTLNVTDCSNLKYLLSFSMAESLVNLQSIFVSECAIM
EDIFRPEDAEYIDVFPKLKKMEIICMDKLCTIWKSDIGLHSFSCLNTLMIRECHKLVTIFPNYMGQRLQSLQSLTVTDCKLV
ENIFDFANIPHTYDIIETNLGKIILEDLPNLVNVWKGYTGEILKYNNLQSIRVYESPKLKYLFPVSIANDLEKQEVLEVRNCG
AMTEIIALDKHSSETFITFKFPHLNTLSLIDLHDLRSFYSGIHTLEWPPLKKLDIINCSMLEGLTSEITNSKEQPIVLATKKAIY
NLEYMSVSLKEAEWLQKYIVNVHRMHKLEELTLYGLKNNEILFWFLHRLPNLKRLTLELCHLKRIWALKSLISREKIGGVV
QLKELKLESMWSLEEIGFEHEVLLQRVERISIHHCTKLKNLVSSSVTFSYLTYLEVMNCKSMRSLMTCSAAKTLVQLTTLK
VCSCPMIVEIVADNKVEKVREIEFKQLKSLELVSLQNLTSFSKVEKCDLKFPLLEKLVVSECPQMTKLSEVQSAPNLQKVH
VEAGEKDKWYWEGDLNATLQTHFTNQVSFEYSKYINLVDYPEKKVPHDKFAFPDNFFGYLKKLEFDEACKRDTLIPSHV
LPYLKNIEELNVEKCKSAQLIFDIDESEIQTYGMVFRLKNLTIKNLSNLKCVWKENLEGIVSFSNLQKVDVDGCGSLLTLFPL
SVAKDLGKLKTVDIKECEKMIEIVGREDEREHGTTIMFEFPCLSYLNLDNMPLLSCFYPGKHHLECPLLEKLYVACCPKLKL
FRSSFDDDSKKEVLEAPTNLLQQPLFSIQKVSPKPVGLTLNEENIKLMSDARLPQDLLCKLKFLILSFEDDNNGTDSLPFDF
FHKLPNLESLTVQKCSGLKEIFPSQKLQVHDSILVGLKGLFLFELSELESIGLEHTWVQPYTEKLELLYLLLTPQVENIVSCAV
SFINLKDLFVMFCEKMEYLFTSTTLKSLVKLETLLIGYCGSIKEIARNEEEDGCDEIIFGRLRSIKLEYLPRLISFYSGNATLQCP
CLQNVMITECPNMITFSEGVIKLAMFSGIQTFEDSDFTFHDDLNTTVESLFHEKEFFNHSKHMILDEYLEITGAQHIKPDI
ADNFFGSFKELEFNAACKRAIVIPFHLLPYLKNLEKLNVHSSDAVNVIFDFDESEDKTKGIVSSLKELTLKNLSNLKCVWKE
NLEGIVSFPNLEKVTVTGCRSLVTLLSSSLAKSLEKLKTLHMARCEKLEEIVGEEDEREHGMTLTFEFPCLTILFLFDMPLLSC

FYPGKHYLECPILDTLYVSYCPKLKLFTSDTDDSHKEEVIEAPISPLQQPLFLVEKVSPKLKKLALNEKNIMLLRDGCLLHDLL
CKLSHLWFLFEDYKIENDTLPFDFFHKLPGLEYLLLQNCFGLKVIFPSQKLQVHNKVLAGLKQLSLLRLRELESIGLENEWV
QSYSRKLEVLKLDTCPQVENIVSCSVSFINLTKLSVKLCEKMEYLFTFATLKSLVKLQTLSIKNCESIKEIVKKEDEDACDEIVF
EQLRSIKLNSLPNLLSFYSGNATLKCLCLQSVMVAKCPNMITFSEGVINMPILSGIKTSKDSVVVFHDNLNTTIETLLHEQE
FMEYSKRMILEDYLGMSGVHHRKPVVSDNFFGSFKKLEFDGACNRTILIPSHVLPYLKNLEELNVKNSDAMQIIFDIDESK
VKTKGVVFGLKKLTLNKLSNLKHVWKENSTGIISFHNLQEVVVNGCGGLIRLFSSSLARNLWKLEELRIKECGKLVEIVEKE
DGTENGTKIMFEFPCLTSLYLKNMPLLSCFYPGKHDLDCPLLEILLVCFCPKLKLFTSDFDENQKGAIEAQISPLQQPMFSV
EKEIFPSQKLQVHDGGLAGLKELFLVDLKELELVGLEHPWVQPYSEKLQVLSLKRCPQLQKLVYCAVSFINLKKLRVKLCER
MEYLFTFATVKSLVKLETLIINSCESIKEIIKHENEDGCAEMVFGRLKSIKLKSLPRLVRFYSGKATLQCSYLKIVMVVKCPSM
KTFSEGVMKVPKFSGIQTSKDSDLIFHEDLNTTIKKLFHEEVEKSACDLEHLKFGDHPNLEVIWLGVVPIPRNDSFNNLQS
LAVVECESLSNVIPFYLLRFLSNLKEIEVSNCQSVKAIFDVKGEGADMKPISLSLKKLILNQLPNLEHIWNLNPDEILSLEDLQ
QVSISNCQTLKSLFPTSVANHLIKLHVRACATLVEIFVEAETAFEGETKQFNFHCLTSITLWELPELKYLYPGKHTLEWPML
AHLDIYHCDKLKLFKIEHHSDEFSDTKDQLGISIHQQPAFSVKKVFPKLVQLSLKKEDAMAILQGQLQVMPSIEHQAITW
KDTRIGQGQFGANVAYLLQNLKLLKLMCYHEDDKSNIFSSGLLEEIPNIENLEVVCSSFTEIFCSQGPTHDCSKVLSKLKRL
HLKNLPQLNAIGLENSWVEPLLKTLETLEVFSCPAMKILVPSTVSFSNLTSLSVDECHGLLYLFTSSTAKRLGQLKHISIRDC
QAIQEIVCKEEDHESEDEDITFDQLSLLSLQSLPNIVVIYSRTFKLKFPCLDQVIIKECPQMKYSYVPDLHEFKPQEQA

>XP_022642225.1

MGGMGKTTLTQHVYNDPKTDEAKFDEKAWVCVSDAFDALRVSKAIIGAFTNSRDDSGDLEMIHGKLKKRLSGRKFLLV
LDDVWNEDRNQWKALQTPLTFGAKGSKIIVTTRSHKVASIMQSTYIHQLKQLDEDHSWQTFAKHAFQDEKLKSELKEI
GTKIVKKCQGLPLALETIGCLLQSKSSVSEWEGVLKSEIWDLPIEDSKIVPTLLLSYYHLPSHLKRCFAYCALFPKDHRFDKE
SLILLWMAQNFVHCSQQSKPPEEVGENYFNDLVSRSFFKQITWYNETYFVMHDLLNDLAKYVSGEICYRLGVDGEKIVS
RKTRHIYVSCPIQYYTTLCDAKGLRTFIAIQKRCKMPIEELISNFKFLRVLSMRWCIKVPGTIGNLIHLRSLDLLGTDIERLPD
SICSLYNLQELKLNNCVKLKELPLTLHGLTNLRRLELIGTTLTMVPHRLGKLKNLQVWMDNFEVGKSSEFSIQQLGELDLH
AEXFSIKNLENISNPYEMNLKNKTRIMSLRLQWNLERNDDDSIKEREVLENLQPSRHLKHLSIDGYGGTQFPRWLSNNSL
SNLVSLTLKNCKYCLWLPSLGLLTFLEELRIDGLDWIGRIYADFYGNSSSAFASLKTLSFKDMKEWEEWQFMTGAFPSLQ
SLFLTNCPKLKELPNILCHLKRLFINNCRQLGAPIPVAGEIPRQVIYFGPGISIPISHWYHSLVELDIGHGCDSLTTFPLDLFPK
LGELSLIHCYNLRIISQEHPHNHLKYLMIRHCPEFESFPNEGLFVPQLXXXXIXGXEKLKSMPKCMSALLPSLNCLYIRNCPA
LELSEECLPSNVKLLYLVNCSKLVASLKGAWGINPSLKDLHISKVDLECFPGKGLLPLSLVKLEIDNYPNLKKLDYRGLCHLS
SLQILTLYNCPRLQCLPEEGLPKSISTLEIEDCPLLKQRCKKEGKDWEKIAHIKSICVD

>XP_022642230.1

MEFASSSLTSSSSSFLTSEPHFIHDVFINFGGEEIGRRFVSHLHSVLLQAQVETLISQENLHEGMKLEEHMRAIGHTKITIIV
FSKSYTESASCLLELEKIIECHETFGQIVVPVFYEIDPSDVRYQKDDFGKALEEAAHKSYSGEQLKHALSKSSRALTTTAGIPS
CDFRNFRHDAELVERIVSRVKTLLDYKDLFITQYPVGLESRVEDMIKGIEKQSTKVCMIGIWGMGGSGKTTIAKATYNRIC
REFIGKCFIENISYAWDPENKNEWYVALQENLLSDVLKSKLKVKSVGMGRTMIENGFSEKKLLIVLDDVNDYGQLENLC
GSREWFGQGTVIIITTRNISLLKRREVSYIYETQLLNENKSLELLSWHVFGEAKPRKGLNELARDIVAYSGGLPLALEFLGSY
LWNRTKEEWETVLRKLQRIPPDKLLKVLKISFDDLNATEKDIFLDVCCFFIGKERDYVTEILNGCGLYADIGIIILIERGLIKIE
GNNKLQMHPLLRDMGREIIHQECSEEPGKRSRLWLQDDVKDVLKEKSGTRAIEGLSLKLHSSSRDCLEACAFKEMKRLR
LLQLDNVQLSGDYGHISKQLRWICWRGFPYKYIPINFHLENVIAIDFKXSRLQLVWEQPVVLERLKFLNLSHSKYLKETPD
FSGLPSLEKLILKDCPNLCEVHQSIGRLRNLLLINLKDCTSLSYLPEEVYELRSLKILILSGCLKFPPIDIAKVKSLATIIAENTKPS
AL

>XP_022642231.1

MKQEEHMRAIGHTKITIIVFSKSYTESACSLLELEKIIECHETFGQIVLPVFYEIDPLDVCHQKDDFGKALEDTAHRSYAGE
QLQHARSRWSSALKRVAGMTGWDHRDFRHDAELVEQIVSRVKPIDLFITEYPVGLESRVEEVIKCIENQSTKVCMIGIW
GMGGSGKTTIAKAICNRIYRKFLANSFIQNIKEAWDPEYEENVRLQEYLLSDVLKSKFKIQSVRIGRTMIQNELSRRKLLIVL
DDVNEFGQLEILCGCREWFGQGTVIIITTRDVRLLNQLKVNYVHKMDFLDENKSLELFSWHAFRDAKPRKEFKELARDT
VDYCGGLPLALEVLGSYLCYKTIEVWKSVLLKLQRIPPDDLVRALKISFEDLRDTEKDIFLDVCCFFIGKDRDYVTEILNDCG
LHADIGITVLIERGLIKVESNNKLEMHILLQEMGREIIRQVYPEEPGKRSRLWLQDDVKDVLKENTGTEAIQGLSLELHSTS
RDCFKTHAFKEMKKLRLLRLDHVQVVGDYGHISKELRWICWKGFPSKYIPNNFYMGNVIAIDLRHSHLQLVWKQPQYV
CRF

>XP_022642232.1

MKQEEHMRAIGHTKITIIVFSKSYTESACSLLELEKIIECHETFGQIVLPVFYEIDPLDVCHQKDDFGKALEDTAHRSYAGE
QLQHARSRWSSALKRVAGMTGWDHRDFRHDAELVEQIVSRVKPIDLFITEYPVGLESRVEEVIKCIENQSTKVCMIGIW
GMGGSGKTTIAKAICNRIYRKFLANSFIQNIKEAWDPEYEENVRLQEYLLSDVLKSKFKIQSVRIGRTMIQNELSRRKLLIVL
DDVNEFGQLEILCGCREWFGQGTVIIITTRDVRLLNQLKVNYVHKMDFLDENKSLELFSWHAFRDAKPRKEFKELARDT
VDYCGGLPLALEVLGSYLCYKTIEVWKSVLLKLQRIPPDDLVRALKISFEDLRDTEKDIFLDVCCFFIGKDRDYVTEILNDCG
LHADIGITVLIERGLIKVESNNKLEMHILLQEMGREIIRQVYPEEPGKRSRLWLQDDVKDVLKENTGTEAIQGLSLELHSTS
RDCFKTHAFKEMKKLRLLRLDHVQVVGDYGHISKELRWICWKGFPSKYIPNNFYMGNVIAIDLRHSHLQLVWKQPQLK
K

>XP_022642233.1

MKLEEHMRAIGHTKITIIVFSKSYTESACSLLELEKIIECYETFGQIVVPVFYEIDPLDVRHQKDDFGKTLEDTAHRSYAGKQ
LQHARSRWSSALNRVAGMTGWDVRYFRHDAQLVEVIVRRVKELLDYKDMFITQYPVALLESRVEDVIKYIENQPSKVCI
IGIWGFRGSGYVALQENLLSDVLKSKLMVGGVGIGRIYIQNEFCRRKLLIVLDDVNEFGQLENLCGSREWFGQGTVIIITT
RDLHLLKQLENNYVYQNESLEAKPRKKLKELDRDIVNYCEGLPLALQFLGSYLCDKTIEGWRSLLRKLQRIHGDNLLKVLKI
SFDGLRDTEKDILLDLCCFFKGKEKDYVTDILNGCGLRADIGITVLIQRGLIKVDRNNKLQMHPLLQYMGREIIRQVCPEE
PGKRSRLWLQDDVKDVLIQKTGTEAIQGLSLKLHSTSRDCFKTHAFKEMKKLRLLRLDHVQLVGDYGHISKELRWICWK
GFPSKYIPKNLYMGNVIAIDLRHSHLQYVWKPPQILEQLQFLNLSHSKYLKETPDFSRLPSLEQLILKDCPSLLKVHPSIADL
SNIRVINLKDCKSLRYLPREVYKLKSLKTLILSGCSKVGPIDISQVKSLVNIICLKQNCETNIDEL

>XP_022642239.1

MAETAVSFASKHVLPKFLEAVKMLRDLPKEVAEVADELESFQDFIHDADKVAEAEEDKNRRDRIRKRLMKLREAAFRM
EDVIDDYVICDEKQPEEDPRCATLLCETVEIIKTQILRLQIAYQIQDVKSLVRAERDSFKNHFPIGPRSDGSRRNENFTWQK
LRIDPLFIKEDEVVGFEGPIHTLKKWLTEGRKERTVISIVGMAGLGKTTLSKQVFDRVHTDFECHALITVSRSYTVEELLRD
MTNKLCKERMEDPPRDVATMNQMSLIEEVRNRLCNKRYVVLFDDVWNETFWDDIELALIDNKNGSRILITTRDEKVV
NFCKKALFFEVHKLQPLSKAKSLELLCKKAFGYGFDGCCPKDYEEAGLDIIRKCECLPLAIVAIGSLLYRKCKSSSEWHLFSQ
NLSLELQSNSELHSVTKILSLSYDDLPHNLRSCLLYFGMYPEDYEVKCGRIIKQWIAEGFVKHESGRNLEEVAQQHLMELI
SRSLVLVASFTTDDKAKTCRVHDSIHEMIRGKIKNTGFCEYIDEHNHLESSGIIRRLTIARNSNGLSGSIEESQNIRSILIFTNE
VSSEDFTRRLLAKYMRLRVLDFGYAPLHDVPEDLGSLIHLKYLSFRGTFISGLPKSISKLQNLETLDVRAFGEIEVPKEITKLR
KLRYLLGNPISSIAVKDSLGSITSLEKMHVLIIDPDGVVIRELGKLKQLRDLRLTMLRGDQADTLCSSINEMPLLERLHISLKY
GTETIDLHIRLSLSKLRKLRLYGSLKELPNWIPRLQNLVKLSLVESRLTNIRLADLGSMPNLLLLSFDSNSYDGETLHFENGG
FQKLKELHLNGLQQLSSIFIDSGALQSLEKLQIMSILQLKAVPCGIQHLKKLQVLDILYMPTEFQQRIDPNGGKEHWMIKH
VPDVHFVTKNRALLLAERAAEIFSSRL

>XP_022642604.1

MSGSSSSFAMCTSLTRYDVFLNFRGDDTRDNFISHLYAALQRKHVEGYIDYRLLRGEEISPALQTAIEESKIYVLVFSENYA
SSTWCLNELTKILDCKKKYGRDVIPVFYKVDPSTVRKQEKRYKEAFEEHELRFRENMNKVQGWKDALTEAAGLSGWDS
NVTRPEYKLVEEIVEDILRKLNRYSISYDKKIIGIEKHIEGIQSLLHLESPDVRIIGICGMGGIGKTTISEQLYHTLAMQFDSHS
LILDVQEKLQKEGIYNIINKYKSELLKETSSTHLSYYNGRLKRTKVLLILDDVTDSTQLNKLMGERDSFGQGSRIIMTSRDRQ
VLKNAGADDIYELKELNFHDSLKLFNLHAFXQNSSXEITXMDLSVKVLRYAXGVPLVLQILGSLLYGRQREAWESQLQNL
EKCQDHNIFNVLKLSYDGLREEEKNIFLDIACFYRGHGEIVVAETLDDCGFSSKIGMDVLKDKCLISISDGRIVMHDLIQEIV
RKECPEHPGKRSRLFKAEEIRDVLRKNKVHFIITQLNKGQMMLMIFPYCWGQIISNVVRESSLVSLPHTLKILCWNNFPQ
RSLPSNLCLQNLVRLEMPKCRLEQLWERDQYLPKLKRLNLSYSPKLTRIPDLSLSPNIKEIILSHCERLIQVHSSRFLSKLSCLL
LDGCYGLKSVTIPSNILTRSPGLILLSDCHELEMFQTYETQPWPFTLLSSMWERDNPRYGRYIPFTRLDPSEIFSITFDRYEE
EEKEVINNHIYLQCDEVLTKLREGVPLNFQSLKKFCFLDVSYCSSLSTFPCDLSETKFLKQLCLRGCSKLENFPEIEDTVEGLA
VLILDXTAIQALPSSLWRLVGLEQLSLRGCKNLEIIPSSIGSLTRLCKLDLTSCQSLQTFPNTIFKLKLRILDXCGCLRLSTFPEIT
EQAQTFSHINLKETAIKELPSSFGNLVNLRSLQLNXCTDLESLPNSIVNLKRLCKLDCSGCAKLTEIPTHIGRLSSLMEMSLR
KSGIVNLPESMAHLSSLKSLDLSDCKKLECIPPIPPFLKQLVALDCPSIRRVMPNSLVRNLSESKEGVFKFHFTNTQQLDSG
ARANIEEDARLRMTDDAYRSVFFCFPGTAVPHWFPFRGNGPSVSINEDLSFCSDDRLIGFALCVVFELLDTNDIEGRYGS
FSYNLKFECDDDGTQIIPNNDVLNSYFEWIHKDRLLDQNHTFVWKFNLEPLRTSGMNLRLCDA

>XP_022642778.1

MVDQKVIGVVAASSYSPSVVSSKRYDVFLSFRGEDTRKKFTSHLYDALKQKKVETFIDNRLEKGEEISTTLIQVIEDSHISIVI
FSENYASSKWCLGELSKIMECKKEKGQIVIPVFYDIDPSHVRKQTGSYEKLFVTHKGEPMCNKWKAALTEAANLAAWDS
QTYRVESELLKDIVEDVLQKLAPIYPNCHKGLVGIEENYEKIESLLKVGSNEVKILGIWGMGGIGKTTLARALYDKLSHEFE
GHCFLENVREESDKHGVKALRNKLFSELLGNKNHCFDVAFSVTKFVLSRLGRKKVFIVLDDVATSEQLENLIEDFDFMGL
GSRVVVTSRNKQIFSQDKIYEVKELSFHHSLQLFSLTVFREKQPKYGYEDLSRSATYYCKGVPLALKVLGATLRSRSKGAW
ECELRKLQKFPNMKIHSVLKLSYDGLDHTQKDIFLDIACFFRGNQRDHVTNMLEAFDFSAISGIEALLDRALITVSGCNQL
EMHDLIQEMGWEIVHQECVKDPGRRSRLWKHEEVHEVFKYNKGTDIVEGIILDLSKLIEDLYLSSDFLAKMTNVRFFKIH
SWSKFNIFNVYLPNGLNTLSHKMRYLHWDGFCLESLPANFCAEKLVELCMRCSKLKKLWDGVQNLVNLKTIDLWGSRD
LIEIPDLSLAEKLENVSLCYCESLREVHVHSKSLRVLNLYGCSSLRKFSVTSEELTRLSLAFTSICSIPTSIWHKRKLKALYLTGC
RNLGKLTEEPRIHGSHKHSLTALASNAERFSMNIKSLSTLRMLWLDDCKKLVSLPKLPPSLGKLSASNCTSLDSYMTQRLV
LQHMLQSRIPYLRTNDLRCYDEEYLFPGNHVIEECVFNTTETSMTIPYLWKTELYGFIYCIILSKGSLLQSDVSCSVYQDGIR
VGWLQRLLEYESLTSNHVLCMYHDINEFDAINEEGHGHFFSNVTFIFENSKASIEEFGVFPIYGSESGLKLVGSR

>XP_022642779.1

MVDQKVIGVVAASSYSPSVVSSKRYDVFLSFRGEDTRKKFTSHLYDALKQKKVETFIDNRLEKGEEISTTLIQVIEDSHISIVI
FSENYASSKWCLGELSKIMECKKEKGQIVIPVFYDIDPSHVRKQTGSYEKLFVTHKGEPMCNKWKAALTEAANLAAWDS
QTYRVESELLKDIVEDVLQKLAPIYPNCHKGLVGIEENYEKIESLLKVGSNEVKILGIWGMGGIGKTTLARALYDKLSHEFE
GHCFLENVREESDKHGVKALRNKLFSELLGNKNHCFDVAFSVTKFVLSRLGRKKVFIVLDDVATSEQLENLIEDFDFMGL
GSRVVVTSRNKQIFSQDKIYEVKELSFHHSLQLFSLTVFREKQPKYGYEDLSRSATYYCKGVPLALKVLGATLRSRSKGAW
ECELRKLQKFPNMKIHSVLKLSYDGLDHTQKDIFLDIACFFRGNQRDHVTNMLEAFDFSAISGIEALLDRALITVSGCNQL
EMHDLIQEMGWEIVHQECVKDPGRRSRLWKHEEVHEVFKYNKGTDIVEGIILDLSKLIEDLYLSSDFLAKMTNVRFFKIH
SWSKFNIFNVYLPNGLNTLSHKMRYLHWDGFCLESLPANFCAEKLVELCMRCSKLKKLWDGVQNLVNLKTIDLWGSRD
LIEIPDLSLAEKLENVSLCYCESLREVHVHSKSLRVLNLYGCSSLRKFSVTSEELTRLSLAFTSICSIPTSIWHKRKLKALYLTGC
RNLGKLTEEPRIHGSHKHSLTALASNAERFSMNIKSLSTLRMLWLDDCKKLVSLPKLPPSLGKLSASNCTSLDSYMTQRLV
LQHMLQSRIPYLRTNDLRCYDEEYLFPGNHVIEECVFNTTETSMTIPYLWKTELYGFIYCIILSKGSLLQSDVSCSVYQDGIR
VGWLQRLLEYESLTSNHVLCMYHDINEFDAINEEGHGHFFSNVTFIFENSKASIEEFGVFPIYGSESGLKLVGSR

>XP_022642938.1

MEDVALAAGECILKLLWETVKRLKDLPDDVREIRDELDKLKNFIDLEDKEADGENDGTKRNKKKQDLMQLRKATFLTED
VIDEYLIRIEEKQPQDDRGWAAFHIQTLIPRFKISFKIESNLSVVRRTNERFRNNRPQSTQNNNNVTFHQCRMDPLFMEE
QEVVGLAGPTEILKDWLKNGQRERTVIFVVGVPGVGKTTLAKHVFNKVCGDFEHHARFTVSQSNTVEELWRVMYKHY
MKSEPPSDNSILIEEVRGYLRGKRKCRALPLAIAVAGGVLKEKDGSAHEWGLFSEELKRNPDLNIITKIVGLSYDNLPSHLR
LCLLYFGMYPKDYEIKYGRLVRQWVAEGFVTDEGRKTLEEVAHGYLLYLVERSLVQVTSYPRKEKVKKCRVTDSIHDMIL
TKVKDTCFGEYIGGGHDQSESSDLVRRLTIETNNDLNRRFKRSHIRSIISIPGKQEESWSVDLERNILKDDMPLKVLDFEGC
GLFCVPKKLGNLIYLRYLSFRGTQITVLPKSIGKLVNLKTLDIRQTQVLVLPKEITKLRKLRHLLTPSSAFSSIEWKDIGGMTM
LQKIPQVRMEDDGEAIKEVGKLKQLRNLRVLCFGGEHITLLFSSINEMEHLQALRIEKSGDRSVVDLNTTLPRFPWSKLRK
LFLDMKLERLPNWIPQLKNLERLTLRHSDLTNDPLESLKDMPCLLVLSLSHAYEGQTLHFQPGGFKKLRMLNLQHLWNL
NSILIAETALQALEDFELTCLDDLRTVPDGIQHLMKLKSVRVPYMPTQFVEEIVRIGKDKPWRINYGYM

>XP_022642993.1

MKAAVVAHQLGNLISNRSILENLETGLKTLQDQRKIVQENLIWEDEEFQTQNDWLKRVNEILGQGDKLLNSYEGKSTCT
NILLRYKVGKQSRKMQPEISALILEGHSHISAKTRKHYERPASRDETIGDIMEALKNTDIQAVGVWGLGGLGTTSLAENIR
KKAKEQKLFDAMVFITVTDKPNQEQVQNAIADELGVQFTNGESLVKRRNKLRQRIKKEQSTLIIVDDXWGELNPQEFDL
EEFGVPPGNEHEGCKVLLTSGNLNFIQYLMGASKHNKVFQLEELQKEEAQMLFEKMVGSFDGDQSSIVEEIVRICEGSIS
LIYALAKALENKGIDALMQLKENISPPKLLSYCLEKNEEYKAFLYLLTIRGRRFINSYSIYIDMWTGVFKNLETADSARKKRE
SLISDLKAYGLVVENGKDWVKVDDYIYQTAYRMAQHDRRASVISREWPPEELLTDLHFCNLHPVGDLELRATLQCPNLK
HLLISRENSTIDVPNSFFEETKLLKVLDFVSFHCPNLPSSFVVLKDLEALSMCKCGLGDITEVCELTNLRMLGLLESRIQQLP
AQIVKLEKLLFLDLRDTNLQVIPPNVLSKLASLEELYLRNSFCNWEIEMSTSENKNASLKELTDLEHLAYIEDMYVPDPQA
WPVDLFFGNLRSYTIFIGKGWDRAHSGDHELKTLKLKLNRRFQSENGIKKMLKEVQVLYLDTLNGVQNVVNDMECDG
FPQLQSLFIQHNAEVKCIAKGSGNDPLDTFPNLESLSLTILSNLEYICHAGSLTEKSFFKLRVIKIEKCNAMLCLFSVSMINGI
PHLATLEVSQCTSIKAIVLFEGAENRPIEFPELCSLTLQGLPALISFCSSEGSSSATLFHDKVSCPKLETMVISEVSILTTIWNE
EYDAENSFGKLKNVIIKDCEKLRTVFPVNLSKNLDNLKTLEVRNCSSMTSIFTVMRQDSTKPGLQLSIPMIEITLTGLPKLEY
VCVTTGFEALKKKFEEEWYAGLPGLSGNARIEHGRKMKKYLEEYLNM

3. NLR genes of Vigna angularis

>Vang09g07260.1

MKDKAVSDNSVPRIKHDVFVSFRSEDIRRGFLTNLTNTFQEKKIKAFVDGQTLKGEAIWPSIVGAIEGSSISVIIFSPGYVSS
EWCLEELAKIFECRVKYGHTIIPVFYHVEQADVLYRSGRYRSPFGKDESKHKSKLQSWRSALEKSAQLSGIESSKIRDDDKL
VKEIVNHVLTRLRMVNSKGQVGIDKKIEEIVSWIKENPKQTRLIGIWGKGGVGKTTLVEEVYNKLQSQYEGCYFLAHVTE
KLSRHGILSLKDNCFSKLLGYDVKIVSPNSLPKDIVRRIESMKVLIVLDDVNDSEHLEKWLEILGNFGSGSRIIVTTRHLEVLE
ANKAYKKYQLGELSSDEALTLFNLNAYNQSDYRREYNRLSECVILYAKGIPLVIKVLAGHLRGKKKEEWKRELDKLEKMP
QTKVYEVMKLSYDGLEHKQQQMFLDLACFFLRTHERVNVGYLKSLLKDNESDNSIDFEFGRLKDKALITFSEDNNFVSM
HDNLQQMAWEIVRQESIEDPGKRIRLWNPSETYEALKYVKGIDSIRSIVLHWPAIKKERLRPHIFAKMSRLQFLEIDDLYH
HNSVEPFDILGDNTCWPKWQKARIADILVEGLHFLATELRFLCWYHYPLESLPENFSAEKLVILRLPHSRMEKLWLGVKN
LVNLKELDLSKSKKLKGLPDLSKAINLELLILRSCSMLTSVHPSIFSLPKLEKLDLERCESLTILASCSNLHGLSYLNLDFCENL
MKFSLISDNMKELRLRCSKIKALPLSFGHQRKLEFLHLEESGIERLPSLKNLTQLLHLDVSFCDKLQTIAELPPFLENLNLQY
CTLLQTIPKLPSSLKTLNTEACTSLETLPELPTSIEILNVESCSSLGTIPELPPCLQTLNVQCCHLLRTIPKLPTCLKTLHTKNCTS
LQNLPQLPPFLETLNATYCKSLKGVLFPTAKDEKLKENMKKLLFWNCSNLDKHSLVAIRLNMKINMMKFVANHISAPDH
SHVEDYSDYEYNYHSYQAVYAYPGSNFPEWLEYKTTEDYIIIDVPSSMSSSLVGFIFCFVLGEFQHTDIIRRVEFKITVSDG
GGKDEGERESVRMYMDYWDDTIESDNICVMYDQRCSHFLLSRSRRLTSFKIQVTMEARISFDETFYVVLQKVLKGFGVS
PISFDKLRSSNYKFR

>Vang0304s00040.1

MMMMMTDKLGFRRFWGLLGRRSSSTDVISSESASSSSGHSSDTIQNQDHRYDVFISFRGSDTRNSFVDHLYSHLLRKG
IFVFKDEHKLRKGESISSQLLQAIRGSRISIIVFSPDYPSSSWCLDEMATIADCKQQSNQTVFPVFYDVDPSHVRRQSGAY
ENAFVLHRSKFKEEPNKVLRWEKAMTDLANSAGWDIRNKAEFGEIENIVQAVIQTLGHKFSGFVNDLIGIQPRVQTLED
KLRLSSKSDDVQVLGIWGMGGIGKTTHAAVLYDKMSHRFDASCFIEDVSKLYRDGGHTAVQKQIIHQTLGEKCLDAYSP
IEISGIVRNRLHKIKVLLVLDNVDELEQLQELAINSKLLFGGSRMIIISRDEHILKVYGADVIHKVSLMSDKDARELFYTKAFK
SEEQNSSCVELIPEVLKYAQCLPLAVRVIGCFLCRRNSREWRDTLDRFENNPDNNKIMDVLQISFDGLHYMEKEIFLHIAC
FFKEEREDYVKRILECCTLHPHIGIRRMIEKSLITIKDQQIHMHDMLQELGKKIVRNEYPEEPQSWSRIWLYEDFFHVLTT
QTGTNDVKAIVLNKKEAISEFSIDGLSKMKNLRLLILYHKSFSGSLSFLSQKLRYLLWHDYPFSSLPSSFAAPGLVELNMPN
SSINCLWEGRTKEMLISGCFNSPQHFPCLKRMDLSNSKYLTETPDFTGVPNLERLDLSGCTDLSFVHPSIGLLQQLAFLSL
RNCSNLISIKFGNGFNVSSLRILHFSGCTKLENTPDFTWTTNLEYLDFNGCTSLSSVHESIGVLAKLTFLSLRNCTSLVSIPSN
NNIMKSLQTLDFSGCFQLTDYSLGRSFISLSMISLILLDIGFCNLLKVPDAIGDLLYLERLNLQGNNFVFIPSSISKLCSLAYLN
VSHCHRLQRLPYLSTRSSSSTGRYFKTVSGSRDHRSGLYFFDCPEINWIDPPYYQELTWLLRLAKNPCYFRCGFDIVVPW
GLELPGWLKQRFKGDSVIRIVEFNEDDDWIGFVFGVIFERKNGHVVARSSSHPFYLSFESEETEEYFDMQLNLERDKVD
GSKHLWIIYISRKHCHFLKTGSHISFKVHPSLEINAWGMSSIFREDEEINFECKGRHVNFDFVEKSSTKSGPKFKLSYNWLV
TDEDEVENINAKAKENNLSYAGL

>Vang0114s00480.1

MKFYPGSARLHRTTKPIFVTLQRPLPLKARGSHMSSRVWPCSKKKTKNDDIRKWLHELPTRDTNVKDWDSMWSRVPT
QIFNFGLQHDRNPNGGMKADVYCFGAALMELLTGKESTTERWQRVKEGQKLMAESAVGFLLQRLAPVFENKVKLFTG
VQAEVIYLKGQLELIRAFLRVADALEESDEELKVWVKQVRDVVHEAEDLLDELELVQVHNLTNGFSIYLRIRNMKARYRIA
HELKSINSRLKAISSSRKRFLSKLDSSSVASSSINTGNAWHDQRGDALLLDNTDLVGIDRPKKQVIGWLINGCPGRKVISV
TGMGGIGKTTLVKKVYDDPDVKKHFKACAWVTVSQSCKIEELLKDLAKKLFSEIRRPIPEGMESMCSDKLKMIIKDLLQK
KSYLVVFDDVWHMYEWEAVKYALPNNNCSSRIMITTRRSDLAFTSTIESNGKVYNLQPLKQDEAWDLFCRNTFQGDS
CPSYLIDICKYILRKCEGLPLAIVAISGVLATKDKRRIDEWGMICHSLGAEIQGNGKLDNFKTVLNLSFNDLPYHLKYCFLYL
SIFPQDYLIQRMRLIRLWIAEGFIEAKEGKTKEDVAHDYLKELLNRNLIQVAGTTTDGRVKTLRVHDLLREIIILKSKDQNFA
SIVKEQSAAWPEKIRRLSVHGTLPYRQQHRSVSQLRSFLMFGVGEYVPLGKLFPSGFKLLSVLDYQDAPLKKFPLAVIDLY
HLRYLSLRNTKVKTVPGHIIGKLHNLETFDLKKTSVRELPVDILKLQKLRHLLVYQLKFKGYAQFHSKDGLKAPSEIGKLKSL
QKLCFVEANQDCGMIIRQLGELSQLRRLGILKLREEDGMAFCLSIERLTNLHALSVASEGESKVIDLTFLCSPPPFLQRLYLS
GRLQELPSWIQSLHSLARLFLKWSCLRYDPLVYLQDLPNLAHLELLKVYDGDTLHFRSGKFKKLKVLGLDKFDGLEEVTVG
KDAMNCLEKLSIGRCELLKKVPSGIENLTKLKVLEFFDMPDELMKTICPHGPGKDYCKVLHIPDVYSTYWRDGGWDVYA
LDTFSRDCSPRSGTLIRSHEPRIQWKV

>Vang0570s00020.3

MASVREAPSWKFHVFLSFRGEDTRNGFTDHLYAAFRGRGFAVFKDDEELERGEVISEALLKAIDESLCSVVVLSPHYASS
RWCLDELLRILESRANFGRNVLPIFYNVDPADVRHQRGTFAEAFAKHGERFGSDEVRMWRQALKDVAALSGWTSKDT
RETELIEEIVADVWEKLQTKLPSYDDELVGIDSRINSIYAFLRTDSQEVRFMSIWGMGGIGKTTLARFVYNKIHDQYDISCF
LENVREVSSERDGLLCLQRKLLSHLKIRSMRIESLDQGKETIRNLLFNKKVLLVLDDLSSDIQVENLAGKPEWFGQGSRVII
TTRDKHQLKSLHVCENYDVQVLNNYESLQLFCQKAFRGEKPEEAYLELSRSVVQYAGGVPLALKVLGSFLCGRSASVWE
DALKMLRKDAQNDICKTLRISYDGLRDNEKAIFLDIACFFKGNTKDDVTRILENCDFNPLIGIEVLIEKSLVTCDGLHLGMH
DLLQEMGRNIVFQESPNDASKRSRLWTLKDVDQVLRNNNGTESIQAIVLNLPEPYEACWNPDAFSKMSNLRMLMILN
KLQLPLGLKCLPSRLKVLIWKEYPLESLPVGAQLDELVELHMCQSKIKHLWGGNKGNISIQYLFLENLKIVNLRNCTNVHR
TPDFTGIPNLEKLDLEGCVNLVEVHASLGLLKKLSYLTFEDCKNLKILPRKLLLDSLKRLVLSGCSAVRKLPEFGESMKSLSEL
ALEETSIAELPVSVGHLTGLTNLLLKGCKNIVCLPNTISNLKSLKRLNISGCSKISKLPDNLNENEALEFLNASETAIRELPSSIV

LLKNLRLLLLRGCKDLASNSWSSLLLPFENILRFNSHPTPKRLILPSFSGLSSLRKLDLSYCNLHDGSIPEDLGCLSSLVTLDLS
GNNFVCFPGSISELLKLERLLLKCCPRLESFPKLPPEVHYVNASDCGSMKPLSDPQQIWGHLASFAFDKLQDASNFRTLLV
SPGNEIPSFFFYQKHLNQVQDIEYLKENYIWADSTVSIPMDLAQLRHRYHRSEWWGILVSLVVEDVESSPSQEYRIGWIS
KVPSFKNILQQLCHKTEQGFISGMHNHKYPHLLILYIPVHRARSFYVHDKFQLIFYCSSLKSKLVIKKCGRHILSKEDAENW
RTKLSEWNTNSTNQCVENPRDCRLSPHFSSWRWISCLMVPQHCKTFLLAILCDRLPANERCSFCCLEGTVIHVLRDCTR
ATAIWVQMVPPEVCDEFFSTSLHDWMHRFLLKLWFPDRDYYADCLRFTITIWLLWKDRNRSIFKRNSPTDNDGLYSMI
QSLVKEYSILLHLKGEEGTSAVNSVSQNSRLKLFVRLNVDGCCNGNPGNAGYGGLFRDVEGKWLGGFYGSLGLATNVK
AELYAICQGLIAAWDLGYRTVLVETDSLEAINLIKEANIEDCAYGGLLADIRSLMQRNWSLDLIHSLRQDNACADMLSKL
GTEQHEVYCFLAHPPQQLQLALVADALQKMQCGLMLKQNCKVLDKALDTNAYVSGSIVCLFRLINQRRLEKLKAQIASE
RKMGDTLTHFRGGLSMASMTSYEYEPCGPKHHLYRLCFKVS

>Vang0570s00020.2

MASVREAPSWKFHVFLSFRGEDTRNGFTDHLYAAFRGRGFAVFKDDEELERGEVISEALLKAIDESLCSVVVLSPHYASS
RWCLDELLRILESRANFGRNVLPIFYNVDPADVRHQRGTFAEAFAKHGERFGSDEVRMWRQALKDVAALSGWTSKDT
RETELIEEIVADVWEKLQTKLPSYDDELVGIDSRINSIYAFLRTDSQEVRFMSIWGMGGIGKTTLARFVYNKIHDQYDISCF
LENVREVSSERDGLLCLQRKLLSHLKIRSMRIESLDQGKETIRNLLFNKKVLLVLDDLSSDIQVENLAGKPEWFGQGSRVII
TTRDKHQLKSLHVCENYDVQVLNNYESLQLFCQKAFRGEKPEEAYLELSRSVVQYAGGVPLALKVLGSFLCGRSASVWE
DALKMLRKDAQNDICKTLRISYDGLRDNEKAIFLDIACFFKGNTKDDVTRILENCDFNPLIGIEVLIEKSLVTCDGLHLGMH
DLLQEMGRNIVFQESPNDASKRSRLWTLKDVDQVLRNNNGTESIQAIVLNLPEPYEACWNPDAFSKMSNLRMLMILN
KLQLPLGLKCLPSRLKVLIWKEYPLESLPVGAQLDELVELHMCQSKIKHLWGGNKGNISIQYLFLENLKIVNLRNCTNVHR
TPDFTGIPNLEKLDLEGCVNLVEVHASLGLLKKLSYLTFEDCKNLKILPRKLLLDSLKRLVLSGCSAVRKLPEFGESMKSLSEL
ALEETSIAELPVSVGHLTGLTNLLLKGCKNIVCLPNTISNLKSLKRLNISGCSKISKLPDNLNENEALEFLNASETAIRELPSSIV
LLKNLRLLLLRGCKDLASNSWSSLLLPFENILRFNSHPTPKRLILPSFSGLSSLRKLDLSYCNLHDGSIPEDLGCLSSLVTLDLS
GNNFVCFPGSISELLKLERLLLKCCPRLESFPKLPPEVHYVNASDCGSMKPLSDPQQIWGHLASFAFDKLQDASNFRTLLV
SPGNEIPSFFFYQKHLNQVQDIEYLKENYIWADSTVSIPMDLAQLRHRYHRSEWWGILVSLVVEDVESSPSQEYRIGWIS
KVPSFKNILQQLCHKTEQGFISGMHNHKYPHLLILYIPVHRARSFYVHDKFQLIFYCSSLKSKLVIKKCGRHILSKEDAENW
RTKLSEWNTNSTNQCVENPRDCRLSPHFSSWRWISCLMVPQHCKTFLLAILCDRLPANERCSFCCLEGTVIHVLRDCTR
ATAIWVQMVPPEVCDEFFSTSLHDWMHRFLLKLWFPDRDYYADCLRFTITIWLLWKDRNRSIFKRNSPTDNDGLYSMI
QSLVKEYSILLHLKGEEGTSAVNSVSQNSRLKLFVRLNVDGCCNGNPGNAGYGGLFRDVEGKWLGGFYGSLGLATNVK
AELYAICQGLIAAWDLGYRTVLVETDSLEAINLIKEANIEDCAYGGLLADIRSLMQRNWSLDLIHSLRQDNACADMLSKL
GTEQHEVYCFLAHPPQQLQLALVADALQKMQCGLMLKQNCKVLDKALDTNAYVSGSIVCLFRLINQRRLEKLKAQIASE
RKMGDTLTHFRGGLSMASMTSYEYEPCGPKHHLYRLCFKEEEMGWRGGGGGGRKYEGAVVEEERR

>Vang0570s00020.1

MASVREAPSWKFHVFLSFRGEDTRNGFTDHLYAAFRGRGFAVFKDDEELERGEVISEALLKAIDESLCSVVVLSPHYASS
RWCLDELLRILESRANFGRNVLPIFYNVDPADVRHQRGTFAEAFAKHGERFGSDEVRMWRQALKDVAALSGWTSKDT
RETELIEEIVADVWEKLQTKLPSYDDELVGIDSRINSIYAFLRTDSQEVRFMSIWGMGGIGKTTLASSDIQVENLAGKPEW
FGQGSRVIITTRDKHQLKSLHVCENYDVQVLNNYESLQLFCQKAFRGEKPEEAYLELSRSVVQYAGGVPLALKVLGSFLC
GRSASVWEDALKMLRKDAQNDICKTLRISYDGLRDNEKAIFLDIACFFKGNTKDDVTRILENCDFNPLIGIEVLIEKSLVTC
DGLHLGMHDLLQEMGRNIVFQESPNDASKRSRLWTLKDVDQVLRNNNGTESIQAIVLNLPEPYEACWNPDAFSKMS
NLRMLMILNKLQLPLGLKCLPSRLKVLIWKEYPLESLPVGAQLDELVELHMCQSKIKHLWGGNKGNISIQYLFLENLKIVN
LRNCTNVHRTPDFTGIPNLEKLDLEGCVNLVEVHASLGLLKKLSYLTFEDCKNLKILPRKLLLDSLKRLVLSGCSAVRKLPEF
GESMKSLSELALEETSIAELPVSVGHLTGLTNLLLKGCKNIVCLPNTISNLKSLKRLNISGCSKISKLPDNLNENEALEFLNAS
ETAIRELPSSIVLLKNLRLLLLRGCKDLASNSWSSLLLPFENILRFNSHPTPKRLILPSFSGLSSLRKLDLSYCNLHDGSIPEDLG

CLSSLVTLDLSGNNFVCFPGSISELLKLERLLLKCCPRLESFPKLPPEVHYVNASDCGSMKPLSDPQQIWGHLASFAFDKLQ
DASNFRTLLVSPGNEIPSFFFYQKHLNQVQDIEYLKENYIWADSTVSIPMDLAQLRHRYHRSEWWGILVSLVVEDVESSP
SQEYRIGWISKVPSFKNILQQLCHKTEQGFISGMHNHKYPHLLILYIPVHRARSFYVHDKFQLIFYCSSLKSKLVIKKCGRHI
LSKEDAENWRTKLSEWNTNSTNQCVENPRDCRLSPHFSSWRWISCLMVPQHCKTFLLAILCDRLPANERCSFCCLEGT
VIHVLRDCTRATAIWVQMVPPEVCDEFFSTSLHDWMHRFLLKLWFPDRDYYADCLRFTITIWLLWKDRNRSIFKRNSPT
DNDGLYSMIQSLVKEYSILLHLKGEEGTSAVNSVSQNSRLKLFVRLNVDGCCNGNPGNAGYGGLFRDVEGKWLGGFYG
SLGLATNVKAELYAICQGLIAAWDLGYRTVLVETDSLEAINLIKEANIEDCAYGGLLADIRSLMQRNWSLDLIHSLRQDNA
CADMLSKLGTEQHEVYCFLAHPPQQLQLALVADALQKMQCGLMLKQNCKVLDKALDTNAYVSGSIVCLFRLINQRRLE
KLKAQIASERKMGDTLTHFRGGLSMASMTSYEYEPCGPKHHLYRLCFKEEEMGWRGGGGGGRKYEGAVVEEERR

>Vang0070s00540.1

MPSNAIVQYSSSSSHVINTYDVFVSFRGEDTRNNFTGFLFQALRRKGIHAFKDDQDLKKGESIAPELLQAIQASRLFIIVFS
NNYASSIWCLRELAEIRNCAQTSARRIIPIFYDVDPSVVRKQSGCYDVSFAEHEYRFRENKVKMEEAKRWREALTEVANL
SGWDIRNKPQYAQIEEIVQNITNILGPKISSLPKDELVGIESRVQELKDLLCCGSLNDVRVVGISGMGGIGKTSLAWTLYE
RICHQYDFHCFIDDVSKIYRDSSSLGLQKQLISQSLNEKNLEICNGFEGTCLVWSRLHNARALIVLDNVDQVEQLRMFTG
NRDTLLRECLGEGSKIIIISRDEHILRTHGVDDVYQVQPLSWENAVQLFCRNALKVNYILSDYEKLARDVLSHAQGHPLAI
EVIGSSLFGRSVSQWKSALARLKENKSKSIMDVLRISFDELDEGDKETFLDIACFYNGYEEIYVKEILIDDFTGYVSLDNLLG
VVTRVM

>Vang0304s00010.1

MTHNPTNYVIVNISKSVLLGPNHPAGENFQLLHRNFAGLFSYTIGTKEESLMFNGSNSFCAGLKRFWDRFGGRSSGSTS
SSSYDSSDSIQNQNYVYDVFISFGGPDTRNSFVDHLCSHLLRKGIFVFKDDHNLQKGESISPQLLEAIQLSRLSIIVFSKNYA
SSTWCLDEMTAIASCKQQSSQIVFPIFYDVDPSHVRHQNGVYENDFVSHRCKFQKDRDKVPGWERAMTDLANSAGI
WGMNGIGKTTQAAVLFDKISHRAIGKFGHRTMLLKGSRMVITTTDMHILKVVYEGGVIHKVPLLNDNDARELFCRKAFK
SEEQSSSCEALIPEVLKYAQCLPLAIRVLGSFLCTRDAVEWRDVLNRLQSSLDKKIMITFQISVDGLNHEEKQIFLHIACFFK
GERVDYVKRILDCCELYPHIGISRLVEKSLITISNEEIHMHELLQELGKKMVWDQSPQEPRFWSRIWLHKDFLQVLTAET
GTEKVKAIVLNKEEEMSECSIGGLSRMKELTLLILYHTKVSGSLEFLSDRLRYLLWHDYPFDSLPPYFTVSNLVELNMPNSH
IISLWHGNKVIYSHSFHFRLGLNITKR

>Vang0693s00060.1

MKGGDISFRGADTRFGFTGNLYSALSQRGIFTFINDEALRKGEEITASLRKTIQESRISIIVFSKNYAFSTFCLDELLQIIECHT
KQNMWILPVFYDVESSQVRHQTGSYQEAFSNHGERFKDDIQKLQQWRLALRHAADLAGFPFKTGEKYESEILKKITEEI
STKLNRPPLHIADYPVGLKVRMQHIQEFMGGEFDKKVTMLGIHGMRGIGKSTLSRAMYNLMAHQFEASHFLANVREK
SYKDGLMHIQETMMSELMGERNIKLGDVHRGIPILQHRLCGKKVLLVLDDISKKEQLHTTAGGLDWFGSALELFKWNA
FKNKEVDPCYKEVTERAMYYANGLPLAWEAIGSNLFGKTLDEWESTLETYERIPNRDVQEVLKDSYDRLEASQQEMFLE
TTCFFSGRSRRYVRNTLEARGGVCPQFGLKVLEERSLIKIRKCPHDTMEMHHIWEKK

>Vang02g14420.6

LVIEQVPSILSIVGMGGLGKTTLAQHVYNDTEIEEAKFDIKAWVCVSDHFDILTVTKTVLEAITKSKDDSGDLEMVHGRLK
EKVSGKKFLLVLDDVWSERREEWEAVRTPLCHGAPGSRILVTTRVEKVASNMRSKVHHLKQLEEDECWKVFEEQALKD
DDLELNDEKKEFGRRIVEKCKGLPLALKTIGSLLRTKSSISDWKSVLESDIWDLPKEVEIIPALLLSYQNLPSHLKRCFAYCAL
FPKNYEFNNNLEEIGEQYFDDLLTRSFFLRSNIKMHFSMHDLLNDLAKYVCADNFVHGAFPCQIKILIRELFSKWKFLRVL
SLNGNYDLEEVPDSVGDLKHLHSLDLSRTMIRKLPDSVGLLYNLLILKLNDCSFLKELPSNLHKLTNLRCLEFEDTEVTKMP
MHFGELKNLHVLNMFCVDRNSEFSAKQLGGINLHGRLSINELQNIVNPLDALEANLKNKQLVELKLIWNWNHIPDDP

MKNKKVLENLQPSNQLEHLSIRSYCGTQFPSWVFDNSLSNLVSLELEDCKYCLCLPPLGLLSSLKTLKIRGFDGIVSIGAEFY
GSSSSSFKSLEILKFYNMKEWEEWECKTTSFPRLQHLVIVRCPKLKGLSEQLLHLKELFIESCPNLIVSEHSEDTSALDLLRTR
SCPLVNIPMTRYDFIEQITIDKSCNSLTIFQLNFFPILRLLRLEGCQNLQRISQEHPHNHLKKMSVCACPQFESFPSEGLSAA
FPFLTELEIIWCRKVEKFPDGGLPSNVKHMSLSSIKLIASLRETLDVNTCLESLSMKYLDVESFPDEVLLPPSLTSLTISNCRN
LKRLGHKVLYNLSSLTLFDCRNLQCLPEEGLPESISSLQILNCPLLKQRCQKPEGKDWRKIAHIQNLRV

>Vang02g14420.4

MAAELVGGALLSAFLQVAFERLASPQFLDFFRGRKLDEKLLSNLNIMLHSINALAHDAERKQFTDPHVKAWLFSVKEAV
FDAEDLFGEIDYELTRCQVEAESEPQTFTYKVSNFFNSTFKTFNKKIESELKELLEKLEYLAKQKGALGLREGTYSGDRTGG
KVSQKLPSSSLVVESVIYGRDADKQMICNWLTSETDNHNHPSILSIVGMGGLGKTTLAQHVYNDTEIEEAKFDIKAWVC
VSDHFDILTVTKTVLEAITKSKDDSGDLEMVHGRLKEKVSGKKFLLVLDDVWSERREEWEAVRTPLCHGAPGSRILVTTR
VEKVASNMRSKVHHLKQLEEDECWKVFEEQALKDDDLELNDEKKEFGRRIVEKCKGLPLALKTIGSLLRTKSSISDWKSV
LESDIWDLPKEVEIIPALLLSYQNLPSHLKRHFSMHDLLNDLAKYVCADFCFRLKFDKGNCIPKTTRHFLFAFDDLRYFDGS
GSLTDAKRLRSFVPITNNFVHGAFPCQIKILIRELFSKWKFLRVLSLNGNYDLEEVPDSVGDLKHLHSLDLSRTMIRKLPDS
VGLLYNLLILKLNDCSFLKELPSNLHKLTNLRCLEFEDTEVTKMPMHFGELKNLHVLNMFCVDRNSEFSGRLSINELQNIV
NPLDALEANLKNKQLVELKLIWNWNHIPDDPMKNKKVLENLQPSNQLEHLSIRSYCGTQFPSWVFDNSLSNLVSLELED
CKYCLCLPPLGLLSSLKTLKIRGFDGIVSIGAEFYGSSSSSFKSLEILKFYNMKEWEEWECKTTSFPRLQHLVIVRCPKLKGLS
EQLLHLKELFIESCPNLIIPMTRYDFIEQITIDKSCNSLTIFQLNFFPILRLLRLEGCQNLQRISQEHPHNHLKKMSVCACPQF
ESFPSEGLSAAFPFLTELEIIWCRKVEKFPDGGLPSNVKHMSLSSIKLIASLRETLDVNTCLESLSMKYLDVESFPDEVLLPPS
LTSLTISNCRNLKRLGHKVLYNLSSLTLFDCRNLQCLPEEGLPESISSLQILNCPLLKQRCQKPEGKDWRKIAHIQNLRV

>Vang02g14420.5

MICNWLTSETDNHNHPSILSIVGMGGLGKTTLAQHVYNDTEIEEAKFDIKAWVCVSDHFDILTVTKTVLEAITKSKDDSG
DLEMVHGRLKEKVSGKKFLLVLDDVWSERREEWEAVRTPLCHGAPGSRILVTTRVEKVASNMRSKVHHLKQLEEDEC
WKVFEEQALKDDDLELNDEKKEFGRRIVEKCKGLPLALKTIGSLLRTKSSISDWKSVLESDIWDLPKEVEIIPALLLSYQNLP
SHLKRCFAYCALFPKNYEFEKEKLILLWMAEGFLHYSSQNNNLEEIGEQYFDDLLTRSFFLRSNIKMHFSMHDLLNDLAKY
VCADFCFRLKFDKGNCIPKTTRHFLFAFDDLRYFDGSGSLTDAKRLRSFVPITNNFVHGAFPCQIKILIRELFSKWKFLRLN
DCSFLKELPSNLHKLTNLRCLEFEDTEVTKMPMHFGELKNLHVLNMFCVDRNSEFSAKQLGGINLHGRLSINELQNIVNP
LDALEANLKNKQLVELKLIWNWNHIPDDPMKNKKVLENLQPSNQLEHLSIRSYCGTQFPSWVFDNSLSNLVSLELETLKI
RGFDGIVSIGAEFYGSSSSSFKSLEILKFYNMKEWEEWECKTTSFPRLQHLVIVRCPKLKGLSEQLLHLKELFIESCPNLIVSE
HRTRSCPLVNIPMTRYDFIEQITIDKSCNSLTIFQLNFFPILRLLRLEGCQNLQRISQEHPHNHLKKMSVCACPQFESFPSE
GLSAAFPFLTELEIIWCRKVEKFPDGGLPSNVKHMSLSSIKLIASLRETLDVNTCLESLSMKYLDVESFPDEVLLPPSLTSLTI
SNCRNLKRLGHKVLYNLSSLTLFDCRNLQCLPEEGLPESISSLQILNCPLLKQRCQKPEGKDWRKIAHIQNLRV

>Vang02g14420.2

LNIMLHSINALAHDAERKQFTDPHVKAWLFSVKEAVFDAEDLFGEIDYELTRCQEYLAKQKGALGLREGTYSGDRTGGK
VSQKLPSSSLVVESVIYGRDADKQMICNWLTSETDNHNHPSILSIVGMGGLGKTTLAQHVREEWEAVRTPLCHGAPGS
RILVTTRVEKVASNMRSKVHHLKQLEEDECWKVFEKTIGSLLRTKSSISDWKSVLESDIWDLPKEVEIIPALLLSYQNLPSH
LKRCFAYCALFPKNYEFEKEKLILLWMAEGFLHYSSQNNNLEEIGEQYFDDLLTRSFFLRSNIKMHFSMHDLLNDLAKYVC
ADFCFRLKFDKGNCIPKTTRHFLFAFDDELPSNLHKLTNLRCLEFEDTEVTKMPMHFGELKNLHNIVNPLDALEANLKNK
QLVELKLIWNWNHIPDDPMKNKKVLENLQPSNQLEHLSIRSYCGTQFPSWVFDNSLSNLVSLELEDCKYCLCLPPLGLLS
SLKTLKIRGFDGIVSIGAEFYGSSSSSFKSLEILKFYNMKEWEEWECKTTSFPRLQHLVIVRCPKLKGLSEQLLHLKELFIESC
PNLIVSEHSEDTSALDLLRTRSCPLVNIPMTRYDFIEQITIDKSCNSLTIFQLNFFPILRLLRLEGCQNLQRISQEHPHNHLKK
MSVCACPQFESFPSEGLSAAFPFLTELEIIWCRKVEKFPDGGLPSNVKHMSLSSIKLIASLRETLDVNTCLESLSMKYLDVE

SFPDEVLLPPSLTSLTISNCRNLKRLGHKVLYNLSSLTLFDCRNLQCLPEEGLPESISSLQILNCPLLKQRCQKPEGKDWRKIAHIQNLRV

>Vang02g14420.3

LNIMLHSINALAHDAERKQFTDPHVKAWLFSVKEAVFDAEDLFGEIDYELTRCQVEAESEPQTFTYKVSNFFNSTFKTFNKKIESELKELLEKLEYLAKQKGALGLREGTYSGDRTGGKVSQKLPSSSLVVESVIYGRDADKQMICNWLTSETDNHNHPSILSIVGMGGLGKTTLAQHVYNDTEIEEAKFDIKATGGKVSQKLPSSSLVVESVIYGRDADKQMICNWLTSETDNHNHPSILSIVGMGGLGKTTLAQHVYNDTEIEEAKFDIKAWVCVSDHFDILTVTKTVLEAITKSKDDSGDLEMVHGRLKEKVREEWEAVRTPLCHGAPGSRILVTTRVEKVASNMRSKVHHLKQLEEDECWKVFEEQALKDDDLELNDEKKEFGRRIVEKCKGLPLALKTIGSLLRTKSSISDWKSVLESDIWDLPKEVEIIPALLLSYQNLPSHLKRCFAYCALFPKNYEFEKEKLILLWMAEGFLHYSSQNNNLEEIGEQYFDDLLTRSFFLRSNIKMHFSMHDLLNDLAKYVCADFCFRLKFDKGNCIPKTTRHFLFAFDKELPSNLHKLTNLRCLEFEDTEVTKMPMHFGELKNLHVLNMFCVDRNSEFSAKQLGGINLHGRLSINELQNIVNPLDALEANLKNKQLVELKLIWNWNHIPDDPMKNKKVLENLQPSNQLEHLSIRSYCGTQFPSWVFDNSLSNLVSLELEDCKYCLCLPPLGLLSSLKTLKIRGFDGIVSIGAEFYGSSSSSFKSLEILKFYNMKEWEEWECKTTSFPRLQHLVIVRCPKLKGLSEQLLHLKELFIESCPNLIVSEHSEDTSALDLLRTRSCPLVNIPMTRYDFIEQITIDKSCNSLTIFQLNFFPILRLLRLEGCQNLQRISQEHPHNHLKKMSVCACPQFESFPSEGLSAAFPFLTELEIIWCRKVEKFPDGGLPSNVKHMSLSSIKLIASLRETLDVNTCLESLSMKYLDVESFPDEVLLPPSLTSLTISNCRNLKRLGHKVLYNLSSLTLFDCRNLQCLPEEGLPESISSLQILNCPLLKQRCQKPEGKDWRKIAHIQNLRV

>Vang02g14420.1

MAAELVGGALLSAFLQVAFERLASPQFLDFFRGRKLDEKLLSNLNIMLHSINALAHDAERKQFTDPHVKAWLFSVKEVDYELTRCQVEAESEPQTFTYKVSNFFNSTFKTFNKKIESELKELLEKLEYLAKQKGALGLREGTYSGDRTGGKVSQDADKQMICNWLTSETDNHNHPSILSIVGMGGLGKTTLAQHVYNDTEIEEAKFDIKAWVCVSDHFDILTVTKTVLEAITKSKDDSGDLEMVHGRLKEKVSGKKFLLVLDDVWSERREEWKVASNMRSKVHHLKQLEEDECWKVFEEQALKDDDLELNDEKKEFGRRIVEKCKGLPLALKTIGSLLRTKSSISDWKSVLESDIWDLPKEVKNYEFEKEKLILLWMAEGFLHYSSQNNNLEEIGEQYFDDLLTRSFFLRSNIKMHFSMHDLLNDLAKYVCADFCFRLKFDKGNCIPKTTRHFLFAFDDLRYFDGSGSLTDAKRLRSFVPITNNFVHGAFPCQIKILIRELFSKWKFLRVLSLNGNYDLEEVPDSLNDCSFLKELPSNLHKLTNLRCLEFEDTEVTKMPMHFGELKNLHVLNMFCVDRNSEFSAKQLGGINLHGRLSINELQNIVNPLDALEANLKNKQLVELKLIWNWNHIPDDPMKNKKVLENLQPSNQLEHLSIRSYCGTQFPSWVFDNSLSNLVSLELEDCKYCLCLPPLGLLSSLKTLKIRGFDGIVSIGAEFYGSSSSSFKSLEILKFYNMKEWEEWECKTTSFPRLQHLVIVRCPKLKGLSEQLLHLKELFIESCPNLIVSEHSEDTSALDLLRTRSCPLVNIPMTRYDFIEQITIDKSCNSLTIFQLNFFPILRLLRLEGCQNLQRISQEHPHNHLKKMSVCACPQFESFPSEGLSAAFPFLTELEIIWCRKVEKFPDGGLPSNVKHMSLSSIKLIASLRETLDVNTCLESLSMKYLDVESFPDEVLLPPSLTSLTISNCRNLKRLGHKVLYNLSSLTLFDCRNLQCLPEEGLPESISSLQILNCPLLKQRCQKPEGKDWRKIAHIQNLRV

>Vang02g14420.8

LVVESVIYGRDADKQMICNWLTSETDNHNHPSILSIVGMGGLGKTTLAQHVYNDTEIEEAKFDIKAWVCVSDHFDILTVTKTVLEAITKSKDDSGDLEMVHGRLKEKVSGKKFLLVLDDVWSERREEWEAVRTPLCHGAPGSRILVTTRVEKVASFAYCALFPKNYEFEKEKLILLWMAEGFLHYSSQNNNLEEIGEQYFDDLLTRSFFLRSNIKMHFSMHDLLNDLAKYVCADFCFRLKFDKGNCIPKTTRHFLFAFDDLRYFDGSGSLTDAKRLRSFVPITNNFVHGAFPCQIKILIRELFSKWKFLRVLSLNGNYDLEEVPDSVGDLKHLHSLDLSRTMIRKLPDSVGLLYNLLILKLNDCSFLKELPSNLHKLTNLRCLEFEDTEVTKMPMHFGELKNLHVLNMFCVDRNSEFSAKQLGGINLHGRLSINELQNIVNPLDALEANLKNKQLVELKLIWNWNHIPDDPMKNKKVLENLQPSNQLEHLSIRSYCGTQFPSWVFDNSLSNLVSLELEDCKYCLCLPPLGLLSSLKTLKIRGFDGIVSIGAEFYGSSSSSFKSLEILKFYNMKEWEEWECKTTSFPRLQHLVIVRCPKLKGLSEQLLHLKELFIESCPNLIVSEHSEDTSALDLLRTRSCPLVNIPMTRYDFIEQITIDKSCNSLTIFQLNFFPILRLLRLEGCQNLQRISQEHPHNHLKKMSVCACPQFESFPSEGLSAAFPFLTELEIIW

CRKVEKFPDGGLPSNVKHMSLSSIKLIASLRETLDVNTCLESLSMKYLDVESFPDEVLLPPSLTSLTISNCRNLKRLGHKVLY
NLSSLTLFDCRNLQCLPEEGLPESISSLQILNCPLLKQRCQKPEGKDWRKIAHIQNLRV

>Vang02g14420.9

LKELLEKLEYLAKQKGALGLREGTYSGDRTGGKVSQKLPSSSLVVESVIYGRDADKQMICNWLTSETDNHNHPSILSIVG
MGGLGKTTLAQHVYNDTEIEEAKFDIKAWVCVSDHFDILTVTKTVLEAITKSKDDSGDLEMVHGRLKEKVSGKKFLLVLD
DVWSERREEWEAVRTPLCHGAPGSRILVTTRVEKVASNMRSKVHHLKQLEEDECWKVFEEQALKDDDLELNDEKKEF
GRRIVEKCKGLPLALKTIGSLLRTKSSISDWKSVLESDIWDLPKEVEIIPALLLSYQNLPSHLKRCFAYCALFPKNYEFEKEKLI
LLWMAEGFLHYSSQNNNLEEIGEQYFDDLLTRSFFLRSNIKMHFSMHDLLNDLAKYVCADFCFRLKFDKGNCIPKTTRH
FLFAFDDLRYFDGSGSLTDAKRLRSFVPITNNFVHGAFPCQIKILIRELFSKWKFLRVLSLNGNYDLEEVPDSVGDLKHLHS
LDLSRTMIRKLPDSVGLLYNLLILKLNDCSFLKELPSNLHKLTNLRCLEFEDTEVTKMPMHFGELKNLHVLNMFCVDRNSE
FSAKQLGGINLHGRLSINELQNIVNPLDALEANLKNKQLVELKLIWNWNHIPDDPMKNKKVLENLQPSNQLEHLSIRSY
CGTQFPSWVFDNSLSNLVSLELEDCKYCLCLPPLGLLSSLKTLKIRGFDGIVSIGAEFYGSSSSSFKSLEILKFYNMKEWEEW
ECKTTSFPRLQHLVIVRCPKLKGLSEQLLHLKELFIESCPNLIVSEHSEDTSALDLLRTRSCPLVNIPMTRYDFIEQITIDKSCN
SLTIFQLNFFPILRLLRLEGCQNLQRISQEHPHNHLKKMSVCACPQFESFPSEGLSAAFPFLTELEIIWCRKVEKFPDGGLPS
NVKHMSLSSIKLIASLRETLDVNTCLESLSMKYLDVESFPDEVLLPPSLTSLTISNCRNLKRLGHKVLYNLSSLTLFDCRNLQ
CLPEEGLPESISSLQILNCPLLKQRCQKPEGKDWRKIAHIQNLRV

>Vang0039ss00520.1

MESEREDNNNGTDSTHAAISSTLKIKILSCSKSGESLDNSNLPSPNINNGTESSPYGSPLVSPPSSAFVSALQSPYISPRAIIP
DPPNGSPLENQPPLLTITTSTTNPSTPEDVPSSSYTPPSDQYEFSDDPADTRLKYVTCVPEPAPPRISFSFPVPRISFAKGPI
SPATNAKLRSCDVYIGFHGQNPNLVRFCRWLKSELELQGIDCMLADRAKYSDSQSHEIADGVICSVAFGVVVVTSSSFLN
HFSMEEVRFFAQKKNLIPLLFDTGPAEIMALLNCKSIDKECKEAIDGLMKCNEFNIEANDSNWRSCIAKAAGILRARLGR
KNAEQKDNVQGLGNLPFPRNTYFVGREKEIMEIEGLFFGRGNCMEQVQDHCRVFTKGEASGSGQSEGLADEESEPVIA
RCGRYISLEMGRSKEPTLEAWVEPIMGNNSLKRLKNKKSKSGSYKSVCSSVICINGVSGIGKSELALEFAHRYHQRYKMV
LWVGGEARYLRQNLLNLSLNLGLDVGADSEMERGRIRSFEDQEFEAFKRVKRELFGETPYLLIIDNLETEVEWWEGKDL
YDLIPRNTGGTHVIVTTRLSKVMSYDTIQLPPLPLSDAMILMIGRKRKEYSADEIDLLEKINEKLGRLSIGLWMIGSLLAELSI
GPSCLYEAINQVPLDEDSNSCYMSIAEGQWCKSNPFLMKTLLFCLETLEKTKAKGNLLALRMLLVSGWFSPSPISSTLLAN
AAKSIPTVESRLKKWTKSLSLTTSCLSPRTWKNEEEPAMILVKMGLARRANQHDGCWLHFHPITQAFAKRKGGLQYAK
AAIQGVRKMGSQVNSDHLWASAFLVFGFKSEPPLVQLKAIDMVLYIKRTALPLAIQAFTIFSRCNSSLELLRVCTNALEEV
EKSFVSQIQDWSSHGSVCWKRRLQRGQKVDEYVWQDVTLLKATLLETRAKLLARGGHLDSAKELCRTCISIRTVMLGH
NHAQTLAAQDTLARLVRMRSKI

>Vang01g19280.2

MALSDFFAGEIATELLKMLISISRKSLLCRASADQLITYIHELLPTIEEIKYSGVELPALRQSQLHLLSEILRSGVELSHKVLASS
RWNVYRNLHLAKKMDKLEKTVSKFLLGPMQAHIMADVHHTRFEMAERFDRVDNSVRRLEQYFGNIKIGVGGGGWV
EEAVRSVDENVVEGSSAVGLEFGKIKVREMIIGREDLWVVGISGIGGSGKTTLAREVCKDDQVRRYFRERILFLTVSQSPN
VEQLRTKIWGYIMGNERLDSNYVVPQWVPQFECKSEAARTLIVLDDLWTFSVMEQLVCRIPGCKYLVVSRTKFQTVLN
YEVELLSEEDALSLFCHHAFGQKSIPLGANENLAKQVVTECGRLPLALKVIGASLRDQPEMFWLSVKNRLSQGQSIGESH
EINLIERMEISINYLPEKVKECFLDLCSFPEDKKIPLDVLINMWVEIHDIPETEAYAIVVELSNKNLLTLMKEPRAGGMYSSC
FEISVTQHDILRNLAINLSNRASINERRRLTEVLIINFTSTEYFLPLFINTMPNLRALIIINYSATYACLHNISVFKNLPNLRSLW
LEKVSTPELSGTVLENLGKLFIVLCKINNRLVEKEVDIAKVFPNLFDLTLDHCDDLTQLPSSICGMKSLQNLSLTNCHNLTQ
LPLELGKLKSLEILRLYACPDLKTLPNSICEMMRLKYIDISQCVNLTCFPEEIGRLVSLEKIDMRECSMIRNVPKSALSLQSLR
LVICDEELSGIWKEVEKAKPNVHIQALRCKSSAEQLITYVREILPTIEEIKYSGVELPAPRQSQLDRLSEILRSGVELSHQALS

SSRWNVYRNFQLAKKMEKLEKHVTRFLQVPMQAHILADVHHVRFEMAERFDRVEASNRRMERFLEEMKIGVNGGG
WVEEAVKSMQEDETWVEGCNGNNGFGVGLDFGKKNVMEMVFSNNDADWIVGICYFKDRILFLTVSQSPNVEQLRA
RIWGHIMGNQGLNGNYEVPQWMPQFECKGEAQALVVLDDVWSFSVLEQLVWKIPGCKFLVVSRFRFPTFFSATYHV
ELLGEEDALSLFCHHAFGQKSIPLGANVSLVKQVVAECGKLPLALKVIGASLRDQNEMFWLSVKSNLSQGHSIGESYEINL
IDRMAISTNYLPEKIKECFLDLCSFPEDRKIPLEILINMWVEIHDIREAEAYAIAVELSNKNLLTLVKEARAGGMYSSCFEISV
TQHDTLRDLALILSKRGSIHEHRRLVMAQREENGLLPREWSRYQDRPFEAQIVSINTGEMTEMDWFELDFPKAEVLIIN
FTSSDYFLPPFISKMPNLRALIIVNYSTSYARLHNVSVLRNLTNLRSLWLEKVSTPQLSGTVLKNLSKLFVVLCQINNSLDGK
QFPNLSELTLDHCNDLTYLPSSICGIKSLRNMSLTDCHNLSELPVEFGNLKSLEILRLYACPDLETLPPSMCEMKKLKYIDIS
QCTNLTCFPKEIGRLVSLEKIDMRECPMIRYLPKSAVSLHSLQLVICDEEVYGTWRDVAEMAKSNVHIQVPEQHFDLDW
LQE

>Vang01g19280.1

MALSDFFAGEIATELLKMLISISRKSLLCRASADQLITYIHELLPTIEEIKYSGVELPALRQSQLHLLSEILRSGVELSHKVLASS
RWNVYRNLHLAKKMDKLEKTVSKFLLGPMQAHIMADVHHTRFEMAERFDRVDNSVRRLEQYFGNIKIGVGGGGWV
EEAVRSVDENVVEGSSAVGLEFGKIKVREMIIGREDLWVVGISGIGGSGKTTLAREVCKDDQVRRYFRERILFLTVSQSPN
VEQLRTKIWGYIMGNERLDSNYVVPQWVPQFECKSEAARTLIVLDDLWTFSVMEQLVCRIPGCKYLVVSRTKFQTVLN
YEVELLSEEDALSLFCHHAFGQKSIPLGANENLAKQVVTECGRLPLALKVIGASLRDQPEMFWLSVKNRLSQGQSIGESH
EINLIERMEISINYLPEKVKECFLDLCSFPEDKKIPLDVLINMWVEIHDIPETEAYAIVVELSNKNLLTLMKEPRAGGMYSSC
FEISVTQHDILRNLAINLSNRASINERRRLTEVLIINFTSTEYFLPLFINTMPNLRALIIINYSATYACLHNISVFKNLPNLRSLW
LEKVSTPELSGTVLENLGKLFIVLCKINNRLVEKEVDIAKVFPNLFDLTLDHCDDLTQLPSSICGMKSLQNLSLTNCHNLTQ
LPLELGKLKSLEILRLYACPDLKTLPNSICEMMRLKYIDISQCVNLTCFPEEIGRLVSLEKIDMRECSMIRNVPKSALSLQSLR
LVICDEELSGIWKEVEKAKPNVHIQALRCKSSAEQLITYVREILPTIEEIKYSGVELPAPRQSQLDRLSEILRSGVELSHQALS
SSRWNVYRNFQLAKKMEKLEKHVTRFLQVPMQAHILADVHHVRFEMAERFDRVEASNRRMERFLEEMKIGVNGGG
WVEEAVKSMQEDETWVEGCNGNNGFGVGLDFGKKNVMEMVFSNNDADWILLFAGYFKDRILFLTVSQSPNVEQLR
ARIWGHIMGNQGLNGNYEVPQWMPQFECKGEAQALVVLDDVWSFSVLEQLVWKIPGCKFLVVSRFRFPTFFSATYH
VELLGEEDALSLFCHHAFGQKSIPLGANVSLVKQVVAECGKLPLALKVIGASLRDQNEMFWLSVKSNLSQGHSIGESYEI
NLIDRMAISTNYLPEKIKECFLDLCSFPEDRKIPLEILINMWVEIHDIREAEAYAIAVELSNKNLLTLVKEARAGGMYSSCFEI
SVTQHDTLRDLALILSKRGSIHEHRRLVMAQREENGLLPREWSRYQDRPFEAQIVSINTGEMTEMDWFELDFPKAEVLII
NFTSSDYFLPPFISKMPNLRALIIVNYSTSYARLHNVSVLRNLTNLRSLWLEKVSTPQLSGTVLKNLSKLFVVLCQINNSLD
GKQFPNLSELTLDHCNDLTYLPSSICGIKSLRNMSLTDCHNLSELPVEFGNLKSLEILRLYACPDLETLPPSMCEMKKLKYID
ISQCTNLTCFPKEIGRLVSLEKIDMRECPMIRYLPKSAVSLHSLQLVICDEEVYGTWRDVAEMAKSNVHIQVPEQHFDLD
WLQE

>Vang0103s00400.2

LTRRDNQAPRMDCLGPFNKSIEEVITFVWKHGVRHLTYIIHYNKNVLELKDSVKGLIFEQERIDHECDQAAKNLQNIEGK
VIEWIQKVREIRTTVEDFENNNGHQKARFLNCYVFPYLWNRHRLGRKAKKFELDVKKLIDESPKFDEISYRQDVASNDTT
LYNYGYVEFGSTKSIMEKIMTELKDSSVRMIGIYGPGGVGKSTLIKEIGRKAKDSKLFDLVVKVEITANPNLQKIQEEIAYVL
GLRLEGEGENALDEKDALVLFQKLSGIHNKMSDSKQEIVMKYCAGLPMAIVSVARALRNTSESVWEATMEQLKKQYLV
GEQTPMDISVKVSYDHLENEEIKSIFLLCAQMGHQPLIMDLLKYSFGLGILEGVSSIWEARDIIKTSIQKLKDSGLLLDESSN
NHFNMHDMVRDAALSIAHKHHNVFTLRNGKLDDWPELESCTSISICNSDIIDEFPAFINCPQLKFFQIDTKDPSLKIPEGF
FTAMKNMRVLIMTGFHMSKLPYSIQCLFKLRMLCLEQCTLDCNLSMVGQLKKLRILSFSGSQLKSLPAELQCLDKLRLLDI
SDCSKLNIIPPNLISSLTCLEELYIRKSLIKVLEEEETNKGQDLFLSELRNLHQLKVVDLSISCVSLLPNHLFFDSLKDYKIVIGHF
ESFSVGEFRMPDKFEAFRVLALQLKDDIDIHSQESIKFLFKTVQSLLLGKIAGVENVVNDLNIEGFPDLKHLSIVNNNEIKY
VNSTKLSNYANVFPNLESLRIYNLENLEMICNGPVTVASFDKLKAIKVEMCYQLKNLFSFNMIKYPIGAEISEISECSSYMN

KFLTSIEMIEVCECGSLKEILQIPMDYGKVELLKLHTLSLQSLPKFTCFYTEVKKSFWPPLTEAQTTNRDVTESTIQDDHSEQ
KPLFSGELVEIPNLEKLTLSLLNIRKIWNNQHSSSSYFQNLIKLVVKDCDKLTYLCSSSMASSLKKLKSLVISGCSSKEKIFEIET
ISEDKVCVFPKLEEICLNQMNRLTNIWQTEVSVDSFSSLTLVKIENCNKLENIFPSHMEGWFESLINVEVSKCKSVKEIFEI
NDLQEIDEFGGIDTNLHVILLENLPKLKKLWSKDPNGILNFHKLRTVEVSHCDELRNLFPASMAKDISNLERMSVLYCEK
MVEIVASKGASEVNNDPLVFPELSYVRLYSLSNIKHFYKERHLIKCPNLKEFSVVQCVKLKIFSKEISRTTNKEERCIFSAEEV
LSNLEYMEINFNQAQNLLPNYQMHNLKELSLIYVTSVDLFYQFSYIMPNLEKLKLTSYYPDEESESNGDIAHKKRLVIVLQL
EELVLLFSTIKDLGLERVPILQRLKLLKLEYCEKLSNLGLPFVSLSYLTYLELKSCQGLRNLMASSTAQSMVQLKTMKVSFAY
SEDLWLDEFIIEQLWHSSDWVRQKSFGYLKKLSAWGCDSLVHIIPSHLLSCFHNLEELEVGYCDAQVIFNISDENRVTKAS
GIFHLKKLSVEFLPELEHVWDKDPEGIIGLQFLKEISVSYCCSLRSLFPASVAKDLTRLQVLEVRKCEELEEIFMKNERSEEEE
GSTQESVFHRLTTLRLEELPSLKYSIHYSKKQCLSVGENGMKMILRGEFERKLLDNLKALTLCFGSDVFRCKILEQVPNIEKI
VVCDGSFKKMFCRESPDNVLQQLKVLQLESLGELVSIGLENSWTDSFVRNLETFEVISCSSLKNLVACTVYFSNLTCLKVY
DCRNLSYLLTSSTAKCLGKLKRMEIKGCNSIEEIVCKEDGEESDEDEIIFPQLISLNLDSLWKLKRFYRGSLASVAKDLWKLE
DLVVEDCEGLMAIVADESDEDEEIIFDRLQVLELKKLQELRCFYAGNFTLRFPSLKEVHVIECSSMRTFSAVSKIDHLIKWY
YSEHARPRKEDNLNYAVRRTSEEEV

>Vang0103s00400.1

DSVKGLIFEQERIDHECDQAAKNLQNIEGKVIEWIQKVREIRTTVEDFENNNGHQKARFLNCYVFPYLWNRHRQDVAS
NDTTLYNYGYVEFGSTKSIMEKIMTELKDSSVRMIGIYGPGGVGKSTLIKEIGRKAKDSKLFDLVVKVEITANPNLQKIQEE
IAYVLGLRLEGEGENALDEKDALVLFQKLSGIHNKMSDSKQEIVMKYCAGLPMAIVSVARALRNTSESVWEATMEQLKK
QYLVGEQTPMDISVKVSYDHLENEEIKSIFLLCAQMGHQPLIMDLLKYSFGLGILEGVSSIWEARDIIKTSIQKLKDSGLLLD
ESSNNHFNMHDMVRDAALSIAHKHHNVFTLRNGKLDDWPELESCTSISICNSDIIDEFPAFINCPQLKFFQIDTKDPSLKI
PEGFFTAMKNMRVLIMTGFHMSKLPYSIQCLFKLRMLCLEQCTLDCNLSMVGQLKKLRILSFSGSQLKSLPAELQCLDKL
RLLDISDCSKLNIIPPNLISSLTCLEELYIRKSLIKVLEEEETNKGQDLFLSELRNLHQLKVVDLSISCVSLLPNHLFFDSLKDYKI
VIGHFESFSVGEFRMPDKFEAFRVLALQLKDDIDIHSQESIKFLFKTVQSLLLGKIAGVENVVNDLNIEGFPDLKHLSIVNN
NEIKYVNSTKLSNYANVFPNLESLRIYNLENLEMICNGPVTVASFDKLKAIKVEMCYQLKNLFSFNMIKYPIGAEISEISECS
SYMNKFLTSIEMIEVCECGSLKEILQIPMDYGKVELLKLHTLSLQSLPKFTCFYTEVKKSFWPPLTEAQTTNRDVTESTIQD
DHSEQKPLFSGELVEIPNLEKLTLSLLNIRKIWNNQHSSSSYFQNLIKLVVKDCDKLTYLCSSSMASSLKKLKSLVISGCSSKE
KIFEIETISEDKVCVFPKLEEICLNQMNRLTNIWQTEVSVDSFSSLTLVKIENCNKLENIFPSHMEGWFESLINVEVSKCKSV
KEIFEINDLQEIDEFGGIDTNLHVILLENLPKLKKLWSKDPNGILNFHKLRTVEVSHCDELRNLFPASMAKDISNLERMSVL
YCEKMVEIVASKGASEVNNDPLVFPELSYVRLYSLSNIKHFYKERHLIKCPNLKEFSVVQCVKLKIFSKEISRTTNKEERCIFS
AEEVLSNLEYMEINFNQAQNLLPNYQMHNLKELSLIYVTSVDLFYQFSYIMPNLEKLKLTSYYPDEESESNGDIAHKKRLVI
VLQLEELVLLFSTIKDLGLERVPILQRLKLLKLEYCEKLSNLGLPFVSLSYLTYLELKSCQGLRNLMASSTAQSMVQLKTMKV
SFAYSEDLWLDEFIIEQLWHSSDWVRQKSFGYLKKLSAWGCDSLVHIIPSHLLSCFHNLEELEVGYCDAQVIFNISDENRV
TKASGIFHLKKLSVEFLPELEHVWDKDPEGIIGLQFLKEISVSYCCSLRSLFPASVAKDLTRLQVLEVRKCEELEEIFMKNERS
EEEEGSTQESVFHRLTTLRLEELPSLKYSIHYSKKQCLSVGENGMKMILRGEFERKLLDNLKALTLCFGSDVFRCKILEQVP
NIEKIVVCDGSFKKMFCRESPDNVLQQLKVLQLESLGELVSIGLENSWTDSFVRNLETFEVISCSSLKNLVACTVYFSNLTC
LKVYDCRNLSYLLTSSTAKCLGKLKRMEIKGCNSIEEIVCKEDGEESDEDEIIFPQLISLNLDSLWKLKRFYRGSLASVAKDL
WKLEDLVVEDCEGLMAIVADESDEDEEIIFDRLQVLELKKLQELRCFYAGNFTLRFPSLKEVHVIECSSMRTFSAVSKIDHL
IKWYYSEHARPRKEDNLNYAVRRTSEEEV

>Vang06g21880.12

LVDLVLTHNEKIGIIPIVGMGGIGKTTLAQLIYNDQRVQKEFDVKAWIYVSEEFDICKITKTLLEAVTSCSCDVEDLNFLQR
DLKMHVMNKKFLFVLDDVWNENYDNWDKFRSPFKHAGEHGSKIIVTTRSGCVASIMQTVSPYNLRELSNEDSWNLFS
KHAFDYGDSSLQLHQSLDKVGREIVRKCKGLPLAVKTLAGLLRCKSDRQEWCKVLDSEMWDLQDSESNILPALRLSYHY

LPSHLKRCFAYCSIFPKDYEFEKENLVLLWMAEGFLQQSKRHRRIEEVGNEYFCELVSRSFFQQPRRGKSCFLMHHLVND
LAQFVSGTFSIRMECSNSNEIKERTRHLSHIIADSSSYVNLKDVSKANCLRTFLQIRPVGTSIDLFNNMPNDLLTKLRSLRVL
SLVGTHIYSLPNSVGELKHLRYLEVADTEIVRLPESICSLFNLQTLKLVGCHNLIELPASIHKLVNLRHLDIRGTSLRWMPLQI
NELNSLQNLSDFFVGKGCGSSLGELGELICLHGELFIHCLEHIVSDKDCEKAKLKEKHGLEKLSLDWCRNGETENSQKEKTI
LNSLQPHTNLKKLDIYDYPGTEFPEWLGDHSFYNLVSLMLNGCKYCYRLPPLGQLPMLKELQISKFEGLVSVGSEFLGNR
TSYLTNCFPALEILRIESMPLWEKWYPNAENAGSKAFFHLREIHIGNCPKLRGNLPDNLPSLTLLVIRDCKRLLCSLPNSPSL
RVLNIQNCESLEFKVHSPLCHQSLTSLFLHGSCDSLVFLPLDLFPNIKSLDIWGCKNLEALTVSESDATRPNLKSLHSLRIRH
CPNFTSFPKGGFAASKLTLLTINYCQKLNSLPEQMHDLMPSLKEVQLRGCPKIESSSTRPLRIRICSKHMEGKQNLSDPLF
ARLKGLATDQSPSSS

>Vang06g21880.13

LQRDLKMHVMNKKFLFVLDDVWNENYDNWDKFRSPFKHAGEHGSKIIVTTRSGCVASIMQTVSPYNLRELSNEDSW
NLFSKHAFDYGDSSLQLHQSLDKVGREIVRKCKGLPLAVKTLAGLLRCKSDRQEWCKVLDSEMWDLQDSESNILPALRL
SYHYLPSHLKRCFAYCSIFPKDYEFEKENLVLLWMAEGFLQQSKRHRRIEEVGNEYFCELVSRSFFQQPRRGKSCFLMHH
LVNDLAQFVSGTFSIRMECSNSNEIKERTRHLSHIIADSSSYVNLKDVSKANCLRTFLQIRPVGTSIDLFNNMPNDLLTKLR
SLRVLSLVGTHIYSLPNSVGELKHLRYLEVADTEIVRLPESICSLFNLQTLKLVGCHNLIELPASIHKLVNLRHLDIRGTSLRW
MPLQINELNSLQNLSDFFVGKGCGSSLGELGELICLHGELFIHCLEHIVSDKDCEKAKLKEKHGLEKLSLDWCRNGETENS
QKEKTILNSLQPHTNLKKLDIYDYPGTEFPEWLGDHSFYNLVSLMLNGCKYCYRLPPLGQLPMLKELQISKFEGLVSVGSE
FLGNRTSYLTNCFPALEILRIESMPLWEKWYPNAENAGSKAFFHLREIHIGNCPKLRGNLPDNLPSLTLLVIRDCKRLLCSL
PNSPSLRVLNIQNCESLEFKVHSPLCHQSLTSLFLHGSCDSLVFLPLDLFPNIKSLDIWGCKNLEALTVSESDATRPNLKSLH
SLRIRHCPNFTSFPKGGFAASKLTLLTINYCQKLNSLPEQMHDLMPSLKEVQLRGCPKIESSSTRPLRIRICSKHMEGKQNL
SDPLFARLKGLATDQSPSSS

>Vang06g21880.10

MRQKEVLDLKVGKEVKMAHKTPTSSVMEACDVFGRDNDKESLVDLVLTHNEKIGIIPIVGMGGIGKTTLAQLIYNDQR
VQKEFDVKAWIYVSEEFDICKITKTLLEAVTSCSCDVEDLNFLQRDLKMHVMNKKFLFVLDDVWNENYDNWDKFRSPF
KHAGEHGSKIIVTTRSGCVASIMQTVSPYNLRELSNEDSWNLFSKHAFDYGDSSLQLHQSLDKVGREIVRKCKGLPLAVK
TLAGLLRCKSDRQEWCKVLDSEMWDLQDSESNILPALRLSYHYLPSHLKRCFAYCSIFPKDYEFEKENLVLLWMAEGFLQ
QSKRHRRIEEVGNEYFCELVSRSFFQQPRRGKSCFLMHHLVNDLAQFVSGTFSIRMECSNSNEIKERTRHLSHIIADSSSY
VNLKDVSKANCLRTFLQIRPVGTSIDLFNNMPNDLLTKLRSLRVLSLVGTHIYSLPNSVGELKHLRYLEVADTEIVRLPESIC
SLFNLQTLKLVGCHNLIELPASIHKLVNLRHLDIRGTSLRWMPLQINELNSLQNLSDFFVGKGCGSSLGELGELICLHGELFI
HCLEHIVSDKDCEKAKLKEKHGLEKLSLDWCRNGETENSQKEKTILNSLQPHTNLKKLDIYDYPGTEFPEWLGDHSFYNL
VSLMLNGCKYCYRLPPLGQLPMLKELQISKFEGLVSVGSEFLGNRTSYLTNCFPALEILRIESMPLWEKWYPNAENAGSK
AFFHLREIHIGNCPKLRGNLPDNLPSLTLLVIRDCKRLLCSLPNSPSLRVLNIQNCESLEFKVHSPLCHQSLTSLFLHGSCDSL
VFLPLDLFPNIKSLDIWGCKNLEALTVSESDATRPNLKSLHSLRIRHCPNFTSFPKGGFAASKLTLLTINYCQKLNSLPEQM
HDLMPSLKEVQLRGCPKIESSSTRPLRIRICSKHMEGKQNLSDPLFARLKGLATDQSPSSS

>Vang06g21880.11

MAHKTPTSSVMEACDVFGRDNDKESLVDLVLTHNEKIGIIPIVGMGGIGKTTLAQLIYNDQRVQKEFDVKAWIYVSEEF
DICKITKTLLEAVTSCSCDVEDLNFLQRDLKMHVMNKKFLFVLDDVWNENYDNWDKFRSPFKHAGEHGSKIIVTTRSGC
VASIMQTVSPYNLRELSNEDSWNLFSKHAFDYGDSSLQLHQSLDKVGREIVRKCKGLPLAVKTLAGLLRCKSDRQEWCK
VLDSEMWDLQDSESNILPALRLSYHYLPSHLKRCFAYCSIFPKDYEFEKENLVLLWMAEGFLQQSKRHRRIEEVGNEYFC
ELVSRSFFQQPRRGKSCFLMHHLVNDLAQFVSGTFSIRMECSNSNEIKERTRHLSHIIADSSSYVNLKDVSKANCLRTFLQ
IRPVGTSIDLFNNMPNDLLTKLRSLRVLSLVGTHIYSLPNSVGELKHLRYLEVADTEIVRLPESICSLFNLQTLKLVGCHNLIE

LPASIHKLVNLRHLDIRGTSLRWMPLQINELNSLQNLSDFFVGKGCGSSLGELGELICLHGELFIHCLEHIVSDKDCEKAKL
KEKHGLEKLSLDWCRNGETENSQKEKTILNSLQPHTNLKKLDIYDYPGTEFPEWLGDHSFYNLVSLMLNGCKYCYRLPPL
GQLPMLKELQISKFEGLVSVGSEFLGNRTSYLTNCFPALEILRIESMPLWEKWYPNAENAGSKAFFHLREIHIGNCPKLRG
NLPDNLPSLTLLVIRDCKRLLCSLPNSPSLRVLNIQNCESLEFKVHSPLCHQSLTSLFLHGSCDSLVFLPLDLFPNIKSLDIWG
CKNLEALTVSESDATRPNLKSLHSLRIRHCPNFTSFPKGGFAASKLTLLTINYCQKLNSLPEQMHDLMPSLKEVQLRGCPK
IESSSTRPLRIRICSKHMEGKQNLSDPLFARLKGLATDQSPSSS

>Vang0984s00020.1

MAEQLMGVGECILKLVWKAFQRLQNLPEGVKEIQDELGMLKNFMEDKEADGENDATERNKKMQLRKITFLTEDVIDE
YLIRIREKRPQDGRGWPAFPSKAAYHIQTLIPRFKISYKIESMLSVVRRTNERCTRDRPKSTQNEANNTISSQREDPLIKDD
EIVGLAEPTQILENLLRNGQEERTVLFVEGAPGVGKTTLARHVFDNVHNDFKCHALITVSQSYVVEKVLKDMIYILCKERG
EDPPNNLPSLLPTLKTEVRNYLRGKRYVLLFDDVWDENFWVEIENALIDDKNSSRIIITTRIENVANSCKNSTFFVEEYKLE
NPLTEEESLRLLYKKARYNSDYPEELRDIALEIVRTCSALPLAIVVAGGLLSEKGGSAHEWRLFSEELKRNSELNIVTKIIGLSY
DNLLSHLRSCLLYFGMYPKDYEIESERLVRQWVAEGFVTHEEGKTLEEVAHKYLLELVRRSLVQVLSFSKKEKVKKCRVSD
SIHNMIRTKMKDTWFGGGGHDQSESSELVRRLTIQANNDLNRRIKRSHIRSIISIPGKQEESWYSDLERNILKDDMPLKV
LDFEGCGLPCVPKMLGNLIYLRYLSFRGTQIKVLPKSIGKLVNLETLDIRQTQVCKVPKEITKLRKLLHLLTPDSAFSSIEWK
DIGGMTRLQKIPQVRMEGDGVAIKEVGKLKQLRVLRVLCFGAEHITSLFNSINEMKHLQALRIEKSGDRIDLNTETMSGE
CRLRKLFLDMKLETLPNWIPQLRHLERLTLRHSDLTNDPLESLKDMPSLLVLSLSHAYEGQTLHFQPGGFKTLRKLNLEHL
WNLNSILIEETALQALEYFELTCLDDLRTVPDGIQHLRKLVRVSYMPTQFVEEIMAICDSRLHSFCTIVNFREAVTEDSLEV
FEVFKKFRVMVEKETSKHIKAVEFTLPLVHKAIP

>Vang04g01790.1

MADSIVVFLIDKLTRLLVEEAKLLTGVRDQVASLQSELKFMNLFLRNSQGKRKEHDMVAELVSQIRDVAHEAEDVIDTYV
ACIIKQSRRNVIGKVGFRVVSVVGMGGLGKTTLARKVYNSNRVKNVFPCRAWGYVSNDYRPREFFLSLLKCLMSTSKYS
NLFKKREETSVSDEELKMKVRECLNRSKYLVVVDDVWQKQVWNEVKGAFPDDQNGSRILMTTRSAEVASHAGPVPPY
ALPFLTKEESWELLSKKVFRGEDCPSDLESLGKLIADSCDGLPLALIVMAGILANKKSPRDWSRIKDHVNWHLGRDNTLK
DILKLSYDSLPARLKPCFLYFGMYPEDYRIPVKQLIQLWISEGLLTQETSGGQDIPEPEYIAEEYLDELVDRSLIQVVSRTND
GGVKTCRIHDLLRDLCISESREDKFFEVCGEIDIQNLNSCPRKLSLQGTLFHFSSSIVSDYTISATRSLLCFGQEVYKVKANH
WRWLLKSFRLARVLDLGRMNVNSIPTDLEKLIHLRYLRIHSHNLETIPPSICRLWNLETLDLRGSPIKSFSGELWQLKQLR
HLLLFGPVGLPEMPSESKTMPNLQTLSTVALDPRTASLLDSRRFPGMTKLGIHYERRDKCNAKIQLQSLHRLSHLRKLKVI
GTTEIPQNANVFPSNITKISLTKFGFFNSTVMHTLGKVPNLQVLKLSSQTNDTRFDLHCATGGFLQLQVFEMIAIKVKM
WRVDRGSMPRVRRLVVRSCKSLTQLPKEVWSLNTLREVQVLWPCTELAKGLQNLVMNNACKLVVYPLSANDELDFL

>Vang04g06010.2

MEAVVSTTTESALQIAGRAVKRQLSYFFNYNDKFEEVKRYTEMLDNTRKRIQHQVNNAEMNAEEIEDDVQHCLKQLD
EKIEKYEKFIHDECHSKTRCSIGFFPRNLSLRYRLGRNATKMVEEMKVEELWNKMFDEVSYRVLPSINVSLTNISYESFASR
TKTIDMFMQALEDSTVNMIGLYGVGGVGKTTLVKEVAKKAQEKKLFTVVVMANITRNPNIITIQGQIAEILGMRLEEESE
IVRADRIRKRLKKEKENTLIILDDLWDRLDLNRLGIPNSDEDDGSQRDVNDISNSGYHKMEKEELSSDFNNMTEEKLSGN
HKRCKILLTSRRKQVLCNQMDVKERSTFSIGVLDENEAKTLLKKVAGIQIQNFVYDDKAIEIARMCDGLPIALVSIGRTLKN
KSSFVWEDVYQQMKKQSYIEGKEPIEFSIKLSYDHLENELLKSVFLQCARMGNDALVMDLVKFCIGLGLLQGVHTIREAR
NKVNMLIEELKESSLLLESYSSNRFNMHDIVRDVALSISSKEKQVFFMKNSILDEWPHKNQLESYTAIFVHSCYIVDDLPG
SIYCPRLEVLHIESKDQFLKIPDDFFKDMIELRVLILTGLNLPCLPSSLICLTKLRMLSLEKCTLGQNLSIIGELKKLRILTLSGSN
IECFPFEFGQLDKLQLLDLSSCSKLRLIPSNVISRMNILEEFYMRDSLIRWETEENIQSQNGSLCELSRLNQLRNLDIHIQNV
VCVPQNLFFDELDSYKIIIGEFNMLTEGEFKIPDKYEVVKLLVLNLKEGIDIHSEIWIKMLLKNVEYLWLGELINVHDVFYEL

NVEGFLKLKHLSIVNNFGIQYMINSMEQLHPLLAFPKLESLYLYKLENLEKICNNQLLEASFSRLKIIKIKSCGKLENIFPFSM
VGHLTKLETIEVCDCDSLEDIISVESQTHINNGDSIEFPELRLLTLKSLHAFTSLYTNDKMPCAQSFEDKGRNMNKDIITEVE
QDGTSSCHSLFNEKVLIPKLEWLELSSINIQKIWSDQSQHCFKNLLTLNVRDCGNLKYLLSFSMAKHLENLQSLFVGECE
MMEDIFCPEDVEGNIDCVFPKLKKMEIMCMEKLNTIWQSHIGLHSFRNLDYLIIRECHKLETIFPSFMRQRFQNLQSLTI
TNCKLVENIFDFANISQTCDKNETNLHNIILQGLPNLVSIWKDDTGEILKHNNLKSIKIVGSPNLKYVFPLSVTYDLENLESL
EVFNCRTMKEIVASDKGSNENVITFKFPHLKTVSLRSLLELVSFYGGTHTLEWPSLKDLSILRCGKLEGITTKISNSQAKPIVL
ATEKVIYNLEYMAMSLGEVEWLQKYIINVHKMHNLQSVVLHGLKNVEVLFWFLHRLPNLKRLIMRFCQMRRIWAPLT
DISREKIGVVMQLKELELRDMWSLEEIGFEHDMLLQRVQHLIIERCTKLKTLASSSVSFRRLTYLEVVNCMMKNLMTYST
AKTLDQLTTMKVSSCPMIVAIVENEEENVQEIEFKQLRSLELFALPNLLSFLLSISELKFPLLEKSIVSECPQMTKFSEFLSAP
HLQKVHAVAGEKDKWYWEGDLNATLQKYFPYQVFFEHSKDMKLVDYPEMKEVRYGKPVFSDNFFGSLKKLEFDAVSK
REIVLPSHVLPYLKNLEELNVHSCKLARTRGIGFVKQAFQGKSFGNIKKLEFDGKSKGDTVIPSNVLSHLKSLEELNVHNSD
EVQVIFGMNDSHTETKETVFHLKKLILKDLSNLKCILNKNPQESVSFPNLHELFVDGCGSLVTLFARNLGKLKTHEKQRYD
KLVEIAGKEDAIENGTTEVLMFEFPCLSLLTLYNLTNLNCFCPEKHHLECPKLEIMHVAYCPKLKLFTSKIHDSHKEAIAEAPI
SCLQQPLFIVEKVVPKLKGLTLNEKNMMLMSDAHVPEAYLSKLNLLRLCFEDDKNEKGTLPFDFLHKVPNLEHFQVQRC
FGIKEIFSSQKLQVHDGIPATLNALTLFELNELESIGFEHPWVKPFSEKLQTLTVTSCPRLEKLGRGAMSFINLKELYVKDCG
RIEYLFTFSTAKSLVQLETLIVKNCESIKEIAMKEDEDDCDEIIFERLTTLTLNCLPRLQSFLSGNATVHFSCLKNAYAINCPN
MKTFSEGVLTAPRFLGIKTSYEDSDLFFHDDLNTSFQRLFQRQVEKSACDIEHLKFGDHSHIQEIWLGVAPIPTNNSFNNL
KSLTVVECESFPNVIPFYLLPFLCNLKEIEVSNCQSVKAIFDVNGAAADMKPISLPLKKLILNQLPNLEHIWNLNPDEILSLQ
DLQQVSISNCQTLKSLFPTSVANHLVKLHVRACATLVEIFAVADAAINGETKQFNFHCLTSLTLWELPELKYLYPGKHTLE
WPMLTHLDIYHCDQLKLFKTEHHSDEFADTEDQLGISIHQQAAFSVEKVFPKLVQLSLKKEDAMAISQGQLQARTVSKIL
LVMPSIERQEITWKDTMIGQGQFGANVAHLLQNLKLLKLMCYHEDDKSNIFSGGLLEEIPNIENLEVVCSSFTEIFCSQGP
TTDCSKVLSKLKRLHLKNLPQLNAIGLEHSWVEPLLKTLETLEVFSCPTMKILVSSTLSFSNLTSLNVGECHGLIYVFTSSTAK
RLGQLKHISIRDCQAIQEIVSKEEDRESEDEDEDITFDQLSLLSLESLPNIVGIYSGTSKLKFPCLDQVTLKECPQMKYSYVP
DLHEFKPQEQT

>Vang04g06010.1

MEAVVSTTTESALQIAGRAVKRQLSYFFNYNDKFEEVKRYTEMLDNTRKRIQHQVNNAEMNAEEIEDDVQHCLKQLD
EKIEKYEKFIHDECHSKTRCSIGFFPRNLSLRYRLGRNATKMVEEMKVEELWNKMFDEVSYRVLPSINVSLTNISYESFASR
TKTIDMFMQALEDSTVNMIGLYGVGGVGKTTLVKEVAKKAQEKKLFTVVVMANITRNPNIITIQGQIAEILGMRLEEESE
IVRADRIRKRLKKEKENTLIILDDLWDRLDLNRLGIPNSDEDDGSQRDVNDISNSGYHKMEKEELSSDFNNMTEEKLSGN
HKRCKILLTSRRKQVLCNQMDVKERSTFSIGVLDENEAKTLLKKVAGIQIQNFVYDDKAIEIARMCDGLPIALVSIGRTLKN
KSSFVWEDVYQQMKKQSYIEGKEPIEFSIKLSYDHLENELLKSVFLQCARMGNDALVMDLVKFCIGLGLLQGVHTIREAR
NKVNMLIEELKESSLLLESYSSNRFNMHDIVRDVALSISSKEKQVFFMKNSILDEWPHKNQLESYTAIFVHSCYIVDDLPG
SIYCPRLEVLHIESKDQFLKIPDDFFKDMIELRVLILTGLNLPCLPSSLICLTKLRMLSLEKCTLGQNLSIIGELKKLRILTLSGSN
IECFPFEFGQLDKLQLLDLSSCSKLRLIPSNVISRMNILEEFYMRDSLIRWETEENIQSQNGSLCELSRLNQLRNLDIHIQNV
VCVPQNLFFDELDSYKIIIGEFNMLTEGEFKIPDKYEVVKLLVLNLKEGIDIHSEIWIKMLLKNVEYLWLGELINVHDVFYEL
NVEGFLKLKHLSIVNNFGIQYMINSMEQLHPLLAFPKLESLYLYKLENLEKICNNQLLEASFSRLKIIKIKSCGKLENIFPFSM
VGHLTKLETIEVCDCDSLEDIISVESQTHINNGDSIEFPELRLLTLKSLHAFTSLYTNDKMPCAQSFEDKGRNMNKDIITEVE
QDGTSSCHSLFNEKVLIPKLEWLELSSINIQKIWSDQSQHCFKNLLTLNVRDCGNLKYLLSFSMAKHLENLQSLFVGECE
MMEDIFCPEDVEGNIDCVFPKLKKMEIMCMEKLNTIWQSHIGLHSFRNLDYLIIRECHKLETIFPSFMRQRFQNLQSLTI
TNCKLVENIFDFANISQTCDKNETNLHNIILQGLPNLVSIWKDDTGEILKHNNLKSIKIVGSPNLKYVFPLSVTYDLENLESL
EVFNCRTMKEIVASDKGSNENVITFKFPHLKTVSLRSLLELVSFYGGTHTLEWPSLKDLSILRCGKLEGITTKISNSQAKPIVL
ATEKVIYNLEYMAMSLGEVEWLQKYIINVHKMHNLQSVVLHGLKNVEVLFWFLHRLPNLKRLIMRFCQMRRIWAPLT
DISREKIGVVMQLKELELRDMWSLEEIGFEHDMLLQRVQHLIIERCTKLKTLASSSVSFRRLTYLEVVNCMMKNLMTYST

AKTLDQLTTMKVSSCPMIVAIVENEEENVQEIEFKQLRSLELFALPNLLSFLLSISELKFPLLEKSIVSECPQMTKFSEFLSAP
HLQKVHAVAGEKDKWYWEGDLNATLQKYFPYQVFFEHSKDMKLVDYPEMKEVRYGKPVFSDNFFGSLKKLEFDAVSK
REIVLPSHVLPYLKNLEELNVHSCKLARTRGIGFVKQAFQGKSFGNIKKLEFDGKSKGDTVIPSNVLSHLKSLEELNVHNSD
EVQVIFGMNDSHTETKETVFHLKKLILKDLSNLKCILNKNPQESVSFPNLHELFVDGCGSLVTLFARNLGKLKTHEKQRYD
KLVEIAGKEDAIENGTTEVLMFEFPCLSLLTLYNLTNLNCFCPEKHHLECPKLEIMHVAYCPKLKLFTSKIHDSHKEAIAEAPI
SCLQQPLFIVEKVVPKLKGLTLNEKNMMLMSDAHVPEAYLSKLNLLRLCFEDDKNEKGTLPFDFLHKVPNLEHFQVQRC
FGIKEIFSSQKLQVHDGIPATLNALTLFELNELESIGFEHPWVKPFSEKLQTLTVTSCPRLEKLGRGAMSFINLKELYVKDCG
RIEYLFTFSTAKSLVQLETLIVKNCESIKEIAMKEDEDDCDEIIFERLTTLTLNCLPRLQSFLSGNATVHFSCLKNAYAINCPN
MKTFSEGVLTAPRFLGIKTSYEDSDLFFHDDLNTSFQRLFQRQVEKSACDIEHLKFGDHSHIQEIWLGVAPIPTNNSFNNL
KSLTVVECESFPNVIPFYLLPFLCNLKEIEVSNCQSVKAIFDVNGAAADMKPISLPLKKLILNQLPNLEHIWNLNPDEILSLQ
DLQQVSISNCQTLKSLFPTSVANHLVKLHVRACATLVEIFAVADAAINGETKQFNFHCLTSLTLWELPELKYLYPGKHTLE
WPMLTHLDIYHCDQLKLFKTEHHSDEFADTEDQLGISIHQQAAFSVEKVFPKLVQLSLKKEDAMAISQGQLQARTVSKIL
LVMPSIERQEITWKDTMIGQGQFGANVAHLLQNLKLLKLMCYHEDDKSNIFSGGLLEEIPNIENLEVVCSSFTEIFCSQGP
TTDCSKVLSKLKRLHLKNLPQLNAIGLEHSWVEPLLKTLETLEVFSCPTMKILVSSTLSFSNLTSLNVGECHGLIYVFTSSTAK
RLGQLKHISIRDCQAIQEIVSKEEDRESEDEDEDITFDQLSLLSLESLPNIVGIYSGTSKLKFPCLDQVTLKECPQMKYSYVP
DLHEFKPQEQT

>Vang0103s00030.1

MALAVVGGALLSAFFDVLFDRLASPEVLNFIRGKKPDKLLQKVKTQLIVVRVVLADAEKRQITDSNVKEWLDLLRDVVYE
VDDLLDEXSTKAATQKEEIXVESYQPWKAQPTSLEDGYAMYGRXKDKEAIMKMVLEDSTNGETVSVIPIVGMGGVGKT
TLARSVFNDGKLQQHIFDLKAWQWMAEDLIKAVKKGKTLEEVGQEYFDDLVSRCFFQYSSRSGGAYFVMHDLIHDLAT
FLGGDFYFRTDELGKETNIDRKTRHLSLTRFSDPVSDIEAFDTVKFPRTFLLIDYKDSPFNNDKAPSIIVSRLKYLRVLSLWN
FRSQLALPDSVGELIHLRYLNLSXXSIETLPESLCNLCNLQTLKLYHCSRLTKLPSAMQNLVNLRHLEILGTLITEMPKRMGK
LNQLRNLDCYVVGKHIENSIKELGGLPNLHGLFYIMKLENVTKGEEALEARIMDKKHISNLFLEWSIANDNSIDFQVELDV
LSKLEPHQDLEALSIIGYKGTRFPEWVGNFSYSYMTSIYLCNCNNCCVLPSLGQLPSLSNLRISKMNSVKTIDAGFYKKDD
CSSVXPFPSLESLHISNMPCWEVWNSLDSEAFPVLKNLYIDNCPKLKGDLPNHLPALQTLRIRNCELLVSSIPGPLTLRTVD
IRKCKKVAFREFPLLVESIEVEGGPMVESMMEAISNIQPTCLQSLKLQNCSSDISFRGGRLPASLKTLDIRGINKLKFPLQH
KHELLESLSINNSDDSLTSLPLAIFPNLTSLRITNCENMESLLVSGSEISKRLNTFTIDHCPNFVSFPXEGLCMPNLTSXTVCN
CDKLKSLPDQMGTLVPKMEYLSISNCQQIESFPGGGMPPNLRTVFIENCVKLLSNQAWVCMDMITSLSVWGPCDGIKS
FPKESLLPPSLVSLHLSNLSSLETLDCKGLLHLTSLQQLNMERCQKLENIAGEKLPLSLIKLMIYECPLLKQRCHKTDRQIWP
KVCHIRGINIEDMLDWLGETAAVHCNVLPSTRTSRAQPNDVKSPTERHPESK

>Vang1009s00010.1

MAESAVTLLAVLAEKLLTLLKEEGKTLSGVHKDVERIKNLVKNIKPFMKNAEEKVLIEESVKNWMNGLREVMFRMEDV
VDLYLFKVAKRDGVRYGMRKKIKSIKHRHRISSEIKDIRQTLDDLFSIRTRLQLLSSHGDTLPNTIPRAHFVKESQLVGIEHN
MQKFRDWLAKASSPLLVVVGPAGIGKTSIVKNVYNKQTKLNQPKKKKDFDFCVWITMTQADSWYPIMQIKEEILMAD
PRGSSSLRSATRENLTEKLGEYFIDKRCLIVLDDVKELKVWDVIQFAIPQHRVIITTQRDDFPNNIGSDDTSVEKIKLEPLSL
EDALKLFHQKVKHVQFPELSQLSKEFMEKCNGVPLAIVAISSLLSTMKSAIEWRRVRDDLGSLLRSHHHLETVRHVLLQS
YQELPYRLKQCFLYFGHFPQGYSISRKRLIRLWIAEDFVEGDTQNKSMEELGGEYLAELICRGLVHASRVDFDGIPRSCHV
YNLMHEIISSICKDQMFCHVMEDVSTPVNSNMDFIHRRLSIIKKTDSATMERDQKWGKVRSCFVFDDAKKWQVNNHF
FSSFEFLIRLDLSDACLSVDVLPEQVGNLLNLKYLSLRNTNIMSLPESIGNLVNLQTLDLKQTKLHEVKIDKLVKLRHLLAYY
VSDQSSEFYCLEGLRLSEGVQNLESLQNLSYLDVTGGRIIKGLQKLTKLRKLGIIKLETTHGEALCNSIEHMINLCSLSIGALG
KQDMLKLQSLNPPLSLKRLYLYGRLGELPTWISTVPNLIRLYLKWSDLKQDPLHYLKDLPQLLYLELYDAYKGERLDFGNG

WLKLKVLYLGLLPNLKTIEIGKGKVPCLEILKIGRCHQMIRLPKDIQNLKHLEKLYLYDMHEQFIKRLCDERSEDYWIINKIPL
VEYSNNDHFASFS

>Vang0022ss04710.1

MKRTVSAIKAVLVDAEGKANNLQISNWLEELKDVLYDADDLLNDISSEAMKRKVIGARKILRKIQVFFSQENQIVYSFKL
GHQMKEIQKRLDAIAKNKITLQLTDRPMETSIAYRRQRQTYSFVREDDVIGRKEEKKLLESYLLDTKVSVIDNVSVLAIVGF
GGLGKTTLAQLVFNDNAVQCSFEKKMWVCVSDEFDMRKIAEKMIGNDXNSEIEQLQQDLRNKVXGKKFLLVLDDVW
NEDRELWLKFKSLVMEGGKGSAIIVTTRSRTVAKIVATHPPLFLKGLDLERSWKLFSRVAFDGGKEPNDLELLAMGRDIV
KKCAGVPLTIRTIGSLLYSRNMGRSDWLYFSEVEFSKIDQHKDKIFAILKLSYDHLPSFLKKCFAYCSLFPKDFVFDKKTLVQ
LWVAEGFIQPSRDNRXEEXVGHEYFMNLLSMSLFQDVTLDDCGDILTCKMHDLIHDLAQLVVGKEYAFVEGKKEHIEN
RTRYLSSCTSLHFSEKTSSSSNKLRTFILLGQPLYGSQNFCPPPSLQFPFLLSIKCLRVLTLCGLHLITIPNNIRDLKQLRYLDLS
MNRFLARLPPDVTSLHNLQTLKLSGCGKLKELPSDINKSLRHLELNNCGELRCMPCGLGRLTNLQTLTHFLLDSKSKNVDI
SELSGLNNLRGKLVIKWLDSLRENVAVVESANILLEKQHLQDLELRWGLGKTKYWRDTIEKRRKEEENTWVEIETILYIEE
TGFENQMKDEKILQGLQPHHSIKRLVIDGYCGNSLPDWIGNLSSLLSLEISNCYGLKSIPDGFRNLVSLQRLCIYNCSRLEG
GCAKGHGGEWSKIAHIPEVLVSAFYPTDLRLVNDGRLRLVNDGRLNNGRLVNDGRLYNWRLVNDGRLYNWRLVNDG
RLYNWRLVNDGRFYNGRLYNGRLCRYCGGIVVAAFIPSSHVEAFLVALLGCSSVVGRARGVVGHAEASLCGVVGCGPS
KAIMVWWTATHGVFWIILL

>Vang01g07720.4

LCCFNNFALNLSNVKEELELTRDRVKERIREAINKDEKVESTDVKWLKDVEKVLEEVQVLEKRIMSVNKSYFRRQCQYSL
AKEIEKKIIEMIQLDFNSEYTQFSRITKLLGMEYYYSNDFSVFNPIKASYKKLLEALMNKSASIIGLVGFGGSGKTTLAKEVC
KKAEDMKLFEKVVLATVSRLLNIRSIQDQIADQLTFELKEGSEIGRAQRLSERLRKGTTLVILDDVWERLNFEALGIPLDES
SKACCVLITTRSKEVCTSMKCQSIIELNLLTHEEAWTLFKHYANINDDSSEALKGVARKIVSECKGLPIAIVTVGSTLKDKTIT
NFELALSRLESSKPLDIPEGLISPYVCLELSYNNLTNQLAQSLLLLCSMFPEDCEIILEDLFRFGRAFDTIGTFETMGNARRE
MDAAIDMLKNCFLLMHAKEKLKVKMDNLVRDVALWIAFKTGKAIFTRTELDPRALADDKTLKDMKAIAIWGLKSYDVL
NYKINCPILEILVLSFNVSGDVKVSYECLQSLEKLKTLSILNLDEWDKGILPLPESLKSLKNIQTLCLRGYDLGDISVVEQLQA
LEILDLRSSSFDDLPVGIVGLKKLKLLDLYKCVIKKNTTVEAYKVVGKCLQIEELYLYLSNYVMDFPHEVSYSKLQRYAIINTD
YYYHDCDFQDNTIMKKYAKSRSLLIKSFDITTQNLISLPIKDLFIRAEYLCLRNLRGDYKNIIPSMDQQGMNQLIALVLEHC
SKIECLIDNTINTSTSVDFRQTEDVFSTLVYLSLRGLHNLQEMFCDPCYRCSLQNLEELLIEDCSKFNNISFPKSSNLCNLKIL
SISKCPVLTYLFMPSIVQTLSLLEVLKISSCSALRHIIEEVEEENYALASTKRLQKLRILEIEDCHNLEYIFTVILAQGLVSLENVK
IVSNMKLKYVFGSEKEHNLAVYQSFHQQTNISINLPNLDTLNLMFLPNVIRIWPEYCPLRLPCLEESLIILCPKLPNSSIDMA
ETASNLEQHTISMVMYYFQTTILLLHNYNYLKWIIFYLVEQIFIYFINYIFITCIHSY

>Vang01g07720.1

LCCFNNFALNLSNVKEELELTRDRVKERIREAINKDEKVESTDVKWLKDVEKVLEEVQVLEKRIMSVNKSYFRRQCQYSL
AKEIEKKIIEMIQLDFNSEYTQFSRITKLLGMEYYYSNDFSVFNPIKASYKKLLEALMNKSASIIGLVGFGGSGKTTLAKEVC
KKAEDMEEVEEENYALASTKRLQKLRILEIEDCHNLEYIFTVILAQGLVSLENVKIVSNMKLKYVFGSEKEHNLAVYQSFH
QQTNISINLPNLDTLNLMFLPNVIRIWPEYCPLRLPCLEESLIILCPKLPNSSIDMAETASNLEQHTISMVMYYFCSELNDV
YFLMNSKLCNLTVLTISECPVLTSFFVSTIVQALEFLEVLRLSHCSTLTHIIERGNDDILRTRNHSSLTLSKLRILEIEECDNLKY
VFSLSLAQGLKSLKSVKIVGNSKLKYVFGSEKEHILMYPSFQQNIVTFHRLQNLMDFNLSKLTNLINIWPEYCRPHLPNLEK
LHCTYCPELSDLCNLTVLTISECPVLTSFFVSTIVQALEFLEVLRLSHCSTLTHIIERGNDDILRTRNHSSLTLSKLRILEIEECDN
LKYVFSLSLAQGLKSLKSVKIVGNSKLKYVFGSEKEHILMYPSFQQNIVTFHRLQNLMDFNLSKLTNLINIWPEYCRPHLPN
LEKLHCTYCPELSDLSKVMTVSDLGQLTTSMVIK

>Vang09g06870.1

MMAQAVVSFIVQSLGDLLIQEAVFLYGVEDQVLQLQTELRMMRSYLQDADRRQDENESLRSWISEIREAAYDSDDVIE
SYALREASRRNLPGVSNLIRRYASIINRFIEIHMVGSHVGNVKARISSLTRSLKTYGIKPEKEEPSNSMHGRQNLRRSYSHVI
EEDIIGVDDDVKKLESYLVDPSKRVVAICGMGGLGKTTLAKKVYHSVDVRNSFKSRAWAYISQHCQARDVWIGILFRLIS
PSQEQRQEIDNMRDEELAKMLYQVQMEKSCLVVLDDIWNAETWSKLKPAFPHGTSVSAVGSKILLTSRNIDVAFQMD
PSCYLHTLKCLNEVDSWELFQKKSFLKIDDPDYGEKEKLGREMVGRCGGLPLAIIVLGGLLASKPTFFEWDTVCQNINSYL
RRANGKEQRLEEVLALSYYELPYQLKPCFLHLAHFPENLEIPTKKLIRIWVAEGIISLAHSEGEGEEALEDVAQRYLTELVER
CMIQVVEKSSSGRIRSCQMHNLMRELCVERAHLENFLQEINSRNVDESRGTSRARSVGKVRRIALFLDQDVDRFFPSEL
KSHHHLRSLLCFHEKTARLSEWGLMKSFFKKCRLLRVLNLEGMQGLGGKLPKEIGYLIHLRFLSLRNTKIDELPTSIGNLKC
LMTLDLLTGNSTVQIPNVIGNMQKMRHLYLPESCGNGIERWQLDNLKNLQTLINFPAEKCHVTDLMKLTNLRKLVIDD
PNFGGIFRYPNVQFRHLESLFFVSYEDISVVHVALGCPNLYKLHIEGPIKNFPEPHQLSSKLLKLKLTGSGLLVDPMPTLEKL
PNLRLLELQLDSFVGKQLHCSSKGFAQLKSLVIHDLFNLEEWRLDKGAMPCLRELKIENCTKLEKVPDGLRFLTTLQHLEIR
SMFAAFRTKLEKGGEDHYKIQHVSAVVFCYCDY

>Vang07g00560.1

LVFDFEFREQVWIFVIWVLLIMADLVSGAAVGEALKLAISTIKKGLDFESTLESNIETLNSLNPLVEKMKTYNTMLDSPSKE
IEKLESLVRNSQELVRKCSKLGRWKMLSFPHYQSKLRSKDGALQTHLSMNMTAQNTVNLLKVMVDMRKVLEILLKEGF
GRYPEYQFMDICGAPQEPECLGMDVPLRKLRIELLKGGVSVLVLTGLGGSGKSTLAKKICWYPQVKDKFGKNIFFVTVSK
TPDLKTIVETLFEHCGFKVPKFQTDEDAVNRLEVLLRLLGQHPILLVLDDVWPGSEDLVEKFKIQIPDYKILVTSRVSFPRFG
TSYQLDKLDDVHAESLFRHFAQLKDKSSYIPEKNLVDEIVKGCKGSPLALKVIAGSLCNKSFEVWRDMMEHLQKQSILES
DTDLRFRLQQSLDILETKFSVNEKEFFMDLGLFPEDQRIPVAALIDMWAELYNLNEDGSNAMSIIHDLTTRNLINVIVTRK
VAKDTDMYYNNHFVLVHDLLRELAIHLSKGEKTFEQRERVMIELKGDNRPEWWVGLNPLGIIGRSFSYILGMFHRQKQ
PKVAARILSISTDETFNSDWCDMQPDEAEVLVLNLLSSQYSLPEFTEKMHKLKVLIVTNYGFHRSELNMFERLGSLTNLK
RIRLEKVSIPPLCILKNLRKLSIHMCNTRQAFERYSISDAMPNLVEMSIDYCKDLIKLPDGLCNITPLKKLSITNCHGLSALPQ
DLAKLENLEVLRLCSCSDLADMPDSVKELNKLSCLDISDCLSLTKLPDDIGELKLQKLYLKGCSKLRELPDSVDKFENLEHEI
HVICDEEMASLWKNSTIRKLKIEIPTVEVNLNWLPGVHS

>Vang0022ss04730.1

MLVPNFIWQRMEVIAQMVLQNLISFAQEEFGIIWNLKDDVQQMKRTVSAIKAVLVDAEGKANNLQISNWLEELKDVL
YDADDLLNDISSEAMKRKVIGARKILRKIQVFFSQENQIVYSFKLGHQMKEIQKRLDAIAKNKITLQLTDRXMETXXAYRX
QRQTYSFVREDDVIGREEEKKLLESYLLDTKVSAIDNVSVLAIVGFGGLDEFDMRKIXEKMIGNDXNSEIEQLQQDLRNK
VXGKKFLLVLDDVWNEDRELWLKFKSLVMEGGKGSAIIVTTRSRTVAKIMATHPPLFLKGLDLERSWKLFSRVAFDGRK
EPNDMELLAMGRDIVKKCAGVPLAIRTIGSLLYSRNLGRSDWLYFSEVEFSKIDQHKDKIFAILKLSYDHLPSFLKKCFAYC
SLFPKDFVFDKKTLIQLWVAEGFIQPSRDNRXEEXVGHEYFMNLLSMSLFQDVTLDDCGDILTCKMHDLIHDLAQLVVG
KEYAFVEGKREHIENRTRYLSSCTSLHFSEKTSSSSNKLRTFIFLGQSLYARQNLGPPPSLQFPFLLSIKCLRVLTLCWLNLITI
PHSIRELKQLRYLDLSMNRFLVSLPPDVTSLHNLQTLKLSGCGKLEELPSDINKSLRHLELNDCGELRCMPCGLGQLTNLQ
TLTHFLLDSKSKNVDISALSALNNLRGKLVIKRLNSLRENAAVVESANILLEKQHLQDLELRWGLGETNYWTDRIEKRRKE
EKNRWVEDENQMKDEKILQGLQPHHSIKRLVIDGYCGNSLPDWIGNLSSLLSLEISNCYGLKSIPDGFRNLVSLQRLCIYN
CSRLEGRCARGHGGEWSKIAHIPLVLVSAFNPNDLRYIN

>Vang03g17620.1

LALKVLGASLRQKSKEVWESELRKLQKIPDIEIHKVLKLSYIYDGLDHYQENIVLDIACFLKGENRDWVTRLLEDFDFFATS
GIEVRLDKALLSTISDGNTIEMHDFMIQEMVWEIVHQECIKNPGKRSRMWRPEEVRNVLKHSKVEDATSFV

>Vang02g03190.1

MALRSCSSSFIYEVFLSFRGEDTRHGFTGNLYKALDDKGIHTFIDDEKLQSGEEITPALLNAIEESRIGITVLSKDYASSSFCL
DELTTILDCRTKGLLVIPVFYMMDPSDVRHQKGTYGEALAKHQKRFKAEKLQKWKMALQQVADLSGYHFKHGNEYEH
EFIGRIVERVSREISSVYLPVDDYLVGLESKVQEVKKLLDLGSHDGVVIGIHGMGGIGKTTLALAVYNLIAANFDESCFLQN
VREESNKYGLKHLQSILLSKILRQKNIILTSWQEGASMIQQRLRRKKVLLILDDVDKIEQLKTFVGRCNWFGPGSRVIITTR
DQHLLTSYDVKRTYVVNKLNYYDALQLLTWKTFKRGKADPSYEEVLHRAVTYASGLPLALEVIGSNLVGKSVEEWDSAIE
HYKRIPSDEILKILKVSFDTLGREEKNVFLDIACCLKGCKLTEIEHMLRALYDDSMKHHISVLVEKSLIKISQSSTVEMHDLIH
AMGRQIDQHESPKEPGKHKRLWLPKDIIQVLKYNTGTGKIDIICLDISISKREETIYWNRNAFRKMKKLKILIIRNGCKKLR
RFPPLNLTSLEQLELSYCSSLENFPEILGKMGNIRKLSLFELPIKNLPVSFHNLTGLQELEMSCDFVQLSSTVLTPELSDFRVH
KGKGWQWVKSQEASFKQLAQVSHLRLRESNVTFLPECIKEFHGLYSLDVNDCKHIQEIRGVPPNLKYFRAINCISLTSTGS
SMLLNQQLHEAGGTDFIFPGGSIPQWFHKQRKEPSISFWFRNKFPAKVLCLLIAPVLGDNVTGLVADKGHDFSVNRM
WDRFLWVVKLKRVDKQEEGLEDVLGIELMSNVIEKRTVFMDLRKECIYTDMASGWG

>Vang0663s00010.1

MVSIVSDLAKSTLEKLINXTIEQSRYICCFTCITDDFEKERENLIAKKETWEEYARVATRRGDNIRKXVXXWQKQAXELIEE
DTKKKVTCFFGWCPNCKWQYSRGKELESKTKEIRRLVERNFENXGISRDVPDIEYHSSENYISFKSRKLKFEELFNALKDD
NNYMIGLQGMGGTGKTTLAKEVGKELKKSKCYNEVIDTTISNTPDIKEIQDDIAGPLGLLLKDCTESERPKKLKDRLTNGE
KILLILDDVWGDINFEEIGIPFKGNHNNCRILVTTRDIRICNKMECEKIIQLDILPEEEGWILFQKHAALSNKSSKSILDIGRKI
SKECKGLPIAIVVIARSLKGQTRQKEWDLALKSLQKSMHQPGDENWSEVYTCLKYSYDNMKNKTAKNLFLLCSLFRDDE
EIPEELLVRFAIGAGLVEQIDVDYNYDAYRNEVIVAKYKLIDSCLLLNEEFESVKMHDLVREMALSIANKEIVAVNTSNKNA
MTMVEKGKNIKYLLCEGKSKDLFSLKFDGSKLDILIIYLNEMEDYYLKVPDPFFENVSRIRVLYLSNDTYEESMMSLPQSIQ
LLTNLRSLLLENFILGDISILGKLQGLETLELVKCWFLELPREIAKLGKLQLLKLNDCMFKRNNSFEVIESCSSLEELYFIHNGIS
KTSHKVQLPNYQRFCILDFSCFLMSKEFFARNSLNMFMADEVFSKETFKNLVQKAEVLCLNGLEGVWKSLIPDIIFPDDE
EGMNNLVEIALKDISQLACLIDNNDSRVQNALSKLVVLKLKSMENLKEICSGPLPFEFLKNLEKIILSDCIHFEGTLFKSNVS
LCNLNHLSISECPMLTSLFELSTAQTLVLLENLSITRCRQLKSIVKDESKRKDSREDIIDAHNDNKSIVSVFPNLTTLHVAHCP
LLHSILPVVVPRNVPKLKSIYVVYCEGLKYIFCPFQDKHEEEESHQELKDLLFTTLKRLVLEYLPNFLDIFMCPSVKKSSSKDE
SKEQKESNPMKCEILHWIHKYRNKRKTTKISLDSTDQLQDSSNSMVNDSNANDVIAQEMSVILTKVAFNNTPQMPVG
SNIFVTLQNVTELLIDKCEKVEVLFCASMLECLPYLHAITIFGCNELKQIIGEDTKNHRKPFFPRLKALVIEECNKLKSVFPISI
SKTLPKLEVLVIVKASMLEEVFEGNSDEKVEIPNLKTAVFAELPSLRQKIEFLTVKHSWVKNGPKFSFSSSLPSIGDILASAK

>Vang08g00920.1

RTVYCAFSHILSALFKALFRVSDWFSYPQDGQVNTLENLILFHLKFSIARCYDLEQWQLKANHSIRCSILQLLEFIDSVSSSS
DKQYLEMDFLGPFGKVVEGLVDFVWKHGVRQVTYIVNYNNNVVELKDSVKDLALEKERINHQRDEAEKNLNNIEGKVI
EWDRKVSEIETTVEVFKNDDGHTRARSPNCFVFPYLWNRHRLGRQAHKMKEDVKRLIGESPELDEVFYRQNVTSNDA
TLSNCGFVEFSSIKSIIEKVMIQLQDSAVRMIGLYGRGGVGKSTLVKEIARKAKEKKLFDVVVKVEITADPNPHKIQEEIAY
VLGLRLEGEGENVRADCLRRRELDDKDALMLFQKLTGIHNEMPSSKQEIVKKYCEGLPMAIVVVARALRNKSESVWEA
TIKRHKKHELVGDGTSMDISVKMSYEHLENEEIKYIFLLCAQMGRRALIMDLVKYSFGLGILEGVSSLWEAREKIKTSIQKL
KDSGLLLDESSNNHFNMHDMVRDTALSIAHKYHNTFNLRNGKLDDWPELEKCTSIFMCNSDIIDGLEVINCPQLKLFQI
DTNDPYLKIPKSFFRRMKNLRVLIMTGFCVSNLPSSIQYLSKLRMLCLQRCTLDCNLSIIGKLKKLRILSFSGSILKSLPIELQC
LDKLRMLDISDCSELKIIPPNVISSLTCLEELYIRESLIKMLVERETNKGQDLFLSELKNLHQLKVVELSIPCVSNFPNHLFFDK
LRDYNIVIGDFDFFSLGEFKMLNKHETFRVLAVQLKDNTIIHSQENIKLLFKTVQTLLLGKTNGVREVVNDLNIDGFQDLE
HLSIINNNDFKYVNSTKLCNYVNVFPNLESLRLYNLGKLDMICYGPVTVVSFAKLRIIKVEMCHRLKNLYSLDMIMFPIGS
QTCDISECNSYMDIFLSSLEIIEVSECKSLKEILQIPKHYGKVEFLKLHTLTLRLLPLLSSFYTKVDKFCWPDLTKAQTRIMGH
KDLTSEEDKQSDEEPPLFGELVEIPNLETLNLSSLKIHKIWSDQNSSSFIFQNLIKLVVKDCDKLTHLCSLSMARSLKKLKSLV

ISECPVMEKIFETERNSADKVCIFPKLEEIHLTKMNKLTDIWQTKVSADSFSSLISVKIEECNKLDKIFPSHMEGWFETLDN
LKVYRCESVEVIFEINDSQEIDEFGGIDTKLQIILLEHLPKLKQLWSTDPNGILNFKKLRTIDVCFCHELKNLFPVSVAKDVPK
LEHMSVLYCDKMEEIVASQDALETNKDLLVFPELTSVRLHCLPDMKYFYKKKYPIKCPKLKELSVTICLKLKTFVKDTINTT
NKVGNFVFSIDEFPYRIPNLEKLKFNSCYFKELAPKANIGRQKGLGIVLQLKELIFSYSNIKDLGFERGQVLQRLEILRLEGCY
KLSNLAPPSVSLIYLTHLELKDCHRLKNLMASSTAKSMVQLKTMKVIDCHKIEQIISMEESEEGKVMKIVFSKLISIELVGLK
KLASFCSHKECEFEFSSLEILIVRECEKMEKFSEKRSIAPKLKNIFGVEGDEKTKWQWEGDLNATIHKIFNDKITFTYSELLD
TEDSTEFIEQLWQGRHWVQQNSFGYLKRFNAEVIFKMNDDNRVIKKPSGLFRLKSLYLSNLPQLKHVWDKDPKEIIDLD
VLEKMRVNICRSLTTLFPASVAKDLTGLEVFKVTE

>Vang0029ss00280.1

MATRIVEGLVKTSEIYSLILDILSGFNGFNDNVQMLEMKLEELCCLEHDINKELEIEELEHGKKRKREVENWLRNVKRKKT
EVHGMVQELRDCGMFRHLKLIVQVRKLTGQVTDLVERGRFPEGIVGSAQESRGCALLTTELAGAMFQKNVRKIWDWL
MNDGVLMIGVYGMGGVGKTSVLMHIHNMLLTAVTNFESVFWVTISKSFSIHKLQCDVAKIVGIDISKESDERKRAARLS
RALVRRKRCVLFLDDVWNHFPLERVGIPVRADGLKLVLTSRSLDVCRRMNCKSSVKVEPLSMEEAWTLFVDNLGQQTT
LSPEVKQVARSVAKKCAGLPLAIITLARSMRGVEEICEWRHALEELRNTEAKHEEMEMEVLRVLRFSYDHLNDKMVQQ
CFLCCALYPEDFEIDRDVLIESFVDEGLVNGMKSLEAMFDEGNSIVNKLENICLLGKVENFVGGKKCMGLQDASSHIAIS
MMKRGCQHVEPMNNVEGYYVGSQLVKMHDLVRAMAINVIKVNNNFLVKAGLQLTKIPDEVEWSEDLEKVSLMCN
WIHEIPTKISPRCPKLRTLILKHNESLTRISDSFFVHMAALEVLDLSFTDIEVLPKSVSDLSTLTALLLTSCKRLKHMPSLAKLQ
ALLRLDLSFTAITEMPQGLEMLVNLKWLNLYAKDLVSSGKEVAKLTSLQFLILHWWSRKIKVKVEYTSCLRKLETFAANLY
NMHHFNSYVKTMNIYGPRSYLLLLDTEESLGNSPWCCFAEVCFRKDVIISNCKIRTGESPLMLPLDIQRLKVERCHDIRSL
CDVMSLKNATSLKRCEIADCDGPEYMFSLSCSSSCCTSLHSLESLELYSLKNLHGLCKEGEVAAQTFPPGRAYTCLKYFFIY
RCPLIKKLLTPRLLAYLPNLEEITVHNCKSMEEIISVDGIDYESFGGKKSYVTNRDTIVVTHSKLVSLSLKHLPELKSISSAQM
ECESLKNFRIFKCPKLARFPETATPVQILYDSF

>Vang04g03010.1

MGCEVLPHDFVLEGDRIIKMVEAVVSFAVDRLGDLLIEEARLLSGVSNKVKSMQNELKRMQCFLRDAESRQDESDRIKN
YISEVRKLAYDAEDVIEIYAIKVAFGISIGTKNPLSRAKNIHKVGSELITINSRISDLTRSLQTYGLTATKDNEEASEVKRQLR
WSYSHIVDEFIVGLDKDINKVAEWLINENQDCRFVYICGMGGLGKTTLAKSIYHYNTIRRNFDGFAWAYISQQCKKRDV
WEGILLKLISPTKEERDEITKMKDDELARKLFKVQQEKKCLIILDDIWSNEAWDILSPAFPSQNTRSKIVFTSRNKDISLHV
NPEGLLHEPSCLNAQDSWSLFKKKAFPRQDNPESTTSDDFKRLGREMVAKCAGLPLAIIVLGGLLATKESVSEWEKIHRH
LSSYLIGAKVRDRRRLDEVLDLSYQDLPCQLKPCFLYLSQFPEDSEIPKTKLLQLWVAEGVVSSQYESDRDETMEDVAERY
LGNLISRCMVQIGQMGSTGRIKTYRLHDLMRDLCLSKARKENFLYIINGSQQNSTVIATGSSNVSDARQINEVRRLAVYL
DQHVDQLIPQDKQVNERLRSLVFFHDKKCRMENWDLVRGVFVKFKLLRVLDLEGIKGLKGQSLPKEVGNLLWLKFLSLK
RTRIQVLPSSLGNLENLQFLNLQTVNKVSWDSTVEIPNVICKLKRLRHLYLPNWCGNIVNNLQLDNLTNLQTLVNFPASK
CDVKDLLKLRRLRKLVLNDPRHFQKFSESFSPPNKRLDCLQSLSLRTDMLSFPENVVDVEKLVLGCPFLRKLQVEGRMER
LPDASLFPRHISKLTLWGCRLVEDPMVTLEKLPNLKFLNGWDMFVGKKMTCSPNGFLQLKVLVLRGLPKLDDWTIENQ
AMPSLYRLSISDCNNLKTVPDGLKYITSLRELEIRWMPKSFKTRLGTAGEDYPKVQHVPSIIFLN

>Vang03g18140.12

LKNNLRDKKFLLVLDDLWNEKYNDWHNLIAPFRSGRKGSKIIITTRQQRVAQVTHTFPTYELKPLSDENCWRILARHAFG
SENFHNYPILEEVGKKIARKCNGLPLAAKTLGGLLRSNVDVGEWNRILNSNMWAHDDVLPALRISYFHLPAHLKRCFAY
CSIFPKQHLLDRKELILLWMAEGFLQHIHEDEEMESVGNDCFNELLSRSLIQKENAVAEENFRMHDLIYDLARLVSGRSS
YHFDGSEIPRTVRHISFLREMFDISEKFEGLYELKCLRTFLPRLSYPFVQCYLTKMVSHGWLPKLRCLRILSLSKYTNITELPN
SIGNLLHLRYLDLSYTSIESLPDETFMLYNLQTLILSNCESLIQLPQKIGNLTNLRHLDISDTNLTEMPTQICNLQELRTLTVFI

VGRQDGLSIRDLSKFPYLQGKLSIMNLQNVVNLVDVFGANLNKKEQIEELILGWGSDPQEPQFEKDVLDNLQPSTNLKK
LSVKYYGGTSFPNWIGNFSFSKITVLTVSDCNNCLSLPPFGQLPSLKELVIKRMKMVKKVGHEFYGSNVDSQLFQPFQSL
ENLEFEDMSEWQEWLPCESEGRNFIFPCLKTLYLCKCPKLRGTLPTHLPSLTNVIFSECNQLVTELADVHWNKSIEAIHIA
EGQEALLSMLDNFSYCELLIEKCDSLLCLPRMLLAANCLQKLTLTNIPSLIYFPADCLLTSLRSLEIWHCRNLEFISHNTCPKF
TSLETLRIWNSCCSMTCFSLGCLPVLQELNIRFIPNLAAITTRGGEAAPKLVDFIVTDCEKLRSLPNQIDLPSLEHLDLSGLP
MLESLSPRCLPSCLRSLHVDVGILSSVSKKELSVLFQRLSSLSHLLLKGLGEEDLVNTLLKQQSLPTSLEYMFLHNFNDLKLL
EGKGLQNLTSLQMLQMYNCPSIESLPEGQLPHSLQVLSLRECPLLEARYQNHNGKYWYKIAHIPAIKINEKVII

>Vang03g18140.10

LNISLLDELKTKLLVLNAVLNDAEEKQITDSAVKQWLDELRDVVLDAEDLLDEINTHALRCKGEGKSRKFATKVRSLLSSSF
KNFYRGMNSKFEAISRRLEQFVRQKDILGLQSVSRRVSYRAVTDSLVDSVVVARENDRERLLSMLLCDDGDGMSNDVE
VITVLGMGGLGKTTLVQCLYNDCEVQSHFDMTGWACVSDDFDILKVTKKIVESLTSKDCHITNLDVLRVELKNNLRDKK
FLLVLDDLWNEKYNDWHNLIAPFRSGRKGSKIIITTRQQRVAQVTHTFPTYELKPLSDENCWRILARHAFGSENFHNYPI
LEEVGKKIARKCNGLPLAAKTLGGLLRSNVDVGEWNRILNSNMWAHDDVLPALRISYFHLPAHLKRCFAYCSIFPKQHLL
DRKELILLWMAEGFLQHIHEDEEMESVGNDCFNELLSRSLIQKENAVAEENFRMHDLIYDLARLVSGRSSYHFDGSEIPR
TVRHISFLREMFDISEKFEGLYELKCLRTFLPRLSYPFVQCYLTKMVSHGWLPKLRCLRILSLSKYTNITELPNSIGNLLHLRY
LDLSYTSIESLPDETFMLYNLQTLILSNCESLIQLPQKIGNLTNLRHLDISDTNLTEMPTQICNLQELRTLTVFIVGRQDGLSI
RDLSKFPYLQGKLSIMNLQNVVNLVDVFGANLNKKEQIEELILGWGSDPQEPQFEKDVLDNLQPSTNLKKLSVKYYGGT
SFPNWIGNFSFSKITVLTVSDCNNCLSLPPFGQLPSLKELVIKRMKMVKKVGHEFYGSNVDSQLFQPFQSLENLEFEDMS
EWQEWLPCESEGRNFIFPCLKTLYLCKCPKLRGTLPTHLPSLTNVIFSECNQLVTELADVHWNKSIEAIHIAEGQEALLSM
LDNFSYCELLIEKCDSLLCLPRMLLAANCLQKLTLTNIPSLIYFPADCLLTSLRSLEIWHCRNLEFISHNTCPKFTSLETLRIWN
SCCSMTCFSLGCLPVLQELNIRFIPNLAAITTRGGEAAPKLVDFIVTDCEKLRSLPNQIDLPSLEHLDLSGLPMLESLSPRCL
PSCLRSLHVDVGILSSVSKKELSVLFQRLSSLSHLLLKGLGEEDLVNTLLKQQSLPTSLEYMFLHNFNDLKLLEGKGLQNLTS
LQMLQMYNCPSIESLPEGQLPHSLQVLSLRECPLLEARYQNHNGKYWYKIAHIPAIKINEKVII

>Vang03g18140.11

MLLCDDGDGMSNDVEVITVLGMGGLGKTTLVQCLYNDCEVQSHFDMTGWACVSDDFDILKVTKKIVESLTSKDCHIT
NLDVLRVELKNNLRDKKFLLVLDDLWNEKYNDWHNLIAPFRSGRKGSKIIITTRQQRVAQVTHTFPTYELKPLSDENCW
RILARHAFGSENFHNYPILEEVGKKIARKCNGLPLAAKTLGGLLRSNVDVGEWNRILNSNMWAHDDVLPALRISYFHLP
AHLKRCFAYCSIFPKQHLLDRKELILLWMAEGFLQHIHEDEEMESVGNDCFNELLSRSLIQKENAVAEENFRMHDLIYDL
ARLVSGRSSYHFDGSEIPRTVRHISFLREMFDISEKFEGLYELKCLRTFLPRLSYPFVQCYLTKMVSHGWLPKLRCLRILSLS
KYTNITELPNSIGNLLHLRYLDLSYTSIESLPDETFMLYNLQTLILSNCESLIQLPQKIGNLTNLRHLDISDTNLTEMPTQICNL
QELRTLTVFIVGRQDGLSIRDLSKFPYLQGKLSIMNLQNVVNLVDVFGANLNKKEQIEELILGWGSDPQEPQFEKDVLDN
LQPSTNLKKLSVKYYGGTSFPNWIGNFSFSKITVLTVSDCNNCLSLPPFGQLPSLKELVIKRMKMVKKVGHEFYGSNVDS
QLFQPFQSLENLEFEDMSEWQEWLPCESEGRNFIFPCLKTLYLCKCPKLRGTLPTHLPSLTNVIFSECNQLVTELADVHW
NKSIEAIHIAEGQEALLSMLDNFSYCELLIEKCDSLLCLPRMLLAANCLQKLTLTNIPSLIYFPADCLLTSLRSLEIWHCRNLEF
ISHNTCPKFTSLETLRIWNSCCSMTCFSLGCLPVLQELNIRFIPNLAAITTRGGEAAPKLVDFIVTDCEKLRSLPNQIDLPSLE
HLDLSGLPMLESLSPRCLPSCLRSLHVDVGILSSVSKKELSVLFQRLSSLSHLLLKGLGEEDLVNTLLKQQSLPTSLEYMFLH
NFNDLKLLEGKGLQNLTSLQMLQMYNCPSIESLPEGQLPHSLQVLSLRECPLLEARYQNHNGKYWYKIAHIPAIKINEKVI
I

>Vang08g00970.1

MDTVLKIFEPVIEYVRDHGINQVIYIFHYTKNIEELNKTVKRLGEVKESLDSQCDEAKRKGHIVKPRVEEWFGEVGEFENR
VENYRKNAGHKKTRGLYYFLPYYRHKLGRKAKKMEVEALKLTNESPKVDEVSHAEKISSFDITSSNSGYIEFNSRKSIMEDI

MTKLKDPNMKIIGLHGSQGMGKSTFIKKVANKAKDEGLFERVVEIDVTISPNPLKIQEEIAYVLNLPLAGESENGYKVLLS
SCDKKVFPDEVDAESSFCLKELDDSDALMLFEKVTGGCNKMSMPKEEIQNYCKGLPMRIVKFAMAYKNWSESESEPTL
DKFKKQGLVEWKKSSDIPNKIKYDLPKNKELKFIFLLCAQMGHLPLVNDLVKYCFGLGIFEGVSSLSAAREKINEAIQELKN
LSLVSFESPNIHFSMSHMVRDDALSNALTDNNVFAFRDGELDYWPNLEKCISISICNSDIIDRFPQVINCPQLNFLQIETN
NPSLEIPDIFFSSMKNLLVLILTGFHLSSLPYSIEDLLNLRMLCLERCTLDCNLSILRKFKKLRILSFSGSQLKKLPFELRFLDKLR
LLDISDCFELKIIPSNLLSSLTCLEELYISKSLIKMLVETDTNKGQNSFLSELTNLHQLKVVDISIPCVSCIPNQLIFDKLTYYKIEI
GEFEMISVGEFWMSNKYEELKVLALQLKNDTDIRSKEGIMLLLKTADRLMLGEASVQSVVNDLNMDGFLNLKHLSIINN
KDIKYVNSTKLSNCGNVFPNLEYICLYNMMNLKMICYGEVTVTSFAKLKTIKVKMCYRLENLFSFYTIKISTCGEETSEISDC
NSYMNEFLASLEMIEVCECESLKEILRIPVNYDKVEFLMLRTLTLQSLPSLTCFSTKVESSRESNLTQAQTTNRGHTEISTEE
DDHSDMAPPLFGELVEVPNLENLNLSSLNIHKIWSNQHSSSVCFQNLIKLVVKDCDKLTHLCSLPMASHLKKLKSLVISGC
PIMEKIFETEGISAENVYVFPKLEEIHLSKMNGLIDIWQTKVSADSFSNLIFVRIEECNQLNKIFPSQMQGWFESLINLEVS
ECKSVIEIFEINDSHEINASGIDTNLQVILLEGLPKLKQLWSKDPDGILNFKKLQSIDVRNCDELTNLFPTSVAKDVSKLERM
SVFYCKKMVEIVASKDAAEDMKDPLKFPELTYVRLYGLSNMKQFYEGRHPIKCPKLKELSIDKCVKLQTFLQQKSETRNE
ENFIFSAEEVSFAYTEILWLDDNIKKQLWDDTQVQQNSFGYLKRLWVWDCHTLKHIIPSHLLSCFHNLEELNVHHCSNA
EVIFNMNDENRVMTKPSGIFRLKILSLDSLPQLKHVWDKDPEGVMGLQLLKEMRVENCKSLKSLFPASVVKDLTRLEVL
EVTNCKKLVEIFWKDEKGGEGETTTQEFVFRDLTSLTPKELPALKYSIHCSKQQVIFKTLPLCDHSKQIRL

>Vang0103s00290.1

MALAVVGGALLSAFIDVLFDRLASPEFVNLIRGKKPDKLLQKMKSQXLVVKVVLADAEKRQITDSNVKEWLDLLNDVVY
RADDLLDEVYTKAATQKEVSSSFSHLFKRNKVVNVSKLEDIVERLDDILKQKESLDLKEIAVENNQPWNAQTTSLEDRYG
MYGRDEDKEAIMKLLLEDSSDGEEVSVIPIVGMGGVGKTTLTRSVYNDGKLNXIFKLKAWICVSDIFDIVKVTKTMLEEIT
QKPCKLSDLNLIQLDLLEKLKGKKFLIVLDDVWIEDCDSWSSLTKPFLSGISGSKVIVTTRNEXVAAVVPFHYVKVYHLNKL
SNEDCWLVFASHALPLSEDSENRETLEKIGKEIVKKCNGLPLAAQSLGGMLRRKHEIKDWNXVLESDIWELPESQCKIIPA
LRISYNYLPPQLKRCFVYCSLYPKDYEFQKHXLILLWMAEDLVKASKKGKTLEEVGQDYFDDLVSRSFFQCSSSRDWGDY
FVMHDLMHDLATFLGGEFYFRADEHGKETKIDRKARHLSFTRFGDSISDTEVLDRVKFSRTFLPTYDEYSPFKNRNTPCII
VSMLKYLRVLSFSNLQCQLVLPDSIGELIHLRYLDLSFTGIETLPESLCNLYNLQTLKLCCCFRLTKLPSAMQNLENLRHLEIR
DSSIKEMPKRMGKLNQLLTLDLYIVGKHKENSIKELGGLPNLHGRVSIEKLENVTNGEEAFEARIMDKKYINSLELQWSLC
DDSSIDFQIELDVLGKLQPHQDLKSLSIKGYKGKRFPKWMDNFSYRYMKYLYLQNCNNCCMLPSLGQLPSLKHILISDM
NSVKTIDAGFCKKEDCSSMAPFPSLESLYIFNMPCWEMWSSVDSKAFPVLQELYIENCPKLKGDLPNHLPSLQTLKIKSC
QLLVSSFPMAPALRTLKICESNKLELHAFPQSVECIKISGRPMVESMMEAITDIPPTCLKELSLIDCSSAISFPGDRLPSSLKT
LDIRGLNKLSFPVLHKHKLLESLSINNSCDSLKSFPPAIFPNLTRLIIENCENLESLLLLGSESLKSLNSFVIGDCPNFVSFPGEG
FSAPNLTFFSVYGCAKLKSLPHQMGTLLPKMEYLSISNCQQIECFPEGGMPPNLTTVTIRNCEKLLISLGWISIDMVTSLE
VYGTCDSINAFPKEGLLTPSLTSLYLIDFSSLDTLDCKGLLHLTSLQTLIIKNCKKLKNIAGERLPVSLVKLIISECPLLQKRCHV
KDRQIWPKICHVRGIKIDGRWI

>Vang0306s00030.1

MPVIETLGGALFGAVLQVLFDKLDSRQVLDYFRRRKFDEKLLKKLKRKLVSINAGVDDAEKKQFRNAYVKAWLDEVRDV
LLDTEDLMDEIYYEFSRYGLEVESHSSSSQVCIFESRIKEVLDDLECLLNQMDDLGLKNASGVGVGLGLNSNVSQKLPSTS
LVVENIIYGRDDEKEMILKWMTSDTEKHSQLSILSVVGMGGLGKTTLAQHVYNDPRIEGKFAIKGWVYVSDEFDVLMLT
KTIFGVLTKSKDDSVDLEMVQGRLKEKLTGRKFLVVLDDVWNEDRAQWKALQTPLNYGAKGSKILVTTRSNKVASIVQS
NKLHELKQLGGDHSWQVFAKHAFQDDNSQMNAEVKEIGTKIVEKCRGLPLALETVGCLLRSKSSVAEWKSVLSSEIWD
FPEEDSKIIPALLLSYYHLPSYLKRCFSYCAMFPKDHQFDKKNLIQLWMAENFLQRSQQSKSQEEVGEHYFNVLLSRCFF
QQSSEGFKSCFVMHGLLNDLAKYVSGDICFRFGIDRAKRTLKETRHFSFVIDDYGVSCNEYENLYDAKGLRTFLPVTRISY
WSWYCETLTLELIFKLKCLHVLSFCGCVNLKEVPETIGNLIHLRFLDLSNTGIQKLPDTMCSLCNLQTLKLNSCVNLKELPC

NLHKLTNLCCPELMKNSLTKMPMHIGKLKNLEIFMSPFNVGKSSELCIQQLGELLSKTCLVRLDLHWDLEQNLVNFMKE
REILENLQPSRHLEELSINDYGGIQFPHWLSDNSLSNLVSLSLINCKHCLLLPSLEFLTFLRHLTISGHDWIRTIDADFYRNSC
SAFASLQTLNFADMKEWEEWQCRTGDFPSLQSLFVTKCPKLKGQLPKQLSHLRKLIIEDCKQLVTLAPRTLEICELQLRDC
GKLQIDYNPTTLKRLQIGGDNMEASLLERLGHIISHTSLESFTIFSCPNMNIPINHCFDFLEKLHIGGGCDSLTNFPLDFFPK
LSELELSECPNLQMIAQGHPLNHLKILRIGKCSRFEYFPNEGLFARQLESFYIIGLEKLKSLPKRMSVLLPSLNLLYINDCPDV
EVSDGCLPSNLNEMCLFNCSKLIASLKGPWGTNPSLKSLSIGKVDEDCFPSEGLLPLSVTNLEIYDCPNLKKLDYRGLCHLF
SLEKLFLYKCPILQCLPEEGLPKSISKLRVEGCPLIKQRCKKQEGEDWQKIAHIKYIMVDRERVNI

>Vang06g21880.9

MSFYCKGVEEKIEDVHERLEFIMRQKEVLDLKVGKEVKMAHKTPTSSVMEACDVFGRDNDKESLVDLVLTHNEKIGIIPI
VGMGGIGKTTLAQLIYNDQRVQKEFDVKAWIYVSEEFDICKITKTLLEAVTSCSCDVEDLNFLQRDLKMHVMNKKFLFV
LDDVWNENYDNWDKFRSPFKHAGEHGSKIIVTTRSGCVASIMQTVSPYNLRELSNEDSWNLFSKHAFDYGDSSLQLH
QSLDKVGREIVRKCKGLPLAVKTLAGLLRCKSDRQEWCKVLDSEMWDLQDSESNILPALRLSYHYLPSHLKRCFAYCSIFP
KDYEFEKENLVLLWMAEGFLQQSKRHRRIEEVGNEYFCELVSRSFFQQPRRGKSCFLMHHLVNDLAQFVSGTFSIRMEC
SNSNEIKERTRHLSHIIADSSSYVNLKDVSKANCLRTFLQIRPVGTSIDLFNNMPNDLLTKLRSLRVLSLVGTHIYSLPNSVG
ELKHLRYLEVADTEIVRLPESICSLFNLQTLKLVGCHNLIELPASIHKLVNLRHLDIRGTSLRWMPLQINELNSLQNLSDFFV
GKGCGSSLGELGELICLHGELFIHCLEHIVSDKDCEKAKLKEKHGLEKLSLDWCRNGETENSQKEKTILNSLQPHTNLKKLD
IYDYPGTEFPEWLGDHSFYNLVSLMLNGCKYCYRLPPLGQLPMLKELQISKFEGLVSVGSEFLGNRTSYLTNCFPALEILRI
ESMPLWEKWYPNAENAGSKAFFHLREIHIGNCPKLRGNLPDNLPSLTLLVIRDCKRLLCSLPNSPSLRVLNIQNCESLEFK
VHSPLCHQSLTSLFLHGSCDSLVFLPLDLFPNIKSLDIWGCKNLEALTVSESDATRPNLKSLHSLRIRHCPNFTSFPKGGFA
ASKLTLLTINYCQKLNSLPEQMHDLMPSLKEVQLRGCPKIESSSTRPLRIRICSKHMEGKQNLSDPLFARLKGLATDQSPS
SS

>Vang06g21880.5

MELVAGPLMGAIFNVLLERIASTEVVNFFKNKNCEKLLKRLKIILLSVNVVLNDAEEKQMKNGAVKEWLEELKDVAFAAE
DLLDEIYTDAKIKAKQVNTLHSGPMSFYCKGVEEKIEDVHERLEFIMRQKEVLDLKVGKEVKMAHKTPTSSVMEACDVF
GRDNDKESLVDLVLTHNEKIGIIPIVGMGGIGKTTLAQLIYNDQRVQKEFDVKAWIYVSEEFDICKITKTLLEAVTSCSCDV
EDLNFLQRDLKMHVMNKKFLFVLDDVWNENYDNWDKFRSPFKHAGEHGSKIIVTTRSGCVASIMQTVSPYNLRELSN
EDSWNLFSKHAFDYGDSSLQLHQSLDKVGREIVRKCKGLPLAVKTLAGLLRCKSDRQEWCKVLDSEMWDLQDSESNIL
PALRLSYHYLPSHLKRCFAYCSIFPKDYEFEKENLVLLWMAEGFLQQSKRHRRIEEVGNEYFCELVSRSFFQQPRRGKSCF
LMHHLVNDLAQFVSGTFSIRMECSNSNEIKERTRHLSHIIADSSSYVNLKDVSKANCLRTFLQIRPVGTSIDLFNNMPNDL
LTKLRSLRVLSLVGTHIYSLPNSVGELKHLRYLEVADTEIVRLPESICSLFNLQTLKLVGCHNLIELPASIHKLVNLRHLDIRGT
SLRWMPLQINELNSLQNLSDFFVGKGCGSSLGELGELICLHGELFIHCLEHIVSDKDCEKAKLKEKHGLEKLSLDWCRNGE
TENSQKEKTILNSLQPHTNLKKLDIYDYPGTEFPEWLGDHSFYNLVSLMLNGCKYCYRLPPLGQLPMLKELQISKFEGLVS
VGSEFLGNRTSYLTNCFPALEILRIESMPLWEKWYPNAENAGSKAFFHLREIHIGNCPKLRGNLPDNLPSLTLLVIRDCKRL
LCSLPNSPSLRVLNIQNCESLEFKVHSPLCHQSLTSLFLHGSCDSLVFLPLDLFPNIKSLDIWGCKNLEALTVSESDATRPNL
KSLHSLRIRHCPNFTSFPKGGFAASKLTLLTINYCQKLNSLPEQMHDLMPSLKEVQLRGCPKIESSSTRPLRIRICSKHMEG
KQNLSDPLFARLKGLATDQSPSSS

>Vang11g03500.1

MDQTTQRVVLPALFQERLRMVSEIVEKGKSSDSNKQILRSTLKDMNPVVQEIKQYNEHLNPPREEIKTLISEKDAKEELV
GKCLSKKFCLDKCLSLFLCRFGHKRDGSFAGGDKQALVAKDIEEKLYKVREILELLSKENFEQKLGGVGGPIRLPFGVPDN
PDFTVGLDVPFSKLKMEVLRDGVSVIMLTGLGGMGKTTLATKLCWDEQVKGKFGGNILFVTVSKTAKLKIIVERLFQHC
GYQVPDFLSDEDAANQLGLLLKQIGRSSMLLVLDDVWPGSEALVEKFKVQIPDYKILVISRVALLRSDMQCILKPLGHDD

AETLFRHYTHLEESGSSIPDEVIQKVVRNCKGLPLAIKVVGRSLCNQRSEMWLQMMEELSQGRSILDSNVELITCLQKILD
VLEDNLVIKGCFMDLGLFPEEQRIPVTALIDMWAELYRLDDDGKEAMTIINKLDSMNLANVMVARKNATNTDSYYYN
NHFIVLHDLLRELAIYQCSQEPMEQRKRLIIEINKNKHGEKTKFLSWCVKQKPQQVTAHTLSISTDENCSSDWPQMQLA
QVEVLIFNLQTKQYSFPDCMEGMNKLKVLIVTNYSYYPSEINNFELLGSSSNLRRIRLERISVPSFVAMKNLKKLSLYFCNM
KQAFQNKDLPISYAFPNLEDLNIDYCKDMVGFPKGVCDIISLKKLSITNCHKLSALPQDIGKLENLELLRLSSCTDLEGIPDSI
GRLSNLRLLDISNCISLPNLPDDFGNLSNLQNLYMTSCARCELPLSVTNLGNLKVVICDEETAASWENFKPMLPNLRIDVS
QVDVNLNWLHTTFS

>Vang03g18140.4

LNISLLDELKTKLLVLNAVLNDAEEKQITDSAVKQWLDELRDVVLDAEDLLDEINTHALRCKGEGKSRKFATKVRSLLSSSF
KNFYRGMNSKFEAISRRLEQFVRQKDILGLQSVSRRVSYRAVTDSLVDSVVVARENDRERLLSMLLCDDGDGMSNDVE
VITVLGMGGLGKTTLVQCLYNDCEVQSHFDMTGWACVSDDLSYTSIESLPDETFMLYNLQTLILSNCESLIQLPQKIGNL
TNLRHLDISDTNLTEMPTQICNLQELRTLTVFIVGRQDGLSIRDLSKFPYLQGKLSIMNLQNVVNLVDVFGANLNKKEQIE
ELILGWGSDPQEPQFEKDVLDNLQPSTNLKKLSVKYYGGTSFPNWIGNFSFSKITVLTVSDCNNCLSLPPFGQLPSLKELV
IKRMKMVKKVGHEFYGSNVDSQLFQPFQSLENLEFEDMSEWQEWLPCESEGRNFIFPCLKTLYLCKCPKLRGTLPTHLP
SLTNVIFSECNQLVTELADVHWNKSIEAIHIAEGQEALLSMLDNFSYCELLIEKCDSLLCLPRMLLAANCLQKLTLTNIPSLI
YFPADCLLTSLRSLEIWHCRNLEQIDLPSLEHLDLSGLPMLESLSPRCLPSCLRSLHVDVGILSSVSKKELSVLFQRLSSLSHLL
LKGLGEEDLVNTLLKQQSLPTSLEYMFLHNFNDLKLLEGKGLQNLTSLQMLQMYNCPSIESLPEGQLPHSLQVLSLRECP
LLEARYQNHNGKYWYKIAHIPAIKINEKVII

>Vang03g18140.5

LIKRIASREFRDFFSSRKLNISLLDELKTKLLVLNAVLNDAEEKQITDSAVKQWLDELRDVVLDAEDLLDEINTHALRCKGE
GKSRKFATKVRSLLSSSFKNFYRGMNSKFEAISRRLEQFVRQKDILGLQSVSRRVSYRAVTDSLVDSVVVARENDRERLLS
MLLCDDGDGMSNDVEVITVLGMGGLGKTTLVQCLYNDCEVQSHFDMTGWACVSDDFDILKVTKKIVESLTSKDCHIT
NLDVLRVELKNNLRDKKFLLVLDDLWNEKYNDWHNLIAPFRSGRKGSKIIITTRQQRVAQVTHTFPTYELFAYCSIFPKQ
HLLDRKELILLWMAEGFLQHIHEDEEMESVGNDCFNELLSRSLIQKENAVAEENFRMHDLIYDLARLVSGRSSYHFDGSE
IPRTVRHISFLREMFDISEKFEGLYELKCLRTFLPRLSYPFVQCYLTKMVSHGWLPKLRCLRILSLSKYTNITELPNSIGNLLHL
RYLDLSYTSIESLPDETFMLYNLQTLILSNCESLIQLPQKIGNLTNLRHLDISDTNLTEMPTQICNLQELRTLTVFIVGRQDGL
SIRDLSKFPYLQGKLSIMNLQNVVNLVDVFGANLNKKEQIEELILGWGSDPQEPQFEKDVLDNLQPSTNLKKLSVKYYGG
TSFPNWIGNFSFSKITVLTVSDCNNCLSLPPFGQLPSLKELVIKRMKMVKKVGHEFYGSNVDSQLFQPFQSLENLEFEDM
SEWQEWLPCESEGRNFIFPCLKTLYLCKCPKLRGTLPTHLPSLTNVIFSECNQLVTELADVHWNKSIEAIHIAEGQEALLS
MLDNFSYCELLIEKCDSLLCLPRMLLAANCLQKLTLTNIPSLIYFPADCLLTSLRSLEIWHCRNLEFISHNTCPKFTSLETLRI
WNSCCSMTCFSLGCLPVLQELNIRFIPNLAAITTRGGEAAPKLVDFIVTDCEKLRSLPNQIDLPSLEHLDLSGLPMLESLSP
RCLPSCLRSLHVDVGILSSVSKKELSVLFQRLSSLSHLLLKGLGEEDLVNTLLKQQSLPTSLEYMFLHNFNDLKLLEGKGLQN
LTSLQMLQMYNCPSIESLPEGQLPHSLQVLSLRECPLLEARYQNHNGKYWYKIAHIPAIKINEKVII

>Vang03g18140.7

MLLCDDGDGMSNDVEVITVLGMGGLGKTTLVQCLYNDCEVQSHFDMTGWACVSDDFDILKVTKKIVESLTSKDCHIT
NLDVLRVELKNNLRDKKFLLVLDDLWNEKYNDWHNLIAPFRSGRKGSKIIITTRQQRVAQVTHTFPTSIFPKQHLLDRKE
LILLWMAEGFLQHIHEDEEMESVGNDCFNELLSRSLIQKENAVAEENFRMHDLIYDLARLVSGRSSYHFDGSEIPRTVRH
ISFLREMFDISEKFEGLYELKCLRTFLPRLSYPFVQCYLTKMVSHGWLPKLRCLRILSLSKYTNITELPNSIGNLLHLRYLDLSY
TSIESLPDETFMLYNLQTLILSNCESLIQLPQKIGNLTNLRHLDISDTNLTEMPTQICNLQELRTLTVFIVGRQDGLSIRDLSK
FPYLQGKLSIMNLQNVVNLVDVFGANLNKKEQIEELILGWGSDPQEPQFEKDVLDNLQPSTNLKKLSVKYYGGTSFPN
WIGNFSFSKITVLTVSDCNNCLSLPPFGQLPSLKELVIKRMKMVKKVGHEFYGSNVDSQLFQPFQSLENLEFEDMSEWQ

EWLPCESEGRNFIFPCLKTLYLCKCPKLRGTLPTHLPSLTNVIFSECNQLVTELADVHWNKSIEAIHIAEGQEALLSMLDNF
SYCELLIEKCDSLLCLPRMLLAANCLQKLTLTNIPSLIYFPADCLLTSLRSLEIWHCRNLEFISHNTCPKFTSLETLRIWNSCCS
MTCFSLGCLPVLQELNIRFIPNLAAITTRGGEAAPKLVDFIVTDCEKLRSLPNQIDLPSLEHLDLSGLPMLESLSPRCLPSCL
RSLHVDVGILSSVSKKELSVLFQRLSSLSHLLLKGLGEEDLVNTLLKQQSLPTSLEYMFLHNFNDLKLLEGKGLQNLTSLQM
LQMYNCPSIESLPEGQLPHSLQVLSLRECPLLEARYQNHNGKYWYKIAHIPAIKINEKVII

>Vang03g18140.1

LNISLLDELKTKLLVLNAVLNDAEEKQITDSAVKQWLDELRDVVLDAEDFVSRRVSYRAVTDSLVDSVVVARENDRERLL
SMLLCDDGDGMSNDVEVITVLGMGGLGKTTLVQCLYNDCEVQKEMESVGNDCFNELLSRSLIQKENAVAEENFRMH
DLIYDLARLVSGRSSYHLYLDLSYTSIESLPDETFMLYNLQTLILSNCESLIQLPQKIGNLTNLRHLDISDTNLTEMPTQICNL
QELRTLTVFIVGRQDGLSIRDLSKFPYLQGKLSIMNLQNVVNLVDVFGANLNKKEQIEELILGCT

>Vang03g18140.2

MALCMVGEALISACVEILIKRIASREFRDFFSSRKLNISLLDELKTKLLVLNAVLNDAEEKQITDSAVKQWLDELRDVVLDA
EDLLDEINTHALRCKGEGKSRKFATKVRSLLSSSFKNFYRGMNSKFEAISRRLEQFVRQKDILGLQSVSRRVSYRAVTDSLV
DSVVVARENDRERLLSMLLCDDGDGMSNDVEVITVLGMGVQSHFDMTGWACVSDDFDILKVTKKIVESLTSKDCHIT
NLDVLRVELKNNLRDKKFLLVLDDLWNEKYNDWHNLIAPFRSGRKGSKIIITTRQQRVAQVTHTFPTYELKPLSDENCW
RILARHAFGSENFHNYPILEEVAAKTLGGLLRSNVDVGEWNRILNSNMWAHDDVLPALRISYFHLPAHLKRCFAYCSIFP
KQHLLDRKELILLWMAEGFLQHIHEDEEMESVGNDCFNELLSRSLIQKENAVAEENFRMHDLIYDLARLVSGRSSYHFD
GSEIPRTVRHISFLREMFDISEKFEGLYELKCLRTFLPRLSYPFVQCYLTKMVSHGWLPKLRCLRILSLSKYTNITELPNSIGN
LLHLRYLDLSYTSIESLPDETFMLYNLQTLILSNCESLIQLPQKIGNLTNLRHLDISDTNLTEMPTQICNLQELRTLTVFIVGR
QDGLSIRDLSKFPYLQGKLSIMNLQNVVNLVDVFGANLNKKEQIEELILGWGSDPQEPQFEKDVLDNLQPSTNLKKLSV
KYYGGTSFPNWIGNFSFSKITVLTVSDCNNCLSLPPFGQLPSLKELVIKRMKMVKKVGHEFYGSNVDSQLFQPFQSLENL
EFEDMSEWQEWLPCESEGRNFIFPCLKTLYLCKCPKLRGTLPTHLPSLTNVIFSECNQLVTELADVHWNKSIEAIHIAEGQ
EALLSMLDNFSYCELLIEKCDSLLCLPRMLLAANCLQKLTLTNIPSLIYFPADCLLTSLRSLEIWHCRNLEFISHNTCPKFTSLE
TLRIWNSCCSMTCFSLGCLPVLQELNIRFIPNLAAITTRGGEAAPKLVDFIVTDCEKLRSLPNQIDLPSLEHLDLSGLPMLES
LSPRCLPSCLRSLHVDVGILSSVSKKELSVLFQRLSSLSHLLLKGLGEEDLVNTLLKQQSLPTSLEYMFLHNFNDLKLLEGKG
LQNLTSLQMLQMYNCPSIESLPEGQLPHSLQVLSLRECPLLEARYQNHNGKYWYKIAHIPAIKINEKVII

>Vang03g18140.3

LIKRIASREFRDFFSSRKLNISLLDELKTKLLVLNAVLNDAEEKQITDSAVKQWLDELRDVVLDAEDLLDEINTHALRCKGE
GKSRKFATKRGMNSKFEAISRRLEQFVRQKDILGLQSVSRRVSYRAVTDSLVDSVVVARENDRERLLSMLLCDDGDGM
SNDVEVITVLGMGGLGKTTLVQCLYNDCEVQSHFDMTGWACVSDDFDILKVTKKIVESLTSKDCHITNLDVLRVELKNN
LRDKKFLLVLDDLWNEKYNDWHNLIAPFRSGRKGSKIIITTRQQRVAQVTHTFPTSIFPKQHLLDRKELILLWMAEGFLQ
HIHEDEEMESVGNDCFNELLSRSLIQKENAVAEENFRMHDLIYDLARLVSGRSSYHFDGSEIPRTVRHISFLREMFDISEK
FEGLYELKCLRTFLPRLSYPFVQCYLTKMVSHGWLPKLRCLRILSLSKYTNITELPNSIGNLLHLRYLDLSYTSIESLPDETFML
YNLQTLILSNCESLIQLPQKIGNLTNLRHLDISDTNLTEMPTQICNLQELRTLTVFIVGRQDGLSIRDLSKFPYLQGKLSIMN
LQNVVNLVDVFGANLNKKEQIEELILGWGSDPQEPQFEKDVLDNLQPSTNLKKLSVKYYGGTSFPNWIGNFSFSKITVLT
VSDCNNCLSLPPFGQLPSLKELVIKRMKMVKKVGHEFYGSNVDSQLFQPFQSLENLEFEDMSEWQEWLPCESEGRNFI
FPCLKTLYLCKCPKLRGTLPTHLPSLTNVIFSECNQLVTELADVHWNKSIEAIHIAEGQEALLSMLDNFSYCELLIEKCDSLL
CLPRMLLAANCLQKLTLTNIPSLIYFPADCLLTSLRSLEIWHCRNLEFISHNTCPKFTSLETLRIWNSCCSMTCFSLGCLPVL
QELNIRFIPNLAAITTRGGEAAPKLVDFIVTDCEKLRSLPNQIDLPSLEHLDLSGLPMLESLSPRCLPSCLRSLHVDVGILSSV
SKKELSVLFQRLSSLSHLLLKGLGEEDLVNTLLKQQSLPTSLEYMFLHNFNDLKLLEGKGLQNLTSLQMLQMYNCPSIESL
PEGQLPHSLQVLSLRECPLLEARYQNHNGKYWYKIAHIPAIKINEKVII

>Vang03g18140.9

MALCMVGEALISACVEILIKRIASREFRDFFSSRKLNISLLDELKTKLLVLNAVLNDAEEKQITDSAVKQWLDELRDVVLDA
EDLLDEINTHALRCKGEGKSRKFATKVRSLLSSSFKNFYRGMNSKFEAISRRLEQFVRQKDILGLQSVSRRVSYRAVTDSLV
DSVVVARENDRERLLSMLLCDDGDGMSNDVEVITVLGMGGLGKTTLVQCLYNDCEVQSHFDMTGWACVSDDFDILK
VTKKIVESLTSKDCHITNLDVLRVELKNNLRDKKFLLVLDDLWNEKYNDWHNLIAPFRSGRKGSKIIITTRQQRVAQVTHT
FPTYELKPLSDENCWRILARHAFGSENFHNYPILEEVGKKIARKCNGLPLAAKTLGGLLRSNVDVGEWNRILNSNMWAH
DDVLPALRISYFHLPAHLKRCFAYCSIFPKQHLLDRKELILLWMAEGFLQHIHEDEEMESVGNDCFNELLSRSLIQKENAV
AEENFRMHDLIYDLARLVSGRSSYHFDGSEIPRTVRHISFLREMFDISEKFEGLYELKCLRTFLPRLSYPFVQCYLTKMVSH
GWLPKLRCLRILSLSKYTNITELPNSIGNLLHLRYLDLSYTSIESLPDETFMLYNLQTLILSNCESLIQLPQKIGNLTNLRHLDIS
DTNLTEMPTQICNLQELRTLTVFIVGRQDGLSIRDLSKFPYLQGKLSIMNLQNVVNLVDVFGANLNKKEQIEELILGWGS
DPQEPQFEKDVLDNLQPSTNLKKLSVKYYGGTSFPNWIGNFSFSKITVLTVSDCNNCLSLPPFGQLPSLKELVIKRMKMV
KKVGHEFYGSNVDSQLFQPFQSLENLEFEDMSEWQEWLPCESEGRNFIFPCLKTLYLCKCPKLRGTLPTHLPSLTNVIFSE
CNQLVTELADVHWNKSIEAIHIAEGQEALLSMLDNFSYCELLIEKCDSLLCLPRMLLAANCLQKLTLTNIPSLIYFPADCLLT
SLRSLEIWHCRNLEFISHNTCPKFTSLETLRIWNSCCSMTCFSLGCLPVLQELNIRFIPNLAAITTRGGEAAPKLVDFIVTDC
EKLRSLPNQIDLPSLEHLDLSGLPMLESLSPRCLPSCLRSLHVDVGILSSVSKKELSVLFQRLSSLSHLLLKGLGEEDLVNTLLK
QQSLPTSLEYMFLHNFNDLKLLEGKGLQNLTSLQMLQMYNCPSIESLPEGQLPHSLQVLSLRECPLLEARYQNHNGKY
WYKIAHIPAIKINEKVII

>Vang0870s00010.1

MADSSVSFLLDKLNALLQEEVNLQRGVREDVQYIKYELERHKAILRVADAMEDRDPELKAWVKGVRCVAHDMEDAID
EFNLRLVDQHGQGNGSSLHRFTFGLKTMKARRRIALDMQSIKSKVNVISQGRPELPGIGSRSSQRLSSSLDSQGDALLLE
EADLVGIDKPKRQLCDFLFNEEPGRAVIPIYGMGGLGKTTLAKQVYDDPKVKKRFRIHAWINVSQSFKLQELLKDLVQQL
HNVIGKPAPEAVGQMKSEELKELIKNLLQSSRYLIVLDDVWHVKVWDSVKLALPSSSRGSRVMITTRKKDIALYSCAELG
KDFDHEFLPEEEAWYLFCKKTFQGNSCPPHLEEVCRKILKMCGGLPLAIVAIGGALATRQRANIEEWQMVCRSFGSEIE
GNDKLEDMKKVLSLSFNELPYYLKSCLLYLSIFPEFHAIEHMRLIRLWIAEGFVNGEDGKTREEVADSYLKELLDRSLLQVV
AKTSDGRMKTCRMHDLLREIVNLKAKDQNFATIAKDQDIIWPDKVRRLSIINTLDNVRQNRASFQLRSLLMFDLSDPLE
HFSIRGLCSTGYKLIRVLDLQDAPLEVFPAEIVNLYLLKYLSLKNTKVKSIPGTIKKLQQLETLDLKQSLVTVLPVEIVELQQLR
HLLVYRYEIESYAYFHSRHGFKVAAPIGLMRSLQKLCFIEADQALMVELGKLTQLRRLGIRKMKQQDGAALSLSIEKMINL
RSLSITAIEKHEIIDIHNIFRPPPYLHQLYLSGRLDIFPHWISSMKNLVRVFLKWSRLTEDPLVHLQDLPNLRHLEFLQVYVG
ETLNFKDKGFPSLKVLGLDDLDELKSMTVEEGAMPGLKKLIIQRCGSLKQLPFGIEHLTKLKSIEFFDMPEELITRLHPKGG
QDYLRAKNVPAVYSSYWRDGGWDVYSLETLEERETDFNRSTAVRSLENCPLWKV

>Vang11g13870.1

MAVSAATTMAMVVWCNGGGLISLSVIMANHGDFTYDVFMSFKGENGTRYSFTDHLYRALLRHGINAFRDEQSLRSG
DEIRPSLFQAIEASRISLVVLCQNYASSSWCLDELAKILHCYENKGKHVVAIFYLVEPSDVRYQKNSYATAMSKHENRYGK
DSEKVKAWRSALSRVCDLTGIHYRNHMYETEVIEKIVKDTSAKLPPMPLQIKHLVGLDSRFERVKSLIDVESNDEVCILGIY
GVGGIGKTTFAVDLYNKIRHHFEAASFLANVREKTNKSIKGLEDLQRTLLSEMGEETETIIGSTFKGSSEIKCRLGHKRVLLI
VDDVDSVKQLEALAGGYDWFGPGSRIIITTRDKDVLHKHHVEIKGYKMEELNYQESLELFCWYAFNRSRAAENFANIST
SAVRYAKGIPLALRVIGSNLKGRGPEECETELQKYRKVPDSEIQGVLEISYTSLSNLDQKIFLDIACFFKGERWDYVKRILDA
CDFNPDIKVFVSKCLITVDENGCLEMHDLIQDMGREIVRKESPSNPGYRSRLWCHKDVHEVLKENSGSDTVEGIMLYPP
KQEKIDYWTMTAFKKMKNLRILIVRNAIFSSGPSYLPNSLRLIDWKGYPSKSFPPDFYPHRIVDFKLPHSSLIFKKPFQRFE
DLTFINLSHCQFITQIPDLSGAKSLKVFTLDKCYRLTRFEKSIGFMPNLVYLSASECTLLTSFVPTMYLPSLEVLSFNFCRRLE
HFPHVMQKMDKPLKIYMMSTAIKKIPESIGNLTGLEHIDMSVCKELKNLPSSFFLLPKLVTLKVDECSQLGESFQRFQKSR
HSVANGSSNLVTLHFCETNPSYEDLCAILETFPKLEDLNVSHNGFVALPNIIGGSSHLKSLDVSFCRNLKEIPELPLSIQKVD

ARYCQSLTSEASSMLWSKVSEEIQRIQVVMPMLKREIPEWFDCIGTKEIPHFWARRKFPVVALALVFQESKKKLSDFEHA
FQSAVESFTGFVNWHTVSLHLFIDGQEICGRDYHCFNVGEDHVLFCDLRVLFRDEEWQGLDASLGDDWKAIQVQYES
DLILSHWGVNVYEQETNTDDIQFRFPTPSSRRNLIPSSLLVPKGCPKQKMKHMLESFDPRDIFNKNLSLIESEEGPSRAGK
VLLRTWRNAKAEITEEASISVYGASLKQEHEESVDDVVQVLEMIKENVPKHFSDSNPEEMQSFGGFVERLLRARVEVMK
EKGLDMGMPILLEYTDGGGSKYRRFWGVLQLKVGDPFYKAVLRKYIQLSLEFSASNKAASSSGSWFENLRITIVLLKCLD
PAMDEALGFGYEESLEEGYYDPELEELMMRIEQDGMGFNKSYGKMKASIVRTDESVSPEYLFETLIFRRLIALGKLSMFG
SATKFKITPYGNIRVEDDPFRIPKTCFWSLILVLHLLFFIIIWLFCSVGYVGLLICRIPVIGKILVCGWWLVMQILVSCKYLYHR
MGKIMKIKKKDL

>Vang07g00820.1

LQQTMAEVAVSTVATKLTELLVEQAAVAVSQLAGVRGQVENLKNELGWMQSFLRDADAKQEGSDRVRLWVSEIRDV
AFEAEELIETYVVYNTTMQRQLDKVFRPFHLYKVRTRIDKILSKIKSISGRRETYGVVMTGHDGNNSNERLRQWRQPSPSS
EEEYLIELEDDMELFLSQLLALEPNPYVVSIVGMGGLGKTTLAKKLYNHNKITNHFDCKAWVYVSKEYRRIDVLQGILRDV
DGAPRHEMGRIPEEEFINKLRTVLSEKRYLVVLDDIWGMEVWDGLKSAFPRGKMGSKILLTTRNWDVALHADACSNP
HQLRPLTADESLRLLSNKAFPGTNGIPAELKDLATEIVVKCGGLPLAVVVVGGLLSRKLKSSGEWKRVLQNISWYLLEEQE
KIARILALSYNDLPSHLKSCFLYLGLFPEGMNIQTKKLIRLWVAEGFLPQEGEETAEGVAQKYLNELIGRCMIQVGTVSSLG
RVKTIRIHHLLRDLSLSKGKEEYFLKIFQGDVTGPSTKARRQSMHSCDERYDFLKHNAHHSRSLLFFNREYNADIARKPWL
PLNFQQEKKLNFIYRKFKLLRVLELDGVRVVSLPSTIGDLIQLRYLGLRKTNLEEELPLSIGNLLNLQTLDLSYCVFLKKIPNVI
WKLVNLRHLLLYTPFDSPDSGHLRLDTLTNLQTLPHIEAGNWIVDGGLANMVNLRQLGICELSGQLVNSVLSTAQGLRN
LCSLSLSLQSEEDEFPIFMQLSQCTHLQKLSLNGKIKKLPDPHEFPPNLLKLTLHNSHLQKESIAKLERLPNLKMLVLGKRAY
NWPKLTFNSEGFSQLHILRLHLKELEDWTVEQSSMPRLEYIVIDRCEKLKTIPEGLKAITSLKKLKIIGMPVEFEHKLRTKDIS
EFTNTPVIESTTDILAFGSYSFSKSLGMASGNYSCKQYRDLPQESISLRLNS

>Vang0747s00020.1

EESEIARVDRIRKRLKNEKENTLIILDDLWGGLDFNKLGIPCNDDASQQEVNDMSDFGSNNDISDFGSNNDISDYGYNK
TEMKELSKVDLDKMKKEKLSKDYRGGKILLTSRNKQVLCNEMDVQQRSIFSVGVLDEKESESLLKKVAGVKNSEFDKNA
TEIAKWSAGFPIALVSIGRTLKNKSLSTWEDVCQQIKRQKFTSEWGFTDFSIKLSYDHLKNEELKCIFLHCARMGNDALIM
DLVKFCVGLNLLPGVHTITDARKRVKEMIQELEESSLLVKSYSIDRFNMHDIVRDVALSISSKEKHVLYMKNAILDEWPHE
NDFERYSAIFLHFCDINDELPESIHCPRLEVLHIDNKNECFEIPDEFFKSMVRLRVLAEENMKSESASLSELRLNSYKIVIGEF
NLFNLLKVGEFKVPDKYEEVSTPKLEWLELSSINIHKIWSDQCNHYFQNLLTLNVTDCGNLKYLLSFSVARSLVNLQSLSV
RDCEMMEDIFRPDDEEFPHLHTLSFIDLYDLRSFYSGIHTLEWPSLKKLDIADCSMLEGFTLEIINSQHQPITLATRKIWAL
KGLISPEKIGGVVQLRELKLESMWSLEEIGFEHE

>Vang0071ss00490.1

LNHVHQVNSKGLVGIGKRIAQVESLLQLETKDVRMIGIWGMGGIGKTTIAQEVYNKLCFKYDSCCFLANIREESWRHGI
NSLKEKLFSTLLREEHLKIGRPFGFQKLVERRLHRMKVLIILDDVGDAEQLENLARTDWFGSGSRIIVTTRDKQVLAEESAS
VYHVEALNFDESLRLFNLNAFKQNHIETEYEELSKKAQLQVKYIDFLLKDRDYSVVAGLERLKDKAFISISQENTVSMHDII
QETAWQIAGEESIENPRRSQIRLFVPDDIYDVLTHNEGNEAIRSIVVNLLRIKQLHLKPQVFTKMSKLHFLNIYTAGTRDT
RFYERWGLYLHQGLESLPNELRYLGWMHYPLESLPSNFSAENLVELHLPYSRVKKLWHKVPDLVNLKVLMLYSSSNIKEL
PDFSKAPNLEVIDLRSCVGLTSVHPSVFSLKKLEKLDLDECRSLTSLRSNVKMESLRYLSLFKCMELKDFSVTSKNMIMLNL
EKTDIKQLPSSFGSQSKLEHLNLKFSSIERLPADMKDLKGLQHLDLRYCRNLSFLPELPPSIETLDCRECVSLGSVAFPSIAE
QWKENKKKAVFWNCLNLDERSLKAIETNARINMVKFAHRHLSTSVQYSERVLKLTVSTEGEDEGDSYSSNTNILINIHGG
AVKRVWG

>Vang0248s00060.1

KVIGVVASSSYSPSVLSSKRYDVFLSFRGEDTRKKFTSHLYDALKQKKVETFIDNCLEKGEEISTTLIQVIEDSHISIVIFSENY
ASSKWCLGELSKIMECKKERGQIVIPVFYDIDPSHVRKQTGSYEKLFVTHKGEPMCNKWKAALTEAANLAAWDSRTYR
VESELLKHIVEDVLEKLAPIYPNWHKGLVGIEENYEKIESLLKVGSNEVKILGIWGMGGIGKTTLACALYDKLSHEFEGHCF
LENVREESDKYGVKALRNKLFSELLGNKNHCFDVAFSVTKFVLSRLGRKKVFIVLDDVATSEQLENLIEDFDFMGLGSRVV
VTSRNKQIFSQVDKICEVKELSFHHSLQLFSLTVFREKQPKYGYEDLSRSATSYCKGVPLALKVLGASLRSRSKGAWECELR
KLQKFPNMKIHTVLKLSYDGLDHSQKDIFLDIACFFRGDQRDHVTNMLEAFDFSAISGIEVLLDRALITISGGNQLEMHDL
IQEMGRQIVHQECVKDPGRRSRLWKHEEVHEVFKYNKGTDIVEGIILDLSKLIEDLYLSSDFLAKMTNVRFFKIHSWSKF
NIFNVYLPNGLNTLSHKMRYLHWDGFCLESLPANFSAEKLVELCMRCSKLKKLWDGVQNLVNLKTIDLWGSRDLIEIPD
LSLAEKLENVSLCYCESLHEVHVHSKSLRVLNLYGCSSLRKFSVTSEELTRLSLAFTSICSIPSSIWHKRKLKALYLTGCRNLGE
LTDEPIIHGSHKHSLTELASNAERFSMKNIKSLSTLRMLWLDDCKKLVSLPKLPPSLEKLSACNCTSLDSYMTQRLVLQHM
LQSRIPYLRKNDLRCYDEEYLFPGNHVIDECVFNTTETSITIPYLWKTELYGFIYCIILSKGSLLQSGISCSVYQDGIRVGWLQ
RLLEYESLTSNHVLCMYHDINEFDAITEEGHGHFFSNVTFIFENSEASIEEFGIFPIYGSESGLKLVGRR

>Vang0693s00030.2

MAQKQAPSSASFMSKYSYDVFLSFRGADTRFGFTGNLYSALSQRGIFTFIDDEALRKGEEITPSLRKAIQESRISIIVFSKNY
AFSAFCLDELLHIIECHKNQNMWILPVFYDVEPSQVRHQTGSYQEAFAKHGERFKDDIQKLQQWRLALRHAADLAGFP
FKTGKEYESEIVKKITEEISTKLNRPPLHIADYPVGLKVRMQQIQELMGGEFDKKVTMLGIHGMGGIGKSTLSRAMYNL
MAHQFEASHFLANVREKSDKDGLVHIQETMLSELVGERNIKLGDVHRGIPILQHRLCGKKVLLVLDDISKKEQLHATAG
GLDWFGPGSIIIITTRDKHLLDVHGVQKQYMVGGINFMEALELFKWNAFKNKEVDPCYKEVTERAMYYANGLPLALETI
GSNLFGKTLDEWESALETYERIPNRDVQEVLKVSYDSLDAYEKEIFLDIACFFRRCSLGYVTDKLEARGFPPKFGLRVLEEK
SLIKIRECPHETVTMHDMIRCMGKEIVRQQKPLPHKRNRMWFYEDVVCVLEKNMENDKIEAVMLEMPEHQEEMQCT
PKFGKMKSLRMLIIEKKDSFLRSPATLPNSMRVLEWRGYPATSLQESCHTQLVTQLLWMGQTTTGIGCKNIRRIPDISGF
PNLTKLCVGECTNLFEIHDSVGSLLNLKEFCAEGCTKLTIGPSRIKLISLEHLCLRGCSSLVMFPEVLAPMHKLKYVNLGGT
GIRNLPLSTQNLEGIRVLSLGKGKMLEINESSNFFQNLPMFFPNLIKLCLQNLDITILPASIEECHFLKILHAHDCKKLQEIRG
LPLSIKDFSAANSSVEANSLTLKLRQAIDYGAMGICVLPGRKIPELFDHSSRGNSVSFWFRKELPTLAVCAIIGVWDNVKP
PFVAFFSFVVGVEKNIYKCVCCFRCGNIRWTTEDSHIIILNFQNDFQHTLNTDIQTVLLTNEWIPGEILLTIHPKSDLGKSG
EIRRTGVYVNRTLSRMEDVRFEDPYDLNKASTMENKLVLLGEASDTQQQAQSSALFNLTTSLESVIGEQPLNYEYNNSD
SKESASTVDSNNQGVLVDDQPILAPSSAETQNAEGKREMAQLILEVSREFTAQVSMKIESTASQSPKVDEVEAGLETQN
QFRTLENQIHQRLTRIKKMKEELGEKFSAIKADISAIEANNSSIKAKISDIKAIFLQTDQSQTEAYKAASHLASVTPPPVMLS
RGIGWQDCDRENVELLGSNRIGNRKVIEVSASFMIFFSMLGKFGALFASIPFPMFVAIYYVLFGIVENTLSGSFMVLQFN
DFLNTIFYSSPTVALIVAVFLDNTLDYKDNVKDRGMPWWTKFRTFK

>Vang11g03570.2

LAIMGETDQLVILTRFQDAMRMVMDIVEKGRKNKRSKRILRSTLKNMTLVVQEIKQYNEHLNPPREEIITLVKENDPGE
DLVCNSCSRSLWWTKFLSWFSLYGEGFLHEKNDLLTSDDKQVKDIKNTLYKVREIIELLDIENFEQKLKGAGTPIKCPYGV
PENPEFTVGFVPVLSKLKMEVLQEGVFTLLLSGLGGSGKTTLATMLCKDKEVKGKFKNNILFVNFSQTPKLKNIVERLFEH
CGYHVPEFVSDEDAIKRLEILMRKIEGSPLLLVLDDVWPGSEALVDKFKFQMSDYKILVISRVAFPKFGTQIILKSLVHEDA
MTLFRHHALLEENSSKCSLSIPNEEIVQKVVRYCKGLPLAIKVIGRSLSHQSTELWQKMVEELSKGHCILDSNTELLTSLQKI
LDVLEDNPIIKECFMDLGLFPEDQRIPLPALIDMWAELYKLDDDGIEAMEIIKKLDSMNLANLLVARRNTGDSDNYCYN
NHFVVLHDLLRDLAIYQNNREPIEQRKRIITGINENQSQWWLGEKQQGMMSRLLSKYRGWCVKQTIQQVSALILSLST
DETCASYWSDIQPSQAEVLILNLQTKKYTFPEFMEKMIKLKVLIVTNYGFHHSELDNFQLLGSVSNLKRIRLERVSVPHLS
ALKNLKKLSLYMCSNISQVFENGTILVSDSFPSLLDLNIDYCKDMVRLPNGICDISSLKKLSITNCHKLCSLPQEIGQLLNLEL

LNLSTCTDLEEIPDSIQNLSKLRLLNISNCISLSNLPEDIGNLCNLRNLNMTSCARCELPYSITNLENLKVVVCDEETAASWE
AFEALLPNLKVEVPQVDVNLNWLHSISS

>Vang01g01600.1

MKAACQCESFDMLGGKSSSSSSDASSRRQWRSTLLPLSILSENFAASTWCLEELRKILDVGKPVIPVFYEVVPSDVRHQR
NSFAKAFEEHERRSEEDQLKVQQWRKSLKEVADFSGWESKDRRLEELIEDIIKSVWTKIRLKLPSYEEERVGIDSRVEKIRS
LLKLELKDVVHFTGIWGMGGIGKTTLARVVFKKICSQFDISCFLENVREISGKTHDMLTLQITLLSHMEVKEFTIQNLDEG
KIVIGGILRNNKVLLVLDDVDDARQLDSLGVNDQGNGTKVRLENLKELSFGGGKLINIYCDLNDESMPDDLGSLSSLLGL
DLSGNNFVRPTYCIRNLHALKSLTLIDCPRLESLPMLPPNVQCLCTANSTQTKPLNSDAYMLWKIFELHMNQVYSLYTPH
SLPHLPPIHPNHFHKVCVYQMEGRPHFMLIIPGRETQKWNEECFLVDPSHHPYNMLGCDSAASIKVEAPQYCESSGWL
GIAICLALEHSNMQPSPQSHVSQHSKGNEETRIYFWACKAHDGEPDLIFPIVPEHSHSVHECNEEKCLLQLIFYVENHSK
AWKPSLEIRDLDEGKNTIRELLFKKKVLLVIDDVDDTSQLESLAESKKAFKRDKPDEHYLELSKAVAKYAGGLPLALELLGSF
LCGRSESQWKEVVDMIKEVPPSHIAMKSLRISYNGLPLRYKTLFLDIACFFKGRIKELVIQALEICERYPPVGIDLLVEKSLAT
YDGFTIGMHDLLQESAREIVKEESYVDPAKRSRLWTLEETNEANESIEGIVLNSPEKEEAIWDPEAFSRMYNLQLLIINYR
VNLPTSLKCLCSSLKFLQWMNYPLEYLPLGVLLDELVELKMHSSRIKRIWNGNQDFAKLKFIDLSYSEDLIQTPINCINLLSL
PNSSCNLRSLRKLYVSGCSRISTLPDGMNENESLEELYVSGTDIREIPSRLEKLRELSFGGRKETTPKSQNFMPPLPSLLALE
SLDLNYGGLTDESIPSDLGPLSLLKRLDLSGNNFVNPPAQFIISLSMLHTLSFNDCPRLESLPLLPPNLQSLYTTNCPKLKPF
HLVEDTLWKIFESHSHEDPIEGPELWFITPGNEIPSWFNNQNFLEIDSSDQTYEKLCCDSVTSITVDVSEDFQLSEWWGI
AVCLVIEPLNMDVPSSSNARSTSTVNEEIGIYYWVCKAPDKDPDPNFPIAPKFGHMLYKFKDPYIHIIFLNADHVYIQHYL
SGEQTQLEVILFVENFSESCKARIKKCGCRVICKEKIEEWRKLSDGLNISRITETNQDEERHELQVEEPTSPNTFSPVEKTEG
QTTLGK

>Vang11g01630.1

MATSTCCDVSEIRHDVFISFTGIDVRRGLISHLKRELHRKHIDAYVDERLDKGGEISSSLLRAIEGSKILLVVFSKHYASSHW
CLEELAKMIECMETNKQIVLPVFFNIDPSHVRHQYGDYGYALAEHEEEFKENMLKVQIDGFRHKEIVLKKKSCMNYIYYIS
AYFLLNARNESDLVDEIVEDISVKLSELYPSESNGLVGIDQNVTQVQSLLRVESNEVLFVGIWGMGGIGKTTIARAIFDKC
SLQYDGCCFFNVREELEQHGFSNLRERLIYELLEGEGLHTNKARFFSSALRMLGRKKVMVVLDDVNTSEELRYLVTKPVC
FGAGSRVIITSRDQDVLTSGGLDQIHEVKEMKPLDSLKLFCLIAFNESQPKMGYEKLTEEVLKIAPGNPLALKVLGAEFHS
RSAINTWNCALSKFKKYPNEKIQSVLRFSYDGLHEVEKKAFLDIAFFFQEDTKAYVIEQLDAWGLHGASGVEVLQRKALIT
VSNENIIQMHDLIRQMGCEIVRQECITHPGRRTRLRDKEEVYKVLRNKLGSDKVEGMQVDVFRIKDLSLKVGTFKKMPC
LRFLKFYLPDDNLFLPPNPDGTLRYEKRHFPLLLSAWCKDLMRVACEIQIKCVDYVYFDGYSHSSLTSTLRNRGREIRSSTSI
SLTELTGSLGDLECSDMLDQQFKILPDGLLCLRSTYYLKLSKSTGQDSGKPKLHVLFDSLRFYERISVGKLKNSDIEGNRML
FLYFAGFNFLFSPFLLRRPWFHFLFSFRFAFFCTLFLYLSWKMIL

>Vang2562s00010.1

LNHDSISYDQGIVGTEKYMNEIKSLLHLESPDIRIIGICGMGGIGKTTISKQIYHTLALKFDSSSLVLDIHEKIRRDGIEYIIIKYL
SELLKEAPSSQLFYYRERLKRTKVFLILDDV

>Vang2562s00010.2

LNHDSISYDQGIVGTEKYMNEIKSLLHLESPDIRIIGICGMGGIGKTTISKQIYHTLALKFDSSSLVLDIHEKIRRDGIEYIIIKYL
SEL

>Vang02g12360.1

LSFFFFLYSDSYLDFPIPFLLLLIPCNHLLLPSLTDSDMMCSSVFEGKIPAKVSQEISTNLCGRGIHTFIDDHHLPRGDQISSEL
ENAIQDSRIFIIVLSQNYASSSFCLNELHYILRFIKHKGRLVFPVFYGVDPSHIRHHTGTFREALAHHQNNSYYNWEKFET
WKMALHQVANLSGYHFKHGEGYEYEFIERIVELVCSKINRAALHVADYPVGLESQVLEVKLLLDVGCDDVVHMVGFHG
LGGVGKTTLAAAVYNSIADHFEALCFLENVRETSSKHGLLHLQSNLLSETVGEIKLTSVKKGISVIQHRLQQKKVLLILDDV
DKEEQLQALAGRPHWFGLGSRVIITTRDKQLLKCHRVKRTYEVKELNEENALELLTWKAFKFEQFDPSYKDVLNLAVTYA
SGLPLALEVIGSNLFGRNIEQWKYALDQYKKIPKMDIQDTLKVSYDALEEDEQSVFLDIACFFKNYDLAEVEDILRAHHGH
SIKHHIDVLVEKSFIKISLDGKVTLHNLIEDMGREIVRRESPKEPGKRSRLWFPQDIVQVLEDNKGTAQIEIICVDFPSVEEV
EIEWDGNAFKKMRNLRTLIIRNGHFSKGPKHLPNSLRVMEWWRYPSQNFPQDFHPKKLAIFKLPYCEFTSLELTDLLRQ
ASKFVIMTSLNFDECRYLKQIPDVSCLIHLENLSFRWCPKLSSLHYSVGLLEKLKILDAEGCSRLKSFPPIKLTSLEQLKLRYC
HSLQNFPEVLGNMEYVRELDLKETPVKKFPPSFRNLTRLQKLHLCLSVRVMKSGCDGLPLSSICMMPELVDIAANEWEG
GLFREANEGAEKVSSILSTNVQYLQLRCCNLTDDFFPTLLPWFANMKNLDLSGNNFTVIPECIKEFHFLTRLNLNFCERLQ
EIRGIPPNLKYFFAIDCQSLTSSCRSMLLNQELHEAGSTFFYLPGAKIAEWFEFQTLELPISFWFRGKLPAMAICLAMERVC
EYSTSEGGKYRPLVIHSTFRFMSPIVIINGNEHLLETWEMLDDCTCVFDLRETKLKNDLDEELVENEWNHVEVTCRYVSL
GQTLIKHGIHVFKQESSVEEIRFTDPSRKRNLDMMNSIAQNHSSNSC

>Vang06g20640.1

MKRICERLEEIADERTRFHLAEMVPERRSGNLSVYPIIGLGGLGKTTLSQLILNHERVRKHFEPRVWICVSEDFGLRRMTK
AIIEAVSGHACEDLDLDPLQRKLQDLLQNKSWERLKSVLACGALGASVLVTTRLTKVAAIMGTVPPLELSNLSDNDCWR
LFKHRAFVADEVEQEKCGGVPLAAKVLGGLLRFKREVREWLKVKESNIWGLTHNIMPALRLSYLNLPIHLKQCFAYCAIF
PKDERIEKQYLVELWMTNGFISSDGKLDAEDVGDGIWNELYWRSFFQDIEKDEFGEVESFKMHDLVCNFQTLPESLCKL
LNLQILKLDYCYTFKKLPDSLVRLKALQQLSLKECKLLSRLTPHMGKLTSLRNLSMYIVGEGRGFLLAELGPLKLKGCIDIKH
LERVKSINDAKESNMSSKQLNKLTLRWEGFREGELARNDEEVLEALKPCSQTLQSLRLEGYQGVNFPQWMSSPCFKSLT
YLKLWFCRNCIKLPVLRKLPSLKRLLIAGAKYVKYVQEESNDDDDHVGFMALEYLSLESLPSLIRLSSEDGENQFPCLFTLK
VNDCPNFSLHGLHSLKTLEIMRLPKLKVWEGLQCLTSLEVLGITGCDEVEGLQYMTALKKLSLRNLPNMESLPDCFGELA
LLRELNIVGCYKLMRLPTSLSLSSVEVLCILDCNLELSKRCEKENGEDWPIIAHIPHLYV

>Vang06g20640.3

MGTVPPLELSNLSDNDCWRLFKHRAFVADEVEQEELVVIGKEIVKKCGGVPLAAKVLGGLLRFKREVREWLKVKESNIW
GLTHNIMPALRLSYLNLPIHLKQCFAYCAIFPKDERIEKQYLVELWMTNGFISSDGKLDAEDVGDGIWNELYWRSFFQDI
EKDEFGEVESFKMHDLVCDLAQFVAEEVCCITKDDDIRKEFLSAIGDLKHLRYLNLSHGNFQTLPESLCKLLNLQILKLDYC
YTFKKLPDSLVRLKALQQLSLKECKLLSRLTPHMGKLTSLRNLSMYIVGEGRGFLLAELGPLKLKGCIDIKHLESNMSSKQL
NKLTLRWEGFREGELARNRLLIAGAKYVKYVQEESNDDDDHVGFMALEYLSLESLPSLIRLSSEDGENQFPCLFTLKVND
CPNFSLHGLHSLKTLEIMRLPKLKVWEGLQCLTSLEVLGITGCDEVEGLQYMTALKKLSLRNLPNMESLPDCFGELALLRE
LNIVGCYKLMRLPTSLSLSSVEVLCILDCNLELSKRCEKENGEDWPIIAHIPHLYV

>Vang06g20640.2

LGLFLGFDQDLERLASVFTTIKATLADAEEQQFSNRSIKDWLQKLKEAAYILDDILDECAYEALRTRFHLAEMVPERRSGVI
EWRQTSSFISEPHVYGREEDRDKLIDFLVHDASHDENLSVYPIIGLGKEIVKKCGGVPLAAKVLGGLLRFKREVREWLKVK
ESNIWGLTHNIMPALRLSYLNLPIHLKQYERIEKQYLVELWMTNGFISSDGKLDAEDVGDGIWNELYWRSFFQDIEKDE
FGEVESFKMHDLVCDLAQFVAEEVCCITKDDDIHALFERKRIHHFSDYGWELHPAQLHQVKSLRTYLGKNVQLSPDVVK
RYSLRLLQFKPRKEFLSAIGDLKHLRYLNLSHGNFQTLPESLCKLLNLQILKLDYCYTFKKLPDSLVRLKALQQLSLKECKLLS
RLTPHMGKLTSLRNLSMYIVGEGRGFLLAELGPLKLKGCIDIKHLERVKSINDAKESNMSSKQLNKLTLRWEGFREGELA
RNDEEVLEALKPCSQTLQSLRLEGYQGVNFPQWMSSPCFKSLTYLKLWFCRNCIKLPVLRKLPSLKRLLIAGAKYVKYVQE
ESNDDDDHVGFMALEYLSLESLPSLIRLSSEDGENQFPCLFTLKVNDCPNFSLHGLHSLKTLEIMRLPKLKVWEGLQCLTS

LEVLGITGCDEVEGLQYMTALKKLSLRNLPNMESLPDCFGELALLRELNIVGCYKLMRLPTSLSLSSVEVLCILDCNLELSKR
CEKENGEDWPIIAHIPHLYV

>Vang06g20640.5

MAEAVIEVVLGNLNSLVQKELGLFLGFDQDLERLASVFTTIKATLADAEEQQFSNRSIKDWLQKLKEAAYILDDILDELCP
SNKVKCSGFSTFHPKRTVFRYKIAKKMKRICERLEEIADERTRFHLAEMVPERRSGVIEWRQTSSFISEPHVYGREEDRDK
LIDFLVHDASHDENLSVYPIIGLGGLGKTTLSQLILNHERVRKHFEPRVWICVSEDFGLRRMTKAIIEAVSGHACEDLDLDP
LQRKLQDLLQNKRYLLVLDDVWDDDPESWERLKSVLACGALGASVLVTTRLTKVAAIMGTVPPLELSNLSDNDCWRLF
KHRAFVADEVEQEELVVIGKEIVKKCGGVPLAAKVLGGLLRFKREVREWLKVKESNIWGLTHNIMPALRLSYLNLPIHLK
QCFAYCAIFPKDERIEKQYLVELWMTNGFISSDGKLDAEDVGDGIWNELYWRSFFQDIEKDEFGEVESFKMHDLVCDL
AQFVAEEVCCITKDDDIHALFERKRIHHFSDYGWELHPAQLHQVKSLRTYLGKNVQLSPDVVKRYSLRLLQFKPRKEFLS
AIGDLKHLRYLNLSHGNFQTLPESLCKLLNLQILKLDYCYTFKKLPDSLVRLKALQQLSLKECKLLSRLTPHMGKLTSLRNLS
MYIVGEGRGFLLAELGPLKLKGCIDIKHLERVKSINDAKESNMSSKQLNKLTLRWEGFREGELARNDEEVLEALKPCSQTL
QSLRLEGYQGVNFPQWMSSPCFKSLTYLKLWFCRNCIKLPVLRKLPSLKRLLIAGAKYVKYVQEESNDDDDHVGFMALE
YLSLESLPSLIRLSSEDGENQFPCLFTLKVNDCPNFSLHGLHSLKTLEIMRLPKLKVWEGLQCLTSLEVLGITGCDEVEGLQY
MTALKKLSLRNLPNMESLPDCFGELALLRELNIVGCYKLMRLPTSLSLSSVEVLCILDCNLELSKRCEKENGEDWPIIAHIP
HLYV

>Vang06g20640.4

MAEAVIEVVLGNLNSLVQKELGLFLGFDQDLERLASVFTTIKATLADAEEQQFSNRSIKDWLQKLKEAAYILDDILDECAY
EALRLEYQGPKRTVFRYKIAKKMKRICERLEEIADERTRFHLAEMVPERRSGVIEWRQTSSFISEPHVYGREEDRDKLIDFL
VHDASHDENLSVYPIIGLGGLGKTTLSQLILNHERVRKHFEPRVWICVSEDFGLRRMTKAIIEAVSGHACEDLDLDPLQRK
LQDLLQNKRYLLNYQIYLTMIVGDCLNTELL

>Vang06g20640.7

MAEAVIEVVLGNLNSLVQKELGLFLGFDQDLERLASVFTTIKATLADAEEQQFSNRSIKDWLQKLKEAAYILDDILDECAY
EALRLEYQGVKLCPSNKVKCSGFSTFHPKRTVFRYKIAKKMKRICERLEEIADERTRFHLAEMVPERRSGVIEWRQTSSFIS
EPHVYGREEDRDKLIDFLVHDASHDENLSVYPIIGLGGLGKTTLSQLILNHERVRKHFEPRVWICVSEDFGLRRMTKAIIEA
VSGHACEDLDLDPLQRKLQDLLQNKRYLLVLDDVWDDDPESWERLKSVLACGALGASVLVTTRLTKVAAIMGTVPPLE
LSNLSDNDCWRLFKHRAFVADEVEQEELVVIGKEIVKKCGGVPLAAKVLGGLLRFKREVREWLKVKESNIWGLTHNIMP
ALRLSYLNLPIHLKQCFAYCAIFPKDERIEKQYLVELWMTNGFISSDGKLDAEDVGDGIWNELYWRSFFQDIEKDEFGEV
ESFKMHDLVCDLAQFVAEEVCCITKDDDIHALFERKRIHHFSDYGWELHPAQLHQVKSLRTYLGKNVQLSPDVVKRYSL
RLLQFKPRKEFLSAIGDLKHLRYLNLSHGNFQTLPESLCKLLNLQILKLDYCYTFKKLPDSLVRLKALQQLSLKECKLLSRLTP
HMGKLTSLRNLSMYIVGEGRGFLLAELGPLKLKGCIDIKHLERVKSINDAKESNMSSKQLNKLTLRWEGFREGELARNDE
EVLEALKPCSQTLQSLRLEGYQGVNFPQWMSSPCFKSLTYLKLWFCRNCIKLPVLRKLPSLKRLLIAGAKYVKYVQEESND
DDDHVGFMALEYLSLESLPSLIRLSSEDGENQFPCLFTLKVNDCPNFSLHGLHSLKTLEIMRLPKLKVWEGLQCLTSLEVL
GITGCDEVEGLQYMTALKKLSLRNLPNMESLPDCFGELALLRELNIVGCYKLMRLPTSLSLSSVEVLCILDCNLELSKRCEK
ENGEDWPIIAHIPHLYV

>Vang06g20640.9

LQRKLQDLLQNKRYLLVLDDVWDDDPESWERLKSVLACGALGASVLVTTRLTKVAAIMGTVPPLELSNLSDNDCWRLF
KHRAFVADEVEQEELVVIGKEIVKKCGGVPLAAKVLGGLLRFKREVREWLKVKESNIWGLTHNIMPALRLSYLNLPIHLK
QCFAYCAIFPKDERIEKQYLVELWMTNGFISSDGKLDAEDVGDGIWNELYWRSFFQDIEKDEFGEVESFKMHDLVCDL
AQFVAEEVCCITKDDDIHALFERKRIHHFSDYGWELHPAQLHQVKSLRTYLGKNVQLSPDVVKRYSLRLLQFKPRKEFLS

AIGDLKHLRYLNLSHGNFQTLPESLCKLLNLQILKLDYCYTFKKLPDSLVRLKALQQLSLKECKLLSRLTPHMGKLTSLRNLS
MYIVGEGRGFLLAELGPLKLKGCIDIKHLERVKSINDAKESNMSSKQLNKLTLRWEGFREGELARNDEEVLEALKPCSQTL
QSLRLEGYQGVNFPQWMSSPCFKSLTYLKLWFCRNCIKLPVLRKLPSLKRLLIAGAKYVKYVQEESNDDDDHVGFMALE
YLSLESLPSLIRLSSEDGENQFPCLFTLKVNDCPNFSLHGLHSLKTLEIMRLPKLKVWEGLQCLTSLEVLGITGCDEVEGLQY
MTALKKLSLRNLPNMESLPDCFGELALLRELNIVGCYKLMRLPTSLSLSSVEVLCILDCNLELSKRCEKENGEDWPIIAHIP
HLYV

>Vang06g20640.8

LVHDASHDENLSVYPIIGLGGLGKTTLSQLILNHERVRKHFEPRVWICVSEDFGLRRMTKAIIEAVSGHACEDLDLDPLQR
KLQDLLQNKRYLLVLDDVWDDDPESWERLKSVLACGALGASVLVTTRLTKVAAIMGTVPPLELSNLSDNDCWRLFKHR
AFVADEVEQEELVVIGKEIVKKCGGVPLAAKVLGGLLRFKREVREWLKVKESNIWGLTHNIMPALRLSYLNLPIHLKQCF
AYCAIFPKDERIEKQYLVELWMTNGFISSDGKLDAEDVGDGIWNELYWRSFFQDIEKDEFGEVESFKMHDLVCDLAQF
VAEEVCCITKDDDIHALFERKRIHHFSDYGWELHPAQLHQVKSLRTYLGKNVQLSPDVVKRYSLRLLQFKPRKEFLSAIGD
LKHLRYLNLSHGNFQTLPESLCKLLNLQILKLDYCYTFKKLPDSLVRLKALQQLSLKECKLLSRLTPHMGKLTSLRNLSMYIV
GEGRGFLLAELGPLKLKGCIDIKHLERVKSINDAKESNMSSKQLNKLTLRWEGFREGELARNDEEVLEALKPCSQTLQSLR
LEGYQGVNFPQWMSSPCFKSLTYLKLWFCRNCIKLPVLRKLPSLKRLLIAGAKYVKYVQEESNDDDDHVGFMALEYLSLE
SLPSLIRLSSEDGENQFPCLFTLKVNDCPNFSLHGLHSLKTLEIMRLPKLKVWEGLQCLTSLEVLGITGCDEVEGLQYMTAL
KKLSLRNLPNMESLPDCFGELALLRELNIVGCYKLMRLPTSLSLSSVEVLCILDCNLELSKRCEKENGEDWPIIAHIPHLYV

>Vang0250s00120.1

LLDKTEFFVAKNPVGVESRVQEMVQLLEQKQSNDVLILGVWGMGGIGKTTIAKAIYNKIGRNFEGRSFLADIREVWGQ
EAGHVCLQEQLLFDIHKENSTKIHNTESGKVILRKRLQGKRILLILDDVNKLQQLNDLCGNRKWFGSGSRIIITTRDIHLLR
GKRVDQVFAMTGMNIDESIELFSWHAFKQASPKEDFMELSRNVVAYAGGLPLALEVLGSYLFDMEVTEWKSVLEKLRK
IPNDEVQEKLKISYDGLSDDTEKGIFLDIACFFIGKDRNDVIHILNGCGLFAENGIRVLVERSLVTVDDRNQLGMHDLLRD
MGREIIRSKSPMELEERSRLWFHEDVLDVLSKETGTKFIEGLTLKLPRSNTKTLCTKAFVNMKKLRLLHLSGVELVGDFEYL
SKDLRWLCWHGFPFSFIPTGFYQGSLVSIELENSKITMVWKETQVSSFFNFMRCSLFI

>Vang0698s00020.1

LQKLIFLNMLSATQSERLQHQVDSALRNGEEIESDVQHCLMLMDEKIKEYKSYINDECHAKTICSIGFFPNNFRMRYQLG
RNATKMVEEITGGELWKTSFENVSYQEFPSIDATFSNNGYESFASRTKTMELIMKALQDSRVGMIGVYGPGGVGKTTL
VKEIAKKAREKLFKIVIIANITRNPDFKKIQEQIAGILGMKLEGESEIAITDRIRKRLKNEKQNTLIILDDLWGGLDFNKLGIPC
SDDASQQEVNDMSDFGSNNDISDFGYNKTKMKELSKVDLDKMKREKLSNDYRGGKILLTSRYKQVLCNEMDVQESSIF
PVGVLDEKESETYLKKVAGVKNSEFDRNAIEIAKWSAGFPIALVSIGRTLKNKSLSTWEDVCQQIKRENFTSEWGFTDFSI
KLSYDYLKNEELKCIFLQCARMGNDALIMDLVKFCVGLKLLPGVHTFTDAKRRIKEMIQELEEWSLLVKSYSIDRFNMHDI
VRDVALSISSKEKHVLYMKNAILDEWPHENDFERYSAIFLHFCDINDELPESIHCPRLEVLYIDNKNESFEIPDEFFKSMVR
LRVLILTGINLSCLPSSIKCLKKLRMLCLEQCTLGENLSIIGELKNLRILTFSGSNIENLPLEFGQLDKLQFLDISNCLKLRQITS
NIIPRMAILEEFYIRDNLIIWEAEDNMKSESASLSELRRLNQLQNLDIHIHSSSHFPQNLFFDRLNSYKIVIGEFNLFNLLKV
GEFKVPDKYEEVKFLALNLKEGIDIHSEKWIKMLFKSVECLLLGELNDVEDIFYELNVEGFPNLKHLSIVNNFGIKYIINPRE
RFHSLAAFPKLESIWLYKLDNLEIICDNQLVETSFRNLKMLGSVPLLQGHENTNIVTNAEHGVTNSCLPLFNEK

>Vang0033ss01420.1

MGIGFLQKYLTMGIHTFIDDEGLRKGEEIRPALFKAIEQSRIAIVVFSENYADSTYCLEELVVILECIMRKGRLVWPVFYGV
TPSSVRFQKGSYGKALAKHEERFKNDQEKLQKWKLALQVAAGLSGSHFKLKQGYEHELIRTIVEEVSKKINRSPLHVANY
PIGLESRVQEVKLLLDVGSNRGVSMVGIFGIGGIGKTAIACAVYNAIADQFDVQCFLGDIRQKSMKYDLVQLQETVLSE

MVGEKSIKLGSINRGMAVMKSKFQRKKVLLILDDVDKLEQLKALAGDPSWFGDGSKIIVTTRNRRFLRVHGVERTYEAK
GLDDKEALELFSWHAFKSNEVGPGYLDISKRAVFHCNGLPLALEIIGSNLNGITMSEWEAALDTIERIPDEDIQEKLKVSY
DGLKGNEKEVFLDMACFFRGYHLKDVINLLLQSRGFSPEYVIRMLVDKSLIKIDQYGFVQMHNLVEDMGREIVRQESPS
EPGKRSRLWLYEDIVDVLENDKGSDTIEVVMLHLPKNREVLWNGSELKKMTNLKMLTIENADFSRGPEYLPSSLRVLKW
RGYPTQSLPPEYDPRRLVIFIKQAPDMSGAKNLRKLCLDNCKNLVEVHNSIGLLDKLTWFTAIGCTSLRTLPHSFKLTSLEY
LSLRKCSSLQRLPNISEEMKHMKNLDLCGTAIEQLPYSFRKLTGLKYLVLDKCKRLNQIPINILMLPKLERLTAVKCGRYVN
LILGKSEEQVRLASSESLRDFRLNYNDLTPTSFPNVEFLVLTGCAFKVLPECISQCRFLKNLVLDNCKELQEIRVVPPKIKYLS
AINCTLLSHESQNMLLNQKLHEGGGTDFSLPGTRLPEWFDHCTRGPSLSFWFRNKFPRMTLAVVGVLDKQGSFPMSR
FHFLSNGIQKLHCHFTVQSKLITYHIFLSDVLLKSYNGGLESVYGEDGWNHVEVSYVGPRVFSHSCRTKKGTIKWMGVH
VHKQKTNMQDIRFINPWFPKRAHSEVSKADLQESFQPLPKRIRVSHRKEICEAPEMKQHEANSSYHGVSQRLWLAICSI
AAPLNVKVLMWNICQDDLPTFEYLFRRKLVLSPLCPICGTEPETVEHVFLFCPWTRPLWFGSDFQWCVDVKEVQSFQL
WLWHKLMEIQRVYPENANQISAQVGSICWSIWKGRNEFVLEGKPVNPLILR

>Vang08g02100.1

MALAVVGGALLSAFLDVLFDRLASPELVSLIHGKKHDKLLQKVENQLIVLRVVLADAENRQITDSNVKKWLDVLRDIVYE
VDDLLDEVSTKAATQKEVSNSFSHIFNRKRIVSINKLEDIAERLDDILKQKESLNLKEIPVESNQPWKDQPTSLEDRYGMY
GRDKDKEAIMKMVLEDNTDGEEVSVIPIVGMGGVGKTTLARSVYNDGKLKQIFDLKTWVCVSDIFDTVKVTKTMVEEI
TKRPCNLNDLNLLQLELMDKLKGKRFFIVLDDVWIEDFDSWNSLTKPFLSGIRGSKVLVTTRNESVAAVVPFNVVKVYHL
NQLSNEDCWLVFANHAFPLSEDSENRGTLEKIGKEIVKKCNGLPLAAQSLGGMLRRKHALRDWINVLESDIWKLPESQC
KIIPALRISYNHLPPHLKRCFVYCSLYPKDYEFRKDELILLWMAEDLLKAPRKEKTLEEVGEEYFDDLVSRSFFQCSTLRPSS
NYFIMHDLMHDLATFLGGEFYFRANELGKETKIHRKTRHLSFARFSDPVSDIEVFETAKFPRTFLQIHDEDSPFNNEKAPR
IIVSTLKYLRVLKFGDYQSKLVLPDSIGELIHLRYLNLNVTSIAMLPESLCNVYNLQTLKLESCFNLTELPRNIQNLVNLRHLRI
FNTPIKEMPKRMGKLNQLHDLDCYVVGEHKENSIKELGGLPNLHGSFSIEKLENITKGEEALEARIMDKKHITHLSLIWSE
GNDDIIDFQIELDVLGKLQPHQDLKLLKIIGYRGTRFPEWVGNFSYKNITSLYLHRCNNCCMLPSLGQLPSLSDLVISNMN
SVKAIDAGFYKKDDCSSVTPFPSLEYLHIYDMPCWEMWNAFDSEAFPVLENLYIERCPKLRGGLPDHLPALETLIIKNCEL
LVSSVPMAPTLQRLTIGNSNKVAFREFPLLVKDLRIEGRTMVEFMMEAITNIQPTCLQSLSLKDCSTAISFPGDRLPASLKS
LYISGLNKLKFPVQHKHELLESLTIINSCGSLKSLPLINFPNLIILEIKDCENMESLLVLESESLKSLNYFAIERCPNFVSFPGEGL
CMPNLTCLIVYDCDKLKSLPNQMETVFPKMEYLHICNCQQIESFPGGGMPPNLRTVDIKNCVKLLSNQAWVCMNMV
TSLEVCGPCDGIKSFPNESLLPPSLVSLHLRYLSSLETLDCKGLLHLTSLQELNIESVQKEIEEEEESPFSQSF

>Vang07g06330.1

MDIQEESPVFGSLTAMTTRNMSSSSSVFFSANQSPFFSPRSPSSCQLSHSARLDTQSNTVHLGLTPSSTTSEIPEPNSTVN
VRCNVSDVSASPAGCNSGGLMKLDRKSSPVGISSTSISSYSNCHDDGYSGQRERRIKKDRNHRTSSTPGSTSFSSYRLRSC
DVFIGLHGRKPPLLRFAKWLCGELEIQGISCFVSDRARSRSSRKLGIAERAMDAASFGIVIITKKSFKNQYTIEELNFFCRRK
NLIPIYFDLSPADCLVRDIIEKRGELWEKHGGELWLSYEGLEQEWKDAVHGLSRVDECKLEAQDGNWRDCILRAVTLIA
MRLGRRSVAERVTKWREKVEKEEFPFIRNENFIGRKKELSQLEFILFGDVTGDAEQHYIELKARPRRKSVRIGWGKSNMI
DERWNDRRKEKEPVVWKESEKDIEMQGVEFSHRHNHPRLKRGKYTKRKNGMKILYGKGIACVSGDSGIGKTELILEFAY
RFHQRYKMVLWIGGESRYIRQNYLNIRSFLEVDVGVENSLDKTKIRSFEEQEVAAISRVRKELMKNIPYLVIIDNLESEKD
WWDHKLVMDLLPRFGVETHVIVSTRLPRIMNLEPLKLSYLSGVEAMSLMVGSSKDYSVAEVDALRSIEEKVGRLTLGLAI
ISAILSELPITPSRLLDTINRMPLKEMPWSDKEALSFTKNAFLLQLFDVCFSIFDHADGPRSLATRMVLVSGWFAPGAIPIS
LLALAAEKVPERCQGTCFWRKMLQLLSCGFPSSYAKKPELEASSLLLRFNIARNSTKQGYIHINEVFKLYARKRENTGAAQ
AMIQAIISNGSISQNLDHLWAACFLLFGFGHDPVIVELKVSELLYLVKRVVLPLAIHTFITYSRCTAALELLRLCTNALEAAD
QAFVTPVDKWFDKSLCWRSIQTNAQLNPCLWQELALTRATVLETRAKLMLRGAQFDVGDDLIRKAVFIRTSICGEDHP
DTVSARETLSKLTRLNANVQIHAST

>Vang04g03590.1

MMDFVGPVLDIIIRLWDCCAHVRDYEENLSCLRDMASDLLGLWVDVSVKVQMAEDQHLRRLNEVNDWLVKVEAMQ
REVQAIQQRVAHAQETRSRCLTNFPTNFRMGRIVSRKIGEIRELIDKGHFDVVAQEMPHAVVDEIPLEVTIGLESSFDELS
ECFDDNNVGIIGLFGMGGVGKTTLLKKFNNEFLPTKFYDVVIWVVVSKEADVGSVQQSIGNKLNVPVGKWGGKSIDDR
AIVLYNFLKRKKFVLLLDDLWERIDLLKLGIPLPDTENGSKVIFTTRSMEVCRSMEANTCIKVECLAPNEAFALFREKVGEE
TLNSHPEIFHLAKIVAKECEGLPLALITVGRSMARKTLPEWKRAARTLKIYPSRFSGMVDYVYCLLEFSYDSLPSASHKSCFL
YCSIFPEDYDIRKDELIQLWIGEGLLAEFGDDVYEARIQGEEIIASLKFACLLEDSERENRIKMHDVIRDMALWLACDHGS
NTRFVVRDGACSGSVETYNQAKWKEVEKLSMWRHSIQKLSGKQDCSNLLTMLVRNTEITSFPEEIFLTANHLRVLDLSG
NKRVRELPSSIGELVQLQHLDLSGTDIQKLPRELQNLKNLRCLLLNYICNRLVFPRNLISSLVSLQVFSKLPWEDQFILPDLG
EPEEAVLLKELECLEYLQDISIALFCFSSVQVLLNSPKLQRCIRHLRVLSPFTSTPHIILFSLLTKMQHLEVLSMSVSSPSSLDH
VRKKGSPSSQCSITECIPMSSKLTEHGYIVGLRELSLEGCDMLNLNWLTRAQSLQLLRIYNCPSLEEVIGEEFGHSENVFSS
LEIVDLDSLPKLRSICSQVLQFPCLKEICVADCPKLIKLPFDSNSARNSLKHINGQKSWWRKLQWEDEATRDHFASRYVPL
RKIRKIR

>Vang02g14420.10

LREGTYSGDRTGGKVSQKLPSSSLVVESVIYGRDADKQMICNWLTSETDNHNHPSILSIVGMGGLGKTTLAQHVYNDT
EIEEAKFDIKAWVCVSDHFDILTVTKTVLEAITKSKDDSGDLEMVHGRLKEKVSGKKFLLVLDDVWSERREEWEAVRTPL
CHGAPGSRILVTTRVEKVASNMRSKVHHLKQLEEDECWKVFEEQALKDDDLELNDEKKEFGRRIVEKCKGLPLALKTIGS
LLRTKSSISDWKSVLESDIWDLPKEVEIIPALLLSYQNLPSHLKRCFAYCALFPKNYEFEKEKLILLWMAEGFLHYSSQNNNL
EEIGEQYFDDLLTRSFFLRSNIKMHFSMHDLLNDLAKYVCADFCFRLKFDKGNCIPKTTRHFLFAFDDLRYFDGSGSLTDA
KRLRSFVPITNNFVHGAFPCQIKILIRELFSKWKFLRVLSLNGNYDLEEVPDSVGDLKHLHSLDLSRTMIRKLPDSVGLLYN
LLILKLNDCSFLKELPSNLHKLTNLRCLEFEDTEVTKMPMHFGELKNLHVLNMFCVDRNSEFSAKQLGGINLHGRLSINEL
QNIVNPLDALEANLKNKQLVELKLIWNWNHIPDDPMKNKKVLENLQPSNQLEHLSIRSYCGTQFPSWVFDNSLSNLVS
LELEDCKYCLCLPPLGLLSSLKTLKIRGFDGIVSIGAEFYGSSSSSFKSLEILKFYNMKEWEEWECKTTSFPRLQHLVIVRCPK
LKGLSEQLLHLKELFIESCPNLIVSEHSEDTSALDLLRTRSCPLVNIPMTRYDFIEQITIDKSCNSLTIFQLNFFPILRLLRLEGC
QNLQRISQEHPHNHLKKMSVCACPQFESFPSEGLSAAFPFLTELEIIWCRKVEKFPDGGLPSNVKHMSLSSIKLIASLRETL
DVNTCLESLSMKYLDVESFPDEVLLPPSLTSLTISNCRNLKRLGHKVLYNLSSLTLFDCRNLQCLPEEGLPESISSLQILNCPL
LKQRCQKPEGKDWRKIAHIQNLRV

>Vang02g14420.11

MICNWLTSETDNHNHPSILSIVGMGGLGKTTLAQHVYNDTEIEEAKFDIKAWVCVSDHFDILTVTKTVLEAITKSKDDSG
DLEMVHGRLKEKVSGKKFLLVLDDVWSERREEWEAVRTPLCHGAPGSRILVTTRVEKVASNMRSKVHHLKQLEEDEC
WKVFEEQALKDDDLELNDEKKEFGRRIVEKCKGLPLALKTIGSLLRTKSSISDWKSVLESDIWDLPKEVEIIPALLLSYQNLP
SHLKRCFAYCALFPKNYEFEKEKLILLWMAEGFLHYSSQNNNLEEIGEQYFDDLLTRSFFLRSNIKMHFSMHDLLNDLAKY
VCADFCFRLKFDKGNCIPKTTRHFLFAFDDLRYFDGSGSLTDAKRLRSFVPITNNFVHGAFPCQIKILIRELFSKWKFLRVLS
LNGNYDLEEVPDSVGDLKHLHSLDLSRTMIRKLPDSVGLLYNLLILKLNDCSFLKELPSNLHKLTNLRCLEFEDTEVTKMP
MHFGELKNLHVLNMFCVDRNSEFSAKQLGGINLHGRLSINELQNIVNPLDALEANLKNKQLVELKLIWNWNHIPDDP
MKNKKVLENLQPSNQLEHLSIRSYCGTQFPSWVFDNSLSNLVSLELEDCKYCLCLPPLGLLSSLKTLKIRGFDGIVSIGAEFY
GSSSSSFKSLEILKFYNMKEWEEWECKTTSFPRLQHLVIVRCPKLKGLSEQLLHLKELFIESCPNLIVSEHSEDTSALDLLRTR
SCPLVNIPMTRYDFIEQITIDKSCNSLTIFQLNFFPILRLLRLEGCQNLQRISQEHPHNHLKKMSVCACPQFESFPSEGLSAA
FPFLTELEIIWCRKVEKFPDGGLPSNVKHMSLSSIKLIASLRETLDVNTCLESLSMKYLDVESFPDEVLLPPSLTSLTISNCRN
LKRLGHKVLYNLSSLTLFDCRNLQCLPEEGLPESISSLQILNCPLLKQRCQKPEGKDWRKIAHIQNLRV

>Vang02g14420.12

MGGLGKTTLAQHVYNDTEIEEAKFDIKAWVCVSDHFDILTVTKTVLEAITKSKDDSGDLEMVHGRLKEKVSGKKFLLVLD
DVWSERREEWEAVRTPLCHGAPGSRILVTTRVEKVASNMRSKVHHLKQLEEDECWKVFEEQALKDDDLELNDEKKEF
GRRIVEKCKGLPLALKTIGSLLRTKSSISDWKSVLESDIWDLPKEVEIIPALLLSYQNLPSHLKRCFAYCALFPKNYEFEKEKLI
LLWMAEGFLHYSSQNNNLEEIGEQYFDDLLTRSFFLRSNIKMHFSMHDLLNDLAKYVCADFCFRLKFDKGNCIPKTTRH
FLFAFDDLRYFDGSGSLTDAKRLRSFVPITNNFVHGAFPCQIKILIRELFSKWKFLRVLSLNGNYDLEEVPDSVGDLKHLHS
LDLSRTMIRKLPDSVGLLYNLLILKLNDCSFLKELPSNLHKLTNLRCLEFEDTEVTKMPMHFGELKNLHVLNMFCVDRNSE
FSAKQLGGINLHGRLSINELQNIVNPLDALEANLKNKQLVELKLIWNWNHIPDDPMKNKKVLENLQPSNQLEHLSIRSY
CGTQFPSWVFDNSLSNLVSLELEDCKYCLCLPPLGLLSSLKTLKIRGFDGIVSIGAEFYGSSSSSFKSLEILKFYNMKEWEEW
ECKTTSFPRLQHLVIVRCPKLKGLSEQLLHLKELFIESCPNLIVSEHSEDTSALDLLRTRSCPLVNIPMTRYDFIEQITIDKSCN
SLTIFQLNFFPILRLLRLEGCQNLQRISQEHPHNHLKKMSVCACPQFESFPSEGLSAAFPFLTELEIIWCRKVEKFPDGGLPS
NVKHMSLSSIKLIASLRETLDVNTCLESLSMKYLDVESFPDEVLLPPSLTSLTISNCRNLKRLGHKVLYNLSSLTLFDCRNLQ
CLPEEGLPESISSLQILNCPLLKQRCQKPEGKDWRKIAHIQNLRV

>Vang02g14420.13

MVHGRLKEKVSGKKFLLVLDDVWSERREEWEAVRTPLCHGAPGSRILVTTRVEKVASNMRSKVHHLKQLEEDECWKV
FEEQALKDDDLELNDEKKEFGRRIVEKCKGLPLALKTIGSLLRTKSSISDWKSVLESDIWDLPKEVEIIPALLLSYQNLPSHLK
RCFAYCALFPKNYEFEKEKLILLWMAEGFLHYSSQNNNLEEIGEQYFDDLLTRSFFLRSNIKMHFSMHDLLNDLAKYVCA
DFCFRLKFDKGNCIPKTTRHFLFAFDDLRYFDGSGSLTDAKRLRSFVPITNNFVHGAFPCQIKILIRELFSKWKFLRVLSLNG
NYDLEEVPDSVGDLKHLHSLDLSRTMIRKLPDSVGLLYNLLILKLNDCSFLKELPSNLHKLTNLRCLEFEDTEVTKMPMHF
GELKNLHVLNMFCVDRNSEFSAKQLGGINLHGRLSINELQNIVNPLDALEANLKNKQLVELKLIWNWNHIPDDPMKNK
KVLENLQPSNQLEHLSIRSYCGTQFPSWVFDNSLSNLVSLELEDCKYCLCLPPLGLLSSLKTLKIRGFDGIVSIGAEFYGSSSS
SFKSLEILKFYNMKEWEEWECKTTSFPRLQHLVIVRCPKLKGLSEQLLHLKELFIESCPNLIVSEHSEDTSALDLLRTRSCPLV
NIPMTRYDFIEQITIDKSCNSLTIFQLNFFPILRLLRLEGCQNLQRISQEHPHNHLKKMSVCACPQFESFPSEGLSAAFPFLT
ELEIIWCRKVEKFPDGGLPSNVKHMSLSSIKLIASLRETLDVNTCLESLSMKYLDVESFPDEVLLPPSLTSLTISNCRNLKRLG
HKVLYNLSSLTLFDCRNLQCLPEEGLPESISSLQILNCPLLKQRCQKPEGKDWRKIAHIQNLRV

>Vang0103s00210.1

LFVPEIMALAVVGGALLSAFLDVLFDRLASPELVSLIRGKKPDKLLRKVENQLIVLRVVLADAENRQVTDSNVKKWLDVLR
DIVYEVDDLLDEISTKAATRKEVSNSFSHIFNRKRIVSINKLEDIAERLDDILKQKESLNLKEIPVESNQPWKDQPTSLQDRY
GMYGRDKDKEAIMKLVLEDNTDGEEMSVIPIVGMGGVGKTTLARSVYNDGKLKQIFDLKTWVCVSDIFDTVKVTKTM
MEEITKMPCNLNDLNLLQLELMDKLKGKRFLIVLDDVWIENCDGWNSLTKPFLSGIRGSKVLVTTRNESVAAVIPFHVV
KVYHLNQLSNEDCWLVFANHAFPLSEGSENRGTLEKIGKEIVKKCDGLPLAAQSLGGMLRRKQAIRDWNNVLESDIWE
LPQSQCIIIPALRISYNHLPSHLKRCFVYCSLYPKDYEFQKDELILLWMAEDLLKAPRKEKTLEEVGEEYFDDLVSRSFFQYS
TSGIRGIDFVMHDLMHDLATFLGGEFYFRANELGKETKIDRKTRHLSFARFSDPVSDIEVFETAKFPRTFLQIHDEDSPFN
NEKAPRIIVSTLKYLRVLKFGDYQSELVLTDSIGELIHLRYLNLSHTSIAILPESLCNVYNLQTLKLESCFNLTELPKDIQNLVNL
RHLEIFNTPIKEMPKRMGKLNQLHNLDCYVVGKHKENSIEELGGLPNLHGSFSIEKLENVTKGEEALEARIMDKKHITHLL
LKWSVRKDDIIDFQIELDVLDKLQPHQDLKSLEISGYRGTTFPEWVGNFSYQNITNLHLQNCNNCCMLPSLGQLPSLNN
LIISTMSSVKTIDTDFYKKDDCSSVTPFPSLQYLSIYNMAGWEVWNAFDSEAFPVLKNLCIEQCPKLMGDLPNHLPALQK
LTIINCKLLVSSIHGPPTLRTLKILNSNKVAFHEFPLLVESIDVEGGPMVESMMEAISNIQPTCLQTLELQNCSSDISFRGGR
LPASLKALDIRGINKLKFPLQHKHELLESLSIDNSCDLLSSLPLAIFPNLTSLEIRNCENMESLLVSGSELPKRLHSFTIGHCPN
FVSFPGEGLCMPNLTTFIVYDCDKLKWLPDQMGTLVPKMEYLGISNCQQIESFPGGGMPPNLKTVEIRNCEKLLRGLG
WKSMDMVTSLIVCGPCDGIKSFPKESLLPPSLVSLDLIDLSSLETLDCKGLLHLTSLQQLNIEWCEKLENIAGEKLPLSLIKLT
IDECPLLKQRCHKKDRQIWPKICHVRGIKIADEDGRLADIRGEGAMKNRRE

>Vang03g16290.1

LSFFFLSPSGTLKASFTSELGLHSGEVDLYSKEVGLHSEEVNLHSEEFILQSEELGLNSEKVDLHNEEIGLHNVELNLYNGEA
SLHNGEVDLHSGELNLHSEELSLHNEKHDPHSKEVNLYSEEVRLYSEEVGLHNGMFGLHNGVVGLHCEGRPPHCAYRG
STVEDKLLLKSHPFCCRDYLKEVSTPLQKPTQEQRKERSFGTLFQKMKAAVVAHQLGNLISNRSILEDLKNSLQTLQDKR
QKVQENLIWEDEEFQTQSDWLKRVDEILGQGDKLLNSYEGSTCTNILLRYKVGKQSRKMQPEISVLINKGESHVSAKTR
KHERPASRDETIADIMDALRNPDIQAVGVWGLGGLGTTSLAENIREKTKEQNLFDAMVFITVTDKPNQEQVQNAIANA
LGVQFTNGESLVKRRNKLRQRIKKEESTLIIVDDTWGELNPEEFDLEEFGVPPGNEHEGCKVFLTSGNLKFIQYLEDASKL
NKVFQLEELQKEEARKLFEKKVGSFDEDQSSIVEEIVRSCEGSISLIYALAKALENKGEDALMQFKENSSPAKLLSYCLEENE
EHKALLYLLAIRGRRFINSYSMYIDMWTGVFKNLETADAARKKRESLISDLKAYGLVVEKGKDWVKVDDYLYHTAYRMA
LHDKRASVISTEWPPEELLTDLHFCNLHPVGDLKLPATLQCPNLKHLLISRENSTIDVPDSFFEETKLLKVLDFVSFHCPKLP
LSFVVLKDLEALSMYHCELGDIKEVCELTNLRMLGLLGSSIRQLPAQIVKLQKLLFLDLRDTNLQVIPPNVLSKLTSLEELYL
RNSFCNWEIETSTSENKNASLKELTDLEHLAYIEDMYVPDPQAWPVDLFFGNLRSYTIFIGNGWDRAYDGDHELKTLKL
KLNRRFQSENGIKKMLKEVQVLYLDTLNGVQNVVNDMECDGFPQLQSLFIQHNAEVKFIATGSGNDPLDTFPNLESLSL
TILSYLEYIYHGDSLTEKSFFKLRVIKIEKCNAMRCLFSVSMINGIPHLATLEVSQCTSIKAIMLFEGAENRPIEFPELCSLTLK
GLPALISFCSTEGSSSATLFHDKVSCPKLETMVISEVSELTTIWNEEYDAENSFGKLKNVIIKDCEKLRTVFPVNLSKNLDNL
KTLEVRNCSLMTSIFTVMRQDSTKPGLQLSIPMIEITLTGLPKLEYVCVTTGFEALKKKFEEEWYAGLPGLSGG

>Vang0335s00100.1

MALAVVGGALLSAFIDVVFDRLASPEVLNFIRGKKPDKLLQKVKTQLIVVRVVLADAENRQITDPNVKEWLDLIRDVVYE
VDDLLDEVSTKAATQKEVSNAFSLLFKTKKVVSISKLEDIVERLDDILKQKESLDLKEIPVESYRPWKAQPTSLEDGYAIYGR
DKDKETIMKLVLEDNTNGEKVSMIPIVGMGGVGKTTLARSVFNDDKLKQQNFDLKAWVCVSDIFDIVKVTRSMIEEITR
KPCKLSDLNALQLELMDKLKGKRLLIVLDDVWIEDCDNWRSLTKPFLSGIRGSKVLITTRNENVAAAVPFHNVEVYHLSK
LSNEDCWLVFANHAFPLSEASETRGTLEKIGKEIVKKCNGLPLAAQSLGGMLRRKQTIRDWNNVLQSDIWELPGGQCEI
IPALRISYNYLPPHLKQCFVYCSLYPKDYEFLKDELIQLWMAEDIVKPPKNGKTLEEVGHEYFDDLVSRSFFQCTNPKGGF
FVMHDLMHDLAAFLGREFYFRADELGKKTKINRKTRHLSFTRFSDPVSDIEVFDTVKFPRTFLLISFKDSPFNNETAPRIIV
SRLKYLRVLSLCDFQSQLALPDSVGELIHLRYLNLSYTSIETLPESLCNLCNLQTLKLSCCSELTKLPSAMQNLVNLRHLEIHA
TSLKEMPKRMGKLNQLQNLDFYIVGKNIENSIKELGGLSNLRGSFSIKALENVTKGEEALEARIIDKNYINHLSLKWSRAN
DSSIDVQNELDVLNRLEPHQDLELLSINGYKGSRFPEWVGSISYRYMTFIGLYNCKNCCILPSLGQLPSLKDLIISDMNSVK
TIDAGFYRKVDGSSVIPFASLESLRISRMPCWEEWNAFDSEAFPVLKDLYITDCHNLRGDLPKHLPALQTLRIRNCEFLAS
SVPMAPSLRELDIRNGNKVEFHAFPLLVESIHVEGGPVVESMMEAITIIQPTCLHSLFLMNCSSPISFPGDRLTASLNSLHI
SGLTKMKFPMQHKHELLKSLSINNSCDSLTSLPLAIFPNLTSLQITDCENMESVLVSESELSKSLNSFDIGHCPNFVSFPGE
GLCMPNLTRFCVYNCDKLKSLPDQMGTFLPKIEYLGISNCHQIESFPGGGMPPNLRTVSIKDCVKLLSNPAWECMDMV
TSVDVWGPCDGIKSFPKDSLLPPSLIHLDLCDLSSLETLDCQGLLHLTSLQELNIQRQAGLVWRNCTCPL

>Vang0137s00140.1

MACNKNQGSSSSHTNNFDVFVSFRGADTRNGFTNHLFAALQRKGVVAFTDDQTIQKGDFLESELLLAIEGSRVFIVVFS
KDYASSTWCMKELTKIVDWVEVTGRSLLPIFYDVTPSEVRKQSGEFAKAFAEHEERFKDDLEMVKEWRAALKTSCDRC
GWDVQNKQQYEEIENVVEEVINILGRDQIWNFGDDLVDMHSRVKKLEELLDLSANDIVHLVGIVGMGGIGKTTLATAL
FNKISPQFNACCYHDDLSKIYCNFGAASAQKQLLCQALNQGNIEIHNASHGTMLIRTRLCHLKALVVVDNVDQVEQLKK
LGLQSEYLGAGSRIIIISRNCRILQNYGVNKVYEVEVLDKTQSLQLLXXKAFRSNDIGKEHKGLTLDILKYVNGLXLAIEVLGS
FLLDRDVCEWRSALTRMEENPSKDIMDXLRISYDGLENIEKEIFLDIACFFSNNNSYSWEPTVKKFLDYRQFXPDIGMKVL
IEKSLISRQNGDIKMHGLLKELGKSIVREKAPKEPRKWSRLWNYKDLQKVMKINKEAENVEAIFIEQHEKEFLQGRIRVDS
LSKMDHLELLILKNVNCYGTLNFISNELRYLFWNHFPWLSLPSTFFLDQLVELILPHSNIKQLWEGKKELKYLPELPTIQEKT
IDRYSRRLYTFDCPMLSDMEHCYSIVFSWMTKNLEVYLVPRMEIVIPGSEIPKWLISKGKYPPSKHCNKMAMAATLVLIL

LNVAVVCHGGKTISFIRKVNRTEDMLLQSDVFASSSSYNASQQVHIIIGDRVGSAMIVSWVTMDESENNLVRYWNETY
KENLLNYEDEEEKEPKGVHKVNT

>Vang0039ss01170.1

MADSAVSFVVEQLYQLLREEGNLLKGLGNDFSDIKHELESIKAFLKDADRRAGDEGGEGDTHEGIKTWVKQLREISFCIE
DVIDEYIMDVAYRANHHPPCIASLQKIAHQIKSLKSRHRIASNIQDIKSAVQGIKERSERYKFQSTFEDGSLNSSKGAKDFK
WDDPRMASHFIEETEVVGFELPRDELIGCLIKGTDQLSLISVVGMGGLGKSTLAKHVFDNQNVKRHFYCRSFITVSQSYT
VRELLTEMVQKFCKDANEPIPKGLHNMDDQTLVTELRQYLQSKRYLVLFDDVWKENFSDEIEHALPKNKKGSRIIITTRN
MHVAEYFKKSVVVHVHKLQHLSPDKAWELFCKKAFRFEPSEQCPTELEDMSKEIVQKCKGLPLAIVCMGGLLATKEKSIL
EWRKVCQNLRMELERNTHLNSLKWILSLSYDDLPHNLKSCMLYFGVYPEDYSISRKRLTRQWMAEGFIKNEERRPMED
VAEEYLTQLISRSLVQVSRVGFDGKVKSCQVHDLLRDIIIRKMNELSFCHLMREDDELDTVEKTRRFSIASCSKNVLRETSN
SGIRAIYVFKKSELPEDFVGSLSAKFKLLKVLDFESTMLNSVPNNLGNLFHLRYLNLSHTKVKILPRSVGKLLNLETLDLRQT
QVQVLPREIKNLTKLRLLPVYYRKYEGQYSMLNFTTGVKMQKGIGCLKSLQKLYFLEADHGGLELMQELKMLKQLRKLGI
RRVKTEYADALSSAIGEMNHLESLNVSAKDQDEIIDLKFLSTPTSLLVLNLKARVTKFPDWIPKLKYLVKLRLGLSNLEGYPL
DSLKDLPSLLRLNMWDNAYIGEILHFKRGGFPRLKELDLTRLSRLNSISIDEGALLGLEHFRFKDNPQMKVVPHGLKHLKN
LQFLGFADMPAELVESIDPEKDGQDYSVIKHIPLVLIQAAISFALGEVFQILKEEKSLLSGINKEFLDIRDELESIQAFLKDAD
RKAADEANTNDGIRTWVKQVRQVSVRIEDVIDEYLRVIHQVPRHGFGASICKITNLIRTSLSRHQIAVEIQDIKLSLSLIKER
SERYKFQVLREKPSSSSTGRIEGSGWNDHRMGSLFIEETEIVGFELPRDELLSLLLEGKKERTLISVVGMGGLGKTTLAKHV
FDSENVKIHFHCRACITVSQSYTVRGIFTDMIKQFCRETKDPLPEMLEEMDEKTLISELRQYLEHKRYLIFFDDVWHEHFC
DQVELAMPSNNRSSRIIITTRMIHVVEFFKKSFPLHVHNLQPLPSDKAWELFCKKAFKFELDGQCPAELKGMSNEIVGKC
KGLPLAIVAIGGLLSTKSKTVFEWRKVSQNLNLELHRNAHLTGLTKILSLSYDDLPYYLKPCILYFGIYPEDSSINHKRLTRQ
WIAEGFVKSDGRTLEQVADEYLSELIYRSLVQVSWVGFEGKVKSCRVHDLLHELIVRKMKDLCFCHFVHEGDDESATSA
STRRLSIDTSINNVLKSTNFTHIRALHAFGKGGTVEPFTGLLASKSRVLKVLDLESTSLNHVPRNLGNIFHLKYLNLKNTKIR
SIPKSVGRLQNLETLDIRETLVHELPSEINKLKKLRHLLAFHRNYEAEYSLLGFTTGVLMKKGIKNLTSLQNLCYVEVEHGGI
DLIQELRFLKQLRKLGLRRVRREHGKAICASVAEMTHLESLNITAIGEGEIIDLNSISSIPQLQRLHLKARLERMPNWISKLE
FLVKMRLALSNLKDDPLRSLENLPNLLKLTIWDNAYGGEILHFQSGGFRKLKELNLARLNTVSAILIDKGALLSLEHVKITKI
THLKKVPSGIKALYNLKVIDFCDMPTELVESIDPQNGQDYWIINHVPLVFIRRW

>Vang0564s00010.1

MAAALVGGALLSSFLNVLFERLATREFVNFIRGKKADKWLQKMKNQLLVVKVVLDDAEKKQITDSNIKEWLDLLSDVVY
EVDDILDEVSTKAATQKKVSYSFCDLFKKKKIVSKLEDIVGRLDDILKQKESLGLKLNEAENDQPPEPQPTSLEGRYGMYG
RDKDKEAIMKLVSEDSSDSEAVSVISIVGMGGVGKTTLARSVYNDDKLTQIFDLKAWVRVSDRFDIVKVTKAMLEEITQK
PCTLSGLNSIQHDLLDKLKGKRFLIVLDDVWIEDCDVWSSLTKPFLSGIRGSKVLVTTRNESVAAGVPFHSAKYDLKILLDE
DCWLVFESIAFPLSEVSENRGTLENIGKNIVKKCDGLPLAAQSLGGMLRRKHDIRDWNNVLESDIWELPRSIIPALRISYN
HLPPHLKRCFVYCSLYPKDFEFRKDELILLWMAEDLIKTPRRGKTLEEVGHDYFDDLVSRSFFQCSTYQALGKYFGNLYGN
CFVMHDLMHDLATFLGGEFYFRANELGKETKIDIKTRHLSFRRFSDPVSDIEVFDTVKFLRTFLPINYEDSPFNNEKAPCVI
VSMLKYLRVLSFRAFQSQLYLPDSIGELIHLRYLNLSSTSIAALPESLCNLYNLQTLKLYGCSNLTKLPRAMQNLVNLRHLIIR
NTPIEEMPQRMGKLNQLHNLDCYTVGKHKENSIKELGGLPNLHGWFCVQKLENVTTGEEALEARIMDKKHIYHLSLEW
SDRNDNSIDFQIELDVLGKLQPHQNLQSLSINGYQGTRFPEWMRNFSTPNITNLYLTNCSNCSKLPSLGQLPSLKLLRISD
MNSVKTIDASFYKEDCSSLTPFPALQSLHIVRMPYWEVWSSFVSEAFPVLNDLLISECPNLRGDFPDHLPALQTLVVTNC
DLLVSSVQRAPTLRTLEIYKTNKIVFHEFPLLVETIEIEGRPMVESMIEVITNIQLPCLHYLSLQDCSLALSFPGDCLPASLKTL
DISGLSNLKFPMLHKHKLLESLSINNSCDSLKSLPLVNFPNLISLQIRNCENLNSLLILRSESLKSLNSFELGDCPNFASFPEEG
FSAPNLTRFSVYGCVKLNSLPYQMETHLPKMEYLSISNCQQIEGFPEEGMPPNLTTVKIRNCEKLLSSLGWISMDMVTSL

KVCGPCDGMNSFPKEGLLLPSLTSLHLCDFSSLETLDCKGILIHLMLLQELHIENCQKLENITSNTFKNHEISSENWKNAFS
KKKIVSLLFVTNLGSVRDVITPSWQGVERLLID

>Vang0114s00280.1

MDMVILSEAISYTLQCATTFLSPQATRLSSNDIQQFEDNLKRILHIVQKAMHSNIQDPSVLSIWLKNVKDVVNDLNDFM
EDHRHNKETAATISLIKAGQNMAHRHKFKHQIKDATEELKRLSNEAENLVISEEARQNERKLTRKSEELEYVEVVVRENV
KKDIIDQLMKMFVNSNVVSVPVVTIVGVAGIGKTKLARLVYGDEQVKGLFASRIWVNLETFNVESIATRVIETTNKGKRF
LLVLDDLRVENGEGCLQKLQDRLAEAGVGGAVVVTTRSNFVAKKISEIGTVKLKPHVLQELNEEESWSLFQKIHGPGSGK
INEDVGRRVVREYCGGVPMKIIAIARLLEDLDSPVPEIELKKKFLREIRFTYYDELSPQQKLCFAYCSLFPQEQEIDAGSLIRL
WMAEGFLWRNLYSDPQEFGLACFNDFVPFVFQEMGSDEFGVVKRYKMNRLMHELARTVAWDENIIVDSAEVEVQE
RVVRSSFHFALDVQCGIPKALFENAKKLRSILLLGKTNKSRLPHEVKMTISTCEKILETFKCLRVLDFHDLGIKMVPSSIGEL
KYLRLLDLSHNNIKKLPSSITKLFHLQTLKLSQCHVLEELPKDLENLSSLIHLYLEGCLDLTQMPRGIGKLSSLQTLSLFVVGK
NYQVGGLRELTDLDGLRGHLEILHLEQLNFSAPLEAKDKYLRDKKHLHCLTLRWDHEEKEEEDEKKRNVIAKKDKESLECL
DPNPNLAVLSVVGYYGKTFSNWLSSIKCLVKFSLNDCYNCQYLPALDHLQHLRVLELRRLDSLEYVSKNSDQISADTEASS
SSSSTPFFPSLKELTISDCPKLRSWWETAKWKPNRPFFTRISKLDVQCCPELHCMPLYPYLDEELVVVDSSVKSMRDTVH
ASISADFLPFSKLKTMLISRITQTPPERWLKNFISLQTLQIRDCSKLLYLPQGFKSLSSLQSLTIERCAELDLDRSKTEWEGLK
RLRFLIIKEIPKLKSLPWGVEDVTSLEELELHECPALLNLPETIANLTSLTKLVICKCVELDSLPKGIGKIESLHTLAITDCPLLTP
RCQPETGDDWPQIGNIRNIILKQSSQDLRDLWSHGRIGKGRYF

>Vang01g07720.2

LCCFNNFALNLSNVKEELELTRDRVKERIREAINKDEKVESTDVKWLKDVEKVLEEVQVQCQYSLAKEIEKKIIEMIQLDFN
SEYTQFSRITKLLGMEYYYSNDFSVFNPIKASYKKLLEALMNKSASIIGLVGFGGSGKTTLAKEVCKKAEDMKLFEKVVLAT
VSRLLNIRSIQDQIADQLTFELKEGSEIGRAQRLSERLRKGTTLVILDDVWERLNFEALGIPLDESSKACCVLITTRSKEVCTS
MKCQSIIELNLLTHEEAWTLFKHYANINDDSSEALKGVARKIVSECKGLPIAIVTVGSTLKDKTITNFELALSRLESSKPLDIP
EGLISPYVCLELSYNNLTNQLAQSLLLLCSMFPEDCEIILEDLFRFGRAFDTIGTFETMGNARREMDAAIDMLKNCFLLMH
AKEKLKVKMDNLVRDVALWIAFKTGKAIFTRTELDPRALADDKTLKDMKAIAIWGLKSYDVLNYKINCPILEILVLSFNVS
GLEKLKTLSILNLDEWDKGILPLPESLKSLKNIQTLCLRGYDLGDISVVEQLQALEILDLRSSSFDDLPVGIVGLKKLKLLDLYK
CVIKKNTTVEAYKVVGKCLQIEELYLYLSNYVMDFPHEVSYSKLQRYAIINTDYYYHDCDFQDNTIMKKYAKSRSLLIKSFDI
TTQNLISLPIKDLFIRAEYLCLRNLRGDYKNIIPSMDQQGMNQLIALVLEHCSKIECLIDNTINTSTSVDFRQTEDVFSTLVY
LSLRGLHNLQEMFCDPCYRCSLQNLEELLIEDCSKFNNISFPKSSNLCNLKILSISKCPVLTYLFMPSIVQTLSLLEVLKISSCS
ALRHIIEEVEEENYALASTKRLQKLRILEIEDCHNLEYIFTVILAQGLVSLENVKIVSNMKLKYVFGSEKEHNLAVYQSFHQQ
TNISINLPNLDTLNLMFLPNVIRIWPEYCPLRLPCLEESLIILCPKLPNSSIDMAETASNLEQHTISMVMYYFQTTILLLHNY
NYLKWIIFYLVEQIFIYFINYIFITCIHSY

>Vang0821s00010.1

MASSSSNVISSESASSSSDHSSDTIQNQDHRYDVFISFRGSDTRNSFVDHLYHHLLRKGIFVFKDDLDLRKGESISSELLQAI
RGSRISIIVFSQDYPSSSWCLDEMATIADCKQQSNQTVFPVFYDVYPSHVRHQIGPTEFGEIDKIVEAVIETLSHKFSGFVN
DLIGIQPRVQALEDKLRLNSKPDDVQVLGIWGMGGIGKTTHAAVLYDKMSHRFDGSCFIEDVSKLYRDGGHTAVQKQII
QQTLKEKSLDTCSPSEVSGIVRNRLRKKNILVVLDNVDELEQLEHLEINSKLLCKGSRVIIISRDEHILKVYGADVIHKVSLM
NDKDARELFYTKAFKSEEQNSSCVELIPEVLKYAQCLPLAVRVVGSFLCGRNSREWRDTLNRFENNPDNNKIMDVLQISF
DGLHCTEKEIYLHIACFFNEESEDYVKRILECCNLHPHIGIQIMIEKSLITIKDQQIHMHDMLQELGKKIVRNQCPEEPESW
SRIWLYKDLFHVLTTETGTNRVKAIVLNEKEAISECSVDGLSKMKNLRLLILYHKSFSGSLNFLSQKLQYLLWHDYPFSSLPS
SFAAYDLVELNMPNSSINCLWEGRKKEMLISGCFNSPQHFPCLKRMDLSNSKYLRETPDFTKVPNLERLDLSGCTDLSFV
HPSIGLLQQLAFLSLRNCSNLISIEFGNGFSLSSLRVLHFSGCTKLENTPDFTWTTNLEYLDFDGCTSLSSVHESIGVLTKLTF

LSLRDCTSLVSIPSNNNIMKSLQTLDFSGCFQLTDLSLGKSFRISLILLDISFCNLLKVPDAIGDLLCLERLNMEGNNFFFIPSS
IHKLRSLTYLNVSHCHWLHRLPYLSTRISSSTGRYFKTVSGSRDHRSGLYFFDCPKIDWMDRPSYQEWLLRLAKNPCSFR
CGFDIVVPWGLELPGWLKQRFKGDSVIRIVEFNEDDDWIGFVFGVIFERKNGAVVARSSSHPFYLSFESEETEEYFDMQL
NLERDKVDGSKHLWVIYISRKHCHFVKTGAHISFKAHPSLEINAWGMKSMFRKDSMFLERHVNFDFVEKSSTNSGPKF
KLPYNWLVTDEDEVENIKAKAKENNLSYAGLY

>Vang09g04870.1

MRGLGHKRVFIVLDDVATSEQLERLITDYGLLGPGSRVFVTTRDKQIFRPNDEIYEVKELSIHHSLELFSLTAFEEKIPKHGY
EDLSRRAISYCKDIACFLKGEDKDRVTNLLEACDFFAASGIEVLLDKALVTISEFNNIEMHDLIQEIGREIIDQESIKDPGRGS
RFRRKSDIQSSSKGGLCYATHAPYSQGSNLS

>Vang08g00920.2

RTVYCAFSHILSALFKALFRVSDWFSYPQDGQVNTLENLILFHLKFSIARCYDLEQWQLKANHSIRCSILQLLEFIDSVSSSS
DKQYLEMDFLGPFGKVVEGLVDFVWKHGVRQVTYIVNYNNNVVELKDSVKDLALEKERINHQRDEAEKNLNNIEGKVI
EWDRKVSEIETTVEVFKNDDGHTRARSPNCFVFPYLWNRHRLGRQAHKMKEDVKRLIGESPELDEVFYRQNVTSNDA
TLSNCGFVEFSSIKSIIEKVMIQLQDSAVRMIGLYGRGGVGKSTLVKEIARKAKEKKLFDVVVKVEITADPNPHKIQEEIAY
VLGLRLEGEGENVRADCLRRRELDDKDALMLFQKLTGIHNEMPSSKQEIVKKYCEGLPMAIVVVARALRNKSESVWEA
TIKRHKKHELVGDGTSMDISVKMSYEHLENEEIKYIFLLCAQMGRRALIMDLVKYSFGLGILEGVSSLWEAREKIKTSIQKL
KDSGLLLDESSNNHFNMHDMVRDTALSIAHKYHNTFNLRNGKLDDWPELEKCTSIFMCNSDIIDGLEVINCPQLKLFQI
DTNDPYLKIPKSFFRRMKNLRVLIMTGFCVSNLPSSIQYLSKLRMLCLQRCTLDCNLSIIGKLKKLRILSFSGSILKSLPIELQC
LDKLRMLDISDCSELKIIPPNVISSLTCLEELYIRESLIKMLVERETNKGQDLFLSELKNLHQLKVVELSIPCVSNFPNHLFFDK
LRDYNIVIGDFDFFSLGEFKMLNKHETFRVLAVQLKDNTIIHSQENIKLLFKTVQTLLLGKTNGVREVVNDLNIDGFQDLE
HLSIINNNDFKYVNSTKLCNYVNVFPNLESLRLYNLGKLDMICYGPVTVVSFAKLRIIKVEMCHRLKNLYSLDMIMFPIGS
QTCDISECNSYMDIFLSSLEIIEVSECKSLKEILQIPKHYGKVEFLKLHTLTLRLLPLLSSFYTKVDKFCWPDLTKAQTRIMGH
KDLTSEEDKQSDEEPPLFGELVEIPNLETLNLSSLKIHKIWSDQNSSSFIFQNLIKLVVKDCDKLTHLCSLSMARSLKKLKSLV
ISECPVMEKIFETERNSADKVCIFPKLEEIHLTKMNKLTDIWQTKVSADSFSSLISVKIEECNKLDKIFPSHMEGWFETLDN
LKVYRCESVEVIFEINDSQEIDEFGGIDTKLQIILLEHLPKLKQLWSTDPNGILNFKKLRTIDVCFCHELKNLFPVSVAKDVPK
LEHMSVLYCDKMEEIVASQDALETNKDLLVFPELTSVRLHCLPDMKYFYKKKYPIKCPKLKELSVTICLKLKTFVKDTINTT
NKVGNFVFSIDEFPYRIPNLEKLKFNSCYFKELAPKANIGRQKGLGIVLQLKELIFSYSNIKDLGFERGQVLQRLEILRLEGCY
KLSNLAPPSVSLIYLTHLELKDCHRLKNLMASSTAKSMVQLKTMKVIDCHKIEQIISMEESEEGKVMKIVFSKLISIELVGLK
KLASFCSHKECEFEFSSLEILIVRECEKMEKFSEKRSIAPKLKNIFGVEGDEKTKWQWEGDLNATIHKIFNDKITFTYSELLD
TEDSTEFIEQLWQGRHWVQQNSFGYLKRLCVWECHTIVHVIPSHLLSCFHNLEELYVYYCSNAEVIFKMNDDNRVIKKP
SGLFRLKSLYLSNLPQLKHVWDKDPKEIIDLDVLEKMRVNICRSLTTLFPASVAKDLTGLEVFKVTE

>Vang0197s00010.1

MDTIASVASSVAAPLLRNITYVLMYSTYLTELEAEIKRLQSEEKEVRHTVEAAKRGGEEIEDTVRDWFDRVRAAVEQGQA
FLEEEERERVGCMDVYSKYTNSQRARTLVEIVREVRKETFDRVSYRRALRCNVGPAARESYVAIQSRTVMLNDVVKMLKD
GGVDIVGVYGVAGVGKTAMVKELAWQVEKDGLFDVVVVATVTDSLDVGRIRNEIADGLGLKFDELTELGRASRLRQRI
QQEQRILVVLDDLWGKLDLTKIGVPFGEDNKGCRCQLLVTSRNRNVLSSNFGSGKFCRLEVLSDDESWELFEKRAGDAV
RDPSIQSVAEKVAKSCGGLPLLIVTVVEELKNKDLYAWKDALEQITSFELEGCLYSPLRSAIELSYDHLESQELKTFFLLLGS
MGNGCSTRDLLVFGWCLGLHKQVDSLADGRNRLYKLIDNLRAACLLLDEGKRDSVVALEVVRHVANSIATRVKPFFTV
QRNKEFKWPRMDFLRSCHHIFLDWCYIRELPEVLECPKLKILQINSKGNYLKIPDEFFVHMKELKVLSLGGLNCTPSLPPSL
SLLTDLQALYLCECKLEDIATVGEITSLEILNLEKSELKELPAKIGGLSNLRLLDLTDCPTLGGIPGNVISRLTSLEELYMGNCD
VQLEAKESKSQNNDSSLSELKHLNQVTILNVQIEDTSVFPRDMLSFGRLESYKILIGDGWKWSAVESENYKTSRLLKLNSG

ADPTILKDYGIKMLMNKAEELYLAELKGVREVLYELNDEGFSQLKHLCILNCAEMESIIGPTEWAYSDHAFPNLESLILHNL
INMERICSDPLPAQAFRKLQVIKVKDCDRMEFVFSHSMVKHLSELVEIEISECKSMTNILSGQRQEDADAGQTNMIRLIN
LRSLTLQCLPSLVSLSPDSSTEASENGNGFSSQLFSNKVEFPNLETLKLYSINIHMIWINHHHSYFENLTSLTVDGCERLTYI
FSYPVAVKLVKLQHLLLSSCKFVEKIFVPDENLGHIHHFRKSTQTELVPIFPNLETFVISQMDNLKAIWPALLPQNSFCKLK
KMEITSCNNLLNVFPCHVLDKLQSLESLNVWKCMALEVVYEIDGINTEQEGSSQEGLDIPLRTLSLGNLPKLKHLWNKDP
QGNIKFQNLFMVQASKCQSLEYVFPLSLAKDLLHLQFLEISDCGVEEIIASDKGGIGAALGFVFPKLVSIKFFNLPDLRCFC
DGNHNLRFPLLNQFFAVECPRMETFSRGILRASILRKIHLTRKGDQWYWQGDLNTTIRKLFNRGSCALMKGPRLSHEKG
HGEFRGSTAKVRDWIMVVASGVMLHG

>Vang10g00830.1

LIPMAESSSSLAVPTSPTKYDVFLSFRGEDTRHNFVSHLHAALHRNHIEAYIDEREQKGEEISPALQTAIEESKIYVLVFSEN
YASSTWCLNELSSILNCKKRYRRDVIPIFYKVDPSTVRKQEKRYKAAFEEYEQRFKDDMDKVQGWKDALTEAAGLSGW
DSNVIRSENTLLEQIVKDILKKLNLDSISYDQGIIGIEKHIEKIRFLMHFESSDIRIIGIRGMGGIGKTTISEQIYYKLAMQFDSR
SLVLDTQKKIERDGIDTVRKKYMSELLNEVPSPSLYYSDRLKRMRILIILDDVTDSVKVKQLVGRLDSFGQGSRIIITSRDGQ
VLKNAGADDIYEVKELNYLDSLQLFNWHAFKQNSSKANAYMNLSIEMLRYAKGIPLALQVVGSLLYDRETEVWESQLQ
KLEKCQDLDIFNVLKLSYDELDEEQKNIFLDMACFYRGHEEIVVAERLEDCGFSSLIGIDVLKDKCLISVLDGRIMMHDLIQ
EMGQEIVRKECPQYPGKRSRLWKADEINEVLKKNKGSDAIQSILVDVGKMEEVEVHAQTFEVMNNLRMLMLYYSTDI
NETKVFRESSLVGLPDTLKILYWTGFPQRSLPPNFYPENLVRLEMPNCHLEQLWEGDQRKERQLISMAESSSSFAASASP
TRYDVFLNFRGEDTRENFISHLYAALQRKHIETYIDYRLQRGEEISPALQTAIEESKIYVLVFSENYASSTWCLNELTKILDCK
KRYERDVIPVFYKVHPATVRKQEERYKEAFEEHELRFKEDMQKVQGWKDALTEAAGLCGWDSNVISFNQGIIGIEKHIE
EIRSLLDLESPDIRIIGICGMGGMGKTTISEQLYHTLTMQFDSHSLILDVQEKLQREGIYNIIKKYKSDLLKEASSTRLSYYNE
RLKRAKVLLILDDVTDSIQLRKLMEGCDSFGQGSRIIMTSRDRQVLKNAGADDIYELKELNFYDSLKLFNLHAFKPNSPEEI
TYMDLSVKVLKYAKGIPLALQILGSLLYDRQREAWESQLQNLEKCQDHDIFNVLKLSYDGLREEEKNIFLDIACFYGGHNE
IVVAETLNDCGFSSKIGMDVLKDKCLISILDGRIVMHDLIQEMGQEIVRKECPQHPGKRSRLFKADEIHEILRNNKGSDAI
QCILQDIDEMEEVKVHAKAFKMMNNLRILMLYSYSNYWYRNVFLESSLVSLPDTLKILYWTGFHKKSLPSNICPQNLVRL
EMPGCHLEQLWKGDQVFQVIYVPLCFRLLNVKSMPQNVYV

>Vang04g02230.1

MVFMLNRDMLIDDIFPRLRKLVLHYPFHRPSHEQLLSNLHSLKIIDFLELPPHKNDFPYHLTKITWKQIRVGTDFSLFSTES
SHNFKCLRLRGMKVRSWRLDECAMPNLQHLLIEACEYLNDLPQRLWSLATLRQVHALLPSETLANNLQHVKLTTAYTEI
TKMADSVVGFLVQRLSQLLESEIKLLSGVEEKIKSLSNELKFMNKFIKSSEGKYKDAVVKEVVTQIRDVAHQAEDVVDTYI
LNIAKHKRRNKLCRLFHLKEKLVVPHEVDAEIEKIRSRIVDIYENQKRYGIKEGEFQSKEAVATEWCRKRRMDVEEEDVVG
LVNETNIVIQQLQKDDVCLNVASIVGMGGLGKTTLARKIYNKDNVKRIFPCRAWGNVSNDYRPKELFQSLLRSLNLSGFE
NLSEEGLKEEVAKGLKEKKYLIVLDDIWETRVWDDIKGAFPDDNKGSRVLITSREMSVARYAGTPSPYQLPFLTQDQSW
ELFCKKVFRGEECPSELEPLGRSIVKATCGGLPLAVVILGGLFAKKEKSQREWSRMKKMSWNSTADKSEVMDILRLSYD
NLPPTLKPCFLYFGIYPEDHEISAREVIRLWGAEGFLQSQENAEPEEVGDFYLDELVDRSLVQVTRRRTDGGVKICQVHD
VLRDFCISERKSCKFMDVCRESNIDTWSDTNPRRLSLQWQRDSNISAMSFDKSMPSTRSVFIFTWRLHYVNDLVKRLM
LARVIHGFDSPLDPYHLKRMIHLRYLKIIVASLPACVCNLWNLETLDVNYEYTASSKIWKLKRLRRLYLRRGECPVLPEGTR
MENLQSLWLCGRSSAIKSLINNGTFPRLVKLVLSPFSEGGNEVDLLSSVHRLNHLGSLKINKIPKLSSDKNVLPSKLTKITFR
NFLNWSFMKTLGQLPNLQILKLEKTNIWKRENFYGFGAVVNRQFYEDIMPEDEPHNLDIGKGEFNELQVFHLKGVGVK
SWRLEEGAMPRLRHLLIIKCPILFELPQQLWSLETLQLVHIEGPSQQLAGSLQIVEVKYNCKIKLENENLLSSEEEY

>Vang0198s00270.2

LVLDCSSLHCVHKAIKNLVMFKAIAPAQMLKTKSGLQRFWDRFGTRSSGSTSSSYDSSDTIQNQHYRYDVFISFRGPDAR
NSFVDHLCSHLLRKGIFVFKDDHNLQKGESISPQLLQAIQLSRLSIIVFSKNYASSTWCLDEMTAIASCKQQSSQIVFPIFYD
VDPSHVRHQNGVYKNNFLSHRWKFGKDRDKVLGWKRAMTDLANSAGWDMRDKPEFAQIQSIVQAVIKKLGHKFSR
SVNDLIGIQPRVQALEDKLRLSSKSDDVRVLGIWGMNGIGKTTHAAVLYDKISHMFDASCFIEDVNKLYRDGGHTAVQK
QIIHQTFRENIDMSNPIEISGIVENRLHSIKVLIVLDNVDELEQLENLAIKPRLLLKGSRMVITTTDEHILKVYEGDVLRHKLP
LLNDADARELFCRNAFKCEDQSSNCAALIPEVLKYAQCLPLAIKVLGSFLCTRDADDWRDVLNRLENSPDDRIMNVLQL
SVDGLQREEKQIFLHIACFFKGERVDYVKRILKCCGLHPDIGISRLTEKSLITISDEEIHMHELLQELGKKMVRDQSPEEPGS
WSRIWLHKDFLHALTAETGTEKVKAIVLNKKEEMSECIVDGLSRMKELTLLILYHTRVSGRLEVLSDRLQYLLWHDYPFAS
LPPYFTAFNLVELNMPNSHITHLWEGGKRPEKKNKLQHSTRQETAWEFDSFSSQASILSPPFCSQTMSHSSGLDAFANR
FCHALSYNNDASKINKPDFRELDLGSPMSPLHTTRTCGEYHNARWPEGGVVVMAVVMAVAIRKSLRSCPNLKRIDLSN
SKYLSETPDFSRIIKLERLDLSGCSSLSYVHSSIGLLKKLAFLNLRNCCNLVCIDFGCVWNMSSLRVLHLSGCSKLESTPDFTR
ATHLEYLDMDECTSLSTIHESIGVLSSLTFLSLRGCIKLVSIPNDINSLVSLQTLDLSCCYNGWNPIPRQALSSHFKSLIFLDIS
NSYLEEVPDAIGDLRCLERLNLQGNRLFSIPDSFRQLHCLAYLNLSHCRYLKNLSGLPTEGDKSGGKYFKTVSGSRDHRSG
FYLFNCPDVSHDRLEFAGLERFFHLIKEPCNFRCGFDLILPWDMGFRPVFGDTFLGNSIIRILQCVMNDNWIGFGFYVIFS
RGIDFSSSHCSLSHLLYLSFESEYTEEYFDMRFNSERDEYFRSRHIWIIYMSREHCHFVKTGAHITFKAQPNVKIDALGLRPI
LKQDISDSKGKKYIKFSKLNHDHLDFEYVEKSNSGSGPKIQLPYNWYVTEEEQVENIDAKAKENNLSNAGL

>Vang0229s00130.1

MDPIVSTTTESALNITASVVKRQVGYLFNYKDKFKELKSYIEKLENNKERLQHQVDSALRSGDEIEKDVQHCLTFIDDKIKE
YKSFINDDCHAKTICSIGFFPNNFRLRYQLGRKATKMVEEIIGDELWKTFFDNVSYQEFPSIDATFSNNVYESFASRTKTM
EMIMKALQDSTVGMIGVYGPGGVGKTTLVKEIANKAREKNLFEIIIIANITGNPDFKKIQEQIAVMLGMKLEEESEIARVE
RIRKRLKNEKENTLIILDDLWGGLDFDKLGIPCNDDASQQEVNDISDFGYNKTEIKELSKVDLDKMKKEKLSNDYRGGKIL
LTSRNKQVLCNEMDVQQRSIFSVGVLDEKESETLLKKVAGVKNSEFDRSATEIAKWSAGFPIALVSIGRTLKNKSLSTWE
DVCQQIKRQNFTSEWGFTDFSIKLSYDHLKNEELKCIFLHCARMGSDALIMDLVKFCVGLDLLPGVHTITDARKRVKEMI
QELEESSLLVKSYSIDRFNMHDIVRDVALSISSKEKHVLYKKNAILYEWPHENDFERYSAIFVHFCDINDELPESIHCPQLEV
LHIDNKNESFEIPDEFFKSMVRLRVLVLTGINLSCLPSSIKCLKKLRMLCLERCTLGENLSIIGELKNLRILTLSGSNIENLPLEF
GQLDKLQFLDISNCLKLRQITSNIIPRMGILEEFYIRDNLIIWEAEENMKSENASLSELMHLNQLQNLDIHIHCSSYFPQNL
FFDRLNSYKIVIGEFNLSNLLKVGEFKVPDKYEEVKFLALNLKEGIDIHSEKWIKMLFKSVECLLLGELDDVQDIFYELNVEG
FPNLKHLSIVNNFGINYIINPKERFHSLLAFPKLESIWLYKLDNLEIICDNQLVETSFRNLKVIKIQTCLKLVNLFLFSMVRLLT
LLETIEVCDCDSLKEIVSKESQTNTISDDKIQFPQLRLLKLKYLPTFIYLYNVDKTPGSPQSLQDQVFQQRNKDIVVDVEHM
VTNSCLPLFNEKVSTPKLEWLELSSINIHKIWSDQCNHCFQNLLTLNVTDCSNLKYLLSFSMAESLVNLQSIFVSECAMME
DIFRQEDAEYIDVFPKLKKMEIICMDKLCTIWKSDIGLHSFSSLNSLMIRECHKLVTIFPNYMGQILQSLQSLTVTDCKWV
ENIFDFENIPHTCDIIETNLGKIILENLPNLVNVWKGYTGEILKCNNLQSIRVYESPKLKYLFPVSIANDLEKQEVLEVRNCG
VTEIIALDKHSCETAISFKFPHLNTLSLIDLHDLRSFYSGIHTLELPPLKKLDIINCSMLQGLTSEITDSKEQPIVLAAKKAIYNL
EYMSLSLKEAEWLQKYIVNVHRXHKLEELTLYGLKNNEILFWFLHRLPNLKRLTLELCHLKRIWALKSLISREKIGGVVQXK
ELKLESMWSLEEIGFEHEVLLQMVERLSIQHCTKLKNLVSSSVTFSYXTYLEVMNCKSMRSLMTCSTAKTLVQLTTLKVCS
CPMXVEIVAENKVEKVREIEFKQLKSLELVSLQNLTSFSRVEKCDLKFPLLEKLVVSECPQMTKLSEVQSAPNLQKVHVEA
GEKDQWYWEGDLNATLQTHFTNQVSFEYSKYINLVDYPEKKVRHDKFAFP

>Vang0229s00130.2

MDPIVSTTTESALNITASVVKRQVGYLFNYKDKFKELKSYIEKLENNKERLQHQVDSALRSGDEIEKDVQHCLTFIDDKIKE
YKSFINDDCHAKTICSIGFFPNNFRLRYQLGRKATKMVEEIIGDELWKTFFDNVSYQEFPSIDATFSNNVYESFASRTKTM
EMIMKALQDSTVGMIGVYGPGGVGKTTLVKEIANKAREKNLFEIIIIANITGNPDFKKIQEQIAVMLGMKLEEESEIARVE
RIRKRLKNEKENTLIILDDLWGGLDFDKLGIPCNDDASQQEVNDISDFGYNKTEIKELSKVDLDKMKKEKLSNDYRGGKIL

LTSRNKQVLCNEMDVQQRSIFSVGVLDEKESETLLKKVAGVKNSEFDRSATEIAKWSAGFPIALVSIGRTLKNKSLSTWE
DVCQQIKRQNFTSEWGFTDFSIKLSYDHLKNEELKCIFLHCARMGSDALIMDLVKFCVGLDLLPGVHTITDARKRVKEMI
QELEESSLLVKSYSIDRFNMHDIVRDVALSISSKEKHVLYKKNAILYEWPHENDFERYSAIFVHFCDINDELPESIHCPQLEV
LHIDNKNESFEIPDEFFKSMVRLRVLVLTGINLSCLPSSIKCLKKLRMLCLERCTLGENLSIIGELKNLRILTLSGSNIENLPLEF
GQLDKLQFLDISNCLKLRQITSNIIPRMGILEEFYIRDNLIIWEAEENMKSENASLSELMHLNQLQNLDIHIHCSSYFPQNL
FFDRLNSYKIVIGEFNLSNLLKVGEFKVPDKYEEVKFLALNLKEGIDIHSEKWIKMLFKSVECLLLGELDDVQDIFYELNVEG
FPNLKHLSIVNNFGINYIINPKERFHSLLAFPKLESIWLYKLDNLEIICDNQLVETSFRNLKVIKIQTCLKLVNLFLFSMVRLLT
LLETIEVCDCDSLKEIVSKESQTNTISDDKIQFPQLRLLKLKYLPTFIYLYNVDKTPGSPQSLQDQVFQQRNKDIVVDVEHM
VTNSCLPLFNEKVSTPKLEWLELSSINIHKIWSDQCNHCFQNLLTLNVTDCSNLKYLLSFSMAESLVNLQSIFVSECAMME
DIFRQEDAEYIDVFPKLKKMEIICMDKLCTIWKSDIGLHSFSSLNSLMIRECHKLVTIFPNYMGQILQSLQSLTVTDCKWV
ENIFDFENIPHTCDIIETNLGKIILENLPNLVNVWKGYTGEILKCNNLQSIRVYESPKLKYLFPVSIANDLEKQEVLEVRNCG
VTEIIALDKHSCETAISFKFPHLNTLSLIDLHDLRSFYSGIHTLELPPLKKLDIINCSMLQGLTSEITDSKEQPIVLAAKKAIYNL
EYMSLSLKEAEWLQKYIVNVHRXHKLEELTLYGLKNNEILFWFLHRLPNLKRLTLELCHLKRIWALKSLISREKIGGVVQXK
ELKLESMWSLEEIGFEHEVLLQMVERLSIQHCTKLKNLVSSSVTFSYXTYLEVMNCKSMRSLMTCSTAKTLVQLTTLKVCS
CPMXVEIVAENKVEKVREIEFKQLKSLELVSLQNLTSFSRVEKCDLKFPLLEKLVVSECPQMTKLSEVQSAPNLQKVHVEA
GEKDQWYWEGDLNATLQTHFTNQVSFEYSKYINLVDYPEKKVRHDKFAFP

>Vang03g18140.6

LVDSVVVARENDRERLLSMLLCDDGDGMSNDVEVITVLGMGGLGKTTLVQCLYNDCEVQSHFDMTGWACVSDDFDI
LKVTKKIVESLTSKDCHITNLDVLRVELKNNLRDKKFLLVLDDLWNEKYNDWHNLIAPFRSGRKGSKIIITTRQQRVAQVT
HTFPTYELFAYCSIFPKQHLLDRKELILLWMAEGFLQHIHEDEEMESVGNDCFNELLSRSLIQKENAVAEENFRMHDLIYD
LARLVSGRSSYHFDGSEIPRTVRHISFLREMFDISEKFEGLYELKCLRTFLPRLSYPFVQCYLTKMVSHGWLPKLRCLRILSLS
KYTNITELPNSIGNLLHLRYLDLSYTSIESLPDETFMLYNLQTLILSNCESLIQLPQKIGNLTNLRHLDISDTNLTEMPTQICNL
QELRTLTVFIVGRQDGLSIRDLSKFPYLQGKLSIMNLQNVVNLVDVFGANLNKKEQIEELILGWGSDPQEPQFEKDVLDN
LQPSTNLKKLSVKYYGGTSFPNWIGNFSFSKITVLTVSDCNNCLSLPPFGQLPSLKELVIKRMKMVKKVGHEFYGSNVDS
QLFQPFQSLENLEFEDMSEWQEWLPCESEGRNFIFPCLKTLYLCKCPKLRGTLPTHLPSLTNVIFSECNQLVTELADVHW
NKSIEAIHIAEGQEALLSMLDNFSYCELLIEKCDSLLCLPRMLLAANCLQKLTLTNIPSLIYFPADCLLTSLRSLEIWHCRNLEF
ISHNTCPKFTSLETLRIWNSCCSMTCFSLGCLPVLQELNIRFIPNLAAITTRGGEAAPKLVDFIVTDCEKLRSLPNQIDLPSLE
HLDLSGLPMLESLSPRCLPSCLRSLHVDVGILSSVSKKELSVLFQRLSSLSHLLLKGLGEEDLVNTLLKQQSLPTSLEYMFLH
NFNDLKLLEGKGLQNLTSLQMLQMYNCPSIESLPEGQLPHSLQVLSLRECPLLEARYQNHNGKYWYKIAHIPAIKINEKVI
I

>Vang0984s00030.1

MENYTKVAETSMPFARRYLPPKPFEALKMMRDFSKEVAEITDEHGSLLDFIDDADKVADAEEDVKRRDRMKERLMRL
REAAFRMEDVIDEYCIRVKEKQPQHGPRWAASLSKTVHSIKTFILRLQIACKIPDEKSVVRDEKVGFERQYFLEKKLNSSRE
NKNVTFNQLRMDPLFMKEEEVVGLAEPTERLTDWLAMGREERTVISVVGIAGVGKTTLAKHVFDRVHQYFECHALITV
SQSYTVEELLRDLVRKLCKERKEDPPCKISTMDRGFLIEEVRDRLHKKRYVVLFDDVWNEKFWDDIQSALIDDKNKSRIIIT
TRLEKVAVFCKKSSFVKVHKLEEPLKDDSFRLFCRKAFKYGSSGGCPEELKDISLEIVRKCKGLPLAIVAIGGVLSQKDDSEK
EWRLFSQNLSSELKRNPDLNIITKIIGLSYDNLPSHLRLCLLYFGMYPKDYEIESERLVRQWVAEGFVTHEEGKTLEEVAHE
YLLGLVRRSLVQVSSFSMKGKVKKCRVHDLIHDMIRTKVKDTCFGEYIGGGHDQSESSESVRRLTIQANNDLNRRIKRSH
IRSIIVIPGKKEELLSVHLVKKIPKDYILLKVLDFEDCGLLCVPKKLGNLIYLRYLSFRGTQIKVLPKSIGKLVNLETLDIRQTQVC
KVPKEIKKLRKLRHLLTPDSVSSSIEWKDIGGMTRLQKIPQVRMEGDGVAIREVGKLKQLRVLRVLCFGGEHITLLFSSINE
MEHLQALRIEKSDDRNVVDLTTTSIISELRKLFLDLKLEKLPNWIPQLLHLERLTLRHSDLTNDPLESLKDMPSLLVLSLSHA

YEGKTLHFQPGGFKKLRKLNLEHLWNLNSILMDETALQSLEHLNLKGLDELRTVPDGLQHLKKLKFLLVLSMPTLFVKEID
RIGKDQHWSINYGYM

>Vang0103s00320.1

METVFKIIDPVIVFLWGHGIKQVSYIFRYTKNLEELNKKVKRLGEEKERLDRQCSEARKKGHIVEARVEEWFGEVDEFETS
VENYRKNAGHKKTRGLYYLLPYYRHTLGRQAKKMEIEALRLTEESKVDEVSHAEKVTSFDLTSSNSGYIEFDSRKSIVEDI
MTKLKDPNMKIIGLHGSQGMGKSTLIRKIANKAKDEGLFDRVAEIDVTVNPNPLKIQEDIAHVLGLSLAGESENVRAYYL
RRWLKIENPSILIILDNLHERLDLNRLGIPVDDDYDLRKKNQLSIRSSRQGSYSNHEDTEGSKKISGHTKEAARVKQDVGK
YLEKANFLGDYKGYKVLLSSRYKEVFLDEVDVESKFSLQELDDNDALILFEKVTGGDNKISMSKEEIQNYCTGLPMRIVKF
ALAFKKWSESENKPTMDKFKNQGLVEWNKSLEIPNNIKNDLPNNKELKFIFLLCAQMGHLPLVNDLVKYCFGLGILEGV
SSLSAAREKINESIQELKNLSLVSYKNPNVHFNMSHMVRDDALSNALMDHNIFVFRDGKLDYWPNLEKCISISICNSDIT
DGFPQVINCPQLKFLQIETKDPSLEIPKNFFSSMKNLLVLILTGFHLSSLPNSIEDLLNLRMLCLERCTLDCNLSVVGKFKKL
RILSFSGSQLKNLPIELRYLDKLRLLDISDCFELKIIPPNLISNLTCLEELYIRKSLIEMLKEREESKGHNAFLSELANLHQLKVV
DLSIPCVSILPNHLFFDRLKDNKIEIGDFEIFSVGEFKMPNKYEELKVLALQVKDDTDIHSYEGIKLLFETTQSLLLGNVGVQ
NVVNELNIDGFQDLKHLSIINNNDIKYVNSTDLSNCVNIFPNLESLCLYNMMNLKMICDGPMTLESFAKLKTIKVEMCY
QLETLFSFYAIKTSTSSRTSEIFECNSYTKNFLASLEMIEVCECKSLKEILQIPIDFDKVEFLKLRTLTLQSLPSFTCFNTKVKRSC
WPHAMEAQTTNRGHTEISTEEDDHSDNASPLFGELVEVPNIENLNLSSLNIHKIWSNQHLSSFQNLIRLVVKDCDKLTYL
CSLPMASSLKKLKSLIISGCLIMENIFEIEGINAHKVCVFPKLEEIHLSDMKRLRDIWHTKVNIDSFSSLISVNIEECNQLDKIF
PSHMEGWFESLINLKVSNCKYVKVIFEVSDSEEIDVSGASIAKDVSKLERISIFNCERMEEIVSSKDASEANNDPLEFPELTY
ARLYELQNIKQFYEGRHPIKCPKLKELSMGKCMKLKTFSQKTSETTSEEKFVFSAEAVFPNLEYMEIDFEEPQILLSKYQM
HNLKELIILNAIQPLDLFYPFLYKMPNLEKLTLTSYYSESLESLQSTNIGQQDRLGVVLRLKQLFIRSSNIKDLGFERYKVLQR
LEVLSLEFCYKLETLAPSSLSLSYLTYLELKSCNRLRNLMASSTAQSMVQLKTMKVINCYNLKEIVSNEKNEEGKVKKIVFSK
LISIELVRLKNLGSFCSYKDYEFEFPSLEILIVRECLKMEKFSEKRAIAPKLKDVFGVEGDQKAKWQWKGDLNATIQKVFDD
KVSFAYSEHMRLDEFIIEQPWHSSDWVRQKSFGYLKKLSVWRCKTLVHIIPSHLLSCFHNLEELQVMQCKAAQVIFNISD
ENRVMTKPSGIFRLKRLSLTFLPKLEHVWDKDPEGIISLQFLKEISVYSCERLKSLFPASVAKDLTRLQVLNVTKCKELAEIFK
DGADEEGERTTQELVLGGLTTLKLAELPSLKYSFHCSKQQVILTTLPLCDHSKLAFIQN

>Vang0663s00020.1

MVSIVSDLAKSNLEKLINATVEXSRYICCFTCITDNFEKERENLKAKKKTWEESARLATRRGDNIREDFTLWQKQTKELIEE
NTKKKVTCFFGWCPNYKWQYSRGKELESKTKEIRRLVECNFENVGITRDVLDIEYHSSQNYISLESRKLKFEELFNALKDD
NNYMIGLQGMGGTGKTTLAIEVGKELKKSKCFNQVIDTTISNTPDIKEIQDDIAGPLGLLLKDCTESERPKKLKDRLTNGE
KILLILDDVWGDINFEEIGIPFKGNHNNCRILVTTRNMKICQQMDCDKTIELHILPEKEGWILFQKYAGLSDNSSKSILDRG
RKISKECKGLPIAIAFIARSLKGPRPLEEWDVALXSLQKSMHVXDNEDESRKKVYTCLKYSYDNMKDETAKKLFLLCSLFRE
DEEISEELLVRLAKGATLIXKIDDDDSYDECRKKVIVAKNKLIDSCLLLNCKYERVKMHDLVREMALWIANEENVAVNTSK
KNEMTKVEKGKDIKYLLCEGKIKNLFSSKFDGSKLVILIVYMKTHHHVEVPNSFFENKPGLQVLILSNSFVPRPSLSLPQSIQ
RLTNIKSLYLKRFKLGNISIIGNLQALETLELETLKNLVQXAEILRLSGPLGVWKNLIPDIIFPEDESTSNLMEIELADNSQLTC
LIDNNDSLVQSALSGLVMLRLERMKNLKEICKISLCKLRCLCILHCPMLTSLFELSTTQSLSSLETLRIGSCEQLKSIIRDENK
WKDSGEEIVDTHNNNKSTISIFPNLTTLHVSECHLLQFVLPVTQKIPKLEDTSIGDCDGLKYLFGRYQHEHEEEDLHQELK
DVIIKDVYLWNLPNFVDIFQKCDESLCSLVKKSTXKDESKGQIESKSITYKVNEPNANDVIAQNMSVELKNLNDTSKILFTL
QNVTVLRIESCEKVEVLFCASMLECLPYLGALNIYRCNELKQIIDEDAKNQRKPFFPRLKALIIRECNKLKCIFPVSTSKMVP
MLEALLIMEASMLEEVFEGNSDEKVEIPNLKTAGFVELPSLRQKIEFLTVKHGLVRNCPKPYISSFPQDLKHFSYSLTEYFK
NRDLHEQISGALDNLKPEEIGNENPRREDSGVEAASCRGSELTSSQNNANQSTLAKIDEDNKQREDVVKQSKERVEEEQ
LIVVGKASSMPISPTDFPLDKTHSPVSFLF

>Vang04g06570.1

MDPSIIVSTATESVFKFGENLVTRHLGYFCNYNGKFEEVKHRIEMLDDTRKRVQNDVMVAEMNAEEIEEDVKHWLKH
VDEKIKEYENFLCDKRHEKTRGSIGFFPNNLQLRYRLGRKATKIVEEIIADELLNKKFNKVSYHMGPSMDAALSNTGYESF
TSRKKIMGMIMQALEDSTISMIGVYGVGGVGKTTLVKEVAKQAKERKLFNKVVMANITRNPDIKKVQGQIAEMLGMR
LEEESEIVRADRIRKRLKKEKENTLIILDDLWNGLDLNRLGIQRNEDDGVSQKVAKDVADFGYKKVETEKLPADSNKMKK
EKLSSDYNKIKKEKLSVDHKGFKIFLTSRNKEVLCNQMDVQERSTFPLGVLDQKEGEALLKKMAEISVTNSAFDDKVIEISK
MCAGLPIALISIGKTLKNKNHYVWEDVRRQIERQNFTGGQKPIEFSARLSYDHLKTEELKLIFLQCSRMGSDFSIMDLVKF
CIGLDMLQGVFTIRETKYRVNVLMEELTESSLLMKSYSNDCFNMHDIVRDVALSISSKEKNVFFMKNGKLNEWPHKDKL
ERYTAIDLHYCEIVGLPESKYCSRLEVFHIDSKDDLLKIPDDLFKYMIELKVLILTGVNLSRLPSSITCLTNLKMLCLERCTLRN
NLSIMGELKKLRILSLSGSNIENLPVELRQLDKLQLLDLSNCSQLRAIPSNMILGMNSLEEFYMRDDLILRETNEEIQSKNSS
LSELRHLKQLRSLDIHIPSVAHFPQNLFFDKLDSYKIIIGEINMLSVGEFKIPDKYEVVKFLALNLKDGINIHSEKWIKMLFKR
VEYLLLGELNDVHDVFYELNVEGFPNLKHLFIVNNVGLLYIINSVKRFHPLLAFPKLESMCLYKLENLEKICDNQLTEASFCR
LKIIKIKTCGQLESIFSFFMLSRLTMLETIEVCDCDSLKEIVYVERESDIVSDVQTDKIEFPQLRFLTLQSLPAFFCLYTNDKMP
SISESSEDQVKNRELKEITAVAGQDTNACFSLFNGKSLFVSGCELMEDIFCVEDAMVGLLFDHSIYFLLQQNIDIFPKLKK
MEVIHNLEYMSISLKEAQWLRDYVFSVHRMHKLQSLVLSGLENTEILFWLLHRLPNLESITLKNCLFEGVWASTSLVVHE
KVGVVVQLKELIIDNLRYLQNIGFEHDLLLQRIERLVISECLNLKSLLPSSVSFSYLTYLEVTNCSGLRNLITSSTAMTLVQLAI
MKVSLCQGIEKIVAEEEKTQVIEFRHLKAIELVSLPSLTCFCSSEKCDLKFPSLENLVVSDCLLMETFSEVQSAPNLRKIHVLV
GEKDRWYWEGDLNSTLQKLSTDKVSFKHSKHLTITEDSDLEEIWRSKAAFQDNYFHSLKTLLVMDITKDHVIPSHLLPCL
KNLEELEVGSCGAVEVIFDVNDIDTKRKGTVARLKKLTLTTLPNLSRVVPKLKELTVNEESITLLNHALPQDLFCKLNFLQLC
FEDENNKKYTFPFHFLHKVPSLEHLQVYECFGLMEIFPSQTLQYHERILVRLRKLTLNNLPELDTIGLEHSWIKPYTKKLDFL
KLEECPRLEKLVSDVVSFSNLKQLAVESCEEMKNLFTFSTAKSLVQLEILTVLNCESMKEIVKNEDEDAYEEIILGRLKKLKL
NFLSKLVSFYSGNAMLQLPCLSTVTIVKCPKMNTFSKGGLNAPMFSGIKTSLKDSDFHFHNDLNSTVRWLHQHVSDEH
SKHLTLADDSKLEKILHSKAAFHDNNFSSLKSLVVNNVTKDHLIPSQVLLCLKSLEELEVKSCKEMGKIFDVNDLDTEKKGI
VSRLKKLTLDTLPNLKCVWNKNPLGIVSFPNLEEVFVSDCGELEALFPSSLARNLVKLDELDIENCEKLVDIVGKDDEIELET
TKMFKFP

>Vang0162s00090.1

VEAGFIRNSQIAAAFSNQNVGRSNADTCAVSKCYQIWLFEYHFPFQFLTSSPRAKAWLKKQLILLDLYETRVGKVKDVVE
KLKSKRDAIQHTVDEEERRHGRRIHDEVKEWMESVDKLIRAYEDFHNDEICHKCAVFDFFNSGYLPKPGIRYRRSRKAN
DITKQANGLLQNAKFDILSYWSGPPSMATFFSNLGYESYSSRKETVKKITAEFQKPDVRTIGLHGLSGVGKTSLVKEVVKK
ALKDKMFEVVTMASVTKSPDVRKIQGQIADMLGIVLEEESDIARAARIHQILNNENNSTLIILDDLWEQVNFNLLGIPCEL
EKEDGLTKVQGKPLDVDSLKNVVSDGKSLGTADWVNVKRGGFLGGSSKNVSEGKSPLDASERVKAEHSVPQYKGCKIL
MISEIKQVLLKQMEGKEESIFPVEVLKEKEAEMLFKKKAGISGKNSEYDKLAAQIASKCKGLPMTIVTTARALKNKSLSVW
DETNRKLESQNLTGAPEFSTKLSYELLVDEELKDTFLLCARMGHDALIMDLVKYCIGFGFLRGVNTARQTRDRVYTLVGK
LKESGLLSDSYSSDHFTMPDTVRRAALSIEHEENQLFTMTKGKLDEWPDKLERYTAISLHHCDFIEDFSGRIEYPRLRVLQI
VNNIPRPNIPKNFFKGVKELRVLILTGIHLSLIDSSISSLHKLRMLCLEQCCILDEELSILGELKRLRVLSFSGSDIKSLPDELNEL
KMLQIFDISNCSKLKKIPRGVMSSLVSLEELYMRNTLIQWEDEEQTCQSKTALLSDLKHLNQLTTLDIQIPNVLYLPKNLFF
DKLDSYKIAIGDLSSFLETGFQMPEIYETLKFLAVQLVNGSDIHSLLGIKMLFEGVENLFLELNTVHEKHSVREARNIVHDLF
YRLNLKGFPCLRYLWIVNNSTIQSLIHPKDRQHHEKAFPKLESLHLYNLKMDEICSCKLSEPSFGKLKVIKINLCRELKNVFSI
SMVGLLKVLETIEVSECSSLKEIINVGPPINLENTELLMLPELRYLKLQSLSEFIGFDAIPSIHEGERKLFHEKVGVSKLERLELS
SIQIDVIWSVNQSSERLSFENLTHLDVNGCWNLKSLMSFTMAKSLVNLRSLYVSECGKMTSIFLSEQDTEKDIMVRRLGS
IFPKLKNMKLCSIISLSKIWYPKLPSDSCNKLETLIIEECHRLENALEGIFGSLCNLRVRNCRSMQAIFNIGEQVGDVANNLQ
DVHLETLPKLELVWKSNDEDLVGIPKFNNLKKIWAQVCDNLEYIFPFYVAKNLDNLESLVVCDCRGLSKIVAEKHVTNTD
TAKFIFPKLSTIKFSHLPKLTSFYPTSYDLSCPLNELSIEFCNNLEPFNNGTEHAQTNPVHALFPEEVINNLKSMQIEFWHAK
SPNSYMGKGNHRRDNLEELSLSRLTNTEILYSFLYRNPNLKSLSLNYCFFEKIVPPKEDTEIENLGVVPNLKSLMLIDLFNLK

ELSFEPDIILERLEFLILKNCCLMITIAPSSVSFTRLTNLEVVNCDRLQSLMTASTAKSLVQLNTMKVVKCESLMEIVRKDGE
QSDRIVFQQLKALELVSLKNLKSLCVSDCDFEFPSLEKLVVSACYNMDKFSNRVTISPILQNVHVVHGKENKRCCWEGDI
NATVQKIFKEKKFFEGMEEISLSEHQELQETWQPGADLQKKNSWFYSLKILKLENCVIQPCAIPSNILPYLRSLKELQVQG
CNNIEVIFEMNAEEGTGSTFHLQKLTLQKLPKLKDVWERNGKGTENFQNLKLVNVKNCENLQTVFPLTLAKSLKKVDEL
EIVDCRELYEIVRKEDDTAAVFVFPCLTTLALGELPKLTYFYPESFTLECSALHEMNVWNCPELKLFESTNRQSIFFHLKVIN
MQYVLG

>Vang06g21880.8

MKNGAVKEWLEELKDVAFAAEDLLDEIYTDAKIKAKQVNTLHSGPMSFYCKGVEEKIEDVHERLEFIMRQKEVLDLKVG
KEVKMAHKTPTSSVMEACDVFGRDNDKESLVDLVLTHNEKIGIIPIVGMGGIGKTTLAQLIYNDQRVQKEFDVKAWIYV
SEEFDICKITKTLLEAVTSCSCDVEDLNFLQRDLKMHVMNKKFLFVLDDVWNENYDNWDKFRSPFKHAGEHGSKIIVTT
RSGCVASIMQTVSPYNLRELSNEDSWNLFSKHAFDYGDSSLQLHQSLDKVGREIVRKCKGLPLAVKTLAGLLRCKSDRQ
EWCKVLDSEMWDLQDSESNILPALRLSYHYLPSHLKRCFAYCSIFPKDYEFEKENLVLLWMAEGFLQQSKRHRRIEEVG
NEYFCELVSRSFFQQPRRGKSCFLMHHLVNDLAQFVSGTFSIRMECSNSNEIKERTRHLSHIIADSSSYVNLKDVSKANCL
RTFLQIRPVGTSIDLFNNMPNDLLTKLRSLRVLSLVGTHIYSLPNSVGELKHLRYLEVADTEIVRLPESICSLFNLQTLKLVGC
HNLIELPASIHKLVNLRHLDIRGTSLRWMPLQINELNSLQNLSDFFVGKGCGSSLGELGELICLHGELFIHCLEHIVSDKDC
EKAKLKEKHGLEKLSLDWCRNGETENSQKEKTILNSLQPHTNLKKLDIYDYPGTEFPEWLGDHSFYNLVSLMLNGCKYCY
RLPPLGQLPMLKELQISKFEGLVSVGSEFLGNRTSYLTNCFPALEILRIESMPLWEKWYPNAENAGSKAFFHLREIHIGNC
PKLRGNLPDNLPSLTLLVIRDCKRLLCSLPNSPSLRVLNIQNCESLEFKVHSPLCHQSLTSLFLHGSCDSLVFLPLDLFPNIKS
LDIWGCKNLEALTVSESDATRPNLKSLHSLRIRHCPNFTSFPKGGFAASKLTLLTINYCQKLNSLPEQMHDLMPSLKEVQL
RGCPKIESSSTRPLRIRICSKHMEGKQNLSDPLFARLKGLATDQSPSSS

>Vang06g21880.4

MGGIGKTTLAQLIYNDQRVQKEFDVKAWIYVSEEFDICKITKTLLEAVTSSGEHGSKIIVTTRSGCVASIMQTVSPYNLREL
SNEDSWNLFSKHAFDYGDSSLQLHQSLDKVGREIVRKCKGLPLAVKTLAGLLRCKSDRQEWCKVLDSEMWDLQDSES
NILPALRLSYHYLPSHLKRCFAYCSIFPKDYEFEKENLVLLWMAEGFLQQSKRHRRIEEVGNEYFCELVSRSFFQQPRRGKS
CFLMHHLVNDLAQFVSGTFSIRMECSNSNEIKERTRHLSHIIADSSSYVNLKDVSKANCLRTFLQIRPVGTSIDLFNNMPN
DLLTKLRSLRVLSLVGTHIYSLPNSVGELKHLRYLEVADTEIVRLPESICSLFNLQTLKLVGCHNLIELPASIHKLVNLRHLDIR
GTSLRWMPLQINELNSLQNLSDFFVGKGCGSSLGELGELICLHGELFIHCLEHIVSDKDCEKAKLKEKHGLEKLSLDWCRN
GETENSQKEKTILNSLQPHTNLKKLDIYDYPGTEFPEWLGDHSFYNLVSLMLNGCKYCYRLPPLGQLPMLKELQISKFEGL
VSVGSEFLGNRTSYLTNCFPALEILRIESMPLWEKWYPNAENAGSKAFFHLREIHIGNCPKLRGNLPDNLPSLTLLVIRDC
KRLLCSLPNSPSLRVLNIQNCESLEFKVHSPLCHQSLTSLFLHGSCDSLVFLPLDLFPNIKSLDIWGCKNLEALTVSESDATR
PNLKSLHSLRIRHCPNFTSFPKGGFAASKLTLLTINYCQKLNSLPEQMHDLMPSLKEVQLRGCPKIESSSTRPLRIRICSKH
MEGKQNLSDPLFARLKGLATDQSPSSS

>Vang06g21880.6

MGAIFNVLLERIASTEVVNFFKNKNCEKLLKRLKIILLSVNVVLNDAEEKQMKNGAVKEWLEELKDVAFAAEDLLDEIYTD
AKIKAKQVNTLHSGPMSFYCKGVEEKIEDVHERLEFIMRQKEVLDLKVGKEVKMAHKTPTSSVMEACDVFGRDNDKES
LVDLVLTHNEKIGIIPIVGMGGIGKTTLAQLIYNDQRVQKEFDVKAWIYVSEEFDICKITKTLLEAVTSCSCDVEDLNFLQR
DLKMHVMNKKFLFVLDDVWNENYDNWDKFRSPFKHAGEHGSKIIVTTRSGCVASIMQTVSPYNLRELSNEDSWNLFS
KHAFDYGDSSLQLHQSLDKVGREIVRKCKGLPLAVKTLAGLLRCKSDRQEWCKVLDSEMWDLQDSESNILPALRLSYHY
LPSHLKRCFAYCSIFPKDYEFEKENLVLLWMAEGFLQQSKRHRRIEEVGNEYFCELVSRSFFQQPRRGKSCFLMHHLVND
LAQFVSGTFSIRMECSNSNEIKERTRHLSHIIADSSSYVNLKDVSKANCLRTFLQIRPVGTSIDLFNNMPNDLLTKLRSLRVL
SLVGTHIYSLPNSVGELKHLRYLEVADTEIVRLPESICSLFNLQTLKLVGCHNLIELPASIHKLVNLRHLDIRGTSLRWMPLQI

NELNSLQNLSDFFVGKGCGSSLGELGELICLHGELFIHCLEHIVSDKDCEKAKLKEKHGLEKLSLDWCRNGETENSQKEKTI
LNSLQPHTNLKKLDIYDYPGTEFPEWLGDHSFYNLVSLMLNGCKYCYRLPPLGQLPMLKELQISKFEGLVSVGSEFLGNR
TSYLTNCFPALEILRIESMPLWEKWYPNAENAGSKAFFHLREIHIGNCPKLRGNLPDNLPSLTLLVIRDCKRLLCSLPNSPSL
RVLNIQNCESLEFKVHSPLCHQSLTSLFLHGSCDSLVFLPLDLFPNIKSLDIWGCKNLEALTVSESDATRPNLKSLHSLRIRH
CPNFTSFPKGGFAASKLTLLTINYCQKLNSLPEQMHDLMPSLKEVQLRGCPKIESSSTRPLRIRICSKHMEGKQNLSDPLF
ARLKGLATDQSPSSS

>Vang06g21880.7

LLKRLKIILLSVNVVLNDAEEKQMKNGAVKEWLEELKDVAFAAEDLLDEIYTDAKIKAKQVNTLHSGPMSFYCKGVEEKIE
DVHERLEFIMRQKEVLDLKVGKEVKMAHKTPTSSVMEACDVFGRDNDKESLVDLVLTHNEKIGIIPIVGMGGIGKTTLA
QLIYNDQRVQKEFDVKAWIYVSEEFDICKITKTLLEAVTSCSCDVEDLNFLQRDLKMHVMNKKFLFVLDDVWNENYDN
WDKFRSPFKHAGEHGSKIIVTTRSGCVASIMQTVSPYNLRELSNEDSWNLFSKHAFDYGDSSLQLHQSLDKVGREIVRK
CKGLPLAVKTLAGLLRCKSDRQEWCKVLDSEMWDLQDSESNILPALRLSYHYLPSHLKRCFAYCSIFPKDYEFEKENLVLL
WMAEGFLQQSKRHRRIEEVGNEYFCELVSRSFFQQPRRGKSCFLMHHLVNDLAQFVSGTFSIRMECSNSNEIKERTRH
LSHIIADSSSYVNLKDVSKANCLRTFLQIRPVGTSIDLFNNMPNDLLTKLRSLRVLSLVGTHIYSLPNSVGELKHLRYLEVAD
TEIVRLPESICSLFNLQTLKLVGCHNLIELPASIHKLVNLRHLDIRGTSLRWMPLQINELNSLQNLSDFFVGKGCGSSLGELG
ELICLHGELFIHCLEHIVSDKDCEKAKLKEKHGLEKLSLDWCRNGETENSQKEKTILNSLQPHTNLKKLDIYDYPGTEFPEW
LGDHSFYNLVSLMLNGCKYCYRLPPLGQLPMLKELQISKFEGLVSVGSEFLGNRTSYLTNCFPALEILRIESMPLWEKWYP
NAENAGSKAFFHLREIHIGNCPKLRGNLPDNLPSLTLLVIRDCKRLLCSLPNSPSLRVLNIQNCESLEFKVHSPLCHQSLTSL
FLHGSCDSLVFLPLDLFPNIKSLDIWGCKNLEALTVSESDATRPNLKSLHSLRIRHCPNFTSFPKGGFAASKLTLLTINYCQK
LNSLPEQMHDLMPSLKEVQLRGCPKIESSSTRPLRIRICSKHMEGKQNLSDPLFARLKGLATDQSPSSS

>Vang06g21880.1

MELVAGPLMGAIFNVLLERIASTEVVNFFKNKNCEKLLKRLKIILLSVNVVLNDAEEKQMKNGAVKEWLEELKDVAFAAE
DLLDEIYTDAKIKAKQVNTLHSGPMSFYCKGVEEKIEDVHERLEFIMRQKEVLDLKVGKEVKMAHKTPTSSVMEACDVF
GRDNDKESLVDLVLTHNEKIGIIPIVGMGGIGKTTLAQLIYNDQRVQKEFDVKAWIYVSEEFDICKITKTLLEAVTSCSCDV
EDLNFLQRDLKMHVMNKKFLFVLDDVWNENYDNWDKFRSPFKHAGEHGSKIIVTTRSGCVASIMQTVSPYNLRELSN
EDSWNLFSKHAFDYGDSSLQLHQSLDKVGREIVRKCKGLPLAVKTLAGLLRCKSDRQEWCKVLDSEMWDLQDSESNIL
PALRLSYHYLPSHLKRCFAYCSIFPKDYEFEKENLVLLWMAEGFLQQSKRHRRIEEVGNEYFCELVSRSFFQQPRRGKSCF
LMHHLVNDLAQFVSGTFSIRMECSNSNEIKERTRHLSHIIADSSSYVNLKDVSKANCLRTFLQIRPVGTSIDLFNNMPNDL
LTKLRSLRVLSLVGTHIYSLPNSVGELKHLRYLEVADTEIVRLPESICSLFNLQTLKLVGCHNLIELPASIHKLVNLRHLDIRGT
SLRWMPLQINELNSLQNLSDFFVGKGCGSSLGELGELICLHGELFIHCLEHIVSDKDCEKAKLKEKHGLEKLSLDWCRNGE
TENSQKEKTILNSLQPHTNLKKLDIYDYPGTEFPEWLGDHSFYNLVSLMLNGCKYCYRLPPLGQLPMLKELQISKFEGLVS
VGSEFLGNRTSYLTNCFPALEILRIESMPLWEKWYPNAENAGSKAFFHLREIHIGNCELVAGPLMGAIFNVLLERIASTEV
VNFFKNKNCEKLLKRLKIILLSVNVVLNDAEEKQMKNGAVKEWLEELKDVAFAAEDLLDEIYTDAKIKAKQVNTLHSGP
MSFYCKGVEEKIEDVHERLEFIMRQKEVLDLKVGKEVKMAHKTPTSSVMEACDVFGRDNDKESLVDLVLTHNEKIGIIPI
VGMGGIGKTTLAQLIYNDQRVQKEFDVKAWIYVSEEFDICKITKTLLEAVTSCSCDVEDLNFLQRDLKMHVMNKKFLFV
LDDVWNENYDNWDKFRSPFKHAGEHGSKIIVTTRSGCVASIMQTVSPYNLRELSNEDSWNLFSKHAFDYGDSSLQLH
QSLDKVGREIVRKCKGLPLAVKTLAGLLRCKSDRQEWCKVLDSEMWDLQDSESNILPALRLSYHYLPSHLKRCFAYCSIFP
KDYEFEKENLVLLWMAEGFLQQSKRHRRIEEVGNEYFCELVSRSFFQQPRRGKSCFLMHHLVNDLAQFVSGTFSIRMEC
SNSNEIKERTRHLSHIIADSSSYVNLKDVSKANCLRTFLQIRPVGTSIDLFNNMPNDLLTKLRSLRVLSLVGTHIYSLPNSVG
ELKHLRYLEVADTEIVRLPESICSLFNLQTLKLVGCHNLIELPASIHKLVNLRHLDIRGTSLRWMPLQINELNSLQNLSDFFV
GKGCGSSLGELGELICLHGELFIHCLEHIVSDKDCEKAKLKEKHGLEKLSLDWCRNGETENSQKEKTILNSLQPHTNLKKLD
IYDYPGTEFPEWLGDHSFYNLVSLMLNGCKYCYRLPPLGQLPMLKELQISKFEGLVSVGSEFLGNRTSYLTNCFPALEILRI

ESMPLWEKWYPNAENAGSKAFFHLREIHIGNCPKLRGNLPDNLPSLTLLVIRDCKRLLCSLPNSPSLRVLNIQNCESLEFK
VHSPLCHQSLTSLFLHGSCDSLVFLPLDLFPNIKSLDIWGCKNLEALTVSESDATRPNLKSLHSLRIRHCPNFTSFPKGGFA
ASKLTLLTINYCQKLNSLPEQMHDLMPSLKEVQLRGCPKIEELQISKFEGLVSVGSEFLGNRTSYLTNCFPALEILRIESMPL
WEKWYPNAENAGSKAFFHLREIHIGNCPKLRGNLPDNLPSLTLLVIRDCKRLLCSLPNSPSLRVLNIQNCESLEFKVHSPLC
HQSLTSLFLHGSCDSLVFLPLDLFPNIKSLDIWGCKNLEALTVSESDATRPNLKSLHSLRIRHCPNFTSFPKGGFAASKLTLL
TINYCQKLNSLPEQMHDLMPSLKEVQLRGCPKIESSSTRPLRIRICSKHMEGKQNLSDPLFARLKGLATDQSPSSS

>Vang06g21880.2

LKVGKEVKMAHKTPTSSVMEACDVFGRDNDKESLVDLVLTHNEKIGIIPIVGMGGIGKTTLAQLIYNDQRVQKEFDVKA
WIYVSEEFDICKITKTLLEAVTSCSCDVEDLNFLQRDLKMHVMNKKFLFVLDDVWNENYDNWDKFRSPFKHAGEHGSK
IIVTTRSGCVASIICFAYCSIFPKDYEFEKENLVLLWMAEGFLQQSKRHRRIEEVGNEYFCELVSRSFFQQPRRGKSCFLMH
HLVNDLAQFVSGTFSIRMECSNSNEIKERTRHLSHIIADSSSYVNLKDVSKANCLRTFLQIRPVGTSIDLFNNMPNDLLTKL
RSLRVLSLVGTHIYSLPNSVGELKHLRYLEVADTEIVRLPESICSLFNLQTLKLVGCHNLIELPASIHKLVNLRHLDIRGTSLR
WMPLQINELNSLQNLSDFFVGKGCGSSLGELGELICLHGELFIHCLEHIVSDKDCEKAKLKEKHGLEKLSLDWCRNGETE
NSQKEKTILNSLQPHTNLKKLDIYDYPGTEFPEWLGDHSFYNLVSLMLNGCKYCYRLPPLGQLPMLKELQISKFEGLVSV
GSEFLGNRTSYLTNCFPALEILRIESMPLWEKWYPNAENAGSKAFFHLREIHIGNCPKLRGNLPDNLPSLTLLVIRDCKRLL
CSLPNSPSLRVLNIQNCESLEFKVHSPLCHQSLTSLFLHGSCDSLVFLPLDLFPNIKSLDIWHCPNFTSFPKGGFAASKLTLL
TINYCQKLNSLPEQMHDLMPSLKEVQLRGCPKIESSSTRPLRIRICSKHMEGKQNLSDPLFARLKGLATDQSPSSS

>Vang06g21880.3

MGAIFNVLLERIASTEVVNFFKNKNCEKLLKRLKIILLSVNVVLNDAEEKQMKNGAVKEWLEELKDVAFAAEDLLDEIYTD
AKIKAKQVNTLHSGPMSFYCKGVEEKIEDVHERLEFIMRQKEVLDLKVGKEVKMAHKTPTSSVMEACDVFGRDNDKES
LVDLVLTHNEKIGIIPIVGMGGIGKTTLAQLIYNDQRVQKEFDVKAWIYVSEEFDICKITKTLLEAVTSCSCDVEDLNFLQR
DLKMHVMNKKFLFVLDDVWNENYDNWDKFRSPFKHAGEHGSKIIVTTRSGCVASIMQTVSPYNLRELSNEDSWNLFS
KHAFDYGDSSLQLHQSLDKVGREIVRKCKGLPLAVKTLAGLLRCKSDRQEWCKVLDSEMWDLQDSESNILPALRLSYHY
LPSHLKRCFAYCSIFPKDYEFEKENLVLLWMAEGFLQQSKRHRRIEEVGNEYFCELVSRSFFQQPRRGKSCFLMHHLVND
LAQFVSGTFSIRMECSNSNEIKERTRHLSHIIADSSSYVNLKDVSKANCLRTFLQIRPVGTSIDLFNNMPNDLLTKLRSLRVL
SLVGTHIYSLPNSVGELKHLRYLEVADTEIVRLPESICSLFNLQTLKLVGCHNLIELPASIHKLVNLRHLDIRGTSLRWMPLQI
NELNSLQNLSDFFVGKGCGSSLGELGELICLHGELFIHCLEHIVSDKDCEKAKLKEKHGLEKLSLDWCRNGETENSQKEKTI
LNSLQPHTNLKKLDIYDYPGTEFPEWLGDHSFYNLVSLMLNGCKYCYRLPPLGQLPMLKELQISKFEGLVSVGSEFLGNR
TSYLTNCFPALEILRIESMPLWEKWYPNAENAGSKAFFHLREIHIGNCPKLRGNLPDNLPSLTLLVIRDCKRLLCSLPNSPSL
RVLNIQNCESLEFKVHSPLCHQSLTSLFLHGSCDSLVFLPLDLFPNIKSLDIWGCKNLEALTVSESDATRPNLKSLHSLRIRH
CPNFTSFPKGGFAASKLTLLTINYCQKLNSLPEQMHDLMPSLKLNSLQPHTNLKKLDIYDYPGTEFPEWLGDHSFYNLVS
LMLNGCKYCYRLPPLGQLPMLKELQISKFEGLVSVGSEFLGNRTSYLTNCFPALEILRIESMPLWEKWYPNAENAGSKAF
FHLREIHIGNCPKLRGNLPDNLPSLTLLVIRDCKRLLCSLPNSPSLRVLNIQNCESLEFKVHSPLCHQSLTSLFLHGSCDSLVF
LPLDLFPNIKSLDIWGCKNLEALTVSESDATRPNLKSLHSLRIRHCPNFTSFPKGGFAASKLTLLTINYCQKLNSLPEQMHD
LMPSLKEVQLRGCPKIESSSTRPLRIRICSKHMEGKQNLSDPLFARLKGLATDQSPSSS

>Vang0250s00070.1

MVDSEIWCLRDHWREKVREAYGRYGDNQLYSRRKIDIELHLENCTKALQAAIAIPRKAVSIPSFQKLKAYNRIESLVEYW
SIALCEAAEISRLAVQHYRGITDNEINYIKKHASDALHEAAGISGVVILNSRNESEAVKNIVKNVTSLLDKTEFFVAKNPVG
VESRVQEMVQLLEQKQSNDVLILGVWGMGGIGKTTIAKAIYNKIGRNFEGRSFLADIREQLNALCGNREWFGSGSRIIIT
TRDIHLLRAKRVDQVCAMTGMNVDESIELFSWHAFKQASPKEDFIELSRNVVAYAGGLPLALEVLGSYLFDMEITEWKS
VLEKLRKIPNDEVQEKLKISYDGLSDDTEKGIFLDIACFFIGKDRNDVIHILNGCGLFAENGIRVLVERSLVTVDDKNQLGM

HDLLRDMGREIIRSKSPMELEERSRLWFHEDVLDVLSKETGTKFIEGLTLKLPRTNTKSLCTKAFMNMKKLRLLQLSGVEL
VGDFEYLSKDLIWLCWHGFPFAFIPTSFYQGSLVSIELENSKITMVWKATQIDKLEEDIEQMESLTTLVADKTAIRRVPFSI
VRSKSIGYISLCGYEGFSRDVFPSIIWSWMSPVNSLSSRVETLVDMSSLVSLDVQNSSSNQLSYIYEELPKLQSLWIECGSD
LQLSRDITSILDVLNATNSEESESYGTTSQMHNVFTLIECNSRSKLFEKRLLIQMGRSSEITHILKQGILQNITTSDGGDCLLP
GDYYPDWLTFSNEGSSVTFEIPEVNGRSLKTMMCHIHYSSSDSITSDGLKNLSVINHTKSTIQLYKRNALASFDDEEWQR
VLSNIEPGNKVQIIVVFWSRITVNKTSIYLIYEAIDEKVEHYHAPNMNVPRSISPGVESMEDLRGSSVKSLTKRLLNKFLSRK
YFCKGKVEK

>Vang0693s00030.1

MAQKQAPSSASFMSKYSYDVFLSFRGADTRFGFTGNLYSALSQRGIFTFIDDEALRKGEEITPSLRKAIQESRISIIVFSKNY
AFSAFCLDELLHIIECHKNQNMWILPVFYDVEPSQVRHQTGSYQEAFAKHGERFKDDIQKLQQWRLALRHAADLAGFP
FKTGKEYESEIVKKITEEISTKLNRPPLHIADYPVGLKVRMQQIQELMGGEFDKKVTMLGIHGMGGIGKSTLSRAMYNL
MAHQFEASHFLANVREKSDKDGLVHIQETMLSELVGERNIKLGDVHRGIPILQHRLCGKKVLLVLDDISKKEQLHATAG
GLDWFGPGSIIIITTRDKHLLDVHGVQKQYMVGGINFMEALELFKWNAFKNKEVDPCYKEVTERAMYYANGLPLALETI
GSNLFGKTLDEWESALETYERIPNRDVQEVLKVSYDSLDAYEKEIFLDIACFFRRCSLGYVTDKLEARGFPPKFGLRVLEEK
SLIKIRECPHETVTMHDMIRCMGKEIVRQQKPLPHKRNRMWFYEDVVCVLEKNMENDKIEAVMLEMPEHQEEMQCT
PKFGKMKSLRMLIIEKKDSFLRSPATLPNSMRVLEWRGYPATSLQESCHTQLVTQLLWMGQTTTGILVILQNSKVLRHLI
LRGCKNIRRIPDISGFPNLTKLCVGECTNLFEIHDSVGSLLNLKEFCAEGCTKLTIGPSRIKLISLEHLCLRGCSSLVMFPEVLA
PMHKLKYVNLGGTGIRNLPLSTQNLEGIRVLSLGKGKMLEINESSNFFQNLPMFFPNLIKLCLQNLDITILPASIEECHFLKI
LHAHDCKKLQEIRGLPLSIKDFSAANSSVEANSLTLKLRQAIDYGAMGICVLPGRKIPELFDHSSRGNSVSFWFRKELPTLA
VCAIIGVWDNVKPPFVAFFSFVVGVEKNIYKCVCCFRCGNIRWTTEDSHIIILNFQNDFQHTLNTDIQTVLLTNEWIPGEI
LLTIHPKSDLGKSGEIRRTGVYVNRTLSRMEDVRFEDPYDLNKASTMENKLVLLGEASDTQQQAQSSALFNLTTSLESVI
GEQPLNYEYNNSDSKESASTVDSNNQGVLVDDQPILAPSSAETQNAEGKREMAQLILEVSREFTAQVSMKIESTASQSP
KVDEVEAGLETQNQFRTLENQIHQRLTRIKKMKEELGEKFSAIKADISAIEANNSSIKAKISDIKAIFLQTDQSQTEAYKAAS
HLASVTPPPVMLSRGIGWQDCDRENVELLGSNRIGNRKVIEVSASFMIFFSMLGKFGALFASIPFPMFVAIYYVLFGIVE
NTLSGSFMVLQFNDFLNTIFYSSPTVALIVAVFLDNTLDYKDNVKDRGMPWWTKFRTFK

>Vang0103s00270.1

MALSVVGGALLSAFIEVVFDKLASPELVNFIRWKKPDKLLQKMRSQLLVVKVVLDDAEKRQITDSNVKEWLDLLNDVVY
EVDDLLDEVSTKAATQKEVTNSFSHLFNRKKIFSISKLEDILGRLDDILRQKQNLDLKDIPVENNQPWKPQPTSLEDRYHIY
GRYEDKETIMKLVLEDSSDGEEVSVIPIVGMGGVGKTTLARSVYNDGKLKQIFDLKAWVCVSDIFDIVKVTKTMIEEIIQK
PCKLNDLNSIQLDLWDKLRGKRFLIVLDDVWMEDCDSWSSLTKPFLSGIRGSKVLMTTRNENVAAAVPFHSVKVYHLN
KLSNEDCWLVFANHAFPVSRGSGNRGTLEKIGKEIVKKCNGLPLAAQSLGGMLRRKHAIRDWNNVLESDIWELPESQC
KIIPALRISYNYLPPHLKRCFVYCSLYPKDYKIQKDELILLWMAEDLVKAPKKGKTLEQVGKEYFDELVSRSLFQSSSHRTSG
NYFLMHDLMHDLATFLGGEFYFRADELSKGTKINRKTRHLSFTRFSDPVSDIEIFETVKFSRTFLPINYKDSPFNNEKAPRII
GSMLKYLRVLSFRDFQSMLALPDSIGELIHLRYLNLSYTGIATLPESLCNLYNLQTLKLYCCSELTKLPGAMQNLVNLRHLEI
LNTSIKEMPKAMRKLNQMQNLDFYIAGKHIENSIKELRGLPNLHGSFCIQKLENVTQGDEALEARLMDKKHINDLSLEW
SIRNDNSTNFQIELDVLSNLQPHRDLKSLSISGYKGTRFPEWMENCSYYCMTILSLHNCNNCSKLPSLGQLLSLRRLHISN
MISVKTIDAGFYKKNDCSSVTPFPSLESLYIYNMPCWEAWVAFHSEAFLVLKDLYIQNCPKLKGDLPDHLPALQTLAIRN
CELLVSSVPGTPTLRTLEISESNKVAFYSFPLLVERIEIEGSPVVESMMEAITNIQPTCLHHLSIKDCSSDISFPGDHLPISLKT
LIISGLNKLKFPMQHKHELLESLSVNSSCDSLTSLPLVSFPNLIRLQITNCENMESLSVLGSDSFKSLSSFEIGRCPNFVSFSG
EALSAPNLTRFIVYDCDKLKSLPNQMCTLLPKMEYLSISNCQQLESFPEGGMPPNLRIVEINNCEKLLSGQPWVSKDIFTY
LKVWGPCDGINSFPKEGLLPPSLTSLQLFGFSSLETLECKGLLHLTSLRELHIQSCKKLANIVGERLPISLIKLSINRCPLLQKR
CHIKDRQIWPKISHVRGINIDGRWIQ

>Vang0039ss01030.1

MAITLGEGASSSSGFDRGWTYDVFLSFRGEDTRRSFTGFLYHGLCQRGINVFIDDEKLRRGEDLSPALLGAIQESRIAIIVF
SQNYAFSTWCLDELAKIIDCYKTRGQLVWPVFFHVDPSVVRHQRGTFQTAMAQHEVRFKGNVQKLQKWKKALFEAS
NFSGWTLENGYEFQIIQDIVEEASRKLSHTILHIAEYPVGIETRISEVMPLLQIEPGEDIRVIGIYGLGGIGKTTIARALYNMI
AGQFEATSFLADIRESSSQRQGLVQLQESLLFDTVGDKNIKLGSIYKGIPIIKKRLCCKKVLLIIDDVDRLEQLQALAGGRD
WFGFGSVIIITTRDKHLLSAHQVDKTYEVKKLNYGEAFELFTWSAFKRKTPDAGYLEVSNHVVLYAEGLPLALKIMGSNLF
GKTVEEWKSALGKYEKIPNKEVQNVLRVTYDNLEENEKGIFLDVACFFKGETVEYVEKTLQARGFYPTIGISVLIDRSLVSV
DEYNRLRMHDLIQDMGKEIVREVSPLEPGKRSRLWYHEDVLEVLTENKGTYRIQGMMVDLPDGYMVHLKDDSFKKM
KNLKILIVRNGNFFGSPQHLPNSLRLLDWMKYPSSLPSSFQPKKLVVLNLSGSRFTMQEPFMYLDSLTSMDLSSCELLTKL
PEIAGVPNLTQLTLDYCTNLEEVHESVGFLEKLVEFRAYGCTKLKVFPNAIRLTSLRSLILNWCSSLQNFPAILGKMDNLITI
SIEGTGIKELPPSIGNLVGLQELSMTSCLSLKELPHNFDMLQSLTNLDMDGCPQLRNFLTKLANMGESTHTFGNILSLNLE
NCGLIDEDLPIIFNSFPNLASAVLSRNNFEALPSCIQQCPCLELLHLDNCKKLQEIPAFPPNMQYINAQNCTSLSVESSNLLL
NKETFEGWELQAMVPGTVVPEWFDHITKGEYMTFWVREKFPAIIICFVLEVESEMKKIFNCEICFYINGEEIYELEIPRGFS
DMVTDHVWLYDLRTHSSINWRSLDLYLMDDWNQVEISCEKISGASNVTVSWCGVHVVKQEANMKDILLTDPDPDLD
SVIASGSNTLVFDHPVKAQLQSQVTSFILQTPQNNNSSTIVLPTTVQTSLTVNDADMEAFYAVLDDEISVVSLNNDSTVV
SKLTNQRPSEETKKALKSLQAHVTKEFSALLGPNEYSTMNDTLEYLTNLPAEDGISVEIRSLIIQVSRQFTRWSRDYTSENK
KIESTTSKLLKADELEKCLEANKTNFKQVMCMENELCNDLAYLEQRKRELEEQIKAVKANISASEAAKNMATQIKRKIFGE
AKILKAQRDELREQVPHLRDEQELAKKIQSNIRDEWSKLGEKFNYELRHGKID

>Vang0137s00090.1

MACNKRQRSSSSHTNNFDVFVSFRGADTRNGFTNHLFAALQRKGVVAFTDDQTIQKGDFLESELLLAIEGSRVFIVVFS
KDYASSTWCMKELTKIVDWVEVTGRSLLPIFYDVTPSEVRKQSGEFAKAFAEHEERFKDDLEMVKEWRAALKTSCDRC
GWDVQNKQQYEEIENVVEEVINILGRDQIWNFGDDLVDMHSRVKKLEELLDLSANDIVHLVGIVGMGGIGKTTLATAL
FNKISPQFNACCYHDDLSKIYCNFGAASAQKQLLCQALNQGNIEIHNASHGTMLIRTRLCHLKALVVADNVDQVEQLKK
LGLQSEYLGAGSRIIIISRNCRILQNYGVNKVYEVEVLDKTQSLQLLRXKAFRSNDIGKEHKGLTLDILKYVNGLXLAIEVLGS
FLLDRDVCEWRSALTRMEENPSKDIMDXLRISYDGLENIEKEIFLDIACFFSNNNSYSWEPTVKKFLDYRQFYPDIGMKVL
IEKSLISRQNGNIKMHDLLKELGKSIVREKAPKEPRKWSRLWNYKDLQKVMKINKEAKNVEAIFIEQHEKEFLQGRIRVDT
LSKMDQLELLILKNVNCYGTLNFISNELRYLFWNHFPWLSLPSTFFPDQLVELILPHSNIKQLWEGKKDYLVPRMEIVIPGI
EIPKWFSKQNASTSISMDPSAVIDDPNWIGVSICALFVTHEGPVNLDERDYPTNDTLYYGVNNFINFGLKMYSRVSLKFK
KDLVTVGLDYLLIVFYSRQDFIHLLNGHSDTMHDLQAVKFETWVESLPGLRFMVKKCGYRWVFKEDLQQFNSDKFFSR
NSSSRKRKLLTSE

>Vang0291s00070.1

MALAAVGGALLSAFFDVLFDRLASPEVLNFIRGKKPDKLLRKVKTQLIVVRVVLADAEERQITDSNVKEWLXLLRDVVYE
VDDLLDEVSTKAATQKEVSNSFSRLFKTKKIVSISKLEDIVERLDDILKQKESLDLKEIPVESYRPWKAQPTSLEDGYAMYG
RDNDKEAIMKMVSVDSINGERVSMISIVGMGGVGKTTLARSVFNDVKLNQQIFDLKAWVCVSDIFDIVKVTRSMIEEIT
RKPCKLSDLNALQLELMDKLKGKKLLIVLDDVWIEDCDNWSSLTKPFLSGNRGSKVLITTRNENVAAAVPYHTVEVYHL
NKLSNEDCWLVFANHAFPLSEASETRGTLEKIGKEIVKKCNGLPLAAQSLGGMLRRKQTIRDWNNVLQSDIWELPESQC
KIIPALRISYNYLPPHLKWCFVYCSLYPKDYEFKKDELIQLWMAEDFVKPAKKGKTSEEVGQEYFDDLVSRCFFQFASRSL
GDYFVMHDLIHDLATFLGGDFYFRTDELGKETKIDRKTRHLSFTRFSDPVSDIEVFDTVKFPRTFLLISYKDSPFNNENAPH
IIVSRLKYLRVLSLINFQSQRALPDSVGELIHLRYLNLSRTSIETLPESLCNLCNLQTLKLSFCSKLTKLPSAMQNLVNLRHLEI
GDSSIKEMPKRMGKLNQLQNLEFYIVGNYKENSIKELGGLPNLGGSFSIKALENVTNGEEALEARIMDKNHISHLSLEWSI
ANDNSIDFKIELDVLSNLEPHQDLKSLSIKGYKGTRFPEWVGNFSYRYMTRIDLYNCNNCCMLPLLGQLPSLCELIISNMN
SVKTIDAGFYKNDDCSSVTPFPSLEDLCIYNMPCWEVWNDFDSEAFPLLKNLYIEECPKLKGDLPNHLPALQTLTIRNCEL

LVYSVPGPLTLRTLEIRKSDKVTFHEFPLLVEGIDLEGGPVVESMMEAITIIQPTCLESLFLQNCSSAISFPSDLLAASLKSLNI
SGLTKMKFPMQHKHKLLKSLSINNSCDSLTSLPLAIFPNLTSLKITNCENMESLLVSGSELSMIEVVARSYEYLYVSNCQQI
ESFPGGGMPPNLRTVEISNCEKLLRGQGWKSMDMVTSLDVWGPCDGIKSFPKESLLPPSLVSLHLFDLSSLETLDCKGL
QHLTSLQELNIE

>Vang0197s00010.3

MDTIASVASSVAAPLLRNITYVLMYSTYLTELEAEIKRLQSEEKEVRHTVEAAKRGGEEIEDTVRDWFDRVRAAVEQGQA
FLEEEERERVGCMDVYSKYTNSQRARTLVEIVREVRKETFDRVSYRRALRCNVGPAAREYVAIQSRTVMLNDVVKMLKD
GGVDIVGVYGVAGVGKTAMVKELAWQVEKDGLFDVVVVATVTDSLDVGRIRNEIADGLGLKFDELTELGRASRLRQRI
QQEQRILVVLDDLWGKLDLTKIGVPFGEDNKGCRCQLLVTSRNRNVLSSNFGSGKFCRLEVLSDDESWELFEKRAGDAV
RDPSIQSVAEKVAKSCGGLPLLIVTVVEELKNKDLYAWKDALEQITSFELEGCLYSPLRSAIELSYDHLESQELKTFFLLLGS
MGNGCSTRDLLVFGWCLGLHKQVDSLADGRNRLYKLIDNLRAACLLLDEGKRDSVVALEVVRHVANSIATRVKPFFTV
QRNKEFKWPRMDFLRSCHHIFLDWCYIRELPEVLECPKLKILQINSKGNYLKIPDEFFVHMKELKVLSLGGLNCTPSLPPSL
SLLTDLQALYLCECKLEDIATVGEITSLEILNLEKSELKELPAKIGGLSNLRLLDLTDCPTLGGIPGNVISRLTSLEELYMGNCD
VQLEAKESKSQNNDSSLSELKHLNQVTILNVQIEDTSVFPRDMLSFGRLESYKILIGDGWKWSAVESENYKTSRLLKLNSG
ADPTILKDYGIKMLMNKAEELYLAELKGVREVLYELNDEGFSQLKHLCILNCAEMESIIGPTEWAYSDHAFPNLESLILHNL
INMERICSDPLPAQAFRKLQVIKVKDCDRMEFVFSHSMVKHLSELVEIEISECKSMTNILSGQRQEDADAGQTNMIRLIN
LRSLTLQCLPSLVSLSPDSSTEASENGNGFSSQLFSNKVEFPNLETLKLYSINIHMIWINHHHSYFENLTSLTVDGCERLTYI
FSYPVAVKLVKLQHLLLSSCKFVEKIFVPDENLGHIHHFRKSTQTELVPIFPNLETFVISQMDNLKAIWPALLPQNSFCKLK
KMEITSCNNLLNVFPCHVLDKLQSLESLNVWKCMALEVVYEIDGINTEQEGSSQEGLDIPLRTLSLGNLPKLKHLWNKDP
QGNIKFQNLFMVQASKCQSLEYVFPLSLAKDLLHLQFLEISDCGVEEIIASDKGGIGAALGFVFPKLVSIKFFNLPDLRCFC
DGNHNLRFPLLNQFFAVECPRMETFSRGILRASILRKIHLTRKGDQWYWQGDLNTTIRKLFNRDPQT

>Vang0197s00010.2

MDTIASVASSVAAPLLRNITYVLMYSTYLTELEAEIKRLQSEEKEVRHTVEAAKRGGEEIEDTVRDWFDRVRAAVEQGQA
FLEEEERERVGCMDVYSKYTNSQRARTLVEIVREVRKETFDRVSYRRALRCNVGPAAREYVAIQSRTVMLNDVVKMLKD
GGVDIVGVYGVAGVGKTAMVKELAWQVEKDGLFDVVVVATVTDSLDVGRIRNEIADGLGLKFDELTELGRASRLRQRI
QQEQRILVVLDDLWGKLDLTKIGVPFGEDNKGCRCQLLVTSRNRNVLSSNFGSGKFCRLEVLSDDESWELFEKRAGDAV
RDPSIQSVAEKVAKSCGGLPLLIVTVVEELKNKDLYAWKDALEQITSFELEGCLYSPLRSAIELSYDHLESQELKTFFLLLGS
MGNGCSTRDLLVFGWCLGLHKQVDSLADGRNRLYKLIDNLRAACLLLDEGKRDSVVALEVVRHVANSIATRVKPFFTV
QRNKEFKWPRMDFLRSCHHIFLDWCYIRELPEVLECPKLKILQINSKGNYLKIPDEFFVHMKELKVLSLGGLNCTPSLPPSL
SLLTDLQALYLCECKLEDIATVGEITSLEILNLEKSELKELPAKIGGLSNLRLLDLTDCPTLGGIPGNVISRLTSLEELYMGNCD
VQLEAKESKSQNNDSSLSELKHLNQVTILNVQIEDTSVFPRDMLSFGRLESYKILIGDGWKWSAVESENYKTSRLLKLNSG
ADPTILKDYGIKMLMNKAEELYLAELKGVREVLYELNDEGFSQLKHLCILNCAEMESIIGPTEWAYSDHAFPNLESLILHNL
INMERICSDPLPAQAFRKLQVIKVKDCDRMEFVFSHSMVKHLSELVEIEISECKSMTNILSGQRQEDADAGQTNMIRLIN
LRSLTLQCLPSLVSLSPDSSTEASENGNGFSSQLFSNKVEFPNLETLKLYSINIHMIWINHHHSYFENLTSLTVDGCERLTYI
FSYPVAVKLVKLQHLLLSSCKFVEKIFVPDENLGHIHHFRKSTQTELVPIFPNLETFVISQMDNLKAIWPALLPQNSFCKLK
KMEITSCNNLLNVFPCHVLDKLQSLESLNVWKCMALEVVYEIDGINTEQEGSSQEGLDIPLRTLSLGNLPKLKHLWNKDP
QGNIKFQNLFMVQASKCQSLEYVFPLSLAKDLLHLQFLEISDCGVEEIIASDKGGIGAALGFVFPKLVSIKFFNLPDLRCFC
DGNHNLRFPLLNQFFAVECPRMETFSRGILRASILRKIHLTRKGDQWYWQGDLNTTIRKLFNRDPQT

>Vang08g00650.1

MGSKKDNQAPRMNWSTPFSKVVVEVLSFVWRHSVRHVTYIVRYKQSVHELKDSVKDLENEKDKIDHQSDEADKNLK
NIEGKVTEWSRKVSEIKTTVEVFENDDGHKRARSLNCYVFPYLWNRHRLGRQATKMEMGVKKIIDESPKLDEISYRENV

TSNDATLSNYGFEEFGSTKSTMEKVMRQLENSSVRMVGLYGEGGVGKSALIKEIARIARDKKLFNVVVKVEITANPNLQ
SIQEEIAYVFGLQLEGEGENKLSGIHNMSDSRKEIVKKYCAGLPMAIITVAKALRGKSELVWEAALGKLEKKELVGVQTY
MDISVKMSYDHLENEEIKSIFLLCAQMGHQPLIMDLVKCCFGLGILEGVFSLWEARDKIKITIQKLKDSGLLLDGNSDIHF
NMHDIVRDAALSIANKEKNVFTLRNGKLDEWPELKRCTSMSICNCDIIDELPIVNCSRLKFFQIDTNNQSLTIPDKFFEGM
KNLKVLILAGFHLKRFPPSIKSLLKLRMLCLERCTLEDNLSIGELKKLRILSFSGSQLKSLPTELGYLDKLQLLDITDCSILEINIP
PNILSSLTHLEELYIRKSLIKMLVEGGQSHGQNSFLSQLKNLHQLKVVDLSIPCFSVLPNHLFYDKLKDYKIVVGDVEMFSVI
GFKMLDKYETFRVLALQLNHDTNIDSQEDINLLFKTAQSLLLGKVEVVKVVNELNIDGFPDLKHLSIIDSNAIIYVNSMKLS
NCINVFPNLESLCLYNLKNLEMIFYGPLTVASFAKLKSIKVNMCVRLVTLYSVYIVELPNSEEPCEIIECNSYLDKFCATLEIVE
VSECEALKEILLIPMNCDKVKFLKLHTLTFQSLPSFTCFYTEVEESCWPHPTKPQAKNSGSEEDQKSFNPPPLFGEQVEIPN
LESLNLCSLNIHKIWSDQLSSSFCFQSLIKLVVKDCDKLTYLCSLSVASSLKKLKSLIISECPIMEKIFETKENNADKVCVFPKL
EEIHLSKMRRLRDIWHTKVNIDSFSSLISVNIEECNKLDKIFPINMEGWFESLDNLKVYKCQSVEVIFEIKDYEEIDASGGIS
TNLQVILLQDLPKLKELWSKDPHGVIHFKKLRTIDVSNCHELWNLFPTSMAKDVSKLEHISIWSCKKMVEIVSSKDTSEA
NNHPLEFSELTYVRLYLLPNIKHFYKGRHPIKCPKLKELIVSNCRKLKTFSKEISKTTEEEESFVFSAPKVLSKLEYMQIDFKEA
QNLLPKYPMHRLKELSLTSVESVDFLNRFPYRTPNLEKLKLICSSKELEPRVNFASEERLRITLELKELVLWRARINDLTGVSI
VRKLELLSLVSCNQLNNLGPSSVSLTYLTYLELYHCEKLKYLMTPSTAKNMVQLKTMKVINCPKVKEIVSNELSEEGTEMKI
VFSKLITIELVKLVNLATFCSYKDCEFEFPSLEILIVRECLKMEKFSEREAITPKLKNVFGVEGDEKSKWHWEGNLNDTIHKIF
FDKISKLNELDLKSRPGLQEIWRGALHIPDFCFSELDTLIVEDCQFLSDAVLPFHLLSLLPKLKTLEVRNCDSVKTIFPESVAK
DIVKLEALIVEHCQSLRAIVAKEYSEGEELVEDEMIFSQLIYLKVESCNSLPYLFRSSTAKCLGKLEIMAINKCKSVEEIISKKG
EESDENVEIKFEQLRDLYLEKLDGLRCFYDGNFTLSFPSLEEVHIIKCSSMKTFSAFNKIDNPLYSDLNSVLHRTSEEEAPDA
STTSNTSDIEDTGYGDD

>Vang0033ss01460.1

MSPERDVILATPGAFRLRWDVFLSFRGTDTRXTITKGLYKSLQTRGVRVFLDDEGLERGEAVANGLMKGIDDSAAFIVIL
SENYASSHWCLEELTKICDTGRLLLPVFYRVDPXQVRHVSGPFGSGFESHEKRFEKNTVSKWKEALKKVGGIAGWVFNH
RLTAGTPCRRLLICDSEEXDLIQRLVRRVLKELSNTPMGVPEFAVGLDERVEKVMEVLQVQSNGVKVLGLYGMGGVGK
TTLAKALFNALVNRFEHRCFISDVRQVSSKHDGLVSLQSKIIKDLFPGAGSPSIGDVNVGISAIKXXVSENRVLLVLDDXDE
VKQXDALIGKREWFYDGSCVXITTRDTKVLTQNHVTVSYEVRELYASEARELFSYHALRRSAPPENLLSLSEEIIFLTGKMP
LALEVFGSFLFGKRREEEWEDAVKKLRLIRPHDLQDVLKISYDGLDEEEKCIFLDIACLFVQKKMKRDGVIDVLRGCGFRG
EIAITVLVQKCLMKITPENTVWMHDQIRDMGRQIVMDESFVDPGSRSRLWDRAQIMTVLKGHKGTRCVQXIVLDFEE
ERFYKGKFGSVFPKKFQWRPSLRNISCYIKQCLKNHPEPQAEEXTEFVLHTKSFEPMVNLRXLQINNLKLQGKFLPSELK
WLQWQGCPLERMPLKSWPGELAVLDLKNSKKMETLWGWNGHNKVCFSYLVYLPESFGMLSSLRTLKMAKRPELDT
NESSFLAEPEXNHSPFVLTSSFCNLTLLTELDARAWKISGKIPDEFEKLSLLETLKLDXNDFHSLXSSLKGLSILXVALRNLQN
LSMPGSKLPEWFXGQTVSFSKRKNLELKSVLVGVIISINHSIDIPNMKRDDMPGLIDVEANVLKGGRTLFRTVLNICGVPR
TDEEHMHLCRFHDYHQLVAFLKDADTFSVSKXNPPFDTGLELRKCXVYLIFEGDDDYDGGEESLDXGLQSVSEKLAXFFS
TREGGDQVSVNGIGHAGT

>Vang0033ss01460.2

MSPERDVILATPGAFRLRWDVFLSFRGTDTRXTITKGLYKSLQTRGVRVFLDDEGLERGEAVANGLMKGIDDSAAFIVIL
SENYASSHWCLEELTKICDTGRLLLPVFYRVDPXQVRHVSGPFGSGFESHEKRFEKNTVSKWKEALKKVGGIAGWVFNH
SEEXDLIQRLVRRVLKELSNTPMGVPEFAVGLDERVEKVMEVLQVQSNGVKVLGLYGMGGVGKTTLAKALFNALVNRF
EHRCFISDVRQVSSKHDGLVSLQSKIIKDLFPGAGSPSIGDVNVGISAIKXXVSENRVLLVLDDXDEVKQXDALIGKREWF
YDGSCVXITTRDTKVLTQNHVTVSYEVRELYASEARELFSYHALRRSAPPENLLSLSEEIIFLTGKMPLALEVFGSFLFGKRR
EEEWEDAVKKLRLIRPHDLQDVLKISYDGLDEEEKCIFLDIACLFVQKKMKRDGVIDVLRGCGFRGEIAITVLVQKCLMKIT
PENTVWMHDQIRDMGRQIVMDESFVDPGSRSRLWDRAQIMTVLKGHKGTRCVQXIVLDFEEERFYKGKFGSVFPKK

FQWRPSLRNISCYIKQCLKNHPEPQAEEXTEFVLHTKSFEPMVNLRXLQINNLKLQGKFLPSELKWLQWQGCPLERMPL
KSWPGELAVLDLKNSKKMETLWGWNGHNKVCFSYLVYLPESFGMLSSLRTLKMAKRPELDTNESSFLAEPEXNHSPFV
LTSSFCNLTLLTELDARAWKISGKIPDEFEKLSLLETLKLDXNDFHSLXSSLKGLSILXVALRNLQNLSMPGSKLPEWFXGQT
VSFSKRKNLELKSVLVGVIISINHSIDIPNMKRDDMPGLIDVEANVLKGGRTLFRTVLNICGVPRTDEEHMHLCRFHDYH
QLVAFLKDADTFSVSKXNPPFDTGLELRKCXVYLIFEGDDDYDGGEESLDXGLQSVSEKLAXFFSTREGGDQVSVNGIGH
AGT

>Vang0033ss01460.3

MSPERDVILATPGAFRLRWDVFLSFRGTDTRXTITKGLYKSLQTRGVRVFLDDEGLERGEAVANGLMKGIDDSAAFIVIL
SENYASSHWCLEELTKICDTGRLLLPVFYRVDPXQVRHVSGPFGSGFESHEKRFEKNTVSKWKEALKKVGGIAGWVFNH
SEEXDLIQRLVRRVLKELSNTPMGVPEFAVGLDERVEKVMEVLQVQSNGVKVLGLYGMGGVGKTTLAKALFNALVNRF
EHRCFISDVRQVSSKHDGLVSLQSKIIKDLFPGAGSPSIGDVNVGISAIKXXVSENRVLLVLDDXDEVKQXDALIGKREWF
YDGSCVXITTRDTKVLTQNHVTVSYEVRELYASEARELFSYHALRRSAPPENLLSLSEEIIFLTGKMPLALEVFGSFLFGKRR
EEEWEDAVKKLRLIRPHDLQDVLKISYDGLDEEEKCIFLDIACLFVQKKMKRDGVIDVLRGCGFRGEIAITVLVQKCLMKIT
PENTVWMHDQIRDMGRQIVMDESFVDPGSRSRLWDRAQIMTVLKGHKGTRCVQXIVLDFEEERFYKGKFGSVFPKK
FQWRPSLRNISCYIKQCLKNHPEPQAEEXTEFVLHTKSFEPMVNLRXLQINNLKLQGKFLPSELKWLQWQGCPLERMPL
KSWPGELAVLDLKNSKKMETLWGWNGHNKVALRNLQNLSMPGSKLPEWFXGQTVSFSKRKNLELKSVLVGVIISINHS
IDIPNMKRDDMPGLIDVEANVLKGGRTLFRTVLNICGVPRTDEEHMHLCRFHDYHQLVAFLKDADTFSVSKXNPPFDT
GLELRKCXVYLIFEGDDDYDGGEESLDXGLQSVSEKLAXFFSTREGGDQVSVNGIGHAGT

>Vang0911s00020.1

MICSVMNHTIYEVTLSSSNYSGLGKKLLSLNKVEQRALNDCLDLFDETVEELKTTVADLSQSTIGSKNIWYGGIGKTTIAC
AVYKTIFWHYQGSCYLADIREKAINKHGIVQLQEILLSETLNEDIKVGDVNRGIALIKRRLQQKKVLLVLDDVDKLEQLKVL
AGGYDWFGSGSMIIITTRDKHLLDAHGVVNLYEVKPLNVENALELFTWHAFKNDKVDPPYMNISKRVSYACGLPLALE
VIGSHLFGKSLDECSSALDKYESIPHEKIHGILKVSYDGLEENEKGIFLDIACFFSTCELGNVRPMLKVHGFHVEDGLRVLV
DRFLIKIDSSCFVRMHDLIRDTGREIVRQESTLEPGMRSRLWFNEDIVHVLEENRGSDKIEFIKFEGYNNIQVQWNGKAF
KEMKNLRILIIKGSSFSIGPEHLPNSLRVLDWTCYPSPSLPSDFNPKRFEILLMPESCLQMFKPQKASLSIKDFALNMSLYN
LRETPFLTSLCLDKCSNLVNLDESIGFLDKLRFLSAKHCIKLKTLAPCIMLTSLETLDLWRCVSLEYFPEVLGKMEKIRTIYLD
GTAIDKLPFSIGNFVGLELLSLEGCKRLLQLPGSISIMSKVKVVSCGRVGYQIFEEELSPKVSPRVMLIGDSDLYLDVCYLYIS
PHNAIQVCSPNPLMHSDFRLLFQKLGREEDWLCLRREPSMHFAFRNKFPKIALCCSISLPVMTMVMVLNFKFRVSINDT
MQFSALCNFMHKEWETILWCDLEGKVEGVFTEHEWNKAEIVFELDFPMRGNGNTAGSTGIGSPSWSLIGVYEEGNN
KEDIEFKVPMSVFPLCNIEPPSSSPSSLYYDVIRGTTE

>Vang0045ss00880.1

LVSHLAFCPQIPKSTFAIAMASSVFVSFNDEATRDITAFVLDPLRSRGIQVFVKGESTTFDLFQVIERSRLFIVVLSKNYASSI
CCLRELVAIINAVESSPRFLLPIFYGVHQYGHAISKHEERFREHKERMEEVQRWREALTRVACFPGWHMKNAKGVEVR
GFINYAVDILNREFSTPQNETILNRYGKFEDIVQYKDGDEVSYKELRGLIRLRDFISLVLTYPVKLEEDGVLRSWIGAFPEW
KAQLLSTDGFSVICKSWSLLTEKALRCMKGFMPEEIMAEIESGERSLFNRHNDVIPEGLLPLSVDLAVDQLIQTLFSGDYC
ARYIRLLTRNSSEKESVVNKIHTALEDKHNMFGNDKDFLMAAWINALTCETDAEVHLQEEINKIMVSISMTEGDDMLTT
VDTDKKKRSNRLLVIVVDADSNRKLDLQKVQFPSGIVVLIATESSTQAVKDDDFGIACTMDLNIWTQDHMLPWKLFYTY
VGSCISCSTVSSSMTIQKIAVEIVKKSHGHLLAIVLVAKHLRYVKDDKYWELVLDKLSNPNPFYDYQDCDRIGISRVMVNA
FVNIIWQDIDDELKLCLQLSLPVHNIKNGVRDDILVSYWANTLRYTQELGEYKRQLQYYLEELLDCFLLLKFESRDVCLPIET
YDIIKSLHISEPSIIWNGALGLTEIGQWHSLIQIELVNNKICELPQSPDCPKLKVLLLQGNADLLDIPDLFFDHMPLLQHLDL
SYTSIRDLPPSLAKLMQLKKLYLKGCDLFMEISPQIFQLKNLEELDLHGTLVTHLPKDIQELINLQRLALCFDAYHHVLSRGK

KGKQIFNTMIIPRGVISNLTQLNYLSLDVDPEDEQWSENVNSVLVEILGLEKLKTVSMYVPKADLLELIPAEKYLNFRLVVG
HHMRRLISRVTPELETKFKDFDYSMKFVNGVNVPKGVKMNLGRFKALYLDRHMTIKSLSDFNLNNVRRLKVCILAECNE
METIVDGDEPFSLKLELLSVFYMKNLRSICQGCGSFSYLKYMTLHTCPILTTIFTLRTFISLPFLEEIHVEDCPKVTTLISDDSP
KRKAAFFLPKLRVISLLYLPNLVNIFNGLPVEHVLEEMIFYCCPKLQSLSSSELAWEYLKFIKGERMWWEALEWSVSEWG
NAGRPKFIEQFFKPINVEVDMMNPPSAHQETQLNKYIGGMYQGVSSSTKLMTKLHLETPLLLPSTPFPLSDSKEGEAQK
RKAVIEPIVVPPLLRRKPNQV

>Vang0198s00320.1

MVITTTDEHILKVYEGYVLIHKVPLLNDKDAGELFYRKAFKSEEQNSNCAAMIPEVLKYAQCLPLAIRVLGSFLCTRDADE
WRDVLNRLENSPDDKIMNVLQISVDGLQREEKQIFLHIACFFKGERVDYVKRILDGCGLHPRIGISRLIEKSLITISNEEILM
HELLRKLGKKMVRDESPEEPGSWSRIWLHQDFFQALTRETCREHEFSESYISLVVVGVKARQILQGQHILKVPDAIGELR
CLERLNLQGNNFVSIPDSFRVLHCLAYINLSHCHELKSLSNLPFEGAASGGKYFRAVSGSRDHRSGLYLFNCIKMVDILSKP
WDCWSLELAWLFRLIKESCHFRCGFDIVVPWGLEIPRWFTKRFEGDSVIRIEEFNVDENWMGFAFCVIFEGNIAPVVGD
SSSHPLYLSFVRARITFKTLSRVKIHAWGMRSIFKKDVHDFKRMQQGQPLPFPRNEVHPFNFVFVKKSKTNSGPIFRLPY
NWLLTREDEVEKHDAEVKESNLSYAGF

>Vang01g07720.3

LCCFNNFALNLSNVKEELELTRDRVKERIREAINKDEKVESTDVKWLKDVEKVLEEVQVLEKRIMSVNKSYFRRQCQYSL
AKEIEKKIIEMIQLDFNSEYTQFSRITKLLGMEYYYSNDFSVFNPIKASYKKLLEALMNKSASIIGLVGFGGSGKTTLAKEVC
KKAEDMRLQKLRILEIEDCHNLEYIFTVILAQGLVSLENVKIVSNMKLKYVFGSEKEHNLAVYQSFHQQTNISINLPNLDTL
NLMFLPNVIRIWPEYCPLRLPCLEESLIILCPKLPNSSIDMAETASNLEQHTISMVMYYFQALEFLEVLRLSHCSTLTHIIERG
NDDILRTRNHSSLTLSKLRILEIEECDNLKYVFSLSLAQGLKSLKSVKIVGNSKLKYVFGSEKEHILMYPSFQQNIVTFHRLQ
NLMDFNLSKLTNLINIWPEYCRPHLPNLEKLHCTYCPELSDLSKVMTVSDLGQLTTSMVIK

>Vang0537s00090.1

MPLESDVTISSYRLCWDVFLSFRGTHTGHTFTMRLYHALHGRGVRVFRNDDGLERRGEIQKKLLEAVEDSAAAVVVISP
DYASSHWCLEELAKICEVGRLILPVFYWVDPSHVRKQEGPFEEWFVWHAQRFPTERVEQWRDAMKKVGGLAGFVLD
EKSDGDKSDELIQILVQNLMKQLRNTPLSVAPFTVGLDDRVEVLKNLLDLKSNDVRVLGLYGMGGIGKTTLAKSLFNNLV
VHSFERRSFIPNVRSQVSKHHGLVSLQNKIHGDLCGRKEDLITDVSDGISAIQKIVQENRVLLILDDVDDVEQLNFLMGKR
EWFYKGSRVVITTRDKEILHGSYVDVDFEVKELEFSEAMELFCFHAIRRKEPAEGFLDVSKQIVEKTGGLPLALEVFGSFLF
DKRTEREWKDALEKLKQIRPPCLQEVLKISFDALDEQKQCIFLDIACLFVQMEMKRDDVVDILNGCDFRGEIAVAVLTAR
CLIKIIGDGKVWMHDQVRDMGRQIVRSESLTDPGLRSRLWDRDEILTVLKNMKGTRNVQGVVLDCVKRRMSIPRDRS
ADEITRENFRRKPSCKSAFEYIKERYKKYVEDRKEKAKEVILQSKHFQPMVSLRMLQINYSRLEGQFICLPPKLKWLQWK
QCSLRYMPSSYNPLELAVMDLSESLIETLWKGRSNKVAVHLTVLNLSRCHRLTATPDLSGYLSLKKLNLEECSHLTRIHESL
GNLNSLVHLNFRLCYNLIELPSDVSGLKHLEDLVLSDCWKLKTLPKDLSCMVSLRQLLLDSTSITELPLSIFHLTKLEKLSAN
GCHLLKKLPTCTGKLCSLQELSLNHTALEELPDSVGSLEKLEMLSLTGCKSLSVIPNSTGKLISLTQLYLDGSGIKELPASIGAL
SYLRKLSVGDCTSLDKFPVSMEALVSIVELKLDGTKVSNFPDEIFVGMKMLEKLEMGKVQHLKFVPVSLGYLSALTILDM
HDANITELPESIGMLENLIRLRLDKCKQLQRLPDSIGNLKSLRWLMMKETALTRLPDSFGMLRSLVELDMKRMPYLNGA
GNNMSTGTIIPEIREQPSSEAILTSFCNLSLLEKLNAHGWGIYGKIPDEFEKLSSLETLSLGHNNICSLPASMTGLSCLKKLLL
SDCRELMFLPPLPSSLEELNLENCVAVQYIHDISNLERLEEFNLTNCEKVVDVPGLEHLKSLRRLYMSGCIGCSLAVKRRFS
KVLLKKLEILIMPGSRVPDWLTAEPVVFSKRSNRELKGVIFFGVISFKNIPENQREGLELVDVQGKIFNLTSEVFSTTFRLLR
VPRTNEDHIFLRRFGARTPLVFQLKDRYTLHLQRRNPPRIERLELNNCRIHLVFYGDDDYEGDEGSLEESQYSVSQKLAKF
FNFAADDPGV

>Vang03g16290.2

MYYFPTNLNFQEISEEISAYEYTIDRDFYTGTLKASFTSELGLHSGEVDLYSKEVGLHSEEVNLHSEEFILQSEELGLNSEKV
DLHNEEIGLHNVELNLYNGEASLHNGEVDLHSGELNLHSEELSLHNEKHDPHSKEVNLYSEEVRLYSEEVGLHNGMFGL
HNGVVGLHCEGRPPHCAYRGSTVEDKLLLKSHPFCCRDYLKEVSTPLQKPTQEQRKERSFGTLFQKMKAAVVAHQLGN
LISNRSILEDLKNSLQTLQDKRQKVQENLIWEDEEFQTQSDWLKRVDEILGQGDKLLNSYEGSTCTNILLRYKVGKQSRK
MQPEISVLINKGESHVSAKTRKHERPASRDETIADIMDALRNPDIQAVGVWGLGGLGTTSLAENIREKTKEQNLFDAM
VFITVTDKPNQEQVQNAIANALGVQFTNGESLVKRRNKLRQRIKKEESTLIIVDDTWGELNPEEFDLEEFGVPPGNEHEG
CKVFLTSGNLKFIQYLEDASKLNKVFQLEELQKEEARKLFEKKVGSFDEDQSSIVEEIVRSCEGSISLIYALAKALENKGEDAL
MQFKENSSPAKLLSYCLEENEEHKALLYLLAIRGRRFINSYSMYIDMWTGVFKNLETADAARKKRESLISDLKAYGLVVEK
GKDWVKVDDYLYHTAYRMALHDKRASVISTEWPPEELLTDLHFCNLHPVGDLKLPATLQCPNLKHLLISRENSTIDVPDS
FFEETKLLKVLDFVSFHCPKLPLSFVVLKDLEALSMYHCELGDIKEVCELTNLRMLGLLGSSIRQLPAQIVKLQKLLFLDLRD
TNLQVIPPNVLSKLTSLEELYLRNSFCNWEIETSTSENKNASLKELTDLEHLAYIEDMYVPDPQAWPVDLFFGNLRSYTIFI
GNGWDRAYDGDHELKTLKLKLNRRFQSENGIKKMLKEVQVLYLDTLNGVQNVVNDMECDGFPQLQSLFIQHNAEVK
FIATGSGNDPLDTFPNLESLSLTILSYLEYIYHGDSLTEKSFFKLRVIKIEKCNAMRCLFSVSMINGIPHLATLEVSQCTSIKAI
MLFEGAENRPIEFPELCSLTLKGLPALISFCSTEGSSSATLFHDKVSCPKLETMVISEVSELTTIWNEEYDAENSFGKLKNVII
KDCEKLRTVFPVNLSKNLDNLKTLEVRNCSLMTSIFTVMRQDSTKPGLQLSIPMIEITLTGLPKLEYVCVTTGFEALKKKFE
EEWYAGLPGLSGG

>Vang11g03590.1

MEERRKGVGFGRMKGPFGVPEKPEFCVGLDTPLTKVKIDLLNRPKSIIVVTGFGGSGKTTLATLLCWDQHIKSKFRENIFF
VTFSKTPKLKIMVEKLFEHLGYEAPKFQNDEEGINQLGLLLRKLKGSPTLMILDDVWPDSEDLVEKFKFHLSDYKLLVTSR
VAFPRFGTPCVLKPLLHQDAMTFFRHCARLDSNGLNIPDEDTIQKIVKGCMGLPLAIKVIGRSLSHGPNELWQKMVLEL
SLGNSILLDCNTELLTYLPKILDVLEDNTVIKECFMDLGLFPEDQRIPVTALIDIWAELYGLDNDGIEAMAIINKLESMNLA
NVLITRKNTSDTDNYYYNNHFIVLHDLLRELAIYESTLEPIEERKRLIIRTNENESEGGLDDKQQGFVTRILSNCFKYCCKHK
PQQITARTLSISIDENVSSYWSHMHPTQAKVLIFNLRVNQYSFPESMQKMRKLKALIITNYNSHPSELTNIELLGSLSNLKR
IRLERIGVLSSFVTLKSLRKLSIYMSQAFQNGILLTSDAFPNLVDLDIDYCKDMVVLPSGLCDIIPLKKLSVTNCHKLLALPQE
IGQLVNLELLRLSSCTDLEALPDCIGRLQNLRHLDISNCISLSNLPEDVGKLCNLRNLSMINCERCELPYSVVNLVNLKTVSC
DEETATSWDGFKAMLPNLLIEVSQVDKGYGNLSSKKRKTKSLNFVNDVARGRLSTAVTHVVSSLISFCISLVEDTASTSFS
TFSIIVAPSSASANNLDNALTAPRLTSFSLFHFERTRKVGLFMEETAQQMKRPFGIPEKPEFSVGLDVPLKKLKLNVLSEGA
SVIVLTGLGGSGKTTLATMLCWDEQVIGKFKENILFVTFSKAPQLKIIVERLLERFGYQVPELQSDGDAIDQLVLLLRKINA
NPMLLVLDDVWSCSEAVVEKLKFQISDYKILVTSRIAFPTFGTSLVLKPLVPEDAITLFRHHAFLKRASSNIPDEDLVQKVV
RHCKGLPLAITAIGRSLGHQSIELWQKMVGELSEGHCILDSNTELLSSLQKILDVLEDNPIIKECFMDLALFPEHQRIPVAA
LVDMWVELYGLDNYGLKAIAIVNRLDSMNLANVTVTRKNLSDTDSYYYNNHFIVLHDILRDLAIYQSNKAQIELRKRLMI
GINETKPEVCLGEKQQGIMIRTLSNNFRWCDEQKTQKIPSGTLSISSDETWTSYWSHLRPAQAEVLILNIRTNLYSFPKYL
KEMSKLKVLIVMNYGFHPCELFDFELLGTLSVLKRLRLERISIPSFVSLKNLKKLSLYLCDTREAFENSNILISDAFPNLEDLNI
EYSKDMVGLPKGLCNITSLNMLSISNCHKLSALPQEIGNLENLKLLRLSSCTDLQVIPNSVGRLSNLRHMDISNCINLPNLP
EEFGNLCNLRILYMTSCERCELPSSIINLKNLKEVVCDEETAFQWEAFKPMLPNLKIDVPQLDVVRYCKGLLLAVKAIGTSV
SSRPIELWQKMVEELSQGSLQKMLDVLEDNSIIKVIAP

>Vang02g13290.1

MQCLIALATGTLTKLGESLVAPIGNQFGYLVHYKKNIKDLKRELKTLEGRKQGVQGVVDEDRRNGRQIVSIVQDWLFKV
ERIIDEIEKINDFQVENNECLDIWSPNLVSRYSLSKKAKILIMSVTKLNEEKFDIISYSLPTPKLGSTFSNVIKSFPSRKSIITEVL
ETLKDEDFKILSICGMGGVGKTTLVKEVIKILEVCKLFDEVVMVVVSQNLDYVKIQGQIADALGLRFEKETIQGKACQLHE
RLKGVDNILIVLDDVWVNFDFESIGIPSNEHHKNCKILFTSRNEDVCYKMGSQKNFTVSILSPNESWDLFHDIVSRNLSTK

LDILHIAKEVSNECGGLPIAIVTMAKALANKEKHVWEDALDQLKRSSIPFLLEMQACVYSSIKLSYNFLDSAEEKYVLFLCC
LFPEDFDIPIEVLLRHGMGLQLFNNINALWKVRNRVHTIVDKLKRRFMLLDGNVEECVKMHDVIRDVIISVVSTEEYDF
MIQCDGYQMEQSKRETCYHSSAISLISKEAKEHPKVLNCPKLKLLQIASKKKGLVPDNFFQCVNKLMVLSLQNVHIHSTA
LVFEALDNIHTLLLEDCLVSDISIIGKKLKRLEILSFSNSNIKELPEEIGQLSSLRLLDLTECNDLIQISTNVFASLSKLEELYIRVR
SLSYKETNHILFELQSLSHHLKVIEIIILTNEDLPIDLFFKSIERFWVYLGDSSTLSHGIVRKGYLHPNILKLNNVYYKYIKRSVT
VQQLLEKVEILNLVAIKNMKSVILELNDRGFPFLKHLSIEFCNNLEYIVDVVDGFIFPQLISISLSNLDDLKGIFNVSSYMSQT
SDFAKVKSITSSQCLGTLTNLKIEYCNKLKTVFTLFPRTISLATLQCLHVVESYGIECIISSNNKYDEKSLIEFSNLVELKLQELP
NLIGYTNVIREHCSSTIQIHESSEQLIEDGQLLSSKCVDQIMPIASLFESNCMQLFPKLEKLFLHACSSLDMVFDLQKSQFH
GESMAFLFPQLKEIEISWLSKLRHIWGNVPSYIQGFQNVKSIKVKKCDSLGFLLTPNIARALTQLQKMVIHSCHSMEKIVG
KEENLNGDDEEKNVETLVFGQLESLTLIDLPNLMSIISDSYEVMWPSLRFVCIDGCPQLVTSSMFTQTVARQENFNGSSS
CSTRCDVANGTFKEDSPKFLQCCLGCTPHVFSNLKFKASSTEKVPSVSNTYSKAETVSSIPILEQMQVKGWDSLEVMFLL
KQNQLSDASNTNCLVKLILAQTPKSSVEITAFNNLTVLTIDSCHKLRYLFSYSIAKLLVKLQEVKMSNCKVIKQLVQRDGED
SLTLFLPQSSSVKVFENSSSSDHATSSQEACALEWSSLKRISISHCGVLEVVVGEIGEKIDTIIASFAQLQSLTLSHLPNAVSF
CLRHCTSESPSIENIHGSGYQNHEATSNEEMRIMRVDPLINGFIFPNLTYLAITSCNKVRNLFSPSTSTSFVRLVELDISGCR
EIEEIVSAEETQGNVIKIVFHSLQRLKLENLPKLKAFCQSSYGFDFPSLHQVLIKNCHMMQTFSHDPLYTPKLETVTMEIGI
HHLLTMIKLDNCLLKPWPIVSFTDEYDFQLAFQTSETLGWIKQDTCIQRYFTHEKHLTVESFQRLLKLVPSNVIHIFQNLKE
LTIKNCGSLVEIFESHGVDAKQMHAMVHYKLEALNLYCLPKLINLWKNYDGVLGFQKLRILNVQHCGNLCNLFPPSIARS
LVQLRHLRVHSCHMMEEITTKEDEESEGSNNAMIVFPLLNKLELRYVPNLKCFSSGNFNIDLPSCEEMIIEKCPKMTTFCY
GSVTAAKLPHIYKGSYEYVDIMGDLNMTIYHANESLKVAQQTSETISCIEHGQQLQPYLRSDTELVVQGSENLLYCIPSN
MLHRFQHLKQLKLHDCGSLVEIFESEEVDGNEDEGGTTTPYNFDLQELHLYDLPKLMHIWKYDGRILSFMNLKKLKIQH
CHSLKNVLSPEMAISLSQLQELSVHECELMEEIITRDEKLSEEPNKVKIIFQALQWLTLYRLPSLRCFCSSTYHFELPSCHDIT
ITECPKMEACHGNKGTSELPSVSFNNGLRAGKRSK

>Vang03g15210.1

MLIDLPQEVAEVRDELESFQYFIHEEDKVAEAEEDKVAEAEEDNKRSHGIRKRLMKLRKAAYRMEDVIDDYEIFKENQPE
EDPRCAALLRETVKIIKTQILLIPIVFRIQDVKKLIREERDGFEKSFPRGSTSDGSRGNENSTWLKLRMDPFFMKEDEVVGF
EEPIQRLTKWLTEGGKERTVISIVGMAGLGKTTLSKQVFDRVKDFDCHALITVSRSYTVERLLRDLMKELCNDRAEKPPP
DLATMNRMSLIKEVINRLRNKRYVVLFDDVWKEKFWDDIESALIDDKNKSRILITTRDEKVVEFCKKALSFKVHKLQPLSD
AKSLELLFKKAFGYGFDERSPKDYEKVGLDIVRKCGYLPLAIVAIGSLLSCLLYFGMYPEDYEVKCGTLIQLWIAEGFVKQES
GRSLEEVARQHLMELISRSLVQVASFTTDGKAKACRVHDLIHEMIREKIKNTGFCHYSDGHNHSGSSDIIRRLTIGRSSNC
LSGSMEESQNVRSILIFKKEASSEDFTRPLLAKYMRLKVLDFANAPLEDIPENLGSLIHLKYLSFRGTSITSLPKSISELQNLET
LDVRADGEIKVPEEITKLKKLRYLLGKPISSIEVKDSLGSMTSLEKMHVLKIDPDGDVIRELGKLTQLRDLRLISLPGDHADT
LCSSINTMPLLERLHISIIDLTQVDLHIPSSLSKLRKLLLSGNLKELPDLSSFQNLVKLSLASSRLTKNPLIPLGQMPNLLTLFFG
SSSYEGETLHFKNGGFLKLKELQFDSLLYLRFISIDSGALQALEKLKILNIFPLKAVPGIQHLKKLQFLEIIHVSAEFHRKIDPNE
GEEYFMIKHVPHLRILPFPEKWQHIQSLTAALITFYQRQDSFINTRNLPTRATTTASTSSSQESNI

>Vang11g03570.1

LAIMGETDQLVILTRFQDAMRMVMDIVEKGRKNKRSKRILRSTLKNMTLVVQEIKQYNEHLNPPREEIITLVKENDPGE
DLVCNSCSRSLWWTKFLSWFSLYGEGFLHEKNDLLTSDDKQVKDIKNTLYKVREIIELLDIENFEQKLKGAGTPIKCPYGV
PENPEFTVGFVPVLSKLKMEVLQEGVFTLLLSGLGGSGKTTLATMLCKDKEVKGKFKNNILFVNFSQTPKLKNIVERLFEH
CGYHVPEFVSDEDAIKRLEILMRKIEGSPLLLVLDDVWPGSEALVDKFKFQMSDYKILVISRVAFPKFGTQIILKSLVHEDA
MTLFRHHALLEENSSKCSLSIPNEEIVQKVVRYCKGLPLAIKVIGRSLSHQSTELWQKMVEELSKGHCILDSNTELLTSLQKI
LDVLEDNPIIKECFMDLGLFPEDQRIPLPALIDMWAELYKLDDDGIEAMEIIKKLDSMNLANLLVARRNTGDSDNYCYN
NHFVVLHDLLRDLAIYQNNREPIEQRKRIITGINENQSQWWLGEKQQGMMSRLLSKYRGWCVKQTIQQVSALILSLST

DETCASYWSDIQPSQAEVLILNLQTKKYTFPEFMEKMIKLKVLIVTNYGFHHSELDNFQLLGSVSNLKRIRLERVSVPHLS
ALKNLKKLSLYMCSNISQVFENGTILVSDSFPSLLDLNIDYCKDMVRLPNGICDISSLKKLSITNCHKLCSLPQEIGQLLNLEL
LNLSTCTDLEEIPDSIQNLSKLRLLNISNCISLSNLPEDIGNLCNLRNLNMTSCARCELPYSITNLENLKVVVCDEETAASWE
AFEALLPNLKVEVPQVDVNLNWLHSISS

>Vang11g03570.3

LAIMGETDQLVILTRFQDAMRMVMDIVEKGRKNKRSKRILRSTLKNMTLVVQEIKQYNEHLNPPREEIITLVKENDPGE
DLVCNSCSRSLWWTKFLSWFSLYGEGFLHEKNDLLTSDDKQVKDIKNTLYKVREIIELLDIENFEQKLKGAGTPIKCPYGV
PENPEFTVGFVPVLSKLKMEVLQEGVFTLLLSGLGGSGKTTLATMLCKDKEVKGKFKNNILFVNFSQTPKLKNIVERLFEH
CGYHVPEFVSDEDAIKRLEILMRKIEGSPLLLVLDDVWPGSEALVDKFKFQMSDYKILVISRVAFPKFGTQIILKSLVHEDA
MTLFRHHALLEENSSKCSLSIPNEEIVQKVVRYCKGLPLAIKVIGRSLSHQSTELWQKMVEELSKGHCILDSNTELLTSLQKI
LDVLEDNPIIKECFMDLGLFPEDQRIPLPALIDMWAELYKLDDDGIEAMEIIKKLDSMNLANLLVARRNTGDSDNYCYN
NHFVVLHDLLRDLAIYQNNREPIEQRKRIITGINENQSQWWLGEKQQGMMSRLLSKYRGWCVKQTIQQVSALILSLST
DETCASYWSDIQPSQAEVLILNLQTKKYTFPEFMEKMIKLKVLIVTNYGFHHSELDNFQLLGSVSNLKRIRLERVSVPHLS
ALKNLKKLSLYMCSNISQVFENGTILVSDSFPSLLDLNIDYCKDMVRLPNGICDISSLKKLSITNCHKLCSLPQEIGQLLNLEL
LNLSTCTDLEEIPDSIQNLSKLRLLNISNCISLSNLPEDIGNLCNLRNLNMTSCARCELPYSITNLENLKVVVCDEETAASWE
AFEALLPNLKVEVPQVDVNLNWLHSISS

>Vang08g04630.1

LYRVAIVVFTKTYSQSPWCLHQLQQIIQWHQTYSRHVLPVYYEIQPFDVRSQKGDFGKAFKKAAHQTFSQQQLEHGMS
KWTHALTKAANFFGWNESNYRSDAELVDKIVKSVLNLPVLSATKFPVGLQSKVEDLIQTIKDKSSEVCIIGISGAGGSGKT
TLAKAIYNKIHETFTEKSFIEDIGQVSRTRGHHRLQEQFLLDVLKTRVEIPSVDMGRRMIRERLTGKRVLIVLDDVTENFTL
LDLWGCREWFGGGTVIIITTRDVDLPRILKVDSVFGIRLMNANESLELLSWHAFREVKPKEEYNYLAKGVVTHCGGLPLA
LEVIGNCLFKKTKEEWNSILLKLEKIPLHNVQGKLKISFDGLRNQIEKDLFLDICCSFVGEGRDYVTKMLNGCGVDADSGIR
VLIELSLIKVKRNNKLGMHLLLQEMGRNIINEIYEKEFRRERRLRFDDSEYVLTDNTGRRAIERVPVKLRSVRREPSRLLTYI
ENSDYISKKLRWISLDWFSLEYLPDKFYLHDAITIDLKHSSLRFLLKEPQDLRWLKVLNLSHSKYLTETPDFSGLPSLEQLILK
DCTGLREVHPSIGCLCNLTLLNLKDCTSLSNLPREIYKLISLETLILSGCSMIDLLEKDVVQMESLVTLIAENTIVNHVPFSILS
SKSIGHISLRGFERLSRNIFPSIIRSWMSTVMNPISYIHSLCMDIDNSWENIGPLLSSLENLRSVLVQCDTEYQLSKQVKSIL
VEYFANFAESGISKQFRSSFIRLGTYHEFFNAVSDNISEVLLNSESCDVSLPVDNLPNWLAYMGEGNSVSFSVPWDRDM
KGMALSVVYLSTGEIVATECLRSVLIVNYTKSTLQIHKYGTIISFNDIDWQGIMSNLAPEDKVEIFVTFGHGLVVKNTILYLV
CGEPNYLKKELESKKNSLLRSIMKIVM

>Vang02g14420.7

MAAELVGGALLSAFLQVAFERLASPQFLDFFRGRKLDEKLLSNLNIMLHSINALAHDAERKQFTDPHVKAWLFSVKEAV
FDAEDLFGEIDYELTRCQVEAESEPQTFTYKVSNFFNSTFKTFNKKIESELKELLEKLEYLAKQKGALGLREGTYSGDRTGG
KVSQKLPSSSLVVESVIYGRDADKQMICNWLTSETDNHNHPSILSIVGMGGLGKTTLAQHVYNDTEIEEAKFDIKAWVC
VSDHFDILTVTKTVLEAITKSKDDSGDLEMVHGRLKEKVSGKKFLLVLDDVWSERREEWEAVRTPLCHGAPGSRILVTTR
VEKVASNMRSKVHHLKQLEEDECWKVFEEQALKDDDLELNDEKKEFGRRIVEKCKGLPLALKTIGSLLRTKSSISDWKSV
LESDIWDLPKEVEIIPALLLSYQNLPSHLKRCFAYCALFPKNYEFEKEKLILLWMAEGFLHYSSQNNNLEEIGEQYFDDLLTR
SFFLRSNIKMHFSMHDLLNDLAKYVCADFCFRLKFDKGNCIPKTTRHFLFAFDDLRYFDGSGSLTDAKRLRSFVPITNNFV
HGAFPCQIKILIRELFSKWKFLRVLSLNGNYDLEEVPDSVGDLKHLHSLDLSRTMIRKLPDSVGLLYNLLILKLNDCSFLKEL
PSNLHKLTNLRCLEFEDTEVTKMPMHFGELKNLHVLNMFCVDRNSEFSAKQLGGINLHGRLSINELQNIVNPLDALEAN
LKNKQLVELKLIWNWNHIPDDPMKNKKVLENLQPSNQLEHLSIRSYCGTQFPSWVFDNSLSNLVSLELEDCKYCLCLPPL
GLLSSLKTLKIRGFDGIVSIGAEFYGSSSSSFKSLEILKFYNMKEWEEWECKTTSFPRLQHLVIVRCPKLKGLSEQLLHLKELFI

ESCPNLIVSEHSEDTSALDLLRTRSCPLVNIPMTRYDFIEQITIDKSCNSLTIFQLNFFPILRLLRLEGCQNLQRISQEHPHNH
LKKMSVCACPQFESFPSEGLSAAFPFLTELEIIWCRKVEKFPDGGLPSNVKHMSLSSIKLIASLRETLDVNTCLESLSMKYL
DVESFPDEVLLPPSLTSLTISNCRNLKRLGHKVLYNLSSLTLFDCRNLQCLPEEGLPESISSLQILNCPLLKQRCQKPEGKDW
RKIAHIQNLMAAELVGGALLSAFLQVAFERLASPQFLDFFRGRKLDEKLLSNLNIMLHSINALAHDAERKQFTDPHVKA
WLFSVKEAVFDAEDLFGEIDYELTRCQVEAESEPQTFTYKVSNFFNSTFKTFNKKIESELKELLEKLEYLAKQKGALGLREG
TYSGDRTGGKVSQKLPSSSLVVESVIYGRDADKQMICNWLTSETDNHNHPSILSIVGMGGLGKTTLAQHVYNDTEIEEA
KFDIKAWVCVSDHFDILTVTKTVLEAITKSKDDSGDLEMVHGRLKEKVSGKKFLLVLDDVWSERREEWEAVRTPLCHGA
PGSRILVTTRVEKVASNMRSKVHHLKQLEEDECWKVFEEQALKDDDLELNDEKKEFGRRIVEKCKGLPLALKTIGSLLRTK
SSISDWKSVLESDIWDLPKEVEIIPALLLSYQNLPSHLKRCFAYCALFPKNYEFEKEKLILLWMAEGFLHYSSQNNNLEEIGE
QYFDDLLTRSFFLRSNIKMHFSMHDLLNDLAKYVCADFCFRLKFDKGNCIPKTTRHFLFAFDDLRYFDGSGSLTDAKRLRS
FVPITNNFVHGAFPCQIKILIRELFSKWKFLRVLSLNGNYDLEEVPDSVGDLKHLHSLDLSRTMIRKLPDSVGLLYNLLILKL
NDCSFLKELPSNLHKLTNLRCLEFEDTEVTKMPMHFGELKNLHVLNMFCVDRNSEFSAKQLGGINLHGRLSINELQNIV
NPLDALEANLKNKQLVELKLIWNWNHIPDDPMKNKKVLENLQPSNQLEHLSIRSYCGTQFPSWVFDNSLSNLVSLELED
CKYCLCLPPLGLLSSLKTLKIRGFDGIVSIGAEFYGSSSSSFKSLEILKFYNMKEWEEWECKTTSFPRLQHLVIVRCPKLKGLS
EQLLHLKELFIESCPNLIVSEHSEDTSALDLLRTRSCPLVNIPMTRYDFIEQITIDKSCNSLTIFQLNFFPILRLLRLEGCQNLQ
RISQEHPHNHLKKMSVCACPQFESFPSEGLSAAFPFLTELEIIWCRKVEKFPDGGLPSNVKHMSLSSIKLIASLRETLDVNT
CLESLSMKYLDVESFPDEVLLPPSLTSLTISNCRNLKRLGHKVLYNLSSLTLFDCRNLQCLPEEGLPESISSLQILNCPLLKQR
CQKPEGKDWRKIAHIQNLRV

>Vang02g09720.1

LCQKGIHTFIDQEGLRKGEEITPALFHAIQNSRISIVVFSKNYASSTFCLNELVKILECAKEEGRSIYPIFYVVDPSEIRYQTGT
YAEVLSKHEAKSGSEYEFIEKIVVEIFTKINYIPLYVPNNGIGLENAVQGVKSLLGDGSAVNMIGIYGIGGIGECDWFGSGSI
IIITTRDKHLLEAHGVVNLYEVKPLHVEKALELFNRHAFKNGKVDPAHMNISKRAVSYACGLPLALEVIGSHLFGKSLDEC
RSALDKYESIPHEKIHEILKISYDGLEENEKGIFLDIACFFNTCELGNVTPLLKAHGFHVEDGLRVLVDRSLIKIDSSDFLRMH
DLIRDTGREIILESLSVINLEDCKYLTDLPSLREASLLTTLRLDRCYNLVNIDESIGFLDKLSFLSAKHCTKLKTLAPCIMLTSLET
LDLRKCVSLESFPEVLGKMDKIRTICLDHTAIDKLPFPIGNFVWLELLSLKGCVRLHQLPGSICMMPNVNMIIGYGHEGY
NFFEKELRSEVSPMAMRIGGSNRYPKIVLCCCLYFPATKRVMIMTFNFRVFINDTLQFNGMCNSLFGEPEQILWCDLEG
KVEGVFSEQEWNKAEIVFELDFPMRRNSRNENTTRIFDRGFLPWSLIGVYEEGNNKEDIQFQDPMSIFPLSNTEPPSSLP
ASLYYAVSRGIPEA

>Vang1033s00010.1

MKKERENLKAKNKTLEESARVATRRGDNIRKDVTHWQKQAKELIEEDTKKKVTCFFGWCPNCKWQYSRGKELESKTKE
IRRLVERNFENVGISRDDNNYISLESRKLKFEELFNALKDDNNYMIGLQGMGGTGKTTLVIEVGKELKKSKCYNQVIDTTI
SNTPDITKIQDDIAGPLGLPLKDFNESERPKKLKDRLTNGEKIILILDDVWGDINFEEIGIPFKGNHNNCRILVITRNMKICQ
QMDCDKTIELHILPEKEGWILFQKYAGLSDNSSKSILDRGRKISKECKGLPIAIAFIARSLKGPRPLEEWDLRNCSFYVLCFE
KMKKYLKNF

>Vang0250s00170.1

LLLRLHFRRQQRRLVSSMSLSGSDDEMEMDFLRDRYQEMDGGNFEVFLSFRGEDTRASFTSHLYTALQNAGIFVFKDD
ESLSRGKQISPSLRLAIEESRISIVVFSKNYAESIWCMKELEKIMECHRTIGNVVIPVFYDVDPSEVRHQRGDFGKAFRRLLS
KFSIEKEVKVLDWKQLWWKTLREICDISALQIIFSSWTERRDKLDNRWKKTLLVQISKILDLNPRWKMKIADSIESIVSQST
RGLLEGDEIPFRKEKVDDVKDFLVKQWTEVFFEPVSISEVALLDPCGETKIANAIKLNLEHCREALFEAAGISRNLVLNFCE
ETEITNAIELHMKHWMDAFSKDDGTVLHSGYPCVHIQGLGNSITFMESDFQSRLETVYSEISSLKLHWREKVLKAYGNE
LYSRRKSDIERHLERCASALTKAARISTGFQSSLRKLKAYNRIESLVEYWRGALCETAEISRLVVQYYRGLTDNEINYMEKN

ARDALREAAGISGLVILNSRNESEAVKNIVKNVTSLLNKTELFVAKNPVGVESRVQEMVQLLEQKQSNDVLLLGIWGMG
GIGKTTIAKAIYNKIGRNFEERSFLADIREAWGQEAGHVCLQQQLLYDIHKENNTKIHNSESGKVILRERLRHKRILLILDD
VNKLQQLNALCGSREWFGSGSRIIITTRDIHLLRGKRVDQVFAMTEMNVDESIELFSWHAFKQASPEEDFIELSRNVVAY
AGGLPLALEVLGSYLFDMEVTEWKSVLEKLRKIPNDEVQEKLKISYDGLSDDTEKGIFLDIACFFIGKDRNDVIHILNGCGL
FAENGIRVLVERSLVTVDDKNQLGMHDLLRDMGREIIRSKSPMELEERSRLWFHEDVLDVLSKETGTKFIEGLTLKLPRT
NTKSLCTKAFMNMKKLRLLQLSGVELVGDFEYLSKDLRWLCWHGFPFAFIPTSFYQGSLVSIELENSKITMVWKATQIDK
LEEDIEQMESLTTLVADKTAIRRVPFSIVRSKNVQNSSSNQLSYISEELPKLQSLWIECGSDLQLSRDTTSILDALNATNSEE
SESYGTTSQMQNNVTTSDGGDLLPGDCYPDWLTFSSEGSSVTFEIPEVNGRSLKTMMCHIHYSSSDSITSDGLKNLLVIN
HTKSTIQLYKRNALASFDDEEWQRVLSNIDPGNKVQIVVVFWNRITVNKTSIYLIYEAIVDEKVEHYHASNMNVPRSISP
GVESMEDLRGASVKSLTKRLFNKFLSCKYFCKGKVEKKKN

>Vang0198s00270.1

MLKTKSGLQRFWDRFGTRSSGSTSSSYDSSDTIQNQHYRYDVFISFRGPDARNSFVDHLCSHLLRKGIFVFKDDHNLQK
GESISPQLLQAIQLSRLSIIVFSKNYASSTWCLDEMTAIASCKQQSSQIVFPIFYDVDPSHVRHQNGVYKNNFLSHRWKFG
KDRDKVLGWKRAMTDLANSAGWDMRDKPEFAQIQSIVQAVIKKLGHKFSRSVNDLIGIQPRVQALEDKLRLSSKSDDV
RVLGIWGMNGIGKTTHAAVLYDKISHMFDASCFIEDVNKLYRDGGHTAVQKQIIHQTFRENIDMSNPIEISGIVENRLHS
IKVLIVLDNVDELEQLENLAIKPRLLLKGSRMVITTTDEHILKVYEGDVLRHKLPLLNDADARELFCRNAFKCEDQSSNCAA
LIPEVLKYAQCLPLAIKVLGSFLCTRDADDWRDVLNRLENSPDDRIMNVLQLSVDGLQREEKQIFLHIACFFKGERVDYVK
RILKCCGLHPDIGISRLTEKSLITISDEEIHMHELLQELGKKMVRDQSPEEPGSWSRIWLHKDFLHALTAETGTEKVKAIVL
NKKEEMSECIVDGLSRMKELTLLILYHTRVSGRLEVLSDRLQYLLWHDYPFASLPPYFTAFNLVELNMPNSHITHLWEGG
KRPEKKNKLQHSTRQETAWEFDSFSSQASILSPPFCSQTMSHSSGLDAFANRFCHALSYNNDASKINKPDFRELDLGSP
MSPLHTTRTCGEYHNARWPEGGVVVMAVVMAVAIRKSLRSCPNLKRIDLSNSKYLSETPDFSRIIKLERLDLSGCSSLSYV
HSSIGLLKKLAFLNLRNCCNLVCIDFGCVWNMSSLRVLHLSGCSKLESTPDFTRATHLEYLDMDECTSLSTIHESIGVLSSL
TFLSLRGCIKLVSIPNDINSLVSLQTLDLSCCYNGWNPIPRQALSSHFKSLIFLDISNSYLEEVPDAIGDLRCLERLNLQGNRL
FSIPDSFRQLHCLAYLNLSHCRYLKNLSGLPTEGDKSGGKYFKTVSGSRDHRSGFYLFNCPDVSHDRLEFAGLERFFHLIKE
PCNFRCGFDLILPWDMGFRPVFGDTFLGNSIIRILQCVMNDNWIGFGFYVIFSRGIDFSSSHCSLSHLLYLSFESEYTEEYF
DMRFNSERDEYFRSRHIWIIYMSREHCHFVKTGAHITFKAQPNVKIDALGLRPILKQDISDSKGKKYIKFSKLNHDHLDFE
YVEKSNSGSGPKIQLPYNWYVTEEEQVENIDAKAKENNLSNAGL

>Vang10g00850.1

MSGGSSSSFPVSTSPTKYDVFLSFRGEDTRYNFISHLYDALHRNRIQAYRDDRLQRGEEISPALQTAIEESKIYVLVFSENY
ASSTWCLNELTKILNCKKIYERDVIPVFYKVDPSTVRKQEERYKAAFDEHEQRFKDNMDKVQGWKDALTEAAGFSGWD
SNVIRSENTLVEEIVKDILKKLDLYSNSYDPGIIGIEKHIERIRFMMHFESTDIRIIGICGMGGIGKTTISEQIYHTFAMQFVSR
SLVLDTQKKIERDGIDTVREKYMSELLNPSLLYYNERLKRMRILIILDDVTDSVQVKQLLGRRDSFGQGSRIILTSRDKQVLK
NAGADDIYEVKELNELDSLKLFSLHAFKQNSSQETKYMDLVEEVLGYAKGIPLALQILGSLLYGRTREAWKSQLQKLKKC
QDLKIFSVLKLSYDGLDDEEKNIFLDIVCFYRGHEEIVVVERLDDCGFSSKIGMEILKDKCLISIFNGRIVMHDLIQEMGQEI
VRQECPHYPGKRSRLFKDEEIHEVLKKKKGSDAIQCILLDLKNIKEVIVHGQAFQKMDNLRMLMVDNFGYGRVFESKVS
LASSLVILPDTLKILYWNGFPQRSLSPNFCPQNLVRLEMPNCRLKQLWEEDQRYLCMGILF

>Vang03g15160.1

MAETAMSFASQHVLPKFLEAVKMLKDLPKEVSEVTDELESFQDFIHDADKMAEVEEDKNRRERIKKRLMRLRKAAVC
MEDVIDDYVICNEKQSEEDPRCAALLCEAAEFIKTQIFRLKIAYQIQDVKSHVRAERDGFENHFPIGSRSDEGRKERTVISI
VGMAGLGKTTISKQVFDRVHTDFECHALITVSRSYTIEELLRDMTNKLCKERREDPPRDVATMNQMSLIEEVRNRLCNK
RYVVLFDDVWNETFWDDIELALIDNKNGSRVLITTRDEKAFGYGFDGCCPKDYEAVGLDIVRKCECLPLAIVAIGSLLYRK

CKSPSEWHLFSQNLSSELQSNSELHSVTKILSLSYDDLPQNLRSCLLYFGMYPEDYEVKCGRLIQHWIAEGFVKHENGRN
LEEVAQQHLMELISRRLVLVASFTIDDRAKACRVHDSIHEMIRGKIKNTGFCEYIDEHNHLESSGIIRRLTIARNSNGLSGSI
EESQHVRSILIFTDEVSSKDFTRRLLEKYMRLKVLDFEYAPLYDVPENLGSLIHLKYLSFRGTSITSLPKSINKLQNLETLDVR
AFGGIEVPKEITKLRKLRCLLGDPISSVAVKDSLGSITSLEKMYVLIIDPDGVVIRELGKLKQLRDLRLSNLSGDHDLGSMPN
LLLLSFDSNSYDGETLHFENRGFQKLKELKLNGLQQLSSIFIDSGALQSLEKLQIMSILQLKAVPCGIQHLKKLQVLDILYMP
TEFQQRIDPNGGKEHWMIKHVPDVHFVTKNRALILAEIAAEIFSSRL

>Vang0045ss00890.1

MSELELVGPLVGGMGSIGVGEMHTTIASKIAFSKNLDDNCNILVKDTEMLHAIKKDKEMKAQRNSHKDTTNAYKLWT
NRVSEATKEVQKLKLKYEEETLPWWRILKRSDLSEEMEKKSNCVRQLMNDECLKDFLVDKPPEPVLKELNVPQISGYPTL
QGALDNTLVLLKNNKIKIIGVCGTKGVGKTTIMRNLNNNEEIAKLFEIVIFVKVTPNDRKLQEKIAHRLMLDKGIDKEDSD
DIARRIHRELENKRYLLILDEVEDAINLELLGIPSDNNNGSKVVIVTRFPLVYKLNRVQRVIKVAELSPDEAWKMFRDTVYA
FNPKIDSPDIQPTAKLVCKRCSRLPLLIYNIANSFKLKESASSWWAGLEDLKPWPELQSQGLEELYSCLKFCYDELNDKRK
QKCFLYTSLYPADSKVYSDYLVECWAAQGLLGDINDTRSYQSARNCGIDILEHLANVSLLEKGEAMIYVNMNHCMRQL
ALHISSKDPECSFYLQDGEESENLSNSRAWQQARWVSMRKVHELRRSQDCSTILTLLLRKNPELTALPECFFENMSSLLL
LDLYSSMITQLPSSLSKLTGLRGLFLNRCELLESLSSEIGLLQFLEVLDIRDTKVIFIPLQIGFLTKLRCLRIPFIASEDNEAQNV
HVISKLHRLEELTIQIISYEQWCNDANNVLAHVASLENVTHLRCCFPSSIILGEFLSRSKSWHNKQNSFRFTVGCQNSRRP
QILEFFEYKITNYLRYCNGGQRDDPAIIEVLPKTDAFELVCHKDIRKLSNFAGIACLERIRGLLIKRCNQVLTIVSGETSSNV
MNGIQIETAVILPNLEQLYLENLLNLKCAFRGPLHSGTFSRLQTLSLKNCPRLSQIFSNGAIQHFSELQKLKLEDCSIIEELIG
EDIERERDVLPKLEILLLVNLPNFKSICTTHTLAWSSLELLRIHNCHKFKTLPLDSVNAVNLKSIKGQQEWWANLDWTNN
EEVLQRLQPIFVASNEYFS

>Vang0071ss00440.1

LRLESHNCSLVLGVDTKKDPEDIRSNPMTYSNSFRVFDRIKRVIFVCVLLAAIDNNNSNNSSYPVTDFAYDNDSPQIKFDV
FVSFRGTDIRQDFLSHLIEAFSQRHINAFVDNNVVRGDGLSEALIRAIEGSSVSLIIFSQDYASSHWCLSELVKIVECRKKNG
QIVLPVFYKVDPAHVRHQKGTYEHAFAKHQVRYSLTTMQIWRTALTEAANLAGFHSSTFRDEAEFIKEIVKCVLNRLNQ
VQQGKSKGLVGVGKRIAHVESLLQSEEPDVRVMGIWGMGGIGKTTIAQEVYDKLCFEYEGCCFLANIREESGRLGIISLK
KKLFSTLLGGEDLKIDTPNGLPQYIERRLRRMKVLIILDDVNDSDQLEVLAGTRDWFGSGSRIIITTRDKQVLARQFASIYE
VEALNFDESLRLFNLNAFKQNHLEIEYHELSKKVVNYAKGIPLVLKVLGHLLHGKDKETWESQLERLKKVQNRKVHDIIKL
SYNDLDRDEKKIFLDIACFFDGLNLKVKRMNFLLKDHDYSVVAGLERLKDKALISVSQENGVSMHNIIQETAWQIAREESI
ENPRSQIRLLDPEDIYHVLNYNKGDEAIRSIVINLSRIKQLQLNPQVFARMSKLHFLDFYSKGSCSCLGDQGGLYLPQGLES
LSNELRYLRWTHYPLESLPSKFSAENLVELNLPNSRLKKVWQETPDLVNLRVLILHSSTRLKELPNFSKATNLKVIDLRFCV
RLTSVHSSIFSVRNLEKLYLGGCLSLRSLRSNVHLDSLRYLSLYGCMSLKDFSVTSKNMVKLNLELTGIKQLPSSFGLQSNL
QKLRLAYTYIDHLPTSIKHLTRLRHLDLRYCRELRTLPELPASLETLDARGCISLETVTFPSTAGEQLKENKKRVAFWNCLKL
DEPSLTAIELNAQINMMKFAHQHFSLFGDAQSTYVYPGSKVPEWLAHKTTHDDFVIIDLSSVLSPQSSHIGFIFGFVVPE
VPFGGSALEFKISTSGEGSHINVYMDRPRHRITSDHVYLMYDQACSRYLNGRAKHEPRIKIKVALASRTLTSKYVPLKLRA
FGIRFSVVLSDMCPLISGITTKDAVLSFELGMRALDLALGNKIHLEPIDDDPSCSDDGK

>Vang08g00840.1

MDFLRPFGRVVEGLVDFVWKHGVRQVTYIVNYNKNVFELKDSVKDLALEKERINHQREEAEKNLNNIEGKVIEWDRKV
CEIETTVEVFTNDDGHTKARSPNCFVFPYLWNRHRLGRQAHKMKDDVKRLIGESPELDEVFYRQNVTSNDATLSNCGF
VEFSSIKSIIEKVMIQLQDSAVRMIGLYGRGGVGKSTLVKEIARKAKEKKLFDVVVKVEITADPNPHKIQEEIAYVLGLRLEG
EGENVRADCLRRRLKKDRGNILLIFDDLWHKLDLNKLGIPIEDYHDDEDFNRQKADNKDGNNDPSSKVLKKENILGGHK
GCKILLTSRDKNVLCVEMDVKSTFCVRELDDKDSLILFQKLAGIHNEMSSSKQEIVKKYCEGLPMAIVVARALRNKSESV

WEATMKRHKKHELVGEGRSMDISVKMSYEHLENEEIKSIFLLCAQMGRRALIMDLVKYCFGLGILEGVSSLWEAREKIK
TSIEKLKDSGLLLDESSNNHFYMHDMVRDTALSIAHKDHNAFNLRNGKLDDWPELEKCTSIFMCNSDIIDGLEVINCPQL
KLFQIDTNDPYLKIPKSFFRRMTNLRVLIMTGFRVSNLPSSIQYLSKLRMLCLQQCTLDCNLSIIGKLKKLRILSFSGSILKNLP
IELQCLDKLRMLDISDCSELKIIPPNVISSLTCLEELYIRESLIKMLVERETNKGHYLFLSELKNLHQLKVVELSIPCVSNFPNHL
FFDKLRDYNIVIGDFGSFSLGKSRMFDKHETFRVLAVQLKDNTNIHSQENIKLLFKTVQTLLLGNINGVREVVNDLDIDGF
QDLKHLSIINNNDIKYVNSTKLCNYVNVFPNLESLCLYNMGKLDMICYGPVIAVSFAKLRTIKVEMCHRLKNLYSLDMVK
FPIAAQTCDISECSSYMDIFLSSLEIIEVSECKSLKEIFQIPKNYGKVEFPKLHTLTLRLLPLFSCFYTKVDKFCWPHLAKAQTTI
RGHKELTSAEDKQSDEEPLFGELVEIPNLETLNLSSLKIHKIWNYQNSSSFTFQNLIKLVVKDCDKLTHLCLLSIARSLNKLKS
LVISECPVMEKIFETERNNVDKVCIFPKLEEIHLTKMNSLIDIWPTKMSADSFSSLISVKIEECNKLDKIFPSHMEGWFDSL
DNLKVNRCKSVEVIFEINDSQEIDEFGGIDTKLQIILLEGLPKLKQLWSTDPNGILNFKKLRTIDVGFCHELKNLFPVSIAKD
VPKLEHMSILECDKMEEIVVSQDASEANKDLLAFPELTFVRLHRLSNMKYFYKKKYPIKCPKLKELSVGQCVQLKTFPKDTI
KTTNKEGNFVFSIEEVSFAYTEFLWLKKDTLVQLRHGKHWEEQNSFSYLKYLDAWGCHTVVHIIPSHLLSCFHNLEELYV
RNCSNAEVIFNMNEENRVMTKPSRLKTLYLDYLPQLKHVWDKDPKGITGLHVLEEVHVSRCGRLKSLFPASVAKDLTGL
EVFKVKRCEKLEEIFRKDERIGDEEGSTQESVFHRLTTLTLQELPRLNYSIHCSNQQAILTLFTLSILTLIISIL

>Vang0063ss00420.1

MISSSIFEAIKHSNVSLVVLSKYYASSTWCLRELAKILQLRKRGEHIVIPVFYKIDPSHVRKQTGTYGMAFQKYKEDVKQN
MAMLQKWKAALTEVADIVGWESKNFRTENELIQEIVKDVMQKLNHMYPTEVKTLVGIDQNLAPIESLLRLRSKEVRIIGI
WGMGGLGKTTIARALFDKLSSQFEASCFLANVMAEHEKQGLDYLRNKLLSEILEDVNPRISTSKVRSTFVMKRLRQKKVL
IVLDDVDDPKKLEDLAAQHDCLGSGSRVIVTTRDKHVLSKGVDAIYEVKGLSLDHAVRLFSLKAFGKTYPERGFEMLSKQ
AVDHANGNPLALKVLGSVLHSRNEQQWDNAMRKFKKVPNAEIQNVLRWSYDGLDYEQKNMFLDIACFFRGENKEN
VIRLLDICGFYAYIGIKILLEKGLITFSDDGDVCMHELIQEMGWEIVHQESIKDPGSRSRLWDLKEVYDVLKNNRGTEAVE
GIILDVSQIRVLLLSCETFSRMINMRFLKFYMGKGRKCNLHLPSGLQSLPNKLMYLQWDGYPSKSLPSTFCPENLVVLSM
MESHVEKLWDGTKCLPSLKEMNLHASKKLTNLPDLSQAPNLETIDVSNCTSLLHVPLSIQYVKKLLLFNLESCKSLKSLPR
NIHLASLEMFILRRCSSLDEFSLTSENMTRLDLRETKIADFPESVWQHLNKLVYLNLESCNKLKSLTSKIHLKSLQRLNLRDC
SILEEFSVTSESMEYLNLRGTSIRELPTSVWRNNKLYTLVLHSCKKLVNFPERPKVEDLPLVSSSERPNMDELWTLSSLADL
SLKGSTVENLPASIKDLPSLKKLTLTECKKLRSLPSLPPSLEDLSLDESNIVCLPVSIKDLSHLRKLALINHKKLLTPQDLPPSLK
APLLTESKVDPHLVSMKGLSQLQMFPQVKWKMFHSLPELPPFLEEFSLSESNIKFIPESIKNLSHMRKLAFTKCTRLQYLP
ELPPNLEDLFVSGCDIESLPTSIRDLVHLRKITLIECKKLKALPELPQCLQSLCAADCTSLKIIRSTKNILIEDRYTFYWNCINLD
QKSRNNIIADAPFEAAFTSLKERTPLGPLISICLPGTEIPDWFSHQSTNSSLDMEIPLEWFVDSMFLGFALCLVIGGFQRNS
NEGYDPDINCYHFVKSASYSDPSVPFLGHCTTVMQVPRGFNSDHIFICYYPSFNASILQDFKDLSLYYDANDLRLRVIFKF
KGPSQRLDIVKKCGVRPLLIANTERLHIESELQPE

>Vang0279s00180.1

MIPALLVEKPWLCYNAFLVGNHMLSAHKICSLLIFFPVASYNPSSSVSILQSCNMAGAILEIVLENLNSLEVGLCLGFNLD
MKRLASLLTTIKETLEDAEQKQLSNRAVKDWLEKLKDAAHVLDDILDEFAYESLRLEHQGVKCGPSNKVQMSCLSSIHPK
NVAFRCKIAKKMKKITERLEEIAEGSTNFHFTKMEMFPWRNSAVTEWHQTKPFITDSKVYGREGDTDKFIDFLTGYVSH
SENASVYTIVGEGGLGKTTLAKLIFKHERIINHFELRIWVRVSEDFRLKRVIKAIIEVASRHPCEDMDIEPLQIRLVDLLQRKR
YLLVLDDVWIDELESWSQLKYVLLSCGAKGSSILVTTRLPNTVAASIGTVPPHVLSRLSDNDCWEIFKEHAFGLNEVEQEV
LVATGMEIVKKCRGVPLAVKIMGGMFSFKREQKEWLYVKESNLWSLPQHEKNSVIPILRLSYLDLPMKLRQCFAYCARF
PKGETISKEDLIEQWMANGFIPSSETFDAEDTGDSMWNELYWRSFFQDIETDELGKVKTFQIHDPVHDLTQFLAEDVC
CIAHDNDVTTLSDTIHHLADHRAAWDVSEESINSMQLHQVKSLRTYIGQLYPVVFKCYSLRVLWCTAVKELSNSICHLKH
LRYLNLSQGNFKTLPESLGKLWNLHILKLDYCYRLKQLPNRLTRLKALQQLSLKDCYNLSSLPPHLGKLTSLKKLSAYLVGSE
RGFHLAELGSLHLKGYLHIKHLEKVKSVTDAKEANMSNKQLHKLYLSWDENEESELQQNVEEILEALQPDTQQLQNLTL

QGYKGENFPLWLSSSSSLKDLAIVGCRKFILSSGFRCVEDLLVYRCKEVEHLHESLQRMSSLHSLRLWDLPNVESLSGCFA
NLPLLQSLSLLDLPKLKSLPDCFGRVPLLRSLTLRDLPNVESLPDCFGNFRLLREMVIGDCSKLACLPTSLNLKSLEKVEIYNC
PSLYKRREEEN

>Vang0103s00300.1

MDFVSGSTVTTITKQIMALAVVGGALLSAFIDVLFDRLASPEFVNLIRGKKPDKLLQKMKSQLLVVKVVLADAEKRQITD
SNVKEWLDLLNDVVYRADDLLDEVYTKAATQKEVSSSFSHLFKRNKVVNVSKLEDIVERLDDILKQKESLDLKEIAVENN
QPWNAQTTSLEDRYGMYGRDEDKEAIMKLLLEDSSDGEEVSVIPIVGMGGVGKTTLTRSVYNDGKLNEIFKLKAWICV
SDIFDIVKVTKTMLEEITQKPCKLSDLNLIQLDLLEKLKGKKFLIVLDDVWIEDCDSWSSLTKPFLSGISGSKVIVTTRNESVA
AVVPFHYVKVYHLNKLSNEDCWLVFASHALPLSEDSENRETLEKIGKEIVKKCNGLPLAAQSLGGMLRRKHEIKDWNXV
LESDIWELPESQCKIIPALRISYNYLPPQLKRCFVYCSLYPKDYEFQKHXLILLWMAEDLVKASKKGKTLEEVGQXYFDDLV
SRSFFQCSSSRDWGDYFVMHDLMHDLATFLGGEFYFRADEHGKETKIDRKTRHLSFTRFGDSVSDTEVLDRVKFSRTFL
PTYDEYSPFKNRNTPCIIVSMLKYLRVLSFFNLQCQLVLPDSIGELIHLRYLDLSSTGIETLPESLCNLYNLQTLRLCCCFRLTK
LPSAMQNLENLRHLEIRDSSIKEMPKRMGKLNQLLTLDLYIVGKHKENSIKELGGFPNLHGWVSIQKLENVTNGEEALEA
GILNKKYIKTLDLQWSLCNDSSIDFQIELDVLDKLQPHQDLKSLRVDGYNGTRFPKWVENFSYRNMNSLFLKNCNNCC
MLPSLGQLPSLKHILISKMNSLKTIDAGFCKKEDCSSMAPFPSLESLYILDMPCWEMWSSVDSKAFPVLQELYIENCPKLK
GDLPNHLPSLQTLKIKSCQLLVSSFPMAPALRTLKICESNKVELHAFPQSVESIKISGRPMVEFMMEAITDIPPTCLKKLSLI
DCSSAISFPGDRLPSSLKTLDIRGLNKLSFPVLHKHKLLEFLSINNSCDSLKSFPPAIFPNLTSLIIKNCENLESLLLLGSESLKSL
NSFVIKDCPNFVSFPGEGFSAPNLTRFNVYGCAKLKSLPHQMGTLLPKMEYLSISNCQQIECFPEGGMPPNLTTVTIRNC
EKLLISLGWIPIDMVTSLQVYGPCDGINAFPMEGLLPSSITSLYLSDFSGLNTLECKGLLHLTSLQELHLVNCEKLENIAGER
LPVSLIKLSIRECPLLQKRCHRKDRQIWPKICHVRGIKVDGRWI

>Vang10g06640.1

MDIQEESSTTVGPLTTPSLRNMSSSSSAFFSANQSPFFSPRSSSCQLSESLQPDAPSDRIHLDVAAPSSSSGIPEPMSLVN
VGCTFSEVAASPAGCNAGLHGRKPPLIRFANWLRVELEIQGISCFVSDRAGYRNSCKLSIAEKAMNVASYGIVIITRKSFK
NPYTIEELQFFSGKKNLVPIYFDLSPADCLVRDIIEKRGELWEKHGGELWLLYGGLEQEWKDVVHGLSRVEERKLEAQDG
NWRDCILRTVTLLAMKLGRRSAAEHLTKWREKVKEEELPFTRNENFIGRKKELSQLEFMLFGDVTGDSRQDYIDLKARP
KRRHLTICRSKSNVQEERHVGNGSREEKTPVLWKESEKEIEMQNIEFSHRRSRLKRGGKYTRRKKGMRILYGKGIACISG
DSGIGKTELILEFAYRFHQRYKMVLWIGGESRYIRQNYLNLRSFLEVDASVENSLEKTRIKGFEEQEEAAVSGIRKELMRNI
PYLVIIDNLESEKDWWDHKLVMDLLPRFGGETHVIISTCLPRVMNLEPLKLSYLSGVEAMSLMLGSGKEYPVAEVDALRT
IEEKLGRLTLGLAIVSGILSELPITPSRLLDTINRMPLKDMSWCNKKAHSFRQNTFLLQLLDVCFSIFDHADGARSLATRMV
LVSGWFAPCAVSVSLLALAAQKIPEKQKGTCFWRKLLQSLTCGFTSSHTKKSELEACSLLLRFNIARSSTKQGHIHFNEMI
KLYARKREVTGSAQAMVQAVMNQGSISESIEHLWAACFLLFAFGHNPAAVEVEVSELLCLVKKVLLPLAIHTFITYSRCS
AALELLHLCTNALEAADQALVTPVDKWFDKSLCWRSIQTNAQLNPCLWQELALCRATVLETRGKLMLRGAQFDIGDDL
IRKAVFIRTSICGEDHPDTISARETLSKLTRLIANVQIRASA

4. NLR genes of *Vigna umbellata*

>XP_047146929.1

MVQELRDCGMFRHLKLIVQVRKLTGQVTDLVERGRFPEGIVGSAQESRGCALLTTELAGAMFQKNVSKIWDWLMND
GVLMIGVYGMGGVGKTSVLKHIHNMLLTAVTNFESVFWVTISKSFSIHKLQCDVAKIVGIDISKESDERKRAARLSRALV
RRKRCVLFLDDVWNHFPLERVGIPVRADGLKLVLTSRSLDVCRRMNCKNSVKVEPLSMEEAWTLFVDNLGQQTTLSPE

VKQVARSVAKKCAGLPLAIITLARSMRGVEEICEWRHALEELRNTEAKHEEMEMEVLRVLRFSYDHLNDKMVQQCFLC
CALYPEDFEIDRDVLIESFVDEGLVNGMKSLEAMFDEGNSIVNKLENICLLGKVENFVGGKKCMGLQDASSHIAISMMK
RGCQHVEPMNNVEGYYVGSQLVKMHDLVRAMAINVIKVNNNFLVKAGLQLTKIPDEVEWSEDLEKVSLMCNWIHEI
PTEISPRCPKLRTLILKHNESLTRISDSFFVHMAALEVLDLSFTDIEVLPKSVSDLSTLTALLLTSCKRLKHMPSLAKLQALLRL
DLSFTAITEMPQGLEMLVNLKWLNLYAKDLVSSGKEVAKLTSLQFLILHWWSRKIKVKVEYTSCLRKLETFAANLYNMH
HFNSYVKTMNIYGPRSYLLLLDTEESLGNSPWCCFAEVCFRKDVIISNCKIRTGESPLMLPLDIQRLKVERCHDIRSLCDV
MSLKNATSLKRCEIADCDGPEYMFSLSCSSSCCTSLHSLESLELYSLKNLHGLCKEGEVAAQTFPPGRAFTCLKYFFIYSCPL
IKKLITPRLLAYLPNLEEITVHNCKSMEEIISVDGIDYESFGGKKSYVTNRDTIVVTHSKLVSLSLKHLPELKSISSAQMECESL
KNFRIFKCPKLARFPETATPVQILYDSF

>XP_047146930.1

MIGIHGMGGLGKTTLSLAVYNLIADDFDSSCFLHNVREESNKHGLKHLQTVLLSEILGEKDINLASVQRGISVIQQRLRRK
KVLLILDDVDNRKQLQAFAGRSDWFGPGSRVIITTRDKQLLKSHEIERTYVVKELNKNDSLQLLVWNAFKREKVDPRYED
VLKRVVTYASGLPLAIEVIGSNLVGKSVEEWESAIEHYKRIPNGQILEILKVSFDALGKEEKYVFLDIACCFKGSSLKEVERIL
GVLYDNNMKHHIGVLVEKSLIKVGRGWFDVIEMHDLIEDMGRQIDLQKSPTEPGKRRRLWLGKDIIHVLKDNKVSKLLR
PLICFLNLI

>XP_047146931.1

MATPSFRRFTYDVFLSFRGEDTRYGFTGNLYKALCDRGLHTFMDDDKLQSGEEITPALQKAIEESRIAIIVLSHNYASSSFC
LDELATILHCQSKGLLVIPVFYKVDPSNVRHQKDSYQEALAKHQKRFKGQKEKLHKWKMALHQVADFSGYHFKDGYPP
YQSFYFIENYNVWNRDAYQYEFIGRIVERVSRVINHARLHVADYPVGLLSQVLKVKKLLDVGSDDVVHMIGIHGMGGL
GKTTLSLAVYNLIADDFDSSCFLHNVREESNKHGLKHLQTVLLSELLGEKDINLASVQRGISVIQQRLGRKKVLLILDDVDN
RKQLQAFAGRSDWFAPGSRVIVTTRDEQLLKCHEIERTYEVKELNKNDSLQLLIWNAFKREKVDPSYKDVLKRVVTYASG
LPLALEVIGSNLVGKSVEEWESAIEHYKRIPNGEIIEILKVSFDALGEEEKNVFLDIACCLKGSSLKEVEQILGVLYDNNMKH
HIGVLVKKSLIKVGRERFNVIEMHDLIEDMGRQIDLQKSPKEVGKRRRLWLGKDIIHVLKDNKGTSETEIICLDLSISEKEET
IEWNANAFRRMKNLKILIIRNGKFFKGPNYFPESLRVLEWHGYPSNCLPSNFDPNKLVTCKLPHSYFTSFGFLGSSKKFEN
LTVLNFDRCKFLTQTPDMSDLGNLEEVSFKDCESLVEVHDSVGFMSKLKILNAEGCIKLMSFPPLNLPTLERLELSXDAYQ
YEFIGRIVERVSRVINHARLHVADYPVGLLSQVLKVKKLLDVGSDDVVHMIGIHGMGGLGKTTLSLAVYNLIADDFDSSC
FLHNVREESNKHGLKHLQTVLLSEILGEKDINLASVQRGISVIQQRLRRKKVLLILDDVDNRKQLQAFAGRSDWFGPGSR
VIITTRDKQLLKSHEIERTYVVKELNKNDSLQLLVWNAFKREKVDPRYEDVLKRVVTYASGLPLAIEVIGSNLVGKSVEEW
ESAIEHYKRIPNGQILEILKVSFDALGKEEKYVFLDIACCFKGSSLKEVERILGVLYDNNMKHHIGVLVEKSLIKVGRGWFD
VIEMHDLIEDMGRQIDLQKSPTEPGKRRRLWLGKDIIHVLKDNKGTRETEIICLDLSISEKEETLEWNTNAFIRMKNLKILII
RNVSLFSLQKFENLTVLNFDWCIFLTQTPDMSDLGNLEEVSFKGCESLVEVHDSVGFMNKLKILNAEGCIKLMSFPPLNL
PTLERLELSFCFSLEKFPEILGKMGNIRVLQLQDLPIKELPLSFQNLDGLEELSLSCENVHFSSSIATMPKLFRFSVTNCRGW
QWVKSEDAEDNFGSMVPSKVEWFAAWSCNLDDDFFSGGLMRLAHVRCLFLRDNNFKHLPECIKEFHNLWALDVSHC
KHLEEIRGFPPKLEHFKAINCISLTSSSLSMLLNKVLSCF

>XP_047147332.1

MAESFLFSIADSLVAKLASDAFQEAFRLVGLYDNLRHFRKTLSLVKAVLLDAQQKQDHNHELREWLTQLKNIFLDAEDVL
DEFECETLRNKVVKGHGSTKDKVSHFFSTSNPLLFRYQMVQQIKDISNRLDKIASDRHKFILQIVDVDTRVVHRRDMTYS
RVSDSDVIGRKNDKQKIIELLMQQNPNDDDTSLSVIPIVGIGGLGKTTLAMFVFNDNKIRECFPLRMWVCVSDEFDIKQL
IIKIINSASDLASADAPSRQHKWKTLDLEQLQNQLTNKLFGQKFLLVLDDVWNENRVKWVDLRNLIQVSAAGSKILVTTR
SHSIASMMGTLPSHNLEGLSEEDSLSLFVKWAFKEGEEEKHPHLINIGRQIVKKCRGVPLAVRTLGCLLFSKFEASEWEYV
SHNEIWNLPQKNDDILPALKLSYDLLPSYLRQCFALFSLYPKDYVFNSYEMTSLWGALGLIALPEKNRTLEYVTNQYLDEL

QSRSFLQDFVKFGTTYKFRIHDLVHDLALFVSKDECVHLSSNIKYIPDNVRHLSFAESSLFGNLFTKKLVALRTILFPEVFNT
CLSKFKYLRVLDLRSSTIETLPRNFSKLKHLRYLDLGYNDNIKRLPESICKLQSLQVLKLDGCMELEALPKKLRKLISLRYFNFS
TTQSVFPNKEIASLGSLEIMSIKSCHNVESIFGGVKFPALKTLNVFDCPSLKSLSLDSQNFPQLETFFVGGCGNLDLQLWKS
HHEKESPKLKLKVVAFSRLSQLVALPEWLQEAANSLQYLLVLDCPNIETFPEWLSTLTGLKALRLIDCPNLISLPDNIDHLT
ALESLKIGSCPKLCKKYQPHVGELWSKISHIKNVMIDELEEPEEL

>XP_047147335.1

MAESFLFSIAESLIEKLASHAFQEASRLVGLYHDLRDLTKTLSLVKAVLLDAQQKQDHNHQLREWLTQLKIVFSEAEDVLD
EFECQTLRNKVVKAHDSTKDKVSHFLSTSNPLVFRCKMARQIKHINNRLDKVAADRHKFSLQIIDVDTRVVHRRDMTHS
RVSDSDVIGRKHDKEKIIELLMQQNPNDDDTILSVIPIVGIGGLGKTTLAKFVFNDNRIQECFPWKMWVCVSDDFDIRQL
VIKIINSANDVDVPPHQQNLNKMDLEQLQNQLKRKLSGKKFLLVLDDVWNEDRVKWVELRNLIQVSGAGSKVVVTTR
SHSIASMMGTVPSHILEGLSKEESLSLFVKWAFKEGEEEKHPHLVNIGREIVKKCRGVPLAVRTLGSLLFSKFEDSEWEYV
SDSEIWNLPQKKDDILPALKLSYDLLPSYLRQCFALFSLYPKDYLFNSYKMTALWEAHGLITLPKTNKTLEDVANXSHSIAS
MMGTVPSHILEGLSKEESLSLFVKWAFKEGEEEKHPHLVNIGREIVKKCRGVPLAVRTLGSLLFSKFEDSEWEYVSDSEIW
NLPQKKDDILPALKLSYDLLPSYLRQCFALFSLYPKDYLFNSYKMTALWEAHGLITLPKTNKTLEDVANQYLHELLSRSFLQ
DFENFGTSYIFRIHDLVHDLALFVATDECLHVRFNIQNIPENVRHLSFAGNSLFDNLVIKKSAAVRTVLFPNGAVGANGEA
ILNTCLSKFKCLRVLDLRGLTLETLPRTIAKMKHLRYLDISKNLNIKRLPDSICKLQSLQVLIIRGCVELEVLPKGLRKLISLRYLF
FSTKQIVLPVNEIAKLGSLEYLSIEWCHNVESIFGGVKFPSLKTLCVGDCQSLKSLSLDGQNFPELETLLVKNCGNLDLGQS
KGHHKEESPKLKLKLVLFFGLSQMVALPKWLQEASKSLQRLYVLNCDNIERLPYWLTTLTDLKALDLINCPNLVSLPDNIH
HLTALESLRIRGCPNLHKIYEPMLEGFGPKYHTSKTL

>XP_047147542.1

MKDSLNQFGCFNFHVPQGEMYLAEQSQKKIQYLTEEIQEHKKWKPLVLSNEYFGREFEKNVEKLWKLMRDDRVFIIGI
HGMGGVGKTFLANYMQNEIKRIKTFKDVLWVTVSHYFTIFKLQERIAKIIKVESFTQTMRGKEQCDDPDNDSHNDFDN
DDDDDEDYCDDYEEDWMLFLLKLKSYGTPLTLSPQMRNIARYVVKKCYGLPLGISMMARTMKGKTDIHWWRHVLNK
FDKLEMGVDMEEKVFTVLRRSYDNLSGKDLQKCFLYVALLPNSMFRNCLIGKLVNTELLDGNKSVEEIFDETNVIVDELI
NHSLLEAKEARVPSSVEVLSMHGLVRKMALNILRESVSKMMIKCNGNMPDTQKWAIDLEVVSLARNKIKTIPEGTSPN
CPRLLTLLLFENYITHIPECFFTHMNALTTLDLSENFFLRCLPHSLSNLRSLTSLMLNECNKLEDIPPLGELQSLLRLEISNCSI
RAPPEGLENLVNLKWLDLSMNKNLKLVTGSFLPSLTKIRYLDLSGCIGGIGVEDVKEMTMLECFAGTFVDHDNFDNYVE
EILNSTNGPQTYFIHMDHGSKMSLYSESCLSTFNGRIISFRNCKEVLYVLPLNIMKLLVYCNDYWV

>XP_047147745.1

QVKDKFGKNIFFVTVSKTPNLKTIVETLFEHCGCKVPKFQTDEDAVNRLEVLLRVLGKHPILLVLDDVWPGSEDLVEKFKI
KIPDYKILVTSRVSFPRFGTSYQLDKLDHVHAESLFRHFAQLKDKSSDIPENLVDEIVKGCKGSPLALKVIAGSLCNQPFVV
WQNMMERLQGQSILESDSTDLLSRLQQSLDILETKFSINEKECFMDLGLFPEDQRIPVVALIDMWAELYNLNEDGSNA
MSIIHDLVSRNLINFIVTRYMASPPFSLLVI

>XP_047147873.1

MPVLETLGGALFGVVLQMLFDKLDSHQVLGFFRGRNLDEKLLKKLKRKLMDINAVIDDAEQKQFTNSLVKEWLDEVRD
VLYDAEDLLEQIDYEFSKTKLEAEFQTSSSKVYSFESKIIALLDDLESLLNHKIVKDFKIYSGVRSELGNKVSDKKVESTSLVAE
EVIYGRNEEKEMIFTWLRSDTDDNKQSILSIVGMGGMGKTTLAQHVYNDPKTEEAKFDEKAWVCVSDAFDALRVSKAI
IGAFTNSRDDSDDLEMIHGKFKKRLSGRKFLLVLDDVWNEDRNQWKALQTPLTFGAKGSKILVTTRSHKVASIMQSTYI
HQLKQLDEDHSWQTFAKHAFHDQDSELKSELKEIGMKIVKKCQGLPLALETIGCLLQSKSSVSEWEGVLTSEIWDLPIED
SKIVPTLLLSYYHLPSHLKRCFAYCALFPKDHRFDKESLILLWMAQNFLHCSQQSKSPEEVGEDYFNDLVSRSFFKQITWD

KEIYFVMHDLLNDLAKYVSGEICYRLGVDGEKGVSRKTRHISYVSGLSQCYTNLCDAKGLRTFITFQGWDCPMSIEESISN
FKFLLVLSWRWCIKVPDTIGDLIHLRSLDLSGADIKRLPDSTCSLYNLQELKLNNCFKLKELPLTLHELTNLRRLELKGITLTK
VPHRLGELKNLHVWINKFAVEKSSEFSIQQLGELDLRAEELSINNLENIANPCETNLKNKTHIVSLSLRWNLKRNNDDSVK
EREVLENLQPSRHLKHLSIDGYLQMISQEQPHNHLKYLWIEECSEFESFPNEGLFAPQLVSFNIRGLKKLKSMPKCMSALL
PSLNRVDIFYCPALEMSEGYLPSNVKKMFLGDCSKLVASLKGAWGTNPSLKILYICNVDLECFPGEGLLPLSLDHLEIYDCP
NLKKLHYKGLCHLSSLQTLVLRDCPILQCLPEEGLPKSISKLEIVYCLLLKQRCKKQGEDWEKIAHIKSICVDYVNEKRL

>XP_047147874.1

MPVLETLGGALFGVVLQMLFDKLDSHQVLGFFRGRNLDEKLLKKLKRKLMDINAVIDDAEQKQFTNSLVKEWLDEVRD
VLYDAEDLLEQIDYEFSKTKLEAEFQTSSSKVYSFESKIIALLDDLESLLNHKIVKDFKIYSGVRSELGNKVSDKKVESTSLVAE
EVIYGRNEEKEMIFTWLRSDTDDNKQSILSIVGMGGMGKTTLAQHVYNDPKTEEAKFDEKAWVCVSDAFDALRVSKAI
IGAFTNSRDDSDDLEMIHGKFKKRLSGRKFLLVLDDVWNEDRNQWKALQTPLTFGAKGSKILVTTRSHKVASIMQSTYI
HQLKQLDEDHSWQTFAKHAFHDQDSELKSELKEIGMKIVKKCQGLPLALETIGCLLQSKSSVSEWEGVLTSEIWDLPIED
SKIVPTLLLSYYHLPSHLKRCFAYCALFPKDHRFDKESLILLWMAQNFLHCSQQSKSPEEVGEDYFNDLVSRSFFKQITWD
KEIYFVMHDLLNDLAKYVSGEICYRLGVDGEKGVSRKTRHISYVSGLSQCYTNLCDAKGLRTFITFQGWDCPMSIEESISN
FKFLLVLSWRWCIKVPDTIGDLIHLRSLDLSGADIKRLPDSTCSLYNLQELKLNNCFKLKELPLTLHELTNLRRLELKGITLTK
VPHRLGELKNLHVWINKFAVEKSSEFSIQQLGELDLRAEELSINNLENIANPCETNLKNKTHIVSLSLRWNLKRNNDDSVK
EREVLENLQPSRHLKHLSIDGYLQMISQEQPHNHLKYLWIEECSEFESFPNEGLFAPQLVSFNIRGLKKLKSMPKCMSALL
PSLNRVDIFYCPALEMSEGYLPSNVKKMFLGDCSKLVASLKGAWGTNPSLKILYICNVDLECFPGEGLLPLSLDHLEIYDCP
NLKKLHYKGLCHLSSLQTLVLRDCPILQCLPEEGLPKSISKLEIVYCLLLKQRCKKQGEDWEKIAHIKSICVDYVNEKRL

>XP_047148180.1

MAGAILKIVLENLNSLEVGLCLGFNLDMKRLASLLTTIKETLEDAEQKQLSNRAVKDWLEKLKDAAHVLDDILDEFAYESL
RLEHQGVKCGPSNKVQRSCLSSIHPKNVAFRCKIAKKMKKITERLEEIAEGSTNFHFTKMEMFPWRNSAVTEWHQTKP
FITDSKVYGREGDTDKFIDFLTGYVSHSENASVYTIVGEGGLGKTTLAKLIFKHERIINHFELRIWVRVSEDFRLKSVIKAIIEV
ASRHPCEDMDIEPLQIRLDDLLQRKRYLLVLDDVWIDELESWSQLKYVLLSCGAKGSSILVTTRLPNTVAASIGTVPPHVL
SRLSDNDCWEIFKEHAFGLNEVEKEVLVATGMEIVKKCRGVPLAVKIMGGMFSFKREQKEWLYVKESNLWSLPQHEK
NSVIPILRLSYLDLPMKLRQCFAYCARFPKGETISKEDLIEQWMANGFIPSSETFDAEDTGDSMWNELYWRSFFQDIETD
ELGKVKTFQIHDPVHDLTQFLAEDVCCIAHDKDVTTLSDTIHHLADHRAAWDVSEESINSMQLHQVKSLRTYIGQLYPV
VFKCYSLRVLWCTAVKELSNSICHLKHLRYLNLSQGNFKTLPESLGKLWNLHILKLDYCYRLKQLPNRLTRLKALQQLSLKD
CYNLSSLPPHLGKLTSLKKLSAYLVGSERGFHLAELGSLHLKGYLHIKHLEKVKSVTDAKEANMSNKQLHKLYLSWDENEE
SELQQNVEEILEALQPDTQQLQNLTLQGYKGENFPLWLSSSSSLKDLAIVGCRKFILSSGFRCVEDLLVYRCKEVEHLHESL
QRMSSLHSLRLWDLPNVESLSGCFANLPLLQSLSLLDLPKLKSLPDCFGRVPLLRSLTLRDLPNVESLPDCFGNFRLLREM
VIGDCSKLACLPKSLNLKSLEKVEIYNCPSLYKRREEEN

>XP_047148252.1

MAVRSHSQEFTYDVFLNFRGSDTRYGFAGNLYKALDDRGIHTFIDDKKIRGGDELAPTLVKAIQGSMIAITVLSITYASSSF
CLDELVYILERAEEKKLLVLPVFYDVDPSFVRHQKGSFGEALAWHEERLKADNKERLNHHMEKLEKWKMALHQVANLS
GFHYKYGEGYEYKFGIKIVEWVSSEINRTPLHVADYPVGLDSQELKVMKLLDVGSSDGVLMIGIYGMGGIGKTSLASAVY
NLIASHFDGSCFLQNVREKSRKHGLEHLQSIILSEILGDKRIMFASEQQGISMIQHRLQNKRLLLILDDVDKHELLQTLVGR
PDCFGPGSKVIITTRDKQLLASHNVQKTYKVKKLNKNHALQLLTWKVFKSEQVYPEYEEVLNRAVTYACGLPLALEVIGA
NLCGKSIQECKSVIDQYKRIPNNRIQETLKVSFDALQEEEKRVFLDISCCFKGYKLTEVEDILHAHHGACMKYHIGVLAEKS
LIKIGQYDCVTLHDLVEDMGKEIVRQESPEEPGKRSRLWSPKDIIQVLEDNTGTSEIKIICLDFPLFEKKMVEWNGMAFQ
KMQNLKTLIIRSGIFSDDPKCFPNSLRVLEWWRYPSHCLPSYFQPKQLAICKLPHSLFMSFEMDGLSKKLRNLRVLNFDC

CEGLTQIPDAVSDLQNLEELSFQNCVNLVRVHNSVGLLEKLRILEASGCIKLRNFPPLKLTSLEKLELSHCSSLERFPEIIGKM
ENIRELRLLGTLIKGLPLSFQNLTRLKKLSLLFCGIVQLQSSIVMMPELTLIEAWGWEGWQWIKWEEDEEKDGSMVSSK
VELLWAPKCNLRDDFFQIGFTRFAHVKDLDLSNNNFTQLPECIKECQFLKKLDVSCCKHLREIRGIPPKLKHFNATNCLSL
TPSSISMFLNEDLYENRKTMLVLPGSRKPEWFNYFCYERSSSFWIRNKFPGKVLCLFVAPKDGDISDYVKLMLVINDKFY
ACFFDIKFLKLGAEHTFLFDLRNLIFTDNLYKVPLVNEWNXRAEEKKLLVLPVFYDVDPSFVRHQKGSFGEALAWHEERL
KADNKERLNHHMEKLEKWKMALHQVANLSGFHYKYGEGYEYKFIGKIVEWVSSEINRTPLHVADYPVGLDSQELKVM
KLLDVGSSDGVLMIGIYGMGGIGKTSLASAVYNLIASHFDGSCFLQNVREKSRKHGLEHLQSIILSEILGDKRIMFASEQQ
GISMIQHRLQNKRLLLILDDVDKHELLQTLVGRPDCFGPGSKVIITTRDKQLLASHNVQKTYKVKKLNKNHALQLLTWKV
FKSEQVYPEYEEVLNRAVTYACGLPLALEVIGANLCGKSIQECKSVIDQYKRIPNNRIQETLKVSFDALQEEEKRVFLDISCC
FKGYKLTEVEDILHAHHGACMKYHIGVLAEKSLIKIGQYDCVTLHDLVEDMGKEIVRQESPEEPGKRSRLWSPKDIIQVLE
DNTGTSEIKIICLDFPLFEKKMVEWNGMAFQKMQNLKTLIIRIGIFSDDPKCFPNSLRVLEWWRYPSHCLPSYFQPKQLA
ICKLPHSLFMSFEMDGLSKKLRNLRVLNFDCCEGLTQIPDAVSDLQNLEELSFQNCVNLVRVHNSVGLLEKLRILEASGCI
KLRNFPPLKLTSLEKLELSHCSSLERFPEIIGKMENIRELRLLGTLIKGLPLSFQNLTRLKKLSLLFCGIVQLQSSIVMMPELTLI
EAWGWEGWQWIKWEEDEEKDGSMVSSKVELLWAPKCNLRDDFFQIGFTRFAHVKDLDLSNNNFTQLPECIKECQFL
KKLDVSCCKHLREIRGIPPKLKHFNATNCLSLTPSSISMFLNXDLYENRKTMLVLPGSRNPKWFNCLSNEPSSSFWIRNKF
PGKVLCLFVAPKDGDISDYVKVMLVINDKVFVCFFDRLKFLKLGVGHTFLFDLRNLIFTNNLYEVPLENEWNHVKVIYFDR
KQPQCXIKSGIHVFKQESSDEDIMFTDPYAKIREKEKEERHNVRMQSWQGYKIVGNPSVSLSPTLERNEKVEWFRRRKT
H

>XP_047148419.1

TYILFYKSWVRLEHILVEEIIDDILKKLNRYSVSYNQGIIGIEKHIEEIRSLLDLESPDIRIIGICGMGGMGKTTISEQLYHTLAV
QFDSHSLVLDVQEKLQREGIHNIIAKYWSELLKETSSSHLSYYNERMKRTKVLLILDDVTDSTLLKKLMEGCDRFGLGSRII
MTSRDRQVLKNAGTDDIYELKELNFYDSLKLFNLHAFKQNSSKEITYMDLSIKVLRYAKGVPLALQILGSLLYDRQREAWE
SQLQNLEKCQDHDIFNVLKLSYDGLREAEKNIFLDIACFYRGHNEIVVAETLNDCGFSSKIGMDVLKDKCLISILDGRIVM
HDLIQEMGQEIVRKECPQHPGKRSRLFKADEIHEILKKNKMQYNVYYKI

>XP_047148520.1

MALAVVGGALLSAFFDVLFDRLASPELLSLIRGKKPDKLLQKVENHLIVLRVVLADAENRQITDSNVKKWLDVLRDIVYEV
DDLLDEVSTKAATQKEVSNSFSHIFNRKRIVSINKLEDIAERLDDILKQKESLNLKEIPVESNQPWKDQPTSLEDRYGMYG
RDKDKEAIMKMVLEDNTDGEEVSVIPIVGMGGVGKTTLARSVYNDGKLKQIFDLKTWVCVSDIFDTVKVTKTMVEEIIK
MPCNLYDLNLLQLELMDKLKGKRFFIVLDDVWIQDFDSWNSLTKPFLSGIRGSKVLVTTRNESVAAVVPFNVVKVYHLN
QLSNEDCWLVFANHAFPLSEDSENRGTLEKIGKEIVKKCNGLPLAAQSLGGMLRRKHALRDWINVLESDIWKLPESQCK
IIPALDXFIIIPPVXKRCFVYCSLYPKDFEFRKDELILLWMAEDLLKAQRKEKTLEEVGEEYFDDLVSRSFFQCSSLRPSSNYFI
MHDLMHDLATFLGGEFYFRANELGKETKIDRKTRHLSIERFSDPVSDIEVFERANFSKTFLLTL

>XP_047148609.1

MATPSSRRFTYNVFLSFRGKDTRSGFTGYLYDALCDKGVHTFMDDEELQSGEEITPALQKAIEESRIAIVVLSHNYASSSF
CLDELATIIHCQSKGLLVIPVFYKVDPSNVRHQKGSYEEALAKHQKRFKGQEEKLNKWKMALRQVGDLSGYHLGDGNE
YEYKFIESIVKRVSRVINRVPLHVVDYPVGLSARVLKVKKLLDVGSDGVVHMIGIHGMGGLGKTTLALAVYNSIADDFDQ
SCFLLNVREESNKHELKDLQSILLSKMLREKNIILTSWQEGASMIQERLGRKKVLLILDDVDNREQLQAFAGRADWFGPG
SRVIITTRDKQLLKSYEIERTYEVEELNENDSLKLLIWNAFKRENVDPSYQDVLKRVVTYASGLPLALEVIGSNLVGKSVEE
WESAIEHYKRIPNGEILEILKVSYDALGKEEQSVFLDIACFFKGGSSLEEVEHILRVLYDHSMKHHIGVLVDKSLIKVLWDDE
GFGEIEMHDLIEDMGRHIDRQKSPEKPGKRRRLWLGKDILHVLKHNKGTGKTEIIRLDLSISEREETLEWNPNAFRRMK
NLKMLIIRNGKFSEGPSYFPKSLRVLEWHAYPSNCLPSNFDPSKLVTCMLPYGQFTSFGFLGSSKASLKSVFLIL

>XP_047148768.1

SLPKLKRLNLSFSQKLTRIPDLSLSPNIEEIILNDCPELIKVHSSILLTKLIFLRLDNCDNLKSVTVPSNILSRSPGCILLYSCCKLE
MFSTTQTQICYSPPSSSLIGRQNFSGVPLNTGSLKRLIPIRPYSSEIFSITFDRYWGTDDEKEVTNNNVYADDEVLLQLTRY
MSLNTPSPKNLCFINLSKCSSLTIFLFDLSEMKFLKKLCLSGCSKLENFPEIEDTMENLAVLILDHTAIQALPSSLWRLVGLQ
QLSLRSCMXIERDPIDTVREKYMSEVVNMSEYRLKRMRILIILDDVTDSVQVKELLGRHDSFGQGSRIIITSRDKQVLRNA
GADDIYDVEELNKDDSLKLFSLHAFQQNSSQEITYKDLTKKVLRYAKGIPLVLQILGSLLFGRTREAWESLLQKLKKFQHSK
IFSVLKLSYDGLDDEEKNIFLDIACFYRGCKESEVVKRLDDCGFSSKIGMDVLKDRCLISIVDGRTKMHDLMREMGEEVD
GRIEMHDLIQEMGQEIVRQECPQYPEKRTRLFNADEIDEVLKKNKVCYIVTQLKESHGFTFLKLQTTYYLEQLWEGDQFI
YFLWQSLPKLKRLNLSFSQKLTRIPDLSLSPNIEEIILNDCPELIKVHSSILLTKLIFLRLDNCDNLKSVTVPSNILSRSPGCILLY
SCCKLEMFSTTQTQICYSPPSSSLIGRQNFSGVPLNTGSLKRLIPIRPYSSEIFSITFDRYWGTDDEKEVTNNNVYADDEVLL
QLTRYMSLNTPSPKNLCFINLSKCSSLTIFLFDLSEMKFLKKLCLSGCSKLENFPEIEDTMENLAVLILDHTAIQALPSSLWRL
VGLQQLSLRSCMNLEIIPSSIGSLTRLCKLDLTKCESLQTFPSTIFKLKLRKLDLCGCLRLRTFPEIMEPTQTFAHINLTETAIK
ELPSSFGNLVKLRSLQLNNSSDLESLPNSIVNLKHLCKLDCSGCAKLTEIPTHIGCLSSLMEMSLSESGIVNLPESIAHLSSLK
SLDLSYCKKLECIPQIPPCLKQLVALDCPSIKRVMLNSLVRNLSESKEGVFKFHFTNAQQLDSGARANIEEDARLRMTDDA
YRSVFFCFPGTAVPHWFPFRSEGSSVSINEDLSFCSDDRLIGFALCVVFELLDTNQIIGRRDSFRYCLKFECDDDGTQIILKE
DLLHNYFEWNGKERVVNQDHTFMWKFNLESLRRSGMNLRLSDARSFTFEITPYYVYFVGSNFYSVANELKSVVTIKECG
LCPLYRSGSNVGESSRETTEHRKRKAESHLVG

>XP_047149246.1

VLLVLDDISKKEQLHATAGGLDWFGPGSIIIITTRDKHLLDVHGVQKQYMVGGINFMEALELFKWNAFKNKEVDPCYKE
VTERAMYYANGLPLALETIGSNLFGKTLDEWESALETYERIPNRDVQEVLKVSYDSLDAYEKEIFLDIACFFRRCSLGYVTD
KLEARGFPPKFGLRVLEEKSLIKIRECPHETVTMHDMIRCMGKEIVRQQKPLPHKRNRIWFYEDVVCVLEKNMENDKIE
AVMLEMPEHQEEMQCTPKFGKMKSLRMLIIEKKVSFLGSPATLPNSLRVLEWRGYPATSLPSNFHFKNLVILNLSHSYF
GWYKPLQNSKVLRHLIIIGCKNIRRIPDISGFPNLTKLCVRECTNLFEIHDSVGSLLHLKEFCAEGCTKLTIGPSRIKLISLEHL
CFRGCSSLVMFPEVLAPMHKLKYVDLGGTGIRNLPPSMQNLKGIKVLSMGKGQMLEINESSNFFQNLPMFFPNLETLY
LQNLDITILPASIEECHSLKFLHVTNCKKLQEIRGLPLNINQFYAANCSVKANSLILKLRQAIDCGAMGICVLPGRKIPELFD
HSSRGNSVSFWFRKELPTLAVCAIIGVWDNVKPPFVAFFRFVVGVEKNIYKCVCCFRCRNIRWTTEDSHIIILNSRNDFQ
HTLNTDIQTVLLTNEWIPGKILLTIRPKSDLGKSGEIRRTGVYVNRTLSSMEDVRFEDPYDLNKASAIENKLVLLGEASDTQ
QQAQSSALFNLTTSLESLIGEQPLNYEYNNSYSKESASTVDSNNQGVLVDDQPILAPSSAETQNAEAKREMAQLISEVSS
EFTAQVSMKIESTASQSPKVDEVEAGLETQNQFRNTMVLENQIHQRLTRIKKMKEELGEKISAIKADISAIEANNSSIKAN
ISDIKAIFLQIDQRFIR

>XP_047149268.1

MGGIGKTTIARAIFDKCSLQYDGCCFFNVREELERHGFSNLRERLIYELLEGEGLHTNKARFFSSALRMLGRKKVMVVLD
DVNTSEELRYLVTKPVCFGAGSRVIMTSRDQDVLTSGGLDQIHEVKEMKPLDSLKLFCLIAFNESQPKMGYEKLTEEVLKI
AQGNPLALKVLGAEFHSRSTINTWKCALSKFKKYPNEKIQSVLRFSYDGLHEVEKKAFLDIAFFFQEDTKAYVIEQLDAW
GLHGASGVEVLQRKALITVSNENIIQMHDLIRQMGCEIVRQECITHPGRRTRLRDKEEVYNVLRYKLGTDKVEGMQVD
VFRIKDLSLKVGTFKKMPCLRFLKFYLPDDNLFLPPNPDGTLWYEKRHFPLLLSAWCKDLMRVACEIQIKCVDYVYFDGY
SHSSLTSTLRNRGREIRSSTSISLTELTGSPGDLECSDMLDQQFKILTDGLLCLRSTYYLKLSKSTGQDSGKPKLHVLFDSLRF
YERISVGKLKNSDIEGNRMLFLYFAGFNFLFRPFLLRRPWFHFLFSFRFAFFCTLFLYLLWKMIL

>XP_047149310.1

MIRETLYGKKVLIVLGDVPNNYELLALWTYNHYWFSKGTVIIITTSDEDLLMNQPIDSIFRIERMNAEESLELFSWHAFRE
PKPKDEYEDLARRVVRYCGGLPLALEIVGKIIKVSIEGSLNEMEKDIFLDVCCFFVGKSKAYVRKILNGCGVDADIGIRVLM
ERNLIKINKNNKFGIHPLLQEIGEQIIREKSGNDLGKNRRLWLDKDAKYGTEALQWFPVKLPLVNNGFQDVQPT

>XP_047149435.1

MPVLETLGGALFGAVLQVLFDKLDSHQVLDFFRGRNLDDKLLKKLKRKLMDVNSVIDDAEQKQFSNSLVKEWVDEVRD
VLYDAEDLLEQIHYEFFKSESEAELHASASKVRNFESKMIEVLDDLESLLSQKVVHDFKISTGVRSGFGNKVSAKKVESSSL
MAKDAVIYGRDKDKEMIFSWLTSDTNDNKLSILSIVGMGGMGKTTLAQHVYNDPKTEEANFDEKAWVCVSDAFDAL
RVSKAIIGAFTNSRDDSGDLEMVHGKLKKKLSERKFLLVLDDVWNEDRNQWKALQTPLTFGAKGSKILVTTRSHKVASI
MQSTYIHQLKQLDEDRSWQIFAKHAFQDENTKLNSELKEIGIKIVEKCQGLPLALETIGCLLQSKSSVSEWEGVLTSEIWD
LPIEDSKIVPTLLLSYYHLPSQLKRCFAYCALFPKDHRFDKES

>XP_047149436.1

MAQNFLDCSQQSKSPEEVGENYFNDLVSRSFFKQIIRNKKTYFVMHDLLNDLAKYVSGEICYRLGVDGGKRVSRKTRHL
SYVSGPIQYCTSLCDAKGLRTFITFRWREMSIEELISNFKFLRVLAWRWCIKVPDSIGDLIHLRSLDLSGTDIERLPDSTCSLF
NLQELKLNNCVKLKELPLTLHELTNLRRLELKGTTLTKAPLHLGKMKNLHVWMDKFEVGKSREFSIQQLGELDLHGELSIK
NLEKITNPYETNLKNKTHIVWLSLQWNLKRNNADSMKQSEVLENLQPSRHLKQLVIYCYGGTKFPRWLSDNSLTNVLSL
TLKNCKYCLFLPSLGLLTFLKHLRIDGLDQIVKIDAEFYGNSCSAFASLETLRFTDMKEWEEWQCMTGAFPSLQSLSLTNC
PKLKELPDKLCHLKKLTVKNCGKFGAPIPRAVESQCVNMRPSSFDMTWLPHISDIINHWYYSLVELYINGCDSLTTFPLDL
FPKLGKLSLVECCNLQMISQGHPHNHLKRLSIEKCSEFESFPNEGLSASQLETFFIEGLEKLKLMPKCMSALLPSLNDLYIR
NCPVVEFSEGCLPSNVKTMRLRYCSKLVASLKGAWGTNPSLKVLNIGNVDLECFPGEGLLPFSLPEIFIRDCPNLKKLDYR
GLCHLSSLKKLTLDNCPILQGMPEEGLPKSISTLIIEDCPLLKQRCKKQEGEDWEKIAHIKSIIVDNKEVNI

>XP_047149437.1

MPVLETLGGALFGAVLQVLFQKLDSHQVLDYFRGRKLDEKLLKILRRKLVSMNAVVDHAELKQFRNEYVKTWLDDVRD
VLLDTEDLLDEIELEYQRSASKVHTFESKLKEVLDDLEFLLNQKDDLGLKNAGGIGYELGNKVLERKNESSSLVAEHIICGR
DEDKEIILNWLTSGNGNLNQLSILAIVGMGGMGKTTLAQHVYNDPKMKEAGFDEKAWVCVSDEFDVLKVSKAIIGALT
KSKDDSEDIEMVHGKLKEKLTGRKFLLVLDDVWNEDRNLWKTLQTPLRYGAKGSKIILTTRSNKVASIMQSSYVHQLKQ
LQKHYSWQVFAKNALQDDNSMLNCELEEIGMKIVEKCKGLPLALETLGCLLHTKSSVSEWEGVLTSEIWDLSIEDSKIIPA
LLLSYYHLPSHLKRCFAYCALFPKDHKFDKETLILLWMAENFLQCSQQSKCPEEVGELYFNDLLSRSFFQQSIKDNETCFL
MHDLLNDLAKYVSGEICFRLGVDKAERVPKTTRHFSTVINPIKYRKSLCDAKGLRTFISFWADCEISIQELISNFKFLRVLSL
HCFKVKEVSDTIADLKHLRSLDLSNTGITKLPDSTCSLCNLQVLKLNKCFNLQELPSNLHQLTSLRCLELVGTTLRKAPVHL
GKLKNLRVWMGRFEVGKSSQSNVQLGEFELHGQLSIRSLENIVNPYDAMAANLKNKKHLVGLNLEWSLNRNNDDSIIE
REVLENLQPSRQLKHLLIDGYGGTQFPRWLSDNSLLNVESLSLNNCRHCQLLPSLGLLTFLKHLTIHGLDCIVRIDADFYG
NSSFAFASLETLSFNDMKEWEEWQCMTGAFPSLHCLSVMNCPKLKGNLPVQLSRLKKLTIGQCKQLVASIPKAIEIEGV
KMEPSSFDTPLESLSIYSCPGMNIPTNHWYSLHVELDISQCCDSLTNIPLDIFPKLCDLCLEECHNLKMISQEHCHNDLKTL
SIEKCSQFESFPNEGLFAQKLERFRIEGLEKLKSMPKSMSVLLPSLNYLSIRNCPEVELSEGCLPTNLKEMRLKNCSKLVASL
KGVWGTNTSLKSLYIGEVDVEFFPCEGLLPLSLTNLAIYDCPNLKKLDCNALSHLSSLEKLDLLSCPSLQCLSEQALPKSISEL
RIKNCPLLKQRCKKQEGEDWEKIAHIKYIDFD

>XP_047149498.1

MAMGSRSSSFTYDVFLSFRGEDTRHGFTGHLYKALHDRGIYTFIDDEELQRGEEITPALVKAIEESRIAITVLSINYASSSFC
LNELDYILECFNKKYMLVLPVFFKVDPSDVRHQKGSYGEALAKHEQRFNHSMEKLENWKKALHQVANLSGFHLKHGD
GYEHEFIGKIVELVSSKINHAPLPVADYPVGLESQALAVRKLLDVGSDDVHMIGIYGTGGIGKSTLALAVYNLIVHHFDCS

CFLQNVREKSNKHGLQHLQSILLREMLGEKEANFASVEQGASVIHHRFQRKKVILILDDVDKYEQLQAIVGRPCLFGPGS
RVIITTRDKQLLSSYGVIRTYEVKLLDKNNALDLLSWKAFKTKKIDASYKEVLNDVVIYASGLPLALEVIGSNLFGKSIEEWK
SAIKQYKRIPNNQILEILKVSFDSLEEEEKSVFLDICWLNRYPLSKVEDLLLAHYGDCMKYHIGVLVDKSLIKLSQYTLETRIA
LHSLIQDMGKEIVRQESPKDPGKRSRLWLPEDIIQVLEDDKGTSEIEIICLDLPKFDKEVIVEFNTKAFKTMKNLRTLILRNG
NFSEGPKHLPNSLRVLEWWGYPSHCLPSDFHPKKLALCKLSRSSISSLEFSKDFMNLRVLNFDWCKCLTQIPDMSVLQN
LEELSFKCCVNLITVHNSVGFLDKLKTLSAFSCEKLRSFPPIKLTSLEKLDLSGCHSLKGFPEVLVKMENIRELNLEGCPIAELP
LSFQNLTGLPSLEMFFSNSAIVKVPSSITMMPKLADIYARGLKGLQWLKQEDGEEQMGPGVVPSKVEWLTASDCNLYD
DFFSIDLTRFARLKNLWLPKNNFTILPECLKQCQFLSNLNVSDCKHLREIRGIPPNLKRFFAINCKSLASSSKRMLLNQELH
EAGNTVFCLPGFSIPKWFNHQNRGTSISFWFRNKFPDKVQCVLVAPMQHRYFRPRVFINGKQYTFYDHYYLTGNHHK
YLFDLRKTSFRKSPYEVPFDSEWNHAKVTFPEGKYTSINRAKIGIHIVKQENNMEDVRFTDPCSKRKSDVDISISDSESIPV
AKEHRFLDFDL

>XP_047149735.1

MALCMVGEALISACVEILIKRIASREFRDFFSSRKLNISLLDELKTKLLVLNAVLNDAEEKQITDSAVKQWLDEFRDVILDAE
DLLDEINTHALRCKGEGKSRKFATKVRSLLSSRFKNFYRGMNSKFEAISRRLEQFVRQKDILGLQSVSRRVSYRAVTDSLV
DSVVVARENDRERLLSMLLCDDGDGMSNDVEVITVLGMGGLGKTTLVQCLYNDCEVQSHFDMTAWACVSDDFDILK
VTKKIVESLTSKDCDITNLDVLRVELKNNLRDKKFLLVLDDLWNEKYNDWHNLIAPFRSGRKGSKIIITTRQQRVAQVTHT
FPTYELKPLSDENCWIILARHAFGSENFHNYPILEEVGKKIARKCNGLPLAAKTLGGLLRSNVDVGEWNRILNSNMWAH
DDVLPALRISYFHLPAHLKRCFAYCSIFPKQHLLDRKELILLWMAEGFLQHIHEDEEMESVGNDCFNELLSRSLIQKENAV
AEENFRMHDLIYDLARLVSGRSSYHFDGSEIPRTVRHISFLREMFDISEKFEGLYELKCLRTFLPRLSYPFVQCYLTKMVSH
GWLPKLRCLRILSLSKYTNITELPNSIGNLLHLRYLDLSYTSIESLPDETFMLYNLQTLILSNCESLIQLPQKIGNLTNLRHLDIS
DTNLTEMPTQICNLQELRTLTVFIVGRQDGLSIRDLSKFPYLQGKLSIMNLQNVVNLVDVFGANLNKKEQIEELILGWGS
DPQEPQFEKDVLDNLQPSTNLKKLSVKYYGGTSFPNWIGNFSFSKITVLTVSDCNNCLSLPPFGQLPSLKELVIKRMKMV
KKVGHEFYGSNVDSQLFQPFQSLENLEFEDMSEWQEWLPCESEGRNFIFPCLKTLYLCKCPKLRGTLPTHLPSLTNVIFSE
CNQLVTELSDVHWNKSIEAIHIAEGQEALLSMLDNFSYCELLIEKCDSLLCLPRMLLAANCLQKLTLTNIPSLIYFPADCLLT
SLRSLEIWHCRNLEFISHNTCPKFTSLETLRIWNSCCSMTCFSLGCLPVLQELNIRFIPNLAAITTQGGEAAPKLVDFIVTDC
EKLRSLPNQIDLPSLEHLDLSGLPMLESLSPRCLPSCLRSLHVDVGILSSVSKKELSVLFQRLSSLSHLLLKGLGEEDLVNTLLK
QQSLPTSLEYMFLHNFSDLKLLEGKGLQNLTSLQMLQMYNCPSIESLPEGQLPHSLQVLSLRECPLLEARYQNHNGKYW
YKIAHIPAIKINEKVII

>XP_047149960.1

MGEFGKAFEKSGPRTFSGEELEHGMSRWSHALTKAANFFGWDESNYRSDAELVDKIVKSVLNLPVLSATKFPVGLQSN
VEDLIQTIKDKSSEVCIIGISGAGGSGKTTLAKAIYNKIHETFTEKSFIENIGQVSRRRGHHRLQEQFLLDVLKPKVEIPSVD
MGTRMIRERLTGKRVLIVLDDVTENFTLLDLWGCREWFGGGTVIIITTRDVRLLNVLEVDHVYEVEEMNEIESLELFSWH
AFKEANPPEDFLELSKQVVTYCGALPLALEVIGNCLFKKTKEEWNSVMLKLEKTPLYNVRQKLEISFDGLRNQIEKDLFLDI
CCSFVGEGRDYVTKMLNGCGVDADSGIRVLIELSLIKVKRKNKLGMHLLLQEMGRNIINEIYEKEFRRERRLRFDDSEYVL
TDTIEVVQSNNGIIKS

>XP_047150254.1

MATPSSRRFTYHVFLSFRGEDTRRGFTGYLYKALCDRGLHTFMDDDKLQSGEEITPALLKAIEESRIAIVVLSHNYASSSFC
LDELATIIHCQSKGLLVIPVFYKVDPSNVRHQKDSYEEALAKHQKRFKGQEEKLNKWKMALRQVADLSGYHLGDGDEYE
YKFIESILERVSTVINHAPLHVADYTVGLPSRVLKVQILLDVGSEDVVHMIGIHGMGGLGKTTLSLVVYNSIAAYFDESCFL
QNVRKNSKNKDELQHLQSIFLSKMLREKNIILTSWQEGASMIQQRLGRKKVLLILDDVDNREQLEAFAGSADWFGPGS
RVIITTRDEQLLKSHGIKRTYEMEALNFNDSLQLLIWNAFKRENXVDFAEKKEKSFTGNI

>XP_047150305.1

MVTGVLVSTFLEKTIDTLASRLVHILRQRKHKKQLNNLKMKFLAIDVVALDAEQKQFKDPRVRDWLLRAKDVVFDVEDL
LDEIDYELSKSQAEAESQSATNKVWNSLNSSFVSFFEIEIESRMEQVIEDLEDLANESYILGLEKGGGVGIGSGSGSKLTYTS
LPNESVIYGRDDDKEFVFNWLTSDTPNNLSILSIVGMGGMGKTSLAQHVFNDPRLEGKFDTNVWVSVPHEFDVLKVSR
AILDTITASTDHSIQQEVIQRRLKEELMGKKFLLVLDDVWNERPSKWEDVQKPLVFGGQGSRILVTTRSEKVAVTMRSE
KHLLQALKEDHCWDLFAKHAFENADPQPDPDFIEIGKKIVQKCNGLPLALKTLGSLLHNKSSLWEWESVMKSEIWHFSE
NESDILPALRLSYFHLPSHLKKCFAFCALFPRGYRFDKECLIQLWMAQNFLANPLQKKSPKEVGEEYFNDLLSWSFFQQA
SNEEEKRFIMHDLLNDLAKYVCEDICIRLGVDEPKGIPKTTRHCSFSSSKLCFDGFGSSIDTQKLHTFTPTDRGWVWVCK
MSINDLFSRFKLIRVLSLCHCRSLVEVPKSVGNLKHLLSLDLSYTQIEKLPESISLLFKLQILKLNNCRKLKELPSCLHQLDSLR
CLELVGIGVKNVVEHLGKLKNPQVSMSSFHVDKSKKINIQQLGKLNLLGSLTIDDLQNIENPSYALEVDLKNKTHLVELRL
KWNFIGSSSVDSEKAEDVIENLRPSKYLKKLSVSNYIGKQFPNWLLHNSLPNLVSLVLEECESCQRLPPLGLLPFLKYLEISG
LDEIVSIDADFNGNNSCSFKSLQKLYFSNMRQWEKWDCQAVTGAFPRLEFFSIGNCPKLKGHLPKFVALKSLYVFLCEQL
EALMVSALELLLQYCGKLQLERSTMKKLTMDGHDKAASLVAMVGRMLLDSSLENLSICSLLHTTITRVACSRERTAKVEK
TYQFQNSKMFGSNLSLGRRKYSYGA

>XP_047150307.1

MKFLAIDVVALDAEQKQFKDPRVRDWLLRAKDVVFDVEDLLDEIDYELSKSQAEAESQSATNKVWNSLNSSFVSFFEIEI
ESRMEQVIEDLEDLANESYILGLEKGGGVGIGSGSGSKLTYTSLPNESVIYGRDDDKEFVFNWLTSDTPNNLSILSIVGMG
GMGKTSLAQHVFNDPRLEGKFDTNVWVSVPHEFDVLKVSRAILDTITASTDHSIQQEVIQRRLKEELMGKKFLLVLDDV
WNERPSKWEDVQKPLVFGGQGSRILVTTRSEKVAVTMRSEKHLLQALKEDHCWDLFAKHAFENADPQPDPDFIEIGK
KIVQKCNGLPLALKTLGSLLHNKSSLWEWESVMKSEIWHFSENESDILPALRLSYFHLPSHLKKCFAFCALFPRGYRFDKE
CLIQLWMAQNFLANPLQKKSPKEVGEEYFNDLLSWSFFQQASNEEEKRFIMHDLLNDLAKYVCEDICIRLGVDEPKGIP
KTTRHCSFSSSKLCFDGFGSSIDTQKLHTFTPTDRGWVWVCKMSINDLFSRFKLIRVLSLCHCRSLVEVPKSVGNLKHLLS
LDLSYTQIEKLPESISLLFKLQILKLNNCRKLKELPSCLHQLDSLRCLELVGIGVKNVVEHLGKLKNPQVSMSSFHVDKSKKI
NIQQLGKLNLLGSLTIDDLQNIENPSYALEVDLKNKTHLVELRLKWNFIGSSSVDSEKAEDVIENLRPSKYLKKLSVSNYIGK
QFPNWLLHNSLPNLVSLVLEECESCQRLPPLGLLPFLKYLEISGLDEIVSIDADFNGNNSCSFKSLQKLYFSNMRQWEKW
DCQAVTGAFPRLEFFSIGNCPKLKGHLPKFVALKSLYVFLCEQLEALMVSALELLLQYCGKLQLERSTMKKLTMDGHDKA
ASLVAMVGRMLLDSSLENLSICSLLESISDDCVSLRIFPLDFFPTLRTLELSGFPNLHLISQDQVHNHLQHLTIKDCPRLESY
GDTVLPWKA

>XP_047150322.1

MAESLLFSFAESLLRKLATAAVQEASLGFGVDRQLQQMKATTALIKAVLLDAEQKNPHSSALSEWLVQVKRVFSEAEDI
VDDFDCEALRKHVANTYGGSSRKVRRFFSTSNPVVYRLRMAHHIQDINTRLAKLADQRSMFGLQIIDHDTRVVQLREM
THSHVNPSNVTGREHDKKEIVKLLVQDDDRESLSIIPIVGMGGLGKTTLAKLVFNDTTIHACFQLRMWVCVSNDFELRN
VLIKILSSAPNPASVNFNSFETEQLQNHLRNKLQDQKFLLVLDDVWNEDPARWDELKEIMDLSVKGSKILVTTRSHAVV
AAMHTKSSNSYLLECLSEEDSLSLFLKSAFEDGDEKKHPQFSEIGKEIVKKCGGLPLAVKTLGSSLFSKFDKKEWESIRDNEI
WNSKQNEKGIFPALKLSYDQLPSYLKPCFASFSLYQQDSVFTAAEVCMLWGALGFLPPPKQGESMTDISTQYLHELWT
RSFLLDFVDLGGDCYFKLHDLVSDLAVFVGKGEFERIDHRNPKISENIQHLAFEENNFYGETLLPTSPRTVIFLNGGSDKDF
LKTLLSRCKGLRYLNLKNCEYESLPRCIGKLKHLRFLSLAKNEKLKELPYSLCKLQNLQTLFLNGCIKLQMLPKGIENLISLRHL
AITTTQAAFPEEMIAKLTSLENLYISYCDNLRSLFEEVQLFTLKTFHLCNCGSLKSVSFHAIRNLEGLVIKNCDNLDLSLGLGS
QISNSRLKYVVLTDLPQLLTLPQWLQGCANTLRSLLIQQCMNLKEFPEWLPTLIHLKVLEIGHCPNLLSFPLHHLTNLEDL
QIIGCPALSRRFHPIIGYDWHRISYVKKVYIGRSL

>XP_047150464.1

MAAALVGGALLSSFLNVLFERLATREFVNFIRGKKADKWLQKMKNQLLVVKVVLDDAEKKQITDSNIKEWLDLLSDVVY
EVDDILDEVSTKAATQKKVSYSFCDLFKKKKIVSKLEDIVGRLDDILKQKESLGLKLNEAENDQPPEPQPTSLEGRYGMYG
RDEDKEAIMKLVSEDSSDSEAVSVISIVGMPGVGKTTLARSVYNDDKLTQIFDLKAWVRVSDRFDIVKVTKAMLEEITQK
PCTLSDLNSIQHDLLDKLKGKRFLIVLDDVWIEDCDVWSSLTKPFLSGIRGSKVLVTTRNESVAAGVPFHSAKYDLKILLDE
DCWLVFESIAFPLSEVSENRGTLENIGKKIVQKCDGLPLAAQSLGGMLRRKHDIRDWNNVLESDIWALPGSIIPALRISYN
HLPPHLKRCFVYCSLYPKDFEFRKDELILLWMAEDLIKTPRKGKTLEEVGHDYFDDLVSRSFFQCSTYQALGKYFGNRYG
NCFVMHDLMHDLATFLGGEFYFRANELGKETKIDIKTRHLSFRRFSDPVSDIEVFDTVKFLRTFLPINYEDSPFNNEKAPCI
IVSMLKYLRVLSFRAFQSQLYLPDSIGELIHLRYLNLSSTSIAALPESLCNLYNLQTLKLYGCSNLTKLPRAMQNLVNLRHLII
LNTPIEEMPHRMGKLNQLHNLDCYTVGKHKENSIKELGGLPNLHGWFCVQKLENVTTGEEALEARIMDKKHIYHLSLE
WSERNDNSIDFQIELDVLGKLQPHQNLESLSINGYQGTRFPEWMRNFSIPNITNLYLTNCSNCSKLPSLGQLPSLKLLRIS
EMNSVKTIDASFYKEDCSSLTPFPALQSLHIVRMPYWEVWSSFVSEAFPVLNDLLISECPNLRGDFPDHLPALQTLVVTN
CDLLVSSVQRAPTLRTLEIYKTNKIVFHEFPLLVETIEIEGRPMVESMMEVITSIQLPCLHYLSLQDCSLALSFPGDCLPASLK
TLDISGLSNLKFPMLHKHKLLESLSINNSCDSLKSLPLVNFPNLISLQIRNCENLESLLILRSESLKSLNSFELGDCPNFASFPE
EGFSAPNLTRFSVYGCVKLNSLPYQMETHLPKMEYLSISNCQQIEGFPEEGMPPNLTTVKIRNCEKLLSSLEWISMVMVT
SLKVCGPCDGMNSFPKEGLLLPSLTSLHLCDFSSLETLDCKGILHLTLLQELHIENCQKLENIVGERLPVSLIKLRIEGCPLLQ
KLCHRKHRKIWPKICHVRGINIDGRWI

>XP_047150473.1

MAEAVIEVVLGNLNSLVQKELGLFLDFDQDLERLASLFTTIKATLADAEEQQFSNRSIKDWLQKLKEATYILDDILDECAYE
ALRLEYQGVKLCPSNKVKCSGFSTFHPKRTVFRYKIAKKMKRICERLEEIADERTRFHLAEMVPERRSGVIEWRQTSSFISE
PHVYGREQDRDKLIDFLVHDASHDDNLSVYPIIGLGGLGKTTLSQLIFNHERVRKHFEPRVWICVSEDFGLRRMTKAIIEA
VSGHACEDLDLDPLQRKLQDLLQNKRYLLVLDDVWDDDPESWERLKSVLACGALGASVLVTTRLTKVAAIMGTVPPHE
LSNLSDNDCWRLFKRRAFVADEVQQEELVVIGKEIVKKCGGVPLAAKVLGGLLRFKREVREWLKVKESNIWGLTHNIMP
ALRLSYLNLPIHLKQCFAYCAIFPKDERIEKQYLVELWMTNGFISSDGKLDAEDVGDGIWNELYWRSFFQDIEKDEFGEV
ESFKMHDLVCDLAQFVAEEVCCITKDDDIHALFERKRIHHISDYGWELHPAQLHQVKSLRTYLGKNVQLSPDVVKRYSLR
LLQFKPRKEFLSAIGDLKHLRYLNLSRGNFQTLPESLCKLLNLQILKLDYCYTFKKLPDSLVRLKALQQLSLKECKLLSRLTPH
MGKLTSLRNLSMYIVGEGRGFLLAELGPLKLKGCIDIKHLERVKSMNDAKESNMSSKQLNKLTLRWEGFREGELARNDE
EVLEALKPCSQTLQSLRLEGYQGVNFPQWMSSPCFKSLTYLKLWFCRNCIKLPVLRKLPSLKRLLIAGAKYVKYVQEESND
DDDHVGFMALEYLSFESLPSLMRLSSEDGENQFPCLFTLKVSDCPNFSLHGLHSLKTLEIMRLPKVKVWAGLQCLTSLEV
LGITGCDEVEGLQYMTALKKLSLRNLPNMESLPDCFGELALLRELNIVGCYKLMRLPTSLSLSSVEVLCILDCNLELSKRCEK
ENGEDWPIIAHIPHLYV

>XP_047150567.1

MSASFSYDVFLSFRGSDTRYGFTGNLYKALCDKGIHAFIDDEELQRGDEIGEALIEAIKQSRMAIVVFSKNYASSSFCLDEL
VKIIDCVKEKGRLILPIFYDVRPSHVRGQSGSYAEALAMHQERFKSSNQSLSDNMGRLQKWKMALNQAANVSGKHYKL
GNEYEHEFIGKIVKEVCNKINRRPLHVADYPVGMECRVQKVKSLLQFGSDTGVRIVGIHGIGGMGKTTLARAVYNSIAD
QFEGLCFLDGVRENAVKQGLVHLQEMLLSEIVGEKDIRIGSVSKGISIIKHRLHRKKVLLILDDVDKLEQVRATAGERNWF
GSGSRVIITTRDKHLLQGVDGKYEVEDLNEEEALELLSWNAFKDHRVDPCYKDISNQAVAYASGLPLALEVIGSLLFGKGT
REWESALDQFKKIPNRRIQEILKVSYNALEENQQRIFLDIACCLKGYELEEVEDILGVHYGVCMKYDIGVLVDKSLIKIKNG
CVTLHELIEVMGKEIDRQESPKELGKHRRLWFHKDIIQVLAENTGTSEIEIICLDFPLFEEDEEVFVEWDGEAFKKMENLK
TLIIRNSHFSKGPAYLPNSLRVLEWWTYPLQDLPTDFHPSKLAICKLPRSCFTSLELATISKKFMNLTVLNFDGTECLTKIPDI
SSLQNLEKLTFECCENLVAIHGSVGFLDKLKILSAFGCSKLTSFPPIKLISLEQLDISSCSSLESFPEILGKMENITQLELKYTPLK
EFPFSFRNLARLQDLVLVDCGNVQLPSSIVMLPELAEIFALGCKGWLLPKQDENDEEKVSLVSSNVKCLCLSGCNLSDEYF
PMVLAWFGNVKELELSSNSFTFLPECIKQCRSLKLLNLDNCEHLREIRGTPPNLEYFSAGNCKSLSFCCSAMLLDQELHVA

GNTMFCLPGSRIPEWLEQQSIGPSLSFWFREKFPVMDLCFVIGPMGKDSILFRPIMTINGNTMEIQSLTDKRFCFDFPAL
DYHILIIGTKYMKFGDNLDKPLSKNEWNHVVVSIALDFEPTPKEIIVKQTALHVIKPESSMDDIQFTDPCKQPSFKEKQRL
VDTMQHQTTLVSLEPHVRQGRNSLSLIPPQACKNNLNWDSFSTGTSSIASVQEYEIASKKLRLDMGILQFVQQRKRLAIL
GLLQQRRMASLDLLQRRGRELLSLLCSPSLELMVSWQRRCITTFQGLQEQHLPSTIKQNFEGCNGINEAKVNEVIRCNN
NYGNDNERNPLVEQLLLRKVSV

>XP_047150568.1

MAEYFVFEIAESLLGKLASNLYEEVSRAFDLYEDVQSFRETLSIVNGVLLDAEEKKEKKHGLREWLRQIQNVCLDAEDVLD
GFECQNLRKQVLKASGTTRMKVNHFFSSSNSLVFRFRMARKIKNVTRRLNKIAADGNKFGLERIDVDNRLVQRREMTY
SHVDASGVIGRESDREEIIKLLMQPHPNGDGYGDQSVCVIPIVGIGGLGKTTLAKLVFKDNRIDDLFQLKMWVCISDDFD
IRQILIKIINSASDPIISVVPQESMNNLDIEQLQGRLRHELSCQKYLLVLDDVWNDNRAKWIELKDLIKVGAVGSKILVTTR
STSIASMMGTVPSYVLEGLSVENCLSLFLKWAFREGEEKEHPYLVDIGKEIVKKCRGVPLAVKTSGSSLFSVFDSQRWEIM
RDHELWNRKQQKDDILPSLKLSYDQMPSYLRHCFAFFSLYPKDFGFTSAEIANFWATLGLLRSPFGSQKIENIGKAYINEL
YSRSFLEDFEDFGTVYYFKLHDLVHDLSLYVAKEEFLMVNSNSRNIPEQVRHISVVENDSLSHSLFPKSRVVRTIIFPVNGV
GVGSESLLETWIERYKYLRHLDLSNSSFEKLPNSIAKLEHLRALSLDNNSKIKRLPNSFCGLQNLQMLSLRRCLGLETLPKRL
GMLISLR

>XP_047150574.1

MSASFSYDVFLSFRGSDTRYGFTGNLYKALCDKGIHAFIDDEELQRGDEIGEALIEAIKQSRMAIVVFSKNYASSSFCLDEL
VKIIDCVKEKGRLILPIFYDVRPSHVRGQSGSYAEALAMHQERFKSSNQSLSDNMGRLQKWKMALNQAANVSGKHYKL
GNEYEHEFIGKIVKEVCNKINRRPLHVADYPVGMECRVQKVKSLLQFGSDTGVRIVGIHGIGGMGKTTLARAVYNSIAD
QFEGLCFLDGVRENAVKQGLVHLQEMLLSEIVGEKDIRIGSVSKGISIIKHRLHRKKVLLILDDVDKLEQVRATAGERNWF
GSGSRVIITTRDKHLLQGVDGKYEVEDLNEEEALELLSWNAFKDHRVDPCYKDISNQAVAYASGLPLALEVIGSLLFGKGT
REWESALDQFKKIPNRRIQEILKVSYNALEENQQRIFLDIACCLKGYELEEVEDILGVHYGVCMKYDIGVLVDKSLIKIKNG
CVTLHELIEVMGKEIDRQESPKELGKHRRLWFHKDIIQVLAENTGTSEIEIICLDFPLFEEDEEVFVEWDGEAFKKMENLK
TLIIRNSHFSKGPAYLPNSLRVLEWWTYPLQDLPTDFHPSKLAICKLPRSCFTSLELATISKKFMNLTVLNFDGTECLTKIPDI
SSLQNLEKLTFECCENLVAIHGSVGFLDKLKILSAFGCSKLTSFPPIKLISLEQLDISSCSSLESFPEILGKMENITQLELKYTPLK
EFPFSFRNLARLQDLVLVDCGNVQLPSSIVMLPELAEIFALGCKGWLLPKQDENDEEKELHVAGNTMFCLPGSRIPEWL
EQQSIGPSLSFWFREKFPVMDLCFVIGPMGKDSILFRPIMTINGNTMEIQSLTDKRFCFDFPALDYHILIIGTKYMKFGDN
LDKPLSKNEWNHVVVSIALDFEPTPKEIIVKQTALHVIKPESSMDDIQFTDPCKQPSFKEKQRLVDTMQHQTTLVSLEPH
VRQGRNSLSLIPPQACKNNLNWDSFSTGTSSIASVQEYEIASKKLRLDMGILQFVQQRKRLAILGLLQQRRMASLDLLQR
RGRELLSLLCSPSLELMVSWQRRCITTFQGLQEQHLPSTIKQNFEGCNGINEAKVNEVIRCNNNYGNDNERNPLVEQLL
LRKVSV

>XP_047150641.1

MPVLETLGGALFGAVLQMIFDKLDSHQVLHYFRGRNLDEKLLKKLKRNLMDINAVIDDAEQKQFSNSLVKEWIDEVRD
VLYDAEDLLEQIDYEFSKTMLKAEFQTSSSKVHSFESKIIALIDDLEYLLNQKIVKNLKVYSGDRSGFGNKVSEKKVESTSLV
AEEVIYGRDKDKEIIFSWLTSDTNDNKLSILSIVGMGGMGKTTLAQHVYNDPKTEEANFDEKAWVCVSDAFDALRVSKT
IIGAFTNSIYGSGDLEMVHGKLKTWLPGKKFLLVLDDVWNEDRNQWKALQTPLTFGAKGSKIIVTTRSHKVASIMQSTYI
LQLKQLDEDHSWETFAKHAFQDENSKLNSELKEIGMKIVEKCQGLPLALETIGCLLQSKSSVSEWEGVLTSEIWDLPIEDS
KIVPTLLLSYYHLPSHLKRCFAYCAIFPKDYRFDKESLILLWMAQNFLYCSQQSKSLEEVGENYFNDLVSRSFFKQIIRNKKT
YFVMHDLLNDLAKYVSGEICYRLGVDDEKRVSRKTRHLSYESGPIQCDTSLCDAKGLRTFITLSCDRKISIEELISNCKFLRVL
SLHWCFTVPDSIGDLIHLRLLDLSNSNIKRLPDSTCSLYNLQELKLNDCMRLKELPLTLHELTNLRRLELMGNTLIKALPHL
GKLKNLHVWMGMFEVGKNSEFSIRQLGELDLHGRLSIKSLENIANPYEMNLKNKRHIVRLSLQWNLKENNADSMKQR

EVLENLQPSRHLKYLSIDGYGGTQFPRWLYDNSLSNVVSLTLKHCKYCLLLPSLGLLTFLKHLAIDGLDQILRIDADFYGNSS
SAFPSLEILSFTNMKEWEEWQCMKGAFPCLQSLSLMNCPKLKGNLPEQLSLLKRLSIRRCEQLGASIPLAIEMEGVKMEP
SSFDMIGPLVSDTPLEFLYIDFCPGRNIPINHCYYFLVDLNIKKCCDSLTNFSLDLFPKLSHLSLNRCHNLQMISQGHTHGH
LKSLEIKKCPQFESFPNDGLFAPQLETFSIKGLEKLKSMPKCMSALLPSLNQLYIHSCPVVELSEGCLPSNVKKMSLLHCSKL
VASLKGAWGTNHSLKVLDIGNVDVECFPGEGLLPLSLSYLEISDCPNLKKLDFRGLCQLSSLQNLTLLDCPILQYLPEEGLP
KSISLLVIENCPLLK

>XP_047150642.1

MPVLETLGGALFGAVLQMIFDKLDSHQVLHYFRGRNLDEKLLKKLKRNLMDINAVIDDAEQKQFSNSLVKEWIDEVRD
VLYDAEDLLEQIDYEFSKTMLKAEFQTSSSKVHSFESKIIALIDDLEYLLNQKIVKNLKVYSGDRSGFGNKVSEKKVESTSLV
AEEVIYGRDKDKEIIFSWLTSDTNDNKLSILSIVGMGGMGKTTLAQHVYNDPKTEEANFDEKAWVCVSDAFDALRVSKT
IIGAFTNSIYGSGDLEMVHGKLKTWLPGKKFLLVLDDVWNEDRNQWKALQTPLTFGAKGSKIIVTTRSHKVASIMQSTYI
LQLKQLDEDHSWETFAKHAFQDENSKLNSELKEIGMKIVEKCQGLPLALETIGCLLQSKSSVSEWEGVLTSEIWDLPIEDS
KIVPTLLLSYYHLPSHLKRCFAYCAIFPKDYRFDKESLILLWMAQNFLYCSQQSKSLEEVGENYFNDLVSRSFFKQIIRNKKT
YFVMHDLLNDLAKYVSGEICYRLGVDDEKRVSRKTRHLSYESGPIQCDTSLCDAKGLRTFITLSCDRKISIEELISNCKFLRVL
SLHWCFTVPDSIGDLIHLRLLDLSNSNIKRLPDSTCSLYNLQELKLNDCMRLKELPLTLHELTNLRRLELMGNTLIKALPHL
GKLKNLHVWMGMFEVGKNSEFSIRQLGELDLHGRLSIKSLENIANPYEMNLKNKRHIVRLSLQWNLKENNADSMKQR
EVLENLQPSRHLKYLSIDGYGGTQFPRWLYDNSLSNVVSLTLKHCKYCLLLPSLGLLTFLKHLAIDGLDQILRIDADFYGNSS
SAFPSLEILSFTNMKEWEEWQCMKGAFPCLQSLSLMNCPKLKGNLPEQLSLLKRLSIRRCEQLGASIPLAIEMEGVKMEP
SSFDMIGPLVSDTPLEFLYIDFCPGRNIPINHCYYFLVDLNIKKCCDSLTNFSLDLFPKLSHLSLNRCHNLQMISQGHTHGH
LKSLEIKKCPQFESFPNDGLFAPQLETFSIKGLEKLKSMPKCMSALLPSLNQLYIHSCPVVELSEGCLPSNVKKMSLLHCSKL
VASLKGAWGTNHSLKVLDIGNVDVECFPGEGLLPLSLSYLEISDCPNLKKLDFRGLCQLSSLQNLTLLDCPILQYLPEEGLP
KSISLLVIENCPLLK

>XP_047151209.1

MLNFCGSYSFIRVLFSFVSWLFGGLKGAEEKPTTDNNDPTIDNNDPQIKHDVFVSFRGKDVHRGFLSHLTEAFQRRNID
AFVADNNLEKGEEIWPSLVEAIEGSSISLVIFSQNYASSRWCLEELVTILECREKYGTYVLPVFYYVEPTYVRHQSEVQRW
RRALNLASCLAGINSSKFSGDAELVKEIVNEVLKRLVKQSINTKGLVGIDEKIAAIESLMTEEPKQTRLIGIWGMGGIGKTT
LAEEVFNKLQSEYEGSYFAASETDRSNKHELISLKEKMFSKLLGYDVKIDTPNSLPKGIVRRIGCMKVLIVLDGVNESEQLE
KLLGTLDNFGSGSRIIVTTRDEQVLRANKADKIYQLKEFNFDKALELFHLNAFKTNDRVQEYKELSKRVVDYAQGIPLVVK
VLAGLLHGKNKEEWESMLGKLKKMPPKKVYKVMKLSYDSLDRKGKQIFLDLACFFLRTHVAVNVDYLKSLLKDDDSDN
SVAFELGRLKDKALITISDDNTVSIHDSLQEMAWEIVRQESVEHPGNRSRLWELNDICEALKNDKVKEAIRSIRIHLPTIKE
QKLVPQILEINMSKLEVFVCGRYDELTLLKGPQFLATELRFLSWYQYPLKSLPENFSAEKLVILKLRNGRMEKLWDGVKN
MVNLKQVDLSRSQNLKKLPDLSKATNLEVLLLMSCSSLTSVHPSIYSLPKLVKLDLLCCTSLTTLGSSNFNQLLHLDISFCSK
LRTIPKLPLSLETLNARECQSLKTILFPSPAVEQLKENRKGILFWKCTNLDNQTLEAIGLNVRMNVMNYANQNISTPCHN
HVEHCDDDNCDSYSGVYVYPGIRVPKWLEYKTRKDSIIIDLSSAPPSLLIGFIFCFVLGKYNNTDVDRFEVMITIDGEDEGK
NISVPIYIDYGYEKTESDHVCVMYEQRCSTFLNNRAKNQTRFKIQVTVEEALSHKFLKGFGVSLINRSTYKKFIQEMQLRE
SQYQFR

>XP_047151210.1

MLNFCGSYSFIRVLFSFVSWLFGGLKGAEEKPTTDNNDPTIDNNDPQIKHDVFVSFRGKDVHRGFLSHLTEAFQRRNID
AFVADNNLEKGEEIWPSLVEAIEGSSISLVIFSQNYASSRWCLEELVTILECREKYGTYVLPVFYYVEPTYVRHQSEVQRW
RRALNLASCLAGINSSKFSGDAELVKEIVNEVLKRLVKQSINTKGLVGIDEKIAAIESLMTEEPKQTRLIGIWGMGGIGKTT
LAEEVFNKLQSEYEGSYFAASETDRSNKHELISLKEKMFSKLLGYDVKIDTPNSLPKGIVRRIGCMKVLIVLDGVNESEQLE

KLLGTLDNFGSGSRIIVTTRDEQVLRANKADKIYQLKEFNFDKALELFHLNAFKTNDRVQEYKELSKRVVDYAQGIPLVVK
VLAGLLHGKNKEEWESMLGKLKKMPPKKVYKVMKLSYDSLDRKGKQIFLDLACFFLRTHVAVNVDYLKSLLKDDDSDN
SVAFELGRLKDKALITISDDNTVSIHDSLQEMAWEIVRQESVEHPGNRSRLWELNDICEALKNDKVKEAIRSIRIHLPTIKE
QKLVPQILEINMSKLEVFVCGRYDELTLLKGPQFLATELRFLSWYQYPLKSLPENFSAEKLVILKLRNGRMEKLWDGVKN
MVNLKQVDLSRSQNLKKLPDLSKATNLEVLLLMSCSSLTSVHPSIYSLPKLVKLDLLCCTSLTTLGSSNFNQLLHLDISFCSK
LRTIPKLPLSLETLNARECQSLKTILFPSPAVEQLKENRKGILFWKCTNLDNQTLEAIGLNVRMNVMNYANQNISTPCHN
HVEHCDDDNCDSYSGVYVYPGIRVPKWLEYKTRKDSIIIDLSSAPPSLLIGFIFCFVLGKYNNTDVDRFEVMITIDGEDEGK
NISVPIYIDYGYEKTESDHVCVMYEQRCSTFLNNRAKNQTRFKIQVTVEEALSHKFLKGFGVSLINRSTYKKFIQEMQLRE
SQYQFR

>XP_047151593.1

MALSVVGGALLSAFIEVVFDKLASPELVNFIRWKKPDKLLQKMRSQLLVVKVVLDDAEKRQITDSNVKEWLDLLNDVVY
EVDDLLDEVSTKAATQKEVTNSFSHLFNRKKIFSISKLEDILGRLDDILRQKQNLDLKDIPVENNQPWKPQPTSLEDRYHIY
GRYEDKETIMKLVLEDSSDGEEVSVIPIVGMGGVGKTTLARSVYNDGKLKQIFDLKAWVCVSDIFDIVKVTKTMIEEIIQK
PCKLNDLNSIQLDLWDKLRGKRFLIVLDDVWMEDCDSWSSLTKPFLSGIRGSKVLMTTRNENVAAAVPFHSVKVYHLN
KLSNEDCWLVFANHAFSVSRGSGNRGTLEKIGKEIVKKCNGLPLAAQSLGGMLRRKHAIRDWNNVLESDIWELPESQC
KIIPALRISYNYLPPHLKRCFVYCSLYPKDYKIQKDELILLWMAEDLVKAPXKGKTLEQVGKEYFDELVSRSLFQSSSHRTIG
NCFLMHDLMHDLATFLGGEFYFRADELSKGTKINRKTRHLSFTRFSDPVSDIEIFETVKFSRTFLPINYKDSPFNNEKAPRII
GSMLKYLRVLSFRDFQSVLALPDSIGELIHLRYLNLSYTGIATLPESLCNLYNLQTLKLYCCSELTKLPGAMQNLVNLRHLEI
LNTSIKEMPKAMRKLNQMQNLDFYIAGKHIENSIKELRGLPNLHGSFCIQKLENVTQGEEALEARLMDKKHINDLSLEW
SIRNDNSTNFQIELDVFQNCGALLSAFIEVVFDKLASPELVNFIRWKKPDKLLQKMRSQLLVVKVVLDDAEKRQITDSNV
KEWLDLLNDVLYEVDDLLDEVSTKAATQKEVTNSFSHLFNRKKIFSISKLEDILGRLDDILRQKQNLDLKDIPVENNQPWK
PQPTSLEDRYHIYGRYEDKETIMKLVLEDSSDGEEV

>XP_047151601.1

MDIQEESSTTVGPLTTPSLRNMSSSSSAFFSANQSPFFSPRSSSCQLSESLQPDAPSDRIHLDVAAPSSSSGIPEPKSLVKV
GCTFSEVAASPAGCNAGDLQKLDRISSSVGISSCTVSGHFHPYDDNYSGQKDKRSKKGRNKRISSTAGSRSVSSYRLKSC
DVYIGLHGRKPPIIRFANWLRVELEIQGISCFVSDRAGYRNSCKLSIAEKAMDVASYGIVIITRKSFKNPYTIEELQFFSGKK
NLVPIYFDLSPADCLVRDIIEKRGELWEKHGGELWLLYGGLEQEWKDVVHGLSRVEERKLEAQDGNWRDCILRTVTLLA
MKLGRRSAAEHLTKWREKVKEEELPFTRNENFIGRKKELSQLEFMLFGDVTGDSRQDYIDLKARPKRRHLTICRSKSNVQ
EERHVGNGSREEKTPVLWKESEKEIEMQNIEFSHRRSRLKRGGKYTRRKKGMRILYGKGIACISGDSGIGKTELILEFAYRF
HQRYKMVLWIGGESRYIRQNYLNLRSFLEVDASVENSLEKTRIKGFEEQEEAAVSGIRKELMRNIPYLVIIDNLESEKDW
WDHKLVMDLLPRFGGETHVIISTCLPRVMNLEPLKLSYLSGVEAMSLMLGSGKEYPVAEVDALRTIEEKLGRLTLGLAIVS
GILSELPITPSRLLDTINRMPLKDMSWCNKKAHSFRQNTFLLQLLDVCFSIFDHADGARSLATRMVLVSGWFAPCAVSV
SLLALAAQKIPEKQKGTCFWRKLLQSLTCGFTSSHTKKSELEACSLLLRFNIARSSTKQGHIHFNEMIKLYARKREVTGSAQ
AMVQAVMNQGSISESIEHLWAACFLLFAFGHNPAAVEVEVSELLCLVKKVLLPLAIHTFITYSRCSAALELLHLCTNALEA
ADQALVTPVDKWFDKSLCWRSIQTNAQLNPCLWQELALCRATVLETRGKLMLRGAQFDIGDDLIRKAVFIRTSICGED
HPDTISARETLSKLTRLIANVQIRASA

>XP_047151603.1

MDIQEESSTTVGPLTTPSLRNMSSSSSAFFSANQSPFFSPRSSSCQLSESLQPDAPSDRIHLDVAAPSSSSGIPEPKSLVKV
GCTFSEVAASPAGCNAGDLQKLDRISSSVGISSCTVSGHFHPYDDNYSGQKDKRSKKGRNKRISSTAGSRSVSSYRLKSC
DVYIGLHGRKPPIIRFANWLRVELEIQGISCFVSDRAGYRNSCKLSIAEKAMDVASYGIVIITRKSFKNPYTIEELQFFSGKK
NLVPIYFDLSPADCLVRDIIEKRGELWEKHGGELWLLYGGLEQEWKDVVHGLSRVEERKLEAQDGNWRDCILRTVTLLA

MKLGRRSAAEHLTKWREKVKEEELPFTRNENFIGRKKELSQLEFMLFGDVTGDSRQDYIDLKARPKRRHLTICRSKSNVQ
EERHVGNGSREEKTPVLWKESEKEIEMQNIEFSHRRSRLKRGGKYTRRKKGMRILYGKGIACISGDSGIGKTELILEFAYRF
HQRYKMVLWIGGESRYIRQNYLNLRSFLEVDASVENSLEKTRIKGFEEQEEAAVSGIRKELMRNIPYLVIIDNLESEKDW
WDHKLVMDLLPRFGGETHVIISTCLPRVMNLEPLKLSYLSGVEAMSLMLGSGKEYPVAEVDALRTIEEKLGRLTLGLAIVS
GILSELPITPSRLLDTINRMPLKDMSWCNKKAHSFRQNTFLLQLLDVCFSIFDHADGARSLATRMVLVSGWFAPCAVSV
SLLALAAQKIPEKQKGTCFWRKLLQSLTCGFTSSHTKKSELEACSLLLRFNIARSSTKQGHIHFNEMIKLYARKREVTGSAQ
AMVQAVMNQGSISESIEHLWAACFLLFAFGHNPAAVEVEVSELLCLVKKVLLPLAIHTFITYSRCSAALELLHLCTNALEA
ADQALVTPVDKWFDKSLCWRSIQTNAQLNPCLWQELALCRATVLETRGKLMLRGAQFDIGDDLIRKAVFIRTSICGED
HPDTISARETLSKLTRLIANVQIRASA

>XP_047151604.1

MDIQEESSTTVGPLTTPSLRNMSSSSSAFFSANQSPFFSPRSSSCQLSESLQPDAPSDRIHLDVAAPSSSSGIPEPKSLVKV
GCTFSEVAASPAGCNAGDLQKLDRISSSVGISSCTVSGHFHPYDDNYSGQKDKRSKKGRNKRISSTAGSRSVSSYRLKSC
DVYIGLHGRKPPIIRFANWLRVELEIQGISCFVSDRAGYRNSCKLSIAEKAMDVASYGIVIITRKSFKNPYTIEELQFFSGKK
NLVPIYFDLSPADCLVRDIIEKRGELWEKHGGELWLLYGGLEQEWKDVVHGLSRVEERKLEAQDGNWRDCILRTVTLLA
MKLGRRSAAEHLTKWREKVKEEELPFTRNENFIGRKKELSQLEFMLFGDVTGDSRQDYIDLKARPKRRHLTICRSKSNVQ
EERHVGNGSREEKTPVLWKESEKEIEMQNIEFSHRRSRLKRGGKYTRRKKGMRILYGKGIACISGDSGIGKTELILEFAYRF
HQRYKMVLWIGGESRYIRQNYLNLRSFLEVDASVENSLEKTRIKGFEEQEEAAVSGIRKELMRNIPYLVIIDNLESEKDW
WDHKLVMDLLPRFGGETHVIISTCLPRVMNLEPLKLSYLSGVEAMSLMLGSGKEYPVAEVDALRTIEEKLGRLTLGLAIVS
GILSELPITPSRLLDTINRMPLKDMSWCNKKAHSFRQNTFLLQLLDVCFSIFDHADGARSLATRMVLVSGWFAPCAVSV
SLLALAAQKIPEKQKGTCFWRKLLQSLTCGFTSSHTKKSELEACSLLLRFNIARSSTKQGHIHFNEMIKLYARKREVTGSAQ
AMVQAVMNQGSISESIEHLWAACFLLFAFGHNPAAVEVEVSELLCLVKKVLLPLAIHTFITYSRCSAALELLHLCTNALEA
ADQALVTPVDKWFDKSLCWRSIQTNAQLNPCLWQELALCRATVLETRGKLMLRGAQFDIGDDLIRKAVFIRTSICGED
HPDTISARETLSKLTRLIANVQIRASA

>XP_047152029.1

MSYSTKKHDVFVSFRGEDTRTNFTSHLYKALEDKSIGAYIDRQLDRGESVWPALAKAIRDSHVSIVVFSENYACSKWCLE
ELVKILECRKELGLVVIPVFYNIDPSDIRNQKGTYEKALAELLESDEEKGPKWKASLTEAANISGWDSRTHRDEAHVIENV
VNDVLQKLHLRCPTELKGPVENEENCRNVELLLKSCRVIGIWGMGGIGKSTIAKILFAKHFPQYDHVCFVTNAKEYSLDK
LFSTILKEEVSAKNVVGSSFHMRRLRSKKVFIVLDDVDMDSFEPLEYLCGVYEGQHSDSKLVITTRDRQLLVGRVDAIYEV
QKWKKTESLKLFCSESFKKSYPEGGYESLSESAVEYAGGVPFALKVLGSYLRSKGISFWESTIRKLSLYPNERIQKVLEMSYT
GLHDLEKNIFLDIVFFFREKQEDHATRILDACGFEATSGIEVLADKSLLTISYTNIIQMHDLLQQMGLEIVRQECRADPGRR
SRLKDNEAREVIEENKGTEAIQGIELDLSQVKNLRLRSDTLTKMKALRFLRFYNSSGQSSWNTYLDLPATLEPFSDKLRYTE
WIGYPFECLPSPFCAKFLVEIHMQHGKVKQLWQGIQELDNLEGIDLSGCKQFEELPDLSKAPRLKWVNLSCCESLRYLHP
SVLSSATLVTLILNGCTKLESVKGEKRLKSLEKISVNGCLSLEEFAVSLDLIKILNVSNAPVRRMGKLKSLNLEEFPRSLRRWL
ELVKTTVSHHLKHSYVLKLDQYDGTTLPEFIKNSENLRILSVENCDLLMHLQKLPSSIGYLGVINCTSLVSVSDLVNLANVM
RGSTRFITFKNCLKLDEHSCRLIMKSVKLIMVCSAFDNLVRKSCDVRDYSYNSVELCLPGRKVPQEIKYRSTESFITIDLPKLS
NLRGFIYSVVLSPSGEMKKHDTKIICKRHLRQNTRESWVYSDIEGLNTDHVYIWYDPFHSDGILKYNEPSVSFEFCVRND
KGEVDGSMFIKECGVGLISVSELPSVLEELDWHSDKKKDLVNRVELITGQRITLTSIEQSDERKNHFSALEEIINSTHKEAKT
DSGTDYGQNTTKSTDAFVQVKYEGNRETSIKQDATLPETVESELDKEHESKEKSKSDSEIVKPVSAENGKSTHKETKTNS
GTSVVKYKGNKEVPKSDATLHETMESHSDNENQFREKSVTQSEVVEMIELENTESTCKETLSIAGRGPKEKSRESTKIVA
DEHLQSTSLQASSQGGSEHLHDRLEESNKQVVQTYDTEKFVTKYSYFDLESCLQQLHENPFAILDLLSNELSPPLKQSETC
VQKVAQANDATTVLDEFRTLVFSTSLLDKLPDQSYRQQIEESLRKLHAYRREITKEQEGVDKFIELYDKAANISQEKMLTE

DKQIKLASKKRDLYNKLQDSKLKVQQFDTAISTSKSEIENLQKRQREIQGAINKLQQENEALEKERSAQEVLYSEKQTKKD
ETLELVKYMSTSVVYTIKQLEELEEKRLSLALAYEDLKEPYGIMKTKPPF

>XP_047152030.1

MSYSTKKHDVFVSFRGEDTRTNFTSHLYKALEDKSIGAYIDRQLDRGESVWPALAKAIRDSHVSIVVFSENYACSKWCLE
ELVKILECRKELGLVVIPVFYNIDPSDIRNQKGTYEKALAELLESDEEKGPKWKASLTEAANISGWDSRTHRDEAHVIENV
VNDVLQKLHLRCPTELKGPVENEENCRNVELLLKSCRVIGIWGMGGIGKSTIAKILFAKHFPQYDHVCFVTNAKEYSLDK
LFSTILKEEVSAKNVVGSSFHMRRLRSKKVFIVLDDVDMDSFEPLEYLCGVYEGQHSDSKLVITTRDRQLLVGRVDAIYEV
QKWKKTESLKLFCSESFKKSYPEGGYESLSESAVEYAGGVPFALKVLGSYLRSKGISFWESTIRKLSLYPNERIQKVLEMSYT
GLHDLEKNIFLDIVFFFREKQEDHATRILDACGFEATSGIEVLADKSLLTISYTNIIQMHDLLQQMGLEIVRQECRADPGRR
SRLKDNEAREVIEENKGTEAIQGIELDLSQVKNLRLRSDTLTKMKALRFLRFYNSSGQSSWNTYLDLPATLEPFSDKLRYTE
WIGYPFECLPSPFCAKFLVEIHMQHGKVKQLWQGIQELDNLEGIDLSGCKQFEELPDLSKAPRLKWVNLSCCESLRYLHP
SVLSSATLVTLILNGCTKLESVKGEKRLKSLEKISVNGCLSLEEFAVSLDLIKILNVSNAPVRRMGKLKSLNLEEFPRSLRRWL
ELVKTTVSHHLKHSYVLKLDQYDGTTLPEFIKNSENLRILSVENCDLLMHLQKLPSSIGYLGVINCTSLVSVSDLVNLANVM
RGSTRFITFKNCLKLDEHSCRLIMKSVKLIMVCSAFDNLVRKSCDVRDYSYNSVELCLPGRKVPQEIKYRSTESFITIDLPKLS
NLRGFIYSVVLSPSGEMKKHDTKIICKRHLRQNTRESWVYSDIEGLNTDHVYIWYDPFHSDGILKYNEPSVSFEFCVRND
KGEVDGSMFIKECGVGLISVSELPSVLEELDWHSDKKKDLVNRVELITGQRITLTSIEQSDERKNHFSALEEIINSTHKEAKT
DSGTDYGQNTTKSTDAVKYEGNRETSIKQDATLPETVESELDKEHESKEKSKSDSEIVKPVSAENGKSTHKETKTNSGTSV
VKYKGNKEVPKSDATLHETMESHSDNENQFREKSVTQSEVVEMIELENTESTCKETLSIAGRGPKEKSRESTKIVADEHL
QSTSLQASSQGGSEHLHDRLEESNKQVVQTYDTEKFVTKYSYFDLESCLQQLHENPFAILDLLSNELSPPLKQSETCVQKV
AQANDATTVLDEFRTLVFSTSLLDKLPDQSYRQQIEESLRKLHAYRREITKEQEGVDKFIELYDKAANISQEKMLTEDKQIK
LASKKRDLYNKLQDSKLKVQQFDTAISTSKSEIENLQKRQREIQGAINKLQQENEALEKERSAQEVLYSEKQTKKDETLEL
VKYMSTSVVYTIKQLEELEEKRLSLALAYEDLKEPYGIMKTKPPF

>XP_047152432.1

MDTIKFDIKVWVCVSDHFDVLTVTKTILEAIDNKKDDNLEMVHKKLKEKLSGRKFLLVLDDVWNEKREEWEAVRTPLSY
GAPGSRIIVTTRAERVASNMRSEVHSLKQLEEDECWKVFIKHALKDDDLKLNDEQKEIGRRIVEKCKGFPLALKTIGSLLH
TKSSISDWKI

>XP_047152827.1

MASASSSSSFSKFRIDRDVFIHCLGYDIRRNFVSHLSSALLQAGVKPCLLAVEMQQEQFVASIEGFQIGIVVLTKTYFESFR
CVDELVRIIECHETHGLMVMPVFYEMDRSDFENRLKATAREVMKVEHIIWSGEYRKTWFQRWNVALTKAGTLPTWEE
SQHRSDAELVEEIVKSVLAKLDCPLGITKFPVGLKPHVKNVMGLFENQPTRVCTVGIWGMGGSGKTTVAKAIYNQIPCA
FDDKSFIQGIREVCETDGRGLVHLQEKLPSDALKTNVKIESGEIKKITFEDRLFGKKLFIVLDDVKEIDQLKDLLGNNKRLGQ
RFGQRFGQGSVIIITTRNLDLLYQLNVDYVYEMDTMDENDSVELFNWHAFGEAKPREDFVKLARSAVSYCGGLPLALEV
LGSYLSKRSVNEWRSVLSKXELIPNTQVQNILRISFDGLCLMEKDIFLNVCCFFIGKDRDYVTEILNGCGLHADIGTKVLIER
GLIKIEKNNELGMHPLLRDMGREIVRQTSTIQPGKRRRLWLRKDVLDVLTKNTGTEAIEGLSLNCQLTSSDFVKACAFEK
MKRLRFLQLDHVEVNGDYGYLSKQLRWIYWRGFPLKYIPNNFYLKKAIVIDFQHSNLRLMWNEPQVLRWLKILNLSHS
MFLIETPDFSELRSLEKLIVKNCPRLRKVHQSIGDLHNLVLINLKGCRRLRNLPTEAYKLKSLKTLILSGCLKIHIYEKDILCMG
SLTTLISENTAVKQVPFSIVSSKSIGHILLGGNNGLSFTVFQSIISSWISPKINFLSGIRPFRGISSSLVSMSMEDNDLGDLAPI
LSSDLNILNVLVQCDVEFQRYQQVMAILDEIRGLNLTEFEIRPSTPETSKHRLTPHLIEFGSFQEEVFDILSKSIFEGSENSAS

>XP_047152833.1

MASVSSSLSFFFKLVSGEVFIHCLEDDIHENFVSHLRSGLLQAGVEPSLVAVGVLSEKFMPSITRFQIGIVVFTKAYIESSRC
VEDLERIIECHETYGLMIIPVFYDIDSSYDQKSDIDPSHVRDRNRDVDLSVARVSKEKFRNATRAQRTTGAETKIPMVKILK
ICKRIRQKAKMRRALNRVRNLPAWDESKHRDDAELVEEIVKSVLVKLDRALPVTKFPVELEIHVKNVIELFENQPNKVCKI
GIWGMGGSGKTTLAKAIYNKIPFTFGDKSFIQDIRKVCQTDGRSGLVQLQEQLLSDVLKYVKIESGEMEKTTIENRLSGKK
CFIVLDDVNEIDQLKHLCENGIRFRAGSVIIITTRHLDLLCQHKVDYVYEMDELNENDSAELFSWHAFRQAKPREDFNKL
ARSAVAYCGGLPLALEVLGSYLSKRSENEWRSVLSKLEIIPNTQIQNILRISFDGLCNEMEKDIFLDVCCFFIGKDRDYVTEI
LNGCGLHADIGLKVLIERGLVKIEKKNKLGMHHLLRDMGREIVRQTSTMQPGKRSRLWVPKDVLDVLTKNTGTEAIVGL
SLNTKLTNSDSFKADAFKEMKTLRFLQLDHVRLTGDYGYLSKQLRCISWQGFSLEHIPNNFYLEGAIVMDFQHSNLRLL
WKEPTVLPWLKILNLSHSKYLTETPDFSKLPSLEKLILKHCVSLGKVHQSIGDLHNLLLINLKGCTNLSNLPSETYKLKSLKTLI
LSGCLKIDIFKEDIMHMKSLKTLISHNTAVKQVPISVVSSKSIGYIQVDEGKGLSRTVLHSIILSWMSHTFNPLYHMRPFRG
ISPSLASMNVEHNDLVDLAPVLRSILNLRTVLVQCVTEYPILQQVTAILEEVRCVTWTTSTQLSNHPFSPYLIQIGGYQEEV
FNTLRKSIYDEELAARQTRKVFVPSDNYPHWLAYKNEGHSAKFTVPDNFHMNGMILCVEHLSRLGDPTQYLSCVLMVN
YTKCIIQLFNRETLTSLNDVDWQGVISHLGYGDKLEIFIIFEKGFEVQKTAVYLVCNDQLTESQCNEP

>XP_047152835.1

MDFTSSPSFSKAKHQRIHDVFINFRGEDTRRKFVSHLHYALSNAGVNTFFDEENLVKGMQLQELMRAVEGSQIAIVVFS
QTYTESTWCLDELEQIIKCNQTQGQSVLPVFYEIDPSDVRHQKGDFGKSLEEAARRTYSGEQLERALSRWSRALNKAAGI
SGWDVKNFRNEAELVRQIVDRVQKLLDYEVLSITEYPVGLESRAQDVIGLIETRSTQVCMIGIWGMGGSGKTTVAKAVY
NQIHRRFMDKSFIENIRETCENHGRGYVPLQEQLLSNVLKTKVEIHSVGMGTTMIESRLDGKTALIVLDDVNEYNQLKA
VCGNRKWIGQGSVIIITTRDVGLLTRLDVDYIYGMDKMDEDESLQLFSFHCFGDAKPKEDFSELSRNVVAYCGGLPLALE
VLGSYLFDKTSKKVWEGVLSILEKIPNDEVQRKLRISFDSLSNHMEKDIFLDVCCFFIGKDRGYVTEILNGCELCADVGIPVL
IERSLIKVEKNNKLGMHPLLQEMGREIIRENSRKEPGKHSRLWFQKEVVEVLTKNTGTEDIEGLVLKMHLTIRDCFKADSF
QKMERLRLLQLHHVQLDGNYGYLSKQLKWISWHGFPSNCLPNSFCMNDVIAIDLKYSHLRFVWKQSQDLKWLKVLNL
SHSRYLTETPDFSRLPSLEQLILKDCPSLLAIHKSIGDLCNILLINLKDCTSLNNLPKEIYKLKSVKTFILSGCSKIDKLEEDIAQM
ESLTTLIADNTAVKQVPVSIVSSKSIAYISLCGFEGLARNVFPSIIQSWMSPTMNPLLFIRPYSGTSCSLVSMNTQNNTLGE
LAPMLRSLPYLRSVLLRYETESQLSKLVETFLVEHVINVAELGISRHHLRSSLIGVGSYKAHFDILNDKISKGLVTNEACEVSL
PIDNKPFWLAHTGEGHSVFFTVPEDCGMKGMTLCIVYLSNPEIKPTECLTSVLIANYTKRTLQIHRQETVISFNDEDWQV
IISHLAGGDKVEIFVTFGHDLVVKKTAVYLMYGESNDIESGPTNCESNDVEIEPPHCESNNIEIKAICIPNDIESKPMPCEA
NAIKIAAMNSESNVLEMKSDPKPNRNGCIRFFKKFAMCDWNTSG

>XP_047152837.1

MTLSIPSMELVSSTSKLPRMYDVLINFTGEDIRRKFVSHLDYVLSSVGLTTFLQHDNAVQPKHIQEPILNLCRVAIVVFTET
YSQSACCLHQLQQIIEWHETYGRHVLPVYYEIQPSDVRLQKGDFGKAFRETAHQTFSAQELEHGMFRWSHAITKAANF
FGWDDCNYRSDAELVDTIVKSIINLPVLSATKFPVELHSLAEEVIQIIKDKSTGLCRIGICGMGGSGKTTLAKAIYSQIHGTF
VEKSFIEDILEVSRTRGDVHLQRQLLSDVLKTKVEIHNVEMGRSMILERLYRKRVLIVLDDVNEHCPLDLWESRAWFGEG
TVIIITTRDERLLRKHEVRSVYRIDPMNENKSLELLSWHAFREAKPKAEYIDLAKRVVSRCGGLPLALEVIGSSLFERTKEEW
KSVVSKLDNIPPHEVLQKLKVSFDSLHNQMEKDLFLDVCCFFVGKGKAYVRKILNGCGVDADSAIRVLIERNFIKVKKNN
KFGVHPLLRKMGREVILEISRKEPRNKNRLWLDKDMHHALLENTLFSSRENKIIHRWPMEEDLFERYPPLEISGPLTLVKI
TRDSEFHPKKLKWISRQGFPTEYLPNELYLHDAMVIDLKYNLLRFFWMEPQVFIKRHFLLKLN

>XP_047153497.1

MLGSSSSFPVSTSTTTRYHVFLSFRGEDTRKNFISHLYAALQRKRIQAYIDERAQKGEEISPALQTAIEESKIYVLVFSENYASS
TWCLKELTMILNCKKRYGRDVIPVFYKVDPSTVRKQEERYKEAFEEHEQLFKDDMEKVQGWKDALTEAAGLSGWDSN
VIRSENTLVEGIVEDIMRKLNLYSISYDPGTIGIEKHIESIQLLMHFESSDIRIIGIWGMGGIGKTTISEQIYHTFTMQFDSRS

LVLDTQEKIKRDGIDAVRKKYMPELLNEVPSLKGMRILIILDDVTDSVQLKQLLGRCDSFGQGSRIIITSRDKQVLKNAGADDI

>XP_047153498.1

MAESSSSLAVPTSPTKYDVFLSFRGEDTRHNFVSHLHAALHRNHIEAYIDEREQKGEEISPALQTAIEESKIYVLVFSENYASSTWCLNELTSILNCKKRYRRDVIPIFYKVDPSTVRKQEKRYKAAFEEYEQRFKDDMDKVQGWKDALTEAAGLSGLEQIVKDILKKMNLDSISYDQGIIGIEKHIEKIRFLMHFESSDIRIIGICGMGGIGKTTISEQIYHTFAKQFESRSLVLDTKXKIERDGIATVREKYMSELLNEVPSFRLYYSERLKRMRILIILDDVTDSVQVKQLVGRLDSFGQGSRIIITSRDGQVLKNAGADDIYEVKELNYLDSLQLFNWHAFKQNSSKANAYMNLSIEMLRYAKGIPLALQVVGSLLYDRETEVWESQLQKLEKCQDLDIFNVLKLSYDELDEEQKNIFLDMACFYRGHEEIVVAERLEDCGFSSLIGIDVLKDKCLISVLDGRIMMHDLIQDMGQEIIRKECPQHPGKRSRLLKVDEIHEVLKLEEGVPLNFPSLKKLCFINFFNCSSLTTFPCDLSETKFLKQLCLCLCLCLKLENFPEIEDTVEGLAVLILDGTAIQALPSSLWRLVGLEQLSLRDCKNLEIIPSSIGSLTRLCKVDLTYCKSLQTFPSTIFNLKLRKLDLCGCLKLKTFPEITEQAQTFAHINLTETAIKDLPSSFGNLINLRSLQLNKCRDLESLPNSIVNLKHLCKLDCSGCAKLTEIPTHIGSLSSLMELSLSESGIVNLPESIANLTSLKSLDLSGCKNLECIPKIPTFLKQLVALDCPSIRQVRSNSLVQNLSNSKESFFNFHLTNAQQLDSGARANIEEDARLRMSMSWAQRRIFLFR

>XP_047154364.1

MASSVFVSFHDEDIHFITGFVLDPLRSSGIHVFVKGESRTFDLFQAIDRSRLFIVVLSKNYASSICCLRELVAIIDAVESSPRSVLPIFDDVQQSEVLFLNGCYGQIFSKHEERFREHKQRMEEMQRWREALTRVVGFPGLNMENVTEHGDFDFVRYAAHILGREFLTSQNETISSTYGKFDDIVQYKDGDEVSYKELRGLIRLRDFISLVLTYPVKLEEDGVLRSWISAFPEWKAQLFSTDGFPGICKPWSLLTEKALGCMKGFMPVEIMAEIESGERSLFNRHNDVIPEGLLPLSVDLAVDQLIQTLFTVDYCARYIRLLTRNSSEKESVVNKIHTALEAKHNMFGNDQDFLMAVWINALTCETDAEVHVQEEINKIMVSISMTEGDDMLTTLDTDKKKSSNRLLVIVVDADSNRKLDLQKVQFPSGIVVLIATESSTQAMKDDDFGIACTMDLNIWTQDHMLPWKLFYTYVRSCISCSTVDSSMTIQKIAVEIVKKSHGHLLAIVLVAKHLRYVKDDKYWELVLDKLSNPNPFYDYQDCDRIGISRVMVNAFVNIIWEDIDDELKLCLQLSLPVHNIKNGVRDDILVSYWANTLRYTQELGEYKRQLQYYLEELLDCFLLLKFESRDVCLPIETYDIIKSLQISKPSIIWHGALGLTDIGQWHSLIQIELVNNKICELPQSPDCPKLKVLLLQGNADLLDIPDLFFDHMPLLQHLDLSYTSIRDLPPSLAKLMQLKKLYLKGCDLFMEISPQIFQLKNLEELDLHGTLITHLPKDIRELINLQRLVLCFDAYHQVLSRGKKGKQISNTMIIPPGVISNLTQLNYLSLDVDPEDEQWSENVNGVLMEILGLEELKTVSIYVPEADLLELIPAKKFLNFRLVVGHHMRRLISRVTPELETKFKHFDYSMKFVNGVNVPNGVKMNLGRFKALYLDRHMTSKSLSDFNLNNVRMLKVCILAECNEMETIVDGDYSPDEDFSLMLELLSVFYMKNLRSICQGCGPFSYLKYMALHTCPMLTTIFNLRNFISLPFLEEIHVEDCPKVTTLISDDSPKRKPAFSLPKLRVMSLLYLPNLVNIFNGLRVEHVLEEMIFYCCPKLQSLSRSELPWVFLKFIKGESIWWEALKWRVSEWGYGGRPKFFEQFFKPINVEVGMMNPPSAHQETQLNKYLGGMYQGVSSSTELMTKLHLETPRLLPSTPFPLSDSKEGEAQKRKAVIEPIVVPPLLRRKPNQGIYKRRLLRQAVKEIRTDQTLDDGFSWRKYGQKNVLGAKYPRAYYRCTHKSTQGCPATKQVQRLDGDPTTFELTYIGTHSCTQKQDLIAESGDAEVLVAESRDAKVLVAESGDAEVLEDFWGKANSASSSNKSSITVLEGTSPEPKQLQMSLPSEDDLDFGLFTFLDDLI

>XP_047154414.1

MELASSSSKLQRKYDVLINFTEIEDIRKKFVSHLDSALSANGLTTLLHHENALQPLHIQQPILHLCRVAIVVFTKTYSQSAWCLHQLQQIIEWHQTYSRHVLPVYYEIEPSDVRLQKGDFGEAFKATAHQTFSEQQLEDGVSRWSHALTKAANFFGWDESNHRSDAELVDTIVKSVLNLPVLSVTKFPVGLQSQVEDVIRTIKNKSMEVCRIGICGMEGSGKTTLAKAIYNQIHGTFMEKSFIEDVSEVNRTRGCIHLQAQLLSDVLKSRVEIHSVEMGTSMIQERLHGKRMLIVLDDMINEYYPLFDLRKCCAWFGKGTVIIITTKDVELLRKHQVDSIFRMNLMNANESLELFSWHAFREAKPKEYSDLAKTVVTYCGGLPLALELIGACLFERTKEEWNRVLLRLDNKIYSLMYVVPLILKISFDGLRNKIEKDLFLDICCFFVGKGRAYVKKMLNGCGVDPDSGIRVLIERNLIKVKKNNKFGMHPLLRDMGRGIIRENSKEEPGKNSRMWYDSDAEYALSENTLLSSQRTKVIQGLPEKLFLTSTDGFKPSPLKETNT

SRMLKLTGDSGYVSKKLRWISLRGFPSEYLRNNFYLHDAIAIDLKHSLLRFVWKQPQVLLWLKVLNLSHSKYLTKTPDFIG
LPRLEQLILKDCPRLNEVHQSIGCLCYLTLLNLKDCTRLSNLPKEIYELTTLQTLILSGCSKIDLLKKDIMQMESLVTLIAENTV
VKQLPFSMVSSKSIGYISLCGLEGLSHNLFPSIIRSWMSSTMNKLSHKHSFCMDMEDNSWDDFVPFLSSLANLRSVLVQ
CDTEFQLSKQVETIMIEYGANITESGTSKQHFKSSLIGVGRCKDFFNAVSDRISEVFASSESCDISLPSDNDPYWLAHRGE
GHSVSFTVPQNRVLKGMALCVVYLSNPEILTTECFRSVLIVNYTKCTLQMHNHGMVIIFNDIDWQGIISNLGSGDKVDIF
VIFGHGLLVKRTIVYLICGESNDIQKEPAPKKNSLTRFIKKKEK

>XP_047154415.1

MELASSSSKLQRKYDVLINFTEIEDIRKKFVSHLDSALSANGLTTLLHHENALQPLHIQQPILHLCRVAIVVFTKTYSQSAW
CLHQLQQIIEWHQTYSRHVLPVYYEIEPSDVRLQKGDFGEAFKATAHQTFSEQQLEDGVSRWSHALTKAANFFGWDES
NHRSDAELVDTIVKSVLNLPVLSVTKFPVGLQSQVEDVIRTIKNKSMEVCRIGICGMEGSGKTTLAKAIYNQIHGTFMEKS
FIEDVSEVNRTRGCIHLQAQLLSDVLKSRVEIHSVEMGTSMIQERLHGKRMLIVLDDMINEYYPLFDLRKCCAWFGKGT
VIIITTKDVELLRKHQVDSIFRMNLMNANESLELFSWHAFREAKPKEYSDLAKTVVTYCGGLPLALELIGACLFERTKEEW
NRVLLRLDNKIYSLMYVVPLILKISFDGLRNKIEKDLFLDICCFFVGKGRAYVKKMLNGCGVDPDSGIRVLIERNLIKVKKN
NKFGMHPLLRDMGRGIIRENSKEEPGKNSRMWYDSDAEYALSENTVRTISIYGFETSFWK

>XP_047154446.1

MAMVLDAVIGQVLDELLSTVIAMKDRAVKFRATLENLHSILKKVEPMAREIDGLNKRLDKPATETQKLIDEMEKGKELVI
ECSKVDWWNCCYKASSQEKLQDVIDSITRYFQLDMQGNINVIVLENQQLLKKLVESVPRRIAGLCSPPEPPAFTVGLDV
HLGALKFKLLENHHEGSVLTVTGAGGSGKSTLAKKFCSDEEVNGKFKDNIFFISLVESPKLSTIVERLFEHNGYEKPQFQSD
EGAVYRLENLLKQIGKKPILLVLDGVLPESASVVEKFVFQIPNYKILVTSRFTIKGFGQPYLLKSLNEADALHLFRHSASLDQ
TSSNIPDTIVQKIAKGCSGSPLALIVTGKSLSLEEPVVWHNRARILSRGHSVLYYSSSSDGLLTCLQKSFDDLDAKLAESFM
DLSLFPEAQKIPAAALVDIYAEQRDEDDDIAMENIHDLVKRNVADLVVTRNTTSGTVDYNYHYVTQHGLLRDLAIHQTR
NLPTEKKHRLIIDLRGNNIPKWWTTQNEYHIAAHSLSISTDEEFTSEWCNLQPNEVKVLVMNLREKKRSLPPFMKKMNK
LKVLTITNYDVNRAELENLELLDYLSDLKRIRLEKVSIPFLSKTGVPLKNLHKFSFFMCNVNEAFKNSTIKVSDVLPNLKEM
NIDYCDMEELPAGLSDTVSLKKLSITNCHKLSKLPTGIGKLVNLESLRLTSCTKLEELPDSITSLHKLNFLDISDCVSLRMLPE
NIGELGSLERLNCRGCNRLSELPYSVKDLESLRVVVCDEERAALWEPIRSMFSDLKLEVVLTDFKLDPLL

>XP_047154447.1

MKDRAVKFRATLENLHSILKKVEPMAREIDGLNKRLDKPATETQKLIDEMEKGKELVIECSKVDWWNCCYKASSQEKLQ
DLIDSITLYFQLDMQGNINVIVLENQMLLHQIHEKLVENVPRRIAGLCSPPEPPAFTVGLDVHLRALKFKLLENHHEGSVL
TVTGAGGSGKSTLAKKFCSDEEVNGKFKDNIFFISLVESPKLSTIVERLFEHNGYEKPQFQSDEGAVNRLENLLKQIGKKPI
LLVLDGVLPESASVVEKFVFQIPNYKILVTSRFTIKGFGQPYMLKSLNEADALHLFRHSASLDQTSSNIPDTIVQKIAKGCS
GSPLALIVTGKSLSLEEPVVWHNRARILSRGHSVLYYSSSSDGLLTCLQKSFDDLDAKLAESFMDLSLFPEAQKIPAAALVD
IYAEQRDEDDDIAMENIHDLVKRNVADLVVTRNTTSGTVDYNYHYVTQHGLLRDLAIHQTRNLPTEKKHRLIIDLRGNNI
PKWWTTQNEYHIAAHSLSISTDEEFTSEWCNLQPNEVKVLVMNLREKKRSLPPFMKKMNKLKVLTITNYDVNRAELEN
LELLDYLSDLKRIRLEKVSIPFLSKTGVPLKNLHKFSFFMCNVNEAFKNSTIKVSDVLPNLKEMNIDYCDMEELPAGLSDTV
SLKKLSITNCHKLSKLPTGIGKLVNLESLRLTSCTKLEELPDSITSLHKLNFLDISDCVSLRMLPENIGELGSLERLNCRGCNRL
SELPYSVKDLESLRVVVCDEERAALWEPIRSMFSDLKLEVVLTDFKLDPLL

>XP_047154809.1

MALNDFFAGEIATELLKMLISISRKSLLCRASADQLITYIHELLPTIEEIKYSGVELPALRQSQLHRLSEILRSGVELSHKVLASS
RWNVYRNLHLAKKMDKLEKTVSKFLLGPMQAHIMADVHHTRFEMAERFDRVDNSVRRLEQYFGNIKIGVGGGGWV
EEAVRSVDENVVEGSSAVGLEFGKIKVREMIIGREDLWVVGISGIGGSGKTTLAREVCKDDQVRRYFRERILFLTVSQSPN

VEQLRTKIWGYIMGNERLDSNYVVPQWVPQFECKSEAARTLIVLDDLWTFSVMEQLVCRIPGCKYLVVSRTKFQMVLN
YEVELLSEEDALSLFCHHAFGQKSIPLGANENLAKQVVTECGRLPLALKVIGASLRDQPEMFWLSVKNRLSQGQSIGESH
EINLIERMEISINYLPEKVKECFLDLCSFPEDKKIPLDVLINMWVEIHDIPETEAYAIVVELSNKNLLTLMKEPRAGGMYSSC
FEISVTQHDILRNLAINLSNRASINERRRLVMPKRENAIPKEWLRYKHKPFEAQIVSIHTGEMKDVYWCNMEFPKTEVLII
NFTSTEYFLPLFINTMPNLRALIIINYSATYACLHNISVFKNLPNLRSLWLEKVSTPELSGTVLENLGKLFIVLCKINNRLVEKE
VDIAKVFPNLFDLTLDHCDDLTQLPSSICGMKSLQNLSLTNCHNLTQLPLELGKLKSLEILRLYACPDLKTLPNSICEMMRL
KYIDISQCVNLTCFPEEIGRLVSLEKIDMRECSMIRNVPKSALSLQSLRLVICDEELSGIWKEVEKAKPNVHIQVSEQHFDL
DWLKE

>XP_047154810.1

MALTDLFTGEIASDLWKMLITISRKALRCKSSAEQLITYVREILPTIEEIKYSGVELPAPRQSQLDRLSEILRSGVELSHQALS
SSRWNVYRNFQLAKKMEKLEKHVTRFLQVPMQAHILADVHHVRFEMAERFDRVEASNRRMERFLEEMKIGVNGGG
WVEEAVKSMQEDETWVEGCNGNNGFGVGLDFGKKKVMEMVFSNNDADWIVGICGIGGSGKTTLARELCRDDQVR
CYFKDRILFLTVSQSPNVEQLRARIWGHIMGNQGLNGNYEVPQWMPQFECKGEAQALVVLDDVWSFSVLEQLVWKI
PGCKFLVVSRFRFPTFFSATYHVELLGEEDALSLFCHHAFGQKAIPMGANVSLVKQVVAECGKLPLALKVIGASLRDQNE
MFWLSVKSNLSQGHSIGESYEINLIDRMAISTNYLPEKIKECFLDLCSFPEDRKIPLEILINMWVEIHDIREAEAYAIAVELS
NKNLLTLVKEARAGGMYSSCFEISVTQHDTLRDLALILSKRGSIHEHRRLVMAQREENGLLPREWSRYQDRPFEAQIVSI
NTGEMTEMDWFELDFPKAEVLIINFTSSDYFLPPFISKMPNLRALIIVNYSTSYARLHNVSVLRNLTNLRSLWLEKVSTPQ
LSGTVLKNLSKLFVVFCQINNSLDGKQFPNLSELTLDHCNDLTYLPSSICGIKSLRNMSLTDCHNLSELPVEFGNLKSLEILR
LYACPDLETLPPSMCEMKKLKYIDISQCTNLTCFPKEIGRLVSLEKIDMRECPMIRYLPKSAVSLHSLQLVICDEEVYGTWR
NVAEIAKSKVHIQVPEQHFDLDWLQE

>XP_047155270.1

MAELFLFSIAESLIAKLASHAFQEASRVVGLYHDLRNLRKTLYLVKAVLLDAQQKQDHNHQLREWLTHLKTVFSEAEDVL
DEFECQTLQNKVVKAHGNTKEKVSHFLSTSNPLLFRYKMTQQIKYINKRLDKVAAHRHKFSLQIIDVDTRVVYRRDMTH
SRVSDSDVIGRKNDKEKIIELLMQQNPNDDDTSLSVIPIVGIGGLGKTTLAKFVFNDNRIQECFPLKMWVCVSDDFDIKQ
LIIKIINSANDEDVPPHQQNLNMLDLEQLQKQLKRTLSGQKFLLVLDDVWNEDRVKWVELRNLIQVSGAGSKVVVTTRS
HSIASMMGTVPSHILEGLSEQESLSVFVKWAFKEGEEEKHPHLVNIGREIVKKCRGVPLAVRTLGSLLFSKFEDSEWEYVS
DSEIWNLPQKKDDILPALKLSYDLLPSYLRQCFALFSLYPKDYSFPSYEIAAIWGSLGLIALPKTSRTLEDVANQYLHELLSRS
FLQDFETFGTKFRFRIHDLVHDLALFVATDECLHVNFKIQNIPDNVRHLSFAESSLFGNLVTKKSAVVRSVLFSNGAAAAK
DEALLNTCLSKFKCLRVLDLHGSTCKTLPRAIAKMKHLRYLDISENLNIKRLPDSICKLQSLQVLSIKRCKKLEILPKGLRKLISL
RSLDFSTKQTILPLNEIAQLGSLEYLVIDSCHNVESVFGGVKFHALKTLVVRNCQSLKSLWLDGQNFPELETLIVADCGDL
DLDLWKGDHEKERPKLKLKFIGFNSLSQLVALPKWLQEAAKSLQCLFVSNCHNIETLPDWLTTLTDLKALGLINCPNLVS
MSDNIHYLTALESLKIRGCANLYKKYEPNVGEFWPKISHIKNIVIDEKEEPMKKSQT

>XP_047155271.1

MAELFLFSIAESLIAKLASHAFQEAYRVVGLYHDLRNLRKTLSLVKAVLLDAQQKQDHNHQLREWLTHLKTVFSEAEDVL
DEFECQTLQNKVVKAHGNTKDKVSHFFSTSNPLLFRYKMTQQIKYINKRLDKVAAHRHKFSLQIIDVDTRVVYRRDMTH
SRVSDSDVIGRKNDKEKMIELLMQQNPNDDDTSLSVIPIVGIGGLGKTTLAKFVFNDNRIQECFPLKMWVCVSDDFDIK
QLIIKIINSANDEDVPPHQQNLNMLDLEQLQKQLKRTLSGQKFLLVLDDVWNEDRVKWVELRNLIQVSGAGSKVVVTT
RSHSIASMMGTVPSHILEGLSEQESLSVFVKWAFKEGEEEKHPHLVNIGREIVKKCRGVPLAVRTLGSLLFSKFEDNEWE
YVSKNEIWNLPQKKDDILPALKLSYDLLPSYLRQCFALFSLYPKDYSFTSYEIAAIWGSLGLIALPKTSRTIEDVANQYLHEL
MSRSFLQDFETFGTKFRFRIHDLVHDLALFVATDECLHVNFKIQNIPDNVRHLSFAESSLFGNLVTKKSAVVRSVLFSNGA
AAAKDEALLNTCLSKFKCLRVLDLHGSTCKTLPRAIAKMKHLRYLDISENLNIKRLPDSICKLQSLQVLSIKRCKKLEILPKGL

RKLISLRSLDFSTKQTILPLNEIAQLGSLEYLVIDSCHNVESVFGGVKFHALKTLVVRNCQSLKSLWLDGQNFPELETLIVAD
CGDLDLDLWKGDHEKERPKLKLKFIGFNSLSQLVALPKWLQEAAKSLQCLFVSNCHNIETLPDWLTTMTDLKALGLINCP
NLVSMSDNIHYLTALESLKIRGCANLYKKYEPNVGEFWPKISHIKNIVIDEKEEPMKKSQT

>XP_047155408.1

MQSVVNPMSFQVSHRIKYVVFISLVFVTCSTSSNDTSQIKYDVFVSFRGEDVRRGFLSHLIEAFSQKQIAFFVDDSIQKGE
ELSEALFGAIEESYISLVIFSDNYASSRWCLSELEKILECRRKNGQTVVPIFYKVDPSDIRHQRNTYGDAFVKHERNYSLTTV
QTWRSALSESANLSGFHLSQFLDEAELVKKIVKSVWRTLNHVHQVNSKGLVGIGKRIAQVESLLQLETKDVRMIGIWG
MGGIGKTTIAQEVYNKLCFKYDSCCFLANIREESWRHGINSLKEKLFSTLLREEHLKIGRPFGFQKLVERRLHRMKVLIILD
DVGDAEQLENLARTDWFGSGSRIIVTTRDKQVLAEESASVYHVEALNFDESLRLFNLNAFKQNHIETEYEELSKKAVDYA
KGIPFVLKVLGHRLHGKDKETWESELERQQVHNKKVHDIIKSSYYDLEEDEKRIFLDIACFFYGQQLQVKYIDFLLKDRDYS
VAAGLERLKDKAFISISQENTVSMHDIIQETAWQIAGEESIENPRRSQIRLFVPDDIYDVLTHNEGNEAIRSIVVNLLRIKQL
HLKPQVFTKMSKLHFLNIYTAGTRDTRFYERWGLYLHQGLESLPNELRYLGWMHYPLESLPSNFSAENLVELHLPYSRVK
KLWHKVPDLVNLKVLMLYSSSNIKELPDFSKASNLEVIDLRSCVGLTSVHPSVFSLKKLEKLDLDECRSLTSLRSNVQMESL
RYLSLFKCMELKDFSVTSKNMIMLNLEKTDIKQLPSSFGSQSKLEHLNLKFSSIESLPADMKDLKGLQHLDLRYCRNLSFLP
ELPPSIETLDCRECVSLGSVAFPSIAEQWKENKKKALFWNCLNLDERSLKAIETNARINMVKFAHRHLSTSGDAHAIYVYP
GSQVPEWLTHKTTHDDDYYDDDDGGGGDDCITFAPNPSHLGYIFCLILPAVQYSERVLKLTVSTEGEDEGDSMIVYLDR
PRHTIKSDHVYLMYNEALSRFLTSRAKQQPMLKIKVTVATLTFSSTYTEVQLRGFGVSTISNLLQKRQLYDTPIPKEHSEVC
PDALWNS

>XP_047155409.1

MTYSNSFRVFDRIKRVIFVCVLLAAIDNNNSNNSSYPVTDFAYDNDSPQIKFDVFVSFRGTDIRQDFLSHLIEAFSQRHIN
AFVDNKVVRGDGLSEALIRAIEGSSVSLIIFSQDYASSHWCLSELVKIVECRKKNGQIVLPVFYKVDPAHVRHQKGTYEHA
FAKHQIRYSLTTMQIWRTALTEAANLAGFHSSTFRDEAEFIKEIVKCVLSRLNQVQQGKSKGLVGVGKRIAHVESLLQSE
EPDVRVMGIWGMGGIGKTTIAQEVYDKLCFEYEGCCFLANIREESGRLGIISLKKKLFSTLLGGEDLKIDTPNGLPQYIERR
LRRMKVLIILDDVNDSDQLEVLAGTRDWFGSGSRIIITTRDKQVLARQFASIYEVEALNFDESLRLFNLNAFKQNHLESEY
HELSKKVVNYAKGIPLVLKVLGHLLHGKDKETWESQLERLKKVQNRKVHDIIKLSYNDLDRDEKKIFLDIACFFDGLNLKV
KHMNFLLKDHDYSVVAGLERLKDKALISVSQENGVSMHNIIQETAWQIAREESIENPRSQIRLLDPEDIYHVLNYNKGDE
AIRSIVINLSRIKQLQLNPQVFARMSKLHFLDFYSKGSCSCLGDQGGLYLPQGLESLSNELRYLRWTHYPLESLPSKFSAEN
LVELNLPNSRLKKVWQETPDLVNLRVLILHSSTRLKELPNFSKATNLKVIDLRFCVRLTSVHSSIFSVRNLEKLYLGGCLSLRS
LRSNVHLDSLRYLSLYGCMSLKDFSVTSKNMVKLNLELTGIKQLPSSFGLQSNLQKLRLAYTYIDHLPTSIKHLTRLRHLDLR
YCRELRTLPELPASLETLDARGCISLETVTFPSTAGEQLKENKKRVAFWNCLKLDEPSLTAIELNAQINMMKFAHQHFSLF
GDAQSTYVYPGSRVPEWLAHKTTHDDFVIIDLSSVLSPQSSHIGFIFGFVVPEVPFGGSALEFKISTSGEGSHINVYMDRP
RHRITSDHVYLMYDQACSRYLNGRTKHEPRIKIKVALASRTLTSKYVPLKLRAFGISVINTTDFHSFVQKVKFGDNVPNVPI
LSRFFCTFCIVVFVGTFNICIRRLV

>XP_047155851.1

MVEQKVIGVVASSSYSPSVLSSKRYDVFLSFRGEDTRKKFTSHLYDALKQKKVETFIDNRLEKGEEISTTLIQVIEDSHISIVIF
SENYASSKWCLGELSKIMECKKERGQIVIPVFYDIDPSHVRKQTGSYEKLFVTHKGEPMCNKWKAALTEAANLTAWDS
RTYRVESELLKDIVEDVLQKLAPIYPNWHKGLVGIEENYEKIESLLKVGSNEVKILGIWGMGGIGKTTLACALYDKLSHEFE
GHCFLENVREESDKHGVKALRNKLFSELLGNKNHCFDVAFSVTKFVLSRLGRKKVFIVLDDVATSEQLENLIEYFDFMGL
GSRVVVTSRNKQIFSQVDKIYEVKELSFHHSLQLFSLTVFREKQPKYGYEDLSRSATSYCKGVPLALKVLGASLRSRSKGA
WECELRKLQKFPNMKIHTVLKLSYDDLDHSQKDIFLDIACFFRGNQRDHVTNMLEAFDFSAISGIEVLLDRALITISGGNR
LEMHDLIQEMGWEIVHQECVKDPGRRSRLWKHEEVHEVFKYNKGTDIVEGIILDLSKLIEDIYLSSDFLAKMTNVRFFKI

HSWSKFNIFNVYLPNGLNTVSHKMRYLHWDGFCLESLPANFSAEKLVELCMRCSKLKKLWDGVQNLVNLKTIDLWGS
RDLIEIPDLSLAEKLENVSLCYCESLHEVHVHSKSLRVLNLYGCSSLRKFSVTSEELTRLSLAFTSICSIPSSIWHKRKLKALYLT
GCRNLGELTDEPIIHGSHKHSLTELASNAERFSMKNIKSLSTLRMLWLDDCKKLVSLPKLPPSLEKLSACNCTSLDSYMTQ
RLVLQHMLQSRIPYLRKNDLRCYEEEYLFPGNHVIDECVFNRTGTSITIPYLWKTELYGFIYFIILSKGSLLQSGVSCSVYQD
GIRVGWLQRLLEYESLTSNHVLCMYHDITEFDAITEDGHGHFFSNVTFIFENSEASIQEFGIFPIYGSESGLKLVGSR

>XP_047155852.1

MVEQKVIGVVASSSYSPSVLSSKRYDVFLSFRGEDTRKKFTSHLYDALKQKKVETFIDNRLEKGEEISTTLIQVIEDSHISIVIF
SENYASSKWCLGELSKIMECKKERGQIVIPVFYDIDPSHVRKQTGSYEKLFVTHKGEPMCNKWKAALTEAANLTAWDS
RTYRVESELLKDIVEDVLQKLAPIYPNWHKGLVGIEENYEKIESLLKVGSNEVKILGIWGMGGIGKTTLACALYDKLSHEFE
GHCFLENVREESDKHGVKALRNKLFSELLGNKNHCFDVAFSVTKFVLSRLGRKKVFIVLDDVATSEQLENLIEYFDFMGL
GSRVVVTSRNKQIFSQVDKIYEVKELSFHHSLQLFSLTVFREKQPKYGYEDLSRSATSYCKGVPLALKVLGASLRSRSKGA
WECELRKLQKFPNMKIHTVLKLSYDDLDHSQKDIFLDIACFFRGNQRDHVTNMLEAFDFSAISGIEVLLDRALITISGGNR
LEMHDLIQEMGWEIVHQECVKDPGRRSRLWKHEEVHEVFKYNKGTDIVEGIILDLSKLIEDIYLSSDFLAKMTNVRFFKI
HSWSKFNIFNVYLPNGLNTVSHKMRYLHWDGFCLESLPANFSAEKLVELCMRCSKLKKLWDGVQNLVNLKTIDLWGS
RDLIEIPDLSLAEKLENVSLCYCESLHE

>XP_047156009.1

MAEIGTAVASTLIERVVDAIIGRARCLFYFKKSVVELQKSKRELEKSLLHMRERVKEATTNAEKIVQPVEEWLKDVERVLE
DVQGLEERVEESECCLNMTFKYSLAKETLWHWILGSQFMKSYCRRIQDERSNVIGMVGVEGSGKSTLARVVGKQLEES
KLFDNVVMTIVSQDVKLRDIQGQIADHLSFSFDGRDRTGRALRLSHRLKTEKILIILDGVWRNWTWKLIGIPLNENEKRV
ASSFTTRNQEVCTSKLPNSDLNSPCLDEDEGWDLFKQRAQIHDDSPEELREVAKRVFDKCKGLLVTIVAVARTLKGKTFT
NWELAII

>XP_047156323.1

MATLSSRRFTYDVFLSFRGEDTRYGFTGNLYRALCDRGLHTFIDDDKLQSGDEITPALLKAIEESRIAIVVLSHNYASSSFCL
DELTTILHCQSEGLLVIPVFYKVDPSYVRHQKGTYEEALAKHQKRFKAQKEKLQKWKIALRQVADFSGYHFKDGHEYQYE
FTERIVERVSREINRAPLHVADYPVGLLSQVLQVKKLLDVGSDDVVHMIGIHGMGGLGKTTLSLAVYNLIADDFDSSCFL
QNVREESNQKHLQTVLLSELLGEKDINLASVQRGISVIQQRLGRKKVLLILDDVDNRKQLQAFAGRSDWFAPGSRVIVTT
RDEQLLKCHEIERTYEVKELNKNDSLQLLIWNAFKREKVDPSYKDVLKRVVTYASGLPLALEVIGSNLVGKSVEEWESAIE
HYKRIPNGEIIEILKVSFDALGEEEKNVFLDIACCLKGSSLKEVEQILGVLYDNNMKHHIGVLVKKSLIKVGRERFNVIEMH
DLIEDMGRQIDLQKSPKEVGKRRRLWLGKDIIHVLKDNKGTSETEIICLDLSISEKEETIEWNANAFRRMKNLKILIIRNGK
FYKGPNYFPESLRVLEWHGYPSNCLPSNFDPNKLVTCKLPHSYFTSFGFLGSSKKFENLTVLNFDRCKFLTQTPDMSDLG
NLEEVSFKDCESLVEVHDSVGFMSKLKILNAEGCIKLMSFPPLNLPTLERLELSYCSNLEKFPEILGKMGNIRVLQLEELPIK
ELPLSFQNLIGLEDLFLSCEIVHLPSSIATMPNLFDFTVTNCKGWQWVKSEDAEDNVGSMVPSKVDWFSAFSCNLDDD
FFSVGFMRLAQVRSLFLRNNNFTQLPECLKEFHNLCTLDVSHCKHLQEIRGFPPKLKLFKAINCISLTSSSLSMLLNKQLHE
ARKTEFWFPGASFPEWVDLQSSGPSCSFWFRNKFPAKVLSLLIAPVGDKDEFGFIRPMVFIDGKVQESRAFYFKEIERMF
EFDQTYLFDLQKLPTYSNLFEQPLQKEWKHVKVTYEGVIESSIVKATGIHVFKKENSMMEDIRFDDPYTNNKLDKDLNAS
QSQNHLLRTIGFFMCKFFIGLFLFSCCAFIFYFIYRP

>XP_047156388.1

MDFVGTVLEIIIRLWDCCAHVRDYEENLSCLRDMASDLLGLWVDVSVKVQMAEDQHLRRLNEVNDWLVKVEAMQR
EVQAIQQRVAHAQETRSRCLTNFPTNFRMGRIVSRKIGEIRELIDKGHFDVVAREMPHAVVDEIPLEVTIGLESSFDELSE
CFDDNNVGIIGLFGMGGVGKTTLLKKFNNEFLPTKFYDVVIWVVVSKEADVGSVQQSIGNKLNVPVGKWGGKSIDDR

AIVLYNFLKRKKFVLLLDDLWERIDLLKLGIPLPDTENGSKVIFTTRSMEVCRSMEANTCIKVECLATNEAFALFREKVGEE
TLNSHPEIFHLAKIVAKECEGLPLALITVGRSMARKTLPEWKRAARTLKIYPSRFSGMVDYVYCLLEFSYDSLPSASHKSCFL
YCSIFPEDYDIRKDELIQLWIGEGLLAEFGDDVYEARIQGEEIIASLKFACLLEDSERENRIKMHDVIRDMALWLACDHGS
NTRFVVRDGACSGSVETYNQAKWKEVEKLSMWRHSIQKLSGKQDCSNLLTMLVRNTEITSFPEEIFLTANHLRVLDLSG
NKRVRELPSSIGELVQLQHLDLSGTDIQNLPRELQNLKNLRCLLLNYICNRLVFPRNLISSLVSLQVFSKLPWEDQFILPDLG
EPEEAVLLKELECLEYLQDISIALFCFSSVQVLLNSPKLQRCIRHLRVLSPFTSTPHIILFSLLTKMQHLEVLSMSVSSPSSLDH
VRKKGSPSSQCSITECIPMSSKLTEHGYIVGLRELSLEGCDMLNLNWLTRAQSLQLLRIYNCPSLEEVIGEEFGHSENVFSS
LEIVDLDSLPKLRSICSQVLQFPCLKEICVADCPKLIKLPFDSNSARNSLKHINGQKSWWRKLQWEDEATRDHFASRYVPL
RKIRKIR

>XP_047156455.1

MTRLLHEFSSTLSSVLLSDVHPHQVQHTRISIVCLCRYETEVIEKIVKDTSAKLPPMPLQIKHLVGLDSRFERVKSLIDVESN
DEVCILGIYGVGGIGKTTFAVDLYNKIRHHFEAASFLANVREKTNKSIKGLEDLQRTLLSEMGEETETIIGSTFKGSSEIKCRL
GHKRVLLIVDDVDSVKQLEALAGGYDWFGPGSRIIITTRDKDVLHKHDVEIKGYKMEELNYQESLELFCWYAFNRSRAA
ENFANISTSAVRYAKGIPLALRVIGSNLKGRGPEECETELQKYRKVPDSEIQGVLEISYTSLSNLDQKIFLDIACFFKGERWD
YVKRILDACDFNPDIKVFVSKCLITVDENGCLEMHDLIQEMGREIVRKESPSNPGYRSRLWCHKDVHEVLKENSGSDTVE
GIMLYPPKQEKIDYWTMTAFKKMKNLRILIVRNAIFSSGPSYLPNSLRLIDWKGYPSKSFPPDFYPHRIVDFKLPHSSLIFK
KPFQRFEDLTFINLSHCQFITQIPDLSGAKSLKVFTLDKCYRLTRFDKSIGFMPNLVYLSASECTLLTSFVPTMYLPSLEVLSF
NFCRRLEHFPHVMQKMDKPLKIYMMSTAIKKIPESIGNLTGLEHIDMSVCKELKNLPSSFFLLPKLVTLKVDECSQLGESF
QRFQKSRHSVVNGSSNLVTLHFCETNPSYEDLCAILETFPKLEDLNVSHNGFVALPNIIGGSSHLKSLDVSFCRNLKEIPEL
PLSIQKVDARYCQSLTSEASSMLWSKVSEEIQRIQVVMPMLKREIPEWFDCIGTKEIPHFWARRKFPVVALALVFQESKK
KLSDFEHAFQSAVESFTGFVNWHTVSLHLFIDGQEICGRDYHCFNVGEDHVLFCDLRVLFRDEEWQGLDASLGDDWK
AIQVQYESDLILSHWGVNVYEQETNTDDIQFRFPTPSSRRNLIPSSLLVPKGCPKQKMKHMLESFDPRDMFNKNLSLIES
EEGPSRAGKVLLRTWRNAKAEITEEASISVYGASLKQEHEESVDDVVQVLEMIKENVPKHFSDSNPEEMQSFGGFVERL
LRARVEVMKENGLDMGMPILLEYTDGGGSKYRRFWGVLQLKVGDPFYKAVLRKYIQLSLEFSASNKAASSSGSWFENL
RITIVLLKCLDPAMDEALGFGYEESLEEGYYDPELEELMMRIEQDGMGFNKSYGKMKASIVRTDESVSPEYLFETLIFRRLI
ALGKLSMFGSATKFKITPYGNIRVEDDAFRIPKTCFWSLILVLHLLFFIIIWLFCLVGYVGLLICRIPVIGKILVCGWWLVMQI
LVSCKYLYHRMGKIMKIKKKDL

>XP_047156501.1

MALRSCSSSFIYEVFLSFRGEDTRHGFTGNLYKALDDKGIHTFIDDEKLQSGEEITPALLNAIEESRIGITVLSKDYASSSFCL
DELTTILDCRTKGLLVIPVFYMMDPSDVRHQKGTYGEALAKHQKRFKAEKLQKWKMALQQVADLSGYHFKHGNEYEH
EFIGRIVERVSREISSVHLPVDDYLVGLESKVQEVKKLLDLGSHDGVVIGIHGMGGIGKTTLALAVYNLIAANFDESCFLQN
VREESNKYGLKHLQSILLSKILRQKNIILTSWQEGASMIQQRLRRKKVLLILDDVDKIEQLKTFVGRCNWFGPGSRVIITTR
DQHLLTSYDVKRTYVVNKLNYYDALQLLTWKNFKREKADPSYQEVLHRAVTYASGLPLALEVIGSNLVGKSVEEWDSAIE
HYKRIPSDEILKILKVSFDTLGREEKNVFLDIACCLKGCKLTEIEHMLRALYDDSMKHHISVLVEKSLIKISQSSTVEMHDLIH
AMGRQIDQHESPEEPGKHKRLWLPKDIIQVLKYNTGTGKIDIICLDISISKREETIHWNRNAFRKMKNLKILIIRNGKFSEG
PNYFPESLKVLEWHRYPSNCLPSNFDPRKLVICKLPHSPFTSFGFHGSSKKLENLTVLNFDKCNYLTQIPNLSDLLNLEVLSF
KKCGRLIVVHDSIGFLNKLKILKSKGCKKLRRFPPLNLTSLEQLELSYCSSLENFPEILGKMGNIRKLSLFELPIKNLPVSFHNL
TGLQELEMSCDFVQLSSTVLTPELSDFRVHKGKGWQWVKSQEGEEKEGSMVSPKIHMFWVLSCNLNDDFFSASFKQL
AQVSHLRLRESNVTFLPECIKEFHSLFSLDVNDCKHLQEIRGVPPNLKYFRAINCTSLTSTGSSMLLNQQLHEAGGTDFIFP
GGSIPEWFHKQRKEPSISFWFRNKFPAKVLCLLIAPVLGDNVTGLVRYIMFINGRVRKQYLLHYLNRQVKMLESDYTHLF
DLQHSHFSTDDDLMEEVSLEKEWNQVTITYEGLIESSLIKIIGIHVVKEENSSMEDIRYDDPYANTNEDNHLNTSQSQPFC
ERGLMLTCKFFIGSFMLFSCFVLLILSNLTNNPT

>XP_047156507.1

MANHGDFTYDVFMSFKGENGTRYSFTDHLYRALLRHGINAFRDEQSLRSGDEIRPSLFQAIEASRISLVVLCQNYASSSW
CLDELAKILHCYENKGKHVVAIFYLVEPSDVRYQKNSYATAMSKHENRYGKDSKKVKAWRSALSRVCDLTGIHYRNHMY
ETEVIEKIVKDTSAKLPPMPLQIKHLVGLDSRFERVKSLIDVESNDEVCILGIYGVGGIGKTTFAVDLYNKIRHHFEAASFLA
NVREKTNKSIKGLEDLQRTLLSEMGEETETIIGSTFKGSSEIKCRLGHKRVLLIVDDVDSVKQLEALAGGYDWFGPGSRIIIT
TRDKDVLHKHDVEIKGYKMEELNYQESLELFCWYAFNRSRAAENFANISTSAVRYAKGIPLALRVIGSNLKGRGPEECET
ELQKYRKVPDSEIQGVLEISYTSLSNLDQKIFLDIACFFKGERWDYVKRILDACDFNPDIKVFVSKCLITVDENGCLEMHDLI
QDMGREIVRKESPSNPGYRSRLWCHKDVHEVLKENSGSDTVEGIMLYPPKQEKIDYWTMTAFKKMKNLRILIVRNAIFS
SGPSYLPNSLRLIDWKGYPSKSFPPDFYPHRIVDFKLPHSSLIFKKPFQRFEDLTFINLSHCQFITQIPDLSGAKSLKVFTLDK
CYRLTRFDKSIGFMPNLVYLSASECTLLTSFVPTMYLPSLEVLSFNFCRRLEHFPHVMQKMDKPLKIYMMSTAIKKIPESIG
NLTGLEHIDMSVCKELKNLPSSFFLLPKLVTLKVDECSQLGESFQRFQKSRHSVVNGSSNLVTLHFCETNPSYEDLCAILET
FPKLEDLNVSHNGFVALPNIIGGSSHLKSLDVSFCRNLKEIPELPLSIQKVDARYCQSLTSEASSMLWSKV

>XP_047156755.1

MDMVILSEAISYTLQCATTFLSPPATRLSSNDIQQFEDNLKRILHIVQKAMHSNIQDPSVLSIWLKNVKDVVNDLNDFM
EDHRHNKETAATISLIKVGQNMAHRHKFKHQIKDATEELKRLSNEAENLVISEEARQNERKLTRKSEELEYVEVVVRENV
KKDIIDQLVKMFVNSNVVSVPVVTIVGVAGIGKTKLARLVYGDEQVKGLFASRIWVNLETFNVESIATRVIETTNKGKRFL
LVLDDLRVENGEGCLQKLQDRLAEAGVGGAVVVTTRSNFVAKKISEIGTVKLKPHVLQELNEEESWSLFQKVHGPGSGK
INEDVGRRVVREYCGGVPMKIIAIARLLEDLDSPVPEIELKKKFLREIRFTYYDELSPQQKLCFAYCSLFPQEQEIDAGRLIRL
WMAEGFLWRNLYSDPQEFGLACFNDFVPFVFQEMGSDEFGVVKRYKMNRLMHELARTVAWDENIIVDSAEVEVQE
RVVRSSFHFALDVQCGIPKSLFENAKKLRSILLLGKTNKSRLPHEVKMTISTCEKILETFKCLRVLDFHDLGIKMVPSSIGELK
YLRLLDLSHNNIKKLPSSITKLFHLQTLKLSQCHVLEELPKDLENLSSLIHLYLEGCLDLTQMPRGIGKLSSLQTLSLFVVGKN
YQVGGLRELTHLDGLRGHLEILHLEQLNFSSPLEAKDKYLRDKKHIHCLTLRWDHEEKEEEDEKKRNVIAKKDKESLECLD
PNPNLAVLSVVGYYGKTFSNWLSSIKCLVKFSLNDCYNCQYLPALDHLQNLRVLELRRLDSLEYVSKNSDQISADTEASSS
SSSTPFFPSLKELTISDCPKLRSWWETANWKPNRPFFTRISKLDVQCCPELHCMPLYPYLDEELVVVDSSVKSMRDTVHA
SISADFLPFSKLKTMLISRITQTPPERWLKNFISLQTLQIRDCSKLLYLPQGFKSLSSLQSLTIERCAELDLDRSKTEWEGLKRL
RFLIIKEIPKLKSLPWGVEDVTSLEELELHECPALLNLPETIANLTSLTKLVICKCGELDSLPKGIGKIESLHTLAITDCPLLTPRC
QPETGDDWPQIGNIRNIILKQSSQDLRDLWSHGRIGKGRYF

>XP_047156924.1

MADSSVSFLLDKLNALLQEEVNLQRGVREDVQYIKYELERHKAILRVADAMEDRDPELKAWVKGVRCVAHDMEDAID
EFNLRLVDQHGQGNGSSLHRFTFGLKTMKARRRIALDMQSIKSKVNVISQGRPELPGIGSRSSQRLSSSLDSQGDALLLE
EADLVGIDKPKRQLCDLLFNEEPGRAVIPIYGMGGLGKTTLAKQVYDDPKVKKRFRIHAWINVSQSFKLQELLKDLVQQL
HNVIGKPAPEAVGQMKREELKELIKNLLQSSRYLIVLDDVWHVKVWDSVKLALPSSSRGSRVMITTRKKDIALYSCAELG
KDFDHEFLPEEEAWYLFCKKTFQGNSCPPHLEEVCRKILKMCGGLPLAIVAIGGALATRQRANIEEWQMVCRSFGSEIE
GNDKLEDMKKVLSLSFNELPYYLKSCLLYLSIFPEFHAIEHMRLIRLWIAEGFVNGEDGKTREEVADSYLKELLDRSLLQVV
AKTSDGRMKTCRMHDLLREIVNLKAKDQNFATIAKDQEIIWPDKVRRLSIINTLDNVRQNRASFQLRSLLMFDLSDPLE
HFSIRGLCSTGYKLIRVLDLQDAPLEVFPAEIVNLYLLKYLSLKNTKVKSIPGTIKKLQQLETLDLKQSLVTVLPVEIVELQQLR
HLLVYRYEIESYAYFHSRHGFKVAAPIGLMRSLQKLCFIEADQALMVELGKLTQLRRLGIRKMRQQDGAALSLSIEKMINL
RSLSITAIEKHEIIDIHNIFRPPPYLHQLYLSGRLDIFPHWISSMKNLVRVFLKWSRLTEDPLVHLQDLPNLRHLEFLQVYVG
ETLNFKAKGFPSLKVLGLDDLDELKSMTVEEGAMPGLKRLIIQRCGSLKQLPFGIEHLTKLKSIEFFDMPEELITRLHPKGG
QDYLRAKNVPAVYSSYWRDGGWDVYSLETLEERETDFNRSTAVRSLENCPLWKV

>XP_047156926.1

MAESAVRFLLKRLTPVFENKVKLFTGVQAEVIYLKGQLELIRAFLRVADALEESDEELKVWVKQVRDVVHEAEDLLDELEL
VQVHNLTNGFSIYLRIRNMKARYRIAHELKSINSRLKAISSSRKRFLSKLDSSSVASSSINTGNAWHDQRGDALLLDNTDL
VGIDRPKKKVIGWLINGCPGRKVISVTGMGGIGKTTLVKKVYDDPDVKKHFKACAWVTVSQSCKIEELLKDLAKKLFSEIR
RPIPEGMESMCSDKLKMIIKDLLQKKRYLVVFDDVWHMYEWEAVKYALPNNNCSSRIMITTRRSDLAFTSTIESNGKVY
NLQPLKQDEAWDLFCRNTFQSDSCPSYLIDICKYILRKCEGLPLAIVAISGVLATKDKRRIDEWGMICHSFGAEIQGNGKL
DNFKTVLNLSFNDLPYHLKYCFLYLSIFPQDYLIQRMRLIRLWIAEGFIEAKEGKTKEDVAHDYLKELLNRNLIQVAGTTTD
GRVKTLRVHDLLREIIILKSKDQNFASIVKEQSAAWPEKIRRLSVHGTLPYRQQHRSVSQLRSFLMFGVGEYVPLGKLFPS
GFKLLSVLDYQDAPLKKFPLAVIDLYHLRYLSLRNTKVKTVPGHIIGKLHNLETFDLKKTSVRELPVDILKLQKLRHLLVYQLK
FKGYAQFHSKDGLKAPSEIGKLKSLQKLCFVEANQDCGMIIRQLGELSQLRRLGILKLREEDGMAFCLSIERLTNLHALSIA
SEGESKVIDLTFLCSPPPFLQRLYLSGRLQELPSWIQSLHSLARLFLKWSCLRYDPLVYLQDLPNLAHLELLKVYDGDTLHF
RSGKFKKLKVLGLDKFDGLKEVTVGKDAMNCLEKLSIGRCELLKKVPSGIENLTKLKVLEFFDMPDELMKTICPHGPGKD
YCKVLHIPDVYSTYWRDGGWDVYALDTFSRDCSPRSGTLIRSHEPRIQWKV

>XP_047157419.1

MMAQAVVSFIVQSLGDLLIQEAVFLYGVEDQVLQLQTELRMMRSYLQDADRRQDENESLRSWISEIREAAYDSDDVIE
SYALREASRRNLPGVSNLIRRYASIINRFIEIHMVGSHVGNVKARISSLTRSLKTYGIKPEKEEPSNSMHGRQNLRRSYSHVI
EEDIIGVDDDVKKLESYLVDPSKRVVAICGMGGLGKTTLAKKVYHSVDVRNSFKSRAWAYISQHCQARDVWIGILFRLIS
PSQEQRQEIDNMRDEELAKMLYQVQMEKSCLVVLDDIWNAETWSKLKPAFPHGTSVSAVGSKILLTSRNIDVAFQMD
PSCYLHTLKCLNEVDSWELFQKKSFLKIDDPDYGEKEKLGREMVGRCGGLPLAIIVLGGLLASKPTFFEWDTVCQNINSYL
RRANGKEQRLEEVLALSYYELPYQLKPCFLHLAHFPENLEIPTKKLIRIWVAEGIISLAHSEGEGEEALEDVAQRYLTELVER
CMIQVVEKSSSGRIRSCQMHNLMRELCVERAHLENFLQEINSRNVDESRGTSRARSVGKVRRIALFLDQDVDRFFPSEL
KSHHHLRSLLCFHEKTARLSEWGLMKSFFKKCRLLRVLNLEGMQGLGGKLPKEIGYLIHLRFLSLRNTKIDELPTSIGNLKC
LMTLDLLTGNSTVQIPNVIGNMQKMRHLYLPESCGNGIERWQLDNLKNLQTLINFPAEKCHVTDLMKLTNLRKLVIDD
PNFGGIFRYPNVQFGHLESLFFVSYEDISVVHVALGCPNLYKLHIEGPIKNFPEPHQLSSKLLKLKLTGSGLLVDPMPTLEKL
PNLRLLELQLDSFVGKQLHCSSKGFAQLKSLVIHDLFNLEEWRLDKGAMPCLRELKIENCTKLEKVPDGLRFLTTLQHLEIR
SMFAAFRTNLEKGGEDHYKIQHVSAVVFCYCDY

>XP_047158692.1

MAVEMVTGVLVSTFLERTIDTLAFRLVDIFHQRKHKKQLSNLKMKLLAIDVVAFEAEQKQFTDPRVRDWLLRAKDVVID
AEDLLDEIDYELSKTQVEAESQSASKKVWNSLDSSFFEIEFEPMMEQVIEDLEELAIQSDFLGLKKVGGVGVGSGSGSKLA
HTSLPNESVIYGRDDDKEFVLNWLTSDTHNNLSILSIVGMGGMGKTSLAQHVFNDPRLEEANFDTKVWVSVPQEFDVL
KVSRAILDTITGSTDHSIQHEVIQKRLKEELMGKKFLLKFLLVLDDVWNERPSKWEDVQKPLVFGGQGSRILVTTRSEKVA
VTMRSEKRLLQVLKKDYCWDLFAKHAFQNANPQPDSDFIEIGKKIVEKCNGLPLALKTMGSLLHNKSSLWEWESIMKS
EIWDFLENESDILPALRLSYFHLPSHLKKCFAFCALFPKGYWFEKEWLIQLWLAENFLENPLQKKSPKEVGEEYCNDLLSW
SFFQQSSNEKEKCFIMHDLINDLAKYVCEDICIRLGVDEPKGIPKTTRHCLFSYSKLGFDGFGSSINTQKLHTFTTTKRIWG
WDCKMSIDDLFSRFKLIRVLSLYNCRSLTEVPKSIGNLKHLRSLDLSLTQIEKLPDSISLLYKLQILQLNYCRKLKELPSCLHQL
HNLRRLELVHVGVKNVIAHLGKLKNVQVSMSSFHVEKSKEINFQQLGELDLHGSLTIDELQNIENPSFALEVDLKNKPHL
TELRLEWNFIGSSFVDSEKAENVIENLRPTKHLKKLSIRNYIGKQFPDWLLHNSLPNLVSLKLEGCESCQRLPPLGLLPFLNY
LEISGFDEIVSIDADFHGNNSSSFKSLQTLYFSDMWQWEKWDCQAVTGAFPCLRDISIENCPKLKGHLPKFVALKFLKLIH
CEQFEAVRVEELSICSPLESISDDCVCLRVFPLDFFPTLGVLLLSGFPNLQMISHNHVHNHLYYMIIKECPKFESLPANMH
MLLPSLEHLKIEDCPRLVSFSDEGLPLNLKHITLNCFRLVGSLKRVLEDRPSSLESLTIEKVEAECFPDEGLLPLCLFSLTISDC
RNLKKLNYKGLLELSYLGRLYLWNCPNLQCLPEEGLPKSISSLEIINCPLLKQPCHKEGGEDXEKISHIQPINRIV

>XP_047158696.1

MAQNQASSSTSIVSKYRYDVFLSFCGDDTRFGFTGNLYSALSERGILTFIDDEALRKGEEITPSLRKAIQESRISIIVFSQNYA
FSTFCLDELLHIIECRTKQNMLIFPVFYDVEPSQVRNQTGSYQEAFAKLGERFKDDIQKLQQWRRALYDAANLSGLHFKS
GEKYESNIVKKIIKEISDSFKRSPLHIAEYPVGLKDRMQQIQELMGGEFDKKVTMLGIHGMGGIGKSTLARAMYNLMED
QFEASQFLANVREKGLVHIQETMLSELVGERNIKLGDVHRGIPILQHRLCGKKVLLVLDDISKKEQLQATVGGLDWFGP
GSIVIITTRDKHLLDVHGVQKQYMVGEINHMEALELYKWNAFKNKEVDPCYKEVIKRAVWYADGLPLALETIGSHLFGK
XYALQHTSQTRG

>XP_047158698.1

MAQNQASSSTSIVSKYRYDVFLSFCGDDTRFGFTGNLYSALSERGILTFIDDEALRKGEEITPSLRKAIQESRISIIVFSQNYA
FSTFCLDELLHIIECRTKQNMLIFPVFYDVEPSQVRNQTGSYQEAFAKLGERFKDDIQKLQQWRRALYDAANLSGLHFKS
GEKYESNIVKKIIKEISDSFKRSPLHIAEYPVGLKDRMQQIQELMGGEFDKKVTMLGIHGMGGIGKSTLARAMYNLMED
QFEASQFLANVREKGLVHIQETMLSELVGERNIKLGDVHRGIPILQHRLCGKKVLLVLDDISKKEQLQATVGGLDWFGP
GSIVIITTRDKHLLDVHGVQKQYMVGEINHMEALELYKWNAFKNKEVDPCYKEVIKRAVWYADGLPLALETIGSHFEKY
ESNIVKKIIKEISDNFKRSPLHIAEYPVGLKDRMQQIQELMGGEFDKKVTMLGIHGMGGIGKSTLARAMYNLMEDQFEA
SQFLANVREKGLVHIQETMLSELVGERNIKLGDVHRGIPILQHRLCGKKVLLVLDDISKKEQLQATDGGLDWFGPGSIVII
TTRDKHLLDVHGVQKQYMVGEINHMEALKLYKWNAFKNKEVDPCYKEVIKRAVWYADGLPLALETIGSHLFGKTLDE
WESALETYERIPNRGVQDVLKVSYDSLEVSHQEMFLDIACFFRGRSRGYVTNTLEARGGVPPHFGLKVLEERSLIKIRKCP
HDAVEMHDMIRCMGKEIARQQSTLRRNGPRLWIYEDIVCVLEKNKENYKIEAMMLEMPEHQEEMQCNLEFGKIQSL
RMLLVEKNVGFLRIPATLPNSLRVLEWRGYPEPSLPSNFHFKNLVILNLSHSYFRWDKPLQNSKVLRHLILRGCKNIRRIPD
ISGFPNLTILSVAECTNLSEIDDSVGSLLHLKVFCAGGCTKLKIGPSRIELKSLEHLCFGGCSSLVMFPEVLAPMHKLKQVYL
GGTGIRNLPLSMQDLRGIEVFSLGKGQMLEINESSNFFQNLPMFFPNLKTLYLQNLDITILPASIEECHSLKYLHVTNCKKL
QEIRGLPLNIYQFYAANCSVEVNSLILKLRQAIDSAAMRICLLPGREIPELFDHSSGGNSVRFWFRKELPSLAVCTIIGASN
NVKPPFAARIDYYVRVKNNFECVRYIVFINIEWITEDSHIIIVNIQNDFHRQLNSDVQKALLTNKWIPGIIRLTINPESDSSK
LGEIRTTGVYVHRKLSRMEDVRFSNPRRR

>XP_047158810.1

MSPERDVILATPGAFRLRWDVFLSFRGTDTRGTITKGLYKSLQTRGVRVFLDDEGLERGEAVANGLMKGIDDSAAFIVIL
SENYASSHWCLEELTKICDTGRLLLPVFYRVDPSQVRHVSGPFGSGFESHEKRFEKNTVSKWKEALKKVGGIAGWVFNH
SEENDLIQRLVRRVLKELSNTPMGVPEFAVGLDERVEKVMEVLQVQSNGVKVLGLYGMGGVGKTTLAKALFNALVNR
FEHRCFISDVRQVSSKHDGLVSLQSKIIKDLFPGAGSPSIGDVNVGISAIKGRVSENRVLLVLDDVDEVKQLDALIGKREW
FYDGSCVIITTRDTEVLTQNHVTVSYEVRELYASEARELFSYHALRRSAPPENLLSLSEEIISLTGKMPLALEVFGSFLFGKRR
EEEWEDAVKKLRLIRPHHLQDVLKISYDCLDEEEKCIFLDIACLFVQKKMKRDGVIDVLRGCGFRGEIAITVLVQKCLMKIT
PDNTVWMHDQIRDMGRQIVMDESFVDPGSRSRLWDRAQIMTVLKGHKGTRCVQGIVLDFEEERFYKGKFGSVFPKK
FQWRPSLRNISCYIKQCLKNHPEPQAEENTEFVLHTKSFEPMVNLRQLQINNLKLQGKFLPSELKWLQWQGCPLERMP
LKSWPGELAVLDLKNSKKMETLWGWNGHNKVPQKLMVLNLSNCIQLTAIPDLSGCQCLEKIDLENCINLTKIHESIGCLS
TLRSLNLTRCSSLINLPIDVSGLKQLESLFLSGCSKLKSLPENIGILKSLKALHANDTAIAELPQSIFRLTKLEQLVLERCQYLRR
LPNSLGHLCSLQVLSLYHSGLEELPESVGSLNNLETLNLMGCESLTVIPDSVGNLMSLTELLVDRTAIKELPTTVGSLSYLRE
LSVGNCKLLTQLPNSVKTLASVVELQLDGTAITNLPDEIGEMKLLRVLKLMNCKNLEYLPESIGHLASLTTLNTVNGNIKEL
PESIGQLENLVNLRLNKCRMLRKLPASIGDLKSLYHIFMEETTVSSLPESFGMLSSLRTLKMAKRPELDTNESSFLAEPEEN
HSPFVLTSSFCNLTLLTELDARAWKISGKIPDEFEKLSLLETLKLDRNDFHSLPSSLKGLSILKVLSLSNCTQLSSLPSLPSNLIK
LNVQNCSSLETIHDMSNLESLQELNLTNCVKVGDIPGLESLKSLRRLYMSGCIACSSQIQKRLSKVALRNLQNLSMPGSKL
PEWFSGQTVSFSKRKNLELKSVLVGVIISINHSIDIPNMKRDDMPGLIDVEANVLKGGRTLFKTVLNICGVPRTDEEHMH
LCRFHDYHQLVAFLKDADKFSVSKRNPPFDTGLELRKCGVYLIFEGDDDYDGGEESLDTGLQSVSEKLANFFSTREGGDQ
VSVNGIGHAGT

>XP_047158840.1

MAVQLITDAALSKFFEKTFDILFSRFEGIFRGDNKSKKKQISNLKGKLLAIDVVADDAEQKQFTNPRVRDWLLAAKDAVY
DAEDLLEEIDHDAKEVCNPLTSSFISFFENKFESRMEKLIEDLEDLATQSHVLGLKKADDVGVSGWVSKLRSTYLPNESVIY
GRDDDKKFVFEWLTSNTHNNLSILSIVGMPGVGKTTLAQHLFNDPSMNKDKFEVKVWVCVSDEFDVFKVSRAILEAVT
KSTDDSRDLEMVHRRLKEKLLGKKFLLVLDDVWNENQSKWEEVQKPLVFGAQGSRILVTTRNKEVARIMQSEEHSLEQ
LQYEHSWRLFAKHAFGNDDTKPNPKYNEIDVKIVKKCKGLPLALKTMGSLLYNKSSVPEWETVLQSEIWEFSQERCDIV
PALALSYIHLPSHLKVCFAYCALFPKDYEFRKEDLIQLWMTENFLHCHQHSKSPEEACQQYFNDLKSRSLFQPLTEKKEVF
VMHDLLNDLAKYVAGGIYFRGNIDQTKKIQEVTRHFSFELGYDRHFEGFGTLYKRQRLRTFMPTGRSMDQLPCYWNCK
ISIPELFAMFSLLRILSLSHCSDLKEVPDCVGNLKYLRSLDLSRTAIKKLSESICSLSRLQILKLNYCGDLVELPSNLNSLTSLCRL
EFIETKVIKVPDLKNLKNLKLKMSSFNVGCSSELGIQRLGELNNLHESLSIDELEKIEKPRDASEANLKNNTHLVKLELKWN
SRRKDNPIDSKKEEDVIDKLQPSKNLKELSIFSYGGTQFPDWLLEDSSWEMVSLAFKECKSCQRLPPLGLWKDLKDLKIG
GLDGIVSIDADFYGNNSSSFNSLENLEISDMKQLEKWECKAVRGAFPSLKRLSISNCPKLKGDLPEQLVPLNDLKISHCDQ
ALTVTALKVRWPTMKRLKIDGDYLEASMVKIVWHFTSLEVLDINSHSKTNSDESVSPWTFPLHFFPTLSALNLRGFLNLQ
MISQDDRHNHLKYLTIEKCPKFESLPESMHTLLPSLMRLSIYDCQKLESFPHGGLPSNLNFVKLQNCCSTLIGSLKGALRA
NTLKSLWIGLVDAECFPDEGLLPLSLTSLCIYCFENLKKLDYEGLRQLSSLEKLSLRDCPNLESLPEEGLPESISQFSISGNCPL
LKHRCQKEVGEDWEKISQIQNLDVE

>XP_047158841.1

MAAEKIAGALVSTFVERTIDNLASRFVDIFRGNKSHKKQLSNLKVKLLAVDVVADDAEQKQFTDPCVRDWLLAAKDAV
YDAEDLLEEIDHDAKEVGNPLTSFISFFENKFESRMEKLIEDLEDLATQSHLLGLERGHDFGEGSRSGSKLTSTYLPNDSVIY
GRDDDKEFVFNWLTSDIPDNLSILSIVGMGGVGKTTLAQHVFNDPRVDEAKFDVRAWVCVSVESDVFKVSRKILEDVT
RSPDHSSDTDMVHRRLKEKLTGKKFLVVLDDVWNETLSNWKEVQKPLLLGAQGSRIVVTTRSKEVASTMFSEERFLKQL
QEDDSWELFAKHAFRDDTQPNPECREIGKKIVKKCKGLPLALKAIGSLLYNKSSVSEWDTVFQNEIWEIPKDRCDIVPAL
ALSYVHLPSHLKTCFAYCALFRKDYEFKKEELTQLWMTENFQQHSKTPDETCQQYFNDLLSRSFFQPSGNGKELFVMHD
LLNDLARYIAGDIFFRCKDSQTNNIQKVTRHFLFELPNFGRFHEFGTLCKTESLRTFLPTPDRKLDYFHWFCSMSIHELFSE
FMFLRILSLSHCSNLLELPDSVGNLKLLRSLDLSHTAIRKLPEKICSLSHLQILELNYCTYLEELPINLHLLTNLCSLEFRFTKVR
KVPPGLEKLKNLVVMMNVFYVDHSMESGIQRLGKLNNLSEHLEIWGLQCIKNPEDALEVDLKNKTHIVRLTLALERTGN
SIDSKKEEDVIENLQPSKNLKELSILNYGGSKFPNWLLEDSLPNLVSLVLLNCKSCQHLPPLGLLPFLKSLYISGFDETVSIDA
YFHGNNSSSFQSLKKLEFSDMKQWEKWECQAVTGAFPNLQILSLKKCPKLKGQLPELLVPLKTLKITRCQQLEAFPPRTL
ELDLRHCGKLQLDWAPMEWPRMDGHHMTAFFSESDGSHTLDDLEIVEVISDGSIPLTTFPLDSFPTVKRLVLSWLRNLE
MISQDQAHHDHLLDLTITRCPKFESLSGNMRVLSLTSLWIEDCPRFKSIPYGGLPSNLENLTIKGCPKFESLPDSVGNLKLL
RSLDLSDTAIRKLPENICSLSHLQILELNYCTYLEELPINLHLLTNLCRLEFRFTKVRKVPPGLEKLKNLKVIMSNFNVDLIMES
GIQRLGKLNNLYEDLSIGELHDIENPQDAFEADLKNKTHIESLTLGWERTGNSIDSKKAEDVLENLQPSKTLKELSIFNHGE
NKFPNWLQQTSIWNMVSLELDKCKSCQSLPPLGLLPFLKKLVISGFDQIVNVDADFHGKNSSSFKSLETLYFSDMRQWE
KWECKVVTGAYPCLQHLSISFCPKLKGQLPEQLVPLETLHITNCEQLEAFAPRALDLELRNCGKLQLDSAMKRLVMGGH
NTEASLLEMVGSNTLEHLDISSSLESMSDDCVFVRTISLDFFPILRTLNLRGFGNLQKVLQDHAHYHLQDLTIKKCPKFESL
SGIMHMLSLKSLWIEDCPRLVSFPDGGLPSNLNDMRLSNCSRLVCSLKGAFGDRSSLESLWIEGMDAECFPGEGLLPLSL
TSLTICDCPNLEKLDYKGLYQLSSLRRLTLVSCPNLQCLPEEGLPRSISYLCIGDCPLLEQRCQTEGGEDWEKIAHIQNLNIL

>XP_047159334.1

MKIWVYVSVDFNVKAILQSIIEYATGQNPNLHTLETMRKKVEEVLQSKRYLLVLDDVWNEDQEKWKHLEGMLRFARG
AKGATVLVTTRLEEVASTMETHPAYHLTELSEYDSWSLFKSYAFGPNREEKEELVTIGKDIVKKCVGSPLAIKTLGSLLRDQ
TAVTQWENIKESEIWNIRSESSSKTDKENSIMRALKLSYFNLELSLRRCFSFCAIFPKGFEIVKEELIHLWMANGFIKSEGN
VEVEDVANNVWRKLYRRSFFQEAKSDKFGMITSCKIHDLFYDLAKSIMGEECVMVEEGRLTQLSTRVHHLHFLSYNMS

VDVAAFKKVESMRTFLFGNIRQLPSNRCLRALCIRSSMLPPLNDLAHLRYLSVHDSSVRNLNDSICQLSKLQILKLESCFDF
YEIPKKLTQLQDLRHILINKCASIPDMPPNIGKLRHLRTLSLFDAGSTPGYGLTELSGLRLGGKLHIRGLGNVSNEWDAKEA
NLRSKKELNRLYLSWGGGANSESNNVSAERVLEALEPPSTLKSFGMNEYQGKQLSSWMRSVVHLRDLVEVILFDCDNC
EELPPLGKLPHLKRVHVRGMKNVKWIDGESYEGVEEKAFPSLEKLSVENLPKLERMLRDEGVEMVPHLSQLTIDGVFNF
KVPHLPCVEELHARKVVAATSFIEGVGDNMVCLKTLRVSSIKELKVLPDQLRRLGALEYLRIGFWYDLEHFPEHVSEGLTS
LRSLSIYCCENLKSLPEGVRHLTCLRHLNIGNCPQLVALPSNMSKLTSLRTVYIYDFSTLPYGLQFVPSLRTLNIERCMFTSLP
DWLGDMTTVEELSIWFCEELRSLPSSIQRLTNLSYLSIDRCPHLRKRCMRETGEDWQYIKHIPEIKLDSYSLPEEKSLTLSG
ESIRRLSCSWNCFKTARQPAKGVVSSNSFDRVLEE

>XP_047159344.1

MADALLGIVFQDLQSVVQSQLATYWGIDQQAQKLSSNLTAIRAVLRDAERKQIASHAVKDWLQKLTDAAYVLDDILDE
CSIHSREVLSDDGHTSCLARAHPKDILFRFHIGKRMKNITQRFHDINEERRMFELRVGVTEKPTVDDGEDYQTTSVITEPI
FCGRDEDREKVVKLLLEANNNEDLTIYSIVGMGGLGKTTLAKHVFHDHRV

>XP_047159375.1

ILDNWRQTSSDITEPIVYGRDQDREQIVKFLLEDVSDSEELSIYPIVGMGGLGKTTLAKQVFNDNRVCKHFDLTIWVCVS
DDFNTEVILQSIIECITGQNPNLNSLESMRKKVEEVLHGSRYLLVLDDVWNEDQEKWKQLKGKLQCARAAKGAFILVTT
RLKEVASSMQTHPAYHLKELSGEDSWSLFKHHAFGPNREEKEELVSIGKEIVRKCVGSPLAIKTLGSIIRDESDVKQWQN
VKESEIWDIREESSSATGEENSIMRALKLSYFNLELSLRRCFSFCAIFPKDFEIDKEELIHLWMANGFIKCEGNVEVEDVGN
KVWKKLYSRSFFQEAKYDEFGMIKNFKIHDLFHDLAQSIMGEECVVIVKGRLTPLPTRVHYSSLFNSGVSDDMTAFKQR
TTTDLKKVESLQTFLDIGGIGPVPSNHCLRALQTSSSLLSPLKDLTLLRYLSLRFCRCSEESLNSSICQLPKLQILKLFHCQELH
GLPKDLTRLQDLRHIVINCCHLIQETPPNIGKLRHLRTLNTFVVGLKGGCGLAELHSLKLGGTLSIRGLENVPNEWDAKQA
NLMSKKELNRLHLSWGSSANSEGSNVSVERVLEALEPPSALKSFGMRGYQGRQLSNWMKSSVVLRDLVEVELLDCDN
CEELPPLGKLAHLKRLEVSGMKNVKWIDGDTYDGVEEKAFPSLEKLTMSNLPNLERMLREGGVEMLPRLSELRIFCDSN
LKFPRLSSVEKLVAMSIDEVASFMEGVLGNLPSLKTLGIYCIKGVVVLPDQLSGLHALQELRIEGWYDVEYFPEHVLEGLTS
LRTLSIKNCKKLKSLSEGVRHLACLESLTIGNCPELVTLPSNMNQLTALRNVSIIQGYSTLPYGLQRVPSLRVLGIYACTCTSL
PDWLGDMTTLEELRIHCCWELRSLPSSIXKGLNIENK

>XP_047159384.1

MAEALLGIVIQNLQSFGQDQLATFWGVHQQTQKLSSNLTAIRAVLRDAERKQITSHAVKDWLQKLTDAAYVLDDILDE
CSIHSTKVNFDDRHTSCLSRLHPKDILFRFNIGKRMKDITQRFHDIHEERSMFNLVPGVTEVQTILDNWRQTSSDITEPIV
YGRDQDREQIVKFLLEDVSDSEELSIYPIVGMGGLGKTTLAKQVFNDNRVCKHFDLTIWVCVSDDFNTEVILQSIIECITG
QNPNLNSLESMRKKVEEVLHGSRYLLVLDDVWNEDQEKWKQLKGKLQCARAAKGAFILVTTRLKEVASSMQTHPAYH
LKELSGEDSWSLFKHHAFGPNREEMEELVSIGKEIVRKCVGSPLAIKTLGSIIRDESDVKQWQNVKESEIWDIREETSSAT
GEENSIMRALKLSYFNLELSLRRCFSFCAIFPKDFEIDKEELIHLWMANGFIKCERNVEVEDVGNKVWKKLYSRSFFQEAK
YDEFGMITTFKIHDLFHDLAQSIMGEECVVIVKGRLTPLPTRVHYSSLFNSGVSDDMTAFKQRTTTDLKKVESLQTFLDIG
GIGPVPSNHCLRALQTSSSLVSPLKDLTLLRYLSLRFCRCSEESLNSSICQLPKLQILKLFHCQELHGLPKDLTRLQDLRHIVI
NCCHLIQETPPNIGKLRHLRTLNTFVVGLKGGCGLAELHSLKLGGTLSIRGLENVPNEWDAKQANLMSKKELNRLHLSW
GSSANSEVLRDESDVKQWQNVKESEIWDIREESSSATGEENSIMRALKLSYFNLELSLRRCFSFCAIFPKDFEIDKEELIHL
WMANGFIKCEGNVEVEDVGNKVWKKLYSRSFFQEAKYDEFGMITTFKIHDLFHDLAQSIMGEECVVIVKGRLTPLPTR
VHYSSLFNSGVSDDMTAFKQRLTTDLKKVESLQTFLDIGGIGPVPSNHCLRALQTSSSLLSPLKDLTLLRYLSLRFCRCSEES
LNSSICQLPKLQILKLFHCQELHGLPKDLTRLQDLRHIVINCCHLIQETPPNIGKLRHLRTLNTFVVGLKGGCGLAELHSLKL
GGTLSIRGLENVPNEWDAKQANLMSKKELNRLHLSWGSSANSEGSNVSVERVLEALEPPSALKSFGMRGYQGRQLSN
WMKSSVVLRDLVEVELLDCDNCEELPPLGKLAHLKRLEVSGMKNVKWIDGDTYDGVEEKAFPSLEKLTMSNLPNLERM

LREGGVEMLPRLSELRIFCDSNLKFPRLSSVEKLVAMSIDEVASFMEGVLGNLPSLKTLGIYCIKGVVVLPDQLSGLHALQE
LRIEGWYDVEYFPEHVLEGLTSLRTLSIKNCKKLKSLSEGVRHLACLESLTIGNCPELVTLPSNMNQLTALRNVSIIQGYSTL
PYGLQRVPSLRVLGIYACTCTSLPDWLGDMTTLEELRIHCCWELRSLPSSIQRLTNLSHLAIRECPHLKKRCKRETGEDWQ
YIKHIPKIQLYFWREPTFCDEFKSILFTSRNV

>XP_047159852.1

MASSITATILRYVILCHAQYLCCFNNFALNLSNVKEELELTRDRVKEQIREAINKVEKVESTDVKWLKDVKKVLEEVQVLEK
RIMSVNKSYFRRQCQYSLAKEIEKKXTIEMIDFNSEYTQFSRITKLLGMEYFSSNDFSMLNPIKASYKKLLEALMNKSASIIG
LVRLGGSGKTTLAKEVCKKAEDMKLFEKVVFARVSRLPNIRSIQDQIADQLTFELKEGSEIGRAQRLSERLRKGTTLVILDD
VWERLNFEALGIPLDESSKACCVLITTRSKEVCPSMKCQSIIELNLLTHEEAWTLFKHYANINDDSSAALKGVARKIVIKID
NECKGFPIAIVTLGSTLKDKTIANFESALSRLESSKPLDIPKDLTSPYVCLELSYNNLTNQLAQSLLLLCSMFPEDCEIILEDLF
RFGRAFDTIGTFETMGNARREMDAAIDVLKNCFLLMHAKEKLKVKMDNLVRDVALWIAFKTGKAIFTRTKVDPRALAD
DETLKDMKAIAIWGLKSYDVLNYKINCPILEILVLSFNVSGDVKVLDGCLQSLEKLKTLSILNLDEWDRGIFPLPESVKSLKII
QTLCLRGYDLGDISVVEQLQALEILDLRGCSFDDLPVGIVGLKKLKLLDLYKCVIKKNTIVEAYKVLGKCLQIEELYLYLSNYV
MDFQHEVSYSKLQRHAIINTDYYYHGYDFEDNTIMKKYAQSRSLLIEREGLNLDITTQNLISLPIKDLFIRAEYLCLRNLRGD
YKNIIPSMDQQGMNQLIALVLEHCSKIECLIDNTINTSTSVDFRQTEDVFSTLVYLSLRGLHNLQEMFCDPCYRCSLQNLE
ELLIEDCSKFNNISFPKSSNLCNLKILSISKCPVLTYLFMPSIVQTLSLLEILKISSCSALRHIIEEVEEENYVLASTKRLQKLRILEI
EDCNNLEYIFTMILVRGLVSLESVRIASNMKLKYVFGSEKEHNLAMYQSFRQQTLNLLNLGTLNLIKLTNLINIWPEYCHP
HLSNLEKLHCTYCPELSDLSKVMTVSDLGQLTTSMKTEILCLITNIFQPQLKAFLKCRELWLRAVRMKGIFQFQMGEQRG
TTELILLNLDINYLRLENLPELNFIWKGPIGFLNLQKLYDIRVDGCPKLKTIFSTTVVTSLPRLTYLSIINCDELEQIFDSECSKL
HKAFGFEHELDDRGLRXMDKDGKQVLLQNLKSTQLINLPNFEEIHHGFKLKEDVEQTIKECPMYSPSLYLQPGKIHVFFS
RHNYNVFGLL

>XP_047160255.1

MASLVQKPSNDYRPRELFLSLLKCLLSASEYNDFFKKTTVGGEELSEEELKKRVAEVLKGKKYLVVLDDIWETQQWDKVK
GAFPDHQTGSRILITSRDKEVAHYTGTTSPYYLPLLDKHESWELFSKKVFRGEECPSNLEPLGRSIVENCGGLPLAIVVLAG
LVARKE

>XP_047161034.1

MPSNAIVQYSSSSSHAINTYDVFVSFRGEDTRNNFTGFLFQALRRKGIHAFKDDQDLKKGESIAPELLQAIQASRLFIIVFS
NNYASSIWCLRELAEIRNCAQTSARRVIPIFYDVDPSVVRKQSGCYDISFAQHEYRFGENKAKMEEAKRWREALTEVAN
LSGWDIRNKPQYAQIEEIVESITNILGPKISSLPKDELVGIESRVQELKDLLCCGSLNDVRVVGISGMGGIGKTSLAWTLYE
RICHQYDFHCFIDDVSKIYRDSSSLGLQKQLISQSLNEKNLEICNGFEGTCLVWSRLHNARALIVLDNVDQVEQLRMFTG
NRDTLLRECLGEGSKIIIISRDEHILRTHGVDDVYQVQPLSWENAVQLFCRNALKVNYILSDYEKLARDVLSHAQGHPLAI
EVIGSSLFGRSVSQWKSALARLKENKSKSIMDVLRISFDELDEEDKETFLDIACFYNGYEEIYVKEILSFRGFHPEYGMQVL
VDKSLITNNYGRIYMHSLLMDLGRSIVREKSPKEPRKWSRLWDYQDFHNVMLHNQEIDNLEAIVVKNEEWMFGKTT
MKADGLSNIRQLKLLKLENVNFSGSLNHLSNELGYLTWNKYPFKCLPPSFRPDILVELKLRWSSIQRLWEGTMLLHNLKR
LDLSYSKDLIEMPDVGEALNLERVDLEGCIKLQKINPSIGLLRRLAILNLKNCKELVSLPRSIFGLNCLEYLSISGCSKLYNNQL
FDEPRNTEYLKKLYLVEAPIHLRSTPSFIKEMLSWPSDLLYLRSQSSCLLPSSPSHPCLRELDLSFCNLVQIPDAIGKLHCLEK
LNLKGNNFTTLPNLKDLFKLYYLNLQHCKRLKYLPDLPSRTHLPSKVYMLPFPYSPSILGRLVEDDEDKAGLIIFNCPELVDR
ERCSSMSISWMIQIVQANHQYGSLTSTTIGPDVESIIPGSEIPEWFNNQFQSRLNLIEINSSPVENDNDWIGVVCCVIFGR
GNEMEMRYPRPYYPRPDNPIKKPPLDLRRDPTMELSDHMWLFYFSRKQFIEQRDWLGLRFIIPEMKKFRYAVYVKKYG
YRWVYEQDLIL

>XP_047161091.1

MVEEIIEDGLYEKKFDKVSYQEYPSDDYAFSNSGYVSFASRTRTLEKIMKELKDSTVDMIGVYGPSGMGKTFLVKEIAKKA
KEKLFKRVIIANITGNPDFEKIQGQIAGMLDMTLEETNEFARANRIRKKLKKDKKNTLIILDDLWDELDLNRLGIPCNDDD
GDEEDDASRRDANDIDNKFDYNKKENNELLKVDLNKMKKEKSSNSYKGCKILLTSRKKEVLCDEMNVQLTFSVENLNEK
EAATLVKKVADVKTSEFDRNAIEIAKWSGLPMALVSIGKTLKNKSLSAWEDICQKIKTEIFTEEWGFTDFSIKLSYNELKSE
QLKRVQTITHARKRVQRMIEELEESSLLVRSYSTDRFNMHDIVRGIALSKSKEKQVFFMKNDILDEWPHEDDFERYTAIFL
HSCDIKDELPKRLHCPRVEVLHIDNITESFKIPDYLFKFMIRLRVLVLTGVNLSRLPSSIKCLKKLRMLCLERCTLGKNLSIIGE
LKNLRILSLSGSNIESLPLGFGQLDKLQLFDISNCPKLREIPSNIIPRMNILEEFYIRESLILWEAEETESKNASLSELRHLNHLQ
NLDIHIQSYAHFPQNFFLDKLNSYKIVIGEFNLLNLLTVGEFKVPDKYEEVKFLALKLKEGIDIHSDKWIKMLFKTVECLLLG
ELNDVRDIFYELNVEGFPNLKHLSIVNNFGIKYIINPRERFHSLVAFTKLESIWLYKLDNLEIICDNQLEETSFRNLKVIKIKTCI
KLVNLFPFCMVRLLTELETIEVCDCDSLKEIVSKERQTQTMSDEMIEFPKLRQLTLKSLPTFICLYDVDEMLGSVPLLQGHE
NTNIVTNAEHGVTNSCLPLFNEKVSIPKLESLELSSINIQKIWSDEYDHCFENLLTLNVTDCGNLKYLLSFSIAESLVNLESIF
VSGCEMMEDIFRPEDAEYIDVFPKLKKMEIICMEKLCTIWKSDIGLHSFSNLNSLMIRECHKLVTIFPNYMGQRLQSLQSL
TVKDCKLVENIFDFANIPHTCDIIEANLGNIFLENLPNLVNVWKGDTGEILKCNNLQSIRVNVSPKLKYLFPVSIANDLQKL
EVLEIWDCWAMTEIIALDKHSSATAITFKFPHLNTLSLIDLHELRSFYSGIHTLEWPPLKKLKIVDCSMLKGLTSEITNSSEQ
PIVLATKKAIYNLEYMSVSLKEAEWLQKYIVNVHRMHKLEELNLYRLKNNEVLFWFLHRLPNLKSLTLGLCHIKRICTRESL
SSPEKIGGVMQLKELELRSMWFLEEIGFEHDVLLQRVERLIISGCKKLRNLASSSVSFSYLTSLEVVNCMMRNLMTLSTAK
TLNQLTTMKVSSCPLIVEIVAENEEEKVQEVEFKQLKSLELVSLQNLTNFSNVEKCDLKFPLLEKLVVSECPQLKKLSEVQS
APKLQKVHVEAGEKDKWYWEGDLNATLQTHFTNQVSFEYSKDINLVDYPERKDRRGKFAFPDNFFGCLKKLEFDEAYK
KDTLIPSHVLPYLKNLEELNVEKCESAQLIFDIDESKIQTYGMVFRLKNLTLQQLSNLKCVWKENRKGIVSFPNLNTVVVT
DCEGLVTLFPSSLARNFKKLKNLIIWRCEKLVAIVGKEDGTEHETTIMFEFPCLAQLKILNMTLLSCFYPGKHQLECPLLKVL
YVAGCPKLKLFTSDSKKGDMEAPIRSLQQALFLVEKVSPKLTDLALDEENIMLFREENLPQNLLCNLSRLCLCFEDDYNEK
DSLPFELFHKLPNLKHLMVDNGFGLKEIFPSQKLQVHDKVLAGLEVLILLQLKDLECIGLENMWVQPYSRKLELLQLYKCP
LVERIAYCAVSFINLKYLCVTYCERMECLFTFATLKSLVKLETLTIKNCESIKEIARTEEEDGCDEIIFGRLTSINLEYLPRLISFYS
GNATFQCPCLQNVIVAECPNMTTFSEGVIKLAMFLGIQTSEDSDFTFHVDLNTTVERYTNTN

>XP_047161168.1

MAESSSSFAASASPTRYDVFLNFRGEDTRENFISHLYAALERKHIETYIDYRLQRGEEISPALQTAIEESKIYVLVFSENYASS
TWCLNELTKILDCKKTYGRDVIPVFYKVHPATVRKQEERYKEAFEEHELRFKEDMGKVQGWKDALTEAAELCGWDSNV
IRLEHILVEEIIDDILKKLNRYSVSYNQGIIGIEKHIEEIRSLLDLESPDIRIIGIYGMGGMGKSTISEQLFHTLAVQFDSHSLVL
DVQEKLQREGIHNIIAKYRSELLKEEASSFTDLSYSNERLKRAKVLLILDDVTNSTQLRKLMERHDSFGQGSRIIMTSRDRQ
VLKNAGADDIYELQELNFYDSLKLFNLHAFKQNSPEEITYMDLSVQVLKYAKGIPLALQILGSLLYGRQREAWESQLQNLE
KCQDDDIFNVFKLSYDGLREEEKNIFLDIACFYRGHNEIVVAEILNECGFSSKIGMDVLKDKXLISILDGRIVMHDLIQEMG
QEIVRKECPQHPGKRSRLFKADEIHEILKKNKGVPLNFQSLKKLCFIDLSNCSSLTIFPFDLSEIKFLKKLCLGGCSKLENFPEI
EDTMEDLEVLILDRTAIQALPSSLWRLVGLQELSLSGCFNLEIIPSFIGSLTRLCKLDLTYCESLQTFPSTIFKLKLRKFDLCGC
LRLRTFPEITDQVQTFSHINLKETAIKELPSSFGNLVSLRSLQLNKCTNLESLPDSVANLKHLCKLDCSGCAKLTEIPRHIGRL
TSLMELSLSESGIVNLPVSIAHLSNLKSFDLSDCKKLECIPQIPPCLKQLVALDCTSIRRVMSNSPVRNLSNSKEGVFKFHFT
NAQQLDSGARANIEEDARLRMTDDAYRSVFFCFPCTAVPHWFPFRGNGPSVSINEDLSFCSDDRLIGFALCVVFELLDT
NDFEGRYGSFSYNLKFECDDNGTQIIPNNHVLDNYFEWNDKDRVVNQDHTFMWKFNLESVRTSGMSLRLRDARNFT
FEIDPKHYDFEFELPDYDSVVNELKSVVTIKECGMCPLYRSGSNVGESSRETKEDRKRKAESYLVG

>XP_047161527.1

IQLWMAENFLESSSSPKEVGEQYFNDLLSLSFFQQSNKEAKITFIMHDLLIDLAKYLCQDSCIRFGVDEPQGIHKRTRHFS
FATDLIQIFDGFGNLIDSQKLHTFVQTSWRRYPPSMSCWRCNMSIDDLFSKFKYIRVLSLNGFLRFTEVPKSIGNLKHLRS

LDLSYTEIEKLPDSISSLYKLQTLKLNYCERLKELPSGLLQLDRLCFLELLNTKVKNVHILGKLKNLQVLMNSFCVDMHKELSI
QQLGQINLHGSLSISGLQNIEDSSHASEAYLKNKPQLVELVFNWKWSDSSSVDSTKAGDVIENLQPSKHLKKLSIRSYMG
AQFPNWLLDNSLPYLVSLVLKECKYCKRLPPLGLLPSLKDLKIKKLHGIVNIDADFHGNNSSSFKSLETLYFSNMGEWEKW
ECKDLTGAFPCLQMLSIMYCPKLKGQLPELLVPLEKLCISYCQKLEAFAPRALDIKLEESAKVLFDWATVKSLRLAGYIMEA
SFLDMVRDNIPDNSIQHLKINGSSWGRPPIIGDSVSLWNFPLDFFPTLKSLTLWRLNSLRMISQNHAQNHLDWLQIGN
CPKIESLPENWDMLKSLHISDCPKLEPFTEGGLPSNLKEMTLSKCSRLVGSLKVAFGDNPSLKTLHIEHLDAECFPDEGLLP
LSLTELVIRNFPNLQKLDYRVLNQLSFLQSLTLSNCPKLQQLPEEGLPKSISDLSIHLCFLLEERCRKGGKDWEKVAHIPRLR
TSYLELPFFP

>XP_047161538.1

MFKEDILSAICINFSXNSSFVSFFEIEIESRMEQVIEDLEDLANESYILGLEKCGGAVVGSGSGSNITYTSLPNESVIYGRDDD
KEFVFNWLTSDTHNNLSILSIVGMGGMGKTSLVQHVFNDPRLEGKFDTKVWVSVPQKFDVLKLSRAILDTITGSADHSI
HQEVIQKRLKEELMGKKFLLVLDDVWNERPSKWEDVQKTLVFGGQGSRLLVTTRSEKVAITMRSEKHLLQVLKEDYCW
NLFAKHAFENANPQPDPDFIEIGKKIVEKCNGLPLALKTMGSLLHNKSSLWEWESIMKSEIWHLSENESDILPALKLSYFH
LPSHLKKCFAFCALFPRGYRFDKEYLIQLWMAQNFLENPLQKKSPKEVGDEYFNDLLSWSFFQRSSNEEKKRFIMHDLL
NDLAKYVCEDICIRLGVDEPKGIPKTTRHCSFSTSKLCFDGFGSSIDTQKLHTFTPRDWGWDWNCKMSIVDLFSKFKFIR
VLSLSHYRNLREVPESVGNLKHLRSLDLSYTDIEKLPDSISLLYKLQILQLNYCQRLKQLPLYLHQLDNLRCLEFIKTAVKNVP
AHLGKLKNLQVLMSSFYIEKGKEFSIKHLRELSLHGSLTLDDLQNIENPSYALEVDLKNKPHLVDLRLKWNFIGSSSVDPAK
VGSVIENLRPSKYLKKLSIKNYIGKQFPNWLLHNSLPNLVSLVLEECESCQRLPPLGLLPFLKKLEISGFDEIVSIDADFHGND
SCSFKSLQTLYFSNMRQWEKWDCQAVTGAFPRLQLFSIENCPKLKGYLPKFVALKTLSVIHCQQLEALIVSAVELRLEDC
GKLDEHGMAASLVAIVGHMLFDTSLENLSISSALESKSDDYVYLRIFPLDFFPTLKSLKLSRFPNLLMISQDQVHNHLQYLE
IKECPKFESLPANMHMLLPSLTRLWIEDCPRLASFPEGSLPSNLKYITLYNCFRLVGLLKGALGDSSSLERLGISTPDAECFP
DEGLLPLSLSFLIISDCRNLKKLNYKGLLELSSLRRLNLWKCPNLQCLPEEGLPKSISFLEIRECSLLEQRCHKRGKDRGKIAHI
RKVFMSRIL

>XP_047161549.1

MAAEMVTGALVSTFLERTIDTLASRLLYIFPQRKHKKQLSNLKMKLLAIDVVALDAEQKQFTDPRVRNWLLKTKDVVFD
AEDILDEMDYELSKSQAEAESQSVTKKVWNSFVGPXFNWLTSDTHNNLSILSIVGMGGMGKTSLVQHVFNDPRLEGK
FDTKVWVSVPQKFDVLKLSRAILDTITGSADHSIHQEVIQKRLKEELMGKKFLLVLDDVWNERPSKWEDVQKTLVFGGQ
GSRLLVTTRSEKVAITMRSEKHLLQVLKEDYCWNLFAKHAFENANPQPDPDFIEIGKKIVEKCNGLPLALKTMGSLLHNK
SSLWEWESIMKSEIWHLSENESDILPALKLSYFHLPSHLKKCFAFCALFPRGYRFDKEYLIQLWMAQNFLENPLQKKSPKE
VGDEYFNDLLSWSFFQRSSNEEKKRFIMHDLLNDLAKYVCEDICIRLGVDEPKGIPKTTRHCSFSSSKLCFDGFGSSIDTQK
LHTFKPTDEGWVWDCKMSIDDLFSRFKFIRVISLNHCRNLTEVPQSIGNLIHLRSIDLSWTEIEKLPDSISSLYKLQTLKLNY
CHRLKELPSCLHQLDNLRCLELVAIGVKNVVAYLGKLKNVQVSMSSFHVEKSKEMNIQQLGELNLHGSLTIDDLQNIENP
SDTMKVDLKNKPHLVDLRLVWNFIGSSSVDSEKAEDVIENLRPSKYLKELSISNYIGKQFPNWLLDNSLPNLVSLVLKGCE
SCQRLPPLGLLPFLNYLSLSGFNEIVSIDADFHGNNSSSFKSLQELYLYDMRQWEKWECQAVTGAFPRLQRLWIQNCPK
LKGHLPKFVALKSLYVFNCEQLEVLIVSVIELRLEECGKLQLERSTMKKLTTDGHGMAASLVATVGHMLFDTSLEILYIGSA
LESKSHDCVSLKIFPLDFFPTLRTLKLGGFPDLQIISQDHVHNHLQDLTIKDCPRLELFPEGGLPSNLMDIQLINCFRLVGSL
KRVFGDSSSLESLRIEKVEAECFPDEGLLPLSLSSLIISDCPNLNKLNYKALLELSSLRTLRLWHCPNLQCLPEEGLPKSISSLHI
HDCPLLKQRYHEGGEDRKKIAHIRYIFIW

>XP_047161756.1

MSIRTNKMKAVAVLMKQLTIARNKFHEKGRDESFDKKLEKLRLDLKRIKDVFVEVKKNEEKLLDKLAEVYGHLQRLDRKK
LDEDMEGICKRIRDSAHNLLPTLVFDDSYKEEDDRGAKISHSSKQLVQPHQRQSWTAGDFNPLDNPFNDFLLRLLIFPEN

AIIRKRIAINLWIAEGLIENTEKKTAEELGEDVVHDLLKFKVIVRYGSTKDPHVNKFRISPDIKFYVDRYINFIRPPCLLLDRKK
FTIGGIDTKGVTLINFFNIGSSYLNFKPQWVTDQLKDLKVLQLGRWQDSALHLIEVGSEEFLKELIKLKRLKYLSLRGISRIFE
IPSSVAKLESLLILDLKACHNLETLPDDISSMKSLTHLIMSKCCLLEGMPKGIEKLTNLQVLKGFLITTSEKTPCTISDLANNLT
NLRRLSILIGSEAVIRDGEFRSLENFPALKHLKISWSVSDPRYAKIGIRLPVSLKKLHLECFPGKSVVEFLPEEIFAEILELNITG
GKLENIFVNIIRWRVKILRLKYLKQLNVDIDDLKIFYPSLQYLEIKQISNHSYIEHRYE

>XP_047161959.1

MPLESDVTISSYRLCWDVFLSFRGTHTGHTFTMRLYHALHGRGVRVFRNDDGLERRGEIQKKLLEAVEDSAAAVVVISP
DYASSHWCLEELAKICEVGRLILPVFYWVDPSHVRKQEGPFEEWFVWHAQRFPTERIEQWRDAMKKVGGLAGFVLDE
KSDKSDELIQILVQNLMKQLRNTPLSVAPFTVGLDDRVEVLKNLLDLKSNDVRVLGLYGMGGVGKTTLAKSLFNNLVVH
SFERRSFIPNVRSQVSKHHGLVSLQNKIHGDLCGRKEDLITDVSDGISAIQKIVQENRVLLILDDVDDVEQLNFLMGKRE
WFYKGSRVVITTRDKEILHGSYVDVDFEVKELEFSAAMELFCFHAIRRKEPAEGFLDVSKQIVEKTGGLPLALEVFGSFLFD
KRTEREWKDALEKLKQIRPPCLQEVLKISFDALDEQKQCIFLDIACLLVQMEMKRDDVVDILNGCDFRGEIAVDVLTARC
LIKIIGDGKVWMHDQVRDMGRQIVRSESLTDPGLRSRLWDRDEILTVLKNMKGTRNVQGVVLDCVKRRMSIPRDRSA
DEITRENFRRKPSCKSAFEYIKERYKKYVEDRKEKAKEVILQSKHFQPMVSLRMLQINYSRLEGKFICLPPKLKWLQWKQC
SLRYMPSSYNPLELAVMDLSESLIETLWKERSNKVAVHLTVLNLSRCHRLTATPDLSGYLSLKKLNLEECSHLTRIHESLGN
LNSLVHLNFRLCYNLIELPSDVSGLKHLEDLVLSDCWKLKTLPKDLSCMVSLRQLLLDSTSITELPLSIFHLTKLEKLSANGCH
LLKKLPTCTGKLCSLQELSLNHTALEELPDSVGSLEKLEMLSLTGCKSLSVIPNSTGKLISLTQLYLDGSGIKELPASIGALSYL
RKLSVEDCTSLDKFPVSMEALVSIVELKLDGTKVSNFPEEIFVGMKMLEKLEMGKVQHLKFVPVSLGYLSALTILDMHDA
NITELPESIGMLENLIRLRLDKCKQLQRLPDSIGNLKSLRWLMMK

>XP_047162027.1

MELVAGPLMGAIFNVLLERIASTEVVNFFKNKNCEKLLKRLKIILLSVNVVLNDAEEKQMKNGAVKEWLEELKDVAFAAE
DLLDEIYTDAKIKAKQVNTLHSGPMSFYCKGVEEKIEDVHERLEFIMRQKEVLDLKVGKEVKMAHKTPTSSVMEACDVF
GRDNDKESLVDLVLTHNEKIGVIPIVGMGGIGKTTLAQLIYNDQRVQKEFDVKAWIYVSEEFDICKITKTLLEAVTSCSCD
VEDLNFLQRDLKMHVMNKKFLFVLDDVWNENYDNWDKFRSPFKHAGEHGSKIIVTTRSGCVASIMQTVSPYNLRELS
NEDSWNLFSKHAFDYGDSSLQLHQSLDKVGREIVRKCKGLPLAVKTLAGLLRCKSDRQEWCKVLDSEMWDLQDSESNI
LPALRLSYHYLPSHLKRCFAYCSIFPKDYEFEKENLVLLWMAEGFLQQSKRHRRIEEVGNEYFCELVSRSFFQQPRRGKSC
FLMHHLVNDLAQFVSGTFSLRMECSNSNEIKERTRHLSHIIADSSSYVNLKDVSKANCLRTFLQIRPVGTSIDLFNNMPN
DLLTKLRSLRVLSLVGTHIYSLPNSVGELKHLRYLEVADTEIVRLPESICSLFNLQTLKLVGCHNLIELPASIHKLVNLRHLDIR
GTSLRWMPLQINELNSLQNLSDFFVGKGCGSSLGELGELICLHGELFIHCLEHIVSDKDCEKAKLKEKHGLEKLSLDWCG
NGETENSQKEKTILNSLQPHTNLKKLDIYDYPGTEFPEWLGDHSFYNLVSLMLNGCKYCYRLPPLGQLPMLKELQISKFE
GLVSVGSEFLGNRTSYLTDCFPSLEILRIESIPLWEKWYPNAENAGSKAFFYLREIHIGNCPKLRGDLPDNLPSLTLLVIRDC
KRLLCSLPNSPSLRVLNIQNCESLEFKVHSPLCHQSLTSLFLHGSCDSLVFLPLDLFPNIKSLDIWGCKNLEALTVSESDATR
PNLKSLHSLRIRHCPNFTSFPKGGFAASKLTLLTINYCQKLNSLPEQMHDLMPSLKEVQLRGCPKIESSSTRPLRIRICSKH
MEGKQNLSDPLFARLKGLATDQSPSSS

>XP_047162166.1

MAAACVGGALLSAFLQVAFDRLASPKVLHFFRRRKLDEALLSKLNIKMLSINSLADDAEQMQFRDTRVKAWLFAVKDA
VLDAEDLLDEIDYELTKCEVEAQSESQSLSNKVSSFFHSTFSSFNRKIDSGLKQVLEKLEYLASQKDYLGLKEATYSGLRPGS
VVQQKLASTSLVAENVIYGRDYDKETIFNWITYETDNRNQLSILSIVGMGGVGKTTLAQHVYNDPRMEEADFSIKAWV
CVSDDFDVMVVTSTILEAITKSKDDSRNLEMAHGRLKEKLSGKKFFLVLDDVWNERREKWEAVQTPLNFGASGSKILVT
TRSEKVASTMRSSKVHRLMHLQEDHCWDVFAKHALQDDHPQLNAELKDIGIKIVKKCKGLPLALKAIGSLLHTKSLFSE

WQCVLVSKIWDTPIEENEIMPALLLSYHHLPSHLKRCFAYFALFPKDYKFDKQSIILLWIAENFLECPQHNKSPEEIGELYF
DDLLSRSFFQQSSGLESCFVMHDLLN

>XP_047162167.1

MAAACVGGALLSAFLQVAFDRLASPKVLHFFRRRKLDEALLSKLNIKMLSINSLADDAEQMQFRDTRVKAWLFAVKDA
VLDAEDLLDEIDYELTKCEVEAQSESQSLSNKVSSFFHSTFSSFNRKIDSGLKQVLEKLEYLASQKDYLGLKEATYSGLRPGS
VVQQKLASTSLVAENVIYGRDYDKETIFNWITYETDNRNQLSILSIVGMGGVGKTTLAQHVYNDPRMEEADFSIKAWV
CVSDDFDVMVVTSTILEAITKSKDDSRNLEMAHGRLKEKLSGKKFFLVLDDVWNERREKWEAVQTPLNFGASGSKILVT
TRSEKVASTMRSSKVHRLMHLQEDHCWDVFAKHALQDDHPQLNAELKDIGIKIVKKCKGLPLALKAIGSLLHTKSLFSE
WQCVLVSKIWDTPIEENEIMPALLLSYHHLPSHLKRCFAYFALFPKDYKFDKQSIILLWIAENFLECPQHNKSPEEIGELYF
DDLLSRSFFQQSSGLESCFVMHDLLNDLAKYVCGDICFNLEVDKALCIPKMARHFSFAIKDIKYFDSLHDAKRLRTFIPLPA
YVRVSSLNDPWQCKIPVHELFSKFKFIHTLSLLCCSGLLEVPDSIGDLKHLRSLDLSRTDIRKLPDSSCLLYNLQILKLNFCLLL
KELPSNLYKLNNLRCLEFIATSVRKVPMHMGKMKNLQVLSSFYVGKSNEFGIQQLVGLNLHGGLSIGDMQNIVNPLDA
LQVDLKNKKHLVKLELEWNSHHIPDNPRNEKQVLEYLQPPKHLKNFSINHYGGTQFPSWLFDNTLSNLVSLSLIGCKFCL
HLPPLGLLPFLKQLVIIELDGIVGVGAEFHGSSSSSFTCLETLYFFNMKEWEEWDCETDFPRLQHLSIIHCPKLKGLPKQLL
HVKQIIICECETLTISGHNMESSTLERIGDTITNNSLEVLHIYSCLSMNIPLRLCYNLLVTLEIDGAFDTPITFPLDFFPKLFSLK
LGCCNLQMISQDHTHNHLKDLCISNSPQFESFPMEGLSAPRLVKFSIKELMNLKLLPKRMDILLPSLTDLQILDCPQVELF
SDGGFPSNLNTMDLSDCSKFMASMKMALGANNSLEVLSVRKLNVESFPDEGFLPFSLTCLEIRNCSDLKNLDYKGLCHL
SSLQKLLLFNCSNLQCLPEEGLPNSILELKIVGCPLLEQCQDYKTGN

>XP_047162413.1

MAAISCSYAFSHDVFLSFRGSDTRHGFVGNLYKALDDKGIHTFIDDEKLQRGEEITPALMKAIEESRIAITVLSHNYASSSF
CLDELVHIIACAKEKGLLVLPVFYDLNPSDVRHQKGSYAEALTRHEERFKDKKESFSHNMERLEKWKMALHHVASLSGY
HFKQGYEYEYEFIRRIVELVSSKINRTPLHVADYPVGLEAQMVEVMKLLDVGTDDGVHMVGIHGIGGIGKTTLALAIYNL
VFDHFDGLCFLENVRENSDKHGLQNLQSILLAELVKEKRINIASVQEGISMIQHRLQRKKVLLIVDDVDKHEQMQAIVGR
SDWFSSGSRIIITTRDEQLLASHEVKRMYEVKELNKKDALQLLTWKAFRTDEADPSYEEVLKRVVAYASGLPLALKVIGSN
LFGKGIEEWKSAIKQYERIPNNQILKILKVSFDALEEEEKNVFLDIACCFKGYELEEVQDILHAHYGDCMKYHIGVLVDKSL
LKFNMQCMMVTMHDLVEDMGKEIVRKESPKDPGKRSRLWLHEDIIQVLEDNTGTSEIEIIHLDFPLLDKEEMVEWNRK
AFKKMRNLKTLIIKSGNFSAGPKYLPNSLRVLEWWRYPSHDLPSDFHSKKLDMXKLPQSCFTSHELVSLLKKFMGMRFL
NLDKSKSLTQIPDVSGLPNLEKLSFQHCQNLTAIHNSIGFLCKLKILSAFGCTKLVSFPPIKLTALEKLNLSRCYSLENFPEILG
KMENIRELQLEYTAIKEFPYSFQNLTRLQKLQLSNCGVVRLPSSIAVMPELTDLIGWKWKGWQWLKQEEDEEKEGSSIV
SSNIEYLWASECNLCDDFFSIGFMKFAHVKDLDLSKNNFTMLPECIKEFQFLRKLNVSDCKLLQEIRGIPQSLKHFLATNC
KSLTPSSTSMFLNQELHEAGKTQFYLPGERVPEWFDHQSNGPSVSFWFRNRFPEKVLCLVIGPVNDSGMFRPMVVIN
GNKCFLGSDYFMMGMDHTYIFDLLTIKFEDNLYGVPLENEWNHAEVKYVGLEETSILKQSGIHVFKQESGLEAVWFSDP
YGKRKLEDDLNSFESQNQQLLKKHRFVDMEAYT

>XP_047162417.1

MALAVVGGALLSAFLDVLFDRLASPELVSLIRGKKPDKLLRKVENQLIVLRVVLADAENRQVTDSNVKKWLDVLRDIVYE
VDDLLDEISTKAATRKEVSNSFSHIFNRKRIVSINKLEDIAERLDDILKQKESLNLKEIPVESNQPWKDQPTSLQDRYGMYG
RDKDKEAIMKLVLEDNTDGEEVSVIPIVGMGGVGKTTLARSVYNDGKLKQIFDLKTWVCVSDIFDTLKVTKTMMEEITK
MPCNFNDLNLLQLELMDKLKGKRFLIVLDDVWIENCDGWNSLTKPFLSGIRGSKVLVTTRNESVAAVIPFHVVKVYHLN
QLSNEDCWLVFANHAFPLSEGSENRGTLEKIGKEIVKKCNGLPLAAQSLGGMLRRKHEIKDWNDVLESDIWELPESQCK
IIPALRISYNYLPSQLKRCFVYCSLYPKKDYEFEKDELIQLWMAEDLVKAAKKGKTLEEVGQEYFEDLVSRCFFQSSSRRGG
DYFVMHDFIHDLATFLGGDFYFRTDELGKETNIDRKTRHLSLTRFSDPVSDIEAFDTVKFPRTFLLIDYKDSPFNNDKAPSII

VSRLKYLRVLSLWKFRSQLALPDSVGELIHLRYLNLSRTSIETLPESLCNLCNLQTLKLSYCSELTKLPSSMQNLVNLRHLEIV
DTPIKEMPKRMGKLNQLRTLDCYVVGKHIENSIKELGGLQNLHGRFYIKKLENVTKGEEALEARIMDKNHISHLHLVWSI
ANDNSIDFQVELDVLSKLEPHQDLEALSIIRYKGTRFPEWVGNFSYLYMTSIGLYNCNNCCMLPSLGQLPSLRNLRISNM
NSVKTIDAGFYKKDDCSSVTPFPSLEYLLIYNMPGWEVWNSFDSEAFPVLKNLCIENCPKLKGDLPNHLPALQTLGIRNC
ELLVSSVPGPLSLRTVEIHKCKKVAFREFPLLVKSIEAEGGPMVESMMEAISNIQPTCLQTLKLQNCSSDISFRGGLPTSLK
TLDIRGINKLKFPLQHKHELLESLLIINSCDSLTSLPLAIFPNLTSLQITNCENMESLLVSGSEISKRLNTFAIDHCPNFVSFPGE
GLCMPNLTRFNVYNCENLKSLPDQMGTLVPKMEYLKISNCQQIESFPGGGMPPNLKTVEIRNCEKLLRGLGWKSMDM
VTSLIVCGPCDGIKSFTKESLLPPSLVSLDLIDLSSLETLDCKGLLHLTSLQQLNIEWCQKLENIAGEKLPLSLIKLTIYGCPLLK
QRCHKKDRQIWPKICHVRGIKIAGRYI

>XP_047162549.1

MAERVVTFVLDHLAQLAAREVNLLYGVEDRVQSLQYELQMIKELLNTTKKKKGMEHTVFNHIRDVAHLAEDVIDTFVA
KVSIYKRRTIMGRMLCGFGQARLLHNVTDKIDKLKTTLNEIRDNKDKYDAFKETTNQSTAEAEEEEEKKRLQSLQKLRRD
VEEEHVVGFVQDSKDVVKRLVGGGSTRKVVSIVGMGGLGKTTLARKVYNSSQVKNHFDCRAWVYVSNECRVHDLLID
LLKHLMPNFEQQRRGNKRGKKSVGDINDLSNEELKKLVRNCLEWKRYLVVVDDLWKKQDWDEVQDAFPDNNKGSIIL
ITSRLKEVALHAGDDVPHYLQFLSEEESWELFGRKVFRGENLPSDLEPLGKQMVQSCRGLPLSIIVLAGLLANKEKSHRE
WSKVVDHVNWYLTRDETQVKDIVLKLSYDNLPSRLKPCFLYLGLFPEDFEIPVTPLLQKWVAEGFIQDTGNRDPDDVAE
DYLYELINRSLVQVTGVKFNGGLEMCKVHDLLRDLCISESKEDKVFEVCTNNNILIPTKPRRLSIHSDIGHYISSSNNDHSCI
RSLFFFGPHYEVHGREWKWLLDDFKLVRVLEFGPNSCHKIPSNLGNFIHLSFPQLEVLEIKYMRLRKWEIGNGAMRRLQ
NVIIHKCPLLDNLPTELFFLNELRKVNITESPSEQMAHILQILETNNRVEVVIGTTS

>XP_047162551.1

MAESVVTFVLDHLAQLASREANLLCGVEDRVQSLQYELQMIKELLNTTKRKKGMEHTVLNQIRDVAHLAEDVIDTFVA
KVSIYKRRTILGRMLRGFGQERLLHHVAHKIDKLKTTLNEIRDNKDKYDAFKETTDQSAVEEEEKERLQLLHKLRRDVEEE
NVVGFVHDSKDVVKRLLGGGSNREVVSVVGMGGLGKTTLARKVYNSSQVMSHFDCHAWVYVSNECRVHDLLIDLHK
HLMPDFEQQRRGNKRGNKSAGDIKDLSKEELKKHVWNYLERKRYLVVVDDLWKRQDWDDVQDAFPDNNRGSRILIT
SRLKEVALHAGDDDPHHLQFLSEKESWKLFCRKAFRGKNCPSDLEPLGKQMVQSCRGLPLSIVVLAGLLAKKEKSDREW
SKVVGHVNWYLTRDETQVKDIVLKLSYDNLPRRLKPCFLYLGLFPEDFEIPVTPLLQKWVAEGFIQDTGNRDPDDVAED
YLYELIDRSLVQVARMDFNRGLETCKVHDLLRDLCISESKEDKVFEVCTNNNILLPTKPRRLSIHVDMGHYISSSSNDHSCI
RSLFFFGPHYKVRGREWKWLLDDFKLIRVLEFGPNDRNKIPSNLGNFIHLRYLKILSSYDTFVPDSILNLWNLQTLDLGSW
DHLMPVSFPGQIWKLKHLRHLYTQGPIKLRGRCSEPDEKMWNLQTISLVSNTQAISLIKKGIFPNIKRLRLREEHGFVGEL
PNLLENLQQLNHLNKLLILSPFRQGYKLQERVQSLGQLSCLTILIISDVVDLLTSMLILPPNITELTLSQINWITDEGMNSLA
NHSKLKVLRLRGTGLFQEDSFDLNCADGGFPQLEVFKMRDIKLRKWRLGNCAMQKLQNVFIQRCQPLDHLPTELCSLN
ELREVHITEDPSKQMTHILEILKTNNGVRVIIGVFPTPDVEWDHVSRVTYEIYYSK

>XP_047163027.1

MEFASSSLSSSSSSFLTSEPHFIHDVFINFGGEEIGRRFVSHLQSVLLQAQVKTFISQENLQEGMKLEEQLRAIGGTKITIIVF
SKSYAETTRCLLELQKIIECHETFGQIVLPVFYEIGPSDLRYQMYDFGKTLEETAHRSYSGEQLKYALSKWNHALTTAAVM
TGWDVRNFRHDAELVDVIVNRVKTLLDYKDLFITEYPVGLESRVEDVIKCIQNQSTKVCMIGIWGMGGSGKTTIAKAIY
NRIYREFIGKSFIENISSEGYFALQENLLSDVLKSNLEVKSVGMGRTMIENRFSRKKLLIVLDDVNHIGQLENLCGSREWFG
QGTVIIITTRNVSLLNQIRVNYVYQTHLLNENESLELFSWHAFRDAIPKKEWNELARNVVVYCGGLPLALQFLGSCLCDRT
IEVWENILLKLQRIPPDELLSVLKISFEDLRDTEKDIFLDVCCFFIGKERDYVTEILNGCGLHADIGITVLIERGLIKVERNNKIQ
MHNMLQEMEREINRQE

>XP_047163454.1

MVHSEIWYLQHHWWEKVREAYGSRYLDNLLYSGTKIDIEVHLENCTKALHEAIAIPRGAVSISSFQKLKAYNRIESLVEF
WRIALCEAAEISRLVVQHYRGITDNEINYIEKHARDALHEAAGISGVVILNSRNESEAVKNIVKNVTSLLDKIELFVAHNPV
GVESRVQEMVQLLQQKQSNDVLLLGVWGMGGIGKTTIAKAIYNKIGQNFEGRSFLADIRDVWGQEAGHVCLQQQLL
FDIHKENNIKIHNIESGKVILRERLRHKRILLILDDVNKLQQLNALCGNREWFGSGSRIIITTRDIHLLRGKRVDQVFAMTG
MNVDESIELFSWHAFKQASPKEDFIELSRNVVAYAGGLPLALEVLGSYLFDMEVTEWKTVLEKLRKIPNDEVQEKLKISY
DGLSDDTEKGIFLDIACFFIGKDRNDVIHILNGCGLFAENGIRVLVERSLVTVDDKNQLGMHDLLRDMGREIIRSKSPMEL
EERSRLWFHEDVLDVLSKETGTKFIEGLTLKLPRSNTKSLCTKAFMNMKKLRLLQLSGVELVGDFEYLSKDLRWLCWHGF
PFAFIPTSFYQGSLVSIELENSKITMVWKATQLMEKLKILNLSHSHYLTKTPDFLNLPNLEKLILMDCPRLSEVSYTIGHLTK
VLLINFQDCISLRNLPRSIYKLKSLKTLILSGCLKIDKLEEDIEQMESLTTLVADKTAIRRVPFSIVRSKSIGYISLCGYEGFSCDV
FPSIIWSWMSPVNSLSSRVQTLVDMSSLVSLDVQNSSSNQLSYISEELPKFQSLWIECGSDLQLSRDTTSILDALNATNSE
ESESYGTTSQMQNVFTLIESNSRSKLFEKTLLIQMGSSWEITHILKQRILQNMTTSNGGDCLLPGDCLLPGDCYPDWLTF
SSEGSSVTFEIPEVNGRSLKTMMCHIHYSSSDSITSDGLKNLLVINHTKSTIQLYKRNALASFDDEEWQRVLSNIEPGNKV
QIVVVFWNRITVNKTSIYLIYEAIDEKVEHYHASNMNVPRSISPGVESMEDLRGASVKSLTKRLLNEFLSCKYFCKGKVDK
KKNQG

>XP_047163682.1

MQSSPSSFPYRFRYDVFLSFRGKDTRQGFTGNLYKSLCDRGIHTFIDDHHLPRGDQISSELENAIQDSRIFIIVLSQNYASS
SFCLNELHYILRFIKHKGRLVFPVFYGVDPSHVRHHTGTFGEALAHHQNNSYYNWEKLETWKMALHQVANLSGYHFKH
GEGYEYEFIERIVELVCSKINRAALHVADYPVGLESQVLEVKLLLDVGCDDVVHMVGFHGLGGVGKTTLAAAVYNSIAD
HFEALCFLENVRETSSKHGLLHLQSNLLSETVGEIKLTSVKKGISVIQHRLQQKKVLLILDDVDKEEQLKALAGRPHWFGL
GSRVIITTRDKQLLKCHRVKRTYEVNELNEENALELLTWKAFKFEQFDPSYKDVLNLAVTYASGLPLALEVIGSNLCGRNIE
QWKYALDKYKKIPKMDIQDTLKVSYDALEEDEQSVFLDIACFFKNYDLAEVEDILRAHHGHSIKHHIDVLVEKSFIKISLDG
KVTLHNLIEDMGREIVRRESPKEPGKRSRLWFPQDIVQVLEDNKGTGQIEIICVDFPSFEEVEIEWDGDAFKKMKNLRTLI
IRNCHFSKGPKHLPNSLRVMEWWRYPSQNFPQDFHPKKLAVFKLPYCEFTSLELTDLLRQKFVNMTSLNFDECRYLKQI
PDVSCLIHLENLSFRWCPKLSSLHYSVGFLEKLKILDAEGCSRLKSFPPIKLTSLEQLKLRYCHSLQNFPEVLGNMEYVRELD
LKETPVKKFPPSFRNLTRLQKLHLCLSVRVMKSGCDGLPLSSICMMPELVDIAANEWEGGLFREANEGAEKVSSILSTNV
QYLQLRCCNLTDDFFPTLLPWFANMKNLDLSGNNFTVIPECIKEFHFLTRLNLNFCERLQEIRGIPPNLKHFFAIDCQSLTS
SCRSMLLNQELHEAGSTFFYLPGAKIAEWFEIQTLELPISFWFRGKLPAMAICLAMERVWEYSSSEGGKYRPLVIHSTFRL
MSPIVIINGNEHLLETWEMLDDCTCVFDLRETKLKNDLDEELVENEWNHAEVTCRYVSLDQTLIKHGIHVFKQESSVEEI
RFTDPSRKRNLDDELNSSKSQQQQLLKKQR

>XP_047163771.1

MAEALLGLVIQNLQSFGQDQLATFWGVHQQTQKLSSNLTAIRAVLRDADRKQIKSHAVKDWLQKLTDAAYVLDDILDE
CSIHSTKVNFDDRHTSCLSRLHPKDILFRFNIGKRMKDISQRFHDIHEERSMFNLEPGVTEVQTIHDNWRQTSSDITEHIV
YGRGQDREQILKFLLEDVSDNEDLSIYPIVGMGGLGKTTLAKQVFNDHRVCKHYDLTIWVCVSDDFNTMTILQSIIECIT
GQNPHLNSLEAMRRKVEEVLHGMRYLLVLDDVWNEDPEKWKQLKGKLQCARAAKGATILVTTRLEEVASTMQTHPA
YHLKELSGDDCWSLFKHHAFGPNREEMEELVSIGKEIVRKCVGSPLAIKTLGRLLRDESDVKQWQNVKESEIWDIREESS
SATGEENSIMRALKLSYFNLELSLRRCFSFCAIFPKDFEIDKEELIHLWMANGFIKCEGNVEVEDVGNKVWKKLYSRSFFQ
EAKYDEFGMITTFKMHDLFHDLAQSIMGEECVVIMKGRLTPLSTTVHYSSLFNSGVSSVDMTGQRFMTALKKVESLRTF
LDYGGIGLMPTNHCVRALQISSSFLSPLKDLTLLRYLNLRCSRSSEENLNNSICQLLKLQILKLFHLPKLHGLPKDLTQLQDL
RHIVIDGMDITQEMSPNIGKLRHLRTLSIFVVGSKPGCGLAELHSLNLGGTLRIGGLENVPNERDAKQANLIGKKDLNILR
LSWDGNANPKGSNLSVERVLEALEPPSTLKSFEMNGYKGRQISSWMRNRAVLKDLVDVTLSNCDNCEDLPPLGKLPHL
KRLSVSGMKNVKWIDGDTYDGVEEKAFPSLENLGVKNLRNLERLLRDEGVEMLPRLSQLTIEGVLNFKVPRLPCVEELSV

ERIGEAASFMEVVGNMAFLKTLSIEIIKGVVVLPDEFRRLGALQELYIAEWYDVEYFPEHVLEGLTSLRTLTIHYCKKLKSLS
EGVRHLACLVSLAISNCPELVALPSNMSQLSALRKVSINYCCTLPNGLQRIPSLRVLYIDECTCTSLPDWLGDMTTLEELTI
WYCKELRSLPSSIQRLANLSYLTIHYCPHLKKRCNRETGEDWQYIKHIQRLELLFTRKQTFCGKFNSILRSVHIK

>XP_047163772.1

MAEALLGIVLDNLGSFLQDQLATYWGVDQHAHKLSSNLTTIRAVLRDAERKQITSHAVKDWLQKLTDAAYVLDDILDEC
SVQSKKLHSVDGHTSRLSHLHPKDILFRFRIGKRMKDITQRFHDIHEERLTFELRVGVTEKQAVNDDDNDWRQTSSVITX
PIFCGRDKDREKIVKFLLEDASNSEELSIYPIVGMGGLGKTTLAKRVFNDHEISKHFDLRIWICVSGDFNVKRILQSIIECSIG
QNPNLGDLEARRKRVEEALQRKRYLLVLDDVWNEDREKWKELKGMLEWAKGAKGATILVTTRLQEVASIMGTHPAYS
LTALSEDDSWSLFKHHAFELNREKREELVAIGKEIVRKCVGSPLAIKTLGSCLRDVSEIKQWQNIKESEIWNIQEVSSSLISD
ENSIMRALKLSYFNLEWSLRRCFSFCAIFPKDFEIDKEELIHLWMANGFIKQEGNVEVEDVGNKVWKRLYDRSFFQEAK
ANRIGMIKTFKMHDLFHDLAQSIMGEECVIYGERKLTRLPNRVHYLRLLNSNYFVDMAAFKKVESFRTVIDFGQGLHCV
NIGLLPSNHCLRALHIRSSLFSPLNNLSHLRYLSLDMCSVLHNSICGLQKLQILKLEYCGGLHNFFPENMTQLQDLRHLVIN
KCPSIAEMPRNIGKLRHLRTLNTFVVGSKLEYGLAELHGLSLVGKLHMRGLENVSNEWEAKEANLMSKKELNRLYLSWG
GSANSQGSNVSVERVLEDLEPPSTLKSFGMQGYQGRKLSSWMRSVVVLKDLVEVILVDCKNCEELPPLGKLPHLKKIDV
RGMKNVKWIDGESYEEGVEEKAFPSLEKLSVKNLPNLERLLRDEGVEMVPRLSQLRIDGVLNFKVPRLPCVETVHARGIE
AVTSFMEGVAENMTCLKSLQIAFMKDLKVLPDKLSSLSALQKLEILGCEELRSLPSSFQRLTNLSKLSIFNCPWLMERYRR
ETGNDSQLIAHIPNIELEYIFAFEETEALPFSVESWLRSSCSWNCFKATRPPAKIEKFYFDYMDEMAEPLLGIVIQNLQSFG
QDQLATFWGVHQHTQKLSSNLTAIRAVLRDAERKQITSHAVKDWLQKLTDAAYVLDDILDECSIHSTKVNFGDGDTSC
LSRLHPKDIFFRFNIGKRMKDITQRFHDIHEERSMFNLVPGVTEVQTIHDNWRQTSSDITEPIVYGRDQDREQILKFLLED
VRDSEDLSVYPIVGMGGLGKTTLAKQVFNDNRVCKHFDLTIWVCVSDDFNTKTILQSIIECITGQNFNLNSLEAMRKKVE
EVLHGMRYLLVLDDVWNQDPEKWEQLKGKLQCARAAKGATILVTTRLKEVASTMQTHPAYDLKELSGDDSWSLFKH
HAFGPNREETEELVSIGKEIMTKCVGSPLAIKTLGSLLRDESDVKQWQNVKESEIWDIREESSSATGEENSIMRALKLSYF
NLELSLRRCFSFCAIFPKDFEIDKEELIHLWMANGFIKCEGNVEVEDVGNKVWKKLYSRSFFQEAKYDEFGMITTFKIHDL
FHDLAQSIMGEECVVIVKGRLTPLPTRVHYSSLFNSGVSDDMTAFKQRLTTDLKKVESLQTFLDIGGIGPVPSNHCLRAL
QTSSSLLSPLKDLTLLRYLSLRFCSCSEESLNSSICQLPKLQILKLFHCQELHGLPKDLTRLQDLRHIVIDDMDTIQEMPPNIG
KLRHLRTLSIFVVGSKPGCGLAELHSLNLGGTLSIRGLENISDEWDAKQTNLIGKKELNILHLSWDGNANPKGSNVSVER
VLEALEPPSTLKSFEMNGYQGRHLSSWMRNNAVLRDLVKVTLSDCDNCEELSPLGKLPHLKRLNVSGMKNVKWIDGE
TYDGVEEKAFPSLEELIVDNLPNMERLLRDEGVEMLPRLSQLRIEGVLNFKVPRLPCVEELSVERIGEAASFMEVVGNMA
FLKTLSIEIIKGVVVLPDEFRRLGALQELYIAEWYDVEYFPEHVLEGLTSLRTLSINYCKKLKSLSEGVRHLARLESLMISDCPE
LVALPSNMSQLTALREVSINYCCTLPDGLQRVPSLRVLYIYECTCTSLPDWLGDMTTLEQLRIAYCKELRSLPSSIKRLTNLS
YLSICRCPHLKMETGEDWQYIKHIPKLELLP

>XP_047163848.1

MQWLPSSVSRDLVYGALNQLRYPCCFNNFVKRLEKEESNLIRTRDSVQKSVTDDKKQAKTPSAIVDKWLEDAVNDVDN
VNKLLKEARTQKHCCFGHCPNWIWRYQVGKKLANKTIDLKKFIEKGTKYVPFNSPAPLPLNTLDMLSEKCVYFDSTQSA
YEKLLDAVKNNDVSMIGLYGMGGCGKTTLAMEVRKLVEAEHLFEKVLFVPVSSSVEVRSIQEKIASSLQFKFPETEEMQR
AQRLCSRLTQEEHIFIILDDVWENLDFGRIGIPSSEHHKGCKILITTRSEAVCTL

>XP_047163994.1

MAVPSFSSSFTYDVFLSFRGEDTRYSFTGNLYRALRDRGIHTFIDDEKLPKGDEITSALEKAIEGSRIFIIVFSRNYASSSFCLN
ELAYILPYANKNGLLVLPLFYDVVPSHVRHHTGSFGEALDTHENRFKATSQGFELNMEKLNKWKMALRGTANLSGYHF
KHGEEYEYEFIKRIVDLVSNKINRAPLHVADYPVGLETRVLEVKLLLDIGSDDGVHMVGIHGLGGVGKTTLALAVYNSIAD
HFEGLCFLENVRENSNKHGLQHLQRILLSQMIGENNVNITSARQGISMMEHRLRQKKILLILDDVDKHEQLQAIVGRPD

WFGPGSRVIITTRDKHLLSCHLIEKLYKVKKLEKNNALRLLSWKAFRTEEVDTSYLNVMDRVLAYASGHPLALEVIGSKLF
RKSVKEWESAIKQYEKIPNNQILEVLKISFDALEEVEKSVFLDISCCFKAYALSEVEDILRAHYGDCMKYHIGVLVEKSLIKYG
YNSVVTMHDLIEDMGKEIVRQKSPDKPGKRSRLWSPEEIIKVLEDNLGSGEIEIICLNSSLPDKEEIVEWNRKAFKKMKNL
KTLIIKNGNFSEGPEYLPNSLRVLEWLKYPSQGLPPDFRSKQLSLCKLPSSCFGSLGLAEFSKKFMNMTLLNFDECEGLTQI
PDLSGLPNLERFSFKNCKSLITIHDSIGFLGKLNSLNAVGCSKLRSFPPLKLTSLENLELSYCYSLERFPEILGKMGQITELVLE
DCHIKELPISFQNLTELQTLQLRLCPRLRLPSSIVMMPKLANIIAWESKGWLFPKQVEGEEKIGSMVSSNVDCLYLPGCKL
SDDFFPIILEWFANVKDLDLSRNNFTVLPECIANCHLLCKLTLDACDSLREIRGIPPNIRQLLARNCKSFTSSCGRTLLNQKL
HEAGNTMFSFSGARFPEWFDHHSRGPSCSFWVGKKFPSIALCIAIGPTHLEHVEIVGPIMIINSIECSFDGEENPYLYMFP
HHTYIFDLQHIVFSDYLDRFVSENEWNHVEITYSVEQRFKEKDKHAVTPISIENGIYVFKQRSNMEDIQFTDPHKKRRLDV
DPE

>XP_047164227.1

MWICVSNDFELRNVLIKILNSASNPIGENFNNLDIEQLQIHLRNTFHGQKFLLVLDDVWNEDRARWDELKEIIDVGVERS
KILVTTRSHKVAAIMHTKSSNSYLLGCLSEKDSLSLFVKYAFEDGDEMKHPQLLKIGEEIVNKCGGLPLAVKTVGSSLFSRV
DEKEWESVRDNEIWNLKQNEKDILPALKLSYDQLPSYLKPCFASFSLFPEDTFLFSSQVCMLWGALGFLPPPKAGESMID
VATQLLHELWSRSFLSEYEDHGGECRFKLHDLVIELAVYIAKGKFEIIKNHNPKSYKNAHHLTFAANNLLDQTLLPRSLRSI
TFPRGANNEVFLNTLVSRCKFLRVLNLDFSEYASLPRCIGKLKHLRSLSLLENENLTELPDSICKLQNLQSLILNGCIKLQKLP
EGLGNLISLRHLFITTKQPVFPEKEVASLTSLEQLGFY

>XP_047164329.1

MAEVAVQTVATKLTELLVEQAAVAVSQLAGVRGQVENLKNELGWMQSFLRDADAKQEGSDRVRLWVSEIRDVAFEA
EELIETYVYNTTMQRQLDKVFRPFHLYKVRTRIDKILSKIKSISVRRETYGVVMTGHDGNNSNERLRQWRQPSPSSEEEYL
IELEDDMELFLSQLLALEPNPYVVSIVGMGGLGKTTLAKKLYNHNKITNHFDCKAWVYVSKEYRRIDVLQGILRDVDGAP
RHEMGRIPEEEFINKLRTVLSEKRYLVVLDDIWGMEVWDGLKSAFPRGKMGSKILLTTRNWDVALHADACSNPHQLRP
LTADESLRLLSNKAFPGTNGIPAELKDLATEIVVKCGGLPLAVVVVGGLLSRKLKSSGEWKRVLQNISWYLLEEQEKIARIL
ALSYNDLPSHLKSCFLYLGLFPEGVNIQTKKLIRLWVAEGFLPQEGEETAEGVAQKYLNELIGRCMIQVGTVSSLGRVKTI
RIHHLLRDLSLSKGKEEYFLKIFQGDVTGPSTKARRQSMHSCDERYDFLKHNAHHSRSLLFFNREYNADIARKPWLPLNF
QQEKKLNFIYRKFKLLRVLELDGVRVVSLPSTIGDLIQLRYLGLRKTNLEEELPLSIGNLLNLQTLDLSYCCFLKKIPNVIWKL
VNLRHLLLYTPFDSPDSGHLRLDTLTNLQTLPHIEAGNWIVDGGLANMVNLRQLGICELSGQLVNSVLSTAQGLRNLCSL
SLSLQSEEDEFPIFMQLSQCTHLQKLSLNGKIKKLPDPHEFPPNLLKLTLHNSHLQKESIAKLERLPNLKMLVLGKRAYNW
PELTFNSEGFSQLHILRLHLKELEDWTVEQSSMPRLEYIVIDRCEKLKTIPEGLKAITSLKKLKIIGMPVEFEHKLRTKDISEFT
NTPVIESTTDILAFD

>XP_047164500.1

MHWETSPVDSTNLEGVHKKLKEKLTGKKFLLVLDDNWNENKSKWEQVQKALEFGAPGSRLLVTTRSEKVVVTMRSEK
HLLQVLRKDYCWDLFEKHAFQSANSQLDPDFMEIGKKIVEKCNGLPLALKTMGSLLYNKPSLREWESIMKSEMWDFSE
NESDILPALRLSYLHLPPHLKKCFAFCGLFPKGHRFDRDLLIQLWMAENFLESSSSPKEVGEQYFNDLLSRSFFQQSNEEE
ENAFIMHDLLLDLAKYLCQDSCIRLGVDEPQGVHKRTRHFSFATDQIRTFDGFGNFIDSRKLHTFVQTSWTGYPRVMLY
WRCKISLDDLFSKFKYIRVLSLNVFRILTEVPKSIGNLKHLRSLDLSYTEIEELPDSIGLLYKLQILKLNHCKSLTELPSCLLQLDK
LCFLELLNTEVKNVHILGELKNLQVLMNSFCVDIHEELSIQQLGQINLHGSLSISGLQSIEDPSHASEAYLKNKPHIVELEFN
WNWSCSSSVDSSDSSSDDSTKDEDVIENLQPSKHLKKLSIRSYMGEHFPNWLFDNSLPNLVSLVLKECEYCKRLPPLGLL
PSLKDLRIEKLHGIVSIDADFYGNNSSSFKSLEKLYFFDMKEWEKWECKVVTGAFPRLQKLSIKGCPKLKGQLPELLVPLEK
LCIRYCQKLEAFAPRALDIKLEESAKVLLDWATVKSLLLAGDIMEASFLDMVRDNIPDNSIQHLKIYGSSFRPSPNIGLWTF
PLHFFPTLKTLVLWRLKSLQMISQNHALECLDIGWCPKIESLPENKDMLKSLYISDCPRLEPFTEGGLPSNLKEMTLSNCP

RLVGSLKGTFGDNPSLKTLHIEHLDAECFPDEGLLPLSLTELGIRNFPNLQELDYTVLNQLSSLKSLTLWNCPKLQRLPEEG
LPKSISDLCIYLCLSLEDRIREGGEDWEKVAHIPRLRI

>XP_047164501.1

MASEMVIGVLVSTFVQRTIDTLAARLVHIFRQRKHKKQLGNLKMKLLAIDVVALDAEQKQFKDPQVRDWLLRAKDDVF
DAEDLLDEIDYELSKSQAEAESQSATNKVWNSLNSSFVSFFEIEIESKMEQVIEDLEDLAKESYILGLEKSAGVGVGSRSGS
KLTYTSLPNESVIYGRDDDKESIFNWLTSELTSDTDINLSILSIVGMGGLGKTSLAQHVFNDPRLEGKFNIKAWVSVPQEF
DVLNVSKAILEGVSTSPVDSTNLEGVHKKLKEKLTGKKFLLVLDDNWNENKSKWEQVQKALEFGAPGSRLLVTTRSEKV
VVTMRSEKQLLQVLRKDYCWDLFEKHAFQSANSQLDPDFMEIGKKIVEKCNGLPLALKTMGSLLYNKPSLREWESIMK
SEMWDFSENESDILPALRLSYLHLPPHLKKCFAFCGLFPKGHRFDRDLLIQLWMAENFLESSSSPKEVGEQYFNDLLSRSF
FQQSNEEEENAFIMHDLLLDLAKYLCQDSCIRLGVDEPQGVHKRTRHFSFATDQIRTFDGFGNFIDSRKLHTFVQTSWT
GYPRVMLYWRCKISLDDLFSKFKYIRVLSLNVFRILTEVPKSIGNLKHLRSLDLSYTEIEELPDSIGLLYKLQILKLNHCKSLTEL
PSCLLQLDKLCFLELLNTEVKNVHILGELKNLQVLMNSFCVDIHEELSIQQLGQINLHGSLSISGLQSIEDPSHASEAYLKNK
PHIVELEFNWNWSCSSSVDSSDSSSDDSTKDEDVIENLQPSKHLKKLSIRSYMGEHFPNWLFDNSLPNLVSLVLKECEYC
KRLPPLGLLPSLKDLRIEKLHGIVSIDADFYGNNSSSFKSLEKLYFFDMKEWEKWECKVVTGAFPRLQKLSIKGCPKLKGQL
PELLVPLEKLCIRYCQKLEAFAPRALDIKLEESXKERMVTRGRKRNIRITFLTMVASGGGSGEA

>XP_047164917.1

MVNSKGQVGIDKKIEEVVSWIKENPKQTRLIGIWGKGGVGKTTRVEEVYNKLQSQYEGCYFLAHVTEELSRHGILSLKD
NCFSKLLGYDVKIVSPNSLPKDIVRRIESMKVLIVLDDVNDSSKHLEKMVETILGNFWIITTLFNLNAYNQSDYRREYNRLS
ERVILYAKGIPLVIKVLAGHLRGKKKEEWKRELDKLEKMPQRKVYEVMKLSYDGLEHKQQQMFLDLACFFLRTHERVNV
GYLKSLLKDNESDNSIDFEFVRLKDKALITFSEDNNFVSMHDNLQQMAWEIVRQESIEDPGKRIRLWNPSETYEALKYVK
GIDSIRSIVLHWPAIKKERLRPHIFAKMSRLQFLEIDDLYHHNSVEPFDILGDNTCWPKWQKARIADILVEGLHFLATELRF
LCWYHYPLESLPENFSAEKLVILRLPHSRMEKLWLGVKNLVNLKELDLSKSKKLKGLPDLSKAINLELLILRSCSMLTSVHPS
IFSLCESLTILASCSNLHGLSYLNLDFCENLMKFSLISDNMKELRLRCSKIKVLPLSFGHQRKLEFLHLEESGIERLPSLKNLTQ
LLHLDVSFCDKLQTIAELPPFLENLNLQYCTLLQTIPKLPSSLKTLNTEACTSLETLPELPPSIEILNVESCSSLGTIPELPQCLQ
TLNVQCCHLLRTIPKLPTCLKTLHTKNCTSLQNLPQLSPFLETLNATYCKSLKRVLFPTVKDEKLKENMKKLLFWNCSNLD
KHSLVAIRSNMKINMMKFVANHIFAPDHSHVEDYSDYEYNYHSYQAVYAYPGSIFPEWLEYKTTEDYIIIDVPSSMSSSL
VGFIFCFVLGEFQHTDIIRRVEFKITVSDGGGKDEGERESVRMYMDYWDDTIESDNICVMYDQRCSHFLLSRPRRLTSFR
IQVTMEARISFDETFNVLLQKVLKGFGVSPISFDKLRSSNYTFR

>XP_047165861.1

MAENAVTLLAVLAENLLMLLKEEGKTLSGVHKDVEHIKNLVKNIKPFMKNAEEKVLIEESVKNWMNGLREVMFRMED
VVDLYLFKVAKRDGMRKKIKSIKHRHRISSEIKDIRQTLDDLFISISTRLQLLPSHGDTLPNTIPRAHFVKESQLVGIEQNMQ
KFRDWLAKANSPLLVVVGAAGIGKTSIVKNVYNKQTKLNQPKKKKDFDFCVWITMTQADSWYPIMQIKEKILMADPL
GSSSLRSATRENLTEKLREYFIDKRCLIVLDDVKELKVWDVIQFAIPQHRVIITTQRDDFPNNIGGDTSVEKIKLEPLSLEDA
LKLFHQKVKHVQFPELSQLSKEFMEKCNGVPLAIVAISSLLSTVKSAIEWRRVRDDLGSLLRSHHHLETVRHVLLQSYQEL
PYRLKQCFLYFGHFPQGYSISCKRLIRLWIAEDFVEGDTQNKSMEELGGEYLAELICRGLVHASRVDFDGIPRSCHVYNL
MHEIISSICKDQMFCHVMEDVSTPVNSNMDFIHRRLSIIKKNDSATMERDQKWGKVRSCFVFDDAKKWQVNNHFFSS
FEFLIRLDLSDACLSVDVLPEQVGNLLNLKYLSLRNTNIMSLPESIGNLVNLQTLDLKQTKLHEVKIDKLVKLRHLLAYYVSD
QSSEWYCLEGLRLSEGVQNLESLQNLSYLDVTGGRIIKGLQKLTKLRKLGIIKLEAKHCEALCNSIEHMINLCSLSIGALGKQ
DMLKLQSLNPPLSLKRLYLYGRLGELPSWISTLPNLIRLYLKWSDLKQDPLHYLKDLPQLLYLELYDAYKGERLDFGNGWL
KLKVLYLGLLPNLKTIEIGKGKVPCLEILKIGRCHQMIRLPKDIQNLKHLEKLYLYDMHEQFIKRLCDERSEDYWIINKIPLVE
YSNNDHFASFS

>XP_047166174.1

MGETDQLVILTRFQDAMRMVMDIVEKGRKNKRSKRILRSTLKNMTLVVQEIKQYNEHLNPPREEIITLVKENDPGEDLV
CNSCSRSLWWTKFLSWFSLYGEGLLHEKNDSLTSDDKQVKDIKNTLYKVREIIELLDIENFEQKLKGDGTPIKCPYGVPEN
PEFTVGFVPVLSKLKMEVLQEGVFTLLLSGMGGSGKTTLATMLCKDKEVKGKFKNNILFVNFSQTPKLKNIVERLFEHCG
YHVPEFVSDEDAIKRLEILMRKIEGSPLLLVLDDVWPGSEALVDKFKFQMSDYKILVISRVAFPKFGTQIILKSLVHEDAMT
LFRHHALLEENSSKCSLSIPNEEIVQKVVRYCKGLPLAIKVIGRSLSHQSTELWQKMVEELSKGHCILDSNTELLTSLQKILD
VLEDNPIIKECFMDLGLFPEDQRIPLPALIDMWAELYKLDDDGIEAMEIIKKLDSMNLANLLVARRNTGDSDNHCYNNH
FVVLHDLLRDLAIYQNNREPIEQRKRIITGINENQSQWWLGEKQQGMMSRLLSKYRGWCVKQTIQQVSALILSLSTDET
CASYWSDIQPSQAEVLILNLQTKKYTFPEFMEKMIKLKVLIVTNYGFHHSELDNFQLLGSVSNLKRIRLERISVPHLSALKN
LKKLSLYMCSNISQVFENGTILVSDSFPSLLDLNIDYCKDMVRLPNGICDISSLKKLSITNCHKLCSLPQEIGQLLNLELLNLS
TCTDLEEIPDSIQNLSKLRLLNISNCISLSNLPEDIGNLCNLRNLNMTSCARCELPYSITNLENLKVVVCDEETAASWEAFEA
LLPNLKVEVPQVDVNLNWLHSISS

>XP_047166180.1

MGETDQLVILTRFQDAMRMVMDIVEKGRKNKRSKRILRSTLKNMTLVVQEIKQYNEHLNPPREEIITLVKENDPGEDLV
CNSCSRSLWWTKFLSWFSLYGEGLLHEKNDSLTSDDKQVKDIKNTLYKVREIIELLDIENFEQKLKGDGTPIKCPYGVPEN
PEFTVGFVPVLSKLKMEVLQEGVFTLLLSGMGGSGKTTLATMLCKDKEVKGKFKNNILFVNFSQTPKLKNIVERLFEHCG
YHVPEFVSDEDAIKRLEILMRKIEGSPLLLVLDDVWPGSEALVDKFKFQMSDYKILVISRVAFPKFGTQIILKSLVHEDAMT
LFRHHALLEENSSKCSLSIPNEEIVQKVVRYCKGLPLAIKVIGRSLSHQSTELWQKMVEELSKGHCILDSNTELLTSLQKILD
VLEDNPIIKECFMDLGLFPEDQRIPLPALIDMWAELYKLDDDGIEAMEIIKKLDSMNLANLLVARRNTGDSDNHCYNNH
FVVLHDLLRDLAIYQNNREPIEQRKRIITGINENQSQWWLGEKQQGMMSRLLSKYRGWCVKQTIQQVSALILSLSTDET
CASYWSDIQPSQAEVLILNLQTKKYTFPEFMEKMIKLKVLIVTNYGFHHSELDNFQLLGSVSNLKRIRLERISVPHLSALKN
LKKLSLYMCSNISQVFENGTILVSDSFPSLLDLNIDYCKDMVRLPNGICDISSLKKLSITNCHKLCSLPQEIGQLLNLELLNLS
TCTDLEEIPDSIQNLSKLRLLNISNCISLSNLPEDIGNLCNLRNLNMTSCARCELPYSITNLENLKVVVCDEETAASWEAFEA
LLPNLKVEVPQVDVNLNWLHSISS

>XP_047166192.1

MGETDQLVILTRFQDAMRMVMDIVEKGRKNKRSKRILRSTLKNMTLVVQEIKQYNEHLNPPREEIITLVKENDPGEDLV
CNSCSRSLWWTKFLSWFSLYGEGLLHEKNDSLTSDDKQVKDIKNTLYKVREIIELLDIENFEQKLKGDGTPIKCPYGVPEN
PEFTVGFVPVLSKLKMEVLQEGVFTLLLSGMGGSGKTTLATMLCKDKEVKGKFKNNILFVNFSQTPKLKNIVERLFEHCG
YHVPEFVSDEDAIKRLEILMRKIEGSPLLLVLDDVWPGSEALVDKFKFQMSDYKILVISRVAFPKFGTQIILKSLVHEDAMT
LFRHHALLEENSSKCSLSIPNEEIVQKVVRYCKGLPLAIKVIGRSLSHQSTELWQKMVEELSKGHCILDSNTELLTSLQKILD
VLEDNPIIKECFMDLGLFPEDQRIPLPALIDMWAELYKLDDDGIEAMEIIKKLDSMNLANLLVARRNTGDSDNHCYNNH
FVVLHDLLRDLAIYQNNREPIEQRKRIITGINENQSQWWLGEKQQGMMSRLLSKYRGWCVKQTIQQVSALILSLSTDET
CASYWSDIQPSQAEVLILNLQTKKYTFPEFMEKMIKLKVLIVTNYGFHHSELDNFQLLGSVSNLKRIRLERISVPHLSALKN
LKKLSLYMCSNISQVFENGTILVSDSFPSLLDLNIDYCKDMVRLPNGICDISSLKKLSITNCHKLCSLPQEIGQLLNLELLNLS
TCTDLEEIPDSIQNLSKLRLLNISNCISLSNLPEDIGNLCNLRNLNMTSCARCELPYSITNLENLKVVVCDEETAASWEAFEA
LLPNLKVEVPQVDVNLNWLHSISS

>XP_047166201.1

MGETDQLVILTRFQDAMRMVMDIVEKGRKNKRSKRILRSTLKNMTLVVQEIKQYNEHLNPPREEIITLVKENDPGEDLV
CNSCSRSLWWTKFLSWFSLYGEGLLHEKNDSLTSDDKQVKDIKNTLYKVREIIELLDIENFEQKLKGDGTPIKCPYGVPEN
PEFTVGFVPVLSKLKMEVLQEGVFTLLLSGMGGSGKTTLATMLCKDKEVKGKFKNNILFVNFSQTPKLKNIVERLFEHCG

YHVPEFVSDEDAIKRLEILMRKIEGSPLLLVLDDVWPGSEALVDKFKFQMSDYKILVISRVAFPKFGTQIILKSLVHEDAMT
LFRHHALLEENSSKCSLSIPNEEIVQKVVRYCKGLPLAIKVIGRSLSHQSTELWQKMVEELSKGHCILDSNTELLTSLQKILD
VLEDNPIIKECFMDLGLFPEDQRIPLPALIDMWAELYKLDDDGIEAMEIIKKLDSMNLANLLVARRNTGDSDNHCYNNH
FVVLHDLLRDLAIYQNNREPIEQRKRIITGINENQSQWWLGEKQQGMMSRLLSKYRGWCVKQTIQQVSALILSLSTDET
CASYWSDIQPSQAEVLILNLQTKKYTFPEFMEKMIKLKVLIVTNYGFHHSELDNFQLLGSVSNLKRIRLERISVPHLSALKN
LKKLSLYMCSNISQVFENGTILVSDSFPSLLDLNIDYCKDMVRLPNGICDISSLKKLSITNCHKLCSLPQEIGQLLNLELLNLS
TCTDLEEIPDSIQNLSKLRLLNISNCISLSNLPEDIGNLCNLRNLNMTSCARCELPYSITNLENLKVVVCDEETAASWEAFEA
LLPNLKVEVPQVDVNLNWLHSISS

>XP_047166213.1

MEETAQQMKRPFGIPEKPEFSVGLDVPLKKLKLNVLSEGASVIVLTGLGGSGKTTLATMLCWDEQVIGKFKENILFVTFS
KAPQLKIIVERLLERFGYQVPELQSDGDAIDQLVLLLRKINANPMLLVLDDVWSCSEAVVEKLKFQISDYKILVTSRIAFPTF
GTSLVLKPLVPEDAITLFRHHAFLKRASSNIPDEDLVQKVVRHCKGLPLAITVIGRSLSHQSIELWQKMVGELSEGHCILDS
NTELLSSLQKILDVLEDNPIIKECFMDLALFPEHQRIPVAALVDMWVELYGLDNYGLKAIAIVNRLYSMNLANVTVTRKN
LSDTDSYYYNNHFIVLHDILRDLAIYQSNKAQIELRKRLMIGMNETKTEVCLREKQQGIMIRTLSNNFKWCDEQKTQKIP
SGTLSISSDETWTSYWSHLRPAQAEVLILNIRTNQYSFPKYLKEMSNLKVLIVMNYGFHPCELFDFYLLGTLSVLKRLRLERI
SIPSFFSLKNLKKLSLYLCDTREAFENSNILISDAFPNLEDLNIEYSKDMVGLPKGLCNITSLNMLSISNCHKLSALPQEIGNL
ENLKLLRLSSCTDLQVIPNSVGRLSNLRHMDISNCINLPNLPEEFGNLCNLRILYMTSCERCELPSSIINLKNLKEVVCDEET
AFQWEAFKPMLPNLKIDVPQLDVNLNWLHAIHS

>XP_047166587.1

MRLQATLYCFFCCLSKSLSLFFHHHPQTRIKVCRHVSSVMAESVVTFVLDHLAQLSAREANLLYGVEDRVQFLKSELEMI
KELLDTTTRKKGMEHIVLNQIKDVAHLAEDVIDTFVAKVSIHKRRTILGRMLCGFGQVRLLHHVAHKIDKLKTTLNEIRDN
KDKYDAFRETTNQSATEEEEEKERLQSLHKLRRDVEEEHVVGFVQDSKDLVKQLLEGGSNRKVVSIVGMGGLGKTTLAR
KVYSSGEVKNHFDCRAWVYVSNEGRVRELLHDLLKHLMQNVEEQRRGHEKDKERAEDVNSLSEEELKKRVWKCLERE
RYLVVIDDLWKRQDWNEVQDAFPDNNRGSRILITSRLTEVAKHAGHDVPHNLPFLSQEESWELFCRKVFRGENCPSDL
ETLGKQMVQSCHGLPLSIMVLAGLLANKEKSSEEWSKVVGHVNWYITRDETQVNDIVLKLSYDNLPRRLKSCFLYLGLF
PQDFEIHVTPLLQKWVAEGFIQDTRDRDPYDVAEDYLYELIDRSLVQITRVKFNRGLDRCQVHDLLRDLCMQKIKEDKV
FEVCTDNNIVIPTKPRRLSIHSDMGHYISSSNNDHSCIRSLFFFGPHYDVHGKEWKWLLDDFKLVRVLDFGPNSYQKIPSS
LGNFIHLRYLRIHSKLISFVPDSILNLWNLQTIDLGFSTDIIIVSFPIQMWKLKYLRHLNTQGPIKLRGTCSQSDEKMWNLQ
TISPLILNKQATSLITKGTFPNIKRMGLVEAHDYEGKLLNLLQNLQQLKHLNKLVIVPQLWLSCKPKELVQSLGQLSCLTIL
MIDNVVDLLTSELIFPPNIIELMLSGIKWITNEGMNSLGNHSKLKILKLLGDITWSGDSIDLNCVDESFPQLEIFRMSHLAV
RKWELGNGAMQRLQNVLIDNCKELDNLPSELCSLNGLRKMHITESPSEQMAHILRILKTNNGVEVVTGTYQSRDYDIFD
IF

>XP_047166606.1

MAESVVSFVLDHLAQLASREANLLYGVEDRVQSLQYELQMIKDLLNTTKRKKGIEHTVFNQIRDVAHLAEDVIDTFVAK
VSIHKRRTILGRMLHGFGQARLIHHVAKKIDKIKTTLNEIRDNKDKYDAFKETNNQSESEEAEEKERLQSLHKLRKDVEEE
DVVGFVQDSTDVVKRLEEGGSNRKVVSIVGMGGLGKTTLARKVYNSNQVKNHFDCRAWVYVSNECRVDELLLGLLKN
SMPKFEQQRRGNKKVKKSAKDVNSLNEEELKKHVWNCLERKRYLVVIDDLWKGRDWDEVQDAFPDNNRGSRILITSR
LKEVALHAGDDVPHYLQFLSEEESWELFCRKVFRGEDYPSDLEPLGKQMVQSCRGLPLSIIFLGGLLAKKEKSHREWSKV
VGHVNWYLTRDETQVKDIVLKLSYDNLPRRLKPCFLYLGLFPEDFEIPVIPLLQKWVAEGYIQDTGNRDPDDVAEDYLYE
LIDRSLIQVTRVNFNGGLKTCQVHDLLRDLCIAESKDGKMFEVCTDNNILILTKPRRLSIHSDMGHYISSSDNDHSCIRSLF
FCGPRYHVRRREWEWLLDNFKLVRVLEFGPNRCQKIPSNLGNFIHLRYLRIDSKHAKFVPDSILNLWNLQTIDLGIWRYD

VPISFPIQLWKLKYLRHLNTQGPIKLRGRCSPSSYEKMWNLQTISPLILNVQATLLIGKGIFPNIKSMGLDVEDHGYEELTL
SWIKCITDEGMNSLANHSKLKFLRLLGNPLKSEDSIDLNCVSGSFPQLEVLQMEYLKLRKWELGNGALRRLQNVIIHDCQ
SLDNLPNELCSLNRLRKVHITDSPSKQTTHILRILETNNGVQVVIGDFSPWNDDIYSKRHISFFMYKAAEDFIQFISQS

>XP_047166615.1

MEESVVTFVLDHLAQLVAREANLLYGVEDRVQSLQNELQMIKDLLNTTKRKKGMEHTVLNQIRDVAHVAEDVIDTFVA
KVAIYKRRTILGRMLRGFGQLRLFHHVAEKIDTIKATLNDIRDNKDKYDAFKETNNQSAADLEAEEEKRAQSLHILRRNVE
EEDVVGFIHDSKDVINLLLEGGSNRKAVSIVGMGGLGKTTLARKVYNSSQVMNHFDCRAWVYVSNECRVKDLLIGLFK
HLMPNFEQQQPRGNKRGKKSAGDINDLSEEELKKLVRNCLEWKKYLVVVDDLWKKQDWDEVLDAFPDNNRGSRILIT
SRLKEVALHAGHDVPHYLQFLNEEESWELFRRKVFRGEDYPSDLEPLGKQMVRSCRGLPLSIIVLAGLLANKEKSHKEWS
KVVGHVNWYLTQDETQVKDIVLKLSYDNLPRRLKPCFLYLGLFPEDFEIPVTPLLQKWVAEGFIQNTGNRDPDDVAEDY
FYELIDRSLVQVVRVETNAGVETCQVHDLLRDLCILESKEDKVFEVCTDHNILISSKPRRLSIHGKTDHYISLSNNDHSCVRS
LFFFGSHYYIRGRDWKWIFKNLKLVRVLEFGLNGSDKIPSNVGNFIHLKYLRIDIAYVRFVPDSILKLWNLQTIDLSPPRGN
VPISFPAKIWKLRHLRHLNTPRPIKLGGSCSGSYEKMWNAQTISSLVLNSQAMSLIKRGTFPNVKRLGLRVTSECEAELPK
LLQSLQQLSYLNKLVIVLRDRDDESVKKLTDESLKRNNGFKPQEVLQSLGQFNCLTILTIENAFDLLTCELTFPPTVTELTLS
EIDCISDEGINGLGNHTKLKKLRLLGDVTWSRESFDLNCVGGGFSQLEVIEMENLTLQKWKLDNGAMSRLQSVTINHCE
RLDDLPNELWSLSGLRKVQVMKPSKEMAHTLRNLEMKNGVQLVTEDYQPRFQSRLDSIDSMDSNTFEYISEI

>XP_047166985.1

MAESFIFSIAESLIANLTSRTFQEASRLVGLYDDLQDLTNTLSLVKDVLLDAQQKQEHNHELRRWLSQLKTVFSDAEDVL
DEFECQTLRKKVVKAHGSTKDKVSHFFSSSNPLVFRYKMAQQIKDINNRLDKVAANKRKFSLQMSDVDTRVVHRRDM
THSRVSDSDVIGRKHDKEKIIELLMQQNPDDDDDTSLSVIPIVGIGGLGKTTLAKFVFNDKKIQECFPLKMWVCVSDDFD
IKQLIIKIINSVNDSESDHAPIHQMNLNMLDLEQLQINIGREIVKKCGGVPLAVRTLGSLLFSKFEANEWEYVSQNEIWILP
QKKDDILPALKLSYDLMPSYLRQCFALFSLYPKDYEYISYEIIWLWGALGLIALPKTDRKREDVANQYLHELLSRSFLQDFQ
NFGTVYRFRIHDLVHDLALFVAMDECLHINSNIQNIPDNIRHLSFAERGLFSNLVTKKSTAVRTVLFPNEVAAANGKAILK
TCLEKFKCLRVLDLSCATFETLPRKISKLKHLRYLDISKNPNIKRLPDSICKLQCLEVLNLGGCVELEALPKGLRKLISLYCFEFS
IKQTVLPLNEIANLGSLELLTVGSCHNVESVFGGMKFPTLKTLIVQDCQSLKFLLLECKNFPQLETLYVVECSNLDLELWKG
DHEEQSPKLKLKFVIFSTLSQLVTLPKWFEEVVNSLQCLIVVDCHNFESFPDWLPVLTDMKTLAIKNCPKLVALPDNIHLF
TTLEYLTIVDCAADLHKKYEPHVGEFWPKISHISTILTWMNQKI

>XP_047166987.1

MAESFIFSIAESLIAKLASRTFQEASRLVGLYDDLQDLTNTLSLVKDVLLDAQQKQEHNHELRRWLSQLKTVFSDAEDVLD
EFECQTLRKKVVKAHGSTKDKVSHFFSSSNPLVFRYKMAQQIKDINNRLDKVAANKHKFSLQTSDVDTRVVHRRDMTH
SRVTDSDVIGRKHDKEKIIELLMQQNPDDDDDTSLSVIPIVGIGGLGKTTLAKFVFNDKKIQECFPLKMWVCVSDDFDIK
QLIIKIINSVNDSESAHAPIHQMNLNMLDLEQLQNQLKNKLSGQKFLLVLDDVWNEDRVKWYELRNLIQVTAGSKILVT
TRSHSIASMMGTIPSHILEGLSEEDSISVLVKWAFKEGEEQKYPHLVNIGREIVKKCGGVPLAVRTLGSLLFSKFEANEWE
YVSQNEIWNLPQKKDDILPALKLSYDLMPSYLRQCFALFSLYPKDYEYTSYEIIWLWRALGLIALPKTDRTREDVANQYLH
ELLSRSFLQDFQNFGTVYRFRIHDLVHDLALFVAMDECLHINSNIQNIPDNIRHLSFAESGLFSNLVTKKSAAVRTVLFPNE
VAAANGKAILKTCLEKFKCLRVLDLSGATFETLPRRIGKLKHLRYLDISKNPNIKRLPDSICKLQSLLVLSLEGCVELEALPKGL
KKLISLYCFEFSTEQTVLPMNEIANLGSLEVLSIGSCHNVESIFGGMKFPTLKTLFVYDCQSLKSLLLEGKNFPQLKTLIIDGC
SNLDLELWKGDHEEQSPKLKLKFVLFSTLSQLVTLPKWFQEAANSLQCLLVYDCHNFESFPDWLPVLTDLKTLEIRNCPKL
VSLPDNIHLFSALESLRIEGCAADLCRKYESHVGEFWPKISHISNIFIDEAEDLDEE

>XP_047167243.1

MDFLGPFGKVVEGLVDFVWKHGVRQVTYIVNYNNNVVELKDSVKDLALEKERINHQRDEAEKNLNNIEGKVIEWDRK
VSEIETTVEVFKNDDGHTRARSPNCFVFPYLWNRHRLGRQAHKMKEDVKRLIGESPELDEVFYRQNVTSNDATLSNCG
FVEFSSIKSIIEKVMIQLQDSAVRMIGLYGRGGVGKSTLVKEIARKAKEKKLFDVVVKVEITADPNPHKIQEEIAYVLGLRLE
GEGENVRADCLRRRLKKEKGNILLILDDLWHKLDLNKLGIPVEDYDDDEDFNQNVLCAEMDVKSTFCVRELDDKDALM
LFQKLTGIHNEMPSSKQEIVKKYCEGLPMAIVVVARALRNKSESVWEATIKRHKKHELVGEGTSMDISVKMSYEHLENE
EIKSIFLLCAQMGRRALIMDLVKYSFGLGILEGVSSLWEAREKIKTSIQKLKDSGLLLDESSNNHFNMHDMVRDTALSIAH
KYHNTFNLRNGKLDDWPELEKCTSIFMCNSDIIDGLEVINCPQLKLFQIDTNDPYLKIPKSFFRRMKNLRVLIMTGFCVSN
LPSSIQYLSKLRMLCLQRCTLDCNLSIIGKLKKLRILSFSGSILKSLPIELQCLDKLRMLDISDCSELKIIPPNVISSLTCLEELYIRE
SLIKMLVERETNKGQDLFLSELKNLHQLKVVELSIPCVSNFPNHLFFDKLRDYNIVIGDFDFFSLGEFKMLNKHETFRVLAV
QLKDNTIIHSHENIKLLFKTVQTLLLGKTNGVREVVNDLNIDGFQDLKHLSIINNNDFKYVNSTKLCNYVNVFPNLESLRLY
NLGKLDMICYGPVTVGSFAKLRTIKVEMCHRLKNLYSLDMIMFPIGAQTCDIFECNSYMDIFLSSLEIIEVSECKSLKEILQI
PKHHGKVEFLKLHTLTLRLLPLLSSFYTKVDKFCWPHLTKAQTTIMGHKDLTSEEDKQSDEEPPLFGELVEIPNLETLNLSS
LKIHKIWNDQNSSSFIFQNLIKLVVKDCDKLTHLCSLSMARSLKKLKSLVISECPVMEKIFETERNSADKVCIFPKLEEIHLTK
MNKLTDIWQTKVSADSFSSLISVKIEECNKLDKIFPSHMEGWFESLDNLKVYWCESVEVIFEINDSQEIDEFGGIDTKLQII
LLEELPKLKQLWSTDPNGILNFKKLRTIDVDSCDELKNLFPVSVAKDVPKLEHMSVLYCDKMEEIVASQDALETNKDLLVF
PELTSVRLHCLPDMKYFYKKKYPIKCPKLKELSVTKCLKLKTFVKDTINTTNKVGNFVFSIDE

>XP_047167815.1

MEFASSSSSSSSSFLKSEPHFIYDVFINFGGEDIGRRFLSHLHCALLQAQVKTFISEENGQEETKLEEHMRAIAASKIAIIVFS
KTYAESNSCLLELEENIECLKTFGQIVLPVFYEIDPLDVRDQTNDFGKALGQTANKSYSGEQLEHALSRWNRALAIASGIT
GWDLRNFRHDAEFVEVIVNRVQTLLDYKDLVITQFPVGLEFHVEKVIGCIENHSTKVCMIGIWEMVGSGKTTIAKAIYNR
IYHLFIGKSFVENLKKVWEPVDRWHLHLQEQFLYDVLKSKFRLQTTWMGRVMIENELSRKKLLIVLDDVNEIGQLQNLC
GSHHWFGKGTVIIVTTRDVHLLNRLKLNYVYRMSDMKENESLGCHGFSEAKTRKDLNELVRNVVVYCGGLSLAL

>XP_047168778.1

MDPIVSTTTESALNITASVVKRQVGYFFNYKDKFKELKSYIEKLENNKERLQHQVDSALRSGDEIEKDVQHCLTFMDDKIK
EYKSFINDDCHAKTICSIGFFPNNFRLRYKLGRKATKMVEEIIGDELWKAFFDNVSYQEFPSIDATFSNNVYESFASRTKT
MEMIMKALQDSTVGMIGVYGPGGVGKTTLVKEIANKAREKNLFEIIIIANITGNPDFKKIQEQIAVMLGMKLEEESEIAR
VDRIRKRLKNEKENTLIILDDLWGGLDFNKLGIPCNDDASQQEVNDISDFGYNKTEIKELSKVDLDKMKKEKLSNDYRGG
KKGLIVIDLDKMKKEKLSNDYRGGKILLTSRNKQVLCNEMDVQQRSIFSVGVLDEKESETLLKKVAGVKNSEFDRSATEIA
KWSAGFPIALVSIGRTLKNKSLSTWEDVCQQIKRQNFTSEWGFTDFSIKLSYDHLKNEELKCIFLHCARMGSDALIMDLV
KFCVGLDLLPGVHTITDARKRVKEMIQELEESSLLVKSYSIDRFNMHDIVRDVALSISSKEKHVLYKKNAILYEWPHENDFE
RYSAIFVHFCDINDELPESIHCPRLEVLHIDNKNESFEIPDEFFKSMVRLRVLVLTGINLSCLPSSIKCLKKLRMLCLERCTLGE
NLSMIGELKNLRILTLSGSNIENLPLEFGQLDKLQFLDISNCLKLRQITSNIIPRMGILEEFYIRDNLIIWEAEENMKSENASL
SELMHLNQLQNLDIHIHCSSYFPQNLFFDRLNSYKIVIGEFNLSNLLKVGEFKVPDKYEEVKFLALNLKEGIDIHSEKWIKM
LFKSVECLLLGELDDVKDIFYELNVEGFPNLKHLSIVNNFGIKYIINPRERFHSLVAFPKLESIWLYKLDNLEIICDNQLVETSF
RNLKVIKIQTCIKLVNLFLFSMVRLLTLLETIEVCDCDSLKEIVSKESQTNTISDDKIQFPQLRLLKLKYLPTFIYLYNADKIPGS
PHSLQDQVFQQRNKDIVVDVEHMVTNSCLPLFNEKVSTPKLEWLELSSINIHKIWSDQCNHCFQNLLTLNVTDCSNLKY
LLSFSMAESLVNLQSIFVSECAMMEDIFRQEDAEYIDVFPKLKKMEIICMDKLCTIWKSDIGLHSFSSLNSLMIRECHKLVT
IFPNYMGQRLQSLQSLTVTDCKLVENIFDFENIPHTCDIIEANLGNIFLENLPNLVNVWKGYTGEILKCNNLQSIRVYESPK
LKYLFPVSIANDLEKQEVLEVRNCGAMTEIIALDKHSSATAITFKFPHLNTLSLIDLHDLRSFYSGIHTLELPPLKKLDIINCSM
LEGLTSEITDSKEQPIVLAAKKAIYNLEYMSLSLKEAEWLQKYIVNVHRMHKLEELTLYGLKNNEILFWFLHRLPNLKRLTL
ELCHLKRIWALKSLISREKIGGVVQLKELKLERMRSLEEIGFEHEVLLQRVERLSIQHCTKLKNLVSSSVTFSYLTYLEVMNC
KSMRSLMTCSTAKTLVQLTTLKVCSCPMIVEIVAENKVEKVREIEFKQLKSLELVSLQNLTSFSNVEKCDLKFPLLEKLVVSE

CPQMTKLSEIQSAPNLQKVHVEAGEKDKWYWEGDLNATLQTHFTNQVSFEYSKYINLVDYPEKKVRHDKFAFPDNFFG
CLKKLEFNEACKRDTLIPSHVLPYLKNLEELNVEKCESAKLIFDIDERKIQMYGMVFRLKTLTLKQLSNLKCVWKENLEGIV
SFSNLQRVDVDGCRSLLTLFPLSLAKDLGKLETLDIKECEKMIEIVGREDEMEHGTTIMFEFPCLSYLNLENMPLLSCFYLG
KHHLECPLLDRLYVACCPKLKVFRSSFDDDSKKEVLEAPTDLLQQPLFSIQKVSPKPVGLTLNEENIKLMSDARLPQDLLCK
LKSLILSFEDDNNGKDSLPFSFFHKLPNLESLTVQKCSGLKEIFPSQKLQVHDNVLAELKRLLLLELSELESIGLEHTWVQPY
SKKLDWLFLYSCHRVENIVSCAVSFINLKDLFVMGCERMEYLFTSATLKCLVKLETLFIGYCGSIKEIARNEEEDDCDEIIFG
RLRSIELEYLPRLISFYSGNATLQCPCLQNVMVTECPNMITFSEGVIKLAMFSGIQTSEDSDFTFHVDLNTTVESLFHEKVS
FEYSKYINLVDYPEKKVRHDKFAFPDNFFGCLKKLEFNEACKRDTLIPSHVLPYLKNLEELNVEKCESAKLIFDIDERKIQMY
GMVFRLKTLTLKQLSNLKCVWKENLEGIVSFSNLQRVDVDGCRSLLTLFPLSLAKDLGKLETLDIKECEKMIEIVGREDEM
EHGTTIMFEFPCLSYLNLENMPLLSCFYLGKHHLECPLLDRLYVACCPKLKVFRSSFDDDSKKEVLEAPTDLLQQPLFSIQK
VSPKPVGLTLNEENIKLMSDARLPQDLLCKIKYLILSFEDDNNGKDSLPFSFFHKLPNLESLTVQKCSGLKEIFPSQKLQVH
DNVLAELKRLLLLELSELESIGLEHTWVQPYSKKLDWLFLYSCHRVENIVSCAVSFINLKDLFVMGCERMEYLFTSATLKCL
VKLETLFIGYCGSIKEIARNEEEDDCDEIIFGRLRSIELEYLPRLISFYSGNATLQCPCLQNVMVTECPNMITFSEGVIKLAMF
SGIQTSEDSDFTFHVDLNTTVESLFHEKEFFNHSKHMILDEYLEITGVQHIKPDIADNFFGSFKEMEFHAACKRAVVIPFH
LLPYLKNLEKLNVHSSDAVKVIFDFDESEDKTKGIVSSLKELTLKNLSNLKCVWKENLGGIVSFPNLEEVIVTDCRSLVTLLSS
SLAKSVEKLKTLRMERCEKLEEIVGEEDEREHGMTLTFEFPCLTVLFLLDMPLLSCFYPGKHYLECPILDSLFVAYCPKLKLF
TSDTGDSHKEEVIEAPISPLQQPLFLVEKVSPKLKKLALNEKNIMLLRDGCLPHDLLCKLSHLWFLFEDYEIENDTLPFDFFH
KLPCLEYLHLEKCFGLKVIFPSQKLQVHDKVLAGLKQLCLIKVSELESIGLEHEWVQPYSRKLELLKLHTCPQVENIVSCSVS
FINLTKLYVKLCEKMEYLFTFATLKSLVKLKTLSIKNCESIKEIVKKEDEDACDEIVFGQLRSIKLNSLPKLLSFYSGNATLKFLC
LQSFMVAKCPNMITFSEGVINMPILSGIKTSKDSVVVFHDNLNTTIETSLHEQEFMEYSKRMILEDYLGMSGVHHRKPIV
SDNFFGSFKKLEFDAACNRTVLIPSHVLPYLKNLEELNVKKSDAMQIIFDIDESEVKTKGVVFGLKKLTLRNLSNLKRVWKE
NSTGMISFHNLQEVVVNGCGSLITLFSSSLARNLGKLEKLSIKECGKLVEIVEKEDGTENGTKIMFEFPCLTWLYLKNMPLL
SCFYPGKHDLDCPLLETLFVCFCPKLKLFTSDFDENQKGAIEAQISPLQQPMFSVERVSPKIKALALNEENIMLFREVQLLQ
DILCNIVILGLCFEDDNIEKDSLPFDFFNTLPNVFYLAIKKCFGLKEIFPSQKLQVHDGGLAGLKQLFLVDLKELESVGLEHP
WVQPYSEKLQALSLKRCPQLQKLVYCAVSFINLEVLCVKLCERMEYLFTFATVKSLVKLETLIINSCESMKEIIKHENEDGC
AEMVFGRLKSIKLKSLPRLVRFYSGKATLQCSYLKVVMVVKCPSMKTFSEGVMKVPKFSGIQTSKDSDLIFHEDLNTTIKK
LFHEEVEKSACDLEHLKFGDHPNLEEIWLGVVPIPRNDCFNNLQSIAVVQCESLSNVIPFYLLRFLSNLKEIEVSNCQSVKA
IFDVRGEGAYMKPISLPLKKLILNQLPNLEHIWNLNPDEILSLQDLQQVSISNCQTLKSLFPTSVANHLVKLHVRACATLVK
IFVETETASEGETKQFNFHCLTSITLWELPELKYLYPGKHTQEWPMLTHLDIYHCDQLKLFKTENHSDEFSDTKDQLGISIH
QQPAFSVKKVFPKLVQLSLKKEDAMAILQGQLQVIPSIEHQAITRKDTRIGQGQFGANVAQLLQNLKLLKLMCYHEDDK
SNIFSSGLLEEIPNIENLEVVCSSFTEIFCSQGPTNDCSKVLLKLKRLHLKNLPQLNTIGLEHSWVEPLLKTLETLEVFSCPNM
KILGPSTLSFSSLTSLSVDECHGLLYLFTSSTAKRLGQLKHFSIRDCQAIQEIVSKEEDHESEDEDITFDQLSFLSLQSLPNIVVI
YSGTFKLKFPCLDQVTIKECPQMKYSYVPDLHEFKPQERTWKATLQCSYLKIVMVVKCPSMKTFSEGVMKVPKFSGIQT
SKDSDLIFHEDLNTTIKKLFHEEVEKSACDLEHLKFGDHPNLEEIWLGVVPIPRNDCFNNLQSIAVVQCESLSNVIPFYLLRF
LSNLKEIEVSNCQSVKAIFDVRGEGAYMKPISLPLKKLILNQLPNLEHIWNLNPDEILSLQDLQQVSISNCQTLKSLFPTSVA
NHLVKLHVRACATLVKIFVETETASEGETKQFNFHCLTSITLWELPELKYLYPGKHTQEWPMLTHLDIYHCDQLKLFKTEN
HSDEFSDTKDQLGISIHQQPAFSVKKVSPKLKKLALNEKNIMLLRDGCLPHDLLCKLSHLWFLFEDYEIENDTLPFDFFHKL
PCLEYLHLEKCFGLKVIFPSQKLQVHDKVLAGLKQLCLIKVSELESIGLEHEWVQPYSRKLELLKLHTCPQVENIVSCSVSFI
NLTKLYVKLCEKMEYLFTFATLKSLVKLKTLSIKNCESIKEIVKKEDEDACDEIVFGQLRSIKLNSLPKLLSFYSGNATLKFLCL
QSFMVAKCPNMITFSEGVINMPILSGIKTSKDSVVVFHDNLNTTIETSLHEQEFMEYSKRMILEDYLGMSGVHHRKPIVS
DNFFGSFKKLEFDAACNRTVLIPSHVLPYLKNLEELNVKKSDAMQIIFDIDESEVKTKGVVFGLKKLTLRNLSNLKRVWKE
NSTGMISFHNLQEVVVNGCGSLITLFSSSLARNLGKLEKLSIKECGKLVEIVEKEDGTENGTKIMFEFPCLTWLYLKNMPLL
SCFYPGKHDLDCPLLETLFVCFCPKLKLFTSDFDENQKGAIEAQISPLQQPMFSVEKVSPKIKALALNEENIMLFREVQLLQ
DILCNIVILGLCFEDDNIEKDSLPFDFFNTLPNVFYLAIKKCFGLKEIFPSQKLQVHDGGLAGLKQLFLVDLKELESVGLEHP

WVQPYSEKLQALSLKRCPQLQKLVYCAVSFINLEVLCVKLCERMEYLFTFATVKSLVKLETLIINSCESMKEIIKHENEDGC
AEMVFGRLKSIKLKSLPRLVRFYSGKATLQCSYLKVVMVVKCPSMKTFSEGVMKVPKFSGIQTSKDSDLIFHEDLNTTIKK
LFHEEVEKSACDLEHLKFGDHPNLEEIWLGVVPIPRNDCFNNLQSIAVVQCESLSNVIPFYLLRFLSNLKEIEVSNCQSVKA
IFDVNGAAADMKPISLPLKKLILNQLPNLEHIWNLNPDEILSLQDLQQVSISNCQTLKSLFPTSVANHLVKLHVRACATLV
EIFAVADAAINGETKKFNFHCLTSLTLWELPELKYLYPGKHTLEWPMLTHLDIYHCDQLKLFKTEHHSDEFADTEDQLGIS
IHQQAAFSVEKVIPSIEHQEITRKDTKIGQGQFGANVAQLLQNLKLLKLMCYHEDDKSNIFSRGLLEEITNIENLEVVCSSF
TEIFCSQGPTNDCSKVLLKLKRLHLKNLPQLNTIGLEHSWVEPLLKTLETLEVFSCPNMKILGPSTLSFSSLTSLSVDECHGL
LYLFTSSTAKRLGQLKHFSIRDCQAIQEIVSKEEDHESEDEDITFDQLSILSLQSLPNIVVIYSGTFKLKFPCLDQVTIKECPQ
MKYSYVPDLHEFKPQERT

>XP_047168903.1

MIQLQKPLSIPFLFLPLLKLMAESLLFSSAESLLGKLASAALQEASLAFGVHRHLQHMKETMGLIRGVLLDAEKKNPQSSA
LSEWLTQIKRVFSDAEDIVDDFECEALRKHVVNTYGSCSRKVRRFFSSSNPVVYRLRMAHHIQDINTRLAKLAAQRSMF
GLQVIHLDTRVVHVREMTHSHVNPSNVIGREDDKQEIIKLLLKDDHGQSLSVIPIVGMGGSGKTTLAKLVFNDATINACF
PLRMWVCVSNDFELRNVLIKILNSAPNPNRENFNNFETEQLQIHLRNTLEGQKSLLVLDDVWNEDRARWDELREIIDV
GVEGSKILVTTRSHKVAAIMHTKSSNSYLLGCLSDENSRSLFVKYAFEDGDEMKHPQLLKIGEEIVNKCGGLPLAVKTVGS
SLFSRVEEREWESVRDNEIWNLQQNEKDILPALKLSYDQLPSYLKPCFASFSLFPEDTFLFSSQVCMLWGALGFLPPPKAS
ESMTDVTTQLLHELWSRSFLSEYEDLGGDCRFKLHDLVVDLAVYIAKGEFEIIQNHNPNLYKNAHHLLLMNNNLLDQAL
LPSSLRSIFFPNGANNEDFLNTLVSRCKFLSVLILDLCEYESLPRCIGKLKHLRYLSLSNNENLTELPDSVCKLQNLQVLNLKG
CIKLQKLPEGLGNLISLRHLFITTKQPVFPEKEVASLTSIEDLRFYHCDNLESLFKGILLLTLKGLTLIDCKSLKTVPFHAIKNLE
VLVIAECNKLELSMGLSNEILYSRLKLLILRDLPSLVRLPRWLQGSANSLQSLGIVDCLNLEELPDWLPTLNCLQRLSVLNCP
NLLSVPHNMLHLTNLKVINVTGSFELWKRYRPEIGQDWHNISHVNLVWDDYQSENEKELTVKI

>XP_047168905.1

VFNDATINACFPLRMWVCVSNDFELRNVLIKILNSAPNPNRENFNNFETEQLQIHLRNTLEGQKSLLVLDDVWNEDRA
RWDELREIIDVGVEGSKILVTTRSHKVAAIMHTKSSNSYLLGCLSDENSRSLFVKYAFEDGDEMKHPQLLKIGEEIVNKCG
GLPLAVKTVGSSLFSRVEEREWESVRDNEIWNLQQNEKDILPALKLSYDQLPSYLKPCFASFSLFPEDTFLFSSQVCMLW
GALGFLPPPKASESMTDVTTQLLHELWSRSFLSEYEDLGGDCRFKLHDLVVDLAVYIAKGEFEIIQNHNPNLYKNAHHLL
LMNNNLLDQALLPSSLRSIFFPNGANNEDFLNTLVSRCKFLSVLILDLCEYESLPRCIGKLKHLRYLSLSNNENLTELPDSVC
KLQNLQVLNLKGCIKLQKLPEGLGNLISLRHLFITTKQPVFPEKEVASLTSIEDLRFYHCDNLESLFKGILLLTLKGLTLIDCKS
LKTVPFHAIKNLEVLVIAECNKLELSMGLSNEILYSRLKLLILRDLPSLVRLPRWLQGSANSLQSLGIVDCLNLEELPDWLPT
LNCLQGLSVLNCPNLLSVPHNMLHLTNLKVINVTGSFELWKRYRPEIGQDWHNISHVNLVWDDYQSENEKELTVKI

>XP_047168906.1

MAESLLFSSAESLLGKLASAALQEASLAFGVHRHLQHMKETMGLIRGVLLDAEKKNPQSSALSEWLTQIKRVFSDAEDIV
DDFECEALRKHVVNTYGSCSRKVRRFFSSSNPVVYRLRMAHHIQDINTRLAKLAAQRSMFGLQVIHLDTRVVHVREMT
HSHVNPSNVIGREDDKQEIIKLLLKDDHGQSLSVIPIVGMGGLGKTTLAKLVFNDATINACFPLRMWVCVSNDFELRNV
LIKILNSAPNPNRENFNNFETEQLQIHLRNTLEGQKSLLVLDDVWNEDRARWDELREIIDVGVEGSKILVTTRSHKVAAI
MHTKSSNSYLLGCLSDHF

>XP_047168908.1

MIGIWEMVGSGKTTIAKAIYNRIYHLFIGKSFVENLKKVWEPVDRWHLHLQEQFLYDVLKSKFRLQTTWMGRVMIENE
LSRKKLLIVLDDVNEIGQLQNLCGSHHWFGKGTVIIVTTRDVHLLNRLKLNYVYRMSDMKENESLGCHGFSEAKTRKDL
NELVRNVVVYCGGLSLAL

>XP_047168973.1

MSIRTNKMKAVAVLLKQLMTARSKFDERGRDESFDRKLEKLRLDLNKIKDVFVRVKKNEEDLLDILAEVYGHLRKLDRGK
LNQDMDDICQRIRDSAHNLLPKHAFDESSEKEDHKGGEIFPLPEESVQLQQKKRWTLEDFNLLDDSLKACLLSLSVFPEN
AVIRKRNAINLWIGKGLIKNTMEKTAENIGEDVIDDLLEFQVIVPYGSRKDALVKKFQILPDIRHGAFYPQIKVDRLELEQK
RVKVGDRYFGHTIRTVFNIGSSYLNFKPQWVTDELKDLEVLQLGRWQDSALHHIEVGSEEFLKELRYLKKLKYLSLRGISNI
FELPSSIGELESLIILDLKACHNLERLPNDISSMKSLTHLIMSDCCLLEGMPKGIEKLTNLQVLKGFLTTTSEKTPCTLSDLAN
NLTNLRRLSIRIGSEAMIRDGEFQSLKNFRKLKHLKISWSVSDPRYAEIPILWPVSLRKLHLECFPGKSLLEFLPGEEIFPEYL
HIQSEYEILELNITGGKLENIFFHRIGRNVNILRLKYLKQLNVDIDDLKIFFPFLQYLEIKQISNHSYIECRYENFANFANFANS
AYVLEVRP

>XP_047169028.1

QQRKSSKKGKKSSGDINNMNEEELKKQVWNCLERKRYLVVVDDLWKRRDWDEVQDAFPDNNNGSRILITSRLKEVAL
HAGDDVPHYLQFLSEEESWKLFCRKVFKGENRPSDLETLGKQMVQSCRGLPLSTIVLAGLLAKKEKSQREWSKVVGHV
NWYLTRDETQVKDIVLKLSYDNLPRRLKPCFLYLGLFPEDFGIPVTPLLQKWVAEGFIQDIGNRDPDDVAEDYLYELIDRS
LVAGVKLNGRLAMCQVHDLLRDLCISESKEEKVFEVCTDNNILIPTKPRRLSIHSDMGHYISSSNNDHSCIRSLFFFGPYYD
VGGRQWKWLLDECKLVRVLDFGSNSCRKIPSNLGNFTHLRYLRIESGNIITFVPDSILNLWNLETIELSNWWPYNIDSISF
PVQMWKLKHLRHLSVRGSIELRGRCSELDEKMWNLQTIYCLVLNEQAISLIKKGTFPNIKRMEINDDEDELPNLLQSLELL
KHLNKLVIYTRKPFSEQVTHIFQNLETNGVEVVIDYKEQYFYRPWAQIWKR

>XP_047169029.1

MADSVVSFVLDHLAQLAAREANLLYGVEDRVQSLQYELQMIKELLNTTKRKKGMEHTVVNQIRDVAHLAEDVIDTFVA
KVSIYKRRTLLGRMLRGFGQARLLRHVAHKIDKLKTTLNEIRDNKDKYDAFREATDQYAAEEEEEKERLQSLHKLRRDVEE
EHVVGFVKDSKDVVKRLQEGGSNRKVVSIVGMGGLGKTTLARKVYNSSQAKHHFDCCAWVYVSNECRVKELLLGLLK
HLMPNFQQQRSGNKKGKKSVADVNNLSEEQLKKQVLKCLEKKRYLVVIDDLWKRRDWDEVQDAFPDNNNGSRILITS
RLKEVALHAGDDVPHYLRFLSKKESWKLFCRKVFKGENCPSDLETLGKQMVQSCRGLPLSVIVLAGLLAKKEKSQREWS
KVVGHVNWYLTRDETQVKDIVLKLSYENLPRALKPCFLYLGLFPEDFEIPVMPLLKKWVAEGFIQDTGNRDPDDVAEDY
LYELMDRSLVQAGVKLNGRLAKCQVHDLLRDLCISESKEEKVFEVCTDNNILIPTKPRRLSIHIYMGHYISSSNNDHSCIRS
LFFFGPYYKVGGREWKWLLDECKLVRVLEFGSNCCHKIPSNLENFIHLRYLRVEISTEPFVPDSILNLWNLQTIHLSFYPIRT
PISFPVQIWKLKHLRHLSVVGSMELRGRCSELDEKMWNLQTISGLKINKQATSLIKKGTFPNIKRMELYVKDYQGELPNL
LQNLAPLKHLNKLVILTSKEFLSEQVTDILQNLETNNGVKVHVDDLF

>XP_047169033.1

MAENVVTFVLDHLAQLAAREANLLYGVEDRVQSLQYELQMIKELLNTTKRKKGMEHTVLNQIRDVAHLAEDVIDTFVA
KVAIYKRRTILGRMLRGFGQARLLHDVAQKIDKLKTTLNEIRDNKEKYDAFRETTNQSAAEEEEEKDRLQSLLKLRRDVEE
EHVVGFVQDSEDVVKRLQEGGSNRKVVSIVGMGGLGKTTLARKVYNSDQVKNHFDCHAWVYVSNLCRVKELLLGLLK
HLMPKSEQQRRGNKKGKKSVEVIISLSEEELKKQVRNCLERKMYLVVIDDLWKTQDWDEVQDAFPNNNRGSRILITSRL
KDVALHAGDDVPHYLQFLSEEESWELFCRKVFKGENCPSDLETLGKQMVQSCRGLPLSIIVLAGMLAKKEKSDREWSKV
VGHVNWYLTRDETQVKDIVLKLSYDNLPRRLKSCFLYLGLFPEDFEIPVMPLLQKWVAEGFIQDTGNRDPDDVAEDYLY
ELIDRSLVQVARMKSNGGLARCQIHDLLRDLCISESKEDKVFEIFTDNNILIPIKPRRLSIQSDMGHYISSSNNDHSCIRSLFF
FGSEYNVGGREWKWLLDDFKLVRVLDFGPNRCKNIPYNLGNFNHLRYLKINSRNVNFVPDSILNLWNLQTIELGTYRFE
SPISFPAQIWKLKHLRHLNTSRPIKLRGRCSESDEKMWNLQTISLLIPNTQTTFLIKKGTFLNVKRMRLYANIFGFEGELPN
LLQNLQQLKHLNILMIDNVFNLLTSEVIFPPSITELSLSKIRCITNEGMNGLRNHSKIKILKLLGDDCHVSSIDLNCVVVGFP

QLEVLEMKYLSLGKCKLGNGAMQRLQNVIIHDCQQLDNLLIDFCSLNVLRKVQISKTPLEQIAHILQILETRVQVVRGNHPPWDESDWILNIY

>XP_047169034.1

MAENVVSFVIDHLGQLAAREAYLLYGVEDRVQSLQYELQMIKDLLNTTKRKKGMEHTVLNQIRDVAQVAEDVIDTFVAKVAIYNRRTVLGRMLHGFGQARLLHDVAQKIDKLKTTLNEIRDNKDKYDAFREATNQSATEDEEEEKRLQSLHKLRRDVEEEHAVGFDQDSKDVVKRLLEGDSNCKAISIVGMGGLGKTTLARKVYNSDQVKNHFDCHAWVYVSNECRVKELLLGLLKHLMPKSEQQRRGNKKGKKSVEDINSLSEEELKKQVRNCLERKMYLVVIDDLWKTQDWDEVQDAFPNNNRGSRILITSRLKEVALHAGDDVPHYLQFLSEEESWELFCRKVFKGENCPSDLETLGKQMVQSCRGLPLSIIVLAGMLAKKEKSNREWSKVVGHVNWYLTRDETQVKDIVLKLSYDNLPRRLKPCFLYLGLFPEDFEIPVTPLLQKWVAEGFIQDTGNRDPDDVAEDYLYELIDRSLVQVARVKFNGGYERCQVHDLLRDLCISESKEDKVFDVCTNNNILIPTKPRILSIHSDMGHYISSSNNNHSCMRSLFFFGPEYDVGGREWKWLLDDFKLVRVLEFGHNRCPEIPSNLGNFVHLRYLRINSTRATFVPDSILDLWNLQTIDLGILGYDSPISFPIQMWKLKHLRHLNTSRPIKLRGGYSESNEKMWDLQTISPLILNTQATSLIKKGTFPNIKKMGLYMETDGFEGELPNLLQYLQQLKHLNTLMIYNVLDLLTSEVIFPPSITELWLSRIKCITDEGMNGLRNHSKIKILRLLGVEWSKSSIDLNCGDGGFPQLEVLEMKYLSIRKCKLCNGAMHRLQNVSIQCC

>XP_047169110.1

MQDNCIESSYIENTMMGNDGAMFETKCRYDVFLSFRGEDTRLTFTCHLYHALCRKGIITFMDDGELKLGEQIGPTLLRAIEESSISIVVLSENYADSSWCLDELVKILECKESKNQLIWPIFYKVNPSDVRHQNGSYGEAMTKHEKMFGTGSEKVLQWRSTLNVIADMTGEHLKSEQGRDESKFIEELVSKIFMIVSPKYLSSDEHIVGREYRVKELKSLLNLESPNITLLGIHGTGGIGKTTLAKALYDSIYKQFERSCFLFNVRENSNQINGIEFLQQRLLSEILEDSKIQLASKAEGTNRIKRSLYSKRVLIVLDDVDDIEQFKNLAGKRFWYGKGSRIIVTTRDKQLLDVGRINNRYEVKVLNNQESLELFCQSAFGNSCPETNYEDLSNRAIGCCKGLPLALKVLGSHMIGKDFVGWEDALKRYGKSPHRDVQKVLRISYDSLPFNEKNIFLDIACFFKGQRSEYVKRVLDACDFNSGDGIDTLVNKSLLTVDSQNKCLEMHDLIQDMGKEIVKEESWTEIGERSRLWFHEDVLQVLADDMGSGKIQGLMLDPPHREEINCTETVFEKMKNVRILIVRNTSFSHEPSYLPNNLRLLDWKNYPSQSFPSEFYPRKIGAFNLSRSPVLALEEPFKVQVXRSRLWFHEDVLQVLADDMGSGKIQGLMLDPPHREEINCTETVFEKMKNVRILIVRNTSFSHEPSYLPNNLRLLDWKNYPSQSFPSEFYPRKIGAFNLSRSPVLALEEPFKRFQLLTYMNISYCNMVTEFPDVSGAKNLRELRLDGCEKLVTIHESVGLLANLVFLSASECILLSKFVPTMYLPSLEYLSLNLCIKLAFFPEISGTMNSKLKINMLDTAITELPESIEKLTGLNYLDMTDCKELQHIPGSLFMLPNFVTLKIGGCRRLRESFTRFKGSHSVRPKLETLHFDNADLSEKDVRTIIYHFPNLKDLNISCNPVVYLPACIKESTNLTSLHLRYCDVLQEIPELPSRVQKVDAKYCYSLSSKSSNILWSQVRKEMKRLEVGMPESDIPEWFDHVNEGGFPVFTARRKFPAVVLALVFGVVDAKADREIMEGRWRTVGMHLFIEGERRRYHNFSVAENHVLLCNLRVLFSLEEWEDVGFGVDEDWKTVQVFCETNLSLCSWGVYVYKTETNMDDINFMTQHPSSSLVLSRHKMISDMDKIESKIISLNIPQMHREFHMWSTAARKLRSFKGESSRVLEKASRRVMALRGEEGRSGMNIEDERDKELDAQLDMLATAELDKHFHYPNNHTAEPFTSQYCRIL

>XP_047169400.1

MDIASSSYKLPRKYDVLINFTGEDINRKFVSHLNSVLSTVGLTTFLHHHNAVKSTHIQEPILSHCRVAIIVFTQTYSQSAWCLNQLEQIIKWHETYFRHVMPVYYEIQPSDVRLQKGDFGKVLKATAQQTFSGEELEHGMSRWSHALTKAANFFGWDESNHRSDAELVDKIVKSVFNLQVLSATKFPVGLQSHVEDLIRTIKNKSTEVCMIEICGEEGSGKTTLAKAIYNQIQWTFKDKSFIENISQFRGIRGLLRLQEQLLLDILKQKVEIPSVDVGRTMIRERLLGKRVLIVLDDVNYFNLFDLFDCLKLLVEGSVIIVTSIYGIGLGDQANFDFQVERMNAEESLELLSWHAFREAKPKEEYKDLARRVVSYCRGLPLALEVVGSSLFEKKKEEWDTVLLKFAIVSRHHVSEIIKISIEGSLNEIEKDIFLDICCFFVGKSRAYVTKILNGCGVDADIGIRVLMERNLIKINKNNKFGMHPLLQEIGIRITQENLGKEHWKNRRLWFGMDARYVRTFFTCGLKLLLKVFSFSCAVHLFLFALFNTGDRSHAVVAWETAFSSHCHRKCTTHGKFSVPSKTILVQVLISLKVLNLSHSKHLTTTPDFTGLPSLEHLIFKYCSRLSRVHQSIGSLNSLILLNLKDCTSLNNLP

TEIYELKSLRTFILSGCSKIDIMDKDIAKLKSLITLIAENTAVKEVPFSIVSSKSIGYISLRQFEGLSHNLFPSIIRSWMSPIMNPI
SYIHSFCMDMEDNTIDIAPLLSTLTNIQSVLVACDTEFELSKRVKNILVEYFANIIESGISKQHFRCSLIGVGAYHQFFNAVS
DNISQVLLYDSCDVCLPAVNDPYCLAHMGEGHSVSFVVPEDGDMKGMILCVVYLSTPKIIEPEFTTVVIVNYTKCTFHIH
NHGTVISFNDEDWHGIMSNLESGDNVEIFVNFGNGLVVKNTTVSPAPWIKREIXVVSYCGGLPLALEVVGSSLFEKTKK
EWDTILSKFAIVGRHRVSEIIKISIEGSLNEMEKDIFLDICCFFVGKSRAYVRKILNGCGVDADIGIRVLIERNLITINKNNKFG
MHPLLQKIGIQIIQENFVKDLGKNRRLWFDKDTKYGTEDMQWMPVKLPSVLIALKTVHSTVNSQYLIKKLRWISLRAFSS
ECLPNNLYEPDAIAIDLKRSLLRFLWKTPQVLRSVKVLNLSHSKHLTATPDFTGLPSLEHLIFKYCSRLSKVHRSIGSLNRLILL
NLKDCTSLNNLPTEIYDMKSLRTLILSGCSKIDIKDKDITKLESLITLIAENTAVKEVPFSIVTSKSIGYISLRGFEGLSHNLFPSII
RSWMSPIMNPISYIHSFCMDMEDDTIDIAPMLSTLANIRSVLVACDTEFQLSNQVKNILVEYFANITESGILKQHCRCSLI
GVGAYHQFFNAVSENIYEVLSSSECGDVCLPAVNDPYCLAHMGEGHSVSFVVPEDGDMKGMILCVVYLSTPKIIEPEFT
TVVIVNYTKCTFHIHNHGTVISFNDEDWHGIMSNLESGDNVEIFVNFGNGLVVKNTTVYLICGESKNMEKRLSQRNILSL
DS

>XP_047169402.1

MDIASSSYKLPRKYDVLINFTGEDINRKFVSHLNSVLSTVGLTTFLHHHNAVKSTHIQEPILSHCRVAIIVFTQTYSQSAWC
LNQLEQIIKWHETYFRHVMPVYYEIQPSDVRLQKGDFGKVLKATAQQTFSGEELEHGMSRWSHALTKAANFFGWDES
NHRSDAELVDKIVKSVFNLQVLSATKFPVGLQSHVEDLIRTIKNKSTEVCMIEICGEEGSGKTTLAKAIYNQIQWTFKDKS
FIENISQFRGIRGLLRLQEQLLLDILKQKVEIPSVDVGRTMIRERLLGKRVLIVLDDVNYFNLFDLFDCLKLLVEGSVIIVTSIY
GIGLGDQANFDFQVERMNAEESLELLSWHAFREAKPKEEYKDLARRVVSYCRGLPLALEVVGSSLFEKKKEEWDTVLLK
FAIVSRHHVSEIIKISIEGSLNEIEKDIFLDICCFFVGKSRAYVTKILNGCGVDADIGIRVLMERNLIKINKNNKFGMHPLLQEI
GIRITQENLGKEHWKNRRLWFGMDARYVRTFFTCGLKLLLKVFSFSCAVHLFLFALFNTGDRSHAVVAWETAFSSHCHR
KCTTHGKFSVPS

>XP_047169844.1

MPVIETXGGALFGAVLQVLFDKLDSRQVLDYFRRRKFDEKLLKKLKRKLVSINAGVDDAEKKQFRNAYVKAWLDEVRDV
LLDTEDLMDEIYYEFSRYGLEVESHSSSSQVCIFISRIKEVLDDLECLLNQMDDLGLKNASGVGVGLGLNSNVSQKLPSTSL
VVENIIYGRDDEKEMILKWMTSDTEKHSWPHNSQLSILSVVGMGGLGKTTLAQHVYNDPRIEGKFAIKGWVYVSDEFD
VLMLTKTIFGVLTKSKDDSVDLEMVQGRLKEKLTGRRFLVVLDDVWNEDRAQWKALQTPLNYGAKGSKILVTTRSNRV
ASIVQSNKLHELKQLGGDHSWQVFAKHAFQDDNSQMNAEVKEIGTKIVEKCRGLPLALETVGCLLRSKSSVAEWKSVL
SSEIWDFPEEDSKIIPALLLSYYHLPSYLKRCFSYCAMFPKDHEFDKKNLIQLWMAENFLQRSQQSKSQEEVGEHYFNVLL
SRCFFQQSSEGFKSCFVMHDLLNDLAKYVSGDICFRFGIDRAKRTLKETRHFSFVIDDYGVSCNEYENLYDAKGLRTFLPV
TRISYWSWYCETLTLELIFKLKCLHVLSFCGCVNLKEVPETIGNLIHLRFLDLSNTGIQKLPDTMCSLCNLQTLKLNSCVNLK
ELPCNLHKLTNLCCLELMKNSLTKMPMHIGKLKNLEIFMSPFNVGKSSELCIQQLGELSLHGDLLIKDLQNTVNPMDALA
ADLKSKTCLVRLNLHWDLEQNLVNFMKEREILENLQPSRHLEELSISDYGGIQFPHWLSDNSLSNLVSLSLINCKHCLLLPS
LEFLTFLRHLTISGHDWIRTIDADFYRNSCSAFASLQTLNFADMKEWEEWQCRTGDFXQSLSVTNCPKLKGQLPKQLSH
LRKLIIEDCKQLVTLAPRTLEICELQLRDCGKLQIDYNPTTLKRLQIGGDNMEASLLERLGHIISHASLESFTIFSCPNMNIPI
NHCFDFLEKLHIGGGCDSLTNFPLDFFPKLSELELSECPNLQMITQGHPLNHLKILRIGKCSRFEYFPNEGLFARKLESFYIIG
LEKLKSLPKRMSVLLPSLNLLYINNCPDVEVSDGCLPSNLSEMCLFNCSKLIASLKGPWGTNPSLKSLSIGKVDEDCFPSDG
LLPLSVTNLEIYDCPNLKKLDYRGLCHLLSLEKLFLYKCPILQCLPEEGLPKSISKLRVEGCPLIKQRCKKQEGEDWQKIAHIK
YIMVDRERVNI

>XP_047169931.1

MAAEMVTGVLVSTFLERTIDTLASRLVDIFHQRKHKKQLSNLKMKLLAIDVVAFEAEQKQFTDPRVRDWLLRAKDVVID
AEDLLDEIDYELSKTQVEAESQSASKKVWNSLDSSFFEIEFDSMREKVIEDLEELAIESDLLGLKKGGGVGVGSGSGSKLTH

TSLPNERVIFGRDDDKEFVLNWLTSDTHNNLSILSIVGMGGMGKTSLAQHVFNDPRLQEANFHTKVWVSVPQEFDVL
KVSRAILDTITGSTDHTIQHEVIQKRLKEELMGKKFLLVLDDVWNERPSKWEDVQKPLVFGCQGSRIIVTTRSEKVAVAM
RSEKRLLQVLKKDYCWDLFAKHAFENANPQPDSEFIEIGKKIVEKCNGLPLALKTMGSLLHNKSSLSEWKSIMKSEIWDF
SENESDILPALRLSYFHLPSHLKKCFAFCALFPKGYWFDKEWLIQLWMAENFLENPLQKKSPKEVGEEYCNDLLSWSFFQ
QDSRFGGEKCFIMHDLLNDLAKYVCEDICIRLGVDEPKGIPKTTRHCSFSGSGFDGFGSSIDTQKLHTFTRTNRDWRWD
WRWDCKMSINDLFSRFKLIRVLSLCNCRSLTELPKSVGNLKHLRSLDLSHTAIEKLPDSISLLYKLQILQLNYCEELKELPSCL
HELNNLRRLELVGTGVENVIAYLAKLKNLQVTMDSFQVEKSKEIYFQQLGELDLHGSLTIDDLQNIENPSYALEVDLKNKP
HLVELRLEWNFIGSSSVDSEKAEDVIENLRPSKYLKKLSIRNYIGKQFPDWLLHNSLPNLVSLELEGCESCQRLPPLGLFLFL
KDLRIARLDGIVSIDADFHGNNSSSFKSLQTLYFYDMRQWEKWDCQAVTGAFPCLQYFSIEKCPKLKGHLPKFVALKTLI
VIHCEQLEALIVSAVELRLQDCGKLPLERSTMKNLTMDGQGMAASLVAMVGHMLFDTSLEKLSICSESISDDCVSLRIFP
LDFFPTLRTLELIGFPNLQMISQNHVHNHLWHLKIKECPKFESLPANMHMLLPSLISLEIKDCPRLESFSEGGLPLNLKDITL
NNCFRLVGSLKRALGDSPSLRSLRIEKVKAKCFPDEGLLPPSLTQLIIRDYRNLKKLNYKGLLELSSLETLKLLKCPNLKCLPEE
GLPKSISFLEIMNCPLLKQRCHKEGGEDWKKISHIRNLLIL

>XP_047169932.1

MAAEMVTGVLVSTFLERTIDTLASRLVDIFHQRKHKKQLSNLKMKLLAIDVVAFEAEQKQFTDPRVRDWLLRAKDVVID
AEDLLDEIDYELSKTQVEAESQSASKKVWNSLDSSFFEIEFDSMREKVIEDLEELAIESDLLGLKKGGGVGVGSGSGSKLTH
TSLPNERVIFGRDDDKEFVLNWLTSDTHNNLSILSIVGMGGMGKTSLAQHVFNDPRLQEANFHTKVWVSVPQEFDVL
KVSRAILDTITGSTDHTIQHEVIQKRLKEELMGKKFLLVLDDVWNERPSKWEDVQKPLVFGCQGSRIIVTTRSEKVAVAM
RSEKRLLQVLKKDYCWDLFAKHAFENANPQPDSEFIEIGKKIVEKCNGLPLALKTMGSLLHNKSSLSEWKSIMKSEIWDF
SENESDILPALRLSYFHLPSHLKKCFAFCALFPKGYWFDKEWLIQLWMAENFLENPLQKKSPKEVGEEYCNDLLSWSFFQ
QDSRFGGEKCFIMHDLLNDLAKYVCEDICIRLGVDEPKGIPKTTRHCSFSGSGFDGFGSSIDTQKLHTFTRTNRDWRWD
WRWDCKMSINDLFSRFKLIRVLSLCNCRSLTELPKSVGNLKHLRSLDLSHTAIEKLPDSISLLYKLQILQLNYCEELKELPSCL
HELNNLRRLELVGTGVENVIAYLAKLKNLQVTMDSFQVEKSKEIYFQQLGELDLHGSLTIDDLQNIENPSYALEVDLKNKP
HLVELRLEWNFIGSSSVDSEKAEDVIENLRPSKYLKKLSIRNYIGKQFPDWLLHNSLPNLVSLELEGCESCQRLPPLGLFLFL
KDLRIARLDGIVSIDADFHGNNSSSFKSLQTLYFYDMRQWEKWDCQAVTGAFPCLQYFSIEKCPKLKGHLPKFVALKTLI
VIHCEQLEALIVSAVELRLQDCGKLPLERSLVAMVGHMLFDTSLEKLSICSESISDDCVSLRIFPLDFFPTLRTLELIGFPNLQ
MISQNHVHNHLWHLKIKECPKFESLPANMHMLLPSLISLEIKDCPRLESFSEGGLPLNLKDITLNNCFRLVGSLKRALGDS
PSLRSLRIEKVKAKCFPDEGLLPPSLTQLIIRDYRNLKKLNYKGLLELSSLETLKLLKCPNLKCLPEEGLPKSISFLEIMNCPLLK
QRCHKEGGEDWKKISHIRNLLIL

>XP_047169933.1

MAAEMVTGVLVSTFLERTIDTLASRLVDIFHQRKHKKQLSNLKMKLLAIDVVAFEAEQKQFTDPRVRDWLLRAKDVVID
AEDLLDEIDYELSKTQVEAESQSASKKVWNSLDSSFFEIEFDSMREKVIEDLEELAIESDLLGLKKGGGVGVGSGSGSKLTH
TSLPNERVIFGRDDDKEFVLNWLTSDTHNNLSILSIVGMGGMGKTSLAQHVFNDPRLQEANFHTKVWVSVPQEFDVL
KVSRAILDTITGSTDHTIQHEVIQKRLKEELMGKKFLLVLDDVWNERPSKWEDVQKPLVFGCQGSRIIVTTRSEKVAVAM
RSEKRLLQVLKKDYCWDLFAKHAFENANPQPDSEFIEIGKKIVEKCNGLPLALKTMGSLLHNKSSLSEWKSIMKSEIWDF
SENESDILPALRLSYFHLPSHLKKCFAFCALFPKGYWFDKEWLIQLWMAENFLENPLQKKSPKEVGEEYCNDLLSWSFFQ
QDSRFGGEKCFIMHDLLNDLAKYVCEDICIRLGVDEPKGIPKTTRHCSFSGSGFDGFGSSIDTQKLHTFTRTNRDWRWD
WRWDCKMSINDLFSRFKLIRVLSLCNCRSLTELPKSVGNLKHLRSLDLSHTAIEKLPDSISLLYKLQILQLNYCEELKELPSCL
HELNNLRRLELVGTGVENVIAYLAKLKNLQVTMDSFQVEKSKEIYFQQLGELDLHGSLTIDDLQNIENPSYALEVDLKNKP
HLVELRLEWNFIGSSSVDSEKAEDVIENLRPSKYLKKLSIRNYIGKQFPDWLLHNSLPNLVSLELEGCESCQRLPPLGLFLFL
KDLRIARLDGIVSIDADFHGNNSSSFKSLQTLYFYDMRQWEKWDCQAVTGAFPCLQYFSIEKCPKLKGHLPKFVALKTLI
VIHCEQLEALIVSAVELRLQDCGKLPLERSLVAMVGHMLFDTSLEKLSICSESISDDCVSLRIFPLDFFPTLRTLELIGFPNLQ

MISQNHVHNHLWHLKIKECPKFESLPANMHMLLPSLISLEIKDCPRLESFSEGGLPLNLKDITLNNCFRLVGSLKRALGDS
PSLRSLRIEKVKAKCFPDEGLLPPSLTQLIIRDYRNLKKLNYKGLLELSSLETLKLLKCPNLKCLPEEGLPKSISFLEIMNCPLLK
QRCHKEGGEDWKKISHIRNLLIL

>XP_047169990.1

MRKQRHLEGLVATVAQLKNENQRIIQSINIANQRMKNSEIDNSVLRAEMTKLSSRLWSLRFYDVFLSFRGEDTPVSFVS
HLYASLQNAGIIVFKDDESLPRGDHISYSLLQAIEQSRVFVVVFSRNYAESQWCLNELVSIMQRHRTTGHVVLPVFYGVD
PSEVRHQTGEFGKAFQSLSNRTFTEESEKQRWMETLREAASISGIVVLNSRNESEAIKSIIENVTRLLDKTELFIADNPVGV
ESRVQDTIQLLDLKRSNDVLLLGMWGMGGIGKTTIAKAIYNKIGRNFEGRSFLAQIREVWGKDGGQVCLQEQVLFDIN
KETKTKIHNVEFGKNILMQRLRHKRVLLILDDVNNLHQLNTLCGNREWFGSGSRIIITTRDMHILRGSRVDKLYMMERM
DESESVELFSWHAFKQATLEVLGSYLFDMKVTEWKSVLEKLQKIPNDEVQEKLKISYDGLSDNTEKEIFLDIACFFIGMDR
NDAIHILNGCGLCAENGIRVLIERSLVTVDHNTLGMHDLLRDMGREIIRGKSPKEPEERSRLWFHEDVLDVLSKETGTKAI
EGLALKLPRTSTKCLNTKAFKKMKKLRLLQLAGVDLIGDYKYLSKDLRWLCWREFPLTSFPANFYTRSLVSVELEHSNVTH
VWKEAQVMENLKILNLSHSHSLTHSPDFSNMPNLEKLVLVGCPRLSNVSPTIGHLDKVLQINLQDCISLRNLPRSIYKLKS
LKTLILSGCLMIDKLEEDIEQMESLTTLVADKTAITKIPFSIVRSKSIAYISLCGYEGFLRDVLPSIIWSWMSPTNSLSSHMQR
FAGISSLASLDVPNNSSHHLASISKDLPKLQSLWVECGSKLQLSQDTKIILDALHDTNSGESETTATTSQMSNINAFTLIEC
NSQVHLSGSNRSLLIQMGMSSKVSYNLKESILQVVIEVYNIMSV

>XP_047170620.1

MADSIVVFLIDKLTRLLVEEAKLLTGVRDQVTSLQSELRFMNIFLRNSSKGKRKEHDMVAELVSQIRDVAHEAEDVIDTY
VACIIKQSRRNVIGKVGFRGVDHALMLHQVAVKVDGIKARIKEIFDNKERYGIDDGKRGSEEEAERIRRQRREVEEEEVV
GFALDSKVVIEKLTVSDSRLKVVSVVGMGGLGKTTLARKVYNSNRVKNMFPCRAWGYVSNDYRPREFFLSLLKCLMSTS
KYSNLFKKREETSVSDEELKMKVRECLNRSKYLVVVDDVWQKQVWNEVKGAFPDDQNGSRILMTTRSAEVASHAGPV
PPYALPFLTKEESWELLSKKVFRGEDCPCDLESLGKLIADSCDGLPLALIVMAGILANKKSPRDWSRIKDHVNWHLGRDN
TLKDILKLSYDSLPARLKPCFLYFGMYPEDYRIPVKQLIQLWISEGLLTQETSGGQDIPEPEYIAEEYLDELVDRSLIQVVSRT
NDGGVKTCRIHDLLRDLCISESREDKFFEVCGEIDIQNLNSCPRKLSLQGTLFHFSSSIVSDYTISATRSLLCFGQEVYKVKA
NHWRWLLKSFRLARVLDLGRMNVNSIPTDLEKLIHLRYLRIHSHNLETIPPSICRLWNLETLDLRGSPIKSFSGELWQLKQ
LRHLLLFGPVGLPEMPSESKTMPNLQTLSTVALDPRTASLLDSRRFPGMTKLGIHYERRDKCNAKIQLQSLHRLSHLQKL
KVIGTTEIPQNANVFPSNITKISLTKFGFFNSTVMHTLGKVPNLQVLKLSSQTNDTRFDLHCATGGFLQLQVFEMIAIKVK
MWRVDRGSMPRVRRLVVRSCKSLTQLPKEVWSLNTLREVQVLWPCTELAKGLQNLVMNNACKLVVYPLSANDELDF
L

>XP_047170941.1

MAASSTYDVFLSFRRGYTGDSFVSNLYKSLCKRGIHTFKDDEMLLKRKESIATLLNAIEESRIAIVVLSHNYASSYFCLDELA
RILHCQTKGLLVIPVFYKVHPSDVRHQKGSYGEALTMNQKKFEDMEKVQKWKMALHQVANLSGYHIEDGVGYEYKFI
KRIVDDVYDKITRDSFTHVSDYPIGLQSQVLEVRKLLDFGSEDGVHMIGIHGMGGIGKSILARAVYNDLIGENFDGLCFLE
NVREKTNKHGLKHLQSILLSQILEEKDINFTSTQQGISKIKYRLKMKKVLLVLDDVNKPDQLQAVAGGCDWFGPGSRIIIT
TRDNKLLENHRVTMTYEVKGLNDNDALQLLKWKAFRNEKADPDYELALNRLVTYASGLPLALEVIGGSLVGKSIEGWKT
AIQQYEMIRKRDILNMLKVSFDALEEEEKSVFLNIAFFLEGHALTLVEYILHACYDRSMKHHIGMLVERSLLKFDYFDNRL
KMHDLLRDMSRIVWKELQKEPGNNSRLWLHKDVIHVSNNNRIFESEINHLVFSISAIQRIVGSYANTFKRMKTPEKHDK
KFQFLNHFLQSLRALIWFRISSNCLPSSLDNLLKKYETLTVMNFDECRILTQLPDQAY

>XP_047170943.1

MLLACHCFEEEWKCAINENDPVGLESRSDAFCYDVFLSFRDLDTLYGFTGYLFKALHDSGIHTFIDDENLQRGEEITPTTV
KAIEESRIAITVLSINYASSTWCLDELATILDCLKRERLLVLPVFYYVDPTQVQLQKGRFGEALTKHEERLKHNMEKSLKWK
MALHQVANLFFFHIDHGDGYEYELIGKIVEWVSKKINRANYPIGLESQVQEVMKLLDVGCDGGAHMIGIHGTGGIGKS
TLVQDVYNNLISERFDASCFIENVSEKSNKHGLQYLQSTILENLLGEKDIKLTSVEQGILMIQRRLQKKKVLLILDDVDRQE
QLQAVVGRADWFGRGSRVIITTRDEQLLASHDVQITYEVKKLSNKDALVLLKWKAFKKHDFDPRYKELLNCAVTFSDGI
PLALEVIGSNLCGKSVEEWKSVIHQLEKCPDNPVEAILKSSFDSLEEKERSVFLDLACCYKGYELAEVEDILQAHYGQNMR
CYIDLLVDKSLVKLSHGTKPCYDRVTLHDLIEDMGKDIVRQESLIGPGERTRLWLLEDVRQLLENNRGTSKIEIICLDFSIFD
QEGKVEWDGKAFQNMQNLKTLIIRHGRFSKGPKYLPDNMRILEWWRYPSNSLPSDFHSKELAICKLPCSSISTIELTNLL
KASLISAFSLSCKLINPL

>XP_047171908.1

MGCVGFSYDVFISFRGEDTRDNFVGHLRKELGRRGILTFHDDRDMGIGESLSPALNNAMEESRIFIVVFSENYASSTWCL
DELVRIIQLSKKREKKQVVFPVFYHVHSSDILQDRNSFGKHMRAHEITFGKQSQRMQAWRSALSEAVHLPRKHITTECE
NNFIEEIVREVYKNIAPKPLYTGQNPLGLEPHIEEVKSLLDIKPDDNTVRMLGIYGLGGIGKTELAKALYDKIVQHFDAASF
LAGVREKSNTINGMEDLQKTLLSEMLEESETTLGSTDKGIYEIKRKLRRKKVLLVLDDVDDKEELEKLAGGCDWFGPGSRI
LITTREKDVLIAHHVGNIYEMKELDEQHSLELFCWNAFGQGCPKTGFRDVSLRAVDYAKGLPLALKVIGSDLATLHEEGL
DAWKDALEEYEKTPPNKKIQDVLKISYERLDDDAKQVFLDIACFFKGERMEYVSKILDEFGSAFKIKVLVNKSLITVDKGCL
KMHDLIQDMGRQIIRQEAPNPGERSRIWDYEDVIEILNEDYGSDKIQGIMLDPPQQEMVKWSGTEFEKMKCLRILIVR
NTSFSSEPEHLPNHLRLLDWDNYPSKSFPPKFHPKKIVLFNLPRSCLTLEEPFKKFPCLTNMDFSYNQHIIEIPDVSELQNL
RELRLDHCRNLIAVHQSVGFLKRLSHLSVSECTKLQNFLSRMFLPSLQIFNLNFCESLGYFPEIMKEMTKPLKIHMINTAV
QELPESISKLTGLVSIDISNNRELKYLPRSLFMLPNVDSFIIKACSKLGESIRSLVQHPLKANVHQKLRLLNFENGNLSDADLL
AIICYFPKLEELIVSENNFVSIPSCIKECGDLTSLDLNGCKKLKKIPELTSLRILDVHDCLYLEEISELPSTVQKVDARFCFKLTKE
TSDMLWYQVKKGVGGIEMVMPFITEIPEWFNFVGVGRIPSFWVRGKFPKIVVAMIFHFQNESQRDRYDGRGLVDLRL
LINGRYAPRKGYRNFRIEAEHILLCDVGVLCSEKEWVGNAVMEHEWNLVEVSYDATSSLMISGWGAFVFEEGTNMED
LLFASPNNLIVNGDSTSVHKGSLEDDENYVALPEGIAWESLFKGIKDGIVEAWKKFPSMDAAEISVAALKKNRKIEWTVE
GMEGIPSAENRTYMSGLYGVLLEAKLRFPDLDVGAALTNVANRKGIKGTFGSPAKPEMTIPKLDWSTVTLPPSHDPLM
QIFMMMMKQQSTSESELKTKTFWKLKESHQILRNRLSHKTAAQNSGSSSKNQYDELIQKFNMQYDAFVGKRVDNLY
GVAKHEKDSGVLKERAKEIERELDAVVGWLQKSEEFGDVMTAMFLNGLRDGILEARAILLALRTDTQTHERVTDEAPNS
ENIA

>XP_047172082.1

MDWSSVVVQGIRFVWRHGVRHVTYIVCYKQSVHELKDSVKDLENEKDKIDHQCDEAYKNLKNIEGKVIEWSRKVSEIK
TIVEEFENDDGHKRARSLNCYVFSYLWNRHRLGRQAKKMEVGVKQLINESPGLDEISYRENVTSNDTTLSNSGFEEFSST
KSTMEKVMRQLENSSVRMIGLYGEGGVGKSALIKEIARIAKDKLFNVVVKVEITANPNLQSIQEEIAYMLQLQLEGEGEN
VRADCLRRRLKKEKGNTLLILDDLWHKLDLNKLGIPSDDNDDLSNDTRGLNDKFLKKDKNNMDHHKTVLKREKIIGGHK
ECKILLTARQKRVLEIEMDVKSTFRVEPLDDKDALMFFQKLSGIHNMSDSRKEIVTKYCAGLPMAIVTVAKALKGKSELV
WEAALEKLKKQELVGVQTYMDISVKMSYEHLENEEIKSIFLLCAQMGHQPLIMDLVKCCFGLGILEGVSSLWEARDKIKI
TIQKLKDSGLLLDGNSDIHFNMHDIVRDAALSIANKDKNVFTLRNGKVDEWPELRRCTSISICNCDIIDELPTVKCSRLNFF
QIDTNNQSLTIPEKFFEGMKNLKVLILTGFHLKRFPPSIKSLLKLRMLCLERCTLEDNLSIGELKKLRILSFSGSQLKSLPTELG
CLDKLQLLDINDCSILEINIPPNILSSLTHLEELYIRKSLTKMLVEGEPSHGQNSILCELKNLHQLKVVDLSIPCFSLLPNHLFF
AKLKDYQIVVGDVEMFSIIGFKMFDKYETFRVLALQLNHDTNIHFQEDIKLLFKTVQSLLLGKVDDVVEAVNKLNMDGF
PDLKHLSIIDSNAIKYVNSMELSNCINAFPNLESLCLYNLKNLEMISNGPLTVASFAKLKSIKVNMCDRLVSLYSVYMVEFA
NSEVPCKICKYNSYLDKFCASLKTIEVSECESLKEIFRIPKKCYKVKFLKLQTLTFQKLPSLTCFYTKVEKSCWLHPTKPQTKSS
GSEEDQKSYQAPPLFGEQVEIPNLESLNLCSLNIHKIWSDQLSSSFCFQNLIKLVVKECDKLTYLCSLSVASSLKKLKSLIISEC

PIMEKIFETNENNADKVCVFPKLEEIHLSKMRRLRDIWHTKVNIDSFSSLISANIDECNKLDMIFPSNMEGWFESLINLKIS
RCKSVKEIFEVRDSKEIDVSGGIDTNLQVILLENLPKLKELWSKDPHGILNFKKLRTIDVSYCDELKNLFPASMVKDVSKLE
HMSILHCERMKEIVSSKDASEANNDPLEFPELTYVRLYSLPNIKHFYKGRHMIKCPKLKELAVNRYLKLKAFSKEIVKTTEE
EESFVFSAQKVFPNLEYMEIDFEEAQNLLPKYQMHRLKELNLISVESVDFLNQFPYRMPNLEKLKLAFPYSSKVLNPRANL
AQQEGLVIALELKELVVMYSGITDLGFPILGRLEVLSLIGCDKLSNLDPPSLSFTYLTCLKLTNCSGLRNLMTFSTAKNMVQ
LKTLKVIRCSNVEEIVSNELSEEGIEMKIEFSKLITIELVKLDNLASFCSCKHSEFEFPSLEILIVRECCKMEKFSERDPITPKLKN
IFGVEGDEKAKWLWEGNLNDTIHKIFTDKVYFTYSDDLWLDSSTEYIIEQLWHGSDWMQQNCFDYLKTLTIFGCDSVV
HVIPSHLLSCFHNLEELEVVNCAAAQVIFNIGDENRVMTKPSGIFRPKKLFLDLLPKLEHVWDKDPEGIIGLQLLKKMSVR
SCECLKSLFPASVAKDLARLEVLEVNDCRELAEIVKDEKDEEGEGTTHESVFPCLTTLILEELQDLKYSIHSKQQVSTSNLSE
RDIQELCLGSRCIPNSYFSLLESLILDGCQFLSDVLLPFNLLPFLTNLETLEVRYCDSVKTIFDVKCTTQDREVASMGPTLPFS
LNKLTLFNLPKLENVCNEDHCSPFVRSLDVQRVPNLKYFYYSSQYCEIFTDLESHAENQVGTEKLFKCLSLWENGVNMIL
RGEFERNLLDSLKALTLCFGSDVFRCKILEQVPNIEKLEVRDCSFKQMFCCESLNNVLQKLKVLRLESLREMVSIGLEKSWS
DSFVRNLEIFEVINCRSLKNLVACTVSFSNLTCLKVNDCGSLSYLLTSSTAKRLGKLKRMEIKDCSSIEEIVCKEDGEESDEDE
IIFPQLTCLNLDNLLNLKRFYGGSLGFPSLEELSVTYCNKMITLGVGTIVAGKLCQVKVGEFSDAIQLKTELNSTTRKEYLKKI
ASKWSLELTERADLEEIWCLSLQIPHFSFTDLKTLIVNKCKIPSDAVLPFTLLPLLPKLETLKVQNCDSVKIIFDIKCTTQDTTT
FSLKKLVLRKLPNLETVCSEVKEANPAHPEGRNPKLILPCVTSLALSDLPNFEHNSIHYIHDDAAPTFELQLIIPNLEHLTVG
KNELKKIVDGEFQRNLLHKLKVLGLCFDIECDEFPEYGFLQQLPNVKKLVVWSSSFKVIFCHQRPNNNELLLQLNELRLES
LGELVSVGLENSWTESFVRNLETFEVIRCSRLENLVTCTMSFSNLTCLNVENCDGLSYLFTSSTAKSLGQLQRMEIKQCKSI
KEIVSKESEESDEDEIIFSQLSCLKLDKLLNLQWFYRGSLSFPSLQQLSVTLCTEMVTLCPSTLKAEKLSQATIGNKDVILVET
DLYTTMQKEFGRKVQTQRQSVIDLKSRRGLHEIWRGSLSIPNFCFSGLVTLIVEDCPFLSDAVLPFHLLSLLPKLKTLEVRN
CDYVKTIFDVKCTTKATSITFPLQKLVLSKLTNLKNVWNEDPRGILGMPSLEKVFVDTCKCLKGVFPASIAKELVKLEDLVV
EDCEGLMAIIADESHENQETIFERLRVLDLKRLKELTCFCVGNFTLSFPSLEEVHVIKCSSMKTFSAVTKIDNPTKWYYSEY
ERPRKETDLNSALHRNFEEEAPDASRAIISVL

>XP_047172537.1

MVKTKSGLKRFWNCFCGRSSGSTSSSSSYDSSHTIQNQHYIYDVFISFRGPDTRNSFVDHLCSHLLRKGIFVFKDDHQLQ
KGKSISPQLLQAIQLSRLSIIVFSKNYASSTWCLEEMAAIASCKQQSSQIVFPIFYDVDPSHVRRQNGVYKNDFVSHRCKF
QKDLDKVLGWERAMTDLANSAGWDVRDKPEFEQIQHIVQAVIKKLGHKFSGFIDDLVGIQPRVQALEDKLRLSSNKVQ
VLGVWGMNGWWEVMVKTKSGLKRFWNCFCGRSSGSTSSSSSYDSSHTIQNQHYIYDVFISFRGPDTRNSFVDHLCSH
LLRKGIFVFKDDHQLQKGKSISPQLLQAIQLSRLSIIVFSKNYASSTWCLEEMAAIASCKQQSSQIVFPIFYDVDPSHVRRQ
NGVYKNDFVSHRCKFQKDLDKVLGWERAMTDLANSAGWDVRDKPEFEQIQHIVQAVIKKLGHKFSGFIDDLVGIQPR
VQALEDKLRLSSNKVQVLGVWGMNGIGKTTHAAVLYDKISHRFDASCFIENVNKLYRDGGHTAVQKQIIHQTLRENID
MSNPIEISGIVENRLHRIKVLIVLDNVDELEQLENLAIKPKLLSKGSRMVITTTDEHILKAYEGEVLIHKVSLLNDKDARELLC
RKAFKSEEQSSRCAALIPEVVKYAQRLPLAIRVLGSFLCDRDGVQWRDVLNRLENSPNNKIMNVLRISVDGLQREEKQIF
LHIACFFKGEREDYVKRILDCCGLYPHIGIQRLVEKSLITISSEEIVMHELLQELGKKMVRDQSPEEPGSWSRIWLLKDFLH
ALKAETVTSYIFYFEGTEKVKAIVLNKKEEMSECSVEGLSRMKELTLLILYHTRISGRLEFLSDRLQYLLWHDYPFASLPPYFT
ASNIVELNMPNSRIRHLWEGRKSCPNLKRIDLSNSKYLSETPDFSRIVKLERLDLSGCTSLSYVHSTIGLLKKLAFLNLRNCC
NLVFIDFGCVGNMSSLRVIHLSGCSKLESTPDFTRATHLEYLDMNECTSLSTIHESIGVLSSLTFLSLRGCIKLVSIPNAIHSL
ASLQTLDLYGCFSLTDLLPKQASSSHLKYLIFLDISNYKLKEVPDAIGDLRSLERLNLQGNNFVSIPDSFHQLHCLAYLNLSH
CDKLIYLPDLPTEGDTSGGKYFKTVSGSRDHRSGFYLFDCPKVSYGKVEFDRLERFFHLIKVHILQPSLFVFG

>XP_047172672.1

MECLLGFASSLSRDLVCGALNQLRYPCCFNNFVKRLEQEESDLIITRDSVQKFVAQSKKQTKKPSEIVDKWLEDANDDVY
NVNQLLKEAKTKNHCCFGHCPNWIWRYHVGKNLANKTMDLEKFIESGKKYVPFDRITTLPSNTLDTLLEKCMNFESRQ

SAYEQLLDAVKNKDVSMIGLYGMGGCGKTTLAMETRKFVEAEHLFDKVLFVPISSTVEVRRIQEKIASSIQFEFPEAEEM
ERAQRLCLRLTQEKNIFIILDDVWEKLDFGRIGIPSSNPGCKILITTRSDSVCSLMDCQRKIYLPILTEEEAWTLFQNKAFISE
GTPDTLKGLGRLISNECKGLPVAIAAVACSLKGKEEPVWRVALNKLKHSKPINIERGLTDPYKCLQLSYDNLDTEEAKSLLL
LCSVFPEDFEIEVELLTRCAIGVGVVGETLSYEEARNEVIAAKIKLVSCCLLLDADYXCVKMHDLVRDVAHIIAKNENMMI
KCEEQKDVIVEQNSIRYLWCVKFPKDLDCSNLEFLFLRTKMKELDGIFERMGVLKVLILVNDEDGKTPLSTVSFNTLTNLR
DLLIYNWELSDFSFLGGMKNLQSLSLFYCLLPSFPELQTSVAITPTALKLLELNECDIKVKNFEVVKRIPLLEELYIIDIEGQW
YANSEDNIEFFKTFSVPETLQRYGIVLGSRYFDGGNFKEFSSYDRTLLLNYFDVSNEVMKGLAKNAKDLFVRNIHRGAKSII
PDIFQIEGGGLNELNTLEICDSEELECLIVTSSHSSEVVTLLSNLHTLKINCMANLKAIWHCFLPVNGPFKNLEKLDLSVCPR
LTSLFTYVVARSLVQLKILKISRCDELKHIVADDDKTGKGQDEFTTGHPVQIFQNLQEVEVYSCPELKHIFPANIVGGLTQL
KVLEIKECDMLDQIISDIVPLTDQDRKEELDEILEQGTLSNLANLKITCCGNLDSIFTASIAKTLTSLEELYIHGCKSLKDIVTH
KRVNKNQEEIIVEDEHDCQSDISIFQSLKKLHISECDLLEGIFPVSFVGELNDITNKEDADLKDFSSRNNTQIELPALQVLEL
DHIRDRIIVGSYHVICPSLRTLSLNIGRYVGFFNINCSTDASEATKRDFIAIKISNSDVVSPVESVEECLSKQPHGLNLIMIHNI
REIKLKGFDKARYLFKLSIASSLMLEILRISECDGLEYIIDTDDEYGTENMKAIFPNLKELSVYKCLRLKYMFGQYHEANKDY
KEIHIQLSALEILSLNDLPNFVSICSTYTLTVTWPSLKNFHCNSCFYPFYGSVNCLTIITIYIFFLNGHEMTEQQVSLRLRYLSL
EYLPQMTYIWVGPKNSFTLQHLTTLRIRGCGMLEVIFPSCVLRSLPELKSLTISECMELKQIVGCEKEESKNSFTFPNLKRLE
IFGCPKLEVIFSKYVLRCLPELNLLQIRKCEKLKQIIEEDEKLSNHISPQPCFPKLEALYVGHCHKMKRLFSGSASNDLPNLHL
LIINGAFELEDLVGCEQGNCDEIGKPKAELPKLKLLIFMHLSNFHLDTHLSNLKNCVVYNCPKLSFTSTTTLGKLIENFPYEE
DFKNTVVERWEFEGIIRLLNEDSTMSGSSEFRSSQETGDIGNESIKAAAEGAKTKSLSNGVEDISIEGGAATHIESSGMDI
PALDSNLVEQDDKMNEGKPGIVLSKGIQIEEGLNLLDKQEGIDTVSNNNSDISPDIRIRLGEYKHFVELDDGQISLLVEAIT
TYPHLWNACERFSERFQAWRLKILADMLLLLQKESGDSMIPQREKEFHKLCEEAIEVGFESSWVEEMRQRVVARDHKL
GEDIANRQIDENSKRYDYLNS

>XP_047173008.1

MRALKLSYSNLELSLRRCFSFCAIFPKDFEIDKEELIHLWMANGFIESERNIEVEDVGNKVWNKLYSRSFFQEAKCDEFG
MVKSFKMHDLFHDLAQSIMGEECAAFVEGRLTPLSSRVHYSTLLSSDVSFGRFKHAFKKVESLRTFLDLRTSFVLGNVAP
VPSNHSLRALCTRSSLLSPLKDLTHLRYLSLNCGYKANLNNVICQMRKLQILKLKRSTYLHGLPKNLTQLQDLRHIVIDYCN
SVVETLPKISKLRHLRTLSIFVVGSKPGCGLAELHGLNLGGRLRIRGLENVPNEWDAKQANLIGKKDLNILHLSWDGNAN
PKGSYVSVERVLEALEPPSTLKSFGMNGYKGRQLPSWMRSSLVLRDLVEVELSGCDNCEELPPLGKLAHLKRLKVSGMK
NVKWIDGETYDGVEEKAFPSLEELIVDNLPNLERLLRDEGVEMVPRLSQLRIDDVLNFKVPRLPCVETVHARGIEAVTSF
MEGVGESMACLKTLSIKFIKGVVVLPDEFRRLGALQELYIREWYDVEYFPEHVLEGLTSLRTLSVEYCEKLKSLSEGVRHLA
CLENLRIDNCPELVALPSNMSQLTALRYVSISYCCTLPDGLQGVPSLGSLDISCCKCTSLPDWLGDMTSLQKLSIVYCTELR
SVPSSLQHLTNLSHLRIDGCPHLKKRCKRETGEDWQYIKHIPKIQLYFWREPTFCDEFKSILFTSPRNVSLCL

>XP_047173009.1

MRALKLSYSNLELSLRRCFSFCAIFPKDFEIDKEELIHLWMANGFIESERNIEVEDVGNKVWNKLYSRSFFQEAKCDEFG
MVKSFKMHDLFHDLAQSIMGEECAAFVEGRLTPLSSRVHYSTLLSSDVSFGRFKHAFKKVESLRTFLDLRTSFVLGNVAP
VPSNHSLRALCTRSSLLSPLKDLTHLRYLSLNCGYKANLNNVICQMRKLQILKLKRSTYLHGLPKNLTQLQDLRHIVIDYCN
SVVETLPKISKLRHLRTLSIFVVGSKPGCGLAELHGLNLGGRLRIRGLENVPNEWDAKQANLIGKKDLNILHLSWDGNAN
PKGSYVSVERVLEALEPPSTLKSFGMNGYKGRQLPSWMRSSLVLRDLVEVELSGCDNCEELPPLGKLAHLKRLKVSGMK
NVKWIDGETYDGVEEKAFPSLEELIVDNLPNLERLLRDEGVEMVPRLSQLRIDDVLNFKVPRLPCVETVHARGIEAVTSF
MEGVGESMACLKTLSIKFIKGVVVLPDEFRRLGALQELYIREWYDVEYFPEHVLEGLTSLRTLSVEYCEKLKSLSEGVRHLA
CLENLRIDNCPELVALPSNMSQLTALRYVSISYCCTLPDGLQGVPSLGSLDISCCKCTSLPDWLGDMTSLQKLSIVYCTELR
SVPSSLQHLTNLSHLRIDGCPHLKKRCKRETGEDWQYIKHIPKIQLYFWREPTFCDEFKSILFTSPRNVSLCL

>XP_047173460.1

MLRGESSSSSSDESSRWSYHVYLSFGMEGTHVDFANTLCVSLQRKGILTFRYDKLVERDVMLKKVQKAMEEYLVAIVLL
SENFAASTWCLEELRKILDVGKPVIPVFYEVVPSDVRHQRNSFAKAFEEHERRLEEDQLKVQQWRKSLKEVVDFSGWES
KDRGREELIEDIIKSVWTKIRLKLPSYEEERVGIDSRVEKIRSLLKLELKDVVHFTGIWGMGGIGKTTLARVVFKKICSQFDIS
CFLENVREISGKTHDMLTLQITLLSHMEVKEFTIQNLDEGKIVIGGILRNNKVLLVLDDVDDARQLDSLGVNDQGKFGPG
SRIIVTTRDMEVLKPLGNFEICKIDLLNSDESLQLFCHKAFKRDKPPEQLLQLSKVAVQQAGGLPLALEMMGSSFFGRNE
SQWKELLDMKEYSKKDIVMEKLTISYHGLPETNGIDVLIGKSLKKESIQGIVLKSSTEPYIANWDPEAFSKMYNLKFLIINF
HNIQFPHGLKCLSSSLEVLQWTKCTLEALPLGVKLEKLVVLKMRYSKIKKIWSDSQHFKRLKFIDLSHSEDLIESPIVSEVPC
LEILLLEGCKNLVKKSENLICLPKSVCYVSGTKVRLENLKELSFGGGKELTSNSSICRKKLVSKELILPPLSSLTSLKFLNLSYCD
LNDESMPGELGSLSSLLGLDLSGNNFVRPTYCIRNLHALKSLTLIDCPRLESLPMLPPNVQCLCTANSTQTKPLNSDAYML
WKIFELHMNQGQILELFEHKLNCFCPRVHVKASMLSGESSSNSSRWTYHDFLSFRGQDTRLGFTDHLYAALVRKGMITF
RDDKNLEKGDKIDKELFKAIKESLGAIVILSENYASSSWCLDXLNKILESNKALGRKVFPVFCGVSPSEVQHQTTKSFEEAF
QKHERRFEKDTEKVQQWRDSLKEVSQIAGWESENYQHQTELIENIVESVWTKLRPEMPSFNDGLVGIVSRVKKMDSLL
RIESKDDARFIGIWAXGGIGKTTLARVVFGKIQHHFDISCFLDNVREISKESGGRLRLQGKLLSHLGIKGLEIRDLDEGKNTI
RELLFKKKVLLVIDDVDDTSQLESLAERLEWFGPGSRVIITTRDTHVLISHGIVENYKIDILNSDESLQLLSQKAFKRDKPDE
HYLELSKVLAKYAGGLPLALELLGSFLCGRSESQWKVVDMIKEVPPSHIAMKSLRISYNGLPLRYKTLFLDIACFFKGRIKEL
VIQALEICERYPPVGIDLLVEKSLATYDGFTIGMHDLLQESAKGNCQRRKLCGSGKTK

>XP_047173576.1

MPVLETLGGALFGAVLQVLFDKLDSHQVLGFFRGRNLDEKLLKKLKRKLMDVNSVIDDAEQKQFTNSLVKEWLDEVRD
VLYDAEDLLEQIDYEFSKTKLEAEFQTSSSKVHNFESKIIALLDDLESLLNQKIVKDYKIYSGVRSGLGNKVAEKKVESTSLVA
EEVIYGRNEDKEIIFTWLRSDTNDNKLSILSIVGMGGMGKTTLAQHVYNDPKTEEAKFEEKAWVCVSDAFDALRVSKAII
GSFTNSRDDSGDLEMVHGKLKTRLFGKKFLLVLDDVWNEDRNQWKALQTPLTFGAKGSKILVTTRSHKVASIMQSTYIL
QLKQLDEDHSWQTFAKHAFQDENSKLNSELKEIGMKIVEKCQGLPLALETIGCVLQSKSSVSEWEGVLTSEIWDLTIEDS
KIVPTLLLSYYHLPSHLKRCFAYCALFPKDHRFDKESLILLWMAQNFLHCSQQSKSPEEVGEDYFNDLVSRSFFKQITWDK
EIYFVMHDLLNDLAKYVSGEICYRLGVDGEKGVSRKTRHISYVSDPIQDYTSLRDATRLRTFITFGWRRELPIEELIPNFKFI
RVLSLRLCFEVPDTIGDLIHLRSLDFSGSAIRRLPDSICSLYNLQELKLNNCFKLKELPLTLHELTNLRRLELMGTTLTKGPIGL
GKLKNLHVWINNFAVGKSSEFSIQQLGELHFHGEQLSIQNLENISNPYETNLKNETHIVTLSLQWNLARNNDDSVKERE
VLENLQPSIHLKHLSIDGYGGTQFPRWLSNNSLSNVVSLTLNNCKHCLWLPCLGLLTFLKNLRIDGLDWIGRIDADFYGD
SSSAFASLKRLSFIDMKEWEEWQCVTGAFPSLKSLFLANCPKLKELPNILCHLKKFIIHECGQLGAPITRAVEIQCVNTRPN
IFDTLEYLPFSFGPGISIPISNWYHSLVELRIRDDCDSLTTFPLDLFPKLRDLCLSGCRNLQMISQGHPHNRLENLFIDQCSE
FESFPNEGLFAPQLVSLIIAGLEKLKSMPKCMSALLPSLNNLYISNCPALELSEECLPSNVKEMFVENCSKVLASLKGAWG
TNPSLIVLRIGKVDLECFPGEGLLPLSLGRLEIYDCPNLKKLDYRGLCHLSSLRILILRNCPVLQCLPEEGLPKSISDLAICDCLL
LKQRCQKQESEDWEKIAHIKSISIDDY

>XP_047173583.1

MALRIAPSLSSSRPTWIYDVFLSFRGEDTRFHFTDNLYHSLCQKGIRTFIDQEGLRKGEEITPALFHAIQNSRISIIVFSENYA
SSTYCLNELVKILECAKKEGRSIYPIFYGVDPSEIRHQTGICAEALSKHETKFYNDADNEKGQKWRKALHEAANLSGWHF
QHGSQPEYEFISKIVEEISEKVNCIPLHVADNAIGLEYAVQGVKSLLGDGSDVNMIGIYGIGDIGKTTIARAVYNTIFWHY
QGSCFLSDIREKAINKHGIVQLQEILLSETFKEGIKVGDVNRAIPILKGRLQQKKVLLVLDDVDELEQLKALAGGYDWFGS
GSIIIITTRNKHLLDAHGVVNLYEVKPLNVEKAFELFNWHAFKNGKVDPSYMNISKRAVSYACGLPLALEVIGSHLFGKNI
NECSSALDKYESIPHEKIHEILKVSYDGLEENEKGIFLDIACFFNTWELGNVRPMLKAHGFHVEDGLRVLVDRSLIKIDYSD
FVRIHDLIRDTGREIVRQESTIEPGRRSRLWFNEDIVHVLEENTGSDKIEFIKLEGYNNVQVQWDGKAFKEMKNLRILIIEG
SRFSTGLKHLPNSLRVLDWSCYPSPSLPSDFNPKRFEILLLPESCLQMLKPQNMLESLSVISLQDCKFLTDLPSLRDAPFLTT
LRLDNCCNLVNIDESIGFLDKLRFLSASYCTKLXCIMLTSLETLDLGRCFSLESFPEVLGKMEKIRTIYLDCTGIDKLPLSIGNF

VWLELLSLTECERLHQLPGSISIMPKVKVLVGYGHGAYQIFQEQLSSEVSPRAMLIGDSDLYLDVYYSYISPNNSIQVCSPD
PLFHSDFNLLFPKLGREEDWLCFRRESSMHFSFRNKFPKIALCCSILLPLLKIIMVMNLKFRVFINDTLQFSALCNFILREW
DTILWCDLEGKVEGVFSDEEWNKAEIVFELDFPMRRNSRNGNTRRSIGIGSLGWSLMGVYEEGNNKEDIEFKDPVSVF
PLCNRQPPSSLPTSLYYVVSRGKP

>XP_047174271.1

MSVTKLNEEKFDIISYSLPTPKLGSTFSNVIKSFPSRKSIITEVLETLKDEDFKILSICGMGGVGKTTLVKEVIKILEVCKLFDEV
VMVVVSQNLDYVKIQGQIADALGLRFEKETIQGKACQLHERLKGVDNILIVLDDVWVYFDFESIGIPSNEHHKNCKILFTS
RNEDVCYKMGSQKNFTVSILSPNESWDLFHDIVCRNLSTKLDILHIAKEVSNECGGLPIAIVTMAKALANKEKHVWEDAL
DQLKRSSIPFLLEMQACVYSSIKLSYNFLDSAEEKYVLFLCCLFPEDFDIPIEVLLWHGMGLQLFININALWKVRNRVHTIV
DKLKRRFMLLDGNVEECVKMHDVIRDVIISVVSTEEYDFMIQCDGYQMEQSKRETCYHSSAISLISKEAKEHPKVLNCPK
LKLLQIASKKKGLVPDNFFQCLNKLMVLSLQNVHIHSTALVFEALDNIHTLLLEDCLVSDISIIGKKLKMLEILSFSNSNIKEL
PEEIGQLSSLRLLDLTECNDLIQISTNVFASLSKL

>XP_047174804.1

MDIQEESPVFGSLTAMTTRNMSSSSSVFFSANQSPFFSPRSPSSCQLSHSARLDTQSNTVHLGLTPSSTTLEIPEPNSTVN
VRCNVSDVSASPAGCNSGGLMKLDRKSSPVGISSSSISSYSNCHDDGYSGQRERRIKKDRNHRTSSTPGSTSFSSYRLRSC
DVFIGLHGSKPPLLRFAKWLCGELEIQGISCFVSDRARSRSSRKLGIAERAMDAASFGIVIITKKSFKNQYTIEELNFFYRRK
NLIPIYFDLSPADCLVRDIIEKRGELWEKHGGELWLSYEGLEQEWKDAVHGLSRVDECKLEAQDGNWRDCILRAVTLIA
MRLGRRSVAERVTKWREKVGKEEFPFIRNDNFIGRKKELSQLEFILFGDVTGDAEQHYIELKARPRRKSVRIGWGKSNMI
DERWNDRRKEKEPVVWKESEKDIEMQGVEFSHRHNHPRLKRGKYTKRKNGMKILYGKGIACVSGDSGIGKTELILEFAY
RFHQRYKMVLWIGGESRYIRQNYLNIRSFLEVDVGVENSLDKTKIRSFEEQEVAAISRVRKELMKNIPYLVIIDNLESEKD
WWDHKLVMDLLPRFGVETHVIVSTRLPRIMNLEPLKLSYLSGVEAMSLMVGSSKDYSVAEVDALRSIEEKVGRLTLGLAI
ISAILSELPITPSRLLDTINRMPLKEMPWSDKEALSFTKNAFLLQLFDVCFSIFDHADGPRSLATRMVLVSGWFAPGAIPIS
LLALAAEKVPERCQGTCFWRKMLQLLSCGFPSSYAKKPELEASSLLLRFNIARNSTKQGYIHINEVFKLYARKRENTGAAQ
AMIQAIISNGSISQNLDHLWAACFLLFGFGHDPVIVELKVSELLYFVKRVVLPLAIHTFITYSRCTAALELLRLCTNALEAAD
QAFVTPVDKWFDKSLCWRSIQTNAQLNPCLWQELALTRATVLETRAKLMLRGAQFDVGDDLIRKAVFIRTSICGEDHP
DTVSARETLSKLTRLNANVQIHAST

>XP_047174805.1

MDIQEESPVFGSLTAMTTRNMSSSSSVFFSANQSPFFSPRSPSSCQLSHSARLDTQSNTVHLGLTPSSTTLEIPEPNSTVN
VRCNVSDVSASPAGCNSGGLMKLDRKSSPVGISSSSISSYSNCHDDGYSGQRERRIKKDRNHRTSSTPGSTSFSSYRLRSC
DVFIGLHGSKPPLLRFAKWLCGELEIQGISCFVSDRARSRSSRKLGIAERAMDAASFGIVIITKKSFKNQYTIEELNFFYRRK
NLIPIYFDLSPADCLVRDIIEKRGELWEKHGGELWLSYEGLEQEWKDAVHGLSRVDECKLEAQDGNWRDCILRAVTLIA
MRLGRRSVAERVTKWREKVGKEEFPFIRNDNFIGRKKELSQLEFILFGDVTGDAEQHYIELKARPRRKSVRIGWGKSNMI
DERWNDRRKEKEPVVWKESEKDIEMQGVEFSHRHNHPRLKRGKYTKRKNGMKILYGKGIACVSGDSGIGKTELILEFAY
RFHQRYKMVLWIGGESRYIRQNYLNIRSFLEVDVGVENSLDKTKIRSFEEQEVAAISRVRKELMKNIPYLVIIDNLESEKD
WWDHKLVMDLLPRFGVETHVIVSTRLPRIMNLEPLKLSYLSGVEAMSLMVGSSKDYSVAEVDALRSIEEKVGRLTLGLAI
ISAILSELPITPSRLLDTINRMPLKEMPWSDKEALSFTKNAFLLQLFDVCFSIFDHADGPRSLATRMVLVSGWFAPGAIPIS
LLALAAEKVPERCQGTCFWRKMLQLLSCGFPSSYAKKPELEASSLLLRFNIARNSTKQGYIHINEVFKLYARKRENTGAAQ
AMIQAIISNGSISQNLDHLWAACFLLFGFGHDPVIVELKVSELLYFVKRVVLPLAIHTFITYSRCTAALELLRLCTNALEAAD
QAFVTPVDKWFDKSLCWRSIQTNAQLNPCLWQELALTRATVLETRAKLMLRGAQFDVGDDLIRKAVFIRTSICGEDHP
DTVSARETLSKLTRLNANVQIHAST

>XP_047174806.1

MDIQEESPVFGSLTAMTTRNMSSSSSVFFSANQSPFFSPRSPSSCQLSHSARLDTQSNTVHLGLTPSSTTLEIPEPNSTVN
VRCNVSDVSASPAGCNSGGLMKLDRKSSPVGISSSSISSYSNCHDDGYSGQRERRIKKDRNHRTSSTPGSTSFSSYRLRSC
DVFIGLHGSKPPLLRFAKWLCGELEIQGISCFVSDRARSRSSRKLGIAERAMDAASFGIVIITKKSFKNQYTIEELNFFYRRK
NLIPIYFDLSPADCLVRDIIEKRGELWEKHGGELWLSYEGLEQEWKDAVHGLSRVDECKLEAQDGNWRDCILRAVTLIA
MRLGRRSVAERVTKWREKVGKEEFPFIRNDNFIGRKKELSQLEFILFGDVTGDAEQHYIELKARPRRKSVRIGWGKSNMI
DERWNDRRKEKEPVVWKESEKDIEMQGVEFSHRHNHPRLKRGKYTKRKNGMKILYGKGIACVSGDSGIGKTELILEFAY
RFHQRYKMVLWIGGESRYIRQNYLNIRSFLEVDVGVENSLDKTKIRSFEEQEVAAISRVRKELMKNIPYLVIIDNLESEKD
WWDHKLVMDLLPRFGVETHVIVSTRLPRIMNLEPLKLSYLSGVEAMSLMVGSSKDYSVAEVDALRSIEEKVGRLTLGLAI
ISAILSELPITPSRLLDTINRMPLKEMPWSDKEALSFTKNAFLLQLFDVCFSIFDHADGPRSLATRMVLVSGWFAPGAIPIS
LLALAAEKVPERCQGTCFWRKMLQLLSCGFPSSYAKKPELEASSLLLRFNIARNSTKQGYIHINEVFKLYARKRENTGAAQ
AMIQAIISNGSISQNLDHLWAACFLLFGFGHDPVIVELKVSELLYFVKRVVLPLAIHTFITYSRCTAALELLRLCTNALEAAD
QAFVTPVDKWFDKSLCWRSIQTNAQLNPCLWQELALTRATVLETRAKLMLRGAQFDVGDDLIRKAVFIRTSICGEDHP
DTVSARETLSKLTRLNANVQIHAST

>XP_047174807.1

MDIQEESPVFGSLTAMTTRNMSSSSSVFFSANQSPFFSPRSPSSCQLSHSARLDTQSNTVHLGLTPSSTTLEIPEPNSTVN
VRCNVSDVSASPAGCNSGGLMKLDRKSSPVGISSSSISSYSNCHDDGYSGQRERRIKKDRNHRTSSTPGSTSFSSYRLRSC
DVFIGLHGSKPPLLRFAKWLCGELEIQGISCFVSDRARSRSSRKLGIAERAMDAASFGIVIITKKSFKNQYTIEELNFFYRRK
NLIPIYFDLSPADCLVRDIIEKRGELWEKHGGELWLSYEGLEQEWKDAVHGLSRVDECKLEAQDGNWRDCILRAVTLIA
MRLGRRSVAERVTKWREKVGKEEFPFIRNDNFIGRKKELSQLEFILFGDVTGDAEQHYIELKARPRRKSVRIGWGKSNMI
DERWNDRRKEKEPVVWKESEKDIEMQGVEFSHRHNHPRLKRGKYTKRKNGMKILYGKGIACVSGDSGIGKTELILEFAY
RFHQRYKMVLWIGGESRYIRQNYLNIRSFLEVDVGVENSLDKTKIRSFEEQEVAAISRVRKELMKNIPYLVIIDNLESEKD
WWDHKLVMDLLPRFGVETHVIVSTRLPRIMNLEPLKLSYLSGVEAMSLMVGSSKDYSVAEVDALRSIEEKVGRLTLGLAI
ISAILSELPITPSRLLDTINRMPLKEMPWSDKEALSFTKNAFLLQLFDVCFSIFDHADGPRSLATRMVLVSGWFAPGAIPIS
LLALAAEKVPERCQGTCFWRKMLQLLSCGFPSSYAKKPELEASSLLLRFNIARNSTKQGYIHINEVFKLYARKRENTGAAQ
AMIQAIISNGSISQNLDHLWAACFLLFGFGHDPVIVELKVSELLYFVKRVVLPLAIHTFITYSRCTAALELLRLCTNALEAAD
QAFVTPVDKWFDKSLCWRSIQTNAQLNPCLWQELALTRATVLETRAKLMLRGAQFDVGDDLIRKAVFIRTSICGEDHP
DTVSARETLSKLTRLNANVQIHAST

>XP_047174808.1

MDIQEESPVFGSLTAMTTRNMSSSSSVFFSANQSPFFSPRSPSSCQLSHSARLDTQSNTVHLGLTPSSTTLEIPEPNSTVN
VRCNVSDVSASPAGCNSGGLMKLDRKSSPVGISSSSISSYSNCHDDGYSGQRERRIKKDRNHRTSSTPGSTSFSSYRLRSC
DVFIGLHGSKPPLLRFAKWLCGELEIQGISCFVSDRARSRSSRKLGIAERAMDAASFGIVIITKKSFKNQYTIEELNFFYRRK
NLIPIYFDLSPADCLVRDIIEKRGELWEKHGGELWLSYEGLEQEWKDAVHGLSRVDECKLEAQDGNWRDCILRAVTLIA
MRLGRRSVAERVTKWREKVGKEEFPFIRNDNFIGRKKELSQLEFILFGDVTGDAEQHYIELKARPRRKSVRIGWGKSNMI
DERWNDRRKEKEPVVWKESEKDIEMQGVEFSHRHNHPRLKRGKYTKRKNGMKILYGKGIACVSGDSGIGKTELILEFAY
RFHQRYKMVLWIGGESRYIRQNYLNIRSFLEVDVGVENSLDKTKIRSFEEQEVAAISRVRKELMKNIPYLVIIDNLESEKD
WWDHKLVMDLLPRFGVETHVIVSTRLPRIMNLEPLKLSYLSGVEAMSLMVGSSKDYSVAEVDALRSIEEKVGRLTLGLAI
ISAILSELPITPSRLLDTINRMPLKEMPWSDKEALSFTKNAFLLQLFDVCFSIFDHADGPRSLATRMVLVSGWFAPGAIPIS
LLALAAEKVPERCQGTCFWRKMLQLLSCGFPSSYAKKPELEASSLLLRFNIARNSTKQGYIHINEVFKLYARKRENTGAAQ
AMIQAIISNGSISQNLDHLWAACFLLFGFGHDPVIVELKVSELLYFVKRVVLPLAIHTFITYSRCTAALELLRLCTNALEAAD
QAFVTPVDKWFDKSLCWRSIQTNAQLNPCLWQELALTRATVLETRAKLMLRGAQFDVGDDLIRKAVFIRTSICGEDHP
DTVSARETLSKLTRLNANVQIHAST

>XP_047174909.1

MDVEEEDVVGLVNESDIVIQQLQKDDVRLNFASIVGMGGLGKTTLARKIYNKDNVKRIFPCRAWGNVSNDYRPKELFQ
SLLRSLNLSGFENLSEEDLKKEVVKGLKGKTYLIVLDDIWETRVWDDIKGAFPDDNIGSRVY

>XP_047175007.1

MLKPKSGLQRFWDRFGTRSSGSTSSSYDSSDTIQNQHYRYDVFISFRGPDARNSFVDHLCSHLLRKGIFVFKDDHDLQK
GESISPQLLQAIQLSRLSIIVFSKNYASSSWCLDEMSAIASCKQQSSQIVFPIFYDVDPSHVRHQNGVYKNNFFSHRRKFG
KDRDKVLRWKRAMTDLANSAGWDMRDKPEFAQIQSIVQAVINKLGHKFSRSVNDLIGIQPRVQALEDKLRLSSNSDD
VRVLGIWGMNGIGKTTHAAVLYDKISHRFDASCFIEDVNKLYRDGGYTAVQKQIIHQTFRENIDMSNPIEISGIVENRLH
SIKVLIVLDNVDELEQLENLAIKPRLLLKGSRMVITTTDEHILKVYEGDVLRHKLPLLNDADARELFCRNAFKCEDQSSNCA
ALIPEVLKYAQCLPLAIKVLGSFLCTRDADDWRDVLNRLENSPDDKIMNVLQISVDGLQREEKQIFLHIACFFKGERVDYV
KRILKCCGLHPDIGISRLTEKSLITISDEEIHMHELLQELGKKMVRDQSPEEPGSWSRIWLHKDFLHALTAETGTEKVKAIV
LNKKEEMSECIVDGLSRMKELTLLILYHTRVSGRLEVLSDRLQYLLWHDYPFASLPPYFTAFNLVELNMPNSHITHLWAG
GKSCPNLKRIDLSNSKYLSETPDFSRIIKLERLDLSGCSSLSYVHSSIGLLKKLAFLNLRNCYNLVCIDFGGVWNMSSLRVLH
LSGCSKLESTPDFTRATDLEYLDMDECTSLSTIHQSIGVLSNLTFLSLRRCRKLVSIPNDINSLVSLQTLDLSGCYNSMNPM
PRQALSSHLKSLIFLDISNSNLKEVPDAIGDLRCLERLNLQGNNFLSIPDSFRQLHCLAYLNLSHCHKLIYLPDLPTEGDKSG
GKYFKTVSGSRDHRSGFYLVNCTNIKLSRLEFLGLERFFHLIKEPCNFRCGFDLILPWDMGFRPVFGETFLGNSIIRILQCV
MNDNWIGFGFYVTFSRGIDFSSSHCSLSHPLYLSFESEYTEEYFDMRFNSERDECFMSRHIWIIYISREHCHFVKTGAHITF
KAQPYLKIEEWGMRPILKQDISDSKGKKYIKFSKLNHDHVDFEYVEKSNSGSGPKIQLPYNWCVTEEEQVENTDAKAKE
NNLSNAGL

>XP_047175008.1

MLKPKSGLQRFWDRFGTRSSGSTSSSYDSSDTIQNQHYRYDVFISFRGPDARNSFVDHLCSHLLRKGIFVFKDDHDLQK
GESISPQLLQAIQLSRLSIIVFSKNYASSSWCLDEMSAIASCKQQSSQIVFPIFYDVDPSHVRHQNGVYKNNFFSHRRKFG
KDRDKVLRWKRAMTDLANSAGWDMRDKPEFAQIQSIVQAVINKLGHKFSRSVNDLIGIQPRVQALEDKLRLSSNSDD
VRVLGIWGMNGIGKTTHAAVLYDKISHRFDASCFIEDVNKLYRDGGYTAVQKQIIHQTFRENIDMSNPIEISGIVENRLH
SIKVLIVLDNVDELEQLENLAIKPRLLLKGSRMVITTTDEHILKVYEGDVLRHKLPLLNDADARELFCRNAFKCEDQSSNCA
ALIPEVLKYAQCLPLAIKVLGSFLCTRDADDWRDVLNRLENSPDDKIMNVLQISVDGLQREEKQIFLHIACFFKGERVDYV
KRILKCCGLHPDIGISRLTEKSLITISDEEIHMHELLQELGKKMVRDQSPEEPGSWSRIWLHKDFLHALTAETGTEKVKAIV
LNKKEEMSECIVDGLSRMKELTLLILYHTRVSGRLEVLSDRLQYLLWHDYPFASLPPYFTAFNLVELNMPNSHITHLWAG
GKSCPNLKRIDLSNSKYLSETPDFSRIIKLERLDLSGCSSLSYVHSSIGLLKKLAFLNLRNCYNLVCIDFGGVWNMSSLRVLH
LSGCSKLESTPDFTRATDLEYLDMDECTSLSTIHQSIGVLSNLTFLSLRRCRKLVSIPNDINSLVSLQTLDLSGCYNSMNPM
PRQALSSHLKSLIFLDISNSNLKEVPDAIGDLRCLERLNLQGNNFLSIPDSFRQLHCLAYLNLSHCHKLIYLPDLPTEGDKSG
GKYFKTVSGSRDHRSGFYLVNCTNIKLSRLEFLGLERFFHLIKEPCNFRCGFDLILPWDMGFRPVFGETFLGNSIIRILQCV
MNDNWIGFGFYVTFSRGIDFSSSHCSLSHPLYLSFESEYTEEYFDMRFNSERDECFMSRHIWIIYISREHCHFVKTGAHITF
KAQPYLKIEEWGMRPILKQDISDSKGKKYIKFSKLNHDHVDFEYVEKSNSGSGPKIQLPYNWCVTEEEQVENTDAKAKE
NNLSNAGL

>XP_047175009.1

MVETKPGLKRFWDRFGGRSSGSTSSSSYDSSDSIQNQNYIYDVFISFRGPDTRNSFVDHLCSHLLRKGIFVFKDDHNLQK
GESISPQLLQAIQLSRLSIIVFSKNYASSSWCLDEMSAIASCKQQSSQIVFPIFYDVDPSHVRHQNGVYENDFVSHRCKFQ
KDRDKVPGWERAMTDLANSAGWDVRDKPEFEQIQNIVQAVIKKLGHKFSWFVNDLIGIQPRVQALEDKLRLNSNSDD
VQVLGIWGMNGIGKTTQAAVLFDKISHRFDASCFIEDVSKLYRDGGNTAVQKQIIHQTFGQKGLDMCSPFQISGIVRTR

IHDIRVLIVLDNVDELEQLENLAIKPKLLLKGSRMVITTTDMHILKVYEGGVIHKVPLLNDNDARELFCRKAFKSEEQSSSC
EALIPEVLKYAQCLPLAIRVLGSFLCTRDAVEWRDVLNRLQSSPDKKIMNVLQISVDGLNHEEKQIFLHIACFFKRERVDY
VKRILDCCELYPHIGISRLVEKSLITISNEEIHMHELLQELGKKMVWDQSPQEPRFWSRIWLHKDFLQVLTAETGTEKVKA
IVLNKEEEMSECSIGGLSRMKELTLLILYHTKVSGSLEFLSDRLRYLFWHDYPFDSLPPYFTVSNLVELNMPNSHIISLWHG
NKVIYSHSFHFRLGLNKSPKDRISFLVI

>XP_047175011.1

MVETKPGLKRFWDRFGGRSSGSTSSSSYDSSDSIQNQNYIYDVFISFRGPDTRNSFVDHLCSHLLRKGIFVFKDDHNLQK
GESISPQLLQAIQLSRLSIIVFSKNYASSSWCLDEMSAIASCKQQSSQIVFPIFYDVDPSHVRHQNGVYENDFVSHRCKFQ
KDRDKVPGWERAMTDLANSAGWDVRDKPEFEQIQNIVQAVIKKLGHKFSWFVNDLIGIQPRVQALEDKLRLNSNSDD
VQVLGIWGMNGIGKTTQAAVLFDKISHRFDASCFIEDVSKLYRDGGNTAVQKQIIHQTFGQKGLDMCSPFQISGIVRTR
IHDIRVLIVLDNVDELEQLENLAIKPKLLLKGSRMVITTTDMHILKVYEGGVIHKVPLLNDNDARELFCRKAFKSEEQSSSC
EALIPEVLKYAQCLPLAIRVLGSFLCTRDAVEWRDVLNRLQSSPDKKIMNVLQISVDGLNHEEKQIFLHIACFFKRERVDY
VKRILDCCELYPHIGISRLVEKSLITISNEEIHMHELLQELGKKMVWDQSPQEPRFWSRIWLHKDFLQVLTAETGTEKVKA
IVLNKEEEMSECSIGGLSRMKELTLLILYHTKVSGSLEFLSDRLRYLFWHDYPFDSLPPYFTVSNLVELNMPNSHIISLWHG
NKVIYSHSFHFRLGLNKSPKDRISFLVI

>XP_047175014.1

MLPVELGELSMRRQLQDPLLNNEELRVELNTVDERRDRAFLRAKAYKRMVERKYNTKVQPQSFKEGDLVWRKTGGAG
RAATHGKLAAKWEGPFRVTENLQNGAYTVFFYAQLLLRGATLSSNSHYHPPIPKKKSGRRWFWSHFCGRSSSSDVISSV
STSSTHHSSDTVQNQDYRYDVFISFRGPDNRNSFVDHLYYHLLRKGIFVFKDDRKLRKGESISSQLPQAIRGSRISIIVFSXD
YPSSSWCLDEMATIADCKQQSNQTVFPVFYDVDPSHVRHQNGVYEKAFVSLRQKFKGKPDKVYRWERAMNWFGTL
AGWDVRNRSESEVIEDIVQTVIKNLGHKFSWFVDDLIGIQPRVQALEDKLRLSSKSDVVQVLGIWGMNGIGKTTHAAV
LYDKISHRFDASCFIEDVSKLYRDGGHTAVHKDIINQTLRENIDMSSPIEISGIVKNRLCNIKLLIVLDNINELEQLENLAVKP
KLLLKGSRMVITTTDEHILKVYEGYVLIHKVPLLNDKDAGELFYRRAFKSEEQNSNCAAMIPEVLKYAQCLPLAIRVLGSFL
CTRDADEWRDVLNRLENGPDDKIMNVLQISVDGLQREEKQIFLHIACFFKGERVDYVKRILDGCGLHPRIGISRLIEKSLIT
ISNEEILMHELLRKLGKKMVRDESPEEPGSWSRIWLHQDFFQALTRETGTERVRAIVLNKKEELSECNVNGLSRMKELTL
LILYHTKLSGRLEFLSDRLQYVLWHDYPFASLPPYFTVSNLVELNMPNSHITRLWEGHKSCPNLKRIDLSNSSYLIETPDFS
RIIKLERLDLSGCTSLSHVHSSIGLLKKLAFLSLRNCCNLFIIDFGSVGNMSSLKVLHLSGCSRLESSQDFTRTTHLEYLDMDE
CTSLSTIHESIGVLSSLTFLGLRGCTKLVSIPNDINSLVSLQTLYLCRCFKLTNLLPRQASKSHLECLIFLDLSFCNLLEVPDAIG
ELRCLERLNLQGNNFVSIPDSFRVLHCLAYINLSHCHELKSLSNLPFEGAASGGKYFRAVSGSRDHRSGLYLFNCIKMVDIL
SKPWDCWSLELAWLFRLIKESCHFRCGFDIGVPWGLEIPRWFTKRFEGDSVIRIEEFNVDEDWMGFAFCVIFEGNIAPV
VGDSSSHPLYLSFEVNIQKNTVICHII

>XP_047175362.1

MIYRPIQTTPRYPCISLPHISLKNIMAESLLFTFAESLIGKLASGAVHEASLALGVQADLQQMKKSMSLIKAFLLDAEQKKP
RSNSLSEWLIQIKQVFSDAEDIVDDFQCEALRKHVVNTHGGLTRQVRRFFSISNPIIYRIRIAHEIKDIKERLQKVAADGNT
FGLQIIDQDTRVVNVTDTTYSHVNPSNVIGRQDEKQEILKLLLQHDHDGHDKSLSVVSILGFGGMGKTTLAKLVFNDTTI
DECFPLKMWVCVSGDFELRNVLIKILNSALNPTNENFKNFETEQLQNRLRNTLQRQKFLLVLDDVWNEYQARWDDLK
EILDVGVEGCKILVTTRSHLTATMMCTKSSNSYLLERLSEDDSLFLFVKTAFKEGKEERYPELLEIGKEIVIKCGGIPLAVKTL
ASSLFSVVGKSKWEAMRDDKIWNLPQKEKDILPALEISYNQLPSHLKPCFVCFSLFCEGSEFFSFYVAKLWEALGFLPPPK
ENETMHEVAIQFLRELWSRSFLTDFIELSHGYSFKLHDLVHDLAMYAAKGEFQTIYPRSSKISPNARHLAFSDNNLLDQA
VIPTGLRTIIFPDEATNEAFFNTLVSRCKYLRFLELSNSIYESLPPSIGKLKHLRYLSLFGNKNLKGLPRSVCNLQNLETLNLN
QCTEIQQLPKGISKLISLRQLHITTRQLHFPDQEISTLTSLETLTFNSCDNLESLLKGIQLSSLKNLSLHDCGKLKSLSSHVISNL

ENLVIENCCELELSMGFGNQIPDLRLKSLAFKSLQQLVTLPQWLQGSVNKLHSLAIADCNNLKELPEWLSSAICLKLLVIEY
CSSLQSLPDNLKNLENLIINSCPELCKRYQPWVGQDYHKISHIQKVFVGELEE

>XP_047175430.1

MAAELVGGALLSAFLQVAFDRLASPQFVDFFRGRKLDDKLLRNLNIMLHSLNALADDAEQKQFRDPHVKSWLFSVKEA
VFDSEDLLDEIDYEITRCQVEAESEPQTIIYKVSNFFNATFRSFNRKIDSGLKEVLEKLEYLTRQKGALGLKEGTYSGDRSGS
AVSQKLPSTSLVAESVIYGRDADKEMIINWLTSETDICNQPSILSIVGMGGLGKTTLVQHVYNDPKVDDAKFDSKAWVC
VSDHFNALTVAKTILEAITDEKDESGNLEMVHKKLKEKLKGKKFLLVLDDIWNQRRDEWEAVQTPLSYGAQGSKILVTTR
DEKVASNMQSKVHRLKQLREDECWKVFEKHASKDYNLELNDELKEIGSRIVDKCKGLPLALKTIGCLLRTKSSISDWKSVL
VSDIWDLPNEDNEIIPALFLSYHHLPSHLKRCFAYCALFPKDYEFVKEELILLWMAESFLQCPQTRHPEEVGEQYFNDLLS
RSFFQQSTTEKRFVMHDLLNDLAKYVCGDICFRLKFDKGKYIPKTTRHFSFEFDHVKCFDGFGSLTDAKRLRSFLPITEIER
TYIGYYPWQFKISVHDLFSMFKFLRILSFYNCLGLTKLPDSIGDLKHLRSLDFSHTAIQKLPDSTCLLYNLLILKLNHCLRLEKL
PSNLHKLTKLRCLEFKNTKVTKMPMHFGELKNLQVLNMFFVDRNNEFSTKQLGRLRLHGRLSINEVQNITNPLDALEAN
LKNQHLVELELKWNSKHILNDPKKEKKILENLQPPKQLEGLGISNYGSTHFPSWLFNNSLTNLVFLRLEDCKYCIFLPPLGL
LSSLKTLEIVGLDGIVSIGDEFYGSNASSFISLERLELYDMKELREWKCKTTSFPRLQHLSMDHCPELKGLSEHLLHLKKLVI
GYCDKLIISRNNMDTSSLELLKICSCPLTNIPMTHYDFLEEMDIDGGCDFLTTFSLDFFPNLRSLQLTRCRNLQRFSHEHTH
NHLKYFIIEKCPLVESFFSEGLSAPLLQRIEIRGAENLKLLPKQMKIQLPSLIELLIIDCPKVETFPEGGLPSNVKHVSLSSLKLIA
SLRESLDANTCLESLSIGKLDVESFPDEVLLPHSLTSLQIFDCPNLKKMEYKGLRDLSSLTLLHCPGLQCLPDEGLSKAISSLTI
WDCPLLKQRCQNPEGVDWGKICHIEKLIIR

>XP_047175431.1

MAAELVGGALLSAFLQVAFDRLASPQFLDFFRGRKLDEKLLGNLNIMLHSINALAHDAELKQFTDPHVKAWLFSVKEAV
FDAEDLLGEIDYELTRSQVEAQSEPQTFTYKVSNFFNSTFNSFNKKIESEMKEVLQKLEYLAKQKGALGLKEGTYYGDRSA
GKVSQKLPSSSLVVESVIYGRDADKEKIFNWLTSETDTHNHPSILSIVGMGGLGKTSLAQHVYNDPKIEEAQFDIKAWVC
ISDHFDVLTVTKTILEAITKSKDDSGDLEMVHGRLKEKISGRKFLLVLDDVWNERQEEWEAVRTPLSYGAPGSRILVTTRI
EKVASNMRSEVHHLKQLEEDECWKVFEKQALKDDDLRLNGEKKEIGRRIVEKCKGLPLALKTIGSLLRTKSSSSYWKSVLE
SDIWQLPKEVEIIPALLLSYQHLPSHLKRCFAYCALFPKDYEFDKKELILLWMAEGFLHHSKQIKNVEEIGEQYFDDLLTRSF
FLQSSLEMRHKNVQEIGEQCFYDLLTRSFDLESSFEMRFVMHDLLNDLAKYVCADFCFRLKFDKGSCIPNTTRHFSFSLD
DVEYFDGLGSLTDAERLRSFHSITNNCGYEFNPCQFNILVHELFSKFKFLRVLSLNGYSELSEVHDSVGDLKHLHSIDLSYTR
IQNLPNSIGFLYNLLVLKLNFCSFLEELPSNLHKLTKLHCLEFEHTKVTKMPMHFGELKNLQVLSTFFVNRNNEVISIKQLG
GLNLHGRLSINELQNISNPLDALEANLKNKPLVQLKLIWNGNHIPDDPRKEKKVLENLQPSNQLEHFSIYNYGGTQFPG
WVFDNSLSNLVSLWLADCKYCLCLPPFGLLSSLRILQIIGFDGIVSIGAEFCGSTSSSFKSLKILEFYNMKEWEEWECKTTSF
PLLQHLSIHNCPKLKSLPEQLLHLKNLDINRCDKLVISVNSMFISSLQLWSIILCPLVEIPMTHYDCIEAMEINNDCVSSTIFP
LDCFPKLHLLQLSWCHNLRRVSQGHTHNHLKVLRISECSQFESFPSEGLSAPWLQIISIREAENLKLLPKHMQILLPSLTEL
EIIDCPKVEKFPEGGLPSNIKRMSLSSLKLIASLRDTLDVNTCLESLTIEKLEVESFPGEVLLPRSLTSLRFLFCLNLKKIDYRGL
CNVSSFTHYGCPNLHPRKFLIS

>XP_047175432.1

MAAELVGGALISAFLQVVFDRVCSPEVLDFFIGRKLDEKLLSNLNILLHSVNSLADDAEQKQFRDPHVKAWLLAVNEAVF
DAEDLLDEIDYELTKCKVEAESSPQNLTYKVSNFFYSTFRSFNKKVNSWMEDVITKLEYLAKKKDALGLQRYSYSEVPQKL
PSSSLVVESVIYGRDADKQIIFNWLTSKTDNHNHPSILSIVGMGGLGKTTLAQHVYNDPKIDDAKFDIKGWVCVSDHFD
VLTVTKTILESITGKKDDSGNLNMVHKNLKENLSGKKFLLVLDDVWNERREEWEAVQTPLSYGAPGSRILVTTRAEKVAS
NMRSKVHHLKQLEEDECWKVFEKQALKEDDLELNDEKKKIGRSIVEKCKGLPLALKTIGCLLRTKSSISYWKSVLESDIWN
LPKELEIIPALLLSYQHLPSHLKRCFAYCALFPKGYVFNKKELILLWMAEGFLRHSQHIENVEEIGEQYFDDLLTRSFFLQSSI

KMQFVMHDLLNDLAKYVCGDFCFRLKFDKGNYIPKTTRHFSFSFDDLEYFDGLGNLTNAKRLRSFLPITSNISFSGIPNQF
KILIHELFSKFKFLRVLSFNGCSELTEVPNTICNLKHLHSIDLSYTHIQKLPDSICLLYNLLILKLNYCVYMEELPSNLHKLTKLC
CLEFKRTNVTKMPMHFGELKNLHVLSTFCVERNGEVSIRELGGLNLHGRLSINEVQNIVNPLDALEANLKNQHLVELELK
WNSNRIPDDPRKEKEVLENLHPSNHLEHLSIRSYYGTQFPNWVFDNSLSNLVFLQLENCKYCLCLPPLGLLSSLKSLLIRGL
DGIVSIGDEFFGSNSSSFTSLERLEFYQMKEWEEWECKTTSFQRLQQLSTNQCPKLKGMPERLLHLKNLFIDSCDKLIISV
NTMDTKSLQILSIRSCPLVNIPVTHYNCLERMTIDDGCDSLTIFPLDFFPKLRLLRLRRCQNLRRISQEHAHDHLNELTITDC
PQFESFPTENLKLLPKRMKILLPSLTALQIIDCPQVEMFPRGGLPSNIKHVSLSSLKLITSLRETLDVNICLERLYIEKVDEDCF
PDEGLLPPSLASVAIYDCPNLKTLSYNGLCHLSSLTLHKCPSLQCLPKEGLPKSISALEIWNCPLLELRCKNPEGEDWGKIA
HIEKLMVW

>XP_047175433.1

MAAELVGGALLSAFLQVAFDRLASPQFIDFFRERKLDEKLLGNLNIMLHSINALAHDAEQKQFTDPNVKAWLFSVKEAE
FDAEDLLVEIDYELTRYQVEAESEPRTFTYKVSNFFNSTFNSFNKKIESEMKEVLEKLEYLAKQKGALGLKDGTYSGDRSG
GKVSQKLPSSSLMVESVIYGRDADKEMIFNWLTSETDNHNHLSILSIVGMGGLGKTTLAQHVYNDPKMETKFDIRAWV
CVSDHFDILTVTKTILETITKSKDDSGDLEMVHGRLKEKVSGKKFLLILDDVWSERREEWEVVRTPLSYGAPGSRILVTTRV
EKVASNMRSRVHRLKQLEEDEGWKVFEEQALKDDDLEWNDEKKEIGRRIVEKCKGLPLALKTIGSVLRTKSSILDWKSVL
ESHIWELPKEVEIMPALLLSYQHLPSHLKRCFAYCALFPKDYEFDKTELILLWMAEGFLHHSQSNKNVQEVGEQYFDDLL
MRSFFLQSSSEMRFVMHDLLNDLGKYVCADFCFRLKFDKGNCIPKTTRHFSFAFRDVRYFDGFGSLTDAKRLRSFVQIT
NNTLYSAFPCQIEILIRELFSKFKFLRVLSLTGIYGLKEVPDSVGDLKHLHSFDLSHTNIQKLPDSIGFLYNLLILRLNGCFYFKE
LPSSLHKLTKLRCLEFERTQVTKMPMHFGELKNLHVVNMFCVNENNEVISIKQLGGLTLHGKLSINELQNIVNPLDALEA
NLKNKPLVELNLIWYRNQIPDDPRKEKKVLENLQPSNQLEHLSIRSYCGTQFPSWVFDNSLSNLVSLYLEDCKYCLCLPPL
GLLSSLKTLRIIGFDGIVSIGTEFYGSNSSSFKSLERLEFYNMKEWEEWECKTTSFPRLRGLFIYECPKLKGLSKQLLHLKELDI
ESCDNLIISEHSEDTSALEVLRTRSCPLVNIPMTRYDFIEQITIDNSCNSLTIFQLNFFPMLRYLFLKGCHNLQRISQKHAHN
HLKKMRIRECPQFESFPGEGLSAAFPSLTELHIVNCPKVEKFPDGGSPSKVKYMCLSSLKLIASLRETLDVNTCLQSLTIENL
EVESFPGEVLLPPSLTSLSINHCRNLKKLDYNFLYNLSSLTLFDCGNLQFLPEEGLPKSISSLEIWDCPLLEQRCQKSEGKE

>XP_047175434.1

MAAELVGGALLSAFLQAAFDRLASPQFVDFFRRRKFDEKLLENLNIMLHSINSLADDAEQKQFRDPHVKAWLFAVKEA
VFDAEDLLDEINYELTRCEVEAQSQSQSITYKVSNFFNSTFSSFNKKTDSGLKEVLEKLEYLSRQKGALGLKECTYSSVGSCS
NISRKLPSTSLVVESVIYGRDADKEIISNWLTSETENHNQPSILSIVGMGGLGKTTLAQHVYNDPKMDTVKFDIKVWVCV
SDHFDVLTVTKTILEAIDNKKDDSGNLEMVHKKLKEKLSGRKFLLVLDDVWNEKREEWEAVRTPLSYGAPGSRVIVTTRA
ERVASNMRSEVHRLKQLQEDECWKVFIKHALKDDDLKLNDEQKEIGRRIVEKCKGLPLALKTIGSLLHTKSSISDWKSVLA
SDIWDLPKEDSEIIPALFLSYHYLPSHLKRCFAYCAIFPKDYEFVKKELIFLWMAENILQCPQQIRQPVEVGEEYFNDLLSRS
FFQQSRSKRHFVIHDLLNDLAKYVCADFCFRLKFDKGKCIPKATRHFSFAFDDVKCFDGFGSLTEAKRLRSFIPLTKFGKGY
YLDYSWKFKISIHDLCSKMKFLRVLSFNCCSDLREVPDSVGDLKHLRSLDLSSTEIQKLPDSTCLLFNLLILKLNYCLNIEELPS
NLHKLAKLCCLEFKRTKVTKMPMHFGELKNLQVLNTFIVDKNSEFSTKQLGGLDLHGRLSIKELQNITSSSDALVMDLKN
KTHLVTVKFKWNKNHILDDPRKEKKVFENLQPSKQLETLGINNYGGTEFPNWVFDNSLSNLVFLQLKDCKYCLCLPPLGL
LSSLKTLKIVGLDGIVSIGYEFYGSESSSFKSLERLEFYYMKEWEEWECKTDSFPRLQTLSMNECPKMKGLSEQLLHLKKLII
RSCEILIISEHNMDPSTLKILRIYSCPLTNVPITLYNFLEEMEINGGCDFLTIFPLDLFPKLCSLKLTRCRNLERISQEQTHNHL
KYLIVEKCPQFESFPTMGLSAPWLQTFEIRGAENLKLLPRHMQILLPSLTDLHIIDCPQVEMFPDGGLPSNVKYVSLSSFKL
IASLRETLNADTCLERLCITNVDVESFPGEVLLPQSLTSLQISCCPNLKKIDYKGLPKSISALNV

>XP_047175438.1

MKEVLKKLEYLAKQKGALGLKEGTYYDDRSDGKVSQKLPSSSLVVERDIYGRDADKEKIYKWLTSETDTHNHPSILSIVG
MGGLGKTSLAQHVYNDPKIEEAQFDIKAWVCISDHFDVLTVTKTILEAITKSKDDSGDLEMVHGRLKEKISGRKFLLVLD
DVWNERQEEWEAVRTPLSYGAPGSRILVTTRIEKVASNMRSEVHHLKQLEEDECWKVFEKQALKDDDLRLNGEKKEIG
RRIVEKCKGLPLALKTIGSLLRTKSSSSYWKSVLESDIWQLPKEVEIIPALLLSYQHLPSHLKRCFAYCALFPKDYEFDKKELIL
LWMAEGFLHHSKQIKNVEEIGEQYFDDLLTRSFFLQSSLEMRHKNVQEIGEQCFYDLLTRSFDLESSFEMRFVMHDLLN
DLAKYVCADFCFRLKFDKGSCIPNTTRHFSFSLDDVEYFDGLGSLTDAERLRSFHSITNNCGYEFNPCQFNILVHELFSKFK
FLRVLSLNGYSELSEVHDSVGDLKHLHSIDLSYTRIQNLPNSIGFLYNLLVLKLNFCSFLEELPSNLHKLTKLHCLEFEHTKVT
KMPMHFGELKNLQVLSTFFVNRNNEVISIKQLGGLNLHGRLSINELQNISNPLDALEANLKNKPLVQLKLIWNGNHIPD
DPRKEKKVLENLQPSNQLEHFSIYNYGGTQFPGWVFDNSLSNLVSLWLADCKYCLCLPPFGLLSSLRILQIIGFDGIVSIGA
EFCGSTSSSFKSLKILEFYNMKEWEEWECKTTSFPLLQHLSIHNCPKLKSLPEQLLHLKNLDINRCDKLVISVNSMFISSLQL
WSIILCPLVEIPMTHYDCIEAMEINNDCVSSTIFPLDCFPKLHLLQLSWCHNLRRVSQGHTHNHLKVLRISECSQFESFPSE
GLSAPWLQIISIREAENLKLLPKHMQILLPSLTELEIIDCPKVEKFPEGGLPSNIKRMSLSSLKLIASLRDTLDVNTCLESLTIEK
LEVESFPGEVLLPRSLTSLRFLFCLNLKKIDYRGLCNVSSFTHYGCPNLHPRKFLIS

>XP_047175439.1

MAAQLVGGALLSAFLQVAFDRLASPQFLDFFRGRKLDDKLLGNLSIMLHSINALAHDAEQKQFTDPNVKAWLFSVKEA
VFDAEDLLGEIDYELTRCQVEAKSEPQTFTYKVSNFFNSTFNSFNKKIESEMKEVLEKLEYLAKQKGALGLKEGTYSADSS
GGKVSQKLPSSSLVVEGGFYGRDADKEKICNWLTSETDTHNHPSILSIVGMGGLGKTSLAQHVYNDPKIEEAQFDIKAW
VCISDHFDVLTVTKTILEAITKSKDDSGDLEMVHGRLKEKISGRKFLLVLDDVWNERQEEWEAVRTPLSYGAPESRILVTT
RIEKVASNMRSEVYHLKELEEDECWKVFEKQALKDDDLELNDEKKKIGRSIVKKCKGLPLALKTIGSLLRTKSSSSYWKSVS
ESDIWQLPKEVEIIPALLLSYQHLPSHLKRCFAYCALFPKDYEFDKKELILLWMAEGFLHYSPQNNNLEEIGEQYFDDLLTR
SFFLRSNIKMHFSMHDLLNDLAKYVVYAEFCFRLKFDKGDCVPKTTRHFSFALRDLYYCDVKYFDGLGSLRDAKRLRSFLPI
VYDGKYGNVLSSRFKILIREFSKLKFLRVLSLNGYYHFKEIPDSVGDLKHLHSLDLSRTRIRKLPYSVGLLYNLLILKLNYCSYL
KELPSSLHRLTKLRCLEFEDTKVKKMPMHFGELKNLHVLNKFCVGRNNELSIKHLGGINLHERLSINELQNIENPLDALEA
NLKDKHLVGLKLLWNSNHIPNDRRKVKKVLENLQPSNQLEHLLIRSYCGTKFPSWVFDNSLSNLVSLELKNCKYCLCLPSL
GLLSSLKTLQITRLDGIVRIGAEVFGSNSSSFKSLEILKFYKMKKWEEWECKTTSFPRLRHLVIVRCPKLKGLSEQLLHLKELF
IESCGNLVISEHSEDTSALELLRTRSCRLVNIPMTHYDFIEEMTINSGCDSLTIFQLNSFPMLRNLSLHRCKNLQRVSQEHA
HNHLKVMSVGECPQFESFPGEGLSAAFPSLTHLHIVNCPKVEKFPDGGLPSNVKHMSLSSLKLIVSLRETLDVNTCLQSLS
IYSLDVKSFPDEVLLPPSLTSLRIDRCRNLKKLNYKDFYNLSSLTLSGCPKLQCLPEEGLPKSISSLYIWRCPLLEQRCQKPEG
KDWRKIAHIQNLSVG

>XP_047175442.1

MAAELVGGALLSAFLQVAFDRLASPQFIDLFRERKLDEKLLGNLNIMLHSIKALAHDAEQKQFTDPNVKAWLFSVKEAV
FDAEDLLVEIDYELTRSQVEAESEPRTFTYKVSNFFNSTFNSFNKKIESEMKEVLGKLEYLAKQKGALGLKEGTYSGDRSG
GKVSQKLPSSSLVVESVIYGRDADKEMIFNWLTSETDNHNHLSILSIVGMGGLGKTTLAQHVYNDPKMETEFDIRAWV
CVSDHFDILTVTKTILEAITKFRDDSRDLEMVHGRLKEKVSGKKFLLVLDDVWSERREEWEAVRTPLSYGAPGSRILVTTR
ALKVASNMRSKVHRLKHLEEDECWKVFEEQAVKDDDLEMNDEKKEIGRRIVEKCKGLPLALKTIGSLLRTKSSISDWKSV
LESDIWDLPKEVEIMPALLLSYQHLPSHLKRCFAYCALFPKDYEFYKKELILLWMGEGFLHHSQQNKNVEEIGEQYFDDLL
TRSFFLQSSSELQFVMHDLLNDLAKYVCADFCFRLKFDKGNCIPKKTRHFSFANNDIRYLDGFGSLTDAKRLRSFVQITNY
CKYHGLRGPFEILICEVFSKLKFLRVISLKGYYRLKEVPDSVGDLKHLHSLDLSHTKIPKLPNTVGLLYNLLILRLNGCSYLKEL
PSSLRKLTKLRCMEFEDTKVTKMPMHFEQVKNLHVLNMYCVSRNSEFSIKQLGGINLHERLSINELQNIENPLDALEANL
KNKQLVGLKLIWNPNHIPDNPMKEKKVLENLQPSNQLEHLSIRSYCGIEFPSWVFDNSLSNLVSLELIDCKYCLYLPSLGLL
SSLKTLDI

>XP_047175590.1

LPNXFNKLARSAVAYCGGLPLALEVLGSYLSKRSENEWRSVLSKLEIIPNTQIQNILRISFDGLREKEKDIFLDVCCFFIGKDR
GYVTEILNGCGLDADFGIKVLIKRGLLKIEKKNKLGMHHLLRDMGREIVRQTSTMQPGKRSRLWLPKDVLDVLTKNTGT
EAIVGLSLNTKLTNSDSFKADAFKEMKTLRFLQLGHVRLTGDYGYLSKQLRCISWQGFSLEHIPNNFYLEGAIVMDFQHS
NLRLLWKEPTVLPWLKILNLSHSKYLTETPDFSKLPSLEKLILKHCVSLGKVHQSIGDLHNLLLINLKGCTNLSNLPSETYKLK
SLKTLILSGCLKIDIFKEDIMHMKSLKTLISHNTAVKQVPISVVSSKSIGYIQVDEGKGLSRTVLHSIILSWMSHTFNPLYHM
RPFRGISPSLASMNVEHNDLVDLAPVLRSILNLRTVLVQCVTEYPILQQVTAILEEVRCVTWTTSTQLSNHPFSPYLIQIGG
YQEEVFNTLRKSIYDEELAARQTRKVFVPSDNYPHWLAYKNEGHSAKFTVPDNFHMNGMILCVEHLSRLGDPTQYLSC
VLMVNYTKCIIQLFNRETLTSLNDVDWQGVISHLGYGDKLEIFIIFEKGFEVQKTAVYLVCNDQLTESQCNEP

>XP_047175592.1

MDVASSLSIFSKFRVSGEVFIHCLGDDIHRNFVSHLSSSLLQAGVKPSVVAVEILSEKFMPSITRFQIGIVVFTTAYIESYRCV
EDLLRIIECHENHGLIVMPVFYDIDPSDLFDLEMDSSYDIDPSDLLDLEMVSSYDDPSDLFDLEMDSSYVRDPIKGGFKKD
SSKVYNLRSARRSVKTTVEGTQMEAERFNYPIILYTSAGSHALNRVANLPTWDESKHRNDAELVEEIVKSVLAKLDRALT
VTKFPVELETQVKNVIGLFENQPNKVCMIGIWGMGGSGKTTLAKAIYNKIPFTFGDKSFILDIRKVCQTYGRRGLVQLQE
QLLSDVLKYVKIGRGEIEKPTIENRLSGKKLFIVLDDVNEINQLKQLCGSGKWFRAGSVIIITTRHLDLLYQQKVDYVYEMD
ELNENDSVELFSWHAFREAKPRENFNKLARSAVAYCGGLPLALEVLGSYLSKRSENEWRSVLSKLEIIPNTQIQNILRISFD
GLREKEKDIFLDVCCFFIGKDRGYVTEILNGCGLDADFGIKVLIKRGLLKIEKKNKLGMHHLLRDMGREIVRQTSTMQPGK
RSRLWLPKDVLDVLTKNTGTEAIVGLSLNTKLTNSDSFKADAFKEMKTLRFLQLGHVRLTGDYGYLSKQLRCISWQGFSL
EHIPNNFYLEGAIVMDFQHSNLRLLWKEPTVLPWLKILNLSHSKYLTETPDFSKLPSLEKLILKHCVSLGKVHQSIGDLHNL
LLINLKGCTNLSNLPSETYKLKSLKTLILSGCLKIDIFKEDIMHMKSLKTLISHNTAVKQVPISVVSSKSIGYIQVDEGKGLSRT
VLHSIILSWMSHTFNPLYHMRPFRGISPSLASMNVEHNDLVDLAPVLRSILNLRTVLVQCVTEYPILQQVTAILEEVRCVT
WTTSTQLSNHPFSPYLIQIGGYQEEVFNTLRKSIYDEELAARQTRKVFVPSDNYPHWLAYKNEGHSAKFTVPDNFHMN
GMILCVEHLSRLGDPTQYLSCVLMVNYTKCIIQLFNRETLTSLNDVDWQGVISHLGYGDKLEIFIIFEKGFEVQKTAVYLVC
NDQLTESQCNEP

>XP_047175646.1

MTQLPSLSRSSSSSSTCEGTHDVFLSFRGDDTRSGFTGNLYKSLCDRGIHTFIDDEGLRKGEEIRPALFKAIEQSRIAIVVFS
ENYADSTYCLEELVVILECIMRKGRLVWPVFYGVTPSSVRFQKGSYGKALAKHGERFKNDQEKLQKWKLALQVAAGLS
GSHFKLKQGYEHELIRTIVEEVSKKINRSPLHVANYPIGLESRVQELKLLLDVGSNWGVSMVGIFGIGGIGKTAIACAVYN
AIADKFDVQCFLGDIRQKSMKYDLVQLQETVLSEMVGEKSIKLGSINRGMAVMKSKLQRKKVLLILDDVDKLEQLKALA
GDPSWFGDGSKIIVTTRNRRFLRVHGVERTYEAKGLDDKEALELFSWHAFKSNEVGPGYLDISKRAVFHCNGLPLALEII
GSNLNGITMSEWEAALDTIERIPDEDIQEKLKVSYDGLKGNEKEVFLDMACFFRGYHLKDVINLLLQSRGFSPEYVIRML
VDKSLIKIDQYGFVQMHNLVEDMGREIVRQESPSEPGKRSRLWLYEDIVDVLENDKGSDTIEVVMLHLPKNREVLWNG
SELKKMTNLKMLTIENADFSRGPEYLPSSLRVLKWRGYPTQSLPPEYDPRRLVMLDLSMSRNILGKQLNLMKFESLSEM
VLRGCRFIKQAPDMSGAKNLRKLCLDNCKNLVEVHNSIGLLDKLTWFTAIGCTSLRTLPHSFKLTSLEYLSLRKCSSLQRLP
NISEEMKHMKNLDLCGTAIEQLPYSFRKLTGLKYLVLDKCKRLNQIPINILMLPKLERLTAVKCGRYVNLILGKSEEQVRLA
SSESLRDFRLNYNDLTPTSFPNVEFLVLTGCAFKVLPECIGQCRFLKNLVLDNCKELQEIRVVPPKIKYLSAINCTLLSHESQ
NMLLNQKLHEGGGTDFSLPGTRLPEWFDHCTRGPSLSFWFRNKFPRMTLAVVGVLDKQGSFPMSRFHFLSNGIQKLH
CHFTVQSKLITYHIFLSDVLLKSYNGGLESVYGEDGWNHVEVSYVGPRVFSHSCRTKKGTIKWMGVHVHKQKTNMQDI
RFINPWFPKRAHSEVSKADLQESFQPLPKRIRVSHRKEICEAPQMKQHEANSSYHGVSQRLWLAICSIAAPLNVKVLM
WNICQDDLPTFEYLFRRKLVLSPLCPICGTEPETVEHVFLFCPWTRPLWFGSDFQWCVDVKEVQSFQLWLWHKLMEIQ
RVYPENANQISAQVGSICWSIWKGRNEFVLEGKPVNPLILR

>XP_047175792.1

MPSSTQEVQDKESVEKDVSDDEKVESLKSERDNEKADLVTLKKNKENLLKHTNKKVSDENVKRKDSKEEVVEKVVEAIN
KKIKKSTKKLGVSEIETEAKEKGKTPKTVHYPTLPLSRDDETLKLVLKRCQASYDALNSHSKVCCLSLSIFPENVVIMKRHIIY
WWIGEGFVKRSTEKTAEKEGENVFDELLNSNLIVPQGXAKCPVVNKFKINPWIRHMLVSSVLGENKQPFGFYPQITTSS
YGCLVLDQQKVEIGGDFAPKSDQWRSVFNLSAKHLTIEPQWMAKMKKLVVLQLGRWQESPKYHIEVAGTEFMTDTK
AQKHLKYLSLRGISRISALPPSIAQLVSLEILDLKACHNLETLPNEITSLKKLTHLDVSQCYLLESMPKGIGKLTELQVLKGFV
VGSSNKTPTISDLANFKKLKRLSIHIGSEAVIQDKEFENLRKSQVKCLKISWGVSSNTKYKDIDIVLPQGLEKLNLEGFPGEN
TPTWLKLLPSELKRLYIIGGKLRSIEELKDSTINCKVEILRLKYLKNLKIDIDHLRKLFPSLKYAEVKKVKEDSYEWSIMW

>XP_047176918.1

MAESLLFSFAESVLGKLATVVVQEASLALGVHSELQQMTESMALIRGVLSDAEQKTPQSSALSQWLRQVKRVFCDAEDI
VDDFECEALRKHVVKTYGSCSRKVSRFFSKSNPAVYRLRMAHHIQDINTRLTKLASQRTLFALQVIDRDTRVVHVRKMT
HSHVNPSNVTGREHDRNEIIKLLVQDGDCQSLSVISIVGMGGLGKTTLAKLVFNDTNIDECFQLKMWVCVSNDFELRN
VLIKILNSAPNPTRENFNDFETEQLQIRLRETLEGQKFLLVLDDVWNEDPEKWDELKEIIDLNVEGSKVLVTTRSRTVTAT
MRNKSSNLYLLGCLSEEDSLSLFVKYAFDDGEEKKHPQLLEIGKEIVEKCGGLPLAVKTVGSSLFSRFDKKEWESIRDNEIW
NLQQNERGILPALKLSYDQLPSYLKPCFASFSLFPEDTSIHCSHISTLWEALGFLPPPKESESMMEVANHLVHELWSRSFL
SDYLDYGSDCSFTLHDLVHDLATYIAKGEFERIDLRNKKNSENAQHLAFMENNLLAQAFPHKGLRSVCLPEGMNNEAFL
ITLVSRCKYLRVLELCSCELESLPYFIGKLKHLRYLNLQCSEKLRRLPENFKICKL

>XP_047176920.1

MAESLIVAVAESLITKLASRAVEQASLALGVYQELQQMKKTMALVKAFLLDAEQKKQQNNALSEWLRQIRQVFAHAED
IVDNFECEVLRNHVVSSHGSFCRKVCRLFSTSNPVVYRYRMGREIKDIKKQLEKVATDGHMFGLQSSDKDTKVLNARE
MTHSHVNVSNVVGREHDKQKIIELLLQDNHHDRSLSVISIVGFGGLGKTTLAKVVFNDTSIEECFTLKMWVCVSNDFEL
RNVLIKILNSAPNPTNEKFKNLDTDQLQIRLRSSLQSEKFLLVLDDVWNENRIKWDELKEIIEMVGNKGSKILVTTRSHSID
AMMRTKTSNSYILKGLSEEDSMSLFVKSAFDDGEGKNHPELMEIGRQIVRKCGGIPLAVRTLGSSLFSRVDRKEWENIR
DNEIWNLKQNENDILPALELSYDQLPSHLKRCFACFSLASKDFDVSSSYVALLWEALGFLSPPKQNETTHDVANEYLREL
WSRSFLTDFLDMGSTCRFKLHDLVRDLAVYVAKGEFQILYPHSTSISEHAQHLSFIENDMLGQDLVPMGARTIIFPMEAT
NDAFLNTLVSRCKYLRVLDLSYSEYESLPSCIGKLKHLRYLNLSGNKKLKGLPDSLCKLQNLQTLDLRGCIKLQNLPKGIRKL
VSLRRLLVTTRQPDFPDKEISKLACIETVELYSCDNLESLFQAIQPRSLKFLHLSGCGGLKSLSFHVITNLESLVIFKCSKMELS
MGLSNLNSIPDSRLKLLVLQSLPQLVTLPEWLQGSVNTLHSLLLVDCNNLEELPEWLSTLTSLKLLIIEHCPRLISLPDSTHH
LRNLEHLEINDCPELCKRCQPGVGLDWHKISHIKEVIIGEPEE

>XP_047176922.1

MAESLIVAVAESLITKLASRAVEQASLALGVYQELQQMKKTMALVKAFLLDAEQKKQQNNALSEWLRQIRQVFAHAED
IVDNFECEVLRNHVVSSHGSFCRKVCRLFSTSNPVVYRYRMGREIKDIKKQLEKVATDGHMFGLQSSDKDTKVLNARE
MTHSHVNVSNVVGREHDKQKIIELLLQDNHHDRSLSVISIVGFGGLGKTTLAKVVFNDTSIEECFTLKMWVCVSNDFEL
RNVLIKILNSAPNPTNEKFKNLDTDQLQIRLRSSLQSEKFLLVLDDVWNENRIKWDELKEIIEMVGNKGSKILVTTRSHSID
AMMRTKTSNSYILKGLSEEDSMSLFVKSAFDDGEGKNHPELMEIGRQIVRKCGGIPLAVRTLGSSLFSRVDRKEWENIR
DNEIWNLKQNENDILPALELSYDQLPSHLKRCFACFSLASKDFDVSSSYVALLWEALGFLSPPKQNETTHDVANEYLREL
WSRSFLTDFLDMGSTCRFKLHDLVRDLAVYVAKGEFQILYPHSTSISEHAQHLSFIENDMLGQDLVPMGARTIIFPMEAT
NDAFLNTLVSRCKYLRVLDLSYSEYESLPSCIGKLKHLRYLNLSGNKKLKGLPDSLCKLQNLQTLDLRGCIKLQNLPKGIRKL
VSLRRLLVTTRQPDFPDKEISKLACIETVELYSCDNLESLFQAIQPRSLKFLHLSGCGGLKSLSFHVITNLESLVIFKCSKMELS
MGLSNLNSIPDSRLKLLVLQSLPQLVTLPEWLQGSVNTLHSLLLVDCNNLEELPEWLSTLTSLKLLIIEHCPRLISLPDSTHH
LRNLEHLEINDCPELCKRCQPGVGLDWHKISHIKEVIIGEPEE

>XP_047177198.1

RILVTTRSKEVASTMRSEVHSLKQLHEDHCWKLFAKHAFQDDDTKLNPECREIGMKIVQKCKGLPLXLLYNKSSVSEWN
TVFRSEIWELSKERCDIIPALALSYIHLPSHLKVCFAYCALFPKDYKFEKECLIQLWMTENLFNCQHSRTPEEVGQQYFNDL
LSRSFFQQSVEDEEVFVMHDLLNDLAKYVGGGIYFMWEFDQREKIQKVTRHFSIELGYRQYFDGFGKLCNTERLRTFTQ
MGEHVFRMNMSIHELLSKFKFLRILSLFNCYALEELPDSVGNLEHLRSLDLSFTAIKKLTEKICSLTHLQILKLNYCRDLEELP
SDLHLLTNLCRLEFMKTKVRKVPLHLRKLKNLKVMMSPFIVGHSKEFGIHRLGELNLDGSLSIEELQNIENSIDALEADLKN
KTCLVKLKLRWDSRRNIDSKKEEDVIENMQPSKNLKELSIFSYGGKQFPNWLLENSLWNMMSLVLEECESCQRLPPLGL
LPFLKDLRIARLDGIVSIDADFHGNNSSSFKSLETLSFSSMKQWEKWECQVVAGVFPNLQRLYINDCPKLKGELPEQLVPL
EILEITDCQQLEASAPRAVELRIQDYGKLQINGATLKELSIGGHNMEAWFVEMAGHIVSCSFALCSDCQISDESVSLWTF
PLHFFPTLTMLSLKGFTNLQMISQDQVHNHLQYLKIRDCPKFESLPANMHMLLPSLTMLSIKDCPRLESFPDGGLPPNL
NEMSLKSCFRLIGSLKGALGDNPSLKNLWIENVDAECFPDEGLLPFSLTSLMITKSPNLIKLDYKGLYQLSSSLETLTLYACP
NLQCLPKEGLPKSLSCLE

>XP_047177569.1

MTTPQRNLDEFISNLTREEDDLKQQLQYLKSKGKKRKRIVDEWFDKLQNMKQRCIYMKNSLNESGRPNFNVQGEIRDL
IKGMQNHKKCKPMVLSNEFVGKKFEGNVKKMWKLLRDDQVFIIGIYGMGGVGKTFLATYIQCEIIRSKIFEDVLRVTVS
HDFNIFKLQQQIAEKIKVNLCKSDNETSRAIILESELEKRKNTVIILDDIWEYVDLEKVGIPLGVGVKGIKVIMTSRLRHVCQ
QMKCLSDNRIEVGAFNADDEYGEAWELFWLGLQNFGTRPKLSPEVRNIAKCVVSKCDGLPLAISVMARIMIGNTSIHE
WKYVLDEFDEWLMKAEMKKEVITVLRRSYDNLAEKKVKKCFLYSALLPKSFVREDLIMMLVDMGLLNGRRSLKKIFNKG
NNIVKKLINHSLLLEHNSTLRMQGLVKKMAWNILKESDANTMLKCNEYMKSISELEWPTDLETVSLANNTIEEIPYGTSF
NCPRLSTLLLFDNSIGHIPEDFFTYMNALKTLGLSEKHNLTSLPRSLSNVRSLTSLMLYECSELNDIPPLGELQSLLRLQISGC
SIEALPEGLENLINLKWLDLSNNVNLELVPGSFLSSLTNIQYLNLWLCSGGIEVEDVEKG

>XP_047177848.1

MEVVVSTAAENAMQIAVRVVKRQFSYFFNYNDKFEEVKCYIERLDNTRKRIQHQVNNAEMNAEEIEDDVQHCLKQLD
EKIEKYEQFIHDEYHSKTRCSIGFFPNNLSLRYRLGRNATKMVEEMEAEELWNKKFDEVSYRVLPSINSALTNTSYESFAS
REKTINVCMQALEDSTINIIGLYGVGGVGKTTLVKEVAKKAQEKKLFNVVVMANITRNPNIIKIQGQIAEMLGMRLEEES
EIVRADRIRKRLKKEKENTLIILDDLWDGLDLNRLGIPISDENEGSEQDVNDISDSSYDRMEKEELSSDFNNMTEENLSED
HKRCKILLTSRRKQVLCNQMDVKERSTFSVGVLNETEGKTLLKRLAGIHIQNLVYDEKAIEIARMCDGLPIALVSIGRTLKN
KSSFVWEDVYQQMKKKSFMEGQEPIEFSIKLSYDHLKNEQLKCIFLHCARMGNDALVMDLVKFCIGLGLIQGVHTIGEA
RNKVNMLIEELKESSLLLESYSSNRFNMHDIVRDVALSISSKEKQMFFMKNGIIDEWPHKDQLERYTAIFLHYCYINDDLP
GSIYCPRLEVLHIDNKDQFLKIPDDFFKDIIELRVLILTGFYFPCLPSSIICLSKLRMLSLEKCTLGQNLSIIGELKKLRILTLSGSN
IESFPFEFGQLDKLQLLDLSNCSKLSVIPSNVISRMNILEELYMRDTLILWEAKEMIQNKNASLSELRPLNQLRNLDLHIQN
VAHVPQNLFFDKLDSYKIIIGEFNLLIEGEFKIPDKYEVVKLLVLNLKEGIDIHSETWVKMLFKNVEYLLLGELIDVHDVFYEL
NVEGFQKLKHLSIVNNIGLQYIINSVERFHPLLAFPKLESLYLYKLYNLEKLCNNQLLEASFCRLKTIKIKSCDKLESLFTFFMV
GLLTMLENIEVCDCDYLKDIVSIERQPHTDSDDNIEFPQLRTLTLKSLPAFTSLYTNDKIPCSARSLEEKGRNRNRDIIVEVE
QDGTNSCLSLFNEKISIPKLESLELSSVNIQKIWSDQSQHCFQNLLTLNVTDCGNLKYLLSFSMAVSLVNLQSLSVSECEM
MEDIFRPKDDEGNIDYVFPKLKKMEITCMEKLNSIWQPHIGLHSFNSLDSLIIRECHKLVTIFPSFMGQKFQSLQSLTITNC
KLVENIFDFEMIPQTSDINETQLHKIVLQNLSNLVSIWKDDTCQILKYNNLQSITVAGSPNLNYLFPVSITKDLENLESLDV
RNCRAMKEIVGWDKGTNENVITFKFPHLKTVSLRSLLELVSFYGGTHTLEWPSLKRLLILRCGKLEGINTDISNSQVKPIGL
ATEKVIYNLEFMAMSFREVEWLQNYIINVHRMQNLQSVVLHGLKNVEVLFWLLHRLPNLKRLTLGFCHLKRIWAPASLI
SREKIGVVMQLQELELKNIWPLEEIGFEHEVLLQRVERLIIQSCTKLKILASSSVSFSYLTYLEVENCMLRNLMTCSTAKTLV
QLKTMKVSSCPMIVEIISENEGEEIQEIEFKLLRSLELVSLQNLTSFMNVDKCDLKFPVMENLVVSECPKMTKFSKVQSAP
NLQKVQVVATEKDKWYWEGDLNATLKKHFTHQVSFEYSKHMKLKDYPEMKEVCQGKPVFQDNFFGSLRKLEIDAASK

REIVLPSYVLPYLKNLEELNIESCKSARVIFDMDDCQIKETVFRLKKLTLKDLSNMKWIWNKNPEGIVSFPNLEEVFVSSCG
TLVTLFPSTLARNLSKLKALTIHNCSKLVEIVEKKEEMEDEITEIFEFPCLSKLFLWNLPMLICFYPRQHLLKCPILERLHVAYC
RKLKLFTSQPHHSLPHPMFLIEEVVPKLKEVILNEKNITLLKDGHSRDLLHKLNYLDLASEDYGNKKDSLPFDFLQKVPNLE
YLVVRQCFGLKEIFPSKKLDGDHDGILLAGLNKLSLNKLLELESIGLDHPWVKPYTEKLQGLAVIKCPRLERLVNCVTSFINL
KQLIVKNCKRMKYLFTFSTAKSLGKLETLRIENCESMKEIIEKEDENGYDEIIFGRLTKLWLYSLPRLVSFYSGNDTLQFSSLQ
IMRLFKCPNMKSFSQGNTNAPMFYGIKSSSDSDLTFHSDLNMTVESLFHEQGFFEYSKQMILLDYLEMRGFGPVKDVF
PRKFFGSLKKLEVDGASKGDTVIPSNVLSHLKSLEELNVHSSDEVQVIFGMDHNSRAKSKETVFHLKKLSLKDLSNLKCILN
KNLQGSVSFPNLQELFVDGCGSLVTLFARKLQTLEMQKSEKLVEIVGNEDATTETLVFEFPCLSSLTLYNLTHLSCFYPGKH
HLECPQLEILHVAYCPKLNLFTSKIHDSHKETAVEAPINWLQQPLFMVEKVVPKLRGLTLNEKNMMLLSDEHVPEVNLT
KLNLLRLCFEDDKNEKDSLPFEFMNKVLNLEHLRVQRCFGVKEIFPSQKLQVHDGIPASLNGLTLFELNELESIGFEHPWV
SPYSEKLQILRVVNCPLLQKLGCHAMSFFNLKELYVKDCDRMEYLFTCSTAKCLVKLETLIIKNCESIKEIANIEDEDGCDKII
FGKLTTLRLYSLPRLQSFLSGNVTLQFSCLRNATVIDCPNMKTFAERVLNVPRILSIQTSLEDSDLFLHNDLPEDASKTGKSI
LVRLASFFKQRNRL

>XP_047177849.1

MEVVVSTAAENAMQIAVRVVKRQFSYFFNYNDKFEEVKCYIERLDNTRKRIQHQVNNAEMNAEEIEDDVQHCLKQLD
EKIEKYEQFIHDEYHSKTRCSIGFFPNNLSLRYRLGRNATKMVEEMEAEELWNKKFDEVSYRVLPSINSALTNTSYESFAS
REKTINVCMQALEDSTINIIGLYGVGGVGKTTLVKEVAKKAQEKKLFNVVVMANITRNPNIIKIQGQIAEMLGMRLEEES
EIVRADRIRKRLKKEKENTLIILDDLWDGLDLNRLGIPISDENEGSEQDVNDISDSSYDRMEKEELSSDFNNMTEENLSED
HKRCKILLTSRRKQVLCNQMDVKERSTFSVGVLNETEGKTLLKRLAGIHIQNLVYDEKAIEIARMCDGLPIALVSIGRTLKN
KSSFVWEDVYQQMKKKSFMEGQEPIEFSIKLSYDHLKNEQLKCIFLHCARMGNDALVMDLVKFCIGLGLIQGVHTIGEA
RNKVNMLIEELKESSLLLESYSSNRFNMHDIVRDVALSISSKEKQMFFMKNGIIDEWPHKDQLERYTAIFLHYCYINDDLP
GSIYCPRLEVLHIDNKDQFLKIPDDFFKDIIELRVLILTGFYFPCLPSSIICLSKLRMLSLEKCTLGQNLSIIGELKKLRILTLSGSN
IESFPFEFGQLDKLQLLDLSNCSKLSVIPSNVISRMNILEELYMRDTLILWEAKEMIQNKNASLSELRPLNQLRNLDLHIQN
VAHVPQNLFFDKLDSYKIIIGEFNLLIEGEFKIPDKYEVVKLLVLNLKEGIDIHSETWVKMLFKNVEYLLLGELIDVHDVFYEL
NVEGFQKLKHLSIVNNIGLQYIINSVERFHPLLAFPKLESLYLYKLYNLEKLCNNQLLEASFCRLKTIKIKSCDKLESLFTFFMV
GLLTMLENIEVCDCDYLKDIVSIERQPHTDSDDNIEFPQLRTLTLKSLPAFTSLYTNDKIPCSARSLEEKGRNRNRDIIVEVE
QDGTNSCLSLFNEKISIPKLESLELSSVNIQKIWSDQSQHCFQNLLTLNVTDCGNLKYLLSFSMAVSLVNLQSLSVSECEM
MEDIFRPKDDEGNIDYVFPKLKKMEITCMEKLNSIWQPHIGLHSFNSLDSLIIRECHKLVTIFPSFMGQKFQSLQSLTITNC
KLVENIFDFEMIPQTSDINETQLHKIVLQNLSNLVSIWKDDTCQILKYNNLQSITVAGSPNLNYLFPVSITKDLENLESLDV
RNCRAMKEIVGWDKGTNENVITFKFPHLKTVSLRSLLELVSFYGGTHTLEWPSLKRLLILRCGKLEGINTDISNSQVKPIGL
ATEKVIYNLEFMAMSFREVEWLQNYIINVHRMQNLQSVVLHGLKNVEVLFWLLHRLPNLKRLTLGFCHLKRIWAPASLI
SREKIGVVMQLQELELKNIWPLEEIGFEHEVLLQRVERLIIQSCTKLKILASSSVSFSYLTYLEVENCMLRNLMTCSTAKTLV
QLKTMKVSSCPMIVEIISENEGEEIQEIEFKLLRSLELVSLQNLTSFMNVDKCDLKFPVMENLVVSECPKMTKFSKVQSAP
NLQKVQVVATEKDKWYWEGDLNATLKKHFTHQVSFEYSKHMKLKDYPEMKEVCQGKPVFQDNFFGSLRKLEIDAASK
REIVLPSYVLPYLKNLEELNIESCKSARVIFDMDDCQIKETVFRLKKLTLKDLSNMKWIWNKNPEGIVSFPNLEEVFVSSCG
TLVTLFPSTLARNLSKLKALTIHNCSKLVEIVEKKEEMEDEITEIFEFPCLSKLFLWNLPMLICFYPRQHLLKCPILERLHVAYC
RKLKLFTSQPHHSLPHPMFLIEEVVPKLKEVILNEKNITLLKDGHSRDLLHKLNYLDLASEDYGNKKDSLPFDFLQKVPNLE
YLVVRQCFGLKEIFPSKKLDGDHDGILLAGLNKLSLNKLLELESIGLDHPWVKPYTEKLQGLAVIKCPRLERLVNCVTSFINL
KQLIVKNCKRMKYLFTFSTAKSLGKLETLRIENCESMKEIIEKEDENGYDEIIFGRLTKLWLYSLPRLVSFYSGNDTLQFSSLQ
IMRLFKCPNMKSFSQGNTNAPMFYGIKSSSDSDLTFHSDLNMTVESLFHEQGFFEYSKQMILLDYLEMRGFGPVKDVF
PRKFFGSLKKLEVDGASKGDTVIPSNVLSHLKSLEELNVHSSDEVQVIFGMDHNSRAKSKETVFHLKKLSLKDLSNLKCILN
KNLQGSVSFPNLQELFVDGCGSLVTLFARKLQTLEMQKSEKLVEIVGNEDATTETLVFEFPCLSSLTLYNLTHLSCFYPGKH
HLECPQLEILHVAYCPKLNLFTSKIHDSHKETAVEAPINWLQQPLFMVEKVVPKLRGLTLNEKNMMLLSDEHVPEVNLT

KLNLLRLCFEDDKNEKDSLPFEFMNKVLNLEHLRVQRCFGVKEIFPSQKLQVHDGIPASLNGLTLFELNELESIGFEHPWV
SPYSEKLQILRVVNCPLLQKLGCHAMSFFNLKELYVKDCDRMEYLFTCSTAKCLVKLETLIIKNCESIKEIANIEDEDGCDKII
FGKLTTLRLYSLPRLQSFLSGNVTLQFSCLRNATVIDCPNMKTFAERVLNVPRILSIQTSLEDSDLFLHNDLPEDASKTGKSI
LVRLASFFKQRNRL

>XP_047177850.1

MEVVVSTAAENAMQIAVRVVKRQFSYFFNYNDKFEEVKCYIERLDNTRKRIQHQVNNAEMNAEEIEDDVQHCLKQLD
EKIEKYEQFIHDEYHSKTRCSIGFFPNNLSLRYRLGRNATKMVEEMEAEELWNKKFDEVSYRVLPSINSALTNTSYESFAS
REKTINVCMQALEDSTINIIGLYGVGGVGKTTLVKEVAKKAQEKKLFNVVVMANITRNPNIIKIQGQIAEMLGMRLEEES
EIVRADRIRKRLKKEKENTLIILDDLWDGLDLNRLGIPISDENEGSEQDVNDISDSSYDRMEKEELSSDFNNMTEENLSED
HKRCKILLTSRRKQVLCNQMDVKERSTFSVGVLNETEGKTLLKRLAGIHIQNLVYDEKAIEIARMCDGLPIALVSIGRTLKN
KSSFVWEDVYQQMKKKSFMEGQEPIEFSIKLSYDHLKNEQLKCIFLHCARMGNDALVMDLVKFCIGLGLIQGVHTIGEA
RNKVNMLIEELKESSLLLESYSSNRFNMHDIVRDVALSISSKEKQMFFMKNGIIDEWPHKDQLERYTAIFLHYCYINDDLP
GSIYCPRLEVLHIDNKDQFLKIPDDFFKDIIELRVLILTGFYFPCLPSSIICLSKLRMLSLEKCTLGQNLSIIGELKKLRILTLSGSN
IESFPFEFGQLDKLQLLDLSNCSKLSVIPSNVISRMNILEELYMRDTLILWEAKEMIQNKNASLSELRPLNQLRNLDLHIQN
VAHVPQNLFFDKLDSYKIIIGEFNLLIEGEFKIPDKYEVVKLLVLNLKEGIDIHSETWVKMLFKNVEYLLLGELIDVHDVFYEL
NVEGFQKLKHLSIVNNIGLQYIINSVERFHPLLAFPKLESLYLYKLYNLEKLCNNQLLEASFCRLKTIKIKSCDKLESLFTFFMV
GLLTMLENIEVCDCDYLKDIVSIERQPHTDSDDNIEFPQLRTLTLKSLPAFTSLYTNDKIPCSARSLEEKGRNRNRDIIVEVE
QDGTNSCLSLFNEKISIPKLESLELSSVNIQKIWSDQSQHCFQNLLTLNVTDCGNLKYLLSFSMAVSLVNLQSLSVSECEM
MEDIFRPKDDEGNIDYVFPKLKKMEITCMEKLNSIWQPHIGLHSFNSLDSLIIRECHKLVTIFPSFMGQKFQSLQSLTITNC
KLVENIFDFEMIPQTSDINETQLHKIVLQNLSNLVSIWKDDTCQILKYNNLQSITVAGSPNLNYLFPVSITKDLENLESLDV
RNCRAMKEIVGWDKGTNENVITFKFPHLKTVSLRSLLELVSFYGGTHTLEWPSLKRLLILRCGKLEGINTDISNSQVKPIGL
ATEKVIYNLEFMAMSFREVEWLQNYIINVHRMQNLQSVVLHGLKNVEVLFWLLHRLPNLKRLTLGFCHLKRIWAPASLI
SREKIGVVMQLQELELKNIWPLEEIGFEHEVLLQRVERLIIQSCTKLKILASSSVSFSYLTYLEVENCMLRNLMTCSTAKTLV
QLKTMKVSSCPMIVEIISENEGEEIQEIEFKLLRSLELVSLQNLTSFMNVDKCDLKFPVMENLVVSECPKMTKFSKVQSAP
NLQKVQVVATEKDKWYWEGDLNATLKKHFTHQVVPKLKEVILNEKNITLLKDGHSRDLLHKLNYLDLASEDYGNKKDSL
PFDFLQKVPNLEYLVVRQCFGLKEIFPSKKLDGDHDGILLAGLNKLSLNKLLELESIGLDHPWVKPYTEKLQGLAVIKCPRL
ERLVNCVTSFINLKQLIVKNCKRMKYLFTFSTAKSLGKLETLRIENCESMKEIIEKEDENGYDEIIFGRLTKLWLYSLPRLVSF
YSGNDTLQFSSLQIMRLFKCPNMKSFSQGNTNAPMFYGIKSSSDSDLTFHSDLNMTVESLFHEQGFFEYSKQMILLDYL
EMRGFGPVKDVFPRKFFGSLKKLEVDGASKGDTVIPSNVLSHLKSLEELNVHSSDEVQVIFGMDHNSRAKSKETVFHLK
KLSLKDLSNLKCILNKNLQGSVSFPNLQELFVDGCGSLVTLFARKLQTLEMQKSEKLVEIVGNEDATTETLVFEFPCLSSLTL
YNLTHLSCFYPGKHHLECPQLEILHVAYCPKLNLFTSKIHDSHKETAVEAPINWLQQPLFMVEKVVPKLRGLTLNEKNM
MLLSDEHVPEVNLTKLNLLRLCFEDDKNEKDSLPFEFMNKVLNLEHLRVQRCFGVKEIFPSQKLQVHDGIPASLNGLTLF
ELNELESIGFEHPWVSPYSEKLQILRVVNCPLLQKLGCHAMSFFNLKELYVKDCDRMEYLFTCSTAKCLVKLETLIIKNCESI
KEIANIEDEDGCDKIIFGKLTTLRLYSLPRLQSFLSGNVTLQFSCLRNATVIDCPNMKTFAERVLNVPRILSIQTSLEDSDLFL
HNDLPEDASKTGKSILVRLASFFKQRNRL

>XP_047177951.1

MAMILDAVVGRVLDELLSTVIAMKDRAVKFRATLENLHSILKKVEPMAREIDGLNKRLDKPATETQKLIDEMEKGKELVI
ECSKVDWWNCCYKASSQEKLQDLIDSITLYFQLDMQGNINVIVLENQMLLHQIHEKLVENVPRRIAGLCSPPEPPAFTV
GLDVHLRALKFKLLNNHHVGSVLTVTGTGGSGKSTLAKKFCSDEEVKGEFKDNIFFISLAEVPKLSTIVERLFEHNGYEKPQ
FQSDEGAVDRLENLLKQIGKNPILLVLDGVLPESASLVEKFVFQIPNYKILVTSRFTIKGFGQPYVLKSLNEADALNLFRHH
ASLDQTSFKIPDTIVKKIAKGCSGSPLALIVTGKSLSLEEPVVWHNRARTLSRGQSVLSYSSNSDGLLTCLQKSFDDLDAKL
AESFMDLSLFPEAQKIPAAALVDIYAEQRDEDDDIAMENIHDLVKRNVADLVVTRNTTSGTVDYNYHYVTQHGLLRDLA

IHQTRNLPTEKKHRLIIDLRGNNIPKWWTTQNEYHIAAHSLSISTDEEFTSEWCNLQPNEVKVLVMNLREKKRSLPPFMK
KMNKLKVLTITNYDVNRAELENLELLDYLSDLKRIRLEKVSIPFLSKTGVPLKNLHKFSFFMCNVNEAFKNSTIKVSDVLPN
LKEMNIDYCDMEELPAGLSDTVSLKKLSITNCHKLSKLPTGIGKLVNLESLRLTSCTKLEELPDSITSLHKLNFLDISDCVSLR
MLPENIGELGSLERLNCRGCNRLSELPYSVKDLESLRVVVCDEERAALWEPIRSMFSDLKLEVVLTDFKLDPLL

>XP_047177952.1

MAMILDAVVGRVLDELLSTVIAMKDRAVKFRATLENLHSILKKVEPMAREIDGLNKRLDKPATETQKLIDEMEKGKELVI
ECSKVDWWNCCYKASSQEKLQDLIDSITLYFQLDMQGNINVIVLENQMLLHQIHEKLVENVPRRIAGLCSPPEPPAFTV
GLDVHLRALKFKLLNNHHVGSVLTVTGTGGSGKSTLAKKFCSDEEVKGEFKDNIFFISLAEVPKLSTIVERLFEHNGYEKPQ
FQSDEGAVDRLENLLKQIGKNPILLVLDGVLPESASLVEKFVFQIPNYKILVTSRFTIKGFGQPYVLKSLNEADALNLFRHH
ASLDQTSFKIPDTIVKKIAKGCSGSPLALIVTGKSLSLEEPVVWHNRARTLSRGQSVLSYSSNSDGLLTCLQKSFDDLDAKL
AESFMDLSLFPEAQKIPAAALVDIYAEQRDEDDDIAMENIHDLVKRNVADLVVTRNTTSGTVDYNYHYVTQHGLLRDLA
IHQTRNLPTEKKHRLIIDLRGNNIPKWWTTQNEYHIAAHSLSISTDEEFTSEWCNLQPNEVKVLVMNLREKKRSLPPFMK
KMNKLKVLTITNYDVNRAELENLELLDYLSDLKRIRLEKVSIPFLSKTGVPLKNLHKFSFFMCNVNEAFKNSTIKVSDVLPN
LKEMNIDYCDMEELPAGLSDTVSLKKLSITNCHKLSKLPTGIGKLVNLESLRLTSCTKLEELPDSITSLHKLNFLDISDCVSLR
MLPENIGELGSLERLNCRGCNRLSELPYSVKDLESLRVVVCDEERAALWEPIISMFSDLKLEVVLTDFKLDPLL

>XP_047178135.1

MAECFVFDITESLLSKLASYVYEEASRAYGVYDDLQGIKDTLSIVKGVLLDAEEKKEQNHGLREWLRQIRNVCFDAEDVL
DGLDCQNLRKQVLKASGNTRMKVDHFFSLSNSLVFRFRMAHQIKHVRRRLDKIAVDGNKFGLERIDVDNRLVQRREM
TYSHVDASGVIGRESDREEIIKLLMQPHPNGDGYGDQSVCVIPIVGIGGLGKTTLAKLVFNDNRIDDLFQLKMCGPMILS
MWVCISDDFDIRQVLIKIINSASASAPTIAHAHQENIKNFDIEQLQSRLRHRLSGQKYLLVLDDVWNDNRVKWTQLKDLI
KVGAVGSKILVTTRSNSIASMMGTVPSYVLEGLSLENCYALFNKWAFKEGEEKKYSNLVEIGKEIVKKCRGVPLAVRTLGS
SLFLNFNLERWEFVRDHGIRNLKQEKDDILPALKLSYDQMPSYLRHCFAFFSLYPKDYGFTGAEVVNLWMSLGLLRSEV
GSQKLENAARLYIDELHSRSFLEDFEDFGNIYYFKVHDLVHDLAQYVAKEEILVVDSGTRNIPEQVRHLSVVENDSLRDAL
FPKSSRVRTILFPTDGVGVDSVALLDAWITRYKFLRILDLSDSSFETFPNSIAKLEHLRALSLENNRRIKRLPQSICKLQNLQV
LTLRGCVELETLPKGFGMLISLRKFFITTKQSILSEDEFASLTDVHNLSFEFCDNLMFLFRGAQQLKSLEVLIVQSCRRLESFP
FHILSKLEVLFVIRCEMLNLSLNCENPIARLRMKFLHIEECPRQQTLPQWIQGAAETLQTLFISNLYSLEMLPEWLTAMTH
LKMLHIVNCHKLLCLPSEMHSLTALDDLTIEGCPELCRKCEPQNGEYWSFIAHIKHLSIGETREGKLLFQMQKKIRLKLQD
LQN

>XP_047178136.1

QESINNLDIEQLQSRLRHRLSCQKYLLVLDDVWNNNRAKWIELKDLIKVGAVGSKILVTTRSTSIASMMGTVPSYVLEGL
SVENCLSLFLKWAFREGEEKEHPYLVDIGKEIVKKCRGVPLALKTSGSSLFSIFDLERWKILRDHELWNRKQQKDDILPSLK
LSYDQMPSYLRHCFALFSLYPKDFGFTCAEIANFWATLGLLRSPFGSQKIENVGKLYINELYSRSFLEDFGTVYYFKLHDLV
HDLSLYVAKEEFLMVNSHTRSIPEQVRDISVVENDSLRHTVFPKSRGVRTIIFPVDGVGVGSESLLETWIKRYKYLRHLNLS
NSSFATLPNSIAKLEHLRALILDKKFKIKRLPNSICRLQNLQKLSLRRCLGLETLPKRLGMLISLRKLYITTKQSILSEDEFASLN
HLHTLVFEYCDNLKFLFRGAQTQLPSLEVLIIQSCGKLECLPFHILPKIEVLIVTRCLMLILSLNSEIPIQR

>XP_047178137.1

MAEYFVFDIAESLVRKLASFVCEEASRVYEVYDDVKGIKDTLSIVKGVLLDAEQKKEQRHGLREWIRQIQTVCLDAQDVL
DGLDCQNVRKQFLKASGSTRMKVGHFFSPSNSLVFRFQMARQIKNIRRRLDKIAADGNKFGLERIEVDSRLVQRREMT
YSHVDASGVIGRESDREEVIKLLMQPHPHGDGYGDQSVCVIPIVGIGGMGKTTLAKLMFNDNRMDYLFQLKMWVCIS
DDFDIRQILINIINSASVSASATTTAIAHQENIKNFDIEQLQTVLKLKLSGQKYLLVLDDIWNDNRAKWTELKDLIKVGAVG

SKILVTTRSNSISSMMGTVPSYVLEGLSMENCYALFNKWAFKEGEEKEYSNLVKIGKEIVKKCRGVPMAVRTLGSSLFLVF
DLERWEFVRDHEIWNIKQNKDDILPALKLSYEQMPSCLRHVFAFFSLYPKDYGFCSAEILILWESLGLLQSLGGNRKLENV
ARQYIDELHARSFLEDFEDFGYYYYFKVHDLVHDLALFVAKEELLMVNSFTRYIPEQIRHLSVVENDSLNHVLFPKSNRVR
TILFPLKGTGIESKTLLHTWTTRYKHLRFLDLSDSSFDTLPNSIAELKHLRTLFSDKCKIKRLPHSLCKLHNLQILSLRGCMELE
TLPERLGMLISLRKLYITTKQSILPENEFASLRNLHTLSFEYCGNLKLLLRREQLAFLEVLIKDQKASSKNGWRNWRNFRGP
LPVFDGKMSDWRIKMWLCLLQDVLESLKDGVPVLIEGPQTQKKGTQDTGENVTARPGFLD

>XP_047178256.1

MAQNQASSSTSIVSKYRYDVFLSFCGDDTRFGFTGNLYSALSERGILTFIDDEALRKGEEITPSLRKAIQESRISIIVFSQNYA
FSTFCLDELLHIIECRTKQNMLIFPVFYDVEPSQVRNQTGSYQEAFAKLGERFKDDIQKLQQWRRALYDAANLSGLHFKS
GEKYESNIVKKIIKEISDSFKRSPLHIAEYPVGLKDRMQQIQELMGGEFDKKVTMLGIHGMGGIGKSTLARAMYNLMED
QFEASQFLANVREKGLVHIQETMLSELVGERNIKLGDVHRGIPILQHRLCGKKVLLVLDDISKKEQLQATVGGLDWFGP
GSIVIITTRDKHLLDVHGVQKQYMVGEINHMEALELYKWNAFKNKEVDPCYKEVIKRAVWYADGLPLALETIGSHLSGK
KGKKTLINAFIEIITFSVTWQEKV

>XP_047178260.1

AMYNLMAHQFEALHFLANVREKSDKDGLVHIQETMLSELVGERNIKLGDVHRGIPILQHRLCGKKVFLVLDDISKKEQL
HATAGGLDWFGPGSIIIITTRDKHMLDVHGIQKQYMVDEINFVEALELFKWNAFKIKEVDPCYKEVTERAMYYANGLPL
ALETIGSNLFGKTLDAWESALETYERIPNRDVQEVLRVSYYRLDAYEDTLQISLKLEDFLKNLV

>XP_047178345.1

MSELELVGPLVGEMGSIGVGEMHTTIASKIAFSKNLDDNCNILVKDTEMLHAIKKDKEMKAQRNSHKDTTNAYKLWTN
RVSEATKEVQKLKLKYEEETLPWWRILKRSDLSEEMEKKSNCVRQLMNDECLKDFLVDKPPEPVLKELNVPQISGYPTLQ
GALDNTLVLLKNNKIKIIGVCRTKGVGKTTIMRNLNNNEEIAKLFEIVIFVKVTPNDRKLQEKIAHRLMLDKGINKEDSDDI
ARRIHRELEKKRYLLILDEVEDAINLELLGIPSDNNNGSKVVIVTRFPLVYKLNRVQRVIKVAELSPDEAWKMFRDTVYAF
NPKIDSPDIQPTAKLVCKRCSRLPLLIYNIANSFKLKESASSWWAGLEDLKPWPELQSQGLEELYSCLKFCYDELNDKRKQ
KCFLYTSLYPADSKVYSDYLVECWAAQGLLGDINDTRSYQSARNCGIDILEHLANVSLLEKGGAMIYVNMNHCMRQLA
LHISSKDPECSFYLQDGEESENLSKSRAWQQARWVSMRKVHELRRSQDCSTILTLLLRKNPELTALPECFFENMSSLLLLD
LYSSMITQLPSSLSKLTGLRGLFLNRCELLESLSSEIGLLQFLEVLDIRDTKVIFIPLQIGFLTKLRCLRIPFIASEDNEAQNVHVI
SKLHRLEELTIQIISYEQWCNDANNVLAHVASLENVTHLRCCFPSSIILGEFLSRSKSWHNKQNSFRFTVGCQNSRRPQIL
EFFEYKITNYLRYCNGGQRDDPAIIEVLPKTDAFELVCHKDIRKLSNFAGIACLERIRGLLIKRCNQVLTIVSGETSSNVMNG
IQIKTAVILPNLEQLYLENLLNLKCAFRGPLHSGTFSRLQTLSLKNCPRLSQIFSNGAIQHFSELQKLKLEDCSIIEELIGEDIER
ERDVLPKLEILLLVNLPNFKSICTTHTLAWSSLELLRIHNCHKFKTLPLDSVNAVNLKSIKGQQEWWANLDWTNNEEVLQ
RLQPIFVASNEYFS

>XP_047178364.1

MASAVFVSFNDEDTRDITAFVLDPLRSRGIQVFVKGESTTFDLFQAIQRSRLLIVVFSKNYASSICCLRELVAIINVVESSPRI
VLPIFFGVHQSNVLFQNGCYVQVFSKHEERFREHKQRMEEVQRWREALTRVACFPGWHMKNAKGVEVRGFINYAVD
ILNREFSTPQNETILNRYGKFEDIVQYKDGDEVSYKELRGLIRLRDFISLVLTYPVKLEEDGVLRSWISAFAEWKAQLLSTD
GFSVICKPWSLLTEKALRCMKGFMPEEIMAEIESGERSLFNRHNDVIPEGLLPLSVDLAVDQLIQTLFSGDYCARYIRLLTR
NSSEKESVVNKIHTALEDKHNMFGNDKDFLMAVWINALTCETDAEVHVQEEINKIMVSISMTEGDDMLTTLDTDKKKS
SNRLLVIVVDADSNRKLDLQKVQFPSGIVVLIATESSTQAAVKDDDFGIACTMDLNIWTQDHMLPWKLFYTYVGSFISCS
TVGSSMTIQKIAVEIVKKSHGHLLAIVLVAKHLRYVKDDKYWELVLDKLSNPNPLYDYQDCDRIGISRVMVNAFVNIIWE
DIDDELKLCLQLSLPVHNIKNGVRDDILVSYWANILRYTQELGEYKRQLQYYLEELLDCFLLLKFESRDVCLPIETYDIIKSLHI

SEPSIIWDGALGLTEIGQWHSLIQIELVNNKICELPQSPGCPKLKVLLLQGNADLLDIPDSFFDHMPLLQHLDLSYTSIRDL
PPSLAKLMQLKKLYLKGCDLFMEISPQIFQLKNLEELDLHGTLITHLPKDIQELINLQRLALCFDAYHHVLSRGKRGKQIFN
TTIIPRGVISNLTQLNYLSLDVDPEDEQWSENVNSVLVEILGLEKLKTVSMYVPKADLLELVPAEKSLNFRLVVGHHMRRL
ISRVTPELETKFKDFDHSMKFVNGVNVPKGVKMNLGRFKALYLDRHMTIKSLSDFNLNNVRRLKVCILAECNEMETIVD
GDEPFSLKLELLSVFYMKNLRSICQGCGSFSYLKYMALHTCPMLTTIFTLRTFISLPFLEEIHVEDCPKVTTLISDDSPKRKAA
FFLPLRVISLLYLPNLVNIFNGLPVEHVLEEMIFYCCPKLQSLSRSELAWEYLKFIKGERMWWEALDWSVSEWGNAGRPF
FEQFFKPINVEADMMNPSAAQLNKYHGGMYQGVSSSTELTTKLHLETPLWLPSTPFPLSSSKEGEAQKRKAVIEPIIVPP
LLRRKPNQDREAVKILIHNDQSLDDGFTWRKYGQKDILGSKYPRSYYRCNERGCKATRQVQRLDDQRTIEVNYIGTHSC
PRKQDLIAESGDIKVLQDFRGKPDSPSSSNKNAIIVLKEIPENREVSAERGVSAERRVSDERMNEEYQLN

>XP_047178392.1

MADLFSGAAVGALMGEALKGAISIIKKGVAFKSTLDSNIETLESLAPLVEEMKMYNMRLDRPSKEIEKLESLMRNSQELV
RKCSKLGRSKMWSFPYYQSKLRSKDGALQTHLSMNMTAQNTANLMKVMDDMRKVMEILLKEGFGRYPEYQFMGIC
GAPQEPECLGMVVPLSKLRIELLKDGVSVLVLTGLGGSGKSTLAKKICWVPQVKDKFGKNIFFVTVSKTPDLKTIVETLFE
HCGFKVPKFQTDEDAVNRLEVLLRLLGQHPILLVLDDVWPGSEDLVEKFKIQIPDYKILVTSRVSFPRFGTSYQLDKLDDV
HAESLFRHFAQLKDKSSYIPEKNLVDEIVKGCKGSPLALKVIAGSLCNKSFEVWRDMMEHLQKQSILESDTDLRFRLQQS
LDILETKFSVNGKEFFMDLGLFPEDQRIPVAALIDMWAELYNLNEDGSNAMSIIHDLTTRNLINVIVTRKVAQDTDMYY
NNHFVLVHDLLRELAIHLSKGEKTFEQRERVMIELKGDNRPEWWVGLNPLGIIGRSFSYILGMLHRQKQPKVAARILSIS
TDETFNSDWCDMQPDEAEVLVLNLLSSQYSLPEFTEKMHKLKVLIVTNYGFHRSELNMFERLGSLTNLKRIRLEKVSIPPL
CILKNLRKLSIHMCNTRQAFERYSISDAMPNLVEMSIDYCKDLVKLPDGLCNITPLKKLSITNCHGLSALPQDLAKLENLEV
LRLCSCSDLAYMPDSVKELNKLSCLDISDCLSLTKLPDDIGELKLQKLYLKGCSKLRELPDSVDKFENLEHEIHVICDEEMAS
LWKNSTIRNLKIEIPTVEVNLNWLPRVHS

>XP_047178514.1

MEFASSASKLSRYYDVLINFNGEDIRRKFVSHLDSALSSFGLTTFLHHQNAVESTHVQQPILDLCRVVIVVFTKTYSESAW
CLYQLQQIIQWHQTYCRHVLPVYYEIQPSDVRFQKGDFGKAFKETAHQTFSAQQLKHGMSRWSHALTKAANLFGWD
ESNHRSDAELVHEIVKSVRNSSVFSATKFPVGLQYPVEELIQTIKDKSMDVCTIGICGMRGSGKTTLAKAIYHQIHGTFM
HKSFIEGIAQFSEPRGGIHFQEQLLSDVIKTKVKIDTLEMGKDMIRDRLLGKRILIVLDDMDDYLLLLSLRESLSWLSKGTVII
MTTTDDDLFNEYQVQSVFRINPMNEHRSLELLSWHAFREAKPKEEYDYLARRLISLCGGLPLVLEVIGSTLFERTEVEWY
SVLFKLEKIPSYNVQQKLKISLDRLSNQLERNLFVDLCCFFVGKDRAYATKILSSSVVEADSETGVLHRFRVDADRGIRVLM
EGNLIKVKRNNKLGMHPLLQELGKTIFHETSRRKLWNNGQLLFYVDAEYALKVNDGRKDYEVLLSTRREPSRLPKDGVN
SENLSPKLRWISFRGFSTEYLPNDFNAHDIIAIDLKHSLLQFFLEKPQVLKSLKVLNLSHSMYLTETPNFWRLPNLEELILKD
CPRLQEVHQSIGHLCYLILLNLKDCKSLSNLPQEIYRLKSLRTLILSGCSKIDPMEKDIVQMKSLITLAAENTTVKKVPFSIVSS
KAIGYISLRGFERLSCNPFPSIIIRSWMSPTINSISYINLNCVDMEDNSWDDIAPLLGNLANLRTVLVQCNTEFQLSKQVKN
ILVDYFSNITESEISKQHFRSSLIGVGAYHEFFNAVSNNISEVLASSESSDVSLPGDNPPYWLAYMGQGDSVSFTVPPDND
MKGMVMFLCVFNVSTPEIVASEGIRSVLIVNYTKCTLQIHMHGKRISFNDIDWQAIRSNLGSGDKVEIFVTFSQGVVVK
NTHVYLIFGESHYLEKVPTPKKK

>XP_047178515.1

MEFASSSSKLPRYYDVLINFNGEDILTKFVSHLDSVLSSVGLTTFLHHQNAVESTHVQQPILDLCRVVIVVFTKTYSESAW
CLHQLQQIIQWHQTYCRHVLPVYYEIQPSDVRFQKGDFGKAFKETAHQTFSAQQLKHGMSRWSHALTKAANLFGWD
ESNHRSDAELVHEIVKSVRNSSVFSATKFPVGLQYPVEELIQTIKDKSMDVCTIGICGMRGSGKTTLAKAIYHQIHGTFM
HKSFIEGIAQFSEPRGGIHFQEQLLSDVIKTKVKIDTLEMGRNMIRDRLSGKRVLIVLDDMDDYRSLLYLRESRSWLSKGT
VIIMTTRDDDLFRQYQVQSVFRINPMNENRSLELLSWHAFREPKPKEEFDYLARRLISRCGGLPLVLEVIGSTLFERTEEE

WHSVLFELENIPLYNVQLKLKISLNRLRNQLEKDLFLDLCCFFVGKDRAYATKILSSSVVEADIEAGVLHDFRVDADSGIRA
LMDRNLIKIKRNNKLGMHPLLQEMGKTIFRETSRRKLWKNGQLLFYVDAEYALKGRKDYEVLPLILLSTRREPSRLPKDG
VNSENLSPKLRRISFRGFSIEYITNVFNAHDTIAIDLKHSLLQFFWDAPQVLRSLKVLNLSHSMYLTETPDFSRLPYLEELVL
KDCPRLCKVHQSIGHLSYLIVLNLKGCKCLSNLPRGIYKLKSLRTLILSGCSKIEPMEKDIVQMKSLITLIAENTTAKQVPFSIV
SSNATGYISSQGFERLSFNSFPSIIIRSWMSPTMNPIFYIHSLCADVDNSWEDIGPLLSSLKNLRSVLVQYDSDFQLSKQVK
NILVDYFSNITESEISKQHYRSSLIGVGAYHEFFHAVSDNISEVLLSLFLCLIHSPSF

>XP_047178789.1

MADFFISIAAKIAEYAVDPILHHTQYLCGFNDFAPNLSNAKIRLELTRDGVKERIRKAINMVEKVEPTVENWLKDVEKVLE
EVQMLEERILSVNKSYFRKQCQYSLTKQIKRKTTEMIQLLHNRKFELFWRITELPGTKYYSSNNFFMFNSTEESYKKLLEAL
KNKSAFIIGLVGLGGSGKTTLAKEVGKKVEEMKLFEKAVLATVSQPLNIISIQDQIADQLSVELKEASEISRAQRLSERLRKG
TTLVILDDVWEKLNFEALGIPFDENSKACCIFLTTGSREVCTSMKCQNIIELNPLTDGEAWTLFTFHANISNDSPEALKVM
AERIVTKCNGSSTAIATLGSTLKEKTIEEFESAWLRLQNSERLSSLKGLTSHQVCLKVSYDNLTNQLAKSLLLLCSIFPKNHEI
DLEDLFRFGRGLGVIWRFGRIEKERRVMRAAINILKNSYMLAYVKEKEKVKMRDSIRDVALWIAAESGQAILTCTAVDPR
VLVDDEITKDKNVIALWDMKNGELLNYEWNCPSLEILLLHSPQVGFTISNAFIERLKKLKLLAFLKFEYRWKLPLETETPS
WYASPLSQSIESLKNLNTLSLRGYKLGDISVLESLEALEILDLRGSSFKELPNGVVALQKLKLLDLYGCLIEKNNAYEVIGRCL
RLEELYLYLFPSKEEFPHDVSFSRLQRYVIIQYHSESFSYYIHADILEKHRPSRALCIEGFNASTQTFISLPIKDLFMRAVYLHL
KYIEGGYKNVIPSMRSQGMNQIVALILEHCLDIEFLFDGTFTNNNVDVLHTKTVFSNLCTVRLHQMHGLREVFHDPSSQ
CSLEKLEELSIDSCNQLYNISFPRNSNLCSLKELRIISCPVLTCLFMPFIVQTLKLLEVLQIYECSELMHIIPAEGNDYVGTQDH
TSLMLPKLRIIEIAGCDKLKYIFPVCFGRLPSLERLITKNCDKLKYVFGTEKERHLSMYHEYPDLLNLEVLLLVSLPNLVDIWP
SYCHPRLPNLKELQCTECSTLSNSSLRKMAIDSGLYHQGAAAMKKLLDEAALKFTKLIFSHLGVKCLFQFQIGEPGTNREL
PLRLSTLDLLALPQLKFIWKSPTNFLSLQLLQFLHVDRCPKLKSIFSSAIIRSLPVLRKLEILNCEELEQIFDSECYQIEKCFSFEC
EADMDDKVGRDKDSEQVLLQNLRNITLTSLPNFKEIHRGFQLKDHVEQIIEDCPEYSRIME

>XP_047178969.1

MAETAVSFASQHVLPKFLKAVKMLRDLPKEVAEVTDELESFQDFIQDADKVAEADDDKNRRDRIRKRMMRLRKAAVC
MEDVIDDYVICNEKQSEQDPRCADLLCEAVEFIKTQILRLQIAYRIQDVKTLVHAERDGFKNHFPIGPRSDGSRGNENFT
WQKLRIDPLFIKEDEVVGFEGPIHTLKKWLTEGRKEQRTVISIVGMAGSGKTTLSKQVFDRVKDFECHALITVSRSYTVEG
LLRDMIKELCEERRENPPPAVQTMNRISLIKEIRNRLCHKRYVILFDDVWNETFWDDIELALIDNENGSRILITTQDDKVV
KFCKKAVFFEEHKLQPLSQEKSLVLLCKKAFGKDFDGCCPKDYEEVGLDIVRKCGCLPLAIVAIGSLLYRKCKSPSDWGLFR
QNLSLEMERNPELDSVKKILSLSYEDLPQNLRSCLLYFGMYPEDCEVKCGRLIEQWIAEGFVKHESGRNLEEVAQQHLM
ELISRSLVLVSSFTTDGKAKACRVHDLIHEMIRGKMKNTDFCHNIDEHNHLESSGIIRRLTIGRNSNCLSGSIEESQYVRSILI
FTDEVSSKDFTRRLVAKYMRLKVLDFEYAPLYDVPENLGSLIHLKYLSFRGTSIKGLPKSISKLQNLETLDVRTDKEIEIPKEIT
KLRKLRCLLGSLLSSIEVKDSLGSITSLEKMHLLIIDPNGVVIRELGKLKQLRDLRLVNLRGDHADTLCSSINEMPLLERLHISL
QYGTEAIDLHIRLSLFKLRELLLWGKLKEFPNGIPLFQNLVSLTLIHSALTNIPLISLGNMPNLLILVFGLSSYEGETLHFQNE
GFLKLKELGFTSLYDLRSVFIDSGALQSLEKLTIMNIPQLNAVPSGIQHLKKLQYLWICYMSTAFHQRIDPNGGEEYFMVK
HVPYLHIETHCPVL

>XP_047179032.1

MNQDNYFEKALKMRNLWEEYRHYYGIRRPTTLGVRENIFTGSVSSLAWFMSAQETSXVTLGQRVLANPLKVRMHYG
HPDVFDRFWFLTRGGISKASRVISISEDIFSGFNCTLRGGNVTHHEYIQVGKGRDVGLNQVSMFEARVASGNGEQILSR
DVYRLGHKLDFFRMLSFFYTTAGFFFNTMMVILTLYAFLWGRLYLALSGVENAMESNSNNNEALGTILNQQFIIQLGLFT
ALPMIVENSLEHGFLQAIWDFLTMQLQLSSVFYTFSMGTRSHFFGRTVLHGGIEHKLEELIGIERRLQSVNNVRVVGISG
MGGIGKTTLARALYERIYHQYDFHCFIDDVSKIYRDSSSLGVQKQVLSQSLNEKNLEISNSYEGTCLIWSRLHNVRALVVL

DNVDEVEQLRMFTGKRDTLLRECLGGGSISIVVSRDEHILRTHGVNDIYRVQPLNGENAMQLFCRNAFKVNHILSDYEK
LAWNVLSYAEGHPLAIEVIGSSLFGRNVSQWESALARLKEKKSKNIMDVLRISFDQLDEEDKEIFLDIACALCHHDEEYVT
EVLKFRGFHPEYGLQVLLDKSLISKRSGIIDMHRLLKDLGRYIVKEESPEEPLKRSRLCDYQDFCKAMSNNQTTEIVEAIIAV
NSHGSSKTVRVDGLSKIRHLKFLRLGKMNCSGCLSHLSSELGYLTWYNYPFECLPQSFQPHKLVELILRGSSIQRVWSDTK
VLPNLKRLDLSYSRKLVEMPDVAEALNLEGIILEECIELRKLSPSIGFLSKLTILSLRNCKNLVSLPNSILGLNSLEYLGVYGCSK
LFKNELLDEARNTKHLKKRCLVEGPIHSHSKSHLIKKMLLWPLGLLYSRAQRESVSCLLPSSPTLPCLRVLDLRFCNLVKIPD
AIGKLSCLEKLNLKGNNFVTLPNLKDLFRLYYLNLHNCRRLKYLPDLPSRTHLPLNLYSYPIQYFASFRDRIIEDYKDMTGLM
MFNCPKIVERERCTSMTVSWMLQILQSWYNSECFIPSIGSIIPGSEIPMWFNNQVVSMDNSIIIDVPPFVHDNNWVGV
VCCVLFSENFSSNTVYNADHMWLFYYSRQQFRRKQGGKLDIRNLRLTFTLEDVKILCIDSYLDLANFEVKKYGYRLVNKQ
DLQ

>XP_047179397.1

MESEREDNNNGTDSTHAAISSTLKIKIVSCSKSGESLDNSNLPSPNINNGTESSPYGSPLVSPPSSAFVSALQSPYISPRAIIP
DPPNGSPLENQPPLLTITTSTTNPSTPEDVPSSSYTPPSDQYEFSDDPADTRLKYVTCVPEPAPPRISFSFPVPRISFAKGPI
SPATNAKLRSCDVYIGFHGQNPNLVRFCRWLKSELELQGIDCMLADRAKYSDSQSHEIADGVICSVAFGVVVVTSSSFLN
HFSMEEVRFFAQKKNLIPLLFDTGPAEIMALLNCKSIDKECKEAIDGLMKCNEFNIEANDSNWRSCIAKAAGILRARLGR
KNAEQKDNVQGLENLPFPRNTYFVGREKEIMEIEGLFFGRGNCMEQVQDHCRVFTKGEASGSGQSEGLADEESEPVIA
RCGRYISLDMGRSKEPTLEAWVEPIMGNNSLKRLKNKKSKSGSYKSVCSSVICINGVSGIGKSELALEFAHRYHQRYKMV
LWVGGEARYLRQNLLNLSLNLGLDVGADSEMERGRIRSFEDQEFEAFKRVKRELFGETPYLLIIDNLETEVEWWEGKDL
YDLIPRNTGGTHVIVTTRLSKVMSYDTIQLPPLPLSDAMILMIGRKRKEYSADEIDLLEKINEKLGRLSIGLWMIGSLLAELSI
GPSCLYEAINQVPLDEDSNSCYMSIAEGQWCKSNPFLMKTLLFCLETLEKTKAKGNLLALRMLLVSGWFSPSPISSTLLAN
AAKSIPTVESRLKKWTKSLSLTTSCISPRTWKNEEEPAMILVKMGLARRANQHDGCWLHFHPITQAFAKRKGGLQYAK
AAIQGVRKMGSQVNSDHLWASAFLVFGFKSEPPLVQLKAIDMVLYIKRTALPLAIQAFTIFSRCNSSLELLRVCTNALEEV
EKSFVSQIQDWSSHGSVCWKRRLQRGQKVDEYVWQDVTLLKATLLETRAKLLARGGHLDSAKELCRTCISIRTVMLGH
NHAQTLAAQDTLARLVRMRSKI

>XP_047179438.1

MQKLNHMYPTEVKTLVGIDQNLAPIESLLRLRSKEVRIIGIWGMGGLGKTTIARALFDKLSSQFEASCFLANVMAEHEK
QGLDYLRNKLLSEILEDVNPRISTSKVRSTFVMKRLRQKKVLIVLDDVDDPKKLEDLAAQHDCLGSGSRVIVTTRDKHVLS
KGVDAIYEVKGLSLDHAVRLFSLKAFGKTYPERGFEMLSKQAVDHANGNPLALKVLGSVLHSRNEQQWDNAMRKFKK
VPNAEIQNVLRWSYDGLDYEQKNMFLDIACFFRGENKENVIRLLDICGFYAYIGIKILLEKGLITFSDDGDVCMHELIQEM
GWEIVHQESIKDPGSRSRLWDLKEVYDVLKNNRGTEAVEGIILDVSQIRVLLLSCETFSRMINMRFLKFYMGKGRKCNL
HLPSGLQSLPNKLMYLQWDGYPSKSLPSTFCTENLVVLSMMESHVEKLWDGTKCLPSLKEMNLHASKKLTNLPDLSQA
PNLETIDVSNCTSLIHVPLSIQYVNKLLLFNLESCKSLKSLPRNIHLSSLEMFILRRCTSLDEFSLTSENMTRLDLRETKIADFP
ESVWQHLNKLVYLNLESCNKLKSLTSKIHLKSLQRLNLRDCSILEEFSVTSESMEYLNLRGTSIRELPTSVWRNNKLYTLVL
HSCKKLVNFPERPKVEDLPLVSSSERPNMDELWTLSSLADLSLKGSTVENLPASIKDLPSLKKLTLTECKKLRSLPSLPPSLE
DLSLDESNIVCLPVSIKDLSHLRKLALINHKKLLTPQDLPPSLKAPLLTESKVDPHLVSMKGLSQLQMFPQVKWKMFHSLP
ELPPFLEEFSLSESNIKFIPESIKNLSHMRKLAFTKCTRLQYLPELPPNLEDLFVSGCDIESLPTSIRDLVHLRKITLIECKKLKAL
PELPQCLQSLCAADCTSLKIVRSTKNILIEDRYTFYWNCINLDQKSRNNIIADAPFEAAFTSLKERTPLGPLISICLPGTEIPD
WFSHQSTNSSLDMEIPLEWFVDSMFLGFALCLVIGGFQRNSNEGYDPDINCYHFVKSASYSDPSVPFLGHCTTVMQVP
RGFNSDHIFICYYPSFNASILQDFKDLSLYYDANDLRLRVIFKFKGPSQRLDIVKKCGVRPLLIANTERLHIESELQPE

>XP_047179439.1

SSSSSSSFSSTARLKRHDVFISFRGEDTRNSFTSHLYAAFQHKNIQAFIDNRFHKGDMISSSIFEAIKHSNVSLVVLSKYYAS
STWCLRELAKILQLRKRGGHIVIPVFYKIDPSHVRKQTGTYGMAFQKYKEDVKQNMAMLQKWKAALTEVADIVGWES
KNFRTENELIQEIVKDVMQKLNHMYPTEVKTLVGIDQNLAPIESLLRLRSKEVRIIGIWGMGGLGKTTIARALFDKLSSQF
EASCFLANVMAEHEKQGLDYLRNKLLSEILEDVNPRISTSKVRSTFVMKRLRQKKVLIVLDDVDDPKKLEDLAAQHDCLG
SGSRVIVTTRDKHVLSKGVDAIYEVKGLSLDHAVRLFSLKAFGKTYPERGFEMLSKQAVDHANGNPLALKVLGSVLHSRN
EQQWDNAMRKFKKVPNAEIQNVLRWSYDGLDYEQKNMFLDIACFFRGENKENVIRLLDICGFYAYIGIKILLEKGLITFS
DDGDVCMHELIQEMGWEIVHQESIKDPGSRSRLWDLKEVYDVLKNNRGTEAVEGIILDVSQIRVLLLSCETFSRMINMR
FLKFYMGKGRKCNLHLPSGLQSLPNKLMYLQWDGYPSKSLPSTFCTENLVVLSMMESHVEKLWDGTKCLPSLKEMNL
HASKKLTNLPDLSQAPNLETIDVSNCTSLFHVPLSIQYVNKLLLFNLESCKSLKSLPRNIHLSSLEMFILRRCSSLDEFSLTSEN
MTRLDLRETKIADFPESVWQHLNKLVYLNLESCNKLKSLTSKIHLKSLQRLNLRDCSILEEFSVTSESMEYLNLRGTSIRELP
TSVWRNNKLYTLVLHSCKKLVNFPERPKVEDLPLVSSSERPNMDELWTLSSLADLSLKGSTVENLPASIKDLPSLKKLTLTE
CKKLRSLPSLPPSLEDLSLDESNIVCLPVSIKDLSHLRKLALINHKKLLTPQDLPPFLKAPLLTESKVDPHLVSMKGLSQLQM
FPQVKWKMFHSLPELPPFLEEFSLSESNIKFIPESIKNLSHMRKLAFTKCTRLQYLPELPPNLEDLFVSGCDIESLPTSIRDLV
HLRKITLIECKKLKALPELPQCLQSLCAADCTSLKIVRSTKNILIEDRYTFYWNCINLDQKSRNNIIADAPFEAAFTSLKERTPL
GPLISICLPGTEIPDWFSHQSTNSSLDMEIPLEWFVDSMFLGFALCLVIGGFQRNSNEGYDPDINCYHFVKSASYSDPSVP
FLGHCTTVMQVPRGFNSDHIFICYYPSFNASILQDFKDLSLYYDANDLRLRVIFKFKGPSQRLDIVKKCGVRPLLIANTERL
HIESELQPE

>XP_047179573.1

MADSVVTFVLDHLAQLAAREANLLYGVEDRVQSLQYELELIKELLDTTKRKKGMEYTVLKQIRDVAHLAEDVIDTFVAKV
SIYKKRTIMGRMLRGFGQARLLHHVADKIDKLKTTLNEIRDNKDRHDAFKEITNQSAVKEEEEKERAQSQLKKLRRNVEE
EDVVGFDQDSKDIIKRLLEGDSNRKAVSIIGMGGLGKTTLARKVYNNSQVKQHFMCRAWVYVSNECRVKELLVSLLKHL
MPNFKQQSKGNKKGKKRDGDINSLSEEELKEQVRSCLERKRYLVIVDDLWKRQDWDEVQDAFPDNNGGSRILITSRLK
EVALHAGDDVPHYLQFLNNEESWELFRRKVFRGEDYPSDLEPLGKQMVQSCRGLPLSIIVLAGLLANKEKSRREWSKVV
GHVNWYLTQDETQVKDIVLKLSYDNLPRRLKPCFLYLGLFPEDFEIPVTPLLQKWVAEGFIQDTGNRDPDDVAEDYLYEL
IDRSLVQIARLESNADVKICQVHDLLRDLCISESKEDKAFEVCTDNNILVPTKPRRLSIHSKMDHYISSSNNDHSCIRSLFFF
GPDYDVHKREWKWLLKDFKLVRVLELAPKSRGKIPSNIGNFMHLKYLRIDSQYITFVPDSILHLWSLQTIDLGPWSRNVSI
SFPVQVWKLKHLRHLSTRGPIKLRGSCLGSCEKMCNLQTISPLVLNKQATSLIEKGTFPNIKRIGLSVVFGKGQLSKLLQSL
QLLRYLNEFVIVLRDKVYGNFVTVHEGVERKNGCKPQELFQSLGQFNCLTVLEINNVIDLLTCALTLPSNVTELKLAGIRCI
SDEGMKGLGNHARLKILRLFGFMIFSTLNSYDLTCVGGSFPQLEVFQMEFLKIEKWNLGKGAMPKLQSLVINFCGRLDD
LPNELWSLSDLKKVHVTKPSRQMIGVLQNLEINNAVQLVTEDLPPWMDETDWEVFEYLMEG

>XP_047179754.1

MIEICGEEGSGKTTLAKAIYNQIHWTFKEKSFIENISQVSGIRGYLRLLEQLLLDVLKQKVEIPSIDVGRRMIRETLNGKRVLI
VLDDVSYFSLLDLWNCGKWFGEGTVIIVTSTFERVLKDHANSFFWVERMNAEESLELLSWHAFIEPKPKEEYYYLAIRVV
SYCGGLPLALEVVGSTLFEKTKKEWDTILSKFAIVGRHRVSEIIKISIEGSLNEMEKDIFLDICCFFVGKSRAYVRKILNGCGV
DADIGIRVLIERNLITINKNNKFGMHPLLQKIGIQIIQENFVKDLGKNRRLWFDKDTKYGTEDMQWMPVKLPSVLIALKS
VQPTVNSQYLIKKLRWISLHAFSSECLPNNFYQHDAIAIDLKRSLLRFVWKTPQVMRSVKVLNLSHSKYLTTTPDFTGLPS
LEHLIFKYCSRLRKLHPSIGSLNSLILLNLKDCTSLYNLPTEIYDLKSLRTFILSGCSNIDIKDKDIAKLKSLLTLIAENTAVKHVPF
SIVNSKDIGYMSLRGFEGLSHNFFPSIIRSWMSPTMNPISYIHSFCEDMEDNTIDIAPLLSTLSNIRSILVACDTEFQLTKQV
KNILVEYFANITQSGISKQYFRCSLIGVGAYHQFFNAVSENIYEVLSSSECGDVCLPAVNDPYCLAHMGEGHSVSFVVPED
GDMKGMILCVVYLSTPKIIEPEFTTVVIVNYTKCTFHIHNHGTVISFKDEDWQDIMSNLESGDNVEIFANFGNGLAVKNT
AVYLICGESKNMKKTFEAKKHVSLDS

>XP_047179755.1

MSRWSHALTKAANFFGWAESNHWSDAELVDKIVKSVVNLPVLSATEFPVEILQSRVDAMFLTIKNKSTREVCMIEIWG
DQGHGKTTLPNAIYNQIHCTFKEKSFIENILQVSGIRGYLPLLEELYESGRIIDWKESMIRETLSGKRVLIVLDDVPYNYELLA
LWTYNHYWFSEGTVIIITTSDEDLLICQRTDSLFWIERRKGERSLVYSWPFRSCTQSYESPYKIPYGSPYQSLAHIDLDTTYV
HLHTLLIHDFFYNCY

>XP_047179764.1

MDNASSSYKLPRMYDVLINFNGEDIHRNFVSHLNSVLSTVGLTTFLHHHNAVNSTHIQEPILSRCRVAIVVFTQTYSQSA
WCLHQLLQIIKWHETYCRHVLPVYYEIQPSDVRLQKGDFGKALKATAQQTFSGQELEDGMSRWSHALTKAANFFGWD
ESNHRSDAELVEKIVKSVVNLPVLSATKFPVGLQSHVEDLIRTIKNKSTEVCMIEICGEEGSGKTTLAKAIYNQIHWTFKEK
SFMENISQVRRIRGYLRLQEQLLLDVLKQKVEIPNIDLGRSMIRERLSGKRVLIVLDGVSCFSLFDLWNCGKWFGEGTVII
VTSTFEGVLNDANSFFWVERMNAEESLELLSWHAFREAKPKEEYYYLATKVVSYCGGLPLALEVVGSSLFEKTKKEWDTI
LSKFAIVGRHRFSEIIKISIEGSLNEMEKDIFLDICCFFVGKSRAYVRKILNGCGVDADIGIRVLIERNLITINKNNKFGMHPLL
QKIGIQIIQENFVKDLGKNRRLWFDKDTKYGTEDMQWMPVKLPSVLIALKSVQPTVNSQYLIKKLRWI

>XP_047180205.1

MPVIETLGGALFGAVLQLLFDRLDSHHVFDYFRRRKLNEKLLYKLKVKLLSINNVIDDAELKQFTNSYVKAWLDEVKDAV
FDAEDLLDEIDYEFCKYKLKAESHTRSRKVWKFFKPSSKSFFDKKIESRMEQVLEQIEFLSSQKGDLGLEYVSCVGVGPESV
SKVTQKLPSTSLVVESVIYGREDEKEMILNWLSSDTDNHSQPAVLSIVAMGGMGKTTLAQHVYNDPRIEEAKFDLKAW
VCVSDEYDAFKVAREILQAIHNSIDDSRNLEMVQVRLKEKLTGKKFLLVLDDVWNEDRDRWKPLQTPLKYGAKGSKILV
TTRSSKVATTVQSHKVHELKQLEEDHSWQVFAKHAFQDNNHHLNVEAEKIGKKIVEKCKGLPLALETVGCLLHTKSSVS
EWDSVLISKIWDLPREDSKIIPALLLSYYHLPSHLKRCFAYCALFPKDQEFHKESLILLWMAENFLQCSQQGRSPEVVGELY
FDELLSRSFFQKSIQDNKTCFVMHDLLNDLAKYVCGDICFRFGVDEEKKTLVKTRHLSLVTNQDQYSDGFRSLYDAKGLR
TIMPTSGRINIDYYWDCKMSVDELFSKFKFLRVLSLSWCRGLKEVPGLVGDLKHLRSLDLSGTLIEKVPDSTCSLYNLQILK
LNSCFNLKVLPSNLHQLTNLRRLEVMKTNVIKMPVHLGKLNNLQVLMSSFRVGESSEFTIQQLGDLSLRGGLTIEDLQNI
LNPLDAVAADLKSKRNLVELTLKWNNSWNLADLIKETEVLENLQPSKHLEKLSIWNYGGTQFPSWLANTSLSSLVSLSLE
NCNFCTRLPPLGLFPYLKDLSISGLGEVVSINADFFGSTSSSFSSLETLKFSFMYEWENWECQAVTVTDAFPRLQHLSIRH
CPKLKGNLPEKLLQLRKLLICECKELVASAPVAQEIFELDLQNCGKLQFDYHPTALRRLTVIGDSSMQLSLDFLCKSKTNIP
VSYYDFLETLDINDGCDSLMNISLDCFPKLLRLCLKYCHNLHTISQGRIHNHLKDLQITGCPQFQSFPDEGLSAPRLESFA
MKRLGKLKSLPQHMHVLLPSLTSLLIHDCPQMKILSDGGLPSNLENMNISNCSRLVASLKGPLGANTSLQTLSVQEVDVE
SFPSEGFLPISLTCLIIRDCPHLKTLNYKGLCNPSSLKKLTLFDCPNIRCLPEEGLPKSISTLRILGNCPLLKESSQKPQGQD

>XP_047180273.1

MEFASFSSSSSSLSFLKSEPQFIYDVFINFGGEDIGRKFVSHLHSALLLAQVKTFISKENLQEGIKLEEHMRAIASSKIAIIVFS
KTYTESTCSRLELEKIIECRQTFGQIVLPVIYDIARLDEHLQKVVRVLEKAAPGSYSGEQLDHAMSRWSRALTKAAGVTG
WDFRDFRHDAEFVEEIVRRIQTLLDYTDMSITQFPVGLESHVEKVIALVEEQSTEVCMIGIWGMGGSGKTTLAKAIYNRI
YHSFIGKSFIENIGQFWNLVNKRHVHLQENLLYDVLKSKFEVESDRVGRTMIETKLSRKKLLIVLDGVNEFGQLENLCGNR
EWFGQGTVIILTTRDVTVLNRLKVNHVYKIDVMNENDSIELLSWHAFREAKPRKELNEHARSIVAYCGGLPLALQFLGSY
LCDRTKEEWESVSSKLKVNPFNQILEKLEISFDGLHDMEKDIFLDICCFFIGKERSYVTKILNGCGLYADIGITVLIERGLIKVE
RYNKLQMHPLLRDMGREIIRRRCPKERGKRSRLWFQDDIKDVLKKNTVRTFFICL

>XP_047180434.1

MDGGGASTSLAALFEGNEEEYDNGVLTTLQSKLDELRSDLTREEKEIQGQLQLLESHGKKCKRKVALWLNEIQNIKQRAI
DMKDSLNQFGCSDFYVPQGEMYSAEESQKKIEHLTEEIQKHKKLKPLVLSNEYVGRKFEKNVETLWKLMRDDRVFIIGIY
GMGGVGKTFLANYMQSEIKRTKTFEDVLWVNVPRYFNLDNLQELIAKMIKVKFYYTYNETDRAIILAKELEKRKNIILILD

NVWKYVDLEMLGISLRINGIKLIMTSRLKDVFQQMDCLSINIIEVKPFYYYHHYNSCDIYYRNDYDDDSDEDDDKYDEA
WKLFLLKLGSYGTPLTLSPEERNSARYVVKECDGLPLGISVMAQSMKGKTNIHWWRHVLNKFDKLEMGVEMQQKVFT
TLKHSYYNLNEEDWQKCFLYIAFLPNFMHRNSLIQKLVDTGLLEQNRSLQQIFDEANVLVDKLVNDSLLEANKEKEIKHLS
SNKNDLVLSMHGLVRKMALNIWKERVSNMMIKCNGNMTKIPYTDKWAIDLEVVSLAHNNIQVIPESTSPNCPRLSTLL
LFENSIDHIPECFFAQMNALKTLDLSKNKSLTCLPRSLSNLRSLTSLMLHECSELKDIPPLGELHSLLRLQISSCSIKAPPQGL
ENLINLKWLDLSMNKNLKLVPGSFLPSLTKIHYLDLLGCAGGIGVEDVKGMTMLECFAGTFILQDNYKDNFTRYVQEILD
SANGLQTYFINLDNKSKMSTLIEYPFSRFKDRTMGFSDCKELRHFLPRDLLKLELKYNDYWVCLCYGLLSCGNSLLEEIRIY
DWKKLWSLFCSSCYLCTNLKNLQSLNLNCLESLTVICQKLPESDMFSHLKTLCVHKCHEMKILLTSKFVQQLQNLESITVS
HCNSIEQIFEDEEDDEGDSNKIILRKLTWLSITYLPQLKTVYKGILICKSGFKSSIDNCPQLCKPGTEYVVS

>XP_047180606.1

MAEALLGIVIKNLQSFGQDQLATFWGVHRQTQKLSSNLTAIRAVLRDAERKQITSHAVKDWLQKLTDAAYVLDDILDEC
SIHSTKVNFDDAHTSCLSRLHPNDILFRFNIGKRMKDITRRFHDIHEERSMFNLEPGVTEVQTIHDNWRQTSSDITEPVIY
GRDQDREQIVKFLLEDASNSEEVSIFPIVGMGGLGKTTLTKKVFNDHRIRKHFELTIWVCVSDDFNTMTILQSIIECIAGQ
NPNINSLEAMRKKVEDVLHGKRYLLVLDDVWNEDPEKWEQLKGKLQCARAAKGATILVTTRLEEVASTMQTHPAYRL
KELPGDESWSLFKYHAFGPNREEKEELVAIGKEIVRNCIGLPLAIKTLGSLLRDQSEVRQWIYVKESSSKTNEENSIMRALK
LSYSNLELSLRRCFSFCAIFPKDFEIDKEELIHLWMANGFIESERNIEVEDVGNKVWNKLYSRSFFQEAKCDEFGMVKSFK
MHDLFHDLAQSIMGEECAAFVEGRLTPLSSRVHYSTLLSSDVSFGRFKHAFKKVESLRTFLDLRTSFVLGNVAPVPSNHSL
RALCTRSSLLSPLKDLTHLRYLSLNCGYKANLNNVICQMRKLQILKLKRSTYLHGLPKNLTQLQDLRHIVMDYCNSVVETL
PKISKLRHLRTLSIFVVGSKPGCGLAELHGLNLGGRLRIRGLENVPNEWDAKQANLIGKKDLNILHLSWDGNANPKGSD
VSVERVLEALEPPSTLKSFGMNGYKGRQLPSWMRSSLVLRDLVEVELSGCDNCEELPPLGKLAHLKRLKVSGMKNVKWI
DGETYDGVEEKAFPSLEELIVDNLPNLERLLRDEGVEMVPRLSQLRIDDVLNFKVPRLPCVETVHARGIEAVTSFMEGVG
ESMACLKTLSIKFIKGVVVLPDEFRRLGALQELYITEWYDVEYFPEHVLEGLTSLRTLSIQYCEKLKSLSEGVRHLACLENLTI
GNCPELVALPSNMSQLTALRKVSISYCSTLPDGLQGVPSLGSLDISCCKCSSLPDWLGDMTSLQKLSIVYCKELRSVPSSL
QHLTNLSHLRIDGCPHLKKRCKRETGEDWQYIKHIPQLELYFRRETTLSVEGAHG

>XP_047180608.1

MAEALLGIVIKNLQSFGQDQLATFWGVHRQTQKLSSNLTAIRAVLRDAERKQITSHAVKDWLQKLTDAAYVLDDILDEC
SIHSTKVNFDDAHTSCLSRLHPNDILFRFNIGKRMKDITRRFHDIHEERSMFNLEPGVTEVQTIHDNWRQTSSDITEPVIY
GRDQDREQIVKFLLEDASNSEEVSIFPIVGMGGLGKTTLTKKVFNDHRIRKHFELTIWVCVSDDFNTMTILQSIIECIAGQ
NPNINSLEAMRKKVEDVLHGKRYLLVLDDVWNEDPEKWEQLKGKLQCARAAKGATILVTTRLEEVASTMQTHPAYRL
KELPGDESWSLFKHHAFGPNREETEELVSIGKEIVTKCVGSPLAIKTLGSLLRDESDVKQWQNVRESEIWDIREESSSATG
EENSIMRALKLSYFNLDLSLRRCFSFCAIFPKDFEIDKEELIHLWMANGFIKCEGNVEVEDVGNKVWKKLYSRSFFQEAKY
DEFGMITTFKIHDLFHDLAQSIMGEECVVIVKGRLTPLSSRVHYLSSYAYVRGFKYAFKKVESLRTSIGTYCVVPSNHSLRA
LCTSSSLLSPLKDLTRLRYLSLTCYSKANLNNFICQMPKLQILKLQNSACLHGLPKNLTQLQDLRHIVMDYCNSVVETLPKI
SKLRHLRTLSIFVVGSKPGCGLAELHSLNLGGRLRIRGLENVPNEWDAKQANLIGKKDLNILQLSWDGNANPKGSNVSV
ERVLEALEPPSTLKSFEMNGYQGRHLSSWMRNNSVLRDLVKVTLSDFDNCEELFPLGKLAHLKRLNVSEMKNVKWIDG
ETYDGVEEKAFPSLEKLIVDNLPNLERLLRDEGVEMLPRLSQLTIEGVLNFKVPRLPCVEELSVERIGEAASFMEVVGNMA
CLKTLSIIGVKGVVVLPVEFSRLGALQTLFIANWYDVEYFPEHVLEGLTSLRILDISNCKKLKSLSEGVRHLARLERLMISGCP
ALVALPSNMSQLSALRYVLIEYCFTLPDGLQRVPSLRVLYIYECTCTSLPDWLGDMTTLEELRIHCCWELRSLPSSIQRLTN
LSRLNIRECPHLKKRCKRETGEDWQYIKHIPKIELYFWREPTVL

>XP_047180842.1

MEEEALEVGKCILKLLWEAFKRMKDLPKVVDEIQTELDELKNLINLEEQRLMKERKTAFLAEDVIDEFETRIKEKQPQDDR
GWAAFPAKAVYHIQTLIPRFLISYKVNDILSDVRNTKERLQRNRPQSTQNNNNVTFHHCRMDPLFMEEEEVVGLAGPT
KILTDWLENGQRERTVIFVVGVAGVGKTTLAKHVYDKVICSNFVHHEFFTVSQSNTVEEFLGVMYRHFMKSEPPSDYSIL
KNEVRGYLRGKRYVLLFDDVWEDKFWETIETALIKNENKSRIIITTRKKSVAKSFTNSTLFKKYNLEEPLTEEECLRLLHKKA
GYSSDCPEELKDISLEIVSKCSALPLAIEVVGRVLSQKDRSAHEWGLFSEELNWNLTNPTNPEFNIIEKVIGLSYDNLPTHLR
LCLLYFGMYPKHYEIKSGRLVRQWVAEGFVTHEERKTLEEVAQEYLLGLVHTSLVQVASFSMKGKVKTCRVHDLIHNMI
RTKVKHTYFGEYIGGGHDQSKSSELVRRLAIETNNDLNRPIERSHIRSIISIPGKQEESWSSDLERNVLKDDMPLKVLDFEG
CGLLCVPKMLGNFIYLRYLSFRGTQITVLPKSIGKLVNLETLDIRQTQVLELPKEITKLRKLLHLLTPDSAFSSIEWKDIGGMT
KLQKIPQVRMEDDGVAITEVGKLKQLRVLRVLCFGAEHITLLFFSINEMEHLQALRIEKSGDRIVVDLNTASMSGGSRLRK
LFLDMKLERLPNWFPQLRHLERLTLRHSDLSSDPLESLKDMPSLLVLSLSHAYEGQTLHFQSGGFKKLRKLNLEHLWNLN
SILIDETALQALEYFELTCLDDLRTVPDGIQHLNKLKFVRVPYMPTQFVEEIDRIGKDKPWRINYGYM

>XP_047180843.1

MENYTKVAETSMPFARRYLPPKPFEALKMMRDFSKEVADITDEHGSLLDFIDDANKVADAEEDVKRRDRMKERLMRL
REAAFRMEDVIDEYCIRVKEKQPQHGPPWAASLSKTVHSIKTFILRLQIACKIQDEKSVVRDEKVGFESQYFLDKKLNSSR
ENKNVTFNQLRMDPLFMKEEEVVGLAEPTERLTDWLTMGREERTVISVVGIAGVGKTTLAKHVFDRVHQYFECHALIT
VSQSYTVEELLRDLVRKLCKERKEDPPCKISTMDRGSLIEEVRDRLHNKRYVVLFDDVWNEKFWDDIQSALIDDKNNSRII
ITTRNEKVAVFCKKSSFVEVHKLEEPLKDESFRLFCRKAFKYGSAGGCPEELKDISLEIVRKCKGLPLAIVAIGGVLSQKDDS
EKEWRLFSQNLSSELKRNPDLNIITKIIGLSYDNLPSHLRLCLLYFGMYPKDYEIESERLVRQWVAEGFVTHEEGKTLEEVA
HEYLLGLVRRSLVQVSSFSMKGKVKKCRVHDLIHDMIRTKVKDTCFGEYIGGGHDQSESSESVRRLTIQANNDLNRRIKR
SHIRSIIVIPGKKELLSVHLVKKIPKDYILLKVLDFEDCGLLCVPKKLGNLIYLRYLSFRGTQIKVLPKSIGKLVNLETLDIRQTQ
VCKVPKEIKKLRKLRHLLTPDSVSSSIEWKDIGGMTRLQKIPQVRMEGDGVAIREVGKLKQLRVLRVLCFGGEHITLLFSSI
NEMEHLQALRIEKSDDRNVVDLTTTSTISELRKLFLDLKLEKLPNWIPQLLHLERLTLRHSDLNNDPLESLKDMPSLLVLSL
SHAYEGQTLHFQPGGFKKLRKLNLEHLWNLNSILMDETALQSLEHLNLKGLDELRTVPDGIQHLKKLKFVLVLSMPTLFV
KEIDRIGKDQHWSINYGCM

>XP_047181033.1

MEKVQGWKDALTEAAGLSGWDSNVIRSENTLVEGIVEDIMRKLNLYSISYDPGTIGIEKHIESIQLLMHFESSDIRIIGIW
GMGGIGKTTISEQIYHTFTMQFDSRSLVLDTQEKIKRDGIDAVRKKYMPELLNEVPSLKGMRILIILDDVTDSVQLKQLLG
RCDSFGQ

>XP_047181197.1

MIQLQKPLSIPFLFLPLLKLMAESLLFSSAESLLGKLASAALQEASLAFGVHRHLQHMKETMGLIRGVLLDAEKKNPQSSA
LSEWLTQIKRVFSDAEDIVDDFECEALRKHVVNTYGSCSRKVRRFFSSSNPVVYRLRMAHHIQDINTRLAKLAAQRSMF
GLQVIHLDTRVVHVREMTHSHVNPSNVIGREDDKQEIIKLLLKDDHGQSLSVIPIVGMGGSGKTTLAKLVFNDATINACF
PLRMWVCVSNDFELRNVLIKILNSAPNPNRENFNNFETEQLQIHLRNTLEGQKSLLVLDDVWNEDRARWDELREIIDV
GVEGSKILVTTRSHKVAAIMHTKSSNSYLLGCLSEKDSLSLFVKYAFEDGDEMKHPQLLKIGEEIVRKCGGLPLAVKTVGSS
LLSRVDEREWESVRDNEIWNLKQNEKDILPALKLSYDQLPSYLRPCFASFSLYPEDTFLFTSQVCRLWGGLGFLPPPKASE
SMFDVATQLLHELWSRSFLSEYEDFGGDFRFKLHDLVVDLAKYIAKGEFEIIQKHNPNLYKNAHHLLLMNNNLHDQALL
PSSLRSIIFPNGANNEDFLNTLVSRCKFLRVLILDICEYESLPRCIGKLKHLRYLSLSNNENLTELPDSVCKLQNLQVLNLKGCI
KLQKLPEGLGNLISLRHLFITTKQPVFPEKEVASLTSIKNLRFYNCDNLESLFKGILLPTLKRLTLIDCKSLKTVPFHAIKNLEVL
VISNCKKLKLSMGLSDEILYSRLKLLILVDLPSLVRLPRWLQGSANSLQSLAIEHCLNLEELPDWLPTLNCLQRLSVLYCPNL
SSLPHNMLHLTNLKEIDVTGSSELWKRYRPGVGQDWHNISHVNLVWQDYRSENEKELTVKS

>XP_047181198.1

MIQLQTPLSIPFLFLPLLKLMAESLLFSSAESLLGKLASAAFQEASLAFGVHRHLQQMKETMELIRGVLLDAEKKNPQSSA
LSEWLIQIKHVFSDAEDIVDDFECEALRKQVVNTYGSCSRKVRRFFSSSNPVVYRLRMAHHIQDINTRLAKLAYQRNVFG
LQVIHQDTRVVHVREMTHSHVNPSNVIGREHDKQEIIKLLLKDDHGQSLSVIPIVGMGGSGKTTLAKLVFNDATINACF
PLRMWVCVSNDFELRNVLIKILNSAPNPNRENFNNFETEQLQIHLRNTLEGQKSLLVLDDVWNEDRARWDELREIIDV
GVEGSKILVTTRSHKVAAIMHTKSSNSYLLGCLSEKDSLSLFVKYAFEDGDEMKHPQLLKIGEEIVRKCGGLPLAVKTVGSS
LLSRVDEREWESVRDNEIWNLKQNEKDILPALKLSYDQLPSYLRPCFASFSLYPEDTFLFTSQVCRLWGGLGFLPPPKASE
SMFDVATQLLHELWSRSFLSEYEDFGGDFRFKLHDLVVDLAKYIAKGEFEIIQKHNPNLYKNAHHLLLMNNNLPDQALL
PSSLRSIIFPNGANNEDFLNTLVSRCKFLRVLILDICDYESLPRCIGKLKHLRYLSLSNNENLTELPDSVCKLQNLQVLNL

>XP_047181297.1

LSLTRYKFQSTFEDGSLNSSKGAKDFKWDDPRMASHFIEETEVVGFELPRDELIGCLIKGTDQLSLISVVGMGGLGKSTLA
KHVFDNQNVKRHFYCRSFITVSQSYTVRELLTEMVQKFCKDANDPIPKGLHNMDDQTLVTELRQYLQSKRYLVLFDDV
WKENFSDEIEHALPKNKKGSRIIITTRNMHVAEYFKKSVVVHVHKLQHLSPDKAWELFCKKAFRFEPSEQCPTELEDMSK
EIVQKCKGLPLAIVCMGGLLATKEKSILEWRKVCQNLRMELERNTHLNSLKWILSLSYDDLPHNLKSCMLYFGVYPEDYSI
SRKRLTRQWMAEGFIKNEERRPMEDVAEEYLTQLISRSLVQVSRVGFDGKVKSCQVHDLLRDIIIRKMNELSFCHLMRE
DDELVTVEKTRRFSIASCSKNVLRETSNSGIRAIYVFKKSELPEDFVGSLSAKFKLLKVLDFESTMLNSVPNNLGNLFHLRYL
NLSHTKVKILPRSVGKLLNLETLDLRQTXVQVLPREIKNLTKLRLLPVYYRKYEGQYSMLNFTTGVKMQKGIGCLKSLQKL
YFLEADHGGLELMQELKMLKQLRKLGIRHVKTEYADALSSAIGEMNHLESLNVSAKDQDEIIDLNFLSTPTSLLVLNLKAR
VTKFPDWIPKLKYLVKLRLGLSNLEGDPLDSLKDLPSLLRLNMWDNAYIGEILHFKRGGFPRLKELDLTRLSRLNSISIDEG
ALLGLEHFRFKDNPQMKVVPHGLKHLKNLQFLGFADMPAELVESIDPEKDGQDYSVIKHIPLVLIRQNVGPKFHDYELR
AIPTLATKFKFAHDELDTVEKTRRFSIASCSKNVLRETSNSGIRAIYVFKKSELPEDFVGSLSAKFKLLKVLDFESTMLNSVPN
NLGNLFHLRYLNLSHTKVKILPRSVGKLLNLETLDLRQTKVQVLPREIKNLTKLRLLPVYYRKYEGQYSMLNFTTGVKMQK
GIGCLKSLQKLYFLEADHGGLELMQELKMLKQLRKLGIRHVKTEYADALSSAIGEMNHLESLNVSAKDQDEIIDLNFLSTP
TSLLVLNLKARVTKFPDWIPKLKYLVKLRLGLSNLEGDPLDSLKDLPSLLRLNMWDNAYIGEILHFKRGGFPRLKELDLTRL
SRLNSISIDEGALLGLEHFRFKDNPQMKVVPHGLKHLKNLQFLGFADMPAELVESIDPEKDGQDYSVIKHIPLVLIRQNV
GPKFHDYELRAIPTLATESTTEMAEAAISFALGEVFQILKEEKSLLSGINKEFLDIRDELESIQAFLKDADRKAADEANTNDG
IRTWVKQVRQVSVRIEDVIDEYLRVIHQVPRHGFGASICKITNLIRTSLSRHQIAVEIQDIKLSLSLIKERSERYKFQVLQEKP
SSSSTGRIEGSGWNDHRMGSLFIEETEIVGFELPRDELLSLLLEGKKERTLISVVGMGGLGKTTLAKHVFDSENVKIHFHC
RACITVSQSYTVRGIFTDMIKQFCRETKDPLPEMLEEMDEKTLISELRQYLEHKRYLIFFDDVWHEDFCDQVELAMPSNN
RSSRIIITTRMIHVVEFFKKSFPLHVHNLQPLPSDKAWELFCKKAFKFELDGQCPAELKEMSNEIVGKCKGLPLAIVAIGGL
LSTKSKTVFEWLKVSQNLNLELHRNAHLTXLTKILSLSYDDLPYYLNPCILYFGIYPEDSSINHKRLTRQWIAEGFVKSDGRT
FEQVADEYLSELIYRSLVQVSWVGFEGKVKSCRVHDLLHELIVRKMKDLCFCHFVHEGDDESATSASTRRLSIDTSINNVL
KSTNFTHIRALHAFGKGGTVEPFTGLLASKSRVLKVLDLESTSLNHVPRNLGNIFHLKYLNLKNTKIRSIPKSVGRLQNLETL
DIRETLVHELPSEINKLKKLRHLLAFHRNYEAEYSLLGFTTGVLMKKGIKNLTSLQNLCYVEVEHGGIDLIQELRFLKQLRKL
GLRRVRREHGKAICASVAEMTHLESLNITAIGEGEIIDLNSISFIPQLQRLHLKTRLERMPNWISKLEFLVKMRLALSNLKD
DPLRSLENLPNLLKLTIWDNAYGGEILHFQSGGFRKLKELNLARLNTVSAILIDKGALLSLEHVKITKITHLKKVPSGIKDLYN
LKVIDFCDMPTELVESIDPKNGQDYWIINHVPLVFIRRWVGPKLNDFEVRIVHSSTKESLTN

>XP_047181793.1

MEKIVTWLESNNGFKAIGVHGMCGTGKTTLAKMVLSDPRVKDKYKKPIWVCLYDLQSKEEMDIRIVKEMLALLDDDP
DLLAEEPEDKWLVKNLHDKLLDQKKYLIVLDAVWHCNDWFNNLFCVDQDGGDTSKELFSQALPKETGGAVIVTSRQKE
VTTKLVREENLIHLKPWGDEKLKNFVKKCLEKQDKITEENINCVAYHCHGIPYVAATMSGWIAKQITKKSDSN

>XP_047181831.1

MAITLGEGASSSSGFDRGWTYDVFLSFRGEDTRRSFTGFLYHGLCQRGINVFIDDEKLRRGEDLSPALLGVIQESRIAIIVF
SQNYAFSTWCLDELAKIIDCYKTRGQLVWPVFFHVDPSVVRHQRGTFQTAMAQHEVRFMGNVQKLQKWKKSLFEAS
NFSGWTLENGYEFQIIQDIVEEASRKLSHTILHIAEYPVGIETRISEVMPLLQIXRIKSISFPYLDQNERLELRDLPVSEPGSIR
FRLKLTTQGVLLWFLKQTLLLMAITLGEGASSSSGFDRGWTYDVFLSFRGEDTRRSFTGFLYHGLCQRGINVFIDDEKLRR
GEDLSPALLGAIQESRIAIIVFSQNYAFSTWCLDELAKIIDCYKTRGQLVWPVFFHVDPSVVRHQRGTFQTAMAQHEVRF
MGNVQKLQKWKKALFEASNFSGWTLENGYEFQIIQDIVEEASRKLSHTILHIAEYPVGIETRISEVMPLLQIEPGEDIRVIG
IYGLGGIGKTTIARALYNMIAGQFEATSFLADIRESSSQRQGLVQLQESLLFDTVGDKNIKLGSIYKGIPIIKKRLCCKKVLLII
DDVDRLEQLQALAGGRDWFGFGSVIIITTRDKHLLSAHQVDKTYEVKKLNYGEAFELFTWSAFKRKTPDAGYLEVSNHV
VLYAEGLPLALKIMGSNLFGKTVEEWKSALGKYEKIPNKEVQNVLRVTYDNLEENEKEIFLDVACFFKGETVEYVEKTLQA
CGFYPTIGISVLIDRSLVSVDEYNRLRMHDLIQDMGREIVREVSPLEPGKRSRLWYHEDVLEVLTENKGTYRIQGMMVD
LPDGYMVHLKDDSFKKMKNLKILIVRNGNFFGSPQHLPNNLRLLDWMKYPSSLPSSFQPKKLVVLNLSGSRFTMQEPF
MYLDSLTSMDLSSCELLTKLPEIAGVPNLTQLTLDYCTNLEEVHESVGFLEKLVEFRAYGCTKLKVFPNAIRLTSLRSLILNW
CSSLQNFPAILGKMDNLITISIEGTGIKELPPSIGNLVGLQELSMTSCLSLKELPHNFDMLQSLTNLDMDGCPQLRNFLTKL
ANMGESTHTFGNILSLNLENCGLIDEDLPIIFNSFPNLASAVLSRNIFEALPSCIQQCPCLELLHLDNCKNLQEIPAFPPNM
QYINAQNCTSLSVDSSNLLLNKVFIFFLKSSYFETFEGWELQAMVPGTVVPEWFDHITKGEYMTFWVREKFPAIIICFVLE
VESEMKKIFNCEIRFYINGEEVYELEIPRGFSDMVTDHVWLYDLRTHSSINWRSLDLYLMDDWNQVEISCEKISGASNVT
VSWCGVHVVKQEANMKDILLTDPDPDLDSVIASGSNTLVFDHPVKAQLQSQVTSFILQTPQNXI

>XP_047181832.1

MAITLGEGASSSSGFDRGWTYDVFLSFRGEDTRRSFTGFLYHGLCQRGINVFIDDEKLRRGEDLSPALLGAIQESRIAIIVF
SQNYAFSTWCLDELAKIIDCYKTRGQLVWPVFFHVDPSVVRHQRGTFQTAMAQHEVRFMGNVQKLQKWKKALFEAS
NFSGWTLENGYEFQIIQDIVEEASRKLSHTILHIAEYPVGIETRISEVMPLLQIEPGEDIRVIGIYGLGGIGKTTIARALYNMI
AGQFEATSFLADIRESSSQRQGLVQLQESLLFDTVGDKNIKLGSIYKGIPIIKKRLCCKKVLLIIDDVDRLEQLQALAGGRD
WFGFGSVIIITTRDKHLLSAHQVDKTYEVKKLNYGEAFELFTWSAFKRKTPDAGYLEVSNHVVLYAEGLPLALKIMGSNLF
GKTVEEWKSALGKYEKIPNKEVQNVLRVTYDNLEENEKEIFLDVACFFKGETVEYVEKTLQACGFYPTIGISVLIDRSLVSV
DEYNRLRMHDLIQDMGREIVREVSPLEPGKRSRLWYHEDVLEVLTENKGTYRIQGMMVDLPDGYMVHLKDDSFKKM
KNLKILIVRNGNFFGSPQHLPNNLRLLDWMKYPSSLPSSFQPKKLVVLNLSGSRFTMQEPFMYLDSLTSMDLSSCELLTK
LPEIAGVPNLTQLTLDYCTNLEEVHESVGFLEKLVEFRAYGCTKLKVFPNAIRLTSLRSLILNWCSSLQNFPAILGKMDNLIT
ISIEGTGIKELPPSIGNLVGLQELSMTSCLSLKELPHNFDMLQSLTNLDMDGCPQLRNFLTKLANMGESTHTFGNILSLNL
ENCGLIDEDLPIIFNSFPNLASAVLSRNNFEALPSCIQQCPCLELLHLDNCKKLQEIPAFPPNMQYINAQNCTSLSVESSNL
LLNKETFEGWELQAMVPGTVVPEWFDHITKGEYMTFWVREKFPAIIICFVLEVESEMKKIFNCEIRFYINGEEVYELEIPR
GFSDMVTDHVWLYDLRTHSSINWRSLDLYLMDDWNQVEISCEKISGASNVTVSWCGVHVVKQEANMKDILLTDPDP
DLDSVIASGSNTLVFDHPVKAQLQSQVTSFILQTPQNNNSSTIVLPTTVQTSLTVNDADMEAFYAVLDDEISVVSLNNDS
TVVSKLTNQRPSEETKKALKALQAHVTKEFSALLGPNEYSTMNDTLEYLTNLPAEDGISVEIRSLIIQVSRQFTRWSRDYTS
ENKKVESTTAKLLKADELEKCLEANKTNFKQVMCMENELCNDLAYLEQRKRELEEQIKAVKANISASEAAKNMATQIKR
KIFGEAKILKAQRDELREQVPHLRDEQELAKKIQSNIRDEWSKLGEKFNYELRHGKID

>XP_047182513.1

MGYEVLPHDFVLEGDRIIKMVEAVVSFAVDRLGDLLIEEARLLSGVSNKVKSMQNELKRMQCFLRDAESRQDESDTIKN
YISEVRKLAYDAEDVIEIYAIKVAFGISIGTKNPLSRAKNIHKVGSELITINSRISDLTRSLQTYGLTATKDNEEASEVKRQLR
WSYSHIVDEFIVGLDKDINKVAEWLINENQDCRFVYICGMGGLGKTTLAKSIYHYNTIRRNFDGFAWAYISQQCKKRDV
WEGILLKLISPTKEERDEITKMKDDELARKLFKVQQEKKCLIILDDIWSNEAWDILSPAFPSQNTRSKIVFTSRNKDISLHV
NPEGFLHEPSCLNAEDSWALFKKKAFPRQDNPDSTTSDDFKRLGREMVAKCAGLPLAIIVLGGLLATKESVSEWEKIHR

HLSSYLIGAXVRDRRRLDEVLDLSYQDLPCQLKPCFLYLSQFPEDSEIPKTKLLQLWVAEGVVSSQYESDRDETMEDVAER
YLGNLISRCMVQIGQMGSTGRIKTYRLHDLMRDLCLSKARKENFLYIINGSQQNSTVIATGSSNVSDARQINEVRRLAVY
LDQHVDQLIPQDKQVNERLRSLVFFHDKKCRMENWDLVRGVFVKFKLLRVLDLEGIKGLKGQSLPKEVGNLLWLKFLSL
KRTRIQVLPSSLGNLENLQFLNLQTVNKVSWDSTVEIPNVICKLKRLRHLYLPNWCGNIVNNLQLDNLTNLQTLVNFPAS
KCDVKDLLKLRRLRKLVLNDPRHFQKFSESFSPPNKRLDCLQSLSLRTDMLSFPENVVDVEKLVLGCPFLRKLQVEGRME
RLPDASLFPRHISKLTLWGCRLVEDPMVTLEKLPNLKFLNGWDMFVGKKMTCSLNGFLQLKVLVLRGLPKLDDWTIEN
QAMPSLYRLSISDCNNLKTVPDGLKYITSLRELEIRWMPKSFKTRLGTAGEDYPKVQHVPSIIFLN

>XP_047182836.1

MQNHKKCKPLVLSSEFVGKKFEVNVKKTWKFLQDDRVFTIGIYGMGGVRKTFITTYIYNGIKTSKIFEDVLLVTVSHNFN
VSKLQENIVEEIKVNLCGYINKKSRAIILVSKLRKRKNTILIFDDTWKYIDLKIVGIPLQVKSIKVVMTTRLRHVCQEMQCLS
DNMIEVGTFYDDEYEETLELFLQQLQHFGIPSKLHPQVXDIARCVVSKCDDLSLAISVMARIMRGKTNIHKGRYALNQFD
KWEMRQEMEEEMLTVLRRSYDNLTKSEVKNCFLYNALLPKLFC

>XP_047182914.1

MEFASSSSSSSFLKSEPHFIYDVFINFGGEDIGRKLVSHLHSVLLQAQVKTLVNEENLQEGMKLEEHMGAIAVSKIAIIVFS
KTYTESTCCLLELEKIIECLETFGQVVLSVFYEIEPLDVRDQKDDFGKALEETAKKSYLGEQLEHALSRWRRALTTAASMTS
WDVRDFRDDAELVEVIVRHVHTLLDYKDLFITEFPVGLESRVEKVIGCIQNHDTKVCIIGIWGMVGTGKTTIAKAIYNRIY
RLFIGKSFIETIREFWCLLYRTYVDLQEHLLDDVLKYKFELENDEMGRTMIKTKFSRKKLLIVLDDVSEFGQLENLCGNREW
FGRGTVIIITTRDAKMLNRLKVNYVYKMDAMNKNDSLELLSWHAFGEAKPRKELNEIARNMVANCGGLPLALKVLGG
WTMQIPYVQVQEWKYVSLRLPNAFARVREKLKISFDGLGDMEKDIFLDVCCFFIGKERGYVTEILNGCGLHADIGITVLIE
RDFIKVERNNKLEMHPLLRDMGREITRRDWPQEPGKRSRLWFHEDVKDILKTKSVRTFFI

5. NLR genes of Vigna unguiculata

>XP_027901769.1

MMAQAIVNFIVQSLGDLLIQEAVFLYGVEDQVLQLQTELRMMRSYLQDADRRQDENESLRSWISEIREAAYDSDDVIE
SYALREASRRNLPGVWNLVRRYVSIINRFIEIHIVGSHVDNVIARISSLTRSLKTYGIKPEKGEPSNSVHGRQILRRSYSHVIE
EDIIGVDDDVKSLETCLLDPSKRVVAICGMGGLGKTTLAKKVYHSVDVRNSFESLAWAYISQHCQARDVWIGILFRLISPS
QEQRQEIENMRDEELAKMLYEVQVEKSCLVVLDDIWNADSWNKLKPAFPHGTSVSAVGSKILLTSRNIDVAFQMDPSC
YLHTPEFLNEVDSWELFQKKALLKIHDPDYREKEKLGREMVGRCGGLPLAIIVLGGLLASKPTFNEWDTVCKNINSYLRR
ANGQEQRLGEVLALSYYELPYQLKPCFLHLAHFPENLEIPTKKLIRIWVAEGIISLDHSEGEGEEALEDVAQRYLTELVERC
MIQVVEKSSSGRIRTCQMHNLMRELCVERAYRENFLLEINSRNVDESRGTSRARPVGKVRRIALFLDHDVDRFFPSQLKS
HHHLRSLLCFHEKPAKLSEWGLMKPFFKKCRLLRVLNLEGMQGLGGKLPKEIGYLIHLRFLSLRNTKIDELPASIGNLKCL
MTLDLLTGNSTVQIPNVIGNMQKMRHLYLPESCGNSIERWQLDNLKNLQTLINFPAEKCHVRDLMKLTNLRKLVIDDP
NFGGIFRYPNVQFKHLESLFFVSYEDISVVHVALGCPNLYKLHIEGPIKNFPEPHQLSSKLQKLKLSGSGLVVDPMPTLEKL
PNLRLLELQLDSFVGKQLHCSGTGFAQLKSLVIHDLFNLEEWRLDKGAMTCLRELKIENCTKLEKVPEGLRFLTSIQHLEIR
SMFAAFRTKLEKGGEDHYKIQHVPTVVFCYCDY

>XP_027901987.1

MAEAVLELVLENLSSLVGKELALFLGFHDDLERLASLLTTIKATLEDAEHKQFSDVAIQIWLQKLKDAALTLDDIMDECGC
ELLGMEFQRIRGVSNKVQIFGSLSSFNPKHVAFRYKIAKKMRRISKRLEEIGEERTKFHFTETVSERSGDSDWRQTTSFITE
PQVYGREEDKDQIINFLVGVASRSENLSVYPILGLGGLGKTTLAQLIFNHERVVNHFELRIWVCVSDDFSLTRITKAIIEAVT

QRSCDDLDLEPLQRMLQDLLRSKRYLLVLDDVWDDKQENWQKLKSVLACGTMGASILVTTRLSKVAAIMGTIPPHALS
ELPDSYCWELFRSRAFGANEVEPEELVFIGKEIVKKCRGEPLAAKALGGLLRFKREEKEWLNVKENNLWSLPQDEDSIMP
ALRLSYLNLPIKLRTCFAYCAIFPKDEIIKKENLIELWMTNGFISSSEILNAEDVGESVWNELYWRSFFQDIKTDECGKVKSF
KIHDLVHDLAQFVAIDVCCVSKQNHVTTLSERIHHLSIYGKDSIPLHQVNSLRTYIKPSQRNYFGQICDDVLKCHSLRVLHY
ERWEPFIPSSIGHLKHLRYLNLSLGKFQTLPISVCKLWNLQILKLDYCKHLRKLPDNLVGLKGLQQLSLKGCFSLSRLPPHV
GNLTSLRILNMYIVGKQRGSLLAELGPLKLKGDLLIKHMGKVKSVKDAKEANMFGKKLNKLRLSWDRYDEELELQENVE
EILEVLQPDPKQLERVTIDGYKGAYFPKWMSSSSLHLLNFLELNDCGSCLKLPQLGKLPSLKIICLRNISNVKYLYEESDDGG
VVFMSLELLSLRHLPNLTRISREGGENMFPHLSTLEIIECPKLLNVSAGFECFTCVKDILITGCREKVEGVNEALRHMTALG
KLTLDDLPNLESLPHCLGNLSLLHDLTIHNCSKLRCLPTCLSRSLQYLTISGYTHPELKKRCEKEKGEDWPTIAHIPHLDLST
WR

>XP_027902400.1

MGGIGKTTLAEVVFHRLRSQYQGSYFLANERDQSNKHGLIHLKKIMFSRLLGHEVDIDTPNSLPENIVRRIGCMKVLVVL
DDVNDSDHIEKLLGSLENFGMGSTVIVTTRDEQVLRINRVSKTYKVKEFSSDEALELFNLNAFSESNEAKEYGEISKLVVDY
AKGNPLVVKVLAHLLQGRNKEEWESLLGKLKTMPPREVYDVMKLSYDSLDRKEQQMFLDLACFFLRMLVPVQVGDLK
CLLKDNESDNSVAFELRRLEDKALITISEDNTVSMHDSLQEMAWEIVRHECVEDPGRRSRLWDPNDISEALKNEKVKAS
LNTISCS

>XP_027902404.1

MTRLVKPFIQSKGLVGIEEKIVEVELLIRKDPEDTTFIGIWGMAGIGKTTLAEQVFNKLRSEYEGCYFLANEKEQSHRHGII
SLKNEMFSALLGHAVKIDTPTSLPQEILRRISCMKVLIVLDDVNDLDHTEKLLGTLDNFGSGTRIIVTTRDKQVLKANKVD
KIYQVNEFSSKEALELFNLIAFNQSDHEMEFNELSQRVVDYAHGIPLVVKVLARLLCGRNRQVWESELHKLKKMSLTEVY
DVMKLSYNGLDRKEKQIFLDLACFFLGSHVRVNAGDLKYLLKEDENDDTIVVGLERLKDKALITSFDDNFVSMHDALQE
MAWEIVHQESRKPGSSTWLLDPNDDVYKALKNDKCLCAIRSLRIHLPTTGKKKLIPGIFAEMSSRLQFLEISVENNDDLFD
QVYPLAEELQFLETELRFLCGLHYPLKSLPENFSTDKLVILKLQHGRMEKLWEGLKNLVNLKELDLMHSKELKKLPDLSQA
TNLEVLVLLGCSMLTSVDSSIFSLPKLESIDLWGCESLTLLTSNSQFCNFSYLNLDFCNNLREFSLISQNMKELRLGFTKVKV
LPSSFECHSKLKSLHLTRSDIEMLPSSFKNLTQLQHLDMNNCNKLRTIPELPPSLKTLEVSKCNSLQNLPKLPSSLKTLNAV
DCKSLETVSFHSTADEQLTENKKRYLFWNCRNLDESSAEAIVVNAEVNLMELANQPLPTPSQEHEFYNDYEYNYHSYQG
VYVYPGSSVPAWFKHTETNGDIIIDLSSASPFELFGFIFCFVLNKFHETDITGRLEFNITISNVDVGEGKIGSVKIYTDYYSD
WSYHVCVMFDQRCSVTLNNIAREQKRFKINVTVGARIEFYDNYHELPQEVLKGFGVSISAYNTQQIEL

>XP_027902740.1

MEQPCSMKKYTYDVFLSFRGDTRFGFTGHLYNALRQRGISTFMDHEALERGEQISATIFKAIEESRMAIVVFSKSYASST
WCLEELLQILDCKKTKKLKVYPIFYNVDPSEIRHQTGSYGQQLANHENKMRYKKEKVENWRLALHEASNLVGWSFKDG
YGYEYELITQIVDTVGIPKRKLLTVDENLVGVELRIPQIEFRLQISDPAIIMMGICGVSGIGKTTLAQVLYNYISQQFEGSCFL
NDVRGNSAKYGLAYLQEAIISDIAGDSTKVVNENQGIPILIRKLQGKRVLLILDNVDKLEQLEYLAGECNWFGLGSRIVITS
SCKDVLASHGVKNVYDVPKLDNYEAIQLLSSMVTVGPVPGYYYGVWKRVVDCSNGLPLALKYIGSDLLEKMKAVDCYLS
ETSVDELEIALERYEGVCDGGEIQSLHKVIYFSLNECEKRIFLDIACFFIGETLSYVEEILSACGFDPKYSISRLIDRSLLSVTPSG
NLMMHDNIKDMAMKIVEQESPLHPGKRSRLWYPQDVIHVLNENEGTDQIEVMMLVDLPQGNEVLKLSDKAFKDMK
NLRILIIKDAIYSGVPQYLSNSLRVLIWSGYPSDCLPLDFLNLPSDCLILNNFKNMQCLTKLDFTDCEFLTEVPDISGISDLRV
LNVDNCINLIKIHDSVGFLGNLEVLTASGCSCLEIIPSTFKLASLRELTFSECLRLVRFPKILCEIQNLCYLNLWQTAIEELPFSI
GNLRGLESLNLMDCARLDKLPSSLFTLPRLMEIQADSCRRFDISVECENHEQLKSTASSNTVYLYLSSCNLTTEHLVTCLSG
FAGVVYLDISYNNFTVLPACIKECVHLKTILLSNCKQLQHISVIPPKLKDVNALNCTSLTSQSSNVLLNQTFHATRQSTVILP
GSRIPEWFDHFSSERSIIFWGRERFPRICVCVSFGMVGNPLHHFRVRIIINGYKSILSQHCYDWSIETDHVWLFDLTAFVN

HNDLIGTFVKSDWNSVEIEVERNACMLDENARIMATVKWYGIHVYREESKMEDISFTKPKNLQENSTSSKRVGSQVLD
SLKRQKGNLEVISLWD

>XP_027903516.1

MSNKSSTQIKYDVFVSFRGEDIRDGFLSHLTEAFDVKKINAFVDDKLEKGEELWTSLVAAIEGSSISLIIFSPDYASSRWCLK
ELVTIFECKEKYGHTVIPVFYHVTPTDVRHQSSDSYRKAFAEHARKHKNEVQLWRRVFKKSADLSGIESSKFRNDADLLK
AIVDLVITKLRKSLVNSKVLVGIDKRIADVESLIHRESEKARLIGLWGMGGIGKTTLAEEVFNKLTSKYEGCYFLANEREQLS
RHGKVSLKKEIFSALLGGVKIDTPNSLPEDIVRRIRQMKVLIVLDDVYDSDHITDLLGAVDNFGSGSSIIVTTRDEQVLKAN
KADEIYHVRELTSDEALELFNLNAFNQSVHQREYNELSKRIVHYAKGLPLILKFLAHRLHGKNVEVWESELDKLKKVPPAK
VYDVIKLSYDNLDRKEKEVFLDLACFLCRSREKITIGYLKYLLKDGERDNSVVVALERLKDKALVTFSKDNVVCMHDSIQE
MAWEIVRQESTEDPGKRSRLLDPDDIYEALENDKVSEAIRSIQIDLKELKEQKLMPHIFAKMSRLRFMEIYGECNYNCYS
QLVAEGLQLLGTELRFLCWDFYPLKSLPDKFSGEKLVILKLESGGMEKLWDGVKNLANLKELDLRNSQNLKELPDLSKAT
NIEVLCLMRCLSLNSVHPSVFSLPKLEILNLCGCESLTILATDLHLCRLSYLNLSRCYNLKEFTLKSDNLKELRLELTRMKALPS
SLGHQRRLELLSLGRSLIERLPAFFSNTQLLHLDISFCWNLVTIPELPLSLQTLYAAGCESLKTVFFHSTAVEQLKENRKQVL
FFKCRNLDEHSLEAIGLNARINVMKFANQHLSAPKQDDFENYNDYDKKYESYQAFYGYPGSSVPEWLEFKSKKDYVIIDL
SSAPPSPVYGFILGFVLFEELDKLEFSIAITDCEGKGIFDEVRLKLSYMFWSVKSCEVVVMYDQRCSNFLNSIAKNLTRFKIN
VRSNGKYYRAFPSRGFGVSIVRTSSYSSFMQQMELGDSMYQFH

>XP_027903517.1

MLYFFVSCSFLCVFLFIFSRLFPKLNGVEKKPISNNNSPKIKYDVFVSFRGEDIRDGFLSHLTEAFDVKKINAFVDDKLEKGE
ELWPSLVAAIGGSSISLIIFSPEYASSRWCLKELVTILECKEKYGHTVIPVFYHVTPTDVRHQSSQGYKEAFSEHARKRKSEV
QLWRRVFEKSADLSGIESSKFRNDADMLKAIIDLVLMRLRKSVVISKGLVGIDKRIADVESLIHRDVESEKARLIGLWGMG
GIGKTTLAEEVFNKLRSKYEGCYFLANEREQSSRKGKVSLKEEIFSALLGDVKIDTPNSLPEDIVRRIRQMKVLIILDDVNDS
DHITELLGAVDNFGSGSSIIVTTRDEQVLKASKADEIYHLRELTSDEALELFNLNAFNQSDHQREYDELSKRIVHYAKGLPLI
LKVLAHRLHGKNEEVWESEIDKLKMVPPTKVYDVIKLSYDDLDRKEKQIFLDLACFVCRSRLKITIDCLKYLLKDGERDNSV
VVALERLKDKALITFSKDNLLCMHDSIQEMAWEIVRQESTENPVNRSRLWDPDDTYEALENDKVSEAIRSIQIDLKALKE
QKLMPHIFAKMCRLRFMEIHGYDNYNCYSQLVAEEELQFLATELRFLYWDFYPLKSLPDKFSGEKLVILILGSGRMEKLW
DGVKNLVNLKELDLRNSLRLKELPDLSKATNIEVLCLRECISLTSLHPSVFSLPNLETLNLCGCMRLTIPATDIHLRSFSYLDL
DGCCNLMEFTLTSDNMKELNLELTSMKALRSSFGHQRKLEFLCLGGSHIERLPSSFVNLIQLLHLDITFCGKLLEIPELPLSL
QTLYAGGCESLKTVFFHSTAVEQIKENRKQVLFDNCMNLDERSLEAIGLNARINIMKFANQHLSAPRQDDFQNYNNHY
DSFQAFYAYPGSSVPEWLEYKTKIFCNYRSLFCSTFPCIWLHILLRTPRRS

>XP_027903518.1

MLYFFVSCSFLCVFLFIFSRLFPKLNGVEKKPISNNNSPKIKYDVFVSFRGEDIRDGFLSHLTEAFDVKKINAFVDDKLEKGE
ELWPSLVAAIGGSSISLIIFSPEYASSRWCLKELVTILECKEKYGHTVIPVFYHVTPTDVRHQSSQGYKEAFSEHARKRKSEV
QLWRRVFEKSADLSGIESSKFRNDADMLKAIIDLVLMRLRKSVVISKGLVGIDKRIADVESLIHRDVESEKARLIGLWGMG
GIGKTTLAEEVFNKLRSKYEGCYFLANEREQSSRKGKVSLKEEIFSALLGDVKIDTPNSLPEDIVRRIRQMKVLIILDDVNDS
DHITELLGAVDNFGSGSSIIVTTRDEQVLKASKADEIYHLRELTSDEALELFNLNAFNQSDHQREYDELSKRIVHYAKGLPLI
LKVLAHRLHGKNEEVWESEIDKLKMVPPTKVYDVIKLSYDDLDRKEKQIFLDLACFVCRSRLKITIDCLKYLLKDGERDNSV
VVALERLKDKALITFSKDNLLCMHDSIQEMAWEIVRQESTENPVNRSRLWDPDDTYEALENDKVSEAIRSIQIDLKALKE
QKLMPHIFAKMCRLRFMEIHGYDNYNCYSQLVAEEELQFLATELRFLYWDFYPLKSLPDKFSGEKLVILILGSGRMEKLW
DGVKNLVNLKELDLRNSLRLKELPDLSKATNIEVLCLRECISLTSLHPSVFSLPNLETLNLCGCMRLTIPATDIHLRSFSYLDL
DGCCNLMEFTLTSDNMKELNLELTSMKALRSSFGHQRKLEFLCLGGSHIERLPSSFVNLIQLLHLDITFCGKLLEIPELPLSL

QTLYAGGCESLKTVFFHSTAVEQIKENRKQVLFDNCMNLDERSLEAIGLNARINIMKFANQHLSAPRQDDFQNYNNHY
DSFQAFYAYPGSSVPEWLEYKTKIFCNYRSLFCSTFPCIWLHILLRTPRRS

>XP_027903519.1

MLYFFVSCSFLCVFLFIFSRLFPKLNGVEKKPISNNNSPKIKYDVFVSFRGEDIRDGFLSHLTEAFDVKKINAFVDDKLEKGE
ELWPSLVAAIGGSSISLIIFSPEYASSRWCLKELVTILECKEKYGHTVIPVFYHVTPTDVRHQSSQGYKEAFSEHARKRKSEV
QLWRRVFEKSADLSGIESSKFRNDADMLKAIIDLVLMRLRKSVVISKGLVGIDKRIADVESLIHRDVESEKARLIGLWGMG
GIGKTTLAEEVFNKLRSKYEGCYFLANEREQSSRKGKVSLKEEIFSALLGDVKIDTPNSLPEDIVRRIRQMKVLIILDDVNDS
DHITELLGAVDNFGSGSSIIVTTRDEQVLKASKADEIYHLRELTSDEALELFNLNAFNQSDHQREYDELSKRIVHYAKGLPLI
LKVLAHRLHGKNEEVWESEIDKLKMVPPTKVYDVIKLSYDDLDRKEKQIFLDLACFVCRSRLKITIDCLKYLLKDGERDNSV
VVALERLKDKALITFSKDNLLCMHDSIQEMAWEIVRQESTENPVNRSRLWDPDDTYEALENDKVSEAIRSIQIDLKALKE
QKLMPHIFAKMCRLRFMEIHGQIFWGKACDIDIGIGSNGKTLGWSEESGEFKRT

>XP_027903611.1

MEETAQQMKRPFGIPEKPEFTVGLDEALKKLKMNVLSEGVSVMVLTGVGGSGKTTLATMLCWDEQVIGKFKENILFVT
FSKAPELKIIVERLSEHLGYQKHLQTLKFSCIDGDQTLLLLESIILAIPFVNQRIYVRA

>XP_027903700.1

MTEAVVELVLGNLSSLVGKELALFLGFHDDLERLASLLNMIKATLEDAEHKQFSDGAIQIWLQNLKYAALTLDDIIDEYGY
EVLGMEFQTLRNVSNKVQNFGSSSSFHPKHVFSRYKIAKKMKRISERLEEIAEERAKFHLTEMVSERRSGISEWRQTTSF
VSEPQLYGREEEKDQIINLLVGVASRSENLSVYPILGLGGLGKTTLAQLIFNHEKVVNHFELRIWVCVSDDFSLKRITKAVIE
AVTQRSCDDLDPEPLQRMLRDLLRSKRYLLILDDVWDDKQENWQKLKSVLACGTMGTSILITTRLSKSWEQYLLAAKLS
TIFNYQLSELSDSYCWELFRSRAFGANEVEPEELVFIGKEIVKKCRGVPLAAKALGGLLRFKREEKEWLNVKENNLWSLPQ
DEDSIMPTLRLSYLNLPIKLRPCFAYCAIFPKDKIIEKQYLIELWMANGFISSSEILEAEDVGEGVWNEFYWRSFFQDIETDE
YGKVKSFKIHDLVHDLAQFVATNLFFCSVCCVSKDNHIATLSERIHHLSIYGNDSIQLHQLNCLRTYISPQLSHQRFHDVLK
CHSLRALYCELWKSLFPSSIGHLKHLRYLNLSEGTFKTLPKSVCKLWNLQILKLDNCRHLKKLPHNLVLLKGLQQLSLKGCL
FLSSLPPHVGNLTSLRILSMYIVGKERGSLLAELGPLKLKGDLEIKYMGKVKSVKDAEEANMSSKQLNTLRLSWHRLFWD
WISKDMLSMDRYDKDLEVQENVEEILEVLQPDPKQLESMTVLGYKGAYFPQWMSSSSLHLLNFIELTDCRNCVKLPQL
GKLHSLKTIRLCNISNVKYLYEESCDGGVVFMSLEILSLRYLPNLTRLSREGGETMFPHLSTLEIIECPKLLLTSDETQIRTRIR
RYEL

>XP_027904257.1

MADYFVFDIVNSLLGKIATYAYEEVSQAYGLYEDVQGIKDTLSIVKGVLLDAEEKKEHNQALREWLRQIQNVCTDAEDVL
DGFEYQSSRKQVVKASGSIRVKVDHFFSSSNSLVFRLRMAHQINDVRERLDKIAADGNKFGLERIDVDNRLVQRREMT
HSHVDASDVVGRESDRDEIIKLLMQSHPDGDKSVCVIPIVGIGGLGKTTLAKLVFNDKRVDELFKLKMWVCVSVDFDIK
QIIIKIINSASASSSAPTMALGHQENINSLDIDQLQSRLRHKLSGEKFILVLDDIWNDDYAKWIELKNLIKVGAVGSKIIVTT
RSNSIGSMMGTISPYILEGLSPQNCLSLFVRWAFKDGQEKQHPDLMEIGKEIVKKCRGVPLAVRTLGTSLFSASDIEKWE
FVRDHEIWNLKQKREDILPALKLSYDQMPFHLKHCFAYFSLFPKDFRFINTEITNLWASLGLLQSPDGSQKVDKIARQYIE
ELHSRSFLQDFTDYGLFYFFNVHDLVHDLALYVAKEEFLMVDSRTQRIAEQVRHLSIVETDSLDHTLFPKSKSLRTIIFPVE
GVGLARETLLQTWLSRYRYLRILDLSDSSFETLPNIISKLVHLRVLDLSNNRQIKKLPHSIGKLQNLQVFSVSGCMQLEALP
KGLGMLKKLRELKITTKQSFLSQEEVAKLSNLQTLSFEYCVNLKFLLEKEQLAQLSSLQILVVRSCGSLESLPFYILTKLEALFV
ADCEKLNLFLDEEAQTLRMKFLHVENFPWLTRLPTWIEGAADTLQTLIIYNLSILCLLPECLPEMTHLKRLHISECPSLLFPS
THMHRLTTIEDLSIEGCPAWNKIRDKPYFT

>XP_027904444.1

MLFFSVSCLCLCVMFYFVLRIVKRQKPVEEELSLDNSTPQIKYDVFVSFRGEDIRDGFLSHLVMDFERKKINAFVDVKLER
GDEVWSSLVRAIKGSSISLIIFSQHYASSRWCLQELVTILQCRDKFGQILIPVFYKVEPTDVRHQSSKSYQDAFAKHQRKY
QTNRVQIWRDSLKKSTIISGIASSKYQDDAELVKEIVKVVLEKLAKPSVNLKRLVGIDEKIATVESLIGKEPEDTRLIGVWGT
GGMGKTTLVEEVFNKLKFKYDGSYFLANERDQSNKHGIIPLKKSIFSKLLGYDVDIDTPNSLPEDVVRRIGGMKVLIVLDD
VNDLEDLEKLLGSVDNFGSGSRIIVTTRNRQVLKANKFDEEYQLRKFSSEEALELFNLNVFNQKDHQREYNELSERVVNY
AQGVPLVVKVLAGRLRGNEREVWESELDRLKKMPLREVYDVLKWSYNDLDRKEQQVFLDLACFFLRLRKSVNVGSLKS
LLKDGESDNSVAVDLKRLEDRALITISKDHSLHEMALEIIQSKSSEESFVFMHDSVQEMAWEIVRRESSTQGNRSRLWDP
DDVYEALKNNKGNEAIRSIRIQLSTIKKQKLSPHIFAKMSILQFLEISGNCNDDLFHQHYILAEGLQLLVTELKFLYWDRYPL
KSLPENFSTEKLVILSLQQANIEKLWDGVKNLVNLRELDLNSSRMLKELPDLSKATNLEVLDLMCCSMVNALPSSFKHQS
KLRILDMSYCSKLQNISDLPLLLKTLDVNSCKSLQTLPEFPVSLETLDVRGCNSLQTLPNLPLSLQVLDIEFCTSLLTLPKLPPS
LKILKATKCESLQVLPKLPLFLETLEATNCESLQTLPELPQLLETLDVNGCKSLQTLPQLPMNLKIMDVRHCEYLQALLNLPL
SLESLDCTGLESLQALPKFPQFLKTLDVSGCKSLQTLPEFPLFLENLDVSGCEALQTLPKLPMGLKSLDVSGCVSLQMLPEL
PLSLETLDVRGCESLQILPKLPISLETIDVRGCESLQTLPELPLSPKALDVKDFELLQTLPELPLSLETLEVMNGCEFVQAITDL
SLSIETPDTKLSISLKNAKNLPSSLKTLQVTDCESLQTLPKLPHSLITLNTQCCSSLQTLPELPQSLKTLSVKSCQSFLCLPELPL
CLETLEVKNCEALQNFPRLPRFLKTLDVNGCKSLQTLPNLPLRLETLDVRDCESLQAIPKLPLSLETLDLRGCESLQKLQNLP
LSLETLDVTGCESLQILPEFPQSLKTLQCTKCESLQTLPQLPQSLKTLDANGCKSLQTLPKLPLSLETLDVRCCDSLLTLPELP
LSLQNLDVRGCESLETLPKLPMTLKSLDFRACESLQTLPELPLSLENLDVRGCEALQSLPKLPFSLKILEATECESLLTLPELPP
SLKILDVNGCKSLQFLPHLPQSIEILDIRGCESLETIQEFPLSLEILDTELCTSLQTFPKLPSSLKTLEATVCESLQTLPELPWSL
QTLNVQHCSSLQNLPELPISLKTLNVSFCQSLESLPKLPLSLETLDVSGCISIQTLPKLPLSLKALNAKDCESLKTALFPSTTVE
QLKENRKWLLFWNCLNLDEHSLLSIGLNARINVMKFANHHLSSPNHDDLENYNDYDDKYGFYQAGYMYPGSSILEWL
ENKTTEDYLVIDLSSHAFSHRLGFIFCFILGQCEETEMFEKLEVNLIVSDGDGEGQKDSVGMYIDIGSWTIESDHVCVIYD
QRCSEMLNKRAKIQIRLKIQVSIQTSDTGTTILSNPALKGFGVSPVSTSAYKSFIQQMGFHDSMFQFHQSN

>XP_027904445.1

MSDSNVPEIKYDVFVSFRGKEIRDGFLSHLTEAFDMKKINGFVDDKLERGEEIWPSLVTAIERSAISLIIFSPDYASSRWCLE
EVLKIVECKEKYERIVIPVFYKVAPTDVRHQSGSYENAFANHRLNYTRQVQMWKDALKKSADLSGIESSKFRNDAEVVKE
IVDLVLKRLDKHQVIPKGLVGIDEKIATLESWICNEPKATHLIGIWGMGGIGKTTLAEVVFHRLRSQYQGSYFLANERDQS
TKHGIIHLKKIIFSKLLGHEVDIDTPNSLPENIVRRIGYMKVLVVFDDVNDSDHIGKLLGSLDNFGMGSTVIVTTRDEQVLR
INRVSKTYKVKEFSSDEALELFNLNAFSESNEAKEYDEISKLVVHYAKGNPLVVKVLAHLLHGRNKEEWEGLLGKLKTMPP
REVYDVMKLSYDSLDRKEQQIFLDLACFFLRLLVPVEVGDLKCLLKDNESDNSVAFELRRLEDKALITISEDNTVSMHDSL
QEMAWEIVRQECVEDPGSRSRLWDPNDINEALKNEKVSEDIKSIQVQLTSNKKQMLSPQIFSLRKLKFLEISGVDGNTY
HQVILVEGAPFLATELRFVCWEDFPMKCLPESFNAEKLVILELRMSRMRKLWDGVKNLANLKRLNLHWAMKLKELPDL
SGAIRLEELYLCGCYRLRSLHSSIFSLPKLKTLELSRCMSFTILPSSPWRNSELKCLLMRACDNLQTISELPPFLTTLDVSFSAS
LKTLPNLPLSLQALNAAFCESLENLPELPPLLSTLNVSACYSLQIPPNLPFSLQIVNLCESCSLRSILEHIRSIKTTDTKYFESQQ
TLLNFPTDLQMPCTCNSIETPQELSPSLSSQFDDFSAYLDTCIRNFLKA

>XP_027905501.1

MDWSGLFGKATEGVIDFVWKHGLQQVTYIIHYKQNVLELKDSARDLRFEKERINHQCDEALKNLNNIEGKVIEWVRKV
GEIETIVEEFENGDGHKRAQSLSCNILSYLLNRHRLGRQAKKMEVDVRKLIDECPNLDEVSYREDITSNDATLSNYGFIEFS
STKSTMEKVIAQLEDSTVRMVGLYGPGGVGKSTLVKEIARKVKDNKVFDVAVRVKITANPNLQNVQEEIAYVLGLRLER
EGENVRADCLRRSLKKEKRNILLILDDLWDKLDLNKLGIPVEDYDDDDDDFGNGNKDLNHQMLEKNNDNKDPSYKVLN
KEKILGSHKGCKILLTSRDKKVLCDKMDIKSTFCVKELDEKDALMLFQKLARNRDEMSDYKQEIVKKYCAGLPIAIVMVAR
ALRSKSESVWEATLEKLKKQELVEVQTSMDISVKMSYDNLENEEIKSIFLLCAQMGHQPLIMDLVKYCFGLGILNGISSIW

EARDRIXTSIQKLKDSGLLLDGSSNNHFNMHDMVRDAALSIANKDRNVFTLRNVKLDYWPELKSCTSISISNCDITDGLP
DDINCSQLQFFRIDTNDSSLVIPDNFFEGMKNLEVLILTGFRLSRLPSSIQFLLKLRMLCLERCTLDENLSIGKLKKLRILSFSG
SQLKNLPSDLGCLDKLQLLDIDDCSLLNIHIPPNLLSCLTYLEELYIRKSLIKTLVEGERNQGQNLFLSELQNLHQLKVVDLSI
PCLSVLPNHLFFDTLKDYKIVVGDLEVFSVGDFRMPDKYETFRVLALQLNDAIDIHSHEGVKMLFKTVQCLLLGKVDRVQ
SVVNDLNIDGFPDLKHLYIVNNNNIKYVNATKLSNCVDIFPNLESLCLHNLVNLEMICYGLLTVASFAKLKAIKVEMCNRL
ENLYSFYTVKFPTGSKPCEISECNSFMDKFPTNVEIIEVCECGSLKEILQIPMDYGKFEFLKLHTLSLQSLPSLTSFYTKVDRS
CRPHLTEVETTNPGSREIINEEDKQNNKTLPLFDELVEIPNLEILNISSLNIYKIWSNKHSSSFWGQNLIKLVVKDCDKLTYL
CSLSMARSLKKLKSLVISECLNMEKIFETEENSADKVCVFPKLEEIHLSKMNRLTDIWQTEVSVDSFSNIISVKIEECYKLNKI
FPSHMKGWVECLDNLTVCSCESVEVIFEINDYEKIDEFGGINTNLQVILLENLPKLKQLWSTDPGGILNFKKLRAIDVCDC
DELINLFPASVAEDVPKLERISTLYCEKMVEIVTSQDASYANNDPLVFPELTCVRLEWLPNIKRFCKGKHLIKCPKLKQFSIH
KRVKLNTFLKEINETTNKEEKCVFSAEEVLPNLEYMEIDFNEVQELLPKYQMQRLKELSLMSAQSLNLLYQFPYSMPNLE
KLTMTYPYFLEELERRANFAQQEKIGIPLQLKELVLVLSKIKDLNFGRVPVLQRLELLSLKHCNNLNNLGPPSVSLTYLTHLE
LKYCKGLRNLMASSTAQSMVQLKTMKIINCPEVEQIVSNEGNEKGKVMKIVFSKLISIELVRLNYMRSFCGDNECEFEFP
SLEILIVRECPKMKKFSERRSITQKLKNVFGVEGDEKSRCQWEGDLNATLQKVFNDKVSFAYTEDLRLDNDIIEQLWRDT
DWVHQNSFRSLRRLNAWGCDTGEHVIPSHLLSCFHNLEELEVVDCKAARFLFGINDENRVRKGSGIFRLKSLSLSKLSNL
KDVWEKDPEGIIGLQLLKEIRVEECGRLQSLFPASVAKDLTRLQVLQVTKCEELTEIFRKDEKGGEGPTQVFPRLTTLKLEK
LPGLEYSIHRSKQQVILTNLSSAQEQLCLGSWPIPSSSFGLLDSLTVDGCHFFSDVLLPFNLLPFLSNLKELTIRNCTFVKTIF
GVKCTTQDTITFGLKKLSLSNLRTLQNVWNEDPRGILNMNHLQEVNVNQCKGLKNVFPASIAQDLVKLKSLVVEDCEEL
ITIVAEDDTDPSRRNQDLPCPCVRSLKLRRLPMFKYFYYCSLQSNNFTHRKSHIEMLFKCLSLGKNGVEMILRGEFQGNFL
HNLKVLTLLGCESDIFAYKVLKQVPNIEKLVVCDGSFKGIFKCQSPNNVDYSELQLQLKELHLEFLRESVCIGLENFWIQPF
VRNLETFEVISCRSLENLVGCKVSFFNLTYLKVESCDSLSYLFTSSTAKSLSQLKKMEINRCKSIEEIVSKEKGEEESEGNEIIFP
KLDH

>XP_027905505.1

MEIGSSSSKPPRNYDVLINFNGEDIRRKFVSHLDSAFSSVGLTTFLHHQNAVESMHVQQPILNLCRVVIVVFTKTYSQSA
WCLHQLQQIIAWHESYCRHVLPVYYEIQPSDVRLQKGDFGKAFKETAQQTFSAQQLEDGMSRWSHALTKVASLFGW
DESNHRSDAELVDKIVKSVLNLSVLSATKFPVGLQDQVEHLIRTIRNKSSDVCIIGFCGMGGSGKTTLAKAIYHQIHGTFM
QKSFIEDIAQVSEPRGHIHLQQQLLSDVLKIKVEIHSVEMGRSIIRDRLFRKRVLIVLDDMDDYLPLLDLRKSRSWLSEGTVI
IITTRDEDLLRKHQVDSVFRINEMNDNESLELFSWHAFREPKPRKEYDYLARRVVSCCGGLPLALELIGSSLFERREKEWH
SVLVGLEGIHMYDVEQKLKISFDLCNQMERDLFLVLCRYFVGKDRSYATKILNASGINADSGIRVLMERNLIKVKANNKF
GIHYLLQGMGIEIIREILRKEPWKHNRLWVDEDGKYALKVDTTLPVKLISTSREPSSRPKVAVNSGYLSAKLRWINLHGFS
SEHLPEDFNVHGAVAIDLKHSLLRFVWKEPQILKWLKVLNLSHSEYLTETPDFSGIPSLQRLILKDCPRLCKIHPSIGFLCYLT
LLNLKDCECLSNLPREIYKLKSLRTLILSGCSKIDLVEKDIVPMKSLITVIAETKALKQVPFLILSSKAIGYMSQRGFERLSSNLF
PSIIRFWMSATMNPIFYIHSIGMDIDNIWDDIAPLLGSLANLRSVLVQCNTEFQLSKLVKNILVEYFANVTESGISKQHLRF
SLIGIGAYYEFFNAVSDNISQVLAGSESCDVSVLGDNHPYWLAYMGEGHSVSFTVPPDTDLKGMILCVVYLSTLEIEATEC
LRIVLIVNYTKCRFHIHMHGTGISFNDIDWEGIMSNFGYGDKVEIFVTSGHGLVVKNTILYLIWGESNYYEKEPMSKKNS
VLRFIKKIVE

>XP_027905521.1

MARNLLSTCSAVYYEIEPSECTSFRRVIWKAFKATAQQTFSGQQLEHGMSSWSHALTKAANFFGWDERNYRSDAELV
DTIVKRILYLPALSATKFPVGLQSYVEDVIRTIENKSTEVCMIGIYGMDGSGKTTVAKAIYNEIHDTFTEKSFIEDNAQVSRT
RGYVHLQEKLLSDVLKTKVEIHSDEMGRSMIRERLFQKRVLIVLDDLNEHGPLLDLWESRAWSGKGTVIIVTTRHEHLLR
ARQVDAVFRINPMNENESLELLSWHAFREAKPKEEFHDLAKTIVTRCGGLPLALEVIGTYLCERTKEEWQRVLFKLVKIP
QHEVLQILKICFDGLPNQMEKDLFLDICCFFVGKGRAYFTKILNGCGIDADTAIRVLIERSLIKVKKNNKFGMHPLLRDMG

REIIREISRNEPENSRLWLDEDMKHAPSKNTVRDTSRMLKLAGHSEYLFKKLRCISLQGFSSEYLPNDFYLHDAIVVDLKYS
LLRLVWKESQVLVSLKVLNLSHSKYLTKTPDFSRLPSLEQLILKDCPSLREVHRSIGCLYNLTLLNLKDCTGLRNLPREIYMLK
SLKTLIVSGCSKIDLLEKDIVQMQCLITLIAENTVVKQVPFSLVSSKTTAYISLRGCEGLSHNLFLFIIRSWISPSMHPLSYIRSF
CMDMEVNSWDDIAPLLSSLANLRSVLVQCDTEFQLSKQVEIIVVEYRVNITESDTSKPHFRSSLIGVGRCKEFFDAFSDSIS
QVLARSESCDVSLPVGNGPYWLAHMGEGHSVSFTVPQDRVINGMALCFVYLSTFEIVATECLRSVLIVNHTKCTLQIHN
HGTVISFNDIDWQGIISNLESGDKVEIFLTFSQELVVKNTVVYLICGESNDLETKPVPKKNSLIRFIKNIV

>XP_027905522.1

MARNLLSTCSAVYYEIEPSECTSFRRVIWKAFKATAQQTFSGQQLEHGMSSWSHALTKAANFFGWDERNYRSDAELV
DTIVKRILYLPALSATKFPVGLQSYVEDVIRTIENKSTEVCMIGIYGMDGSGKTTVAKAIYNEIHDTFTEKSFIEDNAQVSRT
RGYVHLQEKLLSDVLKTKVEIHSDEMGRSMIRERLFQKRVLIVLDDLNEHGPLLDLWESRAWSGKGTVIIVTTRHEHLLR
ARQVDAVFRINPMNENESLELLSWHAFREAKPKEEFHDLAKTIVTRCGGLPLALEVIGTYLCERTKEEWQRVLFKLVKIP
QHEVLQILKICFDGLPNQMEKDLFLDICCFFVGKGRAYFTKILNGCGIDADTAIRVLIERSLIKVKKNNKFGMHPLLRDMG
REIIREISRNEPENSRLWLDEDMKHAPSKNTVRDTSRMLKLAGHSEYLFKKLRCISLQGFSSEYLPNDFYLHDAIVVDLKYS
LLRLVWKESQVLVSLKVLNLSHSKYLTKTPDFSRLPSLEQLILKDCPSLREVHRSIGCLYNLTLLNLKDCTGLRNLPREIYMLK
SLKTLIVSGCSKIDLLEKDIVQMQCLITLIAENTVVKQVPFSLVSSKTTAYISLRGCEGLSHNLFLFIIRSWISPSMHPLSYIRSF
CMDMEVNSWDDIAPLLSSLANLRSVLVQCDTEFQLSKQVEIIVVEYRVNITESDTSKPHFRSSLIGVGRCKEFFDAFSDSIS
QVLARSESCDVSLPVGNGPYWLAHMGEGHSVSFTVPQDRVINGMALCFVYLSTFEIVATECLRSVLIVNHTKCTLQIHN
HGTVISFNDIDWQGIISNLESGDKVEIFLTFSQELVVKNTVVYLICGESNDLETKSLPKKNSLIRFLKNIV

>XP_027905544.1

MESASSSLKHQRMYDVLINFAGEDIRRKFVSHLDSVLTSVGISTFLHHQNAVKEMYIEEPILNMCQVVIVVFTKTYSQSA
WCLHQLQQIIQWHETYRRHVLPVYYEIQPSDVRLQKGDFGKAFTATAHQTFSGKQLEHGMSRWRYALTKAANFFGW
DESNHRSDAELVDTIVKSVLNLSVLSATNFPVGLQSHVEDVIRTIKKKSTEVCTIGICGMEGSGKTTIAKAIYNHIHGTFKE
KSFIEDIAQVSRTRGYAHLQGKLLSDILKTKVEIHSVEMGRSMLQQRLLGKRVLIVLDNIDEKAPLLDLWRNHAWFNKGT
VIIITTAHEHLLRTRTPLDDSIFRINIMNASESLEFLSWHAFREAKPKAEYDDLAKEVVAYCGGLPLALEVIGSSLFQSTKEE
WKWVVSKLEKIPMYDVQQKLKIIFGGLHNELEIDLFLDVCCSFVGKDRAYVTKILKSCGVDADNGIRVLIDRNLIKVKRNN
KLTMHPLLQNMGRESFNKFIQEEFWTKSLRFYDSYVLTNNTGKKSSKELPVKLLSVRRKLSGLLKLAQNSEFRYKKVRW
MSLQGFSSKNLSIDFDMKDAIAIDLKRSLLRLVWKQPQILRWLKVLNLSHSRYLKKTPDFIGLPHLEQLILRDCPRLLEVHQ
SIGFLYNLTLLNLKDCTSLTNLPREIYKLKSLKALILSGCSMIDLLEKDIVQMKSLITLITENTTVKEVPFSIVNSESIGYISLRRF
EGLLRNLFPSIIRSWMSPTMNPISYIHSLYMDIDNTWDDIAPLLGSLKNLRSVLVECDTEFQLSKQVQNILVEYFANIIEPEI
SKQQLRWSLIGVGAYHEFFNVFSDNIPKDLACIEWCDVSLPVVNDTYWLAQMGEGHSVSFTVPENRVMKGIALCVFYL
STSEIVAAECLRSVLIVNYTKCTLHIHNNDTIISFNDIDWQGIKSNLGSGDKVEIFLTFAHGLLVKNTIVYSICAE

>XP_027905556.1

MELASSSSSSFLKSEPHFIYDVFINFWGEDIRRKFVSHLHSVLLQAQVKTLINEENLQEGITLEENMRAIAASKIAIIVFSKTY
TESTCCLRELEKIIECRETFGQIVLPVFYELYPFDVRYQQDDFGKSLEETGHKSYSGEQLEHALSSWSRALNIAASITGWDV
KDFRHDAELVEEIVSRVQALLDYKDLFITQFPVGLESHVEKVIGCIENHSTKVCMIGIWGMGGSGKTTLAKAIYNQIYHPF
IGKSFIENVREVRDQVNRHVDLQEKFLYEVLKSKFEVKTIGMGRSIIENELSKKRLLIVLDDANEFCHLENLCGNRGWFGQ
GTVIIITTRDVRVLNQLKVDFVYKMDVMNENDSLELLCWHAFRDAKPSEDFNELARNIVTYCGGLPLALVVLGSHLYDK
TLEMWESVTLKLQIFPDERILRNLEKGFEALCDDMEKDIFLDVCCFFIGKDRGYVTEILNGCGLHADIGMTILIERGLIKVE
RNNKLEMHPLLRDMGRDIIRRRWPKEPGKRSRLWFHEDVKHVLQNKTGSKATEGLSLKLHSTNRDCFKAHAFKQMKR
LRLLQLDHVQLVGDYGYLSKQLRWICWQGFPSQYIPNNFHMENVIAIDLKHSHLQLVWRQPRVLEGLKFLNLSHSKYLR
ETPDFSGLPSLEQLILKDCTSLCKVHPSIGDLCNLLLINLRDCTSLTSLPKEVYKLKSLKTFNLSGCFKIDILKEDIMQMESLITL

ISENTVVKQVPCSIVSSKHMAYISLHGFEGLSHNFFSSTIRSWMSPTMNSQSYISPLCLDTNNDNWGELAPLHSCLTNLR
SMLVQCDTEFQLSMQLNTILVEYGANFIESRISNHRLRFSLIGVGSCNEFFNTLSDNVSKGFARSKCCDVPLPGDNHPYW
LSHIGDGNSVSFTMPKDCDMKGIALCVAYLSTPEMVATECFRSVLIVNYTKCTCHIHNHGTVMSFNDEDWHGIMSNLE
SGDKVEFFVSCGHGLVFKNTAVYLIYGD

>XP_027905588.1

MEFACSSSSSSPSFLKSEPQFKYDVFINFGGEDMGRKFVSHLHYALLQAQFKNLISTEDVQEEMKLEEHMRAIASSKIAII
VFFPLPVFTGENFDLWKLKLKTYFISQKLWDIVQSGYTKPDNIETLSEEERKKLEDSEQKDAQALFVLQQAVGETIARRIM
DAETAKRAWDILEEEFEGNEQSSHAEKDCWHRGKPICHYCKKPGHVEKYCRNKNKHQANFAEEHNQEQRLFYANQES
HSGEGNWYLDSGCSNHMAKDQSIFKDIDKSINVKVRLGNGATVESQGKGTVMVETKKGTKFIKDVLLVPNLKENLLSIG
QMMENGYSLHFEKDTCKIYDNRRIEIGEVKMEKRNRSFPISFKPGTNIAMKAEVDDSWLWMSSRQATPIAILNRQSME
SKRFTRINPHRCLWTDEDAFTSQQQKFKALVDKQSGKQIKVLRSDRGKEYTSHEFDKFCEDEGIERQLTVAYTPQQNGV
SERKNRTVMEMARSMLKEKGLPNTFWAEAVYTAVYLLNRCPTKAVQDKTPIEAWTQLHNQPLKQNMWQPLKQQV
RLYGFEEYSKIWEKNKLEKIIECRQTFGQIVLPVIYDIVQLEERNRKDDLVKALEEVAQSSYSGEQLEHALSRWSRALTNAA
GITGWDFRDFRHDAEFVEKIVRRVQTLLDYTNMSITAFPVGLESHVEKVIGLIEEQSTEVCMIGIWGMGGSGKTTLARAI
YNRIYHPFIGKSFIENIGQFQVNKRHVHLQENLLYDVLKSKFKVESDGVGRTMIETKLSQKKLLIVLDAVDEFGQLENLCG
NRQWFGQGTVIIVTTRDVKVLNRLKVNYVHTMDVMNENDSLELLSWHAFREAKPRKEFNELSRNIVDYCGGLPLALQ
FLGSYLCDRTKEEWESVSSKLKVNPINQIQEKLEISFDGLHDMEKDIFLDICCFFIGKERGYVTEILNACGLYADIGITVLIER
GLIKVERNNKLEMHPLLRDMGREIIRQRCPNEPRKRSRLWFEDDIKDVLKRNTGTKATQGLSLKLHSTGKDYFEAHAFK
EMKRLRLLQLHHVQLTGDYEYLSKQLRWICWKGFPLKYIPNNFHMENVIAINLKHSYLQLVWQETPDFSGLPSLQQLIL
KDCPSLCMVHESIGDLCNLLLINLKDCTSLSCLPRNVYKLRSLRTFILSGCSKIDILEEDIVGLHSLITIVTENTIVKQVPCSIVK
SIAYISLRRFKGMLHNTFASVIQSWMSSTMKQQYCNSPFFMDMENNNWRVLVPLHNGLANLRSVLVQCKTEFQLYKK
VKKILVESPLVNFSESRISNHHLRFSLIGVGSYNKFLSTLSDSISEELVSSESCDVSLPGDNHPYWLAHRGEGHSVSFTVPRD
SDMKGMIVCVVYLSTSEIVATECLTSVSIVNYTKCTLHIHRHGTVISFNDEDWHNIISNLGSGDKVEIFVTFDHGLVLKNT
VVYLMCGESNGLEMVPFPEAEENALNKFFKKIVMCDFW

>XP_027905589.1

MTERSSGTTDKFALYFNKDIAQDNKKKWLHFKEKLLSDVLKPKVEVRNIEKGRNIRERHFRKRVLIVLDNVNDHSLLINLS
DSLLWFDKGTVIIITTKHEQLLKTHDVNAVFRINLLNAKESLELLSWHAFREAKPKEEYHDLAKAIVTHCGGLPLALEVIGT
YLYERTKEEWHRVLFKLGKTPQHDVLPVLKICFEGLPNQIERNLFLDIYCFFVGKDRAYVTKILNGCGVDADSGIGILIERSL
IIVKKNNKFGLHPLLREMAREIIGEITSGMEAKKTSRLWFDMDADYVLLEHILFSSQEKKFIQRFPPKWFPTVKDFFERDY
LEVRDAIRRMKLGGHCEYRSKELGLIRLEKFSSEYHPIGFQHDAIAIDLKHRLPRLVWKEPQVLA

>XP_027905591.1

MAPELPDLTPDTPSPTFKRGSKGLVSKVLTRFQYSKIINIKDLATSNAFVSDTDHLFRLLTSLEFASSTSKLPDMYDVLIHFT
GEDIRRKFVSHLESALSAVGFTTFLLHQNAVNPMHIEEHNLNLCRVAIVVFTKTYSQSAWCLHQLQQIIQWHQTYCRHV
LPVYYEIQPSDVRLQNGDFGKAFEATAHQTFSEQQLEHGMSRWSHALTKAAHFYGWDESNYRSDAELVDTIVKGVLN
LPLLSATEFPVGLQSYVERVIRTIENESTGVCMIGISGMEGSGKTTVAKAVYNQIHDRFIEKSFIEDNPEVSRTRGHLHLLK
KLLLDILKIKVEIQNVEMGRIMIRERLYGKRMLIVLDDLNQYKYHELSDLWECRAWSGRGSVIIITSTYENLLRTSQVDAVF
RINPMNPNESLELLSWHAFKEAKPKEEYHDLAKTIVTHCGGLPLALEVLGSYLYERTKKEWHTVLSKLVRIPQLEVLPILKIS
FNGLRNQMEKDLFLDICCFFVGKSRAYVTKILNGCGIDADSGIRVLIERSLIKVKKNNKFGMHSVLRDMGQQIVREISGK
EPENCRRWYDERMKHALSKNNHTKVMQTLRDFFKPYPLEVRDTSRMLKLAGHSEYLSKKLGCISLQGFSSDYLPNDLHL
HDAIVIDIKHSLLRLVCKKPQVLASLKVLNLSHSKYLTKVLDFSRLPSLEQLILKDCPRLREVHKSIGCLCNLTLLNLKDCTSLR
NLPGEIYMLKSLKTLILSGCPKIDLLETDIVQMKSLVILITENTASKQVPFSIVTSKSIGYISLCGLEGLSHNLFPSIIRFWMSPT

MNTLSYKHSFCMDMENNSWDDFAPMLSSLANLRSVLVQCDTEFQLSKQLETIMIEYGANSTKPGTSKQHFKSSLIGVG
RCKYFFNAVSNSISQVFASIESCDASLPGDNDPYWLAHTGEGHSVSFIVPQNRVLNGLTLCVVYLSIPEIVASECLRSVLIV
NYTKCTLHLHNHGTAIFFNDIDWRGIISNLGSGDKVEIFLIFCHGLVVTRTIVYLIFAESNDLEMEPVPTKNSHVRFIKKV

>XP_027905595.1

MEFEYFSSASSDRMHDVFINFRGKDTRRNFVSHLYAALSNAGVNTFLDEADFPKGADLKDGLLDRIASSRICVVVFSRNY
TESSWCLNELEKIIECHKTYGHIVLPVFYHVVPSQVRHQTGDFGNVLKAFAQERSWGESVMSRWNTALTAATNFSGW
NARNTRNQAQLVGEIVEVVLKKLDNASMSITEFPVGLESRIQEVIGFTKREFSTVCIIGIWGMGGLGKTTTAKATFNRIHR
RFTDKCFIEDIREVCETDRRGHVHLQEQLLSGILKAKVNITSVGMGRDMLENKLSVRKALIVLDDVNEFGQLKDLCGNRK
WFGHGSVIIITTRDVRLLKKLKVDYVYKIEEMNENESLELFSWHAFREEKPKEDFNEVARSVVGYCGGLPLALEVLGSYLS
ERTKKEWKSVLSKLKKIPNNQVQEKLRISFDGLRDQMEKDIFLDVCCFFIGKDRGYVTDILNGCGLHADIGITVLIERSLIKV
VKSNKLAMHDLLRDMGREIIRESSAKRLGKRSRLWFHEDVVEVLTKNTGTEAIEGLTLKLQLTSRGCFNTSVFKKMQRLR
LLQLDHVNLTEDYRYLPKQLRWIYWKRFPLKYIPHDFYLERVIAIDLRHSNLRLVWKEPQVFPWMKVLNLSHSKYLIKTP
DFSELPSLEKLILKDCPSLCDVHQSVGNLQNLQLINLKDCTRLSSLPRQTYKLKSLKTLILSGCLKIDKLEEDIGQMESLRTLL
AKNTAVKQVPFSVVRSKSIGYISLCGFEGLSRNVFPSIIWSWMSPTMNPLSRIHSFSGTSSSLVSMDMQNNDLSDLAPV
LSNLSNLRSLLVQFNTEVQLSKQLRTVLDGVYDVNFPGLEVTSYTSQISKDCLKSYLIGIGSYEEVFNTICKSVSEGLATSESC
DVLLPGDNYPYWLAQTGEGRSVSFIVPDDCCIKGMALCVVYFSTPENTMTEYLVSVLMVNYTRCTIQIYKHDTVISFND
EDWRCIMSHLGSGEKVEIFVTFGHGFVVKKTAVYLMRDESSDMEVGLSPKPNKNLFVRFMKKIVSSKNQEHVPTNFKFI
GSMPLLLTITEFDPSVEPLQVMDSRTTMRHSQLIPQVLIQSLDSSNASWEDVKKIKEIFPKFNLEDKVAFGGNGIVIVAAE
IKKLLLFIYNDFITSIHRLVINHIHLLLQLYRYQIESTFFLAFTVLVLFHRSASTMSYTSSSSKSKPRWLYDVFISFRGEDTRKNF
VSHLYSALSNAGVNTFLDDEKLAKGQQLKTELFHAIEGSQISIVVLSENYIYSTWCLDELVKIMECHAFRGQVVLPVFYGV
FPWFLRSLYDVSFEVILEKASDLHRVKQWKKALGEAAGFAGWDVSNYRNENFVVKEIVSEVLERLDRTYMSITDFPVGL
DCRVEHCIGFLRKETRGAYILGIWGMGGIGKTTIAKAIYNEIRCEFKHKSFLANIREVWQRDQGQTDLQERLLSDILKTEK
VKVYSSDWGKAMIKETLCTKRVLVVLDDVNTLEQLSALCGNGNGIVQGSVIIITTRDVRLLNVLDVDQVYEVEEMNEIES
LELFSWHAFKEANPPEAFLELSKQVVTYCGALPLALEVLGSYLYKRREKQWQSVLSKLKEIPNDKIQEKLKISYDGLTDHTE
KDIFLDICCFFIGKDRGYVTEILNGCGLHAEIGITVLVERSLIKIEKNNKFGIHDLLRDMGREIVRQSSPLEPQKRSRLWVHD
DVLDILSEQTGTGVIEGLALKMQTTGGVCFSTETFEKMKRLRLLQLDHVQLAGDYVHLPKQLRWVHWKAFSLTHIPENF
YQENIVAIDLKYSYLKLVWKVPPFLERLKFLNLSHSKYLSKTPDFSKLPNLEKLILKDCPSLYEVHHSIGDLNNLLLLNLKDCT
CLGNLPMVIYKLKSLQTLILSGCSNIDKLEEDIGQMESLTTLMAENTSLTQVPFAIVRSKKIGYISLCGYEGLARNVFPSLIW
SWMSHTRGTLSSIQPFGVMPTSIVSMDIQDNNLANLLSNFSEFSKLRSISVQCDSDFQLTQELRIMLHGLCNVNSSGSE
NAYQSPITENSMVSNLIGMGSYQQVFDMLSNSISKVLRTSSSADFVLPGDKYPYWLAYTGEGHSVPFQVPEDSDCRMK
GMLLCVVYSSTPENMATQALTNVFIFNYTKCTIQIYKQATTMFFSDEDWQGVISNLGPGDNVEIFVGVGDGITAKKTAV
YLIYGQSITMRMELLGPSAQASPELSVTLSPKLSAQASSELSVTLSPKSCAQPITHVGEPTKKPQENIFAKFRNKVRECSCL
N

>XP_027905597.1

MDVCTIGICGIEGSGKTTLAKAIYHQIHGTFTKKIFIEDVSEVSRTRSHVSLQEQLLLDILKIKVEIRSVEMGRRMIRERLSGK
RLLIVLDDMNAYGPFLDLYRCRSRFSRGTVIIITTRDEDLLRIHQVDSVLQLKLMSAKESLELLSWHAFREAIKV

>XP_027905599.1

MYDVLINFNGEDIRNKFISHLDSALSTVGFTTFLHHENAVKAMHIQQPILNLCRVAIVVFTKTYSESCWCLHQLQQIIEW
NETYCRHVLPVYYEIQPSDVRLQKGEFGKAFKATAEQTFSGQQLEHGMSMWSHTLTKAANLFGWDESNYRSDAELLE
KIVKSVSNLPVLLATKFPVGLHSQVEDVIQTIKNKSTEVCTIGICGEEGSGKTTLAKAIYHLVHGTFMDKSFIEDIEQISETR
GYVHLQEQLLSDLLKTKVEILNVEMGRSMIRERVSGKKVLIVLDDVPNVR

>XP_027905606.1

MDFPSSTSKLPRKYDVLINFTGEDIHRKFVSHLHYALSAVGLTTFLHHQDAVQPMNIQQPILNLCRIAIVVFTKTYSESAW
CLHQLQQIIKCHETYCQHVLPVYYEIQPSDVRLQQGRFGKAFKETAHQTFSEQELEHGMSRWSHALTKAANCFGYDER
NYRSDAELVEKIAKFVVNLPVLSATKFPVGLQSRMEDVIQTIKNKSTEICKIGICGEGGSGKTTLAKAIYHQIHDTFKEKSFL
EDIRQVSGIREDLRLQEQLLLDVLKTKVEIPSVDIGRKMIRERLFGKRMLIVLDDLPDFCELLDILKCNHLFSGGTVIIITTRD
EHILRQHGIDSIFQSKLMYENESLELLSWHAFREAKPKKPHYEHAIDIVHYCGGLPLALEVIGSSLFERTGNEWYSILCYED
PKWLWSIR

>XP_027905831.1

MALAVVGGALLSAFIDVLFDRLASPEFVNFIRGKKPERLLQKMKSQLLVVKVVLADAEKRQISNSDVKDWLDLLRDLVYE
VDDLLDEVSTKAATQKEVSNSFSRIFNRKKIVSISKLEEMVERLDDILKQKESLDLKDIPVENYHSWKAQPTSLEDGYGMY
GRDKDKEAIMKMVLEDVTDTEQLCVIPIVGMGGVGKTTLARSVFNDGKLKQQIFDLKAWVCVSDIFDIVKVTRTMIEEI
TRKPCKLSDLNALQLVLRDKLKGKRFFIVLDDVWIEDCDNWSCLTKPFLSGSKGSKVVVTTRNENVAAAVPFHSVEVYRL
NKLTNEDCWLVFANHAFPPSEACEDRETLRMIGKEIVKKCNGLPLAAQSLGGMLRRKHAVRDWNNVLESDIWELPES
QCKIIPALRISYHYLPPHLKRCFVYCSLYPKDYEFDKDELIQLWMAEELVKAPKKEKTLEEVGHEYFDDLVSRSFFHRVSHA
TWGDYFLMHDLMHDLATFTGGEFYFRADELGKETKISRKTRHLSFTRFSEPVSDIEGFDTVKFSRTFLLINYKDSPFNNEK
APYIVVSMLKYLRVLSFCSFKSLFALPDSIGELIHLRYLNLSHTSIETLPESLCNLCNLQTLKLAFCSKLTQLPIAMQNLVNLR
HLEIHESRIKEMPKRMGKLNQLQKLDLYIVAKDKENSIKELGGLPNLCGSFCITALENVTKGEEAIEASIMDKKHINHLSLE
WSISNDNGIDFQIELHVLRKLQPHRDLQTLLIIGYKGTIFPEWVGNFSYRYMTSVSLYNCNNCCMLPSMGQLPSLKFLYIS
EINSVKTIDAGFYKTEDCSSAIPFPSLEFLHIFRMPRWEVWTAFASEAFPVLKDICISDCPKLRGAFPNHLPALQTLSITNCE
LLVSSVLRVPNLRSIEICNTNKVMFHEFPLLVKFIDVEGPMVESMMEAITNIQSTCLQSLRLQKCSSAIWFPGDRLPASLT
ALDVSCLNNLKFPMQHKHELLESLRIKNSCNSLTSLPLAIFPSLTHLDIISCENMESLLVSRSESLKSLKSLEIEHCPNFVSFLG
EGLCAPNLIRFRVCDCEKLKLLPDEMGTLLPKMEYLNISNCQQIESFPVGGMPPNLRTVEIENCEKLLSSKAWVCMDMV
TSLIVSGPCDSIDSFPEEALLPPSLTSLSLYNFTSLETLECKGFLHLTSLRELDIQKCEKLKNIAGESLPVSLMKLSINGCPLLQE
RCHKKDREIWPKICHVHVLEIDDRYI

>XP_027905832.1

MALAVVGGALLSAFIDVLFDRLASPEFVNFIRGKKPERLLQKMKSQLLVVKVVLADAEKRQISNSDVKDWLDLLRDLVYE
VDDLLDEVSTKAATQKEVSNSFSRIFNRKKIVSISKLEEMVERLDDILKQKESLDLKDIPVENYHSWKAQPTSLEDGYGMY
GRDKDKEAIMKMVLEDVTDTEQLCVIPIVGMGGVGKTTLARSVFNDGKLKQQIFDLKAWVCVSDIFDIVKVTRTMIEEI
TRKPCKLSDLNALQLVLRDKLKGKRFFIVLDDVWIEDCDNWSCLTKPFLSGSKGSKVVVTTRNENVAAAVPFHSVEVYRL
NKLTNEDCWLVFANHAFPPSEACEDRETLRMIGKEIVKKCNGLPLAAQSLGGMLRRKHAVRDWNNVLESDIWELPES
QCKIIPALRISYHYLPPHLKRCFVYCSLYPKDYEFDKDELIQLWMAEELVKAPKKEKTLEEVGHEYFDDLVSRSFFHRVSHA
TWGDYFLMHDLMHDLATFTGGEFYFRADELGKETKISRKTRHLSFTRFSEPVSDIEGFDTVKFSRTFLLINYKDSPFNNEK
APYIVVSMLKYLRVLSFCSFKSLFALPDSIGELIHLRYLNLSHTSIETLPESLCNLCNLQTLKLAFCSKLTQLPIAMQNLVNLR
HLEIHESRIKEMPKRMGKLNQLQKLDLYIVAKDKENSIKELGGLPNLCGSFCITALENVTKGEEAIEASIMDKKHINHLSLE
WSISNDNGIDFQIELHVLRKLQPHRDLQTLLIIGYKGTIFPEWVGNFSYRYMTSVSLYNCNNCCMLPSMGQLPSLKFLYIS
EINSVKTIDAGFYKTEDCSSAIPFPSLEFLHIFRMPRWEVWTAFASEAFPVLKDICISDCPKLRGAFPNHLPALQTLSITNCE
LLVSSVLRVPNLRSIEICNTNKVMFHEFPLLVKFIDVEGPMVESMMEAITNIQSTCLQSLRLQKCSSAIWFPGDRLPASLT
ALDVSCLNNLKFPMQHKHELLESLRIKNSCNSLTSLPLAIFPSLTHLDIISCENMESLLVSRSESLKSLKSLEIEHCPNFVSFLG
EGLCAPNLIRFRVCDCEKLKLLPDEMGTLLPKMEYLNISNCQQIESFPVGGMPPNLRTVEIENCEKLLSSKAWVCMDMV
TSLIVSGPCDSIDSFPEEALLPPSLTSLSLYNFTSLETLECKGFLHLTSLRELDIQKCEKLKNIAGESLPVSLMKLSINGCPLLQE
RCHKKDREIWPKICHVHVLEIDDRYI

>XP_027905833.1

MALAVVGGALLSAFIDVLFDRLASPEFVNFIRGKKPERLLQKMKSQLLVVKVVLADAEKRQISNSDVKDWLDLLRDLVYE
VDDLLDEVSTKAATQKEVSNSFSRIFNRKKIVSISKLEEMVERLDDILKQKESLDLKDIPVENYHSWKAQPTSLEDGYGMY
GRDKDKEAIMKMVLEDVTDTEQLCVIPIVGMGGVGKTTLARSVFNDGKLKQQIFDLKAWVCVSDIFDIVKVTRTMIEEI
TRKPCKLSDLNALQLVLRDKLKGKRFFIVLDDVWIEDCDNWSCLTKPFLSGSKGSKVVVTTRNENVAAAVPFHSVEVYRL
NKLTNEDCWLVFANHAFPPSEACEDRETLRMIGKEIVKKCNGLPLAAQSLGGMLRRKHAVRDWNNVLESDIWELPES
QCKIIPALRISYHYLPPHLKRCFVYCSLYPKDYEFDKDELIQLWMAEELVKAPKKEKTLEEVGHEYFDDLVSRSFFHRVSHA
TWGDYFLMHDLMHDLATFTGGEFYFRADELGKETKISRKTRHLSFTRFSEPVSDIEGFDTVKFSRTFLLINYKDSPFNNEK
APYIVVSMLKYLRVLSFCSFKSLFALPDSIGELIHLRYLNLSHTSIETLPESLCNLCNLQTLKLAFCSKLTQLPIAMQNLVNLR
HLEIHESRIKEMPKRMGKLNQLQKLDLYIVAKDKENSIKELGGLPNLCGSFCITALENVTKGEEAIEASIMDKKHINHLSLE
WSISNDNGIDFQIELHVLRKLQPHRDLQTLLIIGYKGTIFPEWVGNFSYRYMTSVSLYNCNNCCMLPSMGQLPSLKFLYIS
EINSVKTIDAGFYKTEDCSSAIPFPSLEFLHIFRMPRWEVWTAFASEAFPVLKDICISDCPKLRGAFPNHLPALQTLSITNCE
LLVSSVLRVPNLRSIEICNTNKVMFHEFPLLVKFIDVEGPMVESMMEAITNIQSTCLQSLRLQKCSSAIWFPGDRLPASLT
ALDVSCLNNLKFPMQHKHELLESLRIKNSCNSLTSLPLAIFPSLTHLDIISCENMESLLVSRSESLKSLKSLEIEHCPNFVSFLG
EGLCAPNLIRFRVCDCEKLKLLPDEMGTLLPKMEYLNISNCQQIESFPVGGMPPNLRTVEIENCEKLLSSKAWVCMDMV
TSLIVSGPCDSIDSFPEEALLPPSLTSLSLYNFTSLETLECKGFLHLTSLRELDIQKCEKLKNIAGESLPVSLMKLSINGCPLLQE
RCHKKDREIWPKICHVHVLEIDDRYI

>XP_027905834.1

MALAVVGGALLSAFIDVLFDRLASPEFVNFIRGKKPERLLQKMKSQLLVVKVVLADAEKRQISNSDVKDWLDLLRDLVYE
VDDLLDEVSTKAATQKEVSNSFSRIFNRKKIVSISKLEEMVERLDDILKQKESLDLKDIPVENYHSWKAQPTSLEDGYGMG
GVGKTTLARSVFNDGKLKQQIFDLKAWVCVSDIFDIVKVTRTMIEEITRKPCKLSDLNALQLVLRDKLKGKRFFIVLDDVW
IEDCDNWSCLTKPFLSGSKGSKVVVTTRNENVAAAVPFHSVEVYRLNKLTNEDCWLVFANHAFPPSEACEDRETLRMIG
KEIVKKCNGLPLAAQSLGGMLRRKHAVRDWNNVLESDIWELPESQCKIIPALRISYHYLPPHLKRCFVYCSLYPKDYEFDK
DELIQLWMAEELVKAPKKEKTLEEVGHEYFDDLVSRSFFHRVSHATWGDYFLMHDLMHDLATFTGGEFYFRADELGKE
TKISRKTRHLSFTRFSEPVSDIEGFDTVKFSRTFLLINYKDSPFNNEKAPYIVVSMLKYLRVLSFCSFKSLFALPDSIGELIHLRY
LNLSHTSIETLPESLCNLCNLQTLKLAFCSKLTQLPIAMQNLVNLRHLEIHESRIKEMPKRMGKLNQLQKLDLYIVAKDKEN
SIKELGGLPNLCGSFCITALENVTKGEEAIEASIMDKKHINHLSLEWSISNDNGIDFQIELHVLRKLQPHRDLQTLLIIGYKG
TIFPEWVGNFSYRYMTSVSLYNCNNCCMLPSMGQLPSLKFLYISEINSVKTIDAGFYKTEDCSSAIPFPSLEFLHIFRMPR
WEVWTAFASEAFPVLKDICISDCPKLRGAFPNHLPALQTLSITNCELLVSSVLRVPNLRSIEICNTNKVMFHEFPLLVKFID
VEGPMVESMMEAITNIQSTCLQSLRLQKCSSAIWFPGDRLPASLTALDVSCLNNLKFPMQHKHELLESLRIKNSCNSLTS
LPLAIFPSLTHLDIISCENMESLLVSRSESLKSLKSLEIEHCPNFVSFLGEGLCAPNLIRFRVCDCEKLKLLPDEMGTLLPKME
YLNISNCQQIESFPVGGMPPNLRTVEIENCEKLLSSKAWVCMDMVTSLIVSGPCDSIDSFPEEALLPPSLTSLSLYNFTSLE
TLECKGFLHLTSLRELDIQKCEKLKNIAGESLPVSLMKLSINGCPLLQERCHKKDREIWPKICHVHVLEIDDRYI

>XP_027905869.1

HRSRTNPVYCNQFLSFLRNSHLFAVEILALAVVGGALLSAFIDVLFDRLASPEFVNYIRGKKPDKLLQKMKSQLLVVKVVL
ADAEKRQITDSNVKEWLDLLNDLVYEVDDLLDEVSTKVATKKEVSNPFSHLFKRNKVVSISKLEDIVGRLDEILKQKESLDL
REIPVENNQSWKAQTTSLEDRYEIYGRDKDRETIMKLVLEDSSDGEEVSVIPIVGMGGVGKTTLTRSVYNDGNLNQIFEL
KAWVCVSDIFDIVKVTKTMIEEITQKPCKLSDLNSIQLDLLDKLKGKKFLIVLDDVWIEDCESWSSLTKPFIRGIRGSKVLM
TTRNESVAAVVPFHSVKVYHLSKLSNEDCWSVFASHAFPLSEGSENRGALEKIGKEIVKKCNGLPLAAQSLGGMLRRKH
DIRDWNNILESDIWELSESRCKIIPALRISYNYLPSHLKRCFVYCSLYPKDYEFHKDELIPLWMAEDLLKATKKGKTLEEVGH
EYFDDLVSRSFFQCSSRRNWGSYFVMHDLMHDLATFLGREFYFRADELGKETKIDRKTRHLSFKRFSDSVLDTEVLDKVK
FSRTFLPINFKDSPFNNEKATRIIVSLKYLRVLSFSYFQSQLALPDSIGELIHLRYLNLSGTSIATLPDSLCNLNNLQTLMLSSC

LNLTKLPSAMQNLVNLRHLEIIGTPIKEMPKRMGKLNQLQNLDIYIVGKHVENSIKELGGIPNLHGYFCIKNLENVTKGEE
ALEARIMEKKHINTLRLEWSACNDNCTEFQIELDVLSNLQPHQDLKWLSISGYKGTRFPEWIGNFSYQNMTNLSLRNCK
SCCKLPSLGQLPSLKKLEISDMNSVKTIDAGFYKKEDCSSMIPFPSLESLHIYDMPCWEEWSGFDSKAFPVLKVLYIFSCPK
LKGDLPNHLPALGKLRITNCELLVSSVPRAPTLQVLEILESNKLAFDVFPLMVESIEIKGRPMVESVMETITNIQPTCLRYLA
LEDCSSVIGLCIYFSYGRLPASLKNLHISGLKKLKFPMQQKHELLESLRMNNSCDSLTSLPLATFPNLIRLQITNCEYMESLS
VLASESFKSLSSFDIGGCPNFVSFPGEGLCAPNLTRFSVYDCAKLKSLPYQMRTLLPKMEYLNISNCQQIEWFPGGGMPP
NLRTLDLRNCEKLLSGLEWMDMVTSLNVHGPCDAINSFPKEGLLPPSLTSLSLIDLSSLKTLECKGLLHLSSLQQLQIQNCK
KLENIAGERLPVSLLKLSIGGCPLLQKLCHKKDRQIWPKICHVRGIKIDGRWI

>XP_027905874.1

MDSLGPFRKAVEGVINFIWKHGVRHVTYIVHYKQNVLELNDSVRNLVSEKERIKHQSDEAEKNLNKIEGKVTEWVRKV
GEIETIVKEFENDNGHKRARSPNYVFHXLWNRHKLGRKAKKMEAGVKKLIDESPKLDEVSYRQNVTSNDATLSNFDFEE
FGSTKSTMEKVMRQLEDSTVRMIGLYGPGGVGKSTLVKEIARKAKEKKLFDVVVKVEITANPNLQNIQEEIAYVLGLRLE
EEGENVRADCLRRRLKAEKRNILLILDDLWDKLDLNKLGIPVVDDDDDLSNDNRDLKDKDLEKNDDNNKDLNRKKMKK
EKFLGSHKGCKILLTSRNKRVLCDEMDIKSTFCVQKLDDKDALMLFQKLVGIKNEMSDSKQEIVKKYCAGLPLAIVTVAR
ALRSKSESVWETTLEKLKKQELVGVQTSMDISVKMSYDHLENEELKSIFLXCAQMGHQPLVMDLVRYCFGLSILEGISSL
WEARDRINTSIQKLKDSGLVLDGSSNNHFNMHDMVRDSTLSIAKKHRNVFTLRNGKLDDWPELESCASISICNCDITDG
LPEVINCSQLKFFQIDTNDASLIIPETFFAGMKNLKLRMLCLERCTLDDNLSIGELKKLRILSFSGSQLKSLPIELGCLHKLQLL
DISDCSIMEMNIPHDLLSRLTHLEELYIRKSLIKMFGETNQGQNLFLSELRNLHQLKVVDLSIPHVSLLPNHLFFDRLKDYKI
VVGNLEMFGGFRMSDKYETFRVLALQLNDDTNIHSQEDIKLLFKTVQSLLLGNMDGVQNFVGELNIDGFPDLKHLSIIN
NNHIKYVNSTELSNCVDVLPNLESLCLYNLGSLEMISYGPITDVSFAKLKAIKVEMCYRLENLYSFYRVKFCANVEIIEVCEC
GSLKEILQIPVDYGKVEFLKLHTLTLQSLPSFTCFYTKVEDSCWPHLREAQSTNRCDREITNEGDGESDKTFPIFGELVEIPN
LESLNISSLNIRKIWSNQPSSSFCFQNLIKLVVKGCHKLTYLCSLSMASSLKKLKTLVISECSIMENIFEAKGSSANKVCVFPK
LEEIHLSKMNKLTNIWQTKMSVDSFSSLISVNIEECNELDKIFPRHMEGWFENLDNLKVSKCQLVEVIFEGILNFKKLRTIE
VGDCNELRNLFPTSIGKDVPKLERMSVLHCWKIEEIVASQDASEANKDPLVFPELTYIRLYDLPNIKYFYKWGHAIKCPKL
KELNVKLKMFLKETCKTTDEEEEFVFSSKEVFPNLEYMELDFEEAQELLPKYQMHRLKELSLISVKVDLLSQFPYRIPNLEKL
KLIFSNIEVLVPKENFAQQKRLGITLELKELVLLHSTIKDLGLGRVPILRKLKLLSLESCSDLSNLGPPSVSLTYLTYLEVKFCRE
LSSLMASSTAKSMVQLKTMKVIYCPKVEQIVSNEGSKEDEVIKIVFNKLISIELVGLKNMRSFCSYKDCEFEFPSLEILIVREC
PKMEKFSERRSITPKLKDVFGVEGEEKAKWQWEGDLNATIQKIFNDKVTYSYIEDLYLGMGFGYNQYFELVNQLLHDSH
WVQQNSFRSLKRLSVEGCDTLVHVIPSHLFSCFHNLEVLQVVNCRNVEVIFNINDENRLKKASGIFRLKKLSLLDLPKLELL
WDKDPKGISLKVLEEVCVSRCNVLRSLFPASVAKDLTRLEVLQVTECGQLAEIFWKDEKGLFRARVVFLSIWYGELAERFR
KDEKGDEGEGTTQQFVFAHLTSLRLEKLPRLQYSIHCSKEEESISNLSERDIQELCLGSWPIPNSSFGLLESLIVDGCKFLSD
VFLPFSLLPFLTNLRKLDVANCEFVKTIFDITYATQEKDVTSTISTLLFSLENLTLSKLPNLENVWNKDPRSILSMRYLKEVHV
KKCKDLTSVFPASIAKDLVELENLMVEDCEGLMTIIAEDNTDLSGTHQQLLCLPIRSLVLRALPKLKYFYYCSLPGDNFAPL
ESHNDNQVCTQKCLSLGEKGMEMVLRGEFQKKLLNNIKFLTLYLQKDVFRYEILKELPNIEKLVVREGSLQEMFCCESLN
NVDYNGLLLQLKVLDLESIKKLISIGSNLVACKVSFFGLTYLKVQSCDNLLYLFTSSTTKSLGQLKRMEIKCCDSIEETVFKEG
EESNEDEIIFPKLNYLNFERLENLSWFCSGSLSLPSLEELSVTDCHRLISLCTGTLEADKLSQVTMNCEETIPLKTDLNSIMM
KTFLTEVCV

>XP_027905883.1

MALAVVGGALLSAFFDVLFDRLASPQVLNFFRGKKPDKLLQKMKNQLIVVRVVLADAEKTQITDSTVKEWLDLLRDLVY
EVDDLLDEVSTKAAIQKKKVSNSFSRIFKRKKIVSISKLEDIVERLDDILKQKESLDLKDIPVESYQSWKAQPTSLEDGYGMY
GRDKDKEAIMNMVLQDVTDDSEQVSVIPIVGMGGVGKTTLARSVFNDCKLNQQIFNSKAWVCVSDIFDIVKVTRTMV
EEITRKPCKLSDLNVLQLELMDKLKGKRFFIVLDDVWIEDCDNWTTLTKPFVSGIRGSKVLITTRNENVAAAVPFHTVEIY

HLNKLSNEDCWLVFANHAFPLQEASERRGTLEKIGKEIVKKCNGLPLAAQSLGGMLRRKHAVRDWNNVLESDIWELPG
GQCKIIPALTISYNYLPPHLKRCFLYCSLYPKDYGFQDNELIQLWMAEDLVKAPKKGKTLEEVGHEYFYDLVSRSFFQCAS
RWTGDGDDCFVMHDLMHDLATFISGEFYLRADELGKETKIDRKTRHLSFATFSDPVSDIEVFDTVTFPRILDLSS

>XP_027905952.1

MAALIACFHFLTLSPRAKLWLKKQFIQLDLYKARFDEVKDVVERLKNKRDAIRHTTDEEERRHGRRIHIEVKEWMERVDK
LILEYRVFNEDEICHKCALFDFFDSGYLPKPGIRYRRSRKANDITKQANGLLQDAKFDILSYWSGPPSMAAFFSNLGYESYS
SRNDTVKKIADEFQKPGVRMIGLHGLSGVGKTTLVKEVVKKALKDKMFEVVTMASVTKNPDIRKIQGQIADMLGVVLE
EESDIARAARIHQILNNENKSTLIILDDLWEEVNFDLLGIPYELEKEDGVKNIQGKSPDVYSLKNVSDGKSPILDGSASFKK
GRSHDVDGSKNVKKGEVLGGGFKNVNEGKSPIDAFGRVKAENIVPQYKGCKILMISEIKQVLLSQMEGKEESIFPVEVLK
EKEAEMLFKKKAGISGKSFEYDKLGAQIASKCKGLPMTIVTTARALKNKSLSVWEETNRKLESQNLTGAPEYSTKLSYELL
EDEELKHIFLLCARMGHDALIVDLVKYCIGFGSLRGFNTARETRDRVYTLVAKLKESGLLSDSYSSDHFTMPDTVRRAALSI
AYKENHLFTMTKGKVDEWPEKLQRYVALSLHHCDFVDDFPGRLNYPRLRVLQIVNNIPHLKIPKNFFKGMKELRVLILIGI
QLPLIDSSISSLHKLRMLSLEQCCMLDEELSIVGEMKRLRVLSFSGSDIKSLPYELNELKRLQIFDISNCSKLKNIPHGVVSSL
VSLEELYMRNTLIQLKDEEQTRQSQIALLSDLKHLNQLTTLDIQIPNVSNLPKNLFFDKLDNYKIVIGDLSSFSDTGFQMPE
KYETLKFLAVQLKNGCDIHSLKEIKMLFEGVENLFLELNTVHEKPTSAREAHNIVHDLFYRLNLKGFPYLKHLWIVNNSTIQ
SLIHPKDRQHPEKAFPKLESLCLYNLKMDELCSCKLSEPSFGKLKVIKINLCGELKNVFSISVVGLLKVLETIEVSECNSLKEII
NVGPPNNFENIELLMLPELRYLKLQSLSEFIGLDAIPHIEGEERKLFHEKVGVSKLQRLELSSIQIDVIWSVDQSSERSSFENL
THLDVNGCWKLKYLMSSTMAKCLVNLQSLYVSECEKMKNIFLPEQDREKDIMGNIFPKLKNMKLRSMKSLSKIWNPKL
PSDSFSKLDTLIIEECHKLENAMERIFGRLCNLRVTNCRSMQAIFNICEKVGDVANNLQDVHLETLPKLKLVWKMNNED
QVGIPKFNNLKRILAKDCDNLEYIFPFYVANSLDNLESLVVCGCYGLNEIVAEREATNTVRVRFNFPKLSTIKFSELPKLKSF
CPTAYDLSCPLKELSIELCNNLEPFNKGTQHEQANHVHVFFPEEVLVINNLKTMQIESRYAKSSSSYMGKRNHRRDNLEE
LSLSRLIDTEILYSFLYRNPNLKSLSLNNCFFEKIVPPKEDTEIENLGVVPNLKSLMLIDLPNLEEIGFEQDIILERLELLILKNCP
RMITIAPSSVSFTRLTNLEVVNCDGLQSLMSASTAKSLVQLNTMKVVKCESLMEIVRKDGEKSDRVVFQQLKALELVSLK
NLKSFCVSDCDFEFPSLEKLVVSSCYNMDRFSETVTSSPILQNVHVVHGKENKRFFWEGDINATIQKIFEEKKFFEGMEE
MSLPEHQETWQRGVGLQKQNSCFYSLKVLKLENCVIQPCAIPSDILPYLRSLKELQVRGCNNVEVIFEMNGEEGIGSTFH
LHKLTLEKLPKLKDVWERNGKGTESFQNLKLVNISECDNLQTVFPLSLAKNLKKLDELQIVHCHRLQEIVRTEDDTSVVFV
LPCLSKLCLVDLPELIYFYPESFTLECSALNKLFVWSCPELELFGSANRQPIFFDLKDVCNLKELILDWKHILVLRKKLGEPM
NNLKYLNYFKFFFDVDENERPDLPIQILQKMPNLRKMTICYCNCLEVFQTQIPEIVEKKVLTYLKTLKLDSVSKLQSIGSEDS
PWLNVICDSEKLQKLHVVDCPDLKTLVHSTPSVSFTYVKKLYIERCQELKYLFTLSAVNKLENLEYIQVKDCESMEAIILKED
DISEEIKLQHLKRIDLNLLSSLECFYSGNDTLRVPSLMQVDIWECPKMKFFSRGEIHLNSSFRGIQASNVSSDDLVFYHDLN
SSVEKVFLQQEFFQAVDKDYHSDQLQTLSELYCKISMENKWLANLETLKLQNCTLLYAIPSSILALLKNLKELEVRDSDQIE
AIFYINDDTEIKETESQLKILTLNGLSKLTHVWEKDTNKILIFQNLLEVVVSDCAKLQTLFPASLAKSLKDLKKLKIDTCENLQ
DFIEHEETTFVTEKFVFPCLEDLELCDLPRITCPKMFTLEFPSVKFLDVRGCDGLGLFQSVYDPMEGTSSRKLPLISDPNVIS
NLEKLTLDCKQILALSLWFKSQKSIERLTNLNSISLCFFRAKENEMPMLPVEILKAPNLIEMDISSCVSLKNFLAKNPKIGEE
EMLGKLTIVKLCNVSTTQLFELEYSSSLNIFERLHKLFVSDSHYLTTLGVHFTSTVSFSCLKEVNIYKCQKLKYLFTSSAAKML
MNLEEISVVECESLTEIVVKEGYTTSEAINFERLHSIYLQSLTSLVCFYSGSDTLQLSSLKIVTIWRCPNMEIFSQGIESLNGIT
LSTDLEPDDLPSPQDLNTRIKGISQRKEFIEAVDKKCFSYYLELQEDPHCKFGLQNQWLRDLVTLKLQKCTLPCAIPSAILAL
LKSLKELEVRDSNTVEVLFNMNDTEITPMASQLKMLTLEKLSKMRRVWEKRKNGVVIFPNLQEVIVRSCENLQTLFPASL
AKNLKSLKTLEIHNCDKFHEIVEKEEDTEAKFALPCLEMLDLYSLPQLTCFYAQTFTLEGPALNKISVVKCGKLELFTSVNRK
PLISSLEVISNLRKLNLDWKQIMALRARFRSEKFTRVFKFVTEMKLALDGNVSEMPIVLNEILHTTPNLIKMTMFIYNCNS
TEIFLAQNYKIVEDGMLLNLRMLTLSHVSTIRSIQTQNSSWLNTICEKVHELTVFRCHHVETIGVHSTSTMSFSFLKKLYAY
RCPQLRYLFTSSVAKKLVNLKEIRVTECKSLKEIVSKEGDEHEQKGEGEDEDENEMITMKLEILTLISLGKFERFYSGSSTLN
VPFLRRVRVDKCFHTKIFRHRDKLRPEVSVVIDEIRRKGDTKALIMQQFEEEAS

>XP_027905980.1

MDWSGPFGKVVEGVISFVWRHSVRHVTYIVRYKQSVLELKDSVKDLGYEKDKIDHQCDEEAVRNLNNIEGKVTEWNR
KVSEIETKVEKFENDDGHKRARSINCYVIPYFWNRHRLGRQATKMEVDVKNLIDKSPKFDEISYRQNLTSNEATLSNFDFI
EFDSTKSTMEKVMRQLEDSNVRMVGLYGRGGVGKSALIKEIARIARDKKLFNVVIKVEITINPDLQKIQEEIAYVLGLRLE
GEGENVRADCLRRRLKKEKGNTLLILDDLWHKLDLNKLGIPIDDNDDNDDFSSDNKDPNHKGLKREKIIGGHKGCKILLT
SREKRVLDVEMDVKPTFCVEQLDDKDALKLFQKLAGIHNVMPDSRQEIVKYCAGLPMAIVTVAKALRGKSELVWEAAL
GKLKKQELVGVQTYMDISVKMSYDHLENEEIKSIFLLCAQLGHQPLIMDLVKCCFGLGILEGVSSLWEARDKIKITIQKLK
DSGLLLDGNSDIHFNMHDIVRDAALSIANKEKNVFTLRNGKLDDWPELSRCTSISLCNCDIIDELPLVNCSKLIFFQIDTNN
PSLTIPEKFFVGMKNLKVLILTGFHLQRLPPSIKYLLKLRMLCLERCTLEDTLPIGELKKLRILSFFGSQLKNLPTELGCLDKLQ
LLDINDCSILETNIPPNILSGLKNLEELYIRKSLIKMLVEGEPNHGQNSFLSELKNLHQLKVLDLSIPCFSVFPNHLFFDKLKN
YKIVVGDLEMFSVIGFKMIDKYETYRVLALQLSHDTSFHSQEDIKLLFKTVQSLLLGKVDDVVKVVNELNIDGFPDLKHLSI
TDSKAIKYVNSMELSNCVNVFTNLESLCLYNLKNLEMISYGPLTVASFSKLKSIKVNMCDQLVTLYSVYMVEFANSEESCE
ICKYNSYLDKFCASLETIEVSECETLKGIIQIPMNYDNVKFLKLQTLSFQSLPSFTCFYTEVEESCWPHPTKPQSENSSSEED
QKSDKAPPLFGELV

>XP_027905984.1

MDVLSKPLDPIINFVWGKGVKHVTYIFSYTKNFEELNKRVKRLGEEKQRLDGKRDKAKRKGDIVEDRVEEWFGEVGEFE
SRVEKYRNNAGHKKTRGLYYLFPYHRHKLGRQAKKMEMEALRLKDECPKDDQVSHADKVTSFDRPNPGYIEFDSRKFI
VEDFMTKLKDPNIKIIGLHGAQGMGKSTLIKEIVKKAEDEGLLVAEIDVTENPNPLKIQEDIAHVLGLPLAGESENVRADY
LRRWLKVENVSILIILDNLHERLDLNRLGIPVDDDYGLRKKNELSIMSSKQGPDRTQDTAGNTRGTDEKVPRKKNESSTK
PSDDKQGSSGLKQDPDHTQYTAGDTRGIDEKVLKKGNFVGDYQGCKVLLSSRDKKVFPRDVGPTFNLKEIDENEALQLF
EKVTGGGDKMSMPKEEIQNYCTGLPITIVTFAEAFKNWIKSESKPTLDKFKKQGLVEWQKSSETPNKKKYDLPKNKELKF
IYLLCAQMGHLPLVNDLVKYCFGLGILEGVSSLSTARGKINESLQELKNLSLVSYENPNIHFHMSHMVRDDALSNALMD
HNVFVLRDGKLDYWPDLEKCISISICNSYITDGFPQVINCPQLQFLQIETNDPSLEIPQRFFSSMKNLLVLILTGFHLSSLPYS
IKDLLNLRMLCLERCTLDCNLSILRKFKKLRILSFSGSQLKNLPVELRYLDKLRMLDISDCFKLKIIPLDLFSNLTCLEELYIRKSL
IKMLVEKGENKGHNSFLSELKNLHQLKVVDLSIPCVSILPNHLFFDRLKGYKIEIGDVEMFSVGEFRMPNKYEELKVLALQ
VKDDTDIQSHQGIKLLFKTSQSLFLGNVCVQNVVNELNIDGFQNLKHLSIINNNDVEYFNSTGLSYCENIFSNLESLWLCN
MMNLKMICRGPITLESFAKLKTIKVEMCCQLENLFSFYAIKISTSTGTSEIFKCNSNMKKFLASLEMIEVCECVSLKEILQIP
PDCVKDCECESLKEILQIPQDYGKVEFLKLRTLTLQSLPSFTCFYTKVERSCWPHSIEAQTTNRGHTEISTEQAGHNDNAP
PLFGEPVEVPNLENLNLSSLNIRKIWSDQHLSSFYFQNLIKLVVKDCDKLTHLCSLPMASSLKKLKSLVISGCLKMKTIFEIE
GVSANKVCVFPKLEEIHLSKMKRLTDMWQTEVGIDSFSSLISVRIEECDELDKIFPSHMEGWFESLINLKVSNCKSVKVIFE
VSDSEEIDVSSGIDTNLQVILLDELPKLKELWSKDPYGILNFKKLRTIDVSKCDELRNLFPASMVNDVSKLERMSVLHCER
MVEIVSSKDVLEVDNDPLEFPELTFVRLYELPNMKQFYKGRHPIKCPKLKELSMGKCMKLKRFETSDEKFVFSAEVVFPKL
EYMEIDFLEAQNWLSNYKMLKLKELIILNSVHRPDLLYPFLYKMPNLEKLKLTSSYSGSLQSTNIGQHDRLRVVLQLKQLFI
CSSKLKDIGFERDQVLERLELLKLKDCDNLCNLGPSSVSLSYLTCLKLKRCNGLKNLMASSTAKSMVQLKTMKVIDCGQV
EQIVSNDGSEEGKGIKIVFSKLISIELVGLMNMTSFCGYKNCEFEFPLLEILIVRTCPKMEKFSERGLIAPKLKDVYGVEGDK
KAQWQWEGDLNDTIQEIFHDKL

>XP_027905992.1

MDVLSKPLDPIINFVWGKSVKHVTYIFSYTKNFEELNKRVKRLGEEKQRLDGKRDKAKRKGDIVEDRVEEWFGEVGEFES
RVEKYRNNAGHKKTRGFYYLFPYQRHKLGRQAKKMEMEALRLKDECPKDDQVSHADKVTSFDRPNPGYIEFDSRKFIV
EDFMTKLKDPNIKIVGLLGAQGMGKSTLIKEIVRKAKDEGFLVAEIDVTENPNPLKIQEDIAYVLGLPLAGESENVRADYL
RRWLKVENVSILIILDNLHERLDLNRLGIPVDDDYGLRKKNELSIMSSKQGPDRTQDTASDTRGTDEKMLKKGNFLGDYK
GCKVLLSSRNKKVFPCDVGPTFNLKEIDENEALQLFEKVTGGGDKMSMPKEEIQNYCTGLPITIVTFAEAFKNWSESESE

PTLEKFKRQGLVEWQKSLETPNKKKYDLPKSKELKFIYLLCAQMGHLPLVNDLVKYCFGLGILEGVSSLSVAREKINESLQE
LKNLSLVSYENSNIHFHISRMVRDDALSNALMDHNVFALRDGKLDYWPDLEKCISISICNSYITDGFPQVINCPQLQFLQI
ETNDPSLEIPEIF

>XP_027905993.1

MEPKALRLKDECPKDEEVSHAENVTSFDLTSSYSGYIEFDSRKSIVQDIMRKLKDPNIKIIGLHGAQGMGKSTLIKKIANKA
KDEGLFDRVAEIDVTVNPNPLTIQADIAHVLGLPLVGESENVRADYLRRWLKIENVSILIILDNLHERLDLNRLGIPVDLDY
DLRKKNELSIMSSNQGTNPNPGGGAQGTGEKVLKKGNFLGDYKGCKVLLSSRDKKVFHDEVDVESNFCLKELDGNDAL
MLFEKVIGGGNKMSMPKEEIQNYCTGLPMRIVTFGVAFKNWIESESKPTLDKFKKQGLVEWQKSSETPNKKKYDLPKN
KELKFIYLLCAQMGHLPLVNDLVKYCFGLGIFEGVSSLSAAREKINESIQELKNLSLVSYENPNIHFHMSHMVRDDALSNA
LMDHNVFALRDGKLDYWPDLEKCISISICNSYITDGFPQVINCPQLKFLQIETNDPSLEIPQRFFSSMKNLLVLILTDFHLSS
LPYSIKDLLNLRMLCLERCTLDCNLSVLRKFKKLRILSFSGSQLKNLPVELRYLDKLRMLDISDCFKLKIIPPDLFSNLRCLEEL
YIRKSLIKMLVEKGENKGHNSFLSELKNLHQLKVVDLSIPCVSILPSHLFFDRLKDYKIEIGDFEMFSVGEFRMPNKYEELKV
LALQLKDDTDLNSHQGIKLLFKTAQSLFLGNVCVQNVLNELNIDGFQNLKHLSIINNNDVEYFNSTDLSYCVNIFSNLESL
WLCNMMNLKMICRGPITLESFAKLKTIKVEMCCQLENLFSFYAIKISTSTGTSEIFKCNSIMKKFLASLEMIEVCECESLKEI
LQIPLDCGEVCEVCECESLKEILQIPQDYGKVEFLKLRTLTLQSLPSFTCFYTKVERSCWPHLTEAQTTNWGHTEISTEQDG
HSDNAPPLFGELV

>XP_027905997.1

MDVKLTFCVKELDDKDALVLFQKLAGIHSEMSDSKQEIVKKYCAGLPVAIVSVARALRNKSESVWEATIEKLRKQELTGV
QTPMDISMKMSYDHLENEEIKSIFLLCAQMGHQPLITDLVKYCFGLGILDGISSIWEARDRIKTSIQKLKDLGLVSDGISN
NHFNMHDMVRDAVLFIANKDRNVFTLRNGKLDDWPELEKCTSISICNCDIIDGLPQVINCPQLRFFQIDTNDPSLKIPKN
SFEVMKNLRVLILNGFHLPRLPSSVKCLLKLRMLCLERCTLDDNLDIIGELKKLRILSFSGSQLKSFSSQLGCLDKLQLLDISD
CSIVEIDMLSNILSRLTNLEELYIRKSLVKMFVEGETNQGQTLFLSELKNLHKLKVVDLSIPCVSVLPNHLFFDKLKDYKIVV
GDLEIFSVGNFRMPEKYEAFKVLALQLNDDTDIHSQAGIKLLFKRVESLLLGKVDGVKNIVNELNIDGFPYLKHLSIINNNH
IKYVNSTKLFNGVKVFSNLESLFLYNLGNIEMISYGPVTLASFAKLKSIKVEMCCRLENLYSFFTITFPTGAETSETLECSSYV
DKFCASLETVEVSECQSLKEILQIPIDYGKVEFLKLNTLTLQSLRSFTCFYNIVERPCKPHMSGAKTTNTSLGEIANEEDEQS
DKAPPLFASSLKKLKSLIISECSIMERIFDSKGDNVDKVCVFPKLEEIQLRKMNRLTDIWQSKVSVDSFSSLTSVNIEECNKL
DKIFPSHMKGWIENLDNLKVSRCESVEMIFEINDSQERDASGGTDTNLQVILLEELPKLKQLWNIDPDGILNFKKLRTIEV
GTCGELRNLFPASVAKDVSNLERMSALNCQKMVEIVASHDTSKANNDLLVFPELICVRLYLLPNVRHFYKGRHPIKCPKL
KELSVNNCEELKTFFEETTEATNEEENFVFSANEVLPNLEYMEIDFNQAQNLLSKYQMHRLKELSLISGRRGNILYQFPYK
MPNLEKLNLTSFWPEESVPRANISRQKRLGIVLQLKQLCFFYSWMEDLGLERDQVLQRLELLRLEYCDKLSTLAPSSVSLT
YLTHLELKRCLRLRNLMASSTAKTMVQLKTLKVIGCTRIEEIVRKEGIEENKVVKIVFSKLITIELVGLEDLTCFCKYNECEFEF
PSLEILMVRECPKMKKFSEGEAIAPKVKNIFGREGDGKAKWMWEGDLNATIQKVYTDKVTFKYSELVDLNVIEQLLNDN
HWVQQNRFGYLKRLIACECESVVDVIPSHLLSCFHNLEELEVVNCSNAEVIFNINDENRLTKASGIFRLKSLYLSNLPKLEHI
WKKDPEGIIGLQLLKEVRVQSCERLENLFPASTAKDLTRLGLLEVTDCKELAEIFRKDEKSGEGEGEGKTQHSLFPRLTTLIL
GQLPRLKYSINSFQHQEIILSWSQRDIQELCLGSRTIPNSCLDFLESLTLCDCQSLSDVLLPFNLLSFLTNLRTLTVKNCNFVK
TIFDVTCATQDSEITSVGPALPFSLKKLTLSDLQNLKNVWSEDPHGILSMHHLQELLVDNCKGLKSVFPASIAKNLLELENL
RVEDCEGLMTVVAEDNTDPTVEVTFTCPSVRSLELRGLPKFKYFYYCSPNSDLESHTKDQLATGKSLQCLAVGENGVEM
ILRGEFERNFLYNLKVLTLYFSSDSDVFPHGILRQVPNIEKFVVCDGSLFCCQSSNNVDYSGLLLQLKELHLESLEELVCIGLE
NCWTEPFVRNLETFKVISCGSLKSLVACTLSFSNLICLKVEDCNSLSYLFTSSTAKSLGKLKSMEIKNCYSIEEIVSKENGEES
DEDEIMFSQLSFLNLESLFELRRFYKGSLSFPSLGELSVIDCHQMITLCPGTLKADRLSQVTIDYENILLETDITSTIQKTFLEK
AQTQSWIELKSSPVLQEIWHSPVHLPDLCFSELAALYVEDCQFLSDAVLPSDLLPLLPKLETLEVENCDHVKVIFDVKSAQ
GTVTFPLKKLTLSRLPSPLENVWNKDPHGILNMSRLEEVHVKELM

>XP_027906042.1

MASSSERETSSGSSTIQKSGSEEDAEAQMDERKRKRMTSNQESARKYRMRKQKHLEDLVSTVAQLKNENQHILQSINL
TSQNYMNFEAENSILRAEMSELSSRLSNLRFYDVFLSFRGEDTRASFVSHLHASLQNAGIIVFKDDESLPRGDQISYSLFQ
AIEQSRISIVVFSRNYSQSTWCLNELVRIMQCHSTIGQVVLPVFYDVDPSEVRHQTGEFGRAFQSLSSRTFTEVSKKRRW
MKTLREAASISGIVVLNSRNESEAIKSIIENVTRLLDKTELFIADNPVGVESRVQDTIQLLDLKRSNDVLLLGMWGMGGIG
KTTIAKAIYNKIGRNFEGRSFLAQIRDVWGKDGGQVCLQEQLLFDINKETKTKIHNVEFGKNILMQRLHRKRVLLILDDV
NNLHQLNALCGNRGWFGSGSRIIITTRDMHILRGSRVDKLYMMERMDESESVELFSWHAFKQASPREDFAELSRSVIA
YSGGLPLALEVLGSYLFDMEVTEWKSVLEKLQKIPNDEVQEKLKISYDGLSDNTEKEIFLDIACFFIGMDRNDVIHILNGCG
LCAENGIRVLTERSLVTIDHNNTLGMHDLLRDMGREIIRGKSPKEPEERSRLWFLEDVLDVLSKETGTKAIEGLALKLPRT
NTKSLNTKAFKKMKKLRLLQLAGVDLIGDYKYLSKDLRWLCWREFPLTSFPANFYQGSLVSVELENSNVTHVWKEAQV
MENLKILNLSHSHCLTHSPDFSNMPNLEKLVLVDCPRLLKISRTIGHLNKVLHINLQDCISLRKLPRSIYKLKSLKTLILSGCL
MIDKLQEDIEQMESLTTLVADKTAITKIPFSIARSKSIAYISLCGYEGFLRDVFPSIIWSWMSPTNSLSSHMQRFAGISSLAS
LDVPNNSSHHLASISKDLPKLQSLWVECGSKLQLSQDTKIIFDALHDTNSGESETTATTSQMSNINAFTLIECNSQVHLSG
SNRSLLIQMGVSSEVSYNLKENILKNNQDCLVSDNCYPHWLAFDSNGSSVTFEIPEVNGYKMKTIMCHVHYSSPANITS
DGLKNLLVINHTKGTIQLHKRNALAVFEDEDWERVISNIEPGNKVEVVVIFGNSLIVNHTKIYVTYEPNDNKSEVPCTT

>XP_027906043.1

MASSSERETSSGSSTIQKSGSEEDAEAQMDERKRKRMTSNQESARKYRMRKQKHLEDLVSTVAQLKNENQHILQSINL
TSQNYMNFEAENSILRAEMSELSSRLSNLRFYDVFLSFRGEDTRASFVSHLHASLQNAGIIVFKDDESLPRGDQISYSLFQ
AIEQSRISIVVFSRNYSQSTWCLNELVRIMQCHSTIGQVVLPVFYDVDPSEVRHQTGEFGRAFQSLSSRTFTEVSKKRRW
MKTLREAASISGIVVLNSRNESEAIKSIIENVTRLLDKTELFIADNPVGVESRVQDTIQLLDLKRSNDVLLLGMWGMGGIG
KTTIAKAIYNKIGRNFEGRSFLAQIRDVWGKDGGQVCLQEQLLFDINKETKTKIHNVEFGKNILMQRLHRKRVLLILDDV
NNLHQLNALCGNRGWFGSGSRIIITTRDMHILRGSRVDKLYMMERMDESESVELFSWHAFKQASPREDFAELSRSVIA
YSGGLPLALEVLGSYLFDMEVTEWKSVLEKLQKIPNDEVQEKLKISYDGLSDNTEKEIFLDIACFFIGMDRNDVIHILNGCG
LCAENGIRVLTERSLVTIDHNNTLGMHDLLRDMGREIIRGKSPKEPEERSRLWFLEDVLDVLSKETGTKAIEGLALKLPRT
NTKSLNTKAFKKMKKLRLLQLAGVDLIGDYKYLSKDLRWLCWREFPLTSFPANFYQGSLVSVELENSNVTHVWKEAQV
MENLKILNLSHSHCLTHSPDFSNMPNLEKLVLVDCPRLLKISRTIGHLNKVLHINLQDCISLRKLPRSIYKLKSLKTLILSGCL
MIDKLQEDIEQMESLTTLVADKTAITKIPFSIARSKSIAYISLCGYEGFLRDVFPSIIWSWMSPTNSLSSHMQRFAGISSLAS
LDVPNNSSHHLASISKDLPKLQSLWVECGSKLQLSQDTKIIFDALHDTNSGESETTATTSQMSNINAFTLIECNSQVHLSG
SNRSLLIQMGVSSEVSYNLKENILKNNQDCLVSDNCYPHWLAFDSNGSSVTFEIPEVNGYKMKTIMCHVHYSSPANITS
DGLKNLLVINHTKGTIQLHKRNALAVFEDEDWERVISNIEPGNKVEVVVIFGNSLIVNHTKIYVTYEPNDNKSEVPCTT

>XP_027906044.1

MASSSERETSSGSSTIQKSGSEEDAEAQMDERKRKRMTSNQESARKYRMRKQKHLEDLVSTVAQLKNENQHILQSINL
TSQNYMNFEAENSILRAEMSELSSRLSNLRFYDVFLSFRGEDTRASFVSHLHASLQNAGIIVFKDDESLPRGDQISYSLFQ
AIEQSRISIVVFSRNYSQSTWCLNELVRIMQCHSTIGQVVLPVFYDVDPSEVRHQTGEFGRAFQSLSSRTFTEVSKKRRW
MKTLREAASISGIVVLNSRNESEAIKSIIENVTRLLDKTELFIADNPVGVESRVQDTIQLLDLKRSNDVLLLGMWGMGGIG
KTTIAKAIYNKIGRNFEGRSFLAQIRDVWGKDGGQVCLQEQLLFDINKETKTKIHNVEFGKNILMQRLHRKRVLLILDDV
NNLHQLNALCGNRGWFGSGSRIIITTRDMHILRGSRVDKLYMMERMDESESVELFSWHAFKQASPREDFAELSRSVIA
YSGGLPLALEVLGSYLFDMEVTEWKSVLEKLQKIPNDEVQEKLKISYDGLSDNTEKEIFLDIACFFIGMDRNDVIHILNGCG
LCAENGIRVLTERSLVTIDHNNTLGMHDLLRDMGREIIRGKSPKEPEERSRLWFLEDVLDVLSKETGTKAIEGLALKLPRT
NTKSLNTKAFKKMKKLRLLQLAGVDLIGDYKYLSKDLRWLCWREFPLTSFPANFYQGSLVSVELENSNVTHVWKEAQV
MENLKILNLSHSHCLTHSPDFSNMPNLEKLVLVDCPRLSND

>XP_027906045.1

MASSSERETSSGSFSIQNSGWEEDSEAQMDERERKRKRMTSNQESSSKYRMRNQKHLEDLVATVAMLKNENQRILQS
INITSKNYMNAEAEKVLEAQISELRSRISSIRFYDVFLSFRGEDTRASFVSHLHASLQNAGIIVFKDDESLPRGDQISYSLFQ
AIEQSRISIVVFSRNYSQSPWCLNELVRIMQCHRTIGQVVLPVFYGVDPSEVRNQTGEFGRAFQRLSNRIFTEEKLDAMR
WMETLHEAASISGIVVLNSRNESEAIKSIIENVTRLLDKTELFIADNPVGVESRVHDTIQLWDLERSNDVLLLGMWGMG
GIGKTTIAKAIYNKIGRNFEGRSFLAQIREVWGKDGGQVCLQEQLLFDINKETKTKIHNIEFGKNILMQSLHHKRVLVILD
DVNNLHQLNALCGNREWFGLGSRIIITTRDMHILRGSRVDKLYTMERMDESESIELFSSHAFKQASPRKDFAKLSRSVVA
YSGDCHCVALEVLGSYLFDLEVTEWKSVLEKLHKIPNDEVQEKLKISYDGLSDNTEKEIFLDIACFFIGMDRNDVIHILNGC
GLCAENGIRVLTERSLVTIDHNNTLGTHDLLQDMGREIIRGKSPKEPEERSRLWFHEDVLDVLSKETGTKAIEGLALMLPR
TNTKCLNTKSFKKMKKLRLLQLAGVDLIGDYKYLSKDLRWICWREFPLTSFPANFYQGSLVSVELENSNVTHVWKEAQV
MENLKILNLSHSHYLTHSPDFSNMPNLEKLVLLDCPRLLKVSWTIGHLNKVLHINLQDCISLRNLPRSIYKLKSLKTLILSGC
LMIDKLEEDIEQMESLTTLVADKTAITKIPFSIVRSKSISFISLCGYEGFLRDVFPSIIWSWMSPMNSLSSHMQRFAGISFLA
SLDVSNKSSHHLASISKDLPKLQSLWVECGSKLQLSHDTKIILDALNDTNSGKSETTGTTSQMSNINVFTLIKCNSQVHVS
RSNRSLLIQMGRSYEVSYNLKESILQNNQDCLVSDNCYPHWLAFDSNGSSVTFEIPQVNGYKMKTIMCHVHYSSPANIT
SDGLKNLLVINHTKDTIQLHKRNALAVFEDEDWERVISNIEPGNKVEVVVIFGNRLIVNQTKIYVTYEPNDNKSEVPCTT

>XP_027906093.1

MALAVVGGALLSAFIEVVFDKLASPEVVNFIRWKKPDKLLQKMRSQLLVVKVVLADAEKRQITDSNVKEWLDLLNDVV
YDVDDLLDEVSTKAATQKKVTNSFPHLFKRKKIVSISKLEDIVERLDDILKQKENLDLKEIPVENNQPSKPQSTSLEDRYDM
YGRDKDKETIMKLVLEDSSDGEEVSVIPIVGMGGVGKTTLARSVYNDGKLKQIFDLKAWVCVSDIFDIVKVTKTMIEEIIQ
KPCKLSDLNSIQLDLLDKLRGKRFLIVLDDVWIEDCDNWSSLTKPFLGGIRGSKILVTTRNENVAAVVPFHSVNVYHLNRL
SNEDCWLVFANHAFHPSGGTENRGTLEKIGKEIVKKCNGLPLAAQSLGGMLRRKHAIGDWNNVLKSDIWELPESQCKII
PALRISYNHLPPHLKRCFVYCSLYPKDYEFQKDELILLWMAEDLVKAPKTEKSIEEVGDEYFDDLVSRSFFQCSSHRTWGN
YFVMHDLMHDLATFLGGEFYFRADELGKGTKINRKTRHLSFTRFSDPVSDIEVFETVKFSRTLLPINYKDYPFNNEKATRII
GSMLKYLRVLSFRDFRSVLALPDSIGELIHLRYLNLSYTSIASLPESLCNLYNLQTLKLYCCFKLTKLPGAMQNLENLRRLEIL
NTAIKEMPKGMGKLNQMQNLDFYIVGKHIENSIKELRGLPNLHGSFCIQKIENVTTGEEALEARIMDKKHIHDLSLEWSI
CNDNSTNFQIELDVLSNLQPHQDLKSLSISGYKGTRFPEWMENCSYYYMTVLSLNNCNNCCMLPSLGQLLSLKRLHISN
MISVKTIDAGFYKKNDSSSVTPFPSLESLYIYNMPFWEMWVAFDSEAFLVLKDLYIQNCPKLKGDLPYHLPALQTLAIRN
CELLVSSVPGASTLRTLEISESNKLAFHTFPLSVERIEIEGSPVVESMMEAITNIQPTCLRYLTLRDCSSSISFPGDRLPASLKT
LQISGLKKLKFPMQQKHELLESLTINNSCDSLTSLPLDSFPNLIRLQITNCENMESLSVLGSDSFKSLSSFEIDRCPNFVSFLG
GLSAPNLTRFIVYDCDKLKSLPDQICTLLPKMEYLSISNCQQIESFPEGGMPPNLRIVEINNCEKLLSGQQWVSKDIFTYLK
VWGPCDGINSFPKEGLLPPSLTYLQLFGFSSLETLECKGLLHLTSLRELHIQSCKKLENITGERLPISLIKLSINRCPLLQKRCH
RKDRGIWHKICHVRGINIDGRWIQ

>XP_027906094.1

MALAVVGGALLSAFIEVVFDKLASPEVVNFIRWKKPDKLLQKMRSQLLVVKVVLADAEKRQITDSNVKEWLDLLNDVV
YDVDDLLDEVSTKAATQKKVTNSFPHLFKRKKIVSISKLEDIVERLDDILKQKENLDLKEIPVENNQPSKPQSTSLEDRYDM
YGRDKDKETIMKLVLEDSSDGEEVSVIPIVGMGGVGKTTLARSVYNDGKLKQIFDLKAWVCVSDIFDIVKVTKTMIEEIIQ
KPCKLSDLNSIQLDLLDKLRGKRFLIVLDDVWIEDCDNWSSLTKPFLGGIRGSKILVTTRNENVAAVVPFHSVNVYHLNRL
SNEDCWLVFANHAFHPSGGTENRGTLEKIGKEIVKKCNGLPLAAQSLGGMLRRKHAIGDWNNVLKSDIWELPESQCKII
PALRISYNHLPPHLKRCFVYCSLYPKDYEFQKDELILLWMAEDLVKAPKTEKSIEEVGDEYFDDLVSRSFFQCSSHRTWGN
YFVMHDLMHDLATFLGGEFYFRADELGKGTKINRKTRHLSFTRFSDPVSDIEVFETVKFSRTLLPINYKDYPFNNEKATRII
GSMLKYLRVLSFRDFRSVLALPDSIGELIHLRYLNLSYTSIASLPESLCNLYNLQTLKLYCCFKLTKLPGAMQNLENLRRLEIL
NTAIKEMPKGMGKLNQMQNLDFYIVGKHIENSIKELRGLPNLHGSFCIQKIENVTTGEEALEARIMDKKHIHDLSLEWSI

CNDNSTNFQIELDVLSNLQPHQDLKSLSISGYKGTRFPEWMENCSYYYMTVLSLNNCNNCCMLPSLGQLLSLKRLHISN
MISVKTIDAGFYKKNDSSSVTPFPSLESLYIYNMPFWEMWVAFDSEAFLVLKDLYIQNCPKLKGDLPYHLPALQTLAIRN
CELLVSSVPGASTLRTLEISESNKLAFHTFPLSVERIEIEGSPVVESMMEAITNIQPTCLRYLTLRDCSSSISFPGDRLPASLKT
LQISGLKKLKFPMQQKHELLESLTINNSCDSLTSLPLDSFPNLIRLQITNCENMESLSVLGSDSFKSLSSFEIDRCPNFVSFLG
GLSAPNLTRFIVYDCDKLKSLPDQICTLLPKMEYLSISNCQQIESFPEGGMPPNLRIVEINNCEKLLSGQQWVSKDIFTYLK
VWGPCDGINSFPKEGLLPPSLTYLQLFGFSSLETLECKGLLHLTSLRELHIQSCKKLENITGERLPISLIKLSINRCPLLQKRCH
RKDRGIWHKICHVRGINIDGRWIQ

>XP_027906169.1

MASSIPSMEFASSSSKRQRRYDVLINFSGEDIRKKFVSHLDSALSAVGLTTIHHHHNAVHIQQPILKLCRVAIVVFTQTYSE
SAWCLHQLQQIIQWHETYCQHVLPVYYEIRPSDVRLQKGNFGETLKATAQQAFSGQQLEHGMSMWNLALTKAANLF
GWDDSNYRSDAEVVDNIVKTVLHLPALSATKFPVGLQSHMEDLIQTIKNKSSEVCIIGIYGGGGLGKTTLAKAIYHQIHGT
FKEKCFIEDVAQVSKIRGHAHLQEQLLSNVLKTKVEIHSVEMGGRMIRESLSGKRVLIVLDDMNEYSTLLDLCRCRARFSE
GTVIIITTTNEGLLIRHPVDSVFRIERMNAKESLELLSWHAFREAKPKEDYNDLARKVVTYSGELPLLLEVTGSSLFERTKRE
WNGVLFEFAKRPQNNVPRKLKISFDVLCNQIEKDLFLDVCRFFVGKGRVYATNILNGFGVDVDNGIRVLTERSLIQVEKN
NKLGMHSLLREMGREIIREITGKEPGKISQLWLDKDVEYVLTENTFFSSQRTKVIQRLPVKMLLNMRDFFDPYPEVREPL
LKLTENSEYLSKKLRWISLQGFSSKYLPNDFYLHDAMVIDLKHSLLRVVWKETQVLASSESYDISLPVVNDPYCLVHMGE
GHSIFFTVPRDLEMKGMTLCVVYLSTPMIFEPEVSTISIVNYTKCTCQIHKHDPIISFNDEDWHRIVSNLGSGDKVEIFVSF
SHRLVVKNTIVYLICGESNNLEKEPEQMKNYLSKFIRKL

>XP_027906170.1

MASSIPSMEFASSSSKRQRRYDVLINFSGEDIRKKFVSHLDSALSAVGLTTIHHHHNAVHIQQPILKLCRVAIVVFTQTYSE
SAWCLHQLQQIIQWHETYCQHVLPVYYEIRPSDVRLQKGNFGETLKATAQQAFSGQQLEHGMSMWNLALTKAANLF
GWDDSNYRSDAEVVDNIVKTVLHLPALSATKFPVGLQSHMEDLIQTIKNKSSEVCIIGIYGGGGLGKTTLAKAIYHQIHGT
FKEKCFIEDVAQVSKIRGHAHLQEQLLSNVLKTKVEIHSVEMGGRMIRESLSGKRVLIVLDDMNEYSTLLDLCRCRARFSE
GTVIIITTTNEGLLIRHPVDSVFRIERMNAKESLELLSWHAFREAKPKEDYNDLARKVVTYSGELPLLLEVTGSSLFERTKRE
WNGVLFEFAKRPQNNVPRKLKISFDVLCNQIEKDLFLDVCRFFVGKGRVYATNILNGFGVDVDNGIRVLTERSLIQVEKN
NKLGMHSLLREMGREIIREITGKEPGKISQLWLDKDVEYVLTENTFFSSQRTKVIQRLPVKMLLNMRDFFDPYPEVREPL
LKLTENSEYLSKKLRWISLQGFSSKYLPNDFYLHDAMVIDLKHSLLRVVWKETQILRSLKVLNLSHSMHLIETPDFSRIQCL
EQLILKDCPRLRKVHQSIGCLYNLILLNLKDCTSLRNLPVGIYMLKSLRTLILSGCSKIDLSEKDIVL

>XP_027906171.1

MASSIPSMEFASSSSKRQRRYDVLINFSGEDIRKKFVSHLDSALSAVGLTTIHHHHNAVHIQQPILKLCRVAIVVFTQTYSE
SAWCLHQLQQIIQWHETYCQHVLPVYYEIRPSDVRLQKGNFGETLKATAQQAFSGQQLEHGMSMWNLALTKAANLF
GWDDSNYRSDAEVVDNIVKTVLHLPALSATKFPVGLQSHMEDLIQTIKNKSSEVCIIGIYGGGGLGKTTLAKAIYHQIHGT
FKEKCFIEDVAQVSKIRGHAHLQEQLLSNVLKTKVEIHSVEMGGRMIRESLSGKRVLIVLDDMNEYSTLLDLCRCRARFSE
GTVIIITTTNEGLLIRHPVDSVFRIERMNAKESLELLSWHAFREAKPKEDYNDLARKVVTYSGELPLLLEVTGSSLFERTKRE
WNGVLFEFAKRPQNNVPRKLKISFDVLCNQIEKDLFLDVCRFFVGKGRVYATNILNGFGVDVDNGIRVLTERSLIQVEKN
NKLGMHSLLREMGREIIREITGKEPGKISQLWLDKDVEYVLTENTVLASSESYDISLPVVNDPYCLVHMGEGHSIFFTVPR
DLEMKGMTLCVVYLSTPMIFEPEVSTISIVNYTKCTCQIHKHDPIISFNDEDWHRIVSNLGSGDKVEIFVSFSHRLVVKNTI
VYLICGESNNLEKEPEQMKNYLSKFIRKL

>XP_027906172.1

MEFVSSSSKLPRNYDVLINFTGEDIHRKFVSHLEFAFSTVGLTTFLHHQNAVKSTHIQQPILNLCRVAIVVFTKTYSQSAW
CLNQLQQIIKWHQTYCRHVLPVYYEIQPSDVRLQKGDFGKTLKATARQTFLGQQLEDGLSRWSQALTKAANFFGWDE
SNHWSDAELVDNIVKSVLNLPVLSVTKFPVGLQYRVQDVIQTIKDKSEEVCIIGIWGERGSGKTTLARAIYHQIHGTFTEK
SFVEDISEVNLTRGYVSLQRQLLSDVLKRKVEIHSVEMGRKMTMERLSGKRVLIVLDDMNEYGPFFDLCRYRKRLSGGT
VIIITTTDRDLLVNHSDSVFSIQLMNDMESIELLSWHAFREAKPKQEYEDFARRVVRYCGGLPLALEVIGSTLFETTEEEW
CSVLFELEENPQHSVIHKLKISFDCLRNQMEKDLFLDVCCFFVGKDRAYAMKILNGCVVDADSGIRVLIERSLIKVSKNNK
FGMHPLLRKMGRQISLDILGNKHGKNIELWFDKDVEYALPHNTLLSSQKTEVIQRLVLTLRDFSELNPLDLRDPQTKFTE
DPDYLYEKLRWIRLQGFSSEYLPNDYGRGDTIAIDLKHSLLQRVWKQPQVLRSLKFLNLSHSMHLKETPNLSGLPCLEQLI
LKCCPKLRKVHQSIGCLCNLLLLNLKDCTSLSNLPRGIYKSKSLRTLILSGCSKIEIMDKDITQMESLITLIAENTAVKSLPFSIV
SSKSIGYISTHQFEGLSRSFFLLSFGLGCRQQ

>XP_027906177.1

MDTVLKIFDPVIEFVRDHGINQLTYIFCYTKNFKELNKRVKRLGEEKERLDRQRGEAKRKGHIVEDRVEEWFEEVGEFEIR
VEKYMNNAGHKKTRGLYYLFPYYRHKLGRQAKKMEPEALRLKDECPKDEEVSHAENVTSFDLTSSYSGYIEFDSRKSIVQ
DIMRKLKDPNMKIIGLHGAQGMGKSTLIKKIANKAKDEGLFDRVAEIDVTVNPNPLTIQADIAHVLGLPLVGESENVRA
DYLRRWLKTENVSILIILDNLHERLDLNRLGIPVDLDYDLRKKNELSIMSSNQGTNPNPGGDAQGTGEKVLKKGNFLGDY
KGCKVLLSSRDKKVFRDEVDVESNFCLKELDGNDAMMLFEKVIGGGNKMSMPKEEIQNYCTGLPIRIVTFAVAFKNWIE
SESKPTLAKFKKQGLVEWQKSSETPNKKKYDLPENKELKFIFLLCAQMGHLPLVNDLVKYCFGLGIFEGVSSLSAAREKIN
ESIQELKNLSLVSYENPNIHFHMSHMVRDDALSNALMDHNIFALRDGKLDDWPELESCISISLCNSDITDGFPQVINCPQ
LKFLQIETNEPSLEIPECFFSRMENLRVLILAGFHLLSLPYSIEDLLNLRMLCLERCTLDCNLSVLRKFKKLRILSFSGSQLKNL
PVELRYLDKLQMLDISDCFKLKIIPLDLFSNLTCLEELYIRKSLIKMLVEKGENKGHNSFLSKLKNLHQLKVVDLSIPCVSILPS
HLFFDRLKDYKIEIGDFEMFSVGEFRMPNKYEELKVLALHLKDDTDIHSHKGIKLLFKTAQSLFLGKICVQNVVNELNIDGF
ENLKHLSIISNKDVEYVNSTDLSNYVNIFSNLESLCLCNMMNLKMICRGPITLESFAKLKTIKVEMCCQLESLFSFYAIKISTS
TGTSEIFKCNSNMKKFLASLEMIEVCECESLKEILQIPPDCVKDCECESLKEILQIPHDYGKVEFLKLRTLTLQSLPSFTCFYTK
VERSCWPHLTEVQTTNRGLTEISIEQDGHSDNAPPLFGELVEVPNLENLNLSSLNIHKIWSDQYLSSFYFQNLIKLAVKDC
DKLTHLCSLPMASSLKKLKSLVISGCLKMEQIFEIEGSSENKVCVFPKLEEIHLSKMNRLKDVWQTKVSVDSFSSLISVSIEE
CNELDKIFPSHMEGWFESLINLKVSKCKSVKEIFEINDSQEIDASGGIATNLQVILLEELPKLKELWSKDPDGILNFKKLRTI
DVSYCHELRNLFPASVAKDVPKLERMSVLYCERMMEIVSNKDTSEADNDH

>XP_027906178.1

MDTVLKIFDPVIEFVRDHGINQLTYIFCYTKNFKELNKRVKRLGEEKERLDRQRGEAKRKGHIVEDRVEEWFEEVGEFEIR
VEKYMNNAGHKKTRGLYYLFPYYRHKLGRQAKKMEPEALRLKDECPKDEEVSHAENVTSFDLTSSYSGYIEFDSRKSIVQ
DIMRKLKDPNMKIIGLHGAQGMGKSTLIKKIANKAKDEGLFDRVAEIDVTVNPNPLTIQADIAHVLGLPLVGESENVRA
DYLRRWLKTENVSILIILDNLHERLDLNRLGIPVDLDYDLRKKNELSIMSSNQGTNPNPGGDAQGTGEKVLKKGNFLGDY
KGCKVLLSSRDKKVFRDEVDVESNFCLKELDGNDAMMLFEKVIGGGNKMSMPKEEIQNYCTGLPIRIVTFAVAFKNWIE
SESKPTLAKFKKQGLVEWQKSSETPNKKKYDLPENKELKFIFLLCAQMGHLPLVNDLVKYCFGLGIFEGVSSLSAAREKIN
ESIQELKNLSLVSYENPNIHFHMSHMVRDDALSNALMDHNIFALRDGKLDDWPELESCISISLCNSDITDGFPQVINCPQ
LKFLQIETNEPSLEIPECFFSRMENLRVLILAGFHLLSLPYSIEDLLNLRMLCLERCTLDCNLSVLRKFKKLRILSFSGSQLKNL
PVELRYLDKLQMLDISDCFKLKIIPLDLFSNLTCLEELYIRKSLIKMLVEKGENKGHNSFLSKLKNLHQLKVVDLSIPCVSILPS
HLFFDRLKDYKIEIGDFEMFSVGEFRMPNKYEELKVLALHLKDDTDIHSHKGIKLLFKTAQSLFLGKICVQNVVNELNIDGF
ENLKHLSIISNKDVEYVNSTDLSNYVNIFSNLESLCLCNMMNLKMICRGPITLESFAKLKTIKVEMCCQLESLFSFYAIKISTS
TGTSEIFKCNSNMKKFLASLEMIEVCECESLKEILQIPPDCVKDCECESLKEILQIPHDYGKVEFLKLRTLTLQSLPSFTCFYTK
VERSCWPHLTEVQTTNRGLTEISIEQDGHSDNAPPLFGELVEVPNLENLNLSSLNIHKIWSDQYLSSFYFQNLIKLAVKDC
DKLTHLCSLPMASSLKKLKSLVISGCLKMEQIFEIEGSSENKVCVFPKLEEIHLSKMNRLKDVWQTKVSVDSFSSLISVSIEE

CNELDKIFPSHMEGWFESLINLKVSKCKSVKEIFEINDSQEIDASGGIATNLQVILLEELPKLKELWSKDPDGILNFKKLRTI
DVSYCHELRNLFPASVAKDVPKLERMSVLYCERMMEIVSNKDTSEADNDH

>XP_027906192.1

MESASSSFQHQRMYDVLINFAGEDIQRKFVSHLDSVLTSVGISTFLHHQNAVKEMYTEEPILNMCQVVIVVFTKTYSQS
AWCLHQLQQIIQWHETYRRHVLPVYYEIQPSDVRLQKGDFGKAFTETAHQTFSGKQLEHGMSRWRYALTKAANFFG
WDESNHRSDAELVDAIVKSVLNLSVLSATNFPVGLQSHVEDVIRTIKNKSTEVCTIGICGMEGSGKTTIAKAIYNHIHGTF
KEKSFIEDIARVSRTKGYAHLQGQLLSDILKSKVEIHSVEMGRRMLQQRLLGKRVLIVLDNIDEKAPLLDLWRNHAWFNK
GTVIIITTAHEHLLRTRIAQDDSIFRINVMNENESLELLSWHAFREAKPKAEYDDLAKEVVAYCGGLPLALEVIGSSLFERTK
EEWRWKVSLLEKIPMYDVQKKLKTSFGGLHNLMEIDLFLDVCCSFVGKDRAYVTKILKSCGVDADNGIRFLIDRNLIKAK
RNNKLTMHPLLQDMGREIVHKFIQEEVWMKSPRFDHSYVLTNNTGKKSSRELPVKLLSSIREPSGLLKLAQNSECRSKKL
RWMSLQGFSSKNLSIDFDLNDAIAIDLKRSLLRLVWKQPQFLMWLKVLNLSHSKNLTQTPDFIGLPRLERLILKDCPRLHE
LHHSIGFLRNLILLNLKDCTGLRYLPKEIRKLKSLKSLILSGCLKILLLEKDMMQMKSLITLVAENKPMKQVPFSIVNSESIGYI
SLRRFEGLLRNLFPSIIRSWMSPTMNPISYIHSMCMDIDNSWDDIEPLHGSLKNLRSVLVQCDTEFQLSKQVQNILVEYF
ASIIEPEISKQQLRCSLIGVGAYDEFFNVFSDNIPKDLAYSEWCDVSLPVVNETYWLAHMGEGHSVSFTVPENRVMKGM
ALCVFYLSTSEIVAAECLRSVLIVNYTKCTLHIHNNDTIISFNDIDWQGIKSNLGSGDKVEIFLTFAHGLLVKNTIFYSICAE

>XP_027906193.1

MESASSSFQHQRMYDVLINFAGEDIQRKFVSHLDSVLTSVGISTFLHHQNAVKEMYTEEPILNMCQVVIVVFTKTYSQS
AWCLHQLQQIIQWHETYRRHVLPVYYEIQPSDVRLQKGDFGKAFTETAHQTFSGKQLEHGMSRWRYALTKAANFFG
WDESNHRSDAELVDAIVKSVLNLSVLSATNFPVGLQSHVEDVIRTIKNKSTEVCTIGICGMEGSGKTTIAKAIYNHIHGTF
KEKSFIEDIARVSRTKGYAHLQGQLLSDILKSKVEIHSVEMGRRMLQQRLLGKRVLIVLDNIDEKAPLLDLWRNHAWFNK
GTVIIITTAHEHLLRTRIAQDDSIFRINVMNENESLELLSWHAFREAKPKAEYDDLAKEVVAYCGGLPLALEVIGSSLFERTK
EEWRWKVSLLEKIPIAYVTKILKSCGVDADNGIRFLIDRNLIKAKRNNKLTMHPLLQDMGREIVHKFIQEEVWMKSPRFD
HSYVLTNNTGKKSSRELPVKLLSSIREPSGLLKLAQNSECRSKKLRWMSLQGFSSKNLSIDFDLNDAIAIDLKRSLLRLVWK
QPQFLMWLKVLNLSHSKNLTQTPDFIGLPRLERLILKDCPRLHELHHSIGFLRNLILLNLKDCTGLRYLPKEIRKLKSLKSLILS
GCLKILLLEKDMMQMKSLITLVAENKPMKQVPFSIVNSESIGYISLRRFEGLLRNLFPSIIRSWMSPTMNPISYIHSMCMD
IDNSWDDIEPLHGSLKNLRSVLVQCDTEFQLSKQVQNILVEYFASIIEPEISKQQLRCSLIGVGAYDEFFNVFSDNIPKDLA
YSEWCDVSLPVVNETYWLAHMGEGHSVSFTVPENRVMKGMALCVFYLSTSEIVAAECLRSVLIVNYTKCTLHIHNNDTI
ISFNDIDWQGIKSNLGSGDKVEIFLTFAHGLLVKNTIFYSICAE

>XP_027906194.1

MEGSGKTTIAKAIYNHIHGTFKEKSFIEDIARVSRTKGYAHLQGQLLSDILKSKVEIHSVEMGRRMLQQRLLGKRVLIVLD
NIDEKAPLLDLWRNHAWFNKGTVIIITTAHEHLLRTRIAQDDSIFRINVMNENESLELLSWHAFREAKPKAEYDDLAKEV
VAYCGGLPLALEVIGSSLFERTKEEWRWKVSLLEKIPMYDVQKKLKTSFGGLHNLMEIDLFLDVCCSFVGKDRAYVTKILK
SCGVDADNGIRFLIDRNLIKAKRNNKLTMHPLLQDMGREIVHKFIQEEVWMKSPRFDHSYVLTNNTGKKSSRELPVKLL
SSIREPSGLLKLAQNSECRSKKLRWMSLQGFSSKNLSIDFDLNDAIAIDLKRSLLRLVWKQPQFLMWLKVLNLSHSKNLT
QTPDFIGLPRLERLILKDCPRLHELHHSIGFLRNLILLNLKDCTGLRYLPKEIRKLKSLKSLILSGCLKILLLEKDMMQMKSLITL
VAENKPMKQVPFSIVNSESIGYISLRRFEGLLRNLFPSIIRSWMSPTMNPISYIHSMCMDIDNSWDDIEPLHGSLKNLRSV
LVQCDTEFQLSKQVQNILVEYFASIIEPEISKQQLRCSLIGVGAYDEFFNVFSDNIPKDLAYSEWCDVSLPVVNETYWLAH
MGEGHSVSFTVPENRVMKGMALCVFYLSTSEIVAAECLRSVLIVNYTKCTLHIHNNDTIISFNDIDWQGIKSNLGSGDKV
EIFLTFAHGLLVKNTIFYSICAE

>XP_027906210.1

MEFASSSSSSSSFLTLEPHFIYDVFINFWGEDIGRRFISHLHYALLQAQVKTLIKEENLPEGPKREEHMRAIGGTKATIIVFTK
SYAESTCCLRELEKIIECHETFGQILMPVFYEIDKLDVRHQKYDFGQALEEAAHKSYSGEQVEHALSRWSRALTKAASITC
WDLRNFRHDAELVEGIVSRVKMILDYKDLFITQYPVGLESHVEKVIACIENHSTKVCMIGIWGMGGSGKTTIAKAIYNRI
YREFIGKSFIENISDVWDPKNERYVDLQENLLSDILKSKLEVESVGMGRTMIENGLTRKKLFIVLDDVSEFGQLENLCGNR
EWFGQGTVIIITTRDVNLLNQIKVNYVYKMDVMNENDSLELLSWHAFREAKPRKELHEHGRNVTPYCGGLPLALEVLG
SFLCEKTMEEWESVSSKLKIIPIDQIEEKLKISFDGLHDTEKDIFIDVCCFFIGKERGHVTRILNDCGLHGDIGITVLIERGLIKV
DRHNKLQVHPLLKHMGREIIRQRCQKEPDKWSRLWFQDDVKDVPKDNTATEAIQGLNRKVYYLEPMVLSKGGD

>XP_027906211.1

MEFASSSSSSSSFLTLEPHFIYDVFINFWGEDIGRRFISHLHYALLQAQVKTLIKEENLPEGPKREEHMRAIGGTKATIIVFTK
SYAESTCCLRELEKIIECHETFGQILMPVFYEIDKLDVRHQKYDFGQALEEAAHKSYSGEQVEHALSRWSRALTKAASITC
WDLRNFRHDAELVEGIVSRVKMILDYKDLFITQYPVGLESHVEKVIACIENHSTKVCMIGIWGMGGSGKTTIAKAIYNRI
YREFIGKSFIENISDVWDPKNERYVDLQENLLSDILKSKLEVESVGMGRTMIENGLTRKKLFIVLDDVSEFGQLENLCGNR
EWFGQGTVIIITTRDVNLLNQIKVNYVYKMDVMNENDSLELLSWHAFREAKPRKELHEHGRNVTPYCGGLPLALEVLG
SFLCEKTMEEWESVSSKLKIIPIDQIEEKLKISFDGLHDTEKDIFIDVCCFFIGKERGHVTRILNDCGLHGDIGITVLIERGLIKV
DRHNKLQVHPLLKHMGREIIRQRCQKEPDKWSRLWFQDDVKDVPKDNTATEAIQGLNRKVYYLEPMVLSKGGD

>XP_027906212.1

MEFASSSSSSSSFLTLEPHFIYDVFINFWGEDIGRRFISHLHYALLQAQVKTLIKEENLPEGPKREEHMRAIGGTKATIIVFTK
SYAESTCCLRELEKIIECHETFGQILMPVFYEIDKLDVRHQKYDFGQALEEAAHKSYSGEQVEHALSRWSRALTKAASITC
WDLRNFRHDAELVEGIVSRVKMILDYKDLFITQYPVGLESHVEKVIACIENHSTKVCMIGIWGMGGSGKTTIAKAIYNRI
YREFIGKSFIENISDVWDPKNERYVDLQENLLSDILKSKLEVESVGMGRTMIENGLTRKKLFIVLDDVSEFGQLENLCGNR
EWFGQGTVIIITTRDVNLLNQIKVNYVYKMDVMNENDSLELLSWHAFREAKPRKELHEHGRNVTPYCGGLPLALEVLG
SFLCEKTMEEWESVSSKLKIIPIDQIEEKLKISFDGLHDTEKDIFIDVCCFFIGKERGHVTRILNDCGLHGDIGITVLIERGLIKV
DRHNKLQVHPLLKHMGREIIRQRCQKEPDKWSRLWFQDDVKDVPKDNTATEAIQGLNRKVYYLEPMVLSKGGD

>XP_027906213.1

MEFASSSSSSSSFLTLEPHFIYDVFINFWGEDIGRRFISHLHYALLQAQVKTLIKEENLPEGPKREEHMRAIGGTKATIIVFTK
SYAESTCCLRELEKIIECHETFGQILMPVFYEIDKLDVRHQKYDFGQALEEAAHKSYSGEQVEHALSRWSRALTKAASITC
WDLRNFRHDAELVEGIVSRVKMILDYKDLFITQYPVGLESHVEKVIACIENHSTKVCMIGIWGMGGSGKTTIAKAIYNRI
YREFIGKSFIENISDVWDPKNERYVDLQENLLSDILKSKLEVESVGMGRTMIENGLTRKKLFIVLDDVSEFGQLENLCGNR
EWFGQGTVIIITTRDVNLLNQIKVNYVYKMDVMNENDSLELLSWHAFREAKPRKELHEHGRNVTPYCGGLPLALEVLG
SFLCEKTMEEWESVSSKLKIIPIDQIEEKLKISFDGLHDTEKDIFIDVCCFFIGKERGHVTRILNDCGLHGDIGITVLIERGLIKV
DRHNKLQVHPLLKHMGREIIRQRCQKEPDKWSRLWFQDDVKDVPKDNTATEAIQGLNRKVYYLEPMVLSKGGD

>XP_027906214.1

MEFASSSSSSSSFLTLEPHFIYDVFINFWGEDIGRRFISHLHYALLQAQVKTLIKEENLPEGPKREEHMRAIGGTKATIIVFTK
SYAESTCCLRELEKIIECHETFGQILMPVFYEIDKLDVRHQKYDFGQALEEAAHKSYSGEQVEHALSRWSRALTKAASITC
WDLRNFRHDAELVEGIVSRVKMILDYKDLFITQYPVGLESHVEKVIACIENHSTKVCMIGIWGMGGSGKTTIAKAIYNRI
YREFIGKSFIENISDVWDPKNERYVDLQENLLSDILKSKLEVESVGMGRTMIENGLTRKKLFIVLDDVSEFGQLENLCGNR
EWFGQGTVIIITTRDVNLLNQIKVNYVYKMDVMNENDSLELLSWHAFREAKPRKELHEHGRNVTPYCGGLPLALEVLG
SFLCEKTMEEWESVSSKLKIIPIDQIEEKLKISFDGLHDTEKDIFIDVCCFFIGKERGHVTRILNDCGLHGDIGITVLIERGLIKV
DRHNKLQVHPLLKHMGREIIRQRCQKEPDKWSRLWFQDDVKDVPKDNTATEAIQGLNRKVYYLEPMVLSKGGD

>XP_027906215.1

MEFASSSSSSSFLTLEPHFIYDVFINFWGEDIGRRFISHLHYALLQAQVKTLIKEENLPEGPKREEHMRAIGGTKATIIVFTK
SYAESTCCLRELEKIIECHETFGQILMPVFYEIDKLDVRHQKYDFGQALEEAAHKSYSGEQVEHALSRWSRALTKAASITC
WDLRNFRHDAELVEGIVSRVKMILDYKDLFITQYPVGLESHVEKVIACIENHSTKVCMIGIWGMGGSGKTTIAKAIYNRI
YREFIGKSFIENISDVWDPKNERYVDLQENLLSDILKSKLEVESVGMGRTMIENGLTRKKLFIVLDDVSEFGQLENLCGNR
EWFGQGTVIIITTRDVNLLNQIKVNYVYKMDVMNENDSLELLSWHAFREAKPRKELHEHGRNVTPYCGGLPLALEVLG
SFLCEKTMEEWESVSSKLKIIPIDQIEEKLKISFDGLHDTEKDIFIDVCCFFIGKERGHVTRILNDCGLHGDIGITVLIERGLIKV
DRHNKLQVHPLLKHMGREIIRQRCQKEPDKWSRLWFQDDVKDVPKDNTATEAIQGLNRKVYYLEPMVLSKGGD

>XP_027906216.1

MEFASSSSSSSFLTLEPHFIYDVFINFWGEDIGRRFISHLHYALLQAQVKTLIKEENLPEGPKREEHMRAIGGTKATIIVFTK
SYAESTCCLRELEKIIECHETFGQILMPVFYEIDKLDVRHQKYDFGQALEEAAHKSYSGEQVEHALSRWSRALTKAASITC
WDLRNFRHDAELVEGIVSRVKMILDYKDLFITQYPVGLESHVEKVIACIENHSTKVCMIGIWGMGGSGKTTIAKAIYNRI
YREFIGKSFIENISDVWDPKNERYVDLQENLLSDILKSKLEVESVGMGRTMIENGLTRKKLFIVLDDVSEFGQLENLCGNR
EWFGQGTVIIITTRDVNLLNQIKVNYVYKMDVMNENDSLELLSWHAFREAKPRKELHEHGRNVTPYCGGLPLALEVLG
SFLCEKTMEEWESVSSKLKIIPIDQIEEKLKISFDGLHDTEKDIFIDVCCFFIGKERGHVTRILNDCGLHGDIGITVLIERGLIKV
DRHNKLQVHPLLKHMGREIIRQRCQKEPDKWSRLWFQDDVKDVPKDNTATEAIQGLNRKVYYLEPMVLSKGGD

>XP_027906217.1

MEFASSSSSSSFLTLEPHFIYDVFINFWGEDIGRRFISHLHYALLQAQVKTLIKEENLPEGPKREEHMRAIGGTKATIIVFTK
SYAESTCCLRELEKIIECHETFGQILMPVFYEIDKLDVRHQKYDFGQALEEAAHKSYSGEQVEHALSRWSRALTKAASITC
WDLRNFRHDAELVEGIVSRVKMILDYKDLFITQYPVGLESHVEKVIACIENHSTKVCMIGIWGMGGSGKTTIAKAIYNRI
YREFIGKSFIENISDVWDPKNERYVDLQENLLSDILKSKLEVESVGMGRTMIENGLTRKKLFIVLDDVSEFGQLENLCGNR
EWFGQGTVIIITTRDVNLLNQIKVNYVYKMDVMNENDSLELLSWHAFREAKPRKELHEHGRNVTPYCGGLPLALEVLG
SFLCEKTMEEWESVSSKLKIIPIDQIEEKLKISFDGLHDTEKDIFIDVCCFFIGKERGHVTRILNDCGLHGDIGITVLIERGLIKV
DRHNKLQVHPLLKHMGREIIRQRCQKEPDKWSRLWFQDDVKDVPKDNTATEAIQGLNRKVYYLEPMVLSKGGD

>XP_027906218.1

MEFASSSSSSSFLTLEPHFIYDVFINFWGEDIGRRFISHLHYALLQAQVKTLIKEENLPEGPKREEHMRAIGGTKATIIVFTK
SYAESTCCLRELEKIIECHETFGQILMPVFYEIDKLDVRHQKYDFGQALEEAAHKSYSGEQVEHALSRWSRALTKAASITC
WDLRNFRHDAELVEGIVSRVKMILDYKDLFITQYPVGLESHVEKVIACIENHSTKVCMIGIWGMGGSGKTTIAKAIYNRI
YREFIGKSFIENISDVWDPKNERYVDLQENLLSDILKSKLEVESVGMGRTMIENGLTRKKLFIVLDDVSEFGQLENLCGNR
EWFGQGTVIIITTRDVNLLNQIKVNYVYKMDVMNENDSLELLSWHAFREAKPRKELHEHGRNVTPYCGGLPLALEVLG
SFLCEKTMEEWESVSSKLKIIPIDQIEEKLKISFDGLHDTEKDIFIDVCCFFIGKERGHVTRILNDCGLHGDIGITVLIERGLIKV
DRHNKLQVHPLLKHMGREIIRQRCQKEPDKWSRLWFQDDVKDVPKDNTATEAIQGLNRKVYYLEPMVLSKGGD

>XP_027906219.1

MEFASSSSSSSFLTLEPHFIYDVFINFWGEDIGRRFISHLHYALLQAQVKTLIKEENLPEGPKREEHMRAIGGTKATIIVFTK
SYAESTCCLRELEKIIECHETFGQILMPVFYEIDKLDVRHQKYDFGQALEEAAHKSYSGEQVEHALSRWSRALTKAASITC
WDLRNFRHDAELVEGIVSRVKMILDYKDLFITQYPVGLESHVEKVIACIENHSTKVCMIGIWGMGGSGKTTIAKAIYNRI
YREFIGKSFIENISDVWDPKNERYVDLQENLLSDILKSKLEVESVGMGRTMIENGLTRKKLFIVLDDVSEFGQLENLCGNR
EWFGQGTVIIITTRDVNLLNQIKVNYVYKMDVMNENDSLELLSWHAFREAKPRKELHEHGRNVTPYCGGLPLALEVLG

SFLCEKTMEEWESVSSKLKIIPIDQIEEKLKISFDGLHDTEKDIFIDVCCFFIGKERGHVTRILNDCGLHGDIGITVLIERGLIKV
DRHNKLQVHPLLKHMGREIIRQRCQKEPDKWSRLWFQDDVKDVPKDNTATEAIQGLNRKVYYLEPMVLSKGGD

>XP_027906220.1

MEFASSSSSSSFLTLEPHFIYDVFINFWGEDIGRRFISHLHYALLQAQVKTLIKEENLPEGPKREEHMRAIGGTKATIIVFTK
SYAESTCCLRELEKIIECHETFGQILMPVFYEIDKLDVRHQKYDFGQALEEAAHKSYSGEQVEHALSRWSRALTKAASITC
WDLRNFRHDAELVEGIVSRVKMILDYKDLFITQYPVGLESHVEKVIACIENHSTKVCMIGIWGMGGSGKTTIAKAIYNRI
YREFIGKSFIENISDVWDPKNERYVDLQENLLSDILKSKLEVESVGMGRTMIENGLTRKKLFIVLDDVSEFGQLENLCGNR
EWFGQGTVIIITTRDVNLLNQIKVNYVYKMDVMNENDSLELLSWHAFREAKPRKELHEHGRNVTPYCGGLPLALEVLG
SFLCEKTMEEWESVSSKLKIIPIDQIEEKLKISFDGLHDTEKDIFIDVCCFFIGKERGHVTRILNDCGLHGDIGITVLIERGLIKV
DRHNKLQVHPLLKHMGREIIRQRCQKEPDKWSRLWFQDDVKDVPKDNTATEAIQGLNRKVYYLEPMVLSKGGD

>XP_027906221.1

MEFASSSSSSSFLTLEPHFIYDVFINFWGEDIGRRFISHLHYALLQAQVKTLIKEENLPEGPKREEHMRAIGGTKATIIVFTK
SYAESTCCLRELEKIIECHETFGQILMPVFYEIDKLDVRHQKYDFGQALEEAAHKSYSGEQVEHALSRWSRALTKAASITC
WDLRNFRHDAELVEGIVSRVKMILDYKDLFITQYPVGLESHVEKVIACIENHSTKVCMIGIWGMGGSGKTTIAKAIYNRI
YREFIGKSFIENISDVWDPKNERYVDLQENLLSDILKSKLEVESVGMGRTMIENGLTRKKLFIVLDDVSEFGQLENLCGNR
EWFGQGTVIIITTRDVNLLNQIKVNYVYKMDVMNENDSLELLSWHAFREAKPRKELHEHGRNVTPYCGGLPLALEVLG
SFLCEKTMEEWESVSSKLKIIPIDQIEEKLKISFDGLHDTEKDIFIDVCCFFIGKERGHVTRILNDCGLHGDIGITVLIERGLIKV
DRHNKLQVHPLLKHMGREIIRQRCQKEPDKWSRLWFQDDVKDVPKDNTATEAIQGLNRKVYYLEPMVLSKGGD

>XP_027906222.1

MEFASSSSSSSFLTLEPHFIYDVFINFWGEDIGRRFISHLHYALLQAQVKTLIKEENLPEGPKREEHMRAIGGTKATIIVFTK
SYAESTCCLRELEKIIECHETFGQILMPVFYEIDKLDVRHQKYDFGQALEEAAHKSYSGEQVEHALSRWSRALTKAASITC
WDLRNFRHDAELVEGIVSRVKMILDYKDLFITQYPVGLESHVEKVIACIENHSTKVCMIGIWGMGGSGKTTIAKAIYNRI
YREFIGKSFIENISDVWDPKNERYVDLQENLLSDILKSKLEVESVGMGRTMIENGLTRKKLFIVLDDVSEFGQLENLCGNR
EWFGQGTVIIITTRDVNLLNQIKVNYVYKMDVMNENDSLELLSWHAFREAKPRKELHEHGRNVTPYCGGLPLALEVLG
SFLCEKTMEEWESVSSKLKIIPIDQIEEKLKISFDGLHDTEKDIFIDVCCFFIGKERGHVTRILNDCGLHGDIGITVLIERGLIKV
DRHNKLQVHPLLKHMGREIIRQRCQKEPDKWSRLWFQDDVKDVPKDNTATEAIQGLNRKVYYLEPMVLSKGGD

>XP_027906227.1

MDRSSTFPIKYEVFLSFRGADTRTNFTSHLLNALTQKSINAFIDYELTRGHYTWPTLATAIEESLLSIVVLSENYASSTWCLK
ELAHILECRKKRGLVVIPVFYEVDPSHVRKLSGSFETSFAKHERDSTSWDRISRRKDVSVWKAALKEVANISGWDSRSYR
DEAQVIQNLVNDVLQKLHLRCPTELKGVVRIKKTYGKLELLLQKSRVIGIWGMGGMGKSTIAKALFARHFPYFDHVCFV
ANAKEFSLDKLFSQLFREEVSASNVVGSTFDMSRLRRKKVFIVLDDVDCLDPLEYLCREYENLDPRSKLIITTRDKQLLEGR
VDQIYEVKKWETRASLKLFCLEAFKKRHPKKGYESLSQSAVEYAGGVPLALKVLGSSLRSKGINFWESTIRKLSMYPNERI
QKLLEVTYTGLHDLEKNIFLDVVFFFKEKQKDHVTRILDACGFEATSGIEVLADKALLTISYRNIIHMHDLLQQMGLEIVRQ
ESSGDPGRRSRLKDNEAREVIEENKGTSAIQGIALDLSQIKGLILHADTFXKMKTLRLLKFYNTLDQSARDTYLDLPATLEP
FSDKLRYIEWIGYPFESLPSPFCAKLLVEIHMPHSKVKQLWQGIQELDYLEGINLRQCKQFEELPDLSKAPRLKWVNLSCC
ESLRYLHPSVLSSDSLVTLILDKCTNLKSVKSEKHLKSLEKISVNGCLNLVEFAVSSDLIENLDLSNTGIQTLGTSIGSMHKLK
SLNLEGLKLKHILKELSWLTYLKVLKISDNGLVIDKQQILFLFNGLRYLQILYLKDCSKLFELPDNISLLTQLKELRLDRSDLKRL
PENIKNLQMLEILSLEDCKELLCLPKFPSLIKYLRVINCTSLVSLSNLKTLAIEMLGMTKRITFKNSGKLDEHSLRIIMESLHLT
MMSAAYHNVLVRKTFGRYNSCNYTSVELCLPGGNVPEQILYRSTKSSITIVLPPRSELLGFIYSVVLSPAGGKKTYGTKIFC
KCHLPEEGIKVTWLYGDIRGLKSDHVYVWYDPLHCDNILKYHKQSKVCFEFCVANDKGEVDGSICIKECGVGLVNVLEV

HSVLQELDFDSDKKKELVEGVESESKLRNQQQDWSESSSSDSPRSSSESDSEIVKPVSAEKGKSIHKETKTDSGTNAVKYK
SNKETPKSDATLHETMESHSDNENESREKSITQSEVVPVELESTSLSTTKRGPKEKSKESTQIVANEHLQSTPLQASSQGG
SEKVNDRLEESNKQVVETYDTEKFVTKYSYFDLESCLQQLDENPFAILHLLSNELSLPLKQSQTCVQKVAQAKDATTVLNE
FRTLVFSTSLLEKLPDQSYRQQIDESLRKLHTHRREITKEQEEGVDKFIELYNKAVDIYQEKMLTKDKQANLASQKRDLYN
KLQDSKLKVQQFDTTISTSKYEIENLQKRQREIQEAINKLHQENEALEKERSALEVLYSEKQTKKNETLELVKYISTSVVYTT
KQLEELEEKRLSLASALDDLKEPYQRMKTKPPF

>XP_027906229.1

MELGSSSSSSSSSFLKSEPQFIYDVFINFWGENMSRKFVSHLHSVLLQAQVKTLINEENLQQGKKLEEHMRAIAASKIAIIV
FSQTYTESTCCLRELEKIVECRETFGQIVLPIFYEIYPFDVRHQKGDFRKALEEAAHKSYSGEQVEHAFSRWSNALTTVAGI
TGWNVRDFRHDAELVEVTVSRVQTILDYADLSLTRFPVGLDPHVRKVIECIENHSSKVCMIGIWGMVGSGKTTIAKAIY
NRIYRPFIGKSFIGNIREDWNNNPVHLQENLLYDVLKSKFEVESVGLGRTMIENEFSRKKLLIVLDDVNEFSQLENLCGNR
DWFGQGTVIIITSRDVHVLNRLKVNYIYKMNGMNENDSLELFSCHAFRDSKPRKGFDEFARSITAHCGGLPLALEVLGSY
LCGRTKDWWESALAILKVLPTIQVQDTLRISFKSLCDDMEKDIFLDICCFFIGEDRGYVTQILNGCGLHADIGITVLIERGLI
EVGRNNKLEMHPLLRDMGREIIRRRCPEKPGERSRLWFEDDIKDVLKRNTGTKGTQGLSLKHKSTRGDCFEAHAFKKM
KRLKLLQLDHVQLTGDYGYLSKQLRWICWKGFPSKYIPNNFHMENVIAIDLKHSHLQLVWKQPQVLERLKFLNLSHSKY
LIETPDFSGLPSLEKLILKDCPSLCKVHQSIGHLCNLLLINLKDCTNLSSLPREVYKLTSLRTFILSGCFKIHILEEDIMQMKSLIT
LVTENTAVKKVPCSIVSSKSIGYISLRGFEGLPHNIFPSIIRSWMSPTMNPQSYISLLCLDMENDNLSDLVPLYRSLANLRSI
LIECNTDFQLSEQVKAILVEYGVNFTESRISNHPLRFSLIGVGSYNEFFNTLNDSISEVLARSESCDVSLPGDNYPFWLANM
GDGHSVSFTVPRDRNMKGMIFCVVYLSTPEIVATECLTSVFIVNYTKCTLQMHNHDTVISFKDEDWHGIMSNLGSGDK
VEIFVNCSNGLVIKNTTIYLIYGESNDLEMEPCLEPKENALNKFIMKMVMCDFW

>XP_027906283.1

MTSPIPSIEVASSTTKLPRKYDVLINFNGEDIRRKFVSHLDSVLSAVGLTTFLHHDNGVKPMHEPILNLCRVAIVVFTQTYS
QSAWCLHQLQQIIQWHQTYCKHVLPVYYEIQPSDIRLQKGDFGKAFETTAHQTFSAQELEHGMSRWSHALTKAANFF
GWDESNHRSDAELVDKIVKSVLNLPVMSATKFPVGLQSNVEDLIRTIKDKSSEVCIVGITGAGGSGKTTLAKAIYNQIHDT
FREKSFIEDIGQVSRPRGHRGLQEQFLFDVLKTKVEITSVDMGRRMIRERLTGRRVFLVLDDLTENFTLFDLWECREWFS
GGTVIIITTRDVDLPRILKVDSVFGIKLMSAKESLELLSWHAFREPKPKEEYNDLAESVVTHCGGLPLALEVVGNCLFERTK
KEWKSVLLKLEKIPLHNVQQKLKISFDGLRNEIEKDLFIDICCSFVGEGRAYVTKILNDCGVDADSGIRVLIECSLITVKKNN
KLGMHPLLQEMGREIIREIDEEEFWREWPPGFDDAEYVLTDNTGRRAIERLPVKLRSVRREPSRLLKHTENSDSLSKKLR
WISLDWFSSEYLPDEFYLHEAIAIDLKHSDLRFVLKEPQDLRWLKVLNLSHSKYLIETPDFSGLPSLEQLILKDCTGLREVHQ
SIGCLCNLTLLNLKDCTRLRNLPRDICKMKSLKTLILSGCPMIDLLEKDVVQMESLRTLIAENTTMKHVPFSIVSSKSIGHISL
CGFERLSRNFFPSIIRSWMSPVMNPISYIHSLCMDMNNNWDDIAPLLSSLTNLRSILVQCDSEFQLSEQVKFILVEYFANF
TESGISKQQFRSPLIGLGTYQEFFNSVSDNISEVLANSESCDVSLPSDNLPYWLAYMGEGNSVSFTVPWDRDMKGMAL
SVVYLSTPEIVATECLTGVLIVNYTKCTLQIHNYDTITSFNDIDWQCIMSNLGPKDKVEIFVTFGDELVVKNTILYLICGESN
YLKKEIESKKNCLLRFITKIVMCDFW

>XP_027906284.1

MTSPIPSIEVASSTTKLPRKYDVLINFNGEDIRRKFVSHLDSVLSAVGLTTFLHHDNGVKPMHEPILNLCRVAIVVFTQTYS
QSAWCLHQLQQIIQWHQTYCKHVLPVYYEIQPSDIRLQKGDFGKAFETTAHQTFSAQELEHGMSRWSHALTKAANFF
GWDESNHRSDAELVDKIVKSVLNLPVMSATKFPVGLQSNVEDLIRTIKDKSSEVCIVGITGAGGSGKTTLAKAIYNQIHDT
FREKSFIEDIGQVSRPRGHRGLQEQFLFDVLKTKVEITSVDMGRRMIRERLTGRRVFLVLDDLTENFTLFDLWECREWFS
GGTVIIITTRDVDLPRILKVDSVFGIKLMSAKESLELLSWHAFREPKPKEEYNDLAESVVTHCGGLPLALEVVGNCLFERTK
KEWKSVLLKLEKIPLHNVQQKLKISFDGLRNEIEKDLFIDICCSFVGEGRAYVTKILNDCGVDADSGIRVLIECSLITVKKNN

KLGMHPLLQEMGREIIREIDEEEFWREWPPGFDDAEYVLTDNTGRRAIERLPVKLRSVRREPSRLLKHTENSDSLSKKLR
WISLDWFSSEYLPDEFYLHEAIAIDLKHSDLRFVLKEPQVLANSESCDVSLPSDNLPYWLAYMGEGNSVSFTVPWDRDM
KGMALSVVYLSTPEIVATECLTGVLIVNYTKCTLQIHNYDTITSFNDIDWQCIMSNLGPKDKVEIFVTFGDELVVKNTILYLI
CGESNYLKKEIESKKNCLLRFITKIVMCDFW

>XP_027906298.1

MEFATSSSKLPRKYDVLISFNGDDIRRKFVSHLDSALSAVGLTTLLHYENAVKSTHIQQPILNLCRVVIVVFTKTYSQSAWC
LHQLQQIIQWHQTYCRHVLPVYYEIQPSHVRIQQGDFGMAFKTTAQQTFSEHELEHGLSSWSHALTKAANFFGWDES
NHRSDAELVDKIVKSVLNLSALSATKFPVGLQDPVELLLQAINNKSMDVCTIGVCGMGGSGKTTLAKAIYHQIHGTFMQ
KSFIEDIAQVSKPRGRIHLQQQLLSDVLKTKVEIHSVEMGRSMIRERLFRKRVLIVLDDMDDYLPLLDLRNSRSWLSEGTV
IIITTRDRHLLRKHQVDFVFEMNLLNENESLELLSWHAFREAKPKEEYHDLAKAIVTHCGGLPLALEVIGSTLFESTEEEWQ
TVLFELEEIPMDDVQLKLKISLDRLHSQMEKDLFLDVCCYFVGKDRAYATKILNGSGVDADNEIRTQHGYIVDADTGIRVL
MERNLIKVKSNNKFGMHPLLQEMGIGFFREISGEKRLKNSQLLFDVDVEYALKVNSVRTFFVFGLKLLLKVFNFMRPIHC
FSLVFKSQGRKSFEVLPVKLLSTRREISRQTKDALNSEYLPPKLRWITLHGFSSQYLPNGFNVQDAIAIDLKHSLLRFFWEE
PQVLRSLKVLNLSHSMYLTETPDFSRLPSLEQLILKDCPRLRIVHSSIGDLCYLILLNLKDCQCLSNLPREIYKLKSLRTLILSGC
SKICRMEKDILRMKSLITLIAENTAAKQVPFSIVSSKAIGYISLRGFERLSCNLFPSIIIRSWMSHIINPISYVHSLCMDIDNSW
DDIVPLLGSLTNLRSVLVQCDTEFQLSKQVQNILVEYFSNITESGISMQQFRCSLIGVGAYHEFFNVVSDNISEVLAGGEC
CDASLQCDNHSYWLAYIGEGDSVSFTMPPHHDVKGMILCVIYLSIPEIVATEGLRSVLIVNYTKCTLHVHMHGTIICFNDI
DWEGIKSNLRSGDKVEIFVTFGHGLVVKNTRLYLICGESNYLETKPRLKKNLVIRFIKKVVE

>XP_027906299.1

MEFATSSSKLPRKYDVLISFNGDDIRRKFVSHLDSALSAVGLTTLLHYENAVKSTHIQQPILNLCRVVIVVFTKTYSQSAWC
LHQLQQIIQWHQTYCRHVLPVYYEIQPSHVRIQQGDFGMAFKTTAQQTFSEHELEHGLSSWSHALTKAANFFGWDES
NHRSDAELVDKIVKSVLNLSALSATKFPVGLQDPVELLLQAINNKSMDVCTIGVCGMGGSGKTTLAKAIYHQIHGTFMQ
KSFIEDIAQVSKPRGRIHLQQQLLSDVLKTKVEIHSVEMGRSMIRERLFRKRVLIVLDDMDDYLPLLDLRNSRSWLSEGTV
IIITTRDRHLLRKHQVDFVFEMNLLNENESLELLSWHAFREAKPKEEYHDLAKAIVTHCGGLPLALEVIGSTLFESTEEEWQ
TVLFELEEIPMDDVQLKLKISLDRLHSQMEKDLFLDVCCYFVGKDRAYATKILNGSGVDADNEIRTQHGYIVDADTGIRVL
MERNLIKVKSNNKFGMHPLLQEMGIGFFREISGEKRLKNSQLLFDVDVEYALKVNSVRTFFVFGLKLLLKVFNFMRPIHC
FSLVFKSQGRKSFEVLPVKLLSTRREISRQTKDALNSEYLPPKLRWITLHGFSSQYLPNGFNVQDAIAIDLKHSLLRFFWEE
PQVLRSLKVLNLSHSMYLTETPDFSRLPSLEQLILKDCPRLRIVHSSIGDLCYLILLNLKDCQCLSNLPREIYKLKSLRTLILSGC
SKICRMEKDILRMKSLITLIAENTAAKQVPFSIVSSKAIGYISLRGFERLSYNSWDDIVPLLGSLTNLRSVLVQCDTEFQLSK
QVQNILVEYFSNITESGISMQQFRCSLIGVGAYHEFFNVVSDNISEVLAGGECCDASLQCDNHSYWLAYIGEGDSVSFT
MPPHHDVKGMILCVIYLSIPEIVATEGLRSVLIVNYTKCTLHVHMHGTIICFNDIDWEGIKSNLRSGDKVEIFVTFGHGLV
VKNTRLYLICGESNYLETKPRLKKNLVIRFIKKVVE

>XP_027906300.1

MEFATSSSKLPRKYDVLISFNGDDIRRKFVSHLDSALSAVGLTTLLHYENAVKSTHIQQPILNLCRVVIVVFTKTYSQSAWC
LHQLQQIIQWHQTYCRHVLPVYYEIQPSHVRIQQGDFGMAFKTTAQQTFSEHELEHGLSSWSHALTKAANFFGWDES
NHRSDAELVDKIVKSVLNLSALSATKFPVGLQDPVELLLQAINNKSMDVCTIGVCGMGGSGKTTLAKAIYHQIHGTFMQ
KSFIEDIAQVSKPRGRIHLQQQLLSDVLKTKVEIHSVEMGRSMIRERLFRKRVLIVLDDMDDYLPLLDLRNSRSWLSEGTV
IIITTRDRHLLRKHQVDFVFEMNLLNENESLELLSWHAFREAKPKEEYHDLAKAIVTHCGGLPLALEVIGSTLFESTEEEWQ
TVLFELEEIPMDDVQLKLKISLDRLHSQMEKDLFLDVCCYFVGKDRAYATKILNGSGVDADNEIRTQHGYIVDADTGIRVL
MERNLIKVKSNNKFGMHPLLQEMGIGFFREISGEKRLKNSQLLFDVDVEYALKVNSGRKSFEVLPVKLLSTRREISRQTKD
ALNSEYLPPKLRWITLHGFSSQYLPNGFNVQDAIAIDLKHSLLRFFWEEPQVLRSLKVLNLSHSMYLTETPDFSRLPSLEQL

ILKDCPRLRIVHSSIGDLCYLILLNLKDCQCLSNLPREIYKLKSLRTLILSGCSKICRMEKDILRMKSLITLIAENTAAKQVPFSIV
SSKAIGYISLRGFERLSCNLFPSIIIRSWMSHIINPISYVHSLCMDIDNSWDDIVPLLGSLTNLRSVLVQCDTEFQLSKQVQN
ILVEYFSNITESGISMQQFRCSLIGVGAYHEFFNVVSDNISEVLAGGECCDASLQCDNHSYWLAYIGEGDSVSFTMPPHH
DVKGMILCVIYLSIPEIVATEGLRSVLIVNYTKCTLHVHMHGTIICFNDIDWEGIKSNLRSGDKVEIFVTFGHGLVVKNTRL
YLICGESNYLETKPRLKKNLVIRFIKKVVE

>XP_027906301.1

MEFATSSSKLPRKYDVLISFNGDDIRRKFVSHLDSALSAVGLTTLLHYENAVKSTHIQQPILNLCRVVIVVFTKTYSQSAWC
LHQLQQIIQWHQTYCRHVLPVYYEIQPSHVRIQQGDFGMAFKTTAQQTFSEHELEHGLSSWSHALTKAANFFGWDES
NHRSDAELVDKIVKSVLNLSALSATKFPVGLQDPVELLLQAINNKSMDVCTIGVCGMGGSGKTTLAKAIYHQIHGTFMQ
KSFIEDIAQVSKPRGRIHLQQQLLSDVLKTKVEIHSVEMGRSMIRERLFRKRVLIVLDDMDDYLPLLDLRNSRSWLSEGTV
IIITTRDRHLLRKHQVDFVFEMNLLNENESLELLSWHAFREAKPKEEYHDLAKAIVTHCGGLPLALEVIGSTLFESTEEEWQ
TVLFELEEIPMDDVQLKLKISLDRLHSQMEKDLFLDVCCYFVGKDRAYATKILNGSGVDADNEIRTQHGYIVDADTGIRVL
MERNLIKVKSNNKFGMHPLLQEMGIGFFREISGEKRLKNSQLLFDVDVEYALKVNSVRTFFVFGLKLLLKVFNFMRPIHC
FSLVFKSQGRKSFEVLPVKLLSTRREISRQTKDALNSEYLPPKLRWITLHGFSSQYLPNGFNVQDAIAIDLKHSLLRFFWEE
PQVLRSLKVLNLSHSMYLTETPDFSRLPSLEQLILKDCPRLRIVHSSIGDLCYLILLNLKDCQCLSNLPREIYKLKSLRTLILSGC
SKICRMEKDILRMKSLITLIAENTAAKQVPFSIVLAGGECCDASLQCDNHSYWLAYIGEGDSVSFTMPPHHDVKGMILCV
IYLSIPEIVATEGLRSVLIVNYTKCTLHVHMHGTIICFNDIDWEGIKSNLRSGDKVEIFVTFGHGLVVKNTRLYLICGESNYLE
TKPRLKKNLVIRFIKKVVE

>XP_027906375.1

MALAVVGGALLSAFVDVLFEKLASPEVVNLIRGKKPDKLLQKVKNQLIVVRVVLADAEKRQITDSTVKEWLDLLKDVIYE
VDDLLDEVSTKAATQKELSNSFSRIFNRKKIVSISKLEEMVERLDDILKQKESLDLKDIPVESYQQWKAQPTSLEDGYGMY
GRDKDKEAIMQLVLEDRTDGEQVSVIPIVGMGGVGKTTLARSVFNDGKLKEQIFDLKAWVCVSDIFDMVKVTKTMIEEI
TKKPCKLSDLNALQLDLMDKLKGKRFLIVLDDVWIEDFDNWSSLTKPFLSGIRGSKILVTTRNESVVVRVPFQTVNVHHL
NQLSNEDCWLVFANHAFPLSEANEKRGTLEKIGKEIVKKCNGLPLAAQSLGGMLRRKQAIRDWINVLESEIWELPEGQC
KIIPALRISYHHLPPHLKRCFVYCSLYPKDYEFIKDELIQLWMAEDIVKAPQKGKTLEEVCHEYFDDLLSRSFFQCSSSRIWG
NCFVMHDLMHDLATSIGGEFYFRTDEFGKETKISKKTRHLSFTRFSDPVSDIEVFDTVKSPRTFLLTNYVFSPFNNEMAP
HIIVSMLKYLRVLSFCHFQSPFALPDSIGQLIHLRYVNLTGTSIKSLPESVCNLYNLQTLKLSFCYKLTKLPSAMQNLVNLRYL
EILDIPIKEMPKRMGKLNQLQKLDFYIAGKRAENSIKELGGLPNLRGAFSIKGLENVTKGEEALEARIRDKKHITHLSLEWSI
VNDNIIDFQIELDVLSKLQPHQDLKSLSIDGYKGTRFPKWMGNFCYCHMTRLSLYNCNNCCMLPSMGQLPSLKFLNISD
MNSVKTIDAEFFKMENCSSVIPFPSLERLEISDMPCWEVWNSFDSKAFPVLKCFYITDCPKLKGALPNHLPALQNLSIVN
CELLVSSVPRAPTLRSLEIRKSDKVMFHEFPLLVESIDVEGGPMVESMMEAITNIQPTCLKYLTLQNYSSALLFPGDRLPAS
LKTLRISGLTKMKFPMQHKHELLESLSINNSCESLTSLQLAIFPNLTSLRITNCENMESLLVSGSDSLKSLNCVEIGHCPNFV
SFLGEGLSAPNLTCFSFYDCDKLKSLPDQMGTLFPKMEYLIISNCQQIESFPVGGMPPNLIKVSISNCEKLLSGKAWVCM
DMVTSLDVWGPCDGINSFPEEGLLPPSLKSLRLCNLSSLETLDCMGFLHLTSLRELYIENCKTLENVAGERLPVSLTKLIIM
GCPLLQKRCHKKDRHIWPKICHVRGIKIDGRWI

>XP_027906376.1

MALAVVGGALLSAFVDVLFEKLASPEVVNLIRGKKPDKLLQKVKNQLIVVRVVLADAEKRQITDSTVKEWLDLLKDVIYE
VDDLLDEVSTKAATQKELSNSFSRIFNRKKIVSISKLEEMVERLDDILKQKESLDLKDIPVESYQQWKAQPTSLEDGYGMY
GRDKDKEAIMQLVLEDRTDGEQVSVIPIVGMGGVGKTTLARSVFNDGKLKEQIFDLKAWVCVSDIFDMVKVTKTMIEEI
TKKPCKLSDLNALQLDLMDKLKGKRFLIVLDDVWIEDFDNWSSLTKPFLSGIRGSKILVTTRNESVVVRVPFQTVNVHHL
NQLSNEDCWLVFANHAFPLSEANEKRGTLEKIGKEIVKKCNGLPLAAQSLGGMLRRKQAIRDWINVLESEIWELPEGQC

KIIPALRISYHHLPPHLKRCFVYCSLYPKDYEFIKDELIQLWMAEDIVKAPQKGKTLEEVCHEYFDDLLSRSFFQCSSSRIWG
NCFVMHDLMHDLATSIGGEFYFRTDEFGKETKISKKTRHLSFTRFSDPVSDIEVFDTVKSPRTFLLTNYVFSPFNNEMAP
HIIVSMLKYLRVLSFCHFQSPFALPDSIGQLIHLRYVNLTGTSIKSLPESVCNLYNLQTLKLSFCYKLTKLPSAMQNLVNLRYL
EILDIPIKEMPKRMGKLNQLQKLDFYIAGKRAENSIKELGGLPNLRGAFSIKGLENVTKGEEALEARIRDKKHITHLSLEWSI
VNDNIIDFQIELDVLSKLQPHQDLKSLSIDGYKGTRFPKWMGNFCYCHMTRLSLYNCNNCCMLPSMGQLPSLKFLNISD
MNSVKTIDAEFFKMENCSSVIPFPSLERLEISDMPCWEVWNSFDSKAFPVLKCFYITDCPKLKGALPNHLPALQNLSIVN
CELLVSSVPRAPTLRSLEIRKSDKVMFHEFPLLVESIDVEGGPMVESMMEAITNIQPTCLKYLTLQNYSSALLFPGDRLPAS
LKTLRISGLTKMKFPMQHKHELLESLSINNSCESLTSLQLAIFPNLTSLRITNCENMESLLVSGSDSLKSLNCVEIGHCPNFV
SFLGEGLSAPNLTCFSFYDCDKLKSLPDQMGTLFPKMEYLIISNCQQIESFPVGGMPPNLIKVSISNCEKLLSGKAWVCM
DMVTSLDVWGPCDGINSFPEEGLLPPSLKSLRLCNLSSLETLDCMGFLHLTSLRELYIENCKTLENVAGERLPVSLTKLIIM
GCPLLQKRCHKKDRHIWPKICHVRGIKIDGRWI

>XP_027906377.1

MALAVVGGALLSAFVDVLFEKLASPEVVNLIRGKKPDKLLQKVKNQLIVVRVVLADAEKRQITDSTVKEWLDLLKDVIYE
VDDLLDEVSTKAATQKELSNSFSRIFNRKKIVSISKLEEMVERLDDILKQKESLDLKDIPVESYQQWKAQPTSLEDGYGMY
GRDKDKEAIMQLVLEDRTDGEQVSVIPIVGMGGVGKTTLARSVFNDGKLKEQIFDLKAWVCVSDIFDMVKVTKTMIEEI
TKKPCKLSDLNALQLDLMDKLKGKRFLIVLDDVWIEDFDNWSSLTKPFLSGIRGSKILVTTRNESVVVRVPFQTVNVHHL
NQLSNEDCWLVFANHAFPLSEANEKRGTLEKIGKEIVKKCNGLPLAAQSLGGMLRRKQAIRDWINVLESEIWELPEGQC
KIIPALRISYHHLPPHLKRCFVYCSLYPKDYEFIKDELIQLWMAEDIVKAPQKGKTLEEVCHEYFDDLLSRSFFQCSSSRIWG
NCFVMHDLMHDLATSIGGEFYFRTDEFGKETKISKKTRHLSFTRFSDPVSDIEVFDTVKSPRTFLLTNYVFSPFNNEMAP
HIIVSMLKYLRVLSFCHFQSPFALPDSIGQLIHLRYVNLTGTSIKSLPESVCNLYNLQTLKLSFCYKLTKLPSAMQNLVNLRYL
EILDIPIKEMPKRMGKLNQLQKLDFYIAGKRAENSIKELGGLPNLRGAFSIKGLENVTKGEEALEARIRDKKHITHLSLEWSI
VNDNIIDFQIELDVLSKLQPHQDLKSLSIDGYKGTRFPKWMGNFCYCHMTRLSLYNCNNCCMLPSMGQLPSLKFLNISD
MNSVKTIDAEFFKMENCSSVIPFPSLERLEISDMPCWEVWNSFDSKAFPVLKCFYITDCPKLKGALPNHLPALQNLSIVN
CELLVSSVPRAPTLRSLEIRKSDKVMFHEFPLLVESIDVEGGPMVESMMEAITNIQPTCLKYLTLQNYSSALLFPGDRLPAS
LKTLRISGLTKMKFPMQHKHELLESLSINNSCESLTSLQLAIFPNLTSLRITNCENMESLLVSGSDSLKSLNCVEIGHCPNFV
SFLGEGLSAPNLTCFSFYDCDKLKSLPDQMGTLFPKMEYLIISNCQQIESFPVGGMPPNLIKVSISNCEKLLSGKAWVCM
DMVTSLDVWGPCDGINSFPEEGLLPPSLKSLRLCNLSSLETLDCMGFLHLTSLRELYIENCKTLENVAGERLPVSLTKLIIM
GCPLLQKRCHKKDRHIWPKICHVRGIKIDGRWI

>XP_027906378.1

MALAVVGGALLSAFVDVLFEKLASPEVVNLIRGKKPDKLLQKVKNQLIVVRVVLADAEKRQITDSTVKEWLDLLKDVIYE
VDDLLDEVSTKAATQKELSNSFSRIFNRKKIVSISKLEEMVERLDDILKQKESLDLKDIPVESYQQWKAQPTSLEDGYGMY
GRDKDKEAIMQLVLEDRTDGEQVSVIPIVGMGGVGKTTLARSVFNDGKLKEQIFDLKAWVCVSDIFDMVKVTKTMIEEI
TKKPCKLSDLNALQLDLMDKLKGKRFLIVLDDVWIEDFDNWSSLTKPFLSGIRGSKILVTTRNESVVVRVPFQTVNVHHL
NQLSNEDCWLVFANHAFPLSEANEKRGTLEKIGKEIVKKCNGLPLAAQSLGGMLRRKQAIRDWINVLESEIWELPEGQC
KIIPALRISYHHLPPHLKRCFVYCSLYPKDYEFIKDELIQLWMAEDIVKAPQKGKTLEEVCHEYFDDLLSRSFFQCSSSRIWG
NCFVMHDLMHDLATSIGGEFYFRTDEFGKETKISKKTRHLSFTRFSDPVSDIEVFDTVKSPRTFLLTNYVFSPFNNEMAP
HIIVSMLKYLRVLSFCHFQSPFALPDSIGQLIHLRYVNLTGTSIKSLPESVCNLYNLQTLKLSFCYKLTKLPSAMQNLVNLRYL
EILDIPIKEMPKRMGKLNQLQKLDFYIAGKRAENSIKELGGLPNLRGAFSIKGLENVTKGEEALEARIRDKKHITHLSLEWSI
VNDNIIDFQIELDVLSKLQPHQDLKSLSIDGYKGTRFPKWMGNFCYCHMTRLSLYNCNNCCMLPSMGQLPSLKFLNISD
MNSVKTIDAEFFKMENCSSVIPFPSLERLEISDMPCWEVWNSFDSKAFPVLKCFYITDCPKLKGALPNHLPALQNLSIVN
CELLVSSVPRAPTLRSLEIRKSDKVMFHEFPLLVESIDVEGGPMVESMMEAITNIQPTCLKYLTLQNYSSALLFPGDRLPAS
LKTLRISGLTKMKFPMQHKHELLESLSINNSCESLTSLQLAIFPNLTSLRITNCENMESLLVSGSDSLKSLNCVEIGHCPNFV

SFLGEGLSAPNLTCFSFYDCDKLKSLPDQMGTLFPKMEYLIISNCQQIESFPVGGMPPNLIKVSISNCEKLLSGKAWVCM
DMVTSLDVWGPCDGINSFPEEGLLPPSLKSLRLCNLSSLETLDCMGFLHLTSLRELYIENCKTLENVAGERLPVSLTKLIIM
GCPLLQKRCHKKDRHIWPKICHVRGIKIDGRWI

>XP_027906379.1

MALAVVGGALLSAFVDVLFEKLASPEVVNLIRGKKPDKLLQKVKNQLIVVRVVLADAEKRQITDSTVKEWLDLLKDVIYE
VDDLLDEVSTKAATQKELSNSFSRIFNRKKIVSISKLEEMVERLDDILKQKESLDLKDIPVESYQQWKAQPTSLEDGYGMY
GRDKDKEAIMQLVLEDRTDGEQVSVIPIVGMGGVGKTTLARSVFNDGKLKEQIFDLKAWVCVSDIFDMVKVTKTMIEEI
TKKPCKLSDLNALQLDLMDKLKGKRFLIVLDDVWIEDFDNWSSLTKPFLSGIRGSKILVTTRNESVVVRVPFQTVNVHHL
NQLSNEDCWLVFANHAFPLSEANEKRGTLEKIGKEIVKKCNGLPLAAQSLGGMLRRKQAIRDWINVLESEIWELPEGQC
KIIPALRISYHHLPPHLKRCFVYCSLYPKDYEFIKDELIQLWMAEDIVKAPQKGKTLEEVCHEYFDDLLSRSFFQCSSSRIWG
NCFVMHDLMHDLATSIGGEFYFRTDEFGKETKISKKTRHLSFTRFSDPVSDIEVFDTVKSPRTFLLTNYVFSPFNNEMAP
HIIVSMLKYLRVLSFCHFQSPFALPDSIGQLIHLRYVNLTGTSIKSLPESVCNLYNLQTLKLSFCYKLTKLPSAMQNLVNLRYL
EILDIPIKEMPKRMGKLNQLQKLDFYIAGKRAENSIKELGGLPNLRGAFSIKGLENVTKGEEALEARIRDKKHITHLSLEWSI
VNDNIIDFQIELDVLSKLQPHQDLKSLSIDGYKGTRFPKWMGNFCYCHMTRLSLYNCNNCCMLPSMGQLPSLKFLNISD
MNSVKTIDAEFFKMENCSSVIPFPSLERLEISDMPCWEVWNSFDSKAFPVLKCFYITDCPKLKGALPNHLPALQNLSIVN
CELLVSSVPRAPTLRSLEIRKSDKVMFHEFPLLVESIDVEGGPMVESMMEAITNIQPTCLKYLTLQNYSSALLFPGDRLPAS
LKTLRISGLTKMKFPMQHKHELLESLSINNSCESLTSLQLAIFPNLTSLRITNCENMESLLVSGSDSLKSLNCVEIGHCPNFV
SFLGEGLSAPNLTCFSFYDCDKLKSLPDQMGTLFPKMEYLIISNCQQIESFPVGGMPPNLIKVSISNCEKLLSGKAWVCM
DMVTSLDVWGPCDGINSFPEEGLLPPSLKSLRLCNLSSLETLDCMGFLHLTSLRELYIENCKTLENVAGERLPVSLTKLIIM
GCPLLQKRCHKKDRHIWPKICHVRGIKIDGRWI

>XP_027906390.1

MEFASSSSSSSSSSSSSFLKSEPVFINDVFINFGGEDIGRRFVSHLHSALLQAQVKTFISQENLQEGMEQEEHLRAIGGTKI
AIMVFSKSYAECTCCLHELEKIIECHQTFGQMVVPVYYEIDPLDVQHQKNDFGKALEEAAHKSYAEDLLEYALSKWSRAL
TTATGVTGWDVRDFRHDAELVERIVSLVESLLDYKDLFTTQYPVGLESPVEDVIKCIENQSTKVCVIGIWGMGGSGKTTI
AKAIYNRIYHQFIGKSFIENIREAWASEYDRYVDLQANLLSDVLKSKLEVKSVGMGRAMIHDELSRRKLLIVLDDVNDFVQ
LENLCRSREWFGQGTVIIITTRDVHLLNLLKVNYVYKMHLLNKNDSLELLSWHAFREAKPIKEWNEHARDIVDYCGGLPL
ALQFLGSYLCDITKEAWERVFSNLNSIPTYQVQKVLKISFDALRDMEKDIFLDVCCFFIGKQREYVTEILNGCGLCADIGIT
VLIERGLIKVERNNKLEMHPLLRDMGREFIRELHPKEPGKRSRLWFQEDVQDVLEENSGTEAIEGLSLKLHSSNRDSLEAL
AFKKMKRLRLLQLDHVRLSGDYGHISKELRWICWRGFPYKYIPNSFHLENVIAIDFKHSHLQLLSKQHLVLERLKFLNLSHS
KYLIETPDFSGLPSLEQLILKDCPSLREVHESIGRLRNLLLINLKDCTSLCYLPREVYRLRSLKTLVLSGCLKFGPIDKAWVKSF
VTIITENTTVKQAPFDQL

>XP_027906411.1

MTSHQFLSMDNASSSSSKLPQKYDVLINCTGEDIRRKFVSHLDSALSTAGLTTFLHHQNATKPTHIQQPILNLCRVVIVVF
TKTYSESAWCLLQLQQIIKWHETYSRYVLPVYYEIQPSDVRLQEGDFGKAFKATAHQTFSAQQLDHGMSSWSHALTKA
ANFFGYDESNYRSDAEVVDKIVKNILNLSVLSATKFPIGLPSHVEDLIQTIKCKSAEVCTIGICGEVGYGKTTLAKAIYNQIH
GIFAEKSFIENISQVIQTRGYAKLQEKLLSDVLNTKVEIQDVDMGRRMIQDKFYGKRLLIVLDDMNESGPFLELCTYRARF
SGGTVIIITTTDERILRTYAVDSVFQIKLMNEKESLELLSWHAFSEAKPKEEYNDLAERVVSYCGGLPLALELIGSTLFERTKE
EWNSVLFKFEKIPQHHVVHKLKISFDGLKNQIEKDLFLDVCCFFVGKDRSSATKILNGSGVDADSGIRVLIERNLIKVKKNN
KIEMHPLLQEMGIRIIREISIMEPWKTNQLWFDKDAEYALIENKGKNAMKWLPLKLDQVKIAVNSEYLLQKLRWISLHG
FPSEYVHNKYCVHDAITIDLKHSLVRFVWKAPQVLRSLKVLNLSHSKYLTITPDFTGLPNLEQLILKYCPRLYIVHRSIRCLC
NLILLNLKNCKSLTNFPTEIYELKSLRTLILTGCSEIDLMGNDIAQMESLITLIAENTAVKEVPFSMVSSKSIGYISLRGMERLS

RNLFPSIICSWMLPTVNRTSYIHSFFMDTKDNSWDDIAPLLSTLRNLRSVLVQCDSEFQLSKQVKNILSEDFANITESEISK
FHTVSSLIGVGRYNAFFNSVNYSISEVMTSSDSCEVFYCLAHIGEGHSVSFSVPQDRDMKGMAFRVVYLSTHEIIEPEFTT
PLIVNYTKCTFHIHNHGTVISFNDEDWHGIVSNLRCGDKVEIFVSFGNGLVVNNTVFYLICGESHNFRKRL

>XP_027906463.1

MELASSSSKLQRKYDVLINFNGEDIRNKFVSHLDSVLSSAGLTTFLHHPNSVPPMDIQQPILDLCRVAIVVFTKTYSQSAW
CLHELQQIIKWHQTYCRHVLPVYYEVEPSDVRLQKGNFGKDLKTTAQQTFSGQQLEDGISRWSHALTKAANFFGWDES
NYRSDAELVDKIVKSVLNLSVLSATKFPVGLQSRVEDVMQIINNKSTKVCIIGIGGREGSGKTTLAKAIYNTIHGTFMEKSF
IKDIAQVSQTRGHVPLQEQLLSDVLKTKVEIRSVEMGSRMIREILSVKRMLIVLDDMIEKGSPFLDLIRKSGTWFGAGTVII
MTTKEEGLLSMREVDFVFRIKLMNPNESLELLSWHAFREAKPKEEYNDLAKGIVTRCQGLPLALEVIGSYLYERTKEEWN
RIFLKLDKIPMLREVDGIFTISFDGLGNQMEKYLFLDLCSSFMGKGRASATNILNNCGLDADSGIRVLIERNLIHVERNNKL
GMHPLLQKMGMEIIFDIPRKEPEVVWFDRDAEYARSENTGTKVIHRLPGKMFFSRRDFFKPYPLEVRDPSRLLKLAEDS
EYLSKKLRRINLQGFSQEYIPNAFYLHDAIVINLKHSHLRFFWKQPQDLTWLKVLNLSHSKYLMETPDFSRLPSLEQLILKD
CPGLCEVHQSIGCLNNLILLNLKDCTSLSNLPIEIYKLKSLKTLILCGCSKVDLLERDILKMESLIILIAQNTTVKQVPFSIVSSQ
SIGYISLRRFQGLSHNLFPSIIRFRMPPTLNPQSYIHSFMNVEDNIWHDIAPFLSNLAHLRSVLVQYDAEFQLSKQVKAILV
EYGVNITEPGILKHQFRSSLIGVGRYRELFNMYQVLASSELSDVSLPGDNNPYWLAHMGEGDSVSFTVPQDRVMKGM
ALSVVYLSTSKTIEPEFTSVFIVNYTKCTCQIHNHDTVISFNDEDWHGIISNLESGDKVEIFVTVVHGLVAKNTVVYLIYGES
NDFEKVPEPKKNSLVRFIKKIAI

>XP_027906464.1

MRAITVGLQSRVEDVMQIINNKSTKVCIIGIGGREGSGKTTLAKAIYNTIHGTFMEKSFIKDIAQVSQTRGHVPLQEQLLS
DVLKTKVEIRSVEMGSRMIREILSVKRMLIVLDDMIEKGSPFLDLIRKSGTWFGAGTVIIMTTKEEGLLSMREVDFVFRIKL
MNPNESLELLSWHAFREAKPKEEYNDLAKGIVTRCQGLPLALEVIGSYLYERTKEEWNRIFLKLDKIPMLREVDGIFTISFD
GLGNQMEKYLFLDLCSSFMGKGRASATNILNNCGLDADSGIRVLIERNLIHVERNNKLGMHPLLQKMGMEIIFDIPRKE
PEVVWFDRDAEYARSENTGTKVIHRLPGKMFFSRRDFFKPYPLEVRDPSRLLKLAEDSEYLSKKLRRINLQGFSQEYIPNA
FYLHDAIVINLKHSHLRFFWKQPQDLTWLKVLNLSHSKYLMETPDFSRLPSLEQLILKDCPGLCEVHQSIGCLNNLILLNLK
DCTSLSNLPIEIYKLKSLKTLILCGCSKVDLLERDILKMESLIILIAQNTTVKQVPFSIVSSQSIGYISLRRFQGLSHNLFPSIIRFR
MPPTLNPQSYIHSFMNVEDNIWHDIAPFLSNLAHLRSVLVQYDAEFQLSKQVKAILVEYGVNITEPGILKHQFRSSLIGV
GRYRELFNMYQVLASSELSDVSLPGDNNPYWLAHMGEGDSVSFTVPQDRVMKGMALSVVYLSTSKTIEPEFTSVFIVN
YTKCTCQIHNHDTVISFNDEDWHGIISNLESGDKVEIFVTVVHGLVAKNTVVYLIYGESNDFEKVPEPKKNSLVRFIKKIAI

>XP_027906480.1

MAALIACCQFLTLSPRAKAWLKKQLIQLDLYETRVGKLEDVVEKLKKKRDSIQNTVDEEERRHGRKIHVEVKEWMDKVD
KLILTYRGFHNDEICHKCAMFEFFNSGFLPKPGIRYRRSRKADDITKQANGLLQNAKFDILSYWSGPPSMAAFFSNLGYE
SYSSRNDTVKKITDEFQKPGVRMIGLHGLSGVGKTSLVKEVVKKALKDKMFEVVTMASVTKSPDVRKIQGQIADMLGV
VLEEESNIARAARIHQILNDENKSTLIILDDLWEEVDFNLLGIPCELEKDDGVTSVKGKSVDVDILKNVSDGKSPVLDNSSS
FRKGTLRGVDGSKNVNKAKSPVLDGSISFRKGTLYGVDGSNNVNKGKFSGAANSMNVKKGEFLGGGLKNVNEGKSSV
DASDTVKTEKIVPQYKGCKILMISEIRQVLLSQMEGKEESIFPVEVLKEKEAEMLFKKKAGIGGKNSEYDKLAAQIANKCK
GLPMTIITTARALKNKSLSVWDETNRKLDSQNLAGAPEFSTKLSYELLEDEELKHTFLLCARMGQDALVMDLVKYCIGFG
FLRGINTARQTRDKVHTLVSKLKESGLLSDSYSSDYFTMPDSVRTAALSIAYKENHLFTMTKGKLDEWPDNLEMYAAISL
HHCDFIEEFPGRINYPRLRVLQIVNNIPRPKIPEKFFETMKELRVLILTGIYLSLVGSSLSSLHKLRMLCLEQCCMLDDELYII
GELKRLRILSFSGSDIESLPTKLNELKMLQIFDISNCSKLKKIPNGVIPSLVSLEELYMRNTLIQWEDEEQTRQSKIALLSGLK
HLNQLTTLDIQIRNVSYLPKNLFFDKLFSYKIVIGDLSSVLDIDFKMPEKYEALKFLAIRLKNGSDIHSLKGIKMLFEGVENLF
LELNTVDDKQNSEADNIVHDLFYRLNLKGFPYLKHLWIVNNSTIQSLIYPKDRQLPEKAFPKLESLCLYNLKIDKICSCNLSE

PSFGKLKVIKINHCGELKNVFSISVVGLLKVIETIEVSECNSLKEVIDVEPQNDPDKTELLMLPELRYLKLQSLSEFIGFDAIPH
REGQERKLFHEKVGVSKLERLELSSLHIDVIWSVDQSSKRLSFESLTHLDVNGCWKLKYLMSFTMAKILVNLQSLYVSDCE
KMRSIFLPDQDKEKDIMASIFPKLKNMKLLNMNSLSKIWNLKLPLDSFNKLDTLIIEECHKLGNAMEGIFVSLCNLRVTNC
RSMQAIFNIRDQVGDAANNLQDVHLETLPELKLVWKMNNEDRIGIPKFNNVKKIWAQDCDNLEYIFPFSIAKSLGNLES
LVVCDCYGLSEIVAQREATHTGRARFNFPKLSTVKFSKLPKLTSFYPTAYDLSCPLNELSIELCKNLEPFNKGTQHAERNPI
HVCFPEEVINNLKSMQIESWHAKSPSSYMGKRNHRRDNLEELSLSRLIDTGILYSFLHRSPNLKSLSLSNCYFEKIVPPKED
TEIENLGVVPNLKSLMLIDLLNLKEIGFEPDIILERLEFLILKNCPRMITMAPSSVSFTRLTNLEVINCDGLKSLMSASTAQSL
VQLNTMKVVKCESLVEIVRKDGENSDMVLFQQLKALELVSLKKLKSFCVSDCAFEFPSLEKLVVSACYNMAKFSETVTSS
PLLQNIHVVHGKENKRFCWEGDINATIQKIFEEMKFFEGMEEMSLSQHRELEESWERGTILQKQNSWFYSLKILKLENC
DIEPCAIPSNILPYLRSLKELQVRGCDNVEVIFEMNAKEGTGTTFQLQKLTLSKLPKLEDVWERNGKGTESFQNLKMVHV
SECENLQTVFPLTLAKNLKKLVKLEIIGCHALREIVRKEKNVSAVFVFPCLITLGLFDLPELIYFYPESFTLECSALNKLIVCYCP
DLELFGSANRQSIFFDLKDICNLEELTIDWEQTLVLRTKLGEPTDNLKYLNHIQLFLEVDENERPDLPIQILLKMPNLTKMSI
HYSNCLEVFQTQIPEVVEKRVLTHLKTLRLNCVSKLQSIGSEDSPWLNVICDSENLQKLVVLKCPDLKTLVHSPASVSFTYV
KEVYIDRCKELKYLFTLASVNKLENLERIKVNDCESMEAIVLKEEDDISEEIKLQQLKHIDLNGLSSLECFYSGNDSLQLPSLV
QVDIWKCPKMEFFSRGEIHLNSSFRGIQASNVSSDDFVFYHDLNFSVEKVFLQQEFFQAVDKECFSDNLDLQAEPRGKI
GLQNKWLANLETLKLQNCTQSYAIPSFILCLLKNLKELEVRDSDQVKAIFDMNDDTEIKETESQLKILTLNGLSELTHVWE
KDTHRILIFRNLQEVVVSDCSKLQTLFPASLAKSLKDLKKLKIDSCENLHDFVEQEETTFVTKKFVFPCLEDLELNDLPLVTC
PKTFTLEFPSVKFLSVRNCDELGLFQSVYDPMGEGTSSSRLPLISDPKVISNLEKLTLDCKQILALSLWFKSQKSTEGLTNFN
TISLSFFGIDGNEVPMLPIEILKAPSLIELDMNNCNNIENFLAQNPKIGEEEMLRQLTILRLCNVSTTQFFELEYCLSLNIICE
RLHKLTLSQCPHLTTILRVHSAVSFSCLKELNIYKCPNLKYLFTTSAAKKLMNLEEIRVTECETLTEIVSKEGDASSEGIKFDRL
HTIYLQSLTSLVCFYSGSDSIELSSLKTVAIRSCPNMEIFSHGNESLMGVALSTDQGADDVHPPQDLNTRIKGIIQRKEFFE
AVDKECFSDNIELQEDPHCKFGLQNQWLRDLVSLKLQNCTLLCAIPFSILALLQSLKELEVQDSTTIEVLFYMNDNEIMGI
TSQLRILTLKGLSKLTRVWEKNKNGVLNFSSLEQVVVSNCENLQTLFPASLARNLKSLKGIDIESCVEFQEIVEKEDDTEAK
FVLPCLEELNLSFLPQLTCFYPQTFTLECPTLNLLSVFECERLELFQSEHSMGEGTSVKRQPLISSLEVISNLKELELDWKHIL
ALRSRLRSEKFTGIFKFVNKMNLLLNADVSEMLIVVNEIVHTTPNLIEMIVMIDNCNSMEIFLAQNPKIGEDGMLLQLRT
LNLFRISDIRSNQPENSSWSDTFSEKIHELHVFECPHVETIGVHPTFSVSSSFLKQVFVQKCPQMQYLFTFSVAKELVNLEE
ITVIECESLKEIVSKEGDEDEPKGEGDDKYENEMIFMKLENLILASLDNLESFYSGSCILNFPSLRKVAVNECLNSKIFRHRD
KVPPKFTVIIDEILGKGDKKALVTQQFEEEAS

>XP_027906481.1

MAALIACCQFLTLSPRAKAWLKKQLIQLDLYETRVGKLEDVVEKLKKKRDSIQNTVDEEERRHGRKIHVEVKEWMDKVD
KLILTYRGFHNDEICHKCAMFEFFNSGFLPKPGIRYRRSRKADDITKQANGLLQNAKFDILSYWSGPPSMAAFFSNLGYE
SYSSRNDTVKKITDEFQKPGVRMIGLHGLSGVGKTSLVKEVVKKALKDKMFEVVTMASVTKSPDVRKIQGQIADMLGV
VLEEESNIARAARIHQILNDENKSTLIILDDLWEEVDFNLLGIPCELEKDDGVTSVKGKSVDVDILKNVSDGKSPVLDNSSS
FRKGTLRGVDGSKNVNKAKSPVLDGSISFRKGTLYGVDGSNNVNKGKFSGAANSMNVKKGEFLGGGLKNVNEGKSSV
DASDTVKTEKIVPQYKGCKILMISEIRQVLLSQMEGKEESIFPVEVLKEKEAEMLFKKKAGIGGKNSEYDKLAAQIANKCK
GLPMTIITTARALKNKSLSVWDETNRKLDSQNLAGAPEFSTKLSYELLEDEELKHTFLLCARMGQDALVMDLVKYCIGFG
FLRGINTARQTRDKVHTLVSKLKESGLLSDSYSSDYFTMPDSVRTAALSIAYKENHLFTMTKGKLDEWPDNLEMYAAISL
HHCDFIEEFPGRINYPRLRVLQIVNNIPRPKIPEKFFETMKELRVLILTGIYLSLVGSSLSSLHKLRMLCLEQCCMLDDELYII
GELKRLRILSFSGSDIESLPTKLNELKMLQIFDISNCSKLKKIPNGVIPSLVSLEELYMRNTLIQWEDEEQTRQSKIALLSGLK
HLNQLTTLDIQIRNVSYLPKNLFFDKLFSYKIVIGDLSSVLDIDFKMPEKYEALKFLAIRLKNGSDIHSLKGIKMLFEGVENLF
LELNTVDDKQNSEADNIVHDLFYRLNLKGFPYLKHLWIVNNSTIQSLIYPKDRQLPEKAFPKLESLCLYNLKIDKICSCNLSE
PSFGKLKVIKINHCGELKNVFSISVVGLLKVIETIEVSECNSLKEVIDVEPQNDPDKTELLMLPELRYLKLQSLSEFIGFDAIPH
REGQERKLFHEKVGVSKLERLELSSLHIDVIWSVDQSSKRLSFESLTHLDVNGCWKLKYLMSFTMAKILVNLQSLYVSDCE

KMRSIFLPDQDKEKDIMASIFPKLKNMKLLNMNSLSKIWNLKLPLDSFNKLDTLIIEECHKLGNAMEGIFVSLCNLRVTNC
RSMQAIFNIRDQVGDAANNLQDVHLETLPELKLVWKMNNEDRIGIPKFNNVKKIWAQDCDNLEYIFPFSIAKSLGNLES
LVVCDCYGLSEIVAQREATHTGRARFNFPKLSTVKFSKLPKLTSFYPTAYDLSCPLNELSIELCKNLEPFNKGTQHAERNPI
HVCFPEEVINNLKSMQIESWHAKSPSSYMGKRNHRRDNLEELSLSRLIDTGILYSFLHRSPNLKSLSLSNCYFEKIVPPKED
TEIENLGVVPNLKSLMLIDLLNLKEIGFEPDIILERLEFLILKNCPRMITMAPSSVSFTRLTNLEVINCDGLKSLMSASTAQSL
VQLNTMKVVKCESLVEIVRKDGENSDMVLFQQLKALELVSLKKLKSFCVSDCAFEFPSLEKLVVSACYNMAKFSETVTSS
PLLQNIHVVHGKENKRFCWEGDINATIQKIFEEMKFFEGMEEMSLSQHRELEESWERGTILQKQNSWFYSLKILKLENC
DIEPCAIPSNILPYLRSLKELQVRGCDNVEVIFEMNAKEGTGTTFQLQKLTLSKLPKLEDVWERNGKGTESFQNLKMVHV
SECENLQTVFPLTLAKNLKKLVKLEIIGCHALREIVRKEKNVSAVFVFPCLITLGLFDLPELIYFYPESFTLECSALNKLIVCYCP
DLELFGSANRQSIFFDLKDICNLEELTIDWEQTLVLRTKLGEPTDNLKYLNHIQLFLEVDENERPDLPIQILLKMPNLTKMSI
HYSNCLEVFQTQIPEVVEKRVLTHLKTLRLNCVSKLQSIGSEDSPWLNVICDSENLQKLVVLKCPDLKTLVHSPASVSFTYV
KEVYIDRCKELKYLFTLASVNKLENLERIKVNDCESMEAIVLKEEDDISEEIKLQQLKHIDLNGLSSLECFYSGNDSLQLPSLV
QVDIWKCPKMEFFSRGEIHLNSSFRGIQASNVSSDDFVFYHDLNFSVEKVFLQQEFFQAVDKECFSDNLDLQAEPRGKI
GLQNKWLANLETLKLQNCTQSYAIPSFILCLLKNLKELEVRDSDQVKAIFDMNDDTEIKETESQLKILTLNGLSELTHVWE
KDTHRILIFRNLQEVVVSDCSKLQTLFPASLAKSLKDLKKLKIDSCENLHDFVEQEETTFVTKKFVFPCLEDLELNDLPLVTC
PKTFTLEFPSVKFLSVRNCDELGLFQSVYDPMGEGTSSSRLPLISDPKVISNLEKLTLDCKQILALSLWFKSQKSTEGLTNFN
TISLSFFGIDGNEVPMLPIEILKAPSLIELDMNNCNNIENFLAQNPKIGEEEMLRQLTILRLCNVSTTQFFELEYCLSLNIICE
RLHKLTLSQCPHLTTILRVHSAVSFSCLKELNIYKCPNLKYLFTTSAAKKLMNLEEIRVTECETLTEIVSKEGDASSEGIKFDRL
HTIYLQSLTSLVCFYSGSDSIELSSLKTVAIRSCPNMEIFSHGNESLMGVALSTDQGADDVHPPQDLNTRIKGIIQRKEFFE
AVDKECFSDNIELQEDPHCKFGLQNQWLRDLVSLKLQNCTLLCAIPFSILALLQSLKELEVQDSTTIEVLFYMNDNEIMGI
TSQLRILTLKGLSKLTRVWEKNKNGVLNFSSLEQVVVSNCENLQTLFPASLARNLKSLKGIDIESCVEFQEIVEKEDDTEAK
FVLPCLEELNLSFLPQLTCFYPQTFTLECPTLNLLSVFECERLELFQSEHSMGEGTSVKRQPLISSLEVISNLKELELDWKHIL
ALRSRLRSEKFTGIFKFVNKMNLLLNADVSEMLIVVNEIVHTTPNLIEMIVMIDNCNSMEIFLAQNPKIGEDGMLLQLRT
LNLFRISDIRSNQPENSSWSDTFSEKIHELHVFECPHVETIGVHPTFSVSSSFLKQVFVQKCPQMQYLFTFSVAKELVNLEE
ITVIECESLKEIVSKEGDEDEPKGEGDDKYENEMIFMKLENLILASLDNLESFYSGSCILNFPSLRKVAVNECLNSKIFRHRD
KVPPKFTVIIDEILGKGDKKALVTQQFEEEAS

>XP_027906482.1

MAALIACCQFLTLSPRAKAWLKKQLIQLDLYETRVGKLEDVVEKLKKKRDSIQNTVDEEERRHGRKIHVEVKEWMDKVD
KLILTYRGFHNDEICHKCAMFEFFNSGFLPKPGIRYRRSRKADDITKQANGLLQNAKFDILSYWSGPPSMAAFFSNLGYE
SYSSRNDTVKKITDEFQKPGVRMIGLHGLSGVGKTSLVKEVVKKALKDKMFEVVTMASVTKSPDVRKIQGQIADMLGV
VLEEESNIARAARIHQILNDENKSTLIILDDLWEEVDFNLLGIPCELEKDDGVTSVKGKSVDVDILKNVSDGKSPVLDNSSS
FRKGTLRGVDGSKNVNKAKSPVLDGSISFRKGTLYGVDGSNNVNKGKFSGAANSMNVKKGEFLGGGLKNVNEGKSSV
DASDTVKTEKIVPQYKGCKILMISEIRQVLLSQMEGKEESIFPVEVLKEKEAEMLFKKKAGIGGKNSEYDKLAAQIANKCK
GLPMTIITTARALKNKSLSVWDETNRKLDSQNLAGAPEFSTKLSYELLEDEELKHTFLLCARMGQDALVMDLVKYCIGFG
FLRGINTARQTRDKVHTLVSKLKESGLLSDSYSSDYFTMPDSVRTAALSIAYKENHLFTMTKGKLDEWPDNLEMYAAISL
HHCDFIEEFPGRINYPRLRVLQIVNNIPRPKIPEKFFETMKELRVLILTGIYLSLVGSSLSSLHKLRMLCLEQCCMLDDELYII
GELKRLRILSFSGSDIESLPTKLNELKMLQIFDISNCSKLKKIPNGVIPSLVSLEELYMRNTLIQWEDEEQTRQSKIALLSGLK
HLNQLTTLDIQIRNVSYLPKNLFFDKLFSYKIVIGDLSSVLDIDFKMPEKYEALKFLAIRLKNGSDIHSLKGIKMLFEGVENLF
LELNTVDDKQNSEADNIVHDLFYRLNLKGFPYLKHLWIVNNSTIQSLIYPKDRQLPEKAFPKLESLCLYNLKIDKICSCNLSE
PSFGKLKVIKINHCGELKNVFSISVVGLLKVIETIEVSECNSLKEVIDVEPQNDPDKTELLMLPELRYLKLQSLSEFIGFDAIPH
REGQERKLFHEKVGVSKLERLELSSLHIDVIWSVDQSSKRLSFESLTHLDVNGCWKLKYLMSFTMAKILVNLQSLYVSDCE
KMRSIFLPDQDKEKDIMASIFPKLKNMKLLNMNSLSKIWNLKLPLDSFNKLDTLIIEECHKLGNAMEGIFVSLCNLRVTNC
RSMQAIFNIRDQVGDAANNLQDVHLETLPELKLVWKMNNEDRIGIPKFNNVKKIWAQDCDNLEYIFPFSIAKSLGNLES

LVVCDCYGLSEIVAQREATHTGRARFNFPKLSTVKFSKLPKLTSFYPTAYDLSCPLNELSIELCKNLEPFNKGTQHAERNPI
HVCFPEEVINNLKSMQIESWHAKSPSSYMGKRNHRRDNLEELSLSRLIDTGILYSFLHRSPNLKSLSLSNCYFEKIVPPKED
TEIENLGVVPNLKSLMLIDLLNLKEIGFEPDIILERLEFLILKNCPRMITMAPSSVSFTRLTNLEVINCDGLKSLMSASTAQSL
VQLNTMKVVKCESLVEIVRKDGENSDMVLFQQLKALELVSLKKLKSFCVSDCAFEFPSLEKLVVSACYNMAKFSETVTSS
PLLQNIHVVHGKENKRFCWEGDINATIQKIFEEMKFFEGMEEMSLSQHRELEESWERGTILQKQNSWFYSLKILKLENC
DIEPCAIPSNILPYLRSLKELQVRGCDNVEVIFEMNAKEGTGTTFQLQKLTLSKLPKLEDVWERNGKGTESFQNLKMVHV
SECENLQTVFPLTLAKNLKKLVKLEIIGCHALREIVRKEKNVSAVFVFPCLITLGLFDLPELIYFYPESFTLECSALNKLIVCYCP
DLELFGSANRQSIFFDLKDICNLEELTIDWEQTLVLRTKLGEPTDNLKYLNHIQLFLEVDENERPDLPIQILLKMPNLTKMSI
HYSNCLEVFQTQIPEVVEKRVLTHLKTLRLNCVSKLQSIGSEDSPWLNVICDSENLQKLVVLKCPDLKTLVHSPASVSFTYV
KEVYIDRCKELKYLFTLASVNKLENLERIKVNDCESMEAIVLKEEDDISEEIKLQQLKHIDLNGLSSLECFYSGNDSLQLPSLV
QVDIWKCPKMEFFSRGEIHLNSSFRGIQASNVSSDDFVFYHDLNFSVEKVFLQQEFFQAVDKECFSDNLDLQAEPRGKI
GLQNKWLANLETLKLQNCTQSYAIPSFILCLLKNLKELEVRDSDQVKAIFDMNDDTEIKETESQLKILTLNGLSELTHVWE
KDTHRILIFRNLQEVVVSDCSKLQTLFPASLAKSLKDLKKLKIDSCENLHDFVEQEETTFVTKKFVFPCLEDLELNDLPLVTC
PKTFTLEFPSVKFLSVRNCDELGLFQSVYDPMGEGTSSSRLPLISDPKVISNLEKLTLDCKQILALSLWFKSQKSTEGLTNFN
TISLSFFGIDGNEVPMLPIEILKAPSLIELDMNNCNNIENFLAQNPKIGEEEMLRQLTILRLCNVSTTQFFELEYCLSLNIICE
RLHKLTLSQCPHLTTILRVHSAVSFSCLKELNIYKCPNLKYLFTTSAAKKLMNLEEIRVTECETLTEIVSKEGDASSEGIKFDRL
HTIYLQSLTSLVCFYSGSDSIELSSLKTVAIRSCPNMEIFSHGNESLMGVALSTDQGADDVHPPQDLNTRIKGIIQRKEFFE
AVDKECFSDNIELQEDPHCKFGLQNQWLRDLVSLKLQNCTLLCAIPFSILALLQSLKELEVQDSTTIEVLFYMNDNEIMGI
TSQLRILTLKGLSKLTRVWEKNKNGVLNFSSLEQVVVSNCENLQTLFPASLARNLKSLKGIDIESCVEFQEIVEKEDDTEAK
FVLPCLEELNLSFLPQLTCFYPQTFTLECPTLNLLSVFECERLELFQSEHSMGEGTSVKRQPLISSLEVISNLKELELDWKHIL
ALRSRLRSEKFTGIFKFVNKMNLLLNADVSEMLIVVNEIVHTTPNLIEMIVMIDNCNSMEIFLAQNPKIGEDGMLLQLRT
LNLFRISDIRSNQPENSSWSDTFSEKIHELHVFECPHVETIGVHPTFSVSSSFLKQVFVQKCPQMQYLFTFSVAKELVNLEE
ITVIECESLKEIVSKEGDEDEPKGEGDDKYENEMIFMKLENLILASLDNLESFYSGSCILNFPSLRKVAVNECLNSKIFRHRD
KVPPKFTVIIDEILGKGDKKALVTQQFEEEAS

>XP_027906483.1

MAALIACCQFLTLSPRAKAWLKKQLIQLDLYETRVGKLEDVVEKLKKKRDSIQNTVDEEERRHGRKIHVEVKEWMDKVD
KLILTYRGFHNDEICHKCAMFEFFNSGFLPKPGIRYRRSRKADDITKQANGLLQNAKFDILSYWSGPPSMAAFFSNLGYE
SYSSRNDTVKKITDEFQKPGVRMIGLHGLSGVGKTSLVKEVVKKALKDKMFEVVTMASVTKSPDVRKIQGQIADMLGV
VLEEESNIARAARIHQILNDENKSTLIILDDLWEEVDFNLLGIPCELEKDDGVTSVKGKSVDVDILKNVSDGKSPVLDNSSS
FRKGTLRGVDGSKNVNKAKSPVLDGSISFRKGTLYGVDGSNNVNKGKFSGAANSMNVKKGEFLGGGLKNVNEGKSSV
DASDTVKTEKIVPQYKGCKILMISEIRQVLLSQMEGKEESIFPVEVLKEKEAEMLFKKKAGIGGKNSEYDKLAAQIANKCK
GLPMTIITTARALKNKSLSVWDETNRKLDSQNLAGAPEFSTKLSYELLEDEELKHTFLLCARMGQDALVMDLVKYCIGFG
FLRGINTARQTRDKVHTLVSKLKESGLLSDSYSSDYFTMPDSVRTAALSIAYKENHLFTMTKGKLDEWPDNLEMYAAISL
HHCDFIEEFPGRINYPRLRVLQIVNNIPRPKIPEKFFETMKELRVLILTGIYLSLVGSSLSSLHKLRMLCLEQCCMLDDELYII
GELKRLRILSFSGSDIESLPTKLNELKMLQIFDISNCSKLKKIPNGVIPSLVSLEELYMRNTLIQWEDEEQTRQSKIALLSGLK
HLNQLTTLDIQIRNVSYLPKNLFFDKLFSYKIVIGDLSSVLDIDFKMPEKYEALKFLAIRLKNGSDIHSLKGIKMLFEGVENLF
LELNTVDDKQNSEADNIVHDLFYRLNLKGFPYLKHLWIVNNSTIQSLIYPKDRQLPEKAFPKLESLCLYNLKIDKICSCNLSE
PSFGKLKVIKINHCGELKNVFSISVVGLLKVIETIEVSECNSLKEVIDVEPQNDPDKTELLMLPELRYLKLQSLSEFIGFDAIPH
REGQERKLFHEKVGVSKLERLELSSLHIDVIWSVDQSSKRLSFESLTHLDVNGCWKLKYLMSFTMAKILVNLQSLYVSDCE
KMRSIFLPDQDKEKDIMASIFPKLKNMKLLNMNSLSKIWNLKLPLDSFNKLDTLIIEECHKLGNAMEGIFVSLCNLRVTNC
RSMQAIFNIRDQVGDAANNLQDVHLETLPELKLVWKMNNEDRIGIPKFNNVKKIWAQDCDNLEYIFPFSIAKSLGNLES
LVVCDCYGLSEIVAQREATHTGRARFNFPKLSTVKFSKLPKLTSFYPTAYDLSCPLNELSIELCKNLEPFNKGTQHAERNPI
HVCFPEEVINNLKSMQIESWHAKSPSSYMGKRNHRRDNLEELSLSRLIDTGILYSFLHRSPNLKSLSLSNCYFEKIVPPKED

TEIENLGVVPNLKSLMLIDLLNLKEIGFEPDIILERLEFLILKNCPRMITMAPSSVSFTRLTNLEVINCDGLKSLMSASTAQSL
VQLNTMKVVKCESLVEIVRKDGENSDMVLFQQLKALELVSLKKLKSFCVSDCAFEFPSLEKLVVSACYNMAKFSETVTSS
PLLQNIHVVHGKENKRFCWEGDINATIQKIFEEMKFFEGMEEMSLSQHRELEESWERGTILQKQNSWFYSLKILKLENC
DIEPCAIPSNILPYLRSLKELQVRGCDNVEVIFEMNAKEGTGTTFQLQKLTLSKLPKLEDVWERNGKGTESFQNLKMVHV
SECENLQTVFPLTLAKNLKKLVKLEIIGCHALREIVRKEKNVSAVFVFPCLITLGLFDLPELIYFYPESFTLECSALNKLIVCYCP
DLELFGSANRQSIFFDLKDICNLEELTIDWEQTLVLRTKLGEPTDNLKYLNHIQLFLEVDENERPDLPIQILLKMPNLTKMSI
HYSNCLEVFQTQIPEVVEKRVLTHLKTLRLNCVSKLQSIGSEDSPWLNVICDSENLQKLVVLKCPDLKTLVHSPASVSFTYV
KEVYIDRCKELKYLFTLASVNKLENLERIKVNDCESMEAIVLKEEDDISEEIKLQQLKHIDLNGLSSLECFYSGNDSLQLPSLV
QVDIWKCPKMEFFSRGEIHLNSSFRGIQASNVSSDDFVFYHDLNFSVEKVFLQQEFFQAVDKECFSDNLDLQAEPRGKI
GLQNKWLANLETLKLQNCTQSYAIPSFILCLLKNLKELEVRDSDQVKAIFDMNDDTEIKETESQLKILTLNGLSELTHVWE
KDTHRILIFRNLQEVVVSDCSKLQTLFPASLAKSLKDLKKLKIDSCENLHDFVEQEETTFVTKKFVFPCLEDLELNDLPLVTC
PKTFTLEFPSVKFLSVRNCDELGLFQSVYDPMGEGTSSSRLPLISDPKVISNLEKLTLDCKQILALSLWFKSQKSTEGLTNFN
TISLSFFGIDGNEVPMLPIEILKAPSLIELDMNNCNNIENFLAQNPKIGEEEMLRQLTILRLCNVSTTQFFELEYCLSLNIICE
RLHKLTLSQCPHLTTILRVHSAVSFSCLKELNIYKCPNLKYLFTTSAAKKLMNLEEIRVTECETLTEIVSKEGDASSEGIKFDRL
HTIYLQSLTSLVCFYSGSDSIELSSLKTVAIRSCPNMEIFSHGNESLMGVALSTDQGADDVHPPQDLNTRIKGIIQRKEFFE
AVDKECFSDNIELQEDPHCKFGLQNQWLRDLVSLKLQNCTLLCAIPFSILALLQSLKELEVQDSTTIEVLFYMNDNEIMGI
TSQLRILTLKGLSKLTRVWEKNKNGVLNFSSLEQVVVSNCENLQTLFPASLARNLKSLKGIDIESCVEFQEIVEKEDDTEAK
FVLPCLEELNLSFLPQLTCFYPQTFTLECPTLNLLSVFECERLELFQSEHSMGEGTSVKRQPLISSLEVISNLKELELDWKHIL
ALRSRLRSEKFTGIFKFVNKMNLLLNADVSEMLIVVNEIVHTTPNLIEMIVMIDNCNSMEIFLAQNPKIGEDGMLLQLRT
LNLFRISDIRSNQPENSSWSDTFSEKIHELHVFECPHVETIGVHPTFSVSSSFLKQVFVQKCPQMQYLFTFSVAKELVNLEE
ITVIECESLKEIVSKEGDEDEPKGEGDDKYENEMIFMKLENLILASLDNLESFYSGSCILNFPSLRKVAVNECLNSKIFRHRD
KVPPKFTVIIDEILGKGDKKALVTQQFEEEAS

>XP_027906484.1

MAALIACCQFLTLSPRAKAWLKKQLIQLDLYETRVGKVEDVVEKLKKKRDSIQNTVDEEERRHGRKIHVEVKEWMHKV
DKLILIYRNFHNDEICHKCAVFEFFESGFLPKPGIRYRRSRKADDITKQANGLLQNAKFDILSYWSGPPSMAAFFSNLGYES
YSSRNDTVKKITDEFQKPGVRMIGLHGLSGVGKTSLVKEVVKKALKDKMFEVVTMASMTKNPDVSKIQGQIADMLGV
VLEEESDIARAARIHQILNDENKSTLIILDDLWEEVNFNLLGIPRELEKDDGIINVKGKSLDVNSLKNFSDGKSPVLDGSASF
KKKVKSPDYADSVDVKKGEFFEGALKNVNEGKPPIDASDRAKIEKTVPRYKGCKILMISEIKQVLLNQMEGKEEYIFPVEV
LKEKEAEMLFKKKAGIDAKNSEYDKLAAQIASKCKGLPMTIVTTARALKNKSLSIWDETNRKLESQNLTGAPEFSTRLSYE
LLEDAELKYTFLLCARMGHDALIMNLVKYCIGFGFLRGINTARQTRDKVHTLVAKLKELGMLSDSYSSDHFTMPDTVRR
AALSIAYEENHLFTMTKEKVDEWPDELEQYAAISLHHCNFIEEFPATINYPRLRVLEIVNNISRSKMPKNFFKGVKELRVLI
LTGIHLPLIDSSISSLHKLRMLCLEQCRMQDEELSIIGELNRLRILSFSGSNIKSLPNELNELKMLQIFDISNCSKLKKIPYGVIS
SLVSLEELYMRNTSIQWEDEDQSRQSKITLLSDLKHLNQLTALDIQIPNVSYLPKNLFFDNLYSYKIIIGDLSSFSETDFKMP
EKYEMIKFLAIQLKNGSDIHSLKGIKMLFEGVENLFLELNSVHEKRNSVHEAHNIVHDLFYRLNLKGFPCLKHLWIVNNST
LQSLIHPKDRQEHEKAFPKLESLSLHNVKMDEICSCKLSEPSFGKLKVIKISLCGELKNVFSISMVILLKFLETIEVSECNSLKEI
IDMGPQSNPEKTELLMLPELRHLKLQSLCEFVGFDAIPQTGGEERKLFHEKVRVSKLERLELSSIQIDVIWSVIDQSSKRLS
FENLTHLDVNGCWKLKYLMSLTMAKSLVNLQSLYVSDCEKMRSIFLLEQNREKDVTGNIFPKLKNMKLCSMKSLSQIW
NPKLASDSFSKLDTLIIEECDKLENVMEGIFVSICNLRVTNCMSMQAIFNIREQVGDAANNLQDVHLETLPKLKLVWKM
NYEDRVGIPKFNNLKKIWAQDCHSLEYIFPFSVAMSLDNLESLVVCDCYGLSVIVAERETTNTDRARFNFAKLSTIKFSKLP
ELTSFCPTTYDLSCPLNELSIELCDNLEPFSKGTEHAQRNHGHVFFPKEVINNLKSMQIESWHAKSPSSYMGKRNHRRN
NLEELSLSRLMDTDILYSFLHRNPNLKSLLLNNCFFEKIVPPKEGTEIENLGVVPNLKSLTLIDLPNLKEIGFEPDIILERLELLIL
KNCPCMITIAPSSVSFTRLTNLEVVNCDRLQSLMSASTAKTLVQLKTMKVVKCESMMEIVRKDGEKFDRVVFQQLKTLE
LVSLKKLKSFCVSDCGFEFPSLEKLVVSACYNMAKFSETVTISPILQNIHVVHGKENKRLCWEGDINATIKKIFEEKKFFEG

MEEMSLSQHKELHKSWKRGAGLQEQNSQFYSLKILKLENCVIRPCAIPSNILPYLRSVKELQVRGCNNVRVIFEMNAKE
GTGTTFQLQKLILEQLPKLENVWESNGKGTESFQNLKLVHVSKCEKLQTVFPFTLAKNLKKLVKLEIVSCGGLYEIVKNEG
DTTTMFVLPCLTTLYLCDMPELIYFYRQSFTLDCSALNTLAVVGCPELELFGSANRQSIFFDLKDICNIEVLLLDWQHILVLR
TKLGEPMDNLESLNHIHLCFLVDENERPYLPIQILQKMPNLTQMSIYYCSCLEVFQTQISEIDEKGVLTHLKTLTLDSVSKL
QSIGSEDSPWLNVICDSEKLQELHVIDCPDLKTLVHSTPSVSFTYVKKMYINNCKELKYLFTLSSVNKLENLEHIEINGCES
MEAIVLKEEKDIATEIKLQKLRRMDLTLLPKLEYFYSSNDTLHLPCLTQVGIWMCPKMEFFSGGEIHLNSSFRGIQASNGS
SDDLVFYHDLNSSVEMVFLQQEFFKAVGKECFSDNLELQTDIRCKTGLQNNWLANLETLKLQNCKLSYAIPSSILCLLNNL
KELEVRDSDKVKAIFDMNDDTEIKETESQLKILTLNRLSELTHVWEKDTHRILIFRNLQEVVVSDCSKLPTLFPASLAKCLD
DLKKLKIDFCENLHDFVEQEETTFVTEKFVFPCLEDLELNDLPQVTCPKTFTLEFPSIKFLSVRNCDELGLFQSVYDPMGEG
TSSSRLPLISDPKVISNLEKLTLDCKQILALSLWFKSQKSTEGLTNFNTISLSFFGIDGNEVPMLPNEILKAPNLIELDMNNG
NNIENFLAQNPKIGEEEMLRQLTILRLCNVSTTQFFELEYCSSLNIICERLHKLTLSQCPHLTTILRVHSAVSFSCLKELNIYRC
PNLKYLFTSSAAKDLMNLEEIRVTECETLTEIVSKEGDASSEGIKFDRLHTIYLQSLTSLVCFYSGSDNIELSSLKTVAIRSCPN
MEIFSHGNESLMGVALSTDQGADDVHPPQDLNTRIKGISQRKKFFEAVDKECFSDNIELQEDLQCNFGLQNQWLGDL
VTLKLQNCTLPCAIPSVILSLLKSLKELEVRDSATIEVLFYMNDNEIIKIASQLRILTLERLSKLTQVWEKKKNGVLMFPNLQR
VIVRSCKNLQTLFPASLAKNLRSLKNLKIECCAEFREIVEKEEDTEANFVLPCLEKLALLSLPQLNCFYAHTFTLECPALNKIY
VSDCDKLELFQGADLMGEVTSVNRKPLISSLEVISNLKSLQLDWKHILVLRSRLRSKQFSGVFKFVNEMILVLDGDKSEIPI
VLNEILHTTPNLKKMGMIIDNCNTTDTFLGQYPKIGEDGMLLQLRELTLFCVSAIRTNQSENSSWLNTIFEKVHEMHTFE
CPNVEKIGVHSTSTMSFCFLKEVCAYQCPQFQYLFTSSVAKELVNLKEITVVECESLKEIVAKEEDEDEPKGEGEYKYENE
MIFMKLEELTLVSLDKLESFYTGSSTLNFPSLRKVVVRKCLKAKIFRHPDKVPLKFRVIIDGICCSGDKNALIMQQFEEEAS

>XP_027906485.1

MAALIACCQFLTLSPRAKAWLKKQLIQLDLYETRVGKVEDVVEKLKKKRDSIQNTVDEEERRHGRKIHVEVKEWMHKV
DKLILIYRNFHNDEICHKCAVFEFFESGFLPKPGIRYRRSRKADDITKQANGLLQNAKFDILSYWSGPPSMAAFFSNLGYES
YSSRNDTVKKITDEFQKPGVRMIGLHGLSGVGKTSLVKEVVKKALKDKMFEVVTMASMTKNPDVSKIQGQIADMLGV
VLEEESDIARAARIHQILNDENKSTLIILDDLWEEVNFNLLGIPRELEKDDGIINVKGKSLDVNSLKNFSDGKSPVLDGSASF
KKKVKSPDYADSVDVKKGEFFEGALKNVNEGKPPIDASDRAKIEKTVPRYKGCKILMISEIKQVLLNQMEGKEEYIFPVEV
LKEKEAEMLFKKKAGIDAKNSEYDKLAAQIASKCKGLPMTIVTTARALKNKSLSIWDETNRKLESQNLTGAPEFSTRLSYE
LLEDAELKYTFLLCARMGHDALIMNLVKYCIGFGFLRGINTARQTRDKVHTLVAKLKELGMLSDSYSSDHFTMPDTVRR
AALSIAYEENHLFTMTKEKVDEWPDELEQYAAISLHHCNFIEEFPATINYPRLRVLEIVNNISRSKMPKNFFKGVKELRVLI
LTGIHLPLIDSSISSLHKLRMLCLEQCRMQDEELSIIGELNRLRILSFSGSNIKSLPNELNELKMLQIFDISNCSKLKKIPYGVIS
SLVSLEELYMRNTSIQWEDEDQSRQSKITLLSDLKHLNQLTALDIQIPNVSYLPKNLFFDNLYSYKIIIGDLSSFSETDFKMP
EKYEMIKFLAIQLKNGSDIHSLKGIKMLFEGVENLFLELNSVHEKRNSVHEAHNIVHDLFYRLNLKGFPCLKHLWIVNNST
LQSLIHPKDRQEHEKAFPKLESLSLHNVKMDEICSCKLSEPSFGKLKVIKISLCGELKNVFSISMVILLKFLETIEVSECNSLKEI
IDMGPQSNPEKTELLMLPELRHLKLQSLCEFVGFDAIPQTGGEERKLFHEKVRVSKLERLELSSIQIDVIWSVIDQSSKRLS
FENLTHLDVNGCWKLKYLMSLTMAKSLVNLQSLYVSDCEKMRSIFLLEQNREKDVTGNIFPKLKNMKLCSMKSLSQIW
NPKLASDSFSKLDTLIIEECDKLENVMEGIFVSICNLRVTNCMSMQAIFNIREQVGDAANNLQDVHLETLPKLKLVWKM
NYEDRVGIPKFNNLKKIWAQDCHSLEYIFPFSVAMSLDNLESLVVCDCYGLSVIVAERETTNTDRARFNFAKLSTIKFSKLP
ELTSFCPTTYDLSCPLNELSIELCDNLEPFSKGTEHAQRNHGHVFFPKEVINNLKSMQIESWHAKSPSSYMGKRNHRRN
NLEELSLSRLMDTDILYSFLHRNPNLKSLLLNNCFFEKIVPPKEGTEIENLGVVPNLKSLTLIDLPNLKEIGFEPDIILERLELLIL
KNCPCMITIAPSSVSFTRLTNLEVVNCDRLQSLMSASTAKTLVQLKTMKVVKCESMMEIVRKDGEKFDRVVFQQLKTLE
LVSLKKLKSFCVSDCGFEFPSLEKLVVSACYNMAKFSETVTISPILQNIHVVHGKENKRLCWEGDINATIKKIFEEKKFFEG
MEEMSLSQHKELHKSWKRGAGLQEQNSQFYSLKILKLENCVIRPCAIPSNILPYLRSVKELQVRGCNNVRVIFEMNAKE
GTGTTFQLQKLILEQLPKLENVWESNGKGTESFQNLKLVHVSKCEKLQTVFPFTLAKNLKKLVKLEIVSCGGLYEIVKNEG
DTTTMFVLPCLTTLYLCDMPELIYFYRQSFTLDCSALNTLAVVGCPELELFGSANRQSIFFDLKDICNIEVLLLDWQHILVLR

TKLGEPMDNLESLNHIHLCFLVDENERPYLPIQILQKMPNLTQMSIYYCSCLEVFQTQISEIDEKGVLTHLKTLTLDSVSKL
QSIGSEDSPWLNVICDSEKLQELHVIDCPDLKTLVHSTPSVSFTYVKKMYINNCKELKYLFTLSSVNKLENLEHIEINGCES
MEAIVLKEEKDIATEIKLQKLRRMDLTLLPKLEYFYSSNDTLHLPCLTQVGIWMCPKMEFFSGGEIHLNSSFRGIQASNGS
SDDLVFYHDLNSSVEMVFLQQEFFKAVGKECFSDNLELQTDIRCKTGLQNNWLANLETLKLQNCKLSYAIPSSILCLLNNL
KELEVRDSDKVKAIFDMNDDTEIKETESQLKILTLNRLSELTHVWEKDTHRILIFRNLQEVVVSDCSKLPTLFPASLAKCLD
DLKKLKIDFCENLHDFVEQEETTFVTEKFVFPCLEDLELNDLPQVTCPKTFTLEFPSIKFLSVRNCDELGLFQSVYDPMGEG
TSSSRLPLISDPKVISNLEKLTLDCKQILALSLWFKSQKSTEGLTNFNTISLSFFGIDGNEVPMLPNEILKAPNLIELDMNNG
NNIENFLAQNPKIGEEEMLRQLTILRLCNVSTTQFFELEYCSSLNIICERLHKLTLSQCPHLTTILRVHSAVSFSCLKELNIYRC
PNLKYLFTSSAAKDLMNLEEIRVTECETLTEIVSKEGDASSEGIKFDRLHTIYLQSLTSLVCFYSGSDNIELSSLKTVAIRSCPN
MEIFSHGNESLMGVALSTDQGADDVHPPQDLNTRIKGISQRKKFFEAVDKECFSDNIELQEDLQCNFGLQNQWLGDL
VTLKLQNCTLPCAIPSVILSLLKSLKELEVRDSATIEVLFYMNDNEIIKIASQLRILTLERLSKLTQVWEKKKNGVLMFPNLQR
VIVRSCKNLQTLFPASLAKNLRSLKNLKIECCAEFREIVEKEEDTEANFVLPCLEKLALLSLPQLNCFYAHTFTLECPALNKIY
VSDCDKLELFQGADLMGEVTSVNRKPLISSLEVISNLKSLQLDWKHILVLRSRLRSKQFSGVFKFVNEMILVLDGDKSEIPI
VLNEILHTTPNLKKMGMIIDNCNTTDTFLGQYPKIGEDGMLLQLRELTLFCVSAIRTNQSENSSWLNTIFEKVHEMHTFE
CPNVEKIGVHSTSTMSFCFLKEVCAYQCPQFQYLFTSSVAKELVNLKEITVVECESLKEIVAKEEDEDEPKGEGEYKYENE
MIFMKLEELTLVSLDKLESFYTGSSTLNFPSLRKVVVRKCLKAKIFRHPDKVPLKFRVIIDGICCSGDKNALIMQQFEEEAS

>XP_027906625.1

MTYSNSFRVFDRIKRVIFVCVLLAAIDNNNSNNSSYPVTDFAYHNDSLQIKYDVFVSFRGTDIRQDFLSHLIEAFSQRHIN
AFVDNKIVKGDVLSEALIRAIEGSSISLIIFSQDYASSHWCLSELVKIVECRRTNGQIVLPIFYKVDPSHVRYQKGSYEHAFA
KHQIRYSLSTMQIWRTALTEAANLSGFHSSTFRDEAEFMKEIVKCVLMRLNQVQQVKSRGLVGIGKRIAHVELLLQSEEP
DVRIIGIWGMGGIGKTTIAEEVYNKLCFEYEGCCFLANIREESGRHGIMSLKKKLFSTLLGGEDLKIDTPNGLPQYIERRLR
RMKVLIILDDVNDSDQLEVLAGTHDWFGSGSRIIITTRDKQVLAKEFASIYKVEALNFDESLRLFNLNAFKQNHLESEYHE
LSKKVVNYAKGIPLVLKVLGHLLHGKDKETWESQLERLKKVQNRKVHDMIKLSYNDLDRDEKKIFLDIACFFDGLNLKVK
HINFLLKDHDYSVVAGLERLKDKALITVSQENGVSMHNIIQETAWQIAREESIEDPRSQIQLLDPEDIYHVLNYNKGDEAI
RSIVINLSRIKQLQLNPQVFARMSKLHFLDFYSKGSCSCLGDQGGLYLPQGLESLSNELRYLRWTHYPLESLPSKFSAENLV
ELNLPHSRLKKLWQEAPDLVNLRVLILHSSTRLKELPNFSKATNLKAIDLRFCVGLTSVHSSIFSLRNLEKLDLGGCISLRSLR
SNVHLDSLRYLSLYGCMSLKDFSVTSKNMVKLNLELTGIKQLPSSFGLQSNLQKLRLAYTYIDNLPTSIKHLTRLRHLDLRY
CRELRTLPELPPSLETLDARGCVSLESVMFPFAVSEQLKENKKRVAFWNCLKLDEPSLNAIELNAQINMMKFAHQHFSLF
GDAQSTYVYPGSKVPKWLVHKTTHDDFVIMDLSSVLSPHSSHIGFIFGFVVPEVPFGGLALEFRISTGGEGGGEGSHINVY
VDRPRHGITSDHVYLMYDQACSHYLNSRAKHEPRLKIKVALASRTLTSKYVPLELKAFGISLINTTDFLSFVQNVKFGDNV
PNVPILPKFFCPFCIVVFVGTLNICIRRLV

>XP_027906626.1

MTYSNSFRVFDRIKRVIFVCVLLAAIDNNNSNNSSYPVTDFAYHNDSLQIKYDVFVSFRGTDIRQDFLSHLIEAFSQRHIN
AFVDNKIVKGDVLSEALIRAIEGSSISLIIFSQDYASSHWCLSELVKIVECRRTNGQIVLPIFYKVDPSHVRYQKGSYEHAFA
KHQIRYSLSTMQIWRTALTEAANLSGFHSSTFRDEAEFMKEIVKCVLMRLNQVQQVKSRGLVGIGKRIAHVELLLQSEEP
DVRIIGIWGMGGIGKTTIAEEVYNKLCFEYEGCCFLANIREESGRHGIMSLKKKLFSTLLGGEDLKIDTPNGLPQYIERRLR
RMKVLIILDDVNDSDQLEVLAGTHDWFGSGSRIIITTRDKQVLAKEFASIYKVEALNFDESLRLFNLNAFKQNHLESEYHE
LSKKVVNYAKGIPLVLKVLGHLLHGKDKETWESQLERLKKVQNRKVHDMIKLSYNDLDRDEKKIFLDIACFFDGLNLKVK
HINFLLKDHDYSVVAGLERLKDKALITVSQENGVSMHNIIQETAWQIAREESIEDPRSQIQLLDPEDIYHVLNYNKGDEAI
RSIVINLSRIKQLQLNPQVFARMSKLHFLDFYSKGSCSCLGDQGGLYLPQGLESLSNELRYLRWTHYPLESLPSKFSAENLV
ELNLPHSRLKKLWQEAPDLVNLRVLILHSSTRLKELPNFSKATNLKAIDLRFCVGLTSVHSSIFSLRNLEKLDLGGCISLRSLR
SNVHLDSLRYLSLYGCMSLKDFSVTSKNMVKLNLELTGIKQLPSSFGLQSNLQKLRLAYTYIDNLPTSIKHLTRLRHLDLRY

CRELRTLPELPPSLETLDARGCVSLESVMFPFAVSEQLKENKKRVAFWNCLKLDEPSLNAIELNAQINMMKFAHQHFSLF
GDAQSTYVYPGSKVPKWLVHKTTHDDFVIMDLSSVLSPHSSHIGFIFGFVVPEVPFGGLALEFRISTGGEGGEGSHINVY
VDRPRHGITSDHVYLMYDQACSHYLNSRAKHEPRLKIKVALASRTLTSKYVPLELKAFGISLINTTDFLSFVQNVKFGDNV
PNVPILPKFFCPFCIVVFVGTLNICIRRLV

>XP_027906627.1

MTYSNSFRVFDRIKRVIFVCVLLAAIDNNNSNNSSYPVTDFAYHNDSLQIKYDVFVSFRGTDIRQDFLSHLIEAFSQRHIN
AFVDNKIVKGDVLSEALIRAIEGSSISLIIFSQDYASSHWCLSELVKIVECRRTNGQIVLPIFYKVDPSHVRYQKGSYEHAFA
KHQIRYSLSTMQIWRTALTEAANLSGFHSSTFRDEAEFMKEIVKCVLMRLNQVQQVKSRGLVGIGKRIAHVELLLQSEEP
DVRIIGIWGMGGIGKTTIAEEVYNKLCFEYEGCCFLANIREESGRHGIMSLKKKLFSTLLGGEDLKIDTPNGLPQYIERRLR
RMKVLIILDDVNDSDQLEVLAGTHDWFGSGSRIIITTRDKQVLAKEFASIYKVEALNFDESLRLFNLNAFKQNHLESEYHE
LSKKVVNYAKGIPLVLKVLGHLLHGKDKETWESQLERLKKVQNRKVHDMIKLSYNDLDRDEKKIFLDIACFFDGLNLKVK
HINFLLKDHDYSVVAGLERLKDKALITVSQENGVSMHNIIQETAWQIAREESIEDPRSQIQLLDPEDIYHVLNYNKGDEAI
RSIVINLSRIKQLQLNPQVFARMSKLHFLDFYSKGSCSCLGDQGGLYLPQGLESLSNELRYLRWTHYPLESLPSKFSAENLV
ELNLPHSRLKKLWQEAPDLVNLRVLILHSSTRLKELPNFSKATNLKAIDLRFCVGLTSVHSSIFSLRNLEKLDLGGCISLRSLR
SNVHLDSLRYLSLYGCMSLKDFSVTSKNMVKLNLELTGIKQLPSSFGLQSNLQKLRLAYTYIDNLPTSIKHLTRLRHLDLRY
CRELRTLPELPPSLETLDARGCVSLESVMFPFAVSEQLKENKKRVAFWNCLKLDEPSLNAIELNAQINMMKFAHQHFSLF
GDAQSTYVYPGSKVPKWLVHKTTHDDFVIMDLSSVLSPHSSHIGFIFGFVVPEVPFGGLALEFRISTGGEGGEGSHINVY
VDRPRHGITSDHVYLMYDQACSHYLNSRAKHEPRLKIKVALASRTLTSKYVPLELKAFGISLINTTDFLSFVQNVKFGDNV
PNVPILPKFFCPFCIVVFVGTLNICIRRLV

>XP_027906629.1

MTYSNSFRVFDRIKRVIFVCVLLAAIDNNNSNNSSYPVTDFAYHNDSLQIKYDVFVSFRGTDIRQDFLSHLIEAFSQRHIN
AFVDNKIVKGDVLSEALIRAIEGSSISLIIFSQDYASSHWCLSELVKIVECRRTNGQIVLPIFYKVDPSHVRYQKGSYEHAFA
KHQIRYSLSTMQIWRTALTEAANLSGFHSSTFRDEAEFMKEIVKCVLMRLNQVQQVKSRGLVGIGKRIAHVELLLQSEEP
DVRIIGIWGMGGIGKTTIAEEVYNKLCFEYEGCCFLANIREESGRHGIMSLKKKLFSTLLGGEDLKIDTPNGLPQYIERRLR
RMKVLIILDDVNDSDQLEVLAGTHDWFGSGSRIIITTRDKQVLAKEFASIYKVEALNFDESLRLFNLNAFKQNHLESEYHE
LSKKVVNYAKGIPLVLKVLGHLLHGKDKETWESQLERLKKVQNRKVHDMIKLSYNDLDRDEKKIFLDIACFFDGLNLKVK
HINFLLKDHDYSVVAGLERLKDKALITVSQENGVSMHNIIQETAWQIAREESIEDPRSQIQLLDPEDIYHVLNYNKGDEAI
RSIVINLSRIKQLQLNPQVFARMSKLHFLDFYSKGSCSCLGDQGGLYLPQGLESLSNELRYLRWTHYPLESLPSKFSAENLV
ELNLPHSRLKKLWQEAPDLVNLRVLILHSSTRLKELPNFSKATNLKAIDLRFCVGLTSVHSSIFSLRNLEKLDLGGCISLRSLR
SNVHLDSLRYLSLYGCMSLKDFSVTSKNMVKLNLELTGIKQLPSSFGLQSNLQKLRLAYTYIDNLPTSIKHLTRLRHLDLRY
CRELRTLPELPPSLETLDARGCVSLESVMFPFAVSEQLKENKKRVAFWNCLKLDEPSLNAIELNAQINMMKFAHQHFSLF
GDAQSTYVYPGSKVPKWLVHKTTHDDFVIMDLSSVLSPHSSHIGFIFGFVVPEVPFGGLALEFRISTGGEGGEGSHINVY
VDRPRHGITSDHVYLMYDQACSHYLNSRAKHEPRLKIKVALASRTLTSKYVPLELKAFGISLINTTDFLSFVQNVKFGDNV
PNVPILPKFFCPFCIVVFVGTLNICIRRLV

>XP_027906630.1

MTYSNSFRVFDRIKRVIFVCVLLAAIDNNNSNNSSYPVTDFAYHNDSLQIKYDVFVSFRGTDIRQDFLSHLIEAFSQRHIN
AFVDNKIVKGDVLSEALIRAIEGSSISLIIFSQDYASSHWCLSELVKIVECRRTNGQIVLPIFYKVDPSHVRYQKGSYEHAFA
KHQIRYSLSTMQIWRTALTEAANLSGFHSSTFRDEAEFMKEIVKCVLMRLNQVQQVKSRGLVGIGKRIAHVELLLQSEEP
DVRIIGIWGMGGIGKTTIAEEVYNKLCFEYEGCCFLANIREESGRHGIMSLKKKLFSTLLGGEDLKIDTPNGLPQYIERRLR
RMKVLIILDDVNDSDQLEVLAGTHDWFGSGSRIIITTRDKQVLAKEFASIYKVEALNFDESLRLFNLNAFKQNHLESEYHE
LSKKVVNYAKGIPLVLKVLGHLLHGKDKETWESQLERLKKVQNRKVHDMIKLSYNDLDRDEKKIFLDIACFFDGLNLKVK

HINFLLKDHDYSVVAGLERLKDKALITVSQENGVSMHNIIQETAWQIAREESIEDPRSQIQLLDPEDIYHVLNYNKGDEAI
RSIVINLSRIKQLQLNPQVFARMSKLHFLDFYSKGSCSCLGDQGGLYLPQGLESLSNELRYLRWTHYPLESLPSKFSAENLV
ELNLPHSRLKKLWQEAPDLVNLRVLILHSSTRLKELPNFSKATNLKAIDLRFCVGLTSVHSSIFSLRNLEKLDLGGCISLRSLR
SNVHLDSLRYLSLYGCMSLKDFSVTSKNMVKLNLELTGIKQLPSSFGLQSNLQKLRLAYTYIDNLPTSIKHLTRLRHLDLRY
CRELRTLPELPPSLETLDARGCVSLESVMFPFAVSEQLKENKKRVAFWNCLKLDEPSLNAIELNAQINMMKFAHQHFSLF
GDAQSTYVYPGSKVPKWLVHKTTHDDFVIMDLSSVLSPHSSHIGFIFGFVVPEVPFGGLALEFRISTGGEGGEGSHINVY
VDRPRHGITSDHVYLMYDQACSHYLNSRAKHEPRLKIKVALASRTLTSKYVPLELKAFGISLINTTDFLSFVQNVKFGDNV
PNVPILPKFFCPFCIVVFVGTLNICIRRLV

>XP_027906631.1

MTYSNSFRVFDRIKRVIFVCVLLAAIDNNNSNNSSYPVTDFAYHNDSLQIKYDVFVSFRGTDIRQDFLSHLIEAFSQRHIN
AFVDNKIVKGDVLSEALIRAIEGSSISLIIFSQDYASSHWCLSELVKIVECRRTNGQIVLPIFYKVDPSHVRYQKGSYEHAFA
KHQIRYSLSTMQIWRTALTEAANLSGFHSSTFRDEAEFMKEIVKCVLMRLNQVQQVKSRGLVGIGKRIAHVELLLQSEEP
DVRIIGIWGMGGIGKTTIAEEVYNKLCFEYEGCCFLANIREESGRHGIMSLKKKLFSTLLGGEDLKIDTPNGLPQYIERRLR
RMKVLIILDDVNDSDQLEVLAGTHDWFGSGSRIIITTRDKQVLAKEFASIYKVEALNFDESLRLFNLNAFKQNHLESEYHE
LSKKVVNYAKGIPLVLKVLGHLLHGKDKETWESQLERLKKVQNRKVHDMIKLSYNDLDRDEKKIFLDIACFFDGLNLKVK
HINFLLKDHDYSVVAGLERLKDKALITVSQENGVSMHNIIQETAWQIAREESIEDPRSQIQLLDPEDIYHVLNYNKGDEAI
RSIVINLSRIKQLQLNPQVFARMSKLHFLDFYSKGSCSCLGDQGGLYLPQGLESLSNELRYLRWTHYPLESLPSKFSAENLV
ELNLPHSRLKKLWQEAPDLVNLRVLILHSSTRLKELPNFSKATNLKAIDLRFCVGLTSVHSSIFSLRNLEKLDLGGCISLRSLR
SNVHLDSLRYLSLYGCMSLKDFSVTSKNMVKLNLELTGIKQLPSSFGLQSNLQKLRLAYTYIDNLPTSIKHLTRLRHLDLRY
CRELRTLPELPPSLETLDARGCVSLESVMFPFAVSEQLKENKKRVAFWNCLKLDEPSLNAIELNAQINMMKFAHQHFSLF
GDAQSTYVYPGSKVPKWLVHKTTHDDFVIMDLSSVLSPHSSHIGFIFGFVVPEVPFGGLALEFRISTGGEGGEGSHINVY
VDRPRHGITSDHVYLMYDQACSHYLNSRAKHEPRLKIKVALASRTLTSKYVPLELKAFGISLINTTDFLSFVQNVKFGDNV
PNVPILPKFFCPFCIVVFVGTLNICIRRLV

>XP_027906632.1

MQCVVVNPTMSFQVSHKIKYVAFMCLVFVTSSICSNDTAQIKYDVFVSFRGEDVRRGFLSHLIELFSQKQIAFFVDDGIP
KGEEISEALFGAIEESYISLVIFSENYAFSRWCLSELEKILECRRKNGQTVMPIFYKVDPSDVRHQRRTYGDAFVKHETKYSL
TTVQTWRSALSESANLSGFHLSNFRDEAELVKGIVKFVWRTLNHVHHVNSKGLVGIGKRIADVESLMELEATDVRMIGI
WGMGGIGKTTIAQEVYDKMCFKYDSCCFLANIREESGRHGMNILKEKLFSKLLREEHLNIGTPDGFQKLVERRLHRMKV
LIVLDDVDDSEQLEKLARTDWFGYGSRIIVTTRDKQVLATESANVYHAEALNFDESLRLFNLNAFKKKHVEAEYEELSKKA
VDYAKGIPLVLKVLGHRLHGKDKEIWESELEREGVHNKKVHDIIKSSYYDLEEDEKRMFLDIACFFYGQQLQVKYIDFLLK
DRDYSVVAGLERLKDKALISISQENTVSMHDIIRETAWQIAGQESIEDPRSQVRLFDPDDIYRVLTYNKGNEAIRSIVVNLL
RIKQLHLKPQVFTKMSKLHFLNIYTAGTRDIRFYEPWGLYLHQGLESLPNELRYLGWAHYPLESLPSNFSAENLVELHLPY
SRLKKLWQEVPDLVNLRVLMLYSSSNIKELPDFSKAPNLEVIDLRSCVGLTSVHPSIFSLKKLEILDLEDCRSLTSIRSNVHM
DSLRYLSLYKCMELKDFSVTSKNMIMVNLEHTSIKQLPSSMGSQSKLEKINLAFSYIESLPASMKDLKGLRHLDLRHCRNL
SSLPELPPSIETLDARECVSLESVTFPSITKQWNENKKKIVFWNCLNLDERSLKAIETNARINMVKFAHRHLSTSGDAQAIY
VYPGSQVPEWLTHKTTLYDDDDDDDDYISFAPHSSHSGYILCFILPGVQYAERVLKLKVSTEGEGEDEGDSTIVYLDRPH
HSIKSDHVYLMYNQECSRFLTSRAKQQPMLKIKVTVASLGFSFEYIQVQLRGFGVSTISNFLQNQQLCDAAKEHCEVCP
DAL

>XP_027906633.1

MQLEATDVRMIGIWGMGGIGKTTIAQEVYDKMCFKYDSCCFLANIREESGRHGMNFLKEKLFSKLLREENMNIGTPD
GFQKLVERRFHRMKVLIVLDDVDDSEQLEKLARTDWFVYGSRIIVTTRDKQVLATESANVYHAEALNFDESLRLFNLNAF

KKKHVEAEYEELSKKAVNYAKGISLVLKVLGHRLHGKDKEIWESELEREGVHNKKVHDIIKSSYYDLEEDEKRMFLDIACFF
YGQQLQVKYIDFLLKDRDYSVVAGLERLKDKAFISISQENTVSMHDIIRETAWQIAGQESIEDPRSQVRLFDPDDIYRVLT
YNKGNEAIRSIVVNLLRIKQLHLKPHVFTKMSKLHFLNIYTAGTRDIRFYEPWGLYLHQGLESLPNELRYLGWAHYPLESL
PSNFSAENLVELHLPYSRLKKLWQEVPVNNIRIPFNLKLTLIQAKS

>XP_027906666.1

MSSSTKKHDVFVSFRGEETRANFTSHLYKALEDKSIGAYIDCQLDRGVDVWRGLAKAIQDSHVSIVVFSENYACSKWCL
DELVEILECRKKLGLVVIPIFYNVDPSDIRNQKGTFEKELTELLESNEEKGPKWKAALTEAANISGWDARTHRDEAHVIEN
VVNDVLQKLHLRCPTELKGLVGNEENCRNVELLLKSCRVIGIWGMGGIGKSTIAKILFAKHFPQYDHVCFVTNAKEYSLD
KFFSTILKEEVSAKNVVGSSFHMRRLRSKKVFIVLDDVDMDSFEPLEYLCGEYEGQHGDSKLVITTRDRQLLVGRVDAIYE
VQKWKKTESLKLFCSEAFKKSYPEGGYESLSESAVEYAGGVPLALKVLGSYLRSKGICFWESTIRKLSFYPNERIQKVLEVSY
TALHDLEKNIFLDIVFFFREKQKDHVTRILDACGFEATSGIEVLVDKAMLTISYRNIQMHDLLQQMGLEIVRQECTADPG
RRSRLKDNEARQVIEENKGTDAVQGIELDLSQVQNLRLRSDTMAKMKSLRFLRFYNSSGQSSRNTYLDLPTTLEPFSDQL
RYIEWIGYPFECLPSPFCAKFLVEIHMKHGKVKQLWQGIQELDNLVGIDLSGCKQFEELPDLSKAPRLKWVNLSCCESLR
YLHSSVLSSAALVTLILNGCTKLQSVKGEKRLKSLEKISVNGCSSLEEFAVSLDLFKMFNLSHKGIQMPGTPVRRKGKRKSH
NLEEDLRPLTSLKEIKLSYCLQRDMDWIKYNLPSLQHMYMLKLDQCKVTTLPENIKNLGNLRVLSLENCDELLRLPKLPSSI
NYFSAINCTSLVSVSDLVNLANEMWGSTRFLTFKNCLKLDEHSCKLIMKSVQLVMVCAAFDNMVRKSSDLHDYSYNSV
QLCLPGSKVPQEIKYRSTESFITIDLPKLSNLRGFIYSVVLSPSGETKKHGTKIICKRHLRENTRESWVSSDIEGLNTDHVYIW
YDPFHCDGILKYNAPSVCFEFCVTNDKGEVDDSMCIKECGVGLISVSELPSVLEELDWDSDKKKDLVKRVELITGQRITLT
SIEQSDEEENNGMKNQMGNQQGNLSEHSHKIVGTELDSKMVDSDSGEEGNERKNHYSAIEEIINSTQKEVKTDSVSNY
RQNTESANVVKYEGNTERPIKQDATLHDTVESELDKENESKEKSMMVELDSVEDNRGSKEYPSSVEESIEYSTCSATKTT
AKRGPKEKSKKSTEIVANEHSQNAPLQASSEGDSEDLHNRLEESIKQVVETHDTGNFATKYSSLDLENCLQQLNENPFAI
LDLLSYELYPSLKQLETCVQKVAQANDATTVLNEFRTLVFSNSLLKKLQDQSYRQQIAESLQKLHTYRREITKEQEAGLDKF
LELYNKAVDISQDKMLTEDKQAKLAYEKRDLYNKLQNSKLKVQQFDTTISTCKSQRENLKKRQREIQEAIKKLQLENEALE
KESTTLEVLYSEQQTKKNETLESVKCISISVVQTTKQLEELEEKSLSLASAYEGLKEPYERMKTKPPF

>XP_027906667.1

MSSSTKKHDVFVSFRGEETRANFTSHLYKALEDKSIGAYIDCQLDRGVDVWRGLAKAIQDSHVSIVVFSENYACSKWCL
DELVEILECRKKLGLVVIPIFYNVDPSDIRNQKGTFEKELTELLESNEEKGPKWKAALTEAANISGWDARTHRDEAHVIEN
VVNDVLQKLHLRCPTELKGLVGNEENCRNVELLLKSCRVIGIWGMGGIGKSTIAKILFAKHFPQYDHVCFVTNAKEYSLD
KFFSTILKEEVSAKNVVGSSFHMRRLRSKKVFIVLDDVDMDSFEPLEYLCGEYEGQHGDSKLVITTRDRQLLVGRVDAIYE
VQKWKKTESLKLFCSEAFKKSYPEGGYESLSESAVEYAGGVPLALKVLGSYLRSKGICFWESTIRKLSFYPNERIQKVLEVSY
TALHDLEKNIFLDIVFFFREKQKDHVTRILDACGFEATSGIEVLVDKAMLTISYRNIQMHDLLQQMGLEIVRQECTADPG
RRSRLKDNEARQVIEENKGTDAVQGIELDLSQVQNLRLRSDTMAKMKSLRFLRFYNSSGQSSRNTYLDLPTTLEPFSDQL
RYIEWIGYPFECLPSPFCAKFLVEIHMKHGKVKQLWQGIQELDNLVGIDLSGCKQFEELPDLSKAPRLKWVNLSCCESLR
YLHSSVLSSAALVTLILNGCTKLQSVKGEKRLKSLEKISVNGCSSLEEFAVSLDLFKMFNLSHKGIQMPGTPVRRKGKRKSH
NLEEDLRPLTSLKEIKLSYCLQRDMDWIKYNLPSLQHMYMLKLDQCKVTTLPENIKNLGNLRVLSLENCDELLRLPKLPSSI
NYFSAINCTSLVSVSDLVNLANEMWGSTRFLTFKNCLKLDEHSCKLIMKSVQLVMVCAAFDNMVRKSSDLHDYSYNSV
QLCLPGSKVPQEIKYRSTESFITIDLPKLSNLRGFIYSVVLSPSGETKKHGTKIICKRHLRENTRESWVSSDIEGLNTDHVYIW
YDPFHCDGILKYNAPSVCFEFCVTNDKGEVDDSMCIKECGVGLISVSELPSVLEELDWDSDKKKDLVKRVELITGQRITLT
SIEQSDEEENNGMKNQMGNQQGNLSEHSHKIVGTELDSKMVDSDSGEEGNERKNHYSAIEEIINSTQKEVKTDSVSNY
RQNTESANVVKYEGNTERPIKQDATLHDTVESELDKENESKEKSNSVEDNRGSKEYPSSVEESIEYSTCSATKTTAKRGPK
EKSKKSTEIVANEHSQNAPLQASSEGDSEDLHNRLEESIKQVVETHDTGNFATKYSSLDLENCLQQLNENPFAILDLLSYE
LYPSLKQLETCVQKVAQANDATTVLNEFRTLVFSNSLLKKLQDQSYRQQIAESLQKLHTYRREITKEQEAGLDKFLELYNK

AVDISQDKMLTEDKQAKLAYEKRDLYNKLQNSKLKVQQFDTTISTCKSQRENLKKRQREIQEAIKKLQLENEALEKESTTL
EVLYSEQQTKKNETLESVKCISISVVQTTKQLEELEEKSLSLASAYEGLKEPYERMKTKPPF

>XP_027906709.1

MASSIPSMEFTALSSSLPRNYDVLINFTGEDIHRKFVSHLDSALSAVGFTTFLHHQNAVKPMHIQEPIVNLCRVAIVVFTK
TYSESAWCLHQLQQIVKWHETCCQHVLPVYYEIAPSDVRLQQGDFGKAFKATAHQTFSEQELEHGMSRWSHALTKVA
NCFGWDESNYRSDAEVVNKIVKSLLNLSVFSATKFPVGLQSRVENVIRTIKSKSTKVCIIGICGKGGSGKTTLAKAIYNQIQI
TFRDKSFIEDITRVSGIRGTLPLQEQLLLDVLKTRVEIPSLDMGSRMIQERLSGKKVLIVLDDVPEFCKLLDLLKCRNWFSG
GTVIIVTAEDLELLEKHRVDYVFRMKPMNDNESLELLSWHALREAKPKQKEHDYLARRIAIYCLRLPLILEVIGSNLFERTK
EEWYGVLRDLEKIGSPSVENKLNICFKGLQNQMVKDLFLYVSRFFVGKGRNYATKILNGCQIDVDIGIRVLLERNLLKVTK
NNKFGMHPLLQEMGLTIIREISEKDPSKNRPWLFDKDTKYGTKATQLLLQKLDQVEMSLNPEYLLQKLRWISPYGFYSEY
LDNKFCADDAIAFDQFAPQVLRSLKVLNLSHSNYLTTTPDFTRLPCLEQLILKCCRRLRKVHQSIGCLCNLLLLDLKDCTSLS
NLPKEIYKLKSLRTLNLSGCSKIDLMDKDIAQLESLTILIAENTTVKHVPFSVISSKSIGYICLRRFEGLSHNLFPSIIRSWMSQ
NMNLISYMHLFCMDKEDNSWDDIVPSVSSSLRNLRSVLVQCNTEFQLSKQVKSILVEYFANTTESGISKHRVRSSLIGVG
RCNEFFNSVSYSISEVLRSSEWCEVSLPTVNDPCCLAHMGEGHSVSFIMPQDRDMKGMALCVVYLSTPEVIEPEFTTILI
VNHTKCTFHIHNLDTIICFNDEDWNGIRSNLGCGDRVEMFVSFGNGLVVKKTAVYLICGESKNMEKASEPKKHSLTRFIK
KVVM

>XP_027906712.1

MEFASSSSKLSRKYDVLINFNGEDIRRKFVSHLDSAFSAVGLTTFLPHDNAMKSTHIQQPIVDLCRIAIVVFTKTYSQSARC
LHQLQQIIEWHETYCRHVLPVYYETQPSDVRLQKGDFGEAFKATAHQTFSGQELEHGMSRWSYALTKAANFFGLDDS
NYRSDAELVEKIVKSVLNLPILSAAKFPVGLQSRVEDVIQIMEKKSWRVFTIVIFGRGGSGKTTLAKAIYNQIHGTFTEKSFI
EDIAQVRRNRAYAHLQKLLSDVLKTKVEMHSFEMGANMIRERLSGKRMLIVLDDLNEYTPFLELCACFGGGTVVIITTRN
ESIRNIYPVGSVFRIKLLNANESLELLSWNAFREAKPKEEYNDLAKSVAFHCGGLPLALEIIGNCLFQSTKEEWNSILCKLAR
IPQHHVEPKLKISFEGLRNQMEKDLFLDVCCSFVGEGRTFVKKILNGCGIYDDDSGIRVLIERGLVKVKKNNKLGMHHLL
QKMGRQIIHEIDKDEMWKERQMRFDGAGYVLTNNRGTRVTDRLPVKLRSTRREPSRLQKLDGNSEYHSKKLRWISLQ
GFHENYQIFNEFSLHDAIAIDLDHNLLQFDWKQPQVLRWLKVLNLSHSKYLTETPDFSGLQSLEQLFLKNCQRLRKVHRS
IGCLCYLILLNLKNCTSLSNLPREIYTLNSLRTLILSGCSKIDLMEKDIVRMKSLITLIAENTVVKQVPFSIVSSKAIGYISLRGFE
RFSCNLFPSIIIRS

>XP_027906713.1

MFCLYITKLSHLMYVFRRVILERPSKQLHTKHSRDKNWSMVCPGGATHSPKLQISLDWMTAITVGLQSRVEDVIQIMEK
KSWRVFTIVIFGRGGSGKTTLAKAIYNQIHGTFTEKSFIEDIAQVRRNRAYAHLQKLLSDVLKTKVEMHSFEMGANMIRE
RLSGKRMLIVLDDLNEYTPFLELCACFGGGTVVIITTRNESIRNIYPVGSVFRIKLLNANESLELLSWNAFREAKPKEEYNDL
AKSVAFHCGGLPLALEIIGNCLFQSTKEEWNSILCKLARIPQHHVEPKLKISFEGLRNQMEKDLFLDVCCSFVGEGRTFVK
KILNGCGIYDDDSGIRVLIERGLVKVKKNNKLGMHHLLQKMGRQIIHEIDKDEMWKERQMRFDGAGYVLTNNRGTRV
TDRLPVKLRSTRREPSRLQKLDGNSEYHSKKLRWISLQGFHENYQIFNEFSLHDAIAIDLDHNLLQFDWKQPQVLRWLK
VLNLSHSKYLTETPDFSGLQSLEQLFLKNCQRLRKVHRSIGCLCYLILLNLKNCTSLSNLPREIYTLNSLRTLILSGCSKIDLME
KDIVRMKSLITLIAENTVVKQVPFSIVSSKAIGYISLRGFERFSCNLFPSIIIRS

>XP_027906787.1

MECLFGFASSVARDLVCGALNQLRYPCCFNNFVKKLEQEKDDLIVTRDSVQKFVTHTKRQARKTSEVVDKWLQDAISD
NVDQMLEEARTKKGCCFGHCPNWIWRYRVGKKLANKTVDLEKFIEEGRKYVPFDRIATLPLGTLDVLSEKCMNFESRQ
SAYKQLLDAVKSNDVAMIGLYGMGGCGKTTLAMDIRKSVEAEHLFDKVLSVPISRTVEVRRIQEKIASSLQFVFPESEEM

ERAQRLCLRLTQKEKFLIILDDVWQKLDFGAIGIPSPEHRSSCKILIITRLEDVCISMDCQRKIFLRVLTDEEAWTLFQSKAFI
SENTPDTIKHLGRLISNECKGLPVAIAAVASSLKEKAETIWRVALTRLRSSKPINIQKGLADPYECLQLSYDNLDTKEAKSLF
LLCSVFPEDSAIQVEHLARCAMGLGLVEEAQSYEEARNEVLAAKIKLISCCLLLDADDECVKMHDLLRDVAHLIAKNENKI
IRCELEKYVTVEQNSVRYLWCAKFPNDLDCSNLEFLCLYTKLEELDGIFERMGMLKVLILIDKEEGEIPLSTMFLKTLTNLRY
LLIDGYELNDFSFLSYMKNLQSLSLHRCSLSSLPELQTDAAITQLTTLKLLELYKCDIKVKNFEVIKRIPLLEELYIVDIKGEWD
TKSEDTVEFFNTFSVPQTLQRYGIVLGSHNFAFSHHRTLVFNHFDISNEVIKGLAKKAENLYVANIHGGVKNIIPDIFQIEG
ECLDELNKFEIRDSKEIECLIDTNNNSSEEITLLSKLHMLIIDNMENLRVIWHCFQPVNGPFGNLEKLYLSDCPQLTSLFTYV
VARSLVQLKILVISKCDGLKHILAGDDKMEKIQDEYSNENPIQIFQSLQKVKINRCKELKHIFSANIVGGLGQLKVLKIEKCH
KLDQIIGDIVPLEDQDQKEEVDEIIEEGTISSLASLKKNKANLNGIFPNLRKLSVRDCGQLKYMLGQYDVANQDSKEIHIQF
SALERLSLHNLPNFVSICSTNTLIVTCPSLKDFDCYRCFYRFYDSDPKWIQNHLPTLQTLSVRYSEVECIFCLNGHGMIGQQ
VNLRLSHLSFENLPQMTYIWVAFKNSVTLQHLTTLEITRCAKLEVIFPPSVLRSLPELKFLTIIECMELKQIIEEDEDDGKLSN
LISTQPCFPKLESLYVEECHKLKCLSSSSNNLPDLRILTIIGASELKEIIGCEQGASDNSSTFPNLGKKMSNLLSTQPCFPKLES
LYIQDCHKLKCLSSSSNDLRNLRFLTINGASELEEIIICEQGASRNSLTFSNLGTLKLSGCAKLEVIFPKSVLGCLPELKSLIITEC
TNLKQIVGCEQGASKYSFTFPSLERLEIIGCAKLEVIFSWSVLRCLPELNLLKIINCTELRQIIEDDVEDKKLSNLLSRQPCFPK
LEAMHVDHCQKLKRFFPGSTSNYLPNLHLLIINGASELEELVGCKQGKCDEIRNTEVELPRLKLLIFFHLSSFCQEIELPNLN
NSIVYECPNFSFTSTTTIGKLCQNFPHEDFKNTALYMWDIVRVIRNCDEDSTVGGSNEFTSSEESHSEGGLADQHALEET
TEIGDIGNGSIKDGPASECAKTKSSSTGVEDISIGGGVATHIESSEDSKLVEQDDTAQTQIDENSKRSSSMDMVRHYEVV
EQTDGPNEKWERPSLHLKVEHVNSTFGEGSNLVDKEGEIGVVSGDCIVPARNEEPKREFVAKVSTSETSRIAPPLTNSQP
VERPTPSCSDISLRHTLSNSIERVVVEETTAKNTNMTTSSIHFSEIKSSQTEALNAKESEEHPNKIIQDFGANDMMSLFAPV
VVGKEGEDNLVGKTLAELEKYLKMPLKDIVSSETNALRLLSALNFLSNLPFKDQTVSDGLQHIIGTMHQEFPTILCSFKQC
FATTNKLAELEARANEVTIKRNLYEEAQRKEVVLKEQIIRLKEEIRVCEVALSSLEEEKNKCIAETVGYKTELQNVRKYESQ
MLEVAYKWSVLCSQYQLNRMPARNPS

>XP_027906849.1

MEIATSSSSSSFLKSEPHFINDVFINFREEEISRKFVWHLKNVLLQAQVKTLVEEENLQERMKLEEHMGAIACSKIAIIVFSE
TYSESALCLRELEKIIECHETFGQIVMPVFYQVNPFDVCTKCDFGKALEEAAEESYSEEQLKHALSRWSHALTKAALITGW
DVKDFRHDGELVEVIVSRIQTLLDYKDLFITQFPVGLESRVEKVIGCIENHSTKVCMIGIWGMGGSGKTTIAKAIYNRIYRI
FIGKSFIENIREVYDGVSRSNIHLQEHLLYDVLKSKLQLESISLGRTKIENELSRKRLLIVLDDVNEFDQLDSLFGKDEWFGQ
GSVIIITTRDVRHLRRNNVDYVYKTEEMNETESLELFSWHAFGEEKPIEDFNDVARNVVGYCGGLPLALEVLGSYLSERTK
KEWKSVFSKLKIIPNTKVQEKLRISFDDLRDEVEQEIFLDICCFFIGKERGYVTEILNGCGLRGDIEIRVLIERALISVERNNKL
GMHPLLRDMGREIIRERWPVETENRSRLWFDDDVKHILTNNTGTEATEGLSLKLHSTSTDCFKTRAFKEMKRLRFLQLD
NVQLAGEYGYLSKQLRWICWQGFPFKYIPNNFHMKSVIAIDLKHSYLHLVWKKAQILEWLKILDLSHSMYLRETPDFSRL
PSLEQLILEDCPSLCKVHPSIGDLGNLQLINLKDCTSLSNLPREVYKLKSLTTFVLSGCLKIDILEEDIAQMESLIILVAENTAV
KQIPFSIVSSQSTGYIFPEGFESEISHFIIQSWMSPTLNPHSSCLYWMEIEDYNWHVHGPLHTGLANLRSILVKCDTEFKLS
FLVKSIVVEYGVNFTESRISNQRLRRFSLIGVGRYNEFLNTLSTRVSEELASSESCEFSVPGDNHPYWFAHMGNGYSVSFT
VPQDCDIKGMILCFVYLSTPVIIATECVTSVLIVNYTKCTLHMHNHDTVNSLNDEDWRGIISNLGPGDKVEIFLTFGHGLV
VKNTAVYLIYGESNDMEIEPCRAKGKCSA

>XP_027906860.1

MDCLGPFGKPPRAMINFIWEHGVRQMTYIIYYKQNVLQLSSSLKDLGFEKEKVDHQCDEAEKNLNKVERKVTEWVEKV
SEIETTVAKYENRNGHKRARSPNCFIFPYLWNRHKLGRQAKKLEVNVTKLIDDCPKFDEVAYRENVTSNDTTLSNYGFIEF
GSTKSTMDKVMRQLEDSTVRMIGLYGPGGVGKSTLVKEIARKAKENKLFDVMAVVEITANPNPQKIQEEIAYVLGLKLE
GKGENVRADCLRRRLKREKGNTLLIFDDLWDKLDLNKLGIPLDEDEDDDDDFNSGNKDLSRKVFKKKKSXGGHKGCKIL
LTSRDKKVLCIEMDVKSTFCVKELDDRDALMLFKNLAGIHNEMSSSKQEIVKKYCAGLPMAIVTVARALRNKSELVWEA

TLEKLKKQELVGVQTSMDVSVKMSYDHLENEEIKSIFLLCAQMGHQPLTMDLVKYCFGLGILEGVSSLLEARDRIKTSIQK
LKDSGLLLDENSNNHFSMHDMVRDAALSIAHKDHNVFTLRNGKLDDWPELEKCTSISICDSDIIDELPEVLNCSQLKLFQI
DTNNPSLEIPERFFSRMENMKVLVMTGFHLSSLPHSIQFLSKLRMLCLERCTLDCNLSILGKLKKLRILSFSGSQLTSFPSEL
KFLDKLRLLDINDCSELKIIPPNLISSLTCLEELYIRKSLIKMLVEGETKKGQASFLIELKNLQNLKVLDLSIPYASIFRNHFFFDK
LKDYKIVIGDLDMFSVGEFRMPDKYEALRVLALQPKDDTNIHSQKSIKLLFKTVQSLLLGEMDGVGDVVNELNIDGFPDL
KHLSIINNNDIKYVNSTQLSTYVNAFPNLESLCLYNLGKLDMISFGPVTVVSFAKLKIIKVEMCNRLKNLYSFYKVKFPTSA
QTCEISECNSYMDKFLPSLETIEVFECGSLKEIFQIPMHYGKVEFLKLHTLTLQSLPSFKCFYTKVEEYCGSHMTEPQTTNR
GHGEITNEEVNQSYGRSHLFGEMVEFPNLENLTLSSLKIHGIWDQHSTSFIFQNLIKLVVKDCHKLRYLCSLSMASNLKNL
KNLVISDCPIMEKIFKVEENSANEVCIFPKLEEIHLSKMDKLTDIWQSKVSVDSFSSLISVNIEKCNELEKIFPSHMEGWFES
LENLKVSKCHSVKVIFEINDSQEIDTFGGIETNLQVLLLEHLSMLKQLWSTDPYGILKFKKLRTIEVLDCDELRSLFPTSVAK
DASKLEGISALQCENMVEIVASKDAFEANNDPLEFPELNYVRLYKLPKIKHFYRQRHPIKCPKLKELSVRDVKLETILKETSE
TTNKEEKCVFPAEEVLPNLECMEIDFYQAQELLRKHQMHRLKELSLISVQSFDLSQFSYRMPNLEKLKFISSKFRELVQRE
KIARKKRLGIVLQLKELVFVGSKINHLGFGRDQVLEKLELLTLEYCNKLSNLGPPSVSLTYLTYLEVKSCKRLRNLMASCTAQ
SMVQLKTMKVIGCCEVEEIVSNKGSEEGKVMKIIFNKLISIELAELNNMKRFCSYKGCEFEFPSLEILIVRECPKMEKFSERE
PITPKLKNIFGVEGDEKTKWQWEGNLNTTIQKILNDKVIFTDYLRLHNYSESIIERLWRGSHSVHQNSFGYLKNLSVYECD
TLVHVIPSHLLYCFQNLEELEVKSCKAAQVIFNMNDEKRLMTKASGILRLNTLSLDNLPKLEHIWNKDPEGIIDHQLLKKM
RVEHCKRLTSLFPASVAKDLAELQVLEVRKCEELAEIFRKDEKGGEGTTQESVFPRLTSLKLYQLPRLKYSIHRSKRQESISKL
SMIDMQELCLGSQPIPNSFFGLLGSLTVDGCQFLSDDVLPFNVLRFLTNLETLEVENCDYVKTIFDVKCTGQDIMTFPLK
NLSLFRLPNLKNVWNEDPHEILRMPHLQEVHVERCNDLTSVFPKSVAKYLVLVENLSVKNCEGLMTIVAEDNIDPSLDT
MFPCPCVRSLKLKGLPKFKYFYHCSPRSDTHTHLESHTENQLSSKKLVKPFSLLENGVEMILRGEFQRNLLPNLKFLILWFE
SDVFRPEILKQAPNIEKLVVCNGSFNEMFCSKSPNNVDYSGLQLKKLQLNSLGELVSIGLENSWTEPFVRNLETFKVTGC
GSLKNLVTCRVSFSNLTYLKVHDCSSLSYLFSSSTVKSLGQLKRMEIKNCTSITEIVPREADESDEDEIIFPKLSLLNLEDLWD
LQKFYRGSLSFPSLEKVSITSCTWLPYLFSSSTVKSLGQLKRMEIKYCGSIEEIVSREKDESDEDEIIFPELSLLNLEGLGLRKFY
RGSLSFPSLEKVSITGCERMTSFCLGTLEAGNLSHVKVGEFQEVAPLQTDLNSTIQKEYLRRISELKHRSLKYMPELQDIW
DGSIEIPNFCFSGLVTLNVDDCKFLTDAVLPFHLLPSLPRLETLRVENCDLIKTIFDVKCTTEDTLITFPLKNLFLFKLSKLKNV
WNEDPHGILSMDHLQQVIVENCKRLKSVFPTSVAKDLNLEDLKVGDCEGLMTIVAENNTNRSLELTFPCPRVSSFQLRR
LPNFKYFYYCSLKSDIYTHLESHTKDQLGTEKRLLLWEEGVEMIFRGKFQRNLLHNLKFLILWYQPDVFRQEILEQVPNME
KLVVQNGSFKEMFCCKSPNNVDYSELQLKKLHLNSLKKLVSIGLENSWTEPFVRNLETFKVFDCDSLKNLVTCRVSFSNLT
YLKVQSCYTLSYLFTSSTAKSLVQLKRMEIKNCFSIQEIVSKEEDESDEDEIIFPKLSLLNLEHLGDLRKFYRGSLRFPSLEELSI
TSCYEMVTLCTGTLEAGKLSHVRLEEPDAIPLETDLNSILTNEFIKRARWEMPQRSLEFRYGADLQVIWGLSLQIPHFRFR
KLETLFLNGCHSLSYVLPSTLLPLLSELKTLKVVNCDSVKTIFDVKSAQGTLTCPLEKLVLWKLSNLEALSNEDTAEIVPLDN
PKQTNPKFTLPCVTSLSLSDLPKFKLNTTFQLITPNLQHLTVGKYESKMIVDGEFQRNHELKFLTFCSDIKCDEYPEYGLLQ
QLPNVKKLVVCHRSFKVIFSDQRPNNSKLLLQLKALSSESLEELVPIGFKNSWTEPFIRNLESFEVISCSSLKNLVTCTVSFSN
MVSLTIENCHNLSYLFTSSTARSLDKLQRMEIKWCKSIEEIVFKEEESDEGEIMFLQLRCLNLDNLLNLRRFYGGSLSFPSLE
ELSVTNCVEMVILCPTTVKADKLTRVTIDYRTVIPPENINSVVRKRFHRQISELFHFDLKSRPALQELWHDSLHMPDFCFS
CLYELTVEDCQFLSDAVLPFHLLPLLPRLRRMAVRNCDFVKYIFEVNCTTKDTLVTLPLEKLTLSNLSNLENVWNEDPTGIL
SVHNLEEVFVDRCKCLGSVFPASVAKDLLKLERLAVENCEKLMTIVAEECDGDEEIIFERLQVLDLKMLEELRCFYTGNFTL
SFPSLKKVHVIKCSSMKSFSAVNKIDHPIKWYYSEYAKPRKETDLNSAVCRTFEKEAPDASGVIISVLQ

>XP_027906872.1

MDTVLKIVDPVIEFVRDHGINQLTYIFCYTKYFEELNKRVKRLGEEKERLDRQRDEAKRKGDIVEDRVEEWFEEVGEFESR
VEKYMNNAGHKKTRGLYYLFPYYRHKLGRQAKKMEPEALRLKDECPKDDEVSHAEKVTSFDLTSSYSGYIEFDSRKSIVD
DIMTKLKDPNMKIIGLHGAQGMGKSTLIKKIANKAKDEGLFDRVAEIDVTEYPNPLTIQADIAHVLGLPLAGESENVRAD
YLRRWLKIENVSILIILDNLHERLDLNRLGIPVDDDYDLRKKNELSISNQKTASDKQGTSGDKQGTGAKVLKKTNFLGDYK

GCKVLLSSRDKNVFRDEVDVESNFCLKELDGNDALMLFEKVTGGGNKMSMPKEEIQNYCTGLPMRIVTLAVAFKNWIE
SESKPTLDKFKKQGLVEWQKSSETPNKKKYDLPKNKELKFIYLLCAQMGHLPLVNDLVKYCFGLGIFEGVSSLSAAREKIN
ESLQELKNLGLVSYENPNIHFHMSHMVRDDALSNALMDHNVFAFRDGKLDYWPDLEKCISISICNSYITDGFPHVINCP
QLQFLQIETIDPSLEIPQRFFSSMKNLLVLILAGFHLSSLPYSIEDLLNLRMLCLERCTLDCNLSVLRKFKKLRILSFSGSQLKN
LPVELRYLDKLRMLDISDCFKLEIIPLDLFSNLTCLEELYIRKSLIKMLVEKWENEGHNSFLSELKNLHQLKVVDLSIPCVSILP
NHLFFDRLKDYKIEIGDFEMFSVGEFRMPNKYEELKVLALQLKDDTDIHSHQGIKLLFKTTQSLFLGNVCVQNVVNELNI
DEFQNLKHLSIINNNDVEYFNSTDLSYCVNIFSNLESLYLYNMMKLKMICRGPITLESFAKLKTIKVEMCCQLENLFSFYAI
KISTSTGTSEIFKCNSNMKKFLASLEMIEVCECESLKEILQIPPDCVKDCECESLKEILQIPQDYGKVEFLKLRTLTLQSLPSFT
CFYTKVERYCWPYSTEAQTTNRCHTEISTEEDGHSDNAPPLFGEPVEVPNLENLNLSSLNIRKIWSDQHLSSFYFQNLIKL
VVKDCDKLTHLCSLPMASSMKKLKSLVISGCLKMEKIFEIEGISANKVCVFPKLEEIHLSKMKRLTDMWQTEVGIDSFSSLI
SVRIEECDQLDKIFPSHMEGWFESLINLQISKCKSVEEIFEINDSQEIDASGGIDTNLQVILLEELPKLKELWSKDPDGILNFK
KLRTIDVSYCDELRNLFPASVAKDVPKLERMSVLYCERMMEIVSNKDTSEADNDPLEFPELTFVRLYGLPTMKQFYKGRH
PIKCPKLKELSMGKCTKLKTFLQETSDEKFVFSAEEVSMILSEFQKQKV

>XP_027906893.1

MVLTRFHGYKQILIFCYFEWFCVCKEIISHVLHYCFLFQHFRTSPVSSMEFASSTSKLPQMYDMLINFNGEDIHRKFVSHL
DSVLSAAGLTTLLHHQNAVNDMDIQQPILNLCRVAIVVFTKTYSQSAWCLHQLQQIIEWHKTYYRHVLPVYYEIQPSDV
RLQRGDFGKTLKATAQQSFSAQQLEHGMSGWNHALSKTADFFGWDESNYRSDAEVVDKIVKSVLNLAVLSATKFPVG
LQSRMEEVIQIIKKESTKVCRIAICGKGGSGKTTLAKAIYDQIHDTFTEKFFIEDFEQKQLCSIEMGRSLIPERIYGKKVLIVLD
DVNFSYYLEMIVRSRFGEGTVIIVTTTKQNPPFTRDLYSIFQINLMNPNESLELLSWHAFREAKPKDEYHFLAKRVVAYCG
GLPLLLEVIGSCLYERTKEEWNNVLSRLERLPQHKVLEILKISFDGLPNQYERNLFLDICCFFVGKDRVYVTKILNGCGVNA
ESGIRILIERSLIIVKNNKFGLHPLLREMAREIVAKITSGKEPEKTSRLWFDKDVLLEHILFSSQDKKVIQRFPPKWFLTVRDF
FKHDYLEVRDAIRRMKLGGHCEYRSKELGWIRLENFSSEFLPIGFLRDAIAIDLKHSLPRLVWKEPQVLASLTVLNLSHSKY
LTETPDFSRLPSLEQLILKDCPRLCEVHQSIGGLCNLTLLNLKDCTRIKNLPREIYMLKSLKTLILSGCSGIHLMEKDIRQMES
LITLITESTVMKQVPFSIVSSKSIGYLSLRGFEGLSHNLFPFIMRSWMLPSMNPLSYYHSFCMDVEVNSWDDIAPLLRILV
NLRSVLLQCETEFQLSKQVQDILVEYGVNITESHTSKQHFRSLLIGDGRCKEFLDAFSDSISEVFAGSESCDVSLPGDNNP
NCLADMGEGYSVSFTVPRDRDIKGMALCAVYLSTPEIVATEDLRSVLIVNYTKCTLHIHNHGTVIFFNDKDWEGIISNLGS
GDKVEIFVIFGHGLVVRRTIVYLIFGESYDIEKESTSKKNSLIRFIKKL

>XP_027906894.1

MVLTRFHGYKQILIFCYFEWFCVCKEIISHVLHYCFLFQHFRTSPVSSMEFASSTSKLPQMYDMLINFNGEDIHRKFVSHL
DSVLSAAGLTTLLHHQNAVNDMDIQQPILNLCRVAIVVFTKTYSQSAWCLHQLQQIIEWHKTYYRHVLPVYYEIQPSDV
RLQRGDFGKTLKATAQQSFSAQQLEHGMSGWNHALSKTADFFGWDESNYRSDAEVVDKIVKSVLNLAVLSATKFPVG
LQSRMEEVIQIIKKESTKVCRIAICGKGGSGKTTLAKAIYDQIHDTFTEKFFIEDFEQKQLCSIEMGRSLIPERIYGKKINLMN
PNESLELLSWHAFREAKPKDEYHFLAKRVVAYCGGLPLLLEVIGSCLYERTKEEWNNVLSRLERLPQHKVLEILKISFDGLP
NQYERNLFLDICCFFVGKDRVYVTKILNGCGVNAESGIRILIERSLIIVKNNKFGLHPLLREMAREIVAKITSGKEPEKTSRL
WFDKDVLLEHILFSSQDKKVIQRFPPKWFLTVRDFFKHDYLEVRDAIRRMKLGGHCEYRSKELGWIRLENFSSEFLPIGFL
RDAIAIDLKHSLPRLVWKEPQVLASLTVLNLSHSKYLTETPDFSRLPSLEQLILKDCPRLCEVHQSIGGLCNLTLLNLKDCTRI
KNLPREIYMLKSLKTLILSGCSGIHLMEKDIRQMESLITLITESTVMKQVPFSIVSSKSIGYLSLRGFEGLSHNLFPFIMRSW
MLPSMNPLSYYHSFCMDVEVNSWDDIAPLLRILVNLRSVLLQCETEFQLSKQVQDILVEYGVNITESHTSKQHFRSLLIG
DGRCKEFLDAFSDSISEVFAGSESCDVSLPGDNNPNCLADMGEGYSVSFTVPRDRDIKGMALCAVYLSTPEIVATEDLRS
VLIVNYTKCTLHIHNHGTVIFFNDKDWEGIISNLGSGDKVEIFVIFGHGLVVRRTIVYLIFGESYDIEKESTSKKNSLIRFIKKL

>XP_027906895.1

MSLRISSMGFASSSSKLPLMFDVIINFHGEEVRKKFISHLESVLSTVGITTYLHHENEANAMPIQQPILDLCRIAIVVFTKTY
SQSAWCLHQLHQIIKWHETYCRHVLPVYYEIQPSDVRLQKGDFGEAFKATAQQTFSAQQLEHGMSRWSHALAKAAN
FYGWDESNYRSDAELVDKIVKSVLNLPLLSATKFPVGLQSHVEDVIHTIKNNSTEVCIIGIYGMEGSGKTTVAKAIYNQIH
DTFMGKIFIEDIAHANRTKFLHLIEKILSDVQTPKVEERNIEKGRSIPERHFQKRVLIVLDNVKHHSLLIALSESRLRFDKGSV
IIITSKTEQLLKTHEVDAVFGINLMNAKESLELLSWHAFREAKPKEEYHDLAKAIVTHCGGLPLALEVIGTYLYERTKEEWH
RVLFKLGKTPQHDVLPVLKICFEGLPNQIERNLFLDIYCFFVGKDRAYVTKILNGCGVDADSGIRILIERSLILVKKNKFGLHP
LLREMAREIIGEITSGEEPRKTSQVWFDKNAHCVLLENILFSSQEKKVIQIFHWGSFSTARHFFKADPVEVRMMKLERHS
EYSSKILRWICLQGFSSEYLPLGFLHNAIAIDLKHSLLRLVWKEPQVLASLKVLNLSRSKYLRKTPDFSRLPSLEHLILKDCPRL
CEVHQSIGGLCNLTLLNLKDCTRIKNLPREIYMLKSLKTLILSGCSRIHLLEKHIVQMESLITLITENTAMKQVPFSIVSSKSIG
YLSLQGFEGLSHNLFPFIIRSWMLPLMNPQPYHCSFCTDMEVHSCDDIGPLLSIISNIRSVLVQCDTEFQLSKQVQIILVEY
GVNSTESDTSKQHFRSSLIGVGRCKEFFDAFSERVSKVYAGSEYCDVSLLGDNGPNWLAHMGEGYSVSFTVPRDRDIKG
MALCVVYLSTTEIVATACLRSILIVNYTKCTLHIHNHDSVISLNDKDWEGIISNLGSGDRVEIFVNFGHGLVVKNTSVYLIYG
DSKSLEMGPHHEPKENALNKFIEKIVMCDFCKLLFFFGNK

>XP_027906896.1

MFCLYITRFSHLMCVFRREILEKHSKQLHNKHFQHNNWSMACQGGATRSPKQQISMAGMRAITDVQTPKVEERNIEK
GRSIPERHFQKRVLIVLDNVKHHSLLIALSESRLRFDKGSVIIITSKTEQLLKTHEVDAVFGINLMNAKESLELLSWHAFREA
KPKEEYHDLAKAIVTHCGGLPLALEVIGTYLYERTKEEWHRVLFKLGKTPQHDVLPVLKICFEGLPNQIERNLFLDIYCFFV
GKDRAYVTKILNGCGVDADSGIRILIERSLILVKKNKFGLHPLLREMAREIIGEITSGEEPRKTSQVWFDKNAHCVLLENILF
SSQEKKVIQIFHWGSFSTARHFFKADPVEVRMMKLERHSEYSSKILRWICLQGFSSEYLPLGFLHNAIAIDLKHSLLRLVW
KEPQVLASLKVLNLSRSKYLRKTPDFSRLPSLEHLILKDCPRLCEVHQSIGGLCNLTLLNLKDCTRIKNLPREIYMLKSLKTLIL
SGCSRIHLLEKHIVQMESLITLITENTAMKQVPFSIVSSKSIGYLSLQGFEGLSHNLFPFIIRSWMLPLMNPQPYHCSFCTD
MEVHSCDDIGPLLSIISNIRSVLVQCDTEFQLSKQVQIILVEYGVNSTESDTSKQHFRSSLIGVGRCKEFFDAFSERVSKVYA
GSEYCDVSLLGDNGPNWLAHMGEGYSVSFTVPRDRDIKGMALCVVYLSTTEIVATACLRSILIVNYTKCTLHIHNHDSVI
SLNDKDWEGIISNLGSGDRVEIFVNFGHGLVVKNTSVYLIYGDSKSLEMGPHHEPKENALNKFIEKIVMCDFCKLLFFFG
NK

>XP_027906898.1

MEFASSTSKLPHMYDVLINFNGEDIHRKFVSHLDSVLSAAGLTTFLHHQNAVNDMDIQQPILNLCRVAIVVFTKTYSQS
AWCLHQLQQIIEWHKTYYRHVLPVYYEIQPSDVRLQTEDFGKDLKATAQQTFSAQQLEHGRSMWSHALSKTAEFFGW
DESNYRSDAELVDTIVKSILNLPVLSATKFPIGLQPHVEDVIQIIKNKSNGVCTVGICGIGGSGKTTLAKAIYNQIHAKFLEKS
FIEDISEVSRTRMHAHLQQRLLSDLLKTKVEIRRVEMGRNMIRERLYGKKVLIVLDDVNEYGPLDLWESSARFGEGTVIIIT
SRDESLLRTHQVDSIFRMNLMNPNKSLELLSWHAFRDAKPKEECHFLAKMVVDYCGGLPLALEVIGSFLYERTKEEWNK
VLSRLECLPQHEVIEILKISFDGLPNEIERNLFLDICCFFVGNDRVYVTKILNDCGVDPDRGIRVLIKRNLIKVRKNNKFGMH
PLLRQMARQIISEILSKEPGKISGLWLDEDMEHALSWYTLFSSQQTKVIERLPSGRDFFERYPYPSEVREPSRTPVNLAGV
SWSLYEKVRWVSLKGFSSEYLPNDFYLHDAIVIDLKHNLLRLVWKEPQVLARLKVLNLSHSIYLTETPDFSRLPALQQLILK
NCQRLREVHRSIGFLYNLTLLNLKGCTGLSNLPREIYMLKSLKALVLSGCSKIVLLEKDIVQMESLITLISENTVVKQVPFSIA
SSKSIGYISLCGLEEVSHNLFPFIIRSQMSPTMNPLSYIHTFSDMEDNSWDDIVPFFNSLAILRSVLVQCDPEFQLSKQLKAI
LVDYCVNITESRISKHHLRSCLVGVGRYEEFFNSVNDGISEVLASSGSCDVCLPGDNYPYWSAHRGEGHSASFTVPRDCV
MKGMILCVVCLSTPEIIEPELTTVLIGNYTRCTLQIHNHGTVISFNDEDWHHIISNLGSGDRVEIFVSSAYGLVVKETAVYL
MHGEPKKNSLIRSIKKIIM

>XP_027906915.1

MEFGSSTSKLQWKFDVLINFTGEDIHKKFVSHLDSVLSANGLTTFLHHQNALQPMHIQQPILNLCRVAIVVFTKTYSQSA
WCLHQLQQIIQWHQTYTRHVLPVYYEIQPSDVRLQKGDFGKAFKATAHQTFSRPQLEHGMSRWSHALTQAANFFG
WDESNYRSDAELVDTIVKSILNLPVLSATKFPVGLQSRVEDVIQIIKDRSSEVCRIGICGEGGSGKTTLAKAIYNQIEGTFM
EKSFIEDIAQVSERRGILHLQKQLLSDVLKSKVEIHSDEMGKSMIRERLYRKRLLIVLDDVDDYCPIELRDSNSCFAEGTVIIL
TIRNEDSWITKQVHPIVHTNLMNPNESLELLSWHAFREARPTEEFHFLAKTIVAYCEGLALALEVIGSYLYEKTEEEWNRV
FLRLPNTPENKFSEILEISFDGLHNQMEKDIFFDVCCFFVGTSRAYVTKILNGCGVDSDSGIRVLIERHLIKIKKNNKLTMHP
VLQEMGRRIIVQNSLENELGSIKPVWIDIDGKYTLLENMLFSSRGAKVIRGLPSGRDLFERYPLQVRDPPKWTKRTGNSE
NLSQRWISLQGFPSEYLPQDFYLHQAIIIEIKHNLLRFVWKEPQVLTLLKVLNLSHSKYLRKTPDFSGLPSLEQLILKDCPRL
RQVHQSIGCLYNLTLLNLKDCPRLNNLPREIYMLKSLKTLIVSGCSKIDLLEKDIVQMESLITLVTENTAVKEVPFSIVSSKNI
GYISLRRFEGLSRNLLPSIIRSRMSSTMNPLSYIHSFKEMEDNNWDDIAPSFSNLANLRSVFVQCDPEFQLSNQLQTILVE
YGVNITESVISQHHFRSSFIGVRRYKEIFDAVSDSMSQVLASSESCDVCLPGDNDPYWLAYTGEGHSVSFTVPPDRDIKGI
AFCVVYLSTSKIIEPELNTVLIVNYTKSTLHIHNHCTVITFSDEDWHDILSNLGSGDKIEIFVAFDHGLVVKNTAVYLIYGEPK
KNSLIRFIKKIVM

>XP_027906916.1

MEFGSSTSKLQWKFDVLINFTGEDIHKKFVSHLDSVLSANGLTTFLHHQNALQPMHIQQPILNLCRVAIVVFTKTYSQSA
WCLHQLQQIIQWHQTYTRHVLPVYYEIQPSDVRLQKGDFGKAFKATAHQTFSRPQLEHGMSRWSHALTQAANFFG
WDESNYRSDAELVDTIVKSILNLPVLSATKFPVGLQSRVEDVIQIIKDRSSEVCRIGICGEGGSGKTTLAKAIYNQIEGTFM
EKSFIEDIAQVSERRGILHLQKQLLSDVLKSKVEIHSDEMGKSMIRERLYRKRLLIVLDDVDDYCPIELRDSNSCFAEGTVIIL
TIRNEDSWITKQVHPIVHTNLMNPNESLELLSWHAFREARPTEEFHFLAKTIVAYCEGLALALEVIGSYLYEKTEEEWNRV
FLRLPNTPENKFSEILEISFDGLHNQMEKDIFFDVCCFFVGTSRAYVTKILNGCGVDSDSGIRVLIERHLIKIKKNNKLTMHP
VLQEMGRRIIVQNSLENELGSIKPVWIDIDGKYTLLENMVRDPPKWTKRTGNSENLSQRWISLQGFPSEYLPQDFYLHQ
AIIIEIKHNLLRFVWKEPQVLTLLKVLNLSHSKYLRKTPDFSGLPSLEQLILKDCPRLRQVHQSIGCLYNLTLLNLKDCPRLNN
LPREIYMLKSLKTLIVSGCSKIDLLEKDIVQMESLITLVTENTAVKEVPFSIVSSKNIGYISLRRFEGLSRNLLPSIIRSRMSSTM
NPLSYIHSFKEMEDNNWDDIAPSFSNLANLRSVFVQCDPEFQLSNQLQTILVEYGVNITESVISQHHFRSSFIGVRRYKEIF
DAVSDSMSQVLASSESCDVCLPGDNDPYWLAYTGEGHSVSFTVPPDRDIKGIAFCVVYLSTSKIIEPELNTVLIVNYTKSTL
HIHNHCTVITFSDEDWHDILSNLGSGDKIEIFVAFDHGLVVKNTAVYLIYGEPKKNSLIRFIKKIVM

>XP_027906917.1

MEFSSSSSSSSSSSFLKSGPHFIYDVFINFWGEDIGKKFISHLYYALLEAQVKTFINEESLPKELELKEHMRAIAASKIAIIVFS
KTYAESSSCLLELEEIIECLQTFGQIVLHVFYDVDPLDVRDQVSDFGKALQETAEKSYSGEQVEHVLSRWSRALTTASDST
GWDVRYFEHDAQLVERIVSDVKTFLDYKDLLITPFPVGIDSRVEDVVKCIESQSTKVCMIGICGMRGSGKTTIAKAIYNRI
YREFIGKSFTERIRLLSWDTAYKTNIRSQLLHNVLKFKVPDVRSRRTMVDHELSGRKLLIVFDDVNEFGQLEYLCKNREWF
GQGTVIIITTRYVRLLNRFKVNYVYKMDVMNKNESLELFSWHAFREPKPRKEFGEVARNTVAYCGGLPLALEVLGSSLYD
RIMVEWESISSQIPIDKVQEILKITFDDLTDMEKNIFLDVCCFFIGKERGYVTEILNDCGLLGNYPIEVLLRLGLVKVERNNK
LQMHPLLHDIGKEIVRQSCPEEAEKRSRLWFQDDIKYVLKNNTGTEAIEGLSLKLHSTSRDCFETHAFKK

>XP_027906918.1

MEFSSSSSSSSSSSFLKSGPHFIYDVFINFWGEDIGKKFISHLYYALLEAQVKTFINEESLPKELELKEHMRAIAASKIAIIVFS
KTYAESSSCLLELEEIIECLQTFGQIVLHVFYDVDPLDVRDQVSDFGKALQETAEKSYSGEQVEHVLSRWSRALTTASDST
GWDVRYFEHDAQLVERIVSDVKTFLDYKDLLITPFPVGIDSRVEDVVKCIESQSTKVCMIGICGMRGSGKTTIAKAIYNRI
YREFIGKSFTERIRLLSWDTAYKTNIRSQLLHNVLKFKVPDVRSRRTMVDHELSGRKLLIVFDDVNEFGQLEYLCKNREWF
GQGTVIIITTRYVRLLNRFKVNYVYKMDVMNKNESLELFSWHAFREPKPRKEFGEVARNTVAYCGGLPLALEVLGSSLYD

RIMVEWESISSQIPIDKVQEILKITFDDLTDMEKNIFLDVCCFFIGKERGYVTEILNDCGLLGNYPIEVLLRLGLVKVERNNK
LQMHPLLHDIGKEIVRQSCPEEAEKRSRLWFQDDIKYVLKNNTGTEAIEGLSLKLHSTSRDCFETHAFKK

>XP_027906920.1

MEFDSSTSKLPGMYDVLINFTGEDIRRKFVSHLDYALSIAGLTTFLHEENAVKGRQIQQPILNLCRVAIVVFSKTYSQSAW
CLHQLQQIIKWQETYSRHVLPIYYEIQPSDVRLQKGDFGEAFKATAHKTFSRQQLQHGMSRWSHALTKAANFFGWDG
SNYRSDAELVDKIVKSVLNLAVLSATNFPVGLQSRMEEVIQIIKNESRDVCRIGICGMGGSGKTTLAKAIYNQIHGTFTDKI
FIEDVAQVTQTRGHAHLKKQLLSSVLKTDIHSVKGTRGNMIRERLRKRVLIVLDDVDDHYFSYLEETRYGPALWPFFSEG
SVIIITTRDVVVDIHFFSVFQINKMNPDESVELLSWHAFREAKPKEECHFLAKMIVDYCGGLPLSLEVIGSCLYERPKEEW
NKVLSRLESIPPHEVLQILKISFDGLLNQSEKDLFLDVCCFFVGKDITYVTKILNVCGVDPDRGIRLLIERSLIKVEKNNKVG
MHPLLQIMGIKIVCDFSRKRNLRGTIIFWLMKISNMRCQRNIKNIERKILIQVN

>XP_027906921.1

MEFDSSTSKLPGMYDVLINFTGEDIRRKFVSHLDYALSIAGLTTFLHEENAVKGRQIQQPILNLCRVAIVVFSKTYSQSAW
CLHQLQQIIKWQETYSRHVLPIYYEIQPSDVRLQKGDFGEAFKATAHKTFSRQQLQHGMSRWSHALTKAANFFGWDG
SNYRSDAELVDKIVKSVLNLAVLSATNFPVGLQSRMEEVIQIIKNESRDVCRIGICGMGGSGKTTLAKAIYNQIHGTFTDKI
FIEDVAQVTQTRGHAHLKKQLLSSVLKTDIHSVKGTRGNMIRERLRKRVLIVLDDVDDHYFSYLEETR

>XP_027906922.1

MELASSSSSSSASSSSSFLKSEPVFIYDVFINFGGEDIGRRFVSHLHSALLQAQVKTFISQENVQDGLNLEEHMREIAVSKI
AIIVFSKTYAESTCCLLELEKVIECHQTFGQIVLPIFYEIDPFDVRDQKNDFGKALEETAEKSYLGEQGKHALSRWSHALNT
ASGITGWDLRNFRHDAEFVEVIVNRVQTLLDYKDLFITEFPVGLEFHVEKVIGCIENHSTKVCMIGIWEMVGSGKITIAKA
IYDQIYHLFIGKSFVENLRKVWDPVSRWHLHLQEQLLYDVLKSKISLKSTWMERVMIENELSRKKLLIVLDDVNELGQLR
NLCGSRDWFGKGTVIIFTTRDIHLLNRLKLNYVYSMDDMKENESLGCDGFGEAKPRKDLYELVRNVVVYCGGLPLAL

>XP_027907096.1

MEFASSSSSSSSSFLKSEPQFIYDVFINFGGEDICRKFVSHLHSAFSLSQVKTLISKENPQEGMKLEEHMRAIESSKITIIVFS
KTYTESSCWRLELEKIIECRQTFGQIVLPVIYDIVRLHESHQKVVKVLEKATPGSYSGEQLEHAMSRWSRALTKAAGITGW
DFRDFRHDAELVDEIVRRVQTLLDYTDLSITRFPVGLESHVEKVIGIIEEQSTKVCMIGIWGIGGSGKTTIAKAIYNRIYHPFI
GKSFIENIGQFQNRVNRMHVHLQENLLYDVLKSKFEVESDRVGRTMIETKLSRKKLLIVLDGVNEFGQLENLCGNREWF
GQGTVIIITTRDVTVLNRLKVNHVYKMDVMNENDSLELLSWHAFGEAKPRKELNELARNIVAYCAGLPLALQFLGSYLC
DRTKEEWESVSSKLNVNPFNQIQEKLEISFDGLNHMEKDIFLDICCFFIGKDRGYVTEILNGCGLYADMGITVLIERGLIKV
ERNNKLEMHPLLRDMGREIIRQRCPKEPGKRSRLLFEDDIKDVLKRNTGTKATQGLSLKQHSTSKDCFEAHAFKEMKRL
RLLQLDHVQLTGDYGYLSKQLRWICWKGFPSKYMPNNFHMENVIAIDLKHSYLQLVWQQPQVLEGLKFLNLSHSKYLR
ETPDFSGLPNLEQLILKDCPSLCKVHQSIGDLRNLLLINLKDCTSLSSLPRKVYKLKSLRTFILSGCFKIDILEEDIVRMESLIAIV
SENTVVKQVPCSIVKSIAYMSLRGFKGLLRNTFPSIIRSWMSPTMNDQYLNTPFCMDMENNDWRVLALLHRSLASLRSI
LVQCKTEFQLSKKVKTILVEYPVNFTESRISNHHLRFSLIGVGSYIKFLNTLNDRISEGLASSGSCKVSLPSDNHPYWLARM
GEGHSVSFIVPQDCHMKGIIMCVVYLSTHGIVATEYLASVSIVNYTKCTLHIHKHGTVISFNDEDWHGIISNLESGDKVEIF
VTFDHGLVVKNTIVYLIYGDSNGLEMGPCPEPEENGLNKFIKKIVMCDFW

>XP_027907126.1

MELGSSSSSSSSSFLKSEPQFIYDVFINFWGENMSRKFVSHLHSVLLQAQVKTLINEENLQQGKKLEEHMRAIAASKIAIIV
FSQTYTESTCCLRELEKIVECRETFGQIVLPIFYEIYPFDVRHQKGDFRKALEEAAHKSYSGEQVEHAFSRWSNALTTVAGI
TGWNVRDFRHDAELVEVTVSRVQTILDYADLSLTRFPVGLDPHVRKVIECIENHSSKVCMIGIWGMVGSGKTTIAKAIY

NRIYRPFIGKSFIGNIREDWNNNPVHLQENLLYDVLKSKFEVESVGLGRTMIENEFSRKKLLIVLDDVNEFSQLENLCGNR
DWFGQGTVIIITSRDVHVLNRLKVNYIYKMNGMNENDSLELFSCHAFRDSKPRKGFDEFARNIVAYCGGLPLALEVLGS
YLCGRTKEH

>XP_027907127.1

MALVGEALISASVHILVEKIMSPEFRHLFCSKELDPLIMNKLKTTLLTLQAVLNDAEEKQITNPAVQDWLHELRDVVYDA
DNLVDVINTRVLQQKVEADSQTFSYHMMNFLSSSFIPFSGGINSEVQNLLQRLEQFVKEKDILGLKEGVSGALWIGKTTT
SVVDQSTIYGRDGDVEKLKKYLLSDDVCNDHVEVITIVGMGGLGKTTLAQLLYNDIEVKEHFNLNAWSCVTKDFDVIKTT
KTLLESVNSKAVTTNNLDALQVELQQSLHGRKFLFVLDDLWETYNDWNRLKAILKCGDVGSKVIITTRQENVALAMHTF
PIHNMMPLSNQDCWDIAAKHAFGSNQSNKDPKLEKIGREIAAKCNGLSLAAEALGGLLRIKLSHKDWNKILKSNIWDLP
GNKVLPPLLLSYHYLPVPLKGCFAFCSIFPKNYELHKETVVRLWMAEGLIQFNRNKSLEETGDEYFDELVSRSLIQKSTTYY
QYKFKMHDLINDLATVVSGKYCYLLV

>XP_027907142.1

MTSSIPSMEFESSSSKLPWMYDVLINFTGDDIRKKFVSHLDSALTSVGFTTFLHHEPMHTQEPIMNLCRVAIVVFTKTYS
QSAWCLDQLQQIIQWHRTYYKHVLPVYYEIQPSDVRLQKGDFGKAFRATVGQNFSRQEQVACVYRWSHALTKATNLF
GWDESNYRSDAELVDKIVKTVLNLSVLSATKFPVGLQPNVEDLVRTIKNKSGEVCIIGICGEGGSGKTTLAKAIYNQIHDT
FTEKSFIEDIAQVSRTRSHVSLQEQLLSDVLKVKVDIRSDEMGTSMIRKRLSGKRVLIVLDDMNDYSPLLDLRKSRAWFSQ
GTVIIITTRDEDLLRIHHVDSVFRLKPMSANESLELLSWHAFREAKPKKTYIYIAKKVVTLCGGLPLTLEVIGTYLSKRTKEE
WEIVVSKLQEIPQHDVQQKLKISFDGLDKQMEKDLFLQICCFFVGKDRAYVTKILKDFVVDVDSGIRVLIVRCLIKLKANN
KFGVHPLLQEMGRKIMYYTREDALITFPATRRCCFELYLSEIRRRSRQVFSWLKVHLNQWKVVKEVPFSIVRSKNIGYISL
RRFEQLWPTIFPYIIRSRMSPTMNPLTYIHPLCMDMEDNSWNDIAPLLSSLANLRSILVECDTNFQLYKQVKTILVEYGV
NISESRISKHHFRSSLIGVGRYNTFINSVSNTISEVLGSNESCDVYLPGDNYPYWLAHIGEGHSVSFTVPQDRDMKGMAF
CVVYLSTPGIVTTECFTSVLIVNYTKCTLQMHNHGTIISFNDEDWDNIMSNLRSGDKVEIFVTFGHELVIKNIGVYLIYGES
NDLEREPAPMENSLIRFIKKIVMCDFCLFMYFL

>XP_027907173.1

MESPSSSSKSQPMYDVLINFTGDDIRRKFVSHLDSALSSVGFTTFLHHNNAVPVTPKHIQEPILNNCRVAIVVFTTTYSQS
AWCLHQLQQIIKWHETYCRQFLPVYYEIQPSDVRLQKGHFGKSFKATAQQTFSAQQLEHGMSRWSHALRKAADFFG
WDESNYRSDAELVEEIVKSVLNLPVLSATKFPVGLQSQVEDVIQTIKNKSAEVCTIAICGMEGSGKTTIAKAIYHQIHDRFK
QKSFVEDIAQVSQTRGYVHLQEQLLSEVLKTKVEIHSVEMGTSMIRARLLRKRVLIVLDNINDYDPLLDLWENRAWFDK
GTVIIITTRHEHLLGIRRVDSIFRINLLNSNQSLELLSWHAFREAKPDEEYNFLARRIVAYCGGLPLVLEVIGSCLFERTKEEW
NNVLFKLAKIPQNDVQQKLKISYDYLHNHLEKDLFLDVCCLFVGKGRAYAVKILYGCGVDADSGIRVLIQRNLIKVKKNNK
FGMHPLLHEMGRKIVRDFEIGKNSRRGIEEEAEYVLLSDNRRTEAIEGYCLKLCSARRDCFEQLAVNSEYLSPKLRWISLH
GFSSEYLHNFYLHEVVAIDLKRSLLRLVWKEPQVLRSLKVLNLSHSVYLTETPDFSRLPNLEQLILKDCSRLHEIHHSIGCLC
NLTLLNLKDCISLSNLPEEIYKLKSLRTLILSGCSKIDLRETDIVQMKSLITLISENIAVKQVPLSIVSSESMGYISLLGFERLTDN
IFPSILRYWMVPTMNPISYIHSFCMDMEHNSWDDIVPLLSSLENLRSVLVQCDTEFQLSKQVKNIMVDYFANITESGISK
EHLRFSLIGVGRYNEFFNAANDILSEVSSELCDVSLPGDNDPYWLAHMGEGRSVSFTVPQNRDLKGMALCVFYLSTPKII
EPEFTTVLIVNYTKCTLHMHKHDTVISFNDEDWHGIMSNLGSGDKVEIFVSFGHGLLIKNTAIYLIFDESNNMKKMLEKK
KFSLFRFIKKNCNSSSSKILSRIFV

>XP_027907175.1

MASPVPSMEFASSTSKLPQMYDVLINFNGEDIRRKFVSHLDSVLSAAGLTTFLHHQNAVNDMDIQQPILNLCRVAIVVF
TKTYSQSAWCLHQLHQIIKWHETYCRHVLPVYYEIQPSDVRLQKGDFGEAFKATAQQTFSPQQLEHGMSRWSHALAK

AANFYGWDESTYRSDAELVDKIVKSVLNLSVLSATNFPVGLQSRMEEVIQLIKNESREVCRIGICGMGGSGKTTLAKAIY
NQILGTFTVKIFIEDVAQVIQTRGHAHLRKQLLSNVLKTDIHSVKGMRGNMIRKRLSKRVLIVLDDVDDHYFSCLDETEYG
PALWYHFAEGSVIIITTRDVVDIHLKKNCSVFQINKMNPDESVELLSWHAFREAKPKEECHFLAKMVVDYCGGLPLALEV
IGSCLYERPKEEWNKVLSRLESIPRHEVLQILKISFDGLLNQSEKDLFLDVCCFFVGKDITYVTKILNVCGVDPDRGIRVLIER
SLIKVEKNNKVGMHPLLQEMGTKIVRDFSRKRNLRGTIIFWLMKISNMRCQRNIKNIEPKILIQVN

>XP_027907227.1

MDTVLKIVDPVIEFVRDHGINQLTYIFCYTKYFEELNKRVKRLGEEKERLDRQRDKAKRKGDIVEDRVEEWFEEVGEFEIR
VEKYMNNAGHKKTRGLYYLFPYYRHKLGRQAKKMEPEALRLKDECPKDDEVSHAENVTSFDLTSSYSGYIEFDSRKSIVD
DIMTKLKDPNMKIIGLHGAQGMGKSTLIKKIANKAKDEGLFDRVAEIDVTEYPNPLTIQADIAHVLGLPLAGESENVRAD
YLRRWLKIENVSILIILDNLHERLDLNRLGIPVDDDYDLRKKNELSLSNQETAGGKKGTSGGAQSTGAKVLKKTNFLGDYK
GCKVLLSSRDKNVFRDKVDVESNFCLKELDGNDALMLFEKVIGGGNKMSMPKEEIQNYCTGLPMRIVTLAVAFKNWIE
SESKPTLDKFKKQGLIEWQKSSETPNKKKYDIPKNKELKFIYLLCAQMGHLPLVNDLVKYCFGLGIFEGVSSLSAAREKINE
SLQELKHLGLVSYENPNIHFHMSYMVRDDALSNALMDHNVFAFRDGKLDYWPNLEKCISISICNSYITDGFPQVINCPQ
LQFLQIETIDPSLEIPQRFFSSMKNLLVLILTGFHVSSLPYSIKDLLNLRMLCLERCTLDCNLSVLRMFKKLRILSFSGSQLKNL
PVELRYLDKLRMLDISDCFKLKIIPPDIFSNLTCLEDLYIRKSLIKMLVEERENKGHNSFLSELKNLHQLKVVDLSIPCVSILPN
HLFFDGLKDYKIEIGDFEMFSVGEFRMPNKYEELKVLALQVKDDTDIHSHKGIKLLFKTAQSLLLGKGCVQKVVNELNIDG
FQNLKHLCIINNNDVEYVNSTDLSNYVNIFPNLESLCLCSMMNLKMICRGPIILESFAKLKTIKVEMCYRLENLFSFYAIKIS
TSTGTSEIFKCNSNMKKFLANLEMIEVCECESLKEILQIPPDCGEVCECESLKEILQIPQDYGKVEFLKLRTLTLQSLPSFTCF
YTKVERSCWPHSTEAQTTNRSHTEISAEQDCHSDNAPPLFGEPVEVPNLENLNLSSLNIRKIWSDQHLSSFYFQNLIKLVV
KDCDKLTHLCSLPMASSLKKLKSLVISGCLKMKKIFEIEGISANKVCVFPKLEEIHLSKMKRLTDMWQTEVGIDSFSSLISVR
IEECDELDKIFPSHMEGWFESLINLKVSKCKSVKEIFEINDSQEIDASGGIDTNLQVILLEELPKLKELWSKDPDGILNFKKLR
TIDVSKCDELRNLFPVSMVKDVSKLERMSILHCERMVEIVSNKDASEANNDPLEFPELTFVRLYELPNMKQFYKGRHPIK
CPKLKELSMGKCMKLKTFPQETSDEKFVFSTEAVFPNLEYMETDFEEAQNWFSKYQMQNLKELIILNSVQRPDLLYPFLY
KMPNLEKLKLTSSFSESLETLPSTNNWQQDRLGVVLQLKQLLICSSNIKDIGFERDQVLERLELLRLKDCDNLSNLGPSSVS
LNYLTCLKLVRCDGLKNLVASSTAKTMVQLKTMKVIDCRKVEQIVSNDGSEEGKGIKIVFSKLISIELVGLMNMTSFCGYK
NCEFEFPLLEILIVRTCPKMEKFSERGLIAPKLKEVFGVEGDKKAKWQWEGDLNDTIQKVFHDKLSFTYTEDLFLKDDSIA
QLWQASGWVQQNSFGYLKSLTVWRCDSVVHVIPSHLLSCFHNLEELQVVSCRNAEVIFSINDENRVITKASPIFRLKTLIL
ANLGKLEHVWEKDPEGIMGLQALEEMKVIDCERLKSLFPASLATRDLTRLQVLEVEECAELTEIFKKDEKVEEGEEGTTQ
HSAFPSLTTLELKYLPSLEYSIHCSKQQESTSNLRDLSEGDIEELCLGSRSIPNSYFGLLESLTLDGCKVLSDVLLPFNLLPFLT
NLETLEVRNLDSVKTIFDVKCTTQDREVAYMGQTLLKKLIVSKLPNLKNVWNEDPRGIISMSNLREVYVEECKTLSSVFPP
RLAVEDVLLPFSLLPFLTNLETLQVRNSDSVKAIFDVKCSTQDRDVISVGQTLPFSLKKLVLSKLPNLKNVWNEDPREIISM
SNLREVYVEECKTLSSVFPPRLAVEDVLLPFSLLPFLTNLETLQVRNSDSVKAIFDVKCSTQDRDVISVGQTLPFSLKKLVLS
KLPNLKIVWNEDPQVILSLHHLQEVCVEECEGLTSVFPASKDKYILKLENLEVKDCKGLMTIFAEDPRTKLELTCPFVRSLEL
EGLPNFKYFYYSSPYTDIFTDLESHTENEVGTEKWMSVGENGMKMILRGELERKVLDSLKALTLCFGSDVFGCKILEEVR
NIEKLVVCDGSFKEMFSCESPNNVLQHLKVLRLESLRKLVSIGLENSWTDSFVRNLETFEVISCESLKSLVLCGVCFSNLTCL
KVENCKRLSHLFSSSTAKSLLQLKRMEIKGCESIKEVVSNKEESDEDEIIFPKLSCLNLEDLKNLRRFYGGSLSFPSLEEFSVR
GCSQLMSLCTVVSGKLSEAIDLNSVTRKRFLKERASWYKSLEFRDRADLQEMWSLALQIPDFCFTNLKKLIVNECNISSDA
VLPFTLLPLLPKLETLEVQNCDSVKTIFDVKCTTQDTTTSLKKLVLRKLPNLETIWNEDTDEIVTEPNPAHPEVTNPKLTFPT
VTSFTLCDLPNFNHNTIYCINEAIATSELIIPNVEDLTVGKNELKMIVDGEFQTNLLHNLKVLGLSFDNECDEFPEYGFLQQ
LPNVEKLMVWSSSFKLIFCHQRPNNSELLLQLKELRLECLEKLVFIGLENPWTELFVSNLETFEVITCSSLENLATCTVSFTN
LRCLKVENCDGLSYLFTSSTAKSLGRLERMEIKQCKSIEEILCGEKSDDEEDEIIFSQLSCLELASLPNLRRFYRGNLSFPSLEE
LSVTHCNDMVTLCPSTLKADKLTQVRIEYGEVISLDTDLNSTTRKEFGRKISKLQELKLKSRPKLPEIWHDPLYIPDLCFSKL
VTLTVKNCQFLSDAVLPFHLLPLLPKLETLEVRKCDYVKTIFNLKRATKDTLVTLPPKKLTLSNLPNLENIWSEDPPGILIMH

HLKEVYVKECKGLTSVFPASAAKDLVKLKDLVVDDCEGLKAIVGEESKEDEIIFPQLMYLKVQSCNSLPYLFTSSTAKSLGEL
KSMKIKECKSIEEIISKEGEESDEKVEIKFEQLQDLYLEKLDELRCFYDGNFTLNFPSLEEVHVIKCSSMKTFSAFNKIDNPW
YYSEYARPRKITHLNSALHRTSEEEAPNASSAIISVLQ

>XP_027907228.1

MDTVLKIVDPVIEFVRDHGINQLTYIFCYTKYFEELNKRVKRLGEEKERLDRQRDKAKRKGDIVEDRVEEWFEEVGEFEIR
VEKYMNNAGHKKTRGLYYLFPYYRHKLGRQAKKMEPEALRLKDECPKDDEVSHAENVTSFDLTSSYSGYIEFDSRKSIVD
DIMTKLKDPNMKIIGLHGAQGMGKSTLIKKIANKAKDEGLFDRVAEIDVTEYPNPLTIQADIAHVLGLPLAGESENVRAD
YLRRWLKIENVSILIILDNLHERLDLNRLGIPVDDDYDLRKKNELSLSNQETAGGKKGTSGGAQSTGAKVLKKTNFLGDYK
GCKVLLSSRDKNVFRDKVDVESNFCLKELDGNDALMLFEKVIGGGNKMSMPKEEIQNYCTGLPMRIVTLAVAFKNWIE
SESKPTLDKFKKQGLIEWQKSSETPNKKKYDIPKNKELKFIYLLCAQMGHLPLVNDLVKYCFGLGIFEGVSSLSAAREKINE
SLQELKHLGLVSYENPNIHFHMSYMVRDDALSNALMDHNVFAFRDGKLDYWPNLEKCISISICNSYITDGFPQVINCPQ
LQFLQIETIDPSLEIPQRFFSSMKNLLVLILTGFHVSSLPYSIKDLLNLRMLCLERCTLDCNLSVLRMFKKLRILSFSGSQLKNL
PVELRYLDKLRMLDISDCFKLKIIPPDIFSNLTCLEDLYIRKSLIKMLVEERENKGHNSFLSELKNLHQLKVVDLSIPCVSILPN
HLFFDGLKDYKIEIGDFEMFSVGEFRMPNKYEELKVLALQVKDDTDIHSHKGIKLLFKTAQSLLLGKGCVQKVVNELNIDG
FQNLKHLCIINNNDVEYVNSTDLSNYVNIFPNLESLCLCSMMNLKMICRGPIILESFAKLKTIKVEMCYRLENLFSFYAIKIS
TSTGTSEIFKCNSNMKKFLANLEMIEVCECESLKEILQIPPDCGEVCECESLKEILQIPQDYGKVEFLKLRTLTLQSLPSFTCF
YTKVERSCWPHSTEAQTTNRSHTEISAEQDCHSDNAPPLFGEPVEVPNLENLNLSSLNIRKIWSDQHLSSFYFQNLIKLVV
KDCDKLTHLCSLPMASSLKKLKSLVISGCLKMKKIFEIEGISANKVCVFPKLEEIHLSKMKRLTDMWQTEVGIDSFSSLISVR
IEECDELDKIFPSHMEGWFESLINLKVSKCKSVKEIFEINDSQEIDASGGIDTNLQVILLEELPKLKELWSKDPDGILNFKKLR
TIDVSKCDELRNLFPVSMVKDVSKLERMSILHCERMVEIVSNKDASEANNDPLEFPELTFVRLYELPNMKQFYKGRHPIK
CPKLKELSMGKCMKLKTFPQETSDEKFVFSTEAVFPNLEYMETDFEEAQNWFSKYQMQNLKELIILNSVQRPDLLYPFLY
KMPNLEKLKLTSSFSESLETLPSTNNWQQDRLGVVLQLKQLLICSSNIKDIGFERDQVLERLELLRLKDCDNLSNLGPSSVS
LNYLTCLKLVRCDGLKNLVASSTAKTMVQLKTMKVIDCRKVEQIVSNDGSEEGKGIKIVFSKLISIELVGLMNMTSFCGYK
NCEFEFPLLEILIVRTCPKMEKFSERGLIAPKLKEVFGVEGDKKAKWQWEGDLNDTIQKVFHDKLSFTYTEDLFLKDDSIA
QLWQASGWVQQNSFGYLKSLTVWRCDSVVHVIPSHLLSCFHNLEELQVVSCRNAEVIFSINDENRVITKASPIFRLKTLIL
ANLGKLEHVWEKDPEGIMGLQALEEMKVIDCERLKSLFPASLATRDLTRLQVLEVEECAELTEIFKKDEKVEEGEEGTTQ
HSAFPSLTTLELKYLPSLEYSIHCSKQQESTSNLRDLSEGDIEELCLGSRSIPNSYFGLLESLTLDGCKVLSDVLLPFNLLPFLT
NLETLEVRNLDSVKTIFDVKCTTQDREVAYMGQTLLKKLIVSKLPNLKNVWNEDPRGIISMSNLREVYVEECKTLSSVFPP
RLAVEDVLLPFSLLPFLTNLETLQVRNSDSVKAIFDVKCSTQDRDVISVGQTLPFSLKKLVLSKLPNLKNVWNEDPREIISM
SNLREVYVEECKTLSSVFPPRLAVEDVLLPFSLLPFLTNLETLQVRNSDSVKAIFDVKCSTQDRDVISVGQTLPFSLKKLVLS
KLPNLKIVWNEDPQVILSLHHLQEVCVEECEGLTSVFPASKDKYILKLENLEVKDCKGLMTIFAEDPRTKLELTCPFVRSLEL
EGLPNFKYFYYSSPYTDIFTDLESHTENEVGTEKWMSVGENGMKMILRGELERKVLDSLKALTLCFGSDVFGCKILEEVR
NIEKLVVCDGSFKEMFSCESPNNVLQHLKVLRLESLRKLVSIGLENSWTDSFVRNLETFEVISCESLKSLVLCGVCFSNLTCL
KVENCKRLSHLFSSSTAKSLLQLKRMEIKGCESIKEVVSNKEESDEDEIIFPKLSCLNLEDLKNLRRFYGGSLSFPSLEEFSVR
GCSQLMSLCTVVSGKLSEAIDLNSVTRKRFLKERASWYKSLEFRDRADLQEMWSLALQIPDFCFTNLKKLIVNECNISSDA
VLPFTLLPLLPKLETLEVQNCDSVKTIFDVKCTTQDTTTSLKKLVLRKLPNLETIWNEDTDEIVTEPNPAHPEVTNPKLTFPT
VTSFTLCDLPNFNHNTIYCINEAIATSELIIPNVEDLTVGKNELKMIVDGEFQTNLLHNLKVLGLSFDNECDEFPEYGFLQQ
LPNVEKLMVWSSSFKLIFCHQRPNNSELLLQLKELRLECLEKLVFIGLENPWTELFVSNLETFEVITCSSLENLATCTVSFTN
LRCLKVENCDGLSYLFTSSTAKSLGRLERMEIKQCKSIEEILCGEKSDDEEDEIIFSQLSCLELASLPNLRRFYRGNLSFPSLEE
LSVTHCNDMVTLCPSTLKADKLTQVRIEYGEVISLDTDLNSTTRKEFGRKISKLQELKLKSRPKLPEIWHDPLYIPDLCFSKL
VTLTVKNCQFLSDAVLPFHLLPLLPKLETLEVRKCDYVKTIFNLKRATKDTLVTLPPKKLTLSNLPNLENIWSEDPPGILIMH
HLKEVYVKECKGLTSVFPASAAKDLVKLKDLVVDDCEGLKAIVGEESKEDEIIFPQLMYLKVQSCNSLPYLFTSSTAKSLGEL

KSMKIKECKSIEEIISKEGEESDEKVEIKFEQLQDLYLEKLDELRCFYDGNFTLNFPSLEEVHVIKCSSMKTFSAFNKIDNPW
YYSEYARPRKITHLNSALHRTSEEEAPNASSAIISVLQ

>XP_027907238.1

MEHASSTSKLPQMYDVLIDFTGEEIRRKFVSHLDSAFTSVGLTTFLHQENAMKEMQIQEHSLDLCRVAIVIFTKTYSQSA
WSLRQLQQIIKWHEAYYRHVLPVYNEIQPSDVRLQKCDFGKSFKATAHQTFPAQQLEHGISRWSHALTKAADFFGWD
ESNCRSDAELVDKIVKSVLNLPVLSATKFPVGLQSYVEDVIRTIEDKSSQVCTIVICGMEGSGKSTIAKAIYNRINGTFTEKS
FIEDVAQISQTRGYVHLQERLLSDVLKTKVKIRSVEMGRRMIRERLFKKRMLIVLDEYSPFLDLRKSRAWFGKGTVIIITTR
NEDLPGIREVDSVLQINLMNPDESLELLSWHAFREPKPKEEYYDLAKAVAAYCGGLPLVLEVIGSTLFESTKEEWKSVLLE
LEKLHKNDVHHKLKISFDSLRSEKEKDLFLDVCCFPIGLCCFPIGKGTTYVMNILNVFGLDADSGIRVLIQRNLIKVKNNNK
FVIHPLLQKMGRKISQQEFWREKRLQFDDAECLFTYNRGTKSAQSLPMKLRSANREPSTLLKLAGNSEYLPKKLRWINLR
EFSSPYLLNDFYLDDAIAIDLKHSLLRVVWKKTQVSITCHFLLKHKV

>XP_027907239.1

MEHASSTSKLPQMYDVLIDFTGEEIRRKFVSHLDSAFTSVGLTTFLHQENAMKEMQIQEHSLDLCRVAIVIFTKTYSQSA
WSLRQLQQIIKWHEAYYRHVLPVYNEIQPSDVRLQKCDFGKSFKATAHQTFPAQQLEHGISRWSHALTKAADFFGWD
ESNCRSDAELVDKIVKSVLNLPVLSATKFPVGLQSYVEDVIRTIEDKSSQVCTIVICGMEGSGKSTIAKAIYNRINGTFTEKS
FIEDVAQISQTRGYVHLQERLLSDVLKTKVKIRSVEMGRRMIRERLFKKRMLIVLDEYSPFLDLRKSRAWFGKGTVIIITTR
NEDLPGIREVDSVLQINLMNPDESLELLSWHAFREPKPKEEYYDLAKAVAAYCGGLPLVLEVIGSTLFESTKEEWKSVLLE
LEKLHKNDVHHKLKISFDSLRSEKEKDLFLDVCCFPIGLCCFPIGKGTTYVMNILNVFGLDADSGIRVLIQRNLIKVKNNNK
FVIHPLLQKMGRKISQQEFWREKRLQFDDAECLFTYNRGTKSAQSLPMKLRSANREPSTLLKLAGNSEYLPKKLRWINLR
EFSSPYLLNDFYLDDAIAIDLKHSLLRVVWKKTQVFLMKRVTTKI

>XP_027907246.1

MSLSGGDDDMEMDFLRDRYQDSRNFEVFLSFRGEDTRFSFTSHLYTALQNAGIVVFKDDEALPRGNQISPSLRLAIEES
RISVVVFSKNYAESWWCLKELEKIMECHRTIGQVVIPVFYDVDPSEVRHQRGDFGKAFQRLLNKVSIEEEEKVLDWKLR
WRTTLGETSGISAVEILNFSFERVDEHVERWKEALSEVAQISGVVDLDPRINFLVKQWVEGLCKGAEIPIREMEIDDKMD
FLLKQWRVFCKPVCMLEVAFFDPSGDMEISDVIEFNVKRCREALFKAAGISGGAILNSCEEMEISDAIKLHIKYWSEAFSE
KAGAIDYFSFDEGTIDCEISNLENHWREKLCEAYGISMDKDLQWRYVLKIWMKALYGISKDAVYTPSVQKLKAYNRIESL
VKHWRIALSEAVSISRLVVQHYRGITDNEINYIEKRARDTFREAAGISAVVILNSRNESEAVKNIVKNVTSLLDKTELFVAN
NPVGVESRVQEMVQLLEQKQSNNVLLLGVWGMGGIGKTTIAKAIYNKIGRNFQGRSFLADIREVWGQEAGPICLQER
LLFDIHQENNTKIHNIESGKIILRERLHRKRILLILDDVNKLQQLNALCGNHEWFGSGSRIIITTRDIHLLRGKRVDQVFAMT
GMDVDESIELFSWHAFKQASPKEDFIELSRNVVAYAGGLPLALEVLGSYLFDMEVKEWKIVLEKLRKIPNDEVQEKLKISY
DGLSDDTEKGIFLDIACFFIGKDRNDVIHILNGCGLFAENGIRVLVERSLVTVDDKNRIGMHDLLRDMGREIIRSKSPMEL
EERSRLWFHEDVLDVLSKETGTKFIEGLTLKLPRSNTKSLSTKAFMNMKKLRLLQLSGVELVGDFEYLSKDLRWLCWDGF
PFSFIPASFYQGSLVSIELENSKITMVWKGTQLMEKLKILNLSHSHYLTKTPDFLNLPNLEKLVFMDCPRLSEVSYTIGHLTK
LLLINFQDCISLRNLPRSIYKLKSLKTLILSGCLKIDKLEEDIEQMESLTTLVADNTAIARLPFSIVRSKSIGYISLCGYEGFSRDV
FPSIIWSWMSPVNSLSSRVHTFVDMSSLVSLDVQNSSSNQLSYISEEFPKLRSLWIECGSDLQLSRDTTSILDALNATNYE
ESESSATASQMQNVFTLIECNSRSKLFEKTLLIQMGRSWEITHILKQRILQNMTTSDDGHCLLPGDCYPDWLTFNSEGSS
VTFDIPQVNGRNLKTMMCHIHYSSSDNITSDGLKNLLVINHTKSTIQLYKRHALASFDDEEWQRVLSNIEAGNKVQFVV
VFWSRLTVIKTSIYLIYEAINEKEEHYHAPNMTIPSYESSCAVGSISPPVESMEDLRGASVKSLTKRLLNKFFSCKGKVEKKK
NEG

>XP_027907247.1

MEIDDKMDFLLKQWRVFCKPVCMLEVAFFDPSGDMEISDVIEFNVKRCREALFKAAGISGGAILNSCEEMEISDAIKLHI
KYWSEAFSEKAGAIDYFSFDEGTIDCEISNLENHWREKLCEAYGISMDKDLQWRYVLKIWMKALYGISKDAVYTPSVQK
LKAYNRIESLVKHWRIALSEAVSISRLVVQHYRGITDNEINYIEKRARDTFREAAGISAVVILNSRNESEAVKNIVKNVTSLL
DKTELFVANNPVGVESRVQEMVQLLEQKQSNNVLLLGVWGMGGIGKTTIAKAIYNKIGRNFQGRSFLADIREVWGQE
AGPICLQERLLFDIHQENNTKIHNIESGKIILRERLHRKRILLILDDVNKLQQLNALCGNHEWFGSGSRIIITTRDIHLLRGKR
VDQVFAMTGMDVDESIELFSWHAFKQASPKEDFIELSRNVVAYAGGLPLALEVLGSYLFDMEVKEWKIVLEKLRKIPND
EVQEKLKISYDGLSDDTEKGIFLDIACFFIGKDRNDVIHILNGCGLFAENGIRVLVERSLVTVDDKNRIGMHDLLRDMGRE
IIRSKSPMELEERSRLWFHEDVLDVLSKETGTKFIEGLTLKLPRSNTKSLSTKAFMNMKKLRLLQLSGVELVGDFEYLSKDL
RWLCWDGFPFSFIPASFYQGSLVSIELENSKITMVWKGTQLMEKLKILNLSHSHYLTKTPDFLNLPNLEKLVFMDCPRLS
EVSYTIGHLTKLLLINFQDCISLRNLPRSIYKLKSLKTLILSGCLKIDKLEEDIEQMESLTTLVADNTAIARLPFSIVRSKSIGYISL
CGYEGFSRDVFPSIIWSWMSPVNSLSSRVHTFVDMSSLVSLDVQNSSSNQLSYISEEFPKLRSLWIECGSDLQLSRDTTSI
LDALNATNYEESESSATASQMQNVFTLIECNSRSKLFEKTLLIQMGRSWEITHILKQRILQNMTTSDDGHCLLPGDCYPD
WLTFNSEGSSVTFDIPQVNGRNLKTMMCHIHYSSSDNITSDGLKNLLVINHTKSTIQLYKRHALASFDDEEWQRVLSNIE
AGNKVQFVVVFWSRLTVIKTSIYLIYEAINEKEEHYHAPNMTIPSYESSCAVGSISPPVESMEDLRGASVKSLTKRLLNKFF
SCKGKVEKKKNEG

>XP_027907355.1

MTSPVPSMEFASSTSKLPQMYDVLINFNGEDIRRKFVSHLDSVLLAAGLTTFLHHQISVNDMDIQQPILDLCRIVIVVFTK
TYSQSAWCLHQLHQIIKWHETYCRHVLPVYYEIQPSDVRLQKGDFGKDLRATAQQTFSAQQLEHGMSRWSHALSKTA
DFFGWDVSNYRSDAELVDTIVKSILNLPVLSATKFPIGLQSHVEDGIQIIKNKATGVCRLGICGIGGSGKTTLAKAIYNQIH
GTFLKKSFIENISGVSRTQKYANLQQRLLSDLLKTKVEIRSVEMGRSMIEERLYGKKVLIVLDDVNDYWPLDLLESSAWFG
EGTVIIITTRDESLLRIHHQVDSIFRMNLMNPNDSLELLSWHAFREAKPKEECHFLAKMVVDYCGGLPLALEVIGSLLYER
TKEEWNKVLLRLESVPQYEVLEILKISFDGLRNQIERNLFLDICRFFVGKDRVYVTKILNGCGVDADSGIRILIERSLIIVKRN
NKFGLHPLLKEMGREIIGEITTGKESEKASRLWFDKDEDYALIEHILFSSQEKNVIQRFPPKWFLTVRDFFDRDYLEVRDAI
SRMKLGGHCECRSKELGWIILEKFSSDFLPVGLLHDAIAIDLKHSLPRLVWKEPQVLASLKVLNLSHSKYLTETPDFSRLPG
LEHLILKDCPRLCEVHPSIGGLCNLTLLNLKDCTKINNLPREIYMLKSLKTLILSGCSGIHLMEKDIVQMESLITLITENRVVKE
VPFSIVSSKSIGYLSLRGFEGLSHNIFPFIIRSWMLPSMNPLSYNHSFCMDMEVNSWDDITPLLRILVNLRSILVQCETEFQ
LSKQVQDILVEYGVNITESHTSKQHFRFSLIGVGRCKEFFDGFSDSISEVFAGSESCDVSLPGDNNPNCLAHMGEGYSVS
FTVPRDRDMKGMALCVVYLSTPEIVATECLRSVLIVNYTKCTLHIHNHGTVIFFNDKDWEGIISNLGSGDKVEIFVIFGHG
LVVRRTIVYLVCGESNESSSKKNSLIRFIKKL

>XP_027907356.1

MEFASSSSSSSSSFLKSEPHFIYDVFINFGAKDIGRRFVSHLHSALLQAEVKTLINEENPQEGMNLEEHLRAIAGTKITIIVFT
KTYTESTRCLLELEKIIECHQTFGQIVLPVFYEIDQLDVRYQEDDFGKALEEAVYKSYSGEQLEHALSRWTDALTTAAGIDG
WDLRDFRHDAQLVDVIVSRVQTLLDYAELGITRFPVGLQSQVEKVIECIENHSTKVCVIGIWGMGGSGKTTIAKAIYNRI
YRLFIGKCFIENIEEVRNRVYRTNVHLQEKLINNVLKSEVVVTGLGMGKLVIKTELSRKKLLIVLDDVNKFGQLKHLCGNPE
WFGQGTVIIITTRDVSLLNRLKVNYVYQMNGMNDNDSLELLSWHAFGEEKPRKELNELARNIVAYCGGLPLALKVFGG
MTMKEWESVSSKLPVIPFAQVKEKLKISFDGLGDMEKDIFLDVCCFFIGKERGYVTEILNGCGLHATIGITTLIERNLIKVER
NNKLEMHPLFRDMGREIIFRRWPEEPGKRSRLWFHEDVKDVLEKNTGTKATQGLSLKLPSTNRNCFEAHAFKKMKRLR
LLQLDHVQLTGDYGHLSKQLRLICWQGFHSKYIPNNFHMENVIAMDLKHSHLQLVWKQPTWKSPLWKQPQVLEHLK
FLNLSHSKYLRETPDFSRLPNLRRLILKDCPSLCKIHSSIGDLCNLLLINLKDCTSLSSLPRELYNLKSLRTFILTGCFKIDILEEDI
VQMESLITLVTENTAVKHVPCSIVSSKSIGYISLCGLEGLSLNLFPSLIRSWMPPTMNPQSYISLYCMDMENNNWHDLAP
LFGGLGNIRSVLVQCDTEFQLFKQVKTLLVEYDDNFTESRISKHQLRFSLIGVGSYNELCNTLSNSVSEGLASCESCDVCLP

GDNHPYWLAHIGEGRSVSFTVPQDRELKGMVLCVVYLSAPEFMANECFQRVLVVNYTKCTLQIHNHGTVISFNDRDW
EGIISNLGSGDKVEFFVSFGHGLVVKNTAIYLNIW

>XP_027907357.1

MEFASSSSSSSSSFLKSEPHFIYDVFINFGAKDIGRRFVSHLHSALLQAEVKTLINEENPQEGMNLEEHLRAIAGTKITIIVFT
KTYTESTRCLLELEKIIECHQTFGQIVLPVFYEIDQLDVRYQEDDFGKALEEAVYKSYSGEQLEHALSRWTDALTTAAGIDG
WDLRDFRHDAQLVDVIVSRVQTLLDYAELGITRFPVGLQSQVEKVIECIENHSTKVCVIGIWGMGGSGKTTIAKAIYNRI
YRLFIGKCFIENIEEVRNRVYRTNVHLQEKLINNVLKSEVVVTGLGMGKLVIKTELSRKKLLIVLDDVNKFGQLKHLCGNPE
WFGQGTVIIITTRDVSLLNRLKVNYVYQMNGMNDNDSLELLSWHAFGEEKPRKELNELARNIVAYCGGLPLALKVFGG
MTMKEWESVSSKLPVIPFAQVKEKLKISFDGLGDMEKDIFLDVCCFFIGKERGYVTEILNGCGLHATIGITTLIERNLIKVER
NNKLEMHPLFRDMGREIIFRRWPEEPGKRSRLWFHEDVKDVLEKNTGTKATQGLSLKLPSTNRNCFEAHAFKKMKRLR
LLQLDHVQLTGDYGHLSKQLRLICWQGFHSKYIPNNFHMENVIAMDLKHSHLQLVWKQPTWKSPLWKQPQVLEHLK
FLNLSHSKYLRETPDFSRLPNLRRLILKDCPSLCKIHSSIGDLCNLLLINLKDCTSLSSLPRELYNLKSLRTFILTGCFKIDILEEDI
VQMESLITLVTENTAVKHVPCSIVSSKSIGYISLCGLEGLSLNLFPSLIRSWMPPTMNPQSYISLYCMDMENNNWHDLAP
LFGGLGNIRSVLVQCDTEFQLFKQVKTLLVEYDDNFTESRISKHQLRFSLIGVGSYNELCNTLSNSVSEVFESSESCHISLSS
GNDPYWLAHRGEGNDFMCRLFITS

>XP_027907358.1

MEFASSSSSSSSSFLKSEPHFIYDVFINFGAKDIGRRFVSHLHSALLQAEVKTLINEENPQEGMNLEEHLRAIAGTKITIIVFT
KTYTESTRCLLELEKIIECHQTFGQIVLPVFYEIDQLDVRYQEDDFGKALEEAVYKSYSGEQLEHALSRWTDALTTAAGIDG
WDLRDFRHDAQLVDVIVSRVQTLLDYAELGITRFPVGLQSQVEKVIECIENHSTKVCVIGIWGMGGSGKTTIAKAIYNRI
YRLFIGKCFIENIEEVRNRVYRTNVHLQEKLINNVLKSEVVVTGLGMGKLVIKTELSRKKLLIVLDDVNKFGQLKHLCGNPE
WFGQGTVIIITTRDVSLLNRLKVNYVYQMNGMNDNDSLELLSWHAFGEEKPRKELNELARNIVAYCGGLPLALKVFGG
MTMKEWESVSSKLPVIPFAQVKEKLKISFDGLGDMEKDIFLDVCCFFIGKERGYVTEILNGCGLHATIGITTLIERNLIKVER
NNKLEMHPLFRDMGREIIFRRWPEEPGKRSRLWFHEDVKDVLEKNTGTKATQGLSLKLPSTNRNCFEAHAFKKMKRLR
LLQLDHVQLTGDYGHLSKQLRLICWQGFHSKYIPNNFHMENVIAMDLKHSHLQLVWKQPTWKSPLWKQPQVLEHLK
FLNLSHSKYLRETPDFSRLPNLRRLILKDCPSLCKIHSSIGDLCNLLLINLKDCTSLSSLPRELYNLKSLRTFILTGCFKIDILEEDI
VQMESLITLVTENTAVKHVPCSIVSSKSIGYISLCGLEGLSLNLFPSLIRSWMPPTMNPQSYISLYCMDMENNNWHDLAP
LFGGLGNIRSVLVQCDTEFQLFKQVKTLLVEYDDNFTESRISKHQLRFSLIGVGSYNELCNTLSNSVSEVTTILTGSPISVRD
VLFLSLCLKIVS

>XP_027907359.1

MEFASSSSSSSSSFLKSEPHFIYDVFINFGAKDIGRRFVSHLHSALLQAEVKTLINEENPQEGMNLEEHLRAIAGTKITIIVFT
KTYTESTRCLLELEKIIECHQTFGQIVLPVFYEIDQLDVRYQEDDFGKALEEAVYKSYSGEQLEHALSRWTDALTTAAGIDG
WDLRDFRHDAQLVDVIVSRVQTLLDYAELGITRFPVGLQSQVEKVIECIENHSTKVCVIGIWGMGGSGKTTIAKAIYNRI
YRLFIGKCFIENIEEVRNRVYRTNVHLQEKLINNVLKSEVVVTGLGMGKLVIKTELSRKKLLIVLDDVNKFGQLKHLCGNPE
WFGQGTVIIITTRDVSLLNRLKVNYVYQMNGMNDNDSLELLSWHAFGEEKPRKELNELARNIVAYCGGLPLALKVFGG
MTMKEWESVSSKLPVIPFAQVKEKLKISFDGLGDMEKDIFLDVCCFFIGKERGYVTEILNGCGLHATIGITTLIERNLIKVER
NNKLEMHPLFRDMGREIIFRRWPEEPGKRSRLWFHEDVKDVLEKNTGTKATQGLSLKLPSTNRNCFEAHAFKKMKRLR
LLQLDHVQLTGDYGHLSKQLRLICWQGFHSKYIPNNFHMENVIAMDLKHSHLQLVWKQPTWKSPLWKQPQVLEHLK
FLNLSHSKYLRETPDFSRLPNLRRLILKDCPSLCKIHSSIGDLCNLLLINLKDCTSLSSLPRELYNLKSLRTFILTGCFKIDILEEDI
VQMESLITLVTENTAVKHVPCSIVSSKSIGYISLCGLEGLSLNLFPSLIRSWMPPTMNPQSYISLYCMDMENNNWHDLAP
LFGGLGNIRSVLVQCDTEFQLFKQVKTLLVEYDDNFTESRISKHQLRFSLIGVGSYNELCNTLSNSVSEKMVQILQNRELQ
SSTSSLY

>XP_027907360.1

MEFASSTSKLPQMYDLLINFNGEDIHRKFVSHLDSVLSAAGLTTFLHHQNAVNDMDIQQPILNLCRVAIVVFTKTYSESA
WCLHQLQQIIEWHKTYSRHVLPVYYEIQPSDVRLQKGDFGKTLKATAQKSFSAQQMEHGMSRWNHALSKTADFFGW
DESNYRSDAELVDTIVKSILNLPVLSATKFPIGLQPHVEDVIQVIKNKSTGVCTVGICGMEGSGKTTLAKAIYNQIHGTFLK
KSFIEDISEVSRTGKHANLHERLLSDLLKTKLEIHKVEMGRRMIGERLYGKKVLIVLDDVNEYGPLDLWESSAWFGEGTVII
ITTTDARLLRIYQVDYIFQMNVMNPNKSLELFSWHAFREAKPKKEYHFLARRVVAYCGGLPLLLEVIGSCLYERTKEEWN
RLLLQLDNSAQHEVDQILKISYEDLLNQMEKDLFLDVCCFFIGKCKFYVTKILNDCGVDPDSGIRVLIKRNLVKIRKNNKVG
MHPLLRQMGREISHEILRKEPEKISGLWLDEDMEHALSRNSQTNVIQRFSRRLVNLAGFSWSLCEKLRWVSLKGFSSQY
LPNDFYLRDAIGIDLKHSLLRLVWKESQVLARLKVLNLSHSIYLTETPDFSRLPALEQLILKNCQSLRQVHQSIGFLYNLTLL
NLKDCTGLTNLPREIYMLKSLKALVLSGCSKIVLLEKDIVQMESLITLISENTVLKQVPFSIASSKSIGYISLCGLEERSNNLFPS
IIRSRMSPTTNPLSYIHTFSDTEDNSWDDVVPFFSSLAILRSVLVQCDPEFQLSVQLKAILMDYCVNITKSRISKHHFRSCLI
GVGRYEEFFNTVSDGISEVLASSGSCDVCLPGDNYPYWSAHRGEGHSVSFTVPRDCVMKGLILCVVCLSTPEIIEPELTTV
LIVNYTRCTLQIHNHGTVISFNDEDWDHIVSNLGSGDRVEIFVSSAYGLVVKETAVYLMYGEPKKNSLIRSIKKIIM

>XP_027907361.1

MKMSSVVSSKAFLEASFDWNWKLPRILQYISEMYDLLINFNGEDIHRKFVSHLDSVLSAAGLTTFLHHQNAVNDMDIQ
QPILNLCRVAIVVFTKTYSESAWCLHQLQQIIEWHKTYSRHVLPVYYEIQPSDVRLQKGDFGKTLKATAQKSFSAQQME
HGMSRWNHALSKTADFFGWDESNYRSDAELVDTIVKSILNLPVLSATKFPIGLQPHVEDVIQVIKNKSTGVCTVGICGM
EGSGKTTLAKAIYNQIHDLLKTKLEIHKVEMGRRMIGERLYGKKVLIVLDDVNEYGPLDLWESSAWFGEGTVIIITTTDAR
LLRIYQVDYIFQMNVMNPNKSLELFSWHAFREAKPKKEYHFLARRVVAYCGGLPLLLEVIGSCLYERTKEEWNRLLLQLD
NSAQHEVDQILKISYEDLLNQMEKDLFLDVCCFFIGKCKFYVTKILNDCGVDPDSGIRVLIKRNLVKIRKNNKVGMHPLLR
QMGREISHEILRKEPEKISGLWLDEDMEHALSRNSQTNVIQRFSRRLVNLAGFSWSLCEKLRWVSLKGFSSQYLPNDFYL
RDAIGIDLKHSLLRLVWKESQVLARLKVLNLSHSIYLTETPDFSRLPALEQLILKNCQSLRQVHQSIGFLYNLTLLNLKDCTG
LTNLPREIYMLKSLKALVLSGCSKIVLLEKDIVQMESLITLISENTVLKQVPFSIASSKSIGYISLCGLEERSNNLFPSIIRSRMSP
TTNPLSYIHTFSDTEDNSWDDVVPFFSSLAILRSVLVQCDPEFQLSVQLKAILMDYCVNITKSRISKHHFRSCLIGVGRYEE
FFNTVSDGISEVLASSGSCDVCLPGDNYPYWSAHRGEGHSVSFTVPRDCVMKGLILCVVCLSTPEIIEPELTTVLIVNYTRC
TLQIHNHGTVISFNDEDWDHIVSNLGSGDRVEIFVSSAYGLVVKETAVYLMYGEPKKNSLIRSIKKIIM

>XP_027907362.1

MKMSSVVSSKAFLEASFDWNWKLPRILQYISEMYDLLINFNGEDIHRKFVSHLDSVLSAAGLTTFLHHQNAVNDMDIQ
QPILNLCRVAIVVFTKTYSESAWCLHQLQQIIEWHKTYSRHVLPVYYEIQPSDVRLQKGDFGKTLKATAQKSFSAQQME
HGMSRWNHALSKTADFFGWDESNYRSDAELVDTIVKSILNLPVLSATKFPIGLQPHVEDVIQVIKNKSTGVCTVGICGM
EGSGKTTLAKAIYNQIHGTFLKKSFIEDISEVSRTGKHANLHERLLSDLLKTKLEIHKVEMGRRMIGERLYGKKVLIVLDDV
NEYGPLDLWESSAWFGEGTVIIITTTDARLLRIYQVDYIFQMNVMNPNKSLELFSWHAFREAKPKKEYHFLARRVVAYC
GGLPLLLEVIGSCLYERTKEEWNRLLLQLDNSAQHEVDQILKISYEDLLNQMEKDLFLDVCCFFIGKCKFYVTKILNDCGVD
PDSGIRVLIKRNLVKIRKNNKVGMHPLLRQMGREISHEILRKEPEKISGLWLDEDMEHALSRNSQTNVIQRFSRRLVNLA
GFSWSLCEKLRWVSLKGFSSQYLPNDFYLRDAIGIDLKHSLLRLVWKESQVLARLKVLNLSHSIYLTETPDFSRLPALEQLIL
KNCQSLRQVHQSIGFLYNLTLLNLKDCTGLTNLPREIYMLKSLKALVLSGCSKIVLLEKDIVQMESLITLISENTVLKQVPFSI
ASSKSIGYISLCGLEERSNNLFPSIIRSRMSPTTNPLSYIHTFSDTEDNSWDDVVPFFSSLAILRSVLVQCDPEFQLSVQLKAI
LMDYCVNITKSRISKHHFRSCLIGVGRYEEFFNTVSDGISEFPAKHSLRPHLIGIGSYQEFFNTYQRFFLSIFF

>XP_027907364.1

MFCPYITKFSHLMFVFKRVILEKPSKQLHKNHFQHNKWSMACPGGTTHSAKLQIFLDGMRAITGICGMEGSGKTTLAK
AIYNQIHGTFLKKSFIEDISEVSRTGKHANLHERLLSDLLKTKLEIHKVEMGRRMIGERLYGKKVLIVLDDVNEYGPLDLWE
SSAWFGEGTVIIITTTDARLLRIYQVDYIFQMNVMNPNKSLELFSWHAFREAKPKKEYHFLARRVVAYCGGLPLLLEVIGS
CLYERTKEEWNRLLLQLDNSAQHEVDQILKISYEDLLNQMEKDLFLDVCCFFIGKCKFYVTKILNDCGVDPDSGIRVLIKR
NLVKIRKNNKVGMHPLLRQMGREISHEILRKEPEKISGLWLDEDMEHALSRNSQTNVIQRFSRRLVNLAGFSWSLCEKL
RWVSLKGFSSQYLPNDFYLRDAIGIDLKHSLLRLVWKESQVLARLKVLNLSHSIYLTETPDFSRLPALEQLILKNCQSLRQV
HQSIGFLYNLTLLNLKDCTGLTNLPREIYMLKSLKALVLSGCSKIVLLEKDIVQMESLITLISENTVLKQVPFSIASSKSIGYISL
CGLEERSNNLFPSIIRSRMSPTTNPLSYIHTFSDTEDNSWDDVVPFFSSLAILRSVLVQCDPEFQLSVQLKAILMDYCVNIT
KSRISKHHFRSCLIGVGRYEEFFNTVSDGISEVLASSGSCDVCLPGDNYPYWSAHRGEGHSVSFTVPRDCVMKGLILCVV
CLSTPEIIEPELTTVLIVNYTRCTLQIHNHGTVISFNDEDWDHIVSNLGSGDRVEIFVSSAYGLVVKETAVYLMYGEPKKNS
LIRSIKKIIM

>XP_027907365.1

MFCPYITKFSHLMFVFKRVILEKPSKQLHKNHFQHNKWSMACPGGTTHSAKLQIFLDGMRAITGICGMEGSGKTTLAK
AIYNQIHDLLKTKLEIHKVEMGRRMIGERLYGKKVLIVLDDVNEYGPLDLWESSAWFGEGTVIIITTTDARLLRIYQVDYIF
QMNVMNPNKSLELFSWHAFREAKPKKEYHFLARRVVAYCGGLPLLLEVIGSCLYERTKEEWNRLLLQLDNSAQHEVDQ
ILKISYEDLLNQMEKDLFLDVCCFFIGKCKFYVTKILNDCGVDPDSGIRVLIKRNLVKIRKNNKVGMHPLLRQMGREISHEI
LRKEPEKISGLWLDEDMEHALSRNSQTNVIQRFSRRLVNLAGFSWSLCEKLRWVSLKGFSSQYLPNDFYLRDAIGIDLKH
SLLRLVWKESQVLARLKVLNLSHSIYLTETPDFSRLPALEQLILKNCQSLRQVHQSIGFLYNLTLLNLKDCTGLTNLPREIYM
LKSLKALVLSGCSKIVLLEKDIVQMESLITLISENTVLKQVPFSIASSKSIGYISLCGLEERSNNLFPSIIRSRMSPTTNPLSYIHT
FSDTEDNSWDDVVPFFSSLAILRSVLVQCDPEFQLSVQLKAILMDYCVNITKSRISKHHFRSCLIGVGRYEEFFNTVSDGIS
EVLASSGSCDVCLPGDNYPYWSAHRGEGHSVSFTVPRDCVMKGLILCVVCLSTPEIIEPELTTVLIVNYTRCTLQIHNHGT
VISFNDEDWDHIVSNLGSGDRVEIFVSSAYGLVVKETAVYLMYGEPKKNSLIRSIKKIIM

>XP_027907366.1

MFCPYITKFSHLMFVFKRVILEKPSKQLHKNHFQHNKWSMACPGGTTHSAKLQIFLDGMRAITDLLKTKLEIHKVEMGR
RMIGERLYGKKVLIVLDDVNEYGPLDLWESSAWFGEGTVIIITTTDARLLRIYQVDYIFQMNVMNPNKSLELFSWHAFRE
AKPKKEYHFLARRVVAYCGGLPLLLEVIGSCLYERTKEEWNRLLLQLDNSAQHEVDQILKISYEDLLNQMEKDLFLDVCCF
FIGKCKFYVTKILNDCGVDPDSGIRVLIKRNLVKIRKNNKVGMHPLLRQMGREISHEILRKEPEKISGLWLDEDMEHALSR
NSQTNVIQRFSRRLVNLAGFSWSLCEKLRWVSLKGFSSQYLPNDFYLRDAIGIDLKHSLLRLVWKESQVLARLKVLNLSH
SIYLTETPDFSRLPALEQLILKNCQSLRQVHQSIGFLYNLTLLNLKDCTGLTNLPREIYMLKSLKALVLSGCSKIVLLEKDIVQ
MESLITLISENTVLKQVPFSIASSKSIGYISLCGLEERSNNLFPSIIRSRMSPTTNPLSYIHTFSDTEDNSWDDVVPFFSSLAIL
RSVLVQCDPEFQLSVQLKAILMDYCVNITKSRISKHHFRSCLIGVGRYEEFFNTVSDGISEVLASSGSCDVCLPGDNYPYW
SAHRGEGHSVSFTVPRDCVMKGLILCVVCLSTPEIIEPELTTVLIVNYTRCTLQIHNHGTVISFNDEDWDHIVSNLGSGDR
VEIFVSSAYGLVVKETAVYLMYGEPKKNSLIRSIKKIIM

>XP_027907367.1

MEFASSTSKLPQMYDLLINFNGEDIHRKFVSHLDSVLSAAGLTTFLHHQNAVNDMDIQQPILNLCRVAIVVFTKTYSESA
WCLHQLQQIIEWHKTYSRHVLPVYYEIQPSDVRLQKGDFGKTLKATAQKSFSAQQMEHGMSRWNHALSKTADFFGW
DESNYRSDAELVDTIVKSILNLPVLSATKFPIGLQPHVEDVIQVIKNKSTGVCTVGICGMEGSGKTTLAKAIYNQIHGTFLK
KSFIEDISEVSRTGKHANLHERLLSDLLKTKLEIHKVEMGRRMIGERLYGKKVLIVLDDVNEYGPLDLWESSAWFGEGTVII
ITTTDARLLRIYQVDYIFQMNVMNPNKSLELFSWHAFREAKPKKEYHFLARRVVAYCGGLPLLLEVIGSCLYERTKEEWN
RLLLQLDNSAQHEVDQILKISYEDLLNQMEKDLFLDVCCFFIGKCKFYVTKILNDCGVDPDSGIRVLIKRNLVKIRKNNKVG
MHPLLRQMGREISHEILRKEPEKISGLWLDEDMEHALSRNSQTNVIQRFSRRLVNLAGFSWSLCEKLRWVSLKGFSSQY

LPNDFYLRDAIGIDLKHSLLRLVWKESQVLARLKVLNLSHSIYLTETPDFSRLPALEQLILKNCQSLRQVHQSIGFLYNLTLL
NLKDCTGLTNLPREIYMLKSLKALVLSGCSKIVLLEKDIVQMESLITLISENTVLKQVPFSIASSKSIGYISLCGLEERSNNLFPS
IIRSRMSPTTNPLSYIHTFSDTEDNSWDDVVPFFSSLAILRSVLVQCDPEFQLSVQLKAILMDYCVNITKSRISKHHFRSCLI
GVGRYEEFFNTVSDGISEVLASSGSCDVCLPGDNYPYWSAHRGEGHSVSFTVPRDCVMKGLILCVVCLSTPEIIEPELTTV
LIVNYTRCTLQIHNHGTVISFNDEDWDHIVSNLGSGDRVEIFVSSAYGLVVKETAVYLMYGEPKKNSLIRSIKKIIM

>XP_027907368.1

MEFASSTSKLPQMYDLLINFNGEDIHRKFVSHLDSVLSAAGLTTFLHHQNAVNDMDIQQPILNLCRVAIVVFTKTYSESA
WCLHQLQQIIEWHKTYSRHVLPVYYEIQPSDVRLQKGDFGKTLKATAQKSFSAQQMEHGMSRWNHALSKTADFFGW
DESNYRSDAELVDTIVKSILNLPVLSATKFPIGLQPHVEDVIQVIKNKSTGVCTVGICGMEGSGKTTLAKAIYNQIHGTFLK
KSFIEDISEVSRTGKHANLHERLLSDLLKTKLEIHKVEMGRRMIGERLYGKKVLIVLDDVNEYGPLDLWESSAWFGEGTVII
ITTTDARLLRIYQVDYIFQMNVMNPNKSLELFSWHAFREAKPKKEYHFLARRVVAYCGGLPLLLEVIGSCLYERTKEEWN
RLLLQLDNSAQHEVDQILKISYEDLLNQMEKDLFLDVCCFFIGKCKFYVTKILNDCGVDPDSGIRVLIKRNLVKIRKNNKVG
MHPLLRQMGREISHEILRKEPEKISGLWLDEDMEHALSRNSQTNVIQRFSRRLVNLAGFSWSLCEKLRWVSLKGFSSQY
LPNDFYLRDAIGIDLKHSLLRLVWKESQVLARLKVLNLSHSIYLTETPDFSRLPALEQLILKNCQSLRQVHQSIGFLYNLTLL
NLKDCTGLTNLPREIYMLKSLKALVLSGCSKIVLLEKDIVQMESLITLISENTVLKQVPFSIASSKSIGYISLCGLEERSNNLFPS
IIRSRMSPTTNPLSYIHTFSDTEDNSWDDVVPFFSSLAILRSVLVQCDPEFQLSVQLKAILMDYCVNITKSRISKHHFRSCLI
GVGRYEEFFNTVSDGISEVLASSGSCDVCLPGDNYPYWSAHRGEGHSVSFTVPRDCVMKGLILCVVCLSTPEIIEPELTTV
LIVNYTRCTLQIHNHGTVISFNDEDWDHIVSNLGSGDRVEIFVSSAYGLVVKETAVYLMYGEPKKNSLIRSIKKIIM

>XP_027907369.1

MEFASSTSKLPQMYDLLINFNGEDIHRKFVSHLDSVLSAAGLTTFLHHQNAVNDMDIQQPILNLCRVAIVVFTKTYSESA
WCLHQLQQIIEWHKTYSRHVLPVYYEIQPSDVRLQKGDFGKTLKATAQKSFSAQQMEHGMSRWNHALSKTADFFGW
DESNYRSDAELVDTIVKSILNLPVLSATKFPIGLQPHVEDVIQVIKNKSTGVCTVGICGMEGSGKTTLAKAIYNQIHGTFLK
KSFIEDISEVSRTGKHANLHERLLSDLLKTKLEIHKVEMGRRMIGERLYGKKVLIVLDDVNEYGPLDLWESSAWFGEGTVII
ITTTDARLLRIYQVDYIFQMNVMNPNKSLELFSWHAFREAKPKKEYHFLARRVVAYCGGLPLLLEVIGSCLYERTKEEWN
RLLLQLDNSAQHEVDQILKISYEDLLNQMEKDLFLDVCCFFIGKCKFYVTKILNDCGVDPDSGIRVLIKRNLVKIRKNNKVG
MHPLLRQMGREISHEILRKEPEKISGLWLDEDMEHALSRNSTNVIQRFSRRLVNLAGFSWSLCEKLRWVSLKGFSSQYL
PNDFYLRDAIGIDLKHSLLRLVWKESQVLARLKVLNLSHSIYLTETPDFSRLPALEQLILKNCQSLRQVHQSIGFLYNLTLLN
LKDCTGLTNLPREIYMLKSLKALVLSGCSKIVLLEKDIVQMESLITLISENTVLKQVPFSIASSKSIGYISLCGLEERSNNLFPSII
RSRMSPTTNPLSYIHTFSDTEDNSWDDVVPFFSSLAILRSVLVQCDPEFQLSVQLKAILMDYCVNITKSRISKHHFRSCLIG
VGRYEEFFNTVSDGISEVLASSGSCDVCLPGDNYPYWSAHRGEGHSVSFTVPRDCVMKGLILCVVCLSTPEIIEPELTTVLI
VNYTRCTLQIHNHGTVISFNDEDWDHIVSNLGSGDRVEIFVSSAYGLVVKETAVYLMYGEPKKNSLIRSIKKIIM

>XP_027907370.1

MEFASSTSKLPQMYDLLINFNGEDIHRKFVSHLDSVLSAAGLTTFLHHQNAVNDMDIQQPILNLCRVAIVVFTKTYSESA
WCLHQLQQIIEWHKTYSRHVLPVYYEIQPSDVRLQKGDFGKTLKATAQKSFSAQQMEHGMSRWNHALSKTADFFGW
DESNYRSDAELVDTIVKSILNLPVLSATKFPIGLQPHVEDVIQVIKNKSTGVCTVGICGMEGSGKTTLAKAIYNQIHDLLKT
KLEIHKVEMGRRMIGERLYGKKVLIVLDDVNEYGPLDLWESSAWFGEGTVIIITTTDARLLRIYQVDYIFQMNVMNPNK
SLELFSWHAFREAKPKKEYHFLARRVVAYCGGLPLLLEVIGSCLYERTKEEWNRLLLQLDNSAQHEVDQILKISYEDLLNQ
MEKDLFLDVCCFFIGKCKFYVTKILNDCGVDPDSGIRVLIKRNLVKIRKNNKVGMHPLLRQMGREISHEILRKEPEKISGL
WLDEDMEHALSRNSQTNVIQRFSRRLVNLAGFSWSLCEKLRWVSLKGFSSQYLPNDFYLRDAIGIDLKHSLLRLVWKES
QVLARLKVLNLSHSIYLTETPDFSRLPALEQLILKNCQSLRQVHQSIGFLYNLTLLNLKDCTGLTNLPREIYMLKSLKALVLSG
CSKIVLLEKDIVQMESLITLISENTVLKQVPFSIASSKSIGYISLCGLEERSNNLFPSIIRSRMSPTTNPLSYIHTFSDTEDNSW

DDVVPFFSSLAILRSVLVQCDPEFQLSVQLKAILMDYCVNITKSRISKHHFRSCLIGVGRYEEFFNTVSDGISEVLASSGSCD
VCLPGDNYPYWSAHRGEGHSVSFTVPRDCVMKGLILCVVCLSTPEIIEPELTTVLIVNYTRCTLQIHNHGTVISFNDEDW
DHIVSNLGSGDRVEIFVSSAYGLVVKETAVYLMYGEPKKNSLIRSIKKIIM

>XP_027907371.1

MEFASSTSKLPQMYDLLINFNGEDIHRKFVSHLDSVLSAAGLTTFLHHQNAVNDMDIQQPILNLCRVAIVVFTKTYSESA
WCLHQLQQIIEWHKTYSRHVLPVYYEIQPSDVRLQKGDFGKTLKATAQKSFSAQQMEHGMSRWNHALSKTADFFGW
DESNYRSDAELVDTIVKSILNLPVLSATKFPIGLQPHVEDVIQVIKNKSTGVCTVGICGMEGSGKTTLAKAIYNQIHGTFLK
KSFIEDISEVSRTGKHANLHERLLSDLLKTKLEIHKVEMGRRMIGERLYGKKVLIVLDDVNEYGPLDLWESSAWFGEGTVII
ITTTDARLLRIYQVDYIFQMNVMNPNKSLELFSWHAFREAKPKKEYHFLARRVVAYCGGLPLLLEVIGSCLYERTKEEWN
RLLLQLDNSAQHEVDQILKISYEDLLNQMEKDLFLDVCCFFIGKCKFYVTKILNDCGVDPDSGIRVLIKRNLVKIRKNNKVG
MHPLLRQMGREISHEILRKEPEKISGLWLDEDMEHALSRNSQTNVIQRFSRRLVNLAGFSWSLCEKLRWVSLKGFSSQY
LPNDFYLRDAIGIDLKHSLLRLVWKESQVLARLKVLNLSHSIYLTETPDFSRLPALEQLILKNCQSLRQVHQSIGFLYNLTLL
NLKDCTGLTNLPREIYMLKSLKALVLSGCSKIVLLEKDIVQMESLITLISENTVLKQVPFSIASSKSIGYISLCGLEERSNNLFPS
IIRSRMSPTTNPLSYIHTFSDTEDNSWDDVVPFFSSLAILRSVLVQCDPEFQLSVQLKAILMDYCVNITKSRISKHHFRSCLI
GVGRYEEFFNTVSDGISEFPAKHSLRPHLIGIGSYQEFFNTYQRRYRAAFF

>XP_027907372.1

MFCPYITKFSHLMFVFKRVILEKPSKQLHKNHFQHNKWSMACPGGTTHSAKLQIFLDGMRAITGICGMEGSGKTTLAK
AIYNQIHGTFLKKSFIEDISEVSRTGKHANLHERLLSDLLKTKLEIHKVEMGRRMIGERLYGKKVLIVLDDVNEYGPLDLWE
SSAWFGEGTVIIITTTDARLLRIYQVDYIFQMNVMNPNKSLELFSWHAFREAKPKKEYHFLARRVVAYCGGLPLLLEVIGS
CLYERTKEEWNRLLLQLDNSAQHEVDQILKISYEDLLNQMEKDLFLDVCCFFIGKCKFYVTKILNDCGVDPDSGIRVLIKR
NLVKIRKNNKVGMHPLLRQMGREISHEILRKEPEKISGLWLDEDMEHALSRNSQTNVIQRFSRRLVNLAGFSWSLCEKL
RWVSLKGFSSQYLPNDFYLRDAIGIDLKHSLLRLVWKESQVLARLKVLNLSHSIYLTETPDFSRLPALEQLILKNCQSLRQV
HQSIGFLYNLTLLNLKDCTGLTNLPREIYMLKSLKALVLSGCSKIVLLEKDIVQMESLITLISENTVLKQVPFSIASSKSIGYISL
CGLEERSNNLFPSIIRSRMSPTTNPLSYIHTFSDTEDNSWDDVVPFFSSLAILRSVLVQCDPEFQLSVQLKAILMDYCVNIT
KSRISKHHFRSCLIGVGRYEEFFNTVSDGISEVLASSGSCDVCLPGDNYPYWSAHRGEGHSVSFTVPRDCVMKGLILCVV
CLSTPEIIEPELTTVLIVNYTRCTLQIHNHGTVISFNDEDWDHIVSNLGSGDRVEIFVSSAYGLVVKETAVYLMYGEPKKNS
LIRSIKKIIM

>XP_027907373.1

MFCPYITKFSHLMFVFKRVILEKPSKQLHKNHFQHNKWSMACPGGTTHSAKLQIFLDGMRAITGICGMEGSGKTTLAK
AIYNQIHDLLKTKLEIHKVEMGRRMIGERLYGKKVLIVLDDVNEYGPLDLWESSAWFGEGTVIIITTTDARLLRIYQVDYIF
QMNVMNPNKSLELFSWHAFREAKPKKEYHFLARRVVAYCGGLPLLLEVIGSCLYERTKEEWNRLLLQLDNSAQHEVDQ
ILKISYEDLLNQMEKDLFLDVCCFFIGKCKFYVTKILNDCGVDPDSGIRVLIKRNLVKIRKNNKVGMHPLLRQMGREISHEI
LRKEPEKISGLWLDEDMEHALSRNSQTNVIQRFSRRLVNLAGFSWSLCEKLRWVSLKGFSSQYLPNDFYLRDAIGIDLKH
SLLRLVWKESQVLARLKVLNLSHSIYLTETPDFSRLPALEQLILKNCQSLRQVHQSIGFLYNLTLLNLKDCTGLTNLPREIYM
LKSLKALVLSGCSKIVLLEKDIVQMESLITLISENTVLKQVPFSIASSKSIGYISLCGLEERSNNLFPSIIRSRMSPTTNPLSYIHT
FSDTEDNSWDDVVPFFSSLAILRSVLVQCDPEFQLSVQLKAILMDYCVNITKSRISKHHFRSCLIGVGRYEEFFNTVSDGIS
EVLASSGSCDVCLPGDNYPYWSAHRGEGHSVSFTVPRDCVMKGLILCVVCLSTPEIIEPELTTVLIVNYTRCTLQIHNHGT
VISFNDEDWDHIVSNLGSGDRVEIFVSSAYGLVVKETAVYLMYGEPKKNSLIRSIKKIIM

>XP_027907375.1

MFCPYITKFSHLMFVFKRVILEKPSKQLHKNHFQHNKWSMACPGGTTHSAKLQIFLDGMRAITDLLKTKLEIHKVEMGR
RMIGERLYGKKVLIVLDDVNEYGPLDLWESSAWFGEGTVIIITTTDARLLRIYQVDYIFQMNVMNPNKSLELFSWHAFRE
AKPKKEYHFLARRVVAYCGGLPLLLEVIGSCLYERTKEEWNRLLLQLDNSAQHEVDQILKISYEDLLNQMEKDLFLDVCCF
FIGKCKFYVTKILNDCGVDPDSGIRVLIKRNLVKIRKNNKVGMHPLLRQMGREISHEILRKEPEKISGLWLDEDMEHALSR
NSQTNVIQRFSRRLVNLAGFSWSLCEKLRWVSLKGFSSQYLPNDFYLRDAIGIDLKHSLLRLVWKESQVLARLKVLNLSH
SIYLTETPDFSRLPALEQLILKNCQSLRQVHQSIGFLYNLTLLNLKDCTGLTNLPREIYMLKSLKALVLSGCSKIVLLEKDIVQ
MESLITLISENTVLKQVPFSIASSKSIGYISLCGLEERSNNLFPSIIRSRMSPTTNPLSYIHTFSDTEDNSWDDVVPFFSSLAIL
RSVLVQCDPEFQLSVQLKAILMDYCVNITKSRISKHHFRSCLIGVGRYEEFFNTVSDGISEVLASSGSCDVCLPGDNYPYW
SAHRGEGHSVSFTVPRDCVMKGLILCVVCLSTPEIIEPELTTVLIVNYTRCTLQIHNHGTVISFNDEDWDHIVSNLGSGDR
VEIFVSSAYGLVVKETAVYLMYGEPKKNSLIRSIKKIIM

>XP_027907376.1

MEFASSTSKLPKMYDVLINFNGEDIRRKFVSHLDSVLSAAGLTTFLHHQNSVNDMDIQQPILNLCRVAIVVFTKTYSQSA
WCLHQLHQIIEWHKTYCRHVLPVYYDIQPSDVRLQKGDFGKTLKATAQQSFSAQQLEHGMSRWNHALSKTADFFGW
DESNYRSDAELVDTIVKSILNLPVLSATKFPIGLQPHVEDVIQIIKNKSTGVCTVGICGMGGSGKTTLAKAIYNQIHGTFLEK
SFIEDISEVSRTRKQANLQERLLSDLLKTKVEIHRVEMGRSMIRENLYGKKVLIVLDDVSEYGPLDLWESSARFGEGTVIIIT
TRDDRLLRIHQVDSIFRMNLMNPNESLELLSWHAFREPKLKEEYHFLAERVVAYCGGLPLLLEVIGSCLYERTKEEWNRLL
LQLDNSPQHEVSQILKISYEGLLNQMEKDLFLDVCCFFIGKCRFYVTKILNGCGVDPDRGIRVLIKRNLVKVRKNNKFGM
HPLLRQMGREISHEILSKEPEKISGLWLDEDAEHALSRNTLFSSQQTKVIQRFSRRLVNLAGVSWSLCEKLRWVSLKGFFS
QYLPNDFYLRDAIVIDLKHSLLRLVWKQPQKPLTFRDYQLSNNSFSKIAKVCVKYNLTLLNLKGCTGLGNLPREIYMLKSLK
ALVLSGCSKIILLEKDIVQMESLITLISENTVVKQVPFSIASSKSIGYISLCGLEERSHNLFPSIIRSRMSPTTNPLSYIHTFSGM
EDNSWDDIVPFFSSLTILRSVLVQCDPEFQLSKQLQAILVDYCANITESRISKHHFRSCLVGVGRYEEFFNTVSYGIYEVLAS
SGSCDVCLPGDNYPYWSAHSGEGHSVSFTVPRDCVMKGLILCVVCLSTPEIIEPELTTVIIVNYTRCTLQIHNHGTVISFND
EDWDHIVSNLGSGDRVEIFVSSAYGLVVKETAVYLMYGEPKKNSLIRSIKKIIM

>XP_027907397.1

MDFLGPFGKVVEGVIDFVSKNGVRHMTYIVHYNENVRELKDSVRDLKFEKQRIIHQCEEGTKNLNNIEEKVTEWVRKVS
EIDTTIEVFENDDGHTKARSPNCFVFPYLWNRHRLGRQAYKEKVNVKRLIDESPKVDEVLYRQNVTSNDATLSNCGFVE
FSSIKSTMEKVMIELQDSSVRMIGLYGRGGVGKSTLVKEIARKAKEKKLFDVVVKVEITADPNPQKIQEEIAYVLGLRLEGE
GENVRADCLRRRLKKESGNILLILDDLWDKLDLNKLGIPLDDDEDDDDFNSGNKDLSRQKNKDNKDPNQKTQEKKDDN
RTVLKKEKFLGGHKGCKILLTSRDKKVLCVEMDVKTTFCVRELDDKDALILFKKLAGIHNEMSSSKQEIVKKYCEGLPMAI
VVVARALRNKSESVWEATIKKLKKHELVGEGTSMDISVKMSYHHLENEEIKSIFLLCAQMGRRPLIMDLVKYCFGLGILE
GVSSLWEAREKIKTSIQKLKDSGLLLDESSNNHFNMHGMVRDTALSIAHKDHNAFNLRNGKLDDWPELEKCTSISICNS
DIIDGLPEVINCPQLKLFQIDTNDPSLEIPEGFFRRMKNLNVLIMTGFRLSSLPYSIQCLLKLRMLCLERCTLRCNLSIIGKLK
KLRILSFSGSLLQNLPAELQCLDKLRMLDISDCSELKIIPPNLISSLTCLEELYIRESLIKMLVDRETNKGQDLFLSELKNLHQL
KVVELSIPCVSIFPNHLFFDKLKDYKIMIGDFDFFSLGEFRMPDKYETLRVLALQLKDDTNIHSQESIKLLLKTVQILLLGKIN
GVQSVVNELNIDGFPDLKHLSIINNSDIKYVNSTELCNYVNVFPNLESLCLNNLGNLDMICYGPITVVSFAKLKTIKVEMCY
RLKNLYSLDMVMFPTGAQTCEISEYNSYMDKFLSSLEIIEVSECGSLKEILQIPKHYGKVEFLKLHTLTLRLLPLFTCFYTKVE
KFCCPHLTEAQTTNRGLTELSEEDEQSDKEPPLFGELVEIPNLETLNLSSLNIHKIWGDQHSSSFIFQNLIKLVVKDCDKLTY
LCSLSMARSLKKLKSLVISECPIMEKIFKTERNSADKVCIFPMLEEIHLSKMNRLTDMWQTKVSADSFSSLISVKIEECNKL
DKIFPSHMEGWFESLDNLKVSWCQSVEVIFEINVSQEKDEFGGIDTNLQVILLEGLPNLKQLWSTDPNGILNFKKLQSIE
VGSCDELRNLFPVSVAKDIPKLEHMSALHCEKMEEIVASQDASEAKKDLLLFPKLTSVRLYDLPNMKYFYKKRYPIKCPKL
KELSVRRCVSLKHFSKIPLKQQTKSKISFSQLKRYSPTWSTWKLTSKKHKNCYRSTKCTA

>XP_027907398.1

MDFLGPFGKVVEGVIDFVSKNGVRHMTYIVHYNENVRELKDSVRDLKFEKQRIIHQCEEGTKNLNNIEEKVTEWVRKVS
EIDTTIEVFENDDGHTKARSPNCFVFPYLWNRHRLGRQAYKEKVNVKRLIDESPKVDEVLYRQNVTSNDATLSNCGFVE
FSSIKSTMEKVMIELQDSSVRMIGLYGRGGVGKSTLVKEIARKAKEKKLFDVVVKVEITADPNPQKIQEEIAYVLGLRLEGE
GENVRADCLRRRLKKESGNILLILDDLWDKLDLNKLGIPLDDDEDDDDFNSGNKDLSRQKNKDNKDPNQKTQEKKDDN
RTVLKKEKFLGGHKGCKILLTSRDKKVLCVEMDVKTTFCVRELDDKDALILFKKLAGIHNEMSSSKQEIVKKYCEGLPMAI
VVVARALRNKSESVWEATIKKLKKHELVGEGTSMDISVKMSYHHLENEEIKSIFLLCAQMGRRPLIMDLVKYCFGLGILE
GVSSLWEAREKIKTSIQKLKDSGLLLDESSNNHFNMHGMVRDTALSIAHKDHNAFNLRNGKLDDWPELEKCTSISICNS
DIIDGLPEVINCPQLKLFQIDTNDPSLEIPEGFFRRMKNLNVLIMTGFRLSSLPYSIQCLLKLRMLCLERCTLRCNLSIIGKLK
KLRILSFSGSLLQNLPAELQCLDKLRMLDISDCSELKIIPPNLISSLTCLEELYIRESLIKMLVDRETNKGQDLFLSELKNLHQL
KVVELSIPCVSIFPNHLFFDKLKDYKIMIGDFDFFSLGEFRMPDKYETLRVLALQLKDDTNIHSQESIKLLLKTVQILLLGKIN
GVQSVVNELNIDGFPDLKHLSIINNSDIKYVNSTELCNYVNVFPNLESLCLNNLGNLDMICYGPITVVSFAKLKTIKVEMCY
RLKNLYSLDMVMFPTGAQTCEISEYNSYMDKFLSSLEIIEVSECGSLKEILQIPKHYGKVEFLKLHTLTLRLLPLFTCFYTKVE
KFCCPHLTEAQTTNRGLTELSEEDEQSDKEPPLFGELVEIPNLETLNLSSLNIHKIWGDQHSSSFIFQNLIKLVVKDCDKLTY
LCSLSMARSLKKLKSLVISECPIMEKIFKTERNSADKVCIFPMLEEIHLSKMNRLTDMWQTKVSADSFSSLISVKIEECNKL
DKIFPSHMEGWFESLDNLKVSWCQSVEVIFEINVSQEKDEFGGIDTNLQVILLEGLPNLKQLWSTDPNGILNFKKLQSIE
VGSCDELRNLFPVSVAKDIPKLEHMSALHCEKMEEIVASQDASEAKKDLLLFPKLTSVRLYDLPNMKYFYKKRYPIKCPKL
KELSVRRCVSLKHFSKIPLKQQTKSKISFSQLKRYSPTWSTWKLTSKKHKNCYRSTKCTA

>XP_027907399.1

MDFLGPFGKVVEGVIDFVSKNGVRHMTYIVHYNENVRELKDSVRDLKFEKQRIIHQCEEGTKNLNNIEEKVTEWVRKVS
EIDTTIEVFENDDGHTKARSPNCFVFPYLWNRHRLGRQAYKEKVNVKRLIDESPKVDEVLYRQNVTSNDATLSNCGFVE
FSSIKSTMEKVMIELQDSSVRMIGLYGRGGVGKSTLVKEIARKAKEKKLFDVVVKVEITADPNPQKIQEEIAYVLGLRLEGE
GENVRADCLRRRLKKESGNILLILDDLWDKLDLNKLGIPLDDDEDDDDFNSGNKDLSRQKNKDNKDPNQKTQEKKDDN
RTVLKKEKFLGGHKGCKILLTSRDKKVLCVEMDVKTTFCVRELDDKDALILFKKLAGIHNEMSSSKQEIVKKYCEGLPMAI
VVVARALRNKSESVWEATIKKLKKHELVGEGTSMDISVKMSYHHLENEEIKSIFLLCAQMGRRPLIMDLVKYCFGLGILE
GVSSLWEAREKIKTSIQKLKDSGLLLDESSNNHFNMHGMVRDTALSIAHKDHNAFNLRNGKLDDWPELEKCTSISICNS
DIIDGLPEVINCPQLKLFQIDTNDPSLEIPEGFFRRMKNLNVLIMTGFRLSSLPYSIQCLLKLRMLCLERCTLRCNLSIIGKLK
KLRILSFSGSLLQNLPAELQCLDKLRMLDISDCSELKIIPPNLISSLTCLEELYIRESLIKMLVDRETNKGQDLFLSELKNLHQL
KVVELSIPCVSIFPNHLFFDKLKDYKIMIGDFDFFSLGEFRMPDKYETLRVLALQLKDDTNIHSQESIKLLLKTVQILLLGKIN
GVQSVVNELNIDGFPDLKHLSIINNSDIKYVNSTELCNYVNVFPNLESLCLNNLGNLDMICYGPITVVSFAKLKTIKVEMCY
RLKNLYSLDMVMFPTGAQTCEISEYNSYMDKFLSSLEIIEVSECGSLKEILQIPKHYGKVEFLKLHTLTLRLLPLFTCFYTKVE
KFCCPHLTEAQTTNRGLTELSEEDEQSDKEPPLFGELVEIPNLETLNLSSLNIHKIWGDQHSSSFIFQNLIKLVVKDCDKLTY
LCSLSMARSLKKLKSLVISECPIMEKIFKTERNSADKVCIFPMLEEIHLSKMNRLTDMWQTKVSADSFSSLISVKIEECNKL
DKIFPSHMEGWFESLDNLKVSWCQSVEVIFEINVSQEKDEFGGIDTNLQVILLEGLPNLKQLWSTDPNGILNFKKLQSIE
VGSCDELRNLFPVSVAKDIPKLEHMSALHCEKMEEIVASQDASEAKKDLLLFPKLTSVRLYDLPNMKYFYKKRYPIKCPKL
KELSVRRCVSLKHFSKIPLKQQTKSKISFSQLKRYSPTWSTWKLTSKKHKNCYRSTKCTA

>XP_027907400.1

MDFLGPFGKVVEGVIDFVSKNGVRHMTYIVHYNENVRELKDSVRDLKFEKQRIIHQCEEGTKNLNNIEEKVTEWVRKVS
EIDTTIEVFENDDGHTKARSPNCFVFPYLWNRHRLGRQAYKEKVNVKRLIDESPKVDEVLYRQNVTSNDATLSNCGFVE
FSSIKSTMEKVMIELQDSSVRMIGLYGRGGVGKSTLVKEIARKAKEKKLFDVVVKVEITADPNPQKIQEEIAYVLGLRLEGE
GENVRADCLRRRLKKESGNILLILDDLWDKLDLNKLGIPLDDDEDDDDFNSGNKDLSRQKNKDNKDPNQKTQEKKDDN
RTVLKKEKFLGGHKGCKILLTSRDKKVLCVEMDVKTTFCVRELDDKDALILFKKLAGIHNEMSSSKQEIVKKYCEGLPMAI
VVVARALRNKSESVWEATIKKLKKHELVGEGTSMDISVKMSYHHLENEEIKSIFLLCAQMGRRPLIMDLVKYCFGLGILE

GVSSLWEAREKIKTSIQKLKDSGLLLDESSNNHFNMHGMVRDTALSIAHKDHNAFNLRNGKLDDWPELEKCTSISICNS
DIIDGLPEVINCPQLKLFQIDTNDPSLEIPEGFFRRMKNLNVLIMTGFRLSSLPYSIQCLLKLRMLCLERCTLRCNLSIIGKLK
KLRILSFSGSLLQNLPAELQCLDKLRMLDISDCSELKIIPPNLISSLTCLEELYIRESLIKMLVDRETNKGQDLFLSELKNLHQL
KVVELSIPCVSIFPNHLFFDKLKDYKIMIGDFDFFSLGEFRMPDKYETLRVLALQLKDDTNIHSQESIKLLLKTVQILLLGKIN
GVQSVVNELNIDGFPDLKHLSIINNSDIKYVNSTELCNYVNVFPNLESLCLNNLGNLDMICYGPITVVSFAKLKTIKVEMCY
RLKNLYSLDMVMFPTGAQTCEISEYNSYMDKFLSSLEIIEVSECGSLKEILQIPKHYGKVEFLKLHTLTLRLLPLFTCFYTKVE
KFCCPHLTEAQTTNRGLTELSEEDEQSDKEPPLFGELVEIPNLETLNLSSLNIHKIWGDQHSSSFIFQNLIKLVVKDCDKLTY
LCSLSMARSLKKLKSLVISECPIMEKIFKTERNSADKVCIFPMLEEIHLSKMNRLTDMWQTKVSADSFSSLISVKIEECNKL
DKIFPSHMEGWFESLDNLKVSWCQSVEVIFEINVSQEKDEFGGIDTNLQVILLEGLPNLKQLWSTDPNGILNFKKLQSIE
VGSCDELRNLFPVSVAKDIPKLEHMSALHCEKMEEIVASQDASEAKKDLLLFPKLTSVRLYDLPNMKYFYKKRYPIKCPKL
KELSVRRCVSLKHFSKIPLKQQTKSKISFSQLKRYSPTWSTWKLTSKKHKNCYRSTKCTA

>XP_027907418.1

MEFASSSSSSSSLLKSEPHFIYDVFINFGGEEIGKKFVSHLHSVLLQAEFETLINEENVQEGMKVEEHMRSIAGSKISIIIFSK
TYTESTCCLLELEKIVECYQTFGQIVLPVFYEIDPLDVHHQKGDFGKALEEAAHKSYSGEQVEHALFRWSRALATAAGITC
WDARYFRHDAELVEVIVSRVQTLLDYSELSITRFPVGLESHMEKLIGCIENHSAEVCMIGIWGTGGSGKTTIAKAIYNRIY
RQFIGKSFIENIAEVRNRVYRTDVDLQKNLIYDVLKSTLKVESHGMGRTMIETELSLKKLLIVVDDVNEFSQLENLCGNRE
WFGQGSVIIITARDVRLLNRLKVDYVYKMDGMNENDSFELFSCHAFGEAKPRKDVKELARNIVAQCGGLPLALQVLGSF
LCDRTIEEWESVLSKLKIIPTGVVQKKLKISFDGLSDMEQNIFLDVCFFFIGRERGYVTEMLNNWEEHADIGITVLIKCGLI
QVDRNNKLEMHPLLQDMGREIIRQRWPKESAKRSLLWFQGDVKDVLTKNIETSYPGVVLETAFNQQSSLQTSYAFDG
NEELT

>XP_027907419.1

MCSLTLGEKKSEGMKVEEHMRSIAGSKISIIIFSKTYTESTCCLLELEKIVECYQTFGQIVLPVFYEIDPLDVHHQKGDFGKA
LEEAAHKSYSGEQVEHALFRWSRALATAAGITCWDARYFRHDAELVEVIVSRVQTLLDYSELSITRFPVGLESHMEKLIGC
IENHSAEVCMIGIWGTGGSGKTTIAKAIYNRIYRQFIGKSFIENIAEVRNRVYRTDVDLQKNLIYDVLKSTLKVESHGMGR
TMIETELSLKKLLIVVDDVNEFSQLENLCGNREWFGQGSVIIITARDVRLLNRLKVDYVYKMDGMNENDSFELFSCHAFG
EAKPRKDVKELARNIVAQCGGLPLALQVLGSFLCDRTIEEWESVLSKLKIIPTGVVQKKLKISFDGLSDMEQNIFLDVCFFFI
GRERGYVTEMLNNWEEHADIGITVLIKCGLIQVDRNNKLEMHPLLQDMGREIIRQRWPKESAKRSLLWFQGDVKDVL
TKNIETSYPGVVLETAFNQQSSLQTSYAFDGNEELT

>XP_027907420.1

MSFPSKLMNASYMKWGADLKKDKDKDEDDDEEEEANSIDELEVNSSQLTSILLMEFASSSSSSSSSSFIKSEPHFIYDAFI
NFWGEDIGRKFVSHLHSAFLQAQVKTFISQESLPKELELEEHMRAIGGTKIAIIVFSKTYTESACCLLELEKIIECLETFGQIVL
PVFYENEPLYVRDEKNDFGKAMEETAHKSYSGEQREQVLSRWRRALNKAAGITGLNVREFRHDAQLVAVTVRRVQSLL
GYQDLSVIPFPVELEPHVEKVIGCIENISTKVFMIGICGKEGSGKTAIAKAIYNRIYRLFIAKNFFDYIKGVWDPVDKRYVDL
KEFVNDVLKDKLEFERTVMERVIMDYNEHSRRKLLIVFDDVTVFGQLKNLYRNRTRFGQGTLIIITTRDVRILKRLKVDYV
YKMNVMN

>XP_027907421.1

MSFPSKLMNASYMKWGADLKKDKDKDEDDDEEEEANSIDELEVNSSQLTSILLMEFASSSSSSSSSSFIKSEPHFIYDAFI
NFWGEDIGRKFVSHLHSAFLQAQVKTFISQESLPKELELEEHMRAIGGTKIAIIVFSKTYTESACCLLELEKIIECLETFGQIVL
PVFYENEPLYVRDEKNDFGKAMEETAHKSYSGEQREQVLSRWRRALNKAAGITGLNVREFRHDAQLVAVTVRRVQSLL

GYQDLSVIPFPVELEPHVEKFSCFQISKPRKELKELARNIVVYRGGLPVALKFLGSFLCVRTMEKWDCVFSNLKLIPTGILLD
ACIVKERETMSQRYKLKGTTLEMHSLLRDMGRDIIRKRWPKQQRKRSRLWFQDDVKDVVLTKNIERTFLI

>XP_027907422.1

MSFPSKLMNASYMKWGADLKKDKDKDEDDDEEEEANSIDELEVNSSQLTSILLMEFASSSSSSSSSSFIKSEPHFIYDAFI
NFWGEDIGRKFVSHLHSAFLQAQVKTFISQESLPKELELEEHMRAIGGTKIAIIVFSKTYTESACCLLELEKIIECLETFGQIVL
PVFYENEPLYVRDEKNDFGKAMEETAHKSYSGEQREQVLSRWRRALNKAAGITGLNVREFRHDAQLVAVTVRRVQSLL
GYQDLSVIPFPVELEPHVEKISKPRKELKELARNIVVYRGGLPVALKFLGSFLCVRTMEKWDCVFSNLKLIPTGILLDACIVK
ERETMSQRYKLKGTTLEMHSLLRDMGRDIIRKRWPKQQRKRSRLWFQDDVKDVVLTKNIERTFLI

>XP_027907423.1

MEFASSSSSSSSSFIKSEPRFIYDAFINFGGEDIGRKFASHLTYALLQAQVKTFISDESLPKGLELEEHMRAIGGTKIAIIVFSK
TYTESACCLLELEKIIECLETFGQIVLPVFYDNGPRYVRDHDRNDFGKAMEETARKSYSGEQLKQVLSRWRRALNKAAGII
HGLYGRNFRHDAELVEVTVRRVQSLLDYEDLSVFPFPVELESHVKKVIKCIENHSTKVFMIGIWGKEGSGKTILAKVIYNRI
YRRFIGKNYFEYIKGVWDRVDRRDVDLKEFVNDVLKDKFEIESIRRRRVIMEKTELFRRKLLIVFDGVTEFGQLESLYRNRK
WFGQGTVIIITTRDVQILKRLKVDYVYKMNVMNENNSLLTFQFSCLQISITKKRTE

>XP_027907490.1

MASPVGCCQFLTSSTRAKVWLKKQFIQLYLYETRVAELKDVVDKLEKKKDSIQHTVDEEERRHGRRIHVEVKEWMDRV
NKLILAYKDFDNDEIYHKCAVFDFFDSGYLPKPGIRYRRSRKAYDITEQANELLQNAKFDIFFNWSGPPSTAAFFSNLGYE
SYSSRNDTVKNVIDEFQKPGVRMIGLYGLSGVGKTSLVKEVVKKALKDKMFEVVTMASVTKNPDIRKIQGQIADMLGV
VLEEESDIARAARIHQILNDENKSTLIILDDLWEEVNFNLLGIPHELEKDDDVTIDRRKSLDVDILKNVNDRKSSDLDGSTSF
RKGMLHGVGGLKNINEGKSHVDAFDVVKTKKNVTQYKGCKILMISEIKPVLLSQMEGKEESIFPVEALKENEAEMLFKK
KAGICGNNYEYDKLAAQIASKCKGLPMTIVTTARALKNKSLSVWDETNRKLESQNLARAPEFSTKLSYELLEDEELKYTFLL
CARMGHDGLVMDLVKYCIGFGFLQGINTARQTRDKVYMLVAKLKESGLLSDSYSSDHFTMPDTIRTAALSIAYKENHLF
TMTKRKLDEWPNNLERYAAISLHHCDFIEEFPGRINYPRLRVLQIVNNIPRPKIPEKFFETMKELRVLILTGIHLPLVGSSISS
LHKLRMLCLEQCCMLDKELYIIGELKRLRILSFSGSDIESLPAELNELKMLQIFDISNCSKLKKIPNGVISSLVSLEELYMRNTL
IQWEDEEQTRQSKITLLSDLKHLNQLTTLDIQIPNVSYLPKNLFFDKLFSYKIVIGNLSSVLETDFKMPEKYETLKFLAIQLKN
GSDIHSLKGIKMLFEEVENLFLELNSVHEAQNIVHDLFYRLNLNGFPYLKHLWMVNNSTIQSLIYPKDRQLSEIAFPKLKSL
YLYNLKIDKICSCELSKPSFGKLKVIKINLCGELKNVFSISVVGLLKVLETIEVSECNSLKEIIDVEPQSDPEKTELHMLPELRYL
KLQSLSEFIGFDAIPHIEGNERKLFHEKVAVSKLERLELSSIQIDTIWSVDPSSKRLSFESLTHLDVNGCWKLKYLMSFTMA
KSLVNLQSLYVSDCVKMGSIFLEQVIEKDITGSIFPKLKNMKLRNIKSLSKIWYPKLPSGSFNKLDTLIIEECHRLENALDGIF
GSLCNLRITNCRSMQAIFNILDQVGDVVNNLQDVHLETLPKLKLVWKMNYKDGVGIPKFNNLKKIWAQDCDNLKYIFP
FFVAKSLHNLEFLVVCDCYGLSEIVAEREVTNTDEVNFNFPKLSIVKFSELPKLTSFCPTAYTLSCPLKELSIELCYNLEPFNN
KTDHVHECFPQKVINNLKSMQIEFWHAKSPSSYIGKVNHRRDNLEELSLSRIMDTEILYSFLHRIPNLKSLSLNNCFFEKIV
PPKEDTEFENLGVVPNLKRLMLIDLPNLIEIGFEPDIILERLEFLILKNCPRMITVAPSYVSFTRLTNLEVVSCDGLQSLMSAS
TAKGLVQLNTMKVEKCESLVEIVRKDGEKSDRVVFQQLKALELVSLKKLKSFCVSDCGFEFPSLEKLVVSACYNMDKFSET
VPSSPILQNVDVVHGKENKRLCWEGDINATIKEIFREMKFFEGLEEMSLSQHQELQGSWQHGVGLQEKNSWFYSLKIL
KIENCEIQPCAIPSNILPCLRSLKELHVQACNNVEVIFEMNVEEGTGTTFNLEILTLQKLPKLKDVWERNGKGTESFQNLKL
VNVSECKNLQSVFPLTLAKNLKKLAELQITRCHVLQEIVRKEEDTTTVFAFPCLTTLHLGDLPELIYFYPQSFTLECSTLNSLF
VWNCQELELFGSAYRQLIFLDLKNIFNLEELLLDWEHTMVLTKLGKSMDNLNYLNHIQLFFDADENERPYFPIQILQKMP
NLTDMGIYYCSCLEVFQTKISEIAEKKVLTNLETLTLDNVSKLQSIGSKDSWLNVICDSEKLQHLYISNCPDLKTLVHSTPLV
SFTYVKEMYIHRCQELKYLFTLSSLKKLKNLEHIEVSDCESMEVIVFKERDDTSEEIKLQQLKSINLIRLSSLKCFYSDNDTLRL
PSLMLVDIWICPKMKFFSGGVIHLNSSFKGTLASNVSSDDLVFCRDLNSSVEKDSLQLEFFEAVDKECFSDNFELQVDPLC

KNGLLYKWLVNLETLKLQNCMLSYAIPSSILSALKNLKELEVRDSKQVKAIFYINDDTDIKETELQLKILTLDGLSELTHVWK
NDTNKILIFRNLQEVVVSDCAKLQTLFPASLSKSLKDLKKLEIYFCKNLQDLVEQEETTLVTEKFVFPCLEDLEFKDLPQVTC
PKTFTLEFPSVKFLSVRNCDELRLFQSVYDPTGEGTSSKTLPLISDPKVMSNLKKLTLDWKQILALSLWFKSQQTTEGLTNL
DRISLCFFGAKENEMPMLPVEILKAPNLTEMDIINCESLENFLAQNPMIGDEEMLGQLTILMLYNVATTQLFELEYSSSLN
IMCGRLHELNVVQCLHLTTLGVLSTSTVSFSCLKEVSIHKCPNLKYLFTTSAAKKLANLEKILVIKCESITEIVAKEGDATSEAI
KFERLHAIHLQSLTSLKCFYSGSDTLQLPSLKFVAIWNCPNMEIFSHGIESLMEITLSMDQEAGYLPPPQDLNTRIKGISQR
KEFFEAVDKECFSDNLELQENPHCKFGLQNQWLGDLVTLKLKKCTLSCAIPSAILALLKNLKELEVRDSPTVEVLFCMNDT
EILETASQLRFLTLKRLSKLTHFWEKKKNGVLIFPNLQQVVVRKCEKLETLFPASLAKNLKSLKAIKIEDCAKFQEIVEKEEAT
EAKFVLPCLQKLDLFCLPQLTCFYPQTFTLECSALNELSILKCDKLELFQSAPSMGEVTSVNRQPLISSLEVISNLKILKLDWK
QILALRSRLKSEKFTGIFKCVNKMKLVLDGGESEMHVVLNEILHTTPNLIEMIMGIYNCNSPEIFLAQNPKIAEDGMLLHL
RILALSYVSTIRSIQSQNSSWLNTICEKVHDLNVFQCRHLETIGVHSTSTLSFSLLKKVDVLRCRRLQYLFTSSVAKELVNLKE
IIVQECKSLKEIIAKEGDEEGEGEDNSENEIIFVKLEKLSLGSLGKLESFYTGSCTLNFPSLRKVEVNKCFNSKIFRHRDKVPPK
FTVIIDRIGCRSDKKPLIMQQVEEEVSRVSLVTTNSLDFCRESGDSKKEMIAT

>XP_027907491.1

MASPVGCCQFLTSSTRAKVWLKKQFIQLYLYETRVAELKDVVDKLEKKKDSIQHTVDEEERRHGRRIHVEVKEWMDRV
NKLILAYKDFDNDEIYHKCAVFDFFDSGYLPKPGIRYRRSRKAYDITEQANELLQNAKFDIFFNWSGPPSTAAFFSNLGYE
SYSSRNDTVKNVIDEFQKPGVRMIGLYGLSGVGKTSLVKEVVKKALKDKMFEVVTMASVTKNPDIRKIQGQIADMLGV
VLEEESDIARAARIHQILNDENKSTLIILDDLWEEVNFNLLGIPHELEKDDDVTIDRRKSLDVDILKNVNDRKSSDLDGSTSF
RKGMLHGVGGLKNINEGKSHVDAFDVVKTKKNVTQYKGCKILMISEIKPVLLSQMEGKEESIFPVEALKENEAEMLFKK
KAGICGNNYEYDKLAAQIASKCKGLPMTIVTTARALKNKSLSVWDETNRKLESQNLARAPEFSTKLSYELLEDEELKYTFLL
CARMGHDGLVMDLVKYCIGFGFLQGINTARQTRDKVYMLVAKLKESGLLSDSYSSDHFTMPDTIRTAALSIAYKENHLF
TMTKRKLDEWPNNLERYAAISLHHCDFIEEFPGRINYPRLRVLQIVNNIPRPKIPEKFFETMKELRVLILTGIHLPLVGSSISS
LHKLRMLCLEQCCMLDKELYIIGELKRLRILSFSGSDIESLPAELNELKMLQIFDISNCSKLKKIPNGVISSLVSLEELYMRNTL
IQWEDEEQTRQSKITLLSDLKHLNQLTTLDIQIPNVSYLPKNLFFDKLFSYKIVIGNLSSVLETDFKMPEKYETLKFLAIQLKN
GSDIHSLKGIKMLFEEVENLFLELNSVHEAQNIVHDLFYRLNLNGFPYLKHLWMVNNSTIQSLIYPKDRQLSEIAFPKLKSL
YLYNLKIDKICSCELSKPSFGKLKVIKINLCGELKNVFSISVVGLLKVLETIEVSECNSLKEIIDVEPQSDPEKTELHMLPELRYL
KLQSLSEFIGFDAIPHIEGNERKLFHEKVAVSKLERLELSSIQIDTIWSVDPSSKRLSFESLTHLDVNGCWKLKYLMSFTMA
KSLVNLQSLYVSDCVKMGSIFLEQVIEKDITGSIFPKLKNMKLRNIKSLSKIWYPKLPSGSFNKLDTLIIEECHRLENALDGIF
GSLCNLRITNCRSMQAIFNILDQVGDVVNNLQDVHLETLPKLKLVWKMNYKDGVGIPKFNNLKKIWAQDCDNLKYIFP
FFVAKSLHNLEFLVVCDCYGLSEIVAEREVTNTDEVNFNFPKLSIVKFSELPKLTSFCPTAYTLSCPLKELSIELCYNLEPFNN
KTDHVHECFPQKVINNLKSMQIEFWHAKSPSSYIGKVNHRRDNLEELSLSRIMDTEILYSFLHRIPNLKSLSLNNCFFEKIV
PPKEDTEFENLGVVPNLKRLMLIDLPNLIEIGFEPDIILERLEFLILKNCPRMITVAPSYVSFTRLTNLEVVSCDGLQSLMSAS
TAKGLVQLNTMKVEKCESLVEIVRKDGEKSDRVVFQQLKALELVSLKKLKSFCVSDCGFEFPSLEKLVVSACYNMDKFSET
VPSSPILQNVDVVHGKENKRLCWEGDINATIKEIFREMKFFEGLEEMSLSQHQELQGSWQHGVGLQEKNSWFYSLKIL
KIENCEIQPCAIPSNILPCLRSLKELHVQACNNVEVIFEMNVEEGTGTTFNLEILTLQKLPKLKDVWERNGKGTESFQNLKL
VNVSECKNLQSVFPLTLAKNLKKLAELQITRCHVLQEIVRKEEDTTTVFAFPCLTTLHLGDLPELIYFYPQSFTLECSTLNSLF
VWNCQELELFGSAYRQLIFLDLKNIFNLEELLLDWEHTMVLTKLGKSMDNLNYLNHIQLFFDADENERPYFPIQILQKMP
NLTDMGIYYCSCLEVFQTKISEIAEKKVLTNLETLTLDNVSKLQSIGSKDSWLNVICDSEKLQHLYISNCPDLKTLVHSTPLV
SFTYVKEMYIHRCQELKYLFTLSSLKKLKNLEHIEVSDCESMEVIVFKERDDTSEEIKLQQLKSINLIRLSSLKCFYSDNDTLRL
PSLMLVDIWICPKMKFFSGGVIHLNSSFKGTLASNVSSDDLVFCRDLNSSVEKDSLQLEFFEAVDKECFSDNFELQVDPLC
KNGLLYKWLVNLETLKLQNCMLSYAIPSSILSALKNLKELEVRDSKQVKAIFYINDDTDIKETELQLKILTLDGLSELTHVWK
NDTNKILIFRNLQEVVVSDCAKLQTLFPASLSKSLKDLKKLEIYFCKNLQDLVEQEETTLVTEKFVFPCLEDLEFKDLPQVTC
PKTFTLEFPSVKFLSVRNCDELRLFQSVYDPTGEGTSSKTLPLISDPKVMSNLKKLTLDWKQILALSLWFKSQQTTEGLTNL

DRISLCFFGAKENEMPMLPVEILKAPNLTEMDIINCESLENFLAQNPMIGDEEMLGQLTILMLYNVATTQLFELEYSSSLN
IMCGRLHELNVVQCLHLTTLGVLSTSTVSFSCLKEVSIHKCPNLKYLFTTSAAKKLANLEKILVIKCESITEIVAKEGDATSEAI
KFERLHAIHLQSLTSLKCFYSGSDTLQLPSLKFVAIWNCPNMEIFSHGIESLMEITLSMDQEAGYLPPPQDLNTRIKGISQR
KEFFEAVDKECFSDNLELQENPHCKFGLQNQWLGDLVTLKLKKCTLSCAIPSAILALLKNLKELEVRDSPTVEVLFCMNDT
EILETASQLRFLTLKRLSKLTHFWEKKKNGVLIFPNLQQVVVRKCEKLETLFPASLAKNLKSLKAIKIEDCAKFQEIVEKEEAT
EAKFVLPCLQKLDLFCLPQLTCFYPQTFTLECSALNELSILKCDKLELFQSAPSMGEVTSVNRQPLISSLEVISNLKILKLDWK
QILALRSRLKSEKFTGIFKCVNKMKLVLDGGESEMHVVLNEILHTTPNLIEMIMGIYNCNSPEIFLAQNPKIAEDGMLLHL
RILALSYVSTIRSIQSQNSSWLNTICEKVHDLNVFQCRHLETIGVHSTSTLSFSLLKKVDVLRCRRLQYLFTSSVAKELVNLKE
IIVQECKSLKEIIAKEGDEEGEGEDNSENEIIFVKLEKLSLGSLGKLESFYTGSCTLNFPSLRKVEVNKCFNSKIFRHRDKVPPK
FTVIIDRIGCRSDKKPLIMQQVEEEVSRVSLVTTNSLDFCRESGDSKKEMIAT

>XP_027907492.1

MASPVGCCQFLTSSTRAKVWLKKQFIQLYLYETRVAELKDVVDKLEKKKDSIQHTVDEEERRHGRRIHVEVKEWMDRV
NKLILAYKDFDNDEIYHKCAVFDFFDSGYLPKPGIRYRRSRKAYDITEQANELLQNAKFDIFFNWSGPPSTAAFFSNLGYE
SYSSRNDTVKNVIDEFQKPGVRMIGLYGLSGVGKTSLVKEVVKKALKDKMFEVVTMASVTKNPDIRKIQGQIADMLGV
VLEEESDIARAARIHQILNDENKSTLIILDDLWEEVNFNLLGIPHELEKDDDVTIDRRKSLDVDILKNVNDRKSSDLDGSTSF
RKGMLHGVGGLKNINEGKSHVDAFDVVKTKKNVTQYKGCKILMISEIKPVLLSQMEGKEESIFPVEALKENEAEMLFKK
KAGICGNNYEYDKLAAQIASKCKGLPMTIVTTARALKNKSLSVWDETNRKLESQNLARAPEFSTKLSYELLEDEELKYTFLL
CARMGHDGLVMDLVKYCIGFGFLQGINTARQTRDKVYMLVAKLKESGLLSDSYSSDHFTMPDTIRTAALSIAYKENHLF
TMTKRKLDEWPNNLERYAAISLHHCDFIEEFPGRINYPRLRVLQIVNNIPRPKIPEKFFETMKELRVLILTGIHLPLVGSSISS
LHKLRMLCLEQCCMLDKELYIIGELKRLRILSFSGSDIESLPAELNELKMLQIFDISNCSKLKKIPNGVISSLVSLEELYMRNTL
IQWEDEEQTRQSKITLLSDLKHLNQLTTLDIQIPNVSYLPKNLFFDKLFSYKIVIGNLSSVLETDFKMPEKYETLKFLAIQLKN
GSDIHSLKGIKMLFEEVENLFLELNSVHEAQNIVHDLFYRLNLNGFPYLKHLWMVNNSTIQSLIYPKDRQLSEIAFPKLKSL
YLYNLKIDKICSCELSKPSFGKLKVIKINLCGELKNVFSISVVGLLKVLETIEVSECNSLKEIIDVEPQSDPEKTELHMLPELRYL
KLQSLSEFIGFDAIPHIEGNERKLFHEKVAVSKLERLELSSIQIDTIWSVDPSSKRLSFESLTHLDVNGCWKLKYLMSFTMA
KSLVNLQSLYVSDCVKMGSIFLEQVIEKDITGSIFPKLKNMKLRNIKSLSKIWYPKLPSGSFNKLDTLIIEECHRLENALDGIF
GSLCNLRITNCRSMQAIFNILDQVGDVVNNLQDVHLETLPKLKLVWKMNYKDGVGIPKFNNLKKIWAQDCDNLKYIFP
FFVAKSLHNLEFLVVCDCYGLSEIVAEREVTNTDEVNFNFPKLSIVKFSELPKLTSFCPTAYTLSCPLKELSIELCYNLEPFNN
KTDHVHECFPQKVINNLKSMQIEFWHAKSPSSYIGKVNHRRDNLEELSLSRIMDTEILYSFLHRIPNLKSLSLNNCFFEKIV
PPKEDTEFENLGVVPNLKRLMLIDLPNLIEIGFEPDIILERLEFLILKNCPRMITVAPSYVSFTRLTNLEVVSCDGLQSLMSAS
TAKGLVQLNTMKVEKCESLVEIVRKDGEKSDRVVFQQLKALELVSLKKLKSFCVSDCGFEFPSLEKLVVSACYNMDKFSET
VPSSPILQNVDVVHGKENKRLCWEGDINATIKEIFREMKFFEGLEEMSLSQHQELQGSWQHGVGLQEKNSWFYSLKIL
KIENCEIQPCAIPSNILPCLRSLKELHVQACNNVEVIFEMNVEEGTGTTFNLEILTLQKLPKLKDVWERNGKGTESFQNLKL
VNVSECKNLQSVFPLTLAKNLKKLAELQITRCHVLQEIVRKEEDTTTVFAFPCLTTLHLGDLPELIYFYPQSFTLECSTLNSLF
VWNCQELELFGSAYRQLIFLDLKNIFNLEELLLDWEHTMVLTKLGKSMDNLNYLNHIQLFFDADENERPYFPIQILQKMP
NLTDMGIYYCSCLEVFQTKISEIAEKKVLTNLETLTLDNVSKLQSIGSKDSWLNVICDSEKLQHLYISNCPDLKTLVHSTPLV
SFTYVKEMYIHRCQELKYLFTLSSLKKLKNLEHIEVSDCESMEVIVFKERDDTSEEIKLQQLKSINLIRLSSLKCFYSDNDTLRL
PSLMLVDIWICPKMKFFSGGVIHLNSSFKGTLASNVSSDDLVFCRDLNSSVEKDSLQLEFFEAVDKECFSDNFELQVDPLC
KNGLLYKWLVNLETLKLQNCMLSYAIPSSILSALKNLKELEVRDSKQVKAIFYINDDTDIKETELQLKILTLDGLSELTHVWK
NDTNKILIFRNLQEVVVSDCAKLQTLFPASLSKSLKDLKKLEIYFCKNLQDLVEQEETTLVTEKFVFPCLEDLEFKDLPQVTC
PKTFTLEFPSVKFLSVRNCDELRLFQSVYDPTGEGTSSKTLPLISDPKVMSNLKKLTLDWKQILALSLWFKSQQTTEGLTNL
DRISLCFFGAKENEMPMLPVEILKAPNLTEMDIINCESLENFLAQNPMIGDEEMLGQLTILMLYNVATTQLFELEYSSSLN
IMCGRLHELNVVQCLHLTTLGVLSTSTVSFSCLKEVSIHKCPNLKYLFTTSAAKKLANLEKILVIKCESITEIVAKEGDATSEAI
KFERLHAIHLQSLTSLKCFYSGSDTLQLPSLKFVAIWNCPNMEIFSHGIESLMEITLSMDQEAGYLPPPQDLNTRIKGISQR

KEFFEAVDKECFSDNLELQENPHCKFGLQNQWLGDLVTLKLKKCTLSCAIPSAILALLKNLKELEVRDSPTVEVLFCMNDT
EILETASQLRFLTLKRLSKLTHFWEKKKNGVLIFPNLQQVVVRKCEKLETLFPASLAKNLKSLKAIKIEDCAKFQEIVEKEEAT
EAKFVLPCLQKLDLFCLPQLTCFYPQTFTLECSALNELSILKCDKLELFQSAPSMGEVTSVNRQPLISSLEVISNLKILKLDWK
QILALRSRLKSEKFTGIFKCVNKMKLVLDGGESEMHVVLNEILHTTPNLIEMIMGIYNCNSPEIFLAQNPKIAEDGMLLHL
RILALSYVSTIRSIQSQNSSWLNTICEKVHDLNVFQCRHLETIGVHSTSTLSFSLLKKVDVLRCRRLQYLFTSSVAKELVNLKE
IIVQECKSLKEIIAKEGDEEGEGEDNSENEIIFVKLEKLSLGSLGKLESFYTGSCTLNFPSLRKVEVNKCFNSKIFRHRDKVPPK
FTVIIDRIGCRSDKKPLIMQQVEEEVSRVSLVTTNSLDFCRESGDSKKEMIAT

>XP_027907493.1

MASPVGCCQFLTSSTRAKVWLKKQFIQLYLYETRVAELKDVVDKLEKKKDSIQHTVDEEERRHGRRIHVEVKEWMDRV
NKLILAYKDFDNDEIYHKCAVFDFFDSGYLPKPGIRYRRSRKAYDITEQANELLQNAKFDIFFNWSGPPSTAAFFSNLGYE
SYSSRNDTVKNVIDEFQKPGVRMIGLYGLSGVGKTSLVKEVVKKALKDKMFEVVTMASVTKNPDIRKIQGQIADMLGV
VLEEESDIARAARIHQILNDENKSTLIILDDLWEEVNFNLLGIPHELEKDDDVTIDRRKSLDVDILKNVNDRKSSDLDGSTSF
RKGMLHGVGGLKNINEGKSHVDAFDVVKTKKNVTQYKGCKILMISEIKPVLLSQMEGKEESIFPVEALKENEAEMLFKK
KAGICGNNYEYDKLAAQIASKCKGLPMTIVTTARALKNKSLSVWDETNRKLESQNLARAPEFSTKLSYELLEDEELKYTFLL
CARMGHDGLVMDLVKYCIGFGFLQGINTARQTRDKVYMLVAKLKESGLLSDSYSSDHFTMPDTIRTAALSIAYKENHLF
TMTKRKLDEWPNNLERYAAISLHHCDFIEEFPGRINYPRLRVLQIVNNIPRPKIPEKFFETMKELRVLILTGIHLPLVGSSISS
LHKLRMLCLEQCCMLDKELYIIGELKRLRILSFSGSDIESLPAELNELKMLQIFDISNCSKLKKIPNGVISSLVSLEELYMRNTL
IQWEDEEQTRQSKITLLSDLKHLNQLTTLDIQIPNVSYLPKNLFFDKLFSYKIVIGNLSSVLETDFKMPEKYETLKFLAIQLKN
GSDIHSLKGIKMLFEEVENLFLELNSVHEAQNIVHDLFYRLNLNGFPYLKHLWMVNNSTIQSLIYPKDRQLSEIAFPKLKSL
YLYNLKIDKICSCELSKPSFGKLKVIKINLCGELKNVFSISVVGLLKVLETIEVSECNSLKEIIDVEPQSDPEKTELHMLPELRYL
KLQSLSEFIGFDAIPHIEGNERKLFHEKVAVSKLERLELSSIQIDTIWSVDPSSKRLSFESLTHLDVNGCWKLKYLMSFTMA
KSLVNLQSLYVSDCVKMGSIFLEQVIEKDITGSIFPKLKNMKLRNIKSLSKIWYPKLPSGSFNKLDTLIIEECHRLENALDGIF
GSLCNLRITNCRSMQAIFNILDQVGDVVNNLQDVHLETLPKLKLVWKMNYKDGVGIPKFNNLKKIWAQDCDNLKYIFP
FFVAKSLHNLEFLVVCDCYGLSEIVAEREVTNTDEVNFNFPKLSIVKFSELPKLTSFCPTAYTLSCPLKELSIELCYNLEPFNN
KTDHVHECFPQKVINNLKSMQIEFWHAKSPSSYIGKVNHRRDNLEELSLSRIMDTEILYSFLHRIPNLKSLSLNNCFFEKIV
PPKEDTEFENLGVVPNLKRLMLIDLPNLIEIGFEPDIILERLEFLILKNCPRMITVAPSYVSFTRLTNLEVVSCDGLQSLMSAS
TAKGLVQLNTMKVEKCESLVEIVRKDGEKSDRVVFQQLKALELVSLKKLKSFCVSDCGFEFPSLEKLVVSACYNMDKFSET
VPSSPILQNVDVVHGKENKRLCWEGDINATIKEIFREMKFFEGLEEMSLSQHQELQGSWQHGVGLQEKNSWFYSLKIL
KIENCEIQPCAIPSNILPCLRSLKELHVQACNNVEVIFEMNVEEGTGTTFNLEILTLQKLPKLKDVWERNGKGTESFQNLKL
VNVSECKNLQSVFPLTLAKNLKKLAELQITRCHVLQEIVRKEEDTTTVFAFPCLTTLHLGDLPELIYFYPQSFTLECSTLNSLF
VWNCQELELFGSAYRQLIFLDLKNIFNLEELLLDWEHTMVLTKLGKSMDNLNYLNHIQLFFDADENERPYFPIQILQKMP
NLTDMGIYYCSCLEVFQTKISEIAEKKVLTNLETLTLDNVSKLQSIGSKDSWLNVICDSEKLQHLYISNCPDLKTLVHSTPLV
SFTYVKEMYIHRCQELKYLFTLSSLKKLKNLEHIEVSDCESMEVIVFKERDDTSEEIKLQQLKSINLIRLSSLKCFYSDNDTLRL
PSLMLVDIWICPKMKFFSGGVIHLNSSFKGTLASNVSSDDLVFCRDLNSSVEKDSLQLEFFEAVDKECFSDNFELQVDPLC
KNGLLYKWLVNLETLKLQNCMLSYAIPSSILSALKNLKELEVRDSKQVKAIFYINDDTDIKETELQLKILTLDGLSELTHVWK
NDTNKILIFRNLQEVVVSDCAKLQTLFPASLSKSLKDLKKLEIYFCKNLQDLVEQEETTLVTEKFVFPCLEDLEFKDLPQVTC
PKTFTLEFPSVKFLSVRNCDELRLFQSVYDPTGEGTSSKTLPLISDPKVMSNLKKLTLDWKQILALSLWFKSQQTTEGLTNL
DRISLCFFGAKENEMPMLPVEILKAPNLTEMDIINCESLENFLAQNPMIGDEEMLGQLTILMLYNVATTQLFELEYSSSLN
IMCGRLHELNVVQCLHLTTLGVLSTSTVSFSCLKEVSIHKCPNLKYLFTTSAAKKLANLEKILVIKCESITEIVAKEGDATSEAI
KFERLHAIHLQSLTSLKCFYSGSDTLQLPSLKFVAIWNCPNMEIFSHGIESLMEITLSMDQEAGYLPPPQDLNTRIKGISQR
KEFFEAVDKECFSDNLELQENPHCKFGLQNQWLGDLVTLKLKKCTLSCAIPSAILALLKNLKELEVRDSPTVEVLFCMNDT
EILETASQLRFLTLKRLSKLTHFWEKKKNGVLIFPNLQQVVVRKCEKLETLFPASLAKNLKSLKAIKIEDCAKFQEIVEKEEAT
EAKFVLPCLQKLDLFCLPQLTCFYPQTFTLECSALNELSILKCDKLELFQSAPSMGEVTSVNRQPLISSLEVISNLKILKLDWK

QILALRSRLKSEKFTGIFKCVNKMKLVLDGGESEMHVVLNEILHTTPNLIEMIMGIYNCNSPEIFLAQNPKIAEDGMLLHL
RILALSYVSTIRSIQSQNSSWLNTICEKVHDLNVFQCRHLETIGVHSTSTLSFSLLKKVDVLRCRRLQYLFTSSVAKELVNLKE
IIVQECKSLKEIIAKEGDEEGEGEDNSENEIIFVKLEKLSLGSLGKLESFYTGSCTLNFPSLRKVEVNKCFNSKIFRHRDKVPPK
FTVIIDRIGCRSDKKPLIMQQVEEEVSRVSLVTTNSLDFCRESGDSKKEMIAT

>XP_027907494.1

MAAIIGCYQFLTSSPRAKEWLKKQLIQLNLYEAKVSEVEDVVEKLKKKRDTIQHIVEEEERRHGRRIHVEVKEWIENVDKL
ILAYKDFHEDEICHKYAVFDFLDSGYLPKPGIRYHRSRKADDITKQANGLLQNAKFDILSYWSGPPSMAAFFSNLGYESYP
SRNDTVKKITDEFQKPGVRMIGLHGLSGVGKTSLVKEVVKKALKDKMFEVVTMASVTKNPDIRKIQGQIADMLGAVLE
EESDISRAARIHQILNDENKSTLIILDDLWEKVNFNLLGIPHELEKDDGVTNVKGKSLDVDILKNVNDGESPVLDGSTSFRR
GTLQGEGSKNVNKGKSSSVADSMNVKKGEFFGGDLNNVNEGKSLLDASGRLKTEKTVPQYKGCKILMISEIKQVLLSQ
MEGKEKSIFPVEVLKEKEAEMLFNKKAGIGDNNSEYDKLAAQIASKCKGLPMTIVTTARALKNKSLSVWEETNRKLESQ
NLAGAPEFSTKLSYDLLEDEELRYTFLLCARMGHDALIMDLVKYCIGLDFLRGINTARETRDRVYTLVAKLKESGLLSDSYS
SDHFTMPDTVRRAALSIAYKENHLFTMTKEKVDEWPDELERYAAISLHHCNFTKEFPGTINYPRLRVLEIVNSIPRPKIPK
NFFKGVKELRVLLLIGIHLPLIDSSISSLHKLRMLCLEQCCMLDEELSIIGELEKLRILSFSGSDIKNLPDELNELKMLQIFDISN
CSKLKKIPYGVISSLVSLEELYMRNTLIQWEDEEQTRQSKIAILSDLKHLNQLTTLDIQIPNVSYLPKNLFFDKLDSYKIVIGDL
SSFLETDFKMPEKYETLKLLAVQLKNGSDIHSLKGIKMLFEGVENLFLELNTVHEKHNSVREAHNIVHDLFYRLNLKGFSYL
KHLWIVNNSTIQSLINPKDRQQPEKAFPKLESLSLHNLKIYEICSCKLSEPSFGKLKVIKINLCGELKNVFSISMVRLLKFLETI
EVSECNSLKEIIDVGPQNKIELPMLPELRNLRLQSLSEFVGFDVIPQIEREERKLFHEKVGISKLERLELSSIQIDVIWSVEQSS
KRSSFENLTHLDVNGCWKLKYLMSFTMAKSLVNLQSLYVSDCEKMGSIFLLEQDREKDITDNIFPKLKNMKLRNMNSLS
KIWNPKLPSDSFSKLDTLIIEECHRLENVMEGTFVSICNLRVTNCRFMQAIFNIREQVGDVANNLQNVHLEALPKLKLVW
KMNNEDRLGIPKFNNLKKILAQDCESLEYIFPFSVAKSLDNLESLVVCDCYGLSEIITKREANHMNRVNFNFPKLSTIKFSN
LLKLTSFYPSTYDLSCPLNELSIDLCNNLEPFNSVHVCFPEEVINNLKSMQIESWHAKSPSSYMGKRNHRRDNLEELSLFRL
MDTEILYSFLHRNPNLRSLSLNYCFFEKIVPPKEDTEIENLGVVPNLKSLMLIDLPNLKEIGFEPDIILERLEFLILRNCPCMIT
VAPSSVSFTRLTNLEVVDCDGLQSLMSASTAKTLVQLNILKVVKCEFLMEIVRKDGEKSDRVVFKKLKALELVSLKKLKSFC
VSDCDFEFPSLEKLVVSACYNMAKFSETVTSSPLLQNIHVVHGKENKRFCWEGDINATIQKIFEEMKFFEGMEEMSLSE
HQELQETWQHGGGMQKQNSWFYSLKILKLENCEIQPCAIPSNILPYLRTLKELQVRGCNNVEVIFEMNAEEGTGSTFHL
EKLALEKLPKLKDVWESNGKGTESFQNLRLVNVNECEKLQSVFPLTLAKNLKKLEKLEILYCHRLHEIVRKEDDTEAVFFFP
CVTTLYLFDLPDLVYFYPEAFTLDCSALNSLIVLSCPALKLFGSANKQSIFFDLKDICNLEVLILDWEHTLVLRTKLGEPKDNL
KYLNDIKLFFGDDENERPDLPIQILQKMPNLTKMSINYCSCLEVFQTQIPKNVKERVLTHLKTLKLYNVSKLQSIGSEDSP
WLNVICDSEKLQKLYVFTCPDLKTLVHSTPLVSFRYVKEMYIDRCKELKYLFTLSSVNKLENLEHIEVSNCKSMQAIVFKED
DDISEEIKLQQLKRVHIYCLSSLECFYSGYETLRLPSLMLVDIWKCPKMEFFSGGEIHLNSSFRGIRVSNVLSDDMVFYHDL
NSSVDKVFLQQEFFQAVDKKCFSDNLELQADPHCKTGLQNKWLANLETLKLENCKLSYAIPSFILCLLKNLKELEVRDSD
QVKAIFDMNDDTEIKETESQLKILTLNGLSELKHVWEKYTSRILIFPNLQEVVVSDCAKLQTLFPASLAKSLKDLKKVKIDSC
ENLQDFVEQEETTFVTEKFVFPCVEDLELNDLPQVTCPKTFTLEFPSVKFVSVRNCDELGLFQSVYDPMGEGTSSSRLPLI
SDPKVISNLEKLTLDWKQVLALSLGFTSQKSTKGLRNLNCISLSFFRAKENEMPVLPVEILKAPNLIEMGINNCDSIENFLA
QNPNIGEEEMLRQLTILRLCNVSTTQFFELNYCSSLNMICERLHKLIVSQCPHLTAILGVRSAVSFSCLKELNIYKCSNLKYL
FTTSAAKRFMNLEEISVIECESLTKIVAKEGNATSEAIKFERLHTIHLQSLTSLVCFYSGSDTLQLSSLKTVAVWSCPNMEFF
SHRIQSLMGVTVSMDPEADDLPPSRDINTIIKRISERKEFFEAVDKECFLDNLELQEDPHCKSGLQNKWLRDLISLKLQNC
TLPCAIPSVILSLLKCLKELEVRDSATVEVLFYMNDNEIVQIASQLRILALKGLSKLTHVWEKKKNGVLMFPNLQQVVVSN
CKKLETLFPASLAKNLKSLKGIEIKDCSKFREIVEKEEATEAKFVLPCLERLILSSLPQLSCFYPQTFTLECPALNILSVFECERLE
LFQSEHSMGEGTSVKREPLISSLEVISNLKELELDWKQIL ALRSRFRSEKFTGVFKCINKMHLLSYGDKSEMTIVLNEILHKA
PNLTEMIIQVLNCKNPEIFHAQNPKVCEDGMLLQLRILTLFNVSAIMSIQSENSSWLNTICENLHELNVPSC

>XP_027907509.1

MASSIPSMEFTSSSSKLQQRYDVLINFSGEDIRKKFVSHLDSALSAVGFTTFFHEENAVNEMHIQEPILDLYQVAIVVFTKT
YAQSAWCLHQLRQIIRWHQTYSRHLLPIYYEVEPSDVRLQKGDFGKAFKATAYQTFSTQQLELGMSRWSQALTKAADF
FGWDESNHRSDAELVDKIVKSVLNLPVMSATKFPVGLHSRVEDVIRTIRSKSTEVCIIGIYGEGGSGKTTLATAIYNQIQDT
FTKKSFIEGQVGEIRGDLRLREQLLLDILKAKVEIPNADIGRSMIRKRLSGKRMLIVLDDVPYFSIFLDLWDYCKWFGEGTV
IIVTTRYEILELRRGQPDSVFRVKLMNEKESLELLSWHAFREPKPKEEYNELAKRVVHYCGGLPLALEVIGSCLFERTKEEW
HSVLFELEKIPSHNAEQKLKIGFDGLRNQIEKDLFLDVCCFFVGKGRTYATKILNGCGVDADSGIRVLIERSLIKVKKNRKFG
MHPLVEEMGRAIIHEISRNESLMDNQLCVDDAEYVLIDNTETKVSQALLMKLRSARIEPSRLLKLDGNSENTSKKLRGISL
HGVSSQYVPDDFCLHDVIAIYLKHCFLRLWKQPQVLRWLKVLNLSHSKYLRETPDFSGLPSLEQLILKDCPRLCQVHKSIG
CLCYLLLLNLKDCTSISNLPKGIYKLKSLRTLILSGCSKIDLMEKDIVQMKSLITLIAENAAVRQVPFSIVSSKSIGYISLLGFERL
PHNLFSSIIRSWISPTMNPISNIHSLWMDTDNSWDDIAPLLSSLANLRSVLVQCDTEFQLSKQIKNDLVEYFSNITHSGISK
QHFRCSLIGVGRYHEFFNAVGDNIFEILASRESCDVFLPIVNDPYCLAHIGEGHSVFFTVPRDLDMKGMALCIYLSTLEIVV
SECLRSVLIVNYTKCTFQIHYHDPVISFNDDDWNAINSNLESEDKIEIFVSFGPGLVVKNTVVYLICGESNNMDKESLPKK
HSLIRFIKKIVK

>XP_027907534.1

MVLTRFHGYKQILIFCYFEWFCVCKEIISHVLHYCFLFQHFRTSPVSSMEFASSTSKLPQMYDMLINFNGEDIHRKFVSHL
DSVLSAAGLTTLLHHQNAVNDMDIQQPILNLCRVAIVVFTKTYSQSAWCLHQLQQIIEWHKTYYRHVLPVYYEIQPSDV
RLQRGDFGKTLKATAQQSFSAQQLEHGMSGWNHALSKTADFFGWDESNYRSDAEVVDKIVKSVLNLAVLSATKFPVG
LQSRMEEVIQIIKKESTKVCRIAICGKGGSGKTTLAKAIYDQIHDTFTEKFFIEDFEQKQLCSIEMGRSLIPERIYGKKVLIVLD
DVNFSYYLEMIVRSRFGEGTVIIVTTTKQNPPFTRDLYSIFQINLMNPNESLELLSWHAFREAKPKDEYHFLAKRVVAYCG
GLPLLLEVIGSCLYERTKEEWNNVLSRLERLPQHKVLEILKISFDGLPNQYERNLFLDICCFFVGKDRVYYVTKILNGCGVNA
ESGIRILIERSLIIVKNNKFGLHPLLREMAREIVAKITSGKEPEKTSRLWFDKDVLLEHILFSSQDKKVIQRFPPKWFLTVRDF
FKHDYLEVRDAIRRMKLGGHCEYRSKELGWIRLENFSSEFLPIGFLRDAIAIDLKHSLPRLVWKEPQVLASLTVLNLSHSKY
LTETPDFSRLPSLEQLILKDCPRLCEVHQSIGGLCNLTLLNLKDCTRIKNLPREIYMLKSLKTLILSGCSGIHLMEKDIRQMES
LITLITESTVMKQVPFSIVSSKSIGYLSLRGFEGLSHNLFPFIMRSWMLPSMNPLSYYHSFCMDVEVNSWDDIAPLLRILV
NLRSVLLQCETEFQLSKQVQDILVEYGVNITESHTSKQHFRSLLIGDGRCKEFLDAFSDSISEVFAGSESCDVSLPGDNNP
NCLADMGEGYSVSFTVPRDRDIKGMALCAVYLSTPEIVATEDLRSVLIVNYTKCTLHIHNHGTVIFFNDKDWEGIISNLGS
GDKVEIFVIFGHGLVVRRTIVYLIFGESYDIEKESTSKKNSLIRFIKKL

>XP_027907535.1

MVLTRFHGYKQILIFCYFEWFCVCKEIISHVLHYCFLFQHFRTSPVSSMEFASSTSKLPQMYDMLINFNGEDIHRKFVSHL
DSVLSAAGLTTLLHHQNAVNDMDIQQPILNLCRVAIVVFTKTYSQSAWCLHQLQQIIEWHKTYYRHVLPVYYEIQPSDV
RLQRGDFGKTLKATAQQSFSAQQLEHGMSGWNHALSKTADFFGWDESNYRSDAEVVDKIVKSVLNLAVLSATKFPVG
LQSRMEEVIQIIKKESTKVCRIAICGKGGSGKTTLAKAIYDQIHDTFTEKFFIEDFEQKQLCSIEMGRSLIPERIYGKKINLMN
PNESLELLSWHAFREAKPKDEYHFLAKRVVAYCGGLPLLLEVIGSCLYERTKEEWNNVLSRLERLPQHKVLEILKISFDGLP
NQYERNLFLDICCFFVGKDRVYYVTKILNGCGVNAESGIRILIERSLIIVKNNKFGLHPLLREMAREIVAKITSGKEPEKTSRL
WFDKDVLLEHILFSSQDKKVIQRFPPKWFLTVRDFFKHDYLEVRDAIRRMKLGGHCEYRSKELGWIRLENFSSEFLPIGFL
RDAIAIDLKHSLPRLVWKEPQVLASLTVLNLSHSKYLTETPDFSRLPSLEQLILKDCPRLCEVHQSIGGLCNLTLLNLKDCTRI
KNLPREIYMLKSLKTLILSGCSGIHLMEKDIRQMESLITLITESTVMKQVPFSIVSSKSIGYLSLRGFEGLSHNLFPFIMRSW
MLPSMNPLSYYHSFCMDVEVNSWDDIAPLLRILVNLRSVLLQCETEFQLSKQVQDILVEYGVNITESHTSKQHFRSLLIG
DGRCKEFLDAFSDSISEVFAGSESCDVSLPGDNNPNCLADMGEGYSVSFTVPRDRDIKGMALCAVYLSTPEIVATEDLRS
VLIVNYTKCTLHIHNHGTVIFFNDKDWEGIISNLGSGDKVEIFVIFGHGLVVRRTIVYLIFGESYDIEKESTSKKNSLIRFIKKL

>XP_027907568.1

MDYLGPFSKPVEAVIDFISKLGVRHVTYIICYKQNVLELSNSVKDLGFEKERINHQCDDTQKNLNSIEGKVIEWVRKVNEI
ETLMNVFENDDGHKRARSLSCYVFSYLLNRHRLGRRAKKMEVDVKKLIDESPNFDEVSYREDVTSNDATLFNSDFVEFG
STKSIMEKVMTQLEDSTVRIIGLYGPAGVGKSTLIKEIARKAKDKKVFDVVVKVEITANPNLQNVQEEIAYVLGLRLEGEG
ENVRADCLRRRLKKKKXNTLLILDDLWDKLDLNKLGIPLFDDEDDYEDVSISDNKDHNHQKFEDYNHQNFEDHNHQKL
AKKNDNKDHSDQKLAKNNDNKDHTYKVLNKEKILGSHKGCKILLTSRDKKILCDKMDIKSTFCVKELDDKDALMLFQKL
AGIRNEMSDSKQEIVKKYCAGLPMAIVTVARALRSKGEPVWEATLEKLKRQELVEVQTSMDISVKMSYDHLENEEIKSIF
LLCAQMGHQPLIMDLVKYCFGLGILEGVSSIWEARDRIKTSIQKLKDSGLLLDGSSNNHFNMHDLVRDAALSIANNDRN
VFTLRNGKLDDWPELESCTSMSICSCDIVDGLPKEINCSQLKFFQIDTNDPSLIIPENFFEGMKNLKVLILIGFRLSRLPSSIK
CLLKLRMLCLERCTLDDNLHIGDLKKLRILSFSGSQLKTLPTELGCLDKLQLLDISDCFIVDTNIPPHLLSSLTHLEELYIRKSVI
RMLGEGETNQGQNLFLSEIKNLYQLKVVDLSIPCVSVLPDHLFFDRLKDYKIVVGDLEMFSVGDFRMPDKYETFRVLALQ
LNDASDIHSYEGIKMLFKTVQCLLLGNVDGVQNVVNELNVDGFPYLKHLHIVNNNNIKYLNSTTLSNCVDIFPNLESLCLY
NLVNLEVICYGLLTAASFAKLKAIKVEVCNRLKNLHSFYALKVLTSSKTCEISECNSFMDKFPTSVEIIEVCECGSLKEILQIPV
DYGQLEFHKLQSLTLQSLPSFTSFYTIVDGSYGSHVIELQTTNGGSREIVGEDVQNDKTPLFGELVATPNLESLNISSLNIHK
IWSDKHSACFGGQNLIKIVVKHCDKLTYLCSLSMANSLKKLKSLVISECRIMEKIFETEENSADKVCVFPMLEEIHLSKMNR
LTNIWQTKVSVDSFSNLASVKIEECNKLNKIFPSHMKGWVECLDNLTVCSCLSVEEIFEINDYKEIDEFGGIDTNLQAILLE
NLPKLKQLWSTDPNGILNFKKLRTIDIYNCNELRNLFPSSVAKDVPKLERISTLHCEKMVEIVASQVASGVNNDPLVFPEL
VCVRLQWLPNIKHFYKGKHLIKCPKLKELSIGTRVTLKTFLKETSETTKKEEECVFSAEEVLPNLEYMEIDFDEAQELLPKYQ
MQRLKELTLMSVESVDLLWQFPYCMSNLEKLKMVYPSFLQEVEPRANFARQERFGITLQLKELVLVHSKIRDLGLGRVP
VLRRLELLSLKNCDLLNNLAPPSVSLTYLTYLELKHCSRLRNLMAFSTAQSMVQLKTMKVINCGNVKEIVSNEGSENGKE
MKIVFSKLISIELVGLNYMRSFCSSRECEFDFPSLEILIVRECPNMEKFSERRPITPKLKNVFGVEGDEKTRWQWEHNLNA
TIQKVFNDKVTFRYTEDLLYNYGTEFIIKCKIR

>XP_027907673.1

MGRTMIQNRLSRKKLLIVVDDVNEFGQLENLCLNREWFGRGTVMIITTRDIHLLNLLKVDYVYKMHLLNEDDSFELFSW
HAFREAKPREEWSDLARNTVAYCEGLPLALQFHERYYVTEILNACELDADFGITVLIERGLIKVERNNKLEMHPLLRDMG
REFIRELHPKEPGKRSRLWFQEDVQDVLEENSGTEAIEGLSLKLHSSNRDSLEALAFKKMKRLRLPQLDHVQLSGDYGHI
SKQLRWICWRGFPYKYIPNNFHLENVIAIDFKHSHLQLLSKQHLVFITTKIV

>XP_027907674.1

MGGSGKTTLVKAIYNRTYHQFIGKSFIENISEAWDSENERYVDLQENLLSDVLKSKLDVESVGMGRTLIENRLSRKKLFIV
LDDRSNNSLELLSWHAFREAKPRKEWNDLARCIVDYCRGLPLALQSLGSHLFDRTIEVWKSLLSKLKQFPTNQVQSVLKI
SFDGLNDTEKDIFLDVCCFFIGKQRNYVIDILNGCGLRADIGITILIERGLIKVERSNKLEMHPLLRDMGREIICQECPKEPG
KRSRLWFQEDVQEVLKENTGTKAVEGLSLKLHSSSKDCLEAHAFKEMKRLRLLQLDHVQLSGDYGHISKQLRWICWRG
FPYKYIPNNFHLENVIAIDFKHSHLQLLWEQPLVLQRLKFLNLSHSKFLRETPDFSGLPSLEQLILKDCAWA

>XP_027907681.1

MALAVVGGALLSAFIDVLFDRLASPELVNFIRGKKPDKLLQKMKSQLLVVKVVLADAEKKQITNSNVKEWLDLLSDVVY
QADDLLDEVSTKAATQKGVSHSFSHLFQRNKLVNVSKLEGIVERLDDILKQKESLDLKEIAVENNQQWNAQTTSLEGRY
GMYGRDKDKDAIMKLVLEDSSDGEEVSVIPIVGMGGVGKTTLIRSVYNDGKLNGTFKLKAWICVSDIFDIVKVTKTMLE
EITQKPCKLNDLNLIQLDLLEKLKGKKFLIVLDDVWIEDCDSWSSLTKPFLSGIRGSKVLVTTRNESVAAVVPLHTVEVYHL
NKLSDEDCWLVFASHAFPLSEGSHNRESLEKIGKEIVKKCNGLPLAAQSLGGMLKRKHAIRDWNNVLESDIWELPESQC
KIIPALRISYNYLPPQLKRCFVYCSLFPKDYEFRKDKLILLWMAEDLVKPSRKRKTLEEVGQEYFDDLVSRSFFQCSGSWN
WGNHFVMHDLMHDLATFLGGDFYFRADEHGKETKIDRKTRHLSFTRLSDPVSDTEVLDRVKFSRTFLPTYFHYSPFKNR
KTPCIIVSMLKYLRVLSFSDLECQLVLPDSIGELIHLRYLDLTSTGIEKLPESLCNLYNLQTLRLSNCSRLTKLPSAMQNLVNL

RHLEIFESSIEEMPKGMGKLNQLQRLDLYIVGKHKENSIKELGGFPNLHGRFSIEKLENVTNGEEALEAGILNKKYISTLDLR
WSLSNDSSIDFQIELDVLGKLQPHPDLKSLRIKGYNGTRFPKWMDNFSYRYMKSLFLQNCNNCCMLPSLGQLPSLKHILI
SEMNSVKTIDAGFYKKEDCSSMAPFPSLESLYIFNMPCWEMWSSDSKAFPVLEELYIKNCPKLKGDLPNHLPTLQTLKIK
SCQLLASSFPRTPALRTLRICESNKVRLHAFPQSVESIKVIGSPMVESMMEAITEIQPTCLNDLSLNDCSSAISFPGDRLPSS
LKILYISGLNKLNLPVLHKHELLESLSINNSCDSLTSFPPAIFPNLTGLKIQNCENLESLLVLGSESMKSLNYFVIGDCPNFVSF
PGEGFSAPNLTRLSVYACAKLKSLPHQMGTLLPKMEYLGISNCQQIECFPGGGMPPNLTTVRIENCEKLVMSLGGISID
MVTSLDVYGPCDGINAFPKEGLLPPSLTSLFLYDLSSLDTLLCKGLLHLTSLKTLVIRNCKKLENIAGERLPVSLVKLIIKDCPL
LQKRCHVKDRQIWPKICHVRGIEVDGRWI

>XP_027907682.1

MALAVVGGALLSAFIDVLFDRLASPELVNFIRGKKPDKLLQKMKSQLLVVKVVLADAEKKQITNSNVKEWLDLLSDVVY
QADDLLDEVSTKAATQKGVSHSFSHLFQRNKLVNVSKLEGIVERLDDILKQKESLDLKEIAVENNQQWNAQTTSLEGRY
GMYGRDKDKDAIMKLVLEDSSDGEEVSVIPIVGMGGVGKTTLIRSVYNDGKLNGTFKLKAWICVSDIFDIVKVTKTMLE
EITQKPCKLNDLNLIQLDLLEKLKGKKFLIVLDDVWIEDCDSWSSLTKPFLSGIRGSKVLVTTRNESVAAVVPLHTVEVYHL
NKLSDEDCWLVFASHAFPLSEGSHNRESLEKIGKEIVKKCNGLPLAAQSLGGMLKRKHAIRDWNNVLESDIWELPESQC
KIIPALRISYNYLPPQLKRCFVYCSLFPKDYEFRKDKLILLWMAEDLVKPSRKRKTLEEVGQEYFDDLVSRSFFQCSGSWN
WGNHFVMHDLMHDLATFLGGDFYFRADEHGKETKIDRKTRHLSFTRLSDPVSDTEVLDRVKFSRTFLPTYFHYSPFKNR
KTPCIIVSMLKYLRVLSFSDLECQLVLPDSIGELIHLRYLDLTSTGIEKLPESLCNLYNLQTLRLSNCSRLTKLPSAMQNLVNL
RHLEIFESSIEEMPKGMGKLNQLQRLDLYIVGKHKENSIKELGGFPNLHGRFSIEKLENVTNGEEALEAGILNKKYISTLDLR
WSLSNDSSIDFQIELDVLGKLQPHPDLKSLRIKGYNGTRFPKWMDNFSYRYMKSLFLQNCNNCCMLPSLGQLPSLKHILI
SEMNSVKTIDAGFYKKEDCSSMAPFPSLESLYIFNMPCWEMWSSDSKAFPVLEELYIKNCPKLKGDLPNHLPTLQTLKIK
SCQLLASSFPRTPALRTLRICESNKVRLHAFPQSVESIKVIGSPMVESMMEAITEIQPTCLNDLSLNDCSSAISFPGDRLPSS
LKILYISGLNKLNLPVLHKHELLESLSINNSCDSLTSFPPAIFPNLTGLKIQNCENLESLLVLGSESMKSLNYFVIGDCPNFVSF
PGEGFSAPNLTRLSVYACAKLKSLPHQMGTLLPKMEYLGISNCQQIECFPGGGMPPNLTTVRIENCEKLVMSLGGISID
MVTSLDVYGPCDGINAFPKEGLLPPSLTSLFLYDLSSLDTLLCKGLLHLTSLKTLVIRNCKKLENIAGERLPVSLVKLIIKDCPL
LQKRCHVKDRQIWPKICHVRGIEVDGRWI

>XP_027907703.1

MVLTRFHYYKTNIKASATLNAFVSVPRSSLPIPSMDFASSTSELPKTYDVLIHFTGEDIRRKFVSHLDSALSAVGFSTFLLHQ
NAVNPMHIEEHNLNLCRVAIVVFTETYSESAWCLHQLQQIIQWHQTYCRPVLPVYYESQPSDVRLQNGDFGKAFEATA
HQTFSEQQLEHGMSRWSHALTKAAHFYGWDESNYRSDAELVDTIVKGVLNLPLLSATEFPVGLQSYVEDVIRTIENKST
GVCMIGIYGMEGSGKTTVAKAVYNQIHDRFVEKSFIEDNAEVSRTKGHVYLPEKLLSDLLKTKVEIHSVEMGRSMIWGR
LYGKRVLIVLDNVNEYEYGALSDLWECRGWSGKGSVIIITSTHESVLRTRQVDAVFRINPMNANESLELLSWHAFKEAKP
KEEYCDLAKALVTHCEGLPLALEVIGSYLYERTKKEWHGVLSKLVRIPQLAVLQKLKISFNGLHNQMEKDLFLDICCFFVGK
GRAYVTKILNGGGIDADCGIRVLIERSLIKVKKNNKFGMHPLLGDMGREIIREISKKEPENSRRWFDEGMKHAMPKTTV
RTFLIYGFETSFWKGLLSQVRDTSRMLKLARHSEYLSKKLRCISLQGFSSEYLPNDFYLHEAIVIDFKTQSSSTYLERTPGFGI
GKSP

>XP_027907704.1

MVLTRFHYYKTNIKASATLNAFVSVPRSSLPIPSMDFASSTSELPKTYDVLIHFTGEDIRRKFVSHLDSALSAVGFSTFLLHQ
NAVNPMHIEEHNLNLCRVAIVVFTETYSESAWCLHQLQQIIQWHQTYCRPVLPVYYESQPSDVRLQNGDFGKAFEATA
HQTFSEQQLEHGMSRWSHALTKAAHFYGWDESNYRSDAELVDTIVKGVLNLPLLSATEFPVGLQSYVEDVIRTIENKST
GVCMIGIYGMEGSGKTTVAKAVYNQIHDRFVEKSFIEDNAEVSRTKGHVYLPEKLLSDLLKTKVEIHSVEMGRSMIWGR
LYGKRVLIVLDNVNEYEYGALSDLWECRGWSGKGSVIIITSTHESVLRTRQVDAVFRINPMNANESLELLSWHAFKEAKP

KEEYCDLAKALVTHCEGLPLALEVIGSYLYERTKKEWHGVLSKLVRIPQLAVLQKLKISFNGLHNQMEKDLFLDICCFFVGK
GRAYVTKILNGGGIDADCGIRVLIERSLIKVKKNNKFGMHPLLGDMGREIIREISKKEPENSRRWFDEGMKHAMPKTTV
RTFLIYGFETSFWKGLLSQVRDTSRMLKLARHSEYLSKKLRCISLQGFSSEYLPNDFYLHEAIVIDFKTQSSSTYLERTPGICQ
Q

>XP_027907710.1

MTSSIPSMEFASSTSKLSQIYDVLIHFTGEDIRKKFVSHLDSALSGVGLTTFLHHDNGVKPIQIQEPILNLCRVAIVVFTKTY
SQSDWCLDQLQHIIKWHETYARHVLPVYYEIQPSDVRLQKGDFGEAFKETAQQTFSAQQLEHGMSRWSHALTKAANF
FGWDESNYRSDAELVDTIVKSILTLPVLSATKFPVGLQSHVEDAIQIIKNKSSEVCRIGICGMGGSGKTTLAKAIYNQIQGT
FMEKSFIEDIAQVTQTRGRVHLLEQLLHDVLKTNVDSVQMGRSMIRERLYGKRVLIVLDDVNEYFCFSYMNEYGPLDL
WNSPWFGKGTVIIITTRDAALLTMEHVYSIIRINEMNPNASLKLLSWHAFREAKPKEEYHLLAKRVVDYCGGLPLTLEVIG
SYLYERSIEEWTKVLSRLENIPQHEVPQILKIGFDGLRNQMEKYLFLDICCFFVGKDRTYVTKILNGCGVDPDSGIRVLIECS
LIKVTKNNKFGVHPLLQDMGREVIREISRKEPRKNIELVLDKDTKYALLENTLFSSQGTNVIQGLSEKRFLTSTGCFKPYSL
QATKMLKFIGDSEYVSKKLRWFILRGFPLEYLPNDFYLHDAIAIDLKHNLLRFVWKQPQVLTCLKVLNLSHSRYLQQTPDF
SRLPSLEQLILKDCPRLREVHHSIGCLCYLTLLNLKDCTSLINLTIEIYKLKSLKILILSGCSKVSLLEKDIVQMESLITLIAESTAV
KEVPFSIVSSKSIGYISLRGFQGLSHNLLPSIIRSWMLPTMKSLSYIHSFCMDMEDNSWDDIAPLLSTLVNLRSVLVQCNT
EFQLSKQVETILNEYSLNITKSGTSTHHLKFSLIGVGRCKDIFNAISDSISEALASSKSSVVSLPGDNDPDWLAHIGEGPFVS
FTVPQDCEVKGMALCVVYLSTPGIVTTEYLTSVLIVNYTKCTLQIHNHGTVISFNDKDWEGIISNLGSGDKVEIFYTFNLEL
VIKNTAVYLIYGETNDLEKEFVPKKKSLIRFMKKSVM

>XP_027907711.1

MSRWSHAITKAANFFGWDESNYRSDAELVDTIVKSILTLPVLSATKFPVGLQSHVEDAIQIIKNKSSEVCRIGICGMGGS
GKTTLAKAIYNQIQGTFMEKSFIEDIAQVTQTRGRVHLLEQLLHDVLKTNVDSVQMGRSMIRERLYGKRVLIVLDDVNE
YFCFSYMNEYGPLDLWNSPWFGKGTVIIITTRDAALLTMEHVYSIIRINEMNPNASLKLLSWHAFREAKPKEEYHLLAKR
VVDYCGGLPLTLEVIGSYLYERSIEEWTKVLSRLENIPQHEVPQILKIGFDGLRNQMEKYLFLDICCFFVGKDRTYVTKILNG
CGVDPDSGIRVLIECSLIKVTKNNKFGVHPLLQDMGREVIREISRKEPRKNIELVLDKDTKYALLENTLFSSQGTNVIQGLS
EKRFLTSTGCFKPYSLQATKMLKFIGDSEYVSKKLRWFILRGFPLEYLPNDFYLHDAIAIDLKHNLLRFVWKQPQVLTCLKV
LNLSHSRYLQQTPDFSRLPSLEQLILKDCPRLREVHHSIGCLCYLTLLNLKDCTSLINLTIEIYKLKSLKILILSGCSKVSLLEKDIV
QMESLITLIAESTAVKEVPFSIVSSKSIGYISLRGFQGLSHNLLPSIIRSWMLPTMKSLSYIHSFCMDMEDNSWDDIAPLLS
TLVNLRSVLVQCNTEFQLSKQVETILNEYSLNITKSGTSTHHLKFSLIGVGRCKDIFNAISDSISEALASSKSSVVSLPGDNDP
DWLAHIGEGPFVSFTVPQDCEVKGMALCVVYLSTPGIVTTEYLTSVLIVNYTKCTLQIHNHGTVISFNDKDWEGIISNLGS
GDKVEIFYTFNLELVIKNTAVYLIYGETNDLEKEFVPKKKSLIRFMKKSVM

>XP_027907712.1

MACPGGATHSPKLQISLDGMRAITVGLQSHVEDAIQIIKNKSSEVCRIGICGMGGSGKTTLAKAIYNQIQGTFMEKSFIE
DIAQVTQTRGRVHLLEQLLHDVLKTNVDSVQMGRSMIRERLYGKRVLIVLDDVNEYFCFSYMNEYGPLDLWNSPWFG
KGTVIIITTRDAALLTMEHVYSIIRINEMNPNASLKLLSWHAFREAKPKEEYHLLAKRVVDYCGGLPLTLEVIGSYLYERSIEE
WTKVLSRLENIPQHEVPQILKIGFDGLRNQMEKYLFLDICCFFVGKDRTYVTKILNGCGVDPDSGIRVLIECSLIKVTKNNK
FGVHPLLQDMGREVIREISRKEPRKNIELVLDKDTKYALLENTLFSSQGTNVIQGLSEKRFLTSTGCFKPYSLQATKMLKFI
GDSEYVSKKLRWFILRGFPLEYLPNDFYLHDAIAIDLKHNLLRFVWKQPQVLTCLKVLNLSHSRYLQQTPDFSRLPSLEQLI
LKDCPRLREVHHSIGCLCYLTLLNLKDCTSLINLTIEIYKLKSLKILILSGCSKVSLLEKDIVQMESLITLIAESTAVKEVPFSIVSS
KSIGYISLRGFQGLSHNLLPSIIRSWMLPTMKSLSYIHSFCMDMEDNSWDDIAPLLSTLVNLRSVLVQCNTEFQLSKQVE
TILNEYSLNITKSGTSTHHLKFSLIGVGRCKDIFNAISDSISEALASSKSSVVSLPGDNDPDWLAHIGEGPFVSFTVPQDCEV

KGMALCVVYLSTPGIVTTEYLTSVLIVNYTKCTLQIHNHGTVISFNDKDWEGIISNLGSGDKVEIFYTFNLELVIKNTAVYLI
YGETNDLEKEFVPKKKSLIRFMKKSVM

>XP_027907713.1

MASSIPPTEFASSTSKLPRKYDVLINFTGEDIRRKFVSHLDYALSTVGLTTFLHEENAVNDMHIQQPILNLCRVAIVVFTKT
YSQSAWCLHQLQQIIKWQETYSRHVFPVYYEIQPSDVRLQKGDFGETFKETAQQTFSAQQLEHGMSRWSHTLTKAAN
FFGWDESNYRSDAELVDKIVEGVLNLPVLFATKFPVGLQSRLNDVIQIIKDKSREVCIIGIWGEGGSGKTTLAKAIYNQLH
GTFTHKSFIEDISQVIQTKGHVHLQEQLLSDVLNTKMEIHSVEMGKNKIREKLCGKKLLIVLDDTKYDPLLNLRDSHVWFA
KGTVIIITAREEHLLRIPQNGSAFPVNLLSTNESLELLSWHAFREAKPKQEYNDLAKRVVVYCGGLPLALEVIGSSLFERTKE
EWKSVLLELKEIPEHDVHRKLKISFNGLRNEMEKDLFLDVCCFFVGKGRTFVTKILNDCGVDADSGLRILIERSLVQVKKN
KKLGMQQLLQKMGRKIICEISRKELGKNPPLWSGQDAEYERLENTLFSSQQTKVIQKRLSLKMFLIATREFERYPSGVRD
TSRLLRLFKVFGKLRWISLQGFSSEYLPKDIYLHDAMAIDLKHSLLRFVWKEPQVLSWLKVLNLSHSMYLRETPDFSGLPR
LEQLVLKDCPSLHKVHHSIGCLNNLLLLNLKDCTSLSNLPREVYKLKSLNTLILSGCSKIDLLEKYIVQVESLLILIAENAAVKQ
VPFSIVSSKNIGYIFLRGCEGLSCNLFPSIIRSWISPIMNPLSYVHSFCMDIEDNGWNDFDPLLSTLANLRSVLVQCDTEFQ
LSKLVETILIEYGVNISKSEISQQHFKYSLIGVGRCKDFFNAVSDIISKVFASNESRDVSLPGDNDPYWFGHMGEGRSVFFT
VPRDHDLKGMALCVVYLSTPEIVAPVCLRSVLIVNYTKCTLQIHNHGRVISFNDIDWQGIISNLGPGDKVEICVTFAQELV
VTNTILYLICDELNDLQKEPAPKKNSLIRFVKKVVM

>XP_027907714.1

MHIQQPILNLCRVAIVVFTKTYSQSAWCLHQLQQIIKWQETYSRHVFPVYYEIQPSDVRLQKGDFGETFKETAQQTFSA
QQLEHGMSRWSHTLTKAANFFGWDESNYRSDAELVDKIVEGVLNLPVLFATKFPVGLQSRLNDVIQIIKDKSREVCIIGI
WGEGGSGKTTLAKAIYNQLHGTFTHKSFIEDISQVIQTKGHVHLQEQLLSDVLNTKMEIHSVEMGKNKIREKLCGKKLLI
VLDDTKYDPLLNLRDSHVWFAKGTVIIITAREEHLLRIPQNGSAFPVNLLSTNESLELLSWHAFREAKPKQEYNDLAKRVV
VYCGGLPLALEVIGSSLFERTKEEWKSVLLELKEIPEHDVHRKLKISFNGLRNEMEKDLFLDVCCFFVGKGRTFVTKILNDC
GVDADSGLRILIERSLVQVKKNKKLGMQQLLQKMGRKIICEISRKELGKNPPLWSGQDAEYERLENTLFSSQQTKVIQKR
LSLKMFLIATREFERYPSGVRDTSRLLRLFKVFGKLRWISLQGFSSEYLPKDIYLHDAMAIDLKHSLLRFVWKEPQVLSWLK
VLNLSHSMYLRETPDFSGLPRLEQLVLKDCPSLHKVHHSIGCLNNLLLLNLKDCTSLSNLPREVYKLKSLNTLILSGCSKIDLL
EKYIVQVESLLILIAENAAVKQVPFSIVSSKNIGYIFLRGCEGLSCNLFPSIIRSWISPIMNPLSYVHSFCMDIEDNGWNDFD
PLLSTLANLRSVLVQCDTEFQLSKLVETILIEYGVNISKSEISQQHFKYSLIGVGRCKDFFNAVSDIISKVFASNESRDVSLPG
DNDPYWFGHMGEGRSVFFTVPRDHDLKGMALCVVYLSTPEIVAPVCLRSVLIVNYTKCTLQIHNHGRVISFNDIDWQG
IISNLGPGDKVEICVTFAQELVVTNTILYLICDELNDLQKEPAPKKNSLIRFVKKVVM

>XP_027907715.1

MASSIPPTEFASSTSKLPRKYDVLINFTGEDIRRKFVSHLDYALSTVGLTTFLHEENAVNDMHIQQPILNLCRVAIVVFTKT
YSQSAWCLHQLQQIIKWQETYSRHVFPVYYEIQPSDVRLQKGDFGETFKETAQQTFSAQQLEHGMSRWSHTLTKAAN
FFGWDESNYRSDAELVDKIVEGVLNLPVLFATKFPVGLQSRLNDVIQIIKDKSREVCIIGIWGEGGSGKTTLAKAIYNQLH
GTFTHKSFIEDISQVIQTKGHVHLQEQLLSDVLNTKMEIHSVEMGKNKIREKLCGKKLLIVLDDTKYDPLLNLRDSHVWFA
KGTVIIITAREEHLLRIPQNGSAFPVNLLSTNESLELLSWHAFREAKPKQEYNDLAKRVVVYCGGLPLALEVIGSSLFERTKE
EWKSVLLELKEIPEHDVHRKLKISFNGLRNEMEKDLFLDVCCFFVGKGRTFVTKILNDCGVDADSGLRILIERSLVQVKKN
KKLGMQQLLQKMGRKIICEISRKELGKNPPLWSGQDAEYERLENTLFSSQQTKVIQKRLSLKMFLIATREFERYPSGVRD
TSRLLRLFKVFGKLRWISLQGFSSEYLPKDIYLHDAMAIDLKHSLLRFVWKEPQVLSWLKVLNLSHSMYLRETPDFSGLPR
LEQLVLKDCPSLHKVHHSIGCLNNLLLLNLKDCTSLSNLPREVYKLKSLNTLILSGCSKIDLLEKYIVQVESLLILIAENAAVKQ
VPFSIVSSKNIGYIFLRGCEGLSCNLFPSIIRSWISPIMNPLSYVHSFCMDIEDNGWNDFDPLLSTLANLRSVLVQCDTEFQ
LSKLVETILIEYGVNISKSEISQQHFKYSLIGVGRCKDFFNAVSDIISKVIMILIGSATWVRVVLFFSPCLEIMT

>XP_027907716.1

MACPGGATHSPKQQISLDGMRAITVGLQSRLNDVIQIIKDKSREVCIIGIWGEGGSGKTTLAKAIYNQLHGTFTHKSFIED
ISQVIQTKGHVHLQEQLLSDVLNTKMEIHSVEMGKNKIREKLCGKKLLIVLDDTKYDPLLNLRDSHVWFAKGTVIIITARE
EHLLRIPQNGSAFPVNLLSTNESLELLSWHAFREAKPKQEYNDLAKRVVVYCGGLPLALEVIGSSLFERTKEEWKSVLLELK
EIPEHDVHRKLKISFNGLRNEMEKDLFLDVCCFFVGKGRTFVTKILNDCGVDADSGLRILIERSLVQVKKNKKLGMQQLL
QKMGRKIICEISRKELGKNPPLWSGQDAEYERLENTLFSSQQTKVIQKRLSLKMFLIATREFERYPSGVRDTSRLLRLFKVF
GKLRWISLQGFSSEYLPKDIYLHDAMAIDLKHSLLRFVWKEPQVLSWLKVLNLSHSMYLRETPDFSGLPRLEQLVLKDCP
SLHKVHHSIGCLNNLLLLNLKDCTSLSNLPREVYKLKSLNTLILSGCSKIDLLEKYIVQVESLLILIAENAAVKQVPFSIVSSKNI
GYIFLRGCEGLSCNLFPSIIRSWISPIMNPLSYVHSFCMDIEDNGWNDFDPLLSTLANLRSVLVQCDTEFQLSKLVETILIEY
GVNISKSEISQQHFKYSLIGVGRCKDFFNAVSDIISKVFASNESRDVSLPGDNDPYWFGHMGEGRSVFFTVPRDHDLKG
MALCVVYLSTPEIVAPVCLRSVLIVNYTKCTLQIHNHGRVISFNDIDWQGIISNLGPGDKVEICVTFAQELVVTNTILYLICD
ELNDLQKEPAPKKNSLIRFVKKVVM

>XP_027907718.1

MASSIPPTEFASSTSKLPRKYDVLINFTGEDIRRKFVSHLDYALSTVGLTTFLHEENAVNDMHIQQPILNLCRVAIVVFTKT
YSQSAWCLHQLQQIIKWQETYSRHVFPVYYEIQPSDVRLQKGDFGETFKETAQQTFSAQQLEHGMSRWSHTLTKAAN
FFGWDESNYRSDAELVDKIVEGVLNLPVLFATKFPVGLQSRLNDVIQIIKDKSREVCIIGIWGEGGSGKTTLAKAIYNQLH
GTFTHKSFIEDISQVIQTKGHVHLQEQLLSDVLNTKMEIHSVEMGKNKIREKLCGKKLLIVLDDTKYDPLLNLRDSHVWFA
KGTVIIITAREEHLLRIPQNGSAFPVNLLSTNESLELLSWHAFREAKPKQEYNDLAKRVVVYCGGLPLALEVIGSSLFERTKE
EWKSVLLELKEIPEHDVHRKLKISFNGLRNEMEKDLFLDVCCFFVGKGRTFVTKILNDCGVDADSGLRILIERSLVQVKKN
KKLGMQQLLQKMGRKIICEISRKELGKNPPLWSGQDAEYERLENTLFSSQQTKVIQKRLSLKMFLIATREFERYPSGVRD
TSRLLRLFKVFGKLRWISLQGFSSEYLPKDIYLHDAMAIDLKHSLLRFVWKEPQVLSWLKVLNLSHSMYLRETPDFSGLPR
LEQLVLKDCPSLHKVHHSIGCLNNLLLLNLKDCTSLSNLPREL

>XP_027907719.1

MTSSIPSMELPSSSSKLPPMYDVLINFTGEDIRRKFVSHLDSALSANGLTTFLHHDNAIKPVHIQQPILNLCRVAIVVFTET
YSQSAWCLHQLQQIIQWHETYCRHVLPVYYEIQPSDVRLQKGDFGKAFKATAHQTFSAQELEHAMSRWSHAITKTAN
FFGWDDCNYRSDAELVDTIVKSVLNLSVLSATKFPVGLQSRVEDVIEIIKNKSTEVCRIGICGMGGSGKTTLAKAIYSQIHG
TFVEKSFVEDISEVSRTRGHVHLQRQLLSDVLKTKVKIHNVEMGRCMILEKLYRRRVLIVLDDVNEHCPLDIWESKGWFS
EGTVIIITTRDEYLLRKHEVRSVFRINRMNENQSLELFSWHAFREAKPKEEYYSLAKRLVAYCGGLPLALEVVGTYLYERTK
EEWNRVLLKLDNVPRHEILQILKISFDGLPNQMERDLFLDICCFFVGKGKVYATKILNGCGVDADSGIRVLIERNLIKVKN
NNKFGMHPLLRDMGREIILEISSKEPWNKNRVCLENDMHHALSENILFSSQETKVVHRSPTGRDSYERYPLEVSDPSRLV
KINADSKYRTKKLIWISWQGFPSEYLPNDFYLHDAIAIDLKYNLLRFLWKQPQVLESLKVLNLSHSMYLTKTPDFSRLPRLE
QLILKDCPRLHKVDQSVGCLCNLTLLNLKDCTSLSNLPREIYKLKSLKALILSGCSKLSLMEKDIGQMESLISLIAENAVVKQ
VPFSIVSSKCIGHISLHRFEGLSHNPFPSIIWCWMSPTMNPLSYIHSFCMDKEHSSGDDIMPLFNTLANLRSVVVQCDTE
FPLSTQVKTTLVEYVVNISESGISKHHFRLSLIGFGRHGEFFNTVSDGISEVLASSELSEVSLPGDNDPFWLAHMGEGHYV
SFTLPQDRVMKGMALCVVYLSTSVPAENK

>XP_027907726.1

MDCLGPFGKAAEGAIDIVWKHGVRHVTYIINYKQNVLELNDSAKDLGFEKERINHQRDEAEKNLNNIEGKVTEWVRKV
SEIETVIDEFENDDGHKRARSPNCYVFPYLWNRHKLGRKAKKMEVDVKKLIDESPEFDEVSYRQNITSNDATLYNYGFVE
FGSTKSTMEKVMRQLEDSSVRMIGLYGPGGVGKSTLVKEIARKAKDEKLFDVVVKVEITVNPNVQKIQEEIAYVLGLRLE
GEGENVRADCLRRRLKIKKGSTLLILDDLWDKLDLSKLGVPLDDDDDDDLSNDKVLEKDDNNKDPNRKVLKKEKILGGV

KGCKILLTSRDKKVLCDEMDVKSTFCVKELDDKDALMLFQKLVGIHNEISDSKQEIVKKYCAGLPMAIVTVARALKSKSES
VWEATLEKLKKQELVGVQISMDISVKMSYDHLENEEIKSIFLLCAQMGHQPLVMDLVRYCFGLGLLEGVSSLWEARDRI
KTSIQKLKDSGLLLDGSSNNHFNMHDMVRDAALSIASKDRNVFTLRNAKLDDWPEFESCTSISICNCDIIDGLPEVINSP
QLKFFQIETKDTSLIIPENFFEGMKDLKVLILTGFRLSRLPSSIKCLLKLRMLCLERCYVDDNLSIGELKKLRILSFSGSQLQNF
PIELGCLDKLQLLDISDCSIETNIPLNLLSSLKHLEELYIRKSLIKMLAEGETSQYQKLFFSELKNLCELKVVDLSIPCATVLPNH
LFFDKLKDYKIVIGDLEMFGDFIMPDKYETFRVLALQLHDNTNIHSQEDIKSLFKTVQSLLLGKVNGVQNVVSDLNIDGFP
DLKHLSIINNNHIKCVSSSKFFNSADVFPNLESLCLYGLGNLEMISYGPITVASFAKLKAINVKMCYQLKNLYSVYNFKFSTS
GKICEISECNFMDQFRASVEIIEVCECGSLKEILQIPMDYGKVEFLKLHTLTLQSLPSFTSFYTEVKTSCSPHPAEPQTTSYG
NREITSEDEQSVKTPTVFGELVEIPNLESLNLSALNIHKIWSDQPSSSFCFQNLIKLVVKECDKLTYLCSLSVAHSLKKLKSLV
IIECAIMEKIIETKGNILEKVCVFPKLEEIHLTKMNALTDVWQTNASVDSFSSLISVTIEECNKLDKIFPSHMEGWFESLENL
RVSRCESVEVTFEINDSQEIDASGGIDTSLQVILLNDLPKLKQLWSIDPDGILNFKKLRTIEVYSCGELRNLFPASIANDVPK
LERMSALYCENMVEIIASEDASEDDKDPLVFPELTYMRLYWLRNIKHFYKGRRPIKCPKLKEFSIDNYVNKFIKETSKTNEE
ENFVFSAQEVFSNLECMEIDFYQAQKLLPKFQMHRLKELSLISVKSVNLLHQFPYAMPNLQKLKLFSSYGEDLVASANSV
PQNGLGIVLELKELVMQFLGIKDLGLGQLPVLRKLEFLSLEHCDELKNLGPSSVSLTYLTHLELKSCQGLRNLMASSTAKS
MVQLKTMKVIDCDGVDQIVSNEGSEEVNEMKIVFPKLISIGLVGLKNMTSFCRSKEFEFEFPSLEILIVRECPKMERFSER
KSITPKLKNVFGVEGDEKTKWQWEGDLNATIQKVFNDKVTYAYTEQLALGRFSYTEFIQQIWHDSHSVHQSSFRSLKRL
SAYKVNLLRVIPSHLLSCFENLEELDIRSCSATQFIFNINENRVRKPSGIFRLKSLYLFNLPKLEHVWDKDPKGIIGLKVLKEV
RVSRCECLKSLFPASVAKDLTRLEVLEVTECEELAEIFWKDEKGEEGEGTTPQFVFARLTSLTLKQLPGFKYSIRCSKEEEIIS
NLSERDIEELCLGSRPIPNSCFGLLESLTVDGCQFLSNVLLPFNLLISLTNLETLEVRNSDFVKTIFDVKCTTQNRDVTSVGQ
TLPFSLKKLTLSMLPNLENVWNEDPHRILSMHHLKQVLVDNCKCLTSLFPASVAKDFVELEHLEVKDCEGLMTIVAEDNT
DPSGTKQELPCPPVWSLLLRGLPKFKYFYYSSLQCDNFATENEVYVEKCLSLGEKGLEMILSGEFQRDLLYNLKVLALWFE
RDVFPHEILEQVPNIEKLLLCGGSFREMFCCESSNNVDYNGLLLQLKVLHLESLGNLISIAACKLTFSNLTYLNVKGCNNLR
YLFTSSAAKNLGQLKRMEIKWCESIEEIVSKEVEDSSNEDEIIFPQLNCLNFEYLENLRIFYQGNLSFPSLEEFSVTDCHQLIT
LSTGTLEVHKLSHVTIDSKEIIFPLKSDLNTIMRKTFLAEISELEQLDLESSPKLQKIWHDLVYIPDLCFSELTTLIVYNCQFLSD
VVLPFHLLPSLPKLETLRVQNCHYVKAIFDVKCAQEIVTFPLKKMVLWELQNLKNVWNEDPHEILTMHQLQNVYIKKCK
GLTSVFPASVAKGIVKLKKLTVKRCKELMTIVADMKGTNVEVKFPCPSVRSLKLRRLPRFKYFYYCSLKSDVYTHLGSHNE
DRVATEKCLSLGEKGMEMILYGEFQKSLLYNLKVLTLYFQSDVFRYEIIEQVPNIKKLVVRDGSFKEMFCSQNPNYNVEYS
GVLSQLKELRLDSLKELVSIGLDNSWTHPFVRTLETFEVIKCSSLETLVACKVAFSNLTYLNVESCNSLSYLFTSLTAKSLLQL
KRMVIKQCESIEEIVLFMEADEADEDKIIFPHLNCLNIEYLTNLRRFYRGSLSFPSLQELSVTECDKMITLCTGTLEADKLSQ
VTVDISETIPLQTDLHLTMRKKFLRKNGWQQSLEFRDRADLHEIWRVSLQIPYFCFRGLSILVVDGCQFLSDVLPFSLLPLL
PGLKTLEVQYCYSVKTIFDVKCTTQAALITFPPLRKLVLWKLPNLETVWNEDPAEIVNPAHPKHTNLKLTFPSVTSLTLWD
LPKLKRNSIYCIHDSTPTFELITPNLQRLSVGEDELKMIVDGEFQENHFNKLKILTLCFLTESGVFLEFLQLVPNVENLIVYGG
SLKEIFCSQSSNNVDYSGLPLQLKGLLFESLGELISIGFENSWTEPFVRNLETFEVISCSSLKNLVASKVFLSNLTYLKIESCDN
LSYLFTSSTAKSLRELKEMVIKRCKSIEEIVSKEGEEWCEDKEIIFEKLQVLYLKSLDELRCFYPGNFTLSFPSLERIHVINCSSM
KTFSAFNEIDHFTQWYNAEYAIPFEETDLNSAVHRTHEEEVIPPEEGDLNTVVHRTLEEEGLDHWSFSSLSSYSDGYKDG
DSVYDSIP

>XP_027907727.1

MDCLGPFGKAAEGAIDIVWKHGVRHVTYIINYKQNVLELNDSAKDLGFEKERINHQRDEAEKNLNNIEGKVTEWVRKV
SEIETVIDEFENDDGHKRARSPNCYVFPYLWNRHKLGRKAKKMEVDVKKLIDESPEFDEVSYRQNITSNDATLYNYGFVE
FGSTKSTMEKVMRQLEDSSVRMIGLYGPGGVGKSTLVKEIARKAKDEKLFDVVVKVEITVNPNVQKIQEEIAYVLGLRLE
GEGENVRADCLRRRLKIKKGSTLLILDDLWDKLDLSKLGVPLDDDDDDDLSNDKVLEKDDNNKDPNRKVLKKEKILGGV
KGCKILLTSRDKKVLCDEMDVKSTFCVKELDDKDALMLFQKLVGIHNEISDSKQEIVKKYCAGLPMAIVTVARALKSKSES
VWEATLEKLKKQELVGVQISMDISVKMSYDHLENEEIKSIFLLCAQMGHQPLVMDLVRYCFGLGLLEGVSSLWEARDRI

KTSIQKLKDSGLLLDGSSNNHFNMHDMVRDAALSIASKDRNVFTLRNAKLDDWPEFESCTSISICNCDIIDGLPEVINSP
QLKFFQIETKDTSLIIPENFFEGMKDLKVLILTGFRLSRLPSSIKCLLKLRMLCLERCYVDDNLSIGELKKLRILSFSGSQLQNF
PIELGCLDKLQLLDISDCSIETNIPLNLLSSLKHLEELYIRKSLIKMLAEGETSQYQKLFFSELKNLCELKVVDLSIPCATVLPNH
LFFDKLKDYKIVIGDLEMFGDFIMPDKYETFRVLALQLHDNTNIHSQEDIKSLFKTVQSLLLGKVNGVQNVVSDLNIDGFP
DLKHLSIINNNHIKCVSSSKFFNSADVFPNLESLCLYGLGNLEMISYGPITVASFAKLKAINVKMCYQLKNLYSVYNFKFSTS
GKICEISECNFMDQFRASVEIIEVCECGSLKEILQIPMDYGKVEFLKLHTLTLQSLPSFTSFYTEVKTSCSPHPAEPQTTSYG
NREITSEDEQSVKTPTVFGELVEIPNLESLNLSALNIHKIWSDQPSSSFCFQNLIKLVVKECDKLTYLCSLSVAHSLKKLKSLV
IIECAIMEKIIETKGNILEKVCVFPKLEEIHLTKMNALTDVWQTNASVDSFSSLISVTIEECNKLDKIFPSHMEGWFESLENL
RVSRCESVEVTFEINDSQEIDASGGIDTSLQVILLNDLPKLKQLWSIDPDGILNFKKLRTIEVYSCGELRNLFPASIANDVPK
LERMSALYCENMVEIIASEDASEDDKDPLVFPELTYMRLYWLRNIKHFYKGRRPIKCPKLKEFSIDNYVNKFIKETSKTNEE
ENFVFSAQEVFSNLECMEIDFYQAQKLLPKFQMHRLKELSLISVKSVNLLHQFPYAMPNLQKLKLFSSYGEDLVASANSV
PQNGLGIVLELKELVMQFLGIKDLGLGQLPVLRKLEFLSLEHCDELKNLGPSSVSLTYLTHLELKSCQGLRNLMASSTAKS
MVQLKTMKVIDCDGVDQIVSNEGSEEVNEMKIVFPKLISIGLVGLKNMTSFCRSKEFEFEFPSLEILIVRECPKMERFSER
KSITPKLKNVFGVEGDEKTKWQWEGDLNATIQKVFNDKVTYAYTEQLALGRFSYTEFIQQIWHDSHSVHQSSFRSLKRL
SAYKVNLLRVIPSHLLSCFENLEELDIRSCSATQFIFNINENRVRKPSGIFRLKSLYLFNLPKLEHVWDKDPKGIIGLKVLKEV
RVSRCECLKSLFPASVAKDLTRLEVLEVTECEELAEIFWKDEKGEEGEGTTPQFVFARLTSLTLKQLPGFKYSIRCSKEEEIIS
NLSERDIEELCLGSRPIPNSCFGLLESLTVDGCQFLSNVLLPFNLLISLTNLETLEVRNSDFVKTIFDVKCTTQNRDVTSVGQ
TLPFSLKKLTLSMLPNLENVWNEDPHRILSMHHLKQVLVDNCKCLTSLFPASVAKDFVELEHLEVKDCEGLMTIVAEDNT
DPSGTKQELPCPPVWSLLLRGLPKFKYFYYSSLQCDNFATENEVYVEKCLSLGEKGLEMILSGEFQRDLLYNLKVLALWFE
RDVFPHEILEQVPNIEKLLLCGGSFREMFCCESSNNVDYNGLLLQLKVLHLESLGNLISIAACKLTFSNLTYLNVKGCNNLR
YLFTSSAAKNLGQLKRMEIKWCESIEEIVSKEVEDSSNEDEIIFPQLNCLNFEYLENLRIFYQGNLSFPSLEEFSVTDCHQLIT
LSTGTLEVHKLSHVTIDSKEIIFPLKSDLNTIMRKTFLAEISELEQLDLESSPKLQKIWHDLVYIPDLCFSELTTLIVYNCQFLSD
VVLPFHLLPSLPKLETLRVQNCHYVKAIFDVKCAQEIVTFPLKKMVLWELQNLKNVWNEDPHEILTMHQLQNVYIKKCK
GLTSVFPASVAKGIVKLKKLTVKRCKELMTIVADMKGTNVEVKFPCPSVRSLKLRRLPRFKYFYYCSLKSDVYTHLGSHNE
DRVATEKCLSLGEKGMEMILYGEFQKSLLYNLKVLTLYFQSDVFRYEIIEQVPNIKKLVVRDGSFKEMFCSQNPNYNVEYS
GVLSQLKELRLDSLKELVSIGLDNSWTHPFVRTLETFEVIKCSSLETLVACKVAFSNLTYLNVESCNSLSYLFTSLTAKSLLQL
KRMVIKQCESIEEIVLFMEADEADEDKIIFPHLNCLNIEYLTNLRRFYRGSLSFPSLQELSVTECDKMITLCTGTLEADKLSQ
VTVDISETIPLQTDLHLTMRKKFLRKNGWQQSLEFRDRADLHEIWRVSLQIPYFCFRGLSILVVDGCQFLSDVLPFSLLPLL
PGLKTLEVQYCYSVKTIFDVKCTTQAALITFPPLRKLVLWKLPNLETVWNEDPAEIVNPAHPKHTNLKLTFPSVTSLTLWD
LPKLKRNSIYCIHDSTPTFELITPNLQRLSVGEDELKMIVDGEFQENHFNKLKILTLCFLTESGVFLEFLQLVPNVENLIVYGG
SLKEIFCSQSSNNVDYSGLPLQLKGLLFESLGELISIGFENSWTEPFVRNLETFEVISCSSLKNLVASKVFLSNLTYLKIESCDN
LSYLFTSSTAKSLRELKEMVIKRCKSIEEIVSKEGEEWCEDKEIIFEKLQVLYLKSLDELRCFYPGNFTLSFPSLERIHVINCSSM
KTFSAFNEIDHFTQWYNAEYAIPFEETDLNSAVHRTHEEEVIPPEEGDLNTVVHRTLEEEGLDHWSFSSLSSYSDGYKDG
DSVYDSIP

>XP_027907728.1

MDCLGPFGKAAEGAIDIVWKHGVRHVTYIINYKQNVLELNDSAKDLGFEKERINHQRDEAEKNLNNIEGKVTEWVRKV
SEIETVIDEFENDDGHKRARSPNCYVFPYLWNRHKLGRKAKKMEVDVKKLIDESPEFDEVSYRQNITSNDATLYNYGFVE
FGSTKSTMEKVMRQLEDSSVRMIGLYGPGGVGKSTLVKEIARKAKDEKLFDVVVKVEITVNPNVQKIQEEIAYVLGLRLE
GEGENVRADCLRRRLKIKKGSTLLILDDLWDKLDLSKLGVPLDDDDDDDLSNDKVLEKDDNNKDPNRKVLKKEKILGGV
KGCKILLTSRDKKVLCDEMDVKSTFCVKELDDKDALMLFQKLVGIHNEISDSKQEIVKKYCAGLPMAIVTVARALKSKSES
VWEATLEKLKKQELVGVQISMDISVKMSYDHLENEEIKSIFLLCAQMGHQPLVMDLVRYCFGLGLLEGVSSLWEARDRI
KTSIQKLKDSGLLLDGSSNNHFNMHDMVRDAALSIASKDRNVFTLRNAKLDDWPEFESCTSISICNCDIIDGLPEVINSP
QLKFFQIETKDTSLIIPENFFEGMKDLKVLILTGFRLSRLPSSIKCLLKLRMLCLERCYVDDNLSIGELKKLRILSFSGSQLQNF

PIELGCLDKLQLLDISDCSIETNIPLNLLSSLKHLEELYIRKSLIKMLAEGETSQYQKLFFSELKNLCELKVVDLSIPCATVLPNH
LFFDKLKDYKIVIGDLEMFGDFIMPDKYETFRVLALQLHDNTNIHSQEDIKSLFKTVQSLLLGKVNGVQNVVSDLNIDGFP
DLKHLSIINNNHIKCVSSSKFFNSADVFPNLESLCLYGLGNLEMISYGPITVASFAKLKAINVKMCYQLKNLYSVYNFKFSTS
GKICEISECNFMDQFRASVEIIEVCECGSLKEILQIPMDYGKVEFLKLHTLTLQSLPSFTSFYTEVKTSCSPHPAEPQTTSYG
NREITSEDEQSVKTPTVFGELVEIPNLESLNLSALNIHKIWSDQPSSSFCFQNLIKLVVKECDKLTYLCSLSVAHSLKKLKSLV
IIECAIMEKIIETKGNILEKVCVFPKLEEIHLTKMNALTDVWQTNASVDSFSSLISVTIEECNKLDKIFPSHMEGWFESLENL
RVSRCESVEVTFEINDSQEIDASGGIDTSLQVILLNDLPKLKQLWSIDPDGILNFKKLRTIEVYSCGELRNLFPASIANDVPK
LERMSALYCENMVEIIASEDASEDDKDPLVFPELTYMRLYWLRNIKHFYKGRRPIKCPKLKEFSIDNYVNKFIKETSKTNEE
ENFVFSAQEVFSNLECMEIDFYQAQKLLPKFQMHRLKELSLISVKSVNLLHQFPYAMPNLQKLKLFSSYGEDLVASANSV
PQNGLGIVLELKELVMQFLGIKDLGLGQLPVLRKLEFLSLEHCDELKNLGPSSVSLTYLTHLELKSCQGLRNLMASSTAKS
MVQLKTMKVIDCDGVDQIVSNEGSEEVNEMKIVFPKLISIGLVGLKNMTSFCRSKEFEFEFPSLEILIVRECPKMERFSER
KSITPKLKNVFGVEGDEKTKWQWEGDLNATIQKVFNDKVTYAYTEQLALGRFSYTEFIQQIWHDSHSVHQSSFRSLKRL
SAYKVNLLRVIPSHLLSCFENLEELDIRSCSATQFIFNINENRVRKPSGIFRLKSLYLFNLPKLEHVWDKDPKGIIGLKVLKEV
RVSRCECLKSLFPASVAKDLTRLEVLEVTECEELAEIFWKDEKGEEGEGTTPQFVFARLTSLTLKQLPGFKYSIRCSKEEEIIS
NLSERDIEELCLGSRPIPNSCFGLLESLTVDGCQFLSNVLLPFNLLISLTNLETLEVRNSDFVKTIFDVKCTTQNRDVTSVGQ
TLPFSLKKLTLSMLPNLENVWNEDPHRILSMHHLKQVLVDNCKCLTSLFPASVAKDFVELEHLEVKDCEGLMTIVAEDNT
DPSGTKQELPCPPVWSLLLRGLPKFKYFYYSSLQCDNFATENEVYYVEKCLSLGEKGLEMILSGEFQRDLLYNLKVLALWFE
RDVFPHEILEQVPNIEKLLLCGGSFREMFCCESSNNVDYNGLLLQLKVLHLESLGNLISIAACKLTFSNLTYLNVKGCNNLR
YLFTSSAAKNLGQLKRMEIKWCESIEEIVSKEVEDSSNEDEIIFPQLNCLNFEYLENLRIFYQGNLSFPSLEEFSVTDCHQLIT
LSTGTLEVHKLSHVTIDSKEIIFPLKSDLNTIMRKTFLAEISELEQLDLESSPKLQKIWHDLVYIPDLCFSELTTLIVYNCQFLSD
VVLPFHLLPSLPKLETLRVQNCHYVKAIFDVKCAQEIVTFPLKKMVLWELQNLKNVWNEDPHEILTMHQLQNVYIKKCK
GLTSVFPASVAKGIVKLKKLTVKRCKELMTIVADMKGTNVEVKFPCPSVRSLKLRRLPRFKYFYYCSLKSDVYTHLGSHNE
DRVATEKCLSLGEKGMEMILYGEFQKSLLYNLKVLTLYFQSDVFRYEIIEQVPNIKKLVVRDGSFKEMFCSQNPNYNVEYS
GVLSQLKELRLDSLKELVSIGLDNSWTHPFVRTLETFEVIKCSSLETLVACKVAFSNLTYLNVESCNSLSYLFTSLTAKSLLQL
KRMVIKQCESIEEIVLFMEADEADEDKIIFPHLNCLNIEYLTNLRRFYRGSLSFPSLQELSVTECDKMITLCTGTLEADKLSQ
VTVDISETIPLQTDLHLTMRKKFLRKNGWQQSLEFRDRADLHEIWRVSLQIPYFCFRGLSILVVDGCQFLSDVLPFSLLPLL
PGLKTLEVQYCYSVKTIFDVKCTTQAALITFPPLRKLVLWKLPNLETVWNEDPAEIVNPAHPKHTNLKLTFPSVTSLTLWD
LPKLKRNSIYCIHDSTPTFELITPNLQRLSVGEDELKMIVDGEFQENHFNKLKILTLCFLTESGVFLEFLQLVPNVENLIVYGG
SLKEIFCSQSSNNVDYSGLPLQLKGLLFESLGELISIGFENSWTEPFVRNLETFEVISCSSLKNLVASKVFLSNLTYLKIESCDN
LSYLFTSSTAKSLRELKEMVIKRCKSIEEIVSKEGEEWCEDKEIIFEKLQVLYLKSLDELRCFYPGNFTLSFPSLERIHVINCSSM
KTFSAFNEIDHFTQWYNAEYAIPFEETDLNSAVHRTHEEEVIPPEEGDLNTVVHRTLEEEGLDHWSFSSLSSYSDGYKDG
DSVYDSIP

>XP_027907729.1

MDCLGPFGKAAEGAIDIVWKHGVRHVTYIINYKQNVLELNDSAKDLGFEKERINHQRDEAEKNLNNIEGKVTEWVRKV
SEIETVIDEFENDDGHKRARSPNCYVFPYLWNRHKLGRKAKKMEVDVKKLIDESPEFDEVSYRQNITSNDATLYNYGFVE
FGSTKSTMEKVMRQLEDSSVRMIGLYGPGGVGKSTLVKEIARKAKDEKLFDVVVKVEITVNPNVQKIQEEIAYVLGLRLE
GEGENVRADCLRRRLKIKKGSTLLILDDLWDKLDLSKLGVPLDDDDDDDLSNDKVLEKDDNNKDPNRKVLKKEKILGGV
KGCKILLTSRDKKVLCDEMDVKSTFCVKELDDKDALMLFQKLVGIHNEISDSKQEIVKKYCAGLPMAIVTVARALKSKSES
VWEATLEKLKKQELVGVQISMDISVKMSYDHLENEEIKSIFLLCAQMGHQPLVMDLVRYCFGLGLLEGVSSLWEARDRI
KTSIQKLKDSGLLLDGSSNNHFNMHDMVRDAALSIASKDRNVFTLRNAKLDDWPEFESCTSISICNCDIIDGLPEVINSP
QLKFFQIETKDTSLIIPENFFEGMKDLKVLILTGFRLSRLPSSIKCLLKLRMLCLERCYVDDNLSIGELKKLRILSFSGSQLQNF
PIELGCLDKLQLLDISDCSIETNIPLNLLSSLKHLEELYIRKSLIKMLAEGETSQYQKLFFSELKNLCELKVVDLSIPCATVLPNH
LFFDKLKDYKIVIGDLEMFGDFIMPDKYETFRVLALQLHDNTNIHSQEDIKSLFKTVQSLLLGKVNGVQNVVSDLNIDGFP

DLKHLSIINNNHIKCVSSSKFFNSADVFPNLESLCLYGLGNLEMISYGPITVASFAKLKAINVKMCYQLKNLYSVYNFKFSTS
GKICEISECNFMDQFRASVEIIEVCECGSLKEILQIPMDYGKVEFLKLHTLTLQSLPSFTSFYTEVKTSCSPHPAEPQTTSYG
NREITSEDEQSVKTPTVFGELVEIPNLESLNLSALNIHKIWSDQPSSSFCFQNLIKLVVKECDKLTYLCSLSVAHSLKKLKSLV
IIECAIMEKIIETKGNILEKVCVFPKLEEIHLTKMNALTDVWQTNASVDSFSSLISVTIEECNKLDKIFPSHMEGWFESLENL
RVSRCESVEVTFEINDSQEIDASGGIDTSLQVILLNDLPKLKQLWSIDPDGILNFKKLRTIEVYSCGELRNLFPASIANDVPK
LERMSALYCENMVEIIASEDASEDDKDPLVFPELTYMRLYWLRNIKHFYKGRRPIKCPKLKEFSIDNYVNKFIKETSKTNEE
ENFVFSAQEVFSNLECMEIDFYQAQKLLPKFQMHRLKELSLISVKSVNLLHQFPYAMPNLQKLKLFSSYGEDLVASANSV
PQNGLGIVLELKELVMQFLGIKDLGLGQLPVLRKLEFLSLEHCDELKNLGPSSVSLTYLTHLELKSCQGLRNLMASSTAKS
MVQLKTMKVIDCDGVDQIVSNEGSEEVNEMKIVFPKLISIGLVGLKNMTSFCRSKEFEFEFPSLEILIVRECPKMERFSER
KSITPKLKNVFGVEGDEKTKWQWEGDLNATIQKVFNDKVTYAYTEQLALGRFSYTEFIQQIWHDSHSVHQSSFRSLKRL
SAYKVNLLRVIPSHLLSCFENLEELDIRSCSATQFIFNINENRVRKPSGIFRLKSLYLFNLPKLEHVWDKDPKGIIGLKVLKEV
RVSRCECLKSLFPASVAKDLTRLEVLEVTECEELAEIFWKDEKGEEGEGTTPQFVFARLTSLTLKQLPGFKYSIRCSKEEEIIS
NLSERDIEELCLGSRPIPNSCFGLLESLTVDGCQFLSNVLLPFNLLISLTNLETLEVRNSDFVKTIFDVKCTTQNRDVTSVGQ
TLPFSLKKLTLSMLPNLENVWNEDPHRILSMHHLKQVLVDNCKCLTSLFPASVAKDFVELEHLEVKDCEGLMTIVAEDNT
DPSGTKQELPCPPVWSLLLRGLPKFKYFYYSSLQCDNFATENEVYVEKCLSLGEKGLEMILSGEFQRDLLYNLKVLALWFE
RDVFPHEILEQVPNIEKLLLCGGSFREMFCCESSNNVDYNGLLLQLKVLHLESLGNLISIAACKLTFSNLTYLNVKGCNNLR
YLFTSSAAKNLGQLKRMEIKWCESIEEIVSKEVEDSSNEDEIIFPQLNCLNFEYLENLRIFYQGNLSFPSLEEFSVTDCHQLIT
LSTGTLEVHKLSHVTIDSKEIIFPLKSDLNTIMRKTFLAEISELEQLDLESSPKLQKIWHDLVYIPDLCFSELTTLIVYNCQFLSD
VVLPFHLLPSLPKLETLRVQNCHYVKAIFDVKCAQEIVTFPLKKMVLWELQNLKNVWNEDPHEILTMHQLQNVYIKKCK
GLTSVFPASVAKGIVKLKKLTVKRCKELMTIVADMKGTNVEVKFPCPSVRSLKLRRLPRFKYFYYCSLKSDVYTHLGSHNE
DRVATEKCLSLGEKGMEMILYGEFQKSLLYNLKVLTLYFQSDVFRYEIIEQVPNIKKLVVRDGSFKEMFCSQNPNYNVEYS
GVLSQLKELRLDSLKELVSIGLDNSWTHPFVRTLETFEVIKCSSLETLVACKVAFSNLTYLNVESCNSLSYLFTSLTAKSLLQL
KRMVIKQCESIEEIVLFMEADEADEDKIIFPHLNCLNIEYLTNLRRFYRGSLSFPSLQELSVTECDKMITLCTGTLEADKLSQ
VTVDISETIPLQTDLHLTMRKKFLRKNGWQQSLEFRDRADLHEIWRVSLQIPYFCFRGLSILVVDGCQFLSDVLPFSLLPLL
PGLKTLEVQYCYSVKTIFDVKCTTQAALITFPPLRKLVLWKLPNLETVWNEDPAEIVNPAHPKHTNLKLTFPSVTSLTLWD
LPKLKRNSIYCIHDSTPTFELITPNLQRLSVGEDELKMIVDGEFQENHFNKLKILTLCFLTESGVFLEFLQLVPNVENLIVYGG
SLKEIFCSQSSNNVDYSGLPLQLKGLLFESLGELISIGFENSWTEPFVRNLETFEVISCSSLKNLVASKVFLSNLTYLKIESCDN
LSYLFTSSTAKSLRELKEMVIKRCKSIEEIVSKEGEEWCEDKEIIFEKLQVLYLKSLDELRCFYPGNFTLSFPSLERIHVINCSSM
KTFSAFNEIDHFTQWYNAEYAIPFEETDLNSAVHRTHEEEVIPPEEGDLNTVVHRTLEEEGLDHWSFSSLSSYSDGYKDG
DSVYDSIP

>XP_027907735.1

MEFASSSSKLPRKYDVLINFDGEEIGRKFVSHLDSVLSAVGLTTLLHYDNAVKSTHIQQPILNLCRIVIVVFTKTYSESAWCL
HQLQQIIEWHQTYCRHVLPVYYEIQPSDVRLQKGDFGKAFKATAQRTFSEQELEHGMSRWSHALTKAANFFGWDESN
HRSDAELVDKIVKSILNLSALSATKFPVGLQDRVELLILAINNKSKEVCTIGICGMGGSGKTTLAKAIYHQIHGRFMQKSFIE
DIAQVSEPRGRIHLQEQLLSDILNTKVEIHSVEMGRNMIRDRLFQKRVLIVLDDMDDYLPLLDLRKSRSWLSEGTVIIMTT
RDEDLLRKHQVDSVFRTNLMNEKESLELLSWHAFREPKPKEEYDYLARRVISCCGGLPLALEVIGSTLFERTEEEWHSILFE
LEETPMYDVQLKLKISLGGLRNQMERDLFLDVCCFFVGKNRAYATKILNGSGVDADSGIRVLMERNLIKVKRNNKLGM
HPLLQQIGITIFQLNSTEIRLLYGDVIEYALKVNTSQGRKDSELLPVRLLSSIRKPSRLPNDVENSENLSQKLRWISLHGCSSE
YIPFAFNVHDTITIDLKHSLLRFFWGEPQVLRSLKVLNLSHSMYLTETPDFSRLPSLEQLILKDCPRLRKVHRSIGHLCYLILL
NLKDCKCLSNLPREIYKLKSLRTLILSGCSKIGRMGKDIVRMKSLITLIAENTAVKQVPFSIVTSKAIGYLSLPGFERLSCNLFP
SIIIRSWMSPTMNPVSYTYSLSMDIDNSWDDIVPLLGSLANLRSVLVQCDTEFQLSKQVQNILVEYFSNITESRISKQHFR
SSLIGVGAYYEFFNAVSGNISEVLASRESCDVSLLGDNHPYWLAYMGEGDSVSFTVPPHNDVKGMILCVVYLSTLEIEAT

ECLRSVLIVNYTKCTFHIHMHGTGISFNDIDWEGIMSNFGYGDKVEIFVTFGHGLVVKNTFVYLIYGESNYLEKEPTPQKN
SVLRFIKKIVE

>XP_027907739.1

MALAVVGGALLSAFIDVLFDRLASPEFVNFIRGKKPDKLLQKMKSQLLVVKVVLADAEKRQISNSDVKDWLDLLRDLVYE
VDDLLDEVSTKAATQKEVSNSFSRLFIKKKIVSISKLEEMVEKLDDLLKQKECLDLKDIPVECYQPWKAHQTSLEDGYGMY
GRDKDKEAILKMVLEDSTDGEPVSVIPIVGMGGVGKTTLARSVFNDDKLKQQIFDLKAWVCVSDLFDIVKVTRTMIEEIT
RKACKLSDLNALQLELTEKLKGKRFLIVLDDVWIEDCDNWSFLTKPFLSGSKGSKVVVTTRNENVAAAVPFHRVEVYRLN
KLTNEDCWLVFANHAFPPSEACEDRETLEKIGKEIVKKCNGLPLAAQSLGGMLRRKNAVRDWNNVLESDIWELPESQC
KIIPALRISYHYLPPHLKRCFVYCSLYPKDYAFDKDELIQLWMAEDLVKAPKKGKTLEEVGHEYFYDLVSRSFFQCGSGSN
GDDCFVMHDLMHDLATFIGGEFYFRADEIGKETKINRKTRHLSFTRFSEPVSDIEGFDTVKFTRTFLVINYKDSPLNNEKA
PYSVVSMLKYLRVLSFCVFKSLFVLPDSIGELIHLRYLNLSHTSIETLPDSLCSLCNLQTLKLVYCPKLIQLPIAMQNLVNLRH
LEIHQSPIKEMPKRMGKLNQLQKLDLYIVGKRKENSIKELGGLPNLCGSFCIKALENVTKGEEAIEASIMDKKHINHLSLK
WSIGNDNSIDFQIELDVLGKLQPHRDLQTLLIIGYKGTIFPEWVGNFSYRYMTSVSLYNCNNCCMLPSMGQLPSLKRLCI
SDMNSVKTIDAGFYKTEDCSSAIPFPSLESLHIFRMPSWEVWTAFASEAFPVLKDIFIFDCPKLRGAFPNHLPALQRLIIRN
CELLVSSVPRAPSLRTIEICNTNKVTFHEFPLLVKFIDVEGGPTVESMMEAMTNSQPTCLKYLTLRKCSSAISFPGDRLPAS
LKALDVRDLNKLKFPMQHKHELLESLKVKNSCDSLMSLPLAIFPSLTHLDIRSCENMESVLVSGSESLKSLKSLMIQHCPSF
VSFLGEGLSAPNLRGFSVFDCEKLKSLPNQMGSLLPNLEYLNISNCQLIESFPEGGMPPNITVWIENCEKLLSSKAWVCM
DMVTSLGVCGPCDGINSFPEEALLPPSLTSLSLHNFTSLETLECKGFLHLTSLRELDIQNCEKLKNIGGESLPVSLMKLSING
CPLLHERCHKKDCEIWPKICHVRVLEIDGR

>XP_027907740.1

MALAVVGGALLSAFIDVLFDRLASPEFVNFIRGKKPDKLLQKMKSQLLVVKVVLADAEKRQISNSDVKDFDDLLDEVSTK
AATQKEVSNSFSRLFIKKKIVSISKLEEMVEKLDDLLKQKECLDLKDIPVECYQPWKAHQTSLEDGYGMYGRDKDKEAILK
MVLEDSTDGEPVSVIPIVGMGGVGKTTLARSVFNDDKLKQQIFDLKAWVCVSDLFDIVKVTRTMIEEITRKACKLSDLNA
LQLELTEKLKGKRFLIVLDDVWIEDCDNWSFLTKPFLSGSKGSKVVVTTRNENVAAAVPFHRVEVYRLNKLTNEDCWLV
FANHAFPPSEACEDRETLEKIGKEIVKKCNGLPLAAQSLGGMLRRKNAVRDWNNVLESDIWELPESQCKIIPALRISYHYL
PPHLKRCFVYCSLYPKDYAFDKDELIQLWMAEDLVKAPKKGKTLEEVGHEYFYDLVSRSFFQCGSGSNGDDCFVMHDL
MHDLATFIGGEFYFRADEIGKETKINRKTRHLSFTRFSEPVSDIEGFDTVKFTRTFLVINYKDSPLNNEKAPYSVVSMLKYL
RVLSFCVFKSLFVLPDSIGELIHLRYLNLSHTSIETLPDSLCSLCNLQTLKLVYCPKLIQLPIAMQNLVNLRHLEIHQSPIKEMP
KRMGKLNQLQKLDLYIVGKRKENSIKELGGLPNLCGSFCIKALENVTKGEEAIEASIMDKKHINHLSLKWSIGNDNSIDFQ
IELDVLGKLQPHRDLQTLLIIGYKGTIFPEWVGNFSYRYMTSVSLYNCNNCCMLPSMGQLPSLKRLCISDMNSVKTIDAG
FYKTEDCSSAIPFPSLESLHIFRMPSWEVWTAFASEAFPVLKDIFIFDCPKLRGAFPNHLPALQRLIIRNCELLVSSVPRAPS
LRTIEICNTNKVTFHEFPLLVKFIDVEGGPTVESMMEAMTNSQPTCLKYLTLRKCSSAISFPGDRLPASLKALDVRDLNKLK
FPMQHKHELLESLKVKNSCDSLMSLPLAIFPSLTHLDIRSCENMESVLVSGSESLKSLKSLMIQHCPSFVSFLGEGLSAPNL
RGFSVFDCEKLKSLPNQMGSLLPNLEYLNISNCQLIESFPEGGMPPNITVWIENCEKLLSSKAWVCMDMVTSLGVCGPC
DGINSFPEEALLPPSLTSLSLHNFTSLETLECKGFLHLTSLRELDIQNCEKLKNIGGESLPVSLMKLSINGCPLLHERCHKKDC
EIWPKICHVRVLEIDGR

>XP_027907741.1

MALAVVGGALLSAFIDVLFDRLASPELVNLIRGKKPDKLLQKVEKQLIVIRVVLADAENRQITDPNVKKWLDVLKDLVYE
VDDLLDEVSTKAAAQKELRNSFSRLFKRNKIVSISKIEDIVERLDDILKQKESLGLKDIPVECYQPWKAHQTSLEDGYGMY
GRDKDKEAILKMVLEDSTDGEPVSVIPIVGMGGVGKTTLARSVFNDDKLKQQIFDLKAWVCVSDLFDIVKVTRTMIEEIT
RKACKLSDLNALQLELTDKLKGKRFLIVLDDVWIEDCDNWSCLTKPFLSGIRGSKVLVTTRNENVAVAVSFHTVEVYRLN

KLSTEDCWLVFANHAFPLSVDSRSRGTLEKIGKEIVKKCNGLPLAAQSLGGMLRRKHTVRDWNNVLESDIWKLPEGQC
KIIPALSISYNYLPPHLKRCFVYCSLYPKDFNFKKDELIQLCMAEDLVIAPNKGKTLEEVGDEYFDDLVLRSFFQLSYSWARG
SYFVMHDLMHDLAAFLGGKFYFRADELGKKTKIDRKTRHLSFTRFSDPVSDIEVFDIVKFPRTFLLFKFKDSLFNNEKAPRI
VVSMLKYLRVLSFSYFQGLFALPDSIGELIHLRYLNLSFTSIKTLPESLCNLWNLQTLKLSFCRELTKLPKLTKLPSDMQNLV
NLCYLEILNTPIKEMPKRMGKLNKLQRLDFYIVGKHIENSIKELGGLPNLSGSFAIKALENVTKGEEAIEANIMDKKHIYDLS
LEWSIGNDNSINFETELDVLSKLKPHQDLKSLLIAGYNGAKFPDWVGNFSYGNMTSVSLYNCDNCCMLPSMGQLPSLK
SLWISRMNSVKTIEEGFHKNEDGSSVTLFPSLEWLQISYMPCLEVLNFFDSEAFPVLESLYINYCPNLRGDLPENLPALKSL
NIKICELLVSSVTRAPTLRRLEIHKSNNVVFHEFPLLVESIDVEGGPMVESMMEAITNIQPTCLQSLTLQNCSSAISFPGDC
LPAFLKTLEISGLKKLKFPTQHKHESLTSLSINNSCESLTSLQLAIFPSCENMESLLVSGSESLKSLNSLEIEQCPNFVSFPGEG
FCAPNLTRFLICDCEKLKSLPDQMRTLLPKMEYLNISNCQQIESFPEEGMPPNMRTVWIENCEKLLRSKTWVCMDMVT
SLYLCGPCDGLNSFPEEALLPPSLTSLVLRDFSSLETLDSKGFLHLTSLRELDIVNCKKLENITGERLPSALIKLSIRDCPLLQKR
CHKKDRKIWPKICHVRGIKMDDRWIQ

>XP_027907749.1

MDFTSSPSFSKSKHERIHDVFINFRGEDTRKKFVSHLHYALSNAGINTFLDNESLFKGMQPHHERMRAVEGSQIAIVVFS
QTYTESAWCLHELEQIIKCNETQGQSILPVFYKIDPSDVRYQSGHFGKLLEETARRRTYSGEHLEHTLSRWRCALNKAASF
YGWDVSSFRNEAELVRQIVDHVQKLLSYEVLSITEHPVGLESRTQEVIGLIETRSSQVCMIGIWGMGGSGKTTVAKAIYN
HIHRAFMNKSFIENIRQTCENHGRGYLPLQEQLLSNVLRKKMDIHSVGMGITLIENILAGKRALIVLDDVNEYNQLQAVC
GNRKWIGQGSVIIVTTRDVSLLYRLEVDYVYEMDKMDEDESLQLFSFHCFRDAKPKEDFSELSRNVVAYCGGLPLALEVL
GSYLFDKTSKREWEGVLSMLEKIPNDEIQRKLRISFDNLSNDMEKDIFLDVCCFFIGKDIGYVTDILNGCDLCADVGIPVLI
ERGLIKVRKNNKLEMDSLLQEMGREIIREDSRKEPGKQSRLWFQKDVVEVLTKNTGTEAIEGLALKMHLTSGDFFKADS
FQKMERLRLLQLHHVQLAGNYGYLSKQLRWISWQGFPSKFLPNNFYMDHVIAIDLKHSHLRFLWKQSPVLKWLKVLNL
SHSRFLIETPDFSRLPSLEQLILKDCPSLLAIHKSIGDLRNILLINLRDCTSLTNLPKEIYELKSVKVLILSGCSKIDKLGEDMAQ
MESLVTLIADDIYVKQVPFSIVSSKSIRYISLSGFEGLARNAFPVIIRSWMSPTMNPLLYTHPFYVTSCYLVSMITQNNTFG
ELAPMLTSLPNLRSVLVQCETESQLSKHVKTILVEDALSFRESGISRHHLRSSLIGVGSYKAFFDILSNRISEGLASNEACEVV
LPGDNYPYWSAHTGDGHSVYFTVPEDRGMKGMALCIVYFSNPEMKPTECFTSVLIANYTKRTLQIHKQDTVISFNDED
WKEIISHLGGGDKVEIFVTFGDDLVVKKTAVYLIYSESNDIEIEPTHGESNDIEIEPMDCEANDIKIESMNSESNLLEMYGE
SNDMDMTETGIQGDVFIHFSGDEIRRNFVSHLNSALLQAGVEPCLLAMRMKWEHFVASIKMFQIGIVVLTKEYSESLG
CLDELERIIECHKIHGLMVMPVFYEIDPSDAHIQNGGFGDALKATAQGIFTREYLETGLSTLSSPLTEAGKLHKWDETKHS
GRNDAELVEEIVKSVLAKLDRVLSITKFPVGLESHVKNVIRLFENQPTEFCMIGIWGIGGSGKTTLAKAIYNQIPYTFGGKS
FIQDIREVCETDGRGLVHLQEQLLSDVLKTKWKIERGEMEETMNEIRHSGKRLFIVLDDVNEIDQLKQLCGNGKWFSRG
SVVIITTRNLDLLYQFNVDYVYEMDELDESDSVELFSWHAFGEAKPREDFNELAKSAVTYCGGLPLALEILGSFLRKRSEN
EWKSVLSKLEIIPNTQVQNILRISFDGLCVMEKDIFLDVCCFFIGKDRDYVTEILNGCGLHADIGIIVLIKRGLIKIEKNNKLG
MHRLLLDMGREIVRQSSTMQPGKRSRLWLRKDVLDVLKKNTGTEAIVGLSLNCQLPNSDFFEAYAFEKMKMLRFLQLD
HVQMTGDYGYLSKQLRWIYWQGFPLESIPNNFYLKGAIVMDFQRSNLRLVWKEPEVLPLLKILNLSHSKYLTETPDFSKL
PNLEKLILKHCPSLRKVHQSIGDLHNLLLVNLKGCINLSNLPSETYKLKSLKTLILSGCLKIGIFTQDILHLESLITLISEDTGVKQ
VPISVVSSKSIGYILLDEKKGLLLADFRSIIWSWMSHTFNPLYHIRPFRGISLSLISMNTENNDLGDLAPILSSILNLRTVLVQ
CVTEYQISQVRTILEEIRGATWTTLKIRPSTPEISNHPLRSYLIEFGGYQEEVFNTLRKSIYEGLAARQTGNVSLPSDNYPH
WLTYMDEGHSVYFTMPKNFHTDGMILCVEHSSTFGDPTDCLDSVLIVNYTKCTIQLFKRDTITSFNDVDWQGMISHLA
YGDKVGIFVIFRDGFEVRKTSVYLVCDGSIDRMSM

>XP_027907750.1

MAFDSSSPSSKSERIYDVFINFRGVDTRKKFVSHLHSSLSKAGVKTFLDEENLLKGMELKELLRAIEVSQIAIVVFSKRYADS
SWCLEELQKIFECRQTCGLRVVPVFYYVEPSEVRQQKGDFGDALRAAARSGYAGEHLEFALSSWRRTLTDAANLSGWN

PKDWRTEAELVRDIVNYVIANLDYNALPITKFPVGLDHPVQEVIRFIERTRNSCKIGIWGMGGSGKTTIAKTIYNKLHRLF
ENKSFIENIREVCQTDRRRGLVRLQEKLLSDILKVKVEIQSNGIGQGMIENRFIGKRAVIVLDDVNKFDQLQALCGNTQW
MGERSVIIITTRDLRLLKSFEVDFVYEMKEMEANESLELFCRHAFREEKPREDFKELAKDAVAYCGGLPLALEVLGSYLSKR
TMIEWRSVLSKLKISPNTQVQEKLRISFDSLCDQMEKEIFLDVCCVFIGKDRGCVTEVLNGCGLFADIGITVLLERSLIKVEK
NNKLRMHHLLQDMGREIIREGSNKEPGKRSRLWFQEEVRDVLTNSTGTDAIEGLTLKLNLTNRECFKADAFEEMSSLRL
LQLHHVELTGDYGYLSKQLRWIYWQGFPSKYIPDNFYLEDAIAINFKHSNLRQLWKEPKVLFMLKFLNLSHSKYLTETPN
FSGLPYLEKLILKYCPSLRCVHKSIGDLCKIVLINLKDCTSLSSLPREIYKLKSLKTLILSGCSKIDTLEEDIVEMKSLTTLIAENAV
AKQVPFSIVSSKSIGYLFPCGYEGLSHDVLPSIIRSWMSLTMNPLSCIHPLCGISASLVSMNMQNIDLGDLAPILTNLLNLR
SVWVQCDTEFQITKQVRKILNDVQGVHFTDLEIASCTSEISDNSLRSHWIRIGSYQEEDFNTLNKSITKGLAASGSCNAFL
LGGNYPLLLAHTGEGHSVDFTVPEDWDMKGMALCFVYLSTPETVATKCLISVVLVNYTKCSIQIYKRDTVISFNDADWQ
GIISHLEAGDKVEIFLSFRNELVIKNTAVYLLKKPVMKVYSRKRNLKDQESSRKRQG

>XP_027907751.1

MAFDSSSPSSKSERIYDVFINFRGVDTRKKFVSHLHSSLSKAGVKTFLDEENLLKGMELKELLRAIEVSQIAIVVFSKRYADS
SWCLEELQKIFECRQTCGLRVVPVFYYVEPSEVRQQKGDFGDALRAAARSGYAGEHLEFALSSWRRTLTDAANLSGWN
PKDWRTEAELVRDIVNYVIANLDYNALPITKFPVGLDHPVQEVIRFIERTRNSCKIGIWGMGGSGKTTIAKTIYNKLHRLF
ENKSFIENIREVCQTDRRRGLVRLQEKLLSDILKVKVEIQSNGIGQGMIENRFIGKRAVIVLDDVNKFDQLQALCGNTQW
MGERSVIIITTRDLRLLKSFEVDFVYEMKEMEANESLELFCRHAFREEKPREDFKELAKDAVAYCGGLPLALEVLGSYLSKR
TMIEWRSVLSKLKISPNTQVQEKLRISFDSLCDQMEKEIFLDVCCVFIGKDRGCVTEVLNGCGLFADIGITVLLERSLIKVEK
NNKLRMHHLLQDMGREIIREGSNKEPGKRSRLWFQEEVRDVLTNSTGTDAIEGLTLKLNLTNRECFKADAFEEMSSLRL
LQLHHVELTGDYGYLSKQLRWIYWQGFPSKYIPDNFYLEDAIAINFKHSNLRQLWKEPKCDTEFQITKQVRKILNDVQG
VHFTDLEIASCTSEISDNSLRSHWIRIGSYQEEDFNTLNKSITKGLAASGSCNAFLLGGNYPLLLAHTGEGHSVDFTVPED
WDMKGMALCFVYLSTPETVATKCLISVVLVNYTKCSIQIYKRDTVISFNDADWQGIISHLEAGDKVEIFLSFRNELVIKNT
AVYLLKKPVMKVYSRKRNLKDQESSRKRQG

>XP_027907752.1

MAFDSSSPSSKSERIYDVFINFRGVDTRKKFVSHLHSSLSKAGVKTFLDEENLLKGMELKELLRAIEVSQIAIVVFSKRYADS
SWCLEELQKIFECRQTCGLRVVPVFYYVEPSEVRQQKGDFGDALRAAARSGYAGEHLEFALSSWRRTLTDAANLSGWN
PKDWRTEAELVRDIVNYVIANLDYNALPITKFPVGLDHPVQEVIRFIERTRNSCKIGIWGMGGSGKTTIAKTIYNKLHRLF
ENKSFIENIREVCQTDRRRGLVRLQEKLLSDILKVKVEIQSNGIGQGMIENRFIGKRAVIVLDDVNKFDQLQALCGNTQW
MGERSVIIITTRDLRLLKSFEVDFVYEMKEMEANESLELFCRHAFREEKPREDFKELAKDAVAYCGGLPLALEVLGSYLSKR
TMIEWRSVLSKLKISPNTQVQEKLRISFDSLCDQMEKEIFLDVCCVFIGKDRGCVTEVLNGCGLFADIGITVLLERSLIKVEK
NNKLRMHHLLQDMGREIIREGSNKEPGKRSRLWFQEEVRDVLTNSTGTDAIEGLTLKLNLTNRECFKADAFEEMSSLRL
LQLHHVELTGDYGYLSKQLRWIYWQGFPSKYIPDNFYLEDAIAINFKHSNLRQLWKEPKGLAASGSCNAFLLGGNYPLLL
AHTGEGHSVDFTVPEDWDMKGMALCFVYLSTPETVATKCLISVVLVNYTKCSIQIYKRDTVISFNDADWQGIISHLEAG
DKVEIFLSFRNELVIKNTAVYLLKKPVMKVYSRKRNLKDQESSRKRQG

>XP_027907764.1

MAAIIGCYQFLTSAPRAKEWVKKKLIQLNLYEARVGEVENVVEKLKKKRDTIQHTVEEEERRHGRIIHVDVKEWIESVDKL
ILAYKDFHEDEICHKCAVFDFLDNGYLPKPGIRYRRSRKANDITKQANGLLQNAKFDILSYWSGPPSMAAFFSNLGYESYS
SRNDIVKKITDEFQKPGVRMIGLHGLSGVGKTSLVKEVVKKALKDKMFEVVTMASVTKNPDIRKIQGQIVDMLGVVLEE
ESDIARAARIHQILNDENKSTLIILDDLWEEVNFNLLGIPCELEKDDGITNVKGKSVDVDILKNVSDGKSPVLDGSTSFRKG
TLQGVDGSKNVNKGKSPILEGSISFRKGTLHGADDSKNINKGKSLSDADSVNVKKGEFFGGGLRNVNEGKSAIDGSDRV
KIEKVVPQYKGCKVLMISEIKQVLLSQMEGKEECIFPVEVLKEKEAETLFKKKAGIGGKNSEYEKLAAQIASKCKGLPMTIV

TTARALKNKSLSVWEETNRKLESKNLAGAPEFSTKLSYELLEDEELKHTFLLCARMDHDALIMDLVKYSIGFGFLQGINTA
RQTRDKVYTLVAKLKESGLLSDSYSSDHFTMPDVVRAAALSIAYKENHLFTMTKGKVDEWPDKLERYAAISLHHCDFIEE
FPGRVNYPRLRVLQIVNNIPRPKIPKNFFKGVKELRVLVLIGIHLPLFDSSISSLHKLRMLCLEQCCMLDEELSIIGELKRLRIL
SFSGSDIKSLPNELNELKMLQIFDISNCSKLNKIPYGVISSLVSLEELYMRNTLIQWEDEEQTRQSQIALLSDLKHLNQLTTL
DIHIPNVSYLPNKLYFDKLFSYKIVIGDLSSILEIDFKMPEKYETLKFLAVQLKNGYDIHSLKGIKMLFEGVENLFLELNTVPEK
QNSVHEARNIVHDLFYRLNLKGFPYLKHLWIVNNSTIQSLIHPKDRQHPEKAFPKLESLCFYNLKMDEICSCKLSEPSFGKL
KVIRIYLCGELKNVFPISVVGLLKVLETIEVSECNSLKEIINVGRQSNPENPELLTLPDLRYLKLQSLYEFIGFDVIPQVEGEER
KLFYGKVGVSKLERLELSSIHIDIIWSVDQSSKRSSFENLTHLDVNGCWNLKYLMSSTMAKGLVNLQSLYVSECEKMSDIF
LLEQDREKDIMGNIFPKLKNMKLRSMKSLSKIWNLKHPSDSFKKLDTLIIEECDKLENAMEGIFGSLCNLRVTNCRYMQA
IFNISEQVGDVASNLQDVHLETLPKLELVWRMNNKDLVGIPKFKNLKRILVQDCDSLEYIFPFYVAKNLDNLESLVVCDCY
ELTKIVAESEVTNTDKARFNFPKLSTIKFSNMPTLTSFYPTVYDLSCPLNELSIELCNYLEPFNKRTEHAQRNHVHVFFPEEV
INNLKSMQIESWHTKSPSSYMGKRNHRRDNLEELSLFRSMDTEILYSFLHRNPNLKSLSLNNCFFQKITSPKEDTEIENLG
VVPSLKSLKLIDLPNLEEIGFEDIILDRLEFLILRNCSCMITVAPSSVSFTRLTHLEVVNCERLQSLMSASTAQSLVQLSTMKV
VKCESLMEIVRKDGEKSDRVVFQQLKVLELVSLKKIKSFSVSDCDFEFPSLEKLVVSACYNMAKFSETVPSSPILQNIHVVH
GKENKRFCWEGDINATIQKIFKEMKFFEGTEEMNLSEHQKLQETWQCRVGMQKQNSWFYSLKILKLENCEIQPCAIPS
NILPYLRTLKELQVRGCNNVEVIFEMNGEEGIGSTFHLQKLILEKLPKLKDVWERNGKGTESFQNLKLVDVSECDNLEIVF
PLSLAKNLKKLDELKIISCESLHEIVRKQEETIAMFVFPCLTTLTLGYLPDLIYFYPEPFTLECSTLNKLSVWNCPELELFGSGN
RQSFFFDLKDIYNLEVLVLDWEHTLVLRTKLGEPMDNLKYLNHIQLYFDDEKEIPDLPIQILQKMPNLTKMTISECSCLEVF
QTQIPEIVEKRVLTHLKILKLDNVSKLQSIGSEDSPWLNVICDSEKLQQLYVINCPDLKTLVHSTPSVTFTYVKEMYIDNCN
ELTYLFTLSSVNKLENLEHIEVTDCESMEAIVLKEEDDISEEIKLQKLKRVELNDLSSLECFYSGNDTLRLPSLMQVDIWICPK
MEFFSRGDIYLNSSFKGIQASNVSSDDLVFHHDLNSSVQKVFLQQEFFQAVDKECFSNNLELQEELSWKIGLQNKWLA
NLETLKLQNCTLSYAIPSSILSLLKNLKELQVQDSDQVETIFDMNDDTEIMESESQLKILTLNGLSKLTRVWEKDSHRILIFR
NLQEVVVSDCAKLQTLFPASLAKSLKDLKKLKIDSCENLQDFVEQEETTFATEKFVFPCLENLELNDLTHVTCPKNFILEFPS
VKFLSVRDCDELGLFQSVYDPMGKGTSNNRLPLISDPKVISNLEKLTLDWKQILSLSLWFKSQKSSKGLTNLNSIDLFFGV
DGNKMPMLPIEILKAPNLIELNINNCDSIQNFLAQNPKIGEEEMLRQLTILKLCNVSTTQFFELEHCSSLNIICERLHKLTVS
QCPHLTTLGVHSMVSFSCLKEVNIYKCSNLKYIFTTSASKKLMNLEEIRVIECESLTEILAKEGEATFEAIKFERLHTIHLQSLT
SLVCFYSGSDTLQLSSLKIVAIWNCPNMEIFSQGIESLMGITLSMDQQADDLPPPQDLNTRIKGISQRKEFFKTVDKQCFP
DYVELQENLHCEFGLHNQWFGDLVSLKLQNCTMPYAIPSDIIALLKSLRELEVRDSTTIEVLFFMNDSESMEIASRLRILTI
EGLPKLTRVWEKNKNGVLIFPNLQQIFVSNCEKLETLFHASMAKNLKRLKRIEIEYCAELREIVQKEEDIEEKFLLPCLEKLYL
WTLPQLTCFYPQTFALECPALNELYVVECEELELFQSENSMGEGNSVNKPPLFSSLEVISNLKELELDWKQSLPLRSRFKS
EKFTGVFKSINKIGLCLGTDQCEMPIVLDEIVLKAPNLIEMCIQIPNCNNSEVFLAQNPKIGEDGVLIQLRILEIIQVSAIMSI
LSENSSWLNPICEKVHELNVLKCPDVEAVGVHSTMSFSFLKKLVVSHCPQLQYLFTTSVAKKLVNLEEIAVLKCESLKEIVA
KEGDEEERKVKGEDKYENEIIFMKLEKLMLGLLGKLESFYTGSCTLNFPSLRNVVVIQCLNTKILRHRDKVPPKFRVVIDKIR
CKGDKKALITQQFEEEAS

>XP_027907765.1

MAALITCYHFLTSSPRAKAWLKKQLIQLDLYEARVDQVKDVVEKLKKKRDSIQHTVDEEERRHGRKIHVEVKEWMESVD
KLIRAYKDFDDDEICHKRAVFEFFDSGYLPRPGIRYRRSIKAKDITKQANGLLQNAKFDILSYWSGPPSMAAFFSNLGYES
YTSRNDTVKKITDEFQKPGVRMIGLHGLSGVGKTSLVKEVVKKALKDKMFEVVTMASVTKNPDVRKIQGQIADMLGFV
LEEESDIARAARIHKILNNENKSTLIILDDLWEEVNFNLLGIPCELEKEDGVTNVQGKSLDVDSLKNVSDGKSPDVDILMN
VDGSKNINKGKSPVDASDRVKAENFVPQYKGCKILMISEIKQVLLTQMEGKEESIFPVDVLKEEEAEMLFKKKAGISDKN
SEYDKLAAQIASKCKGLPMTIVTTARALKNKSLSVWDQTNRKLESQNLTGVPEFSTKLSYELLEDEELKYTFLLCARMGH
DALIMDLVKYCIGFGFLQGINTARQTRDKLFMLVAKLKEAGLLSDSYSSDHFTMPDTVRRAALSIAYKENQLFTMTNGR
LDEWPDKLQRYAAISLHHCDFIEDFPGKLNYPRLRVLEIVNNIPRPKIPKNFFKGVKELRVLILTGIDLPLIDSSVSSLYKLRM

LCLEQCSMLDEELSIIGVLKRLRVLSLSGSDIKSLPNELNELKMLQIFDISNCPKLKKIPHGVISSMVSLEELYMRNTLIQWE
DEEQTRQSKIALLSDLNHLNQLTTLDIQIPNVSYLPKNLYFDKLDSYKIVIGDLSSFLETGFQMPEKYETLKFLAVQLENGSD
IHSLMGIKMLFEGVENLFLELNTVHEKHNSVHEAHNIVHDLFYRLNLKGFPYLKHLWIVNNSTIQSLIHPKDRQYPEKAFP
KLESLSLYNLKMDEICSCKLSEPSFGKLKVIKINLCGELKNVFSISMVGLLKVLETIEVSECSSLKEVIYVGPPINPEKTVIPML
PELRYLKLHSLSKFIGFDATPYIEGEERKLFHEKVGVSKLQRLELSSIQIDVIWSVRSQSSEILSFENLTHLDVNGCWKLKSL
MSFTMANCLVNLQSLYVSDCKKMSCIFLPKQDTEKDIMGSIFPKLKNMKLSNMISLSKIWYPKLPSDSFYTLETLIIEECHK
LENAVERIFGSLCNLRVTNCRSMEAIFNICEQVGDVANNLQDVHLETLPKLEIVWRINNKDLVGIPKFNNLKRILVQDCE
SLEYIFPFYVAKNLDNLESLVVCDCCGLNEIVAKREVTNTDREKFNFPKLSTIKFSDLPKLTSFYPTAYDLSCPLNELSIKFCN
NLEPFNNGTEHAQRNPVHAFFPEKVINNLKSMQIEFWHAKSPRSYMGKGNHRRDNLEELSLSRLMNTEILYSFLHRNP
NLKSLSLNNCYFENILPLKEDTEIENLGVVPNLKSLMLIDLLNLKEISFEPAIILERLEFLILKNCCNMITIAPSSVSFTRLKNLE
VVKCDRLQSLMSASTAKSLVQLNTMKVVKCESLMEIVRKDGQKSDRVVFQQLKALELVSLKNLKSFCVSDCDFEFPSLEK
LVVSACYNMDKFSETVTSSPILQNVHVVHGKENKRFCWEGDINATIQKMFEEMKFFEGMEEISLSEHQELHETWQRG
AGLQKKNSWFYSLKILKLENCVIQPCAIPSNILPYLMSLKELQVRGCNNVEVIFEMNAEESTASAIHLQKLTLEKLPHLKDV
WERNDKGTKSFQNLKLVNVSECRDLQTVFPFTLAKSLKKLDELKIMHCHGLHEIVRKEEDTTAVFVFPCLTTLKLADLPEL
LYFYPESFTLECSTLKELVVWKCPKMELFGSANRQSIFFDLKDISNLEVLILGWEHTLALRTILGEPMDNLKYLNDIQLVFII
DENERPDLPIQILQKMPNLTKLSIHHCSWLEVFQTQIPEIVEKRVLTHLKSLRLNGVSKLQSIGSEDSPWLNLLCDSEKLQ
HLEVVNCPDLKSLVHSTPSPSVSFKHVKEMYITNCQDMKYLFTLPAVNNLESLEYIEVNNCESMEAIVLKVEDDISKEIQL
QRLKHIDLNHLSSLKCFYSGDATLQLPSLIQVDIWMCPKMEFFSRGRIQLNSSFRGIEALNVSRDELVFYLDLNSSVKKVFL
QQEFFQALDPVCKLGLENKWWANMETLKLQNCTLSYAIPSYILALLKNLKELEVRESNQVKAIFDINDDTEINETESQLKI
LTLIGLSELTHVWEKDTHRILIFRNLQQVVVSNCAKLQILFPTYLAKSLKDLNKLKINNCKNLQDLVEQEETTCVTEKFVFP
CLEDLELRNLPRVTCPKMFTLEFPSVKFLDVRSCDGLGLFKSVYDPMGEGTSSSRLPLISDPKVISNLEKLTLEWKQILPLSL
WLKSQQSTEGLPNLNSIYVSFFGAKENEIPMLPIEILKAPNLIEMNVMHCESLENFLVQNAKIGEEEMLGKLTMLRLYDV
STTQLFELEYSSSLNIFERLHRLFVSHCPHLTTLGVHSTSIVSFYCLKELFIYKCPNLKYLFTSSAAKMLMNLEEISVIECESLTK
IVVKEGDATSEAIKFERLHTIYLKSLTSLVCFYSGSETLQLSSLKIVTIWSCPNMEIFSQGIESLMGITLSIDLEPNDLPPPQDL
NTRIKGISQRKEFIESVDKECFSDYLKLQEDPHCNFRLQNRWLSDLVSLKLQNCTLSCAIPSPILALLKSLEELEVRDSTTVE
VIFYMNDSDSMEIASRLRILTLEGLSKLTRVWENKKNGVLIFPNLQQIVVSNCEKLETLFPASMAKNLKSLKGIKINFCDEL
REIVEKEEDREEKFVLPCLEKLDLSSLSKLTCFYPETFALECPALNELSVFDCNELELFQCAHSTGEGTSVNRRPLISSLDVIS
NLRELNLDWKHILALRSRFRSEKFKGVFKFVNKMLLDLDGDVGEMPIVLNEILHKAPNLIAMIMVMQKCNNPEIFLAQN
PKIGEDGMLLQLRKLILVEVSAIRSIQSENSSWLNTICEKVHELHVFECPDIETIGVHSTSTMSFSFLKKVFASDCPQLQYLF
TSSVAKKLVNLKEIMVTECKSLKEIVSKEGDEHEQKGEGEDEDENEMIFLKLEILTLISLGKFEMFYTGSSTLNFPSLRRVRV
DKCFSTKIFRRRDKVPPKFNVMIDEIRCKGDTKALIMQQFEEEAS

>XP_027907766.1

MAALITCYHFLTSSPRAKAWLKKQLIQLDLYEARVDQVKDVVEKLKKKRDSIQHTVDEEERRHGRKIHVEVKEWMESVD
KLIRAYKDFDDDEICHKRAVFEFFDSGYLPRPGIRYRRSIKAKDITKQANGLLQNAKFDILSYWSGPPSMAAFFSNLGYES
YTSRNDTVKKITDEFQKPGVRMIGLHGLSGVGKTSLVKEVVKKALKDKMFEVVTMASVTKNPDVRKIQGQIADMLGFV
LEEESDIARAARIHKILNNENKSTLIILDDLWEEVNFNLLGIPCELEKEDGVTNVQGKSLDVDSLKNVSDGKSPDVDILMN
VDGSKNINKGKSPVDASDRVKAENFVPQYKGCKILMISEIKQVLLTQMEGKEESIFPVDVLKEEEAEMLFKKKAGISDKN
SEYDKLAAQIASKCKGLPMTIVTTARALKNKSLSVWDQTNRKLESQNLTGVPEFSTKLSYELLEDEELKYTFLLCARMGH
DALIMDLVKYCIGFGFLQGINTARQTRDKLFMLVAKLKEAGLLSDSYSSDHFTMPDTVRRAALSIAYKENQLFTMTNGR
LDEWPDKLQRYAAISLHHCDFIEDFPGKLNYPRLRVLEIVNNIPRPKIPKNFFKGVKELRVLILTGIDLPLIDSSVSSLYKLRM
LCLEQCSMLDEELSIIGVLKRLRVLSLSGSDIKSLPNELNELKMLQIFDISNCPKLKKIPHGVISSMVSLEELYMRNTLIQWE
DEEQTRQSKIALLSDLNHLNQLTTLDIQIPNVSYLPKNLYFDKLDSYKIVIGDLSSFLETGFQMPEKYETLKFLAVQLENGSD
IHSLMGIKMLFEGVENLFLELNTVHEKHNSVHEAHNIVHDLFYRLNLKGFPYLKHLWIVNNSTIQSLIHPKDRQYPEKAFP

KLESLSLYNLKMDEICSCKLSEPSFGKLKVIKINLCGELKNVFSISMVGLLKVLETIEVSECSSLKEVIYVGPPINPEKTVIPML
PELRYLKLHSLSKFIGFDATPYIEGEERKLFHEKVGVSKLQRLELSSIQIDVIWSVRSQSSEILSFENLTHLDVNGCWKLKSL
MSFTMANCLVNLQSLYVSDCKKMSCIFLPKQDTEKDIMGSIFPKLKNMKLSNMISLSKIWYPKLPSDSFYTLETLIIEECHK
LENAVERIFGSLCNLRVTNCRSMEAIFNICEQVGDVANNLQDVHLETLPKLEIVWRINNKDLVGIPKFNNLKRILVQDCE
SLEYIFPFYVAKNLDNLESLVVCDCCGLNEIVAKREVTNTDREKFNFPKLSTIKFSDLPKLTSFYPTAYDLSCPLNELSIKFCN
NLEPFNNGTEHAQRNPVHAFFPEKVINNLKSMQIEFWHAKSPRSYMGKGNHRRDNLEELSLSRLMNTEILYSFLHRNP
NLKSLSLNNCYFENILPLKEDTEIENLGVVPNLKSLMLIDLLNLKEISFEPAIILERLEFLILKNCCNMITIAPSSVSFTRLKNLE
VVKCDRLQSLMSASTAKSLVQLNTMKVVKCESLMEIVRKDGQKSDRVVFQQLKALELVSLKNLKSFCVSDCDFEFPSLEK
LVVSACYNMDKFSETVTSSPILQNVHVVHGKENKRFCWEGDINATIQKMFEEMKFFEGMEEISLSEHQELHETWQRG
AGLQKKNSWFYSLKILKLENCVIQPCAIPSNILPYLMSLKELQVRGCNNVEVIFEMNAEESTASAIHLQKLTLEKLPHLKDV
WERNDKGTKSFQNLKLVNVSECRDLQTVFPFTLAKSLKKLDELKIMHCHGLHEIVRKEEDTTAVFVFPCLTTLKLADLPEL
LYFYPESFTLECSTLKELVVWKCPKMELFGSANRQSIFFDLKDISNLEVLILGWEHTLALRTILGEPMDNLKYLNDIQLVFII
DENERPDLPIQILQKMPNLTKLSIHHCSWLEVFQTQIPEIVEKRVLTHLKSLRLNGVSKLQSIGSEDSPWLNLLCDSEKLQ
HLEVVNCPDLKSLVHSTPSPSVSFKHVKEMYITNCQDMKYLFTLPAVNNLESLEYIEVNNCESMEAIVLKVEDDISKEIQL
QRLKHIDLNHLSSLKCFYSGDATLQLPSLIQVDIWMCPKMEFFSRGRIQLNSSFRGIEALNVSRDELVFYLDLNSSVKKVFL
QQEFFQALDPVCKLGLENKWWANMETLKLQNCTLSYAIPSYILALLKNLKELEVRESNQVKAIFDINDDTEINETESQLKI
LTLIGLSELTHVWEKDTHRILIFRNLQQVVVSNCAKLQILFPTYLAKSLKDLNKLKINNCKNLQDLVEQEETTCVTEKFVFP
CLEDLELRNLPRVTCPKMFTLEFPSVKFLDVRSCDGLGLFKSVYDPMGEGTSSSRLPLISDPKVISNLEKLTLEWKQILPLSL
WLKSQQSTEGLPNLNSIYVSFFGAKENEIPMLPIEILKAPNLIEMNVMHCESLENFLVQNAKIGEEEMLGKLTMLRLYDV
STTQLFELEYSSSLNIFERLHRLFVSHCPHLTTLGVHSTSIVSFYCLKELFIYKCPNLKYLFTSSAAKMLMNLEEISVIECESLTK
IVVKEGDATSEAIKFERLHTIYLKSLTSLVCFYSGSETLQLSSLKIVTIWSCPNMEIFSQGIESLMGITLSIDLEPNDLPPPQDL
NTRIKGISQRKEFIESVDKECFSDYLKLQEDPHCNFRLQNRWLSDLVSLKLQNCTLSCAIPSPILALLKSLEELEVRDSTTVE
VIFYMNDSDSMEIASRLRILTLEGLSKLTRVWENKKNGVLIFPNLQQIVVSNCEKLETLFPASMAKNLKSLKGIKINFCDEL
REIVEKEEDREEKFVLPCLEKLDLSSLSKLTCFYPETFALECPALNELSVFDCNELELFQCAHSTGEGTSVNRRPLISSLDVIS
NLRELNLDWKHILALRSRFRSEKFKGVFKFVNKMLLDLDGDVGEMPIVLNEILHKAPNLIAMIMVMQKCNNPEIFLAQN
PKIGEDGMLLQLRKLILVEVSAIRSIQSENSSWLNTICEKVHELHVFECPDIETIGVHSTSTMSFSFLKKVFASDCPQLQYLF
TSSVAKKLVNLKEIMVTECKSLKEIVSKEGDEHEQKGEGEDEDENEMIFLKLEILTLISLGKFEMFYTGSSTLNFPSLRRVRV
DKCFSTKIFRRRDKVPPKFNVMIDEIRCKGDTKALIMQQFEEEAS

>XP_027907767.1

MAALITCYHFLTSSPRAKAWLKKQLIQLDLYEARVDQVKDVVEKLKKKRDSIQHTVDEEERRHGRKIHVEVKEWMESVD
KLIRAYKDFDDDEICHKRAVFEFFDSGYLPRPGIRYRRSIKAKDITKQANGLLQNAKFDILSYWSGPPSMAAFFSNLGYES
YTSRNDTVKKITDEFQKPGVRMIGLHGLSGVGKTSLVKEVVKKALKDKMFEVVTMASVTKNPDVRKIQGQIADMLGFV
LEEESDIARAARIHKILNNENKSTLIILDDLWEEVNFNLLGIPCELEKEDGVTNVQGKSLDVDSLKNVSDGKSPDVDILMN
VDGSKNINKGKSPVDASDRVKAENFVPQYKGCKILMISEIKQVLLTQMEGKEESIFPVDVLKEEEAEMLFKKKAGISDKN
SEYDKLAAQIASKCKGLPMTIVTTARALKNKSLSVWDQTNRKLESQNLTGVPEFSTKLSYELLEDEELKYTFLLCARMGH
DALIMDLVKYCIGFGFLQGINTARQTRDKLFMLVAKLKEAGLLSDSYSSDHFTMPDTVRRAALSIAYKENQLFTMTNGR
LDEWPDKLQRYAAISLHHCDFIEDFPGKLNYPRLRVLEIVNNIPRPKIPKNFFKGVKELRVLILTGIDLPLIDSSVSSLYKLRM
LCLEQCSMLDEELSIIGVLKRLRVLSLSGSDIKSLPNELNELKMLQIFDISNCPKLKKIPHGVISSMVSLEELYMRNTLIQWE
DEEQTRQSKIALLSDLNHLNQLTTLDIQIPNVSYLPKNLYFDKLDSYKIVIGDLSSFLETGFQMPEKYETLKFLAVQLENGSD
IHSLMGIKMLFEGVENLFLELNTVHEKHNSVHEAHNIVHDLFYRLNLKGFPYLKHLWIVNNSTIQSLIHPKDRQYPEKAFP
KLESLSLYNLKMDEICSCKLSEPSFGKLKVIKINLCGELKNVFSISMVGLLKVLETIEVSECSSLKEVIYVGPPINPEKTVIPML
PELRYLKLHSLSKFIGFDATPYIEGEERKLFHEKVGVSKLQRLELSSIQIDVIWSVRSQSSEILSFENLTHLDVNGCWKLKSL
MSFTMANCLVNLQSLYVSDCKKMSCIFLPKQDTEKDIMGSIFPKLKNMKLSNMISLSKIWYPKLPSDSFYTLETLIIEECHK

LENAVERIFGSLCNLRVTNCRSMEAIFNICEQVGDVANNLQDVHLETLPKLEIVWRINNKDLVGIPKFNNLKRILVQDCE
SLEYIFPFYVAKNLDNLESLVVCDCCGLNEIVAKREVTNTDREKFNFPKLSTIKFSDLPKLTSFYPTAYDLSCPLNELSIKFCN
NLEPFNNGTEHAQRNPVHAFFPEKVINNLKSMQIEFWHAKSPRSYMGKGNHRRDNLEELSLSRLMNTEILYSFLHRNP
NLKSLSLNNCYFENILPLKEDTEIENLGVVPNLKSLMLIDLLNLKEISFEPAIILERLEFLILKNCCNMITIAPSSVSFTRLKNLE
VVKCDRLQSLMSASTAKSLVQLNTMKVVKCESLMEIVRKDGQKSDRVVFQQLKALELVSLKNLKSFCVSDCDFEFPSLEK
LVVSACYNMDKFSETVTSSPILQNVHVVHGKENKRFCWEGDINATIQKMFEEMKFFEGMEEISLSEHQELHETWQRG
AGLQKKNSWFYSLKILKLENCVIQPCAIPSNILPYLMSLKELQVRGCNNVEVIFEMNAEESTASAIHLQKLTLEKLPHLKDV
WERNDKGTKSFQNLKLVNVSECRDLQTVFPFTLAKSLKKLDELKIMHCHGLHEIVRKEEDTTAVFVFPCLTTLKLADLPEL
LYFYPESFTLECSTLKELVVWKCPKMELFGSANRQSIFFDLKDISNLEVLILGWEHTLALRTILGEPMDNLKYLNDIQLVFII
DENERPDLPIQILQKMPNLTKLSIHHCSWLEVFQTQIPEIVEKRVLTHLKSLRLNGVSKLQSIGSEDSPWLNLLCDSEKLQ
HLEVVNCPDLKSLVHSTPSPSVSFKHVKEMYITNCQDMKYLFTLPAVNNLESLEYIEVNNCESMEAIVLKVEDDISKEIQL
QRLKHIDLNHLSSLKCFYSGDATLQLPSLIQVDIWMCPKMEFFSRGRIQLNSSFRGIEALNVSRDELVFYLDLNSSVKKVFL
QQEFFQALDPVCKLGLENKWWANMETLKLQNCTLSYAIPSYILALLKNLKELEVRESNQVKAIFDINDDTEINETESQLKI
LTLIGLSELTHVWEKDTHRILIFRNLQQVVVSNCAKLQILFPTYLAKSLKDLNKLKINNCKNLQDLVEQEETTCVTEKFVFP
CLEDLELRNLPRVTCPKMFTLEFPSVKFLDVRSCDGLGLFKSVYDPMGEGTSSSRLPLISDPKVISNLEKLTLEWKQILPLSL
WLKSQQSTEGLPNLNSIYVSFFGAKENEIPMLPIEILKAPNLIEMNVMHCESLENFLVQNAKIGEEEMLGKLTMLRLYDV
STTQLFELEYSSSLNIFERLHRLFVSHCPHLTTLGVHSTSIVSFYCLKELFIYKCPNLKYLFTSSAAKMLMNLEEISVIECESLTK
IVVKEGDATSEAIKFERLHTIYLKSLTSLVCFYSGSETLQLSSLKIVTIWSCPNMEIFSQGIESLMGITLSIDLEPNDLPPPQDL
NTRIKGISQRKEFIESVDKECFSDYLKLQEDPHCNFRLQNRWLSDLVSLKLQNCTLSCAIPSPILALLKSLEELEVRDSTTVE
VIFYMNDSDSMEIASRLRILTLEGLSKLTRVWENKKNGVLIFPNLQQIVVSNCEKLETLFPASMAKNLKSLKGIKINFCDEL
REIVEKEEDREEKFVLPCLEKLDLSSLSKLTCFYPETFALECPALNELSVFDCNELELFQCAHSTGEGTSVNRRPLISSLDVIS
NLRELNLDWKHILALRSRFRSEKFKGVFKFVNKMLLDLDGDVGEMPIVLNEILHKAPNLIAMIMVMQKCNNPEIFLAQN
PKIGEDGMLLQLRKLILVEVSAIRSIQSENSSWLNTICEKVHELHVFECPDIETIGVHSTSTMSFSFLKKVFASDCPQLQYLF
TSSVAKKLVNLKEIMVTECKSLKEIVSKEGDEHEQKGEGEDEDENEMIFLKLEILTLISLGKFEMFYTGSSTLNFPSLRRVRV
DKCFSTKIFRRRDKVPPKFNVMIDEIRCKGDTKALIMQQFEEEAS

>XP_027907779.1

MFPMYDSDCGNDEMEMDFLRDRYEEMDNRNFEVFLSFRGEDTRASFTSHLYAALQNAGIIVFKDDESLPRGKQISPSL
RLAIENSPISIVVFSKNYAESRWCLKELEKIMECHRTIGHVVLPVFYDVDPSEVRHQRGDFGKAFQRLLGKIWNEDEEKEL
YWKQLWRKTLGEIGGFSAVETLSSRFERDISMVYWKFALAQTPSSYVESNPRDETEIADSINFLVKQWMERICMGAEIP
FREMEIDDKIDFLVKQWAVVFPKPICRFYPRFEMETADAIEFQVKHCREALFKAAGISRGAILNSRREMEITEAIELQLKY
WRDAFSKEAGGVLDSKGYGKRVVDSEIRNLQNHWREKLLEAYSILEDKRVYSSREIQIEADLERWMKVLVEAANVATD
AVYVSCIIMLKAYNRIEKLVNYWRMALCHAIGISSFTVQQHRVMKDYEINDIKKHRDALREAAGISGVVILNSRNENEAV
KTIVKNVTSLLDKTELFVANNPVGVESRVQEMVQLLEQKQSNDVLLLGIWGMGGIGKTTIAKAIYNKIGRNFQGRSFLA
DIREVWGQEAGHICLQQQLLFDIHKENNTKIHNTESGKMILRERLRHKRILLILDDVNKLQQLNALCGNREWFGSGSRIII
TTRDIHLLRGKRVDQVFAMTGMDVDESIELFSWHAFKQASPEEDFIELSRNVIAYAGGLPLALEVLGSYLFDMEVTEWKI
VLEKLRKIPNDEVQEKLKISYDGLSDDTEKGIFLDIACFFIGKDRNDVIHILNGCGLFAENGIRVLVERSLVIVNDKNQLGM
HDLVRDMGREIIRSKSPMEVEERSRLWFDEDVLDVLSKETGTKYIEGLRLKLPRSNTKSLSTKAFMNMKKLRLLQLSDVE
LVGDFEYISKDLRWLCWHGFPFSFIPTSFYQGRLVSIELENSKITMVWKETQLMEKLKILNLSHSHYLTKTPDFLNLPNLEK
LVLMDCPRLSEVSYTIGHLTKVLLINFQDCVSLRNLPRSIYKLKSLKTLILSGCLKIDKLEEDIEQMESLTTLVADKTGITRVPF
SIVRSKSIGYISLCGYEGFSRNVFPSIIWSWMSPVNSLSSRVQTFVDMSSLVSLDVQNSSSNQLSYISEELPKLQSLWIECG
SDLQLSRDTTSILEALNATNSVESESSATASQLHNVFTLIECNSGSKLFEKTLLIQMGRSWEITHILKQRILQNMTTSDGGD
CLLPGDCYPDWLTFSSEGSSVTFEIAQVNGRNLKTMMCHIHYSSSDSITSDGLKNLLVINHTKSTIQLFKRNALASFDDEE

WQRVLSNIEPGNKVQIVVVFWSRLIVIKTSIYLIYEAIEEKEEHYHAPNMTIPSYESSCAVGSISPPVESMEDLRGASVKSLT
KRILNKFFSCKGKVEKKKNQG

>XP_027907780.1

MFPMYDSDCGNDEMEMDFLRDRYEEMDNRNFEVFLSFRGEDTRASFTSHLYAALQNAGIIVFKDDESLPRGKQISPSL
RLAIENSPISIVVFSKNYAESRWCLKELEKIMECHRTIGHVVLPVFYDVDPSEVRHQRGDFGKAFQRLLGKIWNEDEEKEL
YWKQLWRKTLGEIGGFSAVETLSSRFERDISMVYWKFALAQTPSSYVESNPRDETEIADSINFLVKQWMERICMGAEIP
FSFEMETADAIEFQVKHCREALFKAAGISRGAILNSRREMEITEAIELQLKYWRDAFSKEAGGVLDSKGYGKRVVDSEIRN
LQNHWREKLLEAYSILEDKRVYSSREIQIEADLERWMKVLVEAANVATDAVYVSCIIMLKAYNRIEKLVNYWRMALCHA
IGISSFTVQQHRVMKDYEINDIKKHRDALREAAGISGVVILNSRNENEAVKTIVKNVTSLLDKTELFVANNPVGVESRVQE
MVQLLEQKQSNDVLLLGIWGMGGIGKTTIAKAIYNKIGRNFQGRSFLADIREVWGQEAGHICLQQQLLFDIHKENNTKI
HNTESGKMILRERLRHKRILLILDDVNKLQQLNALCGNREWFGSGSRIIITTRDIHLLRGKRVDQVFAMTGMDVDESIEL
FSWHAFKQASPEEDFIELSRNVIAYAGGLPLALEVLGSYLFDMEVTEWKIVLEKLRKIPNDEVQEKLKISYDGLSDDTEKGI
FLDIACFFIGKDRNDVIHILNGCGLFAENGIRVLVERSLVIVNDKNQLGMHDLVRDMGREIIRSKSPMEVEERSRLWFDE
DVLDVLSKETGTKYIEGLRLKLPRSNTKSLSTKAFMNMKKLRLLQLSDVELVGDFEYISKDLRWLCWHGFPFSFIPTSFYQ
GRLVSIELENSKITMVWKETQLMEKLKILNLSHSHYLTKTPDFLNLPNLEKLVLMDCPRLSEVSYTIGHLTKVLLINFQDCV
SLRNLPRSIYKLKSLKTLILSGCLKIDKLEEDIEQMESLTTLVADKTGITRVPFSIVRSKSIGYISLCGYEGFSRNVFPSIIWSW
MSPVNSLSSRVQTFVDMSSLVSLDVQNSSSNQLSYISEELPKLQSLWIECGSDLQLSRDTTSILEALNATNSVESESSATAS
QLHNVFTLIECNSGSKLFEKTLLIQMGRSWEITHILKQRILQNMTTSDGGDCLLPGDCYPDWLTFSSEGSSVTFEIAQVN
GRNLKTMMCHIHYSSSDSITSDGLKNLLVINHTKSTIQLFKRNALASFDDEEWQRVLSNIEPGNKVQIVVVFWSRLIVIKT
SIYLIYEAIEEKEEHYHAPNMTIPSYESSCAVGSISPPVESMEDLRGASVKSLTKRILNKFFSCKGKVEKKKNQG

>XP_027907781.1

MEFTSASSSSSSSSSSSSFLKSEPHFIYDVFINHSGENISRKFVSHLHSAFLEAQVKTLINEENLQEGMKLEEHMRAIAGCKIA
IIVFSKTYAESTNCLLELEKIIACHQTFGQTILPIFYEIDPLDVRDQKDDFGKSLEETAHKSYSGEQVEHALSSWTRSLTTVAS
ITGLDVRDFRHDAQLVEITVRRVKTLLDYAELSTTLFPVGLESHVEKVIGCIEKHSTKLCMIGIWGMGGSGKTTLAKAIYN
RIYRTFIGKSFSENIREACSRVNRSYVHLQENLLYDVLKSKLEVESVGMGRTMIQNRLSRKKLLIVVDDVNEFGQLENLCL
NREWFGRGTVIIITTRDVHLLNRLKVNYIYKMEGMNENDSLELLSWHAFVAAKPRKELNELARNVVAYCGGLPLALKVL
GSFLCGRRKKEWESIPSKLNLIPIDQFQEKLKISFDGLYNEMEKDIFLDVCCFFIGKERGYVTEILNACGLRADMGIKVLIER
ALIRVKRNNKLEMHPLIRDMGREIIRQRSPTELGGRSRLWFHEDLKDILEKNTGTKAIEGLSLKLPSISQDFFECHAFKEM
KRLRILELDHVRLTGNYRYLSKQLRWICWKGFPSKYIPNNFYMKNVIAIDFKHSHLQPVWQQPKVLKWLKFLNLSHSKYL
RETPDFSTLPSLQRLILKDCPSLCKVHPSIGDLYNIQLINLKDCTSLSSLPREIYKLKSLTTFILSGCFKIHILEEDILQMESLITLV
TENITVKQVPCSIVNSKSIGYISLRGFEGFPPNIFPSIIRSWMSSTMNHQSYFSPFCMDTHNWRDFAPLCSSLANIRSVLL
QCDTMFQLSEQVKKIVVEYSVNFTEPRISNHQLRFSLIAVRRYNQFLNSLRDSISEGLESSESCDVCLPHDNHPNWLAYM
GEGHSVSFTVPEDDDLKGMVLCVVYLSAPEIMANECLQSVLVVNYTKCTLQIHNHGTIISFNDKDWEGIISNLGSGDKV
EFFVSFGHGLVVKNTAIYLNIW

>XP_027907782.1

MEFASSSSNLQRKYDVLIHFDGEDIRRKFVSHLDSALSAVGFTTFLHEENAVKEMQIQEPILDLCRVVIVVFTKSYSQSAW
CLHQLQQIIQWQETYSRHVLPVYYEIQPSDVRLQKGNFGTAFKATAHQTFSDKQLEHGMCMWSHALTKTANFFGWD
ESNYRSDAELVDKIVKSILNLPVLSATKFPVGLQSYVEDVIQIIKDKSAEVCRIGICGVGGSGKTTLATAIYNQIHSTFTHQSF
IEDISEVSVTKRDYYIQKQLFSDVLKTNLKIHCIEMGRRMIQERLYGKKMLIVLDNVKDDISLYISECSALLGEGSVIIMTTR
NEDLLKKLQVDSIFRIKLGNPKKSIELLSWHAFREAKPKEEYYSLAKRLVAYCGGLPLALEVVGTYLYERTEEEWNTVLLKL
DNAPQHKVLQILKISFEGLPNQMERDLFLDICCFFVGKGRSYVRKILNGCGVDADSGIKVLIERNLIKVKKNNKFGVHPLL

RDMGREIISDISRNETRKLLENTLFSSQQTKIIHRLRGTGDFFERHPLKVRDPSRLLELLGASEYHPKKLTWISWQGFPSEY
LPNDFYLHDAIAIDLKYNLLRFLWKQPQVLESLKVLNLSHSIYLTKTPDFSTLPSLEQLILKDCPRLREVHQSVGCLYNLTLL
NLKDCTSLSNLPREIYKLKSLKTLILSGCSKLGLMEKDIGQMESLITLIAENTVVKQVPFSIVCSKSIGYISLHRFEGLSHYLFPS
IIRCWMSPTMNPLSYIHSFCMDKEHSSGDDIMPLFNTLPNLRSVVVQCDSEFPLSKQVKSTLVEYVVNISESGISKHHFRL
SLIGFGRHGEFFNTMNESISKVLTNNKSGDVSLPGDNDPYWLTHMGEGHSVSFTLPQDRVMKGMTLCVVYLSTSKIIEP
ELTAVLIVNYTKCTLQMHNHGSTMSFNDEDWDCIMSNLGSGDKVEIFVTFGHGLMIKSTSVYLIYAELNDFEMEHCPE
PKENTLNKFIKKMLMSHFW

>XP_027907783.1

MEFASSSSNLQRKYDVLIHFDGEDIRRKFVSHLDFTLSAVGLTTFLHQENAVKGMKIQEPILDFCRVAIVVFTKTYSESAW
CLHQLQQIIQWHETYSRHVLPVYYEIQPSDVRLQKGHFGKAFKATAHQTFSGQELEHGMCRWSRALTKVANFFGWD
DSNYRSDAELVDRIVKSVLNLPILSATEFPVGLESQVKDVIRTIKSNPRKVCIIGIWGMRGSGKTTLAKAIYNQIHGTFTEKS
FIENISDFSQRRHLNLQKRLLSDILKTKINIHSVEMGRSMIRERLYGKRVLIVLDDMIDEHYPLLDLRICRASLGGGSVIILTT
RYKDILRKHGVDAVFGKNLLNSNESLELFSWHAFREAKPKEEYHFLAKSVVAYCEGNPLSLEVIGSYLYERTKIEWHRVLL
KLIEVPQFEILPILKISFDGLRDQTEKDLFLDICCFFVGKGRAYVTKILNGCGIDADCGIRVLLERSLIKVKRNNKFGIHPLVRD
MGREIIDDISRKEPRKNRWLWLDDDMKHAPSKYTTGYSKYLSKKLRCISLQCFSSEYLCNDFYLHDAIVVDLKRSLLGIIW
KQPQVLAFLKVLNLSHSKYLRKTPDFSGLQNLEHLILKDCPRLREVHRSIGCLCNLTLLNLKDCTSLSNLPREIYMLKSLKTLI
LSGCSKINLLEKDIVQMESLITLIAENTVVKQVPFSIVSSKGIGYISLRGFEGLSHNLFPFIIQSWMSPSMNSLSYNPSFCMD
VEVKSWDDITPLLSSLGNLRSVLVQCDTEFQLSKQVKTILVEYRGNITEIGTSKPHFRYCLIGVGSCKEFFNAFSDGTSEGL
ASSESCDVFLPGGNDPYWLVHMGEGHSVSFTLPKDHVLKGMALCAIYSPNPEILTTECLRSVLIVNYTKCTLQIHYHGTII
SFNDIDWQGIISNLGSGDRVEIFVIFCDGLVVKRTVVYLICGESNDLEKEPG

>XP_027907784.1

MEFASSSSNLQRKYDVLIHFDGEDIRRKFVSHLDFTLSAVGLTTFLHQENAVKGMKIQEPILDFCRVAIVVFTKTYSESAW
CLHQLQQIIQWHETYSRHVLPVYYEIQPSDVRLQKGHFGKAFKATAHQTFSGQELEHGMCRWSRALTKVANFFGWD
DSNYRSDAELVDRIVKSVLNLPILSATEFPVGLESQVKDVIRTIKSNPRKVCIIGIWGMRGSGKTTLAKAIYNQIHGTFTEKS
FIENISDFSQRRHLNLQKRLLSDILKTKINIHSVEMGRSMIRERLYGKRVLIVLDDMIDEHYPLLDLRICRASLGGGSVIILTT
RYKDILRKHGVDAVFGKNLLNSNESLELFSWHAFREAKPKEEYHFLAKSVVAYCEGNPLSLEVIGSYLYERTKIEWHRVLL
KLIEVPQFEILPILKISFDGLRDQTEKDLFLDICCFFVGKGRAYVTKILNGCGIDADCGIRVLLERSLIKVKRNNKFGIHPLVRD
MGREIIDDISRKEPRKNRWLWLDDDMKHAPSKYTTGYSKYLSKKLRCISLQCFSSEYLCNDFYLHDAIVVDLKRSLLGIIW
KQPQGLASSESCDVFLPGGNDPYWLVHMGEGHSVSFTLPKDHVLKGMALCAIYSPNPEILTTECLRSVLIVNYTKCTLQI
HYHGTIISFNDIDWQGIISNLGSGDRVEIFVIFCDGLVVKRTVVYLICGESNDLEKEPG

>XP_027907786.1

MSSSSKLQRRYDVLINFNGEDIHRKFVSHLDSALSTVGFTTFLHEENTMKGMNIQEPILNLCRVAIVVFTKTYSQSSWCL
NQLQQIIKWNETYCRHVLPVYYEIQPSDVRLQKGDFGKALEVTAQQTFSGHHQERGMSKWSQALTKAANFFGWDES
NHRSDAELVEKIVKSVLNLPILSATKFPVGLHSRVEKVIGTIKNNSTKVCIIGICGEGGSGKTTLARAIYHQIQLRFTEKSFIE
DIGQVGGIKGDLRLREQLLLDILKTKVEIPSVDMGRSMIRERLSGKRMLIVLDDVPYCSILLDLWDRFKWLGGGTVVIITT
RDESILTIPQGYSVFRTKLMNAKESLELLSWHAFREAKPKEEYYGLAKRVVHNCGGLPLALEVIGSTLFERTKYIWHSVLFK
LEKIPRYNVIEKLKISFDSLSNQMERYIFLDVCCFFVGKGIAYATKILNGCGVEANNGIRVLIERSLIKVKKNNKCGMHPLLQ
EMGITLSREISLKEPGENRRLWFHKDEKYGTKAMHWLPLNQDQVQLAVNSEYLFQKLRWISLHGFSSEYLHNKFYVHD
AIAIDLKHSLLRLVWKEPQVLRSLKVLNLSHCKYLTTTPDFTGLQSLEQLILKCCPRLRKVHQSIGCLGNLLLLNLKDCTNLS
NLPRGIYKLKSLKTLILSGCSKIDLMEKDTAEMESLITLIAENTAVKELPISIVSSKSIGYISLGRFERLSPNIFPSVIRSWISPTM
NPISYMHSFCMDIEDNNWDDIAPLLSTLRNLRSVLV

>XP_027907787.1

MGIRRMNCEASSSWSNHVFLSFRGEDTRRGFTDHLFASLERRGIKTFKDDHDLERGKVISVELMKAIEDSMFALIILSPN
YASSTWCLDELQKIVDCKKEAFPIFHGVDPSDVRHQRGSFAKAFEEHEEKFREDKEKVERWRHALRQVASYSGWDSKD
QSEATLIETIVGHIEKKIIPRLPCFTDNLVGIDSRMKEVISLMGIGLNDIRFIGIWGMGGIGKTTLARLVYEKIKEKFKVCCFL
ENIRELSKTNGLVHIQKEILSHLNVRSNDFYNLYDGKKIIANSLSNKKVLLVLDDVSDISQLENLTGKREWFGPGSRLIITTR
DKHLLKTYGVDVTYKARGLAQNEALQLFCLKAFKQDQPKKEYLNLCKGVVEYARGLPLALEVLGSHLYGRTFEVWHSAL
EQIRSFPHSKIQDTLKISYDSLEPLEKKMFLDIACFFVGMDIDEVMNILENCGDHPRIGIDILTERSLVTLDRVSNKLGMHD
LLQEMGRNIVYQESPNDPGKRSRLWSQKDIDYVLTKNKGTDEIRGIVLNLVQPYDCEARWNTESFSKISQLRFLKLCDV
QLPRGLSCLPSALNVVHWRGCPLKTLPLSNQLDEVVDLKLPHSRIEQLWHGTKLLDKLKFINLSFSKNLKQSPDFVGVPN
LESLVLEGCTSLTEVHPSLVRHKKLVSLNLKDCKKLKSLPSKMETSSLNDLNLSGCSQFKCLPEFAESMEHLSVLSLEGTAIT
KLPTSLGCLTGLSHLDMKNCKNLVCIPDTIHKLRSLIVLNVSGCSKLSSLPEGLKEIKCLEELNASETAIQELPSFVFYLENLN
DISFAGCKGPVSKSANGFFLPFKWLFGNHQTSIGFRLPPSALSLPSLKRINLSYCNLSEESFPDDFYCLSSLVILDLTGNNFV
SLPSSISKLARLERLILNYCKKLQRLPELPSNMRGLDASNCTSFEISKFNPSKPCSLFASPAKWHLPRQLKGVLEKLRLPRER
FDMLIRGSEIPPWFAPNKCVSFAKISVPHNCPINEWVGFALCFLLVSYVVPPDVCHHEVDCYFFGPHGKVCISSRCLPPM
EPCDPHLYIIYLSFDELRDIICKGGDCREIEFVLKTYCCHSLQIERCGSRLVCKQDVEDIYGNGL

>XP_027907788.1

MKAIEDSMFALIILSPNYASSTWCLDELQKIVDCKKEAFPIFHGVDPSDVRHQRGSFAKAFEEHEEKFREDKEKVERWRH
ALRQVASYSGWDSKDQSEATLIETIVGHIEKKIIPRLPCFTDNLVGIDSRMKEVISLMGIGLNDIRFIGIWGMGGIGKTTLA
RLVYEKIKEKFKVCCFLENIRELSKTNGLVHIQKEILSHLNVRSNDFYNLYDGKKIIANSLSNKKVLLVLDDVSDISQLENLTG
KREWFGPGSRLIITTRDKHLLKTYGVDVTYKARGLAQNEALQLFCLKAFKQDQPKKEYLNLCKGVVEYARGLPLALEVLG
SHLYGRTFEVWHSALEQIRSFPHSKIQDTLKISYDSLEPLEKKMFLDIACFFVGMDIDEVMNILENCGDHPRIGIDILTERS
LVTLDRVSNKLGMHDLLQEMGRNIVYQESPNDPGKRSRLWSQKDIDYVLTKNKGTDEIRGIVLNLVQPYDCEARWNTE
SFSKISQLRFLKLCDVQLPRGLSCLPSALNVVHWRGCPLKTLPLSNQLDEVVDLKLPHSRIEQLWHGTKLLDKLKFINLSFS
KNLKQSPDFVGVPNLESLVLEGCTSLTEVHPSLVRHKKLVSLNLKDCKKLKSLPSKMETSSLNDLNLSGCSQFKCLPEFAES
MEHLSVLSLEGTAITKLPTSLGCLTGLSHLDMKNCKNLVCIPDTIHKLRSLIVLNVSGCSKLSSLPEGLKEIKCLEELNASETAI
QELPSFVFYLENLNDISFAGCKGPVSKSANGFFLPFKWLFGNHQTSIGFRLPPSALSLPSLKRINLSYCNLSEESFPDDFYCL
SSLVILDLTGNNFVSLPSSISKLARLERLILNYCKKLQRLPELPSNMRGLDASNCTSFEISKFNPSKPCSLFASPAKWHLPRQ
LKGVLEKLRLPRERFDMLIRGSEIPPWFAPNKCVSFAKISVPHNCPINEWVGFALCFLLVSYVVPPDVCHHEVDCYFFGP
HGKVCISSRCLPPMEPCDPHLYIIYLSFDELRDIICKGGDCREIEFVLKTYCCHSLQIERCGSRLVCKQDVEDIYGNGL

>XP_027907799.1

MEFTSSTSKIPQMYDVLINFNGEDIHRKFVSHLDSVLSAAGLTTFLHHQNAVNDMDIQQPILNLCRVAIVVFTKTYSQSA
WCLHQLHQIIEWHQTYSRHVLPVYYELQPSDVRLQKGDFGKTLKATAQQSFSAQQLKHGMSRWKHALSKTADFFGW
DESNYRSDAEVADKIVKSVLNLAVLSATKFPVGLQSRMEEVIQIIKKESTKVCTIAICGKGGSGKTTLAKAIYNQIHDTFTQ
KFFIEDIEQKQLCNNEMGRSLIPKIIYGKRVLIVLDDVRFSYYLEMIVRSHLREGTVIIITTTNQNPPLIRYPYSIFQINLMNP
NESLELLSWHAFREPKPKEECHFLAKMVVDYCGGLPLSLEVIGSFLYEKTKEEWNNVLSRLERVPQHEVLQILKLSFDGLP
NEIERNLFLDICCFFVGKDRVYVTKILNGCGVNAESGIRILIERRLIIVKKNNKFGLHPLLREMGREIIGEITSGKEPKKTSRL
WFDKDEDYVLLEHILFSSQEKKVIQRFPPKWSLTIRDFFERDYLEVRDAIGRRKLGGHCEYRSKELGWIRLAKFSSKDLPIG
FLHDAIAIDLKHSLPRLVWKKPQVLASLKVLNLSHSKYLTETPDFSRLPGLEHLILKDCPRLCEVHPSIGGLCNLTLLNLKDC
TKINNLPREIYMLKSLKTLILSGCSGIHLMEKDIVQMESLITLITENTVVKQVPFSIESSKSIGYLSLRGFEGLSHNLFHFIIRS
WKLPSMTPLSYNHSFCMDMELNSWDDIAPLRRIFVNLRSVLLQCETEFQLSKQVQDILVEYGVYITESDTSKQHFRSSLI
GVGRCKEFLDAFSDSISEVIAGSESCDVSLPGDNDPNCLAHMGEGYSVSFTVPRNGDIKGMALCIVYLSTPEIVATEGLTS
VLIVNYTKCTLHIHNHATIISFNDKDWEGIISNLGSGDKVEIFVIFGDGLVVRRTIVYLICGESNDLEKESPSKKNSPIRFIKNL

>XP_027907851.1

MAEEKSKGSMEYVKLVGSLVGPLLGPGWPYLKPLLYKLVPYEIRVGHLAKDVNKLRLVKSRVQDKVKDEENRNERAISG
GVKKWLDEVDEVIFDYEEFLEDEDRSYAVYSDGYLPKPSIRYRLRKMVNDIGSRVSVLLQTSNDDNFSCWLGPPSYDAD
FDNIRYQMFESRNQTTGNIIAALANSSVGMIGVYGLNGVGKTSLIKEVVKKVKDNMFDVVIMVNVTSRPDIRRIQGQIA
KKLGMKLKGESESERAVHLRDRLKDPKLKTLIILDNLEVKLDFNMLGISSENNDDSQMNSRMKDLSAHHNYALKNKELD
ASILRKVEDPLARYKGCKILMISKNEQLLVRQMDGKAIKTFCVTPLTEKEAESMFKTMSEIDNENSLYKALAAQISKKCKG
LPMTIVATAKALKNKSLLVWEDAYRNLERQNLTAVQEFSTKLSYNLLENDELKHTLLVCARMSNDALLTDLVRNCIGLGL
LQGIYMVKEARDRIHMLVAELKELSLLSDSFSSDRFTMQDNIRDAVLSIASQEMHAFALTKGKLEEWPDKDKLERCTAIS
LQNCDVTDIMNKFPETINCFRLRVFHLENKDPHLKIPDNFFIGMKELRVLILIGVSLSFLPSSIMYLKKLRMLCLERCKLDKH
LSIIGELETLRVLSLSGSDVEKLPTELSQLTKLQIFDISNCFKLREIPVHVLSSLIDLEELYVGNSPIQWKYEGHVNASLSELRQ
LYQLTTLDIEIPGTTHMPENLFFDKLDSYNIVIRDVGAYSIWDIKMLERRETSRFLALQLENGFDIHHQKDIKILFERVENLL
LGQLNDVEDIFYELNYEGFPYLKYLSIVSNSKIKSVINSKNQKHLEKVFPRLESLFLYEVNHMEHICYNQLTTDSFGKLKIIKL
NMCGQLKNVFFSSTIKLLSALEAVEVSECDTLKEIVTSEAENREQIIFPELRSVTLQSLSELIGFYGALPGEQESNKLFDEKV
VISKLERMKLSSIKIQKIWSDQYWTSFQNLIKLDVTDCWNLKNLLSFTMSKSLMNLQSLLVSECGMMESIFEFIETEVSM
FEIEPERIFPKLKNINLGSMKRLKEIWHPKFPLHSFGKLDELIIEGCNKLKNVFPSYMIGRFHSLCNLKVTNCLSMKEIFDLQ
DCQKQDFEDMTRLQSVHAEALPKLEHVWNKDPEGILNLKNLKKIWIQECLNLEHIFPVSTAKDLQELEYLEVWNCGKLK
KIVSKGETNNTSSISFKFPKLTTVRFSKLPSLEGFYEGEHELHYSALNNLCVESCPKLELFSGENTNSEIKSVFFPEKAIYNLKS
MQIESENAIWLRRYMGNYRMHKLEEFQLFGLPDTEILYFFIHRNPNMKSLLLSNCSFKELVPPRSHSEEKSGVVPKLKSLK
VMNLQSLKMIDFKDDTILFQRLECLILKECPCLNTIAPSSISFTYLTTLEVGNCNKLACLMTPSTAKSLVQLTTMKVIQCEQ
MKTIVSELEHKEHIIFRKLKEIELVALQNLLSFCSSNHCAFDFPLLEKFVVSACSNMRKFSQHANSTPILRQILIGNGKEEKR
YHWKGDLNVTISYMHQIWALHATEVVDSNPYKPLENSRLKILKLANCELGSHAIPTVVFSSLKNLEELEVSNTNVETIFDI
MDEEMKGYTFLLKKMTLNNLPNLKKVWRDENPEGIFSSQNLQEVVVNNCEILETLFPIKLAKIVKKLKKLEIRCCKKFLKIV
EQENAITEATAEFSFPRLTSLNLRMLPQLSCFYPERFTLECPHLSHLEVVSCGDFVTFEIHQAHGSTSVNRKPLFSEENANF
ILESLKLDWKNTTMLCNGKFPDEMLHQVIKFELDLDKDNDKEVADVILKKMPHAECIRIKGYSGLKELTSSQHEHGESSH
PPEQGDSSHHEQGGRNQNLGRSRPPENKKKNKKIK

>XP_027907852.1

MAEEKSKGSMEYVKLVGSLVGPLLGPGWPYLKPLLYKLVPYEIRVGHLAKDVNKLRLVKSRVQDKVKDEENRNERAISG
GVKKWLDEVDEVIFDYEEFLEDEDRSYAVYSDGYLPKPSIRYRLRKMVNDIGSRVSVLLQTSNDDNFSCWLGPPSYDAD
FDNIRYQMFESRNQTTGNIIAALANSSVGMIGVYGLNGVGKTSLIKEVVKKVKDNMFDVVIMVNVTSRPDIRRIQGQIA
KKLGMKLKGESESERAVHLRDRLKDPKLKTLIILDNLEVKLDFNMLGISSENNDDSQMNSRMKDLSAHHNYALKNKELD
ASILRKVEDPLARYKGCKILMISKNEQLLVRQMDGKAIKTFCVTPLTEKEAESMFKTMSEIDNENSLYKALAAQISKKCKG
LPMTIVATAKALKNKSLLVWEDAYRNLERQNLTAVQEFSTKLSYNLLENDELKHTLLVCARMSNDALLTDLVRNCIGLGL
LQGIYMVKEARDRIHMLVAELKELSLLSDSFSSDRFTMQDNIRDAVLSIASQEMHAFALTKGKLEEWPDKDKLERCTAIS
LQNCDVTDIMNKFPETINCFRLRVFHLENKDPHLKIPDNFFIGMKELRVLILIGVSLSFLPSSIMYLKKLRMLCLERCKLDKH
LSIIGELETLRVLSLSGSDVEKLPTELSQLTKLQIFDISNCFKLREIPVHVLSSLIDLEELYVGNSPIQWKYEGHVNASLSELRQ
LYQLTTLDIEIPGTTHMPENLFFDKLDSYNIVIRDVGAYSIWDIKMLERRETSRFLALQLENGFDIHHQKDIKILFERVENLL
LGQLNDVEDIFYELNYEGFPYLKYLSIVSNSKIKSVINSKNQKHLEKVFPRLESLFLYEVNHMEHICYNQLTTDSFGKLKIIKL
NMCGQLKNVFFSSTIKLLSALEAVEVSECDTLKEIVTSEAENREQIIFPELRSVTLQSLSELIGFYGALPGEQESNKLFDEKV
VISKLERMKLSSIKIQKIWSDQYWTSFQNLIKLDVTDCWNLKNLLSFTMSKSLMNLQSLLVSECGMMESIFEFIETEVSM
FEIEPERIFPKLKNINLGSMKRLKEIWHPKFPLHSFGKLDELIIEGCNKLKNVFPSYMIGRFHSLCNLKVTNCLSMKEIFDLQ
DCQKQDFEDMTRLQSVHAEALPKLEHVWNKDPEGILNLKNLKKIWIQECLNLEHIFPVSTAKDLQELEYLEVWNCGKLK
KIVSKGETNNTSSISFKFPKLTTVRFSKLPSLEGFYEGEHELHYSALNNLCVESCPKLELFSGENTNSEIKSVFFPEKAIYNLKS
MQIESENAIWLRRYMGNYRMHKLEEFQLFGLPDTEILYFFIHRNPNMKSLLLSNCSFKELVPPRSHSEEKSGVVPKLKSLK

VMNLQSLKMIDFKDDTILFQRLECLILKECPCLNTIAPSSISFTYLTTLEVGNCNKLACLMTPSTAKSLVQLTTMKVIQCEQ
MKTIVSELEHKEHIIFRKLKEIELVALQNLLSFCSSNHCAFDFPLLEKFVVSACSNMRKFSQHANSTPILRQILIGNGKEEKR
YHWKGDLNVTISYMHQIWALHATEVVDSNPYKPLENSRLKILKLANCELGSHAIPTVVFSSLKNLEELEVSNTNVETIFDI
MDEEMKGYTFLLKKMTLNNLPNLKKVWRDENPEGIFSSQNLQEVVVNNCEILETLFPIKLAKIVKKLKKLEIRCCKKFLKIV
EQENAITEATAEFSFPRLTSLNLRMLPQLSCFYPERFTLECPHLSHLEVVSCGDFVTFEIHQAHGSTSVNRKPLFSEENANF
ILESLKLDWKNTTMLCNGKFPDEMLHQVIKFELDLDKDNDKEVADVILKKMPHAECIRIKGYSGLKELTSSQHEHGESSH
PPEQGGRNQNLGRSRPPENKKKNKKIK

>XP_027907853.1

MAEEKSKGSMEYVKLVGSLVGPLLGPGWPYLKPLLYKLVPYEIRVGHLAKDVNKLRLVKSRVQDKVKDEENRNERAISG
GVKKWLDEVDEVIFDYEEFLEDEDRSYAVYSDGYLPKPSIRYRLRKMVNDIGSRVSVLLQTSNDDNFSCWLGPPSYDAD
FDNIRYQMFESRNQTTGNIIAALANSSVGMIGVYGLNGVGKTSLIKEVVKKVKDNMFDVVIMVNVTSRPDIRRIQGQIA
KKLGMKLKGESESERAVHLRDRLKDPKLKTLIILDNLEVKLDFNMLGISSENNDDSQMNSRMKDLSAHHNYALKNKELD
ASILRKVEDPLARYKGCKILMISKNEQLLVRQMDGKAIKTFCVTPLTEKEAESMFKTMSEIDNENSLYKALAAQISKKCKG
LPMTIVATAKALKNKSLLVWEDAYRNLERQNLTAVQEFSTKLSYNLLENDELKHTLLVCARMSNDALLTDLVRNCIGLGL
LQGIYMVKEARDRIHMLVAELKELSLLSDSFSSDRFTMQDNIRDAVLSIASQEMHAFALTKGKLEEWPDKDKLERCTAIS
LQNCDVTDIMNKFPETINCFRLRVFHLENKDPHLKIPDNFFIGMKELRVLILIGVSLSFLPSSIMYLKKLRMLCLERCKLDKH
LSIIGELETLRVLSLSGSDVEKLPTELSQLTKLQIFDISNCFKLREIPVHVLSSLIDLEELYVGNSPIQWKYEGHVNASLSELRQ
LYQLTTLDIEIPGTTHMPENLFFDKLDSYNIVIRDVGAYSIWDIKMLERRETSRFLALQLENGFDIHHQKDIKILFERVENLL
LGQLNDVEDIFYELNYEGFPYLKYLSIVSNSKIKSVINSKNQKHLEKVFPRLESLFLYEVNHMEHICYNQLTTDSFGKLKIIKL
NMCGQLKNVFFSSTIKLLSALEAVEVSECDTLKEIVTSEAENREQIIFPELRSVTLQSLSELIGFYGALPGEQESNKLFDEKV
VISKLERMKLSSIKIQKIWSDQYWTSFQNLIKLDVTDCWNLKNLLSFTMSKSLMNLQSLLVSECGMMESIFEFIETEVSM
FEIEPERIFPKLKNINLGSMKRLKEIWHPKFPLHSFGKLDELIIEGCNKLKNVFPSYMIGRFHSLCNLKVTNCLSMKEIFDLQ
DCQKQDFEDMTRLQSVHAEALPKLEHVWNKDPEGILNLKNLKKIWIQECLNLEHIFPVSTAKDLQELEYLEVWNCGKLK
KIVSKGETNNTSSISFKFPKLTTVRFSKLPSLEGFYEGEHELHYSALNNLCVESCPKLELFSGENTNSEIKSVFFPEKAIYNLKS
MQIESENAIWLRRYMGNYRMHKLEEFQLFGLPDTEILYFFIHRNPNMKSLLLSNCSFKELVPPRSHSEEKSGVVPKLKSLK
VMNLQSLKMIDFKDDTILFQRLECLILKECPCLNTIAPSSISFTYLTTLEVGNCNKLACLMTPSTAKSLVQLTTMKVIQCEQ
MKTIVSELEHKEHIIFRKLKEIELVALQNLLSFCSSNHCAFDFPLLEKFVVSACSNMRKFSQHANSTPILRQILIGNGKEEKR
YHWKGDLNVTISYMHQIWALHATEVVDSNPYKPLENSRLKILKLANCELGSHAIPTVVFSSLKNLEELEANFILESLKLDW
KNTTMLCNGKFPDEMLHQVIKFELDLDKDNDKEVADVILKKMPHAECIRIKGYSGLKELTSSQHEHGESSHPPEQGDSS
HHEQGGRNQNLGRSRPPENKKKNKKIK

>XP_027907854.1

MAEEKSKGSMEYVKLVGSLVGPLLGPGWPYLKPLLYKLVPYEIRVGHLAKDVNKLRLVKSRVQDKVKDEENRNERAISG
GVKKWLDEVDEVIFDYEEFLEDEDRSYAVYSDGYLPKPSIRYRLRKMVNDIGSRVSVLLQTSNDDNFSCWLGPPSYDAD
FDNIRYQMFESRNQTTGNIIAALANSSVGMIGVYGLNGVGKTSLIKEVVKKVKDNMFDVVIMVNVTSRPDIRRIQGQIA
KKLGMKLKGESESERAVHLRDRLKDPKLKTLIILDNLEVKLDFNMLGISSENNDDSQMNSRMKDLSAHHNYALKNKELD
ASILRKVEDPLARYKGCKILMISKNEQLLVRQMDGKAIKTFCVTPLTEKEAESMFKTMSEIDNENSLYKALAAQISKKCKG
LPMTIVATAKALKNKSLLVWEDAYRNLERQNLTAVQEFSTKLSYNLLENDELKHTLLVCARMSNDALLTDLVRNCIGLGL
LQGIYMVKEARDRIHMLVAELKELSLLSDSFSSDRFTMQDNIRDAVLSIASQEMHAFALTKGKLEEWPDKDKLERCTAIS
LQNCDVTDIMNKFPETINCFRLRVFHLENKDPHLKIPDNFFIGMKELRVLILIGVSLSFLPSSIMYLKKLRMLCLERCKLDKH
LSIIGELETLRVLSLSGSDVEKLPTELSQLTKLQIFDISNCFKLREIPVHVLSSLIDLEELYVGNSPIQWKYEGHVNASLSELRQ
LYQLTTLDIEIPGTTHMPENLFFDKLDSYNIVIRDVGAYSIWDIKMLERRETSRFLALQLENGFDIHHQKDIKILFERVENLL
LGQLNDVEDIFYELNYEGFPYLKYLSIVSNSKIKSVINSKNQKHLEKVFPRLESLFLYEVNHMEHICYNQLTTDSFGKLKIIKL

NMCGQLKNVFFSSTIKLLSALEAVEVSECDTLKEIVTSEAENREQIIFPELRSVTLQSLSELIGFYGALPGEQESNKLFDEKV
VISKLERMKLSSIKIQKIWSDQYWTSFQNLIKLDVTDCWNLKNLLSFTMSKSLMNLQSLLVSECGMMESIFEFIETEVSM
FEIEPERIFPKLKNINLGSMKRLKEIWHPKFPLHSFGKLDELIIEGCNKLKNVFPSYMIGRFHSLCNLKVTNCLSMKEIFDLQ
DCQKQDFEDMTRLQSVHAEALPKLEHVWNKDPEGILNLKNLKKIWIQECLNLEHIFPVSTAKDLQELEYLEVWNCGKLK
KIVSKGETNNTSSISFKFPKLTTVRFSKLPSLEGFYEGEHELHYSALNNLCVESCPKLELFSGENTNSEIKSVFFPEKAIYNLKS
MQIESENAIWLRRYMGNYRMHKLEEFQLFGLPDTEILYFFIHRNPNMKSLLLSNCSFKELVPPRSHSEEKSGVVPKLKSLK
VMNLQSLKMIDFKDDTILFQRLECLILKECPCLNTIAPSSISFTYLTTLEVGNCNKLACLMTPSTAKSLVQLTTMKVIQCEQ
MKTIVSELEHKEHIIFRKLKEIELVALQNLLSFCSSNHCAFDFPLLEKFVVSACSNMRKFSQHANSTPILRQILIGNGKEEKR
YHWKGDLNVTISYMHQIWALHATEVVDSNPYKPLENSRLKILKLANCELGSHAIPTVVFSSLKNLEELEANFILESLKLDW
KNTTMLCNGKFPDEMLHQVIKFELDLDKDNDKEVADVILKKMPHAECIRIKGYSGLKELTSSQHEHGESSHPPEQGGRN
QNLGRSRPPENKKKNKKIK

>XP_027907894.1

MNQKESLELLSWHAFREAKPKEEYHYLARSVLSYCRGLPLALEVVGSCLFERTEEEWDIVLSRLRKMTYHIWKISFYCLGN
QMERDLFLDVCCCFVGEDIAYATRILNGCGVDADSGIRVLIDRNLIKLKKNNKLGMHPLLQEMGRHIIREIRQEEMWKE
RRLRFDGAEYVLTDNTKTGTIERLPLKLRSGRRELSRLLKAAVNSDYLSPKLRWIRLQGFSSQHLSNSFNVDHAIAIDLKHS
LLRFVWTEPQVLRGLKVLILSHSKNLTQTPDFSGLPSLEKLILKDCSGLRKVHQSIGCLSHLTLLNLKDCTSLRNLPRDICKLK
SLKTLILSGCPMIDLSEKDVVEMESLITLIAENTTMKHVPFSILNSKSIGHISLRGFEGLPRNLFPSIIRSWLSPEMNSISYIHS
SFMDIDNCWDDIASLLSSLKNLRSILVQCDTDFQLSNQVKSIVVEYFANFTESGISKQQFRSPLIGLGTYHEFFNAVTDNIS
EALVNSECSNVSLPGDNLPYWLAYMGEGYSVSFTVPRDRDIKGLALCVVYLSTPEIVAIDYLRSVLIVNHTKCTLQIHNHG
TVISFNDTDWHGIMSNLGAGDKVEIFVAFGHGLVVKNTILYLICDESDYLRKELESKMNSLLRFIMKIVMCDFS

>XP_027907927.1

MSRWSHALTKAANFFGWDESNYRSDAELVDKIVQGVLNLPVLSATKFPVGLQSHVEDLIRIIKSKSMTVCKIGICGAGG
SGKTTLAKAIYHQIQGTFRNKSFIEDVGQVGGIRGDIHVQEKLLLDILKTKVKIPSDDVGRTMIWERLYGKRVLIVVDDVP
NYFEILALRNCSRWFSGGSVIMFTVRDESLLKHYLLDSVYRIELMNANKSLELLSWHAFREAKPKEEYNDLAKAVVAYCG
GLPLALEVIGNSLFERTKEKWNTVLFELKKIPPHNVIEKLKISFNGLRNEMEKEFFLDVCCFFIGKGIAYATKILNGCGVDVD
TGIRVLIERGLIKVKNNKFGMHPLLREMGREIICEISGKESGKNSELWFDKDVEYALPDNTLFSSKQTKVIQRLPVKMVLT
TKDFFEPYLSDRQLKLAENPEYLSQKLRWITWQGFSSEYLPNDFYLHDAIAVDLKHSLLRLVWKEPQVLRSLKFLNLSHS
MHLTETPDFSGLPTLEHLILKDCPRLCKVHQSIGCLRNLILLNLKDCECLNNLPREIYKLKSSRTFILSGCSIIDLMEKDTVRM
KSLITVITENTVVKEVPFSMVSSKRIGYISLRGLEGLSHNIFPSIIRSWMSPTMNSISYVHSFCMDMEDNSWDDIVPLLSSL
KNLRSILVQCDTEFQLSLQLETILAEYGVNIIESGISKQHFRSSLIGVGRYNEFFSTVSDSISEVLVSSESCDVSLAAVNNCLA
HMGEGHSVSFTVPRDRNLKGMAFCVVYLSTPKIIEPEFTTVVIVNYTKCTCHIHNHGTIISFSDEDWRGIMSNLEYGDKV
EIFVTFGHGLVLKNTVVYLICGKSHNLKKEPESKKNCLIRFIKKVVMCDFW

>XP_027907928.1

MSRWSHALTKAANFFGWDESNYRSDAELVDKIVQGVLNLPVLSATKFPVGLQSHVEDLIRIIKSKSMTVCKIGICGAGG
SGKTTLAKAIYHQIQGTFRNKSFIEDVGQVGGIRGDIHVQEKLLLDILKTKVKIPSDDVGRTMIWERLYGKRVLIVVDDVP
NYFEILALRNCSRWFSGGSVIMFTVRDESLLKHYLLDSVYRIELMNANKSLELLSWHAFREAKPKEEYNDLAKAVVAYCG
GLPLALEVIGNSLFERTKEKWNTVLFELKKIPPHNVIEKLKISFNGLRNEMEKEFFLDVCCFFIGKGIAYATKILNGCGVDVD
TGIRVLIERGLIKVKNNKFGMHPLLREMGREIICEISGKESGKNSELWFDKDVEYALPDNTVLRSLKFLNLSHSMHLTETP
DFSGLPTLEHLILKDCPRLCKVHQSIGCLRNLILLNLKDCECLNNLPREIYKLKSSRTFILSGCSIIDLMEKDTVRMKSLITVITE
NTVVKEVPFSMVSSKRIGYISLRGLEGLSHNIFPSIIRSWMSPTMNSISYVHSFCMDMEDNSWDDIVPLLSSLKNLRSILV
QCDTEFQLSLQLETILAEYGVNIIESGISKQHFRSSLIGVGRYNEFFSTVSDSISEVLVSSESCDVSLAAVNNCLAHMGEGH

SVSFTVPRDRNLKGMAFCVVYLSTPKIIEPEFTTVVIVNYTKCTCHIHNHGTIISFSDEDWRGIMSNLEYGDKVEIFVTFGH
GLVLKNTVVYLICGKSHNLKKEPESKKNCLIRFIKKVVMCDFW

>XP_027907935.1

MEFASSSSKLPRNYDVLISFNGEDIRRKFVSHLDSALSSVGLTTFLHYQNAVESMHVQQPILNLCRVVIVVFTKTYSQSA
WCLHQLQQIIAWHESYCRHVLPVYYEIQPSDVRLQKGDFGKAFKETAQQTFSAQQLEDGMSRWSHALTKVASLFGW
DESNHRSDAELVGKIVKSVLNLSALTATKFPVGLQDQVEDLIRTIRNKSTDVCTIGICGMGGSGKTTLAKAIYYQIHGTFM
EKSFIEDIAQVSEPRERIHLQQQLLSDVLKTKVEIHSVEMGRSMIRDRLFRKRVLIVLDDIDDYFPLLDLRKSRSWLSEGTVI
IMTARDEDLLRKHQVDSVFRINLMNEKESLELLSWHAFREPKPKKEYDYLARRVISCCGGLPLALELIGSSLFERREKEWH
SVLVGLEGIHMDDVEQKLKISFDLCNQMEKALFLIVCRYYIGKDRSYAMKILNGPVVNADSGIRVLMERNLIKVKANNKF
GIHRLLQQMGVEIFRENLKEEPWKHGRLWFDKGGEYALEVDTVRAFFICGLKLPLKVFAFSCSQYIRFSLLLNSQGRNAS
QTFPPILIPSREPSGFPEVVTVSKYIYTKLIWINLQGFSSEHLPKDFNMHGAVAIDLKHSLLRFFWKEPQVLRSLKVLNLSH
SMYLTETPDFSRLPSLEQLILKDCPRLRKVHRSIGHLCYLILLNLKDCKCLSNLPREIYKLKSLRTLILSGCSKVGRMGKDIVR
MKSLITLIAENTAVKQVPFSIVTSKAIGYMSIPGFKRLSCNLFPSIIIRSWMSPTMNPVSYIHSLCMDINNSWDDIVPLLGS
VANLRSVLVQCETEFQLSKQVQYILAEYFSNITESGISKQHFRSSFIGVGAYYEFFNAVSGNISEVLLASSESSDISLLGDTHP
YWFAYMGEGDSVSFTVPPDIDVKGMILCVVYLSTLEIEATECLRSVLIVNYTNCTLQIYKHSPVISFNDIDWHDIMSNLGP
GDKVEIFVTFGHRLVVKNTILYLICGESNYLRKEPESKMNFLLRFIMKIVMCDF

>XP_027907936.1

MEFASSSSKLPRNYDVLISFNGEDIRRKFVSHLDSALSSVGLTTFLHYQNAVESMHVQQPILNLCRVVIVVFTKTYSQSA
WCLHQLQQIIAWHESYCRHVLPVYYEIQPSDVRLQKGDFGKAFKETAQQTFSAQQLEDGMSRWSHALTKVASLFGW
DESNHRSDAELVGKIVKSVLNLSALTATKFPVGLQDQVEDLIRTIRNKSTDVCTIGICGMGGSGKTTLAKAIYYQIHGTFM
EKSFIEDIAQVSEPRERIHLQQQLLSDVLKTKVEIHSVEMGRSMIRDRLFRKRVLIVLDDIDDYFPLLDLRKSRSWLSEGTVI
IMTARDEDLLRKHQVDSVFRINLMNEKESLELLSWHAFREPKPKKEYDYLARRVISCCGGLPLALELIGSSLFERREKEWH
SVLVGLEGIHMDDVEQKLKISFDLCNQMEKALFLIVCRYYIGKDRSYAMKILNGPVVNADSGIRVLMERNLIKVKANNKF
GIHRLLQQMGVEIFRENLKEEPWKHGRLWFDKGGEYALEVDTGRNASQTFPPILIPSREPSGFPEVVTVSKYIYTKLIWIN
LQGFSSEHLPKDFNMHGAVAIDLKHSLLRFFWKEPQVLRSLKVLNLSHSMYLTETPDFSRLPSLEQLILKDCPRLRKVHRS
IGHLCYLILLNLKDCKCLSNLPREIYKLKSLRTLILSGCSKVGRMGKDIVRMKSLITLIAENTAVKQVPFSIVTSKAIGYMSIPG
FKRLSCNLFPSIIIRSWMSPTMNPVSYIHSLCMDINNSWDDIVPLLGSVANLRSVLVQCETEFQLSKQVQYILAEYFSNITE
SGISKQHFRSSFIGVGAYYEFFNAVSGNISEVLLASSESSDISLLGDTHPYWFAYMGEGDSVSFTVPPDIDVKGMILCVVY
LSTLEIEATECLRSVLIVNYTNCTLQIYKHSPVISFNDIDWHDIMSNLGPGDKVEIFVTFGHRLVVKNTILYLICGESNYLRKE
PESKMNFLLRFIMKIVMCDF

>XP_027907959.1

MASASSSSSFSKFRIEGDVFIHCMGDDIRRNFVSHLSSALLQAGVKPYLLAVDMQREQFVASIEGFQIGIVVFTKTYFESF
RSVDELVRIIECHETHGLMVMPVFYEIDRSDFGNTLKATAREVMKIEHIIWSGEYQKTWFQRWNSALIKAGTLPNWEES
EHRSDAELVEEIVKSVLAKLDCPLCTTKFPVGLEPHVKNVIGLFENQPTKVCMIGIWGMGGSGKTTVAKAIYNQIPYTFG
DKNFIQGIKEVCETDGRDLVHLQEKLPSGSLKTNVTTESGEMKKITTENRLFGKKLFIVLDDVNEIDQLKELRRNDKQFGQ
GSVIIITTRNLNLLYQLNVDYVYEMDKLDENDSLELFSWHAFGEAKPKEDFIKFARSAVAYCGGLPLALEVLGSYLRKRSV
NEWRSVLSKLEIIPNTQVQNILRISFDGLCLMEKDIFLDVCCFFIGKDRDYVTEILNGCGLHADIGMKVLIERGLLKIEKNNE
LGMHCLLRDMGREIVRQTSTMQPGRRRRLWLHKDVLDVLTKNSGTEAIEGLSLNCQFTRSEFVKACAFEKMKRLRFLQ
LDNVQVTGDYGYLSKQLRWIYWQGFPFKYIPNNFYLKEAIVIDFQQSNLRQMWKEPQVLPWLKILNLSHSKFLIETPDF
SKLPSLEKLIVKHCPRLCKVHQSIGDLHNLLLINLKGCSRLWNLPTETYKLKSLKTLILSGCLKIHILEEDILRMESLTTLISEST
VVKQVPFSIVSSKSIGYILLGGNNGLSFTVFNSIISSWISPKINLLSGIRPFRGLSSSLVSMSMENNDLGDLAPILSSDLNILNI

LVQCDAEFQRYQQVIALLDEIRDLNLTEFEIRSTPETSKHPLRRCLIKFGSDQEKVFDILSESILQGSENSASCDVFLPGDKD
PYWLAHMGEAHSVTFTVPEDRHIKGMTLCTVNLYNPRHFTTSEYLISILMVNYTKCTIQIYRRKTVQSFSDVDWQGIISN
LGPGDKVEVFVIFGDKFLVKKTALYLMYHGLIDKEVDPSLDPKNIDLCSTDTDTVTQDDTDADTDTDTVTQDDTDADTD
TGTCTSTSTSTIRHV

>XP_027907971.1

MEFGSSSSSASSSSSFLKSEPVFIYDVFINFGGEDIGRRFVSHLHSSLLQAQVKTFISQENLQEGMKLEEHMGEIAASKIAII
VFSKTYAESTCCLFELEKVIECHQTFGQIVLPIFYEIDPLDVYLQKGDFGKALEEAAHKSYSGEQLEHVLFRWSCALTKAAG
ISGWDLRNFRHDAELVEVTVNRVQKLLDYQGLFITQFPIGLESHVEEVIGCIENHSTKVCMIGIWGMVGSGKTTIAKAIY
NRIYRPFIGKSFIENIGEVWDQVYRTHVDLQENFLYDVLKSKLKLESVGMGRTMIKNELSRRKLLIVLDDVNEFGQLENLC
GNPEWFSQGTVIIITTRDVRLLKRIEVNYVYKMDGMNENDSLELFCCHAFGEARTRNLNEIARNRVSYCGGLPLALEVLG
SFSCKKTMREWESVLSKVKLIPLEVEKKLKLSFDGLNKEEMDIFLDVCCFFIGKERGYVTEILNGCELHADIGITVLIECGLIK
FGRNNKLEMHPLFRDMGREYICQRFLKEPWKRKRLGFQGDAKYVLKKNIGTEATEGLFLKMHSTSRDYFEAHAFKKMK
RLRLLQLDHVQLAGDYGYLSKQLRWICWKGFPSKYIPNNFHMENLIAMDLKYSNLLLVWKQSQVLEQLKFLNLSHSKYL
RETPDFSGLPSLERLILKDCPCLCKVHPSIGDLCNLQLINLKDCASLSNIPREVYKLKSLKSFILSGCFKIEILEEDIVQMKSLITL
VTENTAVRRVPCSVVSSKSIGYISLRGFEGLSQNLFPSIIRSWMRPMVNPQSYFSPFCMDMDNNNWRDLAPLHSGLAN
IRSLLVQCDTTFQLSEQVKTILVEYSLNFTEQRISNHYLRFSLIGVGSYSEFLNTLSDSISKGLASSESCDVCLPGDNHPYWL
AHIGEGRSVSFTVPQERDLKRMALCVVYLSAPEFMANECFQSVLVVNYTKCTLQIHNHGTVISFNDRDWEGIISNLGSG
DKVEFFVTFGHGLVVKNTAIYLNIW

>XP_027907972.1

MEFGSSSSSASSSSSFLKSEPVFIYDVFINFGGEDIGRRFVSHLHSSLLQAQVKTFISQENLQEGMKLEEHMGEIAASKIAII
VFSKTYAESTCCLFELEKKGDFGKALEEAAHKSYSGEQLEHVLFRWSCALTKAAGISGWDLRNFRHDAELVEVTVNRVQ
KLLDYQGLFITQFPIGLESHVEEVIGCIENHSTKVCMIGIWGMVGSGKTTIAKAIYNRIYRPFIGKSFIENIGEVWDQVYRT
HVDLQENFLYDVLKSKLKLESVGMGRTMIKNELSRRKLLIVLDDVNEFGQLENLCGNPEWFSQGTVIIITTRDVRLLKRIE
VNYVYKMDGMNENDSLELFCCHAFGEARTRNLNEIARNRVSYCGGLPLALEVLGSFSCKKTMREWESVLSKVKLIPLEV
EKKLKLSFDGLNKEEMDIFLDVCCFFIGKERGYVTEILNGCELHADIGITVLIECGLIKFGRNNKLEMHPLFRDMGREYICQ
RFLKEPWKRKRLGFQGDAKYVLKKNIGTEATEGLFLKMHSTSRDYFEAHAFKKMKRLRLLQLDHVQLAGDYGYLSKQLR
WICWKGFPSKYIPNNFHMENLIAMDLKYSNLLLVWKQSQVLEQLKFLNLSHSKYLRETPDFSGLPSLERLILKDCPCLCKV
HPSIGDLCNLQLINLKDCASLSNIPREVYKLKSLKSFILSGCFKIEILEEDIVQMKSLITLVTENTAVRRVPCSVVSSKSIGYISL
RGFEGLSQNLFPSIIRSWMRPMVNPQSYFSPFCMDMDNNNWRDLAPLHSGLANIRSLLVQCDTTFQLSEQVKTILVEY
SLNFTEQRISNHYLRFSLIGVGSYSEFLNTLSDSISKGLASSESCDVCLPGDNHPYWLAHIGEGRSVSFTVPQERDLKRMA
LCVVYLSAPEFMANECFQSVLVVNYTKCTLQIHNHGTVISFNDRDWEGIISNLGSGDKVEFFVTFGHGLVVKNTAIYLNI
W

>XP_027908023.1

MEFGSSSSSASSSSSFLKSEPVFIYDVFINFGGEDIGRRFVSHLHSSLLQAQVKTFISQENLQEGMKLEEHMGEIAASKIAII
VFSKTYAESTCCLFELEKVIECHQTFGQIVLPIFYEIDPLDVYLQKGDFGKALEEAAHKSYSGEQLEHVLFRWSCALTKAAG
ISGWDLRNFRHDAELVEVTVNRVQKLLDYQGLFITQFPIGLESHVEEVIGCIENHSTKVCMIGIWGMVGSGKTTIAKAIY
NRIYRPFIGKSFIENIGEVWDQVYRTHVDLQENFLYDVLKSKLKLESVGMGRTMIKNELSRRKLLIVLDDVNEFGQLENLC
GNPEWFSQGTVIIITTRDVRLLKRIEVNYVYKMDGMNENDSLELFCCHAFGEARTRNLNEIARNRVSYCGGLPLALEVLG
SFSCKKTMREWESVLSKVKLIPLEVEKKLKLSFDGLNKEEMDIFLDVCCFFIGKERGYVTEILNGCELHADIGITVLIECGLIK
FGRNNKLEMHPLFRDMGREYICQRFLKEPWKRKRLGFQGDAKYVLKKNIGTEATEGLFLKMHSTSRDYFEAHAFKKMK
RLRLLQLDHVQLAGDYGYLSKQLRWICWKGFPSKYIPNNFHMENLIAMDLKYSNLLLVWKQSQVLEQLKFLNLSHSKYL

RETPDFSGLPSLERLILKDCPCLCKVHPSIGDLCNLQLINLKDCASLSNIPREVYKLKSLKSFILSGCFKIEILEEDIVQMKSLITL
VTENTAVRRVPCSVVSSKSIGYISLRGFEGLSQNLFPSIIRSWMRPMVNPQSYFSPFCMDMDNNNWRDLAPLHSGLAN
IRSLLVQCDTTFQLSEQVKTILVEYSLNFTEQRISNHYLRFSLIGVGSYSEFLNTLSDSISKGLASSESCDVCLPGDNHPYWL
AHIGEGRSVSFTVPQERDLKRMALCVVYLSAPEFMANECFQSVLVVNYTKCTLQIHNHGTVISFNDRDWEGIISNLGSG
DKVEFFVTFGHGLVVKNTAIYLNIW

>XP_027908025.1

MEFGSSSSSASSSSSFLKSEPVFIYDVFINFGGEDIGRRFVSHLHSSLLQAQVKTFISQENLQEGMKLEEHMGEIAASKIAII
VFSKTYAESTCCLFELEKKGDFGKALEEAAHKSYSGEQLEHVLFRWSCALTKAAGISGWDLRNFRHDAELVEVTVNRVQ
KLLDYQGLFITQFPIGLESHVEEVIGCIENHSTKVCMIGIWGMVGSGKTTIAKAIYNRIYRPFIGKSFIENIGEVWDQVYRT
HVDLQENFLYDVLKSKLKLESVGMGRTMIKNELSRRKLLIVLDDVNEFGQLENLCGNPEWFSQGTVIIITTRDVRLLKRIE
VNYVYKMDGMNENDSLELFCCHAFGEARTRNLNEIARNRVSYCGGLPLALEVLGSFSCKKTMREWESVLSKVKLIPLEV
EKKLKLSFDGLNKEEMDIFLDVCCFFIGKERGYVTEILNGCELHADIGITVLIECGLIKFGRNNKLEMHPLFRDMGREYICQ
RFLKEPWKRKRLGFQGDAKYVLKKNIGTEATEGLFLKMHSTSRDYFEAHAFKKMKRLRLLQLDHVQLAGDYGYLSKQLR
WICWKGFPSKYIPNNFHMENLIAMDLKYSNLLLVWKQSQVLEQLKFLNLSHSKYLRETPDFSGLPSLERLILKDCPCLCKV
HPSIGDLCNLQLINLKDCASLSNIPREVYKLKSLKSFILSGCFKIEILEEDIVQMKSLITLVTENTAVRRVPCSVVSSKSIGYISL
RGFEGLSQNLFPSIIRSWMRPMVNPQSYFSPFCMDMDNNNWRDLAPLHSGLANIRSLLVQCDTTFQLSEQVKTILVEY
SLNFTEQRISNHYLRFSLIGVGSYSEFLNTLSDSISKGLASSESCDVCLPGDNHPYWLAHIGEGRSVSFTVPQERDLKRMA
LCVVYLSAPEFMANECFQSVLVVNYTKCTLQIHNHGTVISFNDRDWEGIISNLGSGDKVEFFVTFGHGLVVKNTAIYLNI
W

>XP_027908038.1

MASSIPPTEFASSTSKLPRKYDVLINFTGEDIRRKFVSHLDYALSTVGLTTFLHEENAVNDMHIQQPILNLCRVAIVVFTKT
YSQSAWCLHQLQQIIKWQETYSRHVFPVYYEIQPSDVRFQKGDFGETFKATAQKTFSGQQLEHGMSRWSHALTKAAN
LFGWDESNYRSDAELVDKIVKGVLNLPVFSATKFPVGLQSCVKDVIQIIKNKSREVCIIGIWGEGGSGKTTLAKAIYHQLH
GTFTQKSFIEDIAQVIQTRGHVHLQEQLLSDVLNTKMEIRSVEMGKKMIRVKLSGKKLLIVLDNTKYDPLLDLYDSHVWF
AKGTVILITAREEHLLRIHQVDSIFRMNLLSTNESLELLSWHAFREEKPKEEYNDLAKRVVVYCGGLPLVLEVIGSCLYERTK
EEWNRLLLQLDKSPQHEVSQTLKISYEGLLNQTEKDLFLDVCCFFVGKGRTFVTKILNDCGVDADSGIRILIERNLIQVKKN
NKLGMQPLLQKMGRKIIREISGKELGKNARLWFGQDAENDLLENTLFSSQQTKVIQRLSLKMFLIATRELLERYPSMVR
DTSRLLRLSRDFGKLRWISLHGFSSENLPKDIYLHDAIAIDLKNSFLRFVWKEPQVLRSLRVLNLSHSMYLRETPDFSGLPR
LEQLILKDCPSLRKVHHSIGCLNNLVLLNLKDCTSLSNLPRELYKLKSLNTLILSGCSKIDLLEKYIVQMESLIILIAENAAVKQ
VPISIVSSKNIGYIFLRGCEGFSFNLFPSIIRSWISPIMNPLSYIHSICMDIEDNGWNDFAPLLSTLANLRSVSVQCDTEFQLS
KLVETILIEYGVNISKSEISQQHFKYSLIGVGRCKDFFNAVSDIISKVFASNESRDVSLPGDNDPYWFGHMGEGRSVFFTV
PRDHDLKGMALCVFYLSTPEIVAPVCLRSVLIVNYTKCTFQIHNHGTVISFNDIDWQGIISNLESGDKVEICVTSAHELVVK
NTIVYLICDELNDLQKEPAPKKNSLVRFVKKVVM

>XP_027908039.1

MASSIPPTEFASSTSKLPRKYDVLINFTGEDIRRKFVSHLDYALSTVGLTTFLHEENAVNDMHIQQPILNLCRVAIVVFTKT
YSQSAWCLHQLQQIIKWQETYSRHVFPVYYEIQPSDVRFQKGDFGETFKATAQKTFSGQQLEHGMSRWSHALTKAAN
LFGWDESNYRSDAELVDKIVKGVLNLPVFSATKFPVGLQSCVKDVIQIIKNKSREVCIIGIWGEGGSGKTTLAKAIYHQLH
GTFTQKSFIEDIAQVIQTRGHVHLQEQLLSDVLNTKMEIRSVEMGKKMIRVKLSGKKLLIVLDNTKYDPLLDLYDSHVWF
AKGTVILITAREEHLLRIHQVDSIFRMNLLSTNESLELLSWHAFREEKPKEEYNDLAKRVVVYCGGLPLVLEVIGSCLYERTK
EEWNRLLLQLDKSPQHEVSQTLKISYEGLLNQTEKDLFLDVCCFFVGKGRTFVTKILNDCGVDADSGIRILIERNLIQVKKN
NKLGMQPLLQKMGRKIIREISGKELGKNARLWFGQDAENDLLENTLFSSQQTKVIQRLSLKMFLIATRELLERYPSMVLR

SLRVLNLSHSMYLRETPDFSGLPRLEQLILKDCPSLRKVHHSIGCLNNLVLLNLKDCTSLSNLPRELYKLKSLNTLILSGCSKI
DLLEKYIVQMESLIILIAENAAVKQVPISIVSSKNIGYIFLRGCEGFSFNLFPSIIRSWISPIMNPLSYIHSICMDIEDNGWNDF
APLLSTLANLRSVSVQCDTEFQLSKLVETILIEYGVNISKSEISQQHFKYSLIGVGRCKDFFNAVSDIISKVFASNESRDVSLP
GDNDPYWFGHMGEGRSVFFTVPRDHDLKGMALCVFYLSTPEIVAPVCLRSVLIVNYTKCTFQIHNHGTVISFNDIDWQ
GIISNLESGDKVEICVTSAHELVVKNTIVYLICDELNDLQKEPAPKKNSLVRFVKKVVM

>XP_027908040.1

MASSIPPTEFASSTSKLPRKYDVLINFTGEDIRRKFVSHLDYALSTVGLTTFLHEENAVNDMHIQQPILNLCRVAIVVFTKT
YSQSAWCLHQLQQIIKWQETYSRHVFPVYYEIQPSDVRFQKGDFGETFKATAQKTFSGQQLEHGMSRWSHALTKAAN
LFGWDESNYRSDAELVDKIVKGVLNLPVFSATKFPVGLQSCVKDVIQIIKNKSREVCIIGIWGEGGSGKTTLAKAIYHQLH
GTFTQKSFIEDIAQVIQTRGHVHLQEQLLSDVLNTKMEIRSVEMGKKMIRVKLSGKKLLIVLDNTKYDPLLDLYDSHVWF
AKGTVILITAREEHLLRIHQVDSIFRMNLLSTNESLELLSWHAFREEKPKEEYNDLAKRVVVYCGGLPLVLEVIGSCLYERTK
EEWNRLLLQLDKSPQHEVSQTLKISYEGLLNQTEKDLFLDVCCFFVGKGRTFVTKILNDCGVDADSGIRILIERNLIQVKKN
NKLGMQPLLQKMGRKIIREISGKELGKNARLWFGQDAENDLLENTVLRSLRVLNLSHSMYLRETPDFSGLPRLEQLILKD
CPSLRKVHHSIGCLNNLVLLNLKDCTSLSNLPRELYKLKSLNTLILSGCSKIDLLEKYIVQMESLIILIAENAAVKQVPISIVSSK
NIGYIFLRGCEGFSFNLFPSIIRSWISPIMNPLSYIHSICMDIEDNGWNDFAPLLSTLANLRSVSVQCDTEFQLSKLVETILIE
YGVNISKSEISQQHFKYSLIGVGRCKDFFNAVSDIISKVFASNESRDVSLPGDNDPYWFGHMGEGRSVFFTVPRDHDLK
GMALCVFYLSTPEIVAPVCLRSVLIVNYTKCTFQIHNHGTVISFNDIDWQGIISNLESGDKVEICVTSAHELVVKNTIVYLIC
DELNDLQKEPAPKKNSLVRFVKKVVM

>XP_027908041.1

MASSIPPTEFASSTSKLPRKYDVLINFTGEDIRRKFVSHLDYALSTVGLTTFLHEENAVNDMHIQQPILNLCRVAIVVFTKT
YSQSAWCLHQLQQIIKWQETYSRHVFPVYYEIQPSDVRFQKGDFGETFKATAQKTFSGQQLEHGMSRWSHALTKAAN
LFGWDESNYRSDAELVDKIVKGVLNLPVFSATKFPVGLQSCVKDVIQIIKNKSREVCIIGIWGEGGSGKTTLAKAIYHQLH
GTFTQKSFIEDIAQVIQTRGHVHLQEQLLSDVLNTKMEIRSVEMGKKMIRVKLSGKKLLIVLDNTKYDPLLDLYDSHVWF
AKGTVILITAREEHLLRIHQVDSIFRMNLLSTNESLELLSWHAFREEKPKEEYNDLAKRVVVYCGGLPLVLEVIGSCLYERTK
EEWNRLLLQLDKSPQHEVSQTLKISYEGLLNQTEKDLFLDVCCFFVGKGRTFVTKILNDCGVDADSGIRILIERNLIQVKKN
NKLGMQPLLQKMGRKIIREISGKELGKNARLWFGQDAENDLLENTLFSSQQTKVIQRLSLKMFLIATRELLERYPSMVR
DTSRLLRLSRDFGKLRWISLHGFSSENLPKDIYLHDAIAIDLKNSFLRFVWKEPQVLRSLRVLNLSHSMYLRETPDFSGLPR
LEQLILKDCPSLRKVHHSIGCLNNLVLLNLKDCTSLSNLPRELYKLKSLNTLILSGCSKIDLLEKYIVQMESLIILIAENAAVKQ
VPISIVSSKNIGYIFLRGCEGFSFNLFPSIIRSWISPIMNPLSYIHSICMDIEDNGWNDFAPLLSTLANLRSVSVQCDTEFQLS
KLVETILIEYGVNISKSEISQQHFKYSLIGVGRCKDFFNAVSDIISKVIMILIGSATWVRVVLFFSPCLEIMT

>XP_027908214.1

MFSQPTCMSLNDKMANIAPDLAKSILEKLINATLEQSRYIFCLTCITEDFEKERENLIAKEETVKERVRAANRRGDKIQRD
VVLWQKQAKDLLEEDTKKKVTCFFGWCPNCKRQYSRGKELESKTKEIKRLIMESNFETVGIIRDVPGTEYHSSQNYISFKS
RESKIEELCNALRDHNKYMIGLQGMGGTGKTTLAKEMGKKLKQSNIFDQVVDTTVSNTPNTKKIQDEIAGPLGLSLENC
TESERPKRLWNRLTDGEKILVILDDVWGDISFEEIGIPYKDNHKGCRILVTTRDVNICHKMECEETIQLDILPEEDVWKLF
QKHAGLNDSYPKSVLDTGRKISKECKGLPIAIVVIAGSLKGKRHLEQWDVALKSLQNFKSVAGDDENKRKIYSCLKYSYD
NMQNKTAKKLFLLCSLFREDEEIYEELLVRLAIGGGLIEKNNDDYNYDAYRKEVIAAKYELIDSCLLLNCASRKVKMHDLVR
EVALWIANKEILAVNTSKKNEMTMVDKGKHIEYLLCEGRSMDVFSLKFDASKLDILIVYLNGNFHAEVPNSFLENMISLR
VLYLSNMTYGASKATLSLPQSIQLLTNLKSLYLESFILGDISIFGNLHGLETLELVTCQLDKLPREIIKLVKLKLLMLSYCDVVA
YDFFEIIVRCTSLEELYFVHRDFSETCHSVKLPNYQRFCISEYFNSSRSEWFLWSSIELSEADSVFSEETFKNLVQKAEIVSLK
RLQGVWKNLIPEIVSPDDEGMTNLVEIGLKDISTMRCLIDNTDSLSQSVLSRLVNLELRTMENLKEICNGPLPFELLKNLEY

FILYDCMHLEGSLFKSKISLCHLKQLYIEGCPMLTSLFEWSTTQSLLLLVELHISNCEQLKSIVRDENRRKDSIEEIVDDSIISR
FPNLTTLEIWECPQLLFIFPMAFTRNIPKLERINVFRCDGLKYLFGPYQHKHEEEDLYEELKDVIFTNLELMILEDLPNFVDI
FPKCGESKCLPVKRSTSKDETKAQIESNPMKCKILHWIDKCRIKRGTTKIPLGSKNQLQDSSLSPVNESNANDVIPQDMSL
TLTNIGIDVSSNIFVTLQNFTELTIEGYVKAKVLFCASMLECFPYLHTLVISGCNELEQIIGEDTKNQRKPFFPRLKLLAINKC
NKLKCIFPISTSKMLPNLEVLIIIQACMLEEVFKGNSDEKVEIPNLKIAVFAELPSLRQEIQFLTVKHCIVKNCPKLSLSSSLQS
SHDFRTSIAGSLGGDIPFILDKVLGELDEMIRNENARTEDSASGIEVGTTLLEGSELTSSHNNDEDKKHHENGIESKELEDA
VKQSRERVEEEQQILGKAPPFGISSMPISQENEEGQNMEPATNKDVNDGDFEGTSNTVVIHSESSNAPGSEVTNQHIK
NILQETMDDLHEDPKTRESNQNEMTKVTEDFPSDTKPQVASGSELTSSQYLEERVEQRNEESPLSEKSLKSTLSSYPKIRE
TPREPVLTPPQKESKRKFEEGTTSNLAETITLSTDSESEHGAGQISLPSFSNVGVGDIQKASESTIALFETKNSPENLSQITE
DSTFTSVIRRELEKLVSNKHLALENQPLLVDFLVKHPSVRLTDNTVSDRYKGFAYTCLAELLKFLQTHSVLDVLGSSHSEFV
ELLQDMRRFSFDRVWLDGVERRALFPGLLLSQDALQKLSHSKNTLIQHLEEVKDQLELSITEQEEQVLRVKATLSTPLGY

>XP_027908215.1

MFSQPTCMSLNDKMANIAPDLAKSILEKLINATLEQSRYIFCLTCITEDFEKERENLIAKEETVKERVRAANRRGDKIQRD
VVLWQKQAKDLLEEDTKKKVTCFFGWCPNCKRQYSRGKELESKTKEIKRLIMESNFETVGIIRDVPGTEYHSSQNYISFKS
RESKIEELCNALRDHNKYMIGLQGMGGTGKTTLAKEMGKKLKQSNIFDQVVDTTVSNTPNTKKIQDEIAGPLGLSLENC
TESERPKRLWNRLTDGEKILVILDDVWGDISFEEIGIPYKDNHKGCRILVTTRDVNICHKMECEETIQLDILPEEDVWKLF
QKHAGLNDSYPKSVLDTGRKISKECKGLPIAIVVIAGSLKGKRHLEQWDVALKSLQNFKSVAGDDENKRKIYSCLKYSYD
NMQNKTAKKLFLLCSLFREDEEIYEELLVRLAIGGGLIEKNNDDYNYDAYRKEVIAAKYELIDSCLLLNCASRKVKMHDLVR
EVALWIANKEILAVNTSKKNEMTMVDKGKHIEYLLCEGRSMDVFSLKFDASKLDILIVYLNGNFHAEVPNSFLENMISLR
VLYLSNMTYGASKATLSLPQSIQLLTNLKSLYLESFILGDISIFGNLHGLETLELVTCQLDKLPREIIKLVKLKLLMLSYCDVVA
YDFFEIIVRCTSLEELYFVHRDFSETCHSVKLPNYQRFCISEYFNSSRSEWFLWSSIELSEADSVFSEETFKNLVQKAEIVSLK
RLQGVWKNLIPEIVSPDDEGMTNLVEIGLKDISTMRCLIDNTDSLSQSVLSRLVNLELRTMENLKEICNGPLPFELLKNLEY
FILYDCMHLEGSLFKSKISLCHLKQLYIEGCPMLTSLFEWSTTQSLLLLVELHISNCEQLKSIVRDENRRKDSIEEIVDDSIISR
FPNLTTLEIWECPQLLFIFPMAFTRNIPKLERINVFRCDGLKYLFGPYQHKHEEEDLYEELKDVIFTNLELMILEDLPNFVDI
FPKCGESKCLPVKRSTSKDETKAQIESNPMKCKILHWIDKCRIKRGTTKIPLGSKNQLQDSSLSPVNESNANDVIPQDMSL
TLTNIGIDVSSNIFVTLQNFTELTIEGYVKAKVLFCASMLECFPYLHTLVISGCNELEQIIGEDTKNQRKPFFPRLKLLAINKC
NKLKCIFPISTSKMLPNLEVLIIIQACMLEEVFKGNSDEKVEIPNLKIAVFAELPSLRQEIQFLTVKHCIVKNCPKLSLSSSLQS
SHDFRTSIAGSLGGDIPFILDKVLGELDEMIRNENARTEDSASGIEVGTTLLEGSELTSSHNNDEDKKHHENGIESKELEDA
VKQSRERVEEEQQILGKAPPFGISSMPISQENEEGQNMEPATNKDVNDGDFEGTSNTVVIHSESSNAPGSEVTNQHIK
NILQETMDDLHEDPKTRESNQNEMTKVTEDFPSDTKPQVASGSELTSSQYLEERVEQRNEESPLSEKSLKSTPREPVLTP
PQKESKRKFEEGTTSNLAETITLSTDSESEHGAGQISLPSFSNVGVGDIQKASESTIALFETKNSPENLSQITEDSTFTSVIRR
ELEKLVSNKHLALENQPLLVDFLVKHPSVRLTDNTVSDRYKGFAYTCLAELLKFLQTHSVLDVLGSSHSEFVELLQDMRRF
SFDRVWLDGVERRALFPGLLLSQDALQKLSHSKNTLIQHLEEVKDQLELSITEQEEQVLRVKATLSTPLGY

>XP_027908216.1

MANIAPDLAKSILEKLINATLEQSRYIFCLTCITEDFEKERENLIAKEETVKERVRAANRRGDKIQRDVVLWQKQAKDLLEE
DTKKKVTCFFGWCPNCKRQYSRGKELESKTKEIKRLIMESNFETVGIIRDVPGTEYHSSQNYISFKSRESKIEELCNALRDH
NKYMIGLQGMGGTGKTTLAKEMGKKLKQSNIFDQVVDTTVSNTPNTKKIQDEIAGPLGLSLENCTESERPKRLWNRLT
DGEKILVILDDVWGDISFEEIGIPYKDNHKGCRILVTTRDVNICHKMECEETIQLDILPEEDVWKLFQKHAGLNDSYPKSV
LDTGRKISKECKGLPIAIVVIAGSLKGKRHLEQWDVALKSLQNFKSVAGDDENKRKIYSCLKYSYDNMQNKTAKKLFLLCS
LFREDEEIYEELLVRLAIGGGLIEKNNDDYNYDAYRKEVIAAKYELIDSCLLLNCASRKVKMHDLVREVALWIANKEILAVN
TSKKNEMTMVDKGKHIEYLLCEGRSMDVFSLKFDASKLDILIVYLNGNFHAEVPNSFLENMISLRVLYLSNMTYGASKAT
LSLPQSIQLLTNLKSLYLESFILGDISIFGNLHGLETLELVTCQLDKLPREIIKLVKLKLLMLSYCDVVAYDFFEIIVRCTSLEELY

FVHRDFSETCHSVKLPNYQRFCISEYFNSSRSEWFLWSSIELSEADSVFSEETFKNLVQKAEIVSLKRLQGVWKNLIPEIVS
PDDEGMTNLVEIGLKDISTMRCLIDNTDSLSQSVLSRLVNLELRTMENLKEICNGPLPFELLKNLEYFILYDCMHLEGSLFK
SKISLCHLKQLYIEGCPMLTSLFEWSTTQSLLLLVELHISNCEQLKSIVRDENRRKDSIEEIVDDSIISRFPNLTTLEIWECPQL
LFIFPMAFTRNIPKLERINVFRCDGLKYLFGPYQHKHEEEDLYEELKDVIFTNLELMILEDLPNFVDIFPKCGESKCLPVKRS
TSKDETKAQIESNPMKCKILHWIDKCRIKRGTTKIPLGSKNQLQDSSLSPVNESNANDVIPQDMSLTLTNIGIDVSSNIFVT
LQNFTELTIEGYVKAKVLFCASMLECFPYLHTLVISGCNELEQIIGEDTKNQRKPFFPRLKLLAINKCNKLKCIFPISTSKMLP
NLEVLIIIQACMLEEVFKGNSDEKVEIPNLKIAVFAELPSLRQEIQFLTVKHCIVKNCPKLSLSSSLQSSHDFRTSIAGSLGGDI
PFILDKVLGELDEMIRNENARTEDSASGIEVGTTLLEGSELTSSHNNDEDKKHHENGIESKELEDAVKQSRERVEEEQQIL
GKAPPFGISSMPISQENEEGQNMEPATNKDVNDGDFEGTSNTVVIHSESSNAPGSEVTNQHIKNILQETMDDLHEDPK
TRESNQNEMTKVTEDFPSDTKPQVASGSELTSSQYLEERVEQRNEESPLSEKSLKSTLSSYPKIRETPREPVLTPPQKESKR
KFEEGTTSNLAETITLSTDSESEHGAGQISLPSFSNVGVGDIQKASESTIALFETKNSPENLSQITEDSTFTSVIRRELEKLVS
NKHLALENQPLLVDFLVKHPSVRLTDNTVSDRYKGFAYTCLAELLKFLQTHSVLDVLGSSHSEFVELLQDMRRFSFDRVW
LDGVERRALFPGLLLSQDALQKLSHSKNTLIQHLEEVKDQLELSITEQEEQVLRVKATLSTPLGY

>XP_027908217.1

MANIAPDLAKSILEKLINATLEQSRYIFCLTCITEDFEKERENLIAKEETVKERVRAANRRGDKIQRDVVLWQKQAKDLLEE
DTKKKVTCFFGWCPNCKRQYSRGKELESKTKEIKRLIMESNFETVGIIRDVPGTEYHSSQNYISFKSRESKIEELCNALRDH
NKYMIGLQGMGGTGKTTLAKEMGKKLKQSNIFDQVVDTTVSNTPNTKKIQDEIAGPLGLSLENCTESERPKRLWNRLT
DGEKILVILDDVWGDISFEEIGIPYKDNHKGCRILVTTRDVNICHKMECEETIQLDILPEEDVWKLFQKHAGLNDSYPKSV
LDTGRKISKECKGLPIAIVVIAGSLKGKRHLEQWDVALKSLQNFKSVAGDDENKRKIYSCLKYSYDNMQNKTAKKLFLLCS
LFREDEEIYEELLVRLAIGGGLIEKNNDDYNYDAYRKEVIAAKYELIDSCLLLNCASRKVKMHDLVREVALWIANKEILAVN
TSKKNEMTMVDKGKHIEYLLCEGRSMDVFSLKFDASKLDILIVYLNGNFHAEVPNSFLENMISLRVLYLSNMTYGASKAT
LSLPQSIQLLTNLKSLYLESFILGDISIFGNLHGLETLELVTCQLDKLPREIIKLVKLKLLMLSYCDVVAYDFFEIIVRCTSLEELY
FVHRDFSETCHSVKLPNYQRFCISEYFNSSRSEWFLWSSIELSEADSVFSEETFKNLVQKAEIVSLKRLQGVWKNLIPEIVS
PDDEGMTNLVEIGLKDISTMRCLIDNTDSLSQSVLSRLVNLELRTMENLKEICNGPLPFELLKNLEYFILYDCMHLEGSLFK
SKISLCHLKQLYIEGCPMLTSLFEWSTTQSLLLLVELHISNCEQLKSIVRDENRRKDSIEEIVDDSIISRFPNLTTLEIWECPQL
LFIFPMAFTRNIPKLERINVFRCDGLKYLFGPYQHKHEEEDLYEELKDVIFTNLELMILEDLPNFVDIFPKCGESKCLPVKRS
TSKDETKAQIESNPMKCKILHWIDKCRIKRGTTKIPLGSKNQLQDSSLSPVNESNANDVIPQDMSLTLTNIGIDVSSNIFVT
LQNFTELTIEGYVKAKVLFCASMLECFPYLHTLVISGCNELEQIIGEDTKNQRKPFFPRLKLLAINKCNKLKCIFPISTSKMLP
NLEVLIIIQACMLEEVFKGNSDEKVEIPNLKIAVFAELPSLRQEIQFLTVKHCIVKNCPKLSLSSSLQSSHDFRTSIAGSLGGDI
PFILDKVLGELDEMIRNENARTEDSASGIEVGTTLLEGSELTSSHNNDEDKKHHENGIESKELEDAVKQSRERVEEEQQIL
GKAPPFGISSMPISQENEEGQNMEPATNKDVNDGDFEGTSNTVVIHSESSNAPGSEVTNQHIKNILQETMDDLHEDPK
TRESNQNEMTKVTEDFPSDTKPQVASGSELTSSQYLEERVEQRNEESPLSEKSLKSTLSSYPKIRETPREPVLTPPQKESKR
KFEEGTTSNLAETITLSTDSESEHGAGQISLPSFSNVGVGDIQKASESTIALFETKNSPENLSQITEDSTFTSVIRRELEKLVS
NKHLALENQPLLVDFLVKHPSVRLTDNTVSDRYKGFAYTCLAELLKFLQTHSVLDVLGSSHSEFVELLQDMRRFSFDRVW
LDGVERRALFPGLLLSQDALQKLSHSKNTLIQHLEEVKDQLELSITEQEEQVLRVKATLSTPLGY

>XP_027908282.1

MSYTSSSSKSKPQWVYDVFISFRGEDTRKNFVSHLYSALTNAGVNTFLDDKKLAKGQQLKPELLHSIEGSQISIVVFSENYI
FSKWCLDELVKIMECHAFRGQVVLPVFYDVFPSFLRNLNDVSFEEDSDLNRVKQWKKALSEAAGFVGWDVSNYRNEN
FVVKEMVTAVLERLDRTYMSITDFPVGLEYRVEHCIGFLRKQTKGAYILGIWGMGGIGKTTIAKAIYNEIRYEFKHKSFLA
NIREVWQRDFGRIDLQERLLSDILKTEKVKVYSIDWGKAMIKETLCTKRVLVVLDDVNTFEQLNALSANSNGIAQGSVIII
TTRDVRLLNMLDVEHVYEVEEMNEIESLELFSWHAFKEANPPEGFLELSKQVVTYCGALPLALEILGSYLYKRRVKEWQS
VLSKLKEIPNDKIQEKLKISYDGLTDHTEKDIFLDVCCFFIGKDRGYVTEILNGCGLHAEIGITVLVERSLIKIEKNNKLGIHDL

LRDMGREIVRGSSPLEPQKRSRLWVHDDVLDILTEHTGTGVIEGLALKMQRTSGVCFGTETFEKMKRLRLLQLDHVQLA
GDYGRLPKQLRWVHWKAFSLTHMPENFYQENIVAIDLKYSYLKLVWKVPPFLERLKFLNLSHSRYLSKTPDFSKLPNLEK
LILKDCPSLYEVHHSIGDLNNLLLLNLKDCTCLGNLPMVIYKLKSLQTLILSGCSNIDKLEEAIGKMESLTTLIADNTSIKQVP
FAIVRSKKIGYISLCGYEGLTRNVFPSLIWSWMSHTRGALSCIQPFGITPTSIVALDIQDTNLVNLLSKLSEFSKLRSISVQCD
SDFQLTQELRSILHELCNVNSSELENAYLSPISENSMVSCLIGMGSYQQVFDMLSNSISEVLRTNSSNDFVLPGDNYPYW
LAYTGEGHSVPFQVPEDGDCRMNGMLLCIVYSSTPENMATQALTNVFIFNYTKCTIEIFKHATTMLFSDEDWQGVISSL
GPGDNVEIFVGVGDGITAKKTAVYLIYGQSITMRLESLGLNAQESPELSVILSPKMSAQPTTDVEMEAKKPKKNTFAKIRK
KVRVCSCLK

>XP_027908286.1

MTSPIPSTEVASSTTKLPRKYDVLINFNGEEICRKFVSHLDSVLSDVGLTTFLHHDNGVKPMHEPIPNLCRVAIVVFTKTYS
QSAWCLHQLQQIIEWHETYCKHVLPVYYEIQPSDIRLQKGDFGKAFETTAHQTFSAQQLEHGMSRWSHALTKAANFF
GWDESNHRSDAELVDKIVKSVLNLPILSATKFPVGLQSNVEDLIRTIKDKSSEVCIIGITGAGGSGKTTLAKSIYNKIHGTFK
KKSFIEDIGQVSRTIGHRRLEEQFISDVLKTEVEIPSDYMESRTIREILTGKKVLIVLDDVTEDFTLLDLWECREWLSGGTVIII
TTRDIDLPRILKVDTVFGIKLMNANESLELLSWHAFREPKPKEEYNDLAKSVVTHCGGLPLALEVIGNCLFKREKEEWNSV
LLKLEQIPLHNVQQKLKISFDGLRNQIEKYLFLDICCFFVGEGRACVTKILNGCEVDADSGIRVLIECSLIKIKKNNKLGMHP
LLQEMGREIIREISRKESWMNNRLWFDDAEYVLTDNLGREGVLKLRSVRREPSRLLKHTGNSDYLSKKLKWISLDRFSSK
YLSGDFYLHDAIVIDLKHTDLKHDDPRLVWKEPQALRWLKVLNLSHSMYFTETPDFSGLPSLEQLILKDCTRLRNVHRSIR
CLCNLTLLNLKDCTSLSNLPIEIYKLQSLKTLILSGCSMIDLLEKDVVQMGSLITLIAENNAVKHVPFSILSSKSIGHISLRGFEK
LPLNLFPSIIRSWMSPIMNPVSYIHSLCMDINNNWDDIAPLLSSLTNLRSVLVQCDIEFQLSKLVKSIMVEYFTNFTESGIS
KQQFRSSLIGLGTYHEFFNAVSDNISEVLANSESCDVSLPSDNLPYWLAYMGEGNSVSFTVPWDRDMKGMALSVVYLS
TPEIVATECLRSVLIVNYTKCTLQIHNYDTIISFNDIDWQCIMSNLGPKDKVEIFVTFGHELVVKNTILYLICGESNYLKKEIES
KKNCLLRFITKIVMCDFW

>XP_027908293.1

MALAVVGGALLSAFVDVLFERLASPEVVNLIRGKKPDKLLQKVENQLIVVRVVLADAENRQITDPNVKKWLDVLKDLVY
EVDDLLDEVSTKAAAQKEVRNSFSRLFNKRKIVSISKLEDIVERLDDILKQKESLDLKEIPVERNQPWKAQPTSLEDGYGM
YGRDKDKDAIMKMVLEDGTDGEQVSVIPIVGMGGVGKTTLARSVFNDDKLKQQIFDLKAWVCVSDIFDMVKVTKTMI
EEITKKPCKLSDLNALQLELTDRMKGKRFLIVLDDVWIEDCDNWCSLTKPFLSGISGSKVLVTTRNENVAAIVPLRTVKVY
HLDKLSNEDCWLVFANHAFPLPEASEKRGTLEKIGKEIVKKCNGLPLAAQSLGGMLRRKEAIRDWNNVLESDIWELPES
QCKIIPALRISYHYLPPQLKRCFVYCSLYPKDCQFKKDELIQLWMAEDLVKAAKKGKTLEEVGQEYFDDLVSRCFFQSTSR
SCGDYFVMHDLIHDLATFLGGEFYFRANELGKETKIDRKTRHLSFTRFSDPVSDIEVFDTLKFPRTFLLINYKDSPFNNEKA
PRIVVSMLKYLRVLSFCDFRSLFALPDTIGELIHLRYLNLSNTGIITLPESLCNLCNLQTLKLFSCFNLTKLPSAMQNLVNLRH
LEILDIFIEEMPKRMGKLSQLQKLDFYIAGKHIENSIKELGGLPNLHGSFSIEELENVTKGEEALEARIMDKKHITHLSLEWS
TGNDNSIYSQIELDVLSNLQPHQDLESLSIKGYKGTKFPKWVGNLSYRYMTSMSLYNCKNCCMLPSMGQLPSLKRLCIA
DMNSVKTIDAGFYKTEDCSSVIPFPSLECLEIFRMPSWEVWSAFGSDAFPVLRDLYIFDCIKLKGDLPNHLPALQRLIIRNC
ELLVSSVPRAPTLRTLKIRNSNKVTFHEFPLLVESIDVEGGPMVESMMEAITNVQPTCLQSLTLQNCSSAISFPGERLPASL
KALLMSGLKKLKFPIQHKHELLESLSIMNSCDSLMSLPLATFPNLSSLKIKDCENMKSLLLSGSDSLKSLSYFQISNCPNFVS
FPEEGLSAPNLTLFRVYGCEKLKSLPEQMRTLLPKMEYLNISNCQQIESFPVGGMPPNLKTVEISNCEKLVSGKAWVCTD
MVTSLIVWGPCDGINSFPKEDLLPPSLVSLHLCDLSSLETLECKGLLHLTSLQELRIQNCEKLQNIAGERLPVSLIKLIIVGCP
LLQTRCHSKDREIWSKICHVRGIKIDGRWI

>XP_027908330.1

MEFASSTSKLPQMYDVLINFNGEDIHRKFVSHLNSVLSAAGLTTFLHHQNAVNDMDIQQPILNLCRVAIVVFTKTYSKSD
WCLDQLQHIIKWHETYSRYVLPVYYEIQPSDVRLQKGDFGKAFKETAQQTFSAQQLEHGMSRWSHALTKAANLFGW
DESNYRSDADLVDTIVKSILTLPVLSATKFPVGLQSHVEDAIQIIKNKSSEVCRIGICGMGGSGKTTLAKAIYNQIQGTFME
KSFIEDIAQVRQTRGLLHLQEQLLSDVLKTKVEIHSDEMGRSMIRERLYRKRLLIVLDDVNDYGPLELWGRGSSSWLAEG
TVIIITLRDEDLLKMQQVYSIVRINVMNPNESLELLSWHAFREAKPKMEYHFLAKRVVDYCGGLPLALEVIGSYLYERTKE
EWNRVLLRLDNIPQNEFLDILKISFDGLHNQREKDLFLDICCFFVGTSRAYVSKILNACGVDPDRGIRVLIKRNLVKVRKN
NKFGMHPLVRDMGIREISRKQFVTNKRLWFDEDMNYALSENTLFSSQGAKVIQRLPTGRDLFERHPSYPLKVRDLLQLA
GNSGNLKWISLQGFSSEYLPNDFYLHDTIVIELKHSLLRFVWKEPQVLASLKVLNLSYSMYLSKTPAFWGLPGLEQLILKD
CPNLCHVHRSIGFLCSLRLLNLKGCTSLSNLPREIYKSKSLNSLILSGCSKIFLLEKDIVQMKSLITLIAENTAVKQVPFSITSSK
SIGYISLPQLEGRSRNLFPSIIRSRLSTTMNPQSYIHSFMDMEDNSWDYVAPFLSSLANLRSVFVQCDPEFEISKQLKPILV
EYGMNITESRIPKHNIRSSLIGVGRYQEFFNTVSDSISKALAKSESCDVSLPSVNDPNWLAQMGDGQSVSFTVPRDRVV
KEMVLCVFYLSTSKIIEPELTTVLIVNYTKCTLVIHNHGTVISFSDEDWHDIMSNFGSGDKVEIFVSFGHGLLVKNTVVYLM
YGEPNKNSLIGFIKKIVM

>XP_027908331.1

MEFASSTSKLPQMYDVLINFNGEDIHRKFVSHLNSVLSAAGLTTFLHHQNAVNDMDIQQPILNLCRVAIVVFTKTYSKSD
WCLDQLQHIIKWHETYSRYVLPVYYEIQPSDVRLQKGDFGKAFKETAQQTFSAQQLEHGMSRWSHALTKAANLFGW
DESNYRSDADLVDTIVKSILTLPVLSATKFPVGLQSHVEDAIQIIKNKSSEVCRIGICGMGGSGKTTLAKAIYNQIQGTFME
KSFIEDIAQVRQTRGLLHLQEQLLSDVLKTKVEIHSDEMGRSMIRERLYRKRLLIVLDDVNDYGPLELWGRGSSSWLAEG
TVIIITLRDEDLLKMQQVYSIVRINVMNPNESLELLSWHAFREAKPKMEYHFLAKRVVDYCGGLPLALEVIGSYLYERTKE
EWNRVLLRLDNIPQNEFLDILKISFDGLHNQREKDLFLDICCFFVGTSRAYVSKILNACGVDPDRGIRVLIKRNLVKVRKN
NKFGMHPLVRDMGIREISRKQFVTNKRLWFDEDMNYALSENTLFSSQGAKVIQRLPTGRDLFERHPSYPLKVRDLLQLA
GNSGNLKWISLQGFSSEYLPNDFYLHDTIVIELKHSLLRFVWKEPQVLASLKVLNLSYSMYLSKTPAFWGLPGLEQLILKD
CPNLCHVHRSIGFLCSLRLLNLKGCTSLSNLPREIYKSKSLNSLILSGCSKIFLLEKDIVQMKSLITLIAENTAVKQVPFSITSSK
SIGYISLPQLEGRSRNLFPSIIRSRLSTTMNPQSYIHSFMDMEDNSWDYVAPFLSSLANLRSVFVQCDPEFEISKQLKPILV
EYGMNITESRIPKHNIRSSLIGVGRYQEFFNTVSDSISKALAKSESCDVSLPSVNDPNWLAQMGDGQSVSFTVPRDRVV
KEMVLCVFYLSTSKIIEPELTTVLIVNYTKCTLVIHNHGTVISFSDEDWHDIMSNFGSGDKVEIFVSFGHGLLVKNTVVYLM
YGEPNKNSLIGFIKKIVM

>XP_027908349.1

MELATSSSKRQRKYDVLINFSEIEDIGKKFVSHLDSALSANGLTTFLHHQNAVQPKDIQQPILHLCRVAIVVFTKTYSESA
WCLHQLQQIIDWHETYSRHVLPVYYEIQPSDVRLQKGDFGKAFKATAHQTFSEQQLEHGMSRWSHALTKVANFFGW
DESNHRSDAELVDTIVKSVLNLPVLSVTNFPVRLQSHVQDVIGTIKNKSMEVCRIGICGMEGSGKTTLAKAIYNQIHGTFT
EKSFIEDISEVNRTRGCIYLQEQLLSDVLKIKVKIDHSVEMGTGMIQQRLHGKRMLIVLDDMINEYYPLFDLRKCCAWFG
KGTVIIITTKDEELLRKHQIDSIFRINLMNANESLELFSWHAFREAKPNEEYSDLAKTVVTYCGGLPLALELIGACLFERTKEE
WNTVLLRLDNKIYSLMYIVPLILKISFDSLPNKIEKDLFLDACCFFVGKGRAYVKKMLNGCGVDPNSGIRVLIERNLIKVRK
NNKFGIHPLLRDMGSGIIREISGKEHGKNSRMWYDSDAEYALSENTLFSSQRAKVIQGLPEKLFLTNTDGLKPCPLKETN
TSRIC

>XP_027908359.1

MALAVVGGALLSAFLDVLFERLASPELVSLIRGKKPDKLLQKVENQLIVLRVVLADAENRQITDSNVKKWLDVLIDVVYEV
DDLLDEVSTKAATQKEVSNSFSHIFNRKTIVSISKLEDIAERLDEILKQKESLNLKEIPVESNQPWKPQPTSLEDRYGMYGR
DADKEAIMKLLLEDSIDGEEVSVIPIVGMGGVGKTTLARSVYNDGKLTQIFDLKTWVCVSDIFDTAKVTKTMIEEITKMP
CNLNDLNLLQLELMDKLKGKRFFIVLDDVWIEDSDNWNSLTKPFLSGIRGSKVLVTTRNESVAAVVPFHVVKVYHLNQL

SNEDCWLVFANHAFPFSEETENRETLEKIGKEIVKKCNGLPLAAQSLGGMLRRKHAIRDWNNVLESDIWELPESQCKIIP
ALRISYNHLPPHLKRCFVYCSLYPKDYEFQKNELILRWMAEDLVKAPRKEKTLEEVGQEYFDDLVSRSFFQCSRIQSPGYF
VMHDLMHDLATFLGGEFYFRANELGKETKIDRKTRHLSFGRFSDPVSDIEAFETAKFPRTFLPINYKESPFNNEKAPHIIVS
MLKYLRVLKFGDYKSKLALPDSIGELIHLRYLDLSYTTIAMLPESLCNLYNLQTLKLRSCLSLTKLPRDMQNLVNLRHLQIYG
TPISEMPKRMGKLNHMQKLDFYVVGKHKENSIKELGGLPNLHGYFNIKKLESVSEGEEALEARIMDKKHITNLLLEWSVP
NDDIIDFQNELDVLDKLQPHYDLKWLSIIGYKGTRFPEWVGTFSYQHIINLYFKNCNNCSKLPSLGHLPSLKVLRISDMNS
LKTIDVGFYKKDDFSSMTPFPSLESLFINNMPCWEVWSAFDSEAFPVLKELDIENCPKLRGDLPDHIPALKILTIRNCELLV
SSVPGAPALQTLNISKSNKLAFHVFPILVERIEIEGRPMVESVMEAITNIQPTSLRHLSLEDCSSSISFPGDRLPASLKTLYIM
SLSELKFPVLEKHELLESLTIIDSCDSLKSLSLVIFPNLTSLKINNCENMESLSISGSESLKSLDLFEIAQCPNFVSFPREGMSAP
NLTRFIVYDCDKLKSLPDHMGTLFPKLEYLRITNCQQIECFPTGVMPSNLRTIEIGNCEKLLSDLRWISMDMVTSLNVLGP
CDGINSFPMEGVLPPSLTSLHLFGFSSLEMLECKGLLHLKSLQELGIFNCKNLQNISGERLPVSLTKLSIAKCPLLQKRCNRK
DRQIWPKICHVRGINIDRRWI

>XP_027908422.1

MEIAAAASSSSSSSSSSFLNSEPYFIYDVFISHSGEEISRNFVSHLHNVLLQAQVQTLVDEGNLQEGMNLEEHMRAISESKI
IIIVFSKTYIESTCSFLEFEKIIECHKTFGQIVLPVFYQIDPFEVCNEGYFGKALEADAQKSYSEEQLEHSLSWWVCALTIAAGI
NGWNVRDFRYDAELVELIASRVQTLLDYKELFITRFPVGLDSHVKKVIGCIENHSTKVCMIGIWGMGGSGKTTIAKAIYN
RIYREFIGKSFIENIREACSSRGVHRLQENLLYDVLKSKFKVESVGLGRTMIENELSRKKLLIVLDDVNEFIQLQYLCGNCEW
VGQGSVIIITTRDVHLLNLLEVTYVFKMDDMNENESLELFSWHAFGEAKLRKELNELAKNIVAYCGGLPLALEVLGSYLCD
RTKEQWKSVFSKLKIIPNYEVLHNLRASFDGLHDDTEKEIFLDVCCFFIGKERGYVTQILNGCGLHADIGIRVLIERDLIKVE
KNNKLEMHPLLRDMGREIIRQRWPVELENRSRLWFHDDVKEVLTNNTGTEAIEGLSLKLQLNSRDCFEAHAFEKMERL
KLLQLDHVQLTGDYGYLSKQLRWICWKGFPSKYIQNNFHMENVIAIELKHSHLQLVWKQSQVLKWLKFLNLSHSKYLRE
TPDFSGLPSLEKLILKDCPSLCKVHQSIGDLCNLLLINLKDCTSLSSLPREVYKLKSLRTFILSGCFKIHIMEEDIVQMESLITLV
TENTAVKQVPCSIVRSKSIGYISLRRFEGLSNSIFPSIIQYWISPTMNPESYIRPFCMDIENYYWRDLPPLHKSLANIRSVLV
QCDADFQLSKQVKTILVEYGVNFTESRLSNRGLRFSLIGVGRYNEFFSTLRDSVSEGLACSESCEVSLPGDNHPYWLSHV
GEGHSVCFNLPLDCDMKGMILCVVYLSTPEIVAAECLRSVLIVNYTKCTFQIHNHDTLIPFNDIDWQVIISNLGCGDRVEI
FLTFGHGLMVKNTAVYLICGQSNDLEMESCPEPNENTLNNFIKKMVMCDFW

>XP_027908440.1

MTSHQFLSMDNSSSSYKLPEKYDVLINFTGDDIHRKFVSHLDSVLTSVGISTFLHHQNSVKATHIQQPILKNCRVAIVVFT
QTYSQSAWCLNQLQQIIKWHETYLRHVLPVYYEIQPSDVRLQKGDFGKALKATAQQTFSGQQLEDAMSRWSYALTKA
ANFFGWDENNHRSDAELVDKIVKSLLNLPVLSATRFPVGLQSHVEDLIRTINSKSREVCTIRICGQGGSGKTTLAKAIYHQ
IQCKFRMKSFIEDIEQLSGTRRDLRLQEQLLLDVLKTKVEIPSVDVGRTMIQERLSGKRVLIVLDHVYYFCELLNLLKYRHSF
SEGTVIIITAEKEMLLGEYQTETVFWVEQMDINESLELLSWHAFREAKPIQEYDDLAKQVVNYCGGLPLTLELIGSSLFERT
KEEWQRVIFGLEDTRKYAVQQKFEVIFHALLNEMEKNLFLDVCCFFVGKGRGYVTKILNGWGVNADIGIRVLMERNLIK
IKKNNKLGMHPLLQQMGITISVKEPGKNRRLWFDKDKKYGTQNMQWLPVEDPFRRVHPTENSQYRHKKLGWISSLLF
SSQGTKANKRFSPKRDEVGMSVNSEYLLQKLKWTIVHGFPSEYLHRKFYVHDAIGIDLKHSLLRLVWKKPQVLRWLKVL
NLSHSKYLKETPDFSGLPSLEQLILKHCSRLRKVHRSIGSLNNLILLNLKDCTSLSNLPREIFELKSLRTLILSGCSMIDQMEN
DLVQMESLITLIAENTAVKEVPFSIVSSRSIGYISLHRFEGLSSNLFPSIIGSWMSPTMNPISCIHSFCMDMADNSWDDM
APLLSTLANLRSVLVACDTEFQLSEQVKNILVEYFANITEAGISKQHFRCSLIGVGAYHQFFNAVGDNIYEVLASSESCDVS
LPVVNDPYCLAHMGEGHSVSFTVPQDCDMKGMVLCVVYLSTPKIIEPEFTTVLIVNYSKCTFQIHNHGTIICFNDEDWH
GIMSNLESGDTVEIFVNFGNGLVVKNTAVYLICGESKNMEKASEPKHSLIRFIKKVVM

>XP_027908441.1

MTSHQFLSMDNSSSSYKLPEKYDVLINFTGDDIHRKFVSHLDSVLTSVGISTFLHHQNSVKATHIQQPILKNCRVAIVVFT
QTYSQSAWCLNQLQQIIKWHETYLRHVLPVYYEIQPSDVRLQKGDFGKALKATAQQTFSGQQLEDAMSRWSYALTKA
ANFFGWDENNHRSDAELVDKIVKSLLNLPVLSATRFPVGLQSHVEDLIRTINSKSREVCTIRICGQGGSGKTTLAKAIYHQ
IQCKFRMKSFIEDIEQLSGTRRDLRLQEQLLLDVLKTKVEIPSVDVGRTMIQERLSGKRVLIVLDHVYYFCELLNLLKYRHSF
SEGTVIIITAEKEMLLGEYQTETVFWVEQMDINESLELLSWHAFREAKPIQEYDDLAKQVVNYCGGLPLTLELIGSSLFERT
KEEWQRVIFGLEDTRKGYVTKILNGWGVNADIGIRVLMERNLIKIKKNNKLGMHPLLQQMGITISVKEPGKNRRLWFD
KDKKYGTQNMQWLPVEDPFRRVHPTENSQYRHKKLGWISSLLFSSQGTKANKRFSPKRDEVGMSVNSEYLLQKLKWT
IVHGFPSEYLHRKFYVHDAIGIDLKHSLLRLVWKKPQVLRWLKVLNLSHSKYLKETPDFSGLPSLEQLILKHCSRLRKVHRSI
GSLNNLILLNLKDCTSLSNLPREIFELKSLRTLILSGCSMIDQMENDLVQMESLITLIAENTAVKEVPFSIVSSRSIGYISLHRF
EGLSSNLFPSIIGSWMSPTMNPISCIHSFCMDMADNSWDDMAPLLSTLANLRSVLVACDTEFQLSEQVKNILVEYFANI
TEAGISKQHFRCSLIGVGAYHQFFNAVGDNIYEVLASSESCDVSLPVVNDPYCLAHMGEGHSVSFTVPQDCDMKGMVL
CVVYLSTPKIIEPEFTTVLIVNYSKCTFQIHNHGTIICFNDEDWHGIMSNLESGDTVEIFVNFGNGLVVKNTAVYLICGESK
NMEKASEPKHSLIRFIKKVVM

>XP_027908484.1

MAEALVESVIENLGSLVKDQLAIYWGVDEQTEKLSSNLKAIRAVLRDAERKQITSYAVKDWLQKLTDAAYVLDDILDECSI
HSTKMHFVDGHTSLLSRLHPKDILFRFHIGKRMREITQRFHSIHEERLTFELRVSVTEEQTVDDDDWRQTSSVITEPILYG
RDEDREKIVKFLLKDANNNEDLTVYPIVGMGGLGKTTLAKQVFNDHEISKHFDLRIWICVSDDFNLKRILQSIIECCIGQN
PNLGDLEARRKKVEEALHNKRYLLVLDDVWNENPEKWKELKGMLECARGAKGATILVTTRLEEVVSIMGTHSAYRLTAL
SEDDSWSLFKHHAFGPNREEREELVTIGKEIMRKCVGSPLAIKTLASCLRDESEVSQWENVKKSEIWNIREESSSVTGDE
NSIMRVLKLSYSNLKSSVKRCFSFCAIFPKDFEIEKEELIHLWMANGFIKCEGDVEVEDVGNKVWRKLYSRSFFQEAKYDE
FGMITSFKMHDLFHDLAQSIMGEECVVIEASSMTMLSARVHYSSLFSSDFLFDRPAFSRRLMPAFKKVESLRTFLDFCHI
SSGPSNHYLRALCLRPIPYFSPYKDLANKDLAHLRYLSLSFCFEKACLNSIICQMPKLQILKLNSCTEVELPKNLTQLQDLRH
VLIDDCASIAEMPPNISKLRHLRTLGIFVVGSKPGCGLGELQSLKLGGTLRIKGLENVSSEWDAKQANLIGKNLNMLWLS
WDGRSSSEGSNVSVERVLEALEPPSTLKSFQMNGYEGRHLSSWMRSLVALRDLVEVKVLECDNVEELPPLGKLAQLKRL
EVSGMKNVKCIDGETYEGVEEKAFPSLEELILKNLPNLERLLRDEGVEMLPRLSQLTIKKVSNFKFPRLPSVEKLDVESIDD
VEGVVGNTPCLKTLKISSIKGVKTLPDQLGTLDALEVLEIEFWYDLEYFPEHVLEGLTSLRTLEIRHCEKLKSLSEGVRHLACL
ESLTIRECPELMVLPSNMSQLTALRVVAIEYCSTLPDGLQRVPSLRSLYISEYKSTSLPDWLGDISTLEELIFNYCRELRSLPSS
IQRLTNLSRLIIGGCPHLQKRCKRETGEDWQYINHIPNIKLYGF

>XP_027908542.1

MSSSSSSSSKPQWIYDVFINFRGGDTRRDFVSHLYCALSNAGVNTFFDDENLLKGTPLEELTRAIEASQIAIVVFSETYTES
TWCLTELEKIIDCHESYGQIVVPIFHGIEPSILRNPKGRFRDALEAAAQKKYSAEHSEYGLSRWKNVLNKAANFSGWDVK
NHRNTAKLVKEIVEDILTKLDYALLSITEFPVGLESRVQEVVGIVENQSAKVCIIGIWGMGGSGKTTMAKAIYNQIHRRFN
DKSFIENIREVREIDGRGNVHLQEQLLSDVLKTKVKIHSVGMGTTMIENRLSRKKVFIVLDDVNDFGQLKDLCGNQKWF
GKESVIIITTRDLHLLDLLKVDYVYKMEEMDKNESLELFSWHAFREAKPREDFDELARNVVAYCGGLPLALEVLGSYLIERT
KKDWESVLLKLERIPNDKVQEKLRISFDGLCDEMEKDIFLDVCCFFIGKDRAYVTEILNGCGLHADIGITVLIERSLLKVEKN
NKLGMHQLLRDMGREIICESSRKDPGKRSRLWFHEDVLDVLTKNTGTETIEGLALKLHFIGKDCFKAYAFEEMKRLRLLQ
LDHVQLTGNYGYLSKQLRWICWQGFPSKYIPKSFYLDGAVVIDLKHSNLQLFWKEPQVLGWLKILNLSHSKYLTVTPDFS
KLPKLEKLILKDCPRLCKVHKSIGGLRNLLLINLKDCKSLENLPKGVYKLKSVKTLILSGCSKIDKLEEDIVQMESLITLIAENTA
MKQVPFSIVKSKSIGYISLCGFEGFSRNVFPSIIRSWMSPTMNPLSYVRPFCSTSSYLVSMDMRSYNLGDLEPMLSGLSN
LRSVLVQCDRESQVSMQVRTILDDSVNFTQFEITSQISNHYLRSYLIGIGSYQEVFNILSASISEGLATSDSCDAFLPGDND
PFWLAHMGEGNSVYFTVPEDCRMKGMTLCVVYLSAPEITATEYLISVLMVNYTRYTIQVFKRETVFSFNDVDWQGIISH
WSPGDKVEIFVNFGNGLEVKKTAIYLMCDESIDKEHNPSPEPKKEPKKNVFERFIKKILI

>XP_027908543.1

MSSSSSSSSKPQWIYDVFINFRGGDTRRDFVSHLYCALSNAGVNTFFDDENLLKGTPLEELTRAIEASQIAIVVFSETYTES
TWCLTELEKIIDCHESYGQIVVPIFHGIEPSILRNPKGRFRDALEAAAQKKYSAEHSEYGLSRWKNVLNKAANFSGWDVK
NHRNTAKLVKEIVEDILTKLDYALLSITEFPVGLESRVQEVVGIVENQSAKVCIIGIWGMGGSGKTTMAKAIYNQIHRRFN
DKSFIENIREVREIDGRGNVHLQEQLLSDVLKTKVKIHSVGMGTTMIENRLSRKKVFIVLDDVNDFGQLKDLCGNQKWF
GKESVIIITTRDLHLLDLLKVDYVYKMEEMDKNESLELFSWHAFREAKPREDFDELARNVVAYCGGLPLALEVLGSYLIERT
KKDWESVLLKLERIPNDKVQEKLRISFDGLCDEMEKDIFLDVCCFFIGKDRAYVTEILNGCGLHADIGITVLIERSLLKVEKN
NKLGMHQLLRDMGREIICESSRKDPGKRSRLWFHEDVLDVLTKNTGTETIEGLALKLHFIGKDCFKAYAFEEMKRLRLLQ
LDHVQLTGNYGYLSKQLRWICWQGFPSKYIPKSFYLDGAVVIDLKHSNLQLFWKEPQVLGWLKILNLSHSKYLTVTPDFS
KLPKLEKLILKDCPRLCKVHKSIGGLRNLLLINLKDCKSLENLPKGVYKLKSVKTLILSGCSKIDKLEEDIVQMESLITLIAENTA
MKQVPFSIVKSKSIGYISLCGFEGFSRNVFPSIIRSWMSPTMNPLSYVRPFCSTSSYLVSMDMRSYNLGDLEPMLSGLSN
LRSVLVQCDRESQVSMQVRTILDDSVNFTQFEITSQISNHYLRSYLIGIGSYQEVFNILSASISEGLATSDSCDAFLPGDND
PFWLAHMGEGNSVYFTVPEDCRMKGMTLCVVYLSAPEITATEYLISVLMVNYTRYTIQVFKRETVFSFNDVDWQGIISH
WSPGDKVEIFVNFGNGLEVKKTAIYLMCDESIDKEHNPSPEPKKEPKKNVFERFIKKILI

>XP_027908544.1

MSSSSSSSSKPQWIYDVFINFRGGDTRRDFVSHLYCALSNAGVNTFFDDENLLKGTPLEELTRAIEASQIAIVVFSETYTES
TWCLTELEKIIDCHESYGQIVVPIFHGIEPSILRNPKGRFRDALEAAAQKKYSAEHSEYGLSRWKNVLNKAANFSGWDVK
NHRNTAKLVKEIVEDILTKLDYALLSITEFPVGLESRVQEVVGIVENQSAKVCIIGIWGMGGSGKTTMAKAIYNQIHRRFN
DKSFIENIREVREIDGRGNVHLQEQLLSDVLKTKVKIHSVGMGTTMIENRLSRKKVFIVLDDVNDFGQLKDLCGNQKWF
GKESVIIITTRDLHLLDLLKVDYVYKMEEMDKNESLELFSWHAFREAKPREDFDELARNVVAYCGGLPLALEVLGSYLIERT
KKDWESVLLKLERIPNDKVQEKLRISFDGLCDEMEKDIFLDVCCFFIGKDRAYVTEILNGCGLHADIGITVLIERSLLKVEKN
NKLGMHQLLRDMGREIICESSRKDPGKRSRLWFHEDVLDVLTKNTGTETIEGLALKLHFIGKDCFKAYAFEEMKRLRLLQ
LDHVQLTGNYGYLSKQLRWICWQGFPSKYIPKSFYLDGAVVIDLKHSNLQLFWKEPQVLGWLKILNLSHSKYLTVTPDFS
KLPKLEKLILKDCPRLCKVHKSIGGLRNLLLINLKDCKSLENLPKGVYKLKSVKTLILSGCSKIDKLEEDIVQMESLITLIAENTA
MKQVPFSIVKSKSIGYISLCGFEGFSRNVFPSIIRSWMSPTMNPLSYVRPFCSTSSYLVSMDMRSYNLGDLEPMLSGLSN
LRSVLVQCDRESQVSMQVRTILDDSVNFTQFEITSQISNHYLRSYLIGIGSYQEVFNILSASISEGLATSDSCDAFLPGDND
PFWLAHMGEGNSVYFTVPEDCRMKGMTLCVVYLSAPEITATEYLISVLMVNYTRYTIQVFKRETVFSFNDVDWQGIISH
WSPGDKVEIFVNFGNGLEVKKTAIYLMCDESIDKEHNPSPEPKKEPKKNVFERFIKKILI

>XP_027908545.1

MSSSSSSSSKPQWIYDVFINFRGGDTRRDFVSHLYCALSNAGVNTFFDDENLLKGTPLEELTRAIEASQIAIVVFSETYTES
TWCLTELEKIIDCHESYGQIVVPIFHGIEPSILRNPKGRFRDALEAAAQKKYSAEHSEYGLSRWKNVLNKAANFSGWDVK
NHRNTAKLVKEIVEDILTKLDYALLSITEFPVGLESRVQEVVGIVENQSAKVCIIGIWGMGGSGKTTMAKAIYNQIHRRFN
DKSFIENIREVREIDGRGNVHLQEQLLSDVLKTKVKIHSVGMGTTMIENRLSRKKVFIVLDDVNDFGQLKDLCGNQKWF
GKESVIIITTRDLHLLDLLKVDYVYKMEEMDKNESLELFSWHAFREAKPREDFDELARNVVAYCGGLPLALEVLGSYLIERT
KKDWESVLLKLERIPNDKVQEKLRISFDGLCDEMEKDIFLDVCCFFIGKDRAYVTEILNGCGLHADIGITVLIERSLLKVEKN
NKLGMHQLLRDMGREIICESSRKDPGKRSRLWFHEDVLDVLTKNTGTETIEGLALKLHFIGKDCFKAYAFEEMKRLRLLQ
LDHVQLTGNYGYLSKQLRWICWQGFPSKYIPKSFYLDGAVVIDLKHSNLQLFWKEPQVLGWLKILNLSHSKYLTVTPDFS
KLPKLEKLILKDCPRLCKVHKSIGGLRNLLLINLKDCKSLENLPKGVYKLKSVKTLILSGCSKIDKLEEDIVQMESLITLIAENTA
MKQVPFSIVKSKSIGYISLCGFEGFSRNVFPSIIRSWMSPTMNPLSYVRPFCSTSSYLVSMDMRSYNLGDLEPMLSGLSN
LRSVLVQCDRESQVSMQGLATSDSCDAFLPGDNDPFWLAHMGEGNSVYFTVPEDCRMKGMTLCVVYLSAPEITATEY
LISVLMVNYTRYTIQVFKRETVFSFNDVDWQGIISHWSPGDKVEIFVNFGNGLEVKKTAIYLMCDESIDKEHNPSPEPKK
EPKKNVFERFIKKILI

>XP_027908546.1

MSSSSSSSSKPQWIYDVFINFRGGDTRRDFVSHLYCALSNAGVNTFFDDENLLKGTPLEELTRAIEASQIAIVVFSETYTES
TWCLTELEKIIDCHESYGQIVVPIFHGIEPSILRNPKGRFRDALEAAAQKKYSAEHSEYGLSRWKNVLNKAANFSGWDVK
NHRNTAKLVKEIVEDILTKLDYALLSITEFPVGLESRVQEVVGIVENQSAKVCIIGIWGMGGSGKTTMAKAIYNQIHRRFN
DKSFIENIREVREIDGRGNVHLQEQLLSDVLKTKVKIHSVGMGTTMIENRLSRKKVFIVLDDVNDFGQLKDLCGNQKWF
GKESVIIITTRDLHLLDLLKVDYVYKMEEMDKNESLELFSWHAFREAKPREDFDELARNVVAYCGGLPLALEVLGSYLIERT
KKDWESVLLKLERIPNDKVQEKLRISFDGLCDEMEKDIFLDVCCFFIGKDRAYVTEILNGCGLHADIGITVLIERSLLKVEKN
NKLGMHQLLRDMGREIICESSRKDPGKRSRLWFHEDVLDVLTKNTGTETIEGLALKLHFIGKDCFKAYAFEEMKRLRLLQ
LDHVQLTGNYGYLSKQLRWICWQGFPSKYIPKSFYLDGAVVIDLKHSNLQLFWKEPQVLGWLKILNLSHSKYLTVTPDFS
KLPKLEKLILKDCPRLCKVHKSIGGLRNLLLINLKDCKSLENLPKGVYKLKSGLATSDSCDAFLPGDNDPFWLAHMGEGNS
VYFTVPEDCRMKGMTLCVVYLSAPEITATEYLISVLMVNYTRYTIQVFKRETVFSFNDVDWQGIISHWSPGDKVEIFVNF
GNGLEVKKTAIYLMCDESIDKEHNPSPEPKKEPKKNVFERFIKKILI

>XP_027908547.1

MSSSSSSSSKPQWIYDVFINFRGGDTRRDFVSHLYCALSNAGVNTFFDDENLLKGTPLEELTRAIEASQIAIVVFSETYTES
TWCLTELEKIIDCHESYGQIVVPIFHGIEPSILRNPKGRFRDALEAAAQKKYSAEHSEYGLSRWKNVLNKAANFSGWDVK
NHRNTAKLVKEIVEDILTKLDYALLSITEFPVGLESRVQEVVGIVENQSAKVCIIGIWGMGGSGKTTMAKAIYNQIHRRFN
DKSFIENIREVREIDGRGNVHLQEQLLSDVLKTKVKIHSVGMGTTMIENRLSRKKVFIVLDDVNDFGQLKDLCGNQKWF
GKESVIIITTRDLHLLDLLKVDYVYKMEEMDKNESLELFSWHAFREAKPREDFDELARNVVAYCGGLPLALEVLGSYLIERT
KKDWESVLLKLERIPNDKVQEKLRISFDGLCDEMEKDIFLDVCCFFIGKDRAYVTEILNGCGLHADIGITVLIERSLLKVEKN
NKLGMHQLLRDMGREIICESSRKDPGKRSRLWFHEDVLDVLTKNTGTETIEGLALKLHFIGKDCFKAYAFEEMKRLRLLQ
LDHVQLTGNYGYLSKQLRWICWQGFPSKYIPKSFYLDGAVVIDLKHSNLQLFWKEPQVLGWLKILNLSHSKYLTVTPDFS
KLPKLEKLILKDCPRLCKVVRRLTN

>XP_027908548.1

MKHAFSLLLTWSFFIEFSQPMPSYSSSSSFSKSRSKPKSKSQWTYDVFINFRGEDTRRNFVSHLYSALKDSGVNAFLDDEK
LDKGGDLKSELLHAIEGSQITIVVFSQNYIHSTWCLDELHKIMECNAFRGQVVMPVFYDIGPSFLRDTQHISFEVISDQHS
RIKQWKKALTQAANLAGWDLRNYRNENDVMKEIVCEVLKRLDRTYLSITNFPVGLECRLQHGIDFIRNKKIGTCILGIWG
MGGIGKTTIAKSIYNEIRHEFKYRSFLANIREVWGGDRGPIDLQEQLLSDILKTTKTNVHSIDWGKGKIKEMLCTKKVLVV
LDDVSTSDQLKALCGNRNEISRGSVIIVTTRNACLLREIGVDCVYGIEKMNKIESLELFSWHAFRRANPTRDFFELSKEVIT
YCGGLPLALEVLGSYLYKRRKEEWQSVLSKLKEIPHNKIQEKLKISYDGLADHMEKDIFLDICCFFIGKDRGYVTEILNGCGL
HADIGIPVLVERSLIKVEKNNKLGIHDLLRDMGREIVRQSSPLPQKRSRLWVHDHVLDILTEHTGTEAIEGLALKLHRPSR
VHFSAETFENMKSLRILQFDHVQLAGDYGHLSKHLTWVYWRGFSLKYIPDNFYQGNVVAIDMKHSNLKLVWKEPQQ
QSLERLKFINLSHSKLLSKTPDFSKLPNLERVILKDCPNLYEVHHSIGDLPNLLILNLKDCTCLRNLPVIKSKSLRVLILSGCLKI
DKLEEDIVQMESLTTLKADNTNLKQVPFSIIRFKKIGFISLCGYEGLAGDLFPSIIWSWMSPTTGRVSSMQSFGSTSTSLVS
VHIQDNNLGNLLSKLTEFSKLRSICVQCNSDLQLTQELRRIVDDFYKVYSAMETTYAPQISENSVVSRLIGIGSHHQVMD
MLSNDMTEVGSISLISRLSFFLCV

>XP_027908788.1

MDFDFDCRALLSVFLHVVLEKLAFTEILDFFRARKLDHKLLNKLKTKLNSIDSLVDDAERKQFTDPHIRKWLLKVKDAVFD
AEDLLDDIQKLSKREVNSESEPQSTSGCTFKVLNFFKSSPISSFDQEIESRMQEVIDNLEYLLSQKGYLGSKTTRGDRYGLS
NERPQKFPSTSLDVGTDIYGKDHVKEKILDWLISKNNDPNPLSILSIVGMGGVGKTTLAQHVYNDPRVNEAKFDVKAW
VYVSDEFDVFKVSKTILEVVTMSIDDSRDLEMVHRRLKKELSEKRFLLVLDDVWNKNKFKWEELREPLLSGAQGSKILVT

TRNKDVACTMQSEELSLELLQEDDGWKLFAEHAFRDYDIQSYAEFREIGMKIVKQCKGLPITLKTIGSLLYNKASVSEWE
SVFQSEIWEFSQEHCDIIPALALSYIHLPSHLKICFAYCALLPKDYEFKKEHLMHLWMTENLLHYCQHSKTPEEVCEGYFN
DLLSRSFLQKSSKKELFVIHDLLIDLAKYVGGDMYFKWKVDKEEKIQKETCHFSVDLGSNQYFDGFGTLCNIERLRTFMPT
GRSIDYFSHWSINMLINELFSKFKFLRILSLSHCSDIKEVPDSIGNLEYLRSLDLSNTTIKKLTEKICSLSHLQILKLNYCTKLEEL
PSNLHLLTNLCRLEFMKTKVRKVPLHLEKLKNLKVVINSFNVGHGIDFGIQRLGELNLEGSLSIEELQNIENPLHALQADLG
NKTFLEKIKLRWGGDRNSVNSEKEGGVIENLQPSKELKELSIFSYGGKQFPNWLLENSLRNMVLLELDECESCQRLPPLG
LLLLLKVLKIRKLDGIVRIDADFHGNTSSSFKSLKTLEFSDMSQWEKWDCQAVIGAFPRLQHLSISSCPKLKGHLPEQFVPL
KTLRITDCQGLQSSAPRALDLELRDCGNLQLNWATMKRLKMGGHNMEASLLEIVGSDTLQHLHIYSVSKPTGDGYVSL
WTFPLDFFPTLRTLNLSGFGNLQIISQDDVHNHLDDLTIKNCPKFESLPANMHTLLPSLSWLYLEDCPRLKSFPDRGLPSN
LYDITLNNCSRLVDSLKFRDRSTLKRLSIKELDVECFPREALLPHSLTSLAIRDCPNLKTLNYKGLYQLSSLNSLVLVKCPNLQ
CLPEEGLPKSISYLCIDECPLLNRRCKKERGKDWKKIAHIQDLDIW

>XP_027908932.1

MADSVVSFVLNHLSQLVAREANLLYGVEDRVQSLQYELQMIKELLSSTKSKKGTEHTVLNQIRDVAHVAEDVIDTFVAK
VAIYKRRTILGRMLLGFGQARLLRDVTEKIDKIKATLNEIRDNKDKYDAFKETNNQSAAEEEEEEKRVQSLHKLRRNVEEE
DVVGFVEDSKDVINRLLEGSLNRKAVSIVGMGGLGKTTLARKVYNSSQVKQHFMCHAWVYVSNECRLRELLLDLLKHL
MPDFEQQCRGKKKGKKNTLDINSLSEEELKKLVRNCLERKRYLVVVDDLWKRQDWDEVQDAFPENNRGSRILITSRLKE
VALHAAHDVPHYLQFLNEEESWELFRRKVFRGEDYPSDLESLGKQMVQSCRGLPLSITVLAGMLANKEKSYREWSKVV
GHVNSYLTQDETQVKDIVLNLSYDNLPRKLKPCFLYFGIYPEDSEILVRPLLQKWVAEGFIQNTGSRDPNDVAEDYLYELI
DRSLAQAARVDTNGDVMVIRVHDLLRDLCISESKEDKVFEVCTDNNILIPTKPRRLSVHSKMDHYISSSNNDHSCIRSLFF
FGPLYDVRNRDWKWLFERLKLVRVLDFGLIPSSKIPSDIGNFIHLRYLKIQSDYVTFVPDSILNLWNLETVELGPSRFNIFPI
SFPAQIWKLKHLRHLNISWAIRLRGSCLGSNEKMWNVQTVSTLMLNSQATSLIKKGTFPNVKKLRLRVTSECEDELPKLL
QSLQELSYLNMLVIVLRDRDDAGVEHSTDESVKRNSGFKPQELLRNLGQFNCLTILTIENAFDLLTCAFAFPPNVTELTLF
RIKCISDEGMNGLGNHTKLKILRLLGD

>XP_027908933.1

MAESVVSFVLDHLAQLAAREANLLCGVEDRIQSLQHELQMINELLNTTKSKKGMEHTVLNQIRDVAHLAEDVIDTFVAK
VSIYKRRTILGRMLRGFGQARLLHHVAEKIDKIKTTLNEIRENKNKYDAFKETNSQSAAEEEEEEKRAKLLHKLRRNVEEEG
VVGFVHDSKDVINRLLEGGSNRKAVSIVGMGGLGKTTLARKVYNSTQVKQHFMCQAWVYVSNECRVRELLFGLLKHLI
PNFEQQWRGNKEGKKIARDINNLSEEELKKLVQNCLESERYLVVVDDLWKTQDWEEVQDAFPDNNRGSRILITSRLKE
VALHAAHDVPHYLQFLSEEESWELFCKKVFRGEDCPFDLETLGKQMVQSCRGLPLSIIVLAGLLGNKEKSHREWSKVVG
HVNWYLTQDETQVKDTVLKLSYDNLPKRLKPCFLYLGIFPGDIEIPVRPLLQKWIAEGFIQNTGNRDPDDVAEDYLYELID
RSLVQIGRVESNGSLETCQIHELLRDLCISESKEDKVFDVCIDNNILIPTKPRRLSIHSDMGHYISSSNNDHSHIRSLFFFGQ
DYYVRERDWKWLLECFKLVRVLELRPTSCRKIPSNLGNFIHLRNSNI

>XP_027908934.1

MAESVVSFVLDHLAQLAAREANLLYGVEDRVQSLQYELQMIKELLSSTRSKKGMEHTVLNQIRDVSHLAQDVIDTFVAK
VSIYKRRTILGRMLLGFGQAKLLRDVAEKIDKIKATLNEIRDNKSKYDAFKETNNQSAAEEEEEEEKERAKSLHKLRRYVEE
DDVVGFVHDSKDLIKRLLEGGSNRNAVSTIGMGGLGKTTLARKVYNSTQVKQHFKCRAWVYVSNECRVKELLLGLLKH
LKPNFEQQRRGNKKGKKGTGDISSLSEEEMKKLVRECLERERYLVVVDDLWKRKDWDEVQDAFPDNNRGSRILITSRLK
EVAFHTAHDVPHYLQFLNEEESWELFRRKVFRGEDYPSDLEPLGKQMVKSCRGLPLSIIVLAGLLANKEKSHREWSKVV
GHVNWYLTRDETQVKDIVLKLSYDNLPRRLKPCFLYLGIFLEDCEIPVRPLLQKWVAEGFIQDTGNRDPEDVAEDYLYEL
VDRSLVQVARVDTNGDVKAIQVHDLLRDLCISESKEDKVFEVCIDNNILIPTKPRRLSIHNSMGHYISSSNNDHSCIRSLFF
FGPDYSVRGREWKWLLDNFKLIRVLEFVPHSCGKIHSDIGNFIVRIIRRGFTGENKHQ

>XP_027909220.1

MTAEMVTGALVSAFLERTIDTLASRFMDIFRARKHKKKQLSDLKMKLFTIDVVAYDAEQKQFTDPRVRDWLLRAKDVV
FDAEDLLDEIDYELLTNQVVEAESQSATKKVWNSLKSSFVSFFENEIESKMDQVIEDLEDLATQSDFLGLKKASGAGIGSG
SGSKLTYTSLPNETVIYGRDDDKEFVFNWLTSDTDNKLSILSIVGMGGLGKTSLAQHVFNDPRLEAKFDIKAWISVPQEF
DVLNVSRAILDTITGSTEHSMQQEVIQRKLKEKIMGKKFLLILDDVWNEKRSKWEDVQKPLTFGGQGSRILVTTRSEKVV
ATMRSEKHLLQVLKEDYCWDLFAKHAFQSYNPQTDPDFVEIGKNIVKKCNALPLALKTTGSLLHNKSSLWEWESIMKSE
IWDFSENESDILPALRLSCLHLPSHLKKCFAFCAMFPKGYRFDKDLLIQLWMAEMFLESPLKEKSPEEVGEQYFNDLVSW
SFFQLSGDDEENYFIMHDLLNDLAKYVSEDICIRLGFDEPKGISNITRHCSFSTAKFFFDGFGSSINTQKLRTFIQTDWRTN
FPFPLRSWHCTTSIDDLFSKFKSIRVLYLSHCTNLREVPKSVGNLKHFRSLDLSHTDIEKLPDSMSLLYKLQILKLNKCEKLKE
LPSYLHELDNLRCLELVNTGVKNVPAHLGKMKNVQVLMSPFYVEKSKEFSIQQLGDLNLHGSLTIDELQNIENPSYALEA
NLKSKAHLVELVLNWNFIENSSVDSAKAGDVIENLRPSKYLKKLAIKNYVGNQLPNWLLNNSLLNMVSLVLRRCKSCQR
FPPLGLLPFLKNLEISGFEEIVNIDADFHGNNSSSFKSLERLEFSSMSQWEKWDCQDVTGAFPRLHYFSISSCPKLKGHLPE
FVALKTLRVIRCEHVEALIVFAIELRLQDCGKLQLELSTTNKLRMGGHTMEASMVKTVGHIIFNTSLEYLSIYSPLKSISDDC
VSLRTFPLHFFPKLKNLYLGGFCNLQRISQHEPHNHLKDLKIKNCPKFESFPENMHRMSLWELWIEECPKLESFPNGGLP
SNLSFMDLKDCSKLIGSLRGAFRDNPSLRCLWIEKVDAKSFPDEGLLPCSLVSLTILDFPNLEKLDHKGLYQLSSLEKLGLW
NCPNLQHLPEEGLPISISRLQIRNCPLLKERCQEEGGEDWQKIAHIQDLEIQE

>XP_027909234.1

MALEFVGSALLSAFLQVAFEKLASPQILDFFRARKLDEKLLNKLETNLHSIHSLADDAERKQFTDPHVRNWLLKVKDAVL
DAEDLLDDLQNLSKSQVDVESESQTFAYCALFPKDYKFEKECLIQLWMTENLLHCQHSRTPEEVGQQYFNDLLSRSFFQ
QLARNEEVFVMHDLLNDLAKYVGGGIYFMWEFDKTEKIQKVTRHFSVELGYKQHFDGFGKLCNTEKLRTFIMPKGREL
PDSIGNLEHLRSLDLSYTPIKKLTETICSLSHLQILKLNYCRDLEELPSDLHLLTNLCRLEFMKTKVRKVPLHLGKLKSLKVM
MSPFIVGHSKEFGIHRLGELNLDGSLSIEELQNIENSLDALEADLKNKTNLVKLKLRWDSRRNGNSIDSKKEENVIENLQP
SKNLKELSIFSYGGKQLPNWLLENSLRNMMSLVLEECESCQRLPPLGLLPFLKDLRIARIDGIVSIDADFHGNNSSSFKSLE
TLEFSSMKQWEKWECQVVKGVFPNLQRLYINDCPKLKGELPEQLFPLEILDIRDCQQLDASAPRAVVLRIKDYGKLKING
ATLTELSIGGHNKEVWFMEMVGHIVSCSFDLCNACQISDDSVSLWTFPLHFFPTLTMLSLKGFSNLHMISHDQAHNHL
QYLTIRDCPKFESLPANMHMLLPSLTMLSIKDCPRLESFPDGGLPPNLCEMRLMNCSRLIGLLKGALGDNPSLKNLWIEN
VDAECFPDEGLLPFSLTSLIITYSPNLIQLDYKGLYQLSSLETLTLYSCPNLRRLPKEGLPKSVSCLEIDDCPLLEQRCKTGRRL
LTFKNCTYCSDDVS

>XP_027909268.1

MALELVGGALLSVFLDVAFNKLASSQILDFFRARKLDEKLLNNLKTKLNSIHSLADDAERKQFTDPHVRNWLLEVKDAVL
DAEDLLDDIQMLSKRQADADSESQTSACCTFKVLNFFKSSPITSYNKEIESRMQEVLDKLEFLSSQRGDLGLKTARAVRS
GLSSELPQKSQTTSLVVGTDIYGRDDDKKHIVDWLISDTNNSNQPSILSIVGMGGLGKTTLAQHVFNDPRINEAKFDVK
VWVCVSDEFDVFKVSRAILEAVTKSTDDSRDLEMVHTRLKENLTKKKFLLVLDDVWNENQHNWEEVQKPLMFGVQG
SRILVTARSKEVASTMRSEVHSLKQLQEDDCWNLFVKYAFKDIDTQQNPECTEIGKKIVEKCKGLPLALKTMGSLLYNKSS
VSEWESVFQSEIWEFSQDRCDIIPALAMSYIHLPSHLKVCFAYCALFPKDYEFKKEHLMHLWMTENLLHCPSEKVCQQY
FNDLLSRSFFQQSGKNEVFVMHDLLNDLAKYVGGGIYFRWEVGQKEKIQKATRHYSIKLGHNQYFDGFETSCNTKRLRT
FIPTGRRIHSLARWGVNMSMHEFFSKFRHLRILSLSYCSKIRELPDSIANLEYLRSLDLSHTSIRILTEKICSLSHLQILKLNYCS
DLEELPSNLHLLTNLCLLELMENIVKKLPPSLGKLKNLKVVMRSFHVGHGREFGIQQLGELNLDGSLSIMELQNIENSLDA
SEAYLKNKTLLVRLELRWEWNRDSIDSKKEEEVIKNLQPSENLKELEILFYGGKQFPNWLHSLPNLVALELFGCESCEHLP
PLGLLPFLKDLSIDRLDGIVSIDADFYGSNSSSFKSLQSLEFSNMEQWKKWECKAGVFPRLQYLSIISCPKLKGELPEQLVPL
NKIHIRDCQQLEASAPRALDLKLYDCGKLHLDWATMKSLLMEASLTEIVRSDTVKYLKITLETISDDCVSLRIFPLDFFPTLC
NLYLSGFPNLQTISQDHIHHHLEHMTIKECPKFELLPANMHTLLPSLNALNIEDCPKLESFLKGVLPSNLKFMRLKSCSRLL

VDSLKGAFRDNPSLERLSIEKVDAKCFPDEGLLPLSLTQLSISNSPNLEKLDYKGLYQLSSLQSLRLDNCPNLQCLPEEGLPK
SISHLHIGRCPLLEQRCQKERGEDWEKISHIQDLYIH

>XP_027909297.1

MATEMVTGALVSAFVERTIDTLASRFVDIFRGRKHNKKQLSDLKMKLLAIDVVAFDAEQKQFTNPRVRDWLLRAKDAV
LDAEDLLDEIECELPKSQVEAESQSAAKKVWNSVRSFVGFFENKIDSRMEQVIENLEHLATQSDLLGLKKGSGVGVGSGS
GSKLTYTSLPNETVIYGRDDDREFIFNWFTSDTNKSLSILSIVGMGGLGKTSLAQHVFNDPRIEENFDIKAWVSVPQEFD
VFNVSRAILGGVATLTDDSTNLEMVHRKLKENLTGKKFLLVLDDNWNENQSKWEQVQKALGFGATGSRLLVTTRSEKV
AVTMRSEKHLLQVIKEDDCWELFEKHAFQSVQPDPDFMEIGKKIVEKCEGLPVALKTMGSLLRNKSSLCEWENIMKSEI
WDFSENESNILPALKLSYLHLPSHLKKCFASCALFPKGCVFDKEGLIQLWMAENFLESLPQTKSSKEVGEQYFNDLLSWSF
FQQSNKEEKNCFIMHDLLIDLAKYVCGDICIRLEVDEPQELPKRTRHISFTTDLLQNFDGFENLIDTQKLHTFVQTSWRKY
PPTFVSSWYCKMSIDDFFSKFKYIRVLSLYDFLNLNEVPKSIGNLKHLRSLDLSYTRIENLPDSVGLLYKLQILKLNNCERLKE
LPSCLLQLNKLCFLELLNTKVKNVHILGNLKNLQVLMNLFCVDIHEELSIQQLGQINIHGSLTISGLQNIENPSHASQACLE
NKPHLVELVLDWNWSNNSFADSSDSSSVIENLQPSKHLKKLSIRSHVGEQFPNWLLNNSLPNLVSLVLELCYSCKRLPPL
GLLPFLKDLKIKSLYGIVSIDADFHGSNSSSFKSLETLHFSDMGEWEKWECKAVTDAFPRLQHLYIHHCPKLKGQLPELLV
PLENLHIEDCKELEAFAPRAVDLKLEYSEKVLFDWATVKSLKLAGYNIEASFMEMVTDIVPHNSIQHLQINDFDHHPCPN
VSDFVSLWTFPLHFFPTLRTLTLLRLNNLQKISQNHIHNHLEGLTICCCPKIESISENWDMLKSLLIQDCPRLEPFTEGGLPS
NLKKMTLSKCSTLVDSLKGALGDNPSLKILRVDSLDDECFPRKDWLPLSLTDLTIYNFPNLEKLDFRLLNQLSSLKILTLVNC
PKLLPLPDEVFLNQYQILF

>XP_027909316.1

MAVELITDAALSKFFEKTFDNVFSRLGDIFRGDKSKKKQLSNLKVKLLAVEVVTDDAEQKQFTDQRVREWLLSAKGFMF
DVEDLLEEINHALSKSQVEAESHSAAKKVWNSLKSPFVSFFKNEIESRMEKLIENLEDLETRSHVLGLKRRDDVGEGSRSG
RKLRSTYLPNDSVIYGRDDDKEFVLNWLTSHTHKNLSILSIVGMGGVGKTTLVQHVFNDPRLDEAKYDVKAWVCVSDE
FDVFKVSKTILEHVTRSTDNSGDIERVHQSLKETLTGKKFLLILDDVWNEDKSKWEEVQKPLLYGAQGSRIVVTTRSKEVA
STMRSKERFLEQLPEGPSLELFAKHAFADDYDAQSNPECNKIGEKIIKKCKGLPLALKTIGSLLYNKLSVSEWEFVFRSEIW
DLPKERCNIVPALALSYIHLPSHLKVCFAYCALFPKDYKFQKEHLIELWMTEDFLQHGKSPEETGQQYFNELLSRSFFQRS
GDAEEVFVMHDLLNDLAKYVAGDIYFRHELGQTNEIQKVCRHFLFELGYIERFHGFGTLCKTQRLRTFLPTPDRKIVLFH
WFCNMSIHELFTKFKFLRILSLSRCSNLEELPDSVGNLEHLRSLDLSRTNIKKLSERICSLSHLQILKLNRCMNLEELPSNLHLI
TTLCRLEFTYTKVRKVPPGLEELKNLKVRMDIFKVDHSMESGIQRLGKLNNLHERLSIQGLQDIENPRDALKADLENKTHL
MRLVLVWKRTGNCIDSKKEEDVIENLKPPKNLKGLSIFNYGGKQLPNWLLENSLWNMVSLKLDGCESCQRLPPLGLLPF
LKYLDISGFDEIVSVDVDFHGNNSSSFQSLERLEFSNMRQWEKWECQAVTGAFPNLRILSIKDCPKLKGQLPELPAPLETL
EMRDCQQLEESINDDSVFVSIFPLDSFPTLEFLSLSGLNNLQMISLDQAHHHLEDLIISKCPKLESLPGSMHMLLPSLMSL
CIKDCPRLESFPDGGLPSNLNEMTLENCSRLVGSLKGAFRDGSYLGRLSIKELDAKRFPEEGLLPASLNHLTIGDCPNLEEL
DYKGLSQLSYLQSLTLEHCPKLQCLPEQGLPESISNLTIKNCPLLKQRCQKGGEDREKIAQIQYICFFN

>XP_027909349.1

MPVLETLGGALFGAVLQVLFHKLDSHQVLDYFRGRKLNEKLLKNLRRKLVSINAVVDHAEQKKFRNAYVKTWLDDVRD
VLLDTEDLLDEIHYEFLKSESELEYQSSAIKVRSFECKLKEVLDDLEFLLNQKDDLGLKNVCGVVSELGNKLLEGKNESSSLV
VEDIIYGRDEDKEIILNWLTSDNGNRNHLSILAIVGMGGMGKTTLAQHVYNDPKMKEVGFDEKAWVCVSDEFDVLKVS
KAIIGAFTKSRDDSEDIEMVHGKLKEKLTGRKFLLVLDDVWNEDRNQWKTLQTPLRYGAKGSKVLVTTRSNKVASIMQS
SSVHQLKQLQKYYSWQVFAKHALQDDNSTLSCELEEIGTKIVEKCKGMPLALETLGCLLRTKSSVSEWEGVLTSEIWDLSI
EDSKIIPALLLSYYHLPSHLKRCFAYCALFPKDHKFDRETLIQLWMAESFLQCSRQSKCAEEVGEQYFNDLLSRSFFQQSIN
DNETCFVMHDLHNDLAKYVCGEICFRLGVDRAERVPKTTRHFSTVIDPVQDRKSLCDAKGLRTFISFSADCEISIQELVSN

FKFLRVLSLSCYKVKEVSDIIGNLIHLRSLDLSNTGIKKLPDSTCSLCNLQVLKLNNCFNLQELPSSLHKLTNLRRFELVGTTL
RKAPILLGKLKNLQVWMGRFDVGKNSQFGIQLGELDLYGQLSIRNLENIMNPYDALAADLKNKTHLVALYLEWSLRRN
NEDSIKEREVLENLQPSRHLKHLLIDGYGGTHFPRWLSDNSLSNVESLTLNNCRYCQRLPSLGLLTFLKHLTIRGLDWLVRI
DADFYGNSCSAFASLETLSFIDMKEWEEWECMTGAFLSLQHLSVTNCPKLKGHLPEQLSHLKILTIDQCEQIEASIPKGVE
IEDVKMEASSFDMIGPLVFDTPLESLSIYSCPGINMPLNHWYSLLVELDISESCDSLTNFPLDIFPKLCDLCLNECHNLQMI
SQGHPHSHLKSLTIQNCYKFESFPCEGLFATQLESFCIEELDRLKSMPRFMSVLLPSLNYLSIRDCPGVEFSDGCLPSNLKE
MRLFNCSKLVASLKGVWGTNPSLKSLYIREVDVEFFQSEGFLPLSLTNLEIYDCPNLKKLDYEALSPLSSLEKLDIVNCPSLH
CLPEEGLPKSILELGIKSCPLLKQRCKKEEGEDWAKIAHIKTIWVDFEQVNIEDEARVGKY

>XP_027909448.1

MALELVGGTLLSVFLNVAFEKLASPQILDFFRTRKLDEELLNKLKTKLNSIHSLVDDAERKQFTNPHVKNWLLEVKDAVLD
AEDLLDDLQMLYKRQVDADSESQTSACCTFKVLNFFKSSPISSYNKEIESRMQKVLDKLDTLLSQRGNLGLKTVRVVGSG
LSNELPQKSQTTSLVVGTDIYGRDDDKKQIVDWLISDTNNSNQPSILSIVGMGGVGKTTLAQHVFNDPNVDEAKFDVK
AWVCVSDEFDVFKVSRAILEAITKSTDDSRDLEMVHTRLKEQLTKKKFLLVLDDLWNENKPKWEELQKPLMFGVQGSKI
IVTTRSKEVASTMRSEVHSLKQLQEDDCWNLFVKYAFKDDDTQQNLECTEIGKKIVEKCKGLPLALKTMGSLLYNKSSVS
EWESVFQNEIWEFSQERCDIIPALALSYIHLPSHLKVCFAYCALFPKDYEFKKEHLMHLWMTENLLHYPSENFCKQYFND
LLSRSFFQQSGEKEEVFVMHDLLNDLAKYVGGGIYFRWEVNQEGKIQKVTRHFSVEVGYNQYFDGFGTSCNTKRLRTF
MSTGLGKLKNLKVVMESFNVGHGREFGIQQLGELNLDGSLSIKALENIENSVDASEAYLKNKTLLVKLKLRWTRNRDSID
SKKEEEVIKNLQPSENLKELVILHYGGKEFPNWLHSLLNLVSLELNGCDSCERLPPLGLLPFLEDLNIIGLDGIVSIDADFYGS
NSSSFKSLQNLRFFNMGQWKKWECKAGIFPNLQTLYISDCPKLKGELPEQLVPLKKYKLQSANYLRLPLQEL

>XP_027909472.1

MPPESDVTVSSYRLRWDVFLSFRGTDTGHTFTMRLYHALHGRGVRVFRKEDGLERRDEIQKKLLEAVEDSAAAVVVISS
DYASSHWCLEELAKICDVGRLILPVFYWVDPSHVRKQEGPFEDWFLRHAERFPKERVEQWKNAMKKVGGLAGFVLDE
KSDKSDELIQILVQNLMKQLRNTPLSVAPFTVGLEDRVEVLKNLLDLKSSDVRVLGLYGMGGVGKTTLAKSLFNNLVVH
NFERRSFIPNVRSQVSKHHGLVSLQNKIRGDLCGRKEDLINDISDGISAIQKIVQENRVLLILDDVDDVEQLNFLMGKRE
WFYKGSRVVITTRDKEILHESYVDVDYEVKELEFSEAMELFCFHAIRRKEPAEGFLDVSKQIVEKTGGLPLALEVFGSYLFD
KRTKREWKDALEKLKQIRPACLQDVLKISFDALDEEEQCIFLDMACLFVQMEMKRDDVVDILNGCDFSGEIAVAVLTAR
CLIKIIGDGKVWMHDQVRDMGRQIVRSESLTDPGLRSRLWDRDEILTVLKNMKGTRNVQGIVLDCVKRRMSIPRDSTA
EEITWENFRRKPSCKSAFEYIKERYKKYVEDRKERAKEVILQPKHFQPMVSLRMLQINYSRLEGQFRFLPPKLKWLQWKQ
CPLRYMPSSYNPLELAVMDLSESLIETLWKGRSNKVAEHLMVLNLSSCHRLTATPDLSGYLSLKKLNLEECSHLTRIHESLG
NLNSLVHLNLRLCYNLIELPGEVSGLKQLEDLVLSGCWKLKALPKDLSCMVSLKQLLLDSTSITELPESIFHLTKLEKLSANG
CHSLKRLPTCTGKLCSLQELSLNHTALEELPDSVGSLEKLEMLSLMGCKSLSVIPNSTGKLISMTQLFLNGSGIKELPASIGS
LSYLRKLSVGDCTSLDKFPVSMELLVSIVELKLDGTKVSNFPDEIFVGMKMLEKLEMGKVQLLKFVPESFGCLSALTILDM
HESNITELPESIGMLENLIGLRLDKCKQLQRLPNSIGNLKSLQWLMMKETEVTRLPDSFGMLRSLVELDMKRMPYLNNN
GAGTNMSTVTIIPNIQEQPNSEAILSSFCNLTFLEKLNAHGWGIYGKIPDEFEKLSSLETLSLGHNNICSLPANMMSLSYLK
KLLLSNCRELMFLPSLPSSLEELNLENCVAVQYIHDISNLERLEEFNLTNCEKVVDVPGLEHLKSLRRLYMSGCIGCSLAVK
RRFSKVVLKKLEILIMPGSRVPDWFTAEPVVFSKRRNRELKGVIFFGVLSFKNIPENQRERLQLEDVQGKIFNLSSEVFSTT
FRLLGVPGTNEDHIFLRRFGARTPLVFQLKDRYTLHLQMRNPPRVNGLELNNCRIHLVYYGDDDYEGDEGSLEESQFSV
SQKLAKFFNFAAEDHACI

>XP_027909529.1

MIFSWMMTSNTNDNKLSICSIVGMGGMGKTTLAQHVFNDPKIEEVGFDEKAWVCVSDEFDVLKVSKAIIGAFTKSKD
DSEDIEIVHGKLKEKLTGRKFFLVLDDVWNEDQHLWKSLQTPLKYGAMGSKILVTTRNSNVASIMESSKIHQLEQLRED

HSWQVFAKHSVKDNSSTLNSELKEIGMKIVEKCKGLPLALQTVGSLLHSKSSVSDWEGVLRSNIWDLPIKDSKIIPALLLSY
FHLPSHLKRCFAYCALFPKGHEFDKESLILLWMAQNFLHCSEQSKSQEEVGEQYFNDLLSRSLFQKSIIFNERYFIMHDLL
NDLAKYVSGEVCFRLGVDKEERIPKTTRHFSTKENPVIYHEFKSLCEAKGLRTFLSINGGIGMSMQELISNFKFLRNLSVSY
TNDTEEVHDSIEVPESIGNLIHLHLLDLSNSNIEEVPDTIGDLVHLCLLNLSATKLKEVPDTISNLIHLRSLDLSGTYIDILPVST
CLLCNLQVLKLNHCYLLEELPSNLHELTDLRRLELMGTYLRKVPPLLGKLKNLQVWMNSFEFGIQQLRELDLHGELSITNL
ENIVNPCDALAANFKNKTHLHGIHLEWRRNNDDSIKEREVLENLQPSIHLKDLSIQGYNGTQFPRWLSDNCLLNMVSLT
LKRCKYCLWLPSLGLLKFLKHLTVEGLNWMRSIDADFYGKSSHAFASLETLSFTNMKEWEEWQCMTGAFPSLQRLSVE
HCPKLKGHLPEQHSHLKELTIKCCKQLVASIPRAVEIEDVKMKPSSVDMIESLVSDTPLECLIIAFCPGMTIPINHCYHSLA
QLVITHGCDSLTTLPLDLFPKLRMLTLHECRNLQMISQGHLHRHLESLTIRNCSEFESFPTEGLLAPQLQRFWIEGSEKLKS
MPKHMNSLLPSLNNLSIFNCPEVEFPEGCLPSNLMEMRLQNCSKLVAALKGVWGTNPSLEILSIGKVDVEFFPGEGLLPL
SLTHLIIDNCQNVEKLDYKGLCHLSSLEKLDLFDCPLLQCLPEKGLPKSISDLEIRNCPVLKQRCKKLEGEDWEKIAHIKTIW
VDHEDVNINDEAMMYLE

>XP_027909540.1

MCKIRSRIIIISRNCHILQNYGVNEVYKVQVLDDTQSLQLLCKKAFKSNDIGEEYKELTLDILKYVNGLPLALEVLGSFLLDR
DVCEWRYALTRMEENPSKDIMDVLRISFDGLENLEKEIFLDIACFFSNKTHHSSQQTMKKLLHYRQFYPDIGMKVLIEKS
LISCQDGYIGMHDLLKELGKSIVREKAPKEPRKWSRLWNYKDLQKVMKINKEAKNVEAIIIKQYPKEFLKAIRVDALSKM
NQLELLILKNVQCFGTLDCISNELRYLKWNHFPCMSFPSTFHPDQLVELILRHSNIKQLWEGKKCLPNLRNMDLSHSKNL
IEVPDLSEAPRLKNINLGGCIQLVQIHPSIGILRELQHLYLNNCKNLVLNLIILFGISSLKTLDLSGCSKLLNSKMLMEPRDTK
HLEEVVKNRNIIQLPTSSVYEFLMLPFKFFYPPKAEASLGLVFSSLSSVPCLEYLDISFGNLLQIPDGIGNLRSLRGLNLGGNK
FVTLPSTIKKLSNLQHLNLEHCKEMKYLPELPTMKEETIDGYYGQLLIFYCPKFRDMEHCYSTVFSWMTQNLQVYLQPR
MEIVIPGSEIPKWFNKQNASTSIIMDPYDVIDDPNWIGVAICALFVTHQDPMNLGEIYDFLNTIAYGVNNINGCGKYYQ
SIPIHLTTDLVTVELNHLFIVFYSQQGLTSLLSSRSNTMHDLHGIEFGTWIRSPKGLRVVVKNCGYRWVFKKDLQPLNST
MFFSGNSSSRKRKFLTSD

>XP_027909541.1

MCKIRSRIIIISRNCHILQNYGVNEVYKVQVLDDTQSLQLLCKKAFKSNDIGEEYKELTLDILKYVNGLPLALEVLGSFLLDR
DVCEWRYALTRMEENPSKDIMDVLRISFDGLENLEKEIFLDIACFFSNKTHHSSQQTMKKLLHYRQFYPDIGMKVLIEKS
LISCQDGYIGMHDLLKELGKSIVREKAPKEPRKWSRLWNYKDLQKVMKINKEAKNVEAIIIKQYPKEFLKAIRVDALSKM
NQLELLILKNVQCFGTLDCISNELRYLKWNHFPCMSFPSTFHPDQLVELILRHSNIKQLWEGKKCLPNLRNMDLSHSKNL
IEVPDLSEAPRLKNINLGGCIQLVQIHPSIGILRELQHLYLNNCKNLVLNLIILFGISSLKTLDLSGCSKLLNSKMLMEPRDTK
HLEEVVKNRNIIQLPTSSVYEFLMLPFKFFYPPKAEASLGLVFSSLSSVPCLEYLDISFGNLLQIPDGIGNLRSLRGLNLGGNK
FVTLPSTIKKLSNLQHLNLEHCKEMKYLPELPTMKEETIDGYYGQLLIFYCPKFRDMEHCYSTVFSWMTQNLQVYLQPR
MEIVIPGSEIPKWFNKQNASTSIIMDPYDVIDDPNWIGVAICALFVTHQDPMNLGEIYDFLNTIAYGVNNINGCGKYYQ
SIPIHLTTDLVTVELNHLFIVFYSQQGLTSLLSSRSNTMHDLHGIEFGTWIRSPKGLRVVVKNCGYRWVFKKDLQPLNST
MFFSGNSSSRKRKFLTSD

>XP_027909542.1

MCKIRSRIIIISRNCHILQNYGVNEVYKVQVLDDTQSLQLLCKKAFKSNDIGEEYKELTLDILKYVNGLPLALEVLGSFLLDR
DVCEWRYALTRMEENPSKDIMDVLRISFDGLENLEKEIFLDIACFFSNKTHHSSQQTMKKLLHYRQFYPDIGMKVLIEKS
LISCQDGYIGMHDLLKELGKSIVREKAPKEPRKWSRLWNYKDLQKVMKINKEAKNVEAIIIKQYPKEFLKAIRVDALSKM
NQLELLILKNVQCFGTLDCISNELRYLKWNHFPCMSFPSTFHPDQLVELILRHSNIKQLWEGKKCLPNLRNMDLSHSKNL
IEVPDLSEAPRLKNINLGGCIQLVQIHPSIGILRELQHLYLNNCKNLVLNLIILFGISSLKTLDLSGCSKLLNSKMLMEPRDTK
HLEEVVKNRNIIQLPTSSVYEFLMLPFKFFYPPKAEASLGLVFSSLSSVPCLEYLDISFGNLLQIPDGIGNLRSLRGLNLGGNK

FVTLPSTIKKLSNLQHLNLEHCKEMKYLPELPTMKEETIDGYYGQLLIFYCPKFRDMEHCYSTVFSWMTQNLQVYLQPR
MEIVIPGSEIPKWFNKQNASTSIIMDPYDVIDDPNWIGVAICALFVTHQDPMNLGEIYDFLNTIAYGVNNINGCGKYYQ
SIPIHLTTDLVTVELNHLFIVFYSQQGLTSLLSSRSNTMHDLHGIEFGTWIRSPKGLRVVVKNCGYRWVFKKDLQPLNST
MFFSGNSSSRKRKFLTSD

>XP_027909543.1

MCKIRSRIIIISRNCHILQNYGVNEVYKVQVLDDTQSLQLLCKKAFKSNDIGEEYKELTLDILKYVNGLPLALEVLGSFLLDR
DVCEWRYALTRMEENPSKDIMDVLRISFDGLENLEKEIFLDIACFFSNKTHHSSQQTMKKLLHYRQFYPDIGMKVLIEKS
LISCQDGYIGMHDLLKELGKSIVREKAPKEPRKWSRLWNYKDLQKVMKINKEAKNVEAIIIKQYPKEFLKAIRVDALSKM
NQLELLILKNVQCFGTLDCISNELRYLKWNHFPCMSFPSTFHPDQLVELILRHSNIKQLWEGKKCLPNLRNMDLSHSKNL
IEVPDLSEAPRLKNINLGGCIQLVQIHPSIGILRELQHLYLNNCKNLVLNLIILFGISSLKTLDLSGCSKLLNSKMLMEPRDTK
HLEEVVKNRNIIQLPTSSVYEFLMLPFKFFYPPKAEASLGLVFSSLSSVPCLEYLDISFGNLLQIPDGIGNLRSLRGLNLGGNK
FVTLPSTIKKLSNLQHLNLEHCKEMKYLPELPTMKEETIDGYYGQLLIFYCPKFRDMEHCYSTVFSWMTQNLQVYLQPR
MEIVIPGSEIPKWFNKQNASTSIIMDPYDVIDDPNWIGVAICALFVTHQDPMNLVFYSQQGLTSLLSSRSNTMHDLHGI
EFGTWIRSPKGLRVVVKNCGYRWVFKKDLQPLNSTMFFSGNSSSRKRKFLTSD

>XP_027909544.1

MCKIRSRIIIISRNCHILQNYGVNEVYKVQVLDDTQSLQLLCKKAFKSNDIGEEYKELTLDILKYVNGLPLALEVLGSFLLDR
DVCEWRYALTRMEENPSKDIMDVLRISFDGLENLEKEIFLDIACFFSNKTHHSSQQTMKKLLHYRQFYPDIGMKVLIEKS
LISCQDGYIGMHDLLKELGKSIVREKAPKEPRKWSRLWNYKDLQKVMKINKEAKNVEAIIIKQYPKEFLKAIRVDALSKM
NQLELLILKNVQCFGTLDCISNELRYLKWNHFPCMSFPSTFHPDQLVELILRHSNIKQLWEGKKCLPNLRNMDLSHSKNL
IEVPDLSEAPRLKNINLGGCIQLVQIHPSIGILRELQHLYLNNCKNLVLNLIILFGISSLKTLDLSGCSKLLNSKMLMEPRDTK
HLEEVVKNRNIIQLPTSSVYEFLMLPFKFFYPPKAEASLGLVFSSLSSVPCLEYLDISFGNLLQIPDGIGNLRSLRGLNLGGNK
FVTLPSTIKKLSNLQHLNLEHCKEMKYLPELPTMKEETIDGYYGQLLIFYCPKFRDMEHCYSTVFSWMTQNLQGLTSLLSS
RSNTMHDLHGIEFGTWIRSPKGLRVVVKNCGYRWVFKKDLQPLNSTMFFSGNSSSRKRKFLTSD

>XP_027909545.1

MCKIRSRIIIISRNCHILQNYGVNEVYKVQVLDDTQSLQLLCKKAFKSNDIGEEYKELTLDILKYVNGLPLALEVLGSFLLDR
DVCEWRYALTRMEENPSKDIMDVLRISFDGLENLEKEIFLDIACFFSNKTHHSSQQTMKKLLHYRQFYPDIGMKVLIEKS
LISCQDGYIGMHDLLKELGKSIVREKAPKEPRKWSRLWNYKDLQKVMKINKEAKNVEAIIIKQYPKEFLKAIRVDALSKM
NQLELLILKNVQCFGTLDCISNELRYLKWNHFPCMSFPSTFHPDQLVELILRHSNIKQLWEGKKCLPNLRNMDLSHSKNL
IEVPDLSEAPRLKNINLGGCIQLVQIHPSIGILRELQHLYLNNCKNLVLNLIILFGISSLKTLDLSGCSKLLNSKMLMEPRDTK
HLEEVVKNRNIIQLPTSSVYEFLMLPFKFFYPPKAEASLGLVFSSLSSVPCLEYLDISFGNLLQIPDGIGNLRSLRGLNLGGNK
FVTLPSTIKKLSNLQHLNLEHCKEMKYLPELPTMKEETIDGYYGQLLIFYCPKFRDMEHCYSTVFSWMTQNLQVYLQPR
MEIVIPGSEIPKWFNKQNASTSIIMDPYDVIDDPNWIGVAICALFVTHQDPMNLGID

>XP_027909547.1

MKKLLDYRQFYPDIGMKVLIEKSLISCTRQYIQMHDLLRELGKSIVREKSPKEPRKWNRLWSYKDLQKVMKVNKESENV
EAIVIDQHPDEFLLSRLRVDALSKMNHLELLVLNNVNCFGTLNYISNELKYLYWSNFSWMSLPSTFHLDQLVELILPHSNI
KKLWEGKKCLPNLKTLDLSHSRNLVGLPNFSEIPHLTKLILEGCIQIMQIDPSIDILKELDHLNLRNCKNLVLNLNILFGISSL
GSLNVSGCSKLLNSKMLMEPRNTEHLEKADKNKNIIQLPTSSVYKLLMLPFHFFYPLEAEDSLGLLMSSFSFSAPCLFELDI
SFCGLLRIPDEIGNLRSLAKLKLGGNKFVTLPSTIKQLSNLQSFNLEHCKQLKYLPELPTITGKNGRYPMGLYVFDCPKLSD
MEHCYSMVFPWMTQTLKVYLQPTVSPARMEIVTPGSQIPKWFNKQYSTRSVRMDPSAIIDDPNWIGVAICVLFVTHQ

DPMNLGEIYGHPYRGIEYGFNNVFHLSKYYLVVPIHFKNDLVTIGLDHLLTVFCCRQEFIHLLGRHPNTMHDLHSIEFETSIRSPKGLRVVVKNCGYRWVFQEDLQQLNSNMFFGENSSYRTRKLLTID

>XP_027909618.1

MAAMAAEMIIGALVSSSVQMTIDNLASRFMDICCGNKSNMKLLSSLKLKLLAVDVVADDAEQKQFTNPRVRDWLLAVKDVVFDVEDLLEETDHTLSKTQVEAQSQYAAKKVWNFLISCFVSSLQNEIGSRLEKLIEDLENLATKSQILGLQKAHDVGVRSGWGTKLRSTYLPKESVIYGRDNDKTFVFNWLTSKTHKNLSILSIVGMGGVGKTALAQHVFNDPRMDEAKFDVKAWVCVSDEFDVFKVSRAILEHVTGSIDNSRDTEMVHKSLKEKLTGKKYLLILDDVWNENPSKWEEVQKALVFGAEGSRILVTTRSREVASIMRSEEHSLKQLRDNHSCELFAKHAFRDVDIQANPDCWEIGRKIVKKCKGLPLALKTMGSLLYNKSSVSEWESVLQSEIWELPEEHCGIIPALALSYIHLPSDLKVCFAYCALFPKDHEFKKEHLMHLWMTKNRLNCPQEEVCQQYFNELLSRSFFEQSSKKEEVFGMHDLLNDLAKYVGQGIHFRCEAGQIENIQKVTRHYSVEFGYNRDFDGFGTLCDIEKFRTFMPTHRSTDSTDNLWSWYINMPIHELLSKFKFLRILSLSHLTLLTELPDSISDLEYLRSLDLSYTSIRILTEKTCLLSYLQILKLNYCRDLEELPTNLHLLTNLCRLEFKGTKVGKVPPHLEKLKNLKVVMDFNVGQGRDFGIQQLGELNLDGSVSIGELQNVENSVDALEADLKNKTHLVELELEWTRNGNSIDSEKVEDVIEKLEPPKNLKVLSIYDYAGKQFPNWLLKNSLLNLESLELCRCAPCHRLPPLGLLPFLKNLKISSCFEIVSIDADFHGNNFSSFKSLQTLYFSNMRQWEKWDCQSVTSAFPRLQHLSISYCPKLKGYLPKQLVPLETLEIKYCQKLEASAPKALCLDLCNCGKLHLDGTTIKKLKMEETSLEIVRSDTLKHLEIEHLEASIGDDDSVSLWTFPLHFFPTLRILYLRRLGNLQMISHFGAHNYLHYLHISECPKLESFPGNIPFLKSLYIKDCPTLEAVLPSSNPFLNSALSTRWGLCGCPKLPNLREEALSQSISIFHVRGFPLVEEFSEEEGEEWEKGSKRCY

>XP_027909634.1

MACNKIQRNSSSRTKNFDVFVSFRGEDTRNGFTNHLFAALQRKGVVAFRDDYTIQKGEFLESELLQAIEGSRVFIVVFSKDYASSTWCMQELTKIVGWVQQTGRSLLPIFYDVDPSEVRKQSGEFEKAFAEHEQRFKDDMEMIKTWRAALKTSCDRCGWDLKNKKQYEEIENVVEKVIDILGRNQIWSFGDDLVDMHSRVKQLEEVLDLSANDTVRLVGLCGMGGIGKTTLATALFNKISPQYNASCYIDDVSKTYYNFGATNAQKQLLCQILNQGNMEIHNVSHGTMLIRTRLCHLKTLVVLDNVDEVEQLEKLGLHPEYLGAGSRLIIISRDSRILKKYGVSEVYNVQVLDETQALQLFSKIAFRSNNIRKEYKELTFDVLKYVRGLPLAIKVLGTFLHDRDVCEWRSALIRMKENPKKDIMDVLRISFDGLENLEKEIFLDIACFFNKYGVWEVKKLLDYRQFYPDIGMQVLIEKSLISYQDEEIEMHDLLKELGRIIVREKAPKEPRKWSRVWSYKDFQEVMEINKEAENVEAIVIEQYPEEFLQGRIRVDALSKMDHLKLLILKNVNCFGTLNYISNELRYLFWNHFPWMFLPSTFHLDQLVELIMPHSNMKKLWEGKKCLPNLRRMDLSHSKNLIGVPDLSEVQRLTFLDLEGCIKIVGIHPSIGILRELHYLNLKNCKNLVLNLNILFALSSLRTLILSGCLKLLNSKMLMDPSDTKHLEEVVKITNVIQFPTSSVYRLLMLPFHIFYPSKPEDSLGMVLSSLSCVPCLVHLDISFCNLLQVPDEIKNLRSLVGLNLGGNKFVTLPSSIKLLSNLQRLNLEHCKQLKYLPELPTMKEKQIGTYYVGLYIFDCPKLCDMEHCYSTVFSWMTQNLQVCLQPTISSAHVAIVIPGSEIPKWFNKQKASTSISMDPSAVIDDPNWIGVAICVLFVTHQDPTNLVERYDNSSHTFSYGVNNVNLWPKSYSVAPMHFKKDLVTAGFDHLLTVFYSRQEFIHLLSSHPNTMHDLHGIEFETWIQSPKGLCVVVKNCGYRWVFKEDLQQFNSNMFFSGNSSSRKRKLLTRH

>XP_027909655.1

MPVIETLGGALFGAVLQVLLDRLDSRQVLDYFRRRKLDEKLLKKLKRKLVSINAVVDNAEKNQFRTAYMKAWLDEVRDVLLDTEDLMDEIHYEFSRYGLEAESQSSSSKVCIFESRIKEVLEDLESLLNQKDDLGLKNASRVGVGLGLSSNVSQKLPSTSLVVENTIYGRDDEKEMILKWMTSDTEKHSQLSILSVVGMGGLGKTTLAQHVYNDSRIEGKFSIKGWVCVSDEFDVLMVTKAIVGVLTKSKDDSVDLEMVQGRLKEKLTGRKFLVVLDDVWNEDRDQWKALQTPLNYGAKGSKILVTTRSNKVASIVQSNKVHELKQLGGDHSWKVFAKHAFQDDNPQINAEVKEIGTKIVEKCRGLPLALETVGCLLRSKSSVAEWKSVLSSEIWDFPEEDSKIIPALLLSYYHLPSHLKRCFSYCAMFPKDHEFDKKNLIQLWMAENFLPRSEQSKSQEEVGEHYFNVLLSRCFFQQSSGGLKSCFVMHDLLNDLAKYISGDICFRFGVDRAKRTLKETRHISFVIDDYGVSCNEYENLYDAKRLRTFLPVTRISYWSWYCETLTLELIFKLKCLHVLSFCGCVNLKEVPETIGDLMHLRFLDLSNTGIQKLPNTMCSLCDLQTLKLNSCVNLKELPCNF

HKLTNLRCLELIKNSLTKMPMHIGKLKNLEIFMMSPFNVGKSSELCIHQLGELSLHGDLVIKDLQNTVNPMDALAADLKS
KTCLVRLDLNWDLERNLDNFMKEKEILENLQPSKHLKELSISDYGGIQFPHWLSDNSLSNLVSLSLINCKHCLLLPSLEFLT
FLRHLTISGHDWIGTIDADFYRNSCSAFASLETLSFADMKEWEEWQCTTGDFPSLQSLSVTNCPKLKGHLPEQLSHLKKL
IIEDCKKLVTVAPRTLEICELHLRDCGKLQIDYNPTTLKMLRIGGDNMEASLLERLGHIISHTSLESFTIFSCPNMNIPINHCF
DFLEKLHICGGCDSMTNFPLDFFPRLSEIDLSECPNLQMITQRHPLNHLKILRIGKCSRFEYFPNEGLFARQLESFYIIGLEN
LKSLPKHMSDLLPSLNLLYINDCPEVEVSDGYLPSNLDEMCLFNCSKLIVSLKGPWGTNPSLKSLSIGKVDEDCFPGEGLLP
LSITNLEIYDCPNLKKLDYRGLCHLSSLEKLFLYKCPILQCLPEEGLPKSISKLRVEGCPLLKKRCKKQEGEDWEKIAHIKYIIVD
RERVNI

>XP_027909713.1

MPVLETLGGALFGAVLQVLFDKLDSHQVLDYFRGRKLDEKLLKNLRRKLVSINAVVDDAELKQFTDAYVKAWLDEVRD
VLFDAEDLLEEIDYEFSKSASQLEYQSSASKVCSFESKLKEVLDDLESLLNQKDDLGLKKAASVRSELGNKVLERKNESSSLV
ADDVFYGRDDDKEIILNWLTSDSNNHNQLSILSIVGMGGMGKTTLAQHVYNDPKIKEARFHEKAWVCISDEFDVLKAS
KAIIGAFTKSKDDSETIEMVHGKMKEKLTGKKFLLVLDDVWNEDRNQWKALQTPLRCGAKGSKILVTTRSNKVASIIQS
GYIHQLKQLQEDHSWHVFAKHAFQDENSMLNSELEEIGMKIVEKCKGLPLALETLGCLLHTKSSVSEWEGVLKSETWDL
AIEDSKIIPALLLSYYHLPSNLKRCFAFCALLPKDRMFDKESIILLWMAQNFLQCSQWSKSPEEVGEQYFNDLLSRSFFQQL
DNEMSYETHFIKLNKKLFIMHDLLNDLAKYVSGEMCFRLGVDRAEKVPKTTRHFSSVFNLLKYSECRSLCDAKRLRTFLSI
NGNCGISIQELISNFKFLRVLSLERCFNIEEVPNTIGNLIHLRSLNLSGTKIERLPDSTCSLCNLQLLKLNHCFNLQELPSTLHE
LTNLRRFEFEGTPLRKAPLLLGKLKNLQVWMGEFEVGKSNESSIQQLGELNLHGRLSISHLENIVNPSDALAVNLKDKTHL
IRLCLEWDFKQNIDDSRKEREILENLQPSRHLEHLSIVGYGGTGFPRWLSDNSLSNVVSLSLSNCKYCQSLPSLGILTFLKHL
TIDGLHHIVKINADFYGNSSSSFASLKMLDISNMKELEEWQCMTGAFPNLQSLYVRYCPKLKGHLPEQLSCLKELTIESCE
QLVASIPRAAIEIHNVNMQPSSFDMTGPLLSDIPLELLRIVFCPGMNIPINHCYHSLKELEIIHGCDSLTTFPLDLFPKLCNLE
LDVCRNLQMISQGHPHNNLKSLKIEKCSQFQSFPNEGLFAPELKILFIEGLEKLKSMPKRMSTLLPSLNHVNINDCPGVEL
SEGCLPSNLKEMNLWNCSKLVASLKGAWGTNPSLECLHIAEMDVEFFPGEGLLPLSLSTLGIYCCPNLKKLDYMGLCHLS
SLQKLDLSNCPSLQCLPEEGLPKSILELRISHCPLLKQCCRKQEGEDWEKIAHIKTIWVDDELQVEN

>XP_027909714.1

MPVLETLGGALFGAVLQVLFDKLDSHQVLDYFRGRKLDEKLLKNLRRKLVSINAVVDDAELKQFTDAYVKAWLDEVRD
VLFDAEDLLEEIDYEFSKSASQLEYQSSASKVCSFESKLKEVLDDLESLLNQKDDLGLKKAASVRSELGNKVLERKNESSSLV
ADDVFYGRDDDKEIILNWLTSDSNNHNQLSILSIVGMGGMGKTTLAQHVYNDPKIKEARFHEKAWVCISDEFDVLKAS
KAIIGAFTKSKDDSETIEMVHGKMKEKLTGKKFLLVLDDVWNEDRNQWKALQTPLRCGAKGSKILVTTRSNKVASIIQS
GYIHQLKQLQEDHSWHVFAKHAFQDENSMLNSELEEIGMKIVEKCKGLPLALETLGCLLHTKSSVSEWEGVLKSETWDL
AIEDSKIIPALLLSYYHLPSNLKRCFAFCALLPKDRMFDKESIILLWMAQNFLQCSQWSKSPEEVGEQYFNDLLSRSFFQQL
DNEMSYETHFIKLNKKLFIMHDLLNDLAKYVSGEMCFRLGVDRAEKVPKTTRHFSSVFNLLKYSECRSLCDAKRLRTFLSI
NGNCGISIQELISNFKFLRVLSLERCFNIEEVPNTIGNLIHLRSLNLSGTKIERLPDSTCSLCNLQLLKLNHCFNLQELPSTLHE
LTNLRRFEFEGTPLRKAPLLLGKLKNLQVWMGEFEVGKSNESSIQQLGELNLHGRLSISHLENIVNPSDALAVNLKDKTHL
IRLCLEWDFKQNIDDSRKEREILENLQPSRHLEHLSIVGYGGTGFPRWLSDNSLSNVVSLSLSNCKYCQSLPSLGILTFLKHL
TIDGLHHIVKINADFYGNSSSSFASLKMLDISNMKELEEWQCMTGAFPNLQSLYVRYCPKLKGHLPEQLSCLKELTIESCE
QLVASIPRAAIEIHNVNMQPSSFDMTGPLLSDIPLELLRIVFCPGMNIPINHCYHSLKELEIIHGCDSLTTFPLDLFPKLCNLE
LDVCRNLQMISQGHPHNNLKSLKIEKCSQFQSFPNEGLFAPELKILFIEGLEKLKSMPKRMSTLLPSLNHVNINDCPGVEL
SEGCLPSNLKEMNLWNCSKLVASLKGAWGTNPSLECLHIAEMDVEFFPGEGLLPLSLSTLGIYCCPNLKKLDYMGLCHLS
SLQKLDLSNCPSLQCLPEEGLPKSILELRISHCPLLKQCCRKQEGEDWEKIAHIKTIWVD

>XP_027909774.1

MRLNPGYKLSNTSLPNESVMYGRDDDKEFVFNWLTSHTDNKLSILCIVGMGGLGKTSLAQHVFNDPMIDGKFDIKAWI
SVPQEFDVLNVSKAILDTIAGSTDHSIQQELVQRRLKEKLTGKKFLFVLDDVWNERQSKWEDVQKPLLFGGQGSRILVTT
RSEKVAITMRSEKYLLQVLREDYCWDLFAKHAFQSGTNPQPDPEFMEIGKKIVGKCNGLPLALKTMGSLLHNKSSLCEW
ESIMKSEIWDFSENESDILPALRLSYLYLPSHLKKCFEFCALFPKGYIFNKKCLIELWMAENLLESPVQKKSPEEVGKQYFSD
LLSLSFFQQLGKEEGVCFIMHDLLNDLAKYVSDGIYIRLGVDELKGIHKTTRHLSFSTTRSLFDGFGSLIDTQKLHTFIPTSW
YWSWKMLADDNFVSKFKFMRVLSLSHCHNLREVPKSVENLKHLRSLDLSYTSIEKLPESISLLYKLQILKLNGRRRLKELPS
YLYQLHNLCCLEFIASGVRNFPTHLGNLKNLQVWMSSFCVKKSKEFNIQLLGELNLHGSLTIDELQNIENPSYALEADLKN
KPHLVGLRLIWNFVGCSSFDSIKVRDVIENLQPSKHLKTLSIVNFCGKQFPNWLLNNSVPNLVSLVLRKCKSCQRLPPLGV
LPFLKRLEISGFDEIVNIDADFHGNNSSSFKSLQTLEFSYMTQWEKWECQAVTGAFPRLQRLSISSCPKLKGQLPEFVALK
KLEVFDCEQLEDLNVSAPELHIQDFRKLQLDWTTIKRDIIFDTSSEHLHISSPLKSVRDDPDPLRNFSLNFFPSWTTLTINGC
ANVEEMISHDHTNNLLEDLTIKKCPKLESLPANMHMLLPSLKRLSIYDCPRLESFPDRGLPSNISYMTLINCSRLIDSLKGA
LGGNPYCLRSLWIGKMDAECFPNEGLLPLSLTSLAISHCRNLKELDYKGLHQLSSLKTLSLCVCSNLQCLPEEGLPKSVSYL
EIGECPLLKERCQKEGGKDWKKIAHIVTVKIW

>XP_027909775.1

MAAEMVTGALVSTFAERTIDTLASRFVDIFRARKNNKKQLSHLKMKLLAIDVVAFDAEQKQFIDPRVRDWLLRAKDVVF
DAEDLLDEIDYELSKSQVKAESHSTSKKVWNSFKSSFVSLFVNEIESRLEQVIEDLEDLATQSEVIGLKKACHARVGSGSGY
KLSNTSLPNESVMYGRDDDKEFVFNWLTSHTDNKLSILCIVGMGGLGKTSLAQHVFNDPMIDGKFDIKAWISVPQEFD
VLNVSKAILDTIAGSTDHSIQQELVQRRLKEKLTGKKFLFVLDDVWNERQSKWEDVQKPLLFGGQGSRILVTTRSEKVAI
TMRSEKYLLQVLREDYCWDLFAKHAFQSGTNPQPDPEFMEIGKKIVGKCNGLPLALKTMGSLLHNKSSLCEWESIMKS
EIWDFSENESDILPALRLSYLYLPSHLKKCFEFCALFPKGYIFNKKCLIELWMAENLLESPVQKKSPEEVGKQYFSDLLSLSFF
QQLGKEEGVCFIMHDLLNDLAKYVSDGIYIRLGVDELKGIHKTTRHLSFSTTRSLFDGFGSLIDTQKLHTFIPTSWYWSWK
MLADDNFVSKFKFMRVLSLSHCHNLREVPKSVENLKHLRSLDLSYTSIEKLPESISLLYKLQILKLNGRRRLKELPSYLYQLH
NLCCLEFIASGVRNFPTHLGNLKNLQVWMSSFCVKKSKEFNIQLLGELNLHGSLTIDELQNIENPSYALEADLKNKPHLVG
LRLIWNFVGCSSFDSIKVRDVIENLQPSKHLKTLSIVNFCGKQFPNWLLNNSVPNLVSLVLRKCKSCQRLPPLGVLPFLKRL
EISGFDEIVNIDADFHGNNSSSFKSLQTLEFSYMTQWEKWECQAVTGAFPRLQRLSISSCPKLKGQLPEFVALKKLEVFDY
DPDPLRNFSLNFFPSWTTLTINGCANVEEMISHDHTNNLLEDLTIKKCPKLESLPANMHMLLPSLKRLSIYDCPRLESFPD
RGLPSNISYMTLINCSRLIDSLKGALGGNPYCLRSLWIGKMDAECFPNEGLLPLSLTSLAISHCRNLKELDYKGLHQLSSLK
TLSLCVCSNLQCLPEEGLPKSVSYLEIGECPLLKERCQKEGGKDWKKIAHIVTVKIW

>XP_027910047.1

MPVLETLGGALCGAVLQVLLDKLDSHKVVDYFRGRKLDEKLLKYLRRKLVSINAVVDDAELKQFVDTYVKSWLDEVRDV
LFDAEDLLDEIDYEFSKSASELEYRSSASKVSSLESKLKEVVDDLESLLNQKDDLGLKNAASVRSELGNKVLERKNESSSLVA
EDVIYGRDDDKEIILNWLTSDSNNHNRLSILSIVGMGGMGKTTIAQHVYNDPKMKEVRFDEKAWVCVSDEFDVLKVSK
AIIGAFTKVKDDSETIEMVHGKLKEKLTGRKFLLVLDDVWNEDRKDWKTLKTPLRCGAKGSKITRSHKVASIMQSTYIHH
LKQLDEDHSWQVFAKHAFQDENSRLNYELKEIGMKIVGKCKGLPLGLETLGCLLHTKSFTSEWEGVLRSEIWDLAIEESKI
IPALLLSYYHLPSHLKRCFAFCALFPKDHKFDKESLILLWMAQNFLQCSQRSKSPEEVGEHYFNDLLSRSFFQPSLIYNYPC
FVMHDLLNDLAKYVSGEMCFRLGVDRAERVPKTIRHFSTVIDPVRCHKSLCDAKGLRTFISIRGGCGMALQELISNFKFL
RLLSLPWCCNIKEMSDSIGNLIHLRSLDLSNTKIKEVSDTLGNLIYLCSLDLSGTRIERLPNSTYLPCNLQVLKLNKCLYLKELP
STLHELTNLCRLELVGTTLRKDPLHLGKLENLQLWMDRFEVGKSNESIIQLGEIDLHGKLSIRNVENIVNPCDALLVNLKD
KTHLKLLQTFCLTERGFGNQPFPRIFVYWRCGCGAFSG

>XP_027910048.1

MPVLETLGGALCGAVLQVLLDKLDSHKVVDYFRGRKLDEKLLKYLRRKLVSINAVVDDAELKQFVDTYVKSWLDEVRDV
LFDAEDLLDEIDYEFSKSASELEYRSSASKVSSLESKLKEVVDDLESLLNQKDDLGLKNAASVRSELGNKVLERKNESSSLVA
EDVIYGRDDDKEIILNWLTSDSNNHNRLSILSIVGMGGMGKTTIAQHVYNDPKMKEVRFDEKAWVCVSDEFDVLKVSK
AIIGAFTKVKDDSETIEMVHGKLKEKLTGRKFLLVLDDVWNEDRKDWKTLKTPLRCGAKGSKITRSHKVASIMQSTYIHH
LKQLDEDHSWQVFAKHAFQDENSRLNYELKEIGMKIVGKCKGLPLGLETLGCLLHTKSFTSEWEGVLRSEIWDLAIEESKI
IPALLLSYYHLPSHLKRCFAFCALFPKDHKFDKESLILLWMAQNFLQCSQRSWELIEQKEYQRQSVTFPL

>XP_027910233.1

MADSVVSFVLDHLAQLVAREANLLYGVEDRVQSLQYELQRMKELLSSTKSKKGMEHTVLNQIRDVSHLAEDVIDTFVA
KVSIHKRRTIMGRMLLGFGQAKLLRDVAEKIDKIKATLKEIRDNKSKYDAFKETNNQSAAEEEEEEEKEGAKSLHKLRRYV
EEDDVVGFVHDSKDLIKRLLEGGSNRNAVSTIGMGGLGKTTLARKVYNSTQVKQHFKCRAWVYVSNECRVKELLLGLLK
T

>XP_027910316.1

MASNKIQRCSSSQTKNFDVFVSFRGEDTRNGFTDHLFAALQRKGVVAFRDDQTIEKGGFLESELLQAIEGSRVFIVVFSK
DYASSTWCMKELTKILDLVEETGRSMLPIFYDVDPSEVRKQSSEFAKAFAEHEERFKDDLEMVEKWREALKASCDRCG
WDVQNKKQYEEIENVVEKVIDILGRNQIWSFGDDLVDMHSRVEELEELLDLSANDIVRIVGICGMGGIGKTTVATSLFK
KISPQYNSCCYIDDLNKIYCNFGATSAQKQLLSQALNEIKNMEIHNVFHGTMLIRTRLRHLKMLVVLDNVDEVEQLEKLG
LRPEYLGAGSRLVIISRDRHILQNYGVNEVYNVEVLDETQALQLFCKKVFKSHDIPKEYKELTLEALKYANGLPLAIKVLGSF
LHDRDVSEWRNALARMKENPSKHIMDVLRISYDALENLEKEIFLDIACFFSNRNNYSYETRVKRLLEYRQFYPDIGMKVLI
EKSLISCQDGEIEMHDLLKELGKSIVREKAPKEPRKWNRLWNYKDLQKVMKINKETENVEAIVIHQSEKEFLEDVDALSK
MNQLELLILENVNCSGTLDCISNELRYLQWNHFPLMSLPSNFHPDQLVELILPHSNIKKLWEGKKFLPNLTMLDMSHSK
YLIAVPDLSEVPRLESLDLEGCIQLVEIHPSIGILRELRCLNLKNCKNLVLNLNMLFGISSLSGLYLSGCSKLLNSKMLMEPRD
TKHLEEIVKITNAIQFPTSSVYKLLMLPFNFLYPPKPEDSLGLVLSLSSVPCLVYLDISFCNLLRIPDEIGNLHSLVGLNLGGNK
FVTLPSTIKQLSNLERLYLEYCKQLKYLPELPTIKQKNIGGYYGLGLYIFDCTKLSDMEHCYSMVFSWMTQNLQVFFQPTIS
SARIEMEIVIPGSEIPKWFNKQSASTSISMDPSDVIDDPNWIGVAICVLFVTLQDPMNLGERDDDHLSNLWFGVNNSKR
RWYIGVPIYFKKDLVTVGLDHLVTVFYSRQQFIHLLSTPPNTMHDLYQTEFGTFIHHPKGLRIVVKKCGYRWVFKEDLQQ
FNSNMFFSTNSSSRKRKLLTSD

>XP_027910317.1

MGGIGKTTLATALFNKISPQYDACCYIDDLSKIYFNFGATSAQKQLLCQALNQGNMEIHNVSRGTMLMKSRLCRLKTLL
VLDNVDEVEHLEKLGLRPEYLGAGSRLIIISRDRCILQSYGVKEVYDVQLLDKIEALQLFCKKAFKSNDIGREHEELTLDVLQ
YANGLPLAIKVLGSLLHDRDVSEWRNALARMKENPSKHIMDVLRISYDALENLEKEIFLDIACFFSNRNNYSYETRVKRLL
EYRQFYPDIGMKVLIEKSLISCQDGEIEMHDLLKELGKSIVREKAPKEPRKWNRLWNYKDLQKVMKINKETENVEAIVIH
QSEKEFLEDVDALSKMNQLELLILENVNCSGTLDCISNELRYLQWNHFPLMSLPSNFHPDQLVELILPHSNIKKLWEGKK
FLPNLTMLDMSHSKYLIAVPDLSEVPRLESLDLEGCIQLVEIHPSIGILRELRCLNLKNCKNLVLNLNMLFGISSLSGLYLSGC
SKLLNSKMLMEPRDTKHLEEIVKITNAIQFPTSSVYKLLMLPFNFLYPPKPEDSLGLVLSLSSVPCLVYLDISFCNLLRIPDEI
GNLHSLVGLNLGGNKFVTLPSTIKQLSNLERLYLEYCKQLKYLPELPTIKQKNIGGYYGLGLYIFDCTKLSDMEHCYSMVFS
WMTQNLQVFFQPTISSARIEMEIVIPGSEIPKWFNKQSASTSISMDPSDVIDDPNWIGVAICVLFVTLQDPMNLGERDD
DHLSNLWFGVNNSKRRWYIGVPIYFKKDLVTVGLDHLVTVFYSRQQFIHLLSTPPNTMHDLYQTEFGTFIHHPKGLRIVV
KKCGYRWVFKEDLQQFNSNMFFSTNSSSRKRKLLTSD

>XP_027910318.1

MGGIGKTTLATALFNKISPQYDACCYIDDLSKIYFNFGATSAQKQLLCQALNQGNMEIHNVSRGTMLMKSRLCRLKTLL
VLDNVDEVEHLEKLGLRPEYLGAGSRLIIISRDRCILQSYGVKEVYDVQLLDKIEALQLFCKKAFKSNDIGREHEELTLDVLQ
YANGLPLAIKVLGSLLHDRDVSEWRNALARMKENPSKHIMDVLRISYDALENLEKEIFLDIACFFSNRNNYSYETRVKRLL
EYRQFYPDIGMKVLIEKSLISCQDGEIEMHDLLKELGKSIVREKAPKEPRKWNRLWNYKDLQKVMKINKETENVEAIVIH
QSEKEFLEDVDALSKMNQLELLILENVNCSGTLDCISNELRYLQWNHFPLMSLPSNFHPDQLVELILPHSNIKKLWEGKK
FLPNLTMLDMSHSKYLIAVPDLSEVPRLESLDLEGCIQLVEIHPSIGILRELRCLNLKNCKNLVLNLNMLFGISSLSGLYLSGC
SKLLNSKMLMEPRDTKHLEEIVKITNAIQFPTSSVYKLLMLPFNFLYPPKPEDSLGLVLSLSSVPCLVYLDISFCNLLRIPDEI
GNLHSLVGLNLGGNKFVTLPSTIKQLSNLERLYLEYCKQLKYLPELPTIKQKNIGGYYGLGLYIFDCTKLSDMEHCYSMVFS
WMTQNLQVFFQPTISSARIEMEIVIPGSEIPKWFNKQSASTSISMDPSDVIDDPNWIGVAICVLFVTHQDPMNLGERD
DDHLSTFSFGVNNSKRRVYIGVPIYFKKDLVTVGLDHLVKVFYSRQQFIHLLSTPPNTMHDLYQTEFGTLIHHPKGLRIVV
KKCGYRWVFKEDLQQFNSNMFFSTNSSSPKRKLLTRD

>XP_027910331.1

MVISEVVKPLIAPVCMAIFTFFTRNDVDAAILEELKTQVLCLNSFLGDAEEKQVSNPGVKIWVDELREAVYDADDVMDQ
LAAKHLIGSTIVLSKVTDYLNCIIEPFDEGMKSKLNRINYRLKSLAHNRSLLGLKEGGGTCLINEAEVYGRDGDRDHVVDTL
LSEPTCLPNVIAVVGMAGMGKTTLSQMVYHHSAVKSNFNVRSWVYISDGSNVFEVTKKIYESLTYCNCEIRDLNTLQM
KLQLVLHNKKFLLVLDDYWTGGFLDWDLLKRPLESGKHGSCIIVTTRNRSVALTIHAAETYMLPRLEDEACRKLFTHHAF
RSKDPEKTPALKDIGEEIVKKCKGHPLSVKALASLLTFNAEVKDWNGVLQSKVLNIPANQSNILHSLMLSYRYLPGHMKQ
CFAYCSLFPKGHKESKRNCIHLWMAEGLLPESKNEETTEEVGEKYFNEFSSRSILQVENSLFYMYDLMNDLAQYVGGQF
FHKMEPNDHQSQNKVRHLSYLLEEFEGVEKFVSIFQLPHLRTFLPFAN

>XP_027910332.1

MMTKNLSLTGSHSNTAKNLSVLSIVGMPGLGKTALAQHVFNDPRMSEAKFDIKVWVCVSDEFDVFNISRAILEGVTRS
TDDSRDTEMVHRRLKEKLMGKKFLLVLDDVLNKNQSKWEEVQKVLVFGAQGSRILVTTRNKDVASTMRSEEYFPKLLQ
EDDCWKLFAKYAFRDDDTQPNPECRDIAMEIVKKCKGLPLALKTMGSLLYNKSSVSEWETVFRSEIWELPKVRCDIVPTL
ALSYIHLPSHLKACFSYFALFPKDYEFKKEHLMHLWMTKNLCCRQHSRTPEEVCQQYFNDLLSRSFIQQSGQEEEVFVM
HDLLNDLAKYVAGDIYLRCEVGQIEKIQKETRHFSVELEYDDQYFDGFGTLCNTERLRTFMPISRSRFSYHWHCGINMLI
HELFSKFKFIRILSLSGYSDIKEIPDSIDNLKHLRSLDLSHTAIEKLTGKICLLSHLQILKLNFCKNLELSSNLHLLTNLCRLEFIST
TLAKVPPHLGKLNNLKVMMDSFNVDHGRRVGCSLKEAF

>XP_027910336.1

MAAELITGALVSTFVEKTIDNLASRFGDIFRGNKSNKKMLSNLKVKLLAIDVVADDAEQKQFTNPRVRDWLLAAKDVVF
DAEDLLEEIDDALSKSQAEAESQTAAKKVWNFLKSSFVSFFENEIESRMEKLIENLEDLATQNHVLGLKRNDDVEKEEDC
WNLFAKHAFRAPPNQECREIGMKIVEKCKGLPLVLKTMGSLLYKISSVSEWETVFQSKIWEFSEDSCDIVPALALSYIHLP
SHLKICFAYCGLFPKDYI

>XP_027910338.1

MVTGALVSTFVERTIDTLASLFGDVFRARKHNKKQLSDLKMKLLAIDVVAFDAEQKQFTDSRVRNWLLKAKDVVLDAE
DLLEEIDYELSKSQVETESQSATNKVWISLRSSFFSFFENEIKSMMEQVIEDLEHLATQSCFLGLKKGSGGVGSGSGIIYTFY
GGLVKTSLAQHAFNDPRLEAKFDIKAWINVPQEFDVLNVSRAILDTITGSTERSMQQEVIQRKLKKKIMGKKFLLVLDDV
WNEKQSKWEEVQKPLAFAVQSSRILVTTQSDKVAITMRSEKHLLQVLKEDYCWELFAKHAFQGPNPQPDLEFIVIGEKI
VEKCNGLPLALKTMGSLLHNKSLIREWENIMKSGIWDFQKMKVIYYLL

>XP_027910357.1

MPVLETLGGALFGAVLQVLFDKLDSDQVLGFFRGRNLEEKLLKKLKRKLMDVNAVIDDAEQKQFSNSLVREWLDELRD
VLYDAEDLLEQIDYEYSKTKLEAEFHTSSSKVHSFESKIIALRDDLKSLLKQKIVEDFKICISVRSGLGNKVSEKRNESSSLVAE
EVIFGREEEKEMIFSWLTSETNDNNVSILSIVGMGGIGKTTLAQHVYDDPKTKEAKFDEKAWVCVSDEFDVLKVSKAIIG
AFTESKDDSEDLQMAHGKLKNKLSGRKFLLVLDNVWNEDRNQWKSLQTPLKYGAKGSKILVTTRSNKVASAMGSNNT
HELKQLGEDHSWQVFAKHAFQDENCELKEIGMEIVEKCKGLPLALETLGCLLRTKSSVSDWEGVLRSNIWDLPIENSKI
MPALLLSYYHLPSYLKRCFAYCALYPKDYKFDKESFILLWMAQNFLHCSQESKSLEDVGEHYFNDLLSRSFFQQIITDIETY
FVMHDLLNDLAKYVSGVICYNLVDREERISRKTRHFVMVFDDVQYHESLHDAKGLRTFISISEYYMISIQELISSFKFLRVLS
LSECSEVPDTIGDLIHLCSLDFSGARISRLPDSISSLYNLQVLKLNECSYLTELPSSLYELTNLRRLELIGTTLRKVPPLLGDLKN
FQVWMDKFHVGKSCEFSIHHLQEVDHHGELSVRNLENMVNCCALVADFKNKTHLAGLRLEWDLERNIEDSTTEREVL
ENLQPSRHLEQLSINGYGGTQFPHWLCDNSLLNMVSLTLKHCKHCLWLPSLGLLTFLKHLTIEDLDWIESIDVDFYGNSS
WAFASLEMLSFTNMKEWKEWRCMLGAFCWISWQAYQKSTLV

>XP_027910359.1

MPVLETLGGALFGAVLQLLFDKLDSHQVLGYFRQRDLDEKQLKKLKRKLMDINAVIDDAEQKQFTNSLVKEWLDEVRDI
LYDAEDLLEQIHYEYSKTKLEAEFHTSSSKVHIFESKIIALLDDLESLLNQRIVRDFKIYSGDRSGLGSKVSEKKVESTSLVVEE
DIYGRDEDKEMIFNWLMSDTNDNKLSILSIVGMGGMGKTTLAQHVYNDMNTKEVKFDEKAWVCVSDEFDVFKLTRA
IFEAIHKSTDDSRNLDTVQGRLKEKLTGRKFLLVLDDVWNEDRDQWKSLQTPLKYGAKGSKILITTRSNKVASAVGSNNT
HELKQLREDHSWQVFAKHAFQDENCELNSELKEIGMQIVEKCKGLPLALETLGCLLRTKSSVSDWEGVLRSNIWDLPIE
NSKIMPALLLSYYHLPSHLKRCFAYCALYPKDHHFDKEKLIFSWMSQNFLHCSQESKSLEDVGEHYFNDLLSRSFFQQIIT
DIETYFVMHDLLNDLAKYVSGEICYSLDDREKRISRKTRHFLMAFDHVQYRESLYDAKGLRTFICISHYCMISIQELISNFKF
LRVLSLYRCSEVPDTIGNLIHLRSLDLSYTYIERLPDSTSSLCNLQVLKLNGCFYLKELPSTLYELTNLRRLELMGTTLIKVPPLL
GNLKNLQVWMDTFEVGKSCEFSVHQLQEVDLHGELSVRNLENMVNCCALGADFKNKTHLAELALEWDLERDIEDSTK
ERQVLEDLQPSRHLEELSINGYGGTQFPRWLSDNSLLNMVSLTLKHCKHCLWLPSLGLLTFLKHLTIEDLDGIESIGVDFY
GNSSCAFASLEMLSF

>XP_027910373.1

MPVLETLGGALFGAVLQVLFDKLDSHHVLNYFRGRNLDEKLLKKLKRKLMDINAVIDDAEQKQFSNSLVKEWLDEVRDT
LYDAEDILDEIDYEFSKTVLEAESQTSSSKVRSLESKMIEVLDDLESLSNQKVVQDFKISSSVRPGLDNKVSEKKVESTSLVA
EEVIYGRDEDKEMILSLLTSDTNDNKLSILSIVGMGGVGKTTLAQHLYNNPKTNEAKFDEKAWVCVSDVFDVLTVSKAV
FGAFTNSRDDSQGLEMVHRKLKERLSGRKFLLVLDDVWNEDRNQWKALQTPLMCGAKGSKILVTTRSSKVASIMQSS
YIHQLKQLRKDHSWQVLAKHAFQDENSKINSELEEIGMKIVEKCKGLPLALETLGCLLHTKSSVSEWESVLRSEIWDIPTE
DSQIIPALLLSYYHLPSHLKRCFSYCALFPKDYEFDKKSLILLWMAENFLQCSKHSKSPEEVGEQYFNDLLSRSFFQQIVTYN
KIYFVMHDLLNDLAKYVFGEICHRLGVDRVEKVSKKTRHLSTIIDPVQYYTSLRNAEGLRTFIFIDVDREMSIQREMSIQELI
SNLKFLRLLSLSSCYNIEEVPDSIGNLIHLRLLDLSNTPIKRLPKPVSSLCNLLVLRLNHCYDLKELPSTLHELTNLRCLELLGTT
LRKAPVLLGKLKNLQVWMKEFEFSIRHLGELDLHGQLSIQNLENIVNPCDILAAENSKL

>XP_027910467.1

MAESVVSFVLDHLSQLVAREANLLYGVEDRIQSLQHELQMMKELLSSTRSKKGMEHTVLNQIRDVSHLAEDLIDTFVAK
VSIYKRRTILGRMLRGFHQARLLHDVAEKIDKIKTTLNEIRENKDKYDAFKETSNQSAAEEEEEEKRAQSVQKLRRNVEEE
DVVGFVQDSKDVINRLLEGGSNRKVVSIIGMGGLGKTTLARKVYNSTQVKQHFMCHAWVYVSNECRLRELLLDLLKRL
MPDFEQQCRGKKKGKKNTLDINSLSEEELKKHVWNCLERKRYLVVVDDLWKRQDWDEVQDAFPDNNRGSRILITSRL
KEVALHAAHDVPHYLQFLNEEESWELFRRKVFRGEDYPSDLESLGKQMVQSCRGLPLSIIVLGGLLASKEKSHREWSKVV
GHVNWYLTQDETQVKDIVLNLSYDNLPRKLKPCFLYLGIFLEDSEIPVRPLLQKWVAEGFIQDTGSRDPDDVAEDYLYELI
DRSLVQVAKVETNGCVETCQVHDLLRDLCISESKEDKVFEVCTDNNILIPTKPRRLSIQSDMGHYISSSNNDHSCIRSLFFF

GPLYDVHGREWKWLLDDFKLVRVLEFGPNNFLKIPSKLGNFIHLRYLRMDATYIRFVPDSILNLWNLQTIDFGPWVYKIP
ISFPIQMWKLKYLRHLSTLGPIRLRGSCSGSDEKMWNLQTISALLLNRQATSLIKKETFPNLKKIGLKVAHAYEGELPKLLQ
TLQQSSHLNEFEIHFEDRYERRNGCTPQELFQSLGHFSSITVLEIQNHSELLTSVVTFPPNVTELTLSCIMCITDEGMIGLE
NHTKLKILRLWGYAFRFGDSFDLNCTRGGFPQLEVFQMTYMRVGKWKLENGAMLKLQSLIINKCKMLDDLPKELWCL
SGLKKVHVTNPSNQLGCMLRNLELNNGVQLVIDEPQIVIC

>XP_027910469.1

MGGMGKTTLAQHIFNDPKIKEARFDEKVWVWVSDDFDVLKVSKAIIRAFTKSKDDSEDLEMVHGKLKEKLTGRKFLLV
LDDVWNEDRDQWKSLQTPLKYGAKGSKILVTTRSSKVASTMESNTIHQLKQLRENHSWKVFAKHAVQDDNSKLNSK
MKEIGMKIVEKCKGLPLALETVGSLLHSKSTVSDWEGVLRSNIWYSKIIPTLLLSYYHLPSHLKRCFAYCALFPKNYEFEKES
LILLWMAQNFLHCSQQSKSHEVGEQYFNDLLSRSFFQQSIISDETRFVMHDFVNDLAKYVSGEVCFRLGVDKAERVPKT
TRHFSTMEDPFEYREFRSLSDAKGLRTFLTIHGYCRMSIQDLISNFKFLRVLSLSFCRNIEEVPDTVDNLIHLRLLDLSGTKIE
RLPDTTCSLCNLKVLKLNNCLYLGELPSTLHELTNLRLLELMGTTLRKVPLLLGELKNLQLWMGSFEVGNNVSEFNIQQLE
ELDLHGELSIKNIENIVNPCDALAADLKNKTHLVGLHLERDLKRNIDDSIKEREVLENLQPSKHLEKLKIDGYCGTEFPNWL
SDNSLSNVVSLVLMRCKHCLWLPSLGLFTFLKDLTIDGLDSIGRIDADFYGNSGSVFACLETLSFTDMKEWEEWHCIPGA
FPNLQRLTVKNCPKLKGHLPEQLSHLMELTIKHCDQLVGWIPRAVEIEDVKMEPSSVDMIGSGLSDTPLEYLIITSCPSINI
PINHCYHFLVELEIRHGCDSLKTFPLDLFPKLRKLILEECCNLQVIPQEHPHSHLKLLEIIKCSEFESFSNEGLFAPQLESFRIV
GLEKLKSMPKRMSSLLPSLNNLFIFDCAEVEFSEGCLPSNLNVMFLLNCFKLVASLKGAWGTNPSLKSLYIHKVDLECFPD
EGLLPPSLTKLSIDNCPNLKKLDYRGLCHLSSLQRLSLYNCPILQCLPEEGLPESISELEIKDCPLLK

>XP_027910513.1

MADSVVSFVLDHLAQLVAREANLLYGVEDRVQSLQYELQMMKELLSSTKRKKGMEHTVLNQIRDVSHLAEDLIDTFVA
KVSIYKRRTILGRMLRSFGQARSLHHVAEKIDNIKTTLNEIRDNKDKYDAFKESSNQSAAEEEEEEKRAQSLHKLRKNVEE
EDVVGFVQDSKDVINRLLEGDSNRKAVSIVGMGGLGKTTLARKVYNSSQVKQHFMCRAWVYVSNECRAKELLIGLLKH
LMPNFEQQGRGKKKGKKSAGDINELREEELKKLVRNCLEWKRYLVVVDDLWKRQDWDEVQDAFPDNNRGSRILITSR
LKEVALHAAHDVPHYLQFLSEEESWELFRRKVFRAENCPSDLEALGKQMVRSCRGLPLSIIVLAGLLANKEKSHREWSKV
VGHVNWYLTRDETQVKDIVLRLSYDNLPRRLKPCFLYLGLFPEDFEIPVMPLLQKWVAEGFIQDTGSRDPDDVAEDYLY
ELIDRSLVQAARVDTNGDVLVIRVHDLLRDLCILESKEDKVFEVCTDHNILIPTKPRRLSIHSKMDHYISSSYNDHSCVRSM
FFFGSIDYVGSRDWKWLFERLKLVRVLDFGLTPSSKIPSDIGNFIHLRYLKIQSHYVTFVVPDSILNLWNLETVELGPSRHNI
PISFPAQIWKLKHLRHLNISWAIGLRGSCLGSDEKMWNVQTVSTLVLNSQATSLIKKGTFPNVKKLGLRLTSECEGELPKL
LQSLQQLSYLNKLVIVLRDRDDAGVEHSSDESVKRNNGLKPQELLRSLGQFNCLTILTIDNVFDLLTCGLAFPPNVTELTLS
EIKCISDEGMNGLGNHTKLKILRLLGDRASCGDSFVVNCGRGSFPELEVVEMEILKVREWKLENGAMSRLQNVLINRCE
RLDDLPNELWSLSGLRKVQVKYPSLQMAHMLRNLEINSGCQLVIEN

>XP_027910556.1

MAESVVSFVLDHLVQLAAREANLLYGVEDRVQSLQYELQMIKELLNTTKRKKGMEHTVLNQIRDMAHVAEDVIDTFVA
KVAIYKRRTILGKMLRGFAQARLLHHVADKIDNIKATLNEIRDNKDRYGAFKETNNQSAAEEEEEEKRVQSQLKKLRRNV
EEEDVVGFVQESKDIIKRLLEGGSNRKAVSIIGMGGLGKTTLARKVYNSTQVEQHFMYRAWVYVSNECRVKDLLVGLLK
HLMPNFEKQSRGNKKGKKGTGDIDSLNEEKLKIPVQNWSKRKMYLLMPNLEQQSRANKKGKKGTGDIDNLSEEELKIL
VRNCLERKRYLVVVDDLWKRQDWDELQEAFPDNNSGSRILITSRLKEAALHTANDVPHYLQFLSEEESWELFCKRVFRG
EDCPSDLEALGKQMVQSCRGLPLSIIVLAGMLANKEKSHREWSKVVGHVNWYLTQDETQVKDIVLKLSYDNLPRRLKP
CFLYLGLFPEDFEIPVTPLLRKWVAEGFIQYTRNRDPDDIAEDYLYELIDRSLVQVARVETNAGVKTCQVHDLLRDLCISES
KEDKVFEVCTDQNILVPTKPRRLSIHSKMDHYISSSNNDHLCIRSLFFFGSDYYVDRREQKWLLKDFKLVRVLELAPKSCG
KIPSNIGNLMHLRYLRIDSQHITFVPDSILNLCSLQTIDLGDWTQNDPISFPIKMWKLKHLRHLNTRGPIKLRGSCLGSGEK

MWNLQTISPLVLNKQATSLIEKGTFPNIKRIGLNVVSGKGELSKLLQSLLLLRYLNKLVIVLRDSYDTTITVHEGVERKNGC
KPQELFQSLGQFSSLTVLEIGNVMDLLTCALIFPPNITELTLSKIMYITDEGMNGLGNHAKLKILRLLGDLIFWPFNSYDLT
CVGGSFPQLEVFQMEYLKVEKWNLGNGAMPRLQSLVINFCGKLDNLPNELWSLSDLKKVHVTKPSEQMTRILQNLEIN
NAVKLVTEDHPPEMDERDLNHLNYILKGFGYNDP

>XP_027910565.1

MMKEVLSSTRSKKGMKHTVLNQIRDVSHLAEDLIDTFVAKVSIYKRRTILGRMLRGFGQLRLLHRVADKIDKIETTLNEIR
DNKDKYDAFKETKNQSAAEEEEEEEKERAKSVQKLRRNVEEDDVVGFVQDSKDLIKRLLEGGSDRNAVSIIGMGGLGKT
ILARKVYNDTQVKQHFKCRAWVYVSNECRVKELLLGLLKT

>XP_027910568.1

MDTIASVASSVAVPLLRNITYVLMYSTYLTELETEIKKLQSEEKEVRHTVEAAKRGGEEIEDTVRDWFDRVRAAVEQGQT
FLEEEERERVGCMDVYSKYMNSQRARTLVEIIREVRKETFDRVSYRRALRCNVGPASREYVAIQSRTVMLNEVVKMLKD
GGVDIVGVYGVAGVGKTAMVKELAWQVEKDGLFDAVVVATVTDSPDVGRIRNEIADGLGLKFDELTELGRASRLRQRI
RQEQRILVVLDDVWGKLDLTKIGVPFGEDYKGCRCQLLVTSRNRNVLSSNFGSGKFYRLEVLSEDESWELFEKRAGDAV
SDPSIQSMAEKVAKSCAGLPLLIVTVVEELKNKDLYAWKDALEQITSFELEGDLYSPFRSAIELSYDHLESQELKTFFLLLGS
MGNGCSTRDLLVFGWCLGLHKHVDSLADGRNRLHKLIDNLRAACLLLDEGKRDSVVALEVVRHVAASIASRVKPFFTV
QRNKEFKWPRMDFFRTCHHIFLDRCYIRELPEVLECPKLKILQINSQGNYLKIPDDFFVEMKELKVLSLGGLNGTPSLPPSL
SLLTDLQALFLCKCKLEDIATVGSITSLEILNLEKSELKELPAEIGGLNNLRLLDLTDCPTLGGIPGNVISRLTSLEELYMGNCD
VQGEAKESKTQDNDSSLGELKHLNQITTLNVQIEDTSVFPRDMLSFGRLESYKILIGDGWKWSEVESESYKTSRLLKLNSG
ADPSILKDFGIKMLMNKAEDLYLAELKGVREVLYELNDEGFSQLKYLCILNCDEMESIIGSTEWSYSDHAFPNLESLILHNL
INMERICSDPLPAQAFRKLQVIKVKGCDRMEFLFSHSMVKHLSELVEIEISECKSMTNILSGQRQEDADAGQTDKIRLINL
SSLTLQCLPSLVSISPDSSTEASENGNGFSSQLFNNKVEFPNLETLKLYSINIHKIWNHHHSYFENLTSLTVDGCERLTYIFSY
PVAIKLVKLEHLILSSCKFVENIFVPDENLGHTHIHHFRRSAPTELIPIFTNLETFVISHMDNLKAIWPALLPENSFCKLKKME
ITSCNNLLNVFPCHVLDKLQSLESLNVWNCMALEVVYEIDAINREQEGSSQRVLDIPLRTLSLGNLPKLKHLWNKDPQG
NIKFQNLFMVKASKCQSLKYVFPLSLAKDLLHLQFLEISDCGVEEIIASDKGGVGALGFVFPKLVSIKLFNLPDLQCFCNGN
HNLRFPLLNQFYAVECPRMETFSGGILRASILRKIFMTREGDQWYWEGDLNTTIRKLVNRDLQTRLSIL

>XP_027910569.1

MDTIASVASSVAVPLLRNITYVLMYSTYLTELETEIKKLQSEEKEVRHTVEAAKRGGEEIEDTVRDWFDRVRAAVEQGQT
FLEEEERERVGCMDVYSKYMNSQRARTLVEIIREVRKETFDRVSYRRALRCNVGPASREYVAIQSRTVMLNEVVKMLKD
GGVDIVGVYGVAGVGKTAMVKELAWQVEKDGLFDAVVVATVTDSPDVGRIRNEIADGLGLKFDELTELGRASRLRQRI
RQEQRILVVLDDVWGKLDLTKIGVPFGEDYKGCRCQLLVTSRNRNVLSSNFGSGKFYRLEVLSEDESWELFEKRAGDAV
SDPSIQSMAEKVAKSCAGLPLLIVTVVEELKNKDLYAWKDALEQITSFELEGDLYSPFRSAIELSYDHLESQELKTFFLLLGS
MGNGCSTRDLLVFGWCLGLHKHVDSLADGRNRLHKLIDNLRAACLLLDEGKRDSVVALEVVRHVAASIASRVKPFFTV
QRNKEFKWPRMDFFRTCHHIFLDRCYIRELPEVLECPKLKILQINSQGNYLKIPDDFFVEMKELKVLSLGGLNGTPSLPPSL
SLLTDLQALFLCKCKLEDIATVGSITSLEILNLEKSELKELPAEIGGLNNLRLLDLTDCPTLGGIPGNVISRLTSLEELYMGNCD
VQGEAKESKTQDNDSSLGELKHLNQITTLNVQIEDTSVFPRDMLSFGRLESYKILIGDGWKWSEVESESYKTSRLLKLNSG
ADPSILKDFGIKMLMNKAEDLYLAELKGVREVLYELNDEGFSQLKYLCILNCDEMESIIGSTEWSYSDHAFPNLESLILHNL
INMERICSDPLPAQAFRKLQVIKVKGCDRMEFLFSHSMVKHLSELVEIEISECKSMTNILSGQRQEDADAGQTDKIRLINL
SSLTLQCLPSLVSISPDSSTEASENGNGFSSQLFNNKVEFPNLETLKLYSINIHKIWNHHHSYFENLTSLTVDGCERLTYIFSY
PVAIKLVKLEHLILSSCKFVENIFVPDENLGHTHIHHFRRSAPTELIPIFTNLETFVISHMDNLKAIWPALLPENSFCKLKKME
ITSCNNLLNVFPCHVLDKLQSLESLNVWNCMALEVVYEIDAINREQEGSSQRVLDIPLRTLSLGNLPKLKHLWNKDPQG

NIKFQNLFMVKASKCQSLKYVFPLSLAKDLLHLQFLEISDCGVEEIIASDKGGVGALGFVFPKLVSIKLFNLPDLQCFCNGN
HNLRFPLLNQFYAVECPRMETFSGGILRASILRKIFMTREGDQWYWEGDLNTTIRKLVNRDLQTRLSIL

>XP_027910598.1

MADTVVSFVLDHLAQLVAREANLLYGVEDRVQSLQYELQMIKQLLNTTKRKKGMEHTVLNQIRDMAHLAEDVIDTFV
AKVSIYKRRTILGKMLRGFHQARLLRDVAEKVDNIKITLNEIRDNKSKYEAFKETNNQSAAEEEEEEEKERAKALQKLRRN
VEEDDVVGFVHDSNDVIKRLLEGGSNRKAVSIIGMGGLGKTTLARKVYNSSQVTQHFMCRAWVYMSNECRVRELLLG
LLKHLMPDFEQQCRGNKKGKKSAGNISNLSEEELKILVRNCLESERYLVVVDDLWKKQDWDEVQDAFPDNNRGSRILIT
SRLKEVALHAAHDVPHYLQFLNEEESWKLFSRRVFRGEDYPSDLESLGKQMVQSCRGLPLSITVLAGMLANKEKSYREW
SKVVGHVNSYLTQDETQVKDIVLKLSYDNLPRRLKPCFLYLGIFPEDFEIPVVPLLQKWVAEGFIQDKGNRDPDDIAEDYL
YELIDRSLVQVAIVDTNGSVKTCQVHDLLRDLCISESKEDKVFEVCTDNNILIPTKPRRLSIHSSMGHYISSNGNDHSCIRSL
FFFGPDYHVHMKEWKWLSEGFKLVRVLEIGPTRWGKIPSNLGNFIHLRYLRMNSFNVKFVPTSILTLWNLQTIDIGPWR
RSVPISFPVQMWKLKHLRHLNTPQPIELRGSYSGSDEKMWNLQTISAILLNSQATSLIKKGTFPNLKSLGLKVAYGYKGEL
PKLLQSLQQLSYLNKLVIVLRDRHDTEVFVDESVERNNGCKPQELLRCLGQFNCLTILSIENCLDLLTCAVTLPPNVTELTLS
GIDYISDQGMNGLGNHTKLKILTLWGIWSCDTFNLNCVGGSFPQLEVFQMGHLEVGNWRLGNGAMPKLQKVVIFNC
DRLNDLPNELWSLSGLRKVQVRKPSEQMACMLRNLEVNNGVQLFINGININNF

>XP_027910607.1

MARAFGLLRLFEKLNSVQILGYFRGRDFHEKLLKKLKRKLMNINAVIDDAEQKQLSNSLVKKWLDEARGVLYDAEDLLEK
IDYELSKSELKSGFQTSESKERSFESKMREVLDDLDSLSNQQIVKDFKISSGDISGLGNEVLEKKVESSSLVAEDHVMYGRD
EDKEVIFSWLTSDTNGNKLSILSIVGMGGVGKTTLAQHVYNDPKTKEAKFDEKAWVCVSDEFDVFTMSKAIIEALSKSKY
DTGDLEMVHGKLKEKLSGRKFLLVLDDVWIVDPNQWKALQTPLTCGAMGSKILVTTRGNVVASILQSDYIHQLKQLNE
DHSWQAFGKHAFQDGNSKLNSELKEIGKKIVGKCAGLPLALQMVGCLLQSKSSVSEWEAVLRSSIWDLTIEEDSTIFPVL
LLSYYHLPSHLKRCFVYCALFPKNYEFDKESLILLWMAQNFLQYSQQSQSPEEVGEQYFNDLLSRSFFQQIIRYNKTYFVM
HDLLNDLAIYMSGEMCYRLGVDRVETLSKKTRHFLIVRGSKYSTNLHDAKGLRTFISCRWHRYMSIQEVISNFKSLRVLSL
HLCFEVPDTIGDLTHLRILDLSHTYIERLPDSICSLRNLQVLKLNDCTCLKELPSTLHELTDLCRLELMETTLTKAPLLLGKLN
NLQIWMNSFDFSIQELRELDLHGELSIKNLENILNPGDALAVDFKNKTHLVRLHLEWNLMQNNEDLTKEREVLENLQPS
CHLKELSMDGYSGTRFPHWLSDNSLSNVVSLTLKRCKQCLWLPSLGLLTFLKHLTIDGLDLIGRIDADFYANSSSAFASLET
LSFTDMKEWEEWQCMTGAFSNLQSLTVKNCPKLRGNLPEQISHLKELTIEQCQQLVASIQDVKMEQSSVDVIRSVVSD
TPFKFLSITFCPGMTIPIYQTYHFLLELRIIHGCDSLTTFPLDLFPKLCKLELGECRNLQMISQEHPHIHLKFLKIEKCYEFESFP
NEGLFAPELKRFQILGLEKLKSMPKRMSALLPSLNDMLISNCPEVELSDRSLPSNLKCMSLWSCSKLVASLKGAWGTEPS
L

>XP_027910635.1

MEESVVSFVLDHLAQLVAREANLLYGVEDRVQSLQYELQMIKELLSSTRSKKGMEHTVLNQIRDVSHLAEDVIDTFVAK
VSIYKRRTILGRMLRGFGQLRLLHRVADKIDKIKTTLNEIRDNKDKYDAFKETKNQSAAEEEEEEKRAQSVQKLRRNVEEE
DVVGFVHDSKDIIKRLLEGGSNRKAVSIVGMGGLGKTTLARKVYNSSQVKQRFMCRAWVYVSNECRAKELLIGLLKHL
MPNFEQQGRGKKKGKKSAGDINELSEEELKKLVRNCLEWKRYLVVVDDLWKKQDWDEVQDAFPDNNRGSRILITSRL
KEVALHATHDVPHYLQFLNEKESWELFHRKVFRGEDYPSDLESLGKQMVKSCRGLPLSIIVLAGLLANKEKSHREWSKVV
GHVNWYLTQDETQVKDIVLKLSYDNLPRRLKPCFLYLGLFPEDFEIPVTPLLQKWVAEGFIQDTGSRDPDDVAEDYLYELI
DRSLVQVAKVDTNASAETCRVHDLLRDLCILESKEDRVFEVCTDHNILIPTKPRRLSIHSKMDHYISSSNNDHSCVRSMFF
FGSIYYVRSWDWKWLFESLKLVRVLEFGVNGSNKIPSDIGNFIHLRYLRIDIEYVMFVPDSILNLWNLQTIDLGPPRRNVP
ISFPAKIWKLKHLRHLNTLRAIELRGSCSGSDVKMWNVQTVSSLVINSQATSLIKKGTFPNVKRLGLRVTSESEGELPKLL
QSLQQLSYLNKLVIVLRDRDDAGAEHSTDESVKRNSGFKPQELLRNLGQFNCLTILTIENAFDLLTYALTFPPNVTELTLSEI

NCISDEGMNGLGNHTKLKKLRLLGDVTWSGESFDLNCVEGGFSQLEVIEMEDLKVGKWKLGNGGMLRLHSVMIQNC
ARLDDLPNEIWSLSSLRKVQVMKPSEEMARMLRNLEIKNGVQLVTEDHQSRTDWTDFNILDCISEI

>XP_027910642.1

MTAEMIKGALVSSFVQITIDNLASRFGDIFRGDKSNKKMLSNLKVKLLAVDVVADDAEQKQFTDGRVREWLLQAKDAV
FDAEDLLEEIDHALSKTQVEAQSHSTATKVWNSLKSPFVSFFKNEIESRMEKLIENLEYLETQSHVLGLKRNDDVGEGSRS
GSKLRSTYLPNDSVIYGRDDDKEFVFNWLTSHTHNNLSILSIVGMGGVGKTLLAQHVINDPRTDEAKFDVKAWVCVSD
EFDVFKVSKTILEHVTRSTNNSRDIEMVHQSLKETLTGKKFLLILDDVWNENQSKWEEVQKPLLYGAQGSRIVVTTRSKE
VASTMRSKERFLEQLPEGPSLDLFAKHAFADDYDAQSNPECNKIGEKIVKKCKGLPLALKTIGSLLYNKLSVSEWEFVFQS
EIWDLPKERCNIVPALALSYIHLPPHLKVCFAYCALFPKDYKFKKEHLIELWITENFLQHGKSPEETGQQYFNELLSRSFFQ
RSGDAEEVFVMHDLLNDLAKYVAGDIYFRCEVGQTNEIQKVSRHVLFELRNHGCFSGFGTLCKTQRLRTFLLTPDRKMV
FFWKMIFFWSCNMSIHKLFTKFKFLRILSLSRCYSLKELPDSVGILEHLRSLDLSRTYIKKLSESICSLSHLQILKLNYCMDLEE
LPSNLHLITTLCRLEFTSTKVRKVPPGLEELKNLKVRMDIFKVDHSMESGIQRLGKLNNLHESLAIEGLQNIENPREALKAD
LKNKTHLLKLALGWERTGNSIDSKKEEDVIENLKPPKNLKELSIFNYGGKQLPNWLLENSLWNMVLLKLDGCESCQSLPP
LGLLPFLKDLYISGFDEIVSIDVGFHGNNSSSFQSLERLEFSNMRQWEKWECQAVTGAFPNLRILSIKDCPKLKGQLPELP
APLGMLEMRDCQQLEGFAPRALELKLHNCGKVQLDWATMEWLRMGGHHMKALFCERDGSHTLDELEIEESINDDSF
FLSIFPLDSFPTLEVLTLSRLKNLQMISLDQAHHHLDDLTISKCPKLESLPGSMHMLLPSLRSLCIKDCPRLELLSGGDLPSNL
TEMRLENCSRLVGSLKGGFRDGSYLGRLSIRELDAKCFPEEGLLPTSLTYLTIGDCPNLEELDYKGLSQLSSLRSLTLEHCPK
LQCLPEQGLPESISNLTIQNCPLLKQRCQRGGEDREKIAQIRYIYLFN

>XP_027910643.1

MTAEMIKGALVSSFVQITIDNLASRFGDIFRGDKSNKKMLSNLKVKLLAVDVVADDAEQKQFTDGRVREWLLQAKDAV
FDAEDLLEEIDHALSKTQVEAQSHSTATKVWNSLKSPFVSFFKNEIESRMEKLIENLEYLETQSHVLGLKRNDDVGEGSRS
GSKLRSTYLPNDSVIYGRDDDKEFVFNWLTSHTHNNLSILSIVGMGGVGKTLLAQHVINDPRTDEAKFDVKAWVCVSD
EFDVFKVSKTILEHVTRSTNNSRDIEMVHQSLKETLTGKKFLLILDDVWNENQSKWEEVQKPLLYGAQGSRIVVTTRSKE
VASTMRSKERFLEQLPEGPSLDLFAKHAFADDYDAQSNPECNKIGEKIVKKCKGLPLALKTIGSLLYNKLSVSEWEFVFQS
EIWDLPKERCNIVPALALSYIHLPPHLKVCFAYCALFPKDYKFKKEHLIELWITENFLQHGKSPEETGQQYFNELLSRSFFQ
RSGDAEEVFVMHDLLNDLAKYVAGDIYFRCEVGQTNEIQKVSRHVLFELRNHGCFSGFGTLCKTQRLRTFLLTPDRKMV
FFWKMIFFWSCNMSIHKLFTKFKFLRILSLSRCYSLKELPDSVGILEHLRSLDLSRTYIKKLSESICSLSHLQILKLNYCMDLEE
LPSNLHLITTLCRLEFTSTKVRKVPPGLEELKNLKVRMDIFKVDHSMESGIQRLGKLNNLHESLAIEGLQNIENPREALKAD
LKNKTHLLKLALGWERTGNSIDSKKEEDVIENLKPPKNLKELSIFNYGGKQLPNWLLENSLWNMVLLKLDGCESCQSLPP
LGLLPFLKDLYISGFDEIVSIDVGFHGNNSSSFQSLERLEFSNMRQWEKWECQAVTGAFPNLRILSIKDCPKLKGQLPELP
APLGMLEMRDCQQLEGFAPRALELKLHNCGKVQLDWATMEWLRMGGHHMKALFCERDGSHTLDELEIEESINDDSF
FLSIFPLDSFPTLEVLTLSRLKNLQMISLDQAHHHLDDLTISKCPKLESLPGSMHMLLPSLRSLCIKDCPRLELLSGGDLPSNL
TEMRLENCSRLVGSLKGGFRDGSYLGRLSIRELDAKCFPEEGLLPTSLTYLTIGDCPNLEELDYKGLSQLSSLRSLTLEHCPK
LQCLPEQGLPESISNLTIQNCPLLKQRCQRGGEDREKIAQIRYIYLFN

>XP_027910740.1

MKHTISHTFLSCDSETTCKMAEAVLKSVLGSLASPAVAELKPFLCFRREKEKLESMFTAIKATLEDAEEKQFSDRAIKDWV
GKLKDAAYELDDILDEFAYEQMRLEEEESEEVKCCISEMVLRSSLASFHPMNLYFRYNIVRRMNIVSERLDQIASEKNQLH
LTSTIQEETRGVPEWRQTFSFITEPKVYGREQDIKKIVEFLAGAASRPENLPVYPIVGQGGLGKTTLAKLIFNHNDLKDFQL
KIWVCVSEDFGLERILKAIIEAASEDVRKDLGLEATQRRLRKLLTGKRYLLVLDDVWDVMKQNWKDNWQVLRSLLDCG
EKGSSVLVTTRFSNVAQIMGTIKHPHMLPELSVDYCWELFKHQAFEADEVEPEELVVIGREIVKKCGGVPLAANTIGGLL
RFYRNKDKWLNILESNLLALSLNEKSIMPVLRLSYLNLPIELRQCFAYCAIFPKDERIEKQYLIEFWMANGLISSDGRLDAE

DVGDEVWNELYRRSLFQDIETDEFGKVTSFKMHDLVHDLAQVVADEEVCCITAEDYAPVLFERKRIHHLSDCRWWLNS
TELHQVKSLRTYIKSTATELSSDVLKCYSLRMLHVSGLEELSSSIDNLKYLSYLNLSRGRFKTLPESLCKLLNLKILKLDSCRRL
QKLPDGLVRLKALQQLSLKSCRSLSRLAPYIGKLNSLRSLSMYFVGEEKGFLLAELGQLKLKRDLEIKHLERVKNINDAKES
NMSSKQLNNLMLRWRTVREGELEGNDEEVLEALEPCTETLQSLRVEGYQGVRFPEWMSSPSLKKLTHLELWSCTNCIK
LPVLRNLRSLKWLEITKAKYVKYVQEECYDNDVGFMRLEYLSLRNLPSLIRLSSEDGENQFPCLSALDIEDCPHFSLQGLPS
LKLLRMSRNPKLKVCPGLECLPCVEDLTIESCDEVEGLQFMTGLKKLALIHLMNITLPDCFGDLPLLRELHIYGLLVDASSN
KP

>XP_027910797.1

MLERFIHCRNNIGMGEHQMISAVASFSNSSSMVSSKNYDVFLSFRGEDTRMNFTSHLHEALKEKKVETYIDYQLEKGDE
ISPAVFKAIEDSRVSIVILSENYASSKWCLEELSKILECHRNQEQIVIPVFYNVDPSHVRKQTGCYEQSFATHEEELRCNKW
RSALTAVANLAGWDSRNRIESQFIKDVVKDVIGKLTPRYPNELKGLVGIEKSFKQIESLLKIGSSEVRTVGIWGMGGIGKT
TLAIALYDKLSHEFEGRCVLTNIREKSDKLEGLRDELLSKLLGNKIHGIDYFDMRRLQRKKVFIVLDDVDTSEQIEKLILEYEF
LGPGSKVIVTTRNKQILSLVDEIYQVEELSSYHSLQLFCLTISGEKQPKDGYEDLSKRAILYCKGLPMALKVLGSNLRKKSKV
VWECELRKLQKIPNVQIHNVLKLSYECLDRSQKDIFLDIVCFFNGWERDRVTYILEACDFFAASGIETLLDKTLVTISNYSHI
KVNDLIQKMGWEIVHQESIKDLGRRSRLWKQEEVYDVLKYNKGTDFVEGMVLDLEQLAGDLYLSCDSLAKMTNMRIL
RIHRRKWGCRFSVHLPDGIELPYKLRYLEWEGFCLRSLPSNFCAEQLVELHMWNSKLKMLWDGVQNLVNLKTIDLDDS
RDLIEIPDLSMAEKLERVSLYDCESLCKLHPSISSLPKLKYLILSGCKVIQSVNVHSKSLNVLRLRGCSSLSEFSVTSEEMTHLD
LSQTAIRELLSPMISLPKLTYLYLSGCRHIENVNLHLRSLRVLTLVGCSCLKEFSVASDKLTLLELPDTAIGVLPSSIGHLLSLEE
LDLRGTNIECLPASIKSLSMLRVLWLNDCKKLVSVQELPPSLRELHINDCWKLVSLPELPPSVKEVSAFNCRSLEENITQEL
VLRHMLQSCIPDKHQQYPYNPVYFDGGYFIFPGDHITDNCAFHATESSITIPSLPRSHLWGYIFCIIIAKGPMSDHQFSCSI
YQEDILVGCGHRRFIGCENLISDHVLFFYHDVINFGGTDEVYGPFNSFTFMFEFNGDKHTIKGCGVFPVYLTSSGFKFCN
VDSQPRANGPKIGGCDKENLEQLFAAKRRKTA

>XP_027910834.1

MAESVVTFVFDHLAQLAAREANLLYGVEDRVQSLQYELQMMKELLSSTKSKKGMEHTVLNQIRDVSHLAEDVIDTFVA
KVAIYKRRSILGRMLRGFGQARLLRDVAEKIDKIKNTLNEIRDNKDKYDAFKENNNRSAAEEEEEEKRAQSVQKLRRNVE
EEDVVGFVQDSKDVINRLLEGSLNRKALSIIGMGGLGKTTLARKVYNNTQVKKHFTCRAWVYVSNECRVREILLGLHKH
LMPDFEQQSRSSKKGKKSSRDNSSLSEEELKILVRNDLEGERYLVVVDDLWKRQDWDAVQDVFPDNHRGSRILITSRLK
EVALHAAHDVPHYLRFLNEEESWELFRRKVFRAEDYPSDLEAMGKQMVQSCRGLPLSIIVLAGLLANKEKSFREWSKVV
GHVNWYLTQDETQVKDIVLKLSYDDLPRRLKPCFLYLGIYPEDMEIPVRPLLQKWVAEGFIQDTRNRDSDDVAEDYLYEL
MDRSLVQVAEVKTNGGVKVCHVHDLLRDLCISESKEEKVFEVCTDNNILIPVNSRRLSIHSNISHYISSSNNDHSCIRSLFF
FGPRCYVHEKEWKCLLEGFKLVRVLEVGPTKCEKTPSNLREFIHLRYLRIDSCFVEFIPSSILNLWNLQTIDIVSGSYSPISFP
VQMWKLKYLRHLNTSKPIKLRGNCSGSDEKIWNLQTISAILLNRQATTLIKKGAFPNLKRIGLKVDSAYKAELPKLLQSLQ
QSSHLNKLVISLQHRYTGVEECANESVETCNGCKPQELLQSLEQFNSLTVLKIENVLDLLTYALTFPPNVTELTFSRIKCITY
VGMKCLGNHTKLKILRLLGDVISVDSFDLNCIAGSFPQLEVFDMEHLRVGKWKLGNGAMPRLQRLVIYYCDWLDDLPN
QLWSLSHLRKVHIINPSEQITHRLRNLEINNEIQLVLCVEDYNEKVSKTLKHWERSII

>XP_027910835.1

MAESVVTFVFDHLAQLAAREANLLYGVEDRVQSLQYELQMMKELLSSTKSKKGMEHTVLNQIRDVSHLAEDVIDTFVA
KVAIYKRRSILGRMLRGFGQARLLRDVAEKIDKIKNTLNEIRDNKDKYDAFKENNNRSAAEEEEEEKRAQSVQKLRRNVE
EEDVVGFVQDSKDVINRLLEGSLNRKALSIIGMGGLGKTTLARKVYNNTQVKKHFTCRAWVYVSNECRVREILLGLHKH
LMPDFEQQSRSSKKGKKSSRDNSSLSEEELKILVRNDLEGERYLVVVDDLWKRQDWDAVQDVFPDNHRGSRILITSRLK
EVALHAAHDVPHYLRFLNEEESWELFRRKVFRAEDYPSDLEAMGKQMVQSCRGLPLSIIVLAGLLANKEKSFREWSKVV

GHVNWYLTQDETQVKDIVLKLSYDDLPRRLKPCFLYLGIYPEDMEIPVRPLLQKWVAEGFIQDTRNRDSDDVAEDYLYEL
MDRSLVQVAEVKTNGGVKVCHVHDLLRDLCISESKEEKVFEVCTDNNILIPVNSRRLSIHSNISHYISSSNNDHSCIRSLFF
FGPRCYVHEKEWKCLLEGFKLVRVLEVGPTKCEKTPSNLREFIHLRHCFWELFSNFFPCANVETQIFKAFEYIKAYQVARQ
LFRIR

>XP_027910842.1

MALEFVGGALLSAFLQVAFQKLASPQILDFFRVRKLDQKLLFKLETKLHSIHSLADDAERKQFTYSHVRSWLLKVKDAVLD
AEDLLDDIQMLSKRELDAETESQVFTGCTCKVFNFFKSSSISSRNKEIESRMEEILEILEFLSNQKGALRLKTASGVRSGLSN
ELPQKSQTTSSVVGTDIYGRDDDKKQIVDWLISDNNNSNQPSILSIVGMGGLGKTTLARHVFSDPRINKAKFDVKAWV
CVSAEFDVFKVSKVILEAVTKSTDDSGNLEMVHTRLKENLTEKKFLLVLDDVWNENQCNWEEVHKPLMFGVQGSRILV
TTRSKEVASTMRSEIHSLKQLQENDCWNLFVKYAFKDDDTQQNLEFTEIGKKIVEKCKGLPLALKTMGNLLYNKSSVSE
WESVFQSEIWEFSQERCDIIPALAVSYIHLPSHLKVCFAYCALFPKDYEFKKEHLMHLWMTENLLQCPSEKVCQQYFNDL
LSRSFFQQSDEEEEVFMMHDLLNDLAKYVGGGIYFRWEVGQKEKIQKVTRHYSVELGHNQYFNGFETPCNTKRLRTF
MLTSRGIDPFTRWGANMSIHEFFSKFRLLRILSLSCCDIKELPDSVANLEHLQLLDLSFTVLKTLSEKICSLCHLQILKLNYCR
YLEELPSNLHLLTSLCHLELISNNLKKLPPSLGKLKNLKVVMESFNVGRGGEFSIQQLGELNLDGSLSIGELQNIENSVDALE
AYLKNKTLLVKLKLQWRRNRDSIDSKKEEEVIKNLQPSKNLKELSILNYGGKQFPNWLHLLSNLVSLELDRCKSCERLPPLG
LLSNLKDLIIHRLDGIVSIDADFYGSNSSSFKSLQSLTFANMGQWEKWECKAGGFPNLQTLYISDCPKLKGELPEQLVPLKI
LHITHCEQLEASAPKALDLIAVYCGKLHLDWATMKSLMMQAPLLEIVWSNTVEYLHINWMSISDDCVAGRIFSLDSLPR
LRELYLGRFPNLEMISQDHVHDHLEGMTIVECPKFESLPANMHTLLPSLNELHIGDCPKLESFPEGGLPSNLKFMKLNNC
SRLVVGSLKGAFRDDPSLKRLSIEKVDVECFSDEGVFPLSLTELYIRDCPNLEKLDYKVSQLSSLKSLTLSICPNLQCLPEEGL
PSSISDLHIISCPLLKQRYQKGSEDWEKIAHIQNLLIE

>XP_027910845.1

MAVELITDAALSKFFEKTFDNVFSRLGDIFRGDKSKKKQLSNLKVKLLAVEVVTDDAEQKQFTDQRVREWLLSAKGFMF
DVEDLLEEINHALSKSQVEAESHSAAKKVWNSLKSPFVSFFKNEIESRMEKLIENLEYLETQSHVLGLKRNADVGEGSRSG
SKLRSTYLPNDSVIYGRDDDKEFVLNWLTSHTHNNLSILSIVGMGGVGKTTLAQHVFNDPRTDEAKFDVKAWVCVSDE
FDVFKVSKTILEHVTRSTDNSGDIEMVHQSLKETLTGKKFLLILDDVWNEDKSKWEEVQKPLLYGAQGSRIVVTTRSKEV
ASTIRSKERFLEQLPEGPSLELFAKHAFPDDYDAQSNPECNKIGEKIVKKCKGLPLALKTMGSLLYNKLSVSEWEFVFQSEI
WDLPEERCNIVPALALSYIHLPSHLKVCFAYCALFPKDYKFKKEHLIELWITENFLQHGKSPEETGQQYFNELLSRSFFQRS
GDAEEVFVMHDLLNDLAKYVAGDIYFRCELSQTNEIQKASRHFFFEPGDDGGFPGFGTLCKTQRLRTFLPTPNSFWICN
MSIHELFTKFKFLRILSLSNCCDIEELPDSVGNLQHLRSLDLSGTDIKKLSESICSLSHLQILKLNYCMDLEELPSNLHLITTLCR
LEFTFTKVRKVPPGLEELKNLKVMMDIFKVDHSMESGIQRLGKLNNLHESLSIQGLQDIENPRDALEADLKNKTHLTGLA
LVWERTGNFIDSKREEDVIENLKPPKNLKELSIFNYGGKQLPNWLLENSLWNMVLLKLDGCESCQRLPPLGLLPFLKKLDI
SGFDEIVSIDVDFHGNNSSSFQSLERLKFSNMRQWEKWECQAVTGAFPNLLMLSIKDCPKLKGQLPELPAPLGMLEMI
DCQQLEGFAPRVLKLELHNCGKVQLDWATMEWFRMGGHHMKALFSERDGSHTLDELEIVESITDDSKTPLMTFPLDS
FPTVTKLVLSGFGNLQMISLDQAHHHLDVLTISKCPKLESLPGSMHMLLPSLTSLCIKDCPRLESFTDGGLPSNLESLRIED
CPRLESLPDGGLLSNLREMRIIKCSKLKSLPGNMHMLLPSLKHIWIEDCPRLESFTDGGLPSNLESLIRIEDCPRLESLPDGG
LLSNLREMRIIKCSKLKSLPGNMHMLLPSLKHLWIEDCPRLESFPDGGLPSGLHEMTLKNCSRLVGSLKGAFRDGSYLGD
LSIKELDAKCFPEEGLLPASLTDLTISACPNLEELDYKGLSQLSSLRSLTLECCPKLQCLPKEGLPQSISYLFIIGCPLLEQRCQE
GGKDRKKIAHIQHSNLY

>XP_027910846.1

MAVELITDAALSKFFEKTFDNVFSRLGDIFRGDKSKKKQLSNLKVKLLAVEVVTDDAEQKQFTDQRVREWLLSAKGFMF
DVEDLLEEINHALSKSQVEAESHSAAKKVWNSLKSPFVSFFKNEIESRMEKLIENLEYLETQSHVLGLKRNADVGEGSRSG

SKLRSTYLPNDSVIYGRDDDKEFVLNWLTSHTHNNLSILSIVGMGGVGKTTLAQHVFNDPRTDEAKFDVKAWVCVSDE
FDVFKVSKTILEHVTRSTDNSGDIEMVHQSLKETLTGKKFLLILDDVWNEDKSKWEEVQKPLLYGAQGSRIVVTTRSKEV
ASTIRSKERFLEQLPEGPSLELFAKHAFPDDYDAQSNPECNKIGEKIVKKCKGLPLALKTMGSLLYNKLSVSEWEFVFQSEI
WDLPEERCNIVPALALSYIHLPSHLKVCFAYCALFPKDYKFKKEHLIELWITENFLQHGKSPEETGQQYFNELLSRSFFQRS
GDAEEVFVMHDLLNDLAKYVAGDIYFRCELSQTNEIQKASRHFFFEPGDDGGFPGFGTLCKTQRLRTFLPTPNSFWICN
MSIHELFTKFKFLRILSLSNCCDIEELPDSVGNLQHLRSLDLSGTDIKKLSESICSLSHLQILKLNYCMDLEELPSNLHLITTLCR
LEFTFTKVRKVPPGLEELKNLKVMMDIFKVDHSMESGIQRLGKLNNLHESLSIQGLQDIENPRDALEADLKNKTHLTGLA
LVWERTGNFIDSKREEDVIENLKPPKNLKELSIFNYGGKQLPNWLLENSLWNMVLLKLDGCESCQRLPPLGLLPFLKKLDI
SGFDEIVSIDVDFHGNNSSSFQSLERLKFSNMRQWEKWECQAVTGAFPNLLMLSIKDCPKLKGQLPELPAPLGMLEMI
DCQQLEGFAPRVLKLELHNCGKVQLDWATMEWFRMGGHHMKALFSERDGSHTLDELEIVESITDDSKTPLMTFPLDS
FPTVTKLVLSGFGNLQMISLDQAHHHLDVLTISKCPKLESLPGSMHMLLPSLTSLCIKDCPRLESFTDGGLPSNLESLRIED
CPRLESLPDGGLLSNLREMRIIKCSKLKSLPGNMHMLLPSLKHIWIEDCPRLESFTDGGLPSNLESLIRIEDCPRLESLPDGG
LLSNLREMRIIKCSKLKSLPGNMHMLLPSLKHLWIEDCPRLESFPDGGLPSGLHEMTLKNCSRLVGSLKGAFRDGSYLGD
LSIKELDAKCFPEEGLLPASLTDLTISACPNLEELDYKGLSQLSSLRSLTLECCPKLQCLPKEGLPQSISYLFIIGCPLLEQRCQE
GGKDRKKIAHIQHSNLY

>XP_027910847.1

MAVELITDAALSKFFEKTFDNVFSRLGDIFRGDKSKKKQLSNLKVKLLAVEVVTDDAEQKQFTDQRVREWLLSAKGFMF
DVEDLLEEINHALSKSQVEAESHSAAKKVWNSLKSPFVSFFKNEIESRMEKLIENLEYLETQSHVLGLKRNADVGEGSRSG
SKLRSTYLPNDSVIYGRDDDKEFVLNWLTSHTHNNLSILSIVGMGGVGKTTLAQHVFNDPRTDEAKFDVKAWVCVSDE
FDVFKVSKTILEHVTRSTDNSGDIEMVHQSLKETLTGKKFLLILDDVWNEDKSKWEEVQKPLLYGAQGSRIVVTTRSKEV
ASTIRSKERFLEQLPEGPSLELFAKHAFPDDYDAQSNPECNKIGEKIVKKCKGLPLALKTMGSLLYNKLSVSEWEFVFQSEI
WDLPEERCNIVPALALSYIHLPSHLKVCFAYCALFPKDYKFKKEHLIELWITENFLQHGKSPEETGQQYFNELLSRSFFQRS
GDAEEVFVMHDLLNDLAKYVAGDIYFRCELSQTNEIQKASRHFFFEPGDDGGFPGFGTLCKTQRLRTFLPTPNSFWICN
MSIHELFTKFKFLRILSLSNCCDIEELPDSVGNLQHLRSLDLSGTDIKKLSESICSLSHLQILKLNYCMDLEELPSNLHLITTLCR
LEFTFTKVRKVPPGLEELKNLKVMMDIFKVDHSMESGIQRLGKLNNLHESLSIQGLQDIENPRDALEADLKNKTHLTGLA
LVWERTGNFIDSKREEDVIENLKPPKNLKELSIFNYGGKQLPNWLLENSLWNMVLLKLDGCESCQRLPPLGLLPFLKKLDI
SGFDEIVSIDVDFHGNNSSSFQSLERLKFSNMRQWEKWECQAVTGAFPNLLMLSIKDCPKLKGQLPELPAPLGMLEMI
DCQQLEGFAPRVLKLELHNCGKVQLDWATMEWFRMGGHHMKALFSERDGSHTLDELEIVESITDDSKTPLMTFPLDS
FPTVTKLVLSGFGNLQMISLDQAHHHLDVLTISKCPKLESLPGSMHMLLPSLTSLCIKDCPRLESFTDGGLPSNLESLRIED
CPRLESLPDGGLLSNLREMRIIKCSKLKSLPGNMHMLLPSLKHIWIEDCPRLESFTDGGLPSNLESLIRIEDCPRLESLPDGG
LLSNLREMRIIKCSKLKSLPGNMHMLLPSLKHLWIEDCPRLESFPDGGLPSGLHEMTLKNCSRLVGSLKGAFRDGSYLGD
LSIKELDAKCFPEEGLLPASLTDLTISACPNLEELDYKGLSQLSSLRSLTLECCPKLQCLPKEGLPQSISYLFIIGCPLLEQRCQE
GGKDRKKIAHIQHSNLY

>XP_027910848.1

MAVELITDAALSKFFEKTFDNVFSRLGDIFRGDKSKKKQLSNLKVKLLAVEVVTDDAEQKQFTDQRVREWLLSAKGFMF
DVEDLLEEINHALSKSQVEAESHSAAKKVWNSLKSPFVSFFKNEIESRMEKLIENLEYLETQSHVLGLKRNADVGEGSRSG
SKLRSTYLPNDSVIYGRDDDKEFVLNWLTSHTHNNLSILSIVGMGGVGKTTLAQHVFNDPRTDEAKFDVKAWVCVSDE
FDVFKVSKTILEHVTRSTDNSGDIEMVHQSLKETLTGKKFLLILDDVWNEDKSKWEEVQKPLLYGAQGSRIVVTTRSKEV
ASTIRSKERFLEQLPEGPSLELFAKHAFPDDYDAQSNPECNKIGEKIVKKCKGLPLALKTMGSLLYNKLSVSEWEFVFQSEI
WDLPEERCNIVPALALSYIHLPSHLKVCFAYCALFPKDYKFKKEHLIELWITENFLQHGKSPEETGQQYFNELLSRSFFQRS
GDAEEVFVMHDLLNDLAKYVAGDIYFRCELSQTNEIQKASRHFFFEPGDDGGFPGFGTLCKTQRLRTFLPTPNSFWICN
MSIHELFTKFKFLRILSLSNCCDIEELPDSVGNLQHLRSLDLSGTDIKKLSESICSLSHLQILKLNYCMDLEELPSNLHLITTLCR

LEFTFTKVRKVPPGLEELKNLKVMMDIFKVDHSMESGIQRLGKLNNLHESLSIQGLQDIENPRDALEADLKNKTHLTGLA
LVWERTGNFIDSKREEDVIENLKPPKNLKELSIFNYGGKQLPNWLLENSLWNMVLLKLDGCESCQRLPPLGLLPFLKKLDI
SGFDEIVSIDVDFHGNNSSSFQSLERLKFSNMRQWEKWECQAVTGAFPNLLMLSIKDCPKLKGQLPELPAPLGMLEMI
DCQQLEGFAPRVLKLELHNCGKVQLDWATMEWFRMGGHHMKALFSERDGSHTLDELEIVESITDDSKTPLMTFPLDS
FPTVTKLVLSGFGNLQMISLDQAHHHLDVLTISKCPKLESLPGSMHMLLPSLTSLCIKDCPRLESFTDGGLPSNLESLRIED
CPRLESLPDGGLLSNLREMRIIKCSKLKSLPGNMHMLLPSLKHIWIEDCPRLESFTDGGLPSNLESLIRIEDCPRLESLPDGG
LLSNLREMRIIKCSKLKSLPGNMHMLLPSLKHLWIEDCPRLESFPDGGLPSGLHEMTLKNCSRLVGSLKGAFRDGSYLGD
LSIKELDAKCFPEEGLLPASLTDLTISACPNLEELDYKGLSQLSSLRSLTLECCPKLQCLPKEGLPQSISYLFIIGCPLLEQRCQE
GGKDRKKIAHIQHSNLY

>XP_027910850.1

MALELVGGALLSVFLDVAFQKLASPQILDFFHARKLDEKLLNKLETKLHSIHSLADDAEGKQFTDPHVRNWLLEVKDAVL
DAEDLLDDIQMLSKRGVDADSESQTFSGCTCKVLDFFKSSRISSLNKEIESRMEQILDGLEFLSSQKGALGLKKASGVGSG
LSSELPQKSQTTSLVVGTDFYGRDHDKELIFDWLISDDNSTNRPSILSIVGMGGVGKTTLAQHVFNDPRVDEAKFDFKA
WVCVSDEFDVFKVSRAILEAVIGSTDDSRNLEMVHRRLKEKLTEKKFLLVLDDVWNENQSKWEDVQKSLVFGAKGSRIL
VTTRSKEVASTMGSKEHSLKQLQRDDCWKLFAKHAFRDDDTEPNPECREIGMKIVEKCKGLPLALKTMGSLLYRKSSISE
WKSVFQSEIWDFSQDRYDIVPALALSYIHLSSHLKVCFAYCALFPKDYEFRKEDLFQLWMTENFLHCSQHSRTPEDVCQ
QYFNDLLSRSFFQQSDEKEEVFVMHDLIHDLANYVGGGIYFMWEVGKTEKIQKVTRHFSVKLRYNQYFDGFGKLCSTKK
LRTFMPTGGLLLNMSIHELFSKFKLLQMLSLCFVLNLEELPDSVGNLEHLRSLDLSNTAIKKLTEKICSLTLLQILKLNFCRDL
EELPSNLYLLTNLYRLEFIETKVRKVPPHLEKLKNLKVIMNFSVGHGREFGIQHS

>XP_027910872.1

MMKELLSSTKSKKGMEHTVLDQIRDVSHLAEDVIDTFVAKVSIYKTRTILGRMLRGFGQARLLRDVAEKIDKIKATLNEIR
DNKSKYDAFKETKNQSAAEEEEEEEERAQSVQKLKRNVEEDDVVGFVQDSKVVITRLLEGGSDRNAVSIIGMGGLGKTTL
ARKVYNSTQVKQHFKYRAWVYVSNECRVKELLLGLLKT

>XP_027910879.1

MLERFIHCRNNIGMGEHQMISAVASFSNSSSMVSSKNYDVFLSFRGEDTRMNFTSHLHEALKEKKVETYIDYQLEKGDE
ISPAVFKAIEDSRVSIVILSENYASSKWCLEELSKILECHRNQEQIVIPVFYNVDPSHVRKQTGCYEQSFATHEEELRCNKW
RSALTAVANLAGWDSRNRIESQFIKDVVKDVIGKLTPRYPNELKGLVGIEKSFKQIESLLKIGSSEVRTVGIWGMGGIGKT
TLAIALYDKLSHEFEGRCVLTNIREKSDKLEGLRDELLSKLLGNKIHGIDYFDMRRLQRKKVFIVLDDVDTSEQIEKLILEYEF
LGPGSKVIVTTRNKQILSLVDEIYQVEELSSYHSLQLFCLTISGEKQPKDGYEDLSKRAILYCKGLPMALKVLGSNLRKKSKV
VWECELRKLQKIPNVQIHNVLKLSYECLDRSQKDIFLDIVCFFNGWERDRVTYILEACDFFAASGIETLLDKTLVTISNYSHI
KVNDLIQKMGWEIVHQESIKDLGRRSRLWKQEEVYDVLKYNKGTDFVEGMVLDLEQLAGDLYLSCDSLAKMTNMRIL
RIHRRKWGCRFSVHLPDGIELPYKLRYLEWEGFCLRSLPSNFCAEQLVELHMWNSKLKMLWDGVQNLVNLKTIDLDDS
RDLIEIPDLSMAEKLERVSLYDCESLCKLHPSISSLPKLKYLILSGCKVIQSVNVHSKSLNVLRLRGCSSLSEFSVTSEEMTHLD
LSQTAIRELLSPMISLPKLTYLYLSGCRHIENVNLHLRSLRVLTLVGCSCLKEFSVASDKLTLLELPDTAIGVLPSSIGHLLSLEE
LDLRGTNIECLPASIKSLSMLRVLWLNDCKKLVSVQELPPSLRELHINDCWKLVSLPELPPSVKEVSAFNCRSLEENITQEL
VLRHMLQSCIPDKHQQYPYNPVYFDGGYFIFPGDHITDNCAFHATESSITIPSLPRSHLWGYIFCIIIAKGPMSDHQFSCSI
YQEDILVGCGHRRFIGCENLISDHVLFFYHDVINFGGTDEVYGPFNSFTFMFEFNGDKHTIKGCGVFPVYLTSSGFKFCN
VDSQPRANGPKIGGCDKENLEQLFAAKRRKTA

>XP_027910906.1

MPSKAIMQCSSSSSSQVTRIYDVFVSFRGEDTRNNFTDFLFQVLRRKGFHAFKDDADLRKGESIAPELQQAIEGSRIFIVV
FSKNYASSTWCLRELAHICYLVETPGRHVLPIFYDVDPSDVRKQSGYYEKPFVEFEERFREDNEGMEEVQRWREALTQV
ANLSGWDIRNRPQYAVIEEIVQKVTDVLGHKFSSLPNDNLVGMQFRVEELEKLLYMGSSNDVRVFGISGMGGVGKTTL
VRALYERICHQYDFTCYIDDVNQIYGDSSTLGVQKQLLSCSLNEKYEEICNVSEGTYLIWKMLQRKKTLIVLDNVDEVEQL
KIFTGNRDTMLRDCLGRGSIIIIISRDEQILRIHGVDEVHQVQPLEKEEAVQLFCRHAFKANIIMSDYEKMTNEVLWHVQ
GHPLALEILGSTLFGLSVSQWRSALAMLRENKSKKIMDVLRISFEALDSTNKEIFLDISCFLHNYEVENAVEILKFRGFYPDY
GLKVLINKSLLIVKDGRLIMHRLLIDLGRSIVREISPKEPRNWSRVWSSKDLQKILSNNMPAENLEAVVCNKFEYRKTTLN
ADAFSNVSHLKKLLKINGAYFSGILDYLPSELGYLCWIGYPFKSLPSRFVPYKLVSLSLTYSNIEQLWEDTKPLHNLKRLDLSFS
EDLIKMPDLGEAINLEWLSLRSCTKIKEIHPSIGLRRKLSYVTLEGCENLIKLPHFEETQNLEILDLESCIKLEKIHPSIGHIRKLK
FLNLKDCESLTMLPHFREDLNLEILNLQGCMKLRQINPSIHHLKKLTILNMEGCKRLVSLPYTILCLNSLSYLNLSACSNLCYI
QFLEEARDEGHLKRPCIGEASVRSSIMKRWFKWPLHLLHSRRHKDSASHFLPSPYFSCMRELDLSFCALLEIPDVVGSFH
RLERLNLSGNSFSKLPSLKELSKLYHLVLRHCEQLIYLPELPSHTYLPSVIYKRPWPTTETPVWTDGPQKQPGHYIKSGLEVL
NCPKLVEIERERCTRMTVSWMIQILQAQRRVDPLSMPMNPMFSSVIPGSEIPSFFNHQEDSGISCIDASRYGHNYVAAL
LCVVFGAQCRRGIVSPMCPSKARGIYNGSKIQMVLHDDLAIMDEFSDCMWIAFFNEHEFNRCQYSWFYDKILAIIGND
FDINVKKWGYRLIYDLDLELSTLAMMHGGNSSALKNELLAIEENK

>XP_027910908.1

MPSKAIMQCSSSSSSQVTRIYDVFVSFRGEDTRNNFTDFLFQVLRRKGFHAFKDDADLRKGESIAPELQQAIEGSRIFIVV
FSKNYASSTWCLRELAHICYLVETPGRHVLPIFYDVDPSDVRKQSGYYEKPFVEFEERFREDNEGMEEVQRWREALTQV
ANLSGWDIRNRPQYAVIEEIVQKVTDVLGHKFSSLPNDNLVGMQFRVEELEKLLYMGSSNDVRVFGISGMGGVGKTTL
VRALYERICHQYDFTCYIDDVNQIYGDSSTLGVQKQLLSCSLNEKYEEICNVSEGTYLIWKMLQRKKTLIVLDNVDEVEQL
KIFTGNRDTMLRDCLGRGSIIIIISRDEQILRIHGVDEVHQVQPLEKEEAVQLFCRHAFKANIIMSDYEKMTNEVLWHVQ
GHPLALEILGSTLFGLSVSQWRSALAMLRENKSKKIMDVLRISFEALDSTNKEIFLDISCFLHNYEVENAVEILKFRGFYPDY
GLKVLINKSLLIVKDGRLIMHRLLIDLGRSIVREISPKEPRNWSRVWSSKDLQKILSNNMPLHNLKRLDLSFSEDLIKMPDL
GEAINLEWLSLRSCTKIKEIHPSIGLRRKLSYVTLEGCENLIKLPHFEETQNLEILDLESCIKLEKIHPSIGHIRKLKFLNLKDCES
LTMLPHFREDLNLEILNLQGCMKLRQINPSIHHLKKLTILNMEGCKRLVSLPYTILCLNSLSYLNLSACSNLCYIQFLEEARD
EGHLKRPCIGEASVRSSIMKRWFKWPLHLLHSRRHKDSASHFLPSPYFSCMRELDLSFCALLEIPDVVGSFHRLERLNLSG
NSFSKLPSLKELSKLYHLVLRHCEQLIYLPELPSHTYLPSVIYKRPWPTTETPVWTDGPQKQPGHYIKSGLEVLNCPKLVEIE
RERCTRMTVSWMIQILQAQRRVDPLSMPMNPMFSSVIPGSEIPSFFNHQEDSGISCIDASRYGHNYVAALLCVVFGAQ
CRRGIVSPMCPSKARGIYNGSKIQMVLHDDLAIMDEFSDCMWIAFFNEHEFNRCQYSWFYDKILAIIGNDFDINVKKW
GYRLIYDLDLELSTLAMMHGGNSSALKNELLAIEENK

>XP_027910955.1

MLERFIHCRNNIGMGEHQMISAVASFSNSSSMVSSKNYDVFLSFRGEDTRMNFTSHLHEALKEKKVETYIDYQLEKGDE
ISPAVFKAIEDSRVSIVILSENYASSKWCLEELSKILECHRNQEQIVIPVFYNVDPSHVRKQTGCYEQSFATHEEELRCNKW
RSALTAVANLAGWDSRNRIESQFIKDVVKDVIGKLTPRYPNELKGLVGIEKSFKQIESLLKIGSSEVRTVGIWGMGGIGKT
TLAIALYDKLSHEFEGRCVLTNIREKSDKLEGLRDELLSKLLGNKIHGIDYFDMRRLQRKKVFIVLDDVDTSEQIEKLILEYEF
LGPGSKVIVTTRNKQILSLVDEIYQVEELSSYHSLQLFCLTISGEKQPKDGYEDLSKRAILYCKGLPMALKVLGSNLRKKSKV
VWECELRKLQKIPNVQIHNVLKLSYECLDRSQKDIFLDIVCFFNGWERDRVTYILEACDFFAASGIETLLDKTLVTISNYSHI
KVNDLIQKMGWEIVHQESIKDLGRRSRLWKQEEVYDVLKYNKGTDFVEGMVLDLEQLAGDLYLSCDSLAKMTNMRIL
RIHRRKWGCRFSVHLPDGIELPYKLRYLEWEGFCLRSLPSNFCAEQLVELHMWNSKLKMLWDGVQNLVNLKTIDLDDS
RDLIEIPDLSMAEKLERVSLYDCESLCKLHPSISSLPKLKYLILSGCKVIQSVNVHSKSLNVLRLRGCSSLSEFSVTSEEMTHLD
LSQTAIRELLSPMISLPKLTYLYLSGCRHIENVNLHLRSLRVLTLVGCSCLKEFSVASDKLTLLELPDTAIGVLPSSIGHLLSLEE
LDLRGTNIECLPASIKSLSMLRVLWLNDCKKLVSVQELPPSLRELHINDCWKLVSLPELPPSVKEVSAFNCRSLEENITQEL

VLRHMLQSCIPDKHQQYPYNPVYFDGGYFIFPGDHITDNCAFHATESSITIPSLPRSHLWGYIFCIIIAKGPMSDHQFSCSI
YQEDILVGCGHRRFIGCENLISDHVLFFYHDVINFGGTDEVYGPFNSFTFMFEFNGDKHTIKGCGVFPVYLTSSGFKFCN
VDSQPRANGPKIGGCDKENLEQLFAAKRRKTA

>XP_027911018.1

MPSKAIIQCSSSSSSQVTRTYDVFVSFRGEDTRNNFTDFLFQALRRKGFDAFKDDADLRKGESIAPELQQAIEGSRVFIVV
FSKNYASSTWCLRELAHICYLVETPGRHVLPIFYDVDPSDVRKQSGYYEKPFVEFEERFREDNEWMEEVQRWREALTQV
ANLSGWDIRNRPQYEGIEVIVRKVTDILGHKFSSLPNDNLVGMEFRVEELEKLLYSESSNEVRVFGISGMGGVGKTTLVR
ALYERICHQYDFTCYIDDVNQIYGDSSTLGVQKQLLSCSLNEKYEEICNVSEGTYLIWKRLHRKKALIVLDNVDEVEQLKVF
SGNRDTMLRDCLGRGSIIIIISRDEQILRIHGVDEVRHVQPLEEEEAVQLFCRHAFKANVIMSDFEKMTYEVLWHAQGH
PLAIEILGSTLFGLSVSQWRSALAMLRENKSKKIMDVLRISFEALDSTNKEIFLDISCFLHNYIMDHAVEILNIRGFYPDFGL
QVLINKSLLIVKHGIIKMHRLLIDLGQSIVREISPKEPRNWSRVWSCKDLQKILSNNMPAENLEAVVFNQFEFRKRTLSAD
AFSNLSHLKLLKINGPYFSGSLDYLPSELGYLYWNRYPFKSLPPRFLPYKLVSLSLTYSNIERLWEDTKPLHSLKHLDLSYSEK
LIKLPDLGEAINLERLCLRYCTKIREIHPSIGLLRKLSNVTLEGCENLIKLPHFEETQNLEILNLERCIKLEKLHPSIGHIRKLKSLY
LRHCESLTMLPDFGEDLNLERLYLDRCIKLRHINPSIHRLKKLTILCLAGCKRLVSLPITILCLNSLSDLYVSTCSNLSYIQLLEE
ARDEGHLKRPCVGEASVRSSIMKRWFKWPLHLLHSRTHKDSASHLLPSPNFSCIRVLQLSFCALLEIPDVVGSLHSLERLK
LSGNSFSKLPSLKELSKLHYLVLRHCKQLKYLPELPSQTYLPSPTTEFPVCTDTKDKEIDLHFVNSVLDALNCPKLDEIERERC
TKMAVSWMIQILQAQYRRDPLSITNKYFSSLTPGSEIPRLFNHQYKSSYVSSIDASPYRNDYVAAVFCVLFEARCKRGIVS
PMCPQKAKGGSKIPMLLDDDLAVMDNCSDYLWMTFLNKYEFLEYQHSRLYNNISEVVGDGFHISVKKWGYRLIYDLDL
ELSILARMHGGS

>XP_027911031.1

MASNESQRCSSSHTKNFDVFVSFRGTDTRNSFTNHLFEALQRKGVVAFRDDQTIQKGDFLDSELLHAIEGSQVFIVVFSK
NYASSTWCMKELTKIVAWVQQTARTLLPIFYDVDPSEVRKQSGEFAKAFAEHEERFKDDLEMVKEWREALKTSCDRCG
WDLKNKKQYEEIENVVEKVNDILGRNQIWSFGDDLVDMHSRVEELEELLDLSANDIVRIVGICGMGGIGKTTVATSLFK
KISPQYNARCYIDDLSKIYCNFGATSAQKQLLSQALNEIKNMEIHNVFHGTMLIRTRLRHLKMLVVLDNVDEVEQLEKLG
LRPEYLGAGSRLIIISRDCHILQNYGVNEVYNVEVLDETQALQLFCKKAFKSHDIPKEYKELTLEALKYANGLPLAIKVLGSFL
HDRDVCEWRSALARMNENPSKDIMDVLRISFDSLENLEKEIFLDIACFFSNRNGYSWKPTVKRLLEYRQFYPDIGMKVLI
EKSLISCQNKEIEMHDLLKELGKNIVREKAPKEPSKWSRLWSYKDFQKVMKLNKEAKNVEAIVIQQNENEFVQERIRVD
ALSKMGHLELLSLNNVKCFGTLDYISNELRYLYWDPFPWMSLPSTFHLDQLVELILPRSNIKQLWKGKKCVPNLTKLDLN
HSKNLIEVPDLSEVPLLMDLNLEGCIKLVQIHPSIGILRQLRHLDLRNCKNLIEVPNLSEVERLTDLTLEGCIEVVHIDSSIGN
LRQLIYLNLKNCKNLVLNLNILSGITSLRTLILSGCSNLHNSKMLRDSKVKKLLMSPFDFLYRPKPQDSCGLSWSLLSFSVPC
LVYLDISFCSLLQIPDEIGNLSSLIILNLGGNKFVTLPNTIKRLSNLHCLNLEHCKRLEYLPELPTVKERFIDEDMSLYIFDCPKL
RDMEHCYSTVFSWMMQNLQVYLEPGMEIVIPGSEIPKWFNKQNASTSISMDPSDVIDDPNWIGVAICVLFVTQQDP
MNLDENPCPLSTIQYWVDDDWLSTIPIYFEKDLVTVYVLW

>XP_027911032.1

MASNESQRCSSSHTKNFDVFVSFRGTDTRNSFTNHLFEALQRKGVVAFRDDQTIQKGDFLDSELLHAIEGSQVFIVVFSK
NYASSTWCMKELTKIVAWVQQTARTLLPIFYDVDPSEVRKQSGEFAKAFAEHEERFKDDLEMVKEWREALKTSCDRCG
WDLKNKKQYEEIENVVEKVNDILGRNQIWSFGDDLVDMHSRVEELEELLDLSANDIVRIVGICGMGGIGKTTVATSLFK
KISPQYNARCYIDDLSKIYCNFGATSAQKQLLSQALNEIKNMEIHNVFHGTMLIRTRLRHLKMLVVLDNVDEVEQLEKLG
LRPEYLGAGSRLIIISRDCHILQNYGVNEVYNVEVLDETQALQLFCKKAFKSHDIPKEYKELTLEALKYANGLPLAIKVLGSFL
HDRDVCEWRSALARMNENPSKDIMDVLRISFDSLENLEKEIFLDIACFFSNRNGYSWKPTVKRLLEYRQFYPDIGMKVLI
EKSLISCQNKEIEMHDLLKELGKNIVREKAPKEPSKWSRLWSYKDFQKVMKLNKEAKNVEAIVIQQNENEFVQERIRVD

ALSKMGHLELLSLNNVKCFGTLDYISNELRYLYWDPFPWMSLPSTFHLDQLVELILPRSNIKQLWKGKKCVPNLTKLDLN
HSKNLIEVPDLSEVPLLMDLNLEGCIKLVQIHPSIGILRQLRHLDLRNCKNLIEVPNLSEVERLTDLTLEGCIEVVHIDSSIGN
LRQLIYLNLKNCKNLVLNLNILSGITSLRTLILSGCSNLHNSKMLRDSKVKKLLMSPFDFLYRPKPQDSCGLSWSLLSFSVPC
LVYLDISFCSLLQIPDEIGNLSSLIILNLGGNKFVTLPNTIKRLSNLHCLNLEHCKRLEYLPELPTVKERFIDEDMSLYIFDCPKL
RDMEHCYSTVFSWMMQNLQVYVLW

>XP_027911038.1

MGEHQMISAVASFSNSSSMVSSKNYDVFLSFRGEDTRMNFTSHLHEALKEKKVETYIDYQLEKGDEISPAVFKAIEDSRV
SIVILSENYASSKWCLEELSKILECHRNQEQIVIPVFYNVDPSHVRKQTGCYEQSFATHEEELRCNKWRSALTAVANLAG
WDSRNRIESQFIKDVVKDVIGKLTPRYPNELKGLVGIEKSFKQIESLLKIGSSEVRTVGIWGMGGIGKTTLAIALYDKLSHE
FEGRCVLTNIREKSDKLEGLRDELLSKLLGNKIHGIDYFDMRRLQRKKVFIVLDDVDTSEQIEKLILEYEFLGPGSKVIVTTR
NKQILSLVDEIYQVEELSSYHSLQLFCLTISGEKQPKDGYEDLSKRAILYCKGLPMALKVLGSNLRKKSKVVWECELRKLQKI
PNVQIHNVLKLSYECLDRSQKDIFLDIVCFFNGWERDRVTYILEACDFFAASGIETLLDKTLVTISNYSHIKVNDLIQKMG
WEIVHQESIKDLGRRSRLWKQEEVYDVLKYNKGTDFVEGMVLDLEQLAGDLYLSCDSLAKMTNMRILRIHRRKWGCRF
SVHLPDGIELPYKLRYLEWEGFCLRSLPSNFCAEQLVELHMWNSKLKMLWDGVQNLVNLKTIDLDDSRDLIEIPDLSMA
EKLERVSLYDCESLCKLHPSISSLPKLKYLILSGCKVIQSVNVHSKSLNVLRLRGCSSLSEFSVTSEEMTHLDLSQTAIRELLSP
MISLPKLTYLYLSGCRHIENVNLHLRSLRVLTLVGCSCLKEFSVASDKLTLLELPDTAIGVLPSSIGHLLSLEELDLRGTNIECL
PASIKSLSMLRVLWLNDCKKLVSVQELPPSLRELHINDCWKLVSLPELPPSVKEVSAFNCRSLEENITQELVLRHMLQSCIP
DKHQQYPYNPVYFDGGYFIFPGDHITDNCAFHATESSITIPSLPRSHLWGYIFCIIIAKGPMSDHQFSCSIYQEDILVGCGH
RRFIGCENLISDHVLFFYHDVINFGGTDEVYGPFNSFTFMFEFNGDKHTIKGCGVFPVYLTSSGFKFCNVDSQPRANGPK
IGGCDKENLEQLFAAKRRKTA

>XP_027911041.1

MPSNAIIHHTSSSSHAISRNDVFVSFRGEDTRNNFIGFLFQALHRKGIGAFKDDEDLKKGESIAPKLLQSIQDSRVYLVVFS
KNYASSTWCLRELAEILNCVETSVRRVIPIFYDVDPSVVRKQSGCYDKAFAEHEKRFRGDKVKMEEAQRWREALTEVAN
LSGWDVRNKSQHIVIEKIVQEILSILSPNFSSIPNDLVGVESPLEELQQLLLCDPVDDVRIVGICGMGGIGKTTLATVLYGIIS
HQYNARCFIDDVNKIYRDCGTIGVVKQLLHQTLNEENLQIHNLYNAANLMRRRFRCVKTLIVLDHVDEVKEREKLVVNR
EWLGEGSRVIIISRDKHILKEFGVTTVYDVQLLNDANSLKLFCKKAFNSDEIVGGYKVLTHAVLQYAKSLPLAIKVLGSFLFG
RRVSEWRSALVRLKENPNKDILDVLQISYDGLDDLEKQIFLDIACFFSGYEELYVKKVLDCRGFHPEIGIRVLLDKSLVNNF
HGFIKMHDLLKALGRKIVQGNSPKEPAKWSRLWLYEHLFSMSKAKETANTEAIVLDMLREIEVLATDAEELSKMSNLRLL
ILHDVKFMGNLSFLSNKLQFLEWSHYPFSYLPSSFQPNSLVELILPHDNIKQLWKGTKYLPNLRALDLRDCKNLIKIPDFTG
LPNLEWIILDRCTKLVCIHPSVGLLRKLALLSLQNCINLVSLPSNILGLNSLEYLNISGCSRVFSNHLLDKPIHDHSKILDIREN
SMQSQSTSSSIIKKLIVPFNFSYYRGFRNSRGCLLHSLHNFFCMHDLNISFCNLAQIPDAIGSMHSLETLNLGGNHFVTLPS
SINKLSKLVHLNLEHCKQLRFFPEMPSPTALPVTRETYSFGRYERGLLIFNCPKIVDIEGCQRMTFAWFLQILRLSKESRTPI
NLIDIVLPGNQIPRWFNQSLGTSISLDPSPIMHDNNWMGIAFSLVFVAYENQSNLDLDLGSYVGIGFHTNPFFSSIEMPV
LLTDLVTVGMHHLWLLFLTREEFFSYFQIEGTLYVNGMEMHTTAGDSQGLHIEVISCGYQWVFEKDLDNLNPTKIRKEH
INTHFGDEGNSILSLVDGH

>XP_027911042.1

MPSNAIIHHTSSSSHAISRNDVFVSFRGEDTRNNFIGFLFQALHRKGIGAFKDDEDLKKGESIAPKLLQSIQDSRVYLVVFS
KNYASSTWCLRELAEILNCVETSVRRVIPIFYDVDPSVVRKQSGCYDKAFAEHEKRFRGDKVKMEEAQRWREALTEVAN
LSGWDVRNKSQHIVIEKIVQEILSILSPNFSSIPNDLVGVESPLEELQQLLLCDPVDDVRIVGICGMGGIGKTTLATVLYGIIS
HQYNARCFIDDVNKIYRDCGTIGVVKQLLHQTLNEENLQIHNLYNAANLMRRRFRCVKTLIVLDHVDEVKEREKLVVNR
EWLGEGSRVIIISRDKHILKEFGVTTVYDVQLLNDANSLKLFCKKAFNSDEIVGGYKVLTHAVLQYAKSLPLAIKVLGSFLFG

RRVSEWRSALVRLKENPNKDILDVLQISYDGLDDLEKQIFLDIACFFSGYEELYVKKVLDCRGFHPEIGIRVLLDKSLVNNF
HGFIKMHDLLKALGRKIVQGNSPKEPAKWSRLWLYEHLFSMSKAKETANTEAIVLDMLREIEVLATDAEELSKMSNLRLL
ILHDVKFMGNLSFLSNKLQFLEWSHYPFSYLPSSFQPNSLVELILPHDNIKQLWKGTKYLPNLRALDLRDCKNLIKIPDFTG
LPNLEWIILDRCTKLVCIHPSVGLLRKLALLSLQNCINLVSLPSNILGLNSLEYLNISGCSRVFSNHLLDKPIHDHSKILDIREN
SMQSQSTSSSIIKKLIVPFNFSYYRGFRNSRGCLLHSLHNFFCMHDLNISFCNLAQIPDAIGSMHSLETLNLGGNHFVTLPS
SINKLSKLVHLNLEHCKQLRFFPEMPSPTALPVTRETYSFGRYERGLLIFNCPKIVDIEGCQRMTFAWFLQILRIPRWFNQ
SLGTSISLDPSPIMHDNNWMGIAFSLVFVAYENQSNLDLDLGSYVGIGFHTNPFFSSIEMPVLLTDLVTVGMHHLWLLF
LTREEFFSYFQIEGTLYVNGMEMHTTAGDSQGLHIEVISCGYQWVFEKDLDNLNPTKIRKEHINTHFGDEGNSILSLVDG
H

>XP_027911043.1

MEEAQRWREALTEVANLSGWDVRNKSQHIVIEKIVQEILSILSPNFSSIPNDLVGVESPLEELQQLLLCDPVDDVRIVGIC
GMGGIGKTTLATVLYGIISHQYNARCFIDDVNKIYRDCGTIGVVKQLLHQTLNEENLQIHNLYNAANLMRRRFRCVKTLI
VLDHVDEVKEREKLVVNREWLGEGSRVIIISRDKHILKEFGVTTVYDVQLLNDANSLKLFCKKAFNSDEIVGGYKVLTHAV
LQYAKSLPLAIKVLGSFLFGRRVSEWRSALVRLKENPNKDILDVLQISYDGLDDLEKQIFLDIACFFSGYEELYVKKVLDCRG
FHPEIGIRVLLDKSLVNNFHGFIKMHDLLKALGRKIVQGNSPKEPAKWSRLWLYEHLFSMSKAKETANTEAIVLDMLREI
EVLATDAEELSKMSNLRLLILHDVKFMGNLSFLSNKLQFLEWSHYPFSYLPSSFQPNSLVELILPHDNIKQLWKGTKYLPN
LRALDLRDCKNLIKIPDFTGLPNLEWIILDRCTKLVCIHPSVGLLRKLALLSLQNCINLVSLPSNILGLNSLEYLNISGCSRVFS
NHLLDKPIHDHSKILDIRENSMQSQSTSSSIIKKLIVPFNFSYYRGFRNSRGCLLHSLHNFFCMHDLNISFCNLAQIPDAIGS
MHSLETLNLGGNHFVTLPSSINKLSKLVHLNLEHCKQLRFFPEMPSPTALPVTRETYSFGRYERGLLIFNCPKIVDIEGCQR
MTFAWFLQILRLSKESRTPINLIDIVLPGNQIPRWFNQSLGTSISLDPSPIMHDNNWMGIAFSLVFVAYENQSNLDLDLG
SYVGIGFHTNPFFSSIEMPVLLTDLVTVGMHHLWLLFLTREEFFSYFQIEGTLYVNGMEMHTTAGDSQGLHIEVISCGYQ
WVFEKDLDNLNPTKIRKEHINTHFGDEGNSILSLVDGH

>XP_027911076.1

MAAEMVTGVLVSTFLGRTIDTLASRLFDIFHQKKHKKQLRNLKMKLLAIDVVAFDAEQKQFTDSCVRDWLLRAKDVVID
AEDLLDELDYELSKSQVEAESQSAAKKVWSSLNSSFLIENEIESRMAQVIEDLDDLADESNILGLKKGGGVEVGSGSSSKL
TYSSLPNESDICGRDDDKESIFKWLTSDTHNNLSILSIVGMGGLGKTSLAQHVFNDPRLEGKFDINVWVSVPQEFDVLKV
SRAILDTIASSTDHSIPKEVIQKRLKENLMGKKFLLVLDDVWNENSSKWEDVQKPLVFGGQGSRILVTARGEKVADSMR
SEKYRLEVLKEDYCWELFAKHAFQGANPQQDPDFVEIAKKIVKKCDGLPLALKTMGSLLHNKSFLWEWENIMRSDIWD
LSENESGILPALKLSYLHLPSHLKKCFAFCALLPKGYQFDKDILIQWWMAQNFLESHVQIKSPIEVGEQYFNDLLSWSFFQ
QSSNQDEERFIMHDLLNDLAKYVCKDVCIRIGVDEPEGISKTTRHCSFLSSELYFDGFGSSIDTQKLHTFTRTDPNSGWIW
YCKMSIDDLFSRFKLIRILSLNHYLNLTEVPESIGNLKHLRSLDLSCTNIEELPDSMSLLYKLQILKLNKCRRLKQFPSCLHKLQ
NLRCLELVGIGVENVAAHLGKLKNVQVSMSSFHVEKSKEMNIQRLGQFNLYGSLTIDDLQNIENPSDALEADLKSKPHL
MGLHLEWNFIGSSSVDSTKAEDIIENLRPSKYLKKLSIRNYIGKQFPNWLLHNSLPNLVSLVLDDCRSCERLPPLGLLPFLK
ELRIYRLDGIVSIDADFHGNNSSSFKSLQTLWFSDMRQWEKWDCQDVTGAFPRLEDFWIKNCPKLKAYLPKFVALKYLY
VSNCEQLEALIVSAIELLLQDCGKLQLDCSTMEKLTMDGHDMAASSVAMVGHMLFNTSLEDLSICSTLETISDDCVSLRI
FPLDFFPTLKRLELSGFPNLQMISQDHVHNHLQDLTIEKCPKFESLPANMHMLLPSLVGLHIEDCPTLESFPDGGLPSNLN
YIRLGNCFRLVGLLKGALGDSSSLESLGISTPDAECFLDGGLLPTSLTELQFLECQNLEKLDYKGLLQLSSLRRLYLLDCPNLQ
RLPEEGLPKSISSLHIIDCPLLKQRCQEGGEDWEKVAHIRNLYICSRKNLYKIELLK

>XP_027911077.1

MAAEMVTGVLVSTFLERTIDTLASRLFDIFHQRKHKKQLRNLKMKLLAIDVVALDAEQKQFTDSRVRDWLLRAKDVVID
AEDLLDELDYELSKSQVEAESQSAAKKVWSSLNSSFLIEKEIESRMAQVIEDLDDLADESNILGLKKGGGVEVGSGSSSKLT

YSSLPNESVIYGRNDDKEFLIQWLTSDTHNNLSILSIVGMGGLGKTSLAQHVFNDQRIKDKFGIKAWVSVPQEFDVLKVS
RAILDTITGSTERSMQQEVIQRSLKEELAGKKFLLVLDDVWNENPSKWEDVQKPLVFGGQGSRILVTARGEKVADSMR
SEKHLLQVLKEDYCWELFAKHAFQGANPQQDPDFVEIAKKIVKRCDGLPLALKTMGSLLHNKSFLWEWENIMRSDIW
DLSENESGILPALKLSYLHLPSHLKKCFAFCALLPKGYRFDKDILIQWWMAQNFLESHIQKKSAIEVGEQYFNDLLSWSFF
QQSSNEEKERFIMHDLLNDLAKYVCKDVCIRIGVDEQEGISKTTRHCSFLSSGLCFDGFGSSIDTQKLHTFTQTDPNSGW
VWDCKMSIDDLFSRFKLIRVLSLKSCRSLTEVPESIGNLKHLRSLDLSCTRIEKLPESMSLLFKLQILRLNKCRRLKELPSCLH
QLENLRCLELVDIGVENVAAHLGKPKNVQVSMSSFHVEKSKEMNIQRLGQFNLHGRLTIDDLQNIENPSDALEADLKSK
PHLVGLHLEWNFIGSSSVDSTKAEDVIENLRPSKYLKNLSIRNYIGQEFPNWLLHNSLPNLVSLELDDCRSCERLPPLGLLP
FLEELSIDGLDGIVSIDADFHGNNSSSFKSLQTLRFSNMRQWEKWDCQAVTGAFPRLEHFSIRNCAKLKGHLPKFIALKYL
YVRHCKHLEALIVSALELRLQDCGKLQLDCSTMEKLTMDGHGKTTSLLAMVGHMLFDTSLEILTLESISDDCVSLRIFPLD
FFPKLRMLELSGFPNLQMISQDHVQNHLCYLRMEKCPKFESLPANMHMLLPSLDGLHIRDCPTLESFPDGGLPSNLKYIT
LWNCFRLVGLLKGALGDSSSLECLGISTPDAECFLDEGLLPPSLTQLQFLECQNLEKLDYKGLLQLSSLRTLYLRDCPNLQR
LPEEGLPKSISSLHIINCPLLKQRCQKGGEDREKIAHIRNLYIY

>XP_027911078.1

MAAEMVTGVLVSTFLERTIDTLASRLFDIFHQRKHKKQLRNLKMKLLAIDVVALDAEQKQFTDSRVRDWLLRAKDVVID
AEDLLDELDYELSKSQVEAESQSAAKKVWSSLNSSFLIEKEIESRMAQVIEDLDDLADESNILGLKKGGGVEVGSGSSSKLT
YSSLPNESVIYGRNDDKEFLIQWLTSDTHNNLSILSIVGMGGLGKTSLAQHVFNDQRIKDKFGIKAWVSVPQEFDVLKVS
RAILDTITGSTERSMQQEVIQRSLKEELAGKKFLLVLDDVWNENPSKWEDVQKPLVFGGQGSRILVTARGEKVADSMR
SEKHLLQVLKEDYCWELFAKHAFQGANPQQDPDFVEIAKKIVKRCDGLPLALKTMGSLLHNKSFLWEWENIMRSDIW
DLSENESGILPALKLSYLHLPSHLKKCFAFCALLPKGYRFDKDILIQWWMAQNFLESHIQKKSAIEVGEQYFNDLLSWSFF
QQSSNEEKERFIMHDLLNDLAKYVCKDVCIRIGVDEQEGISKTTRHCSFLSSGLCFDGFGSSIDTQKLHTFTQTDPNSGW
VWDCKMSIDDLFSRFKLIRVLSLKSCRSLTEVPESIGNLKHLRSLDLSCTRIEKLPESMSLLFKLQILRLNKCRRLKELPSCLH
QLENLRCLELVDIGVENVAAHLGKPKNVQVSMSSFHVEKSKEMNIQRLGQFNLHGRLTIDDLQNIENPSDALEADLKSK
PHLVGLHLEWNFIGSSSVDSTKAEDVIENLRPSKYLKNLSIRNYIGQEFPNWLLHNSLPNLVSLELDDCRSCERLPPLGLLP
FLEELSIDGLDGIVSIDADFHGNNSSSFKSLQTLRFSNMRQWEKWDCQAVTGAFPRLEHFSIRNCAKLKGHLPKFIALKYL
YVRHCKHLEALIVSALELRLQDCGKLQLDCSTMEKLTMDGHGKTTSLLAMVGHMLFDTSLEILTLESISDDCVSLRIFPLD
FFPKLRMLELSGFPNLQMISQDHVQNHLCYLRMEKCPKFESLPANMHMLLPSLDGLHIRDCPTLESFPDGGLPSNLKYIT
LWNCFRLVGLLKGALGDSSSLECLGISTPDAECFLDEEHCIFETAPTSNAYQRRAFLNQFHLFI

>XP_027911079.1

MAQVIEDLGYLADESNILGLKKGGGVEVGSGSSSKLTYSSLPNESVIYGRDNDKEFLIQWLTSDTHNNLSILSIVGMGGL
GKTSLAQHVFNDQRIKDKFGIKAWVSVPQEFDVLKVSRAILDTITGSTERSMQQEVIQRSLKEELAGKKFLLVLDDVWN
ENPSKWEDVQKPLVFGGQGSRILVTARGEKVADSMRSEKHHLEILKEDYCWELFAKHAFQGANPQPDPDFVEIAKKIV
KKCDGLPLALKTMGSLLHNKSFLWEWENIMRSDIWDLSENESGILPALKLSYIHLPSHLKKCFAFCALLPKGYQFDKDILI
QWWMAQNFLESPIQKKSPTEAGEQYFNDLLSWSFFQQSSNQDEERFIMHDLLNDLAKYVCKDVCISIGVDEQEGISKT
TRHCSFLSEFCFDGFGSSIDTQKLHTFTQTDPNSGWVWDCKMSIDDLFSRFKLIRVLSLNRCRILTDVPESIGNLKHLRSL
DLSWTQIEKLPDSMSLLYKLQILKLNECRRLKQFPSCLHKLQNLRCLELVGIGVENVAAHLGKLKNVQVSMSSFHVEKSK
EINIQQLGQLNLHGSLTIDDLQNIENPSYALEANLESKPHLAELWLTWNFIDSSFVDSEKVEDVVENLRPLKYLKKLSIWN
YIGKHFPNWLLHNSLPNLVSLVFVDCRSCERLPPLGLLPFLNHLSIVGLDGIMSIIDADFHGNNSSSFKSLQRLYFSDMRQ
WEKWDCQAVRGAFPRLQHFSIKNCPKLRAHLPKFVALKDLYVSNCEQLEALIVSAIELRLQDCGKLQLERSTMKKLTMD
GHGMAASSVAMVGHMLFDTSLEYFSICSPLETISDDCVSLKIFPLDFFPKLRTLKLSGFPDLQMISKDHVLNHLRHLTIDN
CPKFESLPANMHMLLPSLIGIDIKDCPRLESFPEGGLPSNLKYITLNNCSRLHVSLKGAFRDGSSLEILTINNVEAECFPEEG
LSFAGTTLW

>XP_027911123.1

MCIHSSLFHEVRLDLCFRGEDTRMNFTSHLHEALKEKKVETYIDYQLEKGDEISPAVFKAIEDSRVSIVILSENYASSKWCL
EELSKILECHRNQEQIVIPVFYNVDPSHVRKQTGCYEQSFATHEEELRCNKWRSALTAVANLAGWDSRNRIESQFIKDVV
KDVIGKLTPRYPNELKGLVGIEKSFKQIESLLKIGSSEVRTVGIWGMGGIGKTTLAIALYDKLSHEFEGRCVLTNIREKSDKL
EGLRDELLSKLLGNKIHGIDYFDMRRLQRKKVFIVLDDVDTSEQIEKLILEYEFLGPGSKVIVTTRNKQILSLVDEIYQVEELS
SYHSLQLFCLTISGEKQPKDGYEDLSKRAILYCKGLPMALKVLGSNLRKKSKVVWECELRKLQKIPNVQIHNVLKLSYECLD
RSQKDIFLDIVCFFNGWERDRVTYILEACDFFAASGIETLLDKTLVTISNYSHIKVNDLIQKMGWEIVHQESIKDLGRRSRL
WKQEEVYDVLKYNKGTDFVEGMVLDLEQLAGDLYLSCDSLAKMTNMRILRIHRRKWGCRFSVHLPDGIELPYKLRYLE
WEGFCLRSLPSNFCAEQLVELHMWNSKLKMLWDGVQNLVNLKTIDLDDSRDLIEIPDLSMAEKLERVSLYDCESLCKLH
PSISSLPKLKYLILSGCKVIQSVNVHSKSLNVLRLRGCSSLSEFSVTSEEMTHLDLSQTAIRELLSPMISLPKLTYLYLSGCRHIE
NVNLHLRSLRVLTLVGCSCLKEFSVASDKLTLLELPDTAIGVLPSSIGHLLSLEELDLRGTNIECLPASIKSLSMLRVLWLNDC
KKLVSVQELPPSLRELHINDCWKLVSLPELPPSVKEVSAFNCRSLEENITQELVLRHMLQSCIPDKHQQYPYNPVYFDGG
YFIFPGDHITDNCAFHATESSITIPSLPRSHLWGYIFCIIIAKGPMSDHQFSCSIYQEDILVGCGHRRFIGCENLISDHVLFFY
HDVINFGGTDEVYGPFNSFTFMFEFNGDKHTIKGCGVFPVYLTSSGFKFCNVDSQPRANGPKIGGCDKENLEQLFAAKR
RKTA

>XP_027911360.1

MALELVGGALLSVFLDVAFKKLASSQILDFFRARKLDEKLLNNLKTKLNSIHSLADDAERKQFTNSYVRNWLVEVKDVVL
DAEDLSDDIQMLSKRQVDADSESQTSACCTFKVLNFFKSSPITSYNKEIESRMQEVLDKLDTLLSQRGNLGLKTGRAIGS
GLGNELPQKSQTTSLVVGTDIYGRDDDKKQIVDWLISDNNNPNLPSILCIVGMGGLGKTTLAQHVFNDPKINEAKFDVK
AWVCVSDEFDVFKVSRAILEAVTESTDDSRDLEMVHKRLKEELTQKKFLLVLDDLWNENQHNWEEVQKPLMFGVQGS
RILVTTRSKEVASTMQSEVYSLKQLQEDDCWNLFSKCAFRDDDTQPNPECREIGMKIVKRCKGLPLALKTMGSLLYNKS
SVSEWDTVFQSEIWEFSQERYDIVPALALSYIHLPSHLKVCFAYCAMFPKDYEFKKEHLMHLWMTENLLHCPSENVCQ
QYFNDLLSRSFFQQSGEKEDVYVIHDLLNDLAKYVGGGIYFRWEVGQNEKIQKVTRHYSVELGHNRYFDGFGFGTSCNT
ERLRTFMLTELPDSVSNLEHLRLLDLSHTILSQLSEKICSLSHLQILKLNYCIYLEELPSNLYLLTNLCRLELIENNLEKLPPGLG
KLKNLTVVMESFNVGDGWREFGIQQLGELNLDGHLSIKELQNIENSVDASKACLRNKRLLMKLELRWRGNRRFIDSKN
EEEVIKNLQPSENLKELAILEYGGKQLPNWLHSLPYLVSLELNGCGYCERLPPLGLLPFLEDLSIDGLDGIVSIDADFYGSNS
CSFKSLQSLVFSHMRQWKKWECKAGAFPRLQYLTITSCPKLKGELPKQLIPLKTLTIRDCQQLEASAPRALDLKLYDCGKL
HFDWATMKGLILEASLLEIVWSDTVEYLCIIFKSMSDDCVAIRIFPLDSLPRLRKLYLGGFPNLQMISQDHVQNHLVGMTI
EKCPKFESLPANIHMMLPSLNDLNIEDCPKLESFPEGGLPSNLEHLTIKRCLKLESLSGNMHMLSLKNLWIEDCPKLESFIK
GGLPSNLRFMKLNNCSTLLVDSLKGAFRDNPSLESLTIEKVDAKYFPAEGLLPLSLTELIMRDCPNLEKLDYKGLYQLSSLQ
SLRLDNCPKLQCLPEEGLPTSISFLYIARCPVLIQRCQKERGEDREKLSHIKHLYIMW

>XP_027911565.1

MVEKWREALKTSCDRCGWDVQNKQQYEEIEKIVEKVIDILGRNQIWSFGDDLVDMHSRVKQLEEVVDLSANDIVRLV
GICGMGGIGKITLATALFNKF

>XP_027911687.1

MAEALLEGVLGSLTSLAEAELGPFLGFSEEKEKLESMFTAIKAALEDAEEKQFSDKSTKDWMGKMKDAAYELDDILDEC
VYEQLRLEQEEVRCCVSKMVLSSSFSSFHPMNIYFRYKIAKRMKILSERLDRIASEKDQLRLNFTVQEETRGVPKWRQTFS
LVTEPKVYGREDDVKKIVVFLAGAASRAENLPVYPIVGQGGLGKTTLAKLIFNHNDLKDFQLRIWVCVSEDFGLERMLKA
IIQAASNKEVCKDLGPEPMQRRLRNLLTGKRYLLVLDDVWDVVGQNWENNWQMLRSVLDCGEKGASVLVTTRFSNV
AEIMGTVKHPHRLSELSVDYCWELFKDQAFGADEVEPEELEMIGREIVKKCGGVPLAANAIGGLLRFHRNKDKWLNIM

RSNLLTLSPNEKSIMPVLRLSYLNLPIELRQCFAYCAIFPKDERIEKQYLIELWMANGFISSYGRLGAEDVGHDVWNELYR
RSFFQDIETDEFGKVTSFKMHDLVHDLAQFVAEEVCCITDDNVAPVFFERKRIHHLSDYRWWIHSTQLHQVKSLRTYVD
SRTTDELSYDVLRCYSLRVLHLSRWKELSSSIGDLKHLRYLNLSYGDFKSLPESLCKLLNLQILKLDYCYSLQRLPNSLVRLKA
LQELSLKNCRSLSRLPPYIGKLTSLRSLSIFFVGKERGFLLAELGQLKLKRNLDIKHLEKVKSGNDAKEINMSIKQLNKLTLT
WGRFREGKLEGNDEEVLEALEPCTETLLSLRVEGYQGSRFPEWMSSPSFRYLTYLVLWSCRNCVQLPLLAKLPSLKRLNI
VWAEYVKYLHEPSCDDDAAFMALEFLSLCRLPSLLRLSSEDGKNLFPCLTTLEIDDCCNLLAEEVWLQGLQSLKKLKAKM
CPKLNVWAGLQCLTCLEDLTIERCEEVEGLQHMTALKKLTLRDVPNIQSLLHELRFGELPLLRELRIGECYKLMCLPTSLRL
SSVEMLSIMGCNPELKKRCEKETGEDWPIIAHIPRVYTH

>XP_027911898.1

MAAEMVTGVLVSTFLERTIDTLASRLFDIFHQRKHKKQLRNLKMKLLAIDVVALDAEQKQFTDSRVRDWLLRAKDVVID
AEDLLDEIDYELSKSQVEAESQSAAKKVWSSLNSSFLIENEIESRMAQVIEDLDDLADESNILGLKKGGGVEVGSGSSSKLT
YSSLPNESVIYGRDNDKEFLIKWLTSDTHNNLSILSIVGMGGMGKTSLAQHVFNDPRLEGKFDINVWVSVPQEFDVLKV
SRAILDTIASSTDHSIPKEVIQKRLKENLMGKKFLLVLDDVWNENSSKWEDVQKPLVFGGQGSRILVTARGEKVADSMR
SEKHHLEILKEDYCWELFAKHAFQGANPQQDPDFVEIAKKIVKKCDGLPLALKTMGSLLHNKSSLWEWENIMKSEIWDL
SENESGILPALKLSYLHLPSHLKKCFAFCALLPKGYRFDKDILIQWWMAQNFLESPIQKKSPTEAGEQYFNDLLSWSFFQP
SSNKFEKLFIMHDLLNDLAKYVCKDVCIRIGVDEQEGISKTTRHCSFLSEFCFDGFGSSIDTQKLHTFTQTDPNSGWVWD
CKMSIDDLFSRFKLIRVLSLNRCCILKDVPESIGNLKHLRSLDLSWTQIEKLPDSMSLLYKLQILKLNECRRLKQFPSCLHKLQ
NLRCLELVGIGVENVAAHLGKLKNVQVSISSFHVEKSKEMNIRRLGQFNLHESLTIDDLQNIENPSDALEADLKSKPHLVG
LQFKWNFIGSSFVDSAKAEDVIENLRPSKYLKKLSIRNYIGKQFPNWLLHNSLPNLVSLVLVDCRSCEHLPPLGLFPFLKELS
IDGLDGIVSIDADFHGNNSSSFKSLQKLWFSNMRQWEKWDCQAVTGAFPRLERFWIKNCPKLKAYLPKFVAFPRLQSF
SIDNCPKLKGHLPKFVALKCLYVRHCEQLEALIVSAIELRLQDCGKLQLDCSTLKNLTMDGHDMAASSVAMVGHMLFNT
SLEILTLESISDDCVSLRIFPLDFFPTLRMLELSGFPNLQMISQDHVHNHLEDLTIKECPKFESLPENMHMLLPSLDGLHIED
CPTLESFPDGGLPSNLKYITLWNCFRLVGLLKGALGDSSSLESLGISTPDAECFLDEGLLPPSLTQLQFLYCQNLEKLDYKGL
LQLSSLRRLYLRDCPNLQRLPEEGLPKSISYLDITNCPLLKQRCQEGGEDREKIAHIRRIHLF

>XP_027912012.1

MTVDHILHLSFLSDEHCWSLFSDHCFGNKSSCNPQLGVLGRRILSKCGGVPLAAKMLGGLLHKKDDKDWMEILNSPQ
WEQTNINFATPFVRLCYLSLPIQLKRCITYLGMFHKGYQFQKRQVVLLWIGKALIQREKNESMENLGNKYFDFLVARSFL
IRSNSGDEADFTMHDTVRDLALAVSRKSHYLSYQSLFGSDYESIDTSFLISNIQNTEESSSELYEPQTEEYAPSKDNVGTAN
ITHGLDQQSPEIDANLPTITNDVVQPSFESGTQFDTV

>XP_027912668.1

MADLFEGGAVGALMGEVLKGAISTIKKGLAFEPTLESNIETLNSLAPLVEEIKKYNMLLDRPREEIERLESHMRAGEELVR
KCSKLGRWRMWSFPYYQSKLQSKDGALQKHLSVNVSVQNRRDLMEVVDGMRKVLEILLNGEFGRYSGYQLMGLCGA
PQEPACMGMEVPLSKLRIELLKDSVSVLVLTGLGGSGKSTLAKKICWDPQVIGKFGKNIFFVTVSKTPNLKTIVETLFEHC
GCRVPKFQTDEDVVNRLEVLLRVLGKHPILLVLDDVWPGSEDLVEKFKIKIPDYKILVTSRVSFPRFGTSYQLDKLDHVHA
ESLFRHIALKDKSSYIPEKNLVDEIVKGCKGSPLALKVIAGSLCNQPFEVWQNMKERLQRESILESDTDLLFRLQQSLDILET
KFSINEKECFMDLGLFPEDQRIPVAALIDMWAELYNLNEDGSNAMSIIHDLITRNLINVIVTRKVAKDTDMYYNNHFVLV
HDLLRELAIHLSKEKTFEQRERVMIELNGDNRPEWWVRLNPLGIIGRSFSYILGMLYTQKQPKVAARILSISTDETFTSDW
CDMQPDEAEVLVLNLLSSQYSLPEFTEKMHKLKVLIVTNYGFHRSELNMFERLGSLTNLKRIRLEKVSIPPLCILKNLRKLSI
HMCNTCHAFDTYSISDAMPNLVEMSIDYCKDLVKFPDGLCNITPLKKLSITNCHSLSALPQDLAKLENLEVLRLCSCSDLV
GMPDSVKGLDKLSCLDISDCLNLTKLPDDIGELKLEKLYLKGCSQLRELPHSVVKFENLKHKIHVICDEEMVASLWENLTIR
NLKIEISTVEVNLNWLPGVHS

>XP_027912908.1

MEFASSTSKLPQMYDLLINFNGEDIHRKFVSHLDSVLSAAGLTTFLHHQNAVNDMDIQQPILNLCRVAIVVFTKTYSESA
WCLHQLQQIIEWHKTYSRHVLPVYYEIQPSDVRLQKGDFGKTLKATAQKSFSAQQMEHGMSRWNHALSKTADFFGW
DESNYRSDAELVDTIVKSILNLPVLSATKFPIGLQPHVEDVIQVIKNKSTGVCTVGICGMEGSGKTTLAKAIYNQIHGTFLK
KSFIEDISEVSRTGKHANLHERLLSDLLKTKLEIHKVEMGRRMIGERLYGKKVLIVLDDVNEYGPLDLWESSAWFGEGTVII
ITTTDARLLRIYQVDYIFQMNVMNPNKSLELFSWHAFREAKPKKEYHFLARRVVAYCGGLPLLLEVIGSCLYERTKEEWN
RLLLQLDNSAQHEVDQILKISYEDLLNQMEKDLFLDVCCFFIGKCKFYVTKILNDCGVDPDSGIRVLIKRNLVKIRKNNKVG
MHPLLRQMGREISHEILRKEPEKISGLWLDEDMEHALSRNSQTNVIQRFSRRLVNLAGFSWSLCEKLRWVSLKGFSSQY
LPNDFYLRDAIGIDLKHSLLRLVWKESQVLARLKVLNLSHSIYLTETPDFSRLPALEQLILKNCQSLRQVHQSIGFLYNLTLL
NLKDCTGLTNLPREIYMLKSLKALVLSGCSKIVLLEKDIVQMESLITLISENTVLKQVPFSIASSKSIGYISLCGLEERSNNLFPS
IIRSRMSPTTNPLSYIHTFSDTEDNSWDDVVPFFSSLAILRSVLVQCDPEFQLSVQLKAILMDYCVNITKSRISKHHFRSCLI
GVGRYEEFFNTVSDGISEVLASSGSCDVCLPGDNYPYWSAHRGEGHSVSFTVPRDCVMKGLILCVVCLSTPEIIEPELTTV
LIVNYTRCTLQIHNHGTVISFNDEDWDHIVSNLGSGDRVEIFVSSAYGLVVKETAVYLMYGEPKKNSLIRSIKKIIM

>XP_027912909.1

MEFASSTSKLPQMYDLLINFNGEDIHRKFVSHLDSVLSAAGLTTFLHHQNAVNDMDIQQPILNLCRVAIVVFTKTYSESA
WCLHQLQQIIEWHKTYSRHVLPVYYEIQPSDVRLQKGDFGKTLKATAQKSFSAQQMEHGMSRWNHALSKTADFFGW
DESNYRSDAELVDTIVKSILNLPVLSATKFPIGLQPHVEDVIQVIKNKSTGVCTVGICGMEGSGKTTLAKAIYNQIHGTFLK
KSFIEDISEVSRTGKHANLHERLLSDLLKTKLEIHKVEMGRRMIGERLYGKKVLIVLDDVNEYGPLDLWESSAWFGEGTVII
ITTTDARLLRIYQVDYIFQMNVMNPNKSLELFSWHAFREAKPKKEYHFLARRVVAYCGGLPLLLEVIGSCLYERTKEEWN
RLLLQLDNSAQHEVDQILKISYEDLLNQMEKDLFLDVCCFFIGKCKFYVTKILNDCGVDPDSGIRVLIKRNLVKIRKNNKVG
MHPLLRQMGREISHEILRKEPEKISGLWLDEDMEHALSRNSQTNVIQRFSRRLVNLAGFSWSLCEKLRWVSLKGFSSQY
LPNDFYLRDAIGIDLKHSLLRLVWKESQVLARLKVLNLSHSIYLTETPDFSRLPALEQLILKNCQSLRQVHQSIGFLYNLTLL
NLKDCTGLTNLPREIYMLKSLKALVLSGCSKIVLLEKDIVQMESLITLISENTVLKQVPFSIASSKSIGYISLCGLEERSNNLFPS
IIRSRMSPTTNPLSYIHTFSDTEDNSWDDVVPFFSSLAILRSVLVQCDPEFQLSVQLKAILMDYCVNITKSRISKHHFRSCLI
GVGRYEEFFNTVSDGISEVLASSGSCDVCLPGDNYPYWSAHRGEGHSVSFTVPRDCVMKGLILCVVCLSTPEIIEPELTTV
LIVNYTRCTLQIHNHGTVISFNDEDWDHIVSNLGSGDRVEIFVSSAYGLVVKETAVYLMYGEPKKNSLIRSIKKIIM

>XP_027912910.1

MEFASSTSKLPQMYDLLINFNGEDIHRKFVSHLDSVLSAAGLTTFLHHQNAVNDMDIQQPILNLCRVAIVVFTKTYSESA
WCLHQLQQIIEWHKTYSRHVLPVYYEIQPSDVRLQKGDFGKTLKATAQKSFSAQQMEHGMSRWNHALSKTADFFGW
DESNYRSDAELVDTIVKSILNLPVLSATKFPIGLQPHVEDVIQVIKNKSTGVCTVGICGMEGSGKTTLAKAIYNQIHGTFLK
KSFIEDISEVSRTGKHANLHERLLSDLLKTKLEIHKVEMGRRMIGERLYGKKVLIVLDDVNEYGPLDLWESSAWFGEGTVII
ITTTDARLLRIYQVDYIFQMNVMNPNKSLELFSWHAFREAKPKKEYHFLARRVVAYCGGLPLLLEVIGSCLYERTKEEWN
RLLLQLDNSAQHEVDQILKISYEDLLNQMEKDLFLDVCCFFIGKCKFYVTKILNDCGVDPDSGIRVLIKRNLVKIRKNNKVG
MHPLLRQMGREISHEILRKEPEKISGLWLDEDMEHALSRNSTNVIQRFSRRLVNLAGFSWSLCEKLRWVSLKGFSSQYL
PNDFYLRDAIGIDLKHSLLRLVWKESQVLARLKVLNLSHSIYLTETPDFSRLPALEQLILKNCQSLRQVHQSIGFLYNLTLLN
LKDCTGLTNLPREIYMLKSLKALVLSGCSKIVLLEKDIVQMESLITLISENTVLKQVPFSIASSKSIGYISLCGLEERSNNLFPSII
RSRMSPTTNPLSYIHTFSDTEDNSWDDVVPFFSSLAILRSVLVQCDPEFQLSVQLKAILMDYCVNITKSRISKHHFRSCLIG
VGRYEEFFNTVSDGISEVLASSGSCDVCLPGDNYPYWSAHRGEGHSVSFTVPRDCVMKGLILCVVCLSTPEIIEPELTTVLI
VNYTRCTLQIHNHGTVISFNDEDWDHIVSNLGSGDRVEIFVSSAYGLVVKETAVYLMYGEPKKNSLIRSIKKIIM

>XP_027912911.1

MEFASSTSKLPQMYDLLINFNGEDIHRKFVSHLDSVLSAAGLTTFLHHQNAVNDMDIQQPILNLCRVAIVVFTKTYSESA
WCLHQLQQIIEWHKTYSRHVLPVYYEIQPSDVRLQKGDFGKTLKATAQKSFSAQQMEHGMSRWNHALSKTADFFGW
DESNYRSDAELVDTIVKSILNLPVLSATKFPIGLQPHVEDVIQVIKNKSTGVCTVGICGMEGSGKTTLAKAIYNQIHDLLKT
KLEIHKVEMGRRMIGERLYGKKVLIVLDDVNEYGPLDLWESSAWFGEGTVIIITTTDARLLRIYQVDYIFQMNVMNPNK
SLELFSWHAFREAKPKKEYHFLARRVVAYCGGLPLLLEVIGSCLYERTKEEWNRLLLQLDNSAQHEVDQILKISYEDLLNQ
MEKDLFLDVCCFFIGKCKFYVTKILNDCGVDPDSGIRVLIKRNLVKIRKNNKVGMHPLLRQMGREISHEILRKEPEKISGL
WLDEDMEHALSRNSQTNVIQRFSRRLVNLAGFSWSLCEKLRWVSLKGFSSQYLPNDFYLRDAIGIDLKHSLLRLVWKES
QVLARLKVLNLSHSIYLTETPDFSRLPALEQLILKNCQSLRQVHQSIGFLYNLTLLNLKDCTGLTNLPREIYMLKSLKALVLSG
CSKIVLLEKDIVQMESLITLISENTVLKQVPFSIASSKSIGYISLCGLEERSNNLFPSIIRSRMSPTTNPLSYIHTFSDTEDNSW
DDVVPFFSSLAILRSVLVQCDPEFQLSVQLKAILMDYCVNITKSRISKHHFRSCLIGVGRYEEFFNTVSDGISEVLASSGSCD
VCLPGDNYPYWSAHRGEGHSVSFTVPRDCVMKGLILCVVCLSTPEIIEPELTTVLIVNYTRCTLQIHNHGTVISFNDEDW
DHIVSNLGSGDRVEIFVSSAYGLVVKETAVYLMYGEPKKNSLIRSIKKIIM

>XP_027912912.1

MEFASSTSKLPQMYDLLINFNGEDIHRKFVSHLDSVLSAAGLTTFLHHQNAVNDMDIQQPILNLCRVAIVVFTKTYSESA
WCLHQLQQIIEWHKTYSRHVLPVYYEIQPSDVRLQKGDFGKTLKATAQKSFSAQQMEHGMSRWNHALSKTADFFGW
DESNYRSDAELVDTIVKSILNLPVLSATKFPIGLQPHVEDVIQVIKNKSTGVCTVGICGMEGSGKTTLAKAIYNQIHGTFLK
KSFIEDISEVSRTGKHANLHERLLSDLLKTKLEIHKVEMGRRMIGERLYGKKVLIVLDDVNEYGPLDLWESSAWFGEGTVII
ITTTDARLLRIYQVDYIFQMNVMNPNKSLELFSWHAFREAKPKKEYHFLARRVVAYCGGLPLLLEVIGSCLYERTKEEWN
RLLLQLDNSAQHEVDQILKISYEDLLNQMEKDLFLDVCCFFIGKCKFYVTKILNDCGVDPDSGIRVLIKRNLVKIRKNNKVG
MHPLLRQMGREISHEILRKEPEKISGLWLDEDMEHALSRNSQTNVIQRFSRRLVNLAGFSWSLCEKLRWVSLKGFSSQY
LPNDFYLRDAIGIDLKHSLLRLVWKESQVLARLKVLNLSHSIYLTETPDFSRLPALEQLILKNCQSLRQVHQSIGFLYNLTLL
NLKDCTGLTNLPREIYMLKSLKALVLSGCSKIVLLEKDIVQMESLITLISENTVLKQVPFSIASSKSIGYISLCGLEERSNNLFPS
IIRSRMSPTTNPLSYIHTFSDTEDNSWDDVVPFFSSLAILRSVLVQCDPEFQLSVQLKAILMDYCVNITKSRISKHHFRSCLI
GVGRYEEFFNTVSDGISEFPAKHSLRPHLIGIGSYQEFFNTYQRRYRAAFF

>XP_027912913.1

MFCPYITKFSHLMFVFKRVILEKPSKQLHKNHFQHNKWSMACPGGTTHSAKLQIFLDGMRAITGICGMEGSGKTTLAK
AIYNQIHGTFLKKSFIEDISEVSRTGKHANLHERLLSDLLKTKLEIHKVEMGRRMIGERLYGKKVLIVLDDVNEYGPLDLWE
SSAWFGEGTVIIITTTDARLLRIYQVDYIFQMNVMNPNKSLELFSWHAFREAKPKKEYHFLARRVVAYCGGLPLLLEVIGS
CLYERTKEEWNRLLLQLDNSAQHEVDQILKISYEDLLNQMEKDLFLDVCCFFIGKCKFYVTKILNDCGVDPDSGIRVLIKR
NLVKIRKNNKVGMHPLLRQMGREISHEILRKEPEKISGLWLDEDMEHALSRNSQTNVIQRFSRRLVNLAGFSWSLCEKL
RWVSLKGFSSQYLPNDFYLRDAIGIDLKHSLLRLVWKESQVLARLKVLNLSHSIYLTETPDFSRLPALEQLILKNCQSLRQV
HQSIGFLYNLTLLNLKDCTGLTNLPREIYMLKSLKALVLSGCSKIVLLEKDIVQMESLITLISENTVLKQVPFSIASSKSIGYISL
CGLEERSNNLFPSIIRSRMSPTTNPLSYIHTFSDTEDNSWDDVVPFFSSLAILRSVLVQCDPEFQLSVQLKAILMDYCVNIT
KSRISKHHFRSCLIGVGRYEEFFNTVSDGISEVLASSGSCDVCLPGDNYPYWSAHRGEGHSVSFTVPRDCVMKGLILCVV
CLSTPEIIEPELTTVLIVNYTRCTLQIHNHGTVISFNDEDWDHIVSNLGSGDRVEIFVSSAYGLVVKETAVYLMYGEPKKNS
LIRSIKKIIM

>XP_027912914.1

MFCPYITKFSHLMFVFKRVILEKPSKQLHKNHFQHNKWSMACPGGTTHSAKLQIFLDGMRAITGICGMEGSGKTTLAK
AIYNQIHDLLKTKLEIHKVEMGRRMIGERLYGKKVLIVLDDVNEYGPLDLWESSAWFGEGTVIIITTTDARLLRIYQVDYIF
QMNVMNPNKSLELFSWHAFREAKPKKEYHFLARRVVAYCGGLPLLLEVIGSCLYERTKEEWNRLLLQLDNSAQHEVDQ
ILKISYEDLLNQMEKDLFLDVCCFFIGKCKFYVTKILNDCGVDPDSGIRVLIKRNLVKIRKNNKVGMHPLLRQMGREISHEI

LRKEPEKISGLWLDEDMEHALSRNSQTNVIQRFSRRLVNLAGFSWSLCEKLRWVSLKGFSSQYLPNDFYLRDAIGIDLKH
SLLRLVWKESQVLARLKVLNLSHSIYLTETPDFSRLPALEQLILKNCQSLRQVHQSIGFLYNLTLLNLKDCTGLTNLPREIYM
LKSLKALVLSGCSKIVLLEKDIVQMESLITLISENTVLKQVPFSIASSKSIGYISLCGLEERSNNLFPSIIRSRMSPTTNPLSYIHT
FSDTEDNSWDDVVPFFSSLAILRSVLVQCDPEFQLSVQLKAILMDYCVNITKSRISKHHFRSCLIGVGRYEEFFNTVSDGIS
EVLASSGSCDVCLPGDNYPYWSAHRGEGHSVSFTVPRDCVMKGLILCVVCLSTPEIIEPELTTVLIVNYTRCTLQIHNHGT
VISFNDEDWDHIVSNLGSGDRVEIFVSSAYGLVVKETAVYLMYGEPKKNSLIRSIKKIIM

>XP_027912915.1

MFCPYITKFSHLMFVFKRVILEKPSKQLHKNHFQHNKWSMACPGGTTHSAKLQIFLDGMRAITDLLKTKLEIHKVEMGR
RMIGERLYGKKVLIVLDDVNEYGPLDLWESSAWFGEGTVIIITTTDARLLRIYQVDYIFQMNVMNPNKSLELFSWHAFRE
AKPKKEYHFLARRVVAYCGGLPLLLEVIGSCLYERTKEEWNRLLLQLDNSAQHEVDQILKISYEDLLNQMEKDLFLDVCCF
FIGKCKFYVTKILNDCGVDPDSGIRVLIKRNLVKIRKNNKVGMHPLLRQMGREISHEILRKEPEKISGLWLDEDMEHALSR
NSQTNVIQRFSRRLVNLAGFSWSLCEKLRWVSLKGFSSQYLPNDFYLRDAIGIDLKHSLLRLVWKESQVLARLKVLNLSH
SIYLTETPDFSRLPALEQLILKNCQSLRQVHQSIGFLYNLTLLNLKDCTGLTNLPREIYMLKSLKALVLSGCSKIVLLEKDIVQ
MESLITLISENTVLKQVPFSIASSKSIGYISLCGLEERSNNLFPSIIRSRMSPTTNPLSYIHTFSDTEDNSWDDVVPFFSSLAIL
RSVLVQCDPEFQLSVQLKAILMDYCVNITKSRISKHHFRSCLIGVGRYEEFFNTVSDGISEVLASSGSCDVCLPGDNYPYW
SAHRGEGHSVSFTVPRDCVMKGLILCVVCLSTPEIIEPELTTVLIVNYTRCTLQIHNHGTVISFNDEDWDHIVSNLGSGDR
VEIFVSSAYGLVVKETAVYLMYGEPKKNSLIRSIKKIIM

>XP_027912917.1

MCVVGSWKRDLFVNNYEIQPSDVRLQKGDFGEAFKATAKQTFSAQQLEHGMSRWSHALTKAANFFGWDESNYRSD
AELVDKIVKSVLNLPVLSATKFPVGLQSHVEDVIHTIKNNSTEVCMIGICGMEGSDVLKPKVEVRNIEKGRNIRERHFRKR
VLIVLDNVNDHSLLINLSDSLLWFDKGTVIIITTKHEQLLKTHDVNAVFRINLLNAKESLELLSWHAFREAKPKEEYHDLAK
AIVTHCGGLPLALEVIGTYLYERTKEEWHRVLFKLGKTPQHDVLPVLKICFEGLPNQIERNLFLDIYCFFVGKDRAYVTKILN
GCGVDADSGIGILIERSLIIVKKNNKFGLHPLLREMAREIIGEITSGMEAKKTSRLWFDMDADYVLLEHILFSSQEKKFIQRF
PPKWFPTVKDFFERDYLEVRDAIRRMKLGGHCEYRSKELGLIRLEKFSSEYHPIGFQHDAIAIDLKHRLPRLVWKEPQQV
QDILVEYGVNITESHTSKQHFRSFSIGVGRWKEFFDAFSDRISKVIAGSESCEVSLPGDNDPNCLAHMGEGYSVSFTVPR
DRDIKGMALCIVYLSNPEIVATEGLRSVLIVNYTKCTLHIHNHGTVIFFNDKDWEGIISNLGSGDKVEIFVIFGDGLVVRRTI
VYLIFGESNDLEKESPSKKNSLIRFMKKL

>XP_027913085.1

MAEVAVSTVATKLAELLMEQAAVAVSQLAGVRGQVENLKNELGWMQSFLRDADAKQEGSDRVRLWVSEIRDVAFE
AEELIETYVYNTTMQRQLDKVFRPWHLYKVRTRIDKIMSKIKSISNKRETYGVVMTGDDGNNSNERLRQWRQPSPSSE
EEYLIELEDDMGLFLTQLLALEPNPYVVSIVGMGGLGKTTLAKKLYNHNKITNHFECRAWVYVSKEYRRRDVLQGILRDV
DGAPRNEMERIPEEEFINKLRNVLSEKRYLVVLDDIWGMEVWDGLKSVFPRGKMGSKILLTTRNWEVALHADACSNP
HQLRPLTEDESLRLLCNKAFPGTNGIPSELKDLATEIVVKCGGLPLAVVVVGGLLSRKLKSSGEWKRVLQNISWYLLEEQE
KIARILALSYNDLPSHLKSCFLYLGLFPEGVNIQTKKLIRLWIAEGFLPQEGEETAEGVAQKYLNELIGRCMIQVGTVSSLGR
VKTIRIHHLLRDLSLSKGKEEYFLKIFQGDVAGPSTKARRQSMHSCDERYDFLKHNAHHSRSLLFFNREYNADIARKPWL
RLNFQQEKKLNFIYRKFKLLRVLELDGVRVVSLPSTIGDLIQLRYLGLRKTNLEEELPLSIGNLLNLQTLDLRYCCFLKKIPNVI
WKLVNLRHLLLYTPFDSPDSGHLRLDTLTNLQTLPHIEAGNWIVDGGLANMANLRQLGICELSGQLVNSVLSTAQGLRN
LYSLSLSLQSEEDEFPIFMQLSQCTHLQKLSLNGKIKKLPDPHEFPPNLLKLTLHNSHLQKESIAKLERLPKLKMLVLGERAY
NWPELTFNSEGFSQLLILRLVLLKELEDWTVEQSSIPRLEYMVIDRCEKLKTIPEGLKAITSLKKLRIIGMPVEFEHKLRTKDI
SEFTNTPVIESTTDILAID

>XP_027913147.1

MESEREDNNKGTDSTHAAISSTLKIKIVSCSKSGESLDNSNLPSPNINNGIESSPYGSPLVSPPSSAFVSALQSPYISPRAIIP
DPPNGSPLENQPELLTIATSTTNPSTPEDVPSSSYTPPSDQYEFSDDAADTRLKYVTCVPEPAPPRISFSFPVPRISFAKGPI
SPATNAKLRSCDVYIGFHGQNPNLVRFCRWLKSELELQGIDCMLADRAKYSDSQSHEIADGVICSVAFGVVVVTSSSFLN
HFSMEEVRFFAQKKNLIPLLFDTGPAEIMALLNCKSIDKECKEAIDGLMKCNEFNLEANDGNWRSCIAKAAGILRARLGR
KNAEQKDNVQGLENLPFPRNTYFVGREKEIMEIEGLFFGRGNGMEQVQDHCKAFTKGEASGSGQSEGLADEESEPVIA
RCGRYISLEMGRSKEPTLEAWVEPIMGNNSLKRLKNKKSKSGNYKSVCSSVICINGVSGIGKSELALEFAHRYHQRYKMV
LWVGGEARYLRQNLLNLSLNLGLDVGADSEMERGRIRSFEDQEFEAFKRVKRELFGETPYLLIIDNLETEEEWWEGKDLY
DLIPRNTGGTHVIVTTRLSKVMSYDTIQLPPLPLSDAMILMIGRKRKEYSADEIDLLEKINEKLGRLSFGLWMIGSLLAELSI
GPSCLYEAINQVPLDEDSNSCYMSIAEGQWCKSNPFLMKTLLFCLETLEKTKAKGNLLAIRMLLVSGWFSPSPISASLLAN
AAKSIPTVENRLKKWTKSLSLTSCLSPRTWKNEEESAMILVKMGLARRANHHDGCWLHFHPITQAFAKRKGGLQYAKA
AIQGVRKMGSQVNSDHLWTSAFLVFGFKSEPPLVQLKAIDMVLYIKRTALPLAIQAFTSFSRCNSSLELLRVCTNALEEVE
KSFVSQIQDWSSHGSVCWKRRLQRGQKVDEYVWQDVTLLKATLLETRAKLLARGGHLDSAKELCRTCISIRTVMLGHN
HAQTLAAQDTLARLVRMRSKI

>XP_027913344.1

MSIRTNPMKAIPLLLKRLRKVEQQANLDDLKSVLINIKDVFSLVKKNEEELLDTLALVDGYIRKSNIRKLMEDKEKLCKKIM
DSTQKLLPREAIEATKSSSKPFQDVNRAPPSSQLQNIEEYKVNYQNLGHLCHKRGFLSLLSFPKNAITSKRNIFTWWMG
MGDIDGEEDGEDVFVELLHCNLVVPHRQGKCPHVNKFKVNPWVRNSILSQNDKNQPFGIYSQIVSSVHANDTSYSNLI
LDQREAKLSDELGFESNHWRSIFNVNTSYVKFGFQWMAKMKHLEVLHLGRWLQHSSSHHIEVESEEFLKELIDHEYLKY
LSLRGISRISNLPTNIFELESLETLDLKACHNLETLPNDIASLKKLKHLNLSQCYLLERMPKGIEKLTELQVLKGLVIDSSSKTP
YKISDLENLKKLEQLSIHIAREGVIEEGDFESLKDLSTLHHLKISWGVSTSMHTDVKIILPPNLEKLHVEGFPGQNFPEWLM
PHKVPLSLKELNISGGKLNRMDHGKIYDTLHWLKILRLRLFIVSFCIFFAPWIKDNLICPTTAHNTRYPCCLHILR

>XP_027913438.1

MAEALLEVVIQNLGSFVQEQLATYWGVDEQIQKLSSNLTAIRAVLRDAEKKQITSHAVKDWLQKLTDAAYVLDDILDEC
SIHFTKMHSHDGHTSCLSRLHPKDIHFRFHIGKRIKDITERFHDIHTERLTFELRVDLTKKQAVDDDDDWRETSSVITEPIL
YGRDEDRDKIVKFLLKDAIYSDELTIYAIVGMGGLGKTTLAKEVFHHDGISKHFDLKIWICVSDDFKVKEILQSIIECTLGPN
HNLDNLEARRKKVEEALQSKRYLLVLDDVWNDNREKWNELKGMLECARAAKGATVLVTTRLQEVVSVMGAHFSFPLK
ALSEEDSWSLFQQHAFGPNTQEREELMVIGEEITRKCVGSPLAIKTLASCLRDENEVRQWENVKESEIWDIREESNSATG
EENSIMRALKLSYSNLEPYMRRCFSLCAIYPKDFEIEKEELIHLWMANGFIRCERNVEVEDIGNKVWKKLYSRSFFQEAKY
DRFGMIRTFKMHDLFHDLAQSIMGEECVVVMKKWLTPSSRIHYLNLKYYGVSVDASSFKFAFKKAESLRTLFYFGRMGL
MLSNHCCLRALRTDFTKLSSLKSLSHLRYLSLSGGMHVSLNNSICELTKLQILKLRNWYKLYELPKNLTQLQDLRHVVIDEC
PSIVEMSPNISKLRHLKTLSIFIVGSKPGYGLAELHSLKLGGTLRIKGLEKVANEWDAKQTNLIDKKDLNILYLSWDGNANS
RGSTVSEEIVLEALEPPSTLKSFHIKGYQGRQLSSWMRSPVTLRELVEVKLLDCENCEELPPLGKLPYLKRLEVRGMKNVK
CIDGETYDGVEEKAFPSLEELILYNLPNLERMLRDEGVEMLPRLSEITIYGVSNVKFPRLPTVEKLRANMIDEVGFFMEGV
VGNTPCLKTLNISSIKGVKTLPDQLGTLDALEVLEIGFWYDLEYFPEHVLEGLTSLRILRICCCEKLKSLSEGVRHLACLERLTI
RECPESMVLPSNMSQLTALRDVSILYCSTLPEGLQRVPSLRSLYILYCKSTSLPDWLGDISTLEELSIDRCRELRSLPSSIQRL
TNLSHLSIISCPYLKKRCKRETGEDWQYINHIPNIKLRDL

>XP_027913439.1

MAEALLEVVIQNLGSFVQDQLATYWGVNQQIQKLSSNLTAIRAVLRDAEKKQITSHAVKDWLQKLTDAAYVLDDILDE
CSIHSTKMHSVDGRTSCLSRLHPKDIHFRFHIGKRIKDITERFHDIHTERLTFELRVDLTKKQAVDDDGWRETSSVITEPILY

GRDGDRAKIVKFFLEDASSSDELTISAIVGMGGLGKTTLAKEVFHDDGISKHFDLKIWICVSDDFKVKEILQSIIECTLGPN
QNVDNLEARRKKVEEALQSKRYLLVLDDVWNDNREKWTELKGMLESARGAKGATVLVTTRLQEVVSVMGTHYSYPLK
ALSEDDSWSLFKQYAFGPNTQERKELTVIGEEIVRKCVGSPLAIKTLGSCLRDENEVTQWKNVKESEIWDIREESNSITGE
ENSIMRALKLSYSNLEPYMRRCFSLCAIYPKDFEIEKEELTYLWMANGFIRCERNVEVEDIGNKVWKKLYSRSFFQEAKYD
KFGMITTFKMHDLFHDLAQSIMGEECVVVTKKWLTPSSRVHYLKLEDYDMSVDASSFKKPESLRTLFYFSHVGLMLSNH
CYLRALSTRFTMLSSLKNLAHLRYLSLSGFGGMHVSLNNSICELTKLQILKLTHWDKLYGLPKNLTQLQDLRHIVICECPSIV
EMSPNTSKLRHLKTLSLFVVGSKAGYGLAELHSLKLGGTLRIKGLENVANEWDAKQANLMSKKELNHLHLSWDGNANS
EGSNVNVERVLEALEPPSTLKSFKIKGYQGRHVSSWMRSRVALRDLVEVKFLDCDYCEELPPFGKLPHLKRLVVSGMKN
VKWIDGETYDGVEEKAFPSLEKLSVENLPNLERLLRDEGVDMLPRLSQLIIDGVSNFKFPRLPFVEEVYYANMIDEVGFF
MEGVVGNTPCLKTLNISSIKGVTTLPDQVGMVDALEVLEIKLWYDLEYFPEHVLEGLTSLRILRIRGCEKLKSLSEGVRHLT
CLQRLRICCCPELVALPNNMSQLTALQHVSIANCSTLPNGLQRVPSLRSLYISDCKSTSLPDWLGDMTSLQKLDIWDCKE
LRSLPSSIQRLTNLSSLSIYNCSHLEKRCKRETGEDWQYINHIPNIEWETF

>XP_027913440.1

MAEALLEVVIQNLGSFVQDQLATYWGVDEQIQKLSSNLTAIRAVLRDAEKKQITSHAVKDWLQKLTDAAYVLDDILDEC
SIHFTKMHSHDGHTSCLSRLHPKDIHFRFHIGKRIKDITERFHDIHTERLTFELRVDLTKKQAVDDDGWRETSSVITEPILY
GRDGDREKIVKFLLEDASSSDELTIYAIVGMGGLGKTTLAKQVFHDDEISKHFDLKIWICVSDDFKVKEILHSIIECTLGPN
QNVDNLEARRKKVEEALQSKRYLVVLDDVWNDNREKWNELKGMLECARGAKGATVLVTTRLQEVVSVMGAHFSYPL
KALSEEDSWSLFKQHAFGPNRQERKELSVIGEDIVRKCVGSPLAIKTLGSCLRDENEVTQWKNVKESEIWGIREESNSAT
GEENSIMRALKLSYSNLEPYMRRCFSLCAIYPKDFEIEKEEIIHLWMANGFIKCEGNVEVEDVGNKVWKKLYSRSFFQEAE
YDKFGMITTFKMHDLFHDLAQSIMGEECVVIMKKLLTPSSRIHYLNLKNYGVSVDASSIKLAFKKAESLRTLFYFGHMGL
MLSNHCCLRALDTNFTKLSSLKSLTLLRYLSLSGGMHVSLNNSICELTKLQILKLRDWYRLYGLPKNLTQLQDLRHVVIDN
CPSMVEMSPNISKLRHLKTLSIFIVGSKPGYGLAELHSLKLGGTLRIKGLENVSSEWDAKQVNLIGKNLNMLCLSWDGRS
SSEGSNVSVERVLEALEPPSTLKSFQMNGYQGRHLSSWMRSLVALRDLVKVKLWNCENCEELPPFGKLPHLKRLEVSG
MKNVKCIDGETYEGVEEMAFPSLEELILKNLPNLERLLRDEGVEMLPRLSQLTIKKVSNFKFPRLPSVEKLDVESIDDVEGV
VGNTPCLKTLNISSIKGVKTLPDQLGILDALEDLDIVGWYDLEYFPEHILEGLTSLRILRIRYCEKLKSLSEGVRHLACLERLTI
WYCPELVVLPSNMSQLTALRAVSIFVCSTLPDGLQRVPSLRSLHISDCKSTSLPDWLGDITSLQELDISYCKELRSLPSSIQR
LTNLSSLGIYNCSHLEKRCKRETGEDWQYINHIPNIEWETF

>XP_027913599.1

MADSSVSFLLDKLNALLQEEVNLQRGVREDVQYIKYELERHKAILRVADAMEDRDPELKAWVKGVRDVAHDMEDAID
EFNLRLVDQHGQGNGSSLHRFTFGLKTMKARRRIALDMQSIKSKVNVISLGRPELPGLGSRSSQRLSSSLDSQGDALLLE
EADLVGIDKPKRQLCDLLFNEEPGRAVIPVYGMGGLGKTTLAKQVYDDPKVKKRFRIHAWINVSQSFKLQELLKDLVQQ
LHNVIGKPAPEAVGQMKSEELKELIKNLLQSSRYLIVLDDVWHVKVWDSVKLALPNSNRGSRVMITTRKKDIALYSCAEL
GKDFDLEFLPEEEAWYLFCKKTFQGNSCPPHLEEVCRKILKMCGGLPLAIVAIGGALATRGRANIEEWQMVCRSFGSEIE
GNDKLEDMKKVLSLSFNELPYYLKSCLLYLSIFPEFHAIEHMRLIRLWIAEGFVNGEDGKTREEVADSYLKELLDRSLLQVV
AKTSDGRMKTCRMHDLLREIVNLKAKDQNFATIAKDQDIIWPDKVRRLSIINTLDNVRQNRTSFQLRSLLMFDLSDPLE
HFSIRGLCSTGYKLIRVLDLQDAPLEVFPAEIVNLYLLKYLSLKNTKVKSIPGSIKKLQQLETLDLKQSLVTVLPAEIVELQQLR
HLLVYRYEIESYAYFHSRHGFKVAAPIGLMKSLQKLCFIEADQALMVELGKLTQLRRLGIRKMRQQDGAALSLSIEKMTN
LRSLSITAIEDDEIIDIHNIFKPPPYLQQLYLSGRLDIFPHWISSLKNLVRVFLKWSRLREDPLVHLQDLPNLRHLEFLQVYVG
ETLNFKAKGFPSLKVLGLDDLDELKCMIVEEGAMPGLKKLIIQRCDSLKQVPFGIEHLTKLKSIEFFDMPEELITRLHPNGG
EDYWRAQHVPAVYSSYWRDGGWDVYSLETLGERETDFSRSTAMRSLEICTLWKV

>XP_027913604.1

MAEALLEIVIQNLGSFVEDQLATYWGVDQQIQKLSSNLTAIRAVLRDAERKQITSHAIKDWLQKLTDAAYVLDDILDECSI
HSTKVHSDDGQTSCLSRLHPKDIHFRFHIRKRMKDITERFHDIHTERLTFELRVDLTKKQAVDDDDWRETSSVITEPILYG
RDEDRAKIVKFLLEDANSSDELTICAIVGMGGLGKTTLAKEVFHDDGISKHFDLKIWICVSDDFKVKEILQSIIECTLGPNH
NLDNLEARRKKVEEALQSKRYLLVLDDVWNENREKWNELKGMLECARGAKGATVLVTTRLQEVVSVMGEHFSYPLKA
LSEDDSWSLFKQHAFGPNRQEREELRVIGEEIVRKCVGSPLAIKTLGSCLRDENEVRQWKNVKESEIWDIREESNSATGK
ENSIMRALKLSYSNLEPYMRRCFSLCAIYPKDSEIEKEEIIHLWMANGFIKCEGNVEVEDVGNKVWKKLYSRSFFQEAEYD
KFGMITTFKMHDLFHDLAQSIMGEECVVIMKKWLTPSSRVHYLKLEDYDVSVDASSFKLAFKKPESLRTLFYFGRMGPM
LSNHCCLRALSTEITMLSSLKSLAHLRYLSLSGFRGTPVSLNNSICELSKLQILKLTNWYRLYGLPKNLTQLQDLRHIVIDECP
SIVEMSPNISKLRHLKTLSLFVVGSKPGYGLAELHSLKLGGTLRIKGLENVANEWDAKQANLIDKKDLNILYLSWDGNAN
SRGSTVSEEIVLEALEPPSTLKSFHIKGYEGRQLSSWMRSPATLRELVEVKLLECDNCEELPPLGKLPYLKRLEVRGMKNVK
CIDGETYEGVEEKAFPSLEKLILKNLPNLERLLRDEGVELLPRLSQLTIDGVSNLKFPRLPFVEEVYYANRIDEVGFFMEGVV
ENTPCLKTLNIDSIKGVKTLPDQLGTLDALEDLVIGYWYDLEYFPEHVLEGLTSLRVLRINYCHKLKSLSEGVRHLACLQRLL
ISACPELVGLPNNMSQLTALQHVSISICSTLPDGLQRVPSLRSLNIWSCKCTSLPDWLGDMTSLQKLDIWDCKELRTLPSS
IQRLTNLSSLSIRNCSHLEKRCKRETGEDWQYINHIPNIKLRAR

>XP_027913605.1

MAEALLGIVIQNLQSLGRDQLSSCWGVDQQTQKLSSNLTAIRAVLRDAERKQITSHAVKDWLQKLTDAAYVLDDILDEC
SIHSTKMHSLDGHTSCLSRLHPKDILFRFHIGKRMKDITQRFDVIHEERRSLGLCVGVTEKQVVDDDDDWRQTTSVITEP
IFCGRDRDREEIVKFLLEEASNSEDLNIYPIVGMGGLGKTTLAKQVFNDHRVCKHFDLTIWVYVSVDFNMKEILQSIIEYV
TGQNPNFQSLETMRKKIEEVLCNKRYLLVLDDVWNEDQEQWKHLKGMLQCARGAKGATVLVTTRLEECASTMETHH
AHHLKELSGEDSWSLFKSFAFGPNREEREELVAIGKEIMKKCVGSPLAIKTLGSILRHQNEVTQWENVKESEIWDIREESS
LMTGEENAIMRVLKLSYFNLELSLRRCFSFCAIFPKGFQIVKEELIHFWMANEFIKCEGSVGVEDVGNYVWRKLYSRSFF
QEAKFDEFGMIISFKMHDLFHDLAQFVMGEECVVIETGRFTELSARVHHLRLLNYDVPVDMSAFKKIESMRTILGYGNF
SQLPSNHGVRALCTKSFPRIPLNDLVHLRYLSMRGRLGASVLNSICGLPKLQILKLKSCTDVELPKNLTQLQDLRHVLIDDC
ASIAEMPPNISKLRHLRTLSIFVVGSKPGCGLAELHSLKLGGALRIRGLNNVPSEWDAKQANLIGKKELNILHLSWDGSAN
SKSSNVSVERVLEALEPPSTLKSFEMNGYEGKQLSSWMRSSIVLRDLVKVKLWNCGNCEELPPFGKLPHLKRLELSGMK
NVKCIDGETYDGAEEMAFPSLEELSVYYLPNLERLLRDEGVEMLPRLSQLTIKKVSNFKFPRLPSVEKLYVESIDDVERVVG
NTPCLKTLNIVSIKGVKTLPDKLGMLNALEVLYIDDWYDLKYFPEHVLEGLTSLRILRIYSCEKLKSLSEGVRHLACLQRLIIC
GCPELVALPNNMSQLTALQHVSIIFSTLPDGLQRVPSLRSLYISDCKSTSLPDWLGDITSLQELDIWYCKELRSLPSSIQRLT
NLSSLSISNCPYLKKRCNRETGKDWQYINHIPNIKCE

>XP_027913640.1

MAESLLGFVIENLESFVQDQLATYWGVDQQTQKLSNNLTAIRAVLRDAERKQITSHAVKNWLQKLTDAAYVLDDILDE
CLIHSTKVQAHDGHISRLSRLHPKDIVFRFNIGKRMKDITQRFHDIHEEKSRFNLEHGVTQVQTVDDDWRQTSSDITEPV
VYGREQDREQIVKFLVEDASNSEDLSIYSIVAMGGLGKTTLVKQVFNDDRVCKHFDLTIWVCVSDDFNTKTILQSMIECI
TGKNPNLNSLEAWRKRVEEVLHGKRYLLVLDDVWNEDQEKWKQLKGKLQCARAAKGATILVTTRLEEVASTMQTHPA
YHLKELSGDDSWSLFKHHAFGPNREEMEELVAIGKEIVRKCIGLPLAIKALGSLLRDQSEVRQWKNVKESEIWDIREENSS
MISEENSIMRALKLSYSNMELSLKRCFSFCAIFPKDFEISKEELIHLWMANGFIECEGNIEVEDVGNKVWNKLYRRSFFQE
AKCDEFGMVTSFKMHDLFHDLARSIMGEECVSFWGGRLTPLSSRVHYSTLLSDGSFRGFMRAFKKVESLRTFIDLHPCV
LIGFSPVPSNPSLRALCTNSCLLSPLKDLTHLRYLSLSKSWKSSLNNSICQMPKLQILKLQSFRFLRGLPKDLTQLQDLRHIV
MNQCNSVEKTPPKISKLRHLRTLSIFVVGSKPGCGLAELHSLNLGGALRIRGLKNVPSEWDAKQANLIGKKELNILHLSW
DGSANSKSSNVSVERVLEALEPPSTLKSFHIKGYEGRQLSSWMRSPVTLRDLVEVKLLDCDNCEELPPLGKLPYLKRLELIR
MKNVKWIDGETYVGVEEKAFPSLEELSVDNLPNLERLLRDERVEMVPHLLQLRIERVSNLKCPRLPSVEKLDAGEIGEAA
SFMEVVGNTACLKTLSIEYIKGVVVLPEQFSGLGALQDLYIAYWYDVEYFPEHVLEGLTSLRTLSINYCEKLKSLSEGVRHL

ACLESLTICGCPELVALPNKMSQLTALQHVSINICSTLPDGLQRVPSLRSLYICDYKSTSLPDWLGDISTLEELIINYCRELRSL
PSSIQRLTNLSHLSICKCPHLKKRCKRETGEDWQYINHIPKIELFSR

>XP_027913641.1

MAEALLGIVLENLGSFVQDQLATYWGVEQQTHKLSSNLTAIRAVLRDAERKQITSHAVKDWLQKLTDAAYVLDDILDEC
SVQSKMLHSDDGQSSCLAYVHPKHILCRFYIGKRMKDITQRFHDIHEERLTFELRVGVTEKQTVNDDDDWRQTSSVITE
PIFCGRDKDREKIVKFLEEDASGSEDLTIYPIVGMGGLGKTTLAKQVFNDHEISKHFDLRIWICVSDDFNVKRILQSIIECSI
GQNPNLGDLEARRKRVEEALQSKRYLLVLDDVWNEDREKWKELKGMLECAKGAKGATILVTTRLQEVASIMGTHPAY
SLTALSEDDSWSLFKHHAFGLNREEREEFVTIGKEITRKCVGSPLAIKTLGSCLRNENEIKQWQNIKESEIWDIREVSSSLT
NDENAIMRALKLSYFNLELSLRRCFSFCAIFPKDFEIDREELIHLWMANGFIKQEGNVEVEDVGNKVWKRLYDRSFFQEA
KANRIGMIKTFKMHDLFHDLAQSIMGEECVVYEEGKLTRLSNRVHYLRLLNSNKFVDMAAFKKVESFRTLLDFGLGLHH
VNIRLLPSNHCLRALHIRSTLFSPLNDLSHLRYLSLHRCSVLHSSICGLQKLQILKLEDCGGSHNFFPEHMTQLQDLRHLVIK
GCPKIAEMLPNIGKLGHLRTLNTFAVGSKLEYGLAELHGLSLVGKLHIRGLENVSNEWEAKEANLMSKKELNRLYLSWSD
SPNSQGSNVSVERVLENLEPPSTLKSFGMKGYLGRKLSSWMRSVVVLKDLVEVMLFDCFFCEELPPLGKLPHLKRVDLR
RIRNVKWIDGESYEGVEEKAFPSLEKLNVENLPNLERLLRDEGVEMLPRLSQVRIDGVLNFKVPHLPCVEELDARDIKAAT
SFMEGVVENVTCLKKLLIASMRDLKVLPEKLSRLSALQDLKILECEELRSLPSSFQRLTNLSKLSILNCDRLEERYKRETGND
WQFIAHIPNVELESFPSHGVKPALSFSDRYRQSSCSWNCFKVKRPPAETERMVEYNFFDYMDEYE

>XP_027913912.1

MAEAILGVVLEKLTSLAVEELRSFMGFNTDLEKLRVMLTSIEAILDDAAEKQFSSQRVKYWLEMLKDASYELNDILDECAE
LTLEYQGIKCGSYHEVCSCLSYFRPKHAAFHYTIAKRMKSICERLDTIAEARHMFQLTAKPPRSGGVYERLQTTSVIVESRV
YGREEDEQNIVDFLMANADVHTSRSNNLSVYPIVGLGGLGKTTLARLIFNHVSVVNHFDARVWICVTEDFDLNKITKAII
GSLSLEICEDLELESLLTKVQDLLQNKRYLLVLDDVCDYQQEKWLMLKSVLACGAMGASILITTRLLRVAAMMGTVSPHE
LSYLSDNDCWKLFKEQAFQAGEIEQEELVNIGMDIVKKCGGVPLAAKVLGAFLRFNREESTWTNVKNSDTWKSSQGEN
SIMPALILSYSNLPHKVRQCLVYCAIFPKDEIIRKQDLIEHWMANGFIPPNETLDVGDSMWNELYWRSFFQDIETDEFSH
VTSFKIHNIVHDLLKFAAEEVCCITNDNDVTVPERIHHISEHSWRSKLDSAQFRFESLRTYLLPPQRYKTGQLSPQVLKCYN
LRVLQYEPTETLSSSISRLKHLRYLNVSGGDFVTIPKSLCRLWNLQILKLDHCHRLQKLPKNLIGLKALQKLSLNGCSSLSSLP
RQMGKLTSLRNLSMYIVCEKKGFLLAELGPLKLKGDLDIKHMEKVRSVEDAKEAKMSEKQLNRLSLSWDNNEATELQE
NVEEILQVLEPSTNQLASLSVVGYKGACFPQWMSSSFLKYLELEDCKMCSKLPQLGKLRFLKSLSISKMTPVEYLYEEDSY
DGEAVFIALEFLFLKELSNLKRLLKREDGENMFPLLNKLEIAECPNLLGLPSLPSIRDLHIQGKCNQHLLTSVKKLSSLQDLW
LEDNEELRFFPDGMFQGLTSLKELYFHRLFKLEIFPTKLPDSLEKLEFVGCHKFVSAGLHEALQNVTALHSLKFEHLPNLAS
LPDCFQKLALLRELTLYNCSKLKCLPASLKFCALKRLDIQGCPELEKRCQEYTGEDWPIISTIPNIRVIPMRR

>XP_027913986.1

MECVLGFASSISRDLVCGAVNQLRYPCCFNDFVKKLEEDEGNLIITRDGVQKFIAYANTQTRKTSEIVDKWLQDAINDVD
NVNELLKEARTKKICCFGHFPNWIWRYRVGKKLGNKSVDLKKFIDEGRQYVPFDRIATLPSNILDILSEKCMNFESRESAY
EQLLDAVKNNDVSMIGLYGMGGCGKTTLAMEVKKLVEAEHLFEKVLFVPVSCTVEVQRIQEKIASSLQYVFP

>XP_027913987.1

MECVLGFASSISRDLVCGAVNQLRYPCCFNDFVKKLEEDEGNLIITRDGVQKFIAYANTQTRKTSEIVDKWLQDAINDVD
NVNELLKEARTKKICCFGHFPNWIWRYRVGKKLGNKSVDLKKFIDEGRQYVPFDRIATLPSNILDILSEKCMNFESRESAY
EQLLDAVKNNDVSMIGLYGMGGCGKTTLAMEVKKLVEAEHLFEKVLFVPVSCTVEVQRIQEKIASSLQYVFP

>XP_027913988.1

MECVLGFASSISRDLVCGAVNQLRYPCCFNDFVKKLEEDEGNLIITRDGVQKFIAYANTQTRKTSEIVDKWLQDAINDVD
NVNELLKEARTKKICCFGHFPNWIWRYRVGKKLGNKSVDLKKFIDEGRQYVPFDRIATLPSNILDILSEKCMNFESRESAY
EQLLDAVKNNDVSMIGLYGMGGCGKTTLAMEVKKLVEAEHLFEKVLFVPVSCTVEVQRIQEKIASSLQYVFP

>XP_027914093.1

MAESAVGFLLQRLAPVFENKVKLFTGVQAEVICLKGQLELIRAFLRVADALEENDEELKVWVKQVRDVVHEAEDLLDEL
ELVQVHNHNNGFSIYLRIRNMKARYRIAHELKTINSRLKAISSSRKRFRSKLDTSSVASNSVHTGNAWHDQRGDALLLDN
TDLVGIDRPKKQVIGWLINGCPGRKVISVTGMGGIGKTTLVKKVYDDPDVKKHFKACAWVTVSQSCKIEELLKDLAKKLF
SEIRRPIPEGMESMCSDKLKMIIKDLLQRKRYLVVFDDVWHMYEWEAVKYALPNSNCCSRIMITTRRSDLAFNSTIESSG
KVYNLQPLKEDEAWDLFCRNTFQGDSCPSYLIDICKYILRKCEGLPLAIVAISGVLATKDKRRIDEWDMICRSLGAEIQGN
GKLDNFKTVLNLSFNDLPYHLKYCFLYLSIFPQDYLIQRMRLIRLWIAEGFVEAKEGKTKEDVAHDYLKELLNRNLIQVAGT
TSDGRVKTLRVHDLLREIIILKSKDQNFASIVKEQSAAWPEKIRRLSVHGTLPYRQQHRSVSQLRSFLMFGVGEYVPLGKL
FPSGFKLLSVLDYQDAPLKKFPLAVIDLYHLRYLSLRNTKVKTVPGHIIGKLHNLETLDLKNTSVRELPVDILKLQKLRHVLVY
QFKFKGYAQFHSKDGLKAPSEIGNLKALQKLCFVEANQDCGMIIRQLGELSQLRRLGILKLREEDGMAFCLSIERLTNLHA
LSVTSEGESKVIDLTFLCSPPPFLQRLYLSGRLQELPCWIQSLHSLARLFLKWSCLKYDPLVYLQDLPNLAHLELLQAYDGD
TLHFRSGKFKKLKVLGLDKFDGLKEVTVGKDAMTRLEKLSIGRCELLKKVPSGIENLTKLKVLEFFDMPDELMKTICPHGP
GKDYGKVLHIPDVYSTYWRDGGWDVYALDTFSRDCSPRSGTLIRSHEPRIQWKV

>XP_027914409.1

MDVAILSDAISHILQCATTILSPPATRLSSNDIQQFEDNLKRILLTVQKAMHSKIQDRSVLSLWLKNVKDMVNDLNDLME
DHRHNTEATTATISLIKAGQNMAHRHKFKHQIKDAIEELKRLSNEAESLVISEEARENERKLTRINEEFENVEAVGRENVK
KDIKDQLKMFVNSHVVSVPVVTIVGVAGIGKSKLARLVYGDEEVKALFPSRIWVNLETFNVESIATRVTETANQGKRFLL
VLDDLRVENGEGCLQKLQGELAEAGVGGAVVVTTRSNFVANKIAESGTVKLKPHVLQGLNEEESWCLFQNRPGSRKIN
EDMGRRVVREYCGGVPMKIIAIARLLEDLDSPVSEIELMEKFLREIRFTYYDELSLQQKLCFAYCSLFPQEHEIDAGRLIHL
WMAEGFLSRNLCSDPQEFGLACFNDFVPFVFQETGSDEFGVVKRYKMNRLMHELARTVAWDENIIVDSAEVKVHERV
VRSSFHFALDVQCGIPKALFEKAKKLRSILLLGKTNKSRLPHEVKLTISTCEKILETFKCLRVLDLHDLGIKMVPSSIGELKHLR
LLDLSHNNIEKLPSSITKLFHLQTLKLSQCHVLKELPKDLENLSCLIHLYLEGCLDLTHMPRGIGKLSSLQTLSLFVVSKNYHL
GGLRELTDLDDLRGHLEILHLEQLNFCAPLEAKDKYLRDKKHLHCLTLRWDHEEKEEEDEKKRNGIAGKDKESLECLDPN
PNLAVLSVVGYYGKTFSNWLSSIKCLVKFSLNDCYNCQYLPPLDHLPHLRVLELRRLDSLVFVSRNSDQISADTEASSSSSS
SSSSSSSTPFFPSLKELTISDCPKLRSWWETANWESNRPFFTRISKLHIQCCPDLHCMPLYPYLDEELVVVDSSVKSMRDT
VHATISDGFLPFSKLKTMLIARITQSPPERWLKNFISLQTLQIRDCSKLFYLPQGFRSLSSLQSLTIERCAELDLDLSRTEWEG
LKHLRFLIIKEIPKLKSLPWGVEDVTSLEKLELHECPALTNLPETIGNLSLLTKLVICKCENLDSLPKGLEKLGSLDTLAITDCPL
LTPRCQPETGDDWPQIAHIKNRILKQSSQDLRDLWSHGRIGLRKYF

>XP_027914493.1

MAEAVLKAVLGSLTSLVATELGSFLAFSGEKEKLESMFTAIKATLEDAEEKQFSDKAIKDWVGKLKDAAYELDDILDEFVY
KQLWLEQKDEEEVKCCESEMVLCSSLSSLHPTNLYFRHNIVRRMKSVSERLDRIAREKDQLRLTSTVEVETRGVPEWRQ
TISLVTEPRVYGREKDIRKIVEFLAGAASRAENLPVYPIVGQGGIGKTTLPKLIFNHKDLIDFQLKIWICVSEDFGLERMLKAI
LHAASEECKDLDLESLQRKVRNLLPRKKYLLVLDDVWDVTRHNWEENWHMLRSVLDCGEKGGSVLVTTRFWNVAEI
MGTVKHPHRLSELSEYYCWELFKHQAFGADEAEPDELVVIGRKIVKKCGGVPLAAKAVGGLLRFHRNKDKWLNIMESN
ILMLSSNEKSVMPVLRLSYVNLPVQLRQCFAYCAIFLKDDLIKKQYLIELWIANGFISSDGRLDAEDVGDEVWNELYRRSL
FQDIETDQFGKVTSFKMHDLVHDLAQFVADEEICCITDEDCAPVLFERKRIHHLSDHRWNLYSAQLDQLTSLRTYLKRTR
SKELSSDVLKCYSLRLLHVNLLEELSSSIGNLKYLTYLNLSRGRFKTLPESLCKLLNLKILKLDYCQSLQKLPDGLVRLKALQQL
SLKVCRSLSTLPPYIGKVNSLRSLSMYFVGEQKGFLLSELGLLKLKRDLEIKHLERVKNINDAKESNMSSKQLNNLMLRWR

TVREGELEGNDKEVLEALEPCTETLQSLRVEGYQGVSFPQWMSSPFFKNLTSLELWSCTNCIKLPVLRNLPSLKRLQITEA
KYVKYVQEECYDNDVGFMGLEYLTLRNLPSLIRLSSEDGENQFPCLSTLDIEDCPHFSLQGLPSLKWLRMSRNPKLKVCP
GLECLPCLEDLIIMRCGEVEGLQHMTGLKKLALINVPKVSLLQELRFEDLPLLRELHIIGCYSLMRLPTNLSLSRLEVLCIKDC
DPELEMRCEKENGEDWPIIAHIPHVYTRQIL

>XP_027914921.1

MQCWGHHVFPGGFLQLVISLLLYLQTRLLLSFNRCDLPRIFSETRLIWLEAKYRIYRRVFLFGILIKIVWLRAWDLVSCITG
NPTSTGMDIQEESPVFGSLTAMTTRNMSSSSSVFFSANQSPFFSPRSPSSCQLSHSARLDTQSNTVHLGLAPSTTTLEIPE
PNSTVNVRCNFSDVSASPAGCNSGGFVKLDRKSSPVGVSSSSISSYSNCHEDGYSGQKERRIKKDRKHRTSSTPGSTSFSS
YRLRSCDVFIGLHGSKPPLLRFAKWLCAELEIQGISCFVSDRARSRSSRKLGIAERAMDAASFGIVIITKKSFKNQYTIEELNF
FCRKKNLIPIFFDLNPADCLVRDIIEKRGELWEKHGGELWLSYEGLEQEWKDAVHGLSRVDECKLEAQDGNWRDCILRA
VTLIATRLGRRSVAERVAKWREKVEKEEFPFIRNENFIGRKKELSQLEFILFGDVTGDAEQDYIELKARPRRKSVRIGWGKS
NMIDERWNDRRKEKEPVVWKESEKEIEMQGIEFSNRHNHPRLKRGKYSKRKNGMKILYGKGVACVSGDSGIGKTELIL
EFAYRFHQRYKMVLWIGGESRYIRQNYLNIRSFLEVDVGVENSLDKTKIRSFEEQEVAAISRVRKELMRNIPYLVIIDNLES
EKDWWDHKLVMDLLPRFGVETHVIVSTRLPRIMNLEPLKLSYLSGVEAMSLMVGSSKEYSVAEVDALRSIEEKVGRLTL
GLAIISAILSELPITPSRLLDTINRMPLKEMPWSGKEALSFTKNAFLLQLFDVCFSIFDHADGPRSLATRMVLVSGWFAPG
AIPISLLALAAEKVPERCQGKCFWRKMLQLLSCGFPSSYAKKPELEASSLLLRFNIARNSTKQGYIHINEVFKLYARKRENT
GAAQAMIQAIMSHGSISQNLDHLWAACFLLFGFGHDPVVVELKVSELLYLVKRVVLPLAIHTFITYSRCSAALELLRLCTN
ALEAADQAFVTPVDKWFDKSLCWRSIQTNAQLNPCLWQELALTRATVLETRAKLMLRGAQFDVADDLIRKAVFIRTSIC
GEDHPDTVSARETLSKLTRLNANVQIHT

>XP_027914922.1

MIVFFSDMVPSSLLLNLVIELTRHQSSASNDCIPQADTVFWDAFTVRNQSLSIKIVWLRAWDLVSCITGNPTSTGMDIQ
EESPVFGSLTAMTTRNMSSSSSVFFSANQSPFFSPRSPSSCQLSHSARLDTQSNTVHLGLAPSTTTLEIPEPNSTVNVRCN
FSDVSASPAGCNSGGFVKLDRKSSPVGVSSSSISSYSNCHEDGYSGQKERRIKKDRKHRTSSTPGSTSFSSYRLRSCDVFIG
LHGSKPPLLRFAKWLCAELEIQGISCFVSDRARSRSSRKLGIAERAMDAASFGIVIITKKSFKNQYTIEELNFFCRKKNLIPIF
FDLNPADCLVRDIIEKRGELWEKHGGELWLSYEGLEQEWKDAVHGLSRVDECKLEAQDGNWRDCILRAVTLIATRLGR
RSVAERVAKWREKVEKEEFPFIRNENFIGRKKELSQLEFILFGDVTGDAEQDYIELKARPRRKSVRIGWGKSNMIDERWN
DRRKEKEPVVWKESEKEIEMQGIEFSNRHNHPRLKRGKYSKRKNGMKILYGKGVACVSGDSGIGKTELILEFAYRFHQRY
KMVLWIGGESRYIRQNYLNIRSFLEVDVGVENSLDKTKIRSFEEQEVAAISRVRKELMRNIPYLVIIDNLESEKDWWDHKL
VMDLLPRFGVETHVIVSTRLPRIMNLEPLKLSYLSGVEAMSLMVGSSKEYSVAEVDALRSIEEKVGRLTLGLAIISAILSELPI
TPSRLLDTINRMPLKEMPWSGKEALSFTKNAFLLQLFDVCFSIFDHADGPRSLATRMVLVSGWFAPGAIPISLLALAAEK
VPERCQGKCFWRKMLQLLSCGFPSSYAKKPELEASSLLLRFNIARNSTKQGYIHINEVFKLYARKRENTGAAQAMIQAIM
SHGSISQNLDHLWAACFLLFGFGHDPVVVELKVSELLYLVKRVVLPLAIHTFITYSRCSAALELLRLCTNALEAADQAFVTP
VDKWFDKSLCWRSIQTNAQLNPCLWQELALTRATVLETRAKLMLRGAQFDVADDLIRKAVFIRTSICGEDHPDTVSAR
ETLSKLTRLNANVQIHT

>XP_027914923.1

MDIQEESPVFGSLTAMTTRNMSSSSSVFFSANQSPFFSPRSPSSCQLSHSARLDTQSNTVHLGLAPSTTTLEIPEPNSTVN
VRCNFSDVSASPAGCNSGGFVKLDRKSSPVGVSSSSISSYSNCHEDGYSGQKERRIKKDRKHRTSSTPGSTSFSSYRLRSC
DVFIGLHGSKPPLLRFAKWLCAELEIQGISCFVSDRARSRSSRKLGIAERAMDAASFGIVIITKKSFKNQYTIEELNFFCRKK
NLIPIFFDLNPADCLVRDIIEKRGELWEKHGGELWLSYEGLEQEWKDAVHGLSRVDECKLEAQDGNWRDCILRAVTLIA
TRLGRRSVAERVAKWREKVEKEEFPFIRNENFIGRKKELSQLEFILFGDVTGDAEQDYIELKARPRRKSVRIGWGKSNMI
DERWNDRRKEKEPVVWKESEKEIEMQGIEFSNRHNHPRLKRGKYSKRKNGMKILYGKGVACVSGDSGIGKTELILEFAY

RFHQRYKMVLWIGGESRYIRQNYLNIRSFLEVDVGVENSLDKTKIRSFEEQEVAAISRVRKELMRNIPYLVIIDNLESEKD
WWDHKLVMDLLPRFGVETHVIVSTRLPRIMNLEPLKLSYLSGVEAMSLMVGSSKEYSVAEVDALRSIEEKVGRLTLGLAI
ISAILSELPITPSRLLDTINRMPLKEMPWSGKEALSFTKNAFLLQLFDVCFSIFDHADGPRSLATRMVLVSGWFAPGAIPIS
LLALAAEKVPERCQGKCFWRKMLQLLSCGFPSSYAKKPELEASSLLLRFNIARNSTKQGYIHINEVFKLYARKRENTGAAQ
AMIQAIMSHGSISQNLDHLWAACFLLFGFGHDPVVVELKVSELLYLVKRVVLPLAIHTFITYSRCSAALELLRLCTNALEAA
DQAFVTPVDKWFDKSLCWRSIQTNAQLNPCLWQELALTRATVLETRAKLMLRGAQFDVADDLIRKAVFIRTSICGEDH
PDTVSARETLSKLTRLNANVQIHT

>XP_027914924.1

MDIQEESPVFGSLTAMTTRNMSSSSSVFFSANQSPFFSPRSPSSCQLSHSARLDTQSNTVHLGLAPSTTTLEIPEPNSTVN
VRCNFSDVSASPAGCNSGGFVKLDRKSSPVGVSSSSISSYSNCHEDGYSGQKERRIKKDRKHRTSSTPGSTSFSSYRLRSC
DVFIGLHGSKPPLLRFAKWLCAELEIQGISCFVSDRARSRSSRKLGIAERAMDAASFGIVIITKKSFKNQYTIEELNFFCRKK
NLIPIFFDLNPADCLVRDIIEKRGELWEKHGGELWLSYEGLEQEWKDAVHGLSRVDECKLEAQDGNWRDCILRAVTLIA
TRLGRRSVAERVAKWREKVEKEEFPFIRNENFIGRKKELSQLEFILFGDVTGDAEQDYIELKARPRRKSVRIGWGKSNMI
DERWNDRRKEKEPVVWKESEKEIEMQGIEFSNRHNHPRLKRGKYSKRKNGMKILYGKGVACVSGDSGIGKTELILEFAY
RFHQRYKMVLWIGGESRYIRQNYLNIRSFLEVDVGVENSLDKTKIRSFEEQEVAAISRVRKELMRNIPYLVIIDNLESEKD
WWDHKLVMDLLPRFGVETHVIVSTRLPRIMNLEPLKLSYLSGVEAMSLMVGSSKEYSVAEVDALRSIEEKVGRLTLGLAI
ISAILSELPITPSRLLDTINRMPLKEMPWSGKEALSFTKNAFLLQLFDVCFSIFDHADGPRSLATRMVLVSGWFAPGAIPIS
LLALAAEKVPERCQGKCFWRKMLQLLSCGFPSSYAKKPELEASSLLLRFNIARNSTKQGYIHINEVFKLYARKRENTGAAQ
AMIQAIMSHGSISQNLDHLWAACFLLFGFGHDPVVVELKVSELLYLVKRVVLPLAIHTFITYSRCSAALELLRLCTNALEAA
DQAFVTPVDKWFDKSLCWRSIQTNAQLNPCLWQELALTRATVLETRAKLMLRGAQFDVADDLIRKAVFIRTSICGEDH
PDTVSARETLSKLTRLNANVQIHT

>XP_027914925.1

MDIQEESPVFGSLTAMTTRNMSSSSSVFFSANQSPFFSPRSPSSCQLSHSARLDTQSNTVHLGLAPSTTTLEIPEPNSTVN
VRCNFSDVSASPAGCNSGGFVKLDRKSSPVGVSSSSISSYSNCHEDGYSGQKERRIKKDRKHRTSSTPGSTSFSSYRLRSC
DVFIGLHGSKPPLLRFAKWLCAELEIQGISCFVSDRARSRSSRKLGIAERAMDAASFGIVIITKKSFKNQYTIEELNFFCRKK
NLIPIFFDLNPADCLVRDIIEKRGELWEKHGGELWLSYEGLEQEWKDAVHGLSRVDECKLEAQDGNWRDCILRAVTLIA
TRLGRRSVAERVAKWREKVEKEEFPFIRNENFIGRKKELSQLEFILFGDVTGDAEQDYIELKARPRRKSVRIGWGKSNMI
DERWNDRRKEKEPVVWKESEKEIEMQGIEFSNRHNHPRLKRGKYSKRKNGMKILYGKGVACVSGDSGIGKTELILEFAY
RFHQRYKMVLWIGGESRYIRQNYLNIRSFLEVDVGVENSLDKTKIRSFEEQEVAAISRVRKELMRNIPYLVIIDNLESEKD
WWDHKLVMDLLPRFGVETHVIVSTRLPRIMNLEPLKLSYLSGVEAMSLMVGSSKEYSVAEVDALRSIEEKVGRLTLGLAI
ISAILSELPITPSRLLDTINRMPLKEMPWSGKEALSFTKNAFLLQLFDVCFSIFDHADGPRSLATRMVLVSGWFAPGAIPIS
LLALAAEKVPERCQGKCFWRKMLQLLSCGFPSSYAKKPELEASSLLLRFNIARNSTKQGYIHINEVFKLYARKRENTGAAQ
AMIQAIMSHGSISQNLDHLWAACFLLFGFGHDPVVVELKVSELLYLVKRVVLPLAIHTFITYSRCSAALELLRLCTNALEAA
DQAFVTPVDKWFDKSLCWRSIQTNAQLNPCLWQELALTRATVLETRAKLMLRGAQFDVADDLIRKAVFIRTSICGEDH
PDTVSARETLSKLTRLNANVQIHT

>XP_027914926.1

MDIQEESPVFGSLTAMTTRNMSSSSSVFFSANQSPFFSPRSPSSCQLSHSARLDTQSNTVHLGLAPSTTTLEIPEPNSTVN
VRCNFSDVSASPAGCNSGGFVKLDRKSSPVGVSSSSISSYSNCHEDGYSGQKERRIKKDRKHRTSSTPGSTSFSSYRLRSC
DVFIGLHGSKPPLLRFAKWLCAELEIQGISCFVSDRARSRSSRKLGIAERAMDAASFGIVIITKKSFKNQYTIEELNFFCRKK
NLIPIFFDLNPADCLVRDIIEKRGELWEKHGGELWLSYEGLEQEWKDAVHGLSRVDECKLEAQDGNWRDCILRAVTLIA
TRLGRRSVAERVAKWREKVEKEEFPFIRNENFIGRKKELSQLEFILFGDVTGDAEQDYIELKARPRRKSVRIGWGKSNMI

DERWNDRRKEKEPVVWKESEKEIEMQGIEFSNRHNHPRLKRGKYSKRKNGMKILYGKGVACVSGDSGIGKTELILEFAY
RFHQRYKMVLWIGGESRYIRQNYLNIRSFLEVDVGVENSLDKTKIRSFEEQEVAAISRVRKELMRNIPYLVIIDNLESEKD
WWDHKLVMDLLPRFGVETHVIVSTRLPRIMNLEPLKLSYLSGVEAMSLMVGSSKEYSVAEVDALRSIEEKVGRLTLGLAI
ISAILSELPITPSRLLDTINRMPLKEMPWSGKEALSFTKNAFLLQLFDVCFSIFDHADGPRSLATRMVLVSGWFAPGAIPIS
LLALAAEKVPERCQGKCFWRKMLQLLSCGFPSSYAKKPELEASSLLLRFNIARNSTKQGYIHINEVFKLYARKRENTGAAQ
AMIQAIMSHGSISQNLDHLWAACFLLFGFGHDPVVVELKVSELLYLVKRVVLPLAIHTFITYSRCSAALELLRLCTNALEAA
DQAFVTPVDKWFDKSLCWRSIQTNAQLNPCLWQELALTRATVLETRAKLMLRGAQFDVADDLIRKAVFIRTSICGEDH
PDTVSARETLSKLTRLNANVQIHT

>XP_027915187.1

MEVIAQIVLQNLSSFAQEEFGIIWNLKDDVQQMKSTVSAIKAVLLDAEAKTNNLQISNWLEELKDVLYDADDLLNDISSE
AMKRKVIGSSTVLRKIQIFFSQENQIAYSFKLGHQMKAIQKRLDAIAKNKITLQLTDRPLETPIAYREQRQTYSFVREDDVI
GREEEKKLLKSYLLDTKVSVIDNVSVLAIVGFGGLGKTALAQLVFNDNAVQCYFEQKMWVCVSDEFDMRKIAEKMIGN
DKNSEIEQLQQDLRNKVRGKKFLLVLDDVWNEDRELWLKLKSLVVEGGKGSAIIVTTRSRTVAKIMATHPPLFLKGLDLE
RSWKLFSRVAFDEGKEPNDLELLAMGRDIVQKCAGVPLAIRTIGSLLYSRNLGRSDWLYFSEVEFSKIDQHKDKIFAILKLS
YDHLPSFLKKCFAYCSLFPKDFEFDKKTLIQLWVAEGFIQSSRDNRCEEDVGHEYFMNLLSMSLFQDVTLDDFGDILTCK
MHDLVHDLAQLVVGKEYAFVEGKKEDIRNRTRYLSSCTSLHFLEKTSSFSNKLRTFIFIGEPLYGSQNFGPPPSLHFPFLLS
MKCLRVLTLSGLHLITIPNSIRELKQLRYLDLSMNKFLVSLPLDVTSLHNLQTLKLSRCGELKELPSDISRSLRHLELNDCGKL
TCMPCGLGQLTNLQTLTHFLLDSKSKNVDISELSGLNNLRGKLVIKWLDSLRENAAVVESANILLEKQHLQELELRWRLG
VNSLWRDPIEERLKMENEKIYIDEPLLKKDEKIMQGLQPHHSIKRLVIDGYCGKSLPDWIGNLSSLLSLEISNCNGLKSLPD
GIRDLVSLQRLCIYNCSLLEKRCARGNGVEWSKIAHIPKVLVSAFTPTDLRYIN

>XP_027915208.1

MAEAVLKSVLGSLASPAVAELKPFLCFRREKEKLESMFTAIKATLEDAEEKQFSDRAIKDWVGKLKDAAYELDDILDEFAY
EQMRLEEEEKPQSLNDYKPNYMKKIYIKSPTFSSHESLFHLTSTIQEETRGVPEWRQTFSFITEPKVYGREQDIKKIVEFLA
GAASRPENLPVYPIVGQGGLGKTTLAKLIFNHNDLKDFQLKIWVGVSEDFGLERILKAIIEAASEDVRKDLGLEATQRRLR
KLLTGKRYLLVLDDVWDVMKQNWKENWQVLRSILDCGEKGSSVLVTTRFSNVAEIMGTIKHPHMLPELSVYYCWELF
KHQAFGADEIEQEELVMIGRKIVRKCGGVPLAAKALGGLLRSHRNKNKWINILQSNLLTLSPNEKSIMPVLRLSYLNLPIE
LRQCFAYCAIFPKDELIGKQYLIELWMANGFISSDGRLDAEDVGDDVWNELYRRSLFQDIEVDEFGKVTSFKMHDLVHD
LAQFVADEEVCCITDEDYAPVLFERKRIQHLSDYRWWLHSTQLHQVKSLRTYIKSKATKELSSDVLKCYSLRLLHVSLREEL
SASIVDLKHLKYLNLSCSDFKTLPESLCVGKLLNLKILKLDYCQRLQKLPDSLVRLKALQQLSLKVCRSLSRLAPYIGKLNSLRS
LSMYFVGEQKGFLLAELGLLKLKRDLEIKHLERVKNITDAKESNMSSKKLNNLMLRWSIVREGELEGNDEEVLEALEPCTE
TLQSLRVEGYQGVRFPPWMSTPFLKNLTSLELWCCTNCIKLPVLRNLRSLKRLEITKAKYVKYVQEECYDNDVGFMALEY
LSLRSLPSLIRLSSEDGENQFPCLSTLDIEDCPHFSLQGLPSLKLLRMSRNPKLKVCPGLECLPCLEDLTIDSCEEVEGLRFMT
ALKKLALVNLINIKSLPECFGDLPLLRELRIFGCYKLMRLPTSLGLSRLEVLYIQDCHPKLQMRCLKESGEDWPIIAHIPHLYF

>XP_027915232.1

MAEALLGIVIQNLQSFGQDQLATFWGVDKQTLKLSSNLSAIRAVLRDAERKQITSHAVKDWLQKLTDAAYVLDDILDEC
SIHFTKMHSDDGHISCLSRLHPSDILFRFNIGKRMEDITQRFHDIHEEKSKFNLEPGVTEVVQTVDDDWRQTSSDITEPV
VYGRDHDREQIVKFLLEDASNSEDLSIYPIVGMGGLGKTTLAKQVFNDHRVCKHFDLTIWVCVSDDFNTKTILQSIMECI
TGKNPNLNSLEAMRKKVEEVLHGMRYLLVLDDVWNEDQEKWEQLKGKLQCARAAKGATVLVTTRLEEVASTMQTQP
AYHLKKLSEDDS

>XP_027915233.1

MANGFIKCEGHVEVEDVGDKVWKKLYRRSFFEEAKSDEFGIIRSFKMHDLFHDLAQSIMGEECMVIEKGSWTPLSTRV
HYLANFNEYVDMPASMQFMTAFKKVESLRTFLNFGTMGQLPSNNCLRALCTTSSLFSRLNDVRHLRYLSLSWSSVAILD
NTICELPKLQTLKLEYYPQISELPKDLTQLQDLRHIVINYCALLQEMPANIGKLRHLRTLSFFSVGSKPGCGLAELHSLNLGG
ELSIRGLKNVSSEWDAKQANLIDKKDLNILYLSWDGNANSRGSTVSEEIVLEALEPPSTLKSFHIKGYQGRQLSSWMRSP
VTLRDLVEVKLLDCDNCEELPPLGKLPHLKRLEVSGMKNVKCIDGETYEGVEEKAFPSLEELILKNLPNLERLLRDEGVEML
PRLSQLTIKKVSNFKFPRLPSVEKLDVESIDDVERVVGNTPCLKTLKISSIKGVKTLPDQLGMLDALEYLDIRYWYDVEYFPE
HVLEGLTSLRTLEIRHCKKLKSLSEGVRHLACLERLTIRECPELMVLPSNMSQLTALRDVSILYCSTLPEGLQRVPSLRSLYIF
NCNSTSLPDWLGDITSLQQLSIDYCMELRSLPSSIQRLTNLSSLRIYNCSHLEKRCKRETGEDWQYINHVPKIELHLTWKP
TFCDELKSVFRTSFRNVSLCP

>XP_027915323.1

MAEALLGFVIQNLGSFVQDQLGPYWGVEQQTQKLSNNLTAIRAVLRDAERKQTTSHAVKDWLQKLTDAAYVLDDILD
ECSIHSTKVHSDGHTSCLSRLHPKDIVFRFNIGKRMKDITQRFHDINEEKSRFNLEHGVTQVQTVDDDWRQTSSDITEPV
VYGRDQDREQIVKFLVEDASNSEDLSIYPIVAMGGLGKTTLAKQVFNDHRVCKHFDLTIWVCVSDDFNTKTILQSMIECI
TGQNPNLNSLEALRKRVEELLHGKRYLLVLDDVWNEDQEKWKQLKGKLQCARAAKGATILITTRLEEVASTMQTHPAY
HLKELSGDDSWSLFKHHAFGPNREEMEELVAIGKEIVRKCIGLPLAIKTLGSLLRDQSEVRQWKNVKESEIWDIREDSNS
MTSEENSIMRVLKLSYSNLELSLKRCFSFCAIFPKDFVIDKEELIHLWMANGFIECEGNIEVEDVGNKVWNKLYRRSFFQE
AKFDEFGIITSFKMHDLFHDLARSIMGEECVAFGKGRLTPLSSRVHYSTLLSDVSFRGFTNAFKKVESLRTFLDLRTSFELN
NLCPVPSNHSLRALCTNSSLLSPLKDLTHLRYLSLSHSDKASLNNSICQMPKLQILKLQDFIFLRGLPKDLTQLQDLRHIVM
NQCNSVVKTPPKISKLRHLRTLSLFVVGSKPGCGLAELHALKLGGALRIRGLKNVPNEWDAKQANLTAKEDLNILHLSW
DGSANSKSSNVSVERVLEALKPPSTLKSFEMNGYEGKQLSSWMRSSIILRDLVKVKLWNCENCEELPPFGKLPHLKRLEV
SGMENVKCIDGETYEGVEEKAFPSLEELSVDNLPNLKRLLRDERVEMVPHLFQLRIQRVSNLKCPRLPAVEKLDARGIGE
AASFMEVVGNTACLKTLRIEFIKGVVVLPDQFSRLGALQELNIADWYDVEYFPEHVLEGLTSLRILRIYRCEKLKSLSEGVR
HLACLERLTIRECPELMVLPSNMSQLTALRDVSILYCSTLPEGLQRVPSLRSLYILDCNSTSLPDWLGDITSLQQLSIDYCM
ELRSLPSSIQRLTNLSHLSISDCPYLYTRCKRETGEDWQYINHVPKIELHLTWKPTFCDELKSVFRTSFRNVSLCP

>XP_027915324.1

MADALVESVIENLGSLVKDQLAIYWGVDEQTKKLSSNLKAIRAVLRDAERKQITSYAVKDWLQKLTDAAYVLDDILDECS
IHSTKMHSVDGHTSRLSRLHPKDILFRFHIGKRMRDITQRFHSIHEERLTFELRESVTEKQAVDDDDWRKTSSDITEPIVY
GRDQGREQILKFLLEDASDSEDLSIYPIVGMGGLGKTTLAKQVFNDDRVCKHFDLTIWVCVSDDFNTKAILQSIIECITGQ
NPNLNSLEAMRKKVEEALHNKRYLLVLDDVWNEDQEKWKELKGKLQCARGAKGATILVTTRLEEVVSIMGTHSAYRLT
ALSEDDSWSLFKHHAFGPNREESEELVTIGKEIMRKCVGSPLAIKTLASCLRDESEVSQWENVKKSEIWNIREESSSVTGD
ENSIMRVLKLSYSNLKSSVKRCFSFCAIFPKDWEIKKEEVIHLWMANGFIKCEGDVEVEDVGNKVWKKLYSRSFFQEAKY
DEFGMITTFKMHDLFHDLAQSIMGEECVVIVEGRLTPLSTRVHYSSLLNSGVSVDVSDFRQRFMTVLKKVESLRTFLDIG
GIGRVPTNHCLQALQTSSSLLSPLKDLTHLRYLSLSWNSEASLNNSICHLPKLQILKLQHLRHLYGLPKDLTQLQDLRHIVII
ECPSIVEMPPKISKLRHLRTLSIFVVGSKPGCGLAELHSLKLGGTLRIRGLENVSNEWDAKEANLKSKKDLNRLHLSWDGS
ANSKGNNVSAEIILEALEPPSTLKSFGMNGYEGRRLSNWMRSVVVLKDLVEVKLFNCDNCEELPPLGKLPHLKRLVVSG
MKNVKWIDGETYDGVEEKAFPSLEKLSVENLPNLERLLRDEGVEMLPRLSQLTIFGVSNLKFPRLPSVEILLARSIDEVTM
EGVVGNMTCLKTLDIQIINGAVVLPDQLSGLDALQDLRVGYWNDLEYFPEHVLEGLTSLRTLSISYCHKLKSLSEGVGHLA
RLESLSIRYCPELMDLPSNMSQLTTLWKVSIAGSSSLPYGLQRAPSLRTLNITFCKCTSLPDWLGDITSLQQLSIDYCMELR
SLPSSIQRLTNLSSLRIYNCSHLEKRCKRETGEDWQYINHIPKIELHFPKKPTFCA

>XP_027915942.1

MAAELVGGALLSSFVNVAFDRLASREVVDFFRGRKLDEKLLHNLNIMLHSINSLADDAEQKQFRDPHVKAWLFAVKEA
VFDAEDLLDEIDYEHTRCEVEAEAGSEPQTITYKVSNFFNATFSSFNKKIDSRLKEVLKKLEYLARQKGALGLKECTYSSVGS
GSNISQKLPSTSLVVESVIYGRDADKEIIFNWLTSETNSRNQPSILSIVGMGGLGKTTLAQHVYNDPKLDNAKFDIKLWVC
VSDHFDVLTVTKTILETIDNKKDDSGNLEMVHKKLKEKLSGRKFLLVLDDVWNEKQKEWEAVRTPLSYGAPGSRILVTTR
AEKVASNMRSEVHRLKQLQEDECWKVFTKHALKDDDLELNDEQKEIGRRIVEKCKGLPLALKTIGSLLHTKSSISYWKSV
LVNDIWDLTKEDSEIIPALFLSYHYLPSHLKRCFAYCAIFPKDYEFLKKELIFLWMAENILQCPQQIRHPVEVGEEYFNDLLS
RSFFQQSSAKRHFVMHDLLNDLAKHVCADFCFRLKFDKGKCIPKATRHFSFAFDDVKCFDGFRSLTEAEKLRSFIPITKIG
KHFYLDYSWKFKISIHDLCSKMKFLRVLSFDCCSDIREVPCSVGDLKHLRLLDLSRTEIQKLPDSTCLLYNLLILKLNYCSNLE
ELPSNLHKLTKLCCLEFKRTKVTKMPMHFGELKNIQVLNTFIVDRTSEFNSKQLGRLNLHGRLSIKELQNIINSSDALVMD
LKNKTHLVTLKLKWNKNHILDNPRKEKKVLENLQPSKYLETLGINNYGGMEFPNWVFDNSLPNLVFLQLKNCKYILCLPP
LGLLSSLKTLKIIGLDGIVSIGAEFYGGSDSSSFKSLERLEFYNMKEWEEWECKTASFPRLQRLSVNKCPKLKGLPEQLHLKK
LIICSCEILTISGHSMDPSTLEVLKIYSCPLTNVPITHYDFLEEMEIDGGCDFLTTFPLDFFPKLRFLKLSRCQNLQRISHEHTH
DHLKYLIVEKCPQFESFPSEGLSAPWLQGIEIRGAKNLKLLPKHMQTLLPSLTDLHIIDCPQVEMFPDGFPSNVKYVSLSSL
KLIASLSEILETNTCLKSLSIEKVDVECFPDEVLLPRSLTSLCICDCPNLKKMDYKGLCHLSSLTLIDCPNLQCLPEEAMLKFIS
SLTVRGCPLLKQYCQNPEGKDWRKVAHIEELLIE

>XP_027916036.1

MEETKKGGVGFPRMMKGPFGVPEKPEFNVGLDAPLTKLKMELLNRPKSIIVLTGFGGSGKTTLATLLCWDQQIRGKFG
ENIYFVTISKTPKLKMMVERLFEHLAYEAPKFQNDEETINQLEILLRKLKGSPMLMVLDDVWPHSEDLVEKFKFHLSDYKL
LVTSRVAFPRFGTPCVLKPLLHQHAITLFRHYAHLDTNSLNIPDQDTIQKVVKSCMGLPFAIKVIGRSLSHGPNELWKKM
VLELSHGNSILHCNTELLTYLHKILDVLEDNTVTKECFLDLGLFPEHQRIPVTALIDMWVELYGLDNDGIEAMAIIDKLESM
NLANVLITRKNTSDTDNYYYNNHFVVLHDLLRELAVYESTLEPIEERKRLIIDTNENQCELGLDEKQQCMVTRILSNCFKYC
VKHKPQMITARTLSTSIDENGPSYWSHMHPTHAKVLILNLRVNQYSFPESMKKMRKLKVLIVTSYNFHPSELTNFELLGS
LSNLKRIRLERIAVLSSFVTLKSLTKLSIYMSQAFQNGLLLISDAFPNLMDLSIDYCKDMVVLPSGLCDIISLKKLSVTNCHKL
LALPQEIGKLVHLELLRLSSCTDLEGLPDSVGRLQNLRHLDISNCISLSNLPEDVGKLSNLRNVSMINCERCELPYSVVNLV
NLKTVSCDEETATSWEGFKAMLPNLLIEVPQVDINLNWLHSVIHHTRTCSEYISNFDSPRTFAALIHKQENIFIESIFQLRK
DSKSWLFMEETAQQMKRPFGIPEKPEFTVGLDEALKKLKMNVLSEGVSVMVLTGVGGSGKTTLATMICWDKQVIGKF
KENILFVTFSKAPKLKIIVERLSEHLGYQVPELQSDGDAIDQLVLLLRKINANPMLLVLDDVWPGSETVVEKLKFQISDYKIV
VTSRVALPRLGTSLVLKPLVPEDATTLFRNHAFLERESSNIPDEDHVQKVLVPSLIDMCYTREDLKVERICIPSSFVTLKNLK
KLSLYLCDTREAFENSNMPISDAFPNLEELNIEYSKDMVGLPKGLCNITSLKMLSISSCHKLSSLPQEIGNLENLKVLRLNSC
TDLEEIPNSVGRLSNLRDMDISNCINLPNLPEEFGNLCNLRILCMRSCARCELPSSIINLKNLKEVVCDEETAFSWEPFKPM
LPNLKIDVPPLDVNLNWLHAIHS

>XP_027916364.1

MAMLQTWKAALTQVADIVGWESKNFRTESELIEEIVKDVMQKLNHMYPAEVKETLVGIDQNLAPIESLLRLRSKEVRII
GIWGMGGLGKTTIASALFAKLSSQFEASCFLANVMLEHEKKGLDYLRNKLLSEILEDVNPHISTSKVRSTFVMKRLRQKK
VLIVLDDVDDSKKLEDLVAQHDCLGSGSRVIVTTRDKHVLSKGVDAIYEVKGLSLDQSVRLFSLNAFGKTYPERGFEMLSK
QAVDHANGNPLALKVLGSVLHSRNEQQWDNALRKFKKVPNAEIQNVLRWSYDGLDYEQKNMFLDIACFFRGENKEN
VIRLLEFCGFYAYIGIKILLEKGLITFSDNGDVCMHELIQEMGWEIVHQESIKDPGRRSRLWDLKEIYDVLKNNRGTEAVE
GIILDVSQIRVLLLSYETFSRMINMRFLKFYMGKSRKCNLHLPSGLESLPNKLMYLQWDGYPSKSLPSTFCPDNLVVLSM
MESYVEKLWDGIKCLPSLKEMNLHACKNLTNLPDLSQAPNLETIDVSNCTSLLHVPLSIQYVKKLLLFNLESCKSLKSLPRN
IHLTSLDMFILRRCSSLDEFSLTSENMTRLDLRETKIADFPETVWQHLNKLVYLNLESCNKLKSLTSKIHFKSLQRLNLRDCS
ILEEFSVTSESMEYLNLRGTSIRELPTSVWRNNKLYTLVLHSCKKLVSFPERPKLEDSSLVSFSERPNMHELWTLSSLADLSL
KGSTIENLPASIKDLPSLKKLTLTECKKLQSLPSLPPSLEDLSLDESNIVCLPVSIKDLSHLRKLALINHKKLLTPQDLPPSLKAP

LLSESKVDPHLVSMKGLSQLQMFPQVKWKMFHSLPELPPFLEEFSLSESNIKFLPESIKNLSHMRKLAFTKCTRLQYLPEL
PPNLEDLFVSGCDIESLPISIRDLVHLRKITLIECKKLKALPELPQCLQSLCAADCRSLKIVQSTKNILIEDRYTFYWNCINLDQ
KSRNNIIADAPFEAAFTSLKERIPLGPLISICLPGTEIPDWFSHQSTNSSLDMEIPLEWFLDSMFLGFALCLVIGGFQQNSYE
GYDPDINCYHFVKSASYSGPSDPFLGHCTTVMQVPRGFNSDHIFICYYPTFNASILQDFKDLSLYYDANNLKLRVIFKFKG
PSQRLDIVKKCGVRPLLIANTERHHIESELQPE

>XP_027916365.1

MAMLQTWKAALTQVADIVGWESKNFRTESELIEEIVKDVMQKLNHMYPAEVKETLVGIDQNLAPIESLLRLRSKEVRII
GIWGMGGLGKTTIASALFAKLSSQFEASCFLANVMLEHEKKGLDYLRNKLLSEILEDVNPHISTSKVRSTFVMKRLRQKK
VLIVLDDVDDSKKLEDLVAQHDCLGSGSRVIVTTRDKHVLSKGVDAIYEVKGLSLDQSVRLFSLNAFGKTYPERGFEMLSK
QAVDHANGNPLALKVLGSVLHSRNEQQWDNALRKFKKVPNAEIQNVLRWSYDGLDYEQKNMFLDIACFFRGENKEN
VIRLLEFCGFYAYIGIKILLEKGLITFSDNGDVCMHELIQEMGWEIVHQESIKDPGRRSRLWDLKEIYDVLKNNRGTEAVE
GIILDVSQIRVLLLSYETFSRMINMRFLKFYMGKSRKCNLHLPSGLESLPNKLMYLQWDGYPSKSLPSTFCPDNLVVLSM
MESYVEKLWDGIKCLPSLKEMNLHACKNLTNLPDLSQAPNLETIDVSNCTSLLHVPLSIQYVKKLLLFNLESCKSLKSLPRN
IHLTSLDMFILRRCSSLDEFSLTSENMTRLDLRETKIADFPETVWQHLNKLVYLNLESCNKLKSLTSKIHFKSLQRLNLRDCS
ILEEFSVTSESMEYLNLRGTSIRELPTSVWRNNKLYTLVLHSCKKLVSFPERPKLEDSSLVSFSERPNMHELWTLSSLADLSL
KGSTIENLPASIKDLPSLKKLTLTECKKLQSLPSLPPSLEDLSLDESNIVCLPDLPPSLKAPLLSESKVDPHLVSMKGLSQLQM
FPQVKWKMFHSLPELPPFLEEFSLSESNIKFLPESIKNLSHMRKLAFTKCTRLQYLPELPPNLEDLFVSGCDIESLPISIRDLV
HLRKITLIECKKLKALPELPQCLQSLCAADCRSLKIVQSTKNILIEDRYTFYWNCINLDQKSRNNIIADAPFEAAFTSLKERIPL
GPLISICLPGTEIPDWFSHQSTNSSLDMEIPLEWFLDSMFLGFALCLVIGGFQQNSYEGYDPDINCYHFVKSASYSGPSDP
FLGHCTTVMQVPRGFNSDHIFICYYPTFNASILQDFKDLSLYYDANNLKLRVIFKFKGPSQRLDIVKKCGVRPLLIANTERH
HIESELQPE

>XP_027916586.1

MGETNQLVILTRFQDAIRMVMDIVEKGRKNKRSKRILRSTLKNMTLVVQEIKQYNEHLNPPREEIITLVKEKDAGEKLVC
NSCSRSLWWTKFLSWFSLYEEGLLHQKNDSRTADDKQVKDIKNTLYKLREIIELLDMENFEQKIKGAGMTIKSPYGVPEN
PEFTVGFGPLLSKLKMEVLQEEGVSTLLLSGLGGSGKTTLATMLCKDKEVKGKFKNNILFVTISQTPKLKNIVERLFEHCGY
HVPEFVSDEDAIKRLEILMRKIEGSPLLLVLDDVWPSSEALVEKFKFQMSDYKILVISRVAFPKFGTQFILKPLAHEDAMTL
FRHHALLDERSSKCSLSIPNEEIVQKVVRYCKGLPLAIKVIGRSLSNQSLELWQKTLDELSQGHSILDSSTELISCFQKLLDVL
EDNPITKECFMDLGLFPEDQRIPLPALIDMWAELYKLDDDGIEAMEIIKKLDSMNLANLLVARRNTSDSDNYYYNNHFV
VLHDLLRDLAIYQNNREPIDHRKRIITGINENQSQRWLGEKQHGMLSRLLSKYRGWCVKQTIQQVSARTLSLSTDETCAS
YWSDLQPSQAEVLILNLQTKKYTFPEFMEKMIKLKVLIMTNYGFHYSEVDNFQLLGSVSNLKRIRLERISVPHLGALKNLK
KLSLYMCSNISQAFENGTILASDSFPSLLDLNIDYCKDMVKLPNGICDITSLKKLSITNCHKLCSLPQEIGQLLNLELLNLSTC
TDLEEIPDSIQNLAKLRLLNISNCISLSNLPEEFGNLCNLRNLNMTSCARCELPYSITNLENLKVVVCDEETAASWEAFEAM
LPNLKVEVPQVDVNLNWLHSISS

>XP_027916587.1

MGETNQLVILTRFQDAIRMVMDIVEKGRKNKRSKRILRSTLKNMTLVVQEIKQYNEHLNPPREEIITLVKEKDAGEKLVC
NSCSRSLWWTKFLSWFSLYEEGLLHQKNDSRTADDKQVKDIKNTLYKLREIIELLDMENFEQKIKGAGMTIKSPYGVPEN
PEFTVGFGPLLSKLKMEVLQEEGVSTLLLSGLGGSGKTTLATMLCKDKEVKGKFKNNILFVTISQTPKLKNIVERLFEHCGY
HVPEFVSDEDAIKRLEILMRKIEGSPLLLVLDDVWPSSEALVEKFKFQMSDYKILVISRVAFPKFGTQFILKPLAHEDAMTL
FRHHALLDERSSKCSLSIPNEEIVQKVVRYCKGLPLAIKVIGRSLSNQSLELWQKTLDELSQGHSILDSSTELISCFQKLLDVL
EDNPITKECFMDLGLFPEDQRIPLPALIDMWAELYKLDDDGIEAMEIIKKLDSMNLANLLVARRNTSDSDNYYYNNHFV
VLHDLLRDLAIYQNNREPIDHRKRIITGINENQSQRWLGEKQHGMLSRLLSKYRGWCVKQTIQQVSARTLSLSTDETCAS

YWSDLQPSQAEVLILNLQTKKYTFPEFMEKMIKLKVLIMTNYGFHYSEVDNFQLLGSVSNLKRIRLERISVPHLGALKNLK
KLSLYMCSNISQAFENGTILASDSFPSLLDLNIDYCKDMVKLPNGICDITSLKKLSITNCHKLCSLPQEIGQLLNLELLNLSTC
TDLEEIPDSIQNLAKLRLLNISNCISLSNLPEEFGNLCNLRNLNMTSCARCELPYSITNLENLKVVVCDEETAASWEAFEAM
LPNLKVEVPQVDVNLNWLHSISS

>XP_027916588.1

MGETNQLVILTRFQDAIRMVMDIVEKGRKNKRSKRILRSTLKNMTLVVQEIKQYNEHLNPPREEIITLVKEKDAGEKLVC
NSCSRSLWWTKFLSWFSLYEEGLLHQKNDSRTADDKQVKDIKNTLYKLREIIELLDMENFEQKIKGAGMTIKSPYGVPEN
PEFTVGFGPLLSKLKMEVLQEEGVSTLLLSGLGGSGKTTLATMLCKDKEVKGKFKNNILFVTISQTPKLKNIVERLFEHCGY
HVPEFVSDEDAIKRLEILMRKIEGSPLLLVLDDVWPSSEALVEKFKFQMSDYKILVISRVAFPKFGTQFILKPLAHEDAMTL
FRHHALLDERSSKCSLSIPNEEIVQKVVRYCKGLPLAIKVIGRSLSNQSLELWQKTLDELSQGHSILDSSTELISCFQKLLDVL
EDNPITKECFMDLGLFPEDQRIPLPALIDMWAELYKLDDDGIEAMEIIKKLDSMNLANLLVARRNTSDSDNYYYNNHFV
VLHDLLRDLAIYQNNREPIDHRKRIITGINENQSQRWLGEKQHGMLSRLLSKYRGWCVKQTIQQVSARTLSLSTDETCAS
YWSDLQPSQAEVLILNLQTKKYTFPEFMEKMIKLKVLIMTNYGFHYSEVDNFQLLGSVSNLKRIRLERISVPHLGALKNLK
KLSLYMCSNISQAFENGTILASDSFPSLLDLNIDYCKDMVKLPNGICDITSLKKLSITNCHKLCSLPQEIGQLLNLELLNLSTC
TDLEEIPDSIQNLAKLRLLNISNCISLSNLPEEFGNLCNLRNLNMTSCARCELPYSITNLENLKVVVCDEETAASWEAFEAM
LPNLKVEVPQVDVNLNWLHSISS

>XP_027916764.1

MDQTTQRVVLPALFQERLRMVSEIVEKGQSSESNKQILRSTLKDMNPVVQEIKHYNEHLNPPREEIKTLISEKDAKEELV
CKCLSKKICLDKCLSWFLCRFGHKRDGSFAGGDKQALVAKDIEEKLYKVREILELLSKENFEPKLGGVGGPIKLPFGVPENP
DFTVGLDVPLSKLKMEVLRDGMSVIMVTGLGGMGKTTLATKLCWDEQVKGKFGGNILFVTVSKTANLKIIVERLFQHC
GYQVPHFLSDEDAANQLGLLLRQIGRSSMLLVLDDVWPGSEALVEKFKVQIPDYKILVISRVALLKSDMQIILKPLGHDD
AETLFRHYTHLEESGASIPDEVIQKIVRNCKGLPLAIKVIGRSLCNQRSELWLKMMEELSQGRSILDSNVELLTCLQKILDVL
EDNIVIKGCFMDLGLFPEDQRIPVAALIDMWAESYRHDDDGKEAMAIINKLDSMNLVNVMVARKNATNTDNYHYNN
HFIVLHDLLRELAIYQCSQEPMEQRKRLIIEINQNKHGEKTKFLSWCEQKPQQVTAHTLSISTDENCPSDWPQMQLAQV
EVLIFNLRTKQYSFPGFMKDMNKLKVLTVTNYSYYPSEINNFELLGSLSNLRRIRLERISVPSFVAMKNLTKLSLYFCNMKQ
AFENNNLLISYAFPNLEDLNIDYCKDMVGFPKGLCDIISLKKLSITNCHKLSALPQDIGKLENLELLRLSSCTDLEGIPDSIGR
LSNLRLLDISNCIILPNLPDDFGNLSNLQNLYMASCARCELPFSVTNLGNLKVVICDEETAASWENFKPMLPNLRIDVPQV
DVNLNWLHPTSS

>XP_027917019.1

MAAECISGAFLAAVFQVTFDKLASRDIEDYFHRRKIKDKMLKKLEIVLNSINQVLEDAEERQYKSPNVMNWLNQLKEA
MYEAELLIDEVANEASRHKLEAEFQPTTSKVRGFFKAFVNPFDIQIASRVEELLENIQFLASQKDMLGLRKGIFAGNEVGV
SWKQSQQLPTTSLVDESNICGREEEKEEIIKILLSDNVTCNQVPIISIVGMGGMGKTTLTQLVYNDQRVLDQFDLKAWVY
VSQDFDVVAVTRAILKALGSKAAEEKDLNLLQLQLKQRLMGKRFLLVLDDVWNEDYASWGVLQIPFIYGPSGSRIISTTR
NEKVALIMNSSHLYHLKPLEKEDCWKLFSDIAFHDKDATKYPYLVSIGSKIVDKCGGLPLALKALGNILRVKFSQHEWVKIL
ESDMWHLSDNEANINPALRLSYHNLPSYLKRCFAHCSIFPKGYEFDRDQLIQLWMAEGLLSCCQINMSEEELGTEFFND
LVARSFFQQSRHRASCFTMHDLLNDLAISVSGEFCSQISDSLEINITKRTRHISWSRKINIDDKFLEHVSKCNKLRCFVAFR
WEFGRGGLINTDKQRDLFSTLKYLRVLSFNDCLLTELVDDIGNLKLLRYLDLSYTKIKTLPDSICRLHNLQTLLLLWCYHLVE
LPIDLHKLVNLRHLDIRMSGINKMPNHIGRLKHLRTLTSFSIGKHDVKELGNLSNLQGTLSIFRLENVTNPRDAVEANLKG
KKHLDGLVLNWGDKFGRCNENEDSILERQVLEALQPNGYLKKLSVLRYDGTSFPRWFGASHLPNLVSIALTKSKFCFVLP
PFGQLPSLKELSISCFYGIEVIGPEFCGSDSSNIPFRSLEILKFEEMSAWKEWCSLEGHNEEGQGLSCLKELSVRRCPWLRS
ALPQHLPSLQKLEICDCQHLEDSVPKAASIHEIKLRLCEKLFLKDLPSSLKKATMHGTCIIESTLHQILVNNPFLEEMKIHNF

HGPNKKWSSLDLHIHDSLVTLSITSWYSSSLPFPLHLFSNLHSLLFHDCPHLESFPEGGLPSSLRKLEIERCPKLVASREKW
GLFKLHSLTELRISDDFENVESFPEDMLLPPTLSVLYLIACSKLKKTNYRGFLHLKSLNSLYILYCPLLQSLPEAALSNSLSVLYI
HECPLLKQRYQRDGKHWHKIHNIPSVIMS

>XP_027917262.1

MEPPYSCLATGLKHDVFISFRGPDVRNGLLSHLNKELCRRKIDVYVDKRIERGDEISSALLRAIEGSQILLVIFSEDYASSPW
CLEELAKMVECNEKNKQILLPVFYNVDPSDVRSKRGNYAVALAKHEERFKENMLMFKENMLMVQSWRSALEKASHV
AGFHYPKNFDDESDLVDEIVERISGTLTNFSPSGSNGLVGIDQNIAQIQSLLLKESSEVRFVGIWGMGGIGKTTIARTIFDK
YSPEYEGCCFLNVKEEVERHGLSNLQEKLISELMEGEGLYTSGTNKATILNSVERRMIRKKVLIVLDDVNTPKQLKYLIGKPI
CFGPGSRVLVTSRDMRVLTTGGVNRIHEVKEMDPQDSLKLFCLNAFNENQPRKGYKELSEEVVKIAQGNPLALKVLGSD
FHSRGIDTWECALSKIKKYPNEEIQSVLRFSYDGLDEVEKKAFLDIAFFFKDDDKDYVIKQLDAWGFHGASGIEALQQKAL
ITISDNRIQIHDLIREMGCEIVRQESTKYPRKRSRLRDPMEVSDVLKQNLRTDKVEGMQIDVSGIKDFPLRLGTFKKMPCL
RFLKFYLPLHAELSLLQSHDGPIWCPEKQDELLLSAGCKELMRVASEIHIKCLHYLVIEDCSDPSRLNELTSTEMKMLQNT
AQDAGVEIILNSSIGHLSSLECSDVVDQQFRNLPNELLYVRYTYYLKISKGKRPDSGKPKLNILFDSLRYYQRVSMDELYNT
VVVFPRSSRGEMWVKGAKKEVKGFVVATFYFHLYTVLFFLSPSSFSITLLIQTVICSFILFFCILLSFFSRSRYLGQSFDNC

>XP_027917263.1

MEPPYSCLATGLKHDVFISFRGPDVRNGLLSHLNKELCRRKIDVYVDKRIERGDEISSALLRAIEGSQILLVIFSEDYASSPW
CLEELAKMVECNEKNKQILLPVFYNVDPSDVRSKRGNYAVALAKHEERFKENMLMFKENMLMVQSWRSALEKASHV
AGFHYPKNFDDESDLVDEIVERISGTLTNFSPSGSNGLVGIDQNIAQIQSLLLKESSEVRFVGIWGMGGIGKTTIARTIFDK
YSPEYEGCCFLNVKEEVERHGLSNLQEKLISELMEGEGLYTSGTNKATILNSVERRMIRKKVLIVLDDVNTPKQLKYLIGKPI
CFGPGSRVLVTSRDMRVLTTGGVNRIHEVKEMDPQDSLKLFCLNAFNENQPRKGYKELSEEVVKIAQGNPLALKVLGSD
FHSRGIDTWECALSKIKKYPNEEIQSVLRFSYDGLDEVEKKAFLDIAFFFKDDDKDYVIKQLDAWGFHGASGIEALQQKAL
ITISDNRIQIHDLIREMGCEIVRQESTKYPRKRSRLRDPMEVSDVLKQNLRTDKVEGMQIDVSGIKDFPLRLGTFKKMPCL
RFLKFYLPLHAELSLLQSHDGPIWCPEKQDELLLSAGCKELMRVASEIHIKCLHYLVIEDCSDPSRLNELTSTEMKMLQNT
AQDAGVEIILNSSIGHLSSLECSDVVDQQFRNLPNELLYVRYTYYLKISKGKRPDSGKPKLNILFDSLRYYQRVSMDELYNT
VVVFPRSSRGEMWVKGAKKEVKGFVVATFYFHLYTVLFFLSPSSFSITLLIQTVICSFILFFCILLSFFSRSRYLGQSFDNC

>XP_027917465.1

MAESVVSFVLDHLSQLVAREANLLYGVEDRIQSLQHELQMINDLLNTSKNKKGTAHTILNQIRDVAHVAEDVIDTFVAK
VSIYKRRTILGKMLRGFGQARLLRDVAEKIDKIKATLTEIRNNKDKYDAFKETSNQSAAEEEEEEKRAKSVQKLRRNVEEE
DVVGFVHDSKDVINRLLEGGSNRKAVSIVGMGGLGKTTLARKVYHSSQVKQRFVCRAWVYVSNECRSKELLIGLLKHL
MPNFEQQGRGKEKGKKSAGDINDLSEEELKKLVRNCLEWKRYLVVVDDLWKKQDWDEVQDAFPDNNRGSRILITSRL
KEVALHAAHDVPHYLQFLNEEESWKLFRRKVFRGEDCPSDLEALGKQMVQSCRGLPLSIIVLAGLLANKKKSHREWSKV
VGHVNWYLTQDETQVKDIVLKLSYDNLPRRLKPCFLYLGLFSEDFEIPVTPLLQKCVAEGFIQDTGSRDLDDVAEDYLYELI
DRSLVQVAKVDTNGDVTAIRVHDLLRDLCILESKEDKVFEVCTDHNILIPAKPRRLSIHSNMDHYISSSNNDHSCVRSLFFF
GSYYFFQSWEWKWLFERLKLVRVLDFGLNTSNKIPSDIGNFIHLRYLRIQALSITFVPNSILNLWNLQTIDLGPWKHRVPI
SFPAQIWKLKHLRHLNTTRAIKLRGSCSGSDERMWNLQTVSTLVLNSQATSLIEKGTFPNVKELGLTVISECEGELPKLLH
SLQQLSYLNMLVIVLRDRDDAGVEHSSDESVKRNNGFKPQELLRNLGQLNCLTILTIENALDLLTCALTFPPNVTELTLSDI
DCISDEGMNGLGKHTKLKILRLCGDNITSSGDSIVLNCGRGSFPQLEVVEMENLKLGKWKLDNGAMSRLQNVMINSCE
WLEDLPNELWSLSGLKKVHVKNPSVQMARMLENLEINSGCQLVIEN

>XP_027918285.1

MASSTSCGVSEIKHDVFISFRGTDVRSGLLSHLKRELHRKHIDAYVDERLDKGGEILPLLLRAIEGSKIFLVVFSKHYASSQS
CLEELAKMVECMETNNQILLPVFFHVDPSHVRHQHGDYGDDLAQHEEKFKENMLKVQSWRSALKKAGRSSGFHYPT
NYKNESDLVDEIVKDISVKLSEFYPSESNGLVGIDQNITQIQSLLRVESNEVVFVGIWGMGGIGKTTIARAIFDKCSLRYDG
CCFFNVREESERHGFSNLRERLIYELLEGEDLHRSGTSKARIFSSALRMLGRRKVMVVIDDVNTSEELQYLVTKPVCFGAG
SRVIVTSRDQNVLTSGELHQIHEVKEMGPLDSLKLFCLNAFNESQPKTGYEKLTEEVLKIAQGNPLALKVLGADFHSRSGK
NTWKCALSKFKKYPNEKIQSVLKFSYDGLHEVEKKAFLDIAFFFQEDTEEYVIKQLDAWGLHGASGVEVLQRKALITVSN
DNIIQMHDLIRQMGWEIVRQECIIHPGRRTRLRDKEEVYNVLRYKLGSDKVEAMQVDVFRIKDLPLKLGTFKKMPRLRF
LKFYLPLHDNLFMPPNQDGNLWYGEHHFPLLLSAWCKELMKVACEIQIKCIEYLYIDGCSHPSQLNKSSVTPTLGNHAM
ETLSLALMSLNEPIGSLRDLECSDMLDQQFKTLPDGLLCLRSTYYLKLSKKSTGQDSGKPKLHVLFDSLRFYERISVSQLDN
SDVGGHRVPFFYVAAFIFLLHLLLKRPWFQFLFSFPFQFSCIFFLYLFSHLTSLFYPPLAFSLSWVEAFFLLLFFNILGKICYWF
LRVFKKY

>XP_027919232.1

MAMILDAVLGKVLDQLLSTVVDMKDRAVKFRDTLNKLHSTLEKVAPMARQIDGLNKRLDKPATETQKLIDQIKQGKEL
VMECSKVDWWNFCYKANSQQKLQDLIDSIYEYFKLDMQGNINIIVLENQIMLSEIHATLMENVPRRTELKGLCSPPEPP
AFTVGLDVHLRALKFKLLNNHHVGSVLTVTGTGGSGKSTLAKKFCSDKEVKGKFKDNIFFISLVDVEAPKFRTIVERLFEH
NGCKKPQFQSDEGAVYRLETLLKEIGKNPMLLVLDGVLPESASLVEKFVFQIPNYKILVTSRFAIKGFGQPYVLKSLNEAD
ALNLFRHSASLDQTSSEIPDNVVKKIAKGCSGSPLALIVTGKSLSLEAPVVWQNRAKTLSKGHSILAYSSSNDVLHTCLQN
CFDDLNAKVAEVFTDLSLFPEAQKIPAAALVDICAEQRDEDDDIAMENILELVKRNVADLIVTRIGTVDYNYHYVTQHGL
LRDLAIHQTRNLPTEKKHRLIIDLRGNNIPKWWTTQNEYHIAARTLSISTDEEFTSDWCNLQPNEVEVLVMNLREKKRSL
PPFMMKMNKLKVLIITNYDVNRAELENLEVLDYLTDLKRIRLEKVSIPFLSKTGVALKNLHKFSFFMCNVNEAFKNSTINV
SDVLPNLKEMNIDYCDVEELPAGLSDIVSLKKLSITNCHNLSKLPEGIGKLVNLESLRLTSCTKLEELPDTITNLHKLNFLDIS
DCVNLGMLPENIGELRSLERFNCRGCTRLSELPYSVTELESLRVVVCNEETAALWEPIKPMFSDLKLEVVLIDFKLESIS

>XP_027919324.1

MTQLPSSSSSSSSSTCEGSHDVFLSFRGDDTRSGFTGNLYKSLCDRGIHTFMDDEGLRKGEKIGPALFKAIEQSRIAIVVLS
ENYADSTYCLEELVVILDCIMKKGRLVWPVFYGVTPSYVRFQKGSYGKALAKHGERFKNDQEKLQKWKLALQEAADLF
GSHFKLKQGYEHELIRTIVEEVSKKINRSPLHVANYPIGLESRVQDVKSLLDVGSNRGVSMVGIYGIGGIGKTAIACAVYN
AIADQFEVQCFLGDIRQKSLKYDLVQLQETVLSEMVGEKSIKLGSINRGMAVMKSKLQRKKVLLILDDVDKLEQLKALAG
DPSWFGDGSKIIVTTRNRHFLRVHGFERTYEAKALDDKEALELFSWHAFKSNEVGPGYLDISKRAVFYSNGLPLALEIIGS
NLNGITMSEWEAALDTIERIPDEDIQEKLKVSYDGLKGDEKEVFLDMACFFRGYHLKDVISLLLQGRGFSPEYAIRMLVD
KSLIKIDQYGFVLMHNLVEDMGREIVRQESPSEPGKRSRLWLYEDIVDVLENDKGTDSIEIIMLHLPKNREVLWSGSELKK
MTNLKMLTIENADFSRGPEHLPSSLRVLKWRGYPTQSLPPEYDPRKLVMLDLSMSRNILGKQLHLMKFESLSEMILRGC
RFIKQAPDMSGAKNLRKLCLDNCKNLVEVHNSIGVLDKLTWFTAIGCTSLRTLPQSFKLTSLEYLSLRKCSSLQRLPNILEE
MKHMKNLDLCGTAIEQLPYSFRKLTGLKYLVLDKCKRLNQIPINILMLPKLERLTAVKCGRYVNLILGKSEGQVRLASSESL
RDFRLNYNDLTPTSFPNVEFLDLTGCAFKVLPECISQCRFLRNLVLDNCKELQEIRGVPPKIKYLSAINCTLLSHESQNMLL
NQRLHEGGGTDFSLPGTRLPEWFDHCTRGPSLSFWFRNKFPRMTLGVVGVLDKQGSFPMSRFHLLINGIQKLHCHFTV
QSKLITYHIFLSDVLLKSYDGGLESVYGEDGWNHVEVSCVGPRVFPHSCRTKKGIIKWMGVHVHKQKTNMHDIRFINP
WSPKRTHSEVSKPDLQESFQSLPKRIRVSHRKEICEAPQSKQHEANSGYHGVSQRLWLAICSIAAPLNVKVLMWNICQD
NLPTFEYLFRRKLVLSPLCPICGKEPETVEHVFLFCPWTRPLWFGSDFQWCVDAKEVQSFQLWLWHKLMEIQRVYPEN
ANQISAQVGSICWSIWKGRNEFVLEGKPVNPLILR

>XP_027919426.1

MSGLEPFVSPLVGAVGTIGIGEVRNTLASKFAFSKNLDDNYHILVKDTEMLQAIMKDKEIEAQRNHHRDTSNAYKLWT
NKVSNVTEEVQELKLKYEIKTLPRWRIQKRSRMSEEMEKKSNWIRQLINDGCLKNFLVDKPPEPVLKELNVPQISGYPTL
QGTLDNILDLLKNNKIKIIGVCGTKGVGKTTIMRNLNNNEEIAKLFEIVIFVKVTSNDHKLQEKIAHRLMLYKGTTNNEDS
DDVARRIYRELDNKRYLLILDEVEDAINLELLGIPSNGSKVVIVTRFPRVYKLNRVQRVINVEKLSLDEAWKMFRDTVHAF
NPKIDSPDIQLPAKLVCKRCCCGLPLLIYNIANSFRLKESASSWWAGLEDLKPWPELQSQGLEELYSCLKFCYDELNDKRK
QKCFLYTSMYPADSKVYSDYLVECWAAQGLLGDINDERSYQSARNRGIDILEHLANVSLLEKGEAMIYVNMNHCMRQL
ALHISSKDPECSFYLQDGEESENLSNSRAWQQARWVSMGQVHDLRTSQDCSTILTLLLRKIPELPESFFENMSSLLLLDLY
SSMITQLPSSLSKLTGLRGLFLNRCELLESLSSEIGLLQFLEVLDIRDTKVTFIPLQIGFLTKLRCLRIPFIASEDNQVQNVHAIS
KLHRLEELTIQVISYEQWCNHAENVLQHVASLENVTHLRCCFPSSTILGEFLSRSKSWHKKQSSFRFTVGCQNSRRPQILE
SFEYKITNYLRYCNGGQKDDPAIIEVLPKTDAFELVCHKDMKKISNFAGIACLERIRGLLIKRCNQVLTIVSGETSSNAMNG
IQIETTVILPNLEQLYLENLLNLKCAFRGPLHSGTFSRLQALSLKNCPRLSQIFSNGAIQHFSELQKLKLEDCSKIEELIGEDIE
RERDVLPKLEILLLVNLPNLKNICATHTLAWSSLELLRIHNCPMFKTLPLDSTNAVNLKSIKGQQEWWTNLDWTNNDKV
HKRFQPIFVASNEYFS

>XP_027919427.1

MSGLEPFVSPLVGAVGTIGIGEVRNTLASKFAFSKNLDDNYHILVKDTEMLQAIMKDKEIEAQRNHHRDTSNAYKLWT
NKVSNVTEEVQELKLKYEIKTLPRWRIQKRSRMSEEMEKKSNWIRQLINDGCLKNFLVDKPPEPVLKELNVPQISGYPTL
QGTLDNILDLLKNNKIKIIGVCGTKGVGKTTIMRNLNNNEEIAKLFEIVIFVKVTSNDHKLQEKIAHRLMLYKGTTNNEDS
DDVARRIYRELDNKRYLLILDEVEDAINLELLGIPSNGSKVVIVTRFPRVYKLNRVQRVINVEKLSLDEAWKMFRDTVHAF
NPKIDSPDIQLPAKLVCKRCCCGLPLLIYNIANSFRLKESASSWWAGLEDLKPWPELQSQGLEELYSCLKFCYDELNDKRK
QKCFLYTSMYPADSKVYSDYLVECWAAQGLLGDINDERSYQSARNRGIDILEHLANVSLLEKGEAMIYVNMNHCMRQL
ALHISSKDPECSFYLQDGEESENLSNSRAWQQARWVSMGQVHDLRTSQDCSTILTLLLRKIPELPESFFENMSSLLLLDLY
SSMITQLPSSLSKLTGLRGLFLNRCELLESLSSEIGLLQFLEVLDIRDTKVTFIPLQIGFLTKLRCLRIPFIASEDNQVQNVHAIS
KLHRLEELTIQVISYEQWCNHAENVLQHVASLENVTHLRCCFPSSTILGEFLSRSKSWHKKQSSFRFTVGCQNSRRPQILE
SFEYKITNYLRYCNGGQKDDPAIIEVLPKTDAFELVCHKDMKKISNFAGIACLERIRGLLIKRCNQVLTIVSGETSSNAMNG
IQIETTVILPNLEQLYLENLLNLKCAFRGPLHSGTFSRLQALSLKNCPRLSQIFSNGAIQHFSELQKLKLEDCSKIEELIGEDIE
RERDVLPKLEILLLVNLPNLKNICATHTLAWSSLELLRIHNCPMFKTLPLDSTNAVNLKSIKGQQEWWTNLDWTNNDKV
HKRFQPIFVASNEYFS

>XP_027919467.1

MSGLEVVGPLVGEMGSIGVGEMHTTITSKIAFSKNLDDNYNILVKDTEMLHAIKKDKEMKAQRNSHKDTTNAYKLWT
NRVSETTEEVQKLKLKYEEKTLPWWRIQKRSHLSEEMEKKSNCIRQLMNDECLKDFLVDKPPEPVLKELNVPQISGYPTL
QGALNSILVLLKNSKIKIIGVCGTKGVGKTTIMRNLNNNEEVAKLFEIVIFVKVTSNDSNLQEKIAHRLMLEKGTNKEDSDY
VARRIHRELENKKYLLILDEVEDAINLELLGIPSDNNNGSKVVIVTRFPLVYKLNRVQRVIKVAELSPDEAWKMFRDTVHA
FNPKIDSPDIQPTAKLVCKRCSRLPLLIYNIANSFKLKESASSWWAGLEDLKPWPELQSQGLEELYSCLKFCYDELNDKRK
QKCFLYTSLYPAESKVYSDYLVECWAAQGLLGDINDKRSYQSARNCGVDILEHLANVSLLEKGEAMIYVNMNHCMRQL
ALHISSKDPECSFYLQDGEESENLSNSRAWQQARWVSMRQVHDLRRSQDCSTILTLLLRKNPELTALPESFFENMNNLL
LLDLYSSMITQLPSSLAKLTCLRGLFLNRCELLESLSSEIGLLQFLEVLDIRDTKVTFIPLQIGLLTKLRCLRIPFTASEDNGVQN
VHAISKLHRLEELTIQVISYEEWCNHAENVLQHVASLENVTHLRCCFPSSIILGEFLSRSKSWHKKQSSFIFTVGCQNSRRP
QILESFEYKITNYVRYYNGGQKDDPAIIEVLPKTDAFELVCHKDIKKLSNFAGIACLERIRGLLIKRCNQVLTIVSGETSNNA
MNGIQVETTVILPNLEQLYLENLLNLKCAFRGPLHSGTFSRLQTLSLKNCPRLSQIFSNGAIQHFSELQKLKLEDCSKIEELI
GEDIERERDVLPKLEILLLVNLPNLKSICATHTLAWSSLELLRIHNCPKFKTLPLDSANAVNLKSIKGPQEWWANLDWTN
NEEVHQRLQPMFVASNEYFS

>XP_027919638.1

MAESSSSFVVSTSPTRHDVFLSFRGEDTRDNFISHLYAALQRKNIEAYIDYRLQRGEEISPALETAIEESKIYVLVFSENYASS
TWCLNELTKILDCKKRYERDVIPVFYKVDPSTVRKQEERYKEAFEEHEQRFKDDMDRVQRWKNALTEAAELSGWDSKV
TRPEYKLVEQIVEDILRKLNRYSIINDQRIVGTEKHISEIKSLLHLESSDVRIIGICGMGGIGKTTISKQIYHALALQFDSSSLVL
DVQEKLQRDGIDNIRIKYMFELLNETPSPRLLYYNERLKRTKVFLILDDITDSAQVRRLMEGLDSFGQGSRIIMTSRDRQV
LKNAGADDIYELKGLNFYDSLKLFNLHAFKQNSSDESTYMDLSIKVLRYAKGIPLVLRLLGSLLYGRKREAWESQLQKLKK
CQDLKIFNVLKLSYDGLDEEQKNIFLDIACFYRGHNESMVVKILDDCGFSSLIGMNILKDRCLISILDGRIVMHDLIQEMG
QEIVRKECPQHPAKHSRLFKADEIHEILRNNKGSDAIQCILQDVDKMKKVKVHAKAFKMMNNLRMLVLYSYSRFSHSN
VFLESSLVSLPDTLKILYWIGFRQKSLPPKFCPQNLVRLEMPRCHLEQLWEGDQVFHVIYVPLCFRLFKIKIKSTSMFSTLYT
YKFSQFLCVFFTLFILILFLLEPHNYTYIIRIIFSMLLNIIINQRVCICLVHIVQCPMIVLPILLMIDISSYCPTCIIYSISTLSSIIRIVH
SVIFLWQSLPKLKRLNLSYSRKLTRIPDLSLSPNIEEIILSSCQKLINVHSSKLLSKLTCLCLDNCYDLKSVTIPSNILSSSPGLILL
SSCDKLEMFSTSQPKSHLKLKRQRGTFSRSHRKKLEYQPGTFSSFPRLKKQFRFFRKSFVSDGLAESTLESYSTISLSIPIFSIIF
MRYEEEEKEVTNNNIYVYDERPIQLTGGVPLNYRSLKKLCFIDLSNCSSLTIFPFDLSEMKFLKQLCLRGCSKLETFPEIEDT
MEDLAVLVLDNTAIQALPSSLWRLVGLQELSLRSCRNLEIIPSSIGSLTRLCKLDLAYCESLQTFPSTIFRLKLKKLDLCGCLRL
TTFPEITEPTQTFAHINLSETLIKELPSSFGNLVKLRSLQLNKCTDLESLPNSIVNLLDLRKLDCSGCVKLTEIPTHIGRLTSLM
ELSLSETGIVNLPESIAHLSSLKSFDLSDCKKLECIPQIPPCLNQLVALDCTSIRRVMSNSLVRNLSDSKEGVFQFHFTNAQQ
LDSGARENIEEDARLRMTDDACTSACFCFPGTEVPNWFPFRNEGASVTINDDLRFCSDDRLIGFALCVVFGMLDTNDIG
GRRGSFRYSLKFESDDDGTQIIPNNDLLNNYFEWIHKDRLLDQNHTFVWKFNLESLRRSGMSIRLCDARSFTFEISRNDY
DFQWPNYDSFVYEFRSVVTIKECGMCPLYSSGCGSNVAQSSRETKEDRKRNAESYLVMKALTNLEKLYLKGQLSIFLFIN
SLFFRCSKRKERKLIPMAESSSSFVVSTSHTRHDVFLSFRGEDTRDNFISHLFAALQRKNIEAYIDDRLQRGEEISPALETAIE
ESKIYVIIFSENYASSTWCLNELTKILDCKKRYGRYVIPVFYKVDPSTVRKQEERYKEAFEVHEQLFKDDMDKVLRWKNAL
TQAAELSGWDSKVTRPEYKLVEQIVEDILRKLNRYSIINDQRIVGTEKHISEIKSLLHLESSDVRIIGICGMGGIGKTTISKQIY
HTLALQFDSTSLVLDVQEKLQRDGIDNIRIKYMSELLNETPSPRLSYYNERLKRTKVFLILDDITDSAQVIRLMEGLDSFGQ
GSRIIMTSRDRQVLKNAGADDIYELKELNFHDSLKLFSLHAFKQNNSQEITYMDLSVKVLIYAKGIPLALQILGSLLYGRER
EAWESHLQKLEKCQDLKIFDVLKLSYDELDEEQKNIFLDIACFYRGHEETVVAERLDDCGFSSKIEMDVLKDKCLISVLDG
RIVMHDLIQEMGQEIVRKECPQHPGKRSRLLKAEEIYEVFRNNKGSDAIQCILANMSKMKEVEVHVQTFEMMNNLRM
LMLYSYCDDLEESNVFLESSLVSLPDTLRILYWTGFYQKSLPPKFCPQNLVRLEMPHCHLEQLWEGDQYLPKLKRLNLSG
SMKLTRIPDLSLSPNIEEIILSSCEKLTNVHSSKLLSKLTCLCLDNCYDLKCVTVPSNILSRSPGLILLSWCCELEMFSTSQSRF
PQVKLGRQRETFSIFPRDEPEHQRRTFLRPSYTMERAYTNQERAVQDYFSTSSEVFSITFDRYEEEENEVTDNNNVYSQS
NEVSRKLIEGVPLNFRSLKKLCFIDLLNCSSLAIFPFNLSEMKFLKQLCLRGCSKLETFPEIEDTMEDLAVLVLVDTAIQTLPS
SLWRLVGLQELSLSMCRYLEIIPSSIGSLTRLCKLDLRYCESLQTFPSTIFKLKLKKLDLCGCLRLRTFPEITEQAQTFAHINLK
QTAIKELPSSFDNLVNLRSLQLNKCTNLESLPNSIVNLKSLCKLDCSGCVKLTEIPTYIGRLSSLTELSLSDTRIVNLPESIAHLS
SLKSLDLSDCKKLECIPQIPPFLKQLVALDCPSIRTVMSNSLVQNLSNSKESVFKFHLTNAQQLDSGARANIEEDARLRMS
DDAYTSVLFCFPGNAVPEWFRFRGKGRSVTINEDLSFCSDDRLIGFALCVVFGLLDTNDVEARRGYFSYSLKFESDDDGT
QNIPNNDVLKSFFEWNGEERPVDEDHTLVWKFTLESLRRSGMSLRLNRARSLSFEMSPYDFDFRWPNYESVVGEFKSV
VTIKECGICPLYGSGGNVAQS

>XP_027919806.1

MDIQEESSTTVGPLTTPSLRNMSSSSSAFFSANQSPFFSPRSSSCQLSESLQPDAPSDRIHLDVAAPSSSSGIPEPKSLVNV
GCTFPEVAASPASCNAGDLQNLDRISSSVGISSCTVSGHFHPYDDSYSGQKDKRSKKSRNKRVSSTPGSRSLSSYRLKSCD
VYIGLHGRKPPLIRFANWLRVELEIQGISCFVSDRAGYRNSCKLSIAEKAMDMASYGIVIITRKSFKNPYTIEELQFFSGKKN
LIPIYFDLSPADCLVRDIIEKRGELWEKHGGELWLLYGGLEQEWKDAVHGLSRVEERKLEAQDGNWRDCILRAVTLLAM
KLGRRSAAEHLTKWREKVKEEELPFTRNENFIGRKKELSQLEFMLFGDVTGDSRQDYIDLKARPKRRHLTICRSKSSVQEE
RHVGNGSREEKTPVLWKESEKEIEMQSIEFSQRRSRLKRGGKYTRRKKGMRILYGKGIACISGDSGIGKTELMLEFAYRF
HQRYKMVLWIGGESRYIRQNYLNLRSFLEVDASVENSLEKTRIKGFEEQEEAAVSGIRKELMRNIPYLVIIDNLESEKDW

WDHKLVMDLLPRFGGETHVIISTCLPRVMNLEPLKLSYLSGVEAMSLMLGSGREYSVAEVDALRTIEEKLGRLTLGLAIVS
GILSELPITPSRLLDTINRMPLKDMSWCGKKAPSFRQNSFLLQLLDVCFSIFDHADGARSLATRMVLVSGWFAPCAVSVS
LLALAAQKIPEKQKGTCFWRKLLQSLTCGFTSSHTKKSELEACSLLLRFNIARSSTKQGHIHFNEMIKLYARKREVTGSAQA
MVQAVMNQGSISESIEHLWAACFLLFAFGHNPAAVELEVSELLYLVKKVVLPLAIHTFITYSRCSAALELLHLCTNALEAA
DQALVTPVDKWFDKSLCWRSIQTNAQLNPCLWQELALCRATVLETRGKLMLRGAQFDIGDDLIRKAVFIRTSICGEDHP
DTVSARETLSKLTRLIANVQIRASA

>XP_027919992.1

MAEYFVFEIAESLLGKLASNLYEEISRAFDLYEDVKSFRDTMSIVKGVLLDAEEKKDKKHGLREWLRQIQNVCLDAEDVLD
GFECQNLRKQVLKASGTTSMKVNHLFSSSNSLVFRFRMAWQIKNVTRRLDKIAADGNKFGLERIVFYHSPMQRREMTY
SHVDASGVIGREKEKEEIIKILMQPHPRGDGYGDQNVCVIPIVGIGGLGKTTLAKLVFNDKRMDDLFQLKMWVCISDDF
NLRQIIIKIINSDSDPTISVVPQESMNNLDIEQLQSRLRHKLSCQKYLLVLDDVWNDNRAKWIELKDLIKVGALGSKIIVTT
RSNSIASMMGTVPSYVLEGLSVENCLSLFLKWAFREGEEKEHPNLVDIGKEIVKKCRGVLLAVKTSGSSLFSIFDSERWEI
MRDHELWNLKQQEDDILPSLKLSYDQMPSYLRHCFAFFSLYPKDFGFTSAEIANFWATLGLLRSPFGSQKIENVGKLYIN
ELHSRSFLEDFEDFGTVYYFKLHDLVHDLSLYVAKEEFLMVNSHTSNIPKQVRHISVVENDSLSHTLFPKSRGVRTIIFPVD
GVGVGSESLLETWIKRYKYLRHLDLSNSTFETLPNSIAKLEHLRAFSLDNNCKIKRLPNSFCKLQNLEMLSLRRCLGLETLPK
GLGMLISLRKLYITTKQSILSEDDFATLNNLHTLIFEYCDNLKFLFQGAEAQLSSLEVLIIQSCGSLESLPLHILPKLHVLIVTRC
VMLNLSLNSETAIQRLKMKYLHIEQCPRQQTLPEWIQAAANTLRTLIILNCHCLEVLPEWLSTLTQLKMLHIVNCPQLLDF
PSNMHCLRALEDLIIDGYPELGRKCEPRSGEYWSFIAHIKCVSIGKTRKMKLLFQMLSRLGLNCTQ

>XP_027920003.1

MAESFLFSIAESLLAKLVSRAFEEASRVVGLYDNLRDLTKTLSLIKAVLLDAQQKQEHNHELREWLTQIKTVFSDAEDLLDE
FECQTLRNKVVKAHGSTKDEVSNFFSTSNPLVFRYKMAQQIKDISSRLDKVAADRHKFSLQTIDVDTRVVHRRDMTHSR
VSDSDVIGRKHDKEKIVELLLQQNPNDDDTSISVIPIVGIGGLGKTTLAKFVFNDSRIQEHFPLKMWVCVSDDFDIKQLTI
KIINSANDSASADAPSHQMNLNMLDLEQLQNQLKNKLFGQKFLLVLDDVWNEDRVKWVELRNLIQVSAAGSKILVTT
RSHSIASMMGTVPSHILEGLSEEDSLSLFVKWAFKEGEEEKHPHLLNIGRQIVKKCKEVPLAVRTLGSLLFLKFEASEWEY
VRDNEIWNLPQKRDDILPALKLSFDLMPSYLRQCFALFSLYPKDHQIDSYKITALWEALGLIALPTTNRTREDVGNQYLHE
LLSRSFLQDFENFDTFYGFRIHDLVHDLALFVAKDECLQVTSDSQNISDNVRHLSFTKSSLFENLVTKETAAVRTVLFPIGA
TVTNNEALLNICLSKFKCLRVLDLHGSTFRTLPRTITKLKHLRYLDISRNPCIKRLPDSICKLQSLQVLSVNGCMELEALPKGL
RKLISLWCFQFSTKQSILPLSEIANLGSLEFLTVESCNNVESIFGGVKFPALKTLLVWHCQTLKYLLLNDQNFPKLETLMVN
NCRNLDLELLKGDHEEESSKLKLKFIGFINLSQLVVLPKWLQEAANSLRCLYVSNCHNIETFPDWLATLIHLKTLTIKNCPKF
VSLPDNILHLSALENLRIEGCPDLCKKYAPHVGKFWPRISHIKNIFIDEPEGLEEERE

>XP_027920016.1

MAESFLFSIAESLLAKLVSRAFEEASRVVGLYDNLRDLTKTLSLIKAVLLDAQQKQEHNHELREWLTQIKTVFSDAEDLLDE
FECQTLRNKVVKAHGSTKDEVSNFFSTSNPLVFRYKMAQQIKDISSRLDKVAADRHKFSLQTIDVDTRVVHRRDMTHSR
VSDSDVIGRKHDKEKIVELLLQQNPNDDDTSISVIPIVGIGGLGKTTLAKFVFNDSRIQEHFPLKMWVCVSDDFDIKQLTI
KIINSANDSASADAPSHQMNLNMLDLEQLQNQLKNKLFGQKFLLVLDDVWNEDRVKWVELRNLIQVSAAGSKILVTT
RSHSIASMMGTVPSHNLEGLSEEDSLSLFVKWAFKEGEEEKHPHLINIGRQIVKKCKEVPLAVRTLGSLLFLKFEVSEWEY
VRDNEIWNLPQKRDDILPALKLSYDLMPSYLRQCFALFSLYPKDYEMDSYNITALWGALGLIALPTTNRAREDVANHYLH
ELLSRSFLQDFENFGTFYQFRIHDLVHDLALFVAKDECLHVTSNSQNISDNVRHLSFSESSLFENLVSKETAAVRTVLFPIG
AAVTNSEALLNTCLSKFKCLRVLDLHGSTFKTLPRAITKLKHLRYLDISRNPYIKRLPDSICKLQSLQVLSVNGCMELEALPK
GLRKLSSVWCFEFSTNQSILPVNEISNLESLEMLNVESCNNVESIFGGVKFPALKTLFVRDCQTLKSLLLNGQDFPELETLIV

YHCRNLDLELWKGAHQEESPKLKLKFIGFLSLSQLVTLPRWLQEAANSLQCLYVSNCHNMERFPDWLTTLTHLKTLTVIY
CPKLVSLPDNILHLSALENLRIEGCPDLCKKYAPHVGEFWPKISHIKNIFMDEPEG

>XP_027920229.1

MSPERDMISATPGAFRLRWDVFLSFRGTDTRGTITKGLYESLQARGVRVFLDDEGLERGEAVAKGLMEGIDDSAAFIVII
SQNYASSHWCLEELTKICGTGRLLLPVFYRVDPSQVRHVSGPFRAGFESHEKRFEKNTVSKWKEALKKVGGIAGWVFN
HSEEDDLIRRLVRRVLKELSNTPMGVPEFAVGLDERVEKVMEVLQVQSNGVKVLGLYGMGGVGKTTLAKALFNALVN
RFEHRCFISNVRQVSSKHDGLVSLQSKIIKDLFPGAGSPSIADVNVGVSAIKGRVGENRVLLVLDDVDEVKQLDALIGKRE
WFYDGSCVIITTRDTKVLTQDHVNVSYEVRELYASEARELFSYHALRRSEPPENLLSLSEEIISLTGRMPLALEVFGSFLFGK
RREEEWEDAVKKLRLIRPHHLQDVLKISYDALDEEEKCIFLDIACLFVQMEMKRDGVIDVLRGCGFRGEIAITVLVQKCL
MKITPENTVWMHDQIRDMGRQIVMDESFVDPGARSRLWDRAQIMTVLKGHKGTRCVQGIVLDFEEERFYKGKVGS
VFPKKLQWRSCLRNISCYIKQCLKNHLEPQAEENKEFVLHAKSFEPMVNLRQLQINNLKLQGKFLPSELKWLQWQGCPL
ERMPLKSWPRELAVLDLKNSKKMETLWGWNGYNKVPQKLMVLNLSYCNQLTAIPDLSGCQCLEKIDLENCINLTKIHE
SIGSLSTLRSLNLTRCSSLVNLPIDVSGLKQLESLYLSGCSKLKALPENIGILNSLKALQANDTAIAELPQSIFRLTKLEQLVLEG
CRYLRRLPNSLGHLCSLQELSLYHSGLEELPESVGSLDNLETFNLMGCESLTVIPDSIGNLMSLTELLVDRTAIKELPTTVGS
LSYLRELSVGNCKLLTQLPNSVKRLASVVELQLDGTAITNLPDEIGEMKLLRILKLMNCKNLEYLPESIGHLASLTTLNTVN
GNIKELPESTGRLENLVNLRLNKCKMLRKLPASIGDLKSLYHFFMEETAVSSLPESFGMLSSLRTLKMAKRPDLHTDESSFL
AEPEENHGPFILSSSFCNLTLLTELDARAWKISGKIPDEFEKLSLLETLKLDRNDFHTLPSSLKGLSILKVLSLSNCTQLNSLPS
LPSSLINLNVQNCSSIETIHDMSNLESLQELNLTNCVKVGDIPGLESLKSLRRLYLSGCIACSSQIRKRLSKVALRNLQNLSM
PGSKLPEWFSGQTVSFSKRKNLELKSVLVGVIVSINHSIDIPNMKRDDMPGLIDVQANILKGDRTLFSTVLNICGVPRTDE
EHMHLCKFHDYHQLVAFLKDADTFCVSKRNPPFDKGLELRKCGVYLIFEGDDDYDGGEESLDKGLQSVSEKLANFFSTSE
DEVSVNGIGIGHAGT

>XP_027920233.1

MGMETFTGAVAGAVIQEGPKQVARLVKKGLNFRESRRNLGSTVHHAIPVAQEIERLDQELGRSEAERAPLLEVLQEGE
KLFNARSNVPWWLCCCLPFFQTQLEEALEGVTRSNVNLLPLVARDTQEILLLSRNSVRGKGLKRWLKPPPKPDLIVGLD
NPLSHFNKLKKQLLQTGNSVLVLSGLAGYGKTTLATLLCWDDHVRGKFGENILFITVSKTCSLKTIVQSLFLQHYGLVVPD
LDLGNDRCAISHLKILAEEIMKSPMLLVLDDVWPNSESLIEAFKVHGLSDYKILVTSRFNVQGFEPACRMEPLSFEDSVTL
LHHLALPNDGRSSSLSDDYEEKVDLIPQIAKACYGSPLALELVGGSLRKERLNVWRQKKKKLSKGHPIVESHTEMLAIFKK
YLDDGLEDKPIIKECFMDLSLFPEDQKIPVSALIDIWTEQQHKLGGDLEQRQNLKEADAVNIVYDLTDRHLANLVVRNVG
IDIDDYCNHRFLLQHDLIKEVVEASQEPYTQRKRLMFDMNENNWSQQRQQNTVATTLSISTSKMSSTERLDNIVKVEV
VQVLVLNLRTKEYTLPEFIRKMNELKVLIITNYFDLNCSLINFELVGSLSSLRRIRLERVSVTTFGKWYNLRRLSLYHCNTREA
FESDSIPISEALPNLVELCIDYCKDLVTLPTGLCDITRIKKLSITKCMNFIALPNDIGNLENLKTLRLSSCAVFEEIPASIGKLFQL
RFLDISGCVSLQNLPEEIGDLQNLERLHMVGCPCKIPSSVSGLENLKNVRCDEETAIPWKEVYKPNLPSLKIEEAYDHSLFL
F

>XP_027920547.1

MASSVFVSFHHEDINFITGCVLDPLRNRGIHVFVKGESKTFDLFQAIERSRLFIVVLSKNYASSICCLRELVAIINGVESSPRS
VLPIFYGVHQSEVLFQNGCYGKAFSKHEERFREHKKRMEEVQTWREALTRVAVFRGLHMKNATGHGGFDFVQYAINI
VGPEFSTPQDEAIYEKFEDIVQSKDGDEVSYKELRGLVRLRDFISLMLTYPVKLEEDGVLRSWISAFPEWKAELFSTGGYP
VIYKPWSLLTEKALRCMKGFMPVEIMAEIESGERSIFNRDNDVILEGLLPLIVDLAVNQLIQTLFSGDYCARYIRLLTRNSA
EKESVVSKIVSALEDKHNLFGIDKDFLMAVWINALTRETDAEVQVQEEINKIMGSISITEGDDMLTTLDTDEKKRRNRLL
VIVVDADSNRKKLDLQKVQFPTGIVVLITTESSTQAVMDDGIASTMDLNIWTQDHLLPWKLFNTYVGSCISCSTVGSSTT
IQTIAVEIVKKIHGHLLAIVLVAKHLRNVKDDKYWELVLDKLSSRNPFYDYQDCDRIGISRVMVNAFVNIIWEDIDDELKLC

LQLSLPVRNIKNGVRDDILVSYWANILRYTQEVGEYKRQLQYYLEELLDCFVLLEFESGDVYLPIEAYDIIKSLHISQPSIIRHS
ALGLTEIGQWHSLIQLELIDNKICELPQSPDCPKLKVLLLQGNADLLDIPDSFFDHMPLLQHLDLSYTSIRDLPPSLTKLIQL
KKLYLKGCDLFMEISPQIFQLKNLEELDLDGTLITHLPKDIQELINLQRLALCFDAYHHVLSRGKKGMQISNTLIIPPGVISNL
TRLNYLSLDVDPEDEQWTENVNSVLLEILGLEKLKTVSIYVPETDLLELIPGTKSLNFRLVVGHHIRRLISRVPPELETKFKQC
NYSMKFVNGVNVPNGVKMNLERFKALYLDRHMTIKSLSDFNLSNLGRLKVCILAECNEMETIFDGGKSSDEPVSLMLEF
LSVFYMKNLRNICQECCPFSSIKYIALHTCPMLTTIFTISTFISIPLLEEISVEDCPKVTTLIIHDSPEERRSAFSLPKLRVISLLYL
PNLVNIFNGLRVEHALEEMIFYCCPKLQSLSRSELPWEYLKFIKGESMWWEALDWSVSEWGYAGRPKFLEQFFKPITME
ADMKSPPAAHQETQLNEYHGTMYQGVSSSTELMTKLHLETPLLSLSGSKEGEAQKRKAVIEPIIFSPPLLKRKPNESFRRR
TPQPSMKVIRTKNLDDGYSWRKYGQKEILGAKYTRGYYRCTYRNTHGCQATKQVQRFDEKPTTFEVNYRGTHTCTQK
QDLIAESGDEEVFEDFIAKPDSSSATNKNDIFSWEEITAELEEPSQISFGSSGVGSSEIVDPSTPSNGSP

>XP_027920549.1

MRHGGFDFVQYAINIVGPEFSTPQDEAIYEKFEDIVQSKDGDEVSYKELRGLVRLRDFISLMLTYPVKLEEDGVLRSWISA
FPEWKAELFSTGGYPVIYKPWSLLTEKALRCMKGFMPVEIMAEIESGERSIFNRDNDVILEGLLPLIVDLAVNQLIQTLFS
GDYCARYIRLLTRNSAEKESVVSKIVSALEDKHNLFGIDKDFLMAVWINALTRETDAEVQVQEEINKIMGSISITEGDDML
TTLDTDEKKRRNRLLVIVVDADSNRKKLDLQKVQFPTGIVVLITTESSTQAVMDDGIASTMDLNIWTQDHLLPWKLFNT
YVGSCISCSTVGSSTTIQTIAVEIVKKIHGHLLAIVLVAKHLRNVKDDKYWELVLDKLSSRNPFYDYQDCDRIGISRVMVNA
FVNIIWEDIDDELKLCLQLSLPVRNIKNGVRDDILVSYWANILRYTQEVGEYKRQLQYYLEELLDCFVLLEFESGDVYLPIEA
YDIIKSLHISQPSIIRHSALGLTEIGQWHSLIQLELIDNKICELPQSPDCPKLKVLLLQGNADLLDIPDSFFDHMPLLQHLDLS
YTSIRDLPPSLTKLIQLKKLYLKGCDLFMEISPQIFQLKNLEELDLDGTLITHLPKDIQELINLQRLALCFDAYHHVLSRGKKG
MQISNTLIIPPGVISNLTRLNYLSLDVDPEDEQWTENVNSVLLEILGLEKLKTVSIYVPETDLLELIPGTKSLNFRLVVGHHIR
RLISRVPPELETKFKQCNYSMKFVNGVNVPNGVKMNLERFKALYLDRHMTIKSLSDFNLSNLGRLKVCILAECNEMETIF
DGGKSSDEPVSLMLEFLSVFYMKNLRNICQECCPFSSIKYIALHTCPMLTTIFTISTFISIPLLEEISVEDCPKVTTLIIHDSPEE
RRSAFSLPKLRVISLLYLPNLVNIFNGLRVEHALEEMIFYCCPKLQSLSRSELPWEYLKFIKGESMWWEALDWSVSEWGY
AGRPKFLEQFFKPITMEADMKSPPAAHQETQLNEYHGTMYQGVSSSTELMTKLHLETPLLSLSGSKEGEAQKRKAVIEPI
IFSPPLLKRKPNESFRRRTPQPSMKVIRTKNLDDGYSWRKYGQKEILGAKYTRGYYRCTYRNTHGCQATKQVQRFDEKP
TTFEVNYRGTHTCTQKQDLIAESGDEEVFEDFIAKPDSSSATNKNDIFSWEEITAELEEPSQISFGSSGVGSSEIVDPSTPS
NGSP

>XP_027920685.1

MNPSHFRFYHKKRQIYTISTFAMAIASPVFVSFNYEDTRHDITGLVLDPLRRKGIQVLVKGESRTFDLFQAIEHSRFFIVVL
SKNYASSICCLRELVAIINGVESSPRSVLSIFYGVHESEVLSQDGCYGKAFSKHEERFREHKQRMEEVQTWRKALKRVCSL
GLHLENATGHGGFEFVQHEINILCPEFSTPENETIYSRYGKFEDIVQYKDGDEVSYKELRGLVRLRDFISLMLTYPVMLEED
GVLRSWISAFPEWKAELFSTGGYSKPWSLLTEKALRCMEGFVPVEIRAEIESGETSIFNRHNDVIPEGLLPLIVDLAVNQLI
QTLFSGDYYARYIRLLTRNSAEKESVVNKIVTALEDKHNMFGIGKEFFKAVWINALTRETDSEVQVQEEINKIMVSISMA
EGDDMLTTLDTDEKERSNRLLVIVVNADSNRELDLQKVQFPPGIVVLITTESSTQEVKDDGFGIACTMDLNIWTQDHLL
PWKLFNTYSGSCISCNTVGSSMTIQKIAVEIVKKSHGHLLAIVLVARHLRYVKDDKYWELVLDKLSRPNPFYDYQDCDRI
GISRVIVNAFVNIIWEDLDDELKFYLQLSLPVPNIKIGVRDDILVSYWANVLRYAEEVVEYKRQLQYYIEELLDCFVLLKFES
GVVYLPIETYDIIKSLRKSKSLGLTEPPYIGQWHKLVQIELIDDKICELPQSPDCPKLKVLLLPGNADLLDIPDSFFDHMPLLQ
HLDLSYTSIRDLPPSLTKLIQLKKLYLKGCDLLMEISPQIFQLKNLEELDLDGTLITHLPKDIRELINLQRLVLCFDAYHRVLSR
GKKGKQISDTMIIPPGVISNLTQLNYLSLDVDPEDEQWSENVNSVLVEILGLEKLKTVSIYVPKADLLELIPAKKSLDFRLVV
GHHMRRLISRVTPELETKFKHFDYSMKFVNGVNIPNGVDMNLGRFKALYLDRHMTIKSLSDFNLSNVRGLKVCILAECN
EMETIVDGGNSHDEPVSLMLEFLIVFYMKNLRSICQDCTPFSYLKYIVLRTCPMLTTIFTLRTLISLPFLEEISVEDCQKVTTL
ISHDSPEERRSAFSLPKLRVISLLYLPNLVNIFNGLRVDHVLEEMIFYCCPKLQSLSRSELPWEYLKFIKGESMWWEALEWS

VSEWGYGGRPKIFEQFFKPINVEADMMNLLAAHQETQLNEYHGTIYQGVSSSTELMTMNIHLERPLLSPSTSVSLLGSS
EGEAQTQKADIEPTIGQQPLSERKIKQGREGTSLHPEAVKMIRTKSEQVSDRFSSIKYEQKDIVGAKYQRFTSGIVQAHFV
ASSGLLLKTWDAISSCDEDVVPRVGNGLFWKVCEKSDWTVVVFEVNNDFDLHQELVSSSDLKEKNNFHCFEFLCTKKIP
EFSVNESAVSLFIDNLDELDELKSKINSSHPLIVTGHGIGGAIASLFTMLLLESIDSGKKRPLCITFGSPLIGDKKLQEAISRSST
WSSCFLHVVSYKDSLLRNFNPHPVDYVPFGTFLFCSGSGSTCLENPESVFELLLVSSINDQSQGFEVVDYGKLVENLNRK
AICKDFTPRGLNLTNSTSLNASIYLLLCAALELTSDLQELQHQNIDINALATKLERLENKFMFQKKVKFDPSKKLNWMKIH
MAKLEWYKKHCKSLGIGYYDCFKNAVSTIDIDAVQWQKSLSNYWRDIVEEAELKPQTEAAAFSTPWLFAGTNYRRMV
EPLAIAEYYAIGLKDYEAEGRSRHYAVLEKWLKEDKKEKGDSYGKTRRNVEWILTSDSCFWAKLEEALLLCGQWENLKE
NEKAKGKLLEFENYVYESLKKYEVSPEIFLKKSSYMTWWNKYKGFADNHRLASFMSNPQHFDQYTKGAYFFP

>XP_027920747.1

MAESSSSFAVSTSPTRHDVFLSFRGEDTRDNFISHLYAALKRKNIETYIDYRLQRGEEISPALETAIEESKIYVIIFSENYASST
WCLNELTKILDCKKRYGRYVIPVFYKVDPSTIRKQKERYKEAFEEHEHRFKEDMEKVQGWKDALTEAAGLSGWDSKVTR
PEYTLVEEIVKDILRKLINRCSISYDQGIIGIEKHIEAIRCLLHLESPYLRIIGICGMGGIGKTTISEQIYHTLAVHFDSRSLVLDV
QEKLQRDGIDSIRIKYMSELLNEAPSPLLLYYKERLKRTRVLLILDDVTDSAQLEKLLRGCDSFGQGSRIIMTGRNKQVLKN
VGADDVYELKGLNFHDSLKLFSLHAFKQNSLREITYMDLSVKVLRYAKGIPLALQILGSLLYDKPREAWESQLQKLERCQD
LKIFNVLKLSYDELDEEQKNIFLDIACFYRGHEETVVAETLDDCGFSSKIEMDVLKDRCLISVLDGRIVMHDLIQEMGQEIV
RKECPQHPGKRSRLLKVEEISEVLRNNKGSEALQSIVADMRKMEEVEVHAQCFEMIKNLRMLMLYYSRFRNESNVFRES
SLIILPETLKILYWTGFPQRSLPPNFCPQNLVRLEMIGSHLEQLWEGDQYLPKLKRLNLSGSLKLTRIPDLSLSPNIEEIILSSC
EKLINVHSSKLLSRLTCLCLDNCYDLKCVTVPSNILSTSPGLILLSHCHELEIFKTVETQLCDLPSLSSSRLRCNCALYGKALRS
RLHHSEIFSITFDRYKEEEVANNDICLQCDGVSRKLTEGVPLDFQSLKKLCCLDLSGCSSLKIFPFNLSEMKFLRQLCLRRCS
KLENFPEIQDTMEDLTVLILDRTAIQALPSSLWRLVGLQELSLCTCRNLEIIPSSIGSLTRLCKLDLSYCDSLQTFPSTIFKLKLR
KLDLCCCFKFRTFPEITESAQTFAHINLTNTAVKELPSSFGNLANLRSLQLNRCTNLESLPNSIVNLKHLCKLDCSGCAKLTE
IPTHIGLLSSLMELSLSVTGIVNLPKSIVHLSSLKSLDLTGCKNLECIPQIPPFLKQLVALDCPSIRQVMSNSLARNISNSKESV
FKFYLTNAQQLDSGARANIEEDARLMMTDDAYKSACFCFPGSEVPNWFPFRSEGASVTINEDLSFCSDDRLIGFALCVV
FGVLDTNDIEGRRGSFGYNLKFESDDDGTQIIPNNDVLKNYFEWNYEDKVVDKDRTFMWKFKLESLRRSGMRLRLCDA
RSFTFEISRNDYDFEQPDYESVVGEFKSVLTIKECGICPLYSNGSNVSQSSRTYRKRQKAESYLVS

>XP_027920759.1

MAEYFVFDIAESLLRKLASFVCEEASRVCEVYEDIKGIKDTLSIVKGVLLDAEHKKEQRHGLREWIRQIQTVCLDAEDVLD
GLECQNLRKQVLKASDSTRMKVAHFFSSSNSLVFRFRMAHQIKHVRRRLDKIAADGNKFGLERIDVDNRLVQRREVTYS
HVDASGVIGRESDREEIIKLLMQPHPHGDGCGDESVCVIPIVGIGGLGKTTLAKLVFNDKRMDDLFQLKMWVCISDDFD
IRQILIKIINSVSASDPAPTIALAHQENIKNFDIEQLQIVLKLKLSGQKYFLVLDDIWNDNRAKWIELKDLIKVGAVGSKILVT
TRSNSIASMMGTVPSYVLEGLSMENCYALFNKWAFKEGEEKAYSNLVEIGKEIVKKCRGVPLAVRTLGSSLFLVFDLERW
EFVRDHEIWNIKQNKDDILPALKLSYDQMPSCLRHIFAFFSLYPKDYGFCSAEILILWESLGLLQSLGGNRKLENIARQYID
ELHARSFLEDFEDFGHYYYFKVHDLVHDLALFVGKEEHLMVNSFTRYIPEQIRYLSVVENDSLNYVLFPKSSRVRTILFPLK
GTAVESKTLLHTWTTRYKHLRFLDLSDSSFDTLPSSIAKLKHLRTLFLDKCKIKRLPHFLCKLHNLQILSLRGCMELETLPKSL
GMLISLRKLYITTKQSILPENEFASLRNLHTLSFEYCPNLKLLFRKEQLALLEVLIIQSCRSLEFLPLHVLPKLEVLIVSRCEMLN
LNYGSPIHRLRMKFLHIEHCPKLHTLPQWIEGSVETLRTLLILNCHTLKMFPEWITTMSGLKRLHIVNCPKLLCLPGEMHS
LTALDDLTIEGCPELCRKCEPKNGEFWSFIAHIKRVSIGETRKGKLLVRMLQQMRLRLRDQ

>XP_027920767.1

MAEYFVFDIAESLLRKLASFVCEEASRVCEVYEDIKGIKDTLSIVKGVLLDAEHKKEQRHGLREWIRQIQTVCLDAEDVLD
GLECQNLRKQVLKASDSTRMKVAHFFSSSNSLVFRFRMAHQIKHVRRRLDKIAADGNKFGLERIDVDNRLVQRREVTYS

HVDASGVIGRESDREEIIKLLMQPHPHGDGCGDESVCVIPIVGIGGLGKTTLAKLVFNDKRMDDLFQLKMWVCISDDFD
IRQILIKIINSVSASDPAPTIALAHQENIKNFDIEQLQIVLKLKLSGQKYFLVLDDIWNDNRAKWIELKDLIKVGAVGSKILVT
TRSNSIASMMGTVPSYVLEGLSMENCYALFNKWAFKEGEEKAYSNLVEIGKEIVKKCRGVPLAVRTLGSSLFLVFDLERW
EFVRDHEIWNIKQNKDDILPALKLSYDQMPSCLRWKSKA

>XP_027920789.1

MANHGDFTYDVFMSFKGENGTRYAFTDHLYRALLRHGINAFRDDQSLRSGDEIRPSLLQAIEASRISLVVLCQNYASSS
WCLDELAKIIHCYENKGKHVVAIFYLVEPSDVRYQKNSYATAMSKHESRYGKDSEKVKTWRSALARVCDLTGIHYRNH
MYETEVIEKIVKDTSAKLPPMPLQIKHLVGLDSRFERVKSLIDVESNDEVCILGIYGVGGIGKTTFAVDLYNKIRHHFEAASF
LANVREKTNKSIKGLEDLQRTLLSEMGEETETIIGSTFKGSSEIKCRLGHKRVLLVVDDVDSVKQLEALAGGYDWFGRGSR
IIITTRDKDVLHKHDVEIKGYEMEELNYHESLELFCWYAFNRSSPAENFASISTSAVRYAKGIPLALRVIGSNLKGRGPEKW
ETELQKYRKVPDSEIQGVLEISYTSLSNLDQKIFLDIACFFKGERWDYVKRILDACDFNPDIQVFVSKCLISVDENGCLEMH
DLLQDMGREIVRKESPSNPGYRSRLWSHKDVHEVLKENSGSYAVEGIMLYPQKQEKIDYWTMTAFKKMKNLRILIVRN
AIFSYGPSYLPNSLRLIDWKGYPSKSFPPDFYPHRIVDFKLPHSSLIFEKPFQRFEDLTFINLCHCQFITQIPDLSGAKSLKVLT
LDQCYKLARFDKSIGFMPNLVYLSASECTQLTSFVPKMYLPSLEVLSFNFCRRLEHFPHVMHKMDKPLKIYMMSTAIKKI
PTSIGNLTGLEHMDMSVCKELKNLPSSFFLLPKLVTLKVDECSQLGESFQRFRESRHSVANGSSNLVTLQFRETNPSYEDL
CAILEIFPKLEDLNVSHNGFVALPNIIRGSSHLKSLDVSFCRNLKEIPELPLSIQKVDARYCQSLTSEASSMLWSKVSEEIQR
MQVVMPMLKREIPEWFDCIGTKDIPHFWARRKFPVVALSLVFQEPKKKLSDFEHAFQSAVESFTGFVNWHTVSLHLFI
DGQEIYGRDYHCFNVGEDHVLFCDLRVLFRDEEWQGLDASLGDDWKSIQVQYESDLILSHWGVNVYEQETNMDDIQ
FRFPIPSSTRNLIPSSLLVPKVCPKQKMKHMLESFDPRDIFNMNLSLIESEEGPSRSGKVLLRTWRNAKAEITEEASVSVYG
ASLKQEHEESVDDVVEVLEMIKENVPKHFSDSNPEEMQLFGGFVERLLRARVEVMKENGLDMGMPILLEYTDVGGSK
YRRFWGVLQLKVGDPFYKAVLRKYNQLSWEFSTSNRASSSGTWFENLRITIVLLKCLDPAMEAASGFGYEESLEEGYYDP
ELAELMMRIEQDAMGFNKSYGKMKACIVRTDESVSPQYLFETLMFRRLIALGKLTTFGFITKFKITPYGNIRVEDDPFRIP
KTCFWSLILVLHLLFFIIIWLFCFVGYVGLFICRIPVIGKILVCGWWLLMQVLVSCKYLYHRMGKIMKIKKKDL

>XP_027921471.1

MAESFLFSIAESLIAKLASRAFEEASRVVGLYDDLRDLTNTLSLVKDVLLDAQQKQEHNHELRQWLTQLKTAFSDAEDVL
DEFECQTLQKKVVKAHGSRKDKVSHFFSTSNPLVFRYKMAQQIQRISTRLDNVATNRNKFTLERIEVDTRVVHRRDMT
HSRVSDSNVIGRKDDKEKVIELLMQQNPNDDDTSLSVIPIVGIGGLGKTTLAKFVFNDNRVQEYFPLKMWVCVSDDFDI
KQKIIKIINSANDSISDDAPSHQPNWNMVDLEQLQNQLKNKLSGQKFLLVLDDVWNEDRVKWVELRDLIQVSAAGSKI
LVTTRNPSIAAMMGTVPFHLLKGLSDEDLLSLLVKWAFKEGEEEKHPHLVNIGREIVKKCGGVPLAVRTLGSLLFSKFEAS
EWEYVRDNEVWNLSQKKDDILPALKLSYDLMPSYLRQCFALLSLYPKDYLYNSYEISWLWGALGLIALPKTNRTREDMA
NQYLHELMSRSFLQDFENYGTMYGFRIHDLVHDLALFVAMDDCLHVNSNIQNIPDKARHLSFVESSLFSNLVTKKSAAV
RTVLFPNGVSTANCESILKTCMTKFKCLRVLDLTGATFETLPRNIAKLRHLRYLNISENPNIKRLPESICKLQSLQALMLDGC
MELEALPKGLRKLISLRDFSFSTKQTVFPVNEIAKLRSLEFLIVESCHNVESIFGGVKFPTLKTLFVSNCQSLKSLRLDDQNFP
ELETLVVDNCNNLDLEMWNGHNEEESSKMKLKLVAFSGLSQLVTWPKWLQEAANSLQSLLVLNCPNIKTLPDWLPTLT
NLKALTIRNCPKLVSLPDNMHHLSALENLRIEGCADLCKKYESYIGEFWPKISHIKNIYINGPEA

>XP_027921965.1

MKRPFGVPEEPEFTVGLDVPFSKLKMELLNEPASIIVLTGFGGSGKTTLATKLCWDQQVMGKFKGNILFITFSKSPKLKIIV
ERLFEHCGDQVPEFQSDEDAVNQLGLLLRKTDAGPVLLVLDDVWPSTEAIVEKFRVQISDYKILVTSRVAFPIFGSPFILKP
LVHEDAITLFRHHALLKKSSSIIPDEDLLQKVLXYTSNPILEHLQLGHLMNSCNRPIELWQKMVEELSQGHSILDFNIDLLTS
LQKILDVLEDNSIIKECFKDLALFPEHQRIPVAALVDIWVELYGLDNNGMQVMAILNKLDSMNLVNVLVTRKSTSDTDSY
YYNNRYVILHDILRDLAIYQSTQEQTEQRKRLMIGMDDNKPEGWLREKQQGVMIRLCSNIFGWCIEQKHRQIRTRTLSI

STDETFTSYCSNLLPAQAEVLILNLRTNRYSFPKLLKKMSILKVLIVMKHGFRPCEMINFEVLGSLNNLKRIRLERIFIPSFVM
LKNLKKLSLYMCDTRQAFENSDMLISDAFPNLEELNIEYSKDMVRLPTGLCNITSLKMLSISNCHKLSELPQEIGSLVNLKL
LRLSSCTDLEGIPNSIVRLSNLRHMDISNCISLRNLPEDFGNLCNLRNLYMTSCARCELPHSIVNLKNLKEVVCDEETAAS
WEPFKHMLPNLNIDIPQLDVNLNWLHAIHS

>XP_027922356.1

MAAELVGGALLSAFLQVAFDRLASPQVLDFFRGRKLDEKLLSKLHITLHSINSLADDAEQKQFRDPYVKAWLLAVNDAV
FDSEVLLDSIDYELIKCNVEAESAPQSLTSKVSNFFDSTFRSFNKKINSGMREVTEKLEYLAKQKGALGLKKYSYSGDGPDS
KVPQKLPSSSLVVESVIYGRDADKEIIFNWLTSETDNHNHLSILSIVDMGGLGKTTLAQHVYNDPKIDDAKFDIKAWVCV
SDHFDVLTVTKTILESITDKKDDSGNLNMVHKNLKEKLSVKKFLLVLDDVWNEKREEWEVVQTPLNYGAPGSRILVTTR
AEKVASNMRSKVHHLKQLEKDECWKVFKKHALKDDDLELNDEKKKIGRSIVENAKDYLSL

>XP_027922453.1

MAAEVVGGALLSAFLQVAFDRLASPQFVDFFRGRKLDEKLLGNLNIMLHSINALADDAEQKQFTDRHVKEWLLSVKEA
VFDAEDLLGEIDYELTRCQVEAGSEHQTFTDKVSTFFNSTFSSFNKKIESEMKEVLEKLKYLEQQKDALGLKNGTSYSDSK
VSQKLPSSSLVVESIIYGRDADKEIIFNWLRSETDNDNHPSIFSIVGMGGLGKTTLAHHVYRDPKMEEAKFDIKAWVCVS
DHFDVLTVTKTILEAITNSKDDSGNLEMVHGRLKDEISGRKFLLVLDDVWNEGGEEWEAVRTPLSYGAPGSKILVTARAE
KVASNMRSKVHLLRELRGDECWNVFKKHALRDDDLELNDEKEEIGRRIVEKCKGLPLALKTIGSLLCKESSISYWKRVLEN
DIWDLPKEVKIIPALLLSYQHLPSHLKRCFAYCALFPKDHEFDKKELILLWMAEGFLHHSQQINNVEEIGEQYFNDLLTRSF
FLQSDFKTYFSMHDLLNDLAKYVCADFCFRLKFDKGNCIPKKTRHFSFAFDDVECFDGFGSFTDAKRLRSFFPYEEFGGR
NIDYYPLQFKILVHELFSNFKFLRVLSLDGYSELKEVPDSVGDLKHLHSLDLSRTGIQKLPESTCLLYNLLILKLNYCSSLEELPL
NLHKLTKLHCIEFENTKVTKMPMHFGELKNLQVLSPVFIDKNKEFSTKHLGCLNLHGRLSINEVQNIVNPLDALEANLKN
KDLVELLLKWESDHIPDDPRKEKKVLENLQPSKIVEYLSIENYGGTEFPSWVFDNSLSNLVSLRLEDCKYCLCLPPLGLLSSL
KTLEIIGFDGIVSIGDEFYGNSSSSFTSLESLKFSKMKELEECERKTAAFPRLEILSVYQCPKLKGLPDQLVNVKNLYIRKASCL
ERCEHTVSHNSLEALTFFVFPIMNISMSRSFDLLEQICIFESCDSLTTFPLDFFPNLKDLTLSSCRNLQMISQDHTHTSLEML
SIRSCSRFDSFPSEGLSAPQLLLIDIDGAENLKLLPKRIRILHPSLYVLNIINCPKVEMFPDGGLPPNVKDVSLSSLKLIASLKET
LGTNTCLQSLSIEYIDVEYFPDEVLLPHSITSLEICRCPNLKKMEYKGLCHLSYLKLYDCPNLQCLPEDGLPESISSLRIWSCPL
LERRCQNPEGQDWNKIAHIEDLSVRSKV

>XP_027922454.1

MAAEVVGGALLSAFLQVAFDRLASPQFVDFFRGRKLDEKLLGNLNIMLHSINALADDAEQKQFTDRHVKEWLLSVKEA
VFDAEDLLGEIDYELTRCQVEAGSEHQTFTDKVSTFFNSTFSSFNKKIESEMKEVLEKLKYLEQQKDALGLKNGTSYSDSK
VSQKLPSSSLVVESIIYGRDADKEIIFNWLRSETDNDNHPSIFSIVGMGGLGKTTLAHHVYRDPKMEEAKFDIKAWVCVS
DHFDVLTVTKTILEAITNSKDDSGNLEMVHGRLKDEISGRKFLLVLDDVWNEGGEEWEAVRTPLSYGAPGSKILVTARAE
KVASNMRSKVHLLRELRGDECWNVFKKHALRDDDLELNDEKEEIGRRIVEKCKGLPLALKTIGSLLCKESSISYWKRVLEN
DIWDLPKEVKIIPALLLSYQHLPSHLKRCFAYCALFPKDHEFDKKELILLWMAEGFLHHSQQINNVEEIGEQYFNDLLTRSF
FLQSDFKTYFSMHDLLNDLAKYVCADFCFRLKFDKGNCIPKKTRHFSFAFDDVECFDGFGSFTDAKRLRSFFPYEEFGGR
NIDYYPLQFKILVHELFSNFKFLRVLSLDGYSELKEVPDSVGDLKHLHSLDLSRTGIQKLPESTCLLYNLLILKLNYCSSLEELPL
NLHKLTKLHCIEFENTKVTKMPMHFGELKNLQVLSPVFIDKNKEFSTKHLGCLNLHGRLSINEVQNIVNPLDALEANLKN
KDLVELLLKWESDHIPDDPRKEKKVLENLQPSKIVEYLSIENYGGTEFPSWVFDNSLSNLVSLRLEDCKYCLCLPPLGLLSSL
KTLEIIGFDGIVSIGDEFYGNSSSSFTSLESLKFSKMKELEECERKTAAFPRLEILSVYQCPKLKGLPDQLVNVKNLYIRKASCL
ERITLILVSRCCQFAVAHDLTHFLVKVYLLRSYC

>XP_027922471.1

MAELVGGALLSAFLQVAFDRLASPQFVDFFRGRKLDEKLLGNLNIMLHSINALADDAEQKQFTDPHVKAWLLSVKEAV
FDAEDLLGEIDYELTRCQVEAGSEPQTFTDKVSTFFNSTFSSFNKKIESEMREVLEKLEYLAKQKGALGLKEGIYSGDSSGS
KVSQKLPSSSLVVETVIYGRDVDKEIIFNWLTSETGNHNHPSVLSIVGMGGLGKTTLAQHVYNDTKLEEAKFDITAWVCV
SDHFNVLTVTKTILEAVTKSKDDSGDLQMVHERLKEKISGKKFFLVLDDVWNERQEKWEAVQTPLSYGAPGSRILVTTR
GEKVASIMRSKVHRLKQLKKDICWNVFEKHALRDDELELNDEKKEIGRRIVQKCKGLPLALKTIGSLLRTKSSISDWQSVL
ESDIWDLPKEVEIMPALLLSYQHLPSHLKRCFAYCALFPKDYKFDKKELILLWIAQDFLHCSQQSNNLEEIGEQYFNDLLM
RSFFLQSDFKTCFFMHDLLNDLAKYVCADFCFRLKFDKGNCISKTTRHFSFGISDVKYFDGLGSLTDAKRLRSFFPYKEFGR
RYIDYYPLQFKILVHELFSNFKFLRVLSLDQYSELREVPDSIGDLKHLHSLDLSGTQIQKLPDSTCLLYNLLILKLNYCSSLKELP
LNLHKLTKLRCLEFENTNVTEMPMHFGELKNLQVLSAVFVDKNKEFSTKHLGGLNLHGGLSINEVQNIVNPVDALEANL
KNKDLVKLELKWKSDYIPDDPRKEKKVLENLQPSKTLEHLSIKSYGGTEFPSWVFDNSLSNLVSLRLEDCKYCLCLPPLGLL
SSLKTLQIIGFDGIVSIGDEFYGNSSSSFTSLERLTFSNMKELEECERKTAAFPRLEFLSVYQCPKLKGLPKELVNVKYLDIRG
SMKAWCLERCEHTVSHNSLEDLNFCAFPIMNIPMSRSFDLLEQINIFRGCDSLTTFPLDFFPNLKALSLYFCRNLQIISQD
HTHNHLESLTIDGCSRFDSFPSEGLSAPRLKRIDIDEAENLKLLPKQMQILLPSLNILKIICCPKVEMFPNGGLPPNVKAVFL
SSLKLMASLRETLGTNTCLQSLYIEKMDVEFFPDEVLLPHSITALRICDCPNLKKMEYKGLCHLSSLLLHNCPNLQCLPEDG
LPKSISSLQILNCPLLKQRCQNPEGQDWNKIAHIEDLIILP

>XP_027922525.1

MMKELLSSTKSKKGMEHTVLNQIRDVSHLAEDVIDIFVAKVSIFKRRTILGRMLLGFGQTRLLHDVAEKIDKIKATLNEIR
DNKSKYDAFKETNNQSAAEEEEEEEKERAKALHKLRRNVEEDDVVGFVQESKVVINRLLEGGSDRNAVSTIGMGGLGK
TTLARKVYNSTQVKQHFKCRAWVYVSNECRVKELLLGLLKT

>XP_027922831.1

NLYKALCDKGIHTFIDDDKVEGGEKITPALMKAIQESMIAITVLSHNNASSSFCLDELAAILDCKNKGLLVIPVFYKVDPSY
VRHQKGSYGEALTKQQRWFNDKEKLRYMQKWKMALRQVADLSCYHFKDDIEYEYKFIGSIVERVSSEINRGSCLHVAD
YPVGLDSQVLEVRKVLEVGCYDGVHMIGIHGMGGVGKSTLARAVYNSLIAENFDSLCFLEDVREKSNKHGLEHLQSILIS
QVLGKEEINLTSKQQGISMIKCRLKRKKILLILDDVDNIEQLRALAGGCDWFGPGSRIIITTRDKQLLATHQVTRTYEVREL
NEKDALRLLTLKAFKKEKADPDYVLVLNRVVTYASGLPLALEVIGSNLFGKSVKEWESATRQYKRIPKKEIIEILKVSYEALEE
EEKVVFLDIACCFKGYAFREVEDILGALYDDCMKHHTGVLAEKSLIKVSLLSTVEMHDLIEDMGRKIDQKESPNEPGKRR
RLWLPKDIIQVLKYNMGTSKIEIMCLDFSISENEETVEFDENTFMEMDNLKILINRNVKSFKCPNYFPQSLKVLEWHRYPS
NCLPSNFHPNKLVICKLPDSCFESFGIHASSKKFENLTSLNFDDCKLLTRIPDVSVSFEWCESLTAVHDSVGFMTKLKILSAE
GCSKLTSFPSLHLTSLERLELSYCSSLENFPEILGNMKNIKRLELIDNKLHEAGGTVFIFPGGSVPEWLDKESKGPSISFWFR
NKFPVKVLCLLIAPVLGPLDLAIPMVSINGKIRKYLFHMNKGVKILELDYTHLFDIRELNFEDDLMGVSTEWKQVEITYEGL
FDTSVIKATGIHVVKEESMNVEDIRYDDPCTNNKEDSHLNTFQSQNYS

>XP_027922881.1

MVESFLFSMAEKLLAKLACRAFEEVSRVVGLYDHLRNLTNTLSFIKAVLLGVQQKQEHNPELREWLTQIKTIFSDAEDVL
DEFECQTLRMKVAKAHGSTKDKVSNFFSSSHPLVFRHKMTQQIKDICNRLDKVAADRDKFSLQIIDVETRVVHRRNMT
HSRVSDSDVIGRKHDKENIIELLMQQNLNHNDTNLSIIPIVGMGGLGKTTLAKFVFNDIRIQECFSLKMWVCVSVDFDIE
QLIIKIINSANYPASADALSHQTNWNMLDLEQLQNQLKNKLSGQKFLLVLDDVWNEDRVKWLELRNLIQVSAAGSKILV
TTRNPSIAAMMGTVPFHLLKGLSDEDSLSLLVKWAFKEGEEEKHPHLINIGRQIVKKCRGIPLAVRTLESLLFSKFEASEW
EYVRDNEVWNLPRKKDDILPSLKLSYDLMPSYLRQCFALFSLYPKDRQFYSDEITRVWGTWLWGALGLIALPKTNRTPEE
VASQYLNELMSRSFLQDFENFGTAYTFRIHDLVHDLALFVALDECLHVNSKIQNIPDNVRHLSFAESILFDNLVTKKSAAV
RTVLFPHGAAEAANGEAILTTCLAKFKCLRVLDLSGAIFETFPRNIAKLRHLRYLDISDNPNIKRLPDSICKLQSLQVLLLGG
CNELEALPKGLRKLISLRYLEFSIKQTVLPVNEIANLVSLQILSIESCINVESIFGGVKFPILTSLFVSGCQSLKSLSLDGQNFPE

LETLFVDDCSNLDLELWKGHHEQESSKLKLKLLAFSSLSQLVALPKGLQEAANSLQCLFISNCHNIEALPDWLTTLTDLKTL
RLIDCPNLVSLPHNFHDLTALETLRMKGCADLCKKYKPHVGEFWPKISHIKNIFIDEPEEPEKN

>XP_027923030.1

MEKCGLGILTEPETVKLNVRLDTLWNPSRGGSNSSRKCEGSVFTKRSKILVTARAEKVASNMRSKVHLLRELRGDECLNV
FEKHALKDDDLELNDDLKEIGRRIVEKCKGLPLALKTIGSLLRTKSSISYWKSVLENDIWDLPKEVKIIPALLLSYQHLPSHLK
RCFAYCALFPKDREFDKKELILLWMAEGFLHHSQQINNVEEIGEQYFEDLLMRLKFDKGNCIPKTTRHFSFAFDDFLRVLS
LNAYSELREVPDSVGDLKHLHSLDLSRTGIQKLPDSTCLLYNLLILKLNYCSRLEELPSNLHKLTKLCFLEFKNTKVTEMPMR
FGELKNLQVLSPVFVNKNNEFNIKHLGGLNLHGRLSINEVQNIVNPLDALEANLKNKDLVELELKWESDHIPDDPRKEKK
VLENLQPSKIVEYLSIENYGGTKFPSWVFDNSLSNLVSLRLEDCKYCLCLPPLGLLSSLKTLKIIGLDGIVSIGDEFYGNSSSSF
TSLESLEFSR

>XP_027923031.1

MGGLGKTTLAQHVYNDPKIDDAKFDIKAWVCVSDHFDVLTVTKTILESIADKKDDSGNLNMVHKNLKEKLSGKKFLLVL
DDVWNEKREEWEVVQTPLNYGAPGSRILVTTRAEKVASNMRSKVHHLKQLEKDECWKVFKKHALKDDDLELNDEKKK
IGKSIVEKCKGLPLALKTIGCLLYTKASISYWKSVVESDIWNLPKELGIIPALLLSYQHLPSHLKRCFAYCALFPKDYEFDKKEL
ILLWMAEDFLRHSQHIENVEEVGEQYFDDLLTRSFFLQSSIKMRFVMHDLLNDLAKYVGGNFCFMFKFDKGVRIPKTTR
HFSFSIDNEVYDDGMGSLIDAKRLRSFIPMTNNIDGLPCELILIDELFSKFKFLRVLSLSGFGDLITEVPDSVGALKHLRSLDL
SYTNIQKLPDSICLLFNLLLLKLNRCLYLEELPSNIHKLTKLHCLEFEHTKVTKMPMHFGELKNLHLLDAFRVHSESSIKQLG
GLNLHGSLSIYQVQNIVNPLDALEANLKDKQLVELGLIWNSNHVPNDAREEKEVLENLQPSIHLEHLSIWSYHGIEFPSW
LFDNSLSNLVFLRLNNCKNCLCLPSLGELSCLKNLEIEGLDGIVSIGVSDGFYGSNSSSFASKERLSFRNMKEWEEWECKTT
SFPRLQYLFVDQCCKLKGLPEQLIHLKNIFIGGCDKLTISVNNMDTWSLQFLNINSCPRVNIPITAFNSLEVMRITHGCPSL
IIFPLDFFPKLRTLLLFHCQNLQRISQEETHNHLKELQILCCPQFESFPSEGLSARWLQKIEIKAAPNLKLLPKRMHTLLPSLS
SLEITYCPQVEMFEEGSLPSNLKEVSLSSFRLITSLREALDAEPCLERLYVENVDTEYFPDEGLLPPSLTFLRIFNCPNLKKLDY
KGLSHLSSLIIIR

>XP_027923032.1

MGGLGKTTLAQHVYHDPKMEEAKFDIRAWVCVSDHFDVLTVTKTILEAITNSKDDSGNLEMVHGRLKDQISGRKFLLV
LDDVWNEGGEEWEAVRTPLSYGAPGSKILVTARAEKVASNMRSKVHLLRELRGDECWNNNECNIKQLGGLNLHGSLS
IKEVQNIVNPLDALEANLKNKHLVNLELEWKRKHIPDDPLKEKKVLENLQPSKHLERLSIENYGGTEFPSWVFNNSLSTLV
FLCLENCKYCLCLPPLGLLSSLKTLKIRGFHGIVSIGAEFCGSNSTSFTSLESLEIDNLKEWEEWECKTTFPCLRYLFINRCPKL
KGTSEQLLNLKELFVSLRGSRNLRGISNEHTHNHLKEMKIDECPQFESFPSEGLSAPQLWKIEIKGARNLKLLPKRMQILLP
SLTELRITDCPQVEMFEEGSLPSNLKEVSLSCFRLIASLREALGADTCLETLSIEEVDVQCFPDEGLLPPSLTSLEIYDCPTLKK
LNYKGLSHLSSLRLLDVPT

>XP_027923033.1

MPALLLSYRHLPSNLKRCFAYCALFPKDYEFDKNELILLWIAQDFLHCSQQSNNLEDIGQQYFNDLLTRSFFLQSDLKTCF
SMHDLLNDLAKYVCADFCFRLKFDKGNCIPKTTRQFSFAFWDVKYFDEFGSLTDAKRLRSFFPIKEIGRRYIGHYPLQFKIL
VHELFSNFKFLRVLSLDKYYELREVPDSIGDLKHLHSLDLSRTGIQKLPDSICLLYNLLILKLNYCSSLEELPLNLHKLTKLRCLE
FKYTKVTKMPMHFGELKNLQVLNTIFFDRNNEFSTKHLGGLNLHGRLSINEVQNIVNPVDALEANMKNKDLVKLELKW
KSDHIPDDPRKEKKVLENLQPSKNVEHLSIENYGGTEFPSWVFDNSLSNLVFLRLEDCKYCLCIPPLGLLSSLKTLEIVGLDG
IVSIGAEFYGSSSSSFASLEKLKFLNMKELEEWEYMIGAFPRLQILRVCVCPKLKGMPEQLLHVKHLYISCSMITSSHKIVSH
NSLEALNCCAFTIMNFPMSRSYDLLAKIEIIGSCDSLTTFPLDFFPNLKALLLVGCRNLQIISQKHTHNRLKHLSISGSSRFDS
FPSEGLSAPRLQTIDIYGAEI

>XP_027923034.1

MGGLGKTTLAQHVYNDPKIDVAKFDIKAWVCVSDHFDVLTVTKTILESIADKKYDSGNLNMVHKNLKEKLSGKKFLLVL
DDVWNEKREEWEVVQTPLNDGAPGSRILVTTRAEKVASNMRSKVHHLKQLEKDECWKVFKKHALKDDDLELNDEKK
KIGRSIVEKCKGLPLALKTIGCLLYTKASISDWKSVLESDIWNLPKELGIIPALLLSYQHLPSHLKRCFAYCALFPKDYEFDKKE
LILLWMAEDFLRHSQHIENVEEVGEQYFDDLLTRSFFLQSSIKMRFVMHDLLNDLAKYVGGNFCFMFKFDKGVRIPKTT
RHFSFSIDNEVYDDGMGSLIDAKRLRSFIPMTNNIDGLPCALILIDELFSKFKFLRVLSLSGFGDLITEVPDSVGALKQLRSL
DLSYTNIQKLPDSICLLFNLLILKLNYCSDLEELPSNIHKLTKLHCLEFENTKVTKMPMHFGELKNLHLLDAFRVHSESSIKQL
GGLNLHGSLSIYQVQNIVNPLDALEANLKDKQLVELGLIWNSNHVPNDAREEKEVLENLQPSIHLEHLSIWSYHGIEFPS
WLFDNSLSNLVFLSLNNCKNCLCLPSLGELSSLKNLEIEGLDGIVSIGVSDGFYGSNSSSFASMERLSFRNMKEWEEWEC
PQVEMFEEGSLPSNLKEVSLSSFRLITSLREALGAEPCLERLYVENVDTEYFPDEGLLPPLLPF

>XP_027923035.1

MAEDFLCHSQHIENVEEVGEQYFDDLLTRSFFLRSSIKMRFVMHDLLNDLAKYVCGNFCFMFKFDKGDRIPETTRHFSF
LIDNEVYDDGMGSLIDAKRLRSFTPITNKINDSFPCELILIDELFSKFKFLRVLSSSGFGDLITEVPDSVGALKHLRSLDLSYTK
IQKLPDSVCFLYNLLVLKLNCCSYLEELPSNIHKLTKLHCLEFEDTKVTKMPMHFGELKNLHLLNAFRVHSESSIKQLGGLN
LHGSLSIYQVQNIVNPLDALEANLKDKHLVELGLIWNSNHVPNDAREEKEVFENLQPSIHLEHLSIWSYHGIEFSSWLFD
NSLSNLVFLRLNNCKNCLCLPSLGELSSLKNLEIEGLDGIVSIGVSDGFYGSNSSSFASMERLSFRNMKEWEEWECKTTSF
PRLQYLFVDQCCKLKGLPEQLIHLKNIFIGGCDKLTISVNNMDTSSLQFLNINSCPRVNIPITAFNSLEVMRITHGCPSLIIFP
LDFFPKLLKDYLHGGYKKSKLKQRQI

>XP_027923037.1

MGGLGKTTLAQHVYNDPKIDDAKFDIKAWVCVSDHFDVLTVTKTILESIADKKDDSGNLNMVHKNLKEKLSGKKFLLVL
DDVWNEKREEWEVVQTPLNYGAPGSRILVTTRAEKVASNMRSKVHHLKQLEKDECWKVFKKHALKDDDLELNDEKKK
IGKSIVEKCKGLPLALKTIGCLLYTKASISYWKSVVESDIWNLPKELGIIPALLLSYQHLPSHLKRCFAYCALFPKDYEFDKKEL
ILLWMAEDFLRHSQHIENVEEVGEQYFDDLLTRSFFLQSSIKMRFVMHDLLNDLAKYVGGNFCFMFKFDKGVRIPKTTR
HFSFSIDNEVYDDGMGSLIDAKRLRSFIPMTNNIDGLPCELILIDELFSKFKFLRVLSLSGFGDLITEVPDSVGALKHLRSLDL
SYTNIQKLPDSICLLFNLLLLKLNRCLYLEELPSNIHKLTKLHCLEFEHTKVTKMPMHFGELKNLHLLDAFRVHSESSIKQLG
GLNLHGSLSIYQVQNIVNPLDALEANLKDKQLVELGLIWNSNHVPNDAREEKEVLENLQPSIHLEHLSIWSYHGIEFPSW
LFDNSLSNLVFLRLNNCKNCLCLPSLGELSCLKNLEIEGLDGIVSIGVSDGFYGSNSSSFASKERLSFRNMKEWEEWECKTT
SFPRLQYLFVDQCCKLKGLPEQLIHLKNIFIGGCDKLTISVNNMDTWSLQFLNINSCPRVNIPITAFNSLEVMRITHGCPSL
IIFPLDFFPKLRTLLLFHCQNLQRISQEETHNHLKELQILCCPQFESFPSEGLSARWLQKIEIKAAPNLKLLPKRMHTLLPSLS
SLEITYCPQVEMFEEGSLPSNLKEVSLSSFRLITSLREALDAEPCLERLYVENVDTEYFPDEGLLPPSLTFLRIFNCPNLKKLDY
KGLSHLSSLIIIR

>XP_027923039.1

MGGLGKTTLAQHVYNDPKIDVAKFDIKAWVCVSDHFDVLTVTKTILESIADKKYDSGNLNMVHKNLKEKLSGKKFLLVL
DDVWNEKREEWEVVQTPLNDGAPGSRILVTTRAEKVASNMRSKVHHLKQLEKDECWKVFKKHALKDDDLELNDEKK
KIGRSIVEKCKGLPLALKTIGCLLYTKASISDWKSVLESDIWNLPKELGIIPALLLSYQHLPSHLKRCFAYCALFPKDYEFDKKE
LILLWMAEDFLRHSQHIENVEEVGEQYFDDLLTRSFFLQSSIKMRFVMHDLLNDLAKYVGGNFCFMFKFDKGVRIPKTT
RHFSFSIDNEVYDDGMGSLIDAKRLRSFIPMTNNIDGLPCALILIDELFSKFKFLRVLSLSGFGDLITEVPDSVGALKQLRSL
DLSYTNIQKLPDSICLLFNLLILKLNYCSDLEELPSNIHKLTKLHCLEFENTKVTKMPMHFGELKNLHLLDAFRVHSESSIKQL
GGLNLHGSLSIYQVQNIVNPLDALEANLKDKQLVELGLIWNSNHVPNDAREEKEVLENLQPSIHLEHLSIWSYHGIEFPS
WLFDNSLSNLVFLSLNNCKNCLCLPSLGELSSLKNLEIEGLDGIVSIGVSDGFYGSNSSSFASMERLSFRNMKEWEEWEC

PQVEMFEEGSLPSNLKEVSLSSFRLITSLREALGAEPCLERLYVENVDTEYFPDEGLLPPSLTFLRIFNCPNLKKLDYKGLSHL
SSLTIIRCFNLQCLPEEGLPKSISFLEIRSCPLISERYQNPQDQDWKKIAHIKEPIIV

>XP_027923104.1

MAAEVVGGALLSAFLQVAFDRLASPQFVDFFRGRKLDEKLLGNLNIMLHSINALADDAEQKQFTDPHVKAWLLSVKEA
VFDAEDLLGEIDYELTRCQVEAGSEPQTFTDKVSNFFNSTFSSFNKKIESEMKEVLEKLKYLEQQKDALGLKNGTSYSDNK
VSQKLPSSSLVVESIIYGRDADKEIIFNWLTSETDNHNHPSILSIVGMGGLGKTTLAQHVYRDPKMEEAKFDIRAWICVSD
HFDVLTVTKTILEAITNSKDDSGNLEMVHGRLKDQISGRKFLLVLDDVWNEGGEEWEAVRTPLSYGAPGSKILVTARAE
KVASNMRSKVHRLKQLEDDESWNVFKKQALKDDDLEWNDEKEEIGRRIVEKCKGLPLALKTIGSLLCKESSISYWKMVL
ENDIWELPKEVKIIPALLLSYQHLPSHLKRCFAYCALFPKDYGFDKKELILLWMAEGFLHHSQHINNVEEIGEQYFNDLLTR
SFFLQSDFKTHFSMHDLLNDLAKYVCAEFCFRLKFDKGNCIPKTIRHFSFAFDDVECFDGFGSLIDAKRLRSFFPYKEFGRR
YIDHYPLQFKILVHELFPNFKFLRVLSLDQYSELREVPDSVGDLKHLHSLDLSSTGIQKLPDSTCLLYNLLILKLNYCSSLEELP
LNLHKLTKLRCLEFISTKVTKMPMHFGELKNLQVLSAVFVDKNNEFSTKDLGVLNLHGRLSINEVQNIVNPVDALEANLK
NKDLVKLELKWKSDHIPDDPRKEKKVLENLQPSKNVEHLSIENYGGTQFPSWVFDNSLSNLVFLMLKDCKYCLCMPPLG
LLSSLKTLEIIGFDGIVSIGAEFYGNSSSSFTSLERLTFSDMKELEECEHKTAAFPRLETLKVYQCPKLKGLPDQLVNVKYLNI
RDSMKASCLERCEHIVSHNSLEDLNFCAFPIMNIPMSRSFDLLEQIEIISGCDSLTTFPLDFFPNLKALSLFLCHNLQIISQKH
THNRLKHLSILKCSRFDSFPSEGLSAPRLQRMYIDGAENLKLLPKRMQILLPSLSELQIINCPKVEDFPDGGLPPNVKKVSLS
SFKLMASLRETLGTNTCLQSLSIEEMDVEFFPDEVLLPQSITSLRIYDCPNLKKMEYKGLCHLSSLTLSNCPNLQCLPEDGL
PKSISCLEIWNCPLLEQRCQNPEGQDWKKIAHIQKLSVRS

>XP_027923119.1

MAAEVVGGALLSAFLQVAFDRLASPQFVDFFRGRKLDEKLLGNLNIMLHSINALADDAEQKQFTDPHVKAWLLSVKEA
VFDAEDLLGEIDYELTRCQVEAGSEPQTFTDKVSNFFNSTFSSFNKKIESEMKEVLEKLKYLEQQKDALGLKNGTSYSDNK
VSQKLPSSSLVVESIIYGRDADKEIIFNWLTSETDNHNHPSILSIVGMGGLGKTTLAQHVYRDPKMEEAKFDIRAWICVSD
HFDVLTVTKTILEAITNSKDDSGNLEMVHGRLKDQISGRKFLLVLDDVWNEGGEEWEAVRTPLSYGAPGSKILVTARAE
KVASNMRSKVHRLKQLEDDESWNVFKKQALKDDDLEWNDEKEEIGRRIVEKCKGLPLALKTIGSLLCKESSISYWKMVL
ENDIWELPKEVKIIPALLLSYQHLPSHLKRCFAYCALFPKDYGFDKKELILLWMAEGFLHHSQHINNVEEIGEQYFNDLLTR
SFFLQSDFKTHFSMHDLLNDLAKYVCAEFCFRLKFDKGNCIPKTIRHFSFAFDDVECFDGFGSLIDAKRLRSFFPYKEFGRR
YIDHYPLQFKILVHELFPNFKFLRVLSLDQYSELREVPDSVGDLKHLHSLDLSSTGIQKLPDSTCLLYNLLILKLNYCSSLEELP
LNLHKLTKLRCLEFISTKVTKMPMHFGELKNLQVLSAVFVDKNNEFSTKDLGVLNLHGRLSINEVQNIVNPVDALEANLK
NKDLVKLELKWKSDHIPDDPRKEKKVLENLQPSKNVEHLSIENYGGTQFPSWVFDNSLSNLVFLMLKDCKYCLCMPPLG
LLSSLKTLEIIGFDGIVSIGAEFYGNSSSSFTSLERLTFSDMKELEECEHKTAAFPRLETLKVYQCPKLKGLPDQLVNVKYLNI
RDSMKASCLERCEHIVSHNSLEDLNFCAFPIMNIPMSRSFDLLEQIEIISGCDSLTTFPLDFFPNLKALSLFLCHNLQIISQKH
THNRLKHLSILKCSRFDSFPSEGLSAPRLQRMYIDGAENLKLLPKRMQILLPSLSELQIINCPKVEDFPDGGLPPNVKKVSLS
SFKLMASLRETLGTNTCLQSLSIEEMDVEFFPDEVLLPQSITSLRIYDCPNLKKMEYKGLCHLSSLTLSNCPNLQCLPEDGL
PKSISCLEIWNCPLLEQRCQNPEGQDWKKIAHIQKLSVRS

>XP_027923239.1

MQPSLSSFCYKFRYDVFLSFRGKDTRHGFTGNLYKSLCHRGIHSFFDDHHLPKGDHISSELENTIQDSRIFIIILSENYASSSF
CLNELDYILRFIKHKGRLVFPVFYGVDPSDVRHHMGTFGEALAHHEKKLNSYYNMEKLERWKMALHQVANLSGYHFK
HGEGYEYEFIERIVELVCSKINRVALHVSDYPVGLESQVLQVKMLLDVGSDDVVHMVGFHGLGGVGKTTLVVAVYNSIA
DHFEALCFLENVREASSKHGLLHLQSTLLSETVGEIKLTSVKKGISIIQHRLQQKKVLLILDDVDKEEQLQALAGRPHWFGL
GSRVIITTRDKQLLKSHGVERTYEVKELNEENALELLTWKAFKFENFDPSYKDVLNLAVTYASGLPLALEVIGSNLFGRNIE
QWKSGLDQYKKIPKMDIQDTLKVSYDALEEDEQSVFLDIACFFKNYDLAEVEDILRAHHGHNIKHHIDVLVEKSLIKISLD

GKVTLHDLIKDMGREIVRRESPKEPGKRSRLWFLEDIVQVLEDNKGSGKIETICMDFPSFEEVEIEWDGDAFKKMKNLRT
LIIRNGHFSTGPKHLPNSLRVMEWWRYPSQNFPQDFHPKKLSIFKLPYCEFTSLELNDLVRQASVLTSFPGYIYVHKFSFTL
LIFFFFFSLLQKFVNMTSLNFDECRYLKQIPDVSCLTHLENLSFRWCPKLSSLHYSVGFLEKLKILDAEGCSKLKSFPPIKLTSL
EQLKLRYCHSLENFPEVLGNMENVRELDLKDTPIKKFPLSFQNLTRLQKLHLCLSCGVMENGCDGVPLSSICMMPELVDI
VANEWKGELFCKANEGVEKVSSIFYTNVQYLQLRCCNLTDDFFLTLLPWFANMKNLDLSGNNFIIIPECIKEFHFLTRLNL
NFCEQLQEIKGIPPNLKYFSAIDCISLTSSCRSMLLNQELHEAGSTFFYLPGAKIVEWFEFQTLEVPICFWFHGKLPAMVIC
LAMERMCEYSSSKGCKYRPLVIHSTFRLMSPIVIINGNEHLLQTWEMMDDCTCVFDLRETKLKNDFDEQLLENEWNHA
EVTCRYVSLGQTLIKHGIHVLKQDSSVEEIRFTDPCKKRNLDIMSSIAQNHSSNNC

>XP_027923240.1

MQPSLSSFCYKFRYDVFLSFRGKDTRHGFTGNLYKSLCHRGIHSFFDDHHLPKGDHISSELENTIQDSRIFIIILSENYASSSF
CLNELDYILRFIKHKGRLVFPVFYGVDPSDVRHHMGTFGEALAHHEKKLNSYYNMEKLERWKMALHQVANLSGYHFK
HGEGYEYEFIERIVELVCSKINRVALHVSDYPVGLESQVLQVKMLLDVGSDDVVHMVGFHGLGGVGKTTLVVAVYNSIA
DHFEALCFLENVREASSKHGLLHLQSTLLSETVGEIKLTSVKKGISIIQHRLQQKKVLLILDDVDKEEQLQALAGRPHWFGL
GSRVIITTRDKQLLKSHGVERTYEVKELNEENALELLTWKAFKFENFDPSYKDVLNLAVTYASGLPLALEVIGSNLFGRNIE
QWKSGLDQYKKIPKMDIQDTLKVSYDALEEDEQSVFLDIACFFKNYDLAEVEDILRAHHGHNIKHHIDVLVEKSLIKISLD
GKVTLHDLIKDMGREIVRRESPKEPGKRSRLWFLEDIVQVLEDNKGSGKIETICMDFPSFEEVEIEWDGDAFKKMKNLRT
LIIRNGHFSTGPKHLPNSLRVMEWWRYPSQNFPQDFHPKKLSIFKLPYCEFTSLELNDLVRQKFVNMTSLNFDECRYLKQ
IPDVSCLTHLENLSFRWCPKLSSLHYSVGFLEKLKILDAEGCSKLKSFPPIKLTSLEQLKLRYCHSLENFPEVLGNMENVREL
DLKDTPIKKFPLSFQNLTRLQKLHLCLSCGVMENGCDGVPLSSICMMPELVDIVANEWKGELFCKANEGVEKVSSIFYTN
VQYLQLRCCNLTDDFFLTLLPWFANMKNLDLSGNNFIIIPECIKEFHFLTRLNLNFCEQLQEIKGIPPNLKYFSAIDCISLTSS
CRSMLLNQELHEAGSTFFYLPGAKIVEWFEFQTLEVPICFWFHGKLPAMVICLAMERMCEYSSSKGCKYRPLVIHSTFRL
MSPIVIINGNEHLLQTWEMMDDCTCVFDLRETKLKNDFDEQLLENEWNHAEVTCRYVSLGQTLIKHGIHVLKQDSSVE
EIRFTDPCKKRNLDIMSSIAQNHSSNNC

>XP_027923241.1

MGTFGEALAHHEKKLNSYYNMEKLERWKMALHQVANLSGYHFKHGEGYEYEFIERIVELVCSKINRVALHVSDYPVGL
ESQVLQVKMLLDVGSDDVVHMVGFHGLGGVGKTTLVVAVYNSIADHFEALCFLENVREASSKHGLLHLQSTLLSETVG
EIKLTSVKKGISIIQHRLQQKKVLLILDDVDKEEQLQALAGRPHWFGLGSRVIITTRDKQLLKSHGVERTYEVKELNEENAL
ELLTWKAFKFENFDPSYKDVLNLAVTYASGLPLALEVIGSNLFGRNIEQWKSGLDQYKKIPKMDIQDTLKVSYDALEEDE
QSVFLDIACFFKNYDLAEVEDILRAHHGHNIKHHIDVLVEKSLIKISLDGKVTLHDLIKDMGREIVRRESPKEPGKRSRLWF
LEDIVQVLEDNKGSGKIETICMDFPSFEEVEIEWDGDAFKKMKNLRTLIIRNGHFSTGPKHLPNSLRVMEWWRYPSQNF
PQDFHPKKLSIFKLPYCEFTSLELNDLVRQKFVNMTSLNFDECRYLKQIPDVSCLTHLENLSFRWCPKLSSLHYSVGFLEKL
KILDAEGCSKLKSFPPIKLTSLEQLKLRYCHSLENFPEVLGNMENVRELDLKDTPIKKFPLSFQNLTRLQKLHLCLSCGVME
NGCDGVPLSSICMMPELVDIVANEWKGELFCKANEGVEKVSSIFYTNVQYLQLRCCNLTDDFFLTLLPWFANMKNLDL
SGNNFIIIPECIKEFHFLTRLNLNFCEQLQEIKGIPPNLKYFSAIDCISLTSSCRSMLLNQELHEAGSTFFYLPGAKIVEWFEFQ
TLEVPICFWFHGKLPAMVICLAMERMCEYSSSKGCKYRPLVIHSTFRLMSPIVIINGNEHLLQTWEMMDDCTCVFDLRE
TKLKNDFDEQLLENEWNHAEVTCRYVSLGQTLIKHGIHVLKQDSSVEEIRFTDPCKKRNLDIMSSIAQNHSSNNC

>XP_027923243.1

MVGLESQVLQVKMLLDVGSDDVVHMVGFHGLGGVGKTTLVVAVYNSIADHFEALCFLENVREASSKHGLLHLQSTLLS
ETVGEIKLTSVKKGISIIQHRLQQKKVLLILDDVDKEEQLQALAGRPHWFGLGSRVIITTRDKQLLKSHGVERTYEVKELNE
ENALELLTWKAFKFENFDPSYKDVLNLAVTYASGLPLALEVIGSNLFGRNIEQWKSGLDQYKKIPKMDIQDTLKVSYDAL
EEDEQSVFLDIACFFKNYDLAEVEDILRAHHGHNIKHHIDVLVEKSLIKISLDGKVTLHDLIKDMGREIVRRESPKEPGKRS

RLWFLEDIVQVLEDNKGSGKIETICMDFPSFEEVEIEWDGDAFKKMKNLRTLIIRNGHFSTGPKHLPNSLRVMEWWRY
PSQNFPQDFHPKKLSIFKLPYCEFTSLELNDLVRQKFVNMTSLNFDECRYLKQIPDVSCLTHLENLSFRWCPKLSSLHYSV
GFLEKLKILDAEGCSKLKSFPPIKLTSLEQLKLRYCHSLENFPEVLGNMENVRELDLKDTPIKKFPLSFQNLTRLQKLHLCLSC
GVMENGCDGVPLSSICMMPELVDIVANEWKGELFCKANEGVEKVSSIFYTNVQYLQLRCCNLTDDFFLTLLPWFANM
KNLDLSGNNFIIIPECIKEFHFLTRLNLNFCEQLQEIKGIPPNLKYFSAIDCISLTSSCRSMLLNQELHEAGSTFFYLPGAKIVE
WFEFQTLEVPICFWFHGKLPAMVICLAMERMCEYSSSKGCKYRPLVIHSTFRLMSPIVIINGNEHLLQTWEMMDDCTC
VFDLRETKLKNDFDEQLLENEWNHAEVTCRYVSLGQTLIKHGIHVLKQDSSVEEIRFTDPCKKRNLDIMSSIAQNHSSNN
C

>XP_027923244.1

MQPSLSSFCYKFRYDVFLSFRGKDTRHGFTGNLYKSLCHRGIHSFFDDHHLPKGDHISSELENTIQDSRIFIIILSENYASSSF
CLNELDYILRFIKHKGRLVFPVFYGVDPSDVRHHMGTFGEALAHHEKKLNSYYNMEKLERWKMALHQVANLSGYHFK
HGEGYEYEFIERIVELVCSKINRVALHVSDYPVGLESQVLQVKMLLDVGSDDVVHMVGFHGLGGVGKTTLVVAVYNSIA
DHFEALCFLENVREASSKHGLLHLQSTLLSETVGEIKLTSVKKGISIIQHRLQQKKVLLILDDVDKEEQLQALAGRPHWFGL
GSRVIITTRDKQLLKSHGVERTYEVKELNEENALELLTWKAFKFENFDPSYKDVLNLAVTYASGLPLALEVIGSNLFGRNIE
QWKSGLDQYKKIPKMDIQDTLKVSYDALEEDEQSVFLDIACFFKNYDLAEVEDILRAHHGHNIKHHIDVLVEKSLIKISLD
GKVTLHDLIKDMGREIVRRESPKEPGKRSRLWFLEDIVQVLEDNKGSGKIETICMDFPSFEEVEIEWDGDAFKKMKNLRT
LIIRNGHFSTGPKHLPNSLRVMEWWRYPSQNFPQDFHPKKLSIFKLPYCEFTSLELNDLVRQKFVNMTSLNFDECRYLKQ
IPDVSCLTHLENLSFRWCPKLSSLHYSVGFLEKLKILDAEGCSKLKSFPPIKLTSLEQLKLRYCHSLENFPEVLGNMENVREL
DLKDTPIKKFPLSFQNLTRLQKLHLCLSCGVMENGCDGVPLSSICMMPELVDIVANEWKGTA

>XP_027923280.1

MAQRISSSLSSPRRTWIYDVFLSFRGEDTRFQFTNNLYHSLCQKGIRTFIDQDGLRRGEEITPALFHAIQNSMISIVVFSES
YASSTYCLNELVKILEGAKEEGRSIYPIFYGVDPSEVRHHTGTYAEALSKHEAKFHNDADTEKVQKWRKALHEAANLSG
WHCKYWYQPEYEFIRKIVEEISEKINYIPLHVADNPIGLEYAVKGVKSLLGDGSDVNMIGIYGIGGIGKTTTARAVYNIIFW
HYEGSCFLPDIREKAVNKNEIVQLQEILLSQILKGEDIKVGDVNRGIPLIKQRLQQKKVLLVLDDVDKLKQLKALAGGCDW
FGSGSIIIITTRDKHLLDAHGVVNLYEVKPLHFEKALELFNLHAFRSGKVGPPYMSISKRAVSYACGLPLALEVIGSHLFGKS
LDECNSALDKYESIPHQKIHEILKVSYDGLEENEKGIFLDIACFFNTYELGNVTPMLKAHGFHVEDGLRVLVDKSLIKIDSFG
FVRIHDLIRDTGREIVRQESTLEPGRRSRLWFDQDIVHVLEENTGSDRIEFIKLEEYNNVQVQWDGKAFKEMKNLKILIIE
DATFSVGPEHLPNS

>XP_027923312.1

MAELVGGALLSAFLQVAFDRLASTQFVDFFRGRKLDEKLLDNLNIMLHSINSLADDAEQKQFRDPHVKAWLLSVKEAVF
DAEDLLGEIDYELTRCKLEAESQPQTFTYKVSNFFNSTFNSFNKKIESEMKEVLEKLKYLEQQKDALGLKKGTSYSDSKASQ
KLPSSSLVVEGIIYGRDADKDIIFNWLRSETDNHNHPSIFSIVGMGGLGKTTLAQHVYHDPKMEEAKFDIKAWVCVSDH
FDVLTVTKTILEAITNSKDDSGNLEMVHGRLKDKISGRKFLLVLDDVWNEGGEEWEAVRTPLSYGAPGSKILVTTRSKKV
ASNMRSRVHLLEELREDKSWNVFEKHALKDDEIELNDDLKEIGRRIVVKCKGLPLALKTIGRLLHTKSSISDWENILESDM
WNLPKEASEIIPALLLSYHYLPTPLKRCFAYCALFPKDYEFVKKELILLWMAENFLHCPQEIRHPEEVGEQYFNDLLSRSFF
QQYKNNFVMHDLLNDLAKYVYGDICFRLKFDKGKYIPKTTRHFLFTPGDVKCFNGLESLSDAKRLRSFIAIVRLRGRYLCN
YPWQFMISLHDLFSKIKFLRVLSLYGFSGLRKVPDSIGNLKHLQSIDLEYTHIQKLPDSIGLLYNLLILKLSNCSYLEELPSNLH
KLTKLRCLEFEGTKVTKMPKHFGELKNLQVLSTFLVDRNNECNIKQLGGLNLQGSLSIKEVQNIVNPLDALEANLKNKHL
VNLELEWKRKHIPDDPLKEKKVLENLQPSKHLERLSIKNYGGTEFPSWVFNNSLSTLVFLCLENCKYCLCLPPLGLLSSLKTL
KIRGFDGIVSIGAEFCGSNSTSFTSLESLEIDNLKEWEEWECKTTFPCLRYLFINRCPKLKGTSEQLLNLKELFVSLRGKLIIYS
CPLVNIPITHYDFLEEVIINGGCDSLTIFPLDFFPKLHLLHITGSRNLRGISNEHTHNHLKEMKIDECPQFESFPSEGLSAPQL

WKIEIKGAKNLKLLPKRMQILLPSLTELRITDCPQVEMFEEGSLPSNLKEVSLSCFRLIASLREALGADTCLETLSIIEVDVQC
FPDEGLLPPSLTSLKIYNCPTLKKLNYKGLSHLSSLRLFRCPSLECLPEEGLPKSISFLQIWYCPLLRPRCQNSEGEDWGKIAH
IQQLSID

>XP_027923315.1

MAELVGGALLSAFLQVAFDKLASTQFVDFFRGRKLDEKLLDNLNIMLHSINSLADDAEQKQFTDPHVKAWLLSVKEAVF
DAEDLLGEIDYELTRCQVEAGSEPQTFTYKVSTFFNSTFSSFNKKIELEMKEVLEKLKYLEQQKDALGLKKGTSYSDSKVSQ
KLPSSSLVVESIIYGRDADKEIIFNWLTSETDNHNHPSILSIVGMGGLGKTTLAQHVYRDPKMEEAKFDIRAWVCVSDHF
DVLTVTKTILEAITKSKDDSGDLEMVHGRLKDEISGRKFLLVLDDVWNEGGEEWEAVRTPLSYGAPGSKILVTTRSKKVA
SNMRSRVHLLEELREDKSWNVFEKHALKDGEIELNDDLKEIGRRIVVKCKGLPLALKTIGRLLHTKSSISDWQNILESDM
WNLPKEASEIIPALLLSYHYLPTPLKRCFAYCALFPKDYEFAKKELILLWMAENFLHCPQEIRHPEEVGEQYFNDLLSRSFF
QQYEGNFVMHDLLNDLAKYVYGDICFRLKFDKGKYIPKTTRHFLFTPGDVKCFNGLESLSDAKRLRSFIAIARLRGRYLGN
YPWQFMISLHDLFSKIKFLRVLSFYGISDLKKVPDSIGNLKHLQSIDLEYTNIQKLPDSIGLLYNLLILKLSNCYDLEELPSNLH
KLTKLRCLEFERTKVTKMPKHFGELKNLQVLSTFLVDRNNECNIKQLGGLNLHGSLSIKEVQNIVNPLDALEANLKNKHLV
NLELEWKRKHIPDDPLKEKKVLENLQPSKHLERLSIENYGGTEFPSWVFNNSLSTLVFLCLENCKYCLCLPPLGLLSSLKTLK
IRGFDGIVSIGAEFCGSNSTSFTSLESLEIDNLKEWEEWECKTTFPCLRYLFINRCPKLKGTSEQLLNLKELFVSLRGKLIIYSC
PLVNIPITHYDFLEEVIINGGCDSLTIFPLDFFPKLHLLHITGLRNLRGISHEHTHNHLKEMEIDECPQFESFPSEGLSAPQL
WKIEIKGPRNLKLLPKRMQILLPSLTELRITDCPQVEMFEEGSLPSNLKEVSLSCFRLIASLREALGANTCLETLSIEEVDVQC
FPDEGLLPPSLTFLKIYDCPTLKKLNYKGLCHLSSLRLFGCPNLECLPEEGLPKSISFLHIWYCPLLRPRCQNSEGEDWGKIA
HIQQLSID

>XP_027923343.1

MVKYLTINFIIVVRKLHFKLYISFHHFSTPTMAAEVVGGALLSAFLQVAFDRLASPQVLQFFRGRKLDEKLLSNLNIMLHSI
NALADDAEQKQFRNPHIKAWLFAVKDVVFDAEDLLKEIEYELTRCQVEARSDPQTLTSKVSNFFNSTFTSFNKKIESEMR
DVLEKLEYLAKQKGALGLKEGIYSGDGSGSKESQKLPSSSLVVESVIYGRDADKEMILNWLTSETENHDQPSILSIVGMG
GLGNKTTLAQHVYNDPKIEEAKFDIRAWVCVSDHFDVLIVTKTILEAITKSKDDSGDLEMVHGRLKEKISGRKFLLVLDDV
WNERREEWEAVRTPLSYGAPGSRIIVTTRGEKVASNMMSEVHRLKQLEEEECWKLLKSMH

>XP_027923344.1

MVKYLTINFIIVVRKLHFKLYISFHHFSTPTMAAEVVGGALLSAFLQVAFDRLASPQVLQFFRGRKLDEKLLSNLNIMLHSI
NALADDAEQKQFRNPHIKAWLFAVKDVVFDAEDLLKEIEYELTRCQVEARSDPQTLTSKVSNFFNSTFTSFNKKIESEMR
DVLEKLEYLAKQKGALGLKEGIYSGDGSGSKESQKLPSSSLVVESVIYGRDADKEMILNWLTSETENHDQPSILSIVGMG
GLGNKTTLAQHVYNDPKIEEAKFDIRAWVCVSDHFDVLIVTKTILEAITKSKDDSGDLEMVHGRLKEKISGRKFLLVLDDV
WNERREEWEAVRTPLSYGAPGSRIIVTTRGEKVASNMMSEVHRLKQLEEEECWKLLKSMH

>XP_027923347.1

MVKYLTINFIIVVRKLHFKLYISFHHFSTPTMAAEVVGGALLSAFLQVAFDRLASPQVLQFFRGRKLDEKLLSNLNIMLHSI
NALADDAEQKQFRNPHIKAWLFAVKDVVFDAEDLLKEIEYELTRCQVEARSDPQTLTSKVSNFFNSTFTSFNKKIESEMR
DVLEKLEYLAKQKGALGLKEGIYSGDGSGSKESQKLPSSSLVVESVIYGRDADKEMILNWLTSETENHDQPSILSIVGMG
GLGNKTTLAQHVYNDPKIEEAKFDIRAWVCVSDHFDVLIVTKTILEAITKSKDDSGDLEMVHGRLKEKISGRKFLLVLDDV
WNERREEWEAVRTPLSYGAPGSRIIVTTRGEKVASNMMSEVHRLKQLEEEECWKLLKSMH

>XP_027923348.1

MVKYLTINFIIVVRKLHFKLYISFHHFSTPTMAAEVVGGALLSAFLQVAFDRLASPQVLQFFRGRKLDEKLLSNLNIMLHSI
NALADDAEQKQFRNPHIKAWLFAVKDVVFDAEDLLKEIEYELTRCQVEARSDPQTLTSKVSNFFNSTFTSFNKKIESEMR
DVLEKLEYLAKQKGALGLKEGIYSGDGSGSKESQKLPSSSLVVESVIYGRDADKEMILNWLTSETENHDQPSILSIVGMG
GLGNKTTLAQHVYNDPKIEEAKFDIRAWVCVSDHFDVLIVTKTILEAITKSKDDSGDLEMVHGRLKEKISGRKFLLVLDDV
WNERREEWEAVRTPLSYGAPGSRIIVTTRGEKVASNMMSEVHRLKQLEEEECWKLLKSMH

>XP_027923349.1

MVKYLTINFIIVVRKLHFKLYISFHHFSTPTMAAEVVGGALLSAFLQVAFDRLASPQVLQFFRGRKLDEKLLSNLNIMLHSI
NALADDAEQKQFRNPHIKAWLFAVKDVVFDAEDLLKEIEYELTRCQVEARSDPQTLTSKVSNFFNSTFTSFNKKIESEMR
DVLEKLEYLAKQKGALGLKEGIYSGDGSGSKESQKLPSSSLVVESVIYGRDADKEMILNWLTSETENHDQPSILSIVGMG
GLGNKTTLAQHVYNDPKIEEAKFDIRAWVCVSDHFDVLIVTKTILEAITKSKDDSGDLEMVHGRLKEKISGRKFLLVLDDV
WNERREEWEAVRTPLSYGAPGSRIIVTTRGEKVASNMMSEVHRLKQLEEEECWKLLKSMH

>XP_027923350.1

MVKYLTINFIIVVRKLHFKLYISFHHFSTPTMAAEVVGGALLSAFLQVAFDRLASPQVLQFFRGRKLDEKLLSNLNIMLHSI
NALADDAEQKQFRNPHIKAWLFAVKDVVFDAEDLLKEIEYELTRCQVEARSDPQTLTSKVSNFFNSTFTSFNKKIESEMR
DVLEKLEYLAKQKGALGLKEGIYSGDGSGSKESQKLPSSSLVVESVIYGRDADKEMILNWLTSETENHDQPSILSIVGMG
GLGNKTTLAQHVYNDPKIEEAKFDIRAWVCVSDHFDVLIVTKTILEAITKSKDDSGDLEMVHGRLKEKISGRKFLLVLDDV
WNERREEWEAVRTPLSYGAPGSRIIVTTRGEKVASNMMSEVHRLKQLEEEECWKLLKSMH

>XP_027923351.1

MVKYLTINFIIVVRKLHFKLYISFHHFSTPTMAAEVVGGALLSAFLQVAFDRLASPQVLQFFRGRKLDEKLLSNLNIMLHSI
NALADDAEQKQFRNPHIKAWLFAVKDVVFDAEDLLKEIEYELTRCQVEARSDPQTLTSKVSNFFNSTFTSFNKKIESEMR
DVLEKLEYLAKQKGALGLKEGIYSGDGSGSKESQKLPSSSLVVESVIYGRDADKEMILNWLTSETENHDQPSILSIVGMG
GLGNKTTLAQHVYNDPKIEEAKFDIRAWVCVSDHFDVLIVTKTILEAITKSKDDSGDLEMVHGRLKEKISGRKFLLVLDDV
WNERREEWEAVRTPLSYGAPGSRIIVTTRGEKVASNMMSEVHRLKQLEEEECWKLLKSMH

>XP_027923352.1

MVKYLTINFIIVVRKLHFKLYISFHHFSTPTMAAEVVGGALLSAFLQVAFDRLASPQVLQFFRGRKLDEKLLSNLNIMLHSI
NALADDAEQKQFRNPHIKAWLFAVKDVVFDAEDLLKEIEYELTRCQVEARSDPQTLTSKVSNFFNSTFTSFNKKIESEMR
DVLEKLEYLAKQKGALGLKEGIYSGDGSGSKESQKLPSSSLVVESVIYGRDADKEMILNWLTSETENHDQPSILSIVGMG
GLGNKTTLAQHVYNDPKIEEAKFDIRAWVCVSDHFDVLIVTKTILEAITKSKDDSGDLEMVHGRLKEKISGRKFLLVLDDV
WNERREEWEAVRTPLSYGAPGSRIIVTTRGEKVASNMMSEVHRLKQLEEEECWKLLKSMH

>XP_027923353.1

MVKYLTINFIIVVRKLHFKLYISFHHFSTPTMAAEVVGGALLSAFLQVAFDRLASPQVLQFFRGRKLDEKLLSNLNIMLHSI
NALADDAEQKQFRNPHIKAWLFAVKDVVFDAEDLLKEIEYELTRCQVEARSDPQTLTSKVSNFFNSTFTSFNKKIESEMR
DVLEKLEYLAKQKGALGLKEGIYSGDGSGSKESQKLPSSSLVVESVIYGRDADKEMILNWLTSETENHDQPSILSIVGMG
GLGNKTTLAQHVYNDPKIEEAKFDIRAWVCVSDHFDVLIVTKTILEAITKSKDDSGDLEMVHGRLKEKISGRKFLLVLDDV
WNERREEWEAVRTPLSYGAPGSRIIVTTRGEKVASNMMSEVHRLKQLEEEECWKLLKSMH

>XP_027923354.1

MPALLLSYQHLPSHLKRCFAYCALFPKDYEFNKKELILLWMAEDFLNHSKQIKSVHEIGEQYFDDLLTRSFFLQLSSEMRQ
IKNVREIGEQYFHDLLTRSFFLESSFGMRFVMHDLLNDLAKYVCADFCFRFKYDKGNIPNTTRHFSFSFDDVEYFDDLGSL

IDAKRLRSFHSITNDCSYDFNPCQFNILVHELFSKLKFLRVLSLNGYSQLSEVPNSLGDLKHLCSIDLSYTHIQKLPDSICLLY
NLLIMKMNFCSFLEELPSNLHKLTKLNCLEFEHTNVRKMPMHFGELKNLQVLSTFSVDRDSKVINIKQLGGLNLHGRLSI
NESITESGMC

>XP_027923472.1

MAAEVVGGALLSAFLQVAFDKLASTQFVDFFRGRKLDEKLLGNLNIMLHSINALADDAEHKQFTDPHVKAWVLSVKEA
VFDAEDLLGEIDYELTRCQVEAGSEPQTFTYKVSTFFNSTFSSFNKKIESEMKEVLEKLKYLEQQKDALGLKNGTSYSDNK
VSQKLPSSSLVVQSIIYGRDADKEIIFNWLRSETDNHNHPSIFSIVGMGGLGKTTLAQHVYHDPKMEEAKFDIRAWVCVS
DHFDVLTVTKTILEAITNSKDDSGNLEMVHGRLKDQISGRKFLLVLDDVWNEGGEEWEAVRTPLSYGAPGSKILVTARA
EKVASNMRSKVHLLRELRGDECWNVFKKHALRDDDLELNDDLKEIGRRIVVKCKGLPLALKTIGRLLHTKSSISDWENILE
SDMWNLPKEASEIIPALLLSYHYLPTPLKRCFAYCALFPKDYEFAKKELILLWMAENFLHCPQEIRHPEEVGEQYFNDLLSR
SFFQQYEGNFVMHDLLNDLAKYVYGDICFRLKFDKGKYIPKTTRHFLFTPGDVKCFNGLESLSDAKRLRSFIAIARLRGRYL
GNYPWQFMISLHDLFSKIKFLRVLSFYGISDLKKVPDSIGNLKHLQSIDLEYTNIQKLPDSIGLLYNLLILKLSNCYDLEELPSN
LHKLTKLRCLEFERTKVTKMPKHFGELKNLQVLSTFLVDRNNECNIKQLGGLNLHGSLSIKEVQNIVNPLDALEANLKNKH
LVNLELEWKRKHIPDDPLKEKKVLENLQPSKHLERLSIENYGGTEFPSWVFNNSLSTLVFLCLENCKYCLCLPPLGLLSSLKT
LKIRGFHGIVSIGAEFCGSNSTSFTSLESLEIDNLKEWEEWECKTTFPCLRYLFINRCPKLKGTSEQLLNLKELFVSLRGKLIIY
SCPLVNIPITHYDFLEEVIIDGGCDSLTIFPLDFFPKLHLLHITGSRNLRGISNEHTHNHLKEMKIDECPQFESFPSEGLSAPQ
LWKIEIKGARNLKLLPKRMQILLPSLTELRITDCPQVEMFEEGSLPSNLKEVSLSCFRLIASLREALGADTCLETLSIEEVDVQ
CFPDEGLLPPSLTSLEIYDCPTLKKLNYKGLSHLSSLRLFGCPNLECLPEEGLPKSISFLEIWNCPLLNPRCQNSESEDWGKIA
HIQQLRVGKWEKDKEVQN

>XP_027923493.1

MAELVGGALLSAFVNVAFDKLASPKVVGFFRGRKLDEKLLGNLNIMLHSINALAHDAEQKQFRDPHVKAWLFAVKEAV
FDAEDLLKEIEYELTRCQVEAGSDPQTLTSKVSNFFNSTFTSFNKKIESEIREVLEKLEYLARQKGALGLKESIYSGDGSGSKE
PQKMPSSSLVVESVIYGRDADKEKILNWLTSETENHDQPSILSIVGMGGLGKTTLAQHVYNDTKLEEAKFDIRAWVCVS
DHFNVLTVTKTILEAVTKSKDDSGDLQMVHERLKEKISGKKFFLVLDDVWNERQEKWEAVRTPLSYGAPGSRILVTTRG
EKVASNMSSKVHRPKHLKEDECWKVFQKHALRGDELELNDEKKEIGRRIVEKCKGLPLALKTIGSVLRTKSSISDWQSVL
ESDIWDLPKEFEIMPALLLSYQHLPSHLKRCFASCALFPKDYEFDKKELILLWIAQGFLHCSPQSNNLEEIGEQYFNDLLMR
SFFLQSDFKTCFFMHDLLNDLAKYVCADFCFRLKFDKGNCISKTTRHFSFAFSDVKYFDGFGSLTDAKRLRSFFPYKEFGR
RDNDYYPLQFKILVHELFSNFKFLRVLSLDAYSELREVPDSVGDLKHLHSLDLSRTGIQKLPDSTCLLYNLLILKLNYCSSLEE
LPLHLHKLTKLRCLEFKKTKVTKMPTHFGELKNLQVLSAVFVDKKKEFSTKHLGGLNLHGRLSINELQNIVNPVDALEANL
NNKHLVKLELRWKSDHIPDDPRKEKKVLENLKPSKKLEHLSIKSYGGPEFPSWVFDNSLSNLVFLKLKDCKYCLCLPPLGLL
SSLKTLEIIGLDGIVSIGAEFYGNSYSSFTSLERLTFSNMKELEECERKTAAFPRLQILKVYQCPKLKGLPDQLVNVKYLYITGS
MKASFLERCEHTVSHNSLEDLNFCAFPIMNIPMGSSYDLLANIKISCGCDSLTTFPLDFFPNLKSLSLLFCRNLQIISQKHTH
NRLKHLSVAGCSRFDSFPSEGLSAPRLQTIDIHGAENLKLLPKRMQILLPSLHVLKIIHCPKVEMFPDGGLPPNVEDVSLSS
FILMASLRETLGTNTCLQSLSIKYMDVEFFPDEVLLPHSITSLKLSDCPNLKKMEYKGLCHLSSLSLHNCPNLQCLPEDGLP
KSISSLEILNCPLLEQRCQNPQGQDWKKIAHIEKLIVRSKV

>XP_027923494.1

MLHSINALAHDAEQKQFRDPHVKAWLFAVKEAVFDAEDLLKEIEYELTRCQVEAGSDPQTLTSKVSNFFNSTFTSFNKKI
ESEIREVLEKLEYLARQKGALGLKESIYSGDGSGSKEPQKMPSSSLVVESVIYGRDADKEKILNWLTSETENHDQPSILSIV
GMGGLGKTTLAQHVYNDTKLEEAKFDIRAWVCVSDHFNVLTVTKTILEAVTKSKDDSGDLQMVHERLKEKISGKKFFLV
LDDVWNERQEKWEAVRTPLSYGAPGSRILVTTRGEKVASNMSSKVHRPKHLKEDECWKVFQKHALRGDELELNDEKK
EIGRRIVEKCKGLPLALKTIGSVLRTKSSISDWQSVLESDIWDLPKEFEIMPALLLSYQHLPSHLKRCFASCALFPKDYEFDK

KELILLWIAQGFLHCSPQSNNLEEIGEQYFNDLLMRSFFLQSDFKTCFFMHDLLNDLAKYVCADFCFRLKFDKGNCISKTT
RHFSFAFSDVKYFDGFGSLTDAKRLRSFFPYKEFGRRDNDYYPLQFKILVHELFSNFKFLRVLSLDAYSELREVPDSVGDLK
HLHSLDLSRTGIQKLPDSTCLLYNLLILKLNYCSSLEELPLHLHKLTKLRCLEFKKTKVTKMPTHFGELKNLQVLSAVFVDKK
KEFSTKHLGGLNLHGRLSINELQNIVNPVDALEANLNNKHLVKLELRWKSDHIPDDPRKEKKVLENLKPSKKLEHLSIKSY
GGPEFPSWVFDNSLSNLVFLKLKDCKYCLCLPPLGLLSSLKTLEIIGLDGIVSIGAEFYGNSYSSFTSLERLTFSNMKELEECE
RKTAAFPRLQILKVYQCPKLKGLPDQLVNVKYLYITGSMKASFLERCEHTVSHNSLEDLNFCAFPIMNIPMGSSYDLLANI
KISCGCDSLTTFPLDFFPNLKSLSLLFCRNLQIISQKHTHNRLKHLSVAGCSRFDSFPSEGLSAPRLQTIDIHGAENLKLLPKR
MQILLPSLHVLKIIHCPKVEMFPDGGLPPNVEDVSLSSFILMASLRETLGTNTCLQSLSIKYMDVEFFPDEVLLPHSITSLKL
SDCPNLKKMEYKGLCHLSSLSLHNCPNLQCLPEDGLPKSISSLEILNCPLLEQRCQNPQGQDWKKIAHIEKLIVRSKV

>XP_027923495.1

MPSGSWIRPTNPYFQGSKEPQKMPSSSLVVESVIYGRDADKEKILNWLTSETENHDQPSILSIVGMGGLGKTTLAQHVY
NDTKLEEAKFDIRAWVCVSDHFNVLTVTKTILEAVTKSKDDSGDLQMVHERLKEKISGKKFFLVLDDVWNERQEKWEA
VRTPLSYGAPGSRILVTTRGEKVASNMSSKVHRPKHLKEDECWKVFQKHALRGDELELNDEKKEIGRRIVEKCKGLPLAL
KTIGSVLRTKSSISDWQSVLESDIWDLPKEFEIMPALLLSYQHLPSHLKRCFASCALFPKDYEFDKKELILLWIAQGFLHCSP
QSNNLEEIGEQYFNDLLMRSFFLQSDFKTCFFMHDLLNDLAKYVCADFCFRLKFDKGNCISKTTRHFSFAFSDVKYFDGF
GSLTDAKRLRSFFPYKEFGRRDNDYYPLQFKILVHELFSNFKFLRVLSLDAYSELREVPDSVGDLKHLHSLDLSRTGIQKLPD
STCLLYNLLILKLNYCSSLEELPLHLHKLTKLRCLEFKKTKVTKMPTHFGELKNLQVLSAVFVDKKKEFSTKHLGGLNLHGRL
SINELQNIVNPVDALEANLNNKHLVKLELRWKSDHIPDDPRKEKKVLENLKPSKKLEHLSIKSYGGPEFPSWVFDNSLSNL
VFLKLKDCKYCLCLPPLGLLSSLKTLEIIGLDGIVSIGAEFYGNSYSSFTSLERLTFSNMKELEECERKTAAFPRLQILKVYQCP
KLKGLPDQLVNVKYLYITGSMKASFLERCEHTVSHNSLEDLNFCAFPIMNIPMGSSYDLLANIKISCGCDSLTTFPLDFFPN
LKSLSLLFCRNLQIISQKHTHNRLKHLSVAGCSRFDSFPSEGLSAPRLQTIDIHGAENLKLLPKRMQILLPSLHVLKIIHCPKV
EMFPDGGLPPNVEDVSLSSFILMASLRETLGTNTCLQSLSIKYMDVEFFPDEVLLPHSITSLKLSDCPNLKKMEYKGLCHLS
SLSLHNCPNLQCLPEDGLPKSISSLEILNCPLLEQRCQNPQGQDWKKIAHIEKLIVRSKV

>XP_027923496.1

MPSGSWIRPTNPYFQGSKEPQKMPSSSLVVESVIYGRDADKEKILNWLTSETENHDQPSILSIVGMGGLGKTTLAQHVY
NDTKLEEAKFDIRAWVCVSDHFNVLTVTKTILEAVTKSKDDSGDLQMVHERLKEKISGKKFFLVLDDVWNERQEKWEA
VRTPLSYGAPGSRILVTTRGEKVASNMSSKVHRPKHLKEDECWKVFQKHALRGDELELNDEKKEIGRRIVEKCKGLPLAL
KTIGSVLRTKSSISDWQSVLESDIWDLPKEFEIMPALLLSYQHLPSHLKRCFASCALFPKDYEFDKKELILLWIAQGFLHCSP
QSNNLEEIGEQYFNDLLMRSFFLQSDFKTCFFMHDLLNDLAKYVCADFCFRLKFDKGNCISKTTRHFSFAFSDVKYFDGF
GSLTDAKRLRSFFPYKEFGRRDNDYYPLQFKILVHELFSNFKFLRVLSLDAYSELREVPDSVGDLKHLHSLDLSRTGIQKLPD
STCLLYNLLILKLNYCSSLEELPLHLHKLTKLRCLEFKKTKVTKMPTHFGELKNLQVLSAVFVDKKKEFSTKHLGGLNLHGRL
SINELQNIVNPVDALEANLNNKHLVKLELRWKSDHIPDDPRKEKKVLENLKPSKKLEHLSIKSYGGPEFPSWVFDNSLSNL
VFLKLKDCKYCLCLPPLGLLSSLKTLEIIGLDGIVSIGAEFYGNSYSSFTSLERLTFSNMKELEECERKTAAFPRLQILKVYQCP
KLKGLPDQLVNVKYLYITGSMKASFLERCEHTVSHNSLEDLNFCAFPIMNIPMGSSYDLLANIKISCGCDSLTTFPLDFFPN
LKSLSLLFCRNLQIISQKHTHNRLKHLSVAGCSRFDSFPSEGLSAPRLQTIDIHGAENLKLLPKRMQILLPSLHVLKIIHCPKV
EMFPDGGLPPNVEDVSLSSFILMASLRETLGTNTCLQSLSIKYMDVEFFPDEVLLPHSITSLKLSDCPNLKKMEYKGLCHLS
SLSLHNCPNLQCLPEDGLPKSISSLEILNCPLLEQRCQNPQGQDWKKIAHIEKLIVRSKV

>XP_027923497.1

MPSGSWIRPTNPYFQGMGGLGKTTLAQHVYNDTKLEEAKFDIRAWVCVSDHFNVLTVTKTILEAVTKSKDDSGDLQM
VHERLKEKISGKKFFLVLDDVWNERQEKWEAVRTPLSYGAPGSRILVTTRGEKVASNMSSKVHRPKHLKEDECWKVFQ
KHALRGDELELNDEKKEIGRRIVEKCKGLPLALKTIGSVLRTKSSISDWQSVLESDIWDLPKEFEIMPALLLSYQHLPSHLKR

CFASCALFPKDYEFDKKELILLWIAQGFLHCSPQSNNLEEIGEQYFNDLLMRSFFLQSDFKTCFFMHDLLNDLAKYVCADF
CFRLKFDKGNCISKTTRHFSFAFSDVKYFDGFGSLTDAKRLRSFFPYKEFGRRDNDYYPLQFKILVHELFSNFKFLRVLSLDA
YSELREVPDSVGDLKHLHSLDLSRTGIQKLPDSTCLLYNLLILKLNYCSSLEELPLHLHKLTKLRCLEFKKTKVTKMPTHFGEL
KNLQVLSAVFVDKKKEFSTKHLGGLNLHGRLSINELQNIVNPVDALEANLNNKHLVKLELRWKSDHIPDDPRKEKKVLEN
LKPSKKLEHLSIKSYGGPEFPSWVFDNSLSNLVFLKLKDCKYCLCLPPLGLLSSLKTLEIIGLDGIVSIGAEFYGNSYSSFTSLER
LTFSNMKELEECERKTAAFPRLQILKVYQCPKLKGLPDQLVNVKYLYITGSMKASFLERCEHTVSHNSLEDLNFCAFPIMN
IPMGSSYDLLANIKISCGCDSLTTFPLDFFPNLKSLSLLFCRNLQIISQKHTHNRLKHLSVAGCSRFDSFPSEGLSAPRLQTID
IHGAENLKLLPKRMQILLPSLHVLKIIHCPKVEMFPDGGLPPNVEDVSLSSFILMASLRETLGTNTCLQSLSIKYMDVEFFP
DEVLLPHSITSLKLSDCPNLKKMEYKGLCHLSSLSLHNCPNLQCLPEDGLPKSISSLEILNCPLLEQRCQNPQGQDWKKIA
HIEKLIVRSKV

>XP_027923499.1

MAELVGGALLSAFVNVAFDKLASPKVVGFFRGRKLDEKLLGNLNIMLHSINALAHDAEQKQFRDPHVKAWLFAVKEAV
FDAEDLLKEIEYELTRCQVEAGSDPQTLTSKVSNFFNSTFTSFNKKIESEIREVLEKLEYLARQKGALGLKESIYSGDGSGSKE
PQKMPSSSLVVESVIYGRDADKEKILNWLTSETENHDQPSILSIVGMGGLGKTTLAQHVYNDTKLEEAKFDIRAWVCVS
DHFNVLTVTKTILEAVTKSKDDSGDLQMVHERLKEKISGKKFFLVLDDVWNERQEKWEAVRTPLSYGAPGSRILVTTRG
EKVASNMSSKVHRPKHLKEDECWKVFQKHALRGDELELNDEKKEIGRRIVEKCKGLPLALKTIGSVLRTKSSISDWQSVL
ESDIWDLPKEFEIMPALLLSYQHLPSHLKRCFASCALFPKDYEFDKKELILLWIAQGFLHCSPQSNNLEEIGEQYFNDLLMR
SFFLQSDFKTCFFMHDLLNDLAKYVCADFCFRLKFDKGNCISKTTRHFSFAFSDVKYFDGFGSLTDAKRLRSFFPYKEFGR
RDNDYYPLQFKILVHELFSNFKFLRVLSLDAYSELREVPDSVGDLKHLHSLDLSRTGIQKLPDSTCLLYNLLILKLNYCSSLEE
LPLHLHKLTKLRCLEFKKTKVTKMPTHFGELKNLQVLSAVFVDKKKEFSTKHLGGLNLHGRLSINELQNIVNPVDALEANL
NNKHLVKLELRWKSDHIPDDPRKEKKVLENLKPSKKLEHLSIKSYGGPEFPSWVFDNSLSNLVFLKLKDCKYCLCLPPLGLL
SSLKTLEIIGLDGIVSIGAEFYGNSYSSFTSLERLTFSNMKELEECERKTAAFPRLQILKVYQCPKLKGLPDQLVNVKYLYITGS
MKASFLERCEHTVSHNSLEDLNFCAFPIMNIPMGSSYDLLANIKISCGCDSLTTFPLDFFPNLKSLSLLFCRNLQIISQKHTH
NRLKHLSVAGCSRFDSFPSEGLSAPRLQTIDIHGAENLKLLPKRMQILLPSLHVLKIIHCPKVEMFPDGGLPPNVEDVSLSS
FILMASLRETLGTNTCLQSLSIKYMDVEFFPDEVLLPHSITSLKLSDCPNLKKMEYKGLCHLSSLSLHNCPNLQCLPEDGLP
KSISSLEILNCPLLEQRCQNPQGQDWKKIAHIEKLIVRSKV

>XP_027923500.1

MLHSINALAHDAEQKQFRDPHVKAWLFAVKEAVFDAEDLLKEIEYELTRCQVEAGSDPQTLTSKVSNFFNSTFTSFNKKI
ESEIREVLEKLEYLARQKGALGLKESIYSGDGSGSKEPQKMPSSSLVVESVIYGRDADKEKILNWLTSETENHDQPSILSIV
GMGGLGKTTLAQHVYNDTKLEEAKFDIRAWVCVSDHFNVLTVTKTILEAVTKSKDDSGDLQMVHERLKEKISGKKFFLV
LDDVWNERQEKWEAVRTPLSYGAPGSRILVTTRGEKVASNMSSKVHRPKHLKEDECWKVFQKHALRGDELELNDEKK
EIGRRIVEKCKGLPLALKTIGSVLRTKSSISDWQSVLESDIWDLPKEFEIMPALLLSYQHLPSHLKRCFASCALFPKDYEFDK
KELILLWIAQGFLHCSPQSNNLEEIGEQYFNDLLMRSFFLQSDFKTCFFMHDLLNDLAKYVCADFCFRLKFDKGNCISKTT
RHFSFAFSDVKYFDGFGSLTDAKRLRSFFPYKEFGRRDNDYYPLQFKILVHELFSNFKFLRVLSLDAYSELREVPDSVGDLK
HLHSLDLSRTGIQKLPDSTCLLYNLLILKLNYCSSLEELPLHLHKLTKLRCLEFKKTKVTKMPTHFGELKNLQVLSAVFVDKK
KEFSTKHLGGLNLHGRLSINELQNIVNPVDALEANLNNKHLVKLELRWKSDHIPDDPRKEKKVLENLKPSKKLEHLSIKSY
GGPEFPSWVFDNSLSNLVFLKLKDCKYCLCLPPLGLLSSLKTLEIIGLDGIVSIGAEFYGNSYSSFTSLERLTFSNMKELEECE
RKTAAFPRLQILKVYQCPKLKGLPDQLVNVKYLYITGSMKASFLERCEHTVSHNSLEDLNFCAFPIMNIPMGSSYDLLANI
KISCGCDSLTTFPLDFFPNLKSLSLLFCRNLQIISQKHTHNRLKHLSVAGCSRFDSFPSEGLSAPRLQTIDIHGAENLKLLPKR
MQILLPSLHVLKIIHCPKVEMFPDGGLPPNVEDVSLSSFILMASLRETLGTNTCLQSLSIKYMDVEFFPDEVLLPHSITSLKL
SDCPNLKKMEYKGLCHLSSLSLHNCPNLQCLPEDGLPKSISSLEILNCPLLEQRCQNPQGQDWKKIAHIEKLIVRSKV

>XP_027923501.1

MPSGSWIRPTNPYFQGSKEPQKMPSSSLVVESVIYGRDADKEKILNWLTSETENHDQPSILSIVGMGGLGKTTLAQHVY
NDTKLEEAKFDIRAWVCVSDHFNVLTVTKTILEAVTKSKDDSGDLQMVHERLKEKISGKKFFLVLDDVWNERQEKWEA
VRTPLSYGAPGSRILVTTRGEKVASNMSSKVHRPKHLKEDECWKVFQKHALRGDELELNDEKKEIGRRIVEKCKGLPLAL
KTIGSVLRTKSSISDWQSVLESDIWDLPKEFEIMPALLLSYQHLPSHLKRCFASCALFPKDYEFDKKELILLWIAQGFLHCSP
QSNNLEEIGEQYFNDLLMRSFFLQSDFKTCFFMHDLLNDLAKYVCADFCFRLKFDKGNCISKTTRHFSFAFSDVKYFDGF
GSLTDAKRLRSFFPYKEFGRRDNDYYPLQFKILVHELFSNFKFLRVLSLDAYSELREVPDSVGDLKHLHSLDLSRTGIQKLPD
STCLLYNLLILKLNYCSSLEELPLHLHKLTKLRCLEFKKTKVTKMPTHFGELKNLQVLSAVFVDKKKEFSTKHLGGLNLHGRL
SINELQNIVNPVDALEANLNNKHLVKLELRWKSDHIPDDPRKEKKVLENLKPSKKLEHLSIKSYGGPEFPSWVFDNSLSNL
VFLKLKDCKYCLCLPPLGLLSSLKTLEIIGLDGIVSIGAEFYGNSYSSFTSLERLTFSNMKELEECERKTAAFPRLQILKVYQCP
KLKGLPDQLVNVKYLYITGSMKASFLERCEHTVSHNSLEDLNFCAFPIMNIPMGSSYDLLANIKISCGCDSLTTFPLDFFPN
LKSLSLLFCRNLQIISQKHTHNRLKHLSVAGCSRFDSFPSEGLSAPRLQTIDIHGAENLKLLPKRMQILLPSLHVLKIIHCPKV
EMFPDGGLPPNVEDVSLSSFILMASLRETLGTNTCLQSLSIKYMDVEFFPDEVLLPHSITSLKLSDCPNLKKMEYKGLCHLS
SLSLHNCPNLQCLPEDGLPKSISSLEILNCPLLEQRCQNPQGQDWKKIAHIEKLIVRSKV

>XP_027923502.1

MPSGSWIRPTNPYFQGSKEPQKMPSSSLVVESVIYGRDADKEKILNWLTSETENHDQPSILSIVGMGGLGKTTLAQHVY
NDTKLEEAKFDIRAWVCVSDHFNVLTVTKTILEAVTKSKDDSGDLQMVHERLKEKISGKKFFLVLDDVWNERQEKWEA
VRTPLSYGAPGSRILVTTRGEKVASNMSSKVHRPKHLKEDECWKVFQKHALRGDELELNDEKKEIGRRIVEKCKGLPLAL
KTIGSVLRTKSSISDWQSVLESDIWDLPKEFEIMPALLLSYQHLPSHLKRCFASCALFPKDYEFDKKELILLWIAQGFLHCSP
QSNNLEEIGEQYFNDLLMRSFFLQSDFKTCFFMHDLLNDLAKYVCADFCFRLKFDKGNCISKTTRHFSFAFSDVKYFDGF
GSLTDAKRLRSFFPYKEFGRRDNDYYPLQFKILVHELFSNFKFLRVLSLDAYSELREVPDSVGDLKHLHSLDLSRTGIQKLPD
STCLLYNLLILKLNYCSSLEELPLHLHKLTKLRCLEFKKTKVTKMPTHFGELKNLQVLSAVFVDKKKEFSTKHLGGLNLHGRL
SINELQNIVNPVDALEANLNNKHLVKLELRWKSDHIPDDPRKEKKVLENLKPSKKLEHLSIKSYGGPEFPSWVFDNSLSNL
VFLKLKDCKYCLCLPPLGLLSSLKTLEIIGLDGIVSIGAEFYGNSYSSFTSLERLTFSNMKELEECERKTAAFPRLQILKVYQCP
KLKGLPDQLVNVKYLYITGSMKASFLERCEHTVSHNSLEDLNFCAFPIMNIPMGSSYDLLANIKISCGCDSLTTFPLDFFPN
LKSLSLLFCRNLQIISQKHTHNRLKHLSVAGCSRFDSFPSEGLSAPRLQTIDIHGAENLKLLPKRMQILLPSLHVLKIIHCPKV
EMFPDGGLPPNVEDVSLSSFILMASLRETLGTNTCLQSLSIKYMDVEFFPDEVLLPHSITSLKLSDCPNLKKMEYKGLCHLS
SLSLHNCPNLQCLPEDGLPKSISSLEILNCPLLEQRCQNPQGQDWKKIAHIEKLIVRSKV

>XP_027923503.1

MPSGSWIRPTNPYFQGMGGLGKTTLAQHVYNDTKLEEAKFDIRAWVCVSDHFNVLTVTKTILEAVTKSKDDSGDLQM
VHERLKEKISGKKFFLVLDDVWNERQEKWEAVRTPLSYGAPGSRILVTTRGEKVASNMSSKVHRPKHLKEDECWKVFQ
KHALRGDELELNDEKKEIGRRIVEKCKGLPLALKTIGSVLRTKSSISDWQSVLESDIWDLPKEFEIMPALLLSYQHLPSHLKR
CFASCALFPKDYEFDKKELILLWIAQGFLHCSPQSNNLEEIGEQYFNDLLMRSFFLQSDFKTCFFMHDLLNDLAKYVCADF
CFRLKFDKGNCISKTTRHFSFAFSDVKYFDGFGSLTDAKRLRSFFPYKEFGRRDNDYYPLQFKILVHELFSNFKFLRVLSLDA
YSELREVPDSVGDLKHLHSLDLSRTGIQKLPDSTCLLYNLLILKLNYCSSLEELPLHLHKLTKLRCLEFKKTKVTKMPTHFGEL
KNLQVLSAVFVDKKKEFSTKHLGGLNLHGRLSINELQNIVNPVDALEANLNNKHLVKLELRWKSDHIPDDPRKEKKVLEN
LKPSKKLEHLSIKSYGGPEFPSWVFDNSLSNLVFLKLKDCKYCLCLPPLGLLSSLKTLEIIGLDGIVSIGAEFYGNSYSSFTSLER
LTFSNMKELEECERKTAAFPRLQILKVYQCPKLKGLPDQLVNVKYLYITGSMKASFLERCEHTVSHNSLEDLNFCAFPIMN
IPMGSSYDLLANIKISCGCDSLTTFPLDFFPNLKSLSLLFCRNLQIISQKHTHNRLKHLSVAGCSRFDSFPSEGLSAPRLQTID
IHGAENLKLLPKRMQILLPSLHVLKIIHCPKVEMFPDGGLPPNVEDVSLSSFILMASLRETLGTNTCLQSLSIKYMDVEFFP
DEVLLPHSITSLKLSDCPNLKKMEYKGLCHLSSLSLHNCPNLQCLPEDGLPKSISSLEILNCPLLEQRCQNPQGQDWKKIA
HIEKLIVRSKV

>XP_027923556.1

MAAEVVGGALLSAFLQVAFDRLASPQFVDFFRGRKLDEKLLGNLNIMLHSINALADDAEQKQFTDPHVKAWLLSVKEA
VFDAEDLLGEIDYELTRCQVEAGSEPQTFTDKVSNFFNSTFSSFNKKIESEMKEVLQKLKYLEQQKDALGLKNGTSYSDNK
VSQKLPSSSLVVESIIYGRDADKEIIFNWLRSETDNDNHPSIFSIVGMGGLGKTTLAHHVYRDPKMEEAKFDIKAWVCVS
DHFDVLTVTKTILEAITNSKDDSGNLEMVHGRLKDEISGRKFLLVLDDVWNEGGEEWEAVRTPLSYGAPGSKILVTARAE
KVASNMRSKVHLLRELRGDECWNVFKKHALKDDDLELNDDLKEIGRRIVEKCKGLPLALKTIGCLLRTKSSISYWKSVLEN
DIWELPKEVKIIPALLLSYQHLPSHLKRCFAYCALFPKDHEFDKKELILLWMAEGFLHHSQQINNVEEIGEQYFNDLLTRSF
FLQSDFKTYFSMHDLLNDLAKYVCADFCFRLKFDKGNCIPKKTRHFSFAFDDVECFDGFGSFTDAKRLRSFFPYEEFGGR
NIDYYPLQFKILVHELFSNFKFLRVLSLDGYSELKEVPDSVGDLKHLHSLDLSRTGIQKLPESTCLLYNLLILKLNYCLSLEELPL
NLHKLTKLHCIEFENTKVTKMPMHFGELKNLQVLSPVFIDKNKEFSTKHLGCLNLHGRLSINEVQNIVNPVDALEANLKN
KDLVELELKWESDHIPDDPRKEKKVLENLQPSKIVEYLSIENYGGTEFPSWVFDNSLSNLVFLRLEDCKYCLCLPPLGLLSSL
KTLEIIGFDGIVSIGDEFYGNSSSSFTSLESLAFSKMKELEECERKTAAFPRLEYLDVYECPKLKGLPDQLVNVKNLYIRKASC
LERCEHTVSHNSLEALTFLVFPIMNISMSRSFDLLEEILIFDSCDSLTTFPLDFFPNLKYLSLCSCRNLQIISQKHTHNRLKHLT
IRSCSRFDSFPSEGLSAPQLLFIDIDGVENLKLLPKRIRILLPSLYILNIINCPKVEMFPDGGLPPYVGQVSLSSLKLIASLKETL
GTNTFMKRLCIENIDVEFFPDEVLLPHSITSLEICRCPNLKKMEYKGLCHLSYLKLYDCPNLQCLPEDGLPKSISSLRIWSCPL
LERRCQNPEGQDWNKIAHIEDLSVRSKV

>XP_027923557.1

MEAAKFDIRAWVCVSDHFDVLTVTKSILEAITKSKDDSADLEMVHGRLKEKVSGKRFLLVLDDVWNERREEWEAVQTP
LRYGAPGSRIIVTTRGEKVASNMKSKVHRLKQLEEDECWKVFEKQALKDDDLELNDEKKEIGRRIVEKCKGLPLALKTIGS
LLCTKSSILDWQSVLESDIWDLPKEVEIIPALLLSYQHLSSHLKRCFAYCALFPKDYEFDKKELILLWMAEGFLHHSQQNKN
VQEIGEQYFDDLLTRSSFFNQPSKCNSSCMTF

>XP_027923642.1

MAAEVVGGALLSAFLQVAFDRLASPQFVDFFRGRKLDEKLLGNLNIMLHSINALADDAEQKQFRDPHVKAWLLSVKEA
VFDAEDLLGEIDYELTRCQVEAGSEPQTFTYKVSTFFNSTFSSFNKKIESEMKEVLEQLKYLEQQKDALGLKNSTSYSDSKA
SQKLPSSSLVVESIIYGRDADKEIIFNWLRSETDNHNHPSIFSIVGMGGLGKTTLATHVYCDPKMEEAKFDIKAWVCVSD
HFDVLTVTKTILEAITKSKDDSGDLEMVHARLKDQISGRKFLLVLDDVWNEGGEEWEAVRTPLSYGAAGSKILVTARAEK
VAYNMRSKVHRLKQLEDDESWNVFKKQALKDDDLELNDEKEEIGRRIVEKCKGLPLALKTIGSLLYKESSTSYWKRVLEN
DIWDLPKEVKIIPALLLSYQHLPSHLKRCFAYCALFPKDYEFDKKELILLWMAEGFLHHSQQINNVEEIGEQYFKDLLTRSF
FLQSRIKKRFSMHDLLNDLAKYVCADFCFRLKFDKGNCIPKTTRHFSFAFDDVECFDGFGSLTDAKRLRSFFPYKEFGRRN
IGYYPLQFKTLIHELFSKLKFLRVLSLDGYSDLREVPDSVGDLKHLHSLDLSRTGIQKLPESTCLLYNLLILKLNYCSSLEELPLN
LHKLTKLHCLEFENTKVTKMPMHFGELKNLQVLSSVFIDKNKEFSTKHLGCLNLHGRLSINEVQNIVNPLDALEANLKNK
DLVELELKWKSDHIPDDPRKEKKVLENLQPSKIVEYLSIENYGGTEFPSWVFDNSLSNLVSLRLEDCKYCLCLPPLGLLSSLE
TLEIIGFDGIVSIGDEFYGNSSSSFTSLESLTFSKMKELEECKRKTAAFPRLEILSVYQCPKLKGLPNQLVNVKNLYIMDSME
ASCLERCEHTVSHNSLEALTFSVFPIMNISMSRSFDLLKKIRIFRSCDSLTTFPLDFFPNLKDLSLFSCRNLQMISQDHAHTS
LEILSIRSCSRFDSFPSEGLSAPQLWTIDIDGAENLKLLPKRIRILLPSLYVLNIINCPKVEMFPDGGLPPYVEQVSLSSFKLIAS
LKETLGTNTFMKRLCIENIDVEFFPDEVLLPHSITSLQIYDCPNLKKMEYKGLCHLSSLTLYDCPNLQCLPEDGLPKSISSLQI
WSCPLLERRCQNPEGQDWNKIAHIEHLSILP

>XP_027923643.1

MAAEVVGGALLSAFLQVAFDRLASPQFVDFFRGRKLDEKLLGNLNIMLHSINALADDAEQKQFRDPHVKAWLLSVKEA
VFDAEDLLGEIDYELTRCQVEAGSEPQTFTYKVSTFFNSTFSSFNKKIESEMKEVLEQLKYLEQQKDALGLKNSTSYSDSKA
SQKLPSSSLVVESIIYGRDADKEIIFNWLRSETDNHNHPSIFSIVGMGGLGKTTLATHVYCDPKMEEAKFDIKAWVCVSD
HFDVLTVTKTILEAITKSKDDSGDLEMVHARLKDQISGRKFLLVLDDVWNEGGEEWEAVRTPLSYGAAGSKILVTARAEK

VAYNMRSKVHRLKQLEDDESWNVFKKQALKDDDLELNDEKEEIGRRIVEKCKGLPLALKTIGSLLYKESSTSYWKRVLEN
DIWDLPKEVKIIPALLLSYQHLPSHLKRCFAYCALFPKDYEFDKKELILLWMAEGFLHHSQQINNVEEIGEQYFKDLLTRSF
FLQSRIKKRFSMHDLLNDLAKYVCADFCFRLKFDKGNCIPKTTRHFSFAFDDVECFDGFGSLTDAKRLRSFFPYKEFGRRN
IGYYPLQFKTLIHELFSKLKFLRVLSLDGYSDLREVPDSVGDLKHLHSLDLSRTGIQKLPESTCLLYNLLILKLNYCSSLEELPLN
LHKLTKLHCLEFENTKVTKMPMHFGELKNLQVLSSVFIDKNKEFSTKHLGCLNLHGRLSINEVQNIVNPLDALEANLKNK
DLVELELKWKSDHIPDDPRKEKKVLENLQPSKIVEYLSIENYGGTEFPSWVFDNSLSNLVSLRLEDCKYCLCLPPLGLLSSLE
TLEIIGFDGIVSIGDEFYGNSSSSFTSLESLTFSKMKELEECKRKTAAFPRLEILSVYQCPKLKGLPNQLVNVKNLYIMDSME
ASCLERCEHTVSHNSLEALTFSVFPIMNISMSRSFDLLKKIRIFRSCDSLTTFPLDFFPNLKDLSLFSCRNLQMISQDHAHTS
LEILSIRSCSRFDSFPSEGLSAPQLWTIDIDGAENLKLLPKRIRILLPSLYVLNIINCPKVEMFPDGGLPPYVEQVSLSSFKLIAS
LKETLGTNTFMKRLCIENIDVEFFPDEVLLPHSITSLQIYDCPNLKKMEYKGLCHLSSLTLYDCPNLQCLPEDGLPKSISSLQI
WSCPLLERRCQNPEGQDWNKIAHIEHLSILP

>XP_027923644.1

MAAEVVGGALLSAFLQVAFDRLASPQFVDFFRGRKLDEKLLGNLNIMLHSINALADDAEQKQFRDPHVKAWLLSVKEA
VFDAEDLLGEIDYELTRCQVEAGSEPQTFTYKVSTFFNSTFSSFNKKIESEMKEVLEQLKYLEQQKDALGLKNSTSYSDSKA
SQKLPSSSLVVESIIYGRDADKEIIFNWLRSETDNHNHPSIFSIVGMGGLGKTTLATHVYCDPKMEEAKFDIKAWVCVSD
HFDVLTVTKTILEAITKSKDDSGDLEMVHARLKDQISGRKFLLVLDDVWNEGGEEWEAVRTPLSYGAAGSKILVTARAEK
VAYNMRSKVHRLKQLEDDESWNVFKKQALKDDDLELNDEKEEIGRRIVEKCKGLPLALKTIGSLLYKESSTSYWKRVLEN
DIWDLPKEVKIIPALLLSYQHLPSHLKRCFAYCALFPKDYEFDKKELILLWMAEGFLHHSQQINNVEEIGEQYFKDLLTRSF
FLQSRIKKRFSMHDLLNDLAKYVCADFCFRLKFDKGNCIPKTTRHFSFAFDDVECFDGFGSLTDAKRLRSFFPYKEFGRRN
IGYYPLQFKTLIHELFSKLKFLRVLSLDGYSDLREVPDSVGDLKHLHSLDLSRTGIQKLPESTCLLYNLLILKLNYCSSLEELPLN
LHKLTKLHCLEFENTKVTKMPMHFGELKNLQVLSSVFIDKNKEFSTKHLGCLNLHGRLSINEVQNIVNPLDALEANLKNK
DLVELELKWKSDHIPDDPRKEKKVLENLQPSKIVEYLSIENYGGTEFPSWVFDNSLSNLVSLRLEDCKYCLCLPPLGLLSSLE
TLEIIGFDGIVSIGDEFYGNSSSSFTSLESLTFSKMKELEECKRKTAAFPRLEILSVYQCPKLKGLPNQLVNVKNLYIMDSME
ASCLERFICTAAMDN

>XP_027923669.1

MAAELVGGALLSGFLQVAFDRLASPQVVQFFRGRKLDEKLLSKLNIVLHSINDLADDAEQKQFRNPHIKAWLFAVKDVV
FDAEDLLKEIEYELTRCQVEAGSDPQTLTSKVSNFFNSTFSSFNKKIESEMKDVLEKLEYLAKQKGALGLKEGIYSGDGSGS
KESQKLPSSSLVVESVIYGRDADKEMILNWLTSETENHDQPSILSIVGMGGLGKTTLAQHVYNDPKIEEAKFDIRAWVCV
SDHFDVLIVTKTILEAITKSKDDSGDLEMVHGRLKEKISGRKFLLVLDDIWNERREEWEAVRTPLSCGAPGSRIIVTTRGEK
VASNMMSEVHRLKQLEEEECWKLFKKHALKNDDDELNDERKEIGKRIVEKCKGLPLALKTIGSVLRTKSSISDWQSVLES
DIWDLSKEVEIMPALLLSYQHLPSHLKRCFAYCALFPKDYEFDKKELILLWMAEGFLHHSQHINNVEEIGEQYFEDLLTRS
FFLQSSIKTRFSMHDLLNDLAKYVCANFCFRFKFDKGNFVPNTTRHFSFRLEDARCFDGLGSLTDAKRMRSFLPIRETATT
KSYHYKLPCQFKTLIHELFSKLKFLRVLSLDGYSDLREVPDSVGDLKHLHSLDLSRTYIQKLPESISVLYNLLILKLNGCSYLEEL
PSNLHKLTKLHCLEFEDTKVTKMPLNFEELKNLHVLNMFLVDRNGESSVKQLGGLNLHGKLSINEVQNIVNPLDALEANL
KDKHLVELQLEWKSNHVCDDSEKEKKVLENLQPSKHLEHLSITNYGGTQLPSWVFNNSLSNLVFLRLKSCKYCVCLPSLG
LLSSLKTLEIIGLKGIYSIGVEFYGSNSSFMSLETLNFIDMKEWEEWECKTTSFPRLQHLFVNRCPKLKGLSNQLLHLKHLVI
CYCDKLIISETNMDTRSLEFLKLNSCPLVSIPTTHYNLLKEMSIDDGCDSLTTFSLDFFPNLCYLYLRRCRNLQRISQEDTHN
HLQRMTIAVCPQFESFPTQNLKLLPKHMQVLLPSLNQLWIIDCPQVEMPPDGGLPSNVKHMHLSNVKLMASMRVNL
DNTLQIGKLDVECFPDEGLLPRSLTTLLITESPNLKRLNYKGLCHLSSLTLNGCTNLECLPEEGIPKSISSLTIWGCPLLQKRC
QNPEGKDWEKIAHIQQLRVGKWE

>XP_027923740.1

MAAACVGGALLSAFLQVAFHRVTSPKLLHFFRGRKLDEALLRKLNIKLLSINSLADDAEQKQFMDTRVKAWLSAVKDVV
FDAEDLLDEIDYELTKRQVEAESECQSLSSKVSSFFNSTFSSFNRKIDSGLKQVLENLEYLASQKGDLGLKEATYFGLQPGS
VSEVQQKLPSTSLVAENVIYGRDDDTETIFNWLTSETHNHSQLSILSIVGMGGVGKTTLAQHVYNDPRMEEVHFSIKAW
VCVSDYFDVMVVTSTILEAITKSKDDSRNLEMAHARLKEKLSGKKFFLVLDDVWNERRDKWEAVQTPLNFGATGSKILV
TTRSEKVATTMWSSKVHRLMHLQEDHCWDVFAKHAFQDDHPQLNAEMKDIGIKIVKKCKGLPIALKAIGSLLHTKSSFS
ERECVLESKIWDLPREENEIMPALLLSYHHLSSHLKRCFTYFALFPKDYKFDKESIILLWLAENFLHCPQHNKSPEEIGELYF
DDLLSRSFFQRSSGLESCFVMHDLLNDLAKYVCGDIYFNLEVDKALFIPERARHISFAINDVKYFDSSYDAKRLRTFIPLPTFI
KLSPLNDPWQCKVSFHELFSKFKFIHTLSLLCCSGLLEVPDSIGDLKHLRSLDLSCTHIRKLPDSSCLLYNLQILKLNYCLLLKE
LPSNLYKLSNLRCLEFIGTSVRKMPMHMGKLKNLQVLSSFYVGKSSGIQQLVGLNLHGGLLIGDMQNILNPSDALQVDL
KNKKHLVKLELEWNSNQIPDNPRKEKQVLEYLQPPKHLKNFSISHYGGTQFPSWLSDSSLSNVVSLRLIGCKYVLQLPPL
GLLPFLKELTIIELDGIVGVGAEFHGSSSSSFTCLETLYFYNMKEWEEWDCETPFPRLQHLSIVHCPKLKGLPNQLLHVKQII
ICECERFTISGHNTESSTLERIGDTINNNSLEVLHIYSCLYINIPLRLRYNLLVTLDIDGGFDSPMTFPLDFFPKLCSLKLRCCN
LQMISQDHTHNHLKDLDISKSPQFESFPMEGLSAPKLVKFSIKELKNLKLLPKRMDILLPSLTDIRILDCPQVELCSDGGLPS
NLNTMDLSDCSKFMASMKMALGANNSLEVLSVQKLDVESFPDEGFLPFSLTSLEISNCLDLKNLDYSGLCHLSTLQELLL
FNCPNLRCLPEEGLPESISELKIVGCPLLEQCGLKPKG

>XP_027923741.1

MAAACVGGALLSAFLQVAFHRVTSPKLLHFFRGRKLDEALLRKLNIKLLSINSLADDAEQKQFMDTRVKAWLSAVKDVV
FDAEDLLDEIDYELTKRQVEAESECQSLSSKVSSFFNSTFSSFNRKIDSGLKQVLENLEYLASQKGDLGLKEATYFGLQPGS
VSEVQQKLPSTSLVAENVIYGRDDDTETIFNWLTSETHNHSQLSILSIVGMGGVGKTTLAQHVYNDPRMEEVHFSIKAW
VCVSDYFDVMVVTSTILEAITKSKDDSRNLEMAHARLKEKLSGKKFFLVLDDVWNERRDKWEAVQTPLNFGATGSKILV
TTRSEKVATTMWSSKVHRLMHLQEDHCWDVFAKHAFQDDHPQLNAEMKDIGIKIVKKCKGLPIALKAIGSLLHTKSSFS
ERECVLESKIWDLPREENEIMPALLLSYHHLSSHLKRCFTYFALFPKDYKFDKESIILLWLAENFLHCPQHNKSPEEIGELYF
DDLLSRSFFQRSSGLESCFVMHDLLNDLAKYVCGDIYFNLEVDKALFIPERARHISFAINDVKYFDSSYDAKRLRTFIPLPTFI
KLSPLNDPWQCKVSFHELFSKFKFIHTLSLLCCSGLLEVPDSIGDLKHLRSLDLSCTHIRKLPDSSCLLYNLQILKLNYCLLLKE
LPSNLYKLSNLRCLEFIGTSVRKMPMHMGKLKNLQVLSSFYVGKSSGIQQLVGLNLHGGLLIGDMQNILNPSDALQVDL
KNKKHLVKLELEWNSNQIPDNPRKEKQVLEYLQPPKHLKNFSISHYGGTQFPSWLSDSSLSNVVSLRLIGCKYVLQLPPL
GLLPFLKELTIIELDGIVGVGAEFHGSSSSSFTCLETLYFYNMKEWEEWDCETPFPRLQHLSIVHCPKLKGLPNQLLHVKQII
ICECERFTISGHNTESSTLERIGDTINNNSLEVLHIYSCLYINIPLRLRYNLLVTLDIDGGFDSPMTFPLDFFPKLCSLKLRCCN
LQMISQDHTHNHLKDLDISKSPQFESFPMEGLSAPKLVKFSIKELKNLKLLPKRMDILLPSLTDIRILDCPQVELCSDGGLPS
NLNTMDLSDCSKFMASMKMALGANNSLEVLSVQKLDVESFPDEGIVVV

>XP_027923846.1

MAESFLFTIAESLIEKLASHVFQEASRVVGLYDDLQELTKNLSLVKAVLLDAEQKQERNHELREWLTHLKTVFSDAEDVLD
EFECQTLRNKVVKVHGSTKDKVSHFFSTSNPLLFRCKMGQQIKDISKRLDKVAADRHKFSLQIIDVDTRVVHPRDMTHS
RVSDSEVIGRKKDKEKIIELLMQQNPNNDDTPLSVIPIVGIGGLGKTTLAKFVFNDSRIQECFPLKMWVCVSVDFDIKQLII
KIINSATDSASADAPSNPPNWNVLDLEQLQNQLKKKMSGQKFLLVLDDVWNEDRVKLIELRDLIHVSAAGSKILVTTRST
SIASMMGTVPFHTLEGLSEEDSLSLFVKWAFKEGQEKRHPHLVNIGRQIVKKCKGVPLAVRTLGSSLFSKFEASDWEYVS
DNEIWNLPQKKNDILPALKLSYDLMPSYLRQCFALFSLYPKDYEFGSYEITFLWGALGLIALPKTKRTLEEVGNQYLHELLS
RSFLQDFVNFGTAYSFRIHDLVHDLAQFIAMDECLYVNSNIQNIPDNVRHLSFAESSLFNNLVTKKSAALRTVLFPNGAA
AANCEALLNTCLPKFKCLRVLDLRGAKFETLPRNIVKLKHLRYLDIGENANIKRLPDSVCKLQSLQVLLLAKCIELEALPKGL
RKLISLRCLEFSIKQTVLPMNEIANLGSLEMLNIESCHNVESIFGGVKFPILNTLCVEDCQSLKSLLLDGQNFPQLETLMVA
NCGNLDLEVWKGHHEEESSKLKLKLLLFFNLSQLVALPKWLQKAANSLQCLTVSNCHNFEILPDWLTTLTHLETLHLSYC
PNLVSLPDNIHHLTVLESLIIVGCPYLCKNYEPHVGEFWPKISHIKNVVIKKPEEPEKN

>XP_027924055.1

MARRISLSLSSSSHTWIYDVFLSFRGEDTRYQFTHNLYHSLCEKGIHTFIDQEGLRRGEEITPALFHAIQNSRISIVVFSKNY
ASSTYCLNELVKILECAKEEGRSIYPIFYGVDPSEVRHQTGSYGEALSKHEARFHNDADNEKTQKWRKALHEAANLSGW
HFRHGSQPEFEFIREIVKEISAKINYIPLHVADKPIGLEYAVQGVKSLLGEGSDVNMIGIYGIGGIGKTTIARALYNTIFWHY
DGSCFLPDIREKAVNKHGNVQLQEILLSQILKGEDIKVGDVNRGIPLIKRRLQQKKVLLVLDDVDKLEQLKALAGGCDWF
GSGSIIIITTRDKHLLDARGVVNLYEVKPLKVERALELFSWHAFKNGKVDPPYMKIAMRAVSYACGLPLALEVIGSQLFGK
SLDECSSALDKYEGIPHEKIQEILKVSYNSLEENEKGIFLDIACFFNTYELSNVTPMLKAHGFHVEDGLRVLADRSLIKIDSSG
FVRMHDLIRDTGREIVRQESTLEPGRRSRLWFDEDIVHVLEENTGSDKIEFIKLEGYNNIQVQWDGKAFKEMKNLRILIIK
GSSFSTVPEHLPNSLRVVDWSCYPSPSLPSDFNPKRFEILLMPESSLRMFKPQKMMESLSVINLEDCTFLTDLRSLREAPF
LTTLRLDRCSNLVNIDESIGFLDKLRLLSAKHCTKLKALAPSIMLTSLETLDLWGSESLESFPEVLGKMEKIRKVYLDGTGIEK
LPFSIGNFVWLELLSLKGCEKLHQVPGSISIMPKVKVVVDYGHEVYQIFEEELSSEVSPRAMLIGDSDVYLDVYNSHHVYL
DVYYSYVSPNNVIRVYPPNQLLHQLLHSDLRLLFSKIQLEDDRLWLNQKNSEINMFRFRNKFPKVALCCSVLAPALKSVV
VMNLKFRVLINNTLQFSALCNFIVGGGSNTILWCDLEGKVDEVIWKVDEVISEREWDKAKIYFQLDFPMQRNCEDKKTK
RSIGGGSLIWSLIGVYEEGNNKEDIRAYDN

>XP_027924148.1

MAIPSSPAFRYDVFLSFRGEDTRYGLTGNLYKALRDRGIHTFIDDEHLQRGDEITAALLKAIEESRIAIVVLSHNYASSSFCL
DELAAILECKNKGLLIIPVFYKVHPSDVRHQKGSYGEAFTKHQRRFKDKKEKLQKWKMALSQVAELSGYHFEEGDGYEY
KFIESVVDRVCHKINPARLHVADYPVGLGPQVLEVRKLLNVECGDGFHMIGIHGMGGVGKTTLALALYNLIADCFDSSC
FVQNVREKSKKHGLEHLQSIILSNLLGAKDINLTSEHQGISMIQQRLQRKKVLLILDDVDRCEQLQALTGSPDWFGPGSR
VIITTRDTQPLASHQVTGTYEVKTLNAKDALQLLTWKAFKTEQVDPSYTEVLNDVVSYASGLPLVLEVIGSNLFAKSVEE
WKSAINQYKRIPNNRILDILKVSFDGLEEEEKSVFLDIACCFEGYELTEVEVMLRALYDDCMKYHIGVLVQKSLIKIYYSSTI
VMHDLIWDMGRQIDRRESSKKPGKGRRLWLPKDIIQVLRDNTGTRETKILCLDLSISEKEETLEWNANAFRRMKNLKILI
IRNGKFSKGPNYFPESLRVLEWHGYPSNCLPSNFHPNKLVTCKLPNSPFTSFGFHGSSKKFENLTDLNFDNCQLLTRMPD
LSDLPNLEKLSFERCESLIALDDSIGFLNKLKILKAQHCTKLQRFPPLNLPSLEVLQFPYCYSLENFPEILGKMGKIRELHLYEL
AIKELPVSFQNLTGLRELYAACDFLQLNSSALMSSLTVVEAYGCKEWKWVNPKDGEEVSLTVSSNIRCFWLSSCNLNDD
FFSADFTQLTTVKSLNLSRSNITFLPECIKEFHHLVALDVSYCKYLQEIRGIPPKLRTFRAIDCRSLTSSSSSMFLNQQIHEAG
KTMFVLPGGSIPRWLDKESRGPSISFWFRNQFPPKVLCFLIASVRDDTFFRFVTFDVLINGKVQEYQAGYDTDVIMEELD
HIHLFDLHVLFSENLIKIPSEKEWKHVEITYDGLFDTSLIKSTGIHIVKSKRRGMKDIRYYDPYTTTKMWPCVMAQKTARE
DYDTAATQVNSPITRRKPKRNNTKPKHLNDYDCRGLLPTPPVISPVPPKLNIFPCSPTVKIKNY

>XP_027924150.1

MIGIHGMGGVGKTTLALAVYNLIADCFDGSCFIQNVREISNKHGLEHLQSILLSKLLGDKDINLASEHEGISVIQQRLQRK
KVLLILDNVDKCEQLQTLAGSPDWFGPGSRVIITTRDTHLLASHQVKTTYEVKTLYAKDALQLLTWKAFKTEHVDPRYVE
VLNDVVIYASGLPLALEVIGSDLFAKTVEEWKSAISQYKRIPNNQILEILKVSFDALEEEEKCVFLDIACCFKGYELTEVEVM
LRALYHDCMKHHIRVLLEKSLIKIGWFTTIEMHDLIEDMGRQIDLKQSAKEPGKRRRLWLPKDIIQVLKDNTETSRIEILCF
HISISEKEETLDWNRNAFGKMEKLKILIIRNGKFCKGPNSFPESLRVLVWHGYPSNCLPSNFDPSKLVTCKLPNSHFTSFGF
LGSSKKLENLTELNFDCCELLTRMPDVSDLPNLQLTTQLVF

>XP_027924154.1

MAIQNSPAFTYEVFLSFRGEDTRHDFTGNLYKALCDRGIHTFIDDEDLQSGEEITPALAKAIEESRIAIVVLSHNYASSSFCL
DELVVILDCKNKGLLVIPVFYKVDPSDVRHQKESYGEALTKRQRRFEDVEKVQKWKMALRQVADLSGYHFKDGDGYEY
KFIESVVDRVCHKINPARLHVADYPVGLGPQVLEVRKLLNVECGDGFHMIGIHGMGGVGKTTLALALYNLIADCFDGSC

FLQNVTEKSNKHGLEHLQNILLSKILGDKDINIASEHEGISMIQQRLQRKKVLLILDNVDKCEQLQTLAGSPDWFGPGSR
VIITTRDTHLLASHQVKTTYEVKTLNKDDALQLLKWKAFKTKHVDPRYVEVLNDVEIYASGLPLALEVIGSNLFAKTVEQW
KSAINQYKRIPNSQILDKLKVSFDALEEEEKGVFLDIACCFKGYILRDVEDILGAIYDDCMKHHISVLVDKSLIKIGQWSTVEI
HDLIEEMGRQIDQQESPEGSGKRRRLWLPKDITEVLKDNTGTSRIEILCFHISISEKEETLDWNRNAFGKMEKLKILIIRNG
KFCKGPNSFPESLRVLEWHGYPSNCLPSNFDPSKLVTCKLPNSHFTSFGFLGSSKKLENLTELNFDNCQLLTRMPDVSDL
PNLEKLSFDLCKSLIAVDDSIGFLNKLKILKARGCTKLRRFPPLNLPSLEKLKLSYCSSLENFPEILGNMGNIEKLKLSQLPMIK
ELPVSFQNLTGLHRLFVACDFIRLNSLALTPSLTDFEAYGCKEWKWVNPKDGEEVGSTVSSNILEFSLTACNLNDDFFSA
GFTQLGTVKNLNLSRSNITFLPGCIKEFHCLNYLNVNRCKRLQEIRGLPPNLKRLRAIKCTSLTSSASSMLLNQQLHEAGET
DFIFPGGSIPRWLDKESRGPSISFWFRNEFPPKVLCLLIAPEPVDITVEFNTPVVLINGKVQEYRWNSMEREVRMLESDHI
HLFDLHVLPFRYKLMEMYSENEWKHVEITYQGLIHTSLIKGMGIHVVKEERRGMKDIRYDDPYTTIKMCITSLPFLFCFYL
ALILFSSHGLLNPTMTTLFGFMLYKIYLKSIDIG

>XP_027924155.1

MATPRFGYDVFLSFKGEDTRYGFTGNLYKALCNRGIHTFIDDEELQSGEKITPALQKSIEESRIAIVVLSQNYASSSFCLDEL
ATILHCHTQGLLVLPVFYKVQPSDVRHQKGSYGEELTKHQRRFKDKEKLQKWKMALRQVADLSGYHFEDCDGYEYKFI
ESVVDRVCHKINPARLHVADYPVGLGPQVLEVRKLLNVECGDGFHMIGIHGMGGVGKTTLALALYNLIADCFDGSCFL
QNVTEKSNKHGLEHLQNILLSKILGDKDINIASEHEGISMIQQRLQRKKVLLILDNVDKCEQLQTLAGSPDWFGPGSRVII
TTRDTHLLASHQVKTTYEVKTLNKDDALQLLKWKAFKTKHVDPRYVEVLNDVEIYASGLPLALEVIGSNLFAKTVEQWKS
AINQYKRIPNSQILDKLKVSFDALEEEEKGVFLDIACCFKGYILRDVEDILGAIYDDCMKHHISVLVDKSLIKIGQWSTVEIH
DLIEEMGRQIDQQESPEGSGKRRRLWLPKDITEVLKDNTGTSRIEILCFHISISEKEETLDWNRNAFGKMEKLKILIIRNGK
FCKGPNSFPESLRVLEWHGYPSNCLPSNFDPSKLVTCKLPNSHFTSFGFLGSSKKLENLTELNFDNCQLLTRMPDVSDLP
NLEKLSFDLCKSLIAVDDSIGFLNKLKILKARGCTKLRRFPPLNLPSLEKLKLSYCSSLENFPEILGNMGNIEKLKLSQLPMIKE
LPVSFQNLTGLHRLFVACDFIRLNSLALTPSLTDFEAYGCKEWKWVNPKDGEEVGSTVSSNILEFSLTACNLNDDFFSAG
FTQLGTVKNLNLSRSNITFLPGCIKEFHCLNYLNVNRCKRLQEIRGLPPNLKRLRAIKCTSLTSSASSMLLNQQLHEAGETD
FIFPGGSIPRWLDKESRGPSISFWFRNEFPPKVLCLLIAPEPVDITVEFNTPVVLINGKVQEYRWNSMEREVRMLESDHIH
LFDLHVLPFRYKLMEMYSENEWKHVEITYQGLIHTSLIKGMGIHVVKEERRGMKDIRYDDPYTTIKMCITSLPFLFCFYLA
LILFSSHGLLNPTMTTLFGFMLYKIYLKSIDIG

>XP_027924157.1

MAIQNSPAFTYEVFLSFRGEDTRHDFTGNLYKALCDRGIHTFIDDEDLQSGEEITPALAKAIEESRIAIVVLSHNYASSSFCL
DELVVILDCKNKGLLVIPVFYKVDPSDVRHQKESYGEALTKRQRRFEDVEKVQKWKMALRQVADLSGYHFKDGDGYEY
KFIESVVDRVCHKINPARLHVADYPVGLGPQVLEVRKLLNVECGDGFHMIGIHGMGGVGKTTLALALYNLIADCFDGSC
FLQNVTEKSNKHGLEHLQNILLSKILGDKDINIASEHEGISMIQQRLQRKKVLLILDNVDKCEQLQTLAGSPDWFGPGSR
VIITTRDTHLLASHQVKTTYEVKTLNKDDALQLLKWKAFKTKHVDPRYVEVLNDVEIYASGLPLALEVIGSNLFAKTVEQW
KSAINQYKRIPNSQILDKLKVSFDALEEEEKGVFLDIACCFKGYILRDVEDILGAIYDDCMKHHISVLVDKSLIKIGQWSTVEI
HDLIEEMGRQIDQQESPEGSGKRRRLWLPKDITEVLKDNTGTSRIEILCFHISISEKEETLDWNRNAFGKMEKLKILIIRNG
KFCKGPNSFPESLRVLEWHGYPSNCLPSNFDPSKLVTCKLPNSHFTSFGFLGSSKKLENLTELNFDNCQLLTRMPDVSDL
PNLEKLSFDLSYGCKEWKWVNPKDGEEVGSTVSSNILEFSLTACNLNDDFFSAGFTQLGTVKNLNLSRSNITFLPGCIKEF
HCLNYLNVNRCKRLQEIRGLPPNLKRLRAIKCTSLTSSASSMLLNQQLHEAGETDFIFPGGSIPRWLDKESRGPSISFWFR
NEFPPKVLCLLIAPEPVDITVEFNTPVVLINGKVQEYRWNSMEREVRMLESDHIHLFDLHVLPFRYKLMEMYSENEWKH
VEITYQGLIHTSLIKGMGIHVVKEERRGMKDIRYDDPYTTIKMCITSLPFLFCFYLALILFSSHGLLNPTMTTLFGFMLYKIYL
KSIDIG

>XP_027924158.1

MAIQNSPAFTYEVFLSFRGEDTRHDFTGNLYKALCDRGIHTFIDDEDLQSGEEITPALAKAIEESRIAIVVLSHNYASSSFCL
DELVVILDCKNKGLLVIPVFYKVDPSDVRHQKESYGEALTKRQRRFEDVEKVQKWKMALRQVADLSGYHFKDGDGYEY
KFIESVVDRVCHKINPARLHVADYPVGLGPQVLEVRKLLNVECGDGFHMIGIHGMGGVGKTTLALALYNLIADCFDGSC
FLQNVTEKSNKHGLEHLQNILLSKILGDKDINIASEHEGISMIQQRLQRKKVLLILDNVDKCEQLQTLAGSPDWFGPGSR
VIITTRDTHLLASHQVKTTYEVKTLNKDDALQLLKWKAFKTKHVDPRYVEVLNDVEIYASGLPLALEVIGSNLFAKTVEQW
KSAINQYKRIPNSQILDKLKVSFDALEEEEKGVFLDIACCFKGYILRDVEDILGAIYDDCMKHHISVLVDKSLIKIGQWSTVEI
HDLIEEMGRQIDQQESPEGSGKRRRLWLPKDITEVLKDNTGTSRIEILCFHISISEKEETLDWNRNAFGKMEKLKILIIRNG
KFCKGPNSFPESLRVLEWHGYPSNCLPSNFDPSKLVTCKLPNSHFTSFGFLGSSKKLENLTELNFDNCQLLTRMPDVSDL
PNLEKLSFDLCFTQLGTVKNLNLSRSNITFLPGCIKEFHCLNYLNVNRCKRLQEIRGLPPNLKRLRAIKCTSLTSSASSMLLN
QQLHEAGETDFIFPGGSIPRWLDKESRGPSISFWFRNEFPPKVLCLLIAPEPVDITVEFNTPVVLINGKVQEYRWNSMER
EVRMLESDHIHLFDLHVLPFRYKLMEMYSENEWKHVEITYQGLIHTSLIKGMGIHVVKEERRGMKDIRYDDPYTTIKMCI
TSLPFLFCFYLALILFSSHGLLNPTMTTLFGFMLYKIYLKSIDIG

>XP_027924159.1

MATQRFGYDVFLSFRGEDTRYGFTGNLYKALCDRGIHTFIDDEELQRGDEITAALMKAIEESRIAIVLLSQNYASSSFCLDE
LATILHCHTKGLLVIPLFYKVNPSDVRHHRGSYGEALTKHQRMFKDKKKLQKWKMALRQVADLSGYHIKDGDGYEYKFI
GSVVDEVCHKINPTRLHVADYPVGLGPQVLEVRKLLNVECGDGFHMIGVHGMGGVGKTTLALAVYNLIADCFDGSCFI
QNVREKSNKHGLEHLQSILLSKILGDKDINIASEHEGISMIQQRLQRKKVLLILDNVDKCEQLQTLAGSPDWFGPGSRVII
TTRDTHLLASHQVKTTYEVKTLNKDDALQLLKWKAFKTKHVDPRYVEVLNDVEIYASGLPLALEVIGSNLFAKTVEQWKS
AINQYKRIPNSQILDKLKVSFDALEEEEKCVFLDIACCFKGYELTEVEVMLRALYDDCMKHHISVLLEKSLIKIGWFNTIEM
HELIEDMGRQIDQKQSSKEPGKRRRLWLSKDITDVLKDNTGTSRIEMLCFDISMSEKEETLDWNGNAFGNMKNLKILVI
RNGKVSGGPNCFPESLRVLEWHEYPSNCLPSNFDPTKLVTCKLPNSHFTSFGFGSSKKLENLTELNFDNCQLLTRMPDVS
DLPNLEKLSFERCKSLIAVEESIGFLNKLKILKAQRCTKLRRFPPLNLPSLEKLELSDCSSLENFPEILGEMGNIGELSLSKLAIK
ELTVSFQNLTGLRELDVKCDFLQLSHIALTPSLCDLNVGKCKEWKWVKSKDGEEEMGSTVFSDLRYFQLNSCNLNDDFF
RAGFTQLTTVISLNLSETDITFIPECIKEFHCLYDLNVNRCTRLQEIRGVPPNLHTFWAIECTSLTSSGSSMLLNQELNQLRR
TTFMYRGGSIPRWFDKESRGPSISFWFRNEFPPKVLCLLIAPVLDDITLNLVIPVVLINGKDLEYTSLSRRKRRVRMVELDH
IHLFDLHDFQFLEDLRKMASEKEWKHVEITYQGLFDTSLIKSMGIHIVKSERRGIKDIRYDDPYTTTKVSPFNFFITFLPFFS
RFLFALILFISIMAYPIQL

>XP_027924160.1

MATQRFGYDVFLSFRGEDTRYGFTGNLYKALCDRGIHTFIDDEELQRGDEITAALMKAIEESRIAIVLLSQNYASSSFCLDE
LATILHCHTKGLLVIPLFYKVNPSDVRHHRGSYGEALTKHQRMFKDKKKLQKWKMALRQVADLSGYHIKDGDGYEYKFI
GSVVDEVCHKINPTRLHVADYPVGLGPQVLEVRKLLNVECGDGFHMIGVHGMGGVGKTTLALAVYNLIADCFDGSCFI
QNVREKSNKHGLEHLQSILLSKILGDKDINIASEHEGISMIQQRLQRKKVLLILDNVDKCEQLQTLAGSPDWFGPGSRVII
TTRDTHLLASHQVKTTYEVKTLNKDDALQLLKWKAFKTKHVDPRYVEVLNDVEIYASGLPLALEVIGSNLFAKTVEQWKS
AINQYKRIPNSQILDKLKVSFDALEEEEKCVFLDIACCFKGYELTEVEVMLRALYDDCMKHHISVLLEKSLIKIGWFNTIEM
HELIEDMGRQIDQKQSSKEPGKRRRLWLSKDITDVLKDNTGTSRIEMLCFDISMSEKEETLDWNGNAFGNMKNLKILVI
RNGKVSGGPNCFPESLRVLEWHEYPSNCLPSNFDPTKLVTCKLPNSHFTSFGFGSSKKLENLTELNFDNCQLLTRMPDVS
DLPNLEKLSFERCKSLIAVEESIGFLNKLKILKAQRCTKLRRFPPLNLPSLEKLELSDCSSLENFPEILGEMGNIGELSLSKLAIK
ELTVSFQNLTGLRELDVKCDFLQLSHIALTPSLCDLNVGKCKEWKWVKSKDGEEEMGSTVFSDLRYFQLNSCNLNDDFF
RAGFTQLTTVISLNLSETDITFIPECIKEFHCLYDLNVNRCTRLQEIRGVPPNLHTFWAIECTSLTSSGSSMLLNQLRRTTFM
YRGGSIPRWFDKESRGPSISFWFRNEFPPKVLCLLIAPVLDDITLNLVIPVVLINGKDLEYTSLSRRKRRVRMVELDHIHLFD
LHDFQFLEDLRKMASEKEWKHVEITYQGLFDTSLIKSMGIHIVKSERRGIKDIRYDDPYTTTKVSPFNFFITFLPFFSRFLFA
LILFISIMAYPIQL

>XP_027924161.1

MAPPRSPAFRYDVFLSFRGEDTRYGFTGNLYKALCNRGIHTFIDDEELQSGEEITPALQKSIEESRIAIVVLSQNYASSSFCL
DELVTILHCHTQGLLVIPLFYKVKPSDVRHHRGSYGEAVSNHQKRFKDKKEKVHKWKMALRHVADLSGCHFKEGDGYE
YKFIGSVVDEVCHKINPTRLHVTDYPVGLGPQVLEVRKLLNVECGDGFHMIGIHGMGGVGKTTLALALYNLIAVCFDGS
CFLQNVTEKSNKHGLEHLQSILLSKILGDKDINIASEHEGISMIHQRLQRKKVLLILDDVDKCEQLQRLVGSPDWFGPGSR
VIITTRDTHLLASHQVKTTYEVKTLNKDDALQLLTWKAFKTEHVNPSYVEVLNGVVTYASGLPLVLEVIGSNLFAKGVEQ
WKSAINQYKRIPNKKILEIMKVSFDALEEEEKSVFLDIACCFKGYKLTEVEIMLRALYDDCMKHHIGVLVEKSLIKVSRGGT
VELHDLIKDMGRQIDQKESPKEPGKRKRLWLPKDIIQVLKCNKGTGEIEMVCLDLSPSDRGEVVEWNGMAFQDMENL
KILIIRNCKFSKGPKYLPNKLTVLEWWRYPSPCLPYDFNANKLVICKLPDSCFTSFGFHGSSKKFENLTVLKFDYCKHLTRIH
DISDLPNLEKLSFKWCESLVSVHNSVGFLTKLKILIAERCEKLRRFLPLNLTSLERLELSYCSSLENFPEILGNMGNIRKLSLLK
LPIKELPDSFQNLTGLQELDLNCDFVHLSGNVLTPELTRLYVLTCKEWKWLKSKEGEEDVGSTVSSNVQSFWGSCNLDD
EFFSAGFTQLAQVRQLWLVNGNVTFLPERMKEFHHLNALDVSYCENLHEIRGLPPNLKSFRAIKCTSLTSLGSSMLLNQ
QQLHEVGGTEFIFPGGRIPEWLDKQSNGPSISFWFRNKFPAKVLCLLIAPVMCSSLFVRPMVFIHGKVTKCLSNPMKTK
VEMLESDYTHLFDLRRFLSHGALMEVGLENEWKHVEVTYEGVFESSVIKAMGIHVVKDKNINMEDIRYDDPYTNIKVEN
QE

>XP_027924162.1

MAPPRSPAFRYDVFLSFRGEDTRYGFTGNLYKALCNRGIHTFIDDEELQSGEEITPALQKSIEESRIAIVVLSQNYASSSFCL
DELVTILHCHTQGLLVIPLFYKVKPSDVRHHRGSYGEAVSNHQKRFKDKKEKVHKWKMALRHVADLSGCHFKEGDGYE
YKFIGSVVDEVCHKINPTRLHVTDYPVGLGPQVLEVRKLLNVECGDGFHMIGIHGMGGVGKTTLALALYNLIAVCFDGS
CFLQNVTEKSNKHGLEHLQSILLSKILGDKDINIASEHEGISMIHQRLQRKKVLLILDDVDKCEQLQRLVGSPDWFGPGSR
VIITTRDTHLLASHQVKTTYEVKTLNKDDALQLLTWKAFKTEHVNPSYVEVLNGVVTYASGLPLVLEVIGSNLFAKGVEQ
WKSAINQYKRIPNKKILEIMKVSFDALEEEEKSVFLDIACCFKGYKLTEVEIMLRALYDDCMKHHIGVLVEKSLIKVSRGGT
VELHDLIKDMGRQIDQKESPKEPGKRKRLWLPKDIIQVLKCNKGTGEIEMVCLDLSPSDRGEVVEWNGMAFQDMENL
KILIIRNCKFSKGPKYLPNKLTVLEWWRYPSPCLPYDFNANKLVICKLPDSCFTSFGFHGSSKKFENLTVLKFDYCKHLTRIH
DISDLPNLEKLSFKWCESLVSVHNSVGFLTKLKILIAERCEKLRRFLPLNLTSLERLELSYCSSLENFPEILGNMGNIRKLSLLK
LPIKELPDSFQNLTGLQELDLNCDFVHLSGNVLTPELTRLYVLTCKEWKWLKSKEGEEDVGSTVSSNVQSFWGSCNLDD
EFFSAGFTQLAQVRQLWLVNGNVTFLPERMKEFHHLNALDVSYCENLHEIRGLPPNLKSFRAIKCTSLTSLGSSMLLNQ
QLHEVGGTEFIFPGGRIPEWLDKQSNGPSISFWFRNKFPAKVLCLLIAPVMCSSLFVRPMVFIHGKVTKCLSNPMKTKVE
MLESDYTHLFDLRRFLSHGALMEVGLENEWKHVEVTYEGVFESSVIKAMGIHVVKDKNINMEDIRYDDPYTNIKVENQ
E

>XP_027924188.1

MVKYLTINFRIVVRKLQFKLYISIHHFSTPAMAAELVGGALLSGFLQVAFDRLASPQVVQFFRGRKLDEKLLSKLNIVLHSI
NDLADDAEQKQFRNPHIKAWLFAVKDVVFDAEDLLKEIEYELTRCQVEAGSDPQTLTSKVSNFFNSTFTSFNKKIESEMR
DVLEKLEYLAKQKGALGLKEGIYSGDRSGSKVSQKLPSSSLVVESVIYGRDVDKEIIFNWLTSETDNHNHPSVLSIVGMGG
LGKTTLAQHVYNDPKIEEAKFDIRAWVCVSDHFDVLIVTKTILEAITKSKDDSGDLEMVHGRLKERISGRKFLLVLDDVW
NERREEWEVVRTPLSYGAPGSRILVTTRGEKVASNMMSEVHRLKQLEEEECWKLFKKHALKDDDHELNDEKKEIGRRIV
DKCKGLPLALKTIGSLLRTKSSISDWQSVLESDIWDLPKEVEIMPALLLSYQHLPSHLKRCFAYCALFPKDYEFDKKELILLW
MAEDFLHHSKQIKNVQEIGEQYFDDLLTRSFFLQLSSEMRQIKNVGEIGEQYFHDLLTRSFFLESSFEMRFVMHDLLNDL
AKYVCADFCFRFKFDKGNIPNTTRHFSFSFDDVEYFDDLGSLIDAKRLRSFHSITNDCSYDFNPCQFNILVHELFSKFKFLR
VLSLNGYSQLSEVPNSVGDLKHLCSIDLSYTHIQKLPDSICLLYNLLIMKMNVCSFLEELPSNLHKLTKLNCLEFEHTNVRK
MPMHFGELKNLQVLSTFCVDRDSKVINIKQLGGLNLHGRLSINEVQNIVNPLDALEANLKNQHLVELKLKWNLNHIPD
DPMKEKKVLENLQPPKHLELLSIENYGGTQFPCWVFDNSLSKLVSLWLNDCKYCLCLPPFGLLSSLKNLEIGGLDGIVSIG

AEFCGSNSSSFKSLEKLEFNNMKEWEEWECKTTSFPRLQLLSIYGCPKLKGLPEQLLYLKSLEINKCDKLGISVNNMFTSSV
QLLSVILCPLVNIPMIHYDFLEAMEINSDCDSLTIFLLDFFPKLHLLQLSRCQNLRRVSQEEHAHNHLKVLRISECPQFESFP
SEGLSAPWLQIISIRGAENLKLMPKRMQILLPSLTELEIIDCPKVEMFPDEGLPSNLKKMSLSSLKLITSLRDTLDANTCLESL
IVEKLDVESFPGEVLLPRSLTTLHIRFCPNLKKLDYKGLSHISSVIYYGCPNLHQRKLQSS

>XP_027924189.1

MVKYLTINFRIVVRKLQFKLYISIHHFSTPAMAAELVGGALLSGFLQVAFDRLASPQVVQFFRGRKLDEKLLSKLNIVLHSI
NDLADDAEQKQFRNPHIKAWLFAVKDVVFDAEDLLKEIEYELTRCQVEAGSDPQTLTSKVSNFFNSTFTSFNKKIESEMR
DVLEKLEYLAKQKGALGLKEGIYSGDRSGSKVSQKLPSSSLVVESVIYGRDVDKEIIFNWLTSETDNHNHPSVLSIVGMGG
LGKTTLAQHVYNDPKIEEAKFDIRAWVCVSDHFDVLIVTKTILEAITKSKDDSGDLEMVHGRLKERISGRKFLLVLDDVW
NERREEWEVVRTPLSYGAPGSRILVTTRGEKVASNMMSEVHRLKQLEEEECWKLFKKHALKDDDHELNDEKKEIGRRIV
DKCKGLPLALKTIGSLLRTKSSISDWQSVLESDIWDLPKEVEIMPALLLSYQHLPSHLKRCFAYCALFPKDYEFDKKELILLW
MAEDFLHHSKQIKNVQEIGEQYFDDLLTRSFFLQLSSEMRQIKNR

>XP_027924193.1

MAAEVVGGALLSAFLQVAFDRLASPQFVDFFRGRKLDEKLLGNLNIMLHSINALADDAEHKQFTDPHVKAWLLSVKEA
VLDAEDLLGEIDYELTRCQVEAGSEPQTFTYKVSTFFNSTFSSFNKKIESEMKEVLEKLKYLEQQKDALGLKNGTSYSDNK
VSQKLPSSSLVVESIIYGRDADKEMIFNWLRSETDNHNHPSIFSIVGMGGLGKTTLAHHVYCDPKMEEAKFDIKAWVCV
SDHFDVLTVTKTILEAITNSKDDSGNLEMVHGRLKDEISGRKFLLVLDDVWNEGGEEWEAVRTPLSYGAPGSKILVTARA
EKVASNMRSKVHRLKQLEDDESWNVFKKQALKDDDLELDNEKEEIGRRIVEKCKGLPLALKTIGSLLRTKSSISYWKSVLE
NDIWDLPKEVKIIPALLLSYQHLPSHLKRCFAYCALFPKDREFDKKELILLWMAEGFLHHSQQINNVEEIGEQYFNDLLTRS
FFLQSDFETYFCMHDLLNDLAKYVCADFCFRLKFDKGNCIPKKTRHFSFAFDDVECFDGFGSFTDAKRLRSFFPYEEFWG
RNIHYYPLQFKILVHELFSNFKFLRLLSLDGYSELKEVPDSVGDLKHLHSLDLSRTGIQKLPESTCLLYNLLILKLNYCLSLEELP
LNLHKLTKLHCIEFENTKVTKMPMHFGELKNLQVLSPVFIDKNKEFSTKHLGCLNLHGRLSINEVQNIVNPLDALEANLK
NKDLVELELKWESDHIPDDPRKEKKVLENLQPSKIVEYLSIENYGGTEFPSWVFDNSLSNLVSLWLEDCKYCLCMPPLGLL
SSLKNLAIIGFDGIVSIGDEFYGNSSSSFTSLESLKFSKMKELEECERKTAAFPRLQTVSVYECPKLKGLPDQLVNVKYLYIRK
ASCLERCEHTVSHNSLEALTFFVFPIMNISMSRSFDLLEQIRIFGICDSLTTFPLDFFPNLKDLSLFSCRNLQMISQDHTHTS
LEILSIRSCSRFDSFPSEGLSAPQLKRIDIDGAENLKLLPKRMQILLPSLIILMTIDCPKVEMFPDGGLPPYVELVSLSSFKLIAS
LKETLGTNTCLQSLRIENIDVEFFPDEVLLPHSITSLDICCCPNLKKMHYKGLCHLSYLKLYDCPNLQCLPEDGLPKSISSLRI
WSCPLLERRCQNPEGQDWNKIAHIEDLSVRSKV

>XP_027924195.1

MAAEVVGGALLSAFLQVAFDRLASPQFVDFFRGRKLDEKLLGNLNIMLHSINALADDAEQKQFRDPHVKAWLLSVKEA
VFDAEDLLGEIDYELTRCQVEAGSEPQTFTDKVSTFFNSTFSSFNKKIESEMKEVLEKLKYLEQQKDALGLKNGTSYSDSK
VSQKLPSSSLVVESIIYGRDADKEIIFNWLRSETDNHNHPSIFSIVGMGGLGKTTLAQHVYHDPKMEEAKFDIRAWVCVS
DHFDVLTVTKTILEAITNSKDDSGNLEMVHGRLKDQISGRKFLLVLDDVWNEGGEEWEAVRTPLSYGAPGSKILVTTRSK
KVASNMRSRVHLLEELREDKSWNVFEKHALKDDEIELNDDLKEIGRRIVVKCKGLPLALKTIGRLLHTKSSISDWENILESD
MWNLPKEASEIIPALLLSYHYLPTPLKRCFAYCALFPKDYEFAKKELILLWMAENFLHCPQEIRHPEEVGEQYFNDLLSRSF
FQQYEGNFVMHDLLNDLAKYVYGDICFRLKFDKGKYIPKTTRHFLFTPGDVKCFNGLESLSDAKRLRSFIAIARLRGRYLG
NYPWQFMISLHDLFSKIKFLRVLSFYGISDLKKVPDSIGNLKHLQSIDLEYTNIQKLPDSIGLLYNLLILKLSNCYDLEELPSNL
HKLTKLRCLEFERTKVTKMPKHFGELKNLQVLSTFLVDRNNECNIKQLGGLNLHGSLSIKEVQNIVNPLDALEANLKNKHL
VNLELEWKRKHIPDDPLKEKKVLENLQPSKHLERLSIENYGGTEFPSWVFNNSLSTLVFLCLENCKYCLCLPPLGLLSSLKTL
KIRGFHGIVSIGAEFCGSNSTSFTSLESLAIDNLKEWEEWECKTTFPCLRYLFINRCPKLKGTSEQLLNLKELFVSLRGKLIIYS
CPLVNIPITHYDFLEEVIIDGGCDSLTIFPLDFFPKLHLLHITGSRNLRGISNEHTHNHLKEMKIDECPQFESFPSEGLSAPQL

WKIEIKGARNLKLLPKRMQILLPSLTELRITDCPQVEMFEEGSLPSNLKEVSLSCFRLIASLREALGADTCLETLTIIEVDVQC
FPDEGLLPPSLTSLEIYNCPTLKKLNYKGLSHLSSLTLFGCPNLECLPEEGLPKSISYLKIWLCPLISKRCRNPEGEDWKKIAHI
KKRIIEPVALQKVFPLP

>XP_027924228.1

MADSAVSFVVEQLYQLLREEGNLLKGLGNDFADIKHELESIKAFLKDADRRAGDEGDTINEGIKTWVKQLREISFCIEDVI
DEYIMDVAYRGNHHPPCIASLQKIAHQIKTLKSRHRIASNIQDIKLAVQGIKERSERYKFQSAFEDGSLSSSRGAKDFKWD
DPRMASHFIEETEVVGFELPRDELIGSSIKGNDQLSLISVVGMGGLGKTTIAKHVFNNQQVKTHFYCRSFVTVSQSYTVR
ELLTEMIQKFCKDANEPTPKGLHNMDDETLVTELRQYLQSKRYLVVFDDVWKENFSDEIEHALPNNNKGSRIIITTRNM
QVAEYFKKSVVVHVHRLQHLSPDKAWELFCKKAFRFEPSEQCPTELEEMSKEIVQKCGGLPLAIVCIGGLLATKEKNILE
WRKVCQRMELERNTHLNSLKWILSLSYDDLPHNLKSCMLYFGVYPEDYSISRKRLTRQWMAEGFIKNEERRPTEDVAE
EYLTQLISRSLVQVSRVGFDGKVKNCQVHDLLRDIIIRKMNELSFCHIMREDDELDTVGITRRFSIASCSNNVLRKTSNSGI
RAIYVFKKSELPEDFAGSLSAKFKLLKVLDFESTMLSSVPNNLGNLFHLRYLNLSHTKIKSIPRSVGNLLNLETLDLRQTNVQ
VLPREIKNLTKLRLLPVYYRKYEGHYSMLNFTTGVKMQKGIGCLKSLQKLYFLEADHGGLELMQELKMLKQLRKLGIRLV
QTEHANALSSAIGEMRHLESLNVGAKDQDEIIDLNFVSTPTSLLVLNLKARVTKFPDWIPRLKYLVKLRLGLSNLEGDPLD
SLKDLPSLLRLNMWDNAYVGESLHFKRGGFPRLKELDLTRLSRLNSISIDEGALLGLEHFRFKDNPQMKVVPHGLKHLKN
LQFLGFADMPAELVESIDPAKDGQDYSVIKHIPLVLIRQNVGPKFHDYELRPIPTLAAKKCLVMVLNKENWKPSNNCTRS
SSLTLEIKFPTNSIPNERKKVRGTIFLKCLCWIHENSIIERCESKTEMAEAAISFALSEVFQILKEEKKLLSGINRDFLDIRDELE
SIQAFLKDADRKAADEANTNEGIRTWVKQVRQVSVRVEDVIDEYLRVIHQVPCHGFGASICKITNLIRTSLSRHRIAVEIQ
DIKLSLSLIKERSERYKFQVSQEKPSSSSRGWTTEGTGWNDPRMGSLFIEETEMVGFELPRDELLCWLLEGEKERTLISVV
GMGGLGKTTLAKHVFDSENVKNHFDCRACITVSQSYTVRRIFRDMIKQFCRETKDPLPEMLEEMDEKTLISELRQYLEH
KRYLIFFDDVWHEDFCDQVELAMPRNNRSSRIIITTRMIHVVEFFKKSFPVHIHSLQPLPSDKSWELFCKKAFKFELDGQC
PAELKGMSDEIVGKCKGLPLAIVAIGGLLSTKSKTVFEWQKVSQNLNLELHRNAHLTRLTKILSLSYDDLPYYLKPCVLYFGI
YPEDSSINHKRLTRQWIAEGFVKSDGRALEQVADEYLSELIYRSLVQVSWVGFEGKVKNCQVHDLLHEVIVRKMKDLSF
CHFVHEGDDESATSASTRRLSIDTSSSSINNVSKSTNFTHIRAIHAFGKGGAVELFSGPLASESRVLKVLDLESTSLNYVPR
NLGNIFHLKYLNLKNTKIRSIPKSVGRLQNLETLDIRETLVHELPSEINKLKKLRHLLAFHRNYEAEYSLLGFTTGVVMKKGIK
NLTSLQNLCYVEVEHGGIDLIQELRFLRQLRKLGLRRVRREHGREICASVTEMTHLESLNITAIGEDEIIDLNSISSIPQLRRL
HLKARLEKMPNWISKLEFLVKMRLALSNLKDDPLRSLEKLPNLLKLTIWDNAYAGEILHFQSGGFRKLKELNLARLNTVSA
ILIDKGALLSLEYVKITKITRLKTVPSGIKAMDNLKVIDFCDMPTELVESIDPQNGQDYWIINHVPLVFIRRWMGPKLNDF
EVRIIHSSTKELLTN

>XP_027924347.1

MFSSSLTYDVFLSFRGEDALHGFTGNLYKALCDRGIHTFIDDDDLESGEEISPALLKVIEESRIAIVVLSQNYASSCFCLDELA
VILNCKNKGLHVIPVFYKVDPSDVRHQKGSYGEALTKHQIRFKDMEKVQKWKMALRQVANLSGYHFEDGVGYEYKFIK
RIAEDVKHKIIRDSFTHVSDYPIGLDSQVLEVRKVLEFGTEDGVHMIGIHGMGGIGKSTLARAVYNDLIGENFDGLCFLEN
VRENTNKHGLKHLQSILLSQILGDKDIYFTSMQQGISTIKCRLKMKKVLLILDDVDKPEQLEAVAGGCDWFGPGSRIIITTR
NKQLLENHRVTMTYEVKELNNNDALQLLKWKAFRNVKVDPEYELVLNSLLTYASGLPLALEVIGGSLVGKSIEGWKTTV
KQYEMIPKMEILSILKVNFDALEEEEKSVFLDISRFLNVYAPTEVEDILNACYGRSMKHHIGMLVEKSLLKYDYFDNRLTM
HDLVRDMCISIAWEELRKEPGKNSGLWLHKDVIHVSNNTRIKGLNASSFKRMKTLQKLHKKFQFFNHFLRSSRALIWFR
FSSNCLPSALDNLLKKFENLTVMSFDDCKILTHIPDVSDLPNLKELSFKECENLITVHDSVGFLTKLKILRVVGCSKLISFPPL
NLNSLERLELSNCSSLENFQRFTTEEWESALNQYDSVGLESRSDAFSYDVFLSFRGLDTRYGFTGYLYKALHDSGIHTFIAD
ENLQRGEEITPTTVKAIKESRIAITVLSVNYASSTCCFDELATILDCLKRKRLLVLPVFYYVDPTLVQLQKDSFREALTKHEKR
LKHDMEKLLKWKMLLHQVAKLFFFHIDHGNGYEYEFIGKIVEWVSKKINPGHYPVGIESKVQEVMKLLDVGCDGGVH
MIGIHGIGGVGKSTLAKEVYNDLISYKFDASCFIENVREKSNKHGLQYLQSIILLNLLGEKDMKLTSVQQGISVIQRRLQKK

KVLLILDDVDKEEQLQAVVGRADWFGPGSRVIITTRDEQLLASHNVQTTYEVKGLNNDEALQLLKWKAFKKHYFDPSYE
ELLNHVVTFAAGIPLALEVIGSNLYGKSVEEWKPVIHKLKKCPNNPVETILKASFDSLEEKERSVFLDFACCFKGYELAEVE
DILQAQYGQNMKCYIDILVDKSLVKLIHGTKPCYDRVTLHDLIEDMGKDIVQQESLLEPGQRSRLWLLEDVREVLENNR
GTNKIEIICLDFPIFDQEEGVEWNGKAFQNMQNLRTLIIRNGSFSKGPEYLPDNLRVLEWWRYPSNSLPSDFHPKELAIC
KLPCSSISTIELTNLLKKSVNLRVFKFSNANV

>XP_027924348.1

MFSSSLTYDVFLSFRGEDALHGFTGNLYKALCDRGIHTFIDDDDLESGEEISPALLKVIEESRIAIVVLSQNYASSCFCLDELA
VILNCKNKGLHVIPVFYKVDPSDVRHQKGSYGEALTKHQIRFKDMEKVQKWKMALRQVANLSGYHFEDGVGYEYKFIK
RIAEDVKHKIIRDSFTHVSDYPIGLDSQVLEVRKVLEFGTEDGVHMIGIHGMGGIGKSTLARAVYNDLIGENFDGLCFLEN
VRENTNKHGLKHLQSILLSQILGDKDIYFTSMQQGISTIKCRLKMKKVLLILDDVDKPEQLEAVAGGCDWFGPGSRIIITTR
NKQLLENHRVTMTYEVKELNNNDALQLLKWKAFRNVKVDPEYELVLNSLLTYASGLPLALEVIGGSLVGKSIEGWKTTV
KQYEMIPKMEILSILKVNFDALEEEEKSVFLDISRFLNVYAPTEVEDILNACYGRSMKHHIGMLVEKSLLKYDYFDNRLTM
HDLVRDMCISIAWEELRKEPGKNSGLWLHKDVIHVSNNTRIKGLNASSFKRMKTLQKLHKKFQFFNHFLRSSRALIWFR
FSSNCLPSALDNLLKKFENLTVMSFDDCKILTHIPDVSDLPNLKELSFKECENLITVHDSVGFLTKLKILRVVGCSKLISFPPL
NLNSLERLELSNCSSLENFQRFTTEWESALNQYDSVGLESRSDAFSYDVFLSFRGLDTRYGFTGYLYKALHDSGIHTFIADE
NLQRGEEITPTTVKAIKESRIAITVLSVNYASSTCCFDELATILDCLKRKRLLVLPVFYYVDPTLVQLQKDSFREALTKHEKRL
KHDMEKLLKWKMLLHQVAKLFFFHIDHGNGYEYEFIGKIVEWVSKKINPGHYPVGIESKVQEVMKLLDVGCDGGVHM
IGIHGIGGVGKSTLAKEVYNDLISYKFDASCFIENVREKSNKHGLQYLQSIILLNLLGEKDMKLTSVQQGISVIQRRLQKKKV
LLILDDVDKEEQLQAVVGRADWFGPGSRVIITTRDEQLLASHNVQTTYEVKGLNNDEALQLLKWKAFKKHYFDPSYEEL
LNHVVTFAAGIPLALEVIGSNLYGKSVEEWKPVIHKLKKCPNNPVETILKASFDSLEEKERSVFLDFACCFKGYELAEVEDIL
QAQYGQNMKCYIDILVDKSLVKLIHGTKPCYDRVTLHDLIEDMGKDIVQQESLLEPGQRSRLWLLEDVREVLENNRGTN
KIEIICLDFPIFDQEEGVEWNGKAFQNMQNLRTLIIRNGSFSKGPEYLPDNLRVLEWWRYPSNSLPSDFHPKELAICKLPC
SSISTIELTNLLKKSVNLRVFKFSNANV

>XP_027924349.1

MATPSSRRFTYDVFLSFRGEDTRYDFTGNLYKALCDRGIHTFIDDEELQSGEEITAALVKAIEESRIAIVVLSHNYASSSFCL
DELATILHCQRKGLLVIPVFYKVDPSYVRHQKGSYEEALAKHQKRFKAQKEKLHKWKMALRQVADFSGYHFKDGDEYQ
YEFIGRIVERVCREINRAPLYVANYPVGLLSQVLKVKKLLDVGSDDVRMIGIHGMGGLGKTTLSVAVYNLIANDFDCLCFL
QNVREESNKHGLKHLQTILLSKTLGEKHINLASEHEGISMIQQRLQRKKVLLILDDVDRCEQLQALAGSPDWFGPGSRVII
TTRDTQPLASHQVKGTYEVKTLNAKDALQLLTWKAFKTEQVDPSYMEVLNDVVTYASGLPLALEVIGSNLFAKSVEEWK
SAINQYKRIPNNQILKILKVSFDALEEEEKCVFLDIACCFKGYKMTKVEVMLRALYDDCMKHHIGALVQKSLIKIYYSTTIV
MHDLIWDMGKQIDQRESSKKPGKGRRLWLPKDIIQVLRDNTGTRETEILCLDLSISEKEETLEWNANAFKRMKNLKILII
RNGKFSKGPNYFPESLRILEWHGYPSNCLPSNFHPNKLVTCKLPNSPFTSFGFNGSSKKFENLTNLNFDNCQLLTRMPDL
SDLPNLEKLYFEQCESLIALDDSIGFLNKLKILKAQRCKKLRRFPPLNLPSLKNLELSFCSSLENFPEILGEMGNIRELSLSKLAI
KELPVSFQNLTGLHRLFVGCYFLQLNSSVLISSLTTFRAFRCKEWKWINSKDGEEVGSTVSSNLRSFGVVDCDLNDDIFSA
GFTQLTTVKSLNLSRTNITFLPECIKEFHHLNDLDVSDCKRLEEIRGIPPNLRKFSAKDCRSLITSGSSMLLNQQLQEAGKT
DFVLPGGSIPEWLDRESRGPSISFWFRNQFPPKVLCFLIAPVRYDVLFHLVRPVVLINGKVQEYKPSYLKDVIMVELDHIH
LFDLHVLPFSEDLMKVASEKEWKHVEITYQGLFDTSFIKSMGIHIVKSERRGMEDIRYDDPNTTTKVCPCNFSVTFLPFFF
RFLFALILFLTLLIHLDFVNLSSLCVFNLRF

>XP_027924350.1

MATPSSRRFTYDVFLSFRGEDTRYDFTGNLYKALCDRGIHTFIDDEELQSGEEITAALVKAIEESRIAIVVLSHNYASSSFCL
DELATILHCQRKGLLVIPVFYKVDPSYVRHQKGSYEEALAKHQKRFKAQKEKLHKWKMALRQVADFSGYHFKDGDEYQ

YEFIGRIVERVCREINRAPLYVANYPVGLLSQVLKVKKLLDVGSDDVRMIGIHGMGGLGKTTLSVAVYNLIANDFDCLCFL
QNVREESNKHGLKHLQTILLSKTLGEKHINLASEHEGISMIQQRLQRKKVLLILDDVDRCEQLQALAGSPDWFGPGSRVII
TTRDTQPLASHQVKGTYEVKTLNAKDALQLLTWKAFKTEQVDPSYMEVLNDVVTYASGLPLALEVIGSNLFAKSVEEWK
SAINQYKRIPNNQILKILKVSFDALEEEEKCVFLDIACCFKGYKMTKVEVMLRALYDDCMKHHIGALVQKSLIKIYYSTTIV
MHDLIWDMGKQIDQRESSKKPGKGRRLWLPKDIIQVLRDNTGTRETEILCLDLSISEKEETLEWNANAFKRMKNLKILII
RNGKFSKGPNYFPESLRILEWHGYPSNCLPSNFHPNKLVTCKLPNSPFTSFGFNGSSKKFENLTNLNFDNCQLLTRMPDL
SDLPNLEKLYFEQYARSGNG

>XP_027924351.1

MGSVVDWVCHKINPGHLHVADYPVGLGWPVVELRKLLNVESDDGFHMIGIHGMGGVGKTTLALAIYNMIAICFEGSC
FLQNVREKSNKHGLEHLQSILLSKILGEKDINLASEHEGISMIRQRLQRKKVLLILDDVDKCEQLRALAGSPDWFGPSSRVI
ITTRDTHLLASHQVKRTYEFNPLNEKDALQLLTWKAFKTEQVDPSYIEVLNHAVTCASGLPLALEVIGSNLFAKSLEQCKS
AVNQYKRIPSSQILDKLKVSFDALEGEEKSVFLDIACCFRGYALAEVEIMLRALYDDCMKHHIGVLIDKSLIKIGWSSTIEM
HDLIEDMGRQIDRKESSKEPGKCRRLWLPKEIIQVLKNNTGTSRIEILCLDISVSEKEETLHCNEDAFEKMKNLKILIIRNGK
VSGAPSCLPESLRVLEWHGYPSNCLPSSFDPNKLVTCKLLKSHFTSFGFLGKKFENLTVLNFDNCQLLTRIPDVSDLSNLKK
LSFEGCKGLIALDDSIGFLNKLIILKAEGCTKLRRFPPLNLPSLEKLELSYCSSLKNFPEILGKMGNIGELSLSKLAIKELPVSFQ
NLTELHRLHISCDFYRLNSIVLTPKLVAIIVINCKDWKWVKSKDGEEVGSTVFSDLRYFELNSCNLNDDFFSAAFMQLATV
TSLHFREVNITFLPECIKEFHCLNYLNVTRCKRLEEIRGVPPNLKIFRAIHCTSLTSSGSSMLLNQQLHEVGETDFIFRGGSIP
SWFDKQSRGPSISFWFRHKFPPKVLCLTVAPLLDLVAEMIRPMVLINDKVQEHTFKPIDEVRLELDHMHLFGVRELHFD
DGLMEMPLEKEWKHVEVTYEGLVDTSLIKAIGIHVVKEESMGMKDIRFDDPYTSTKVWPCNFFIAFLPFFFLFSLALVLFT
SFMAYPTQL

>XP_027924354.1

MAASSTYDVFLSFRGGDTRYGFAGNLYKALRDRGIHTFMDDEMILKGEVPITALLNAIEESRIAIVVLSQNYASSCFCLDE
LAVILNCKNKGLHVIPVFYKVDPSDVRHQKGSYGEALTKHQRRFKDMEKVQKWKMALRQVANLSGYHFEDGVGYEYK
FIKRIAEDVYHKIIRDSFTHVSDYPIGLDSQVLEVRKLLDFGTEDGVHMIGIHGMGGIGKSTLARAVYNDLIGENFDGLCFL
ENVRENTNKHGLKHLQSILLSQILGDKDIYFTSMQQGISTIKCRLKMKKVLLILDDVDNPEQLEAVAGGCDWFGPGSRIII
TSRNKQLLENHRVTMTYEVKGLNNNDALQLLKWKAFRNEKVDPEYELVLNSLLTYASGLPLALEVIGGSLVGKSIEGWK
TTVKQYEMIPKMEILSILKVNFDALEEEEKSVFLDISRFLNVYAPTEVEDILNACYGRSMKHHIGMLVEKSLLKYDYFDNRL
TMHDLVRDMCIRIAWEELRKEPGKNSGLWLHKDVIHVSNNTRICESEFNYLDFSKSVKKRIKGWNASSFKRMKTLQKF
HKKFQFFNHFLRSSRTLIWFRFSSNCLPSALDNLLKKFENITVMSFDDCKILTHIPDVSDLPNLKELSFKECENLITVHDSVG
FLTKLKILRVVGCSKLISFPPLNLNSLEILELSNCSSLENFQRFTTGFKVFFFFVFCFRFYHYKLHDLTQPEYD

>XP_027924355.1

MAVTSLSQAFMYDVFINFRGLDTRQGFTGHLRKALDDSGIHVFVDDEGIQSGKKITPELKKAIEKSRIAITVFSTNYASSSF
CLDELALILDCSKRNGLLVLPVFYKVPPCHVRHQQGNYAEALARLEKRLLEERRHRSMENWKMALNEVADHAGFSFED
GRKEYEHELIEKIVERVFSFINNGEEKLHVADYPVGLGSQVPEIRKLLDVGCDDGAHMIGIHGMGGVGKSTLARAVYNLI
TDKFEGSCFLQNVREESNKHGLKHIHSIILSEVLGMKKINLASEQQGISIIKNRLKRKKVLLVLDDVDQHKQLQGIAGSPD
WFGPGSIVIITTRDKQLLASHEVKTTHEVKELNKEDALKLLKFKAFRMEDVDPTYTEVLNQVVTYASGIPLTLEVIGSNLFG
KSVQEWESAIKQYKRIPSNQILEMLKVSFDSLGEEEKSVFLDIVCCFKGYKLSEIEEKLRALYDNCMKYHIGVLVEKSLIKIN
HDERVTFHDLIEDIGKRIDRQQSPREPGKRRRLWLQEDIIQVLRDNSGTSEIKIICLYFPISDIQVVEWDGNAFTNMKSLKI
LIVRNGIFSQHISYLPESLKVLEWRAQVSF

>XP_027924356.1

MAVTSLSQAFMYDVFINFRGLDTRQGFTGHLRKALDDSGIHVFVDDEGIQSGKKITPELKKAIEKSRIAITVFSTNYASSSF
CLDELALILDCSKRNGLLVLPVFYKVPPCHVRHQQGNYAEALARLEKRLLEERRHRSMENWKMALNEVADHAGFSFED
GKEYEHELIEKIVERVFSFINNGEEKLHVADYPVGLGSQVPEIRKLLDVGCDDGAHMIGIHGMGGVGKSTLARAVYNLIT
DKFEGSCFLQNVREESNKHGLKHIHSIILSEVLGMKKINLASEQQGISIIKNRLKRKKVLLVLDDVDQHKQLQGIAGSPDW
FGPGSIVIITTRDKQLLASHEVKTTHEVKELNKEDALKLLKFKAFRMEDVDPTYTEVLNQVVTYASGIPLTLEVIGSNLFGKS
VQEWESAIKQYKRIPSNQILEMLKVSFDSLGEEEKSVFLDIVCCFKGYKLSEIEEKLRALYDNCMKYHIGVLVEKSLIKINHD
ERVTFHDLIEDIGKRIDRQQSPREPGKRRRLWLQEDIIQVLRDNSGTSEIKIICLYFPISDIQVVEWDGNAFTNMKSLKILIV
RNGIFSQHISYLPESLKVLEWRAQVSF

>XP_027924357.1

MAVTSLSQAFMYDVFINFRGLDTRQGFTGHLRKALDDSGIHVFVDDEGIQSGKKITPELKKAIEKSRIAITVFSTNYASSSF
CLDELALILDCSKRNGLLVLPVFYKVPPCHVRHQQGNYAEALARLEKRLLEERRHRSMENWKMALNEVADHAGFSFED
GRKEYEHELIEKIVERVFSFINNGEEKLHVADYPVGLGSQVPEIRKLLDVGCDDGAHMIGIHGMGGVGKSTLARAVYNLI
TDKFEGSCFLQNVREESNKHGLKHIHSIILSEVLGMKKINLASEQQGISIIKNRLKRKKVLLVLDDVDQHKQLQGIAGSPD
WFGPGSIVIITTRDKQLLASHEVKTTHEVKELNKEDALKLLKFKAFRMEDVDPTYTEVLNQVVTYASGIPLTLEVIGSNLFG
KSVQEWESAIKQYKRIPSNQILEMLKVSFDSLGEEEKSVFLDIVCCFKGYKLSEIEEKLRALYDNCMKYHIGVLVEKSLIKIN
HDERVTFHDLIEDIGKRIDRQQSPREPGKRRRLWLQEDIIQVLRDNSVSF

>XP_027924358.1

LLLLAFHCVEEEWESALNHDSVGLESSSDAFSYDVFLSFRGLDTRYGFTGYLYKALHDSGIHTFIDDENLQRGEEVTPTTV
KAIKESRIAITVLSVNYASSTCCFDELATILDCLKRKRLLVLPVFYYVDPTLVQLQKGSFGEALTKHEKRLKHDMEKLLKWK
MLLHQVAKLFFFHIDHRNAYEYEFIGKIVEWVSKKINPGHYPVGIESKVQDVMKLLDVGCDGGVHMIGIHGIGGVGKST
LAKEVYNDLISYKFDASCFIENVREKSNKHGLQYLQSIILLNLLGEKDMKLTSVQQGISVIQRRLQKKKVLLILDDVDKEEQL
QAVVGRADWFGPGSRVIITTRDEQLLASHNVQTTYEVKGLNNNDALQLLKWKAFKKHYFDPSYEDLLNHVVTFAAGIP
LALEVIGSNLYGKSVEEWKPVIHKLKKCPNNPVETILKASFDSLEEKEKSVFLDFACCFKGYELAEVEDILQAQYGQNMKC
YIDILVDKSLVKLIHGTKPCYDRVTFHDLIEDMGKDIVRQESLIEPGERSRLWLLEDVREVLENNRGTTKTEIICLDFPIFDQ
EEVVEWDGKAFQNMQNLRTLIIRNGSFSKGPEYLPDNLRVLEWWRYPSNSLPSDFHPKELALCKLPCSSISTIELINLLKK
LVNLRVLKFSNANV

>XP_027924359.1

MFYSSVIYDVFLSFRGEDTRHGFTGNLYKALCDKGIHTFIDDEDLQSGEEITPALVKAIEESRIAIVVLSQNYASSSFCLDEL
ATILHCHTQGLLVIPVFYKVNPSVVREHKGSYGDALSEHERRFEDLEKVLKWRMALSQVAEFSGYHVEDGVGYDYKFIK
RIVEEVYHRIIHASFVHIAEAFQMIQTMGMGGVGKSTLARAVYNDLIREMITSFGSHVSLKGLEPTKVKDIPHAHYDRSN
CLPFTFDIFWKELAKLTVLNVEQCEVLTEIPDLSNLPNLEKLSLKELRIFPLRNLTPIKSLQLSNCSILENFPRLTTGFRDFFFLS
ILFPVFIIFILFTS

>XP_027924360.1

MFYSSVIYDVFLSFRGEDTRHGFTGNLYKALCDKGIHTFIDDEDLQSGEEITPALVKAIEESRIAIVVLSQNYASSSFCLDEL
ATILHCHTQGLLVIPVFYKVNPSVVREHKGSYGDALSEHERRFEDLEKVLKWRMALSQVAEFSGYHVEDGVGYDYKFIK
RIVEEVYHRIIHASFVHIAEAFQMIQTMGMGGVGKSTLARAVYNDLIREMITSFGSHVSLKGLEPTKVKDIPHAHYDRSN
CLPFTFDIFWKELAKLTVLNVEQCEVLTEIPDLSNLPNLEKLSLKELRIFPLRNLTPIKSLQLSNCSILENFPRLTTGFRDFFFLS
ILFPVFIIFILFTS

>XP_027924361.1

MFYSSVIYDVFLSFRGEDTRHGFTGNLYKALCDKGIHTFIDDEDLQSGEEITPALVKAIEESRIAIVVLSQNYASSSFCLDEL
ATILHCHTQGLLVIPVFYKVNPSVVREHKGSYGDALSEHERRFEDLEKVLKWRMALSQVAEFSGYHVEDGVGYDYKFIK
RIVEEVYHRIIHASFVHIAEAFQMIQTMGMGGVGKSTLARAVYNDLIREMITSFGSHVSLKGLEPTKVKDIPHAHYDRSN
CLPFTFDIFWKELAKLTVLNVEQCEVLTEIPDLSNLPNLEKLSLKELRIFPLRNLTPIKSLQLSNCSILENFPRLTTGFRDFFFLS
ILFPVFIIFILFTS

>XP_027924363.1

MFYSSVIYDVFLSFRGEDTRHGFTGNLYKALCDKGIHTFIDDEDLQSGEEITPALVKAIEESRIAIVVLSQNYASSSFCLDEL
ATILHCHTQGLLVIPVFYKVNPSVVREHKGSYGDALSEHERRFEDLEKVLKWRMALSQVAEFSGYHVEDGVGYDYKFIK
RIVEEVYHRIIHASFVHIAEAFQMIQTMGMGGVGKSTLARAVYNDLIREMITSFGSHVSLKGLEPTKVKDIPHAHYDRSN
CLPFTFDIFWKELAKLTVLNVEQCEVLTEIPDLSNLPNLEKLSLKELRIFPLRNLTPIKSLQLSNCSILENFPRLTTGFRDFFFLS
ILFPVFIIFILFTS

>XP_027924364.1

MFYSSVIYDVFLSFRGEDTRHGFTGNLYKALCDKGIHTFIDDEDLQSGEEITPALVKAIEESRIAIVVLSQNYASSSFCLDEL
ATILHCHTQGLLVIPVFYKVNPSVVREHKGSYGDALSEHERRFEDLEKVLKWRMALSQVAEFSGYHVEDGVGYDYKFIK
RIVEEVYHRIIHASFVHIAEAFQMIQTMGMGGVGKSTLARAVYNDLIREMITSFGSHVSLKGLEPTKVKDIPHAHYDRSN
CLPFTFDIFWKELAKLTVLNVEQCEVLTEIPDLSNLPNLEKLSLKELRIFPLRNLTPIKSLQLSNCSILENFPRLTTGFRDFFFLS
ILFPVFIIFILFTS

>XP_027924365.1

MFYSSVIYDVFLSFRGEDTRHGFTGNLYKALCDKGIHTFIDDEDLQSGEEITPALVKAIEESRIAIVVLSQNYASSSFCLDEL
ATILHCHTQGLLVIPVFYKVNPSVVREHKGSYGDALSEHERRFEDLEKVLKWRMALSQVAEFSGYHVEDGVGYDYKFIK
RIVEEVYHRIIHASFVHIAEAFQMIQTMGMGGVGKSTLARAVYNDLIREMITSFGSHVSLKGLEPTKVKDIPHAHYDRSN
CLPFTFDIFWKELAKLTVLNVEQCEVLTEIPDLSNLPNLEKLSLKE

>XP_027924367.1

MLHGYVINKCIFIEVWSRSATKTLLLMAVISPSQAFSYDVFLNFRGSDTRQGFTGYLNKALHDSGIHVFIDDEGLQSGKIIT
PELKEAIEKSRIAIPVLSTNYASSSFCLDELAVILDCSRRNGLLVLPVFYKVPPRHVRHQQGSYGEALARLEKRLLEEHNME
NWKMALKEVAGHSGFHFEDGKEYEHKLVEKIVERVFSFINNGEERLHVADYPVGLGSQVLEIRKLLDARKNDASVNMI
GIHGMGGVGKSTLARAVYNLITDQFEGSCFLQNVREESNKHGLKHLQSIILSQVLGMKEINLASEQQGISIIKNRLKRKKV
LLILDDVDQHKQLQGIAGSSDWFGPGSIVIITTRDKQLLASHEVKTTHEMKELNKEDALKLLKFKAFKNGEVYPSYTEVLD
QVVTYASGIPLALEVIGSNLFGKSVQEWESAIKQYKRIPNNQIREILEVSFDSLGEEEKSVFLDIACCFKGYKLWEIEEKLCA
LYDNCMKYHIGVLIEKSLIKISHDERVTFHDLIEDMGKKIDHQQSPREPGKRRRLWLHEDIIQVLKDNSVSD

>XP_027924437.1

MATPRFGYDVFLSFRGEDTRYGFTGNLYKALCNRGIHTFIDDEELQSGEEITPALQKSIEESRIAIVVLSQNYASSSFCLDEL
ATIHYCHTQGQLVLPVFYKVQPSDVRHQKGGYGEALTKHQRRFKDKEKLQKWKMALRQVADLSGYHFKDGDGYEYKF
IESVVDRVCHKINPARLHVADYPVGLGPQVLEVRKLLNVECGDGFHMIGIHGMGGVGKTTLALAVHNLIADRFDGCCF
LQNVREKSKKHGLEHLQSILLSNILGAKDINLTSEHQGISMIQRRKPKKVLLILDDVDRCEQLQALVGSPDWFGPGSRVIIT
TRDIQLLASHQVKRTYSVKTLNKDDALQLLTWKAFKTEQVDPSYMEVLNDVVTYASGLPLALEVIGSNLFEKSVEEWKS
AINQYKRIPNNQILEILKVSFDALEEEEKCVFLDIACCFQGYELTEVEVMLRALYDDCMKYHIGVLVQKSLIKIYYSGTIVMH
DLIGDMGRQIDRRESSKKPGKGRRLWLLKDIIQVLRDNTGTSETEIICLDLSISEKEETLEWNANAFRRMKNLKILIIRNGK
FCKGPNYFPESLRILEWHGYPSNCLPSNFHSNKLVTCKLPNSPFTSFGFHGSSKKFENLTELNFDNCQLLTRMPDLSDLP

NLEKLSFERCESLIALDDSIGFLNKLKILKAQRCTKLRRFPPLNLPSLEVLHFPYCYSLENFPEILGKMGNIRELYLLNLAIKELP
VSFQNLTGLHELYAACDFLQLNSSALMSSLTTFYASRCKEWKWINSKDGEEVGSTLSSNLRSFDFELCDLNDDIFSAGFT
QLTTVTYLNLSYTNITFLPECIKEFQHLDDLEVSYCKYLQEIRGLPPNLREFRAIDCRSLTSSSSSMLLNQQLHEAGKTKFVL
PGGCIPEWLEKESRGPSISFWFRNQFPPKVFCFLIGSVRDDTHFYFIRPVVLINGKVQEYKAGYHTDVIMPELDHMQLFD
LHVLPFRYKLMKMASEKEWKHVEVTYEGLFDTSLIKAMGIHVFKSKRGGMKDIRYDDPYTTTKVCPCNFFIPFLPFFFRF
LFALILFISLMAYPT

>XP_027924588.1

MARRISLSLSSSTHTWIYDVFLSFRGEDTRFQFTHNLYHSLCEAGIHTFIDQEGLRIGEEITPVLFHAIQNSRISIIVFSKSYAS
STYCLNELVRILECAKEEGPSIYPIFYDVDPSEVRHQTGTYAEALSKLETRFHNDADNEKVQKWRKALHEAANLSGWHY
QHGSQPEYEFIRKIVQAISRKINYIPLYVADNPIGLEYALEGVKSLLEDGSEINMIGIYGIGGIGKTTIARAVYNNIFWGFQG
SCFLPDIREKAINKHGIVQLQELVLSEILKEKDIKVGDVNRGIPLIKRRLQQKKVLLVLDNVDKLEQLKALAGGYDWFGSGS
IIIITTRDKHLLDAHGVVNLYEVKPLHVEKALELFNWHAFRSDKVGPPYMSISNRAVSYACGLPLALEVIGSHLFGKSLDEC
HSALDKYESIPHRKIHEILKVSYDGLEENEKGIFLDIACFFNNCELGNVTPMLKAHGFYAEDGLRVLADRSLIKINSSDFVR
MHDLIRDTGREIVRQESTLEPGRRSRLWFNQDIVHVLEGNTGSDKIEFIKLEGYNNIQVQWNGKALKKMKNLRILIVED
ATFSTSPEHLPNSLRVLDWSCYPSPSLPSDFNPKRFEIILMPESSLLMFEPQKMLESLSIINLEDCKFLTNLPSLREASLLTTL
RLDRCCNLVNIDESIGFLDKLRLLSAKGCTKLKTLAPRIMLTSLETLDLAMCYNLESFPEVLGKMEKIKTIYLDDTDIEKLPFS
IGNFVGLELLSLKGCERLHQLPGSICMMPKVRVVIGYGHETYNFFEKELSSDVSPMAMLIGGSNLYLDVYYPYMNPNNG
IQVCSPNPLMHSDFNLLFSKLRREEDWYRRCRVSVMHFSFRKKFPKIALCCSLFFPAMKRVMIMTFNFRVYINDTLQFS
GMCNFMFRGYEKILWCDLEGKVERVFSEQEWNRAEIAFELDFPMRRNTRNGITTNSIGRGNLSWSLIGVYEEGNNKE
DIEFEDPMSIFPLSNTQPLSSLSSSLHYVVSLGFRQGWVDMD

>XP_027924589.1

MARRISLSLSSSTHTWIYDVFLSFRGEDTRFQFTHNLYHSLCEAGIHTFIDQEGLRIGEEITPVLFHAIQNSRISIIVFSKSYAS
STYCLNELVRILECAKEEGPSIYPIFYDVDPSEVRHQTGTYAEALSKLETRFHNDADNEKVQKWRKALHEAANLSGWHY
QHGSQPEYEFIRKIVQAISRKINYIPLYVADNPIGLEYALEGVKSLLEDGSEINMIGIYGIGGIGKTTIARAVYNNIFWGFQG
SCFLPDIREKAINKHGIVQLQELVLSEILKEKDIKVGDVNRGIPLIKRRLQQKKVLLVLDNVDKLEQLKALAGGYDWFGSGS
IIIITTRDKHLLDAHGVVNLYEVKPLHVEKALELFNWHAFRSDKVGPPYMSISNRAVSYACGLPLALEVIGSHLFGKSLDEC
HSALDKYESIPHRKIHEILKVSYDGLEENEKGIFLDIACFFNNCELGNVTPMLKAHGFYAEDGLRVLADRSLIKINSSDFVR
MHDLIRDTGREIVRQESTLEPGRRSRLWFNQDIVHVLEGNTLEGYNNIQVQWNGKALKKMKNLRILIVEDATFSTSPEH
LPNSLRVLDWSCYPSPSLPSDFNPKRFEIILMPESSLLMFEPQKMLESLSIINLEDCKFLTNLPSLREASLLTTLRLDRCCNLV
NIDESIGFLDKLRLLSAKGCTKLKTLAPRIMLTSLETLDLAMCYNLESFPEVLGKMEKIKTIYLDDTDIEKLPFSIGNFVGLELL
SLKGCERLHQLPGSICMMPKVRVVIGYGHETYNFFEKELSSDVSPMAMLIGGSNLYLDVYYPYMNPNNGIQVCSPNPL
MHSDFNLLFSKLRREEDWYRRCRVSVMHFSFRKKFPKIALCCSLFFPAMKRVMIMTFNFRVYINDTLQFSGMCNFMFR
GYEKILWCDLEGKVERVFSEQEWNRAEIAFELDFPMRRNTRNGITTNSIGRGNLSWSLIGVYEEGNNKEDIEFEDPMSIF
PLSNTQPLSSLSSSLHYVVSLGFRQGWVDMD

>XP_027924591.1

LRIKKGSKIRRRSSRDAQLLSSHQVKRAYEVKTLNAKDAPKLLTWKAFKTEQVDPSYVKVLNRVVAYTSGLPLALEVIGSN
LFAKSVEQWKSAVNKYKRIPNNQILEILKVSFDALEEEEKGVFLDIACCFKGYKLTEVEIMLRALYDDCMKHHIGVLIEKSLI
KVSQRGTVELHDLIKDMGREIDQKESPKEAGRRRRLWLPKDIIHVLKHNTGTSEI

>XP_027924702.1

MAAELVGGALLSAFLQVAFEKLASTQFADFFRGRKLDEKLLGNLNIMLHSINALAHDAEQKQFTDPHIKAWLFSVKEAV
FDAEDILDEIDYEITRCKVEAESEPQTFTYKVSNFFTSTFSSFNKKIDSGLKEVLEKLEYLAKQKGALGLKEGTYSDDRSRSKV
SQKLPSSSLVVESIIYGRDADKEIIFNWLRSETDNDNHPSIFSIVGMGGLGKTTLAHHVYRDPKMEEAKFDIKAWVCVSD
HFDVLTVTKTILEAITNSKDDSGNLEMVHGRLKDEISGRKFLLVLDDVWNEGGEEWEAVRTPLSYGAPGSKILVTARAEK
VASNMRSKVHLLRELRGDECWNVFKKHALKDDDLELNDDLKEIGRRIVEKCKGLPLALKTIGCLLRTKSSISYWKSVLEND
IWELPKEVKIIPALLLSYQHLPSHLKRCFAYCALFPKDHEFDKKELILLWMAEGFLHHSQQINNVEEIGEQYFNDLLTRSFF
LQSDFKTYFSMHDLLNDLAKYVCADFCFRLKFDKGNCIPKKTRHFSFAFDDVECFDGFGSFTDAKRLRSFFPYEEFGGRNI
DYYPLQFKILVHELFSNFKFLRVLSLDGYSELKEVPDSVGDLKHLHSLDLSRTGIQKLPESTCLLYNLLILKLNYCLSLEELPLN
LHKLTKLHCIEFENTKVTKMPMHFGELKNLQVLSPVFIDKNKEFSTKHLGCLNLHGRLSINEVQNIVNPVDALEANLKNK
DLVELELKWESDHIPDDPRKEKKVLENLQPSKIVEYLSIENYGGTEFPSWVFDNSLSNLVFLRLEDCKYCLCLPPLGLLSSLK
TLEIIGFDGIVSIGDEFYGNSSSSFTSLESLAFSKMKELEECERKTAAFPRLEYLDVYECPKLKGLPDQLVNVKNLYIRKASCL
ERCEHTVSHNSLEALTFLVFPIMNISMSRSFDLLEEILIFDSCDSLTTFPLDFFPNLKYLSLCSCRNLQIISQKHTHNRLKHLTI
RSCSRFDSFPSEGLSAPQLLFIDIDGVENLKLLPKRIRILLPSLYILNIINCPKVEMFPDGGLPPYVGQVSLSSLKLIASLKETLG
TNTFMKRLCIENIDVEFFPDEVLLPHSITSLEICRCPNLKKMEYKGLCHLSYLKLYDCPNLQCLPEDGLPKSISSLRIWSCPLL
ERRCQNPEGQDWNKIAHIET

>XP_027924836.1

MAIQNSPAFTYDVFLSFRGEDTRHGFTGNLYKALCDRGIHTFIDDEDLQSGEEITPALAKAIEDSRIAIVVLSKNYASSSFCL
DELVVILDCKNKGLLVIPVFYKVDPSDVRHQKESYGEALTKRQRRFEDVEKVQKWKMALRQVADLSGYHFKDCDGYEY
KFIGSVVDQVCQKINPARLHADYTVGLGPQVLRLRKLLNVECGDGFHMIGIHGMGGVGKTTLALALYNLIAGCFDGSCF
LGNVREKSNKDGLEHLQSILLSKILGEKDIKLASKHEGISMIQRRLQRKKVLLILDDVDTCEQLQALAGSPDWFGPGSRVV
ITTRDTQPLASHHVKETYEVNPLNKDDALQLLTRKAFKTEQVDASYVEVLNHAVTYASGLPLALEVIGSNLAGKSVEKWK
SAINQYKRIPNNQILEILKVSFEGLEREEKSVFLDIACCFKGYALREVEDILGAIYDDCMKHHISVLVDKSLIKIGWWSTVEI
HDLIEEMGRQIDQQESPEGLGKRRRISLPKVIIQVLKENTETSMIEILCLDISIYKEVERLDWNGNAFGNMKNLKALIIRNC
KISGGPNCFPESLRVLEWHGYPSNCFPSNFDPNKLLICKLPDSDFTSFKFPGSSKKFENLTELNFDYCNLLTQIPNVSHLPN
LEKLSFKECASLIAVDDSVGFLTKLKILIAEECAELKRFPPLNLPSLEELELSDCFSLENFPEILGKTGKIKKLRLVRLPMIKELPV
SFQNLTGLRYLEMTGCHFLRLNSNILTSALTHFRVFGCKEWKWINSKDGEEVGSTVSSNLRSFGVMYCDLNDDIFSADF
TQLTTVTSLNLSGTNITFLPECIKEFQHLDDLDVSYCKYLQEIRGIPPKLRKFRAKDCRSLTSSSSSMFLNQQLHEARETDFI
FPGGSIPRWFEKQSRGPSICFWFRNKFPAKVVCLLILSLQQPEYAELVKPMVLINGLLRGSYDYSYYLKREEGIVELDHVYL
FDLRVLPFQDDLMEMPLEEEWKHVEVTYQGMFDTSLIKGMGIHVVKTERRSMEDIRYDYPL

>XP_027924838.1

MSSSFSYDVFLSFRGSDTRYGFTGNLYKALCDKGIHAFIDDEELQRGDEIGEALIEAIKQSRMAIVVFSKNYASSSFCLDEL
VKIIDCVKEKSRLLLPIFYDVHPSHVRGQSGSYAEALAMHQERFKSSNQSLNDNMGRLQKWKMALNQAACLSGKHYKL
GNEYEHEFIGKIVKEVSNKINRRPLHVADYPVGMECRVQKVKSLLQFGSDSGVHIVGIYGIGGMGKTTLARAVYNSIAD
QFEGLCFLDGVRENAVKHGLVHLQEMLLSEIVGEKDIKIGSVSKGISIIKHRLHRKKVLLILDDVDKLEQVRATVGVPNWF
GSGSRVIITTRDKHLLQGVDGKYEIEDLNEEEALELLSWNAFKDDKVDPSYKDISNQAVAYASGLPLALEVIGSLLFGKGIR
EWESALDQFKKIPNKRIQEILKVSYNALEENQQRIFLDIACCLKGYEFEEVEDILGAHYGVCMKYDIGVLVDKSLIKIKNGC
VTLHELIEVMGKEIDRQESPKELGKRRRLWFHKDIIQVLAENTGTSEIEIICFDFPLFEEDEEVFVEWDGGAFKKMKNLKT
LIIRNSHFSKGPAYLPNSLRVLEWWTYPLQDLPTDFHPNKLAICKLPRSCFTSLELATISKKFMNLTVLKFDGTECLTKIPDI
SSLQNLEKLTFECCENLVAIHDSVGFLDKLKILSAFGCSKLTSFPPIKLISLEQLDLSSCSSLESFPEILGKMENITQLELKYTPL
KEFPFSFRNLARLQDLVLVDCGNVQLPSSIVMLSELAEIFALGCKGWLLPKQDENDEQKVSLVSSNVKCLCLSGCNLSDE
YFPMVLAWFGNVKELELSSNSFTFLPECIKQCRSLKLLNLDNCEHLREIRGTPPNLEYFSAGNCKSLSFCCSAMLLDQELH
EAGNTMFCLPGSWIPEWLEQQSIGPSLSFWFREKFPVMDLCFVIGPMGTDSILFRPIMTINGNTMEINSLTDKRFCFDF

PASDYHILIIGTKYMKFGDNLDKPLSKNEWNHVVVSIALDFEPTPKEIIVKQTALHVIKPESSMDDIQFTDPCNQPSFKEK
QRLVDTVDCHRQFMQQQTTLVSLEPLSMNPPQACKNNLNWDSFSTGTSSIASVQEYEIASQKLRLDMGILQFVQQRK
RLAILGLLQQRRTASLDLLQRRGRELLSLLSSPSLELMVSWERRCITSVQGLQEQHLPSTIKQNFEGCNGINDAKVNEVIR
CNNNSGNDNERTPLMEELLMTKENDVHSKRYQHVGKMASDPMELEYLLHIQKINFSQR

>XP_027924839.1

MECRVQKVKSLLQFGSDSGVHIVGIYGIGGMGKTTLARAVYNSIADQFEGLCFLDGVRENAVKHGLVHLQEMLLSEIVG
EKDIKIGSVSKGISIIKHRLHRKKVLLILDDVDKLEQVRATVGVPNWFGSGSRVIITTRDKHLLQGVDGKYEIEDLNEEEALE
LLSWNAFKDDKVDPSYKDISNQAVAYASGLPLALEVIGSLLFGKGIREWESALDQFKKIPNKRIQEILKVSYNALEENQQR
IFLDIACCLKGYEFEEVEDILGAHYGVCMKYDIGVLVDKSLIKIKNGCVTLHELIEVMGKEIDRQESPKELGKRRRLWFHKD
IIQVLAENTGTSEIEIICFDFPLFEEDEEVFVEWDGGAFKKMKNLKTLIIRNSHFSKGPAYLPNSLRVLEWWTYPLQDLPTD
FHPNKLAICKLPRSCFTSLELATISKKFMNLTVLKFDGTECLTKIPDISSLQNLEKLTFECCENLVAIHDSVGFLDKLKILSAFG
CSKLTSFPPIKLISLEQLDLSSCSSLESFPEILGKMENITQLELKYTPLKEFPFSFRNLARLQDLVLVDCGNVQLPSSIVMLSEL
AEIFALGCKGWLLPKQDENDEQKVSLVSSNVKCLCLSGCNLSDEYFPMVLAWFGNVKELELSSNSFTFLPECIKQCRSLKL
LNLDNCEHLREIRGTPPNLEYFSAGNCKSLSFCCSAMLLDQELHEAGNTMFCLPGSWIPEWLEQQSIGPSLSFWFREKF
PVMDLCFVIGPMGTDSILFRPIMTINGNTMEINSLTDKRFCFDFPASDYHILIIGTKYMKFGDNLDKPLSKNEWNHVVVS
IALDFEPTPKEIIVKQTALHVIKPESSMDDIQFTDPCNQPSFKEKQRLVDTVDCHRQFMQQQTTLVSLEPLSMNPPQAC
KNNLNWDSFSTGTSSIASVQEYEIASQKLRLDMGILQFVQQRKRLAILGLLQQRRTASLDLLQRRGRELLSLLSSPSLELM
VSWERRCITSVQGLQEQHLPSTIKQNFEGCNGINDAKVNEVIRCNNNSGNDNERTPLMEELLMTKENDVHSKRYQHV
GKMASDPMELEYLLHIQKINFSQR

>XP_027924840.1

MATPSSRRFTYDVFLSFRGEDTRYDFTGNLYKSLCDRGIHTFIDNEELQSGEKITAALVKAIEESRIAIVVLSHNYASSSFCL
DELAAILDCKNKGLLVIPVFYKVDPSYVRHQKGSYGEALTKRQRRFEDTQKVQKWEAALRQVANLSGDHFKEGDEYQY
EFIGRIIERVSREINRAPLHVADYPVGLLSQVLEVKELLDVGSDDVHMIGIHGMGGLGKTTLSVAVYNLIADDFDSSCFLQ
NVREESKKHGLKHLQTILLSKILGEKDINLASEHEGISMIQQRLQRKKVLLILDDVDKWKQLQALAGRSDWFGPGSRIIITT
RDQQLLKSHEIERTYEVEKLNRNDSLQLLKWKAFKKEQVDPSYVEVLDDVVTYASGLPLALEVIGSNLLAKSVEEWKSAI
NQYKRIPSNQILEILKVSFEDLESEEKSVFLDIACCFKGYILREVEDILGAIYDDCMKHHISVLVDKSLIKVKNDWWSKVEIH
DLIEEMGRQIDRQESPEGSGKRRRILLPKDLIQVLKGNTGTSIIEILSLDISIYMGKETLDWNAFGNMKNLKVLIIRNFGISG
GPNCFPESLRVLKWDGYPSNCFPSNFDPNKLLICKLAFGRFTSFKFPDSSKKFENLTELNFDDCNLLEQIPDVSDLPNLEKL
SFQWCGSLVSVHNSVGFLTKLKILIAERCEKLRRFPPLNLTSLETLELSHCLSLENFPEILGKMGNIRKLSLRRLPIKELPDSF
QNLTGLQELELNCDFIQLSGNVLTPELTSLCVVTFKEWKWVKSKEGEEDVGSTVSSNVQSFWGSYNLDDDFFSAVFPQL
AQVKQLWLVRSNVSFLPECMKEFHHLNDLGVSFCKYLKEIRGIPPNLRKFRAVECRSLTSSSSSILLNKQLHEAGKTDFIFQ
GGSIPSWFDKQSRGPSISFWFRNKFPAKVVSLVILALQRPQFAETVRPMVLINGEYRGSYDYSYYLKREEGIVELDHVYLF
DLRVTPFRDDLTEMPLEEEWKHVEVTYEGMYDTSLIKAMGIHVVKTERTSMEDIRYDIPF

>XP_027924841.1

MAMGSRSSSFTYDVFLSFRGEDTRHGFTGHLYKALHDRGIYTFIDDEELQRGEEITPALLKAIQESKIAIIVLSMNYASSSF
CLDELDCILECYNKKDMLVLPVFFKVDPSDVRDQKGSYGEALVKHEQRFNHNMEKLEKWKKALRQVADVSGFHLKHG
DGYEHEFIGRIVELVSSEINHAALPVVDYPVGLESQALEVSKLLEVGSDDDVCMVGIHGIGGIGKSTLALAVYNLIAHHFD
CSCFLQNVRQKSNKHGLQHLQSILLREMLGEKEVNFASIEQGASVIHHRLQRKKFILILDDVDKYGQLQAIVGRPDLLGP
GSRVIITTRDKQLLSSYGVTKTYEVRVLNKNNALDLLSWKAFKTKNIDASYKEVLNDVVIHASGLPLALEVIGSNLFGKTIEE
WKSAIKQYKRIPNNQIMEILRVSFDYLEEEEKSVFLDICCLNIYALSKLENLLHAHYGYCMKYHIGVLVDKSLIKFNCYRQET
RISLHSLIEDMGKEIVRQESPKYPGKRSRLWLPEDIIQVLEDDKGSSEIEIICLDLPEFVEEAIVELNSKAFKKMENLKTLILRN

ANFSKGPKYLPNSLRVLEWWGYPSHCLPSDFHPKKLVICKLPRSSISTLELSKVWQSFVNLRELNFDGCKCLTEIPDISVLQ
NLEELSFEDCVNLITVHNSVGFLDKLKTLSANGCRKLRTFPPIKLTSLEKLELSHCSSLESFPEILIKMENIRELHLCYSLVTELP
LSCQNLTRLRTLEMFSLNSAIVKVPSSIIMMPELTDIFVCGLKGLQWLKQEEGEEQMGQVVVPSKVERLSVLSCNLNDD
FFSIDFTRFALVKELALPENNFTILPECLKQCQFLWILDVSGCKHLQEIRGIPPNLRHFFAIKCISLASSSKRMLLNQELHEAR
NTVFCLPGAELPKWFNHQNRGTSISFWFRNKFPDKVLCVLVAPIENDFFRSRVFINGKAYTGYYSHHHLTGEHHVYLFD
MREELRFRDSPYEVPFESEWNHAKVTFPEGIDTSINHAKIGIHIVKQKNSMEDVKFTDPCSKTKSDEDINSSDSQSTSY

>XP_027924852.1

MALRSCSSSFIYEVFLSFRGEDTRHGFTGNLYKALDDKGIHTFIDDEELQSGEEITPALLNAIEESRIGITVLSKDYASSSFCL
DELTTILECRMKGLLVIPVFYMVDPSDVRQQKSTYGEALAKHQKRFKPEKLQKWKMALRQVADLSGYHFKHGNEYEHE
FIGRIVERVSREINSVHLPVDDYVVGLESKVQEVKKLLDLGSHDGVVIGIHGMGGIGKTTLALAVHNLIAENFKESYFLQD
VREESDKNGLKHLQSILLSKMFREKNIILTSWQEGASMIQQRLRRKKVLLILDDVDKLEQLNAFVGRSNWFGPGSRVIITT
RDQHLLTSYDVKRTYEVKKLKNYDALQLLTWKTFKTGMADPSYEEVLHRAVTYASGLPLALEVIGSNLVGKSVEEWESAI
EHYKRIPSDEILKILKVSFDTLGREEKNVFLDIACCLKGCKLAEIEHMFRALYDDSMKYHIGVLVEKSLIKISQRRTVEMHDLI
QDMSRRIEQQESPKEPGKCKRLWLPKDIIQVLKYNTGTWKIEIICLDISISEREETIYWNGNAFRKMKNLKILIIRNGKFSE
GPNYFPESLRVLEWHGYPSNCLPSNFDTTKLVICKLPHNPFTSFRFHGSSKNFNLTVLNFDKCNYLTQIPNLSDLLNLEVLS
FEKCTSLLAVHDSIGFLNKLKILKAEGCTKLSRFPPLNLTSLEELELSYCSSLENFPEILGKMGNIRELSLFELPIKELPVSFHNLI
GLQELAIQCDFVQLGSIALTPELPDFRVYKGKGWQWVKSEEGEEKVGSMVSPKMHKFWVLSCNLNDDFFSACFKQLA
QVSDLRLRESNVTFLPECIKEFHSLSSLDVNDCKHLQEIRGVPPNLKYFRAINCISLTSTGSSMLLNQHLHEAGGTDFIFPG
GTIPEWFHKQRKGTSISFWFRKKFPAKVLCLLIAPVLGDNIIGLVRHMMLINGKVKKQYLLHYLNREVKKMELDYTHLFD
LRQSHFSPDDDLMEEVSLEKEWNHVEITYEGLIQSSLIKIIGIHVVKEENSSMEDIRYDDPYAITKPFIIKYTALMLTFIFFTG
SFLLFSYF

>XP_027924854.1

MATPRFRYDVFLSFRGEDTRHGFTGNLYKALCNRGIHTFIDDEDLQRGEEITPSLRKAIEESRIAIVVLSQNYASSSFCLDEL
ATILHCHTQGHLVLPVFYKVQPSDVRHQRGSYGEELTKHQRRFKDKEKLQKWKMALRQVADLSGYHFKDADGYEYKFI
ESVVDRVCHKINPARLHVADYPVGLGPQVLELKKLLNVECGDGFHMIGIHGMGGVGKTTLALALYNLIADYFDSSCFVQ
NVREKSKKHGLEHLQSIILSNLLGAKDINLTSEHQGISMIQLLLQRKKVLLILDDVDRCEQLQALTGSPDWFGPGSRVIITT
RDTQLLASHQVKGTYEVDTLDAKDALQLLTWKAFKTEQVDPSYMEVLNDVVTYASGLPLALEVIGSNLFAKSVEQWKS
AINQYKRIPNNKIQEILKVSFDALEEEEKCVFLDIACCFEGYELTKVEVMLRALYDDCMKYHIGVLVQKSLIKINYPGIIVMH
DLIGDMGKQIDQRESSKKPGKGRRLWLPKDIIQVLMDNTGTSETEILCLDLSISEKEETLEWNANAFRRMKNLKILIIRNG
KFSKGPNYFPESLRILEWHGYPSNCLPSNSHPNKLVTCKLPNSSFTSFGFNGSSKKFKNLTELNFDNCKILTRIPDVSDLPN
LEKLSFERCERVIAVDDSIGFLNKLKILKAQHCTKLRRFPPLNLPSLKVLQLSHCYSLENFPEILGKMGNIREPFLFKVGIEELP
VSFQHLTGLHELYAECDFLQLNSSVLTSSLTGFFANGCKEWKWINSKDGEEVGSRVSSTLRSFEQLHEAGKTDFVLPGGS
IPEWLDKESWGPSISFWFRNQFPPKVLCFFIASVRNDIIYHLVRPVVLINGKVQGYKAGYYMHEVMAELDHMQLFDLH
VLPFSEDLMKMASEKEWKHVEITYQGLLINSFIKAMGIHIVKSERRGMEDIRYDDPNTTTKVWPCNFSVTFLPFFFRFLF
ALILFLTLLIHLDFVNLSSLCVFNLRF

>XP_027924874.1

MSHDKATKRLRRGREKPVLDNHAPKKLKYWVEEKSSENNTPQIKYDVFVSFRVEDIRHTLLSHLIGAFQRKKICAFIDAEL
QRGHEIGPSLFQAIERSDILLIIFSPHYASSHWCLEELEKILECRDKYERTVIPVFYNVQPTDVRHQLRTYENAFVEHELNYQ
NKVQIWKDALKHSADLSGIDSSKFPCDFELVEEIATVVLKGLAKPLVVAKELVGIDEKIATIESWIKKDSKGTCLIGIWGMG
GIGKTTLAEEVFNKLHSEYEACYFLAHEREESNKHGIISLKEKIFSELLGCEVKIYTQNSLPGHIARRISRMKVLIVLDDVNNS
DHIKNLLGDFGNFGSGSTIIVTTRNEQVLKTNKVDETYQLTKLSFNEALELFHLLVGKQNDNQREYNELCERVVHYAQGI

PLVLKVLAGHLRGNKKEVWESELDKLKGMPHKEVYDVMTWSFYDLDRKQQQIFLTEWINVGELKFLLKDSERDNSVVA
DLQRLKEKSLITVSKDNFVSMHDNLQDMAWEIVRQESIEDPGRRSRLWDPDDIYEALKSDKVSLNEYISFLLTNFWFTH
TWILNNYEFDCLNQLHILAEGLQFLATELRFIAWLGCPLKSLPENFSAEKLVILKLPDSNMEKLWDGVKNLVNLIEVDLSG
SEKLKELPDLSKATNLEVLYLGGCSALICVHPSIFSLAKLKKLELWGCVSLTTLTSNCHLGSLSFLDLDYCKNLTEFSVISENM
EELRLERTKVKALPSSFEFQSKLRFLDLAGSDIESLPSSLTNLNQLLYLEVSNTSLQVITYNSKSKFLYSTSNSTKSSTLPPSLKT
LSAQYCNSLQILPELPLSLETLNAKFCPSLQTLSKLHQSLTTLDVAGCESLQILPNIPQSLETLDVSFCKSLQTIPKLPLSLKNL
AIRHLSLVKTLPNLPQSLQTLHTEFFLLLRTLPEIPHSLRTLNVVFWTSLQTLPELPPSLETLSAQHCYSLQILPKLPPSLKTLD
VTHNVSLQTLPQLPQSLQTLTAKSCTLLQILPKLPLSLETLKVEYCSSLRTLPNLPQSLTTLDITYCKSLQTLPDIPQFLETLDF
SFCKSLQILPKLPLSLKTLVVGHLSSLQTLPDLPQSLQTLNTKFCSSLLTLPNLPHSLKTLEVPGCKSLQTLPDLP

>XP_027924944.1

MAIRSHSQKFTYDVFLNFRGSDTRYGFAGNLYKALDDRGIHTFIDDEKLQGGDELAPTLVKAIQESRIAITVLSHTYASSSF
CLDELVYILERAEEKKLLVLPVFYNVDPSFVRYQEGSYGEALARHEERLKANNNKEKLEKWKMALHQVANFSGFHYKYG
EEYEYKFIGRIVEWVSGEINRAPLHVVDYPVGLDSQVLKVMKLLDVGSGDGVHLIGFYGMGGVGKTSLASAVYNLIAGH
FDGSCFLQNVREKSNKHGLDHLQSIILSEILGDKRIMFPSEQQGISMIQHRLQKKRLLLILDDVDKHEQLQTLVGRPHWV
GRGSKVIITTRDKHLLTSHHVQETYEVKKLNKNHALQLLTWKVFKSEHVYPAYVEVLNRAVTYACGLPLALEVIGANLCG
KSIQECESVIDQYKIIPNNRIQETLKVSFDALQEEEKRVFLDIACCFKGYKLTEVEDILHAHHGACMKYQICVLAEKSLIKID
QYDRVTLHDLVEDMGKEIVRQESPEEPGKRSRLWLPNAIIQVLEDNTGTSEIKIICLDFPLFEKKMVEWDGMAFQKTEN
LKTLIIRNGIFSDDPKCFPNSLRVLEWWRYPSHCLPSYFQPKQLAICKLPHSLFMSFQMGGLSKKLRNLRVLNFDCCECLT
QIPDAVSNLQNLEELSFKNCVNLVRVHNSVGLLHKLRILEASGCIKLRNFPPLKLSSLEKLELSHCSSLKSFPEIIGKMENIRE
LRLLGTLIKELPLSFQNLTRLRKLSLLFCGIVQLQSSIVMMPELTLIEAWGWKGWQWTKREEDEEKDGSMVPSKVELLW
ASKCNLCDDFFQIGFTRFAHVIDLDLSNNNFTHLPECIKECQFLKKLDVSCCRQLREIRGIPPKLKHFNATNCLSLTSSSISM
FLNQDLHETRKTLLLLPGSRNPEWFNHTSYRPSSSFWIRNKFPGKVLCLFVAPKDRDISDYVKPMLLINDKVYVCFFDRLK
FLKLGAEHTFLFDLRNLIFTNNLYEVPLENEWNHVKVTCFDLTAASMPTPVPIQSGIHVFKQENSDEDIMFTDPYTKKRE
LEMILPLSPSIILDLDLNFDYLDF

>XP_027924961.1

MQCLIALATGTVTKLGESLVAPIGNQFGYLVHYKKNIKDLKRELKTLEGRKQGVQGVVDEDRRNGRQIVSIVQDWLYKV
ERIIDEIEKINDFQVENNKCLHIWSPNLVSRYSLSKKAKILIMSVTRLNEEKFDIISYSLPTPRLGSTFSNVIKSFPSRKSIIIEVL
EKLKDEEFKIIGICGMGGVGKTTLVKEVIKTLDVCKLFDEVVMVVVSQNLDYVKIQGQIADALGLRFDKETIQGRACQLH
ERLKGVNNILIVLDDVWIDFDFESIGIPSNEHHKNCKILFTSRNEDVCYKMGSQKNFTISILSPEESWDLFHDMLGRNLST
KLDILDIAKEVSNECGGLPIAIVTMAKALANKEKYTWEDALHQLKRSSITSLLEMQACVYSSIKLSYDFLDSAEEKKVIFLCC
LFPEDFDIPIEVLLRKGMGLRLFKGIDALWKVRNRVHTIVDKLKRCFMLLDGNVEECVKMHDVVRDVIISVASTKEFGFM
VECDGYQMEQPKEETCCHSTAISLISKEAKEHPKVLNPKLKLLQIASKKKDLVPDNFFQCANKLMVLSLQNVHIHSMSSV
FQALGNIHTLLLEDCHVRDVSIIGKQLKRLEILSFSNSNIKELPEEIGQLSSLRLLDLTECNDLIQISANVLASLSRLEELYLRVR
SLSSKETNHILFELQSLSHHLKVIEIVIWTNEDLPKDLFFKNIERFWVYLGDSSSLFHGIVRKGYLQPNILKLNNAYYKYIKM
SVTIQQLLEKVEILNLVDIKNMKDVISELNERGFPFLKHLSIEFCNNIEYVVNAFEGCIFPQLLSFSLSNLDNLKDIFNVSGS
MSQTNDFAKVKPITSSQCFGILRQLKIEYCNKLKTVFTLFPRTINLATLQCLHVVESYGIECIISSNSKYDGKSIITFSNLVELK
LQELPNLMGMFKTNVIHEHCSSTIQIHESNDQLIDQIKPIATLFKSSCMQLFPKLEKLFLQACSSLEMVFDLQKSQFHGES
MVFLFPQLKEIEISWLSKLRHIWGNVPSYIQGFQNVKSIKVKKCDSLGFLLTPNIARALTQLQKMVIHSCHSMEKIVGKEE
NLNGDDEEKNVETLVFGQLESLTLIDLPNLTSICSDSYEVMMPSLRFLCIDACPQLVTSSMFTQTVSSQENFNASTTCDV
ANGTFEEDSPRFLQCCLKCTPHVFSNLKLKASSTKKDVSSVSNIYSKSETVSHIPILEQMQVKGWDSLEILFLLKQNQLSD
DTDTNCVVKLILAQTLKNPVEITAFNNLTVLTIDSCHKLKHLFSYSIAKLLVKLQEIKMSNCKVIKQLVQREGEDNLTLFLPQ
SSCVKVHENSSSSDHVTSSQEACGLEWLSLKRISISHCGVLEVVIGEIREKVDTIIASFAQLQSLTLSHLPNAASFCLTHCAS

ESPHFENIHGSGYQNHEVTSNEEIRNVDPFINGFIFPNLTYLAITSCNKVRSLFSPSTSTSFVRLVELDISGCREIEEIVSAEET
QGNVIKIVFHSLQRLKLENLPKLKAFCQGSYGFDFPSLHQVFLKNCHMMETFSHDPSYTPKLETVIMEIGSITKNMWMG
DLNATVPLSKGLLAFQTSETLGWIQQDKCMQRYFTHEKHLTVEGFERLLKLVPSNVIHIFQNLKELTIKNCGSLVEVFESH
GVDAKQMHAMIHYKIEALNLYFLPKLINLWKNYGGVLGFQKLRILKVQHCGNLCNLFSPSIARSLVQLRHLRVHSCHM
MEEITTKEDEESEGPNNAKIVFPLLNKLELRYIPNLKCFCSGTFNIDLPSCEEMIIEKCPMMTTFCYGSVTTAKLPHIYKGSY
EYVDIMGDLNMTIYHANESLKVAQQTSETITCIEHGQKLLPYLRSDTELVVQGSEKLLHCIPSSMLHRFQHLKQLKVHDC
GSLIEIFESEKVGENEDEGGTTTPYTFDLQELHLYDLPKLMHIWKYHGGILSFMNLKKLKIQHCNSLKNVLSPSMARSLSQ
LQELSVHECELIEEIITRDEKLSEEPNKVKIIFPALQWLTLYRLPSLRCFCSNTYHFELPSCIDITITECPKMEVCHGNRHLRNS
FCLH

>XP_027924984.1

MALKQAPISSSSSFTSKYSYDVFLSFRGTDTRFGFTGNLYSALSQRGIFTFIDDEALRMGEEITPSLRKAIQESRISIIVFSKNY
ASSTFCLDELLQIIECHTKQNMLILPVFYDVEPSQVRHQRGSYQEAFAKHEGEKFKDDIEKVQQWRLALRHAANLSGLH
FKTGEEYESEIVKRIAEEISTKLNRPPLHIADYPVGLKVQMQQIQQIMGDEFDNKVTMLGIHGMGGIGKSTLSRAMYNL
MANQFEASHFLANVREKSEKDGLVHIQETMLSELVGEKIKLGDVHRGIPILQHRLCGKKVLLVLDDISKREQLHATAGGL
DWFGPGSIIIITTRDKHLLDVHGVQKQYMVGEINEMEALELFKWNAFKNKEVDPCYKEVTKRAMYYANGLPLALETIGS
NLFGKTLDEWESALENYERIPNRDVQEVLRVSYDSLDAYEKEIFLDIACFFRGCSVKYVTYTLEARGFPTKFGLRVLEEKSLI
KIRECRHETVTMHDLIRCMGKEIVRQQSALPHKRNRLWFYEDIVCVLEKNKENDKIEAMMLDMPEHQEIQCKPKLFGK
MKSLRMLIIKEKVCFLRTPPALPNSLRVLEWQGYPTTSLPRNFHLKNLVILNLSYSYFGWEKPLENSKVLRQLILKGCKNIR
RIPDMSGFPNLTELRVGECTNLFEIHDSVGSLLNLKKFCAEGCSKLTIGPSRIKLISLEHLCLRDCSSLVMFPEVLAPMHKLK
YVDLVGTGIRNLPLPMQSLDGIQALSLGKGKMLEINESSNFFQTLPIFFPNLTTLYLRDLDITILPASIEEYHSLKYLHVTNCK
KLQEIRGLPLSINEFSAANSPVKANSLTSKLRQAIHSAAIRIFVLPGRKIPELFDHSSRGNSLLFWFRKELPSLAVCAIIGVWE
NVNPPFVARFNFHVRVNNIYKCVSCFSCGNINWTTEDSHIIILNRQKDFQHPLSSDIQRALLTNEWIPGKILLSIEPDNDS
NKLGEIKRTGVYVNRTCSRMEDVRFRDPYDLHKASTTENKLVLLSEASDTQQQEQSSPLLNSTTPLESAVGEQPLNYVY
NNSDSKESTSPVDSNNQDGVLLDDQPILAPSFAEMQNDEAKRERAPLILEVSSEFTAQSRDYSKVSMKIESTASQSRKV
DEVEAGLETQKQFRNSMVLENQIHQRLTMIKKMEEELGDKLGAIKADISAIEANNSSIKANISDIKSIFLQTDLRYNN

>XP_027925185.1

MAAISCSYDVFLSFRGSDTRHGFVGNLYKALDDKGIHTFIDDEKLQRGEEITPALMKAIEESRIAITVLSHNYASSSFCLDEL
VNIIACAKKKGLLVLPVFYDLNPSDVRHQKGSFAEALARHEERFKDKKESFSHNMDRLEKWKMALHHVASFSGYHFKQ
GYEYEYEFIRRIVEFVSSKINRTPLHVADYPVGLEAQMVEVMKLLDVGSEDGVHMVGIHGIGGIGKTTLALAIYNMVVD
HFDGLCFLENVRENSDRHGLQHLQSILLAELVKEKRINIASVQEGISMIQHRLQRKKVLLIVDDVDKHEQLQAIVGRCEW
FGSGSRIMITTRDEQILASHEVKRMYEVKELNKKDSLQLLTWKAFGTDEADPSYKEVLNRVVAYASGLPLALEVIGSNLF
GKSIEEWKSAIKQYERIPNNQILKILKVSFDALEEEEKSVFLDIACCFKGYELEEVQDILRAHYGDCMKYHIGVLVDKSLLKF
SVHGVMVKMHDLVEDMGKEIVRKESPKDPGKRSRLWLHEDIIQVLEDNTGTSEIEIIHLDFPPLDKEEIVEWNRKAFKK
MRNLKTLIIKSGNFSAGPKYLPNSLRVLEWWRYPSHDLPSDFHSKKLAMCKLPQSCFTSHELVRLLKKFMGMRFLNLDK
SKSLTQIPDVSGLPNLEKLSFQHCQNLTAIHNSIGFLCKLKILSAFGCTKLVRFPPIKLSALEKLNLSRCHSLENFPEILGKME
NIRVLQLEYTAIKELPCSFQNFTRLQELQLSNCGVVQLPSSIAVMPELTDLIGWKWKGWQWLKEEEDEEKHGSCVVSSN
VECLWASECNLCDDFFSIGFMRFAHVKDLDLSKNNFTVLPECIKEFQFLRKLKVSDCKLLQEIRGIPPSLKHFLAKNCKSLT
SSSTSMFLNQELHEDGKTEFYLPGERVPEWFDHKSNGPSISLWFRNRFPDKVVCLVIGAVNDSGMFRPMVVINGNKSF
VGSGYFMMGMDHTYIFDLKTMEFEDDLYGVPLENEWNHAEVKYIGLEETSILKESGIHVFKQESGMEDIWFSDPYGKR
KLEDDLNSLESQNQQLLKKHRFVDMEAL

>XP_027925186.1

MAAISCSYDVFLSFRGSDTRHGFVGNLYKALDDKGIHTFIDDEKLQRGEEITPALMKAIEESRIAITVLSHNYASSSFCLDEL
VNIIACAKKKGLLVLPVFYDLNPSDVRHQKGSFAEALARHEERFKDKKESFSHNMDRLEKWKMALHHVASFSGYHFKQ
GYEYEYEFIRRIVEFVSSKINRTPLHVADYPVGLEAQMVEVMKLLDVGSEDGVHMVGIHGIGGIGKTTLALAIYNMVVD
HFDGLCFLENVRENSDRHGLQHLQSILLAELVKEKRINIASVQEGISMIQHRLQRKKVLLIVDDVDKHEQLQAIVGRCEW
FGSGSRIMITTRDEQILASHEVKRMYEVKELNKKDSLQLLTWKAFGTDEADPSYKEVLNRVVAYASGLPLALEVIGSNLF
GKSIEEWKSAIKQYERIPNNQILKILKVSFDALEEEEKSVFLDIACCFKGYELEEVQDILRAHYGDCMKYHIGVLVDKSLLKF
SVHGVMVKMHDLVEDMGKEIVRKESPKDPGKRSRLWLHEDIIQVLEDNTKFMGMRFLNLDKSKSLTQIPDVSGLPNLE
KLSFQHCQNLTAIHNSIGFLCKLKILSAFGCTKLVRFPPIKLSALEKLNLSRCHSLENFPEILGKMENIRVLQLEYTAIKELPCS
FQNFTRLQELQLSNCGVVQLPSSIAVMPELTDLIGWKWKGWQWLKEEEDEEKHGSCVVSSNVECLWASECNLCDDFF
SIGFMRFAHVKDLDLSKNNFTVLPECIKEFQFLRKLKVSDCKLLQEIRGIPPSLKHFLAKNCKSLTSSSTSMFLNQELHEDG
KTEFYLPGERVPEWFDHKSNGPSISLWFRNRFPDKVVCLVIGAVNDSGMFRPMVVINGNKSFVGSGYFMMGMDHTY
IFDLKTMEFEDDLYGVPLENEWNHAEVKYIGLEETSILKESGIHVFKQESGMEDIWFSDPYGKRKLEDDLNSLESQNQQL
LKKHRFVDMEAL

>XP_027925192.1

MAESFLFSIAESLVAKLVSRAFEEASRVVGLYDNLRDLTKTLSLIKAVLLDAQQKQEHNHELREWLTQIKTVFSDAEDLLD
EFECQTLRNKVVKAHGSTKDKVSHFFSTFNPLVFRYQMAQQIKDISNRLDKVAADRHKFSLQTIDVDTRVVHRRDMTH
SRVSDSDVIGRKHDKEKIVELLLQQNPNDDDTRISVIPIVGIGGLGKTTLAKFVFNDSRIQEHFPLKMWVCVSDDFDIKQL
TIKIINSANDSASADAPSHQMNLNMLDLEQLQNQLKNKLFGQKFLLVLDDVWNEDRVKWVELRNLIQVSAAGSKILVT
TRSHSIASMMGTVPSHILEGLSEEDSLSLFVKWAFKEGEEEKHPHLLNIGRQIVIKCKEVPLAVRTLGSLLFLKFEASEWEY
VRDNEIWNLPQKRDDILPALKLSYDLMPSYLRQCFALFSLYPKDYVMDSYEITALWGALGLLALPTTNRTREDVANQYL
HELLSRSFLQDFEKIGTLYSFRIHDLVHDLALFVAKYECLHVTSNSQNISDNVRHLSFTESSLFENLVTKETAAVRTVLFTIG
ATVTNNEALLNTCLSKFKCLRVLDLNGSTFKTLPRAITKLKHLRYLDISRNPYIKRLPDSICKLQSLQVFSVNGCMELEALPK
GLRKLSNLWGFEFSTKQSILPLSEIANLGSLEVLNIELCNNVESIFGGVKFPALKTLVVSDCRTLKSLLLNSQNFPELESLIVD
KCNNLDLELWKGDHEEESPKLKLKLIGFSSLSQLVTLPKWLQEAANSLQCLYVSSCPNIETFSDWLTTLTHLKTLIISYCPKL
VSLPDNILHLSELENLRIEGCPDLCKKYAPHVGEFWPKISHIKNIFIDEPEGLEEERE

>XP_027925210.1

MARRISLSLSSSSHTWIYDVFLSFRGEDTRFQFTHNLYHSLCEKGIHTFIDQEGLRKGEEITPALFHAIQNSRISIIVFSKNYA
SSTYCLNELVRILECAKEEGRSIYPIFYGVDPSEVRHQTGTYAEALSKHEARFHNDANNEKVQKWRKALHEAANLSGWH
FQHRSQPEYEFIRKIVEAISREINYIPLYVADNPIGLEYARKGVKSLLEDGSEINMIGIYGIGGIGKTTIARDVYNTIFFYFQGS
CFLPDIREKAIDKYGIVQLQELVLSEILEEKDIKVGDVNRGIPLIKRRLQQKKVLLVLDNVDKLEQLKALAGGYDWFGSGSR
IIITTRDKHLLDAHGVVNLYEVKPLHVEKALELFNWHAFRSDKVGPPYMSISNRAVSYACGLPLALEVIGSHLFGKSLDEC
HSALDKYESIPHQKIHEILKVSYDGLEENEKGIFLDIACFFNNCELGNVTPMLKAHGFYAEDGLRVLADRSLIKINSSDFVR
MHDLIRDTGREIVRQESTLEPGRRSRLWFNQDIVHVLEGNTGSDKIEFIKLEGYNNVQVQWNGKAFKKMKNLSILIVED
ATFSLSPEHLPNSLRVLDWSCYPSPSLPSDFNPKRFEIILMPESCLLMFKPQKMLESLSVINLEDCKFLTDLPSLGEASLLTT
LRLDRCFNLVNIDESIGFLDKLRLLSAKGCAKLKTLAPCIMLTCLETLDLRRCVSLESFPEVLGKMEKIRTIYLDHTDIEKLPFS
IGNFVWLELLSLKGCKRLCQLPGSISIMPKVKVLIGYGHEAYNFFEERLSSEVSPMAMRIDGSNRYLDVYYQYISPNNAIQ
VCSPNPLFHSDFNLLFQKLEREANWSSRCTVSRMHFSFRKKFPKIALCCSSYYLAMKSVMIQTYKLRVFINDTMQFSAM
CNFLFKWEEQILWCDLEGKAEEVFSEQEWNNVEIVFELDFPMRRNSIYVNTTMSIGRGILSWSLIGVYEEGNNKEDIKFE
DRLSIFPLSNIEPPSLPSSLYYVVRGMTE

>XP_027925290.1

MAAELVGGALLSGFLQVAFDRLASPQVVQFFRGRKLDEKLLSKLNIVLHSINNLADDAEQKQFRNPHIKAWLFAVKDVV
FDAEDLLKEIEYELTRCQVEARSDPQTLTSKVSNFFNSTFTSFNKKIESEMRDVLEKLEYLAKQKGALGLKEGIYSGDGSGS
KESQKLPSSSLVVESVIYGRDADKEMILNWLTSETENHDQPSILSIVGMGGLGKTTLAQHVYNDPKIDEAKFDIRAWVC
VSDHFDVLIVTKTILEAITKSKDDSGDLEMVHGRLKEKISGRKFLLVLDDVWNERREEWEAVRTPLSCGAPGSRIIVTTRG
EKVASNMMSEVHRLKQLEEEECWKLFKKHALKNDDDELNDERKEIGKRIVEKCKGLPLALKTIGSLLRTKSSISDWQSVL
ESDIWDLPKEVEIMPALLLSYQHLPSHLKRCFAYCALFPKDYEFDKKELILLWMAEGFLHHSQHINNVEEIGEQYFEDLLT
RSFFLQSSIKTRFSMHDLLNDLAKYVCANFCFRFKFDKGNFVPNTTRHFSFRLEDARCFDGLGSLTDAKRMRSFLPIRETA
TTKSYHYKLPCQFKTLIHELFSKLKFLRVLSLDGYSDLREVPDSVGDLKHLHSLDLSRTYIQKLPESISVLYNLLILKLNGCSYL
EELPSNLHKLTKLHCLEFEDTKVTKMPLNFEELKNLHVLNMFLVDRNGESSVKQLGGLNLHGKLSINEVQNIVNPLDALE
ANLKDKHLVELQLEWKSNHVCDDSEKEKKVLENLQPSKHLEHLSITNYGGTQLPSWVFNNSLSNLVFLRLKSCKYCVCLP
SLGLLSSLKTLEIIGLKGISSIGVEFYGSNSSFMSLETLNFMDMKEWEEWECKTTSFPRLQHLFVNRCPKLKGLSNQLLHLK
HLVICYCDKLIISETNMDTRSLEFLKLNSCPLVSIPTTHYNLLKEMSIDDGCDSLTTFSLDFFPNLCYLYLRRCRNLQRISQED
THNHLQRMTIAVCPQFESFPTQNLKLLPKHMQVLLPSLNQLWIIDCPQVEMPPDGGLPSNVKLMYLSNVKLMASMRV
NLDNTLQIGKLDVECFPDEGLLPRSLTTLLITESPNLKRLNYKGLCHLSSLTLNGCTNLECLPEEGIPKSISSLTIWDCPLLQK
RCQNPEGKDWEKIAHIQQLRVGKWE

>XP_027925352.1

MAELVGGALLSAFLQVAFDRLASPQFVDFFRGRKLDEKLLGNLNIMLHSINALADDAEQKQFTDPHVKAWLLSVKEAV
FDAEDLLGEIDYELTRCQVEAGSEPQTFTDKVSTFFNSTFSSFNKKIESEMREVLEKLEYLAKQKGALGLKEGIYSGDSSGS
KVSQKLPSSSLVVETVIYGRDVDKEIIFNWLTSETGNHNHPSVLSIVGMGGLGKTTLAQHVYNDTKLEEAKFDITAWVCV
SDHFNVLTVTKTILEAVTKSKDDSGDLQMVHERLKEKISGKKFFLVLDDVWNERQEKWEAVQTPLSYGAPGSRILVTTR
GEKVASIMRSKVHRLKQLKKDICWNVFEKHALRDDELELNDEKKEIGRRIVQKCKGLPLALKTIGSLLRTKSSISDWQSVL
ESDIWDLPKEVEIMPALLLSYQHLPSHLKRCFAYCALFPKDYKFDKKELILLWIAQDFLHCSQQSNNLEEIGEQYFNDLLM
RSFFLQSDFKTCFFMHDLLNDLAKYVCADFCFRLKFDKGNCISKTTRHFSFGISDVKYFDGLGSLTDAKRLRSFFPYKEFGR
RYIDYYPLQFKILVHELFSNFKFLRVLSLDQYSELREVPDSIGDLKHLHSLDLSGTQIQKLPDSTCLLYNLLILKLNYCSSLKELP
LNLHKLTKLRCLEFENTNVTEMPMHFGELKNLQVLSAVFVDKNKEFSTKHLGGLNLHGGLSINEVQNIVNPVDALEANL
KNKDLVKLELKWKSDYIPDDPRKEKKVLENLQPSKTLEHLSIKSYGGTEFPSWVFDNSLSNLVSLRLEDCKYCLCLPPLGLL
SSLKTLQIIGFDGIVSIGDEFYGNSSSSFTSLERLTFSNMKELEECERKTAAFPRLEFLSVYQCPKLKGLPKELVNVKYLDIRG
SMKAWCLERCEHTVSHNSLEDLNFCAFPIMNIPMSRSFDLLEQINIFRGCDSLTTFPLDFFPNLKALSLYFCRNLQIISQD
HTHNHLESLTIDGCSRFDSFPSEGLSAPRLKRIDIDEAENLKLLPKQMQILLPSLNILKIICCPKVEMFPNGGLPPNVKAVFL
SSLKLMASLRETLGTNTCLQSLYIEKMDVEFFPDEVLLPHSITALRICDCPNLKKMEYKGLCHLSSLLLHNCPNLQCLPEDG
LPKSISSLQILNCPLLKQRCQNPEGQDWNKIAHIEDLIILP

>XP_027925354.1

MAAALVGGALLSAFLQVAFDRLASPNVVDFFRGRKLDEKLLGNLNIMLHSINALAHDAEQKQFTDPHVKAWLFAVKEA
VFDAEDLLNEIDYELTRCQVEAGSDPQTLASKVSNFFNSTFSSFNKKIESEIREVLEKLEYLAKQKGALGLKEGIYSGDSSGS
KEPQKLPSSSLVVESVIYGRDADKEMILNWLTSETENHDQPSILSIVGMGGLGKTTLAQHVYNDPKIDEAKFDIRAWVC
VSDHFDVLIVTKTILEAITKSKDDSGDLEMVHGRLKKKISRRKFLLVLDDVWNERREEWEAVRTPLSYGAPGSKILVTTRG
EKVASNMMSEVHRLKQLEEEECWKLFKKHALKNDDHELNDEKKKIGRRIVEKCKGLPLALKTIGSLLRTKSSISDWQSVL
ESDIWDLPKEVEIMPALLLSYQHLPSHLKRCFAYCALFPKDYEFDKKELILLWMAEDFLHHSNKIKSAQEIGEQYFDDLLT
RSFFLQLSSEMRQIKKVREIGEQYFHDQLTRSFFLESSFEMRFVMHDLLNDLAKYVCADFCFRLKFDKGSCIPNTTRHFSF
SFGDVEYFDGLGNLTDAKRLRSFHSITNNCSYKFNPCQFNILVHELFSKFKFLHVLSLNGYAQLIEVPNSVGDLKHLCSIDL
SYTHIQKLPDSICLLYNLLIMKMNFCSFLEELPSNLHKLTKLNCLEFEHTNVRKMPMHFGELKNIQVLSTFSVDRDSKVINI
KQLGGLNLHGRLSINEVQNIVNPLDALEANLKNQHLVELKLKWTLNHMPDDPKKERKVLENLQPPKHLEHLSIENYGGT

KFPSWVFDNSLSNLVFLHLENCKYCLWLPPFGLLSSLKSLEITGLDGIVSIGAEFYGSDSSSFRSLETLKFYNMKELEEWECK
TTSFPRLQNLHIYHCRKLKCLPNQLLHLKNLDISCCDKLVISVNKMFTSSLQLLSVMLCPLVNMPRTNYDFLEAMEINSNC
VYFTIFPLDFFPNLRLLQLSWCQNLKAVSQEHAHNHLKVLRIGDCPQFESFPSEGLSAPRLQIISIRGAESLKLMPKRMQF
LLPSLTDLVIIDCPKVEMFPDEGLPSNLKKMSLSSLKLIASLRDTLDANTCLESLIVEKLDVESFPGEVLLPRSLTTLHIRFCPN
LKKLDYNGLSHISSVIYYGCPNLHQRKLQSS

>XP_027925510.1

MAEGLLSCCQINMSEEELGTEFFNDLVARSFFQQSRHCASCFTMHDLLNDLAKSVSGEFCSQISDSLEINITKRTRHLSW
SRKINIDDKFFEHVSKCNILRFMAFRWEFGRGGLINTDKQRVLFSTLKYLHVLSFNDCLLTKLVDDIGNLKLLRYLDISYTKI
KTLPDSICRLHNLQTLLLLWCYHLAELPLDWNKLVNLRHLDMRMSGIKKMPNHIGRLKHLRTLTSFSIGKHDVNELGNLS
NLQGTLSIFRLENVTDPADAVEANLKGKKHLDGLVLNWGDKFGRCNENEDSILERQVLEALQPNGNLKKLSILRYDGTS
FPRWFGASHLPNLVSIALTESKFCFVLPSFGQLPFLKELSISCFYGIEAIGPEFCGNDSSNIPFRSLEILKFEEMSAWKEWCS
FEGHSEEGQGLSCLKELSVRRCPWLRRDLPQHLPSLQKLEICDCQHLEDSIPKAASIHEIKLCLCEKLFLKDLPSSLKKATMY
GTCIIESTLHQILVNNPFLEEMKIHNFHGPNKKWSSLDLRIHDSLVTLSITS

>XP_027925606.1

MSIRTNKMKAIPVLLKQLMIARRKFHERGRDESFDRKLEKLRSDLKKIEDVFVRVKKKEEVLLDTLAEVYDHLRKLDRGKL
DEDMDGICQRIRDSAHNLLPILAFDDSSKDEDHIGGQIFHPSEELWQAHERIRNLENLYYSPDNPSTLCLSSLFIFPENAVI
RKRNAINLWIGEGLIGNTENKRAEEEGEDVIDDLLKCGVIVRCGNGKDPSVNRFQIFINVRHQMELYLSKENGKHQGQY
FIPTTYPQIRVERLELERKKATLGGDDWYFGDRTIGTIFNIGASYLNFRPKWVTELKNLLVLQLGRWQDSALHHIEVGSQ
EFLKELRNLKQLRYLSLRGISRIFELPSSIVELESLLVLDLKACHNLETLPDDISTMKSLTHLILSECCFVEGMPKGIEKLTNLQ
VLKGFLISRPGKTPCRISELVKLRKLRGLSIHIGSEAVIRNGEFERLEDFFKLEQLKISWNVSDPKYANINIIVPPHLKKLHLEC
FPGKSFVECFMPSKWERAGLESTLELNITGGKLESIKTHLTLRTLKILRLKYLKQLNVDIDDLKALFPRLKYVEIKQISNHSYIE
HEWGII

>XP_027925612.1

MDFVGPVLDIIIRLWDCCAYVRDYEENISCLRDVASDLLGLWVDVSVKVQLAEAQHLRRLNEVNDWLVKVEAMQREV
QAIQQRVAHAQETRSRCSTNFPTSCRMGRIVSKKIGEIRELIDKGHFDVVAQEMPYAVVDEIPLEVTIGLESTFEELGECF
DDNNVGIIGLFGMGGVGKTTLLKKFNNEFLPTKFYDVVIWVVVSKEADVGSVQQSIGNKLNVPVGKWGGKSIDDRAIV
LYNFLKRKKFVLLLDDLWERIDLLKLGIPLPDTENGSKVIFTTRSMEVCRNMEANRCIKVECLAPNEAFALFREKVGEETLN
SHPEIFLLAQIVAKECEGLPLALITVGRSMARKTLPEWKRAARTLKIYPSRFSGMVDYVYCLLEFSYDSLPSASHKSCFLYCS
IFPEDYDIRKDELIQLWIGEGLLAEFGDDVYEARIQGEEIIASLKFACLLEDSEKENRIKMHDVIRDMALWLACDHGSNTR
FLVRDGASSGSVETYNQAKWKEVEKLSMWRHSIQKLSGKQDCSNLLTMLVRNTEITNFPDEIFLTANHLRVLDLSGNKR
VRELPSSIGELVQLQHLDLSGTDIQKLPRELQNLKKLRCLLLNYICNRLVFPRKLISSLVSLQVFSKLPWEDQFILPDLGEPEE
TVLLKELECLECLQDISIALFCFSSMQVLLNSSKLQRCIRHLRVLSPFNSTPHVILFSLLTKMQHLEVLSMSVSSPSSLDHVR
KKGSPSQVSMTECIPMSSKITEHGYIVGLRELSLEGCGMLNLNWLTRAPSLQLLRIYNCPSLEEVIGEEELGHAETVFSSLE
IVDLDSLPKLRSMCSQVLQFPCLKEICVADCPKLIKLPFDSNSARNSLKHINGQKSWWRKLQWEDEATRDHFASKYVPL
RKIHRIR

>XP_027925688.1

MEAVVSTTTESALQIAGRVVKRQLSYFFNYNDKFEEVKCYIELMDNTRKRIQHQVNNAEMNAEEIEHEVQHCLKQLDE
KIEKYEQFVHDEYHSKTRCSIGFFPSNLSLRYRLGRNATKMVEEMKVEELWNKRFDEVSYRVLPSINVSLTNTSYESFASR
TKTIHMFMQALEDTTVNMIGLYGVGGVGKTTLVKEVAKKAQEKKLFTVVVMANITRNPNIIKIQGQIGEMLGMRLEEE
SEIVRADRIRKRLKKEKENILIILDDLWDRLDLNRLGIPISDEDDGSQQDANDISDSGYHKMEKEELPSDFNNMTEENLSS

NHKRCKILLTSRRKQVLCNQMDVKERSTFSIGVLNENEAKILLKKVAGIEIQNLVYDEKAIEIARMCDGLPIALVSIGRTLK
NKSSFVWEDVYQQMKRQSYIEGKEPIEFSIKLSYDHLENEQLKCIFLQCARMGNDALVMDLVKFCIGLGLLQGVHTIREA
RNKVSMLIEELKESSLVLESYSSNRFNMHDIVRDVALSISSKEKHVFFMKNSILDEWPHKNQLERYTAIFVHSCYIIDDLSG
SIYCPRLEILHIDNKDHFLKIPDEFFKDMIELRVLILIGLNLPCLPSSMICLTKLRMLSLEKCTLGQNLSIIGELKKLRILTLSGSN
IECVPFEFGQLDKLQLLDLSNCSKLRLIPSNVISRMNILEEFYIGDSLIQWETEENIQSQNCSLCELSHLNQLRNLDIHIQNV
VYVPQNLFFDELDSYKIVIGEFNMLTEGEFKIPDKYEVVKLLVLNLKEGIDIHSEIWIKILLKNVEYLWLGELINVHDVFYEL
NVEGFLKLKHLSIVNNFGIQYIINSMEQFHPLLAFPKLESLYLYKLHNLEKICNNQLLEASFCRLKIIKIKSCGKLENIFPYCMV
GHLAMLETIEVCDCDSLEDIISVGRQTHTDNGDNIEFPELRTLTLKSLHAFTCLYTYDKLPCAQSFEDKGQNMNKDIIVEV
DQDGTNSCHSLFNEKVSIPKLEWLELSSINIQKIWSDQTEHCFKNLLTLNVTDCGSLKYLLSFSMAKHLENLQSLFVSECE
MMEDIFFPEDVEGNIDYVFPKLKKMEIMCMEKLNTIWQPNIGLHSFRSLDYLTIKECHKLKTIFPDFMRQRFQSLQSLTIT
NCKLVENIFDFTNISQTCDKNETNLHNIILQGLPNLVSLWKDGTGEILKHNNLQSIKIVGSPNLKYVFPLSVTNDLENLESL
EVWNCRTMKEIVAWDKGSNENAITFKFPHLKTVSLRSLFELVSFYEGTHTLEWPSLKKLSILRCGKLEGITTKISNSQAKPI
VLATEKVIYNLEFMAMSFREVKWLQKYIINVHRMHNLQSVVLHGLKNAEVIFWFLHRLPNLKRLILRFCHMRRIWAPIT
HNSREKIGVVMQLKELELRDMWFLEEIGFEHDMLLQRVQHLIIERCTKLKTLVSSLVSFRRLTYLEVVNCMMRNLMTYS
TAKTLDQLTTMKVSSCPMIVAIVAENEEENVQEIDFKQLRSLELVSLPNLRSFLTADKCVLNFSLLENLVVSECPQMTKFS
DVLSAPQLQKVHVVVGEKDKWYWEGDLNATLQKHFPYQVLFEHSKDMKLVDYPEMKEVRYGKPIFSDNFFGSLKKLE
FDAVSKRDIVLPSHVLPSLKNLEELNVESCKLARVIFDLDESETQTKGIVFRLKKLTLKDLSNLKCVWNKHSQGIVNFSNLK
EVFVYGCGTLVTLFPLTLAKNLGKLKTLTMHQCFKLIAIVEKEEETIHGTTETFEFPCLSKLFLWNMPQLVCFYPRQHHLKC
PMLERLHVAYCRKLKLFNSVFHHSSLQHHMFSIEEVVPKLKELMMSEENIILLNDGHSPQDLLHKLNYLDISFEDHDNEN
DTLPFDFLHKVPNLECFVVRRCFGLKELFPSQKLNGHDGILTELKTLSLHNLFELESIGLEHPWVKPYIEKLKVLGIVKCHRL
DRLVSCATSFINLKQLVVKGCRKMKCLFTFSTAKSLLNLEVLIIENCESIQEIIEKEDEDVNGEIVFGRLINLSMCSLPRLVSFY
SGNATLHFSSLQYVILFKCPNMTTFSEVSINAPMLHGIKPSMNDSDLIFFYDLNTTIQSLFYEKDFFEYSKHTILRDYFEMR
GFGSVKQAFPGKSFGNIKKLEFDGTSKGDTVIPSDVLSHLKSLEELNVHNADEVKVIFGMNDSHTKTKGTVFHLKKLILKD
LSNLKCILNKNPQESVSFPNLHELFVDGCGSLVTLFATKLGMIETHELQRYDKLVEIVGKEDAVENRTPEILMFEFPCLFLL
TLYNLTNLSCFYPEKHHLECPKLEIIHVAYCPKLKLFTSKIHDSHKEAMTEAPISCLQQPLFLVEKVVPKLKGLTLNEKNMM
LFSDAHVQQDYLSKLNLLRLCFEDDKNEKGTLPFDFLHKVPNLEHFQVQRCFGIKEIFSSQKLQVHDGIPATLNELTLFEL
NELESIGFQHPWVKPFCENLQTLKVISCPRLENLGYRAMSFICLKKLFVKDCGRMEYLFTFSTAKSLGQLETLTIKNCESIKE
IAKKEDEDDCDEIIFERLRTLSLNCLPRVQSFLSGNATLQFPCLENANVIDCPNMKTFSEGVLNAPKFLGIKTSLEDSDLFF
NDDLNTSFQRLFQKQVEKSACDIEHLKFSDHSRLEEMWVGVVPIPTNNCFNNLKSLAVVECESLSNVIPFYLLRFLSNLKE
IEVSNCQSVKAIFDVKVEAAKMMSITVPLKKLILNQLPNLEHIWNLNPDEILSLQELQEVSISNCQTLKSLFPTSVANHLVK
LDVRACATLVQIFEEADAAINGETKQFNFHCLTSLTLWELPELKHLYPGKHTLEWPMLTHIDIYHCDQLKLFKTEHHSHE
VAHTDDQLGISIHQQVVFSVEKVFPKLVQLSLKKEDAMAISQRQLQVMPSMEHQAITYNETMIGQGQFGANAAYLLQ
NLKLVKLMCYHEDDDSNIFSSGLLEEIPNIENLEVVCSSFNELFYSQVIPTTDRSKVLSKLKRLHLKNLPQLSAIGLEHSWVE
PLLKTLETLEVFSCPTMKILVPSTLSFSNLTSLSIGECHGMLFLFTSSTAKRLRQLKHISIQDCEAIQEIVSKEGDDESKDEYIT
FDQLSVLSLESLPNIVGIYSGTFKLKFPCLDQVTLKECPQMKYSYVPDLREFKPQGQI

>XP_027925689.1

MEAVVSTTTESALQIAGRVVKRQLSYFFNYNDKFEEVKCYIELMDNTRKRIQHQVNNAEMNAEEIEHEVQHCLKQLDE
KIEKYEQFVHDEYHSKTRCSIGFFPSNLSLRYRLGRNATKMVEEMKVEELWNKRFDEVSYRVLPSINVSLTNTSYESFASR
TKTIHMFMQALEDTTVNMIGLYGVGGVGKTTLVKEVAKKAQEKKLFTVVVMANITRNPNIIKIQGQIGEMLGMRLEEE
SEIVRADRIRKRLKKEKENILIILDDLWDRLDLNRLGIPISDEDDGSQQDANDISDSGYHKMEKEELPSDFNNMTEENLSS
NHKRCKILLTSRRKQVLCNQMDVKERSTFSIGVLNENEAKILLKKVAGIEIQNLVYDEKAIEIARMCDGLPIALVSIGRTLK
NKSSFVWEDVYQQMKRQSYIEGKEPIEFSIKLSYDHLENEQLKCIFLQCARMGNDALVMDLVKFCIGLGLLQGVHTIREA
RNKVSMLIEELKESSLVLESYSSNRFNMHDIVRDVALSISSKEKHVFFMKNSILDEWPHKNQLERYTAIFVHSCYIIDDLSG

SIYCPRLEILHIDNKDHFLKIPDEFFKDMIELRVLILIGLNLPCLPSSMICLTKLRMLSLEKCTLGQNLSIIGELKKLRILTLSGSN
IECVPFEFGQLDKLQLLDLSNCSKLRLIPSNVISRMNILEEFYIGDSLIQWETEENIQSQNCSLCELSHLNQLRNLDIHIQNV
VYVPQNLFFDELDSYKIVIGEFNMLTEGEFKIPDKYEVVKLLVLNLKEGIDIHSEIWIKILLKNVEYLWLGELINVHDVFYEL
NVEGFLKLKHLSIVNNFGIQYIINSMEQFHPLLAFPKLESLYLYKLHNLEKICNNQLLEASFCRLKIIKIKSCGKLENIFPYCMV
GHLAMLETIEVCDCDSLEDIISVGRQTHTDNGDNIEFPELRTLTLKSLHAFTCLYTYDKLPCAQSFEDKGQNMNKDIIVEV
DQDGTNSCHSLFNEKVSIPKLEWLELSSINIQKIWSDQTEHCFKNLLTLNVTDCGSLKYLLSFSMAKHLENLQSLFVSECE
MMEDIFFPEDVEGNIDYVFPKLKKMEIMCMEKLNTIWQPNIGLHSFRSLDYLTIKECHKLKTIFPDFMRQRFQSLQSLTIT
NCKLVENIFDFTNISQTCDKNETNLHNIILQGLPNLVSLWKDGTGEILKHNNLQSIKIVGSPNLKYVFPLSVTNDLENLESL
EVWNCRTMKEIVAWDKGSNENAITFKFPHLKTVSLRSLFELVSFYEGTHTLEWPSLKKLSILRCGKLEGITTKISNSQAKPI
VLATEKVIYNLEFMAMSFREVKWLQKYIINVHRMHNLQSVVLHGLKNAEVIFWFLHRLPNLKRLILRFCHMRRIWAPIT
HNSREKIGVVMQLKELELRDMWFLEEIGFEHDMLLQRVQHLIIERCTKLKTLVSSLVSFRRLTYLEVVNCMMRNLMTYS
TAKTLDQLTTMKVSSCPMIVAIVAENEEENVQEIDFKQLRSLELVSLPNLRSFLTADKCVLNFSLLENLVVSECPQMTKFS
DVLSAPQLQKVHVVVGEKDKWYWEGDLNATLQKHFPYQVLFEHSKDMKLVDYPEMKEVRYGKPIFSDNFFGSLKKLE
FDAVSKRDIVLPSHVLPSLKNLEELNVESCKLARVIFDLDESETQTKGIVFRLKKLTLKDLSNLKCVWNKHSQGIVNFSNLK
EVFVYGCGTLVTLFPLTLAKNLGKLKTLTMHQCFKLIAIVEKEEETIHGTTETFEFPCLSKLFLWNMPQLVCFYPRQHHLKC
PMLERLHVAYCRKLKLFNSVFHHSSLQHHMFSIEEVVPKLKELMMSEENIILLNDGHSPQDLLHKLNYLDISFEDHDNEN
DTLPFDFLHKVPNLECFVVRRCFGLKELFPSQKLNGHDGILTELKTLSLHNLFELESIGLEHPWVKPYIEKLKVLGIVKCHRL
DRLVSCATSFINLKQLVVKGCRKMKCLFTFSTAKSLLNLEVLIIENCESIQEIIEKEDEDVNGEIVFGRLINLSMCSLPRLVSFY
SGNATLHFSSLQYVILFKCPNMTTFSEVSINAPMLHGIKPSMNDSDLIFFYDLNTTIQSLFYEKDFFEYSKHTILRDYFEMR
GFGSVKQAFPGKSFGNIKKLEFDGTSKGDTVIPSDVLSHLKSLEELNVHNADEVKVIFGMNDSHTKTKGTVFHLKKLILKD
LSNLKCILNKNPQESVSFPNLHELFVDGCGSLVTLFATKLGMIETHELQRYDKLVEIVGKEDAVENRTPEILMFEFPCLFLL
TLYNLTNLSCFYPEKHHLECPKLEIIHVAYCPKLKLFTSKIHDSHKEAMTEAPISCLQQPLFLVEKVVPKLKGLTLNEKNMM
LFSDAHVQQDYLSKLNLLRLCFEDDKNEKGTLPFDFLHKVPNLEHFQVQRCFGIKEIFSSQKLQVHDGIPATLNELTLFEL
NELESIGFQHPWVKPFCENLQTLKVISCPRLENLGYRAMSFICLKKLFVKDCGRMEYLFTFSTAKSLGQLETLTIKNCESIKE
IAKKEDEDDCDEIIFERLRTLSLNCLPRVQSFLSGNATLQFPCLENANVIDCPNMKTFSEGVLNAPKFLGIKTSLEDSDLFF
NDDLNTSFQRLFQKQVEKSACDIEHLKFSDHSRLEEMWVGVVPIPTNNCFNNLKSLAVVECESLSNVIPFYLLRFLSNLKE
IEVSNCQSVKAIFDVKVEAAKMMSITVPLKKLILNQLPNLEHIWNLNPDEILSLQELQEVSISNCQTLKSLFPTSVANHLVK
LDVRACATLVQIFEEADAAINGETKQFNFHCLTSLTLWELPELKHLYPGKHTLEWPMLTHIDIYHCDQLKLFKTEHHSHE
VAHTDDQLGISIHQQVVFSVEKVFPKLVQLSLKKEDAMAISQRQLQVMPSMEHQAITYNETMIGQGQFGANAAYLLQ
NLKLVKLMCYHEDDDSNIFSSGLLEEIPNIENLEVVCSSFNELFYSQVIPTTDRSKVLSKLKRLHLKNLPQLSAIGLEHSWVE
PLLKTLETLEVFSCPTMKILVPSTLSFSNLTSLSIGECHGMLFLFTSSTAKRLRQLKHISIQDCEAIQEIVSKEGDDESKDEYIT
FDQLSVLSLESLPNIVGIYSGTFKLKFPCLDQVTLKECPQMKYSYVPDLREFKPQGQI

>XP_027925690.1

MEAVVSTTTESALQIAGRVVKRQLSYFFNYNDKFEEVKCYIELMDNTRKRIQHQVNNAEMNAEEIEHEVQHCLKQLDE
KIEKYEQFVHDEYHSKTRCSIGFFPSNLSLRYRLGRNATKMVEEMKVEELWNKRFDEVSYRVLPSINVSLTNTSYESFASR
TKTIHMFMQALEDTTVNMIGLYGVGGVGKTTLVKEVAKKAQEKKLFTVVVMANITRNPNIIKIQGQIGEMLGMRLEEE
SEIVRADRIRKRLKKEKENILIILDDLWDRLDLNRLGIPISDEDDGSQQDANDISDSGYHKMEKEELPSDFNNMTEENLSS
NHKRCKILLTSRRKQVLCNQMDVKERSTFSIGVLNENEAKILLKKVAGIEIQNLVYDEKAIEIARMCDGLPIALVSIGRTLK
NKSSFVWEDVYQQMKRQSYIEGKEPIEFSIKLSYDHLENEQLKCIFLQCARMGNDALVMDLVKFCIGLGLLQGVHTIREA
RNKVSMLIEELKESSLVLESYSSNRFNMHDIVRDVALSISSKEKHVFFMKNSILDEWPHKNQLERYTAIFVHSCYIIDDLSG
SIYCPRLEILHIDNKDHFLKIPDEFFKDMIELRVLILIGLNLPCLPSSMICLTKLRMLSLEKCTLGQNLSIIGELKKLRILTLSGSN
IECVPFEFGQLDKLQLLDLSNCSKLRLIPSNVISRMNILEEFYIGDSLIQWETEENIQSQNCSLCELSHLNQLRNLDIHIQNV
VYVPQNLFFDELDSYKIVIGEFNMLTEGEFKIPDKYEVVKLLVLNLKEGIDIHSEIWIKILLKNVEYLWLGELINVHDVFYEL

NVEGFLKLKHLSIVNNFGIQYIINSMEQFHPLLAFPKLESLYLYKLHNLEKICNNQLLEASFCRLKIIKIKSCGKLENIFPYCMV
GHLAMLETIEVCDCDSLEDIISVGRQTHTDNGDNIEFPELRTLTLKSLHAFTCLYTYDKLPCAQSFEDKGQNMNKDIIVEV
DQDGTNSCHSLFNEKVSIPKLEWLELSSINIQKIWSDQTEHCFKNLLTLNVTDCGSLKYLLSFSMAKHLENLQSLFVSECE
MMEDIFFPEDVEGNIDYVFPKLKKMEIMCMEKLNTIWQPNIGLHSFRSLDYLTIKECHKLKTIFPDFMRQRFQSLQSLTIT
NCKLVENIFDFTNISQTCDKNETNLHNIILQGLPNLVSLWKDGTGEILKHNNLQSIKIVGSPNLKYVFPLSVTNDLENLESL
EVWNCRTMKEIVAWDKGSNENAITFKFPHLKTVSLRSLFELVSFYEGTHTLEWPSLKKLSILRCGKLEGITTKISNSQAKPI
VLATEKVIYNLEFMAMSFREVKWLQKYIINVHRMHNLQSVVLHGLKNAEVIFWFLHRLPNLKRLILRFCHMRRIWAPIT
HNSREKIGVVMQLKELELRDMWFLEEIGFEHDMLLQRVQHLIIERCTKLKTLVSSLVSFRRLTYLEVVNCMMRNLMTYS
TAKTLDQLTTMKVSSCPMIVAIVAENEEENVQEIDFKQLRSLELVSLPNLRSFLTADKCVLNFSLLENLVVSECPQMTKFS
DVLSAPQLQKVHVVVGEKDKWYWEGDLNATLQKHFPYQVLFEHSKDMKLVDYPEMKEVRYGKPIFSDNFFGSLKKLE
FDAVSKRDIVLPSHVLPSLKNLEELNVESCKLARVIFDLDESETQTKGIVFRLKKLTLKDLSNLKCVWNKHSQGIVNFSNLK
EVFVYGCGTLVTLFPLTLAKNLGKLKTLTMHQCFKLIAIVEKEEETIHGTTETFEFPCLSKLFLWNMPQLVCFYPRQHHLKC
PMLERLHVAYCRKLKLFNSVFHHSSLQHHMFSIEEVVPKLKELMMSEENIILLNDGHSPQDLLHKLNYLDISFEDHDNEN
DTLPFDFLHKVPNLECFVVRRCFGLKELFPSQKLNGHDGILTELKTLSLHNLFELESIGLEHPWVKPYIEKLKVLGIVKCHRL
DRLVSCATSFINLKQLVVKGCRKMKCLFTFSTAKSLLNLEVLIIENCESIQEIIEKEDEDVNGEIVFGRLINLSMCSLPRLVSFY
SGNATLHFSSLQYVILFKCPNMTTFSEVSINAPMLHGIKPSMNDSDLIFFYDLNTTIQSLFYEKDFFEYSKHTILRDYFEMR
GFGSVKQAFPGKSFGNIKKLEFDGTSKGDTVIPSDVLSHLKSLEELNVHNADEVKVIFGMNDSHTKTKGTVFHLKKLILKD
LSNLKCILNKNPQESVSFPNLHELFVDGCGSLVTLFATKLGMIETHELQRYDKLVEIVGKEDAVENRTPEILMFEFPCLFLL
TLYNLTNLSCFYPEKHHLECPKLEIIHVAYCPKLKLFTSKIHDSHKEAMTEAPISCLQQPLFLVEKVVPKLKGLTLNEKNMM
LFSDAHVQQDYLSKLNLLRLCFEDDKNEKGTLPFDFLHKVPNLEHFQVQRCFGIKEIFSSQKLQVHDGIPATLNELTLFEL
NELESIGFQHPWVKPFCENLQTLKVISCPRLENLGYRAMSFICLKKLFVKDCGRMEYLFTFSTAKSLGQLETLTIKNCESIKE
IAKKEDEDDCDEIIFERLRTLSLNCLPRVQSFLSGNATLQFPCLENANVIDCPNMKTFSEGVLNAPKFLGIKTSLEDSDLFF
NDDLNTSFQRLFQKQVEKSACDIEHLKFSDHSRLEEMWVGVVPIPTNNCFNNLKSLAVVECESLSNVIPFYLLRFLSNLKE
IEVSNCQSVKAIFDVKVEAAKMMSITVPLKKLILNQLPNLEHIWNLNPDEILSLQELQEVSISNCQTLKSLFPTSVANHLVK
LDVRACATLVQIFEEADAAINGETKQFNFHCLTSLTLWELPELKHLYPGKHTLEWPMLTHIDIYHCDQLKLFKTEHHSHE
VAHTDDQLGISIHQQVVFSVEKVFPKLVQLSLKKEDAMAISQRQLQVMPSMEHQAITYNETMIGQGQFGANAAYLLQ
NLKLVKLMCYHEDDDSNIFSSGLLEEIPNIENLEVVCSSFNELFYSQVIPTTDRSKVLSKLKRLHLKNLPQLSAIGLEHSWVE
PLLKTLETLEVFSCPTMKILVPSTLSFSNLTSLSIGECHGMLFLFTSSTAKRLRQLKHISIQDCEAIQEIVSKEGDDESKDEYIT
FDQLSVLSLESLPNIVGIYSGTFKLKFPCLDQVTLKECPQMKYSYVPDLREFKPQGQI

>XP_027925691.1

MDAVVSTTTEGGLKIVGHVVKRQLGYFFSYKDKFKELEFYIEKLEHNKERLQHRVDNALRNAEEIENDIQHCLKEMDEKI
KEYKSYINDKCHEKTICSISFFPNNFRSRYQLGRKATKMVDEIIRDELWKTSKIENVSYRESPSTNVVFSNTGYESFASRTRT
MEMIMKALEDSTVDMIGVYGPGGVGKTTLVLEIAKKTREKKLFKTVVMANVTRNPDTKKIQGQIADVLGMRLEEESEI
ARADHIRKRLKNEKENVLIILDDLWDGLDLNKLGIPCNDDNLDDVSQNEVNDIFDGGYNDNISNFGYNKTKPKKLPEVY
LNKMKREKLSSSYKGGKILLTSRNKQVLCNQMDVQLSSTFSVGVLEEKEAETLLKKVADVKNSEFDRNATEIAKWSAGF
PIALVSIGRTLKHKSLSTWDDVCQQIKRQSFTEKWGFTNFSIKLSYDYLENEELKCIFLHCARMGNDALIMDLVKFCIGLN
LLPGVDTITEARKRVKEMIHELEESSLLVRSYSIDRFNMHDIVRDVALLISSKEKHVFFMKDAILDEWPHEDDVKRYTAIFL
LYCDINELPESIHCPRLEVLHIDNKNESLEIPDHFFESMIRLRVLVLTNVNLSCLPSSIKCLKKLRMLCLERCTLEKNLSIIGELK
NLRILSLLGSNIENLPPGFGQLDKLQLFDISNCLKLREIRSNIIPRINTLEEFYTRDSPILWAEENIKSENASLSELRHLNQLQN
LDIRIQSSSHFLQLFFDNLNSYKIVIGEFDFLNLLKVGEFKIPDKYEEVKFLALDLKEGIDIHSETWVKMLFKSVECLLLGELN
DVQDIYYELNVEGFPNLKHLSIVNNFGIKYIINPVDQFQLLFVFPKLESIWLYKMDTLEKICGNWLAETSFRSLKVIKIKTCV
KLVNLFPFSIVRLLIALETIEVYDCDSFKGIVSEERQTHTVIDDKIEFPQLRLLTLNSLPIFTCLYVIDKMFGSPQSLKDLQDIN
KDIVTDVENKVTNSCLPLFNEKVSFPKLEWLELSSLNIKKIWSDQFDHCFQNLLTLNVINCGNLKYLLSCSMAGSLVNLQS

LFVSKCEVMEDIFHSEDVEYIDVFPKLKKMEIICMEKLATIWKSHIGLHSFCSLDSLIIRECHKLVTIFPSYMGQRFQSLHSLI
ITNCNLVENIFDFANIPQTCDLIETNLDNIILEMLPNLVNVWKDDICKILKYNNLRSIRVFMSPNLKYIFPLSVALDLEKLEVL
EVHICWAVKEIIVWDKHSSENVINFKFPRLNTLSLMDLYGLRSFYSRTHTLEWPQLKKLDIVDCSRLEGLTSKITNSQVQPI
VLATKKVIYNLEYMSFSLKEAEWLQKYIVNVHIMHKLEGLSLYGLKNNQILFWFLHRLPNLKRLKLGLSNLKRIWTPQSLIS
REKIGVVMQLKELELKCIWPLEEIGFEHNVLLQRVECLIIQQCTKLRNLASSPISFSYLRYLEVTNCMMRNLMTTSIAKTLV
QLNTMKVISCPMIVEIVADNVEEKVEEIEFKQLKSLELVSLQNLISFSNVKNCDIKFPLLEKLIVSECFQMTKFCELQRTPKL
QKIHVVVEEKEKWYWEGDLNTTLLKHFKVALEYLKHVRLIDYPETKGVRYGKPIFQDNLFNCLQKLEVDAAFKRDILIPRH
VLPYLKNVKELLVHDSDTVHVIFEIDETDVKTNGMVFRLEKLTLTNLPNLKCVWNENPKEIVNFPYLQEVYVKGCGSLVT
LFSLSLAKNLEKLKKLEVQECEKLVEIVEKGDAMKHGTTVIFIFPCLSILILSIMPLLSCFYLGKHHLECPLLDTLYISYCPQLKL
FISNLDDGQKGAIEAPISPAQQPLFSIEILASSKLKNLQLNVKNIMLLSDAQLPPDLLYKLKVLWLCFEDYNNEKGTLPFDF
FHRVPNLEHFILQTCSGVKEIFPAQKPQVHDTIFKGLKQLFLNVVPDLDWVGLEHTWVQPYSEKLELLQLYHCRRVEKIV
YCAVSFINLKELQVVHCERMVYLFTFSTLKTLVKLETLIVGECESIKEIVKNEDEDGCDEIVFERLTLIQLNCLPRLVSFYSGN
ATLQCSCLNNVLVTECPNMTTFSLGIINVTSFKNIHTSIEDSDLLLENDLNTVIEKSFHTKDFFDYSKRMILDDYLERTGVQ
HKNPVISINFFGSFKKLEFDATCKRVIVIPSHILPYLRNLEELNVHSSDVVQVIFDTDETEVETRGIIFGLKKLTLKHLSNLKCV
WKKNLEGIVSFSNLEEVNVDGCGSLVTLFPLSLAKNLGKLWSLELKRCEKMVEIVGREDNGTTIMFEFPRLSFLYLKNMP
LLSCFYPRKHYLECPLLGSLLVECCPKLKLFTSDSDDSHKDEVIEAPIKPLQQPLFSIEKVSPKLMGLTLNEENIKLMSDARW
PQDLSKLFILILSFEDDNNGKDSLPFDFFHKVPNLFYLTVQKWFGMKEIFPSQKLQVHDNVLAGLKQLSLWELSELESIGL
EHTWVQPYSTKLEWLKLGTCPRVEKIVSCAVSFINLKKLSVKHCEKMEYLFTFATLKSLVKLETLSIKECESIKEIAKEEDED
GCDEMVFGRLRSIKLNFLPKLICFYSGNATLQFSYLKTVMVAKCPNMKTFSEGVTKVPIFLEIKTSKDSDLTFHDDLNTTIQ
R

>XP_027925692.1

MDAVVSTTTEGGLKIVGHVVKRQLGYFFSYKDKFKELEFYIEKLEHNKERLQHRVDNALRNAEEIENDIQHCLKEMDEKI
KEYKSYINDKCHEKTICSISFFPNNFRSRYQLGRKATKMVDEIIRDELWKTSKIENVSYRESPSTNVVFSNTGYESFASRTRT
MEMIMKALEDSTVDMIGVYGPGGVGKTTLVLEIAKKTREKKLFKTVVMANVTRNPDTKKIQGQIADVLGMRLEEESEI
ARADHIRKRLKNEKENVLIILDDLWDGLDLNKLGIPCNDDNLDDVSQNEVNDIFDGGYNDNISNFGYNKTKPKKLPEVY
LNKMKREKLSSSYKGGKILLTSRNKQVLCNQMDVQLSSTFSVGVLEEKEAETLLKKVADVKNSEFDRNATEIAKWSAGF
PIALVSIGRTLKHKSLSTWDDVCQQIKRQSFTEKWGFTNFSIKLSYDYLENEELKCIFLHCARMGNDALIMDLVKFCIGLN
LLPGVDTITEARKRVKEMIHELEESSLLVRSYSIDRFNMHDIVRDVALLISSKEKHVFFMKDAILDEWPHEDDVKRYTAIFL
LYCDINELPESIHCPRLEVLHIDNKNESLEIPDHFFESMIRLRVLVLTNVNLSCLPSSIKCLKKLRMLCLERCTLEKNLSIIGELK
NLRILSLLGSNIENLPPGFGQLDKLQLFDISNCLKLREIRSNIIPRINTLEEFYTRDSPILWAEENIKSENASLSELRHLNQLQN
LDIRIQSSSHFLQLFFDNLNSYKIVIGEFDFLNLLKVGEFKIPDKYEEVKFLALDLKEGIDIHSETWVKMLFKSVECLLLGELN
DVQDIYYELNVEGFPNLKHLSIVNNFGIKYIINPVDQFQLLFVFPKLESIWLYKMDTLEKICGNWLAETSFRSLKVIKIKTCV
KLVNLFPFSIVRLLIALETIEVYDCDSFKGIVSEERQTHTVIDDKIEFPQLRLLTLNSLPIFTCLYVIDKMFGSPQSLKDLQDIN
KDIVTDVENKVTNSCLPLFNEKVSFPKLEWLELSSLNIKKIWSDQFDHCFQNLLTLNVINCGNLKYLLSCSMAGSLVNLQS
LFVSKCEVMEDIFHSEDVEYIDVFPKLKKMEIICMEKLATIWKSHIGLHSFCSLDSLIIRECHKLVTIFPSYMGQRFQSLHSLI
ITNCNLVENIFDFANIPQTCDLIETNLDNIILEMLPNLVNVWKDDICKILKYNNLRSIRVFMSPNLKYIFPLSVALDLEKLEVL
EVHICWAVKEIIVWDKHSSENVINFKFPRLNTLSLMDLYGLRSFYSRTHTLEWPQLKKLDIVDCSRLEGLTSKITNSQVQPI
VLATKKVIYNLEYMSFSLKEAEWLQKYIVNVHIMHKLEGLSLYGLKNNQILFWFLHRLPNLKRLKLGLSNLKRIWTPQSLIS
REKIGVVMQLKELELKCIWPLEEIGFEHNVLLQRVECLIIQQCTKLRNLASSPISFSYLRYLEVTNCMMRNLMTTSIAKTLV
QLNTMKVISCPMIVEIVADNVEEKVEEIEFKQLKSLELVSLQNLISFSNVKNCDIKFPLLEKLIVSECFQMTKFCELQRTPKL
QKIHVVVEEKEKWYWEGDLNTTLLKHFKVALEYLKHVRLIDYPETKGVRYGKPIFQDNLFNCLQKLEVDAAFKRDILIPRH
VLPYLKNVKELLVHDSDTVHVIFEIDETDVKTNGMVFRLEKLTLTNLPNLKCVWNENPKEIVNFPYLQEVYVKGCGSLVT
LFSLSLAKNLEKLKKLEVQECEKLVEIVEKGDAMKHGTTVIFIFPCLSILILSIMPLLSCFYLGKHHLECPLLDTLYISYCPQLKL

FISNLDDGQKGAIEAPISPAQQPLFSIEILASSKLKNLQLNVKNIMLLSDAQLPPDLLYKLKVLWLCFEDYNNEKGTLPFDF
FHRVPNLEHFILQTCSGVKEIFPAQKPQVHDTIFKGLKQLFLNVVPDLDWVGLEHTWVQPYSEKLELLQLYHCRRVEKIV
YCAVSFINLKELQVVHCERMVYLFTFSTLKTLVKLETLIVGECESIKEIVKNEDEDGCDEIVFERLTLIQLNCLPRLVSFYSGN
ATLQCSCLNNVLVTECPNMTTFSLGIINVTSFKNIHTSIEDSDLLLENDLNTVIEKSFHTKDFFDYSKRMILDDYLERTGVQ
HKNPVISINFFGSFKKLEFDATCKRVIVIPSHILPYLRNLEELNVHSSDVVQVIFDTDETEVETRGIIFGLKKLTLKHLSNLKCV
WKKNLEGIVSFSNLEEVNVDGCGSLVTLFPLSLAKNLGKLWSLELKRCEKMVEIVGREDNGTTIMFEFPRLSFLYLKNMP
LLSCFYPRKHYLECPLLGSLLVECCPKLKLFTSDSDDSHKDEVIEAPIKPLQQPLFSIEKVSPKLMGLTLNEENIKLMSDARW
PQDLSKLFILILSFEDDNNGKDSLPFDFFHKVPNLFYLTVQKWFGMKEIFPSQKLQVHDNVLAGLKQLSLWELSELESIGL
EHTWVQPYSTKLEWLKLGTCPRVEKIVSCAVSFINLKKLSVKHCEKMEYLFTFATLKSLVKLETLSIKECESIKEIAKEEDED
GCDEMVFGRLRSIKLNFLPKLICFYSGNATLQFSYLKTVMVAKCPNMKTFSEGVTKVPIFLEIKTSKDSDLTFHDDLNTTIQ
R

>XP_027925694.1

MDAVVSTTTEGGLKIVGHVVKRQLGYFFSYKDKFKELEFYIEKLEHNKERLQHRVDNALRNAEEIENDIQHCLKEMDEKI
KEYKSYINDKCHEKTICSISFFPNNFRSRYQLGRKATKMVDEIIRDELWKTSKIENVSYRESPSTNVVFSNTGYESFASRTRT
MEMIMKALEDSTVDMIGVYGPGGVGKTTLVLEIAKKTREKKLFKTVVMANVTRNPDTKKIQGQIADVLGMRLEEESEI
ARADHIRKRLKNEKENVLIILDDLWDGLDLNKLGIPCNDDNLDDVSQNEVNDIFDGGYNDNISNFGYNKTKPKKLPEVY
LNKMKREKLSSSYKGGKILLTSRNKQVLCNQMDVQLSSTFSVGVLEEKEAETLLKKVADVKNSEFDRNATEIAKWSAGF
PIALVSIGRTLKHKSLSTWDDVCQQIKRQSFTEKWGFTNFSIKLSYDYLENEELKCIFLHCARMGNDALIMDLVKFCIGLN
LLPGVDTITEARKRVKEMIHELEESSLLVRSYSIDRFNMHDIVRDVALLISSKEKHVFFMKDAILDEWPHEDDVKRYTAIFL
LYCDINELPESIHCPRLEVLHIDNKNESLEIPDHFFESMIRLRVLVLTNVNLSCLPSSIKCLKKLRMLCLERCTLEKNLSIIGELK
NLRILSLLGSNIENLPPGFGQLDKLQLFDISNCLKLREIRSNIIPRINTLEEFYTRDSPILWAEENIKSENASLSELRHLNQLQN
LDIRIQSSSHFLQLFFDNLNSYKIVIGEFDFLNLLKVGEFKIPDKYEEVKFLALDLKEGIDIHSETWVKMLFKSVECLLLGELN
DVQDIYYELNVEGFPNLKHLSIVNNFGIKYIINPVDQFQLLFVFPKLESIWLYKMDTLEKICGNWLAETSFRSLKVIKIKTCV
KLVNLFPFSIVRLLIALETIEVYDCDSFKGIVSEERQTHTVIDDKIEFPQLRLLTLNSLPIFTCLYVIDKMFGSPQSLKDLQDIN
KDIVTDVENKVTNSCLPLFNEKVSFPKLEWLELSSLNIKKIWSDQFDHCFQNLLTLNVINCGNLKYLLSCSMAGSLVNLQS
LFVSKCEVMEDIFHSEDVEYIDVFPKLKKMEIICMEKLATIWKSHIGLHSFCSLDSLIIRECHKLVTIFPSYMGQRFQSLHSLI
ITNCNLVENIFDFANIPQTCDLIETNLDNIILEMLPNLVNVWKDDICKILKYNNLRSIRVFMSPNLKYIFPLSVALDLEKLEVL
EVHICWAVKEIIVWDKHSSENVINFKFPRLNTLSLMDLYGLRSFYSRTHTLEWPQLKKLDIVDCSRLEGLTSKITNSQVQPI
VLATKKVIYNLEYMSFSLKEAEWLQKYIVNVHIMHKLEGLSLYGLKNNQILFWFLHRLPNLKRLKLGLSNLKRIWTPQSLIS
REKIGVVMQLKELELKCIWPLEEIGFEHNVLLQRVECLIIQQCTKLRNLASSPISFSYLRYLEVTNCMMRNLMTTSIAKTLV
QLNTMKVISCPMIVEIVADNVEEKVEEIEFKQLKSLELVSLQNLISFSNVKNCDIKFPLLEKLIVSECFQMTKFCELQRTPKL
QKIHVVVEEKEKWYWEGDLNTTLLKHFKVALEYLKHVRLIDYPETKGVRYGKPIFQDNLFNCLQKLEVDAAFKRDILIPRH
VLPYLKNVKELLVHDSDTVHVIFEIDETDVKTNGMVFRLEKLTLTNLPNLKCVWNENPKEIVNFPYLQEVYVKGCGSLVT
LFSLSLAKNLEKLKKLEVQECEKLVEIVEKGDAMKHGTTVIFIFPCLSILILSIMPLLSCFYLGKHHLECPLLDTLYISYCPQLKL
FISNLDDGQKGAIEAPISPAQQPLFSIEILASSKLKNLQLNVKNIMLLSDAQLPPDLLYKLKVLWLCFEDYNNEKGTLPFDF
FHRVPNLEHFILQTCSGVKEIFPAQKPQVHDTIFKGLKQLFLNVVPDLDWVGLEHTWVQPYSEKLELLQLYHCRRVEKIV
YCAVSFINLKELQVVHCERMVYLFTFSTLKTLVKLETLIVGECESIKEIVKNEDEDGCDEIVFERLTLIQLNCLPRLVSFYSGN
ATLQCSCLNNVLVTECPNMTTFSLGIINVTSFKNIHTSIEDSDLLLENDLNTVIEKSFHTKDFFDYSKRMILDDYLERTGVQ
HKNPVISINFFGSFKKLEFDATCKRVIVIPSHILPYLRNLEELNVHSSDVVQVIFDTDETEVETRGIIFGLKKLTLKHLSNLKCV
WKKNLEGIVSFSNLEEVNVDGCGSLVTLFPLSLAKNLGKLWSLELKRCEKMVEIVGREDNGTTIMFEFPRLSFLYLKNMP
LLSCFYPRKHYLECPLLGSLLVECCPKLKLFTSDSDDSHKDEVIEAPIKPLQQPLFSIEKVSPKLMGLTLNEENIKLMSDARW
PQDLSKLFILILSFEDDNNGKDSLPFDFFHKVPNLFYLTVQKWFGMKEIFPSQKLQVHDNVLAGLKQLSLWELSELESIGL
EHTWVQPYSTKLEWLKLGTCPRVEKIVSCAVSFINLKKLSVKHCEKMEYLFTFATLKSLVKLETLSIKECESIKEIAKEEDED

GCDEMVFGRLRSIKLNFLPKLICFYSGNATLQFSYLKTVMVAKCPNMKTFSEGVTKVPIFLEIKTSKDSDLTFHDDLNTTIQ
R

>XP_027925695.1

MDAVVSTTTEGGLKIVGHVVKRQLGYFFSYKDKFKELEFYIEKLEHNKERLQHRVDNALRNAEEIENDIQHCLKEMDEKI
KEYKSYINDKCHEKTICSISFFPNNFRSRYQLGRKATKMVDEIIRDELWKTSKIENVSYRESPSTNVVFSNTGYESFASRTRT
MEMIMKALEDSTVDMIGVYGPGGVGKTTLVLEIAKKTREKKLFKTVVMANVTRNPDTKKIQGQIADVLGMRLEEESEI
ARADHIRKRLKNEKENVLIILDDLWDGLDLNKLGIPCNDDNLDDVSQNEVNDIFDGGYNDNISNFGYNKTKPKKLPEVY
LNKMKREKLSSSYKGGKILLTSRNKQVLCNQMDVQLSSTFSVGVLEEKEAETLLKKVADVKNSEFDRNATEIAKWSAGF
PIALVSIGRTLKHKSLSTWDDVCQQIKRQSFTEKWGFTNFSIKLSYDYLENEELKCIFLHCARMGNDALIMDLVKFCIGLN
LLPGVDTITEARKRVKEMIHELEESSLLVRSYSIDRFNMHDIVRDVALLISSKEKHVFFMKDAILDEWPHEDDVKRYTAIFL
LYCDINELPESIHCPRLEVLHIDNKNESLEIPDHFFESMIRLRVLVLTNVNLSCLPSSIKCLKKLRMLCLERCTLEKNLSIIGELK
NLRILSLLGSNIENLPPGFGQLDKLQLFDISNCLKLREIRSNIIPRINTLEEFYTRDSPILWAEENIKSENASLSELRHLNQLQN
LDIRIQSSSHFLQLFFDNLNSYKIVIGEFDFLNLLKVGEFKIPDKYEEVKFLALDLKEGIDIHSETWVKMLFKSVECLLLGELN
DVQDIYYELNVEGFPNLKHLSIVNNFGIKYIINPVDQFQLLFVFPKLESIWLYKMDTLEKICGNWLAETSFRSLKVIKIKTCV
KLVNLFPFSIVRLLIALETIEVYDCDSFKGIVSEERQTHTVIDDKIEFPQLRLLTLNSLPIFTCLYVIDKMFGSPQSLKDLQDIN
KDIVTDVENKVTNSCLPLFNEKVSFPKLEWLELSSLNIKKIWSDQFDHCFQNLLTLNVINCGNLKYLLSCSMAGSLVNLQS
LFVSKCEVMEDIFHSEDVEYIDVFPKLKKMEIICMEKLATIWKSHIGLHSFCSLDSLIIRECHKLVTIFPSYMGQRFQSLHSLI
ITNCNLVENIFDFANIPQTCDLIETNLDNIILEMLPNLVNVWKDDICKILKYNNLRSIRVFMSPNLKYIFPLSVALDLEKLEVL
EVHICWAVKEIIVWDKHSSENVINFKFPRLNTLSLMDLYGLRSFYSRTHTLEWPQLKKLDIVDCSRLEGLTSKITNSQVQPI
VLATKKVALEYLKHVRLIDYPETKGVRYGKPIFQDNLFNCLQKLEVDAAFKRDILIPRHVLPYLKNVKELLVHDSDTVHVIF
EIDETDVKTNGMVFRLEKLTLTNLPNLKCVWNENPKEIVNFPYLQEVYVKGCGSLVTLFSLSLAKNLEKLKKLEVQECEKL
VEIVEKGDAMKHGTTVIFIFPCLSILILSIMPLLSCFYLGKHHLECPLLDTLYISYCPQLKLFISNLDDGQKGAIEAPISPAQQP
LFSIEILASSKLKNLQLNVKNIMLLSDAQLPPDLLYKLKVLWLCFEDYNNEKGTLPFDFFHRVPNLEHFILQTCSGVKEIFPA
QKPQVHDTIFKGLKQLFLNVVPDLDWVGLEHTWVQPYSEKLELLQLYHCRRVEKIVYCAVSFINLKELQVVHCERMVYL
FTFSTLKTLVKLETLIVGECESIKEIVKNEDEDGCDEIVFERLTLIQLNCLPRLVSFYSGNATLQCSCLNNVLVTECPNMTTFS
LGIINVTSFKNIHTSIEDSDLLLENDLNTVIEKSFHTKDFFDYSKRMILDDYLERTGVQHKNPVISINFFGSFKKLEFDATCKR
VIVIPSHILPYLRNLEELNVHSSDVVQVIFDTDETEVETRGIIFGLKKLTLKHLSNLKCVWKKNLEGIVSFSNLEEVNVDGCG
SLVTLFPLSLAKNLGKLWSLELKRCEKMVEIVGREDNGTTIMFEFPRLSFLYLKNMPLLSCFYPRKHYLECPLLGSLLVECCP
KLKLFTSDSDDSHKDEVIEAPIKPLQQPLFSIEKVSPKLMGLTLNEENIKLMSDARWPQDLSKLFILILSFEDDNNGKDSLP
FDFFHKVPNLFYLTVQKWFGMKEIFPSQKLQVHDNVLAGLKQLSLWELSELESIGLEHTWVQPYSTKLEWLKLGTCPRV
EKIVSCAVSFINLKKLSVKHCEKMEYLFTFATLKSLVKLETLSIKECESIKEIAKEEDEDGCDEMVFGRLRSIKLNFLPKLICFYS
GNATLQFSYLKTVMVAKCPNMKTFSEGVTKVPIFLEIKTSKDSDLTFHDDLNTTIQR

>XP_027925696.1

MIHELEESSLLVRSYSIDRFNMHDIVRDVALLISSKEKHVFFMKDAILDEWPHEDDVKRYTAIFLLYCDINELPESIHCPRLE
VLHIDNKNESLEIPDHFFESMIRLRVLVLTNVNLSCLPSSIKCLKKLRMLCLERCTLEKNLSIIGELKNLRILSLLGSNIENLPPG
FGQLDKLQLFDISNCLKLREIRSNIIPRINTLEEFYTRDSPILWAEENIKSENASLSELRHLNQLQNLDIRIQSSSHFLQLFFDN
LNSYKIVIGEFDFLNLLKVGEFKIPDKYEEVKFLALDLKEGIDIHSETWVKMLFKSVECLLLGELNDVQDIYYELNVEGFPNL
KHLSIVNNFGIKYIINPVDQFQLLFVFPKLESIWLYKMDTLEKICGNWLAETSFRSLKVIKIKTCVKLVNLFPFSIVRLLIALETI
EVYDCDSFKGIVSEERQTHTVIDDKIEFPQLRLLTLNSLPIFTCLYVIDKMFGSPQSLKDLQDINKDIVTDVENKVTNSCLPL
FNEKVSFPKLEWLELSSLNIKKIWSDQFDHCFQNLLTLNVINCGNLKYLLSCSMAGSLVNLQSLFVSKCEVMEDIFHSEDV
EYIDVFPKLKKMEIICMEKLATIWKSHIGLHSFCSLDSLIIRECHKLVTIFPSYMGQRFQSLHSLIITNCNLVENIFDFANIPQT
CDLIETNLDNIILEMLPNLVNVWKDDICKILKYNNLRSIRVFMSPNLKYIFPLSVALDLEKLEVLEVHICWAVKEIIVWDKHS

SENVINFKFPRLNTLSLMDLYGLRSFYSRTHTLEWPQLKKLDIVDCSRLEGLTSKITNSQVQPIVLATKKVIYNLEYMSFSLK
EAEWLQKYIVNVHIMHKLEGLSLYGLKNNQILFWFLHRLPNLKRLKLGLSNLKRIWTPQSLISREKIGVVMQLKELELKCI
WPLEEIGFEHNVLLQRVECLIIQQCTKLRNLASSPISFSYLRYLEVTNCMMRNLMTTSIAKTLVQLNTMKVISCPMIVEIV
ADNVEEKVEEIEFKQLKSLELVSLQNLISFSNVKNCDIKFPLLEKLIVSECFQMTKFCELQRTPKLQKIHVVVEEKEKWYWE
GDLNTTLLKHFKVALEYLKHVRLIDYPETKGVRYGKPIFQDNLFNCLQKLEVDAAFKRDILIPRHVLPYLKNVKELLVHDSD
TVHVIFEIDETDVKTNGMVFRLEKLTLTNLPNLKCVWNENPKEIVNFPYLQEVYVKGCGSLVTLFSLSLAKNLEKLKKLEV
QECEKLVEIVEKGDAMKHGTTVIFIFPCLSILILSIMPLLSCFYLGKHHLECPLLDTLYISYCPQLKLFISNLDDGQKGAIEAPIS
PAQQPLFSIEILASSKLKNLQLNVKNIMLLSDAQLPPDLLYKLKVLWLCFEDYNNEKGTLPFDFFHRVPNLEHFILQTCSGV
KEIFPAQKPQVHDTIFKGLKQLFLNVVPDLDWVGLEHTWVQPYSEKLELLQLYHCRRVEKIVYCAVSFINLKELQVVHCE
RMVYLFTFSTLKTLVKLETLIVGECESIKEIVKNEDEDGCDEIVFERLTLIQLNCLPRLVSFYSGNATLQCSCLNNVLVTECPN
MTTFSLGIINVTSFKNIHTSIEDSDLLLENDLNTVIEKSFHTKDFFDYSKRMILDDYLERTGVQHKNPVISINFFGSFKKLEFD
ATCKRVIVIPSHILPYLRNLEELNVHSSDVVQVIFDTDETEVETRGIIFGLKKLTLKHLSNLKCVWKKNLEGIVSFSNLEEVN
VDGCGSLVTLFPLSLAKNLGKLWSLELKRCEKMVEIVGREDNGTTIMFEFPRLSFLYLKNMPLLSCFYPRKHYLECPLLGSL
LVECCPKLKLFTSDSDDSHKDEVIEAPIKPLQQPLFSIEKVSPKLMGLTLNEENIKLMSDARWPQDLSKLFILILSFEDDNN
GKDSLPFDFFHKVPNLFYLTVQKWFGMKEIFPSQKLQVHDNVLAGLKQLSLWELSELESIGLEHTWVQPYSTKLEWLKL
GTCPRVEKIVSCAVSFINLKKLSVKHCEKMEYLFTFATLKSLVKLETLSIKECESIKEIAKEEDEDGCDEMVFGRLRSIKLNFL
PKLICFYSGNATLQFSYLKTVMVAKCPNMKTFSEGVTKVPIFLEIKTSKDSDLTFHDDLNTTIQR

>XP_027925697.1

MEVVVSTATENALQIATRVVKRQFSYFFNYNDKFEEVKCYIEMLDNTRKRIQHQVNNAEMNAEEIEDDVQHCLKQLDE
KIEKYELFINDEYHSKTRCSIGFFPNNLSLRYRLGRNATKMVEEMKADELWNKKFDEVSYRVLPSINAALTNTSYESFASR
EKTINVCMQALEDSTINMIGLYGVGGVGKTTLVKEVAKKAQEKKLFNVVVIANITRNPNILKIQGQIAEMLGMRLEEESE
IVRADRIRKRLKNEKENTLIILDDLWDGLDLNRLGIPISDENNGSQQDVNDISDSSFDKMEKEELSSDFNNMTEENISEDP
KRCKILLTSRRKQVLCNQMDVKERSTFSVGVLNETEGKTLLKKLAGIHSQNLVYDEKAVEIARMCDGLPIALVSIGRALKN
KSSFVWEDIYQQMKKKSFMEGQEPIEFSIKLSYDHLKNEQLKCIFLHCARMGNDALVMDLVKFCIGLGLIQGVHTIREAR
NKVNMLIEELKESSLVGASYSGDRFNMHDIVRDVALSISSKEKQMFFMKNGILDEWPHKYQLERYTAIFLHYCYINDDLP
GSIYCPRLEVLHIDNKDQFLKIPDDFFKDMIELRVLILTGFNLPCLPSSIICLSKLRMLCLEKCTLGQNLSIIGELKKLRILTLSG
SNIESLPFEFGQLDKLQLLDLSNCSKLSVIPSNVISRMNILEEFYMRDTLILWEAKEMIQNKNASLSELRHLNQLRNLDLHI
QNVAHVPQNLFFDKLDSYKIIIGEFNMLIEGEFKIPDKYEVVKLLVLNLKEGIDIHSETWVKMLFKNVEYLLLGELIDVHDV
FYELNVEGFQNLKHLSIVNNIGLQYIINSVDRFHPLLAFPKLESLYLYKLYSLEKLCNNQLLEASFCRLKTIKIKSCDKLENLFP
LFMVRLLTVLENIEVCDCDSLKDIVAIERQPHNDSDDNIEFPQLRLLTLKSLPAFTCLYYNYKMPCSAQLLEEKGRSMNRD
IIIEVEKDGTNSCLSLFNEKISIPKLESLELSSINIQKIWSDQCQHCFQNLLTLNVTDCGNLKYLLSFSMAVSLVNLQSLSVSD
CEMMEDIFRPEDDEGNIDNVFPKLKKMEITCMEKLNSIWQPHIGLHSFHSLDSLVIKECHKLVTIFPSFMGQKFQSLQSL
TITNCKLVENIFDFEMIPQTSDINETQLHKIVLQNLSNLVSIWKDDTCEILKYNNLQNITVSGSPNLSYLFPLSITNDLENLE
VLDVRSCRAMKEIVAVDKGTNENVITFKFPRLNYLSLQSLFELMSFYGRAHTLEWPSLKRLLILRCGKLEGINTDISNSQVK
PIVLATEKVIYNLEYMAMSFREVEWLQNYIYNVHRMQNLQTVVLHGLKNVEVLFWILHRLPNLKRLTLGFCHLKRIWAP
ASLISREKIGVVMQLQALELKNIWPLEEIGFEHEVLLQRVERLIIQRCTKLKFLASSSVSFSFLTYLEVTNCMLKNLMTCSTA
KTLIQLKTMKVSSCPMIAEIISENEGEKVQEIEFKLLRSLELISLQNLTSFMSVDKCDLKFPVLENLVVSECPKMTKFSKVQS
APNLQKIQVVATEKDKWYWEGDLNATLKKHFTHQVSFEYSKHMKLKDYPEMKEICQGKPVFQDNFFGSLKKLEFDATS
KREIVLPSYVLPYLKNLEELNVESCKSARVIFDIDDCEIKETVFRLKKLTLKDLSNMKWIWNKNPQGIVNFPNLEEVLVNNC
GTLVTLFPSTLARNLSKLKTLTIHNCCKLVEIVEKKEEREEEITEMFEFPCLSKLFLWNLPMLVCFYPRQHNLKCPTLERLHV
AYCRKLKLFTSQTQHSEQHHMFLIEEVVPKLKEVILNEENMTLLNDGHSPDLLHRVNYLDLSSEDYENKKDSLPFDFLKKV
PNLEYLVVRQCFGLKEIFPSEKLDGDDHDGILLAGLNKLSLNKLLELESIGLDHPWIKPYTEKLQGLAVIKCPRLDKLVNCVT
SFINLKQLIVKNCKRMKYLFTFSTAKSLGKLETLRIENCELMKEIIEKEDENGCHEIIFGRLTKLWLYSLPRLVSFYSGNGTLK

FSSLQIMRLFKCPNMKSFSQRDTNAPMFYGIKSSTDSDITFHSDLNMTVESLFHEQGFFEYSKQMILGDYLETRGFFPPK
FFGSLKKLEFDGSSKGDTVIPSNVLPHLKSLEELTVHSSDEVQVIFGMDDSRAKSKHIVFHLKKLILKDLSNLKCILNKNLEG
SVSFPNLQELFVDACGSLVTLFARNLETLEMHKCDKLVEIVGNEEATENGTTEILMFEFPCLSSLTLYNLTQLSCFYSGKHH
LECPQLEILHVAYCPKLNLFSSKIHGSHKETAAEAPINCLQQPLFMVEKVVPKLRGLTLNEKNMMLLSDEHVPENYLTKL
NLLRLCFEDDKNEKDSLPLDFLHKVPNLEHFRMQRCFGVKEIFPSQKLQVYDGIPASLKGLTLFELNELESIGLEHPWVSP
YSEKLQILRVVNCPLLQNLGCGAMSFINLKELYVKDCDRMEYLFTFSTAKCLGQLQTLIIKKCESIKEIAKIEDADGCDDKIIF
EKLTALKLYSLPRLQSFLSGNATLQFSCLKYANVIDCPNMKTFAEGVLNAPRILRIETQFEDSDSFLGSNDLPKVASKTAKSI
VGRLASFYKQRNRL

>XP_027925715.1

MDPIVSATTESALKILGHLVKRHWGYIFNYKSKFEKLKSYVEYLEDNRERLQHDVDKALENAKEIENDVQRCLKLMDEKI
KEYRSYINDECHEKKICSTGFLPNNFHLRYQLGRKATKMVEEIIEDGLWKKKFDKVSYQEYPSPDYAFSNSGYVSFASRTR
TLETIMKALENSTVDMVGVYGHSGMGKTFLVKEIAKKAREKLFKIVIIANITENPDFEKIQGQIADMLGMTLEEKSEIGRA
NRIRKRLKKEKKNTLIILDDLWDGLDLNRIGIPCDDDDQDDASRQDVSHISNDFGYGNTENSEWSKVDLNKMKKEKSSK
SYKGGKILLTSRKKEVLCDQMDVQQSSTFRVEGLDEKEAQTLLKKVADVKNSDFDSNAIEIAKWSGGIPIALVSIGRTLKN
KSLSTWEDICKQIKRQSSTEEWGFTDFSIKLSYNQLKTEQLKCVFLHCARMGNDALIMDLVKFCIGLNLLQGVHTITDAR
KSVQRMIKELEESSLLVRSYSSDRFNMHDIVRGIALSISSKEKQVLFMKNDILDEWPHEDDFERYTAIFLHSCDIKYEFPKSI
SCPRVEVLHIDNNTESFEIPDDLFKFMIRLKVLVLTGVNLSCLPSSIKCLKKLRMLCLERCTLEKNLSIIGELKNLRILSLSGSNI
ESLPLEFGQLDKLQLFDISNCPNLRKITSNIIARMNILEEFYIRDNLILWEAEENMKSENASLSELRHLNHLQNLEIHIQSFA
HFPQNLFLDKLNSYKIVIGEFNLLNLLTVGKFKIPEKYEEVKFLALNLNENVDIHSKKWVKMFFKSVECLLLGELNDVQDIF
FELNVEGFPNLKHLSIVNNFGINYVINPMERFHPLLAFPKLESIWLYKLYNLERICDNELVDASFCSLKVIKIKTCAKLVNLFP
FCMVRLLTVLETIEVCDCDSLKEIVSKERQTQNVSDDKIEFPQLRHLTLKSLPTFTCLYTVDEMLGSVPLLQGQILQHETTN
IVTNAEHGVTNSCLPLFNEKVSIPKLESLELSSIKIQKIWCDQYDHNFQNIEKIWCDQYDHNFQNLLTLNVTDCGNLKYLL
SFSMAERLVNLQSIFVSECEMMEDIFRQEDAEYIDVFPKLTKMEIICMGKLSTIWKFDIGLHSFKNLSSLIIRECHKLVTIFP
NYMGQRLQSLQSLTVTNCKLVENIFDLANIPHTCDIIETNLENIFLEDLPNLVNVWKGVTGEILKCNNLQSIRVDESPKLK
YLFPVSISNDLEKLEVLEVWDCWAMKEIVSLDKHSSENAISFKFPHLNTVSLIDLHELRSFYSGIHTLEWPPLKKLKIVDCS
MLEGFTSEITNSQEQPIVLATKKVIYNLEYMSVSLKEAEWLQKYIVNIHRMHKLEELTCYRLKNNEILFWFLHRLPNLKSLT
LGLCHMKRIWIPESLSSREKIGGVVELKELELRSMWSLEDIGFEHDVLLQRIECLIILRCKKLTKLASSSVSFSYLTYLEVVNC
MMRNLMTLSTAKTLVQLTTMKVGSCPLIVEIVVENEEEKVQEVEFKQLKSLELVSLKNLTSFSSVEKCDLKFPLLENLVVS
ECPQMKKLSKVQSVPNLQKVHVEAGEKNKWYWEGDLNATLQTHFKDQVYFEYSKEINLVDYPERKVQHGKFAFPDNF
FGCLKKLEFDEACERDTLIPSHVLPYLKNLEELNVHGCKSAKLIFDIDESEMMTYGIVFRLKKLILKNLLNLKCVWKETPRGI
VSFPNLNIVIVHGCEGLVTLFPSSLARNLKKLETLLISGCEKLVEIAGKEDGMETTIMFEFPCLSYLKLVNMPLLSCFYPGKH
HLECPLLEILFVANCPKLKLFTSATEAATEAATEAPISLLQHPMFSIEILASHKLKRLALNQKNFTLLSDTRLPQDLLWKLNFL
WLCFEDDSNEKDTLHFDLFHKVPSLEYLIVQKWFGLKEIFPSQKLQVHHNVLAGLKQLFLFQLSELECIGLEHAWVQPYS
QKLELLKLYECPLVERIVYGAVSFINLKELYVERCEKMEYLFTFATLKSLVKLETLFIENCESIKEIARNEEEDGCDEMVFGRL
RWIYLICLPRLISFYSGNATLQCPCLEWVVVDECPNMITFSEGLIKLPIFSGIQTSKHPDFTFHVDLNTTVQTLFHEKEFFN
HSKHMILDDYLELTGVQHIKPAISENFFGSFKELEFDAACKRAIVIPFHVLPYLKNLEKLNVHSSDAIKVIFDIDESEIKTEGII
SNLKKLTLNNLSNLKCVWKENVGGIVSFPNLEEVVVNGCGSLVTLLSSSLAKSLQKLKKLHMEMCESLVEIVGDEDEREQ
GRTLTFEFPCLILVDLWNMPLLSCFYPGKHYLECPILEALWVAYCPKLKLFTSDLDDSHKDEVTEAPITPLQQPLFLLEKVS
PKLKQLSVNEKNMMLLRDGCLPHDLLCQLSHLLLCFEDYEIQNDTLPFDLFHMLPSLEYLRLQKCFGLKEIFSSQKFQVED
KVLAGLKKLFLVELSELESIGLEHTWAQPYTKKLEMLTLFTCPRVENIVSCAVSFINLKELSVKHCAKMEYLFTFATLKSLVK
LETLSIKKCESIKEIIKKDDEDGCDEMVFGRLRSIKLNSLTKLVSFYSGNATLQCSYLKNLMIVECPNMITFSQGVVKVSMLL
EIQPSKDSDSTFQGDLNTTIEILFHEQDFFNYSKLRILDDYLGMMGVLHTKPTLSDNFFGSFRKLEFDTTCNRSFVIPYHVL
PYLKNLKELNVHSSDAVQVIFDSDETEVETKGIIFGLEKLTLKHLSNLKCVWKKKLKGIVSFSNLEEVNVDGCGSLVTLFPLS

IAKNLKKLETFDIEECEKMEEIVGREDEMEHGTTITFEFPCLSYLILVNLPLLSCFYSGKHHLECPLLDKLYVGYCPKLKLFRP
SFDDSHKDEVIEAPINRLQQPLFSIEKVSPTPVGLTLNEENIKLMSDARWPQDLSKLFILILSFEDDNNGKDSLPFDFFHKV
PNLFSLAVQKWFGLKEIFPSQKLQVHNNVLAGLKRLSLWELSELECIGLEHTWVQPYSTKLELLKLYECPLVERIVYGAVSF
INLKELYVERCEKMEYLFTFATLKSLVKLETLFIENCESIKEIIKKDDEDGCDEMVFGRLRSIKLNSLPKLVSFYSGNATLQCS
YLKTLMIAECPNMITFSQGVIKVSRFLEIQTSKDSDSTFHGDLNTTIQKLFHNQVIHLS

>XP_027925719.1

MSIRTNKMKAVPVLLKQLMKARSKFRERGRDELFDKKLEKLRLDLNKIKDVFVRVKKKEEELLDTLAEVYNHLRKLDRRK
LDEGMDGMCQRIRDSAHNLLPNDGFDDSSKDEDHIGGQIFHSSQELWKARDKIRYPGNHYYTPDHLSITCLWSLLVFP
ENAVIRKRIAINLWIGEGLLENTKNKTTEKLGEDVISDLFQLNLIEGKSLLANKFQILPSVRHQMESYLLKENAEHRGQYFI
PGIYLQKRPWRLQLERRKVTLGGDDWYFGGRTIGTVFNNGASYLNFRPQWVTELMNLEVLQLGGWQDSALHHIEVG
SQEFLKDMRYLEELKYLSLRGISRIFELPSSTAELEKLLILDVKACHNLERLPDDISSMKSLTHLIMSDCCLLEGMPKGIEKLT
NMRVLKGLLISTYEKTPCRISDLVKLRKLRRLSIRIGSEAEMRDGEFEGLEDFSALEKLKISWSVSDPKYANINVALPLRLKK
LHLECFPGKSSEECFMPRQVGHGGIIFTSRELNITGGKLENMKKKILIGGT

>XP_027926693.1

MSILTNKMKAVPVLLKQLMTTRRKFHERGRDESLDKKLEKLRLDLNRIKDVFVRVKKNEEELLDTLAEVYGHLRRLDRGK
LDEDMDGICKRIRDCSRKLLPKDGFDESYKEEDEKGVKISHSSQELVQPHLNKSWTLEDYYRLSLPSRFCLLSLQIFPENAV
IRKRNAINFWIGEGLITNTEKKTAEEMGEDVIDDLLKCKVIVRYGNEKSPLVKKFQILSGDLRHLEPNLDGDFDYIRPSSLQ
LDSKKVTVGGVHSKNVTIRNIFNIGASYLNFRPQWTTELRNLEVLQLGRWQDSALHHIEVGSQEFLKDLRYLKELKYLSLR
GISRIFEVPSSIAELEKLLILDVKACHNLERLPDDISSMKSLTHLIMSDCCLLEGMPKGIEKLRNLEVVKGFLISTSEKTPCRIS
DLVNLEKLRRLSILIGSEAEIRDGEFESLKDFLALENLKISWSVSDPKYAKIKVFLPLRLKKLHLECFPGKSLQEYFTFMPSAW
FGSTSTELNITGGKLERMVDIQWWRVDILRLKYLKQMNIEVDDLKAMFPLLKYVEIKQISNHSYIKHEWRI

>XP_027927142.1

MSIRTNKMKAVAVLLKQLTTARRKFYERGRDESFDGKLEKLRLDLNKIKDVFVEVKKKEEELLDTLAQVYDHLRKLERVKL
DEDMDGICNRIRDSALMLLPTLVFDDSYKDDDNKGGKISHSSEDLLQLQHQNIWTLEDYYRLHDPSKCCLWSLLIFPVN
AVIRKRNAINLWIGEGLIGNTENKTAEEEGEDVIDDLLKCGVIVRCGNGNDPFVHRFRILPGDLHGVSNLIRDSENIISSPL
QLDSKKVTVGGVHTKNVTLRNIFNIGASYLYFSPQWTTELRNLEVLQLGRWQDSALHHIEVGSQEFLKDLRYLKELKYLS
LRGMSRIFEVPSSIAELEKLLILDVKACHNLERLTDDISSMKSLTHLIMSDCCLLEGMPKGIEKLTNLEVLKGFLISTPEKTPC
RISDLKNLEKLRRLSIRIGSEAQIRDEEFEGLKDFLALEKLKISWSVSDPKYANIGVILPRGLRKFHLECFPGKFLEKCFHVFGL
KLTELKITGGKLESMKVDFSWWWRVEIMRVKYLKQLKVDIDDLKALFPELRYVEVKQVSNISYVPHQWTDE

>XP_027927222.1

MGRPKHEHYWKKVDKEKDGSLKCKKCGHKFKGGVSRIEAHIKGTGGIRKCSSPLNDTTSSNHSEQHMNVIDTSPVGER
TEEMTDSVGRSINNGRVIQSSLGGAYWNEVVEGDQGMVEMVGGAANPENSPSLTHLLEGQEAENGDGVVTTLESDC
DELISDLTSGEEDIQGQLQLMESRGKERNRHVDWWLKELQNMKQRAINVKKTLNEFSCSNFNVQQGQMYLVEELEN
EIQDLFEEMVGHMEGKPLMLSNEFLGRQFEENVKKMWDLLREDKVFSIGIHGMGGVGKTFLATYMESEIKRTKTFKDV
VWVTVSHDFTIFKLQQHIAEILKIKLYGDDERERALILASELEKRENIVLILDDVWKYIDMEKVGIPLRMKGNKLIITSRLRH
VCQQMDCLPSNMIEVMPFKIGINFDDDWELFLLKLGDHGTPSTLPSQVLDIARSMVGKCDGLPLGISVMARTMKGET
RIHWWRHALNKLDKLEMGVEMQEEVLTVLRRSYDNLTEKDVQKCFLYGALLPNPVRSNPVRRDLLIMNHVDMVLLN
GKRRLEEIFDEANVIVDKLINHSLLLEDNRRLMMHPLVRKMALNIIKESGSNLMVKCGESVEKIPDIEEWTIDLEVVSLAN
YKIQKIPDGTSPNCPRLSTLLLFDNRITDIPECFFMHMNALTTLDLSGNDGLTRLPHSLSKLRTLTSLMLNDCSNLKYIRPL
GELQSLLRLEISGCSIQVPPEGLENLVNLKWLDMSNDVNLKLVPGSFLPSLTNIQYLDLYGCSGIKAEDIQGMNFLECFAG

TFVDRENLNRYVQQTLNSDYGPQTYSIHYQDQSHKGLWEEFWNREPLSEYKCRTMCIKDCEELSYVLPRDLVKLSVEDN
GQWVCLCAALSSDDSSTLKEINIQYCAKLKSLFCLSCSLCANIQSLQSLRLSHLESLTTICNKDIVNLIQPWLPSGMFSQLK
HFHISYCHGIKTLMTSSLVSDFQNLVSIIVSNCDSMEQIFALTSDDNDSIKITLPKLTTLCVSFLPQIKTVSERILVCKYGFRP
VFFGCPKLCEPIIESII

>XP_027927223.1

MGRPKHEHYWKKVDKEKDGSLKCKKCGHKFKGGVSRIEAHIKGTGGIRKCSSPLNDTTSSNHSEQHMNVIDTSPVGER
TEEMTDSVGRSINNGRVIQSSLGGGDQGMVEMVGGAANPENSPSLTHLLEGQEAENGDGVVTTLESDCDELISDLTS
GEEDIQGQLQLMESRGKERNRHVDWWLKELQNMKQRAINVKKTLNEFSCSNFNVQQGQMYLVEELENEIQDLFEE
MVGHMEGKPLMLSNEFLGRQFEENVKKMWDLLREDKVFSIGIHGMGGVGKTFLATYMESEIKRTKTFKDVVWVTVS
HDFTIFKLQQHIAEILKIKLYGDDERERALILASELEKRENIVLILDDVWKYIDMEKVGIPLRMKGNKLIITSRLRHVCQQM
DCLPSNMIEVMPFKIGINFDDDWELFLLKLGDHGTPSTLPSQVLDIARSMVGKCDGLPLGISVMARTMKGETRIHWW
RHALNKLDKLEMGVEMQEEVLTVLRRSYDNLTEKDVQKCFLYGALLPNPVRSNPVRRDLLIMNHVDMVLLNGKRRLEE
IFDEANVIVDKLINHSLLLEDNRRLMMHPLVRKMALNIIKESGSNLMVKCGESVEKIPDIEEWTIDLEVVSLANYKIQKIPD
GTSPNCPRLSTLLLFDNRITDIPECFFMHMNALTTLDLSGNDGLTRLPHSLSKLRTLTSLMLNDCSNLKYIRPLGELQSLLR
LEISGCSIQVPPEGLENLVNLKWLDMSNDVNLKLVPGSFLPSLTNIQYLDLYGCSGIKAEDIQGMNFLECFAGTFVDREN
LNRYVQQTLNSDYGPQTYSIHYQDQSHKGLWEEFWNREPLSEYKCRTMCIKDCEELSYVLPRDLVKLSVEDNGQWVCL
CAALSSDDSSTLKEINIQYCAKLKSLFCLSCSLCANIQSLQSLRLSHLESLTTICNKDIVNLIQPWLPSGMFSQLKHFHISYCH
GIKTLMTSSLVSDFQNLVSIIVSNCDSMEQIFALTSDDNDSIKITLPKLTTLCVSFLPQIKTVSERILVCKYGFRPVFFGCPKL
CEPIIESII

>XP_027927224.1

MGRPKHDHYWNEVDEEKDGKLKCKKCGHKFKGGVSRIEAHIKGTGGIRKCSPPLNDTTFSNHSEQHMNVIDTSPAGE
RTEEMTDGRSINHGGVQLSSLGGVVEGDQGMVEMVGGAANPENSPSLTLLSEGLDAENGHGAVTTLESDCDELISDL
TSKEEDIQGQLQLMESRGKKRKGKVDGWLKELQDMKQRAIEVKTTLNNEMYLVEELQEQMYLVEELKKKIPHLNNEM
PLVLSNEFVGREFEENVEKMWDLLREDKVFIIGIHGMGGVGKTFLATYMESEIKRTKTFKDVVWVTVSHDFTIFKLQQHI
AEILKIKLYGDDERERAVILASELEKRENIVLILDDVWKYIDMEKVGIPLRIKGNKLIITSRLRHVCRQMDCLPSNMIGVMP
FNYGNIFDDDHFELFLLKLGDHGTPSTLPSQVLGIARSMVGKCEGLPLGISVMARTMKGETRIHWWRHALNKLDKLEM
GVEMQEEVLTVLRRSYDNLTEKDVQKCFLYSALLPNPLRRDLLIMNRVNMVLLNGKRRLEEIFDEANVIVDKLINHSLLLE
DLEDNSELTMHALVRKMALNIIKENGSNLMVKCDESMEKIPDIEE

>XP_027927335.1

MSIRTNKMKAVAVLLKQLTTTRRKFHERGRDESFDGKLEKLRLDLNKIKDVFVEVKKKEEELLDTLAQVYDHLRKLERVKL
DEDMNGICQRIRDSAHNLLPTLVFDDSSKEEDDKIFHSSKDLVQPHQDSWTMEDYDQLSLPSRNCLLSLFIFPENAVIKK
RNAINLWIGEGLITNNKKETAEEEGEDVIDDLLKCGVIVRCGNGNDPFANRFRILPGDLHGVSNLIRDFDYIRSSPLQLDS
KKVTVGGVHTKNVTLRNIFNIGASYLNFRPQWTTELRNLEVLQLGRWQDSALHHIEVGSQEFLKDLRYLKELKYLSLRG
MSRIFEVPSSIAELEKLLILDVKACHNLERLPDDISSMKSLTHLIMSDCCLLEGMPKGIEKLTNLEVLKGLLISTPEKTPCKISD
LVNLGKLRQLSIRIGSEAEIRDGEFKGLEDFLALEKLKISWSVSDPKYAYIGVILPSSLRKLHLECFPGKSLEGWVLPIIHRFRF
ISTELKITGGKLESMEVNVERGTVEIVRLKYLKQLKVDIDYLKALFPELRYVEVKQVSNISYLQHQWPWPYELAFKP

>XP_027927359.1

MSIRTNKMKAVAVLMKQLTTARRKFDESGRDESFDGELEKLRSVLNKIKDVFMEVKKKEEKLLDTLAEVYDHLRRLNRR
KLHEDMHSICESIRDSALMLLPTLVFDDSSKDEDHKGGKISHSLEELVQPHHQNNWTVEDYYLLHDRFKRCLWSLLIFPV
NAVIRKRNAINLWIGEGLIGNTYNKTAEKEGEDVIDDLLKCNMIVRCDNGKGPFVNRFRILPGDPRGVLNLIRDLENIKSS

PSPLQLDIKNVTVGRFDAKDHTLRNIFNIGASYLNFRPQWATELRNLEVLQLGRWQDSALHHIEVGSQEFLKDLRYLEEL
KYLSLRGISRIFELPSSIAELEKLLILDVKACHNLERLPDDISSMKSLTHLIMSECWLLEGMPKGIENLSNLEVLKGFLISTPEK
TPCRISDLVNLRKLRRLSIRIGSEAEIRDGEFENLKYFSALEHLKISWSVSDPKYGYIRVILPPSLIKLHLECFPGNSFVDCFMP
GSYDSGLSELNITGGKLESMNPSMLLWKVKILRLKYLKQLHVNMDDLKACFPGLKYVQIKQTSNVSYIERELNF

>XP_027927610.1

MVLTVKLITMVSTCHKSHKEAYITSLLILLLVYSALQHHIESKEAMAFVGEAFLSAAVEVLLNRIISCEFQDFFHSKKLDFLPL
RKLRITLLSLQAVLNDAEERQITNPAVKEWLDELTQAVFDAEDLLDEINTEALRCKLEANSLSQSTTDQVLNFFSSPFNNF
QKVTNSKIQELFQRLEQFTLQKDILQLKEGVSSRVWHRTPTSSVVDESTIYGRDGDRQKLVSYLLSEDAGGCKVGVISIVG
MGGLGKTTLAKLLYNDPNAQEKFDLKAWTHISDDFDVCKVTKTILESVSFKSNDINNLNILQVELKQSLSNKRFLLVLDDI
WDGSYVDWNNLMDIFSAGKMGSRIIITTRDESVARAMQTFLPIHHLTSLTSEDCWSLLARHAFGANNCSQHSTLEEIG
KEIAKKCDGLPLAAVTVGGLLRTKLSENYWTKVLKSNIWDLPNVKVLPALLLSYHYLPGPLKRCFAYLSIFPKNSKIEKEMV
VRLWIAEGLVCQSKSDKTMEEIGDEYFDELVSRSLIHRSLTWHAKFKMHDLINGLATMISSTYCVRREDTMSHASVERIR
HFSYNRGEYDSFNKFDHFYESKGLRSFIALPLRLWWIRNGRFSAQHLSNKVVHDLLPAMTKLRVLSLSHYVNITELPNCL
GNLIFLRYLDLSNTRIQRLPEVTCKLYNMQTLLLSNCWSLTELPEDIGNLVNLQHLDNSGTRLNKMPKQIATLQNLQTLST
FVISKPQDGLMVGELKNFPRLQGKLSILKLQNVVDPSEAFQANLKNREQINELALEWDCGTTEDTQTERLVLEQLQPPA
NLKKLTIKCYGGTSFPNWLGDPAFDNMVHLRISGCDHCWSLPPLGRLVSLKELYISDMKLVKTVDTEFYGSTSPSFQPFP
SLVILSFEGMLGWEEWNMIGGTVIEFPSLSDLSLKNCPKLKGTLPTNCPYPIDFELSGCPLLFAMVCPELKESNLHSSIELQ
STSNILNLTISGVTSPASLRDGLPTTLKYLTLRNCKKLEFLPHETLNNYTSLKELTIFNSCHSLTSFTLGCLPVLESLSISGCKNL
KSISIAENSSPSLSLLQWLSIRSCPELDSFPAGGLLIPNLTTLAMSSCDKLNSLPVPINTLAPLQTLSIQNLPNLESFAQEGLPI
RLRSFTVWSRGSFLTTTIGEWGLQKLTCLSSLRIGGDEILNAFMRMKMPLLPTSLVSLSICNSFGIKCLDGKWLQHLTSLE
KLEIQCFRQLEFLPEEGLPSTLSVLTIWKCPSLEVSCSNGGKNWPKISHIPCIIINKEVII

>XP_027927715.1

MTQEIYRVLSEGNFDKISKPPTIKEIQQVLKDPNICKIGLYGIDGVGKTTLMKELAKEVLKDGSFDVVAMAEVTDSPNVE
NIQGQIADALALKFDEETKEERMQKLRGRISKEKNILVILDDIWGQVDLAELGIPFGDDHKGCKLLLTSERLNVLKRQMG
TQNNFRLEVLSDEDSWKLFEKIADQAIKSISTNKSVVELDEINSIVQNVPKCCNGFPLFIVVVAKALRTKDLSTWKDALKQ
LKGMNEKGHFEKVVCPLELGYRYLESDELRTLFLFIVSLGPGRIHTGELFSCYWGLYGDSHQLTKARNNYYKFIDDLRASS
LLLEVEIEYVRMHDSVRDTAKAISSRTHLTYEVPKFTQKEQWDIDQLKKCHYINLPCYSLDEVPKKLDCPELKQMSLKSNL
GQLTIPDNFFAGMGEVKVLNLHRMSFAPSPPPSFRLLKNLRSLNLYECVLDDITMVAELTSLEILSLERSKLQELPKEIGQL
TRLRMLNLTNCYQLKSVPRYLIYSLVYLEELYMGNCNIQWEAEGVESQSNNPSLGELRKLDRLTTLDLSIHDASVLPTDM
DVFKQLQRYNISIGNMWKWSSFWSCDAREISTTLKLVDSLNTEIFLNHGIQMLFTTIKDLSLAKINFSDDVFYELNREAFQ
HLRHLYVQNNTDSLESA

>XP_027927912.1

MSIRTNKMKAVAVLMKQLTTARRKFHERGRDESFDGKLEKLRLDLNKIKDVFVRVKKKEEELLDTLAEVYDHLRKLDCRK
LDQDMNGICQRIRDSAHNLLPTLVFDDSSKEEDDKIFHSSKDLVQPHQDICTKEDYDQLSLPSRNCLLSLFIFPENAVIKKR
NAINLWIGEGLITNNKKKTAEEMGEDVIDDLLKFNVIVRYGNWKSPVVNKFQILPGVRDLIEGHVSKQYVEHHGTYFSIS
LLRLLRMEGLELDQKRVTLGGRYYIDRTIPTVFNIGSNYLNFRPQWVSELKNLVVFQLGRWQDSALHHIEVGSQEFLKEL
RYLKKLKYVSLRGISRIFELPSSITELENLLILDVKACHNLERLPDDISSMKSLTHLIMSECWLLEGMPKGIENLSNLEVLKGF
LISTPEKTPCRISDLVNLRKLRRLSIHIGSEAEMRDGEFENLKDFLALKHLKISWSVSDPKCANIHGLLPYGLRKLHLECFPG
KSFKECFMLKHSYAHYFTLLDIKITGGKLESMKEDLEWRMGSLRLKYLKQLNVDIDDLKAYFPDLRYVEVKQVSNISYLQH
QWAD

>XP_027927913.1

MSIRTNKMKAVAVLMKQLTTARRKFHERGRDESFDGKLEKLRLDLNKIKDVFVRVKKKEEELLDTLAEVYDHLRKLDCRK
LDQDMNGICQRIRDSAHNLLPTLVFDDSSKEEDDKIFHSSKDLVQPHQDICTKEDYDQLSLPSRNCLLSLFIFPENAVIKKR
NAINLWIGEGLITNNKKKTAEEMGEDVIDDLLKFNVIVRYGNWKSPVVNKFQILPGVRDLIEGHVSKQYVEHHGTYFSIS
LLRLLRMEGLELDQKRVTLGGRYYIDRTIPTVFNIGSNYLNFRPQWVSELKNLVVFQLGRWQDSALHHIEVGSQEFLKEL
RYLKKLKYVSLRGISRIFELPSSITELENLLILDVKACHNLERLPDDISSMKSLTHLIMSECWLLEGMPKGIENLSNLEVLKGF
LISTPEKTPCRISDLVNLRKLRRLSIHIGSEAEMRDGEFENLKDFLALKHLKISWSVSDPKCANIHGLLPYGLRKLHLECFPG
KSFKECFMLKHSYAHYFTLLDIKITGGKLESMKEDLEWRMGSLRLKYLKQLNVDIDDLKAYFPDLRYVEVKQVSNISYLQH
QWAD

>XP_027927914.1

MSIRTNKMKAVAVLMKQLTTARRKFHERGRDESFDGKLEKLRLDLNKIKDVFVRVKKKEEELLDTLAEVYDHLRKLDCRK
LDQDMNGICQRIRDSAHNLLPTLVFDDSSKEEDDKIFHSSKDLVQPHQDICTKEDYDQLSLPSRNCLLSLFIFPENAVIKKR
NAINLWIGEGLITNNKKKTAEEMGEDVIDDLLKFNVIVRYGNWKSPVVNKFQILPGVRDLIEGHVSKQYVEHHGTYFSIS
LLRLLRMEGLELDQKRVTLGGRYYIDRTIPTVFNIGSNYLNFRPQWVSELKNLVVFQLGRWQDSALHHIEVGSQEFLKEL
RYLKKLKYVSLRGISRIFELPSSITELENLLILDVKACHNLERLPDDISSMKSLTHLIMSECWLLEGMPKGIENLSNLEVLKGF
LISTPEKTPCRISDLVNLRKLRRLSIHIGSEAEMRDGEFENLKDFLALKHLKISWSVSDPKCANIHGLLPYGLRKLHLECFPG
KSFKECFMLKHSYAHYFTLLDIKITGGKLESMKEDLEWRMGSLRLKYLKQLNVDIDDLKAYFPDLRYVEVKQVSNISYLQH
QWAD

>XP_027927980.1

MADTIVVFLIDKLTRLLVEEAKLLAGVRDQVASLQSELRFMNLFLRNSQGKRKEHDMVAELVSQIRDVAHEAEDVIDTY
VAGVIKQSRRNVIGKVGFRGVDHALMLHQVAGKVDGIKARIKEIFDNKQRYGIEDGKRGGEEEAERIRKQRREVEEEEV
VGFALDSKVVIEKLTVSDSRLKVVSIVGMGGLGKTTLARKVYNSNRVKNMFPCRAWGYVSNDYRPREFFLSLLKCLLSTS
KYSGLFKKREETSVSDEELKMKVRECLNRSKYLVVVDDVWQKQVWNEVKGAFPDDQNGSRILMTTRWAEVASHAGP
VPPYALPFLTKEESWELLSKKVFRGEECPSDLESLGKLIAESCDGLPLALIVMAGILGNKKSPRDWSRIKDHVNWHLGRD
NTLKDILKLSYDSLPARLKPCFLYFGMYPEDYRIPVKQLIQLWISEGLLTQETSVSQDIPEPEYIAEEYLDELVDRSLIQVVSR
TNDGGVKTCRIHDLLRDLCISESREDKFFEVCGEIDFQNLNSCPRKLSLQGTLFHFSSSIVSDYTISATRSLLCFGQEVYKVK
ANHWRWLLKSFRLARVLDLGRMNVNSIPTDLEKLIHLRYLRIHSHNLETIPPSICRLWNLETLDLRGSPIKSFSGELWQLK
QLRHLLLFGPVGLPEMPSESKTMPNLQTLSTVALDPRTTSLLDSRRFPGMTKLGIHYERRDKCNARIQLQSLHRLSHLRKL
KVIGTTEIPQNANMFPSNITKISLTKFGFFNSTVMHMLGKLPNLQVLKLSSQTNDTRFDLHCATGGFLQLQVFEMVAIK
VKVWRVDRGSMPRVRRLVVRSCKSLTQLPKEVWSLNTLREVQVLWPCTELAKGLQNLVMNNACKLVVYPLSANDEL
DFLEING

>XP_027928050.1

MASVREAPSWKFHVFLSFRGEDTRNGFTDHLYAAFRGRGFAVFRDDEELERGEVISHALLKAIDESLCSVVVLSPHYASS
RWCLDELLRILESRANFGRNVLPIFYDVDPADVRHQRGTFAEAFAKHVERFGSDEVRMWRQALKDVAALSGWTSKDK
RETELIEEIVAEVWKNLQSKLPSYDDELVGIDYRINSIYSLLRTNSQEVRFMSIWGMGGIGKTTLARFVYNKIHDQYDISCF
LENVREVSNERDGLLCLQRKLLSHLKIRSMRIESLDQGKETIRNLLFNKKVLLVLDDLSSDIQVENLAGKPEWFGQGSRVII
TTRDKHLLKSLHVCENYDVQVLNSYESLQLFCQKAFRGEKPEEAYLELSRSVVQYAGGVPLALKVLGSFLCGRSASIWED
ALKMLRKDAQNDICKTLRISYDGLRDNEKAIFLDIACFFKGNTKDDVTRILENCDFNPLIGIEVLIEKSLVTYDGLHLGMHD
LLQEMGRNIVLQQSPNDASKRSRLWTLKDIDQVLRNNNGTESIQAIVLNLPEPYETCWNPDAFSKMSNLRLLMILNKL
QLPLGLKCLPSGLKVLVWKEYPLESLPVGAQLDELVELHMCQSKIKHLWGGTKFVENLKIINLRNCNNLHRTPDFTGIPN

LEKLDLEGCVNLVEVHASLGLLKKLSYLTFEDCRNLKILPRKLLLDSLNRLVLSGCSAVRNLPEFGESMKSLSVLALEETSIAE
LPVSVGHLTSLTNLLLEGCKNIVRLPNTISNLKSLRRLNISGCSKISKLPDNLNENEALESLNASETAIREVPSSIVLLKNLRLLL
FRGCQDLASNSWSSLLPFEKILRFNSHPTTKRLVLPSFSGLSSLRKLDLSYCNLHDGSIPEDLGCLSSLVTLDLSGNNFVCFP
GCISELLKLERLLLKCCPRLESFPKLPPEVHYVNASDCGSMKPLSDPQQIWGHLVSFAFDKLQDASNFKTLLVSPGNEIPSF
FFYQKHLNQVQDIEYLKENYIWADSTVSIPMDIAPLRHRYHRSEWWGILVSLVVEDVESSPSQEYRIGWISKVPSLKNIL
QQLCHKTEQGLISGIQNHKYPHLLILYIPIYRARWFYVHDKFQLIFYSSSLKSKLVIKKCGWRILCKEDAENWRTNLSECNT
NSANQCVANPRDGRLSPHFSSWRWISRLKVPQHCKTFLLAILCDRLPANERCSFCCLEGTVIHVLRDCTRATAIWVQM
VPPEVCDEFFSTSLHDWMHRFLLKLWFPDRDYYADCLRFTITIWLLWKDRNSSIFKRNSPTPTDNDGLYSLIQSLVKEYAI
LLHLKGEEGTSAANSLSQNSRLKLFVKLNVDGCCNGNPGNAGYGGLFRDVEGKWLGGFYGSLGLATNVRAELYAICQG
LIAAWDLGYRTMLVETDSLEAINLIKEANIEDCAYSGLLADIRSLMQRNWSLDLIHSLRQDNACANMLSKLGAEQHEVY
CFLAHPPQQLQPALVADALQVQLPCL

>XP_027928160.1

MVEAVVSFAIDRLYDLLIEEARLLNGVSDKVKSMQNELKRMQCFLRDAESKQDEGDIIKNYISEVRKLAYDAEDVIEIYAI
KVAFSISIGTKNPLSRVKSIHKVGSELITINSRISDLTRSLQTYGLTATKDNEESSNMKRQLRWSYSHIVDEFIVGLDKDINK
VTEWLLNENENEGCRFVYISGMGGLGKTTLAKSIYHYNAIRRNFDGFAWAYISQQCKKRDVWEGILLKLISPTKEERDEI
TKMKDDELARKLFKVQQEKKCLIILDDIWSNEAWDILSPAFPSQNTRTKIVFTSRNKDISLHVNPEGLLHEPSCLNAEDS
WALFKKKAFPRQDDPESTISDDFKRLGREMVAKCAGLPLAIIVLGGLLATKESVSEWEKIHRHLSSYLIGAEVRDSRRLDE
VLDLSYQDLPCQLKPCFLYLSQFPEDSEIPKTKLLQLWVAEGVVSSQFESERDETMEDVAERYLGNLISRCMVQIGQMG
STGRIKTYRLHDLMRDLCLSKARKENFLYIINGSEQNSTTDATRSSNVSDARQIGEVRRLAVYLDQHVDQLIPQDKQVNE
RLRSLVFFHDKKCRMENWDLVRGVFVKFKLLRVLDLEGIKGLKGQSLSKEVGNLLWLKFLSLKRTRIQILPSSLGNLENLQ
FLNLQTVNKVSWDSTVEIPNVICKLKRLRHLYLPNWCGNIVNNLQLDNLTNLQTLVNFPASKCDVKDLLKLKKLRKLVLN
DPRHFQKFSESFSPPNKRLDCLLSLSLRTDMLSFPENVVDVEKLVLGCPSLRKLQVEGRMERLPDASLFPPHLSKLTLWG
CRLVEDPMVTLEKLPNLKFLNGWDLFIGNKMTCSPNGFPQLKVLVLRGLPKLDEWMIENQAMPNLYRLSISDCNNLKT
VPDGLKYITSLRELEIRWMPKSFKTRLGTAGEDYHKVQHVPSIIFLN

>XP_027928161.1

MVEAVVSFAIDRLYDLLIEEARLLNGVSDKVKSMQNELKRMQCFLRDAESKQDEGDIIKNYISEVRKLAYDAEDVIEIYAI
KVAFSISIGTKNPLSRVKSIHKVGSELITINSRISDLTRSLQTYGLTATKDNEESSNMKRQLRWSYSHIVDEFIVGLDKDINK
VTEWLLNENENEGCRFVYISGMGGLGKTTLAKSIYHYNAIRRNFDGFAWAYISQQCKKRDVWEGILLKLISPTKEERDEI
TKMKDDELARKLFKVQQEKKCLIILDDIWSNEAWDILSPAFPSQNTRTKIVFTSRNKDISLHVNPEGLLHEPSCLNAEDS
WALFKKKAFPRQDDPESTISDDFKRLGREMVAKCAGLPLAIIVLGGLLATKESVSEWEKIHRHLSSYLIGAEVRDSRRLDE
VLDLSYQDLPCQLKPCFLYLSQFPEDSEIPKTKLLQLWVAEGVVSSQFESERDETMEDVAERYLGNLISRCMVQIGQMG
STGRIKTYRLHDLMRDLCLSKARKENFLYIINGSEQNSTTDATRSSNVSDARQIGEVRRLAVYLDQHVDQLIPQDKQVNE
RLRSLVFFHDKKCRMENWDLVRGVFVKFKLLRVLDLEGIKGLKGQSLSKEVGNLLWLKFLSLKRTRIQILPSSLGNLENLQ
FLNLQTVNKVSWDSTVEIPNVICKLKRLRHLYLPNWCGNIVNNLQLDNLTNLQTLVNFPASKCDVKDLLKLKKLRKLVLN
DPRHFQKFSESFSPPNKRLDCLLSLSLRTDMLSFPENVVDVEKLVLGCPSLRKLQVEGRMERLPDASLFPPHLSKLTLWG
CRLVEDPMVTLEKLPNLKFLNGWDLFIGNKMTCSPNGFPQLKVLVLRGLPKLDEWMIENQAMPNLYRLSISDCNNLKT
VPDGLKYITSLRELEIRWMPKSFKTRLGTAGEDYHKVQHVPSIIFLN

>XP_027928365.1

METVVSTTTENALNIVQSVVKRQVGYFFNYKDKFKELESYIDKLEHNRERLQHQVDIALRSGEKIENDVQHCLILMDEKIK
NYKSYINDEFHAKTICSIGFFPKNFQLRYQLGRKATKMVEEIVGDELWKTSFDNVSYQECPSIDASLSNTGDESFASRTKT
MEMIMKALQDSTVGMIGVYGPGGVGKTTLVKEIANKALEMKLFKIVIIANITGNPDFKKIQEQIAGMLGMKLEEESEIA

RVDRIRNRLKNEKENTXIILDDLWGGLDFNKIGIPCNDDASQQEVNDMSDFASNNDISDFGYNRLEIKELPKVDLDKMK
KEMLSSHYKGGKILLTSRNKQVLCNEMDVQQSSIFSVGVLDEKEAETLLKKVAGVKNSEFDRNATEIAKWSAGFPIALVS
IGRTLKNKSLSTWEDVCQQIKRQNFTSEWGFTDFSIKLSYDHLKNEELKCIFLHCARMGNDALIMDLVKFCVGLNLLPGV
HTITGARKRVKEMIQELEESSLLVKSYSIDRFNMHDIVRDVALSISSKEKDVLYMKNAILDEWPHENDFERYSAIFVHYCDI
NDKLPESIQCPRLEVLQIYNKSESFEIPDDFFKSMIRLRVLVLTGINLSCLPSSIKCLKKLRMLCLEGCTLEENLSIIGELKNLRI
LTFSGSNIESLPLEFGQLDKLQFFDISNCSKLRQITSNIIPRMTILEEFYIRDNLILWEAEDNMKNENASLSELRYLNKLQNL
DIHIHSSSHFSQNLFFDRLNSYKIVIGEFNWFNMLKVEEFKIPDKYEEVKFLALNLKRGIDIHFEKWAKMSFKSVEYLLLGE
LNDVQDIFYELNVEGFPNLKHLSIVNNFDINYIINPRERFHSLSAFPKLESIWLYKLDKLEIICYNQLVETSFCNLKVIKIKTC
MKLVNLFVFSMVRLLTALETIEVCDCDSLKEIVSKERKTHTISDGNIEFPQLRLLTLKYLPTFICLYNVDKIPGSAHSLQDQV
FQQRNKDIVVDVEDMVTNYCLPLFNEKVSTPKLEWLELSSINIHKIWSDQCNHCFQNLLTLNVTDCGNLKYLLSFSMVG
SLVNLQSLFVSECETMEDIFRPEDEEHIDIFPKLKKMEIINMDKLNTIWKSHFGLHSFCSLDSLIIKECHKLIMIIPGYMMQ
RFRSLQSLTIINCKLVENIFDFTNIPQTSDITETNFDNILLEKLPNLVNVWKDDIGEIPKYKNLQRIKIVDSPNLKCLFPLSIAN
DLEKLEILEVWNCWVMKEIVACNKHSSGSDINFRFPHLNTLSLINLYDLRSFFSETHTLEWPSLKKLDIANCSMLEGFTSEI
TNIQEQTIALAIKKVIYNMEYMSMSLKEAEWLQIYIVNVHRMHKLEELTLYGLKDNEILFWFLHRLPNLKRLTLGLCHLKR
IWALQSLISHGKIGGVVQLKELKLESMWSLEEIGFEHEVLLQRVERLSIQRCTKLKTLVSSSVTFSYLTYLEVMNCKLMRNL
MTCSVAKTLVQLTTMKVCSCPMIMEIVAENKVEKVQEIEFKQLKSLELVSLQNLTSFSTITKCELKFPLLEKLVVSECPLMT
KFAEVQSAPNLQKVLVEAGEKDKWYWEGDLNATLQTHFSDQVSFEYSKHARLVDYPERKDVGLGKSAFPDNFFGCLK
KLEFGEACRRNILIPSYLLPYLRNLEELNVENCKATQVIFDMDESELKMMGMIFRLKKLNLKNLSNLKCVWKENSGGIVSF
PNLHRVDVNGCGTLVTLFSSSLAKNIKKLETLTITKCEKLVEIVGKEDGTEDGTRIIFEFPCFLVLCVDNVPLLSCFYPGKHHL
KCPLLQGLFVGNCPKLKLFTSEFDDNQRATFAKTILLQPLFSVEILASPKLVKLLLNEENIMLLRDADLPKDLFCKLNLLWLH
FEDDNEKDISLPFDLFHKLPSLECLVVQNCFGLREIFPSQKLQVHDKVLATLKQLFLLNLKELESIGLEHTWVQPYSQKLES
LRLHFCPRLQNLVSCAPSLINLKELEVMYCDQMEYLFTFTTAKSLVKLETLIVTNCESXQEIAKHEDEDDCDEIVCAQLRSIE
LNSLPRLLRFYSGNATLQFSCLQTVTVVKCPNMVNFSLGIINVPVFSGVNVSSEDSDLTFDHDLNTTIETLFHEQEFLNHS
KHMTLDDYLEKTGVQHRKPAISDSFFGSFKELEFDAACKRAIVIPFHVLPYLKNLEKLNVHSSDAARVIFDIDESEIKTKGM
VSNLKKLTLNNLPNLKCVWKKNLGRIVSFPNLEEVVVNGCGSLVTLLSSSLAKSLEKLKTLHMAECENLVEIVGEEDERGQ
GMTLTFEFPCLTLLYLWNMRLLSSFYPGKHYLECPVLDTLWVAYCPKLKLFTSDFDDSDKDEVIKAPITPLQQPLFLLEKVS
PKLKQLSLNEKSFMLLRDGCLPHDLLCKLRNLLFCFEDYKNENDTLPFDFFHKLPCLEHLHLQNYFGLKEIFPSQKLQVHXS
ELESIGLEHTWVQPISRKLQLLNLHGCYRLVNIVSCAMSFINLKELHVTLCNGMNYLFTSGTLKSLVKLETLIVENCKSIKRI
AKKEDENDCGEIILGRLRSIELNYLPKLESFYSGNATLKCLCXQTVIVANCPNLITFSEGVINVPVLSGIKTSKDSDLIFHVNL
NTTIEMLLHHQEFVEYSERTILEDYLGMSRVHHRKPIVSDNFFGRFKKLEFDAACNRPILIPSHVLPYLKNLEELNVHSSDA
LQVIFNIDESEVKMNGTVFGLKKLTLENLSNLKYVWKENSTGIISFHNLQEVVVNGCGNLITLFSSSLARNLKKLHKLRITE
CGKLVAIVRKEDGIEHGKKIMFEFPFLYYLHLENMPLLSCFYPEKHHLDCPLLQTLILCYCPKLKPFASDFDDNXKEVIEAQI
SPLQQFLFSVEKVSPSIRFLALNEEHIMLYRELHLLQDILSNIVFLWLCFEDDKNKKNSLPFDFFHKVPKLFSLIVQKCFGLK
EIFPSQKPQVHERGLVGLKDLLLIDLKKLECVGLEHPWVQSYSEKLERLKLEKCPLLQKIVSCAAPFINLRDLDVKLCV

>XP_027928366.1

METIISTTTETALQIGGGVVKRQLGYFFNYNEKFQELKDYIVMLGDARKRVQNEVKKAERNAEEIEDDVHNWFKQVDG
KIKKYASFIVDERHSKISSIGFFPNNLHLRYRLGRNATKMIEEIKADEHWKKKFDRVSYRVFPTVDSALTTTGYESFGSRNK
TLEMIMKTLEDSKTNMVGVYGVGGVGKTILVKAIAKKVQEKKLFNMVVMANITRNPDIKNIQGQIAEMLGMRMEEES
ETLRADLIRKRLKKEKENTLIILDDLWDGLDLNKLGIPSSDDEDDDNQWDVKDISDFGYNKREKEDMSIDFDKMKKDKSS
ADSNKVKKEKVPIDHKRCKILLTSRSKEVICNQMDVKDQSTFLVGVIDEKEAETLLKKVAGIHSTNSVFDREVTEIAKMCA
GLPIALVSIGRALKNKSASVWEDVYRQIKRQSFTEEQESIEFSVKLSYDHLKNDELKCLFLQCARMGNDALIMDLVKFCIG
SGLLQGVHTIKEARNRVNVLIEGLKDSSLLVESYSIERFNMHDIVRDVALSISSKEKHVLFMKNGILDEWPHKDELKRYTAI
FLQYYDFNDLPKHIHCPKLQVLHISSKDDLMKIPNNFFEDMFELRVLILTGVNFSRLPSSLKCLKKLRMLSLERCSLGKNLS

DIGMLKKLRILTLSGSNIESLPHEFGQLDKLQLFDLSNCPKLRIIPPNIISRMKSLEEFYMRDYSIQEKEEQNIQSLNATLAEL
MQLNQLRTLDIHIPSVANFPQNMFFDKLESYKIVIGELNMLSLVEFKVLDKYEAVRFLALNLRGNHINIHSQKWIKMLFK
NVEYLLLGDLNDVNDVLYEFNVEGFANLKHLYVVNNFGIQFIINSVEQFQPLLAFPKLESMSLYKLENLEKICDNKLTRDS
FSRLKIINVKTCGQLKNIFSFSMIECFGMLERIEVCDCDSLKEIVSVEGESYNADAIESEKIEFPQLRFLTLQSLPAFCCLSTNC
KMPLISQSFEDQVPNKEIKEITSASEQDNNCFLSLFNGKVSIPKLECLELSSIHIPQIWNDQSLHSFQNLVKLKVSDCDNLK
YLLSFPTAGSLVNLQSLFVSGCKLMEDIFSTKDATVCRNIDIFPKLKEMEINYMKQLNTIWQPHMGFNSFNRLDSLIVRK
CNKLVTIFPNHIEKGFESLQSLVITDCTSVETIFDFGNIPQTYGRSELNLHDVFLKGLPKLVHIWKLDTDEILNINNLQSIVV
HKCKMLEYLFPLSVASGLEKLETLDVSNCWEMKEVVAWNSRSNEEDVTIRFPQLNTLSLQHLFELRNFYPGTHSLEWPL
LRKFSLLVCSNLEETTNSQMNPILLATEKVIHNLEYMSISSKEAKWLQFYIVSVHRMHKLKSLVLSGLKNTEIVFWLLHRLP
NLESLTLMNCLFKEFWASTSLATDEKIGIVVQLKELVFNNVWYLQSIGFEHGLLLQRVEHLVVSGCPKLKSLMPPLASFSY
LTYLEVTDCLGLLNLMTSSTAKSLVQLLTLKVSLCEDMKKIIKKEEKRQVIEFRQLKAIELVSLENLTCFCSSEKCDLKFPSLE
NLLVSDCPKMETFCEIQSAPNLRKVHVAAGEKDRWYWERNLNATLKKISTDQVSFEDSKELTLMEDSLEDIWYKKVVFP
YNYFGNLKKLVVEDIEKEAVIPSQILACLKSLEELQVHKCEAVKFVFDMLDIEMKKTKGIISRLKKLDLDDLTNLTCVWNKN
PQGIVSFPYLQELRLVKLQKLENLFPSSVARNLVKLQKLKILRCDGLVEIVAKEDATEHGTTEIFYFPCLSSFSLYKLSKLSCFY
PGKHLLECPMLETLDVSYCPMLKLFTSEFYGKGPVRESEVSAPNTVSQLQQPLFSVEKVAPKLKQLTLSEENIILLSQGEW
PHLLRTLNKLDLSFEHHDKEKDTLPFDFLFKLPSLQRLVVRRCFGLKEMFSSQQLEVHDRKLPELERLTLLNLQELESIGLEH
PWVKPFSVTLKKLTVRFCENIHYLFTFSTAESLVQLEFLYIKKCGLIREIVKKEDEDASAEIKFRRLTTLEMDSLPMLASFYSG
KATLHFSRLKTVTVNECPNMRTFSEGSINAPMFHGIETLTDDYGLTFHNDLNSTVQWLLVKQENLEMEEFWHGKAALR
DNYFQRVKTLVLKNITENIEISSQILYVLRSLEELQVHSCKAVQVIFDIETMESNGIVSPLKKLTIEKLPNLEWVWRQNTQG
MINFPNLQEVSVGECKRLATLFHSSLSKNLVKLETLEIQNCAKLVSIVGKEVAMEQETTTMFEFSCLSSLLLYKLPQLSCFY
PGKHHLECPILESLDVSYCPELKLFTSEFIDSDTKEITESEVSSPYSSESEVSSSDTLQQPFFFVEKVVPKLKKLTLNEEHFMLL
SNKHLPQDLLGKLNYLGLCFEDADNADSEEDADSEDTDSEEDADCEDADIEDADGEDADSEDADDEDADSEDADSEE
DADSEDTDSEEDADCEDADGEDADSEDADDEDADSEDADSEEDADSEDTDSEEDADCEDADSEDADDEDTDSEEDA
DSEDADSEEDDGEDADSEDDDSEEDTLLFDFLHKVPNLEHLVVRCLGMKEIFPAEKLQVHEKLQILNLESCPRLKNLVPN
SVSFISLKQLSVNFCEKMKYLFKISTAKSLVQLESLIVKSCKSIKEIVKNEAEDDDDDDDDDDDDEIIFGQLTTLRLDSLRGLA
GFYSGKATLQFSCLKEVQIAKCPKMKKFSRGVAKAPLFPHINFENNPILIADNDLNNIVKSLFHKEVSD

>XP_027928367.1

MDAVVTATTESALQIVGGAVKRHVGYIFKYSDKFKEVKHYIKMLRNAKSRVQNEVTKAKMNAEEIEDDVQEWLKQVD
EKIEKYSSFIDDERHEKTRCSFGFFPNNFQLRYRLGRNAAKMIEEIKADELWNKKFDKVSYRVRPSNDAALANIGYESFAS
RTKTMKMIMQALKDSTINLIGLYGVGGVGKTTLVKEVAKQAKEKKLFNTVVMANVTRNLDLKKVQGQIAEMLGMKLE
EEAEIVRADRIRKRIIEEKENTLIILDDLWDGLDMNRLGIPCRDDENAIQQLGYYKKMERQEFSNNFKRMEENMNRLGIP
CRDDENDIQQLGCYKKMERQEFSNNFKRMEENMLTTNFTKTKKEKLLGDHKRCKILLTSRSKEVICNQMNVPETSTFLI
GVIDGKESETLLKKMAGIHLRNSMFDGKANEIAKMCGGLPIALVSIGRALKNKSSFVWNDVCRQIKKQSFTEAHESIEFS
VKLSYDHLKNEELKCIFLQCARMGSDALIIDLVKFCLGLGVLQGVQTISETKYRVNVLIEELKESSLLVECYSTECFNMHDIV
RDVALSISAKERNVLFMKNDILDEWPHEDEQAKYSAIFLHYCDINDELPESIYCPRLEVLHIDNKNECLEIPNNFFKDMIRL
RVLILTGVNLSCLPSSIKSLKKLRMLCLERCILGENLEIIGELKKLRILTLSGSNFECLPLEFGQLDKLQLFDISNCSKLRVIPSNII
SRMKNLEEYYKRDNLITWDFENTIQSGNASFSDLRHLNRLRSVEIHIPSIDDFPQNVFFNKLDSYKIVIGKFNILTQGEFKM
PDKYEVVKFLALDLKEDVDIHSKKWVKMLFKSVENLLLGELNDVHDILNELNVEGFPNLKHLFIVNNSGIQHIINSKEELH
LLCAFPILESMCLYKLENMEKICDTQLAEASFCRLKIIKIKTCDKLENVFTFSMVRSLTMLEQVEVCACDSLKEIVSAERQTS
TTSDNKIEFPQLRFLTLQCLPTFSSLYSNDKLTCSAQLLQNQVPDKNKDITTDACFALFNEKVSIPKLESLQLSMINIHKIWS
DQSLQCSENLTTLNVTDCSNLKYLLSISMAGCLVNLQNLLVSESEKMENIFCPEDPKKNIDVFPKLKNMQIICMEKLETIW
QPHIGLNSFHNLDSLIIRECHKLVTVFPSYMMQILQSLQTLNIINCELVEIIFDFGNIPQTYDRNETNLHSVYIKMLPNLVH
VWKDNTRETKYKNLKRVDVRDNPNLKYLFPLSIVNDLEKLEVLNIWNCGAMKEIVAGDKGSNENAITFKFPHLDVLLFQ

QSPELENFYRGAHTVEWPLLKKLAITNCFKLEGIRTKIENSQGKSIGLATEKVIYNLEWMLMNLGEGGMFQKYAVSVPR
MHKLQSLVTWGLKNTKILFQMLHRLPNLEKLTLAGSSMKRIWTPESLISHEKIGVMQLKELELLQLWCLEEIGFEHDSLL
QSIERLVIVECLKLINLASTSISFTSLTYLEVSDCSGLRNLMTSSSAKSLIQLTTMKISFCPMMVEIIEENEEEKVEEIEFRQLK
VLELVSLQNLTSFCSSDKCDLKFRLLENLVVSGCFKMSKFSKVHKAPNLQKIHVVAGEKDKWYWEGDLNGTLQKMITYK
AHFEYSKHMELVEYPELTRVRHGKPIFPDNFFCCLKTLEFVAASKIQIVIPSYVLPYMKNIEELKVNSCGAAQVIFDIVDTET
RHKSIVFLLKRLTLESLSNLECVWNKNPKGIASFPNLQEVVVNSCDSLKSLFPSSLPRNLGKLKTLRISWCNKLEEIVGKED
AMEHGVSETFEFPSLLSLNLLHLPRLSCFYPGKHHLECPRLETLYVHVCSKLKLFTSEFHCHKETVIHSDQNIVPIMRLQQP
LFSVEKVVPKLKELNVNEENITFLSEAHLPRNLLCKLSILYVIFEGYDNNKGTLLFDFLHKVPNLKKLLVSGCYGLTEIFPHEK
LQVHDGILVGLELEHPWVKSLSEKLESLTLNNCPRLEKLVPCDVSFVNLKELILTHCKRMEYVCTFSTVKSMVQLERLDIQ
GCESMKKVVKEEDENAFDEIIFGRLRELILISLQRLECFYMGNATLQFPCLPLARVAKCPNMKTFSKGIINAPSFLGVVTSK
EVWDCQFDDDINTTMERLFHEQVERFACDIQHLRFGYHPQLEEIWLGVVPMPSENCFNNLKYLIVFEYENLSHVIPFYIL
PLLRNLQEIHVVNCQSVKAIFDMKNMGADNKPASKYSLPLKKLILNRLPNLESVWNLNVDEIIGLQDLQEVYIYKCQRLK
SLFRSSAANHLVILFVRNCPSLEEIFVEDEAALKGATKELIFHCLTSITLWKLPELKYLFPGKHSLEWPMLSRLNIFHCDKLK
WFTIEHHSQVADHQLDFSNDEQAIFVAEKIFPNLEVLSLTKEDVTVISHGLFQGKANPQIAIHDREHQTITKEEAVIGQG
QFDTDAANLLQNLKYLQLQCYHAEDESNTLFSSGLIEKIPNLKTLKVLCSTFNEIFTSQKPNADCAKIVLKLKGLYLDTLKRL
SSIGLEHSWVEPLLKTLETLDVGLCPCLKILVPSNVSFSNLINLTVDGCHGLVYLLTLSTARSLGLLKNLSVQDCQAIEEIVSK
EGDHESIEDEIIFGQLRTLSLQFLPNFVGFHTGTSKFIFPLLSQMNLMGCPQMKYFFEHDLRQFVPSNFATL

>XP_027928368.1

MDAVVTATTESALQIVGGAVKRHVGYIFKYSDKFKEVKHYIKMLRNAKSRVQNEVTKAKMNAEEIEDDVQEWLKQVD
EKIEKYSSFIDDERHEKTRCSFGFFPNNFQLRYRLGRNAAKMIEEIKADELWNKKFDKVSYRVRPSNDAALANIGYESFAS
RTKTMKMIMQALKDSTINLIGLYGVGGVGKTTLVKEVAKQAKEKKLFNTVVMANVTRNLDLKKVQGQIAEMLGMKLE
EEAEIVRADRIRKRIIEEKENTLIILDDLWDGLDMNRLGIPCRDDENAIQQLGYYKKMERQEFSNNFKRMEENMNRLGIP
CRDDENDIQQLGCYKKMERQEFSNNFKRMEENMLTTNFTKTKKEKLLGDHKRCKILLTSRSKEVICNQMNVPETSTFLI
GVIDGKESETLLKKMAGIHLRNSMFDGKANEIAKMCGGLPIALVSIGRALKNKSSFVWNDVCRQIKKQSFTEAHESIEFS
VKLSYDHLKNEELKCIFLQCARMGSDALIIDLVKFCLGLGVLQGVQTISETKYRVNVLIEELKESSLLVECYSTECFNMHDIV
RDVALSISAKERNVLFMKNDILDEWPHEDEQAKYSAIFLHYCDINDELPESIYCPRLEVLHIDNKNECLEIPNNFFKDMIRL
RVLILTGVNLSCLPSSIKSLKKLRMLCLERCILGENLEIIGELKKLRILTLSGSNFECLPLEFGQLDKLQLFDISNCSKLRVIPSNII
SRMKNLEEYYKRDNLITWDFENTIQSGNASFSDLRHLNRLRSVEIHIPSIDDFPQNVFFNKLDSYKIVIGKFNILTQGEFKM
PDKYEVVKFLALDLKEDVDIHSKKWVKMLFKSVENLLLGELNDVHDILNELNVEGFPNLKHLFIVNNSGIQHIINSKEELH
LLCAFPILESMCLYKLENMEKICDTQLAEASFCRLKIIKIKTCDKLENVFTFSMVRSLTMLEQVEVCACDSLKEIVSAERQTS
TTSDNKIEFPQLRFLTLQCLPTFSSLYSNDKLTCSAQLLQNQVPDKNKDITTDACFALFNEKVSIPKLESLQLSMINIHKIWS
DQSLQCSENLTTLNVTDCSNLKYLLSISMAGCLVNLQNLLVSESEKMENIFCPEDPKKNIDVFPKLKNMQIICMEKLETIW
QPHIGLNSFHNLDSLIIRECHKLVTVFPSYMMQILQSLQTLNIINCELVEIIFDFGNIPQTYDRNETNLHSVYIKMLPNLVH
VWKDNTRETKYKNLKRVDVRDNPNLKYLFPLSIVNDLEKLEVLNIWNCGAMKEIVAGDKGSNENAITFKFPHLDVLLFQ
QSPELENFYRGAHTVEWPLLKKLAITNCFKLEGIRTKIENSQGKSIGLATEKVIYNLEWMLMNLGEGGMFQKYAVSVPR
MHKLQSLVTWGLKNTKILFQMLHRLPNLEKLTLAGSSMKRIWTPESLISHEKIGVMQLKELELLQLWCLEEIGFEHDSLL
QSIERLVIVECLKLINLASTSISFTSLTYLEVSDCSGLRNLMTSSSAKSLIQLTTMKISFCPMMVEIIEENEEEKVEEIEFRQLK
VLELVSLQNLTSFCSSDKCDLKFRLLENLVVSGCFKMSKFSKVHKAPNLQKIHVVAGEKDKWYWEGDLNGTLQKMITYK
AHFEYSKHMELVEYPELTRVRHGKPIFPDNFFCCLKTLEFVAASKIQIVIPSYVLPYMKNIEELKVNSCGAAQVIFDIVDTET
RHKSIVFLLKRLTLESLSNLECVWNKNPKGIASFPNLQEVVVNSCDSLKSLFPSSLPRNLGKLKTLRISWCNKLEEIVGKED
AMEHGVSETFEFPSLLSLNLLHLPRLSCFYPGKHHLECPRLETLYVHVCSKLKLFTSEFHCHKETVIHSDQNIVPIMRLQQP
LFSVEKVVPKLKELNVNEENITFLSEAHLPRNLLCKLSILYVIFEGYDNNKGTLLFDFLHKVPNLKKLLVSGCYGLTEIFPHEK
LQVHDGILVGLELEHPWVKSLSEKLESLTLNNCPRLEKLVPCDVSFVNLKELILTHCKRMEYVCTFSTVKSMVQLERLDIQ

GCESMKKVVKEEDENAFDEIIFGRLRELILISLQRLECFYMGNATLQFPCLPLARVAKCPNMKTFSKGIINAPSFLGVVTSK
EVWDCQFDDDINTTMERLFHEQVERFACDIQHLRFGYHPQLEEIWLGVVPMPSENCFNNLKYLIVFEYENLSHVIPFYIL
PLLRNLQEIHVVNCQSVKAIFDMKNMGADNKPASKYSLPLKKLILNRLPNLESVWNLNVDEIIGLQDLQEVYIYKCQRLK
SLFRSSAANHLVILFVRNCPSLEEIFVEDEAALKGATKELIFHCLTSITLWKLPELKYLFPGKHSLEWPMLSRLNIFHCDKLK
WFTIEHHSQVADHQLDFSNDEQAIFVAEKIFPNLEVLSLTKEDVTVISHGLFQGKANPQIAIHDREHQTITKEEAVIGQG
QFDTDAANLLQNLKYLQLQCYHAEDESNTLFSSGLIEKIPNLKTLKVLCSTFNEIFTSQKPNADCAKIVLKLKGLYLDTLKRL
SSIGLEHSWVEPLLKTLETLDVGLCPCLKILVPSNVSFSNLINLTVDGCHGLVYLLTLSTARSLGLLKNLSVQDCQAIEEIVSK
EGDHESIEDEIIFGQLRTLSLQFLPNFVGFHTGTSKFIFPLLSQMNLMGCPQMKYFFEHDLRQFVPSNFATL

>XP_027928369.1

MDAVVTATTESALQIVGGAVKRHVGYIFKYSDKFKEVKHYIKMLRNAKSRVQNEVTKAKMNAEEIEDDVQEWLKQVD
EKIEKYSSFIDDERHEKTRCSFGFFPNNFQLRYRLGRNAAKMIEEIKADELWNKKFDKVSYRVRPSNDAALANIGYESFAS
RTKTMKMIMQALKDSTINLIGLYGVGGVGKTTLVKEVAKQAKEKKLFNTVVMANVTRNLDLKKVQGQIAEMLGMKLE
EEAEIVRADRIRKRIIEEKENTLIILDDLWDGLDMNRLGIPCRDDENAIQQLGYYKKMERQEFSNNFKRMEENMNRLGIP
CRDDENDIQQLGCYKKMERQEFSNNFKRMEENMLTTNFTKTKKEKLLGDHKRCKILLTSRSKEVICNQMNVPETSTFLI
GVIDGKESETLLKKMAGIHLRNSMFDGKANEIAKMCGGLPIALVSIGRALKNKSSFVWNDVCRQIKKQSFTEAHESIEFS
VKLSYDHLKNEELKCIFLQCARMGSDALIIDLVKFCLGLGVLQGVQTISETKYRVNVLIEELKESSLLVECYSTECFNMHDIV
RDVALSISAKERNVLFMKNDILDEWPHEDEQAKYSAIFLHYCDINDELPESIYCPRLEVLHIDNKNECLEIPNNFFKDMIRL
RVLILTGVNLSCLPSSIKSLKKLRMLCLERCILGENLEIIGELKKLRILTLSGSNFECLPLEFGQLDKLQLFDISNCSKLRVIPSNII
SRMKNLEEYYKRDNLITWDFENTIQSGNASFSDLRHLNRLRSVEIHIPSIDDFPQNVFFNKLDSYKIVIGKFNILTQGEFKM
PDKYEVVKFLALDLKEDVDIHSKKWVKMLFKSVENLLLGELNDVHDILNELNVEGFPNLKHLFIVNNSGIQHIINSKEELH
LLCAFPILESMCLYKLENMEKICDTQLAEASFCRLKIIKIKTCDKLENVFTFSMVRSLTMLEQVEVCACDSLKEIVSAERQTS
TTSDNKIEFPQLRFLTLQCLPTFSSLYSNDKLTCSAQLLQNQVPDKNKDITTDACFALFNEKVSIPKLESLQLSMINIHKIWS
DQSLQCSENLTTLNVTDCSNLKYLLSISMAGCLVNLQNLLVSESEKMENIFCPEDPKKNIDVFPKLKNMQIICMEKLETIW
QPHIGLNSFHNLDSLIIRECHKLVTVFPSYMMQILQSLQTLNIINCELVEIIFDFGNIPQTYDRNETNLHSVYIKMLPNLVH
VWKDNTRETKYKNLKRVDVRDNPNLKYLFPLSIVNDLEKLEVLNIWNCGAMKEIVAGDKGSNENAITFKFPHLDVLLFQ
QSPELENFYRGAHTVEWPLLKKLAITNCFKLEGIRTKIENSQGKSIGLATEKVIYNLEWMLMNLGEGGMFQKYAVSVPR
MHKLQSLVTWGLKNTKILFQMLHRLPNLEKLTLAGSSMKRIWTPESLISHEKIGVMQLKELELLQLWCLEEIGFEHDSLL
QSIERLVIVECLKLINLASTSISFTSLTYLEVSDCSGLRNLMTSSSAKSLIQLTTMKISFCPMMVEIIEENEEEKVEEIEFRQLK
VLELVSLQNLTSFCSSDKCDLKFRLLENLVVSGCFKMSKFSKVHKAPNLQKIHVVAGEKDKWYWEGDLNGTLQKMITYK
AHFEYSKHMELVEYPELTRVRHGKPIFPDNFFCCLKTLEFVAASKIQIVIPSYVLPYMKNIEELKVNSCGAAQVIFDIVDTET
RHKSIVFLLKRLTLESLSNLECVWNKNPKGIASFPNLQEVVVNSCDSLKSLFPSSLPRNLGKLKTLRISWCNKLEEIVGKED
AMEHGVSETFEFPSLLSLNLLHLPRLSCFYPGKHHLECPRLETLYVHVCSKLKLFTSEFHCHKETVIHSDQNIVPIMRLQQP
LFSVEKVVPKLKELNVNEENITFLSEAHLPRNLLCKLSILYVIFEGYDNNKGTLLFDFLHKVPNLKKLLVSGCYGLTEIFPHEK
LQVHDGILVGLELEHPWVKSLSEKLESLTLNNCPRLEKLVPCDVSFVNLKELILTHCKRMEYVCTFSTVKSMVQLERLDIQ
GCESMKKVVKEEDENAFDEIIFGRLRELILISLQRLECFYMGNATLQFPCLPLARVAKCPNMKTFSKGIINAPSFLGVVTSK
EVWDCQFDDDINTTMERLFHEQVERFACDIQHLRFGYHPQLEEIWLGVVPMPSENCFNNLKYLIVFEYENLSHVIPFYIL
PLLRNLQEIHVVNCQSVKAIFDMKNMGADNKPASKYSLPLKKLILNRLPNLESVWNLNVDEIIGLQDLQEVYIYKCQRLK
SLFRSSAANHLVILFVRNCPSLEEIFVEDEAALKGATKELIFHCLTSITLWKLPELKYLFPGKHSLEWPMLSRLNIFHCDKLK
WFTIEHHSQVADHQLDFSNDEQAIFVAEKIFPNLEVLSLTKEDVTVISHGLFQGKANPQIAIHDREHQTITKEEAVIGQG
QFDTDAANLLQNLKYLQLQCYHAEDESNTLFSSGLIEKIPNLKTLKVLCSTFNEIFTSQKPNADCAKIVLKLKGLYLDTLKRL
SSIGLEHSWVEPLLKTLETLDVGLCPCLKILVPSNVSFSNLINLTVDGCHGLVYLLTLSTARSLGLLKNLSVQDCQAIEEIVSK
EGDHESIEDEIIFGQLRTLSLQFLPNFVGFHTGTSKFIFPLLSQMNLMGCPQMKYFFEHDLRQFVPSNFATL

>XP_027928370.1

MDAVVTATTESALQIVGGAVKRHVGYIFKYSDKFKEVKHYIKMLRNAKSRVQNEVTKAKMNAEEIEDDVQEWLKQVD
EKIEKYSSFIDDERHEKTRCSFGFFPNNFQLRYRLGRNAAKMIEEIKADELWNKKFDKVSYRVRPSNDAALANIGYESFAS
RTKTMKMIMQALKDSTINLIGLYGVGGVGKTTLVKEVAKQAKEKKLFNTVVMANVTRNLDLKKVQGQIAEMLGMKLE
EEAEIVRADRIRKRIIEEKENTLIILDDLWDGLDMNRLGIPCRDDENAIQQLGYYKKMERQEFSNNFKRMEENMNRLGIP
CRDDENDIQQLGCYKKMERQEFSNNFKRMEENMLTTNFTKTKKEKLLGDHKRCKILLTSRSKEVICNQMNVPETSTFLI
GVIDGKESETLLKKMAGIHLRNSMFDGKANEIAKMCGGLPIALVSIGRALKNKSSFVWNDVCRQIKKQSFTEAHESIEFS
VKLSYDHLKNEELKCIFLQCARMGSDALIIDLVKFCLGLGVLQGVQTISETKYRVNVLIEELKESSLLVECYSTECFNMHDIV
RDVALSISAKERNVLFMKNDILDEWPHEDEQAKYSAIFLHYCDINDELPESIYCPRLEVLHIDNKNECLEIPNNFFKDMIRL
RVLILTGVNLSCLPSSIKSLKKLRMLCLERCILGENLEIIGELKKLRILTLSGSNFECLPLEFGQLDKLQLFDISNCSKLRVIPSNII
SRMKNLEEYYKRDNLITWDFENTIQSGNASFSDLRHLNRLRSVEIHIPSIDDFPQNVFFNKLDSYKIVIGKFNILTQGEFKM
PDKYEVVKFLALDLKEDVDIHSKKWVKMLFKSVENLLLGELNDVHDILNELNVEGFPNLKHLFIVNNSGIQHIINSKEELH
LLCAFPILESMCLYKLENMEKICDTQLAEASFCRLKIIKIKTCDKLENVFTFSMVRSLTMLEQVEVCACDSLKEIVSAERQTS
TTSDNKIEFPQLRFLTLQCLPTFSSLYSNDKLTCSAQLLQNQVPDKNKDITTDACFALFNEKVSIPKLESLQLSMINIHKIWS
DQSLQCSENLTTLNVTDCSNLKYLLSISMAGCLVNLQNLLVSESEKMENIFCPEDPKKNIDVFPKLKNMQIICMEKLETIW
QPHIGLNSFHNLDSLIIRECHKLVTVFPSYMMQILQSLQTLNIINCELVEIIFDFGNIPQTYDRNETNLHSVYIKMLPNLVH
VWKDNTRETKYKNLKRVDVRDNPNLKYLFPLSIVNDLEKLEVLNIWNCGAMKEIVAGDKGSNENAITFKFPHLDVLLFQ
QSPELENFYRGAHTVEWPLLKKLAITNCFKLEGIRTKIENSQGKSIGLATEKVIYNLEWMLMNLGEGGMFQKYAVSVPR
MHKLQSLVTWGLKNTKILFQMLHRLPNLEKLTLAGSSMKRIWTPESLISHEKIGVMQLKELELLQLWCLEEIGFEHDSLL
QSIERLVIVECLKLINLASTSISFTSLTYLEVSDCSGLRNLMTSSSAKSLIQLTTMKISFCPMMVEIIEENEEEKVEEIEFRQLK
VLELVSLQNLTSFCSSDKCDLKFRLLENLVVSGCFKMSKFSKVHKAPNLQKIHVVAGEKDKWYWEGDLNGTLQKMITYK
AHFEYSKHMELVEYPELTRVRHGKPIFPDNFFCCLKTLEFVAASKIQIVIPSYVLPYMKNIEELKVNSCGAAQVIFDIVDTET
RHKSIVFLLKRLTLESLSNLECVWNKNPKGIASFPNLQEVVVNSCDSLKSLFPSSLPRNLGKLKTLRISWCNKLEEIVGKED
AMEHGVSETFEFPSLLSLNLLHLPRLSCFYPGKHHLECPRLETLYVHVCSKLKLFTSEFHCHKETVIHSDQNIVPIMRLQQP
LFSVEKVVPKLKELNVNEENITFLSEAHLPRNLLCKLSILYVIFEGYDNNKGTLLFDFLHKVPNLKKLLVSGCYGLTEIFPHEK
LQVHDGILVGLELEHPWVKSLSEKLESLTLNNCPRLEKLVPCDVSFVNLKELILTHCKRMEYVCTFSTVKSMVQLERLDIQ
GCESMKKVVKEEDENAFDEIIFGRLRELILISLQRLECFYMGNATLQFPCLPLARVAKCPNMKTFSKGIINAPSFLGVVTSK
EVWDCQFDDDINTTMERLFHEQVERFACDIQHLRFGYHPQLEEIWLGVVPMPSENCFNNLKYLIVFEYENLSHVIPFYIL
PLLRNLQEIHVVNCQSVKAIFDMKNMGADNKPASKYSLPLKKLILNRLPNLESVWNLNVDEIIGLQDLQEVYIYKCQRLK
SLFRSSAANHLVILFVRNCPSLEEIFVEDEAALKGATKELIFHCLTSITLWKLPELKYLFPGKHSLEWPMLSRLNIFHCDKLK
WFTIEHHSQVADHQLDFSNDEQAIFVAEKIFPNLEVLSLTKEDVTVISHGLFQGKANPQIAIHDREHQTITKEEAVIGQG
QFDTDAANLLQNLKYLQLQCYHAEDESNTLFSSGLIEKIPNLKTLKVLCSTFNEIFTSQKPNADCAKIVLKLKGLYLDTLKRL
SSIGLEHSWVEPLLKTLETLDVGLCPCLKILVPSNVSFSNLINLTVDGCHGLVYLLTLSTARSLGLLKNLSVQDCQAIEEIVSK
EGDHESIEDEIIFGQLRTLSLQFLPNFVGFHTGTSKFIFPLLSQMNLMGCPQMKYFFEHDLRQFVPSNFATL

>XP_027928371.1

MDPNIFVSTATESVFKFGENLVTRHLGYFYNYNGKFEEVKHRVEMLDDTRKRVQNDVMVAEMNAEEIEEDVKHWLK
HVDEKIKEYENFLCDKRHEKTSCSIGFFPNNLQLRYRLGRKATKIVEEVIADELLNKKFDKVSYHIGPSMDAALSNTGYESF
TSRKKIMGMIMQALEDSTISMIGVYGVGGVGKTTLVKEVAKQAKERKLFNKVVMANITRNPDIKKVQGQIAEMLGMR
LEEESEIVRADRIRKRLKKEKENTLIILDDLWNGLDLNRLGIQRNEVDDVSQKVAKDVADFGYKRVETEKLSADSNKMKK
EKLSSDYNKIKKENLSVDHKGFKIFLTSRNKEVLCNQMDVQERSTFPLGVLDQKEGEALLKKMAEISVTNSAFDDKVTEIS
KMCAGLPIALISIGKTLKNKSPYVWEDVCRQIERQNFTGGQEPIEFSAKLSYDHLKTEELKHIFLQCARMGSDFSIMDLVK
FCIGFDMLQGVYTIRETKSRVNVLMEELTESSLLVKSYSNDCFNMHDIVRDVALSISSKEKHVFFMKNGKLNEWPHKDK
LERYTTIVLHYCDIVELPESKYCSRLEVFHIDSKDDFLKIPDDLFKYMIELKVLILTGVNLSCLPSSITYLTNLKMLCLERCTLRN
NLAIIGELRKLRILSLSGSNIECLPVELRQLDKLQLLDLSNCSQLRAIPSNMILGMNSLEEFYMRDDLILRETNEEIQSKNASL

SELRHLKQLRSLDIHIPSVAHFPQNLFFDKLDSYKIIIGEINMLSVGEFKIPDKYEVVKFLALNLKDGINIHSEKWIKMLFKRV
EYLLLGELNDVHDVFYELNVEGFPHLKHLFIVNNVGLLYIINSVKRFHPLLAFPKLESMCLYKLENLEKICDSQLTEASFCRL
KIIKIKTCGQLESIFSFFMLSRLTMLETIEVCDCDSLKEIVYFEGEFDTVSDVQTDKIQFPQLRFLTLQSLPAFFGLYTNDKMP
SISESSEDQMQNRELKEIGQDTNACFSLFNGKVAMPKLEFLELSSINIPQIWNEKSLHCFQSLLTLNVSDCGNLKYLLSLS
MSESLVNLQSLFVSGCELMEDIFCAEDALHNIDILPKLKKMEINCMEKLSTLWQPYIGFHSFHSLDSLIIRECNKLETIFPSH
TGEGFQSLQSVVITNCMSVETIFDFGNISQTCGTNVTNLHSVFLKGLPKLVHIWKVDTDEILNFSNLQSIVVYESKILKYLF
PLSVAKGLEKLETLDVCNCWEMEEVVACDYQSNENLITFRFPQLNTLSLQHLFELRSFYLGPHDIEWPFLKKLFILFCGKLE
ETTNLQVKSIFLATEKVIHNLEYMSISLTEAEWLRDYIYSVHRMHKLQSLVLSGLENTEILFWLLHRLPNLESITLKNCLFEG
VWASTNLAAHEKVGVVVQLKELIIDKLRYLQNIGFEHDLLLQRVERLVISECLNLKSLLPLSVSFSYLTYLEVSNCSGLRNLIT
SSTAMTLVQLTIMKVSLCQGIEKIVAEEEKTQVIEFRHLKAIELVSLPSLTCFCSSEKCDLKFPSLENLVVSDCLLMETFSEV
QSAPNLRKIHVLVGEKDRWYWEGDLNSTLQKLSTDKVSFKHSKHLTITEDSELEEIWHSKAAFQDNYFHSLKTLVVMDI
TKDHVIPSHVLKNLEELEVESCGAVEVIFDVNDTATKKKGTVARLKKLTLTMLPNLSRVWKKNPQGIVSFPNLQEVSVFD
CGQLASLFPLSLAINLFKLQTLEIQWCDNLVEIVEKDDAIEPGKAEMFKFPCLFSLLLYNLPLLACFYSGKHRLECHMLDVL
DVSCCPMLKLFTSKFHDSYKEAVTESQQPLFLVEEVVPKLKELTVNEESIILLSHAHLPQDLFCRLNLLQLCFEDENNKKDT
FPFHFLHKVPSLEHLQVYECFGLMEIFPSQTLQYHERILIRLRKLTLNNLPELDTIGLEHSWIKPYTEKLEVLKLEECPRLERL
VFDVVSFSNLKQLAVDSCEEMKNLFTFSTAKSLVQLEILTVLNCESMKEIVKDEDEEAYEEIILGRLKTLKLNFLSRLVCFYS
GNAMLQLPCLSTVTIVKCPEMKTFSEGGLNAPMFSGIKTSLKDSDFHFHNDLNSTVQWLHQHVSVEQSKHLTVADDSK
LEKILHSKAAFQDNNFHSLKSLVVKDVTMDINHVIPSQVLPCLKSLEELQVQSCKAVGIIFEVNDIDTKKKGIVSRLKKLTL
DTLPNLKCVWNKNPQGIVSFPNLQEVSVSDCGELTALFPSSLARNLVKLEELQIENCDKLVDIVGKDDEIELETTKVFKFP
CLLFLILFRLPLLSCFYPGKHHLESPLLETLDVSYCPKLKLFTSEFHDSHKESVIEIQVSSTNTITHLQQPLFLIEKVVPKLKELSV
NEENIILLSHANLPQDLFRKLNFLLLCQEDEDEEHRKDTLPFDFLRKVPGLEHLKLLGYFGLKEIFPSKKLRVHDKILSRLKHL
TLDNLEELKSIGLEHPWVKPHSKRLESLELIECPKVVKLVSGAVSFMNMKWLHVTDCKRMEYLFTFSIAKSLVQLLDLSV
QNCGSIKEIVKKENEDASREIIFGWVKTLNLDSLPLLGSFYSGNATLQFSRLKRVTISKCPSMKTFSQGDITAPFFCGVGSSI
EDFDLTFHGDLNTTIKNLSHKQVEGDSVMESTDRGSSDDDNYTSE

>XP_027928396.1

MDISMHILNCSWTFLQRMCRVYLINTTTMMMMMTDKLGFRRFWGGLGLPSSSSNVISSESASSSDHSSGTIQNQDY
RYDVFISFRGSDTRNSFVDHLYSHLLRKGIFVFKDDHKLRKGESISSQLLQAIRGSRISIIVFSKDYPSSSWCLDEMATIADC
KQQSNQTVFPVFYDVDPSIVRHQRGAYKIDFLVHRLKLRNEPNKVLRWEKAMTDLANSTGWDVRYKPEFREIEKIVQA
VIETLGHKFSGFVDDLIGIQPRVQALEDKLRLSSKSDDVQVLGIWGMGGIGKTTHAVVLYDKMSHRFDASCFIEDVSKLY
RDGGHTAVQKQIIHQTLDEKCLDTYSPIEISGIVRNRLHKIKVLIVLDNVDELEQLQELAINSKLLFRGSRMVIISRDEHILKV
YGAHVIHEVSLMNDKDARELFYTKAFESEEQNSSSCVELIPEVLKYAQCLPLAVRVIGSFLCRRDSSEWRDTLDRFENNP
DNKIMDVLQISFDGLHYTEKEIFLHIACFFKEEREDYVKRILECCGLHPHIGIQRMIEKSLITIRDQQIHMHDMLQELGKKI
VRNQCPEEPESWSRIWLYEDFFHVLTTQTGTNDVKAIVLNEKEAISECSIDGLSKMKNLKLLILYHKRFSGSLNFLSQKLRY
FLWHDYPFASLPSSFAASGLVELNMPNSSINCLWEGRKDFPCLKRMDLSNSKYLTETPDFTGVPNLERLDLSGCTDLSFV
HPSIGLLQQLAFLSLRNCTNLISFKLGNGFNLSSLRVLHFSGCTKLENTPDFTGTTILEYLDFDGCTSLSSVHESIGVLAKLTF
LSLRDCTSLASIPSNNNIMKSLQTLDFSGCFQLTDLSLRFFISLSMTSLILLDIGFCNLLEVPNAIGDLLCLERLNLQGNNFVA
IPPSISRLKSLAYLNVSHCHTLQYLPNLSKISTSSTGRYFKTVSGSRDHRSGLYLFDCTMITNYFYPKARFKDLELAWLVRLA
KNPCSFRCGFDIVVPWGLEFPRWLKHRFERDSVIRIVEFNVDDNWIGFVFCVIFERNNGPVVARSSSHPFYLSFESEDTE
EYFDMPLNLERDKVVDSSKHLWIIYISREHCHFVKTGSHISFKAHPSVKINAWGMRSIFREDVIYSELMLEEPRHVKFDFV
EKSSTNSGPKFQLPYNWLVTNEDEVENIDAKAKENNLSYAGL

>XP_027928397.1

MDISMHILNCSWTFLQRMCRVYLINTTTMMMMMTDKLGFRRFWGGLGLPSSSSNVISSESASSSDHSSGTIQNQDY
RYDVFISFRGSDTRNSFVDHLYSHLLRKGIFVFKDDHKLRKGESISSQLLQAIRGSRISIIVFSKDYPSSSWCLDEMATIADC
KQQSNQTVFPVFYDVDPSIVRHQRGAYKIDFLVHRLKLRNEPNKVLRWEKAMTDLANSTGWDVRYKPEFREIEKIVQA
VIETLGHKFSGFVDDLIGIQPRVQALEDKLRLSSKSDDVQVLGIWGMGGIGKTTHAVVLYDKMSHRFDASCFIEDVSKLY
RDGGHTAVQKQIIHQTLDEKCLDTYSPIEISGIVRNRLHKIKVLIVLDNVDELEQLQELAINSKLLFRGSRMVIISRDEHILKV
YGAHVIHEVSLMNDKDARELFYTKAFESEEQNSSSCVELIPEVLKYAQCLPLAVRVIGSFLCRRDSSEWRDTLDRFENNP
DNKIMDVLQISFDGLHYTEKEIFLHIACFFKEEREDYVKRILECCGLHPHIGIQRMIEKSLITIRDQQIHMHDMLQELGKKI
VRNQCPEEPESWSRIWLYEDFFHVLTTQTGTNDVKAIVLNEKEAISECSIDGLSKMKNLKLLILYHKRFSGSLNFLSQKLRY
FLWHDYPFASLPSSFAASGLVELNMPNSSINCLWEGRKDFPCLKRMDLSNSKYLTETPDFTGVPNLERLDLSGCTDLSFV
HPSIGLLQQLAFLSLRNYFTGTTILEYLDFDGCTSLSSVHESIGVLAKLTFLSLRDCTSLASIPSNNNIMKSLQTLDFSGCFQL
TDLSLRFFISLSMTSLILLDIGFCNLLEVPNAIGDLLCLERLNLQGNNFVAIPPSISRLKSLAYLNVSHCHTLQYLPNLSKISTSS
TGRYFKTVSGSRDHRSGLYLFDCTMITNYFYPKARFKDLELAWLVRLAKNPCSFRCGFDIVVPWGLEFPRWLKHRFERD
SVIRIVEFNVDDNWIGFVFCVIFERNNGPVVARSSSHPFYLSFESEDTEEYFDMPLNLERDKVVDSSKHLWIIYISREHCHF
VKTGSHISFKAHPSVKINAWGMRSIFREDVIYSELMLEEPRHVKFDFVEKSSTNSGPKFQLPYNWLVTNEDEVENIDAKA
KENNLSYAGL

>XP_027928398.1

MDISMHILNCSWTFLQRMCRVYLINTTTMMMMMTDKLGFRRFWGGLGLPSSSSNVISSESASSSDHSSGTIQNQDY
RYDVFISFRGSDTRNSFVDHLYSHLLRKGIFVFKDDHKLRKGESISSQLLQAIRGSRISIIVFSKDYPSSSWCLDEMATIADC
KQQSNQTVFPVFYDVDPSIVRHQRGAYKIDFLVHRLKLRNEPNKVLRWEKAMTDLANSTGWDVRYKPEFREIEKIVQA
VIETLGHKFSGFVDDLIGIQPRVQALEDKLRLSSKSDDVQVLGIWGMGGIGKTTHAVVLYDKMSHRFDASCFIEDVSKLY
RDGGHTAVQKQIIHQTLDEKCLDTYSPIEISGIVRNRLHKIKVLIVLDNVDELEQLQELAINSKLLFRGSRMVIISRDEHILKV
YGAHVIHEVSLMNDKDARELFYTKAFESEEQNSSSCVELIPEVLKYAQCLPLAVRVIGSFLCRRDSSEWRDTLDRFENNP
DNKIMDVLQISFDGLHYTEKEIFLHIACFFKEEREDYVKRILECCGLHPHIGIQRMIEKSLITIRDQQIHMHDMLQELGKKI
VRNQCPEEPESWSRIWLYEDFFHVLTTQTGTNDVKAIVLNEKEAISECSIDGLSKMKNLKLLILYHKRFSGSLNFLSQKLRY
FLWHDYPFASLPSSFAASGLVELNMPNSSINCLWEGRKDFPCLKRMDLSNSKYLTETPDFTGVPNLERLDLSGCTDLSFV
HPSIGLLQQLAFLSLRNCTNLISFKLGNGFNLSSLRVLHFSGCTKLENTPDFTGTTILEYLDFDGCTSLSSVHESIGVLAKLTF
LSLRDCTSLASIPSNNNIMKSLQTLDFSGCFQLTDLSLRFFISLSMTSLILLDIGFCNLLEVPNAIGDLLCLERLNLQGNNFVA
IPPSISRLKSLAYLNVSHCHTLQYLPNLSKISTSSTGRYFKTVSGSRDHRSGLYLFDCTMITNYFYPKARFKDLELAWLVRLA
KNPCSFRCGFDIVVPWGLEFPRSSSHPFYLSFESEDTEEYFDMPLNLERDKVVDSSKHLWIIYISREHCHFVKTGSHISFKA
HPSVKINAWGMRSIFREDVIYSELMLEEPRHVKFDFVEKSSTNSGPKFQLPYNWLVTNEDEVENIDAKAKENNLSYAGL

>XP_027928399.1

MDISMHILNCSWTFLQRMCRVYLINTTTMMMMMTDKLGFRRFWGGLGLPSSSSNVISSESASSSDHSSGTIQNQDY
RYDVFISFRGSDTRNSFVDHLYSHLLRKGIFVFKDDHKLRKGESISSQLLQAIRGSRISIIVFSKDYPSSSWCLDEMATIADC
KQQSNQTVFPVFYDVDPSIVRHQRGAYKIDFLVHRLKLRNEPNKVLRWEKAMTDLANSTGWDVRYKPEFREIEKIVQA
VIETLGHKFSGFVDDLIGIQPRVQALEDKLRLSSKSDDVQVLGIWGMGGIGKTTHAVVLYDKMSHRFDASCFIEDVSKLY
RDGGHTAVQKQIIHQTLDEKCLDTYSPIEISGIVRNRLHKIKVLIVLDNVDELEQLQELAINSKLLFRGSRMVIISRDEHILKV
YGAHVIHEVSLMNDKDARELFYTKAFESEEQNSSSCVELIPEVLKYAQCLPLAVRVIGSFLCRRDSSEWRDTLDRFENNP
DNKIMDVLQISFDGLHYTEKEIFLHIACFFKEEREDYVKRILECCGLHPHIGIQRMIEKSLITIRDQQIHMHDMLQELGKKI
VRNQCPEEPESWSRIWLYEDFFHVLTTQTGTNDVKAIVLNEKEAISECSIDGLSKMKNLKLLILYHKRFSGSLNFLSQKLRY
FLWHDYPFASLPSSFAASGLVELNMPNSSINCLWEGRKDFPCLKRMDLSNSKYLTETPDFTGVPNLERLDLSGCTDLSFV
HPSIGLLQQLAFLSLRNCTNLISFKLGNGFNLSSLRVLHFSGCTKLENTPDFTGTTILEYLDFDGCTSLSSVHESIGVLAKLTF
LSLRDCTSLASIPSNNNIMKSLQTLDFSGCFQLTDLSLRFFISLSMTSLILLDIGFCNLLEVPNAIGDLLCLERLNLQGNNFVA

IPPSISRLKSLAYLNVSHCHTLQYLPNLSKISTSSTGRYFKTVSGSRDHRIHVLFGVALTLLFLGVWNFQGLLHIRFIYLLKVK
TQKNTSTCH

>XP_027928401.1

MPKKKSGRRWFWSQFCARSSESASSSYHSSDTIKNQDYRYDVFISFRGPDTRNSFVDHLYSHLLRKGIFVFKDDHKLRK
GESISSQLLQAIRGSRISIIVFSKDYPSSSWCLDEMTTIADCKQQYNQTVFPVFYDVVPSHVRHQNGVYEKAFVSLRQKFK
GNPDKVYRWERAMNGFGKLAGWDVRNKSESEVIEDIVQKVIKKLGHKFSGFVDDLIGIQSRVQALEDKLRLSSKSGVQ
VLGIWGMNGIGKTTHAAVLYDKISHRFDASCFVEDVSKLYRDGGQTAIHKEIINQTLRENIDMSSPIEISGIVKNRLHNIK
VLIVLDNVDELEQLENLAIKPKLLLKGSRMVITTTDEHILKVYEGNVLIHKVSLLNDNDARELFCRKAFKSEEQSSTCAVLIP
EVLKYAQCLPLAIRVLGSFLCTRDTIEWRDAINRLQNSPHKKIMDVLQISIDGLNHEEKQIFLHIACFFKGEREDYVKRILDC
CGLYPHIGISRLIEKSLITISNEEIQMHELLQELGKKMVRGQSPEEPGSWSRIWLHKDFLQVLTTETGTEKVKAIVLNKKEE
LLECSVDGLSRMKELTLLILHHTKLSGSLEFLSDRLRYLLWHDYPFASLPPYFTVSNLVELNMPNSRITHIWEGRKSCPNLK
RIDLSNSKKLTETLDFSRTIKLERLDLSGCTSLSYVHSSIGLLKKLAFLNLRNCRNLVFVDFGCVGNMSSLRVLHLSGCSKLES
TPDFTRATHLEYLDMDECTSLSTIHQSIGVLSNLTFFSLRGCTKLVSIPNDINSLVSLQTLVLCHCYKLTSLFPRQASKSHLEC
LIFLDLSFCNLPEVPDAIGELRCLERLNLQGNNVVSVPDSFRGLHCLGYINLSHCHELKSLSNLPIEGAASGGKYFRKVSGS
RDHRSGLYLFNCANMVDILSKPWDCWSLELAWLFRLIKESCHFRCGFDIVVPWGLEIPRWFTKRFEGDSVIRIVEFSVDE
DWMGFAFCVIFGGNTALVVDDSSPHPLYLSFVSKHTEEYCDVPHNVNIQHFAKSNHFWIIYISREHCHFVKTGAHITFKT
LAGVKIHAWGMRSIFKKDVHDFKRMQQGQPLPFPRNEVHPLNFVFVKKSKTNSGPVFRLPYNWLLTKEDEVEKHDAE
VKENNLSYAGF

>XP_027928402.1

MPKKKSGRRWFWSQFCARSSESASSSYHSSDTIKNQDYRYDVFISFRGPDTRNSFVDHLYSHLLRKGIFVFKDDHKLRK
GESISSQLLQAIRGSRISIIVFSKDYPSSSWCLDEMTTIADCKQQYNQTVFPVFYDVVPSHVRHQNGVYEKAFVSLRQKFK
GNPDKVYRWERAMNGFGKLAGWDVRNKSESEVIEDIVQKVIKKLGHKFSGFVDDLIGIQSRVQALEDKLRLSSKSGVQ
VLGIWGMNGIGKTTHAAVLYDKISHRFDASCFVEDVSKLYRDGGQTAIHKEIINQTLRENIDMSSPIEISGIVKNRLHNIK
VLIVLDNVDELEQLENLAIKPKLLLKGSRMVITTTDEHILKVYEGNVLIHKVSLLNDNDARELFCRKAFKSEEQSSTCAVLIP
EVLKYAQCLPLAIRVLGSFLCTRDTIEWRDAINRLQNSPHKKIMDVLQISIDGLNHEEKQIFLHIACFFKGEREDYVKRILDC
CGLYPHIGISRLIEKSLITISNEEIQMHELLQELGKKMVRGQSPEEPGSWSRIWLHKDFLQVLTTETGTEKVKAIVLNKKEE
LLECSVDGLSRMKELTLLILHHTKLSGSLEFLSDRLRYLLWHDYPFASLPPYFTVSNLVELNMPNSRITHIWEGRKSCPNLK
RIDLSNSKKLTETLDFSRTIKLERLDLSGCTSLSYVHSSIGLLKKLAFLNLRNCRNLVFVDFGCVGNMSSLRVLHLSGCSKLES
TPDFTRATHLEYLDMDECTSLSTIHQSIGVLSNLTFFSLRGCTKLVSIPNDINSLVSLQTLVLCHCYKLTSLFPRQASKSHLEC
LIFLDLSFCNLPEVPDAIGELRCLERLNLQGNNVVSVPDSFRGLHCLGYINLSHCHELKSLSNLPIEGAASGGKYFRKVSGS
RDHRNPVIFDVALTLLFHGVWKFRGGSPKDLKGIQL

>XP_027928403.1

MVKTKSGLQWFWPCFGGRSSSSIVHSQGPTSSSSYQNQDYRYDVFISFRGPDTRNSFVDHLCSHLLQKGLFVLKDDHK
LQKGESISRQLLQAIQQSRLSIIVFSKNYASSTWCLDEMAAIASCKQRSNHIVFPIFYDVDPSHVRHQNGVYKNDFLSHR
WKFGKDRDKVPGWKRAMTDFANSAGCDIRDKPEFEQIQIIVQTVIKKLGHKFSWSVNDLIGIQPRVQALEDKLRLGSN
SDDVQVLGIWGMNGIGKTTHAAVLYDKISHRFDASCFIEDVSKLYREGGHSAVQKEIIYQTLRENIDMSNPIEISRIVNNR
LHSIKVLIVLDNVDELEQLENLAIKPKLLLKGSRLVITTTDEHILKVCEGECLXHKVPLLNDKDARELFCRKAFKSEEQNSNC
AALIPEVLNYAQSLPLAIRVLGSFLCTRDAVEWRDVLNRLKNSPDNKIMSVLQITVDGLHHEEKQIFLHIACFFKGEREDY
VKRILDCCELYPHIGISRLIQKSLITISNEELQMHELLQELGKKMVRDQSPEEPGLWSRIWLHKDFFQVLMTETGTEKVKA
IVLNKKEEMAECSVGGLSRMKDLTLLILYHTRVSGRPEFLSDRLRYVLWHDYPFASLPPYFIASNLVDLNMPNSHITHLW
EGRKSCPNLKRMDLSNSRYLIKTPDFSRITKLERLDLSGCTSLSEIHSSIGLLKKIAFLNLRNCCNLVIIDFGLVGNMSSLRVL

HLSGCSKLESTPDFTRATHLEYIDMDECSSLSTIHQSIGVLSSLTFLSLRKCRKLVSIPNDINSLLSLQTLDLCSCSNLTDLXTK
TSFFISFEILDLSFCNLQEVNEAIRELRCLERLNLQGNNFVSVPDFFSGLPRLAYLNLSYCHKLKSLLRICFKGDGSGGKYFKT
VSGSRDHRSGFYLFDCPNVALEKENLEIFKLEWSLQLMKEPCNFRCGFDVVVPWNLISTFIQXNIKGDSIIRILQCGTNDN
WIGFAFCVVFEGNNGDAAVRSSPHSSFSHPLYLSFENEYTEEYFDMRLNLEALQTNGSAHIWMIYISREHCHFVKTGAH
VTFKAQPNVKINAWGMRAILKQDIHDFKVGKYVLLSQLNRDHVPFEYVEKNIDGSGPKIQLPYNWFVTEEEEVENIDAK
AKENNLSNAGL

>XP_027928500.1

MSIPTNKMKAVALLLKRVKTARRQFRERGRDESLDGNLEKLRRELNRIRKLFEKVKNNEQELLHTLSKVDGHLRKLDSKR
LNQDMNDICKRIRDSAQKLLPKGDFDDSSVEEDDDDRRVILLHPSRPSSKQHKNQLPQHKKLNMNLRVLETYERNCLLS
LLVFPEGAVIKKRQTICWWIGEGFLESTNLKTAEEEGEDVIDKLLKSNVILAYGNSECPIVNKFQINPLVRPKLVILSTENHR
SYPFPLRIPYSHIRSKWSLLEQKKVILGENSLKPESRINTIFNVGASYLNFGSQWLDKSIQNLKVLQLGRWQDSPSHHIEVG
SEKFLQKLQNQKNLTYLSLRGISRISELPPSITQLERLQILDLKACHNLETLPNDISSMKSLTHLILSQCYLLEGMPKGIEKLT
QLQVLEGYVVGNSRKSPQRISELANLKNLKRLSIHIGSEAVIKEGEFESWREMLALEQLKISWGVSCKGYSDIKVILPLRLK
RLHLEGFPGEEIPEWLKPSKLPEGCKELKLMGGMLKSMNHEDNIGWRMEIVRLMYLKNLKLELTNLSKLFPLLRYAEIKQ
NSKRTQMLSLNEDK

>XP_027929042.1

MATRIIEGLVKTSEIYSLILDMLSGFNGFNDNVQMLEMKLEELCSLEHDINKELEIEELERGKKRKREVENWLRNVQRKKT
EVHGMVQELRDCGMFRHLKLIVQVRKLTGQVTDLVARGRFPEGIVGSAQESRGYALLTTELAGAMFQKNVGKIWDW
LMNDGVLMIGVYGMGGVGKTSVLMHIHNMLLTAVTNFESVFWVTISQSFSIHKLQRDVAKIVGIDISKESDERKRAAR
LSWALMRRKRCVLFLDDVWNHFPLERVGIPVRADGLKLVLTSRSLDVCRRMNCQNSVKVEPLSMEEAWTLFVDNLG
QQTTLSPEEKQVARSVAKQCAGLPLAIITMARSMRGVEEISEWRHTLEELRNTEAKQEEMEMEVLRVLRFSYDHLNDK
MVQQCFLCCALYPEDFEIDRDVLIESFVDEGLVNGMTSLEAMFDEGHSIVNKLENICLLGKVENYVGGKKCMGLQDAS
SHIAISMMKRGCQHVEPMNNVEGYYVGSQLVKMHDLVRAMAINVMKVNNNFLVKAGLQLTKIPDEVEWSEDLEKV
SLMCNWIHEIPTEISPRCPKLRTLILKHNESLTRISDSFFVHMSALEVLDLSFTDIEVLPKSVSDLSTLTALLLTSCKRLKHMP
SLAKLQALLRLDLSFTAITEMPQGLEMLVNLKWLNLYAKDLVSSGKEVAKLTSLQFLILHWWSRKIKVKVEHTSCLRKLET
FAANLYNMHHFNAYVKTMHIYGPRSYLLQLDTEESHGNSPWCCFAEVCFRKDVIISNCKIRTGETSLMLPLDIQRLKVER
CHEIRSLCDVMSLKNATSLKRSEIADCDGPEYMFSLSCSSSCCTSLHSLESLELYSLKNLHGLCKEGEVAAQTFPPGRAFTC
LKYFFIYHCPLIKKLLTPRLLAYLPNLEEITVHNCKSMEEIISVDGIDYESFGGKKSFVTNRDTIIVRHSKLVSLSLKHLPELKSIS
SGQMVCESLQNFRIFKCPKLARFPETATPVQILYDSF

>XP_027929801.1

MKSLYPEHNKYHRFSFSIKQRSMKKQTLLLGSLNSKNNVKLLCCTSFLHITIKLEIDAIIYSFPPSQVYFPSKLSVVILSYIWLF
TASKQFELALTTTELSLPQYFVDVFPKFLCQNRILLFKFLLVFGEIKMSIRTNKMKAVPVLLKQLMIARRKFHERGRDESLD
KKLEKLRLDLNRIKDVFVRVKKKEEELLDTLAEVYGHLRKLDRGKLDEDMDGICKRIRDSARKLLPDDGFDESYKKEDEKG
GKISHTSQELVQPHENKSWTAEDFYLLDDRLKVFLGSLQIFPENAVIRKRIAINLWIGEGLLENTESKTTEELGEDVISDLFK
LNLIVRYAKGKSVLLANKFQILPSVRRQMGSYLLKKNVEHRGQYFIASIYLQKGPWRLQLEQNKVTLGDDLHLIRGIIIGSI
FNIGASYLNFRLSWVTEFKNLEVFQLGRWQDSALHHIEVGSEEFLKELRYLKKLKYVSLRGISRIFELPSSIAELEKLLILDVK
ACHNLERLPDDISSMKSLTHLIMSDCCLLEGMPKGIEKLTNLEVLKGFLISTSEKTPCKISDLVKLGKLRRLSIRIGSEAEIRD
GEFEGLKDFSALEHLKISWSVSDPKYANINVLLPSSLKKLHIECFPGKSLEECFMPGEYFPRKSLEECFMPGVHGRFRFILTE
LNITGGKLESMEVDFEWWRVKIVRLKYLKQLNVDIDDLKAMFPLLKYVEIKQVSNISYIQHEWDI

>XP_027929819.1

MEAHMEEKPLVLSNEFDGKKFDKNVKTMWDLLREDKVFIIGIHGMGGVGKTFLATYMESEIKRTKTFKDVVWVTVSH
DFTIFKLQQHIAEILKIKLYGDDERERALILASELEKRENIVLILDDVWKYIDMEKVGIPLRMKGNKLIITSRLRHLFLLKLGD
HGTPSTLPSQVLDIARSMVGKCDGLPLGISVMARTMKGETRIHWWRHALNKLDKLEMGVEMQEEVLTVLRRSYDNLT
EKDVQKCFLYSALLPNPVRRYLLIMNHVDMVLLNGKRRLEEIFDEANVIVDKLINHSLLLEDNSELTMHALVRKMALNIIK
ENGSNLMVKCDESMEKIPDIEEWTIDLEVVSLANDKIQEIPDGTSPNCPRLSTLLLFDNEIRHIPECFFMHMNALTTLDLS
RNDRLTRLPHSLSNLRTLTSLMLNGCSQLKYIPPLGDLQALLRLDISGCFIHVPPEGLENLVNLKWLDMSSNVDLELVPGS
FLPSLTNIQYLDLYGCSGIKVEDIEEMNFLECFAGAFVDRENLNRYVQQTRDSAYGPQNYSIHYQDRSYKGHWEKFWN
REFLSEKYKCRTMCIKDCEELSYVLPRDLVKLLLKIILQLLPVSFTLK

>XP_027930107.1

MAESVVSFLVQNLSNLLDSEIKLLSGVEGKVKSLCNELKFMDIFLKSSEGKYKDAMVKEVVTQIRDVAYKAEDVVDTYILN
IAKHKRRNKLCRLFHLKEKFTIPHEIDAEIEEIKSRIDEIYKNKERYGIKEGEFQSEETEWHRKRRMYVEEEDVVGLVNDSNI
VIEQLQKDDVRLNVASILGMGGLGKTTLARKIFNKDNVKELFHCRAWGNVSNDYRPKELFLSLLRSLNLSEFEKNEEDLN
KKSEEDLKKEVAKWLKGKKYLVVLDDIWETRVWDDIKGAFQDEKRGSRILITSRNKDVARYSGTTSPYELPFLTEDQSWE
LFCKKVFRDEECPSDLEHLGRSIAKSCGGLPLAIVVLGGVYATKEKSEREWLRMKEMRGHPTEDKSEVMDILRLSYDSLP
PKLKPCFLYFGMYPEDYEMNAREMIRLWVAEGLVKPHEDAEPEVVADFYLDELVDRSLVQVTRRRTDGGVKICQLHDL
LRDLCISESKSNKFLQVLKTSNIDTLSDTNPRRLSLQCQPQSNISAVSFHKSMSSTRSVIIFTKQGKLMNDFLKRLLLARVIH
GFFPPFYYISFSHHYKRMIHIRYLKICVISVPACVCNLWNLETLFVRYTKTVSSEIWKLKRLRHLHLEGRGMLPKLPNGTILE
NLRTLVLSRYCGSKMISLLKSGIFPRLVKLDLINREVPDLLREVISLSNLVNLSHFSLRGFDIQRLPSDTNAFPSKLTKITFESIE
GDASPLMKTLSQLPNLQILKLLEDYFRVLLNIDVGKGEFPKLQVFHMRKLHSIKSWKLEEGAMPCLRHLHITDCSSLFELP
QQLWSLRTLQLVRIVRPSPQLASTLQNVEFNNNCKLILEQISLP

>XP_027930108.1

MAESVVSFLVQNLSQLLVSEIKLLSGVEGKVKSLHNDLKLMDIFLRSSEGKYKDAMVKEVVTQIRDVAYRAEDVVDTYIL
NIAKHKRRNKLCRLFHFKEKFIVPHEIDAEIEEIKSRIDEIYKNKERYGIKEGEFQSEEAVTTEWRRKRRINVEEEDVVGLVN
DSNIVIQQLQKDYVRLNVASILGMGGLGKTTLARKIFNKDNVKKLFPCRAWGNVSNDYRPKELFLSLLRSLNLSASENLS
EEDLKKEVAKGLKGKKYLVVLDDIWETRVWDDIKGAFPDEKRGSRILITSRNKDVARYSGTTSPYDLPFLTEDQSWELFC
KKVFRGEECPSDLELLGRSIAKSCGGLPLAIVVLGGVYAMKEKSEREWSRMKKMRWHPTEDKSEVMDILRLSYDNLPLR
LKPCFLYFGMYPEDYEMNAREMIRLWVAEGFVKPHEDAEPEVVADFYLDELVDRSLVQVTRRRTDGGVKICQLHDLLR
DLCISESESSKFLQVWKTSNIDTLSDTNPRRLSLQCQPQSNISAVSFQKSMSSTRSMIIFTDLGKSVNDFVKRLMLARVIG
SFHPAYYVSFSHHYKRMIHIRYLKICVISVPACICNLSNLETLFVIYAKTVSSEIWKLKRLRHLHLERGDVLPMLPNGTIIENL
RTLQLFHGVSEIISLFKSGIFPRLVKLGLRPRGPVFHSFGEVISLSSVVNISHFTLKAFNAGRLPSDTNAFPSKLTKITFESIEG
DASPLMKTLSQLPNLQILKLLEHFGVLLNIDVGNGEFPKLQVFHMRGLDQIKSWKLEEGAMPCLQLLHIKDCPNLFELPQ
QLWSLRTLQLVHIVAPSQQLATTLQNVKFNNNCKLILEQN

>XP_027930109.1

MAESVVSFLVQNLSQLLVSEIKLLSGVEGKVKSLHNDLKLMDIFLRSSEGKYKDAMVKEVVTQIRDVAYRAEDVVDTYIL
NIAKHKRRNKLCRLFHFKEKFIVPHEIDAEIEEIKSRIDEIYKNKERYGIKEGEFQSEEAVTTEWRRKRRINVEEEDVVGLVN
DSNIVIQQLQKDYVRLNVASILGMGGLGKTTLARKIFNKDNVKKLFPCRAWGNVSNDYRPKELFLSLLRSLNLSASENLS
EEDLKKEVAKGLKGKKYLVVLDDIWETRVWDDIKGAFPDEKRGSRILITSRNKDVARYSGTTSPYDLPFLTEDQSWELFC
KKVFRGEECPSDLELLGRSIAKSCGGLPLAIVVLGGVYAMKEKSEREWSRMKKMRWHPTEDKSEVMDILRLSYDNLPLR
LKPCFLYFGMYPEDYEMNAREMIRLWVAEGFVKPHEDAEPEVVADFYLDELVDRSLVQVTRRRTDGGVKICQLHDLLR
DLCISESESSKFLQVWKTSNIDTLSDTNPRRLSLQCQPQSNISAVSFQKSMSSTRSMIIFTDLGKSVNDFVKRLMLARVIG
SFHPAYYVSFSHHYKRMIHIRYLKICVISVPACICNLSNLETLFVIYAKTVSSEIWKLKRLRHLHLERGDVLPMLPNGTIIENL

RTLQLFHGVSEIISLFKSGIFPRLVKLGLRPRGPVFHSFGEVISLSSVVNISHFTLKAFNAGRLPSDTNAFPSKLTKITFESIEG
DASPLMKTLSQLPNLQILKLLEHFGVLLNIDVGNGEFPKLQVFHMRGLDQIKSWKLEEGAMPCLQLLHIKDCPNLFELPQ
QLWSLRTLQLVHIVAPSQQLATTLQNVKFNNNCKLILEQN

>XP_027930110.1

MAESVVSFLVQNLSQLLVSEIKLLSGVEGKVKSLHNDLKLMDIFLRSSEGKYKDAMVKEVVTQIRDVAYRAEDVVDTYIL
NIAKHKRRNKLCRLFHFKEKFIVPHEIDAEIEEIKSRIDEIYKNKERYGIKEGEFQSEEAVTTEWRRKRRINVEEEDVVGLVN
DSNIVIQQLQKDYVRLNVASILGMGGLGKTTLARKIFNKDNVKKLFPCRAWGNVSNDYRPKELFLSLLRSLNLSASENLS
EEDLKKEVAKGLKGKKYLVVLDDIWETRVWDDIKGAFPDEKRGSRILITSRNKDVARYSGTTSPYDLPFLTEDQSWELFC
KKVFRGEECPSDLELLGRSIAKSCGGLPLAIVVLGGVYAMKEKSEREWSRMKKMRWHPTEDKSEVMDILRLSYDNLPLR
LKPCFLYFGMYPEDYEMNAREMIRLWVAEGFVKPHEDAEPEVVADFYLDELVDRSLVQVTRRRTDGGVKICQLHDLLR
DLCISESESSKFLQVWKTSNIDTLSDTNPRRLSLQCQPQSNISAVSFQKSMSSTRSMIIFTDLGKSVNDFVKRLMLARVIG
SFHPAYYVSFSHHYKRMIHIRYLKICVISVPACICNLSNLETLFVIYAKTVSSEIWKLKRLRHLHLERGDVLPMLPNGTIIENL
RTLQLFHGVSEIISLFKSGIFPRLVKLGLRPRGPVFHSFGEVISLSSVVNISHFTLKAFNAGRLPSDTNAFPSKLTKITFESIEG
DASPLMKTLSQLPNLQILKLLEHFGVLLNIDVGNGEFPKLQVFHMRGLDQIKSWKLEEGAMPCLQLLHIKDCPNLFELPQ
QLWSLRTLQLVHIVAPSQQLATTLQNVKFNNNCKLILEQN

>XP_027930112.1

MAESVVSFLVQNLSQLLVSEIKLLSGVEGKVKSLHNDLKLMDIFLRSSEGKYKDAMVKEVVTQIRDVAYRAEDVVDTYIL
NIAKHKRRNKLCRLFHFKEKFIVPHEIDAEIEEIKSRIDEIYKNKERYGIKEGEFQSEEAVTTEWRRKRRINVEEEDVVGLVN
DSNIVIQQLQKDYVRLNVASILGMGGLGKTTLARKIFNKDNVKKLFPCRAWGNVSNDYRPKELFLSLLRSLNLSASENLS
EEDLKKEVAKGLKGKKYLVVLDDIWETRVWDDIKGAFPDEKRGSRILITSRNKDVARYSGTTSPYDLPFLTEDQSWELFC
KKVFRGEECPSDLELLGRSIAKSCGGLPLAIVVLGGVYAMKEKSEREWSRMKKMRWHPTEDKSEVMDILRLSYDNLPLR
LKPCFLYFGMYPEDYEMNAREMIRLWVAEGFVKPHEDAEPEVVADFYLDELVDRSLVQVTRRRTDGGVKICQLHDLLR
DLCISESESSKFLQVWKTSNIDTLSDTNPRRLSLQCQPQSNISAVSFQKSMSSTRSMIIFTDLGKSVNDFVKRLMLARVIG
SFHPAYYVSFSHHYKRMIHIRYLKICVISVPACICNLSNLETLFVIYAKTVSSEIWKLKRLRHLHLERGDVLPMLPNGTIIENL
RTLQLFHGVSEIISLFKSGIFPRLVKLGLRPRGPVFHSFGEVISLSSVVNISHFTLKAFNAGRLPSDTNAFPSKLTKITFESIEG
DASPLMKTLSQLPNLQILKLLEHFGVLLNIDVGNGEFPKLQVFHMRGLDQIKSWKLEEGAMPCLQLLHIKDCPNLFELPQ
QLWSLRTLQLVHIVAPSQQLATTLQNVKFNNNCKLILEQN

>XP_027930113.1

MAESVVSFLVQNLSQLLVSEIKLLSGVEGKVKSLHNDLKLMDIFLRSSEGKYKDAMVKEVVTQIRDVAYRAEDVVDTYIL
NIAKHKRRNKLCRLFHFKEKFIVPHEIDAEIEEIKSRIDEIYKNKERYGIKEGEFQSEEAVTTEWRRKRRINVEEEDVVGLVN
DSNIVIQQLQKDYVRLNVASILGMGGLGKTTLARKIFNKDNVKKLFPCRAWGNVSNDYRPKELFLSLLRSLNLSASENLS
EEDLKKEVAKGLKGKKYLVVLDDIWETRVWDDIKGAFPDEKRGSRILITSRNKDVARYSGTTSPYDLPFLTEDQSWELFC
KKVFRGEECPSDLELLGRSIAKSCGGLPLAIVVLGGVYAMKEKSEREWSRMKKMRWHPTEDKSEVMDILRLSYDNLPLR
LKPCFLYFGMYPEDYEMNAREMIRLWVAEGFVKPHEDAEPEVVADFYLDELVDRSLVQVTRRRTDGGVKICQLHDLLR
DLCISESESSKFLQVWKTSNIDTLSDTNPRRLSLQCQPQSNISAVSFQKSMSSTRSMIIFTDLGKSVNDFVKRLMLARVIG
SFHPAYYVSFSHHYKRMIHIRYLKICVISVPACICNLSNLETLFVIYAKTVSSEIWKLKRLRHLHLERGDVLPMLPNGTIIENL
RTLQLFHGVSEIISLFKSGIFPRLVKLGLRPRGPVFHSFGEVISLSSVVNISHFTLKAFNAGRLPSDTNAFPSKLTKITFESIEG
DASPLMKTLSQLPNLQILKLLEHFGVLLNIDVGNGEFPKLQVFHMRGLDQIKSWKLEEGAMPCLQLLHIKDCPNLFELPQ
QLWSLRTLQLVHIVAPSQQLATTLQNVKFNNNCKLILEQN

>XP_027930114.1

MAESVVSFLVQNLSQLLVSEIKLLSGVEGKVKSLHNDLKLMDIFLRSSEGKYKDAMVKEVVTQIRDVAYRAEDVVDTYIL
NIAKHKRRNKLCRLFHFKEKFIVPHEIDAEIEEIKSRIDEIYKNKERYGIKEGEFQSEEAVTTEWRRKRRINVEEEDVVGLVN
DSNIVIQQLQKDYVRLNVASILGMGGLGKTTLARKIFNKDNVKKLFPCRAWGNVSNDYRPKELFLSLLRSLNLSASENLS
EEDLKKEVAKGLKGKKYLVVLDDIWETRVWDDIKGAFPDEKRGSRILITSRNKDVARYSGTTSPYDLPFLTEDQSWELFC
KKVFRGEECPSDLELLGRSIAKSCGGLPLAIVVLGGVYAMKEKSEREWSRMKKMRWHPTEDKSEVMDILRLSYDNLPLR
LKPCFLYFGMYPEDYEMNAREMIRLWVAEGFVKPHEDAEPEVVADFYLDELVDRSLVQVTRRRTDGGVKICQLHDLLR
DLCISESESSKFLQVWKTSNIDTLSDTNPRRLSLQCQPQSNISAVSFQKSMSSTRSMIIFTDLGKSVNDFVKRLMLARVIG
SFHPAYYVSFSHHYKRMIHIRYLKICVISVPACICNLSNLETLFVIYAKTVSSEIWKLKRLRHLHLERGDVLPMLPNGTIIENL
RTLQLFHGVSEIISLFKSGIFPRLVKLGLRPRGPVFHSFGEVISLSSVVNISHFTLKAFNAGRLPSDTNAFPSKLTKITFESIEG
DASPLMKTLSQLPNLQILKLLEHFGVLLNIDVGNGEFPKLQVFHMRGLDQIKSWKLEEGAMPCLQLLHIKDCPNLFELPQ
QLWSLRTLQLVHIVAPSQQLATTLQNVKFNNNCKLILEQN

>XP_027930115.1

MAESVVSFLVQNLSQLLVSEIKLLSGVEGKVKSLHNDLKLMDIFLRSSEGKYKDAMVKEVVTQIRDVAYRAEDVVDTYIL
NIAKHKRRNKLCRLFHFKEKFIVPHEIDAEIEEIKSRIDEIYKNKERYGIKEGEFQSEEAVTTEWRRKRRINVEEEDVVGLVN
DSNIVIQQLQKDYVRLNVASILGMGGLGKTTLARKIFNKDNVKKLFPCRAWGNVSNDYRPKELFLSLLRSLNLSASENLS
EEDLKKEVAKGLKGKKYLVVLDDIWETRVWDDIKGAFPDEKRGSRILITSRNKDVARYSGTTSPYDLPFLTEDQSWELFC
KKVFRGEECPSDLELLGRSIAKSCGGLPLAIVVLGGVYAMKEKSEREWSRMKKMRWHPTEDKSEVMDILRLSYDNLPLR
LKPCFLYFGMYPEDYEMNAREMIRLWVAEGFVKPHEDAEPEVVADFYLDELVDRSLVQVTRRRTDGGVKICQLHDLLR
DLCISESESSKFLQVWKTSNIDTLSDTNPRRLSLQCQPQSNISAVSFQKSMSSTRSMIIFTDLGKSVNDFVKRLMLARVIG
SFHPAYYVSFSHHYKRMIHIRYLKICVISVPACICNLSNLETLFVIYAKTVSSEIWKLKRLRHLHLERGDVLPMLPNGTIIENL
RTLQLFHGVSEIISLFKSGIFPRLVKLGLRPRGPVFHSFGEVISLSSVVNISHFTLKAFNAGRLPSDTNAFPSKLTKITFESIEG
DASPLMKTLSQLPNLQILKLLEHFGVLLNIDVGNGEFPKLQVFHMRGLDQIKSWKLEEGAMPCLQLLHIKDCPNLFELPQ
QLWSLRTLQLVHIVAPSQQLATTLQNVKFNNNCKLILEQN

>XP_027930116.1

MAESVVSFLVQNLSQLLVSEIKLLSGVEGKVKSLHNDLKLMDIFLRSSEGKYKDAMVKEVVTQIRDVAYRAEDVVDTYIL
NIAKHKRRNKLCRLFHFKEKFIVPHEIDAEIEEIKSRIDEIYKNKERYGIKEGEFQSEEAVTTEWRRKRRINVEEEDVVGLVN
DSNIVIQQLQKDYVRLNVASILGMGGLGKTTLARKIFNKDNVKKLFPCRAWGNVSNDYRPKELFLSLLRSLNLSASENLS
EEDLKKEVAKGLKGKKYLVVLDDIWETRVWDDIKGAFPDEKRGSRILITSRNKDVARYSGTTSPYDLPFLTEDQSWELFC
KKVFRGEECPSDLELLGRSIAKSCGGLPLAIVVLGGVYAMKEKSEREWSRMKKMRWHPTEDKSEVMDILRLSYDNLPLR
LKPCFLYFGMYPEDYEMNAREMIRLWVAEGFVKPHEDAEPEVVADFYLDELVDRSLVQVTRRRTDGGVKICQLHDLLR
DLCISESESSKFLQVWKTSNIDTLSDTNPRRLSLQCQPQSNISAVSFQKSMSSTRSMIIFTDLGKSVNDFVKRLMLARVIG
SFHPAYYVSFSHHYKRMIHIRYLKICVISVPACICNLSNLETLFVIYAKTVSSEIWKLKRLRHLHLERGDVLPMLPNGTIIENL
RTLQLFHGVSEIISLFKSGIFPRLVKLGLRPRGPVFHSFGEVISLSSVVNISHFTLKAFNAGRLPSDTNAFPSKLTKITFESIEG
DASPLMKTLSQLPNLQILKLLEHFGVLLNIDVGNGEFPKLQVFHMRGLDQIKSWKLEEGAMPCLQLLHIKDCPNLFELPQ
QLWSLRTLQLVHIVAPSQQLATTLQNVKFNNNCKLILEQN

>XP_027930118.1

MAESVVSFLVQNLSQLLVSEIKLLSGVEGKVKSLHNDLKLMDIFLRSSEGKYKDAMVKEVVTQIRDVAYRAEDVVDTYIL
NIAKHKRRNKLCRLFHFKEKFIVPHEIDAEIEEIKSRIDEIYKNKERYGIKEGEFQSEEAVTTEWRRKRRINVEEEDVVGLVN
DSNIVIQQLQKDYVRLNVASILGMGGLGKTTLARKIFNKDNVKKLFPCRAWGNVSNDYRPKELFLSLLRSLNLSASENLS
EEDLKKEVAKGLKGKKYLVVLDDIWETRVWDDIKGAFPDEKRGSRILITSRNKDVARYSGTTSPYDLPFLTEDQSWELFC
KKVFRGEECPSDLELLGRSIAKSCGGLPLAIVVLGGVYAMKEKSEREWSRMKKMRWHPTEDKSEVMDILRLSYDNLPLR

LKPCFLYFGMYPEDYEMNAREMIRLWVAEGFVKPHEDAEPEVVADFYLDELVDRSLVQVTRRRTDGGVKICQLHDLLR
DLCISESESSKFLQVWKTSNIDTLSDTNPRRLSLQCQPQSNISAVSFQKSMSSTRSMIIFTDLGKSVNDFVKRLMLARVIG
SFHPAYYVSFSHHYKRMIHIRYLKICVISVPACICNLSNLETLFVIYAKTVSSEIWKLKRLRHLHLERGDVLPMLPNGTIIENL
RTLQLFHGVSEIISLFKSGIFPRLVKLGLRPRGPVFHSFGEVISLSSVVNISHFTLKAFNAGRLPSDTNAFPSKLTKITFESIEG
DASPLMKTLSQLPNLQILKLLEHFGVLLNIDVGNGEFPKLQVFHMRGLDQIKSWKLEEGAMPCLQLLHIKDCPNLFELPQ
QLWSLRTLQLVHIVAPSQQLATTLQNVKFNNNCKLILEQN

>XP_027930119.1

MAESVVSFLVQNLSQLLVSEIKLLSGVEGKVKSLHNDLKLMDIFLRSSEGKYKDAMVKEVVTQIRDVAYRAEDVVDTYIL
NIAKHKRRNKLCRLFHFKEKFIVPHEIDAEIEEIKSRIDEIYKNKERYGIKEGEFQSEEAVTTEWRRKRRINVEEEDVVGLVN
DSNIVIQQLQKDYVRLNVASILGMGGLGKTTLARKIFNKDNVKKLFPCRAWGNVSNDYRPKELFLSLLRSLNLSASENLS
EEDLKKEVAKGLKGKKYLVVLDDIWETRVWDDIKGAFPDEKRGSRILITSRNKDVARYSGTTSPYDLPFLTEDQSWELFC
KKVFRGEECPSDLELLGRSIAKSCGGLPLAIVVLGGVYAMKEKSEREWSRMKKMRWHPTEDKSEVMDILRLSYDNLPLR
LKPCFLYFGMYPEDYEMNAREMIRLWVAEGFVKPHEDAEPEVVADFYLDELVDRSLVQVTRRRTDGGVKICQLHDLLR
DLCISESESSKFLQVWKTSNIDTLSDTNPRRLSLQCQPQSNISAVSFQKSMSSTRSMIIFTDLGKSVNDFVKRLMLARVIG
SFHPAYYVSFSHHYKRMIHIRYLKICVISVPACICNLSNLETLFVIYAKTVSSEIWKLKRLRHLHLERGDVLPMLPNGTIIENL
RTLQLFHGVSEIISLFKSGIFPRLVKLGLRPRGPVFHSFGEVISLSSVVNISHFTLKAFNAGRLPSDTNAFPSKLTKITFESIEG
DASPLMKTLSQLPNLQILKLLEHFGVLLNIDVGNGEFPKLQVFHMRGLDQIKSWKLEEGAMPCLQLLHIKDCPNLFELPQ
QLWSLRTLQLVHIVAPSQQLATTLQNVKFNNNCKLILEQN

>XP_027930120.1

MAESVVSFLVQNLSNLLDSEIKLLSGVEGKVKSLCNELKFMDIFLKSSEGKYKDAMVKEVVTQIRDVAYKAEDVVDTYILN
IAKHKRRNKLCRLFHLKEKFTIPHEIDAEIEEIKSRIDEIYKNKERYGIKEGEFQSEEAVTTEWRRKRRINVEEEDVVGLVNDS
NIVIQQLQKDYVRLNVASILGMGGLGKTTLARKIFNKDNVKKLFPCRAWGNVSNDYRPKELFLSLLRSLNLSASENLSEE
DLKKEVAKGLKGKKYLVVLDDIWETRVWDDIKGAFPDEKRGSRILITSRNKDVARYSGTTSPYDLPFLTEDQSWELFCKK
VFRGEECPSDLELLGRSIAKSCGGLPLAIVVLGGVYAMKEKSEREWSRMKKMRWHPTEDKSEVMDILRLSYDNLPLRLK
PCFLYFGMYPEDYEMNAREMIRLWVAEGFVKPHEDAEPEVVADFYLDELVDRSLVQVTRRRTDGGVKICQLHDLLRDL
CISESESSKFLQVWKTSNIDTLSDTNPRRLSLQCQPQSNISAVSFQKSMSSTRSMIIFTDLGKSVNDFVKRLMLARVIGSF
HPAYYVSFSHHYKRMIHIRYLKICVISVPACICNLSNLETLFVIYAKTVSSEIWKLKRLRHLHLERGDVLPMLPNGTIIENLRT
LQLFHGVSEIISLFKSGIFPRLVKLGLRPRGPVFHSFGEVISLSSVVNISHFTLKAFNAGRLPSDTNAFPSKLTKITFESIEGDA
SPLMKTLSQLPNLQILKLLEHFGVLLNIDVGNGEFPKLQVFHMRGLDQIKSWKLEEGAMPCLQLLHIKDCPNLFELPQQL
WSLRTLQLVHIVAPSQQLATTLQNVKFNNNCKLILEQN

>XP_027930708.1

MAESVVSFASQHVLPKFLEAVKMLRDLPKEVAEVTDELESFQDFIRDANKVAEAEEDINRRDRIRKRLMRLREAAFRME
DVIDDYVICDEKQPEDDPRCAALLSEAIEFIKTQILRLQIAYQIQDVKSLVRAERDGFQNHFPIEPRLHSCIGNEHFTWHKL
RMDPLFIQKDEVVGLDGPIQTLKKWLTEGREERTVISVVGMAGLGKTTLSKQVFDRVHTDFECHALITVSRSYTVEGLLR
DLTNKLCKERLEDPPRDVATMNQMSLIEEVRNRLNNKRYVVLFDDVWNKTFWDDIELALIDNKNGSRILITTRDEKVVD
LCKKCLFFEVHKLQPLSKEKSLELLCKKAFGYGFDGSCPKDYEEVGLEIVRKCECLPLSIVAIGSLLNRKCKSPSEWGLFSQN
LSLELESNSELDSVKKILGLSYDDLPQNLRSCLLYFGMYPENYEVKCDRLIQMWIAEGFVKHESGRNLEEVAQQQLIELISR
SLVLVSSFTIDNRAKLCRVHDSIHEMIRGKTKNTGFCEYIDVNNHLESSGPIRRLTIATSSNDLSGTIEGSHHIRSIIIFGRREV
LSEQFTSHLLSKYTRLKVLDFGYAPLYDVPENLGSLIHLKYLSFWSTFIRSLPKSIGKLQNLETLDVRANREIEVPKEITKLRKL
RCLLGNRMSAIAVKDSIGSMTSLQMMHILIDPDGVVIRELGKLKQLRDMRLSNVMGHHSDTLSSSINQMQLLERLDIFV
QHLSEPIDLHITSSLSKLRKLHLYANLKEFPNWIPRLQNLVKLSLVQSRLTNIPLISIGNMANLLILSFGSTSYEGETLHFENG

GFQKLKELELQRLHQLSSIFIDSGALQSLEKLQIMNIPELKAVPSGIQNLKKLQVLDILYMPTEFLQRIDPNGGEDYFMIKH
VPHLHFVTKNRAFLLAERAANILCGVIE

>XP_027930751.1

MTETVVSFASQHLLSKFLDAVKMVRDLPKEVAEVTDELESFQDFIHDANKVAEAEEDNNKRDRIRKRLTRLTEAAFRME
DVIDDYVLCDNKQPEEDPRCADLLCEAVQIIKTQILRLQIAYQIQDVKSVVRGEKDAFQKHFPIEPRSDGSRENENFTWH
KLRVDPLFKQDEEVVGLEGPIQTLKKGLTEGREERTVISIVGMAGLGKTTLSKQVFDRVHKHFECHALITVSRPYTVERLL
RDLTKELCEERNEQPPQDVETMNQMSLIKEVRKRLCNKRYVVLFDDVWNEKFWDDIELALIDNKNGSRILITTRDERVV
EFCKKALSFEVHKLQPLSKEKSLELLCRRAFGYDFDGRCPEDYEEVGLDIVRKCECLPLAIVAIGSLLYRKCKSSSEWHLFSQ
NLSSELQGNSELHSVTKILSLSYDALPQNLRSCLLYFGMYPEDYEVKCDRLIWQWIAEGFVKHESRKNLEEVAQQYLMEL
ISRSLVLVSSFSTDGKVKACRVHDSMHEMIRLKIKNTEFCEYIDEHNHLEPSGTIRRLAIATSSNGLIGSIEESQHVRSILFFT
NKVLSEDFTIRLLAIAKYMRLKVLDFEFAPLYNVPESLGSLINLKYLSFRDTYIRSLPKSICKLQNLETLDVRANREIAMQNLE
ILDVRANREIEVPKEITKLRKLRCFLGNRISSIALKDSLGSITSLEKMHVLEIDPDGEVIREIGKLKQLRDLRVVSLRGDDADT
LCSSINEMPLLERLHISTKYGTEAIDLQIRSSLSKLRKLHLYGNLKEFPIWIPRLQNLVKLSLVQSRLTNIPLISLGNMPNVLIL
SLSLNSYEGETLHFQKGGFQKLKELELQRLHQLSSIFIDSGTLQSLEKLLIINIPKLKTVPSGIQHLKKLRLLHMLYMPSEFKQ
RIDPNGGKEHWMIKHVPDVRFVPLLLAEGFAKMFSLRL

>XP_027930752.1

MTETVVSFASQHLLSKFLDAVKMVRDLPKEVAEVTDELESFQDFIHDANKVAEAEEDNNKRDRIRKRLTRLTEAAFRME
DVIDDYVLCDNKQPEEDPRCADLLCEAVQIIKTQILRLQIAYQIQDVKSVVRGEKDAFQKHFPIEPRSDGSRENENFTWH
KLRVDPLFKQDEEVVGLEGPIQTLKKGLTEGREERTVISIVGMAGLVERLLRDLTKELCEERNEQPPQDVETMNQMSLIK
EVRKRLCNKRYVVLFDDVWNEKFWDDIELALIDNKNGSRILITTRDERVVEFCKKALSFEVHKLQPLSKEKSLELLCRRAF
GYDFDGRCPEDYEEVGLDIVRKCECLPLAIVAIGSLLYRKCKSSSEWHLFSQNLSSELQGNSELHSVTKILSLSYDALPQNL
RSCLLYFGMYPEDYEVKCDRLIWQWIAEGFVKHESRKNLEEVAQQYLMELISRSLVLVSSFSTDGKVKACRVHDSMHE
MIRLKIKNTEFCEYIDEHNHLEPSGTIRRLAIATSSNGLIGSIEESQHVRSILFFTNKVLSEDFTIRLLAIAKYMRLKVLDFEFA
PLYNVPESLGSLINLKYLSFRDTYIRSLPKSICKLQNLETLDVRANREIAMQNLEILDVRANREIEVPKEITKLRKLRCFLGNRI
SSIALKDSLGSITSLEKMHVLEIDPDGEVIREIGKLKQLRDLRVVSLRGDDADTLCSSINEMPLLERLHISTKYGTEAIDLQIR
SSLSKLRKLHLYGNLKEFPIWIPRLQNLVKLSLVQSRLTNIPLISLGNMPNVLILSLSLNSYEGETLHFQKGGFQKLKELELQR
LHQLSSIFIDSGTLQSLEKLLIINIPKLKTVPSGIQHLKKLRLLHMLYMPSEFKQRIDPNGGKEHWMIKHVPDVRFVPLLLAE
GFAKMFSLRL

>XP_027930824.1

MAETALSLARKYVFPKLLEAVNMIRDLPKEVDDLTDALETFRYFINETEEVVEAEEDSNRRDRMSTRLMKMREATFRME
DVIDDYVICGENQPQEDPRCAALLCEAVEFIKTQISRLQIAHKIRDVKSLASAAKDGFETHFPSKKSPSNSERNQNGNWQ
KLRKDALHVKEDEVVGLDGPRTILTNWLTKGRNGRTVIFVVGIPGVGKTTLAKLVFDKVRNDFDCHASIRVSQSYNVEKL
LRDMIHQLCKASKEDPRRNVSEMGQASLIEEVKDLLHKKRYVVLFDDLWNENFWNDIESALIDDNNKSRIIITTRHEEVA
VFCKKSSFVEVHKLEKPLSEDESFRLFCRKAFMYGSSGGCPEELKDTSLEIVRKCKGLPLAIVAIGGVLAQKDENPHEWKL
VNQNLSLEGNSELNIITKILGLSYHDLPINLKSCLLYFGMYPEDCEIESDRVIRQWVAEGFVRHKTGKTLEEVAEEDLLGLIR
RNLVQVSSSSIDGKVKRCRVHDLIHDMILRKAKDSGFCGYIGRNDECVSSGIVRRLIIIAKDDLIGSVESSLIQSIAINEGRSR
KLTENFVLEFLGDYTLLLSEDLVWKILENYTSLKVLDFEGAPLPCVPENLGHLIHLRYLSFRGTGLEILPKSIGKLQNLETLDIR
KTDVHYIPRDFYKLSKLRHFLADKVSKIEWRYIGGMTSLQTIPPVSRCYHADDIGEVAKLKQLRVLRVLVSDTNLVKSLCS
AINEMQQLEELHIAVDYYISEVIDLNITSPLARLRKLFLDMKLKGLPDWIPQLQKLVKLSLWRTGLANDELQSLKDMPSLL
VLHLWFALEGETLHFQCGGFQKLKKLHLNYLSNLNSILIDRGALHSLEHMRLKGLLKLKTVPDGIQHLGNLKLVELTSMPT
EFKKKIVPIAEQQHCVVQYKTRPRLSATWKKHGK

>XP_027930840.1

MAEIGTAVVSTLIERVVDAIIGRARCLFYFKKSVVDLRKSKRELEKSLQHMRERVKEATTNAEKIVQPVEEWLKDVERVLE
DVQGLEERVEESECCLNMTFKYSLAKEVICMTLQMNVVNNNNKFEPFSHPIKLSSMNYFFPKDFVAFDSRKPVYEELLQ
AIQDERSNVIGVVGVEGSGKSTLARVVGKQVEESKVFDKVVMTIVSQDVKLRDIQGQIADHLSFSLVEETELGRALRLSH
RLKTEKILIIFDGVWQKLDLEAIGIPLNENDKRCCVLLTTRDQEVCTSMNCHTMIELSVLDEDEGWDLFKQRAQIDDDSP
EEMREVAKRVFDKCEGLLVDILAVARTLKGKTFTSWELALFRVETSESIDVQKGLNSTYECNELEEIICLDWKDAGQLRYL
YAPSQPVFPKLKKILIKGCIKLTKIFFPSMVSSLPELRELSVQDCNELVEIVSSEEARQLANLSFQSQQVSFPKLWVIEIERCN
KLKSIFFATIVSSLSELTQLSIRNCNNLEVIISSDSEETKKHRNVNAPFSQFFPKLGRISIENCNKLKTFFSAITVTSLPMLQQLI
VKDCNTWEEIISLDSDQAGQFRNLYTLYNQEDCFPKLGSVQIERCNSLKTIFSMEFVSTLPELLELTVKDCDEWVKIMSLG
LKQAKQAGNLSAHSKQICFTKLQKIEIESCKRLRTVFFTNVVRSLPELEQIVVKNCDEWEGIVSSDSEEERQYRFLSASSQE
VCFPKLRKIEIEKCNKLKAIFSTTIVTSLPELEQLIVKDCDEFEEIISLDSDEAGGLKNSYAPSQQVFFPKLGSISIERCSKIKRM
FYMVIVSILPMLEQLVVKDCNEWEEIISLDSVEASELTNVSAPSHHICFPRLRKIEIEGCNRLKAIFSSTTVTRLPMLEQLFV
KNCSGCEDIISLDSKEASQCTNQLAPFQQVCFPKLMKIVIECCNKLKAIFFTTLVTRLPRLEHLSIDNCNELEEIVSLDSVEIS
QLRNLCDPSQQSFFPVLQRILINRCNKLKKIFSAKIVTTLPELVHVWINNCNEWEEIISSDSEESSKQGNLSAASQQVFFPK
LESLYIEKCNKMKTIFCATVVTSLPKLRRLNVKGCNEWETIISFGSKKVGQNKNFLNPCQSKCFPKLQSIWVEECNKLKTIF
FLSIVSRLPNLESLFVDNCNNLEAIISPYSMEATQSRNLSGRPNQVRFPKLGSIVIRRCSKMKNLFSTITLSRFPKLKQLFVL
DCNELEDIISSEAKPLRNLSTSSQQIYFPTLQNIQIERCNKLKTIFSFIIVRSLPELECVSVEDCNELKEIFFFGSEEEGQVENIS
DPSQHVCLPKVRSIKIKTCNKLKFIFPYSVACQCSSLNLLDVESCPQLAQIVKFEHEGTSEEEGSGVAIDDNHAKHLLFPNL
SSLELKKLPTITGTFPWYEPQNCHLIIQECPKYLWFT

>XP_027931211.1

MAETAASFASRHVFPKFLEAVKMLRDLPKEVVEVADELESFQDFIHDANKVAEAEEDKNRRDRIRKRLMRLREAAFRM
EDAIDDYVICDDEKQPEEDPRCATLLCEAVEFIKTQILRLQVAYQIQDVKSLVRAERNGFENHFPIGSRSNGSRGNENFT
WHKLRMDPLFIKEEEVVGFEGPIHALKKWLTEGQEERTVISIVGMAGLGKTTLSKQVFDRVHTDFECHALITVSRSYTVE
GLLRDLTNKLCKERMEDPPRDVATMNQMSLIEEVRNRLHNKTYVVLFDDVWNETFWDDIELALINNKNGSRILITTRD
EKVAEYCKKALFFEVHKLQPLSKEKSLELLCKKAFGYGFDGCCPKDYEEVGLDIVRKCECLPLAIVAIGSLLYRKCKTPPEWR
RFSQNLSSELESNSELHSVTKILSLSFDDLPQNLRSCLLYFGMYPDDYEVKCGRLIQQWIAEGFVKQESGRNLEEVAQQH
LMELVSRSLVLVSSFTKDDKARACRVHDSIHEMIRGKMKNTGFGEYIDEDNHLESSGIVRRLTIATNSNDLNGSMEESQ
HVRSIIIFRRRDVKFTDRLLAKYRRLKVLDFGYAPLYDVPENFGSLIHLKYLRLRNIMIRSLPKSIGKLKNLEILDVRTHRVIEV
PKEITKLRKLRHLLGSPISSVAVKDSLGSMTSLEKMHVLIVDEDGVVIRELGKLKQLRNLRLSNVMGNHSDTLSFSINQM
QLLERLDISVQYLSEVIDLHITPSLSKIRKLHLYGNLKEFPSWIPRLQNLVKLSLVESRLTNIPLKSLGNMPNLLFISFDYNSYE
GDTLYFENGGFQKLKELELKCLDNLSSIFIDIGALQSLEKLKIMTIPQLKRVPSGIQNLKKLQVLDILYMPREFQKRIDPNGG
EEHWMIKHVPHIHFVMKDRGLLMVHKVAQILSSRLQRKKATVQFEADNETQIKRIT

>XP_027931640.1

MAETALSLARQHVFPKLLEAVNMMRDLPKEVAELKDELESFQYFINETDEVVEAEEDSNRRDRMSKRLMKLREATFRM
EDVIDEYVLIEENQPQEDPRCVALLCEAVEFIKTQISRLQIAHKIRDVKSLASAARDGFETHFPLDPRPSNSRGNENVNWD
KLRMDPLFVKEDEVVGLDGPRTILTNWLREGRNGRTVISVIGIPGVGKTTLAKQVFDKVHNDFECHALITVSQSYSVEEL
LRDMIQKLCKERKEDPPQNVSKMGRSSLIEEVRDRLHEKRYVVLFDDVWNEKFWDEIESALIDDNNKSRIIITTRYEKVA
VVCKKSSFIEVHKLEEPLSEDESFRLFCRKAFMYGSSGGCPEELKDISLEIVRKCKGLPLAIVAIGGVLSQKDESPHEWKLFN
QNLSLELERNSELNNITKILGLSYHDLPMHLRSCLLYFGMYPEDCEIESDRVIRQWMAEGFVRHEIGKTLEEVAEEDLLGLI
RRNLVQVSSSSIDGKVKRCRVHDLIHEMILRKAKDSGFCGYIGRNDECVSSGIVRRLIITAEDDLIASVESSLIQSILFIEERRT
TLTEEDFVRKIISSSVWTEDMVRKILANYRSLKVLDFEGAYISCVPENFEHLIHLRYLSLRRTGAMSLPSIGKLHNLETLDIR
DTNVRYIPTGVYNLRKLRHFLADEVTEIEWMYIGRLKSLQKIPIVSTTDLGEAIREVGKLKQLRVLRVKHIGKDDEKTLCSAI

NEMQQLEALHIEAADYYISDVIDLNITSPLSRLRKLFLDIKLKGLPNWIPQLQNLVKLNLRRTVLTNDELQSLKDMPRLLIL
HLCFGFKGETLHFQCGGFQRLKKMHLDYLLNLNSILIDSGALHSLEYLWLKRLRKLKTVPVGIQHLKNLKLVQITSMPTEF
ELSILPIAEQQQWILQ

>XP_027931835.1

MAENAVTLIALLAEKLLKLVKDEGRILRGVHSDVKHIENLLNQIKPLTKDAVEKVLNEGVKHWMNDLRNVVFRMEDVV
DLYLFKVAKRDGVRFGMTKLKGKIRSVKHRHRISSEIGDIKQTLSNIFILSTSLGLQPFHGQTLPDEIPRLGAHLVEESQLVSI
EHNMQNLTAWLRQNNSPVLVVVGPPGIGKTTIVKNVYNKQTKLNQQKKKHKKDFDFFVWITMPRTQADSLYPIRQIK
ENILLADPHGSTSLRNATMEDLAQRLREYLTGKTCLIVLDDVRELKIWNVIKFAIPQHRVIITTQSANFPNNIGTDTTVEEF
RLGPLSLEHALMLFHQKVKHVKFPELSDLSKKFMEKCNGVPLAIVAISSLLSTKKSAIEWKRVLENLGSLLKSNPHLEMVQ
HVKLQSYQELPFRLKQCFLYFGLFPQGYSISCKRLIRLWIAEDFVEGDTQNQNTSMEEFGDEYLAELICRGLVHGSRLDFD
GRPRSCHVYNLMHETIARICEDQMFCHVMEDASTPVNSNMDFILRRLSVIRKNGSATMERDHKWGKVRSCFVFDDA
KKFQVNNHFFSSFEFLIRLDLSDACLSVDVLPEQVGNLFNLKYLSLRNTNIKSLPKSIGNLENLQTLDLKQTKVHEVKINKLV
KLRHLLAYYVSDQSSELYCLEGLRLSEGVQNLESLQNLSYLDVSGGSIITGLQKLTKLRKLGIIKLEAQHGEALCNSIEHMM
NLCSLSIGALGKEGMLKLESLRYPPLSLKRLYLYGRLGTLPTWISNLPNLIRLYLKWSDLKQDPLDYLKELPQLLHLELYDAY
KGEKLHFRNGWLKLKVLYLGLLPKLKSIEIGKGKVPCLEVLKIGRCHQMIRLPRDIQNLKHLEKLYLYDMHEQFVERLCDE
RSEDYWIINKIPLVEYSNNDHFAFFS

>XP_027931946.1

MVEQKVTGVVASSSYSSSVVSSKRYDVFLSFRGEDTRKKFTSHLYDALKQKKVETFIDNRLEKGEEISTTLIQVIEDSHISIVI
FSENYASSKWCLGELRKIMECKKEKGQIVIPVFYDIDPSHVRKQTGSYEKFFVTHKEEPMCNKWKAALTEAANLAAWDS
QTYRVESELLKDIVEDVLQKLAPIYPNCHKGLVGIEENYEKIESLLKVGSNEVKLLGIWGMGGIGKTTLACALYDKLSHEFE
GHCFLENVREESDKHGVKALRNKLFSELLGKKNHCFDVAFSVTKFVLSRLGRKKVFIVLDDVATSEQLENLIEDFDFLGLG
SRVVVTSRNKQIFSQVDKIYEVKELSFHHSLQLFSLTVFREKQPKHGYEDLSRSATSYCKGVPLALKVLGASLRSRSKGAW
ECELRKLQKFPNKKIHSVLKLSYDGLDHSQKDIFLDIACFFRGNQRDHVTNMLEAFDFSAISGIEVLLDRALITISGGNQLE
MHDLIQEMGWEIVHQECVEDPGRRSRLWKHEEVYEVFKYNKGTDVVEGIILDLSTLIEDLYLGSDFLAKMTNVRFLKIHS
WSKFNIFNVYLPNGLSTLSHKMRYLHWDGFCLESLPSIFCAEKLVELCMRCSKLKKLWDGVQNLVNLKTIDLWGSRDLT
EIPDLSMAEKLENVSLCYCESLCEVQVHSKSLRVLNLYGCSSLRKFSVTSEELTRLSLAFTAICSIPSSIWHKRKLKALYLTGC
RNLHELTDEPRIHGSHKHSHTALASNAERLSMNIKSLSTLRMLWLDDCKKLVSLPKLPPSLEKLSASNCTSLDSYMTQWL
VLQHMLQSRIPYLRKNYLRCYDEEYLFPGDHVIDECAFHTTETSITIPYLWKTELYGFIYCIILSKGSLLQSDVSCSVYQDGIR
VGWLQKLLEYESLTSNHVLYMYHDINEFDAIAEVHGHFFSNVAFIFENSEASIEEFGIFPIYGSESGLKLVGSREIFESKFIDS
QVNICQNST

>XP_027932481.1

MMALAMVGEALISACVEALIKRIASRQFRDFFSSRKLNISLLDELKTTLLALSAVLNDAEEKQITEPAVKEWLDELRDAVL
DAEDLLDEINTHALRCKGVGESTKFVTKVRSLLSSRFKNLYMGMNSKFEAISRRLEQFVRQKDILGLQSVSRRVSYRAVT
DSLVESVVVARENDREKLLSMLVCDDGDGMCNDVEVITVLGMGGLGKTTLVQCLYNDSEVQKHFDLTAWACVSDDF
DILNVTKKIVESLTSKDCHITNLDVLRVELKNNLRDKKFLLVLDDLWNEKYNDWHNLIAPFRSGRKGSKIIVTTRQQRVAQ
VTHTFPTYELKPLSDENCWRILARHAFGSENYHNYPILEEVGKKIARKCNGLPLAAKTLGGLLRSNVDVVEWNRILNSNL
WAHDDVLPALRISYFHLPAHLKRCFAYCSIFPKQHLLNRKELILLWMAEGFLQHIHEDKEMESVGNDCFNELLSRSLIQK
DNAVAEENFRMHDLIYDLARLVSGRSSYHFDGSEIPRTVRHLSFLREMFDISEKFEGLYELKCLRTFLPRLSYPFVQCYLTK
MVSHGWLPKLRCLRILSLSKYTNITELPNSIGNLLHLRYLDLSYTSIESLPDETFMLYNLQTLILSNCESLIQLPQKIGNLTNLR
HLDISDTNLTEMPTQICKLQELRTLTVFIVGRQGGLSIRDLSKLPYLQGKLSIMNLQNVVNLVDVFGANLKKKEQIEELILG
WGSDPHEPQFEKDVLDNLQPSINLKKLSIKCYGGTSFPNWIGHFSFSKITVLTISDCNNCLSLPPFGQLPSLKELVIKRMK

MVKKVGDEFYGSNVGSQLFQPFQSLENLEFEDMSEWQEWLPFESENRNFPFPCLKTLYLYKCPKLRGTLPTHLPSLTNV
IFSECNQLVTKLSDVHWNTSIEAIHIREGQEALLSMLDNFSYCELLIEKCDSLRCLPRMPLAANCLQKLTLTNIPSLIYFPAD
CLLTSLRSLDIWHCRNLEFLSHNTCPKFKSLETLRIWNSCSSLTCFSLAFLPVLQELHIRFIPNLEAITTHGGEAAPKLVDFIV
TDCEKLRSLPNQIDLPSLEHLDLSGLPMLESLSPRCFPSSLKSLHVDIGILSSVSKKELGVLFQRLSSLSHLLVKGLGDEDLVN
TLLKQQSLPTSLEYLFLNNFSGLKLLEGKGLKNLTSLQMLQMYNCPSIESLPEGQLPHSLQVLSLRECPLLEARYQNHKYW
SKIAHIPAIKINEKVII

>XP_027932591.1

MAETAVSFVFPKFLEAVKMLRDLPKEVAEVTDESESFQDFIHDANKIAEAEEDNNRRDRMRKRLMRLRKTAFLMEDIID
DYVICDEKQPEEDPSQEDPRCAVLLCEAVEFIKTQILRLQIAYRIQTVKSLARAERDGFENHFPIGSRSDDSRGNENFTWH
KLRMAPLFNKQDEVVGFEKPIETLKKWLTQGRKERTVISVVGMAGLGKTTLSKHVFDKVDKHFECHAVITVSRPYDVER
LLRDIMKELCQERNEHPAQDVETMNRMSLIKEVRKRLSNKRYVVLFDDVWNETFWDDIELALIDDKNGSRILITTRDEK
VVEFCKQALFFEVYKLQPLSKAKSLELLCKKAFGYAFVGRCPKDYKEVGLDIVRKCECLPLAIVAIGSILYRKCKSPYDWRM
FSQNLSSELQSNYELQSVTKILSLSYDDLQQNLRSCLLYFGMYPEDYEVNCGRLIRQWIAEGFVKHENGRNLEEVAQQYL
MELISRSLVLVSSFTTDGRAKACRVHDLVHEMVRRKIKNTGFCEYIDEHNHLESSGNIRRLTIATSSNGLSGSMEESQHV
RSILIFTNEVSSKDFTSALLAKYMRLKVLDFEFTPLYDVPENLGCLIHLKYLSFRETCIRSLPKSIGKLQNLETLDVRTHMVIEV
PKEITKLRKLRHLWGSPISSTSLKYSVGSMKSLEKMNELRIDRNGEVIREIGKLKQLRDLRVVGFRRGHAETLCSSLNEMP
LLERLHISSSEYVRFSGDFSSSLRKVTVVKLLDRREIDFHITSSLSKLRKLHLYAVLKEFPNWILRLQSLVKLSLVESKLTNIPLK
SLGNMPNLLFLCFDSRCYEGETLHFENGGFQKLKELELKGLEQLRSIFIERRALQYLEKLHIKTVPRLKTVPSGIQHLQRLQ
VLHILYMPREFLHRINPDGGEEHWMIKHVPYVHFGRTYRKKRKEASNCAA

>XP_027932592.1

MAETAVSLASQHVFPKLLEAVKMLRDLPKNVAEVTDELENFQDFIHDANKMADAEEDSNRRDRIRKRLMRLREASFR
MEDVIDDYVMICDENQPEEGPRCAALLCEAVEFIKTQIHHLQIAYQIQDVKSLVRTEREGFINHCPIEPRSHGSRGKENFT
WHKFRMDPLFIKEHEVVGLEGPTQTLKKWLTLTQGREERTVISIVGMAGLGKTTLSKQVFDRVRTDFECHALITVSRSYT
VEELLRTMTNELCKERKEDPPRDVSTMNQLSLIKEVRNRLRNKRYVVLFDDVWNETFWDDVELAMIDDKNGSRIIITTR
EEKVAEFCKSCLLYRLQPLSEEKSLELLCKKAFGYGFHGCCLEDYEKAGLGIVRKCGYLPLAIVAIGSLLYRKCKSPYEWRLFS
QNLSLEMESNSELDSVKKILSLSYDDLSQNLRSCLLYFGMYPEDYEVKCGRLIQQWIAEGFVKHERGRNLEEVAQQQLM
ELISRSLVLVASFTTDGKVKACRVHDSMHEMIRGKMKNTGFCEYIDEHNHLESSGITRRLTIATSSNINGLSGCIEGSHVR
SILILTNEVSSVDFNSRLLAKHTRLKVLDLEFTPLHDVPENLGCLIHLKYLSFRETFIRSLPKSIGKLQNLETLDVRTYMEIEVP
KEITKLRKLRHLLGSTISSSSLKDSLGSMRSLEKMHELGIDEDGVVLRELGKLKKLRDLRLVSLRRDHAETLCSSLNEMPLLE
RLHISFAEYLRLSNFTEDPAALSSSIRKIQVRRPLESGVIDLHITSSLSKLRKLYLYANLKEFPNWISRLQNLVKLSLVESKLTNI
PLTDLGSMPNLLLLSFDSNSFEGETLHFENGGFQKLKELEFKCLYNLSFILIDSGALPSLEKLQIVAIPQLKIVPSGIQHLKKL
QVLYIVHMPTEFLQRIDPEGGDEHWMIKHVPYVHFSHYIS

>XP_027932602.1

MAETAVSFASQHVLPKLLETVKMLRDLPKEVEEVTDELKSFEVYINIDANRVAEAEKDRDTSERIRKRLMRLREAAFRME
NVIDDYVMICDANQPEEGPRCAALLCEAVEFIKTQIHRLQIAYQIQDVKSLVRVERDGFKKDFPTEPISNGSSGNENFTW
HKLRMDPLFFKQDEVVGFEGPIQTLKKWLTEGRKERTVISIVGMAGLGKTTLGKQVFDTVDKHFECHAVVTVSRPYDVL
RFLRELTKKLYRESKEEPPRDVATMDRMSLIEEIRNRLCNKRYVVFFDDVWEEKFWDDIESALIDNKNGSRILITTRVEKV
VDFCKKCLFFEVHKIKPLSKEKSLELLWRKAFGYDFDERCTKDYKEVGLDIVRKCECLPLAIVAIGSLLSTKPKSPSEWGRFS
KNLESNPELDSVKKILSLSYDDLPQNLRSCLLYFGMYPEDCTVKSGRLIQQWIAEGFIRYENGKTLKEVAEKYLMELINRSL
VQVSSFSTGDRAKECRVHDSIHEMIREKMKNTGFCEHSDDEHNQSESSGIIRRLTIAKSSYDLSGPSEGSQYVRSILILTKE
VLSEQFSRALLAKYTRLKVLDFGYARLTEIPENLGSLIHLKYLSFQSTSISSLPKSIGNLQNLETLDIRTGMRIELPKEITKLRKL

RCLLGKPLSSISVKDSLGSITSLEKMHVLLIDPDGVVVRELAKLTKLSDLRVSDVKGDHANTLCASINEMPLLERLHISLNYG
AREIDLHISSSLSELKKLHLCANLKEFPNWILSLENLLKLSLVDSRLTNIPLISLGNMPNLLILSFGPRSFEGETLHFQNGGFR
KLKELKLKRLYQLSSIFIDSGALQSLEKLEIFSIPQLKTVPSGIQHLEKLQVLDILRMPTEFQQSIDPNGGEDHWMIKHVPHL
HFVTKNRGLLKIQELANILASQRKMKQIM

>XP_027932774.1

MLGLRKGIFAGNEVGVSWKQSQQLPTTSLVDESSICGREEEKEEIIKILLSDNVTCNQVPIISIVGMGGMGKTTFTQLVY
NDQRVLDQFDLIAWVYVSQDFDVVAVTRAILKALGSKGAEEKDLNLLQLQLKQTLMGNRFLLVLDDVWNEDYARSRIII
TIRNEKVALIMNSSHLYHLKPLEKEDCWKLFSDVAFHDKDATKYPYLVSIGNKIVDKCGGLPLALKALGNILRVKFSQHE
WVKILESDMWHLFDNDANINPALRLSYHNLPSYLKHCFAHCSIFPKGYEFDRDQLIQLWMAEGLLSCCQINMSEDELG
TEFFNDLVARSFFQQSRHRASCFTMHDLLNDLAKSVSGEFCSQISDSLEKNITKRTCHISWSHKINIDDKFLEHTLLLLWC
YHLVELPIDLHKLVNLRHLDMRMSGINKMPNHIGRLKHLRTLNSFFINKHDVKELGNLNNLQGTLSIFRCNENEDSILER
QVLEALQPNGNLKKLSVLRYDGTSFPGWFGASHLPNLVSIALTESKFCFVLPPFGQLPSLKELSISCFYGIEVIGPEFCGND
SSNIPFRSLEILKFEEMSAWKEWCSFEGHS

>XP_027932848.1

MSFRTNPKKAIPFVLKRLRMTRTTCKTLDKSCDDKLEKLTSQIDQIKDLFMAVKGNEDELLDTLALLDRHLRTIGNIDKEK
FQSDMETISQRIKDSTEKLLPKGWASQGETQASVSPKVTTSSSQDHKTAELHSIPSSSQHKDELHEENLSQKVEVSFEKLE
THLKPCLLSLLVFPEDAVINKRHTIYWWIGEGFVRSSGEKTAEEVGEGVVEELLNCQMIVAYGNGLNPVVKKFKINPRIR
VELMTLDSEKTLSFQEFSNQRAWLEQRKVVLGGDDKDNLKPKQWKTIFNVGASYLSFEPEWLAKMKSLEVLQLGRWQ
DSPSHHIEVASEEFLNELRVHKRLKYLSLRGISRIPELPPSIAQLENLEILDLKACHSLEALPTNIASMKSLTHLDVSECYLLDS
MPKGIEKLTQLEVLKGFVIGSSSKTPCRISDLANLKKLKRFSVHIGSEAVIQEMEFESLKELSAVKCVKISWGVSGEKYSDIQ
VLFPSSLEKLDLEGFPGSAIPEWLKPSRVPGAMRKLYIKGGKLKSLDHGEICHKWHVEILRLRYLKQLQIEERKLHKLFPSLR
YVERTKVLNHSFPEWRLEE

>XP_027932849.1

MSIRTNRMKAVPSLQRRLTSVKSREEPNISDKLDKLMADLTAIKDLFSTVKSNEDELLDTLKLVDDLLRNFKTDKFFDISKR
ILETDKPTNADPQKGINEPSSSQKAEEKSGKRDETIDEKMENLEYTLENLKANLSTLKKNEELRRKLREVENFLQNFNSKL
AEDANENETKGLEQPTQTSREEMNQDLTGGTDKPSSSQESRDKKIVGKVKSSSAISSEDSKQSETKSSELPRQKSRDESD
QDLKATGIGKSPPSEEEEDKKSIVEKDVGIDERFESLKFKLDKMKAMSSTAKEDKELREKLRVMEDLLRNFNAKNLEETLS
SIEDSNQSEKKSSELRSQTSRDELVDQNLKTDGVDKSSSTQESEGKTNLEKDVPIDEKLEVLKSELKTMMTRFSSVKENEE
QRDRLRTLEDLFQNLEEIVKSAEDANQSETKGSEQPNQTSRDELVDQDLNANGIEKPPSSQETQDKKSNVEKDKTIEEKF
ESFMSKLDKIKGMPSTTKESEELKQKLRDVEDLIRKFNSNQSETKNLELPTQRKSDDESMDQGLKATGVDILSSTLESKEE
TLPEKYVPIDEKLESLKLELDNMKAKFSPVKENEEMRKTLRTLEDHLQSFDIPAEDANPSGTKGSELTSQISRGELEDQVLK
DRQQEGRFKKLEENEENLLRNTNKTVSDENVERKVSKEEVVDKVVEAINKKIKKSTKKLGVSETETKANESSEKGKTSKTV
HYPILSLSRRHEDVELVLKRFQASYDALNPHSKVCCLSLSIFPENFVMMKRNVIYWWIGEGFVKKSTEKTSEEEGEDVFD
ELLNSKLIVPHGTGKCPIVNKFKLNPWIRHMLVSSVLAENKQPFRFYSQITTSSHQNHADYGCLVLDQTKVQIGGDFASK
SDHWRSVFNLHASYLTIEPQWMAKMKKLVVLQLGRWQESPKHHIEVAGTEFMTDSKAQKHLKYLSLRGISRISALPPSI
AQLVSLEILDLKACHNLETLPNEITSLKKLTHLDVSQCYLLESMPKGIGKLSELQVLKGFVVGSSNKTPTVSDLANFKKLKRL
SIHIGSEAVIQDKEFESLSNSQVKCLKISWGVSSNTKYKDIEIVLPQGLEKLNIEGFPSEQTPTWLMINHLPLALKKLYIIGGK
LRSIHNENNRALEYCKVEILRLKYLKNLQIDMEHLRELFPSLRYAEVKQVSNLYDEWSIMDG

>XP_027932850.1

MASDTAEILKQNFLEFFKDIELFNLSPPITSLLTEINKEFVENTGFWSPPPVESLCHFYQLDHAIGECRINKKTFSIFSRFDPV
ERTNDVLKDIKKNLQVVRESKNNINEYSTSPLFSSCRYPTRNDAPDLSVFDGEIEKIVKRLESNDGFKAIGVHGMCGTGKT
TLAKMVLSDPRVREKYKKPIWVCLYDMQSEEEMDIRIVKEMLALLDDDPDLLAEEPEDKWLLKNLHDKLLHQEKYLIVL
DSVWHCNDWFNDLFCVDQNDGDASKELFSQALPKDTGGAVIVTSRQKAVTTTLVHEENLIHVKPWDDEKLKKLVKQC
LKRQEKSYVEITEENINCVAYHCHGIPFVAATLSGWIAEQIIKKSDSN

>XP_027932852.1

MVFIPVTDKPNQEQVQNAIADELGVQFTNGESLVKRRNKLRQRIKREQRTLIIVDDTWGELNPQEFDLEEFGVPPGNE
HEGCKVLLTSGNLNFIQYLKGASKLTKVFQLQELQKEEAQMLFEKMVGSVDEDQESSIVEEIVRSCEGSISLIYALAKALQ
NKGPDALMQLKENISPAKLLSYCLEENEELKSLLYLLTIRGRRFINSYSIYIDMWAGVFKNLETADSARKKRESLISDLKAYG
LVVENGKDWVKVDDYIHQTAYRMAQHDRRASVISREWPPEELLKDLYFCNLHPVGDLKLPVRLQCPNLKHLLISRENST
IDVPDSFFEETKLLKVLDFVSFHCPNLPLSFVVLKDLEALSMYQCGLEDITEVCELTNLRMLGLLESRIQQLPAQIVKLEKLL
FLDLRDTNLQVIPPNVLSKLTSLEELYLRNSFCNWKIEMSSSENKNASLKELTDLEHLAYIEDMYVPDPQAWPVDLFFGN
LRSYTIFIGNGWDRAHYGDHELKTLKLKLNRRFQSENGIKKMLKEVQVLYLDTLNGVQNVVNDMECDGFPQVQSLFIQ
HNAEVKCLSTGSGNDSLDTFPNLESLSLTILSNLEYICHGGPLSEKSFFKLRVIKVEKCNAMGCLFSKSMINGLPHIATLKVS
QCTSIKTIVLFEGAENHPIEFPELCSLTLQGLPALISFCSSEGSSSSTDNTATLFHDKVSCPNLETMVISQVSELTTIWNEEYD
AENSFWKLKNVNIKGCEKLRTVFPVNLSKNLDNLKMLEVRNCSSMTSIFTVMRQDSTKPGLQLSFPMIEITLTGLPKLEY
VCVTTGFEALKKKFEEEWYAGLPGLSGNARIEHGRKMKKYLEEYLNM

>XP_027933012.1

MAETAVSFSSQHVLPKLLEAVKMLRNLPKEVAEVIDELESFQDFIDDEDKVAEAEHDHNRRDRIIKGLTRLREAAFRMED
VIDDYVICDEKQPEDPRCAALLCEVVEFIKTQIHRLQIAYQIQDVKALLRAERDGFENRFRTGSRSDGTRGNENFTWQKL
RMDPLFIKEDEVVGFEEDIDTLKKCLTEGRKERTVISIVGMAGLGKTILSKQVFDRVHTDFECHALITVSRSYTVEGLLRD
MTNKLCKERREDPPRDVAFMDQMSLIKEVRNRLHNKRYVVLFDDVWNETFWDDIELALIDNKNGSRILITTRDQKVVD
FCKKCLFFEVHKLQPLSNSKSLELLCKKAFGNGFHGCCPKDYEEVGLDIVRKCGCLPLAIVAIGSLLYRKCKSPSDWGLFSQ
HLSLELESNSELDCVKKILSLSYDDLPQNLRSCLLYFGIYSKDYKVKCSRLFQQWIAEGFVKQERGRNLEEVAEQQIMELIS
RSLVLVSSFTTEGKVKACHVHDSIHEMIREKMKNTGFCHYIDEHNDFESSGMIRRLTIATNSYDLSGSSEGSQYVRSIFIFT
NELSSEGFTRCLLAKYTRLKVLIFEIAPLYDVPENLGSLIHLKYLSFRNTSIRSLPKSIGELQNLESLDVRTNRVIEVPKEITKLR
KLRCLFGYRISTIEVKDSLGKMTSLEKMHELRIDEDGVVIRELGKLNQLRDLRLSNFMGDHSDALSSSINEMQLLERLDIFV
QYMSEPIDLHITSPLYKLRKLHLFGILKEFPSWIPRLQNLVKLSLKLSMLNNIPLKSLGNMPNLLILSFDSGSYEGETLHFEN
GGFQKLKELRFRALYNLRSIFIDKGALQSLEKLHMFDLPQLKAVPSGIQHLKKLHVLDIFHMPTEFHQRIEPETGEDHWM
VKHVADVLAGSDFKNQLMYDLSKNIFMEGVADILRYIDGIEVLASSASGVVGGDDPFQIKL

>XP_027933161.1

MAETAVSFVFPKFLEAVKMLRDLPKEVEELTDELESFQDFIHDANKMAEAEEDNNRRDRMRKRLMRLRKTAFFMEDVI
DDYVICDEKQPEEDSRCAALLCEAVEFIKTQILRLQIAYRIEKVKSLAHTEKDWFENHFPIGSRSDGSIWDNIRMEPHFIKE
DEVVGLEGPIKTLKKWLTEGRKERTVISIVGMAGSGKTTLSKQVFDKVHKHFECHVLITVSRSYDIEKLLRDLTNKICEERK
EDPPRVESMDRMKLIEEVNKLLCKKRYIVLFDDVWNETFWDDIKFALIDNKNGSRILITTRVGKVVDFCKNSSFVEVYKL
QPLSKEKSLELLCKKAFGYGFDGCCPKDYEEVGLDIVRKCECLPLAIVAIGSLLYRKCKSPPEWRRFSQNLSSELQSNSELQ
SVTKILSLSYDDLPKNLKSCLLYFGMYPEDYEVKCGRLIQQWIAEGFVKQDCRRNLEEVAQEQLMELISRSLVLVASFTTD
GKVKACRVHDSIHEMIREKMKNTGFCHYIDEHNDFESSDIIRRLTIATSSKCLSECREESHVRSIIFFTKEDLSEDFTSALLAK
YTRLKVLDFEFTRLWDVPKNLGSLIHLKYLSFRKTSISSLPKSIGELQNLETLDVRTGEEIEIPKEITKLRKLRCLFGYRISTIEVK
DSLGKLTSLEKIHELVIDEDGVVIRELGKLNQLRDLRLSNFLGRHSDSLSSSINEMQLLERLDIFVQYMTEPIDLHITSSLSKL
RKLHLVGTLKAFPSWILQLQSLVKLYLKYSMLNNIPLKSLGNIPNLLILTFASRSYKGETLHFENGGFGKLKELQFKALYKLR

SIVIDSGALQSLEKLHMFRIPKLKTVPSGIQYLQKLQVLDVLYMPTEFQQRIYPETGEDHWMIKHVPDVLRMLNFNDQL
LLDLVVKIGEEREILRAIEWMERASRKSASQVVGGG

>XP_027933268.1

MMSDVASSSNSSKKYDVFLSFRGEDTRMNFTSHLHEVLKQKKVETYIDYRLEKGDEISPALIKAIEDSHVSIVIISENYAFSK
WCLEELSKILECRKIQGQIVIPVFYNIDPSHVRKQTGSYEQAFVKHQEDFRCNKWRAALAEVANFSGWDSRNRIESELL
MDIVGDILRKLTARYPTQLKGLVGIEENYEQIESLLRIGSREVRTLGIWGMGGIGKTTLATALYAKFSPEFEGGCFLTNVRE
NSSRQGGLEALRSKLFTELLENENHCFGSPLLVPQFVMSRLGHKKVFIVLDDVATSEQLECLIIDYGLLGPGSRIIVTTRDK
QIFRPNDEIYEVKELSIYHSLELFSLTAFEEKIPKQGYEDLSRRAISYCKGIPLALKVLGASLCRRSKEAWVSELRKLKKISNME
IHNVLKLSYDGLDRSQKDILLDIACFLKGEHKDRVTNLLEACDFFAASGIEVLLDKALVTISYSNNIQMHDLIQEMGQEIVD
QESIKDSGRRSRLWRPEEVHQVLKHNLGTEVVEAITLDTCDLNRDLNLSSNSFTKMVSMRFLKIHSSYYSSQFNVHLPSG
LESLSDKLRYFRWDGFCHESLSSNFHAEYLVELDMRRSKLRKLWEGVQSLVNLEKIHLEASRDLVEIPDLSKAEKLKRIDLS
DCESLRKLHPSISSLPKLAHLELSGCRKIENLNVHSKYLQRLNLEGCSSLKELSVTSHKMVFLDLSYTAICSLPSSIQYNTELA
RLFLKGCDNLSFVQSPPNIIGHLFSLLLLDLSGTNVESLPASIKNLSMMKLLVLDDCRTLVSLPELPRSLEMLTAYNCTSLET
VFTQLLVSEHMLQSCKPYLSKQYYYPKQFEGGCAVFPGDHIMNDFGFHAEDSSITIPYLSLPELCGFICCFVLSEGSIEGHI
SCSIYQDSEQVGIDEGQLLHTALISDHVVFLFVEICDHFNGIPFKFQFNYYDIFRQKDERVRIKECGVFPVYASESGLKLFGS
DSTEFFELESITQSFDESQSRAIGVGVRCTNGENGLESFVEVSNKESQLREIGVGGSSNENENEWEHLLHVITSSVFF

>XP_027933269.1

MMSDVASSSNSSKKYDVFLSFRGEDTRMNFTSHLHEVLKQKKVETYIDYRLEKGDEISPALIKAIEDSHVSIVIISENYAFSK
WCLEELSKILECRKIQGQIVIPVFYNIDPSHVRKQTGSYEQAFVKHQEDFRCNKWRAALAEVANFSGWDSRNRIESELL
MDIVGDILRKLTARYPTQLKGLVGIEENYEQIESLLRIGSREVRTLGIWGMGGIGKTTLATALYAKFSPEFEGGCFLTNVRE
NSSRQGGLEALRSKLFTELLENENHCFGSPLLVPQFVMSRLGHKKVFIVLDDVATSEQLECLIIDYGLLGPGSRIIVTTRDK
QIFRPNDEIYEVKELSIYHSLELFSLTAFEEKIPKQGYEDLSRRAISYCKGIPLALKVLGASLCRRSKEAWVSELRKLKKISNME
IHNVLKLSYDGLDRSQKDILLDIACFLKGEHKDRVTNLLEACDFFAASGIEVLLDKALVTISYSNNIQMHDLIQEMGQEIVD
QESIKDSGRRSRLWRPEEVHQVLKHNLGTEVVEAITLDTCDLNRDLNLSSNSFTKMVSMRFLKIHSSYYSSQFNVHLPSG
LESLSDKLRYFRWDGFCHESLSSNFHAEYLVELDMRRSKLRKLWEGVQSLVNLEKIHLEASRDLVEIPDLSKAEKLKRIDLS
DCESLRKLHPSISSLPKLAHLELSGCRKIENLNVHSKYLQRLNLEGCSSLKELSVTSHKMVFLDLSYTAICSLPSSIQYNTELA
RLFLKGCDNLSFVQSPPNIIGHLFSLLLLDLSGTNVESLPASIKNLSMMKLLVLDDCRTLVSLPELPRSLEMLTAYNCTSLET
VFTQLLVSEHMLQSCKPYLSKQYYYPKQFEGGCAVFPGDHIMNDFGFHAEDSSITIPYLSLPELCGFICCFVLSEGSIEGHI
SCSIYQDSEQVGIDEGQLLHTALISDHVVFLFVEICDHFNGIPFKFQFNYYDIFRQKDERVRIKECGVFPVYASESGLKLFGS
DSTEFFELESITQSFDESQSRAIGVGVRCTNGENGLESFVEVSNKESQLREIGVGGSSNENENEWEHLLHVITSSVFF

>XP_027933270.1

MMSDVASSSNSSKKYDVFLSFRGEDTRMNFTSHLHEVLKQKKVETYIDYRLEKGDEISPALIKAIEDSHVSIVIISENYAFSK
WCLEELSKILECRKIQGQIVIPVFYNIDPSHVRKQTGSYEQAFVKHQEDFRCNKWRAALAEVANFSGWDSRNRIESELL
MDIVGDILRKLTARYPTQLKGLVGIEENYEQIESLLRIGSREVRTLGIWGMGGIGKTTLATALYAKFSPEFEGGCFLTNVRE
NSSRQGGLEALRSKLFTELLENENHCFGSPLLVPQFVMSRLGHKKVFIVLDDVATSEQLECLIIDYGLLGPGSRIIVTTRDK
QIFRPNDEIYEVKELSIYHSLELFSLTAFEEKIPKQGYEDLSRRAISYCKGIPLALKVLGASLCRRSKEAWVSELRKLKKISNME
IHNVLKLSYDGLDRSQKDILLDIACFLKGEHKDRVTNLLEACDFFAASGIEVLLDKALVTISYSNNIQMHDLIQEMGQEIVD
QESIKDSGRRSRLWRPEEVHQVLKHNLGTEVVEAITLDTCDLNRDLNLSSNSFTKMVSMRFLKIHSSYYSSQFNVHLPSG
LESLSDKLRYFRWDGFCHESLSSNFHAEYLVELDMRRSKLRKLWEGVQSLVNLEKIHLEASRDLVEIPDLSKAEKLKRIDLS
DCESLRKLHPSISSLPKLAHLELSGCRKIENLNVHSKYLQRLNLEGCSSLKELSVTSHKMVFLDLSYTAICSLPSSIQYNTELA
RLFLKGCDNLSFVQSPPNIIGHLFSLLLLDLSGTNVESLPASIKNLSMMKLLVLDDCRTLVSLPELPRSLEMLTAYNCTSLET

VFTQLLVSEHMLQSCKPYLSKQYYYPKQFEGGCAVFPGDHIMNDFGFHAEDSSITIPYLSLPELCGFICCFVLSEGSIEGHI
SCSIYQDSEQVGIDEGQLLHTALISDHVKDERVRIKECGVFPVYASESGLKLFGSDSTEFFELESITQSFDESQSRAIGVGVR
CTNGENGLESFVEVSNKESQLREIGVGGSSNENENEWEHLLHVITSSVFF

>XP_027933275.1

MAETAVSFASQHVLPKFLEAVKMLKDLPKEVADVTDELENFQDFIRDAYKVAEVEKDHNRRERIRKRLIRLREAAFRME
DVIDDYVTCDEKQPEDPRCAALLCEAVEFIKTQIHRLQIAYQIQDVKSLVRAERDGFQNHFPTGSRSDGTRGNENFTWQ
KLRMDPLFIKKDEVVGFEEDIDTLKKWLTEGREERTVISIVGMAGLGKTTLSKQVFDQVHTDFECHALITVSRSYTVEGLL
RDMTNKLCKERREDPPRDVATMDQMSLIEEVRNRLHNKRYVVLFDDVWNETFWDDIELALIDNKNGSRILITTRDQKV
VDFCKKCLFFEVHKLQPLSNSKSLELLCKKAFGNGFHGCCPKDYEEVGLDIVRKCGCLPLAIVAIGSLLYRKCKSPSDWGLF
SQHLSLELESNSELDCVKKILSLSYDDLPQNLRSCLLYFGLYPEDHEVKCGRLFQQWIAEGFVKHERGRNLEEVAEQHLM
ELISRSLVLVSSFTPEDKVKACHVHDSIHEMIRGKMKTTGFCHYINDFESSGIFRRLTMATSSYDLSGTSEGSQYVRSILIFT
NEVLSEEFTKYLLAKYMRLKVLDFEFAALYDVPENLGCLIHLKYLSFRNTSIRSLPKSIGKLKNLESLDVRTNRVIEVPKEITKL
RKLRCLFGYRISTIAVKDSLGKLTSLEKMHELRIDEDGVVIRELGKLNQLRDLRLSNFMGHHSDILCSSVNKMQLLERLDIIL
QYLSEPIPIDLHITSSLSKLRKLHLVAFLKEFPSWIPRLQNLVKLSLKLSMLNNIPLKSLGNMPNLLILSFDSGSYEGETFHFE
NGEFQKLKELRFKSLHKLRSIFIDSGALQSLEKLHMFDIPKLKAVPSGIQHLRKLQVLDVFYMPTEFQKRIDPETGEDHWII
KHVPDVLVDLNFKNQQMYDLGKNIFMERPTFYGFLKGWRYLHRVPVELVGMIHSRSNYGVDFSYSFTPDRLGVAPAA
LDWKSYPFNMKSAFLNLFILAF

>XP_027933276.1

MAETAVSFASQHVLPKFLEAVKMLKDLPKEVADVTDELENFQDFIRDAYKVAEVEKDHNRRERIRKRLIRLREAAFRME
DVIDDYVTCDEKQPEDPRCAALLCEAVEFIKTQIHRLQIAYQIQDVKSLVRAERDGFQNHFPTGSRSDGTRGNENFTWQ
KLRMDPLFIKKDEVVGFEEDIDTLKKWLTEGREERTVISIVGMAGLGKTTLSKQVFDQVHTDFECHALITVSRSYTVEGLL
RDMTNKLCKERREDPPRDVATMDQMSLIEEVRNRLHNKRYVVLFDDVWNETFWDDIELALIDNKNGSRILITTRDQKV
VDFCKKCLFFEVHKLQPLSNSKSLELLCKKAFGNGFHGCCPKDYEEVGLDIVRKCGCLPLAIVAIGSLLYRKCKSPSDWGLF
SQHLSLELESNSELDCVKKILSLSYDDLPQNLRSCLLYFGLYPEDHEVKCGRLFQQWIAEGFVKHERGRNLEEVAEQHLM
ELISRSLVLVSSFTPEDKVKACHVHDSIHEMIRGKMKTTGFCHYINDFESSGIFRRLTMATSSYDLSGTSEGSQYVRSILIFT
NEVLSEEFTKYLLAKYMRLKVLDFEFAALYDVPENLGCLIHLKYLSFRNTSIRSLPKSIGKLKNLESLDVRTNRVIEVPKEITKL
RKLRCLFGYRISTIAVKDSLGKLTSLEKMHELRIDEDGVVIRELGKLNQLRDLRLSNFMGHHSDILCSSVNKMQLLERLDIIL
QYLSEPIPIDLHITSSLSKLRKLHLVAFLKEFPSWIPRLQNLVKLSLKLSMLNNIPLKSLGNMPNLLILSFDSGSYEGETFHFE
NGEFQKLKELRFKSLHKLRSIFIDSGALQSLEKLHMFDIPKLKAVPSGIQHLRKLQVLDVFYMPTEFQKRIDPETGEDHWII
KHVPDVLVDLNFKNQQMYDLGKNIFMEGWRYLHRVPVELVGMIHSRSNYGVDFSYSFTPDRLGVAPAALDWKSYPF
NMKSAFLNLFILAF

>XP_027933277.1

MAETAVSFASQHVLPKFLEAVKMLKDLPKEVADVTDELENFQDFIRDAYKVAEVEKDHNRRERIRKRLIRLREAAFRME
DVIDDYVTCDEKQPEDPRCAALLCEAVEFIKTQIHRLQIAYQIQDVKSLVRAERDGFQNHFPTGSRSDGTRGNENFTWQ
KLRMDPLFIKKDEVVGFEEDIDTLKKWLTEGREERTVISIVGMAGLGKTTLSKQVFDQVHTDFECHALITVSRSYTVEGLL
RDMTNKLCKERREDPPRDVATMDQMSLIEEVRNRLHNKRYVVLFDDVWNETFWDDIELALIDNKNGSRILITTRDQKV
VDFCKKCLFFEVHKLQPLSNSKSLELLCKKAFGNGFHGCCPKDYEEVGLDIVRKCGCLPLAIVAIGSLLYRKCKSPSDWGLF
SQHLSLELESNSELDCVKKILSLSYDDLPQNLRSCLLYFGLYPEDHEVKCGRLFQQWIAEGFVKHERGRNLEEVAEQHLM
ELISRSLVLVSSFTPEDKVKACHVHDSIHEMIRGKMKTTGFCHYINDFESSGIFRRLTMATSSYDLSGTSEGSQYVRSILIFT
NEVLSEEFTKYLLAKYMRLKVLDFEFAALYDVPENLGCLIHLKYLSFRNTSIRSLPKSIGKLKNLESLDVRTNRVIEVPKEITKL
RKLRCLFGYRISTIAVKDSLGKLTSLEKMHELRIDEDGVVIRELGKLNQLRDLRLSNFMGHHSDILCSSVNKMQLLERLDIIL

QYLSEPIPIDLHITSSLSKLRKLHLVAFLKEFPSWIPRLQNLVKLSLKLSMLNNIPLKSLGNMPNLLILSFDSGSYEGETFHFE
NGEFQKLKELRFKSLHKLRSIFIDSGALQSLEKLHMFDIPKLKAVPSGIQHLRKLQVLDVFYMPTEFQKRIDPETGEDHWII
KHVPDVLVDLNFKNQQMYDLGKNIFMERPTFYGFLKGWRYLHRVPVELVGMIHSRQVGSCTGGIGLEKLSF

>XP_027933278.1

MAETAVSFASQHVLPKFLEAVKMLKDLPKEVADVTDELENFQDFIRDAYKVAEVEKDHNRRERIRKRLIRLREAAFRME
DVIDDYVTCDEKQPEDPRCAALLCEAVEFIKTQIHRLQIAYQIQDVKSLVRAERDGFQNHFPTGSRSDGTRGNENFTWQ
KLRMDPLFIKKDEVVGFEEDIDTLKKWLTEGREERTVISIVGMAGLGKTTLSKQVFDQVHTDFECHALITVSRSYTVEGLL
RDMTNKLCKERREDPPRDVATMDQMSLIEEVRNRLHNKRYVVLFDDVWNETFWDDIELALIDNKNGSRILITTRDQKV
VDFCKKCLFFEVHKLQPLSNSKSLELLCKKAFGNGFHGCCPKDYEEVGLDIVRKCGCLPLAIVAIGSLLYRKCKSPSDWGLF
SQHLSLELESNSELDCVKKILSLSYDDLPQNLRSCLLYFGLYPEDHEVKCGRLFQQWIAEGFVKHERGRNLEEVAEQHLM
ELISRSLVLVSSFTPEDKVKACHVHDSIHEMIRGKMKTTGFCHYINDFESSGIFRRLTMATSSYDLSGTSEGSQYVRSILIFT
NEVLSEEFTKYLLAKYMRLKVLDFEFAALYDVPENLGCLIHLKYLSFRNTSIRSLPKSIGKLKNLESLDVRTNRVIEVPKEITKL
RKLRCLFGYRISTIAVKDSLGKLTSLEKMHELRIDEDGVVIRELGKLNQLRDLRLSNFMGHHSDILCSSVNKMQLLERLDIIL
QYLSEPIPIDLHITSSLSKLRKLHLVAFLKEFPSWIPRLQNLVKLSLKLSMLNNIPLKSLGNMPNLLILSFDSGSYEGETFHFE
NGEFQKLKELRFKSLHKLRSIFIDSGALQSLEKLHMFDIPKLKAVPSGIQHLRKLQVLDVFYMPTEFQKRIDPETGEDHWII
KHVPDVLVDLNFKNQQMYDLGKNIFMEGWRYLHRVPVELVGMIHSRQVGSCTGGIGLEKLSF

>XP_027933279.1

MAETAVSFASQHVLPKFLEAVKMLKDLPKEVADVTDELENFQDFIRDAYKVAEVEKDHNRRERIRKRLIRLREAAFRME
DVIDDYVTCDEKQPEDPRCAALLCEAVEFIKTQIHRLQIAYQIQDVKSLVRAERDGFQNHFPTGSRSDGTRGNENFTWQ
KLRMDPLFIKKDEVVGFEEDIDTLKKWLTEGREERTVISIVGMAGLGKTTLSKQVFDQVHTDFECHALITVSRSYTVEGLL
RDMTNKLCKERREDPPRDVATMDQMSLIEEVRNRLHNKRYVVLFDDVWNETFWDDIELALIDNKNGSRILITTRDQKV
VDFCKKCLFFEVHKLQPLSNSKSLELLCKKAFGNGFHGCCPKDYEEVGLDIVRKCGCLPLAIVAIGSLLYRKCKSPSDWGLF
SQHLSLELESNSELDCVKKILSLSYDDLPQNLRSCLLYFGLYPEDHEVKCGRLFQQWIAEGFVKHERGRNLEEVAEQHLM
ELISRSLVLVSSFTPEDKVKACHVHDSIHEMIRGKMKTTGFCHYINDFESSGIFRRLTMATSSYDLSGTSEGSQYVRSILIFT
NEVLSEEFTKYLLAKYMRLKVLDFEFAALYDVPENLGCLIHLKYLSFRNTSIRSLPKSIGKLKNLESLDVRTNRVIEVPKEITKL
RKLRCLFGYRISTIAVKDSLGKLTSLEKMHELRIDEDGVVIRELGKLNQLRDLRLSNFMGHHSDILCSSVNKMQLLERLDIIL
QYLSEPIPIDLHITSSLSKLRKLHLVAFLKEFPSWIPRLQNLVKLSLKLSMLNNIPLKSLGNMPNLLILSFDSGSYEGETFHFE
NGEFQKLKELRFKSLHKLRSIFIDSGALQSLEKLHMFDIPKLKAVPSGIQHLRKLQVLDVFYMPTEFQKRIDPETGEDHWII
KHVPDVLVDLNFKNQQMYDLGKNIFMEVAADFLRFFEGMEVLA

>XP_027933280.1

MAETAVSFASQHVLPKFLEAVKMLKDLPKEVADVTDELENFQDFIRDAYKVAEVEKDHNRRERIRKRLIRLREAAFRME
DVIDDYVTCDEKQPEDPRCAALLCEAVEFIKTQIHRLQIAYQIQDVKSLVRAERDGFQNHFPTGSRSDGTRGNENFTWQ
KLRMDPLFIKKDEVVGFEEDIDTLKKWLTEGREERTVISIVGMAGLGKTTLSKQVFDQVHTDFECHALITVSRSYTVEGLL
RDMTNKLCKERREDPPRDVATMDQMSLIEEVRNRLHNKRYVVLFDDVWNETFWDDIELALIDNKNGSRILITTRDQKV
VDFCKKCLFFEVHKLQPLSNSKSLELLCKKAFGNGFHGCCPKDYEEVGLDIVRKCGCLPLAIVAIGSLLYRKCKSPSDWGLF
SQHLSLELESNSELDCVKKILSLSYDDLPQNLRSCLLYFGLYPEDHEVKCGRLFQQWIAEGFVKHERGRNLEEVAEQHLM
ELISRSLVLVSSFTPEDKVKACHVHDSIHEMIRGKMKTTGFCHYINDFESSGIFRRLTMATSSYDLSGTSEGSQYVRSILIFT
NEVLSEEFTKYLLAKYMRLKVLDFEFAALYDVPENLGCLIHLKYLSFRNTSIRSLPKSIGKLKNLESLDVRTNRVIEVPKEITKL
RKLRCLFGYRISTIAVKDSLGKLTSLEKMHELRIDEDGVVIRELGKLNQLRDLRLSNFMGHHSDILCSSVNKMQLLERLDIIL
QYLSEPIPIDLHITSSLSKLRKLHLVAFLKEFPSWIPRLQNLVKLSLKLSMLNNIPLKSLGNMPNLLILSFDSGSYEGETFHFE

NGEFQKLKELRFKSLHKLRSIFIDSGALQSLEKLHMFDIPKLKAVPSGIQHLRKLQVLDVFYMPTEFQKRIDPETGEDHWII
KHVPDVLVDLNFKNQQMYDLGKNIFMEVAADFLRFFEGMEVLA

>XP_027933351.1

MAETAVSFASQHLLPKFLEAVKMVRDLPKEVAEVTDELESFQEFIHEANKVAEAEEDNSKLNRIRKRLMRLREASFRME
DVIDDYLICDEKQSEEDPRCAALLCEAVEFIKSQIHRLQIAYQIQDVKSLVRAERDGFKNHFPIEPRSDSSRGKENFTWHK
FRMDPLFIKEHEVVGLEGPTQTLKKWLTEGREERTVISIVGMAGLGKTTLSKQVFDKVHKHFECHVLITVSRPYAVEELLR
TMMNELCKEIKEDPPRDISTMNQLSLIKEVRNRLCNKRYVVLFDDVWNETFWNDIELAMIDDKNGSRILITTREEKVAE
FCKNCLLYRLQTLSKEKSLELLYKKAFGYGFHGRCLEDYEEAGLGIVRKCGYLPLAIVAIGSLLYRKCKSPSDWALFSQNLSL
ELESNFELDSVKKILSLSYDDLPQNLRSCLLYFGMYPEDYEVKCGRLIQQWIAEGFVKHELERNLEEVAEQQLMELISRSLV
LVASFTIDGKVKACRVHDSMHEMIRGKIKNTGFCEYIDGHNHLDSSGITRRLTVATSSNDLSGSMEESEHVRSILIFANET
EDFTSRLLGKYIRLKVLDFESAPLYEVPENLGSLIHLKYLSFRSTFIRSLPKSIGRLQNLETLDVRTKGEVQVPKEITKLRKLRH
LLSRSISSTSLKNSLGSMTSLEKMYKLAIDEDGVVIRELGKLNQLRDLRLDDVRGDHVDTLCSSINEMPLLERLDIYVQYGA
VIDLHITLSLCKLRKLHLFGSLKEFPNWISRLQSLVKLSLNQSRLTNIPLKSLGNMPNLLFLCFDAYSYEGETLHFQGFQKLK
ELRLKSLPQLSSIFIDSGALQSLEKLLIMNIRQLKAVPSGIQHLEKLQVLEIFHMPIEFLQSINPNGGEEHWMIQHVPHVSF
VAKNSAMLFAEIAAGIFSSTLYRGVQT

>XP_027933353.1

MAETAVSFASQHLLPKFLEAVKMVRDLPKEVAEVTDELESFQEFIHEANKVAEAEEDNSKLNRIRKRLMRLREASFRME
DVIDDYLICDEKQSEEDPRCAALLCEAVEFIKSQIHRLQIAYQIQDVKSLVRAERDGFKNHFPIEPRSDSSRGKENFTWHK
FRMDPLFIKEHEVVGLEGPTQTLKKWLTEGREERTVISIVGMAGLGKTTLSKQVFDKVHKHFECHVLITVSRPYAVEELLR
TMMNELCKEIKEDPPRDISTMNQLSLIKEVRNRLCNKRYVVLFDDVWNETFWNDIELAMIDDKNGSRILITTREEKVAE
FCKNCLLYRLQTLSKEKSLELLYKKAFGYGFHGRCLEDYEEAGLGIVRKCGYLPLAIVAIGSLLYRKCKSPSDWALFSQNLSL
ELESNFELDSVKKILSLSYDDLPQNLRSCLLYFGMYPEDYEVKCGRLIQQWIAEGFVKHELERNLEEVAEQQLMELISRSLV
LVASFTIDGKVKACRVHDSMHEMIRGKIKNTGFCEYIDGHNHLDSSGITRRLTVATSSNDLSGSMEESEHVRSILIFANET
EDFTSRLLGKYIRLKVLDFESAPLYEVPENLGSLIHLKYLSFRSTFIRSLPKSIGRLQNLETLDVRTKGEVQVPKEITKLRKLRH
LLSRSISSTSLKNSLGSMTSLEKMYKLAIDEDGVVIRELGKLNQLRDLRLDDVRGDHVDTLCSSINEMPLLERLDIYVQYGA
VIDLHITLSLCKLRKLHLFGSLKEFPNWISRLQSLVKLSLNQSRLTNIPLKSLGNMPNLLFLCFDAYSYEGETLHFQGFQKLK
ELRLKSLPQLSSIFIDSGALQSLEKLLIMNIRQLKAVPSGIQHLEKLQVLEIFHMPIEFLQSINPNGGEEHWMIQHVPHVSF
VAKNSAMLFAEIAAGIFSSTLYRRND

>XP_027934626.1

MHQKGIGMTGRCCPFQQHVMELNFMEQMEHINNQFGYALSSRRTFEDGGKIVTFGDGWKPVMEDIIGSRSEPLTST
CFQLFESLWRCLDHNLNTVRRYLAIIKVFLNDIDGAAEDVNQRQKVWVEQLRMVAREGHSIVDAYPDKDGLFFFLRRII
FAKNIDRLLKEIINISKRKEIYGIANIHIRERQSVLEGDNLGLKLVAQPDSCSHRHITEFEKEFQLIKGEKQLMNALFDDLQEI
GYEKLDERYKIWVDQMKEIVPELDLVIKECACELKHVTLLNNILGFKARIHIMDKIKKIRKKIEDAGRGRKACSLVQRQSQ
AESLCRKTQPLLIAKESRMVGFDEDVEILMAQLLSDEKHRCICSIVGTKGTGKTELASFIYRSEAVLSHFDCVIWVTPSSTV
EPLKNQIAKKAAEIIMGGEHDMWTTQVVLTTLATKKYLIVVDGFQTAHALDNLREAIPDRLTASRFLLTTNNAMEALRA
CSKSFFVHPLRLLDDKNSWILLTRELNVKMNLEPKLHENEIEEIGKKIMAKCGGLPLEIQKKSKLLFHKDVTAEEWAKVLE
QPNEDQNTWSETFDTVNNLPSYLRRCLFYFELFPADFEIPVRRLVALWVAEGFVPLGEDQEWPPELVAERYLIELIDLN
MVQIAKRKRNGKVKTCRFPNGLRQIFKSTESRIPQVGGTSTDLEAVPKNFRIRRVADHLEVKDIWHKHIHGKRFTYASDC
DSLRTYYQSVSSFLSFDTRLASKSGHDIRNFLQHCILNNCLLQLRVLVLEGVYKPNLPKDIAKLSRLRYLGLRWTYLELLSPYI
NSLLQLQTLDLKHTYIQTLPRSIWKMKLRHLSLSESNRITFPPKPIGIDIGRSLHDLQTLWGVFVDEETPVKGGLDKLVNIT
KLGITCHSQVSAVADWIAKLQYLQSLRLRSRDKQGRPSKIHLKFLQHHTNLTDLYLFGCLNTPLNQLFLPSTLVVLTLSHSK

LEDDPMQILKDAPNLRSLSLFADSYLGQRIVCESDSFSQLRVLRVWKLEQLEEWKIEEKALPSLRQLEIRLCPRMKVLPDG
LNHVNSLLELKLTNMPMEIHAEKYNIPPNCEVHRDDSPLFQVRFAYTALSEEMSEK

>XP_027935100.1

MVELLLFSFAESLLSKLASGAVQEASLALGVHGELQQMQATMALIRGVLLDAEQKNPQSNALGEWLRQIKQVVSDAED
IVDDFECEALRKHVVNTYGGCSRKVRRFFSTSNPLLYRLRMAHQIQDINTRLATLATQRSIFGLQVIDQDTRVVHVREMT
HSHVKPSSITGREHDKNEIVKLLVQDCDHESLSVIPIVGMGGLGKTTLAKLVFNDTTIHACFTLRMWVCVSNDFELKSVLI
KILNSAPNPTKENFNSFEIERLQNHLRNKLEDQKFLLVLDDMWNEDPARWDELKEIIDLNVKGSKILVTTRSHAVVAAM
RTKSSNSYLLKCLSEEDSLSLFVKYAFEVGDEKQHPQLLKVGRKIVKKCGGLPLAVKTLGSSLFSKFDKKEWECIRDNEIWN
SKQNEKGILPALKLSYDQLPSYLKPCFASFSLFQQDTHISAVDVSMLWGALGFLPPSKQGESMTDIATQLLRELWTRSFLL
DFVDLEGHCYFKLHDLVSDLAVFVGKGEFERIDHSNPKISENVQHLAFEENNFMVKLFPPLISELSFFVME

>XP_027935101.1

MVELLLFSFAESLLSKLASGAVQEASLALGVHGELQQMQATMALIRGVLLDAEQKNPQSNALGEWLRQIKQVVSDAED
IVDDFECEALRKHVVNTYGGCSRKVRRFFSTSNPLLYRLRMAHQIQDINTRLATLATQRSIFGLQVIDQDTRVVHVREMT
HSHVKPSSITGREHDKNEIVKLLVQDCDHESLSVIPIVGMGGLGKTTLAKLVFNDTTIHACFTLRMWVCVSNDFELKSVLI
KILNSAPNPTKENFNSFEIERLQNHLRNKLEDQKFLLVLDDMWNEDPARWDELKEIIDLNVKGSKILVTTRSHAVVAAM
RTKSSNSYLLKCLSEEDSLSLFVKYAFEVGDEKQHPQLLKVGRKIVKKCGGLPLAVKTLGSSLFSKFDKKEWECIRDNEIWN
SKQNEKGILPALKLSYDQLPSYLKPCFASFSLFQQDTHISAVDVSMLWGALGFLPPSKQVFVGKGEFERIDHSNPKISENV
QHLAFEENNFMVKLFPPLISELSFFVME

>XP_027935102.1

MVELLLFSFAESLLSKLASGAVQEASLALGVHGELQQMQATMALIRGVLLDAEQKNPQSNALGEWLRQIKQVVSDAED
IVDDFECEALRKHVVNTYGGCSRKVRRFFSTSNPLLYRLRMAHQIQDINTRLATLATQRSIFGLQVIDQDTRVVHVREMT
HSHVKPSSITGREHDKNEIVKLLVQDCDHESLSVIPIVGMGGLGKTTLAKLVFNDTTIHACFTLRMWVCVSNDFELKSVLI
KILNSAPNPTKENFNSFEIERLQNHLRNKLEDQKFLLVLDDMWNEDPARWDELKEIIDLNVKGSKILVTTRSHAVVAAM
RTKSSNSYLLKCLSEEDSLSLFVKYAFEVGDEKQHPQLLKVGRKIVKKCGGLPLAVKTLGSSLFSKFDKKEWECIRDNEIWN
SKQNEKGILPALKLSYDQLPSYLKPCFASFSLFQQDTHISAVDVSMLWGALGFLPPSKQVICLN

>XP_027935169.1

MAESLLFSFAESLIGKLASGAVHEASLALGVHADLQQMEESMSLIRAFLLDAEQKKPLNNSLSEWLRQIKQVFSDAEDIV
DDFECEALRKHVVNTHGGLTGKVRRFFSTSNPLIYRIRMAHEIKDIKERLQKVAADGNTFGLQIIDQDTRVVHVTDTTHS
HVNPSNVVGRQHEKQEILNLLLQHGHGESLSVVSIQGFGGLGKTTLAKLVFNDTTIDECFPLKMWVCVSGDFELRNVLI
KILNSALNPTNENFKNFETEQLQNRLRSTLQRQKFLLVLDDVWNEYQARWDDLKEILDVGVEGCKIIVTTRSHKTVTIMC
TKSSNSYLLERLSEEDSFSLFVKTAFKEGKEERYPQLLEIGKEIVNKCGGIPLAVKTLASSLFSVVDKTKWEAMRDDKIWNL
PQKEKDILPALEISYNQLPSHLKPCFVCFSLFGEGSEFVSFYVAKLWEALGFLPPPKENETMHDVAIQFLRELWSRSFLTDF
IELSHCYIFKLHDLVHDLAMYAAKGEFQTIYPRSSKISPNARHLAFSDNNLLDQAVLPTGLRTIIFPDEATNEAFLNTLVSRC
KYLRFLELSNSKYETLPRSIGKLKHLRYLCLFGNKNLKGLPCSVCNLQNLENLNLNECTELQELPKGISKLISLRQLHITTKQL
HFPDQEISTLTSLETLTFNSCDNLESLLKGIQLSSLTNLSLHDCGKLKTLSFHVIANLEHLLIDNCCELELSMGFGNGNQIPD
LRLKSLAFKSLQQLVTLPQWLQGSVNTLHSLVIADCNNLKELPKWLSSAVFLKLLVIEYCSSLQSLPDNLINLENLLINSCPE
LCKRYQPGVGQDYHKISHIKKVFVGELEE

>XP_027935361.1

WLRQIKHVFSDVENMLDEFECETLQKQVVQAHGSTTIKVAHFFSSSNPLVFHYRMAQQIKTIKKRLDKVAADRHKFGL
DRIGVDRRFVHRRDMTYSYVIDSDVIGRNQDKENIIQLLVQQNPNNNDKRLSVIPIVGIGGLGKTTLAKIVFNDTRIHELF
PLKMWVCVSNAFEIKQEIIKIINSNNDSAHQQNLDKVDVEQLQCQLRNKLACKKFLLVLDDVWNEDLVKWVELRDLVQ
VGAAGSKILVTTRSHVTASMMGTVPSYLLEGLSEEDSLSLFVKWAFKEGDEKKYPYLVSIGKEIVKKCKGVPLAVRTLGSL
LYLKDQKEEWEFVRDNEIWSSIKHGSAMWPALKLSFDQMPSNLRQCFALFNLYPCGYAFDSFDVTSLWGALGLLPSPN
RNQILKHGGANQYMCELFSRSFLQDVVDYDIGFAFKIHELVHDIACYLGKGSIIVRYPFVFRPEHRYVQHLSFPENVGIEN
FPVHKFVCARTILFPTPGIGANSEVFLLECVSRCNRLRFLDLSDSVSKTLSPYIGKLKHLRYLSLENNKSLKRLPDSLCNLLKLE
VLILSGCTELVALPKGLRKMISLQHLEITTKQCVFPEDEIANLSSLQTLRIEFCNNVESLFGGITLPTLKVLCISCCQSLKSLPL
DIEHFPELETLLVDNCDVLELSKEHNQNFHLRLKIVNFISLPQLLILPQWLQGSTDTLQYLLISSCNNLVGLPQWLSAMNC
LKTLCVTSCPNMLSLHDDIHHLPTLERLEIDGNLESCQHLTFDEPDEEVEEKIEELE

>XP_027936358.1

MKEIAPEIDLVIEECERELKHVTLLNNILQSKARFHIINEIKRIKKKIKDVCRSRKAYSLVQPQSRAESLCRKTQPTLIAKESR
MVGFDEDVEILMAQLLSDEKHRGICSIVGAKGTGKTELARFISRNKVVLSHFDCVIWVTPSSSAERLKNKIAKKAAELIMG
GERDTWTTQDVLTTLATKRYLIVVDGIQTLHVLDTLREAIPDRLTASRFLLTTRNAIILQRAGTTSSFVHPLRLLDDKNSWI
LFTRKLSVEMNSDPKLQEIGKKLVATSGGLPLEILKMNKLLLRKNVAAEDSAKVLKQNPWSETLDTVNTNLPSYLRRCLFY
FELFPANFEIPVRRLVALWVAEGLVTLGEDQEWPPELVAETYLTELIDLDMVQIAKRKPNGKVKTCRFPDDL

>XP_027937676.1

MAITLGEEASSSSGFERGWTHDVFLSFRGEDTRRSFTGFLYHGLCQRGIDVFIDDEKLRRGEDLSPALLGAIEKSRIAIIVFS
KNYAFSTWCLDELAKIIDCYKTRGLLVWPVFFHVDPSAVRHQRGTFQTAMAEHEIRFKGNVEKLQRWKKALFEASNFS
GWAFENGYEFQIIQDIIEEASRKLSHTILHIAEYPVGIETRISEVMPLLQIEPGEDICVIGIYGLGGIGKTTIARALYNMIADQ
FEATSFLADIRESSNQRQGLVQLQESLLFDTVGDKYIKLGSIYKGIPIIKKRLCCKKVLLIIDDVDSLEQLQALAGGRDWFGS
GSVVIITTRDKHLLSAHQVDKTYEVKKLNHGEAFELFTWSAFKRKAPDAGYLEVSNRVVLYAEGLPLALKVMGSNLFGKT
VKEWKSALGKYEKIPSKEVQNVLRVTYDNLEENEKEIFLDVACFFKGETVEYVEKTLQACGFYPTIGISVLIDRSLVSIDEYN
RLRMHDLIQDMGREIVREVSPLEPGKRSRLWYHEDVFEVLTENKGTYRIQGMMVDLPDDYMVHLKDDSFKKMKNLKI
LIVRNGNFFGSPQHLPNNLRLLDWMKYPSSLPSSFQPKKLVVLNVSGSRFTMQEPFKYLDSLTSMDLSSCELLTKLPDIS
GVPNLRELTLDDCTNLEEVHESVGFLEKLVEFRAYGCTKLKVFPSGIRLTSLRSLILNWCSSLQNFPAILGKMDNLISISIEGT
GITELPPSIGNLVGLQELSMTSCLSLKELPHNFDMLQSLTNLDMDGCPQLRNFLTKLANMGESTHTFGNILSLNLQNCG
LVDEDLPIIFNSFPNLASVVLSGNNFEALPSCIQQCPCLELLHLDNCKRLQEIPAFPPNMQYINAQNCTSLSAESSNLLLDK
ETFERWELQAMVPGTMVPEWFDHITKGEYMTFWVREKFPAIIICFVLAVESEMKKIFNCEIRFYINGEEVYELEIRRGFS
DMVTDHLWLYDLRTHSSINWRSLDLYLMDGWNQVEISCEKISGASNVTVSWCGVHVCKQEANMKDILLTDPDPDLD
SVVASVSNTLVSDHPAKAQPQSQVTSFIQTPQKNNSSTIVLPTTVQTSLTVNDADMEAFYAVLDDEVSVVSLDNDSTLV
SKLVNKRPSEETKKALRTLQDHVTKEFYALLGPNEYTTMKDTLEYLTNLPAEDGISVEIRSLIIQVSRQFTHWSMDYTCEN
KKIESTTAKLLKAEELEKCLEANKTNFKQVVCMENELCNDLAYLEQRKRELEEQINAVKANISASEAAKNMATQRKREIF
GEAKILKAERDELREQVPHLRDEQELAKKIQSNIRDEWSKLGEKFNYGLRLGKID

>XP_027937686.1

MVCAKGVSMFSLMMRSLGEGKIYPLLFLEPLKSLELPSLSSLKTMHSPHGVLMSLQRSLTVTKQEGYLCGQFSSMSTLLR
CAIREELFKQQWPNMKSDSRVMLKSCRGGRKPCLKHPISQIIQDIIEEASRKLSHTILHIAEYPVGIETRISEVMPLLQIEPG
EDICVIGIYGLGGIGKTTIARALYNMIADQFEATSFLADIRESSNQRQGLVQLQESLLFDTVGDKYIKLGSIYKGIPIIKKRLC
CKKVLLIIDDVDSLEQLQALAGGRDWFGSGSVVIITTRDKHLLSAHQVDKTYEVKKLNHGEAFELFTWSAFKRKAPDAGY
LEVSNRVVLYAEGLPLALKVMGSNLFGKTVKEWKSALGKYEKIPSKEVQNVLRVTYDNLEENEKEIFLDVACFFKGETVEY
VEKTLQACGFYPTIGISVLIDRSLVSIDEYNRLRMHDLIQDMGREIVREVSPLEPGKRSRLWYHEDVFEVLTENKGTYRIQ

GMMVDLPDDYMVHLKDDSFKKMKNLKILIVRNGNFFGSPQHLPNNLRLLDWMKYPSSLPSSFQPKKLVVLNVSGSRF
TMQEPFKYLDSLTSMDLSSCELLTKLPDISGVPNLRELTLDDCTNLEEVHESVGFLEKLVEFRAYGCTKLKVFPSGIRLTSLR
SLILNWCSSLQNFPAILGKMDNLISISIEGTGITELPPSIGNLVGLQELSMTSCLSLKELPHNFDMLQSLTNLDMDGCPQL
RNFLTKLANMGESTHTFGNILSLNLQNCGLVDEDLPIIFNSFPNLASVVLSGNNFEALPSCIQQCPCLELLHLDNCKRLQEI
PAFPPNMQYINAQNCTSLSAESSNLLLDKETFERWELQAMVPGTMVPEWFDHITKGEYMTFWVREKFPAIIICFVLAV
ESEMKKIFNCEIRFYINGEEVYELEIRRGFSDMVTDHLWLYDLRTHSSINWRSLDLYLMDGWNQVEISCEKISGASNVTV
SWCGVHVCKQEANMKDILLTDPDPDLDSVVASVSNTLVSDHPAKAQPQSQVTSFIQTPQKNNSSTIVLPTTVQTSLTV
NDADMEAFYAVLDDEVSVVSLDNDSTLVSKLVNKRPSEETKKALRTLQDHVTKEFYALLGPNEYTTMKDTLEYLTNLPA
EDGISVEIRSLIIQVSRQFTHWSMDYTCENKKIESTTAKLLKAEELEKCLEANKTNFKQVVCMENELCNDLAYLEQRKREL
EEQINAVKANISASEAAKNMATQRKREIFGEAKILKAERDELREQVPHLRDEQELAKKIQSNIRDEWSKLGEKFNYGLRL
GKID

>XP_027937692.1

MPLLQIEPGEDICVIGIYGLGGIGKTTIARALYNMIADQFEATSFLADIRESSNQRQGLVQLQESLLFDTVGDKYIKLGSIYK
GIPIIKKRLCCKKVLLIIDDVDSLEQLQALAGGRDWFGSGSVVIITTRDKHLLSAHQVDKTYEVKKLNHGEAFELFTWSAFK
RKAPDAGYLEVSNRVVLYAEGLPLALKVMGSNLFGKTVKEWKSALGKYEKIPSKEVQNVLRVTYDNLEENEKEIFLDVAC
FFKGETVEYVEKTLQACGFYPTIGISVLIDRSLVSIDEYNRLRMHDLIQDMGREIVREVSPLEPGKRSRLWYHEDVFEVLTE
NKGTYRIQGMMVDLPDDYMVHLKDDSFKKMKNLKILIVRNGNFFGSPQHLPNNLRLLDWMKYPSSLPSSFQPKKLVV
LNVSGSRFTMQEPFKYLDSLTSMDLSSCELLTKLPDISGVPNLRELTLDDCTNLEEVHESVGFLEKLVEFRAYGCTKLKVFP
SGIRLTSLRSLILNWCSSLQNFPAILGKMDNLISISIEGTGITELPPSIGNLVGLQELSMTSCLSLKELPHNFDMLQSLTNLD
MDGCPQLRNFLTKLANMGESTHTFGNILSLNLQNCGLVDEDLPIIFNSFPNLASVVLSGNNFEALPSCIQQCPCLELLHL
DNCKRLQEIPAFPPNMQYINAQNCTSLSAESSNLLLDKETFERWELQAMVPGTMVPEWFDHITKGEYMTFWVREKFP
AIIICFVLAVESEMKKIFNCEIRFYINGEEVYELEIRRGFSDMVTDHLWLYDLRTHSSINWRSLDLYLMDGWNQVEISCEKI
SGASNVTVSWCGVHVCKQEANMKDILLTDPDPDLDSVVASVSNTLVSDHPAKAQPQSQVTSFIQTPQKNNSSTIVLPT
TVQTSLTVNDADMEAFYAVLDDEVSVVSLDNDSTLVSKLVNKRPSEETKKALRTLQDHVTKEFYALLGPNEYTTMKDTL
EYLTNLPAEDGISVEIRSLIIQVSRQFTHWSMDYTCENKKIESTTAKLLKAEELEKCLEANKTNFKQVVCMENELCNDLAY
LEQRKRELEEQINAVKANISASEAAKNMATQRKREIFGEAKILKAERDELREQVPHLRDEQELAKKIQSNIRDEWSKLGE
KFNYGLRLGKID

>XP_027937701.1

MPLLQIEPGEDICVIGIYGLGGIGKTTIARALYNMIADQFEATSFLADIRESSNQRQGLVQLQESLLFDTVGDKYIKLGSIYK
GIPIIKKRLCCKKVLLIIDDVDSLEQLQALAGGRDWFGSGSVVIITTRDKHLLSAHQVDKTYEVKKLNHGEAFELFTWSAFK
RKAPDAGYLEVSNRVVLYAEGLPLALKVMGSNLFGKTVKEWKSALGKYEKIPSKEVQNVLRVTYDNLEENEKEIFLDVAC
FFKGETVEYVEKTLQACGFYPTIGISVLIDRSLVSIDEYNRLRMHDLIQDMGREIVREVSPLEPGKRSRLWYHEDVFEVLTE
NKGTYRIQGMMVDLPDDYMVHLKDDSFKKMKNLKILIVRNGNFFGSPQHLPNNLRLLDWMKYPSSLPSSFQPKKLVV
LNVSGSRFTMQEPFKYLDSLTSMDLSSCELLTKLPDISGVPNLRELTLDDCTNLEEVHESVGFLEKLVEFRAYGCTKLKVFP
SGIRLTSLRSLILNWCSSLQNFPAILGKMDNLISISIEGTGITELPPSIGNLVGLQELSMTSCLSLKELPHNFDMLQSLTNLD
MDGCPQLRNFLTKLANMGESTHTFGNILSLNLQNCGLVDEDLPIIFNSFPNLASVVLSGNNFEALPSCIQQCPCLELLHL
DNCKRLQEIPAFPPNMQYINAQNCTSLSAESSNLLLDKETFERWELQAMVPGTMVPEWFDHITKGEYMTFWVREKFP
AIIICFVLAVESEMKKIFNCEIRFYINGEEVYELEIRRGFSDMVTDHLWLYDLRTHSSINWRSLDLYLMDGWNQVEISCEKI
SGASNVTVSWCGVHVCKQEANMKDILLTDPDPDLDSVVASVSNTLVSDHPAKAQPQSQVTSFIQTPQKNNSSTIVLPT
TVQTSLTVNDADMEAFYAVLDDEVSVVSLDNDSTLVSKLVNKRPSEETKKALRTLQDHVTKEFYALLGPNEYTTMKDTL
EYLTNLPAEDGISVEIRSLIIQVSRQFTHWSMDYTCENKKIESTTAKLLKAEELEKCLEANKTNFKQVVCMENELCNDLAY

LEQRKRELEEQINAVKANISASEAAKNMATQRKREIFGEAKILKAERDELREQVPHLRDEQELAKKIQSNIRDEWSKLGE
KFNYGLRLGKID

>XP_027937709.1

MIADQFEATSFLADIRESSNQRQGLVQLQESLLFDTVGDKYIKLGSIYKGIPIIKKRLCCKKVLLIIDDVDSLEQLQALAGGR
DWFGSGSVVIITTRDKHLLSAHQVDKTYEVKKLNHGEAFELFTWSAFKRKAPDAGYLEVSNRVVLYAEGLPLALKVMGS
NLFGKTVKEWKSALGKYEKIPSKEVQNVLRVTYDNLEENEKEIFLDVACFFKGETVEYVEKTLQACGFYPTIGISVLIDRSL
VSIDEYNRLRMHDLIQDMGREIVREVSPLEPGKRSRLWYHEDVFEVLTENKGTYRIQGMMVDLPDDYMVHLKDDSFK
KMKNLKILIVRNGNFFGSPQHLPNNLRLLDWMKYPSSLPSSFQPKKLVVLNVSGSRFTMQEPFKYLDSLTSMDLSSCELL
TKLPDISGVPNLRELTLDDCTNLEEVHESVGFLEKLVEFRAYGCTKLKVFPSGIRLTSLRSLILNWCSSLQNFPAILGKMDNL
ISISIEGTGITELPPSIGNLVGLQELSMTSCLSLKELPHNFDMLQSLTNLDMDGCPQLRNFLTKLANMGESTHTFGNILSLN
LQNCGLVDEDLPIIFNSFPNLASVVLSGNNFEALPSCIQQCPCLELLHLDNCKRLQEIPAFPPNMQYINAQNCTSLSAESS
NLLLDKETFERWELQAMVPGTMVPEWFDHITKGEYMTFWVREKFPAIIICFVLAVESEMKKIFNCEIRFYINGEEVYELEI
RRGFSDMVTDHLWLYDLRTHSSINWRSLDLYLMDGWNQVEISCEKISGASNVTVSWCGVHVCKQEANMKDILLTDP
DPDLDSVVASVSNTLVSDHPAKAQPQSQVTSFIQTPQKNNSSTIVLPTTVQTSLTVNDADMEAFYAVLDDEVSVVSLDN
DSTLVSKLVNKRPSEETKKALRTLQDHVTKEFYALLGPNEYTTMKDTLEYLTNLPAEDGISVEIRSLIIQVSRQFTHWSMD
YTCENKKIESTTAKLLKAEELEKCLEANKTNFKQVVCMENELCNDLAYLEQRKRELEEQINAVKANISASEAAKNMATQR
KREIFGEAKILKAERDELREQVPHLRDEQELAKKIQSNIRDEWSKLGEKFNYGLRLGKID

>XP_027937894.1

MAESLLFSFAESLLRKLATVAVQEASLALGVRSELKEMTETMEIIRGVLLDAQQKTPQSTALSEWLKRVKRVFSDAEDIV
DDFECEALRKHVVDTSGSHSMKVRHFFSSSNPVVYRLRMAHRIQDINTRLAKLADQRNMFGLQIINQDMRVVRVREM
THSYVNPTKVTGREHDKNEIVKLLVQDGHHKSLSVIPIVGMGGLGKTTLAKLVFNDTNIHACFPLKMWVCVSNDFELR
NLLVKILNSIPSPTSENFNNFETEQLQIHLRNKLEDQKFLLVLDDVWNEDPARWHDLEEIIDLDVKGSKILVTTRSHAVASI
MHTKSSNSYLLECLSEEDSLSLFVKCAFEDGEEKKHPELLEIGKKIVEKCVGLPLALRTVGSSLFRNVDKKEWESVRDNEIW
NLQKNETGILPALKLSYDQLPSYLKPCFTSFFLYQFDVNIFCYDLTTVWDSLGFLPLPKEGESMSDVLNKVLCELRTRSFLS
DDLDFGIDNVYEIHDLVSDLAVYIGKGEFERVNRRNPKISENAQHLVFEENSFYGEDLLPTGLRSVVFRDGGSNIDFLNAL
VSRCRYLRILDLRYSEYESLPHCIGKLKHLRFLCLAKNEKLKELPDSVCKLQNLKTLVLSGCIRLQKLPKGIKYLISLRHLAITTA
QTDFSEEEIANFTSLENLSFTQCDNLESLFEEVQLSTLQTLTLIDCGNLKSVSLHCIRNLEALTIINCNKLESLSCQIPELKLKYL
FLFDLPLLVKLPQWFQGSANSLQHLAIRNCVNLGELPDWLPTLICLKVLEISDCPKLLSLPDNIHHLTNLEKLDITGCPELRK
RFKPKVGQDWHKISHIKQVDIDDSDDEEDLSE

>XP_027938228.1

MAMELVAGPLMGAVFNVLLERIASTEVVNFFKNKNCEKLLKRLKIILLSVNVVLNDAEEKQMKNGAVKEWLEELKDVA
FAAEDLLDEIYTDAKMKAKQVNTLHTGPVSFYCKGVEEKIEDVHERLEFIMRQKEVLDLKVGKEVKMSQKTPTSSVMEA
CDVYGRDNDKESLVDLVLTHDEKIGVIPIVGMGGIGKTTLAQLIYNDQRVQKEFDLKAWIYVSEEFDICKITKTLLEAVTSC
SCDTEDLNFLQRDLKMHVMKKKFLFVLDDVWNENYDNWDKFRSPFKHAGEHGSKIIVTTRSGCVASIMQTVSPYNLR
ELSNEDSWNLFSKHAFDYGDSSLQLHQSLDKVGREIVRKCKGLPLAVKTLAGLLRCKSDRQEWCKVLNSEIWDLHDSES
NILPALRLSYHYLPSHLKRCFAYCAIFPKDYEFEKENLVLLWMAEGFLQQSKRHRRIEEVGNEYFCELVSRSFFQQSRRGK
SCFLMHHLVNDLAQFVSGTFSIRMECSNTNEIKERTRHLSHIIADSSSYVNLKDASKANCLRTFLQIRPVGTSIDLFNNMP
NDLLTKLRCLRVLSLVGTHIYSLPNSVGELKHLRYLEVADTEIVRLPKSICSLFNLQTLKLVGCHNLIELPASIHKLVNLRHLDI
RGTSLRWMPLQINELNNLQNLSDFFVGKGCGSSIGELGELSCLHGELFIHCLEHIVSEKDCEKAKLKEKQGLEKLSLEWCG
NGETDNSQKEKTILNSLQPHTNLKNLDIYDYPGTEFPEWLGDHSFYNLVSVLLNGSKYCYKLPPLGQLPMLKELQISKFEG
LVSVGSEFLGNRTSYVTDCFPALEILRIEYMPSWEKWYPNAENAGTKAFFHLREFHIGNCPKLRGDLPDKLPSLTLLVIRD

CKRLLCSLPNSPSLRVLNVQNCESLEFQVHSPCCHQSLTSLFLHGSCDSLVFLPLDLFPNIKSLDIWGCKNLEALTVSESDS
TPPTFKSLHSLRIRHCPNFTSFPKGGFAASKLTLLTINYCQKLNSLPEQMHDLMPSLKEVQLRGCPKIESSRMRPLRIRICS
KHMEGKQNLSDPLFARLKGLATDQSPSSS

>XP_027938397.1

MPKVMAESLIFAVAESLISKLASRAVEQASLALGVYQELQQMKKTMALIKAFLLDAEQKKEKNNALSEWLRQIRLVFAH
AEDIVDNFECEVLRKHVVRTHGSFSRKVCRLFSTSNPLVYRYRMGREINDIKKQLEKVAADGHMFGLQSSDKDTKVLHA
REMTHSHVNVSNVVGREHDKQKIIELLLQDDHRDRSLSVISIVGFGGLGKTTLAKVVFNDTKVEEFFTLKMWVCVSNDF
ELRNVLIKILNSAPSATNEKFKNLETDQLQIRLRNTLQREKFLLVLDDVWNENRVKWDALKEIIDMVGNRGSKILVTTRS
HSIDAMMRTKSSNSYTLKGLSEEDSLSLFVKSAFDDGEGKKHPELMEIGRQIVRKCGGIPLAVRTLGSSLFSRVDRKEWE
SIRDNEIWNLKQNENDILPALELSYDQLPSHLKRCFACFSLASKDFDVSSSYVALLWEALGFLPPPKENETTHDVANEYLR
ELWSRSFLTDFLDMGSTCRFKLHDLVRELAVYVAKGEFQILYPHSTTISEHAQHLSFIENDMLGQDLVPMGLRTIIFPME
ATNGAFLNTLVFRCKYLRVLDLSYSEYESLPSYIGKLKHLRYLSLSGNKKLKGLPDSVCKLQNLQTLDLRGCIQLQKLPKGIR
KLISLRRLLVTTMQPDFPDKEISKLASIETLELYSCDNLESLFQAIQPRSLKFLHLSGCGGLRSLSFHVITNLESLVIFKCSKME
LSMGLSNLNSIPDSRLKLLVLQSLPQLVTLPEWLQGSVNTLHSLLVVDCNNLEELPEWLSTLTCLKLLIIEHCPRLISLPETT
HLLRNLEHLEINDCPELCRRCQPGVGLDWHKISHIKQVIIGEPEE

>XP_027938398.1

MPKVMAESLIFAVAESLISKLASRAVEQASLALGVYQELQQMKKTMALIKAFLLDAEQKKEKNNALSEWLRQIRLVFAH
AEDIVDNFECEVLRKHVVRTHGSFSRKVCRLFSTSNPLVYRYRMGREINDIKKQLEKVAADGHMFGLQSSDKDTKVLHA
REMTHSHVNVSNVVGREHDKQKIIELLLQDDHRDRSLSVISIVGFGGLGKTTLAKVVFNDTKVEEFFTLKMWVCVSNDF
ELRNVLIKILNSAPSATNEKFKNLETDQLQIRLRNTLQREKFLLVLDDVWNENRVKWDALKEIIDMVGNRGSKILVTTRS
HSIDAMMRTKSSNSYTLKGLSEEDSLSLFVKSAFDDGEGKKHPELMEIGRQIVRKCGGIPLAVRTLGSSLFSRVDRKEWE
SIRDNEIWNLKQNENDILPALELSYDQLPSHLKRCFACFSLASKDFDVSSSYVALLWEALGFLPPPKENETTHDVANEYLR
ELWSRSFLTDFLDMGSTCRFKLHDLVRELAVYVAKGEFQILYPHSTTISEHAQHLSFIENDMLGQDLVPMGLRTIIFPME
ATNGAFLNTLVFRCKYLRVLDLSYSEYESLPSYIGKLKHLRYLSLSGNKKLKGLPDSVCKLQNLQTLDLRGCIQLQKLPKGIR
KLISLRRLLVTTMQPDFPDKEISKLASIETLELYSCDNLESLFQAIQPRSLKFLHLSGCGGLRSLSFHVITNLESLVIFKCSKME
LSMGLSNLNSIPDSRLKLLVLQSLPQLVTLPEWLQGSVNTLHSLLVVDCNNLEELPEWLSTLTCLKLLIIEHCPRLISLPETT
HLLRNLEHLEINDCPELCRRCQPGVGLDWHKISHIKQVIIGEPEE

>XP_027939129.1

MAESFLFSIAESLIAKLASRAFEEASRVVGLYDDLQDLTNTLSLVKAVLLDAQQKQEHNHQLRQWLTQLKTVFSDAEDLL
DEFECQTLRKKVVKAHGSTKDKVSHFFSSSNPLVFRYQMAQRIKDISNRLDKVATNRNKFTLERIEVDTRVVHRRDMTH
SRVSDSDVIGRKHDKEKIIELLMQQNPNDDDISLSVIPIVGIGGLGKTTLAKFVFNDSRIQECFPLKMWVCVSDDFDIKQLI
IKIINSANDSISDDAPSHQPNWNTLDLEQLQNQLKNKLSGQKFLLVLDDVWNEDRVKWVELRNLIQVSAAGSKIIVTTR
SNSIASMMGTIPPHILGGLSEGDSLSLLVKWAFKEGEEEKHPHLVDIIGREIVRKCGGVPLAVRTLGSLLFSKFEASEWEYV
RDNEVWNLPQKKDDILPALKLSYDLMPSYLRQCFALFSLYPKDHLFISDEITCLWGALGLIALPKTNRTREDVGNQYLHEL
LSRSFLQDFQNYGTMYGFRIHDLVHDLALFVAKDECLHMNSNVQNISDNVRHLSFAESSLFSNLVTKKSAAVRTILFPNG
VAAANSEAILKTCLEKFKCLRVLVLSGATFETLPRNIAKLRHLRYLDISENPNIKRLPDSICKLQSLQVLLVNGCMELEVLPN
GLRKLISLQDFSFSTKQTVFPVNEIAKLRSLEFLIVESCHNVESIFGGVKFPALKTLCVSDCQSLKSLWLDGQNFPKLETLFV
DNCINLDLELWNGHDEEESAKLKLKLVGFYGLSQLVALPRWLQEVANSLQSLLVLNCPNIETLPDWLPTLTNLKALTIRN
CPKLVSLPDSIRHLSTLENLRIEDCADLSEKYELHVGEFWPNISHIKNVFIDEPEA

>XP_027939183.1

MIMAESFLFSIADSLIAKLASQLYEEASRVMGLYQHLQEFTRTLSLVKAVLLDAEQKQEHNHELREWLKQLKRVFSDAQ
DVLDEVECQTLQNQVVKAHGTTKTKVGRFFSTSNPLVFRYKMAQQIKDINNRLDKVAADRHKFGLQTIDVDTRVVHRR
EMTHSHVSDSDVIGREHDKEEIIHLLMQKNPIDVDKSLPVIPIVGMGGLGKTTLAKFVFNDARINECFPLKMWVCVSEN
FDIKQMIVRIINSANDSASHSHAPGFQQNLNILDMEQLQNQLRNKLVGEKFLLVLDDVWNEDRVKWVELRALIQVGA
AAGSAVLVTTRSHSIASMMGTVSSHILEGLSLEDSISLFVKWAFKEGEEENYPHLINIGIDIVKKCRGIPLAVRTIGSLLFSKF
EANEWEYVRDREIWNLPQKKEDILPTLKLSYDLMPSYLRQCFALFSLYPKDYEFVNFEVANLWGALGLLGSLKKNMTQD
VANQYFCELQSRSFIQDFVSYGTVYTFKIHDLVHDLALFVAKDECLLINSHVQSIPESIRHLSFVENDLDDKSISSKSVGVRT
ILFPKDGVGAKSEAFFTLVSRYKYLHILNLSYSSVETLPYFIGKLKHLRSLCLYNNKKIRGLPDSICKLQSLQVLDLGGCMELE
ALPKGLRKLISLRDFVITTKQAVLPENDIANLSSLQYLTIQCCDNVESLFSGIELPYLKALTVNSCKRLEALPLDSKHFPALETL
CIGNCDQLELSKGHEDQKFILKLKAILFTSMPQIETLPHWLQGSIKTLLSLRLEHCHNLEVLPDWLPMVTCLKALDIKDCSK
LHSLPDGINCLTALERLQIIDCPELFRKYTL

>XP_027939215.1

MEFASSSSSSSSSFLKSESNFIYDAFVNFGGEDIRRKFVSHLHYVILQAKVKTLIKEENLHEGMKMEEHLRAIASSKIAIIVF
SKTYTESTCCLLELEQIIECVATFGQIVLPVFYEIDPLDVRHQKDDFGKALEETARKSYSGEQLELSLSKWSRALTTAAGITG
LDLRNFRHDAELVEAIVSRVQTLLDYSELPITRFPVGLESHVKRVIRCIENHSTEVCMIGIWGMGGSGKTTVAKAIYNRIY
RTFIGKSFIENIRDWDLVNSKNVDLQENLLYEVLKSKFGFKSTWMGRTIIENELCRKKVLIVLDDVNEFSQLENLCGSCEW
FGQGSVIIITTRDVQLLNRLKVNYVYEMDVMNQNDSLELFSWHAFREAKPRKDLNELARNIVVYCGGLPLALKVLGSFL
YGRTMEEWENVPSKLKVISIDQVQEKLKISFDDLREMEKDIFLDICCFFIGKERNYVTDVLNGCGLHANIGIKVLIERGLIK
VGRNNKLKMHPLFRDMGREIIFQRCPKEPWKRSRLWFQDDVKDVLKNNTGTEATQGLSLKLHSTSTDCFEAHAFKEM
KRLRLLQLDRVQLNGDYGYVSKQLRWICWQGFLSKYIPNNFHMENVIAIDLKRSHLQLVWKQPQVLEWLKFLNLSHSK
FLRETPDFSGLPSLEKLILKDCPSLYKVHESIGNLCNLLLINLKDCTSLISLPREVYKLKSLRTFILSGCFKIDILKEDIVKMESLIT
LVTENTALKQVPCSILSSKSIAYISLYGFGGLSHHIIPSAIRSWMLPTRNPQSFIRPFCVDKENNNWGDLAPLHNSLANIRS
VLVQFDTEFQLYRHLKTILFECGADFTQSKISKDQLRFSSIGIGSYKEFFNTLSDSVSEGLTNSESCYVSLPSDNYPYWYAH
MGEGHSVSFTVPHNSDMKGMALCVVYSSTSKIIEPECLSSVLIANYTKCTCQIYNHGTIISFKDEDWNGIVSNLGFGDKV
EIFVTFDQGLVVKNTTIYLIYGESNGLEMDPCPEPKVNALDKFIKKMVRCDYW

>XP_027939216.1

MEFASSSSSSSSSFLKSESNFIYDAFVNFGGEDIRRKFVSHLHYVILQAKVKTLIKEENLHEGMKMEEHLRAIASSKIAIIVF
SKTYTESTCCLLELEQIIECVATFGQIVLPVFYEIDPLDVRHQKDDFGKALEETARKSYSGEQLELSLSKWSRALTTAAGITG
LDLRNFRHDAELVEAIVSRVQTLLDYSELPITRFPVGLESHVKRVIRCIENHSTEVCMIGIWGMGGSGKTTVAKAIYNRIY
RTFIGKSFIENIRDWDLVNSKNVDLQENLLYEVLKSKFGFKSTWMGRTIIENELCRKKVLIVLDDVNEFSQLENLCGSCEW
FGQGSVIIITTRDVQLLNRLKVNYVYEMDVMNQNDSLELFSWHAFREAKPRKDLNELARNIVVYCGGLPLALKVLGSFL
YGRTMEEWENVPSKLKVISIDQVQEKLKISFDDLREMEKDIFLDICCFFIGKERNYVTDVLNGCGLHANIGIKVLIERGLIK
VGRNNKLKMHPLFRDMGREIIFQRCPKEPWKRSRLWFQDDVKDVLKNNTGTEATQGLSLKLHSTSTDCFEAHAFKEM
KRLRLLQLDRVQLNGDYGYVSKQLRWICWQGFLSKYIPNNFHMENVIAIDLKRSHLQLVWKQPQVLEWLKFLNLSHSK
FLRETPDFSGLPSLEKLILKDCPSLYKVHESIGNLCNLLLINLKDCTSLISLPREVYKLKSLRTFILSGCFKIDILKEDIVKMESLIT
LVTENTALKQVPCSILSSKSIAYISLYGFGGLSHHIIPSAIRSWMLPTRNPQSFIRPFCVDKENNNWGDLAPLHNSLANIRS
VLVQFDTEFQLYRHLKTILFECGADFTQSKISKDQLRFSSIGIGSYKEFFNTLSDSVSEGLTNSESCYVSLPSDNYPY

>XP_027939217.1

MASSIPSTEFASSNSKLPRKYDVLINFTGEDIRRKFVSHLDSALSNVGLSTFLYVENAVKGMHIQQPILNLCRVAIVVFTKT
YSESAWCLHQLQQIIKWQETYSRHLLPVFYEIEPSDVRLQKGDFGEAFKATAHQTFSGMSMEHDMSSWSHALTKAAS
FFGWDESNYRSDAELVDKIVKSVLNLPVLSATKFPVGLQSRVKGVIEIIQNKSMEVCLIGICGERGSGKTTLAKAIYHQIHS

TFTEKSFIEDIAQVSQTRGHVHLQEQLLSDVLKTKMNIPSVEMGRSIIREKLHGKKLLLVLDDTKYEPLLDLWDSHVWFA
KGTVIIITAREEHLQRIPQIDSVFNVNLLRENESLELLSWHAFREEKPKEEYNYLAKRVADYCRGLPLALEVIGSSLFERTKEE
WESVLLELKEIPRLDVHRKLKISFNNLRNEMEKDLFLDVCCFFVGKGRAFVTKILNDCGVDADSGIRILIERNLIQVRKNNK
LGMQPLLRKMGRKIIREISGKELGKNPQLWFGQDAEYAMLENTLFSSHRTKVIQKLPFKMLVIATRDLFERYPLVIRDTS
RTRKLTEDFGKLRWINLQGFTSEYLPNDFNLHDAMAIDLKHSLLLLVWKEHQVLKWLKVLNLSHSKYLRETPDFSGLPRL
EKLILKDCPRLRKVHPSVGCLSNLILLNLKDCTSLSNLPREVYKLKSLNTLILSGCSKIDLLEKYIVQMESLIILIAENAAVKQVP
FSIVSSKSIGYIFLRGVEGLSRNLFSSIIRSWMSPIMNPLSYIHSFCMDVEDNGWNNIAPLLSTLASLRSVLVQCDTEFQLS
KQVEDILIDYGVNITKSGISKQHFKYSLIGVGRCKDFFNAVSDSISKVVASNESRDVSLPGDVDPYWLGHIGEGHSVSFTV
PPDRDLKGMALCLVYLSTPEIVATECLRSVLIVNYTKCTLHIHNHGRVISFNDIDWQGIISNLGSGDKVEICVTFSHELVVK
NTVVYLICDDQLNDLEKEPAPKKNSLIRFLKKIVM

>XP_027939218.1

MASSIPSTEFASSNSKLPRKYDVLINFTGEDIRRKFVSHLDSALSNVGLSTFLYVENAVKGMHIQQPILNLCRVAIVVFTKT
YSESAWCLHQLQQIIKWQETYSRHLLPVFYEIEPSDVRLQKGDFGEAFKATAHQTFSGMSMEHDMSSWSHALTKAAS
FFGWDESNYRSDAELVDKIVKSVLNLPVLSATKFPVGLQSRVKGVIEIIQNKSMEVCLIGICGERGSGKTTLAKAIYHQIHS
TFTEKSFIEDIAQVSQTRGHVHLQEQLLSDVLKTKMNIPSVEMGRSIIREKLHGKKLLLVLDDTKYEPLLDLWDSHVWFA
KGTVIIITAREEHLQRIPQIDSVFNVNLLRENESLELLSWHAFREEKPKEEYNYLAKRVADYCRGLPLALEVIGSSLFERTKEE
WESVLLELKEIPRLDVHRKLKISFNNLRNEMEKDLFLDVCCFFVGKGRAFVTKILNDCGVDADSGIRILIERNLIQVRKNNK
LGMQPLLRKMGRKIIREISGKELGKNPQLWFGQDAEYAMLENTLFSSHRTKVIQKLPFKMLVIATRDLFERYPLVIRDTS
RTRKLTEDFGKLRWINLQGFTSEYLPNDFNLHDAMAIDLKHSLLLLVWKEHQVLKWLKVLNLSHSKYLRETPDFSGLPRL
EKLILKDCPRLRKVHPSVGCLSNLILLNLKDCTSLSNLPREL

>XP_027939697.1

MGKHCEEEECVGFSYDVFISFRREDTGNSFIGHLRKELGRKGINSFVDESDVSTGEGPSHALCEEAIEESRVFIVVFSENYA
SSTWCLDELVRIMERIDNMNTKQLFFPVFYHVNPSDIRHGKNSFREHMKTHEDKFGKESQRIKAWSSALSKAVNYSGK
HITTTGYENKFIEKIVKKVRKSIAPKPLSTGENLVGLEPHIEEVMSLLDMKPKDKTVRMLGIYGFGGIGKTELAKALYDNIV
QHFDAASFLGGVGEKSNTINGMEDLQKTLLSEMLEELETKLGSTEKGIYEIKRKLHQKKVLLVLDDVDDKEELEKLAGGC
DWFGPGSRIIITTREKDVLIAHHVGNIYEMKELDEQHSLELFCWNAFGQGCPKPGFQDVSVRAVHYAKGLPLALKVIGS
DLATLHEESLEAWEDALEEYENTPPKKDIQDVLKISYDRLDNDAKQVFLDIACFFKGERVEYVNKILDEFGSASKIKLLANK
SLVAVDKGCLKMHDLIQEMGREIVRNEAPNNPGERSRVWDYEDVLEILNEDYGSDKIQGIVLDPPQEEKVNWSGTEFE
KMKWLRILIVRNTSFSSELQHLPNHLRLLHWENYPSKSFPPKFHPKKIVVFNLPRSRLTFQEPFKKFPCLTNMDFSYNQRL
IEIPDVSELENLRELRLDHCRNLIAVHESVGFLKRLAHLSVYACTELQNFVSKMFLPSLKVFDINLCESLGYFPEIMQEMTK
PLKISMINTGIQVLPESIGKLIGLVSIDISNNRKLKYLPTSLFMLPNVDSFKIESCSKLGESFRSLVQHPSEANVRPKLRSLNFE
NGNLSDEDLLAILCYFPKLEELIVAENNFVFIPSCIKECGDLTSLDLHGCKKLKKIPELTGLRILDVHHCFYLEEISELPSTVQK
VDARFCFKLTRETSDMLWCQVKKRAGGIEMVMPFITEIPEWFNFVGVERIPHFWVRGKFPNIVLAMIFHFENESDREK
LVRHRLVDLRILINGRYAPGKGYRSYEIEAEHILICDLGVFLSEKEWLGLDVENEWNLVQVEYEASSSLMISGWGAFVYEE
KEGSNMEDILFTCPNPMYSDKIAAATIPEKDPMEKYKKRIRELRLDQLFKKTLTEWQENRERGGDRSHDDCMRRALGQ
IKKISEDAEDALNSKGSALEDPNSYLRWLLDTSENDDGEPKEIIKGELALIVQKNSPTGKKKDNVGHASCSRHQCRMEEE
EGYDPVVEAPSMPFYTRVMRKRRGNDSVEEDLPEDIVVELFLEGMRDGLVEAQNKFPCLDIAETSNAVMEKGCNVRW
PPEVEAQMSVQSRIYTIGIYSGLSEAMQRFPDLDMWATINTVAKRKGMEGIFVSASQANLGFPHLDWSTVTFSPSQDP
LMQTFMRMKQQSNFEAEVMSKLLWKLKEEHQALRNKLAELDDGNENGGGKIGYDEFVEKREDNLDGVAKYKEVSVV
LRGRGEEIERLYEDGVEGLKRSEEFEDLMGAIYLNGLRAGLLEAHALLLNLLARHRN

>XP_027939698.1

MGCVGFRYDVFISFRGADTRSNFVGHLRKELGRKGIKSFNDDRDMAIGESLSPALKKAIEESRVFIVVFSENYASSTWCL
DELVRIMELKEKKQKQVVLPVFYHVDPSDIVQERNSFGKHMTKHENRFGKEEMKAWRSALSQVLNLPSKHITTGCENS
FIEEIVGEVYKNIAPKPLYTGQNPVGLEPHIEEVMSLLDMNPDDNTVRMLGIYGLGGIGKTELAKALYDNIVQHFDAASF
LAGVREKSNTINGMEDLQKTLLSEMLEESETKLGSSSKGIYQIKQKLRRKKVLLVLDDVDDKKELEKLAGGCDWFGPGSR
IVITTREKDVLIAHHVENIYEMKELDEQHSLKLFCWNAFGQGYPKPGFQDVSVRAVDYAKGLPLALKVIGSDLATLYGAS
LEAWEDALEEYEKTPPNKKIQDVLKISYDRLDNDAKQVFLDIACFFKGERVEYVNKILEEFRSAHKMKELVNKSLITIENGC
LKMHDLIQDMGREIVRQEAPNNPGERSRIWDYEDVLEILNEDYGSEKIQGIMLDPPQEEKVNWSGTEFEKMKWLRILI
VRNTSFSSELQHLPNHLKLLDWENYPSKSFPPKFHPKKIVVFNLPRSCLTFQDPFKKFPCLTNMDFSYNQCITEIPDVSEL
QNLREMRLDHCRNLIAVHESVGVLKRLAHLNLSECRKLQNFMSRMFLPSLEIFNLNFCESLGHFPEIMKEMTKPLKIYMT
NTGIQELPESVSKLTGLVSLDISNNRELKYLPSSLFMLPNVDSFIIKACSKLGESFRSLVQHPSKANVHPKLRILNLENGNLS
DEDLLAILCHFRKLEELIVSENNFVCIPSCIKECGDLTSLDLNGCKKLKKIPELTGLRILDVHDCIYLEEISELPSTVQKVDARFC
FNLTRETSDMLWSQVKKAEGGIEMVMPFISEIPEWFNYVGVERIPRFWVRGKFPNIVVAMIFHFKNERERYIFGGRGL
VDLRLLINGRYAPRKGYQNFQIEEEHILVCDVGALCSEKEWVGDAVMEHEWNLVQVAYDATSSLMISGWGAFVYEEK
EGRSMEDILFACPNNLIVNGDSTCVVKGSVEEDENYVPVPEGVAWELLFEGIKDGIVEAWKMFPSLDIAEVFGAVMKK
NGRIEWTAEGMEGIPSAENRTYFTGLYGALLEANRRFPDLDVGATLSTVANRKGIKGDFKTPLQEKMRIPHLDWTTVTL
PPSHDPLMQIYMMMMKQQSSSESELKTKTLWKLKESHQVLRNGLALRENAAQNAPSCSKNRYDELIQKFHIQYDAFV
GKRVDRVYGVAKYEKDSVVLKERVREIERVFNGVVERLQNSEEFEDVMTAMFLNGLRDGVLEARAILLALCTHTQAHE
RVTDEATNNQNIS

>XP_027939797.1

MDTLYGLLADISKDLMWKGFNQLQYSLCFNGFVKELEKEEDNLTETRKNVEDRVTHARRQTLKTSEVIDKWLENANIDS
EYVNRLLSEANVKKSCFFGYCPNWIWRYRLGKKLANKKAELEKIIQEGRQYIQLERIASIPSNTFDILTEKSMNFESRKYAY
DQVMKALKYDGVGMIGLYGMGGCGKTTLAMEVKKIAEAEHLFDKVIFVSVSSTVAIPRIQEKIASSLQYTFPENEEMER
AQRLCMRLTQEKNILIILDDVWEKIDFGRIGIPSSEHHKGCKILITTRSEEVCTLMDCQKIIYLPILTDEEAWALFQNKALISK
DTSKTIRDLAISISNECKGLPVAIVAVASSLKEKQKDVWRSALNKLRSSKPMNIGKGLQDPYKCLHLSYDNLDTKEAKSLLL
LCSAFPEDYEIPIEGLIRCAIGLGVVDGFDTYEEARTEVTAAKIKLVSSCLLLDVGDKHVKMHDLVRDVAQWIAKNENKAI
KCELEKDVTLDHGLMRYLWCVKFPNDIDCSNLEFLSIQTKLEVSDAIFEKMEKLRVLILSNQNDNNGLQLSTMSFKTLQN
LRCLVLQYWKLSDISFVRDMKKLQSLSLRRCSLPSFLELETDVGVTQLPNLKLLEFSHCDIERRNFEEIKLIRSLEELYILENY
WNGNVEFLNVLPETLKRYGIVLGYNTLYYYSPQLEFCSFHATLALYHFDISNEIIMRMAKSSKELTMGNIEGGAKNIVPDI
FQIGASMSELNQLQIHNSEIECLVDTSNHLNKVGNVFSELCSLTITNMWCLRALWHGCVPVDGSFKKLEKLFIENCPKLT
FLLTCDMARGLVQLKLLEISDCDILKHIVTNDDNFTKKSEDEFAIGHFEHSKIFQNLEDLEVSKCKELKDVFSAGIIGGLPQL
KSLVIQECNMLEQIIGDVIPSVHQDEKEEKDGIIEENEHQLLETNQMIFSSKNSSTPSPPIVNHSSGSFSLSSLAELEIHSCP
MLGSLFTTCVAKTLTSLEELTIEECDGLKHIVTPARVKRNKKENMVEDDHEFDSDLSMFSSLKWVTISNCESLEDIFGMAF
VGGMMGQQVSLKLEYLSLAYLPRMTHIWVATNNSFTLQHLNSLTIMRCQKLNVIFPRSMLRSLPELNYLQVIGCNELR
HVIEDSSIVAYSPRPCFPKLQSLLIERCHKLKCFASVSASNDLSNLKILIIKGATELKAFIGCEYDNTGNTKLQLPQLQLLIFM
HLSNFNQETIFLNVKYRIVRNSSKLSLTSTITPHELPQKFSLLEGLENSGIYSWYIESLMDEIQKLDEVSRSNNSTELPSPQEL
VNATSINSEVDEKSNRVTYSQELVDGKSTTGSYLTDQQNPLGETQSTIKMSHEDPPISEIKSSSSQLNDDNQSMSDSIVE
MVGEHKTIETKTLVSKTQEFQKIHRKSEMTSDPQAMDQNFPLISSPNMTQLTDETEANNIGKTTTSDKLAIPTSVLENIE
EIGRQRKPATEGATIKQDSQVVKQNNEMNEEKTEIGIVSNNRVEVTPASTDPRTRLEKYKKFVDLNDSQISLLVEAIAAY
PHLWNSCEKFNDRFRAWMLKTLADMLLFLRSESVGSVDPEREKEFLELCDEAVQLGFERSWVDDMRQRVVGRDHKL
EHARAQIGELLKRHHHLTQELDSIKVPIGELVKRHDHLTQELHNMKKELRSINDSFDAPTKCFDFL

>XP_027940043.1

MEDFFLSIATKIAEYAVFPILDHAQYLCCFNKFALKLPIAKEQLELTRDSVKERIKEAINKTEKVEPSVEKWVKDVEKVLEEV
KMLEERISSVSKSYFRRQCKYSLVKEIETKTTEMIQLVGNSKFEPFSRITELPGMKYYSSDDFFMFNSTEESYKQLIEALKNK
SVSMIGLVGLGGSGKTTLATEVGKKAEEMKLFEKVVMATVSQPLKIRSIQDQIVDQLGFKLIEESDIGRAQRLSERLRKGT
TLVILDDVWEKVNFQALGIPLDASNKTCCIFITTRTREVCTFMQCQSIVELNLLSDEEAWTLFRNYANITDDSSEGLKGVA
RKIVNECKGLPIAIVTVGSTLKDKTIANFELALSRLENSKPLDVTKGFTSPYDCLELSYNNLTNPLAQSLLLLCSMFPEDCEID
LEDLFRFGRGFDTIGRFGTMENARREMDEAIDMLKNCFLLMHVKEKKRVKMHDLVRDVALWIASKSGQAIFTRTEVD
PKELADDEVMKDMKAIVVWGLNSYHLLNYKINCQILEILLLSFNVSGGVKVLDGCLQSLEKLKTLAIINLAEWNKGPLPL
QESLKSLKNIRTLCLRGHDLGDISFVERLQALEILDLRGSYFDDLPVGMVELKKLKLLDLYKCVIKKNKNVEAYEVVKKCLQ
LEELYLCLIEYEKAFPHDVPFSRLQRYVIMSGYFFSHKHHFCRGDLMIMKKYAQSRSLLIDGFDVGAQNFISSPIKDLFIRA
EYLNLRNLEGDYKNIIPSMDPQGMNELIALSLKYCSKIECLIDSTINSNTSFGLLQTEVVFSTLVYLSLVGLDNLREVFCDPS
SRCSLKNLQELEIERCYKLISVSFPMRSKLCNLKVLTISECPMLTSLFTPSVVQTLELLEVLQIDECNSLRHIIEEENDVLPSTQ
SHFSLTLQKLRTLKISSCHNLEYIFSVFLVEGLVSLERVRIQWNEKLKYVFGSEKEHNLAGYPSFQHTNSERNLPNLKSMEL
RSLPNLIDIWPEYCRAHLPSLNDLYFKGCPKLSNSSIHKVMTVSDIQQHKTPTENDILWLISNTLKQLEDDPLSNPQLKVFL
KFRSLWLSDLRIKGIFKFQMGEEGGTTQPLPLNLDMTWLKLLNLAELNFIWKGPTAFLSFQNLRYITVKECPKLITIFSSTV
VTSLPQLTYLDISTCDELEQIFDLGDGQQLKTLSSTQQLCFPTLSSITLEHCHKLKYIFYNLSVSHFTSVKKLVIKNCSQLHKA
FGFEHEADDDGLEEMGKDGKQLLLHNLKDITLLFLPNFQEIHHGFKLKEDVKQIIAECPKYSPSLYLHPGDT

>XP_027940044.1

MEDFFLSIASKIAEYAVHPIIHHAQYLCCFKNFASSLPNTKEQLELTRDKVKDRIREAINKVEKVEPAVEKWLKDVEKVLEE
VKILEEKILSVNKSVFRRKCQYSLAKEIERKTIEMIELDHNSKFEPFSRITELPGMKYYSSNNFFMFNSTEASCNKLLEELKNK
SVLMIGLVGLGGSGKTTLAKEVGKKAEEMRIFEKVVFATVSQPLNIRSIQDQIVDQLGFKLMEESDIGRAQRLSERLRKG
TTLVILDDVWEKINFQALGIPLDESGKGCCVFITTRSKEVCTYMQCQCTIELDLLSDEEAWTLFTHYSNISDDSSEALKGVA
RKIVNECKGLPIAIVTVGSTLKEKSIEDFELALSRLENSKPLDIPQGLRSPYVCLELSYTNLTNQLSQSLLLLCSMFPEDCEIDL
EDLFRFGRGFGTIGTFGTMENARREMHVAIKLLMNCCLLMHGKEKQRVKLHDLVRDVALWIASKSGQAIFTRTEVDPK
ELADDDVMKDMKAIAIWGLNSYHLLNYKLNYPILEILLLSFEVSGVKVSDGCLQSLAKLKTLAIINEWNEGALPLQESLNS
LKNIRTLCLRGHDLGDISFVERLQALEILDLRGSYFDDLPVGIVELKKLKLLDLYKCVIKKNKNVEAYEVVKKCLQLEELYLCLI
EYEKAFPHDVSFSRLQRYVIMSGYLFSHKHYLCRGDLMVMKKYAQSRSLLIDEFNVGAQSFISSPIKDLFIRAEYLNLRNLK
GDYKNIIPSMDPQGMNQLVALSLKYCSKIECLIDSTINSNTSFGLLQTEVVFSTIVYLSLVDLDNLREVFCDPSSRCSLKNL
QELEIERCYKLISVSFPMSSKLCNLKVLTISECPMLSSLFTPSVVQTLELLEVLRIYRCKSLRHIIEEENDVLSSTQSHSSLTLQK
LRTLQISSCDNLEYIFSVFLVEGLVSLESVEISCNEKLKYVFGNEKEHNLAGYPSFQQTNSERNLPNLKSMELRSLPNLIDF
WPEYCRAHLPSLNGLYCEECPKLSNSSIHKVMTASDIQQQTTPVENDILWLISNTLKQLDDDPLSHPQLKVFLKFRILWLS
DLKIKGIFEFQMGEEGGTAQLLPLNLDMAWLQLLNLPELNFIWKGPTAFLSFQNLRDIDVVSCPKLKTIFSTAVVTSLPM
LEHLRIYNCDELEQIFDLGDAQQLKTLSSSQQLCFPKLSSIRVTNCNMLKYLFYNIPASHFTSVIQLEISNCSQLHKAFGFEH
EADDDGWEEMGKDGKQLLLHNLKFITLTDLPNFQEIHHGFKLKEVVQHIIRECPVYSPSLYLHPGDT

>XP_027940046.1

MEDFFLCIATKIAEYAVFPILDHAQYLCCFNKFALKLPIAKEQLELTRDSVKERIKEAINKTEKVEPSVEKWVKDVEKVLEEV
KMLEERISSVSKSYFRRQCKYSLVKEIETKTTEMIQLVCNSKFEPFSKITELPGMKYYSSDDFFMFNSTEASYNKLLEELKNK
SVFMIGLVGLGGSGKTTLAKEVGKKVEEMKIFEKVVFATVSQPLNIRSIQDQIADQLGFKLMEESDIGRAQRLSERLRKG
TTLVILDDVWEKINFQALGIPLDESGKGCCVFITTRSKEVCTYMQCQCTIELDLLSDEEAWTLFTHYSNISDDSSEALKGVA
RKIVNECKGLPIAIVTVGSTLKEKSIEDFELALSRLENSKPLDIPQGLRSPYVCLELSYTNLTNQLSQSLLLLCSMFPEDCEIGL
EDLFRFGRGFGTIGTFGTMENARREMHVAIKLLMNCCLLMHGKEKQRVKMHDLVRDVALWIASKSGQAIFTRTEVDP
KELADDDVMKDKKAIAIWGLNSYHLLNYKLNYPILEILLLSFEVSGVKVSDGCLQSLAKLKTLAIINKWNEGALPLQESLNS
LKNIRTLCLRGHDLGDISFVERLQALEILDLRGSYFDDLPVGIVELKKLKLLDLYKCVIKKNKNVEAYEVVKKCLQLEELYLCLI

KYEKAFPDDVSFSRLQRYVIMSGYLFSHKHYLCRGDLVVIEKYAQSRSLLIDEFNVGAQSFISSPIKDLFIRAEYLSLSNLKG
DYKNIIPSMDPQGMNQLVALSLKYCSKIECLIDSTINSNTSFGLLQTEVVFSTLVYLSLVDLDNLREVFCDPSSRCSLKNLQ
ELEIERCYKLISVSFPMSSKLCNLKVFTISKCPMLSSLFTPSVVQTLELLEVLRIYRCKSLRHIIEEENDVLSSTQTHSSLTLHKL
RTLQIFSCDNLEYIFSVFLVEGLVSLKSLDISWNPKLKYVFGSEKEHNLAGYPSFQHTNSERNLPNLKSMKLRSLPNLIDIW
PEYCRAHLPSLNDLHCKRCPKLSNSSIHKVMTVSDIQQQTTPVENDILWLISNTLKQLDDDPLSHPQLKVFLKFRILCLND
LKIKGIFEFQMGEEGDTTQLLPLNLDISYLSLSNLAELNFIWKGPTAFLSLQNLETVYVDGCPKLKTIFSTTVVTSLPMLQYL
HIYYCDELEQIFDLGDAQQLKTLSSSQQLCFPKLSSIVVTNCNKLKCLFYNISASHFTNLRYLEITNCSRLHKAFDFEDEADD
GGLERMGKDGKQLLLHNLKFITLTDLPNFQEIHHGFKLKEDVQHTIRECPVYSPSLYLHPGDT

>XP_027940203.1

MALTDLFTGEIASDLWKMLITISRKALRCKSSAEQLITYVREILPTIEEIKYSGVELPAPRQSQLDRLSEILRSGVELSHQALS
SSRWNVYRNFQLAKKMEKLEKHVTRFLQVPMQAHILADVHHARFEMAERFDRVEASNRRMERFLEEMKIGVNGGG
WVEEAVKSMQEDETWVEGCNGNNGFSVGLDFGKKKVMEMVFSNNDADWIVGICGIGGSGKTTLARELCRDDQVR
CYFKDRILFLTVSQSPNVEQLRARIWGHIMGNQGLNGNYVVPQWMPQFECKGEAQVLVVLDDVWSFSVLEQLVWKI
PGCKFLVVSRFRFPTFFSATYHVELLGEEDALSLFCHHAFGEKSIPLGANVSLVKQVVAECGKLPLALKVIGASLRDQNEM
FWLSVKSNLSQGHSIGESYEINLIDRMAISTNYLPEKIKECFLDLCSFPEDRKIPLEILINMWVEIHDIREAEAYAIAVELSNK
NLLTLVKEARAGGMYSSCFEISVTQHDTLRDLALILSKRGSIHEHRRLVMAQREENGLLPKEWSRFQNRPFEAQIVSINT
GEMTEMDWFELDFPKAEVLIINFTSSDYFLPPFISKMPNLRALIIVNYSTSYVRLHNVSVLMNLTNLRSLWLEKVSTPQFS
GTVLKNLSKLFIVLCQINNSLDGKQFPNLSELTLDHCNDLTYLPSSICGIKSLRNMSLTDCHNLSQLPVEFGNLKSLEILRLY
ACPDLETLPPSMCEMKKLKYIDISQCTNLTCFPKEIGRLVNLEKIDMRECPMIRYLPKSAVSLRSLQLVICDEEVYGTWRD
VAEMAKSNVHIQVPEQHFDLDWLQE

>XP_027940721.1

MAKHCEEEECVGFRYDVFISFRGEDTRSNFVGHLRRELGMKGMKSFNDDRDMAIGESLSPALKKAIEESRVFIVVFSEN
YASSTWCLDELVRIMELREKKQKQVVLPVFYHVDPSDIVQERNSFGKHMKARENKFGKESQIMQDWRSALSEAVNLP
WKHIITTGCENNFIKEIVGDIHKYIGPKPLYTGQNPLGLESNIEEVMSLLDMKPDDNTVRMLGIYGLGGIGKTELAKALYD
NIVQHFDAASFLAGVREKSNTINGMEDLQKTLLSEMLEESETKLGSSSKGIYQIKQKLRRKKVLLVLDDVDDKKELEKLAG
WCDWFGPGSRIVITTREKDVLIAHHVKNIYEMKELDEQHSLKLFCWNAFGQGYPKPGFQDVSVRAVDYAKGLPLALKV
IGSDLAIHGASLEAWGDALEEYEKTPPNKKIQDVLKISYDRLDNDAKQVFLDIACFFKGERKEYVNKILEEFRSAHKMEEL
VNKSLITIENGFLKMHDLIQDMGREIVRQEAPNNPGERSRVWDFEDVLEILNEDSGSDKIQGIMLDPPQEEKVNWSGT
EFEKMKWLRILIVRNTSFSSELQHLPNHLRLLHWENYPSKSFPPKFHPKKIVVFNLPRSRLTFQEPFKKFPCLTNMDFSYN
QCITEIPDVSELQNLREMRLDHCRNLIAVHESVGVLKRLAHLNLSECRKLQNFMSRMFLPSLEIFNLNFCESLGHFPEITKE
MTKPLKIYMTNTGIQELPESVSKLTELVSLDISNNRKLKYLPSSLFMLPKLEELIASENNFVSIPSCIKECGDRASLDLNGCKK
LNKVPEPTSLRILDVHDCLHLEEISELSSTVQKVNARSCFKLTEETSDMLWCQVKKGVGGIEMVMPFTTEIPKWFNFVG
VESIPHFWVRGKFPNIVVAMIFHFQNPSEREYGFRQLVDLRLLINGRYVPRKGYQHFRIEAEHMLVCDLRVLCSEEEWF
GLDGLLGNEWNLVEVACDATWSLTISGWGAFVYEEGSNMEDLLFTCPLENENELHDLKGTAIEILYEGIRDGLFEARNR
FPSLDIVEIFTATLEKGPRMLWTAEGMELIPTAENRTYFTGVQAGLLEANRRFPDLDVGATLSTVANRKGIKGDFKTPLQ
EKMRIPHLDWTTVTLPPSHDPLMQIYMMMMKQQSSSESELKTKTLWKLKESHQVLRNGLALRENAAQNAPSCSKNR
YDELIQKFHIQYDALVGKRVDRVYGVAKYENDSVVLKERVKEIERVFNGVVERLQNSEEFEDVMTAMFLNGLRDGVLEA
RAILLALCTHTQAHESVTDEATNNQNIS

>XP_027940772.1

MGKHCEEEECVGFSYDVFISFIGKDTGHNFIGHLRRELGRKGVETFKDDDSDLRIGEGVSAGRSEAIEESRVFIVVFSENY
ASSTWCLDELVSIMEQTDMSRKQVVFPVFYHVDPSDIRKEKNSFGKHMKSHQNKFGKEKMKAWRSALSEAVNFPGK

HITTGYENDIIQEIVEKVRKNIAPKPLCTGDKAVGLERCIEEVISLLDMKDNTVRMLGIYGLGGIGKTELAKALYDKIVQHF
DAASFLADVREKSNRINGMEDLQKALLSEMLEELETELGSAYKGIYEIKRKLHQKKVLLVLDDVDDKEELEKLAGGCDWF
GPGSRIIITTRGKDVLIAHHVENIYEMKGLDAQHSLELFCLNAFGQSCPKTGFHGVSAHAVDYAKGLPLALKVIGSDLAIL
HESLDAWKDALEEYKKTPPNTIQDVLKISYDRLHDYAKQVFLDIACFFKGEIMEYVNNILKEFRSTSNMKILVNKSLITIEN
GCLKMHDLIQDMGREIVRNEAPNYPGERSRVWDYEDVLEILNEDYGSDKIQGIVLDPPQEEKVNWSGTEFEKMKWLR
ILIVRNTSFSSELQHLPNHLRLLHWENYPSKSFPPKFHPKKIVVFNLPRSCLTFQEPFKKFPCLTNMDFSYNQRLIEIPDVSE
LENLRELRLDHCRNLIAVHESVGFLKRLAHLSVYGCTKLQNFVSKMFLPSLKVFDINLCESLGYFPEIMQEMTKPLKISMIN
TGIQVLPESIGKLIGLVSIDISNNRKLKYLPTSLFMLPNVDSFKIESCSKLGESFRSLVQHPSEANVRPKLRSLNFENGNLSDE
DLLAILCYFPKLEELIVAENNFVFIPSCIKECGDLTSLDLHGCKKLKKIPELTGLRILDVHHCFYLEEISELPSTVQKVDARFCFK
LTKETSDMLWCQVKKAEGGIEMVMPFISVIPEWFNYVGVERIPRFWVRGKFPKIVLAMIFHFEIESQRDEFVRGRHVDL
RLLINGRYAPGKGYHNYEIEAEHILICDLGVFLSEKEWLGLDVENEWNLVQVEYEASSSLMISGWGAFVYEEKEGSNME
DIFFICPNPMNSEKIPAATIPEKDPMEKYKKMIRQLGLVDLFQKTLTEWQENRERGGDRSHDDCMRIQLGQLKKISEDA
EDALNSKGSALEDPNSYLRWLLDTLENDDGKPKEIIKGDLALITLEKPLTWKKKDNVGEASCSGHRGSNEEEEGYDPVVE
DPTIPFYTHVMRKRKVNDSVEEDLPEDIVVELFLEGMRDGLVEAQIKFPSLDIAETSNAVMEKGGNVRWAPEGEAQISV
QSRIYMSGIYSGLLEAKLRFPDLDMWATINTVAKRKGMEGIFVSASQANLGFPHLDWSTVTLPPSQDPLMQTFMRMK
QQSNFEAEVMSKLLWKLKEEHQALRNKLAELDEGNENGGGKIGYDEFVEMRQDNLDGVAKYEEVSVVLRGRGEEIER
LYDDGVEGLKRSEEFEDLMGAMYLNGLRAGLLEAHALLRNLLAGHRN

>XP_027940848.1

MALNDFFAGEIATELLKMLISISRKSLLCRASADQLISYIHELLPTIEEIKYSGVELPALRQSQLHRLSELLRSGVELSHKVLAS
SRWNVYRNLHLAKKMDKLEKTVSKFLLGPMQAHIMADVHHTRFEMAERFDRVDNSVRRLEQYFGNIKIGVGGGGW
VEEAVRSVDENVVDGSSAVGLGFGKIKVREMLVGREDLWVVGISGIGGSGKTTLAREVCKDDQVRCYFRERILFLTVSQ
SPNVEQLRTKIWGYIMGNERLDSNYVVPQWVPQFECKIEAARTLIVLDDVWTLSVVEQLVCRIPGCKFLVVSRTKFQTL
VSYEVELLSEEDALSLFCHHAFGQKSIPSAANESLVKQVVTECGRLPLALKVIGASLRDQPEMFWLSVKNRLSQGQSIGES
HEINLIERMGISINYLPEKVKECFLDLCSFPEDKKIPLDVLINMWVEIHDIPETEAYAIVVELSNKNLLTLMKEHRAGGMYS
SCFEISVTQHDILRNLAINLSNRASINERRRLVMPKRENTIPKEWLRYKHKPFEAQIVSVHTGEMKDVDWCNMEFPKAE
VLIINFTSTEYFLPQFINRMPNLRALIIINYSATYACLHNISVFKNLPNLRSLWLEKVSTPELSGTVLENLGKLFIVLCKINNSL
VEKEVDLAKVFPNLFDLTLDHCDDLTQLPSSICGMKSLQNLSLTNCHNLNQLPLELGKLRSLEILRLYACPELKTVPNSICE
MMRLKYIDISQCVNLRCFPEEIGKVVSLEKIDMRECSMIRNVPKSALSLQSLRLVICDEEVSGIWKEVEKAKPNVHIQVSE
QHFDLDWLKE

>XP_027941295.1

MEYIPGILCLYAGKIVEYTLGSVCGCYINDFTSKFQDAKEKLELTRENMKEQIRETTNGTENFEPAFLKHVENLLKVAEKVL
EEVQFLEERISNVNKSYFRRQRLYFLAKEIERETDKMFELLYFTKIESLSRITELSNDLGAFKSTEEAYTEILAALKDRSVSMIG
LVGVGGSGKTSLAKEIGKKAEEMKLFEKVVMATVSQPINIRSIQDQIADQLGFKLMEESDIGRAQRLSERLRKGTTFLILD
DVWEKLNFQALGIPFDNSKACCIFLTTRSREVCTSMKCQNIIELNALTEGEAWALFTFHANISNDSPTDYNARRIVSECN
GSSTAIVTIGSTLKGKTFEEFQLARLMLQQSKQLDIPKGLTSHQVCLKLSYDNLTNQLAKSLLLLCSIFPKNHEIDLEDLFRF
GRGLGVIWRFGTMEKERRVMHAAINILKNSYMLTQVKEKEKMKMRDSIRDLALWIAKAESGQAILTSTAVDPRVLVD
DEVTKDKNVIALWDMTNGQLLNYEMNCPSLEVLLLHSPEVGFTISNAFLVRLKMLKLLAFLTFEYRWKLPLETETPLWYT
SPLSQSIESLKNLNTLSFRGYKLGDISALVSLQALEILDLRGSSFKELPNGIVALQKLKLLDLYCCLIEKNNAYEVIGRCLRLEEL
YLHLFPSKKKFPHDVSFSRLQRYVIIQYRSESCPHYMHADVLEKHRPSRALCIDGFNASTQTFISLPNKNLFLRAVYLHLKYI
EGGYKNVIPSMRSQGMNQIVALILEHCHDIEFLFDGTAITNNNVDMLHTKTVFSNLGTVRLHQMQGLQEVFHDPSSQ
CSLEKLKELSIESCNQLSNISFPKNSNLCSLKELRIISCPVLTSLFMPFIAQTLKLLEVLQILECSELMHVIAVAEGNDDYVRTQ
YHTYLMLPKLRIIEIEGCHKLKYIFPACFGRLPSLERLITKNCDKLKYVFGTEKEHHLSMYHEYPDLLNLEVLMLVSLPNLVDI

WPSHCHPRLPNLKELQCIECSTLSNSSLRKMAIDSGLHHQGTTAMVICYLKFMFQKSKLLRSALKFTKLIFSDLGVKCLFQ
FQIGEPGTNRELPLKLSTLKLLDLPQLKFIWKSPTNFLSLQLLQLIYVDGCPKLKSIFSPTIIRSLPVLRQLEILNCEELEHIFAS
GDAQELKSLFTCSQQVCFPKLEQIKVQTCNLLKFLFHNFEAGHFPSLRILEILQCSQIEKCFSFESEADIDGKVGRDKDSKQ
VLLQNLRNIILIHLPNFKEIHHGFQLKDDVYWTIKDCPEYSPIMGNILIISCLTFSHNYYFCFGQPQLLNSILL

>XP_027941339.1

MLRGESSSSSPDASSRWSYHVYLSFGMEGTHVDFANTLCASLQRKGISTFTYDNQVERDVMLKRVQKAMEEYLVAIVL
LSENYASSTWCLDELQKILAVGKPVIPVFYEVAPSDVRYQINCFAKAFEEHKRSEEDQSKVQQWRESLKEVADFSGWES
KDRRREELIEDIIKSVWTKIRHKLPSYEEEWVGIDSRVEKISSLLKLELKDMVRFIGIWGMGGIGKTTLARVVFKKICSQFDI
SCFLENVREISGKTHDMLTLQITLLSHMEVKEFTIQNLDEGKIVIGGILRNNRVLLVLDDVDDARQLDSLGVNDQGKFGP
GSRIIVTTRDMEVLKPHGKFEICKIGLLNSDESLQLFCQKAFRIEKPSEQLLQLSKVAVQQAGGLPLALEIMGSSFCGRNES
QWKEFLGKKEYSKKDIVMEKLIISYHGLPESYQILFLDIACFFNGWVKEHVEQILTICCRCPANGIDVLIDKSLATCDGSRL
GMHDLLQEMGRKIVVEKCLIDAGKRSRLWSPHDIDQALKRKKKKESIEGIVLKSSTEPYIANWDPEAFSKMYNLKFLIINF
HNIQFPRGLKCLSSSLKVLRWTKCTLEALPLGVKLENLVMLKMRYSKIKKIWSDSQHFRRLKFIDLSHSEDLIETPIVSEVPC
LESLLLEGCKNLVKVHQSVGLHTKLVVLNLKDCINLQILPTKFKMDSLEELILSGCSKLRKRTKFGKNVQCLSLLNLHKSENL
ICQPKSVCNVSGTKVHSENLKELSFCGGKELTSKSTWNLHQWSSICGKKLVSKELNLPPLSSLSSLKFLNLSYCDLNDESIP
DDLGSLSSLLGLDLSGNNFVRPNYCISNLHALKSLTLIDCPRLESLPMLPPNVQCLCTANSTEMKPLNSDAHLLWKIFELH
MNQVYSLYTPYSLPHLPPIHPNYFHKVCVYQMEGRPHFMFIIPGREIQKWNEECFLVDPSHHPYNMLGCVSAASIIVEA
PKYCESSGWLGIAICLALEPSNMQASPQSHVSPHSMGNEEICIYYWACKAHSGEPDLIFPIVPKHSHSVHECNEEKCLLQ
LIFYVENHSKAWKPSIRKCRCSVIFKEDVEEWCCPRVHTKASMLSGESSSNSSRWTYHVFLSFRGEDTRLGFTDHLYAAL
VRKGMITFRDDKNLEKGDKIDKELFKAIKESLGAIVILSENYASSSWCLDELNKILESNRVLGREVFPVFCGVSPSEVQHQT
TTSFEEAFQKHERRFEKDTEKVRQWRDSLKEVSQIPGWESKNYQHQTELIENIVESVWTKLRPKMPSFNDGLVGIGSRV
KKMDSLLSMESKDHARFIGIWGMGGIGKTTLARVVVKKIQHQFDISCFLDNVREISKESDGRIRLQGKLLSHLAIKGLEIR
DLDEGKNTIRELLFKKKVLLVIDDVDDTSQLECLAERLEWFGPGSRVIITTRDTQVLISHGIIENYKINLLNSDESLQLLSQKA
FKRDKPEEHYLELSKAVAKYAGGLPLALELLGSFLCGRRESQWKEVVDMIREVPPSHIAMQSLRISYNGLPLPYKTLFLDIA
CFFRGRIKELVIQALEICERYPPVGIDLLVEKSLATYDGFTIGMHDLLQESAREIVTEESYVDPAKRSRLWTLEDTNEVLKYN
KANESIEGIVLNSPEKEEAIWDPEAFSRMYNLQLLIINYHVNLPTSLKCLCSSLKFLQWMKYPLESLPLGVQLDELVELKM
HSSRIKRIWNGNQDFAKLKFIDLSYSEDLIQTPIVSGAPSLERLLLIGCVNLVEVHPSVGQHKRLDLLMLKDCKNLQIMPR
KLEMDSLEELILSGCSKLEKLPEFGENMKSLSLLNVEHCINLLCLPNSICNLKSLRKLYVSGCSRISTLPDGMNENESLEELD
VSRTEITEISSSKVRLEILRELSFGGRKETTPKSQNLLQWISKFMGQSDMHESIVPPLSSLLALESLDLSYRDLTDESIPSDFG
PLSLLKRLDLSGNNFVNPPAQCIISLSMLHTLSFDHCPRLESLPMLPPNLQALYATNCPKLKPFHLDEDTLWKIFESHSHE
DPIEGPELWFIIPGNEIPSWFNNQNSLAIDSSDQTYQKLCCDSVTSIIVDVPEDCQLSEWWGIAVCLVLEPLNTDVPSSSN
ARPTSTVNEEIGIYYWVCKTPDKDPDPNFPIAPKFGNLLYKFNDPYIHIIFFNADHVYIQHYLSGEQTQLEVVFFVENLSES
CKARIKKCGCRVICKEKIEEWRKHSDGLNISRITETNEDEERHELEVEEPTSPTLGK

>XP_027941379.1

MDALYGLLADISKDLVCKGLNQLQYSLCFNGFVKELEEEENNLTETRENVERRVTDARRQTSKTSEVIDKWLENAKIDSE
YVNRLLREANARKSCFFGYCPNWIWRYRLGKKLANKKAELEKIIQEGRQYIQLERIASIPSNTSDILTEKSMNFESRKYAYD
QVMKALKYDGVGTIGLYGMGGCGKTTLAMEVKKIAEAEHLFDKVIFVPVSSTVEVQRIQEKVASSLQYTFPENQEMER
AQRLCMRLTQEKNILIILDDVWEKLDFGRIGIPSSEHHKSCKILITTRSEEVCTLMDCQKTIYLPILTDEEAWALFQNKALIS
KDTPKTVRDLAISISNECKGLPVAIVAVASSLKEKQKDVWRSTLNKLRSSKPINIGKGLQDPYKCLQLSYDNLDTKEAKSLL
LLCSAFPEDYEIPIEGLIRCAIGLGVVDGFDTYEEARTEVTATKIKLVSSCLLLDVGDKHVKMHDLVRDVAQWIAKNENKV
IKCELEKDVTLEHGSMRYLWCVKFSNDMDCSNLEFLSIQTELEVSDAIFEKMEKLRVLIVSNQSQDNELQLSTMSFKILQ
NLRCLVLQYWKLSDISFVRDMKKLQSLSLRRCSLPSFLELQTDVGFTQLLNLKLLEFSGCYIERENFEEIKLIPSLEELYILKNF

WNGNVEFFSVLPKTLQRYGIVLGYYQLAYYSPQDEFCSYNATLGLSHFDISNEIIMRMAKSAKELIMRNIEGDAKNIVPDI
FQTGASMSELNEFQIYRSEIECLVDTSNHLNEVGNVFSELCSLTIVNMQCLRALWHGCVPVDRSFEKLEKLFIENCPKLKS
LLTCDMARGLVQLKLLKISDCDILKHIVTNDDNFTKKSEDEFAIGHFEQSKIFQNLEDLTVIRCGELKDVFPTGIIGGLPQLK
RLVITKCNMLEQIIGDVVPSSHQDEKEEKDKIIEENEHQHFESNRLIFSSKSTSTPSPPIVNHNSGSFSLSSLAQLEILSCPML
LGSLFTTCVAKTLTSLEELNIDQCDGLKHIVTPARVKRNKKENMVEDDHEFESDISMFSSLKWVTISNCESLEDIFGMAFV
GGMMGQQKLDEVSGSNNSTELPSSQELVNGTSINSEVDEKSNRETYSQELVDGKSTNGSYLTDQQNPLGETQSTIKMS
HEDPPISEIKSSSSQLNDDNQSMLDSRVEMVGEHQTIETKTLVSETQEFQKIHRKSEMTSDPQAMDQNFPLISSPNMT
QHYMVLL

>XP_027941380.1

MDTLYGLLADISKDLVCKGFNQLQYSFCFNEFVKEVEEEENNLTETRENVERRVTHAQRQNIKIDEVIDKWLENAKSDSK
DVDCLLKDAKAKKSCCLGYCPNWIWRYRLGKKLANKKAELEKIIQKGRPYMQLERIASIPSNTFDILTEKSMNFESRKYAY
DQVMKALKYDGVGIIGLYGMGGCGKTTLAMEVKKIAEAEHLFDKVIFVPVSNTVEIPRIQEKIASSLQYTFPENQEMERA
QRLCMRLTQEKNILIILDDVWEKLDFGRIGIPSSEHHKGCKILITTRSEEVCTLMDCQKTINLPILTDEEAWVLFQNKAVIS
KDTPKTITDLARSISNECKGLPVAIVAVASSLKEKQKDIWRSALNKLRSSKQINIGKGLQDPYKCLQLSYDNLDTEEAKSLLL
LCSAFPEDYEIPIEGLIRCAIGLGVVDGFDTYEEARTEVTTAKIKLVSSCLLLDVGDKHVKMHDLVRDIAQWIAKNENKVIK
CELEKDVTLEHGSMRYLWCVKFSNDMDCSNLEFLSIQTQLEVSDAIFEKMEKLRVLIVSNQNYDNELQLSTMSFKTLQN
LRCLVLQY

>XP_027941544.1

MAVPSFSFSFTYDVFLSFRGEDTRYGFTGNLYRALRDRGIHTFIDDEELRKGDEITSALEKAIEGSRIFIIVFSLNYASSSFCL
NELAYILPHAKRNALLVLPLFYDVVPSHVRHHTGSFGEALDAHEKRFRGMSQGFELNIEKLNKWKMALRRAANLSGYH
FKHGEEYEYQFIEKIVKLVSNKINRVPLHVADYQVGLEKRVLEVKMLVDVGSDDCVHMVGIYGLGGVGKTTLALALYNSI
ADHFEGLCFLENVRENSNKQGLHHLQRILLSEMVGENNINIGSVRQGISMMQHRLRQKKILLILDDVDKHEQLQAIVGS
PDWFGPGSRVIITTRDKQLLSCHLVEKLYEVKKLEKNDALRLLSWKGFRTEEVDTSYSNVMDSVLAYASGHPLALEVIGS
KLFRKSVEEWESAIKQYEKIPISQILEVLKISFDALEEVEKSVFLDIACCFKGYALSDIEDILRAQYGHSMKYHIGVLVEKSLIK
YKWKFGVTMHDLIEDMGKEIVRQQSPKRPGKRSRLWSPEDIIQVLEDNSGSGEIEIICLNSSLPDKEEIIEWNRKAFKKM
KNLKTLIIKKGNFSEGPKYLPNSLRVLEWLKYPSQGLPPDFRSKELAICKLPTSCFGSLELAELSKKFMNMTLLDFEECQGLT
QIPDVSGMPNLEKISFKNCKSLNTIHDSIGFLGKLNSLNAVGCSKLRSFPPLKLTSLKNLELSYCYGLESFPEVLGKMGKITK
LVLEDCNIKELPFSFQNLTELQTLQLRFCPMLRLPSSIVMMPKLAEIIVWESKGWLFPKQVEVEEKVSSMVSSNVDCLLLP
RCKLSDDFLPIALAWFANVKELDLSWNNFTVLPECISNCHFLSKLTLDGCHSLREIRGIPPNIQLLLAVDCKSFTSSCRSTLL
NQKLHEAGNTMFRLSGASFPEWFDHHNQGPSCSFWVGNKFPSIALCIAIGPAHLEHVTIDRPIININGVKCSLHGEEKPY
LNMLPHHTYLFDLQHIVFSDYLDRFVTENEWNHVEITYSVKQRFNEKDKHAVTPVSVENGIYVLKQRSSMEDIQFTDPH
KKRRLDVDPE

>XP_027941550.1

MLVDVGSDDCVHMVGIYGLGGVGKTTLALALYNSIADHFEGLCFLENVRENSNKQGLHHLQRILLSEMVGENNINIGS
VRQGISMMQHRLRQKKILLILDDVDKHEQLQAIVGSPDWFGPGSRVIITTRDKQLLSCHLVEKLYEVKKLEKNDALRLLS
WKGFRTEEVDTSYSNVMDSVLAYASGHPLALEVIGSKLFRKSVEEWESAIKQYEKIPISQILEVLKISFDALEEVEKSVFLDI
ACCFKGYALSDIEDILRAQYGHSMKYHIGVLVEKSLIKYKWKFGVTMHDLIEDMGKEIVRQQSPKRPGKRSRLWSPEDII
QVLEDNSGSGEIEIICLNSSLPDKEEIIEWNRKAFKKMKNLKTLIIKKGNFSEGPKYLPNSLRVLEWLKYPSQGLPPDFRSKE
LAICKLPTSCFGSLELAELSKKFMNMTLLDFEECQGLTQIPDVSGMPNLEKISFKNCKSLNTIHDSIGFLGKLNSLNAVGCS
KLRSFPPLKLTSLKNLELSYCYGLESFPEVLGKMGKITKLVLEDCNIKELPFSFQNLTELQTLQLRFCPMLRLPSSIVMMPKL
AEIIVWESKGWLFPKQVEVEEKVSSMVSSNVDCLLLPRCKLSDDFLPIALAWFANVKELDLSWNNFTVLPECISNCHFLS

KLTLDGCHSLREIRGIPPNIQLLLAVDCKSFTSSCRSTLLNQKLHEAGNTMFRLSGASFPEWFDHHNQGPSCSFWVGNK
FPSIALCIAIGPAHLEHVTIDRPIININGVKCSLHGEEKPYLNMLPHHTYLFDLQHIVFSDYLDRFVTENEWNHVEITYSVK
QRFNEKDKHAVTPVSVENGIYVLKQRSSMEDIQFTDPHKKRRLDVDPE

>XP_027941564.1

MAVPSFSFSFTYDVFLSFRGEDTRYGFTGNLYRALRDRGIHTFIDDEELRKGDEITSALEKAIEGSRIFIIVFSLNYASSSFCL
NELAYILPHAKRNALLVLPLFYDVVPSHVRHHTGSFGEALDAHEKRFRGMSQGFELNIEKLNKWKMALRRAANLSGYH
FKHGEEYEYQFIERIVELVSKKINRAPLHVGDYPVGLEARVLEVKLLLEVGSDDVVHLVGIHGLGGIGKTTLALALYNSIAD
HFEGLCFLENVRENSNKHGLHHLQRILLSQVLGENNINITSVRQGISMMQHRLRQKKILLILDDVDKHEQLQAIVGRPD
WFGPGSRVIITTRDKQLLSCHLVEKLYKVKKLEKNNALRLLSWKGFRTEEVDTSYLNVMDRVLAYASGHPLALEVIGSKLF
SKSVKEWESAIKQYEKIPSNQILEVLKVSFDALEEVEKSVFLDIACCFKGYALSEIEDILRAHYGDCMKYHIGVLVEKSLMKY
GYNSVVTLHDLIEDMGKEIVREKSPKNPGKRSRLWSPEDIIQVLEDNSGSGEIEIICLNSSLPDKEEIVGWNRKAFKKMKN
LKTLIIKKGKFSEGPKYLPNSLRVLEWLKYPSQGLPPDFRSKKLAICKLPSSSFGSLELAEFSKKFMNMTLLNFDECEGLTHI
PDVSGLPNLEKVSFKNCKSLVTIHDSFGFLGKLNSLSAVGCSKLRSFPPLKLTSLENLELSYCHSLESFPEILEKMGKITELVLE
DCHIKELPFSFHNLTELQTLQLRWCPILRLPSSIVMMPKLAQIIAWESKGWLFPKQVDGEEKGSSMVSSNVDCLVLSGC
KLSDDFFPVIPEWFSNVKDLDLSRNNFTVLPECISNCHSLCKLTLDSCHSLQEIRGIPPNIRHLSARNCKSFTSSCRSTLLNQ
KLHEAGNTMFWLSGAKFPEWFDHHGRGPSCSFWVGNKFPSIALCIAIGQTHIEQVEIVGPIMIINGIECSFDEEEDPYLY
MLPHHTHIFDLQHIVFSDYLDRYVSENEWNHVEITYSVEQRFNKKDKHAVTPISIENGIYVLKQRSSMEDIQFTDPHKKR
RLDVV

>XP_027941579.1

MAVPSFSFSFTYDVFLSFRGEDTRYGFTGNLYRALRDRGIHTFIDDEELRKGDEITSALEKAIEGSRIFIIVFSLNYASSSFCL
NELAYILPHAKRNALLVLPLFYDVVPSHVRHHTGSFGEALDAHEKRFRGMSQGFELNIEKLNKWKMALRRAANLSGYH
FKHGEEYEYQFIERIVELVSKKINRAPLHVGDYPVGLEARVLEVKLLLEVGSDDVVHLVGIHGLGGIGKTTLALALYNSIAD
HFEGLCFLENVRENSNKHGLHHLQRILLSQVLGENNINITSVRQGISMMQHRLRQKKILLILDDVDKHEQLQAIVGRPD
WFGPGSRVIITTRDKQLLSCHLVEKLYKVKKLEKNNALRLLSWKGFRTEEVDTSYLNVMDRVLAYASGHPLALEVIGSKLF
SKSVKEWESAIKQYEKIPSNQILEVLKVSFDALEEVEKSVFLDIACCFKGYALSEIEDILRAHYGDCMKYHIGVLVEKSLMKY
GYNSVVTLHDLIEDMGKEIVREKSPKNPGKRSRLWSPEDIIQVLEDNSGSGEIEIICLNSSLPDKEEIVGWNRKAFKKMKN
LKTLIIKKGKFSEGPKYLPNSLRVLEWLKYPSQGLPPDFRSKKLAICKLPSSSFGSLELAEFSKKFMNMTLLNFDECEGLTHI
PDVSGLPNLEKVSFKNCKSLVTIHDSFGFLVSRASQKY

>XP_027941590.1

MAVPSFSFSFTYDVFLSFRGEDTRYGFTGNLYRALRDRGIHTFIDDEELRKGDEITSALEKAIEGSRIFIIVFSLNYASSSFCL
NELAYILPHAKRNALLVLPLFYDVVPSHVRHHTGSFGEALDAHEKRFRGMSQGFELNIEKLNKWKMALRRAANLSGYH
FKHGEEYEYQFIERIVELVSKKINRAPLHVGDYPVGLEARVLEVKLLLEVGSDDVVHLVGIHGLGGIGKTTLALALYNSIAD
HFEGLCFLENVRENSNKHGLHHLQRILLSQVLGENNINITSVRQGISMMQHRLRQKKILLILDDVDKHEQLQAIVGRPD
WFGPGSRVIITTRDKQLLSCHLVEKLYKVKKLEKNNALRLLSWKGFRTEEVDTSYLNVMDRVLAYASGHPLALEVIGSKLF
SKSVKEWESAIKQYEKIPSNQILEVLKVSFDALEEVEKSVFLDIACCFKGYALSEIEDILRAHYGDCMKYHIGVLVEKSLMKY
GYNSVVTLHDLIEDMGKEIVREKSPKNPGKRSRLWSPEDIIQVLEDNSGSGEIEIICLNSSLPDKEEIVGWNRKAFKKMKN
LKTLIIKKGKFSEGPKYLPNSLRVLEWLKYPSQGLPPDFRSKKLAICKLPSSSFGSLELAEFSKKFMNMTLLNFDECEGLTHI
PDVSGLPNLEKVSFKNCKSLVTIHDSFGFLG

>XP_027942039.1

MDSLYGLLADISKDLVCKGLNQLQYSLCFNGFVKELEEEENNLTETRENVERRVTDARRQTSKTSEVIDKWLENAKIDSE
YVNRLLREANARKSCFFGYCPNWIWRYRLGKKLANKKAELEKIIQEGRQYIQLERIASIPSNTFDILTEKSMNFGSRKYAY
DQVMKALKYDGVGTIGLYGMGGCGKTTLAMEVKKIAEAGHLFDKVIFVSVSSTVAIPRIQEKIASSLQYTFPENQEMER
AQRLFMRLTQEKNILIILDDVWEKIDFGRIGIPSSEHHKGCKILITTRLEEVCTLMDCQKIIYLPILTDEEAWALFQNKALISK
DTPKTIRDLAISISNECKGLPVAIVAVASSLKEKQKDVWRSALNKLKSSKPINIGKGLQDPYKCLQLSYDNLDTKEAKSLLLL
CSAFPEDYEIPIEGLIRCAIGLGVVDGFDTYEEARTEVTATKIKLVSSCLLLDVGDKHVKMHDLVRDVAQWIAKNENKVIK
CELEKDVTLEHGSMRYLWCVKFPNDMDCSNLEFLSIQTKFEVSDAIFEKMEKLRVLIVSNQNYNNELQLSTMSFKTLQN
LRCLVLQYWKLSDISFVRYMKKLQSLSLRRCSLPSFLELETDVGVTQLSNLKLLEFSKCDIERKNFEEIKLIPSLEELYILEKDW
NGNVELFSVLPETLKRYGIVLGYNELSYYSPQDEFCSYHATLALSHFDISNEIIMRMAKSAKELMMGNIEGGAKNIVPDIF
QIGASMSELNQLKIQSSEIECLIDTSNHLNKVGNLFSELCSLTIAYMQCLRALWHGCVPVDGSFEKLEILSIEYCPKLTSLFT
YDMARGLVHLKSLKIYCCDILKHIVTNDDKTMKSEDEFAIGHFEHSKIFQNLEDLEVTYCRELKDVFSAGIIGGLPQLKSLGI
QECNMLEQIIGDVVPSAHQDEKEAKDEIIEENEHQHFESNQLIFSSKSTSTPSPPIVNHSSGSFSLSSLALLTIRSCPMLESL
FTTSVAKTLTSLEELNIDQCDGLKHIVTPARVKRNKKENMVEDDHEFESDISMFSSLKRVSISNCESLEYIFGMAFVGGVV
NIENHLQTPRLMEQQVSLKLEYLSLAYLPRMTHIWVATNNSFVLQHLYSLFITKCQKLKVIFPWSMLRSLPELDDLYVSEC
DELRHVIEDSNIIAYSPRPCFPKLQSLRIQQCHKLKCFTSVYVSNDLSNLKFLLIEGATALQEFIGCEYDNTGNTKLQLPQLQ
LLIFMHLSNFNQETIFLNVKYRIVRNCPKLSLTSTITPHELPQKTSLLEGLENSGIYSWYIESLMDEIQRLDEVSRSNNSTELP
SSQELVNATSIKSEVDEKSNRVTYSQELVDGKSTTGSYLTDQQNPLGETQSTIKMSHEDPPISEIKSSSSQLNDDNQSMS
DSRVEMVGQHKTIETKTLVSETQEFQKIHRKSEMTSVPQAMDQNFSLISSPNMTQSTDETEANNIGKTTTSDKLSIPTSV
LENIEEIGRQRSRDGPAAEGTTIKTLPIGVDNISLGSGVTIHKSSSADILPQDSQVEKDNEMHEDKSEIAQYKNIKIQEGVS
LLDKTKEIGIVSNNRASADTRTRLEQYKHFVDLNDSQISLLVEAIEAYPHLWNACEKFSDRFQAWMLKTLADMLLFLRSE
SVGSVNPEREKEFLKLCDEAVQLGFERSWVDDMRQRVVTRDHKLDQAKTRIGEFVNRHDHLTQELDNIKKELRILNDF
VEAQTKCFDFL

>XP_027942040.1

MDSLYGLLADISKDLVCKGLNQLQYSLCFNGFVKELEEEENNLTETRENVERRVTDARRQTSKTSEVIDKWLENAKIDSE
YVNRLLREANARKSCFFGYCPNWIWRYRLGKKLANKKAELEKIIQEGRQYIQLERIASIPSNTFDILTEKSMNFGSRKYAY
DQVMKALKYDGVGTIGLYGMGGCGKTTLAMEVKKIAEAGHLFDKVIFVSVSSTVAIPRIQEKIASSLQYTFPENQEMER
AQRLFMRLTQEKNILIILDDVWEKIDFGRIGIPSSEHHKGCKILITTRLEEVCTLMDCQKIIYLPILTDEEAWALFQNKALISK
DTPKTIRDLAISISNECKGLPVAIVAVASSLKEKQKDVWRSALNKLKSSKPINIGKGLQDPYKCLQLSYDNLDTKEAKSLLLL
CSAFPEDYEIPIEGLIRCAIGLGVVDGFDTYEEARTEVTATKIKLVSSCLLLDVGDKHVKMHDLVRDVAQWIAKNENKVIK
CELEKDVTLEHGSMRYLWCVKFPNDMDCSNLEFLSIQTKFEVSDAIFEKMEKLRVLIVSNQNYNNELQLSTMSFKTLQN
LRCLVLQYWKLSDISFVRYMKKLQSLSLRRCSLPSFLELETDVGVTQLSNLKLLEFSKCDIERKNFEEIKLIPSLEELYILEKDW
NGNVELFSVLPETLKRYGIVLGYNELSYYSPQDEFCSYHATLALSHFDISNEIIMRMAKSAKELMMGNIEGGAKNIVPDIF
QIGASMSELNQLKIQSSEIECLIDTSNHLNKVGNLFSELCSLTIAYMQCLRALWHGCVPVDGSFEKLEILSIEYCPKLTSLFT
YDMARGLVHLKSLKIYCCDILKHIVTNDDKTMKSEDEFAIGHFEHSKIFQNLEDLEVTYCRELKDVFSAGIIGGLPQLKSLGI
QECNMLEQIIGDVVPSAHQDEKEAKDEIIEENEHQHFESNQLIFSSKSTSTPSPPIVNHSSGSFSLSSLALLTIRSCPMLESL
FTTSVAKTLTSLEELNIDQCDGLKHIVTPARVKRNKKENMVEDDHEFESDISMFSSLKRVSISNCESLEYIFGMAFVGGVV
NIENHLQTPRLMEQQVSLKLEYLSLAYLPRMTHIWVATNNSFVLQHLYSLFITKCQKLKVIFPWSMLRSLPELDDLYVSEC
DELRHVIEDSNIIAYSPRPCFPKLQSLRIQQCHKLKCFTSVYVSNDLSNLKFLLIEGATALQEFIGCEYDNTGNTKLQLPQLQ
LLIFMHLSNFNQETIFLNVKYRIVRNCPKLSLTSTITPHELPQKTSLLEGLENSGIYSWYIESLMDEIQRLDEVSRSNNSTELP
SSQELVNATSIKSEVDEKSNRVTYSQELVDGKSTTGSYLTDQQNPLGETQSTIKMSHEDPPISEIKSSSSQLNDDNQSMS
DSRVEMVGQHKTIETKTLVSETQEFQKIHRKSEMTSVPQAMDQNFSLISSPNMTQSTDETEANNIGKTTTSDKLSIPTSV
LENIEEIGRQRSRDGPAAEGTTIKTLPIGVDNISLGSGVTIHKSSSADILPQDSQVEKDNEMHEDKSEIAQYKNIKIQEGVS
LLDKTKEIGIVSNNRASADTRTRLEQYKHFVDLNDSQISLLVEAIEAYPHLWNACEKFSDRFQAWMLKTLADMLLFLRSE

SVGSVNPEREKEFLKLCDEAVQLGFERSWVDDMRQRVVTRDHKLDQAKTRIGEFVNRHDHLTQELDNIKKELRILNDF
VEAQTKCFDFL
