# Supplementary figures and images for: Investigation of Resistance Genes in Genus Vigna Reveals Highly Variable NLRome in Parallel Domesticated Member Species
Source: Genes (Basel). 2023 May 23;14(6):1129. doi: 10.3390/genes14061129 (PMC10297842; doi:10.3390/genes14061129)

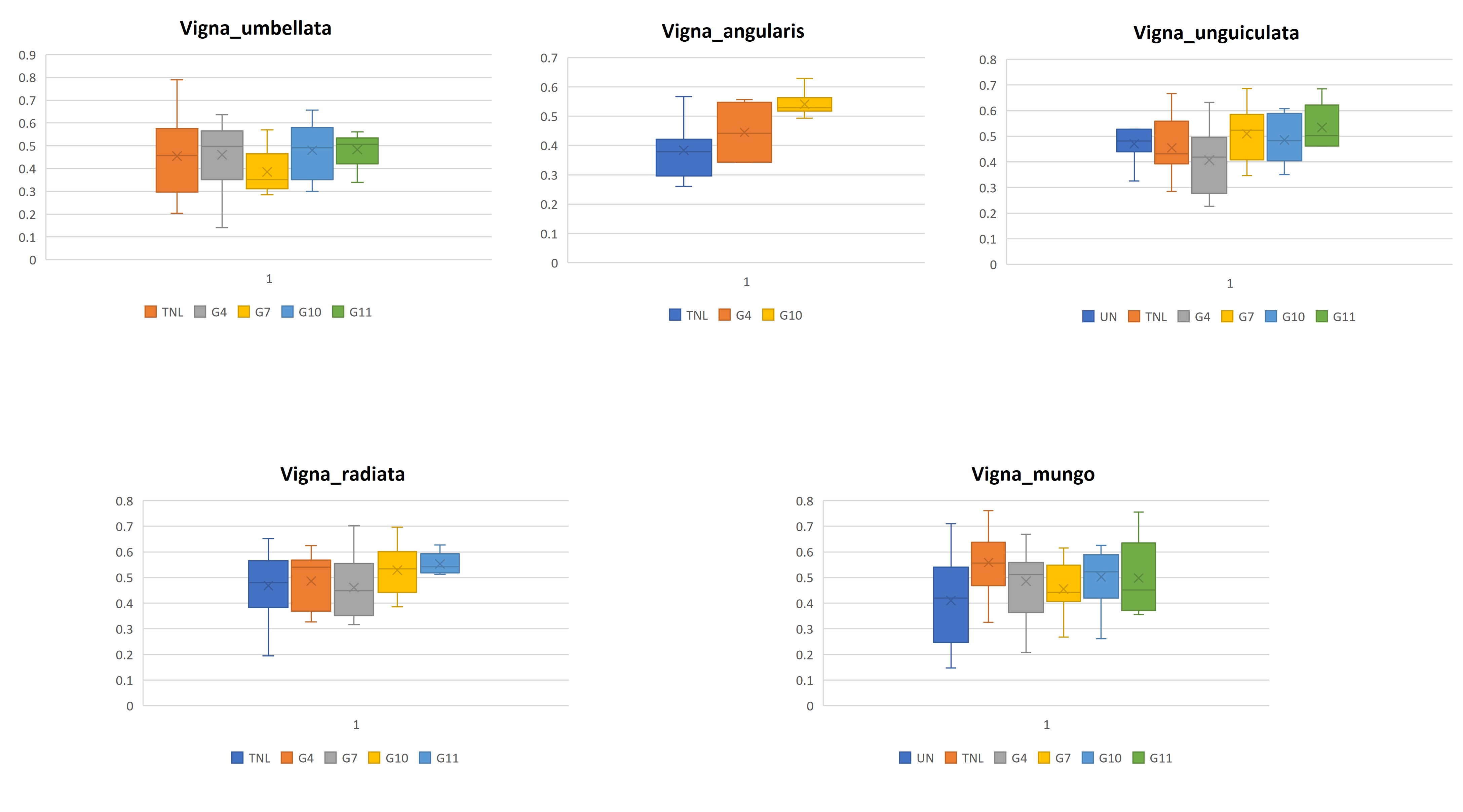

Supplement: Supplementary file 1 [file genes-14-01129-s001.zip › Figure S1 Subgroup_selection.jpg]

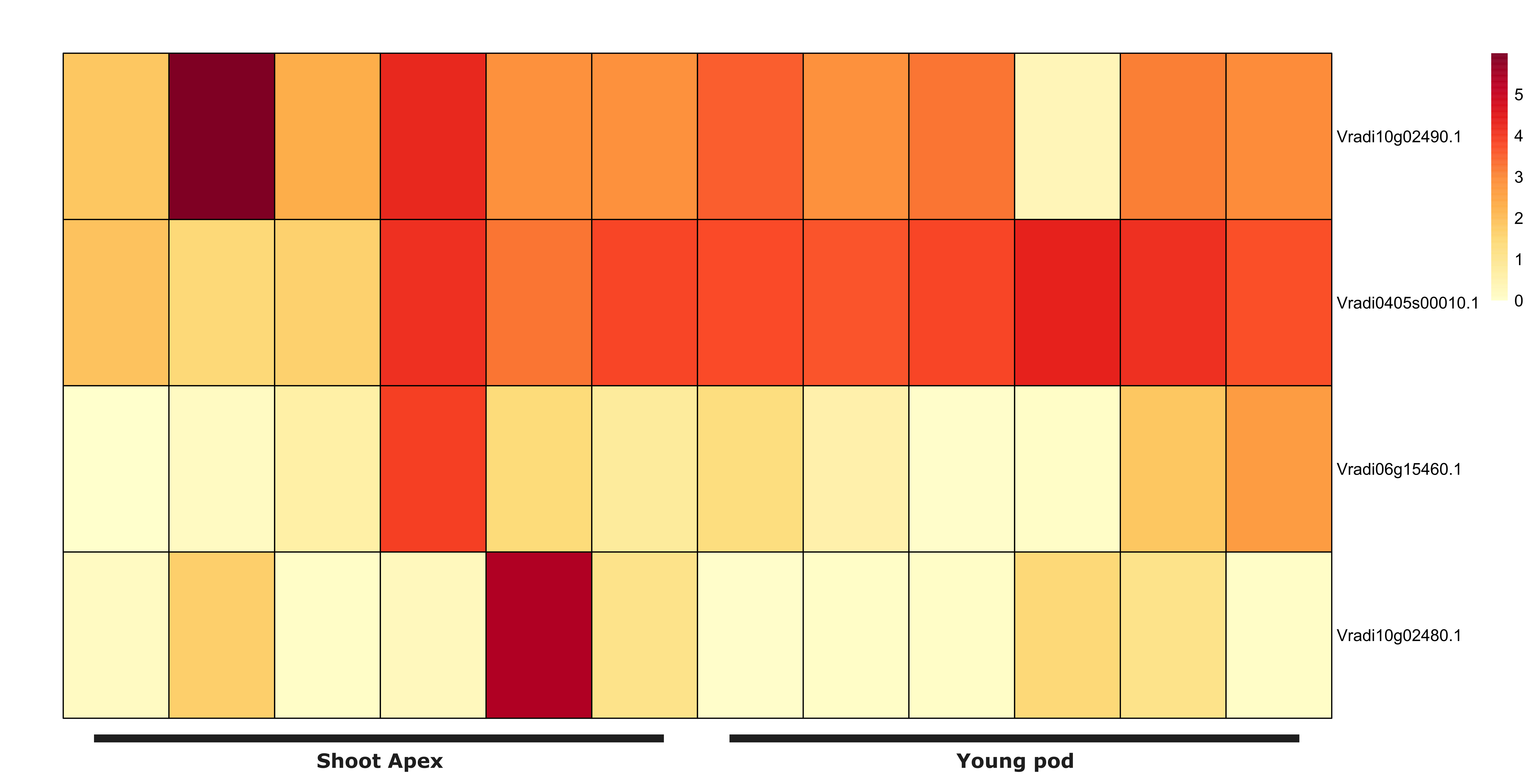

Supplement: Supplementary file 1 [file genes-14-01129-s001.zip › Figure S2.png]
